# Aspirin prevents metastasis by limiting platelet TXA_2_ suppression of T cell immunity

**DOI:** 10.1038/s41586-025-08626-7

**Published:** 2025-03-05

**Authors:** Jie Yang, Yumi Yamashita-Kanemaru, Benjamin I. Morris, Annalisa Contursi, Daniel Trajkovski, Jingru Xu, Ilinca Patrascan, Jayme Benson, Alexander C. Evans, Alberto G. Conti, Aws Al-Deka, Layla Dahmani, Adnan Avdic-Belltheus, Baojie Zhang, Hanneke Okkenhaug, Sarah K. Whiteside, Charlotte J. Imianowski, Alexander J. Wesolowski, Louise V. Webb, Simone Puccio, Stefania Tacconelli, Annalisa Bruno, Sara Di Berardino, Alessandra De Michele, Heidi C. E. Welch, I-Shing Yu, Shu-Wha Lin, Suman Mitra, Enrico Lugli, Louise van der Weyden, Klaus Okkenhaug, Kourosh Saeb-Parsy, Paola Patrignani, David J. Adams, Rahul Roychoudhuri

**Affiliations:** 1 Department of Pathology, University of Cambridge, Cambridge, UK; 2 Systems Pharmacology and Translational Therapeutics Laboratory, Center for Advanced Studies and Technology (CAST), “G. d’Annunzio” University of Chieti-Pescara, Chieti, Italy; 3 Department of Neuroscience, Imaging and Clinical Science, “G. d’Annunzio” University of Chieti-Pescara, Chieti, Italy; 4 Department of Surgery, University of Cambridge and NIHR Cambridge Biomedical Research Centre, Cambridge, UK; 5 Babraham Institute, Babraham, UK; 6 Francis Crick Institute, London, UK; 7 Laboratory of Translational Immunology, IRCCS Humanitas Research Hospital, Rozzano, Italy; 8 Laboratory Animal Center, College of Medicine, National Taiwan University, Taipei, Taiwan; 9 Department of Clinical Laboratory Science and Medical Biotechnology, College of Medicine, National Taiwan University, Taipei, Taiwan; 10 Inserm UMR1277, CNRS UMR9020-CANTHER, Université de Lille, Lille University Hospital, Lille, France; 11 Wellcome Sanger Institute, Wellcome Genome Campus, Cambridge, UK; 12 Present Address: A. B. Department of Innovative Technologies in Medicine and Dentistry, “G. d’Annunzio” University of Chieti-Pescara, Chieti, Italy

**Keywords:** Immunotherapy, Tumour immunology, Adaptive immunity

## Abstract

Metastasis is the spread of cancer cells from primary tumours to distant organs and is the cause of 90% of cancer deaths globally^1,2^. Metastasizing cancer cells are uniquely vulnerable to immune attack, as they are initially deprived of the immunosuppressive microenvironment found within established tumours^3^. There is interest in therapeutically exploiting this immune vulnerability to prevent recurrence in patients with early cancer at risk of metastasis. Here we show that inhibitors of cyclooxygenase 1 (COX-1), including aspirin, enhance immunity to cancer metastasis by releasing T cells from suppression by platelet-derived thromboxane A_2_ (TXA_2_). TXA_2_ acts on T cells to trigger an immunosuppressive pathway that is dependent on the guanine exchange factor ARHGEF1, suppressing T cell receptor-driven kinase signalling, proliferation and effector functions. T cell-specific conditional deletion of *Arhgef1* in mice increases T cell activation at the metastatic site, provoking immune-mediated rejection of lung and liver metastases. Consequently, restricting the availability of TXA_2_ using aspirin, selective COX-1 inhibitors or platelet-specific deletion of COX-1 reduces the rate of metastasis in a manner that is dependent on T cell-intrinsic expression of ARHGEF1 and signalling by TXA_2_ in vivo. These findings reveal a novel immunosuppressive pathway that limits T cell immunity to cancer metastasis, providing mechanistic insights into the anti-metastatic activity of aspirin and paving the way for more effective anti-metastatic immunotherapies.

## Abstract (web summary)

Inhibition of cyclooxygenase 1 releases T cells from immunosuppression by platelet-derived thromboxane A_2_, thereby enhancing the immune response against metastasis.

## Main

Despite advances in primary cancer treatment, many patients treated for early-stage cancers develop metastatic recurrence months to years later owing to the eventual growth of disseminated micrometastases^4^. Micrometastases are vulnerable to immune attack, as they are deprived of the highly immunosuppressive microenvironment found within established tumours^3^. This creates an opportunity for anti-metastatic therapies that utilize the immune system to prevent recurrence in patients with early-stage cancer at risk of metastasis.

Aspirin is an irreversible inhibitor of COX enzymes^5^. Meta-analyses of large randomized controlled trials have shown that daily aspirin treatment is associated with reduction in metastasis at multiple sites in individuals with cancer^6^ (hazard ratio (HR) 0.64, 95% confidence interval (CI) 0.48–0.84). Moreover, low-dose (75–300 mg) aspirin treatment is associated with a reduction in the rate of cancer death in individuals without metastasis at the time of cancer diagnosis^6–8^ (HR 0.49, 95% CI 0.30–0.79). In colorectal cancer, the association of aspirin use with improved survival appears to be restricted to tumours that express high levels of human leukocyte antigen (HLA) class I, suggesting that its effect has an immune component^9^. COX-1 (also known as prostaglandin G/H synthase 1) is constitutively expressed in most tissues, including in platelets, where it is required for TXA_2_ production, whereas COX-2 is predominantly induced during inflammation^5,10,11^. Aspirin has a short half-life (around 20 min), such that only frequent high doses of aspirin can achieve sustained pharmacological inhibition of COX-1 and COX-2 in nucleated cells. By contrast, daily low-dose aspirin primarily targets platelet COX-1, and consequently the production of TXA_2_, since anucleated platelets cannot resynthesize their COX-1 pool, which becomes irreversibly inhibited^5,10^. Collectively, these results suggest a relationship between T cell immunity, suppression of platelet TXA_2_ and the anti-metastatic activity of aspirin, but the direct relation between these has not been established.

In this study, we show that platelet TXA_2_ suppresses immunity to cancer metastasis by activating a T cell-intrinsic immunosuppressive pathway that is dependent on the guanine exchange factor ARHGEF1. Consequently, restricting the availability of TXA_2_ using aspirin, selective COX-1 inhibitors or platelet-specific deletion of COX-1 reduced the rate of metastasis in a manner that was dependent on T cell-intrinsic expression of ARHGEF1 and signalling by TXA_2_ in vivo. These findings reveal a novel immunosuppressive pathway that limits T cell immunity to cancer metastasis, providing a mechanistic basis for the anti-metastatic activity of aspirin and paving the way for the development of more effective anti-metastatic immunotherapies.

## ARHGEF1 suppresses T cell immunity to metastasis

**Fig. 1:**
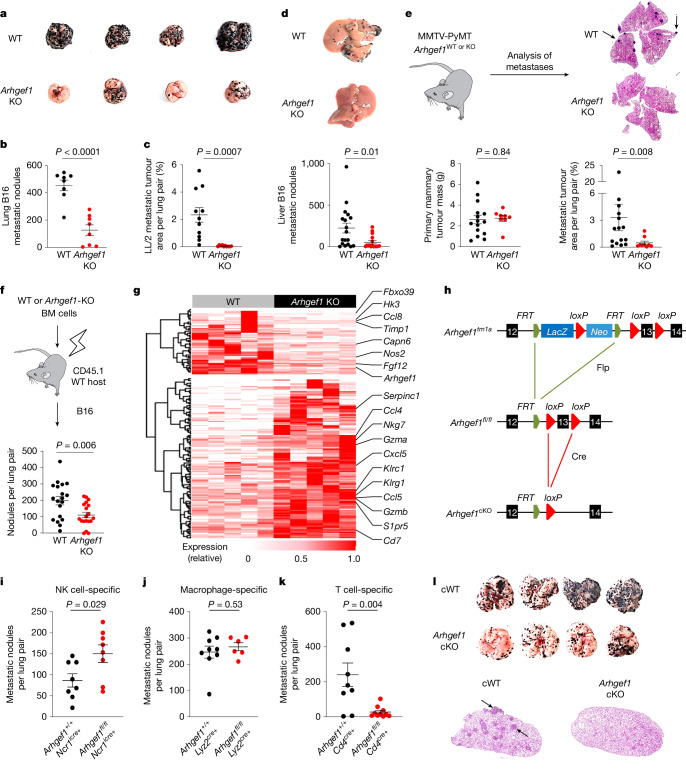
ARHGEF1 suppresses T cell immunity to cancer metastasis. **a**,**b**, Photographs (**a**) and frequency (**b**) of lung metastases from wild-type (WT; *n* = 8) and *Arhgef1*-knockout (KO; *n* = 8) littermates after intravenous injection of B16 melanoma cells. **c**, Quantification of lung metastases relative to total lung area from wild-type (*n* = 11) and *Arhgef1-*KO (*n* = 10) littermates after intravenous injection of LL/2 carcinoma cells. **d**, Photographs (top) and frequency (bottom) of liver metastases from wild-type (*n* = 18) and *Arhgef1*-KO (*n* = 16) littermates after intrasplenic injection of B16 cells. **e**, Schema (top left) showing generation of *Arhgef1*-wild-type or *Arhgef1*-KO MMTV-PyMT mice. Quantification of primary mammary tumour mass (bottom left), haematoxylin and eosin (H&E) staining of lung sections (top right) and lung metastases relative to total lung area (bottom right) in *Arhgef1*-wild-type (*n* = 15) and *Arhgef1*-KO (*n* = 9) MMTV-PyMT mice. Arrows show lung metastases. **f**, Schema (top) and frequency of metastases (bottom) after intravenous injection of B16 cells into bone marrow (BM) chimeras reconstituted with wild-type (*n* = 19) and *Arhgef1*-KO (*n* = 19) bone marrow cells. **g**, Heat map showing differentially expressed genes (*q* < 0.05; fold change (|FC|) > 2) between whole tumour-bearing lungs of wild-type (*n* = 5) and *Arhgef1*-KO (*n* = 5) littermates at day 7 after intravenous tumour injection. **h**, Generation of *Arhgef1* conditional-knockout (cKO) allele. **i**–**k**, Frequency of lung metastases in mice of indicated genotypes: *Ncr1*^*icre*^^*+*^ and *Ncr1*^*icre+*^ *Arhgef1*^*fl/fl*^ (**i**; *n* = 8); *Lyz2*^*cre+*^ (*n* = 9) and *Lyz2*^*cre+*^ *Arhgef1*^*fl/fl*^ (*n* = 6) (**j**); and *Cd4*^*cre*^ (*n* = 9) and *Cd4*^*cre*^ *Arhgef1*^*fl/fl*^ (*n* = 10) (**k**), after intravenous injection of B16 cells. **l**, Photographs (top) and H&E staining (bottom) of *Cd4*^*cre*^ (cWT) and *Cd4*^*cre*^ *Arhgef1*^*fl/fl*^ (*Arhgef1*-cKO) mice from **k**. Data are representative of five (**b**,**k**) or two (**c**,**i**,**j**) independent experiments, or pooled from three (**e**) or two (**d**,**f**) independent experiments. Unpaired two-tailed Student’s *t*-test (**b**,**c**,**f**,**i**–**k**); Two-tailed Mann–Whitney *U*-test (**d**,**e**). Data are mean ± s.e.m. Source data

**Extended Data Fig. 1:**
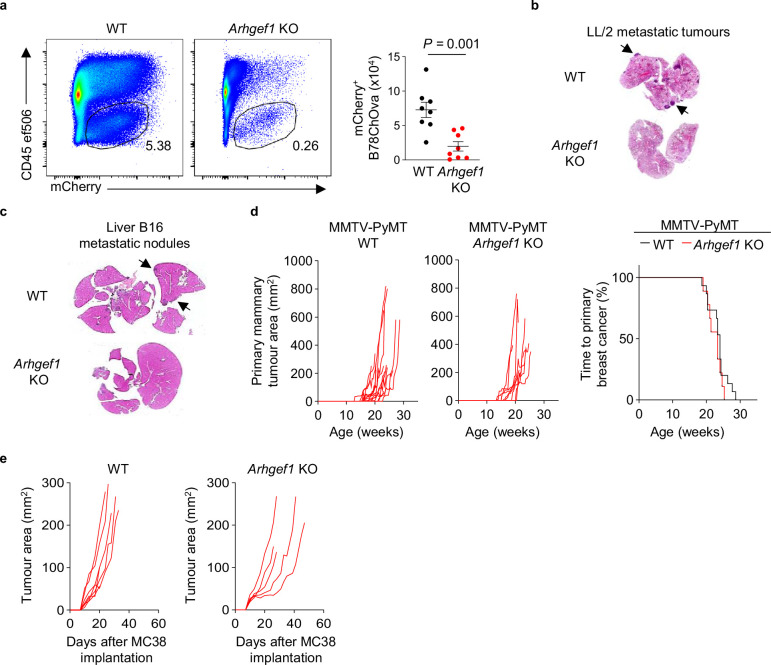
Reduced cancer metastasis in animals lacking ARHGEF1. **a**, Representative flow cytometry plots (left) and number (right) of mCherry^+^ CD45^–^ tumour cells in lungs of WT and *Arhgef1* KO littermates 17 days after intravenous (i.v.) injection of B78ChOva-mCherry cells. **b**, Representative photomicrographs of haematoxylin and eosin (H&E) stains of lung sections from wildtype and *Arhgef1*-deficient animals 17 days after intravenous injection of syngeneic LL/2 lung adenocarcinoma cells. Arrows indicate metastatic deposits. **c**, Representative photomicrographs of H&E stains of liver sections from wildtype and *Arhgef1*-deficient animals 11 days after intrasplenic injection of syngeneic B16-F10 melanoma cells. Arrows indicate metastatic deposits. **d**, Cumulative area of primary mammary tumours arising in *Arhgef1*-WT (*n* = 15) and -KO (*n* = 9) MMTV-PyMT female littermate mice at the indicated ages (left) and survival time to cumulative area of 2.25 cm^2^, at which time point lungs were harvested for histopathology analysis. Log-rank test; *P* = 0.31. **e**, Area of tumours of WT (*n* = 7) and *Arhgef1* KO (*n* = 5) littermates was measured at the indicated time points following subcutaneous implantation of MC38 colorectal adenocarcinoma cells. Data are representative of five (a) and two (e) independent experiments or pooled from three (d) independent experiments. Unpaired two-tailed Student’s t test (a). Graphs show mean ± s.e.m. Source data

We previously performed a large in vivo genetic screen to identify host regulators of cancer metastasis^12^. This screen identified 15 genes whose disruption in host tissues reduced the frequency of lung metastases after intravenous injection of syngeneic B16 melanoma cells, including the gene encoding the 115-kDa RHO guanine exchange factor ARHGEF1^13,14^. Confirming the results of our initial screen using littermate controls, we found a reduced number of metastases in the lungs of *Arhgef1*-deficient mice compared with wild-type controls after intravenous injection of syngeneic B16 melanoma cells (Fig. 1a,b). This corresponded to a reduction in the frequency of mCherry-labelled tumour cells in the lungs of *Arhgef1*-deficient mice upon intravenous injection of B78ChOva melanoma cells (Extended Data Fig. 1a). We similarly observed a decrease in the rate of lung metastasis after intravenous injection of syngeneic LL/2 Lewis lung carcinoma cells into *Arhgef1*-deficient mice (Fig. 1c and Extended Data Fig. 1b). The reduction in the rate of metastasis upon ARHGEF1 loss was not restricted to the lungs, since we also observed reduced numbers of liver metastases upon intrasplenic implantation of B16 cells in *Arhgef1*-deficient mice (Fig. 1d and Extended Data Fig. 1c).

Mice bearing the MMTV-PyMT germline mutation develop primary breast cancers that spontaneously metastasize to the lungs^15^. Although we observed no major difference in the growth of primary mammary tumours in *Arhgef1-*deficient mice compared with wild-type controls when crossed onto the MMTV-PyMT background (Extended Data Fig. 1d), we noted a reduction in the frequency of metastatic nodules in the lungs of *Arhgef1-*deficient mice, when mice with similarly sized primary mammary tumours were analysed (Fig. 1e). Similar to the lack of substantial effect on the growth of primary MMTV-PyMT breast tumours, ARHGEF1 deficiency had minimal effect on the growth of subcutaneously implanted syngeneic MC38 colorectal adenocarcinoma tumours (Extended Data Fig. 1e). These findings showed that loss of ARHGEF1 expression in host tissues reduces the development of cancer metastases at multiple metastatic sites.

**Extended Data Fig. 2:**
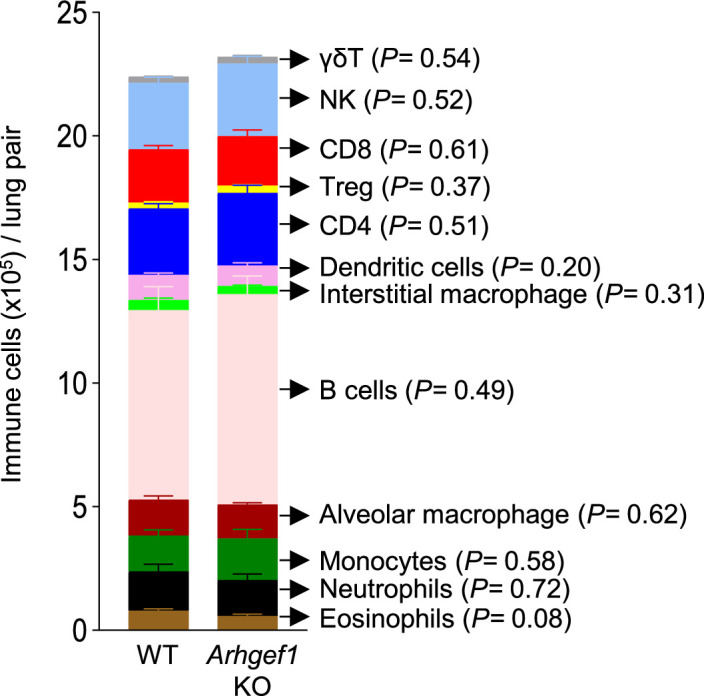
Analysis of haematopoietic lineages within tumour-bearing lungs of WT and *Arhgef1*-deficient animals. Frequency of mature haematopoietic lineages within tumour-bearing lungs of WT (*n* = 9) and *Arhgef1* KO (*n* = 9) littermates 16 days after intravenous injection of B16 cells. Data are pooled from two independent experiments. Unpaired two-tailed Student’s t test. Graphs show mean ± s.e.m. Source data

ARHGEF1 is predominantly expressed in cells of the haematopoietic lineage^13,14,16^. We did not observe major differences in the frequency of mature haematopoietic lineages in tumour-bearing lungs of *Arhgef1*-deficient mice compared with wild-type controls (Extended Data Fig. 2). However, we found that loss of ARHGEF1 in haematopoietic cells was sufficient to confer the observed anti-metastatic effect, since lethally irradiated wild-type mice reconstituted with bone marrow haematopoietic cells from *Arhgef1*-deficient mice exhibited a reduced frequency of B16 lung metastases compared with mice reconstituted with wild-type haematopoietic cells (Fig. 1f). Metastatic colonization of the lungs of *Arhgef1*-deficient mice was associated with increased expression of genes associated with immune activation and cytotoxic function, including *Ccl4*, *Nkg7*, *Gzma*, *Gzmb*, *Cxcl5* and *Klrg1*, compared with wild-type controls (Fig. 1g and Supplementary Table 1). These findings led us to hypothesize that ARHGEF1 exerts an immunosuppressive effect in cells of the haematopoietic lineage.

**Extended Data Fig. 3:**
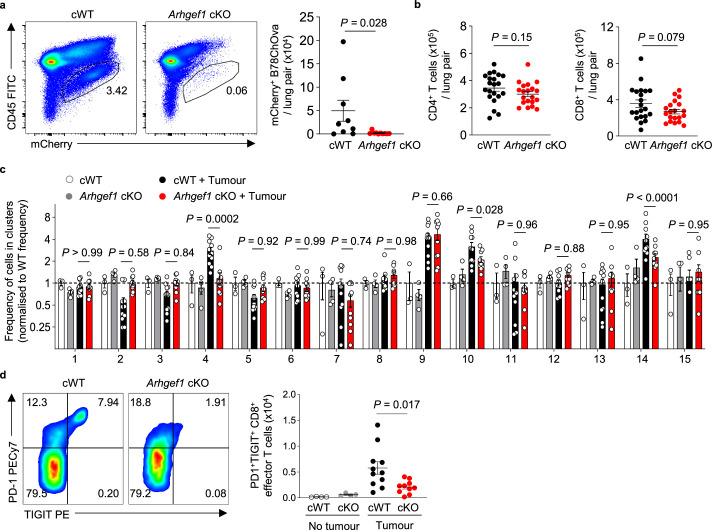
Immune responses in tumour-bearing lungs of animals bearing a conditional deletion of ARHGEF1 in T cells. **a**, Flow cytometry plots (left) and replicate measurements (right) of the number of mCherry^+^ CD45^–^ tumour cells in lungs of *Cd4*^Cre^ (cWT = 9) and *Arhgef1*^fl/fl^ *Cd4*^Cre^ (cKO =10) animals 17 days after intravenous (i.v.) injection of B78ChOva cells. **b**, Quantification of CD4^+^ (left) and CD8^+^ (right) T cells from tumour-bearing lungs of cWT (*n* = 21) and cKO (*n* = 20) mice 17 days after i.v. injection of tumour cells. **c**, Relative frequency of CD8^+^ T cells within all 15 phenograph clusters (data for clusters 4, 9, 10 and 14 also shown in Fig. 2g, as noted in Source Data) from lungs of B78ChOva tumour-bearing and non-tumour-bearing mice of the indicated genotypes (tumour-bearing cWT, *n* = 11; tumour-bearing *Arhgef1* cKO, *n* = 10; cWT, *n* = 3; cKO, *n* = 4). **d**, PD-1 and TIGIT expression on effector CD8^+^ T cells within tumour-bearing lungs of animals in (c) of the indicated genotypes. Representative flow cytometry plots (left) and replicate measurements (right). Data are representative of five (a) and two (c, d) independent experiments or pooled from three (b) independent experiments. Unpaired two-tailed Student’s *t* test (b); 2-way ANOVA with Tukey multiple comparisons test (c); One-way ANOVA with Tukey multiple comparisons test (d) and two-tailed Mann-Whitney U test (a). Graphs show mean ± s.e.m. Source data

To determine the haematopoietic cell types in which ARHGEF1 exerts its immunosuppressive function, we generated a floxed *Arhgef1* mouse allele (hereafter *Arhgef1*^*fl*^), enabling conditional deletion of ARHGEF1 in specific cellular lineages upon Cre-mediated recombination (Fig. 1h). We crossed *Arhgef1*^*fl/fl*^ mice to *Ncr1*^*cre*^, *Lyz2*^*cre*^ and *Cd4*^*cre*^ mice, enabling loss-of-function analysis of ARHGEF1 in haematopoietic cells of the natural killer (NK) cell, macrophage and T cell lineages, respectively^17–19^. Whereas NK cell- and macrophage-specific ablation of ARHGEF1 did not result in reduced frequency of lung metastases after intravenous administration of B16 melanoma cells, we found that T cell-specific ablation of ARHGEF1 in *Arhgef1*^*fl/fl*^ *Cd4*^*cre*^ mice (hereafter *Arhgef1*-cKO mice) resulted in markedly reduced lung metastasis compared with *Arhgef1*^+/+^ *Cd4*^*cre*^ (hereafter cWT) controls (Fig. 1i–l and Extended Data Fig. 3a), recapitulating the resistance to metastasis observed in mice lacking ARHGEF1 in all tissues.

## Loss of ARHGEF1 enhances T cell function

**Fig. 2:**
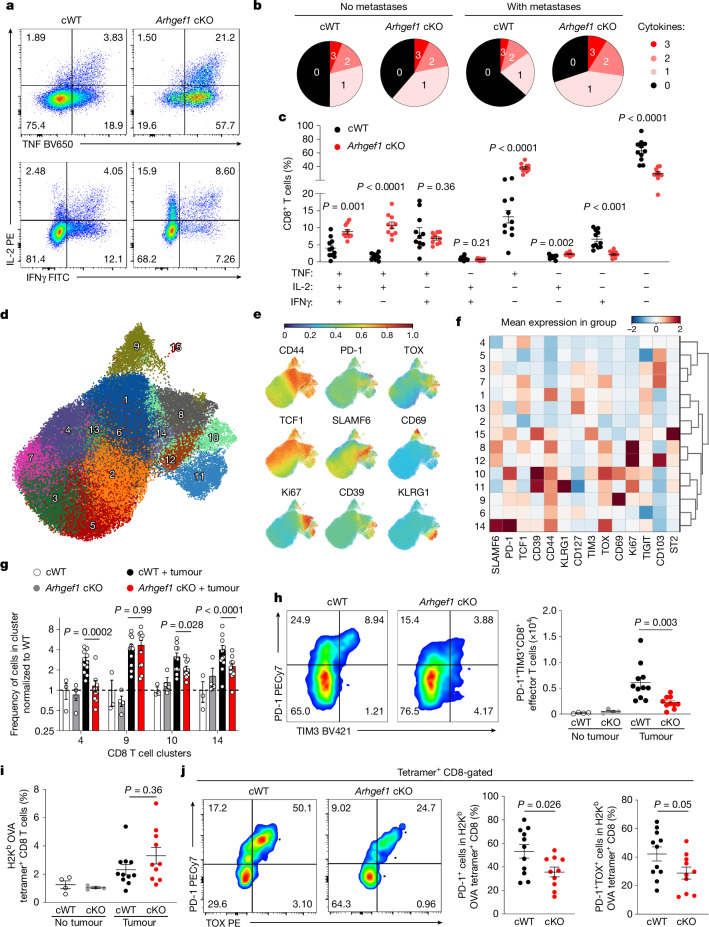
Loss of ARHGEF1 promotes CD8^+^ T cells with enhanced cytokine polyfunctionality. **a**–**c**, Representative flow cytometry plots of TNF, IL-2 and IFNγ expression (**a**) and frequency of cells expressing TNF, IL-2 and IFNγ, presented in terms of cytokine polyfunctionality (**b**) and relative frequency (**c**), following intracellular cytokine staining of CD8^+^ T cells from lungs of *Cd4*^*cre*^ (cWT, *n* = 11) and *Arhgef1*^*fl/fl*^ *Cd4*^*cre*^ (*Arhgef1* cKO, *n* = 10) mice 17 days after intravenous injection of B78ChOva melanoma cells. **d**, Uniform manifold approximation and projection (UMAP) analysis of the phenotype of concatenated CD8^+^ T cells from lungs of B78ChOva tumour-bearing (cWT, *n* = 11; *Arhgef1* cKO, *n* = 10) and non-tumour-bearing mice (cWT, *n* = 3; cKO, *n* = 4). Colours and numbering depict cell clusters identified by Phenograph. **e**, Relative expression of indicated markers by CD8^+^ T cells in **d**. **f**, Mean expression of indicated markers by CD8^+^ T cells in indicated clusters in **d**. **g**, Relative frequency of CD8^+^ T cells in indicated Phenograph clusters in **d**. **h**, PD-1 and TIM3 expression on effector CD8^+^ T cells in tumour-bearing lungs from mice described in **d** of the indicated genotypes. Representative flow cytometry plots (left) and replicate measurements (right). **i**,**j**, Frequency of OVA_257–264_ tumour antigen-specific CD8^+^ T cells as detected by peptide–MHC tetramer staining from tumour-bearing lungs (**i**) of mice in **d** and representative flow cytometry plots and replicate measurements of PD-1 and TOX expression on OVA_257–264_ tumour antigen-specific CD8^+^ T cells (**j**). Data are representative of two independent experiments. Unpaired two-tailed Student *t*-tests with Holm–Šídák correction for multiple hypothesis testing (**c**); two-way analysis of variance (ANOVA) with Tukey multiple comparisons test (**g**); one-way ANOVA with Tukey multiple comparisons test (**h**,**i**); and unpaired two-tailed Student *t*-tests (**j**). Data are mean ± s.e.m. Source data

Cytokine polyfunctionality is a hallmark of effective anti-viral and anti-tumour immune responses^20,21^. To better understand how ARHGEF1-deficient T cells control pulmonary metastasis, we analysed lung-infiltrating T cells using flow cytometry. Although a similar number of CD4^+^ and CD8^+^ T cells were found in tumour-bearing lungs of cWT and *Arhgef1*-cKO mice (Extended Data Fig. 3b), intracellular cytokine staining of T cells from tumour-bearing lungs revealed markedly increased frequencies of polyfunctional cells that co-express two or more cytokines among IFNγ, IL-2 and TNF in *Arhgef1*-cKO mice compared with cWT mice (Fig. 2a–c).

**Extended Data Fig. 4:**
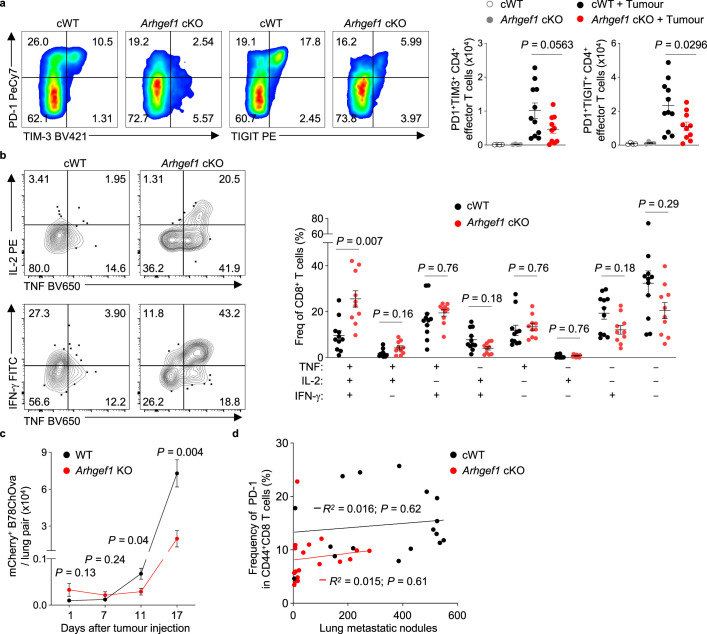
Characterisation of T cell responses and metastasis load in *Arhgef1-*deficient animals. **a**, PD-1, TIM-3 and TIGIT expression on effector CD4^+^ T cells within tumour-bearing lungs of *Cd4*^Cre^ (cWT) and *Arhgef1*^fl/fl^ *Cd4*^Cre^ (*Arhgef1* cKO) animals. Flow cytometry plots (left) show CD4^+^ T cells from tumour-bearing lungs, and graphs show replicate measurements from tumour-bearing (cWT = 11; *Arhgef1* cKO = 10) and non-bearing (cWT = 4; *Arhgef1* cKO = 4) animals (right). **b**, Intracellular cytokine staining of OVA_257-264_-specific CD8^+^ T cells from tumor-bearing lungs of *Cd4*^Cre^ (cWT = 11) and *Arhgef1*^fl/fl^ *Cd4*^Cre^ (*Arhgef1* cKO = 10) animals 17 days after intravenous (i.v.) injection of B78ChOva melanoma cells. Flow cytometry plots of TNF, IL-2 and IFN-γ expression (left) and frequency of cells expressing indicated cytokines upon brief stimulation ex vivo, presented in terms of cytokine polyfunctionality (right). **c**, Kinetic analysis of mCherry^+^ CD45^−^ melanoma cells within lungs from WT (*n* = 6-8) and *Arhgef1* KO mice (*n* = 4-8) at the indicated time points after i.v. injection of B78ChOva cells. **d**, Relationship between tumour burden and PD-1 expression by CD44^+^ CD8^+^ T cells of lungs of *Cd4*^Cre^ (cWT = 17) and *Arhgef1*^fl/fl^ *Cd4*^Cre^ (*Arhgef1* cKO = 18) animals 17 days after i.v. injection of tumour cells. Data are representative of two (a-c) or pooled from two (d) independent experiments. One-way ANOVA with Tukey multiple comparisons test (a); unpaired two-tailed Student *t* tests with Holm-Šídák correction for multiple hypothesis testing (b, c) and Simple Linear Regression (d). Graphs show mean ± s.e.m. Source data

T cell exhaustion is associated with reduced cytokine polyfunctionality and anti-tumour activity^22–24^. Analysing the phenotype of T cells in the lungs by high-dimensional flow cytometry, we found that metastatic colonization induces populations of CD4^+^ and CD8^+^ T cells that express high levels of the exhaustion marker PD-1, including both terminally exhausted TIM3^+^TIGIT^+^TOX^+^CD39^+^TCF1^low^ (cluster 10) cells and progenitor exhausted SLAMF6^+^TIM3^−^TIGIT^−^TCF1^hi^ (cluster 14) cells, whose frequency was reduced in *Arhgef1*-cKO mice compared with cWT mice (Fig. 2d–h and Extended Data Figs. 3c,d and 4a). Of note, we observed increased cytokine polyfunctionality and reduced expression of the exhaustion markers PD-1 and TOX among tumour antigen-specific CD8^+^ T cells responding to ovalbumin (OVA) expressed by B78ChOva metastases in lungs of *Arhgef1*-cKO mice (Fig. 2i,j and Extended Data Fig. 4b).

**Extended Data Fig. 5:**
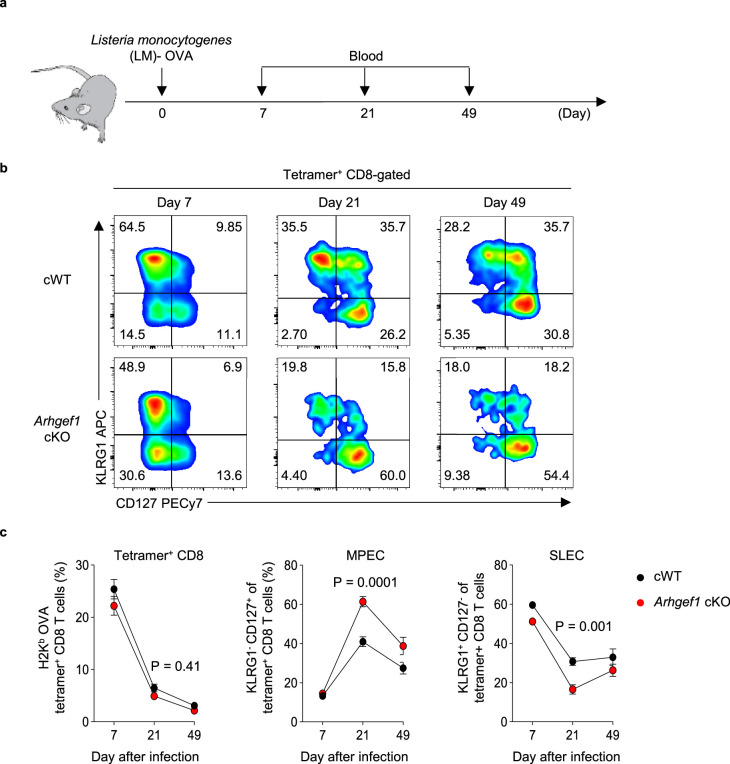
Loss of ARHGEF1 within T cells promotes memory-like CD8^+^ T cell responses following attenuated *Listeria monocytogenes* (LM)-Ova infection. **a**, Experimental schema showing timeline of *Cd4*^Cre^ (cWT) and *Arhgef1*^fl/fl^ *Cd4*^Cre^ (*Arhgef1* cKO) littermates following attenuated LM-Ova infection. **b, c**, Representative flow cytometry plots (**b**) and frequency (**c**) of tetramer^+^ CD8^+^ T cells and its subsets in blood samples of *Cd4*^Cre^ (cWT, *n* = 5-9) and *Arhgef1*^fl/fl^ *Cd4*^Cre^ (*Arhgef1* cKO, *n* = 5-10) littermates at indicated time points following attenuated LM-Ova infection. MPEC, memory precursor effector cells; SLEC, short-lived effector cells; Data are pooled from two independent experiments. Multiple unpaired two-tailed Student’s t-test with Holm-Šídák correction (c). Graphs show mean ± s.e.m. Source data

Corresponding to augmented adaptive immune responses to metastasis, *Arhgef1* deletion reduced the late accumulation of mCherry-labelled cancer cells in the lungs at days 11 and 17 after tumour injection, but did not affect their early accumulation and growth at days 1 and 7 after tumour injection (Extended Data Fig. 4c). PD-1 expression is induced upon sustained exposure to tumour antigen^25^. We found that the frequency of PD-1^+^ cells among antigen-experienced CD8^+^ T cells did not correlate with the number of metastases when either wild-type or *Arhgef1*-deficient mice were analysed, suggesting that reduced PD-1 expression was unlikely to be the consequence of decreased tumour burden in the lungs of *Arhgef1*-deficient mice (Extended Data Fig. 4d). Moreover, the function of ARHGEF1 was not restricted to tumour-specific T cell responses, since OVA antigen-specific CD8^+^ T cells responding to infection with *Listeria monocytogenes* expressing OVA exhibited a less terminally differentiated phenotype with increased levels of the memory marker CD127 in *Arhgef1*-cKO compared with cWT mice^26^ (Extended Data Fig. 5a–c). Collectively, these experiments show that ARHGEF1 functions intrinsically in T cells to limit effector functions and anti-metastatic immunity in vivo.

## TXA_2_ suppresses T cells via ARHGEF1

**Fig. 3:**
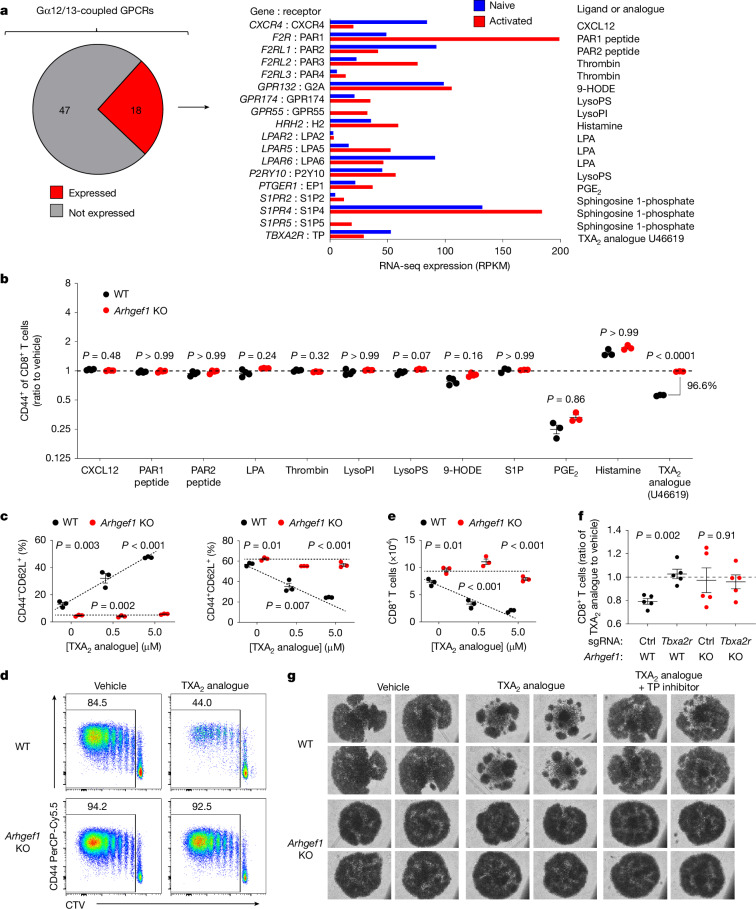
TXA_2_ suppresses activation and proliferation of T cells via ARHGEF1. **a**, Identification of Gα12/13-coupled GPCRs expressed by T cells. Left, expression in naive and activated T cells of genes encoding Gα12/13-coupled GPCRs with moderate to high coupling index to Gα12 or Gα13 (log relative intrinsic activity > –1) identified in ref. ^30^. Right, known ligands or agonists of expressed receptors. 9-HODE, 9-hydroxyoctadecadienoic acid; LPA, lysophosphatidic acid; LysoPI, lysophosphatidylinositol; LysoPS, lysophosphatidylserine; PGE_2_, prostaglandin E_2_. **b**, In vitro ligand screen of identified Gα12/13-coupled GPCRs. Naive FACS-sorted CD8^+^ T cells were stimulated in vitro with anti-CD3/28 antibodies and recombinant human IL-2 (rhIL-2) in the presence of indicated ligands or agonists. The ratio of activated CD44^+^ cells among wild-type and *Arhgef1*-deficient CD8^+^ T cells was measured at day 5. *n* = 3–4 independent replicates per condition. S1P, sphingosine 1-phosphate. **c**, Differentiation state of naive wild-type and *Arhgef1*-deficient CD8^+^ T cells stimulated in vitro with anti-CD3/28 antibodies and rhIL-2 in the presence of indicated concentrations of TXA_2_ analogue U46619. **d**,**e**, CellTrace Violet (CTV) proliferation analysis (**d**) and cell number (**e**) on day 5 for naive wild-type and *Arhgef1*-deficient CD8^+^ T cells stimulated with anti-CD3/28 antibodies and rhIL-2 in the presence or absence of 5μM TXA_2_ analogue U46619. **f**, Naive wild-type and *Arhgef1*-deficient CD8^+^ T cells were electroporated with nucleoprotein complexes of Cas9 and single guide RNAs (sgRNAs) targeting *Tbxa2r* or scrambled sgRNA control (Ctrl) and stimulated with anti-CD3/28 antibodies and rhIL-2 in the presence or absence of 5 μM TXA_2_ analogue U46619. *n* = 5 independent replicates per condition. **g**, Photomicrographs of cells 5 days after stimulation of naive CD8^+^ T cells with anti-CD3/28 antibodies and rhIL-2 in the presence of TXA_2_ analogue U46619 or vehicle control, and treatment with the TXA_2_ receptor inhibitor (SQ 29548, 10 μM). Data are representative of two (**b**,**f**,**g**) or three (**c**–**e**) independent experiments. Two-tailed Student *t*-tests with Bonferroni–Dunn (**b**) and Holm–Šídák (**c**,**e**,**f**) correction for multiple hypothesis testing. Data are mean ± s.e.m. Source data

We sought to define upstream receptors and ligands that drive the immunosuppressive function of ARHGEF1 in T cells so as to reveal extracellular components of the pathway that might be amenable to therapeutic targeting. G-protein-coupled receptors (GPCRs) sense a variety of extracellular signals and control diverse cellular responses. ARHGEF1 is activated by a subset of GPCRs coupled to the G-protein subunits Gα12 and Gα13, and in turn activates the GTPase RHOA to drive intracellular signal transduction^27–29^. To identify candidate GPCRs that act upstream of ARHGEF1 in T cells, we performed a bioinformatic analysis to identify Gα12/13-coupled GPCRs expressed by T cells. Previous work has biochemically quantified ligand-induced interactions between 148 GPCRs and known G-protein subunits^30^. Using these data, we generated a list of Gα12/13-coupled GPCRs and examined their expression at an mRNA level in naive and activated CD8^+^ T cells. This revealed 18 Gα12/13-coupled GPCRs that were expressed at significant mRNA levels by naive or activated CD8^+^ T cells (Fig. 3a).

**Extended Data Fig. 6:**
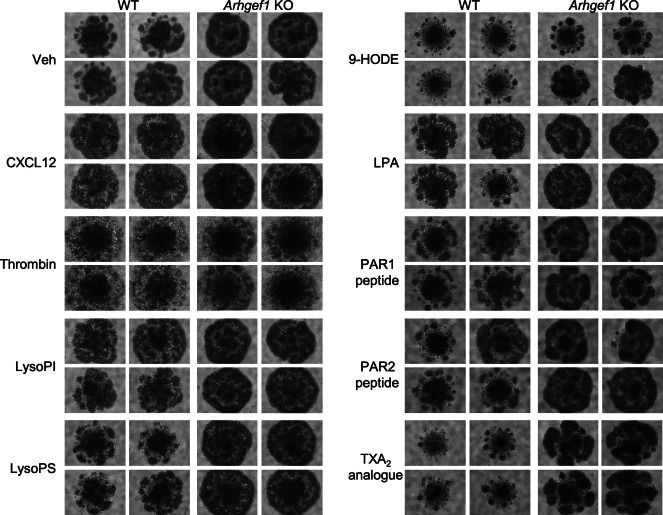
In vitro ligand screen of identified Ga12/13-coupled GPCRs in ARHGEF1-deficient T cells. Naïve FACS-sorted CD8^+^ T cells were stimulated in vitro with anti-CD3/28 antibodies and rhIL-2 in the presence of indicated ligands and photomicrographs of cell culture at day 5 post-stimulation for indicated genotypes were shown. Data are representative of two independent experiments.

**Extended Data Fig. 7:**
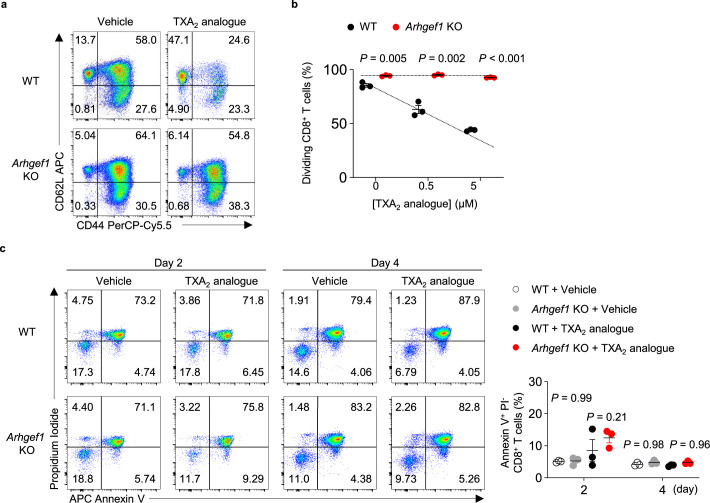
TXA_2_ signaling regulates CD8^+^ T cell differentiation and proliferation in an ARHGEF1-dependent manner in vitro*.* **a-c**, Naïve FACS-sorted CD8^+^ T cells were stimulated in vitro with anti-CD3/28 antibodies and rhIL-2 in the presence of TXA_2_ analogue or vehicle control. The differentiation state based on surface marker expression (a), proliferation measured by CellTrace Violet (CTV) (b), and apoptosis measured by Annexin V and Propidium Iodide (c) of WT and *Arhgef1* KO CD8^+^ T cells at indicated time points post-stimulation were assessed by flow cytometry. *n* = 3 independent replicates per condition. Data are representative of two independent experiments. Multiple unpaired two-tailed Student’s t-test with Holm-Šídák correction (b, c). Graphs show mean ± s.e.m. Source data

**Extended Data Fig. 8:**
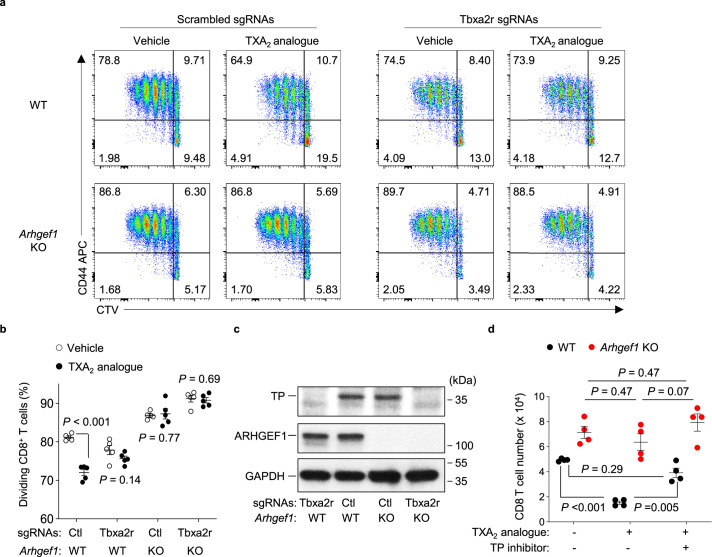
The TXA_2_ receptor TP regulates CD8^+^ T cells in an ARHGEF1-dependent manner*.* **a-b**, Proliferation WT and *Arhgef1*-deficient naïve CD8^+^ T cells electroporated with nucleoprotein complexes of Cas9 and 3 different sgRNAs targeting *Tbxa2r* or scrambled sgRNA control and stimulated in vitro with anti-CD3/28 antibodies and rhIL-2 in the presence or absence of 5 μM TXA_2_ analogue U46619. Proliferation is assessed as dilution of the CellTrace Violet. *n* = 5 independent replicates per condition. **c**, Confirmation of *Tbxa2r* deletion using SDS-PAGE and Western blotting of cell lysates from cells in (a, b). **d**, Number of cells 5 days after stimulation of naïve FACS-sorted CD8^+^ T cells with anti-CD3/28 antibodies and rhIL-2 in the presence of TXA_2_ analogue U46619 or vehicle control and the treatment with TP inhibitor SQ 29548 (10 μM). *n* = 4 independent replicates per condition. Data are representative of two independent experiments (a-d). Multiple two-tailed Student’s t-test with Holm-Šídák correction (b) and 2-way ANOVA with Tukey multiple comparisons test (d). Graphs show mean ± s.e.m. Source data

To determine which among the identified Gα12/13-coupled GPCRs act upstream of ARHGEF1 to suppress T cell activation, we performed a screen to test whether their known ligands or agonists suppress T cells in an ARHGEF1-dependent manner in vitro. The majority of ligands had little effect on T cell activation, or affected T cell activation in a manner that was independent of ARHGEF1. However, T cell activation was suppressed in the presence of a stable analogue of TXA_2_, U46619, which is a ligand of the Gα12/13-coupled TXA_2_ receptor (TP (also known as TXA_2_-R)), in a manner that was almost completely dependent on ARHGEF1 (Fig. 3b and Extended Data Fig. 6). Confirming the results of the screen, we found that U46619 (hereafter TXA_2_ analogue) potently suppressed the activation and proliferation of fluorescence-activated cell sorting (FACS)-purified wild-type naive CD8^+^ T cells upon stimulation, whereas it had a minimal effect on *Arhgef1-*deficient cells (Fig. 3c,d and Extended Data Fig. 7a,b). Moreover, TXA_2_ analogue treatment limited the expansion of wild-type but not *Arhgef1-*deficient CD8^+^ T cells in a manner that was dependent on the activity of its receptor TP (encoded by *Tbxa2r*), since the effect of TXA_2_ was reversed upon genetic or pharmacological disruption of TP in *Arhgef1*-proficient cells (Fig. 3e–g and Extended Data Fig 8a–d). Notably, TXA_2_ receptor signalling suppressed T cell proliferation and activation in the absence of any other cell type, suggesting that TXA_2_ can suppress T cells independently of effects on T cell–dendritic cell interactions^31^. We did not observe differences in apoptosis upon treatment of T cells with the TXA_2_ analogue (Extended Data Fig. 7c). These findings suggest that ARHGEF1 has a critical role in transducing TXA_2_ signalling in T cells, limiting T cell activation and proliferation in response to T cell receptor (TCR) signalling.

## TXA_2_ limits TCR-driven kinase signalling

**Fig. 4:**
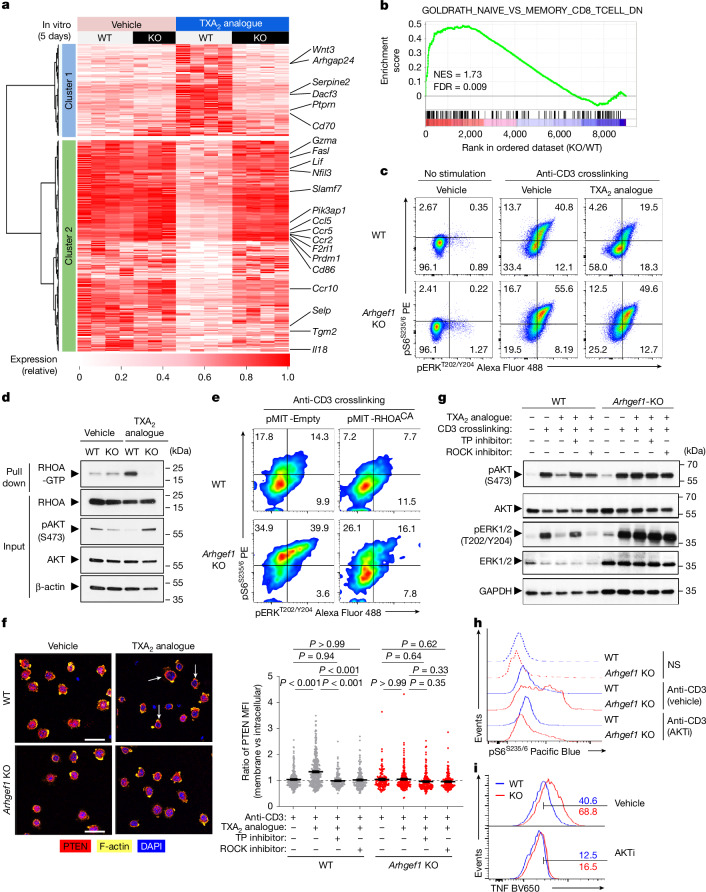
Thromboxane signalling suppresses TCR-driven T cell and kinase pathway activation via TP, ARHGEF1 and RHOA. **a**, Heat map showing differentially expressed genes 5 days after stimulation of naive CD8^+^ T cells with anti-CD3/28 antibodies and rhIL-2 in the presence of TXA_2_ analogue or vehicle (*q* < 0.05; |FC| > 1.5). **b**, Enrichment analysis of indicated gene set in global transcriptional differences between TXA_2_-treated *Arhgef1*-KO versus wild-type CD8^+^ T cells. NES, normalized enrichment score. **c**, S6 and ERK phosphorylation in splenic CD8^+^ T cells stimulated ex vivo with crosslinked anti-CD3 antibodies and TXA_2_ analogue or vehicle (5 min). **d**, Quantity of GTP-bound (active) and total RHOA from wild-type or *Arhgef1*-deficient OT-1 CD8^+^ T cells stimulated with TXA_2_ analogue or vehicle (5 min). **e**, Complementation of *Arhgef1*-deficient OT-1 CD8^+^ T cells with RHOA^CA^ using retroviral expression. S6 and ERK phosphorylation was measured in THY1.1^+^ (transduced) cells after stimulation with crosslinked anti-CD3 antibodies. **f**, Confocal imaging (left) and computational analysis (right) of PTEN, F-actin and DAPI localization in wild-type and *Arhgef1*-deficient OT-1 CD8^+^ T cells stimulated with TXA_2_ analogue after pre-treatment with ROCK inhibitor (Y-27632, 30 μM), TXA_2_ receptor inhibitor (SQ 29548, 10 μM) or vehicle. Arrows show PTEN and F-actin colocalization. *n* = 2 independent replicates per condition. One-way ANOVA with Tukey multiple comparisons test. Scale bars, 20 μm. MFI, mean fluorescence intensity. **g**, AKT and ERK phosphorylation in wild-type and *Arhgef1*-deficient OT-1 CD8^+^ T cells stimulated with TXA_2_ analogue and crosslinked anti-CD3 antibodies following inhibitor pre-treatment as in **f**. **h**, Ex vivo S6 phosphorylation in splenic naive CD8^+^ T cells after anti-CD3 antibody crosslinking (5 min) after AKT inhibitor VIII (AKTi, 1 μM) or vehicle pre-treatment. NS, no stimulation. **i**, TNF expression by splenic naive CD8^+^ T cells 5 h after stimulation in the presence of 1 μM AKTi or vehicle. Data are representative of three (**c**,**d**) and two (**e**–**i**) independent experiments. Data are mean ± s.e.m. Source data

**Extended Data Fig. 9:**
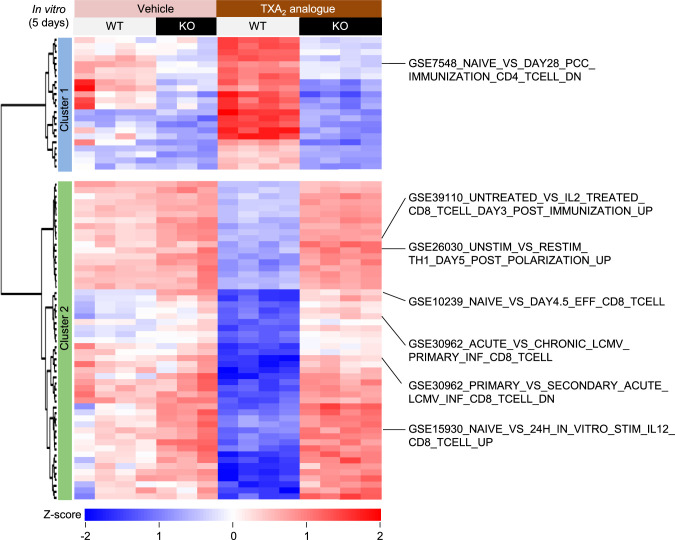
Thromboxane A2 suppresses activation transcriptional programs in T cells via ARHGEF1. Heatmap showing single-sample Gene Set Enrichment Analysis (ssGSEA) of the global gene expression changes between TXA_2_ analogue and Vehicle-treated-WT and *Arhgef1* KO CD8^+^ T cells. Enrichment of all gene sets comprising the MSigDB C7 Immunologic signature gene sets was tested. Significantly differentially enriched gene sets shown (FDR < 0.2, |FC| > 1.5). Data are show results from three to four biological replicates per group.

To better define the effect of TXA_2_-driven ARHGEF1 activity on the global T cell activation programme, we performed whole-transcriptome RNA sequencing (RNA-seq) of naive FACS-sorted wild-type and *Arhgef1*-deficient CD8^+^ T cells undergoing stimulation with anti-CD3ε and anti-CD28 antibodies in the presence of TXA_2_ analogue. Consistent with phenotypic analyses, we observed that treatment of cells with TXA_2_ analogue suppressed the expression of genes involved in T cell activation and effector differentiation, including *Gzma*, *Fasl*, *Ccl5*, *Ccr5* and *Prdm1* in an ARHGEF1-dependent manner (Fig. 4a and Supplementary Table 2). Accordingly, we observed highly significant enrichment in the expression of genes upregulated in memory versus naive CD8^+^ T cells in TXA_2_ analogue-treated *Arhgef1-*deficient cells compared with wild-type cells^32^ (Fig. 4b, Extended Data Fig. 9 and Supplementary Tables 3–5). Together, these findings show that TXA_2_ signalling acts via ARHGEF1 to regulate the global TCR-driven transcriptional programme in T cells.

**Extended Data Fig. 10:**
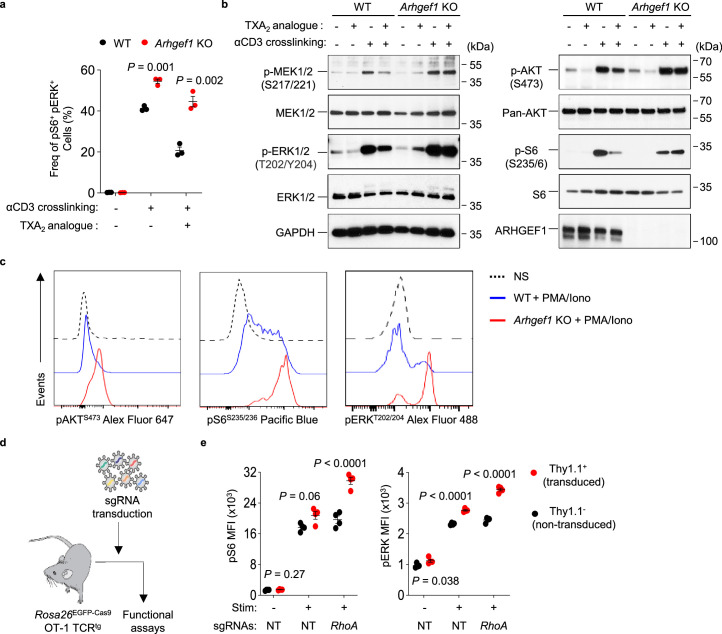
ARHGEF1 suppresses TCR-driven kinase signaling in T cells via RhoA. **a**, Replicate measurements of S6 and ERK phosphorylation within splenic WT (*n* = 3) and *Arhgef1*-deficient (*n* = 3) CD8^+^ T cells stimulated ex vivo with crosslinked anti-CD3 antibodies for 5 min in the presence of TXA_2_ analogue or vehicle control. **b**, Immunoblot analysis of MEK, ERK, AKT and S6 phosphorylation in day 5 stimulated WT or *Arhgef1* KO OT-1 TCR transgenic CD8^+^ T cells stimulated in vitro with crosslinked anti-CD3 antibodies for 5 min in the presence of TXA_2_ analogue or vehicle control. **c**, Representative flow cytometry plots of increased phosphorylation of AKT, S6 and ERK following PMA/Iono stimulation of WT and *Arhgef1* KO cells ex vivo. Gated on CD44^–^ naïve CD8^+^ T cells. NS, no stimulation. **d**, Experimental schema of ablation of *Rhoa* using CRISPR/Cas9 mutagenesis. **e**, Cas9-expressing OT-1 TCR transgenic CD8^+^ T cells were stimulated and transduced with retroviruses expressing sgRNAs targeting *Rhoa* or non-targeting (NT) control. Cells were stimulated with crosslinked anti-CD3 antibodies for 5 min and S6 and ERK phosphorylation was measured on Thy1.1^+^ (transduced) cells. *n* = 4 independent replicates per condition. Data are representative of two independent experiments (a-e). Multiple two-tailed Student’s t-test with Holm-Šídák correction (a, e). Graphs show mean ± s.e.m. Source data

ARHGEF1 activates RHOA to control a variety of processes including cellular differentiation, motility, proliferation and kinase signalling^33–35^. Kinase signalling has a key role in TCR-driven T cell activation, proliferation and differentiation^36^. We found that treatment of T cells with TXA_2_ analogue profoundly inhibited TCR stimulation-driven phosphorylation of S6 and ERK in a manner that was almost entirely dependent on ARHGEF1 (Fig. 4c and Extended Data Fig. 10a,b), suggesting that ARHGEF1 restricts TCR-driven PI3K and MAPK signalling in response to TXA_2_ signalling. We also observed increased phosphorylation of S6 and ERK following in vitro stimulation of *Arhgef1*-deficient cells with phorbol 12-myristate 13-acetate (PMA) plus ionomycin (Extended Data Fig. 10c), suggesting effects on signal transduction downstream of proximal TCR signal transduction^37^. Active RHOA pull-down assays using Rhotekin beads showed that stimulation of wild-type but not *Arhgef1*-deficient T cells with TXA_2_ analogue increased the abundance of GTP-bound active RHOA, demonstrating that ARHGEF1 is critical to TXA_2_-mediated RHOA activation (Fig. 4d). Consistent with a critical role of RHOA activation in the suppressive function of ARHGEF1, complementation of *Arhgef1*-deficient CD8^+^ T cells with a constitutively active mutant RHOA-Q63L (RHOA^CA^)^38^ was sufficient to reverse hyper-phosphorylation of S6 and ERK following anti-CD3 crosslinking (Fig. 4e). Moreover, genetic disruption of *Rhoa* using CRISPR-based mutagenesis in primary Cas9-expressing CD8^+^ T cells resulted in increased stimulation-driven S6 and ERK phosphorylation (Extended Data Fig. 10d,e).

**Extended Data Fig. 11:**
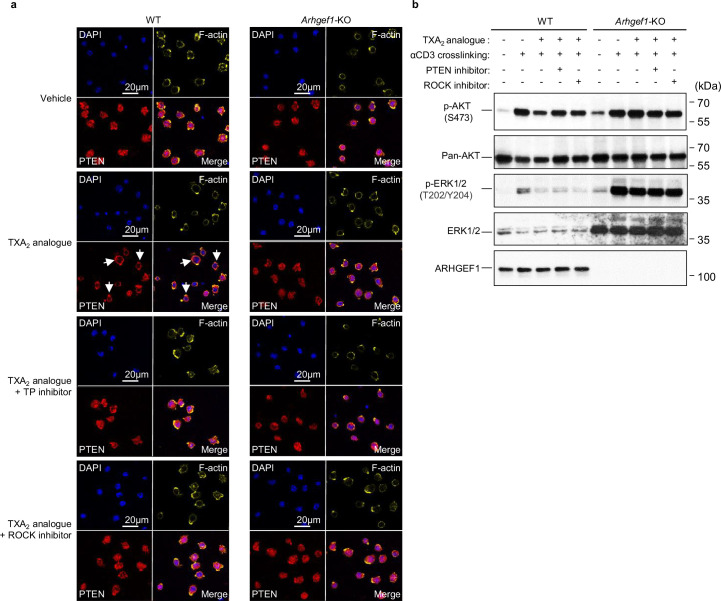
Thromboxane A_2_ induces PTEN recruitment and suppresses AKT phosphorylation in T cells via ROCK kinase. **a**, Confocal immunofluorescence imaging of WT and *Arhgef1*-KO OT-1 CD8 T-cells 5 days after primary stimulation and subsequently seeded on anti-CD3 antibody-coated coverslips with or without 10 min of stimulation with U-46619 (10 μM) and/or 1 h of preincubation with the TP inhibitor (SQ 29548, 10 μM) or ROCK inhibitor (Y-27632, 30 μM). Images were captured on a Leica confocal microscope using a 63x oil objective lens, in three channels, F-actin (yellow) and PTEN (red) plus a DAPI nuclear counter stain (blue) to locate the cells. Images analysed were maximum intensity projections of four consecutive z-slices selected from the centre of a cross-volume image stack. **b**, Immunoblot analysis of protein phosphorylation pre-activated (day 5) WT or *Arhgef1* KO OT-1 TCR transgenic CD8^+^ T cells stimulated in vitro with crosslinked anti-CD3 antibodies for 5 min in the presence of TXA_2_ analogue or vehicle control and treatments with the PTEN inhibitor (bpV(pic), 2.5 μM) or ROCK inhibitor (GSK269962A, 10 μM). Data are representative of two independent experiments.

Activation of PTEN by the RHOA effector ROCK enables RHOA to negatively regulate the AKT–S6 pathway, providing a means for crosstalk between small G-protein and kinase signalling^33,35^. ROCK recruits PTEN to the cell cortex upon activation^33^. Confocal immunofluorescence microscopy of wild-type and *Arhgef1*-deficient T cells in vitro showed that T cells respond rapidly to TXA_2_ sensing by recruiting PTEN to the cell cortex in a manner dependent on both the TXA_2_ receptor TP and ROCK, since pre-treatment of cells with either the ROCK inhibitor Y-27632 or the TP inhibitor SQ 29548 prevented TXA_2_-mediated PTEN recruitment to the cell cortex (Fig. 4f and Extended Data Fig. 11a). Biochemical analysis of cell lysates showed that TXA_2_-driven suppression of AKT phosphorylation is dependent on the activity of TP, ARHGEF1, ROCK and PTEN, whereas TXA_2_-driven suppression of ERK phosphorylation is dependent on TP and ARHGEF1, but not ROCK or PTEN (Fig. 4g and Extended Data Fig. 11b). The PI3K–AKT pathway has a critical role in T cell activation, differentiation and cytokine production^39–41^. Pharmacological inhibition of AKT using the well-characterized allosteric AKT inhibitor, AKT inhibitor VIII, reduced excessive S6 phosphorylation and cytokine production following stimulation of *Arhgef1-*deficient CD8^+^ T cells (Fig. 4h,i). This suggests that inhibition of kinase pathway activation by ARHGEF1 is critical to its suppressive function in T cells. Collectively, these findings show that ARHGEF1 and downstream activation of RHOA are required for suppression of TCR-driven kinase pathway phosphorylation and T cell activation upon TXA_2_ signalling in vitro.

## Aspirin releases T cells from suppression by TXA_2_

The biosynthesis of TXA_2_ is blocked by inhibitors of COX enzymes, including aspirin. Meta-analyses of large randomized controlled trials have shown that aspirin use is associated with protection against metastatic recurrence at multiple sites after primary cancer diagnosis in humans^6,7^. Our observation that TXA_2_ limits T cell activation in an ARHGEF1-dependent manner in vitro led us to hypothesize that aspirin exerts an anti-metastatic effect by releasing T cells from TXA_2_-driven suppression mediated by ARHGEF1 in vivo.

**Fig. 5:**
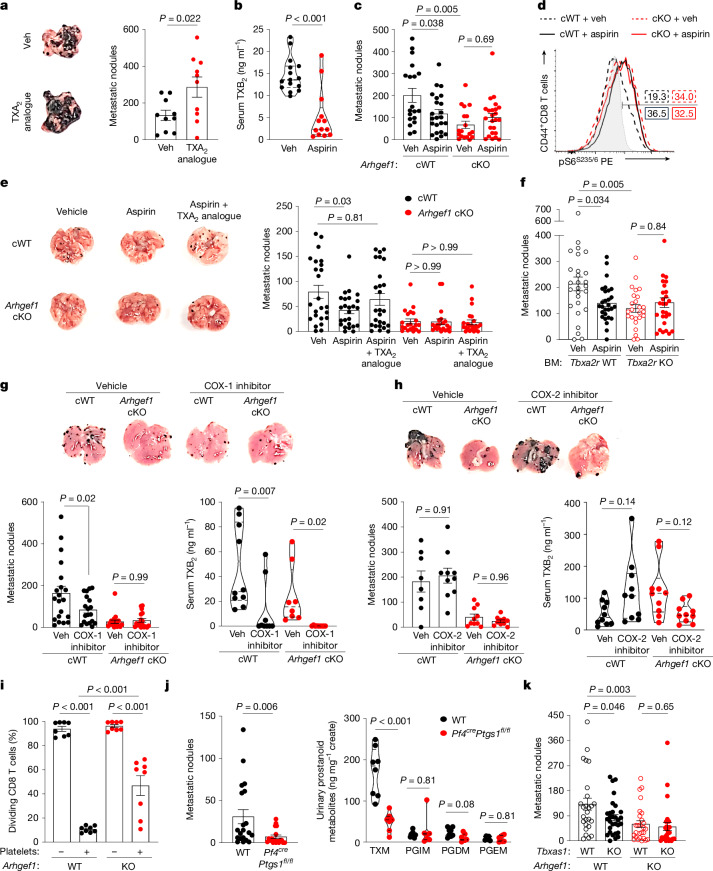
Aspirin promotes anti-metastatic immunity by releasing T cells from ARHGEF1-dependent suppression by TXA_2_. **a**, Photographs (left) and frequency (right) of lung metastases in mice treated with TXA_2_ analogue (*n* = 10) or vehicle (veh; *n* = 10) 17 days after intravenous injection of B16 cells. **b**, Serum TXB_2_ abundance in vehicle (*n* = 15) or aspirin-treated (*n* = 13) B16-bearing mice. **c**, Frequency of B16 lung metastases in *Cd4*^*cre*^ (cWT) and *Arhgef1*^*fl/fl*^ *Cd4*^*cre*^ (cKO) mice treated with vehicle (cWT, *n* = 19; cKO, *n* = 18) or aspirin (cWT, *n* = 23; cKO, *n* = 24). **d**, Ex vivo S6 phosphorylation among CD44^+^ CD8^+^ T cells in lungs of vehicle- or aspirin-treated cWT and *Arhgef1* cKO mice 17 days after intravenous injection of B16 cells. Grey, non-crosslinking control. Numbers show percentages within indicated gate. **e**, Frequency of B16 lung metastases in mice of indicated genotype treated with vehicle (cWT, *n* = 24; cKO, *n* = 21), aspirin (cWT, *n* = 25; cKO, *n* = 21), or aspirin and TXA_2_ analogue (cWT, *n* = 26; cKO, *n* = 23). **f**, Frequency of B16 lung metastases in bone marrow chimeras reconstituted with wild-type or *Tbxa2r-*KO bone marrow cells^49^ and treated with vehicle (wild type, *n* = 27; KO, *n* = 25) or aspirin (wild type, *n* = 28; KO, *n* = 28). **g**,**h**, Frequency of B16 lung metastases and serum TXB_2_ abundance in cWT or cKO mice treated with COX-1 inhibitor SC-560 (cWT, *n* = 19; cKO, *n* = 17) or vehicle (cWT, *n* = 20; cKO, *n* = 19) (**g**), and COX-2 inhibitor celecoxib (cWT, *n* = 10; cKO, *n* = 10) or vehicle (cWT, *n* = 8; cKO, *n* = 10) (**h**). **i**, Proliferation of wild-type and *Arhgef1-*deficient naive CD8^+^ T cells in contact-independent co-culture with platelets. **j**, Frequency of lung B16 metastases (left; wild type, *n* = 20; *Pf4*^*cre*^ *Ptgs1*^*fl/fl*^, *n* = 18) and mass spectrometry of urinary prostanoids (right) in mice of indicated genotypes. PGDM, 11,15-dioxo-9α-hydroxy-2,3,4,5-tetranorprostan-1,20-dioic acid; PGEM, 7-hydroxy-5,11-diketotetranorprostane-1,16-dioic acid; PGIM, 2,3-dinor-6-keto-PGF_1α_; TXM, 2,3-dinor-TXB2. **k**, Frequency of B16 lung metastases in wild-type (*n* = 26), *Tbxas1-*KO (*n* = 30)*, Arhgef1-*KO (*n* = 27) and *Tbxas1-*KO *Arhgef1-*KO (*n* = 28) mice. Data are representative of two (**a**,**b**) or pooled from two (**e**–**j**) or three (**c**,**k**) independent experiments. Unpaired two-tailed Student’s *t*-test (**a**); one-way ANOVA with Tukey (**c**,**e**,**f**–**i**) and Holm–Šídák (**k**) adjustment; two-tailed Mann–Whitney *U*-test (**b**,**j**). Data are mean ± s.e.m. Source data

**Extended Data Fig. 12:**
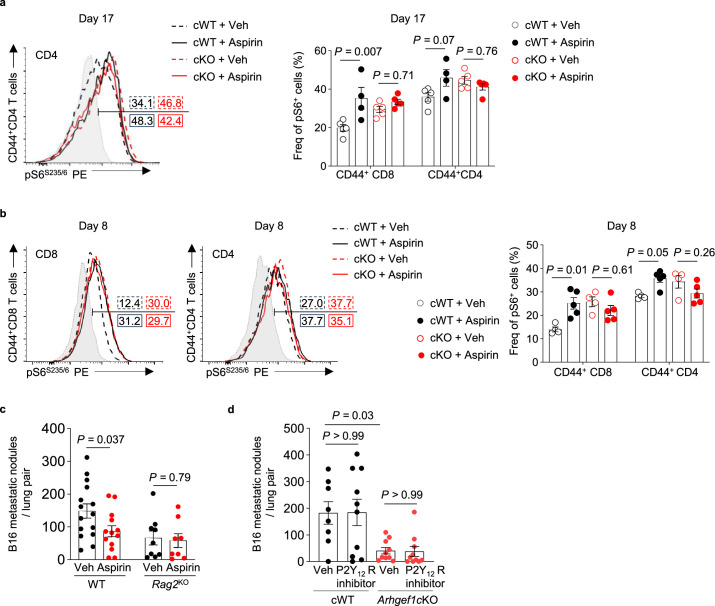
Aspirin treatment increases the signaling capacity of T cells within tumour-bearing lungs of WT but not *Arhgef1* conditional knockout animals. **a-b**, Phosflow analysis of S6 phosphorylation gated on antigen-experienced (CD44^+^) CD4^+^ and CD8^+^ T cells from lungs of vehicle- or aspirin-treated *Cd4*^Cre^ and *Arhgef1*^fl/fl^ *Cd4*^Cre^ mice 17 days (a) and 8 days (b) after i.v. injection of B16 cells upon 5 min stimulation ex vivo with crosslinked anti-CD3 antibodies. Grey, non-crosslinking control. cWT treated with vehicle (n = 5) and aspirin (n = 4); cKO treated with vehicle (n = 5) and aspirin (n = 5) in (a). cWT treated with vehicle (n = 4) and aspirin (n = 5); cKO treated with vehicle (n = 4) and aspirin (n = 5) in (b). **c**, Frequency of metastatic nodules on lungs of vehicle- or aspirin-treated WT and *Rag2*-deficient (KO) mice 17 days after i.v. injection of B16 cells (NK cell activation in *Rag2*-deficient animals results in reduced background rate of metastasis). WT treated with vehicle (n = 15) and aspirin (n = 13); *Rag2* KO treated with vehicle (n = 9) and aspirin (n = 8). **d**, Effect of treatment with the P2Y_12_ inhibitor Ticagrelor on the rate of metastasis to the lungs of *Cd4*^Cre^ (cWT, vehicle = 8 and Ticagrelor = 10) and *Arhgef1*^fl/fl^ *Cd4*^Cre^ (cKO, vehicle = 10 and Ticagrelor = 10) mice 17 days after i.v. injection of B16 cells. Data from vehicle-treated cWT and cKO groups were also used as controls in Fig. 5h (as noted in Source Data). Data are representative of two (a-c) or pooled from two (d) independent experiments. *P* values show the results of unpaired two-tailed Student’s t test (c); One-way ANOVA with Tukey multiple comparisons adjustment (a, b, d). Graphs show mean ± s.e.m. Source data

Supplementation of the drinking water of mice with the TXA_2_ analogue U46619 increased the frequency of pulmonary metastases following intravenous administration of B16 cells (Fig. 5a). Treatment of mice with aspirin decreased the abundance of TXA_2_ as determined by measurement of its stable metabolite TXB_2_ in the serum of mice (Fig. 5b). Of note, aspirin treatment reduced metastasis frequency in control mice but not in mice bearing a T cell-specific conditional deletion of ARHGEF1 (Fig. 5c), suggesting that its activity is immune-dependent. Moreover, aspirin treatment in vivo resulted in increased capacity for TCR-driven S6 phosphorylation among antigen-experienced CD4^+^ and CD8^+^ T cells from tumour-bearing lungs of wild-type mice, to increased levels observed in *Arhgef1*-deficient mice treated with either aspirin or vehicle control (Fig. 5d and Extended Data Fig. 12a,b). Notably, supplementation of aspirin-treated wild-type mice with TXA_2_ analogue U46619 reversed the anti-metastatic activity of aspirin, but neither aspirin nor TXA_2_ analogue affected metastasis frequency in mice carrying a T cell-specific conditional deletion of ARHGEF1 (Fig. 5e), showing that the anti-metastatic activity of aspirin is dependent on its ability to reduce TXA_2_ abundance, releasing T cells from TXA_2_-driven suppression by ARHGEF1. The importance of TXA_2_ receptor signalling to the anti-metastatic activity of aspirin was further supported by bone marrow reconstitution experiments that showed that the anti-metastatic activity of aspirin is dependent on TP expression by cells of the haematopoietic lineage (Fig. 5f). Additionally, the anti-metastatic activity of aspirin was dependent on adaptive immune cells, since we observed no difference in metastasis frequency when *Rag2*-deficient mice (which lack all T cells and B cells, resulting in NK cell expansion and a reduced background rate of metastasis)^12^ were treated with aspirin (Extended Data Fig. 12c).

## Platelet COX-1 suppresses anti-metastatic immunity

Aspirin can inhibit both COX-1 and COX-2^5,10^. We sought to determine their relative contribution to the observed effect of aspirin on metastasis. We found that selective COX-1 inhibition was able to recapitulate the effect of aspirin, reducing metastasis frequency in a manner that was dependent on T cell-restricted expression of ARHGEF1 (Fig. 5g). Reduction in metastasis frequency upon COX-1 inhibition was associated with a decrease in the abundance of the stable TXA_2_ catabolite TXB_2_ in the serum of mice treated with COX-1 inhibitors. By contrast, COX-2 inhibition did not result in a decrease in either metastasis frequency or the systemic abundance of TXB_2_ (Fig. 5h). Moreover, inhibition of P2Y_12_, which is involved in alternative ADP-driven platelet activation, did not affect the rate of pulmonary metastasis (Extended Data Fig. 12d).

**Extended Data Fig. 13:**
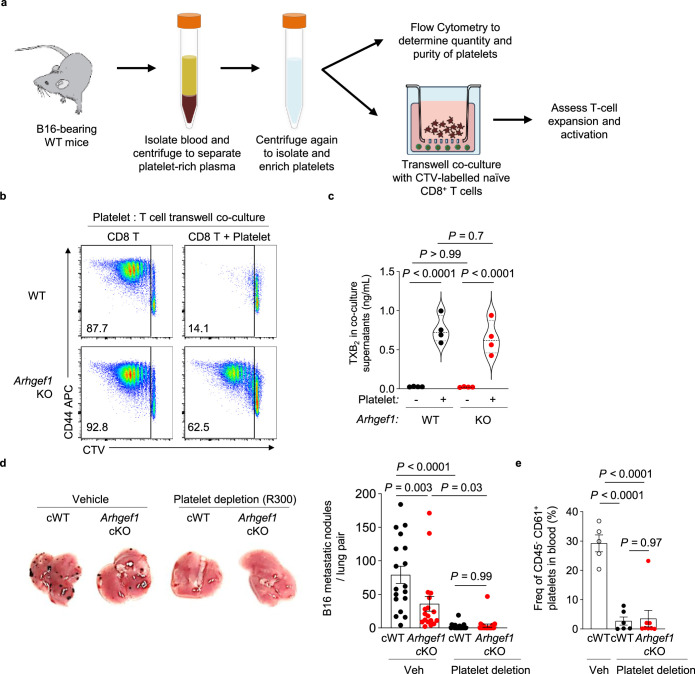
Platelet-mediated suppression of T cells and anti-metastatic immunity. **a**, Schema showing isolation of platelets and transwell co-culture with proliferation dye CellTrace Violet (CTV)-labelled naïve CD8^+^ T cells. Transwell plates were pre-coated with anti-CD3 and soluble anti-CD28 antibodies to stimulate CD8^+^ T cells. **b**, Flow cytometry plots showing proliferation and activation of naïve and *Arhgef1-*decifient T cells in (a) as determined by dilution of CTV dye and expression of CD44. **c**, Abundance of TXB_2_ as determined by enzyme-linked immunosorbent assay (ELISA) of transwell co-culture supernatants of cells in (b) at day 5 post-stimulation. *n* = 4 independent replicates per condition. **d**, Effect of platelet depletion on the frequency of metastases on lungs of *Cd4*^Cre^ (cWT, vehicle = 18 and R300 = 19) and *Arhgef1*^fl/fl^ *Cd4*^Cre^ (cKO, vehicle = 18 and R300 = 20) mice 17 days after i.v. B16 injection. **e**, Frequencies of platelets in blood as determined by flow cytometry from animals in (d). cWT treated with vehicle (*n* = 5) and R300 (*n* = 6), and cKO treated with R300 (*n* = 8). Data are representative of two (b, c, e) or pooled from two (d) independent experiments. One-way ANOVA with Tukey multiple comparisons test. Graphs show mean ± s.e.m. Source data

We next tested whether platelets are the primary source of TXA_2_ responsible for promoting metastasis. Platelet transwell co-culture experiments revealed contact-independent suppression of T cell activation by platelets in a manner that was partially dependent on T cell expression of ARHGEF1 (Fig. 5i and Extended Data Fig. 13a–c). Moreover, antibody-mediated platelet depletion resulted in a profound reduction in metastasis frequency in mice that were inoculated intravenously with B16 cells, in which context ARHGEF1 proficiency was unable to promote metastasis (Extended Data Fig. 13d,e). Platelets produce TXA_2_ in a manner that is dependent on the expression of COX-1. Given that COX-1 inhibition resulted in an anti-metastatic effect dependent on expression of ARHGEF1 in T cells, we tested whether the rate of metastasis is affected in *Pf4*^*cre*^ *Ptgs1*^*fl/fl*^ mice, which harbour a platelet and megakaryocyte-specific conditional deletion of COX-1^42,43^ (Fig. 5j). Of note, platelet and megakaryocyte-specific conditional deletion of COX-1 resulted in a marked reduction in metastasis, accompanied by decreased urinary abundance of the TXA_2_ catabolite TXM, but not of other prostanoid metabolites that we evaluated. To formally test the role of TXA_2_ in ARHGEF1-dependent suppression of anti-metastatic immunity in vivo, we crossed mice with a genetic deficiency in the thromboxane synthase gene *Tbxas1*^44^ with *Arhgef1*-deficient mice. This allowed us to examine the effect of absence of systemic TXA_2_ on metastasis frequency in the presence or absence of ARHGEF1. Among ARHGEF1-proficient mice, genetic absence of thromboxane synthase resulted in a reduction in metastasis frequency, whereas in mice lacking ARHGEF1, loss of thromboxane synthase did not affect the rate of metastasis (Fig. 5k). Collectively, these data show that a critical anti-metastatic function of aspirin and COX-1 inhibitors is to release T cells from suppression by TXA_2_ produced in a platelet COX-1-dependent manner.

## Discussion

**Extended Data Fig. 14:**
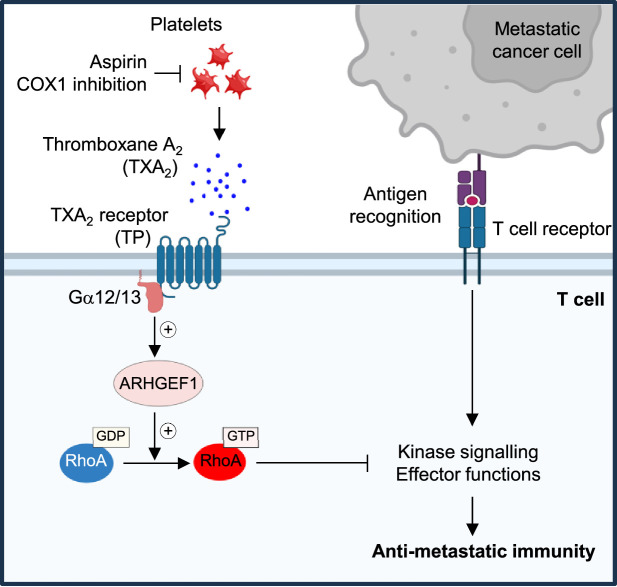
Aspirin promotes immunity to cancer metastasis by releasing T cells from ARHGEF1-dependent suppression by TXA_2_. Graphical schema of study findings. Platelets produce TXA_2_ which binds to its receptor (TP) on T cells, triggering the activation of ARHGEF1, a guanine exchange factor which facilitates the conversion of inactive GDP-bound RhoA into its active GTP-bound conformation. Activation of RhoA inhibits TCR-driven kinase pathways, proliferation and effector functions of T cells, thereby suppressing anti-metastatic immunity. Production of TXA_2_ by platelets is COX1-dependent and inhibited by aspirin and COX-1 selective inhibitors, which release T cells from TXA_2_-mediated suppression. Created in BioRender. Roychoudhuri, R. (2025) https://BioRender.com/u42p292.

Here we show that platelet-derived TXA_2_ functions as a potent immunoregulatory molecule that suppresses T cell immunity to cancer metastasis by inducing the immunosuppressive function of ARHGEF1. Consequently, COX-1 inhibition, including using aspirin, enhances anti-metastatic immunity by releasing T cells from TXA_2_–ARHGEF1-mediated suppression (Extended Data Fig. 14). These findings add to our mechanistic understanding of the observed anti-metastatic effects of aspirin^6–9^.

Although aspirin provides a potentially attractive opportunity for anti-metastatic therapy given its low cost, more selective targeting of the TXA_2_–ARHGEF1 pathway could enable enhanced anti-metastatic activity and/or reduced bleeding risk and gastric toxicity. Understanding of the immunostimulatory effect of aspirin raises the possibility that aspirin may be used to synergize with other adjuvant immunotherapies. These findings build on previous data that show that in patients with colon cancer, the association of aspirin use with improved survival appears to be restricted to cancers with high HLA class I expression^9^, and suggest that additional immune biomarkers may help to stratify patients who are most likely to benefit from the anti-metastatic activity of aspirin. Given contradictory evidence relating to aspirin efficacy in distinct groups of patients^6,8,45^, our findings underscore the need for detailed biomarker identification studies in the context of prospective randomized controlled trials to definitively establish the cancer types and patient populations in which aspirin has the greatest efficacy.

We observed that genetic disruption of ARHGEF1 reduced the frequency of exhausted T cells at the metastatic site and increased the frequency of memory-precursor effector cells (MPECs) in pathogen-specific CD8^+^ T cell responses in vivo. How the observed modulation of kinase pathways by TXA_2_–ARHGEF1 relates to phenotypic changes in exhaustion and MPEC differentiation is unclear and remains an important topic for future investigation. The findings also suggest that the TXA_2_–ARHGEF1 pathway may have broader roles in immune regulation, including in control of T cell memory and exhaustion during chronic viral infection^24,25,46,47^. We observed mild effects of ARHGEF1 deficiency on T cells cultured ex vivo in the absence of TXA_2_, which were more pronounced in short-term assays directly ex vivo than in longer-term culture experiments. Whether ARHGEF1 is mildly basally active in the absence of TXA_2_ signalling, or tonically active after exposure to homeostatic levels of TXA_2_ or a stable metabolite in vivo are interesting questions for future study.

Our findings build on a broader understanding of the importance of prostanoids in cancer development and immune evasion. In particular, COX-2 has well-established and multifaceted roles in carcinogenesis, and prostaglandin E_2_ production by tumour cells has a significant role in immune evasion by established tumours^48^. Recent findings also suggest that platelet TXA_2_ can act directly on cancer cells to promote COX-2-dependent carcinogenesis^43^, although it remains to be determined whether the ability of TXA_2_ to stimulate prostaglandin E_2_ production adds to the immunomodulatory potential of TXA_2_ in the context of metastasis by COX-2-expressing tumours.

This work establishes TXA_2_ as a regulator of T cell immunity, with implications for cancer prevention and therapy. The identification of this pathway provides mechanistic insights into the anti-metastatic effects of aspirin, a potential basis for its more targeted use, and targets for development of new therapeutic strategies for preventing metastatic disease.

## Methods

### Mice and reagents

All animal experiments were conducted in accordance with UK Home Office guidelines and were approved by the University of Cambridge Animal Welfare and Ethics Review Board. Littermate controls or age- and sex-matched mice were used in experiments as indicated. All mice, unless otherwise stated, were housed at the University of Cambridge University Biomedical Services Gurdon Facility or the Babraham Institute Biological Support Unit. Wild-type C57BL/6 mice were obtained from Charles River. *Ptprc*^*a*^ (CD45.1) congenic, OT-1 TCR^tg^, *Rag2*^–/–^ and MMTV-PyMT (B6.FVB-Tg(MMTV-PyVT)634Mul/LellJ) mice were obtained from the Jackson Laboratory. *Arhgef1*-KO (*Arhgef1*^*Tm1a*^) mice have previously been described^12^. *Tbxas1*-KO (*Tbxas1*^*tm1Swl*^) and *Tbxa2R*-KO (*Tbxa2r*^*tm1Cof*^) mice, as previously described^44,49^, were provided by S.-W. Lin. *Arhgef1*^*fl/fl*^ mice were generated by crossing *Arhgef1*^*Tm1a*^ mice with FlpO-deleter mice^50^, and subsequently crossed with Cre-expressing strains, *Ncr1*^*cre*^ (*Ncr1*^*tm1.1(icre)Viv*^), *Lyz2*^*cre*^ (*Lyz2*^*tm1(cre)Ifo*^) and *Cd4*^*cre*^ (*Tg(Cd4-cre)1Cwi*) mice to generate conditional-knockout mice, respectively^17–19^. Mice were genotyped by Transnetyx. *Pf4*^*cre*^ *Ptgs1*^*fl/fl*^ mice have been described previously^42^ and were housed at the animal facility of the “G. d’Annunzio” University of Chieti-Pescara and the animal experiments were performed under the European Communities Council (EEC) Directive of 22 September 2010 (2010/63/EU) and the National Ethical Committee (authorization no. 434/2024-PR).

### Tumour metastasis model

Mouse metastatic melanoma cell lines B16-F10 (purchased from Kerafast) and B78ChOva-mCherry (provided by M. F. Krummel)^51^ were passaged in DMEM (Thermo Fisher Scientific) supplemented with 10% FBS and antibiotics. For lung metastasis model: mice were injected intravenously with 5 × 10^5^ B16-F10 cells or B78ChOva-mCherry cells in 150 µl Dulbecco’s PBS (DPBS) and lungs were dissected between day 14 and 17 for metastasis enumeration and subsequent analysis. LL/2 murine Lewis lung carcinoma cells were passaged in DMEM supplemented with 10% FBS and antibiotics. Mice were injected with 1 × 10^6^ LL/2 cells in 150 μl DPBS and lungs were dissected on day 17. Lungs were fixed in 4% formalin before being embedded in paraffin. Sections were taken from the centre of the lungs and stained with H&E. Slide images were taken using a Pannoramic digital slide scanner (3DHistech) and analysed using QuPath Software. Tumour burden was calculated as a percentage of total tissue area for each sample. For the liver metastasis model: mice were anaesthetized with isoflurane and a small incision was made in the left flank to expose the spleen. 3.5 × 10^5^ B16-F10 cells suspended in 50 μl DPBS were injected into the spleen. The wound was closed with sutures and skin staples. Mice were euthanized and livers were dissected on day 11 for metastasis enumeration and subsequent analyses. Aspirin (Aspégic, Sanofi Aventis and Sigma) was resuspended in drinking water at 600 mg l^−1^. TXA_2_ analogue U46619 (Cayman) was diluted in DMSO and delivered in drinking water at 50 μg kg^−1^. All the drinking water contained 1% sucrose and was replaced 3–4 times per week. For platelet depletion, mice were administered R300 antibody (Emfret) intraperitoneally at 0.25 mg kg^−1^ every 2–3 days from 1 day before intravenous injection of tumour cells. For inhibition studies, COX-1 inhibitor SC-560 (SelleckChem and Abcam), COX-2 inhibitor Celecoxib (SelleckChem), P2Y12 inhibitor Ticagrelor (SelleckChem) or vehicle control were administered at 30 mg kg^−1^ via daily oral gavage 5 days before intravenous injection of tumour cells and continuing throughout the study.

### Spontaneous germline MMTV-PyMT cancer metastasis model

Mice were palpated for mammary tumours once per week. Mammary tumours were assessed by taking length and width measurements with digital callipers three times a week after the first palpable mammary tumour was detected. When the total mammary tumour area exceeded 2.25 cm^2^, mice were euthanized and lungs were collected and fixed in 4% formalin before being embedded in paraffin. Sections were taken from the centre of the lungs and stained with H&E. Slide images were taken using a Pannoramic digital slide scanner (3DHistech) and analysed using QuPath Software. Tumour burden was calculated as a percentage of total tissue area for each sample.

### MC38 subcutaneous tumour model

MC38 mouse colorectal cancer cells were passaged in DMEM supplemented with 10% FBS and antibiotics. Mice were injected subcutaneously into one flank with 2.5 × 10^5^ cells in 100 µl PBS. Tumour growth was assessed by taking length and width measurements with digital calipers 3 times a week starting from day 7 after injection.

### *L. monocytogenes* infection and kinetic analysis

For experiments assessing the response of CD8^+^ T cells to bacterial infections, bacteria were grown in BHI medium to an OD_600_ of 0.1 before each experiment. The mice were infected with a sub-lethal dose of 5 × 10^6^ colony-forming units attenuated (ΔactA) *L. monocytogenes* expressing OVA by intravenous administration^52^. Blood samples were collected via the tail vein at serial time points following infection. CD8^+^ T cell responses were detected and their phenotypes were analysed by flow cytometry.

### Bone marrow reconstitution experiments

For bone marrow reconstitution experiments, C57BL/6 mice were administered 1,000 Gy total-body γ-radiation from a ^137^Cs source before intravenous injection of bone marrow cells from single-cell bone marrow preparations from 8- to 12-week-old mice. Mice were administered neomycin for two weeks following irradiation and reconstitution to limit infection risk, and were used in flow cytometry and cancer metastasis experiments at two to three months after reconstitution.

### Flow cytometry

Single-cell suspensions from lymphoid tissues were prepared by mechanical dissociation through 40-µm cell strainers (BD Biosciences). Lungs were minced in media containing 20 µg ml^−1^ DNase I (Roche) and 1 mg ml^−1^ collagenase (Sigma Aldrich) and incubated with agitation at 37 °C for 40 min before also being dissociated through 40-µm cell strainers. Erythrocytes were lysed using ice-cold ACK Lysing Buffer (Gibco) for 5 min. Cells requiring intracellular staining of cytokines were stimulated prior to flow cytometry analysis using PMA, ionomycin, brefeldin A and monensin for 4 h in complete RPMI 1640 (Thermo Fisher Scientific). Viable cells were discriminated by first staining alone with Zombie UV fixable viability dye (Biolegend) or eFluor 780 fixable viability dye (eBioscience) in PBS, according to the manufacturer’s instructions. Cells were then incubated with specific surface antibodies on ice for 40 min in FACS buffer, in the presence of 2.4G2 monoclonal antibodies to block FcγR binding. Cell surface phosphatidylserine was labelled using the eBioscience Annexin V Apoptosis Detection Set (Thermo Fisher Scientific) according to the manufacturer’s protocol. For intracellular staining, the eBioscience Foxp3/Transcription Factor Staining Buffer Set (Thermo Fisher Scientific) or BD Cytofix/Cytoperm Fixation/Permeabilization Kit was used in accordance with the manufacturer’s instructions followed by intracellular staining with fluorochrome-conjugated antibodies for 40 min. Samples were acquired using Cytek Aurora (Cytek), BD LSR Fortessa (BD Biosciences) or Beckman CytoFLEX (Beckman Coulter) cytometers with their respective software: SpectroFlo (v3.3.0), BD FACSDiva (v8.0.1), and CytExpert (v2.5.0.77). Data were analysed using FlowJo v10.10.0 software (TreeStar LLC).

Antibodies used for flow cytometry are as follows: anti-CD103-Pacific Blue (2E 7, BioLegend, 121418, 1/200), anti-CD127-BV650 (A7R34, BioLegend, 135043, 1/200), anti-CD127-PE-Cy7-A7R34 (BioLegend, 135014, 1/200), anti-CD16/CD32 (93, BioLegend, 101302, 1/200), anti-CD25-PE-Cy7 (PC61, Invitrogen, 25-0251-82, 1/200), anti-CD25-BUV395 (PC61, BD, 564022, 1/200), anti-CD39-AF647 (Duha59, BioLegend, 143808, 1/400), anti-CD4-AF700 (RM4-5, BioLegend, 100536, 1/200), anti-CD4-BV650 (RM4-5, BioLegend, 100546, 1/400), anti-CD44-BV510 (IM7, BioLegend, 103044, 1/200), anti-CD44-BV786 (IM7, BD, 563736, 1/400), anti-CD44-PerCP-Cy5.5 (IM7, Invitrogen, 45-0441-82, 1/400), anti-CD44-APC (IM7, Invitrogen, 17-0441-83, 1/400), anti-CD45.2-ef506 (104, Invitrogen, 69-0454-82, 1/100), anti-CD45.2-FITC (104, BioLegend, 109805, 1/200), anti-CD61-PE (2C9.G3, Invitrogen, 12-0611-82, 1/200), anti-CD62L-BUV737 (MEL-14, BD, 612833, 1/400), anti-CD62L-APC (MEL-14, BioLegend, 104412, 1/400), anti-CD69-PECy5 (H1.2F3, BioLegend, 15-0691-82, 1/300), anti-CD69-PE-Dazzle (H1.2F3, BioLegend, 104536, 1/200), anti-CD8α-BUV395 (53-6.7, BD Horizon, 563786, 1/200), anti-CD8α-BUV805 (53-6.7, BD, 612898, 1/200), anti-CD8α-BV510 (53-6.7, BioLegend, 100752, 1/200), anti-CD8α-FITC, (53-6.7, Invitrogen, 11-0081-86, 1/200), anti-CD90.1-FITC (OX-7, BioLegend, 202503,1/100), anti-CD90.1-PerCP (OX-7, BD, 557266, 1/100), anti-Foxp3-APC (FJK-16S, eBioscience, 17-5773-82, 1/200), anti-IFNγ-BUV737 (XMG1.2, BD, 612769, 1/400), anti-IFNγ-FITC (XMG1.2, BioLegend, 505806, 1/200), anti-IL-2-PE (JES6-5H4, BioLegend, 503808, 1/200), anti-Ki67-PerCP-ef710 (SolA15, Invitrogen, 46-5698-80, 1/200), anti-KLRG1-APC (2F1/KLRG1, BioLegend, 138412, 1/200), anti-KLRG1-BV605(2F1/KLRG1, BioLegend, 138419, 1/200), anti-Ly108-APC (330-AJ, BioLegend, 134610, 1/200), anti-Ly6G-FITC (RB6-8C5, eBioscience, 11-5931-85, 1/400), anti-PD-1-APCef780 (J43, Invitrogen, 47-9985-82, 1/200), anti-PD-1-PE-Cy7 (RMP1-30, BioLegend, 109110, 1/200), anti-pErk T202/Y204-AF488, (197G2, Cell Signalling, 13214S, 1/100), anti-pErk T202/Y204-AF647 (197G2, Cell Signalling, 13148S, 1/100), anti-pS6 S235/6-PE (D57.2.2E, Cell Signalling, 5316S, 1/150), anti-ST2-PerCP-ef710 (RMST2-2, eBioscience, 46-9335-82, 1/200), anti-TCF-1-AF488 (C63D9, Cell Signalling, 6444S, 1/200), anti-TCRβ-BV570, (H57-597, BioLegend, 109231, 1/200), anti-TCRβ-FITC (H57-597, BioLegend, 109206, 1/200), anti-TCRβ-PerCP-Cy5.5 (H57-597, BioLegend, 109228, 1/200), anti-TIGIT-PE-Dazzle (1G9, BioLegend, 142110, 1/100), anti-TIGIT-PE (GIGD7, Invitrogen, 12-9501-82, 1/100), anti-TIM3-BV421 (RMT3-23, BioLegend, 119723, 1/100), anti-TIM3-BV785 (RMT3-23, BioLegend, 119725, 1/100), anti-TNF-APC (MP6-XT22, BioLegend, 506308, 1/200), anti-TNF-BV650 (MP6-XT22, BioLegend, 506333, 1/200), anti-TOX-PE (REA473, Miltenyi, 130-120-716, 1/200) and TER119-FITC (TER119, Invitrogen, MA5-17822, 1/200)

### Computational analysis of flow cytometry data

Data were processed as previously described^53,54^. In brief, Flow Cytometry Standard (FCS) 3.0 files were first imported in FlowJo version 10.10.0 to eliminate dead cells by manual gating, and select CD45^+^ leukocytes, subjected to biexponential transformation, then exported for computational analysis by a custom-made script making use of PhenoGraph (K value set at 30). Here, we modified the Linux-community and the core.py script to fix the seed to “123456” (run in Python version 3.7.3). Data were then converted in comma-separated value (CSV) files and merged into a single file by using the pandas package. The obtained data, exported as new CSV file (one for each cluster), were further imported in FlowJo and analysed to define the percentage of cells positive for each protein as well as their median fluorescent intensity. Data were finally metaclustered using the gplots R package. UMAP was performed with the UMAP Python package.

### Fluorescence-activated cell sorting

Pre-enrichment of CD8^+^ T cells from single-cell suspensions was done using the MagniSort Mouse CD8^+^ T cell Enrichment Kit (Thermo Fisher Scientific) according to the manufacturer’s protocol. Markers required for cell sorting were stained using flow cytometry cell surface antibodies, diluted 1/100, while cell suspensions were being labelled with the Enrichment Antibody Cocktail from the kit. Cells were filtered again and resuspended in RPMI 1640 containing DAPI for live/dead discrimination before sorting. Cell sorting was performed using a BD Fusion or Aria instrument (BD Biosciences). Cells were sorted into solutions of RPMI 1640 supplemented with 25% Charcoal-Stripped FBS (Gibco) before being prepared for experiments as described below.

### Primary T cell culture

Naive CD8^+^ T cells were isolated by FACS from the spleens and lymph nodes of wild-type and *Arhgef1*-KO mice and stimulated with immobilized Ultra-LEAF purified anti-mouse CD3ε (BioLegend) and Ultra-LEAF purified anti-mouse CD28 (37.51; BioLegend) in culture medium containing rhIL-2 (5 ng ml^−1^; Peprotech) for 4–5 days. In some cases, cells were labelled with 2.5 μM CTV proliferation dye (Thermo Fisher) before stimulation, according to the manufacturer’s instructions.

### Gα12/13 GPCR ligand stimulation assays

Naive CD8^+^ T cells were purified as described above by FACS from the spleens and lymph nodes of wild-type and *Arhgef1*-KO mice. Purified naive CD8^+^ T cells (0.8 × 10^5^) labelled with 2.5 μM CTV were activated by plate-bound anti-CD3ε and soluble anti-CD28 (5 µg ml^−1^ each; BioLegend) in lipid-free medium containing 10% charcoal-stripped FBS for 5 days in the presence of rhIL-2 (5 ng ml^−1^) and Gα12/13 GPCR ligands as follows: CXCL12 (1 μg ml^−1^; R&D), Thrombin (10 units ml^−1^; Sigma Aldrich), PAR1 peptide (10 μM; Abcam), PAR2 peptide (10 μM; Abcam), S1P (5 μM; Acros Organics), 20:4 LysoPI (1 μM; Avanti Polar Lipids), 18:1 LysoPS (1 μM; Avanti Polar Lipids), LPA (1 μM; Avanti Polar Lipids), 9-HODE (1 μM; Cayman), histamine dihydrochloride (5 mM; Sigma Aldrich), prostaglandin E_2_ (0.5 μM; Cayman) and U46619 (5 μM; Calbiochem). At the end of the cell culture, cells were collected, and cell proliferation and activation were measured by flow cytometry. For the inhibition study, cells were treated with the TP inhibitor SQ 29548 (Cayman) at the concentration of 10 μM, beginning 1 h before stimulation and continuing until the end of the cell culture.

### Acute T cell stimulation and analysis of protein phosphorylation

CD8^+^ T cells from wild-type and *Arhgef1*-KO OT-1 TCR^tg^ mice were stimulated in vitro with 10 nM OVA_257–264_ peptide in the presence of rhIL-2 for 2 days, followed by a 3-day expansion with rhIL-2. Before TCR crosslinking, cells were serum-starved overnight in RPMI 1640 containing 0.1% fatty acid-free BSA (Sigma Aldrich). Cells were then incubated with soluble anti-CD3ε antibody (Thermo Fisher Scientific) at 4 °C for 20 min. After washing, CD3 molecules bound with anti-CD3ε antibodies were crosslinked using goat anti-Armenian hamster IgG (H+L) (Jackson ImmunoResearch) in a lipid-free medium containing 5 μM U46619 or DMSO control for 5 min. For western blots, following crosslinking, cells were immediately lysed in Pierce RIPA buffer with cOmplete Mini protease and PhosSTOP phosphatase inhibitor cocktails (Roche). Cells were then denatured in 2× Laemmli buffer (Bio-Rad) with 2-mercaptoethanol and boiled for 10 min at 98 °C before gel loading. Western blotting on PVDF membrane was performed using TGX reagents (Bio-Rad Laboratories) and protocols. Following transfer, blots were blocked with 5% BSA then incubated with primary antibodies against pAkt-Ser 473 (193H12, Cell Signaling), pan-Akt (9272, Cell Signaling), pS6-Ser 235/236 (D57.2.2E, Cell Signaling), S6 (5G10, Cell Signaling), pMEK1/2-Ser 217/221 (9121, Cell Signaling), MEK1/2 (9122, Cell Signaling), pERK1/2-Thr202/Tyr204 (D13.14.4E, Cell Signaling), ERK1/2 (3A7, Cell Signaling), ARHGEF1 (D25D2, Cell Signaling), β-actin (AC74, Sigma Aldrich) and GAPDH (1E6D9, Proteintech) with appropriate horseradish peroxidase-conjugated secondary antibodies (Bio-Rad). Horseradish peroxidase-conjugated secondary antibodies blots were developed using chemiluminescence (Thermo Fisher) and gel images were captured in the darkroom using photographic films. For inhibition studies, cells were pre-treated for 1 h with the following inhibitors: AKT inhibitor VIII (1 μM, Calbiochem), TP inhibitor SQ 29548 (10 μM, Cayman), ROCK inhibitors Y-27632 (30 μM, Tocris) and GSK269962A (10 μM, ApexBio) or PTEN inhibitor bpV(pic) (2.5 μM, Sigma Aldrich) before TCR crosslinking.

### Ex vivo phosflow assay

Single-cell suspensions from the spleens and lymph nodes and lungs from mice of indicated genotype and treatments were prepared as described above except for using lipid-free medium throughout the procedure. Two-to-three million cells were incubated with specific surface antibodies and soluble anti-CD3ε antibody (Thermo Fisher Scientific) at 4 °C for 30 min. After washing, CD3 molecules bound with anti-CD3ε antibodies were crosslinked using goat anti-Armenian hamster IgG (H+L) (Jackson ImmunoResearch) in a lipid-free medium containing 5 μM U46619 or vehicle control for 5 min. Following crosslinking, the cells were immediately fixed with ice-cold 2% formaldehyde at 4 °C for 20 min. After fixation, the cells were permeabilized with ice-cold 90% methanol at 4 °C for 20 min. Phosphorylation of S6 and ERK was measured by flow cytometry using anti-pERK T202/Y204 AF488- or AF647-conjugated antibodies (197G2) and anti-pS6 S235/6-PE or -AF647 (D57.2.2E) antibodies.

### Assays for quantification of RHOA activation

The activity of RHOA was measured using the RHOA Pull-Down Activation Assay Biochem Kit (Cytoskeleton). Following overnight serum starvation, 10 × 10^6^ wild-type and *Arhgef1*-KO OT-1 TCR^tg^ CD8^+^ T cells were stimulated with 5 μM U46619 for 5 min at 37 °C. Cells were immediately spun down, washed and lysed in the lysis buffer with the phosphatase and protease inhibitor cocktails at 4 °C. Fifty micrograms of Rhotekin-RBD beads (Cytoskeleton) were added to the lysates and rotated for 1 h at 4 °C. The samples were spun down, washed three times, denatured in 2× Laemmli buffer with 2-mercaptoethanol and boiled for 10 min at 98 °C before gel loading. The primary antibody against RHOA (Abcam) was used for western blotting.

### Plasmids, cloning, retroviral transduction and CRISPR–Cas9 mutagenesis

Platinum-E ecotropic packaging cells (Cell Biolabs) were plated one day before transfections on poly-d-lysine-coated 10-cm plates (Corning) at a concentration of 6 × 10^6^ cells per plate. Packaging cells were transfected with 10 μg of retroviral plasmid DNA encoding MSCV-IRES-Thy1.1 (pMIT) or pMIT-RHOA^CA^ along with 6 μg pCL-Eco plasmid DNA using Transit293 (Sigma Aldrich) in OptiMEM (Thermo Fisher Scientific) for 8 h in antibiotic-free medium. Medium was replaced 8 h after transfection and cells were incubated for a further 48 h. Retroviral supernatants were used to spinfect Cas9-expressing OT-1 TCR^tg^ CD8^+^ T cells following stimulation with 10 nM OVA_257–264_ peptide for 24 h. In brief, CD8^+^ T cells were collected, suspended in viral supernatant and spun at 2,000*g* for 2 h at 32 °C in 24-well plates in the presence of 8 μg ml^−1^ polybrene (Sigma Aldrich) with slow acceleration and deceleration. Cells were cultured for 3–4 further days in rhIL-2-containing media prior to acute restimulation and phospho-flow assays.

For CRISPR–Cas9 mutagenesis, three different pairs of sgRNAs for *Rhoa* were designed using the CRISPick tool (Broad Institute), subcloned into optimized retroviral MSCV-sgRNA-puro-Thy1.1 vectors and retrovirally transduced to Cas9-expressing CD8^+^ T cells as described above. Deletion of *Tbxa2r* in naive T cells was as previously described^55^. In brief, three different sgRNAs targeting *Tbxa2r*: 5′-CCAGAGAAGCTCATGACAGG-3′, 5′-UUAGGAGCCAUGUGGCCCAA-3′ and 5′-CGAGGUGCCAUUGGGCCACA-3′ or their negative control: scrambled sgRNA#1 (Synthego) were incubated with Alt-R S.p.HiFi Cas9 Nuclease V3 Cas9 (Integrated DNA Technologies) to form sgRNA–Cas9 ribonuclear protein complexes before electroporating into naive MACS-sorted CD8^+^ T cells labelled with CTV using a Lonza 4D-Nucleofector system (DS137). Cells were rested overnight before activation in culture for 3 days by plate-bound anti-CD3ε and soluble anti-CD28 (2 µg ml^−1^ each; BioLegend) and rhIL-2 (5 ng ml^−1^) in lipid-free medium containing 5 μM U46619 or vehicle control.

### RNA-seq analysis

Lung tissues were immediately immersed in the RNAlater Stabilization solution (Thermo Fisher Scientific) for storage at −80 °C. The whole tissue was homogenized in 1 ml RLT Plus lysis buffer (Qiagen) by using a Qiagen TissueLyser II homogenizer and RNA was isolated by using the RNeasy Plus Mini kit (Qiagen) according to the manufacturer’s instructions. Naive CD8^+^ T cells (CD62L^+^CD44^−^CD8^+^) were purified by total CD8^+^ T cells enrichment followed by FACS, as described above and activated by plate-bound anti-CD3 and soluble anti-CD28 (5 µg ml^−1^ each) in lipid-free medium containing 10% Charcoal-Stripped FBS for 5 days in the presence of recombinant rhIL-2 (5 ng ml^−1^) and U46619 (1 μM). Cells were then pelleted and resuspended in 40 µl RNAlater Stabilization solution for storage at −80 °C. Processing of cell samples was performed using the QIAshredder Kit (Qiagen) and RNA was then extracted using the RNeasy Plus Mini Plus Kit (Qiagen) according to the manufacturer’s protocol. RNA libraries were prepared by Novogene using the mRNA library preparation kit (poly A enrichment) and sequencing was performed by Novogene on NovaSeq PE150. The FastQ files were then subjected to quality control using FastQC and then alignment to the NCBIM37 *Mus musculus* genome annotation using the STAR workflow. Differential gene expression analysis was performed on all expressed genes (>20 detected reads) using DESeq2^56^, and differentially expressed genes were further analysed and visualized using R. Expression heat maps were generated with the R package heatmap.

### Histopathological analysis of tissues

Lungs were fixed in 4% formalin before being embedded in paraffin. Sections were taken from the centre of the lungs and stained with H&E, using routine histology methods. Slides were genotype-blinded and independently scored for pathological features.

### Quantification of urinary prostanoid metabolites

Urine samples from mice that received intravenous injections of B16-F10 cells were collected using metabolic cages. Systemic biosynthesis of prostaglandin E_2_, prostaglandin D_2_, prostaglandin I_2_ and TXA_2_ was evaluated by quantifying their major urinary enzymatic metabolites: PGEM, PGDM, PGIM and TXM, respectively, using liquid chromatography–tandem mass spectrometry as previously described^42,57,58^. Urine samples (0.2 ml aliquots) were added with internal standards: tetranor PGEM-d_6_ (final concentration of 10 ng ml^−1^, Cayman), tetranor PGDM-d_6_ (final concentration of 10 ng ml^−1^, Cayman), 2,3-dinor-6-keto-PGF_1α_-d_9_ (sodium salt) (final concentration of 10 ng ml^−1^, Cayman) and 2,3-dinor-TXB_2_-d_9_ (final concentration of 10 ng ml^−1^, Cayman). Samples were incubated at room temperature for 15 min, followed by addition of formic acid (5 μl). After 15 min of incubation, methoxyamine HCl (1 g ml^−1^, 0.1 ml) (Sigma Aldrich) was added. Following 30 min of incubation at room temperature, urine samples were diluted to 1 ml with water adjusted to pH 3 with HCl and extracted with Number Strata-X 33u polymeric reversed phase (30 mg ml^−1^, Phenomenex) that had been preconditioned with 1 ml of acetonitrile and 1 ml of water^58,59^. The Number Strata-X 33u polymeric reversed phase column loaded with samples was washed with 1 ml of water (5% acetonitrile) and then eluted with 1 ml of 5% acetonitrile in ethyl acetate. The eluate was evaporated, and the dried residue was resuspended in 100 μl mobile phase (10% acetonitrile in water), and 30 μl was injected into an ACQUITY UPLC I-Class/Xevo TQS micro-IVD System (Waters) equipped with an electrospray ionization source (ESI Z-Spray), under negative ionization conditions, as previously described^58^.

### Platelet and T cell transwell co-cultures

Platelets were isolated as previously described^60^. In brief, blood was collected via cardiac puncture using syringes containing 100 μl of acid citrate dextrose (Sigma Aldrich) from B16-F10 bearing mice. Samples were immediately mixed with modified Tyrode’s buffer to prevent premature activation of platelets. Blood was centrifuged at 300*g* for 5 min and the uppermost platelet-rich plasma (PRP) layer was collected. PRP was subsequently centrifuged at 200*g* for 8 min to increase purity. Prostacyclin I_2_ (Sigma Aldrich) was added at a final concentration of 1 μg ml^−1^ to limit premature platelet activation and the sample was immediately centrifuged for 11 min at 1,000*g*. The cells were resuspended in modified Tyrode’s buffer. Platelet count and purity were determined by flow cytometry using the following antibodies: Armenian hamster anti-mouse/rat CD61-PE antibody (2C9.G2, BioLegend), rat anti-mouse Ter119-FITC antibody (TER119, eBioscience) and anti-mouse CD45.2-PerCp-Cy5.5 antibody (Ly5.2). Platelet purity was greater than 90%.

Platelets and naive FACS-sorted CD8^+^ T cells labelled with 2.5 μM CTV were co-cultured with a ratio of 30:1 in 24-well transwell plates (0.4 μm pore size, Corning). Naive CD8^+^ T cells on the bottom of the transwell were activated by plate-bound anti-CD3ε and soluble anti-CD28 (5 µg ml^−1^ each) in a lipid-free medium in the presence of rhIL-2 (5 ng ml^−1^) as described above. After 5 days, supernatants were carefully collected and TXB_2_ levels were measured using a Thromboxane B_2_ ELISA Kit (Cayman) according to the manufacturer’s instructions. T cells were collected and processed for flow cytometry as previously described.

### Confocal immunofluorescence microscopy

CD8^+^ T cells from wild-type and *Arhgef1*-KO OT-1 TCRtg mice were generated in vitro for 5 days as described above. 5 × 10^5^ serum-starved cells were seeded on 13-mm coverslips (VWR) pre-coated with 10 μg ml^−1^ Ultra-LEAF™ purified anti-mouse CD3ε in a 24-well plate. Cells were stimulated with 10 μM U-46619 or DMSO control for 10 min at 37 °C. After fixation and washing with 15 mM glycine and PBS. Coverslips were blocked with Fc blocking anti-FcR antibody (clone 2.4G2, BD). Cells were then incubated with the rabbit anti-mouse PTEN (138G6, Cell Signalling) antibody, followed by the donkey anti-rabbit IgG-Alexa Fluor 647 (A31573, Thermo-Fischer) antibody and Coralite 594-Phalloidin (Proteintech). Coverslips were mounted onto VWR SuperFrost Microscopy Slides (Appleton Woods MS527) using ProLong Diamond Antifade Mountant with DAPI (Thermo-Fischer P36962). For inhibition experiments, cells were pre-treated with 10 μM TP inhibitor SQ 29548, ROCK inhibitors 30 μM Y-27632 and 10 μM GSK269962A or DMSO for 1 h before seeding.

Images were captured on a Leica TCS SP8 inverted confocal microscope using the Leica Application Suite X (LAS X) software (v1.4.6.28433) with a 63× oil-immersion objective lens, in three channels, F-actin (yellow) and PTEN (red) plus a DAPI nuclear counterstain (blue) to locate the cells. *z*-stacks were obtained to capture the entirety of cells in each region. Three to five distinct locations were imaged for each replicate of each treatment condition. Images analysed were maximum intensity projections of four consecutive *z*-slices selected from the centre of a cross-volume image stack. An image analysis pipeline was constructed using Cellprofiler 4^61^. In brief, cell masks were generated from the F-actin image using as a seed a nuclei mask segmented from the DAPI image. These cell masks could be further compartmentalized into membrane and cytoplasmic regions by using the F-actin image. Ratiometric measurements of mean fluorescence intensity of PTEN on the membrane versus intracellular were used to report its re-localization to the membrane.

### Single-sample gene set enrichment analysis

Every expressed gene detected in the RNA-seq experiment in Fig. 4a (14,025 genes; Supplementary Table 3) was subject to single-sample Gene Set Enrichment Analysis (ssGSEA) analysis^62^. ssGSEA was used to test the relative enrichment of each gene set comprising the MsigDB C7 Immunologic signature gene sets in the total expressed gene transcriptional profile of each replicate sample. ssGSEA first calculates for each sample the differential expression of each expressed gene between that sample and the remainder of the samples. The list of expressed genes in each sample is then ranked by their level of differential expression compared to the other samples. The non-random distribution (enrichment) of genes within each C7 gene set for each sample is then calculated, yielding a sample enrichment score for each gene set per sample. This is achieved by analysing the distribution of the genes in each C7 gene set in the list of expressed genes rank ordered by their differential expression for each sample. The sample enrichment score for each differentially enriched gene set (false discovery rate (FDR) < 0.2, |FC| > 1.5) is represented in the heat map provided and the identity of each gene set in the heat map is given in Supplementary Table 4. Each gene within each of the gene sets included in the analysis can be looked up using the following link: https://www.gsea-msigdb.org/gsea/msigdb/human/genesets.jsp?collection=C7.

### Cell line authentication

Validated B16-F10 melanoma cells were obtained from ATCC. Plat-E cells were obtained from Cell Biolabs. Validated MC38 colorectal adenocarcinoma cells were obtained from Kerafast. All lines were validated as mycoplasma-free by suppliers and expanded at low passage frequency before cryopreservation.

### Statistical testing

Data were analysed using unpaired two-tailed Student’s *t* tests, Mann–Whitney *U-*test, ordinary one-way ANOVA or two-way ANOVA test where stated. Most experiments did not require blinding since objective quantitative assays, such as flow cytometry, were used. For tumour experiments littermate controls or age- and sex-matched mice of different genotypes were randomized and the operator blinded to genotype before injection and again before counting of metastatic nodules or assessment of histology images to allow for objective assessment. Experimental sample sizes were chosen using power calculations, preliminary experiments, or were based on previous experience of variability in similar experiments. Samples which had undergone technical failure during processing were excluded from subsequent analysis.

### Reporting summary

Further information on research design is available in the Nature Portfolio Reporting Summary linked to this article.

## Online content

Any methods, additional references, Nature Portfolio reporting summaries, source data, extended data, supplementary information, acknowledgements, peer review information; details of author contributions and competing interests; and statements of data and code availability are available at 10.1038/s41586-025-08626-7.

## Supplementary information

Supplementary information Supplementary Fig. 1. Western blot source data and flow cytometry gating strategy

Reporting Summary

Supplementary Table 1 Differentially expressed genes in B16 tumour-bearing lungs (*Arhgef1* KO versus WT)

Supplementary Table 2 Differentially expressed genes in TXA2 analogue-treated CD8 T cells (*Arhgef1* KO versus WT)

Supplementary Table 3 Gene expression profiles in treated CD8 T cells

Supplementary Table 4 ssGSEA of gene expression changes in TXA2 analogue-treated CD8 T cells (*Arhgef1* KO versus WT)

Supplementary Table 5 Geneset enrichment analysis genes significantly downregulated in naive versus memory CD8 T cells

## Source data

Source Data Fig. 1

Source Data Fig. 2

Source Data Fig. 3

Source Data Fig. 4

Source Data Fig. 5

Source Data Extended Data Fig. 1

Source Data Extended Data Fig. 2

Source Data Extended Data Fig. 3

Source Data Extended Data Fig. 4

Source Data Extended Data Fig. 5

Source Data Extended Data Fig. 7

Source Data Extended Data Fig. 8

Source Data Extended Data Fig. 10

Source Data Extended Data Fig. 12

Source Data Extended Data Fig. 13

## Data Availability

RNA-seq data are deposited in the Gene Expression Omnibus (GEO) database under the accession numbers GSE281884 and GSE281885. Source data are provided with this paper.
