# Supplementary material for: Aspirin prevents metastasis by limiting platelet TXA2 suppression of T cell immunity
Source: Nature. 2025 Mar 5;640(8060):1052–61. doi: 10.1038/s41586-025-08626-7 (PMC12018268; doi:10.1038/s41586-025-08626-7)
Supplement: Supplementary file 1 — Supplementary Fig. 1. Western blot source data and flow cytometry gating strategy [file 41586_2025_8626_MOESM1_ESM.pdf]

nature portfolio

https://doi.org/10.1038/s41586-025-08626-7

Supplementary information

Aspirin prevents metastasis by limiting platelet TXA_2 suppression of T cell immunity

In the format provided by the authors and unedited

Supplementary Figure 1

a

RhoA-GTP

p-AKT (S473)

Total RhoA

β-actin

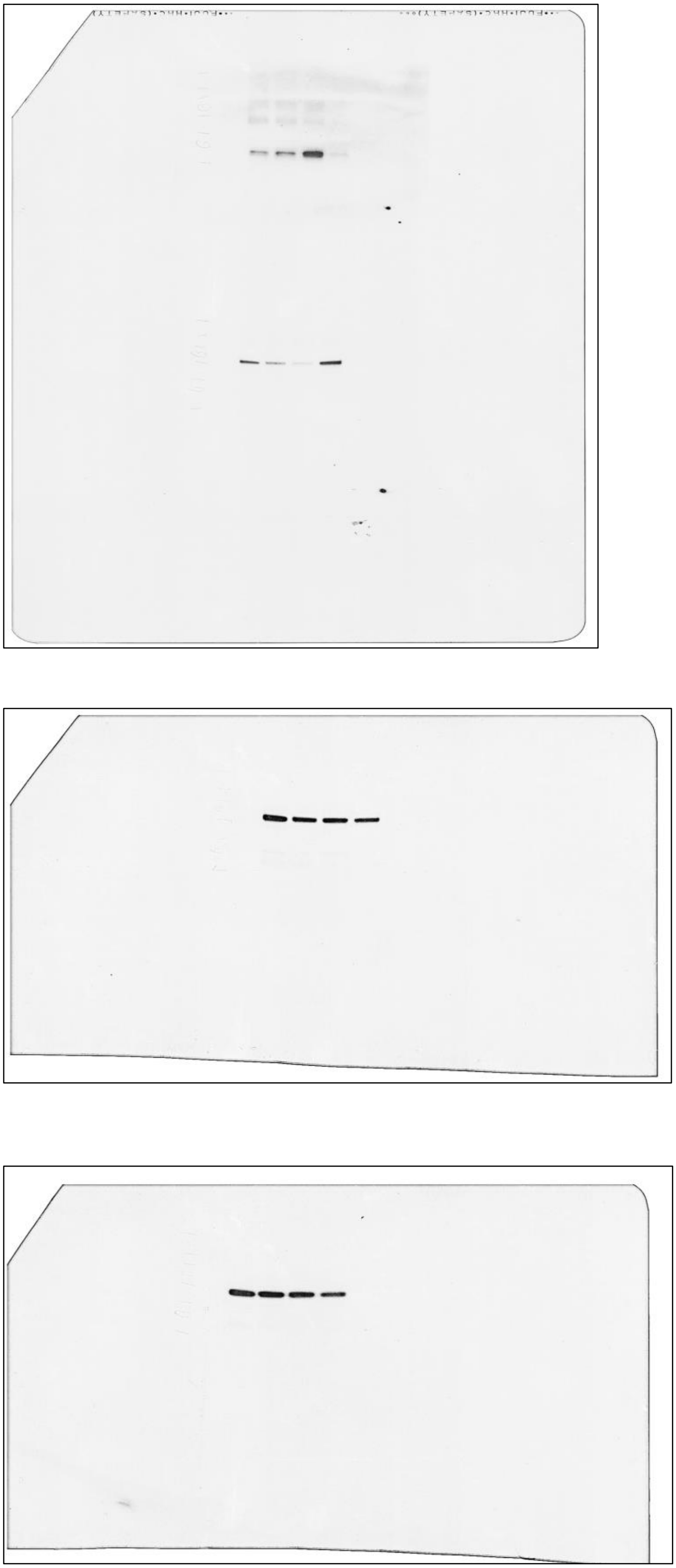

b

p-AKT (S473)

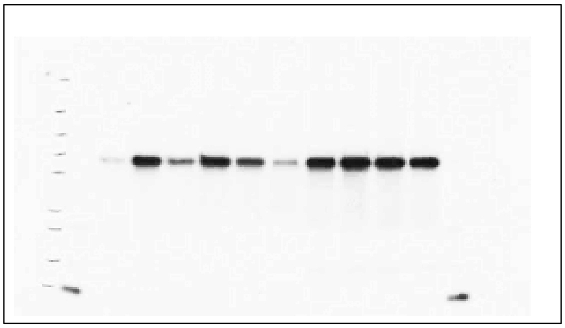

Pan-AKT

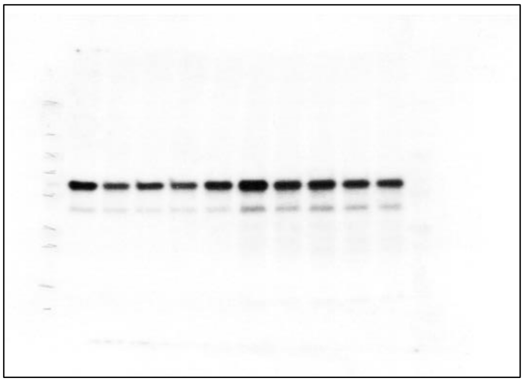

p-ERK1/2 (T202/Y204)

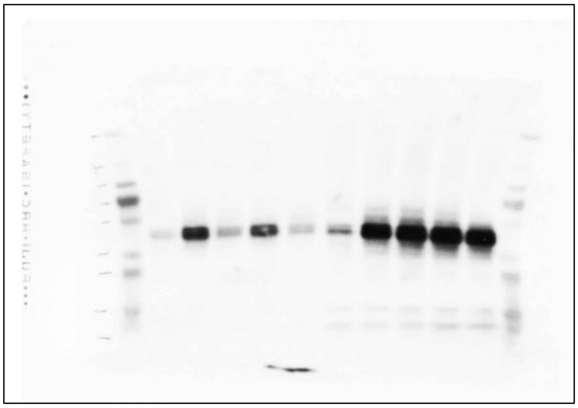

ERK1/2

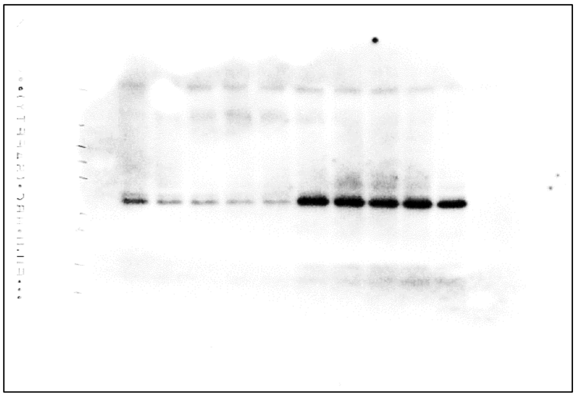

ARHGEF1

GAPDH

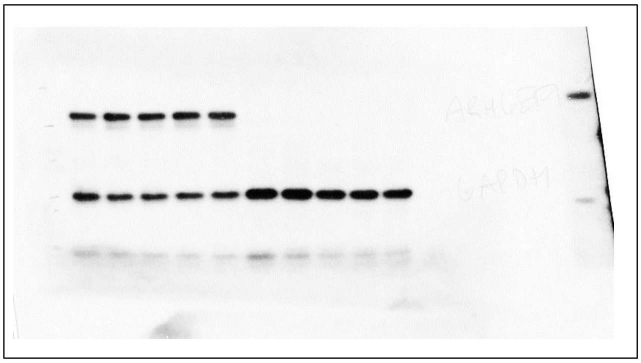

c

ARHGEF1 —
GAPDH —

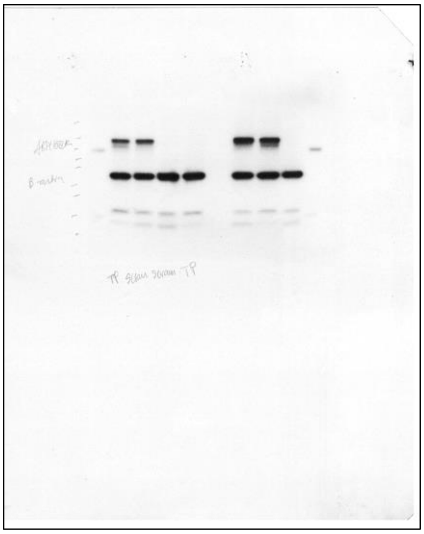

TP —

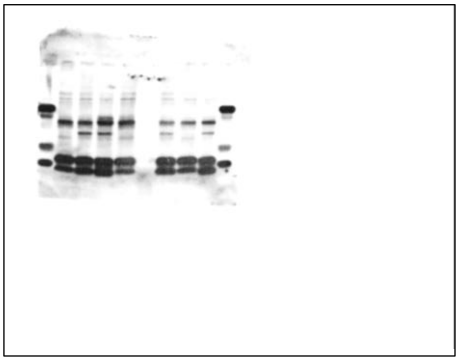

d

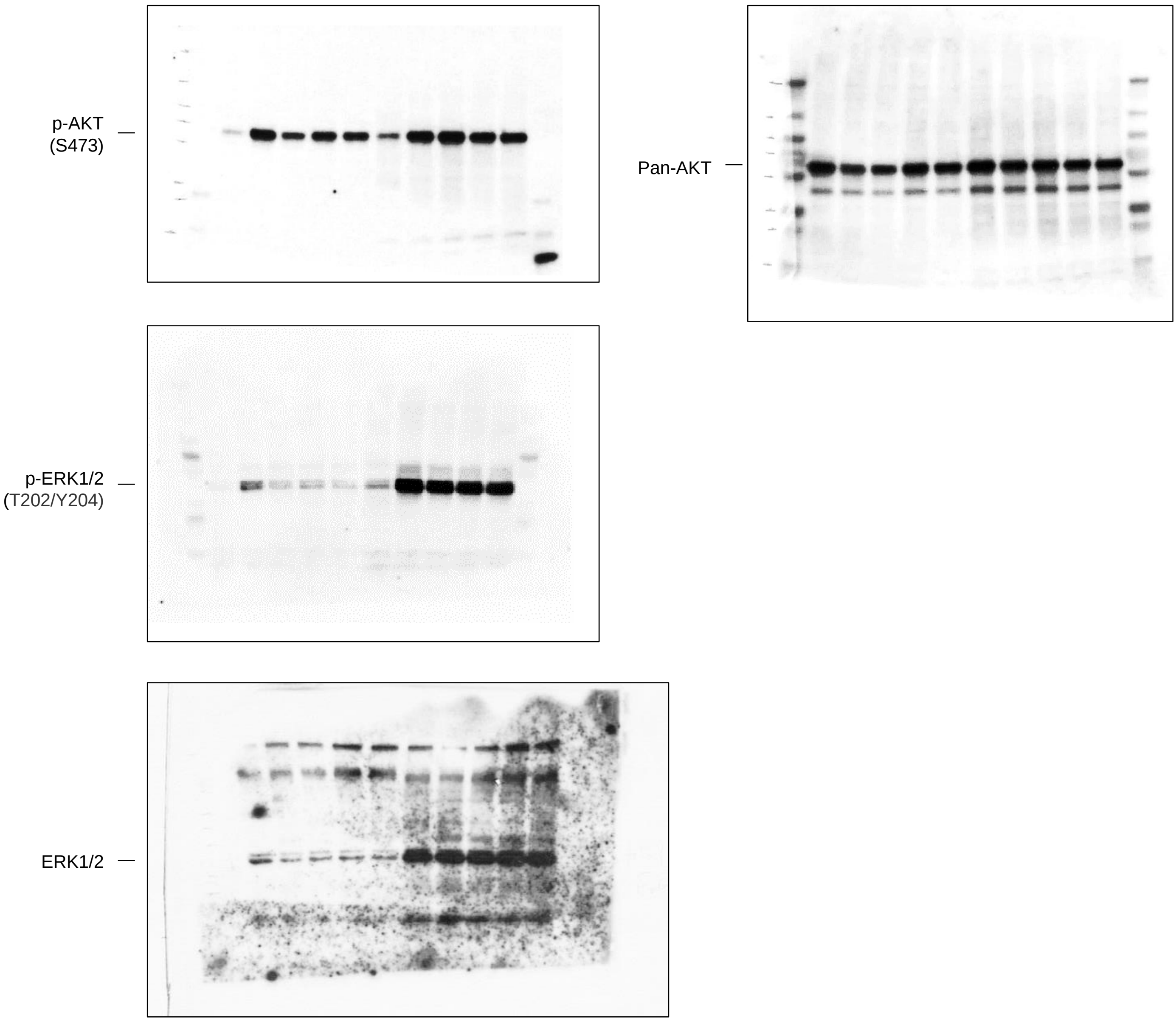

e

Gating strategy:

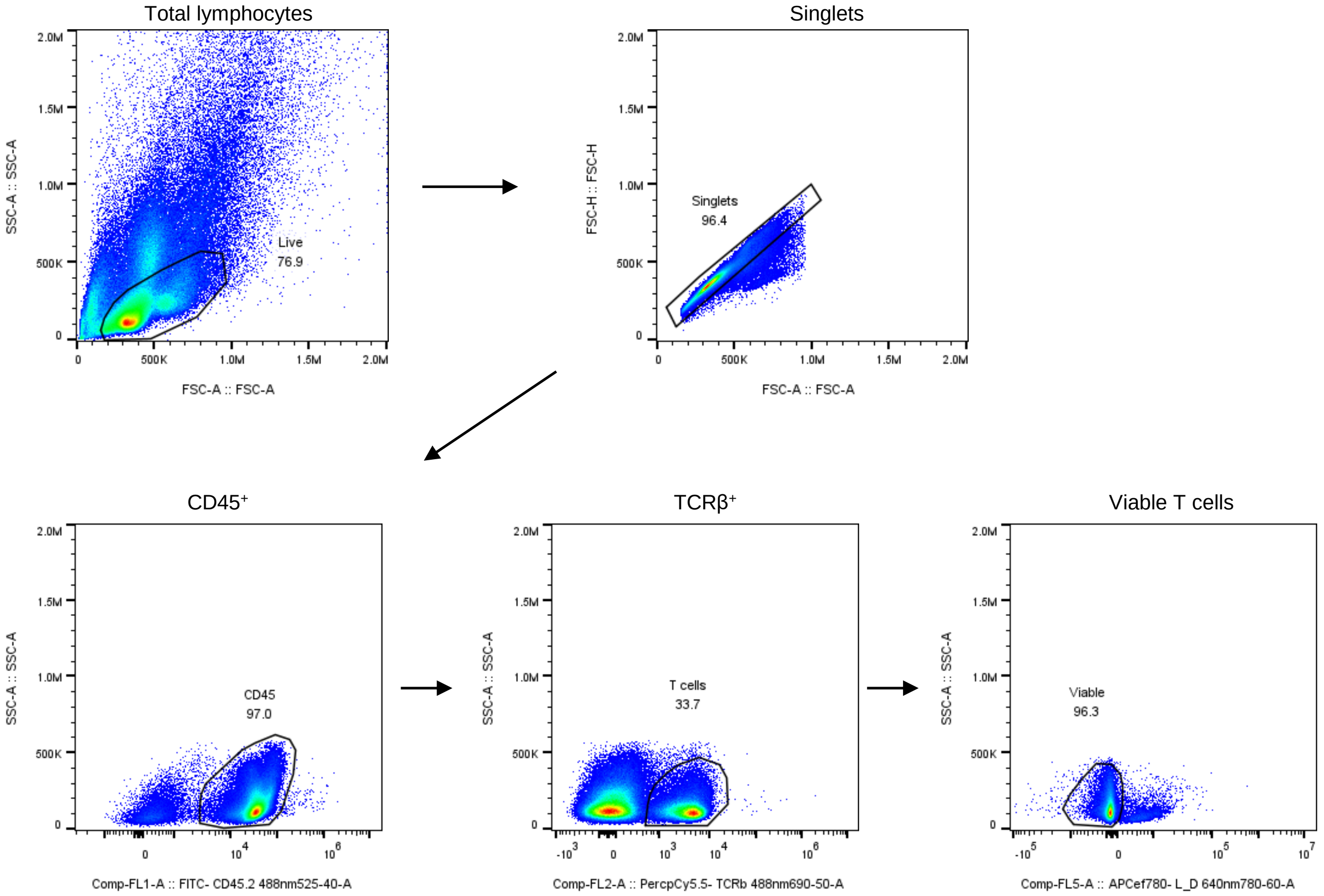

Supplementary Figure 1. Uncropped images for western blot and gating strategy for flow cytometry. **a**, Uncropped blots for Fig. 4d. **b**, Uncropped blots for Fig. 4g. **c**, Uncropped blots for Extended Data Fig. 8c. **d**, Uncropped blots for Extended Data Fig. 11b. **e,** Gating strategy for T lymphocytes in the lungs.
