# Supplementary material for: Aspirin prevents metastasis by limiting platelet TXA2 suppression of T cell immunity
Source: Nature. 2025 Mar 5;640(8060):1052–61. doi: 10.1038/s41586-025-08626-7 (PMC12018268; doi:10.1038/s41586-025-08626-7)
Supplement: Supplementary file 2 — Reporting Summary [file 41586_2025_8626_MOESM2_ESM.pdf]

nature portfolio

Corresponding author(s): Rahul Roychoudhuri and Jie Yang

Last updated by author(s): Dec 10, 2024

Reporting Summary

Nature Portfolio wishes to improve the reproducibility of the work that we publish. This form provides structure for consistency and transparency in reporting. For further information on Nature Portfolio policies, see our Editorial Policies and the Editorial Policy Checklist.

Statistics

For all statistical analyses, confirm that the following items are present in the figure legend, table legend, main text, or Methods section.

n/a	Confirmed	
☐	☒	The exact sample size (n) for each experimental group/condition, given as a discrete number and unit of measurement
☐	☒	A statement on whether measurements were taken from distinct samples or whether the same sample was measured repeatedly
☐	☒	The statistical test(s) used AND whether they are one- or two-sided<br>*Only common tests should be described solely by name; describe more complex techniques in the Methods section.*
☒	☐	A description of all covariates tested
☐	☒	A description of any assumptions or corrections, such as tests of normality and adjustment for multiple comparisons
☐	☒	A full description of the statistical parameters including central tendency (e.g. means) or other basic estimates (e.g. regression coefficient) AND variation (e.g. standard deviation) or associated estimates of uncertainty (e.g. confidence intervals)
☐	☒	For null hypothesis testing, the test statistic (e.g. F, t, r) with confidence intervals, effect sizes, degrees of freedom and P value noted<br>*Give P values as exact values whenever suitable.*
☒	☐	For Bayesian analysis, information on the choice of priors and Markov chain Monte Carlo settings
☒	☐	For hierarchical and complex designs, identification of the appropriate level for tests and full reporting of outcomes
☒	☐	Estimates of effect sizes (e.g. Cohen's d, Pearson's r), indicating how they were calculated

Our web collection on statistics for biologists contains articles on many of the points above.

Software and code

Policy information about availability of computer code

Data collection

Raw uncompensated flow cytometry data were acquired on BD LSR Fortessa or Beckman CytoFLEX instruments and exported as FCS files using BD FACSDiva software (v8.0.1) or CytExpert software (v2.5.0.77), respectively. Alternatively, high-parameter flow cytometry analysis was performed on a Cytek Aurora instrument and exported as FACS files using SpectroFlo software (v3.3.0). Data were compensated and analysed as described below.

RNA-sequencing libraries were prepared as described in the Methods section of the manuscript and sequenced using a NovaSeq PE150 instrument (Illumina) and base calls were acquired using standard Illumina run-time analysis software and stored as FastQ files.

Confocal Images were captured on a Leica TCS SP8 inverted confocal microscope using the Leica Application Suite X (LAS X) software (v1.4.6.28433). Histology images were taken using a Pannoramic digital slide scanner (3DHistech) and analyzed using QuPath v0.5.1 software.

Data analysis	Raw uncompensated flow cytometry data, exported as FCS 3.0 files, were compensated using compensation controls acquired during each acquisition, and analysed using FlowJo v10.10.0 (Treestar).<br><br>The FastQ files were then subjected to quality control using FastQC and then alignment to the NCBIM37 Mus musculus genome annotation using the STAR workflow. Differential gene expression analysis was performed on all expressed genes (> 20 detected reads) using DESeq2, and differentially expressed genes were further analyzed and visualized using R. Expression heatmaps were generated with the R package heatmap.<br><br>Confocal image analysis was performed using Cellprofiler 4.

For manuscripts utilizing custom algorithms or software that are central to the research but not yet described in published literature, software must be made available to editors and reviewers. We strongly encourage code deposition in a community repository (e.g. GitHub). See the Nature Portfolio guidelines for submitting code & software for further information.

Data

Policy information about availability of data

All manuscripts must include a data availability statement. This statement should provide the following information, where applicable:

- Accession codes, unique identifiers, or web links for publicly available datasets
- A description of any restrictions on data availability
- For clinical datasets or third party data, please ensure that the statement adheres to our policy

RNA-Seq data are deposited in the Gene Expression Omnibus (GEO) database under the accession number GSE281884 and GSE281885.

Research involving human participants, their data, or biological material

Policy information about studies with human participants or human data. See also policy information about sex, gender (identity/presentation), and sexual orientation and race, ethnicity and racism.

Reporting on sex and gender	N/A
Reporting on race, ethnicity, or other socially relevant groupings	N/A
Population characteristics	N/A
Recruitment	N/A
Ethics oversight	N/A

Note that full information on the approval of the study protocol must also be provided in the manuscript.

Field-specific reporting

Please select the one below that is the best fit for your research. If you are not sure, read the appropriate sections before making your selection.

☒ Life sciences ☐ Behavioural & social sciences ☐ Ecological, evolutionary & environmental sciences

For a reference copy of the document with all sections, see nature.com/documents/nr-reporting-summary-flat.pdf

Life sciences study design

All studies must disclose on these points even when the disclosure is negative.

Sample size	Where feasible, sample sizes were estimated based on previous experience with similar experiments in our laboratory. For experiments where technical limitations prevented adequate statistical power from single experiments, results from multiple independent experiments were combined. Power calculations were performed using established methods (Dupont, W. D. et al. Power and sample size calculations: a review and computer program. Control. Clin. Trials 11, 116-128, 1990).
Data exclusions	Animals where technical failures occurred during injections were excluded from subsequent analyses. Experiments included positive and negative controls to allow technical failure of experiments to be objectively determined. Pre-established exclusion criteria across samples from a given experiment were used to avoid subjective bias.
Replication	The number of independently repeated experiments for each observation is described in the figure legends throughout the manuscript. Where the results of replicate experiments are pooled this is stated in the figure legends.
Randomization	Sex/aged-matched female or male mice within genotype groups were randomly assigned to control or experimental treatment. Where appropriate, experimental cohorts were composed of random Mendelian segregation of genotypes within litters. Flow cytometry and in vitro measurements reported in the manuscript are in most cases non-subjective and did not require randomisation.

Blinding

Tumor injections and enumeration of metastases were performed by investigators and/or technicians who were blinded to genotype and treatment group. Flow cytometry and in vitro measurements are in most cases non-subjective and did not require blinding.

Reporting for specific materials, systems and methods

We require information from authors about some types of materials, experimental systems and methods used in many studies. Here, indicate whether each material, system or method listed is relevant to your study. If you are not sure if a list item applies to your research, read the appropriate section before selecting a response.

Materials & experimental systems

n/a	Involved in the study	
☐	☒	Antibodies
☐	☒	Eukaryotic cell lines
☒	☐	Palaeontology and archaeology
☐	☒	Animals and other organisms
☒	☐	Clinical data
☒	☐	Dual use research of concern
☒	☐	Plants

Methods

n/a	Involved in the study	
☒	☐	ChIP-seq
☐	☒	Flow cytometry
☒	☐	MRI-based neuroimaging

Antibodies

Antibodies used

Target Fluorophore Clone Company Catalog Dilution
anti-CD103 Pacific Blue 2E 7 BioLegend 121418 1/200
anti-CD127 BV650 A7R34 BioLegend 135043 1/200
anti-CD127 Pecy7 A7R34 BioLegend 135014 1/200
anti-CD16/CD32 93 BioLegend 101302 1/200
anti-CD25 PE-Cy7 PC61 Invitrogen 25-0251-82 1/200
anti-CD25 BUV395 PC61 BD 564022 1/200
anti-CD39 AF647 Duha59 BioLegend 143808 1/400
anti-CD4 AF700 RM4-5 BioLegend 100536 1/200
anti-CD4 BV650 RM4-5 BioLegend 100546 1/400
anti-CD44 BV510 IM7 BioLegend 103044 1/200
anti-CD44 BV786 IM7 BD 563736 1/400
anti-CD44 PerCP-Cy5.5 IM7 Invitrogen 45-0441-82 1/400
anti-CD44 APC IM7 Invitrogen 17-0441-83 1/400
anti-CD45.2 ef506 104 Invitrogen 69-0454-82 1/100
anti-CD45.2 FITC 104 BioLegend 109805 1/200
anti-CD61 PE 2C9.G3 Invitrogen 12-0611-82 1/200
anti-CD62L BUV737 MEL-14 BD 612833 1/400
anti-CD62L APC MEL-14 BioLegend 104412 1/400
anti-CD69 PECy5 H1.2F3 Invitrogen 15-0691-82 1/300
anti-CD69 PE-Dazzle H1.2F3 BioLegend 104536 1/200
anti-CD8α BUV395 53-6.7 BD Horizon 563786 1/200
anti-CD8α BUV805 53-6.7 BD 612898 1/200
anti-CD8α BV510 53-6.7 BioLegend 100752 1/200
anti-CD8α FITC 53-6.7 Invitrogen 11-0081-86 1/200
anti-CD90.1 FITC OX-7 BioLegend 202503 1/100
anti-CD90.1 PerCP OX-7 BD 557266 1/100
anti-Foxp3 APC FJK-16S eBioscience 17-5773-82 1/200
anti-IFN-γ BUV737 XMG1.2 BD 612769 1/400
anti-IFN-γ FITC XMG1.2 BioLegend 505806 1/200
anti-IL-2 PE JES6-5H4 BioLegend 503808 1/200
anti-Ki67 PerCP-ef710 SolA15 Invitrogen 46-5698-80 1/200
anti-KLRG1 APC 2F1/KLRG1 BioLegend 138412 1/200
anti-KLRG1 BV605 2F1/KLRG1 BioLegend 138419 1/200
anti-Ly108 APC 330-AJ BioLegend 134610 1/200
anti-Ly6G FITC RB6-8C5 eBioscience 11-5931-85 1/400
anti-PD-1 APCef780 J43 Invitrogen 47-9985-82 1/200
anti-PD-1 PeCy7 RMP1-30 BioLegend 109110 1/200
anti-pErk T202/Y204 AF488 197G2 Cell Signalling 13214S 1/100
anti-pErk T202/Y204 AF647 197G2 Cell Signalling 13148S 1/100
anti-pS6 S235/6 PE D57.2.2E Cell Signalling 5316S 1/150
anti-ST2 PerCP-ef710 RMST2-2 eBioscience 46-9335-82 1/200
anti-TCF-1 AF488 C63D9 Cell Signalling 6444S 1/200
anti-TCRβ BV570 H57-597 BioLegend 109231 1/200
anti-TCRβ FITC H57-597 BioLegend 109206 1/200
anti-TCRβ PerCP-Cy5.5 H57-597 BioLegend 109228 1/200
anti-TIGIT PE-Dazzle 1G9 BioLegend 142110 1/100

anti-TIGIT PE GIGD7 Invitrogen 12-9501-82 1/100
anti-TIM-3 BV421 RMT3-23 BioLegend 119723 1/100
anti-TIM-3 BV785 RMT3-23 BioLegend 119725 1/100
anti-TNF APC MP6-XT22 BioLegend 506308 1/200
anti-TNF BV650 MP6-XT22 BioLegend 506333 1/200
anti-TOX PE REA473 Miltenyi 130-120-716 1/200
anti-TER-119 FITC TER119 Invitrogen MA5-17822 1/200
anti-p-Akt Ser473 193H12 Cell Signalling 4058 1/1000
anti-panAkt pAb Cell Signalling 9272 1/1000
anti-p-S6 Ser235/236 D57.2.2E Cell Signalling 4858 1/1000
anti-S6 5G10 Cell Signalling 2217 1/1000
anti-p-MEK1/2 Ser217/221 pAb Cell Signalling 9121 1/750
anti-MEK1/2 pAb Cell Signalling 9122 1/750
anti-p-ERK1/2 Thr202/Tyr204 D13.14.4E Cell Signalling 4370 1/1000
anti-ERK1/2 3A7 Cell Signalling 9107 1/1000
anti-RhoA EPR18134 Abcam ab187027 1/2500
anti-ARHGEF1 D25D2 Cell Signalling 3669 1/1000
a-Mouse IgG-HRP N/A Bio-Rad 1721011 1/4000
a-Rabbit IgG-HRP N/A Bio-Rad 1706515 1/4000
anti-b-actin AC74 Sigma A5316 1/4000
anti-GAPDH 1E6D9 Proteintech 60004-1 1/2000

Validation

All antibodies have been validated by the manufacturer. Antibody validation information is available for each of the listed antibodies on the relevant manufacturer's website.
anti-CD103 Pacific Blue BioLegend 121418 https://www.biolegend.com/en-gb/products/pacific-blue-anti-mouse-cd103-antibody-6138
anti-CD127 BV650 BioLegend 135043 https://www.biolegend.com/en-gb/products/brilliant-violet-650-anti-mouse-cd127-il-7ralpha-antibody-13541
anti-CD127 Pecy7 BioLegend 135014 https://www.biolegend.com/en-gb/products/pe-cyanine7-anti-mouse-cd127-il-7ralpha-antibody-6192
anti-CD16/CD32 BioLegend 101302 https://www.biolegend.com/en-gb/products/purified-anti-mouse-cd16-32-antibody-190
anti-CD25 PE-Cy7 Invitrogen 25-0251-82 https://www.thermofisher.com/antibody/product/CD25-Antibody-clone-PC61-5-Monoclonal/25-0251-82
anti-CD25 BUV395 BD 564022 https://www.bdbiosciences.com/en-gb/products/reagents/flow-cytometry-reagents/research-reagents/single-color-antibodies-ruo/buv395-rat-anti-mouse-cd25.564022
anti-CD39 AF647 BioLegend 143808 https://www.biolegend.com/en-gb/products/alexa-fluor-647-anti-mouse-cd39-antibody-9969
anti-CD4 AF700 BioLegend 100536 https://www.biolegend.com/en-gb/products/alexa-fluor-700-anti-mouse-cd4-antibody-3386
anti-CD4 BV650 BioLegend 100546 https://www.biolegend.com/en-gb/products/brilliant-violet-650-anti-mouse-cd4-antibody-7634
anti-CD44 BV510 BioLegend 103044 https://www.biolegend.com/en-gb/products/brilliant-violet-510-anti-mouse-human-cd44-antibody-7994
anti-CD44 BV786 BD 563736 https://www.bdbiosciences.com/en-gb/products/reagents/flow-cytometry-reagents/research-reagents/single-color-antibodies-ruo/bv786-rat-anti-mouse-cd44.563736
anti-CD44 PerCP-Cy5.5 Invitrogen 45-0441-82 https://www.thermofisher.com/antibody/product/CD44-Antibody-clone-IM7-Monoclonal/45-0441-82
anti-CD44 APC Invitrogen 17-0441-83 https://www.thermofisher.com/antibody/product/CD44-Antibody-clone-IM7-Monoclonal/17-0441-83
anti-CD45.2 ef506 Invitrogen 69-0454-82 https://www.thermofisher.com/antibody/product/CD45-2-Antibody-clone-104-Monoclonal/69-0454-82
anti-CD45.2 FITC BioLegend 109805 https://www.biolegend.com/en-gb/products/fitc-anti-mouse-cd45-2-antibody-6
anti-CD61 PE Invitrogen 12-0611-82 https://www.thermofisher.com/antibody/product/CD61-Integrin-beta-3-Antibody-clone-2C9-G3-Monoclonal/12-0611-82
anti-CD62L BUV737 BD 612833 https://www.bdbiosciences.com/en-gb/products/reagents/flow-cytometry-reagents/research-reagents/single-color-antibodies/buv737-rat-anti-mouse-cd62l.612833
anti-CD62L APC BioLegend 104412 https://www.biolegend.com/en-gb/products/apc-anti-mouse-cd62l-antibody-381
anti-CD69 PECy5 Invitrogen 15-0691-82 https://www.thermofisher.com/antibody/product/CD69-Antibody-clone-H1-2F3-Monoclonal/15-0691-82
anti-CD69 PE-Dazzle BioLegend 104536 https://www.biolegend.com/en-gb/products/pe-dazzle-594-anti-mouse-cd69-antibody-11763
anti-CD8α BUV395 BD Horizon 563786 https://www.bdbiosciences.com/en-gb/products/reagents/flow-cytometry-reagents/research-reagents/single-color-antibodies-ruo/buv395-rat-anti-mouse-cd8a.563786
anti-CD8α BUV805 BD 612898 https://www.bdbiosciences.com/en-gb/products/reagents/flow-cytometry-reagents/research-reagents/single-color-antibodies-ruo/buv805-rat-anti-mouse-cd8a.612898
anti-CD8α BV510 BioLegend 100752 https://www.biolegend.com/en-gb/products/brilliant-violet-510-anti-mouse-cd8a-antibody-7992
anti-CD8α FITC Invitrogen 11-0081-86 https://www.thermofisher.com/antibody/product/CD8a-Antibody-clone-53-6-7-Monoclonal/11-0081-86
anti-CD90.1 FITC BioLegend 202503 https://www.biolegend.com/en-gb/products/fitc-anti-rat-cd90-mouse-cd901-thy11-antibody-2412
anti-CD90.1 PerCP BD 557266 https://www.bdbiosciences.com/en-gb/products/reagents/flow-cytometry-reagents/research-reagents/single-color-antibodies-ruo/percp-mouse-anti-rat-cd90-mouse-cd90-1.557266
anti-Foxp3 APC eBioscience 17-5773-82 https://www.thermofisher.com/antibody/product/FOXP3-Antibody-clone-FJK-16s-Monoclonal/17-5773-82
anti-IFN-γ BUV737 BD 612769 https://www.bdbiosciences.com/en-gb/products/reagents/flow-cytometry-reagents/research-reagents/single-color-antibodies-ruo/buv737-rat-anti-mouse-ifn.612769
anti-IFN-γ FITC BioLegend 505806 https://www.biolegend.com/en-gb/products/fitc-anti-mouse-ifn-gamma-antibody-995

anti-IL-2 PE BioLegend 503808 https://www.biolegend.com/en-gb/products/pe-anti-mouse-il-2-antibody-954
anti-Ki67 PerCP-ef710 Invitrogen 46-5698-80 https://www.thermofisher.com/antibody/product/Ki-67-Antibody-clone-SolA15-Monoclonal/46-5698-80
anti-KLRG1 APC BioLegend 138412 https://www.biolegend.com/en-gb/products/apc-anti-mouse-human-klrg1-mafa-antibody-6866
anti-KLRG1 BV605 BioLegend 138419 https://www.biolegend.com/en-gb/products/brilliant-violet-605-anti-mouse-human-klrg1-mafa-antibody-9644
anti-Ly108 APC BioLegend 134610 https://www.biolegend.com/en-gb/products/apc-anti-mouse-ly108-antibody-15660
anti-Ly6G FITC eBioscience 11-5931-85 https://www.thermofisher.com/antibody/product/Ly-6G-Ly-6C-Antibody-clone-RB6-8C5-Monoclonal/11-5931-85
anti-PD-1 APCef780 Invitrogen 47-9985-82 https://www.thermofisher.com/antibody/product/CD279-PD-1-Antibody-clone-J43-Monoclonal/47-9985-82
anti-PD-1 PeCy7 BioLegend 109110 https://www.biolegend.com/en-gb/products/pe-cyanine7-anti-mouse-cd279-pd-1-antibody-3612
anti-pErk T202/Y204 AF488 Cell Signalling 13214S https://www.cellsignal.com/products/antibody-conjugates/phospho-p44-42-mapk-erk1-2-thr202-tyr204-197g2-rabbit-mab-alexa-fluor-488-conjugate/13214
anti-pErk T202/Y204 AF647 Cell Signalling 13148S https://www.cellsignal.com/products/antibody-conjugates/phospho-p44-42-mapk-erk1-2-thr202-tyr204-197g2-rabbit-mab-alexa-fluor-647-conjugate/13148
anti-pS6 S235/6 PE Cell Signalling 5316S https://www.cellsignal.com/products/antibody-conjugates/phospho-s6-ribosomal-protein-ser235-236-d57-2-2e-xp-rabbit-mab-pe-conjugate/5316
anti-ST2 PerCP-ef710 eBioscience 46-9335-82 https://www.thermofisher.com/antibody/product/IL-33R-ST2-Antibody-clone-RMST2-2-Monoclonal/46-9335-82
anti-TCF-1 AF488 Cell Signalling 6444S https://www.cellsignal.com/products/antibody-conjugates/tcf1-tcf7-c63d9-rabbit-mab-alexa-fluor-488-conjugate/6444
anti-TCRβ BV570 BioLegend 109231 https://www.biolegend.com/en-gb/products/brilliant-violet-570-anti-mouse-tcr-beta-chain-antibody-7454
anti-TCRβ FITC BioLegend 109206 https://www.biolegend.com/en-gb/products/fitc-anti-mouse-tcr-beta-chain-antibody-270
anti-TCRβ PerCP-Cy5.5 BioLegend 109228 https://www.biolegend.com/en-gb/products/percp-cyanine5-5-anti-mouse-tcr-beta-chain-antibody-5603
anti-TIGIT PE-Dazzle BioLegend 142110 https://www.biolegend.com/en-gb/products/pe-dazzle-594-anti-mouse-tigit-vstm3-antibody-12500
anti-TIGIT PE Invitrogen 12-9501-82 https://www.thermofisher.com/antibody/product/TIGIT-Antibody-clone-GIGD7-Monoclonal/12-9501-82
anti-TIM-3 BV421 BioLegend 119723 https://www.biolegend.com/en-gb/products/brilliant-violet-421-anti-mouse-cd366-tim-3-antibody-13392
anti-TIM-3 BV785 BioLegend 119725 https://www.biolegend.com/en-gb/products/brilliant-violet-785-anti-mouse-cd366-tim-3-antibody-14928
anti-TNF APC BioLegend 506308 https://www.biolegend.com/en-gb/products/apc-anti-mouse-tnf-alpha-antibody-975
anti-TNF BV650 BioLegend 506333 https://www.biolegend.com/en-gb/products/brilliant-violet-650-anti-mouse-tnf-alpha-antibody-8829
anti-TOX PE Miltenyi 130-120-716 https://www.miltenyibiotec.com/GB-en/products/tox-antibody-anti-human-mouse-reafinity-rea473.html#Conjugate=PE:size=100-tests-in-200-uL
anti-TER-119 FITC Invitrogen MA5-17822 https://www.thermofisher.com/antibody/product/TER-119-Antibody-clone-TER119-Monoclonal/MA5-17822
anti-p-Akt Ser473 Cell Signalling 4058 https://www.cellsignal.com/products/primary-antibodies/phospho-akt-ser473-193h12-rabbit-mab/4058?srsltid=AfmBOooQ8da0rrJAG4BI80cupmclKCQoYVBE7q50g8YlNAPMtqodd-tm
anti-panAkt Cell Signalling 9272 https://www.cellsignal.com/products/primary-antibodies/akt-antibody/9272
anti-p-S6 Ser235/236 Cell Signalling 4858 https://www.cellsignal.com/products/primary-antibodies/phospho-s6-ribosomal-protein-ser235-236-d57-2-2e-xp-rabbit-mab/4858
anti-S6 Cell Signalling 2217 https://www.cellsignal.com/products/primary-antibodies/s6-ribosomal-protein-5g10-rabbit-mab/2217
anti-p-MEK1/2 Ser217/221 Cell Signalling 9121 https://www.cellsignal.com/products/primary-antibodies/phospho-mek1-2-ser217-221-antibody/9121
anti-MEK1/2 Cell Signalling 9122 https://www.cellsignal.com/products/primary-antibodies/mek1-2-antibody/9122
anti-p-ERK1/2 Thr202/Tyr204 Cell Signalling 4370 https://www.cellsignal.com/products/primary-antibodies/phospho-p44-42-mapk-erk1-2-thr202-tyr204-d13-14-4e-xp-rabbit-mab/4370
anti-ERK1/2 Cell Signalling 9107 https://www.cellsignal.com/products/primary-antibodies/p44-42-mapk-erk1-2-3a7-mouse-mab/9107
anti-RhoA Abcam ab187027 https://www.abcam.com/en-us/products/primary-antibodies/rhoa-antibody-epr18134-ab187027
anti-ARHGEF1 Cell Signalling 3669 https://www.cellsignal.com/products/primary-antibodies/p115-rhogef-d25d2-xp-rabbit-mab/3669
a-Mouse IgG-HRP Bio-Rad 1721011 https://www.bio-rad.com/en-uk/sku/1721011-goat-anti-mouse-igg-hl-hrp-conjugate?ID=1721011
a-Rabbit IgG-HRP Bio-Rad 1706515 https://www.bio-rad.com/en-uk/sku/1706515-goat-anti-rabbit-igg-h-l-hrp-conjugate?ID=1706515
anti-b-actin Sigma A5316 https://www.sigmaaldrich.com/GB/en/product/sigma/a5316
anti-GAPDH Proteintech 60004-1 https://www.ptglab.com/products/GAPDH-Antibody-60004-1-Ig.htm?srsltid=AfmBOoq2aF2eXWjAAigzMfbjvQGYiVwa5x_zDVSDGrgA79q_k_0BAPZ5

Eukaryotic cell lines

Policy information about cell lines and Sex and Gender in Research

Cell line source(s)

B16-Fl0 melanoma cells and MC38 colorectal adenocarcinoma cells were obtained from Kerafast Inc. Plat-E cells were obtained from Cell Biolabs. LL/2 murine carcinoma cell line was obtained from CRUK CI.

Authentication

No cell line authentication was performed; Low passage stocks were used.

Mycoplasma contamination	Cell lines were screened and found to be negative for mycoplasma contamination by the manufacturer.
Commonly misidentified lines (See ICLAC register)	No commonly misidentified cell lines were used.

Animals and other research organisms

Policy information about studies involving animals; ARRIVE guidelines recommended for reporting animal research, and Sex and Gender in Research

Laboratory animals	Wildtype C57BL/6 mice (6-8 weeks old) were obtained from Charles River. Ptprca (CD45.1) congenic, OT-1 TCRtg, Rag2–/– and MMTV-PyMT (B6.FVB-Tg(MMTV-PyVT)634Mul/LellJ) mice were obtained from the Jackson Laboratory. Tbxas1 KO (Tbxas1tm1Swl) and Tbxa2R KO (Tbxa2rtm1Cof) mice were kindly provided by Professor Shu-Wha Lin. Arhgef1 KO (Arhgef1Tm1a) mice and Arhgef1fl Ncr1Cre (Ncr1tm1.1(icre)Viv), Lyz2Cre (Lyz2tm1(cre)Ifo) or Cd4Cre (Tg(Cd4-cre)1Cwi) mice were housed at the animal facility within the University of Cambridge. Pf4Cre Ptgs1flox animals were housed at the animal facility within the G. d'Annunzio University School of Medicine. Both male and female animals (12-20 weeks old) were used in the study. Experimental and control groups were comprised of age-matched littermates or age/sex-matched male or female mice.
Wild animals	Wild animals were not used in this study.
Reporting on sex	Both male and female animals were used in the study. Experimental and control groups were comprised of age-matched littermates or age/sex-matched male or female mice.
Field-collected samples	The study did not involve samples collected from the field.
Ethics oversight	All animal experiments were conducted in compliance with applicable ethical regulations and guidelines. Most studies were conducted in accordance with UK Home Office guidelines and were approved by the University of Cambridge Animal Welfare and Ethics Review Board. Experiments involving Pf4Cre Ptgs1flox mice were carried out at the G. d'Annunzio University School of Medicine and were performed under the European Communities Council (EEC) Directive of September 22, 2010 (2010/63/EU) and the National Ethical Committee (authorization n. 434/2024-PR).

Note that full information on the approval of the study protocol must also be provided in the manuscript.

Flow Cytometry

Plots

Confirm that:

☒ The axis labels state the marker and fluorochrome used (e.g. CD4-FITC).

☒ The axis scales are clearly visible. Include numbers along axes only for bottom left plot of group (a 'group' is an analysis of identical markers).

☒ All plots are contour plots with outliers or pseudocolor plots.

☒ A numerical value for number of cells or percentage (with statistics) is provided.

Methodology

Sample preparation	Single-cell suspensions from lymphoid tissues were prepared by mechanical dissociation through 40 µM cell strainers (BD Biosciences). Lungs were minced in media containing 20 µg/ml DNase I (Roche) and 1 mg/ml collagenase (Sigma-Aldrich) and incubated with agitation at 37 °C for 40 minutes before also being dissociated through 40 µM cell strainers. Erythrocytes were lysed using ice cold ACK Lysing Buffer (Gibco) for 5 minutes. Cells requiring intracellular staining of cytokines were stimulated prior to flow cytometry analysis using phorbol 12-myristate 13-acetate (PMA), ionomycin, brefeldin A (BFA) and monensin for 4 hours in complete RPMI 1640 (Thermo Fisher Scientific). Viable cells were discriminated by first staining alone with Zombie UV fixable viability dye (Biolegend) or eFluor 780 fixable viability dye (eBioscience) in PBS, according to manufacturer's instructions. Cells were then incubated with specific surface antibodies on ice for 40 minutes in FACS buffer, in the presence of 2.4G2 monoclonal antibodies to block FcγR binding. For intracellular staining, the eBioscience Foxp3 Transcription Factor Staining Buffer Set (Thermo Fisher Scientific) or BD Cytofix/Cytoperm Fixation/Permeabilization Kit was used in accordance with the manufacturer's instructions followed by intracellular staining with fluorochrome-conjugated antibodies for 40 minutes.
Instrument	Samples were acquired on BD LSR Fortessa or Beckman CytoFLEX instruments and exported as FCS files using BD FACSDiva software or CytExpert software, respectively. Alternatively, high-parameter flow cytometry analysis was performed on a Cytek Aurora instrument and exported as FACS files using SpectroFlo software. Raw data was exported as FCS 3.0 files.
Software	FCS 3.0 files containing uncompensated flow cytometry data were compensated and analysed using FlowJo software (Treestar LLC). Cells were gated as described in the manuscript.
Cell population abundance	For FACS sorting experiments, pre-enriched CD8+ T cells were stained using flow cytometry cell surface antibodies. Cell sorting was performed using a BD Influx or BD Arial III instruments (Becton Dickinson Biosciences). Cells were sorted into solutions of RPMI 1640 medium supplemented with 25% Fetal Bovine Serum. Post-sort samples exceeding >95% purity were

used for subsequent analysis. Purity as assessed by re-running post-sort samples through the FACS instrument and assessing the frequency of cells not falling within sort gates.

Gating strategy

In general, cells were gated based on a viability gate using amine-reactive viability exclusion dye intensity, a lymphocyte gate, a singlet gate and then subsequent gating based on the expression of surface and intracellular proteins as described in the manuscript.

☒ Tick this box to confirm that a figure exemplifying the gating strategy is provided in the Supplementary Information.
