# Supplementary material for: Aspirin prevents metastasis by limiting platelet TXA2 suppression of T cell immunity
Source: Nature. 2025 Mar 5;640(8060):1052–61. doi: 10.1038/s41586-025-08626-7 (PMC12018268; doi:10.1038/s41586-025-08626-7)
Supplement: Supplementary file 3 — Differentially expressed genes in B16 tumour-bearing lungs (Arhgef1 KO versus WT) [file 41586_2025_8626_MOESM3_ESM.pdf]

Suppementary Table 1. Genes differentially expressed (q<0.05; |FC|>2) in the whole B16 tumour-bearing lungs from Arhgef1 KO vs WT mice. Data are representative of five biological replicates per group. Differential expression was determined using DESeq2 with two-sided Wald test and Benjamini-Hochberg correction for multiple comparisons.

Gene name	Log2 foldchange	LfcSE	Stat	p value	Adjusted p value
Xkr4	-0.901771391	1.781679859	-0.50613548	0.612761545	0.974001549
Rp1	-0.54811254	0.395453865	-1.386034094	0.165736493	0.860011234
Sox17	-0.089807609	0.194116174	-0.462648772	0.643616144	0.97889195
Mrpl15	0.064561111	0.16759119	0.385229741	0.70006721	0.988482081
Lypla1	-0.059155523	0.134699911	-0.439165273	0.660541786	0.979924124
Tcea1	0.00819946		0.055991827	0.955348318	0.99614946
Rgs20	0.677264603	1.911645063	0.354283657	0.723126315	0.988585656
Atp6v1h	1813.962102	0.122415573	-0.983470962	0.3253757	0.91640698
Npbwr1	-0.682005779	1.789152902	-0.381189208	0.703062856	0.988585656
Rb1cc1	-0.308552109	0.1309345	-2.356537886	0.018446186	0.585935558
Fam150a	-0.33147744	0.422569694	-0.78443259	0.432786321	0.94605734
Pcmtd1	0.041399983	0.128899643	0.321179964	0.748074015	0.988585656
Sntg1	-0.018076463	1.974231153	-0.009156204	0.992694508	0.998971527
Rrs1	-0.192651598	0.149993143	-1.284402704	0.199001087	0.880661448
Adhfe1	0.049598677	0.295724693	0.167719091	0.866804276	0.992886758
2610203C22Rik	-0.079594866	0.305601542	-0.26045309	0.794514296	0.990988839
3110035E14Rik	0.959453653	1.529275466	0.627390993	0.53040297	0.966026275
Mybl1	0.26411911	0.420145717	0.628636921	0.529586783	0.965637248
Vcpip1	-0.113574399	0.168731071	-0.67310898	0.500877961	0.961175273
1700034P13Rik	-0.172650444	1.179481101	-0.146378305	0.883622753	0.992886758
Sgk3	0.175367498	0.108477268	1.616629011	0.105958386	0.79419908
Snhg6	0.634394185	0.26072176	2.433223005	0.014965081	0.554936045
Tcf24	-0.124029855	0.421266778	-0.294421164	0.768436096	0.989990452
Ppp1r42	0.30848132	0.267731902	1.152202328	0.249237941	0.896939594
Cops5	0.114285505	0.126495907	0.903471956	0.366275465	0.927734956
Cspp1	-0.098035681	0.206744808	-0.474186909	0.635366628	0.977034429
Arfgef1	-0.029512313	0.110040751	-0.268194396	0.788549689	0.990988839
Prex2	0.061577592	0.165414319	0.372262766	0.709697218	0.988585656
A830018L16Rik	-0.709094729	0.264209655	-2.683833527	0.007278334	0.452031612
Sulf1	-0.062367821	0.195850971	-0.318445298	0.750147183	0.988585656
Slco5a1	-0.643078383	1.191543787	-0.539701847	0.589402666	0.974001549
Ncoa2	-0.235219808	0.216543806	-1.086245839	0.277370242	0.901191693
Tram1	-0.140530349	0.090705819	-1.549298053	0.121310088	0.816233249
Lactb2	-0.029586036	0.124446855	-0.237740329	0.812082502	0.991836551
Eya1	-0.680178393	0.463423625	-1.467724899	0.142178964	0.840741274
Msc	0.281481138	0.374871217	0.750874233	0.452728349	0.949967324
Terf1	-0.018474325	0.202936107	-0.091035178	0.927464639	0.994688009
Sbspon	0.131610117	0.436880861	0.301249445	0.763224288	0.989285682
4930444P10Rik	-0.341408041	0.284587737	-1.199658301	0.230272074	0.895404344
Rpl7	0.099120064	0.149570652	0.662697277	0.507524454	0.963011338
Rdh10	0.102953877	0.166146873	0.619655819	0.535484409	0.96674935
Stau2	-0.057756945	0.242101328	-0.238565174	0.811442773	0.991784898
Ube2w	-0.090189104	0.119145687	-0.75696491	0.449070885	0.948476747
Tceb1	0.19484644	0.160234337	1.216009278	0.223981388	0.892989918
Tmem70	-0.009914226	0.144157448	-0.068773595	0.945169836	0.99486515
Ly96	-0.05060561	0.277958311	-0.182061869	0.855534173	0.992886758
Jph1	0.050400792	0.483570888	0.104226274	0.916989784	0.994688009
Gdap1	1.437273346	1.952925477	0.735959135	0.461755567	0.953083512
Pi15	-0.332108185	0.190019279	-1.747760474	0.080505512	0.758011246
Crispld1	-0.097128176	0.346469292	-0.28033704	0.779218935	0.990988839
Pkhd1	-0.42756135	1.204555118	-0.354953744	0.722624244	0.988585656
Mcm3	-0.462990077	0.283935484	-1.630617179	0.102971122	0.79028905
6720483E21Rik	0.445275481	0.928442456	0.479594053	0.631516076	0.977034429
Paqr8	0.067250202	0.248468379	0.270658995	0.786653317	0.990988839
Efhc1	-0.28021163	0.265219653	-1.056526644	0.290727675	0.907334416

Tram2	-0.494901365	0.787458323	-0.628479439	0.529689912	0.965637248
Tmem14a	-0.129811379	0.194321867	-0.6680225	0.50411923	0.96186221
Gsta3	0.365375343	0.190873743	1.914225276	0.055591377	0.714677496
Gm4956	0.208716253	0.180324486	1.157448203	0.24708929	0.896939594
Khdc1a	2.266772773	1.481223723	1.530337881	0.125933116	0.822401003
Khdc1c	1.469193904	1.932133519	0.760399781	0.447015654	0.948476747
Khdc1b	2.73299202	1.608850425	1.698723497	0.089371294	0.769196161
Kcnq5	0.382701496	0.402532633	0.950734089	0.341739379	0.919850248
Rims1	0.13330591	0.897147239	0.148588665	0.881878218	0.992886758
Ogfrl1	0.160608167	0.174368326	0.92108567	0.357005701	0.924354588
B3gat2	0.649831367	0.814493275	0.797835154	0.424966158	0.944290206
Smap1	-0.395973245	0.205771937	-1.924330647	0.054313151	0.707153766
Sdhaf4	0.376624353	0.209252843	1.799852977	0.071883856	0.744227652
Fam135a	-0.220158537	0.207718241	-1.059890243	0.289194535	0.907334416
Lmbrd1	-0.045184064	0.105574876	-0.427981218	0.668664795	0.981863224
Phf3	-0.268015951	0.197032445	-1.360263029	0.173746703	0.863528862
Ptp4a1	-0.329182661	0.208093775	-1.581895758	0.11367337	0.807243653
4931428L18Rik	1.440635196	1.468744051	0.980861979	0.326660809	0.916882624
Prim2	0.237453234	0.255146525	0.930654392	0.352032369	0.923082865
1700001G17Rik	0.417713033	0.626434534	0.666810353	0.504893279	0.962138588
Rab23	-0.114519817	0.167025326	-0.685643428	0.492937989	0.958916246
Bag2	-0.061758949	0.161576157	-0.38222811	0.702292173	0.988482081
Zfp451	-0.082416407	0.229197658	-0.359586602	0.719156305	0.988585656
Bend6	-0.291037226	0.34410454	-0.845781421	0.397674692	0.935327115
Dst	0.090862096	0.207099059	0.438737367	0.660851848	0.979924124
Ccdc115	0.328409363	0.163166499	2.012725435	0.044143522	0.688477147
Imp4	0.124370713	0.133039067	0.934843544	0.349868935	0.922206653
Ptpn18	0.526884967	0.205815684	2.559984534	0.010467682	0.499724698
Arhgef4	-0.031799746	0.457342994	-0.069531504	0.944566557	0.99486515
Fam168b	-0.08302257	0.151770996	-0.547025274	0.584361369	0.97242196
Plekhb2	-0.020228656	0.102025503	-0.19827058	0.842833368	0.992886758
Hs6st1	-0.198814183	0.230031224	-0.864292157	0.387427418	0.932858077
Uggt1	0.141947496	0.18095294	0.784444268	0.432779472	0.94605734
Neurl3	0.071237662	0.239416775	0.29754666	0.76604919	0.989884648
Arid5a	-0.500898107	0.265445067	-1.887012302	0.059158671	0.72285319
Kansl3	-0.308944275	0.179495967	-1.721176691	0.085218766	0.761635307
Fer1l5	1.373369409	0.703881049	1.951138493	0.051040573	0.707153766
Lman2l	-0.011101467	0.19419228	-0.057167395	0.954411851	0.996070133
Cnnm4	0.264979799	0.448728203	0.590512916	0.554846829	0.968926429
Cnnm3	-0.435548888	0.255816913	-1.702580498	0.088646602	0.767814344
Ankrd23	-0.236361016	0.310384619	-0.761510079	0.446352461	0.948476747
Ankrd39	0.040948589	0.169913439	0.240996762	0.809557626	0.991295569
Sema4c	-0.441597546	0.264800014	-1.667664359	0.095382374	0.778239075
Fam178b	0.628376025	0.244625086	2.568730928	0.010207168	0.499427958
Cox5b	0.206742476	0.159718946	1.294414231	0.195522378	0.879601031
Actr1b	0.008233767	0.097887388	0.084114682	0.932965251	0.994688009
Zap70	0.235246748	0.266845832	0.881582995	0.378002354	0.928883222
Tmem131	-0.226207564	0.229727651	-0.98467713	0.32478269	0.91640698
Inpp4a	0.072873467	0.268417025	0.271493459	0.786011529	0.990988839
Coa5	-0.100715739	0.110876997	-0.908355586	0.363690383	0.926455466
Unc50	0.137623739	0.136349766	1.009343421	0.312809963	0.914819219
Mgat4a	-0.321863324	0.224231915	-1.435403719	0.151172087	0.850827708
2010300C02Rik	-0.29809832	0.332629681	-0.896186772	0.370153019	0.928436166
Tsga10	-0.284936606	0.179292914	-1.589224027	0.112009824	0.801067615
Lipt1	0.10954563	0.351269152	0.311856676	0.755149449	0.989196392
Mitd1	0.120307859	0.163501361	0.735821757	0.461839178	0.953083512
Mrpl30	0.30341305	0.154238073	1.967173501	0.049163212	0.707153766
Txndc9	0.086632746	0.132205398	0.655289018	0.51228169	0.963486137
Eif5b	0.310443039	0.172437691	1.800320082	0.071810112	0.744227652
Rev1	-0.42896344	0.250764568	-1.71062221	0.087150876	0.765418158
Aff3	-0.191703263	0.262116694	-0.731366096	0.464555569	0.953083512
Chst10	-0.004492559	0.701124937	-0.006407645	0.994887474	0.999285699

Pdcl3	0.108233773	0.093444545	1.15826744	0.246754914	0.896939594
Npas2	0.477678125	0.524681902	0.910414717	0.362603839	0.926455466
Rpl31	0.329194265	0.158815669	2.072807222	0.038190224	0.679118604
Tbc1d8	-0.34946601	0.270417324	-1.292321087	0.196245972	0.879755417
Cnot11	0.184291228	0.1537777	1.198426225	0.230751128	0.895404344
Snord89	0.400334982	1.250884133	0.320041618	0.748936782	0.988585656
Rnf149	-0.007546286	0.159139063	-0.047419447	0.96217893	0.996474415
Creg2	0.130865614	1.151980215	0.113600574	0.909554432	0.994688009
Rfx8	0.660516444	1.106788588	0.59678646	0.550649959	0.968926429
Map4k4	-0.108020124	0.130765521	-0.826059681	0.408770237	0.940528702
Il1r2	-0.395612793	0.531974614	-0.743668556	0.457077017	0.951874548
Il1r1	-0.191313345	0.13739818	-1.392400871	0.163801041	0.859982992
Il1rl2	0.187238341	0.381585525	0.490685124	0.623649169	0.975691835
Il1rl1	-0.276677182	0.296527068	-0.933058772	0.350789624	0.922338606
Il18r1	0.054645744	0.104211326	0.524374326	0.60001821	0.974001549
Il18rap	0.077750282	0.25906528	0.300118494	0.764086773	0.989384061
Slc9a4	-0.005251674	0.24049088	-0.021837309	0.982577733	0.997390438
Slc9a2	-0.324622781	0.420541611	-0.771915959	0.440164204	0.947849924
Mfsd9	0.100946351	0.307901101	0.327853168	0.743022685	0.988585656
Tmem182	-1.886370976	0.85996341	-2.193547951	0.028267924	0.641905969
Pou3f3	2.730528976	2.274608359	1.200439172	0.229968823	0.895404344
Mrps9	0.209215706	0.148947346	1.404628626	0.16013174	0.857316691
Tgfbrap1	-0.365882197	0.225244385	-1.624378769	0.104294995	0.79155232
AI597479	0.037891428	0.142645085	0.265634303	0.790520865	0.990988839
Fhl2	-0.57233453	0.223381001	-2.562145069	0.010402786	0.499527467
Nck2	-0.064947384	0.132083346	-0.491715163	0.622920718	0.975486913
1500015O10Rik	-0.25687579	0.541394543	-0.474470593	0.635164363	0.977034429
Uxs1	-0.012909793	0.13236317	-0.097533119	0.922303034	0.994688009
Tpp2	-0.392606939	0.161029661	-2.438103249	0.014764556	0.554936045
Tex30	0.112338348	0.214950517	0.522624229	0.601235776	0.974001549
Kdelc1	-0.204045811	0.131351979	-1.553427759	0.120320975	0.81558552
Bivm	0.036240505	0.166336009	0.217875281	0.827526287	0.992886758
Ercc5	0.043047182	0.185523126	0.232031351	0.816513664	0.992676801
Mettl21e	-0.560110019	0.748386648	-0.748423319	0.454204861	0.950990329
Gulp1	-0.06348414	0.14115205	-0.449757124	0.652885577	0.979004493
Col3a1	0.008690975	0.286949462	0.030287477	0.975837784	0.997262526
Col5a2	-0.027873808	0.220878436	-0.126195243	0.899577377	0.99447264
Wdr75	0.025873443	0.187396269	0.13806808	0.890186613	0.993043931
Slc40a1	0.211708358	0.19189069	1.103275815	0.269907406	0.89804647
Dnah7b	-0.119459866	0.375810719	-0.317872429	0.750581707	0.988585656
Slc39a10	0.045752173	0.177543405	0.257695707	0.796641759	0.990988839
Tmeff2	0.355637252	0.205967007	1.72667097	0.084226772	0.76099108
9330175M20Rik	-0.088071927	1.020285535	-0.086320862	0.931211355	0.994688009
Sdpr	0.042784244	0.101563926	0.421254339	0.673569369	0.983375962
Nabp1	0.111273617	0.168551445	0.660175992	0.509140897	0.963452765
Myo1b	-0.049607129	0.161764422	-0.306662786	0.759100058	0.989266039
Stat4	-0.15820555	0.629961717	-0.251135181	0.801709597	0.990988839
Stat1	0.122773446	0.14369938	0.854377004	0.392896133	0.933734231
Gls	-0.082273721	0.122575142	-0.671210487	0.502086449	0.961378071
Nab1	-0.133052793	0.112756189	-1.180004346	0.237998486	0.896609492
Tmem194b	0.065617556	0.213535301	0.307291372	0.758621602	0.989266039
Mfsd6	0.038068594	0.206053252	0.18475124	0.853424154	0.992886758
Inpp1	0.085172956	0.216012082	0.394297185	0.693361642	0.986698113
Hibch	0.082832831	0.152692205	0.542482379	0.587486243	0.973455044
1700019D03Rik	0.393022762	0.749223978	0.524573123	0.599879975	0.974001549
Pms1	0.267316641	0.260214492	1.027293441	0.30428231	0.911879006
Ormdl1	0.092199747	0.198522734	0.464429163	0.6423403	0.978415289
Osgepl1	0.016416856	0.156132816	0.105146739	0.916259373	0.994688009
Asnsd1	0.108679681	0.131323638	0.827571352	0.407913298	0.939557159
Dnah7a	-0.081823826	0.318417082	-0.256970593	0.797201475	0.990988839
Stk17b	-0.015319369	0.16258956	-0.094221109	0.924933517	0.994688009
Hecw2	0.031054206	0.205331512	0.151239359	0.879786904	0.992886758

Ccdc150	-1.049321763	0.802510313	-1.307549256	0.191026238	0.877906723
Gtf3c3	-0.350736543	0.223444591	-1.569680169	0.116489537	0.812418014
Pgap1	-0.155310456	0.323207055	-0.480529289	0.630851082	0.977034429
Ankrd44	-0.099283332	0.196310871	-0.505745459	0.613035351	0.974001549
Sf3b1	-0.161419209	0.103789067	-1.555262171	0.119883643	0.81558552
Coq10b	-0.082814993	0.136955069	-0.604687312	0.54538678	0.967491297
Hspd1	-0.39765306	0.172590504	-2.304026301	0.021221167	0.60743619
Hspe1	0.133110613	0.307890923	0.432330423	0.665501279	0.981727712
Mob4	0.091428605	0.155842688	0.58667241	0.557423741	0.968926429
Rftn2	0.063340231	0.150437556	0.421040014	0.673725863	0.983375962
Mars2	0.155389344	0.233818988	0.664571109	0.506324853	0.962370882
Plcl1	0.284244375	0.270644135	1.050251376	0.293602554	0.907334416
Satb2	1.526557443	2.099335996	0.727162039	0.467126698	0.953239786
4930558J18Rik	-0.533626834	1.743992997	-0.305979918	0.759619936	0.989266039
1700066M21Rik	0.238178144	0.151729174	1.569758394	0.11647133	0.812418014
Tyw5	-0.024613301	0.21205269	-0.116071626	0.907595776	0.994688009
9430016H08Rik	0.11114737	0.135324387	0.821340281	0.411452469	0.941762685
Spats2l	-0.026355975	0.243251404	-0.108348707	0.913719088	0.994688009
Kctd18	0.08677968	0.274787125	0.315806937	0.752149052	0.988585656
Sgol2a	-0.463005849	0.617526227	-0.749775197	0.453390109	0.950146765
Aox1	0.043253194	0.243193176	0.177855295	0.85883662	0.992886758
Aox3	0.068762246	0.125425065	0.548233691	0.583531451	0.972174584
Bzw1	0.000787522	0.115246087	0.006833393	0.994547784	0.999285699
Clk1	0.191723082	0.146128168	1.312020023	0.189513379	0.876091552
Ppil3	0.341158462	0.182784164	1.866455247	0.0619777	0.725730283
Nif3l1	0.147990516	0.149306139	0.991188417	0.321593585	0.91640698
Orc2	-0.107584785	0.121980383	-0.88198432	0.377785287	0.928883222
Fam126b	-0.298556355	0.159035996	-1.87728793	0.060478642	0.725221283
Ndufb3	0.297123432	0.180380739	1.647201544	0.099516627	0.788783372
Gm20257	0.106791498	0.333232127	0.320471794	0.748610709	0.988585656
Als2cr12	0.609463905	0.967672924	0.629824282	0.528809557	0.965637248
Cflar	-0.11283477	0.100407952	-1.123763277	0.261113465	0.897248195
Casp8	0.083565042	0.129863113	0.64348559	0.519909062	0.964665379
Trak2	-0.173648459	0.151150657	-1.148843563	0.250620493	0.896939594
Stradb	0.142632837	0.253102627	0.563537563	0.573068889	0.971613657
Tmem237	0.072302721	0.175115648	0.412885551	0.679690468	0.983903958
Mpp4	0.459556822	0.479172253	0.959063924	0.337526543	0.919246575
Als2	0.34857885	0.32403962	1.075729104	0.282048427	0.90579922
Fzd7	0.173300671	0.183119301	0.946381243	0.343954169	0.920669678
Gm973	-0.089656174	0.292355443	-0.306668393	0.75909579	0.989266039
Sumo1	0.29395071	0.175460074	1.675313955	0.093872649	0.775671949
Nop58	-0.091775835	0.167797875	-0.546942775	0.584418047	0.97242196
Bmpr2	0.015188596	0.102795196	0.147755893	0.88253542	0.992886758
Fam117b	-0.142777603	0.199528495	-0.715574999	0.474253816	0.956061495
Ica1l	-0.035195376	0.687048485	-0.051226918	0.959144703	0.996367077
Wdr12	-0.137128115	0.232533288	-0.589713914	0.555382465	0.968926429
Carf	0.022309224	0.203175982	0.109802468	0.912566034	0.994688009
Nbeal1	-0.193519467	0.194589392	-0.994501631	0.319978712	0.91640698
Cyp20a1	0.03302959	0.119080413	0.277372147	0.781494366	0.990988839
Abi2	-0.330487804	0.227522017	-1.452553069	0.146347884	0.843780493
Raph1	-0.082797494	0.163337175	-0.506911511	0.612216908	0.974001549
Cd28	-0.238567103	0.509658831	-0.468091768	0.639718966	0.977843986
Ctla4	0.370112745	0.601680054	0.615132149	0.538467452	0.966776546
Icos	-0.623317291	0.623895191	-0.999073722	0.31775898	0.91640698
Pard3b	-0.101343576	0.263154704	-0.38511026	0.700155727	0.988482081
Nrp2	-0.082893959	0.196285061	-0.422314149	0.672795732	0.983233856
Ino80d	0.068944483	0.27886398	0.247233375	0.804727617	0.990988839
Ino80dos	0.823284644	0.406679202	2.024408036	0.042928186	0.685039858
Ndufs1	-0.090532373	0.115964099	-0.780693112	0.434983013	0.946762848
Eef1b2	0.296182864	0.230869063	1.282904083	0.199525682	0.880888611
Snora41	-1.015057392	1.976999026	-0.513433431	0.607648166	0.974001549
Zdbf2	-1.124335749	0.874454181	-1.285757188	0.198527816	0.880661448

Adam23	0.092941153	0.47374219	0.196185089	0.844465299	0.992886758
Mdh1b	-0.299502563	0.21414325	-1.398608468	0.161930426	0.858667769
Fastkd2	0.250363988	0.191828851	1.305142505	0.191844328	0.878912371
4933402D24Rik	-1.609345466	2.36234175	-0.681250063	0.495713275	0.959893834
Gm13749	-0.185650306	1.121277508	-0.165570347	0.868495085	0.992886758
Klf7	-0.208109425	0.515728282	-0.40352533	0.686561804	0.98538669
Creb1	-0.044974062	0.127826693	-0.351836236	0.724961081	0.988585656
Mettl21a	0.095743842	0.179771475	0.532586395	0.594319922	0.974001549
2810408I11Rik	0.273644826	1.485641373	0.184193057	0.853862006	0.992886758
Ccnyl1	0.315720884	0.244415063	1.291740699	0.196446958	0.879755417
Fzd5	-0.098784713	0.237988841	-0.415081282	0.678082402	0.983670411
Plekhm3	0.213895431	0.293895307	0.727794647	0.466739302	0.953083512
Akr1cl	0.949934952	1.72747704	0.549897295	0.582389819	0.972017845
D630023F18Rik	-0.801105009	1.757641915	-0.455783969	0.648545333	0.979004493
Idh1	0.157743264	0.14460122	1.090884738	0.275323604	0.900302631
Pikfyve	0.098299933	0.176760578	0.556119097	0.578129441	0.971613657
Map2	-0.134912443	0.187199797	-0.720686907	0.47110217	0.95486913
Unc80	0.992721562	1.388266487	0.715079973	0.474559628	0.956387791
Rpe	-0.030190652	0.118024621	-0.255799612	0.798105573	0.990988839
Kansl1l	-0.185868579	0.178935284	-1.038747497	0.298922185	0.909933614
Acadl	0.18527738	0.118860084	1.558785535	0.119047149	0.814755048
Myl1	0.161675139	0.377720923	0.428028022	0.668630719	0.981863224
Lancl1	0.017051604	0.10866302	0.156921863	0.875306429	0.992886758
Cps1	5.527629518	2.063596125	2.678639221	0.007392199	0.452031612
Ikzf2	-0.134662645	0.380326017	-0.35407161	0.723285218	0.988585656
Spag16	-0.464530404	0.177740026	-2.613538517	0.008960998	0.475600561
Bard1	0.614114645	0.961667573	0.638593483	0.523087422	0.965637248
Atic	-0.369333607	0.187466481	-1.970131435	0.048823309	0.707153766
Apol7d	0.123775488	1.7850421	0.069340375	0.944718689	0.99486515
4933417E11Rik	-0.520668724	0.818452094	-0.636162737	0.524670358	0.965637248
Mreg	0.071452124	0.341447556	0.209262367	0.834243429	0.992886758
Pecr	0.035090065	0.213173044	0.164608357	0.869252253	0.992886758
Xrcc5	-0.207069787	0.190825245	-1.085127847	0.277865035	0.901315669
Mar-04	-0.539562728	0.358122734	-1.506641931	0.131902463	0.827800491
Smarcal1	-0.122468598	0.192721815	-0.635468271	0.525123054	0.965637248
Rpl37a	0.224465407	0.162678831	1.379807106	0.167646044	0.860403806
Igfbp2	0.104803431	0.149993407	0.69872025	0.484726877	0.957710647
Igfbp5	0.005265156	0.278925431	0.018876571	0.98493957	0.998148029
6030407O03Rik	-0.22270294	0.749765143	-0.297030266	0.766443402	0.989884648
Tns1	0.018383681	0.139218192	0.132049415	0.894945206	0.993592437
Rufy4	0.233187116	0.495146829	0.470945388	0.637679734	0.977468848
Cxcr2	0.748376931	0.54934036	1.36231922	0.173097165	0.863084695
Cxcr1	-0.599186924	1.269795002	-0.471876896	0.63701466	0.977284137
Arpc2	-0.069863305	0.130963655	-0.533455672	0.593718188	0.974001549
Aamp	-0.085302068	0.14395614	-0.592555954	0.553478364	0.968926429
Pnkd	0.415062722	0.132330629	3.136558214	0.001709435	0.284736028
Tmbim1	-0.089298684	0.096932324	-0.92124774	0.356921099	0.924354588
Catip	-0.027089561	0.140548323	-0.192741972	0.847161051	0.992886758
Slc11a1	0.120499915	0.215040522	0.560359106	0.575234519	0.971613657
Ctdsp1	-0.029724794	0.138648483	-0.21438961	0.830243244	0.992886758
Vil1	0.533697065	1.266230396	0.421484958	0.673400993	0.983375962
Usp37	-0.226198348	0.272701215	-0.829473195	0.406836698	0.939557159
Rqcd1	-0.091625403	0.16653485	-0.55018756	0.582190735	0.972017845
Plcd4	-1.441901436	1.091305131	-1.321263316	0.186413581	0.872512868
Zfp142	0.420053919	0.432101933	0.972117657	0.330992019	0.917115058
Bcs1l	-0.003100932	0.189048749	-0.016402818	0.986913032	0.998148029
Rnf25	-0.057320916	0.145205574	-0.394756995	0.693022236	0.986599035
Stk36	0.133673521	0.358766232	0.37259226	0.709451934	0.988585656
Ttll4	-0.198827769	0.276807662	-0.718288534	0.472579399	0.955044417
Cyp27a1	-0.070556038	0.205864255	-0.342730885	0.731800926	0.988585656
Prkag3	-0.405498916	0.380690532	-1.065166801	0.286800454	0.906336252
Wnt6	-0.23084588	1.138897859	-0.202692347	0.839375505	0.992886758

Ccdc108	-0.014976245	0.236027007	-0.063451402	0.949407056	0.995624732
Ihh	0.05947707	1.29001868	0.046105588	0.963226092	0.996474415
Nhej1	0.209055145	0.314257792	0.665234563	0.505900477	0.96224682
Slc23a3	1.523270463	0.915272769	1.664280326	0.096056422	0.780429201
Cnppd1	0.147081616	0.156082808	0.942330663	0.346023374	0.921552698
Fam134a	-0.072321933	0.125461775	-0.576445954	0.564313791	0.969658675
Zfand2b	0.260754827	0.188260809	1.385072277	0.166030368	0.860029348
Abcb6	0.017521958	0.17746979	0.098732061	0.921351012	0.994688009
Atg9a	-0.459688034	0.266616398	-1.724155146	0.084679839	0.761325843
Ankzf1	-0.172673433	0.219113777	-0.788053747	0.430665269	0.945321484
Glb1l	0.046080943	0.231318834	0.199209648	0.842098753	0.992886758
Stk16	0.226616469	0.146919568	1.542452596	0.122963649	0.817788082
Tuba4a	-0.000603781	0.161200957	-0.00374552	0.997011514	0.99952149
Dnajb2	-0.038262044	0.127775068	-0.299448436	0.76459791	0.989442146
Ptprn	-0.036073788	0.30688773	-0.117547183	0.906426457	0.994688009
Resp18	-0.33417449	0.438670827	-0.761788725	0.446186111	0.948476747
Dnpep	-0.134516244	0.106898863	-1.258350561	0.208265003	0.884258007
Des	-0.042057008	0.165117473	-0.254709614	0.798947391	0.990988839
Speg	0.159033828	0.293674344	0.541531227	0.58814148	0.973455044
Gmppa	0.024137447	0.143411957	0.168308472	0.866340609	0.992886758
Chpf	-0.086234128	0.223617048	-0.385633068	0.699768439	0.988393777
Tmem198	-0.333799641	0.70174397	-0.475671548	0.634308394	0.977034429
Obsl1	0.679966281	0.456725211	1.488786396	0.136543626	0.832506992
Inha	1.66447857	1.087782304	1.530157794	0.125977675	0.822401003
Stk11ip	-0.234543118	0.264855343	-0.885551771	0.37585911	0.928883222
Slc4a3	-0.024930856	0.381438117	-0.06536016	0.947887244	0.995507207
Epha4	0.422591329	0.320864936	1.317038046	0.187825872	0.874351146
Sgpp2	-0.366254727	0.17643605	-2.07584973	0.037907851	0.679118604
Farsb	-0.170787298	0.221578777	-0.770774625	0.440840531	0.948068187
Mogat1	1.233620168	0.826195284	1.493133877	0.135402146	0.830756223
Acsl3	-0.187394363	0.145444105	-1.288428731	0.197596763	0.880239572
Utp14b	-0.725295779	0.452430723	-1.603109032	0.108910584	0.798400564
Kcne4	0.22599386	0.27506145	0.821612263	0.411297608	0.941762685
Scg2	0.265847341	1.435582391	0.185184315	0.853084472	0.992886758
Ap1s3	-0.145416616	0.185844057	-0.782465784	0.433940886	0.946679964
Wdfy1	0.326408073	0.518652231	0.629338993	0.529127148	0.965637248
Mrpl44	-0.036469718	0.109167556	-0.334071036	0.738325956	0.988585656
Serpine2	-0.260355675	0.150145353	-1.734024195	0.08291379	0.76099108
Fam124b	0.098995899	0.334553305	0.295904711	0.767302853	0.989884648
Cul3	-0.09759719	0.121867335	-0.800847824	0.423219749	0.943509323
Dock10	-0.329478605	0.196739087	-1.674698255	0.09399345	0.776242316
Irs1	0.03727447	0.263832106	0.141281023	0.887647941	0.992886758
Rhbdd1	-0.110999106	0.175650951	-0.631930003	0.527432617	0.965637248
Col4a4	0.036695873	0.241981011	0.15164774	0.879464778	0.992886758
Col4a3	0.188510894	0.167803095	1.123405344	0.261265381	0.897248195
Mff	0.255989742	0.168939253	1.515276871	0.129702303	0.827800491
Agfg1	-0.419379979	0.113545936	-3.693482955	0.000221203	0.092604794
Slc19a3	-0.041149095	0.60766534	-0.067716706	0.946011151	0.995065399
Ccl20	-0.261782031	0.68834342	-0.380307305	0.703717313	0.988585656
Daw1	-0.109125656	0.246122635	-0.443379196	0.657491481	0.979614766
Sphkap	0.24187431	1.395211359	0.173360336	0.862368201	0.992886758
Pid1	-0.069361928	0.129355479	-0.536211754	0.591812211	0.974001549
Dner	2.919010439	1.126329055	2.591614258	0.009552681	0.481378488
Trip12	-0.048807397	0.120497415	-0.405049323	0.685441256	0.98538669
Fbxo36	-0.056100866	0.151659307	-0.369913769	0.711446742	0.988585656
A530032D15Rik	0.554443873	0.720412724	0.769619767	0.441525476	0.948274986
Gm7609	0.189345123	0.681558371	0.277812043	0.781156646	0.990988839
C130026I21Rik	0.57661719	0.307039741	1.87798878	0.0603827	0.725221283
Sp110	0.146514908	0.184640092	0.79351622	0.427477123	0.944824555
Sp140	0.381100223	0.122171332	3.119391572	0.001812249	0.290084285
Sp100	0.223886587	0.189716555	1.180110965	0.237956084	0.896609492
A630001G21Rik	0.040070733	0.277651976	0.144319997	0.885247788	0.992886758

Cab39	-0.00490608	0.0841794	-0.058281238	0.953524612	0.996070133
Itm2c	-0.123425735	0.115517507	-1.068459131	0.285313456	0.905916508
Gpr55	-0.271373655	1.393628706	-0.194724501	0.84560863	0.992886758
2810459M11Rik	0.591164156	1.03869057	0.569143663	0.569258649	0.97144955
Psmd1	-0.226163947	0.127781342	-1.769929346	0.076738912	0.752876039
Htr2b	0.099583578	0.433845128	0.229537158	0.818451444	0.992734473
Armc9	0.091934837	0.244048766	0.376706831	0.706391486	0.988585656
B3gnt7	-0.405111619	0.328141559	-1.234563582	0.21699297	0.887633697
Ncl	-0.057914692	0.178265987	-0.324877971	0.74527343	0.988585656
C130036L24Rik	0.148885181	0.568636119	0.261828568	0.793453613	0.990988839
Nmur1	-0.094396058	1.897326028	-0.049752155	0.960319894	0.996474415
Ptma	0.289699607	0.058793628	4.927397997	8.33E-07	0.001958309
Pde6d	0.284018865	0.148509111	1.912467614	0.055816243	0.715507996
Cops7b	0.106645642	0.154477702	0.690362687	0.489966135	0.958560421
Nppc	4.511719934	2.276068971	1.982242187	0.047452141	0.700850692
Dis3l2	-0.403526082	0.298526814	-1.351724746	0.176463386	0.865862073
Eif4e2	0.294563766	0.10476409	2.811686387	0.004928253	0.399101188
Efhd1	2.237198383	1.664773504	1.343845501	0.178998352	0.867377413
Gigyf2	0.015790561	0.167102049	0.094496514	0.924714751	0.994688009
Kcnj13	-0.234898479	0.202981398	-1.157241406	0.247173745	0.896939594
Ngef	-0.240822787	0.208920815	-1.152698871	0.249034005	0.896939594
Neu2	-0.265361871	0.432826798	-0.613090207	0.539816695	0.966900344
Inpp5d	-0.253072402	0.241790621	-1.046659299	0.295256742	0.907844976
Atg16l1	-0.156674775	0.128867496	-1.215781949	0.224067997	0.892989918
Sag	1.94457864	1.458584662	1.333195591	0.182467626	0.868809836
Dgkd	0.038843379	0.153073982	0.253755595	0.799684381	0.990988839
Usp40	-0.009581409	0.185617966	-0.051618975	0.9588323	0.996236156
Ugt1a7c	0.178106603	0.377434482	0.47188747	0.637007112	0.977284137
Ugt1a6b	-0.054825378	0.693855509	-0.079015554	0.937020251	0.994688009
Ugt1a6a	-0.277036027	0.359237209	-0.77117854	0.440601112	0.94803563
Dnajb3	0.364905024	0.466959156	0.781449553	0.434538136	0.946762848
Mroh2a	1.334138808	1.072142638	1.244366898	0.21336456	0.884945675
Hjurp	0.596032409	1.347440965	0.442343987	0.658240305	0.979697875
Trpm8	1.10024357	1.875894905	0.586516636	0.557528385	0.968926429
Arl4c	-0.280825326	0.143008593	-1.963695465	0.049565419	0.707153766
Sh3bp4	-0.155925799	0.227825182	-0.684409852	0.493716396	0.958916246
Agap1	0.359991074	0.210776239	1.707930061	0.08764932	0.766704005
Iqca	-0.291072873	0.286213532	-1.016978025	0.309163884	0.9139204
Ackr3	-0.154118143	0.176479671	-0.873291197	0.382504378	0.930775109
Cops8	-0.112936324	0.13869247	-0.814293116	0.415477052	0.943415794
Col6a3	0.094188453	0.282251551	0.333703934	0.738602981	0.988585656
Mlph	-0.175578027	0.22199795	-0.790899316	0.429002748	0.945321484
Rab17	0.291180076	0.284350467	1.024018281	0.30582664	0.91333012
Lrrfip1	-0.059674265	0.127460782	-0.468177462	0.639657688	0.977843986
Ramp1	0.14619068	0.191125166	0.76489498	0.444334084	0.948476747
Ube2f	-0.050246692	0.121954715	-0.412011065	0.680331313	0.984207811
Scly	0.13139811	0.142364242	0.922971298	0.356022163	0.924354588
Espnl	-0.694416683	0.964750821	-0.719788641	0.47165514	0.95486913
Klhl30	-0.011598845	1.310295061	-0.008852087	0.992937149	0.999117764
Fam132b	1.208064796	1.592580162	0.758558234	0.448116865	0.948476747
Ilkap	0.204570797	0.094649213	2.161357607	0.030667726	0.657965788
1700020N18Rik	0.201422809	0.528109981	0.381403147	0.702904126	0.988585656
Hes6	-0.189479654	0.144259851	-1.313460766	0.189027733	0.87553356
Per2	-0.698596728	0.405442756	-1.723046516	0.084880114	0.761635307
Traf3ip1	-0.227240429	0.177759921	-1.278355818	0.201123995	0.881109608
Asb1	-0.057488923	0.224774045	-0.255763176	0.79813371	0.990988839
Twist2	0.685596946	0.529179306	1.295585331	0.195118385	0.879588734
Hdac4	-0.196662065	0.713163297	-0.275760216	0.782732242	0.990988839
Ndufa10	-0.127967611	0.110777099	-1.155181101	0.248016272	0.896939594
Myeov2	0.524174166	0.338698407	1.54761332	0.121715423	0.816233249
Otos	3.739695039	1.803609152	2.073450911	0.038130335	0.679118604
Gpc1	-0.026009064	0.226243096	-0.114960697	0.908476274	0.994688009

Ankmy1	-0.875647639	1.154283224	-0.758607264	0.448087526	0.948476747
Dusp28	-0.042431757	0.162935244	-0.260420987	0.794539056	0.990988839
Rnpepl1	-0.131368188	0.180394112	-0.728228802	0.466473537	0.953083512
Capn10	-0.200820952	0.178235514	-1.126716824	0.259862227	0.897248195
Gpr35	0.259876979	0.456449893	0.569343937	0.569122755	0.97144955
Kif1a	-0.775129565	0.541427293	-1.431641099	0.152246561	0.852974739
2310007B03Rik	-0.211199242	0.226858853	-0.930972006	0.351868045	0.923082865
Sned1	-0.220325763	0.501145104	-0.439644648	0.660194498	0.979924124
Mterf4	0.167589684	0.139134573	1.204515029	0.228390577	0.894657617
Pask	-0.66601273	0.603101193	-1.104313402	0.269457211	0.89804647
Ppp1r7	0.072401097	0.198802181	0.364186631	0.71571865	0.988585656
Hdlbp	-0.236327901	0.141255835	-1.673048763	0.094317697	0.777748075
Sep-02	0.045298706	0.089474673	0.506274053	0.612664275	0.974001549
Farp2	-0.293373294	0.45374097	-0.646565581	0.51791314	0.964437658
Stk25	0.180532854	0.100044105	1.804532645	0.071147846	0.744227652
Bok	-0.156249756	0.140288178	-1.113777072	0.265374835	0.89768067
Thap4	-0.131254472	0.141898643	-0.924987506	0.354972406	0.924354588
Atg4b	-0.116030092	0.127629297	-0.909118001	0.363287842	0.926455466
Dtymk	0.237910435	0.182461452	1.303894235	0.192269646	0.879588734
Ing5	0.125526772	0.147876674	0.848861208	0.395958527	0.934066368
D2hgdh	-0.190416994	0.264190323	-0.720756882	0.471059108	0.95486913
Gal3st2	0.013738304	0.758623777	0.018109508	0.985551493	0.998148029
Pdcd1	-0.811640437	0.57160954	-1.419921083	0.155630657	0.855261441
Fam174a	0.211569278	0.177640592	1.190996241	0.23365506	0.89581803
St8sia4	0.117346771	0.227045862	0.516841706	0.605266663	0.974001549
Slco4c1	-0.78206432	0.279613428	-2.79694837	0.005158778	0.406593461
Panct2	-0.058509508	0.240318841	-0.24346617	0.807644289	0.99099448
D1Ertd622e	-0.069825811	0.138022421	-0.50590194	0.612925491	0.974001549
Ppip5k2	-0.027433748	0.192409205	-0.142580226	0.886621715	0.992886758
Gin1	0.22705864	0.209145464	1.085649366	0.27763415	0.901241208
Pam	-0.099323241	0.080070684	-1.240444514	0.214811025	0.88678119
B230216N24Rik	0.893119627	0.997225968	0.895604061	0.370464266	0.928436166
1810006J02Rik	0.778014821	0.857752988	0.90703831	0.364386539	0.926556975
Rnf152	-0.304352658	0.878670089	-0.346378762	0.729058084	0.988585656
Pign	-0.07760315	0.17899205	-0.433556404	0.664610599	0.981378063
2310035C23Rik	0.155397567	0.1838333	0.845317832	0.397933408	0.935327115
Tnfrsf11a	0.228219468	0.462304819	0.493655828	0.621549266	0.97514112
Zcchc2	0.173325949	0.316178303	0.548190522	0.58356109	0.972174584
Gm20753	4.230768141	1.789469859	2.364257839	0.018066234	0.58167504
Phlpp1	-0.218187457	0.191744571	-1.137906828	0.25515939	0.897044747
Bcl2	0.28925824	0.278089752	1.040161451	0.298264898	0.909779403
Kdsr	0.044869754	0.122571713	0.3660694	0.71431329	0.988585656
Vps4b	-0.150737108	0.115866788	-1.300951813	0.193274949	0.879588734
Serpinb5	-0.432677001	1.951918856	-0.221667515	0.824572721	0.992886758
Serpinb12	-1.076760377	1.018117953	-1.057598851	0.290238367	0.907334416
Serpinb7	-0.890204155	1.475869729	-0.603172582	0.546393885	0.96778188
Serpinb2	-1.108047535	0.728899416	-1.520165212	0.128469458	0.826336015
Serpinb10	1.490950538	0.290496583	5.132420222	2.86E-07	0.000934035
Serpinb8	0.13059021	0.21945695	0.595060715	0.551802887	0.968926429
Cdh19	-0.332184164	0.857228107	-0.38750965	0.698378942	0.987736745
Dsel	-0.002884504	0.172483546	-0.01672336	0.986657311	0.998148029
Tsn	0.027799861	0.112358846	0.247420313	0.804582955	0.990988839
Nifk	-0.150803927	0.15508263	-0.972410177	0.330846531	0.917115058
Clasp1	-0.228653526	0.326061621	-0.701258632	0.483141625	0.957463006
Tfcp2l1	-0.265143023	0.30538455	-0.868226712	0.385270234	0.931689378
Gli2	-0.020626479	0.667137514	-0.030917882	0.975335029	0.997262526
Inhbb	-0.156494325	0.148087442	-1.056769722	0.290616696	0.907334416
Ralb	0.092467163	0.114566816	0.807102494	0.419607435	0.943415794
Tmem185b	-0.268706115	0.183552263	-1.463921558	0.143215382	0.840741274
Epb4.1l5	-0.230503282	0.161482169	-1.427422501	0.153458149	0.854159937
Ptpn4	-0.283491292	0.316700772	-0.895139253	0.370712653	0.928436166
Tmem177	-0.058163547	0.221572818	-0.262503082	0.79293361	0.990988839

Cfap221	-0.586582276	0.504644737	-1.162366775	0.245086513	0.896939594
Tmem37	-0.029185168	0.167897644	-0.173827143	0.862001312	0.992886758
Dbi	0.099566623	0.176441187	0.56430488	0.572546661	0.971613657
3110009E18Rik	0.159030523	0.280999994	0.565944933	0.571431215	0.971613657
Steap3	0.094283773	0.233948786	0.403010313	0.686940638	0.98538669
C1ql2	-0.18581533	1.441950714	-0.128863856	0.897465377	0.994101834
Marco	0.538482968	0.442861177	1.215918205	0.224016082	0.892989918
Insig2	0.048131036	0.135479634	0.355263996	0.722391825	0.988585656
Ccdc93	0.116568772	0.245982539	0.473890434	0.635578041	0.977034429
Ddx18	-0.229325327	0.177833346	-1.289551886	0.19720629	0.879755417
Actr3	0.039452954	0.078916637	0.499932026	0.617122941	0.974001549
Slc35f5	0.032896134	0.107942766	0.304755332	0.760552504	0.989266039
Gpr39	-0.579357312	0.697339434	-0.830811056	0.406080376	0.939557159
Nckap5	-0.028998217	0.201721498	-0.143753725	0.885694946	0.992886758
Mgat5	-0.256240232	0.430703646	-0.594933975	0.551887606	0.968926429
Tmem163	0.064581818	0.179529185	0.359728797	0.719049955	0.988585656
Acmsd	0.572391708	0.914027492	0.626230297	0.531163898	0.966166773
Ccnt2	-0.107899997	0.154499383	-0.698384646	0.484936676	0.957710647
Map3k19	-0.344149549	0.375356228	-0.916861167	0.359215395	0.925229839
Rab3gap1	-0.36042896	0.179319465	-2.009982351	0.044433057	0.689308953
Zranb3	-0.377873889	0.537010065	-0.703662581	0.481642928	0.957463006
R3hdm1	0.200691942	0.198477567	1.011156801	0.311941388	0.914819219
Ubxn4	-0.103094704	0.107646209	-0.957717926	0.338205015	0.919246575
Mcm6	-0.30125369	0.200170165	-1.50498797	0.13232717	0.827800491
Dars	0.044124145	0.14360129	0.307268442	0.758639054	0.989266039
Cxcr4	0.205091731	0.150632917	1.361533287	0.173345222	0.863528862
Thsd7b	2.225574024	1.935347928	1.149960682	0.250160066	0.896939594
Cd55b	0.161268312	0.34768769	0.46383095	0.642768867	0.978681333
Cd55	0.032251611	0.1250918	0.257823543	0.796543094	0.990988839
C4bp	0.669601918	1.543288604	0.433879908	0.66437565	0.98119114
C4bp-ps1	-0.024902876	1.489177422	-0.016722572	0.98665794	0.998148029
Pfkfb2	-0.365325166	0.154181448	-2.369449579	0.017814584	0.581000616
Yod1	-0.285790174	0.218687895	-1.306840388	0.191266925	0.878116834
AA986860	-0.297418878	0.197976512	-1.502293763	0.133021259	0.827800491
Fcamr	-0.59786925	0.625810689	-0.955351611	0.339399916	0.919246575
Pigr	-0.34030106	0.195967021	-1.736522087	0.082471563	0.76099108
Fcmr	-0.418968289	0.763521043	-0.548731817	0.583189508	0.972174584
Mapkapk2	-0.078081035	0.134108265	-0.582223884	0.560415887	0.968926429
Dyrk3	-0.230476979	0.162938084	-1.414506501	0.15721324	0.855986074
Eif2d	0.169898314	0.130207688	1.304825514	0.191952269	0.879103702
Rassf5	-0.155556786	0.277718784	-0.560123389	0.575395278	0.971613657
Ikbke	-0.019905125	0.28817582	-0.06907285	0.944931632	0.99486515
Srgap2	-0.085665502	0.186093927	-0.460334756	0.645275958	0.979004493
Fam72a	0.609461249	0.294193378	2.071634832	0.038299509	0.679118604
Ctse	-0.232295477	1.355456682	-0.171378016	0.86392654	0.992886758
Rab7b	0.058782466	0.185741395	0.316474776	0.751642168	0.988585656
Slc26a9	-0.296136913	0.329432901	-0.898929378	0.368690278	0.928436166
Pm20d1	-0.977978199	0.644134074	-1.518283597	0.128942921	0.827295367
Slc41a1	0.135395736	0.227640294	0.594779307	0.551991001	0.968926429
Rab29	0.153992564	0.143226018	1.075171715	0.282297856	0.90579922
Nucks1	-0.071549181	0.129248382	-0.55357893	0.579867049	0.971613657
Slc45a3	0.020876433	0.280280156	0.074484165	0.940625141	0.994688009
Elk4	0.240612182	0.359435705	0.669416475	0.503229845	0.961729397
Mfsd4	0.010411622	0.285565706	0.036459638	0.970915862	0.996733101
Cdk18	-0.11679747	0.403389523	-0.289540168	0.772168046	0.99050004
Lemd1	3.547244703	2.7045525	1.31158286	0.189660919	0.876232344
Klhdc8a	0.161157316	0.275541087	0.584875809	0.558631226	0.968926429
Nuak2	0.068433001	0.276744643	0.247278502	0.804692696	0.990988839
Tmcc2	0.139432671	0.11564267	1.205719923	0.2279255	0.894410876
Dstyk	0.174136247	0.221833159	0.784987456	0.432460923	0.9459793
Rbbp5	-0.172340695	0.231063416	-0.745858855	0.455752682	0.95142233
Tmem81	0.254567962	0.376831425	0.675548655	0.49932725	0.960700483

Lrrn2	-1.114622772	0.835652794	-1.333834793	0.182258005	0.868546072
Mdm4	-0.09780031	0.177411107	-0.551263737	0.581452893	0.971885891
Pik3c2b	0.028299683	0.254301829	0.111283838	0.911391272	0.994688009
Ppp1r15b	-0.2160647	0.097493486	-2.216196264	0.026678064	0.633489138
Plekha6	-0.385502298	0.224313049	-1.718590601	0.085688944	0.763135146
Gm19461	0.022423468	0.254437283	0.088129647	0.929773633	0.994688009
Golt1a	0.303917754	0.28400438	1.070116434	0.284566903	0.905916508
Kiss1	-0.726023177	0.892073319	-0.813860432	0.41572491	0.943415794
Etnk2	-0.114715356	1.295919512	-0.088520432	0.929463045	0.994688009
Sox13	-0.109578223	0.229920388	-0.476592025	0.633652664	0.977034429
Snrpe	0.282771496	0.214020062	1.321238269	0.18642193	0.872512868
Zc3h11a	-0.261406533	0.118775435	-2.200846777	0.027746873	0.637644759
Zbed6	-0.473699529	0.420237107	-1.127219661	0.25964962	0.897248195
Lax1	0.215841289	0.340161237	0.634526412	0.525737335	0.965637248
Atp2b4	-0.412679561	0.285697645	-1.444462591	0.148608895	0.84832848
Optc	0.283166177	0.842859542	0.335958915	0.736901849	0.988585656
Prelp	-0.003283422	0.198648854	-0.016528772	0.986812549	0.998148029
Fmod	-0.455133101	0.415969937	-1.094149025	0.273889624	0.899464226
Btg2	0.205308061	0.385008358	0.533256114	0.593856302	0.974001549
Chil1	-0.276209542	0.340497123	-0.811194935	0.417253734	0.943415794
Adora1	0.228854926	0.468596583	0.488383684	0.625278096	0.976218048
Ppfia4	0.22948045	0.379713307	0.604351878	0.545609722	0.967543166
Tmem183a	0.203468888	0.145511141	1.398304533	0.162021637	0.85887249
Cyb5r1	0.098165042	0.125795331	0.780355206	0.435181826	0.946762848
Adipor1	0.198346109	0.087026078	2.279157165	0.022657724	0.620006604
Klhl12	0.088731429	0.212271514	0.418009122	0.675940448	0.983549699
Rabif	-0.193423281	0.158736433	-1.218518499	0.223026999	0.892391445
Kdm5b	0.249749066	0.218167145	1.144760208	0.252308503	0.897013805
Ppp1r12b	-0.087460702	0.58398015	-0.149766566	0.880948788	0.992886758
Ube2t	0.624675691	0.272875127	2.289236461	0.022065617	0.616892692
Lgr6	-1.359835884	0.667988186	-2.035718464	0.041778634	0.679118604
Ptprv	-0.062831617	1.12121556	-0.056038838	0.955310867	0.99614946
Ptpn7	-0.129522728	0.2760353	-0.469225235	0.63890865	0.977795535
Arl8a	-0.183907358	0.182307424	-1.008776017	0.313082065	0.914925878
Elf3	-0.532397763	0.204663426	-2.60133319	0.009286221	0.478229167
Rnpep	-0.035107747	0.208816015	-0.168127655	0.866482854	0.992886758
Timm17a	0.150320054	0.131766223	1.140808706	0.253949537	0.897044747
Lmod1	-0.062843714	0.280128132	-0.224339174	0.822493396	0.992886758
Shisa4	0.036814588	0.350642034	0.104991942	0.916382203	0.994688009
Ipo9	-0.189464024	0.183719556	-1.03126759	0.302415352	0.911518274
Nav1	-0.223824016	0.388774251	-0.575717182	0.56480636	0.969658675
Csrp1	0.079328831	0.10263001	0.772959397	0.43954641	0.947849924
Phlda3	0.105400777	0.119641483	0.880971837	0.378333064	0.929018488
Tnni1	-0.447923196	1.102767698	-0.406180919	0.684609673	0.985105247
Lad1	0.226507157	0.428521332	0.528578486	0.597097888	0.974001549
Tnnt2	-0.046290421	0.228277869	-0.202781028	0.839306187	0.992886758
Tmem9	0.263225142	0.156675707	1.680063527	0.092944956	0.77525594
Cacna1s	-0.31681107	0.252633644	-1.25403357	0.209829804	0.884258007
Kif21b	-0.723171588	0.666636434	-1.084806577	0.278007332	0.901315669
5730559C18Rik	-0.866342419	0.677638781	-1.278472312	0.201082941	0.881109608
Camsap2	-0.185841625	0.157795496	-1.177737192	0.238901395	0.896609492
9230116N13Rik	0.449878169	0.381873417	1.178081921	0.238763949	0.896609492
Ddx59	0.283365317	0.190815092	1.485025705	0.137537015	0.833085238
Kif14	-0.499161981	0.729923216	-0.683855465	0.494066438	0.958916246
Zfp281	-0.09663189	0.242080824	-0.399172013	0.689766464	0.985803313
Gm19705	0.628665279	0.403140976	1.559417961	0.118897488	0.814755048
Platr22	0.240252158	0.292333038	0.821844017	0.411165679	0.941701842
Nr5a2	0.747379112	1.267498737	0.589648802	0.555426126	0.968926429
Ptprc	0.106330259	0.170379394	0.624079332	0.532575486	0.96674935
Atp6v1g3	-1.015389185	0.592951979	-1.712430722	0.086817321	0.765418158
Nek7	0.054412425	0.118348123	0.459765846	0.645684301	0.979004493
2310009B15Rik	0.019310761	0.230976901	0.083604729	0.933370707	0.994688009

Dennd1b	-0.26811412	0.173590218	-1.54452321	0.122461633	0.817763223
Crb1	0.02423154	0.615126639	0.039392766	0.968577247	0.996474415
Zbtb41	0.052044732	0.186256608	0.279424888	0.779918774	0.990988839
Aspm	-0.53603121	0.79279471	-0.676128641	0.498958973	0.960700483
Cfhr2	3.952215877	2.571389486	1.536996204	0.124294239	0.821164769
Cfh	0.09989961	0.153913248	0.649064401	0.516296751	0.964352198
Kcnt2	-0.75613594	0.963219639	-0.785008849	0.43244838	0.9459793
Cdc73	-0.158371859	0.116004513	-1.365221536	0.172183431	0.86244803
B3galt2	-0.22872626	0.444240763	-0.514870042	0.606643837	0.974001549
Glrx2	0.155858937	0.136910549	1.138399766	0.254953592	0.897044747
Trove2	-0.32678611	0.159193918	-2.05275499	0.040096346	0.679118604
Uchl5	0.126314435	0.156905171	0.805036794	0.420798448	0.943415794
Rgs2	0.619176577	0.303154405	2.042446247	0.041107286	0.679118604
Rgs13	1.844197116	1.096265299	1.682254393	0.092519521	0.775044718
Rgs1	-0.288640659	0.550307236	-0.524508203	0.599925116	0.974001549
Rgs18	0.521517578	0.274415	1.900470377	0.057371419	0.721735927
Pla2g4a	0.025326114	0.144332449	0.175470685	0.860709803	0.992886758
Ptgs2os	0.702531174	1.585815303	0.443009455	0.657758896	0.979614766
Ptgs2	-0.044049851	0.210556392	-0.209206905	0.834286722	0.992886758
Ptgs2os2	0.0783937	0.252770609	0.31013772	0.756456229	0.989266039
BC003331	-0.033771988	0.119890151	-0.281691094	0.778180384	0.990988839
Tpr	0.01985706	0.149916365	0.132454252	0.894625005	0.993556555
Prg4	0.150999584	0.632559346	0.238712123	0.811328816	0.991784898
Hmcn1	-0.038181453	0.201165343	-0.189801347	0.8494648	0.992886758
C730036E19Rik	0.030085807	1.254892558	0.023974807	0.980872704	0.99731481
Ivns1abp	-0.076884702	0.098586499	-0.77987049	0.43546711	0.946762848
Swt1	-0.231475286	0.146351306	-1.581641408	0.113731455	0.807243653
Trmt1l	-0.162426466	0.155025175	-1.047742507	0.294757258	0.907334416
Rnf2	-0.008726296	0.137064413	-0.063665658	0.94923645	0.995624732
Fam129a	0.170765192	0.232231795	0.735322187	0.462143299	0.953083512
Edem3	-0.168558452	0.181774387	-0.927294846	0.353773469	0.92416951
1700025G04Rik	-0.20191892	0.190353835	-1.060755725	0.288800928	0.907334416
Tsen15	0.278693884	0.188176422	1.481024458	0.138600056	0.834874897
Colgalt2	0.020491677	0.276387063	0.074141229	0.94089801	0.994688009
Rgl1	-0.070930584	0.196172491	-0.361572532	0.717671494	0.988585656
Apobec4	0.227621471	0.488025926	0.466412661	0.640920151	0.977921835
Arpc5	0.224474628	0.110477496	2.031858392	0.042167994	0.680982912
Ncf2	0.068476072	0.188665407	0.3629498	0.716642384	0.988585656
Smg7	-0.044122517	0.174126119	-0.253394017	0.799963751	0.990988839
Nmnat2	-0.343809973	0.385053607	-0.89288859	0.371916838	0.928840865
Lamc2	0.020317017	0.141390483	0.143694374	0.885741814	0.992886758
Lamc1	0.167383869	0.23440255	0.714087234	0.47517324	0.956761005
E330020D12Rik	1.114643306	0.515034358	2.16421155	0.030448117	0.657574617
Shcbp1l	1.018852439	1.36434812	0.746768676	0.455203207	0.951224746
Dhx9	-0.295790172	0.187264743	-1.579529429	0.114214672	0.808665631
Npl	-0.185044312	0.237835171	-0.778035944	0.436547824	0.946762848
Rgs16	0.117146087	1.191484837	0.098319411	0.921678665	0.994688009
Rnasel	0.012694744	0.202147928	0.062799276	0.949926342	0.995727234
Rgsl1	0.617250501	1.782870623	0.346211605	0.729183694	0.988585656
Teddm2	-1.905349027	1.911606155	-0.996726769	0.318897152	0.91640698
Glul	-0.141997522	0.197550189	-0.718792137	0.472269003	0.955003345
Cacna1e	-0.305171413	1.654897319	-0.184405044	0.853695713	0.992886758
Ier5	0.073940974	0.125226477	0.590457992	0.554883641	0.968926429
Mr1	0.015796824	0.126010531	0.125361141	0.90023765	0.994688009
Stx6	-0.010359144	0.116515116	-0.088908157	0.929154901	0.994688009
Xpr1	-0.435715598	0.217636436	-2.002034244	0.045281049	0.693980041
Gm5532	1.070695662	0.389619973	2.748051273	0.005995063	0.427133716
Acbd6	0.058596689	0.153835556	0.380904718	0.703273952	0.988585656
Qsox1	-0.154816923	0.149964146	-1.032359578	0.301903701	0.911518274
Cep350	-0.31752322	0.226303855	-1.403083569	0.16059192	0.857316691
Tor1aip1	-0.254070279	0.152362574	-1.667537329	0.095407608	0.778239075
Tor1aip2	-0.165843761	0.133854958	-1.238981083	0.215352504	0.887633697

Tdrd5	1.215166073	1.02898595	1.180935535	0.237628334	0.896609492
Nphs2	-1.100910802	1.309779119	-0.840531648	0.400610365	0.93707241
Soat1	-0.061210166	0.161209009	-0.379694454	0.704172238	0.988585656
Abl2	-0.091120817	0.283762856	-0.321116083	0.748122423	0.988585656
Tor3a	-0.031448087	0.096849015	-0.324712514	0.745398663	0.988585656
Fam20b	-0.019593014	0.133287837	-0.146997766	0.883133784	0.992886758
Ralgps2	0.17944824	0.219805379	0.816396036	0.414273663	0.943415794
Rasal2	0.014391912	0.145789787	0.098716872	0.921363072	0.994688009
2810025M15Rik	0.312535627	0.108207809	2.888290865	0.003873415	0.376754331
BC026585	0.004949231	0.168576741	0.029358918	0.976578337	0.997262526
Sec16b	-0.223470064	0.404325409	-0.55269854	0.580469853	0.971613657
Brinp2	-1.235318438	3.150543481	-0.392096934	0.694986593	0.98715859
Pappa2	-0.177341391	1.435960982	-0.123500146	0.901711058	0.994688009
Rfwd2	-0.046652799	0.139168076	-0.335226301	0.737454384	0.988585656
Tnr	0.991119138	0.880068226	1.126184436	0.26008746	0.897248195
4930523C07Rik	0.029267211	0.153852821	0.190228629	0.849129978	0.992886758
Tnn	0.037782865	0.171970665	0.219705289	0.826100685	0.992886758
Mrps14	0.326427474	0.125398325	2.603124671	0.009237835	0.478229167
Cacybp	-0.028722703	0.202808689	-0.141624619	0.88737652	0.992886758
Rabgap1l	-0.092536992	0.098267282	-0.941686689	0.346353072	0.921552698
Rc3h1	0.068122818	0.151144096	0.450714381	0.652195416	0.979004493
Serpinc1	2.318227104	1.131923365	2.048042452	0.04055584	0.679118604
Zbtb37	-0.257592416	0.383152235	-0.67229783	0.501394111	0.96132573
Gas5	0.327464641	0.156162735	2.096944843	0.035998458	0.674795441
Dars2	0.016068258	0.278769324	0.05763998	0.954035403	0.996070133
Cenpl	0.062846313	0.212110012	0.296291121	0.767007766	0.989884648
Klhl20	0.272965723	0.196256247	1.390863869	0.164266711	0.859982992
Ankrd45	-0.456287	0.316673427	-1.440875555	0.149619843	0.848779008
Prdx6	-0.190805465	0.12371342	-1.542318238	0.122996279	0.817788082
Tnfsf18	-0.205530165	1.167882757	-0.175985272	0.860305513	0.992886758
Fasl	0.713584706	0.97709709	0.730310952	0.465200136	0.953083512
Suco	-0.236037241	0.180038019	-1.311041088	0.189843882	0.876349068
Pigc	0.367328569	0.157162761	2.3372494	0.01942622	0.593178479
Dnm3	-0.398745526	0.324018243	-1.230626776	0.218462486	0.888436964
2810442N19Rik	0.273017624	1.505058286	0.181400034	0.856053593	0.992886758
Dnm3os	-0.243152387	0.412537612	-0.58940659	0.555588557	0.968926429
Mettl13	0.253633794	0.288235606	0.879953027	0.378884757	0.929137643
Vamp4	0.334841041	0.143325156	2.336233574	0.019479071	0.593178479
Myoc	0.31222438	1.643749963	0.189946395	0.849351136	0.992886758
Prrc2c	0.27789941	0.257278678	1.080149401	0.280075656	0.903192609
Fmo4	0.048545986	0.58515867	0.082962089	0.933881684	0.994688009
Fmo1	-0.127492867	0.144072892	-0.884919192	0.376200216	0.928883222
Fmo2	-0.206834805	0.140460325	-1.472549669	0.140872505	0.839671411
Fmo6	-0.302910827	0.521829943	-0.580478049	0.561592286	0.969558766
Fmo3	-0.302079864	0.1820541	-1.659286246	0.097058125	0.782676152
Prrx1	-0.006519663	0.196362671	-0.033202151	0.973513383	0.997148871
Gorab	0.105263831	0.150992523	0.697145985	0.485711438	0.957710647
Kifap3	0.053441479	0.11667856	0.458023128	0.646935825	0.979004493
Scyl3	-0.183637156	0.194038757	-0.946394212	0.343947557	0.920669678
BC055324	-0.087158745	0.666778492	-0.130716192	0.895999823	0.993884714
Mettl18	0.416034291	0.241353584	1.723754354	0.084752198	0.761557038
Sele	0.189744167	0.745544901	0.25450401	0.799106206	0.990988839
Sell	0.130500967	0.292229835	0.44656962	0.655185834	0.979133069
Selp	-0.920676246	0.713198268	-1.290912061	0.196734173	0.879755417
F5	0.469668671	0.527096854	0.89104814	0.372903345	0.928840865
Slc19a2	-0.247863309	0.218092132	-1.13650734	0.255744296	0.897044747
Ccdc181	-0.048240196	0.116218835	-0.415080709	0.678082821	0.983670411
Blzf1	-0.05652541	0.168324644	-0.335811851	0.737012753	0.988585656
Nme7	-0.42061048	0.170903753	-2.461095631	0.013851344	0.545277966
Atp1b1	-0.151115695	0.112380787	-1.344675543	0.178730036	0.867377413
Dpt	0.361191905	0.135887243	2.658026594	0.007859969	0.45506991
Xcl1	0.455967625	0.495354165	0.920488123	0.357317737	0.924354588

Sft2d2	-0.172094545	0.135100365	-1.273827395	0.202724594	0.882297774
Tiprl	-0.028989497	0.115248133	-0.251539843	0.801396762	0.990988839
Gpr161	2.055406226	1.76244334	1.166225421	0.243523317	0.896939594
Dcaf6	-0.070296126	0.117596396	-0.59777449	0.549990414	0.968926429
Mpc2	0.350906795	0.179791741	1.95174035	0.050969039	0.707153766
Mpzl1	-0.084827282	0.162160862	-0.523105766	0.600900653	0.974001549
Rcsd1	-0.035038388	0.127076944	-0.275725766	0.782758704	0.990988839
Creg1	0.181826439	0.161999367	1.122389807	0.261696736	0.897248195
Cd247	0.129096441	0.250207058	0.515958432	0.605883442	0.974001549
Pou2f1	-0.090715724	0.299407975	-0.302983661	0.761902314	0.989285682
Ildr2	0.013824593	0.323340955	0.042755465	0.965896466	0.996474415
Tada1	-0.017668609	0.140121373	-0.126095029	0.899656702	0.99447264
Pogk	-0.331620389	0.243052307	-1.364399268	0.172441938	0.86244803
Gm16701	0.560611538	0.412087114	1.360419969	0.173697063	0.863528862
Fam78b	-0.389913937	0.412713522	-0.944756873	0.344783014	0.92132235
Uck2	-0.088834877	0.275931857	-0.321944984	0.747494374	0.988585656
Tmco1	0.128441792	0.124431293	1.032230628	0.30196409	0.911518274
Aldh9a1	0.232660896	0.12012374	1.936843585	0.052764466	0.707153766
Mgst3	0.149981572	0.164016882	0.914427647	0.36049218	0.925913281
Lrrc52	0.053790683	1.205501955	0.044620984	0.964409417	0.996474415
Rxrg	0.207594773	0.604330172	0.343512176	0.731213182	0.988585656
Pbx1	-0.151188877	0.16821253	-0.89879676	0.368760926	0.928436166
Nuf2	0.100537465	0.344376176	0.291940825	0.770331869	0.989990452
Rgs5	-0.661818956	0.276251727	-2.395709755	0.01658822	0.566772949
Rgs4	0.04262221	0.255558382	0.166780717	0.867542591	0.992886758
1700084C01Rik	-0.571089571	0.585285853	-0.975744703	0.329190993	0.917115058
Hsd17b7	-0.172922975	0.294547746	-0.587079606	0.557150243	0.968926429
3110045C21Rik	0.509732625	0.500670894	1.018099177	0.308630827	0.9139204
Ddr2	-0.219749328	0.169075149	-1.299713941	0.19369903	0.879588734
Uap1	-0.001049575	0.110890859	-0.009464938	0.992448185	0.99886678
Uhmk1	-0.39852386	0.323383169	-1.232358074	0.217815355	0.887633697
Sh2d1b1	0.321940402	0.178453935	1.804053251	0.071222959	0.744227652
Gm7694	-0.41326318	0.413025894	-1.000574505	0.317032561	0.91640698
Nos1ap	-1.00186585	1.049889539	-0.954258342	0.339952889	0.919246575
Olfml2b	-0.420513564	0.34812345	-1.207943802	0.22706888	0.89398447
Atf6	-0.158661338	0.129765251	-1.222679695	0.221450699	0.891315805
Dusp12	0.041647046	0.199170714	0.209102257	0.834368413	0.992886758
Fcrlb	-1.387815064	1.324218772	-1.048025517	0.294626851	0.907334416
Fcrla	-0.164062689	0.296407807	-0.553503266	0.579918845	0.971613657
Fcgr2b	-0.527435392	0.215867179	-2.443332952	0.014552305	0.554936045
Fcgr4	0.10730727	0.262327761	0.409058003	0.682497095	0.984738223
Fcgr3	-0.046821497	0.163755833	-0.285922622	0.774937387	0.990861469
Cfap126	-0.379594595	0.141686151	-2.679122777	0.007381532	0.452031612
Sdhc	-0.121774561	0.087834698	-1.386406097	0.165622936	0.860011234
Mpz	0.084935403	0.424416707	0.200122666	0.841384647	0.992886758
Pcp4l1	-0.048661528	0.102594257	-0.47431045	0.635278541	0.977034429
Tomm40l	0.513206061	0.195561839	2.624264851	0.008683623	0.475600561
Apoa2	0.634576988	1.295283338	0.48991365	0.624195004	0.975969024
Fcer1g	0.119004336	0.147752438	0.805430608	0.420571235	0.943415794
Ndufs2	0.230270279	0.112672099	2.043720508	0.040981166	0.679118604
Adamts4	-0.53738838	1.241533017	-0.432842601	0.665129122	0.981727712
B4galt3	-0.03192052	0.216351795	-0.147539889	0.882705898	0.992886758
Ppox	-0.042813134	0.165091872	-0.259329146	0.795381293	0.990988839
Usp21	-0.093275615	0.226307526	-0.412163117	0.680219869	0.984207811
Ufc1	0.327074282	0.185477476	1.763417794	0.077830051	0.754202
Dedd	-0.015505799	0.146291704	-0.105992332	0.915588436	0.994688009
Nit1	0.312189994	0.169551483	1.841269617	0.065582058	0.736929291
Pfdn2	0.112041371	0.132479944	0.845723263	0.397707143	0.935327115
Klhdc9	0.077080114	0.153083642	0.503516335	0.614601299	0.974001549
Pvrl4	-0.050209324	0.327774985	-0.153182292	0.878254522	0.992886758
Arhgap30	0.19972052	0.19101734	1.045562251	0.295763185	0.908204914
Usf1	0.277255644	0.125186563	2.214739648	0.02677794	0.633489138

Tstd1	0.366929686	0.191045402	1.920641285	0.054776947	0.710924647
F11r	0.028159239	0.098131476	0.286954196	0.774147392	0.990730604
Alyref2	0.000957129	0.278762832	0.00343349	0.997260477	0.99957296
Itln1	-0.575510098	1.312244262	-0.438569339	0.660973618	0.979924124
Cd244	0.376243328	0.290090319	1.296986845	0.194635713	0.879588734
Ly9	0.040289138	0.396454866	0.101623518	0.919055512	0.994688009
Slamf7	-0.029854959	0.295087046	-0.101173396	0.919412815	0.994688009
Cd48	-0.110178598	0.187998273	-0.586061755	0.557834016	0.968926429
Slamf1	0.274371222	0.744928063	0.368319085	0.712635324	0.988585656
Cd84	-0.106620263	0.178243767	-0.59817106	0.549725799	0.968926429
Slamf6	0.191139449	0.326996098	0.584531283	0.558862924	0.968926429
Vangl2	-0.099601484	0.593524329	-0.16781365	0.866729883	0.992886758
Ncstn	-0.219585347	0.207534029	-1.058069117	0.290023933	0.907334416
Copa	-0.327151989	0.19447446	-1.68223626	0.092523035	0.775044718
Pex19	0.032620308	0.097108721	0.335915331	0.736934716	0.988585656
Dcaf8	-0.002960485	0.10735206	-0.027577345	0.97799925	0.997262526
Pea15a	0.08106255	0.091563828	0.885311937	0.375988414	0.928883222
Casq1	0.973112973	0.9960437	0.976978192	0.328579949	0.917115058
Atp1a2	0.307632956	0.189382806	1.624397497	0.104291001	0.79155232
Igsf8	0.09038933	0.263025998	0.343651695	0.731108243	0.988585656
Kcnj10	1.431626554	2.183507988	0.65565437	0.512046534	0.963452765
Pigm	-0.106003581	0.162529681	-0.652210599	0.514265323	0.964352198
Slamf9	0.030113357	0.172345878	0.174726295	0.861294705	0.992886758
Igsf9	-0.177955252	0.509604343	-0.349202778	0.726937081	0.988585656
Tagln2	-0.18142152	0.165602076	-1.095526843	0.273285892	0.89897004
Cfap45	-0.169514614	0.201094255	-0.842960997	0.399250257	0.936273856
4933439K11Rik	-0.483023992	0.555672638	-0.869259991	0.384704941	0.931689378
Vsig8	0.202816672	0.731031124	0.277439175	0.781442905	0.990988839
Slamf8	0.067621884	0.431393685	0.156752142	0.875440191	0.992886758
Dusp23	-0.002532	0.235118794	-0.010769026	0.991407727	0.998770784
Fcer1a	0.184349809	0.891961463	0.206679118	0.836260467	0.992886758
Ackr1	-0.226149816	1.26360387	-0.178972083	0.857959623	0.992886758
Cadm3	0.576291612	0.664371116	0.867424242	0.385709604	0.931855682
Aim2	0.454148255	0.255399712	1.778186249	0.075373271	0.752876039
BC094916	1.16483246	0.522366451	2.229914381	0.025753128	0.631530315
Pydc4	0.024397281	0.460884616	0.052935767	0.957783087	0.996226826
Pyhin1	0.295185479	0.279295415	1.056893391	0.290560245	0.907334416
Pydc3	0.326911245	0.273114475	1.196975168	0.231316234	0.895404344
AI607873	0.133696759	0.422628769	0.316345617	0.75174019	0.988585656
Ifi204	-0.015651084	0.337423433	-0.046384104	0.963004106	0.996474415
Mndal	0.144120192	0.191075393	0.754258252	0.450694165	0.949134998
Mnda	0.098425785	0.35948538	0.273796349	0.784241129	0.990988839
Ifi203	0.099734638	0.215137081	0.463586459	0.642944058	0.978681333
Ifi202b	-0.830926399	1.7979069	-0.462163196	0.643964294	0.97889195
Ifi205	-0.414697874	0.299374811	-1.385212978	0.165987353	0.860029348
Spta1	-0.555437823	1.309582855	-0.424133395	0.671468532	0.98288208
Fmn2	0.07089212	0.819252254	0.086532713	0.931042953	0.994688009
Grem2	-0.055080866	0.184833212	-0.298003079	0.765700814	0.989884648
Rgs7	-1.444939376	1.785209739	-0.809394742	0.418288126	0.943415794
Fh1	0.084420342	0.103457221	0.815992744	0.414504286	0.943415794
Kmo	-0.388170483	0.303576952	-1.278655974	0.20101823	0.881109608
Opn3	0.463672705	0.490250693	0.945786944	0.344257266	0.920669678
Chml	0.218824644	0.256694562	0.852470898	0.393952785	0.933796635
Exo1	0.530832292	0.64696015	0.820502301	0.411929818	0.942121471
Cep170	0.242366351	0.206232023	1.175212014	0.239909905	0.896939594
Sdccag8	0.047217565	0.21200676	0.222717262	0.823755566	0.992886758
Hmga2-ps1	0.257714597	0.384753105	0.669818108	0.502973749	0.961729397
Akt3	-0.107832684	0.128622689	-0.838364402	0.401826076	0.937834293
Zbtb18	0.038014846	0.124545851	0.305227716	0.760192725	0.989266039
1700016C15Rik	0.458937326	0.607441475	0.755525174	0.449933934	0.948896093
Adss	-0.104499823	0.095886156	-1.089832232	0.275787053	0.900302631
Gm16432	0.045791837	0.422731315	0.108323739	0.913738892	0.994688009

Desi2	-0.138095193	0.180014843	-0.767132257	0.443002885	0.948476747
Cox20	0.352835712	0.161116449	2.189942211	0.028528428	0.645679684
Hnrnpu	0.012552111	0.128605627	0.097601568	0.922248679	0.994688009
Efcab2	-0.120766645	0.232887703	-0.518561708	0.604066418	0.974001549
Kif26b	-0.584167785	0.34538872	-1.691334291	0.090772974	0.77029644
Smyd3	-0.215277144	0.191165312	-1.126130795	0.260110161	0.897248195
Tfb2m	-0.065832359	0.158924435	-0.414236863	0.678700648	0.983853811
Cnst	-0.289422641	0.206546498	-1.401246903	0.161140253	0.857316691
Sccpdh	-0.044554304	0.114203365	-0.390131268	0.696439482	0.987490725
Ahctf1	-0.134974035	0.198135852	-0.681219648	0.495732517	0.959893834
Cdc42bpa	0.136668661	0.281641072	0.485258277	0.627493148	0.976931499
Adck3	0.128693163	0.213872875	0.601727373	0.547355626	0.968115622
Psen2	-0.207397524	0.171544768	-1.208999415	0.22666307	0.89398447
Gm5069	0.206045489	0.454609453	0.453236261	0.650378624	0.979004493
Itpkb	-0.155506713	0.26969161	-0.576609385	0.564203358	0.969658675
6330403A02Rik	-0.922739803	0.500156509	-1.844902119	0.065051778	0.734014162
Parp1	0.209309976	0.185176315	1.130328014	0.258338035	0.897248195
Lin9	-0.038349312	0.277801098	-0.138045934	0.890204115	0.993043931
Acbd3	0.096774472	0.129089579	0.749669123	0.453454008	0.950146765
H3f3a	0.155219638	0.156498192	0.991830228	0.321280349	0.91640698
Sde2	-0.31508265	0.161127783	-1.955483059	0.050526081	0.707153766
Pycr2	0.132429667	0.107207513	1.235264801	0.216731969	0.887633697
Lefty1	0.346756538	0.256169038	1.353623925	0.17585639	0.865082036
Tmem63a	0.0110442	0.173486993	0.063660105	0.949240872	0.995624732
Ephx1	-0.164313402	0.148002219	-1.110209047	0.266908955	0.89768067
Nvl	0.062366669	0.227598937	0.274020035	0.784069225	0.990988839
Cnih4	0.200169129	0.167087113	1.197992628	0.230919887	0.895404344
Wdr26	0.024838791	0.105889411	0.234572946	0.814540209	0.992048071
Cnih3	0.854647392	1.318511814	0.648190924	0.516861471	0.964352198
Ccdc121	0.014436051	0.282237522	0.051148587	0.95920712	0.996367077
Lbr	-0.055472656	0.112865531	-0.491493336	0.623077564	0.975545397
Enah	-0.216743835	0.144863337	-1.496195236	0.134602786	0.828680121
Srp9	0.433832786	0.18331991	2.366533929	0.017955528	0.581648476
Degs1	-0.140885056	0.091930167	-1.532522567	0.125393533	0.822401003
Fbxo28	-0.164418855	0.154552984	-1.063834882	0.287403506	0.907334416
Trp53bp2	-0.050057849	0.096781315	-0.517226376	0.604998142	0.974001549
Capn2	-0.031219421	0.102509103	-0.304552669	0.760706873	0.989266039
Capn8	-0.7165775	1.035298074	-0.692146076	0.488845599	0.958560421
Susd4	-0.269650167	0.395167204	-0.682369804	0.495005144	0.959272191
Tlr5	0.05962349	0.558197253	0.106814374	0.914936244	0.994688009
1700056E22Rik	-0.399262935	0.807748497	-0.494291151	0.621100573	0.97514112
Dusp10	0.077834449	0.431189313	0.18051108	0.856751357	0.992886758
Hlx	-0.096333175	0.309006181	-0.311751611	0.755229301	0.98922012
Mar-01	0.373821769	0.447726385	0.834933524	0.403755138	0.937834293
Mar-02	0.00717196	0.101446016	0.070697309	0.943638662	0.99486515
C130074G19Rik	0.066844046	0.179351554	0.372698447	0.709372892	0.988585656
Mark1	-0.468194967	0.46106754	-1.015458531	0.309887305	0.914427982
Rab3gap2	0.031125791	0.187592005	0.165922801	0.868217704	0.992886758
Iars2	-0.18257542	0.194463535	-0.938867129	0.347798968	0.921552698
Bpnt1	-0.192453923	0.134807936	-1.427615676	0.153402509	0.854159937
Eprs	-0.110737313	0.167292034	-0.661940142	0.508009584	0.963106419
Lyplal1	0.338495087	0.233870934	1.447358509	0.14779654	0.846989858
Tgfb2	0.351387759	0.180413699	1.947677814	0.051453526	0.707153766
Rrp15	0.082948397	0.140835984	0.588971619	0.555880312	0.968926429
Spata17	-0.314439942	0.218725305	-1.437602029	0.150547005	0.849879783
Gpatch2	-0.496945541	0.296152185	-1.678007344	0.093345665	0.775558829
Esrrg	-0.427421902	0.445339456	-0.959766523	0.337172734	0.919246575
Kctd3	0.003603471	0.176053935	0.020467994	0.983670044	0.997787078
Kcnk2	0.1471517	0.149167405	0.986486961	0.32389421	0.91640698
Cenpf	-0.301072247	0.62332609	-0.483009218	0.629089191	0.977034429
Ptpn14	-0.105050927	0.20735654	-0.50661979	0.61242162	0.974001549
Smyd2	0.060998214	0.134857586	0.452315777	0.651041511	0.979004493

Prox1	0.615479072	0.930027376	0.661785973	0.508108397	0.963181911
Rps6kc1	-0.513860475	0.279588308	-1.837918326	0.066074441	0.738254074
Angel2	-0.083070391	0.148032898	-0.561161691	0.574687316	0.971613657
Mfsd7b	-0.125128701	0.192194024	-0.65105407	0.515011584	0.964352198
Tatdn3	0.054325346	0.210182206	0.258467866	0.796045846	0.990988839
Nsl1	0.366276221	0.479385224	0.764054048	0.444835035	0.948476747
Batf3	0.015136875	0.443413808	0.034137131	0.972767799	0.996905197
Atf3	-0.120364533	0.258846102	-0.465004234	0.641928425	0.97813956
Nenf	0.349596184	0.175510107	1.991886333	0.046383531	0.698098452
Tmem206	-0.177784457	0.234177924	-0.759185385	0.447741667	0.948476747
Ppp2r5a	-0.057472919	0.146666144	-0.391862209	0.695160027	0.98715859
Dtl	-0.599399748	0.507293018	-1.181565144	0.237378291	0.896609492
Ints7	0.297724523	0.327487204	0.909118032	0.363287825	0.926455466
Lpgat1	-0.209223688	0.119345815	-1.753087768	0.079586943	0.75659671
Nek2	0.328250775	0.270116607	1.215218783	0.224282658	0.892989918
1700034H15Rik	-0.134642479	1.400024591	-0.09617151	0.923384358	0.994688009
Slc30a1	0.134808369	0.18374132	0.733685648	0.463140347	0.953083512
Rd3	-0.048478696	0.188266986	-0.257499718	0.796793033	0.990988839
Traf5	0.061706307	0.143873467	0.428892887	0.668001177	0.981863224
Rcor3	-0.211122414	0.198389414	-1.064181852	0.287246326	0.907190636
Gm10516	0.266139336	0.268743163	0.990311096	0.322022085	0.91640698
Kcnh1	-0.026364408	0.502680252	-0.052447671	0.958171991	0.996226826
Hhat	0.607504166	0.726225073	0.836523261	0.402860597	0.937834293
Sertad4	0.032800962	0.477240374	0.068730484	0.945204153	0.99486515
Gm15867	-0.379868399	0.495115948	-0.767231192	0.44294407	0.948476747
Syt14	0.222619517	0.725172292	0.306988449	0.758852165	0.989266039
Diexf	-0.207343988	0.293104754	-0.707405747	0.479314367	0.957463006
Irf6	-0.203292991	0.155674031	-1.305888908	0.191590339	0.878456795
A130010J15Rik	0.223131429	0.240958116	0.926017487	0.354436893	0.924354588
Traf3ip3	0.329006613	0.303837711	1.082836662	0.278880929	0.90176568
Hsd11b1	0.117414916	0.115355577	1.017852098	0.308748249	0.9139204
G0s2	0.484363533	0.199487809	2.428035752	0.015180847	0.555734718
Lamb3	0.064769279	0.22142356	0.292513042	0.76989439	0.989990452
Plxna2	-0.415607539	0.279012219	-1.489567519	0.136337988	0.832457116
Cd34	0.029756448	0.122449905	0.243009159	0.807998302	0.99099448
Gm16897	-0.319614598	0.71771447	-0.445322774	0.656086512	0.979512392
A330023F24Rik	-1.061665808	0.56720924	-1.871735745	0.061243172	0.725221283
Cd46	0.178763996	0.540120457	0.330970608	0.740666687	0.988585656
Cr1l	0.110685439	0.138229002	0.800739621	0.4232824	0.943509323
Cr2	-0.01754911	0.536406941	-0.032716037	0.973901035	0.997262526
Cnksr3	-0.076062158	0.222050041	-0.342545122	0.731940693	0.988585656
Ipcef1	0.518748683	0.393449483	1.318463248	0.18734862	0.873455432
Rgs17	0.445670245	0.44669164	0.997713422	0.318418343	0.91640698
Mtrf1l	-0.16807207	0.178282538	-0.942728727	0.345819676	0.921552698
Fbxo5	-0.110447418	0.26274028	-0.420367285	0.674217163	0.983375962
Vip	-0.784776751	1.852784017	-0.423566236	0.671882184	0.983223126
Myct1	0.143588113	0.265241164	0.54134928	0.588266859	0.973455044
Syne1	0.15291304	0.222682398	0.686686698	0.492280181	0.958916246
Esr1	0.316812899	0.508387706	0.623171835	0.533171608	0.96674935
Ccdc170	-0.112318704	0.211046818	-0.532198044	0.594588837	0.974001549
Armt1	0.007392888	0.173887661	0.042515313	0.966087905	0.996474415
Zbtb2	-0.072733986	0.185577091	-0.391934076	0.695106924	0.98715859
Akap12	0.052870044	0.227886114	0.232002044	0.816536426	0.992676801
Mthfd1l	0.131228349	0.412121853	0.318421234	0.750165433	0.988585656
Plekhg1	-0.195245077	0.38631659	-0.50540174	0.613276697	0.974001549
Iyd	0.312957657	0.223956815	1.397401804	0.162292777	0.859751515
Ppp1r14c	0.122717146	0.111354312	1.102042152	0.270443347	0.89804647
H60c	0.360124872	0.456029683	0.789696121	0.429705257	0.945321484
Lrp11	-0.152119185	0.332429296	-0.457598615	0.647240839	0.979004493
Pcmt1	0.114615581	0.099411202	1.152944318	0.24893324	0.896939594
A630066F11Rik	0.136795992	0.401927615	0.340349822	0.733593101	0.988585656
Nup43	-0.29779755	0.231321107	-1.287377334	0.197962801	0.880239572

BC020402	-0.618332801	0.356595674	-1.733988511	0.082920122	0.76099108
Lats1	-0.096298571	0.144719473	-0.665415433	0.505784817	0.96224682
Katna1	0.015526205	0.133969233	0.115893811	0.907736701	0.994688009
Ginm1	-0.007446027	0.102395309	-0.072718442	0.942030172	0.994688009
Ppil4	-0.005569251	0.141625648	-0.039323748	0.968632273	0.996474415
Zc3h12d	0.896446881	0.836563265	1.071582889	0.283907422	0.905916508
Tab2	-0.184445333	0.139341294	-1.3236947	0.185604459	0.87179632
Ust	-0.435086607	0.392849073	-1.107515932	0.268070928	0.89804647
Sash1	0.03277938	0.132247435	0.247864016	0.804239622	0.990988839
Samd5	-0.000591574	0.39894798	-0.001482835	0.99881687	0.999739059
Stxbp5	0.032759521	0.227891597	0.143750457	0.885697526	0.992886758
Adgb	0.027557221	0.279369009	0.098640937	0.921423366	0.994688009
Rab32	-0.155274942	0.154291028	-1.006377001	0.314234253	0.91586731
Grm1	-5.11050687	4.919502487	-1.038825955	0.298885688	0.909933614
Shprh	-0.12863638	0.171089233	-0.751867189	0.452130932	0.949574349
Fbxo30	-0.073881368	0.147288908	-0.501608497	0.615942941	0.974001549
Epm2a	-0.090763016	0.356278697	-0.254752856	0.79891399	0.990988839
Utrn	0.060726426	0.146881378	0.413438563	0.679285326	0.983903958
B230208H11Rik	0.077139418	0.396912346	0.194348749	0.845902817	0.992886758
Stx11	0.007437628	0.213022139	0.03491481	0.972147671	0.996750504
Sf3b5	0.140930743	0.133169581	1.058280294	0.289927674	0.907334416
Plagl1	-0.224703404	0.444789563	-0.505190369	0.613425134	0.974001549
Ltv1	-0.076652162	0.153660925	-0.498839648	0.617892354	0.974153
Phactr2	-0.222753943	0.164463096	-1.354431169	0.175598859	0.864596675
Fuca2	-0.098547614	0.11102075	-0.88765041	0.374728829	0.928883222
Pex3	-0.019541852	0.139529176	-0.140055665	0.88861601	0.992886758
Adat2	0.08487622	0.25325618	0.335139778	0.737519648	0.988585656
Aig1	-0.018206336	0.148899493	-0.122272651	0.902683091	0.994688009
Hivep2	0.057691018	0.204044372	0.282737611	0.777377983	0.990962705
Adgrg6	-0.22050448	0.139436455	-1.581397634	0.113787148	0.807243653
Vta1	0.117476006	0.121193607	0.969325102	0.332383009	0.917115058
Cited2	-0.051913829	0.17207721	-0.301689161	0.762889032	0.989285682
Txlnb	-0.381568565	0.377054008	-1.011973237	0.31155085	0.914819219
Heca	-0.161409616	0.181667489	-0.888489274	0.374277623	0.928883222
Abracl	0.19938031	0.168883254	1.180580699	0.237769335	0.896609492
Reps1	-0.284351109	0.190833418	-1.490048818	0.1362114	0.832457116
Ect2l	-0.066869197	0.413479863	-0.161722982	0.871524006	0.992886758
Ccdc28a	0.249062782	0.197769345	1.2593599	0.207900366	0.884225785
Nhsl1	0.197141855	0.371969931	0.529994062	0.596116048	0.974001549
Hebp2	-0.160099436	0.257251605	-0.622345722	0.533714563	0.96674935
Arfgef3	-0.742717625	0.694892701	-1.068823465	0.285149224	0.905916508
4930444F02Rik	-1.472043033	1.241292277	-1.185895587	0.235663536	0.896002526
Perp	-0.207061695	0.1418003	-1.460234535	0.144225627	0.841934045
Tnfaip3	-0.079330698	0.195436564	-0.405915332	0.684804812	0.985105247
Gm20139	1.04717446	2.086470111	0.501888071	0.615746257	0.974001549
Olig3	1.691798173	1.67309886	1.011176455	0.311931983	0.914819219
Ifngr1	-0.103757325	0.098887424	-1.049246927	0.294064486	0.907334416
Il22ra2	-0.118149526	0.530603231	-0.222670197	0.823792198	0.992886758
Il20ra	2.182565193	1.359519523	1.605394521	0.108407009	0.798150849
Slc35d3	0.050891529	0.557874002	0.091224055	0.927314561	0.994688009
Pex7	0.197439279	0.157289126	1.255263377	0.209383165	0.884258007
Map3k5	-0.354160578	0.215680456	-1.642061522	0.100577255	0.788783372
Map7	-0.040597782	0.173306439	-0.234254317	0.814787549	0.992116023
Gm6251	-0.373874645	0.561124643	-0.66629518	0.505222446	0.962138588
Bclaf1	-0.117745988	0.126734485	-0.929076153	0.352849622	0.923529301
Mtfr2	0.246192123	0.797320878	0.308774209	0.757493289	0.989266039
Pde7b	-0.117398833	0.269674266	-0.435335693	0.66331878	0.980889931
Ahi1	-0.770779346	0.281395981	-2.739127057	0.006160256	0.427133716
Myb	-0.358198024	0.213372649	-1.67874386	0.093201974	0.775558829
Hbs1l	0.071206489	0.133403553	0.533767558	0.593502362	0.974001549
Sgk1	-0.154495958	0.156626982	-0.986394272	0.323939675	0.91640698
E030030I06Rik	0.15827723	0.313674091	0.504591342	0.613845892	0.974001549

Raet1a	4.560929274	2.02810638	2.248860967	0.024521343	0.627523461
Raet1e	-3.00137309	1.62857643	-1.842942729	0.065337373	0.735191788
C920009B18Rik	0.390887363	0.74232161	0.52657414	0.598489354	0.974001549
Slc2a12	-0.160183173	0.195572386	-0.819048005	0.412759025	0.942708418
Tbpl1	0.168056666	0.128549183	1.307333604	0.191099436	0.877906723
Tcf21	0.261103779	0.156227529	1.671304544	0.094661539	0.777748075
Eya4	-2.243406588	2.158106987	-1.039525196	0.298560546	0.909779403
Rps12	0.241712409	0.308402613	0.783756033	0.433183277	0.946239431
Snora33	0.793773456	0.626088536	1.267829405	0.204858879	0.883765092
Slc18b1	0.067523465	0.259359138	0.260347354	0.794595848	0.990988839
Vnn3	-0.148370219	0.271108564	-0.547272341	0.584191643	0.972333834
Vnn1	-0.069136699	0.192693968	-0.358790159	0.719752075	0.988585656
Taar2	-0.858269232	0.687169925	-1.248991262	0.21166827	0.884755882
Stx7	0.058861301	0.110743431	0.53151054	0.595065039	0.974001549
Moxd1	-0.662341869	2.208143847	-0.299954131	0.764212143	0.989384061
Ctgf	-0.041563625	0.196328842	-0.21170412	0.832337874	0.992886758
Enpp1	0.018993746	0.37815439	0.05022749	0.959941105	0.996474415
Enpp3	-0.08849571	0.86587935	-0.10220328	0.918595324	0.994688009
Med23	-0.346079715	0.298889508	-1.157885122	0.24691092	0.896939594
Akap7	0.008330319	0.155003903	0.053742641	0.957140209	0.996226826
Epb4.1l2	-0.170531893	0.141459751	-1.205515285	0.228004441	0.894410876
Tmem200a	0.338178668	0.393452505	0.859515859	0.390055974	0.933734231
Samd3	1.593231134	0.520088294	3.063385875	0.002188477	0.308737709
L3mbtl3	0.191505409	0.464020639	0.412708817	0.679819964	0.983903958
Arhgap18	-0.08904199	0.107305488	-0.829799032	0.406652418	0.939557159
Lama2	0.321529222	0.236795564	1.357834654	0.174516156	0.863528862
Ptprk	0.032146996	0.161912806	0.19854511	0.842618594	0.992886758
Themis	0.675358483	0.477615115	1.414022426	0.157355318	0.855986074
9330159F19Rik	-0.317814634	0.459998323	-0.690903897	0.489625937	0.958560421
Echdc1	0.23859191	0.308024466	0.774587528	0.438583429	0.947816234
Rnf146	0.052852142	0.116263832	0.454587993	0.649405674	0.979004493
Rspo3	-0.879619001	0.450248124	-1.953631682	0.050744791	0.707153766
Cenpw	-0.239474347	0.342096798	-0.70001926	0.483915276	0.957710647
Trmt11	-0.04109781	0.130091713	-0.315914127	0.752067688	0.988585656
Gm20300	0.092037091	0.133271119	0.69060042	0.489816683	0.958560421
Hint3	0.284291205	0.207934647	1.367214215	0.171558171	0.86244803
Ncoa7	-0.022358276	0.137873517	-0.162165127	0.871175821	0.992886758
Hey2	-0.238241592	0.311227931	-0.76548911	0.443980349	0.948476747
Hddc2	0.427907682	0.23893339	1.790907844	0.073308085	0.748833631
Tpd52l1	-0.004105015	0.336772483	-0.012189284	0.9902746	0.998417328
Gm3258	1.396751468	0.530674529	2.632030353	0.008487628	0.474579703
Rnf217	-0.265662093	0.36358962	-0.730664679	0.464983995	0.953083512
Nkain2	-1.133090468	1.401485013	-0.808492748	0.418806979	0.943415794
Trdn	0.021321237	0.361705513	0.058946397	0.952994803	0.996070133
D830005E20Rik	0.519632702	1.765249149	0.29436791	0.768476785	0.989990452
Rsph4a	-0.166243391	0.211556658	-0.78581025	0.431978661	0.9459793
Zufsp	0.21098525	0.185896252	1.134962367	0.256391087	0.897248195
A830082N09Rik	-0.128542651	0.812504445	-0.158205474	0.874294891	0.992886758
Rwdd1	0.28865779	0.17524867	1.647132551	0.099530804	0.788783372
Fam26e	-0.109941783	0.193020181	-0.569586983	0.568957859	0.97144955
Fam26f	0.668000978	0.358817017	1.861675857	0.062648794	0.727650961
Dse	0.060790807	0.129764612	0.468469841	0.639448633	0.977843986
Tspyl1	-0.032360405	0.120157655	-0.269316217	0.787686353	0.990988839
Tspyl4	-0.170982911	0.242258391	-0.705787364	0.480320381	0.957463006
Nt5dc1	0.016727832	0.14844573	0.112686517	0.910279091	0.994688009
Col10a1	0.491601396	2.095373269	0.234612803	0.814509271	0.992048071
Frk	-0.225007371	0.268766071	-0.837186666	0.402487652	0.937834293
Hdac2	-0.159791443	0.1117234	-1.430241493	0.15264772	0.853744627
Marcks	-0.093341299	0.1035732	-0.901210919	0.367476186	0.928436166
Lama4	0.127925915	0.165818533	0.771481405	0.44042164	0.947899303
Fam229b	0.500853336	0.258064166	1.940809312	0.052281411	0.707153766
Tube1	-0.381357684	0.92909601	-0.410461008	0.681467801	0.984683806

Fyn	0.080636898	0.178867811	0.450818388	0.652120447	0.979004493
Traf3ip2	-0.027665097	0.171249658	-0.161548334	0.871661546	0.992886758
E130307A14Rik	-0.039124875	0.177752105	-0.220109209	0.825786103	0.992886758
Rev3l	-0.053657896	0.243332381	-0.220512763	0.825471834	0.992886758
AA474331	-0.61458645	0.650973207	-0.944104063	0.345116473	0.92132235
G630090E17Rik	0.011093245	0.297704741	0.037262575	0.970275645	0.996628386
BC021785	-0.835738929	1.501687647	-0.556533132	0.577846452	0.971613657
AI317395	0.094714345	0.263658169	0.359231598	0.719421841	0.988585656
Slc16a10	0.053740202	0.221282427	0.242857975	0.808115422	0.99099448
Rpf2	0.046100982	0.170497403	0.270391107	0.786859381	0.990988839
Gtf3c6	0.101730139	0.146928979	0.692376278	0.488701059	0.958556246
Cdk19	0.071378304	0.210285356	0.339435449	0.734281718	0.988585656
Ddo	-0.081338089	0.165219544	-0.49230307	0.62250511	0.975236037
Mettl24	0.179241367	0.160167486	1.119087102	0.263102982	0.897248195
Cdc40	-0.110683735	0.155483809	-0.711866633	0.476547366	0.95716557
Wasf1	-0.705458335	1.256471022	-0.561460092	0.574483929	0.971613657
Fig4	-0.364789108	0.180390478	-2.022219307	0.043153701	0.685056403
Zbtb24	-0.251701113	0.241442557	-1.042488602	0.297185207	0.909070179
Mical1	0.04330226	0.222455142	0.194656142	0.845662148	0.992886758
Smpd2	0.002895811	0.125527605	0.023069114	0.981595142	0.997390438
Ppil6	0.049495436	0.151850712	0.325947999	0.744463699	0.988585656
Cd164	-0.196587301	0.095303818	-2.06274319	0.039137034	0.679118604
Ccdc162	-0.053294991	0.261759965	-0.20360253	0.838664116	0.992886758
Cep57l1	-0.385787897	0.379531054	-1.016485723	0.309398143	0.9139204
Sesn1	-0.079999174	0.169649326	-0.471556097	0.63724367	0.977284137
Armc2	-0.410681905	0.292466134	-1.40420328	0.160258325	0.857316691
Foxo3	0.079205896	0.411377242	0.192538352	0.847320529	0.992886758
Lace1	0.363724347	0.174380399	2.085809816	0.036995854	0.678243111
Snx3	-0.029056236	0.069003467	-0.421083721	0.673693948	0.983375962
Ostm1	-0.049324482	0.119332422	-0.413336801	0.679359871	0.983903958
Sec63	-0.138373483	0.163408717	-0.846793767	0.397110083	0.934912232
Scml4	0.384038596	0.56472435	0.680046107	0.496475267	0.960362776
Sobp	0.826077085	0.71656538	1.15282863	0.24898073	0.896939594
9030612E09Rik	-0.229459588	1.001828481	-0.229040792	0.818837212	0.992734473
Pdss2	0.226725832	0.295437698	0.767423498	0.442829762	0.948476747
Bend3	0.133987843	0.428506285	0.312685829	0.754519363	0.98902053
1700021F05Rik	0.240143038	0.140805389	1.705496069	0.088101945	0.767228183
Cd24a	-0.056261086	0.107571794	-0.523009646	0.600967541	0.974001549
F830002L21Rik	0.170410692	1.490057008	0.114365217	0.908948286	0.994688009
4933404K13Rik	-0.538570867	1.261864509	-0.426805623	0.669520911	0.981863224
Qrsl1	-0.355155003	0.284896285	-1.246611564	0.212539959	0.884945675
Rtn4ip1	0.342952834	0.231648188	1.480490035	0.138742518	0.834874897
Aim1	0.176236809	0.210103541	0.838809325	0.401576317	0.937834293
Atg5	0.067328546	0.145379187	0.463123694	0.643275707	0.978801899
Prdm1	-0.054788873	0.352570699	-0.155398261	0.876507355	0.992886758
Prep	-0.059102017	0.131404434	-0.449771866	0.652874946	0.979004493
Popdc3	0.150991874	0.146447087	1.031033648	0.302525041	0.911518274
Bves	-0.455714801	0.612213195	-0.744372721	0.456651018	0.951472839
Hace1	-0.038102085	0.161617912	-0.235754095	0.813623491	0.992048071
Grik2	0.203124318	0.786644165	0.258216265	0.796240006	0.990988839
Ascc3	-0.146795235	0.213433904	-0.687778428	0.491592323	0.958916246
Lilr4b	-0.080893926	0.336315754	-0.240529697	0.809919644	0.991419855
Lilrb4a	-0.286134418	0.220084557	-1.300111294	0.193562827	0.879588734
Fam162b	0.37534518	0.223674965	1.678083106	0.093330876	0.775558829
Gprc6a	0.039979249	0.896819629	0.044578918	0.964442947	0.996474415
Ros1	-0.546857288	0.620135767	-0.88183478	0.37786616	0.928883222
Dcbld1	-0.118685984	0.180241849	-0.658481841	0.510228562	0.963452765
Gopc	-0.268276654	0.189336976	-1.416926897	0.156504302	0.855455553
Nus1	-0.037843015	0.108898848	-0.347506113	0.728211127	0.988585656
Cep85l	-0.350214388	0.292041846	-1.199192488	0.230453108	0.895404344
Gm19395	-1.355359514	2.317852276	-0.584748013	0.558717165	0.968926429
Pln	-0.650492527	0.247965164	-2.623322237	0.008707687	0.475600561

Mcm9	-0.075635765	0.278529553	-0.271553822	0.78596511	0.990988839
Asf1a	0.046401007	0.142063142	0.326622418	0.743953485	0.988585656
Fam184a	-0.545533574	0.273995153	-1.991033667	0.046477186	0.698098452
Man1a	0.044538695	0.164084455	0.271437628	0.786054465	0.990988839
Tbc1d32	-0.066796945	0.191803846	-0.348256546	0.727647525	0.988585656
Msl3l2	0.2208555	0.274975224	0.803183273	0.421868812	0.943415794
Gja1	-0.152169948	0.189184577	-0.804346475	0.421196904	0.943415794
Hsf2	-0.260488976	0.184419731	-1.412478888	0.157809003	0.855986074
Serinc1	-0.189375228	0.120192266	-1.575602441	0.115117453	0.81007959
Pkib	-0.246897868	0.253110588	-0.975454521	0.329334851	0.917115058
Fabp7	0.54894074	1.140021473	0.481517895	0.630148465	0.977034429
Smpdl3a	0.001116115	0.094875147	0.01176404	0.99061387	0.998564713
Gcc2	-0.388850057	0.227120665	-1.71208576	0.086880865	0.765418158
Lims1	-0.066059873	0.096571788	-0.684049388	0.49394398	0.958916246
Ranbp2	-0.1316053	0.126242254	-1.04248218	0.297188183	0.909070179
Ccdc138	-0.451532372	0.227322618	-1.986306402	0.046999312	0.698868643
Edar	-0.242535558	0.364914745	-0.664636223	0.506283195	0.962370882
Sh3rf3	-0.169861509	0.381567412	-0.445167757	0.656198526	0.979587943
Sep-10	0.109806443	0.137679906	0.797548796	0.425132375	0.944290206
Sowahc	-0.041070712	0.16330675	-0.25149427	0.801431992	0.990988839
P4ha1	-0.126382378	0.126623595	-0.99809501	0.31823329	0.91640698
Pla2g12b	1.377892984	0.832711571	1.654706183	0.097984112	0.78637468
Oit3	0.043499855	0.435113947	0.099973479	0.920365381	0.994688009
Mcu	-0.017758622	0.242974711	-0.073088355	0.941735808	0.994688009
Micu1	-0.054311811	0.139248956	-0.390033878	0.696511496	0.987490725
Dnajb12	-0.106785414	0.12745345	-0.837838552	0.402121384	0.937834293
Ddit4	-0.100397401	0.413122794	-0.243020726	0.807989342	0.99099448
Anapc16	0.294889894	0.173880124	1.695937913	0.089897639	0.769347579
Ascc1	0.155846936	0.116202916	1.341162008	0.179867859	0.868087032
Spock2	-0.10844903	0.228387847	-0.474845889	0.634896823	0.977034429
Chst3	0.013984516	0.248743693	0.056220586	0.955166082	0.99614946
Psap	-0.222830937	0.160780973	-1.385928522	0.165768731	0.860011234
Cdh23	0.317470497	1.256245329	0.252713773	0.800489404	0.990988839
4632428N05Rik	-0.116437997	0.163964714	-0.710140576	0.477616966	0.95716557
Slc29a3	-0.646224652	0.482569798	-1.339131986	0.180527709	0.868087032
Unc5b	-0.482172107	0.302471839	-1.59410578	0.110912345	0.800111791
Pcbd1	0.122398281	0.17069332	0.717065442	0.473333718	0.95585895
Sgpl1	0.077851778	0.215174794	0.361807146	0.717496152	0.988585656
Tbata	-0.304680631	0.41006484	-0.743005987	0.457478056	0.952102513
Adamts14	2.33367215	0.86399356	2.701029565	0.006912519	0.447859934
Prf1	0.759754249	0.40626416	1.870099125	0.061470054	0.725730283
Pald1	-0.119969766	0.125281617	-0.957600718	0.338264137	0.919246575
Eif4ebp2	-0.059397428	0.361907942	-0.164123031	0.869634292	0.992886758
Lrrc20	0.309687145	0.301056241	1.028668744	0.303635362	0.911786415
Ppa1	-0.04633845	0.144568474	-0.320529424	0.748567029	0.988585656
Sar1a	-0.061451859	0.068350862	-0.899064869	0.368618109	0.928436166
Tysnd1	0.166149143	0.168973334	0.983286174	0.325466613	0.91640698
Aifm2	-0.153599665	0.204375843	-0.751554893	0.452318778	0.94958784
H2afy2	0.056910879	0.317581451	0.17920089	0.857779965	0.992886758
Col13a1	0.035565206	0.240418301	0.147930528	0.882397595	0.992886758
Gm5424	-0.230691949	0.71220655	-0.323911581	0.746004976	0.988585656
2010107G23Rik	0.482058631	0.175077891	2.753395233	0.005898064	0.427133716
Tspan15	-0.007761305	0.108736811	-0.071376977	0.943097732	0.994819052
Tacr2	-0.915188359	1.343915676	-0.680986446	0.495880067	0.960065677
Hk1	0.085251458	0.300220558	0.28396276	0.77643892	0.990861469
Hk1os	-0.537086176	1.144488712	-0.469280448	0.638869189	0.977795535
Hkdc1	-0.523056268	0.447887145	-1.167830498	0.242875144	0.896939594
Supv3l1	0.111228464	0.207515422	0.536000952	0.591957892	0.974001549
Vps26a	-0.054192098	0.102562521	-0.5283811	0.597234853	0.974001549
Srgn	0.122218228	0.112869351	1.08282919	0.278884247	0.90176568
Kif1bp	-0.324742761	0.145402958	-2.233398584	0.025522671	0.631530315
Ddx21	-0.206856017	0.125833692	-1.643884185	0.100200127	0.788783372

Ddx50	0.135003188	0.112635628	1.198583348	0.230689996	0.895404344
Stox1	-0.402818414	0.304941427	-1.320969793	0.186511436	0.872512868
Ccar1	-0.090161583	0.175651929	-0.513296855	0.607743684	0.974001549
Tet1	2.269390934	0.91392298	2.483131492	0.0130233	0.534249815
Slc25a16	-0.174611297	0.140668384	-1.241297382	0.214495911	0.886400046
Dna2	-0.085910516	0.220620791	-0.389403536	0.696977657	0.987518602
Rufy2	-0.325346435	0.177743761	-1.830423936	0.067186571	0.741440336
Hnrnph3	0.181698599	0.29498507	0.615958627	0.537921826	0.96674935
Pbld2	-0.443898876	0.416764936	-1.065106103	0.286827918	0.906336252
Pbld1	-0.382629522	0.546148386	-0.70059627	0.483555007	0.957710647
Mypn	3.748133705	1.797527798	2.085160357	0.037054747	0.678243111
Herc4	0.059611549	0.144404168	0.412810446	0.679745497	0.983903958
Sirt1	-0.217548967	0.169670753	-1.282183074	0.199778432	0.880888611
Dnajc12	0.189328574	0.189204226	1.000657216	0.316992558	0.91640698
Lrrtm3	-0.286452	1.972269367	-0.145239796	0.884521547	0.992886758
Gm31763	1.241566929	1.98137897	0.626617597	0.530909931	0.966027687
Reep3	-0.081909697	0.139974196	-0.585177122	0.558428626	0.968926429
Jmjd1c	-0.048622552	0.136610647	-0.355920661	0.721899982	0.988585656
Nrbf2	-0.031061473	0.184267792	-0.168567021	0.866137222	0.992886758
Egr2	0.373315687	0.447247186	0.83469656	0.403888579	0.937834293
Ado	-0.038990019	0.168934461	-0.23079968	0.81747043	0.992678823
Zfp365	-0.135899149	0.142269654	-0.955222321	0.33946528	0.919246575
Rtkn2	0.123710579	0.124931921	0.990223945	0.322064671	0.91640698
Arid5b	-0.449270497	0.346388543	-1.297013154	0.194626661	0.879588734
1700040L02Rik	-0.171389098	0.256376558	-0.66850534	0.503811075	0.96179897
Tmem26	-1.657467638	0.962216544	-1.72255159	0.084969646	0.761635307
Rhobtb1	-0.296774815	0.246058838	-1.206113209	0.227773842	0.894410876
Cdk1	-0.09482871	0.288225361	-0.329008902	0.742148959	0.988585656
Ank3	-0.170105302	0.222363457	-0.764987665	0.44427889	0.948476747
Ccdc6	-0.07333562	0.209358203	-0.350287778	0.726122736	0.988585656
Mrln	0.44531529	0.553085521	0.805147257	0.420734708	0.943415794
Slc16a9	-0.100558156	0.134060176	-0.75009715	0.453196196	0.950146765
Fam13c	-0.278735759	0.35366948	-0.788124998	0.430623594	0.945321484
Phyhipl	-0.9689662	1.650380932	-0.587116696	0.557125335	0.968926429
D630013N20Rik	2.996464637	1.544027619	1.940680724	0.052297016	0.707153766
Bicc1	-0.340942402	0.299256678	-1.139297556	0.254579067	0.897044747
Tfam	0.149945173	0.137050915	1.094083706	0.273918268	0.899464226
Ube2d1	-0.180770474	0.212611986	-0.850236515	0.395193604	0.933933717
Cisd1	0.157772105	0.133106739	1.185305163	0.235896813	0.896067726
Ipmk	-0.345866618	0.136418485	-2.535335428	0.01123397	0.508080411
Zwint	-0.030666061	0.117186123	-0.261686797	0.793562921	0.990988839
Rsph14	-0.210415939	0.222028919	-0.947696091	0.343284192	0.920191602
Gnaz	-0.501052537	0.65299973	-0.76730895	0.442897849	0.948476747
Rab36	0.14094021	0.327636255	0.430172813	0.667069937	0.981863224
Bcr	-0.220466852	0.203218157	-1.084877725	0.277975815	0.901315669
Specc1l	0.002781919	0.186715524	0.014899236	0.98811257	0.998148029
Adora2a	-0.380568558	0.546616848	-0.696225444	0.486287656	0.957736773
Upb1	0.330923318	0.502871095	0.658067886	0.51049451	0.963452765
Gucd1	0.116536664	0.098204171	1.186677328	0.235354921	0.896002526
Snrpd3	0.408649832	0.124271353	3.288367137	0.001007703	0.240510037
Lrrc75b	0.790507869	0.552366834	1.431128409	0.152393417	0.852974739
Ggt1	0.251230329	0.774943966	0.324191606	0.745792977	0.988585656
Ggt5	0.257971065	0.292055981	0.883293209	0.377077872	0.928883222
Susd2	0.030089893	0.610881878	0.049256484	0.960714898	0.996474415
Cabin1	-0.203102535	0.226829313	-0.895398095	0.370574319	0.928436166
Ddt	0.074004779	0.185061041	0.399893884	0.689234677	0.98547461
Gstt3	0.082519147	0.168764626	0.488959971	0.624870036	0.976148792
Gstt1	0.090507996	0.115282279	0.785098945	0.432395558	0.9459793
Gstt2	0.220673938	0.23122537	0.954367326	0.339897739	0.919246575
Mif	-0.167996109	0.129551575	-1.296750811	0.19471694	0.879588734
Derl3	0.587732392	0.73360253	0.801159167	0.423039507	0.943509323
Smarcb1	-0.091400935	0.146641226	-0.623296311	0.533089822	0.96674935

Mmp11	-0.186768587	0.273633882	-0.6825492	0.494891744	0.959272191
Chchd10	-0.205912342	0.110414143	-1.864909115	0.062194145	0.726656275
Gm867	-0.079801093	0.150530101	-0.530133791	0.596019172	0.974001549
Vpreb3	-0.263532825	0.20494351	-1.285880315	0.198484835	0.880661448
Gm5134	1.851429057	2.413521401	0.767106957	0.443017926	0.948476747
Zfp280b	-0.059458939	0.305439946	-0.194666546	0.845654003	0.992886758
Prmt2	0.05814282	0.127609373	0.455631268	0.648655155	0.979004493
S100b	-0.309377506	0.347902286	-0.889265516	0.3738604	0.928840865
Dip2a	0.029568028	0.25388007	0.116464548	0.907284381	0.994688009
Pcnt	0.259581363	0.222662044	1.165808767	0.243691772	0.896939594
2610028H24Rik	-0.25029962	0.211312733	-1.184498523	0.236215781	0.896358698
Ybey	-0.075856174	0.313191615	-0.24220372	0.808622312	0.991020605
Mcm3ap	0.13206534	0.219866989	0.600660158	0.548066361	0.968738549
Lss	-0.143395939	0.232815184	-0.615921766	0.537946155	0.96674935
Ftcd	0.502227861	1.204702117	0.416889664	0.676759114	0.983549699
Col6a2	-0.231298233	0.19345537	-1.195615473	0.23184665	0.895404344
Col6a1	-0.055736585	0.151180094	-0.368676745	0.712368684	0.988585656
Pcbp3	0.035061069	0.19132984	0.183249349	0.854602375	0.992886758
Slc19a1	-0.110279944	0.326083447	-0.338195469	0.73521589	0.988585656
Col18a1	-0.226582817	0.241392604	-0.938648545	0.34791122	0.921552698
Gm10941	1.552417023	0.66714946	2.326940387	0.019968438	0.595248411
Pofut2	-0.044609222	0.136467563	-0.32688517	0.743754738	0.988585656
Adarb1	0.187516253	0.214096331	0.875849912	0.381111632	0.930523346
Gm17769	-0.041363624	1.470049555	-0.028137571	0.977552429	0.997262526
Fam207a	0.102733691	0.156186866	0.657761398	0.510691462	0.963452765
Itgb2	-0.008195723	0.184605596	-0.044395854	0.964588866	0.996474415
Pttg1ip	-0.20011647	0.081288201	-2.461814471	0.013823615	0.545277966
Sumo3	0.032133763	0.120124051	0.267504823	0.789080502	0.990988839
Ube2g2	0.087859967	0.141428558	0.62123215	0.534446889	0.96674935
Tspear	-1.414981494	1.845634715	-0.766663892	0.443281377	0.948476747
1700009J07Rik	0.174958598	1.954943219	0.089495488	0.928688139	0.994688009
Lrrc3	0.393735216	1.16939983	0.33669854	0.736344164	0.988585656
Trpm2	0.414233105	0.7344856	0.563977164	0.572769674	0.971613657
1810043G02Rik	0.106010666	0.162886045	0.650827186	0.515158049	0.964352198
Pfkl	0.009488886	0.17139277	0.055363395	0.955848958	0.99614946
Dnmt3l	0.662644085	0.670844363	0.987776184	0.323262274	0.91640698
Icosl	-0.159302819	0.23408899	-0.680522475	0.496173696	0.960178728
D10Jhu81e	0.041304297	0.093638649	0.441103089	0.659138367	0.979924124
Pwp2	-0.407857334	0.235756112	-1.729996863	0.083630836	0.76099108
Trappc10	-0.132852168	0.122984401	-1.080235925	0.280037135	0.903192609
Agpat3	-0.040919184	0.171998197	-0.237904727	0.811954989	0.991829202
Rrp1	0.01687654	0.170807477	0.098804456	0.921293531	0.994688009
Cstb	0.067729659	0.190356277	0.355804705	0.721986825	0.988585656
Pdxk	-0.114058565	0.245083684	-0.465386203	0.641654913	0.97813956
Ilvbl	-0.027203521	0.126958181	-0.214271512	0.830335332	0.992886758
Syde1	-0.026617279	0.23486386	-0.11333067	0.909768402	0.994688009
Slc1a6	1.524910261	1.607485373	0.948630878	0.342808381	0.919850248
2610008E11Rik	-0.024949826	0.134101036	-0.186052448	0.852403632	0.992886758
Ppap2c	-0.010887175	0.166437254	-0.06541309	0.947845102	0.995507207
Mier2	-0.141603118	0.287498891	-0.492534486	0.622341549	0.97514112
Shc2	-0.284608272	0.558522966	-0.509573086	0.610350583	0.974001549
Tpgs1	0.224749628	0.134463752	1.671451408	0.094632549	0.777748075
Cdc34	0.23672828	0.188214715	1.257756495	0.208479834	0.884258007
Gzmm	-0.148626174	0.36065514	-0.41210053	0.680265741	0.984207811
Bsg	0.009110704	0.100671985	0.090498902	0.927890766	0.994688009
Hcn2	-0.034376203	0.719348604	-0.047787961	0.961885231	0.996474415
Polrmt	-0.465132774	0.317137474	-1.466659768	0.142468632	0.840741274
Rnf126	-0.032286626	0.187118602	-0.17254632	0.86300805	0.992886758
Fstl3	0.534864974	0.385097279	1.388908733	0.164860505	0.859982992
Prss57	-0.541297061	1.936564454	-0.279514095	0.779850323	0.990988839
Palm	0.121218387	0.208978599	0.580051676	0.56187977	0.969658675
Misp	0.027476304	0.228679646	0.120151943	0.904362786	0.994688009

E130317F20Rik	0.750156034	0.583304685	1.286044932	0.198427381	0.880661448
Ptbp1	-0.154698538	0.116971102	-1.322536374	0.185989605	0.872305715
Lppr3	0.68193392	0.599882676	1.136778819	0.25563076	0.897044747
Prtn3	-0.631867742	0.886561732	-0.71271714	0.476020808	0.95716557
Cfd	-0.354972056	0.359978707	-0.986091812	0.324088061	0.91640698
Med16	-0.133051223	0.235149422	-0.565815649	0.571519108	0.971613657
R3hdm4	0.160284034	0.135047756	1.18686929	0.235279183	0.896002526
Kiss1r	-1.043284435	0.665796034	-1.566973039	0.117120994	0.812679331
Arid3a	0.741951048	0.326087047	2.275315914	0.022886985	0.620006604
Wdr18	-0.021935082	0.122267007	-0.179403115	0.857621185	0.992886758
Grin3b	0.773183099	1.397744445	0.553164852	0.580150532	0.971613657
Tmem259	0.016935852	0.10922881	0.155049313	0.876782441	0.992886758
Cnn2	-0.037843059	0.136861519	-0.276506203	0.782159296	0.990988839
Abca7	0.252955314	0.174979179	1.445630933	0.148280746	0.847956033
Hmha1	0.190357118	0.180276383	1.055918224	0.291005577	0.907334416
Polr2e	0.23293126	0.15286518	1.523769243	0.127566365	0.824941438
Gpx4	0.204165515	0.278261421	0.733718367	0.463120402	0.953083512
Sbno2	-0.279734304	0.1914021	-1.461500705	0.143878082	0.841934045
Stk11	0.088364752	0.181162993	0.48776381	0.625717148	0.97639266
Dos	-0.473660356	0.320647185	-1.477201043	0.139621754	0.836535783
Atp5d	-0.00399107	0.097848939	-0.040788074	0.967464847	0.996474415
Midn	0.096442223	0.239956953	0.401914687	0.687746814	0.98538669
Cirbp	0.341875663	0.206236036	1.657691204	0.09737981	0.78359791
1600002K03Rik	0.159264214	0.187624151	0.848847084	0.395966386	0.934066368
Efna2	0.164906592	0.518431238	0.318087684	0.750418425	0.988585656
Mum1	-0.122495879	0.230749569	-0.530860703	0.595515311	0.974001549
Ndufs7	0.239253717	0.115631841	2.069098909	0.038536806	0.679118604
Gamt	-0.160775376	0.284485307	-0.565144742	0.571975318	0.971613657
Dazap1	-0.047693183	0.151008748	-0.315830599	0.752131091	0.988585656
Rps15	0.275227768	0.241165348	1.141240936	0.253769674	0.897044747
Apc2	-0.005004983	0.950135865	-0.00526765	0.995797043	0.999285699
2310011J03Rik	0.311913575	0.133318741	2.339607858	0.019303997	0.592187315
Pcsk4	-0.740071077	0.462238715	-1.601058182	0.109364033	0.798400564
Reep6	-0.093107021	0.27836704	-0.334475738	0.738020596	0.988585656
Adamtsl5	-0.213629786	0.18616481	-1.147530437	0.251162461	0.896939594
Plk5	-0.767316403	0.453196876	-1.693119355	0.090432751	0.76980997
Mex3d	-0.17508472	0.221323385	-0.79108098	0.428896738	0.945321484
Mbd3	0.201749392	0.129843109	1.553793595	0.120233659	0.81558552
Uqcr11	0.516502756	0.251302693	2.055301319	0.03984991	0.679118604
Tcf3	0.113942683	0.15787211	0.72174042	0.470454086	0.95486913
Rexo1	-0.116689784	0.278279047	-0.419326519	0.674977519	0.983549699
Klf16	-0.136574345	0.21706706	-0.629180425	0.529230943	0.965637248
Abhd17a	0.048331725	0.100827155	0.479352264	0.631688047	0.977034429
Adat3	-0.938975864	0.941491489	-0.997328043	0.318605306	0.91640698
Scamp4	-0.28345794	0.205496443	-1.379381247	0.167777238	0.860602879
Csnk1g2	0.052847151	0.118917486	0.444401854	0.656752073	0.979614766
Btbd2	-0.149807196	0.171414099	-0.873949091	0.382145979	0.930767593
Mknk2	-0.163523019	0.202315145	-0.808258914	0.418941549	0.943415794
Mob3a	-0.191699966	0.145201203	-1.320236764	0.186755981	0.872686005
Izumo4	-0.000931232	0.173953966	-0.005353323	0.995728687	0.999285699
Ap3d1	-0.086510179	0.166746945	-0.518811179	0.603892422	0.974001549
Dot1l	0.024540154	0.370369619	0.06625855	0.947171983	0.995268179
Plekhj1	0.344743291	0.155874215	2.211676188	0.026989047	0.634942608
Sf3a2	0.008216167	0.348232325	0.023593925	0.981176518	0.997364526
Lsm7	0.550535671	0.265371051	2.074588277	0.038024709	0.679118604
Sppl2b	0.09918979	0.230725264	0.42990433	0.667265235	0.981863224
Timm13	0.366522317	0.170693035	2.147259939	0.031772593	0.661395937
Lmnb2	-0.077322496	0.274218879	-0.28197364	0.777963723	0.990988839
Gadd45b	0.217779229	0.115133132	1.891542647	0.058551944	0.72285319
Gng7	0.47661011	0.673007691	0.708179292	0.478833924	0.957463006
Slc39a3	0.226292748	0.129802514	1.743361828	0.081270432	0.760402486
Sgta	0.045384988	0.140673693	0.322625978	0.746978518	0.988585656

Thop1	0.020163973	0.231195461	0.08721613	0.930499718	0.994688009
Map2k2	-0.2334285	0.188064561	-1.241214715	0.21452644	0.886400046
Zbtb7a	0.045388283	0.307218441	0.147739449	0.882548397	0.992886758
Pias4	-0.256442408	0.264275319	-0.970360794	0.331866684	0.917115058
Eef2	0.125462805	0.130538014	0.961120833	0.336491416	0.919242242
Dapk3	0.145572716	0.128120806	1.136214487	0.255866809	0.897044747
Atcayos	-0.583049792	1.215452787	-0.479697606	0.63144243	0.977034429
Nmrk2	0.83950235	1.523208846	0.551140674	0.581537245	0.971915898
Zfr2	0.777145206	0.545185639	1.42546896	0.154021686	0.854159937
Matk	-0.348254141	0.591272321	-0.58899111	0.555867237	0.968926429
Mrpl54	0.304691325	0.198118408	1.537925372	0.124066863	0.820429191
Apba3	-0.288804572	0.191866001	-1.505241002	0.132262127	0.827800491
Tjp3	-0.048886101	0.220437962	-0.221768067	0.82449444	0.992886758
Pip5k1c	0.219549315	0.201004798	1.092259075	0.274719243	0.900302631
Cactin	0.100099568	0.317754384	0.315021831	0.752745078	0.988585656
Tbxa2r	-0.48874161	0.363404428	-1.344897232	0.178658424	0.867377413
Gipc3	-0.140159549	0.749774918	-0.1869355	0.851711205	0.992886758
Hmg20b	-0.028599514	0.124669632	-0.229402412	0.818556162	0.992734473
Mfsd12	0.285965662	0.268216476	1.066174853	0.286344608	0.905916508
4930404N11Rik	0.127786558	0.178058417	0.717666484	0.472962953	0.955346547
Fzr1	-0.032673085	0.21411213	-0.152598011	0.878715294	0.992886758
Dohh	0.299611303	0.143204359	2.09219402	0.036421163	0.677009445
Smim24	0.09348524	0.369604418	0.252933232	0.800319808	0.990988839
Nfic	-0.013225378	0.237996447	-0.055569645	0.955684647	0.99614946
Celf5	5.362527465	1.700511241	3.153479574	0.001613365	0.284736028
Ncln	-0.084158432	0.160427002	-0.524590192	0.599868107	0.974001549
S1pr4	0.075706817	0.180069064	0.42043211	0.674169815	0.983375962
Gna15	-0.199234741	0.329594165	-0.604485035	0.545521215	0.967491297
Gna11	-0.013278435	0.096459287	-0.13765844	0.890510367	0.993043931
Aes	0.077235534	0.129861658	0.594752408	0.552008984	0.968926429
Tle2	-0.426239557	0.23740005	-1.795448475	0.072582275	0.747194706
Tle6	0.040910695	0.286891028	0.142600119	0.886606003	0.992886758
BC025920	0.670901709	0.671770919	0.998706091	0.317937089	0.91640698
Sirt6	0.367918999	0.336145525	1.094522972	0.273725678	0.899400109
Ankrd24	0.550861753	0.257943942	2.135587087	0.032713085	0.665167506
Gm10778	-0.29098601	0.471033147	-0.617761217	0.536732752	0.96674935
Zfp433	0.392952541	0.246537011	1.593888644	0.110960978	0.800111791
Zfp781	-0.025337618	0.258230817	-0.098120041	0.921836974	0.994688009
Zfp873	-0.028547005	0.226664683	-0.125943772	0.899776434	0.994492948
AU041133	0.276333742	0.263875441	1.04721281	0.295001439	0.90740175
Zfp938	0.401152295	0.174205078	2.302758909	0.021292412	0.60743619
1190007I07Rik	0.229119607	0.208683293	1.097929805	0.272235142	0.898370293
Glt8d2	-0.03638437	0.176790812	-0.205804642	0.836943515	0.992886758
Hcfc2	-0.382669734	0.199294766	-1.920119331	0.054842828	0.71121434
Nfyb	0.046523281	0.119181447	0.390356737	0.696272774	0.987490725
Txnrd1	-0.124839924	0.120273969	-1.037962952	0.299287303	0.910105802
Chst11	-0.126944526	0.300033583	-0.423101055	0.672221533	0.983233856
Slc41a2	0.191178296	0.26260745	0.728000275	0.466613418	0.953083512
D10Wsu102e	0.133615447	0.190071394	0.702975046	0.482071301	0.957463006
Aldh1l2	1.062089443	0.871190817	1.219123782	0.222797215	0.892391445
A230046K03Rik	-0.202702549	0.148591839	-1.364156676	0.17251826	0.86244803
Appl2	-0.271498899	0.116486777	-2.330727191	0.019767749	0.594379457
1500009L16Rik	-0.10994102	0.356144908	-0.308697437	0.757551694	0.989266039
Nuak1	-0.01157788	0.331535405	-0.034922003	0.972141935	0.996750504
Ckap4	-0.370120794	0.213391239	-1.734470427	0.082834649	0.76099108
Platr7	-1.193700173	1.932266267	-0.617772091	0.536725583	0.96674935
Tcp11l2	-0.04666163	0.163587326	-0.285239882	0.775460367	0.990861469
Polr3b	-0.092074294	0.232124345	-0.396659358	0.691618674	0.986454135
Ric8b	-0.364132092	0.156194057	-2.331280074	0.019738596	0.594379457
Fhl4	0.927271976	0.768982857	1.205842195	0.227878342	0.894410876
Tmem263	-0.073106347	0.136708438	-0.534761042	0.592815106	0.974001549
Mterf2	0.325043357	0.256054661	1.269429569	0.204287896	0.883765092

Cry1	-0.221391169	0.20680703	-1.07052052	0.284385077	0.905916508
Btbd11	0.533898376	0.842425854	0.633763048	0.526235472	0.965637248
Pwp1	-0.080372332	0.158168598	-0.508143419	0.611352767	0.974001549
Prdm4	-0.133256644	0.194896862	-0.68372904	0.494146282	0.958916246
Ascl4	-0.823206448	1.590852463	-0.517462472	0.604833359	0.974001549
Rtcb	-0.226012161	0.16057207	-1.407543421	0.159266311	0.856502327
Fbxo7	-0.113363471	0.178563807	-0.634862535	0.525518074	0.965637248
Syn3	1.019426953	0.690942748	1.475414506	0.140101138	0.837580108
Timp3	-0.2116051	0.120196523	-1.760492685	0.078324307	0.754732183
1810014B01Rik	0.093271673	0.253503025	0.367931204	0.712924533	0.988585656
Hsp90b1	-0.161643954	0.118440988	-1.364763637	0.172327351	0.86244803
Ttc41	0.241444519	0.409243873	0.589977114	0.555205993	0.968926429
Nt5dc3	-0.199402558	0.323967151	-0.615502398	0.538222986	0.96674935
Stab2	-0.145800379	1.05009991	-0.138844293	0.889573192	0.993043931
Ascl1	1.578527992	1.120006818	1.409391413	0.158719462	0.855986074
Igf1	-0.45425474	0.308556535	-1.472192903	0.140968793	0.839692626
Parpbp	-1.371518888	0.823639312	-1.665193573	0.095874143	0.77993878
Nup37	0.199459641	0.189650677	1.051721218	0.292927473	0.907334416
Ccdc53	0.149155839	0.154039995	0.968292933	0.332898093	0.917115058
Dram1	-0.097050939	0.090537873	-1.071937473	0.283748117	0.905916508
Gnptab	-0.11247838	0.138876348	-0.809917471	0.41798761	0.943415794
Sycp3	-0.166909613	0.252747489	-0.660380896	0.509009429	0.963452765
Chpt1	-0.013000652	0.16060439	-0.080948295	0.935483072	0.994688009
Spic	-0.17033006	1.384822422	-0.122997763	0.90210887	0.994688009
Gm4925	1.019475949	0.775249613	1.31502929	0.188500059	0.874826736
Arl1	0.216243203	0.165221602	1.308807081	0.190599708	0.877833971
Utp20	0.190649186	0.287864515	0.662287904	0.507786728	0.963106419
Slc5a8	-0.552953344	0.418043123	-1.322718432	0.185929032	0.872305715
Gas2l3	-0.216944514	0.82597483	-0.262652693	0.792818282	0.990988839
Nr1h4	0.162074377	1.336838456	0.121237069	0.903503264	0.994688009
Scyl2	-0.531173789	0.193732403	-2.741791159	0.006110518	0.427133716
1500026H17Rik	0.466697987	0.424017482	1.100657417	0.271045786	0.89804647
Actr6	0.347760237	0.193553889	1.796710151	0.072381647	0.74633396
Uhrf1bp1l	-0.011797697	0.161631622	-0.072991265	0.941813068	0.994688009
Anks1b	-0.506441233	1.363854498	-0.371330837	0.710391132	0.988585656
Apaf1	0.119930814	0.185490124	0.646561722	0.517915638	0.964437658
Ikbip	0.108234417	0.10776176	1.004386126	0.315192535	0.91586731
Slc25a3	-0.162620313	0.090021512	-1.806460581	0.070846426	0.744227652
Tmpo	0.052746975	0.083146994	0.634382229	0.525831405	0.965637248
Nedd1	0.07122079	0.164772423	0.43223732	0.665568938	0.981727712
Cdk17	0.016651114	0.123511318	0.134814479	0.892758558	0.993300147
Elk3	-0.160957884	0.174125414	-0.924379049	0.355288999	0.924354588
Gm17745	0.734713677	0.698483486	1.051869789	0.292859294	0.907334416
Mir7688	1.226471192	1.167622235	1.050400683	0.293533932	0.907334416
Lta4h	-0.263700323	0.191966145	-1.373681402	0.169540613	0.861597949
Hal	1.214598594	0.878199361	1.383055658	0.166647801	0.860403806
Amdhd1	-0.250441685	0.857146371	-0.292180768	0.770148415	0.989990452
Ccdc38	1.471194491	1.101144683	1.336059206	0.181529922	0.868446884
Ntn4	-0.154218315	0.18657144	-0.826591221	0.408468795	0.940123943
Gm15915	-1.192234348	1.32048334	-0.902877236	0.366591053	0.927866786
Usp44	1.247849737	0.899937437	1.386596096	0.165564959	0.860011234
Metap2	-0.054062818	0.130870283	-0.413102325	0.679531646	0.983903958
Vezt	0.043487265	0.127613996	0.340771908	0.7332753	0.988585656
Fgd6	-0.680896702	0.256166189	-2.658027213	0.007859955	0.45506991
Nr2c1	-0.325319547	0.255973527	-1.27091091	0.203760345	0.88349335
Ndufa12	0.491559585	0.140078405	3.509174622	0.0004495	0.141134255
Tmcc3	-0.113193446	0.1528963	-0.74032822	0.459100861	0.952683168
Cep83os	0.143021562	0.240647053	0.594320851	0.552297535	0.968926429
Cep83	-0.091181657	0.126473751	-0.72095321	0.470938304	0.95486913
Plxnc1	-0.000917028	0.183769419	-0.004990102	0.996018491	0.999323659
Gm29684	0.612772313	1.382299322	0.443299293	0.657549267	0.979614766
Cradd	0.304040791	0.179410076	1.694669542	0.090138127	0.769347579

2310039L15Rik	0.754912482	1.00147112	0.753803546	0.450967194	0.949134998
Socs2	-0.382781042	0.13586986	-2.817262355	0.004843494	0.395398661
5730420D15Rik	0.9450915	1.322138088	0.714820569	0.474719924	0.956387791
Mrpl42	0.009177281	0.161629181	0.056779854	0.954720562	0.996070133
Ube2n	0.043092551	0.111106285	0.387849808	0.698127184	0.987722923
Nudt4	-0.155433247	0.082000899	-1.895506623	0.058025315	0.72285319
Eea1	-0.112294028	0.208734627	-0.537975083	0.590594251	0.974001549
Btg1	-0.157885838	0.097822124	-1.614009506	0.106525364	0.794899279
Dcn	-0.119598176	0.142415037	-0.839786147	0.401028302	0.937512756
Lum	-0.171366417	0.266517791	-0.64298303	0.520235111	0.964665379
Atp2b1	-0.096694589	0.135997855	-0.711000839	0.477083716	0.95716557
Poc1b	0.04504676	0.26598149	0.169360508	0.865513084	0.992886758
Galnt4	0.002641442	0.179037327	0.014753583	0.988228771	0.998148029
Dusp6	-0.120180968	0.163857496	-0.733448093	0.463285176	0.953083512
Gm20110	-0.087792783	1.604222662	-0.054726058	0.956356709	0.996173928
Kitl	-0.060019258	0.103643877	-0.57909121	0.562527631	0.969658675
Tmtc3	-0.376878885	0.265087743	-1.421713734	0.155109371	0.855261441
Cep290	0.029401083	0.300340119	0.097892627	0.922017555	0.994688009
4930430F08Rik	0.193131292	0.261489962	0.738580135	0.46016199	0.953083512
Nts	-0.341853776	0.503000531	-0.679629056	0.496739367	0.960362776
Rassf9	-0.220837934	0.176459042	-1.251496844	0.210753264	0.884340413
Lrriq1	-0.617027318	0.346149823	-1.782544084	0.074660549	0.75241101
Slc6a15	-0.53332935	0.291984687	-1.826566166	0.067765024	0.742385562
Tmtc2	-0.079352463	0.148873261	-0.533020251	0.594019562	0.974001549
Gm15663	0.132315668	0.433848357	0.304981375	0.760380337	0.989266039
Mettl25	0.18566814	0.187906687	0.988086927	0.323110078	0.91640698
Ccdc59	-0.016246551	0.107864903	-0.150619442	0.880275924	0.992886758
Ppfia2	0.865154655	1.456687964	0.593918997	0.552566292	0.968926429
Acss3	0.060214116	0.277792796	0.216759097	0.82839609	0.992886758
Lin7a	-1.182330929	1.891078962	-0.625214997	0.53182996	0.966606934
Ptprq	0.985085335	1.078631465	0.913273316	0.361098813	0.926251807
Ppp1r12a	-0.396759152	0.227043824	-1.747500307	0.080550592	0.758011246
Pawr	-0.140949923	0.146912865	-0.959411706	0.33735138	0.919246575
Syt1	1.336364751	1.854655649	0.720546022	0.471188874	0.95486913
E2f7	0.724424364	1.397236407	0.518469431	0.604130783	0.974001549
Csrp2	0.156981613	0.18125055	0.866102823	0.386433777	0.932501372
Zdhhc17	0.12427667	0.191915047	0.647560848	0.517269027	0.964352198
Osbpl8	0.012965318	0.147017318	0.08818905	0.92972642	0.994688009
Bbs10	0.451159163	0.283860695	1.589368205	0.111977288	0.801067615
Nap1l1	0.226155201	0.12639785	1.789232972	0.073577304	0.748833631
Phlda1	-0.323524274	0.129285243	-2.502406814	0.012335209	0.525888996
4933440J02Rik	0.419486696	1.727010084	0.242897653	0.808084684	0.99099448
Krr1	-0.135562079	0.158759355	-0.853884036	0.393169245	0.933734231
Glipr1	0.05900574	0.19807992	0.29788855	0.765788227	0.989884648
Caps2	-0.165875113	0.339663408	-0.488351437	0.625300934	0.976218048
Kcnc2	0.686364396	1.457577802	0.470893831	0.637716553	0.977468848
Atxn7l3b	-0.195113147	0.108359535	-1.800608941	0.071764539	0.744227652
Tbc1d15	-0.098447459	0.13467969	-0.730974798	0.464794546	0.953083512
Rab21	-0.11902018	0.111479543	-1.067641446	0.285682281	0.905916508
Tmem19	-0.086237506	0.128124014	-0.673078396	0.500897418	0.961175273
Thap2	-0.173004544	0.227518913	-0.760396316	0.447017725	0.948476747
Zfc3h1	-0.107375211	0.168965609	-0.635485598	0.525111756	0.965637248
Lgr5	-0.751183748	2.167233887	-0.346609451	0.728884745	0.988585656
Tspan8	0.090507337	0.102862459	0.879886971	0.378920544	0.929137643
Ptprr	0.464797119	0.326598397	1.423145745	0.15469391	0.854973821
Ptprb	-0.043122394	0.127213947	-0.338975363	0.73462829	0.988585656
Kcnmb4	0.795488068	1.536609234	0.517690543	0.604674198	0.974001549
Kcnmb4os2	1.437396658	0.875859726	1.641126559	0.100771147	0.788783372
Cnot2	-0.157261159	0.16766656	-0.937939915	0.348275289	0.921552698
Rab3ip	-0.021797878	0.149687978	-0.145622105	0.884219717	0.992886758
4933412E12Rik	-0.087257722	0.259127404	-0.336736756	0.736315352	0.988585656
D630029K05Rik	-0.114133774	0.479965333	-0.237795869	0.812039422	0.991836551

Best3	0.019122697	0.853066919	0.02241641	0.982115791	0.997390438
Lrrc10	0.931828964	1.09769183	0.848898514	0.395937766	0.934066368
Cct2	0.018101546	0.083883941	0.215792745	0.829149304	0.992886758
Frs2	-0.060347032	0.17486075	-0.345114795	0.730008071	0.988585656
Yeats4	0.149142393	0.141211994	1.056159532	0.290895336	0.907334416
9530003J23Rik	-0.163784027	0.583841222	-0.280528372	0.779072162	0.990988839
Lyz2	0.139381894	0.250089802	0.557327381	0.577303771	0.971613657
Lyz1	0.710963247	0.509985345	1.394085642	0.163291744	0.859982992
Cpsf6	-0.188428387	0.133946156	-1.406747254	0.159502348	0.856630464
Cpm	-0.238697601	0.156428878	-1.525917743	0.127030351	0.824450458
Mdm2	-0.039290936	0.114138244	-0.344239887	0.730665888	0.988585656
Slc35e3	-0.10849061	0.169222899	-0.641110691	0.521450769	0.964946923
Nup107	-0.203618875	0.276778555	-0.735674318	0.461928923	0.953083512
Rap1b	0.251426951	0.172318582	1.459082054	0.144542525	0.841977044
4933411E08Rik	0.306031894	0.997217637	0.306885762	0.758930327	0.989266039
Mdm1	-0.376466569	0.137277341	-2.742379523	0.006099582	0.427133716
Ifng	1.121358559	0.92543774	1.211706105	0.225624901	0.89336109
Tmevpg1	0.493550134	1.678576498	0.294028979	0.76873576	0.989990452
Dyrk2	0.094487723	0.282150888	0.334883664	0.737712845	0.988585656
Gm38403	-0.247902633	1.317185978	-0.188206249	0.850714974	0.992886758
Cand1	-0.230104814	0.134823742	-1.70670841	0.087876263	0.7667678
Grip1	-0.003892427	0.387890768	-0.010034854	0.991993479	0.998783765
Helb	0.613331311	0.307148007	1.996859157	0.045840485	0.696869274
Irak3	-0.334645371	0.310053523	-1.07931485	0.280447395	0.903192609
Tmbim4	0.049045	0.106897856	0.458802462	0.646376026	0.979004493
Llph	0.296612982	0.151842012	1.953431583	0.050768477	0.707153766
Hmga2	1.802190312	1.072376271	1.680557806	0.092848837	0.77525594
4921513I03Rik	1.971679	2.181232309	0.903928936	0.366033085	0.927408779
Msrb3	-0.058803936	0.118505435	-0.496212987	0.619744149	0.974471036
Gm15910	-0.581355544	1.486095125	-0.391196724	0.69565183	0.98715859
Lemd3	-0.210543834	0.208765977	-1.008516028	0.313206797	0.915126588
Wif1	-0.145661675	0.238592916	-0.610502931	0.541528702	0.967239812
Tbc1d30	-0.064333012	0.281209982	-0.228772149	0.819046014	0.992886758
Gns	-0.099756278	0.183851343	-0.542592055	0.587410711	0.973455044
Rassf3	-0.172258907	0.093625394	-1.839873778	0.065786771	0.738213474
Tbk1	0.094300499	0.102415546	0.920763522	0.357173904	0.924354588
Xpot	-0.23570501	0.104994802	-2.244920753	0.02477322	0.627796923
D930020B18Rik	-0.384550497	0.547080444	-0.702913988	0.482109353	0.957463006
BC048403	-0.03180302	0.28472277	-0.111698197	0.911062709	0.994688009
Srgap1	-0.169839871	0.63837923	-0.266048554	0.790201816	0.990988839
Gm9079	0.181899525	0.591003062	0.30778102	0.758248965	0.989266039
Tmem5	0.119130808	0.142330903	0.836998894	0.402593189	0.937834293
Avpr1a	0.056781403	0.5314703	0.106838337	0.914917233	0.994688009
A130077B15Rik	0.81130445	0.858380029	0.94515765	0.344578397	0.921226705
Ppm1h	0.02433627	0.20467907	0.118899651	0.905354858	0.994688009
Mirlet7i	-1.283480612	2.386610842	-0.537783785	0.590726328	0.974001549
Mon2	-0.113781819	0.179331411	-0.634477911	0.525768978	0.965637248
Usp15	-0.10905906	0.177406816	-0.614739964	0.538726463	0.966875138
Fam19a2	0.267218001	1.527276164	0.174963773	0.861108099	0.992886758
Slc16a7	-0.193509603	0.225879025	-0.85669576	0.391613047	0.933734231
Lrig3	0.503333931	0.494037249	1.018817777	0.308289485	0.9139204
Xrcc6bp1	0.090862627	0.252064	0.360474431	0.718492375	0.988585656
Ctdsp2	-0.185740448	0.180672889	-1.028048256	0.303927129	0.911786415
Avil	1.087686263	0.953231967	1.141050972	0.253848712	0.897044747
Tsfm	0.102100523	0.184614864	0.553046058	0.580231872	0.971613657
Mettl1	0.060535296	0.151169974	0.400445234	0.688828613	0.98538669
Mar-09	-0.543739182	0.337622148	-1.610496189	0.107289577	0.796597053
Cdk4	-0.038325957	0.087641022	-0.437306139	0.661889344	0.980647283
Tspan31	0.030588731	0.125499756	0.243735379	0.807435771	0.99099448
LOC100504703	-0.06000469	0.340567052	-0.176190533	0.860144258	0.992886758
Agap2	0.484697826	0.876240076	0.553156423	0.580156303	0.971613657
Os9	-0.050878822	0.176542165	-0.288196432	0.77319638	0.990730604

B4galnt1	-0.111452687	0.190361184	-0.585480113	0.558224935	0.968926429
Slc26a10	0.439230433	0.667507	0.65801622	0.510527709	0.963452765
Arhgef25	0.082361212	0.166351968	0.495102117	0.62052804	0.975013118
Dtx3	-0.22270576	0.16141862	-1.379678259	0.16768573	0.860403806
F420014N23Rik	-0.234577355	0.966530476	-0.242700423	0.80823748	0.991020605
Pip4k2c	-0.042890764	0.19752617	-0.217139653	0.828099512	0.992886758
Kif5a	-0.513309849	0.864335247	-0.593878187	0.55259359	0.968926429
Dctn2	0.031006474	0.093431975	0.331861482	0.739993856	0.988585656
Mbd6	-0.443885951	0.308869387	-1.437131585	0.150680608	0.850090631
Ddit3	0.018159152	0.13658128	0.132954911	0.894229039	0.993422442
Mars	0.01446051	0.138267096	0.104583885	0.916706003	0.994688009
Arhgap9	0.256477518	0.175678553	1.459925033	0.144310678	0.841934045
Gli1	-0.385781485	0.519370857	-0.742786162	0.457611155	0.952204734
R3hdm2	-0.041848324	0.222375162	-0.188187942	0.850729325	0.992886758
Stac3	0.67159555	1.115530495	0.602041408	0.547146574	0.968115622
Ndufa4l2	-0.005821366	0.226728778	-0.025675461	0.979516196	0.997262526
Shmt2	0.063834513	0.225558671	0.283006247	0.777172048	0.990861469
Lrp1	0.048396286	0.220693676	0.219291675	0.826422847	0.992886758
Stat6	-0.329795175	0.178431358	-1.848302777	0.064558554	0.73299549
Nab2	-0.025727798	0.266230048	-0.096637468	0.923014301	0.994688009
1700012D01Rik	0.553004881	0.368463469	1.500840456	0.133396832	0.827800491
Tmem194	-0.174906903	0.274977307	-0.636077592	0.52472585	0.965637248
Myo1a	0.978255655	1.967818116	0.497127071	0.619099446	0.974471036
Zbtb39	0.022826155	0.26592226	0.0858377	0.931595437	0.994688009
Gpr182	-0.24279804	0.219970709	-1.103774414	0.269691006	0.89804647
Rdh9	-0.545782079	0.648245623	-0.841937161	0.399823128	0.936395796
BC089597	0.870561363	1.681110737	0.51784891	0.60456369	0.974001549
Sdr9c7	0.032457072	2.095239109	0.015490868	0.98764057	0.998148029
Prim1	0.698464499	0.219543469	3.181440574	0.001465446	0.278213188
Naca	0.194542687	0.177007777	1.099062937	0.271740615	0.89804647
Ptges3	-0.029149678	0.148296774	-0.196563131	0.844169425	0.992886758
Atp5b	-0.030214495	0.083958692	-0.359873337	0.718941857	0.988585656
Baz2a	0.071053482	0.167326046	0.424640893	0.671098478	0.982677283
Rbms2	-0.012964805	0.098036886	-0.132244152	0.894791179	0.993556555
Gls2	-0.032741579	0.687697162	-0.047610462	0.962026694	0.996474415
Spryd4	0.144917936	0.173796999	0.833834513	0.404374244	0.938127315
Timeless	0.036484553	0.167225578	0.218175674	0.827292238	0.992886758
Apon	3.621441221	3.318957615	1.091138135	0.275212105	0.900302631
Apof	0.753458828	4.733791417	0.159166039	0.87353806	0.992886758
Stat2	0.195969316	0.164264955	1.193007461	0.232866451	0.89581803
Pan2	-0.174646331	0.187130485	-0.93328637	0.350672131	0.922338606
Cnpy2	0.32002358	0.183635946	1.742706625	0.081384874	0.760884566
Cs	-0.250093557	0.103452666	-2.417468457	0.015628888	0.557315333
Coq10a	0.051706226	0.160035254	0.323092722	0.746625022	0.988585656
Ankrd52	-0.216432344	0.409159317	-0.528968388	0.596827379	0.974001549
Nabp2	0.217477245	0.109387764	1.988131365	0.046797163	0.698868643
Rnf41	0.107892662	0.128992611	0.836425141	0.402915774	0.937834293
Smarcc2	-0.119017094	0.179913589	-0.661523648	0.508276554	0.963324595
Myl6	0.235055719	0.130474367	1.801547116	0.071616688	0.744227652
Myl6b	0.237627124	0.455201502	0.522026231	0.601652067	0.974001549
A430046D13Rik	0.233018537	0.227275274	1.025270072	0.305235773	0.912948451
Esyt1	0.046412086	0.180808113	0.2566925	0.797416162	0.990988839
Zc3h10	-0.058981325	0.223140763	-0.264323398	0.791530732	0.990988839
Rpl41	0.301652988	0.332599368	0.906955988	0.364430072	0.926556975
Pa2g4	-0.009601308	0.117817271	-0.081493214	0.935049721	0.994688009
Erbb3	0.262375059	0.413981147	0.633785042	0.526221116	0.965637248
Rps26	-0.002942339	0.104646297	-0.028116995	0.97756884	0.997262526
Ikzf4	0.090770426	0.84497035	0.107424392	0.914452305	0.994688009
Suox	-0.237997788	0.200546956	-1.186743456	0.235328828	0.896002526
Rab5b	-0.177470632	0.113309835	-1.566242081	0.117291955	0.812881672
Cdk2	-0.257894701	0.183890479	-1.402436398	0.160784971	0.857316691
Pmel	-1.628956895	1.179334962	-1.381250406	0.167201981	0.860403806

Dgka	-0.054054973	0.216668716	-0.249482132	0.802987861	0.990988839
Wibg	0.246168226	0.124362056	1.979448026	0.047765586	0.701952043
Mmp19	-0.544797963	0.329857534	-1.651615946	0.098612867	0.786907305
Tmem198b	0.125829834	0.341518571	0.368442142	0.712543579	0.988585656
Dnajc14	0.155465653	0.100771249	1.54275803	0.122889496	0.817788082
Ormdl2	0.359784683	0.168669015	2.133081072	0.032918076	0.665167506
Sarnp	0.1932433	0.14313896	1.35003985	0.177003201	0.866546104
Gdf11	-0.067769525	0.910986444	-0.074391365	0.94069898	0.994688009
Cd63	0.152894351	0.171695506	0.890497107	0.373199021	0.928840865
Rdh5	-0.009825401	0.529680628	-0.01854967	0.985200354	0.998148029
Bloc1s1	0.329378767	0.169380086	1.944613288	0.051821539	0.707153766
Itga7	-0.184431427	0.221928973	-0.831038081	0.405952117	0.939557159
Mettl7b	-3.420901167	3.935481791	-0.869245838	0.384712681	0.931689378
Mir684-1	-0.538659438	0.791222176	-0.680794162	0.496001745	0.96007684
Tespa1	0.403887271	0.360276778	1.121047193	0.262267775	0.897248195
Sfi1	0.008389967	0.182771986	0.04590401	0.963386758	0.996474415
Eif4enif1	-0.114581592	0.176183633	-0.650353214	0.515464091	0.964352198
Drg1	0.177700719	0.155704619	1.141268127	0.253758361	0.897044747
Patz1	-0.05111075	0.188265271	-0.271482625	0.786019861	0.990988839
Gm11944	-1.127902356	1.128950232	-0.999071814	0.317759904	0.91640698
Pik3ip1	-0.379903957	0.153863122	-2.469103396	0.013545207	0.540715407
Limk2	-0.061298167	0.144587364	-0.423952445	0.671600495	0.982910363
Rnf185	-0.112691385	0.184804071	-0.609788434	0.542001964	0.967491297
8430429K09Rik	-0.074248636	0.322511438	-0.230220163	0.817920695	0.992734473
Inpp5j	-0.149779574	1.067854852	-0.140262109	0.888452901	0.992886758
Selm	0.246993494	0.17537185	1.408398743	0.159013031	0.855986074
Smtn	-0.273950208	0.247520514	-1.106777789	0.268390014	0.89804647
Tug1	-0.162521755	0.162701203	-0.998897071	0.317844555	0.91640698
Morc2a	-0.034172696	0.152838397	-0.223587115	0.823078591	0.992886758
Osbp2	2.06092684	1.364427148	1.510470414	0.130923433	0.827800491
4921536K21Rik	-0.381337434	0.556180178	-0.685636506	0.492942355	0.958916246
Dusp18	-0.124831082	0.211746875	-0.589529749	0.555505962	0.968926429
Slc35e4	-0.065368135	0.161069917	-0.405837021	0.684862355	0.985105247
Tcn2	-0.389664643	0.134606051	-2.89485234	0.003793369	0.376754331
Pes1	-0.035871407	0.117696082	-0.304779963	0.760533743	0.989266039
Gal3st1	-0.423365299	0.212399952	-1.993245736	0.046234545	0.698098452
Sec14l4	-0.114536798	0.145208267	-0.788776012	0.430242932	0.945321484
Sec14l3	-0.2294108	0.115488841	-1.986432602	0.04698531	0.698868643
Mtfp1	-0.028863934	0.265830434	-0.108580245	0.913535431	0.994688009
Sec14l2	-0.307435515	0.259726338	-1.183690178	0.236535728	0.896358698
Rnf215	-0.365456055	0.124988591	-2.923915305	0.003456588	0.368965929
Mir3060	1.084119941	2.036547663	0.532332221	0.59449592	0.974001549
Ccdc157	-0.207165844	0.191814999	-1.080029431	0.280129075	0.903192609
Sf3a1	-0.0538402	0.181703156	-0.296308559	0.76699445	0.989884648
Tbc1d10a	-0.146202143	0.131490866	-1.111880602	0.26618949	0.89768067
Gatsl3	-0.008496199	0.223915394	-0.03794379	0.969732499	0.996628386
Osm	0.646469688	0.69705534	0.927429504	0.353703578	0.92416951
Lif	0.083166617	0.508927046	0.163415597	0.870191223	0.992886758
Mtmr3	-0.169407867	0.144840654	-1.169615453	0.242155756	0.896939594
Gm11961	1.55872287	1.908174178	0.816866137	0.414004931	0.943415794
Ascc2	-0.053854116	0.263551824	-0.20433976	0.838088001	0.992886758
Uqcr10	0.348175825	0.273028415	1.275236593	0.202225513	0.881638438
Zmat5	0.159324106	0.129142059	1.233711985	0.217310246	0.887633697
Nf2	-0.166458004	0.162757064	-1.022739041	0.306431247	0.913641886
Nipsnap1	0.084045833	0.186706138	0.450150346	0.652602037	0.979004493
Thoc5	0.214445429	0.179222947	1.196528861	0.231490243	0.895404344
Ap1b1	-0.068492256	0.233647478	-0.293143572	0.769412413	0.989990452
Gas2l1	-0.305452043	0.203891769	-1.498108751	0.134104998	0.827800491
Rasl10a	0.529043364	0.367091108	1.441177277	0.149534606	0.848779008
Ewsr1	-0.043249909	0.153087702	-0.282517203	0.777546957	0.990988839
Rhbdd3	0.194814964	0.24583859	0.792450703	0.428097925	0.945321484
Emid1	-0.433291727	0.343398077	-1.2617768	0.207029112	0.883765092

Kremen1	-0.16285143	0.172511631	-0.944002615	0.345168312	0.92132235
Znrf3	0.381899957	0.341665401	1.117760114	0.263669461	0.897248195
Xbp1	-0.176615576	0.105935414	-1.667200501	0.095474543	0.778239075
Ccdc117	-0.074565566	0.119530723	-0.623819248	0.532746297	0.96674935
Mrps24	0.27793072	0.184473709	1.506614261	0.13190956	0.827800491
Urgcp	-0.048056804	0.127809069	-0.376004646	0.706913442	0.988585656
2210015D19Rik	-0.11709639	0.217100092	-0.539365919	0.589634392	0.974001549
Dbnl	-0.069764553	0.113302878	-0.615735049	0.538069401	0.96674935
Pgam2	0.313122476	0.274749881	1.139663738	0.25442642	0.897044747
Polm	0.361147238	0.372362024	0.969882035	0.332105296	0.917115058
Aebp1	0.080691748	0.224243691	0.359839543	0.71896713	0.988585656
Pold2	0.094141851	0.225459332	0.417555797	0.676271921	0.983549699
Myl7	0.227074709	0.26707621	0.850224394	0.395200342	0.933933717
Gck	-0.098497677	0.34305652	-0.287117927	0.774022026	0.990730604
Ykt6	0.076103769	0.089973439	0.845847065	0.397638066	0.935327115
Camk2b	0.138635178	0.401155871	0.345589303	0.729651386	0.988585656
Nudcd3	0.040142773	0.124357331	0.322801823	0.746845332	0.988585656
Rps15a-ps6	-0.019759582	0.447091327	-0.044195852	0.964748288	0.996474415
Ddx56	0.000334421	0.13455198	0.002485442	0.998016906	0.999591134
Tmed4	-0.012257437	0.085126134	-0.143991464	0.88550721	0.992886758
Ogdh	-0.311296316	0.166803716	-1.866243296	0.062007334	0.725730283
Zmiz2	0.354409775	0.258381692	1.371652037	0.170171786	0.861924432
Ppia	-0.07080297	0.14536411	-0.487073251	0.626206422	0.976534277
H2afv	0.287971902	0.164693515	1.748532126	0.080371928	0.75763999
Purb	-0.223409681	0.145314852	-1.537418086	0.12419096	0.820917331
Myo1g	0.424309558	0.197315565	2.15041098	0.031522721	0.661395937
Gm11974	0.224759194	0.299567267	0.75027955	0.453086357	0.950108007
Ccm2	-0.000171645	0.182189447	-0.000942125	0.999248293	0.999886097
Nacad	-0.467361955	0.67069825	-0.696828947	0.485909848	0.957710647
Tbrg4	0.055522801	0.184288055	0.301282688	0.763198941	0.989285682
Ramp3	0.263414611	0.392364187	0.671352329	0.501996106	0.961378071
Adcy1	-0.398112568	0.635912907	-0.626048887	0.531282876	0.96621591
Igfbp3	-0.065288269	0.12595341	-0.518352534	0.604212326	0.974001549
Tns3	-0.069986404	0.192211521	-0.364111387	0.715774834	0.988585656
Hus1	-0.158961257	0.218050871	-0.72900996	0.46599557	0.953083512
Gm11992	-0.19835603	0.177135072	-1.119800998	0.262798574	0.897248195
Upp1	0.193342811	0.166137441	1.163752192	0.244524452	0.896939594
Abca13	1.270447957	0.886814177	1.432597707	0.151972837	0.852666844
Vwc2	0.081315486	0.312637101	0.260095447	0.79479015	0.990988839
Zpbp	0.301746389	0.407386464	0.740688304	0.458882453	0.952683168
4930415F15Rik	-0.347501047	2.232343793	-0.155666456	0.876295939	0.992886758
Ikzf1	-0.037615591	0.217327704	-0.173082354	0.862586695	0.992886758
Fignl1	-0.794600536	0.539496624	-1.472855437	0.14079002	0.839671411
Ddc	0.210671266	0.444538507	0.47391005	0.635564053	0.977034429
Grb10	-0.49361771	0.213647678	-2.310428618	0.020864436	0.604325792
Cobl	0.116781815	0.153352458	0.761525548	0.446343225	0.948476747
Vstm2a	-0.163895369	0.351477329	-0.466304241	0.640997743	0.977921835
Sec61g	0.259676576	0.334213883	0.776977226	0.437172207	0.94702277
Egfr	-0.293161292	0.256972729	-1.140826474	0.253942141	0.897044747
Eldr	-0.261819444	0.740042335	-0.353789819	0.723496404	0.988585656
Fbxo48	0.491759485	1.630993318	0.301509197	0.763026238	0.989285682
Plek	0.056499264	0.160925363	0.351089864	0.725520932	0.988585656
Cnrip1	0.099148577	0.177007471	0.560137807	0.575385444	0.971613657
Ppp3r1	-0.117194343	0.092414984	-1.268131402	0.204751029	0.883765092
Pno1	0.036377712	0.144312565	0.252075849	0.800982437	0.990988839
Wdr92	-0.064994942	0.132823427	-0.489333427	0.624605659	0.976017893
C1d	0.280405923	0.202356906	1.385699794	0.165838591	0.860011234
Etaa1	-0.114425778	0.195942418	-0.583976554	0.559236087	0.968926429
Etaa1os	-0.245972948	0.560888391	-0.438541699	0.660993649	0.979924124
Meis1	0.037869629	0.276515327	0.13695309	0.891067874	0.993043931
Spred2	0.004735014	0.353148352	0.013408002	0.989302283	0.998182772
Actr2	-0.161051471	0.096650983	-1.666320045	0.095649686	0.778888992

Rab1	-0.05160353	0.079912624	-0.645749408	0.518441657	0.96446859
Cep68	-0.087575345	0.147891027	-0.592161314	0.553742572	0.968926429
Slc1a4	-0.133960474	0.212358361	-0.630822699	0.52815646	0.965637248
Sertad2	-0.186595099	0.188824505	-0.988193241	0.323058018	0.91640698
Aftph	-0.152459502	0.11224746	-1.358244565	0.174386094	0.863528862
Lgalsl	-0.040742968	0.121223812	-0.336097074	0.736797665	0.988585656
Peli1	-0.094893516	0.121802332	-0.779077985	0.435933777	0.946762848
Vps54	-0.01070247	0.119535973	-0.089533462	0.928657962	0.994688009
Ugp2	-0.046198223	0.118679735	-0.389267998	0.697077907	0.987521994
Mdh1	-0.087949819	0.118849355	-0.740010905	0.459293378	0.952683168
Wdpcp	0.076739071	0.208437045	0.368164265	0.712750754	0.988585656
Otx1	-1.019678568	1.19737339	-0.851596149	0.394438278	0.933796635
Ehbp1	-0.106862049	0.219616261	-0.48658532	0.626552229	0.97658408
Tmem17	0.033216092	0.224099977	0.148219972	0.88216917	0.992886758
B3gnt2	0.100220549	0.118882559	0.843021468	0.399216438	0.936273856
Commd1	0.249126892	0.167371944	1.488462679	0.136628918	0.832506992
Zrsr1	0.15365911	0.171079222	0.898175173	0.369092167	0.928436166
Cct4	-0.008871449	0.12295586	-0.072151495	0.942481346	0.994688009
Fam161a	0.049792232	0.152180663	0.327191583	0.743522987	0.988585656
Xpo1	0.024887939	0.103675832	0.240055355	0.810287344	0.991438232
Usp34	-0.320122091	0.231504462	-1.382790155	0.166729218	0.860403806
Ahsa2	-0.194414465	0.260751681	-0.745592374	0.455913691	0.951472839
0610010F05Rik	0.052440316	0.159743832	0.328277564	0.742701806	0.988585656
Pex13	0.014654687	0.143153801	0.102370228	0.918462814	0.994688009
Pus10	0.013055039	0.153095374	0.085273896	0.932043644	0.994688009
Rel	-0.769581423	0.511742959	-1.503843695	0.132621617	0.827800491
Papolg	-0.026900008	0.175531922	-0.153248525	0.878202292	0.992886758
Bcl11a	-0.045119507	0.649210623	-0.069499027	0.944592407	0.99486515
Fancl	-0.236369744	0.174244712	-1.356538986	0.17492774	0.864111192
Vrk2	0.337705185	0.187220892	1.803779382	0.071265899	0.744227652
Ccdc85a	-0.047102884	0.179609764	-0.262251245	0.793127747	0.990988839
Efemp1	-0.008699155	0.119992074	-0.072497744	0.942205801	0.994688009
Pnpt1	-0.041824666	0.150598281	-0.277723395	0.7812247	0.990988839
Smek2	-0.043609881	0.099450814	-0.438507026	0.661018778	0.979924124
Cfap36	0.24008037	0.122206278	1.964550218	0.049466319	0.707153766
Ccdc88a	-0.011264304	0.209857574	-0.053675945	0.957193348	0.996226826
Prorsd1	0.281597236	0.188857342	1.49105792	0.135946289	0.832457116
Mtif2	0.081506771	0.162731294	0.500867224	0.616464571	0.974001549
Rps27a	0.467429462	0.245912671	1.900794538	0.05732893	0.721735927
Clhc1	0.618931877	0.977587149	0.633121945	0.526654013	0.965637248
Rtn4	-0.026521996	0.083175556	-0.318867668	0.749826863	0.988585656
Eml6	-0.416346656	0.501172313	-0.830745525	0.406117403	0.939557159
Sptbn1	-0.15588105	0.135119694	-1.153651589	0.248643039	0.896939594
4930505A04Rik	0.003776463	0.373751184	0.010104218	0.991938138	0.998783765
Acyp2	0.593849123	0.276945462	2.144281835	0.032010311	0.664081772
Psme4	-0.026826124	0.164189317	-0.163385321	0.87021506	0.992886758
Erlec1	0.014344629	0.147871263	0.09700755	0.922720398	0.994688009
Asb3	0.27481713	0.196123158	1.40124773	0.161140006	0.857316691
Chac2	0.174566482	0.201517873	0.866258063	0.386348659	0.932501372
Stc2	0.306611044	0.391520725	0.783128516	0.433551647	0.946463129
Bod1	0.240690514	0.151013217	1.5938374	0.110972458	0.800111791
D630024D03Rik	0.215217122	0.255178146	0.843399504	0.399005049	0.936268387
Cpeb4	-0.156752473	0.161465205	-0.970812706	0.331641554	0.917115058
4930524B15Rik	0.665945662	1.988138961	0.334959314	0.737655778	0.988585656
Nsg2	0.718587212	0.310180263	2.316676131	0.020521381	0.603792576
Il9r	0.239806663	0.831738691	0.288319716	0.773102016	0.990730604
Snrnp25	0.597322213	0.280093277	2.132583186	0.032958934	0.665167506
Rhbdf1	-0.07628213	0.191382737	-0.398584174	0.690199625	0.986002801
Mpg	0.3701655	0.180428006	2.051596686	0.040208874	0.679118604
Nprl3	-0.126257802	0.342696612	-0.368424423	0.712556789	0.988585656
Hbq1b	0.240655131	0.605168157	0.397666547	0.690876	0.986094278
Hbq1a	1.165137326	0.555085599	2.099022795	0.035814891	0.674795441

Sh3pxd2b	0.032584651	0.365998861	0.089029377	0.929058563	0.994688009
Ubtd2	0.129813226	0.172521397	0.752447106	0.451782227	0.949134998
Efcab9	1.925405338	1.516900116	1.269302651	0.204333141	0.883765092
Stk10	0.395334516	0.287364087	1.375726939	0.168906189	0.861597949
Fbxw11	-0.254946464	0.128040595	-1.991137762	0.046465744	0.698098452
Fgf18	-0.077854729	0.292085906	-0.266547368	0.789817684	0.990988839
Npm1	0.206756281	0.135011603	1.531396385	0.125671457	0.822401003
Ranbp17	-0.356287348	0.773129475	-0.46083788	0.644914922	0.979004493
Gabrp	-0.419558265	0.148125005	-2.832460761	0.004619124	0.390758702
Kcnmb1	-0.54460232	0.380323834	-1.431943704	0.152159933	0.852974739
Lcp2	0.187709792	0.23798783	0.788736937	0.430265775	0.945321484
Dock2	0.242512803	0.277327403	0.874463903	0.38186567	0.930635634
Spdl1	-0.943029081	0.906984939	-1.039740618	0.298460424	0.909779403
Slit3	0.074794331	0.233846341	0.319843925	0.749086648	0.988585656
Pank3	-0.30608357	0.150407872	-2.035023604	0.041848498	0.679118604
Rars	-0.096349782	0.190725771	-0.505174425	0.613436332	0.974001549
Wwc1	-0.284277821	0.2014369	-1.411249978	0.158170918	0.855986074
Mat2b	0.257324776	0.134679628	1.910643652	0.056050391	0.715507996
Hmmr	0.659835134	0.420280913	1.569985962	0.116418377	0.812418014
Nudcd2	0.134826192	0.187130601	0.720492487	0.471221824	0.95486913
Ccng1	-0.075569459	0.116808708	-0.646950563	0.51766394	0.964437658
Gabra1	-1.163905975	1.863389183	-0.624617759	0.532221961	0.96674935
Atp10b	3.116268325	1.168985886	2.66578781	0.007680816	0.45506991
Mir146	-1.858912394	1.887448646	-0.984881045	0.324682505	0.91640698
Pttg1	-0.004842366	0.123207742	-0.039302452	0.968649252	0.996474415
Slu7	0.023688424	0.223600112	0.105941021	0.915629147	0.994688009
C1qtnf2	0.118860363	0.175056195	0.678984043	0.497147973	0.960475196
Ccnjl	-0.193940678	0.704627062	-0.275238759	0.783132811	0.990988839
Pwwp2a	0.04676775	0.153371361	0.304931437	0.760418372	0.989266039
Ttc1	0.234129246	0.135021601	1.734013253	0.082915732	0.76099108
Adra1b	-0.066423496	0.53685819	-0.123726335	0.901531959	0.994688009
Il12b	0.697571812	0.624241548	1.117470976	0.263793003	0.897248195
Ublcp1	0.125309238	0.108553809	1.154351363	0.248356147	0.896939594
Rnf145	-0.098683599	0.171287827	-0.576127334	0.564529117	0.969658675
Ebf1	-0.184472186	0.267304734	-0.69011941	0.490119098	0.958560421
Gm12159	-1.350035846	0.96124918	-1.404459815	0.16018197	0.857316691
Clint1	-0.263986543	0.118203156	-2.233329048	0.025527253	0.631530315
Lsm11	-0.113181536	0.269878918	-0.419378946	0.674939209	0.983549699
Thg1l	0.235833733	0.194480991	1.212631282	0.225270823	0.893255444
Sox30	-1.693331367	1.664786511	-1.017146256	0.309083858	0.9139204
Adam19	-0.166239809	0.295771728	-0.562054426	0.574078937	0.971613657
Nipal4	-1.548809844	1.852548978	-0.836042589	0.403130944	0.937834293
Cyfip2	0.329730189	0.265677462	1.241092062	0.214571741	0.886400046
Itk	0.059747122	0.397965715	0.15013133	0.880661002	0.992886758
Med7	0.109178259	0.128136999	0.852043207	0.394190111	0.933796635
Havcr2	-0.281276723	0.346827221	-0.810999558	0.417365924	0.943415794
Havcr1	0.606021865	0.609276745	0.994657797	0.319902727	0.91640698
Timd4	-0.302990743	0.648220279	-0.467419415	0.640199835	0.977921835
Sgcd	0.462095099	0.465644526	0.992377389	0.321013465	0.91640698
Gnb2l1	0.151197566	0.229225475	0.659601931	0.509509315	0.963452765
Snord96a	0.312797791	0.451084251	0.693435408	0.488036348	0.958288569
Snord95	0.010151642	1.559593045	0.006509161	0.994806478	0.999285699
Trim41	0.049713881	0.178013032	0.279271019	0.780036845	0.990988839
Trim7	-0.402462625	0.295177296	-1.36346064	0.172737381	0.862471933
Irgm1	0.148209114	0.233314748	0.635232513	0.525276781	0.965637248
Gm5431	-0.166108145	0.427986371	-0.388115502	0.69793056	0.9876159
Gm12185	-0.146379592	0.34960718	-0.418697328	0.675437349	0.983549699
Psme2b	0.297423752	0.206896423	1.437549034	0.150562051	0.849879783
9930111J21Rik1	-0.586208592	0.770671971	-0.760646052	0.446868506	0.948476747
9930111J21Rik2	-0.74741401	0.969828667	-0.770666032	0.440904911	0.948068187
Tgtp2	0.354893646	0.17315018	2.04962909	0.040400639	0.679118604
Olfr56	0.660833785	0.736294574	0.897512772	0.369445361	0.928436166

Ifi47	0.363555551	0.187625909	1.937661769	0.052664501	0.707153766
Btnl9	-0.045377525	1.538940061	-0.02948622	0.976476809	0.997262526
Zfp62	-0.09839866	0.134649391	-0.730776864	0.464915457	0.953083512
Mgat1	-0.100661152	0.086582252	-1.16260722	0.2449889	0.896939594
Flt4	0.271569751	0.348946738	0.778255595	0.436418348	0.946762848
Scgb3a1	0.065398124	0.326285293	0.200432338	0.841142471	0.992886758
Cnot6	-0.145250487	0.113006344	-1.285330375	0.19867686	0.880661448
Gfpt2	-0.37880408	0.25745454	-1.471343565	0.141198227	0.840741274
Mapk9	-0.164472993	0.129427312	-1.270775	0.203808705	0.88349335
Rnf130	0.096939078	0.086159397	1.125113229	0.260541056	0.897248195
Tbc1d9b	0.013039809	0.145354754	0.089710236	0.928517482	0.994688009
3010026O09Rik	0.344510726	0.282283314	1.22044311	0.222296944	0.892391445
Sqstm1	0.021654897	0.110414794	0.196123144	0.844513781	0.992886758
Mgat4b	-0.23334029	0.161787	-1.442268476	0.14922665	0.848779008
Ltc4s	-0.008094363	0.17844157	-0.045361418	0.963819233	0.996474415
Maml1	-0.045204351	0.293109509	-0.154223422	0.877433575	0.992886758
Canx	0.061839746	0.107620841	0.57460753	0.565556758	0.970061308
Hnrnph1	0.104619576	0.103390583	1.011886895	0.311592136	0.914819219
Rufy1	-0.114219202	0.153691959	-0.743169666	0.457378966	0.952102513
Adamts2	0.017962809	0.238378741	0.075354073	0.939933	0.994688009
Zfp354c	0.047784543	0.227496792	0.210044911	0.833632621	0.992886758
Zfp879	-0.374446859	0.649614742	-0.576413734	0.564335564	0.969658675
Grm6	-2.602304319	1.953538817	-1.332097574	0.182828127	0.869258834
Zfp454	-0.070433174	0.701569344	-0.100393745	0.920031736	0.994688009
Zfp2	0.007379194	0.195617623	0.037722544	0.969908901	0.996628386
Zfp354b	0.5170604	0.238159036	2.171071933	0.029925735	0.65030913
Zfp354a	-0.265870827	0.249293129	-1.066498815	0.286198214	0.905916508
BC049762	0.078677377	0.609637211	0.12905606	0.89731329	0.994101834
Clk4	0.236086129	0.140785839	1.676916728	0.093558767	0.775558829
Col23a1	-0.242471984	0.219921173	-1.102540428	0.270226793	0.89804647
Phykpl	-0.379216659	0.256777654	-1.476828894	0.139721509	0.836535783
Hnrnpab	-0.026436256	0.104995246	-0.251785263	0.801207049	0.990988839
Nhp2	0.144399665	0.153490698	0.940771438	0.346822001	0.921552698
Rmnd5b	-0.007228224	0.233184797	-0.030997834	0.975271267	0.997262526
N4bp3	0.153671529	0.149003564	1.031327876	0.302387091	0.911518274
D930048N14Rik	-0.050959914	0.389340822	-0.130887672	0.895864167	0.993801757
0610009B22Rik	0.29433701	0.204524066	1.439131425	0.15011329	0.849826521
Sec24a	0.20844484	0.271511381	0.767720453	0.442653282	0.948476747
Sar1b	0.29532074	0.200561151	1.472472305	0.140893381	0.839671411
Jade2	-0.301819337	0.237440678	-1.27113576	0.203680355	0.88349335
Cdkn2aipnl	0.077038371	0.136796139	0.563161882	0.573324656	0.971613657
Ube2b	0.016560086	0.180818892	0.091583824	0.927028704	0.994688009
Cdkl3	-0.125300637	0.338953961	-0.369668602	0.711629429	0.988585656
Ppp2ca	-0.056746192	0.07850004	-0.722881066	0.469752957	0.95486913
Mir3061	-0.633832132	1.581223539	-0.400849163	0.68853118	0.98538669
Olfr1372-ps1	-0.304808428	0.51034423	-0.597260458	0.5503335	0.968926429
Skp1a	0.075546415	0.133304448	0.566720885	0.570903829	0.971613657
Tcf7	0.176325022	0.256180924	0.688283181	0.491274471	0.958916246
Vdac1	0.072901739	0.108613976	0.671200355	0.502092902	0.961378071
9530068E07Rik	-0.037946793	0.082977293	-0.457315386	0.647444372	0.979004493
Fstl4	-0.899336117	1.287528587	-0.69849798	0.484865821	0.957710647
Hspa4	-0.162023596	0.167457493	-0.96755059	0.333268864	0.917275917
Zcchc10	-0.10733938	0.171222317	-0.62690064	0.530724367	0.966026275
Aff4	-0.392066781	0.130302958	-3.008886264	0.002622073	0.327099208
Uqcrq	0.305849939	0.270906898	1.128985421	0.258903984	0.897248195
Gdf9	0.295921525	0.741056728	0.399323715	0.689654696	0.985729862
Shroom1	-0.473779478	0.584028502	-0.811226639	0.417235531	0.943415794
Sowaha	-0.679218757	0.668556547	-1.015948105	0.309654099	0.9139204
Sep-08	-0.117476352	0.146826312	-0.80010422	0.423650417	0.94351935
Kif3a	-0.039178771	0.226907542	-0.172664031	0.862915518	0.992886758
Il4	-1.577634611	0.830426626	-1.899788087	0.057460935	0.721735927
Rad50	-0.199480503	0.198523594	-1.004820127	0.31498347	0.91586731

Il5	-0.651734138	1.46048774	-0.446244169	0.655420881	0.979133069
Irf1	0.030933145	0.127942049	0.241774658	0.808954776	0.991192232
Gm12216	0.603848143	0.192415702	3.138247747	0.001699612	0.284736028
Slc22a5	-0.160994744	0.179781363	-0.89550297	0.370518279	0.928436166
Slc22a21	-0.282223024	0.508180479	-0.555359828	0.578648564	0.971613657
Slc22a4	-0.051664288	0.382500982	-0.135069687	0.892556777	0.993300147
Pdlim4	0.269435511	0.343570479	0.78422195	0.432909887	0.946074118
P4ha2	-0.342400636	0.142420803	-2.404147628	0.016210226	0.56192007
Csf2	0.291718707	0.288149134	1.012387936	0.311352604	0.914819219
Acsl6	1.285068388	1.498838724	0.857376025	0.391237105	0.933734231
Fnip1	-0.179308368	0.13034692	-1.375624127	0.168938033	0.861597949
Rapgef6	-0.086912222	0.205919283	-0.422069371	0.672974384	0.983235147
Cdc42se2	0.028374495	0.092637997	0.306294344	0.759380546	0.989266039
Lyrm7	-0.145987602	0.308701941	-0.472907949	0.636278853	0.977284137
Hint1	0.324483645	0.255084794	1.272061891	0.20335113	0.882809131
Gpx3	-0.117189826	0.098929771	-1.184575942	0.236185154	0.896358698
Tnip1	-0.087648706	0.175117276	-0.500514332	0.616712967	0.974001549
Anxa6	0.040864766	0.142035589	0.287707932	0.773570318	0.990730604
Ccdc69	-0.116266902	0.229325984	-0.506994016	0.612159017	0.974001549
Gm2a	-0.065898494	0.122184607	-0.53933548	0.589655392	0.974001549
Slc36a3	0.637026056	1.759092981	0.362133249	0.717252459	0.988585656
Slc36a2	-0.228813369	0.203533696	-1.124203872	0.260926547	0.897248195
Slc36a1	-0.274867729	0.305166748	-0.900713236	0.36774081	0.928436166
Fat2	0.318749416	0.818272283	0.389539549	0.69687706	0.987518602
Sparc	0.119474939	0.111762902	1.06900355	0.28506807	0.905916508
Atox1	0.181144291	0.187088097	0.968229908	0.332929561	0.917115058
G3bp1	-0.070074132	0.096426458	-0.726710638	0.467403235	0.953315756
Gria1	0.280427876	0.346884068	0.808419591	0.418849078	0.943415794
Fam114a2	-0.183557855	0.118800725	-1.545090364	0.122324407	0.817515595
Mfap3	-0.455785288	0.170747904	-2.669346319	0.007599904	0.454518821
Galnt10	0.159007819	0.223219149	0.71233951	0.476254563	0.95716557
2010001A14Rik	0.370036218	0.224853811	1.645674653	0.09983076	0.788783372
Sap30l	-0.199436087	0.171172246	-1.165119296	0.243970707	0.896939594
Larp1	0.228404632	0.226833719	1.006925394	0.313970628	0.91586731
Cnot8	0.195793495	0.084595085	2.314478382	0.020641496	0.604325792
Gemin5	-0.068330642	0.257974096	-0.264874045	0.791106494	0.990988839
Mrpl22	0.237462772	0.189453956	1.253406244	0.2100579	0.884258007
Gm12250	0.599998094	0.27651441	2.169861938	0.030017307	0.650824012
Igtp	0.355952757	0.211543818	1.682643153	0.092444193	0.775044718
Irgm2	0.031146755	0.188108598	0.165578581	0.868488604	0.992886758
Zfp692	-0.474293317	0.287529378	-1.649547332	0.099035555	0.787788403
Zfp672	-0.028266798	0.166827645	-0.169437133	0.865452818	0.992886758
Sh3bp5l	0.091414985	0.136181321	0.671274033	0.502045974	0.961378071
Lypd8	-1.677623806	1.51782608	-1.105280657	0.269037997	0.89804647
Fam183b	0.053956853	0.128431181	0.420122693	0.674395826	0.983375962
2810021J22Rik	0.201699161	0.260599652	0.773980929	0.438942069	0.947838535
Zfp39	0.173660696	0.33560781	0.517451295	0.60484116	0.974001549
Rnf187	-0.010626209	0.113517809	-0.093608301	0.925420315	0.994688009
Hist3h2ba	0.891105185	0.442193704	2.01519193	0.043884543	0.688477147
Hist3h2bb-ps	0.2845965	1.449910085	0.19628562	0.844386616	0.992886758
Hist3h2a	0.143439082	0.230982088	0.620996559	0.534601888	0.96674935
Trim17	0.375128209	0.581326995	0.645296387	0.518735134	0.96446859
Trim11	-0.135592731	0.203576682	-0.666052369	0.505377628	0.962138588
Obscn	-0.262419839	0.422077712	-0.621733466	0.534117142	0.96674935
Iba57	-0.089967754	0.334666041	-0.268828452	0.788061697	0.990988839
Gjc2	-0.311222515	0.504958807	-0.616332482	0.537675105	0.96674935
Guk1	0.309911159	0.184357637	1.681032393	0.092756623	0.775044718
2610507I01Rik	0.276787409	0.17148335	1.614077456	0.106510627	0.794899279
Mrpl55	0.027307017	0.160594134	0.170037448	0.864980686	0.992886758
2310033P09Rik	0.249166529	0.240147903	1.037554466	0.299477526	0.910105802
Arf1	0.112963142	0.068801441	1.641871741	0.100616587	0.788783372
Wnt3a	-0.227408226	0.240349114	-0.946157953	0.344068029	0.920669678

Wnt9a	0.05695112	0.300298007	0.189648678	0.84958444	0.992886758
Snap47	-0.271106745	0.134323882	-2.018306355	0.043559365	0.687169821
Jmjd4	-0.080466399	0.286000335	-0.281350717	0.778441414	0.990988839
Zfp867	0.061894155	0.186190188	0.332424363	0.739568844	0.988585656
Zkscan17	-0.149038761	0.256827278	-0.580307366	0.561707361	0.969592734
4933439C10Rik	0.51011548	0.263784006	1.933837791	0.053133075	0.707153766
Nlrp3	-0.534371741	0.8902884	-0.600223187	0.548357503	0.968738549
Mprip	-0.138034838	0.116040341	-1.189541821	0.234226524	0.89581803
Pld6	-0.182967484	0.399033953	-0.458526104	0.646574513	0.979004493
Flcn	-0.116859289	0.141038035	-0.828565771	0.407350163	0.939557159
Gm16062	-2.494863095	1.511332237	-1.650770779	0.098785389	0.786907305
Cops3	0.043715318	0.16071582	0.27200383	0.785619075	0.990988839
Nt5m	0.005413108	0.182280286	0.029696616	0.976309011	0.997262526
1810063I02Rik	0.435475112	0.646581594	0.673503725	0.50062688	0.961175273
Med9os	-0.122066705	0.424280282	-0.287702988	0.773574102	0.990730604
Med9	0.013161302	0.125175964	0.105142403	0.916262813	0.994688009
Rasd1	-0.433934345	0.463063993	-0.937093688	0.348710368	0.921552698
Pemt	-0.390299299	0.266385618	-1.465166558	0.142875481	0.840741274
Rai1	0.026696314	0.187420064	0.142441065	0.886731628	0.992886758
Srebf1	-0.164822011	0.187512652	-0.878991411	0.379405933	0.929137643
Tom1l2	-0.268811931	0.205609938	-1.307387833	0.191081027	0.877906723
Lrrc48	-0.384871723	0.153935846	-2.500208577	0.012412021	0.525888996
Atpaf2	0.183018736	0.176460521	1.037165337	0.29965881	0.910105802
Gid4	-0.14594325	0.11293953	-1.292224694	0.196279342	0.879755417
Drg2	-0.267637404	0.172189117	-1.554322417	0.120107529	0.81558552
Myo15	0.065479312	0.704872577	0.092895246	0.925986782	0.994688009
Alkbh5	-0.049192746	0.170293068	-0.288871098	0.772680022	0.990678393
Llgl1	-0.489422089	0.232532949	-2.104742966	0.035313688	0.674795441
Flii	-0.052870588	0.179192219	-0.295049574	0.76795601	0.989990452
Mief2	-0.089916965	0.144411308	-0.622644903	0.533517897	0.96674935
Top3a	0.073463471	0.441230123	0.166496952	0.867765881	0.992886758
Smcr8	-0.516899992	0.219494101	-2.354960748	0.018524662	0.586147601
Shmt1	0.071993009	0.283126007	0.25427904	0.799279992	0.990988839
Dhrs7b	0.148022116	0.15298025	0.967589712	0.333249317	0.917275917
Tmem11	0.347909698	0.134110079	2.594209915	0.009480858	0.479238301
Natd1	-0.094863533	0.24823188	-0.38215693	0.702344967	0.988482081
Map2k3os	0.301175463	0.33711584	0.893388644	0.371649082	0.928840865
Map2k3	-0.160827591	0.106074048	-1.516182282	0.129473267	0.827800491
Kcnj12	-0.026056676	0.453395119	-0.057470128	0.954170701	0.996070133
Tnfrsf13b	0.582551152	0.52823385	1.102828136	0.270101807	0.89804647
Usp22	-0.057624399	0.18360721	-0.313846057	0.753637962	0.988585656
Aldh3a1	0.167825253	0.194637108	0.862246951	0.388551638	0.933269543
Aldh3a2	-0.096682528	0.102443769	-0.943761919	0.345291325	0.92132235
Slc47a2	1.844447235	1.384388713	1.332318891	0.182755422	0.869194833
Slc47a1	0.067287292	0.438040671	0.153609691	0.877917495	0.992886758
Rnf112	1.636505674	1.090772924	1.50031747	0.133532186	0.827800491
Mfap4	-0.129697058	0.190547979	-0.680653024	0.496091067	0.960132629
Mapk7	0.123608634	0.348196855	0.354996411	0.72259228	0.988585656
B9d1	0.209823328	0.194412158	1.079270608	0.280467111	0.903192609
Epn2	-0.116188957	0.08557051	-1.357815405	0.174522265	0.863528862
Grap	-0.024397231	0.09760053	-0.249970273	0.802610338	0.990988839
Fam83g	0.079647758	0.770573303	0.103361689	0.917675917	0.994688009
Prpsap2	0.082520836	0.162909528	0.506543951	0.612474844	0.974001549
Ulk2	-0.240593241	0.12430861	-1.935451138	0.052934959	0.707153766
Akap10	-0.024306483	0.158811205	-0.153052697	0.878356718	0.992886758
Specc1	0.234027468	0.194821414	1.201240988	0.229657734	0.895404344
Adora2b	-0.184377349	0.190910198	-0.965780515	0.334154021	0.918030088
Zswim7	0.370299019	0.284116749	1.303333999	0.192460759	0.879588734
Ttc19	-0.046950591	0.160728068	-0.292111961	0.770201022	0.989990452
Ncor1	0.065838507	0.123788764	0.531861734	0.594821762	0.974001549
Pigl	-0.060654688	0.269104626	-0.225394446	0.821672432	0.992886758
Cenpv	0.038250321	0.152383945	0.251012799	0.801804213	0.990988839

Ubb	-0.080873387	0.133236714	-0.606990253	0.543857381	0.967491297
Trpv2	0.068870667	0.293096385	0.234976173	0.814227227	0.992048071
2410006H16Rik	0.437314275	0.309213919	1.414277456	0.157280453	0.855986074
Snord49a	-0.347074006	1.482075293	-0.234181089	0.814844396	0.992116023
Lrrc75a	-0.156827582	0.231541928	-0.677318289	0.498204029	0.960700483
Mmgt2	0.026496733	0.190529928	0.139068616	0.889395928	0.993043931
Zfp287	0.032653093	0.376728392	0.086675423	0.930929513	0.994688009
Zfp286	0.561898669	0.323645557	1.736154435	0.082536531	0.76099108
Trim16	-0.21512693	0.14234451	-1.511311744	0.130709043	0.827800491
Fbxw10	1.373627993	0.915576956	1.500286769	0.133540135	0.827800491
Tvp23b	0.247947186	0.137017087	1.809607782	0.070356632	0.744227652
Cdrt4	1.80435273	1.518254034	1.18843928	0.234660389	0.89581803
Tekt3	-0.047057638	0.386591092	-0.121724579	0.903117147	0.994688009
Pmp22	-0.128978789	0.101549208	-1.270111225	0.204045014	0.883671867
Hs3st3b1	-0.195460849	0.318774556	-0.61316327	0.539768389	0.966900344
Cox10	0.362900695	0.230539798	1.574134699	0.115456312	0.81007959
2810001G20Rik	0.476276857	0.306049253	1.556209834	0.119658204	0.81558552
Hs3st3a1	-0.122424814	0.702801268	-0.174195494	0.861711827	0.992886758
Elac2	-0.164207735	0.302819594	-0.542262582	0.587637629	0.973455044
Arhgap44	-0.012111595	0.159774806	-0.075804158	0.939574908	0.994688009
Myocd	2.366491689	1.289266666	1.835533138	0.066426734	0.738254074
Gm12295	0.311673496	1.009812694	0.308644858	0.757591694	0.989266039
Map2k4	0.070426726	0.12317032	0.571783251	0.567468826	0.970955746
Zkscan6	0.119968343	0.173832117	0.690139109	0.490106711	0.958560421
Dnah9	0.153825952	0.250278602	0.61461887	0.538806449	0.966875138
Shisa6	1.445603523	2.438511646	0.592822071	0.553300236	0.968926429
Tmem220	0.140554733	0.204581243	0.687036263	0.492059877	0.958916246
Adprm	0.020694317	0.141835722	0.145903424	0.883997628	0.992886758
Sco1	0.302071168	0.15653708	1.92971	0.05364278	0.707153766
Myh13	0.472661411	0.434973795	1.086643417	0.277194429	0.900939369
Gas7	0.53320351	0.382112987	1.395407974	0.162892848	0.859982992
Dhrs7c	-0.048725274	0.394610962	-0.123476737	0.901729595	0.994688009
Usp43	-0.083278641	0.386863618	-0.215266149	0.82955982	0.992886758
Cfap52	-0.202362411	0.200229762	-1.010651009	0.312183493	0.914819219
Stx8	0.266084962	0.117682213	2.261046543	0.023756375	0.622011301
Ntn1	-0.389127377	0.180243799	-2.158894672	0.030858339	0.659455637
Pik3r5	0.338622556	0.275800426	1.227781121	0.219529145	0.888847883
Pik3r6	0.130387882	0.212639899	0.613186343	0.539753134	0.966900344
Mfsd6l	-0.581031036	0.224518769	-2.587895163	0.009656436	0.483161941
Ccdc42	0.139532908	0.284857971	0.489833256	0.624251896	0.975969024
Myh10	0.297093695	0.16959039	1.75183096	0.079802879	0.75659671
Ndel1	-0.099669347	0.094704683	-1.052422578	0.292605714	0.907334416
Rnf222	3.655491213	2.244362681	1.628743537	0.103367322	0.790925787
Arhgef15	0.045834077	0.140992254	0.325082232	0.745118836	0.988585656
Slc25a35	0.01910873	0.289217758	0.06607039	0.947321785	0.995297476
Rangrf	0.278741423	0.188720964	1.477002966	0.139674842	0.836535783
Pfas	-0.299352852	0.384536649	-0.77847678	0.43628799	0.946762848
Ctc1	-0.369434559	0.424405517	-0.870475393	0.384040661	0.931591679
Aurkb	0.047430749	0.397120873	0.119436554	0.904929503	0.994688009
2310047M10Rik	0.074609541	0.25041301	0.297945943	0.765744422	0.989884648
Tmem107	0.020843058	0.157351021	0.132462172	0.894618742	0.993556555
Vamp2	-0.08573497	0.122161495	-0.701816643	0.482793518	0.957463006
Per1	-0.126354948	0.339863925	-0.371780993	0.710055916	0.988585656
Hes7	1.668017619	1.340493648	1.244330864	0.213377816	0.884945675
Aloxe3	-0.880389672	0.709510333	-1.240841228	0.214664408	0.886400046
Cntrob	-0.333799923	0.361905406	-0.922340251	0.356351125	0.924354588
Trappc1	-0.069873275	0.185705941	-0.376257619	0.706725384	0.988585656
Kcnab3	1.71751345	1.10047236	1.560705668	0.118593213	0.814755048
Chd3os	-0.124439411	0.260828777	-0.477092338	0.63329637	0.977034429
Chd3	0.148481786	0.147599512	1.005977485	0.314426401	0.91586731
Cyb5d1	-0.259989278	0.391711959	-0.663725659	0.506865914	0.96295087
Naa38	0.471337115	0.207078803	2.276124393	0.022838566	0.620006604

Tmem88	-0.341500211	0.228915903	-1.491815147	0.135747612	0.831963687
Kdm6b	-0.055504224	0.237895029	-0.23331393	0.815517642	0.992468437
Dnah2	0.347109025	0.223160443	1.555423624	0.119845211	0.81558552
Efnb3	0.776616092	1.217884257	0.637676435	0.523684328	0.965637248
Wrap53	-0.417133306	0.260540194	-1.601032451	0.109369732	0.798400564
Trp53	-0.020054293	0.264349415	-0.075862824	0.939528233	0.994688009
Atp1b2	0.205089322	0.674380601	0.304115097	0.761040205	0.989266039
Sat2	0.704399167	0.266156746	2.646557626	0.008131565	0.464210002
Fxr2	0.100399116	0.15758393	0.637115189	0.524049815	0.965637248
Mpdu1	0.171330529	0.140920219	1.215798061	0.224061858	0.892989918
Cd68	-0.223471586	0.243403618	-0.918111191	0.358560657	0.925129558
Eif4a1	0.056511291	0.07471348	0.756373428	0.449425335	0.948476747
Senp3	-0.007177912	0.092132307	-0.077908737	0.937900649	0.994688009
Tnfsf13	0.107499256	0.281233678	0.38224176	0.702282049	0.988482081
Tnfsf12	-0.130416889	0.186040224	-0.701014469	0.483293986	0.957614188
Polr2a	-0.034744366	0.292323711	-0.118855793	0.905389606	0.994688009
Slc35g3	-0.482794998	0.372462657	-1.296223899	0.194898358	0.879588734
Zbtb4	-0.010266217	0.144234625	-0.0711772	0.943256727	0.99486515
Chrnb1	-0.401707904	0.249204174	-1.611962987	0.106969994	0.79539432
Fgf11	0.184522678	0.352468744	0.52351501	0.600615909	0.974001549
G630025P09Rik	0.338840373	1.616785752	0.209576545	0.833998187	0.992886758
Nlgn2	0.227801114	0.319158036	0.713756471	0.47537778	0.956791546
Tmem256	0.586611014	0.319947949	1.833457649	0.066734541	0.738254074
Plscr3	-0.084221402	0.204975158	-0.410885899	0.681156203	0.98444165
Tnk1	-0.473090264	0.414439198	-1.141519109	0.253653965	0.897044747
Tmem95	-0.297130709	1.318072969	-0.225428118	0.821646239	0.992886758
Kctd11	-0.075729213	0.190324059	-0.397896166	0.690706727	0.986039532
Acap1	0.197750375	0.270156337	0.731984959	0.464177748	0.953083512
2810408A11Rik	0.011969395	0.429744575	0.027852347	0.977779915	0.997262526
Neurl4	0.027930609	0.162641442	0.171731196	0.86364886	0.992886758
Gps2	-0.38141647	0.156642916	-2.434942342	0.014894163	0.554936045
Eif5a	0.001055959	0.107930701	0.009783672	0.992193884	0.998800761
Ybx2	1.219459577	0.799945518	1.524428288	0.127401758	0.824450458
Slc2a4	0.483176713	0.437801893	1.103642359	0.269748309	0.89804647
Cldn7	-0.227069227	0.121106723	-1.874951453	0.060799403	0.725221283
Elp5	0.094290165	0.114931897	0.820400321	0.411987933	0.942121471
Ctdnep1	0.033917392	0.127222719	0.266598548	0.789778274	0.990988839
Gabarap	-0.061253291	0.111098294	-0.551343217	0.581398418	0.971885891
Phf23	-0.110566608	0.138516865	-0.79821766	0.424744191	0.944280828
Dvl2	-0.099975118	0.333374541	-0.299888281	0.764262373	0.989384061
Acadvl	0.102665645	0.11686322	0.878511178	0.379666374	0.929185635
Mir324	4.732175817	1.813228369	2.60980685	0.009059336	0.475600561
Dlg4	0.033228289	0.291891875	0.113837664	0.909366481	0.994688009
Asgr1	0.468200537	0.259903013	1.801443281	0.07163304	0.744227652
Asgr2	0.557639331	1.12440281	0.49594267	0.61993486	0.974471036
Mgl2	-0.191115826	0.221608148	-0.862404331	0.388465058	0.933269543
Clec10a	0.269739362	0.301365539	0.89505709	0.37075657	0.928436166
Slc16a11	0.182274708	0.136048917	1.339773309	0.180319056	0.868087032
Slc16a13	0.32644657	0.329685152	0.990176744	0.322087737	0.91640698
Bcl6b	-0.093752076	0.245327899	-0.382150078	0.702350049	0.988482081
0610010K14Rik	0.19999928	0.148209487	1.349436427	0.177196827	0.866855369
Rnasek	0.056296012	0.117355286	0.47970581	0.631436596	0.977034429
Alox12	0.317615059	0.439729	0.722297276	0.470111727	0.95486913
Alox12e	-2.369610292	1.679173594	-1.411176486	0.158192582	0.855986074
Alox15	-0.401314197	0.519821935	-0.772022436	0.440101139	0.947849924
Pelp1	0.679092615	0.293885397	2.310739572	0.020847244	0.604325792
Arrb2	0.057729462	0.20383931	0.283210645	0.777015369	0.990861469
Med11	0.225725807	0.18878044	1.19570548	0.231811512	0.895404344
Cxcl16	0.021274901	0.125565842	0.169432229	0.865456675	0.992886758
Zmynd15	-0.665102861	0.385910968	-1.723461931	0.084805024	0.761612554
Tm4sf5	0.279717363	0.846648717	0.330381842	0.74111146	0.988585656
Vmo1	-0.145125547	1.855449606	-0.078215839	0.937656363	0.994688009

Gltpd2	0.046277035	1.184168239	0.039079781	0.968826781	0.996474415
Psmb6	0.278822401	0.164325857	1.696765231	0.089741055	0.769347579
Pld2	0.204594523	0.21269424	0.961918492	0.336090549	0.918908218
Mink1	-0.151971802	0.217069286	-0.700107346	0.483860269	0.957710647
Chrne	1.504985007	1.659838108	0.906705901	0.364562342	0.926556975
Gp1ba	-0.700406241	1.370501153	-0.511058484	0.609310105	0.974001549
Slc25a11	0.046162802	0.094650316	0.487719465	0.625748562	0.97639266
Rnf167	-0.229981824	0.169293801	-1.358477526	0.174312209	0.863528862
Pfn1	0.168199024	0.100338281	1.67631957	0.093675614	0.775558829
Eno3	0.697679424	0.238784273	2.921798058	0.003480171	0.368965929
Spag7	0.108135431	0.135677521	0.797003295	0.425449118	0.944290206
Camta2	-0.095027636	0.313221353	-0.303388114	0.761594103	0.989285682
Inca1	-0.052526823	0.450082517	-0.116704872	0.907093929	0.994688009
Kif1c	-0.014802833	0.114633014	-0.12913237	0.897252909	0.994101834
Zfp3	-0.143452118	0.259861188	-0.552033642	0.580925304	0.971613657
Scimp	0.211165659	0.365624312	0.577548189	0.563569195	0.969658675
Rabep1	-0.215433729	0.13517252	-1.59376869	0.110987853	0.800111791
Nup88	-0.248920479	0.128815608	-1.932378245	0.053312837	0.707153766
Rpain	0.308801862	0.289427352	1.066940841	0.285998551	0.905916508
C1qbp	-0.082363365	0.130853854	-0.629430182	0.529067464	0.965637248
Dhx33	-0.098974088	0.260130001	-0.38047933	0.703589638	0.988585656
Derl2	0.068895142	0.134210767	0.513335431	0.607716703	0.974001549
Mis12	-0.221177815	0.205476109	-1.076416209	0.281741157	0.905331207
6330403K07Rik	-0.115842713	0.268751832	-0.431039716	0.666439493	0.981863224
Nlrp1a	1.566885894	1.146642049	1.366499594	0.171782208	0.86244803
Nlrp1b	-1.194922973	0.666674342	-1.792363825	0.073074706	0.748833631
Nlrp1c-ps	2.553041102	3.818453432	0.668606059	0.503746806	0.96179897
Wscd1	0.062187283	0.261890175	0.237455581	0.812303375	0.991957906
Fam64a	0.424927926	0.400573244	1.060799571	0.288780997	0.907334416
Pitpnm3	-0.196953196	0.218980105	-0.899411366	0.368433587	0.928436166
4933427D14Rik	0.094748275	0.291427512	0.325117811	0.745091909	0.988585656
Txndc17	0.224854605	0.163458722	1.375604814	0.168944015	0.861597949
Med31	0.396343745	0.196439667	2.017636008	0.043629184	0.687580778
4930563E22Rik	-0.293787175	0.670231605	-0.438336797	0.661142155	0.979955675
Xaf1	-0.126891869	0.170001434	-0.746416461	0.455415878	0.951224746
Fbxo39	-4.206945503	1.958379269	-2.148177102	0.031699689	0.661395937
Tekt1	-0.284846642	0.173433506	-1.642396838	0.10050779	0.788783372
Smtnl2	-0.120311658	0.168773179	-0.712860058	0.475932358	0.95716557
Ggt6	-0.255606514	0.450348925	-0.567574385	0.570324005	0.971613657
Mybbp1a	-0.096854834	0.168720773	-0.574053994	0.565931264	0.970278247
Spns2	-0.144663249	0.196894567	-0.734724431	0.462507338	0.953083512
Spns3	1.509841455	1.328556977	1.136452167	0.255767374	0.897044747
Ube2g1	-0.022170965	0.115727104	-0.191579705	0.848071441	0.992886758
Ankfy1	-0.107231577	0.201367336	-0.532517237	0.594367807	0.974001549
Cyb5d2	-0.154260099	0.170961066	-0.902311287	0.366891532	0.927999696
Zzef1	-0.116630262	0.164304088	-0.709843944	0.477800915	0.95730219
Atp2a3	0.012176868	0.188218204	0.064695487	0.948416456	0.99560893
P2rx1	-0.063130944	0.474802523	-0.132962528	0.894223016	0.993422442
Camkk1	-0.167595376	0.258138107	-0.649246941	0.516178775	0.964352198
1200014J11Rik	-0.17459915	0.155665385	-1.121631179	0.262019289	0.897248195
Itgae	-0.125412549	0.278425801	-0.450434363	0.652397272	0.979004493
Gsg2	0.422768742	0.56165493	0.75271972	0.451618357	0.949134998
P2rx5	-0.039770334	0.276542873	-0.143812544	0.885648498	0.992886758
Emc6	0.038054924	0.115062339	0.330733103	0.740846096	0.988585656
Tax1bp3	0.162442129	0.116450897	1.394940985	0.163033637	0.859982992
Ctns	-0.048186871	0.230883042	-0.208706845	0.834677096	0.992886758
Shpk	-0.935418475	0.310663247	-3.011036817	0.002603573	0.327099208
Trpv1	1.87263227	2.504287837	0.747770381	0.454598671	0.951112066
Aspa	-0.236379492	0.171430543	-1.378864511	0.167936532	0.861149421
Spata22	-1.804395959	1.659227078	-1.087491871	0.276819489	0.900604936
Rap1gap2	0.381256981	0.404778368	0.941890703	0.346248601	0.921552698
E130309D14Rik	0.365679514	0.507617901	0.720383408	0.471288963	0.95486913

Cluh	0.170787534	0.1628282	1.048881789	0.294232528	0.907334416
Pafah1b1	-0.113160127	0.130066616	-0.870016688	0.384291284	0.931689378
Mettl16	0.030907351	0.162472681	0.190231064	0.84912807	0.992886758
Mnt	0.227247634	0.419945163	0.541136448	0.588413537	0.973455044
Sgsm2	-0.452114406	0.230054111	-1.965252449	0.049385026	0.707153766
Tsr1	-0.570477185	0.375041333	-1.521104835	0.128233531	0.826336015
Srr	-0.022807654	0.30082169	-0.07581785	0.939564015	0.994688009
Smg6	-0.383265004	0.222620342	-1.721608194	0.085140518	0.761635307
Hic1	-0.007495458	0.478639338	-0.015659928	0.987505696	0.998148029
Ovca2	0.224678036	0.178158085	1.261116139	0.207267007	0.883765092
Dph1	0.171397448	0.297531659	0.576064573	0.564571536	0.969658675
Rtn4rl1	0.026182637	0.277669723	0.094294172	0.924875479	0.994688009
Rpa1	-0.142910168	0.101282318	-1.411008068	0.158242235	0.855986074
Smyd4	-0.035020103	0.245841872	-0.142449706	0.886724803	0.992886758
Serpinf1	0.1650266	0.167133049	0.987396576	0.323448262	0.91640698
Serpinf2	2.343989698	1.377030375	1.702206241	0.088716713	0.767814344
Wdr81	-0.187367129	0.29797851	-0.6287941	0.529483863	0.965637248
Mir22hg	-0.0626616	0.214250907	-0.292468305	0.76992859	0.989990452
Tlcd2	0.386753774	0.245230857	1.57710077	0.11477234	0.809454857
Prpf8	-0.197767305	0.187910103	-1.052457006	0.292589926	0.907334416
Rilp	0.094197312	0.187719768	0.501797507	0.615809967	0.974001549
Scarf1	-0.052489087	0.184361046	-0.284708119	0.77586777	0.990861469
Slc43a2	0.144000417	0.216498871	0.665132415	0.505965804	0.96224682
Pitpna	0.126032181	0.089409617	1.409604311	0.158656553	0.855986074
Gm12338	0.442038827	0.290633354	1.520950094	0.128272362	0.826336015
Inpp5k	-0.198779452	0.15932663	-1.247622276	0.212169417	0.884945675
Myo1c	-0.12023983	0.154250655	-0.77950936	0.435679726	0.946762848
Crk	-0.049514969	0.119521857	-0.414275427	0.678672408	0.983853811
Ywhae	0.011753973	0.077648646	0.151373836	0.879680828	0.992886758
Doc2b	-1.280795028	0.795861371	-1.609319254	0.107546551	0.796929372
Rph3al	-0.113011899	0.265372084	-0.425862049	0.670208371	0.982270383
1700016K19Rik	0.230089572	0.142044565	1.619840734	0.105266494	0.793982106
Fam101b	-0.291056375	0.141749081	-2.053321069	0.040041449	0.679118604
Vps53	0.461144061	0.150644377	3.061143527	0.002204934	0.308737709
Fam57a	-0.344020009	0.197298444	-1.74365293	0.081219628	0.760377276
Gemin4	-0.422710168	0.24453299	-1.728642699	0.083873063	0.76099108
Dbil5	-0.401741901	0.825508912	-0.486659677	0.626499525	0.97658408
Glod4	0.003637625	0.097351157	0.037366021	0.970193165	0.996628386
Rnmtl1	0.197828841	0.387784421	0.510151597	0.609945259	0.974001549
Nxn	0.141806959	0.13588556	1.043576368	0.296681431	0.909070179
Timm22	0.136254919	0.138412272	0.984413576	0.324912206	0.91640698
Abr	0.101467998	0.167541195	0.605630141	0.544760383	0.967491297
Tusc5	-0.020996835	0.273048668	-0.076897774	0.938704868	0.994688009
Gosr1	-0.21126914	0.213088119	-0.991463724	0.321459197	0.91640698
Cpd	-0.190980242	0.158145501	-1.207623621	0.227192069	0.894038302
Tmigd1	-0.509642201	0.641510505	-0.794440929	0.426938785	0.944290206
Blmh	0.246116826	0.109361923	2.250480051	0.024418488	0.626649273
Slc6a4	-0.02513575	0.536827595	-0.04682276	0.962654489	0.996474415
Ccdc55	0.085225734	0.253158173	0.336650139	0.736380655	0.988585656
Efcab5	0.697183812	0.730542851	0.954336643	0.339913265	0.919246575
Ssh2	-0.332796465	0.199603366	-1.667288843	0.095456984	0.778239075
Coro6	0.147187904	0.695584031	0.21160334	0.832416503	0.992886758
Ankrd13b	-0.184173119	0.433997309	-0.424364656	0.671299892	0.982811455
Git1	-0.285501459	0.248515773	-1.148826313	0.250627607	0.896939594
Trp53i13	-0.132025641	0.231648483	-0.569939585	0.568718675	0.97144955
Abhd15	-0.314295953	0.389231907	-0.80747736	0.419391513	0.943415794
Taok1	-0.023715678	0.117335039	-0.202119314	0.839823449	0.992886758
Nufip2	-0.07580385	0.1286218	-0.589354606	0.555623421	0.968926429
Myo18a	0.051799565	0.207332235	0.249838453	0.802712282	0.990988839
Pipox	1.304661487	0.675941102	1.930140782	0.053589397	0.707153766
Phf12	-0.386740079	0.249010348	-1.553108466	0.120397224	0.81558552
Dhrs13	0.069041166	0.15849799	0.435596476	0.663129528	0.980876591

Flot2	-0.016192504	0.118950489	-0.136128096	0.891720017	0.993043931
Eral1	-0.054489981	0.149372649	-0.364792225	0.715266509	0.988585656
Fam222b	-0.209960345	0.395102428	-0.531407376	0.595136511	0.974001549
Traf4	0.108202062	0.214912517	0.503470264	0.614633682	0.974001549
Nek8	0.259817531	0.20425276	1.272039266	0.203359168	0.882809131
Tlcd1	0.402415046	0.242076085	1.662349447	0.096442729	0.781321691
Rpl23a	-0.028213053	0.09768668	-0.288811664	0.772725506	0.990678393
Snord4a	-0.882277911	0.998499084	-0.883604127	0.376909951	0.928883222
Snord42b	0.621361786	0.894547853	0.694609889	0.487299813	0.958288569
Rab34	-0.1902915	0.17141066	-1.110149745	0.266934504	0.89768067
Proca1	0.053453524	0.269239411	0.198535288	0.842626278	0.992886758
Supt6	-0.176705544	0.231311145	-0.763930092	0.444908904	0.948476747
Sdf2	0.140147075	0.158952944	0.881689081	0.377944968	0.928883222
2610507B11Rik	-0.119461308	0.119739377	-0.997677714	0.318435663	0.91640698
BC030499	-0.024113936	2.984921803	-0.008078582	0.993554294	0.999270171
Spag5	0.275040978	0.50105244	0.548926532	0.583055869	0.972174584
Aldoc	0.280631453	0.299623369	0.936614039	0.348957128	0.921552698
Pigs	-0.131254096	0.161436176	-0.813040169	0.416195028	0.943415794
Unc119	0.186875826	0.145917357	1.280696344	0.200300344	0.880888611
Slc13a2	-1.645561797	1.073225856	-1.533285643	0.12520549	0.822296879
Slc46a1	-0.084692092	0.255467118	-0.331518563	0.740252821	0.988585656
Vtn	-0.35732432	0.313328984	-1.140412596	0.254114448	0.897044747
Tmem199	0.201071506	0.160208313	1.255062877	0.209455935	0.884258007
Poldip2	0.021577937	0.112936549	0.191062481	0.848476642	0.992886758
Tnfaip1	-0.030657017	0.152883888	-0.200524841	0.841070132	0.992886758
Ift20	0.34520725	0.178651106	1.932298425	0.053322683	0.707153766
Tmem97	-0.091891675	0.152652081	-0.601968045	0.547195408	0.968115622
Nlk	-0.11671957	0.200228765	-0.582931078	0.559939699	0.968926429
Fam58b	0.00550292	0.140480166	0.039172223	0.968753079	0.996474415
Lyrm9	0.370199425	0.302616708	1.223327776	0.22120592	0.891315805
Nos2	-1.286606623	0.639082687	-2.013208382	0.044092712	0.688477147
Lgals9	-0.103567076	0.129027234	-0.802676094	0.422161974	0.943415794
Ksr1	-0.458182168	0.442001982	-1.036606592	0.29991924	0.910285132
Wsb1	0.067221751	0.162105117	0.414680003	0.678376173	0.983786595
Nf1	-0.421516614	0.271391547	-1.553167809	0.12038305	0.81558552
AU040972	-0.379229722	0.302597553	-1.253247813	0.210115534	0.884258007
Evi2a	0.246905571	0.210181627	1.174724809	0.240104831	0.896939594
Rab11fip4	-0.776523384	0.611244045	-1.270398282	0.203942795	0.883661513
Utp6	0.02499855	0.139735891	0.178898564	0.858017351	0.992886758
Suz12	0.033350322	0.134873184	0.247271706	0.804697954	0.990988839
Crlf3	0.167954171	0.085967031	1.953704452	0.05073618	0.707153766
Atad5	0.03335162	0.526840426	0.063304975	0.949523654	0.995624732
Tefm	0.423562525	0.241120579	1.756641954	0.078978857	0.755854514
Adap2	-0.145125401	0.368436342	-0.393895454	0.693658226	0.986787302
Rnf135	0.109010906	0.196760928	0.554027202	0.57956023	0.971613657
Rhot1	-0.077083806	0.090052976	-0.855982884	0.392007247	0.933734231
Rhbdl3	0.164657994	0.465321122	0.353858842	0.723444673	0.988585656
5730455P16Rik	-0.037333831	0.176558177	-0.211453424	0.832533473	0.992886758
Zfp207	-0.150486999	0.085542336	-1.759210781	0.078541714	0.754732183
Psmd11	-0.103969771	0.11415469	-0.910779675	0.362411474	0.926455466
Cdk5r1	0.358539225	1.307716666	0.274171948	0.783952485	0.990988839
Myo1d	-0.24444096	0.232393233	-1.051841987	0.292872052	0.907334416
Tmem98	0.132656245	0.190871784	0.695001863	0.487054134	0.958288569
Ccl2	0.851334358	0.423589661	2.009809107	0.044451397	0.689308953
Ccl7	-0.010034595	0.430275686	-0.023321314	0.98139397	0.997390438
Ccl11	0.009012471	0.371644787	0.024250228	0.980653014	0.997296835
Ccl12	0.381679757	0.411370222	0.927825439	0.353498126	0.924041611
Ccl8	-1.2111696	0.610130192	-1.985100256	0.047133318	0.69958699
Cct6b	1.059625878	1.022747299	1.036058349	0.300174922	0.910285132
Zfp830	0.072973659	0.162793531	0.448258959	0.653966316	0.979133069
Gm11423	0.107232322	0.296539358	0.361612444	0.717641665	0.988585656
Lig3	-0.314095255	0.230029255	-1.365457863	0.172109188	0.86244803

Rffl	-0.05085325	0.251266507	-0.202387699	0.839613644	0.992886758
Rad51d	0.352278173	0.186684849	1.887020688	0.059157543	0.72285319
Nle1	0.482227264	0.208160082	2.316617379	0.020524584	0.603792576
Unc45b	0.235579059	0.173309706	1.359295246	0.17405305	0.863528862
Slfn5os	0.004819635	0.829618184	0.005809461	0.995364747	0.999285699
Slfn5	-0.036101875	0.192421821	-0.187618403	0.851175799	0.992886758
Slfn9	-0.844047165	1.101965304	-0.765947133	0.44370776	0.948476747
Slfn8	0.030195824	0.502044787	0.060145677	0.952039611	0.996023481
Slfn2	0.289558028	0.14883045	1.945556359	0.051708054	0.707153766
Slfn1	1.257734646	0.277755138	4.528213791	5.95E-06	0.006937154
Slfn4	0.612316469	0.417292197	1.467356623	0.142279068	0.840741274
Slfn3	0.626027658	0.455857313	1.373297391	0.169659913	0.861597949
AI662270	0.298949804	0.198880946	1.503159602	0.132797892	0.827800491
AA465934	0.652854815	0.46468533	1.404939588	0.160039243	0.857316691
AI450353	0.227545938	0.565102863	0.402662865	0.687196256	0.98538669
Pex12	0.304513513	0.172388317	1.766439389	0.077322162	0.753694887
Ap2b1	-0.206780264	0.143342835	-1.442557378	0.149145197	0.848779008
Rasl10b	-0.641522118	0.631441981	-1.015963678	0.309646683	0.9139204
Gas2l2	0.170281817	0.327973248	0.519194227	0.603625305	0.974001549
1700020L24Rik	0.415146528	0.41657412	0.996573018	0.318971808	0.91640698
Mmp28	0.215437296	0.320714183	0.671742342	0.501747741	0.961378071
Taf15	0.256261487	0.265076699	0.966744675	0.333671688	0.917554801
Heatr9	-1.317177964	1.433444215	-0.918890285	0.358152962	0.924515953
Ccl5	1.047664759	0.221538597	4.729039422	2.26E-06	0.003683119
Ccl9	-0.74096191	0.532449681	-1.391609267	0.164040751	0.859982992
E230016K23Rik	0.702376157	1.127144808	0.623146336	0.533188363	0.96674935
Ccl6	-0.12109145	0.397475632	-0.304651256	0.760631778	0.989266039
Ccl3	0.773915466	0.42616625	1.815994265	0.069371252	0.743426713
Ccl4	1.347017443	0.49339285	2.730111396	0.006331293	0.427152964
Wfdc17	-0.109942281	0.513885514	-0.213943142	0.830591397	0.992886758
Wfdc21	-0.089710833	0.449301355	-0.1996674	0.841740712	0.992886758
Heatr6	-0.089167356	0.21095538	-0.422683489	0.672526204	0.983233856
Hnf1b	0.204400277	0.397924491	0.513665988	0.607485535	0.974001549
Ddx52	0.002628401	0.142933541	0.01838897	0.985328552	0.998148029
Synrg	-0.196819556	0.229198508	-0.858729655	0.390489686	0.933734231
Dusp14	-0.221313425	0.14907088	-1.484618754	0.137644845	0.833085238
Tada2a	-0.160339889	0.257284371	-0.623201045	0.533152415	0.96674935
Acaca	-0.149828131	0.233369769	-0.642020308	0.520859997	0.964784335
Aatf	0.150846178	0.220558363	0.683928626	0.494020237	0.958916246
Mrm1	0.44759613	0.166586234	2.68687345	0.007212427	0.452031612
Dhrs11	0.058452693	0.232074737	0.251870127	0.80114145	0.990988839
Ggnbp2	-0.059141662	0.084135698	-0.702931853	0.482098219	0.957463006
Ggnbp2os	0.363141239	0.25070772	1.448464526	0.14748718	0.846658212
Pigw	-0.241919211	0.284813042	-0.849396533	0.395660682	0.934066368
Myo19	-0.02661821	0.470527791	-0.056570963	0.954886966	0.996070133
Znhit3	0.422085397	0.288957428	1.460718281	0.14409277	0.841934045
Car4	0.084135891	0.121946041	0.689943607	0.490229651	0.958560421
Usp32	-0.041573726	0.133984891	-0.310286677	0.756342962	0.989266039
1700125H20Rik	1.150925787	2.186121954	0.526469159	0.598562275	0.974001549
Appbp2	0.03564191	0.194121071	0.183606603	0.854322083	0.992886758
Appbp2os	-0.135459361	0.660810411	-0.204989751	0.837580133	0.992886758
Ppm1d	-0.222086695	0.156562813	-1.418514976	0.15604047	0.855455553
Bcas3	-0.170585437	0.240156365	-0.710309872	0.477511999	0.95716557
Bcas3os1	0.762034288	0.587732519	1.296566488	0.194780389	0.879588734
Bcas3os2	0.202367568	0.377687473	0.535806938	0.592091987	0.974001549
2610027K06Rik	0.608310811	0.372366281	1.633635595	0.102335389	0.789151138
Tbx2	0.028164695	0.172559469	0.163217325	0.870347326	0.992886758
Tbx4	0.171431883	0.16901137	1.014321603	0.310429319	0.914704835
Brip1	-0.401865327	0.525813775	-0.764273105	0.444704509	0.948476747
Brip1os	-0.104894042	0.154494758	-0.678948871	0.497170259	0.960475196
Ints2	-0.29327195	0.263896616	-1.111313798	0.266433302	0.89768067
Med13	0.227420069	0.17408115	1.306402609	0.191415679	0.878250211

Rnft1	0.164127878	0.162583111	1.0095014	0.312734231	0.914819219
Rps6kb1	-0.04360064	0.132146162	-0.329942534	0.741443384	0.988585656
Tubd1	0.146134389	0.185531334	0.787653415	0.430899461	0.945321484
Vmp1	0.137794537	0.240323707	0.573370552	0.566393825	0.970426802
Ptrh2	0.381341345	0.178059445	2.141651873	0.032221506	0.665167506
Cltc	-0.003384826	0.121412148	-0.027878808	0.97775881	0.997262526
Dhx40	0.072883625	0.112212274	0.649515622	0.516005152	0.964352198
Ypel2	-0.199149746	0.230831795	-0.862748333	0.388275852	0.933269543
Gdpd1	-0.387349846	0.1542388	-2.511364496	0.012026545	0.525888996
Smg8	-0.203940503	0.222882664	-0.915012857	0.360184881	0.925913281
Prr11	-0.131555786	0.795955682	-0.16528029	0.868723372	0.992886758
Ska2	0.243423603	0.162541075	1.497612851	0.134233866	0.827986488
Trim37	0.161826046	0.112266538	1.441445063	0.149458987	0.848779008
Ppm1e	-0.214340569	0.892589161	-0.240133511	0.810226756	0.991438232
Rad51c	-0.688750626	0.644557999	-1.068562685	0.28526677	0.905916508
Tex14	0.685059616	0.601770162	1.138407418	0.254950398	0.897044747
Sep-04	0.096415381	0.138452892	0.696376794	0.486192892	0.957736773
Mtmr4	-0.032414644	0.215130486	-0.15067434	0.880232616	0.992886758
Hsf5	0.012509891	1.593561059	0.007850274	0.993736452	0.999270171
Rnf43	-0.014041456	0.39066329	-0.035942604	0.971328125	0.996733101
1110028F11Rik	0.895355382	1.045852552	0.856100968	0.391941933	0.933734231
Supt4a	0.42523525	0.167084573	2.545029994	0.010926835	0.502706181
Mir142b	-0.528198447	0.950368354	-0.555782866	0.5783593	0.971613657
Bzrap1	-0.479612729	0.40293695	-1.190292249	0.233931546	0.89581803
Mks1	-0.139508953	0.234567871	-0.594748771	0.552011416	0.968926429
Dynll2	0.130785162	0.139706799	0.93614028	0.349200966	0.921585102
Srsf1	-0.159654644	0.087623921	-1.82204406	0.068448301	0.743426713
Vezf1	0.017130021	0.115428356	0.148403922	0.882024005	0.992886758
2210416O15Rik	-0.036681085	1.350169925	-0.027167755	0.978325934	0.997262526
Cuedc1	0.12802683	0.188243949	0.680111265	0.496434012	0.960362776
Mrps23	0.200312485	0.16109902	1.243412193	0.213715979	0.884945675
Msi2	-0.063783274	0.102638918	-0.621433619	0.534314358	0.96674935
0610039H22Rik	0.267547644	1.675613981	0.159671408	0.873139925	0.992886758
C030037D09Rik	0.143296926	0.570031075	0.251384412	0.801516919	0.990988839
Akap1	0.103609879	0.171823937	0.603000263	0.546508514	0.967812517
Scpep1	-0.103115525	0.152469286	-0.676303584	0.498847918	0.960700483
Coil	-0.144242007	0.193624496	-0.744957428	0.456297456	0.951472839
Trim25	0.078658075	0.142197485	0.553160802	0.580153306	0.971613657
Dgke	-0.313054359	0.2724788	-1.148912721	0.250591972	0.896939594
Dgkeos	0.720891528	0.731532009	0.985454524	0.324400859	0.91640698
Gm525	0.666944835	0.54918599	1.214424343	0.224585724	0.892989918
4932411E22Rik	-1.263588832	1.22500135	-1.031499951	0.302306431	0.911518274
Ankfn1	3.523981289	2.009217298	1.7539075	0.079446358	0.75659671
Pctp	0.255354597	0.125228506	2.039109183	0.041439132	0.679118604
Tmem100	-0.04158718	0.128312466	-0.324108645	0.745855782	0.988585656
Mmd	0.114368505	0.128511219	0.889949574	0.373492963	0.928840865
Hlf	-0.289804119	0.349417675	-0.829391699	0.406882797	0.939557159
Stxbp4	-0.625523426	0.224018731	-2.792281795	0.005233775	0.406593461
Cox11	-0.050223296	0.182968536	-0.274491433	0.783706986	0.990988839
Tom1l1	-0.16866684	0.168376644	-1.001723496	0.316477155	0.91640698
Utp18	0.133109895	0.136195873	0.977341622	0.328400054	0.917115058
Mbtd1	-0.001778105	0.137021514	-0.012976834	0.989646275	0.998328992
Nme2	0.253153346	0.24634038	1.027656717	0.304111335	0.911786415
Nme1	0.176313786	0.154109917	1.144078132	0.252591236	0.897044747
Spag9	-0.111861885	0.121495824	-0.920705591	0.357204156	0.924354588
Tob1	-0.062489815	0.108394045	-0.576505981	0.564273228	0.969658675
Wfikkn2	0.512826643	0.731161524	0.701386254	0.483061998	0.957463006
Luc7l3	-0.038865755	0.105750793	-0.367522113	0.7132296	0.988585656
Ankrd40	-0.02465241	0.072563085	-0.339737626	0.734054124	0.988585656
Abcc3	0.049921389	0.179343511	0.278356259	0.780738893	0.990988839
Cacna1g	-0.014048204	1.72478395	-0.008144906	0.993501377	0.999270171
Spata20	0.763749714	2.580331861	0.295988948	0.767238521	0.989884648

Epn3	-0.43267566	0.1650654	-2.621237767	0.008761113	0.475600561
Mycbpap	0.104252397	0.214150034	0.486819426	0.626386302	0.97658408
Rsad1	0.059390425	0.466631064	0.127274907	0.89872282	0.994364545
Acsf2	-0.07220398	0.318892531	-0.226421046	0.820873961	0.992886758
Chad	-0.420042119	0.373639114	-1.124192043	0.260931564	0.897248195
Lrrc59	-0.031152703	0.116407863	-0.267616828	0.788994277	0.990988839
Eme1	-0.507582545	1.143032295	-0.44406667	0.656994383	0.979614766
Mrpl27	0.50378676	0.143166626	3.518884068	0.000433366	0.139568962
Xylt2	0.430709726	0.24970457	1.724877226	0.084549601	0.76099108
Gm11545	-0.981361909	0.948936315	-1.034170464	0.30105648	0.911162941
Col1a1	0.283305017	0.229228227	1.235908074	0.216492736	0.887633697
Sgca	-0.356373843	0.861273533	-0.41377545	0.679038565	0.983903958
Ppp1r9b	-0.043343119	0.202693898	-0.213835342	0.830675463	0.992886758
Samd14	0.224881943	0.316847316	0.709748612	0.477860041	0.957303176
Pdk2	-0.110232932	0.261958572	-0.420802919	0.673899	0.983375962
Itga3	-0.086010235	0.154000109	-0.558507626	0.576497799	0.971613657
Dlx4	1.231567752	2.226285246	0.55319405	0.580130541	0.971613657
A730090H04Rik	1.36687748	1.209164158	1.130431688	0.258294368	0.897248195
Tac4	0.596913092	1.619933234	0.368480058	0.712515312	0.988585656
Kat7	-0.205925702	0.249525082	-0.825270549	0.409218008	0.940809748
Fam117a	-0.082576655	0.19535111	-0.42270891	0.672507654	0.983233856
Slc35b1	-0.224957532	0.121651341	-1.849198951	0.06442909	0.73299549
Spop	0.063717045	0.065705718	0.96973363	0.332179283	0.917115058
Nxph3	0.180703497	0.183110809	0.986853247	0.323714587	0.91640698
Ngfr	-2.572651383	2.266269498	-1.135192167	0.256294811	0.897248195
Phb	0.260153731	0.163491797	1.591234151	0.11155689	0.800752782
Zfp652os	0.995317283	1.135633096	0.876442653	0.38078944	0.930293159
Zfp652	0.221522884	0.305759846	0.724499595	0.468759076	0.954173973
Phospho1	-0.161045855	0.994811763	-0.161885756	0.87139582	0.992886758
Abi3	0.097992213	0.204809945	0.478454369	0.632326845	0.977034429
Gngt2	0.62308042	0.168263213	3.703010362	0.000213056	0.091541268
4833417C18Rik	1.962342389	0.743440713	2.639541197	0.008301833	0.468007836
B4galnt2	0.643077246	1.013498729	0.634512139	0.525746647	0.965637248
Igf2bp1	0.766786759	0.75142097	1.020448975	0.307515577	0.9139204
Snf8	0.325778305	0.149800051	2.174754309	0.029648533	0.649459041
Ube2z	0.008646777	0.131134475	0.065938241	0.947426995	0.995343964
Atp5g1	-0.000371707	0.09500824	-0.00391237	0.996878388	0.999449398
Calcoco2	0.693907675	0.568542668	1.220502371	0.222274491	0.892391445
Ttll6	-1.0989943	0.476178818	-2.307944532	0.021002222	0.605836174
Hoxb8	0.950776658	1.368733646	0.694639648	0.487281159	0.958288569
Hoxb7	0.211917612	0.346617968	0.61138669	0.540943612	0.967041099
Hoxb5os	0.927727705	0.345428645	2.685728931	0.007237178	0.452031612
Hoxb6	0.55086461	0.299837017	1.83721348	0.066178386	0.738254074
Hoxb5	0.236219229	0.317130695	0.74486397	0.456353958	0.951472839
Hoxb4	0.087504634	0.262071523	0.333896001	0.738458037	0.988585656
Hoxb3	0.331494671	0.28244795	1.173648709	0.240535763	0.896939594
Hoxb2	0.010417661	0.159004531	0.065518013	0.947761565	0.995503286
Skap1	0.304361791	0.206136432	1.476506543	0.13980796	0.836746541
Snx11	-0.136542851	0.175405487	-0.778441162	0.436308981	0.946762848
Cbx1	0.301213667	0.123455428	2.439857614	0.014693052	0.554936045
Gm11517	-0.081470442	0.343843156	-0.236940712	0.812702785	0.992000477
Nfe2l1	-0.130226994	0.131750562	-0.988435967	0.32293918	0.91640698
Copz2	0.21953221	0.117997428	1.860483006	0.062817221	0.727650961
Cdk5rap3	0.132870815	0.168713938	0.787550904	0.430959442	0.945321484
Prr15l	-0.231199561	0.149541729	-1.546053819	0.122091571	0.816293642
Pnpo	0.012834373	0.170252485	0.075384348	0.939908913	0.994688009
D030028A08Rik	0.022223188	0.524934956	0.042335128	0.966231543	0.996474415
Sp2	-0.106822051	0.374307164	-0.285386072	0.775348377	0.990861469
Sp6	0.777227027	0.438796808	1.771268644	0.076516039	0.752876039
Scrn2	-0.150889193	0.202230799	-0.746123706	0.45559269	0.951331609
Lrrc46	-0.595442814	0.28695595	-2.075032125	0.037983557	0.679118604
Mrpl10	0.027488352	0.118835873	0.231313583	0.817071195	0.992678823

Osbpl7	0.36128517	0.350106222	1.031930163	0.302104835	0.911518274
Tbx21	0.781773434	0.750290974	1.041960334	0.29743007	0.909070179
Tbkbp1	-0.236415818	0.164386466	-1.438170817	0.150385593	0.849879783
Kpnb1	0.00763372	0.144848336	0.052701469	0.957969769	0.996226826
Npepps	-0.373931827	0.165833625	-2.254861315	0.024142035	0.623681975
Mrpl45	-0.206805619	0.123675689	-1.672160636	0.09449265	0.777748075
Gpr179	-0.276577676	1.042018734	-0.26542486	0.790682187	0.990988839
Socs7	-0.105549249	0.215094174	-0.4907118	0.623630299	0.975691835
Arhgap23	0.266866387	0.216995187	1.229826297	0.218762158	0.888671255
Srcin1	-0.139268575	0.476191776	-0.292463209	0.769932485	0.989990452
2410003L11Rik	-1.32154249	1.182542132	-1.117543683	0.263761933	0.897248195
E130012A19Rik	0.052177382	0.28855699	0.180821758	0.856507484	0.992886758
Mllt6	-0.019837644	0.162638992	-0.12197348	0.902920021	0.994688009
Cisd3	-0.051178727	0.147336786	-0.347358781	0.728321795	0.988585656
Pcgf2	-0.200055287	0.268366444	-0.745455669	0.455996301	0.951472839
Psmb3	0.006734862	0.126838638	0.05309787	0.957653928	0.996226826
Pip4k2b	-0.039314926	0.185406712	-0.212046942	0.832070413	0.992886758
Cwc25	-0.132394894	0.255167948	-0.518853935	0.603862603	0.974001549
Rpl23	0.234411799	0.099192147	2.36320924	0.018117437	0.58167504
Lasp1	-0.097457297	0.220459631	-0.44206414	0.658442793	0.979746192
B230217C12Rik	-0.114658852	0.47060046	-0.243643731	0.807506757	0.99099448
Fbxo47	-0.760187876	1.369419811	-0.555116751	0.578814806	0.971613657
Plxdc1	0.002756912	0.319759184	0.008621839	0.993120853	0.999241028
Arl5c	0.403074944	0.282320183	1.427722738	0.153371679	0.854159937
Cacnb1	-0.255695312	0.44679846	-0.572283334	0.567130039	0.970862413
Rpl19	0.223729815	0.18774533	1.191666471	0.23339205	0.89581803
Stac2	0.358996359	0.529148034	0.678442205	0.497491357	0.960690117
Fbxl20	-0.313485485	0.242668393	-1.291826601	0.196417201	0.879755417
Med1	0.022420823	0.191142021	0.11729929	0.906622888	0.994688009
Cdk12	0.195342208	0.352147265	0.554717379	0.579087987	0.971613657
Ppp1r1b	0.365148855	0.285174619	1.280439529	0.200390599	0.880888611
Stard3	-0.070329886	0.120653794	-0.582906542	0.559956217	0.968926429
Tcap	-0.233399598	0.249270463	-0.936330744	0.349102924	0.921552698
Pnmt	-0.530432777	0.506212632	-1.047845794	0.29470966	0.907334416
Pgap3	-0.211630798	0.309066965	-0.684740921	0.493507422	0.958916246
Erbb2	0.059546637	0.230202624	0.258670538	0.795889453	0.990988839
Mien1	0.282866101	0.202874584	1.394290476	0.163229906	0.859982992
Grb7	-0.082344625	0.168760767	-0.487937014	0.625594456	0.97639266
Ikzf3	0.471008102	0.350134397	1.345220882	0.178553915	0.867377413
Zpbp2	-0.405986683	1.027228882	-0.395225145	0.692676737	0.986599035
Ormdl3	-0.017316094	0.133831702	-0.129387087	0.897051365	0.994101834
Psmd3	0.11736841	0.17064363	0.687798362	0.491579767	0.958916246
Csf3	-0.075367138	1.356216819	-0.0555716	0.95568309	0.99614946
Med24	-0.133255109	0.298037013	-0.447109262	0.654796172	0.979133069
Thra	0.10444189	0.145044453	0.720068142	0.471483041	0.95486913
Nr1d1	0.032688905	0.177995308	0.183650375	0.854287741	0.992886758
Msl1	0.185701847	0.132350193	1.403109751	0.160584114	0.857316691
Gm12359	0.654264506	1.548302563	0.422568897	0.672609823	0.983233856
Casc3	-0.1204935	0.186410864	-0.646386685	0.518028961	0.964452679
Rapgefl1	-0.456175093	0.889465364	-0.512864369	0.608046199	0.974001549
Wipf2	0.094488138	0.27172103	0.347739511	0.728035821	0.988585656
Cdc6	-0.30387499	0.503029671	-0.604089595	0.545784077	0.967747244
Rara	0.058070982	0.222916075	0.26050603	0.794473464	0.990988839
Top2a	-0.271969283	0.361349563	-0.752648713	0.451661037	0.949134998
Igfbp4	-0.051455718	0.131908096	-0.390087638	0.696471743	0.987490725
Tns4	0.271164793	0.56860357	0.476896044	0.633436149	0.977034429
Ccr7	-0.541701999	0.303377534	-1.78557058	0.074168814	0.750704981
Smarce1	0.027469536	0.088084276	0.311855163	0.755150599	0.989196392
Krt222	0.799964238	0.647176558	1.236083458	0.216427543	0.887633697
Krt10	0.340005678	0.232017924	1.465428497	0.142804047	0.840741274
Krt23	0.497167213	0.213062974	2.333428493	0.019625667	0.593385684
Krtap17-1	0.388002653	0.299995422	1.293361915	0.195885916	0.879601031

Krt13	-0.250286855	1.176362085	-0.212763449	0.831511474	0.992886758
Krt15	-0.841977641	0.583495773	-1.442988417	0.149023734	0.848779008
Krt19	-0.030513452	0.145596934	-0.209574824	0.833999531	0.992886758
Krt14	-0.160645839	0.888160606	-0.180874763	0.856465878	0.992886758
Krt17	0.379413039	1.955771811	0.193996578	0.846178561	0.992886758
Eif1	0.018208478	0.108589421	0.167681883	0.86683355	0.992886758
Hap1	-0.40034819	0.34632404	-1.155993071	0.247683991	0.896939594
Jup	-0.06156092	0.23692177	-0.259836484	0.794989907	0.990988839
P3h4	0.142111233	0.173278308	0.82013285	0.412140377	0.942121471
Fkbp10	0.0473152	0.287765401	0.164422823	0.869398298	0.992886758
Nt5c3b	0.234673013	0.156118724	1.503170197	0.132795161	0.827800491
Klhl10	-0.257839473	1.18884779	-0.216881821	0.828300445	0.992886758
Klhl11	-0.601114042	0.491980742	-1.221824332	0.221774065	0.891628949
Acly	-0.252987675	0.119084699	-2.124434779	0.033633804	0.671818913
Ttc25	-0.335066528	0.330841944	-1.012769192	0.31117042	0.914819219
Cnp	0.01736823	0.177092621	0.098074272	0.921873317	0.994688009
Dnajc7	0.039189359	0.150146432	0.261007594	0.794086651	0.990988839
Nkiras2	0.073279288	0.14259052	0.513914167	0.607312002	0.974001549
Dhx58	0.086443891	0.295805615	0.292232083	0.770109183	0.989990452
Kat2a	-0.184364752	0.313038233	-0.588952827	0.555892919	0.968926429
Hspb9	0.082594428	0.892448827	0.092548083	0.926262591	0.994688009
Rab5c	0.030768788	0.08801492	0.349586051	0.726649381	0.988585656
Kcnh4	0.261720566	1.750321703	0.149527121	0.88113771	0.992886758
Ghdc	-0.275785648	0.212870321	-1.295557061	0.195128131	0.879588734
Stat5b	-0.169398979	0.164890901	-1.027339763	0.304260505	0.911879006
Stat5a	0.103403541	0.307964101	0.335764917	0.737048148	0.988585656
Stat3	-0.208551292	0.137150755	-1.52059893	0.128360515	0.826336015
Ptrf	0.105840291	0.182316633	0.580530085	0.561557205	0.969558766
Atp6v0a1	-0.19787825	0.239395993	-0.82657294	0.40847916	0.940123943
Naglu	-0.450115146	0.197913439	-2.274303089	0.02294777	0.620006604
Hsd17b1	-0.473956823	1.418827743	-0.334048179	0.738343203	0.988585656
Coasy	0.204339843	0.123172574	1.658971933	0.097121448	0.782676152
Mlx	-0.027407912	0.166044072	-0.165064082	0.868893544	0.992886758
Psmc3ip	0.622938161	0.216131818	2.882214048	0.003948914	0.378480793
Fam134c	0.102688087	0.096130376	1.068216853	0.285422704	0.905916508
Tubg1	0.208693207	0.182998968	1.140406471	0.254116998	0.897044747
Tubg2	0.743580393	0.535752278	1.387918304	0.165161924	0.860011234
Plekhh3	-0.042928321	0.302069917	-0.142113856	0.886990074	0.992886758
Ccr10	-1.716606065	1.801888304	-0.95267063	0.340756979	0.919850248
Cntnap1	0.824166954	1.023392297	0.805328471	0.420630156	0.943415794
Ezh1	-0.220710791	0.156997311	-1.40582529	0.15977601	0.85670375
Ramp2	-0.169820669	0.119024231	-1.426773925	0.153645069	0.854159937
Vps25	-0.006232174	0.103194878	-0.060392283	0.951843205	0.996023481
Wnk4	0.303796025	0.262245184	1.158442723	0.246683413	0.896939594
Coa3	0.343109635	0.242986754	1.412050775	0.157935011	0.855986074
Cntd1	0.053216007	0.515594405	0.103212925	0.917793982	0.994688009
Becn1	0.227422957	0.099577952	2.283868582	0.022379256	0.619833063
Psme3	-0.181758955	0.14410153	-1.261325642	0.207191546	0.883765092
Aoc2	-0.186461767	0.661457826	-0.281895171	0.778023892	0.990988839
Aoc3	-0.808041793	0.681434966	-1.185794439	0.235703488	0.896002526
Aarsd1	0.160186209	0.164214278	0.975470655	0.329326851	0.917115058
Ptges3l	0.331476427	0.239960255	1.381380542	0.167161985	0.860403806
Rundc1	-0.270503271	0.185510261	-1.458157996	0.144796999	0.842516249
Rpl27	0.574273523	0.188682745	3.043593211	0.00233771	0.31402518
Ifi35	0.054487388	0.121213864	0.449514491	0.653060557	0.979004493
Vat1	-0.176787097	0.190315124	-0.92891775	0.352931713	0.923596102
Rnd2	0.333581502	0.180639786	1.846666831	0.064795442	0.733548823
Brca1	-0.113960416	0.616822108	-0.184754104	0.853421908	0.992886758
Nbr1	-0.325093141	0.258985125	-1.255257962	0.20938513	0.884258007
Tmem106a	-0.23185042	0.208317521	-1.112966487	0.265722823	0.89768067
Rdm1	0.225965545	0.196317345	1.151021804	0.249723265	0.896939594
Arl4d	-0.068275256	0.373616052	-0.182741763	0.855000645	0.992886758

Dhx8	-0.181907515	0.296866388	-0.61275888	0.540035783	0.966900344
Etv4	-0.314950118	1.471484984	-0.214035564	0.830519324	0.992886758
Meox1	0.291669175	0.1636204	1.78259664	0.074651987	0.75241101
Sost	0.610009555	1.029265184	0.592665101	0.553405301	0.968926429
Dusp3	-0.192499607	0.112667942	-1.708557064	0.087533028	0.766704005
Mpp3	-0.639811813	0.480230822	-1.332300601	0.18276143	0.869194833
Cd300lg	-0.284279924	0.288077599	-0.986817181	0.32373227	0.91640698
Mpp2	-0.777223653	0.463709678	-1.6760997	0.093718665	0.775558829
Ppy	1.62991552	0.841531453	1.936844446	0.052764361	0.707153766
Nags	-0.028326516	0.931198118	-0.03041943	0.975732549	0.997262526
Tmem101	0.139493488	0.214244224	0.651095679	0.514984726	0.964352198
Lsm12	0.18971124	0.172208856	1.101634637	0.270620544	0.89804647
G6pc3	-0.102828105	0.178327928	-0.576623675	0.564193702	0.969658675
Hdac5	0.280656721	0.133808104	2.097456818	0.035953156	0.674795441
BC030867	-0.610545243	1.78795947	-0.341475997	0.732745271	0.988585656
Tmub2	-0.053358045	0.134349191	-0.39715941	0.691249912	0.986191149
Atxn7l3	-0.212647758	0.168284755	-1.263618668	0.20636693	0.883765092
Ubtf	-0.001580439	0.126378819	-0.012505571	0.990022258	0.998417328
Slc4a1	1.271239219	0.976516345	1.301810487	0.192981178	0.879588734
Rundc3a	0.023488934	0.493727175	0.047574723	0.962055177	0.996474415
Slc25a39	-0.076864003	0.134016093	-0.573543086	0.566277035	0.970426802
Grn	-0.264082657	0.220250839	-1.199008628	0.230524591	0.895404344
Fam171a2	0.4099323	0.290571225	1.410780781	0.158309263	0.855986074
Itga2b	0.450463091	0.346935084	1.298407431	0.194147367	0.879588734
Gpatch8	-0.290234749	0.224803665	-1.291058798	0.19668329	0.879755417
Gm11627	0.205421969	0.243936019	0.842114133	0.399724071	0.936395796
Fzd2	-0.068726706	0.298243601	-0.230438157	0.817751314	0.992734473
2810433D01Rik	0.263672344	0.271292398	0.971912026	0.331094316	0.917115058
Gm1564	-0.359567242	1.576372768	-0.228097852	0.81957017	0.992886758
Ccdc43	-0.089918782	0.14157577	-0.635128327	0.525344724	0.965637248
Adam11	-2.018833407	0.991588259	-2.03595937	0.041754435	0.679118604
Gjc1	0.079242519	0.199605418	0.396995835	0.691370532	0.986196635
Higd1b	0.436002523	0.206551738	2.110863498	0.034784046	0.674795441
Eftud2	0.048856603	0.107189103	0.455798225	0.648535081	0.979004493
Ccdc103	-0.098991988	0.20208291	-0.489858285	0.624234184	0.975969024
Gfap	0.462063041	0.599008922	0.77137923	0.440482182	0.947904651
Kif18b	2.145780491	1.285030503	1.669828449	0.09495331	0.778239075
2410004I01Rik	0.468312908	0.210482589	2.224948442	0.0260847	0.631530315
Dcakd	-0.272035228	0.27974841	-0.972428145	0.330837596	0.917115058
Nmt1	-0.115867095	0.123563559	-0.937712509	0.348392173	0.921552698
Plcd3	0.317063911	0.291163627	1.088954394	0.276173997	0.900302631
Acbd4	0.096415698	0.12536658	0.769070182	0.441851648	0.948476447
Hexim1	-0.049925496	0.165823074	-0.301076894	0.76335586	0.989285682
Hexim2	0.68802539	0.536081488	1.283434339	0.19933995	0.880831248
Fmnl1	0.105406293	0.304277009	0.346415567	0.729030428	0.988585656
1700023F06Rik	-0.287440193	0.864170471	-0.332619781	0.73942131	0.988585656
Map3k14	0.007086326	0.227082247	0.03120599	0.975105263	0.997262526
Arhgap27	0.207730685	0.205816211	1.009301861	0.312829888	0.914819219
Arhgap27os3	0.285745975	1.602802078	0.178279014	0.858503859	0.992886758
Plekhm1	0.03650698	0.195394891	0.186836921	0.851788498	0.992886758
Rprml	-0.195051521	0.403918943	-0.482897681	0.629168389	0.977034429
Gosr2	-0.048202742	0.126194242	-0.38197259	0.702481696	0.988482081
C130046K22Rik	0.086166571	0.282399253	0.305123226	0.760272302	0.989266039
Wnt9b	2.361757082	1.442994335	1.63670572	0.101691973	0.788783372
Nsf	-0.374655224	0.207395601	-1.806476229	0.070843984	0.744227652
Arf2	0.002428626	0.100485261	0.024168973	0.980717827	0.997296835
Mapt	0.176981116	0.165349878	1.070343191	0.28446486	0.905916508
Kansl1	-0.186421044	0.233492283	-0.798403449	0.424636403	0.944280828
Cdc27	-0.369722534	0.130731062	-2.828115428	0.004682292	0.391906015
Myl4	0.50705347	0.29831805	1.699707643	0.089185931	0.768297719
Gm11665	1.620967752	1.639720921	0.988563195	0.322876901	0.91640698
Itgb3	0.013374131	0.221065353	0.060498542	0.951758577	0.996023481

Mettl2	0.023138687	0.134083423	0.172569333	0.862989959	0.992886758
Tlk2	-0.084462803	0.16702421	-0.505691977	0.613072901	0.974001549
1700052K11Rik	0.318198753	0.265630134	1.197901566	0.23095534	0.895404344
Mrc2	0.146907968	0.1568645	0.936527816	0.349001498	0.921552698
Mar-10	1.163214273	1.045399877	1.112697924	0.265838188	0.89768067
Tanc2	0.252604594	0.293663986	0.860182404	0.389688503	0.933734231
Cyb561	-0.102368051	0.172282268	-0.594187969	0.552386398	0.968926429
Ace	-0.167690382	0.206915218	-0.810430394	0.417692855	0.943415794
Ace3	-0.619617431	0.517920115	-1.196357148	0.231557216	0.895404344
Dcaf7	0.072318682	0.225275646	0.32102308	0.7481929	0.988585656
Taco1	0.195825652	0.20816251	0.940734487	0.346840941	0.921552698
Map3k3	-0.223687535	0.170698734	-1.310422927	0.190052802	0.876945711
Limd2	0.12815273	0.109456376	1.17081101	0.241674751	0.896939594
Strada	-0.285017302	0.254059816	-1.121851172	0.261925724	0.897248195
Ccdc47	-0.128500469	0.134509407	-0.955327	0.339412358	0.919246575
Ddx42	0.003301916	0.197809312	0.016692422	0.986681993	0.998148029
Ftsj3	-0.294040521	0.287985324	-1.02102606	0.307242092	0.9139204
Psmc5	0.148907363	0.096743168	1.539202885	0.123754774	0.820382953
Smarcd2	-0.204625513	0.157040868	-1.303008039	0.192572017	0.879588734
Cd79b	0.274795017	0.339512298	0.809381628	0.418295667	0.943415794
Scn4a	0.25774256	1.375576088	0.187370631	0.851370048	0.992886758
Prr29	0.039411572	0.245734515	0.160382728	0.872579594	0.992886758
Icam2	0.025458631	0.112439653	0.226420397	0.820874465	0.992886758
Ern1	-0.049144987	0.414753725	-0.118491973	0.905677855	0.994688009
Snord104	0.360472888	0.353829885	1.018774569	0.308310002	0.9139204
Tex2	-0.003728928	0.162601202	-0.022932965	0.981703745	0.997390438
Pecam1	-0.090546449	0.157364354	-0.575393645	0.565025101	0.969658675
Milr1	0.318356119	0.219109164	1.452956655	0.146235789	0.843780493
Polg2	0.003671271	0.295224869	0.012435507	0.990078157	0.998417328
Ddx5	-0.127347957	0.101007123	-1.260781938	0.207387423	0.883765092
Mir3064	0.478700524	0.585850594	0.817103421	0.413869328	0.943415794
Cep95	0.059559275	0.2321428	0.256563092	0.79751607	0.990988839
Smurf2	-0.193273824	0.129255588	-1.495284097	0.134840314	0.829704995
Kpna2	-0.162829382	0.147925335	-1.100753857	0.271003799	0.89804647
1810010H24Rik	-0.094646746	0.162443289	-0.582644853	0.560132405	0.968926429
Bptf	0.009793362	0.196455716	0.049850229	0.960241739	0.996474415
Nol11	-0.288510667	0.208875757	-1.381254919	0.167200594	0.860403806
Pitpnc1	0.114485198	0.140261944	0.816224237	0.414371897	0.943415794
Psmd12	0.003872125	0.097584171	0.039679852	0.968348365	0.996474415
Helz	-0.304387698	0.613710886	-0.495978978	0.619909243	0.974471036
Prkca	-0.400363147	0.252933902	-1.58287657	0.1134496	0.806530837
Apoh	2.336587974	1.870555975	1.2491409	0.211613544	0.884755882
Cep112	-0.126221567	0.335711862	-0.375981851	0.706930389	0.988585656
Axin2	-0.537467023	0.26215343	-2.050200235	0.040344895	0.679118604
E030025P04Rik	0.614029842	1.632529955	0.376121639	0.706826468	0.988585656
Rgs9	-0.200984018	0.336263857	-0.597697354	0.550041891	0.968926429
1700096J18Rik	-1.680329022	1.606455988	-1.045985097	0.295567913	0.908201248
Gm11696	-0.654347883	0.368697342	-1.77475617	0.075938156	0.752876039
Gna13	-0.079491086	0.157005156	-0.506296024	0.612648854	0.974001549
Amz2	0.087016208	0.121202055	0.717943342	0.472792222	0.955238041
Slc16a6	0.113559455	0.506120314	0.22437245	0.822467506	0.992886758
Arsg	-0.251383281	0.301072083	-0.834960446	0.403739979	0.937834293
Wipi1	-0.315339581	0.229711122	-1.372765839	0.169825154	0.861730117
Prkar1a	-0.114115063	0.086978668	-1.311989086	0.189523817	0.876091552
Fam20a	-0.13603758	0.436189451	-0.311877282	0.755133788	0.989196392
Abca8b	0.030099624	0.282389193	0.106589149	0.915114927	0.994688009
Abca8a	-0.033381346	0.142550384	-0.234172264	0.814851246	0.992116023
Abca9	-0.232169459	0.287952357	-0.806277335	0.420082956	0.943415794
Abca6	-0.551862638	0.465695467	-1.185029011	0.236005976	0.896109204
Abca5	-0.005203629	0.159248471	-0.032676161	0.973932834	0.997262526
Map2k6	-0.496237531	0.280797872	-1.767241065	0.077187865	0.75328528
Kcnj2	-0.319818976	0.225896212	-1.415778394	0.156840397	0.855906801

Sox9	-0.836930885	1.664519312	-0.502806353	0.615100427	0.974001549
2610035D17Rik	0.459717038	0.245997146	1.868790129	0.061652019	0.725730283
Slc39a11	-0.015970693	0.159666387	-0.10002539	0.920324168	0.994688009
Sstr2	0.393763896	0.813637039	0.483955224	0.628417647	0.977034429
Cog1	-0.446774038	0.24665274	-1.811348368	0.070086943	0.744227652
Fam104a	0.209309654	0.165705917	1.263139287	0.206539127	0.883765092
D11Wsu47e	-0.472640538	0.266095159	-1.776208707	0.075698524	0.752876039
Cpsf4l	1.823510256	2.438678002	0.747745399	0.454613742	0.951112066
Cdc42ep4	-0.010044544	0.183497391	-0.05473944	0.956346048	0.996173928
Sdk2	0.415107747	2.265723996	0.183211966	0.854631706	0.992886758
Rpl38	0.132689576	0.171825934	0.77223253	0.439976718	0.947849924
Ttyh2	0.078628992	0.368681889	0.213270558	0.831115937	0.992886758
Dnaic2	-0.38694982	0.163077151	-2.372802188	0.017653716	0.579835303
Kif19a	-0.333051509	0.343409866	-0.969836751	0.332127871	0.917115058
Btbd17	0.618081806	2.353182059	0.262657878	0.792814286	0.990988839
Gprc5c	-0.474854397	0.238631525	-1.989906395	0.046601248	0.698868643
Cd300a	0.801724676	0.211907652	3.78336823	0.00015472	0.077321054
Cd300lb	0.738750371	0.302574771	2.441546487	0.014624505	0.554936045
Cd300c	0.81346634	0.515359681	1.57844389	0.114463669	0.809025248
Cd300ld	0.452365582	0.224502267	2.014971113	0.043907676	0.688477147
AF251705	0.176337871	0.181089962	0.973758394	0.330176516	0.917115058
Cd300e	0.204208574	0.295793129	0.690376327	0.48995756	0.958560421
Rab37	0.772979337	0.392846014	1.967639505	0.049109531	0.707153766
Cd300lf	-0.117806893	0.280252553	-0.42035975	0.674222667	0.983375962
Slc9a3r1	-0.136986792	0.187549497	-0.730403406	0.465143637	0.953083512
Nat9	0.196149771	0.132556641	1.479743073	0.138941826	0.835236816
Tmem104	0.189201871	0.394007181	0.480199042	0.631085868	0.977034429
Grin2c	0.449399274	0.828558393	0.542386967	0.587551956	0.973455044
Fdxr	0.065303217	0.270172205	0.241709604	0.809005187	0.991192232
Fads6	-0.13711614	0.594667228	-0.230576251	0.81764402	0.992688423
Hid1	-0.195983919	0.232746222	-0.842049843	0.399760055	0.936395796
Cdr2l	-0.172875086	0.310916648	-0.556017463	0.578198917	0.971613657
Ict1	0.160289593	0.161751029	0.990964902	0.321702719	0.91640698
Atp5h	0.316163381	0.233402915	1.354581971	0.175550781	0.864596675
Kctd2	-0.086726008	0.118759649	-0.730264939	0.465228255	0.953083512
Slc16a5	-0.757245477	1.420981219	-0.532903227	0.594100571	0.974001549
Armc7	0.188713204	0.363030799	0.519826979	0.603184173	0.974001549
Nt5c	0.168339605	0.152918348	1.10084635	0.270963535	0.89804647
Hn1	0.143512872	0.145805104	0.984278791	0.324978455	0.91640698
Sumo2	0.205016735	0.106108836	1.932136303	0.053342685	0.707153766
Nup85	0.170305648	0.14710847	1.157687576	0.246991556	0.896939594
Gga3	0.060257926	0.325043274	0.185384318	0.852927608	0.992886758
Mrps7	0.098627397	0.116978056	0.843127341	0.399157229	0.936273856
Mif4gd	0.099741518	0.128144993	0.778348926	0.43636334	0.946762848
Slc25a19	-0.046569802	0.199932898	-0.23292716	0.815817968	0.992524109
Grb2	0.077962773	0.0807615	0.965345781	0.334371649	0.918030088
2310067B10Rik	-0.205327401	0.257875724	-0.796226176	0.425900586	0.944290206
Caskin2	0.202386196	0.218533638	0.926110036	0.354388799	0.924354588
Tsen54	0.076119003	0.153359408	0.496343878	0.619651814	0.974471036
Llgl2	0.088831363	0.187650707	0.473386776	0.635937262	0.977218605
Recql5	0.024886273	0.193691276	0.128484223	0.897765783	0.994101834
Smim5	0.034096863	0.157475467	0.216521747	0.828581075	0.992886758
Smim6	0.202422446	0.266550353	0.759415409	0.447604099	0.948476747
Sap30bp	0.146455904	0.117541122	1.245997159	0.212765438	0.884945675
Itgb4	-0.442998739	0.443836709	-0.998111985	0.318225059	0.91640698
Galk1	0.188343912	0.125641476	1.499058413	0.133858479	0.827800491
H3f3b	0.159465168	0.074190899	2.149389888	0.031603506	0.661395937
Unk	-0.082022916	0.322669113	-0.254201325	0.799340027	0.990988839
Unc13d	0.021732291	0.148041854	0.146798292	0.883291233	0.992886758
Wbp2	-0.312770335	0.217574278	-1.437533599	0.150566433	0.849879783
Trim47	0.262022141	0.185890567	1.409550494	0.158672454	0.855986074
Trim65	0.169260537	0.315810508	0.535956001	0.591988959	0.974001549

Mrpl38	0.139160164	0.138627366	1.003843384	0.315454109	0.91586731
Fbf1	-0.114124429	0.230893756	-0.494272476	0.621113761	0.97514112
Acox1	-0.009802665	0.141975014	-0.069045003	0.944953798	0.99486515
Ten1	0.006418703	0.15703116	0.040875348	0.967395271	0.996474415
Cdk3-ps	0.447784008	0.977730817	0.457982913	0.646964717	0.979004493
Evpl	0.275914783	0.478390036	0.576756961	0.564103648	0.969658675
Srp68	0.032555025	0.173146604	0.188020003	0.850860971	0.992886758
Exoc7	-0.01465935	0.166852468	-0.087858153	0.929989417	0.994688009
Foxj1	-0.23441991	0.133189209	-1.76005182	0.078399021	0.754732183
Rnf157	0.329729782	0.594108989	0.55499881	0.578895474	0.971613657
Prpsap1	-0.198881295	0.142462453	-1.396026041	0.162706653	0.859982992
Sphk1	-0.066265209	0.176309384	-0.375846184	0.707031251	0.988585656
Ube2o	-0.30032631	0.25648662	-1.170923887	0.241629373	0.896939594
Rhbdf2	-0.029371484	0.264980228	-0.110844059	0.911740006	0.994688009
Cygb	-0.000475906	0.159580855	-0.002982227	0.99762053	0.99957296
Gm11744	-0.211498829	0.392835629	-0.538390139	0.590307733	0.974001549
1810032O08Rik	0.316949061	0.250937284	1.263060854	0.206567311	0.883765092
St6galnac2	-0.055516436	0.172370146	-0.322076865	0.747394464	0.988585656
Mxra7	0.281178341	0.107913982	2.605578405	0.009171928	0.478229167
Jmjd6	0.030932869	0.14263923	0.216860881	0.828316764	0.992886758
Mettl23	0.282277157	0.260385841	1.084072607	0.278332607	0.9014752
Srsf2	-0.295734556	0.071525864	-4.134652	3.55E-05	0.026382421
Mfsd11	-0.028148759	0.156424791	-0.179950755	0.857191229	0.992886758
Snhg20	0.622046663	0.265383429	2.34395443	0.019080502	0.590246276
Mir6516	-0.168135556	1.159211423	-0.145043046	0.884676885	0.992886758
Sec14l1	-0.286625486	0.158068248	-1.813302096	0.06978524	0.744227652
Sep-09	-0.12795586	0.192084206	-0.66614462	0.505318667	0.962138588
2900041M22Rik	0.400769345	0.415044402	0.965605953	0.334241396	0.918030088
Tnrc6c	0.051128921	0.286988848	0.178156472	0.858600093	0.992886758
Tmc6	0.05106251	0.189300888	0.269742583	0.787358298	0.990988839
Tmc8	0.109737795	0.698666575	0.157067476	0.87519167	0.992886758
Syngr2	-0.22282196	0.140913193	-1.581271099	0.113816065	0.807243653
Tk1	0.430708004	0.276916334	1.555372331	0.11985742	0.81558552
Afmid	0.428997948	0.432622569	0.991621746	0.321382077	0.91640698
Birc5	-0.119969644	0.285389182	-0.42037208	0.674213661	0.983375962
Tha1	0.051874768	0.314589139	0.164896881	0.869025147	0.992886758
Socs3	-0.352693641	0.206707214	-1.706247375	0.087962032	0.7667678
Pgs1	0.017052457	0.141268405	0.12070963	0.903921031	0.994688009
Dnah17	1.516579837	1.438381306	1.054365648	0.291715544	0.907334416
Cyth1	0.047380929	0.148075242	0.319978736	0.74898445	0.988585656
Usp36	-0.226472319	0.229762336	-0.985680783	0.324289782	0.91640698
Timp2	-0.069151793	0.11412944	-0.605906703	0.544576708	0.967491297
BC100451	0.419955664	1.295602921	0.324139176	0.745832668	0.988585656
Lgals3bp	-0.230833252	0.195777146	-1.179061277	0.238373777	0.896609492
Cant1	-0.065716745	0.136596504	-0.481101224	0.630444558	0.977034429
C1qtnf1	-0.720416081	0.291141623	-2.474452377	0.013344061	0.539384266
Gm11747	4.040063128	1.556631858	2.595387669	0.009448428	0.479082259
Engase	0.031855663	0.276770389	0.115097799	0.908367604	0.994688009
Rbfox3	0.096196051	0.537783503	0.178875049	0.858035815	0.992886758
Cbx2	-0.101692322	0.458417749	-0.221833299	0.824443656	0.992886758
Cbx8	0.352657949	0.19116758	1.844758142	0.065072729	0.734014162
Cbx4	-0.202828061	0.304137609	-0.666895692	0.504838763	0.962138588
Tbc1d16	-0.053070707	0.159486027	-0.332760857	0.739314808	0.988585656
Ccdc40	-0.243622335	0.334754587	-0.727763994	0.466758069	0.953083512
Gaa	0.014430873	0.204777463	0.070471001	0.943818781	0.99486515
Eif4a3	-0.007074474	0.126801355	-0.055791782	0.955507681	0.99614946
Sgsh	0.253825479	0.399987325	0.634583805	0.525699893	0.965637248
Slc26a11	0.135334241	0.328440728	0.412050729	0.680302241	0.984207811
Endov	0.129431524	0.202887765	0.637946425	0.523508556	0.965637248
Nptx1	-0.559895745	0.383556102	-1.459749284	0.144358992	0.841934045
Rptor	0.063537731	0.209021245	0.30397738	0.761145124	0.989266039
Rptoros	-0.232856053	0.266051194	-0.87523025	0.381448637	0.930635634

Chmp6	0.171573257	0.123268969	1.391860887	0.163964528	0.859982992
Baiap2	-0.319121335	0.176125605	-1.811896318	0.070002218	0.744227652
Aatk	-0.212146336	0.336975883	-0.629559405	0.52898289	0.965637248
Cep131	0.214526018	0.561448725	0.382093695	0.702391868	0.988482081
Enthd2	-0.341945298	0.281570903	-1.214419863	0.224587434	0.892989918
1810043H04Rik	0.213329721	0.159352748	1.338726341	0.180659778	0.868087032
Slc38a10	0.057068189	0.135832933	0.420135144	0.67438673	0.983375962
2810410L24Rik	0.368602898	0.26970842	1.366671824	0.171728193	0.86244803
Bahcc1	0.622837379	0.437806328	1.422632199	0.154842805	0.854973821
Actg1	-0.148628713	0.15917847	-0.933723715	0.350446429	0.922338606
Mir6935	1.057983731	1.112176009	0.95127365	0.341465479	0.919850248
0610009L18Rik	0.558942215	0.219941836	2.541318312	0.011043533	0.5053707
Fscn2	0.904748108	0.845624237	1.069917427	0.284656478	0.905916508
Faap100	0.067057906	0.249597043	0.268664665	0.788187746	0.990988839
Nploc4	0.026557545	0.167872878	0.158200331	0.874298943	0.992886758
Oxld1	0.191379375	0.220292441	0.868751437	0.3849831	0.931689378
Ccdc137	-0.008874252	0.111884776	-0.079315992	0.936781287	0.994688009
Arl16	0.105498594	0.159147368	0.662898768	0.507395391	0.963011338
Hgs	-0.215843206	0.263980104	-0.817649522	0.413557341	0.94325631
Mrpl12	0.207693278	0.193822889	1.071562182	0.283916727	0.905916508
Slc25a10	-0.032006314	0.147208063	-0.217422287	0.827879264	0.992886758
Gcgr	0.97076849	0.807026634	1.202895232	0.229016866	0.895404344
Fam195b	0.139685343	0.141679855	0.985922403	0.324171192	0.91640698
P4hb	-0.158863289	0.1359225	-1.168778452	0.242492904	0.896939594
Arhgdia	-0.164309849	0.179759217	-0.914055211	0.360687836	0.925913281
Alyref	-0.048001535	0.115905005	-0.414145489	0.678767561	0.983863455
Anapc11	0.250485142	0.173116413	1.446917351	0.147920073	0.847400362
Npb	-1.851800919	1.329735731	-1.392608227	0.163738294	0.859982992
Pcyt2	0.019207363	0.157188261	0.122193368	0.902745879	0.994688009
Sirt7	0.256218007	0.13484707	1.900063588	0.057424775	0.721735927
Mafg	-0.064139087	0.314917955	-0.203669196	0.838612015	0.992886758
Pycr1	-0.037188641	0.697799027	-0.0532942	0.957497501	0.996226826
Myadml2	0.116032854	0.562642522	0.206228376	0.836612523	0.992886758
Notum	-0.142240677	0.29531638	-0.481655222	0.630050891	0.977034429
Aspscr1	0.176962516	0.162602142	1.088316018	0.276455622	0.900604936
Stra13	0.191988253	0.194589568	0.986631788	0.323823181	0.91640698
Lrrc45	0.10693883	0.210298828	0.508508922	0.611096484	0.974001549
Rac3	-0.08182338	0.154524624	-0.529516773	0.596447012	0.974001549
Dcxr	0.185737698	0.146424072	1.26849155	0.204622467	0.883765092
Cbr2	-0.025314203	0.173007761	-0.146318307	0.883670115	0.992886758
Rfng	-0.0958094	0.336106832	-0.28505639	0.77560094	0.990861469
Gps1	-0.047422006	0.080101783	-0.592021851	0.553835957	0.968926429
Dus1l	0.146253263	0.125718356	1.163340562	0.244691355	0.896939594
Fasn	0.010846116	0.181814546	0.059654831	0.952430547	0.996023481
Ccdc57	-0.160858078	0.432829454	-0.371643096	0.710158597	0.988585656
Slc16a3	-0.509984074	0.262555154	-1.942388355	0.052090104	0.707153766
Csnk1d	-0.102863261	0.172545929	-0.596150031	0.551075004	0.968926429
Cd7	1.005393085	0.240556145	4.179452928	2.92E-05	0.022718725
Sectm1b	-1.364729512	1.092132065	-1.249601176	0.211445273	0.884755882
Sectm1a	0.159227977	2.029698859	0.078449065	0.937470846	0.994688009
Ogfod3	0.362367626	0.277176009	1.307355669	0.191091946	0.877906723
Hexdc	0.365266059	0.264685052	1.380002593	0.167585846	0.860403806
BC017643	-0.080537834	0.120248633	-0.669760913	0.503010214	0.961729397
Narf	-0.035382272	0.173529116	-0.203898189	0.83843306	0.992886758
Foxk2	-0.2827216	0.139002548	-2.033931068	0.041958546	0.679118604
Wdr45b	-0.142121711	0.12194937	-1.165415713	0.24385076	0.896939594
Rab40b	0.19190831	0.287672001	0.667108058	0.504703114	0.962138588
Fn3krp	0.026673098	0.193620013	0.137760024	0.890430079	0.993043931
Fn3k	0.527546174	0.271009093	1.946599533	0.051582765	0.707153766
Tbcd	-0.148443254	0.229477049	-0.646876253	0.517712036	0.964437658
Zfp750	-0.994556234	0.634044922	-1.568589542	0.116743611	0.812418014
B3gntl1	-0.005253886	0.358440444	-0.014657627	0.988305325	0.998148029

Metrnl	-0.044979295	0.202236424	-0.222409466	0.823995144	0.992886758
Ptchd3	0.329152605	1.527755216	0.215448523	0.829417642	0.992886758
Rab10os	0.042605466	0.214965571	0.198196697	0.842891172	0.992886758
Rab10	-0.175506501	0.16558395	-1.059924597	0.289178904	0.907334416
Kif3c	-0.113683904	0.236183447	-0.481337303	0.630276789	0.977034429
1110002L01Rik	0.256182182	0.381075779	0.672260468	0.501417892	0.96132573
Asxl2	-0.169920745	0.193520407	-0.878050786	0.379916159	0.929550595
Dtnb	-0.065860366	0.2583197	-0.254956808	0.798756459	0.990988839
Dnmt3a	0.005786764	0.243799788	0.023735723	0.981063412	0.997364526
Pomc	0.522992653	0.551342252	0.94858076	0.34283388	0.919850248
Efr3b	0.218371669	0.448581664	0.486804715	0.626396729	0.97658408
Dnajc27	0.132951212	0.208142477	0.638750982	0.522984941	0.965637248
Adcy3	0.18480394	0.354506236	0.521299547	0.602158117	0.974001549
Cenpo	-0.030848859	0.303849096	-0.101526907	0.919132199	0.994688009
Ptrhd1	0.183473764	0.246647613	0.743870016	0.456955117	0.951742083
Ncoa1	0.032292887	0.20433621	0.158038006	0.874426851	0.992886758
Itsn2	-0.363537853	0.362038769	-1.004140675	0.315310812	0.91586731
Fam228b	3.542787707	1.900312038	1.86431893	0.06227693	0.726801598
Pfn4	-0.457892301	0.917131613	-0.499265639	0.617592258	0.974150208
Gm6682	0.896128718	1.042488527	0.859605353	0.390006623	0.933734231
Sf3b6	0.042939965	0.198464225	0.216361237	0.828706179	0.992886758
Fkbp1b	0.111196181	0.189191813	0.587743095	0.556704745	0.968926429
BC068281	-0.049567242	0.455741728	-0.108761694	0.913391507	0.994688009
Mfsd2b	-0.370971731	0.701819799	-0.528585445	0.597093059	0.974001549
Ubxn2a	-0.236543453	0.131193295	-1.803014801	0.07138589	0.744227652
Atad2b	-0.417535797	0.321277377	-1.299611574	0.193734131	0.879588734
Klhl29	0.166171358	0.535339233	0.310403848	0.756253869	0.989266039
2810032G03Rik	-0.244370811	0.676286122	-0.361342342	0.717843545	0.988585656
Apob	-0.720320002	0.968742487	-0.743561898	0.457141561	0.951887549
Ldah	-0.239497567	0.174128246	-1.375409059	0.169004662	0.861597949
Hs1bp3	-0.439475594	0.222767478	-1.972799609	0.048518398	0.706907143
Rhob	0.04354308	0.171936785	0.253250516	0.800074632	0.990988839
Pum2	-0.082901176	0.108628503	-0.763162272	0.445366626	0.948476747
Sdc1	-0.253802037	0.130835778	-1.939851928	0.052397688	0.707153766
Laptm4a	0.008090357	0.10814297	0.07481168	0.940364549	0.994688009
Matn3	-1.359018574	1.903718612	-0.713875762	0.475304006	0.956761005
Wdr35	-0.041422186	0.151790261	-0.272890934	0.784937056	0.990988839
Ttc32	0.318756598	0.211127067	1.509785567	0.131098149	0.827800491
Osr1	0.516450592	0.453297142	1.139320204	0.254569625	0.897044747
Nt5c1b	-0.036480619	1.251193246	-0.029156663	0.976739645	0.997262526
Rdh14	0.234674093	0.147497372	1.591039145	0.111600766	0.800752782
Gm38407	1.160445752	0.468984022	2.474382277	0.01334668	0.539384266
Kcns3	0.348596098	0.159244468	2.189062527	0.028592296	0.645679684
Gen1	-0.17285184	0.877117588	-0.197068036	0.843774297	0.992886758
Smc6	-0.035109036	0.137303846	-0.255703226	0.798180004	0.990988839
Vsnl1	-0.10792764	0.247746477	-0.435637435	0.663099806	0.980876591
Fam49a	-0.021642692	0.162471224	-0.133209389	0.894027786	0.993422442
Mycn	-0.710800711	1.13079013	-0.62858765	0.529619048	0.965637248
Ddx1	-0.022590337	0.10822156	-0.208741562	0.834649993	0.992886758
Nbas	-0.268796541	0.256227721	-1.049053321	0.294153578	0.907334416
Fam84a	-0.236419504	0.187174751	-1.263095068	0.206555016	0.883765092
Trib2	-0.174783932	0.119536165	-1.462184534	0.143690649	0.841934045
Lpin1	-0.432081465	0.296620525	-1.456680938	0.145204474	0.842729381
E2f6	0.04094099	0.144427322	0.283471225	0.776815637	0.990861469
Rock2	-0.185006276	0.141755716	-1.305106281	0.19185666	0.878912371
Pqlc3	-0.294664506	0.141694563	-2.079575252	0.037564509	0.679118604
2410004P03Rik	-0.120714147	0.137954136	-0.875031013	0.381557031	0.930635634
Kcnf1	0.621164298	1.094361317	0.5676044	0.570303619	0.971613657
Pdia6	-0.121921304	0.105428347	-1.156437598	0.24750221	0.896939594
Atp6v1c2	-0.129705679	0.157007513	-0.826111293	0.408740962	0.940528702
Nol10	-0.092339702	0.243359179	-0.379437925	0.704362692	0.988585656
Odc1	-0.060500708	0.108641014	-0.556886447	0.577605015	0.971613657

Hpcal1	-0.321876591	0.199256922	-1.615384737	0.106227404	0.794491442
5730507C01Rik	0.005070041	0.375447867	0.013503981	0.98922571	0.998182772
1700030C10Rik	-0.437856344	0.511664778	-0.855748456	0.392136931	0.933734231
Asap2	-0.000107046	0.216950653	-0.000493414	0.999606313	0.999950896
Itgb1bp1	0.279960075	0.202907649	1.379741353	0.167666296	0.860403806
Cpsf3	0.07383413	0.145857564	0.506207069	0.612711293	0.974001549
Iah1	-0.075198977	0.122194011	-0.615406404	0.538286364	0.96674935
Adam17	0.039169291	0.179665173	0.218012707	0.827419211	0.992886758
Ywhaq	0.001734566	0.116968785	0.014829308	0.988168358	0.998148029
2410018L13Rik	-1.388125356	1.834630988	-0.756623738	0.449275315	0.948476747
9030624G23Rik	-0.717846794	0.899763704	-0.797817016	0.424976685	0.944290206
Taf1b	0.224859231	0.144299567	1.558280704	0.119166721	0.814755048
Grhl1	-0.154338882	0.362213576	-0.426099109	0.670035629	0.982177283
Klf11	0.194904687	0.223822859	0.870798844	0.383863997	0.931591679
Cys1	0.027895007	0.188147136	0.14826166	0.882136272	0.992886758
Rrm2	-0.254143769	0.27497537	-0.92424194	0.355360364	0.924354588
Mboat2	0.261818116	0.306792867	0.853403531	0.393435564	0.933734231
Kidins220	-0.115912293	0.125903631	-0.920642972	0.357236859	0.924354588
Id2	-0.096621103	0.124174959	-0.77810457	0.436507369	0.946762848
Rnf144a	0.181773363	0.221362152	0.821158276	0.411556119	0.941762685
Rsad2	0.499510332	0.344446732	1.450181653	0.14700787	0.844842482
Cmpk2	0.295090122	0.172389476	1.711764129	0.086940145	0.765418158
Sox11	0.193522083	0.355408203	0.544506517	0.586092965	0.972904784
Colec11	0.470295655	0.521061357	0.902572506	0.366752824	0.927936364
Rps7	0.340166235	0.246154584	1.381921192	0.1669959	0.860403806
Rnaseh1	0.344666041	0.180293807	1.911690957	0.055915845	0.715507996
Adi1	-0.040173851	0.108913724	-0.368859402	0.712232525	0.988585656
Trappc12	0.107105727	0.165219524	0.648263136	0.516814773	0.964352198
Tssc1	0.287026297	0.190423152	1.507307773	0.131731786	0.827800491
Pxdn	0.216223439	0.163985839	1.318549456	0.187319781	0.873455432
Sntg2	0.353442057	0.557621783	0.633838325	0.526186339	0.965637248
Tmem18	-0.11404258	0.113934363	-1.00094982	0.316851069	0.91640698
Fam150b	-3.134947072	2.018173062	-1.553358892	0.120337418	0.81558552
Acp1	0.103626815	0.131325617	0.789083021	0.430063485	0.945321484
Sh3yl1	-0.026464036	0.180984621	-0.146222565	0.883745693	0.992886758
Fam110c	-0.128346396	0.194433163	-0.660105478	0.509186143	0.963452765
Lamb1	0.007750973	0.229649794	0.033751272	0.973075493	0.997013089
Dld	-0.066828181	0.109441731	-0.610627966	0.541445904	0.967217841
Slc26a3	0.452848082	0.752389736	0.601879664	0.547254242	0.968115622
Cbll1	-0.165467677	0.235714458	-0.701983572	0.482689409	0.957463006
Slc26a4	-1.760584418	0.825174842	-2.133589548	0.032876394	0.665167506
Bcap29	0.025111915	0.147844556	0.169853496	0.865125354	0.992886758
Dus4l	-0.03128421	0.205593978	-0.152165012	0.879056789	0.992886758
Cog5	-0.269759711	0.20593527	-1.30992477	0.190221287	0.877080754
Gpr22	2.823350994	4.950687634	0.570294715	0.568477825	0.971380162
Hbp1	-0.219865173	0.105988655	-2.074421779	0.038040156	0.679118604
Prkar2b	-0.007541864	0.137653108	-0.054788914	0.956306632	0.996173928
Pik3cg	-0.101290743	0.373153335	-0.271445364	0.786048516	0.990988839
Ccdc71l	-0.139387382	0.163051275	-0.854868396	0.392624008	0.933734231
Nampt	0.116716498	0.152714356	0.764279802	0.444700519	0.948476747
Gdap10	-1.393702026	1.487934113	-0.93666918	0.348928754	0.921552698
4933406C10Rik	0.494216666	0.393744642	1.255170519	0.209416865	0.884258007
Sypl	-0.183800416	0.087347788	-2.104236647	0.035357809	0.674795441
Cdhr3	-0.180032518	0.148209731	-1.214714555	0.224474979	0.892989918
F730043M19Rik	-0.551228513	0.407440911	-1.352904181	0.176086243	0.865308446
Atxn7l1	0.20278479	0.254923061	0.795474482	0.426337549	0.944290206
Efcab10	0.390586948	0.348149351	1.121894805	0.26190717	0.897248195
Twistnb	-0.060391011	0.1680235	-0.359420029	0.719280894	0.988585656
Twist1	0.599167362	1.25047372	0.479152303	0.631830284	0.977034429
Hdac9	-0.511818767	0.35452481	-1.443675456	0.148830289	0.848743319
Prps1l1	-1.824753031	2.153210614	-0.847456825	0.396740542	0.934469184
Snx13	-0.188366632	0.13110575	-1.436753396	0.150788077	0.850225493

Ahr	-0.163337215	0.198202827	-0.824091249	0.409887712	0.940983181
Agr3	-0.03622506	0.185458867	-0.195326652	0.845137233	0.992886758
Agr2	-0.243586925	0.369214207	-0.659744182	0.509418009	0.963452765
Tspan13	-0.038902099	0.083395246	-0.466478619	0.640872949	0.977921835
Bzw2	-0.324504827	0.161744311	-2.006282788	0.044826085	0.69077168
Ankmy2	0.109550396	0.165801101	0.660733826	0.508783027	0.963452765
Ispd	-0.144000193	0.226778437	-0.634981856	0.525440249	0.965637248
Meox2	0.016418682	0.22492665	0.072995718	0.941809525	0.994688009
Agmo	-0.322530834	0.167231748	-1.928645955	0.05377483	0.707153766
Dgkb	0.621640991	0.623750366	0.996618238	0.31894985	0.91640698
Etv1	-0.47272523	0.337390158	-1.401123355	0.161177189	0.857316691
Arl4a	0.134558179	0.214562989	0.627126696	0.530576189	0.966026275
Scin	0.406535182	0.810920703	0.501325444	0.616142101	0.974001549
Lsmem1	1.112269968	1.75382439	0.634196887	0.525952339	0.965637248
Ifrd1	0.086815318	0.141248686	0.614627441	0.538800788	0.966875138
Gm7008	1.521377354	1.288946403	1.180326311	0.237870457	0.896609492
Zfp277	0.013665235	0.146565638	0.093236281	0.925715852	0.994688009
Dock4	0.139897467	0.188615603	0.741706756	0.458265026	0.952283705
Immp2l	0.11475283	0.24875756	0.461303891	0.644580593	0.97889195
Lrrn3	-0.196277385	0.285099034	-0.688453352	0.491167336	0.958916246
Dnajb9	-0.056743878	0.173565214	-0.326931168	0.743719946	0.988585656
Gm2027	-0.855275752	0.91183348	-0.937973622	0.348257966	0.921552698
Pnpla8	0.016020811	0.087874498	0.182314682	0.855335777	0.992886758
Nrcam	-0.100164417	0.453434319	-0.220901711	0.825168965	0.992886758
Stxbp6	-0.140286632	0.143208666	-0.979595967	0.327285596	0.916882624
Nova1	-0.026939087	0.42980916	-0.062676857	0.950023827	0.99573416
Prkd1	0.078773082	0.184019184	0.428069946	0.668600197	0.981863224
G2e3	-0.125152621	0.171545114	-0.729560982	0.46565858	0.953083512
Scfd1	-0.030503444	0.111118941	-0.274511653	0.78369145	0.990988839
Coch	-1.077753407	0.793765584	-1.35777291	0.174535753	0.863528862
Strn3	-0.041161744	0.108437114	-0.379590917	0.704249104	0.988585656
Ap4s1	0.113528299	0.161618177	0.70244759	0.482400075	0.957463006
Hectd1	0.110814134	0.161977684	0.684132105	0.49389175	0.958916246
Heatr5a	0.24764232	0.206769309	1.197674455	0.231043778	0.895404344
Dtd2	-0.009174676	0.155187739	-0.059119851	0.952856648	0.996070133
Gpr33	-0.803814895	1.615512292	-0.497560371	0.618793942	0.974471036
Nubpl	0.0617675	0.169001931	0.365483991	0.714750155	0.988585656
Arhgap5	-0.184006702	0.173834504	-1.058516567	0.289820002	0.907334416
Akap6	-0.211483869	0.477010633	-0.443352527	0.657510768	0.979614766
Egln3	0.216641501	0.441007651	0.491242047	0.623255263	0.975636499
Sptssa	0.260738162	0.186859387	1.395370954	0.162904006	0.859982992
Eapp	0.265948032	0.15068841	1.764887108	0.077582741	0.754202
Snx6	-0.154139813	0.107386289	-1.435377029	0.151179688	0.850827708
Cfl2	-0.097002395	0.100803034	-0.962296382	0.335900746	0.918908218
Baz1a	-0.175263049	0.211304396	-0.829433995	0.406858872	0.939557159
2700097O09Rik	0.113835576	0.240383396	0.4735584	0.635814847	0.977122459
Srp54a	0.08770119	0.332318108	0.263907345	0.791851316	0.990988839
Srp54c	-0.710240472	0.239458045	-2.966033032	0.003016681	0.346777009
Ppp2r3c	0.05382247	0.125767455	0.427952291	0.668685856	0.981863224
1110008L16Rik	0.077084555	0.22694875	0.339656224	0.734115432	0.988585656
Psma6	0.235546329	0.169104784	1.392901625	0.163649541	0.859982992
Nfkbia	-0.129970562	0.184521578	-0.704365116	0.481205424	0.957463006
Ralgapa1	-0.001799023	0.165845278	-0.010847599	0.991345038	0.998770784
Brms1l	0.229470422	0.104452919	2.196878987	0.02802909	0.640939708
Gm19990	0.301320487	0.202356439	1.48905806	0.136472081	0.832506992
Mbip	0.095666303	0.181342402	0.527545142	0.597815077	0.974001549
E030019B13Rik	0.037151843	0.09246672	0.401786104	0.68784145	0.98538669
Nkx2-1	-0.181870037	0.191168964	-0.951357552	0.3414229	0.919850248
C87198	-0.09088014	0.470002263	-0.193361069	0.846676203	0.992886758
Pax9	-1.282610435	1.095043731	-1.171286953	0.241483454	0.896939594
Slc25a21	1.136412261	1.472526831	0.771742991	0.440266663	0.947849924
Prps1l3	-0.013981017	0.113916369	-0.122730539	0.90232048	0.994688009

Mipol1	-0.039482075	0.282795376	-0.139613579	0.888965311	0.992967239
Foxa1	-0.226213571	0.17660177	-1.280924705	0.200220114	0.880888611
4921506M07Rik	1.191512234	0.890261292	1.338384858	0.180771012	0.868087032
Sstr1	-1.531494761	1.918260877	-0.798376685	0.424651929	0.944280828
Clec14a	0.114959792	0.14146475	0.812639133	0.416424988	0.943415794
Sec23a	-0.035505523	0.166317765	-0.213480039	0.830952557	0.992886758
Gemin2	0.465018714	0.216407646	2.148809083	0.031649537	0.661395937
Trappc6b	0.188312174	0.108299724	1.738805673	0.08206895	0.76099108
Pnn	-0.064371653	0.189270135	-0.340104647	0.733777722	0.988585656
Mia2	-0.272087053	1.369696066	-0.198647758	0.842538292	0.992886758
Ctage5	-0.117477011	0.120627516	-0.973882372	0.330114947	0.917115058
Fbxo33	-0.277724657	0.153030588	-1.814831011	0.069549883	0.743426713
Gm527	-0.126792492	0.268640654	-0.471978048	0.636942457	0.977284137
Klhl28	0.177307428	0.367213792	0.48284523	0.629205633	0.977034429
Fam179b	-0.241220357	0.155790609	-1.548362632	0.121535013	0.816233249
Prpf39	0.048253204	0.178701808	0.270020795	0.787144256	0.990988839
Fkbp3	0.412906802	0.171200462	2.411832285	0.015872581	0.557315333
Fancm	-0.00633958	0.465357489	-0.013623032	0.989130729	0.998182772
Mis18bp1	0.185936634	0.908039459	0.20476713	0.83775407	0.992886758
Wdr20rt	0.749287319	1.552306945	0.482692757	0.629313907	0.977034429
Rps29	0.013336727	0.128387455	0.103878741	0.917265578	0.994688009
Lrr1	-0.034055522	0.910648791	-0.037396988	0.970168474	0.996628386
Rpl36al	0.231279783	0.157247686	1.470799274	0.141345409	0.840741274
Mgat2	0.09945926	0.098367189	1.011101978	0.311967624	0.914819219
Dnaaf2	0.027897678	0.261996175	0.106481242	0.915200537	0.994688009
9330151L19Rik	0.591269194	0.48163798	1.227621612	0.219589045	0.888847883
Pole2	-0.118098538	0.299175193	-0.394747096	0.693029542	0.986599035
Klhdc1	-0.039798376	0.172537794	-0.230664683	0.817575313	0.992678823
Klhdc2	0.233046425	0.138258765	1.685581565	0.091876422	0.772516176
Nemf	-0.121006434	0.186819668	-0.647717851	0.517167456	0.964352198
Arf6	0.072600636	0.134414894	0.54012345	0.5891119	0.974001549
5830428M24Rik	0.411377507	0.260153427	1.581288059	0.113812189	0.807243653
Vcpkmt	0.168096947	0.211254477	0.795708329	0.426201585	0.944290206
Sos2	-0.189609098	0.137661053	-1.37736196	0.168400368	0.861597949
L2hgdh	-0.15714563	0.180714098	-0.869581462	0.384529172	0.931689378
Atp5s	0.111326745	0.179935554	0.618703435	0.536111747	0.96674935
Cdkl1	-0.169659186	0.267161823	-0.635042779	0.525400515	0.965637248
4930512B01Rik	5.00E-05	0.931376403	5.37E-05	0.999957126	0.999957126
Map4k5	-0.109166086	0.12851172	-0.849464045	0.395623129	0.934066368
4931403G20Rik	2.727020536	1.649336984	1.653404103	0.098248648	0.786907305
Atl1	0.141624096	0.478111391	0.296215692	0.767065366	0.989884648
Gm3086	1.254028654	2.065757124	0.607055224	0.543814264	0.967491297
Sav1	0.026302274	0.088221236	0.298139938	0.765596361	0.989884648
Nin	-0.027866152	0.194046607	-0.143605459	0.88581203	0.992886758
Abhd12b	-0.191978552	0.543408616	-0.353285808	0.723874184	0.988585656
Pygl	0.065291539	0.17920437	0.364341222	0.715603222	0.988585656
Trim9	0.240909682	1.585796737	0.151917126	0.879252301	0.992886758
Tmx1	0.045709385	0.11107999	0.411499719	0.680706148	0.984226822
Frmd6	-0.267780853	0.206108973	-1.299219771	0.193868518	0.879588734
Actr10	0.085859321	0.104692335	0.820110864	0.41215291	0.942121471
Psma3	0.215071886	0.168903598	1.273341053	0.202897045	0.882297774
3110056K07Rik	0.310515476	0.219583077	1.414113877	0.157328469	0.855986074
Arid4a	0.093916883	0.206606781	0.454568249	0.649419881	0.979004493
Tomm20l	0.507077532	1.382977086	0.366656495	0.713875261	0.988585656
Timm9	0.17327683	0.205651505	0.842575066	0.399466141	0.936273856
2700049A03Rik	-0.254261518	0.395354138	-0.643123453	0.520143998	0.964665379
Dact1	0.133264236	0.3746737	0.355680785	0.722079637	0.988585656
4930404H11Rik	-1.149925138	1.864600444	-0.616713968	0.537423407	0.96674935
Daam1	-0.143938235	0.154354073	-0.932519834	0.351067941	0.922414914
Gpr135	0.845025386	1.090246345	0.775077477	0.43829388	0.947776577
L3hypdh	0.01071094	0.206219863	0.051939421	0.958576963	0.996226826
Jkamp	0.00903416	0.132475018	0.068195194	0.945630253	0.995032756

Rtn1	0.42326698	0.439927522	0.962128893	0.335984862	0.918908218
Lrrc9	-0.258730217	0.729624623	-0.3546073	0.722883807	0.988585656
Pcnxl4	0.047352648	0.140348062	0.337394385	0.735819617	0.988585656
Dhrs7	0.147293402	0.08411842	1.751024364	0.079941713	0.75659671
Ppm1a	0.007144578	0.095299661	0.074969605	0.940238895	0.994688009
Six1	-0.292975928	0.338148112	-0.866413024	0.386263704	0.932501372
Six4	-0.216251032	0.298362892	-0.724791982	0.468579656	0.953927686
Mnat1	0.160344298	0.112728666	1.422391518	0.154912624	0.854973821
Trmt5	-0.083716197	0.195619333	-0.427954616	0.668684162	0.981863224
Slc38a6	0.049080288	0.240433278	0.204132676	0.83824982	0.992886758
D830013O20Rik	-1.394822421	1.566394746	-0.890466738	0.373215321	0.928840865
Tmem30b	-0.12208896	0.204196421	-0.597899608	0.549906921	0.968926429
2210039B01Rik	-0.046439352	0.774789222	-0.059938047	0.952204978	0.996023481
Prkch	-0.183613538	0.145189823	-1.26464469	0.205998725	0.883765092
Hif1a	-0.252473245	0.14212872	-1.776370358	0.075671894	0.752876039
Snapc1	0.114169062	0.167700485	0.680791485	0.496003439	0.96007684
1700086L19Rik	-0.201636421	1.853494355	-0.108787178	0.913371294	0.994688009
Dbpht2	-3.931724794	2.407192957	-1.633323487	0.10240098	0.789151138
Rhoj	-0.008261875	0.107435045	-0.076901115	0.93870221	0.994688009
Ppp2r5e	0.116914135	0.147506732	0.792602025	0.428009729	0.945321484
Wdr89	-0.615831597	0.630406361	-0.976880367	0.328628383	0.917115058
Sgpp1	-0.050850456	0.112201455	-0.453206741	0.650399879	0.979004493
Syne2	0.114499068	0.135427116	0.845466341	0.397850518	0.935327115
Esr2	-0.678961097	0.39057153	-1.738378363	0.082144167	0.76099108
Mthfd1	0.347120523	0.189688458	1.829950679	0.067257314	0.74146534
Akap5	0.16270013	0.148583321	1.09500938	0.273512527	0.899242656
Zbtb25	0.079280009	0.315143058	0.251568317	0.80137475	0.990988839
Zbtb1	-0.269919298	0.148387295	-1.819018924	0.068908543	0.743426713
Hspa2	-0.242257546	0.283398746	-0.854829279	0.392645666	0.933734231
Ppp1r36	0.010182219	0.157966478	0.064458098	0.948605471	0.99560893
Gm10451	0.05228635	0.616454187	0.084817901	0.932406162	0.994688009
Plekhg3	-0.261113386	0.248118423	-1.052374033	0.292627978	0.907334416
AI463170	0.90004118	0.343863369	2.617438381	0.008859248	0.475600561
Sptb	0.802936418	0.563638154	1.424560089	0.154284404	0.854159937
Churc1	0.603186073	0.269114784	2.241370999	0.025002053	0.627917508
Gpx2	0.2157752	0.374892399	0.575565684	0.564908782	0.969658675
Rab15	0.008569894	0.157991669	0.054242698	0.956741803	0.996226826
Fntb	-0.221637757	0.323946627	-0.684179857	0.4938616	0.958916246
Max	0.122562029	0.109820034	1.116026149	0.264410942	0.89768067
Fut8	-0.111277592	0.113976513	-0.976320381	0.328905723	0.917115058
Gphn	-0.234468435	0.171836905	-1.364482415	0.172415785	0.86244803
Fam71d	-0.955604128	2.111134999	-0.452649465	0.650801174	0.979004493
Mpp5	-0.144899475	0.113492487	-1.276731869	0.201696926	0.881220006
Atp6v1d	-0.030355547	0.111689101	-0.271786113	0.785786483	0.990988839
Eif2s1	0.101737533	0.144794506	0.702633929	0.482283912	0.957463006
Plek2	-0.031568979	0.450263113	-0.070112293	0.944104283	0.99486515
Tmem229b	0.176944302	0.242769861	0.728856134	0.46608967	0.953083512
Plekhh1	-0.740998043	0.39698263	-1.866575482	0.061960894	0.725730283
Pigh	0.278442257	0.197352186	1.410890157	0.158277005	0.855986074
9230116L04Rik	0.467974736	1.696577685	0.275834546	0.782675149	0.990988839
Arg2	-0.043844526	0.322582772	-0.135917133	0.891886791	0.993100705
Vti1b	0.41313028	0.165816453	2.491491475	0.012720802	0.529827892
Rdh11	-0.034216386	0.158987393	-0.215214461	0.829600117	0.992886758
Rdh12	-0.283770384	0.409137841	-0.693581369	0.487944781	0.958288569
Zfyve26	0.239352379	0.340166399	0.703633221	0.481661217	0.957463006
Rad51b	0.21473013	0.301482283	0.712247923	0.476311266	0.95716557
9430078K24Rik	0.492771887	0.783188394	0.629186911	0.529226697	0.965637248
Zfp36l1	-0.014958533	0.131277055	-0.113946285	0.909280374	0.994688009
2310015A10Rik	0.264698533	0.190582136	1.388894775	0.16486475	0.859982992
Actn1	-0.178620602	0.209316511	-0.853351709	0.393464293	0.933734231
Dcaf5	0.068904416	0.213469742	0.322783056	0.746859546	0.988585656
Exd2	-0.142321008	0.151373217	-0.940199405	0.347115287	0.921552698

Galnt16	-0.026779411	0.697550097	-0.038390663	0.969376205	0.996600044
Erh	0.289755523	0.17444886	1.66097688	0.09671809	0.782127914
Slc39a9	-0.133353013	0.179128977	-0.744452489	0.456602774	0.951472839
Plekhd1os	0.062305573	0.35996592	0.173087423	0.862582711	0.992886758
Plekhd1	-0.203136084	0.241200086	-0.842189103	0.399682112	0.936395796
Susd6	-0.337543623	0.129699195	-2.602511324	0.009254376	0.478229167
Gm20337	0.254986309	1.031603586	0.247174702	0.804773023	0.990988839
Srsf5	0.286331153	0.11968439	2.392385126	0.016739267	0.56772809
Smoc1	-0.366118508	0.662033603	-0.553021035	0.580249007	0.971613657
Slc8a3	0.189991853	1.473162631	0.128968689	0.897382424	0.994101834
Cox16	0.02703949	0.361020042	0.074897477	0.940296284	0.994688009
Gm4787	0.702236692	0.597156685	1.175967229	0.239607972	0.896939594
Adam4	-0.045472335	0.768361079	-0.059180946	0.952807986	0.996070133
Synj2bp	0.09732021	0.101021668	0.96335976	0.335367013	0.918714299
Adam21	3.921677243	2.182507017	1.796868103	0.072356561	0.74633396
Med6	0.304612906	0.160952111	1.892568566	0.058415267	0.72285319
Ttc9	-0.056695249	0.135388942	-0.418758342	0.675392752	0.983549699
Map3k9	-0.311615887	0.34996795	-0.890412641	0.373244357	0.928840865
Pcnx	0.027047214	0.1716922	0.157533156	0.87482468	0.992886758
Sipa1l1	-0.177516196	0.218948787	-0.810765836	0.417500157	0.943415794
Rgs6	-0.046269283	0.347164667	-0.133277627	0.893973821	0.993422442
Dpf3	-0.943614417	1.31835696	-0.715750321	0.474145533	0.955974667
Dcaf4	0.027285903	0.251437703	0.108519538	0.913583583	0.994688009
Zfyve1	-0.005890262	0.193910635	-0.030376168	0.975767052	0.997262526
Rbm25	0.046701291	0.158073489	0.295440378	0.767657492	0.989990452
Psen1	-0.150913597	0.234422306	-0.643768078	0.519725836	0.964665379
Papln	1.144735251	0.864547195	1.32408648	0.185474325	0.871716376
Numb	0.116673159	0.127274992	0.916701359	0.359299154	0.925229839
2410016O06Rik	0.061710975	0.228330127	0.27027084	0.786951897	0.990988839
Acot2	0.389183063	0.18345754	2.121379491	0.03388988	0.671818913
Acot1	0.354735342	0.2685324	1.32101505	0.186496346	0.872512868
Acot4	0.353593347	1.153664937	0.306495704	0.75922725	0.989266039
Acot6	-0.469710878	0.791200453	-0.593668617	0.552733777	0.968926429
Dnal1	-0.071814155	0.152308214	-0.471505462	0.63727982	0.977284137
Pnma1	0.046943107	0.332012465	0.141389593	0.887562175	0.992886758
Elmsan1	-0.35528321	0.203722849	-1.743953666	0.08116717	0.760377276
Ptgr2	0.117118217	0.103879418	1.127443906	0.259554844	0.897248195
Zfp410	0.125782731	0.129107735	0.974246286	0.329934268	0.917115058
Fam161b	-0.075220162	0.290490576	-0.258941831	0.795680122	0.990988839
Coq6	0.051627325	0.179272586	0.287982263	0.773360316	0.990730604
Entpd5	-0.181191448	0.157268113	-1.152118151	0.249272525	0.896939594
Ccdc176	-0.398316841	0.229690405	-1.734146623	0.082892071	0.76099108
Rnf113a2	0.132479551	0.182458456	0.72608063	0.467789343	0.953657709
Aldh6a1	-0.137448381	0.132673167	-1.035992317	0.300205727	0.910285132
Lin52	-0.147069688	0.159977831	-0.919312927	0.357931918	0.924354588
Abcd4	-0.067968249	0.236733108	-0.28710918	0.774028724	0.990730604
Npc2	0.097421854	0.176258869	0.552720297	0.580454953	0.971613657
Isca2	0.21349515	0.20316668	1.05083742	0.293333266	0.907334416
Ltbp2	-0.087923922	0.194134313	-0.452902533	0.650618927	0.979004493
D030025P21Rik	3.616687318	2.22490501	1.625546845	0.104046091	0.79155232
Arel1	0.058175389	0.155981048	0.372964472	0.709174886	0.988585656
Fcf1	0.056709394	0.124977705	0.453756086	0.650004394	0.979004493
Ylpm1	-0.04869658	0.250748701	-0.194204716	0.84601559	0.992886758
Prox2	-0.425653396	0.456050935	-0.933346175	0.350641261	0.922338606
Dlst	-0.053673166	0.124611572	-0.430723768	0.666669235	0.981863224
Rps6kl1	-0.526685894	0.659074183	-0.799129912	0.424215087	0.94406679
Pgf	-0.153547495	0.311911048	-0.492279757	0.622521589	0.975236037
Eif2b2	0.090002657	0.128349565	0.701230716	0.483159044	0.957463006
Mlh3	0.211253313	0.150479738	1.403865496	0.160358906	0.857316691
Acyp1	0.559248619	0.316126171	1.769067769	0.076882566	0.752876039
Zc2hc1c	0.022168264	0.201369079	0.110087728	0.912339801	0.994688009
Nek9	-0.048667172	0.097942204	-0.496896847	0.619261795	0.974471036

Tmed10	-0.071050061	0.081728048	-0.869347337	0.384657178	0.931689378
Fos	0.681507345	0.657804003	1.03603405	0.300186257	0.910285132
Jdp2	-0.144663522	0.186073751	-0.777452603	0.436891787	0.946792171
Batf	0.588188528	0.219382804	2.681105885	0.007337929	0.452031612
Mfsd7c	-0.164951453	0.265104779	-0.622212297	0.533802281	0.96674935
0610007P14Rik	0.006470093	0.134031478	0.048272939	0.961498721	0.996474415
Ttll5	-0.313962818	0.271309968	-1.157210772	0.247186258	0.896939594
Tgfb3	0.212808511	0.142965698	1.488528465	0.136611581	0.832506992
Ift43	0.157611812	0.098250068	1.604190366	0.108672098	0.798150849
Gpatch2l	-0.150374102	0.225224932	-0.667661881	0.504349448	0.961863501
Vash1	0.131178432	0.161545088	0.812023651	0.416778061	0.943415794
Angel1	0.15023285	0.402253807	0.373477758	0.708792896	0.988585656
Irf2bpl	-0.009388042	0.1706405	-0.055016492	0.956125325	0.99614946
Cipc	-0.349698365	0.17139681	-2.040285145	0.041321934	0.679118604
Ngb	0.602147393	0.331344461	1.817285222	0.069173452	0.743426713
Pomt2	0.034546691	0.250448608	0.137939242	0.890288436	0.993043931
Gstz1	-0.07433208	0.146705062	-0.506676997	0.612381473	0.974001549
Tmed8	-0.136767692	0.289855527	-0.471847796	0.637035433	0.977284137
Samd15	0.434744373	0.566695612	0.767156767	0.442988314	0.948476747
Noxred1	2.020664993	1.53021799	1.320507932	0.18666549	0.872512868
Vipas39	0.000260121	0.175972581	0.001478191	0.998820575	0.999739059
Ahsa1	-0.159356712	0.111628206	-1.427566724	0.153416607	0.854159937
Ism2	0.53590718	0.825248533	0.64938883	0.516087082	0.964352198
Sptlc2	-0.137725362	0.117171339	-1.175418518	0.239827319	0.896939594
Alkbh1	0.04079161	0.214032523	0.190586037	0.848849931	0.992886758
Slirp	-0.458363103	0.242955353	-1.886614542	0.059212189	0.72285319
Snw1	0.088079435	0.108896559	0.8088358	0.418609601	0.943415794
Gm16381	-0.211043844	2.340120234	-0.090185043	0.92814017	0.994688009
Adck1	-0.070584434	0.245458384	-0.287561717	0.773682253	0.990730604
3200001D21Rik	-0.816546758	1.208020387	-0.675937895	0.499080076	0.960700483
Dio2	-0.295163359	0.65347688	-0.451681411	0.651498512	0.979004493
Cep128	-0.140075741	0.3328597	-0.420825173	0.673882749	0.983375962
Tshr	-2.13478145	2.061265655	-1.035665366	0.300358285	0.910285132
Gtf2a1	0.183846981	0.139075396	1.321923117	0.186193754	0.872305715
Ston2	-0.211413059	0.185396431	-1.140329715	0.254148962	0.897044747
Sel1l	-0.309231865	0.171599792	-1.802052678	0.071537118	0.744227652
Flrt2	-0.550168914	0.242020982	-2.273228171	0.023012434	0.620006604
Galc	-0.308047424	0.175616056	-1.754096016	0.079414056	0.75659671
Gpr65	-0.353106031	0.537257678	-0.657237756	0.511028052	0.963452765
Spata7	-0.186656923	0.183693103	-1.016134629	0.309565281	0.9139204
Ptpn21	-0.309303755	0.195168865	-1.584800707	0.113011622	0.804684147
Zc3h14	-0.115972265	0.125202423	-0.926278117	0.354301465	0.924354588
Eml5	-0.405516975	0.464217227	-0.873550036	0.382363346	0.930775109
Ttc8	0.07117956	0.182390608	0.390258911	0.696345103	0.987490725
Foxn3	0.165005301	0.363930477	0.453397864	0.650262274	0.979004493
1700064M15Rik	-0.255102539	1.150947534	-0.221645671	0.824589726	0.992886758
Efcab11	-0.357816314	0.404394647	-0.884819609	0.376253932	0.928883222
Tdp1	-0.324733585	0.218753965	-1.48446948	0.137684414	0.833085238
Kcnk13	-0.000458657	0.298178621	-0.001538194	0.998772699	0.999739059
Psmc1	0.112757841	0.090138466	1.250940314	0.210956254	0.884390587
Nrde2	0.185772698	0.230331459	0.806545049	0.419928644	0.943415794
Gm10433	0.210472367	0.390283228	0.539281096	0.58969291	0.974001549
Calm1	0.016952881	0.086437235	0.196129382	0.8445089	0.992886758
Gm10432	0.951657651	1.308553126	0.727259469	0.467067022	0.953239786
Ttc7b	-0.013039217	0.223646492	-0.058302801	0.953507436	0.996070133
Rps6ka5	-0.232761379	0.291328722	-0.798964746	0.424310854	0.94406679
9030617O03Rik	-0.487109986	0.257459825	-1.891984456	0.058493052	0.72285319
Gpr68	-0.620776124	0.557746686	-1.113007284	0.265705301	0.89768067
Ccdc88c	-0.325442631	0.275324277	-1.182033909	0.237192247	0.896609492
Smek1	-0.012573562	0.146092835	-0.086065564	0.931414298	0.994688009
D130020L05Rik	0.289893708	0.328653707	0.882064317	0.377742027	0.928883222
Tc2n	-0.002538915	0.17307688	-0.01466929	0.98829602	0.998148029

Fbln5	-0.14907101	0.141161466	-1.056031889	0.290953646	0.907334416
Trip11	0.037184885	0.366468477	0.101468168	0.919178826	0.994688009
Atxn3	-0.053588608	0.1510054	-0.354878755	0.722680425	0.988585656
Cpsf2	-0.241230812	0.131475994	-1.8347898	0.066536841	0.738254074
Slc24a4	0.193890532	0.229528902	0.844732537	0.398260189	0.935327115
Rin3	-0.31058419	0.288361028	-1.077067145	0.28145027	0.904574518
Lgmn	-0.385609002	0.192634839	-2.001761494	0.045310389	0.693980041
Golga5	-0.215686265	0.114446344	-1.884605979	0.059483055	0.724178089
Itpk1	0.168996625	0.214670099	0.787238774	0.431142103	0.945321484
Tmem251	-0.205878036	0.15680888	-1.312923326	0.189208786	0.875548883
AK010878	0.220279306	0.289451403	0.761023453	0.446643059	0.948476747
Ubr7	-0.17051684	0.094617489	-1.802170424	0.071518596	0.744227652
Btbd7	-0.03538474	0.257172069	-0.137591691	0.890563122	0.993043931
Unc79	-0.2661444	0.484028756	-0.549852455	0.582420577	0.972017845
Fam181a	-0.79580815	0.68999325	-1.153356427	0.248764118	0.896939594
Asb2	-0.36303927	0.426687856	-0.850831035	0.394863219	0.933796635
Otub2	-0.13789865	0.206482709	-0.667845993	0.504231905	0.96186221
Ddx24	-0.234394488	0.159192492	-1.472396628	0.140913803	0.839671411
Ifi27l2a	0.553205143	0.319300882	1.732551249	0.083175461	0.76099108
Ifi27	0.02739488	0.145317973	0.188516804	0.850471544	0.992886758
Ifi27l2b	-1.035074318	1.01486935	-1.019908935	0.307771652	0.9139204
Ppp4r4	0.000764698	0.312924827	0.002443712	0.998050202	0.999591134
Serpina10	0.059622858	0.558052187	0.106841007	0.914915114	0.994688009
Serpina1a	-1.220380534	3.279046636	-0.372175412	0.709762251	0.988585656
Serpina1c	0.652789497	4.864957824	0.134181944	0.893258704	0.993341156
Serpina11	-2.46423403	4.707768528	-0.52343993	0.600668143	0.974001549
Serpina9	-0.312185843	0.255374369	-1.22246349	0.221532402	0.891315805
Serpina3c	0.055724272	0.224025504	0.248740749	0.803561326	0.990988839
Serpina3g	0.916865918	0.27146195	3.377511715	0.000731448	0.195776354
Serpina3f	0.677477944	0.589997977	1.148271638	0.250856444	0.896939594
Serpina3h	0.295718471	0.40363273	0.732642448	0.463776534	0.953083512
Serpina3i	0.436345956	0.410696508	1.062453533	0.288029842	0.907334416
Serpina3k	1.834229656	2.7380246	0.669909852	0.502915259	0.961729397
Serpina3m	1.27444965	0.500509313	2.546305567	0.010886984	0.502706181
Serpina3n	-0.116201577	0.23494281	-0.494595163	0.620885917	0.97514112
Dicer1	-0.210870369	0.216006697	-0.976221441	0.32895474	0.917115058
Clmn	-0.026204858	0.341642106	-0.076702659	0.938860089	0.994688009
Syne3	0.578795112	0.526321194	1.099699421	0.271463107	0.89804647
Snhg10	0.442166486	0.334742921	1.320913629	0.186530165	0.872512868
Scarna13	1.119258029	1.785929331	0.626709025	0.530849987	0.966026275
Glrx5	0.320717334	0.169996182	1.886614927	0.059212138	0.72285319
D430019H16Rik	0.503103025	0.350355506	1.435978646	0.151008418	0.850827708
AU015791	-0.311221267	1.65818678	-0.187687703	0.851121471	0.992886758
Bdkrb2	0.056215955	0.731473829	0.076852995	0.938740491	0.994688009
Bdkrb1	0.076038611	0.809110255	0.093978059	0.925126586	0.994688009
Atg2b	-0.133169812	0.252429759	-0.527551952	0.597810349	0.974001549
Gskip	0.10548526	0.139888313	0.754067707	0.450808567	0.949134998
Ak7	-0.384911705	0.21275126	-1.809209989	0.070418386	0.744227652
Papola	-0.032268271	0.102283082	-0.315480045	0.7523972	0.988585656
Vrk1	0.220654149	0.174046254	1.267790282	0.204872854	0.883765092
Bcl11b	0.026651896	0.765798744	0.034802742	0.972237034	0.996779533
Setd3	-0.009241444	0.08189583	-0.112843883	0.910154327	0.994688009
Ccnk	0.029865673	0.248976642	0.119953715	0.904519813	0.994688009
Ccdc85c	0.327036751	0.36538375	0.895050069	0.370760323	0.928436166
Hhipl1	0.469975324	1.928709454	0.243673469	0.807483722	0.99099448
Cyp46a1	-1.939253305	1.549176791	-1.251795997	0.210644209	0.884340413
Eml1	-0.024890695	0.144788299	-0.171910959	0.863507531	0.992886758
Gm16596	1.007819228	1.827264755	0.551545268	0.581259944	0.971861865
Evl	-0.01756054	0.203277119	-0.086387194	0.931158627	0.994688009
Degs2	-0.073537511	0.511968487	-0.143636792	0.885787286	0.992886758
Yy1	0.048942217	0.114172856	0.428667713	0.66816506	0.981863224
Slc25a29	-0.554459813	0.261361806	-2.12142632	0.033885942	0.671818913

Slc25a47	-0.100863776	0.402141562	-0.250816592	0.801955913	0.990988839
Wars	-0.368856004	0.177723224	-2.075451913	0.037944671	0.679118604
Wdr25	0.051502318	0.392530816	0.131205797	0.895612511	0.993657615
Begain	0.385711228	1.09991549	0.350673512	0.725833299	0.988585656
Meg3	-0.476822904	0.570820189	-0.835329432	0.403532251	0.937834293
Rian	0.137787203	0.242452623	0.56830568	0.569827423	0.97144955
AF357359	-0.080591339	0.722318561	-0.111573125	0.911161882	0.994688009
Mirg	0.976354828	1.516294529	0.643908429	0.519634816	0.964665379
Dio3os	-0.823148095	1.325219541	-0.621140928	0.534506902	0.96674935
Ppp2r5c	-0.126913061	0.10292223	-1.233096691	0.217539692	0.887633697
B930059L03Rik	1.930309646	0.823663704	2.343565264	0.01910042	0.590246276
Dync1h1	0.12348927	0.174941422	0.705889253	0.480257011	0.957463006
1700001K19Rik	3.572771576	1.683567883	2.122142869	0.033825742	0.671818913
Hsp90aa1	-0.447421581	0.127206092	-3.517296797	0.000435966	0.139568962
Wdr20	0.282719154	0.14051782	2.011980788	0.044221962	0.688942727
Mok	-0.00898967	0.191712559	-0.0468914	0.962599782	0.996474415
Zfp839	-0.454406975	0.410197686	-1.107775567	0.267958755	0.89804647
Cinp	0.281842074	0.159847236	1.763196416	0.077867368	0.754202
Tecpr2	0.1694897	0.365851479	0.463274607	0.643167545	0.978801899
Ankrd9	-0.32518078	0.346272818	-0.93908838	0.347685369	0.921552698
Rcor1	-0.802685602	0.340166852	-2.35968201	0.018290606	0.582676994
Traf3	0.059509315	0.322330223	0.184622202	0.853525371	0.992886758
Amn	0.828026118	1.136220532	0.728754757	0.466151691	0.953083512
Cdc42bpb	-0.089252437	0.207720229	-0.429676193	0.667431204	0.981863224
A230065H16Rik	0.21691437	1.920070603	0.11297208	0.91005269	0.994688009
Exoc3l4	0.364440087	0.224020001	1.626819415	0.103775457	0.79155232
Tnfaip2	0.263285768	0.181025575	1.454411996	0.145832116	0.842832198
Eif5	0.053185852	0.131577733	0.404216202	0.68605374	0.98538669
Snora28	0.029347094	0.246071257	0.119262582	0.905067327	0.994688009
2810029C07Rik	-0.139978384	0.353330599	-0.396168304	0.691980872	0.986464472
Mark3	-0.164132878	0.126084273	-1.301771224	0.192994604	0.879588734
Ckb	-0.231826432	0.123696157	-1.874160339	0.060908329	0.725221283
Trmt61a	0.388643302	0.24030603	1.617284852	0.105816807	0.79419908
Bag5	0.048688768	0.11877483	0.409924968	0.681860989	0.984683806
Apopt1	0.263986081	0.157890336	1.671958452	0.094532515	0.777748075
Klc1	0.077429849	0.127490242	0.607339412	0.543625688	0.967491297
Xrcc3	-0.125161639	0.611315085	-0.204741617	0.837774004	0.992886758
Zfyve21	0.039505866	0.13058092	0.302539342	0.762240947	0.989285682
Ppp1r13b	0.016590981	0.166086028	0.099893898	0.920428561	0.994688009
5033406O09Rik	0.19993461	0.367542891	0.543976269	0.586457804	0.972904784
2010107E04Rik	0.254073748	0.272315152	0.933013628	0.350812932	0.922338606
Aspg	-1.113555384	0.657331469	-1.694054579	0.090254912	0.769347579
Kif26a	-0.154755151	0.313817272	-0.493137773	0.621915242	0.97514112
A530016L24Rik	-0.284860239	0.563311168	-0.50568896	0.613075019	0.974001549
Inf2	0.397107544	0.290912101	1.365043059	0.172239517	0.86244803
Adssl1	0.424029474	0.231284962	1.83336379	0.066748489	0.738254074
Siva1	0.033740836	0.177736067	0.189836744	0.849437062	0.992886758
Akt1	-0.152914221	0.114268622	-1.338199567	0.18083139	0.868087032
Zbtb42	-0.412822487	0.346216108	-1.192383822	0.233110781	0.89581803
Cep170b	0.042622559	0.290859316	0.146540121	0.88349502	0.992886758
Pld4	0.071296135	0.23114811	0.308443513	0.757744876	0.989266039
BC022687	-0.237185463	0.167884365	-1.412790664	0.157717284	0.855986074
Cdca4	-0.110670057	0.168071426	-0.658470386	0.51023592	0.963452765
Gpr132	-0.37110933	0.452937322	-0.819339259	0.41259288	0.942708418
Jag2	0.09427044	0.292077895	0.322757871	0.74687862	0.988585656
Nudt14	0.111168097	0.178576957	0.622522072	0.533598635	0.96674935
Brf1	0.014314459	0.152250843	0.094018915	0.925094132	0.994688009
Btbd6	-0.311084447	0.222150054	-1.400334784	0.161413089	0.857316691
Pacs2	-0.145042965	0.166083719	-0.873312364	0.382492843	0.930775109
Mta1	-0.141808901	0.19388777	-0.731396832	0.4645368	0.953083512
Crip2	-0.189325466	0.154333171	-1.226732168	0.219923273	0.888847883
Crip1	-0.014374864	0.193317371	-0.074358884	0.940724824	0.994688009

4930427A07Rik	0.344178456	0.883355533	0.389626196	0.696812978	0.987518602
Tmem121	-0.140069998	1.013804287	-0.13816276	0.890111786	0.993043931
Zfp386	0.102779141	0.167013133	0.61539556	0.538293523	0.96674935
Vipr2	-0.076659422	0.202567461	-0.378438971	0.705104523	0.988585656
Wdr60	-0.104092639	0.231325311	-0.449983783	0.652722134	0.979004493
Esyt2	-0.098424547	0.109829513	-0.896157548	0.370168625	0.928436166
D430020J02Rik	0.528966318	0.895450193	0.590726678	0.55470357	0.968926429
Ncapg2	0.066935173	0.473656163	0.141315954	0.887620347	0.992886758
Ptprn2	0.1443553	0.427605271	0.337590085	0.735672115	0.988585656
Gm10421	-0.446166876	1.653462431	-0.269837928	0.787284942	0.990988839
Rapgef5	-0.402711052	0.257154036	-1.566030454	0.117341488	0.812881672
Cdca7l	-0.559859884	0.317738781	-1.762013065	0.07806709	0.754202
Dnah11	-0.09881668	0.482139675	-0.204954466	0.837607701	0.992886758
Sp4	-0.048345029	0.173291409	-0.278981104	0.780259326	0.990988839
Itgb8	-0.923289951	1.330079133	-0.694161669	0.487580828	0.958288569
Macc1	-0.339129341	0.220265924	-1.539635979	0.12364911	0.820382953
Tmem196	1.195510762	1.200120133	0.996159242	0.319172779	0.91640698
2810429I04Rik	0.703276899	1.811094233	0.388316017	0.697782185	0.987577127
Gdi2	-0.129344104	0.074758108	-1.730168243	0.08360022	0.76099108
Fam208b	-0.353251824	0.170129061	-2.076375562	0.03785923	0.679118604
Asb13	0.128646741	0.12771025	1.007332934	0.313774808	0.915639195
Calml3	-0.2213225	0.200233606	-1.105321449	0.269020327	0.89804647
Net1	-0.122475561	0.10297669	-1.189352278	0.234301072	0.89581803
Tubal3	1.518778282	1.437909999	1.056240156	0.290858509	0.907334416
Akr1c14	-0.102929269	0.148289599	-0.694109829	0.487613335	0.958288569
Akr1c18	0.420825996	1.282250606	0.328193252	0.74276555	0.988585656
Akr1c13	0.286913407	0.216533536	1.325029886	0.185161239	0.871716376
Akr1c19	0.530291246	0.234751351	2.258948646	0.02388658	0.622011301
Marcksl1-ps4	0.637854701	1.151206291	0.554075065	0.579527474	0.971613657
Akr1c12	0.160114646	0.637505334	0.251158127	0.801691857	0.990988839
Akr1c6	2.451716058	4.796180316	0.51118096	0.60922435	0.974001549
Akr1e1	0.091821593	0.165768575	0.553914354	0.579637461	0.971613657
Klf6	-0.039986293	0.119328841	-0.335093284	0.737554719	0.988585656
Pitrm1	-0.016899673	0.241541701	-0.06996586	0.944220833	0.99486515
Pfkp	-0.160170519	0.166253566	-0.96341103	0.335341293	0.918714299
Wdr37	-0.167995204	0.146501596	-1.146712446	0.251500485	0.896939594
Idi1	-0.095796275	0.176978962	-0.54128623	0.58831031	0.973455044
Gtpbp4	-0.156189091	0.168661504	-0.926050621	0.354419674	0.924354588
Larp4b	0.026399188	0.197496739	0.133668982	0.893664334	0.993422442
Dip2c	-0.131381003	0.207733311	-0.632450341	0.527092648	0.965637248
Zmynd11	-0.100419003	0.172144745	-0.583340512	0.559664096	0.968926429
Chrm3	-0.892042546	0.483967956	-1.843185144	0.065301983	0.735191788
Ryr2	0.375904874	0.406237641	0.925332455	0.354793002	0.924354588
Gm10336	-0.324192238	0.333680851	-0.971563808	0.331267595	0.917115058
Mtr	0.712419281	0.336090287	2.119725883	0.034029169	0.671818913
Actn2	0.032593139	0.449997258	0.072429639	0.942259998	0.994688009
Heatr1	-0.091796444	0.206677553	-0.444152946	0.656932009	0.979614766
Lgals8	0.168442393	0.145946554	1.154137509	0.248443798	0.896939594
Edaradd	0.238430147	0.910369604	0.261904776	0.793394858	0.990988839
Ero1lb	-0.312251391	0.208309613	-1.498977349	0.133879508	0.827800491
Gpr137b-ps	0.064291264	0.143884394	0.446825832	0.655000818	0.979133069
Gpr137b	-0.140743594	0.162373422	-0.8667896	0.386057301	0.932379553
Nid1	-0.004790962	0.219527906	-0.021823933	0.982588403	0.997390438
Lyst	-0.310013268	0.308101049	-1.006206468	0.314316262	0.91586731
Gng4	0.149088482	0.661833877	0.225265716	0.821772569	0.992886758
B3galnt2	-0.139454703	0.150754835	-0.925042987	0.354943547	0.924354588
Tbce	-0.024170545	0.189836885	-0.127322699	0.898684995	0.994364545
Ggps1	0.159958864	0.144820249	1.104533828	0.269361638	0.89804647
Arid4b	-0.149702821	0.131193044	-1.141088093	0.253833266	0.897044747
Mrpl32	0.133079194	0.188648568	0.705434425	0.480539927	0.957463006
Psma2	0.153368044	0.139845534	1.096696044	0.272774286	0.898806413
AW209491	0.143245013	0.17263425	0.829760105	0.406674431	0.939557159

Gli3	-0.858824184	0.847507567	-1.013352821	0.310891666	0.914819219
Inhba	0.701257593	1.267444163	0.553284802	0.580068406	0.971613657
Sugct	-0.007290825	0.298590592	-0.024417465	0.980519617	0.997296835
Mplkip	0.259805234	0.150096306	1.730923567	0.083465398	0.76099108
Cdk13	-0.088911017	0.154012057	-0.577299069	0.563737443	0.969658675
Mir466i	0.819817982	1.705026082	0.480824305	0.630641374	0.977034429
Rala	-0.121984409	0.106246069	-1.148131033	0.250914475	0.896939594
Yae1d1	-0.044702816	0.112578025	-0.397082961	0.691306284	0.986191149
Vps41	-0.446776099	0.21968516	-2.033710878	0.041980755	0.679118604
Amph	0.291021863	0.239690817	1.214155247	0.224688446	0.892989918
Stard3nl	0.183353595	0.166814717	1.099145198	0.271704738	0.89804647
Epdr1	-0.034454136	0.181242366	-0.190099793	0.849230932	0.992886758
Sfrp4	0.280326752	0.419494372	0.668249137	0.503974575	0.96179897
A530099J19Rik	-0.341419121	0.648814817	-0.526219674	0.598735586	0.974001549
Gpr141	0.837468352	0.32683429	2.562363798	0.010396236	0.499527467
Elmo1	0.012959355	0.285256793	0.045430486	0.963764182	0.996474415
Aoah	-0.436548584	0.549801119	-0.794011814	0.427188554	0.944314584
Trim27	0.081991055	0.146386068	0.560101496	0.57541021	0.971613657
Zscan12	0.226327779	0.251622776	0.899472543	0.368401014	0.928436166
Zkscan3	0.144729026	0.197559572	0.732584224	0.463812056	0.953083512
Pgbd1	0.391679783	0.346599334	1.13006502	0.258448828	0.897248195
Zscan26	-0.154861939	0.12449988	-1.243872192	0.213546605	0.884945675
Nkapl	-0.988371265	0.788002326	-1.254274553	0.20974223	0.884258007
Zkscan4	0.492883751	0.279971903	1.760475766	0.078327173	0.754732183
Zfp389	0.862670352	1.31352827	0.656758116	0.511336459	0.963452765
Zkscan8	-0.161760185	0.294757063	-0.548791549	0.58314851	0.972174584
Hist1h4j	1.133214965	1.005095106	1.127470384	0.259543655	0.897248195
Hist1h4k	0.212900192	0.73028695	0.291529504	0.770646382	0.989990452
Hist1h2bn	1.818963788	0.610719511	2.978394754	0.002897625	0.345325017
Hist1h1b	-0.179156088	1.454916283	-0.123138417	0.90199749	0.994688009
Hist1h3i	-0.931368751	1.505746137	-0.618543012	0.536217454	0.96674935
Hist1h4n	-0.767930218	0.700713059	-1.095926797	0.273110811	0.898826892
Zfp184	-0.82468154	0.391991609	-2.103824473	0.035393761	0.674795441
Prss16	0.996844414	0.915224053	1.089180744	0.276074189	0.900302631
Hist1h4i	-0.046848866	0.366451982	-0.127844487	0.898272043	0.994364545
Hist1h2bj	-0.666302167	1.632210917	-0.408220629	0.683111705	0.984738223
Zfp322a	0.062971236	0.179184568	0.351432247	0.725264093	0.988585656
Abt1	-0.273400255	0.19842206	-1.377872273	0.168242727	0.861597949
C230035I16Rik	0.219335862	0.384501202	0.570442591	0.568377549	0.971380162
Hist1h4h	0.335867018	0.362820433	0.92571142	0.354595971	0.924354588
Hist1h2bh	-0.549895366	1.798472526	-0.30575689	0.759789754	0.989266039
Hist1h3f	-1.917638303	1.487595205	-1.289086102	0.197368155	0.87989762
Hist1h4f	4.673535019	1.881214277	2.484318282	0.012979974	0.533813677
Hist1h1d	1.922171285	1.334012489	1.44089452	0.149614484	0.848779008
Hist1h3e	0.958418112	1.273584369	0.75253602	0.451728777	0.949134998
Hist1h2ae	-1.754518078	0.776821943	-2.258584601	0.023909237	0.622011301
Hist1h2bg	0.260635775	0.715045513	0.364502357	0.715482914	0.988585656
Hist1h2ad	0.358178401	0.694413028	0.515800232	0.605993941	0.974001549
Hist1h3d	1.580711803	1.078840273	1.465195398	0.142867615	0.840741274
Hist1h4d	2.97007219	1.606353363	1.848953199	0.064464571	0.73299549
Hist1h2be	-0.414509919	0.297957599	-1.391170825	0.164173632	0.859982992
Hist1h1e	0.20188427	0.61230566	0.329711586	0.741617898	0.988585656
Hist1h2ac	-0.42731468	0.561843492	-0.760558208	0.44692099	0.948476747
Hist1h2bc	0.058017729	0.140224674	0.413748365	0.679058403	0.983903958
Hist1h4c	-0.19917218	0.593996393	-0.335308736	0.737392204	0.988585656
Hfe	0.143051075	0.121480988	1.177559365	0.238972318	0.896609492
Hist1h1c	0.360565814	0.162612758	2.217327953	0.026600689	0.633489138
Hist1h3c	2.02712497	1.788211604	1.133604639	0.256960426	0.897248195
Hist1h2bb	0.360686971	1.06104561	0.339935406	0.733905172	0.988585656
Hist1h4b	-0.78634372	1.909017968	-0.411910067	0.680405341	0.984226822
Hist1h4a	-0.178891498	1.460396857	-0.122495127	0.902506905	0.994688009
Slc17a2	-0.701488334	1.205502944	-0.581905119	0.560630592	0.968926429

Lrrc16a	-0.206693888	0.233941521	-0.883528018	0.376951051	0.928883222
Cmah	0.036864588	0.225976615	0.163134525	0.870412517	0.992886758
Fam65b	-0.078925062	0.161869535	-0.48758441	0.62584424	0.9764127
Gm11346	0.872480421	0.627971797	1.389362428	0.164722569	0.859982992
Gmnn	0.43275509	0.195871071	2.209387473	0.027147702	0.635926147
BC005537	-0.134696106	0.112123996	-1.201313821	0.229629491	0.895404344
Acot13	0.147602922	0.196445561	0.751368066	0.452431176	0.94958784
Tdp2	-0.063149006	0.138695114	-0.455308082	0.648887613	0.979004493
D130043K22Rik	0.16259553	0.601749363	0.270204739	0.787002747	0.990988839
Aldh5a1	-0.022722	0.381475649	-0.059563435	0.952503341	0.996023481
9330162012Rik	0.243723936	0.366127732	0.665680075	0.505615612	0.96224682
Gpld1	-0.272999951	0.253031233	-1.078917995	0.280624286	0.903520552
Mrs2	0.122167404	0.248421266	0.491775142	0.622878311	0.975486913
Dcdc2a	-0.115154351	0.32022431	-0.359605276	0.719142338	0.988585656
2610307P16Rik	0.018933036	0.238396189	0.079418366	0.936699861	0.994688009
Sox4	-0.004415197	0.288918791	-0.015281794	0.987807367	0.998148029
A330102I10Rik	-1.191348279	1.403004799	-0.849140559	0.395803084	0.934066368
Cdkal1	0.148400639	0.230005722	0.645204118	0.518794918	0.96446859
E2f3	-0.609115447	0.394956129	-1.542235713	0.123016325	0.817788082
Mboat1	-0.120945546	0.171449302	-0.705430381	0.480542443	0.957463006
Agtr1a	-0.168643366	0.355086649	-0.474935812	0.634832725	0.977034429
Uqcrfs1	0.041563589	0.114233015	0.36384918	0.715970635	0.988585656
Dusp22	0.204471814	0.134003792	1.52586588	0.12704327	0.824450458
Irf4	-0.708180115	0.489934036	-1.445460129	0.148328684	0.847956033
Exoc2	-0.114624257	0.143480825	-0.79888206	0.424358802	0.94406679
Hus1b	3.117338115	2.185782557	1.426188577	0.153813916	0.854159937
Foxq1	-0.189036417	0.34533752	-0.547396116	0.584106623	0.972333834
1700018A04Rik	0.625622195	0.656366311	0.953160125	0.340508947	0.919850248
Foxf2	-0.084096766	0.338942124	-0.248115416	0.804045108	0.990988839
Foxc1	-0.421599205	0.476430674	-0.884911967	0.376204113	0.928883222
Gmds	0.007328362	0.154786733	0.047344894	0.962238348	0.996474415
Mylk4	0.069712438	0.664575391	0.104897713	0.916456974	0.994688009
Wrnip1	0.134862514	0.162591368	0.829456786	0.40684598	0.939557159
Serpinb1a	0.102942576	0.146361415	0.703345046	0.481840744	0.957463006
Serpinb1c	0.91296856	1.048292665	0.870909995	0.383803299	0.931591679
Serpinb6b	0.071802815	0.158993576	0.451608279	0.651551205	0.979004493
Serpinb9	0.077629057	0.169570494	0.457798144	0.64709747	0.979004493
Serpinb9b	0.782752717	0.444152498	1.762351267	0.078009967	0.754202
Serpinb1b	0.058620407	0.457926811	0.128012611	0.898138993	0.994364545
Serpinb6c	0.139703712	0.93475715	0.149454553	0.881194968	0.992886758
Serpinb6a	0.13787484	0.186328233	0.739956782	0.459326219	0.952683168
1110046J04Rik	0.155968124	0.247719033	0.629617039	0.528945173	0.965637248
Nqo2	-0.141556275	0.136724564	-1.035339009	0.300510618	0.910285132
Ripk1	-0.08278065	0.139041932	-0.595364641	0.551599756	0.968926429
Bphl	0.021441917	0.125354088	0.171050804	0.864183819	0.992886758
Tubb2a	-0.088439471	0.12508863	-0.707014464	0.479557489	0.957463006
4930447K03Rik	0.590020623	1.000978854	0.589443644	0.555563707	0.968926429
Tubb2b	0.164554845	0.377003434	0.436481023	0.662487765	0.980647283
Psmg4	0.461311996	0.233467301	1.975916947	0.048164184	0.705270529
Slc22a23	0.144229643	0.221562861	0.650964887	0.515069153	0.964352198
Pxdc1	-0.130833371	0.136281204	-0.960025062	0.337042602	0.919246575
Gm15908	-0.063321592	0.439838269	-0.143965627	0.885527612	0.992886758
Prpf4b	-0.267076741	0.146348717	-1.824933941	0.068010999	0.742385562
Fam217a	-0.985700845	0.892779785	-1.104080605	0.269558174	0.89804647
Eci3	-0.329237902	1.06331178	-0.309634397	0.756838996	0.989266039
Eci2	0.084541704	0.100537162	0.840900045	0.400403933	0.936857985
Cdyl	-0.03040461	0.207795189	-0.146320089	0.883668708	0.992886758
Rpp40	0.369914138	0.266274116	1.389223042	0.164764937	0.859982992
Lyrm4	0.021323261	0.168912457	0.126238535	0.899543109	0.99447264
Fars2	0.137227401	0.151074725	0.908341228	0.363697967	0.926455466
Nrn1	0.266205896	0.168775999	1.577273411	0.114732628	0.809454857
F13a1	0.226808164	0.187412123	1.210210744	0.226198036	0.89398447

Ly86	-0.013535913	0.141390124	-0.095734498	0.923731441	0.994688009
Rreb1	0.015435427	0.290847599	0.0530705	0.957675736	0.996226826
Ssr1	-0.06266712	0.083028275	-0.754768419	0.450387946	0.949134998
Cage1	0.229750245	0.727819935	0.315669074	0.752253702	0.988585656
Riok1	0.095970582	0.167396998	0.57331125	0.56643397	0.970426802
Dsp	-0.07319031	0.259664448	-0.281864963	0.778047056	0.990988839
Snrnp48	0.322728481	0.160767088	2.007428796	0.044704025	0.689870152
Bmp6	-0.146178565	0.189640679	-0.770818614	0.440814453	0.948068187
Txndc5	-0.161949451	0.110533072	-1.465167382	0.142875256	0.840741274
Bloc1s5	0.147697811	0.148769056	0.99279927	0.320807786	0.91640698
Eef1e1	0.411445372	0.222394816	1.85006728	0.064303853	0.73299549
Slc35b3	0.044352634	0.200208032	0.22153274	0.824677647	0.992886758
5033403F01Rik	-0.10588982	1.450123231	-0.073021256	0.941789202	0.994688009
Tfap2a	-0.244080761	1.300461115	-0.187687858	0.851121349	0.992886758
Gcnt2	-0.046507001	0.142765953	-0.32575695	0.744608253	0.988585656
Pak1ip1	0.024142458	0.106323909	0.227065183	0.820373057	0.992886758
Tmem14c	0.103341391	0.157152422	0.65758701	0.510803544	0.963452765
Mak	-0.224061526	0.332103627	-0.674673529	0.499883206	0.961017744
Gcm2	-0.768942152	3.308402768	-0.232420961	0.81621107	0.992676801
Smim13	-0.120322314	0.127921248	-0.940596783	0.346911531	0.921552698
Nedd9	-0.137275205	0.142031566	-0.966511948	0.333788071	0.917622131
Tmem170b	-0.301162256	0.211354037	-1.424918398	0.154180791	0.854159937
Adtrp	0.299035759	0.566883273	0.527508525	0.597840498	0.974001549
Gm10790	0.410883809	1.060209381	0.387549682	0.698349312	0.987736745
Hivep1	0.202502214	0.183260873	1.104994267	0.269162073	0.89804647
Edn1	-0.057117299	0.194713584	-0.293340083	0.769262219	0.989990452
Phactr1	-0.386987962	0.180094433	-2.148805791	0.031649798	0.661395937
Tbc1d7	0.032690809	0.242803261	0.134639084	0.892897238	0.993300147
Gfod1	-0.269890162	0.240913484	-1.120278355	0.262595163	0.897248195
Sirt5	0.115429368	0.260388217	0.443297202	0.657550779	0.979614766
Nol7	0.188523634	0.117672919	1.602098723	0.10913378	0.798400564
Ranbp9	-0.005832807	0.189840279	-0.030724814	0.975489002	0.997262526
Mcur1	-0.076379559	0.130392307	-0.58576737	0.558031854	0.968926429
Cd83	0.134944831	0.181855896	0.742042651	0.458061495	0.952235842
1700029N11Rik	-0.614997871	1.679116879	-0.366262694	0.714169064	0.988585656
Jarid2	0.158358991	0.273765342	0.578447915	0.562961753	0.969658675
Dtnbp1	0.051021289	0.138372399	0.368724463	0.712333113	0.988585656
Mylip	0.119381593	0.205299707	0.581499091	0.560904131	0.968985477
Gmpr	-0.017756033	0.194287205	-0.091390646	0.927182194	0.994688009
Atxn1	-0.143431296	0.234898002	-0.610610966	0.541457161	0.967217841
Stmnd1	-0.044897849	0.12375758	-0.36278868	0.716762748	0.988585656
Rbm24	-0.570066614	0.25146819	-2.266953186	0.023393086	0.622011301
Cap2	-0.457923652	0.363757075	-1.258872152	0.208076514	0.884258007
Fam8a1	0.015153873	0.176645014	0.085787155	0.931635617	0.994688009
Nup153	-0.185283719	0.142725335	-1.298183806	0.194224181	0.879588734
Kif13a	0.155626938	0.184430746	0.843823175	0.398768223	0.935847172
Nhlrc1	-0.362275602	0.386166929	-0.938132127	0.348176513	0.921552698
Tpmt	0.030104462	0.386424523	0.077905153	0.9379035	0.994688009
Kdm1b	-0.071388916	0.181789459	-0.392701078	0.694540274	0.987010101
Dek	-0.006626002	0.139709462	-0.047427012	0.962172901	0.996474415
Rnf144b	0.116068464	0.142296486	0.815680465	0.414682914	0.943415794
Id4	-0.13268455	0.310616167	-0.427165628	0.669258695	0.981863224
A330048O09Rik	-0.935571957	1.819086134	-0.514308773	0.607036129	0.974001549
Zfp169	-0.322846905	0.493826187	-0.653766271	0.5132624	0.964352198
Mirlet7d	-0.388610046	1.229997188	-0.315943849	0.752045129	0.988585656
Ptpdc1	-0.205422591	0.29785332	-0.689677021	0.49039732	0.95865865
Phf2	-0.311417079	0.215076553	-1.447935979	0.147634956	0.846658212
Fam120a	0.008094386	0.124198457	0.065172999	0.948036259	0.995535632
Fam120aos	0.224201988	0.167635841	1.337434685	0.181080789	0.868087032
Wnk2	0.611201297	1.320160325	0.462975053	0.643382249	0.978801899
Ninj1	0.102563147	0.132915511	0.771641673	0.440326685	0.947849924
1110007C09Rik	-0.040825196	0.146955007	-0.27780745	0.781160172	0.990988839

Susd3	0.619771225	0.184839677	3.353020493	0.000799348	0.203921137
Fgd3	0.499635532	0.617184708	0.809539714	0.418204769	0.943415794
Bicd2	-0.309801804	0.146998106	-2.107522424	0.03507232	0.674795441
Ippk	0.137709889	0.153438545	0.897492147	0.369456362	0.928436166
Cenpp	0.160563806	0.460278619	0.348840462	0.727209085	0.988585656
Ecm2	-0.191704702	0.21937059	-0.873885152	0.382180801	0.930767593
Aspn	0.142432731	0.159950728	0.890478794	0.37320885	0.928840865
Omd	0.142889618	0.305354582	0.467946534	0.639822825	0.977843986
Ogn	-0.257799932	0.129100741	-1.996889634	0.045837174	0.696869274
Nol8	-0.131013018	0.18909775	-0.692832241	0.488414836	0.958481967
Iars	-0.240323146	0.21845094	-1.100124111	0.27127805	0.89804647
Fbxw17	0.054991826	0.195561473	0.281199691	0.778557242	0.990988839
9430083A17Rik	0.902105252	0.567721602	1.588992295	0.112062132	0.801067615
Spin1	-0.058616525	0.105597457	-0.555094098	0.5788303	0.971613657
Nxnl2	0.180324179	0.485764503	0.371217284	0.710475701	0.988585656
S1pr3	0.181285855	0.275771587	0.657376844	0.510938637	0.963452765
Cks2	0.274723069	0.212690005	1.291659515	0.196475084	0.879755417
Secisbp2	-0.007291802	0.197846898	-0.036855782	0.970599996	0.996733101
Sema4d	-0.056546889	0.286313166	-0.197500136	0.843436175	0.992886758
Gadd45g	0.120114936	0.273467874	0.439228688	0.66049584	0.979924124
Diras2	-0.202564355	0.262040108	-0.773028056	0.439505777	0.947849924
Gm2848	-0.969334857	1.886254184	-0.513894079	0.607326047	0.974001549
Syk	-0.050629505	0.173888778	-0.29116028	0.770928739	0.989990452
BB123696	0.753365069	1.610481807	0.467788624	0.639935757	0.977843986
Auh	0.328350848	0.143350622	2.290543593	0.021989825	0.616886367
Nfil3	0.08743791	0.21827589	0.400584373	0.688726153	0.98538669
Ror2	-0.077597895	0.808737211	-0.095949456	0.923560716	0.994688009
Sptlc1	-0.113087735	0.159037369	-0.711076496	0.477036834	0.95716557
Sfxn1	-0.288184858	0.162177278	-1.776974318	0.075572465	0.752876039
Hrh2	0.38877068	1.272117966	0.305608985	0.759902379	0.989266039
Cplx2	-0.063359257	0.246251904	-0.257294485	0.79695145	0.990988839
Thoc3	0.206948415	0.142119774	1.456154969	0.145349785	0.842729381
Simc1	-0.354568988	0.448976391	-0.789727467	0.429686947	0.945321484
4833439L19Rik	-0.153150023	0.12454978	-1.229629011	0.218836061	0.888671255
Arl10	0.212720816	0.182784368	1.163780136	0.244513124	0.896939594
Nop16	0.103381739	0.165595584	0.624302508	0.532428937	0.96674935
Higd2a	0.180178113	0.120262742	1.498203927	0.134080276	0.827800491
Cltb	0.267682521	0.104792414	2.554407426	0.010636871	0.502706181
Faf2	-0.236188089	0.151488569	-1.55911493	0.118969181	0.814755048
Rnf44	-0.040429686	0.212293104	-0.190442768	0.848962187	0.992886758
Tspan17	0.434607392	0.144454708	3.008606635	0.002624487	0.327099208
Unc5a	1.045956506	0.626333887	1.669966335	0.094926024	0.778239075
Hk3	-1.107534808	0.538101247	-2.058227546	0.039568296	0.679118604
Uimc1	0.097855801	0.121780061	0.803545342	0.4216596	0.943415794
Zfp346	0.010184909	0.182989441	0.055658451	0.955613899	0.99614946
Fgfr4	-0.211921559	0.260768213	-0.812681717	0.416400566	0.943415794
Nsd1	0.053454127	0.147903633	0.36141186	0.717791584	0.988585656
Rab24	0.297671905	0.124609757	2.388833051	0.016901981	0.569373711
Prelid1	-0.077967379	0.154647941	-0.504160472	0.614148615	0.974001549
Mxd3	0.12607899	0.457632023	0.275502989	0.78292983	0.990988839
Lman2	-0.158818608	0.092569877	-1.715661858	0.08622395	0.764681382
Rgs14	-0.215310818	0.209815092	-1.026193187	0.304800532	0.912445598
Grk6	-0.065610916	0.153719414	-0.426822572	0.669508564	0.981863224
Prr7	0.197885334	0.394680667	0.501380866	0.616103103	0.974001549
Dbn1	-0.427707765	0.310815793	-1.376081185	0.1687965	0.861597949
Pdlim7	-0.039597563	0.174519907	-0.226894249	0.820505974	0.992886758
Dok3	0.248845983	0.25166045	0.98881641	0.322752974	0.91640698
Ddx41	0.067772303	0.122984887	0.551062047	0.581591141	0.971915898
Fam193b	0.100935607	0.161921497	0.623361377	0.533047073	0.96674935
Tmed9	0.107255134	0.128294552	0.83600693	0.403151005	0.937834293
B4galt7	0.109245785	0.138233968	0.79029624	0.429354782	0.945321484
Caml	-0.006278761	0.141201258	-0.04446675	0.964532355	0.996474415

Ddx46	-0.089488998	0.128798003	-0.694801129	0.487179941	0.958288569
B230219D22Rik	-0.084841787	0.109694289	-0.773438501	0.439262912	0.947849924
Txndc15	0.114379012	0.125948168	0.908143518	0.363802401	0.926455466
Pcbd2	0.461731111	0.256059192	1.803220213	0.071353637	0.744227652
Catsper3	0.493818136	1.638185269	0.301442178	0.763077335	0.989285682
H2afy	-0.008625352	0.124895822	-0.069060373	0.944941563	0.99486515
Tifab	-0.01312556	0.243309545	-0.053945933	0.956978241	0.996226826
Cxcl14	-0.017850775	0.148777598	-0.119982947	0.904496657	0.994688009
Fbxl21	0.146115964	0.527348877	0.277076468	0.781721391	0.990988839
Tgfbi	-0.117861962	0.179771831	-0.655619744	0.512068819	0.963452765
Smad5	-0.234263793	0.108759467	-2.153962316	0.031243127	0.661395937
Klhl3	-0.296865353	1.206609672	-0.246032632	0.805656972	0.99099448
Hnrnpa0	0.017883525	0.165722043	0.107912774	0.914064884	0.994688009
Idnk	0.374857774	0.186053819	2.014781398	0.043927559	0.688477147
Ubqln1	-0.231706694	0.15197044	-1.524682651	0.127338271	0.824450458
Gkap1	-0.132776256	0.137303911	-0.967024572	0.33353175	0.917554801
Kif27	-0.265147221	0.350376096	-0.756750314	0.449199465	0.948476747
2210016F16Rik	0.134423081	0.129243234	1.040078279	0.298303534	0.909779403
Hnrnpk	0.060616571	0.114668321	0.52862526	0.597065434	0.974001549
Mir7-1	-0.402614482	0.469633874	-0.857294383	0.391282212	0.933734231
Rmi1	0.006797083	0.156537123	0.043421539	0.965365508	0.996474415
Slc28a3	-0.608690223	0.711936333	-0.854978451	0.392563078	0.933734231
Ntrk2	1.025949027	0.373358602	2.747891764	0.005997981	0.427133716
Agtpbp1	-0.089659315	0.146548004	-0.611808502	0.540664464	0.966989525
Naa35	-0.054692565	0.126601328	-0.432006249	0.665736871	0.981798021
Golm1	-0.021583858	0.172474081	-0.125142615	0.900410646	0.994688009
Isca1	0.090266045	0.094225198	0.957982015	0.338071828	0.919246575
Etohd2	0.541081447	0.417297871	1.296631219	0.194758105	0.879588734
Zcchc6	0.074036366	0.167251288	0.442665448	0.658007738	0.979614766
Gas1	-0.217347798	0.22653984	-0.959424171	0.337345103	0.919246575
Gm5084	0.564354363	0.558390598	1.010680275	0.312169481	0.914819219
Dapk1	-0.006769074	0.115395695	-0.058659676	0.953223178	0.996070133
4930486L24Rik	0.069195927	0.263666364	0.262437444	0.792984208	0.990988839
Ctla2b	0.119297545	0.362634636	0.328974492	0.742174968	0.988585656
Ctla2a	0.193531576	0.24973908	0.774935088	0.438378017	0.947776577
Zfp808	-0.385554372	0.231248076	-1.66727602	0.095459533	0.778239075
Gm3604	0.276501334	0.244631953	1.130274809	0.258360446	0.897248195
Zfp935	-0.015143329	0.188423106	-0.080368745	0.935943985	0.994688009
Zfp934	-0.137866217	0.289057174	-0.476951377	0.633396746	0.977034429
6720489N17Rik	0.274825799	0.276966752	0.992270001	0.321065833	0.91640698
Gm5141	-0.021140787	0.330889312	-0.063890812	0.949057168	0.995624732
Fbp2	-0.119223694	0.641501038	-0.185851131	0.852561506	0.992886758
Fbp1	-0.05126303	0.735332638	-0.069714069	0.944421244	0.99486515
2010111I01Rik	0.144921079	0.152228402	0.951997642	0.341098178	0.919850248
Gm16907	-0.997171727	1.366230986	-0.729870525	0.46546933	0.953083512
Fancc	0.170196048	0.153121046	1.1115131	0.266347554	0.89768067
Ptch1	0.015076752	0.369897429	0.040759277	0.967487804	0.996474415
Ercc6l2	-0.099779834	0.163013149	-0.612096843	0.540473686	0.966989525
Slc35d2	-0.113234987	0.163965465	-0.690602664	0.489815273	0.958560421
Zfp367	0.098601268	0.128375999	0.768066221	0.442447844	0.948476747
Habp4	0.039582701	0.109124541	0.362729601	0.716806885	0.988585656
Cdc14b	-0.301545584	0.280473798	-1.075129248	0.282316866	0.90579922
1810034E14Rik	0.129245224	0.247380596	0.522454978	0.601353585	0.974001549
Aaed1	0.089923507	0.148224184	0.6066723	0.544068408	0.967491297
Ctsl	-0.116961914	0.111632141	-1.047744068	0.294756538	0.907334416
Cdk20	0.318098706	0.23792874	1.336949483	0.181239127	0.868087032
Hiatl1	-0.010054867	0.081510688	-0.123356419	0.901824866	0.994688009
Zfp369	-0.174925057	0.634146935	-0.275843101	0.782668578	0.990988839
Uqcrb	0.113435256	0.142693235	0.794958893	0.426637415	0.944290206
Gm10767	0.786665872	0.612296499	1.284779307	0.198869415	0.880661448
Mterf3	-0.060078306	0.142855749	-0.420552246	0.67408207	0.983375962
Ptdss1	0.057785983	0.11893067	0.485879574	0.627052551	0.97671122

4933433G19Rik	0.169568241	2.248087994	0.07542776	0.939874373	0.994688009
Zfp712	-0.105613035	0.234627228	-0.450131196	0.652615844	0.979004493
Zfp708	0.250735078	0.28354868	0.884275244	0.376547649	0.928883222
Rslcan18	-0.529553302	0.472253106	-1.121333656	0.262145865	0.897248195
Zfp759	0.044754245	0.262180229	0.170700304	0.864459424	0.992886758
Rsl1	-0.091130804	0.252000572	-0.361629355	0.717629025	0.988585656
Zfp455	0.021933746	0.283135752	0.077467242	0.93825185	0.994688009
Zfp458	-0.093062696	0.379913913	-0.244957325	0.806489475	0.99099448
F630042J09Rik	-0.490439457	0.550354764	-0.891133301	0.372857661	0.928840865
Zfp457	-0.701484748	1.975216754	-0.355143174	0.722482334	0.988585656
Zfp595	-0.262671907	0.208782198	-1.258114486	0.208350355	0.884258007
Zfp953	0.226446097	0.216054462	1.048097293	0.294593783	0.907334416
Zfp456	0.013741703	0.249060627	0.05517413	0.955999739	0.99614946
Zfp429	-0.050437864	0.208027248	-0.242457969	0.808425321	0.991020605
Zfp459	-0.197441094	0.567739083	-0.347767311	0.728014941	0.988585656
Zfp874a	0.11337465	0.161419608	0.702359842	0.482454783	0.957463006
Zfp874b	0.137841961	0.133991577	1.028736016	0.303603741	0.911786415
Zfp58	-0.180274024	0.183480242	-0.982525538	0.325841008	0.916610292
Zfp87	0.032018469	0.184557166	0.173488085	0.862267793	0.992886758
Zfp748	-0.03848665	0.150789709	-0.255233931	0.798542425	0.990988839
9430065F17Rik	0.624818105	0.51715765	1.208177244	0.226979093	0.89398447
Zfp729b	-0.02667321	0.149476627	-0.178444017	0.858374283	0.992886758
Zfp729a	-0.023892184	0.222026917	-0.107609401	0.914305539	0.994688009
Zfp738	-0.060476633	0.201420929	-0.300249993	0.763986474	0.989384061
Zfp65	0.174793156	0.181420281	0.963470873	0.335311275	0.918714299
Zfp85os	-1.599439375	1.290801506	-1.2391056	0.215306393	0.887633697
Zfp85	0.295812475	0.23407424	1.263754933	0.206318001	0.883765092
Zfp493	-0.271786968	0.346524891	-0.784321633	0.432851409	0.946072952
4930525G20Rik	-0.679699176	1.004034237	-0.676968126	0.498426178	0.960700483
Zfp273	0.027903341	0.238110599	0.117186474	0.906712286	0.994688009
Gm10037	0.820438171	0.734346134	1.117236319	0.263893295	0.897248195
Mtrr	0.068929801	0.180234188	0.38244576	0.702130754	0.988482081
Fastkd3	-0.289757438	0.295363044	-0.981021302	0.326582237	0.916882624
1700001L19Rik	0.20922387	0.196167583	1.066556804	0.286172015	0.905916508
Adcy2	0.142919182	0.275393487	0.518963553	0.603786158	0.974001549
Papd7	-0.236349851	0.248525403	-0.951008823	0.341599897	0.919850248
Srd5a1	-0.883527941	0.568422291	-1.554351323	0.120100637	0.81558552
Nsun2	-0.023794485	0.119886799	-0.198474606	0.842673751	0.992886758
Ube2ql1	-0.372394377	1.850356199	-0.201255508	0.84049879	0.992886758
Med10	0.366145843	0.171534141	2.134536254	0.032798909	0.665167506
Ice1	-0.044571355	0.172736698	-0.258030607	0.796383286	0.990988839
Irx1	-0.320023476	0.182562445	-1.752953501	0.079609989	0.75659671
Gm20554	0.011760715	0.330773943	0.035555143	0.971637077	0.996733101
Irx2	-0.363179521	0.220968113	-1.643583392	0.100262287	0.788783372
Ndufs6	0.540297311	0.173682881	3.110826512	0.001865645	0.292888346
Mrpl36	-0.053038283	0.117785085	-0.450297105	0.652496226	0.979004493
D630045M09Rik	0.100649918	0.424411379	0.23715179	0.812539036	0.992000477
Lpcat1	-0.416061964	0.165729222	-2.510492472	0.012056289	0.525888996
Clptm1l	-0.039987307	0.087129964	-0.458938634	0.646278234	0.979004493
Tert	0.623955567	1.586664734	0.393249786	0.694135002	0.986949593
Slc6a19	-1.206779815	2.085600488	-0.578624632	0.562842481	0.969658675
Slc12a7	-0.355299472	0.253512319	-1.401507722	0.1610623	0.857316691
Nkd2	-0.118843306	0.295990209	-0.401510938	0.688043987	0.98538669
Trip13	-0.060861979	0.435947512	-0.139608502	0.888969323	0.992967239
Brd9	-0.000787344	0.146966219	-0.005357311	0.995725504	0.999285699
Tppp	-0.348725217	0.259030362	-1.346271587	0.178214946	0.867377413
Cep72	-0.195614299	0.530316568	-0.368863262	0.712229648	0.988585656
Exoc3	0.024007597	0.17581819	0.136547856	0.891388195	0.993043931
Ahrr	-0.920659874	1.788397447	-0.514796012	0.606695572	0.974001549
Pdcd6	0.092383894	0.158556697	0.582655259	0.560125398	0.968926429
Sdha	0.003172804	0.117817446	0.026929835	0.978515697	0.997262526
Ccdc127	0.120682224	0.114948166	1.049883853	0.293771516	0.907334416

Lrrc14b	0.518453855	0.745102601	0.695815387	0.486544452	0.958124625
Zfp72	-0.026894699	0.277597985	-0.096883626	0.922818812	0.994688009
Zfp825	0.063121621	0.171368567	0.368338383	0.712620936	0.988585656
Erap1	-0.164179269	0.141120666	-1.163396358	0.244668727	0.896939594
Cast	-0.265572704	0.148630149	-1.786802375	0.073969435	0.750704981
Pcsk1	0.009833379	0.709255794	0.013864361	0.988938195	0.998182772
Mir682	0.276620795	0.220699153	1.253384034	0.210065979	0.884258007
Ell2	-0.202968832	0.140613265	-1.443454368	0.148892518	0.848779008
Glrx	-0.069053167	0.148143314	-0.466124089	0.641126681	0.977921835
Rhobtb3	0.168160958	0.25435803	0.661119124	0.508535922	0.963324595
Spata9	-0.419687241	1.229718997	-0.341287109	0.732887452	0.988585656
Rfesd	0.255196385	0.1766236	1.444860059	0.148497197	0.84832848
Arsk	-0.221987666	0.193954994	-1.144531839	0.252403142	0.897013805
Ttc37	-0.240606828	0.263575263	-0.912858152	0.361317149	0.926371563
Mctp1	0.051941099	0.166244721	0.312437585	0.75470799	0.98902053
Ankrd32	-0.213944518	0.15576502	-1.373508112	0.169594441	0.861597949
2210408I21Rik	-0.065185296	0.200537878	-0.325052289	0.745141497	0.988585656
Fam172a	-0.121958588	0.13514798	-0.902407773	0.366840293	0.927999696
Nr2f1	-0.705445354	0.56077355	-1.257986141	0.208396769	0.884258007
A830082K12Rik	0.132582022	0.50576921	0.26213937	0.793213995	0.990988839
Arrdc3	-0.122225794	0.153191297	-0.797863821	0.42494952	0.944290206
5430425K12Rik	0.19318854	0.2594787	0.744525618	0.456558549	0.951472839
Adgrv1	0.258648085	0.392221108	0.659444583	0.509610321	0.963452765
Lysmd3	-0.133175855	0.216639755	-0.614734147	0.538730305	0.966875138
Polr3g	-0.131375276	0.361416497	-0.363501048	0.71623063	0.988585656
Mblac2	-0.133767668	0.201269247	-0.664620501	0.506293254	0.962370882
Cetn3	0.278422269	0.169506593	1.642545367	0.100477032	0.788783372
Mef2c	-0.098113922	0.248250026	-0.395222205	0.692678907	0.986599035
Tmem161b	0.259256308	0.167346617	1.549217502	0.121329444	0.816233249
Ccnh	-0.051806912	0.141523813	-0.366064984	0.714316586	0.988585656
Rasa1	-0.139455753	0.104816036	-1.330481082	0.183359815	0.869576666
Cox7c	0.153931529	0.173069167	0.889422022	0.373776314	0.928840865
Edil3	-0.125411458	0.187443061	-0.669064284	0.503454472	0.961729397
Hapln1	0.24181189	0.523294828	0.462094937	0.644013241	0.97889195
Vcan	-0.313214194	0.538441687	-0.581704949	0.560765438	0.968926429
Xrcc4	0.26150245	0.188071102	1.390444607	0.164393909	0.859982992
Tmem167	0.056919464	0.149324623	0.381179356	0.703070166	0.988585656
Rps23	0.31528222	0.190788246	1.652524342	0.098427707	0.786907305
Atg10	0.213692335	0.21941166	0.973933357	0.33008963	0.917115058
A830009L08Rik	3.641750003	2.224900733	1.6368146	0.101669214	0.788783372
Ssbp2	0.049339579	0.189153186	0.260844557	0.794212382	0.990988839
4833422C13Rik	0.073659903	0.800175952	0.092054633	0.926654633	0.994688009
Zcchc9	0.093952889	0.143662888	0.653981624	0.513123645	0.964352198
Ckmt2	-0.324766511	0.453664539	-0.715873699	0.47406934	0.955974667
Rasgrf2	-0.111981694	0.167466438	-0.668681409	0.503698729	0.96179897
Msh3	0.199780727	0.319134133	0.626008648	0.531309269	0.96621591
Dhfr	0.313378277	0.232325287	1.348877175	0.177376421	0.866855369
Fam151b	0.181434802	0.207364696	0.874955115	0.381598329	0.930635634
Zfyve16	-0.353722028	0.164114969	-2.155330677	0.031135967	0.661395937
Serinc5	-0.187488085	0.170772795	-1.09788029	0.272256766	0.898370293
Mtx3	0.054308293	0.278679751	0.194877069	0.845489186	0.992886758
Cmya5	-0.027684774	0.460469566	-0.060122918	0.952057737	0.996023481
Papd4	-0.149162883	0.134802963	-1.106525256	0.268499239	0.89804647
Homer1	-0.227361578	0.222123773	-1.023580571	0.306033426	0.91333012
Jmy	-0.063117387	0.180494658	-0.349691165	0.726570485	0.988585656
Arsb	-0.096997949	0.21108904	-0.459512008	0.645866531	0.979004493
Lhfpl2	-0.17383141	0.241459188	-0.71992046	0.47157397	0.95486913
Scamp1	-0.178804679	0.109796908	-1.628503775	0.10341811	0.790925787
Gm9776	0.128903601	0.391623763	0.329151633	0.742041079	0.988585656
Ap3b1	0.004320012	0.140671982	0.030709828	0.975500953	0.997262526
Tbca	0.285456034	0.192495973	1.482919513	0.138095796	0.833928471
Wdr41	0.054804453	0.159570078	0.343450688	0.731259432	0.988585656

Pde8b	-0.104735119	0.208820149	-0.501556577	0.61597947	0.974001549
Zbed3	-0.146084369	0.350279855	-0.417050443	0.676641512	0.983549699
Aggf1	0.005522706	0.214318407	0.025768696	0.97944183	0.997262526
S100z	0.245106618	0.917950515	0.267015066	0.789457561	0.990988839
F2rl1	-0.007982396	0.305143694	-0.026159467	0.979130146	0.997262526
F2r	-0.229413639	0.133619298	-1.716919962	0.085993797	0.763596872
Iqgap2	0.137417082	0.227136838	0.604996896	0.545181059	0.967491297
F2rl2	1.58362713	0.987162107	1.604221959	0.108665136	0.798150849
Sv2c	0.755805303	1.411616635	0.535418246	0.592360676	0.974001549
Poc5	0.163355699	0.195713846	0.834666031	0.403905773	0.937834293
Ankdd1b	-0.463984282	0.523936548	-0.885573421	0.375847439	0.928883222
Polk	-0.29111516	0.196889106	-1.478574236	0.139254143	0.836190654
Col4a3bp	-0.117525661	0.081656168	-1.43927475	0.150072694	0.849826521
Hmgcr	-0.105801007	0.172946881	-0.61175435	0.540700297	0.966989525
Fam169a	0.860125321	1.490075277	0.577236153	0.563779937	0.969658675
Nsa2	0.185263347	0.154343871	1.200328504	0.230011784	0.895404344
Gfm2	0.033882485	0.200369951	0.16909963	0.865718276	0.992886758
Hexb	-0.150891347	0.127484687	-1.183603691	0.236569979	0.896358698
Enc1	-0.037840019	0.31149374	-0.121479227	0.903311467	0.994688009
Gm5086	0.022355951	0.733811021	0.030465543	0.975695774	0.997262526
Arhgef28	-0.075839814	0.173694749	-0.436626985	0.662381889	0.980647283
Utp15	0.127495317	0.23813761	0.535385052	0.592383624	0.974001549
Ankra2	0.100302547	0.137565424	0.729126147	0.465924502	0.953083512
Btf3	0.184386928	0.144414438	1.276790122	0.201676354	0.881220006
Tmem171	-0.039021441	0.314689551	-0.1239998	0.901315433	0.994688009
Fcho2	-0.052888594	0.117735613	-0.449214918	0.653276627	0.979004493
Tnpo1	-0.14441725	0.203875112	-0.708361352	0.478720886	0.957463006
Zfp366	-0.755244551	0.83460787	-0.904909453	0.365513358	0.926667174
Ptcd2	-0.043784478	0.207493584	-0.211016055	0.832874743	0.992886758
Mrps27	0.071989133	0.178011983	0.404406109	0.685914109	0.98538669
6430562O15Rik	-1.085876902	1.212993933	-0.895203902	0.370678099	0.928436166
Map1b	0.530409123	0.429438867	1.235121374	0.216785336	0.887633697
Mccc2	-0.141383063	0.134274578	-1.052939915	0.292368531	0.907334416
Bdp1	-0.043992847	0.226933951	-0.193857494	0.846287467	0.992886758
Serf1	0.382852172	0.248780497	1.53891554	0.123824917	0.820382953
Smn1	-0.049653853	0.191772215	-0.258920998	0.795696196	0.990988839
Naip2	-0.017733494	0.217808213	-0.081417931	0.935109589	0.994688009
Naip5	0.064162065	0.307786736	0.208462735	0.834867677	0.992886758
Naip6	1.051242996	1.537881554	0.683565644	0.494249485	0.958916246
Naip1	0.773977633	2.371208941	0.326406341	0.744116941	0.988585656
Gtf2h2	0.331194188	0.195743054	1.69198437	0.090648954	0.770055369
Ocln	-0.068072079	0.120787136	-0.563570601	0.573046399	0.971613657
Marveld2	0.152151345	0.206026162	0.738504971	0.460207647	0.953083512
Rad17	-0.183740753	0.143907948	-1.276793639	0.201675112	0.881220006
Ak6	0.072066148	0.19686091	0.366076476	0.714308011	0.988585656
Taf9	0.296169227	0.126841909	2.334947723	0.019546152	0.593178479
Ccdc125	0.112461256	0.244946665	0.459125483	0.646144058	0.979004493
Cdk7	0.11783479	0.165870955	0.710400382	0.477455887	0.95716557
Mrps36	0.268078961	0.200701439	1.335710205	0.181644013	0.868546072
Cenph	-0.278401941	0.65572237	-0.424572889	0.67114806	0.982677283
Ccnb1	-0.043960466	0.367929239	-0.119480764	0.904894479	0.994688009
Slc30a5	-0.001849197	0.104259274	-0.017736519	0.985849047	0.998148029
5930438M14Rik	0.273818458	1.332263378	0.205528773	0.83715902	0.992886758
Pik3r1	0.066458166	0.154481588	0.430201211	0.667049281	0.981863224
Cd180	0.557191708	0.524338062	1.062657374	0.287937358	0.907334416
Mast4	-0.149957791	0.285859873	-0.524584965	0.599871741	0.974001549
Srek1	-0.056043948	0.17097405	-0.327792132	0.743068836	0.988585656
Erbb2ip	-0.118920316	0.11338805	-1.048790555	0.294274525	0.907334416
Nln	0.132056309	0.141936811	0.93038802	0.352170219	0.923082865
Sgtb	-0.471936479	0.256778694	-1.83791136	0.066075468	0.738254074
Trappc13	-0.056600659	0.128601556	-0.440124219	0.659847142	0.979924124
Trim23	0.255979386	0.181657598	1.409131181	0.158796382	0.855986074

Ppwd1	0.031135978	0.242508927	0.128391058	0.897839508	0.994101834
Cenpk	0.362845564	0.407994958	0.889338352	0.373821266	0.928840865
Adamts6	0.100428262	0.448027668	0.224156383	0.822635621	0.992886758
Cwc27	0.331768012	0.270108953	1.228274768	0.21934384	0.888847883
Srek1ip1	0.155514057	0.167482102	0.928541345	0.353126831	0.923786159
Rgs7bp	-0.642938644	0.556343937	-1.155649593	0.247824514	0.896939594
Rnf180	-0.270728042	0.445542296	-0.607637131	0.543428169	0.967491297
Ipo11	0.055844505	0.155665627	0.358746541	0.719784708	0.988585656
Dimt1	0.151066166	0.204593787	0.738371227	0.460288895	0.953083512
Kif2a	0.10775204	0.154155059	0.69898478	0.484561543	0.957710647
3830408C21Rik	0.882930111	0.494552208	1.785312243	0.074210684	0.750704981
AI197445	0.250617612	0.503150516	0.498096702	0.618415885	0.974471036
Zswim6	-0.403062512	0.255221773	-1.579263822	0.114275556	0.808745994
Smim15	0.11410384	0.138376035	0.824592499	0.409602981	0.940886864
Ndufaf2	0.395315373	0.234103525	1.688634859	0.091289426	0.771949327
Ercc8	0.123012676	0.186577692	0.65931074	0.509696247	0.963452765
Elovl7	0.048465536	0.190292682	0.254689437	0.798962975	0.990988839
Depdc1b	-1.312334435	0.973461596	-1.348111154	0.177622634	0.866855369
Pde4d	0.05764056	0.274849454	0.209716846	0.833888677	0.992886758
Rab3c	-0.0813206	0.529554764	-0.153564099	0.877953446	0.992886758
Gapt	-0.131969002	0.476262573	-0.277092952	0.781708734	0.990988839
Plk2	0.125611063	0.225478622	0.557086352	0.577468432	0.971613657
Gpbp1	0.027946525	0.103181633	0.270847863	0.786508046	0.990988839
Mier3	-0.151355538	0.166363121	-0.909790206	0.362933161	0.926455466
Map3k1	-0.07489313	0.225803416	-0.331674035	0.740135409	0.988585656
Gm15326	0.444720809	1.213936468	0.366346032	0.714106885	0.988585656
Ankrd55	-1.087478415	0.792874932	-1.371563624	0.170199324	0.861924432
Il6st	-0.085478588	0.12844546	-0.665485476	0.50574003	0.96224682
Il31ra	0.389095122	1.901735589	0.204600011	0.837884647	0.992886758
Ddx4	-0.865089218	1.070927783	-0.807794169	0.419209083	0.943415794
Slc38a9	0.048212011	0.267870727	0.179982381	0.8571664	0.992886758
Ppap2a	-0.328921394	0.122722602	-2.680202241	0.007357769	0.452031612
Skiv2l2	-0.009405725	0.129074094	-0.072870743	0.941908975	0.994688009
Dhx29	0.101048552	0.195814381	0.516042548	0.605824693	0.974001549
Ccno	-0.75912778	0.373274191	-2.033700155	0.041981837	0.679118604
Mcidas	0.832620845	0.847677837	0.982237365	0.325982924	0.916693626
Cdc20b	1.052941508	1.322438683	0.79621197	0.425908841	0.944290206
Gpx8	-0.00963245	0.131061761	-0.073495504	0.941411822	0.994688009
Gzma	1.400709354	0.258143716	5.426083496	5.76E-08	0.0003135
Gzmk	0.388517614	1.478289804	0.262815595	0.792692715	0.990988839
Esm1	-0.725629471	0.239075575	-3.035146823	0.002404186	0.31402518
Snx18	-0.0591853	0.144949935	-0.408315462	0.683042089	0.984738223
Hspb3	-2.812870451	2.143868487	-1.312053639	0.189502037	0.876091552
Arl15	-0.104072769	0.171237327	-0.607769178	0.543340574	0.967491297
Ndufs4	0.284846954	0.200577553	1.420133758	0.155568744	0.855261441
Fst	-0.200910947	0.294901023	-0.681282637	0.495692667	0.959893834
Mocs2	0.147578381	0.119596764	1.233966342	0.217215446	0.887633697
Itga2	-0.501734998	0.551528187	-0.909717781	0.362971364	0.926455466
Itga1	0.137650889	0.149660324	0.919755388	0.357700601	0.924354588
Pelo	0.099607137	0.227605374	0.437630868	0.661653891	0.980470419
Parp8	-0.086440839	0.192890963	-0.448133169	0.654057091	0.979133069
Gm6416	-0.609157614	1.830964846	-0.332697602	0.73936256	0.988585656
Emb	-0.033367705	0.104832434	-0.318295623	0.750260704	0.988585656
Hcn1	0.169839326	1.588832229	0.106895696	0.914871728	0.994688009
Mrps30	-0.12585586	0.136856365	-0.919620075	0.357771332	0.924354588
Fgf10	-0.015357778	0.228551413	-0.067196163	0.946425539	0.995065399
Nnt	-0.216204668	0.178094884	-1.213985839	0.224753132	0.892989918
Paip1	0.09783046	0.093292763	1.048639315	0.294344154	0.907334416
4833420G17Rik	0.089888646	0.159079761	0.565053945	0.572037072	0.971613657
3110070M22Rik	-0.551107651	0.985625076	-0.559145323	0.576062545	0.971613657
Gm7120	-2.050296829	0.350721207	-5.84594484	5.04E-09	4.11E-05
D830030K20Rik	0.260378825	1.114943255	0.233535495	0.81534561	0.992333044

Gm3636	-0.039361603	0.795843607	-0.049458967	0.960553537	0.996474415
Gm3558	-0.278722386	1.471461928	-0.189418687	0.84976468	0.992886758
Flnb	0.217419713	0.214918522	1.01163786	0.311711237	0.914819219
Dnase1l3	0.027294441	1.085705644	0.025139817	0.979943441	0.997296835
Abhd6	0.206045478	0.168313444	1.224177184	0.220885394	0.890248292
Rpp14	0.195563646	0.146376982	1.336027316	0.181540345	0.868446884
Pxk	-0.226032267	0.134068409	-1.685947261	0.091805958	0.772516176
Pdhb	0.117986314	0.104619285	1.12776831	0.25941778	0.897248195
Kctd6	-0.119965802	0.193453698	-0.620126693	0.53517438	0.96674935
Acox2	-0.22436876	0.296307696	-0.757215432	0.448920806	0.948476747
Fam107a	-0.267757162	0.558350702	-0.479550148	0.631547301	0.977034429
Oit1	-0.308452951	0.255838512	-1.205654881	0.227950589	0.894410876
4930452B06Rik	-0.312183691	0.362081396	-0.862192023	0.388581859	0.933269543
Fhit	0.053206155	0.187305509	0.284060812	0.776363778	0.990861469
Ptprg	0.011821644	0.151024608	0.078276274	0.93760829	0.994688009
3830406C13Rik	0.219320067	0.135248979	1.621602388	0.104888511	0.793565672
Synpr	-0.787788855	0.855172076	-0.921205073	0.35694337	0.924354588
Sntn	0.009909384	0.17154712	0.057764793	0.953935983	0.996070133
Thoc7	0.201351575	0.190953737	1.054452135	0.291675964	0.907334416
Atxn7	-0.124275486	0.188535767	-0.659161326	0.509792178	0.963452765
Psmd6	0.073683609	0.108973598	0.676160191	0.498938945	0.960700483
Il3ra	0.357529329	0.162033839	2.20651026	0.027348293	0.636714037
Slc4a7	-0.276045419	0.233470855	-1.182354941	0.237064894	0.896609492
Nek10	-0.245523941	0.482949963	-0.508383807	0.611184206	0.974001549
Lrrc3b	0.057102427	0.706025432	0.080878711	0.935538411	0.994688009
Oxsm	-0.013788777	0.165733369	-0.083198558	0.933693659	0.994688009
Ngly1	0.059840271	0.10810973	0.553514198	0.579911361	0.971613657
Top2b	-0.019213648	0.114259936	-0.168157346	0.866459496	0.992886758
Rarb	-0.664779897	0.249720852	-2.662092058	0.007765664	0.45506991
Thrb	0.302866689	0.234161196	1.293411092	0.195868916	0.879601031
Nr1d2	-0.405357166	0.189975094	-2.133738466	0.032864195	0.665167506
Rpl15	0.084387055	0.1443316	0.584674842	0.558766374	0.968926429
Nkiras1	0.191491222	0.198435894	0.965002944	0.334543337	0.918030088
Ube2e1	-0.209023083	0.134982276	-1.54852244	0.121496563	0.816233249
Ube2e2	-0.014406357	0.145127228	-0.099267088	0.92092621	0.994688009
Nid2	0.628595906	0.560770175	1.120951032	0.262308707	0.897248195
2700060E02Rik	0.065854741	0.138304101	0.476158992	0.633961113	0.977034429
Gng2	-0.241583951	0.16010171	-1.508940476	0.131313995	0.827800491
Saysd1	0.115235023	0.218333034	0.527794723	0.59764182	0.974001549
Kcnk5	0.072822026	0.228949157	0.318070732	0.750431284	0.988585656
Nudt13	0.283703338	0.158526639	1.789625639	0.073514114	0.748833631
Ecd	-0.274728827	0.172400857	-1.593546764	0.111037586	0.800111791
Fam149b	0.230624992	0.150376858	1.53364683	0.125116561	0.822296879
Dnajc9	0.230231255	0.129307662	1.780491987	0.074995479	0.75241101
Mrps16	0.342243357	0.194694647	1.757846774	0.078773584	0.755218033
Cfap70	-0.22869841	0.242939593	-0.941379736	0.346510294	0.921552698
Anxa7	-0.068787147	0.094002822	-0.73175619	0.464317394	0.953083512
Mss51	-0.346880732	0.933735796	-0.371497734	0.710266844	0.988585656
Ppp3cb	-0.072771419	0.123864045	-0.587510435	0.556860944	0.968926429
1810062O18Rik	0.425023538	2.301421781	0.184678681	0.853481069	0.992886758
Usp54	-0.447604165	0.211536359	-2.115967993	0.03434753	0.674629488
Myoz1	-0.046179673	0.649970785	-0.071048844	0.943358882	0.99486515
Synpo2l	0.020117751	1.088507444	0.01848196	0.985254369	0.998148029
Sec24c	-0.08367194	0.143118804	-0.584632752	0.55879468	0.968926429
Fut11	-0.130181329	0.114878326	-1.133210536	0.257125851	0.897248195
6230400D17Rik	0.042170126	0.892009769	0.047275409	0.962293727	0.996474415
Chchd1	0.334896811	0.293385835	1.141489366	0.253666335	0.897044747
Zswim8	-0.165595349	0.193814465	-0.854401393	0.392882624	0.933734231
Ndst2	-0.105721637	0.229567324	-0.460525632	0.645138978	0.979004493
Camk2g	-0.090272779	0.135566493	-0.665893005	0.505479492	0.962220317
Plau	-0.255609896	0.349863527	-0.730598864	0.465024206	0.953083512
Vcl	-0.161616354	0.172430618	-0.937283393	0.348612803	0.921552698

Ap3m1	-0.214889211	0.113459462	-1.893973463	0.058228532	0.72285319
Adk	-0.111214038	0.093200821	-1.193273154	0.232762413	0.89581803
Kat6b	-0.032700636	0.218695775	-0.149525689	0.88113884	0.992886758
Dusp13	3.063951027	1.583252361	1.935225932	0.052962577	0.707153766
Samd8	-0.391668407	0.200869729	-1.949862772	0.051192477	0.707153766
Vdac2	0.006134047	0.115662311	0.053034101	0.957704737	0.996226826
Comtd1	-0.031034776	0.194053436	-0.159929022	0.872936986	0.992886758
Zfp503	-0.061944613	0.248153955	-0.249621705	0.802879912	0.990988839
1700112E06Rik	-0.948458775	0.59108563	-1.604604693	0.108580828	0.798150849
Kcnma1	-1.445831099	1.181027799	-1.224214282	0.220871403	0.890248292
Dlg5	0.085398709	0.361615937	0.236158589	0.813309612	0.992048071
Polr3a	0.173942685	0.371823928	0.467809284	0.639920981	0.977843986
Rps24	0.270662197	0.291614209	0.92815161	0.353328932	0.923786159
Zmiz1	0.031258273	0.25571766	0.122237445	0.902710972	0.994688009
Ppif	-0.062954465	0.187337481	-0.336048423	0.736834352	0.988585656
Ppifos	0.866625164	1.377266547	0.629235616	0.529194815	0.965637248
Zcchc24	-0.183544457	0.15421586	-1.190178863	0.233976099	0.89581803
Anxa11	-0.214592084	0.213461951	-1.005294309	0.314755155	0.91586731
Slmap	-0.143615773	0.101669139	-1.412579806	0.15777931	0.855986074
Dennd6a	-0.116786556	0.123426514	-0.946203153	0.344044978	0.920669678
Arf4	-0.211528132	0.074286538	-2.847462491	0.004406929	0.385355746
Pde12	0.165144052	0.170837503	0.966673295	0.333707381	0.917554801
Asb14	-0.54380676	0.473524554	-1.148423573	0.250793747	0.896939594
Appl1	-0.094983944	0.147690495	-0.643128344	0.520140824	0.964665379
Il17rd	-0.302795171	0.308762776	-0.980672523	0.326754258	0.916882624
Arhgef3	0.022422165	0.109829007	0.204155218	0.838232205	0.992886758
Fam208a	-0.0164254	0.143434049	-0.114515349	0.90882928	0.994688009
Ccdc66	-0.117994656	0.311436004	-0.378872882	0.704782263	0.988585656
Erc2	-0.323129931	0.327645645	-0.986217691	0.3240263	0.91640698
Wnt5a	-0.088847566	0.296623538	-0.299529722	0.764535897	0.989442146
Cacna2d3	-1.211534398	1.045459444	-1.158853559	0.246515882	0.896939594
Lrtm1	0.561724803	1.758875474	0.319365874	0.749449086	0.988585656
Selk	0.280236046	0.166102656	1.687125617	0.0915792	0.772516176
Actr8	-0.133164588	0.187685219	-0.709510258	0.478007888	0.957438234
Il17rb	-0.398353512	1.084731846	-0.367236856	0.71344235	0.988585656
Chdh	-0.026473174	1.073254663	-0.024666256	0.980321171	0.997296835
Cacna1d	-0.171171045	0.310079855	-0.552022461	0.580932965	0.971613657
Dcp1a	0.082344501	0.225518496	0.365134134	0.715011282	0.988585656
Tkt	-0.144810831	0.130180049	-1.112388823	0.265971008	0.89768067
Prkcd	-0.303135725	0.239101099	-1.267814015	0.204864376	0.883765092
Rft1	-0.02308184	0.163968894	-0.140769627	0.888051938	0.992886758
Sfmbt1	-0.244616208	0.21813486	-1.121398975	0.262118072	0.897248195
Tmem110	0.125080367	0.112101629	1.115776535	0.2645178	0.89768067
Mustn1	0.022382735	0.125649854	0.17813578	0.858616343	0.992886758
Itih4	-0.041200521	0.325517004	-0.12656949	0.899281146	0.99447264
Itih3	-0.111714526	0.56815109	-0.196628199	0.844118502	0.992886758
Itih1	-0.376677168	0.681102491	-0.553040361	0.580235773	0.971613657
Nek4	-0.105495942	0.236898741	-0.445320821	0.656087923	0.979512392
Spcs1	0.093870846	0.150304943	0.624535987	0.532275644	0.96674935
Glt8d1	-0.011118532	0.12852253	-0.086510376	0.931060708	0.994688009
Gnl3	0.002200256	0.148469635	0.014819567	0.988176129	0.998148029
Snord69	1.370405232	2.172939314	0.630668893	0.528257043	0.965637248
Pbrm1	0.015781124	0.110650182	0.142621759	0.886588912	0.992886758
Smim4	0.661874163	0.227941203	2.903705666	0.003687747	0.376311503
Nt5dc2	-0.037055119	0.192865768	-0.19212906	0.847641112	0.992886758
Stab1	0.313999453	0.195255902	1.608143215	0.107803816	0.796929372
Nisch	0.074227115	0.126104716	0.588614899	0.556119637	0.968926429
Tnnc1	0.429084351	0.242639064	1.768405898	0.076993071	0.752876039
Sema3g	-0.213155501	0.239461905	-0.89014368	0.373388741	0.928840865
Phf7	-0.121001497	0.163946041	-0.738056837	0.460479911	0.953083512
Bap1	0.071933164	0.108448582	0.663292803	0.507143045	0.963011338
Dnah1	-0.256083531	0.43578006	-0.587643985	0.556771281	0.968926429

Capn7	-0.005036575	0.143115717	-0.035192328	0.97192638	0.996733101
Sh3bp5	0.119426946	0.130543454	0.914844389	0.360273328	0.925913281
Mettl6	0.233057353	0.132665736	1.756726034	0.078964518	0.755854514
Eaf1	-0.143385876	0.13329174	-1.07572964	0.282048187	0.90579922
Colq	-0.254353	0.300304752	-0.846982935	0.397004633	0.934798767
Hacl1	-0.048272165	0.186840047	-0.25836091	0.796128382	0.990988839
Btd	-0.162249417	0.156858795	-1.034366082	0.300965055	0.911154542
Ankrd28	-0.009196677	0.18108032	-0.050787833	0.959494586	0.996367077
Galnt15	-0.15224415	0.29213716	-0.521139282	0.602269748	0.974001549
Dph3	0.235958925	0.181324543	1.301307156	0.193153339	0.879588734
Oxnad1	-0.194030656	0.151473963	-1.280950549	0.200211036	0.880888611
Ncoa4	-0.592986339	0.18435773	-3.21649837	0.001297653	0.26039992
Timm23	0.348006371	0.170034189	2.046684685	0.040689054	0.679118604
Parg	-0.15150297	0.188897678	-0.802037227	0.422531424	0.943509323
Ogdhl	-1.770283936	1.394229374	-1.269722163	0.204183615	0.883765092
1700024G13Rik	0.213251997	0.184274362	1.157252663	0.247169147	0.896939594
Ercc6	-0.545927137	0.27883015	-1.957920034	0.050239397	0.707153766
3425401B19Rik	-1.568948845	1.637783939	-0.95797059	0.338077589	0.919246575
1810011H11Rik	-0.471371266	0.291327426	-1.618011982	0.105660015	0.79419908
Fam170b	0.545929813	1.851981114	0.294781523	0.768160782	0.989990452
Vstm4	0.06612481	0.174658075	0.37859578	0.704988057	0.988585656
Wdfy4	0.12172285	0.252767009	0.481561463	0.630117508	0.977034429
Lrrc18	-0.532188434	0.300007257	-1.773918533	0.076076627	0.752876039
Arhgap22	0.015814809	0.22903979	0.069048304	0.944951171	0.99486515
Mapk8	0.040186979	0.151554449	0.265165285	0.790882136	0.990988839
Ptpn20	-0.770617765	1.14851882	-0.670966597	0.502241809	0.961437685
Gdf10	-0.487055918	0.412718129	-1.180117575	0.237953455	0.896609492
Gdf2	-0.183864875	0.577715867	-0.318261771	0.75028638	0.988585656
Anxa8	-0.217266232	0.122408468	-1.774928117	0.075909757	0.752876039
Syt15	0.331026577	0.301225234	1.098933751	0.271796964	0.89804647
Fam35a	0.198029156	0.204514037	0.968291269	0.332898924	0.917115058
Glud1	0.003118062	0.072040154	0.043282274	0.965476522	0.996474415
Gm3219	0.471715509	0.803748488	0.586894428	0.557274612	0.968926429
Fam25c	0.461406257	0.624483062	0.738861124	0.459991331	0.953083512
Sncg	0.24242595	0.229164043	1.057870803	0.290114348	0.907334416
Mmrn2	0.237377861	0.212626216	1.11640919	0.264247022	0.89768067
Bmpr1a	-0.24204805	0.116136297	-2.084172267	0.0371445	0.678479117
Ldb3	-0.370003855	0.295960631	-1.250179301	0.211234056	0.884390587
Opn4	-1.228741536	1.881335713	-0.65312189	0.513677701	0.964352198
Wapal	-0.011481912	0.126257432	-0.090940484	0.927539882	0.994688009
Ccser2	-0.167872842	0.151127491	-1.110802813	0.266653235	0.89768067
Cdhr1	-0.204330383	1.427707126	-0.143117856	0.886197103	0.992886758
Ghitm	0.030460001	0.099486045	0.306173604	0.759472469	0.989266039
Sh2d4b	0.371854948	1.149132244	0.323596305	0.746243686	0.988585656
Tspan14	-0.158187483	0.179226554	-0.882611861	0.377446018	0.928883222
Fam213a	0.22452499	0.118565177	1.893684091	0.058266954	0.72285319
Dydc2	-0.255594071	0.289776634	-0.882038237	0.37775613	0.928883222
Dydc1	-0.923062377	0.729685335	-1.26501429	0.205866205	0.883765092
Mat1a	-0.095481318	0.227707398	-0.419315839	0.674985323	0.983549699
Sftpa1	0.029858973	0.122763312	0.243223909	0.807831946	0.99099448
Mbl1	-0.539219169	0.539065888	-1.000284345	0.317172921	0.91640698
Sftpd	-0.085856933	0.176576166	-0.486231719	0.626802885	0.976601842
Ear1	0.050056242	0.202290988	0.247446721	0.80456252	0.990988839
Ear2	-0.126126981	0.979406634	-0.128778974	0.897532543	0.994101834
Ptgdr	-3.206728333	2.21455671	-1.448022676	0.147610709	0.846658212
4930431P03Rik	1.854171596	1.397454576	1.326820654	0.184568016	0.871716376
Ptger2	-0.248196972	0.271957371	-0.9126319	0.361436171	0.926371563
Txndc16	-0.176197722	0.20832871	-0.845767834	0.397682273	0.935327115
Gpr137c	-0.728744568	0.611175645	-1.192365196	0.233118081	0.89581803
Ero1l	-0.234647186	0.168858588	-1.389607654	0.16464805	0.859982992
Psmc6	0.120022582	0.149265761	0.804086491	0.421347026	0.943415794
Styx	0.022892283	0.209974865	0.109023922	0.913183517	0.994688009

Gnpnat1	0.135607727	0.173531202	0.781460195	0.434531879	0.946762848
Fermt2	-0.007201316	0.094917103	-0.075869533	0.939522896	0.994688009
4930527F14Rik	1.217568926	1.188836511	1.024168517	0.305755686	0.91333012
Ddhd1	-0.320396316	0.210477936	-1.522232315	0.127950881	0.826336015
Gm1821	0.288303551	0.181177595	1.591275957	0.111547485	0.800752782
Gm15217	-0.107264474	0.718249334	-0.149341557	0.881284125	0.992886758
Bmp4	-0.003187667	0.188339419	-0.016925117	0.986496355	0.998148029
4933425B07Rik	0.704676457	2.431113742	0.289857461	0.771925286	0.990430188
Cdkn3	0.513693467	0.469565621	1.093975887	0.273965554	0.899464226
Cnih1	-0.002373547	0.167375304	-0.014180985	0.98868559	0.998182772
Gmfb	-0.044146253	0.093571068	-0.471793837	0.637073951	0.977284137
Cgrrf1	0.1122108	0.137069914	0.818639166	0.412992314	0.942708418
Samd4	-0.221874827	0.178225896	-1.244907907	0.213165604	0.884945675
Gm10371	0.967787656	1.040776658	0.929870639	0.352438067	0.923152465
Gch1	0.087676029	0.175173335	0.500510133	0.616715923	0.974001549
Wdhd1	-0.100126326	0.278874246	-0.35903755	0.719566998	0.988585656
Socs4	-0.107829299	0.214721378	-0.50218241	0.615539215	0.974001549
Mapk1ip1l	-0.215646918	0.170717085	-1.263182991	0.206523424	0.883765092
Lgals3	-0.116635623	0.174178892	-0.669631215	0.50309291	0.961729397
Dlgap5	-0.375438782	0.522885148	-0.718013857	0.472748743	0.955238041
Fbxo34	-0.149533446	0.171032451	-0.874298679	0.381955618	0.930635634
Atg14	0.02954225	0.143849974	0.205368478	0.837284246	0.992886758
Ktn1	-0.079941459	0.143489676	-0.557123422	0.577443106	0.971613657
Peli2	0.311648695	0.256999357	1.212643871	0.225266008	0.893255444
Gm6498	-1.018039529	1.762574985	-0.577586507	0.563543319	0.969658675
Tmem260	-0.276916339	0.154477881	-1.792595403	0.073037642	0.748833631
Exoc5	-0.056531495	0.123263162	-0.458624412	0.646503903	0.979004493
Ap5m1	0.035873378	0.154830271	0.231694863	0.816775022	0.992678823
Naa30	-0.132324897	0.14139041	-0.935883115	0.349333372	0.921585102
3632451O06Rik	0.315477372	0.21583089	1.461687772	0.14382679	0.841934045
Tlr11	-1.068669963	1.133847293	-0.942516659	0.345928186	0.921552698
Ttc5	0.180113794	0.111372881	1.617214112	0.105832071	0.79419908
Rpph1	0.330523228	0.957463124	0.345207266	0.729938556	0.988585656
Parp2	0.040607241	0.17562484	0.231215816	0.817147143	0.992678823
Tep1	0.268722384	0.196034137	1.370793821	0.170439238	0.862336981
Klhl33	-1.696046512	1.991259691	-0.851745515	0.394355353	0.933796635
Osgep	0.081047185	0.114948248	0.705075422	0.480763301	0.957463006
Apex1	-0.141456127	0.423448714	-0.334057284	0.738336332	0.988585656
Tmem55b	-0.096608919	0.109907666	-0.879000733	0.379400878	0.929137643
Pnp	-0.075109044	0.103175008	-0.72797711	0.466627598	0.953083512
Pnp2	-0.009790701	0.381939986	-0.025634135	0.979549159	0.997262526
Rnase12	2.837181271	1.092280956	2.597483052	0.009390975	0.478229167
Olfr750	0.115252342	0.542337759	0.212510267	0.831708969	0.992886758
Ang	-0.047571227	0.134145146	-0.354625034	0.722870519	0.988585656
Rnase4	-0.072369618	0.125971913	-0.574490104	0.565636195	0.970061308
Rnase6	0.048818065	0.229999073	0.212253311	0.831909418	0.992886758
Rnase1	-3.858552642	1.947076225	-1.981716274	0.047511005	0.700850692
Rnase2b	-1.298535675	2.173100938	-0.597549637	0.550140478	0.968926429
Mettl17	-0.376660711	0.214611596	-1.755080893	0.079245472	0.75659671
Slc39a2	0.042828454	0.509104328	0.084125103	0.932956966	0.994688009
Ndrg2	0.068088839	0.099054142	0.687390127	0.491836918	0.958916246
Arhgef40	0.139243614	0.200994814	0.692772172	0.488452539	0.958481967
Zfp219	0.03967286	0.268041727	0.148010013	0.882334866	0.992886758
Tmem253	0.172414255	1.287888621	0.133873576	0.893502546	0.993422442
Hnrnpc	0.182504593	0.111493955	1.636901241	0.101651107	0.788783372
Rpgrip1	0.743803224	0.789649946	0.941940448	0.34622313	0.921552698
Supt16	-0.020030485	0.232957539	-0.085983416	0.931479601	0.994688009
Chd8	0.154604216	0.16142997	0.957716931	0.338205517	0.919246575
Rab2b	0.205389521	0.280459382	0.7323325	0.463965647	0.953083512
Tox4	0.068096593	0.114039905	0.597129516	0.550420913	0.968926429
Mettl3	-0.056815475	0.228591589	-0.24854578	0.803712154	0.990988839
Sall2	0.212201693	0.368213145	0.576301241	0.564411584	0.969658675

Dad1	-0.161349006	0.099842324	-1.616038173	0.10608606	0.79419908
Abhd4	-0.282602837	0.172239155	-1.640758377	0.100847581	0.788783372
Oxa1l	0.033924755	0.179420392	0.189079706	0.850030347	0.992886758
Slc7a7	0.017523657	0.206441045	0.084884559	0.932353168	0.994688009
Mrpl52	0.153666483	0.145131569	1.058808116	0.289687176	0.907334416
Mmp14	-0.224666657	0.250355818	-0.897389397	0.369511168	0.928436166
Lrp10	-0.172805545	0.200133757	-0.86345026	0.387889956	0.933256603
Rem2	0.003275861	0.490296685	0.006681385	0.994669066	0.999285699
Prmt5	-0.069086702	0.169205259	-0.408301149	0.683052596	0.984738223
Haus4	-0.336106436	0.334711924	-1.004166306	0.31529846	0.91586731
Ajuba	-0.081858421	0.152045548	-0.538380916	0.590314099	0.974001549
4931414P19Rik	0.18498141	0.274066384	0.674951108	0.499706828	0.960979197
Psmb5	0.187233365	0.165807467	1.129221549	0.258804385	0.897248195
Cdh24	0.398429588	1.151888519	0.34589249	0.729423513	0.988585656
Acin1	-0.020357727	0.203237318	-0.10016727	0.92021153	0.994688009
4930579G18Rik	0.100157288	0.942867209	0.106226293	0.91540281	0.994688009
1700123O20Rik	0.165641635	0.137040543	1.208705338	0.22677607	0.89398447
Cebpe	-1.045889402	1.177488197	-0.888237695	0.374412906	0.928883222
Slc7a8	0.260993683	1.036033602	0.251916234	0.801105812	0.990988839
Homez	0.108843674	0.237130029	0.459004177	0.646231167	0.979004493
Ppp1r3e	0.048193879	0.361875019	0.133178242	0.894052418	0.993422442
Bcl2l2	-0.023695122	0.109550624	-0.216293814	0.828758731	0.992886758
Pabpn1	0.192621356	0.09320068	2.066737674	0.038758879	0.679118604
Slc22a17	-0.445374637	0.244014974	-1.825193878	0.067971777	0.742385562
Efs	-0.495016754	0.349032355	-1.418254631	0.156116437	0.855455553
Myh6	-0.095036922	0.385798822	-0.246338029	0.805420574	0.99099448
Myh7	-0.416690656	0.80221996	-0.51942195	0.603466528	0.974001549
Ngdn	0.273968326	0.143822473	1.904906244	0.056792259	0.721593167
Zfhx2	1.086503919	0.825867475	1.315591123	0.188311314	0.874826736
Thtpa	0.077474461	0.167327393	0.463011223	0.643356322	0.978801899
Ap1g2	0.289278961	0.243621304	1.187412414	0.235064986	0.896002526
Dhrs4	0.188918636	0.121146572	1.559422052	0.11889652	0.814755048
Lrrc16b	1.209925371	0.803220525	1.506342697	0.131979223	0.827800491
Pck2	-0.033737718	0.144930625	-0.232785296	0.815928131	0.992524109
Dcaf11	-0.047649466	0.116235748	-0.409938137	0.681851328	0.984683806
Fitm1	-0.284358707	0.518460579	-0.548467363	0.583371033	0.972174584
Psme1	0.093956121	0.138083631	0.680429098	0.496232802	0.960179302
Emc9	0.189728146	0.193519107	0.980410406	0.326883575	0.916882624
Psme2	0.190187856	0.161984519	1.174111307	0.240350445	0.896939594
Rnf31	0.129026976	0.254483606	0.507014884	0.612144375	0.974001549
Irf9	-0.075544632	0.144037711	-0.524478148	0.599946014	0.974001549
Rec8	-0.469246787	0.286710801	-1.636655419	0.101702489	0.788783372
Ipo4	-0.071508554	0.215876318	-0.331247794	0.740457322	0.988585656
Tm9sf1	-0.106817165	0.136100996	-0.784837497	0.432548852	0.946044891
Tssk4	0.218930286	0.750245921	0.291811365	0.770430855	0.989990452
Mdp1	0.04299737	0.142811753	0.301077253	0.763355586	0.989285682
Nedd8	0.146516587	0.121259538	1.208289173	0.226936052	0.89398447
Gmpr2	0.238494346	0.137839304	1.730234686	0.083588354	0.76099108
Tinf2	0.268832322	0.161113099	1.668593828	0.095197903	0.778239075
Tgm1	-0.155967433	0.603807484	-0.258306559	0.796170325	0.990988839
Rabggta	-0.059399508	0.198964779	-0.298542828	0.765288893	0.989884648
Dhrs1	-0.083123357	0.149945196	-0.554358253	0.579333691	0.971613657
Nop9	-0.318778652	0.199723331	-1.59610122	0.110466196	0.800111791
Cideb	0.098184848	1.82795342	0.053712992	0.957163832	0.996226826
Ltb4r2	0.38085083	1.765058415	0.215772366	0.82916519	0.992886758
Ltb4r1	0.258284252	0.342780069	0.753498453	0.45115044	0.949134998
Adcy4	0.004635934	0.176696738	0.026236668	0.979068569	0.997262526
Ripk3	-0.106535374	0.328291227	-0.324514836	0.745548292	0.988585656
Nfatc4	-0.032231038	0.275167165	-0.117132575	0.906754997	0.994688009
Nynrin	-0.113825951	0.509201931	-0.22353794	0.823116858	0.992886758
Khnyn	-0.101166661	0.251666544	-0.40198693	0.687693645	0.98538669
Sdr39u1	0.032324626	0.121472273	0.266107031	0.79015678	0.990988839

Cma1	1.117288554	0.57411759	1.946097059	0.051643082	0.707153766
Gm5801	-0.00326968	0.944359189	-0.003462327	0.997237468	0.99957296
Mcpt4	-0.530484574	0.863302987	-0.614482495	0.538896537	0.966875138
Mcpt8	1.067599933	1.249487941	0.854429961	0.392866801	0.933734231
Gzmb	1.080779642	0.225651977	4.789586406	1.67E-06	0.003031841
Rnf17	0.671067161	1.567892063	0.428005968	0.668646775	0.981863224
Cenpj	0.010695989	0.283798907	0.037688619	0.969935951	0.996628386
Parp4	-0.204084104	0.210537999	-0.969345698	0.332372736	0.917115058
Mphosph8	-0.279076875	0.238379957	-1.170722905	0.241710175	0.896939594
Gm16973	0.106670196	0.328008294	0.325205788	0.745025328	0.988585656
Pspc1	0.0514754	0.165302878	0.311400505	0.75549617	0.989266039
Zmym5	0.034538817	0.12509523	0.27610019	0.782471115	0.990988839
Zmym2	-0.214313871	0.163836439	-1.308096494	0.190840582	0.877906723
Gja3	-1.62003809	1.320496552	-1.226840076	0.219882705	0.888847883
Gjb2	-0.502930499	0.317146564	-1.585798352	0.11278506	0.803772007
Gjb6	0.199707627	0.231223145	0.863700848	0.387752248	0.933137881
Cryl1	0.067819863	0.141873174	0.478031615	0.632627703	0.977034429
Ift88	-0.270424325	0.15657191	-1.727157349	0.084139408	0.76099108
Il17d	-0.267405082	0.229823185	-1.16352526	0.244616456	0.896939594
N6amt2	0.066784061	0.160692432	0.41560178	0.677701424	0.983670411
Xpo4	0.285964539	0.390829435	0.731686289	0.464360067	0.953083512
Lats2	0.025068895	0.12335982	0.203217671	0.838964901	0.992886758
Sap18	0.149555449	0.129562587	1.154310457	0.248372911	0.896939594
Ska3	0.786751314	0.790920946	0.99472813	0.319868509	0.91640698
Mrpl57	0.527391501	0.251025622	2.100946897	0.035645628	0.674795441
Zdhhc20	0.009297573	0.101405092	0.09168744	0.926946377	0.994688009
Micu2	0.158271012	0.100999711	1.567044202	0.11710436	0.812679331
Fgf9	0.460698741	0.761341309	0.605114599	0.545102854	0.967491297
Rcbtb1	0.03897996	0.147601093	0.264089915	0.791710634	0.990988839
Gm6904	2.535447004	0.994991835	2.548208854	0.010827763	0.502706181
Phf11a	-0.052440225	0.455165065	-0.115211445	0.908277528	0.994688009
Phf11b	0.337343255	0.19185925	1.758285073	0.078699016	0.754946439
Phf11d	0.304150115	0.127334939	2.388583346	0.016913472	0.569373711
Phf11c	0.373574613	0.474632335	0.787082095	0.43123381	0.945321484
Setdb2	0.129892217	0.363032167	0.357798091	0.720494419	0.988585656
Cab39l	0.153014189	0.107647393	1.421438877	0.155189211	0.855261441
Cdadc1	0.166051683	0.111736607	1.486099196	0.137252885	0.833085238
Shisa2	0.364576536	0.332848005	1.095324384	0.273374548	0.89897004
Atp8a2	0.432999939	0.547773161	0.790473083	0.429251536	0.945321484
Nupl1	-0.22029856	0.169214009	-1.301893151	0.192952914	0.879588734
Mtmr6	0.032867568	0.103757598	0.316772634	0.751416131	0.988585656
Amer2	-0.184787451	1.664204009	-0.111036538	0.911587373	0.994688009
Spata13	0.187527599	0.248217742	0.755496354	0.449951219	0.948896093
C1qtnf9	0.98691364	0.704036164	1.401793958	0.160976783	0.857316691
Mipep	-0.0393009	0.151504854	-0.25940357	0.795323875	0.990988839
Tnfrsf19	-0.034827671	0.23375627	-0.148991388	0.881560428	0.992886758
Sacs	-1.839801839	1.963009401	-0.937235368	0.3486375	0.921552698
Sgcg	0.135332633	0.427071349	0.316885301	0.751330636	0.988585656
Arl11	-0.291845884	0.201020575	-1.45182096	0.146551394	0.843780493
Ebpl	0.354078456	0.219068699	1.61628958	0.106031719	0.79419908
Kpna3	0.035156914	0.123032797	0.285752377	0.775067785	0.990861469
Spryd7	0.064318958	0.158891834	0.40479713	0.685626638	0.98538669
Trim13	-0.05654115	0.203830248	-0.277393326	0.781478106	0.990988839
Dleu2	0.088446623	0.175414086	0.504216198	0.614109459	0.974001549
Kcnrg	-0.351135422	0.18203726	-1.928920612	0.053740719	0.707153766
Mir16-1	0.695781528	1.37521753	0.505942887	0.612896744	0.974001549
Dleu7	-0.987926461	2.003210375	-0.493171598	0.621891345	0.97514112
Rnaseh2b	0.283774169	0.162424509	1.747114215	0.080617528	0.758204138
Fam124a	-0.389354986	0.190563197	-2.043180382	0.041034585	0.679118604
Serpine3	-1.057507731	0.975330537	-1.084255738	0.278251425	0.901391073
Ints6	-0.070518999	0.208167289	-0.338761192	0.734789639	0.988585656
Wdfy2	0.09596997	0.658699374	0.145696161	0.884161252	0.992886758

2700070H01Rik	1.867707494	0.942619002	1.981402339	0.047546172	0.700850692
Ctsb	-0.084154434	0.092046771	-0.914257318	0.360581652	0.925913281
Fdft1	-0.224956594	0.139974108	-1.607130047	0.108025843	0.797349883
Neil2	0.282537099	0.541772353	0.521505199	0.602014885	0.974001549
Gata4	-0.344097167	0.662989625	-0.519008373	0.603754902	0.974001549
Blk	0.218437301	0.404057338	0.540609662	0.588776657	0.973858421
Fam167a	-0.463642805	0.33131605	-1.399397358	0.161693859	0.858249556
Mtmr9	-0.082533261	0.16861106	-0.489489011	0.624495532	0.975987226
Xkr6	0.2452513	1.062661015	0.230789778	0.817478123	0.992678823
Pinx1	0.014962538	0.232905767	0.064242883	0.948776832	0.99560893
Sox7	-0.041254709	0.270653787	-0.152426129	0.87885085	0.992886758
Msra	-0.07849411	0.129345	-0.60685848	0.543944834	0.967491297
Kif13b	-0.107736031	0.418619673	-0.257360173	0.796900745	0.990988839
Hmbox1	-0.027926186	0.24318683	-0.114834288	0.908576471	0.994688009
Ints9	-0.073909227	0.364494422	-0.202771902	0.83931332	0.992886758
Extl3	-0.057712381	0.197884097	-0.291647391	0.770556237	0.989990452
Fzd3	0.224134851	0.191676142	1.169341415	0.242266103	0.896939594
Fbxo16	-0.507707497	0.27086635	-1.874383797	0.060877545	0.725221283
Zfp395	-0.099279121	0.273192894	-0.363403013	0.716303852	0.988585656
Elp3	0.063620974	0.175008605	0.363530548	0.716208597	0.988585656
Scara5	0.21614154	0.207521046	1.041540335	0.297624844	0.909070179
Pbk	0.029497365	0.350070956	0.084261103	0.932848838	0.994688009
Esco2	0.159746216	0.405954682	0.39350751	0.693944678	0.986936826
Ccdc25	-0.122465137	0.149013083	-0.821841513	0.411167103	0.941701842
Scara3	0.366952299	0.281557693	1.303293457	0.192474594	0.879588734
Clu	-0.301282843	0.17351655	-1.736334906	0.082504635	0.76099108
Ephx2	0.09787865	0.296708009	0.329882064	0.741489077	0.988585656
Ptk2b	0.319708092	0.269202386	1.187612401	0.23498615	0.896002526
1700001G11Rik	0.206180675	1.433176708	0.143862703	0.885608888	0.992886758
Trim35	-0.321739066	0.137953672	-2.332225452	0.019688834	0.594195177
Adra1a	0.878214777	0.385205512	2.279860359	0.022615972	0.620006604
Dpysl2	0.162636768	0.20211423	0.804677475	0.421005821	0.943415794
Gm5464	0.652713188	1.725740453	0.378222106	0.705265605	0.988585656
Pnma2	1.587225366	1.677530351	0.946167898	0.344062957	0.920669678
Bnip3l	0.133463346	0.116848254	1.142193746	0.253373495	0.897044747
Ppp2r2a	-0.089274832	0.120409364	-0.74142765	0.458434185	0.95251399
Cdca2	-0.307964811	0.681188521	-0.452099237	0.651197493	0.979004493
Kctd9	0.075834581	0.162508081	0.466651138	0.640749493	0.977921835
Gnrh1	0.47914071	0.993251786	0.482396022	0.629524647	0.977034429
Dock5	0.001651557	0.298644056	0.005530185	0.995587573	0.999285699
Adam7	-0.837307834	1.292159117	-0.647991275	0.516990593	0.964352198
Adamdec1	-0.138693213	0.326458912	-0.424841253	0.670952402	0.982567035
Adam28	-0.346871134	0.18566097	-1.868304013	0.061719708	0.725730283
Stc1	-0.574490392	0.375694116	-1.52914397	0.126228757	0.822401003
Slc25a37	0.04085483	0.180786046	0.22598442	0.821213538	0.992886758
Synb	-0.361525179	1.913658477	-0.188918338	0.850156821	0.992886758
Entpd4	-0.702752575	0.174435363	-4.028727679	5.61E-05	0.03405023
Loxl2	-0.311932545	0.247967675	-1.257956484	0.208407494	0.884258007
R3hcc1	0.311420092	0.17041532	1.827418403	0.067636885	0.742385562
Chmp7	0.073344968	0.125269301	0.585498342	0.558212681	0.968926429
4930480K23Rik	0.686519753	0.697339273	0.984484568	0.324877315	0.91640698
Tnfrsf10b	-0.248124065	0.264965686	-0.936438483	0.349047472	0.921552698
Rhobtb2	-0.028157135	0.268699597	-0.104790389	0.916542137	0.994688009
Pebp4	2.012699285	1.642222914	1.225594448	0.220351328	0.889301125
Egr3	0.049632215	0.64612982	0.076814618	0.938771021	0.994688009
Bin3	0.273292118	0.148902325	1.83537845	0.066449634	0.738254074
Ccar2	0.050218432	0.142277314	0.352961626	0.724117209	0.988585656
9930012K11Rik	-0.072074935	0.164145088	-0.439092855	0.660594256	0.979924124
Pdlim2	0.044914066	0.112152438	0.400473384	0.688807884	0.98538669
Sorbs3	-0.318033832	0.185759295	-1.712074937	0.086882859	0.765418158
Ppp3cc	0.049338035	0.127465891	0.387068531	0.698705474	0.987770663
Slc39a14	-0.385798872	0.226901401	-1.700293036	0.08907582	0.768297719

Piwil2	0.717476005	0.792207011	0.905667326	0.365111962	0.926556975
Polr3d	0.260705714	0.206848463	1.260370564	0.207535716	0.883846885
Phyhip	-0.251179213	0.819692074	-0.306431184	0.759276368	0.989266039
Bmp1	0.065500133	0.160690112	0.407617696	0.68355437	0.984769452
Sftpc	0.127588073	0.228592038	0.558147492	0.576743673	0.971613657
Lgi3	-0.033185214	0.120705383	-0.274927374	0.783372036	0.990988839
Reep4	-0.170783953	0.211814568	-0.806289932	0.420075694	0.943415794
Hr	-0.147106761	0.389698497	-0.377488654	0.705810494	0.988585656
Nudt18	-0.000379565	0.172487224	-0.00220054	0.998244224	0.999591134
Fam160b2	-0.547216965	0.393710082	-1.389898279	0.164559768	0.859982992
Dmtn	-0.284511494	0.289346723	-0.98328915	0.325465148	0.91640698
Npm2	1.169345324	1.069058603	1.093808442	0.274039001	0.899524481
Xpo7	-0.077224247	0.130018201	-0.593949517	0.552545879	0.968926429
Dok2	-0.020096306	0.263896829	-0.07615213	0.939298067	0.994688009
Gfra2	0.115699692	0.225136369	0.513909384	0.607315346	0.974001549
Fndc3a	-0.065401426	0.139096689	-0.470186791	0.638221571	0.977779844
Gm9199	-0.84151068	0.589002807	-1.428704024	0.153089321	0.854159937
Cysltr2	0.076983301	0.610514751	0.126095727	0.89965615	0.99447264
Rcbtb2	0.131224612	0.13603438	0.964642998	0.334723655	0.918030088
Rb1	-0.071958958	0.167356774	-0.429973378	0.667215007	0.981863224
Lpar6	-0.530017377	0.214164163	-2.474818242	0.0133304	0.539384266
Itm2b	-0.021964303	0.069343977	-0.31674421	0.7514377	0.988585656
Med4	-0.106731307	0.123938322	-0.861164694	0.389147342	0.933734231
Nudt15	0.083820215	0.458184841	0.18293974	0.854845301	0.992886758
Sucla2	-0.057818535	0.107166659	-0.539519803	0.589528237	0.974001549
Htr2a	-0.368181085	1.276510869	-0.288427693	0.773019372	0.990730604
Esd	0.243231887	0.167116427	1.455463665	0.145540942	0.842832198
Lrch1	-0.257115436	0.185714293	-1.384467677	0.166215299	0.860082
5031414D18Rik	-0.165892906	0.83429934	-0.19884099	0.842387131	0.992886758
Lcp1	-0.104280863	0.170360117	-0.612120163	0.540458258	0.966989525
Zc3h13	0.266614358	0.235991975	1.129760273	0.258577252	0.897248195
Cog3	-0.020379087	0.128194477	-0.15897009	0.873692439	0.992886758
Slc25a30	-0.255632623	0.29525989	-0.865788521	0.386606145	0.932626359
Gm4285	0.537731054	0.240439182	2.236453514	0.025322079	0.631196313
Tpt1	0.102518176	0.146134249	0.701534218	0.482969688	0.957463006
Snora31	-0.123741689	1.222551936	-0.101215896	0.919379078	0.994688009
Gtf2f2	-0.267333367	0.170734465	-1.56578443	0.117399092	0.812881672
Gpalpp1	-0.034507497	0.120234631	-0.287001316	0.774111313	0.990730604
Nufip1	0.165514333	0.174428358	0.948895781	0.34267362	0.919850248
Tsc22d1	-0.136525507	0.134298888	-1.016579579	0.309353473	0.9139204
Serp2	0.764834717	0.328532883	2.328030944	0.019910461	0.595248411
1700108F19Rik	-1.283861421	0.940421408	-1.365197994	0.172190829	0.86244803
Lacc1	-0.261041956	0.260228192	-1.003127116	0.315799533	0.915957942
Ccdc122	-0.049858785	0.216585	-0.230204238	0.817933069	0.992734473
Enox1	-0.840279852	1.142828367	-0.735263384	0.462179104	0.953083512
Dnajc15	0.291925625	0.313503134	0.931172909	0.351764129	0.923082865
Epsti1	0.33242206	0.174727513	1.902516979	0.057103601	0.721735927
Fam216b	-0.374660993	0.166242494	-2.253701705	0.02421494	0.624577134
Tnfsf11	0.691144098	0.85261466	0.810617187	0.417585543	0.943415794
Akap11	-0.174013309	0.1795034	-0.969415119	0.332338112	0.917115058
Dgkh	-0.564864286	0.438477393	-1.288240387	0.197662297	0.880239572
Vwa8	0.085943271	0.21904862	0.392347925	0.694801158	0.987037197
Rgcc	-0.151599451	0.386027367	-0.392716848	0.694528625	0.987010101
Naa16	-0.177781652	0.201564004	-0.882010918	0.377770903	0.928883222
Mtrf1	0.325924941	0.218998209	1.48825391	0.136683945	0.832506992
Kbtbd7	0.0096191	0.178381889	0.053924196	0.956995559	0.996226826
Kbtbd6	-0.114782196	1.320772353	-0.086905359	0.93074674	0.994688009
Wbp4	-0.130174462	0.123416939	-1.054753613	0.291538025	0.907334416
Elf1	-0.102367118	0.139474068	-0.733950902	0.462978662	0.953083512
Sugt1	0.19512439	0.137789074	1.416109305	0.156743504	0.855906801
Olfm4	0.735587368	1.317930279	0.558138302	0.576749948	0.971613657
Pcdh17	-0.128792617	0.299309874	-0.430298591	0.666978452	0.981863224

Diap3	0.519371383	0.233309217	2.226107436	0.026006986	0.631530315
Tdrd3	-0.076043119	0.145448146	-0.522819443	0.601099908	0.974001549
Pcdh20	-1.745801359	0.777848531	-2.244397578	0.024806832	0.627796923
Gm5088	-0.498533402	0.961900519	-0.518279585	0.604263215	0.974001549
Dach1	-0.109042944	0.222389237	-0.490324737	0.623904125	0.975903684
Mzt1	0.208640595	0.168933754	1.235043858	0.216814182	0.887633697
Bora	1.049241104	0.526581993	1.992550293	0.046310713	0.698098452
Dis3	-0.112002386	0.185886362	-0.602531483	0.546820413	0.967902958
Pibf1	0.058910628	0.192165893	0.306561311	0.759177306	0.989266039
Klf5	-0.081943829	0.335629977	-0.244149314	0.807115181	0.99099448
Klf12	-0.065931521	0.421062558	-0.156583672	0.875572972	0.992886758
Tbc1d4	-0.066778571	0.208550251	-0.320203741	0.748813887	0.988585656
Commd6	0.430795567	0.202595587	2.126381794	0.033471482	0.671174852
Uchl3	0.098197161	0.167809132	0.585171731	0.558432251	0.968926429
Lmo7	-0.162014079	0.147134959	-1.101125665	0.270841968	0.89804647
Kctd12	-0.146137401	0.154072409	-0.948498188	0.342875895	0.919850248
4933432I03Rik	-0.009482745	1.656545443	-0.00572441	0.995432607	0.999285699
Irg1	0.579338666	1.412416742	0.410175445	0.681677252	0.984683806
Cln5	0.145012033	0.145303562	0.99799366	0.318282433	0.91640698
Fbxl3	0.144599393	0.123666641	1.169267577	0.242295842	0.896939594
Mycbp2	0.027852437	0.167502551	0.166280673	0.867936075	0.992886758
Scel	-0.31622607	0.214028158	-1.47749751	0.139542325	0.836535783
Slain1	0.377665817	0.445655194	0.847439506	0.396750192	0.934469184
Ednrb	-0.063469096	0.165475516	-0.383555812	0.701307697	0.988482081
Rnf219	-0.114581361	0.273684953	-0.418661532	0.675463513	0.983549699
Gm17066	-0.162998085	0.209279772	-0.778852557	0.436066574	0.946762848
Rbm26	-0.273649241	0.183293633	-1.492955518	0.135448831	0.830756223
Ndfip2	0.095169106	0.123578704	0.770109279	0.441235072	0.948068187
9330188P03Rik	0.082866801	0.713452049	0.116149082	0.90753439	0.994688009
Spry2	-0.151241079	0.152856467	-0.989431994	0.322451828	0.91640698
Slitrk6	-0.192241973	0.494614008	-0.3886707	0.697519759	0.987548136
Mir17hg	-0.222991885	0.323080488	-0.690205362	0.490065052	0.958560421
Gpc5	4.442975924	1.98011271	2.243799508	0.024845304	0.627796923
Gpc6	0.028839052	0.616612563	0.046770134	0.962696433	0.996474415
Dct	-3.484232251	0.741766241	-4.697210601	2.64E-06	0.003766595
Tgds	0.074606422	0.168969892	0.441536777	0.658824443	0.979924124
Gpr180	0.046111729	0.141709678	0.325395763	0.744881562	0.988585656
Sox21	0.119771944	0.998535229	0.11994764	0.904524626	0.994688009
LOC105245869	2.142617583	1.753910981	1.221622765	0.221850316	0.891715929
Abcc4	-0.105805397	0.209403416	-0.505270635	0.613368765	0.974001549
Cldn10	0.020685502	0.175291584	0.118006245	0.90606271	0.994688009
Dzip1	-0.328208355	0.221785204	-1.47984784	0.138913858	0.835236816
Dnajc3	-0.330822614	0.110407621	-2.996374798	0.002732104	0.332888529
Uggt2	0.085136504	0.263394504	0.323228095	0.746522505	0.988585656
Hs6st3	-3.050423098	2.273541603	-1.341705423	0.179691528	0.868087032
Mbnl2	-0.338692551	0.109333121	-3.097803738	0.001949605	0.298625366
Rap2a	-0.075750073	0.148601548	-0.509752923	0.610224571	0.974001549
Ipo5	0.055227479	0.140533363	0.392984826	0.694330689	0.987010101
Farp1	0.100658056	0.212960391	0.47266093	0.636455105	0.977284137
Stk24	-0.082313553	0.175964163	-0.467785893	0.63993771	0.977843986
Slc15a1	-0.275112561	0.590040569	-0.466260416	0.641029109	0.977921835
Dock9	-0.084483725	0.169254966	-0.499150646	0.617673261	0.974153
1810041H14Rik	0.109151505	0.408860611	0.266965078	0.78949605	0.990988839
Ubac2	-0.020173224	0.128095084	-0.157486323	0.874861586	0.992886758
Gpr18	-0.700607679	0.722629273	-0.969525737	0.332282945	0.917115058
Gpr183	0.076041933	0.335898351	0.226383763	0.820902956	0.992886758
A330035P11Rik	-0.092124559	0.390778384	-0.235746303	0.813629538	0.992048071
Tm9sf2	-0.285448657	0.105347267	-2.70959718	0.006736497	0.441713985
Clybl	0.315220793	0.249077281	1.265554178	0.20567274	0.883765092
Gm5089	-0.307790563	1.036607038	-0.296921159	0.766526702	0.989884648
Pcca	0.058616695	0.137388121	0.426650386	0.669633993	0.981863224
Ggact	0.164823373	0.149889443	1.099632968	0.271492072	0.89804647

Tmtc4	-0.255015528	0.181347637	-1.406224712	0.159657408	0.856630464
Nalcn	0.893982537	0.86937509	1.028304753	0.303806496	0.911786415
Itgbl1	0.060893966	0.498974869	0.122038142	0.902868811	0.994688009
Fgf14	-0.589719382	1.55995605	-0.378035895	0.705403929	0.988585656
Sepp1	0.087823367	0.177897967	0.493672686	0.621537358	0.97514112
Ccdc152	0.133923396	0.252290941	0.530829191	0.59553715	0.974001549
Ghr	-0.02552654	0.077373837	-0.329911778	0.741466624	0.988585656
Fbxo4	0.125208082	0.147526015	0.848718657	0.396037862	0.934066368
AW549877	-0.532466927	0.144924867	-3.674089457	0.000238699	0.095054639
A630020A06	3.262399647	1.951218081	1.671981045	0.09452806	0.777748075
BC037032	-0.639487489	0.82125065	-0.778675169	0.436171087	0.946762848
Oxct1	-0.260806027	0.10763967	-2.42295453	0.015394853	0.557315333
Plcxd3	-0.821932599	1.058857449	-0.776244809	0.437604453	0.94749626
C6	-0.698321769	0.259790703	-2.688016782	0.007187778	0.452031612
C7	-0.337902835	0.21658132	-1.56016611	0.118720632	0.814755048
Card6	-0.107274356	0.311084922	-0.344839458	0.730215067	0.988585656
Rpl37	0.158740166	0.13361925	1.188003717	0.234831945	0.89581803
Snord72	0.257208287	0.723779344	0.355368372	0.72231364	0.988585656
Prkaa1	-0.026367961	0.139284103	-0.189310631	0.849849363	0.992886758
Ttc33	0.141604293	0.116049345	1.220207605	0.222386185	0.892391445
Ptger4	0.006814867	0.419034419	0.016263264	0.987024365	0.998148029
5430437J10Rik	0.229919765	0.884808045	0.259852706	0.794977393	0.990988839
Dab2	-0.253517029	0.192942397	-1.313951901	0.188862391	0.875363508
Fyb	0.320889769	0.196626286	1.631977981	0.102684125	0.789323778
Rictor	0.200162575	0.211552501	0.946160286	0.344066839	0.920669678
Osmr	-0.178552948	0.179474995	-0.994862532	0.319803128	0.91640698
Lifr	-0.025715663	0.115811204	-0.222048147	0.824276403	0.992886758
Egflam	0.163310427	0.162575971	1.00451762	0.315129182	0.91586731
Gdnf	-0.09578071	0.635809825	-0.150643646	0.88025683	0.992886758
Wdr70	0.275051359	0.227966648	1.206542103	0.227608535	0.894410876
Nup155	-0.512354128	0.200049312	-2.561139163	0.010432956	0.499527467
2410089E03Rik	0.139274766	0.234624083	0.593608143	0.552774233	0.968926429
Nipbl	0.007300028	0.191882787	0.038044203	0.969652439	0.996628386
Slc1a3	0.19985673	0.149491684	1.336908683	0.181252446	0.868087032
Ranbp3l	-2.817356382	2.065077769	-1.364285851	0.172477617	0.86244803
Nadk2	0.164375038	0.123979617	1.325823082	0.184898306	0.871716376
Skp2	-0.164683556	0.276621576	-0.595338795	0.551617028	0.968926429
Lmbrd2	-0.817315227	0.378497363	-2.15936835	0.030821601	0.659455637
Capsl	0.081198299	0.118372467	0.685955964	0.492740878	0.958916246
Il7r	-0.519155332	0.255960036	-2.028267147	0.04253299	0.68118841
Spef2	-0.381309991	0.24264386	-1.571480076	0.116071179	0.811920148
Prlr	-0.318552375	1.003456096	-0.31745522	0.750898213	0.988585656
Dnajc21	-0.126996467	0.185447431	-0.684811141	0.493463104	0.958916246
Brix1	-0.088621097	0.115551731	-0.766938725	0.443117948	0.948476747
Rad1	0.028025602	0.177773381	0.157647908	0.87473425	0.992886758
Rai14	-0.229793134	0.127141528	-1.807380625	0.070702953	0.744227652
4930556M19Rik	0.064345023	0.168742978	0.381319709	0.702966031	0.988585656
C1qtnf3	-0.631147209	0.410117615	-1.538941966	0.123818465	0.820382953
Amacr	-0.045019613	0.206022639	-0.218517796	0.827025694	0.992886758
Slc45a2	-3.921109154	2.139039093	-1.833117107	0.066785158	0.738254074
Adamts12	0.198521603	1.189614433	0.166878946	0.867465299	0.992886758
Tars	-0.189408732	0.17885358	-1.059015607	0.289592672	0.907334416
Npr3	-0.256481676	0.321845156	-0.796910163	0.425503208	0.944290206
Sub1	0.040967092	0.13475472	0.304012298	0.761118522	0.989266039
Zfr	-0.181308223	0.10588583	-1.712299207	0.086841543	0.765418158
Mir1898	0.65883162	1.742314432	0.378135891	0.705329647	0.988585656
Mtmr12	-0.055966473	0.15248446	-0.367030666	0.713596144	0.988585656
Golph3	-0.176905692	0.102510711	-1.725728847	0.084396206	0.76099108
Pdzd2	0.07280536	0.179279489	0.406099774	0.684669292	0.985105247
6030458C11Rik	0.067788569	0.200307405	0.33842268	0.735044686	0.988585656
Drosha	-0.184924136	0.232459293	-0.795511909	0.426315786	0.944290206
Basp1	-0.208230681	0.209802026	-0.992510348	0.320948634	0.91640698

Myo10	0.179524848	0.21270688	0.844001133	0.398668773	0.93579994
Fam134b	-0.0920881	0.126554476	-0.727655815	0.466824304	0.95308746
Zfp622	0.041866344	0.153972793	0.271907414	0.78569321	0.990988839
Fbxl7	-0.1750693	0.529876942	-0.330396146	0.741100653	0.988585656
Ank	-0.623566282	0.139764386	-4.461553459	8.14E-06	0.008748514
Otulin	0.018055567	0.164024459	0.110078505	0.912347115	0.994688009
Fam105a	0.211805585	0.139827523	1.514763199	0.129832383	0.827800491
Trio	-0.18164554	0.219197815	-0.828683166	0.407283714	0.939557159
Dnah5	0.108946323	0.178779494	0.609389367	0.542266384	0.967491297
Ctnnd2	0.425468528	0.501422111	0.848523665	0.396146399	0.934066368
Dap	-0.069917427	0.134432259	-0.520094118	0.602997978	0.974001549
Ankrd33b	-0.046178287	0.139237648	-0.331650873	0.740152901	0.988585656
Ropn1l	-0.034938207	0.216717114	-0.161215728	0.871923494	0.992886758
Mar-06	-0.09971643	0.119603056	-0.833728112	0.404434213	0.938127315
Cmbl	0.028116712	0.156846236	0.179262909	0.85773127	0.992886758
Cct5	0.009150386	0.111634055	0.081967692	0.934672405	0.994688009
Fam173b	0.011347284	0.19696007	0.057612105	0.954057607	0.996070133
Snhg18	0.148772979	0.180658001	0.823506173	0.410220208	0.940983181
Snord123	-1.367819798	1.226576996	-1.115152006	0.264785289	0.89768067
Sema5a	-0.203977544	0.410264872	-0.497185009	0.619058592	0.974471036
Sdc2	-0.301982702	0.1533893	-1.968733815	0.048983666	0.707153766
Cpq	-0.238610246	0.127485642	-1.871663674	0.061253148	0.725221283
Tspyl5	-0.400191035	0.454574172	-0.880364657	0.378661797	0.929137643
Mtdh	0.035183472	0.107410564	0.327560631	0.743243893	0.988585656
Laptm4b	-0.207168291	0.116750296	-1.774456234	0.075987715	0.752876039
Matn2	0.017481358	0.268098226	0.065205048	0.948010742	0.995535632
Rpl30	-0.502686283	1.162429766	-0.432444435	0.665418429	0.981727712
Erich5	-1.637074016	0.833952119	-1.963031184	0.049642551	0.707153766
Hrsp12	0.652002612	0.206101725	3.163499056	0.001558849	0.282792452
Pop1	0.221241512	0.365017946	0.606111328	0.544440829	0.967491297
Nipal2	-0.290221502	0.168889856	-1.718406946	0.085722414	0.763135146
Stk3	-0.062097891	0.178178397	-0.348515264	0.727453253	0.988585656
Vps13b	-0.078686316	0.211637328	-0.371797908	0.710043322	0.988585656
Cox6c	0.151232025	0.198901494	0.760336294	0.447053592	0.948476747
Rgs22	-0.241993922	0.21977945	-1.101076201	0.270863494	0.89804647
Fbxo43	0.05616655	1.805969426	0.031100499	0.975189392	0.997262526
Polr2k	0.350222259	0.223263434	1.568650331	0.116729438	0.812418014
Spag1	-0.303618322	0.253708687	-1.196720244	0.231415614	0.895404344
Rnf19a	-0.064067891	0.128048266	-0.500341729	0.616834476	0.974001549
Ankrd46	0.09139626	0.099797066	0.915821113	0.359760728	0.92584564
Snx31	0.488291409	0.747998161	0.652797606	0.513886767	0.964352198
Pabpc1	-0.157626195	0.101277467	-1.55637972	0.119617825	0.81558552
Ywhaz	-0.103528091	0.101871656	-1.016260021	0.309505581	0.9139204
Zfp706	0.02261905	0.09707003	0.233017857	0.81574754	0.992524109
Grhl2	-0.088913986	0.179274726	-0.495964981	0.619919118	0.974471036
Ncald	0.244672804	0.131914445	1.854784008	0.063627077	0.729199878
Rrm2b	0.106638647	0.144079316	0.740138487	0.459215968	0.952683168
Ubr5	0.18899115	0.210519391	0.897737495	0.369325515	0.928436166
Klf10	0.102230642	0.169377657	0.603566276	0.54613204	0.96778188
Azin1	0.010680719	0.137004536	0.077958871	0.937860769	0.994688009
Atp6v1c1	0.042812476	0.117251042	0.365135143	0.715010529	0.988585656
Baalc	0.167410472	0.870929067	0.192220558	0.847569443	0.992886758
Fzd6	-0.022063618	0.168989695	-0.130561912	0.896121875	0.993952571
Cthrc1	0.112558283	0.18619231	0.604527024	0.545493308	0.967491297
Slc25a32	-0.011836964	0.191454844	-0.061826403	0.950701077	0.99573416
Dcaf13	0.167569483	0.133884039	1.251601639	0.210715057	0.884340413
Rims2	-0.117482575	1.104220066	-0.106394168	0.91526962	0.994688009
Dcstamp	-0.284427149	0.989565636	-0.287426259	0.773785957	0.990730604
Dpys	1.893985398	1.358593123	1.394078452	0.163293915	0.859982992
Lrp12	-0.159659088	0.296182776	-0.539055951	0.589848248	0.974001549
AU022793	0.386473	0.514840197	0.75066594	0.452853726	0.950106559
Zfpm2	0.226190169	0.475601923	0.475587163	0.634368522	0.977034429

Oxr1	0.068618999	0.138890565	0.49405083	0.621270282	0.97514112
Abra	-0.630363565	0.484200506	-1.301864738	0.192962628	0.879588734
Angpt1	0.284602347	0.13601682	2.092405535	0.036402254	0.677009445
Rspo2	0.813368163	1.437428963	0.565849294	0.571496234	0.971613657
Eif3e	0.161257705	0.142318138	1.133079085	0.257181044	0.897248195
Emc2	0.165574189	0.110258803	1.501686803	0.133178014	0.827800491
Tmem74	1.357235886	1.717186667	0.790383429	0.429303877	0.945321484
Nudcd1	-0.145617933	0.156717098	-0.929177065	0.352797331	0.923529301
Eny2	0.094315425	0.172282993	0.547444778	0.584073199	0.972333834
Pkhd1l1	-0.112809537	0.280977796	-0.401489152	0.688060024	0.98538669
Ebag9	-0.049909189	0.123422114	-0.404378012	0.685934767	0.98538669
Sybu	0.048538551	0.30253233	0.160440874	0.872533794	0.992886758
A930017M01Rik	0.018552486	1.349720371	0.013745429	0.98903308	0.998182772
Trps1	-0.164865551	0.25117986	-0.656364529	0.511589608	0.963452765
Eif3h	0.276005078	0.180106601	1.532453984	0.125410445	0.822401003
Utp23	0.286010988	0.190712935	1.499693704	0.133693762	0.827800491
Rad21	-0.212394231	0.101106112	-2.10070614	0.03566677	0.674795441
Aard	0.070123134	0.123200802	0.56917758	0.569235634	0.97144955
Med30	0.336558641	0.139495006	2.412693123	0.015835146	0.557315333
Ext1	-0.157126452	0.168310993	-0.933548363	0.350536912	0.922338606
Samd12	0.897104314	0.31377597	2.859059969	0.004248985	0.385355746
Tnfrsf11b	-0.466432801	0.370175733	-1.260030736	0.207658275	0.883846885
Colec10	-1.714758462	1.5722786	-1.090619984	0.275440132	0.900302631
Mal2	0.003498988	0.128375683	0.027255847	0.978255673	0.997262526
Nov	-0.456071649	0.265102798	-1.720357734	0.085367435	0.761635307
Enpp2	-0.326057484	0.177317418	-1.838835048	0.065939451	0.738254074
Taf2	-0.070311576	0.140540731	-0.500293227	0.616868623	0.974001549
Dscc1	0.671081847	0.603365913	1.112230295	0.266039145	0.89768067
Gm9920	0.215354439	1.612259849	0.133573034	0.893740209	0.993422442
Deptor	-0.038619192	0.213793896	-0.180637488	0.856652129	0.992886758
Col14a1	-0.080313471	0.208990523	-0.384292405	0.700761736	0.988482081
Mrpl13	0.251588705	0.181181735	1.388598606	0.164954841	0.860011234
Mtbp	-0.11788933	0.28907799	-0.407811505	0.683412066	0.984738223
Sntb1	-0.340168262	0.27639004	-1.230754414	0.21841473	0.888436964
Has2	0.114366188	1.279452085	0.089386847	0.928774476	0.994688009
Has2os	-1.533683489	1.924461419	-0.796941666	0.425484911	0.944290206
Slc22a22	1.331226833	1.148958547	1.158637826	0.246603844	0.896939594
Zhx2	0.06935143	0.193411725	0.358568904	0.719917613	0.988585656
Derl1	-0.03620169	0.07537772	-0.480270425	0.631035116	0.977034429
Tbc1d31	0.036921626	0.417524423	0.088429859	0.92953503	0.994688009
Fam83a	-0.151043457	0.759400026	-0.198898409	0.842342214	0.992886758
9130401M01Rik	0.081456618	0.151235673	0.53860717	0.590157939	0.974001549
Zhx1	-0.030004088	0.130466171	-0.229975996	0.818110422	0.992734473
Atad2	0.138356583	0.268054303	0.516151324	0.605748724	0.974001549
Wdyhv1	-0.09259813	0.14678369	-0.630847542	0.528140215	0.965637248
Fbxo32	0.088860876	0.235404711	0.377481297	0.705815961	0.988585656
Klhl38	-0.344946973	0.597234701	-0.577573561	0.563552061	0.969658675
Anxa13	0.060427184	0.429150793	0.140806414	0.888022876	0.992886758
D15Ertd621e	-0.234509299	0.127587792	-1.838023021	0.066059013	0.738254074
Tmem65	-0.007569821	0.094607024	-0.080013312	0.936226668	0.994688009
Trmt12	0.281881519	0.242624675	1.161800709	0.245316426	0.896939594
Rnf139	-0.086869429	0.111663444	-0.777957633	0.43659399	0.946762848
Tatdn1	0.356388785	0.20287486	1.756692698	0.078970203	0.755854514
Ndufb9	0.26747365	0.248212261	1.077600471	0.281212092	0.904300139
Mtss1	-0.084226269	0.181283056	-0.464611921	0.642209394	0.978415289
Zfp572	-0.076799107	1.287408948	-0.059654011	0.952431201	0.996023481
Sqle	-0.134265866	0.155824659	-0.861647104	0.388881742	0.933485538
E430025E21Rik	-0.153293392	0.192576959	-0.796011071	0.426025601	0.944290206
Nsmce2	0.13340413	0.132788873	1.004633349	0.315073433	0.91586731
Trib1	-0.02572736	0.23683439	-0.10863017	0.91349583	0.994688009
Fam84b	-0.262411601	0.127136757	-2.064010491	0.039016722	0.679118604
9930014A18Rik	-0.112944061	0.377194031	-0.299432261	0.76461025	0.989442146

Myc	-0.371479723	0.324333567	-1.145363172	0.252058748	0.896939594
Pvt1	0.513254166	0.321512899	1.596371927	0.11040578	0.800111791
Fam49b	0.058471801	0.125353263	0.466456152	0.640889027	0.977921835
Asap1	-0.196831024	0.227744873	-0.864261053	0.3874445	0.932858077
Adcy8	0.076908514	0.264492172	0.290778035	0.771221085	0.989990452
Efr3a	-0.157931399	0.137656078	-1.147289685	0.251261916	0.896939594
Lrrc6	-0.376311065	0.267598558	-1.406252213	0.159649245	0.856630464
Tmem71	-0.015217318	0.15477185	-0.098320967	0.92167743	0.994688009
Phf20l1	-0.135984681	0.138233488	-0.983731824	0.325247388	0.91640698
Tg	0.841741821	0.7417799	1.134759545	0.256476081	0.897248195
Sla	-0.085793728	0.232630095	-0.368798921	0.712277609	0.988585656
Wisp1	-0.551327718	0.681760482	-0.808682421	0.418697842	0.943415794
Ndrg1	-0.124161774	0.171723986	-0.723031048	0.469660809	0.95486913
St3gal1	0.067537794	0.119908038	0.563246593	0.57326698	0.971613657
1700012I11Rik	0.09738563	1.578287267	0.061703362	0.950799063	0.99573416
Zfat	-0.79966927	0.416569202	-1.919655284	0.054901456	0.711409581
Mir30d	1.136575556	2.080086819	0.546407749	0.584785685	0.972676842
Khdrbs3	0.079957519	0.161232656	0.495913927	0.61995514	0.974471036
Trappc9	-0.155242668	0.379707125	-0.408848445	0.682650885	0.984738223
Peg13	-0.260728762	0.137020675	-1.902842488	0.057061101	0.721735927
Chrac1	0.452827921	0.161087511	2.811067832	0.004937737	0.399101188
Ago2	-0.197909504	0.387202124	-0.511127113	0.609262052	0.974001549
Ptk2	-0.276760086	0.152418248	-1.815793642	0.069402033	0.743426713
Dennd3	-0.308210383	0.195762432	-1.574410272	0.115392631	0.81007959
Slc45a4	-0.405537671	0.249466959	-1.625616762	0.104031207	0.79155232
Ptp4a3	0.131068366	0.146971208	0.891796209	0.372502174	0.928840865
Mroh5	0.33786513	1.509151318	0.22387757	0.822852569	0.992886758
Arc	0.502775169	1.164308178	0.431823102	0.665869987	0.981816968
Jrk	0.15846023	0.388116239	0.40828034	0.683067871	0.984738223
4933427E11Rik	-0.278822483	0.79959272	-0.34870563	0.727310317	0.988585656
Psca	-4.003875694	2.137393342	-1.873251691	0.061033637	0.725221283
Them6	-0.205367663	0.110476194	-1.858931376	0.063036866	0.727650961
Slurp1	0.176291422	0.347847094	0.50680723	0.612290083	0.974001549
Lypd2	0.070449888	0.250615957	0.281106951	0.77862837	0.990988839
2300005B03Rik	0.136689543	0.282062436	0.484607398	0.627954865	0.976968316
Lynx1	-0.014573263	0.202395942	-0.07200373	0.942598939	0.994688009
Ly6d	0.52004491	0.256306594	2.028995437	0.042458755	0.68118841
Ly6k	-0.508548275	0.451455223	-1.126464483	0.259968966	0.897248195
2010109I03Rik	-0.21865011	0.493933574	-0.442671083	0.658003661	0.979614766
Ly6e	-0.205363163	0.183793835	-1.117356101	0.263842097	0.897248195
Ly6i	-0.210946596	0.499655982	-0.422183669	0.672890961	0.983233856
Ly6a	0.086836281	0.137999927	0.629248749	0.529186219	0.965637248
Ly6c1	0.058764202	0.15920656	0.369106665	0.712048221	0.988585656
Ly6c2	0.269077227	0.229437798	1.172767649	0.240888995	0.896939594
Ly6h	-1.022998856	1.818988446	-0.562399864	0.573843611	0.971613657
Gpihbp1	0.246844618	0.237245766	1.040459528	0.298126458	0.909779403
Zfp41	-0.136159767	0.409126013	-0.332806429	0.739280405	0.988585656
Top1mt	0.091074988	0.375976255	0.24223601	0.808597294	0.991020605
Rhpn1	-0.719525025	0.625266376	-1.150749589	0.249835269	0.896939594
Zc3h3	-0.204735908	0.553533603	-0.369870785	0.71147877	0.988585656
Gsdmd	0.081230465	0.131605178	0.617228484	0.537084031	0.96674935
Mroh6	-2.006839573	1.989111163	-1.00891273	0.31301649	0.914898
Naprt	-0.085171968	0.190095925	-0.448047311	0.654119053	0.979133069
Eef1d	0.324208756	0.134401214	2.412245731	0.015854592	0.557315333
Tigd5	-0.254255348	0.582377635	-0.43658158	0.662414824	0.980647283
Pycrl	0.252289298	0.178569519	1.412835175	0.157704193	0.855986074
Tsta3	0.193530652	0.151655709	1.276118472	0.201913643	0.881220006
Zfp623	-0.43480467	0.226341572	-1.921011094	0.054730309	0.710884449
Zfp707	-0.021572307	0.252592631	-0.085403548	0.931940572	0.994688009
Ccdc166	-0.098709978	0.226153982	-0.436472428	0.662494	0.980647283
Mapk15	-0.303080505	0.237557897	-1.275817427	0.202020066	0.881300669
Fam83h	-0.036987344	0.380797521	-0.097131263	0.922622154	0.994688009

Scrib	0.068169392	0.186141525	0.366223453	0.714198343	0.988585656
Puf60	-0.030615371	0.084801466	-0.361024073	0.718081451	0.988585656
Nrbp2	-0.364140154	0.173939999	-2.093481402	0.036306203	0.677009445
Eppk1	0.499085006	0.538351789	0.927061108	0.353894808	0.924338589
Plec	0.126625745	0.327250548	0.386938221	0.698801944	0.987770663
Parp10	0.108944033	0.216672067	0.50280608	0.615100618	0.974001549
Grina	-0.143758698	0.178593236	-0.80495041	0.420848297	0.943415794
Gm10872	0.504843635	1.649471782	0.30606382	0.759556054	0.989266039
Smpd5	-0.076119778	0.389403933	-0.195477682	0.845019007	0.992886758
Oplah	0.070495846	0.21293396	0.331069059	0.740592323	0.988585656
Exosc4	0.146612885	0.169978723	0.862536691	0.388392252	0.933269543
Gpaa1	-0.325837528	0.200564427	-1.624602791	0.104247222	0.79155232
Cyc1	0.155838089	0.125295777	1.243761704	0.213587279	0.884945675
Sharpin	-0.108751853	0.160724768	-0.676634064	0.498638161	0.960700483
Maf1	0.130905869	0.087005701	1.504566576	0.132435545	0.827800491
Hgh1	0.371178771	0.413910299	0.89676138	0.36984626	0.928436166
Mroh1	-0.117792902	0.215395779	-0.546867274	0.584469921	0.97242196
Bop1	-0.17531057	0.120766192	-1.451652708	0.146598195	0.843780493
Scx	0.183831435	0.175811577	1.045616214	0.29573826	0.908204914
Hsf1	-0.206772883	0.219993978	-0.93990247	0.34726759	0.921552698
Dgat1	0.1227779	0.176641376	0.695068752	0.487012217	0.958288569
Fbxl6	-0.221115953	0.244057049	-0.906001091	0.364935275	0.926556975
Slc52a2	-0.091346141	0.265925856	-0.343502291	0.731220617	0.988585656
Adck5	0.234109167	0.174007326	1.345398332	0.178496634	0.867377413
Cpsf1	-0.208363605	0.184100159	-1.131794812	0.25772071	0.897248195
Slc39a4	-0.258663382	0.222831592	-1.160802112	0.245722384	0.896939594
Vps28	-0.03544101	0.099416962	-0.356488562	0.721474717	0.988585656
Tonsl	-0.504887819	0.710918539	-0.710190818	0.477585814	0.95716557
Cyhr1	0.046778796	0.10327334	0.452961003	0.650576823	0.979004493
Kifc2	0.216122377	0.306400929	0.705358101	0.480587412	0.957463006
Ppp1r16a	-0.167132634	0.14411757	-1.159696448	0.246172417	0.896939594
Gpt	-0.013961274	0.1737432	-0.080355801	0.935954279	0.994688009
Mfsd3	0.025413672	0.273159557	0.093035997	0.925874964	0.994688009
Recql4	-0.756420004	1.357525726	-0.557204913	0.577387433	0.971613657
Lrrc14	0.052014711	0.313669639	0.165826414	0.868293559	0.992886758
Lrrc24	0.531662115	1.239104341	0.429069689	0.66787251	0.981863224
C030006K11Rik	0.227201417	0.273699014	0.830114125	0.406474262	0.939557159
Arhgap39	-0.18964536	0.250515476	-0.757020539	0.449037558	0.948476747
Zfp251	0.278306612	0.235173653	1.183408977	0.236647101	0.896358698
Zfp7	0.13691911	0.172370815	0.794328842	0.427004018	0.944290206
Commd5	-0.035545067	0.165155272	-0.215222114	0.829594151	0.992886758
Rpl8	0.239875717	0.123393675	1.943987134	0.051897003	0.707153766
Zfp647	-0.147714063	0.344432854	-0.428861712	0.668023865	0.981863224
1110038F14Rik	0.036585772	0.147110053	0.248696614	0.803595468	0.990988839
Mb	0.061858907	0.299780446	0.206347373	0.836519576	0.992886758
Apol6	-0.633739116	1.066484315	-0.594232008	0.552356948	0.968926429
Rbfox2	-0.126580812	0.159702936	-0.792601657	0.428009943	0.945321484
Apol7a	0.074378634	0.589591563	0.126152813	0.899610963	0.99447264
Apol9a	0.065646521	0.365138301	0.17978536	0.857321077	0.992886758
Apol7b	-0.095665131	1.651869518	-0.057913249	0.953817729	0.996070133
Apol7c	-0.272265571	0.556447598	-0.489292382	0.624634714	0.976017893
Apol10b	0.021565626	0.290563102	0.074220112	0.940835243	0.994688009
Gm8221	-0.679773927	1.313559545	-0.517505224	0.604803523	0.974001549
Apol11b	1.825986044	0.716037608	2.550125893	0.010768403	0.502706181
Apol9b	0.254469139	0.328621793	0.774352599	0.438722306	0.947831419
Myh9	0.275680942	0.151321337	1.821824654	0.068481596	0.743426713
Txn2	0.001564074	0.103877234	0.015056951	0.987986745	0.998148029
Foxred2	1.015959302	0.827437626	1.22783793	0.219507814	0.888847883
Eif3d	-0.005258954	0.100212557	-0.052477995	0.958147828	0.996226826
Ift27	-0.016382299	0.107122395	-0.152930665	0.878452953	0.992886758
Pvalb	-3.459308449	2.217377872	-1.560089732	0.118738678	0.814755048
Ncf4	-0.131996173	0.26342685	-0.501073347	0.616319504	0.974001549

Csf2rb2	-0.865661903	0.35771197	-2.419997021	0.015520634	0.557315333
Csf2rb	0.031095478	0.247388726	0.125694806	0.899973514	0.994508803
Tst	-0.187420345	0.114586862	-1.635618097	0.10191954	0.788783372
Mpst	0.024711482	0.12312976	0.200694636	0.840937354	0.992886758
Kctd17	0.105329667	0.18752423	0.561685638	0.574330221	0.971613657
Tmprss6	0.054157929	1.169079518	0.046325274	0.963050995	0.996474415
Il2rb	0.241897919	0.430837477	0.56145979	0.574484135	0.971613657
C1qtnf6	-0.190650798	0.276379931	-0.68981419	0.490311044	0.958604768
Rac2	-0.250559587	0.25483677	-0.983215988	0.325501148	0.91640698
Cyth4	-0.128627758	0.188089787	-0.683863597	0.494061303	0.958916246
Mfng	0.101918109	0.116008196	0.878542313	0.379649486	0.929185635
Card10	-0.283286923	0.201901391	-1.40309545	0.160588378	0.857316691
Cdc42ep1	-0.048224027	0.19118284	-0.252240351	0.800855291	0.990988839
Lgals2	0.287028021	0.385546288	0.744470976	0.456591594	0.951472839
Gga1	-0.141296941	0.12948168	-1.09125044	0.275162699	0.900302631
Sh3bp1	-0.483282922	0.385392629	-1.254001466	0.209841472	0.884258007
Pdxp	-0.85467757	0.725275058	-1.178418533	0.238629793	0.896609492
Lgals1	0.274491434	0.140809075	1.949387379	0.051249181	0.707153766
Nol12	0.050466559	0.15538085	0.324792659	0.745338001	0.988585656
Triobp	0.309610746	0.161478886	1.917345066	0.055194108	0.712374865
H1f0	0.338297526	0.123979632	2.728654066	0.006359338	0.427279448
Gcat	-0.298541533	0.2305448	-1.294939349	0.195341153	0.879588734
Galr3	-1.126975197	2.125077644	-0.530321892	0.595888771	0.974001549
Ankrd54	-0.335367597	0.159539052	-2.10210348	0.035544212	0.674795441
Eif3l	-0.028206456	0.132633223	-0.212665084	0.831588203	0.992886758
Micall1	0.012706443	0.294750865	0.043109093	0.965614571	0.996474415
1700088E04Rik	-0.167578147	0.324570961	-0.516306653	0.60564025	0.974001549
Polr2f	0.49725075	0.152642113	3.25762491	0.001123488	0.242572819
Gm10863	0.086174998	0.971860218	0.088670156	0.929344051	0.994688009
Pick1	-0.109998913	0.16138644	-0.681587084	0.495500084	0.95978525
Baiap2l2	-1.083694166	1.662149654	-0.65198351	0.51441181	0.964352198
Pla2g6	-0.379453739	0.405118425	-0.936648929	0.348939175	0.921552698
Maff	-0.216763733	0.16431528	-1.319194006	0.187104262	0.873455432
Tmem184b	-0.031510976	0.174249032	-0.180838744	0.856494151	0.992886758
Csnk1e	-0.116716721	0.17803266	-0.655591622	0.512086917	0.963452765
Kdelr3	-0.122001465	0.169357521	-0.720378191	0.471292174	0.95486913
Ddx17	-0.191937982	0.117221911	-1.637389976	0.101549011	0.788783372
Dmc1	-1.072119082	0.851008472	-1.259821867	0.20773363	0.883937183
Fam227a	-0.000590956	0.190332071	-0.003104869	0.997522677	0.99957296
Cby1	0.211500507	0.173969005	1.215736712	0.224085234	0.892989918
Tomm22	0.050884764	0.13276836	0.38325972	0.701527202	0.988482081
Josd1	-0.279414392	0.127361076	-2.193875879	0.028244334	0.641905969
Gtpbp1	-0.128225054	0.232156996	-0.552320437	0.580728831	0.971613657
Sun2	-0.028670041	0.165748269	-0.172973394	0.862672341	0.992886758
Gm16576	0.284706037	0.460013974	0.618907367	0.535977385	0.96674935
Dnal4	0.138098082	0.158515752	0.871194689	0.383647859	0.931591679
Nptxr	-2.232511866	2.38109502	-0.937598814	0.348450621	0.921552698
Cbx6	-0.020251559	0.173673394	-0.116607146	0.907171374	0.994688009
D730005E14Rik	0.42394886	0.281410374	1.506514683	0.131935101	0.827800491
Apobec3	-0.094357926	0.169730975	-0.555926374	0.578261188	0.971613657
Cbx7	0.069869325	0.129338084	0.540206894	0.589054359	0.974001549
Pdgfb	-0.16823783	0.235614022	-0.714039975	0.475202461	0.956761005
Rpl3	0.172489651	0.152693675	1.129645028	0.25862583	0.897248195
Syngr1	0.320696095	0.25552729	1.255036577	0.209465482	0.884258007
Tab1	0.045174669	0.224178252	0.201512271	0.840298035	0.992886758
Mgat3	-0.232133519	0.185661599	-1.250304431	0.21118836	0.884390587
Mief1	-0.190263585	0.16995403	-1.119500289	0.262926768	0.897248195
Atf4	-0.25451548	0.101654788	-2.503723495	0.012289404	0.525888996
Rps19bp1	0.213622768	0.16012265	1.334119619	0.182164657	0.868546072
Cacna1i	0.583965399	1.094538502	0.533526594	0.593669107	0.974001549
Grap2	-0.075816962	0.314134386	-0.241351998	0.809282313	0.991295569
Fam83f	-0.252221656	0.805489591	-0.313128387	0.754183123	0.988804605

A430088P11Rik	-0.163423416	0.624927553	-0.261507778	0.793700952	0.990988839
Tnrc6b	0.324936877	0.354016224	0.917858716	0.358692837	0.925178192
Adsl	0.064182775	0.143826833	0.446250353	0.655416414	0.979133069
Sgsm3	0.055547572	0.176497519	0.314721548	0.752973082	0.988585656
Mkl1	-0.05805523	0.31748496	-0.18285978	0.854908042	0.992886758
8430426J06Rik	0.46318635	0.35211055	1.315457175	0.188356301	0.874826736
Slc25a17	0.113018826	0.116372157	0.971184421	0.33145645	0.917115058
St13	-0.25062248	0.114827861	-2.182592961	0.029065799	0.64858361
Xpnpep3	-0.045030772	0.292515713	-0.153943089	0.877654609	0.992886758
Rbx1	0.164007203	0.14739407	1.112712356	0.265831987	0.89768067
Ep300	0.020975958	0.193776965	0.108247942	0.913799016	0.994688009
L3mbtl2	-0.053716992	0.333564219	-0.16103943	0.872062345	0.992886758
Chadl	0.503775218	0.482694875	1.04367219	0.296637081	0.909070179
Rangap1	-0.280707169	0.140759547	-1.994231831	0.046126725	0.698098452
Zc3h7b	0.082874103	0.209011343	0.396505288	0.691732308	0.986454135
Tef	-0.455279241	0.206411634	-2.205685952	0.027405996	0.636714037
Tob2	-0.226839885	0.244897725	-0.926263749	0.35430893	0.924354588
Phf5a	0.029846885	0.1387462	0.215118575	0.829674872	0.992886758
Aco2	-0.184595238	0.145005404	-1.273023167	0.203009821	0.882297774
Polr3h	0.196095389	0.163974149	1.195892097	0.231738669	0.895404344
Csdc2	0.325564516	0.576224317	0.564996142	0.572076388	0.971613657
Pmm1	0.266621098	0.245239642	1.08718597	0.27695463	0.900604936
Desi1	-0.00993624	0.176218294	-0.056385974	0.955034331	0.99614946
Xrcc6	-0.126713329	0.205647718	-0.616166957	0.537784334	0.96674935
Nhp2l1	-0.004476373	0.10830321	-0.041331856	0.967031337	0.996474415
Ccdc134	-0.058979877	0.146638979	-0.402211457	0.687528411	0.98538669
Srebf2	-0.03400282	0.179740102	-0.189177704	0.849953542	0.992886758
Shisa8	-1.137608745	1.631370992	-0.697332949	0.485594453	0.957710647
Tnfrsf13c	0.117739584	0.519290871	0.226731472	0.820632553	0.992886758
Cenpm	0.11966696	0.698712047	0.171267922	0.864013103	0.992886758
Sep-03	-0.280641385	0.70050169	-0.400629133	0.688693194	0.98538669
Naga	0.014031077	0.148337319	0.09458899	0.924641296	0.994688009
Fam109b	-0.362627685	0.435624803	-0.832431217	0.405165588	0.939383492
Smdt1	0.401989906	0.164404628	2.445125244	0.014480185	0.554936045
Ndufa6	0.363610675	0.140787283	2.582695443	0.009803182	0.487977291
Cyp2d22	-0.138603409	0.168654579	-0.821818235	0.411180354	0.941701842
Tcf20	0.050062841	0.160226768	0.31244992	0.754698617	0.98902053
Gm20324	-0.920596312	0.544686388	-1.690140109	0.091001153	0.771538719
Nfam1	-0.240859291	0.278760457	-0.864036792	0.38756768	0.932893632
Serhl	0.235971292	0.182938596	1.289893424	0.197087664	0.879755417
Rrp7a	0.08897268	0.135150176	0.658324557	0.510329602	0.963452765
Poldip3	-0.034368118	0.137292558	-0.250327613	0.802334004	0.990988839
Cyb5r3	0.004851748	0.118684224	0.040879466	0.967391987	0.996474415
A4galt	-0.17744067	0.326795672	-0.542971296	0.587149566	0.973336477
Arfgap3	-0.141248839	0.142644058	-0.990218875	0.322067149	0.91640698
1700001L05Rik	-0.383297615	0.490004004	-0.782233639	0.434077279	0.946762848
Pacsin2	-0.008123937	0.126748726	-0.064094823	0.948894724	0.995624732
Ttll1	-0.042569549	0.253634902	-0.167837901	0.866710805	0.992886758
Bik	0.426131377	0.214942763	1.982534193	0.047419484	0.700850692
Mcat	0.135868943	0.119151876	1.140300492	0.254161133	0.897044747
Tspo	0.245522006	0.233991722	1.049276463	0.294050896	0.907334416
Ttll12	0.1883195	0.29209139	0.644728007	0.519103463	0.96446859
Scube1	0.14195742	0.392012122	0.362125077	0.717258565	0.988585656
Efcab6	-0.330952521	0.359629502	-0.920259654	0.357437088	0.924354588
Sult4a1	-0.217634089	0.90795706	-0.239696455	0.810565583	0.991438232
Pnpla5	0.50905404	0.560376229	0.908414764	0.363659128	0.926455466
Pnpla3	-0.463635228	0.621941578	-0.745464276	0.4559911	0.951472839
Samm50	0.173680908	0.145103133	1.196948017	0.231326817	0.895404344
Parvb	0.081367159	0.225545037	0.360757921	0.718280422	0.988585656
Parvg	0.030894553	0.236943141	0.130388045	0.896259425	0.993970087
1810041L15Rik	-0.158619123	0.658669827	-0.240817352	0.809696681	0.991295569
Ldoc1l	0.468970904	0.532208601	0.881178738	0.378221085	0.928883222

Prr5	-0.017356357	0.377197534	-0.046013972	0.963299114	0.996474415
Gm20556	-2.311630462	1.976608124	-1.169493555	0.242204836	0.896939594
Arhgap8	-0.148356321	0.260790554	-0.568871528	0.569443329	0.97144955
Phf21b	0.602495232	1.052496771	0.572443782	0.567021362	0.970862413
Nup50	-0.318495339	0.140479785	-2.267196946	0.023378197	0.622011301
5031439G07Rik	0.140124536	0.249084497	0.562558237	0.573735737	0.971613657
Upk3a	-0.277652724	0.690414645	-0.402153584	0.687570999	0.98538669
Fam118a	0.185659028	0.123396368	1.504574496	0.132433507	0.827800491
Ribc2	-0.707743146	0.512926281	-1.37981455	0.167643751	0.860403806
Fbln1	-0.106030798	0.166584918	-0.636496986	0.524452545	0.965637248
Atxn10	-0.136102366	0.110637092	-1.230169404	0.218633675	0.888631319
Wnt7b	-0.576258652	0.366934046	-1.570469292	0.116305973	0.81220172
AU022754	0.509213312	0.964671461	0.527861902	0.597595189	0.974001549
Lincppara	-0.400218166	0.541805317	-0.738675229	0.460104231	0.953083512
Ppara	0.151988634	0.395367443	0.384423748	0.700664402	0.988482081
Cdpf1	0.185667061	0.17011823	1.091400149	0.275096847	0.900302631
Pkdrej	-0.562923985	1.670736656	-0.336931606	0.736168459	0.988585656
Ttc38	-0.269939028	0.251131792	-1.074889907	0.282424021	0.90579922
Gtse1	-1.278330628	1.01950895	-1.253868962	0.209889638	0.884258007
Trmu	0.226481542	0.229856148	0.985318617	0.324467591	0.91640698
Celsr1	0.211746119	0.36741414	0.576314561	0.564402583	0.969658675
Gramd4	-0.100396721	0.211208638	-0.475343822	0.634541929	0.977034429
Cerk	-0.078877508	0.173322522	-0.455090932	0.649043821	0.979004493
Tbc1d22a	-0.150357628	0.182306624	-0.824751314	0.409512792	0.940886864
Fam19a5	-0.154030372	0.592504045	-0.259965097	0.794890696	0.990988839
Brd1	-0.355102934	0.214170977	-1.658034809	0.097310441	0.78359791
Zbed4	0.055420142	0.224228602	0.247159112	0.804785088	0.990988839
Alg12	-0.216435133	0.189649104	-1.141239947	0.253770085	0.897044747
Creld2	-0.109925248	0.149297169	-0.736284877	0.461557348	0.953083512
Pim3	-0.137230866	0.192760352	-0.711924754	0.476511373	0.95716557
Ttll8	-1.65079545	1.013251365	-1.629206244	0.103269366	0.790843776
Mlc1	-0.027388686	0.192388572	-0.142361296	0.886794632	0.992886758
Mov10l1	-0.675664573	1.492600138	-0.45267621	0.650781913	0.979004493
Panx2	-0.195758355	1.541868519	-0.126961769	0.898970658	0.99447264
1810021B22Rik	-0.405117145	0.381395751	-1.062196273	0.288146591	0.907334416
Trabd	-0.075465691	0.114103397	-0.661379882	0.508368724	0.963324595
Selo	-0.18132207	0.125389594	-1.446069514	0.148157705	0.847572129
Tubgcp6	-0.289928336	0.480657936	-0.603190574	0.546381918	0.96778188
Hdac10	0.028865794	0.208027025	0.138759828	0.889639939	0.993043931
Mapk12	0.056811499	0.10495771	0.541279903	0.58831467	0.973455044
Mapk11	0.383584612	0.186794535	2.053510886	0.040023055	0.679118604
Plxnb2	-0.017812825	0.13259385	-0.13434126	0.893132728	0.99333638
Dennd6b	0.019413372	0.301204632	0.064452435	0.94860998	0.99560893
Ppp6r2	-0.128017664	0.197060856	-0.649635176	0.515927906	0.964352198
Sbf1	-0.138811786	0.207959687	-0.667493724	0.504456817	0.961955905
Adm2	0.674681617	1.822290454	0.370238243	0.711204984	0.988585656
Lmf2	-0.243712522	0.203859293	-1.195493806	0.231894155	0.895404344
Ncaph2	0.267500862	0.099376885	2.691781523	0.007107149	0.452031612
Tymp	-0.65159256	0.277813791	-2.345429133	0.019005191	0.590246276
Odf3b	-0.054978268	0.208463446	-0.263730976	0.791987225	0.990988839
Arsa	0.195913304	0.189335245	1.034742921	0.300788986	0.910959335
C230037L18Rik	2.342601818	1.090198415	2.148784832	0.03165146	0.661395937
Shank3	0.083422929	0.218281748	0.382180048	0.70232782	0.988482081
Rabl2	-0.259017742	0.141297089	-1.833142802	0.066781337	0.738254074
Syt10	-0.544361897	0.405376529	-1.342854997	0.179318931	0.867478574
Alg10b	-0.495318629	0.245181332	-2.020213463	0.04336125	0.686006913
Cpne8	-0.243836096	0.178997372	-1.36223283	0.173124418	0.863084695
Kif21a	-0.125419929	0.177453592	-0.706775942	0.479705727	0.957463006
Abcd2	0.007471157	0.212422671	0.035171185	0.971943239	0.996733101
Slc2a13	-0.284528646	0.570918327	-0.49837014	0.618223179	0.974471036
Lrrk2	-0.116167435	0.163502169	-0.71049476	0.477397379	0.95716557
Cntn1	0.173414236	0.659763773	0.26284292	0.792671653	0.990988839

Gxylt1	-0.162541533	0.206969142	-0.785341868	0.432253154	0.9459793
Yaf2	-0.056753133	0.158629532	-0.357771548	0.720514285	0.988585656
Zcrb1	0.307613323	0.157275001	1.955894593	0.050477572	0.707153766
Pphln1	-0.092119769	0.138083658	-0.66713013	0.504689016	0.962138588
Prickle1	0.236926385	0.152629161	1.552300908	0.120590241	0.815945653
D630010B17Rik	1.935350582	1.648584481	1.173946864	0.24041631	0.896939594
Adamts20	-0.645979757	0.817617769	-0.790075487	0.429483685	0.945321484
Pus7l	0.766878334	0.406336252	1.887299818	0.059120011	0.72285319
Irak4	0.003505147	0.224062165	0.015643636	0.987518694	0.998148029
Twf1	0.112843558	0.092636525	1.218132457	0.223173642	0.892421271
Tmem117	-0.101277845	0.322347224	-0.314188668	0.753377749	0.988585656
Ano6	-0.063608244	0.155760602	-0.408371844	0.683000701	0.984738223
D030018L15Rik	0.59371848	0.996597138	0.59574572	0.55134511	0.968926429
E330033B04Rik	-0.537158757	0.706888049	-0.759892259	0.447318989	0.948476747
Arid2	-0.159107692	0.166065794	-0.958100328	0.33801217	0.919246575
Scaf11	-0.064312244	0.174553338	-0.368438926	0.712545977	0.988585656
Slc38a1	-0.030562441	0.330132991	-0.092576149	0.926240293	0.994688009
Slc38a2	-0.303975611	0.144863663	-2.098356522	0.035873663	0.674795441
Slc38a4	-0.684894441	0.219989293	-3.113308073	0.001850028	0.292888346
Amigo2	-0.152210839	0.18818798	-0.808823383	0.418616744	0.943415794
Pced1b	0.087411203	0.422475846	0.206902249	0.8360862	0.992886758
Rpap3	0.179435437	0.155663344	1.152714775	0.249027475	0.896939594
Endou	0.390807541	0.261171835	1.496361737	0.134559415	0.828680121
Rapgef3	-0.19972052	0.223524895	-0.893504591	0.371587015	0.928840865
Slc48a1	0.06429254	0.111325551	0.577518272	0.563589399	0.969658675
Hdac7	0.03620036	0.106441711	0.340095625	0.733784516	0.988585656
Vdr	-0.104700964	0.617274984	-0.169618025	0.865310546	0.992886758
Tmem106c	0.060934083	0.182371296	0.334121016	0.738288242	0.988585656
Senp1	-0.251641238	0.20256737	-1.242259494	0.214140835	0.886067976
Pfkm	0.26657406	0.255133132	1.044842975	0.296095549	0.908883631
Asb8	0.032124057	0.17197181	0.186798387	0.851818712	0.992886758
Ccdc184	0.62276341	0.231982456	2.684528049	0.007263229	0.452031612
Zfp641	0.157393964	0.180737252	0.870844066	0.383839301	0.931591679
Lalba	0.775343426	1.754365983	0.441950787	0.658524819	0.979746192
Kansl2	0.28260298	0.134467477	2.101645584	0.035584334	0.674795441
Mir1291	1.03811983	2.179412221	0.476330187	0.633839163	0.977034429
Ccnt1	0.005539413	0.306280979	0.01808605	0.985570207	0.998148029
9330020H09Rik	0.350017269	0.39041015	0.896537317	0.369965859	0.928436166
4930415O20Rik	0.311685044	0.710845892	0.438470627	0.661045159	0.979924124
Adcy6	-0.083525446	0.212597786	-0.39288013	0.694408018	0.987010101
Cacnb3	-0.117858651	0.255351373	-0.461554797	0.644400617	0.97889195
Ddx23	0.22355121	0.11958704	1.869359838	0.061572768	0.725730283
Rnd1	-0.247142612	0.320973271	-0.769978792	0.441312473	0.948068187
Ccdc65	-0.410677438	0.319660526	-1.284729904	0.198886685	0.880661448
Fkbp11	0.041935895	0.310703158	0.134970932	0.892634857	0.993300147
Arf3	-0.085918424	0.198053851	-0.433813449	0.664423914	0.98119114
Wnt10b	0.034103492	0.270568296	0.126043931	0.89969715	0.99447264
Prkag1	0.110750284	0.100746967	1.099291495	0.271640941	0.89804647
Kmt2d	0.13619083	0.258798013	0.526243721	0.59871888	0.974001549
Rhebl1	0.689532697	0.261799255	2.633822227	0.008442968	0.473705634
Dhh	-0.20247726	0.308697032	-0.655909321	0.511882471	0.963452765
Lmbr1l	0.14891424	0.209667661	0.710239428	0.477555675	0.95716557
Tuba1b	-0.028512563	0.094719599	-0.301020734	0.763398684	0.989285682
Tuba1a	-0.048343903	0.137261773	-0.352202236	0.7246866	0.988585656
4930578M01Rik	0.204177947	0.818260313	0.249526885	0.802953247	0.990988839
Tuba1c	-0.116752171	0.220499963	-0.529488395	0.596466693	0.974001549
Prph	0.758946445	1.066246655	0.711792569	0.476593235	0.95716557
Troap	-2.430197441	1.523290173	-1.595360808	0.110631575	0.800111791
Dnajc22	-0.015958025	0.805509528	-0.019811094	0.984194068	0.998148029
Spats2	-0.136267362	0.229231232	-0.594453736	0.552208677	0.968926429
Kcnh3	0.328451221	0.443200905	0.741088786	0.458639608	0.952683168
Mcrs1	0.021127366	0.139385285	0.1515753	0.879521916	0.992886758

1700120C14Rik	0.160188593	1.317281526	0.121605435	0.903211509	0.994688009
Prpf40b	0.05471176	0.168838889	0.324047143	0.745902343	0.988585656
Fmnl3	0.012234226	0.149082661	0.082063371	0.93459632	0.994688009
Tmbim6	-0.170649904	0.131485387	-1.297862127	0.194334716	0.879588734
Nckap5l	0.702071957	0.638142121	1.10018119	0.271253185	0.89804647
Bcdin3d	0.403306347	0.333403604	1.209664031	0.226407837	0.89398447
Faim2	-0.100698592	0.331153339	-0.304084483	0.761063528	0.989266039
Aqp2	0.091618694	0.712766778	0.128539512	0.897722032	0.994101834
Aqp5	-0.174329116	0.114242679	-1.525954377	0.127021227	0.824450458
Racgap1	0.110220675	0.325702261	0.338409304	0.735054764	0.988585656
Asic1	0.476928189	0.390258396	1.222083098	0.221676204	0.89157489
Smarcd1	-0.31283756	0.276322427	-1.132146831	0.257572709	0.897248195
Gpd1	-0.107569833	0.190787538	-0.563820019	0.572876626	0.971613657
Cox14	0.459232828	0.188843083	2.431822339	0.015023074	0.554936045
Cers5	-0.028905339	0.112402796	-0.257158542	0.797056387	0.990988839
Lima1	-0.028807555	0.143530663	-0.200706623	0.840927981	0.992886758
Larp4	-0.068427939	0.152929398	-0.447447905	0.654551693	0.979133069
2310068J16Rik	-0.003070344	1.756588325	-0.001747902	0.998605377	0.999720579
Dip2b	-0.188199259	0.293819395	-0.64052701	0.521830035	0.965211168
Atf1	-0.033813812	0.100755144	-0.335603825	0.737169639	0.988585656
Mettl7a1	0.214761808	0.114072981	1.882670256	0.059745069	0.725221283
Mettl7a3	-1.358370487	0.596890141	-2.27574623	0.022861203	0.620006604
Slc11a2	-0.150587797	0.147509942	-1.020865402	0.307318213	0.9139204
Letmd1	0.060652234	0.244025334	0.248548923	0.803709722	0.990988839
Csrnp2	0.026896693	0.341772137	0.078697734	0.937273048	0.994688009
Tfcp2	0.106661259	0.22506595	0.473911132	0.635563281	0.977034429
Pou6f1	-0.071441041	0.279841211	-0.255291353	0.798498078	0.990988839
C330013E15Rik	0.052139969	0.451393707	0.115508852	0.908041805	0.994688009
Dazap2	-0.113892654	0.090715241	-1.255496348	0.209298633	0.884258007
Smagp	0.078391936	0.113928763	0.688078531	0.49140333	0.958916246
Bin2	0.834779915	0.311980675	2.675742382	0.007456394	0.452943141
Cela1	0.406400348	0.360061557	1.128696855	0.259025736	0.897248195
Galnt6	-0.056232136	0.328731714	-0.171057836	0.864178289	0.992886758
I730030J21Rik	0.106462901	0.92428603	0.115183934	0.908299332	0.994688009
Slc4a8	-0.001893533	0.320554426	-0.005907056	0.995286879	0.999285699
Fignl2	-0.32562136	0.500557323	-0.650517623	0.515357922	0.964352198
Acvrl1	-0.084088983	0.134989643	-0.622929147	0.533331083	0.96674935
Acvr1b	-0.18265764	0.286611722	-0.637299962	0.523929476	0.965637248
A330009N23Rik	0.821545318	0.482632665	1.702216566	0.088714778	0.767814344
Grasp	0.152796093	0.23992287	0.636855053	0.524219262	0.965637248
Nr4a1	0.689597713	0.592758243	1.163370937	0.244679036	0.896939594
Atg101	0.082432254	0.151045229	0.545745502	0.585240889	0.972904784
6030408B16Rik	0.036594528	0.193342185	0.189273375	0.849878562	0.992886758
Krt80	-0.094261601	0.166286981	-0.566860977	0.570808639	0.971613657
Krt7	0.091006484	0.124469322	0.731155938	0.46468391	0.953083512
Krt83	-0.428710774	0.838649397	-0.511191895	0.609216694	0.974001549
5430421N21Rik	-1.265465535	1.762980778	-0.717798827	0.472881337	0.955299874
Krt5	-0.737515301	1.424670925	-0.517674143	0.604685642	0.974001549
Krt1	-0.838047832	0.4649807	-1.802328209	0.071493783	0.744227652
Krt79	-0.065473298	0.206189911	-0.317538806	0.750834799	0.988585656
Krt8	-0.315837152	0.174018354	-1.814964606	0.069529349	0.743426713
Krt18	-0.057146944	0.119681782	-0.477490751	0.633012706	0.977034429
Eif4b	0.103922528	0.125539621	0.827806606	0.407780033	0.939557159
Tns2	-0.153429323	0.152024062	-1.009243678	0.312857784	0.914819219
Spryd3	-0.047300194	0.155520927	-0.304140381	0.761020944	0.989266039
Igfbp6	0.214124118	0.144890088	1.477838275	0.139451071	0.836535783
Soat2	-0.09810871	1.475477588	-0.06649285	0.94698545	0.995217331
Csad	-0.034689025	0.16190617	-0.21425388	0.830349081	0.992886758
Zfp740	-0.073371562	0.139098485	-0.527479227	0.597860838	0.974001549
Itgb7	0.176987571	0.2414796	0.732929698	0.463601309	0.953083512
Rarg	-0.450569204	0.249766747	-1.803959933	0.071237588	0.744227652
Mfsd5	-0.155000541	0.144206582	-1.074850673	0.282441589	0.90579922

Espl1	-0.208864128	0.529132184	-0.394729587	0.693042465	0.986599035
Pfdn5	0.288867527	0.280112825	1.031254196	0.302421632	0.911518274
Myg1	0.097064454	0.139336397	0.696619515	0.48604094	0.957710647
Aaas	0.10990408	0.263318906	0.417380133	0.676400384	0.983549699
Sp1	-0.118410109	0.185587877	-0.638027174	0.523455991	0.965637248
Amhr2	0.050012579	0.244178632	0.204819637	0.837713044	0.992886758
Prr13	0.029799562	0.148770722	0.200305285	0.841241829	0.992886758
Pcbp2	-0.117414001	0.089881379	-1.306321756	0.191443162	0.878250211
Map3k12	0.176230043	0.27875874	0.632195581	0.527259085	0.965637248
Tarbp2	0.179725962	0.162422428	1.106534145	0.268495393	0.89804647
Npff	0.467989509	0.700398257	0.66817629	0.504021069	0.96179897
Atf7	-0.339770829	0.31144758	-1.090940663	0.275298993	0.900302631
Atp5g2	0.021240113	0.123990956	0.171303727	0.86398495	0.992886758
Calcoco1	-0.300587316	0.214242919	-1.403021007	0.160610575	0.857316691
Hoxc5	-0.516649483	1.209364487	-0.42720742	0.669228257	0.981863224
Hoxc4	-1.480066249	1.066582921	-1.387671057	0.165237233	0.860011234
Smug1	0.005004442	0.140514069	0.035615238	0.971589158	0.996733101
Cbx5	-0.18551089	0.139715963	-1.327771614	0.184253567	0.871468131
Hnrnpa1	0.206977339	0.71754589	0.288451709	0.773000991	0.990730604
Nfe2	0.253101868	0.294372949	0.859800022	0.389899287	0.933734231
Copz1	-0.249967246	0.129979365	-1.923130232	0.054463697	0.708548823
Zfp385a	0.638389114	0.432939313	1.47454642	0.140334529	0.837748392
Itga5	0.031718607	0.264074035	0.120112556	0.904393986	0.994688009
Nckap1l	-0.032716162	0.230384531	-0.142006765	0.887074662	0.992886758
Pde1b	-0.37990271	0.380584088	-0.998209652	0.318177707	0.91640698
Ppp1r1a	0.454308059	0.289018648	1.571898776	0.115974029	0.811920148
Mefv	-0.049014336	0.601534509	-0.081482168	0.935058506	0.994688009
Zfp263	0.025757154	0.184948999	0.139266254	0.889239755	0.993043931
Zfp174	-0.688619686	0.643437168	-1.07022056	0.284520042	0.905916508
Zfp597	-0.138506802	0.152160646	-0.910266911	0.362681764	0.926455466
Naa60	0.054505557	0.154291989	0.353262391	0.723891738	0.988585656
1700037C18Rik	0.038701281	0.184478281	0.209787738	0.833833343	0.992886758
Cluap1	0.096689645	0.140958349	0.685944792	0.492747923	0.958916246
Nlrc3	-0.753099394	1.089785499	-0.691052868	0.489532318	0.958560421
Slx4	0.338468446	0.457062911	0.74052923	0.458978932	0.952683168
Dnase1	-0.071300334	0.460041254	-0.154986827	0.876831702	0.992886758
Trap1	-0.156968663	0.124229249	-1.26354031	0.206395069	0.883765092
Crebbp	0.381216989	0.440462407	0.86549268	0.386768432	0.932626359
Adcy9	-0.154970032	0.284500557	-0.544709062	0.585953631	0.972904784
Srl	-0.071912428	0.342211881	-0.210140067	0.833558355	0.992886758
Tfap4	0.021318995	0.36163601	0.058951527	0.952990717	0.996070133
Glis2	0.01545674	0.280140229	0.05517501	0.955999038	0.99614946
Pam16	0.276072332	0.15674431	1.761290932	0.078189174	0.754732183
Coro7	-0.182877392	0.301569793	-0.606418138	0.544237127	0.967491297
Vasn	-0.360611324	0.212609219	-1.696122702	0.089862645	0.769347579
Dnaja3	0.048963466	0.119856098	0.408518776	0.682892849	0.984738223
Nmral1	0.189939311	0.282383527	0.672628863	0.501183435	0.96121484
Hmox2	-0.011534431	0.152474845	-0.075648089	0.939699076	0.994688009
Cdip1	-0.116971748	0.162067218	-0.721748357	0.470449205	0.95486913
4930562C15Rik	-0.511209989	0.416476272	-1.22746486	0.219647921	0.888847883
Ubald1	-0.218762171	0.20726413	-1.05547531	0.291207996	0.907334416
Mgrn1	-0.159168154	0.178767193	-0.890365574	0.373269622	0.928840865
Gm16861	1.240581598	1.141746309	1.086565018	0.277229092	0.900939369
Nudt16l1	-0.169195063	0.167366475	-1.010925653	0.312052015	0.914819219
Anks3	-0.146899879	0.154637088	-0.949965372	0.342129848	0.919850248
4930451G09Rik	-0.087147022	0.476656081	-0.18282998	0.854931424	0.992886758
Smim22	0.672389957	0.217136803	3.096619038	0.001957413	0.298625366
Rogdi	-0.131775718	0.162040807	-0.813225511	0.416088776	0.943415794
Glyr1	-0.204933601	0.100583816	-2.037441101	0.041605859	0.679118604
Ubn1	0.061737233	0.140303868	0.44002517	0.659918878	0.979924124
Ppl	-0.142861786	0.265794318	-0.537489992	0.590929196	0.974001549
Sec14l5	-0.559731438	1.339611552	-0.417831152	0.676070573	0.983549699

Nagpa	-0.056507147	0.246523302	-0.229216251	0.818700842	0.992734473
AU021092	-0.186009135	0.142204466	-1.308040039	0.190859729	0.877906723
Alg1	-0.254045369	0.172981427	-1.468628006	0.141933714	0.840741274
Eef2kmt	0.161728232	0.163466332	0.989367229	0.322483502	0.91640698
Rbfox1	-0.471215298	1.442910915	-0.326572689	0.743991103	0.988585656
Mettl22	0.107301497	0.22295179	0.48127668	0.630319869	0.977034429
Abat	0.203793952	0.426019196	0.478368002	0.632388304	0.977034429
Tmem186	-0.197050562	0.146664626	-1.343545254	0.179095483	0.867377413
Pmm2	0.003396047	0.182901547	0.018567621	0.985186033	0.998148029
Carhsp1	0.109981364	0.128340849	0.856947459	0.391473922	0.933734231
Usp7	0.049784331	0.108121227	0.460449186	0.645193837	0.979004493
1810013L24Rik	-0.086119187	0.127414083	-0.675900068	0.499104095	0.960700483
Rpl39l	0.666972888	1.072820702	0.62170024	0.534138994	0.96674935
Emp2	-0.218957838	0.106759671	-2.05094148	0.040272646	0.679118604
Gm1600	0.578733576	1.511768095	0.382819017	0.70185396	0.988482081
Tekt5	0.380099568	0.368026113	1.032805973	0.301694708	0.911518274
Nubp1	0.283725317	0.131748293	2.153540754	0.031276205	0.661395937
Tvp23a	0.257051275	0.748310452	0.343508867	0.731215671	0.988585656
Ciita	0.248988236	0.265241287	0.938723525	0.347872712	0.921552698
Dexi	-0.112935275	0.184120046	-0.613378484	0.539626109	0.966900344
Clec16a	-0.444987448	0.288106013	-1.544526765	0.122460772	0.817763223
Socs1	-0.314210749	0.353243727	-0.889501286	0.373733732	0.928840865
Rmi2	0.112318893	0.751496939	0.149460214	0.881190501	0.992886758
Litaf	-0.161787106	0.143910693	-1.124218801	0.260920215	0.897248195
Gm4262	-0.02357403	0.276761606	-0.08517811	0.932119793	0.994688009
Snn	0.07292234	0.118932942	0.613138287	0.539784906	0.966900344
Txndc11	-0.279356703	0.16020082	-1.743790717	0.08119559	0.760377276
Zc3h7a	-0.164175558	0.132926904	-1.235081482	0.216800181	0.887633697
Rsl1d1	0.000239769	0.126214655	0.001899691	0.998484267	0.999708875
2610020C07Rik	0.044744256	1.091185613	0.041005175	0.967291771	0.996474415
Gspt1	-0.226465847	0.098232835	-2.30539867	0.021144256	0.60743619
Tnfrsf17	0.024096065	0.98479255	0.024468163	0.980479178	0.997296835
Snx29	-0.263319979	0.451813198	-0.582807188	0.560023105	0.968926429
Cpped1	-0.313851624	0.163037435	-1.925027975	0.054225859	0.707153766
Gm9961	-0.152451656	1.402083183	-0.108732248	0.913414864	0.994688009
Shisa9	0.437341555	1.407326355	0.31076058	0.755982639	0.989266039
Ercc4	-0.137763827	0.314146612	-0.438533545	0.660999559	0.979924124
Mkl2	-0.200251616	0.271487274	-0.737609588	0.460751727	0.953083512
2310015D24Rik	-0.125143293	2.030573284	-0.061629538	0.950857854	0.99573416
Parn	0.162717215	0.151741548	1.072331325	0.283571242	0.905916508
Bfar	0.025341951	0.103589971	0.244637112	0.806737426	0.99099448
3110001I22Rik	0.080851348	0.292464417	0.276448495	0.782203614	0.990988839
Rrn3	-0.204438517	0.199252044	-1.026029708	0.304877581	0.912452608
Ntan1	0.320254728	0.139268306	2.299552127	0.021473607	0.60763088
Pdxdc1	-0.099637567	0.137306565	-0.725657709	0.468048634	0.953657709
Mpv17l	-0.025019326	0.182079673	-0.13740867	0.890707779	0.993043931
Marf1	0.019459275	0.122729849	0.158553725	0.874020489	0.992886758
Nde1	-0.097828332	0.114461416	-0.854683918	0.392726156	0.933734231
Myh11	0.557118625	0.255475971	2.18070851	0.029204983	0.64858361
Fopnl	0.235497602	0.197687177	1.191263922	0.233549992	0.89581803
Abcc1	-0.263074758	0.255399841	-1.030050595	0.302986255	0.911623139
Snai2	0.090555937	0.297392052	0.304500193	0.760746846	0.989266039
Efcab1	-0.105057387	0.190413774	-0.551732078	0.581131929	0.971846872
Ube2v2	0.365760819	0.170289781	2.147872977	0.031723848	0.661395937
Mcm4	0.075915096	0.221810819	0.342251548	0.732161595	0.988585656
Prkdc	0.054242144	0.281753536	0.192516286	0.847337812	0.992886758
Mzt2	0.266618352	0.206994596	1.288044989	0.197730303	0.880239572
Cebpd	-0.099465818	0.286446882	-0.347240008	0.728411017	0.988585656
Spidr	-0.110327322	0.173688453	-0.635202402	0.525296417	0.965637248
Pkp2	-0.052762786	0.172380952	-0.306082461	0.759541861	0.989266039
Yars2	0.109736791	0.154785048	0.708962474	0.478347764	0.957463006
Dnm1l	-0.21287171	0.1621401	-1.312887497	0.18922086	0.875548883

Fgd4	-0.228932728	0.197160231	-1.161150637	0.245580645	0.896939594
Spag6	-0.323106507	0.167407396	-1.930061129	0.053599264	0.707153766
Rpl31-ps12	-0.424433793	0.697546015	-0.608467089	0.542877727	0.967491297
Igll1	-1.107595742	1.457870468	-0.759735358	0.447412789	0.948476747
Vpreb1	0.512262101	0.643117791	0.796529203	0.425724509	0.944290206
Top3b	-0.302801909	0.225138853	-1.344956255	0.178639362	0.867377413
Ppm1f	0.009129432	0.11231145	0.081286745	0.935213915	0.994688009
Mapk1	-0.197145474	0.092024106	-2.142324251	0.032167399	0.665167506
Ypel1	0.61684516	0.189184245	3.260552493	0.001111954	0.242572819
Ppil2	0.029217432	0.124098867	0.235436738	0.813869774	0.992048071
2610318N02Rik	1.258440805	1.380047167	0.911882459	0.361830596	0.926455466
Sdf2l1	0.067605653	0.194911988	0.346852208	0.728702353	0.988585656
Ccdc116	0.248595092	1.532124424	0.162255159	0.871104925	0.992886758
Ydjc	-0.371523916	0.580631304	-0.639862015	0.522262311	0.965494283
Ube2l3	-0.128174377	0.103838367	-1.234364333	0.217067173	0.887633697
Hic2	-0.079337377	0.641646804	-0.123646492	0.901595179	0.994688009
Tmem191c	0.137066015	0.626332725	0.218838981	0.82677548	0.992886758
Pi4ka	0.03164672	0.191956757	0.1648638	0.869051186	0.992886758
Serpind1	-0.761523322	0.423170843	-1.799564725	0.071929395	0.744227652
Snap29	-0.000566985	0.106786838	-0.005309501	0.995763651	0.999285699
Crkl	-0.237743427	0.217676512	-1.092186863	0.274750976	0.900302631
Aifm3	0.076413133	0.589558108	0.129610859	0.896874312	0.994101834
Lztr1	0.046485902	0.092912073	0.500321441	0.616848759	0.974001549
Thap7	-0.055053153	0.143492422	-0.383665928	0.701226069	0.988482081
Lrrc74b	-0.681349	0.155799635	-4.373238753	1.22E-05	0.010840965
P2rx6	-0.103551149	0.212579845	-0.487116496	0.626175777	0.976534277
Slc7a4	-0.121674854	0.234351579	-0.519197927	0.603622724	0.974001549
Smpd4	-0.299065411	0.25038312	-1.194431201	0.232309338	0.895404344
Ccdc74a	0.196494097	0.222192644	0.884341142	0.376512085	0.928883222
Med15	0.081270081	0.200107697	0.406131709	0.684645829	0.985105247
Klhl22	-0.270356315	0.164982512	-1.638696799	0.101276421	0.788783372
Scarf2	-0.0298121	0.165966421	-0.1796273	0.857445171	0.992886758
Car15	0.139981399	0.644255025	0.217276379	0.827992964	0.992886758
Dgcr2	-0.015472532	0.136083036	-0.113699199	0.909476248	0.994688009
Dgcr14	-0.106433259	0.230394654	-0.461960628	0.644109555	0.97889195
Slc25a1	-0.189567285	0.152407855	-1.243815714	0.213567396	0.884945675
Dgcr6	0.181620548	0.187662925	0.967801968	0.333143281	0.917239519
Prodh	-0.10742594	0.19475137	-0.551605567	0.581218622	0.971861865
Rtn4r	-0.242901186	1.551774776	-0.156531212	0.875614319	0.992886758
Zdhhc8	-0.21506288	0.220469409	-0.975477191	0.329323611	0.917115058
Ranbp1	0.111827477	0.116449898	0.960305499	0.336901483	0.919246575
Trmt2a	0.205961407	0.133446116	1.543405036	0.122732533	0.817788082
Dgcr8	-0.168714083	0.168665451	-1.000288335	0.317170991	0.91640698
Tango2	0.300509681	0.16062812	1.870841058	0.061367115	0.72551838
Arvcf	0.053477023	0.225693467	0.236945376	0.812699168	0.992000477
Comt	-0.089745767	0.091281833	-0.98317227	0.32552266	0.91640698
Txnrd2	0.241938358	0.287647181	0.841094139	0.400295198	0.936803788
Gnb1l	0.559788632	0.265662785	2.107139818	0.035105462	0.674795441
Tbx1	0.116213772	0.274715691	0.423032887	0.672271267	0.983233856
Gp1bb	-0.189849166	0.585469464	-0.324268263	0.745734945	0.988585656
Sep-05	-0.055309026	0.231097231	-0.239332274	0.810847941	0.991589719
Cldn5	-0.065157127	0.090705139	-0.718339975	0.472547688	0.955044417
Cdc45	0.105078849	0.386210403	0.272076692	0.785563051	0.990988839
Ufd1l	0.119016186	0.112540071	1.057544971	0.290262942	0.907334416
2510002D24Rik	0.48770153	0.162093574	3.008765354	0.002623116	0.327099208
Mrpl40	0.06210991	0.148121771	0.419316552	0.674984801	0.983549699
Hira	-0.018593096	0.168015813	-0.110662775	0.911883766	0.994688009
Lamp3	-0.315993554	0.159708035	-1.978570168	0.047864421	0.702771946
B3gnt5	0.040204129	0.284854465	0.141139192	0.887759983	0.992886758
A930003A15Rik	0.521860182	0.439540373	1.187286114	0.235114784	0.896002526
Klhl6	0.624752289	0.228478599	2.734401783	0.006249374	0.427133716
Klhl24	0.005973654	0.131069223	0.04557633	0.963647935	0.996474415

Yeats2	-0.314011345	0.235952247	-1.330825826	0.183246328	0.869576666
Parl	0.318200478	0.121890814	2.610536977	0.00904002	0.475600561
Cyp2ab1	-0.144013045	0.812617332	-0.177221232	0.859334619	0.992886758
Abcc5	-0.058922444	0.11571901	-0.509185521	0.610622191	0.974001549
Eif2b5	-0.01570384	0.118664357	-0.132338308	0.894716709	0.993556555
Dvl3	-0.076702591	0.521496907	-0.147081585	0.883067625	0.992886758
Ap2m1	0.008039558	0.085425474	0.094111948	0.92502023	0.994688009
Gm15760	0.375302226	0.683089342	0.549418946	0.582717975	0.972086107
Abcf3	0.148317728	0.128296152	1.156057495	0.24765764	0.896939594
Alg3	-0.095021898	0.384946702	-0.246844296	0.805028727	0.99099448
Ece2	0.231158644	0.234755855	0.9846768	0.324782852	0.91640698
Camk2n2	-0.363110676	0.535292766	-0.678340339	0.497555928	0.960690117
Psmd2	-0.098665639	0.130371466	-0.756803939	0.449167332	0.948476747
Eif4g1	-0.04835721	0.149607595	-0.323226973	0.746523355	0.988585656
Fam131a	-0.182270901	0.212217344	-0.85888786	0.390402388	0.933734231
Clcn2	-0.256891124	0.412683951	-0.622488768	0.533620527	0.96674935
Polr2h	0.086163315	0.195689771	0.440305665	0.659715738	0.979924124
Thpo	-1.029286375	1.222623183	-0.841867216	0.399862283	0.936395796
Chrd	-0.384192759	0.570662031	-0.673240444	0.500794335	0.961175273
Ephb3	0.050348532	0.205082099	0.245504273	0.806066	0.99099448
Gm16863	1.68392676	2.416762947	0.696769521	0.485947044	0.957710647
Magef1	0.483339346	0.257088709	1.880048908	0.060101413	0.725221283
Vps8	-0.049793964	0.231686139	-0.21491991	0.829829762	0.992886758
2510009E07Rik	-0.402885379	0.170278797	-2.366033742	0.017979806	0.581648476
Ehhadh	-0.016762015	0.216561349	-0.077400768	0.93830473	0.994688009
1300002E11Rik	0.203849648	0.181236321	1.1247726	0.260685408	0.897248195
Map3k13	-0.2021708	0.980260538	-0.206241904	0.836601957	0.992886758
Tmem41a	-0.084262197	0.221770889	-0.379951567	0.703981368	0.988585656
Liph	0.026892531	0.109619437	0.2453263	0.806203789	0.99099448
Senp2	0.091974733	0.118252073	0.777785377	0.43669555	0.946762848
Igf2bp2	-0.206550397	0.346744366	-0.595684938	0.551385722	0.968926429
Tra2b	0.029490117	0.126198545	0.233680323	0.815233165	0.992270157
Etv5	-0.032693126	0.177289866	-0.184404936	0.853695798	0.992886758
Dgkg	0.53181456	0.315615493	1.685007779	0.091987071	0.772965982
9230117E06Rik	0.755944313	0.346730174	2.180209193	0.029241959	0.64858361
Tbccd1	-0.090537072	0.212165624	-0.42672828	0.66957725	0.981863224
Dnajb11	0.050656624	0.119042675	0.425533315	0.670447941	0.982434419
Ahsg	-0.858594596	1.139900959	-0.753218593	0.451318568	0.949134998
Fetub	-0.042586751	0.437919589	-0.097247879	0.922529547	0.994688009
Kng2	-1.429810603	1.203498262	-1.188045424	0.234815514	0.89581803
Eif4a2	0.168159719	0.165434004	1.016476148	0.3094027	0.9139204
Snora81	-0.15694981	0.796911835	-0.196947521	0.843868606	0.992886758
Rfc4	0.020104421	0.2115785	0.095021097	0.92429807	0.994688009
Adipoq	-0.521437488	0.473895339	-1.100322044	0.271191831	0.89804647
St6gal1	-0.137364126	0.186091138	-0.738155115	0.460420195	0.953083512
Masp1	0.576343308	0.825980028	0.697769061	0.485321631	0.957710647
Rtp4	0.265370416	0.163821069	1.619879646	0.105258133	0.793982106
Bcl6	-0.103161895	0.185659136	-0.55565213	0.578448687	0.971613657
Lppos	0.008608047	0.324335621	0.026540554	0.978826188	0.997262526
Lpp	0.004484452	0.207740984	0.021586745	0.982777607	0.997439547
Morf4l1-ps1	0.30809263	0.291711614	1.056154827	0.290897485	0.907334416
Tprg	1.117466996	1.180734319	0.946416969	0.343935954	0.920669678
Trp63	0.205751283	0.843565944	0.243906578	0.807303175	0.99099448
P3h2	-0.264684522	0.506741766	-0.522326243	0.6014432	0.974001549
Cldn1	-0.149720275	0.273417802	-0.547587881	0.583974913	0.972333834
Il1rap	-0.295293189	0.200252436	-1.474604731	0.140318842	0.837748392
Gmnc	-0.034752669	0.308154292	-0.112776847	0.910207475	0.994688009
Uts2b	-0.030378706	1.731544951	-0.017544278	0.986002409	0.998148029
Ccdc50	-0.102034504	0.118238011	-0.862958565	0.388160249	0.933269543
Fgf12	-1.058946102	0.417104716	-2.538801553	0.01112329	0.505877302
Mb21d2	0.060442744	0.199778851	0.30254826	0.76223415	0.989285682
Hrasls	0.222029107	0.649869794	0.34165168	0.73261304	0.988585656

Atp13a4	-0.388695008	0.457291792	-0.849993406	0.395328752	0.934066368
Opa1	-0.113178137	0.180246454	-0.627907703	0.530064404	0.965888564
Gm1968	0.4447196	0.540308098	0.823085203	0.410459543	0.94109998
4632428C04Rik	0.16669334	0.198094918	0.841482162	0.400077874	0.936631983
9030404E10Rik	-0.328921667	0.288578264	-1.13980056	0.2543694	0.897044747
Hes1	-0.045231209	0.169774646	-0.266419104	0.789916454	0.990988839
Gp5	0.523526106	0.748750451	0.69919972	0.484427226	0.957710647
Atp13a3	-0.210944231	0.145683097	-1.44796641	0.147626445	0.846658212
Tmem44	0.41819804	0.174782202	2.392680927	0.016725779	0.56772809
Lsg1	0.012286958	0.140490953	0.087457291	0.930308032	0.994688009
Fam43a	-0.28778692	0.151735486	-1.896635567	0.057876053	0.72285319
Xxylt1	0.078930725	0.239107417	0.330105713	0.741320086	0.988585656
Acap2	-0.28269582	0.157025445	-1.800318542	0.071810355	0.744227652
Ppp1r2	-0.079794618	0.106029757	-0.752568148	0.451709464	0.949134998
Apod	0.098982102	0.320043008	0.309277501	0.757110444	0.989266039
Bdh1	-0.730614392	0.312666895	-2.3367181	0.019453847	0.593178479
Dlg1	0.042164715	0.096550558	0.436711254	0.662320766	0.980647283
Pigz	0.442427447	0.878237387	0.503767493	0.614424773	0.974001549
0610012G03Rik	0.330258412	0.149756133	2.2053081	0.027432482	0.636714037
Ncbp2	0.06206682	0.08888298	0.698298143	0.484990761	0.957710647
Senp5	-0.009856979	0.126635281	-0.077837545	0.93795728	0.994688009
Gm15694	1.300096503	1.149435046	1.131074355	0.258023797	0.897248195
Pak2	-0.120012445	0.12016368	-0.998741425	0.317919968	0.91640698
Pigx	0.246000816	0.197909373	1.242997302	0.213868828	0.885354044
Cep19	-0.011579543	0.149430566	-0.077491127	0.93823285	0.994688009
Nrros	0.002877651	0.166617102	0.017271044	0.986220386	0.998148029
Bex6	1.080014859	2.90063426	0.372337483	0.709641594	0.988585656
Fbxo45	0.330031069	0.215755415	1.529653702	0.126102469	0.822401003
Wdr53	0.160682023	0.180870922	0.888379521	0.374336637	0.928883222
Smco1	0.94933391	1.296448867	0.732257117	0.464011648	0.953083512
Rnf168	-0.003761536	0.100713084	-0.037349029	0.970206713	0.996628386
Ubxn7	-0.025243348	0.128530435	-0.196399771	0.844297275	0.992886758
Tctex1d2	0.084739405	0.20271968	0.418012719	0.675937818	0.983549699
Pcyt1a	-0.197626308	0.182441441	-1.083231454	0.278705702	0.90176568
Tfrc	-0.220811376	0.182647962	-1.208945198	0.2266839	0.89398447
Tnk2	0.199358884	0.258084554	0.772455699	0.439844576	0.947849924
Muc4	0.048558491	0.45112028	0.107639788	0.914281434	0.994688009
Muc20	-0.208717594	0.442110692	-0.472093522	0.636860036	0.977284137
Gm933	-0.312295746	0.650417218	-0.4801468	0.631123012	0.977034429
1700021K19Rik	-0.169237796	0.224505077	-0.753826141	0.450953625	0.949134998
Fyttd1	0.038172578	0.174838961	0.218329931	0.827172055	0.992886758
Lrch3	-0.405572347	0.262089595	-1.547456882	0.121753114	0.816233249
Iqcg	-0.122810507	0.169302956	-0.72538903	0.468213401	0.953657709
Rpl35a	0.54987659	0.291922778	1.883637152	0.059614073	0.724734898
Lmln	-0.184933064	0.588124076	-0.314445661	0.753182581	0.988585656
Osbpl11	0.06823099	0.233024347	0.292806272	0.769670234	0.989990452
Snx4	-0.084349534	0.114911726	-0.734037661	0.462925785	0.953083512
1700007L15Rik	0.051452629	0.327387186	0.157161401	0.875117648	0.992886758
Zfp148	-0.270659499	0.105648015	-2.561898563	0.010410172	0.499527467
Slc12a8	-1.105030327	0.871784519	-1.267549839	0.204958755	0.883765092
Heg1	0.026455799	0.323531263	0.081772	0.934828022	0.994688009
Itgb5	-0.204452425	0.165249267	-1.237236496	0.215999296	0.887633697
Umps	0.188072235	0.157416303	1.194744325	0.232186939	0.895404344
Kalrn	0.21351902	0.336924701	0.633729197	0.526257567	0.965637248
Ccdc14	-0.168829636	0.355330855	-0.475133622	0.634691735	0.977034429
Mylk	-0.106762004	0.1610461	-0.662928216	0.507376529	0.963011338
Hacd2	0.143608597	0.167821337	0.855723118	0.39215095	0.933734231
Adcy5	-0.133983141	0.317004641	-0.422653565	0.672548039	0.983233856
Sec22a	-0.013158353	0.192719467	-0.06827724	0.945564942	0.995032756
Pdia5	-0.470846279	0.200685499	-2.346189839	0.018966444	0.590246276
Dirc2	0.117686761	0.108117908	1.08850386	0.276372733	0.900604936
Hspbap1	-0.037561022	0.259065367	-0.144986661	0.884721404	0.992886758

Parp14	0.121358015	0.179949517	0.67440034	0.500056827	0.9611436
Dtx3l	0.185259179	0.209715697	0.883382513	0.377029636	0.928883222
Parp9	0.068889571	0.123792963	0.556490205	0.577875789	0.971613657
Kpna1	-0.330649748	0.143699274	-2.300984127	0.021392528	0.60743619
Wdr5b	0.362350887	0.270496581	1.339576587	0.18038304	0.868087032
Fam162a	0.414434979	0.158662974	2.612045952	0.009000216	0.475600561
Ccdc58	0.220939752	0.208833105	1.057972831	0.290067829	0.907334416
Stfa2l1	0.28683321	0.687705727	0.417087133	0.676614676	0.983549699
Gm5483	0.891385687	0.863776337	1.031963541	0.302089198	0.911518274
2010005H15Rik	1.040542485	0.57696648	1.803471294	0.07131423	0.744227652
Stfa1	0.178533671	1.650066505	0.108197864	0.913838739	0.994688009
Cd86	0.25534986	0.178412087	1.431236331	0.152362494	0.852974739
Ildr1	-0.174967885	0.192134416	-0.910653535	0.362477953	0.926455466
Slc15a2	-0.125867585	0.151912184	-0.828554903	0.407356315	0.939557159
Eaf2	0.033417624	0.44745728	0.074683385	0.940466628	0.994688009
Iqcb1	0.091271594	0.171927601	0.530872262	0.5955073	0.974001549
4930565N06Rik	-0.098662597	2.633992543	-0.037457432	0.970120281	0.996628386
Golgb1	-0.048243836	0.174278347	-0.276820595	0.781917866	0.990988839
Hcls1	0.111394341	0.289839112	0.38433164	0.70073266	0.988482081
Fbxo40	0.087606281	1.564502196	0.055996266	0.955344782	0.99614946
Polq	0.060896147	1.172564728	0.051934145	0.958581167	0.996226826
Gtf2e1	0.007001752	0.14553263	0.048111216	0.961627608	0.996474415
Rabl3	-0.036012819	0.235272771	-0.15306837	0.878344359	0.992886758
Hgd	-0.026099989	1.881404088	-0.013872612	0.988931612	0.998182772
Ndufb4	0.211741875	0.297983762	0.710581924	0.477343347	0.95716557
Fstl1	-0.109869713	0.103789949	-1.058577589	0.289792198	0.907334416
Lrrc58	0.135266142	0.214506916	0.630591053	0.52830795	0.965637248
Gpr156	0.868691038	1.18375611	0.733842918	0.46304448	0.953083512
BC031361	0.075497796	0.839410217	0.089941479	0.928333719	0.994688009
Gsk3b	-0.050922159	0.201238303	-0.253044067	0.80023416	0.990988839
Nr1i2	-0.039411381	0.390783617	-0.100852182	0.919667802	0.994688009
Maats1	-0.114370504	0.250977367	-0.455700469	0.648605385	0.979004493
Cox17	0.273289568	0.171613486	1.592471397	0.111278825	0.800726918
Popdc2	0.111258851	0.259213263	0.429217431	0.667764999	0.981863224
Pla1a	-0.763870921	0.622135632	-1.227820562	0.219514335	0.888847883
Adprh	-0.077002817	0.117089958	-0.657638096	0.510770709	0.963452765
Cd80	-0.579834019	0.650435604	-0.891454919	0.372685166	0.928840865
Timmdc1	0.33555287	0.171787605	1.953300821	0.05078396	0.707153766
Poglut1	0.140554666	0.122994014	1.142776479	0.253131407	0.897044747
Tmem39a	-0.245429865	0.132913318	-1.846540805	0.06481372	0.733548823
Arhgap31	0.004682885	0.164878385	0.028402052	0.977341487	0.997262526
B4galt4	-0.033791324	0.151685423	-0.222772387	0.82371266	0.992886758
Upk1b	0.037552586	0.179571007	0.209123881	0.834351532	0.992886758
Igsf11	-0.155359022	0.477142781	-0.325602792	0.744724899	0.988585656
Lsamp	0.519691896	0.908371456	0.572113856	0.567244843	0.970899103
Gap43	-0.038516016	0.227050348	-0.169636454	0.865296052	0.992886758
Gm19522	-0.536591659	0.51036691	-1.051384108	0.293082212	0.907334416
Zbtb20	0.211115569	0.513014522	0.411519675	0.680691518	0.984226822
BC002163	0.652765805	0.403226872	1.618854918	0.105478483	0.793982106
Mir568	0.599646143	1.981942876	0.302554705	0.762229237	0.989285682
Tigit	-0.401049391	1.605435908	-0.249807164	0.80273648	0.990988839
Qtrtd1	0.166629652	0.192526893	0.865487669	0.386771181	0.932626359
2610015P09Rik	0.276372382	0.29734616	0.929463431	0.352648968	0.923348232
Zdhhc23	-0.776514233	0.855704141	-0.907456439	0.364165475	0.926556975
Gramd1c	-0.31354044	0.197317365	-1.589015948	0.112056792	0.801067615
Atp6v1a	-0.197727792	0.111511271	-1.773164177	0.076201506	0.752876039
Naa50	0.00393792	0.098065577	0.04015599	0.967968764	0.996474415
Gm608	0.116657047	0.275172316	0.423941802	0.671608257	0.982910363
Sidt1	0.011955433	0.458112423	0.02609716	0.979179842	0.997262526
Spice1	-0.151903071	0.193444763	-0.785252951	0.432305275	0.9459793
Cfap44	-0.276888792	0.220686945	-1.254667747	0.209599399	0.884258007
Boc	0.203671781	0.181740552	1.120673278	0.262426961	0.897248195

BC027231	-0.209786422	0.202330336	-1.036851051	0.299805279	0.910174935
Gtpbp8	0.053736621	0.178272216	0.301430151	0.763086506	0.989285682
Cd200r1	-0.085162068	0.262186514	-0.32481483	0.74532122	0.988585656
Cd200r4	-0.033954419	0.233063405	-0.145687476	0.884168109	0.992886758
Cd200r3	-0.106298334	1.118620327	-0.095026285	0.924293949	0.994688009
Ccdc80	-0.294068915	0.2329256	-1.262501485	0.206768392	0.883765092
Slc35a5	-0.038505768	0.139314494	-0.276394559	0.782245035	0.990988839
Atg3	0.110310932	0.130047893	0.84823314	0.396308144	0.934101786
Btla	0.20234277	0.24803857	0.815771396	0.414630896	0.943415794
Cd200	0.281383827	0.136141677	2.0668456	0.038748705	0.679118604
Gcsam	-0.485796448	0.3744382	-1.297400874	0.194493292	0.879588734
BC016579	0.76085871	0.960600623	0.792065601	0.428322426	0.945321484
Tmprss7	2.29788997	2.185323649	1.051510137	0.293024356	0.907334416
Tagln3	0.661851832	1.268284282	0.52184817	0.601776048	0.974001549
Abhd10	0.317392441	0.139589613	2.273754001	0.022980781	0.620006604
Phldb2	-0.193608711	0.14017248	-1.381217707	0.167212032	0.860403806
Plcxd2	0.034654486	0.737856567	0.046966426	0.962539986	0.996474415
Cd96	0.57893333	0.570399945	1.014960354	0.310124726	0.914704835
Gm4737	0.420451097	0.937627487	0.448420191	0.653849973	0.979133069
Pvrl3	-0.202760193	0.108519431	-1.868422928	0.061703144	0.725730283
Dppa2	-1.313763582	0.982338073	-1.337384367	0.181097205	0.868087032
Morc1	0.364589787	0.935845348	0.389583373	0.696844649	0.987518602
Trat1	0.566396939	0.528346061	1.072018855	0.283711563	0.905916508
Retnla	-1.217157044	0.766336695	-1.588279736	0.112223098	0.801274176
Retnlg	-0.327678927	0.431067039	-0.760157695	0.447160329	0.948476747
Dzip3	-0.348395805	0.204344651	-1.704942129	0.088205218	0.7676581
C330027C09Rik	-0.221470997	0.276546468	-0.80084551	0.423221089	0.943509323
Ift57	0.059604286	0.118437493	0.503255211	0.614784852	0.974001549
Cd47	0.264266081	0.141371447	1.869303078	0.06158066	0.725730283
Gm4827	0.217031656	1.946808742	0.111480728	0.911235147	0.994688009
Bbx	-0.229744254	0.229442656	-1.001314482	0.316674794	0.91640698
G730013B05Rik	-1.29299878	1.695183841	-0.762748411	0.445613453	0.948476747
4930542D17Rik	2.18459082	1.601827478	1.363811553	0.172626883	0.86244803
5330426P16Rik	-0.037569618	0.169773659	-0.221292388	0.824864776	0.992886758
Cblb	-0.207763768	0.24833886	-0.836614001	0.402809573	0.937834293
Alcam	-0.04767242	0.093722394	-0.508655595	0.610993653	0.974001549
Zpld1	2.677350021	2.273645406	1.177558301	0.238972742	0.896609492
Nfkbiz	0.369637144	0.271327454	1.362328578	0.173094212	0.863084695
Nxpe3	0.068675527	0.317288701	0.216444918	0.828640957	0.992886758
Cep97	0.032930428	0.267196859	0.123244068	0.90191383	0.994688009
Rpl24	0.255137767	0.144306741	1.768023907	0.077056907	0.752876039
Zbtb11os1	0.867371777	0.469208103	1.848586527	0.06451754	0.73299549
Zbtb11	-0.151439493	0.12245737	-1.236671119	0.216209205	0.887633697
Pcnp	0.344291925	0.125447407	2.744512093	0.006060092	0.427133716
Trmt10c	-0.009382441	0.145639843	-0.064422215	0.948634041	0.99560893
Senp7	-0.365538191	0.189284075	-1.931161882	0.053463036	0.707153766
Impg2	0.21004229	0.668661118	0.314123678	0.753427106	0.988585656
Abi3bp	0.239358485	0.222606391	1.075254323	0.28226088	0.90579922
Tfg	-0.405481892	0.162226025	-2.499487321	0.012437315	0.525888996
Adgrg7	2.628644106	3.321032784	0.791514049	0.428644081	0.945321484
Tmem45a	-0.099120081	0.214980986	-0.461064407	0.644752397	0.978970741
Tomm70a	-0.076912066	0.095489168	-0.8054533	0.420558145	0.943415794
Nit2	0.189273476	0.206465253	0.91673283	0.359282658	0.925229839
Tbc1d23	-0.047213358	0.122836742	-0.384358601	0.70071268	0.988482081
Cmss1	0.398370384	0.18094241	2.201641856	0.027690617	0.637644759
Filip1l	-0.317369315	0.163764323	-1.937963708	0.05262765	0.707153766
Col8a1	-0.445980009	0.371822005	-1.199444903	0.230354996	0.895404344
Dcbld2	-0.318969196	0.166751414	-1.912842525	0.055768215	0.715507996
St3gal6	0.003412051	0.123081876	0.027721801	0.977884036	0.997262526
Cpox	-0.174394764	0.155749917	-1.119710163	0.262837293	0.897248195
Gpr15	1.142842809	1.268469373	0.900962083	0.36760848	0.928436166
Cldnd1	0.105322949	0.118618495	0.887913387	0.374587343	0.928883222

Mina	0.090711253	0.239797814	0.378282233	0.705220942	0.988585656
Crybg3	0.03476388	0.160120578	0.217110633	0.828122128	0.992886758
Arl6	-0.028737917	0.145501966	-0.197508788	0.843429405	0.992886758
Nsun3	0.084138319	0.309012008	0.272281714	0.785405415	0.990988839
Arl13b	-0.134485418	0.128220398	-1.048861337	0.294241942	0.907334416
Stx19	0.148254959	0.206009613	0.719650685	0.471740097	0.95486913
Pros1	-0.128184757	0.142506725	-0.899499702	0.368386554	0.928436166
Epha3	-0.582157643	0.365923263	-1.590928213	0.111625732	0.800752782
4930453N24Rik	-0.233431641	0.13034097	-1.790930669	0.073304421	0.748833631
Zfp654	-0.24446447	0.144774628	-1.68858641	0.091298717	0.771949327
Cggbp1	-0.11377514	0.105585653	-1.077562503	0.281229044	0.904300139
Chmp2b	0.098649799	0.106941336	0.922466488	0.356285303	0.924354588
Vgll3	0.023707038	0.249553624	0.09499777	0.924316598	0.994688009
Cadm2	-0.127416108	0.374607762	-0.340132055	0.733757083	0.988585656
Gbe1	-0.04087039	0.160984145	-0.253878353	0.799589539	0.990988839
Robo1	0.131077048	0.469616836	0.279114883	0.780156662	0.990988839
Robo2	-0.037469534	0.199733313	-0.187597818	0.851191937	0.992886758
Rbm11	1.431920949	0.831916815	1.721230925	0.085208928	0.761635307
Hspa13	-0.172957744	0.121801372	-1.419998318	0.155608171	0.855261441
Samsn1	0.353114037	0.156939414	2.250002261	0.024448802	0.626649273
Nrip1	-0.500850385	0.489495649	-1.023196807	0.306214802	0.91333012
Usp25	-0.139394677	0.146617917	-0.95073426	0.341739292	0.919850248
Mir99ahg	0.351904488	0.327574915	1.074271785	0.282700887	0.905916508
E330011O21Rik	-0.031523105	0.497228806	-0.063397584	0.949449911	0.995624732
Cxadr	-0.171792169	0.108876738	-1.577859248	0.114597949	0.809432539
Btg3	0.39615152	0.256571008	1.544022932	0.122582777	0.817788082
D16Ertd472e	0.007529411	0.208000489	0.036199008	0.971123677	0.996733101
Chodl	-0.977470668	1.53884972	-0.635195663	0.525300812	0.965637248
Ncam2	-0.308630444	1.146314175	-0.269237222	0.787747138	0.990988839
Mir155hg	0.212091441	0.748634483	0.283304398	0.776943507	0.990861469
Mrpl39	0.130941439	0.126998307	1.031048696	0.302517985	0.911518274
Jam2	-0.111152809	0.12459497	-0.892113135	0.372332295	0.928840865
Atp5j	0.260900822	0.148826664	1.753051608	0.079593149	0.75659671
Gabpa	0.019169416	0.158937472	0.120609794	0.904000111	0.994688009
App	-0.249170988	0.161127729	-1.546419033	0.1220034	0.816233249
Gm10791	0.492481161	1.522033046	0.323567982	0.746265132	0.988585656
Cyyr1	-0.159983239	0.279529663	-0.572330095	0.567098365	0.970862413
Adamts1	-0.117751158	0.260872077	-0.451375092	0.651719232	0.979004493
Adamts5	0.23275412	0.159328995	1.460839692	0.14405944	0.841934045
N6amt1	0.079624664	0.164151173	0.485069117	0.627627318	0.976968316
Ltn1	-0.02381445	0.152622929	-0.156034552	0.876005786	0.992886758
Rwdd2b	0.302536014	0.209773302	1.442204568	0.149244672	0.848779008
Usp16	-0.15888497	0.115864662	-1.371297921	0.170282104	0.86207625
Cct8	-0.13999495	0.113019892	-1.238675307	0.215465767	0.887633697
B130034C11Rik	1.162512743	0.990914713	1.173171342	0.240727102	0.896939594
Map3k7cl	0.682237024	0.399846464	1.706247489	0.08796201	0.7667678
Bach1	-0.150385639	0.143752918	-1.046139735	0.295496522	0.908201248
Cldn8	-0.228404957	0.225804926	-1.011514499	0.311770245	0.914819219
Tiam1	-0.126935217	0.271286669	-0.467900681	0.639855617	0.977843986
Sod1	0.224172496	0.119366754	1.878014505	0.060379181	0.725221283
Scaf4	-0.101392167	0.249395293	-0.406552049	0.684337021	0.985105247
Hunk	0.20719314	0.312703633	0.66258629	0.507595553	0.963011338
Mis18a	0.069046274	0.164934723	0.418627881	0.67548811	0.983549699
Mrap	-0.449503318	0.460247216	-0.976656246	0.328739363	0.917115058
Urb1	-0.31841552	0.357633347	-0.890340686	0.373282981	0.928840865
Eva1c	-0.32511586	0.260745439	-1.246870746	0.212444895	0.884945675
1110004E09Rik	0.076099344	0.112178853	0.678375131	0.497533873	0.960690117
Synj1	-0.342767509	0.183805625	-1.864836885	0.062204271	0.726656275
4930404I05Rik	-0.315392273	0.679757338	-0.463977739	0.642663694	0.978681333
Paxbp1	-0.126008865	0.141548158	-0.890219039	0.373348284	0.928840865
4932438H23Rik	0.075437384	1.457347195	0.051763495	0.958717143	0.996226826
Ifnar2	0.057189237	0.115082055	0.496943131	0.619229155	0.974471036

Il10rb	0.044559138	0.100457102	0.443563841	0.657357953	0.979614766
A930006K02Rik	0.83433474	0.295557814	2.822915521	0.004758911	0.391906015
Ifnar1	-0.067094375	0.140795254	-0.476538614	0.633690705	0.977034429
Ifngr2	-0.129878237	0.136821248	-0.949254878	0.342490997	0.919850248
Tmem50b	-0.240529238	0.084276916	-2.854034647	0.004316782	0.385355746
Dnajc28	-0.496926671	0.301388848	-1.648789179	0.099190833	0.788456049
Gart	-0.11157381	0.130199613	-0.856944255	0.391475693	0.933734231
Son	-0.188105921	0.126491117	-1.487107753	0.136986354	0.833085238
Donson	0.315973727	0.253295661	1.247450213	0.212232465	0.884945675
Gm10785	0.523813715	0.945039682	0.554276953	0.579389321	0.971613657
Cryzl1	0.2042704	0.159030032	1.284476886	0.198975146	0.880661448
Itsn1	0.028841002	0.256772456	0.112321244	0.910568698	0.994688009
Atp5o	0.002975619	0.163527243	0.018196473	0.985482116	0.998148029
Slc5a3	-0.635107138	0.317633679	-1.999495579	0.04555476	0.69515685
Mrps6	-0.125598287	0.193245217	-0.649942537	0.51572934	0.964352198
Kcne2	0.094939524	0.176858509	0.53681061	0.591398442	0.974001549
Smim11	0.255929526	0.173231451	1.477384876	0.139572498	0.836535783
1700048M11Rik	-0.862181381	2.326444623	-0.370600431	0.710935161	0.988585656
Rcan1	-0.046139717	0.112691048	-0.409435518	0.682220079	0.984738223
Clic6	-0.106581517	0.164667138	-0.647254319	0.517467361	0.964352198
Runx1	-0.112152425	0.188683984	-0.594392924	0.55224934	0.968926429
1700029J03Rik	0.816743917	1.357075155	0.601841331	0.54727976	0.968115622
Setd4	0.096297961	0.274891725	0.350312333	0.726104311	0.988585656
Cbr1	0.257358439	0.201460541	1.277463257	0.201438744	0.881220006
Cbr3	0.649477126	0.412096698	1.576030892	0.115018684	0.81007959
Dopey2	0.024835363	0.237593563	0.10452877	0.916749738	0.994688009
Morc3	-0.038610527	0.145745053	-0.264918268	0.791072425	0.990988839
Chaf1b	0.463901536	0.331208354	1.400633562	0.161323679	0.857316691
Hlcs	0.241218533	0.260586058	0.925677049	0.354613839	0.924354588
Ripply3	0.111920681	0.144429951	0.774913237	0.43839093	0.947776577
Pigp	0.149525563	0.200344358	0.746342768	0.455460382	0.951224746
Ttc3	-0.381202385	0.160469479	-2.375544482	0.01752308	0.579835303
Dscr3	0.126225312	0.111854488	1.128477845	0.259118168	0.897248195
Dyrk1a	-0.052698297	0.146119974	-0.360650876	0.718360452	0.988585656
Kcnj15	-0.065580584	0.199926252	-0.328023876	0.74289361	0.988585656
Erg	0.01908109	0.213829695	0.089234986	0.928895162	0.994688009
Ets2	-0.010391317	0.136784418	-0.075968576	0.939444098	0.994688009
1600002D24Rik	-0.62429552	1.326073629	-0.47078496	0.637794306	0.977496258
Psmg1	0.364570596	0.187939626	1.939828249	0.052400566	0.707153766
Brwd1	-0.033460137	0.172040325	-0.194490081	0.845792161	0.992886758
Hmgn1	0.137705799	0.113129615	1.217239177	0.223513228	0.892989918
Wrb	-0.011992666	0.13051244	-0.091889066	0.926786179	0.994688009
Lca5l	0.181569486	0.556455053	0.326296769	0.744199832	0.988585656
Sh3bgr	0.260799027	0.283735816	0.91916146	0.358011126	0.924354588
Igsf5	-0.042870817	0.137931903	-0.310811468	0.755943951	0.989266039
Itgb2l	-0.092654284	1.153654249	-0.080313737	0.935987733	0.994688009
Pcp4	1.240767597	0.84455606	1.469135865	0.141795942	0.840741274
Bace2	-0.178537454	0.169251322	-1.054865935	0.291486644	0.907334416
Mx1	0.751380714	0.339266109	2.214723762	0.026779031	0.633489138
Fam3b	0.124589297	0.430478163	0.289420714	0.772259445	0.99050004
Mx2	-0.216245247	0.413134142	-0.523426233	0.600677673	0.974001549
Tmprss2	-0.191775586	0.114157204	-1.679925386	0.092971834	0.77525594
Ripk4	0.159715738	0.188005127	0.849528632	0.395587205	0.934066368
Prdm15	-0.532491042	0.313873976	-1.696512241	0.089788914	0.769347579
C2cd2	-0.073341348	0.260622914	-0.281407906	0.778397555	0.990988839
Zbtb21	-0.124105471	0.268785782	-0.461726325	0.644277591	0.97889195
B230307C23Rik	0.092788958	0.245722823	0.37761636	0.70571561	0.988585656
A630089N07Rik	0.518043253	0.596715766	0.868157476	0.38530813	0.931689378
Pisd-ps2	-0.321684563	0.18918702	-1.70035219	0.089064699	0.768297719
Scaf8	-0.184437283	0.137730314	-1.339119019	0.18053193	0.868087032
Tiam2	0.247132462	0.544989069	0.453463154	0.650215269	0.979004493
Tfb1m	0.086250586	0.240396661	0.358784458	0.71975634	0.988585656

1700102H20Rik	-0.663682269	1.004036893	-0.661013827	0.508603447	0.96333741
Arid1b	0.024675971	0.224611593	0.109860628	0.912519908	0.994688009
Tmem242	0.464588243	0.256287245	1.812763811	0.069868258	0.744227652
Zdhhc14	-0.13950645	0.253949954	-0.549346232	0.582767866	0.972086107
3300005D01Rik	-0.378480621	0.981248441	-0.385713347	0.699708977	0.988393777
Snx9	-0.22793722	0.155675219	-1.464184352	0.143143585	0.840741274
Synj2	-0.206638202	0.271028866	-0.762421379	0.44580855	0.948476747
Serac1	-0.745436521	0.55389603	-1.345805856	0.178365136	0.867377413
Gtf2h5	0.119057473	0.165608625	0.718908651	0.472197206	0.955003345
Tulp4	0.120754014	0.210904913	0.572551926	0.566948118	0.970862413
Tmem181a	-0.267194335	0.204188171	-1.308569119	0.190680347	0.877906723
Dynlt1a	0.697357028	0.253990552	2.745602234	0.006039995	0.427133716
Dynlt1b	0.471051401	0.378276108	1.245258137	0.213036879	0.884945675
Tmem181b-ps	0.182942291	0.249243883	0.733989091	0.462955387	0.953083512
Sytl3	1.750702307	0.787161173	2.224070961	0.02614367	0.631530315
Ezr	-0.206171421	0.124036067	-1.66218928	0.096474829	0.781321691
Mir692-1	-0.899078694	0.431052262	-2.085776537	0.03699887	0.678243111
Rsph3b	-0.277756582	0.21728068	-1.278330786	0.201132817	0.881109608
Tagap1	-0.119225764	0.139100459	-0.857119845	0.391378655	0.933734231
Rnaset2b	0.708238784	0.144884998	4.888282381	1.02E-06	0.002075969
Rps6ka2	-0.212962247	0.207849923	-1.024596227	0.305553745	0.91333012
Fndc1	0.11241519	0.364908068	0.308064414	0.758033319	0.989266039
Tagap	0.060640475	0.312168862	0.194255359	0.845975938	0.992886758
Rsph3a	-0.186800233	0.181324842	-1.030196584	0.302917733	0.911623139
Fgfr1op	-0.087699793	0.142813531	-0.614086024	0.539158483	0.966900344
Ccr6	-0.323370641	1.342799045	-0.240818343	0.809695912	0.991295569
Mpc1	0.209384031	0.205283169	1.019976609	0.307739554	0.9139204
4930506C21Rik	-0.190524145	0.957543178	-0.198971858	0.842284759	0.992886758
Sft2d1	0.419012541	0.154249012	2.716468233	0.006598253	0.434393837
Prr18	-0.315898264	0.191040024	-1.653571111	0.098214687	0.786907305
T2	0.056603073	0.296538391	0.190879409	0.848620073	0.992886758
Gm16702	2.148684588	1.462189112	1.469498419	0.14169765	0.840741274
Pde10a	-0.448976468	1.198041348	-0.374758741	0.707839901	0.988585656
1700010I14Rik	0.315569256	0.404624579	0.779906294	0.435446033	0.946762848
Qk	-0.090713705	0.097884732	-0.926740081	0.354061502	0.924354588
Cahm	0.211926854	0.522493939	0.40560634	0.685031869	0.985159458
Pacrg	-0.448369231	0.216045387	-2.075347396	0.03795435	0.679118604
Park2	0.165256011	0.246856652	0.669441191	0.503214083	0.961729397
Agpat4	0.011890056	0.146762623	0.081015557	0.93542958	0.994688009
Map3k4	0.168778635	0.217191137	0.777097247	0.437101398	0.94702277
4732491K20Rik	0.787878881	0.419433757	1.878434599	0.060321736	0.725221283
Slc22a3	-0.589224096	0.389343102	-1.513380081	0.130183142	0.827800491
Slc22a1	0.423192802	0.459333889	0.921318483	0.356884174	0.924354588
Igf2r	0.290289613	0.206398542	1.406451858	0.15958999	0.856630464
Airn	0.528607814	0.256719186	2.059089631	0.039485653	0.679118604
Pnldc1	-0.654432601	1.682188672	-0.389036385	0.697249231	0.987548136
Mrpl18	0.194889626	0.161670867	1.205471517	0.228021327	0.894410876
Tcp1	-0.022389754	0.108102456	-0.207116053	0.835919225	0.992886758
Acat3	-0.358131849	0.905895901	-0.39533444	0.692596085	0.986599035
Acat2	-0.005087117	0.158834447	-0.032027792	0.974449888	0.997262526
Wtap	0.213492323	0.137147002	1.556667809	0.119549375	0.81558552
Sod2	0.060886603	0.098524822	0.617982367	0.536586962	0.96674935
Tcp10b	2.114169533	2.288846091	0.923683572	0.355651088	0.924354588
2700054A10Rik	0.740207811	0.643951735	1.149477159	0.25035928	0.896939594
Tcte2	-0.05516539	0.394193059	-0.139945107	0.888703362	0.992886758
Mllt4	0.047410666	0.154376633	0.307110377	0.758759359	0.989266039
Dact2	0.39175134	0.561949794	0.697128719	0.485722243	0.957710647
Smoc2	-0.075600204	0.218937925	-0.345304288	0.729865623	0.988585656
Thbs2	-0.755589949	0.607406193	-1.243961549	0.213513715	0.884945675
Wdr27	0.043584832	0.413873272	0.105309609	0.916130139	0.994688009
1600012H06Rik	0.074970426	0.119701627	0.626310838	0.531111079	0.966166773
Phf10	0.093595922	0.136869475	0.683833424	0.494080358	0.958916246

LOC106740	-0.350960354	0.340520598	-1.030658222	0.302701123	0.911518274
9030025P20Rik	0.018865962	0.486535253	0.038776146	0.969068864	0.996535072
Gm3435	1.051549229	0.72807007	1.444296741	0.148655522	0.84832848
Ermard	0.176723305	0.220162782	0.802693822	0.422151725	0.943415794
Dll1	-0.360824448	0.262928863	-1.372327264	0.169961582	0.861730117
Fam120b	-0.035369639	0.129460587	-0.273207775	0.784693504	0.990988839
Psmb1	0.07874361	0.144723356	0.544097456	0.586374411	0.972904784
Tbp	-0.147492709	0.139899047	-1.054279582	0.291754934	0.907334416
Pdcd2	0.199045535	0.205642867	0.967918498	0.333085075	0.917233939
Prdm9	-0.047856065	0.612133743	-0.0781791	0.937685587	0.994688009
Chd1	0.056209038	0.152455273	0.368691986	0.712357323	0.988585656
Rgmb	-0.345343275	0.219813943	-1.57107084	0.116166195	0.811920148
Zfp960	0.115451008	0.253144677	0.456067293	0.648341589	0.979004493
Zfp97	0.100930775	0.197168599	0.511900856	0.608720399	0.974001549
Riok2	0.109578757	0.139015665	0.788247543	0.430551925	0.945321484
Lix1	0.508096472	0.381971628	1.330194275	0.18345427	0.869576666
Lnpep	-0.38930984	0.226363344	-1.719844887	0.085460641	0.761635307
Spaca6	0.666704648	0.378948341	1.75935497	0.078517235	0.754732183
Has1	-0.413299462	0.519772381	-0.79515472	0.426523508	0.944290206
Fpr1	-0.105928375	0.26033299	-0.406895704	0.684084591	0.985105247
Fpr2	0.189742465	0.274815194	0.690436589	0.489919674	0.958560421
Ppp2r1a	0.018325814	0.180875868	0.101317074	0.919298763	0.994688009
Zfp160	0.173345697	0.161925888	1.070524911	0.284383102	0.905916508
Zfp677	0.208163405	0.190711723	1.091508176	0.275049336	0.900302631
Zfp54	0.013543196	0.184013316	0.073599	0.941329467	0.994688009
Zfp51	-0.02951292	0.165398292	-0.17843546	0.858381003	0.992886758
Zfp53	0.167377562	0.236999911	0.706234705	0.480042191	0.957463006
9330136K24Rik	-0.661306471	0.717093262	-0.922204274	0.356422034	0.924354588
Zfp52	0.185142694	0.196871702	0.94042309	0.347000583	0.921552698
Zfp948	-0.035940911	0.188241283	-0.190930013	0.848580426	0.992886758
3110052M02Rik	0.136698093	0.176337419	0.77520752	0.438217046	0.947776577
Gm10509	-0.005174556	0.216935161	-0.023853006	0.98096986	0.997340737
Zfp760	0.068900403	0.184226932	0.373997449	0.708406214	0.988585656
Zfp229	-0.30871431	0.190283449	-1.622391816	0.10471948	0.793021779
Zfp820	0.001302499	0.186861795	0.006970389	0.994438479	0.999285699
2210404O09Rik	0.124740318	0.240110568	0.519511985	0.603403757	0.974001549
Zfp942	0.067880378	0.159331726	0.426031777	0.670084691	0.982177283
Zfp943	0.097015217	0.158191515	0.613277	0.539693199	0.966900344
Zfp947	0.043585738	0.297093664	0.146707059	0.883363247	0.992886758
Gm4944	0.165532783	0.17609581	0.940015459	0.347209631	0.921552698
Zfp944	0.003477159	0.191521586	0.018155443	0.985514848	0.998148029
Zfp758	-0.154204044	0.159084927	-0.969319011	0.332386047	0.917115058
Zfp946	0.062480824	0.19695531	0.317233511	0.751066424	0.988585656
Gm16386	0.408230544	0.465291973	0.877364252	0.380288823	0.929640874
Zfp945	0.351143896	0.156702191	2.24083591	0.025036706	0.627917508
Zfp40	0.231486538	0.197611433	1.171422798	0.241428873	0.896939594
6330415G19Rik	0.335874832	0.52103733	0.644627193	0.519168808	0.96446859
Zfp213	-0.156742683	0.361014595	-0.434172704	0.664163033	0.981154469
Zfp13	-0.310824238	0.362794171	-0.856750915	0.391582558	0.933734231
Zscan10	4.698681725	1.858472319	2.528249508	0.011463285	0.514178739
Mmp25	-0.921820481	1.318485553	-0.699150991	0.484457675	0.957710647
Ccdc64b	-0.050595134	0.381290916	-0.132694308	0.894435144	0.993556555
Thoc6	0.058069461	0.170937907	0.339710846	0.734074293	0.988585656
Hcfc1r1	-0.061279356	0.119330296	-0.513527224	0.607582572	0.974001549
Tnfrsf12a	0.020876745	0.152617051	0.136791697	0.891195447	0.993043931
Pkmyt1	0.273972399	0.503341336	0.54430737	0.586229976	0.972904784
Paqr4	0.077882193	0.263633571	0.295418342	0.767674323	0.989990452
Kremen2	1.895622829	0.738958599	2.565262563	0.010309776	0.499527467
9530082P21Rik	0.099351243	0.193304472	0.513962465	0.607278234	0.974001549
Flywch1	0.10502375	0.335404663	0.313125493	0.754185322	0.988804605
Flywch2	0.44035123	0.228539996	1.926801596	0.054004361	0.707153766
Srrm2	0.260724559	0.172968837	1.507349904	0.131720992	0.827800491

Mir5125	0.32277883	0.481836316	0.669893114	0.50292593	0.961729397
Tceb2	0.030923475	0.133156147	0.232234679	0.816355744	0.992676801
Prss41	0.541959536	1.346184078	0.402589471	0.687250257	0.98538669
Prss30	-0.587796209	0.525561861	-1.118414887	0.263389839	0.897248195
Prss22	-1.613112853	1.827098609	-0.882882207	0.377299918	0.928883222
Kctd5	-0.176472911	0.13011957	-1.356236506	0.17502393	0.864111192
Pdpk1	-0.441260552	0.165670751	-2.663478909	0.007733726	0.45506991
Amdhd2	0.141170961	0.192017457	0.735198578	0.462218565	0.953083512
Tbc1d24	-0.051625317	0.250057249	-0.206453992	0.8364363	0.992886758
Ntn3	0.252120487	0.495716279	0.508598362	0.611033778	0.974001549
1600002H07Rik	0.075388215	0.292656043	0.257600062	0.796715582	0.990988839
Ccnf	-0.600141768	0.6017731	-0.997289124	0.318624191	0.91640698
Abca17	-0.007874481	0.484218733	-0.016262239	0.987025182	0.998148029
Abca3	-0.09221466	0.238490287	-0.386660023	0.699007916	0.987770663
D330041H03Rik	0.264849264	0.77086475	0.343574232	0.731166506	0.988585656
Rnps1	-0.08305835	0.166934589	-0.497550272	0.618801061	0.974471036
Eci1	0.305555933	0.117635663	2.597477026	0.00939114	0.478229167
Dnase1l2	0.157720835	0.452993685	0.348174467	0.727709162	0.988585656
E4f1	0.031717699	0.211183473	0.150190252	0.880614517	0.992886758
Pgp	0.057865998	0.150615623	0.384196518	0.700832799	0.988482081
Mlst8	-0.063984248	0.238031006	-0.26880636	0.788078699	0.990988839
Caskin1	0.24442441	1.166516396	0.209533626	0.834031688	0.992886758
Traf7	-0.126738129	0.127868514	-0.991159787	0.321607563	0.91640698
Rab26os	0.311423367	0.50971041	0.610980982	0.541212171	0.967217841
Rab26	0.383649	0.727923482	0.527045782	0.598161796	0.974001549
Pkd1	-0.150522212	0.238319313	-0.631598882	0.527649017	0.965637248
Tsc2	0.266943601	0.260370811	1.025243958	0.305248092	0.912948451
Nthl1	0.663758874	0.203844661	3.256199455	0.001129144	0.242572819
Slc9a3r2	0.00633242	0.150004562	0.042214847	0.966327427	0.996474415
Npw	0.1003668	0.172308344	0.582483689	0.560240925	0.968926429
Zfp598	-0.164424449	0.181091575	-0.907962999	0.363897771	0.926455466
Syngr3	-1.012681053	1.372815845	-0.737667078	0.460716783	0.953083512
Gfer	0.085180401	0.178080772	0.478324525	0.632419244	0.977034429
Noxo1	0.036032413	0.325134827	0.110822988	0.911756716	0.994688009
Tbl3	-0.203843646	0.201348922	-1.012390054	0.311351592	0.914819219
Rps2	0.200183149	0.121716094	1.644672806	0.100037304	0.788783372
Ndufb10	0.244244804	0.144839919	1.686308626	0.091736371	0.772516176
Rpl3l	0.612309416	0.40636351	1.506802162	0.131861375	0.827800491
Msrb1	0.088018064	0.112880074	0.779748459	0.435538949	0.946762848
Hs3st6	0.226611943	0.331385235	0.683832347	0.494081038	0.958916246
Meiob	2.604655181	1.462379363	1.781107726	0.074894854	0.75241101
Fahd1	0.120973356	0.154602145	0.782481749	0.433931507	0.946679964
Hagh	0.11007037	0.131532323	0.836831337	0.40268738	0.937834293
Igfals	0.245773221	0.816325016	0.301072754	0.763359017	0.989285682
Nubp2	0.231886217	0.122470499	1.893404686	0.058304073	0.72285319
Spsb3	-0.081365281	0.122557136	-0.663896719	0.506756416	0.962854883
Eme2	0.287221375	0.222856218	1.288819207	0.197460947	0.87989762
Mrps34	-0.02741124	0.146174319	-0.18752432	0.851249557	0.992886758
Nme3	0.341865402	0.252714212	1.352774738	0.176127605	0.865308446
Mapk8ip3	-0.229475286	0.200735222	-1.143174	0.252966355	0.897044747
Hn1l	-0.033258748	0.156206727	-0.212914953	0.831393299	0.992886758
Cramp1l	-0.152780261	0.341197386	-0.447776762	0.654314316	0.979133069
Ift140	-0.266869561	0.195889767	-1.362345593	0.173088845	0.863084695
Tmem204	-0.011697032	0.106959275	-0.109359682	0.912917211	0.994688009
Telo2	0.176114183	0.352365782	0.499805008	0.617212384	0.974001549
Clcn7	0.175116071	0.178184283	0.982780683	0.325715391	0.916443764
Ccdc154	-0.348452368	0.347947709	-1.001450387	0.316609114	0.91640698
BC003965	0.418294374	0.168601291	2.480967801	0.013102621	0.536156618
Unkl	-0.005023073	0.160965089	-0.031205977	0.975105274	0.997262526
Gnptg	0.064217176	0.09507217	0.675457139	0.499385374	0.960700483
Tsr3	0.222788506	0.157364397	1.415749121	0.156848971	0.855906801
Baiap3	0.486356539	0.360835256	1.347863135	0.177702407	0.866855369

Ube2i	-0.109545189	0.093801818	-1.167836524	0.242872712	0.896939594
Tpsb2	-0.711632858	1.293665224	-0.550090429	0.582257351	0.972017845
Cacna1h	0.782672333	1.123971163	0.696345563	0.486212446	0.957736773
Tekt4	-0.171161334	0.148484871	-1.152719014	0.249025735	0.896939594
Sox8	-1.28296042	2.070024728	-0.619780239	0.535402481	0.96674935
2810468N07Rik	0.190366098	0.651803129	0.29206073	0.770240191	0.989990452
Lmf1	-0.435691691	0.283901468	-1.534658114	0.12486783	0.821400908
Gng13	-2.134513243	1.852300772	-1.152357801	0.249174074	0.896939594
Chtf18	0.711899821	0.853520704	0.83407446	0.404239026	0.938127315
Rpusd1	0.08518098	0.265473782	0.320864001	0.748313455	0.988585656
Msln	-0.37864599	0.260292728	-1.454692922	0.145754293	0.842832198
Narfl	0.119619119	0.125753214	0.951221172	0.341492113	0.919850248
Haghl	0.076929299	0.220756819	0.348479831	0.727479858	0.988585656
Ccdc78	-0.352743359	0.193568814	-1.822315027	0.068407199	0.743426713
Fam173a	0.096262277	0.125322592	0.768115911	0.442418325	0.948476747
Metrn	0.000361223	0.208611712	0.001731556	0.998618419	0.999720579
Fbxl16	-0.111288774	0.587303306	-0.189491142	0.849707897	0.992886758
Wdr24	0.102592553	0.156399304	0.655965534	0.5118463	0.963452765
Jmjd8	-0.127022576	0.3165829	-0.401230059	0.688250752	0.98538669
Stub1	0.222320276	0.135994901	1.634769206	0.102097438	0.78889961
Rhbdl1	-0.06000409	0.428441391	-0.140052037	0.888618876	0.992886758
Rhot2	-0.182145208	0.206526974	-0.881943912	0.377807139	0.928883222
Wdr90	-0.090632349	0.283823363	-0.3193266	0.749478864	0.988585656
Fam195a	0.267195371	0.216445622	1.234468815	0.21702826	0.887633697
0610011F06Rik	0.116756758	0.12785827	0.913173296	0.361151406	0.926251807
Wfikkn1	1.329237744	1.794023243	0.740925598	0.458738554	0.952683168
Rab40c	0.01505719	0.122858478	0.122557195	0.902457752	0.994688009
Gm10012	-0.424073328	0.55703003	-0.761311428	0.446471076	0.948476747
Pigq	-0.312029671	0.153613571	-2.031263708	0.042228251	0.680982912
Nhlrc4	-0.216188593	0.224054012	-0.964894984	0.334597414	0.918030088
Capn15	0.389188161	0.418519887	0.929915575	0.352414798	0.923152465
Rab11fip3	-0.167819142	0.294660008	-0.569534846	0.56899323	0.97144955
Decr2	0.07360505	0.131920157	0.557951506	0.576877499	0.971613657
Nme4	0.855967465	0.30834653	2.775991882	0.005503358	0.416706681
Tmem8	-0.218524336	0.227676078	-0.959803675	0.337154032	0.919246575
Mrpl28	0.228336068	0.248268674	0.919713568	0.357722461	0.924354588
Axin1	0.062500577	0.161871458	0.386112395	0.69941343	0.988172644
Pdia2	0.825123312	0.439663439	1.876715777	0.060557059	0.725221283
Arhgdig	-0.169954342	0.161158839	-1.05457661	0.291619006	0.907334416
Rgs11	-0.017375145	0.190686654	-0.091118829	0.927398172	0.994688009
Itfg3	0.100255327	0.193566877	0.517936377	0.60450266	0.974001549
Luc7l	-0.004009753	0.122576064	-0.032712364	0.973903964	0.997262526
Neurl1b	0.146462065	0.408459206	0.358572074	0.719915241	0.988585656
Dusp1	0.114085109	0.43658082	0.261314981	0.793849615	0.990988839
Ergic1	-0.252507694	0.151121971	-1.670886715	0.094744056	0.777748075
Atp6v0e	0.181395863	0.141590745	1.281127965	0.200148723	0.880888611
Crebrf	-0.029409551	0.150096397	-0.195937752	0.844658888	0.992886758
Bnip1	0.101136578	0.151457564	0.667755215	0.504289858	0.96186221
Nkx2-5	-2.542898715	1.905642058	-1.334405223	0.182071089	0.868546072
Kifc5b	0.82374717	0.874503382	0.941959959	0.346213141	0.921552698
Phf1	-0.083581993	0.146148799	-0.571896544	0.567392066	0.970955746
Cuta	-0.02428636	0.124068187	-0.1957501	0.844805769	0.992886758
Syngap1	-0.474521874	0.385605829	-1.230587918	0.218477026	0.888436964
Zbtb9	-0.119951646	0.155903945	-0.769394555	0.44165912	0.948437255
Ggnbp1	0.020991621	0.370198789	0.056703646	0.95478127	0.996070133
Bak1	0.037928615	0.172974904	0.219272357	0.826437894	0.992886758
Itpr3	-0.209354586	0.216118681	-0.968701942	0.332693923	0.917115058
Uqcc2	0.481985969	0.300094387	1.606114575	0.108248738	0.797912932
Ip6k3	-0.406797541	0.424339437	-0.958660699	0.337729703	0.919246575
Lemd2	0.002826824	0.152125052	0.01858224	0.985174371	0.998148029
9630028I04Rik	-0.31047223	0.271117921	-1.145155692	0.252144669	0.896939594
Grm4	-0.159215831	1.272917724	-0.125079436	0.900460663	0.994688009

Hmga1	3.403422213	2.134825904	1.594238765	0.110882567	0.800111791
AI413582	0.539915447	0.297580563	1.814350511	0.06962378	0.743458111
Nudt3	0.128803692	0.124576621	1.033931488	0.301168193	0.911262619
Rps10	0.318631416	0.279133049	1.141503727	0.253660362	0.897044747
Pacsin1	1.458097696	1.206957461	1.208077122	0.227017599	0.89398447
Spdef	-0.642082276	0.440776244	-1.456707988	0.145197004	0.842729381
D17Wsu92e	0.067471172	0.149920156	0.45004737	0.652676284	0.979004493
Snrpc	0.108750182	0.118205534	0.920009229	0.357567936	0.924354588
Uhrf1bp1	0.083948467	0.383731193	0.218768942	0.826830042	0.992886758
Taf11	0.033222572	0.122596978	0.270990136	0.786398619	0.990988839
Anks1	-0.204603079	0.21528928	-0.950363526	0.34192757	0.919850248
Tcp11	-0.20659138	0.250836678	-0.82360914	0.410161681	0.940983181
Zfp523	-0.019318131	0.181119548	-0.10665956	0.915059066	0.994688009
Def6	0.084963106	0.432071754	0.196641196	0.84410833	0.992886758
Ppard	-0.1458714	0.348753511	-0.418265035	0.675753351	0.983549699
Fance	0.24843141	0.169452957	1.466078933	0.142626784	0.840741274
Rpl10a	0.2041528	0.19064759	1.070838608	0.284242004	0.905916508
Tead3	-0.095927116	0.265273653	-0.361615693	0.717639236	0.988585656
Tulp1	0.350566671	0.797626829	0.439512136	0.66029049	0.979924124
Fkbp5	-0.640568064	0.686279672	-0.933392159	0.350617527	0.922338606
Clps	-0.217051718	1.958339208	-0.110834587	0.911747518	0.994688009
Srpk1	-0.065432033	0.187984377	-0.348071655	0.727786371	0.988585656
Slc26a8	-2.439481388	2.069390949	-1.178840271	0.238461785	0.896609492
Mapk14	0.002424561	0.137609126	0.017619184	0.985942652	0.998148029
Mapk13	0.00284601	0.162880442	0.017473	0.986059272	0.998148029
Brpf3	-0.254075811	0.446577731	-0.568939725	0.569397045	0.97144955
4930539E08Rik	-2.524103562	2.117646508	-1.191938103	0.233285516	0.89581803
Kctd20	0.018913977	0.116698542	0.16207552	0.871246384	0.992886758
Stk38	-0.003126875	0.139704703	-0.022382029	0.982143216	0.997390438
Srsf3	0.161626597	0.110260607	1.465859856	0.142686471	0.840741274
Trp53cor1	0.613988873	0.652607626	0.940823933	0.346795094	0.921552698
Cdkn1a	-0.307272347	0.358954661	-0.856019938	0.391986751	0.933734231
Rab44	-0.62275365	0.367000485	-1.69687419	0.089720449	0.769347579
Cpne5	0.574162916	0.661401974	0.868099791	0.385339705	0.931689378
Ppil1	0.169963261	0.127781008	1.330113627	0.183480836	0.869576666
BC004004	0.042220672	0.125446364	0.336563539	0.736445946	0.988585656
Pi16	0.145201125	0.274339419	0.529275471	0.596614369	0.974001549
Mtch1	0.169030192	0.095260852	1.774393035	0.075998161	0.752876039
Fgd2	0.016985836	0.193775902	0.087657112	0.930149208	0.994688009
Pim1	0.165473623	0.174194132	0.949937984	0.342143765	0.919850248
Tbc1d22b	-0.209374179	0.228388844	-0.916744333	0.359276629	0.925229839
Rnf8	-0.115233298	0.361633022	-0.318647056	0.749994167	0.988585656
Ccdc167	-0.546636857	0.653393925	-0.836611478	0.402810992	0.937834293
Mdga1	1.422150006	1.230111208	1.156114989	0.247634126	0.896939594
Zfand3	-0.192683753	0.136785675	-1.408654481	0.158937361	0.855986074
Btbd9	0.043269025	0.211858931	0.204235077	0.838169802	0.992886758
Gm6402	0.766137075	0.575024559	1.332355397	0.182743431	0.869194833
Glo1	0.144821956	0.099709925	1.452432698	0.14638133	0.843780493
1700097N02Rik	-1.950010053	1.594888276	-1.222662479	0.221457204	0.891315805
Dnah8	0.385439115	0.285903249	1.348145276	0.177611662	0.866855369
Glp1r	-0.050761686	0.424231127	-0.119655733	0.904755868	0.994688009
Abcg1	-0.176818875	0.195783171	-0.903136232	0.366453597	0.927834635
Tff2	0.054823994	0.317538053	0.172653303	0.862923951	0.992886758
Ubash3a	-0.359846068	0.964616041	-0.373045909	0.709114275	0.988585656
Rsph1	0.149335293	0.139385331	1.071384573	0.283996546	0.905916508
Slc37a1	0.312275827	0.325006235	0.960830265	0.336637519	0.919242242
Pde9a	0.125821922	0.158606855	0.79329435	0.427606348	0.944982247
Wdr4	0.101074525	0.156685856	0.645077527	0.518876946	0.96446859
Ndufv3	0.230590541	0.127952265	1.802160677	0.07152013	0.744227652
Pknox1	-0.034986104	0.314023606	-0.111412339	0.911289376	0.994688009
Cbs	-0.522016756	0.511919539	-1.019724225	0.307859269	0.9139204
U2af1	0.209814448	0.126974601	1.652412729	0.098450442	0.786907305

Cryaa	0.147037274	0.384056787	0.382852951	0.701828798	0.988482081
Sik1	-0.09962905	0.23162753	-0.430126116	0.667103904	0.981863224
Hsf2bp	-0.085626346	0.477359402	-0.179375007	0.857643255	0.992886758
Rrp1b	0.1079163	0.327506167	0.329509215	0.74177083	0.988585656
Pdxk-ps	1.845251186	2.024934176	0.911264775	0.362155882	0.926455466
Notch3	0.449667786	0.201617081	2.230305997	0.025727135	0.631530315
Ephx3	0.446010804	0.55364311	0.805592621	0.420477782	0.943415794
Brd4	0.641928427	0.267961517	2.395599322	0.016593218	0.566772949
Akap8	0.1277408	0.144956565	0.881235007	0.378190635	0.928883222
Akap8l	0.231373417	0.156270582	1.480594841	0.138714571	0.834874897
Wiz	0.055173503	0.481881703	0.114495949	0.908844657	0.994688009
Rasal3	-0.200327539	0.39489651	-0.507291235	0.611950487	0.974001549
Pglyrp2	0.541625141	0.700714837	0.772960856	0.439545547	0.947849924
Cyp4f39	-0.332411756	0.524778255	-0.633432793	0.526451056	0.965637248
Cyp4f17	0.166879808	0.431130405	0.387075015	0.698700674	0.987770663
Cyp4f16	0.032850763	0.186291034	0.176341085	0.860025987	0.992886758
Cyp4f15	-0.335591953	0.280467889	-1.196543227	0.23148464	0.895404344
Zfp871	-0.50634353	0.206583307	-2.451037975	0.014244492	0.552422395
Zfp811	-0.608841762	0.925792764	-0.657643682	0.510767119	0.963452765
Zfp799	0.212808898	0.187694309	1.133805807	0.256876015	0.897248195
Zfp870	0.215906926	0.152332121	1.417343403	0.156382551	0.855455553
Cyp4f13	0.241948782	0.260218553	0.929790666	0.35247948	0.923152465
Cyp4f41-ps	4.883457131	1.519586517	3.213674954	0.001310479	0.26039992
Zfp472	-0.138840462	0.154013034	-0.901485146	0.367330428	0.928436166
Zfp952	0.376833899	0.154954178	2.431905378	0.015019631	0.554936045
Zfp763	0.259292525	0.177599454	1.459984923	0.144294218	0.841934045
Zfp563	0.207505285	0.203908761	1.017637908	0.308850065	0.9139204
Zfp955a	-0.018581628	0.131728802	-0.141059721	0.887822763	0.992886758
Zfp955b	0.107393561	0.185847097	0.577859774	0.563358796	0.969658675
Zfp81	-0.290539537	0.278495989	-1.043244959	0.296834856	0.909070179
Zfp101	-0.032666331	0.205884173	-0.158663633	0.873933892	0.992886758
Adamts10	0.305680355	0.145200624	2.105227558	0.035271504	0.674795441
Myo1f	0.17239595	0.208535546	0.826698146	0.408408173	0.940123943
Zfp414	-0.345792023	0.249657555	-1.385065328	0.166032492	0.860029348
Pram1	0.973736062	1.013651241	0.960622375	0.336742074	0.919242242
Hnrnpm	-0.146789102	0.168557521	-0.870854652	0.38383352	0.931591679
Mar-02	0.136422351	0.128071489	1.065204696	0.286783309	0.906336252
Rab11b	0.015980059	0.173121006	0.092305721	0.926455143	0.994688009
Angptl4	0.395648819	0.262948295	1.504663945	0.132410497	0.827800491
Kank3	0.05641988	0.107662787	0.524042541	0.600248951	0.974001549
Rps28	0.196846508	0.149819818	1.313888319	0.18888379	0.875363508
Ndufa7	0.259219802	0.274444351	0.944525917	0.344900965	0.92132235
Cd320	0.096014672	0.21360272	0.449501168	0.653070166	0.979004493
Kifc1	0.019372062	0.789726279	0.024530096	0.980429778	0.997296835
Platr17	-0.647734248	0.867225059	-0.746904441	0.455121245	0.951224746
BC051226	0.457763702	0.257052729	1.780816346	0.074942458	0.75241101
Daxx	0.094408518	0.168335014	0.560837082	0.574908605	0.971613657
Zbtb22	-0.10579789	0.12751267	-0.82970492	0.406705639	0.939557159
Tapbp	0.017292723	0.155599938	0.111135796	0.911508664	0.994688009
Rgl2	-0.174559155	0.124054022	-1.407122095	0.159391187	0.856609583
Pfdn6	0.283949849	0.107467398	2.642195256	0.008237055	0.46696668
Wdr46	-0.006595198	0.217726035	-0.030291267	0.975834761	0.997262526
B3galt4	-0.05795339	0.205575831	-0.281907603	0.77801436	0.990988839
Rps18	0.337091433	0.277434867	1.215029087	0.224354998	0.892989918
Vps52	-0.020826233	0.23269495	-0.089500151	0.928684434	0.994688009
H2-K2	0.401473188	0.257978722	1.556225974	0.119654367	0.81558552
AA388235	0.275032626	0.391236741	0.702982613	0.482066585	0.957463006
H2-K1	0.153266806	0.136370771	1.123897776	0.261056395	0.897248195
Ring1	0.084511144	0.154276052	0.54779172	0.583834925	0.972333834
H2-Ke6	0.231088757	0.119799065	1.928969626	0.053734633	0.707153766
Slc39a7	0.2069796	0.114285242	1.811078974	0.070128628	0.744227652
Rxrb	0.151996711	0.106609297	1.425735986	0.153944565	0.854159937

Col11a2	1.521849388	1.144625252	1.329561257	0.18366287	0.869934344
H2-Oa	0.101143144	0.580379025	0.174270847	0.861652609	0.992886758
Brd2	0.090819742	0.08174926	1.110954913	0.266587757	0.89768067
H2-DMa	0.270612732	0.129632551	2.087536892	0.03683963	0.678243111
H2-DMb2	0.346265358	0.213220159	1.623980394	0.104379993	0.79155232
H2-DMb1	0.117048731	0.111200929	1.052587713	0.292529991	0.907334416
Psmb9	0.469086832	0.141885865	3.30608572	0.000946091	0.234042943
Tap1	0.027897195	0.18846357	0.148024335	0.882323563	0.992886758
Psmb8	0.437022242	0.123357806	3.542720616	0.000396022	0.139568962
Tap2	-0.01963355	0.238881785	-0.082189398	0.934496104	0.994688009
H2-Ob	0.476598154	0.193693462	2.460579461	0.013871285	0.545277966
H2-Ab1	0.322262935	0.13698285	2.352578693	0.018643744	0.587637867
H2-Aa	0.089820854	0.115957687	0.774600259	0.438575904	0.947816234
H2-Eb1	0.174349557	0.116134504	1.501272669	0.133285051	0.827800491
H2-Eb2	-0.060499742	0.391706054	-0.154451893	0.87725344	0.992886758
Btnl2	1.488834197	1.154162827	1.289968939	0.197061443	0.879755417
BC051142	-0.034154407	0.950353957	-0.035938617	0.971331304	0.996733101
Notch4	-0.178610946	0.228181749	-0.782757373	0.433769604	0.946679964
Gpsm3	-0.189597291	0.212793605	-0.890991489	0.372933736	0.928840865
Pbx2	-0.057139648	0.132639224	-0.430789972	0.666621092	0.981863224
Ager	0.188126095	0.17012891	1.10578558	0.268819338	0.89804647
Rnf5	0.17675167	0.125123623	1.412616303	0.157768573	0.855986074
Agpat1	-0.294541193	0.188393974	-1.56343213	0.117950988	0.814630194
Egfl8	0.023886069	0.380129663	0.062836635	0.949896593	0.995727234
Ppt2	-0.347960598	0.170222238	-2.044154763	0.04093826	0.679118604
Prrt1	0.302304764	1.588022021	0.1903656	0.849022652	0.992886758
Fkbpl	-0.100301512	0.269755894	-0.371823246	0.710024455	0.988585656
Atf6b	0.130518154	0.144085483	0.905838332	0.365021429	0.926556975
Tnxb	0.081756118	0.200338457	0.408089983	0.683207614	0.984738223
C4b	-0.15384087	0.273963952	-0.561536905	0.574431579	0.971613657
C4a	-0.964542639	1.390445616	-0.693693179	0.487874645	0.958288569
Stk19	0.141530187	0.14728447	0.960930823	0.336586952	0.919242242
Dxo	0.087034196	0.185283742	0.469734662	0.638544601	0.977795535
Skiv2l	-0.17580413	0.174148458	-1.009507243	0.31273143	0.914819219
Nelfe	0.101663614	0.153541876	0.662123043	0.507892369	0.963106419
Cfb	-0.495350737	0.376515009	-1.315620163	0.188301562	0.874826736
C2	-0.139332745	0.237263721	-0.587248418	0.557036879	0.968926429
Zbtb12	0.269350795	0.419730346	0.641723424	0.521052777	0.964784335
Ehmt2	-0.165350006	0.123444233	-1.339471288	0.180417295	0.868087032
Slc44a4	-0.550466588	0.247272433	-2.226154293	0.026003848	0.631530315
Neu1	-0.078586892	0.119425546	-0.658040882	0.510511862	0.963452765
1110038B12Rik	0.091379181	0.214138652	0.426729039	0.669576697	0.981863224
Snord52	0.73810675	1.764234103	0.41837234	0.675674906	0.983549699
Hspa1b	-0.287560395	0.550986622	-0.521900865	0.601739356	0.974001549
Hspa1a	-0.300204	0.640132512	-0.468971649	0.639089901	0.977832483
Hspa1l	-0.614739144	0.62528873	-0.983128457	0.32554422	0.91640698
Lsm2	-0.072173119	0.173627683	-0.415677487	0.677646017	0.983670411
D17H6S56E-5	-0.388118668	0.161591531	-2.401850302	0.016312382	0.563070331
Vars	-0.165742783	0.195491376	-0.84782657	0.396534563	0.934365683
Vwa7	0.0281068	0.721122177	0.038976474	0.968909145	0.996496354
Sapcd1	-0.126217941	2.020996728	-0.062453313	0.95020184	0.99573416
Msh5	0.161572446	0.388274369	0.416129568	0.677315194	0.983670411
Clic1	0.152000399	0.108198889	1.404824022	0.160073614	0.857316691
Ddah2	0.240436627	0.116393966	2.065713847	0.038855507	0.679118604
G6b	0.532087381	1.304541509	0.407873094	0.683366847	0.984738223
Ly6g6c	0.08426773	0.932650139	0.090352991	0.928006711	0.994688009
Ly6g6d	0.500605695	0.347493347	1.440619512	0.149692205	0.848779008
Ly6g6e	0.118921148	0.277292937	0.428864684	0.668021702	0.981863224
Ly6g6f	1.228844868	0.760424928	1.615997612	0.10609483	0.79419908
Abhd16a	0.033000691	0.094176624	0.350412765	0.726028948	0.988585656
Ly6g5b	-0.15008802	1.505062299	-0.099722131	0.92056493	0.994688009
Csnk2b	-0.091719843	0.102486761	-0.894943333	0.370817381	0.928436647

Gpank1	0.189501735	0.138936017	1.363949672	0.172583406	0.86244803
D17H6S53E	0.047984896	0.26250775	0.182794207	0.854959494	0.992886758
Bag6	-0.006027926	0.1342976	-0.044884839	0.964199102	0.996474415
Prrc2a	-0.006348731	0.17909895	-0.035448178	0.971722368	0.996733101
Aif1	0.215032719	0.233360174	0.921462797	0.356808856	0.924354588
Lst1	0.523344995	0.2667818	1.961696769	0.049797798	0.707153766
Ltb	0.321323922	0.233377633	1.376841124	0.168561373	0.861597949
Tnf	1.055036195	0.730574425	1.444118708	0.148705586	0.84832848
Lta	0.223686757	1.107668179	0.201943833	0.839960634	0.992886758
Nfkbil1	0.161542547	0.295793669	0.546132537	0.584974836	0.972892344
Atp6v1g2	0.315524897	0.399954118	0.788902733	0.430168858	0.945321484
Ddx39b	-0.038371491	0.131443521	-0.29192379	0.770344894	0.989990452
H2-D1	-0.090369872	0.147124076	-0.614242583	0.539055038	0.966900344
H2-Q1	0.23793527	0.394736946	0.602769193	0.546662244	0.967902958
H2-Q2	0.185729732	0.207306742	0.895917469	0.370296844	0.928436166
H2-Q4	0.188969473	0.153327771	1.232454316	0.217779422	0.887633697
H2-Q5	0.201302237	0.207411109	0.970547036	0.331773891	0.917115058
H2-Q10	0.420595124	0.396321401	1.06124757	0.288577406	0.907334416
Tcf19	-0.009867334	0.234431079	-0.042090555	0.966426509	0.996474415
Cchcr1	-0.055724442	0.392742643	-0.141885388	0.887170536	0.992886758
Cdsn	-1.171570892	0.844690537	-1.38698238	0.165447136	0.860011234
Dpcr1	-1.528484761	1.260711735	-1.212398298	0.225359952	0.893287677
Sfta2	0.042845977	0.179562551	0.238613098	0.811405608	0.991784898
Vars2	-0.044932223	0.210203954	-0.213755363	0.830737836	0.992886758
Gtf2h4	0.145139864	0.234326887	0.619390571	0.535659091	0.96674935
Ddr1	-0.113343396	0.191817491	-0.590891872	0.554592872	0.968926429
4833427F10Rik	-0.495692276	1.324164561	-0.374343409	0.70814884	0.988585656
Ier3	0.066406618	0.182170777	0.364529477	0.715462666	0.988585656
Flot1	0.106733307	0.106335014	1.003745648	0.315501228	0.91586731
Tubb5	-0.071916298	0.100689959	-0.71423505	0.475081847	0.956761005
Mdc1	0.064302745	0.246057796	0.261331874	0.793836589	0.990988839
Nrm	-0.510128031	0.282346693	-1.806743428	0.070802293	0.744227652
Ppp1r18	-0.132070818	0.1461499	-0.903666837	0.366172089	0.927617021
Dhx16	0.091438028	0.153589958	0.595338579	0.551617173	0.968926429
2310061I04Rik	-0.298484478	0.258392325	-1.15516	0.248024912	0.896939594
Atat1	0.045795316	0.231661987	0.197681618	0.843294173	0.992886758
Mrps18b	0.018811589	0.178106977	0.105619607	0.915884168	0.994688009
Ppp1r10	-0.173499508	0.216896874	-0.799917051	0.423758858	0.943632143
Mir1894	0.642367457	1.59357254	0.403098974	0.686875415	0.98538669
Abcf1	0.063685098	0.155446008	0.40969272	0.682031371	0.984738223
Prr3	-0.058654023	0.238161254	-0.246278613	0.805466564	0.99099448
Gnl1	0.019940366	0.190120452	0.104882804	0.916468804	0.994688009
A930015D03Rik	0.373788023	0.370991542	1.007537857	0.313676375	0.915639195
H2-T24	0.632852314	0.139254947	4.544558947	5.51E-06	0.006913907
H2-T23	0.274910877	0.113932606	2.412925392	0.015825059	0.557315333
C920025E04Rik	0.674390746	0.516429105	1.305872849	0.191595801	0.878456795
H2-T10	0.755541289	0.170010434	4.444087749	8.83E-06	0.008748514
2410017I17Rik	1.039402281	0.848621656	1.224812345	0.220645933	0.889722436
Gm8909	-0.446898803	0.541979941	-0.824567053	0.409617433	0.940886864
Gm6623	0.600718338	0.815676475	0.736466425	0.461446893	0.953083512
Rpp21	0.39051994	0.17732138	2.202328561	0.027642108	0.637644759
Trim39	-0.407234722	0.203671341	-1.999469929	0.045557532	0.69515685
Trim26	-0.036577987	0.173446142	-0.210889594	0.832973424	0.992886758
Trim15	0.669966753	0.40609627	1.649773226	0.098989327	0.787788403
Trim10	0.616693268	0.718491302	0.858316958	0.390717468	0.933734231
Trim40	1.532681878	1.276852166	1.200359696	0.229999675	0.895404344
Rnf39	0.055525702	0.327378295	0.169607157	0.865319093	0.992886758
Ppp1r11	0.29654928	0.169150929	1.753163767	0.0795739	0.75659671
Znrd1	-0.098583812	0.148782721	-0.662602562	0.507585129	0.963011338
Znrd1as	0.141423622	0.196685852	0.719033019	0.472120575	0.955003345
H2-M5	0.119040521	0.667273731	0.178398333	0.858410158	0.992886758
Zfp57	-0.420007159	0.229004913	-1.834053046	0.06664612	0.738254074

Mog	0.833105141	2.075739386	0.401353439	0.688159924	0.98538669
Gabbr1	0.113410747	0.221894524	0.511102056	0.609279596	0.974001549
Ubd	2.134825298	0.725762977	2.941491044	0.003266363	0.357710224
Olfr98	-2.275785993	2.186942328	-1.040624604	0.298049808	0.909779403
H2-M3	0.366894093	0.163470328	2.244407886	0.02480617	0.627796923
Olfr99	0.787170365	0.919414549	0.85616479	0.391906635	0.933734231
H2-M2	-1.498073095	0.940885093	-1.59219559	0.111340764	0.800752782
Rn45s	0.606351409	0.298143913	2.033754114	0.041976393	0.679118604
Crisp3	1.062824503	0.634411347	1.675292394	0.093876877	0.775671949
Crisp2	1.795132441	0.833646718	2.153349136	0.03129125	0.661395937
9130008F23Rik	0.184095176	0.270858758	0.679672228	0.496712024	0.960362776
Glyatl3	0.587264781	1.731563252	0.339152947	0.734494513	0.988585656
Cenpq	-0.000897927	0.216512986	-0.00414722	0.996691007	0.999449398
Mut	-0.265347486	0.150586011	-1.762099179	0.078052542	0.754202
Cd2ap	-0.101469908	0.122587521	-0.827734401	0.407820932	0.939557159
Tnfrsf21	-0.335890753	0.266419252	-1.260760064	0.207395306	0.883765092
E130008D07Rik	1.08385886	1.900482318	0.570307258	0.568469319	0.971380162
Adgrf5	-0.111551434	0.168731916	-0.661116384	0.508537679	0.963324595
Ankrd66	-0.31004	0.175823521	-1.763359067	0.077839949	0.754202
Pla2g7	-0.154206512	0.308605215	-0.499688614	0.617294351	0.974001549
1700071M16Rik	-1.30067025	1.619349134	-0.803205574	0.421855925	0.943415794
Slc25a27	-0.37477819	0.413942185	-0.905387765	0.365259995	0.926556975
Cyp39a1	0.026451749	0.170193755	0.155421383	0.876489128	0.992886758
Rcan2	-0.437364958	0.215658792	-2.028041404	0.042556022	0.68118841
Enpp5	-0.158501064	0.100280681	-1.580574271	0.113975413	0.807965062
Enpp4	-0.348336762	0.135678482	-2.567369246	0.010247343	0.499427958
Clic5	-0.108968564	0.12550731	-0.868224838	0.385271259	0.931689378
Runx2	0.162144169	0.585891489	0.276747781	0.78197378	0.990988839
Supt3	-0.125580392	0.183180256	-0.685556374	0.4929929	0.958916246
Cdc5l	-0.22113187	0.157864669	-1.400768588	0.161283285	0.857316691
B230354K17Rik	0.238666813	0.219179155	1.088912003	0.276192693	0.900302631
Spats1	0.274512148	1.805213492	0.152066307	0.879134639	0.992886758
Aars2	0.277280417	0.447483977	0.619643229	0.5354927	0.96674935
Tcte1	-0.727994532	0.536164237	-1.357782713	0.174532642	0.863528862
Nfkbie	-0.0849395	0.23521418	-0.36111556	0.718013062	0.988585656
Slc35b2	0.102192684	0.124120784	0.823332565	0.4103189	0.940983181
Hsp90ab1	-0.116345252	0.1219131	-0.954329375	0.339916944	0.919246575
Slc29a1	-0.155861416	0.15989392	-0.974780132	0.329669334	0.917115058
Capn11	0.647250921	1.605346774	0.403184491	0.686812508	0.98538669
Tmem63b	-0.055437856	0.176547496	-0.314011003	0.753512682	0.988585656
Mrpl14	0.379985906	0.259643295	1.463492081	0.143332779	0.84088907
Vegfa	-0.054783067	0.12814836	-0.427497215	0.669017213	0.981863224
Mrps18a	0.222853763	0.182237783	1.222873537	0.221377465	0.891315805
Rsph9	-0.052492149	0.122766223	-0.427578106	0.668958309	0.981863224
Mad2l1bp	0.090856403	0.180724087	0.502735436	0.615150292	0.974001549
Gtpbp2	0.081902102	0.134053408	0.610966203	0.541221955	0.967217841
Polh	-0.35538251	0.280395308	-1.267433868	0.205000196	0.883765092
Xpo5	-0.28376258	0.285775707	-0.99295557	0.320731607	0.91640698
Polr1c	0.373232511	0.153318077	2.434367288	0.014917849	0.554936045
Yipf3	-0.161201327	0.213754558	-0.754142172	0.450763857	0.949134998
Lrrc73	0.284938192	0.371152613	0.767711668	0.442658502	0.948476747
Tjap1	-0.151012792	0.268173201	-0.563116639	0.573355462	0.971613657
Dlk2	-1.655973483	1.495423748	-1.107360696	0.268138012	0.89804647
Abcc10	-0.200279975	0.451251037	-0.44383272	0.657163531	0.979614766
Zfp318	-0.055735345	0.247080333	-0.225575807	0.821531359	0.992886758
Crip3	-0.460423421	0.616929608	-0.746314353	0.455477543	0.951224746
Dnph1	0.614664891	0.314290056	1.955724911	0.050497569	0.707153766
Cul9	0.023394479	0.299272852	0.078171071	0.937691973	0.994688009
Srf	-0.230799685	0.1734805	-1.330407075	0.183384185	0.869576666
Ptk7	-0.000546221	0.133795381	-0.004082509	0.996742638	0.999449398
Klc4	-0.041157041	0.160742878	-0.256042701	0.797917865	0.990988839
Mrpl2	0.187343759	0.107512295	1.742533349	0.081415161	0.760884566

Cul7	-0.021041221	0.286288575	-0.073496545	0.941410993	0.994688009
Rrp36	0.282805857	0.125218652	2.258496261	0.023914737	0.622011301
Klhdc3	-0.050874478	0.140674225	-0.361647469	0.717615487	0.988585656
Mea1	0.382316492	0.189955377	2.012664755	0.04414991	0.688477147
Ppp2r5d	0.120861112	0.227596138	0.531033228	0.595395754	0.974001549
Pex6	-0.241983657	0.178879402	-1.352775411	0.17612739	0.865308446
Gnmt	0.408755128	0.265730005	1.538234749	0.123991228	0.820382953
Cnpy3	-0.000778415	0.17279927	-0.004504737	0.996405752	0.999379819
2310039H08Rik	0.404313346	0.191857722	2.107360295	0.035086361	0.674795441
Rpl7l1	0.00534512	0.10331088	0.051738206	0.958737294	0.996226826
Gltscr1l	0.378057112	0.318080784	1.188556907	0.234614074	0.89581803
Tbcc	-0.019919675	0.193242498	-0.103081232	0.9178985	0.994688009
Ubr2	-0.102716117	0.214493899	-0.478876638	0.632026392	0.977034429
Trerf1	-0.529784981	0.516139313	-1.026437956	0.304685195	0.912429485
Mrps10	0.194515671	0.170407958	1.14147058	0.253674148	0.897044747
Guca1b	-0.305285788	0.929596169	-0.328406891	0.742604033	0.988585656
Guca1a	-0.060247539	0.31743259	-0.189796326	0.849468735	0.992886758
1700001C19Rik	1.456959617	1.862805927	0.782131727	0.434137162	0.946762848
AI661453	-0.118452322	0.228230858	-0.519002222	0.603759192	0.974001549
Taf8	-0.070126136	0.162453872	-0.431667985	0.665982739	0.981863224
Ccnd3	-0.254594771	0.184937348	-1.376654168	0.168619195	0.861597949
Bysl	0.213090693	0.229704257	0.927674115	0.35357664	0.924098894
Med20	0.142799396	0.143622238	0.994270792	0.320091051	0.91640698
Usp49	0.195604474	0.418090725	0.467851742	0.639890616	0.977843986
Tomm6	-0.130216741	0.187274071	-0.69532712	0.486850322	0.958288569
Tomm6os	0.737218058	1.646805765	0.447665459	0.654394654	0.979133069
Prickle4	0.269272983	1.081843895	0.248901883	0.803436679	0.990988839
Frs3	-0.916203975	0.471050037	-1.945024741	0.051772001	0.707153766
Pgc	-0.832839046	0.764430475	-1.089489592	0.275938042	0.900302631
Tfeb	0.229918123	0.198939187	1.155720629	0.247795447	0.896939594
Mdfi	0.114781527	0.309362822	0.3710256	0.710618464	0.988585656
Foxp4	-0.160124944	0.250117599	-0.640198629	0.522043474	0.965307763
9830107B12Rik	-0.109481333	1.588688296	-0.068913036	0.945058842	0.99486515
A530064D06Rik	-0.144458003	0.76162682	-0.189670321	0.849567479	0.992886758
Trem1	-0.213131791	0.195930421	-1.087793257	0.276686387	0.900604936
Trem3	0.971890462	0.411444094	2.362144639	0.018169552	0.58167504
Treml4	0.117516935	0.343502283	0.342113986	0.732265113	0.988585656
Treml2	-0.198644615	0.355420117	-0.558900875	0.576229372	0.971613657
B430306N03Rik	-1.742505919	0.882711727	-1.974037352	0.048377496	0.706387941
Trem2	-0.320910429	0.462118974	-0.694432488	0.487411026	0.958288569
Treml1	1.27561335	0.466311079	2.735541592	0.006227772	0.427133716
Nfya	-0.200859237	0.187282943	-1.072490817	0.283499636	0.905916508
Oard1	0.247520086	0.13380074	1.849915672	0.064325705	0.73299549
Apobec2	-0.186763888	0.507752878	-0.367824382	0.713004188	0.988585656
Tspo2	0.426983094	0.804506849	0.530738917	0.595599714	0.974001549
Unc5cl	-0.643438423	0.50764103	-1.267506732	0.204974158	0.883765092
Mocs1	0.045487554	0.226684778	0.200664353	0.840961035	0.992886758
Daam2	-0.094715877	0.338764699	-0.279591932	0.779790598	0.990988839
Kif6	-0.3221531	0.313279116	-1.028326128	0.303796445	0.911786415
Rftn1	0.046244661	0.228309639	0.202552383	0.839484911	0.992886758
Dazl	0.003818775	1.14772356	0.003327261	0.997345235	0.99957296
Plcl2	0.049753291	0.231042216	0.215342857	0.829500019	0.992886758
Tbc1d5	-0.506296733	0.317112012	-1.596586427	0.110357926	0.800111791
Satb1	0.153288889	0.31119612	0.492579692	0.6223096	0.97514112
Efhb	0.20732558	0.367961613	0.563443502	0.573132922	0.971613657
Rab5a	-0.105779348	0.107959806	-0.979803055	0.327183344	0.916882624
Pp2d1	-0.451774796	1.271724995	-0.355245668	0.722405555	0.988585656
Kat2b	-0.083653527	0.107948751	-0.774937423	0.438376638	0.947776577
Sgol1	0.055525162	0.455773184	0.121826303	0.903036582	0.994688009
Sult1c2	1.010162253	1.78149735	0.567029893	0.570693872	0.971613657
Pot1b	0.17012768	0.197717313	0.860459193	0.389535969	0.933734231
Adgre4	0.712465129	0.277412516	2.568251572	0.010221295	0.499427958

Zfp119a	0.165259699	0.19781206	0.835437935	0.403471178	0.937834293
Zfp959	-0.085805298	0.189091751	-0.453775997	0.649990061	0.979004493
Zfp119b	0.096441414	0.175053486	0.550925416	0.581684804	0.971915898
Ebi3	0.108859463	0.988898811	0.110081499	0.912344741	0.994688009
Gm16712	0.297241465	1.41978175	0.209357153	0.834169438	0.992886758
Ccdc94	0.259847359	0.234989471	1.105782985	0.268820461	0.89804647
Shd	1.307369805	1.566281356	0.834696653	0.403888527	0.937834293
Stap2	0.034915031	0.165498221	0.21096922	0.83291129	0.992886758
Mpnd	0.162280603	0.11759271	1.380022651	0.167579671	0.860403806
Sh3gl1	0.007545592	0.1187889	0.063521023	0.949351619	0.995624732
Chaf1a	0.136077575	0.399797112	0.340366579	0.733580484	0.988585656
Ubxn6	0.259081916	0.151188877	1.713630792	0.086596555	0.765418158
Hdgfrp2	0.328237926	0.145495082	2.256007019	0.024070192	0.622811438
Plin4	-0.226560043	0.416374663	-0.54412543	0.586355162	0.972904784
Plin5	-0.050626327	0.269273486	-0.188010812	0.850868176	0.992886758
Lrg1	-0.396163979	0.334226452	-1.185316053	0.235892509	0.896067726
Sema6b	0.065619817	0.388108898	0.169075786	0.865737031	0.992886758
Tnfaip8l1	0.160571495	0.341882834	0.469668198	0.638592092	0.977795535
Mydgf	0.038027985	0.167269981	0.227344947	0.820155525	0.992886758
Dpp9	-0.297490632	0.239735993	-1.240909336	0.214639244	0.886400046
Fem1a	0.056576378	0.125612059	0.45040562	0.652417993	0.979004493
Ticam1	-0.567205034	0.283197255	-2.002862046	0.045192098	0.693980041
Plin3	0.070133669	0.122237906	0.573747308	0.56613881	0.970328402
Uhrf1	-0.689227949	0.497992656	-1.384012274	0.166354696	0.860082
Kdm4b	0.043414612	0.264947084	0.163861444	0.86984022	0.992886758
Ptprs	0.560397594	0.182104933	3.077333408	0.002088615	0.308737709
Gm20219	-0.171454321	0.315694795	-0.543101513	0.587059911	0.973286675
Safb2	-0.129447957	0.246271946	-0.525630138	0.599145212	0.974001549
Safb	0.035550978	0.216995054	0.163833127	0.869862512	0.992886758
2410015M20Rik	0.231702726	0.198178064	1.169164339	0.242337426	0.896939594
Rpl36	0.360566721	0.279488868	1.290093319	0.197018259	0.879755417
Lonp1	0.097369965	0.184686236	0.527218312	0.598041994	0.974001549
Catsperd	-0.609724359	0.556911536	-1.094831619	0.273590412	0.899317626
Ranbp3	-0.070703192	0.16442635	-0.429999161	0.667196252	0.981863224
Vmac	-0.124249852	0.165603907	-0.750283335	0.453084077	0.950108007
Ndufa11	0.172787099	0.187617149	0.920955786	0.357073511	0.924354588
Nrtn	-0.058442905	0.293903468	-0.198850681	0.84237955	0.992886758
Dus3l	0.039188962	0.118485973	0.330747692	0.740835074	0.988585656
Prr22	1.57449496	2.001899877	0.786500353	0.431574412	0.945941124
Rfx2	-0.075662621	0.380292051	-0.198959248	0.842294622	0.992886758
1700061G19Rik	0.04647335	0.686558448	0.067690304	0.946032168	0.995065399
Mllt1	-0.065870235	0.236401751	-0.278636833	0.780523543	0.990988839
Clpp	0.237337695	0.144895366	1.637993691	0.10142301	0.788783372
Alkbh7	0.26346993	0.194231172	1.35647603	0.174947757	0.864111192
Pspn	-0.153094247	1.158833376	-0.132110664	0.894896761	0.993592437
Gtf2f1	-0.225924991	0.188657438	-1.197540866	0.231095809	0.895404344
Khsrp	-0.098706194	0.202660332	-0.487052367	0.626221221	0.976534277
Slc25a23	-0.090215214	0.151842553	-0.594136569	0.552420773	0.968926429
Crb3	0.211735842	0.176990302	1.196313241	0.231574343	0.895404344
Dennd1c	-0.529825204	0.379483463	-1.396174684	0.162661898	0.859982992
Tubb4a	0.090320696	0.370779537	0.243596765	0.807543134	0.99099448
Gm11110	-0.116002218	0.45339503	-0.255852425	0.798064792	0.990988839
Tnfsf9	0.214094919	0.290615425	0.736694961	0.461307872	0.953083512
Tnfsf14	0.67163332	0.627969355	1.069532	0.284830017	0.905916508
C3	-0.329321993	0.298842618	-1.101991394	0.270465413	0.89804647
Gpr108	-0.008921612	0.160817448	-0.055476643	0.955758738	0.99614946
Trip10	0.036705411	0.094236158	0.389504536	0.696902956	0.987518602
Vav1	-0.190559807	0.349436252	-0.545334967	0.585523159	0.972904784
Adgre1	-0.231666604	0.243152147	-0.952763967	0.340709675	0.919850248
Nudt12	-0.073149328	0.210057637	-0.348234554	0.72766404	0.988585656
Efna5	-0.469793161	0.38294594	-1.226787157	0.219902599	0.888847883
Fbxl17	0.01682134	0.143163803	0.117497161	0.906466094	0.994688009

Fer	0.033079195	0.229706131	0.144006582	0.885495272	0.992886758
1110058D11Rik	1.645929441	1.600174322	1.028593834	0.303670577	0.911786415
Pja2	-0.103826328	0.133392015	-0.778354897	0.436359821	0.946762848
Man2a1	-0.21798143	0.156669001	-1.391350094	0.16411929	0.859982992
Tmem232	-0.496219513	0.195813571	-2.534142613	0.011272285	0.508404943
Vapa	-0.168178443	0.107219337	-1.568545818	0.116753806	0.812418014
Txndc2	0.914463227	1.826311318	0.500715961	0.616571037	0.974001549
Rab31	-0.080828202	0.099804958	-0.809861588	0.418019731	0.943415794
Ppp4r1	-0.173769735	0.135272579	-1.284589505	0.198935768	0.880661448
Ralbp1	-0.032973729	0.150918833	-0.218486509	0.827050069	0.992886758
Twsg1	-0.291901659	0.126388747	-2.309554192	0.020912848	0.604325792
Ankrd12	0.263998512	0.22087209	1.195255189	0.231987342	0.895404344
Ndufv2	0.080294642	0.193449382	0.41506797	0.678092146	0.983670411
Wash1	0.169990039	0.144779498	1.174130607	0.240342716	0.896939594
Ddx11	-0.075600148	0.453372055	-0.166750789	0.867566141	0.992886758
Mtcl1	0.207249521	0.157255444	1.317916351	0.187531651	0.873560421
Rab12	0.037261819	0.080248011	0.464333244	0.642409009	0.978415289
Ptprm	0.199999262	0.155373321	1.287217531	0.198018479	0.880239572
Lama1	0.142471304	1.364378809	0.104422103	0.916834383	0.994688009
Arhgap28	-0.482883471	0.249919617	-1.932155139	0.05334036	0.707153766
Epb4.1l3	-0.074301562	0.392892524	-0.189114218	0.850003298	0.992886758
Zbtb14	-0.213757543	0.146031934	-1.463772595	0.143256093	0.840741274
C030034I22Rik	-0.135613967	0.213944822	-0.633873563	0.52616334	0.965637248
Dlgap1	-0.397402369	1.748891284	-0.227231031	0.8202441	0.992886758
Tgif1	-0.318864928	0.196240778	-1.624865795	0.104191158	0.79155232
Myl12b	-0.031186069	0.121979929	-0.255665579	0.798209076	0.990988839
Myl12a	0.014759991	0.09369463	0.157532948	0.874824843	0.992886758
Myom1	0.267158009	0.236983149	1.12732914	0.259603347	0.897248195
Lpin2	-0.076438251	0.164287799	-0.465270408	0.641737823	0.97813956
Emilin2	0.032544652	0.527647576	0.061678766	0.95081865	0.99573416
Smchd1	0.300485757	0.180068708	1.668728341	0.09517123	0.778239075
Ndc80	0.824216403	0.340186038	2.422840186	0.0153997	0.557315333
Spdya	-0.04486519	0.857713242	-0.052307914	0.958283348	0.996226826
Trmt61b	0.093858152	0.264508068	0.354840412	0.722709152	0.988585656
Wdr43	-0.159557346	0.123777415	-1.289066717	0.197374893	0.87989762
Snord53	1.342216471	1.90624643	0.704114877	0.481361236	0.957463006
Fam179a	-0.342785722	0.183594299	-1.867082606	0.061890053	0.725730283
Clip4	-0.293611948	0.2305289	-1.273644858	0.202789307	0.882297774
Ypel5	0.105551185	0.114401047	0.922641771	0.35619392	0.924354588
Lbh	-0.011440058	0.165507968	-0.06912089	0.944893393	0.99486515
Lclat1	-0.204643991	0.254054899	-0.8055109	0.420524919	0.943415794
Capn13	0.100904482	0.837975341	0.12041462	0.904154711	0.994688009
Ehd3	-0.653352557	0.308975063	-2.114580224	0.034465741	0.674726795
Xdh	0.127749055	0.179351599	0.712282779	0.476289686	0.95716557
Memo1	-0.03861903	0.154611289	-0.249781436	0.802756377	0.990988839
Dpy30	0.291796375	0.196416362	1.485601161	0.137384648	0.833085238
Spast	0.035596953	0.185547562	0.191848131	0.847861168	0.992886758
Slc30a6	-0.035319788	0.179054951	-0.197256697	0.843626664	0.992886758
Nlrc4	0.19933002	0.576989393	0.345465658	0.729744324	0.988585656
Yipf4	0.021581339	0.093024064	0.231997376	0.816540052	0.992676801
Birc6	0.070088259	0.160678595	0.436201593	0.662690472	0.980760274
Ttc27	0.219948957	0.182032801	1.208292984	0.226934586	0.89398447
Ltbp1	0.128832512	0.20507462	0.628222604	0.529858125	0.965836062
Rasgrp3	-0.138175582	0.215069092	-0.642470662	0.520567632	0.964784335
Fam98a	-0.150628437	0.142829702	-1.054601632	0.291607557	0.907334416
Crim1	-0.014751475	0.162130994	-0.090984915	0.927504577	0.994688009
Fez2	0.015962775	0.147105001	0.108512799	0.913588929	0.994688009
Vit	-0.123379512	0.506091798	-0.243788799	0.807394396	0.99099448
Strn	-0.811303834	0.393395345	-2.062311728	0.039178067	0.679118604
Heatr5b	-0.124310941	0.38649073	-0.321640163	0.747725314	0.988585656
Gpatch11	0.122175789	0.188967296	0.646544621	0.517926709	0.964437658
Gm6548	0.051823407	0.195077528	0.265655442	0.790504583	0.990988839

Eif2ak2	0.046194708	0.222650245	0.207476563	0.835637694	0.992886758
Sult6b1	-0.390666205	1.496332232	-0.26108253	0.794028864	0.990988839
Cebpzos	0.152079098	0.39304046	0.386929881	0.698808119	0.987770663
Cebpz	-0.144155657	0.181476304	-0.794349753	0.426991847	0.944290206
Ndufaf7	0.121324721	0.125005363	0.970556127	0.331769362	0.917115058
Prkd3	-0.03575415	0.128961293	-0.277247142	0.781590343	0.990988839
Qpct	-0.199174216	0.186073437	-1.070406494	0.284436377	0.905916508
Cdc42ep3	0.111197664	0.112945601	0.984524085	0.324857895	0.91640698
Rmdn2	-0.012294881	0.158030978	-0.07780045	0.937986789	0.994688009
Cyp1b1	0.203153581	0.280523043	0.724195696	0.468945601	0.954315695
Atl2	-0.053899481	0.126240015	-0.426960354	0.669408204	0.981863224
Hnrnpll	-0.187190302	0.108166959	-1.730568227	0.083528803	0.76099108
Galm	0.231097798	0.197130107	1.172311025	0.241072207	0.896939594
Srsf7	0.257414706	0.143727202	1.790995041	0.073294091	0.748833631
Ttc39d	-0.338833591	0.687921205	-0.492547095	0.622332638	0.97514112
Gemin6	0.22483343	0.243116337	0.9247977	0.355071147	0.924354588
Dhx57	-0.229339994	0.23396077	-0.980249782	0.326962837	0.916882624
Morn2	-0.05801148	0.201293351	-0.288193721	0.773198455	0.990730604
Arhgef33	-0.904529043	0.684170222	-1.322081866	0.186140892	0.872305715
Gm10190	0.037370068	1.27847126	0.029230277	0.976680934	0.997262526
Sos1	-0.356801312	0.228847179	-1.559124796	0.118966846	0.814755048
Cdkl4	-0.464516789	0.182089848	-2.551030684	0.010740487	0.502706181
Map4k3	-0.028404284	0.107405692	-0.264457901	0.791427101	0.990988839
C230072F16Rik	0.057298097	0.225809011	0.253745841	0.799691917	0.990988839
Tmem178	-0.10252352	0.260698988	-0.393263972	0.694124525	0.986949593
Thumpd2	0.374807259	0.30886637	1.213493263	0.224941289	0.893236034
Slc8a1	0.095143602	0.176236276	0.539863891	0.589290902	0.974001549
Pkdcc	-0.077502019	0.28314715	-0.2737164	0.784302574	0.990988839
Eml4	-0.017404445	0.249072157	-0.069877119	0.944291465	0.99486515
Cox7a2l	0.250629905	0.149083915	1.681133104	0.092737064	0.775044718
Mta3	0.002213196	0.219427831	0.010086216	0.991952501	0.998783765
Haao	0.460727783	0.256492612	1.796261419	0.072452951	0.74633396
Zfp36l2	0.041648601	0.148016349	0.281378384	0.778420196	0.990988839
Thada	-0.007147866	0.314932821	-0.022696479	0.981892384	0.997390438
Plekhh2	0.273446727	0.25291027	1.081200569	0.279607905	0.902918961
Dync2li1	-0.120194648	0.118491699	-1.014371887	0.310405334	0.914704835
Abcg5	-0.879352557	1.760096525	-0.499604735	0.617353423	0.974001549
Lrpprc	0.28585843	0.248437818	1.150623654	0.249887098	0.896939594
1110020A21Rik	-0.135696164	0.445971234	-0.304271113	0.760921351	0.989266039
Ppm1b	0.096900999	0.119451838	0.811213967	0.417242806	0.943415794
Slc3a1	0.228631079	1.871635642	0.122155762	0.902775661	0.994688009
Prepl	-0.172812091	0.142852554	-1.209723498	0.22638501	0.89398447
Camkmt	-0.092517223	0.534683048	-0.173031899	0.862626354	0.992886758
Srbd1	0.223509466	0.235784103	0.947941203	0.343159387	0.920191602
Prkce	-0.300550578	0.206722427	-1.453884718	0.14597827	0.84337835
Epas1	0.255270136	0.134973541	1.891260572	0.058589569	0.72285319
Rhoq	-0.222261315	0.152806933	-1.454523758	0.145801151	0.842832198
Pigf	0.147851676	0.242646359	0.609329875	0.542305809	0.967491297
Cript	0.086293166	0.187035818	0.46137241	0.644531443	0.97889195
Socs5	-0.245200329	0.105594081	-2.322102978	0.020227391	0.600459306
Mcfd2	0.100037227	0.176166259	0.567856909	0.570132134	0.971613657
4833418N02Rik	-0.180665569	0.353917535	-0.51047363	0.609719684	0.974001549
Ttc7	-0.096233683	0.164438739	-0.585225136	0.558396345	0.968926429
Calm2	0.097983312	0.083614345	1.171848115	0.241258041	0.896939594
Epcam	-0.149814277	0.094963478	-1.577598882	0.114657789	0.809432539
Msh2	0.092204432	0.229779054	0.401274312	0.688218174	0.98538669
Kcnk12	-0.390564764	0.343256156	-1.137823044	0.25519438	0.897044747
Msh6	-0.617231038	0.293147018	-2.105534081	0.035244844	0.674795441
Fbxo11	-0.112444952	0.107831702	-1.042781945	0.297049295	0.909070179
Foxn2	0.12319386	0.192918269	0.638580582	0.523095817	0.965637248
Ppp1r21	-0.111350637	0.178394534	-0.624181888	0.53250814	0.96674935
Ston1	-0.188497401	0.30427381	-0.619499263	0.535587508	0.96674935

Lhcgr	0.469496596	1.364261303	0.344139788	0.730741162	0.988585656
Nrxn1	0.302119274	0.785146786	0.384793365	0.700390515	0.988482081
Mettl4	-0.07886793	0.232714525	-0.338904201	0.7346819	0.988585656
2700099C18Rik	0.003760822	0.549723379	0.006841299	0.994541476	0.999285699
Gm1976	-0.895983002	0.583585854	-1.535306238	0.124708623	0.821164769
2610044O15Rik8	0.114497089	0.188720125	0.606703124	0.544047949	0.967491297
Crem	-0.006401601	0.17901339	-0.035760457	0.971473364	0.996733101
Gm6225	0.54612322	0.707469272	0.771939138	0.440150475	0.947849924
Cul2	-0.086739339	0.128859798	-0.673129558	0.500864871	0.961175273
Bambi	-0.005754235	0.148020417	-0.038874604	0.968990364	0.996517112
Map3k8	0.127052388	0.236829052	0.536472984	0.591631703	0.974001549
4833419F23Rik	0.363824403	0.410869117	0.885499514	0.375887282	0.928883222
Mtpap	0.057419055	0.207193782	0.277127308	0.781682354	0.990988839
9430020K01Rik	-0.030318408	0.172296642	-0.175966332	0.860320393	0.992886758
Svil	-0.135365272	0.210872669	-0.641928955	0.520919313	0.964784335
Zfp438	0.02549716	0.350713985	0.072700722	0.942044274	0.994688009
Gm10125	-0.266336937	0.409378227	-0.650588917	0.515311887	0.964352198
Zeb1	-0.248841122	0.177647956	-1.400754214	0.161287584	0.857316691
Arhgap12	-0.115153924	0.139098644	-0.827857993	0.407750927	0.939557159
Kif5b	-0.298830836	0.136508912	-2.189093973	0.028590011	0.645679684
Epc1	-0.22638713	0.136241436	-1.661661362	0.096580692	0.781791257
Rab18	-0.020532167	0.084704332	-0.242398072	0.808471728	0.991020605
Mkx	0.462975963	0.605755407	0.764295221	0.444691333	0.948476747
Armc4	0.033080586	0.196250444	0.168563111	0.866140298	0.992886758
Mpp7	0.102351077	0.163679865	0.625312571	0.531765931	0.966606934
Wac	-0.064797488	0.098798334	-0.655856078	0.511916731	0.963452765
Fzd8	-0.521942337	0.404753576	-1.289531131	0.197213501	0.879755417
Ccny	-0.29463229	0.11684478	-2.521569978	0.011683243	0.518291414
Colec12	-0.0813287	0.11268554	-0.721731467	0.470459592	0.95486913
Thoc1	0.093883872	0.14453457	0.649559979	0.515976492	0.964352198
Usp14	-0.079991432	0.15246121	-0.524667436	0.599814399	0.974001549
Rock1	-0.089725584	0.12521739	-0.716558486	0.473646567	0.955974667
Greb1l	1.123881499	1.033068763	1.0879058	0.276636696	0.900604936
Esco1	0.097120766	0.105121594	0.923889782	0.355543704	0.924354588
Snrpd1	0.102428099	0.17231322	0.594429719	0.552224736	0.968926429
Abhd3	0.092661461	0.166973352	0.5549476	0.578930502	0.971613657
Mib1	-0.084768419	0.20588453	-0.411727967	0.680538824	0.984226822
1010001N08Rik	-0.033249906	0.359122896	-0.092586428	0.926232127	0.994688009
Gata6	0.0673095	0.227256486	0.296182965	0.767090357	0.989884648
Rbbp8	-0.503469906	0.197764235	-2.545808681	0.010902492	0.502706181
Gm6277	-0.885105055	0.721420942	-1.226891269	0.219863461	0.888847883
Cables1	-0.10211746	0.328883836	-0.310497049	0.756183003	0.989266039
Tmem241	-0.193333287	0.213643007	-0.90493618	0.365499198	0.926667174
Riok3	-0.088126861	0.08105235	-1.087283221	0.276911662	0.900604936
3110002H16Rik	-0.052298936	0.157313513	-0.332450371	0.739549208	0.988585656
Npc1	-0.148484287	0.178661544	-0.8310926	0.40592132	0.939557159
Ankrd29	-0.072335332	0.226438642	-0.319447825	0.74938695	0.988585656
Lama3	0.1552294	0.207806091	0.74699158	0.455068643	0.951224746
Ttc39c	-0.212802167	0.309906052	-0.686666701	0.492292785	0.958916246
Cabyr	-2.095181735	1.468445334	-1.426802677	0.153636779	0.854159937
Osbpl1a	-0.027150652	0.160677049	-0.16897654	0.865815094	0.992886758
Impact	-0.020243817	0.132636875	-0.15262586	0.878693331	0.992886758
Zfp521	-0.11086167	0.342999937	-0.323211924	0.746534751	0.988585656
Ss18	0.026795095	0.086482762	0.309831625	0.756689001	0.989266039
Psma8	-0.214448508	0.504104177	-0.42540514	0.67054136	0.982434419
Taf4b	-0.176821821	0.340240357	-0.51969679	0.603274925	0.974001549
Kctd1	0.180415673	0.228462357	0.789695403	0.429705677	0.945321484
4933424G05Rik	0.316892491	0.693276722	0.457093799	0.647603627	0.979004493
Aqp4	-0.023693707	0.220537505	-0.10743618	0.914442953	0.994688009
Chst9	0.363454393	1.46927062	0.247370626	0.804621405	0.990988839
Cdh2	-0.431205151	0.577533697	-0.746632021	0.455285714	0.951224746
Dsc2	-0.362499814	0.540014834	-0.671277512	0.502043758	0.961378071

Dsg2	-0.156816059	0.346084954	-0.453114351	0.650466402	0.979004493
Ttr	2.848215291	2.716794043	1.048373651	0.294466488	0.907334416
B4galt6	0.002912069	0.351715199	0.008279623	0.993393892	0.999270171
Trappc8	-0.153715281	0.128687383	-1.194486032	0.232287901	0.895404344
Rnf125	-0.484861658	0.326682989	-1.484196222	0.137756872	0.833085238
Rnf138	0.118670515	0.154015574	0.770509841	0.440997519	0.948068187
Garem	0.11539315	0.675822492	0.170744761	0.864424465	0.992886758
Klhl14	-0.62468528	1.560842241	-0.400223202	0.688992127	0.98538669
4930426D05Rik	0.89271053	1.327509582	0.672470122	0.501284455	0.961295666
Dtna	-0.254033071	0.198081366	-1.282468291	0.199678421	0.880888611
Gm15972	1.593879641	0.733239189	2.1737513	0.029723818	0.649459041
Mapre2	-0.05653415	0.178266971	-0.317131936	0.751143493	0.988585656
Zfp397	-0.199951679	0.154216433	-1.296565325	0.19478079	0.879588734
Zfp35	-0.094815198	0.13516718	-0.701466122	0.48301217	0.957463006
Zfp191	-0.100847122	0.102917394	-0.979884135	0.327143315	0.916882624
Ino80c	0.032812631	0.115347068	0.284468703	0.776051215	0.990861469
Galnt1	0.052418146	0.112369144	0.466481665	0.640870768	0.977921835
2700062C07Rik	0.075047964	0.233748303	0.321063142	0.748162541	0.988585656
Rprd1a	-0.319880177	0.158801625	-2.014338187	0.043974039	0.688477147
Slc39a6	-0.098306276	0.204450673	-0.480831267	0.630636426	0.977034429
Elp2	-0.138164153	0.23618044	-0.584994051	0.558551717	0.968926429
Mocos	0.052705893	0.167409754	0.314831673	0.752889462	0.988585656
Fhod3	-0.438012499	0.535485301	-0.817972964	0.413372625	0.943143495
Tpgs2	0.211212174	0.158921585	1.3290339	0.183836786	0.870505569
AW554918	-0.370121712	0.231286199	-1.600275821	0.109537408	0.798400564
Celf4	-0.500477961	0.379551525	-1.31860348	0.18730171	0.873455432
Pik3c3	-0.096633602	0.175663765	-0.550105494	0.582247019	0.972017845
Syt4	-4.581658768	1.674493019	-2.736146831	0.006216328	0.427133716
Slc25a46	-0.300352074	0.139909284	-2.146763007	0.031812154	0.661395937
Sap130	0.09825233	0.266172226	0.369130661	0.712030336	0.988585656
Ammecr1l	-0.353710328	0.15110577	-2.340812855	0.01924181	0.592187315
Polr2d	0.247221603	0.144139195	1.715158765	0.086316123	0.765083244
Wdr33	3.12E-05	0.155903726	0.000200126	0.999840323	0.999957126
Sft2d3	0.066339006	0.19421647	0.341572505	0.732672632	0.988585656
Lims2	-0.321666485	0.185659642	-1.732560086	0.083173889	0.76099108
Gpr17	-0.6368911	1.124277676	-0.566489145	0.57106131	0.971613657
Iws1	-0.166185896	0.207659156	-0.800282051	0.4235474	0.94351935
Proc	0.236088666	0.638473167	0.369770693	0.711553354	0.988585656
Map3k2	-0.071146546	0.158242246	-0.44960526	0.652995095	0.979004493
Ercc3	-0.095499178	0.168368477	-0.567203432	0.570575976	0.971613657
A830052D11Rik	2.200249156	0.970924599	2.266138028	0.023442934	0.622011301
Bin1	0.413677426	0.139871752	2.957548039	0.003100964	0.346777009
Gypc	-0.049271349	0.171601038	-0.287127338	0.77401482	0.990730604
Tslp	0.291910741	0.285583864	1.022154183	0.306707932	0.9139204
Wdr36	-0.053860932	0.139267171	-0.386745357	0.698944734	0.987770663
Camk4	0.381343476	0.563990527	0.676152271	0.498943972	0.960700483
Stard4	-0.115396649	0.237106238	-0.486687527	0.626479785	0.97658408
Nrep	0.297307037	0.158793028	1.872292758	0.061166112	0.725221283
Gm10549	0.304524903	0.99400254	0.306362299	0.75932881	0.989266039
Epb4.1l4aos	0.461109653	0.218411477	2.111196986	0.034755384	0.674795441
Epb4.1l4a	0.026651383	0.160223886	0.166338386	0.867890659	0.992886758
Gm10548	-0.42122559	0.689230808	-0.611153165	0.541098186	0.967211526
Apc	0.180957195	0.196488251	0.920956822	0.35707297	0.924354588
Srp19	0.329525478	0.166956522	1.973720309	0.048413555	0.706387941
Reep5	0.020857418	0.101560516	0.205369355	0.837283561	0.992886758
Pkd2l2	0.319258734	0.282704788	1.129300768	0.258770977	0.897248195
Fam13b	-0.529291777	0.175493273	-3.016023165	0.002561137	0.327099208
Nme5	-0.210702358	0.131503786	-1.602253172	0.109099636	0.798400564
4933408B17Rik	0.493954245	0.353037189	1.399156407	0.161766086	0.858353878
Brd8	0.118234996	0.206593323	0.572307925	0.567113382	0.970862413
Kif20a	-0.05811601	0.406002391	-0.143142038	0.886178005	0.992886758
Cdc23	-0.086387721	0.147461907	-0.585830755	0.557989254	0.968926429

Cdc25c	1.357196037	1.141595801	1.188858644	0.234495296	0.89581803
Fam53c	-0.539414324	0.168016069	-3.210492477	0.001325077	0.26039992
Kdm3b	-0.005931878	0.149571234	-0.039659219	0.968364814	0.996474415
Reep2	0.27534775	0.762680942	0.361026132	0.718079912	0.988585656
Egr1	0.692159258	0.753068951	0.91911804	0.358033834	0.924354588
Etf1	0.064802431	0.092323794	0.701903899	0.482739097	0.957463006
Hspa9	-0.164592224	0.141969366	-1.15935028	0.246313435	0.896939594
Ctnna1	-0.042381692	0.149088244	-0.284272526	0.776201539	0.990861469
Sil1	-0.029952792	0.13910133	-0.215330735	0.829509469	0.992886758
Snhg4	0.554569923	0.295068417	1.879462155	0.060181416	0.725221283
Matr3	-0.041871077	0.109299422	-0.383085989	0.701656008	0.988482081
Paip2	0.235064564	0.145607842	1.614367466	0.106447744	0.794899279
Slc23a1	-0.240670689	0.238145505	-1.010603536	0.312206223	0.914819219
Mzb1	0.358876735	0.255008532	1.407312655	0.159334698	0.856587955
Prob1	-0.128077139	0.27298636	-0.469170472	0.63894779	0.977795535
Spata24	0.286508445	0.226227863	1.266459585	0.20534859	0.883765092
Dnajc18	-0.065399644	0.154544471	-0.423176858	0.67216623	0.983233856
Ecscr	0.060791027	0.131451865	0.46245846	0.643752585	0.97889195
1700066B19Rik	0.676799919	0.486626295	1.390800138	0.164286041	0.859982992
Tmem173	-0.095661136	0.292560457	-0.326979034	0.743683743	0.988585656
Ube2d2a	0.00456811	0.109666417	0.041654589	0.966774055	0.996474415
Cxxc5	-0.001440935	0.239901104	-0.006006371	0.995207638	0.999285699
Nrg2	-0.025250573	0.432600108	-0.058369318	0.953454454	0.996070133
Pura	0.17883055	0.216034997	0.82778509	0.40779222	0.939557159
Igip	0.183065847	0.22974623	0.79681763	0.425556954	0.944290206
Cystm1	0.040352975	0.121079649	0.333276279	0.738925743	0.988585656
Pfdn1	0.244884504	0.167557854	1.461492246	0.143880402	0.841934045
Hbegf	-0.07885273	0.179685677	-0.438837033	0.660779625	0.979924124
Slc4a9	0.074691718	1.115503889	0.066957828	0.946615276	0.995172786
Ankhd1	-0.199980209	0.211419077	-0.945894819	0.344202236	0.920669678
Eif4ebp3	-0.138133355	0.399231768	-0.345997905	0.729344289	0.988585656
Sra1	0.148385018	0.127123893	1.167247274	0.243110525	0.896939594
Apbb3	-0.185801942	0.212426064	-0.874666408	0.381755443	0.930635634
Slc35a4	-0.156577617	0.222023274	-0.705230648	0.480666711	0.957463006
E230025N22Rik	0.4949502	0.524535603	0.943596958	0.345375647	0.921396763
Cd14	-0.023743784	0.166571646	-0.142543972	0.886650349	0.992886758
Tmco6	0.122756323	0.139153097	0.882167376	0.377686302	0.928883222
Ndufa2	0.432384066	0.250217469	1.728033092	0.083982292	0.76099108
Ik	-0.184401083	0.166377593	-1.108328834	0.267719827	0.89804647
Wdr55	0.122746504	0.167387709	0.733306551	0.463371481	0.953083512
Dnd1	0.191397773	0.68080378	0.281135003	0.778606855	0.990988839
Hars	-0.224716948	0.143399197	-1.567072561	0.117097732	0.812679331
Hars2	0.2827172	0.140436524	2.013131565	0.04410079	0.688477147
Zmat2	0.345380418	0.113651844	3.038933681	0.002374171	0.31402518
Pcdhac2	-1.025260367	0.670437748	-1.529240217	0.126204904	0.822401003
Pcdhb1	0.663786409	2.192701005	0.302725455	0.762099097	0.989285682
Pcdhb3	-0.722147644	0.818781922	-0.881978002	0.377788703	0.928883222
Pcdhb4	0.651460018	1.115269981	0.584127636	0.559134443	0.968926429
Pcdhb5	-0.184721476	0.912779261	-0.20237256	0.839625478	0.992886758
Pcdhb6	-1.002030817	1.927729084	-0.519798568	0.603203978	0.974001549
Pcdhb7	-1.331581333	0.477708029	-2.787437622	0.005312668	0.407229728
Pcdhb8	0.223526429	1.275614302	0.175230419	0.860898583	0.992886758
Pcdhb9	0.048963695	0.352040645	0.139085347	0.889382707	0.993043931
Pcdhb10	-0.699579479	1.137189092	-0.615183072	0.538433826	0.966776546
Pcdhb11	-0.267242552	1.037693123	-0.257535244	0.796765612	0.990988839
Pcdhb12	-0.159788441	0.642000875	-0.248891314	0.803444854	0.990988839
Pcdhb13	0.701478275	1.590759829	0.440970574	0.6592343	0.979924124
Pcdhb14	-0.391833896	0.6345736	-0.617475885	0.536920882	0.96674935
Pcdhb15	0.222907711	1.758527727	0.126758144	0.899131825	0.99447264
Pcdhb16	0.10607759	0.485067919	0.218686056	0.826894612	0.992886758
Pcdhb17	-0.77507107	0.407926872	-1.900024546	0.057429899	0.721735927
Pcdhb18	0.143441506	0.802780825	0.178680783	0.858188359	0.992886758

Pcdhb19	-1.09092335	0.792808853	-1.376023169	0.16881446	0.861597949
Pcdhb20	-0.263687188	0.429856485	-0.613430756	0.539591555	0.966900344
Pcdhb21	-0.06964408	1.717747865	-0.040543831	0.967659563	0.996474415
Pcdhb22	-0.780066064	0.815308869	-0.956773677	0.338681501	0.919246575
Slc25a2	0.036688382	1.776342003	0.020653896	0.983521747	0.997698637
Taf7	-0.128177403	0.154240605	-0.831022432	0.405960957	0.939557159
Pcdhga1	0.079516549	1.369019385	0.058082851	0.953682634	0.996070133
Pcdhga2	1.267780086	1.993091048	0.636087392	0.524719463	0.965637248
Pcdhga3	-1.364796467	1.478325237	-0.92320447	0.35590066	0.924354588
Pcdhga4	0.391973536	0.600043502	0.653241863	0.513600365	0.964352198
Pcdhgb2	-0.178373766	0.766734368	-0.23264089	0.816040273	0.99258657
Pcdhga5	-0.074970069	1.276552978	-0.058728522	0.953168342	0.996070133
Pcdhga6	0.068267252	0.97878076	0.069747236	0.944394845	0.99486515
Pcdhga7	0.348256524	1.27621111	0.272883162	0.78494303	0.990988839
Pcdhgb4	0.051730624	1.266723919	0.040838121	0.967424949	0.996474415
Pcdhga8	-0.061587753	1.503289744	-0.040968651	0.967320888	0.996474415
Pcdhgb5	-0.674404162	1.201557616	-0.561274926	0.574610132	0.971613657
Pcdhga9	4.003262114	2.234907169	1.79124313	0.073254288	0.748833631
Pcdhgb6	-0.296776154	0.737967362	-0.402153495	0.687571065	0.98538669
Pcdhga10	0.329278492	0.824083059	0.399569544	0.689473592	0.985586538
Pcdhgb7	0.232249998	0.930109565	0.249701763	0.802817995	0.990988839
Pcdhga11	0.857904483	0.969998137	0.884439311	0.37645911	0.928883222
Pcdhgb8	-0.096996847	3.01897857	-0.032129028	0.974369154	0.997262526
Pcdhga12	-0.040031615	1.268730179	-0.031552504	0.97482892	0.997262526
Pcdhgc3	-0.606264055	0.288914899	-2.09841741	0.035868289	0.674795441
Pcdhgc4	-1.229869361	1.632491692	-0.753369445	0.451227938	0.949134998
Diap1	-0.095650057	0.183453975	-0.521384488	0.602098955	0.974001549
Hdac3	-0.107434021	0.10004754	-1.073829706	0.282899013	0.905916508
Fchsd1	0.804090063	0.420783259	1.910936439	0.05601275	0.715507996
Arap3	-0.052497184	0.143283551	-0.366386678	0.714076559	0.988585656
Pcdh1	0.085644206	0.203707186	0.420428003	0.674172815	0.983375962
1700086O06Rik	-0.086724031	0.448943416	-0.193173634	0.846822987	0.992886758
0610009O20Rik	-0.053048985	0.113625938	-0.466873902	0.640590098	0.977921835
Pcdh12	0.210881992	0.165477005	1.274388497	0.202525767	0.88210274
Rnf14	-0.025577602	0.085086988	-0.300605329	0.763715467	0.989331656
Gnpda1	-0.086369677	0.228853973	-0.377400818	0.705875759	0.988585656
Ndfip1	-0.003943625	0.087879659	-0.044875286	0.964206716	0.996474415
Spry4	-0.552616178	0.282285895	-1.957647151	0.050271431	0.707153766
Fgf1	0.043651695	0.097330463	0.448489545	0.65379993	0.979133069
Gm5820	0.343385797	0.439371776	0.781538133	0.434486058	0.946762848
Arhgap26	0.24323518	0.261210188	0.931185653	0.351757538	0.923082865
Nr3c1	-0.199804891	0.115189877	-1.734569879	0.082817018	0.76099108
Yipf5	-0.048257329	0.108748327	-0.443752379	0.657221622	0.979614766
Prelid2	0.46505446	0.270926667	1.716532617	0.086064603	0.763683033
Sh3rf2	-0.191590095	0.285689411	-0.670623719	0.502460269	0.961630384
Lars	-0.016891207	0.136093833	-0.124114421	0.90122468	0.994688009
Gm4013	0.277044421	0.306056848	0.905205759	0.365356392	0.926556975
Rbm27	0.024950986	0.199944292	0.124789689	0.900690051	0.994688009
Tcerg1	0.205145133	0.173842165	1.18006545	0.237974185	0.896609492
Ppp2r2b	-0.184928836	0.385368716	-0.47987506	0.631316236	0.977034429
Stk32a	-0.265782086	0.408796103	-0.650158071	0.515590122	0.964352198
Dpysl3	-0.032520624	0.231291792	-0.140604313	0.888182541	0.992886758
Scgb3a2	0.113044241	0.255251627	0.442873734	0.657857067	0.979614766
Spink5	0.184177338	0.342851597	0.537192593	0.591134587	0.974001549
Dcp2	0.04801082	0.167415298	0.286776781	0.774283243	0.990730604
Mcc	-0.003036067	0.218846007	-0.013873073	0.988931244	0.998182772
A930012L18Rik	-0.57505329	0.649891021	-0.884845723	0.376239845	0.928883222
Ythdc2	0.01929718	0.221007001	0.087314791	0.930421297	0.994688009
Kcnn2	0.022010029	0.747997386	0.029425275	0.976525415	0.997262526
A330093E20Rik	-0.573098339	1.779108441	-0.322126705	0.747356708	0.988585656
Trim36	-0.103750079	0.397898599	-0.260745023	0.794289143	0.990988839
Pggt1b	0.298538246	0.185106451	1.612792225	0.106789656	0.794908043

Ccdc112	-0.031231622	0.303253176	-0.102988607	0.917972013	0.994688009
Mospd4	1.660096109	1.684291529	0.98563466	0.324312423	0.91640698
Fem1c	-0.443481709	0.225860414	-1.963521194	0.049585644	0.707153766
Ticam2	-0.338424878	0.378386931	-0.89438839	0.371114122	0.928788832
Tmed7	-0.032689255	0.107956203	-0.302801078	0.762041462	0.989285682
Eif1a	-0.110038414	0.13164957	-0.835843321	0.403243052	0.937834293
Cdo1	0.06920746	0.12022355	0.575656435	0.564847427	0.969658675
Atg12	0.09134079	0.129451411	0.705599035	0.480437525	0.957463006
Ap3s1	0.12919774	0.186474889	0.692842562	0.488408359	0.958481967
Lvrn	-1.098222127	0.700727415	-1.567260113	0.117053905	0.812679331
Commd10	0.123554608	0.102770324	1.202240127	0.229270507	0.895404344
Sema6a	0.220087654	0.30792809	0.714737176	0.474771461	0.956387791
Dtwd2	-0.495950774	0.327830048	-1.512828912	0.130323123	0.827800491
Dmxl1	-0.042421818	0.184278835	-0.230204506	0.81793286	0.992734473
Tnfaip8	0.031130641	0.123268218	0.252543934	0.80062066	0.990988839
Hsd17b4	0.048382428	0.155472462	0.311196128	0.755651526	0.989266039
Prr16	0.148380372	0.519415543	0.285667947	0.775132457	0.990861469
Srfbp1	0.140264215	0.216405077	0.648155841	0.51688416	0.964352198
Lox	-0.236685347	0.200882788	-1.178226116	0.238706474	0.896609492
Zfp474	-0.296295922	0.192562513	-1.538699916	0.123877573	0.820382953
Sncaip	-0.271967962	0.599843257	-0.453398382	0.650261901	0.979004493
Snx2	0.10207581	0.101138611	1.009266474	0.312846854	0.914819219
Snx24	0.133026748	0.265423804	0.501186203	0.616240084	0.974001549
Ppic	0.067950019	0.132942287	0.511124187	0.6092641	0.974001549
Prdm6	-0.119800567	0.281809465	-0.425111935	0.670755078	0.982434419
Cep120	-0.10137696	0.165590162	-0.612216082	0.540394803	0.966989525
Csnk1g3	-0.142705473	0.169954845	-0.839666989	0.401095128	0.937534739
Zfp608	0.224718961	0.213063444	1.054704442	0.29156052	0.907334416
Gramd3	-0.003842551	0.176880574	-0.021723984	0.982668131	0.997390438
Aldh7a1	0.073637219	0.161717629	0.455344413	0.648861479	0.979004493
Phax	-0.262912483	0.176985013	-1.485507039	0.13740956	0.833085238
Lmnb1	-0.059280923	0.284392378	-0.208447651	0.834879454	0.992886758
Mar-03	0.617568471	0.275276049	2.24345152	0.024867713	0.627796923
C330018D20Rik	0.192646758	0.187866453	1.025445232	0.305153155	0.912948451
Megf10	-0.367391441	1.020236304	-0.360104261	0.718769166	0.988585656
Prrc1	-0.07060097	0.152045241	-0.464341859	0.642402838	0.978415289
Ctxn3	-0.143826589	1.238800327	-0.11610151	0.907572092	0.994688009
1700011I03Rik	-1.771583523	2.451806211	-0.722562621	0.46994864	0.95486913
4930511M06Rik	-1.144918703	0.934715196	-1.224885085	0.220618521	0.889722436
Slc12a2	-0.038929826	0.117882998	-0.330241226	0.741217699	0.988585656
Slc27a6	-0.19090561	0.356440462	-0.535589055	0.592242595	0.974001549
Isoc1	0.046346374	0.107316295	0.431867071	0.665838029	0.981816968
Adamts19	0.780732518	1.632727843	0.478176765	0.632524399	0.977034429
A730017C20Rik	-0.298246519	0.583383332	-0.511235929	0.609185863	0.974001549
Chsy3	-0.564110909	0.570122658	-0.989455342	0.322440409	0.91640698
Gm4951	0.236764411	0.25757751	0.919196752	0.35799267	0.924354588
Gm4841	0.175628732	1.208334259	0.145347805	0.884436273	0.992886758
F830016B08Rik	0.234431047	1.005346876	0.23318424	0.815618344	0.992517008
Iigp1	0.502052766	0.175674853	2.857852206	0.00426519	0.385355746
Smim3	0.076353297	0.14977586	0.509783733	0.610202984	0.974001549
Dctn4	-0.059098972	0.107996993	-0.547227942	0.584222142	0.972333834
Rbm22	-0.130516921	0.135248663	-0.965014497	0.334537551	0.918030088
Synpo	-0.015349759	0.132725264	-0.115650621	0.907929443	0.994688009
Ndst1	-0.136683864	0.222431778	-0.614497916	0.53888635	0.966875138
Rps14	0.545506618	0.308740764	1.766875911	0.077249012	0.753431673
Cd74	-0.091930111	0.204632257	-0.449245453	0.653254602	0.979004493
Tcof1	-0.474183397	0.249442387	-1.90097362	0.057305468	0.721735927
Arsi	1.037725948	0.94447689	1.098730904	0.271885458	0.89804647
Camk2a	0.192435176	0.883290993	0.217861585	0.827536959	0.992886758
Pdgfrb	-0.122007486	0.248171143	-0.491626401	0.622983476	0.975491629
Csf1r	-0.161894707	0.224932008	-0.719749531	0.471679224	0.95486913
Hmgxb3	-0.152781019	0.179670292	-0.850341015	0.39513552	0.933933717

Slc26a2	-0.214494543	0.280925503	-0.763528198	0.445148452	0.948476747
Ppargc1b	0.329505158	1.783205009	0.184782544	0.8533996	0.992886758
Arhgef37	-1.004422056	0.897300283	-1.119382302	0.262977078	0.897248195
Csnk1a1	-0.030422398	0.088461452	-0.343905709	0.730917199	0.988585656
Bvht	0.448134862	0.217371173	2.061611277	0.03924476	0.679118604
Mir143hg	0.533378675	0.812290525	0.656635352	0.511415412	0.963452765
Il17b	0.177402564	2.200308144	0.080626236	0.935739201	0.994688009
Pcyox1l	0.077812578	0.379223035	0.205189483	0.837424086	0.992886758
Grpel2	-0.095050965	0.134549652	-0.706437838	0.479915897	0.957463006
1500015A07Rik	0.328916857	0.441749602	0.744577597	0.456527116	0.951472839
Afap1l1	-0.121356466	0.139753684	-0.868359692	0.385197454	0.931689378
Ablim3	-0.183486697	0.188410717	-0.9738655	0.330123326	0.917115058
Sh3tc2	-0.124522858	0.183447088	-0.678794413	0.497268134	0.960475196
Adrb2	-0.198497644	0.209071562	-0.949424408	0.342404801	0.919850248
Htr4	-0.248900289	2.121121107	-0.117343743	0.906587663	0.994688009
Fbxo38	-0.188804382	0.19923814	-0.947631723	0.343316971	0.920191602
2700046A07Rik	1.247305375	0.901232789	1.383999106	0.166358728	0.860082
Apcdd1	0.004556982	0.29743288	0.015321043	0.987776054	0.998148029
Napg	0.142702037	0.117601819	1.213433932	0.22496396	0.893236034
Piezo2	0.437379148	0.223052443	1.960880333	0.049892984	0.707153766
Txnl1	0.014975111	0.131697649	0.113708262	0.909469063	0.994688009
Wdr7	0.08022591	0.249609145	0.321406132	0.747902637	0.988585656
Fech	-0.032744917	0.123639182	-0.264842553	0.791130754	0.990988839
Nars	0.107561923	0.118177486	0.910172713	0.362731432	0.926455466
Atp8b1	-0.094532845	0.188283637	-0.502076796	0.615613502	0.974001549
Nedd4l	-0.116614448	0.214065526	-0.544760522	0.585918234	0.972904784
Malt1	-0.090682045	0.205577147	-0.44110956	0.659133683	0.979924124
Zfp532	0.168453043	0.268695994	0.626928002	0.530706431	0.966026275
Oacyl	0.890485567	1.818212321	0.489758846	0.624304556	0.975969024
Sec11c	0.256117876	0.270027165	0.948489296	0.34288042	0.919850248
Grp	-0.197250682	0.318843189	-0.618644803	0.53615038	0.96674935
Lman1	-0.29972977	0.127649575	-2.348067123	0.01887112	0.590246276
Ccbe1	-0.224237103	0.296073321	-0.757370177	0.448828118	0.948476747
Pmaip1	0.113078762	0.228863292	0.494088679	0.621243552	0.97514112
Gnal	-0.142617124	0.211645184	-0.67385008	0.500406632	0.9611436
Chmp1b	-0.211561252	0.11577757	-1.827307768	0.067653508	0.742385562
Mppe1	0.150436002	0.163272115	0.921382087	0.356850977	0.924354588
Impa2	0.270239315	0.220161584	1.22745899	0.219650127	0.888847883
B430212C06Rik	0.680642892	1.170074823	0.58170886	0.560762802	0.968926429
Cidea	0.500214601	0.483140294	1.035340266	0.300510031	0.910285132
Tubb6	0.076734652	0.295832366	0.259385588	0.795337749	0.990988839
Afg3l2	-0.322001864	0.186282769	-1.728564943	0.083886988	0.76099108
Slmo1	-0.924387978	1.688917117	-0.547325839	0.584154895	0.972333834
Spire1	-0.184710096	0.244998398	-0.753923689	0.450895045	0.949134998
Cep76	-0.378972657	0.237822909	-1.593507785	0.111046323	0.800111791
Psmg2	-0.073596408	0.1506856	-0.488410355	0.625259209	0.976218048
Ptpn2	-0.063484505	0.121403191	-0.522922865	0.601027932	0.974001549
Seh1l	0.032529223	0.132047984	0.246343955	0.805415987	0.99099448
Cep192	-0.421481758	0.355200275	-1.18660313	0.235384201	0.896002526
Ldlrad4	-0.112508128	0.242692939	-0.463582208	0.642947104	0.978681333
Fam210a	-0.22516535	0.158806719	-1.417857833	0.156232275	0.855455553
Rnmt	0.060784508	0.168507342	0.360723204	0.718306376	0.988585656
Tcf4	0.091598549	0.160508841	0.570676036	0.568219266	0.971380162
Ccdc68	0.198177529	0.184529324	1.073962256	0.282839598	0.905916508
Rab27b	-0.020105662	0.140179061	-0.143428424	0.885951836	0.992886758
4930503L19Rik	0.052448237	0.208423971	0.251642059	0.801317746	0.990988839
Stard6	0.528343304	0.286080319	1.84683555	0.064770978	0.733548823
Poli	0.318072799	0.352635963	0.901986276	0.367064158	0.928284409
Mbd2	0.034107628	0.084840649	0.402019889	0.687669389	0.98538669
Mex3c	-0.258454281	0.132582312	-1.949387341	0.051249185	0.707153766
Smad4	-0.247998051	0.103192476	-2.403257097	0.016249758	0.562097048
Elac1	0.201798461	0.150543734	1.340464036	0.180094529	0.868087032

Me2	-0.000778922	0.180203493	-0.004322459	0.996551187	0.999428209
Mapk4	0.145888868	1.412193677	0.103306558	0.917719671	0.994688009
Ska1	0.125164699	0.381352126	0.32821293	0.742750672	0.988585656
Cxxc1	-0.208594419	0.175068927	-1.191498814	0.233457822	0.89581803
Mbd1	-0.609120838	0.217893522	-2.795497695	0.005181987	0.406593461
Cfap53	-0.054774791	0.200722505	-0.27288814	0.784939203	0.990988839
Myo5b	0.15538286	0.128806997	1.206323133	0.227692922	0.894410876
Acaa2	0.266858488	0.1173804	2.27345016	0.022999066	0.620006604
Lipg	-0.269947999	0.164232598	-1.64369317	0.100239597	0.788783372
Rpl17	0.288697256	0.303742553	0.950466942	0.341875043	0.919850248
BC031181	0.109868831	0.129579882	0.847884944	0.39650205	0.934365683
Dym	-0.075105291	0.212667604	-0.35315812	0.723969903	0.988585656
Smad7	0.06206209	0.125971473	0.492667807	0.622247328	0.97514112
Ctif	0.009169505	0.333959295	0.027456954	0.978095273	0.997262526
Zbtb7c	0.243126944	0.531391113	0.457529188	0.647290728	0.979004493
Smad2	-0.062842019	0.129005262	-0.487127562	0.626167936	0.976534277
Ier3ip1	0.114949134	0.119748766	0.959919157	0.337095904	0.919246575
Hdhd2	0.065961963	0.110840246	0.595108415	0.551771004	0.968926429
Katnal2	-0.139081758	0.4266681	-0.325971775	0.74444571	0.988585656
Pias2	0.027530469	0.108373378	0.254033508	0.799469671	0.990988839
8030462N17Rik	-0.314966993	0.208106302	-1.513490898	0.130155011	0.827800491
Haus1	0.487337334	0.181685424	2.682313868	0.007311483	0.452031612
Atp5a1	-0.014809184	0.074705234	-0.198234887	0.842861293	0.992886758
Pstpip2	0.389983862	0.329773322	1.182581595	0.23697501	0.896609492
Epg5	0.304570505	0.3515205	0.866437391	0.386250346	0.932501372
Slc14a1	0.029930859	0.485951657	0.061592256	0.950887544	0.99573416
Slc14a2	-3.508327665	2.576726962	-1.361544206	0.173341774	0.863528862
Setbp1	-0.11980189	0.310547058	-0.385776926	0.699661885	0.988393777
Pard6g	-0.078406104	0.114765842	-0.68318328	0.494491036	0.958968423
Adnp2	-0.199185772	0.150430554	-1.32410449	0.185468345	0.871716376
Rbfa	0.254373195	0.177352121	1.434283354	0.151491419	0.851721899
Txnl4a	-0.000703225	0.089944459	-0.007818438	0.993761852	0.999270171
Hsbp1l1	0.470580549	0.428158301	1.099080756	0.271732843	0.89804647
Pqlc1	0.033741683	0.134185013	0.251456422	0.80146125	0.990988839
Kcng2	-0.214952081	0.737831774	-0.29132939	0.770799411	0.989990452
Ctdp1	-0.093683706	0.234183343	-0.400044275	0.689123907	0.98547461
Nfatc1	0.03485917	0.189238133	0.184207956	0.853850318	0.992886758
Atp9b	-0.158917215	0.148666839	-1.068948636	0.285092815	0.905916508
Mbp	-0.056578355	0.189102465	-0.29919417	0.764791897	0.9895982
Zfp236	-0.147534929	0.224022705	-0.658571323	0.510171083	0.963452765
Zfp516	0.44214739	0.285313289	1.54969084	0.121215738	0.816233249
Tshz1	-0.247919887	0.25175408	-0.984770084	0.324737018	0.91640698
Zadh2	0.023110196	0.104835272	0.220442946	0.825526202	0.992886758
Zfp407	-0.338865971	0.320988722	-1.055694323	0.291107892	0.907334416
Cndp2	0.031421175	0.169950186	0.184884618	0.853319536	0.992886758
Cyb5a	0.20774607	0.16098961	1.290431537	0.196900868	0.879755417
Fbxo15	0.137408917	0.62682351	0.219214683	0.826482818	0.992886758
Timm21	0.098354575	0.145661816	0.675225518	0.499532497	0.960847835
Neto1	0.46590698	2.368441472	0.196714584	0.844050896	0.992886758
Socs6	0.026111024	0.25796906	0.101217658	0.919377679	0.994688009
Rttn	0.173169662	0.385471127	0.449241589	0.653257389	0.979004493
Cd226	0.589989366	0.287041714	2.055413332	0.039839099	0.679118604
Tmx3	-0.110545307	0.119102468	-0.928152949	0.353328237	0.923786159
Ighmbp2	-0.15450825	0.330556744	-0.467418236	0.640200678	0.977921835
Mrpl21	0.330598984	0.173016495	1.910794598	0.056030982	0.715507996
Cpt1a	0.26417157	0.193909498	1.362344664	0.173089138	0.863084695
Mtl5	-0.628125428	0.656385497	-0.956945927	0.338594548	0.919246575
Gal	0.052581216	0.390236944	0.134741767	0.892816049	0.993300147
Ppp6r3	-0.116990782	0.12153245	-0.962629993	0.33573324	0.918908218
Lrp5	0.05610627	0.292461452	0.191841589	0.847866293	0.992886758
1810055G02Rik	-0.221602375	0.27557132	-0.804156161	0.421306793	0.943415794
Suv420h1	-0.09667814	0.133812268	-0.722490854	0.469992747	0.95486913

Chka	-0.110148133	0.114248516	-0.964109968	0.334990796	0.918190441
Tcirg1	-0.213061642	0.204232682	-1.04322991	0.296841824	0.909070179
Ndufs8	0.068394733	0.148548847	0.460419146	0.645215394	0.979004493
Aldh3b1	-0.129654244	0.096465669	-1.344045446	0.178933692	0.867377413
Unc93b1	0.005933818	0.140695595	0.042174869	0.966359296	0.996474415
Aldh3b2	0.194612005	1.070838893	0.181737894	0.855788426	0.992886758
Acy3	-0.327282207	0.270499179	-1.20991941	0.226309818	0.89398447
Nudt8	0.246559714	0.204886286	1.203397835	0.228822405	0.895404344
Doc2g	0.116141389	0.330978448	0.350903177	0.725660988	0.988585656
Ndufv1	0.184016881	0.098881544	1.860983081	0.062746566	0.727650961
Gstp1	0.018035192	0.107755332	0.167371687	0.867077602	0.992886758
Gstp2	0.114688629	0.246172024	0.465888149	0.641295565	0.977996701
BC021614	0.738051979	0.270201139	2.731491	0.006304846	0.427133716
Cabp2	1.119115517	0.484536126	2.309663733	0.020906778	0.604325792
Cdk2ap2	-0.088652696	0.132058763	-0.671312482	0.502021485	0.961378071
Pitpnm1	-0.094779941	0.286205042	-0.331160975	0.740522896	0.988585656
Aip	0.235548183	0.121763046	1.934480049	0.053054133	0.707153766
Tmem134	0.288331328	0.141069009	2.043902696	0.040963161	0.679118604
Cabp4	1.531072107	1.154596606	1.326066697	0.184817606	0.871716376
Coro1b	0.1974378	0.090844444	2.173361303	0.029753135	0.649459041
Ptprcap	0.389711507	0.235537913	1.654559566	0.098013871	0.78637468
Rps6kb2	-0.24824656	0.176055477	-1.41004736	0.158525699	0.855986074
Carns1	0.210805503	0.568597369	0.370746533	0.710826328	0.988585656
Tbc1d10c	0.199396083	0.26245956	0.759721169	0.447421272	0.948476747
Ppp1ca	0.276011672	0.137344294	2.009633333	0.044470011	0.689308953
Rad9a	-0.141816971	0.315272599	-0.449823332	0.652837833	0.979004493
Clcf1	0.036168126	0.293787631	0.123109763	0.90202018	0.994688009
Pold4	0.421212834	0.248952731	1.691939001	0.090657605	0.770055369
Ssh3	-0.219459204	0.158324947	-1.386131552	0.165706737	0.860011234
Ankrd13d	0.064114748	0.333949632	0.191989275	0.847750606	0.992886758
Adrbk1	-0.025723718	0.153304789	-0.167794612	0.866744861	0.992886758
Kdm2a	-0.181563428	0.205129145	-0.885117657	0.376093177	0.928883222
Rhod	-0.018588607	0.202225411	-0.091920233	0.926761416	0.994688009
A930001C03Rik	-0.340491564	1.240771076	-0.274419327	0.783762392	0.990988839
Syt12	0.309878844	0.878538972	0.352720657	0.724297871	0.988585656
2010003K11Rik	-1.112809024	1.271980096	-0.874863551	0.381648153	0.930635634
Pcx	-0.286875087	0.184618527	-1.55388027	0.120212979	0.81558552
Lrfn4	0.173538032	0.297649081	0.583028953	0.55987381	0.968926429
Rce1	-0.247553434	0.134205447	-1.844585592	0.065097844	0.734014162
Gm960	-0.117329477	1.936265368	-0.060595763	0.951681148	0.996023481
Sptbn2	-0.472957391	0.595586617	-0.794103456	0.427135206	0.944314584
Rbm4b	0.084097021	0.209325554	0.40175229	0.687866338	0.98538669
Rbm14	-0.26049885	0.286386597	-0.90960559	0.36303055	0.926455466
Ccs	0.374905917	0.123172916	3.043736634	0.002336596	0.31402518
Ccdc87	0.248710978	0.796082187	0.312418721	0.754722325	0.98902053
Ctsf	-0.187736366	0.16832486	-1.11532168	0.264712598	0.89768067
Actn3	1.384873805	1.461492442	0.947575071	0.343345822	0.920191602
Zdhhc24	0.293099378	0.239073219	1.225981645	0.220205582	0.889044644
Bbs1	-0.034361919	0.278788592	-0.123254395	0.901905653	0.994688009
Dpp3	-0.079977585	0.140829038	-0.567905496	0.57009914	0.971613657
Peli3	0.118141583	0.480524901	0.24585944	0.805791042	0.99099448
Mrpl11	0.270670781	0.206724619	1.30933017	0.190422534	0.877513044
Slc29a2	-0.054006699	0.946309104	-0.057070886	0.954488728	0.996070133
B4gat1	-0.161366284	0.166593919	-0.968620495	0.332734574	0.917115058
Brms1	0.2091638	0.127451561	1.641123874	0.100771704	0.788783372
Rin1	0.091566583	0.521320759	0.175643462	0.860574056	0.992886758
Cd248	0.192539997	0.329313022	0.584671677	0.558768502	0.968926429
Tmem151a	-0.041783116	0.395185745	-0.105730322	0.915796321	0.994688009
Yif1a	0.105940922	0.13833717	0.765816747	0.443785348	0.948476747
Cnih2	0.1694861	1.055689575	0.160545395	0.872451465	0.992886758
Rab1b	-0.075823781	0.11713169	-0.647337888	0.517413285	0.964352198
Klc2	0.108677298	0.206002957	0.527552125	0.597810229	0.974001549

Pacs1	-0.063506727	0.288408715	-0.220196977	0.825717751	0.992886758
Sf3b2	-0.076084543	0.132064421	-0.576116884	0.56453618	0.969658675
Gal3st3	-0.422367508	1.20194316	-0.351403895	0.725285361	0.988585656
Cst6	0.760959933	0.607001295	1.253638073	0.209973586	0.884258007
Banf1	0.38806535	0.12777829	3.037020994	0.002389288	0.31402518
Eif1ad	0.053644164	0.142744853	0.375804541	0.707062212	0.988585656
Sart1	0.291851256	0.193820882	1.505778182	0.132124126	0.827800491
D330050I16Rik	-0.750391623	0.84935978	-0.883479111	0.376977464	0.928883222
4930481A15Rik	0.606829754	0.271076547	2.238591868	0.025182484	0.629375214
Drap1	0.495269624	0.172214629	2.875885909	0.004028953	0.378480793
AI837181	-0.184573727	0.175879253	-1.049434338	0.293978261	0.907334416
Ccdc85b	0.377445942	0.12974067	2.909233799	0.003623158	0.374400632
Fibp	0.071990835	0.112241704	0.641391143	0.521268586	0.964828501
Ctsw	0.78020815	0.323218488	2.413872282	0.015783994	0.557315333
Efemp2	-0.072197458	0.172337625	-0.41893033	0.67526705	0.983549699
Mus81	0.071694618	0.1388565	0.516321657	0.605629773	0.974001549
Cfl1	0.058553731	0.12477405	0.46927812	0.638870853	0.977795535
Snx32	-0.585843153	0.357329886	-1.639502252	0.101108702	0.788783372
Ovol1	-0.349092425	0.41028209	-0.850859527	0.39484739	0.933796635
Ap5b1	-1.170974967	0.701765992	-1.668611732	0.095194352	0.778239075
Rnaseh2c	0.28455119	0.135683743	2.097164952	0.035978976	0.674795441
Kat5	0.081963908	0.120710628	0.679011524	0.49713056	0.960475196
Rela	0.057649793	0.230510851	0.250095788	0.802513273	0.990988839
Sipa1	-0.024395993	0.219261983	-0.111264128	0.911406901	0.994688009
Pcnxl3	0.076380585	0.232315666	0.328779313	0.7423225	0.988585656
Map3k11	0.072883907	0.185636851	0.392615509	0.694603483	0.987013595
Kcnk7	-0.06946882	0.506693799	-0.137102172	0.890950036	0.993043931
Ehbp1l1	0.122615983	0.130281249	0.941163701	0.346620975	0.921552698
Fam89b	-0.188251635	0.112032268	-1.680334053	0.092892339	0.77525594
Sssca1	0.038847904	0.181087471	0.214525632	0.830137181	0.992886758
Ltbp3	-0.004993364	0.187850911	-0.026581528	0.978793506	0.997262526
Scyl1	-0.191047976	0.155187772	-1.231076224	0.218294357	0.888436964
Malat1	0.110743756	0.209450181	0.528735546	0.596988915	0.974001549
Neat1	0.083274273	0.141843272	0.587086517	0.557145602	0.968926429
Frmd8	-0.153299025	0.164097529	-0.934194594	0.350203524	0.922338606
Slc25a45	0.052230202	0.276301515	0.189033352	0.850066677	0.992886758
Tigd3	-0.949879758	0.564855356	-1.681633622	0.092639907	0.775044718
Dpf2	-0.307880067	0.183045277	-1.681988584	0.092571054	0.775044718
Cdc42ep2	-0.496280435	0.230528282	-2.15279631	0.03133469	0.661395937
Pola2	-0.016101772	0.182265201	-0.088342547	0.929604423	0.994688009
Slc22a20	3.551034296	2.306063199	1.539868594	0.123592387	0.820382953
Capn1	0.167778855	0.20486583	0.818969445	0.412803847	0.942708418
Gm10814	0.12484613	1.289008421	0.096854395	0.922842026	0.994688009
Syvn1	-0.054857721	0.184258383	-0.297721712	0.76591557	0.989884648
Mrpl49	0.212368267	0.142902609	1.486104894	0.137251378	0.833085238
Fau	0.404966726	0.269325756	1.503631629	0.132676243	0.827800491
Znhit2	-0.05257087	0.142433371	-0.369090962	0.712059926	0.988585656
Tm7sf2	-0.064175593	0.242862236	-0.264246899	0.791589675	0.990988839
Vps51	0.279120617	0.142987155	1.952067767	0.050930159	0.707153766
Zfpl1	0.371600175	0.146498783	2.536541038	0.011195362	0.507740766
Cdca5	1.305806993	0.521627543	2.503332137	0.012303003	0.525888996
Sac3d1	-0.271397202	0.213447249	-1.271495431	0.203552452	0.883178549
Snx15	-0.031715572	0.21060483	-0.150592806	0.880296936	0.992886758
Arl2	0.19850301	0.146003705	1.359575156	0.173964405	0.863528862
Batf2	0.559643675	0.739316171	0.756974752	0.449064989	0.948476747
1700123I01Rik	0.845309169	1.077358175	0.784612943	0.432680539	0.94605734
Gpha2	-0.734962199	0.66869984	-1.099091334	0.27172823	0.89804647
Ppp2r5b	-0.046357283	0.137308706	-0.337613576	0.73565441	0.988585656
Atg2a	0.251695766	0.218930545	1.149660345	0.250283793	0.896939594
Ehd1	0.083542634	0.103880239	0.80422066	0.421269549	0.943415794
Cdc42bpg	-0.310660571	0.170245681	-1.824777985	0.068034539	0.742385562
Men1	-0.025308258	0.213245623	-0.118681253	0.905527889	0.994688009

Map4k2	0.117573228	0.149239153	0.787817576	0.430803419	0.945321484
Sf1	-0.252504846	0.194901727	-1.295549556	0.195130718	0.879588734
Pygm	-0.362335755	0.303269209	-1.194766049	0.232178448	0.895404344
Rasgrp2	0.374874207	0.132012296	2.839691591	0.004515717	0.390758702
Nrxn2	0.457923256	0.164159599	2.789500328	0.005278944	0.406593461
Slc22a12	-0.870065821	1.14833475	-0.757676122	0.448644897	0.948476747
Rps6ka4	-0.184634077	0.158012447	-1.168478058	0.242613985	0.896939594
Ccdc88b	0.088663141	0.353536735	0.250789049	0.801977209	0.990988839
Prdx5	0.216557523	0.154250844	1.403930881	0.160339433	0.857316691
Trmt112	0.041690021	0.108430168	0.384487287	0.700617316	0.988482081
Esrra	0.294634748	0.12177497	2.419501707	0.015541788	0.557315333
Tex40	0.14783393	1.671314612	0.088453682	0.929516097	0.994688009
Gpr137	0.135101934	0.165113732	0.818235599	0.413222672	0.943064937
Bad	0.311128229	0.168292302	1.848737138	0.064495778	0.73299549
Plcb3	0.233765032	0.104833625	2.229866902	0.02575628	0.631530315
Ppp1r14b	0.030906853	0.165333828	0.18693605	0.851710774	0.992886758
Fkbp2	0.271998944	0.133761539	2.033461532	0.042005916	0.679118604
Vegfb	-0.128784328	0.178256143	-0.722467828	0.470006898	0.95486913
Dnajc4	0.159861057	0.130259183	1.227253647	0.219727272	0.888847883
Nudt22	0.372352266	0.182312121	2.04238897	0.041112963	0.679118604
Trpt1	-0.297800595	0.295127284	-1.009058165	0.31294674	0.914857908
Fermt3	0.401162079	0.239218856	1.676966802	0.093548974	0.775558829
Stip1	-0.347823589	0.121265596	-2.86827922	0.004127111	0.38069687
Macrod1	0.114481109	0.14736857	0.776835312	0.43725594	0.9470785
Flrt1	-1.001601954	1.572145483	-0.63709241	0.524064652	0.965637248
Otub1	0.153274094	0.1403603	1.092004608	0.274831076	0.900302631
Cox8a	0.086607743	0.130355788	0.664395069	0.506437488	0.962472921
Naa40	-0.194110514	0.205886087	-0.942805397	0.345780451	0.921552698
Rcor2	-0.007050198	1.053173628	-0.006694241	0.994658809	0.999285699
Mark2	-0.314863049	0.196973686	-1.598503109	0.109931054	0.798948974
AI846148	-0.000822803	0.145693448	-0.005647495	0.995493975	0.999285699
1700105P06Rik	-2.828187951	1.673655714	-1.689826604	0.091061133	0.771538719
2700081O15Rik	-0.011991038	0.198635205	-0.060367133	0.951863235	0.996023481
Rtn3	-0.197304635	0.107518469	-1.835076684	0.066494328	0.738254074
Atl3	-0.252243363	0.119189037	-2.116330236	0.034316731	0.674629488
Pla2g16	0.127595521	0.157663758	0.809288845	0.418349021	0.943415794
Lgals12	0.000408152	0.413332433	0.000987468	0.999212115	0.999886097
Chrm1	-1.22051367	1.580040345	-0.772457282	0.439843638	0.947849924
Slc3a2	-0.34688963	0.156236785	-2.220281411	0.026399671	0.632301593
Snhg1	0.419667066	0.294414642	1.425428649	0.154033332	0.854159937
Snord22	0.978650788	1.589439378	0.615720739	0.538078847	0.96674935
Wdr74	0.106709164	0.132190934	0.807235119	0.419531036	0.943415794
Stx5a	0.059803457	0.109532547	0.545988006	0.585074182	0.972904784
Nxf1	-0.29495448	0.137810574	-2.140289173	0.032331404	0.665167506
Tmem223	0.074463761	0.165352628	0.450333097	0.652470278	0.979004493
Tmem179b	-0.301684676	0.161907057	-1.86332012	0.062417241	0.727399211
Taf6l	0.097085104	0.407929124	0.237995028	0.81188495	0.991817851
Polr2g	0.239658463	0.148512046	1.613730797	0.106585831	0.794908043
Zbtb3	-0.181506701	0.392858425	-0.46201555	0.644070169	0.97889195
Ttc9c	-0.091885917	0.136324841	-0.674021814	0.500297445	0.9611436
Hnrnpul2	0.131183029	0.125493703	1.045335551	0.295867912	0.90835566
Bscl2	0.122074545	0.194233538	0.628493652	0.529680604	0.965637248
Lrrn4cl	0.076804639	0.408397974	0.188063223	0.85082709	0.992886758
Ubxn1	0.171195625	0.08405306	2.03675659	0.04167444	0.679118604
Uqcc3	0.461501782	0.141948097	3.251200901	0.001149186	0.243672251
5730408K05Rik	0.916593496	1.247862223	0.734531008	0.462625169	0.953083512
Ints5	0.026140028	0.340823185	0.076696743	0.938864796	0.994688009
Ganab	-0.338514193	0.12622176	-2.681900441	0.007320524	0.452031612
B3gat3	0.307543647	0.126170987	2.43751478	0.01478861	0.554936045
Rom1	0.284038189	0.223666617	1.269917672	0.204113958	0.883736035
Eml3	0.061644294	0.19818327	0.311046912	0.755764958	0.989266039
Mta2	-0.159408362	0.124218112	-1.28329404	0.19938908	0.880831248

Tut1	0.098711874	0.167443291	0.58952421	0.555509677	0.968926429
Eef1g	-0.031424233	0.096883582	-0.324350444	0.745672734	0.988585656
Ahnak	0.399192001	0.140792596	2.835319568	0.004577987	0.390758702
Scgb1a1	-0.30830056	0.104890284	-2.939267102	0.003289894	0.357710224
Asrgl1	0.016531329	0.177820847	0.092966206	0.925930409	0.994688009
Stxbp3-ps	-0.059930254	1.121367568	-0.053443898	0.957378229	0.996226826
Incenp	0.055006076	0.495117983	0.111096907	0.911539502	0.994688009
Fth1	-0.032452611	0.09893804	-0.328009438	0.742904527	0.988585656
Best1	-0.228026095	0.33385636	-0.683006595	0.494602675	0.959071007
Rab3il1	-0.351951441	0.260633025	-1.350371624	0.176896808	0.866546104
Fads3	-0.178900835	0.131656212	-1.358848412	0.174194629	0.863528862
Fads2	-0.118423182	0.225405465	-0.525378485	0.599320107	0.974001549
Fads1	-0.091344922	0.099943697	-0.913963815	0.36073586	0.925913281
Fen1	-0.15786779	0.343507709	-0.459575682	0.645820818	0.979004493
Tmem258	0.212313008	0.163540152	1.298231689	0.194207732	0.879588734
Myrf	-0.225863863	0.274195845	-0.823731895	0.410091912	0.940983181
Dagla	-0.235843345	0.551099161	-0.427950833	0.668686917	0.981863224
Syt7	0.084707107	0.255767381	0.331188077	0.740502426	0.988585656
Lrrc10b	-0.012135722	0.133135313	-0.091153295	0.927370786	0.994688009
Ppp1r32	-0.292418318	0.314050122	-0.931119899	0.351791547	0.923082865
Sdhaf2	0.116958883	0.15558853	0.751719185	0.452219951	0.949574349
Cpsf7	-0.069142125	0.141359019	-0.489124256	0.62475373	0.976110444
Tmem216	0.216142572	0.128072084	1.687663431	0.091475855	0.772516176
Tmem138	-0.379703827	0.222054848	-1.709955132	0.08727417	0.765677258
Cyb561a3	-0.00476266	0.186914151	-0.025480467	0.979671728	0.997296835
Tkfc	-0.630462091	0.355567849	-1.773113322	0.076209931	0.752876039
Ddb1	-0.093823515	0.106719969	-0.879156135	0.379316624	0.929137643
Vps37c	-0.186865286	0.21757189	-0.858866858	0.390413976	0.933734231
Cd5	-0.409095686	0.797609605	-0.512902156	0.608019765	0.974001549
A430093F15Rik	0.95848703	0.758288818	1.264013139	0.206225311	0.883765092
Cd6	0.968874859	0.642183094	1.508720595	0.1313702	0.827800491
Slc15a3	0.012817718	0.294977574	0.043453194	0.965340275	0.996474415
Tmem132a	0.446095243	0.263663301	1.691912533	0.090662652	0.770055369
Tmem109	-0.220558742	0.15647872	-1.409512697	0.158683622	0.855986074
Prpf19	-0.151654235	0.148209121	-1.023244953	0.306192043	0.91333012
Zp1	-0.164911427	2.102934704	-0.078419661	0.937494234	0.994688009
Ptgdr2	-0.79951499	1.990832557	-0.40159831	0.687979674	0.98538669
Ccdc86	-0.335787709	0.250009965	-1.343097298	0.17924047	0.867377413
Ms4a15	0.847701373	1.491535198	0.568341515	0.569803095	0.97144955
AW112010	0.257502729	0.157563017	1.634284072	0.102199217	0.788963633
Ms4a8a	-0.235910712	0.202619117	-1.164306289	0.244299909	0.896939594
Ms4a1	0.349157422	0.407967099	0.855847011	0.392082408	0.933734231
Ms4a7	0.243627007	0.250794609	0.97142043	0.331338959	0.917115058
Ms4a4c	0.812962884	0.24186806	3.361183298	0.000776093	0.201299795
Ms4a4b	0.51783786	0.208264731	2.486440488	0.012902815	0.532176722
Ms4a6c	0.466276653	0.192838127	2.417969211	0.015607397	0.557315333
Gm8369	0.774446818	0.479098245	1.616467657	0.105993241	0.79419908
Ms4a6b	0.554025538	0.192512848	2.877862659	0.004003794	0.378480793
Ms4a4d	-0.102809838	0.136584015	-0.752722328	0.45161679	0.949134998
Ms4a6d	-0.406754194	0.314460802	-1.293497285	0.195839122	0.879601031
Ms4a2	0.212550114	0.975572161	0.217872262	0.827528639	0.992886758
Oosp1	-2.567552991	1.727273843	-1.486477087	0.137152973	0.833085238
Mrpl16	0.242881153	0.143013586	1.698308246	0.089449599	0.769347579
Stx3	-0.029148136	0.14361727	-0.202957037	0.839168613	0.992886758
Patl1	-0.064717721	0.322572368	-0.200630084	0.840987833	0.992886758
Osbp	-0.297459333	0.147886322	-2.011405306	0.044282663	0.689230739
Mpeg1	-0.099395003	0.14661219	-0.67794501	0.49780656	0.960700483
Dtx4	0.076069811	0.221374586	0.343624862	0.731128425	0.988585656
Fam111a	0.160459292	0.209523509	0.765829535	0.443777738	0.948476747
A330040F15Rik	-1.138891408	2.446390375	-0.465539523	0.64154514	0.97813956
Zfp91	-0.053745976	0.102874027	-0.522444563	0.601360836	0.974001549
Lpxn	-0.124725827	0.244959204	-0.509169791	0.610633216	0.974001549

Tle4	-0.186723519	0.173883489	-1.073842719	0.28289318	0.905916508
Psat1	0.030422822	0.202186558	0.150469061	0.880394559	0.992886758
Cep78	-0.120274958	0.20289261	-0.59280108	0.553314285	0.968926429
C130060C02Rik	0.927062029	1.558982556	0.594658372	0.552071853	0.968926429
Gnaq	-0.113199234	0.143640386	-0.788073863	0.430653502	0.945321484
E030024N20Rik	0.471666752	0.693849291	0.67978271	0.496642056	0.960362776
Gna14	-0.044573551	0.212273475	-0.209981726	0.833681935	0.992886758
Vps13a	-0.233465747	0.197450087	-1.182403867	0.23704549	0.896609492
Prune2	0.184095723	0.441523022	0.416956113	0.676710509	0.983549699
Gcnt1	0.498559471	0.162459824	3.068817015	0.002149082	0.308737709
Rfk	0.091093818	0.111231843	0.818954497	0.412812376	0.942708418
Pcsk5	-0.080100517	0.315200575	-0.254125543	0.79939857	0.990988839
Ostf1	0.018594184	0.104663951	0.177656048	0.858993105	0.992886758
Nmrk1	0.220267662	0.248722297	0.885596767	0.375834854	0.928883222
2410127L17Rik	0.148145513	0.14422436	1.02718787	0.304332009	0.911879006
D030056L22Rik	0.152695439	0.168559573	0.905884114	0.364997193	0.926556975
Trpm6	-0.115825265	0.246544474	-0.469794612	0.638501764	0.977795535
Anxa1	-0.04129587	0.118606445	-0.348175594	0.727708315	0.988585656
E030003E18Rik	0.382083119	0.32292394	1.183198494	0.23673049	0.896358698
C730002L08Rik	-0.225757608	1.371322851	-0.164627613	0.869237096	0.992886758
Aldh1a1	0.053827425	0.168056146	0.320294296	0.748745246	0.988585656
Aldh1a7	0.018727091	0.164016556	0.114178055	0.909096648	0.994688009
Zfand5	-0.169701199	0.087086581	-1.948649243	0.051337327	0.707153766
Gda	-0.153606219	0.191803444	-0.800852246	0.423217189	0.943509323
1110059E24Rik	0.277765777	0.208081952	1.334886445	0.181913515	0.868546072
Abhd17b	-0.150559011	0.142622651	-1.055645859	0.291130041	0.907334416
Tmem2	-0.095560807	0.200475539	-0.476670657	0.633596661	0.977034429
Trpm3	-0.570582362	0.631820641	-0.903076483	0.366485304	0.927834635
Klf9	-0.474884211	0.212826139	-2.231324653	0.025659632	0.631530315
Smc5	-0.057745315	0.178178646	-0.32408662	0.745872456	0.988585656
Mamdc2	0.208933509	0.140605319	1.485957358	0.1372904	0.833085238
1700028P14Rik	-0.005538245	0.159711669	-0.034676522	0.972337682	0.996790728
Ptar1	-0.208775856	0.286684136	-0.728243489	0.466464549	0.953083512
Apba1	-0.568762784	0.649479607	-0.875720774	0.381181849	0.930555629
Fam189a2	-0.211071153	0.128428891	-1.643486536	0.100282309	0.788783372
Tjp2	-0.193682763	0.18764787	-1.032160736	0.301996825	0.911518274
Fxn	0.022260391	0.22099261	0.10072912	0.919765495	0.994688009
Pip5k1b	0.073692755	0.271098433	0.27183025	0.785752545	0.990988839
Fam122a	0.003878256	0.19799874	0.019587278	0.984372613	0.998148029
Tmem252	-0.668221393	0.433801359	-1.540385659	0.123466375	0.820112086
Pgm5	0.146511369	0.12196521	1.201255413	0.22965214	0.895404344
Foxd4	1.460889845	1.71015374	0.854244745	0.392969396	0.933734231
Cbwd1	0.372954854	0.167799672	2.22261969	0.026241455	0.632301593
Dock8	0.444945743	0.187402732	2.374275653	0.017583418	0.579835303
Kank1	-0.197934011	0.205703344	-0.962230401	0.335933881	0.918908218
Dmrt3	-0.608755258	0.98698604	-0.616782035	0.537378503	0.96674935
2610016A17Rik	-0.897717355	0.660548224	-1.359048927	0.174131085	0.863528862
Dmrt2	-0.6933593	0.512424652	-1.353095126	0.176025242	0.865308446
Smarca2	0.087283722	0.111416018	0.783403713	0.433390077	0.946252789
Vldlr	-0.119924152	0.155041125	-0.773498977	0.439227134	0.947849924
D19Bwg1357e	-0.064789241	0.149325861	-0.433878232	0.664376867	0.98119114
C030016D13Rik	-0.512172264	1.122238591	-0.456384469	0.648113532	0.979004493
Rfx3	-0.657924157	0.227497876	-2.892001311	0.003827963	0.376754331
Glis3	-1.018940691	0.471205439	-2.162412841	0.030586369	0.657949462
Slc1a1	-0.121370811	0.273723857	-0.443406039	0.657472068	0.979614766
4430402I18Rik	-0.184386306	0.163760296	-1.125952452	0.260185646	0.897248195
Ppapdc2	-0.067696279	0.259929156	-0.260441269	0.794523413	0.990988839
Cdc37l1	0.113231599	0.112505642	1.006452627	0.314197889	0.91586731
Ak3	-0.247826731	0.121292659	-2.043212945	0.041031363	0.679118604
1700018L02Rik	-0.387693922	1.095676813	-0.35383967	0.723459042	0.988585656
Gm9895	0.8284247	0.653103945	1.268442346	0.204640027	0.883765092
Rcl1	0.047498226	0.139680409	0.340049306	0.733819397	0.988585656

Jak2	-0.193978948	0.117695703	-1.648139599	0.099324028	0.788746793
Insl6	0.857204073	0.471504675	1.818018185	0.069061353	0.743426713
Plgrkt	0.096692755	0.148165378	0.652600201	0.514014056	0.964352198
Cd274	-0.035492877	0.183654483	-0.193258973	0.846756155	0.992886758
Pdcd1lg2	-0.027660839	0.514141592	-0.053800042	0.957094476	0.996226826
A930007I19Rik	-0.214170979	0.763698259	-0.280439265	0.779140516	0.990988839
Ric1	-0.292824286	0.14698487	-1.992207002	0.04634835	0.698098452
Ermp1	0.197114824	0.1584959	1.243658822	0.213625157	0.884945675
Mlana	-0.365438652	0.374453309	-0.975925818	0.329101227	0.917115058
9930021J03Rik	-0.38899538	0.307192995	-1.266289875	0.20540932	0.883765092
Ranbp6	-0.190749471	0.150768495	-1.265181233	0.205806368	0.883765092
Il33	-0.465239991	0.293869563	-1.583151331	0.113386976	0.806530837
Uhrf2	-0.046295403	0.116977464	-0.395763434	0.692279555	0.986599035
Gldc	-2.170588073	1.038934774	-2.089243838	0.036685779	0.677009445
Prkg1	0.18172858	0.244581759	0.743017717	0.457470954	0.952102513
Cstf2t	-0.191027349	0.155001092	-1.232425828	0.217790058	0.887633697
Asah2	0.053882813	0.228314724	0.236002358	0.813430841	0.992048071
Sgms1	-0.087823374	0.179689105	-0.488751804	0.625017423	0.976148792
2700046G09Rik	1.063168736	0.311492661	3.413142168	0.000642184	0.180774885
Minpp1	-0.132238316	0.151447217	-0.873164387	0.382573483	0.930775109
Papss2	0.083661704	0.286857841	0.291648658	0.770555269	0.989990452
Atad1	-0.047357545	0.144160774	-0.32850507	0.742529811	0.988585656
Pten	0.04482052	0.103072737	0.434843604	0.663675953	0.981154469
Rnls	0.234491262	0.216189364	1.084656792	0.278073692	0.901351829
Lipo1	-0.188937165	0.118855966	-1.589631308	0.111917936	0.801067615
Lipf	0.200128849	0.465045407	0.4303426	0.666946443	0.981863224
Lipn	-1.282511625	1.972270255	-0.650271747	0.515516703	0.964352198
Ankrd22	0.511484299	0.858281712	0.595939879	0.551215391	0.968926429
Stambpl1	0.009589382	0.263788232	0.036352578	0.971001227	0.996733101
Acta2	-0.208638446	0.185696671	-1.123544351	0.261206376	0.897248195
Fas	-0.160131346	0.283674769	-0.564489209	0.572421242	0.971613657
Ch25h	-0.480276138	0.299367333	-1.604303761	0.108647112	0.798150849
Lipa	-0.280337061	0.110155723	-2.544915987	0.010930403	0.502706181
Ifit2	0.08568142	0.198198278	0.432301533	0.665522273	0.981727712
Ifit3	-0.049679307	0.160738261	-0.309069581	0.757268598	0.989266039
Ifit1bl1	0.142326935	0.188489623	0.755091621	0.45019401	0.949134998
Ifit3b	0.024048503	0.203585459	0.118124858	0.905968728	0.994688009
Ifit1bl2	0.247786981	0.206672981	1.198932631	0.230554142	0.895404344
Ifit1	-0.222859291	0.221183127	-1.007578177	0.31365701	0.915639195
Slc16a12	-0.336719368	0.174953428	-1.924622868	0.054276556	0.707153766
Pank1	0.03640251	0.255806417	0.142304914	0.886839165	0.992886758
Kif20b	0.076132065	0.502157479	0.151609939	0.879494594	0.992886758
Htr7	0.258284904	1.408965216	0.183315316	0.854550617	0.992886758
Rpp30	-0.057023201	0.146145493	-0.390181043	0.696402679	0.987490725
Ankrd1	-0.365291244	0.215634707	-1.69402806	0.090259951	0.769347579
Pcgf5	-0.213860476	0.172650661	-1.238689007	0.215460691	0.887633697
Hectd2	0.468460429	0.416147945	1.125706457	0.26028979	0.897248195
1500017E21Rik	1.143612673	0.506336437	2.258602364	0.023908131	0.622011301
Ppp1r3c	0.191244688	0.194808926	0.981703928	0.32624573	0.916800143
Tnks2	-0.269951321	0.109181831	-2.472493069	0.013417432	0.540715407
Fgfbp3	-0.265688796	0.310102199	-0.856778174	0.39156749	0.933734231
Btaf1	-0.304605674	0.154534995	-1.971111297	0.048711147	0.707153766
Cpeb3	-0.028001807	0.411361326	-0.068071074	0.945729057	0.995032756
Mar-05	-0.113705522	0.109534244	-1.03808195	0.299231904	0.910105802
4931408D14Rik	-1.014729608	0.924670628	-1.09739574	0.272468435	0.898460682
Ide	-0.709651005	0.223626281	-3.17337928	0.001506755	0.279554507
Kif11	0.244517493	0.321795271	0.759854214	0.447341732	0.948476747
Hhex	0.056413187	0.357566775	0.157769656	0.87463831	0.992886758
Exoc6	-0.001621294	0.149531595	-0.010842488	0.991349116	0.998770784
Myof	0.002481278	0.151977933	0.016326566	0.986973863	0.998148029
Cep55	-0.677530317	0.372396381	-1.819379434	0.068853562	0.743426713
Ffar4	-0.411243776	0.188558329	-2.180989708	0.029184178	0.64858361

Rbp4	0.013170425	0.195763984	0.067277057	0.946361141	0.995065399
Fra10ac1	0.125533881	0.178105552	0.704828569	0.480916927	0.957463006
Lgi1	1.190454203	2.333641392	0.510127309	0.609962274	0.974001549
Slc35g1	-0.02584264	0.281045941	-0.091951657	0.926736449	0.994688009
Plce1	-0.242471911	0.190003199	-1.276146466	0.201903748	0.881220006
Noc3l	-0.176362531	0.228544864	-0.771675757	0.440306493	0.947849924
Tbc1d12	-0.084577966	0.206295682	-0.409984177	0.681817554	0.984683806
Hells	-0.52280018	0.490802961	-1.065193614	0.286788323	0.906336252
Pdlim1	-0.231739849	0.176330628	-1.31423481	0.188767197	0.875363508
Sorbs1	-0.150215858	0.197032484	-0.762391331	0.445826479	0.948476747
Aldh18a1	-0.414793409	0.256364449	-1.617983343	0.105666187	0.79419908
Tctn3	-0.313390149	0.221213366	-1.41668722	0.156574395	0.855552259
Entpd1	-0.26830215	0.126457805	-2.12167331	0.033865181	0.671818913
Ccnj	-0.008792114	0.185483401	-0.047401083	0.962193566	0.996474415
E030044B06Rik	0.340768907	0.300276408	1.134850754	0.256437857	0.897248195
Zfp518a	-0.001113857	0.1893046	-0.005883943	0.99530532	0.999285699
Blnk	-0.24345154	0.361312456	-0.673797807	0.500439869	0.9611436
Dntt	0.69999914	1.379623042	0.507384349	0.611885165	0.974001549
Tm9sf3	-0.077462277	0.088177967	-0.87847655	0.379685158	0.929185635
Pik3ap1	-0.384829187	0.42967455	-0.895629464	0.370450694	0.928436166
Lcor	1.067329024	0.763640565	1.397685081	0.162207656	0.859579489
Arhgap19	-0.102041969	0.347265141	-0.293844551	0.76887669	0.989990452
Frat1	-0.068852464	0.397383565	-0.173264499	0.862443527	0.992886758
Frat2	0.829483866	0.559643066	1.482165894	0.138296159	0.834427712
Rrp12	0.39699188	0.368390527	1.077638678	0.281195034	0.904300139
Pgam1	-0.290770489	0.12023691	-2.418313049	0.015592656	0.557315333
Exosc1	0.152275031	0.157303076	0.968035938	0.333026422	0.917227124
Zdhhc16	0.440587924	0.134419988	3.277696508	0.001046579	0.240510037
Mms19	-0.342815493	0.192781311	-1.778261027	0.075360994	0.752876039
Ubtd1	0.31196262	0.171636496	1.817577417	0.069128746	0.743426713
Ankrd2	-0.461571934	1.559432348	-0.295987149	0.767239896	0.989884648
Hoga1	0.300096424	0.217171188	1.381842717	0.16702	0.860403806
Morn4	-0.271257147	0.302855912	-0.895664031	0.370432227	0.928436166
Pi4k2a	-0.369375814	0.135605012	-2.723909746	0.006451413	0.429927454
Avpi1	0.268492925	0.241613184	1.111251138	0.266460265	0.89768067
Marveld1	-0.167947052	0.152428556	-1.101808322	0.270545012	0.89804647
Zfyve27	0.122591162	0.195357773	0.627521291	0.530317584	0.966026275
Sfrp5	-0.140368246	1.357956922	-0.10336723	0.917671519	0.994688009
Crtac1	2.60936844	2.143301016	1.217453088	0.223431875	0.892989918
R3hcc1l	-0.105026515	0.188668777	-0.556671416	0.57775195	0.971613657
Loxl4	0.28262853	0.367713576	0.768610541	0.442124546	0.948476747
Pyroxd2	0.427273654	0.529941256	0.806266072	0.420089449	0.943415794
Hps1	0.137704654	0.486725836	0.282920372	0.777237878	0.990861469
Hpse2	0.096089291	1.185072548	0.081083045	0.935375909	0.994688009
Got1	0.085005154	0.203784288	0.417133013	0.676581119	0.983549699
Slc25a28	0.06910759	0.182355152	0.378972512	0.704708276	0.988585656
BC037704	0.305910035	0.889071357	0.344078158	0.730787509	0.988585656
Entpd7	-0.091064238	0.494450761	-0.184172511	0.853878124	0.992886758
Cox15	-0.299706443	0.206503422	-1.451338871	0.146685522	0.843881086
Cutc	0.219343068	0.169995428	1.29028804	0.196950668	0.879755417
Dnmbp	-0.661661082	0.41679896	-1.587482564	0.112403393	0.801401832
Cpn1	0.089149685	0.280947185	0.317318307	0.751002088	0.988585656
Cyp2c44	0.048580164	0.899876565	0.053985364	0.956946825	0.996226826
Erlin1	-0.251961093	0.166207938	-1.515938989	0.12953478	0.827800491
Chuk	-0.17934383	0.148073488	-1.211181234	0.225825953	0.893616175
Cwf19l1	0.16800786	0.221915027	0.757081941	0.449000772	0.948476747
Bloc1s2	0.341256307	0.224250458	1.521764151	0.128068187	0.826336015
Scd3	-0.701260668	1.041365126	-0.673405178	0.500689555	0.961175273
Scd2	-0.30025164	0.155975194	-1.924996097	0.054229847	0.707153766
Scd4	-0.27937483	1.263550798	-0.221102967	0.82501226	0.992886758
Scd1	-0.266270646	0.186359451	-1.428801407	0.153061321	0.854159937
Sec31b	-0.135589622	0.577358076	-0.234844939	0.814329087	0.992048071

Ndufb8	0.433629669	0.27441834	1.580177435	0.114066238	0.807965062
Hif1an	-0.124413112	0.21047055	-0.59111886	0.554440784	0.968926429
Fam178a	-0.318097545	0.156117946	-2.037546307	0.041595326	0.679118604
Sema4g	-0.786872837	1.214346441	-0.647980519	0.51699755	0.964352198
Mrpl43	0.077217917	0.118375544	0.652313088	0.514199219	0.964352198
Peo1	0.465071113	0.284298996	1.635852114	0.101870542	0.788783372
Lzts2	0.242685604	0.173194883	1.401228485	0.161145759	0.857316691
Pdzd7	0.148012364	0.861975587	0.171712942	0.863663211	0.992886758
Sfxn3	0.078755933	0.240601976	0.327328702	0.743419286	0.988585656
Kazald1	0.381182329	0.166937547	2.283382839	0.022407827	0.619833063
Btrc	-0.027563551	0.211284281	-0.130457177	0.896204732	0.993970087
Poll	0.36573113	0.24235232	1.509088624	0.131276136	0.827800491
Dpcd	0.105145447	0.11730349	0.896353952	0.370063753	0.928436166
Fbxw4	0.30024464	0.256205824	1.171888427	0.241241854	0.896939594
Npm3	0.302751042	0.205941329	1.470083945	0.14153902	0.840741274
Mgea5	0.110206139	0.109988518	1.001978574	0.316353941	0.91640698
Kcnip2	-0.058538248	0.59117079	-0.099020874	0.921121697	0.994688009
9130011E15Rik	-0.13186117	0.218079123	-0.604648296	0.54541271	0.967491297
Hps6	-0.111642638	0.228388936	-0.488826821	0.624964308	0.976148792
Ldb1	0.018526924	0.153951445	0.120342644	0.904211724	0.994688009
Pprc1	-0.146364976	0.327364157	-0.44710141	0.654801841	0.979133069
Nolc1	-0.279945804	0.201465213	-1.389549089	0.164665845	0.859982992
Gbf1	-0.020658247	0.24870133	-0.083064482	0.933800268	0.994688009
Nfkb2	-0.077727837	0.223329493	-0.348041074	0.727809337	0.988585656
Psd	0.109990016	0.513060056	0.214380391	0.830250432	0.992886758
Fbxl15	0.519204921	0.300870369	1.725676487	0.08440563	0.76099108
Cuedc2	0.322418348	0.166118065	1.940898771	0.052270557	0.707153766
2310034G01Rik	-0.171746122	0.242945876	-0.706931621	0.479608972	0.957463006
Tmem180	-0.460221278	0.342013599	-1.345622745	0.178424213	0.867377413
Actr1a	0.033695974	0.106859766	0.315328912	0.752511936	0.988585656
Sufu	-0.474729726	0.334143046	-1.420738008	0.155392939	0.855261441
Trim8	-0.085847221	0.180140749	-0.476556367	0.63367806	0.977034429
Arl3	0.09032179	0.119768107	0.754138916	0.450765812	0.949134998
Sfxn2	-0.044757449	0.203621188	-0.219807424	0.826021137	0.992886758
Wbp1l	-0.170153993	0.137038518	-1.241650848	0.214365412	0.886400046
2010012O05Rik	0.154677724	0.152898626	1.0116358	0.311712222	0.914819219
As3mt	0.211867438	0.126986326	1.668427175	0.095230957	0.778239075
Cnnm2	-0.068043214	0.423462099	-0.160683127	0.872342979	0.992886758
Nt5c2	-0.261959684	0.134754969	-1.943970505	0.051899008	0.707153766
Pcgf6	0.354923868	0.22053706	1.609361567	0.107537304	0.796929372
Taf5	-0.04457191	0.232922844	-0.191359117	0.848244248	0.992886758
Usmg5	0.415739542	0.310381652	1.339446259	0.180425439	0.868087032
Pdcd11	0.029929907	0.265088184	0.112905472	0.910105498	0.994688009
Calhm2	-0.193011654	0.285498021	-0.676052511	0.499007306	0.960700483
Neurl1a	-0.388003306	0.377448631	-1.02796321	0.303967134	0.911786415
Sh3pxd2a	0.069123469	0.170364057	0.405739743	0.684933837	0.985105247
Obfc1	0.327623125	0.249344228	1.313939077	0.188866707	0.875363508
Gm19557	0.229923587	0.465516001	0.493911244	0.621368864	0.97514112
Slk	0.043427314	0.137293027	0.316311138	0.751766358	0.988585656
Col17a1	0.514196737	0.652920416	0.787533556	0.430969593	0.945321484
Sfr1	0.022241643	0.111493192	0.199488802	0.841880402	0.992886758
Cfap43	-0.260696196	0.192446442	-1.354642844	0.175531376	0.864596675
Gsto1	0.206448199	0.186403339	1.107534875	0.268062743	0.89804647
Gsto2	0.387763795	0.314203665	1.234116079	0.217159653	0.887633697
Itprip	-0.375268055	0.19365579	-1.937809639	0.052646451	0.707153766
Cfap58	-0.257899402	0.25888593	-0.996189332	0.319158162	0.91640698
Xpnpep1	-0.17096444	0.20631839	-0.828643727	0.407306037	0.939557159
Add3	0.035321165	0.110977938	0.318271949	0.75027866	0.988585656
Mxi1	-0.04316305	0.12658756	-0.340973872	0.733123251	0.988585656
Smndc1	-0.229313123	0.112670649	-2.035251646	0.041825559	0.679118604
5830416P10Rik	0.217122544	0.607026897	0.357681917	0.720581367	0.988585656
Dusp5	0.125028901	0.523210942	0.238964614	0.811133022	0.991715504

Smc3	-0.044575993	0.15228771	-0.292709064	0.769744541	0.989990452
Rbm20	0.493126221	0.664060195	0.742592651	0.457728339	0.952222574
Pdcd4	0.195369605	0.186419407	1.048011088	0.294633498	0.907334416
Bbip1	0.019642093	0.200357319	0.098035318	0.921904249	0.994688009
Shoc2	-0.1029384	0.128076399	-0.803726527	0.42155493	0.943415794
Adra2a	2.156027191	0.996616011	2.163347936	0.030514429	0.657949462
Gpam	-0.203738902	0.260070431	-0.78339895	0.433392873	0.946252789
Tectb	0.475026685	1.703871352	0.278792577	0.780404012	0.990988839
Gucy2g	0.167626398	0.600887401	0.27896474	0.780271884	0.990988839
Acsl5	-0.029119457	0.09997658	-0.291262788	0.770850345	0.989990452
Zdhhc6	-0.036778392	0.137897966	-0.266707285	0.789694544	0.990988839
Vti1a	-0.091849478	0.126037109	-0.728749489	0.466154914	0.953083512
Tcf7l2	-0.028147311	0.345637684	-0.081435886	0.935095311	0.994688009
Nrap	0.600417258	0.70773636	0.848362882	0.396235907	0.934066368
Casp7	0.074829053	0.138018521	0.542166748	0.58770364	0.973455044
Plekhs1	0.561186829	1.927632995	0.291127424	0.770953866	0.989990452
Dclre1a	0.239014687	0.252771702	0.945575337	0.344365228	0.92080758
Nhlrc2	0.154630733	0.119842556	1.290282327	0.196952651	0.879755417
Adrb1	-0.150799053	0.218063602	-0.691537019	0.489228125	0.958560421
Ccdc186	-0.100290821	0.287076792	-0.349351893	0.726825145	0.988585656
Vwa2	1.953606192	1.953771087	0.999915601	0.317351354	0.91640698
Afap1l2	0.112043295	0.261713213	0.42811478	0.668567557	0.981863224
Ablim1	0.022917177	0.150488424	0.152285315	0.878961907	0.992886758
B230217O12Rik	0.963888475	0.675581809	1.426753153	0.153651059	0.854159937
Fam160b1	-0.208314328	0.14617243	-1.425127347	0.154120393	0.854159937
Trub1	-0.072353572	0.229606081	-0.315120453	0.7526702	0.988585656
Atrnl1	-0.080188763	0.152554204	-0.525641121	0.59913758	0.974001549
Gfra1	-0.205525278	0.216221262	-0.950532229	0.341841886	0.919850248
Pnliprp1	1.329577313	0.813161629	1.635071387	0.102034083	0.788783372
Hspa12a	0.195804616	0.232006506	0.843961749	0.398690781	0.93579994
Eno4	-0.563461514	0.182119343	-3.093913605	0.00197535	0.298625366
Shtn1	-0.154378278	0.184311742	-0.837593287	0.402259164	0.937834293
Slc18a2	-0.146216379	0.579815056	-0.252177616	0.800903779	0.990988839
Pdzd8	-0.119846701	0.162764451	-0.736319875	0.461536053	0.953083512
Rab11fip2	-0.161929922	0.196546629	-0.823875348	0.410010389	0.940983181
Fam204a	0.16696692	0.129958205	1.284773977	0.198871279	0.880661448
Cacul1	-0.01083754	0.098650488	-0.109857948	0.912522033	0.994688009
Nanos1	0.482299458	0.570915639	0.844782356	0.398232368	0.935327115
Eif3a	-0.130595042	0.145066166	-0.900244665	0.367990062	0.928436166
Fam45a	0.138597212	0.141756591	0.977712654	0.328216462	0.917115058
Sfxn4	0.286716077	0.246823646	1.161623217	0.245388547	0.896939594
Prdx3	0.067375306	0.122461955	0.550173369	0.582200468	0.972017845
Grk5	0.05443554	0.114619196	0.474925162	0.634840316	0.977034429
Zfp950	0.370295422	0.161959397	2.28634725	0.022233952	0.618422027
Csf2ra	0.169720704	0.251860613	0.673867587	0.500395501	0.9611436
Fam171a1	-0.337049396	0.181239953	-1.859685964	0.062929969	0.727650961
Nmt2	-0.121143997	0.159947371	-0.757399118	0.448810785	0.948476747
Rpp38	0.028851834	0.225970226	0.127679802	0.898402375	0.994364545
Acbd7	1.040382657	0.443942353	2.343508453	0.019103329	0.590246276
Olah	-0.986766488	2.2443462	-0.43966768	0.660177814	0.979924124
Meig1	0.243425041	0.211688374	1.149921634	0.250176149	0.896939594
Dclre1c	-0.229681461	0.304470422	-0.754363791	0.450630808	0.949134998
Suv39h2	-0.015006337	0.392943589	-0.038189545	0.969536557	0.996628386
Hspa14	0.068716846	0.107954832	0.636533304	0.524428881	0.965637248
Cdnf	-0.2245847	0.351392155	-0.639128383	0.522739417	0.965637248
Fam107b	-0.030598853	0.160555205	-0.190581509	0.848853478	0.992886758
Frmd4a	-0.001973915	0.177468884	-0.011122599	0.991125633	0.998770784
Prpf18	-0.070781945	0.100794545	-0.702239844	0.482529601	0.957463006
Bend7	-0.15022927	0.158479189	-0.947943203	0.343158369	0.920191602
Sephs1	0.074629579	0.099430452	0.750570652	0.452911089	0.950106559
Phyh	-0.015700528	0.106439584	-0.147506472	0.882732272	0.992886758
Mcm10	-0.550505188	0.781435581	-0.704479296	0.481134339	0.957463006

Optn	-0.223878154	0.195357202	-1.14599386	0.251797693	0.896939594
Ccdc3	-0.009360493	0.609188418	-0.015365514	0.987740576	0.998148029
Camk1d	0.081142837	0.356647387	0.22751558	0.820022855	0.992886758
Cdc123	0.026809718	0.110605192	0.242391132	0.808477105	0.991020605
Nudt5	0.103202783	0.173159676	0.595997781	0.551176709	0.968926429
Sec61a2	-0.077997514	0.200618867	-0.38878454	0.697435538	0.987548136
Dhtkd1	-1.213279738	1.334452994	-0.90919631	0.363246512	0.926455466
Upf2	0.006367306	0.195150159	0.032627728	0.973971458	0.997262526
Proser2	-0.64472262	0.271747701	-2.372504417	0.017667952	0.579835303
Echdc3	0.346407505	0.183280667	1.890038434	0.05875282	0.72285319
A230108P19Rik	-0.284013097	1.652807569	-0.17183676	0.863565865	0.992886758
Usp6nl	-0.25855432	0.214286977	-1.206579717	0.227594042	0.894410876
Celf2	-0.137177987	0.174160117	-0.787654428	0.430898869	0.945321484
Gata3	-0.147614728	0.424585156	-0.347668132	0.728089432	0.988585656
Taf3	-0.328829475	0.324530852	-1.013245654	0.310942839	0.914819219
Atp5c1	0.019947506	0.135751145	0.146941712	0.883178028	0.992886758
Kin	0.193730166	0.275924789	0.702112219	0.482609183	0.957463006
Itih2	-0.489684285	0.423651822	-1.155864932	0.247736409	0.896939594
Itih5	-0.239058179	0.446944837	-0.534871776	0.592738526	0.974001549
Sfmbt2	-0.034150901	0.557501852	-0.061257018	0.951154521	0.995927651
Prkcq	0.192614095	0.230961313	0.83396692	0.404299625	0.938127315
8030442B05Rik	1.257566813	1.472799256	0.853861657	0.393181647	0.933734231
Gm13293	-0.051471076	0.703713718	-0.073142068	0.941693066	0.994688009
Pfkfb3	-0.067949923	0.23387022	-0.29054543	0.771399001	0.99006615
Rbm17	0.051929083	0.152820499	0.339804432	0.73400381	0.988585656
Il2ra	-0.512986531	0.638868174	-0.802961474	0.421997003	0.943415794
Il15ra	0.194857511	0.218715926	0.890915968	0.372974253	0.928840865
Fbxo18	0.108370618	0.145428743	0.745180184	0.4561628	0.951472839
Ankrd16	0.11225394	0.158093356	0.710048433	0.477674102	0.95716557
Itga8	0.105800604	0.106332025	0.995002253	0.319735168	0.91640698
E030013I19Rik	0.286499163	0.245065527	1.16907166	0.242374761	0.896939594
Fam188a	0.017033644	0.138290828	0.123172623	0.901970404	0.994688009
Pter	-0.098531792	0.393955511	-0.250108932	0.802503109	0.990988839
Rsu1	-0.251444672	0.190767487	-1.318068796	0.187480619	0.873560421
Cubn	-0.17980868	0.843305012	-0.213219034	0.831156122	0.992886758
Trdmt1	0.157877921	0.172996855	0.912605727	0.361449941	0.926371563
Vim	-0.026970384	0.151021403	-0.178586499	0.858262396	0.992886758
St8sia6	0.310672818	0.299405784	1.037631318	0.299441732	0.910105802
Hacd1	-0.004813748	0.192909041	-0.024953461	0.980092085	0.997296835
Stamos	-0.06319435	0.675737041	-0.093519145	0.925491141	0.994688009
Stam	-0.26820956	0.121545018	-2.206668485	0.027337229	0.636714037
Mrc1	-0.336215975	0.340411516	-0.987675091	0.323311797	0.91640698
Slc39a12	-0.863471303	0.78318417	-1.102513733	0.270238391	0.89804647
Cacnb2	0.023217586	0.333896865	0.069535202	0.944563613	0.99486515
Nsun6	0.217732103	0.17239268	1.26300086	0.206588871	0.883765092
Arl5b	-0.29756813	0.213184081	-1.395827155	0.162766551	0.859982992
Plxdc2	-0.158787784	0.129573848	-1.225461673	0.220401322	0.889301125
Nebl	-0.19246719	0.26635243	-0.722603468	0.469923537	0.95486913
A930004D18Rik	0.206256789	0.536800993	0.384233247	0.700805578	0.988482081
Gm17762	-0.304426229	0.770472996	-0.395116027	0.692757262	0.986599035
Skida1	0.251096382	0.257387997	0.97555591	0.329284582	0.917115058
Mllt10	-0.398084074	0.132676328	-3.000415225	0.002696118	0.330973815
Dnajc1	-0.133322707	0.144832965	-0.920527358	0.357297243	0.924354588
Commd3	0.202002446	0.166698645	1.21178217	0.225595775	0.89336109
Bmi1	-0.016584049	0.088796517	-0.186764638	0.851845174	0.992886758
Spag6l	0.284683882	1.929419927	0.147548949	0.882698747	0.992886758
Pip4k2a	-0.030904318	0.233570666	-0.132312495	0.894737125	0.993556555
4930426L09Rik	4.638496661	1.773257875	2.615804912	0.00890174	0.475600561
Armc3	-0.273814797	0.230220978	-1.189356412	0.234299445	0.89581803
Msrb2	0.142366206	0.161274085	0.88275935	0.377366308	0.928883222
4921504E06Rik	0.677898553	2.099269315	0.322921194	0.746754925	0.988585656
Gm3230	-0.066990114	0.609633035	-0.109885964	0.912499814	0.994688009

Otud1	-0.00892519	0.149849858	-0.059560881	0.952505375	0.996023481
Etl4	-0.056281778	0.219324614	-0.256614053	0.797476725	0.990988839
Arhgap21	-0.084773765	0.175245718	-0.483742289	0.628568777	0.977034429
Gm13375	-0.300166068	0.27733219	-1.082334032	0.279104129	0.901874653
Enkur	-0.567473242	0.140890263	-4.027767651	5.63E-05	0.03405023
Thnsl1	0.149458981	0.190352242	0.785170584	0.43235356	0.9459793
Apbb1ip	0.065738935	0.311879497	0.210783124	0.833056508	0.992886758
Pdss1	0.636216007	0.399006405	1.594500739	0.110823925	0.800111791
Abi1	0.200190526	0.092618789	2.161446166	0.030660891	0.657965788
Acbd5	-0.083111413	0.086745758	-0.958103482	0.33801058	0.919246575
Mastl	-0.336061497	0.494314924	-0.679853026	0.496597528	0.960362776
Yme1l1	0.088091366	0.119346689	0.738113194	0.460445667	0.953083512
Spopl	-0.027126406	0.184177543	-0.147284006	0.882907857	0.992886758
Hnmt	0.242081391	0.158059771	1.531581309	0.125625788	0.822401003
Il1f9	0.820496513	0.788955308	1.039978444	0.298349916	0.909779403
Il1rn	-0.229101531	0.230039606	-0.995922114	0.31928799	0.91640698
Psd4	0.024358322	0.301732548	0.08072819	0.935658118	0.994688009
Cacna1b	-0.613307011	1.313819907	-0.466812086	0.640634328	0.977921835
Ehmt1	-0.114495187	0.170623754	-0.671038966	0.502195706	0.961437685
Arrdc1	-0.058371883	0.118951052	-0.49072187	0.623623176	0.975691835
Zmynd19	-0.127953414	0.207103966	-0.617822132	0.536692593	0.96674935
Dph7	0.164382397	0.18977382	0.866201653	0.386379587	0.932501372
Mrpl41	0.220993591	0.194205974	1.137934054	0.25514802	0.897044747
Pnpla7	0.339120688	0.197479556	1.717244533	0.085934502	0.763596872
Nsmf	0.020240265	0.153970574	0.131455412	0.895415056	0.993618558
Entpd8	-0.923419644	0.478541154	-1.929655657	0.053649518	0.707153766
Noxa1	-1.339451741	0.592064354	-2.262341469	0.023676314	0.622011301
Nrarp	-0.068749865	0.21896728	-0.313973233	0.753541369	0.988585656
Tor4a	0.045904007	0.139981444	0.327929229	0.742965173	0.988585656
Nelfb	-0.069475463	0.132721644	-0.523467473	0.600648981	0.974001549
Tubb4b	-0.180682556	0.11804105	-1.530675612	0.125849584	0.822401003
Cysrt1	-2.505012808	1.076045902	-2.327979507	0.019913192	0.595248411
Rnf208	0.639055553	0.975113885	0.655365043	0.512232752	0.963486137
Ndor1	0.096744276	0.202106542	0.478679588	0.632166589	0.977034429
Tmem203	0.3265626	0.172409231	1.8941132	0.058209986	0.72285319
Tprn	-0.319727746	0.388360263	-0.82327616	0.410350967	0.940983181
Ssna1	0.232445362	0.170268413	1.365170196	0.172199563	0.86244803
Anapc2	0.011054159	0.152737284	0.072373675	0.942304534	0.994688009
Tmem210	4.382270654	1.888403712	2.320621711	0.020307269	0.601736455
Lrrc26	-0.192539504	0.191600642	-1.004900102	0.314944955	0.91586731
AA543186	0.919405967	0.672395479	1.367358937	0.171512827	0.86244803
Man1b1	-0.130035049	0.113472289	-1.145963037	0.251810446	0.896939594
Dpp7	0.175763116	0.190863817	0.92088233	0.357111865	0.924354588
Uap1l1	0.203193278	0.131030064	1.550737836	0.120964523	0.816233249
Sapcd2	0.641436677	0.386535869	1.659449298	0.097025289	0.782676152
Entpd2	0.112212445	0.313576997	0.357846544	0.720458157	0.988585656
Npdc1	-0.077866029	0.147747457	-0.527021114	0.598178926	0.974001549
Fut7	0.064947421	0.580998994	0.111785772	0.910993269	0.994688009
Abca2	0.191185779	0.228418142	0.8369991	0.402593074	0.937834293
Clic3	-0.035228551	0.132895046	-0.265085506	0.790943593	0.990988839
BC029214	0.091766448	0.146533112	0.62625059	0.53115059	0.966166773
Ptgds	-0.967968656	0.679920287	-1.423650206	0.154547755	0.854776826
C8g	0.277030153	0.460762115	0.60124334	0.547677922	0.96847584
Fbxw5	0.326163954	0.18611959	1.752442897	0.079697681	0.75659671
Traf2	0.036756305	0.123507752	0.29760322	0.766006017	0.989884648
Edf1	0.076976869	0.09473718	0.812530725	0.416487164	0.943415794
Mamdc4	0.444872035	0.432338769	1.028989457	0.303484629	0.911786415
Phpt1	0.446982877	0.182732313	2.446107472	0.014440795	0.554936045
Gm996	0.353763389	1.425430473	0.248180038	0.803995111	0.990988839
Rabl6	0.110844937	0.10757118	1.0304334	0.302806601	0.911623139
Tmem141	0.210759859	0.187508005	1.124004593	0.261011078	0.897248195
Fcna	-0.273613625	0.480953372	-0.568898445	0.569425061	0.97144955

Bmyc	0.164809145	0.270514281	0.609243786	0.542362862	0.967491297
Camsap1	0.005043235	0.236601919	0.021315277	0.982994158	0.997535311
Ubac1	0.065662421	0.140238422	0.468219907	0.639627338	0.977843986
Nacc2	0.49655441	0.246523968	2.014223665	0.043986056	0.688477147
C330006A16Rik	-0.106088367	0.166984201	-0.635319787	0.525219871	0.965637248
Qsox2	-0.106787539	0.131115483	-0.814454073	0.415384873	0.943415794
4932418E24Rik	-0.045198834	0.702842364	-0.064308637	0.948724477	0.99560893
Gpsm1	0.118530676	0.209303051	0.566311267	0.571182202	0.971613657
Dnlz	0.47371884	0.186084478	2.545719269	0.010905285	0.502706181
Card9	0.22765408	0.191128803	1.19110294	0.233613176	0.89581803
Snapc4	0.494920586	0.375253339	1.318897221	0.187203475	0.873455432
Sdccag3	-0.138992796	0.133935054	-1.037762649	0.29938057	0.910105802
Pmpca	-0.026465593	0.138085616	-0.191660753	0.848007951	0.992886758
Inpp5e	-0.307399803	0.303146213	-1.01403148	0.310567731	0.914819219
Sec16a	-0.130876113	0.203003493	-0.644698822	0.51912238	0.96446859
Notch1	0.068970674	0.251705277	0.274013617	0.784074157	0.990988839
Egfl7	0.332852528	0.114139434	2.916192201	0.003543321	0.373237482
Agpat2	-0.148983578	0.135671917	-1.09811656	0.272153595	0.898370293
Fam69b	0.232192584	0.226774351	1.023892617	0.305885998	0.91333012
Snhg7	0.623771214	0.34866802	1.789011834	0.07361291	0.748833631
Lcn4	0.414748811	1.354362785	0.306231695	0.759428242	0.989266039
Surf6	-0.167712481	0.165081552	-1.015937151	0.309659316	0.9139204
Med22	-0.108428568	0.122893653	-0.882295917	0.377616804	0.928883222
Rpl7a	0.185107831	0.16124416	1.147997119	0.250969753	0.896939594
Surf1	0.23656399	0.111209008	2.127201692	0.033403329	0.670819369
Surf2	0.037081423	0.196382873	0.188822083	0.850232263	0.992886758
Surf4	-0.06858352	0.107132661	-0.640173778	0.522059628	0.965307763
Stkld1	-0.285932842	0.419238658	-0.682028809	0.495220735	0.959471809
Rexo4	-0.102323566	0.120762463	-0.84731268	0.396820861	0.934500821
Adamts13	0.495006069	1.693612149	0.292278294	0.770073854	0.989990452
Cacfd1	0.0743456	0.149005559	0.498945141	0.617818032	0.974153
Slc2a6	-0.035598108	0.387351997	-0.091901185	0.92677655	0.994688009
Adamtsl2	-0.227147064	0.393555228	-0.577166933	0.563826693	0.969658675
Sardh	-0.026898342	0.448636041	-0.05995582	0.952190822	0.996023481
Vav2	0.196931196	0.330946007	0.595055362	0.551806465	0.968926429
Brd3	-0.131514962	0.202080835	-0.650803732	0.515173191	0.964352198
Wdr5	-0.022573042	0.162791009	-0.13866271	0.889716687	0.993043931
Rxra	-0.106491152	0.198331997	-0.536933797	0.591313345	0.974001549
Col5a1	-0.125651233	0.305762974	-0.410943259	0.681114141	0.98444165
Olfm1	-0.072089816	0.172576925	-0.417725696	0.676147683	0.983549699
Ppp1r26	-0.291410994	0.385403819	-0.756118595	0.449578094	0.948476747
1700007K13Rik	0.027020771	0.163221869	0.165546265	0.868514038	0.992886758
Mrps2	-0.157000312	0.24597258	-0.638283797	0.523288957	0.965637248
Gbgt1	-0.463835331	0.707080027	-0.655987036	0.511832466	0.963452765
Ralgds	-0.237719573	0.248892213	-0.95511053	0.339521804	0.919246575
Gtf3c5	-0.025656854	0.179491804	-0.142941645	0.886336268	0.992886758
Gfi1b	-0.888087226	1.899448594	-0.467550019	0.640106415	0.977921835
Tsc1	-0.109161686	0.271428857	-0.402174208	0.687555823	0.98538669
1700026L06Rik	-0.32426495	0.159804142	-2.029139828	0.04244405	0.68118841
Ak8	-0.45328237	0.224219143	-2.021604244	0.043217253	0.685056403
Gtf3c4	-0.268136985	0.175257107	-1.529963547	0.126025752	0.822401003
Ddx31	0.048282911	0.21442337	0.225175602	0.821842669	0.992886758
Barhl1	-0.828233649	2.520148693	-0.328644755	0.742424216	0.988585656
1700101E01Rik	-0.013863259	0.339967284	-0.040778216	0.967472706	0.996474415
Ttf1	-0.148074291	0.160333647	-0.923538472	0.355726661	0.924354588
Setx	-0.001799533	0.187508848	-0.009597058	0.992342773	0.998827474
Ntng2	0.187662265	0.844204059	0.22229491	0.824084314	0.992886758
6530402F18Rik	0.099278102	0.500099075	0.198516868	0.842640688	0.992886758
Med27	-0.273804787	0.281437777	-0.972878588	0.330613648	0.917115058
Rapgef1	-0.025588468	0.171794403	-0.148948204	0.881594504	0.992886758
Trub2	0.227770985	0.1318808	1.727097393	0.084150174	0.76099108
Coq4	-0.252727361	0.190624308	-1.325787689	0.184910032	0.871716376

Slc27a4	0.221220598	0.163062615	1.356660437	0.174889129	0.864111192
Urm1	0.314540803	0.154984751	2.029495166	0.042407881	0.68118841
Cercam	-0.194724315	0.38188202	-0.509906998	0.610116619	0.974001549
Odf2	-0.181012789	0.242930131	-0.745122838	0.456197464	0.951472839
Gle1	-0.019954768	0.117380094	-0.170001299	0.865009115	0.992886758
Sptan1	0.025573474	0.205099055	0.124688405	0.900770238	0.994688009
Wdr34	0.067505824	0.138343083	0.487959519	0.625578514	0.97639266
Set	0.099951669	0.095896611	1.042285727	0.297279228	0.909070179
Pkn3	0.151894603	0.259669896	0.584952684	0.558579532	0.968926429
Zdhhc12	0.346667595	0.251895993	1.37623307	0.168749487	0.861597949
Zer1	-0.181299266	0.284804368	-0.636574738	0.524401885	0.965637248
Tbc1d13	0.038149334	0.18312883	0.208319652	0.834979388	0.992886758
Endog	0.222977627	0.202665736	1.100223605	0.271234708	0.89804647
D2Wsu81e	-0.25376958	0.169363087	-1.498375972	0.134035595	0.827800491
Ccbl1	0.058009682	0.258651371	0.224277496	0.822541386	0.992886758
1700084E18Rik	-0.364453858	0.661610707	-0.550858464	0.581730703	0.971915898
Lrrc8a	0.006991033	0.213383744	0.032762724	0.973863804	0.997262526
Phyhd1	0.316140153	0.194453144	1.625790907	0.103994143	0.79155232
Dolk	0.143403	0.194719375	0.736459845	0.461450896	0.953083512
Nup188	-0.05583593	0.304738319	-0.183225825	0.854620832	0.992886758
Sh3glb2	0.09699158	0.132137815	0.734018341	0.46293756	0.953083512
Fam73b	-0.118853545	0.206174591	-0.576470382	0.564297283	0.969658675
Dolpp1	-0.392695123	0.228250504	-1.720456761	0.085349447	0.761635307
Crat	-0.187455872	0.163698608	-1.145128079	0.252156106	0.896939594
Ppp2r4	-0.014073797	0.097810929	-0.143887774	0.88558909	0.992886758
Ier5l	-0.046007124	0.479537495	-0.095940619	0.923567734	0.994688009
Cstad	0.818861159	0.549370181	1.490545332	0.136080906	0.832457116
1700001O22Rik	0.815411687	0.748598562	1.08925094	0.276043241	0.900302631
Ntmt1	0.241562667	0.140310125	1.721633899	0.085135858	0.761635307
Asb6	-0.152340426	0.181711962	-0.838362122	0.401827356	0.937834293
Prrx2	-0.391326181	0.807349044	-0.484705078	0.627885564	0.976968316
Ptges	-0.011989999	0.165023184	-0.07265645	0.942079505	0.994688009
Tor1b	-0.206105977	0.122979602	-1.675936286	0.093750673	0.775558829
Tor1a	0.142306739	0.141802273	1.003557532	0.315591933	0.91586731
BC005624	-0.0982127	0.112481286	-0.873147024	0.382582946	0.930775109
Usp20	0.300581879	0.220378067	1.36393736	0.172587281	0.86244803
Fnbp1	-0.161155914	0.211458161	-0.762117259	0.445990023	0.948476747
D330023K18Rik	0.26077402	0.26660521	0.978127997	0.328011023	0.917115058
Gpr107	0.001175241	0.187005647	0.006284524	0.994985709	0.999285699
Ncs1	-0.146503583	0.417530808	-0.350880894	0.725677706	0.988585656
Ass1	-0.054406005	0.138630894	-0.392452244	0.694724091	0.987013595
Fubp3	0.057920681	0.146953064	0.394144085	0.693474665	0.986698113
Exosc2	0.005308763	0.170576557	0.031122464	0.975171875	0.997262526
Abl1	-0.384457759	0.205258158	-1.873044969	0.061062175	0.725221283
Qrfp	0.341430818	1.150488033	0.296770421	0.766641789	0.989884648
Lamc3	0.165400794	0.542504678	0.304883626	0.760454787	0.989266039
Aif1l	0.151783994	0.244496847	0.620801434	0.534730279	0.96674935
Nup214	0.052411658	0.2416479	0.216892669	0.828291991	0.992886758
Fam78a	-0.311523098	0.413760641	-0.752906555	0.451506069	0.949134998
Ppapdc3	0.239594579	0.448714458	0.533957787	0.59337074	0.974001549
Prrc2b	0.361223866	0.198658589	1.818314865	0.069016022	0.743426713
Pomt1	-0.55948775	0.349681949	-1.599990369	0.10960072	0.798505558
Uck1	-0.119092404	0.104306311	-1.141756455	0.253555267	0.897044747
Swi5	0.239154471	0.152518425	1.568036591	0.116872593	0.812679331
Golga2	-0.12243651	0.177721421	-0.688923764	0.490871244	0.958896244
Dnm1	0.614896079	0.713262702	0.86208921	0.388638429	0.933269543
Ciz1	-0.026147224	0.247249482	-0.10575239	0.915778812	0.994688009
1110008P14Rik	0.0704679	0.209625437	0.336161019	0.736749446	0.988585656
Lcn2	-0.682359798	0.29422924	-2.319143393	0.020387263	0.601921946
Ptges2	0.184534416	0.152351284	1.211242934	0.225802313	0.893616175
Slc25a25	0.034392079	0.243150175	0.141443778	0.887519372	0.992886758
Naif1	-0.517906114	0.45711957	-1.132977339	0.25722377	0.897248195

Fam102a	-0.37956757	0.204124287	-1.859492444	0.062957369	0.727650961
Dpm2	-0.151834889	0.109166517	-1.390855848	0.164269144	0.859982992
Pip5kl1	0.295284217	1.826596241	0.161658176	0.871575042	0.992886758
St6galnac4	-0.249777024	0.217690577	-1.147394744	0.251218512	0.896939594
St6galnac6	0.049691588	0.167116486	0.29734701	0.766201595	0.989884648
Ak1	0.173167149	0.137337924	1.260883691	0.207350755	0.883765092
Eng	-0.179882311	0.176036417	-1.021847149	0.30685325	0.9139204
Fpgs	-0.170536602	0.305753811	-0.557757893	0.577009719	0.971613657
Cdk9	0.05706865	0.107972546	0.528547787	0.597119189	0.974001549
Mir3960	-0.459104309	0.878618932	-0.522529497	0.601301714	0.974001549
Sh2d3c	-0.168688016	0.237829051	-0.709282634	0.478149103	0.957438234
6330409D20Rik	0.314900215	1.409966848	0.223338737	0.823271881	0.992886758
Ttc16	-0.50677629	0.346038575	-1.464508084	0.143055176	0.840741274
Tor2a	-0.182358703	0.137332975	-1.327858103	0.184224988	0.871468131
Ptrh1	0.470527105	0.315998812	1.489015425	0.136483307	0.832506992
1700019L03Rik	-0.113850111	0.255022816	-0.44643108	0.655285886	0.979133069
Stxbp1	0.050368166	0.278262395	0.181009604	0.856360038	0.992886758
Fam129b	-0.00705562	0.174139149	-0.04051714	0.967680842	0.996474415
Lrsam1	0.046512209	0.216547607	0.214789764	0.829931234	0.992886758
Rpl12	0.413756994	0.255075915	1.622093544	0.10478332	0.793137353
Snora65	0.490910274	0.424788336	1.155658555	0.247820847	0.896939594
Slc2a8	-0.071961676	0.241984383	-0.297381487	0.766175276	0.989884648
Garnl3	0.44474807	0.355752671	1.250160874	0.211240786	0.884390587
Ralgps1	-0.436024514	0.449256484	-0.970546958	0.33177393	0.917115058
Angptl2	0.010646316	0.236266307	0.045060661	0.964058957	0.996474415
Zbtb34	0.182688473	0.368337659	0.49598098	0.61990783	0.974471036
Zbtb43	-0.092282824	0.275377403	-0.335114003	0.73753909	0.988585656
C130021I20Rik	-0.209460597	0.822330216	-0.25471592	0.798942519	0.990988839
Mvb12b	-0.223379897	0.183820462	-1.215206915	0.224287184	0.892989918
C230014O12Rik	0.188543401	0.874976506	0.215483958	0.829390018	0.992886758
Pbx3	-0.271201587	0.215841474	-1.256485061	0.208940159	0.884258007
Mapkap1	0.220764996	0.189019987	1.167945249	0.24282885	0.896939594
Gapvd1	-0.129239241	0.140359842	-0.92077078	0.357170113	0.924354588
Hspa5	-0.403301529	0.103898945	-3.881671069	0.000103741	0.056459384
Rabepk	0.122177617	0.186402842	0.655449328	0.512178501	0.963486137
Fbxw2	0.164108427	0.09038674	1.815625035	0.069427911	0.743426713
Psmd5	0.082637612	0.118496711	0.697383166	0.485563034	0.957710647
Cutal	-0.029421489	0.168643685	-0.174459477	0.861504375	0.992886758
Phf19	0.445882636	0.716310923	0.622470805	0.533632335	0.96674935
Traf1	0.122250612	0.168397514	0.72596447	0.467860552	0.953657709
Hc	-0.175766682	0.161245673	-1.090055185	0.275688836	0.900302631
AI182371	0.18835132	0.337581988	0.557942445	0.576883687	0.971613657
Cntrl	-0.017930728	0.186506825	-0.096139793	0.923409547	0.994688009
Rab14	-0.127573877	0.09165091	-1.391954288	0.163936241	0.859982992
Gsn	-0.228574231	0.166978339	-1.368885528	0.17103506	0.86244803
Stom	-0.119131089	0.097092269	-1.226988419	0.219826944	0.888847883
Ggta1	-0.014544901	0.101219732	-0.143696298	0.885740295	0.992886758
Gm13446	-1.579218273	1.868860247	-0.845016783	0.398101469	0.935327115
Dab2ip	0.101581239	0.213882473	0.474939518	0.634830084	0.977034429
Ttll11	0.008798694	0.349632995	0.025165514	0.979922944	0.997296835
Ndufa8	0.336208173	0.119150143	2.821718596	0.004776707	0.391906015
Morn5	0.298528149	0.270381013	1.104101748	0.269549003	0.89804647
Lhx6	0.021493604	0.466129465	0.046110804	0.963221935	0.996474415
Rbm18	-0.062020694	0.099709305	-0.622015108	0.533931934	0.96674935
Mrrf	0.076923446	0.146236604	0.526020465	0.598873988	0.974001549
Ptgs1	-0.417605289	0.172804526	-2.416633984	0.01566476	0.557315333
Pdcl	0.022510065	0.111932433	0.201104041	0.840617222	0.992886758
Rc3h2	-0.090751373	0.161617489	-0.56151951	0.574443434	0.971613657
Zbtb6	0.087947458	0.16541413	0.531680446	0.594947337	0.974001549
Zbtb26	-0.195170096	0.194434746	-1.003781989	0.315483708	0.91586731
Rabgap1	0.029303192	0.123887021	0.236531571	0.813020212	0.992048071
Gpr21	-0.138635376	0.637152344	-0.217585915	0.82775176	0.992886758

Strbp	-0.151106144	0.129476409	-1.167055414	0.243187992	0.896939594
Crb2	-0.169207987	0.651186588	-0.259845626	0.794982855	0.990988839
Dennd1a	-0.446630722	0.275865528	-1.619016068	0.105443806	0.793982106
Nek6	-0.132564972	0.152364894	-0.870049314	0.384273455	0.931689378
Psmb7	0.198268461	0.112416319	1.763698218	0.077782801	0.754202
Nr6a1	-0.234224212	0.415651799	-0.563510642	0.573087216	0.971613657
Olfml2a	-0.050795484	0.340317276	-0.149259198	0.88134911	0.992886758
Wdr38	-0.202626646	0.402518578	-0.503397003	0.614685178	0.974001549
Rpl35	0.481745254	0.262569924	1.834731283	0.066545515	0.738254074
Arpc5l	0.266002523	0.170684728	1.558443611	0.119128125	0.814755048
Golga1	-0.260656793	0.159244454	-1.636834351	0.101665086	0.788783372
Scai	-0.246701897	0.216952726	-1.137122829	0.255486942	0.897044747
Ppp6c	-0.167293572	0.131265328	-1.274468857	0.202497303	0.88210274
Kynu	0.181951645	0.361227574	0.503703644	0.614469647	0.974001549
Arhgap15	0.400967857	0.159440962	2.514835903	0.01190878	0.52408261
Gtdc1	-0.007847043	0.204387078	-0.038393049	0.969374303	0.996600044
Zeb2	0.252137322	0.169148914	1.490623354	0.136060409	0.832457116
Zeb2os	0.142488141	0.279828577	0.509197964	0.61061347	0.974001549
Acvr2a	-0.20154269	0.163576369	-1.2321015	0.217911171	0.887683556
Orc4	0.15947828	0.165312071	0.964710436	0.334689866	0.918030088
Mbd5	0.042465174	0.291739827	0.145558373	0.884270032	0.992886758
Epc2	-0.038708076	0.120965086	-0.319993785	0.748973042	0.988585656
Kif5c	-0.1658054	1.259807459	-0.131611699	0.895291432	0.993618558
Lypd6b	0.027609106	0.299819846	0.092085654	0.926629987	0.994688009
Lypd6	-0.05158477	0.360525495	-0.14308217	0.886225287	0.992886758
Mmadhc	0.135790899	0.100044416	1.357306129	0.174683961	0.864000311
Gm13483	-0.432941589	1.204953885	-0.359301376	0.719369645	0.988585656
Gm13498	0.207672743	1.469042055	0.141366098	0.887580735	0.992886758
Rnd3	-0.033397436	0.22523653	-0.148277176	0.882124027	0.992886758
Gm13490	0.617889869	2.336463181	0.264455213	0.791429171	0.990988839
Rbm43	-0.095119748	0.118404519	-0.803345592	0.421775013	0.943415794
Nmi	0.052642712	0.137427225	0.383058826	0.701676147	0.988482081
Tnfaip6	-0.352563226	0.636923454	-0.553540969	0.579893035	0.971613657
Rif1	-0.309317135	0.166428415	-1.858559636	0.063089583	0.727650961
Neb	0.652789591	1.767631073	0.369301943	0.711902677	0.988585656
Arl5a	-0.069078212	0.112802095	-0.612384126	0.540283642	0.966989525
Cacnb4	0.439214597	0.194174652	2.261956401	0.023700097	0.622011301
Stam2	-0.249789599	0.141115392	-1.770108809	0.076709016	0.752876039
Fmnl2	-0.094844535	0.207815526	-0.456388111	0.648110914	0.979004493
Prpf40a	-0.265067964	0.154096724	-1.720140161	0.085406967	0.761635307
Arl6ip6	-0.125254861	0.143899516	-0.870432813	0.384063921	0.931591679
Rprm	0.20434495	0.292026474	0.699748028	0.484084677	0.957710647
Galnt13	-0.349433263	0.252685518	-1.382878079	0.166702253	0.860403806
Kcnj3	-0.70935166	0.450213272	-1.575590289	0.115120256	0.81007959
Nr4a2	-0.007551013	0.554207527	-0.013624883	0.989129252	0.998182772
BB557941	-3.981352655	1.827218373	-2.178914525	0.029338018	0.64858361
Gpd2	-0.090971311	0.205444807	-0.442801703	0.657909171	0.979614766
Galnt5	-0.375612162	0.475870201	-0.789316417	0.429927094	0.945321484
Cytip	-0.143881028	0.132592083	-1.085140415	0.27785947	0.901315669
Gm13546	-0.027956874	0.567866729	-0.0492314	0.960734888	0.996474415
Acvr1c	-0.05068386	0.837970383	-0.060484071	0.951770102	0.996023481
Acvr1	-0.175166553	0.169631091	-1.032632358	0.301775979	0.911518274
Upp2	0.381323007	0.258431357	1.475529176	0.14007033	0.837580108
Ccdc148	-0.006534732	0.490686348	-0.013317534	0.989374459	0.998182772
Pkp4	-0.422444449	0.142068578	-2.973524858	0.002944006	0.345591998
Dapl1	1.185790373	0.402262052	2.94780571	0.003200381	0.355460045
Tanc1	-0.063228356	0.156502877	-0.404007628	0.68620711	0.98538669
Wdsub1	0.009488602	0.213173264	0.044511218	0.964496911	0.996474415
Baz2b	0.127128464	0.184519665	0.688969732	0.490842315	0.958896244
Mar-07	-0.048387685	0.156710781	-0.30877062	0.757496019	0.989266039
Cd302	0.104510937	0.170726083	0.612155655	0.540434778	0.966989525
Ly75	-0.004536263	0.341315437	-0.01329053	0.989396003	0.998182772

Gm13580	-0.049771068	0.482290211	-0.103197342	0.91780635	0.994688009
Pla2r1	-0.134209394	0.177550694	-0.755893379	0.449713124	0.948639041
Itgb6	-0.06904584	0.206028111	-0.335128247	0.737528346	0.988585656
Rbms1	-0.308406699	0.155949042	-1.977612009	0.047972493	0.703094165
Gm13582	0.080448737	1.915348675	0.042002137	0.966496995	0.996474415
Tank	0.025268421	0.142908981	0.176814785	0.859653875	0.992886758
Psmd14	0.188186752	0.182590077	1.030651583	0.302704237	0.911518274
Slc4a10	0.964753789	1.30774311	0.737724238	0.46068204	0.953083512
Dpp4	-0.056966009	0.13215887	-0.431041889	0.666437913	0.981863224
Fap	1.052473896	0.658317823	1.598732192	0.109880121	0.798948974
Ifih1	0.109612033	0.16261804	0.674045963	0.500282093	0.9611436
Gca	-0.166154444	0.147436759	-1.126953991	0.259761934	0.897248195
Fign	-0.277566632	0.437699185	-0.6341493	0.525983392	0.965637248
Grb14	-0.154078442	0.149201489	-1.032687027	0.301750387	0.911518274
Cobll1	0.276600592	0.221072237	1.251177422	0.210869754	0.884374639
Slc38a11	0.784125906	0.56814034	1.380162349	0.167536664	0.860403806
Scn3a	0.386872991	0.27292947	1.417483391	0.156341647	0.855455553
Scn2a1	-0.408371847	0.715275953	-0.570929087	0.568047713	0.971255107
Galnt3	-0.346747781	0.159839185	-2.169354035	0.030055816	0.650824012
Ttc21b	-0.270604961	0.226209888	-1.196256112	0.23159663	0.895404344
Scn7a	0.036483116	0.113644495	0.321028454	0.748188828	0.988585656
Xirp2	-0.405029772	0.882058264	-0.459187096	0.646099817	0.979004493
B3galt1	-0.309027636	0.639115202	-0.483524152	0.628723615	0.977034429
Stk39	-0.165160599	0.177951216	-0.928122902	0.353343821	0.923786159
Cers6	-0.171464527	0.427750241	-0.400851971	0.688529113	0.98538669
Nostrin	-0.120286525	0.139151733	-0.864427067	0.387353329	0.932858077
Spc25	0.340202224	0.305820079	1.112426053	0.265955008	0.89768067
Dhrs9	-0.562092299	1.1991235	-0.468752633	0.63924646	0.977843986
Lrp2	-0.06499603	0.249165945	-0.260854389	0.7942048	0.990988839
Bbs5	-0.2358238	0.159177532	-1.481514364	0.13846956	0.834856909
Klhl41	0.013256888	0.510784859	0.025953956	0.979294064	0.997262526
Fastkd1	-0.17075628	0.270640686	-0.630933518	0.528083995	0.965637248
Ppig	-0.038509355	0.14217906	-0.270851098	0.786505558	0.990988839
Ccdc173	0.104118039	0.306722783	0.339453227	0.734268327	0.988585656
Phospho2	0.181618334	0.18709525	0.970726591	0.331684447	0.917115058
Klhl23	0.142136749	0.173952748	0.81709976	0.41387142	0.943415794
Ssb	-0.202913532	0.105757539	-1.9186673	0.055026452	0.711899277
Mettl5	0.453895915	0.205778079	2.205754463	0.027401196	0.636714037
Ubr3	-0.122588394	0.182600746	-0.671346621	0.501999741	0.961378071
Myo3b	-2.069381535	1.188657768	-1.740939731	0.081694143	0.76099108
Sp5	-0.346296937	0.50434044	-0.686633293	0.492313843	0.958916246
Gad1os	0.74267277	0.833264057	0.891281418	0.372778215	0.928840865
Erich2	-0.013458275	0.184104337	-0.073101345	0.941725472	0.994688009
Gad1	-0.029520669	1.147831811	-0.025718637	0.979481759	0.997262526
Gorasp2	-0.155989547	0.119782187	-1.30227666	0.192821827	0.879588734
Tlk1	-0.137820275	0.100593524	-1.370071048	0.170664724	0.86244803
Mettl8	-0.017843783	0.18573914	-0.096069054	0.923465729	0.994688009
Dcaf17	0.021094278	0.143910771	0.146578871	0.883464432	0.992886758
Cybrd1	0.163437648	0.288532261	0.566444972	0.571091331	0.971613657
Dync1i2	-0.141613924	0.113070596	-1.252438113	0.210410266	0.884340413
Slc25a12	-0.041774605	0.137767004	-0.303226492	0.761717261	0.989285682
Hat1	0.089906239	0.145164317	0.619341179	0.535691622	0.96674935
Metap1d	0.105854423	0.193112993	0.548147596	0.583590562	0.972174584
Platr26	0.654886672	1.911436691	0.34261489	0.731888199	0.988585656
Itga6	-0.104716587	0.202428849	-0.517300709	0.604946259	0.974001549
Pdk1	0.041565266	0.114696625	0.362393106	0.71705829	0.988585656
Rapgef4	0.131057235	0.217109975	0.603644469	0.546080041	0.96778188
Zak	-0.06899804	0.212338941	-0.324942942	0.745224256	0.988585656
Cdca7	-0.014293048	0.358836782	-0.039831613	0.968227372	0.996474415
Sp3	-0.056430857	0.105843527	-0.533153597	0.59392726	0.974001549
Sp3os	0.068938871	0.267491071	0.257724008	0.796619916	0.990988839
Ola1	-0.042540639	0.108072806	-0.393629453	0.693854633	0.986894729

Cir1	0.039508971	0.136396115	0.289663462	0.772073711	0.99050004
Scrn3	-0.082144981	0.122070325	-0.672931616	0.500990797	0.961184107
Gpr155	0.050948722	0.211817185	0.240531579	0.809918185	0.991419855
Wipf1	-0.064005219	0.160652072	-0.398408923	0.690328781	0.986002801
Chrna1	0.225262313	0.424197171	0.531032096	0.595396538	0.974001549
Chn1os3	2.25624264	1.539450024	1.465616035	0.14275292	0.840741274
Chn1	0.127321391	0.164438214	0.774281037	0.438764615	0.947831419
Atf2	-0.143518617	0.129876597	-1.105038326	0.269142983	0.89804647
Atp5g3	0.125024599	0.127603226	0.979791834	0.327188884	0.916882624
Lnp	0.122620367	0.203615126	0.602216393	0.547030105	0.968115622
Hoxd9	0.329086438	1.371783346	0.239896802	0.81041026	0.991438232
Hoxd8	-0.464858525	0.734582626	-0.632819928	0.526851241	0.965637248
Mtx2	-0.072184399	0.139122522	-0.51885488	0.603861944	0.974001549
Hnrnpa3	-0.050475006	0.118304086	-0.426654794	0.669630781	0.981863224
Nfe2l2	-0.284991774	0.111544977	-2.554949417	0.010620323	0.502706181
Agps	-0.15475822	0.096813771	-1.598514526	0.109928515	0.798948974
Ttc30b	-0.133624901	0.146021679	-0.915103169	0.360137471	0.925913281
Ttc30a2	-0.665935074	1.425920613	-0.467021142	0.640484752	0.977921835
Ttc30a1	0.003092965	0.227581255	0.013590599	0.989156605	0.998182772
Rbm45	0.213311904	0.167375895	1.27444818	0.202504627	0.88210274
Osbpl6	-0.037664462	0.181820156	-0.207152291	0.835890926	0.992886758
Prkra	0.176019797	0.179999182	0.977892205	0.328127641	0.917115058
Fkbp7	-0.01236811	0.114828808	-0.10770912	0.914226435	0.994688009
Plekha3	-0.249432693	0.111943327	-2.228205113	0.02586684	0.631530315
Ttn	0.411013838	0.538898641	0.762692289	0.445646931	0.948476747
Ccdc141	-0.195220006	0.197165521	-0.990132581	0.32210932	0.91640698
Sestd1	-0.15048939	0.156068815	-0.964250224	0.33492049	0.918190441
Zfp385b	-1.187068497	0.495372341	-2.396315658	0.016560821	0.566772949
Cwc22	-0.006183427	0.231578249	-0.026701241	0.978698023	0.997262526
Ube2e3	0.026307756	0.082541578	0.318721258	0.749937893	0.988585656
Itga4	-0.128279351	0.222476732	-0.576596705	0.564211926	0.969658675
Cerkl	-0.067644287	0.790988977	-0.085518622	0.931849092	0.994688009
Ssfa2	-0.046877999	0.159745158	-0.293454901	0.769174466	0.989990452
Pde1a	0.37925778	0.279755475	1.355675992	0.175202281	0.864468918
Dnajc10	-0.151403147	0.097864184	-1.547074133	0.121845371	0.816233249
Nckap1	-0.242798164	0.114204208	-2.126000157	0.033503246	0.671174852
Dusp19	0.400394107	0.201770123	1.984407312	0.047210452	0.700095406
Nup35	0.132286559	0.187326387	0.706182195	0.480074842	0.957463006
Zc3h15	-0.011168074	0.110311749	-0.101241016	0.919359138	0.994688009
Itgav	0.017943164	0.219771174	0.081644757	0.934929209	0.994688009
Fam171b	0.358386258	0.7079718	0.506215442	0.612705416	0.974001549
Zswim2	-2.784176742	2.456117415	-1.133568259	0.256975694	0.897248195
Calcrl	0.132662871	0.138691577	0.956531562	0.338803746	0.919246575
Tfpi	0.053407663	0.109871681	0.486091254	0.626902468	0.976663797
Gm13710	-2.099618664	2.446908841	-0.858069834	0.390853903	0.933734231
Ctnnd1	-0.203391942	0.216485242	-0.939518741	0.347464475	0.921552698
2700094K13Rik	0.180768368	0.196806346	0.918508833	0.358352537	0.924798085
Tmx2	0.06877694	0.120935551	0.568707378	0.56955474	0.97144955
Med19	-0.197772056	0.137933712	-1.433819576	0.151623757	0.85193038
Zdhhc5	-0.003136061	0.141227197	-0.022205788	0.9822838	0.997390438
Clp1	-0.036434724	0.212149881	-0.171740489	0.863641553	0.992886758
Ypel4	0.658626073	1.842741298	0.357416461	0.720780055	0.988585656
Serping1	-0.308557993	0.123018217	-2.508230083	0.012133763	0.525888996
Ube2l6	0.398521635	0.173192886	2.301027748	0.021390063	0.60743619
Smtnl1	-1.597809108	1.426436572	-1.120140313	0.262653974	0.897248195
Timm10	0.278186703	0.224693978	1.238069244	0.215690387	0.887633697
Slc43a1	0.085065246	0.348378205	0.244174993	0.807095293	0.99099448
Rtn4rl2	-2.58447816	0.995152036	-2.597068655	0.009402313	0.478229167
Slc43a3	-0.200399776	0.118282858	-1.69424192	0.090219322	0.769347579
Prg2	-2.173108192	0.990059439	-2.194926998	0.028168835	0.641905969
P2rx3	0.295719873	1.116528804	0.264856466	0.791120036	0.990988839
Ssrp1	-0.247540614	0.183560315	-1.348551913	0.177480935	0.866855369

Tnks1bp1	0.076555792	0.261541157	0.292710305	0.769743592	0.989990452
4930443O20Rik	-1.73494973	0.846423146	-2.049742778	0.040389538	0.679118604
Aplnr	-0.183618221	0.302450816	-0.607101094	0.543783824	0.967491297
Lrrc55	-0.854726852	0.853606459	-1.001312541	0.316675732	0.91640698
4833423E24Rik	0.734464389	1.639306282	0.448033658	0.654128906	0.979133069
Olfr1033	-0.308383305	0.266975754	-1.155098547	0.248050073	0.896939594
Olfr1034	-1.350903626	1.149470661	-1.175239762	0.239898807	0.896939594
Ptprj	0.054002401	0.163015493	0.331271585	0.740439352	0.988585656
Nup160	-0.050886263	0.194054547	-0.262226593	0.793146752	0.990988839
Fnbp4	-0.167055402	0.178506545	-0.935850288	0.349350275	0.921585102
Agbl2	-0.223793285	0.215911468	-1.036504856	0.299966675	0.910285132
Mtch2	-0.035867703	0.136378958	-0.263000269	0.792550372	0.990988839
C1qtnf4	0.175916831	0.2840091	0.619405617	0.535649182	0.96674935
Ndufs3	0.114580886	0.110312107	1.038697284	0.298945545	0.909933614
Kbtbd4	-0.167158807	0.163307474	-1.023583327	0.306032124	0.91333012
Ptpmt1	0.217561382	0.203572064	1.068719247	0.285196196	0.905916508
Celf1	-0.164006716	0.153726511	-1.066873336	0.286029037	0.905916508
Rapsn	0.478310621	0.502439064	0.951977373	0.341108457	0.919850248
Psmc3	0.179988421	0.156364013	1.151085967	0.24969687	0.896939594
Slc39a13	0.175256497	0.21178004	0.8275402	0.407930946	0.939557159
Spi1	0.063714894	0.19886487	0.32039291	0.7486705	0.988585656
Mybpc3	-0.336712777	0.598764734	-0.562345706	0.573880503	0.971613657
Madd	0.091116577	0.229246177	0.397461708	0.691027019	0.986191149
Nr1h3	0.370933044	0.159632628	2.323666842	0.020143357	0.599053895
Acp2	-0.242235694	0.22285801	-1.086950808	0.27705855	0.900743717
Ddb2	0.179397893	0.242154701	0.740840016	0.458790449	0.952683168
A330069E16Rik	1.214166067	0.515542827	2.355121637	0.018516643	0.586147601
Pacsin3	-0.012279105	0.18483946	-0.066431187	0.947034541	0.995217331
Arfgap2	0.098032146	0.087639982	1.118577883	0.263320263	0.897248195
1110051M20Rik	0.055096115	0.143774651	0.383211609	0.701562871	0.988482081
Lrp4	0.097829357	0.191941104	0.509684247	0.610272691	0.974001549
Ckap5	-0.24148585	0.195298483	-1.236496292	0.216274143	0.887633697
F2	0.22416873	0.863255953	0.259678175	0.795112029	0.990988839
Zfp408	-0.237822487	0.259517294	-0.91640323	0.359455442	0.925244994
Arhgap1	-0.299066767	0.209484631	-1.427631066	0.153398077	0.854159937
Atg13	0.089595026	0.19991639	0.448162484	0.654035936	0.979133069
Harbi1	0.062357277	0.217056691	0.287285669	0.773893595	0.990730604
Ambra1	-0.01149276	0.270089853	-0.042551616	0.966058966	0.996474415
Mdk	0.10184457	0.299536419	0.340007303	0.733851028	0.988585656
Dgkz	0.040067651	0.115614786	0.346561646	0.728920664	0.988585656
Creb3l1	-0.287037191	0.364610333	-0.787243711	0.431139214	0.945321484
Phf21a	-0.03157787	0.20873458	-0.15128241	0.879752944	0.992886758
Gyltl1b	-0.427340029	0.609971178	-0.700590528	0.483558592	0.957710647
Pex16	-0.128376784	0.174339293	-0.736361731	0.461510588	0.953083512
1700029I15Rik	2.597134672	1.941544021	1.337664582	0.181005802	0.868087032
Mapk8ip1	-0.15213476	0.16287122	-0.934080064	0.350262595	0.922338606
Cry2	-0.424118967	0.272254272	-1.557804635	0.119279567	0.815185219
Slc35c1	-0.396268552	0.378868565	-1.045926184	0.295595114	0.908201248
Chst1	0.335397654	0.273436548	1.226601407	0.21997244	0.888847883
Syt13	-0.381836929	1.844039458	-0.207065487	0.835958715	0.992886758
Prdm11	-0.605273682	0.751033391	-0.805921134	0.420288325	0.943415794
4631405J19Rik	0.329362776	0.540876504	0.608942658	0.542562448	0.967491297
Trp53i11	-0.207221551	0.328417161	-0.630970532	0.528059793	0.965637248
Tspan18	0.171025106	0.169980155	1.006147489	0.314344628	0.91586731
Gm13807	-0.241216121	0.840936827	-0.286842143	0.774233193	0.990730604
Cd82	0.01244854	0.144972953	0.085868018	0.931571336	0.994688009
Ext2	0.19451824	0.166264147	1.169934973	0.242027138	0.896939594
Accs	0.17543499	0.299087053	0.586568321	0.557493664	0.968926429
Gm13889	0.41968981	0.242409976	1.731322355	0.083394286	0.76099108
4921507L20Rik	1.047034487	1.839624546	0.569156619	0.569249857	0.97144955
Alkbh3	0.132116657	0.159073162	0.830540206	0.406233426	0.939557159
Hsd17b12	-0.148621955	0.116451121	-1.276260405	0.201863482	0.881220006

Ttc17	-0.214413837	0.22505438	-0.952720126	0.340731893	0.919850248
2810002D19Rik	0.100899256	0.209882093	0.480742568	0.630699473	0.977034429
Api5	-0.056454557	0.08739164	-0.645994935	0.518282636	0.96446859
Lrrc4c	1.199166009	0.748945242	1.601139764	0.109345967	0.798400564
B230118H07Rik	0.049232658	0.135347321	0.363750515	0.716044318	0.988585656
Traf6	-0.58676621	0.238602508	-2.459178715	0.013925528	0.545277966
Prr5l	0.040905239	0.402029278	0.101746916	0.918957561	0.994688009
Commd9	0.256047572	0.145276733	1.762481621	0.077987959	0.754202
Ldlrad3	-0.084158699	0.243019643	-0.346304101	0.729114187	0.988585656
Trim44	-0.214313091	0.219178947	-0.977799618	0.32817344	0.917115058
Fjx1	-0.451545545	0.43048225	-1.048929533	0.294210552	0.907334416
Pamr1	0.084485025	0.176400196	0.478939519	0.631981656	0.977034429
Slc1a2	-0.504444802	0.806470325	-0.625497041	0.53164489	0.966606934
Cd44	-0.171606244	0.137906295	-1.244368458	0.213363986	0.884945675
Pdhx	0.048953872	0.165111338	0.29649007	0.766855849	0.989884648
Apip	0.013148815	0.148925711	0.088291102	0.929645311	0.994688009
Ehf	-0.200229934	0.250096456	-0.80061084	0.423356973	0.943509323
BC016548	-1.467207571	0.932046304	-1.574178841	0.11544611	0.81007959
Elf5	-0.017979746	0.200040678	-0.089880447	0.928382219	0.994688009
Cat	0.039704489	0.14710293	0.269909573	0.787229822	0.990988839
Abtb2	-0.053008945	0.45269509	-0.117096355	0.906783698	0.994688009
Nat10	0.491568519	0.250825196	1.959805185	0.050018565	0.707153766
Caprin1	-0.138572492	0.095531434	-1.45054342	0.14690704	0.844560298
Lincred1	-0.131490253	1.131493576	-0.116209456	0.907486542	0.994688009
Lmo2	0.195372447	0.17704859	1.103496206	0.269811738	0.89804647
Fbxo3	-0.153948119	0.086963218	-1.770267052	0.076682664	0.752876039
Cd59b	-0.401819109	0.385697731	-1.04179796	0.29750536	0.909070179
Cd59a	0.014420466	0.340508003	0.042349861	0.966219798	0.996474415
D430041D05Rik	0.161664146	1.018366362	0.158748513	0.873867015	0.992886758
Hipk3	-0.121427347	0.094130088	-1.289995038	0.197052381	0.879755417
Platr14	0.603611971	1.37162183	0.440071715	0.659885167	0.979924124
Cstf3	-0.058771609	0.187001125	-0.314284787	0.753304752	0.988585656
Tcp11l1	0.024957434	0.310089623	0.080484582	0.935851858	0.994688009
Depdc7	-0.022361274	0.378648	-0.05905557	0.952907846	0.996070133
Qser1	-0.347090721	0.19349178	-1.793826697	0.072840834	0.748701713
Prrg4	-0.249266315	0.430710619	-0.578732689	0.562769555	0.969658675
Ccdc73	0.644625116	0.62979694	1.023544376	0.306050529	0.91333012
Eif3m	0.133060764	0.164440527	0.809172574	0.418415888	0.943415794
Wt1os	2.478438373	1.813234356	1.366860475	0.171669043	0.86244803
Wt1	-0.514234947	0.336218627	-1.529465964	0.12614897	0.822401003
Rcn1	-0.020013667	0.165061476	-0.121249778	0.903493198	0.994688009
Elp4	0.029608108	0.244520131	0.121086585	0.903622455	0.994688009
Immp1l	0.369591818	0.273842544	1.349650835	0.17712801	0.866855369
Dnajc24	0.426048924	0.184022286	2.315202863	0.020601833	0.604325792
Mpped2	0.190136052	0.519844313	0.365755761	0.714547333	0.988585656
Arl14ep	-0.144501277	0.189498678	-0.762545041	0.445734772	0.948476747
Mettl15	-0.062106267	0.217906921	-0.285012825	0.775634316	0.990861469
Kif18a	0.011678527	0.265940293	0.043914094	0.964972881	0.996474415
Bdnf	-0.130745634	0.215006164	-0.608101794	0.543119962	0.967491297
Lin7c	0.01678261	0.104279515	0.1609387	0.872141681	0.992886758
Lgr4	-0.164944857	0.358023344	-0.460709783	0.645006835	0.979004493
Ccdc34	-0.009885076	0.159743225	-0.061881032	0.950657573	0.99573416
Bbox1	-0.170201842	0.179095373	-0.950341924	0.341938543	0.919850248
Fibin	-0.091270174	0.122918579	-0.74252546	0.457769032	0.952222574
Slc5a12	-0.412860179	0.314780587	-1.311580815	0.18966161	0.876232344
Ano3	0.745314638	1.8612022	0.400447968	0.6888266	0.98538669
4930430A15Rik	-0.743651392	1.15551028	-0.64356969	0.519854511	0.964665379
Lpcat4	-0.399067843	0.316160509	-1.262231784	0.206865395	0.883765092
Nop10	0.229977336	0.162506407	1.415189349	0.157012987	0.855986074
Slc12a6	-0.342527768	0.155341618	-2.204996787	0.02745432	0.636714037
Emc4	0.182021669	0.124690353	1.459789505	0.144347934	0.841934045
Katnbl1	0.066900917	0.153042674	0.437138974	0.662010564	0.980647283

Emc7	0.225310306	0.144283317	1.561582521	0.11838637	0.814755048
Aven	0.10604765	0.196684322	0.539176936	0.589764773	0.974001549
Ryr3	0.465741764	1.095367683	0.425192172	0.67069659	0.982434419
Fmn1	0.0978508	0.400439054	0.244358783	0.80695296	0.99099448
Grem1	-0.586758091	2.575397632	-0.227832038	0.819776819	0.992886758
Scg5	0.235498049	0.505138076	0.466205302	0.641068555	0.977921835
Arhgap11a	0.240481557	0.629829289	0.381820219	0.702594721	0.988482081
Actc1	-0.012075096	0.224146447	-0.053871456	0.957037578	0.996226826
C130080G10Rik	-0.741069031	0.41004764	-1.807275446	0.070719343	0.744227652
Aqr	0.029424319	0.239162753	0.123030523	0.902082928	0.994688009
Zfp770	-0.023149695	0.144107341	-0.160642022	0.872375356	0.992886758
Dph6	-0.090935026	0.257454994	-0.353207465	0.723932912	0.988585656
BC052040	-0.536210609	0.51029762	-1.05078015	0.293359575	0.907334416
Meis2	-0.360602955	0.258471825	-1.395134479	0.162975291	0.859982992
D330050G23Rik	1.300816968	1.17171091	1.110185932	0.266918914	0.89768067
Spred1	-0.153199555	0.128422266	-1.192936079	0.232894408	0.89581803
Fam98b	0.264782497	0.254914995	1.03870899	0.298940099	0.909933614
Rasgrp1	0.180465128	0.432133585	0.417614215	0.676229202	0.983549699
Gm13986	-4.457162075	1.863673348	-2.391600481	0.016775091	0.56772809
Thbs1	-0.083215481	0.224937103	-0.369949999	0.711419747	0.988585656
Fsip1	0.110352028	0.217767087	0.50674337	0.612334895	0.974001549
Gpr176	-0.19167512	1.290354675	-0.148544523	0.881913051	0.992886758
Eif2ak4	0.080679852	0.212457734	0.379745422	0.704134399	0.988585656
Srp14	0.237949599	0.190949473	1.246139072	0.212713343	0.884945675
Bmf	0.069348377	0.190370428	0.36428125	0.715648	0.988585656
Bub1b	0.119989891	0.395161955	0.303647376	0.761396554	0.989285682
Pak6	-0.914659457	0.711812519	-1.284972422	0.198801921	0.880661448
Ankrd63	-0.032319575	0.390367237	-0.082792744	0.934016339	0.994688009
Plcb2	0.150800769	0.30142843	0.500287145	0.616872905	0.974001549
Inafm2	-0.191207132	0.179065304	-1.067806707	0.285607712	0.905916508
A430105I19Rik	0.040255046	0.163279096	0.246541336	0.805263211	0.99099448
Phgr1	-0.435623981	0.395347786	-1.101875351	0.270515866	0.89804647
Disp2	-0.193219517	0.482362988	-0.400568705	0.68873769	0.98538669
Knstrn	0.065004403	0.28814546	0.225595793	0.821515814	0.992886758
Ivd	-0.208172652	0.181889377	-1.14450143	0.252415745	0.897013805
Bahd1	-0.153012712	0.284722581	-0.537409824	0.590984559	0.974001549
Chst14	-0.26011629	0.32305802	-0.805168958	0.420722187	0.943415794
Ccdc32	0.264058504	0.151926682	1.738065362	0.082199297	0.76099108
Rpusd2	-0.435830809	0.469518597	-0.928250365	0.353277714	0.923786159
Casc5	-0.617385592	0.784359171	-0.787121022	0.431211025	0.945321484
Rad51	-0.094513133	0.411575742	-0.229637277	0.818373638	0.992734473
Rmdn3	-0.037356276	0.164890096	-0.226552575	0.820771673	0.992886758
Gchfr	0.979694047	0.350671412	2.793766508	0.005209808	0.406593461
Dnajc17	0.465072359	0.240699285	1.932171753	0.05333831	0.707153766
Zfyve19	0.025525899	0.151520467	0.168465027	0.866217455	0.992886758
Ppp1r14d	0.09051342	0.311994473	0.290112255	0.771730361	0.990335738
Spint1	-0.285835479	0.142522101	-2.005551963	0.044904071	0.690997903
Rhov	-0.772269523	0.533889795	-1.446496131	0.148038096	0.847481767
Vps18	-0.108310476	0.201958735	-0.536300033	0.591751208	0.974001549
Gm14207	0.099717414	0.241586425	0.41276083	0.679781852	0.983903958
Dll4	-0.008107383	0.183793859	-0.044111281	0.964815701	0.996474415
Chac1	-0.658340441	0.457543349	-1.438859164	0.15019043	0.849830719
Ino80	-0.247686628	0.311270477	-0.795727981	0.42619016	0.944290206
Exd1	0.199912468	0.521153581	0.383596075	0.701277849	0.988482081
Chp1	-0.251332145	0.135420307	-1.855941333	0.063461921	0.728651743
1700020I14Rik	0.135638758	0.127650878	1.06257599	0.28797428	0.907334416
Oip5	0.92522552	0.463234921	1.997313844	0.0457911	0.696869274
Nusap1	0.225000041	0.324327054	0.693744286	0.487842588	0.958288569
Ndufaf1	-0.016758753	0.223914107	-0.074844562	0.940338386	0.994688009
Rtf1	-0.346706236	0.209441524	-1.655384425	0.097846543	0.785804482
Itpka	-0.337962833	0.211183123	-1.600330694	0.109525241	0.798400564
Ltk	-0.447619384	0.412162706	-1.086025925	0.277467523	0.901191693

Rpap1	0.196367614	0.328482346	0.597802641	0.549971628	0.968926429
Tyro3	0.270411057	0.249217655	1.08503973	0.277904059	0.901315669
Mga	-0.092377177	0.155068407	-0.595718872	0.551363049	0.968926429
Mapkbp1	-0.24600013	0.322231464	-0.763426783	0.445208912	0.948476747
Ehd4	-0.031403846	0.142276036	-0.220724771	0.825306742	0.992886758
Pla2g4f	0.240006734	0.570272048	0.420863577	0.673854703	0.983375962
Vps39	-0.233582983	0.143235592	-1.630760758	0.102940811	0.79028905
Tmem87a	0.043272674	0.112552256	0.384467411	0.700632045	0.988482081
Ganc	-0.084460024	0.299989734	-0.281543049	0.778293915	0.990988839
Capn3	-0.482161497	0.530975372	-0.908067535	0.363842542	0.926455466
Zfp106	-0.217031561	0.172134689	-1.260824086	0.207372234	0.883765092
Snap23	0.170057824	0.139872882	1.215802678	0.224060098	0.892989918
Lrrc57	0.060776912	0.122588876	0.495778362	0.620050793	0.974471036
Haus2	0.094732521	0.137850553	0.687211761	0.491949294	0.958916246
Cdan1	0.010498888	0.248062867	0.042323498	0.966240813	0.996474415
Ttbk2	-0.115721534	0.214021308	-0.540700991	0.588713695	0.973852939
AV039307	0.358529659	1.032156017	0.347359947	0.72832092	0.988585656
Ubr1	-0.021117686	0.162229667	-0.130171545	0.896430707	0.994092519
Tmem62	-0.160907627	0.162742173	-0.988727281	0.322796591	0.91640698
Ccndbp1	0.12571252	0.117641792	1.068604258	0.285248029	0.905916508
Epb4.2	-1.115739412	0.748562464	-1.490509431	0.136090339	0.832457116
Lcmt2	0.121186293	0.20815671	0.582187782	0.560440202	0.968926429
Adal	0.189960918	0.252472161	0.752403422	0.451808489	0.949134998
Zscan29	-0.162479138	0.206405327	-0.78718481	0.431173688	0.945321484
Tubgcp4	-0.380506916	0.320225218	-1.188247815	0.234735791	0.89581803
Trp53bp1	-0.23409984	0.241500638	-0.969354955	0.332368119	0.917115058
Map1a	0.357453968	0.714495016	0.50028896	0.616871627	0.974001549
Ppip5k1	-0.126071744	0.176750783	-0.713274038	0.475676199	0.957156188
Ckmt1	-0.105821802	0.210956702	-0.501628065	0.615929174	0.974001549
Catsper2	-0.264620163	0.573176275	-0.461673267	0.644315645	0.97889195
Pdia3	-0.157993399	0.087791444	-1.7996446	0.071916774	0.744227652
Ell3	0.434672658	0.533825027	0.814260547	0.415495706	0.943415794
Serf2	0.500745656	0.190997424	2.62174036	0.008748205	0.475600561
Serinc4	-0.165914333	0.965105935	-0.171913079	0.863505864	0.992886758
Hypk	0.272451155	0.128899956	2.113663681	0.034544003	0.674795441
Mfap1b	-0.025847743	0.169625414	-0.152381308	0.878886199	0.992886758
Mfap1a	0.119738862	0.142522065	0.840142629	0.400828421	0.937313897
Wdr76	-0.166680123	0.41033736	-0.406202649	0.684593707	0.985105247
Frmd5	0.042273336	0.543317129	0.077806006	0.937982369	0.994688009
Casc4	0.152893035	0.157587621	0.970209674	0.33194199	0.917115058
Ctdspl2	-0.127107032	0.125441399	-1.013278178	0.310927308	0.914819219
Spg11	-0.02458257	0.206642525	-0.118961817	0.905305607	0.994688009
B2m	-0.040529102	0.116698386	-0.347297876	0.728367546	0.988585656
Trim69	0.939759337	1.581171918	0.594343554	0.552282353	0.968926429
Sord	0.085274783	0.096223974	0.886211401	0.375503621	0.928883222
Duoxa2	-0.055604458	1.53403143	-0.036247275	0.971085191	0.996733101
Duoxa1	-5.867489894	4.917086709	-1.19328583	0.23275745	0.89581803
Shf	0.12639768	0.488229398	0.25888994	0.79572016	0.990988839
Slc28a2	0.026605852	0.463640244	0.05738469	0.954238758	0.996070133
Bambi-ps1	0.313662191	0.323714225	0.968947816	0.332571226	0.917115058
Gm14085	0.440516735	0.841773117	0.523320032	0.600751563	0.974001549
Gatm	-0.593259649	0.319089456	-1.859226741	0.062995006	0.727650961
Spata5l1	0.355621472	0.360816029	0.985603309	0.324327813	0.91640698
AA467197	-1.7545279	0.932763089	-1.881000567	0.059971842	0.725221283
Slc30a4	-0.115876069	0.161152006	-0.719048258	0.472111186	0.955003345
Bloc1s6	-0.089183434	0.14098313	-0.632582311	0.527006442	0.965637248
Sqrdl	0.035265307	0.103503322	0.340716664	0.733316892	0.988585656
Sema6d	0.161924018	0.239397292	0.676381993	0.498798147	0.960700483
Slc24a5	-0.448090272	0.720504977	-0.621911418	0.534000117	0.96674935
Myef2	-0.055919248	0.218315768	-0.256139299	0.797843278	0.990988839
Dut	0.260945413	0.161074931	1.620024991	0.105226909	0.793982106
Fbn1	0.482428563	0.231480135	2.084103517	0.037150752	0.678479117

Cep152	0.281739832	0.36456594	0.772808979	0.439635439	0.947849924
Shc4	0.208282674	0.885789624	0.235137857	0.814101736	0.992048071
Eid1	0.104058848	0.120384022	0.864390853	0.387373216	0.932858077
Secisbp2l	-0.052402706	0.115706202	-0.452894533	0.650624688	0.979004493
Cops2	-0.15402463	0.123287839	-1.249309194	0.211552006	0.884755882
Galk2	-0.07627796	0.107284522	-0.710987553	0.477091949	0.95716557
Fgf7	0.171560361	0.186965244	0.917605633	0.358825368	0.925199968
Dtwd1	0.041274147	0.193838745	0.212930323	0.83138131	0.992886758
Atp8b4	0.008285971	0.341547657	0.024260072	0.980645162	0.997296835
Slc27a2	-0.023154061	0.404777557	-0.057201939	0.954384333	0.996070133
Hdc	-0.087967513	0.113013783	-0.778378618	0.43634584	0.946762848
Gabpb1	-0.011577643	0.12607964	-0.091828011	0.926834689	0.994688009
Usp8	-0.05654776	0.129528523	-0.436566086	0.662426062	0.980647283
Trpm7	-0.141807148	0.113750235	-1.246653666	0.212524515	0.884945675
Sppl2a	-0.172741376	0.108249584	-1.595769422	0.110540283	0.800111791
Ap4e1	0.016300195	0.213173132	0.076464586	0.939049488	0.994688009
Blvra	0.224314657	0.139096168	1.612658787	0.106818659	0.794908043
Ncaph	0.184289898	0.26518585	0.694946196	0.48708902	0.958288569
Itpripl1	-0.093432698	0.224774016	-0.415673927	0.677648623	0.983670411
1810024B03Rik	0.930475278	0.592097522	1.571489906	0.116068897	0.811920148
Snrnp200	0.303751695	0.199245459	1.524510002	0.12738136	0.824450458
Ciao1	0.03933703	0.111927009	0.351452522	0.725248885	0.988585656
Tmem127	-0.207263767	0.140166463	-1.478697277	0.13922124	0.836190654
Stard7	-0.105927537	0.122089688	-0.867620671	0.385602026	0.931855682
Dusp2	-0.031420008	0.413536048	-0.075978886	0.939435896	0.994688009
Fahd2a	0.327892333	0.200819252	1.6327734	0.102516663	0.789151138
Kcnip3	0.206730194	0.437196777	0.472853884	0.636317428	0.977284137
Prom2	-0.3518355	1.010811535	-0.348072304	0.727785883	0.988585656
Zfp661	-0.398638079	0.449828326	-0.88620048	0.375509505	0.928883222
Mrps5	0.111134253	0.151879476	0.731726602	0.464335456	0.953083512
Mal	-0.174765744	0.135418668	-1.290558726	0.196856736	0.879755417
Mall	-0.078383255	0.222658347	-0.352033759	0.724812945	0.988585656
Nphp1	0.018226727	0.115463213	0.157857442	0.874569134	0.992886758
1500011K16Rik	0.395271036	0.25835385	1.529959922	0.12602665	0.822401003
Bub1	-0.055779045	0.345551931	-0.161420152	0.871762496	0.992886758
Acoxl	0.051210877	0.158182522	0.32374548	0.746130736	0.988585656
Bcl2l11	-0.039764652	0.236038405	-0.168466873	0.866216003	0.992886758
Gm14005	0.527081164	0.205581204	2.563858722	0.010351567	0.499527467
Anapc1	0.188586587	0.168883344	1.11666777	0.264136403	0.897514059
Mertk	-0.161489605	0.22995565	-0.702264133	0.482514457	0.957463006
Tmem87b	0.037635364	0.164314829	0.22904423	0.818834539	0.992734473
Fbln7	-1.340911797	1.17077158	-1.145323153	0.252075318	0.896939594
Zc3h8	-0.160329081	0.228883357	-0.700483787	0.483625227	0.957710647
Zc3h6	-0.061492627	0.273784264	-0.224602488	0.822288529	0.992886758
Ttl	-0.003492312	0.119423909	-0.029242986	0.976670798	0.997262526
Polr1b	0.101300031	0.252697232	0.400875109	0.688512077	0.98538669
Chchd5	0.434243764	0.175825116	2.469748204	0.013520819	0.540715407
AI847159	1.822858108	2.067185743	0.881806637	0.377881382	0.928883222
Slc20a1	-0.091097907	0.164945111	-0.552292255	0.580748136	0.971613657
Ckap2l	0.964570014	0.406594787	2.372312791	0.017677119	0.579835303
Gm14023	2.533174506	1.001810636	2.528596138	0.011451972	0.514178739
Il1a	-0.204276349	0.402378245	-0.50767245	0.611683072	0.974001549
Il1b	0.316229073	0.397920469	0.794704211	0.426785582	0.944290206
Il1bos	1.421247458	0.657218468	2.162519051	0.03057819	0.657949462
Sirpa	-0.008453567	0.141770685	-0.059628456	0.952451554	0.996023481
4932416H05Rik	0.12774969	0.334014141	0.38246791	0.702114327	0.988482081
Stk35	0.076912235	0.292973734	0.26252263	0.792918541	0.990988839
Tgm3	1.549214445	1.766365586	0.877063308	0.380452253	0.929775696
Snrpb	-0.038942824	0.106166022	-0.366810616	0.713760289	0.988585656
Nop56	0.182149546	0.112090018	1.625029139	0.10415635	0.79155232
Idh3b	0.207620049	0.099298025	2.090877939	0.036539007	0.677009445
Ebf4	0.151829861	0.786959186	0.192932319	0.847011974	0.992886758

Cpxm1	-0.181519429	0.239240563	-0.758731823	0.448012997	0.948476747
Pced1a	0.353908719	0.188403456	1.878461927	0.060318001	0.725221283
Vps16	-0.036839215	0.143511018	-0.256699555	0.797410715	0.990988839
Ptpra	-0.073687979	0.12229283	-0.602553553	0.546805727	0.967902958
4930473A02Rik	0.475578638	1.360314579	0.349609307	0.726631925	0.988585656
Mrps26	0.238089424	0.134027688	1.776419686	0.075663769	0.752876039
Ubox5	-0.290386294	0.34081425	-0.852036831	0.39419365	0.933796635
Fastkd5	-0.000101548	0.192266811	-0.000528162	0.999578588	0.999950896
Lzts3	-0.537665663	0.301092608	-1.785715253	0.074145374	0.750704981
Ddrgk1	0.140229483	0.117969219	1.18869553	0.2345595	0.89581803
Itpa	0.159822124	0.134371133	1.189408176	0.234279085	0.89581803
Slc4a11	0.489352721	1.078246177	0.453841369	0.649943006	0.979004493
4930402H24Rik	-0.016392733	0.233991054	-0.070057092	0.944148218	0.99486515
Gm14057	0.838964395	0.825045476	1.016870487	0.309215045	0.9139204
A730017L22Rik	-0.384148379	0.768415919	-0.499922463	0.617129675	0.974001549
Atrn	0.000502904	0.172590101	0.002913865	0.997675075	0.99957296
Gfra4	0.61479067	0.698893686	0.879662648	0.37904209	0.929137643
Adam33	0.08692529	0.428478926	0.202869463	0.839237063	0.992886758
Siglec1	0.134077008	0.527062458	0.254385427	0.799197808	0.990988839
Hspa12b	0.289678095	0.147543314	1.963342753	0.049606361	0.707153766
1700037H04Rik	-0.306259883	0.200712202	-1.525865788	0.127043293	0.824450458
Spef1	0.017968557	0.139945218	0.128397075	0.897834746	0.994101834
Cenpb	0.062290925	0.094994391	0.655732659	0.511996151	0.963452765
Cdc25b	-0.173861749	0.185446806	-0.937528946	0.348486541	0.921552698
Ap5s1	0.080239586	0.264660351	0.303179472	0.761753092	0.989285682
Mavs	-0.252984404	0.279257756	-0.905917199	0.36497968	0.926556975
Pank2	0.107856947	0.123789884	0.871290476	0.38359557	0.931591679
Rnf24	0.108671056	0.55246509	0.196702122	0.844060649	0.992886758
Smox	0.14240445	0.210113298	0.677750775	0.497929727	0.960700483
Adra1d	1.226791115	0.83703198	1.465644258	0.142745227	0.840741274
5330413P13Rik	0.047155143	0.266808026	0.176738099	0.859714113	0.992886758
Prnp	-0.261036679	0.122351545	-2.133497203	0.032883961	0.665167506
Rassf2	-0.283592778	0.186225058	-1.522849726	0.127796306	0.826021492
Slc23a2	-0.328372557	0.195043038	-1.683590249	0.092260884	0.774469646
Tmem230	-0.056494792	0.124997601	-0.451967014	0.651292746	0.979004493
Pcna	0.120997539	0.15488052	0.781231491	0.434666355	0.946762848
Cds2	-0.333594054	0.155968551	-2.138854606	0.032447445	0.665167506
Gpcpd1	-0.134616306	0.135604026	-0.992716143	0.320848306	0.91640698
1110034G24Rik	0.18191753	0.228085114	0.797586157	0.425110687	0.944290206
Chgb	0.136252515	1.512444302	0.090087625	0.928217583	0.994688009
Trmt6	0.278319375	0.184572676	1.50791212	0.13157702	0.827800491
Mcm8	-0.354422008	0.490819312	-0.722102817	0.470231266	0.95486913
Crls1	0.014079663	0.176329984	0.079848374	0.93635785	0.994688009
Lrrn4	0.076227612	0.238370667	0.319786042	0.749130529	0.988585656
Fermt1	-0.461426777	0.410720555	-1.123456744	0.261243562	0.897248195
Bmp2	0.258151546	0.337672423	0.764502897	0.444567611	0.948476747
Tmx4	-0.114081983	0.153147938	-0.744913609	0.456323948	0.951472839
Plcb1	-0.352169677	0.196744828	-1.789981873	0.073456826	0.748833631
Plcb4	-0.110404254	0.243687186	-0.453057279	0.650507496	0.979004493
Ankef1	-0.726648963	0.490095158	-1.482669134	0.138162339	0.833928471
Snap25	-4.075213818	2.448791921	-1.664173172	0.096077828	0.780429201
Mkks	0.176967997	0.165624708	1.068487904	0.285300484	0.905916508
Slx4ip	0.106540677	0.445679853	0.239052038	0.811065232	0.991706885
Jag1	0.201496437	0.206600007	0.975297336	0.329412791	0.917115058
Btbd3	-0.223788113	0.112254901	-1.99357098	0.046198959	0.698098452
Sptlc3	-0.200852825	0.339060022	-0.592381324	0.553595269	0.968926429
Ism1	-0.046054599	0.143557527	-0.320809363	0.748354864	0.988585656
Tasp1	0.123960219	0.180598194	0.686386815	0.492469217	0.958916246
Esf1	-0.295921665	0.239743095	-1.234328208	0.217080629	0.887633697
Ndufaf5	0.28601274	0.21181967	1.350265253	0.176930914	0.866546104
Macrod2	-0.371251192	0.263154083	-1.410774964	0.158310979	0.855986074
Flrt3	-0.097742338	0.266497933	-0.366765841	0.71379369	0.988585656

Kif16b	0.094268447	0.161202097	0.584784249	0.558692797	0.968926429
Snrpb2	0.103633733	0.104599739	0.990764741	0.32180047	0.91640698
Bfsp1	0.195453501	0.560839584	0.348501616	0.727463501	0.988585656
Dstn	-0.239216638	0.107463213	-2.226032812	0.026011984	0.631530315
Rrbp1	0.215823365	0.189155912	1.140981333	0.253877691	0.897044747
Snx5	-0.084359029	0.081094416	-1.040256945	0.298220542	0.909779403
Snord17	1.166835176	2.080476316	0.560850016	0.574899787	0.971613657
Mgme1	0.341423468	0.234363386	1.456812318	0.145168195	0.842729381
Ovol2	-0.192166809	0.374419107	-0.513239857	0.607783549	0.974001549
Csrp2bp	-0.314348157	0.201657866	-1.558819218	0.119039174	0.814755048
Zfp133-ps	0.404533055	0.551208831	0.733901621	0.463008699	0.953083512
Dzank1	-0.208201537	0.323214325	-0.644159374	0.519472092	0.964665379
Polr3f	-0.293931495	0.168573101	-1.743644116	0.081221166	0.760377276
Rbbp9	0.081951936	0.117506166	0.697426692	0.485535802	0.957710647
Sec23b	-0.223474141	0.139456286	-1.602467325	0.109052307	0.798400564
Gm561	0.126264212	0.201327983	0.627156792	0.530556463	0.966026275
Dtd1	0.125997264	0.137320387	0.917542309	0.358858533	0.925199968
Slc24a3	-0.348722655	0.222341089	-1.568413003	0.116784779	0.812418014
BC039771	0.185683519	0.717418493	0.258821763	0.795772765	0.990988839
Rin2	-0.089438447	0.107592135	-0.83127309	0.405819373	0.939557159
Naa20	0.182317734	0.183200592	0.995180923	0.319648278	0.91640698
Crnkl1	-0.218420186	0.169758904	-1.286649364	0.19821653	0.880381196
Cfap61	0.357821499	0.50209145	0.712662004	0.476054934	0.95716557
Ralgapa2	0.243529753	0.239147653	1.018323825	0.30852409	0.9139204
Kiz	-0.010882546	0.131238478	-0.082921915	0.933913629	0.994688009
Xrn2	-0.177031778	0.173234693	-1.021918733	0.306819366	0.9139204
Foxa2	-0.010271265	0.213028202	-0.048215516	0.961544484	0.996474415
Sstr4	1.897451769	0.913933794	2.076137004	0.037881282	0.679118604
Thbd	-0.126028609	0.124594261	-1.011512154	0.311771367	0.914819219
Cd93	-0.355977677	0.177314165	-2.007609924	0.044684759	0.689870152
Nxt1	0.056840837	0.152687247	0.372269707	0.709692051	0.988585656
Gzf1	-0.10938659	0.115819516	-0.944457326	0.344935999	0.92132235
Napb	0.056397049	0.371916269	0.151639102	0.879471591	0.992886758
Cst8	0.554585312	0.268435331	2.065992244	0.038829212	0.679118604
Cst13	1.535573757	1.121761175	1.36889544	0.171031961	0.86244803
Cst9	-0.095231408	0.930137623	-0.102384213	0.918451714	0.994688009
9230104L09Rik	0.25604654	0.202724267	1.263028562	0.206578915	0.883765092
Cst3	0.134308627	0.099745331	1.346515426	0.178136349	0.867377413
Syndig1	-1.316706689	2.417577148	-0.544638954	0.586001858	0.972904784
Zfp120	-0.010865108	0.160253283	-0.067799596	0.945945166	0.995065399
Gm14139	0.363879741	1.66254024	0.218869734	0.826751523	0.992886758
Zfp937	0.025214014	0.216266222	0.116587851	0.907186666	0.994688009
3300002I08Rik	0.097706061	0.556852694	0.175461234	0.860717229	0.992886758
Zfp442	0.017256982	0.296691379	0.058164758	0.953617392	0.996070133
Zfp345	-0.208739938	1.414866578	-0.147533302	0.882711096	0.992886758
Cst7	0.877149626	0.428306082	2.04795043	0.040564857	0.679118604
Apmap	-0.110746493	0.185608605	-0.596666805	0.550729859	0.968926429
Acss1	0.107653005	0.17358043	0.620190912	0.535132104	0.96674935
E130215H24Rik	0.261737098	0.609725641	0.429270282	0.667726541	0.981863224
Entpd6	-0.478005274	0.27115618	-1.762841157	0.077927284	0.754202
Pygb	-0.091680874	0.208603159	-0.439498972	0.660300026	0.979924124
Abhd12	-0.024351025	0.093830515	-0.259521385	0.795232984	0.990988839
Gins1	0.342146312	0.30956977	1.105231664	0.26905922	0.89804647
Ninl	-0.200040226	0.41572435	-0.481184772	0.630385183	0.977034429
Nanp	-0.120514904	0.778261066	-0.154851513	0.876938379	0.992886758
Nsfl1c	0.276790945	0.108443771	2.552391358	0.010698627	0.502706181
Fkbp1a	0.069191676	0.081080136	0.853373951	0.393451962	0.933734231
Sdcbp2	-0.185816079	0.243995998	-0.761553796	0.44632636	0.948476747
Snph	1.037429632	1.998094463	0.519209503	0.603614653	0.974001549
Tmem74b	-0.239396155	1.350772717	-0.177229042	0.859328484	0.992886758
Psmf1	-0.374200715	0.193168229	-1.937175261	0.052723923	0.707153766
Rspo4	0.264799645	1.081321365	0.244885242	0.806545289	0.99099448

Angpt4	-0.246502614	1.32885385	-0.185500169	0.852836747	0.992886758
Fam110a	0.049698286	0.18909698	0.262819036	0.792690063	0.990988839
Slc52a3	-0.066476746	0.247556345	-0.268531779	0.788290017	0.990988839
Srxn1	0.239636026	0.224308158	1.068333974	0.285369888	0.905916508
Tcf15	-0.838747082	1.129272503	-0.742732228	0.457643814	0.952204734
Csnk2a1	-0.001496539	0.117850692	-0.012698598	0.989868257	0.998417328
Tbc1d20	-0.221256279	0.206337535	-1.07230262	0.283584131	0.905916508
Rbck1	-0.085367292	0.162301456	-0.525979827	0.598902224	0.974001549
Trib3	-0.082706937	0.237115218	-0.348804843	0.727235827	0.988585656
Sox12	-0.481370223	0.349846345	-1.375947553	0.168837871	0.861597949
Zcchc3	0.084454395	0.387207985	0.218111191	0.827342478	0.992886758
6820408C15Rik	-0.053930915	0.184220224	-0.29275241	0.769711407	0.989990452
Defb25	0.960165951	0.701683028	1.368375623	0.17119453	0.86244803
Rem1	0.163224052	0.267863379	0.609355607	0.542288756	0.967491297
H13	0.043315964	0.13341647	0.324667292	0.745432892	0.988585656
Mcts2	-0.203616746	0.290251403	-0.701518559	0.482979456	0.957463006
Id1	0.093633639	0.251357304	0.372512109	0.709511598	0.988585656
Cox4i2	0.256554563	0.118153099	2.171373957	0.029902916	0.65030913
Bcl2l1	-0.2402314	0.255859887	-0.938917792	0.347772953	0.921552698
Tpx2	-0.663810852	0.48979219	-1.355290807	0.175324923	0.864596675
Mylk2	-0.347233187	1.940343139	-0.178954526	0.857973408	0.992886758
Foxs1	0.493188064	0.455548098	1.082625668	0.278974609	0.90176568
Ttll9	-0.274564753	0.233231429	-1.177220214	0.239107623	0.896609492
Pdrg1	0.20970542	0.122546325	1.711233857	0.087037951	0.765418158
Ccm2l	0.215000888	0.214927932	1.000339446	0.317146264	0.91640698
Hck	0.184740053	0.144135564	1.281710413	0.199944251	0.880888611
Tm9sf4	-0.250994955	0.214750569	-1.168774342	0.24249456	0.896939594
Tspyl3	-0.156447839	0.310340554	-0.504116646	0.61417941	0.974001549
Plagl2	-0.116339027	0.356326447	-0.326495627	0.744049397	0.988585656
Pofut1	-0.138230567	0.208599612	-0.662659753	0.507548491	0.963011338
Kif3b	-0.289936322	0.17354923	-1.670628684	0.094795043	0.777748075
2500004C02Rik	0.487202808	0.195183213	2.496130687	0.012555633	0.525888996
Asxl1	-0.228148389	0.278361262	-0.819612569	0.412437006	0.942589446
Nol4l	-0.03226424	0.362464948	-0.089013408	0.929071255	0.994688009
Commd7	0.282709086	0.146174385	1.934053541	0.053106545	0.707153766
Dnmt3b	0.079410021	0.611320614	0.129899138	0.896646226	0.994101834
Mapre1	-0.023264248	0.086010263	-0.270482233	0.786789284	0.990988839
Bpifa2	-0.321348163	0.7959753	-0.403716249	0.686421389	0.98538669
Bpifa3	-0.192076821	1.735714432	-0.110661534	0.91188475	0.994688009
Bpifa1	-0.317200738	0.497064268	-0.638148341	0.523377121	0.965637248
Bpifb1	-0.263439101	0.34707166	-0.759033742	0.447832372	0.948476747
Bpifb5	-0.52507702	0.419643948	-1.251244113	0.210845428	0.884374639
Cdk5rap1	0.795572751	0.517801172	1.536444479	0.124429405	0.821164769
Snta1	-0.07089233	0.194081465	-0.365270995	0.714909128	0.988585656
Cbfa2t2	-0.286980855	0.178424964	-1.608412009	0.107744973	0.796929372
Necab3	-0.006325483	0.513776002	-0.012311752	0.990176891	0.998417328
1700003F12Rik	0.073843469	1.632404378	0.045236015	0.963919188	0.996474415
E2f1	0.215130825	0.33365647	0.644767432	0.51907791	0.96446859
Pxmp4	-0.051904348	0.124940012	-0.415434153	0.677824109	0.983670411
Zfp341	1.13444121	0.531183452	2.135686278	0.032704994	0.665167506
Chmp4b	0.054733653	0.115714769	0.473004898	0.636209685	0.977284137
Raly	0.178133708	0.103892984	1.714588425	0.086420712	0.765418158
Eif2s2	0.126169096	0.144051548	0.875860745	0.381105742	0.930523346
Ahcy	-0.016184141	0.154684415	-0.104626836	0.91667192	0.994688009
Itch	-0.190444939	0.156889102	-1.213882526	0.224792587	0.892989918
Dynlrb1	0.116577714	0.102931908	1.13257119	0.257394373	0.897248195
Map1lc3a	0.100973356	0.134971554	0.748108419	0.454394764	0.951019525
Pigu	0.009196933	0.150395782	0.061151534	0.951238528	0.995951741
Trp53inp2	-0.281674693	0.141270015	-1.993874588	0.046165762	0.698098452
Ncoa6	-0.100672851	0.288486647	-0.348968843	0.7271127	0.988585656
Ggt7	-0.875926243	0.421305444	-2.079076486	0.037610321	0.679118604
Acss2	-0.09708948	0.206443323	-0.470296051	0.638143518	0.977779844

Gss	0.115684183	0.157253256	0.735655246	0.461940532	0.953083512
Trpc4ap	-0.276042081	0.186470579	-1.48035193	0.138779351	0.834874897
Edem2	-0.054056668	0.142381472	-0.379660829	0.704197201	0.988585656
Procr	-0.145108388	0.301891874	-0.480663443	0.630755717	0.977034429
BC029722	0.078606925	0.165192423	0.475850672	0.634180767	0.977034429
Eif6	0.157412281	0.111504938	1.411706815	0.158036306	0.855986074
Uqcc1	0.085382022	0.095137885	0.897455539	0.369475888	0.928436166
Gdf5	0.822131352	0.939748004	0.874842349	0.381659691	0.930635634
Cep250	0.457832425	0.399128546	1.147080131	0.251348505	0.896939594
6430550D23Rik	-0.543644538	0.932684483	-0.582881508	0.55997307	0.968926429
Ergic3	0.263880084	0.128301668	2.056715923	0.03971356	0.679118604
Fer1l4	0.004103734	1.256742047	0.003265375	0.997394612	0.99957296
Spag4	-0.840379683	0.974162819	-0.862668608	0.388319697	0.933269543
Cpne1	-0.199540926	0.132601559	-1.50481584	0.13237143	0.827800491
Rbm12	0.004494177	0.243873722	0.018428295	0.98529718	0.998148029
Nfs1	0.067033034	0.121700484	0.550803352	0.581768486	0.971915898
Romo1	0.308082968	0.189885587	1.622466307	0.104703542	0.793021779
Rbm39	-0.021997386	0.091801685	-0.239618548	0.810625985	0.991438232
Phf20	-0.010653725	0.180075346	-0.059162598	0.9528226	0.996070133
Scand1	0.147498099	0.124343842	1.186211523	0.235538777	0.896002526
Cnbd2	0.225450353	0.363856927	0.619612646	0.535512839	0.96674935
2900097C17Rik	0.023060466	0.1118239	0.20622126	0.836618082	0.992886758
Epb4.1l1	0.130658011	0.231448782	0.564522353	0.572398692	0.971613657
Aar2	-0.219127917	0.169115921	-1.29572613	0.195069856	0.879588734
Dlgap4	-0.025726379	0.141633397	-0.181640627	0.855864763	0.992886758
4930405A21Rik	-0.510785556	0.723589192	-0.705905453	0.480246936	0.957463006
Myl9	0.051327297	0.17839047	0.287724436	0.773557683	0.990730604
Tgif2	-0.361913267	0.34261086	-1.056339158	0.290813292	0.907334416
5430405H02Rik	-0.117285959	0.388384277	-0.301984314	0.762664021	0.989285682
1110008F13Rik	0.036478582	0.142046167	0.256807928	0.79732705	0.990988839
Sla2	0.393079521	0.41101017	0.956374197	0.338883216	0.919246575
Ndrg3	-0.26976957	0.150425166	-1.793380573	0.072912092	0.748701713
Dsn1	0.076805037	0.473532781	0.162195819	0.871151652	0.992886758
Soga1	-0.003841534	0.248773688	-0.015441881	0.987679652	0.998148029
Samhd1	0.005233137	0.196913856	0.026575767	0.978798102	0.997262526
Rbl1	-0.168475394	0.157945115	-1.066670497	0.286120654	0.905916508
Mroh8	-1.474527632	2.374818682	-0.620901142	0.53466467	0.96674935
Rpn2	-0.147205486	0.116660757	-1.261825228	0.207011682	0.883765092
Manbal	0.139494016	0.108992206	1.279853131	0.200596794	0.880888611
Src	-0.060449282	0.228099904	-0.265012309	0.790999979	0.990988839
Blcap	-0.106662181	0.12080808	-0.882906019	0.377287051	0.928883222
Nnat	0.04674444	0.203733756	0.229438855	0.81852784	0.992734473
Ctnnbl1	0.204315178	0.151327036	1.350156483	0.176965794	0.866546104
Tti1	-0.195887908	0.299076305	-0.654976356	0.512482977	0.963720538
Rprd1b	-0.390162527	0.177302206	-2.200550885	0.027767834	0.637644759
Tgm2	-0.232744076	0.148588469	-1.566367013	0.117262721	0.812881672
D630003M21Rik	-0.493479448	1.467668586	-0.33623357	0.73669474	0.988585656
Lbp	-0.360791981	0.165649552	-2.178043812	0.029402775	0.64858361
Snhg17	0.139267858	0.21989551	0.633336526	0.526513906	0.965637248
Snhg11	0.030592457	0.243357542	0.12570992	0.899961549	0.994508803
Ralgapb	-0.218604647	0.153996686	-1.419541237	0.155741283	0.855293621
Adig	0.065598949	0.37715216	0.173932317	0.861918655	0.992886758
Arhgap40	0.126807377	1.385819937	0.091503502	0.927092524	0.994688009
Actr5	0.302185594	0.310883272	0.972022686	0.331039262	0.917115058
Ppp1r16b	0.394239683	0.133088022	2.96224768	0.00305402	0.346777009
Fam83d	0.724956279	0.786270149	0.922019334	0.356518491	0.924354588
Dhx35	-0.258126558	0.224324638	-1.150683049	0.249862653	0.896939594
Gm826	0.775236619	0.87880347	0.882150157	0.377695611	0.928883222
Mafb	-0.118779956	0.479545176	-0.247692942	0.804371993	0.990988839
Top1	-0.202393689	0.133871592	-1.511849414	0.130572175	0.827800491
Plcg1	0.14221656	0.19730738	0.720786824	0.471040683	0.95486913
Zhx3	-0.113587129	0.325197133	-0.349286993	0.726873862	0.988585656

Lpin3	0.237201033	0.296035151	0.801259687	0.422981324	0.943509323
Chd6	-0.233286273	0.180170225	-1.294810351	0.19538566	0.879588734
Ptprt	-0.100735211	1.631685521	-0.061736903	0.950772352	0.99573416
Srsf6	0.019828245	0.107147415	0.18505575	0.853185308	0.992886758
Sgk2	-0.364654241	1.624940934	-0.224410767	0.822437693	0.992886758
Ift52	0.028103096	0.106456004	0.263987894	0.791789247	0.990988839
Mybl2	0.83240247	0.908992297	0.91574205	0.359802204	0.92584564
Gtsf1l	0.209643296	0.440684527	0.475721936	0.634272491	0.977034429
Tox2	0.019031969	0.654892422	0.029061215	0.976815769	0.997262526
Jph2	-0.035749273	0.210146913	-0.170115621	0.864919209	0.992886758
Oser1	0.161851202	0.13841235	1.169340752	0.242266371	0.896939594
Fitm2	-0.445583733	0.234763347	-1.898012352	0.057694455	0.72285319
Ttpal	-0.201489971	0.166153756	-1.21267178	0.225255333	0.893255444
Serinc3	-0.310944085	0.12821146	-2.425244086	0.015298097	0.557315333
Pkig	0.206605782	0.177682111	1.162783244	0.244917457	0.896939594
Ada	0.109263265	0.328128731	0.332989022	0.73914257	0.988585656
Wisp2	0.223841307	0.19481175	1.149013377	0.250550465	0.896939594
Ywhab	-0.131386936	0.07871368	-1.669175369	0.09508263	0.778239075
Pabpc1l	-0.97136662	0.763385892	-1.272445077	0.203215027	0.882809131
Tomm34	0.017058727	0.229976458	0.07417597	0.940870366	0.994688009
Stk4	-0.18936401	0.211954864	-0.893416676	0.371634076	0.928840865
Wfdc12	-0.13532047	0.886336906	-0.152673852	0.878655482	0.992886758
Slpi	-0.227896	0.318406331	-0.715739537	0.474152193	0.955974667
Matn4	-0.130116902	0.195598318	-0.665225057	0.505906556	0.96224682
Rbpjl	0.15281721	0.19440263	0.786086124	0.431817033	0.9459793
Sdc4	-0.029202002	0.076273924	-0.382856944	0.701825837	0.988482081
Sys1	0.132332785	0.129940544	1.018410275	0.308483021	0.9139204
Trp53tg5	0.891752566	0.772168746	1.15486747	0.248144703	0.896939594
Dbndd2	0.109754062	0.159071579	0.689966513	0.490215247	0.958560421
Pigt	-0.254430799	0.297247393	-0.85595637	0.392021913	0.933734231
Wfdc2	0.032491631	0.18370619	0.176867373	0.859612567	0.992886758
Spint3	-0.844263999	1.526424605	-0.55309905	0.580195587	0.971613657
Wfdc6a	0.078078983	0.665090166	0.117396086	0.906546185	0.994688009
Wfdc8	1.29702929	0.7198358	1.801840489	0.071570505	0.744227652
Wfdc6b	0.0428627	0.519616052	0.082489176	0.934257725	0.994688009
Wfdc16	-0.410715658	0.535337474	-0.76720887	0.44295734	0.948476747
Wfdc10	-0.063330835	0.393426631	-0.160972416	0.872115126	0.992886758
Wfdc13	-0.827174759	0.488126029	-1.694592604	0.090152731	0.769347579
Wfdc3	0.079213108	0.296505516	0.267155596	0.789349363	0.990988839
Dnttip1	0.196392853	0.16220793	1.210747543	0.225992175	0.893814924
Ube2c	-0.244289299	0.332286322	-0.735177112	0.462231636	0.953083512
Tnnc2	2.266397992	1.872094263	1.210621728	0.226040413	0.893814924
Snx21	-0.239923867	0.172692975	-1.389308786	0.164738873	0.859982992
Acot8	0.133866237	0.171628368	0.779977335	0.435404216	0.946762848
Zswim3	-0.456016554	0.42332043	-1.077237293	0.281374268	0.904508305
Zswim1	0.040497679	0.422495096	0.095853607	0.923636841	0.994688009
Spata25	-2.642405673	4.045435079	-0.653182074	0.513638906	0.964352198
Neurl2	-0.322388603	0.814435601	-0.395842964	0.692220879	0.986599035
Ctsa	-0.074814673	0.121273062	-0.616910895	0.5372935	0.96674935
Pltp	-0.46328912	0.195610295	-2.368429122	0.017863803	0.581000616
Pcif1	0.158385108	0.14786005	1.071182564	0.28408735	0.905916508
Zfp335	-0.04017882	0.202304669	-0.198605499	0.842571352	0.992886758
Mmp9	0.417946422	0.346266625	1.207007524	0.227429247	0.894410876
Slc12a5	0.30104216	0.619532075	0.485918601	0.627024879	0.97671122
Ncoa5	-0.073406925	0.130774417	-0.56132481	0.574576131	0.971613657
Cd40	0.358331846	0.15582705	2.299548415	0.021473817	0.60763088
Cdh22	0.299199493	0.944601544	0.316746775	0.751435754	0.988585656
Slc35c2	-0.047237008	0.218921394	-0.215771549	0.829165827	0.992886758
Elmo2	-0.169968772	0.181240902	-0.937805817	0.348344211	0.921552698
Zfp663	3.457736403	1.355597149	2.550710885	0.010750346	0.502706181
Zfp334	0.166985607	0.426826984	0.391225515	0.69563055	0.98715859
Ocstamp	-4.305872253	2.030843361	-2.120238485	0.033985938	0.671818913

Slc13a3	-0.30882153	0.743491183	-0.415366769	0.67787343	0.983670411
Trp53rka	0.221107949	0.144713946	1.527896624	0.126538207	0.823315433
Slc2a10	-0.419534578	0.446326228	-0.939972942	0.34723144	0.921552698
Eya2	0.037804334	0.146325581	0.258357654	0.796130895	0.990988839
Zmynd8	0.108126507	0.240193811	0.450163584	0.652592492	0.979004493
Ncoa3	-0.230223529	0.274854529	-0.837619561	0.402244403	0.937834293
Sulf2	0.171784596	0.194932744	0.881250591	0.378182203	0.928883222
Gm11468	1.118083701	2.009334722	0.556444722	0.577906873	0.971613657
5031425F14Rik	0.750787559	0.743886501	1.00927703	0.312841793	0.914819219
Prex1	-0.075992542	0.221680947	-0.342801413	0.731747863	0.988585656
Trp53rkb	0.195091579	0.33502103	0.582326364	0.56034687	0.968926429
Arfgef2	-0.374485993	0.20207186	-1.853231775	0.063849145	0.730529076
Cse1l	-0.193041213	0.142790801	-1.351916317	0.176402087	0.865862073
Stau1	-0.215109758	0.13769787	-1.562186534	0.118244052	0.814755048
Ddx27	-0.093309537	0.194504328	-0.479729874	0.631419482	0.977034429
Znfx1	-0.024357209	0.184711754	-0.131866048	0.895090244	0.993604681
1500012F01Rik	0.585031018	0.305937836	1.912254549	0.055843553	0.715507996
Kcnb1	-0.016892452	0.478929106	-0.035271299	0.971863409	0.996733101
Ptgis	-0.013635165	0.162697358	-0.083806924	0.933209943	0.994688009
B4galt5	-0.123556333	0.188008009	-0.657186537	0.511060981	0.963452765
Slc9a8	-0.029357272	0.192043484	-0.152867834	0.878502502	0.992886758
Spata2	-0.10250212	0.258496758	-0.396531548	0.691712939	0.986454135
Rnf114	-0.020332967	0.106868498	-0.190261559	0.849104175	0.992886758
Snai1	-0.342674189	0.270811895	-1.265358705	0.205742771	0.883765092
Ube2v1	0.068381073	0.167049651	0.409345801	0.682285908	0.984738223
Tmem189	-0.095948701	0.164276747	-0.584067455	0.55917493	0.968926429
Cebpb	-0.191046309	0.187820959	-1.01717247	0.30907139	0.9139204
A530013C23Rik	0.093131541	0.481574815	0.193389559	0.846653892	0.992886758
Ptpn1	-0.00845022	0.162067144	-0.052140239	0.95841695	0.996226826
Fam65c	1.048137305	0.715748772	1.464392739	0.143086671	0.840741274
Pard6b	-0.212593833	0.14351696	-1.481315049	0.13852264	0.83486864
E130018N17Rik	4.712374923	2.083418194	2.261847831	0.023706807	0.622011301
Adnp	-1.153567926	0.728266899	-1.583990604	0.113195857	0.805644622
Dpm1	0.045962135	0.459496258	0.100027224	0.920322712	0.994688009
Mocs3	-0.11895714	0.172535142	-0.689466153	0.490529966	0.958803155
Nfatc2	-0.252926147	0.759477918	-0.333026334	0.739114405	0.988585656
Atp9a	0.002859637	0.175608302	0.016284182	0.987007677	0.998148029
Zfp64	0.276623152	0.258804616	1.068849373	0.285137547	0.905916508
Tshz2	-0.010653377	0.326926992	-0.03258641	0.974004407	0.997262526
A630075F10Rik	0.964757441	1.700644308	0.567289372	0.570517597	0.971613657
Zfp217	-0.185148736	0.20203438	-0.916421925	0.35944564	0.925244994
Bcas1	0.116425655	0.190622528	0.610765452	0.541354868	0.967217841
Pfdn4	0.064309173	0.173937836	0.369725034	0.711587377	0.988585656
Fam210b	0.093266414	0.141927276	0.657142285	0.511089432	0.963452765
Aurka	-0.262900821	0.454044969	-0.579019345	0.56257612	0.969658675
Cstf1	0.131929851	0.239579873	0.55067168	0.581858762	0.97196726
Cass4	0.609199447	0.583172399	1.04463011	0.296193958	0.90895519
Rtfdc1	-0.030087044	0.093559132	-0.321583189	0.747768481	0.988585656
Bmp7	-0.54667049	0.704170714	-0.776332328	0.43755279	0.94749626
Spo11	-0.88657697	2.109446988	-0.420288813	0.674274482	0.983375962
Rae1	-0.167844262	0.151393428	-1.1086628	0.267575676	0.89804647
Rbm38	0.310322933	0.350418322	0.885578502	0.3758447	0.928883222
Ctcflos	-3.339552916	2.20782331	-1.512599718	0.130381366	0.827800491
Pck1	0.690430325	0.60816241	1.135272936	0.256260978	0.897248195
Zbp1	0.338172001	0.266636567	1.268288161	0.204695063	0.883765092
Pmepa1	-0.328376517	0.218173371	-1.505117305	0.132293921	0.827800491
Ppp4r1l-ps	0.079663027	0.23580301	0.337837194	0.73548588	0.988585656
Rab22a	0.021576906	0.158098356	0.13647774	0.89144362	0.993043931
Vapb	0.192482757	0.093955163	2.048666092	0.040494777	0.679118604
Stx16	-0.084646498	0.136991397	-0.617896447	0.536643601	0.96674935
Npepl1	0.055487302	0.10744225	0.51643838	0.605548266	0.974001549
Gnas	-0.170211824	0.116680155	-1.458789832	0.144622962	0.841977044

Nelfcd	-0.055401477	0.190466151	-0.290873084	0.771148388	0.989990452
Ctsz	-0.076891676	0.19288582	-0.398638304	0.690159734	0.986002801
Tubb1	0.050087969	0.476434833	0.105130787	0.91627203	0.994688009
Atp5e	0.425514134	0.324775449	1.310179496	0.19013512	0.876945711
Slmo2	-0.005168922	0.126212687	-0.040954057	0.967332522	0.996474415
Zfp831	0.55118461	0.801054287	0.688073978	0.491406197	0.958916246
Edn3	0.028743181	0.19076569	0.150672699	0.880233911	0.992886758
Gm14393	-0.322520865	0.941796294	-0.342452893	0.732010089	0.988585656
0610010B08Rik	0.055423737	1.855543757	0.02986927	0.976171314	0.997262526
Gm14440	-0.568385362	0.814494165	-0.69783847	0.485278218	0.957710647
Gm14295	0.284747191	0.2998248	0.949711936	0.342258643	0.919850248
Gm14420	-0.398120122	0.199325319	-1.997338446	0.04578843	0.696869274
Gm14403	-0.207196795	0.200496281	-1.033419644	0.301407557	0.911518274
Gm14322	-0.03077055	0.216196595	-0.142326708	0.886821951	0.992886758
Gm14325	0.192286803	0.14013007	1.372202292	0.170000472	0.861730117
Gm14327	-0.462802701	1.306342988	-0.354273499	0.723133927	0.988585656
C330013J21Rik	0.590459465	0.772285735	0.764560886	0.444533068	0.948476747
Gm14326	0.329150636	0.198553596	1.657742006	0.097369551	0.78359791
Etohi1	-0.10988829	0.201919771	-0.544217584	0.586291753	0.972904784
Zfp931	0.575650056	0.236612192	2.432884174	0.014979092	0.554936045
Phactr3	0.368271407	1.228816918	0.299695912	0.764409117	0.989417128
Sycp2	0.239232128	1.168803607	0.204681203	0.837821208	0.992886758
Ppp1r3d	-0.313813661	0.320030453	-0.980574375	0.326802676	0.916882624
Fam217b	0.983106786	0.546906707	1.797576761	0.072244104	0.74633396
Cdh26	-0.310189432	0.36483773	-0.850212046	0.395207206	0.933933717
Cdh4	0.677932949	0.744763168	0.910266483	0.36268199	0.926455466
Taf4a	0.132806512	0.204790865	0.648498222	0.516662761	0.964352198
4921531C22Rik	0.54232068	0.303114362	1.789161938	0.07358874	0.748833631
Lsm14b	-0.091705738	0.130473099	-0.702870849	0.48213624	0.957463006
Psma7	0.283202573	0.242078691	1.169878159	0.242050004	0.896939594
Ss18l1	-0.106115105	0.294753866	-0.360012597	0.718837714	0.988585656
Mtg2	0.173733019	0.171692386	1.011885403	0.311592849	0.914819219
Osbpl2	0.146812919	0.106834923	1.374203442	0.169378532	0.861597949
Adrm1	-0.045916191	0.149408393	-0.307320022	0.758599797	0.989266039
Lama5	0.270060936	0.156325516	1.727555057	0.084068026	0.76099108
Rps21	0.50028805	0.28991553	1.725633842	0.084413307	0.76099108
Cables2	-0.495360727	0.278730781	-1.777201374	0.075535114	0.752876039
Rbbp8nl	-0.897400772	1.000399833	-0.897042105	0.36969645	0.928436166
Gata5	-0.119323929	0.284966151	-0.418730186	0.675413333	0.983549699
Slco4a1	0.286528747	0.61593203	0.4651954	0.641791533	0.97813956
Mrgbp	0.353834887	0.197968697	1.787327456	0.073884579	0.750661804
Ogfr	-0.07147298	0.127068158	-0.562477501	0.573790728	0.971613657
Col9a3	-0.464330027	1.242980436	-0.373561814	0.708730348	0.988585656
Dido1	0.075605069	0.175394339	0.431057635	0.666426464	0.981863224
Gid8	-0.021949968	0.10922502	-0.200960984	0.840729082	0.992886758
Slc17a9	-0.541403556	0.434701666	-1.245460043	0.212962694	0.884945675
Ythdf1	-0.229587549	0.120777328	-1.900915942	0.057313024	0.721735927
Nkain4	0.170054372	0.210278938	0.808708537	0.418682817	0.943415794
Arfgap1	0.002239126	0.189447107	0.011819266	0.99056981	0.998564713
Col20a1	-0.293339949	0.615851517	-0.476316029	0.633849247	0.977034429
Eef1a2	0.467417927	0.435630351	1.072969149	0.283284959	0.905916508
Ppdpf	0.115926961	0.126792963	0.914301219	0.36055859	0.925913281
Ptk6	0.372880414	1.774614487	0.210119109	0.833574712	0.992886758
Srms	-0.449348165	0.381029921	-1.179298895	0.238279178	0.896609492
BC051628	1.072234326	0.906088917	1.183365459	0.236664341	0.896358698
Helz2	0.160143973	0.176259182	0.908570954	0.363576644	0.926455466
Gmeb2	-0.152995159	0.145442129	-1.051931513	0.292830972	0.907334416
Stmn3	-0.175155514	1.080560253	-0.162096943	0.871229513	0.992886758
Rtel1	0.154293022	0.274698015	0.561682333	0.574332474	0.971613657
Arfrp1	0.145607837	0.132467568	1.099196124	0.271682529	0.89804647
Zgpat	-0.046314719	0.243465919	-0.190230811	0.849128268	0.992886758
Lime1	0.009423423	0.20097673	0.046888128	0.96260239	0.996474415

Slc2a4rg-ps	0.113702114	0.322659288	0.352390643	0.724545318	0.988585656
Zbtb46	0.594880746	0.168159963	3.537588471	0.000403799	0.139568962
Abhd16b	-3.938421712	2.116621694	-1.860711209	0.06278497	0.727650961
Tpd52l2	0.074298546	0.122404055	0.606994155	0.543854791	0.967491297
Dnajc5	-0.206375287	0.18970134	-1.087895778	0.27664112	0.900604936
Uckl1	0.233894195	0.135037866	1.732063769	0.083262209	0.76099108
Uckl1os	-0.134002365	0.215855104	-0.62079776	0.534732697	0.96674935
Znf512b	-0.067610221	0.200239022	-0.337647581	0.735628781	0.988585656
Samd10	0.091690615	0.230242473	0.398235016	0.690456957	0.986010574
Prpf6	-0.108766647	0.188109932	-0.578207892	0.563123771	0.969658675
Sox18	-0.187753682	0.19104738	-0.982759784	0.325725679	0.916443764
Tcea2	-0.239718151	0.286814866	-0.835794025	0.403270788	0.937834293
Rgs19	-0.120456784	0.166189906	-0.72481408	0.468566097	0.953927686
Pcmtd2	-0.021269575	0.139442503	-0.152532939	0.878766613	0.992886758
Polr3k	0.005813796	0.135918756	0.042774052	0.965881649	0.996474415
Zfhx4	-0.124197783	0.921998325	-0.134704998	0.892845121	0.993300147
Pex2	0.221401116	0.121716874	1.818984575	0.068913783	0.743426713
Pkia	0.080248867	0.157306613	0.510142994	0.609951286	0.974001549
Zc2hc1a	-0.023410609	0.126416461	-0.185186395	0.85308284	0.992886758
Il7	0.068608522	0.228176077	0.300682363	0.763656719	0.989331656
Stmn2	0.275810589	0.152556714	1.807921665	0.070618693	0.744227652
Hey1	0.063548089	0.130910578	0.485431277	0.62737045	0.97683362
Mrps28	0.283024014	0.170381289	1.661121448	0.096689058	0.782127914
Tpd52	0.067403324	0.11123923	0.605931238	0.544560415	0.967491297
Zbtb10	0.236787203	0.232204655	1.019734955	0.307854179	0.9139204
C030034L19Rik	0.035321569	1.022916761	0.034530248	0.972454323	0.996790728
Zfp704	0.092101837	0.236706334	0.389097475	0.697204041	0.987548136
Pag1	0.248840574	0.309686842	0.803523238	0.421672371	0.943415794
Fabp5	-0.148416226	0.1980893	-0.749238987	0.453713174	0.950351284
Gm9833	-0.560924467	0.594617524	-0.943336589	0.345508767	0.921434283
Fabp4	0.008827069	0.39004675	0.022630798	0.981944777	0.997390438
Fabp12	0.371144891	0.267157353	1.389237042	0.164760681	0.859982992
Impa1	-0.092985254	0.144511414	-0.643445745	0.519934909	0.964665379
Slc10a5	-0.378940231	0.410080215	-0.924063676	0.355453164	0.924354588
Zfand1	0.374574677	0.170682462	2.194570394	0.028194429	0.641905969
Chmp4c	-0.205249718	0.214666012	-0.956135145	0.339003961	0.919246575
Snx16	0.086297866	0.174736448	0.493874443	0.621394855	0.97514112
Ralyl	-0.297981127	0.882045176	-0.337829779	0.735491467	0.988585656
Lrrcc1	-0.201859492	0.15763674	-1.280535824	0.200356754	0.880888611
E2f5	0.165128024	0.153154876	1.07817673	0.280954894	0.904228378
1810022K09Rik	0.290137322	0.190725988	1.521225947	0.128203146	0.826336015
Car13	-0.207151764	0.260861354	-0.794106757	0.427133284	0.944314584
Car3	-0.498585524	0.424039836	-1.175798785	0.239675292	0.896939594
Car2	-0.192685927	0.109287	-1.763118463	0.077880512	0.754202
A930001A20Rik	0.227343026	0.284477285	0.799160561	0.424197317	0.94406679
Gm9733	1.101923232	0.550985406	1.999913645	0.045509589	0.69515685
Sirpb1a	0.726722461	0.375874229	1.933419223	0.053184575	0.707153766
Sirpb1b	0.366740681	0.377330265	0.971935505	0.331082635	0.917115058
LOC100038947	0.157447566	0.230765204	0.682284693	0.49505895	0.959272191
Gm5150	0.087074017	0.258612113	0.336697365	0.73634505	0.988585656
Ythdf3	-0.203195314	0.11755549	-1.72850552	0.083897632	0.76099108
Bhlhe22	0.848054062	0.679067057	1.248851719	0.211719313	0.884755882
Cyp7b1	-0.381858087	0.361145782	-1.057351644	0.290351132	0.907334416
Armc1	0.113929328	0.107828757	1.056576473	0.290704923	0.907334416
Mtfr1	-0.100262192	0.12336991	-0.812695675	0.416392561	0.943415794
Pde7a	-0.226053139	0.154847827	-1.459840566	0.144333897	0.841934045
Dnajc5b	-0.027858843	0.69137944	-0.040294578	0.967858276	0.996474415
Trim55	-1.075524372	0.805853251	-1.334640483	0.181994042	0.868546072
4632415L05Rik	-0.233052206	0.15741526	-1.4804931	0.138741701	0.834874897
Cp	-0.343844261	0.140684517	-2.444080327	0.014522193	0.554936045
Hps3	0.151365715	0.187392627	0.807746374	0.419236602	0.943415794
Hltf	-0.157468631	0.154550036	-1.018884467	0.308257819	0.9139204

Gyg	-0.016540962	0.170813192	-0.096836558	0.922856192	0.994688009
Cpa3	0.513659053	0.245500406	2.092294109	0.036412214	0.677009445
Mir7007	1.105314887	1.226305035	0.90133764	0.367408827	0.928436166
Cpb1	0.4778108	0.39862545	1.198645997	0.230665625	0.895404344
Agtr1b	0.342477376	0.745838622	0.459184287	0.646101834	0.979004493
Tbl1xr1	0.091925475	0.171491882	0.536033974	0.591935071	0.974001549
Ect2	-0.222294482	0.36251063	-0.613208177	0.539738699	0.966900344
Nceh1	-0.335750178	0.133953174	-2.506474231	0.012194195	0.525888996
Tnfsf10	0.247950133	0.258881476	0.957774718	0.33817637	0.919246575
Fndc3b	0.105133628	0.159480561	0.659225344	0.509751074	0.963452765
Tmem212	-0.219640447	0.13602217	-1.614740057	0.106366999	0.794899279
Pld1	-0.070661212	0.235191985	-0.300440561	0.763841129	0.989331656
Tnik	0.311043319	0.472521525	0.658262751	0.510369309	0.963452765
Slc2a2	-1.56234639	1.742695497	-0.896511406	0.369979691	0.928436166
Eif5a2	-0.295063008	0.141472095	-2.085662245	0.037009229	0.678243111
Rpl22l1	0.334974028	0.250287567	1.338356643	0.180780205	0.868087032
Egfem1	0.625354377	0.8208833	0.761806674	0.446175397	0.948476747
Mannr	0.781298274	1.510093621	0.517383997	0.604888128	0.974001549
Mecom	-0.235170034	0.188656551	-1.246551118	0.212562135	0.884945675
Mynn	0.030822675	0.114590281	0.268981577	0.78794386	0.990988839
Lrrc34	-0.234508534	0.247224873	-0.948563677	0.342842573	0.919850248
Lrrc31	-0.52936896	0.696067375	-0.760513967	0.446947424	0.948476747
Sec62	-0.128831933	0.075635571	-1.703324662	0.088507328	0.767814344
Gpr160	-0.570772733	0.322836759	-1.767991768	0.07706228	0.752876039
Phc3	0.041432439	0.17612763	0.235240994	0.81402169	0.992048071
Prkci	0.002020101	0.120468431	0.016768715	0.986621128	0.998148029
Skil	-0.025070654	0.144734439	-0.173218301	0.862479839	0.992886758
Cldn11	-0.176569305	1.505640981	-0.117271851	0.906644631	0.994688009
Slc7a14	0.462662386	2.290149967	0.202022746	0.839898942	0.992886758
Kcnmb2	-0.133602672	0.14251514	-0.93746301	0.348520442	0.921552698
Zmat3	-0.045311069	0.235574238	-0.192343055	0.847473495	0.992886758
4930429B21Rik	4.618742608	1.509140847	3.060511295	0.002209594	0.308737709
Pik3ca	-0.088725533	0.152765881	-0.580794168	0.561379186	0.96944293
Zfp639	-0.109958325	0.135089709	-0.813965225	0.415664873	0.943415794
Mfn1	0.032829188	0.145254925	0.226010842	0.821192987	0.992886758
Gnb4	0.29174069	0.134065518	2.176105341	0.029547385	0.64858361
Actl6a	-0.006966076	0.129386076	-0.053839458	0.957063072	0.996226826
Mrpl47	0.044773113	0.2208005	0.20277632	0.839309867	0.992886758
Ndufb5	0.087297924	0.163727354	0.533190829	0.593901489	0.974001549
Usp13	-0.446979788	0.627043488	-0.712836983	0.475946638	0.95716557
Pex5l	-0.435786703	0.818197996	-0.532617661	0.594298274	0.974001549
Ttc14	-0.005918268	0.150094136	-0.039430376	0.968547262	0.996474415
Ccdc39	-0.233631187	0.221112553	-1.056616569	0.290686616	0.907334416
Fxr1	-0.161055762	0.121447855	-1.326130975	0.184796318	0.871716376
Dnajc19	0.184920421	0.197140943	0.938011246	0.348238631	0.921552698
Sox2ot	1.560569873	0.843215979	1.850735651	0.064207593	0.73299549
Sox2	-0.062225781	0.159109896	-0.391086805	0.695733074	0.98715859
Atp11b	0.012603523	0.124409106	0.10130708	0.919306696	0.994688009
Dcun1d1	0.051253283	0.123047685	0.41653188	0.677020844	0.983670411
Mccc1	-0.108074819	0.164735625	-0.656050072	0.511791907	0.963452765
Mccc1os	-0.824149626	0.733502123	-1.123581787	0.261190486	0.897248195
Acad9	-0.143349229	0.147180305	-0.973970186	0.330071343	0.917115058
D3Ertd254e	-0.289079061	0.34067542	-0.848546869	0.396133482	0.934066368
Anxa5	-0.200969556	0.132865497	-1.51257897	0.130386639	0.827800491
1810062G17Rik	0.337866759	0.366932068	0.92078831	0.357160959	0.924354588
Exosc9	-0.182370803	0.190838433	-0.955629327	0.33925954	0.919246575
Ccna2	0.18468423	0.343492732	0.537665613	0.590807924	0.974001549
Bbs7	-0.06060606	0.201668234	-0.300523583	0.76377781	0.989331656
Trpc3	0.700566022	0.523796504	1.337477468	0.181066832	0.868087032
4932438A13Rik	0.091419092	0.173721106	0.526240561	0.598721075	0.974001549
Il2	0.71268804	1.364393731	0.522347783	0.601428206	0.974001549
Il21	-3.706760205	3.7533965	-0.987574909	0.32336088	0.91640698

Cetn4	0.016710024	0.145735777	0.114659725	0.908714838	0.994688009
Bbs12	0.034496368	0.318202038	0.108410268	0.913670256	0.994688009
Fgf2	-0.477842736	0.759572706	-0.629094137	0.529287428	0.965637248
Nudt6	0.031288313	0.193486745	0.161707787	0.871535972	0.992886758
Spata5	0.038410189	0.323143215	0.118864292	0.905382872	0.994688009
Spry1	-0.493851564	0.239835	-2.059130505	0.039481738	0.679118604
Gm5148	0.443426193	0.209805408	2.113511736	0.034556992	0.674795441
Gm20755	0.110237783	0.241909784	0.455697911	0.648607225	0.979004493
Ankrd50	0.123288167	0.231262639	0.53310888	0.593958212	0.974001549
Fat4	0.141961647	0.450493195	0.315124953	0.752666783	0.988585656
Intu	-0.345333757	0.180070602	-1.917768659	0.055140351	0.712244081
Slc25a31	-0.313552269	1.365363735	-0.229647427	0.81836575	0.992734473
Hspa4l	-0.483005393	0.172027972	-2.807714279	0.004989447	0.401294122
Plk4	0.024100181	0.218248064	0.110425636	0.912071823	0.994688009
Mfsd8	-0.050734956	0.138835113	-0.365433173	0.714788083	0.988585656
Gm2011	0.76704149	0.699026808	1.097299103	0.272510663	0.898460682
3110057O12Rik	0.443956172	0.302474104	1.467749357	0.142172318	0.840741274
Larp1b	0.353096941	0.295322358	1.195632267	0.231840094	0.895404344
Pgrmc2	0.162134024	0.145145659	1.117043562	0.2639757	0.897341506
Platr4	-0.396169851	1.31242392	-0.301861194	0.76275788	0.989285682
Jade1	-0.303217054	0.177184928	-1.711302749	0.087025239	0.765418158
Sclt1	-0.067256987	0.262587634	-0.256131585	0.797849234	0.990988839
D3Ertd751e	-0.15807723	0.194173902	-0.814101319	0.415586911	0.943415794
Pabpc4l	0.147191443	0.733731223	0.200606759	0.841006072	0.992886758
Pcdh18	0.632503831	0.308389557	2.050989783	0.040267942	0.679118604
Slc7a11	0.164967247	0.701922889	0.235021894	0.81419174	0.992048071
Ccrn4l	0.066016068	0.344286562	0.191747443	0.847940041	0.992886758
Elf2	-0.096301376	0.130146419	-0.739946413	0.45933251	0.952683168
4930577N17Rik	-0.248834155	0.748689302	-0.332359704	0.739617662	0.988585656
Mgarp	-0.82763105	1.851287538	-0.447056998	0.654833906	0.979133069
Ndufc1	0.164193854	0.151832334	1.08141559	0.27951229	0.902788755
Naa15	-0.160883994	0.124232521	-1.295023173	0.195312236	0.879588734
Rab33b	0.013733413	0.098587562	0.139301683	0.88921176	0.993043931
Setd7	-0.086906236	0.190788063	-0.455511912	0.648741	0.979004493
5031434O11Rik	0.890985468	0.888121795	1.003224415	0.315752596	0.915957942
Mgst2	0.466812839	0.334810939	1.394258027	0.163239701	0.859982992
Maml3	-0.229896172	0.269087878	-0.854353504	0.39290915	0.933734231
Foxo1	0.121051221	0.21771775	0.556000698	0.578210377	0.971613657
Gm2447	0.820982274	1.999587766	0.410575764	0.681383638	0.984683189
Cog6	-0.154586962	0.144738803	-1.068040902	0.285502062	0.905916508
Lhfp	-0.017838778	0.104828149	-0.170171643	0.864875152	0.992886758
Nhlrc3	0.200423464	0.325103607	0.616491048	0.537570478	0.96674935
Proser1	-0.384770378	0.381641193	-1.008199285	0.313358801	0.915243138
Frem2	0.118719743	0.975668241	0.121680442	0.903152103	0.994688009
Ufm1	0.106498811	0.135619422	0.785276987	0.432291185	0.9459793
Postn	-0.039420592	0.150825373	-0.261365786	0.793810439	0.990988839
Supt20	-0.172802655	0.165166887	-1.04623062	0.295454569	0.908201248
Exosc8	-0.062359411	0.148701859	-0.419358652	0.674954038	0.983549699
Alg5	0.125568365	0.131346775	0.956006453	0.339068974	0.919246575
Smad9	0.178590163	0.320737055	0.556811756	0.577656051	0.971613657
Rfxap	-0.0254773	0.238326923	-0.106900639	0.914867806	0.994688009
Ccna1	-0.134853204	0.58870812	-0.229066322	0.818817369	0.992734473
Spg20	-0.085555476	0.192703023	-0.443975788	0.657060089	0.979614766
Dclk1	-0.003165828	0.470487519	-0.006728825	0.994631215	0.999285699
Nbea	0.169019334	0.25853003	0.653770603	0.513259608	0.964352198
Tm4sf1	-0.001597026	0.151826406	-0.010518765	0.991607394	0.998770784
Wwtr1	-0.086606923	0.101309674	-0.854873177	0.392621361	0.933734231
Commd2	0.225495326	0.253463638	0.889655527	0.373650881	0.928840865
Ankub1	-0.220991656	0.603696621	-0.366064092	0.714317251	0.988585656
Rnf13	0.021415008	0.127932362	0.167393203	0.867060673	0.992886758
Pfn2	0.08898564	0.146202876	0.608644938	0.542759811	0.967491297
Tsc22d2	-0.106184882	0.224871893	-0.472201665	0.636782851	0.977284137

Serp1	0.01605487	0.137157904	0.117053914	0.90681733	0.994688009
Eif2a	0.095597646	0.12294349	0.777573872	0.436820269	0.946762848
Selt	0.2013534	0.165087813	1.219674527	0.222588282	0.892391445
Erich6	-1.000115008	1.07679302	-0.928790389	0.352997726	0.923620814
Gm8234	0.374522352	0.568991829	0.658220967	0.510396154	0.963452765
Siah2	-0.170741751	0.214877393	-0.794600814	0.426845745	0.944290206
4930593A02Rik	-0.516990281	1.905366729	-0.27133374	0.786134359	0.990988839
Med12l	-0.355846706	0.525620898	-0.677002583	0.498404315	0.960700483
Gpr171	-0.144915206	0.22772899	-0.636349402	0.524548712	0.965637248
P2ry14	0.029054177	0.192109513	0.151237576	0.87978831	0.992886758
P2ry13	-0.316764483	0.349460803	-0.906437804	0.364704171	0.926556975
P2ry12	0.008662839	0.336889648	0.025714175	0.979485318	0.997262526
Igsf10	0.536752537	0.473849177	1.132749749	0.257319359	0.897248195
Aadac	-1.194903749	0.868088348	-1.37647712	0.168673966	0.861597949
Sucnr1	-1.233043805	1.722469028	-0.715858331	0.47407883	0.955974667
Mbnl1	0.061094353	0.135617536	0.450490069	0.652357113	0.979004493
P2ry1	-0.121508288	0.324573367	-0.374363088	0.708134201	0.988585656
Rap2b	0.283024475	0.145112492	1.950379814	0.051130866	0.707153766
Arhgef26	0.042770551	0.122589328	0.348892938	0.727169687	0.988585656
Dhx36	-0.237878282	0.128280837	-1.854355553	0.063688309	0.729199878
Mme	-0.268722662	0.136870458	-1.963335738	0.049607175	0.707153766
Plch1	-0.344891463	0.513765159	-0.671301775	0.502028304	0.961378071
E130311K13Rik	0.256355619	0.200032203	1.281571748	0.199992916	0.880888611
Slc33a1	0.057303556	0.127181193	0.450566276	0.652302177	0.979004493
Gmps	-0.133153146	0.17693546	-0.752552068	0.45171913	0.949134998
Kcnab1	0.072648196	0.648118637	0.112090892	0.910751339	0.994688009
A730090N16Rik	2.507052166	1.986507238	1.262040288	0.20693429	0.883765092
Ssr3	0.086982527	0.135510916	0.641885757	0.520947363	0.964784335
4931440P22Rik	0.075849164	0.755977779	0.100332531	0.920080332	0.994688009
Tiparp	-0.35807174	0.175824697	-2.036526989	0.041697466	0.679118604
Mir8120	-0.708339688	1.933885183	-0.366278047	0.714157609	0.988585656
Lekr1	0.030127767	0.233455299	0.129051547	0.897316862	0.994101834
Ccnl1	-0.063660737	0.119277297	-0.53372049	0.593534931	0.974001549
Veph1	-0.528209983	0.892094977	-0.592100614	0.553783216	0.968926429
Ptx3	0.307484397	0.916939583	0.335337685	0.737370369	0.988585656
Shox2	-1.654920526	2.100402062	-0.787906542	0.430751374	0.945321484
Rsrc1	0.084847953	0.1400548	0.605819669	0.544634507	0.967491297
Mlf1	-0.013608167	0.160085791	-0.085005465	0.932257046	0.994688009
Gfm1	0.001530728	0.162934391	0.009394753	0.992504182	0.99886678
Lxn	0.03417966	0.180638855	0.189215435	0.849923971	0.992886758
Rarres1	-0.28235255	0.441605907	-0.639376751	0.52257787	0.965637248
Mfsd1	-0.209839774	0.107417882	-1.953490143	0.050761544	0.707153766
Schip1	-0.37728989	0.748401823	-0.504127433	0.614171831	0.974001549
Il12a	-0.190890702	0.451942341	-0.422378442	0.672748811	0.983233856
1110032F04Rik	0.151425863	0.68173548	0.222118208	0.824221864	0.992886758
Ift80	-0.128092337	0.185233196	-0.691519334	0.489239235	0.958560421
Smc4	-0.231982957	0.133702621	-1.735066637	0.082729003	0.76099108
Trim59	0.116842995	0.246046805	0.474881173	0.634871671	0.977034429
Kpna4	0.087347559	0.136274569	0.640967422	0.52154385	0.965009795
Ppm1l	-0.482823285	0.330823702	-1.459457956	0.144439104	0.841934045
B3galnt1	0.185535069	0.211546351	0.877042164	0.380463737	0.929775696
Nmd3	0.091331412	0.130709646	0.698735057	0.484717621	0.957710647
Sptssb	0.328643914	0.322201212	1.019995898	0.307730406	0.9139204
Gm6634	0.635825382	0.387604051	1.640399216	0.100922188	0.788783372
Bche	-0.359891408	0.275362594	-1.306972757	0.191221964	0.878116834
Zbbx	0.633224132	0.603467977	1.04930859	0.294036114	0.907334416
Pdcd10	0.293487209	0.239364071	1.226112204	0.220156454	0.889044644
Serpini1	0.246048709	0.366553786	0.671248584	0.502062183	0.961378071
Golim4	-0.071752771	0.168124826	-0.426782721	0.669537593	0.981863224
Rapgef2	0.052706606	0.233566272	0.225660176	0.821465734	0.992886758
Fnip2	-0.01710437	0.222313363	-0.076938108	0.938672782	0.994688009
Ppid	-0.042222094	0.108623684	-0.388700628	0.697497618	0.987548136

Etfdh	0.114313174	0.173130844	0.660270416	0.509080312	0.963452765
4930579G24Rik	0.170574352	0.296567899	0.575161212	0.565182273	0.969738975
Rxfp1	1.328679872	1.985666749	0.669135378	0.503409124	0.961729397
Tmem144	-0.325815173	0.476884957	-0.683215455	0.494470708	0.958968423
Fam198b	0.052659839	0.140101899	0.375868135	0.707014931	0.988585656
Glrb	-0.081736095	0.603153503	-0.135514582	0.892205036	0.993251849
Pdgfc	-0.191999867	0.305731502	-0.628001584	0.530002903	0.965888564
Ctso	-0.102724238	0.106049768	-0.968641804	0.332723938	0.917115058
Tdo2	-0.348140907	0.551333132	-0.631452904	0.527744434	0.965637248
Gucy1b3	-0.134493819	0.196151958	-0.685661363	0.492926676	0.958916246
Gucy1a3	0.277742751	0.125479739	2.213446997	0.026866844	0.633894445
Map9	-0.492556364	0.34635047	-1.422132798	0.154987703	0.854973821
Rbm46	-0.387002511	0.405894004	-0.953457079	0.340358533	0.919850248
Rbm46os	-3.433732691	2.478166902	-1.385593798	0.165870973	0.860011234
Lrat	-0.885262077	0.332394101	-2.663290576	0.007738056	0.45506991
Fgg	-1.272765098	0.793912488	-1.603155407	0.108900348	0.798400564
Fga	-1.17078929	1.01767432	-1.150455767	0.249956203	0.896939594
Plrg1	-0.135596753	0.138656533	-0.977932666	0.328107627	0.917115058
Sfrp2	-0.02092932	0.283378458	-0.073856427	0.941124627	0.994688009
Tlr2	0.084228719	0.160017051	0.526373401	0.598628793	0.974001549
D930015E06Rik	0.3287879	0.336218423	0.977899715	0.328123926	0.917115058
Mnd1	0.229789328	0.347060167	0.66210228	0.507905675	0.963106419
Trim2	-0.167317852	0.205417846	-0.814524419	0.415344589	0.943415794
Fhdc1	-0.072464595	0.210667944	-0.343975423	0.730864769	0.988585656
Arfip1	0.032477144	0.117545602	0.276293998	0.782322265	0.990988839
Tigd4	2.12228946	1.825237116	1.162747262	0.24493206	0.896939594
Tmem154	-0.28867055	0.158040194	-1.826564137	0.06776533	0.742385562
Fbxw7	-0.07669155	0.191894294	-0.399655187	0.689410503	0.985586538
Gatb	0.144068138	0.168472893	0.855141358	0.392472896	0.933734231
Fam160a1	-0.112287799	0.262342636	-0.428019633	0.668636827	0.981863224
Glt28d2	-0.087308513	0.213030616	-0.409840211	0.681923167	0.984683806
Sh3d19	-0.048448796	0.160779598	-0.301336718	0.763157744	0.989285682
Rps3a1	0.230361109	0.152463626	1.510925033	0.130807552	0.827800491
Rnu73b	2.377526551	2.450247176	0.970321107	0.33188646	0.917115058
Lrba	-0.046873776	0.155018662	-0.302375053	0.76236617	0.989285682
Dclk2	-0.879649869	0.634082548	-1.387279735	0.16535648	0.860011234
Cd1d2	0.587650377	1.063664214	0.552477341	0.580621354	0.971613657
Cd1d1	0.011858409	0.152998558	0.077506673	0.938220483	0.994688009
Kirrel	-0.268426495	0.320648436	-0.837136454	0.402515872	0.937834293
Fcrls	0.094514841	0.298632535	0.31649211	0.751629013	0.988585656
Cd5l	0.074729818	0.83725483	0.089255762	0.928878651	0.994688009
Fcrl1	0.380458413	0.407962784	0.932581176	0.351036256	0.922414914
Fcrl5	-0.459285769	0.914593945	-0.502174513	0.615544769	0.974001549
Etv3	0.005539518	0.221555921	0.0250028	0.98005273	0.997296835
Arhgef11	-0.383346164	0.287142066	-1.335040073	0.181863231	0.868546072
Lrrc71	-0.093353367	0.211633696	-0.441108239	0.659134639	0.979924124
Pear1	0.412776346	0.283365683	1.456691373	0.145201592	0.842729381
Ntrk1	-0.307297214	0.577997598	-0.531658289	0.594962686	0.974001549
Insrr	-3.845775943	2.225178232	-1.728300182	0.083934421	0.76099108
Sh2d2a	0.715567074	0.679160887	1.053604658	0.292063956	0.907334416
Prcc	-0.026557737	0.13826418	-0.192079659	0.847679808	0.992886758
Hdgf	-0.064936657	0.133528063	-0.486314677	0.626744075	0.976601842
Mrpl24	0.443791196	0.141099689	3.145231563	0.001659554	0.284736028
Rrnad1	-0.091913699	0.270871005	-0.339326459	0.734363812	0.988585656
Isg20l2	-0.318739914	0.138993777	-2.293195574	0.021836748	0.614067614
Nes	0.190132695	0.226338363	0.84003742	0.400887406	0.937317582
Bcan	0.175284321	0.779241735	0.224942163	0.822024269	0.992886758
Gpatch4	-0.138120005	0.247453131	-0.558166325	0.576730814	0.971613657
Apoa1bp	0.252758285	0.14474363	1.746248068	0.080767856	0.758307521
Iqgap3	-0.161904837	0.970595179	-0.166809851	0.867519667	0.992886758
Mef2d	0.229407094	0.408108976	0.562122146	0.574032801	0.971613657
1700113A16Rik	0.280063059	0.253904063	1.10302709	0.270015401	0.89804647

Rhbg	0.070480636	0.255509073	0.275843966	0.782667914	0.990988839
Tsacc	0.202014963	0.331986791	0.608503014	0.542853908	0.967491297
Cct3	-0.095925517	0.1151674	-0.832922483	0.404888449	0.938874266
Glmp	-0.036337966	0.165329634	-0.219791003	0.826033927	0.992886758
Tmem79	0.230844746	0.351633537	0.656492405	0.511507353	0.963452765
Smg5	-0.150831172	0.138346609	-1.090241195	0.275606911	0.900302631
Paqr6	0.228290781	0.298256512	0.76541759	0.444022922	0.948476747
Pmf1	-0.003865461	0.225685351	-0.017127655	0.986334777	0.998148029
Slc25a44	-0.128220355	0.312432789	-0.4103934	0.681517387	0.984683806
Sema4a	-0.494492367	0.240967856	-2.052109253	0.040159046	0.679118604
Lmna	-0.006959762	0.15013965	-0.046355256	0.963027099	0.996474415
Mex3a	-0.807312538	0.936406869	-0.862138633	0.388611234	0.933269543
Rab25	0.10867469	0.135651668	0.801130507	0.423056097	0.943509323
Lamtor2	0.332001215	0.118997641	2.789981479	0.005271106	0.406593461
Ubqln4	0.055847674	0.140133107	0.398533047	0.690237303	0.986002801
Ssr2	0.008587701	0.094052488	0.091307533	0.927248233	0.994688009
Arhgef2	-0.091170918	0.144113097	-0.632634507	0.526972348	0.965637248
Rxfp4	-1.187110836	1.506579627	-0.787950941	0.430725402	0.945321484
2810403A07Rik	-0.175584222	0.147448948	-1.190813666	0.233726743	0.89581803
Rit1	0.264507827	0.143595092	1.842039465	0.065469377	0.736169783
Syt11	-0.067921089	0.301347267	-0.225391422	0.821674784	0.992886758
5830417I10Rik	-0.004408436	0.323626692	-0.013621979	0.989131569	0.998182772
1500004A13Rik	-0.025631914	0.95449914	-0.026853784	0.978576355	0.997262526
Gon4l	-0.441916887	0.309050188	-1.429919492	0.152740127	0.853744627
Msto1	0.417456345	0.265141401	1.574466845	0.115379561	0.81007959
Dap3	0.089320795	0.126348721	0.706938656	0.4796046	0.957463006
Ash1l	-0.079776572	0.191495997	-0.41659655	0.676973533	0.983670411
Rusc1	-0.282320663	0.199246506	-1.416941603	0.156500002	0.855455553
Fdps	0.054441272	0.163869916	0.332222492	0.739721261	0.988585656
Pklr	-0.312407221	0.714851488	-0.43702395	0.662093979	0.980647283
Clk2	-0.019740722	0.286925777	-0.068800796	0.945148185	0.99486515
Fam189b	-0.159718554	0.256931002	-0.621639869	0.534178699	0.96674935
Gba	-0.235973479	0.143141591	-1.648531907	0.099243569	0.788491361
Mtx1	-0.045319441	0.163138416	-0.277797484	0.781167822	0.990988839
Thbs3	-0.156454591	0.215766936	-0.725109201	0.46838504	0.953888306
Muc1	-0.238157233	0.175254435	-1.358922718	0.174171079	0.863528862
Trim46	0.471617603	0.518586785	0.909428503	0.363123982	0.926455466
Krtcap2	-0.002088303	0.128614061	-0.016236974	0.987045338	0.998148029
Dpm3	0.386120641	0.205500971	1.878923688	0.060254914	0.725221283
Slc50a1	0.047793882	0.102513755	0.466219215	0.641058597	0.977921835
Efna1	-0.077017813	0.130899614	-0.588373113	0.556281881	0.968926429
Efna3	0.321296646	0.466892797	0.688159353	0.491352437	0.958916246
Efna4	0.077997143	0.354854094	0.2198006	0.826026452	0.992886758
Adam15	-0.021205699	0.160981301	-0.131727718	0.895199661	0.993604681
Dcst1	-0.235183636	0.688124887	-0.34177464	0.732520496	0.988585656
Zbtb7b	-0.026802409	0.192833237	-0.138992685	0.88945593	0.993043931
Gm15417	0.351864521	0.297251624	1.183726153	0.236521483	0.896358698
Lenep	-0.588295189	1.037883279	-0.566822108	0.570835048	0.971613657
Flad1	0.07428635	0.239626497	0.310008914	0.756554177	0.989266039
Cks1b	-0.312804151	0.241547162	-1.295002386	0.195319406	0.879588734
Shc1	-0.073988923	0.119143837	-0.621005038	0.534596309	0.96674935
Pygo2	-0.276402974	0.180042018	-1.535213707	0.124731342	0.821164769
Pbxip1	-0.139767513	0.146012655	-0.957228762	0.338451803	0.919246575
Pmvk	-0.174145973	0.196009091	-0.888458651	0.374294088	0.928883222
Kcnn3	-0.82987173	0.476561345	-1.741374411	0.081617971	0.76099108
Adar	-0.25997008	0.226807353	-1.146215394	0.25170604	0.896939594
Chrnb2	-0.470122551	1.385872594	-0.339224942	0.734440281	0.988585656
4632404H12Rik	0.449818341	0.395763102	1.136584839	0.255711881	0.897044747
Ube2q1	0.091379705	0.092859456	0.984064622	0.32508374	0.91640698
She	-0.054003738	0.168967311	-0.319610568	0.749263562	0.988585656
Il6ra	-0.348217362	0.251069257	-1.386937478	0.165460828	0.860011234
Atp8b2	-0.123658567	0.190916577	-0.647709955	0.517172564	0.964352198

Hax1	0.177363675	0.154064487	1.151230105	0.249637583	0.896939594
Gm19710	-0.630219815	1.650046554	-0.381940627	0.702505405	0.988482081
Ubap2l	-0.228053537	0.19049483	-1.197163919	0.23124267	0.895404344
4933434E20Rik	0.243488782	0.151696258	1.60510737	0.108470178	0.798150849
1700094D03Rik	0.104313753	0.165025467	0.632107	0.527316961	0.965637248
Tpm3	0.088016379	0.087702051	1.003584047	0.315579147	0.91586731
Nup210l	0.024699759	0.561440391	0.043993556	0.964909541	0.996474415
Rps27	0.541505485	0.603207187	0.897710599	0.369339857	0.928436166
Rab13	0.330204395	0.18660547	1.769532235	0.076805096	0.752876039
Jtb	0.141876184	0.158957743	0.892540254	0.372103427	0.928840865
Creb3l4	-0.429130865	0.3739022	-1.147708853	0.251088775	0.896939594
Slc39a1	0.097618446	0.083201643	1.173275459	0.24068536	0.896939594
Crtc2	0.044432025	0.192444396	0.230882407	0.817406159	0.992678823
Dennd4b	-0.05569327	0.294987086	-0.188799012	0.850250346	0.992886758
Gatad2b	-0.272668814	0.409766771	-0.665424416	0.505779073	0.96224682
Slc27a3	0.265263592	0.17889649	1.482776955	0.138133681	0.833928471
Ints3	-0.007876558	0.123760002	-0.063643809	0.949253848	0.995624732
Npr1	-0.198446937	0.171524227	-1.156961555	0.247288068	0.896939594
Ilf2	0.00959456	0.108736058	0.088237155	0.929688187	0.994688009
Snapin	0.015615476	0.107887342	0.144738721	0.884917166	0.992886758
Chtop	0.083621627	0.099784508	0.838022147	0.402018265	0.937834293
S100a1	0.202981383	0.250856258	0.809154153	0.418426482	0.943415794
S100a13	0.184423657	0.151151577	1.220123935	0.222417896	0.892391445
S100a14	0.030025462	0.305060538	0.098424601	0.921595141	0.994688009
S100a16	0.198198878	0.16667187	1.18915614	0.23437823	0.89581803
S100a2	0.997711915	1.360937692	0.73310624	0.463493634	0.953083512
S100a3	0.44767848	0.551665341	0.811503728	0.417076453	0.943415794
S100a4	0.255396649	0.2194625	1.163737082	0.244530577	0.896939594
S100a6	0.095833935	0.17665012	0.542507046	0.587469255	0.973455044
S100a7a	0.190678597	0.629390831	0.30295738	0.761922342	0.989285682
S100a8	0.721365765	0.458880068	1.572013723	0.115947369	0.811920148
S100a9	0.688950726	0.47461883	1.451587427	0.146616357	0.843780493
Lor	1.037686639	1.419660498	0.730939996	0.464815805	0.953083512
Sprr1a	-0.694826553	0.761033325	-0.913004108	0.361240381	0.92633449
Ivl	0.731344112	1.133740353	0.64507196	0.518880554	0.96446859
Rptn	1.012567456	0.703050374	1.440248799	0.149797022	0.848779008
Tchh	0.06423473	0.388622093	0.165288416	0.868716976	0.992886758
S100a11	-0.210150182	0.093679436	-2.24329043	0.024878092	0.627796923
S100a10	0.271125687	0.14329965	1.892019188	0.058488424	0.72285319
Them4	0.149439613	0.213376001	0.700358113	0.483703688	0.957710647
C2cd4d	1.668833279	2.034981565	0.820072923	0.412174537	0.942121471
Rorc	-0.254744385	0.176887102	-1.440152406	0.149824285	0.848779008
Tdrkh	0.234165834	0.317895716	0.736612109	0.461358269	0.953083512
Oaz3	-0.182756853	1.436093248	-0.12725974	0.898734824	0.994364545
Mrpl9	0.240511343	0.113302759	2.122731564	0.033776352	0.671818913
Riiad1	0.188498305	0.194586071	0.968714276	0.332687768	0.917115058
Celf3	0.12934598	1.496898519	0.086409318	0.93114104	0.994688009
Snx27	-0.329876457	0.174695463	-1.88829436	0.058986444	0.72285319
Tuft1	-0.022760325	0.13968799	-0.16293688	0.870568133	0.992886758
BC021767	-0.574218338	1.173070993	-0.489500074	0.624487702	0.975987226
Selenbp2	-0.907271024	0.457884486	-1.981440848	0.047541857	0.700850692
Cgn	-0.231317216	0.247748326	-0.933678219	0.350469904	0.922338606
Pogz	0.270721807	0.254683967	1.062971532	0.287794862	0.907334416
Psmb4	0.317949943	0.159009935	1.999560232	0.045547772	0.69515685
Selenbp1	-0.181785402	0.155581126	-1.168428375	0.242634015	0.896939594
Rfx5	-0.041816794	0.280091933	-0.149296676	0.881319538	0.992886758
Pi4kb	-0.154952052	0.103183924	-1.501707301	0.133172717	0.827800491
Zfp687	0.036264334	0.297243085	0.12200228	0.902897213	0.994688009
Psmd4	0.076382965	0.117719962	0.648853122	0.516433317	0.964352198
Pip5k1a	-0.170743113	0.133008581	-1.283699981	0.199246951	0.880831248
Vps72	0.12806568	0.115687167	1.106999879	0.268293981	0.89804647
Tmod4	0.5953537	0.415511114	1.432822566	0.151908551	0.852599143

Scnm1	0.455979147	0.164830821	2.766346384	0.005668829	0.420704384
Lysmd1	0.21566632	0.376880855	0.572240051	0.567159357	0.970862413
Tnfaip8l2	0.078937432	0.19125027	0.412744159	0.679794068	0.983903958
Sema6c	-0.580241796	0.799463337	-0.725789124	0.467968055	0.953657709
Gabpb2	-0.116257511	0.14422724	-0.8060718	0.420201451	0.943415794
Mllt11	-0.074311104	0.214414792	-0.346576388	0.728909588	0.988585656
Cdc42se1	-0.051725488	0.08622148	-0.599914177	0.548563434	0.968887406
Gm128	0.658422676	1.470339068	0.447803293	0.654295167	0.979133069
Bnipl	0.332074398	0.202983474	1.635967657	0.101846357	0.788783372
Prune	-0.004096586	0.264616989	-0.01548119	0.987648291	0.998148029
Fam63a	0.033252556	0.133637109	0.248827264	0.803494401	0.990988839
Anxa9	-0.182501641	0.279515593	-0.652921144	0.513807117	0.964352198
Cers2	-0.278988877	0.090982568	-3.066399252	0.002166538	0.308737709
Setdb1	0.119186934	0.233068158	0.511382316	0.609083375	0.974001549
4930558C23Rik	-0.821185603	1.210959946	-0.678127799	0.497690666	0.960700483
Arnt	-0.03816212	0.138571783	-0.27539604	0.783011986	0.990988839
Ctsk	-0.495082425	0.416139963	-1.189701707	0.234163654	0.89581803
Ctss	-0.210252611	0.174829381	-1.202616006	0.229124951	0.895404344
Golph3l	-0.267870613	0.120323639	-2.226250922	0.025997379	0.631530315
Ensa	-0.018977904	0.077267333	-0.245613553	0.805981397	0.99099448
Mcl1	-0.061217738	0.111592093	-0.548584911	0.583290343	0.972174584
Adamtsl4	-0.087306848	0.244362085	-0.357284757	0.72087864	0.988585656
Ecm1	-0.10429843	0.174972474	-0.596084785	0.551118588	0.968926429
Tars2	0.067905391	0.122600325	0.553876111	0.579663635	0.971613657
Rprd2	0.433375159	0.216030188	2.006086106	0.044847062	0.69077168
Prpf3	-0.092224166	0.155243205	-0.594062499	0.552470312	0.968926429
Mrps21	0.284343892	0.187476443	1.516691308	0.12934464	0.827800491
C920021L13Rik	-0.310466742	0.453438366	-0.684694472	0.493536738	0.958916246
Ciart	-0.634938231	0.46649987	-1.3610684	0.173492075	0.863528862
BC028528	0.118554603	0.227003657	0.522258561	0.601490317	0.974001549
Aph1a	-0.058740164	0.155207673	-0.378461725	0.705087622	0.988585656
Car14	0.142630086	0.315671538	0.45183068	0.651390966	0.979004493
Anp32e	0.060313022	0.138376443	0.435861923	0.662936913	0.980876591
Gm9054	0.886112967	1.210981481	0.731731228	0.464332632	0.953083512
Plekho1	0.270880008	0.147868669	1.831895889	0.066966933	0.739762596
Vps45	-0.016906768	0.138246889	-0.122294024	0.902666164	0.994688009
Otud7b	-0.206178633	0.149168949	-1.382181977	0.166915832	0.860403806
Mtmr11	0.017884404	0.233782733	0.076500105	0.93902123	0.994688009
Sf3b4	-0.236704277	0.30615827	-0.773143503	0.439437458	0.947849924
Sv2a	1.170843993	1.622747433	0.721519547	0.470589919	0.95486913
Bola1	0.032394535	0.180935805	0.179038833	0.85790721	0.992886758
Hist2h2ac	2.896533792	2.262422651	1.2802797	0.200446785	0.880888611
Hist2h2be	-0.267937018	0.228680049	-1.171667662	0.241330511	0.896939594
Hist2h4	-0.455587928	1.587108198	-0.287055368	0.774069926	0.990730604
Fcgr1	0.110741504	0.224193838	0.49395427	0.621338476	0.97514112
Hfe2	-0.225037919	0.46783343	-0.48102146	0.630501247	0.977034429
Gm15441	0.55078559	0.34238522	1.608672215	0.107688034	0.796929372
Txnip	0.069712231	0.164707597	0.423248423	0.67211402	0.983233856
Polr3gl	0.125905437	0.154814797	0.813264878	0.416066209	0.943415794
Ankrd34a	1.202520316	1.797192958	0.669110298	0.503425121	0.961729397
Lix1l	-0.03806226	0.133521863	-0.285063878	0.775595203	0.990861469
6330549D23Rik	0.015498455	0.520929634	0.029751533	0.976265212	0.997262526
Rbm8a	0.177049332	0.121375084	1.458695854	0.144648838	0.841977044
Pex11b	-0.135732598	0.1113477	-1.218997773	0.222845038	0.892391445
Itga10	-0.627659861	0.456347772	-1.375398104	0.169008057	0.861597949
Ankrd35	0.630164057	0.752805944	0.837086984	0.402543676	0.937834293
Pias3	0.034865693	0.187609409	0.185841921	0.852568729	0.992886758
Nudt17	-0.191517178	0.389272116	-0.491987919	0.622727884	0.97546562
Polr3c	0.186308956	0.158820524	1.173078585	0.240764293	0.896939594
Rnf115	0.110393594	0.10540232	1.047354496	0.294936111	0.90737175
Cd160	0.601349843	0.481030549	1.250128178	0.211252728	0.884390587
Pdzk1	-0.068304964	0.372615944	-0.183311972	0.854553241	0.992886758

Gpr89	0.222666408	0.159426717	1.396669344	0.162513027	0.859982992
Gja5	0.178251882	0.159029719	1.120871513	0.262342558	0.897248195
Acp6	-0.130283623	0.201208684	-0.647504968	0.51730518	0.964352198
Bcl9	0.0751087	0.503815362	0.149079814	0.881490654	0.992886758
Chd1l	0.126219709	0.154627809	0.816280786	0.414339561	0.943415794
Fmo5	-0.198420588	0.255718851	-0.775932581	0.437788794	0.94749626
Prkab2	-0.129476712	0.242239981	-0.534497698	0.592997242	0.974001549
Pde4dip	-0.151244065	0.159826993	-0.946298628	0.343996293	0.920669678
Sec22b	0.067402183	0.092417709	0.72932108	0.46580528	0.953083512
Notch2	0.225431451	0.208205656	1.082734521	0.278926277	0.90176568
Hmgcs2	0.361576414	0.255152611	1.417098624	0.156454095	0.855455553
Phgdh	0.060214935	0.223864087	0.26897988	0.787945166	0.990988839
Zfp697	-0.148552583	0.267213421	-0.555932343	0.578257107	0.971613657
Hsd3b2	1.082476655	1.301435034	0.831756197	0.405546573	0.939557159
Wars2	-0.127075043	0.197586894	-0.643134983	0.520136517	0.964665379
Tbx15	-0.16981332	1.976128081	-0.085932345	0.931520199	0.994688009
Spag17	-0.067185451	0.377342509	-0.178048987	0.858684504	0.992886758
Wdr3	-0.315747231	0.210466918	-1.50022262	0.133556746	0.827800491
Gdap2	-0.30112569	0.195672731	-1.53892517	0.123822566	0.820382953
Fam46c	-0.312663112	0.221968414	-1.408592807	0.158955607	0.855986074
Man1a2	-0.121423943	0.113225661	-1.07240657	0.283537458	0.905916508
Vtcn1	-0.160659627	0.217457348	-0.738809832	0.460022481	0.953083512
Trim45	0.126123545	0.350688509	0.359645502	0.719112252	0.988585656
Ttf2	0.107905384	0.330345864	0.326643664	0.743937414	0.988585656
Cd101	0.611495842	0.371870163	1.644379955	0.100097743	0.788783372
Ptgfrn	0.035552117	0.15474465	0.229746983	0.818288384	0.992734473
Cd2	0.203047033	0.179609706	1.130490312	0.258269678	0.897248195
Igsf3	0.237695067	0.402267005	0.590888798	0.554594932	0.968926429
Atp1a1	0.053527227	0.135033988	0.39639818	0.691811308	0.986464472
Mab21l3	0.487015581	1.108155466	0.439483083	0.660311537	0.979924124
Slc22a15	-0.202206401	0.251976638	-0.802480748	0.42227492	0.943415794
Casq2	0.393103919	0.318686618	1.233512475	0.217384625	0.887633697
Vangl1	-0.248931365	0.325186799	-0.765502678	0.443972272	0.948476747
Ngf	-0.151238024	0.825589606	-0.183187898	0.85465059	0.992886758
Tspan2os	0.086364744	0.799599186	0.108010045	0.913987724	0.994688009
Tspan2	-0.137666641	0.093928458	-1.465654219	0.142742512	0.840741274
Tshb	-0.41880959	0.783131397	-0.534788404	0.592796183	0.974001549
Sycp1	4.008627915	1.835867985	2.18350554	0.028998601	0.64858361
Nr1h5	0.62637584	1.79285362	0.349373665	0.726808801	0.988585656
Sike1	0.252894151	0.15040312	1.681442181	0.092677058	0.775044718
Csde1	0.133985304	0.091991388	1.456498331	0.145254911	0.842729381
Nras	0.012645777	0.099270478	0.127387085	0.898634037	0.994364545
Ampd1	0.423292498	0.35227457	1.201598224	0.22951923	0.895404344
Dennd2c	0.1034761	0.287613771	0.359774497	0.719015776	0.988585656
Bcas2	0.289840791	0.139781595	2.073526136	0.038123341	0.679118604
Trim33	-0.088808609	0.165369448	-0.537031541	0.591245827	0.974001549
Syt6	0.990964173	0.608847213	1.627607308	0.103608178	0.79155232
Olfml3	-0.069877546	0.167016779	-0.41838638	0.675664643	0.983549699
Hipk1	0.011298092	0.130561503	0.086534633	0.931041426	0.994688009
Dclre1b	0.046970474	0.205021745	0.229099964	0.818791221	0.992734473
Ap4b1	0.026170999	0.145125221	0.180333916	0.856890431	0.992886758
Bcl2l15	-1.174560905	1.695451365	-0.692771807	0.488452768	0.958481967
Ptpn22	0.074911332	0.381349716	0.196437361	0.844267856	0.992886758
Rsbn1	-0.54406212	0.249337508	-2.182030789	0.02910726	0.64858361
Phtf1os	-1.024484649	1.233039669	-0.830861062	0.406052123	0.939557159
Phtf1	-0.15510062	0.138973547	-1.116044195	0.264403217	0.89768067
Magi3	-0.074730851	0.152388966	-0.49039542	0.623854117	0.975903684
Lrig2	-0.127580464	0.230109124	-0.554434616	0.579281441	0.971613657
Slc16a1	-0.055199526	0.174939327	-0.315535257	0.752355286	0.988585656
Ppm1j	0.243489383	0.28286462	0.860798296	0.389349144	0.933734231
Rhoc	0.165194523	0.11200621	1.474869317	0.140247681	0.837748392
Mov10	-0.173296203	0.357372095	-0.484918116	0.627734432	0.976968316

Capza1	0.048288942	0.108072061	0.446821696	0.655003805	0.979133069
St7l	-0.048983855	0.178790544	-0.27397341	0.784105056	0.990988839
Wnt2b	-0.224374465	0.505330171	-0.444015571	0.657031326	0.979614766
Cttnbp2nl	-0.308062264	0.123743969	-2.489513354	0.012791811	0.531429778
Kcnd3os	0.783219863	1.57305848	0.497896215	0.618557196	0.974471036
Kcnd3	-0.901924034	1.095294668	-0.823453323	0.410250251	0.940983181
Ddx20	-0.009316354	0.208810211	-0.044616369	0.964413095	0.996474415
Fam212b	0.063789812	0.164574202	0.38760517	0.698308242	0.987736745
Rap1a	0.245753569	0.157146056	1.56385451	0.11785174	0.814289188
Gm5547	0.537520634	0.378356138	1.42067375	0.155411627	0.855261441
Tmigd3	-0.443193821	0.435367358	-1.017976686	0.308689036	0.9139204
Adora3	-1.448229553	1.789934006	-0.809096619	0.418459573	0.943415794
I830077J02Rik	0.182554059	0.558276408	0.326995833	0.743671037	0.988585656
Atp5f1	0.11044913	0.152349466	0.72497222	0.468469073	0.953927686
Wdr77	0.021297676	0.167461115	0.127179828	0.898798071	0.994364545
Ovgp1	-0.075013237	0.619197651	-0.121145869	0.903575499	0.994688009
Pifo	-0.042366295	0.184282493	-0.229898643	0.818170531	0.992734473
Chia1	-0.425573556	0.409293268	-1.039776583	0.298443711	0.909779403
Chil3	-1.091160786	0.578946563	-1.88473489	0.059465639	0.724178089
Chil4	-1.392844657	1.69300054	-0.822707745	0.41067421	0.941195652
2010016I18Rik	-0.824352055	2.440670382	-0.337756405	0.735546765	0.988585656
Dennd2d	-0.202491162	0.151338611	-1.338000662	0.180896221	0.868087032
Cept1	0.040167544	0.104834357	0.383152485	0.701606706	0.988482081
Dram2	0.14858114	0.13621669	1.090770453	0.275373901	0.900302631
Lrif1	-0.071690142	0.159563663	-0.449288645	0.653223448	0.979004493
Cd53	0.099701333	0.122258808	0.815494064	0.414789561	0.943415794
Kcna3	-0.333479204	0.549048006	-0.607377133	0.54360066	0.967491297
AI504432	-0.174219352	0.216508122	-0.804678135	0.42100544	0.943415794
Kcna2	0.097873775	0.533352635	0.183506688	0.854400472	0.992886758
Lamtor5	0.382492957	0.188537035	2.028741765	0.0424846	0.68118841
Slc16a4	0.099537656	0.408416825	0.243715856	0.807450892	0.99099448
Rbm15	-0.044164055	0.22339601	-0.197694017	0.843284471	0.992886758
Kcnc4	2.407289552	1.77099203	1.359288755	0.174055106	0.863528862
Slc6a17	-0.308962853	1.081228507	-0.285751671	0.775068326	0.990861469
Strip1	-0.08898471	0.193785053	-0.459192845	0.646095688	0.979004493
Ahcyl1	0.042702395	0.130795011	0.326483361	0.744058676	0.988585656
Csf1	0.103451631	0.182636489	0.566434628	0.571098361	0.971613657
4933431E20Rik	0.344383383	0.691459128	0.498053129	0.618446595	0.974471036
Gstm5	-0.002545406	0.232390591	-0.010953139	0.991260834	0.998770784
Gstm7	-0.12487334	0.260096035	-0.480104741	0.631152917	0.977034429
Gstm6	0.52845878	0.268026217	1.971668241	0.048647491	0.707153766
Gstm3	0.4042775	0.9767809	0.413887598	0.678956427	0.983903958
Gstm2	0.048923414	0.115857991	0.422270517	0.672827576	0.983233856
Gstm1	-0.127123815	0.201999132	-0.629328519	0.529134004	0.965637248
Gstm4	-0.17623635	0.169526866	-1.039577702	0.298536141	0.909779403
Ampd2	-0.350425311	0.30294622	-1.156724489	0.247384941	0.896939594
Gnat2	1.916636142	1.15519013	1.65915211	0.097085145	0.782676152
Gnai3	-0.090608025	0.079485186	-1.139936001	0.254312965	0.897044747
Gpr61	-1.970681709	1.753373574	-1.123937157	0.261039687	0.897248195
Amigo1	0.497946416	0.295139822	1.687154286	0.091573689	0.772516176
Cyb561d1	0.181329578	0.254478438	0.712553801	0.476121908	0.95716557
Atxn7l2	-0.289222135	0.563761258	-0.513022367	0.607935675	0.974001549
Psma5	0.257581505	0.160089567	1.608983715	0.107619903	0.796929372
Sort1	-0.245284066	0.138158499	-1.775381662	0.07583489	0.752876039
Mybphl	-0.202284504	0.402899979	-0.502071271	0.615617388	0.974001549
Psrc1	-0.516057319	0.386124836	-1.336503823	0.181384652	0.86821085
Celsr2	0.039252248	0.631027587	0.062203695	0.95040062	0.99573416
Sars	0.168394893	0.102390194	1.644638863	0.100044307	0.788783372
5330417C22Rik	-0.413010896	0.348120117	-1.186403417	0.235463023	0.896002526
1700013F07Rik	-0.101350511	0.201042133	-0.504125724	0.614173031	0.974001549
Scarna2	5.016651296	1.603908256	3.127766989	0.001761398	0.284736028
Tmem167b	-0.193413577	0.126844629	-1.524807	0.127307244	0.824450458

Taf13	0.083379645	0.143311173	0.581808405	0.560695742	0.968926429
Wdr47	-0.114986567	0.201225189	-0.571432274	0.567706657	0.971054423
Clcc1	0.009884965	0.115526634	0.085564384	0.931812712	0.994688009
Gpsm2	0.153049905	0.29526463	0.518348253	0.604215312	0.974001549
Stxbp3	-0.261844368	0.113924841	-2.298395731	0.021539276	0.608428657
Fndc7	-0.004783805	0.29886459	-0.016006598	0.987229128	0.998148029
Prpf38b	-0.258360476	0.148926087	-1.734823501	0.082772072	0.76099108
Henmt1	-0.892780625	1.47590783	-0.604902696	0.545243652	0.967491297
Fam102b	-0.297958603	0.126908774	-2.34781721	0.018883786	0.590246276
Slc25a24	0.010999551	0.143371284	0.07672074	0.938845705	0.994688009
Vav3	-0.010523913	0.218573666	-0.048148127	0.961598191	0.996474415
Prmt6	0.002599954	0.227603419	0.011423176	0.990885822	0.998770784
Amy1	-0.422427095	0.396006283	-1.066718162	0.286099123	0.905916508
Rnpc3	0.148016231	0.164311323	0.90082794	0.36767981	0.928436166
S1pr1	-0.350270465	0.142742877	-2.453856005	0.014133356	0.54993854
A930005H10Rik	0.310996444	0.247348376	1.257321552	0.208637223	0.884258007
Dph5	0.178517728	0.197938396	0.901885289	0.367117807	0.928284409
Slc30a7	-0.19371326	0.197093378	-0.982850169	0.325681186	0.916443764
Extl2	0.034351177	0.22755043	0.150960723	0.880006699	0.992886758
Vcam1	0.178299098	0.128124924	1.391603539	0.164042487	0.859982992
Gpr88	0.706521812	0.476104492	1.483963759	0.137818535	0.833085238
Cdc14a	-0.293056874	0.138841353	-2.1107319	0.034795362	0.674795441
Rtca	-0.026122023	0.143589542	-0.181921488	0.855644341	0.992886758
Dbt	-0.028418193	0.154918588	-0.183439529	0.854453162	0.992886758
Lrrc39	-0.103388153	0.602212773	-0.171680438	0.863688765	0.992886758
Trmt13	-0.093610485	0.348729525	-0.268432922	0.788366101	0.990988839
Sass6	-0.211163835	0.384405405	-0.549325875	0.582781833	0.972086107
Hiat1	-0.081322997	0.106396252	-0.764340808	0.444664173	0.948476747
Slc35a3	0.031029003	0.154271838	0.201131997	0.840595363	0.992886758
Agl	-0.110717141	0.251420122	-0.440367065	0.659671274	0.979924124
Frrs1	-0.438275438	0.294088964	-1.490281828	0.136150149	0.832457116
Palmd	-0.023064631	0.154325631	-0.149454313	0.881195157	0.992886758
Lppr4	-0.897389482	1.212293632	-0.740241026	0.459153758	0.952683168
Lppr5	1.729895138	1.667830459	1.037212823	0.299636684	0.910105802
Snx7	0.009486483	0.138687453	0.068401881	0.945465725	0.995012175
Dpyd	0.067922272	0.274692063	0.247266963	0.804701625	0.990988839
Ptbp2	0.130724118	0.161292109	0.810480559	0.417664034	0.943415794
6530403H02Rik	-2.305250913	2.234124141	-1.031836535	0.302148701	0.911518274
Rwdd3	0.134168985	0.255261365	0.52561415	0.599156323	0.974001549
Tmem56	-0.393216816	0.18707838	-2.101882729	0.03556355	0.674795441
Alg14	-0.232806875	0.113219185	-2.056249339	0.039758489	0.679118604
Cnn3	-0.225592437	0.139347849	-1.61891582	0.105465376	0.793982106
Slc44a3	-0.36315327	0.168690983	-2.152772267	0.031336581	0.661395937
A730020M07Rik	0.192140624	1.831212798	0.104925339	0.916435052	0.994688009
F3	-0.459447459	0.176602031	-2.601597827	0.00927906	0.478229167
Abcd3	-0.000664969	0.120485735	-0.005519068	0.995596443	0.999285699
Arhgap29	-0.115158188	0.15994839	-0.719970907	0.471542908	0.95486913
Abca4	-0.191771678	0.307374232	-0.623902911	0.532691348	0.96674935
Gclm	-0.07451856	0.170523941	-0.436997644	0.662113057	0.980647283
Dnttip2	0.003738237	0.108052495	0.034596486	0.972401503	0.996790728
Mir760	-0.181059284	0.954680949	-0.189654234	0.849580086	0.992886758
Bcar3	-0.32417583	0.189820422	-1.707802702	0.087672957	0.766704005
Fnbp1l	-0.100391101	0.128571182	-0.780821167	0.434907683	0.946762848
Pde5a	-0.043931786	0.15403628	-0.285204146	0.775487745	0.990861469
4933405D12Rik	1.158114154	1.244646973	0.930476013	0.352124678	0.923082865
Fabp2	0.125352057	1.330375648	0.094223055	0.924931971	0.994688009
1810037I17Rik	0.462170496	0.252514982	1.830269604	0.067209634	0.741440336
Usp53	-0.480925288	0.254634201	-1.888690858	0.058933264	0.72285319
Myoz2	0.295976115	0.296622639	0.997820382	0.318366465	0.91640698
Synpo2	-0.14305154	0.303408445	-0.471481734	0.637296761	0.977284137
Sec24d	-0.156682919	0.190761963	-0.821353042	0.411445203	0.941762685
Mettl14	0.077766663	0.207648538	0.374511008	0.708024169	0.988585656

Prss12	-0.146245002	0.247344893	-0.591259437	0.554346604	0.968926429
Snhg8	0.409560422	0.216197778	1.894378493	0.058174789	0.72285319
Tram1l1	0.38255671	0.934397945	0.409415188	0.682234996	0.984738223
Ndst4	1.110990163	1.302832215	0.852749994	0.39379796	0.933796635
Ugt8a	-0.859116754	0.451813545	-1.901485165	0.057238494	0.721735927
Arsj	-0.003130691	0.317902293	-0.009847965	0.992142587	0.998800761
Camk2d	-0.033135229	0.15367499	-0.215618879	0.829284839	0.992886758
Ank2	0.775444017	0.899989912	0.861614121	0.388899898	0.933485538
Larp7	-0.539177004	0.256555767	-2.101597675	0.035588534	0.674795441
Zgrf1	0.662648409	0.572073356	1.15832769	0.246730335	0.896939594
Alpk1	-0.195824361	0.340143745	-0.575710604	0.564810807	0.969658675
Tifa	0.067255504	0.165053612	0.407476715	0.683657893	0.984831694
Ap1ar	0.008029307	0.145619676	0.05513889	0.956027814	0.99614946
5730508B09Rik	0.246284386	0.131625173	1.871103982	0.06133067	0.72551838
9830132P13Rik	-0.623976621	1.888346022	-0.330435531	0.741070898	0.988585656
Pitx2	1.069562997	1.288133644	0.830319899	0.406357941	0.939557159
Enpep	-0.162157353	0.138488566	-1.170907878	0.241635808	0.896939594
Elovl6	-0.587715003	0.323530388	-1.816568165	0.069283262	0.743426713
Egf	1.437744845	0.766568055	1.875560605	0.06071564	0.725221283
6330410L21Rik	-0.644112607	1.945186116	-0.331131608	0.740545077	0.988585656
Gar1	-0.432732765	0.269633631	-1.604891658	0.10851765	0.798150849
Cfi	-0.103208199	0.523369248	-0.197199585	0.843671355	0.992886758
Pla2g12a	0.312307476	0.208234407	1.499788053	0.133669313	0.827800491
Casp6	-0.034770641	0.107548888	-0.323300797	0.74646745	0.988585656
Ccdc109b	0.349635019	0.160141434	2.18328892	0.02901454	0.64858361
Sec24b	-0.103417978	0.198806673	-0.520193698	0.602928577	0.974001549
Col25a1	-2.252109282	2.293996849	-0.981740355	0.326227779	0.916800143
Ostc	0.174715369	0.153592316	1.137526752	0.255318148	0.897044747
Rpl34	0.467600357	0.726936178	0.643248158	0.52006309	0.964665379
Lef1	0.573564251	0.515973812	1.111615041	0.266303703	0.89768067
Hadh	0.270432467	0.145113013	1.863599004	0.062378038	0.727399211
Cyp2u1	-0.168501102	0.874191375	-0.192750817	0.847154123	0.992886758
Sgms2	-0.084865297	0.305510343	-0.277782076	0.781179651	0.990988839
Papss1	-0.310960972	0.174052726	-1.786590642	0.074003675	0.750704981
Dkk2	0.223317033	0.744608836	0.299911876	0.764244376	0.989384061
Aimp1	0.276721749	0.157785733	1.753781818	0.0794679	0.75659671
Tbck	0.187695883	0.2345789	0.800139668	0.42362988	0.94351935
Gm29811	-0.82803642	0.992093518	-0.834635451	0.403922996	0.937834293
Npnt	0.093925627	0.114278123	0.821903829	0.411131634	0.941701842
Gstcd	-0.111844628	0.180811169	-0.618571454	0.536198712	0.96674935
Ints12	0.182995386	0.134471538	1.360848457	0.173561585	0.863528862
Arhgef38	-0.429357944	0.335725747	-1.278894895	0.20093407	0.881109608
Ppa2	-0.088451831	0.110402135	-0.801178628	0.423028242	0.943509323
Tet2	0.099634525	0.318950146	0.312382753	0.754749656	0.98902053
Cxxc4	0.316656299	0.383820491	0.825011447	0.409365092	0.940886864
Cenpe	-0.696301165	1.269988375	-0.548273652	0.583504016	0.972174584
Bdh2	-0.130858504	0.484321612	-0.270189273	0.787014645	0.990988839
Slc9b2	1.142958608	0.830157673	1.376797019	0.168575013	0.861597949
Cisd2	0.221836111	0.18294668	1.212572493	0.225293311	0.893255444
4930539J05Rik	-0.209728513	0.354281413	-0.59198283	0.553862086	0.968926429
Ube2d3	0.01577833	0.139937516	0.112752677	0.910226637	0.994688009
Manba	-0.087239166	0.162885666	-0.535585284	0.592245201	0.974001549
Nfkb1	-0.171515595	0.163120141	-1.051467919	0.293043736	0.907334416
Slc39a8	0.071533284	0.103294755	0.692516132	0.488613259	0.95849918
Bank1	0.024182891	0.390904202	0.061863984	0.950671149	0.99573416
Ppp3ca	0.073076184	0.103783216	0.704123337	0.481355968	0.957463006
Emcn	-0.065113123	0.114486424	-0.568740997	0.569531922	0.97144955
Ddit4l	-0.028122006	0.262233556	-0.107240303	0.914598343	0.994688009
H2afz	0.392382746	0.150806072	2.601902829	0.009270812	0.478229167
Dnajb14	-0.233440533	0.213095908	-1.095471682	0.273310045	0.89897004
Lamtor3	0.044325621	0.151909918	0.291788853	0.770448069	0.989990452
Dapp1	0.125097774	0.208420318	0.600218708	0.548360487	0.968738549

Mttp	-0.272358951	0.328628574	-0.828774403	0.407232075	0.939557159
Trmt10a	-0.160103069	0.221713165	-0.722118005	0.470221929	0.95486913
Adh7	-0.023491667	0.16655734	-0.141042518	0.887836353	0.992886758
Adh1	-0.104295105	0.125344757	-0.832065952	0.405371719	0.939557159
Adh5	0.039802344	0.118391979	0.336191223	0.736726671	0.988585656
Metap1	0.167070409	0.153295948	1.08985535	0.275776868	0.900302631
Eif4e	0.158656686	0.127985741	1.239643458	0.215107299	0.887555439
Tspan5	-0.142940027	0.151351261	-0.944425743	0.344952131	0.92132235
Rap1gds1	-0.029965091	0.176904276	-0.169385904	0.86549311	0.992886758
Unc5c	0.787205919	0.484581908	1.624505384	0.104267992	0.79155232
Bmpr1b	0.049397845	0.299100964	0.165154416	0.868822443	0.992886758
Pdlim5	-0.270482766	0.126429365	-2.139398283	0.032403426	0.665167506
Gbp5	0.138186513	0.342568961	0.403383051	0.686666453	0.98538669
Gbp7	0.142932778	0.164811756	0.867248681	0.385805768	0.931950108
Gbp3	0.325470087	0.131383275	2.477256613	0.013239667	0.539384266
Gbp2b	0.504517651	0.7307017	0.690456381	0.489907231	0.958560421
Gbp2	0.146942128	0.157553437	0.932649462	0.351000986	0.922414914
Ccbl2	-0.050796035	0.259441372	-0.195790034	0.844774511	0.992886758
Gtf2b	0.035835257	0.115291778	0.310822314	0.755935705	0.989266039
Pkn2	-0.01390403	0.108106253	-0.128614488	0.897662702	0.994101834
A830019L24Rik	-0.507380926	1.146441114	-0.44257042	0.658076484	0.979614766
Lmo4	0.066523114	0.110736139	0.600735355	0.548016267	0.968738549
Hs2st1	-0.249947628	0.094571827	-2.642939625	0.008218969	0.46696668
Sep-15	0.259663115	0.260755707	0.995809903	0.319342518	0.91640698
Sh3glb1	-0.042465814	0.083550372	-0.508266006	0.611266806	0.974001549
Clca3a1	-0.126801163	0.310777628	-0.408012519	0.683264484	0.984738223
Clca1	-1.071478383	1.01234902	-1.05840808	0.289869438	0.907334416
Clca2	1.671437604	1.902457425	0.878567679	0.379635727	0.929185635
Odf2l	-0.094899882	0.161664124	-0.587018812	0.557191072	0.968926429
Znhit6	-0.02437887	0.235843728	-0.103368746	0.917670316	0.994688009
Cyr61	0.416016347	0.516295012	0.805772547	0.42037401	0.943415794
Ddah1	-0.158622159	0.243665173	-0.650984123	0.515056736	0.964352198
Bcl10	0.063252657	0.093580612	0.675916252	0.499093819	0.960700483
2410004B18Rik	0.203168207	0.162311964	1.251714304	0.210673986	0.884340413
Syde2	-0.162185786	0.236402354	-0.686058254	0.492676375	0.958916246
Wdr63	-0.466413291	0.232761082	-2.00382851	0.045088434	0.693181597
Mcoln3	-0.026301694	0.409216841	-0.064273243	0.948752658	0.99560893
Mcoln2	0.323704863	0.568477054	0.569424677	0.569067974	0.97144955
Lpar3	-0.208384873	0.208212929	-1.000825808	0.31691103	0.91640698
Ssx2ip	-0.056914351	0.200836828	-0.283386029	0.776880937	0.990861469
Ctbs	-0.175177788	0.127630838	-1.372534962	0.169896963	0.861730117
Spata1	-0.173532489	0.300394772	-0.577681454	0.563479202	0.969658675
Gng5	-0.08123388	0.13602132	-0.597214321	0.550364299	0.968926429
Rpf1	0.115451812	0.148463629	0.77764374	0.436779068	0.946762848
Uox	0.210454238	0.314176922	0.669858997	0.50294768	0.961729397
4930503B20Rik	0.361678855	1.225185858	0.295203256	0.767838614	0.989990452
Prkacb	-0.156441728	0.118071923	-1.324969763	0.18518118	0.871716376
Ttll7	-0.072943416	0.148889134	-0.489917662	0.624192165	0.975969024
Adgrl2	0.191332479	0.188228037	1.016492985	0.309394686	0.9139204
Gm1653	1.785507324	1.394809313	1.280108548	0.200506963	0.880888611
Adgrl4	-0.09803182	0.106116475	-0.923813389	0.355583483	0.924354588
Ifi44	0.081012196	0.249765821	0.324352609	0.745671094	0.988585656
Ptgfr	0.036031735	0.1812281	0.198819805	0.842403703	0.992886758
Gipc2	-0.049548229	0.12167668	-0.407212207	0.683852136	0.985024599
Dnajb4	-0.110860402	0.139074557	-0.797129287	0.425375949	0.944290206
Fubp1	-0.123259403	0.108026396	-1.141011893	0.253864974	0.897044747
Nexn	0.084134298	0.230647881	0.364773775	0.715280283	0.988585656
Fam73a	-0.127926136	0.194322109	-0.658320025	0.510332513	0.963452765
Usp33	-0.106971214	0.117357286	-0.911500407	0.362031771	0.926455466
Zzz3	-0.074682932	0.136991953	-0.545162915	0.585641475	0.972904784
Ak5	-1.040163153	0.607933197	-1.710982651	0.087084316	0.765418158
Pigk	-0.016800115	0.123104974	-0.13646983	0.891449873	0.993043931

St6galnac5	-0.601511187	0.859478853	-0.699855715	0.484017416	0.957710647
St6galnac3	-0.040943967	0.147590088	-0.27741678	0.781460099	0.990988839
Rabggtb	0.128069888	0.130168675	0.98387641	0.325176284	0.91640698
Acadm	0.020103275	0.089375103	0.22493149	0.822032572	0.992886758
Slc44a5	1.334645808	1.847770089	0.722300797	0.470109563	0.95486913
Tyw3	0.447580688	0.46846945	0.95541062	0.339370085	0.919246575
Cryz	0.203762922	0.179680099	1.134031668	0.256781264	0.897248195
Erich3	-0.221876563	0.786254433	-0.282194355	0.777794486	0.990988839
Tnni3k	0.301034589	0.897064682	0.33557735	0.737189607	0.988585656
Fpgt	-0.132229785	0.145844431	-0.906649536	0.364592157	0.926556975
Lrriq3	-0.182216931	0.334031431	-0.545508337	0.585403948	0.972904784
4930570G19Rik	1.098778871	1.13228796	0.970405859	0.331844229	0.917115058
Negr1	0.191612258	0.369650051	0.518361239	0.604206253	0.974001549
Zranb2	0.144881061	0.133054907	1.088881758	0.276206032	0.900302631
Mir186	-1.029451068	3.189316771	-0.322781066	0.746861053	0.988585656
Ptger3	-0.01110708	0.599224892	-0.018535746	0.985211462	0.998148029
Cth	-0.333957302	0.249033336	-1.341014446	0.179915763	0.868087032
Ankrd13c	0.000814041	0.117216152	0.006944782	0.994458911	0.999285699
Srsf11	-0.025314144	0.097566403	-0.259455542	0.79528378	0.990988839
Lrrc40	-0.086468355	0.167448652	-0.516387289	0.605583942	0.974001549
Depdc1a	-0.408444012	0.457671295	-0.892439655	0.372157324	0.928840865
Wls	-0.098300753	0.109558444	-0.897244877	0.369588263	0.928436166
Tmem68	0.141209493	0.16659592	0.847616755	0.39665144	0.934469184
Tgs1	0.039160026	0.236222982	0.16577568	0.868333486	0.992886758
Lyn	-0.144414481	0.164590866	-0.877414914	0.380261315	0.929640874
Rps20	0.187713168	0.126212528	1.487278411	0.136941294	0.833085238
Plag1	-0.527037737	1.153437754	-0.456927766	0.647722966	0.979004493
Chchd7	0.402304619	0.144607675	2.782041949	0.005401806	0.412127487
Penk	-0.47988538	0.353344301	-1.358124016	0.174424336	0.863528862
Impad1	-0.258843942	0.157869695	-1.639605001	0.101087323	0.788783372
Fam110b	0.100640477	0.369872099	0.272095348	0.785548707	0.990988839
Ubxn2b	0.034088415	0.169622796	0.200966002	0.840725158	0.992886758
Sdcbp	-0.118275712	0.085088484	-1.390031963	0.164519172	0.859982992
Nsmaf	-0.004944863	0.121629438	-0.040655152	0.967570816	0.996474415
Tox	0.107652052	1.038307308	0.103680338	0.917423031	0.994688009
Car8	-0.048545854	0.165721717	-0.292935981	0.769571085	0.989990452
Rab2a	0.029661361	0.091329682	0.324772412	0.745353325	0.988585656
Chd7	0.418041247	0.376991664	1.108887242	0.267478828	0.89804647
Clvs1	-0.902357635	1.478417853	-0.610353584	0.541627609	0.96731065
Asph	-0.211522054	0.135481453	-1.561262079	0.118461927	0.814755048
4930412C18Rik	0.432874376	0.2580421	1.677533923	0.093438122	0.775558829
Gdf6	1.043299518	1.795350166	0.581111996	0.561164975	0.969333531
2610301B20Rik	-0.337147753	0.142086323	-2.37283749	0.017652029	0.579835303
Plekhf2	-0.023129075	0.122283635	-0.18914285	0.849980858	0.992886758
Ndufaf6	0.299598428	0.245399223	1.220861353	0.222138519	0.892391445
Trp53inp1	-0.348976852	0.183729217	-1.899408582	0.057510776	0.721735927
Ccne2	0.303473418	0.395481723	0.76735131	0.442872669	0.948476747
Ints8	-0.134828013	0.137605601	-0.97981486	0.327177515	0.916882624
Dpy19l4	-0.019307213	0.203865315	-0.094705725	0.92454857	0.994688009
Esrp1	-0.416901884	0.304364556	-1.369745179	0.17076646	0.86244803
1110037F02Rik	-0.297572801	0.163640539	-1.818454051	0.068994764	0.743426713
Rad54b	-0.106975495	0.924527663	-0.115708268	0.907883753	0.994688009
Fsbp	2.705083584	2.352709238	1.149773861	0.250237024	0.896939594
Gem	0.052296794	0.160820798	0.325186759	0.74503973	0.988585656
Cdh17	-0.141957968	1.370559949	-0.103576621	0.917505342	0.994688009
Pdp1	-0.21952865	0.135519236	-1.619907675	0.105252111	0.793982106
1700123M08Rik	0.027656284	0.47560228	0.058150025	0.953629128	0.996070133
Tmem67	-0.096849924	0.203072949	-0.476921837	0.633417782	0.977034429
Rbm12b2	-0.090327561	0.249543512	-0.361971186	0.717373563	0.988585656
Rbm12b1	-0.110280534	0.264207321	-0.417401506	0.676384753	0.983549699
Fam92a	0.146421286	0.180229885	0.812414023	0.416554102	0.943415794
Triqk	0.123678861	0.200533679	0.616748579	0.537400573	0.96674935

Runx1t1	0.818812609	0.522909118	1.565879388	0.117376856	0.812881672
Otud6b	-0.115600185	0.103358985	-1.118433826	0.263381754	0.897248195
Tmem55a	-0.14692948	0.138412864	-1.061530522	0.288448869	0.907334416
Gm11837	-0.133364703	0.485895571	-0.274471946	0.78372196	0.990988839
Tmem64	-0.198820724	0.14868967	-1.33715223	0.181172951	0.868087032
Decr1	0.091125618	0.101871345	0.894516692	0.371045503	0.928788832
Nbn	-0.044428825	0.146107676	-0.304082757	0.761064843	0.989266039
Osgin2	0.02839786	0.163224739	0.173980122	0.861881084	0.992886758
Ripk2	-0.267096164	0.23162122	-1.153159302	0.248845005	0.896939594
A530072M11Rik	0.312566678	0.498263565	0.62731193	0.530454785	0.966026275
Mmp16	1.83325878	1.109717633	1.652004731	0.098533587	0.786907305
Cpne3	-0.272295089	0.147650493	-1.844186795	0.065155922	0.734162002
Rmdn1	0.331801581	0.217339333	1.526652245	0.126847508	0.824450458
Wwp1	-0.174034039	0.111635195	-1.558953159	0.119007467	0.814755048
Slc7a13	-0.869703451	1.513964684	-0.574454253	0.565660449	0.970061308
Atp6v0d2	-0.126620949	0.220680363	-0.573775335	0.566119841	0.970328402
Ttpa	-0.24277245	0.229331199	-1.058610649	0.289777135	0.907334416
Ggh	0.161057912	0.265047226	0.607657413	0.543414714	0.967491297
Ccnc	0.041534818	0.114977917	0.361241694	0.717918776	0.988585656
Tstd3	0.448035643	0.213776647	2.095811915	0.036098879	0.675901831
Usp45	-0.285964487	0.161106983	-1.774997463	0.075898306	0.752876039
Pnisr	-0.027395969	0.159298841	-0.171978457	0.863454465	0.992886758
Coq3	0.265499132	0.215640629	1.231211079	0.218243929	0.888436964
Faxc	0.08833937	0.531804351	0.166112537	0.868068389	0.992886758
Fbxl4	-0.128973556	0.140584844	-0.917407256	0.358929272	0.925199968
Mms22l	0.051634601	0.481404346	0.107258278	0.914584083	0.994688009
Klhl32	-0.409125288	0.581873975	-0.703116663	0.481983049	0.957463006
Ndufaf4	-0.083625367	0.170591541	-0.490208171	0.623986599	0.97593919
Fhl5	3.141001587	2.151748728	1.459743671	0.144360535	0.841934045
Ufl1	-0.028560382	0.128724744	-0.22187173	0.824413738	0.992886758
Fut9	-1.111653495	0.779910962	-1.425359495	0.15405331	0.854159937
Manea	-0.122536402	0.141668145	-0.864953807	0.387064143	0.932858077
Epha7	-0.025813516	0.341102552	-0.075676701	0.939676313	0.994688009
Map3k7	-0.463849275	0.12393247	-3.742758259	0.000182011	0.082547186
Bach2	0.309511939	0.483198991	0.640547568	0.521816675	0.965211168
Casp8ap2	-0.110534678	0.20155415	-0.548411818	0.583409164	0.972174584
Mdn1	-0.214158988	0.311251067	-0.688058646	0.491415851	0.958916246
Lyrm2	0.37203392	0.156001625	2.384807972	0.017088038	0.573539012
Ankrd6	0.3460086	0.470583781	0.735275234	0.462171888	0.953083512
Rragd	-0.217093924	0.195426587	-1.110872004	0.266623447	0.89768067
4933421O10Rik	0.204180443	0.174420449	1.170622161	0.241750686	0.896939594
Ube2j1	-0.333062296	0.115899481	-2.873716883	0.004056724	0.378480793
Pm20d2	-0.491173772	0.313158885	-1.568449102	0.116776359	0.812418014
Srsf12	1.103451623	2.027521074	0.544236821	0.586278517	0.972904784
Pnrc1	0.282097916	0.155716182	1.811615926	0.070045562	0.744227652
Rngtt	0.077510448	0.150302157	0.515697508	0.606065696	0.974001549
Cnr1	-0.146245802	0.528654486	-0.276637777	0.782058254	0.990988839
Spaca1	-0.03020089	0.638377382	-0.047308835	0.962267087	0.996474415
Akirin2	-0.015646274	0.131682461	-0.118818209	0.905419382	0.994688009
Orc3	-0.078220746	0.135019301	-0.579330106	0.562366455	0.969658675
Rars2	-0.007345045	0.134833264	-0.054475025	0.956556706	0.996217474
Slc35a1	0.11751126	0.113633919	1.034121336	0.301079443	0.911162941
1700003M02Rik	-0.225080142	0.167497042	-1.34378577	0.179017672	0.867377413
Smim8	0.217303528	0.196781026	1.104291062	0.269466899	0.89804647
Zfp292	-0.054411553	0.242493911	-0.224383173	0.822459163	0.992886758
Mob3b	-0.453916414	0.263853402	-1.720335652	0.085371446	0.761635307
3110043O21Rik	-0.073790418	0.137094765	-0.538243876	0.590408693	0.974001549
Aco1	-0.060078538	0.160050664	-0.375372001	0.707383825	0.988585656
Ddx58	-0.050021165	0.286226758	-0.174760618	0.861267734	0.992886758
Topors	-0.135398072	0.142355628	-0.951125525	0.341540659	0.919850248
Toporsos	-0.529369506	1.010724353	-0.523752598	0.600450627	0.974001549
Ndufb6	0.430504893	0.193912489	2.22009884	0.026412059	0.632301593

Tmem215	0.293843341	1.037830788	0.283132226	0.777075479	0.990861469
Aptx	0.199670858	0.136089624	1.467201187	0.142321334	0.840741274
Gm6297	0.189583929	0.560685336	0.338128923	0.735266036	0.988585656
Dnaja1	-0.313521498	0.189818974	-1.651686821	0.098598411	0.786907305
Smu1	-0.11897414	0.122053022	-0.974774225	0.329672265	0.917115058
B4galt1	-0.137735592	0.12553511	-1.097187803	0.272559304	0.898460682
Spink4	0.290911933	0.334746098	0.8690525	0.384818416	0.931689378
Bag1	0.138628368	0.119994959	1.155284926	0.247973767	0.896939594
Chmp5	0.177721724	0.100790859	1.763272249	0.077854583	0.754202
Nfx1	0.000607071	0.10547126	0.005755792	0.995407568	0.999285699
Aqp7	0.936753348	1.778542665	0.526697147	0.598403917	0.974001549
Aqp3	-0.796578246	0.770534521	-1.033799557	0.301229879	0.911280384
Nol6	0.245056001	0.353381902	0.693459397	0.488021298	0.958288569
Ube2r2	0.060086549	0.103167959	0.582414825	0.560287298	0.968926429
Ubap2	-0.424964941	0.272665777	-1.558556214	0.119101453	0.814755048
Dcaf12	-0.155718803	0.114128278	-1.364419099	0.1724357	0.86244803
Ubap1	0.14751611	0.113281609	1.302207052	0.192845615	0.879588734
Kif24	-0.655249499	0.823452163	-0.795734747	0.426186226	0.944290206
Nudt2	0.357357972	0.161668245	2.210440106	0.027074633	0.635474249
AI464131	0.260753916	0.292696498	0.890867907	0.373000039	0.928840865
1110017D15Rik	0.13880049	0.165405679	0.839151902	0.401384074	0.937807351
Fam219aos	0.542402141	0.434580184	1.248106013	0.211992236	0.884945675
Fam219a	0.04791073	0.288390716	0.166131318	0.868053608	0.992886758
Dnaic1	-0.273891248	0.266476483	-1.027825216	0.304032053	0.911786415
Enho	-0.00197563	0.183529913	-0.010764621	0.991411241	0.998770784
Cntfr	0.213203928	0.668186454	0.319078495	0.749666991	0.988585656
Rpp25l	-0.087387774	0.231641686	-0.377254092	0.705984785	0.988585656
Dctn3	0.012130527	0.168426419	0.07202271	0.942583834	0.994688009
Sigmar1	0.08449833	0.16321778	0.517702976	0.604665522	0.974001549
Galt	0.04199853	0.152787427	0.27488211	0.783406812	0.990988839
Il11ra1	0.076742409	0.132986177	0.577070573	0.563891782	0.969658675
Ccl27a	0.44905684	0.252735129	1.776788382	0.075603064	0.752876039
Fam205c	-0.673361848	1.353098687	-0.497644299	0.618734774	0.974471036
Phf24	-0.528590924	0.383792659	-1.377282529	0.168424915	0.861597949
Dnajb5	-0.43232791	0.297144973	-1.454939336	0.145686057	0.842832198
Vcp	-0.14344302	0.148364667	-0.966827364	0.333630343	0.917554801
Fancg	-0.124735867	0.35507055	-0.351298825	0.725364176	0.988585656
Pigo	-0.072282661	0.332181513	-0.217599891	0.82774087	0.992886758
Stoml2	0.066095535	0.112375968	0.588164316	0.556422007	0.968926429
Fam214b	-0.097814766	0.150982313	-0.647855791	0.517078227	0.964352198
Unc13b	0.176143595	0.22849683	0.770879821	0.440778169	0.948068187
Atp8b5	0.565881544	0.622607413	0.908889827	0.363408284	0.926455466
Rusc2	-0.208140324	0.162064384	-1.284306386	0.199034773	0.880661448
Fam166b	0.009319243	0.196681273	0.047382463	0.962208405	0.996474415
Tesk1	-0.178860145	0.287150907	-0.622878564	0.533364326	0.96674935
Cd72	0.048350416	0.440203563	0.109836495	0.912539047	0.994688009
Sit1	-0.451925195	0.495235962	-0.912545189	0.361481792	0.926371563
Rmrp	3.471569667	1.287497344	2.696370353	0.007009968	0.452031612
Ccdc107	0.272746255	0.203579961	1.33975001	0.180326633	0.868087032
Arhgef39	-0.176068332	0.533147567	-0.33024315	0.741216245	0.988585656
Car9	-0.498336239	0.380359944	-1.310170132	0.190138287	0.876945711
Tpm2	-0.01821746	0.129149059	-0.141057627	0.887824418	0.992886758
Tln1	0.133037768	0.17698988	0.751668782	0.452250269	0.949574349
Creb3	0.123504722	0.131059573	0.942355594	0.346010614	0.921552698
Gba2	-0.030545616	0.222949636	-0.1370068	0.89102542	0.993043931
Rgp1	0.1195818	0.246677134	0.48477051	0.627839144	0.976968316
Npr2	0.004056498	0.147459098	0.02750931	0.978053515	0.997262526
Spag8	-0.247672369	0.205526052	-1.205065568	0.228177991	0.894468681
Hint2	0.491224491	0.151541804	3.241511431	0.001188977	0.248877204
Tmem8b	-0.039557751	0.329537349	-0.120040266	0.904451251	0.994688009
Hrct1	0.209071219	0.231991879	0.901200595	0.367481675	0.928436166
5430416O09Rik	-0.029875549	0.213445303	-0.139968173	0.888685137	0.992886758

A630077J23Rik	0.764710925	1.12472694	0.679908072	0.49656267	0.960362776
Reck	-0.33869929	0.192465295	-1.759794095	0.078442725	0.754732183
Glipr2	0.12476543	0.136361003	0.91496416	0.360210446	0.925913281
Clta	0.175578629	0.119907801	1.464280282	0.143117382	0.840741274
Gne	0.028645943	0.190673337	0.150235704	0.880578658	0.992886758
Gm12504	-0.641573822	0.953795638	-0.67265334	0.501167859	0.96121484
Rnf38	-0.223008121	0.210952079	-1.057150618	0.290442853	0.907334416
Melk	0.127026011	0.36856424	0.344650939	0.730356806	0.988585656
Pax5	2.026060376	1.1879853	1.70545913	0.088108829	0.767228183
Zcchc7	-0.014642597	0.147190807	-0.099480376	0.920756868	0.994688009
Grhpr	0.016848427	0.164097505	0.102673268	0.918222291	0.994688009
Zbtb5	-0.376288449	0.250444357	-1.502483241	0.132972353	0.827800491
Polr1e	-0.011192929	0.268736293	-0.041650234	0.966777527	0.996474415
Fbxo10	-0.183285607	0.327213121	-0.560141373	0.575383012	0.971613657
Tomm5	0.493516464	0.306177513	1.611863846	0.106991571	0.79539432
Frmpd1	-0.498255034	0.803636264	-0.620000685	0.535257336	0.96674935
Trmt10b	0.226495615	0.188970377	1.198577357	0.230692327	0.895404344
Exosc3	0.206756027	0.124874951	1.655704566	0.097781662	0.785669881
Dcaf10	-0.312188775	0.113071929	-2.76097505	0.005762908	0.423948802
Slc25a51	-0.178180331	0.092427221	-1.927790627	0.053881174	0.707153766
Shb	0.042721067	0.180292127	0.2369547	0.812691934	0.992000477
Aldh1b1	-0.330295382	0.683630212	-0.483149188	0.628989811	0.977034429
Igfbpl1	-0.816117844	1.584451897	-0.515078965	0.606497841	0.974001549
Stra6l	-0.551285334	1.023514377	-0.53862002	0.59014907	0.974001549
Ccdc180	-0.178991463	0.261020128	-0.685738162	0.492878237	0.958916246
Tdrd7	-0.22042305	0.186523814	-1.181742129	0.237308037	0.896609492
Tmod1	-0.1200057	0.208743978	-0.574894191	0.565362859	0.969914825
Tstd2	-0.274302821	0.183198551	-1.497297983	0.134315739	0.828162039
Ncbp1	0.140863566	0.160011911	0.880331752	0.378679616	0.929137643
Xpa	0.072976584	0.143601273	0.508188974	0.611320823	0.974001549
5830415F09Rik	0.182268993	0.198846714	0.916630652	0.359336217	0.925229839
Anp32b	0.182938667	0.104493246	1.750722401	0.079993739	0.75659671
Nans	0.363763727	0.145436474	2.501186373	0.012377802	0.525888996
Trim14	-0.049844515	0.299832411	-0.166241249	0.867967099	0.992886758
Coro2a	-0.113371311	0.313673861	-0.361430534	0.717777625	0.988585656
Tbc1d2	-0.210368188	0.301380201	-0.698015954	0.485167217	0.957710647
Anks6	0.446791081	0.523112085	0.854102007	0.393048472	0.933734231
Galnt12	-0.14569995	0.33493384	-0.435011136	0.663554345	0.98114941
Col15a1	-0.729289856	0.442145306	-1.649434803	0.09905859	0.787788403
Tgfbr1	-0.191499221	0.125908104	-1.520944369	0.128273798	0.826336015
Alg2	-0.431629829	0.174642505	-2.471505031	0.013454566	0.540715407
Sec61b	0.225382225	0.195655588	1.151933493	0.249348404	0.896939594
Nr4a3	-0.350156692	0.398953723	-0.877687491	0.380113335	0.929640874
Stx17	-0.1872534	0.130268088	-1.437446441	0.150591181	0.849879783
Erp44	0.039540408	0.095024426	0.416107836	0.677331096	0.983670411
Invs	-0.222795494	0.197201637	-1.129785217	0.258566739	0.897248195
Tex10	-0.64765898	0.258533553	-2.505125441	0.012240797	0.525888996
Msantd3	0.325261024	0.223629277	1.454465305	0.145817346	0.842832198
Tmeff1	-0.011047553	0.152963188	-0.072223609	0.942423957	0.994688009
Murc	1.549055354	0.752672008	2.058074882	0.039582946	0.679118604
Acnat1	-0.047802487	0.761136054	-0.06280413	0.949922477	0.995727234
Mrpl50	0.181392341	0.14752108	1.229602851	0.218845862	0.888671255
Zfp189	-0.048655383	0.244183461	-0.199257487	0.842061333	0.992886758
Aldob	-0.334749641	2.603788975	-0.128562508	0.897703834	0.994101834
Tmem246	-0.070404269	0.162185979	-0.434095904	0.6642188	0.981154469
Rnf20	-0.236998844	0.137336427	-1.725680856	0.084404844	0.76099108
Ppp3r2	2.315927449	1.698250601	1.363713605	0.17265772	0.86244803
Smc2os	0.447848768	1.102485017	0.406217555	0.684582757	0.985105247
Smc2	-0.029376498	0.240585678	-0.122104103	0.902816573	0.994688009
Nipsnap3b	0.240423785	0.161917204	1.484856335	0.137581885	0.833085238
Abca1	-0.041664209	0.182503188	-0.228293046	0.81941843	0.992886758
4930522O17Rik	0.429526633	0.52301307	0.82125411	0.411501541	0.941762685

AI427809	0.069178966	0.822113899	0.084147666	0.932939027	0.994688009
Slc44a1	-0.226559782	0.182558328	-1.241026819	0.214595842	0.886400046
Fsd1l	-0.402566202	0.182770523	-2.202577287	0.027624556	0.637644759
Fktn	-0.235709576	0.382348035	-0.616479108	0.537578356	0.96674935
Tal2	3.832537457	1.900433637	2.016664714	0.043730515	0.688477147
Tmem38b	0.049257011	0.130575294	0.377230711	0.706002159	0.988585656
Zfp462	0.302521923	0.345871843	0.87466479	0.381756324	0.930635634
Rad23b	-0.120890978	0.129355985	-0.934560372	0.350014909	0.922291372
Klf4	-0.102783291	0.175257171	-0.586471239	0.557558883	0.968926429
Ikbkap	0.032489228	0.219594126	0.147951262	0.882381232	0.992886758
Fam206a	-0.70576496	0.279770638	-2.522655575	0.011647242	0.518291414
Ctnnal1	0.031665949	0.156536076	0.202291701	0.839688687	0.992886758
Tmem245	-0.31880644	0.283731098	-1.123621774	0.261173515	0.897248195
Epb4.1l4b	-0.118842706	0.123507764	-0.962228625	0.335934773	0.918908218
Gm12530	-1.154512817	1.203129194	-0.959591723	0.337260736	0.919246575
Ptpn3	-0.11431035	0.247697181	-0.461492335	0.644445419	0.97889195
Palm2	-0.58333211	1.241875125	-0.469718814	0.638555924	0.977795535
D630039A03Rik	-0.233209313	0.204921127	-1.13804426	0.255102002	0.897044747
Txn1	0.097509838	0.151643111	0.643021879	0.520209903	0.964665379
Svep1	0.248665024	0.272211083	0.913500733	0.360979248	0.926101223
Lpar1	-0.165145124	0.204291916	-0.808378164	0.418872918	0.943415794
AI314180	-0.051834864	0.099396868	-0.521493938	0.602022728	0.974001549
Ptgr1	0.183801994	0.169516225	1.084273758	0.278243437	0.901391073
Dnajc25	0.005596363	0.166562297	0.033599217	0.973196747	0.997074754
Gng10	0.299854613	0.263756631	1.136860943	0.255596422	0.897044747
Ugcg	-0.167888349	0.156751517	-1.071047682	0.284147991	0.905916508
Gm12596	-0.279612875	1.821151799	-0.153536281	0.877975381	0.992886758
Susd1	-0.306442864	0.43140361	-0.710339129	0.47749386	0.95716557
Ptbp3	-0.133656488	0.116487191	-1.147392149	0.251219584	0.896939594
Hsdl2	0.152878328	0.112854285	1.354652401	0.17552833	0.864596675
E130308A19Rik	0.184738278	0.267144766	0.691528723	0.489233337	0.958560421
Inip	0.209218738	0.161302801	1.297055828	0.194611979	0.879588734
Snx30	-0.11657488	0.256822811	-0.453911706	0.649892378	0.979004493
Mup3	0.641337309	2.526009645	0.253893452	0.799577873	0.990988839
Zfp37	0.121911294	0.165888211	0.734900289	0.462400223	0.953083512
Slc31a2	0.14008474	0.139348197	1.005285632	0.314759332	0.91586731
Fkbp15	0.224673227	0.195102737	1.151563687	0.24950041	0.896939594
Slc31a1	-0.045633767	0.112979734	-0.403911085	0.686278104	0.98538669
Cdc26	0.356922797	0.181005533	1.971888869	0.048622294	0.707153766
Prpf4	-0.188163531	0.220124143	-0.85480642	0.392658324	0.933734231
Wdr31	0.032223401	0.187778066	0.171603648	0.863749139	0.992886758
Bspry	0.07666754	0.120616672	0.635629705	0.525017803	0.965637248
Hdhd3	0.212700144	0.24438854	0.870335998	0.384116812	0.931591679
Alad	0.050698522	0.123196393	0.41152603	0.680686858	0.984226822
Pole3	0.488152424	0.158324386	3.083242167	0.002047585	0.306705625
4933430I17Rik	-2.302918118	1.8720321	-1.230170208	0.218633374	0.888631319
Rgs3	0.039536838	0.204275946	0.193546224	0.846531209	0.992886758
Zfp618	-0.37194705	1.68453092	-0.220801557	0.825246951	0.992886758
Ambp	1.336151179	3.933617431	0.339674918	0.734101352	0.988585656
Col27a1	-0.913856492	0.306643322	-2.98019368	0.002880662	0.345325017
Orm1	-0.3254464	0.644306087	-0.505111479	0.61348054	0.974001549
Orm3	0.602157378	0.575215706	1.04683751	0.295174527	0.907763139
Orm2	0.810979592	2.17878027	0.372217246	0.709731107	0.988585656
Akna	-0.067490453	0.317791906	-0.212373103	0.831815968	0.992886758
Whrn	-0.560882039	0.729271274	-0.769099317	0.441834354	0.948476447
Atp6v1g1	0.081675354	0.177773255	0.459435555	0.64592142	0.979004493
6330416G13Rik	-0.084411823	0.195648344	-0.431446651	0.666143636	0.981863224
Tnfsf15	0.155892188	0.36044592	0.432498134	0.665379408	0.981727712
Tnfsf8	-0.0072165	0.683918616	-0.010551694	0.991581122	0.998770784
Tnc	-0.741584177	0.528970539	-1.401938524	0.160933605	0.857316691
Pappa	0.12833163	0.781382555	0.164236621	0.869544873	0.992886758
Astn2	0.423505533	0.316142849	1.339601809	0.180374835	0.868087032

Trim32	-0.410957803	0.354765725	-1.158392069	0.246704074	0.896939594
Tlr4	-0.270576165	0.140445548	-1.926555655	0.05403503	0.707153766
Brinp1	0.573658179	0.703761974	0.815130967	0.414997348	0.943415794
Cdk5rap2	0.577327933	0.372109495	1.551500137	0.120781875	0.816227511
Megf9	-0.025862204	0.25917628	-0.099786153	0.920514102	0.994688009
Tle1	-0.126747011	0.162665406	-0.779188478	0.435868696	0.946762848
C630043F03Rik	0.087516445	0.221247654	0.395558748	0.692430574	0.986599035
Rasef	-0.075166409	0.135217428	-0.555892908	0.578284067	0.971613657
Gm11240	0.135589013	1.423755973	0.095233324	0.924129502	0.994688009
2310002L09Rik	0.289009139	0.514160338	0.562099248	0.574048401	0.971613657
Frmd3	-0.636813025	0.977075688	-0.651754038	0.514559856	0.964352198
Kdm4c	-0.286057963	0.14935807	-1.915249457	0.055460698	0.713559351
Tmem261	0.314752867	0.160510786	1.96094527	0.049885408	0.707153766
Ptprd	-0.021166941	0.196969588	-0.107462988	0.914421686	0.994688009
Tyrp1	-4.24376871	1.8936174	-2.241090893	0.025020188	0.627917508
Lurap1l	0.156897798	0.110964203	1.413949669	0.157376681	0.855986074
Mpdz	0.07577626	0.294863078	0.256987957	0.79718807	0.990988839
Nfib	0.4335692	0.148357441	2.922463454	0.003472744	0.368965929
Zdhhc21	-0.335910374	0.153861778	-2.183195716	0.0290214	0.64858361
Frem1	-0.226568973	0.242456745	-0.934471728	0.350060613	0.922291372
Ttc39b	-0.093146838	0.144112142	-0.646349684	0.518052918	0.964452679
Gm11413	2.466979357	2.429890087	1.015263764	0.309980112	0.914536555
Snapc3	-0.252964563	0.241933613	-1.045594947	0.295748083	0.908204914
Psip1	-0.025866245	0.114293265	-0.226314687	0.820956676	0.992886758
Ccdc171	0.111332076	0.460856745	0.241576319	0.809108473	0.991244394
Bnc2	-0.682472468	0.595623247	-1.145812343	0.251872807	0.896939594
Cntln	-0.205429364	0.17287332	-1.188323127	0.23470613	0.89581803
Sh3gl2	-0.401384292	0.361967875	-1.108894793	0.26747557	0.89804647
Adamtsl1	-0.196666479	0.303283918	-0.648456667	0.516689629	0.964352198
Rraga	0.004577244	0.096982224	0.04719673	0.962356434	0.996474415
Haus6	-0.072037316	0.342495806	-0.210330505	0.833409728	0.992886758
Plin2	0.072135091	0.152881395	0.471836948	0.637043176	0.977284137
Dennd4c	0.005297659	0.209766014	0.025255088	0.979851497	0.997296835
Rps6	0.182288035	0.130056926	1.401601906	0.161034158	0.857316691
Acer2	-0.152119316	0.151822888	-1.001952462	0.316366553	0.91640698
Mllt3	-0.150036582	0.169591593	-0.884693515	0.376321954	0.928883222
Focad	0.007741407	0.240396695	0.032202637	0.974310453	0.997262526
Hacd4	-0.055026951	0.136336997	-0.40360982	0.686499663	0.98538669
Klhl9	-0.109338906	0.136206599	-0.802743087	0.422123243	0.943415794
Mtap	-0.044784221	0.144515545	-0.30989207	0.756643033	0.989266039
Cdkn2a	0.0038357	0.695976549	0.00551125	0.995602681	0.999285699
Cdkn2b	0.296662756	0.167776185	1.768205397	0.077026572	0.752876039
Tusc1	0.008259419	0.218599187	0.037783392	0.969860386	0.996628386
Caap1	-0.019125211	0.158237173	-0.120864211	0.90379859	0.994688009
Plaa	0.081957551	0.133138307	0.61558204	0.538170408	0.96674935
Gm12657	0.285208107	0.442828688	0.64405969	0.519536729	0.964665379
Ift74	-0.010048232	0.105813344	-0.094961858	0.924345123	0.994688009
Lrrc19	-0.029535775	1.377681784	-0.02143875	0.982895663	0.997497357
Tek	-0.111769094	0.171465009	-0.651847825	0.514499346	0.964352198
Eqtn	-1.521661159	2.457124796	-0.619285256	0.535728456	0.96674935
Mysm1	-0.160254519	0.175247926	-0.914444595	0.360483278	0.925913281
Jun	-0.04213697	0.255187908	-0.165121342	0.868848475	0.992886758
Fggy	0.036954095	0.224948815	0.164277795	0.869512461	0.992886758
9530080O11Rik	-1.079354555	2.251204291	-0.479456511	0.631613899	0.977034429
Hook1	-0.310826008	0.276173883	-1.125472127	0.260389022	0.897248195
Cyp2j6	0.042156929	0.15767016	0.267374179	0.789181078	0.990988839
Cyp2j9	-0.009793748	0.244994041	-0.039975455	0.968112695	0.996474415
Gm12695	-0.644776474	0.407989601	-1.580374774	0.114021065	0.807965062
E130114P18Rik	1.500372418	1.213530845	1.236369413	0.21632128	0.887633697
Nfia	-0.26282889	0.213516697	-1.230952399	0.218340668	0.888436964
Tm2d1	0.19062145	0.17107877	1.114232055	0.265179647	0.89768067
Inadl	0.031027464	0.227686335	0.136272842	0.891605593	0.993043931

Kank4	0.653990016	0.292853932	2.233161123	0.025538321	0.631530315
Kank4os	1.078272418	2.271949945	0.474602189	0.635070546	0.977034429
Usp1	-0.051083817	0.12319454	-0.414659753	0.678390999	0.983786595
Dock7	-0.108551922	0.137496481	-0.78948873	0.429826414	0.945321484
Angptl3	-0.366361235	1.126917813	-0.325100225	0.745105218	0.988585656
Atg4c	-0.111890676	0.182915141	-0.611708114	0.540730893	0.966989525
Alg6	-0.156015363	0.15541783	-1.003844685	0.315453482	0.91586731
Itgb3bp	0.275926515	0.232765052	1.185429308	0.235847749	0.896067726
Efcab7	0.509159856	0.251822747	2.021897789	0.043186912	0.685056403
Pgm2	-0.21180614	0.327159964	-0.647408496	0.517367599	0.964352198
Ror1	0.369338633	0.821693141	0.449484868	0.653081922	0.979004493
Ube2u	0.123775627	0.988680925	0.125192693	0.900371002	0.994688009
Cachd1	-0.237644028	0.272413382	-0.872365469	0.383009032	0.931395363
Raver2	0.113082639	0.224168887	0.504452874	0.613943171	0.974001549
Jak1	-0.040697064	0.131598655	-0.309251369	0.757130321	0.989266039
E130102H24Rik	0.524483676	0.381068929	1.376348572	0.168713742	0.861597949
0610043K17Rik	-0.656738884	1.184881299	-0.554265549	0.579397124	0.971613657
Ak4	-0.439607989	0.462689225	-0.950115033	0.342053805	0.919850248
Dnajc6	-0.429743912	0.478999047	-0.897170704	0.369627835	0.928436166
Leprot	0.076078494	0.103638411	0.734076234	0.462902277	0.953083512
Lepr	-0.111296089	0.161038483	-0.691114865	0.489493359	0.958560421
Pde4b	-0.102702211	0.183837496	-0.558657582	0.576395434	0.971613657
Sgip1	-0.005004193	0.241103933	-0.020755335	0.983440828	0.997678538
Wdr78	-0.113194451	0.179212932	-0.631619882	0.527635292	0.965637248
Mier1	-0.033088288	0.12385318	-0.267157354	0.78934801	0.990988839
Slc35d1	-0.278303082	0.349924968	-0.795322163	0.426426125	0.944290206
Oma1	0.252544027	0.131007624	1.927704818	0.053891853	0.707153766
Dab1	0.089628034	1.505504524	0.059533553	0.952527141	0.996023481
1700024P16Rik	0.290311852	0.891953687	0.325478616	0.744818864	0.988585656
Prkaa2	-0.594011759	0.197746486	-3.003905505	0.002665381	0.329679354
Ppap2b	-0.187266584	0.140715923	-1.330813035	0.183250538	0.869576666
Usp24	-0.163651931	0.139614404	-1.172170821	0.241128481	0.896939594
Dhcr24	-0.252027264	0.200516367	-1.256891237	0.208793022	0.884258007
Ttc22	-0.204956845	0.432710247	-0.473658403	0.635743521	0.977104816
Pars2	0.416955436	0.299127612	1.393904871	0.163346333	0.859982992
Ttc4	0.005315905	0.133937868	0.039689337	0.968340803	0.996474415
Mroh7	-0.189070808	1.825570166	-0.103568086	0.917512115	0.994688009
Acot11	0.032493996	0.480849685	0.067576203	0.946122999	0.995065399
Ssbp3	-0.116359439	0.17709879	-0.657031251	0.511160822	0.963452765
Mrpl37	0.093628302	0.122160841	0.766434655	0.443417719	0.948476747
Cyb5rl	-0.001333398	0.294163532	-0.004532846	0.996383324	0.999379819
Tceanc2	0.133437346	0.124071086	1.07549108	0.282154924	0.90579922
Tmem59	-0.067898765	0.084312824	-0.805319551	0.420635302	0.943415794
Ldlrad1	-0.394522552	0.138326228	-2.85211676	0.004342915	0.385355746
Lrrc42	-0.072889073	0.157701983	-0.462195033	0.643941465	0.97889195
Hspb11	0.143950209	0.209503007	0.687103308	0.49201763	0.958916246
Dio1	-0.088476153	0.352429484	-0.251046401	0.801778235	0.990988839
Yipf1	0.22252973	0.146883727	1.515006016	0.129770881	0.827800491
Ndc1	-0.348769962	0.398077071	-0.876136777	0.380955682	0.930420854
Lrp8	-0.432995848	0.951376989	-0.455125416	0.649019014	0.979004493
Magoh	0.118039646	0.142443225	0.828678555	0.407286324	0.939557159
0610037L13Rik	0.32262036	0.111543884	2.892317781	0.003824109	0.376754331
Cpt2	-0.089394517	0.227236048	-0.393399366	0.694024538	0.986949593
Slc1a7	1.54748085	0.820768295	1.885405249	0.059375146	0.723986563
Podn	-0.048138323	0.320421564	-0.150234341	0.880579733	0.992886758
Scp2	0.008694037	0.100220216	0.086749335	0.930870761	0.994688009
Echdc2	-0.046688886	0.194672632	-0.239832817	0.810459865	0.991438232
Zyg11b	0.158559426	0.172866976	0.917233758	0.359020161	0.925199968
Coa7	0.196628153	0.23876418	0.823524506	0.410209787	0.940983181
Fam159a	0.223481732	0.670448202	0.333331839	0.738883808	0.988585656
Gpx7	0.254989812	0.14457879	1.763673714	0.077786929	0.754202
Zcchc11	-0.245000695	0.173633136	-1.411024995	0.158237244	0.855986074

Prpf38a	0.152890079	0.16162156	0.945975766	0.344160947	0.920669678
Orc1	-0.808314635	0.988454568	-0.81775598	0.413496538	0.94325631
Cc2d1b	0.232872414	0.234191865	0.994365938	0.320044745	0.91640698
Zfyve9	0.190390624	0.326982801	0.582264949	0.560388231	0.968926429
3110021N24Rik	0.269416376	0.971508849	0.27731747	0.781536346	0.990988839
Btf3l4	0.058077645	0.126134824	0.460441007	0.645199706	0.979004493
Txndc12	-0.033830658	0.111226256	-0.304160722	0.761005447	0.989266039
Kti12	-0.065222916	0.170680851	-0.38213376	0.702362151	0.988482081
Rab3b	-0.147975998	0.872255678	-0.169647503	0.865287362	0.992886758
Nrd1	-0.132148243	0.135743276	-0.973515937	0.330296943	0.917115058
Osbpl9	-0.111334823	0.074266203	-1.499131765	0.133839452	0.827800491
Calr4	2.785226664	1.972603509	1.411954633	0.15796332	0.855986074
Eps15	-0.038974178	0.087600441	-0.444908463	0.656385906	0.979614766
Ttc39aos1	0.137468231	1.628100441	0.084434736	0.932710791	0.994688009
Ttc39a	-0.234665182	0.138555674	-1.693652631	0.090331311	0.769347579
Rnf11	0.182927275	0.138076157	1.324828839	0.185227927	0.871716376
Cdkn2c	0.416865711	0.175746716	2.37196871	0.017693589	0.579835303
Faf1	0.060021908	0.108220674	0.554625149	0.579151083	0.971613657
Dmrta2	0.385274472	3.252918125	0.118439646	0.905719314	0.994688009
Agbl4	-0.512535341	0.821876313	-0.623616149	0.532879703	0.96674935
Bend5	0.839344973	0.29185828	2.875864866	0.004029222	0.378480793
Spata6	-0.225662319	0.115156843	-1.959608418	0.050041577	0.707153766
Slc5a9	-2.09544609	1.514535524	-1.38355691	0.16649417	0.860403806
Skint3	-0.089603651	0.35498855	-0.252412792	0.800722014	0.990988839
Trabd2b	-0.411861508	0.270889487	-1.52040418	0.128409425	0.826336015
Gm12830	0.501566338	1.585343965	0.316376982	0.751716386	0.988585656
Foxd2	-1.042696075	1.432553856	-0.727858203	0.466700391	0.953083512
Foxd2os	0.042374523	0.301294088	0.140641735	0.888152976	0.992886758
Cmpk1	0.150116479	0.152806509	0.982395841	0.325904874	0.916632021
Stil	1.264926473	1.068464688	1.183872979	0.236463348	0.896358698
Tal1	-0.000363882	0.146843044	-0.002478035	0.998022816	0.999591134
Pdzk1ip1	0.22555576	0.178219894	1.265603714	0.205654996	0.883765092
Cyp4a12b	0.046193948	0.454722143	0.101587196	0.919084343	0.994688009
Cyp4b1-ps2	-0.155762649	0.590306674	-0.263867335	0.791882147	0.990988839
Cyp4a32	-0.624542324	0.568591779	-1.098401959	0.272029007	0.898163317
Cyp4b1	-0.272083952	0.150504828	-1.807808794	0.070636265	0.744227652
Efcab14	-0.11356931	0.150872237	-0.752751548	0.451599227	0.949134998
Atpaf1	0.397294803	0.126200214	3.148130982	0.00164318	0.284736028
Mob3c	-0.15703855	0.178165774	-0.881418171	0.378091526	0.928883222
Mknk1	-0.201599374	0.180507533	-1.11684743	0.264059565	0.897439742
Faah	-0.205070678	0.178293008	-1.150189119	0.250065987	0.896939594
Nsun4	-0.055143313	0.158568616	-0.347756788	0.728022844	0.988585656
Uqcrh	0.230372839	0.29239425	0.787884301	0.430764385	0.945321484
Lrrc41	-0.466160191	0.161261117	-2.890716628	0.003843645	0.376754331
Rad54l	0.56475152	0.808336048	0.698659328	0.484764958	0.957710647
2510003B16Rik	1.059259314	1.5675623	0.675736661	0.499207856	0.960700483
Lurap1	0.276425631	0.363153539	0.761181157	0.446548871	0.948476747
Pomgnt1	-0.076283423	0.105731074	-0.721485368	0.47061094	0.95486913
Tspan1	-0.097211989	0.144424116	-0.673100808	0.50088316	0.961175273
Pik3r3	-0.149771347	0.180675543	-0.82895197	0.407131586	0.939557159
Mast2	-0.14598638	0.270976256	-0.538742331	0.590064661	0.974001549
Ipp	-0.126064229	0.22341424	-0.564262283	0.572575646	0.971613657
Tmem69	-0.02047139	0.180349775	-0.113509371	0.909626734	0.994688009
Gpbp1l1	-0.082812444	0.104675034	-0.791138442	0.428863209	0.945321484
C530005A16Rik	0.106224878	0.908661166	0.116902628	0.906937216	0.994688009
Ccdc17	-0.335658828	0.163006278	-2.059177303	0.039477257	0.679118604
Nasp	-0.027700974	0.195716736	-0.14153605	0.887446483	0.992886758
Akr1a1	0.03241849	0.101211518	0.32030436	0.748737619	0.988585656
AV051173	0.084500853	1.019300744	0.082900805	0.933930414	0.994688009
Prdx1	0.041228278	0.175720336	0.234624401	0.814500269	0.992048071
Mmachc	-0.391730072	0.263944979	-1.484135344	0.137773018	0.833085238
Ccdc163	0.038962573	0.257845411	0.151108266	0.879890312	0.992886758

Tesk2	-0.064861933	0.292937156	-0.221419275	0.824765985	0.992886758
Toe1	-0.055623798	0.154619749	-0.359745752	0.719037274	0.988585656
Mutyh	0.032968752	0.343146333	0.09607782	0.923458767	0.994688009
Hpdl	1.565417672	0.871481995	1.796270813	0.072451457	0.74633396
Zswim5	0.817583022	0.836045622	0.977916756	0.328115497	0.917115058
Urod	-0.11301628	0.132763239	-0.851261844	0.394623915	0.933796635
Hectd3	0.184459014	0.155737279	1.184424281	0.236245153	0.896358698
Eif2b3	-0.063173593	0.208617788	-0.302819783	0.762027206	0.989285682
Ptch2	3.089405863	1.410668502	2.190029663	0.028522086	0.645679684
Btbd19	0.165615427	0.25341681	0.653529761	0.513414809	0.964352198
Tctex1d4	0.038550694	0.18331628	0.210296075	0.833436598	0.992886758
Plk3	-0.159076707	0.338384246	-0.470106717	0.638278776	0.977779844
Rps8	0.301789598	0.185828132	1.624025354	0.104370398	0.79155232
Snord55	1.341680135	0.693515552	1.934607135	0.053038524	0.707153766
Kif2c	-0.399125226	0.86286528	-0.462557986	0.64368123	0.97889195
Gm1661	-0.435710316	0.210238192	-2.072460347	0.038222531	0.679118604
Tmem53	-0.261089945	0.231456211	-1.12803171	0.259306528	0.897248195
Rnf220	0.14719792	0.172952977	0.85108636	0.394721382	0.933796635
Eri3	0.048792562	0.142327425	0.342819118	0.731734542	0.988585656
Dmap1	-0.011445905	0.198824881	-0.057567772	0.954092921	0.996070133
Slc6a9	-0.171336379	0.308342655	-0.555668756	0.57843732	0.971613657
Ccdc24	-0.08337795	0.243905829	-0.341844842	0.732467661	0.988585656
B4galt2	0.068079885	0.221890146	0.306817973	0.758981927	0.989266039
Atp6v0b	0.378899995	0.240798512	1.573514684	0.115599691	0.810389074
Dph2	0.370098042	0.293686665	1.260179932	0.20760446	0.883846885
Ipo13	0.162175115	0.223137386	0.726794904	0.467351605	0.953315756
Artn	-0.294437619	0.715001995	-0.411799717	0.680486229	0.984226822
St3gal3	-0.499468148	0.18102879	-2.759053673	0.005796901	0.424421559
Kdm4a	-0.049656364	0.227967055	-0.217822546	0.827567377	0.992886758
Ptprf	-0.057698704	0.127847864	-0.451307534	0.651767916	0.979004493
Hyi	0.310404608	0.167063857	1.857999767	0.063169047	0.727848297
Szt2	-0.070129689	0.285381453	-0.245740176	0.80588337	0.99099448
Med8	0.103404159	0.117127289	0.882835754	0.37732502	0.928883222
Elovl1	-0.234046711	0.209105732	-1.11927449	0.263023055	0.897248195
Cdc20	0.350185708	0.324624199	1.078741848	0.280702825	0.903595233
Mpl	-1.205387907	0.791673067	-1.522582942	0.12786308	0.826126043
Tie1	-0.117254607	0.205108705	-0.571670555	0.567545186	0.970955746
2610528J11Rik	0.125522974	0.161256837	0.778404045	0.436330855	0.946762848
Tmem125	0.123588378	0.181986112	0.67910884	0.497068902	0.960475196
Cfap57	-0.54655613	0.341460613	-1.600641798	0.109456279	0.798400564
Ebna1bp2	0.059714531	0.115695154	0.516136837	0.605758841	0.974001549
Olfr1342	3.430188664	1.940134245	1.768016143	0.077058205	0.752876039
Slc2a1	-0.216777383	0.18756072	-1.155771761	0.247774527	0.896939594
Zfp691	0.768321119	0.615192104	1.248912517	0.211697073	0.884755882
Ccdc23	0.330631813	0.158408818	2.087205865	0.03686953	0.678243111
4930538K18Rik	0.716771938	1.176755996	0.609108379	0.542452604	0.967491297
AU022252	0.219590144	0.180738807	1.214958465	0.224381933	0.892989918
P3h1	-0.200956857	0.263989877	-0.761229407	0.446520057	0.948476747
Ybx1	0.176350744	0.106499382	1.65588514	0.097745081	0.785669881
Ppih	0.518262614	0.21466511	2.414284349	0.015766153	0.557315333
Ccdc30	-0.051317509	0.194893104	-0.263311057	0.792310838	0.990988839
Ppcs	0.032110657	0.2044703	0.157043135	0.875210853	0.992886758
Zmynd12	-0.096817062	0.201273826	-0.481021622	0.630501131	0.977034429
Rimkla	0.473943953	1.693929808	0.279789606	0.779638927	0.990988839
AA415398	0.014164745	0.51579282	0.027462083	0.978091182	0.997262526
Foxj3	-0.02409243	0.230133055	-0.104689132	0.916622486	0.994688009
Guca2a	-3.03344319	4.413217942	-0.687354042	0.491859651	0.958916246
Hivep3	-0.214103505	0.280550236	-0.763155677	0.445370559	0.948476747
Edn2	0.359518538	1.536059604	0.234052466	0.814944248	0.992140387
Foxo6	-0.103533484	0.442944823	-0.23373901	0.815187601	0.992270157
Scmh1	0.326690181	0.229125837	1.425811185	0.153922851	0.854159937
Ctps	-0.419481661	0.205205333	-2.044204476	0.040933351	0.679118604

Cited4	-1.295025764	0.729548855	-1.775104922	0.075880564	0.752876039
Kcnq4	1.000232281	1.066661476	0.937722327	0.348387127	0.921552698
Nfyc	-0.092890744	0.245101566	-0.378988782	0.704696194	0.988585656
Rims3	0.664842487	1.481231951	0.448844279	0.653543994	0.979133069
Exo5	0.270377696	0.139556739	1.937403374	0.052696055	0.707153766
Zfp69	0.613715427	0.499992881	1.22744833	0.219654131	0.888847883
Smap2	0.207515792	0.192030933	1.080637316	0.279858476	0.903192201
Zmpste24	-0.123873734	0.132709711	-0.93341876	0.350603798	0.922338606
Rlf	-0.113011797	0.156840503	-0.720552372	0.471184966	0.95486913
Ppt1	-0.0700823	0.097071949	-0.721962429	0.470317577	0.95486913
Cap1	-0.02393307	0.150980722	-0.158517394	0.874049116	0.992886758
Mfsd2a	-0.617533876	0.296764381	-2.080889475	0.037444024	0.679118604
Mycl	-0.111744532	0.223032881	-0.501022682	0.61635516	0.974001549
Trit1	0.088553125	0.196714735	0.4501601	0.652595004	0.979004493
Ppie	0.138227677	0.1286777	1.074216255	0.282725769	0.905916508
Hpcal4	0.278363695	0.391412201	0.711177869	0.476974021	0.95716557
Heyl	-0.022153492	0.297864472	-0.074374402	0.940712477	0.994688009
Pabpc4	-0.298217282	0.198430692	-1.502878809	0.132870299	0.827800491
Macf1	0.331496333	0.131460163	2.521648582	0.011680633	0.518291414
D830031N03Rik	-0.333524154	0.450660866	-0.740077916	0.459252718	0.952683168
Akirin1	-0.092897173	0.127731595	-0.727284218	0.467051864	0.953239786
Rhbdl2	0.325221656	0.784367185	0.41462935	0.678413259	0.983786595
Mycbp	0.083467537	0.148654317	0.561487472	0.574465268	0.971613657
Rragc	-0.142356322	0.092236478	-1.543384186	0.122737588	0.817788082
Utp11l	0.322105373	0.133344601	2.415586167	0.015709905	0.557315333
Fhl3	-0.776275007	0.453130616	-1.713137402	0.086687265	0.765418158
Sf3a3	-0.179412426	0.16365759	-1.096267066	0.272961918	0.898826892
Mir697	-1.63415947	2.248058907	-0.726920218	0.467274831	0.953292036
Inpp5b	-0.310453688	0.12426137	-2.498392612	0.012475794	0.525888996
Mtf1	0.053038266	0.168226196	0.315279469	0.752549472	0.988585656
1110065P20Rik	0.460005894	0.215167272	2.13789899	0.032524942	0.665167506
Yrdc	0.105125528	0.143750237	0.731306818	0.464591767	0.953083512
Maneal	-0.479934199	1.35610722	-0.353905791	0.723409487	0.988585656
Cdca8	0.227766451	0.616467211	0.369470504	0.711777055	0.988585656
9930104L06Rik	0.199290641	0.247969653	0.80368964	0.421576239	0.943415794
Rspo1	0.221615963	0.22819121	0.971185365	0.33145598	0.917115058
Gnl2	-0.048555098	0.146285724	-0.331919595	0.739949974	0.988585656
Dnali1	-0.276562239	0.185643602	-1.489748289	0.136290432	0.832457116
Snip1	-0.062632938	0.140072955	-0.447145121	0.654770282	0.979133069
Meaf6	-0.08325869	0.18916488	-0.440138202	0.659837015	0.979924124
Zc3h12a	0.138445316	0.281800672	0.491288097	0.623222697	0.975636499
Csf3r	0.23487263	0.369661816	0.635371628	0.525186068	0.965637248
Mrps15	0.138489622	0.157849293	0.877353448	0.38029469	0.929640874
Oscp1	-0.179467753	0.185222107	-0.968932686	0.332578776	0.917115058
Lsm10	-0.006367286	0.155078983	-0.041058344	0.967249383	0.996474415
Stk40	-0.130279204	0.221293893	-0.588715767	0.556051959	0.968926429
Eva1b	0.149669357	0.17637767	0.848573161	0.396118847	0.934066368
Sh3d21	-0.036261956	0.273415748	-0.132625705	0.894489403	0.993556555
Thrap3	0.116293836	0.140286045	0.828976512	0.407117698	0.939557159
Map7d1	-0.060498641	0.16712386	-0.361998823	0.71735291	0.988585656
Trappc3	0.326591495	0.137566806	2.374057416	0.017593814	0.579835303
Col8a2	-0.459043054	0.186637023	-2.459549804	0.013911139	0.545277966
Adprhl2	0.122451155	0.148698731	0.823484869	0.410232318	0.940983181
Tekt2	-0.298866892	0.17170328	-1.740600945	0.081753552	0.76099108
Ago3	-0.296923904	0.378875428	-0.783697969	0.433217355	0.946239431
Ago1	-0.212094803	0.196027898	-1.08196234	0.279269262	0.90218228
Ago4	-0.003827365	0.266980059	-0.014335771	0.988562102	0.998182772
Clspn	-0.676933311	1.158517121	-0.584310149	0.559011665	0.968926429
5730409E04Rik	0.147957158	0.152083891	0.972865415	0.330620196	0.917115058
Psmb2	0.037768324	0.128794936	0.293243861	0.769335761	0.989990452
Ncdn	-0.488721997	0.37168961	-1.314865911	0.188554971	0.874832912
AU040320	0.056173781	0.210113516	0.267349678	0.789199941	0.990988839

Zmym4	-0.080521039	0.19399316	-0.415071538	0.678089535	0.983670411
Sfpq	-0.232817805	0.163690754	-1.422302721	0.154938389	0.854973821
Zmym1	0.022329475	0.266322671	0.083843688	0.933180713	0.994688009
Zmym6	0.138275111	0.356353737	0.388027672	0.697995556	0.987622276
Gm12942	0.212339908	0.169383935	1.253601223	0.209986986	0.884258007
Smim12	0.264809159	0.165102554	1.603907098	0.108734532	0.798250317
Gja4	0.072206596	0.147477968	0.489609372	0.624410343	0.975987226
Gjb3	0.682105485	0.547614199	1.245594956	0.212913135	0.884945675
Gjb4	-1.645237922	2.841805617	-0.578941048	0.562628952	0.969658675
Gjb5	0.787072484	0.687969393	1.144051599	0.252602239	0.897044747
Zscan20	-0.737736552	0.578271836	-1.275760821	0.202040081	0.881300669
Tlr12	-1.138848048	1.058821223	-1.075581055	0.282114664	0.90579922
Phc2	-0.276456851	0.114957849	-2.404854074	0.016178925	0.56192007
A3galt2	2.154727782	1.860585459	1.158091273	0.246826791	0.896939594
Zfp362	-0.374776408	0.315277003	-1.188721044	0.234549457	0.89581803
Trim62	-0.214974586	0.618754985	-0.347430875	0.728267642	0.988585656
Azin2	-0.008739847	0.149269693	-0.058550715	0.953309967	0.996070133
Ak2	0.152101601	0.123267894	1.233910925	0.217236098	0.887633697
Rnf19b	0.045128544	0.103573681	0.435714391	0.663043963	0.980876591
Tmem54	0.240036622	0.362488526	0.662190952	0.507848852	0.963106419
Fndc5	-0.05915864	0.356829547	-0.165789634	0.868322504	0.992886758
S100pbp	-0.076688236	0.307445674	-0.249436704	0.803022997	0.990988839
Yars	-0.219489084	0.17862829	-1.228747608	0.21916645	0.888847883
C77080	-0.441322848	0.261254131	-1.689247345	0.091172039	0.771949327
Sync	0.158867441	0.629582025	0.252337955	0.800779853	0.990988839
Rbbp4	0.104816394	0.126607118	0.827887056	0.407734466	0.939557159
Zbtb8os	0.154090505	0.148953811	1.034485142	0.300909419	0.911154542
Zbtb8a	0.322933665	0.218802001	1.475917332	0.139966086	0.83738596
Zbtb8b	0.089102316	1.570535954	0.0567337	0.954757329	0.996070133
Bsdc1	0.031500896	0.146931345	0.214391937	0.830241429	0.992886758
Fam229a	0.163337392	0.787100079	0.207517946	0.835605379	0.992886758
Marcksl1	-0.189185251	0.241778231	-0.782474294	0.433935887	0.946679964
Hdac1	0.080781456	0.127899845	0.631599332	0.527648723	0.965637248
Lck	0.66177354	0.208434103	3.174977266	0.001498482	0.279554507
Fam167b	0.329341768	0.798913318	0.412237174	0.680165592	0.984207811
Eif3i	0.129582641	0.165955543	0.780827438	0.434903994	0.946762848
Tmem234	0.436085139	0.102366797	4.260025259	2.04E-05	0.016686504
Dcdc2b	-0.06751181	0.127145873	-0.530979173	0.595433212	0.974001549
Iqcc	0.247442195	0.175220529	1.412175825	0.157898197	0.855986074
Ccdc28b	-0.086957993	0.15568587	-0.558547754	0.576470405	0.971613657
Txlna	-0.274690827	0.158433382	-1.733793866	0.082954664	0.76099108
Kpna6	-0.14014307	0.186218309	-0.752574067	0.451705906	0.949134998
Tmem39b	-0.55916377	0.444133778	-1.258998521	0.208030866	0.884258007
Khdrbs1	0.057604471	0.105397548	0.546544703	0.584691568	0.972619381
Ptp4a2	-0.025809494	0.079211534	-0.325829998	0.744552981	0.988585656
E330017L17Rik	-0.043125005	0.567011018	-0.076056731	0.939373964	0.994688009
1700003M07Rik	-0.30857367	1.787830978	-0.172596668	0.862968471	0.992886758
Adgrb2	-0.028554956	1.261351293	-0.022638385	0.981938725	0.997390438
Col16a1	-0.238798517	0.326239447	-0.731973154	0.464184954	0.953083512
Pef1	-0.054443684	0.09309995	-0.584787472	0.558690629	0.968926429
Tinagl1	-0.259795715	0.183008855	-1.419580025	0.155729984	0.855293621
Serinc2	-0.021718734	0.270661716	-0.080243096	0.936043915	0.994688009
Fabp3	0.360401527	0.221886394	1.6242615	0.10432001	0.79155232
Zcchc17	0.115366911	0.13473594	0.856244526	0.391862538	0.933734231
Snrnp40	0.076318657	0.165601281	0.460857893	0.644900563	0.979004493
Nkain1	-0.154652115	0.351578846	-0.439878898	0.66002482	0.979924124
Pum1	-0.292053295	0.182427379	-1.600929073	0.109392629	0.798400564
Sdc3	0.016506971	0.186624686	0.088450092	0.92951895	0.994688009
Laptm5	0.091496408	0.242398045	0.377463475	0.705829203	0.988585656
Ptpru	-0.297000729	0.229630423	-1.293385803	0.195877658	0.879601031
Mecr	0.157997572	0.153905929	1.026585348	0.304615756	0.912429485
Srsf4	-0.27146966	0.266278178	-1.01949646	0.307967333	0.9139204

Gm12992	1.46491093	1.445025029	1.013761631	0.310696508	0.914819219
Tmem200b	-0.447530226	0.264213715	-1.693819059	0.090299671	0.769347579
Epb4.1	-0.076701082	0.193618926	-0.396144546	0.691998398	0.986464472
Oprd1	-2.643301493	2.09547314	-1.261434205	0.207152451	0.883765092
Ythdf2	0.033996872	0.124177234	0.273777011	0.784255991	0.990988839
Rps15a-ps4	0.726631137	0.738489105	0.983942935	0.325143572	0.91640698
Gmeb1	-0.357013004	0.261422627	-1.365654564	0.172047412	0.86244803
Taf12	0.104205357	0.113130578	0.92110691	0.356994613	0.924354588
Rab42	0.755610335	0.745396135	1.013703049	0.310724469	0.914819219
Snhg12	0.53450615	0.18029812	2.964568624	0.003031076	0.346777009
Snora16a	0.255638698	0.94572452	0.270309898	0.786921851	0.990988839
Snord99	0.252909379	0.718518732	0.351987175	0.724847881	0.988585656
Trnau1ap	0.259232203	0.169462577	1.529731275	0.126083259	0.822401003
Rcc1	-0.151177104	0.204944452	-0.737649162	0.460727673	0.953083512
Snhg3	0.355194477	0.209455413	1.695799942	0.089923774	0.769347579
Phactr4	-0.434981307	0.270776167	-1.606423906	0.108180802	0.797772336
Med18	0.071561675	0.28186579	0.253885634	0.799583914	0.990988839
Sesn2	-0.648878965	0.33274716	-1.950066127	0.051168238	0.707153766
Atpif1	0.254481623	0.190004806	1.339343085	0.180459009	0.868087032
Dnajc8	0.259311879	0.106678631	2.430776207	0.015066517	0.555113898
Ptafr	-0.059775081	0.573809309	-0.10417238	0.917032552	0.994688009
Eya3	-0.051526311	0.319143669	-0.161451774	0.871737592	0.992886758
Xkr8	0.5224635	0.313435106	1.666895285	0.095535229	0.778345149
Smpdl3b	0.249578644	0.444551717	0.561416444	0.574513676	0.971613657
Rpa2	0.091402133	0.178913735	0.510872643	0.609440238	0.974001549
Themis2	0.198590411	0.306333023	0.64828274	0.516802095	0.964352198
Ppp1r8	0.206665193	0.11929415	1.732400063	0.083202357	0.76099108
Stx12	-0.073389751	0.071507446	-1.0263232	0.304739265	0.912429485
Fam76a	0.035768757	0.101647065	0.351891682	0.724919498	0.988585656
Fgr	0.333882627	0.257869491	1.294773668	0.195398318	0.879588734
Ahdc1	0.319018167	0.457698831	0.697004549	0.485799947	0.957710647
Wasf2	-0.023962654	0.25315678	-0.094655391	0.924588552	0.994688009
Cd164l2	-0.818553095	0.494205342	-1.656301593	0.097660758	0.785471525
Map3k6	-0.327351136	0.436612081	-0.749752812	0.453403594	0.950146765
Sytl1	0.427332128	0.26514592	1.611686606	0.107030154	0.79539432
Tmem222	0.015466506	0.142561218	0.10849028	0.913606791	0.994688009
Wdtc1	-0.109226116	0.25066288	-0.435749066	0.663018802	0.980876591
Slc9a1	0.08218944	0.286942166	0.286432076	0.77454721	0.99084133
Fam46b	-0.297814269	0.295361574	-1.008304043	0.313308523	0.915243138
Trnp1	0.076889339	0.214024109	0.359255503	0.719403959	0.988585656
Kdf1	-0.34820364	0.203972837	-1.707107895	0.087802	0.7667678
Nudc	-0.08002634	0.180619699	-0.443065405	0.657718427	0.979614766
Gpatch3	-0.19616314	0.324495268	-0.604517722	0.54549949	0.967491297
Gpn2	0.180310462	0.185855469	0.970164955	0.331964276	0.917115058
Sfn	-0.224641196	0.203731659	-1.102632735	0.270186688	0.89804647
Zdhhc18	-0.141959823	0.218676934	-0.649176028	0.516224604	0.964352198
Pigv	0.197014395	0.18569054	1.0609824	0.2886979	0.907334416
Arid1a	0.050465986	0.238598612	0.211509971	0.832489352	0.992886758
Rps6ka1	-0.172089115	0.198243125	-0.868071035	0.385355446	0.931689378
Hmgn2	0.267162711	0.136405434	1.958592872	0.050160485	0.707153766
Dhdds	0.032938319	0.15887051	0.207328086	0.835753641	0.992886758
Aim1l	-0.018938291	0.431147866	-0.043925281	0.964963964	0.996474415
Cd52	0.024961081	0.186920688	0.133538353	0.893767635	0.993422442
Ubxn11	-0.135273284	0.162565998	-0.832113019	0.405345154	0.939557159
Sh3bgrl3	-0.03408943	0.129752092	-0.262727404	0.792760693	0.990988839
Cep85	-0.023624045	0.238366163	-0.099108217	0.921052348	0.994688009
Catsper4	0.192358308	1.481370439	0.129851591	0.896683845	0.994101834
Cnksr1	-0.06151113	0.275811591	-0.223018653	0.823520989	0.992886758
Zfp593	-0.445634216	0.354269161	-1.257897285	0.208428906	0.884258007
Grrp1	0.132023808	0.215858243	0.611622732	0.540787394	0.966989525
Pdik1l	-0.064711417	0.141909166	-0.456005905	0.648385732	0.979004493
Trim63	0.457778657	0.904554577	0.506081854	0.612799189	0.974001549

Slc30a2	0.558620431	0.936041988	0.596789928	0.550647643	0.968926429
Extl1	0.623932637	0.543166963	1.148694009	0.250682177	0.896939594
Pafah2	-0.053852163	0.202948718	-0.265348622	0.790740911	0.990988839
Stmn1	-0.018160064	0.136422354	-0.133116486	0.894101257	0.993422442
Paqr7	-0.19269015	0.135231298	-1.424893145	0.154188091	0.854159937
Aunip	0.251434544	1.229745634	0.204460611	0.83799357	0.992886758
Mtfr1l	-0.122704372	0.11367204	-1.079459577	0.280382904	0.903192609
Sepn1	-0.315131468	0.168736553	-1.867594559	0.061818605	0.725730283
Man1c1	0.083723333	0.331478191	0.25257569	0.800596117	0.990988839
Ldlrap1	-0.009745505	0.212361859	-0.045891031	0.963397103	0.996474415
Tmem57	-0.19471584	0.188043122	-1.035485041	0.300442449	0.910285132
Rhd	-2.511720691	1.447971073	-1.734648391	0.082803103	0.76099108
Tmem50a	0.091641799	0.113104665	0.810238898	0.417802886	0.943415794
Rsrp1	-0.119723248	0.121734827	-0.983475732	0.325373354	0.91640698
Syf2	0.064432274	0.093029087	0.692603534	0.488558392	0.95849918
Runx3	-0.67944502	0.695574134	-0.976811797	0.328662335	0.917115058
Clic4	-0.235987823	0.12405438	-1.902293359	0.057132813	0.721735927
Srrm1	-0.018278494	0.171999771	-0.106270455	0.915367773	0.994688009
Ncmap	-0.39740597	0.357151509	-1.112709761	0.265833102	0.89768067
Rcan3	-0.196410117	0.190725916	-1.029802982	0.303102501	0.911623139
Nipal3	0.183743657	0.168676507	1.089325714	0.276010277	0.900302631
Stpg1	-0.158285167	0.618307197	-0.255997613	0.79795268	0.990988839
Grhl3	0.27016175	1.120665274	0.241072653	0.809498808	0.991295569
Ifnlr1	-0.407139986	0.209612745	-1.942343656	0.052095511	0.707153766
Il22ra1	-0.15064191	0.386755327	-0.389501835	0.696904953	0.987518602
Myom3	-0.122586664	0.456829444	-0.268342301	0.788435848	0.990988839
Srsf10	0.00975846	0.127463919	0.076558607	0.938974689	0.994688009
Pnrc2	0.161097614	0.120777313	1.333840025	0.182256291	0.868546072
Cnr2	0.157655181	0.26748503	0.589398148	0.555594219	0.968926429
Fuca1	-0.034001325	0.111439209	-0.305110971	0.760281636	0.989266039
Hmgcl	0.260102681	0.165543539	1.571204066	0.116135256	0.811920148
Gale	-0.022283726	0.151011462	-0.147563145	0.882687543	0.992886758
Lypla2	-0.002359121	0.178246678	-0.013235146	0.98944019	0.998182772
Pithd1	0.066430019	0.103823396	0.639836697	0.522278772	0.965494283
Tceb3	-0.183962148	0.107410663	-1.712699117	0.086767908	0.765418158
Rpl11	0.460701598	0.245475066	1.876775532	0.060548865	0.725221283
Id3	0.115192314	0.097844691	1.177297549	0.239076765	0.896609492
E2f2	-0.155408992	0.17000912	-0.914121504	0.360653004	0.925913281
Asap3	0.022989158	0.318930109	0.072082118	0.942536557	0.994688009
Tcea3	-0.142434283	0.114536286	-1.243573435	0.213656598	0.884945675
Zfp46	0.050516963	0.275859218	0.183125884	0.854699246	0.992886758
Hnrnpr	0.143030902	0.146659529	0.975258152	0.329432223	0.917115058
Luzp1	-0.120880879	0.209374314	-0.577343403	0.563707499	0.969658675
Kdm1a	-0.074260625	0.120083742	-0.618406983	0.536307096	0.96674935
Ephb2	-0.540907027	0.593937806	-0.910713245	0.362446484	0.926455466
C1qb	-0.210982932	0.185136461	-1.139607678	0.254449785	0.897044747
C1qc	-0.102540818	0.173961365	-0.589445923	0.555562178	0.968926429
C1qa	-0.057670361	0.16891737	-0.341411667	0.732793693	0.988585656
Zbtb40	0.012114209	0.364437416	0.03324085	0.973482523	0.997148871
Wnt4	-0.382985937	0.193467494	-1.979588037	0.047749838	0.701952043
Cdc42	0.103703618	0.089554004	1.158000908	0.246863666	0.896939594
Hspg2	0.193719837	0.172550148	1.122687163	0.261570382	0.897248195
Usp48	-0.034301708	0.193093649	-0.177642859	0.859003463	0.992886758
Rap1gap	-0.350526897	0.222665369	-1.574231765	0.115433878	0.81007959
Alpl	-0.885844147	0.604952735	-1.4643196	0.143106644	0.840741274
Ece1	-0.230249972	0.183808951	-1.252659196	0.210329762	0.884340413
Eif4g3	-0.039014616	0.131165669	-0.29744533	0.766126541	0.989884648
Hp1bp3	-0.129411374	0.13028976	-0.993258207	0.320584139	0.91640698
Sh2d5	1.327972321	0.826013453	1.607688489	0.107903421	0.796929372
Kif17	-0.002174694	0.944859695	-0.002301605	0.998163586	0.999591134
Ddost	-0.023359392	0.110368291	-0.211649486	0.832380499	0.992886758
Pink1	0.027295712	0.098867398	0.276084054	0.782483508	0.990988839

Cda	0.225002572	0.395744824	0.568554681	0.569658387	0.97144955
Mul1	-0.048868646	0.15190718	-0.321700698	0.747679449	0.988585656
Camk2n1	0.017110455	0.098048083	0.174510859	0.861463997	0.992886758
Ubxn10	-0.242130958	0.179641061	-1.347859761	0.177703492	0.866855369
Pla2g2c	0.517123643	0.558918668	0.925221633	0.354850633	0.924354588
Pla2g2d	-1.066921829	0.606563859	-1.758960433	0.078584229	0.754732183
Pla2g5	0.28563745	0.604023081	0.472891614	0.636290508	0.977284137
Pla2g2e	0.179701425	2.224237576	0.08079237	0.935607076	0.994688009
Otud3	0.049318307	0.376511667	0.13098746	0.895785227	0.993781709
Rnf186	-0.128192529	0.152139978	-0.842595948	0.399454458	0.936273856
Tmco4	-0.035528023	0.258642579	-0.137363394	0.890743564	0.993043931
Nbl1	-0.159954955	0.125742961	-1.2720788	0.203345123	0.882809131
Minos1	0.380619416	0.195076471	1.95112929	0.051041668	0.707153766
Capzb	0.13562942	0.085075637	1.594221629	0.110886404	0.800111791
Pqlc2	-0.404154309	0.315022137	-1.282939393	0.19951331	0.880888611
Akr7a5	-0.057448169	0.173322282	-0.331452879	0.740302428	0.988585656
Mrto4	0.29097189	0.153743001	1.89258626	0.058412912	0.72285319
Emc1	0.238890095	0.201416263	1.186051666	0.235601896	0.896002526
Ubr4	-0.05040799	0.276763646	-0.182133711	0.855477793	0.992886758
Iffo2	0.048550499	0.182345056	0.266256185	0.790041915	0.990988839
Aldh4a1	-0.30608462	0.161405825	-1.896366633	0.057911581	0.72285319
Klhdc7a	-0.321714236	0.195557016	-1.64511733	0.099945617	0.788783372
Arhgef10l	-0.112913205	0.274486048	-0.411362274	0.680806913	0.984285351
Rcc2	0.041201008	0.20595021	0.200053248	0.841438937	0.992886758
Padi4	0.294435432	0.417192887	0.70575372	0.480341307	0.957463006
Padi3	1.101570394	1.633927608	0.674185557	0.500193351	0.9611436
Padi1	-1.307241963	0.997111251	-1.311029198	0.189847899	0.876349068
Padi2	0.573122812	0.780295182	0.734494874	0.462647183	0.953083512
Gm13031	3.53814332	2.464786379	1.435476661	0.151151314	0.850827708
Sdhb	0.136358963	0.118758444	1.148204355	0.250884212	0.896939594
Atp13a2	-0.18748222	0.241023139	-0.777859839	0.436651646	0.946762848
Mfap2	0.14814686	0.179813828	0.823890253	0.410001919	0.940983181
Crocc	1.094479643	0.454784242	2.406590956	0.016102194	0.560555474
Necap2	-0.035545729	0.122226512	-0.290818482	0.771190149	0.989990452
Szrd1	0.194389987	0.092786276	2.095029527	0.036168368	0.676427194
Fbxo42	-0.442354736	0.314167438	-1.408022226	0.159124489	0.856303734
Rsg1	0.14597542	0.872066371	0.167390264	0.867062985	0.992886758
Arhgef19	0.035030005	0.28140737	0.124481477	0.900934066	0.994688009
Epha2	-0.368777395	0.259706705	-1.419976415	0.155614547	0.855261441
Fam131c	-0.153254933	0.619854564	-0.247243372	0.804719881	0.990988839
Hspb7	-0.103908554	0.351843056	-0.29532643	0.767744528	0.989990452
Zbtb17	0.151868796	0.138573611	1.09594313	0.273103663	0.898826892
Spen	0.298318502	0.356371987	0.837098631	0.40253713	0.937834293
B330016D10Rik	0.260475794	0.256084213	1.017148974	0.309082565	0.9139204
Fblim1	-0.234288235	0.21694634	-1.079936331	0.280170533	0.903192609
Tmem82	0.153044181	0.713322003	0.214551325	0.830117147	0.992886758
AI507597	2.08895092	1.534621176	1.361216014	0.173445435	0.863528862
Slc25a34	0.982959757	0.466174538	2.108565948	0.034982065	0.674795441
Plekhm2	0.105719688	0.200574774	0.527083671	0.598135486	0.974001549
Ddi2	-0.142861513	0.359054596	-0.397882423	0.690716857	0.986039532
Agmat	0.098625899	0.692118484	0.142498576	0.886686204	0.992886758
Dnajc16	-0.38018121	0.321280189	-1.183332253	0.236677495	0.896358698
Casp9	0.373649407	0.293248591	1.274172897	0.202602148	0.88210274
Cela2a	-2.16742168	2.152954027	-1.006719908	0.314069392	0.91586731
Efhd2	-0.021197075	0.116363112	-0.182163188	0.855454662	0.992886758
Fhad1	-0.28037259	0.242007572	-1.158528172	0.246648562	0.896939594
4930455G09Rik	-0.060086668	0.607991097	-0.098828203	0.921274675	0.994688009
Tmem51	-0.068079164	0.167009641	-0.407636133	0.683540833	0.984769452
Tmem51os1	-0.376997337	1.100550709	-0.342553355	0.731934498	0.988585656
Kazn	0.14753922	0.242943942	0.607297383	0.543653574	0.967491297
Prdm2	-0.335599373	0.173391708	-1.935498395	0.052929165	0.707153766
Pdpn	-0.018022375	0.111630792	-0.161446267	0.871741929	0.992886758

Pramef8	-0.370638319	0.219867062	-1.68573826	0.091846224	0.772516176
Pramef12	0.826927544	0.724138226	1.141947094	0.253476011	0.897044747
Dhrs3	-0.182201589	0.124289649	-1.46594338	0.142663713	0.840741274
Vps13d	-0.049974282	0.203807036	-0.24520391	0.806298549	0.99099448
Tnfrsf1b	-0.145867344	0.198953283	-0.733173845	0.463452406	0.953083512
Tnfrsf8	-0.453810842	2.011163982	-0.225645868	0.821476863	0.992886758
Smarca5-ps	0.12622442	1.605288878	0.078630346	0.93732665	0.994688009
Gm13212	-0.901235534	0.538678799	-1.673048088	0.09431783	0.777748075
Gm13242	0.543277357	1.616609511	0.336059731	0.736825824	0.988585656
Gm13251	-0.36286283	0.283909207	-1.278094618	0.201216066	0.881187967
Zfp600	-1.555188698	1.748565773	-0.889408178	0.373783752	0.928840865
Gm13139	0.05627236	1.103964533	0.050972978	0.959347053	0.996367077
Znf41-ps	0.40776918	0.512089418	0.796285113	0.425866336	0.944290206
Gm13152	0.661749879	0.387233313	1.708917743	0.087466187	0.766704005
Gm13154	1.417122008	1.122466417	1.262507266	0.206766313	0.883765092
2610305D13Rik	-0.254220195	0.271965037	-0.934753224	0.349915491	0.922206653
Gm13157	0.233360998	0.131555674	1.773857348	0.076086749	0.752876039
Rps19-ps3	0.32369814	0.434504814	0.744981712	0.456282776	0.951472839
Zfp933	0.083505949	0.166358585	0.50196357	0.615693147	0.974001549
Miip	0.218259209	0.17220231	1.267458079	0.204991544	0.883765092
Fv1	-0.012550118	0.422456656	-0.029707469	0.976300355	0.997262526
Mfn2	0.077268287	0.173757292	0.444690903	0.656543145	0.979614766
Plod1	-0.023972935	0.206886539	-0.115874794	0.907751772	0.994688009
2510039O18Rik	0.169035806	0.190766377	0.886088042	0.375570086	0.928883222
Nppb	1.222740944	0.74039146	1.651478994	0.098640807	0.786907305
Nppa	0.481532137	0.551936587	0.872441053	0.382967813	0.931395363
Clcn6	-0.02790437	0.567669613	-0.049156004	0.960794972	0.996474415
Mthfr	-0.106646955	0.240920716	-0.442664113	0.658008703	0.979614766
Agtrap	-0.010045803	0.123357896	-0.081436236	0.935095032	0.994688009
Draxin	0.25926507	0.986705348	0.26275835	0.792736839	0.990988839
Mad2l2	0.267032321	0.205790861	1.29759077	0.194427996	0.879588734
Fbxo6	0.065490536	0.153085933	0.427802441	0.668794959	0.981863224
Fbxo44	-0.019799519	0.238818215	-0.082906236	0.933926096	0.994688009
Fbxo2	-0.058145134	0.350824887	-0.165738338	0.868362874	0.992886758
Ubiad1	-0.478547701	0.25319515	-1.890035025	0.058753276	0.72285319
Mtor	0.47284744	0.160967162	2.937539774	0.003308277	0.357710224
Angptl7	-0.080696619	0.435642175	-0.185236011	0.853043925	0.992886758
Exosc10	-0.003500379	0.158821211	-0.022039748	0.982416249	0.997390438
Srm	0.013860389	0.244019906	0.05680024	0.954704323	0.996070133
Tardbp	0.000489527	0.101249362	0.004834867	0.99614235	0.999379819
Casz1	-0.038748495	0.216293016	-0.179148158	0.857821369	0.992886758
Pex14	-0.177736294	0.150985353	-1.17717574	0.23912537	0.896609492
Dffa	0.093423831	0.175202881	0.533232277	0.593872801	0.974001549
Apitd1	0.2133235	0.398075654	0.535886828	0.592036768	0.974001549
Pgd	-0.049429947	0.189412092	-0.260965105	0.794119417	0.990988839
Kif1b	0.112148323	0.158530766	0.707423081	0.479303597	0.957463006
Ube4b	0.086072998	0.260069813	0.330961126	0.740673849	0.988585656
Rbp7	0.174765803	0.569242403	0.307014731	0.75883216	0.989266039
Nmnat1	0.447222058	0.252167761	1.77351005	0.076144228	0.752876039
Lzic	0.070757543	0.152306417	0.464573613	0.642236832	0.978415289
Ctnnbip1	0.200956486	0.164693563	1.220184219	0.222395048	0.892391445
Clstn1	-0.13887079	0.145811893	-0.952396868	0.340895747	0.919850248
Pik3cd	-0.074654724	0.190728353	-0.391419121	0.695487461	0.98715859
Tmem201	0.620510846	0.364233965	1.703605117	0.088454885	0.767814344
Slc25a33	-0.159305311	0.214726904	-0.7418973	0.458149562	0.952235842
Spsb1	-0.688878317	0.325190779	-2.118382078	0.034142722	0.673246654
H6pd	-0.301200066	0.155225867	-1.940398673	0.052331258	0.707153766
Gpr157	-0.420093846	0.369553228	-1.136761401	0.255638043	0.897044747
Slc2a5	0.605611069	1.622649621	0.373223561	0.708982062	0.988585656
Car6	-4.835114834	2.221229616	-2.176773981	0.029497435	0.64858361
Rere	-0.285161508	0.253105027	-1.126652881	0.259889272	0.897248195
Errfi1	-0.373805647	0.203815163	-1.834042383	0.066647703	0.738254074

Park7	0.157569697	0.091164762	1.728405743	0.083915507	0.76099108
Tnfrsf9	-0.557184279	0.652394908	-0.854059823	0.393071844	0.933734231
Per3	-0.540245808	0.263414404	-2.050934952	0.040273282	0.679118604
Vamp3	0.131633802	0.138892791	0.947736753	0.343263485	0.920191602
Camta1	0.21682726	0.204688372	1.059304235	0.289461247	0.907334416
Dnajc11	-0.144802394	0.210804773	-0.686902827	0.492143966	0.958916246
Thap3	0.232979773	0.154157337	1.511311615	0.130709076	0.827800491
Phf13	-0.418064771	0.210414846	-1.986859669	0.04693795	0.698868643
Klhl21	-0.07990369	0.223665797	-0.357245904	0.720907724	0.988585656
Zbtb48	0.124853997	0.171898982	0.72632191	0.467641451	0.953657709
Tas1r1	1.391715351	1.074048057	1.295766369	0.195055988	0.879588734
Nol9	-0.217597432	0.195932263	-1.11057479	0.266751419	0.89768067
Plekhg5	-0.038059991	0.252806686	-0.150549781	0.880330878	0.992886758
Tnfrsf25	-0.156529262	0.359531527	-0.43537006	0.663293839	0.980889931
Espn	-0.362658997	0.318181133	-1.139787874	0.254374687	0.897044747
Hes2	0.638803321	0.510268012	1.251897643	0.210607164	0.884340413
Acot7	0.066909754	0.123529991	0.541647848	0.588061123	0.973455044
Gpr153	0.105430886	0.233196666	0.452111462	0.651188687	0.979004493
Icmt	-0.114664246	0.19655824	-0.583360155	0.559650875	0.968926429
Rpl22	0.332154899	0.189776134	1.750245889	0.080075894	0.75659671
Chd5	0.796214187	0.967722933	0.822770816	0.410638336	0.941195652
Kcnab2	0.585199696	0.358716691	1.631370134	0.102812243	0.7899367
Nphp4	0.307809351	0.60530353	0.508520661	0.611088254	0.974001549
Ajap1	0.510094985	1.920018207	0.265671952	0.790491866	0.990988839
A430005L14Rik	0.338199741	0.192700042	1.755057947	0.079249397	0.75659671
Dffb	0.08304071	0.227383975	0.365200361	0.714961849	0.988585656
Cep104	-0.116100499	0.20586216	-0.563972025	0.572773171	0.971613657
Lrrc47	-0.157687371	0.44667894	-0.353021727	0.724072152	0.988585656
Smim1	0.243082422	0.211583639	1.148871543	0.250608953	0.896939594
Ccdc27	0.25909875	0.637969725	0.406130166	0.684646962	0.985105247
Trp73	0.077771568	0.322137038	0.241423862	0.80922662	0.991295569
Wdr8	0.015661018	0.179370008	0.087311241	0.930424119	0.994688009
Tprgl	-0.01448841	0.089654979	-0.161601843	0.871619406	0.992886758
Megf6	-0.082391827	0.167102561	-0.493061423	0.621969187	0.97514112
Arhgef16	-0.109758867	0.209730757	-0.523332238	0.60074307	0.974001549
Prdm16	-0.062200073	0.20467122	-0.30390239	0.761202257	0.989266039
5930403L14Rik	-0.023305939	0.440600018	-0.05289591	0.957814843	0.996226826
Ttc34	0.705689188	0.664139749	1.06256129	0.28798095	0.907334416
Fam213b	0.425455042	0.204493668	2.080529172	0.037477023	0.679118604
Tnfrsf14	-0.108425912	0.380132387	-0.285231977	0.775466423	0.990861469
Pank4	0.153109262	0.144824548	1.057205176	0.290417958	0.907334416
Plch2	0.099904191	0.790499451	0.1263811	0.899430262	0.99447264
Pex10	0.291402255	0.143294879	2.033584575	0.041993498	0.679118604
Rer1	0.12967175	0.13089984	0.9906181	0.321872096	0.91640698
Morn1	-0.023454384	0.243652243	-0.09626172	0.923312713	0.994688009
Ski	-0.068534575	0.235384763	-0.291159775	0.770929125	0.989990452
Faap20	-0.058948095	0.162313315	-0.363174736	0.716474359	0.988585656
Prkcz	-0.102565329	0.151261795	-0.678064998	0.497730482	0.960700483
Gabrd	-0.398879535	0.875139928	-0.455789437	0.648541401	0.979004493
5830444B04Rik	0.253473498	0.506107724	0.500829144	0.616491373	0.974001549
Cfap74	-0.310832397	0.326277826	-0.952661729	0.34076149	0.919850248
Tmem52	-0.394813352	0.940018014	-0.420006155	0.674480958	0.983412269
Gnb1	0.062031022	0.09033825	0.686652913	0.492301476	0.958916246
Nadk	-0.061195249	0.087622966	-0.698392804	0.484931575	0.957710647
Slc35e2	-0.22015247	0.273761116	-0.804177282	0.421294597	0.943415794
Gm16023	0.522376998	0.459919414	1.135801149	0.256039796	0.897248195
Cdk11b	-0.122449772	0.173086458	-0.707448597	0.479287746	0.957463006
Mmp23	-0.055347343	0.250995641	-0.220511172	0.825473072	0.992886758
Mib2	0.107949442	0.161658591	0.667761865	0.504285613	0.96186221
B930041F14Rik	0.443083161	0.403233816	1.098824411	0.271844663	0.89804647
Ssu72	0.066466666	0.149049248	0.445937616	0.65564231	0.979294848
Tmem240	0.98659936	0.579899815	1.701327256	0.088881553	0.767814344

Atad3a	0.049765832	0.203813702	0.244173141	0.807096728	0.99099448
Atad3aos	0.629780746	0.445736001	1.412900785	0.157684898	0.855986074
Vwa1	0.005632905	0.245316682	0.022961768	0.98168077	0.997390438
Tmem88b	0.553945697	0.625065368	0.886220427	0.375498758	0.928883222
Mrpl20	0.201068335	0.154808455	1.298820114	0.19400567	0.879588734
Ccnl2	0.044256188	0.125376384	0.352986637	0.724098458	0.988585656
Aurkaip1	0.058367044	0.142971063	0.40824376	0.683094724	0.984738223
Mxra8	-0.301168386	0.174073353	-1.73012343	0.083608225	0.76099108
Dvl1	0.006858243	0.132637102	0.05170682	0.958762303	0.996226826
Tas1r3	0.190098726	1.13506865	0.167477735	0.866994165	0.992886758
Cptp	0.035589368	0.234715432	0.151627728	0.879480563	0.992886758
Cpsf3l	0.180790208	0.116659614	1.54972404	0.121207766	0.816233249
Pusl1	0.212224914	0.203227503	1.044272608	0.296359282	0.909005508
Acap3	-0.298352332	0.231183251	-1.290544753	0.196861584	0.879755417
Ube2j2	0.323527167	0.404208756	0.800396235	0.423481261	0.94351935
Fam132a	0.330535212	0.188657373	1.752039722	0.079766978	0.75659671
B3galt6	-0.318271151	0.200395802	-1.588212665	0.112238258	0.801274176
Sdf4	0.126569	0.092635025	1.366319053	0.171838843	0.86244803
Tnfrsf4	0.003127635	0.396500763	0.007888093	0.993706278	0.999270171
Gm10560	0.438339934	0.470570506	0.931507453	0.351591131	0.923082865
Tnfrsf18	0.099736046	0.257688676	0.387040858	0.69872596	0.987770663
Ttll10	-0.272373863	0.480729392	-0.566584586	0.57099645	0.971613657
9430015G10Rik	-0.016207378	0.223684028	-0.072456572	0.942238565	0.994688009
Agrn	-0.000935377	0.116677061	-0.008016801	0.993603587	0.999270171
Isg15	0.300564683	0.208632268	1.44064332	0.149685475	0.848779008
AW011738	0.569683551	0.38244737	1.489573717	0.136336357	0.832457116
Perm1	-0.15226994	0.658792719	-0.231134825	0.817210061	0.992678823
Plekhn1	1.695402621	1.106927409	1.531629452	0.1256139	0.822401003
Klhl17	-0.036334349	0.18481217	-0.196601496	0.8441394	0.992886758
Cdk6	-0.666513354	0.36358707	-1.833160224	0.066778747	0.738254074
Fam133b	0.127376603	0.145954234	0.872716052	0.382817866	0.931207882
1700109H08Rik	0.159558777	1.615137651	0.098789584	0.921305339	0.994688009
Rbm48	-0.135510998	0.254591359	-0.532268649	0.594539943	0.974001549
Pex1	0.246740939	0.263606917	0.936018452	0.349263688	0.921585102
Gatad1	0.001668642	0.093503134	0.017845835	0.98576184	0.998148029
Ankib1	-0.18526455	0.122019166	-1.518323366	0.1289329	0.827295367
Krit1	-0.407549464	0.130211752	-3.129897719	0.001748672	0.284736028
Mterf1a	0.041103757	0.279320639	0.147156174	0.883008752	0.992886758
Akap9	0.289960168	0.191122564	1.517142518	0.129230705	0.827800491
Cyp51	-0.128267722	0.137836043	-0.930581866	0.352069899	0.923082865
Mterf1b	0.30982662	0.378444767	0.818683852	0.412966812	0.942708418
Fzd1	0.144464835	0.228054875	0.633465233	0.526429878	0.965637248
Cdk14	-0.041721219	0.107272543	-0.388927284	0.697329938	0.987548136
Cldn12	-0.137106146	0.10912077	-1.256462409	0.208948367	0.884258007
Gtpbp10	-0.18306798	0.163230115	-1.121533119	0.262061002	0.897248195
Cfap69	-0.17336378	0.155724423	-1.113272898	0.265591242	0.89768067
Steap2	-0.162000163	0.215689237	-0.751081348	0.4526037	0.949827842
Steap1	-0.385071352	0.341174315	-1.128664541	0.259039373	0.897248195
Steap4	-0.010517758	0.229253058	-0.045878377	0.963407189	0.996474415
Sri	0.341108394	0.076872839	4.437307106	9.11E-06	0.008748514
Adam22	-0.122868468	0.277161721	-0.443309658	0.657541771	0.979614766
Dbf4	-0.038175524	0.229250699	-0.166523041	0.867745352	0.992886758
Slc25a40	-0.006327853	0.167859135	-0.037697401	0.969928948	0.996628386
Rundc3b	-0.181428897	0.417890833	-0.43415381	0.664176752	0.981154469
Abcb1a	0.420403952	0.246877245	1.702886598	0.088589293	0.767814344
Abcb1b	0.262639229	0.215069109	1.221185272	0.222015879	0.892161767
Abcb4	0.460017849	1.103273822	0.416957096	0.67670979	0.983549699
Crot	-0.001047769	0.131728589	-0.007953997	0.993653695	0.999270171
Tmem243	0.023675688	0.160282301	0.147712429	0.882569722	0.992886758
Dmtf1	-0.009786342	0.13692451	-0.071472533	0.943021684	0.994819052
9330182L06Rik	-0.201670331	0.265060535	-0.760846316	0.446748867	0.948476747
Sema3d	-0.140451988	0.2093914	-0.670762927	0.502371568	0.961573341

Sema3a	0.260611845	0.139149749	1.87288764	0.061083902	0.725221283
Sema3e	-0.000299773	0.122637546	-0.00244438	0.998049669	0.999591134
Pclo	-0.829051245	0.572769969	-1.447441887	0.147773201	0.846989858
Speer4e	-0.396688592	0.451085276	-0.87940931	0.379179386	0.929137643
4930572O03Rik	0.159701707	0.645330478	0.247472749	0.804542379	0.990988839
Speer4c	-0.208740719	0.646801123	-0.322727825	0.746901377	0.988585656
Cacna2d1	-0.223515379	0.210987618	-1.059376755	0.289428231	0.907334416
Hgf	-0.547494768	0.30527909	-1.793423744	0.072905194	0.748701713
Speer4f1	0.534044028	0.689821716	0.774176885	0.438826196	0.947838535
Sema3c	0.188112438	0.118440919	1.58823859	0.112232398	0.801274176
Cd36	-0.058191885	0.154864704	-0.375759505	0.707095696	0.988585656
Gnat3	0.349643946	2.548080714	0.137218552	0.890858047	0.993043931
Gnai1	0.067346006	0.143706044	0.468637253	0.639328945	0.977843986
4921504A21Rik	0.151328485	0.616646452	0.245405588	0.806142403	0.99099448
Magi2	-0.365768613	0.355760287	-1.02813222	0.303887636	0.911786415
Phtf2	0.098976108	0.303742648	0.325855157	0.744533945	0.988585656
Tmem60	-0.122352537	0.208554217	-0.586670165	0.557425249	0.968926429
Rsbn1l	0.076927545	0.154144733	0.499060487	0.617736773	0.974153
A630072M18Rik	-0.262914244	0.276529765	-0.950762909	0.341724745	0.919850248
Ptpn12	-0.039327405	0.11670037	-0.336994692	0.736120901	0.988585656
Gsap	0.024352765	0.142347353	0.171079858	0.864160973	0.992886758
Ccdc146	-0.109762684	0.207772377	-0.528283335	0.597302696	0.974001549
Fgl2	-0.050519886	0.138951996	-0.363577982	0.716173171	0.988585656
Fam185a	0.059998166	0.406256891	0.147685289	0.882591142	0.992886758
Fbxl13	0.225716956	0.371282172	0.607939118	0.543227854	0.967491297
Lrrc17	-0.000652595	0.295745204	-0.002206612	0.99823938	0.999591134
Armc10	0.022219079	0.135065225	0.164506289	0.869332596	0.992886758
Napepld	-0.077073287	0.263301537	-0.292718713	0.769737165	0.989990452
Pmpcb	0.131143266	0.113485784	1.155592024	0.247848072	0.896939594
Dnajc2	-0.231760253	0.148395676	-1.56177228	0.118341644	0.814755048
Psmc2	-0.081810127	0.096182125	-0.85057517	0.395005387	0.933933717
Reln	0.324576559	0.644592705	0.503537438	0.614586465	0.974001549
Orc5	-0.005152946	0.165176971	-0.031196517	0.975112817	0.997262526
Gm15421	0.281092562	0.32863857	0.855324322	0.392371627	0.933734231
6030443J06Rik	-0.36966417	0.984794305	-0.375371961	0.707383854	0.988585656
Lhfpl3	-0.824794867	1.061497878	-0.777010378	0.437152648	0.94702277
5031425E22Rik	0.343865676	0.123947515	2.774284543	0.005532327	0.416706681
Kmt2e	-0.121422471	0.242209986	-0.501310756	0.616152436	0.974001549
Srpk2	-0.12112027	0.141742753	-0.854507673	0.39282376	0.933734231
AI506816	-1.061038681	1.233184794	-0.860405258	0.389565688	0.933734231
Pus7	-0.151913769	0.190791439	-0.796229484	0.425898663	0.944290206
Rint1	0.092356958	0.157635519	0.585889261	0.557949935	0.968926429
Tomm7	0.472126003	0.172206987	2.741619327	0.006113715	0.427133716
2700038G22Rik	-1.737545133	0.7428379	-2.339063655	0.019332139	0.592187315
Fam126a	-0.069040472	0.13281442	-0.519826629	0.603184418	0.974001549
Klhl7	-0.110762894	0.13105912	-0.845136863	0.398034429	0.935327115
Nupl2	-0.855274031	0.446252201	-1.916571007	0.055292454	0.713080491
Kcnh2	0.105267535	0.429537991	0.245071535	0.806401043	0.99099448
Nos3	0.116921287	0.359367755	0.325352748	0.744914114	0.988585656
Atg9b	0.809058249	0.691991081	1.169174389	0.242333378	0.896939594
Abcb8	-0.38233699	0.257759515	-1.483308929	0.137992351	0.833827207
Cdk5	0.283301275	0.13769885	2.057397537	0.039648002	0.679118604
Slc4a2	-0.0305182	0.163960862	-0.186131005	0.852342028	0.992886758
Fastk	-0.044872211	0.165695651	-0.270811035	0.786536372	0.990988839
Tmub1	0.254847275	0.163644857	1.557319177	0.119394725	0.81558552
Agap3	-0.035855067	0.190038999	-0.188672153	0.850349778	0.992886758
Asb10	1.638044312	1.04046053	1.574345461	0.115407605	0.81007959
Abcf2	-0.138090876	0.125286687	-1.102199119	0.270375115	0.89804647
Chpf2	-0.139527444	0.22087261	-0.631710034	0.52757637	0.965637248
Smarcd3	0.046117141	0.195641869	0.235722248	0.813648205	0.992048071
Nub1	-0.002681271	0.141425148	-0.018958942	0.984873859	0.998148029
Wdr86	-0.213008038	0.529911639	-0.401968975	0.687706859	0.98538669

Crygn	0.945227588	0.526620655	1.794892735	0.072670792	0.74763454
Rheb	0.1204438	0.114038879	1.056164365	0.290893128	0.907334416
Prkag2	-0.086999946	0.138552535	-0.627920274	0.530056169	0.965888564
2900005J15Rik	-0.635886898	1.11331839	-0.571163563	0.567888775	0.971085047
Galnt11	-0.165440532	0.154935353	-1.067803622	0.285609104	0.905916508
Kmt2c	0.251663146	0.208333918	1.207979708	0.227055068	0.89398447
4831440E17Rik	0.535371501	0.339149038	1.578572959	0.114434041	0.809025248
1700096K18Rik	-0.106090527	0.364327014	-0.291195885	0.770901509	0.989990452
Xrcc2	-0.38386956	0.310307501	-1.237061815	0.216064135	0.887633697
Actr3b	0.711905853	0.415647293	1.71276432	0.086755907	0.765418158
Dpp6	0.117591887	0.829164883	0.141819666	0.88722245	0.992886758
Paxip1	-0.114634681	0.165121923	-0.694242645	0.487530053	0.958288569
Insig1	0.050429462	0.120963189	0.416899244	0.676752106	0.983549699
Rbm33	0.170688027	0.168232232	1.014597647	0.310297661	0.914704835
Shh	0.194263229	0.235403495	0.825235112	0.409238123	0.940809748
9530036O11Rik	-0.184394778	0.260590957	-0.707602367	0.479192223	0.957463006
Rnf32	-0.208388518	0.195707663	-1.064794882	0.286968762	0.906605838
Lmbr1	0.130173455	0.183267607	0.710291668	0.477523285	0.95716557
Nom1	-0.196627805	0.198684067	-0.989650596	0.32234493	0.91640698
Ube3c	-0.154360251	0.133498899	-1.156266103	0.247572329	0.896939594
Gm5129	0.354467582	1.933223584	0.18335571	0.854518925	0.992886758
Dnajb6	0.130686085	0.112246596	1.164276597	0.244311938	0.896939594
Il6	-0.21241968	0.918104796	-0.231367575	0.817029253	0.992678823
Tyms	0.200572593	0.207087738	0.968539204	0.33277515	0.917115058
Gm4961	1.302860796	1.679120314	0.775918667	0.43779701	0.94749626
Hadha	0.008439283	0.145784701	0.057888677	0.953837302	0.996070133
Hadhb	-0.297650683	0.131615779	-2.261512139	0.023727562	0.622011301
Adgrf3	0.513816345	1.690400089	0.30396138	0.761157314	0.989266039
Ept1	-0.159591409	0.180373782	-0.884781631	0.376274418	0.928883222
Drc1	-0.177411957	0.183777458	-0.965362996	0.334363029	0.918030088
Otof	1.302483568	1.717141952	0.758518285	0.448140771	0.948476747
1700001C02Rik	-0.109413334	0.200491149	-0.545726507	0.585253949	0.972904784
Cib4	1.832160209	0.793150307	2.309978567	0.020889341	0.604325792
Gm9899	-0.226721062	1.039358569	-0.218135559	0.827323493	0.992886758
Kcnk3	-0.105972101	0.28581995	-0.370765235	0.710812397	0.988585656
Slc35f6	-0.25697438	0.230712839	-1.113827826	0.265353057	0.89768067
Cenpa	0.339722877	0.207329671	1.638563723	0.101304153	0.788783372
Dpysl5	-0.304973322	0.637628236	-0.478293314	0.632441455	0.977034429
Mapre3	-0.544641208	0.201637974	-2.701084515	0.006911377	0.447859934
Tmem214	-0.203054846	0.169697287	-1.196570965	0.231473823	0.895404344
Agbl5	-0.262809039	0.325913405	-0.806376892	0.420025567	0.943415794
Ost4	0.052486805	0.157376352	0.333511384	0.738748298	0.988585656
Emilin1	0.100472641	0.242436728	0.4144283	0.678560467	0.983853811
Khk	0.408733709	0.207020701	1.974361538	0.048340648	0.706387941
Cgref1	0.44296373	0.418280394	1.059011459	0.289594561	0.907334416
Preb	-0.005123165	0.132730857	-0.038598149	0.969210778	0.996600044
Tcf23	0.848490526	0.426560832	1.989143077	0.046685413	0.698868643
Slc5a6	-0.155873241	0.252378717	-0.617616426	0.536828214	0.96674935
Atraid	-0.071430471	0.130087812	-0.54909426	0.582940764	0.972174584
Cad	-0.080060828	0.302383544	-0.264765823	0.791189867	0.990988839
Trim54	0.382828098	0.670102949	0.571297438	0.567798038	0.971054423
Mpv17	0.073873926	0.109289731	0.675945717	0.49907511	0.960700483
Gtf3c2	-0.199604825	0.164614043	-1.212562561	0.22529711	0.893255444
Eif2b4	0.188920518	0.150586456	1.254565135	0.209636667	0.884258007
Snx17	0.061553215	0.12704151	0.484512619	0.628022111	0.976968316
Zfp513	0.239118796	0.168741725	1.417069759	0.156462533	0.855455553
Ppm1g	-0.039191357	0.18878591	-0.207596831	0.835543779	0.992886758
Nrbp1	0.037557172	0.084564329	0.44412547	0.656951873	0.979614766
Krtcap3	0.099852937	0.177611462	0.562198726	0.573980629	0.971613657
Ift172	0.141810193	0.138000085	1.027609463	0.304133571	0.911786415
Fndc4	0.238281453	0.219061486	1.087737777	0.276710886	0.900604936
Zfp512	-0.071614826	0.195189021	-0.366899869	0.713693709	0.988585656

Gpn1	-0.00190052	0.125007991	-0.01520319	0.987870076	0.998148029
Supt7l	-0.212493072	0.28379031	-0.748767889	0.453997117	0.950796682
Slc4a1ap	-0.120611993	0.229047776	-0.526580068	0.598485237	0.974001549
AI839979	1.080204667	0.363520125	2.971512699	0.002963366	0.345591998
Mrpl33	0.240373864	0.245520029	0.979039735	0.327560347	0.917115058
Rbks	0.18641038	0.213087482	0.87480681	0.381679031	0.930635634
Bre	-0.233116259	0.194633396	-1.197719729	0.231026146	0.895404344
Fosl2	-0.134955117	0.282187935	-0.478245524	0.632475464	0.977034429
Plb1	-0.074252863	0.18810812	-0.394735021	0.693038454	0.986599035
Ppp1cb	0.074727813	0.118843137	0.628793679	0.529484139	0.965637248
Yes1	-0.119683311	0.117951436	-1.014682956	0.310256981	0.914704835
Pisd	-0.156913303	0.13839289	-1.133824889	0.256868009	0.897248195
Prr14l	-0.117536252	0.258208452	-0.455199089	0.648966016	0.979004493
Depdc5	-0.244658378	0.25109907	-0.974349995	0.329882789	0.917115058
Ywhah	0.050344746	0.091299731	0.551422717	0.581343932	0.971885891
Slc5a1	0.218519928	0.459589168	0.475467967	0.63445346	0.977034429
Spon2	0.232910306	0.334328034	0.696652037	0.486020582	0.957710647
Ctbp1	-0.044752416	0.126465315	-0.353871065	0.723435513	0.988585656
Maea	-0.03067663	0.104853921	-0.292565402	0.769854362	0.989990452
Uvssa	0.004618277	0.297760063	0.015510063	0.987625256	0.998148029
Fam53a	0.242357965	0.107097416	2.262967428	0.023637697	0.622011301
Slbp	-0.134612578	0.160032259	-0.84115902	0.400258855	0.936803788
Tmem129	-0.041359655	0.147855579	-0.279730095	0.779684587	0.990988839
Tacc3	-0.019997231	0.224181802	-0.089200956	0.928922206	0.994688009
Fgfr3	-0.19665273	0.173036729	-1.1364797	0.255755857	0.897044747
Letm1	-0.001890973	0.182197754	-0.010378686	0.991719156	0.998770784
Whsc1	-0.100050612	0.176049578	-0.568309296	0.569824968	0.97144955
Nelfa	-0.377352932	0.207552003	-1.818112698	0.06904691	0.743426713
Gm1673	-0.133194086	0.19985953	-0.666438503	0.505130859	0.962138588
Nat8l	-0.276047235	0.71797162	-0.384482099	0.700621161	0.988482081
Poln	0.390616889	0.377185296	1.035610065	0.300384094	0.910285132
Haus3	-0.069558854	0.259385658	-0.268167695	0.78857024	0.990988839
Mxd4	0.091990905	0.129496148	0.710375609	0.477471244	0.95716557
Zfyve28	-0.537029389	0.445524213	-1.205387661	0.228053683	0.894410876
Rnf4	-0.035077687	0.083042397	-0.422406964	0.672727995	0.983233856
Fam193a	-0.427825612	0.312499731	-1.369043135	0.170985792	0.86244803
Tnip2	-0.069192943	0.173783619	-0.398155728	0.690515398	0.986010574
Sh3bp2	0.280424289	0.167005255	1.679134521	0.09312583	0.775558829
Add1	-0.109598779	0.137852015	-0.795046621	0.426586383	0.944290206
Mir7036b	0.101362861	1.767867811	0.057336222	0.954277367	0.996070133
Mfsd10	-0.112327381	0.227931985	-0.492810963	0.622146164	0.97514112
Nop14	-0.242873087	0.200365485	-1.212150319	0.225454845	0.89336109
Grk4	0.405647629	0.262108284	1.547633764	0.121710497	0.816233249
Htt	-0.042689939	0.231791659	-0.184173749	0.853877153	0.992886758
Msantd1	0.956975851	0.755213718	1.267158989	0.205098447	0.883765092
Rgs12	0.059261088	0.161340121	0.367305339	0.713391272	0.988585656
Hgfac	-0.148269145	0.554503548	-0.267390795	0.789168286	0.990988839
Dok7	0.074762519	1.117384161	0.066908519	0.946654531	0.995172786
Lrpap1	-8.28E-05	0.099372835	-0.000833547	0.999334926	0.999886097
Adra2c	1.106696479	1.448333557	0.764117129	0.444797446	0.948476747
Cpz	-0.17773879	0.49573539	-0.358535609	0.719942524	0.988585656
Trmt44	-0.171406014	0.229798615	-0.745896637	0.455729857	0.95142233
Acox3	-0.148637329	0.202559029	-0.733797598	0.463072105	0.953083512
Htra3	-0.236249562	0.24814717	-0.952054229	0.34106948	0.919850248
Sh3tc1	-0.041728267	0.263314075	-0.158473365	0.874083807	0.992886758
Ablim2	-0.030451012	0.584448655	-0.052102116	0.958447327	0.996226826
Afap1	-0.396020911	0.383476532	-1.032712246	0.301738581	0.911518274
Sorcs2	-0.095358096	0.313737604	-0.303942194	0.761171931	0.989266039
Psapl1	-1.052174974	0.77541535	-1.356917907	0.174807296	0.864111192
2210406O10Rik	-1.122438566	2.267065737	-0.495106316	0.620525076	0.975013118
Grpel1	0.271234074	0.164700808	1.646829046	0.099593189	0.788783372
Tada2b	-0.215738377	0.201716592	-1.069512306	0.284838887	0.905916508

Ccdc96	0.021520184	0.349300279	0.061609409	0.950873884	0.99573416
Tbc1d14	0.101015353	0.136100221	0.742212997	0.457958295	0.952235842
D5Ertd579e	-0.302829023	0.184052767	-1.64533806	0.099900115	0.788783372
Bloc1s4	0.064131927	0.118942624	0.53918372	0.589760092	0.974001549
Mrfap1	0.042428961	0.11546251	0.367469588	0.713268773	0.988585656
Man2b2	-0.183741259	0.196371471	-0.935682041	0.34943692	0.921585102
Ppp2r2c	-0.308975622	0.359088821	-0.860443444	0.389544646	0.933734231
Wfs1	-0.395645198	0.386461643	-1.023763173	0.305947149	0.91333012
Jakmip1	0.143565591	1.048525013	0.136921475	0.891092864	0.993043931
Crmp1	3.64533413	1.651618875	2.207127919	0.027305124	0.636714037
Evc	0.047619538	0.329961208	0.144318595	0.885248895	0.992886758
Evc2	0.124467284	0.283707746	0.438716552	0.660866932	0.979924124
Cytl1	0.072387221	0.140042798	0.51689356	0.605230464	0.974001549
Msx1	-0.227353808	0.565559904	-0.401997749	0.687685683	0.98538669
Stx18	0.22627745	0.147563398	1.533425313	0.125171096	0.822296879
Nsg1	-0.035767951	0.119579139	-0.299115308	0.764852066	0.9895982
Zbtb49	-0.169030965	0.358118291	-0.471997577	0.636928518	0.977284137
Lyar	0.318427991	0.197227245	1.614523347	0.106413957	0.794899279
Tmem128	0.030197732	0.151629259	0.199155049	0.842141461	0.992886758
Slc2a9	0.42029672	0.319148846	1.316930094	0.187862058	0.874351146
Wdr1	-0.112512218	0.116632538	-0.964672642	0.334708802	0.918030088
Zfp518b	-0.16590707	0.515588378	-0.321782021	0.747617835	0.988585656
Clnk	-0.449138684	1.329286363	-0.337879554	0.735453956	0.988585656
Hs3st1	0.063644186	0.248008774	0.256620705	0.79747159	0.990988839
Rab28	0.187488865	0.11419016	1.641900362	0.100610655	0.788783372
Bod1l	-0.0388613	0.215001932	-0.180748607	0.856564904	0.992886758
Gm7854	2.683365274	2.210334426	1.214008723	0.224744393	0.892989918
Cpeb2	0.325946354	0.262138478	1.243412858	0.213715735	0.884945675
C1qtnf7	0.35311161	0.128435403	2.749332356	0.00597168	0.427133716
Cc2d2a	-0.308375051	0.25298839	-1.218929655	0.222870893	0.892391445
Fbxl5	0.052763965	0.138563621	0.380792338	0.703357345	0.988585656
Bst1	-0.023962464	0.161921146	-0.147988479	0.882351861	0.992886758
Cd38	-0.511247335	0.179438644	-2.849148456	0.004383642	0.385355746
Fgfbp1	-0.032698251	0.151227005	-0.216219659	0.828816531	0.992886758
Prom1	-0.572656034	0.158247157	-3.618744522	0.000296036	0.112404069
Tapt1	-0.379509586	0.110896439	-3.422198123	0.00062117	0.177927156
Ldb2	0.057022077	0.175045443	0.325755852	0.744609083	0.988585656
D5Ertd615e	-0.547061305	1.694610776	-0.322824163	0.746828413	0.988585656
Qdpr	0.25639947	0.132765016	1.931227651	0.053454906	0.707153766
Lap3	-0.052722552	0.116969029	-0.450739417	0.65217737	0.979004493
Med28	0.300421514	0.176767675	1.699527439	0.089219849	0.768297719
1600023N17Rik	0.009425079	1.824807502	0.005164972	0.995878967	0.99930649
Ncapg	-0.09692322	0.960113864	-0.100949714	0.919590379	0.994688009
Lcorl	-0.315471713	0.247582509	-1.274208402	0.202589568	0.88210274
Gm3414	-0.935398891	0.483191897	-1.935874538	0.052883069	0.707153766
Slit2	0.113805521	0.238006359	0.478161681	0.632535134	0.977034429
Pacrgl	0.186823837	0.170320911	1.096893132	0.272688111	0.898703835
Kcnip4	-0.499812709	0.371883371	-1.344003922	0.178947119	0.867377413
5730480H06Rik	-0.361599598	0.586586412	-0.61644728	0.537599357	0.96674935
Adgra2	-0.093116067	0.185873497	-0.500964733	0.616395943	0.974001549
Ppargc1a	0.045035254	0.394507473	0.114155642	0.909114414	0.994688009
Dhx15	-0.155414341	0.099849793	-1.556481352	0.119593674	0.81558552
9230114K14Rik	-0.040169029	0.383517249	-0.104738519	0.916583297	0.994688009
C130083M11Rik	-0.062373015	0.376348809	-0.16573193	0.868367917	0.992886758
Sod3	-0.193595276	0.212704995	-0.910158581	0.362738884	0.926455466
Ccdc149	-0.50203398	0.227848696	-2.203365605	0.02756899	0.637644759
Lgi2	-0.26354186	0.490171828	-0.537651993	0.590817328	0.974001549
8030423F21Rik	-0.934385993	1.065880354	-0.876633094	0.380685958	0.930179532
Sepsecs	-0.250970589	0.228690709	-1.097423635	0.272456246	0.898460682
Pi4k2b	-0.123282039	0.147454255	-0.836069724	0.40311568	0.937834293
Zcchc4	0.192078468	0.25449844	0.75473338	0.450408974	0.949134998
Anapc4	0.039113672	0.125266845	0.312242813	0.754855997	0.989048541

5033403H07Rik	0.30744856	0.455671561	0.67471527	0.499856681	0.961017744
Slc34a2	-0.135698776	0.177566205	-0.764215104	0.444739067	0.948476747
Sel1l3	-0.251076425	0.477585926	-0.525719899	0.599082836	0.974001549
Smim20	0.308604923	0.195623448	1.577545667	0.114670023	0.809432539
Rbpj	-0.118309715	0.138260638	-0.855700631	0.392163391	0.933734231
Cckar	0.123951569	0.137743057	0.899875255	0.36818664	0.928436166
Tbc1d19	-0.282308616	0.170021976	-1.66042427	0.096829131	0.782250977
Stim2	-0.129778429	0.111237477	-1.166679005	0.243340025	0.896939594
Gm10440	0.259176308	1.112055363	0.233060616	0.815714337	0.992524109
Pcdh7	-0.143345581	0.27643848	-0.518544238	0.604078603	0.974001549
Arap2	-0.214823575	0.167193919	-1.284876723	0.198835366	0.880661448
Dthd1	0.014486536	1.503185296	0.009637226	0.992310725	0.998827474
Nwd2	-1.892089826	0.990665599	-1.909917765	0.056143802	0.716140516
0610040J01Rik	0.00138842	0.140258863	0.009898982	0.992101884	0.998800761
Rell1	-0.261529953	0.118066621	-2.215104917	0.026752864	0.633489138
Pgm1	-0.190061106	0.165412503	-1.149012937	0.250550646	0.896939594
Tbc1d1	-0.256528121	0.216079131	-1.187195261	0.23515061	0.896002526
Gm3716	0.182476299	0.602972604	0.302627843	0.762173493	0.989285682
Klf3	-0.347922838	0.232319882	-1.497602507	0.134236555	0.827986488
Tlr1	0.530017616	0.42862714	1.236547028	0.216255296	0.887633697
Tlr6	0.093295814	0.567495166	0.164399311	0.869416806	0.992886758
Fam114a1	-0.08465677	0.119926996	-0.705902532	0.480248753	0.957463006
Klhl5	-0.204561341	0.087303653	-2.34310174	0.019124167	0.590246276
Wdr19	-0.118066707	0.195810331	-0.602964643	0.546532211	0.967812517
Rfc1	-0.076377111	0.182764349	-0.417899397	0.676020673	0.983549699
Klb	0.807410672	2.331654529	0.346282291	0.729130576	0.988585656
Rpl9	0.297583737	0.312796968	0.95136388	0.341419689	0.919850248
Lias	0.090864094	0.15347624	0.592040137	0.553823711	0.968926429
Ugdh	-0.069342917	0.122787221	-0.564740505	0.57225028	0.971613657
Smim14	0.056864552	0.097913843	0.580761112	0.561401468	0.96944293
Ube2k	0.119274178	0.102479753	1.163880413	0.244472478	0.896939594
Pds5a	-0.454527694	0.221587489	-2.051233557	0.040244207	0.679118604
N4bp2	-0.458801555	0.413274094	-1.110162872	0.266928849	0.89768067
Rhoh	0.112314001	0.24671815	0.455232017	0.648942329	0.979004493
9130230L23Rik	-0.105884107	0.301765701	-0.350881848	0.72567699	0.988585656
Rbm47	0.199286758	0.206894457	0.963229083	0.335432573	0.918739745
Nsun7	0.23352921	0.658102661	0.35485225	0.722700282	0.988585656
Apbb2	0.327776815	0.25210927	1.300137894	0.193553712	0.879588734
Uchl1	-0.268492035	0.227398499	-1.180711553	0.23771733	0.896609492
Limch1	-0.021984215	0.165375189	-0.132935387	0.89424448	0.993422442
Tmem33	-0.272085566	0.126378463	-2.152942517	0.031323196	0.661395937
Slc30a9	-0.068453126	0.093034577	-0.735781553	0.461863649	0.953083512
Bend4	0.446415937	0.858507884	0.519990491	0.603070202	0.974001549
Shisa3	-0.093828206	0.726282833	-0.129189624	0.897207606	0.994101834
Atp8a1	0.059105036	0.106821572	0.553306187	0.580053765	0.971613657
Kctd8	-0.080294552	0.302938467	-0.265052348	0.790969136	0.990988839
Yipf7	-0.752239084	1.12295475	-0.669874796	0.502937608	0.961729397
Guf1	0.249861641	0.139735548	1.78810363	0.07375929	0.749855497
Gnpda2	0.029221102	0.108471869	0.269388759	0.787630536	0.990988839
Gabra4	-0.322150104	0.64829386	-0.496919875	0.619245555	0.974471036
Gabrb1	-0.253495137	2.210567908	-0.114674214	0.908703354	0.994688009
Commd8	0.16014119	0.129955213	1.232279846	0.217844566	0.887633697
Atp10d	-0.674569699	0.238959741	-2.822942877	0.004758505	0.391906015
Corin	-0.186521319	0.93185957	-0.20016033	0.841355192	0.992886758
Nfxl1	-0.142345795	0.18170458	-0.783391342	0.433397339	0.946252789
Cnga1	0.601682064	0.937538341	0.641767955	0.521023859	0.964784335
Nipal1	-0.257241577	0.319158145	-0.806000351	0.420242647	0.943415794
Txk	0.945680943	0.388136158	2.436467006	0.014831522	0.554936045
Tec	-0.046191588	0.231522209	-0.199512555	0.841861823	0.992886758
Slain2	-0.130348974	0.109493567	-1.190471524	0.233861116	0.89581803
Fryl	-0.027128925	0.280108861	-0.096851363	0.922844434	0.994688009
Ociad1	2.48E-05	0.114589536	0.000216685	0.99982711	0.999957126

Ociad2	0.103837242	0.204069821	0.508831937	0.610870031	0.974001549
Dcun1d4	-0.095274082	0.168116778	-0.566713703	0.570908709	0.971613657
Sgcb	-0.024097377	0.115807347	-0.208081592	0.83516526	0.992886758
Spata18	-0.420613936	0.265741533	-1.582793366	0.113468569	0.806530837
Usp46	-0.254911639	0.120243537	-2.119961242	0.034009314	0.671818913
Dancr	0.397037586	0.337441377	1.176612037	0.239350391	0.896918485
Rasl11b	-0.233264189	0.164437238	-1.418560611	0.156027156	0.855455553
Scfd2	0.138917121	0.213197116	0.651590057	0.514665664	0.964352198
Fip1l1	-0.108033472	0.18266019	-0.591445084	0.55422224	0.968926429
Lnx1	-0.016555015	0.191607377	-0.086400718	0.931147876	0.994688009
Gm6116	0.728943085	0.579438501	1.258016311	0.208385857	0.884258007
Chic2	0.291471939	0.161480862	1.804993699	0.071075668	0.744227652
Pdgfra	0.098003958	0.116640665	0.840221193	0.400784378	0.937313897
Kit	-0.024777541	0.188670244	-0.131327232	0.89551645	0.993618558
Kdr	0.112911945	0.189155802	0.596925622	0.55055704	0.968926429
Srd5a3	-0.273628894	0.160821999	-1.701439451	0.088860499	0.767814344
Tmem165	-0.18415822	0.087672979	-2.100512863	0.03568375	0.674795441
Clock	-0.123561572	0.153823367	-0.80326919	0.421819162	0.943415794
Exoc1	-0.068182479	0.20274097	-0.336303405	0.736642083	0.988585656
Cep135	-0.295199627	0.25793048	-1.144492993	0.252419242	0.897013805
C530008M17Rik	0.206012352	0.515149719	0.399907724	0.689224483	0.98547461
Aasdh	-0.229448889	0.288063658	-0.796521472	0.425729	0.944290206
Ppat	-0.190643853	0.179484661	-1.062173515	0.288156921	0.907334416
Paics	0.021535815	0.082193214	0.262014517	0.79331025	0.990988839
Srp72	-0.023546818	0.112152633	-0.209953326	0.833704102	0.992886758
Thegl	0.236680788	0.428682794	0.552111706	0.580871823	0.971613657
Hopx	0.200788935	0.121562254	1.651737518	0.098588071	0.786907305
Spink2	0.151335416	0.288602923	0.524372429	0.600019529	0.974001549
Rest	-0.339266428	0.44536979	-0.761763451	0.446201198	0.948476747
Noa1	-0.241977174	0.213682067	-1.132416854	0.257459223	0.897248195
Polr2b	-0.169517519	0.223307765	-0.759120575	0.447780432	0.948476747
Igfbp7	-0.211016104	0.14904693	-1.415769548	0.156842988	0.855906801
Adgrl3	0.022837624	0.304536509	0.074991416	0.940221542	0.994688009
Tecrl	-0.800113735	0.387990129	-2.062201264	0.039188578	0.679118604
Cenpc1	0.146532817	0.17475358	0.838511105	0.401743714	0.937834293
Stap1	0.503206845	0.32413595	1.552456136	0.12055312	0.815945653
Uba6	-0.134035832	0.262649538	-0.510321977	0.609825908	0.974001549
Ythdc1	-0.129817158	0.138849643	-0.934947727	0.349815239	0.922206653
Ugt2b34	-0.640258021	1.044180671	-0.613167854	0.539765358	0.966900344
Sult1b1	2.052098569	1.336317158	1.535637372	0.124627343	0.821164769
Sult1d1	0.011318162	0.281193864	0.040250388	0.967893506	0.996474415
Sult1e1	0.840434266	1.363685346	0.616296324	0.537698965	0.96674935
Jchain	0.115459088	0.489971129	0.235644675	0.813708404	0.992048071
Utp3	0.192472849	0.134417038	1.431908129	0.152170116	0.852974739
Rufy3	-0.180612481	0.244585133	-0.738444231	0.460244545	0.953083512
Grsf1	-0.034450791	0.105573684	-0.326319869	0.744182357	0.988585656
Mob1b	-0.350766742	0.214806126	-1.632945709	0.102480415	0.789151138
Dck	0.18382119	0.202883824	0.906041625	0.364913821	0.926556975
Slc4a4	0.230228596	0.27789896	0.828461523	0.407409177	0.939557159
Gc	0.231566212	1.109440276	0.208723459	0.834664126	0.992886758
Adamts3	-0.050901209	0.755246622	-0.067396804	0.946265813	0.995065399
Cox18	-0.030518661	0.355831169	-0.085767252	0.93165144	0.994688009
Ankrd17	-0.014294187	0.220161912	-0.064925794	0.948233083	0.99560893
Gm9958	-0.10792699	0.309650692	-0.348544319	0.727431436	0.988585656
Alb	0.932975395	1.356611908	0.68772461	0.491626219	0.958916246
Afp	0.348101353	0.396022853	0.878993093	0.379405021	0.929137643
Rassf6	0.212803574	0.361761031	0.588243497	0.556368866	0.968926429
Cxcl5	1.250720804	0.585809948	2.135028276	0.0327587	0.665167506
Ppbp	0.528552758	0.472006112	1.119800663	0.262798716	0.897248195
Pf4	0.045733811	0.304922587	0.149984991	0.880776457	0.992886758
Cxcl3	-0.99722426	0.987350453	-1.010000306	0.312495144	0.914819219
Cxcl15	0.134813213	0.156826665	0.859631957	0.389991953	0.933734231

Cxcl1	0.586366332	0.510312268	1.149034365	0.250541811	0.896939594
Cxcl2	1.018743625	0.804699528	1.265992573	0.205515741	0.883765092
Mthfd2l	0.557167426	0.229396274	2.428842525	0.01514711	0.555734718
Epgn	-2.536111797	1.625625259	-1.560083902	0.118740055	0.814755048
Ereg	0.426468678	0.655680382	0.650421592	0.515419934	0.964352198
Areg	0.042414027	0.240954512	0.176025039	0.860274272	0.992886758
Btc	-0.135250648	0.208905535	-0.647424914	0.517356976	0.964352198
Parm1	-0.407043419	0.494132626	-0.823753377	0.410079704	0.940983181
Rchy1	0.184903101	0.120432954	1.53531982	0.124705288	0.821164769
Thap6	-0.067171761	0.255229083	-0.263182236	0.792410122	0.990988839
Gm1045	0.82391106	1.20081905	0.686124242	0.492634765	0.958916246
Cdkl2	-0.144214579	0.126845352	-1.136932309	0.255566584	0.897044747
G3bp2	-0.19756868	0.089922697	-2.197094674	0.028013685	0.640939708
Uso1	-0.20413548	0.103023632	-1.981443245	0.047541588	0.700850692
Ppef2	-0.063703864	0.396093418	-0.160830402	0.872226979	0.992886758
Naaa	-0.288530244	0.101334364	-2.847308958	0.004409055	0.385355746
Sdad1	-0.113653299	0.247386216	-0.459416458	0.645935132	0.979004493
Cxcl9	0.560669241	0.504167842	1.11206863	0.266108643	0.89768067
Art3	-0.136494328	0.254078611	-0.53721298	0.591120506	0.974001549
Cxcl10	0.732751842	0.413768631	1.770921689	0.076573725	0.752876039
Nup54	0.105555143	0.150219438	0.702673001	0.482259557	0.957463006
Scarb2	-0.229972118	0.111262736	-2.066928479	0.038740894	0.679118604
Fam47e	-0.32229789	0.151994895	-2.120452076	0.033967939	0.671818913
Stbd1	-0.385898743	0.181215338	-2.129503753	0.033212605	0.668634028
Ccdc158	-1.146139018	0.437120735	-2.622019329	0.008741047	0.475600561
Shroom3	0.051349706	0.193450131	0.265441567	0.790669318	0.990988839
Sowahb	1.155693233	0.458489437	2.520653996	0.011713697	0.518291414
Sep-11	-0.133389399	0.206729567	-0.645236196	0.518774133	0.96446859
Ccni	-0.159167703	0.099507154	-1.599560406	0.109696138	0.798844267
2010109A12Rik	0.743150632	0.878844445	0.845599738	0.397776072	0.935327115
Ccng2	0.285128341	0.148074192	1.92557756	0.054157143	0.707153766
Cxcl13	0.114684099	0.434419409	0.263993958	0.791784575	0.990988839
Cnot6l	-0.225204674	0.114163665	-1.97264754	0.048535733	0.706907143
Mrpl1	0.01068937	0.14759535	0.07242349	0.942264891	0.994688009
Fras1	0.017002247	0.499730265	0.034022849	0.97285893	0.996916322
Anxa3	-0.056234829	0.103088465	-0.545500695	0.585409203	0.972904784
Gm8013	-1.434694263	1.048606878	-1.36819078	0.171252367	0.86244803
Bmp2k	-0.301105074	0.184270449	-1.634038857	0.102250692	0.788963633
Paqr3	0.0116241	0.430107417	0.027026039	0.978438966	0.997262526
Antxr2	-0.130731116	0.150100632	-0.870956465	0.383777924	0.931591679
Prdm8	0.125208701	1.014832642	0.12337867	0.901807247	0.994688009
1700007G11Rik	0.096419644	0.156123436	0.617585971	0.536848295	0.96674935
Mir703	-0.136179261	0.317236964	-0.429266689	0.667729156	0.981863224
Bmp3	-0.009457542	0.259560198	-0.036436797	0.970934074	0.996733101
Prkg2	0.329880721	0.220107822	1.498723301	0.133945429	0.827800491
Rasgef1b	-0.172688351	0.199762037	-0.864470318	0.387329579	0.932858077
Hnrnpd	0.05359637	0.174143822	0.307770721	0.758256802	0.989266039
Hnrnpdl	0.145303781	0.096229159	1.509976638	0.131049385	0.827800491
Enoph1	0.294035222	0.136720163	2.150635403	0.031504989	0.661395937
Tmem150c	0.293946001	0.343109545	0.856711814	0.391604173	0.933734231
Sec31a	-0.279063791	0.202613006	-1.377324175	0.168412044	0.861597949
5430416N02Rik	0.631368327	0.295416798	2.137212006	0.032580752	0.665167506
Lin54	-0.072209063	0.218751317	-0.330096587	0.741326982	0.988585656
Cops4	0.054060229	0.150046674	0.360289421	0.71863071	0.988585656
Plac8	0.807678048	0.302107883	2.673475582	0.007506974	0.453949504
Coq2	0.08644367	0.178467626	0.48436611	0.628126066	0.976968316
Hpse	0.001127929	0.189102289	0.005964653	0.995240924	0.999285699
Helq	-0.290606293	0.226638276	-1.282247191	0.199755946	0.880888611
Mrps18c	0.24916767	0.192556538	1.293997452	0.195666301	0.879601031
Fam175a	0.366699334	0.251889142	1.455796511	0.14544888	0.842832198
Agpat9	-0.250616925	0.233561845	-1.073021687	0.283261386	0.905916508
Cds1	-0.231525828	0.161379305	-1.434668645	0.151381543	0.851397333

Wdfy3	-0.006770188	0.198463389	-0.034113034	0.972787015	0.996905197
Arhgap24	0.192792183	0.335942548	0.573884387	0.566046039	0.970328402
Mapk10	0.699651032	0.585027902	1.19592763	0.231724802	0.895404344
Ptpn13	0.108057651	0.161216859	0.670262726	0.502690324	0.961729397
Slc10a6	-0.280579279	0.185129488	-1.51558394	0.12962459	0.827800491
1700016H13Rik	0.082980415	0.490877998	0.169044885	0.865761336	0.992886758
Aff1	-0.17522858	0.186234775	-0.940901509	0.346755335	0.921552698
Klhl8	0.081517751	0.160326557	0.508448213	0.611139048	0.974001549
Hsd17b11	-0.127082815	0.111610328	-1.138629526	0.254857708	0.897044747
Nudt9	0.139691155	0.098853909	1.413107052	0.15762425	0.855986074
Sparcl1	-0.297892555	0.146498761	-2.033413478	0.042010767	0.679118604
Spp1	-0.355833672	0.325998273	-1.091520114	0.275044086	0.900302631
Pkd2	-0.051675414	0.146998592	-0.3515368	0.725185669	0.988585656
BC005561	-0.426902666	0.335356989	-1.272979777	0.203025218	0.882297774
D930016D06Rik	0.205271093	0.275947564	0.74387717	0.456950788	0.951742083
Zfp951	0.047225131	0.352200863	0.134085789	0.893334737	0.99335805
Abcg3	-0.179472681	0.246601043	-0.727785572	0.466744858	0.953083512
Gbp8	0.492470921	0.237699522	2.071821253	0.038282114	0.679118604
Gbp9	-0.214022778	0.212888856	-1.005326358	0.314739727	0.91586731
Gbp4	0.380466004	0.182117992	2.089118158	0.036697088	0.677009445
Gbp10	0.778588934	0.397540959	1.958512492	0.050169907	0.707153766
Gbp6	0.07597944	0.234184256	0.32444299	0.745602677	0.988585656
Gbp11	0.642729533	0.815698311	0.78795006	0.430725918	0.945321484
Lrrc8b	-0.057959602	0.301979432	-0.191932283	0.847795249	0.992886758
Lrrc8c	-0.285796494	0.206805629	-1.381957036	0.166984893	0.860403806
Lrrc8d	-0.049686376	0.21089673	-0.235595762	0.813746363	0.992048071
Zfp326	-0.068654857	0.161454585	-0.425227052	0.670671165	0.982434419
Zfp644	-0.078707195	0.168546252	-0.466976834	0.640516453	0.977921835
Cdc7	-0.396219519	0.401951313	-0.985740077	0.324260678	0.91640698
Tgfbr3	-0.021903923	0.17772737	-0.12324451	0.901913481	0.994688009
Brdt	-0.130940466	0.269652829	-0.485589069	0.627258548	0.976752533
Ephx4	-0.291170431	0.320061463	-0.909732861	0.36296341	0.926455466
Lpcat2b	-0.08814087	1.475724935	-0.059727168	0.952372934	0.996023481
Btbd8	0.369816755	1.051978341	0.351544077	0.725180211	0.988585656
A830010M20Rik	0.138114254	0.423230291	0.326333574	0.744171989	0.988585656
1700028K03Rik	-3.105971222	2.186816001	-1.420316671	0.15551551	0.855261441
Glmn	-0.085671094	0.231143209	-0.370640756	0.710905122	0.988585656
Rpap2	-0.020949086	0.161348954	-0.129837136	0.896695281	0.994101834
Gfi1	-0.199223487	0.814192066	-0.244688564	0.806697584	0.99099448
Evi5	-0.200177726	0.138144907	-1.449041662	0.147325948	0.846074835
Ube2d2b	0.084448214	1.54524575	0.054650345	0.956417029	0.996173928
Rpl5	0.295297344	0.266948357	1.106196524	0.268641467	0.89804647
Fam69a	0.185258069	0.132863115	1.39435289	0.163211067	0.859982992
Mtf2	-0.11492197	0.150295285	-0.764641223	0.444485215	0.948476747
Tmed5	0.001401963	0.126306196	0.011099716	0.99114389	0.998770784
Ccdc18	3.480876836	1.011297152	3.441992127	0.000577447	0.168356751
Dr1	0.092404259	0.111883192	0.825899383	0.408861171	0.940605373
Pigg	-0.098897585	0.182009491	-0.54336499	0.586878525	0.973183596
Atp5k	0.334071236	0.214237786	1.559347873	0.118914067	0.814755048
Mfsd7a	0.21657991	0.343175807	0.631104832	0.527971982	0.965637248
Pcgf3	-0.164228251	0.160444425	-1.023583402	0.306032088	0.91333012
Gak	-0.075888767	0.174154787	-0.435754702	0.663014712	0.980876591
Tmem175	0.037946133	0.217228794	0.174682796	0.861328886	0.992886758
Dgkq	-0.049198173	0.267115309	-0.184183279	0.853869677	0.992886758
Idua	0.275812921	0.253441919	1.088268752	0.276476481	0.900604936
Slc26a1	-1.294511696	1.664648617	-0.777648618	0.436776191	0.946762848
Fgfrl1	0.035801791	0.142659833	0.250959152	0.801845691	0.990988839
Vmn2r8	0.274460941	1.111590895	0.246908231	0.804979246	0.99099448
Crlf2	-0.024367967	0.185482463	-0.131376122	0.895477777	0.993618558
Gm10416	0.410920425	0.358603814	1.145889725	0.251840783	0.896939594
Gm15446	0.078135663	0.415358315	0.188116286	0.850785495	0.992886758
Zfp932	0.181634146	0.172407285	1.053517815	0.292103734	0.907334416

Plcxd1	-0.176204568	0.812613372	-0.216836905	0.82833545	0.992886758
Gtpbp6	0.327810087	0.241102672	1.359628597	0.173947484	0.863528862
Zfp605	-0.095344944	0.300877005	-0.316890098	0.751326995	0.988585656
Chfr	0.035735516	0.138991299	0.257106137	0.79709684	0.990988839
Gm15787	1.941772401	1.3566241	1.431326777	0.152336583	0.852974739
Golga3	-0.275488658	0.286688769	-0.960932857	0.336585929	0.919242242
Ankle2	0.007521392	0.142466977	0.052793932	0.957896096	0.996226826
Pgam5	-0.031933232	0.114301868	-0.279376288	0.779956067	0.990988839
Pxmp2	0.097557282	0.17474993	0.558267931	0.57666144	0.971613657
Pole	0.922904035	0.950753908	0.970707591	0.331693911	0.917115058
P2rx2	1.100571422	0.913888769	1.204272838	0.228484141	0.894809445
Lrcol1	0.241333268	0.825542906	0.292332799	0.770032184	0.989990452
Fbrsl1	-0.182390981	0.30146394	-0.605017571	0.545167322	0.967491297
Gm29766	-1.085794214	0.567351404	-1.913794884	0.055646369	0.714821616
Galnt9	1.646217937	0.598739574	2.749472405	0.005969129	0.427133716
Noc4l	-0.178358704	0.34426508	-0.518085377	0.604398702	0.974001549
Ddx51	-0.133697125	0.175301945	-0.76266766	0.445661622	0.948476747
Ep400	-0.176718881	0.239707987	-0.737225668	0.460985127	0.953083512
Pus1	0.20999597	0.234799975	0.894361128	0.371128703	0.928788832
Ulk1	0.081608483	0.221985426	0.367629913	0.713149207	0.988585656
Hscb	0.462275644	0.239708133	1.928493785	0.053793737	0.707153766
Chek2	0.027301254	0.169796778	0.160787824	0.872260515	0.992886758
Ttc28	-0.237521729	0.292002406	-0.813423875	0.415975075	0.943415794
Pitpnb	0.067540921	0.112479509	0.600473111	0.548190976	0.968738549
Mn1	-0.24438722	0.704634321	-0.346828437	0.728720212	0.988585656
E130006D01Rik	-0.632365303	1.652668157	-0.382632957	0.701991931	0.988482081
Cryba4	-0.151360554	0.66745188	-0.226773732	0.820599691	0.992886758
Crybb1	1.123541296	1.129392582	0.994819085	0.319824262	0.91640698
Tpst2	0.061274044	0.165069276	0.371201992	0.710487089	0.988585656
Tfip11	-0.047142598	0.254021185	-0.1855853	0.852769982	0.992886758
Srrd	0.236700138	0.256435472	0.923039764	0.355986483	0.924354588
Hps4	-0.066891171	0.296889892	-0.225306326	0.821740978	0.992886758
Myo18b	-0.36750994	1.540923761	-0.238499755	0.811493505	0.991784898
Adrbk2	-0.242006867	0.225566313	-1.07288568	0.283322412	0.905916508
Crybb3	0.453688224	0.865104065	0.524431965	0.599978129	0.974001549
2900026A02Rik	0.076600575	0.316163645	0.242281413	0.808562115	0.991020605
Sgsm1	-0.30502114	0.446151061	-0.68367234	0.494182094	0.958916246
Wscd2	0.347042404	1.156777948	0.300007797	0.764171208	0.989384061
Cmklr1	0.018987068	0.186123456	0.102013302	0.918746117	0.994688009
Ficd	-0.439954896	0.339746447	-1.294950692	0.19533724	0.879588734
Sart3	0.080619906	0.219776632	0.366826561	0.713748394	0.988585656
Iscu	0.084559856	0.094105741	0.898562141	0.368885932	0.928436166
Tmem119	-0.169916982	0.157409717	-1.079456755	0.280384162	0.903192609
Selplg	-0.031490674	0.27960527	-0.112625467	0.910327494	0.994688009
Coro1c	-0.010465837	0.150879384	-0.069365586	0.944698621	0.99486515
Ssh1	-0.309158628	0.329468737	-0.938354976	0.348062016	0.921552698
Dao	-0.853249299	2.196895387	-0.38838868	0.69772842	0.987577127
Svop	-3.905174454	3.602771611	-1.083936168	0.278393102	0.9014923
Usp30	-0.143101644	0.232016947	-0.616772378	0.537384873	0.96674935
Alkbh2	0.307728932	0.264005496	1.165615627	0.243769887	0.896939594
Ung	1.165859175	0.456829261	2.552067641	0.010708573	0.502706181
Acacb	-0.043209627	0.26473861	-0.163216187	0.870348222	0.992886758
Foxn4	2.714914314	1.610717804	1.685530704	0.091886226	0.772516176
Myo1h	-0.10953591	0.368515816	-0.297235302	0.766286872	0.989884648
Kctd10	-0.264245914	0.163461943	-1.616559239	0.105973456	0.79419908
Ube3b	-0.071364744	0.199535637	-0.357654124	0.720602169	0.988585656
Mmab	-0.207978009	0.27668632	-0.751674346	0.452246921	0.949574349
Mvk	-0.06887962	0.135038608	-0.510073531	0.609999948	0.974001549
Fam222a	0.375342697	0.464532419	0.808001081	0.41908996	0.943415794
Trpv4	0.791464644	0.343545318	2.303814382	0.021233065	0.60743619
Gltp	-0.086921778	0.150725328	-0.576689924	0.56414894	0.969658675
Tchp	-0.177483182	0.313222103	-0.566636837	0.570960942	0.971613657

Git2	-0.108213858	0.175655791	-0.616056311	0.537857356	0.96674935
4930515G01Rik	1.494248653	0.791070153	1.888895249	0.058905866	0.72285319
Ankrd13a	-0.213030897	0.164839891	-1.292350391	0.196235829	0.879755417
1500011B03Rik	0.388375106	0.259230287	1.498185688	0.134085013	0.827800491
2610524H06Rik	0.519435489	0.27320385	1.901274409	0.057266079	0.721735927
Oasl2	0.070036603	0.275343856	0.254360507	0.799217059	0.990988839
Oasl1	0.506077685	0.646388443	0.782931208	0.43366751	0.946589497
2210016L21Rik	0.216522704	0.121243892	1.785844228	0.074124483	0.750704981
Hnf1a	-0.408057162	1.12063278	-0.364131025	0.71576017	0.988585656
Sppl3	0.046623156	0.152294439	0.306138272	0.759499369	0.989266039
Rpl37rt	0.319913021	0.207417539	1.54236244	0.122985543	0.817788082
Acads	-0.037473281	0.129045724	-0.29038762	0.771519714	0.990143246
Unc119b	-0.075108914	0.116355039	-0.64551492	0.518593553	0.96446859
Mlec	-0.087026197	0.139649521	-0.623175765	0.533169026	0.96674935
Cabp1	-0.159045525	1.336611019	-0.118991631	0.905281986	0.994688009
Pop5	0.287284012	0.20395373	1.408574445	0.15896104	0.855986074
Rnf10	-0.136723532	0.160465708	-0.852042055	0.394190751	0.933796635
Coq5	-0.102394035	0.191238995	-0.535424456	0.592356383	0.974001549
Dynll1	0.028910422	0.118719498	0.243518735	0.807603574	0.99099448
Srsf9	0.01681188	0.125114827	0.134371602	0.893108736	0.99333638
Gatc	0.092224737	0.206557285	0.446485036	0.65524692	0.979133069
Triap1	0.282889377	0.179641218	1.574746486	0.115314973	0.81007959
Cox6a1	0.185952563	0.125694827	1.479397101	0.139034214	0.835484585
Pla2g1b	-0.170944745	0.258540455	-0.661191475	0.508489527	0.963324595
Sirt4	0.331345359	0.203786914	1.62594031	0.103962354	0.79155232
Pxn	0.034072473	0.164003865	0.207754083	0.835420986	0.992886758
Rplp0	0.195200801	0.172096608	1.134251296	0.256689153	0.897248195
Gcn1l1	0.319732403	0.243103991	1.315208368	0.188439884	0.874826736
1110006O24Rik	0.095391701	0.694896988	0.137274593	0.890813752	0.993043931
Rab35	-0.130921512	0.173076249	-0.756438346	0.449386425	0.948476747
Ccdc64	0.008657887	0.324825438	0.026653968	0.978735728	0.997262526
Cit	-0.200072	1.168504308	-0.171220593	0.864050316	0.992886758
Prkab1	0.065855212	0.122152324	0.539123692	0.589801509	0.974001549
Ccdc60	0.190856983	0.307897881	0.619871051	0.535342687	0.96674935
Hspb8	-0.025874247	0.15754415	-0.164234897	0.869546231	0.992886758
Srrm4	-0.073353754	2.451685053	-0.029919729	0.976131071	0.997262526
Suds3	0.051954471	0.1141556	0.455119778	0.64902307	0.979004493
Taok3	-0.28824882	0.222303831	-1.296643509	0.194753875	0.879588734
Pebp1	0.127103587	0.127029716	1.000581529	0.317029164	0.91640698
Vsig10	0.169015184	0.192162227	0.879544264	0.379106244	0.929137643
Wsb2	-0.100933982	0.11087546	-0.91033653	0.362645059	0.926455466
Rfc5	-0.25933923	0.182767768	-1.418954951	0.15591215	0.855455553
Fbxo21	-0.135049215	0.289332731	-0.466760932	0.64067093	0.977921835
Tesc	0.269115201	0.348012105	0.773292646	0.439349207	0.947849924
Fbxw8	-0.190851454	0.171622477	-1.112042296	0.266119965	0.89768067
Rnft2	0.180027067	1.071910361	0.167949741	0.866622818	0.992886758
2410131K14Rik	-0.117342921	0.227378036	-0.516069726	0.605805711	0.974001549
Med13l	-0.174014498	0.321337787	-0.54153139	0.588141368	0.973455044
AW549542	0.249201907	0.481524376	0.517527085	0.604788266	0.974001549
Tbx3	0.411588117	0.134794144	3.053456952	0.002262212	0.313009588
Tbx3os2	-0.209233694	1.487504247	-0.140660905	0.888137831	0.992886758
Tbx5	-0.458382157	0.247816489	-1.84968385	0.06435913	0.73299549
Rbm19	-0.490661689	0.336876746	-1.456502104	0.145253868	0.842729381
Sdsl	-0.604267824	0.569055201	-1.0618791	0.288290574	0.907334416
Sds	0.033985125	1.418318823	0.023961555	0.980883275	0.99731481
Plbd2	-0.071973339	0.280237443	-0.256829845	0.79731013	0.990988839
Slc8b1	-0.004093196	0.287615771	-0.014231471	0.988645312	0.998182772
Tpcn1	0.140756122	0.230417805	0.610873464	0.541283354	0.967217841
Iqcd	-0.318198881	0.370400703	-0.859066622	0.390303761	0.933734231
Rita1	0.596773716	0.21393815	2.789468431	0.005279464	0.406593461
Ddx54	-0.118757524	0.256098524	-0.463718113	0.642849718	0.978681333
Ccdc42b	-0.509998787	0.700152953	-0.728410535	0.466362316	0.953083512

Dtx1	0.74205411	0.561301818	1.322023349	0.186160376	0.872305715
Oas2	-0.043554034	0.503879485	-0.086437403	0.931118715	0.994688009
Oas3	-1.159538465	1.336116397	-0.867842403	0.385480613	0.931854008
Oas1c	0.087058913	0.230535241	0.3776382	0.705699383	0.988585656
Oas1b	-0.056015437	0.310954938	-0.180140047	0.857042624	0.992886758
Oas1g	0.483242649	0.461167195	1.047868656	0.294699125	0.907334416
Oas1a	0.415931708	0.219608125	1.893972307	0.058228686	0.72285319
Ptpn11	0.010609907	0.142601304	0.074402595	0.940690044	0.994688009
Rpl6	0.06663173	0.132151421	0.504207441	0.614115612	0.974001549
Gm15800	0.201621123	0.326609585	0.617315388	0.537026719	0.96674935
Trafd1	-0.024880521	0.103270739	-0.240925178	0.809613108	0.991295569
Naa25	-0.414447658	0.215007032	-1.927600485	0.053904839	0.707153766
Erp29	0.15821586	0.122472755	1.291845363	0.196410702	0.879755417
Tmem116	-0.092878619	0.20407559	-0.455118709	0.649023839	0.979004493
Adam1a	-0.095026932	0.503711763	-0.188653391	0.850364484	0.992886758
Mapkapk5	-0.026818151	0.122491786	-0.21893836	0.826698064	0.992886758
Aldh2	-0.067015461	0.152268678	-0.440113242	0.659855092	0.979924124
Acad12	0.007899533	0.230935114	0.034206722	0.972712306	0.996905197
Acad10	0.235968813	0.298565203	0.790342647	0.429327687	0.945321484
Brap	0.021414	0.103521731	0.206855115	0.836123012	0.992886758
Atxn2	-0.234306114	0.262264426	-0.893396477	0.371644889	0.928840865
Sh2b3	0.107434055	0.231018841	0.465044559	0.641899547	0.97813956
Fam109a	0.097764546	0.106250907	0.920129053	0.357505324	0.924354588
Cux2	-0.07793151	0.836008579	-0.093218553	0.925729935	0.994688009
Myl2	1.521260258	1.680312128	0.905343854	0.36528325	0.926556975
Ccdc63	1.192277293	2.195300398	0.543104394	0.587057927	0.973286675
Ppp1cc	0.062644507	0.128814443	0.486315864	0.626743233	0.976601842
Hvcn1	-0.172260877	0.282823128	-0.609076344	0.542473837	0.967491297
Tctn1	-0.07990786	0.282176322	-0.283184145	0.777035682	0.990861469
Pptc7	-0.044074616	0.138874844	-0.317369328	0.750963378	0.988585656
Rad9b	0.332218064	0.633150142	0.524706609	0.599787162	0.974001549
Vps29	0.219756785	0.188425583	1.166278915	0.243501695	0.896939594
Fam216a	0.147429685	0.145071451	1.016255674	0.309507651	0.9139204
Gpn3	0.237513363	0.154956476	1.532774683	0.12533138	0.822401003
Arpc3	0.168440203	0.13545327	1.243529992	0.213672596	0.884945675
Anapc7	-0.067203483	0.155188995	-0.43304284	0.664983647	0.981727712
Atp2a2	-0.349866948	0.186242973	-1.878551135	0.060305809	0.725221283
Ift81	-0.134244499	0.131796756	-1.018572105	0.308406153	0.9139204
P2rx7	0.014249419	0.251902949	0.0565671	0.954890043	0.996070133
P2rx4	-0.033057174	0.093184553	-0.354749504	0.722777261	0.988585656
Camkk2	0.230986137	0.174025936	1.327308686	0.184406592	0.871716376
Anapc5	9.84E-05	0.115637419	0.000850876	0.999321099	0.999886097
Rnf34	0.109159064	0.138274399	0.789437992	0.429856058	0.945321484
Kdm2b	0.09572871	0.231399018	0.413695404	0.679097194	0.983903958
A930024E05Rik	0.399402855	0.464251015	0.8603166	0.389614545	0.933734231
Orai1	-0.154603796	0.221173396	-0.699016245	0.484541879	0.957710647
Morn3	-0.119135222	0.249002016	-0.478450834	0.63232936	0.977034429
Tmem120b	0.265836186	0.227382491	1.169114581	0.24235747	0.896939594
Rhof	-0.171943831	0.388496529	-0.442587817	0.658063898	0.979614766
Setd1b	0.059205207	0.408230682	0.145028802	0.884688132	0.992886758
Psmd9	0.292371015	0.084663789	3.453318343	0.000553735	0.164378785
Bcl7a	-0.108959394	0.225285506	-0.483650264	0.628634096	0.977034429
Mlxip	-0.293268533	0.240613612	-1.218836004	0.222906443	0.892391445
Lrrc43	-0.222137986	0.348095475	-0.638152466	0.523374436	0.965637248
B3gnt4	-0.158916444	0.438582488	-0.362341062	0.717097177	0.988585656
Diablo	-0.083722875	0.12227963	-0.684683747	0.493543507	0.958916246
Vps33a	0.050129419	0.151865529	0.330090833	0.741331329	0.988585656
Clip1	-0.214212396	0.161911749	-1.323019467	0.185828904	0.872305715
Zcchc8	-0.173335303	0.141376527	-1.226054336	0.220178228	0.889044644
Rsrc2	-0.214895667	0.134901112	-1.592986629	0.111163191	0.800468873
Kntc1	1.06617004	0.466307345	2.286410564	0.022230251	0.618422027
Hcar2	0.038492873	0.257342316	0.149578481	0.881097187	0.992886758

Hcar1	0.250320853	1.115445954	0.224413251	0.822435761	0.992886758
Denr	0.183743872	0.10496997	1.750442269	0.080042028	0.75659671
Ccdc62	-0.094201412	0.349222426	-0.269746171	0.787355537	0.990988839
Hip1r	0.062499574	0.130564732	0.478686494	0.632161675	0.977034429
Vps37b	0.006611043	0.216043735	0.030600485	0.975588156	0.997262526
Abcb9	-0.286794765	0.508614091	-0.56387499	0.572839212	0.971613657
Ogfod2	0.01640709	0.14995901	0.109410502	0.912876905	0.994688009
Arl6ip4	0.104055921	0.094105243	1.105739889	0.268839119	0.89804647
Pitpnm2	0.060637819	0.238511764	0.254234079	0.799314724	0.990988839
Mphosph9	-0.273910078	0.327937681	-0.835250394	0.403576741	0.937834293
2810006K23Rik	0.383162087	0.203760758	1.880450831	0.060046661	0.725221283
Cdk2ap1	-0.25328495	0.135653323	-1.867148887	0.061880799	0.725730283
Sbno1	-0.10880144	0.157419884	-0.691154361	0.489468541	0.958560421
Setd8	0.150044908	0.147115485	1.019912402	0.307770007	0.9139204
Rilpl2	0.093573773	0.145848359	0.641582626	0.521144217	0.964784335
Snrnp35	0.04316607	0.258253119	0.167146365	0.867254885	0.992886758
Rilpl1	-0.076361766	0.166869747	-0.457613002	0.647230501	0.979004493
Tmed2	0.124285484	0.17092933	0.727116195	0.467154779	0.953239786
Ddx55	-0.080190347	0.20430234	-0.392508215	0.694682743	0.987013595
Eif2b1	-0.013463176	0.154548233	-0.087113104	0.930581609	0.994688009
Gtf2h3	0.032288993	0.148009379	0.218155046	0.827308309	0.992886758
Tctn2	0.189485008	0.179912963	1.053203752	0.292247619	0.907334416
Atp6v0a2	-0.138384507	0.140454544	-0.985261869	0.324495458	0.91640698
Dnah10	-0.058152022	0.260941426	-0.222854697	0.823648596	0.992886758
Ccdc92	0.158980201	0.304047367	0.522879715	0.601057962	0.974001549
Zfp664	-0.112733944	0.123598972	-0.912094516	0.361718965	0.926455466
Fam101a	0.241956449	0.338907297	0.713931068	0.475269805	0.956761005
Ncor2	0.394225257	0.207517834	1.899717483	0.057470205	0.721735927
Scarb1	-0.25329768	0.168874639	-1.499915448	0.133636306	0.827800491
Ubc	-0.173062707	0.198592529	-0.871446211	0.383510563	0.931591679
Dhx37	-0.179076415	0.346037547	-0.517505734	0.604803167	0.974001549
Bri3bp	0.237818995	0.186142416	1.277618503	0.201383973	0.881220006
Aacs	-0.188219173	0.176496763	-1.066417133	0.28623512	0.905916508
Tmem132c	0.020592541	0.220772158	0.093275083	0.925685026	0.994688009
Slc15a4	-0.005501345	0.153911221	-0.035743623	0.971486787	0.996733101
Glt1d1	-0.758082991	1.509362593	-0.502253729	0.615489053	0.974001549
Tmem132d	0.139091754	0.456741531	0.30453056	0.760723714	0.989266039
5930412G12Rik	-0.138763882	0.915805465	-0.151521134	0.879564641	0.992886758
Stx2	0.074257407	0.244321108	0.303933653	0.761178438	0.989266039
Ran	0.146808705	0.185776465	0.790243829	0.429385384	0.945321484
Adgrd1	-0.047395072	0.354293138	-0.133773609	0.893581597	0.993422442
Sfswap	0.108168328	0.197819161	0.546804096	0.584513328	0.97242196
Mmp17	-0.40713633	0.546629137	-0.744812712	0.456384949	0.951472839
Zfp11	-0.166900361	0.418463548	-0.398840858	0.690010469	0.986002801
Mrps17	-0.022832271	0.141974034	-0.160820048	0.872235135	0.992886758
Gbas	-0.017764723	0.080904881	-0.219575415	0.82620184	0.992886758
Psph	0.07931426	0.17699613	0.448112961	0.654071674	0.979133069
Cct6a	-0.064821105	0.112634246	-0.575500853	0.564952614	0.969658675
Sumf2	-0.016588045	0.164132514	-0.101064955	0.919498897	0.994688009
Phkg1	-0.370711206	0.522671884	-0.709261809	0.478162024	0.957438234
Chchd2	0.181903542	0.155945144	1.166458517	0.243429111	0.896939594
Zbed5	0.338964051	0.394857552	0.858446416	0.390646007	0.933734231
Nupr1l	0.329766077	0.324430381	1.01644635	0.309416883	0.9139204
Vkorc1l1	-0.043821473	0.143503219	-0.305369271	0.760084923	0.989266039
Gusb	0.023686651	0.122002988	0.194148123	0.846059902	0.992886758
Asl	0.139012017	0.155702284	0.892806538	0.371960784	0.928840865
Crcp	-0.081423276	0.13721155	-0.593414158	0.552904016	0.968926429
Tpst1	0.078337043	0.144022349	0.543922821	0.586494584	0.972904784
Kctd7	0.587400825	0.336818318	1.743969354	0.081164434	0.760377276
Rabgef1	-0.19645335	0.13076819	-1.502302284	0.133019059	0.827800491
Tmem248	0.088032056	0.139100892	0.632864782	0.526821948	0.965637248
Sbds	0.023949758	0.080747565	0.296600372	0.766771627	0.989884648

Tyw1	-0.069589032	0.276964213	-0.251256404	0.801615879	0.990988839
Wbscr17	0.708723449	0.935230094	0.757806505	0.448566827	0.948476747
Auts2	-0.294427551	0.767954925	-0.383391709	0.70142935	0.988482081
Gatsl2	0.476918356	0.530858541	0.898390662	0.368977313	0.928436166
Wbscr16	0.127317614	0.143177588	0.889228649	0.373880209	0.928840865
Gtf2ird2	-0.248920354	0.347499784	-0.716318011	0.473795008	0.955974667
Ncf1	-0.336922486	0.194393438	-1.733198869	0.083060328	0.76099108
Gtf2i	0.070124034	0.115973299	0.604656718	0.545407112	0.967491297
Gtf2ird1	-0.335460389	0.29557061	-1.134958543	0.256392689	0.897248195
Clip2	-0.274683715	0.252079053	-1.089672908	0.275857254	0.900302631
Syna	0.36026186	1.286430796	0.280047602	0.779440984	0.990988839
Rfc2	0.056634151	0.122769431	0.461304987	0.644579807	0.97889195
Lat2	0.324206341	0.195039679	1.662258375	0.09646098	0.781321691
Eif4h	-0.152067042	0.130538003	-1.16492545	0.244049171	0.896939594
Limk1	-0.347702956	0.271750566	-1.279493033	0.200723492	0.880971088
Eln	0.520536148	0.190378353	2.734219203	0.00625284	0.427133716
Wbscr27	0.152691513	0.300510925	0.508106361	0.611378755	0.974001549
Cldn4	-0.03801979	0.223512801	-0.170101175	0.86493057	0.992886758
Cldn3	-0.280475337	0.112018662	-2.503826878	0.012285813	0.525888996
Wbscr25	-2.14291599	2.166143024	-0.989277239	0.322527517	0.91640698
Abhd11	0.045682256	0.159261552	0.286837942	0.77423641	0.990730604
Abhd11os	0.331993852	0.478514038	0.693801698	0.487806578	0.958288569
Stx1a	-0.140030256	0.392593261	-0.356680232	0.721331206	0.988585656
Wbscr22	0.043491338	0.128478117	0.338511637	0.734977659	0.988585656
Dnajc30	0.260518386	0.208118103	1.251781472	0.210649503	0.884340413
Vps37d	-0.499720813	0.614391512	-0.813358915	0.416012308	0.943415794
Mlxipl	-0.315035127	0.411415087	-0.765735474	0.443833715	0.948476747
Tbl2	-0.218760718	0.29404145	-0.743979184	0.456889069	0.951742083
Bcl7b	0.153779475	0.099024641	1.552941506	0.12043711	0.81558552
Baz1b	-0.058685807	0.144626301	-0.405775484	0.684907574	0.985105247
Nsun5	0.184539791	0.241227611	0.765002771	0.444269895	0.948476747
Pom121	-0.181407062	0.415455629	-0.436646056	0.662368057	0.980647283
Hip1	-0.122377867	0.189799976	-0.644772825	0.519074415	0.96446859
Rhbdd2	-0.246681803	0.186279772	-1.324254375	0.185418578	0.871716376
Por	-0.232998687	0.11768784	-1.979802562	0.047725718	0.701952043
Tmem120a	0.031875385	0.165542819	0.192550695	0.847310862	0.992886758
Styxl1	0.09203878	0.24732304	0.372139936	0.709788663	0.988585656
Mdh2	-0.096644141	0.105016689	-0.920274121	0.357429529	0.924354588
Hspb1	-0.354767601	0.17602411	-2.015448913	0.043857634	0.688477147
Ywhag	-0.231564128	0.159412127	-1.452613002	0.146331234	0.843780493
Ssc4d	0.130887253	0.564767191	0.23175435	0.816728815	0.992678823
Zp3	0.036873674	1.380757984	0.026705385	0.978694718	0.997262526
Dtx2	-0.167373098	0.2040279	-0.820344169	0.412019934	0.942121471
Upk3b	-0.112214088	0.291031163	-0.385574132	0.699812095	0.988393777
Upk3bl	-1.057298086	1.942041366	-0.544426141	0.586148261	0.972904784
Rasa4	-0.133215243	0.30171014	-0.44153386	0.658826554	0.979924124
Polr2j	0.343286919	0.166466798	2.062194523	0.03918922	0.679118604
Lrwd1	0.007874302	0.195646962	0.040247505	0.967895805	0.996474415
Alkbh4	-0.022247511	0.254273978	-0.087494249	0.930278657	0.994688009
Orai2	-0.148505715	0.237556123	-0.625139499	0.531879506	0.966606934
Prkrip1	0.234258736	0.145942568	1.605143299	0.108462272	0.798150849
Sh2b2	-0.255317338	0.622297853	-0.410281567	0.681599412	0.984683806
Cux1	-0.321936546	0.217282647	-1.481648679	0.138433799	0.834856909
Col26a1	-0.075017873	0.697604818	-0.107536346	0.914363493	0.994688009
Ift22	0.235432386	0.135638345	1.735736212	0.082610487	0.76099108
4933404O12Rik	-0.109431247	0.692747734	-0.15796695	0.874482842	0.992886758
Fis1	0.232942109	0.152214313	1.530356146	0.125928598	0.822401003
Cldn15	-0.099789167	0.208262476	-0.479150968	0.631831233	0.977034429
Znhit1	0.188206571	0.169005854	1.113609777	0.26544663	0.89768067
Plod3	-0.204031311	0.237571943	-0.858819054	0.390440354	0.933734231
Ap1s1	0.193748224	0.109311207	1.77244612	0.076320532	0.752876039
Serpine1	0.089133597	0.340098656	0.262081591	0.793258539	0.990988839

Trim56	-0.025205268	0.553544647	-0.045534301	0.963681435	0.996474415
Ache	-0.160315957	1.075204866	-0.149102708	0.881472589	0.992886758
Ufsp1	-0.43806279	0.647506489	-0.67653807	0.498699084	0.960700483
Srrt	-0.100748963	0.109762542	-0.917881097	0.358681119	0.925178192
Trip6	0.117060399	0.130656812	0.895937972	0.370285892	0.928436166
Slc12a9	-0.26038476	0.357792123	-0.727754313	0.466763996	0.953083512
Ephb4	0.133350966	0.106099981	1.256842502	0.208810672	0.884258007
Zan	0.008360016	2.098343415	0.003984103	0.996821154	0.999449398
Pop7	0.167256531	0.155927806	1.072653654	0.283426542	0.905916508
Gigyf1	-0.271053796	0.240058786	-1.129114249	0.258849641	0.897248195
Gnb2	0.019627301	0.113529729	0.172882481	0.862743802	0.992886758
Tfr2	0.281178134	0.590686127	0.476019533	0.634060462	0.977034429
Mospd3	-0.049382485	0.272922748	-0.180939426	0.856415122	0.992886758
Pcolce	-0.127587567	0.167390498	-0.762215111	0.445931629	0.948476747
Fbxo24	0.947877505	0.531945899	1.781905844	0.074764587	0.75241101
Gm20605	-1.457405599	1.10821479	-1.315093077	0.188478623	0.874826736
Irs3	-0.611366873	0.559637161	-1.092434376	0.27464222	0.900302631
Agfg2	-0.2596516	0.181159981	-1.433272395	0.151780008	0.852463772
Nyap1	-0.268174243	0.733917219	-0.365401214	0.714811935	0.988585656
Tsc22d4	-0.176185586	0.181246922	-0.972074912	0.331013282	0.917115058
Ppp1r35	0.2601135	0.180390033	1.44195051	0.149316336	0.848779008
Mepce	-0.104185377	0.176752795	-0.589441186	0.555565355	0.968926429
Zcwpw1	0.017537241	0.715600749	0.024507019	0.980448185	0.997296835
Pilra	-0.059309584	0.241410745	-0.245679139	0.805930622	0.99099448
Pilrb1	0.646178742	0.380133354	1.699873836	0.089154659	0.768297719
Pilrb2	-0.192069349	0.405186504	-0.474027015	0.635480643	0.977034429
Cyp3a13	-0.590441603	0.271739804	-2.172819717	0.029793889	0.649459041
Azgp1	-0.003395972	0.202967496	-0.016731607	0.986650732	0.998148029
Zkscan1	-0.086256213	0.160147566	-0.538604581	0.590159726	0.974001549
Zscan21	0.115074887	0.151178297	0.761186555	0.446545647	0.948476747
Zfp113	-0.001928858	0.211708159	-0.009110929	0.992730631	0.998971527
Cops6	0.260514493	0.115246064	2.260506642	0.023789825	0.622011301
Mcm7	0.140028939	0.120405701	1.162975982	0.244839247	0.896939594
Ap4m1	0.003798653	0.225329572	0.016858207	0.986549734	0.998148029
Taf6	-0.033396871	0.136079654	-0.24542149	0.806130091	0.99099448
Cnpy4	0.04819958	0.222535868	0.21659241	0.828526001	0.992886758
Mblac1	0.482638139	0.276179009	1.747555469	0.080541032	0.758011246
Nxpe5	-0.364023633	1.007281007	-0.361392333	0.717806179	0.988585656
Lamtor4	0.322542747	0.136412746	2.364461953	0.018056282	0.58167504
BC037034	0.08578194	0.220980651	0.388187562	0.697877237	0.9876159
6330418K02Rik	-0.093038457	0.826250934	-0.112603149	0.910345188	0.994688009
Gal3st4	-0.219814632	0.875291668	-0.251133011	0.801711275	0.990988839
Gpc2	-0.366840906	0.858921983	-0.42709456	0.669310455	0.981863224
Zfp157	0.304799206	0.199829501	1.525296338	0.127185201	0.824450458
Zfp68	0.135564224	0.141695679	0.956728002	0.33870456	0.919246575
A430033K04Rik	-0.601789377	0.330614775	-1.820213198	0.068726544	0.743426713
Fam20c	-0.180958249	0.377813268	-0.478962133	0.631965568	0.977034429
Pdgfa	-0.136241536	0.142464026	-0.956322382	0.338909386	0.919246575
Prkar1b	-0.35066614	0.18474789	-1.898079273	0.05768564	0.72285319
Dnaaf5	0.237608435	0.323798808	0.733815038	0.463061474	0.953083512
Sun1	-0.071888276	0.127884165	-0.562135867	0.574023453	0.971613657
Get4	0.16266317	0.13350119	1.218439853	0.223056868	0.892391445
Adap1	0.097865605	0.265447395	0.368681731	0.712364967	0.988585656
Cox19	0.0804698	0.178984771	0.449590207	0.653005951	0.979004493
3110082I17Rik	0.552289256	0.159339431	3.466117916	0.000528032	0.159651346
Mir339	-0.425303325	1.242236358	-0.342369084	0.732073151	0.988585656
D830046C22Rik	-0.241007045	0.29865022	-0.80698767	0.419673587	0.943415794
Gpr146	-0.192307681	0.130151553	-1.477567318	0.139523627	0.836535783
C130050O18Rik	0.18498691	0.427468639	0.432749664	0.665196646	0.981727712
Gper1	0.913256079	0.679252735	1.344501143	0.178786387	0.867377413
Zfand2a	-0.036281481	0.1301744	-0.278714407	0.780464006	0.990988839
Micall2	-0.077126425	0.393826943	-0.195838365	0.84473668	0.992886758

Ints1	0.403097371	0.325442418	1.238613497	0.215488667	0.887633697
Mafk	-0.052598529	0.127954964	-0.411070644	0.681020735	0.984420145
Tmem184a	-0.304188283	0.208536449	-1.458681611	0.14465276	0.841977044
Psmg3	0.339590903	0.161463076	2.103210902	0.035447338	0.674795441
Elfn1	-0.460294214	0.327374544	-1.406017121	0.159719041	0.856679626
Mad1l1	-0.032215291	0.282092612	-0.114201115	0.909078368	0.994688009
Ftsj2	0.249233329	0.14404339	1.730265638	0.083582826	0.76099108
Nudt1	0.210834727	0.209519252	1.006278537	0.314281602	0.91586731
Snx8	0.082875062	0.170632935	0.485692062	0.627185512	0.976752533
Eif3b	-0.028151625	0.118791509	-0.236983475	0.81266961	0.992000477
Chst12	-0.338565807	0.201661148	-1.678884655	0.093174526	0.775558829
Lfng	-0.120576661	0.124329111	-0.969818409	0.332137015	0.917115058
Ttyh3	-0.306730681	0.301244514	-1.018211673	0.308577373	0.9139204
Iqce	-0.224728323	0.21543905	-1.043117866	0.296893707	0.909070179
Brat1	-0.435699295	0.273045996	-1.595699268	0.110555952	0.800111791
Amz1	0.25369752	0.18138386	1.398677478	0.161909721	0.858667769
Gna12	-0.27825913	0.221576683	-1.255814129	0.209183368	0.884258007
Card11	0.454772575	0.288057307	1.578757294	0.114391738	0.809025248
Sdk1	1.309153829	1.124397665	1.164315677	0.244296106	0.896939594
Foxk1	0.030112065	0.336786415	0.089409974	0.928756097	0.994688009
Ap5z1	0.148277967	0.240432782	0.616712771	0.537424196	0.96674935
Radil	-0.081048087	0.40325274	-0.200985831	0.840709653	0.992886758
Papolb	2.472334332	1.681919838	1.46994778	0.141575898	0.840741274
Wipi2	0.120325322	0.11441047	1.05169852	0.29293789	0.907334416
Slc29a4	-0.16778567	1.747187459	-0.096031865	0.923495265	0.994688009
Tnrc18	0.187505902	0.284669127	0.658680147	0.510101184	0.963452765
Cngb1	-0.697550745	1.240180469	-0.562459063	0.573803288	0.971613657
Fbxl18	-0.246956872	0.992156976	-0.248909072	0.803431118	0.990988839
Actb	0.089346679	0.1294595	0.690151584	0.490098867	0.958560421
Fscn1	-0.1809322	0.465309739	-0.388842496	0.697392663	0.987548136
Rnf216	-0.106879463	0.193468198	-0.552439439	0.580647316	0.971613657
Rbakdn	0.520269923	1.002447431	0.518999707	0.603760946	0.974001549
Rbak	0.08358598	0.184561344	0.452889962	0.65062798	0.979004493
Zfp12	0.041609151	0.211153212	0.197056678	0.843783184	0.992886758
Zfp316	-0.473997241	0.425614086	-1.113678462	0.265417152	0.89768067
E130309D02Rik	0.039638075	0.223770807	0.177136937	0.859400829	0.992886758
Zdhhc4	-0.048964592	0.14831845	-0.330131497	0.741300605	0.988585656
0610040B10Rik	0.321944586	0.385103332	0.835995327	0.403157532	0.937834293
Kdelr2	-0.13552884	0.099133066	-1.367140615	0.171581235	0.86244803
Daglb	-0.319939709	0.241153545	-1.326705395	0.184606156	0.871716376
Rac1	-0.239252964	0.090940654	-2.630869184	0.008516681	0.474579703
Fam220a	0.461925488	0.1719613	2.6862177	0.007226599	0.452031612
Cyth3	-0.203440248	0.106913665	-1.902846071	0.057060634	0.721735927
Usp42	0.432554997	0.299453433	1.444481677	0.14860353	0.84832848
D130017N08Rik	0.982724127	0.987461791	0.99520218	0.319637942	0.91640698
Eif2ak1	-0.309290735	0.253646813	-1.219375599	0.222701667	0.892391445
Aimp2	0.141543037	0.178165322	0.794447736	0.426934823	0.944290206
Pms2	0.216696741	0.180606546	1.199827723	0.230206255	0.895404344
Ccz1	0.01882688	0.126338206	0.149019684	0.881538101	0.992886758
Ocm	-0.065957372	1.146400913	-0.057534298	0.954119585	0.996070133
Lmtk2	-0.216541541	0.233682849	-0.926647129	0.354109777	0.924354588
Bhlha15	-0.610254758	0.957176716	-0.637557044	0.523762066	0.965637248
Tecpr1	-0.091917106	0.234952378	-0.391215899	0.695637657	0.98715859
Bri3	0.124580004	0.232942181	0.534810844	0.592780664	0.974001549
Baiap2l1	-0.210817866	0.219986692	-0.958321	0.337900918	0.919246575
Gm15708	0.641674103	1.057565593	0.606746387	0.544019233	0.967491297
Nptx2	-0.361621539	1.608426937	-0.224829323	0.822112054	0.992886758
Trrap	0.303475271	0.206579717	1.469046795	0.141820097	0.840741274
Smurf1	-0.026202834	0.21477564	-0.122000958	0.90289826	0.994688009
1700018F24Rik	0.612870738	2.012604704	0.304516201	0.760734652	0.989266039
Arpc1a	0.075601956	0.13519859	0.559191893	0.576030766	0.971613657
Arpc1b	0.217747405	0.094261065	2.310046111	0.020885601	0.604325792

Pdap1	0.129598215	0.078949914	1.64152446	0.100688594	0.788783372
Bud31	0.244405311	0.16718163	1.461914869	0.14376454	0.841934045
Ptcd1	0.230653247	0.28404412	0.812033168	0.4167726	0.943415794
Cpsf4	0.056781918	0.219932288	0.258179092	0.796268693	0.990988839
Atp5j2	0.269600813	0.192450954	1.400880626	0.161249773	0.857316691
Zkscan14	-0.148739601	0.155849048	-0.95438248	0.339890071	0.919246575
Zkscan5	0.019873181	0.23472589	0.084665485	0.932527336	0.994688009
Zfp655	0.015728102	0.120938163	0.13005078	0.896526251	0.994101834
Zscan25	-0.111402705	0.28450511	-0.391566622	0.695378455	0.98715859
Cyp3a57	0.745302386	1.235644664	0.603168862	0.54639636	0.96778188
1700001J03Rik	0.019591859	1.532198773	0.012786761	0.989797919	0.998417328
Rnf6	-0.30534944	0.180036088	-1.696045736	0.089877219	0.769347579
Cdk8	0.105549908	0.184648498	0.571626139	0.567575283	0.970955746
Wasf3	0.109203764	0.407780741	0.267800201	0.788853116	0.990988839
Usp12	-0.024331931	0.137342468	-0.177162474	0.859380771	0.992886758
Rpl21	0.050834972	0.110138658	0.461554307	0.644400968	0.97889195
Rasl11a	0.158635096	0.167055909	0.949592846	0.342319175	0.919850248
Gtf3a	0.118734774	0.193032156	0.615103599	0.538486306	0.966776546
Mtif3	0.197022974	0.148028331	1.330981531	0.183195088	0.869576666
Lnx2	-0.192033625	0.294522928	-0.652015876	0.514390931	0.964352198
Polr1d	0.200389196	0.172310209	1.16295603	0.244847343	0.896939594
Flt3	0.106411093	0.427995388	0.248626729	0.803649531	0.990988839
D5Ertd605e	1.318482365	1.56841237	0.840647772	0.400545287	0.937054436
Pan3	-0.084864397	0.16975977	-0.499908763	0.617139322	0.974001549
Flt1	0.107932359	0.207454984	0.520268815	0.602876228	0.974001549
Pomp	0.063039547	0.141436038	0.445710639	0.655806278	0.979416887
Slc46a3	-0.483450373	0.217623752	-2.221496364	0.026317361	0.632301593
Mtus2	-0.68580715	0.384349683	-1.78433125	0.074369855	0.751093915
Slc7a1	-0.148109069	0.421849763	-0.351094353	0.725517564	0.988585656
Ubl3	0.074339183	0.124770287	0.595808381	0.551303244	0.968926429
2210417A02Rik	0.17577124	1.790974277	0.098142806	0.921818898	0.994688009
Katnal1	-0.139442259	0.299676235	-0.465309699	0.64170969	0.97813956
5930430L01Rik	0.434295511	0.287120061	1.512592013	0.130383324	0.827800491
Gm15408	0.540183656	0.44729836	1.207658476	0.227178656	0.894038302
Hmgb1	0.124058645	0.073235832	1.693961032	0.090272688	0.769347579
5730422E09Rik	-1.025115992	1.355345121	-0.756350523	0.449439064	0.948476747
Uspl1	-0.112407765	0.163578632	-0.687178782	0.491970074	0.958916246
Alox5ap	0.250112768	0.119012896	2.101560216	0.035591818	0.674795441
Medag	-0.124979413	0.162294001	-0.7700803	0.441252261	0.948068187
Tex26	-0.202856779	0.322513083	-0.628987753	0.529357074	0.965637248
Gm15997	2.322288562	0.951862253	2.439731752	0.014698172	0.554936045
Wdr95	-0.561608072	0.426451006	-1.316934571	0.187860557	0.874351146
Hsph1	-0.694116923	0.204752765	-3.39002466	0.000698863	0.19339563
B3glct	-0.3505692	0.253790042	-1.381335522	0.167175821	0.860403806
Rxfp2	0.897982468	0.473843901	1.89510188	0.058078906	0.72285319
Fry	0.465559626	0.284907617	1.634072233	0.102243684	0.788963633
Brca2	0.46988875	0.647681118	0.725493977	0.468149038	0.953657709
N4bp2l1	0.085347223	0.181856321	0.469311279	0.638847154	0.977795535
N4bp2l2	0.067623064	0.103903173	0.650827711	0.51515771	0.964352198
Pds5b	-0.143963222	0.136329148	-1.055997375	0.290969414	0.907334416
Kl	-0.723497375	1.749561523	-0.413530685	0.679217846	0.983903958
Stard13	0.084452844	0.152982164	0.552043725	0.580918396	0.971613657
1700028E10Rik	1.186354998	1.077428057	1.101099039	0.270853555	0.89804647
Gm3704	0.28255978	0.87991252	0.321122582	0.748117498	0.988585656
Rfc3	0.147938731	0.172517924	0.857526727	0.39115385	0.933734231
Samd9l	-0.076765408	0.114885255	-0.668191999	0.504011042	0.96179897
Hepacam2	-0.445969119	0.532791182	-0.83704298	0.40256841	0.937834293
Ccdc132	-0.200731084	0.173268247	-1.158498961	0.246660475	0.896939594
Tfpi2	-0.568170292	0.353424892	-1.607612552	0.107920061	0.796929372
Gng11	0.467714506	0.187410486	2.495668814	0.012571992	0.525888996
Bet1	0.284825901	0.172532661	1.650852079	0.098768783	0.786907305
Col1a2	-0.017102221	0.192528045	-0.088829764	0.929217203	0.994688009

Casd1	-0.274098369	0.139332073	-1.967230975	0.049156589	0.707153766
Sgce	-0.053385531	0.103281075	-0.516895577	0.605229056	0.974001549
Peg10	-0.683022846	0.56922489	-1.199917394	0.230171424	0.895404344
Ppp1r9a	-0.169549943	0.175378856	-0.966763879	0.333662085	0.917554801
Pon1	0.1852836	0.267473672	0.692717151	0.488487074	0.958481967
Pon3	-0.141754323	0.127799502	-1.109193084	0.267346896	0.89804647
Pon2	-0.023789806	0.094211926	-0.252513742	0.800643994	0.990988839
Asb4	0.374493916	1.320777702	0.283540459	0.776762572	0.990861469
Pdk4	0.302244191	0.293233901	1.030727311	0.302668714	0.911518274
Dync1i1	-1.086336285	1.328716967	-0.81758291	0.413595389	0.94325631
1700019G24Rik	0.496595126	1.694704755	0.293027517	0.769501119	0.989990452
Slc25a13	-0.233344778	0.219899093	-1.061144796	0.288624103	0.907334416
Shfm1	0.409635426	0.125241443	3.270765773	0.001072567	0.240510037
Sdhaf3	0.160597694	0.23689347	0.677932124	0.497814731	0.960700483
Asns	-0.017721419	0.243948084	-0.072644223	0.942089235	0.994688009
C1galt1	-0.639283295	0.203246751	-3.145355541	0.001658851	0.284736028
Col28a1	-0.178026909	1.330511445	-0.133803366	0.893558066	0.993422442
Mios	-0.442233592	0.187891524	-2.353664408	0.018589385	0.58705782
Rpa3	0.405820709	0.214272965	1.89394266	0.058232621	0.72285319
Umad1	0.018181454	0.14839934	0.122517084	0.902489517	0.994688009
A430035B10Rik	0.088867193	0.21400452	0.415258489	0.677952686	0.983670411
Glcci1	-0.332018592	0.269419647	-1.232347367	0.217819353	0.887633697
Ica1	-0.268951184	0.179756592	-1.496196498	0.134602457	0.828680121
Nxph1	-1.106380321	0.435393236	-2.541105901	0.011050244	0.5053707
Ndufa4	0.195990004	0.197495668	0.992376215	0.321014037	0.91640698
Phf14	0.020968753	0.150017215	0.139775643	0.888837258	0.992955588
Thsd7a	0.075743895	0.700182433	0.108177372	0.913854994	0.994688009
Tmem106b	-0.242861134	0.092411371	-2.628043847	0.008587745	0.475600561
Tmem168	0.067107951	0.135264442	0.496124109	0.61980685	0.974471036
B630005N14Rik	0.016066081	0.117266785	0.137004533	0.891027212	0.993043931
Gpr85	-0.208139151	0.924001207	-0.225258527	0.821778161	0.992886758
2610001J05Rik	0.124496355	0.095858161	1.298755929	0.194027704	0.879588734
1110019D14Rik	-0.09085943	0.206814084	-0.439329025	0.660423146	0.979924124
Ppp1r3a	-0.801552412	0.414487389	-1.93384029	0.053132767	0.707153766
Foxp2	-0.068752293	0.343168907	-0.200345344	0.841210502	0.992886758
Mdfic	-0.002614624	0.095590378	-0.027352377	0.978178682	0.997262526
Tfec	-0.265055438	0.260785583	-1.016373045	0.309451776	0.9139204
Tes	0.020187133	0.15040383	0.13421954	0.893228975	0.993341156
D830026I12Rik	0.16901247	0.26606495	0.635230118	0.525278343	0.965637248
Cav2	0.041879554	0.152277757	0.275020824	0.78330024	0.990988839
Cav1	-0.239398607	0.146840672	-1.630329003	0.103031981	0.79028905
Met	-0.117324844	0.143951434	-0.815030743	0.415054713	0.943415794
Capza2	0.055753629	0.102867722	0.541993431	0.587823031	0.973455044
St7	-0.030822306	0.122506159	-0.251598013	0.801351795	0.990988839
Wnt2	0.036410963	0.187610856	0.194077059	0.846115544	0.992886758
Asz1	-1.136288706	0.964755884	-1.177799198	0.238876668	0.896609492
Cftr	-0.043782404	0.283501146	-0.154434663	0.877267025	0.992886758
Cttnbp2	-0.177946182	0.468553842	-0.379777447	0.704110625	0.988585656
Lsm8	0.36109389	0.121193516	2.979481932	0.002887363	0.345325017
Kcnd2	0.365999582	1.812169823	0.201967595	0.839942057	0.992886758
Tspan12	0.085415414	0.118205439	0.722601383	0.469924818	0.95486913
Ing3	-0.020897606	0.224103339	-0.093249863	0.925705062	0.994688009
Cped1	-0.198270037	0.1588318	-1.248301894	0.211920521	0.884945675
Wnt16	-0.57072311	1.95511338	-0.29191305	0.770353105	0.989990452
Fam3c	0.215958577	0.147007712	1.469028901	0.14182495	0.840741274
Ptprz1	0.012445224	0.293532469	0.042398119	0.966181328	0.996474415
Aass	-0.172600973	0.206740649	-0.834867133	0.403792522	0.937834293
Cadps2	0.009472307	0.140425109	0.067454507	0.946219877	0.995065399
Iqub	-0.380432365	0.246573716	-1.542874765	0.122861165	0.817788082
Ndufa5	0.416585665	0.175141096	2.378571766	0.017379853	0.579835303
Asb15	0.053858065	1.252511759	0.043000048	0.965701496	0.996474415
Lmod2	0.006617677	0.419909967	0.015759753	0.987426057	0.998148029

Wasl	0.030270524	0.14992156	0.201909078	0.839987805	0.992886758
Gpr37	0.064959095	1.492664444	0.043518887	0.965287909	0.996474415
Pot1a	-0.195527787	0.207967398	-0.940184803	0.347122775	0.921552698
Zfp800	-0.183516277	0.175120635	-1.047942048	0.294665308	0.907334416
Gcc1	-0.216214996	0.216551532	-0.998445933	0.31806317	0.91640698
Arf5	0.029460066	0.141083307	0.208813268	0.834594013	0.992886758
Snd1	-0.109438382	0.166587306	-0.656943102	0.511217503	0.963452765
Lrrc4	-0.825880516	1.048066559	-0.788003881	0.430694436	0.945321484
Lep	-0.29032917	1.941636117	-0.149528105	0.881136934	0.992886758
Rbm28	-0.170953794	0.212939318	-0.80282869	0.422073757	0.943415794
Prrt4	-0.057173327	0.487340054	-0.117317111	0.906608766	0.994688009
Impdh1	0.168790237	0.189478879	0.890812941	0.373029531	0.928840865
Hilpda	0.545228653	0.17337421	3.144808292	0.001661957	0.284736028
Fam71f2	0.403320306	0.8153724	0.494645521	0.620850363	0.97514112
Calu	-0.251420604	0.111590118	-2.253072299	0.024254591	0.624613096
Opn1sw	1.340819669	3.976958515	0.337147009	0.736006081	0.988585656
Ccdc136	-0.275671039	0.653012013	-0.42215309	0.672913279	0.983233856
Flnc	-0.102767626	0.407559843	-0.252153463	0.800922447	0.990988839
Atp6v1f	0.17276619	0.185404446	0.931834127	0.351422255	0.922900298
Gm9047	-0.17945662	2.17583665	-0.082477065	0.934267356	0.994688009
Kcp	-0.445443445	0.83514998	-0.533369401	0.593777895	0.974001549
Irf5	-0.052852874	0.200776036	-0.263242938	0.792363338	0.990988839
Tnpo3	-0.062984518	0.172473587	-0.365183557	0.714974391	0.988585656
Tspan33	0.212465348	0.347135224	0.612053555	0.540502325	0.966989525
Smo	0.264700082	0.145076565	1.824554379	0.068068303	0.742385562
Ahcyl2	-0.199445919	0.202044128	-0.987140389	0.32357382	0.91640698
Strip2	-0.153610221	0.540687886	-0.284101465	0.776332624	0.990861469
Smkr-ps	0.007684659	0.190524417	0.040334248	0.96782665	0.996474415
Nrf1	0.046518471	0.150619701	0.308847187	0.757437772	0.989266039
Ube2h	-0.062098789	0.136301669	-0.455598155	0.64867897	0.979004493
Zc3hc1	-0.098649341	0.164200364	-0.60078637	0.547982283	0.968738549
Klhdc10	-0.272842402	0.218170806	-1.250590796	0.21108381	0.884390587
Tmem209	-0.027813911	0.242088507	-0.114891496	0.908531126	0.994688009
Ssmem1	0.987883356	1.54213988	0.640592575	0.521787425	0.965211168
Cpa1	-1.308935086	2.008172174	-0.651804215	0.514527482	0.964352198
Cep41	-0.234391245	0.225025393	-1.041621308	0.297587286	0.909070179
Mest	0.196103719	0.329511748	0.595134227	0.551753751	0.968926429
Copg2	-0.160734433	0.164514375	-0.977023641	0.328557449	0.917115058
Lincpint	-0.368088261	1.094245205	-0.336385537	0.736580154	0.988585656
2210408F21Rik	0.30219211	0.287003766	1.052920365	0.292377492	0.907334416
Mkln1os	0.450820763	0.459275738	0.981590634	0.326301564	0.916800143
Mkln1	-0.248840009	0.148479333	-1.675923532	0.093753172	0.775558829
Podxl	0.169977443	0.124506103	1.365213743	0.17218588	0.86244803
Plxna4	-0.128445714	0.32048178	-0.400789443	0.688575152	0.98538669
Plxna4os1	0.265685629	0.2792872	0.951298979	0.341452625	0.919850248
Chchd3	0.261698916	0.109755079	2.384390035	0.01710746	0.573539012
Exoc4	-0.284718378	0.199613754	-1.42634649	0.153768351	0.854159937
Lrguk	-0.010585519	0.253124354	-0.041819439	0.966642638	0.996474415
Slc35b4	-0.15975379	0.154691636	-1.032724164	0.301733002	0.911518274
Akr1b8	-0.107971393	0.185494473	-0.582073368	0.560517263	0.968926429
Akr1b10	-0.230781393	0.156551291	-1.474158357	0.140438959	0.838065382
Akr1b7	0.005893874	0.270876158	0.021758557	0.982640553	0.997390438
Bpgm	0.253008228	0.14454055	1.75043078	0.080044009	0.75659671
Cald1	-0.169205692	0.116109568	-1.45729327	0.145035444	0.842729381
Agbl3	0.266029821	0.215258983	1.235859319	0.216510861	0.887633697
Tmem140	0.873260343	0.499135856	1.749544402	0.080196961	0.757302357
3110062M04Rik	0.329402531	0.292530317	1.126045785	0.26014614	0.897248195
Wdr91	0.147683733	0.233438564	0.632644971	0.526965513	0.965637248
Stra8	2.026187978	2.504052436	0.809163558	0.418421073	0.943415794
2010107G12Rik	-0.674371515	1.243114447	-0.542485462	0.58748412	0.973455044
Cnot4	-0.24437508	0.257492182	-0.949058252	0.342590986	0.919850248
Nup205	-0.027799959	0.249408375	-0.111463613	0.911248718	0.994688009

1810058I24Rik	-0.156033037	0.140403925	-1.111315346	0.266432636	0.89768067
Slc13a4	1.577409304	1.142206579	1.381019277	0.167273033	0.860403806
Fam180a	-0.024610418	0.334951619	-0.073474544	0.9414285	0.994688009
Mtpn	-0.085596996	0.109041257	-0.784996414	0.432455671	0.9459793
9330158H04Rik	0.862959947	0.476650619	1.810466434	0.070223485	0.744227652
Chrm2	-0.036962271	0.609827792	-0.060610998	0.951669014	0.996023481
Ptn	0.264239565	0.396567249	0.666317166	0.505208396	0.962138588
Creb3l2	-0.171887145	0.375230084	-0.458084658	0.646891621	0.979004493
Trim24	-0.3748546	0.19508131	-1.921530051	0.054664918	0.710600406
Svopl	-1.483336191	0.992517866	-1.494518378	0.135040182	0.829809681
Atp6v0a4	-0.239735812	0.308938867	-0.775997576	0.437750417	0.94749626
Tmem213	-0.588740905	0.446519685	-1.318510527	0.187332803	0.873455432
D630045J12Rik	0.394702862	0.451522311	0.874160263	0.382030983	0.930680372
Zc3hav1l	0.048200727	0.447476154	0.107716862	0.914220294	0.994688009
Zc3hav1	-0.140907738	0.205461015	-0.685812527	0.492831336	0.958916246
Ttc26	-0.4342996	0.192927332	-2.251104576	0.024378915	0.626649273
Ubn2	-0.086120429	0.260892024	-0.330099894	0.741324483	0.988585656
1110001J03Rik	0.438185566	0.298705775	1.466947083	0.142390451	0.840741274
Luc7l2	-0.104914755	0.089287568	-1.175020856	0.239986372	0.896939594
Klrg2	-0.231509647	0.423021586	-0.547276203	0.584188991	0.972333834
Clec2l	-1.962668008	2.359753079	-0.831726008	0.405563617	0.939557159
Hipk2	-0.234495782	0.434980136	-0.539095381	0.589821043	0.974001549
Tbxas1	-0.411560448	0.293422639	-1.402619954	0.160730198	0.857316691
Parp12	0.009104371	0.119158578	0.076405501	0.939096494	0.994688009
4930599N23Rik	0.577839223	0.778925963	0.74184101	0.45818367	0.952235842
Kdm7a	-0.245032007	0.213604818	-1.147127713	0.251328842	0.896939594
Slc37a3	0.032517929	0.126655847	0.256742423	0.79737762	0.990988839
Rab19	0.036491647	0.202455011	0.180245709	0.856959676	0.992886758
Mkrn1	0.227784528	0.100536872	2.265681456	0.023470894	0.622011301
Dennd2a	-0.324634409	0.191007982	-1.699585563	0.089208908	0.768297719
Adck2	0.019542167	0.153578522	0.127245441	0.898746141	0.994364545
Ndufb2	0.629519853	0.272279862	2.312032365	0.0207759	0.604325792
Braf	-0.351558585	0.209125693	-1.681087483	0.092745923	0.775044718
Mrps33	0.315149874	0.174997696	1.800880132	0.071721775	0.744227652
Agk	-0.092500868	0.239299068	-0.386549219	0.699089958	0.987801103
E330009J07Rik	-0.281401511	0.393761631	-0.714649395	0.474825714	0.956387791
Ssbp1	0.234188715	0.146812193	1.595158481	0.110676801	0.800111791
Clec5a	0.031971934	0.339750206	0.094104238	0.925026354	0.994688009
Mgam	2.992621131	1.833618018	1.632085365	0.102661504	0.789323778
Ephb6	-0.187291342	0.353933918	-0.52917037	0.596687269	0.974001549
Trpv6	-0.316529356	0.349028459	-0.906886953	0.364466581	0.926556975
Gstk1	0.045547303	0.156151493	0.291686628	0.770526234	0.989990452
Tmem139	0.235978007	0.248094659	0.951161173	0.341522565	0.919850248
Casp2	-0.320888684	0.190592097	-1.683641085	0.092251054	0.774469646
Fam131b	-0.530437472	0.735580182	-0.721114414	0.470839124	0.95486913
Zyx	-0.20603307	0.217158057	-0.948770093	0.342737556	0.919850248
Epha1	-0.465850546	0.277979178	-1.675846908	0.093768183	0.775558829
2010310C07Rik	0.827360034	1.040825085	0.794907853	0.426667106	0.944290206
Olfr456	-0.220380285	0.740648749	-0.297550337	0.766046384	0.989884648
Tcaf2	0.083788509	0.34086989	0.245807893	0.805830947	0.99099448
Tcaf1	-0.069194141	0.229045955	-0.302097196	0.76257797	0.989285682
Arhgef5	-0.054606949	0.174078625	-0.313691295	0.753755513	0.988585656
Tpk1	-0.074950795	0.219097817	-0.34208828	0.732284458	0.988585656
Cul1	-0.037489186	0.099807757	-0.375613948	0.70720392	0.988585656
Ezh2	0.282382899	0.235411401	1.199529407	0.230322157	0.895404344
Pdia4	-0.247869788	0.126375217	-1.961379718	0.049834744	0.707153766
Zfp786	-0.44111466	0.840426178	-0.524870205	0.599673423	0.974001549
Zfp398	-0.093829369	0.580567155	-0.161616737	0.871607676	0.992886758
Zfp282	0.200572634	0.370598585	0.541212629	0.588361034	0.973455044
Zfp212	0.082423555	0.165558345	0.497852011	0.618588354	0.974471036
Zfp783	0.056392991	0.789487754	0.071429849	0.943055654	0.994819052
Zfp956	0.114820355	0.27455991	0.418197817	0.675802492	0.983549699

Zfp777	-0.45300338	0.301321701	-1.503387833	0.132739062	0.827800491
Zfp746	0.031213338	0.210865876	0.148024605	0.88232335	0.992886758
Krba1	-0.178524589	0.346689815	-0.51494039	0.606594675	0.974001549
Zfp467	-0.226384647	0.17297011	-1.308807909	0.190599427	0.877833971
Sspo	-0.093936439	1.087455698	-0.086381854	0.931162872	0.994688009
Zfp862-ps	-0.194204201	0.272407986	-0.712916695	0.475897308	0.95716557
1700026J14Rik	1.076590296	1.10667744	0.972813086	0.330646208	0.917115058
Atp6v0e2	-0.172895593	0.15095613	-1.145336683	0.252069716	0.896939594
Lrrc61	-0.204732186	0.444041224	-0.461065719	0.644751456	0.978970741
Rarres2	-0.077642114	0.198398221	-0.391344809	0.695542381	0.98715859
Repin1	0.037374804	0.266675125	0.140151071	0.88854063	0.992886758
Zfp775	0.081434578	0.358799253	0.226964179	0.820451596	0.992886758
AI854703	-0.459215909	0.89558223	-0.512756834	0.608121428	0.974001549
Gimap8	0.056972234	0.127367858	0.447304638	0.654655119	0.979133069
Gimap9	0.309138069	0.190712818	1.620961155	0.10502597	0.793869912
Gimap4	0.360989621	0.144194395	2.503492739	0.01229742	0.525888996
Gimap6	0.440350727	0.185693267	2.371387687	0.017721432	0.579835303
Gimap7	0.921063764	0.274048474	3.360951992	0.000776743	0.201299795
Gimap1	0.11323028	0.214710682	0.527362119	0.597942144	0.974001549
Gimap5	0.188453687	0.1531081	1.230853802	0.218377549	0.888436964
Gimap3	0.377539648	0.193460557	1.95150708	0.050996755	0.707153766
Tmem176b	-0.192850597	0.111373932	-1.731559564	0.083352011	0.76099108
Tmem176a	-0.272754055	0.113285789	-2.407663457	0.016054973	0.560105876
Aoc1	-0.101001382	0.200399725	-0.503999604	0.614261655	0.974001549
Gpnmb	-0.503990812	0.316989893	-1.58992707	0.111851245	0.801067615
Malsu1	0.143757038	0.182479548	0.787798083	0.430814822	0.945321484
Igf2bp3	-0.954107131	0.587950022	-1.622769104	0.104638773	0.793021779
Tra2a	-0.080406635	0.124860058	-0.643974032	0.519592273	0.964665379
Ccdc126	0.066171106	0.175043498	0.378026646	0.7054108	0.988585656
Fam221a	-0.059998873	0.187803028	-0.319477665	0.749364326	0.988585656
Stk31	-5.518500159	4.918045246	-1.12209219	0.261823244	0.897248195
Mpp6	-0.352728841	0.241670705	-1.459543228	0.144415652	0.841934045
Dfna5	0.25084731	0.505258819	0.496472898	0.619560804	0.974471036
Osbpl3	-0.130271961	0.172532672	-0.755056764	0.450214924	0.949134998
Cycs	0.319603015	0.154945688	2.062677693	0.039143261	0.679118604
5430402O13Rik	-4.85E-05	0.53684068	-9.03E-05	0.999927936	0.999957126
4921507P07Rik	0.042018016	0.96354349	0.043607805	0.96521703	0.996474415
Nfe2l3	-0.452340888	0.298455696	-1.515604807	0.129619311	0.827800491
Hnrnpa2b1	-0.157546334	0.136927601	-1.150581284	0.249904537	0.896939594
Cbx3	0.040186286	0.108727985	0.369603888	0.711677654	0.988585656
Snx10	0.225680912	0.139887431	1.613303713	0.10667854	0.794908043
Skap2	-0.034728171	0.113529298	-0.305896116	0.759683743	0.989266039
Halr1	0.349707797	2.002486128	0.174636814	0.86136502	0.992886758
Hoxa1	-0.623735703	0.703440251	-0.886693222	0.375244089	0.928883222
Hotairm1	0.779127494	0.234211582	3.326596774	0.000879135	0.220825154
Hoxa2	0.466774883	0.504392247	0.925420416	0.354747263	0.924354588
Hoxaas2	0.170103479	0.393739043	0.432020858	0.665726253	0.981798021
Hoxa3	-0.000903739	0.350723305	-0.002576787	0.997944024	0.999591134
Hoxa4	0.139334559	0.325829928	0.427629715	0.668920728	0.981863224
Hoxa5	-0.384826788	0.244126299	-1.57634302	0.114946772	0.81007959
Hoxa6	-0.656913699	1.57598214	-0.416828137	0.67680412	0.983549699
Mira	0.360834733	0.450170156	0.801551875	0.422812225	0.943509323
Hoxa7	0.507203223	0.475463101	1.066756226	0.28608193	0.905916508
Hibadh	-0.102579119	0.076913828	-1.333688893	0.182305836	0.868546072
Tax1bp1	-0.243335509	0.120565158	-2.018290462	0.043561019	0.687169821
Jazf1	-0.288468632	0.303974684	-0.948989002	0.342626206	0.919850248
9430076C15Rik	-0.393551011	1.662295872	-0.236751482	0.812849593	0.992048071
Creb5	-0.162910733	0.818883194	-0.198942576	0.842307664	0.992886758
Tril	0.393570567	0.205462964	1.915530465	0.055424888	0.713559351
Cpvl	0.621486423	1.573977497	0.394850895	0.692952931	0.986599035
Chn2	0.025964988	0.219390621	0.11835049	0.905789953	0.994688009
9130019P16Rik	1.494291733	2.119829989	0.704911121	0.480865549	0.957463006

Prr15	0.073852976	0.308159848	0.239658011	0.810595389	0.991438232
Wipf3	0.488677163	0.383812948	1.273216981	0.202941057	0.882297774
Scrn1	-0.020520083	0.223650438	-0.091750697	0.926896117	0.994688009
Fkbp14	-0.491097865	0.244493312	-2.008635169	0.044575839	0.689848075
Plekha8	-0.056539574	0.219530898	-0.257547228	0.796756362	0.990988839
Mturn	-0.039114996	0.134553673	-0.290701811	0.771279387	0.989990452
Znrf2	0.020807276	0.138389221	0.150353296	0.880485886	0.992886758
Nod1	-0.03075855	0.10806987	-0.284617256	0.775937389	0.990861469
Ggct	0.119738568	0.182977975	0.654387872	0.512861946	0.964133218
Gars	0.018751044	0.120933333	0.155052737	0.876779742	0.992886758
Crhr2	0.323429221	1.639115821	0.197319321	0.84357766	0.992886758
Inmt	0.046575555	0.137188904	0.339499435	0.734233523	0.988585656
Fam188b	0.127921882	0.20917345	0.611558887	0.540829647	0.966989525
Aqp1	-0.173423871	0.115659084	-1.49944012	0.133759492	0.827800491
Ghrhr	-1.721388341	1.804214094	-0.954093168	0.340036484	0.919320363
6430584L05Rik	0.630396588	0.965381904	0.653002284	0.513754806	0.964352198
Adcyap1r1	0.060696816	0.470793528	0.128924492	0.897417397	0.994101834
Ccdc129	-0.286849706	0.203774303	-1.407683413	0.159224836	0.856502327
Pde1c	-0.631095701	0.798726967	-0.790126948	0.429453633	0.945321484
Lsm5	0.321379161	0.221760531	1.44921713	0.147276955	0.846074835
Avl9	-0.280842438	0.12193888	-2.303141033	0.021270909	0.60743619
Kbtbd2	-0.214224087	0.123157359	-1.739433914	0.081958467	0.76099108
Fkbp9	0.032334369	0.119589724	0.270377485	0.78686986	0.990988839
Nt5c3	0.162129672	0.147481373	1.099323047	0.271627183	0.89804647
Ppm1k	-0.056621888	0.163796996	-0.34568331	0.729580729	0.988585656
Herc6	-0.093401438	0.180566583	-0.517268681	0.604968614	0.974001549
Pyurf	0.062862443	0.182343395	0.344747574	0.730284149	0.988585656
Lancl2	-0.108015888	0.158032383	-0.683504772	0.494287936	0.958916246
Vopp1	0.086452286	0.193765095	0.446170587	0.655474028	0.979133069
Abcg2	-0.111077316	0.187084736	-0.593727304	0.552694518	0.968926429
Herc3	-0.483848262	0.282909978	-1.710255201	0.087218692	0.765601928
Nap1l5	0.169753416	0.570248281	0.297683346	0.765944856	0.989884648
Fam13a	-0.270235015	0.147968527	-1.82630063	0.06780499	0.742385562
Tigd2	0.003542013	0.157462484	0.022494329	0.982053635	0.997390438
Gprin3	-0.640874587	0.489974941	-1.307974212	0.190882056	0.877906723
Snca	0.660692436	0.252335749	2.618306916	0.008836729	0.475600561
Mmrn1	0.21461046	0.177270371	1.210639201	0.226033713	0.893814924
A730020E08Rik	0.142155359	0.323976634	0.438782753	0.660818958	0.979924124
Ccser1	-0.294223666	0.196122674	-1.500202193	0.133562035	0.827800491
Smarcad1	-0.063652684	0.135551907	-0.469581617	0.638653961	0.977795535
Hpgds	-0.192001671	0.741946069	-0.258781169	0.795804088	0.990988839
Tnip3	-0.099687597	0.330500854	-0.301625836	0.76293731	0.989285682
Ndnf	-0.25197079	0.16265812	-1.54908215	0.121361973	0.816233249
Prdm5	0.332576228	0.365497901	0.909926507	0.36286127	0.926455466
Mad2l1	0.218051041	0.185826017	1.173415029	0.240629413	0.896939594
Gng12	-0.036931432	0.115231557	-0.320497552	0.748591186	0.988585656
Gadd45a	0.318550407	0.202215296	1.575303225	0.11518647	0.81007959
E230016M11Rik	1.442520983	1.073925143	1.343223029	0.179199767	0.867377413
Serbp1	0.032568073	0.090319464	0.360587539	0.718407806	0.988585656
Il12rb2	0.176894499	1.22674889	0.144197806	0.885344273	0.992886758
Il23r	-0.545656101	1.11833352	-0.487918936	0.625607261	0.97639266
Tacstd2	-0.052084891	0.190617791	-0.273242548	0.784666775	0.990988839
Rpia	-0.044990948	0.157871089	-0.284985355	0.775655362	0.990861469
Eif2ak3	0.128279906	0.223043674	0.575133577	0.565200961	0.969738975
Thnsl2	0.199079453	0.246385343	0.808000389	0.419090358	0.943415794
Fabp1	0.493870723	0.252907584	1.952771503	0.050846677	0.707153766
Smyd1	-0.243459858	0.285965439	-0.851361126	0.394568779	0.933796635
Mir8112	0.607785103	0.7226557	0.841043809	0.400323393	0.936803788
Krcc1	0.060221546	0.133328838	0.451676821	0.651501819	0.979004493
Cd8b1	-0.499783663	0.419590021	-1.191123807	0.233604985	0.89581803
Cd8a	0.390309574	0.414955785	0.940605214	0.346907209	0.921552698
Rmnd5a	-0.159756356	0.153274433	-1.042289656	0.297277406	0.909070179

Rnf103	-0.321122094	0.183181878	-1.753023265	0.079598014	0.75659671
Chmp3	0.102267638	0.105501104	0.969351349	0.332369917	0.917115058
Kdm3a	-0.092607891	0.14112616	-0.656206413	0.511691323	0.963452765
Reep1	-0.073763604	0.253688227	-0.290764791	0.771231215	0.989990452
Mrpl35	-0.219135285	0.163849282	-1.337419868	0.181085623	0.868087032
Immt	-0.352666874	0.121023267	-2.914041922	0.00356782	0.373408948
Ptcd3	-0.240579106	0.146080558	-1.646893396	0.09957996	0.788783372
Polr1a	0.169208518	0.342151996	0.494541958	0.620923482	0.97514112
St3gal5	0.346988971	0.187412517	1.851471699	0.064101723	0.732905339
Atoh8	-0.14373274	0.261445613	-0.549761529	0.582482948	0.972017845
Sftpb	-0.273328794	0.19517368	-1.400438795	0.161381959	0.857316691
Usp39	-0.027317816	0.145960603	-0.18715883	0.851536103	0.992886758
0610030E20Rik	0.120867404	0.155966063	0.774959645	0.438363506	0.947776577
Tmem150a	-0.062057227	0.296465621	-0.209323518	0.834195694	0.992886758
Rnf181	-0.025502801	0.107742306	-0.236701828	0.812888116	0.992048071
Vamp5	0.473030902	0.169550539	2.789910925	0.005272254	0.406593461
Vamp8	0.415502274	0.190416189	2.18207431	0.029104049	0.64858361
Ggcx	-0.061241058	0.19487517	-0.314257883	0.753325184	0.988585656
Mat2a	-0.042137204	0.130499045	-0.322892815	0.746776418	0.988585656
Particl	0.521610066	0.23556852	2.214260486	0.026810865	0.633489138
Capg	0.01877381	0.16322781	0.115016003	0.908432437	0.994688009
Elmod3	-0.071972323	0.275528343	-0.261215678	0.793926189	0.990988839
Retsat	-0.190427032	0.20425448	-0.93230284	0.35118004	0.92247654
Tgoln1	-0.366861921	0.134903535	-2.719438911	0.006539277	0.432254171
Tcf7l1	-0.265528735	0.340964336	-0.778758089	0.43612223	0.946762848
Gm15401	0.561546601	1.244551302	0.451204061	0.651842483	0.979004493
Kcmf1	0.161458894	0.155395662	1.039018028	0.298796352	0.909933614
Tmsb10	0.424721113	0.159671055	2.65997562	0.007814631	0.45506991
Dnah6	-0.02047856	0.23427117	-0.087413916	0.930342509	0.994688009
Suclg1	-0.027487678	0.105880038	-0.259611526	0.795163445	0.990988839
Ctnna2	-2.467908498	1.924952468	-1.282062045	0.199820882	0.880888611
Reg1	-1.037791589	1.086279179	-0.955363601	0.339393854	0.919246575
Reg3g	-0.073601605	0.375334603	-0.196095975	0.844535047	0.992886758
Gcfc2	0.499288802	0.493890668	1.010929815	0.312050023	0.914819219
Mrpl19	0.325351112	0.168965713	1.925545169	0.054161191	0.707153766
Eva1a	0.2755843	0.191820108	1.436680979	0.150808662	0.850225493
Tacr1	-0.12260057	0.895792034	-0.13686276	0.891139275	0.993043931
Pole4	0.133906292	0.188107725	0.711859612	0.476551714	0.95716557
Hk2	-0.308946791	0.408154183	-0.75693648	0.449087918	0.948476747
2310069B03Rik	-0.052239644	1.366444724	-0.038230339	0.969504032	0.996628386
Sema4f	-0.229997878	0.358129779	-0.642219363	0.520730763	0.964784335
M1ap	0.577394063	0.510440542	1.131168109	0.257984342	0.897248195
Dok1	0.319726587	0.130313685	2.453515043	0.014146762	0.54993854
Loxl3	1.059546146	0.512888252	2.065842107	0.038843391	0.679118604
Htra2	0.501717186	0.144226743	3.478669611	0.000503909	0.15523263
Aup1	0.001894793	0.096143674	0.019707932	0.984276363	0.998148029
Dqx1	-0.023294298	0.274184942	-0.084958342	0.932294509	0.994688009
Pcgf1	0.365987516	0.16051421	2.28009418	0.022602103	0.620006604
Lbx2	0.356818956	0.379249522	0.940855387	0.346778973	0.921552698
Ccdc142	0.285539935	0.331144893	0.862280957	0.388532929	0.933269543
Mrpl53	0.198746662	0.272920298	0.728222356	0.466477483	0.953083512
Mogs	-0.212411579	0.180611199	-1.176070918	0.239566538	0.896939594
Wbp1	0.103436356	0.136385511	0.758411614	0.448204607	0.948476747
Ino80b	0.325683025	0.145449748	2.23914465	0.025146506	0.629375214
Rtkn	-0.090527039	0.234942483	-0.38531575	0.700003494	0.988482081
Wdr54	-0.068592323	0.19761261	-0.347104989	0.728512445	0.988585656
1700003E16Rik	-0.015095866	0.265915453	-0.05676942	0.954728874	0.996070133
Dctn1	-0.183187483	0.215099612	-0.851640227	0.394413805	0.933796635
Slc4a5	-0.131219444	0.305566585	-0.429429952	0.667610361	0.981863224
Mthfd2	0.191244209	0.352878956	0.541954134	0.587850103	0.973455044
Mob1a	-0.157113694	0.178155911	-0.881888747	0.377836973	0.928883222
Bola3	0.276920845	0.184484546	1.501051717	0.133342186	0.827800491

Tet3	-0.105428751	0.267402248	-0.39427025	0.693381525	0.986698113
B230319C09Rik	1.060708308	0.978915597	1.083554406	0.278562416	0.901682903
Dguok	-0.062175129	0.128854083	-0.48252355	0.629434073	0.977034429
Actg2	-0.258338897	0.286813694	-0.900720232	0.367737089	0.928436166
Stambp	-0.32953473	0.188172259	-1.751239696	0.07990463	0.75659671
Gm21284	-0.044079367	1.213767971	-0.036316139	0.971030281	0.996733101
Cd207	0.056436721	0.345624981	0.163288894	0.870290978	0.992886758
Atp6v1b1	-0.248274959	0.408922382	-0.607144461	0.543755046	0.967491297
Tex261	-0.046971537	0.124582562	-0.37703139	0.706150277	0.988585656
Nagk	0.091873071	0.134137264	0.68491833	0.493395458	0.958916246
Paip2b	0.014248859	0.097135703	0.146690234	0.883376528	0.992886758
Zfp638	-0.22403188	0.116323555	-1.925937357	0.054112196	0.707153766
Dysf	0.252116489	0.337662131	0.746653137	0.455272964	0.951224746
Cyp26b1	-2.187660758	0.833808365	-2.623697303	0.008698105	0.475600561
Exoc6b	-0.381209713	0.246015729	-1.549533902	0.121253428	0.816233249
Npm3-ps1	0.497908714	0.558058973	0.892215229	0.372277581	0.928840865
Spr	0.221305593	0.117886164	1.877282158	0.060479432	0.725221283
Sfxn5	0.384198309	0.43619226	0.880800381	0.378425873	0.929106652
Rab11fip5	-0.075859605	0.263143034	-0.288282779	0.773130288	0.990730604
Smyd5	0.039517507	0.250051966	0.158037177	0.874427504	0.992886758
Pradc1	0.213663635	0.170637462	1.252149631	0.210515346	0.884340413
Cct7	-0.191843489	0.082146865	-2.335372009	0.019523995	0.593178479
Alms1	-0.332968009	0.434675767	-0.766014657	0.443667581	0.948476747
Gm4477	-0.464350587	0.796581583	-0.582929102	0.559941029	0.968926429
Cml3	-0.291290617	0.412597008	-0.705993044	0.480192463	0.957463006
Alms1-ps2	0.744813606	1.983447466	0.375514662	0.707277744	0.988585656
Cml5	-0.279749552	1.498259857	-0.18671631	0.851883068	0.992886758
Nat8	-0.891258659	1.225843714	-0.727057332	0.467190836	0.953239786
Cml2	2.609521454	1.920658552	1.358659743	0.174254434	0.863528862
1700019G17Rik	-0.252164449	0.369567918	-0.682322347	0.495035145	0.959272191
Cml1	0.242966338	0.267846602	0.907110025	0.364348617	0.926556975
Tprkb	0.00897112	0.136899041	0.065530922	0.947751287	0.995503286
Dusp11	-0.198232534	0.102920054	-1.92608268	0.054094051	0.707153766
Tgfa	-0.107053669	0.28259571	-0.378822697	0.704819532	0.988585656
Fam136a	0.098264569	0.195540632	0.502527624	0.615296426	0.974001549
Snrpg	0.453397483	0.272658577	1.662876289	0.096337202	0.781321691
Pcyox1	-0.102456107	0.097241286	-1.053627642	0.292053429	0.907334416
Tia1	0.043272514	0.133129682	0.325040317	0.745150558	0.988585656
C87436	0.033080342	0.215415753	0.153565104	0.877952654	0.992886758
A430078I02Rik	-0.28372423	1.833498603	-0.154744721	0.877022572	0.992886758
2310040G24Rik	-0.122821012	0.311603123	-0.394158476	0.693464041	0.986698113
Pcbp1	-0.168737773	0.114959578	-1.467800901	0.142158313	0.840741274
1600020E01Rik	0.169545878	0.211259708	0.802547158	0.422236521	0.943415794
Asprv1	0.614170805	0.743010124	0.826598166	0.408464857	0.940123943
Mxd1	-0.140017433	0.200976251	-0.696686463	0.485999033	0.957710647
Snrnp27	0.181206711	0.182040629	0.995419057	0.319532494	0.91640698
Gmcl1	-0.019800637	0.135893138	-0.14570741	0.884152372	0.992886758
Anxa4	-0.112317189	0.120774014	-0.929978109	0.352382419	0.923152465
2610306M01Rik	0.06896229	0.18591822	0.370928091	0.710691092	0.988585656
Aak1	0.209125958	0.217415252	0.961873445	0.336113179	0.918908218
Nfu1	0.191202674	0.138091351	1.384610069	0.166171731	0.860082
Gfpt1	-0.056658718	0.137275088	-0.412738529	0.679798193	0.983903958
D6Ertd527e	-1.773551605	2.345292494	-0.756217661	0.449518705	0.948476747
Antxr1	-0.147769681	0.125253094	-1.179768715	0.238092215	0.896609492
Gkn3	-0.150767704	0.378509518	-0.398319453	0.690394723	0.986010574
Arhgap25	-0.273530574	0.207931755	-1.315482447	0.188347813	0.874826736
Prokr1	-0.125155346	1.024875216	-0.122117643	0.902805849	0.994688009
Aplf	0.135787558	0.441237013	0.307742901	0.758277973	0.989266039
1810020O05Rik	0.886507914	0.994339613	0.891554457	0.37263179	0.928840865
Efcc1	-0.564883102	0.503275305	-1.12241371	0.261686578	0.897248195
Gp9	0.678785107	0.508357741	1.335250853	0.181794258	0.868546072
Rab43	0.02905436	0.173714971	0.167253055	0.867170941	0.992886758

Isy1	0.04808747	0.124351576	0.386705755	0.698974055	0.987770663
Cnbp	0.282178237	0.155474088	1.814953477	0.069531059	0.743426713
Copg1	-0.004233269	0.158331733	-0.026736703	0.978669738	0.997262526
Hmces	-0.114818746	0.189770983	-0.605038474	0.545153433	0.967491297
H1fx	-0.403613605	0.315215016	-1.280439018	0.200390779	0.880888611
Gm5577	-0.898884269	1.4592774	-0.615979025	0.537908364	0.96674935
Rab7	-0.00054856	0.074556447	-0.007357643	0.994129503	0.999285699
Rpn1	-0.168715017	0.097559924	-1.729347564	0.083746909	0.76099108
Gata2	0.006153866	0.181976171	0.033816877	0.973023178	0.997013089
Eefsec	-0.276460104	0.324929509	-0.850831014	0.394863231	0.933796635
Ruvbl1	0.10164611	0.148389813	0.684993853	0.4933478	0.958916246
Sec61a1	-0.16130558	0.138766811	-1.162421901	0.245064131	0.896939594
Kbtbd12	-0.561096183	0.980358248	-0.572337902	0.567093076	0.970862413
Mgll	-0.071814741	0.151105165	-0.47526331	0.634599307	0.977034429
Abtb1	0.278550775	0.167076219	1.667207798	0.095473093	0.778239075
Podxl2	-0.034632671	0.468156778	-0.073976653	0.941028963	0.994688009
Mcm2	-0.496702965	0.329234128	-1.508661836	0.131385223	0.827800491
Tpra1	-0.086490458	0.201281052	-0.429699949	0.66741392	0.981863224
4930512J16Rik	0.478735718	0.845501233	0.56621528	0.571247444	0.971613657
Plxna1	-0.128610162	0.23910634	-0.537878509	0.590660926	0.974001549
Chchd6	0.07966714	0.185475286	0.429529679	0.6675378	0.981863224
Txnrd3	0.039622121	0.275236638	0.143956564	0.885534769	0.992886758
Zxdc	-0.039292793	0.253985766	-0.154704703	0.877054122	0.992886758
Ccdc37	0.038210095	0.173113808	0.220722402	0.825308587	0.992886758
Klf15	-0.361904315	0.368824441	-0.981237343	0.326475713	0.916882624
Aldh1l1	-0.402333096	0.283749188	-1.417918053	0.156214691	0.855455553
Slc41a3	-0.126064394	0.358163545	-0.351974387	0.724857471	0.988585656
Iqsec1	0.024933105	0.196058232	0.127171935	0.898804317	0.994364545
Nup210	-0.050957159	0.320790814	-0.15884856	0.873788189	0.992886758
Hdac11	0.113724755	0.132820489	0.856229003	0.391871123	0.933734231
Fbln2	0.319426391	0.319662125	0.999262554	0.31766752	0.91640698
Wnt7a	-0.000133637	0.373019273	-0.000358258	0.999714152	0.999957126
1810044D09Rik	0.064070542	0.303150906	0.211348673	0.832615205	0.992886758
Chchd4	0.149149924	0.178482657	0.835654997	0.403349019	0.937834293
Tmem43	-0.16033196	0.131578685	-1.21852533	0.223024405	0.892391445
Xpc	-0.055074665	0.148116493	-0.371833436	0.710016867	0.988585656
Lsm3	0.405532249	0.182958718	2.216523234	0.026655689	0.633489138
Slc6a6	-0.208293727	0.230028006	-0.90551464	0.365192808	0.926556975
Grip2	0.158959454	1.32746307	0.1197468	0.904683726	0.994688009
Ccdc174	0.259695027	0.168808969	1.538395908	0.123951842	0.820382953
Fgd5	0.020985577	0.172053739	0.121971062	0.902921936	0.994688009
Nr2c2	-0.286691145	0.262965175	-1.090224762	0.275614148	0.900302631
Mrps25	0.330264427	0.13981028	2.362232781	0.018165232	0.58167504
Rbsn	-0.097894658	0.210352401	-0.465384077	0.641656435	0.97813956
Prickle2	-0.047828932	0.246198097	-0.194270113	0.845964386	0.992886758
Adamts9	0.028172569	0.468957136	0.060074934	0.952095954	0.996023481
9530026P05Rik	0.233514066	0.269119632	0.867696143	0.385560698	0.931855682
Magi1	0.056353819	0.189323937	0.297658182	0.765964063	0.989884648
Slc25a26	0.263031169	0.20886532	1.259333859	0.207909768	0.884225785
Lrig1	-0.044842258	0.199863833	-0.224364047	0.822474044	0.992886758
Kbtbd8	-0.10169391	0.236032436	-0.430847183	0.666579489	0.981863224
Suclg2	-0.221463638	0.105979853	-2.089676777	0.036646845	0.677009445
Fam19a1	0.112563238	0.463304936	0.24295713	0.808038608	0.99099448
Fam19a4	2.419174461	2.044025993	1.183534098	0.236597541	0.896358698
Eogt	-0.099605979	0.138457831	-0.719395775	0.471897099	0.954965783
Tmf1	-0.032862101	0.126212104	-0.260372025	0.79457682	0.990988839
Uba3	0.103571656	0.135025807	0.767050821	0.4430513	0.948476747
Arl6ip5	0.250305332	0.130997169	1.91076902	0.056034271	0.715507996
Lmod3	0.30718635	2.068873784	0.148479986	0.88196398	0.992886758
Frmd4b	-0.19473991	0.180366807	-1.079688179	0.28028106	0.903192609
Mitf	-0.099233445	0.229543875	-0.432307091	0.665518234	0.981727712
Foxp1	0.058297518	0.190219527	0.306474939	0.759243058	0.989266039

Eif4e3	0.018788206	0.084244474	0.223020045	0.823519906	0.992886758
Gpr27	0.485089763	0.82088398	0.590935838	0.554563412	0.968926429
Rybp	-0.302245777	0.270273398	-1.118296432	0.26344041	0.897248195
Shq1	-0.461637464	0.270187596	-1.708581263	0.087528542	0.766704005
Gxylt2	0.677876237	1.163081532	0.582827789	0.560009236	0.968926429
Ppp4r2	0.030347273	0.114017668	0.266162899	0.790113755	0.990988839
Pdzrn3	-0.267493586	0.335429085	-0.797466881	0.42517993	0.944290206
Chl1	-2.102620765	1.508346417	-1.393990626	0.163320435	0.859982992
Cntn6	-0.502290079	0.995219263	-0.504702931	0.613767502	0.974001549
Cntn4	0.044047133	0.987846337	0.044589053	0.964434868	0.996474415
Il5ra	0.178031997	0.727935917	0.244570975	0.806788641	0.99099448
Trnt1	0.19792468	0.141792404	1.395876464	0.162751699	0.859982992
Crbn	0.200993579	0.124090045	1.61973975	0.105288194	0.793982106
Setmar	0.111934064	0.237685117	0.470934258	0.637687683	0.977468848
Sumf1	-0.109683167	0.13069063	-0.839258075	0.401324504	0.937802373
Itpr1	0.078768773	0.1500015	0.5251199	0.599499843	0.974001549
Bhlhe40	-0.494575051	0.236710267	-2.089368815	0.036674536	0.677009445
Arl8b	-0.278670942	0.087318748	-3.191421626	0.001415745	0.27193966
Edem1	-0.289588978	0.162893123	-1.777785166	0.075439146	0.752876039
Lmcd1	-0.093895053	0.186601506	-0.503184858	0.614834309	0.974001549
5031434C07Rik	-1.470307021	1.987816321	-0.739659397	0.459506691	0.952865981
Ssu2	1.41309164	1.01333616	1.394494439	0.163168348	0.859982992
Cav3	0.100230774	0.381538942	0.262701294	0.792780819	0.990988839
Rad18	0.34463785	0.422745381	0.815237412	0.414936427	0.943415794
Srgap3	-0.53863624	0.543210434	-0.991579335	0.321402774	0.91640698
Thumpd3	0.055018157	0.147204462	0.373753324	0.708587849	0.988585656
Gt(ROSA)26Sor	0.428138042	0.248910915	1.720045273	0.085424212	0.761635307
Setd5	-0.27191901	0.142736531	-1.905041457	0.056774682	0.721593167
Lhfpl4	-0.438362769	0.835002814	-0.524983582	0.599594604	0.974001549
Mtmr14	0.077355138	0.16451622	0.470197637	0.638213822	0.977779844
Brpf1	0.148437485	0.223834571	0.663157099	0.507229944	0.963011338
Ogg1	0.08848214	0.141165489	0.626797252	0.530792145	0.966026275
Camk1	0.186561644	0.104773931	1.780611279	0.074975976	0.75241101
Tada3	-0.213761208	0.202596241	-1.055109448	0.291375272	0.907334416
Arpc4	0.225346804	0.121504333	1.854640064	0.063647643	0.729199878
Ttll3	-0.283054725	0.253032664	-1.118648956	0.263289929	0.897248195
Rpusd3	0.00838834	0.263673138	0.031813404	0.974620857	0.997262526
Cidec	-0.24614117	0.240429037	-1.023758085	0.305949553	0.91333012
Jagn1	0.24187683	0.162949183	1.484369697	0.137710869	0.833085238
Il17re	-0.05762604	0.215774818	-0.267065641	0.789418621	0.990988839
Il17rc	0.005103315	0.180715692	0.028239467	0.97747116	0.997262526
Creld1	-0.113689906	0.124349836	-0.914274675	0.360572534	0.925913281
Prrt3	-0.701101062	1.159636451	-0.604586947	0.545453481	0.967491297
Emc3	-0.014668207	0.093655321	-0.156619041	0.875545095	0.992886758
Fancd2	0.71186262	0.873389409	0.815057536	0.415039377	0.943415794
Brk1	0.249384231	0.160705204	1.5518118	0.120707262	0.816092513
Vhl	-0.006631815	0.125943391	-0.052657112	0.958005111	0.996226826
Irak2	0.20097293	0.154154645	1.303709857	0.192332527	0.879588734
Tatdn2	-0.109869846	0.124864877	-0.879909936	0.378908102	0.929137643
Ghrl	0.321617838	0.360965599	0.890993044	0.372932902	0.928840865
Sec13	0.091473108	0.139460092	0.655908845	0.511882777	0.963452765
Atp2b2	-0.073272975	0.437237768	-0.167581533	0.866912501	0.992886758
Hrh1	-0.047045486	1.03164036	-0.045602604	0.963626994	0.996474415
Atg7	0.140606741	0.183698015	0.765423298	0.444019524	0.948476747
Vgll4	-0.353973709	0.295192198	-1.199129625	0.230477546	0.895404344
Tamm41	0.5594719	0.306607755	1.824715427	0.068043984	0.742385562
Syn2	-0.27883945	0.320838416	-0.869096207	0.384794511	0.931689378
Timp4	0.061601746	0.371621179	0.165764895	0.868341973	0.992886758
Pparg	0.142165285	0.271336678	0.523944224	0.600317335	0.974001549
Tsen2	0.248140513	0.233183947	1.064140633	0.287264996	0.907190636
Mkrn2os	-0.474022684	1.09843842	-0.431542338	0.666074075	0.981863224
Mkrn2	0.031670991	0.191087139	0.165741091	0.868360707	0.992886758

Raf1	0.004869279	0.099625246	0.048875956	0.96101815	0.996474415
Tmem40	0.426174008	0.326139309	1.306723832	0.191306522	0.878116834
Cand2	-0.321720838	1.05306803	-0.305508124	0.759979184	0.989266039
Rpl32	0.404009099	0.19401499	2.082360226	0.037309579	0.679118604
Snora7a	0.778908935	1.570152794	0.496072062	0.619843569	0.974471036
Efcab12	0.047086185	0.39291048	0.119839474	0.904610311	0.994688009
Mbd4	-0.059023169	0.345441098	-0.170863193	0.864331339	0.992886758
Ift122	-0.489488112	0.271145249	-1.805261621	0.071033752	0.744227652
Plxnd1	-0.052053714	0.198132599	-0.262721604	0.792765164	0.990988839
Tmcc1	-0.121189402	0.206649946	-0.586447779	0.557574644	0.968926429
9530062K07Rik	-0.112137169	0.562621993	-0.199311741	0.842018895	0.992886758
Fam21	-0.104578584	0.203289933	-0.514430706	0.606950896	0.974001549
Zfand4	-0.191615742	0.188810021	-1.01486002	0.310172558	0.914704835
Mar-08	-0.02755275	0.102381124	-0.269119434	0.787837776	0.990988839
Alox5	0.063983822	0.247697451	0.258314415	0.796164262	0.990988839
Zfp422	-0.34921267	0.170781297	-2.044794582	0.040875114	0.679118604
Rassf4	-0.259060423	0.223113582	-1.161114531	0.245595326	0.896939594
8430408G22Rik	0.293501513	0.413015208	0.71063125	0.477312773	0.95716557
Cxcl12	0.051188595	0.181683416	0.281746107	0.778138197	0.990988839
Zfp637	-0.05396717	0.143193796	-0.37688204	0.706261269	0.988585656
Zfp239	-0.432197385	0.677807541	-0.637640272	0.523707874	0.965637248
Hnrnpf	-0.014926815	0.103265427	-0.144548038	0.885067726	0.992886758
Fxyd4	-0.279459613	0.819475975	-0.341022338	0.733086766	0.988585656
Rasgef1a	0.128691626	0.16529944	0.778536368	0.436252875	0.946762848
Csgalnact2	-0.040062089	0.114963582	-0.348476349	0.727482473	0.988585656
Ret	-0.04592956	0.248500599	-0.184826759	0.853364919	0.992886758
Bms1	0.096160596	0.181019947	0.531215465	0.595269477	0.974001549
Zfp248	-0.036983017	0.263543645	-0.140329764	0.888399449	0.992886758
Zfp9	-0.015079948	0.22816553	-0.066092141	0.947304468	0.995297476
Ankrd26	0.082079414	0.504358062	0.162740363	0.870722866	0.992886758
Cacna1c	-0.365257756	0.376942097	-0.969002294	0.332544045	0.917115058
Dcp1b	-0.04226116	0.25261728	-0.167293229	0.867139332	0.992886758
Cacna2d4	-0.203689689	0.230126271	-0.885121409	0.376091154	0.928883222
Lrtm2	-0.442187986	0.340569933	-1.298376468	0.194158001	0.879588734
Adipor2	-0.207773255	0.137725244	-1.508606911	0.131399267	0.827800491
Wnt5b	-0.161221301	0.309715862	-0.520545831	0.602683193	0.974001549
Fbxl14	-0.244596938	0.155930901	-1.568623896	0.116735601	0.812418014
Erc1	0.052307421	0.377923778	0.13840733	0.889918505	0.993043931
3110021A11Rik	-0.095525764	1.839623605	-0.051926798	0.958587021	0.996226826
Rad52	0.018362323	0.182498912	0.100616071	0.919855239	0.994688009
Wnk1	-0.01865977	0.117037213	-0.159434506	0.873326554	0.992886758
Ninj2	0.307436424	1.2807888	0.240036783	0.810301741	0.991438232
Ccdc77	0.221597137	0.276298647	0.802020347	0.422541189	0.943509323
Kdm5a	-0.049645307	0.162229407	-0.306019163	0.759590055	0.989266039
Il17ra	-0.080718473	0.243054792	-0.332099905	0.739813822	0.988585656
Cecr6	-0.15556212	0.608432331	-0.255676946	0.798200298	0.990988839
Cecr5	0.26304348	0.189756258	1.386217679	0.165680445	0.860011234
Cecr2	-0.308765638	0.349287323	-0.883987532	0.376702944	0.928883222
Slc25a18	0.591495587	0.528932726	1.11828132	0.263446862	0.897248195
Atp6v1e1	0.070018602	0.111304283	0.629073744	0.529300779	0.965637248
Bcl2l13	-0.020461081	0.160263952	-0.127671138	0.898409232	0.994364545
Bid	-0.006789947	0.219481238	-0.030936344	0.975320306	0.997262526
Mical3	-0.437699971	0.325891821	-1.343083634	0.179244894	0.867377413
Pex26	-0.438885263	0.257027995	-1.707538761	0.08772196	0.766721861
Tuba8	-0.282549916	0.24849323	-1.137052772	0.255516225	0.897044747
Usp18	-0.176684174	0.179156685	-0.986199169	0.324035387	0.91640698
Slc6a13	-0.259140322	1.172702548	-0.220977027	0.825110321	0.992886758
Klrg1	1.114314074	0.302930152	3.67845217	0.000234654	0.095054639
M6pr	0.050410917	0.076851104	0.655955668	0.511852649	0.963452765
Phc1	-0.371441802	0.261117829	-1.42250647	0.154879275	0.854973821
Rimklb	0.337272551	1.011375999	0.333478896	0.738772817	0.988585656
Mfap5	-0.046436419	0.116880902	-0.397296888	0.691148542	0.986191149

Apobec1	0.141163582	0.24210337	0.583071528	0.559845149	0.968926429
Gdf3	-0.766259234	1.444928189	-0.530309561	0.595897318	0.974001549
Dppa3	0.20370499	1.490737829	0.136647093	0.891309751	0.993043931
Nanog	-1.037201549	1.070611819	-0.968793292	0.332648334	0.917115058
Slc2a3	0.210354663	0.302136665	0.696223556	0.486288838	0.957736773
Foxj2	0.166356497	0.278723766	0.596850779	0.550607012	0.968926429
C3ar1	-0.621973531	0.596482822	-1.042735027	0.297071031	0.909070179
Necap1	-0.144667884	0.129826371	-1.114318169	0.265142715	0.89768067
Clec4a1	0.13614329	0.362487789	0.375580348	0.707228903	0.988585656
Clec4a3	0.018253052	0.218880925	0.083392611	0.933539364	0.994688009
Clec4a4	1.559406541	2.2807129	0.68373645	0.494141602	0.958916246
Clec4b1	0.086440184	0.303623754	0.284695065	0.775877772	0.990861469
Clec4a2	-0.412277428	0.251946336	-1.636370009	0.101762172	0.788783372
Clec4n	-0.130227076	0.245888527	-0.529618352	0.596376568	0.974001549
Clec4d	0.464374395	0.337712269	1.375059299	0.169113061	0.861597949
Clec4e	0.136162442	0.333861214	0.407841452	0.683390079	0.984738223
Cd163	-0.139411828	0.434836831	-0.320607222	0.748508064	0.988585656
Pex5	0.097447019	0.226474204	0.430278669	0.666992942	0.981863224
Clstn3	0.281002728	0.550588291	0.510368151	0.609793565	0.974001549
C1rl	0.107666104	0.22444532	0.479698592	0.631441729	0.977034429
C1ra	-0.162790678	0.213566599	-0.762247833	0.445912103	0.948476747
C1s1	-0.188933646	0.137452699	-1.374535729	0.169275426	0.861597949
Lpcat3	-0.220329269	0.203574854	-1.082301008	0.279118798	0.901874653
Emg1	0.454719133	0.181923159	2.499512082	0.012436446	0.525888996
Phb2	0.250748585	0.103490906	2.422904528	0.015396973	0.557315333
Ptpn6	0.337139235	0.158004063	2.133737754	0.032864254	0.665167506
Grcc10	0.245537294	0.13855091	1.772181026	0.076364512	0.752876039
Atn1	0.246859363	0.23780692	1.038066357	0.299239163	0.910105802
Eno2	-0.209930449	0.435683764	-0.481841342	0.629918658	0.977034429
Lrrc23	0.085299767	0.135027405	0.631721888	0.527568623	0.965637248
Spsb2	-0.070631031	0.19409392	-0.363901306	0.71593171	0.988585656
Tpi1	-0.017807441	0.102341821	-0.173999646	0.861865741	0.992886758
Usp5	-0.012350144	0.168658417	-0.073225776	0.941626455	0.994688009
Cdca3	0.036384713	0.362296185	0.100428089	0.920004471	0.994688009
P3h3	-0.106673078	0.178055828	-0.599099052	0.549106834	0.968926429
Gpr162	-0.035101337	0.87949247	-0.039910901	0.968164161	0.996474415
Cd4	0.474656504	0.543397349	0.873498013	0.382391689	0.930775109
Lag3	0.544054934	0.439934685	1.236672063	0.216208854	0.887633697
Ptms	-0.000479527	0.152271667	-0.003149151	0.997487345	0.99957296
Mlf2	0.136177252	0.103304276	1.318215054	0.187431667	0.873560421
Cops7a	-0.102497909	0.107249807	-0.955693183	0.339227267	0.919246575
Pianp	-0.853543968	1.330504876	-0.641518857	0.521185633	0.964784335
Zfp384	-0.301229156	0.189726574	-1.587701451	0.112353865	0.801398667
4930557K07Rik	-0.887731444	1.606221831	-0.552682965	0.58048052	0.971613657
Ing4	0.160032565	0.134139078	1.193034628	0.232855812	0.89581803
Acrbp	0.453233432	0.448290599	1.011025959	0.312004006	0.914819219
Lpar5	-0.885972401	2.203284237	-0.402114437	0.687599808	0.98538669
Chd4	-0.103112071	0.128386976	-0.803134978	0.421896723	0.943415794
Nop2	-0.407071095	0.193619224	-2.102431194	0.035515521	0.674795441
Iffo1	0.312518399	0.281109401	1.111732291	0.266253272	0.89768067
Gapdh	-0.030498244	0.163157978	-0.186924624	0.851719732	0.992886758
Ncapd2	0.352675312	0.420140062	0.839423191	0.401231874	0.937720128
Mrpl51	0.417257429	0.130094834	3.207332812	0.001339719	0.26039992
Mir3098	0.007233367	0.653486293	0.011068889	0.991168485	0.998770784
Vamp1	0.283394314	0.428870025	0.660793008	0.508745067	0.963452765
Tapbpl	0.076481628	0.109240668	0.70012047	0.483852073	0.957710647
E130112N10Rik	-0.123441858	0.859446185	-0.143629537	0.885793015	0.992886758
Cd27	0.059747555	0.330816525	0.180606319	0.856676595	0.992886758
Ltbr	-0.063287018	0.108353664	-0.584078244	0.559167671	0.968926429
Scnn1a	-0.381305467	0.217846751	-1.750338093	0.080059991	0.75659671
Tnfrsf1a	-0.143330648	0.172420377	-0.831286017	0.405812072	0.939557159
Plekhg6	-0.590912589	0.625273404	-0.945046736	0.344635016	0.921227228

Cd9	0.065977277	0.132516375	0.497880182	0.618568496	0.974471036
Vwf	0.475011445	0.202067734	2.350753562	0.018735438	0.589390158
Ano2	1.443912583	1.053090439	1.371119259	0.170337782	0.862090815
Ntf3	-0.333895027	0.367658663	-0.90816581	0.363790625	0.926455466
Gm10415	0.720591493	1.17590222	0.612798821	0.540009371	0.966900344
Kcna5	-0.077563099	1.001273859	-0.077464421	0.938254094	0.994688009
Kcna1	-1.809874611	2.049659782	-0.883012208	0.377229675	0.928883222
Kcna6	1.291369006	0.771873619	1.673031665	0.094321063	0.777748075
Ndufa9	0.145918312	0.108216112	1.34839728	0.177530638	0.866855369
Dyrk4	-1.036256165	1.152227981	-0.89934994	0.368466295	0.928436166
Rad51ap1	-0.225582552	0.401921973	-0.561259565	0.574620602	0.971613657
D6Wsu163e	0.221818858	0.161321108	1.375014467	0.16912696	0.861597949
Tigar	0.003809005	0.192390818	0.01979827	0.984204298	0.998148029
Ccnd2	-0.018361063	0.187612357	-0.097867022	0.922037887	0.994688009
9330179D12Rik	-0.020416514	0.499844333	-0.040845745	0.967418871	0.996474415
Parp11	0.115634204	0.209344967	0.552361996	0.580700363	0.971613657
Cracr2a	-0.485513052	0.839355528	-0.57843552	0.562970119	0.969658675
Prmt8	0.217778908	0.202687274	1.074457729	0.282617581	0.905916508
Tspan11	-0.06793544	0.130549585	-0.520380356	0.602798499	0.974001549
Tspan9	-0.162237379	0.159235915	-1.018849162	0.308274582	0.9139204
9330102E08Rik	-0.362987444	1.591076533	-0.228139525	0.819537774	0.992886758
Tead4	0.039061865	0.271461056	0.143894912	0.885583454	0.992886758
Tulp3	-0.263422866	0.202577477	-1.300356141	0.193478935	0.879588734
Rhno1	-0.347384825	0.285916873	-1.214985398	0.224371661	0.892989918
Foxm1	-0.536373587	0.886573221	-0.604996377	0.545181404	0.967491297
Nrip2	-0.732768645	1.694079543	-0.432546777	0.665344063	0.981727712
Itfg2	0.134784159	0.15729902	0.856865853	0.391519026	0.933734231
Fkbp4	-0.114579081	0.118848413	-0.964077501	0.335007072	0.918190441
Gm10069	-0.247546988	0.76076467	-0.325392329	0.74488416	0.988585656
Pzp	-0.208223151	1.546487158	-0.134642664	0.892894408	0.993300147
BC048546	-0.197646841	0.219952964	-0.898586849	0.368872766	0.928436166
Klrb1a	0.175633104	0.610746235	0.287571325	0.773674897	0.990730604
Klrb1c	0.710515935	0.254589643	2.79082812	0.005257338	0.406593461
Klrb1b	-0.523112848	0.273039808	-1.915884911	0.055379746	0.713559351
BC035044	0.109163679	0.266359951	0.409835181	0.681926857	0.984683806
Clec2i	0.223464219	0.388224062	0.575606308	0.564881316	0.969658675
Clec2g	2.140389095	1.928265458	1.110007487	0.2669958	0.897701901
Gm15987	0.378415287	0.536518444	0.705316456	0.480613322	0.957463006
BC064078	0.312393947	0.19955084	1.565485504	0.117469114	0.813021712
Klrb1f	0.144317114	0.45998178	0.313745284	0.753714505	0.988585656
Clec2e	-2.044211374	1.243663174	-1.643701781	0.100237818	0.788783372
Clec2d	0.631047614	0.195867818	3.221803463	0.001273865	0.25997989
2310001H17Rik	0.505695105	0.260683806	1.939879243	0.052394367	0.707153766
Cd69	0.297403975	0.268395264	1.108082053	0.267826381	0.89804647
Clec12a	0.076144006	0.188144474	0.404710299	0.685690471	0.98538669
Clec12b	0.021667323	0.433377953	0.049996366	0.960125284	0.996474415
Clec1b	0.102817496	0.366047667	0.28088554	0.778798192	0.990988839
Clec9a	-0.033570979	0.206404778	-0.162646328	0.870796908	0.992886758
Clec1a	0.061265472	0.127451597	0.480695997	0.630732577	0.977034429
Clec7a	0.022218809	0.30221755	0.073519255	0.941392922	0.994688009
Olr1	-0.58732525	0.392992414	-1.494495134	0.135046253	0.829809681
1700101I11Rik	-0.260391696	0.993844438	-0.26200448	0.793317988	0.990988839
Gabarapl1	0.26992969	0.093443304	2.88870019	0.003868377	0.376754331
Klre1	1.505119556	0.305550338	4.925929933	8.40E-07	0.001958309
Klrd1	0.877336621	0.217662811	4.030714373	5.56E-05	0.03405023
Klrk1	0.586477658	0.237010231	2.474482459	0.013342937	0.539384266
Klrc3	1.696921999	2.085212734	0.813788431	0.415766164	0.943415794
Klrc2	1.785139598	0.629968975	2.833694467	0.00460133	0.390758702
Klrc1	1.398744849	0.320323385	4.366664804	1.26E-05	0.010840965
Klri1	0.268140761	0.422044115	0.63533823	0.525207845	0.965637248
Klri2	0.774271262	0.329591073	2.349187596	0.018814425	0.590246276
Klra17	0.283111321	0.623630687	0.453972722	0.64984846	0.979004493

Klra5	0.465888347	1.442531062	0.322965903	0.746721065	0.988585656
Klra18	0.955753636	1.273645843	0.750407691	0.4530092	0.950108007
Klra8	2.693538635	0.668583697	4.028723177	5.61E-05	0.03405023
Klra14-ps	1.766753517	0.376923723	4.687297209	2.77E-06	0.003766595
Klra9	1.283395753	0.353358765	3.631990711	0.000281243	0.109329964
Klra7	1.275517482	0.35777893	3.565099498	0.000363718	0.134964244
Klra3	1.474487176	0.481838481	3.060127478	0.002212428	0.308737709
Klra1	0.408021836	0.90154613	0.452580098	0.650851132	0.979004493
Klra2	0.663376016	0.171393292	3.870489949	0.000108617	0.057206035
Magohb	0.221342087	0.207102947	1.068753925	0.285180566	0.905916508
Styk1	-0.193690463	0.589251346	-0.328706017	0.742377906	0.988585656
Ybx3	-0.115825383	0.143407272	-0.80766743	0.419282058	0.943415794
Smim10l1	0.06563311	0.098943871	0.663336789	0.50711488	0.963011338
Etv6	0.064363716	0.224331397	0.286913544	0.774178519	0.990730604
Bcl2l14	0.16715903	0.268015011	0.623692789	0.532829359	0.96674935
Lrp6	-0.020984818	0.172696247	-0.121512876	0.903284817	0.994688009
Mansc1	-0.360339499	0.287514995	-1.25328941	0.210100401	0.884258007
Loh12cr1	-0.337059366	0.280998063	-1.19950779	0.230330557	0.895404344
Dusp16	-0.149936913	0.148275515	-1.011204803	0.311918417	0.914819219
Crebl2	0.005539233	0.136231885	0.040660327	0.96756669	0.996474415
Gpr19	-0.213579529	0.338903599	-0.630207321	0.52855895	0.965637248
2810454H06Rik	0.837734467	0.512280669	1.635303687	0.101985401	0.788783372
Cdkn1b	0.234195706	0.131641314	1.779044121	0.075232529	0.752876039
1190002F15Rik	0.410538196	0.305777749	1.342603241	0.17940048	0.867616008
Apold1	0.169015511	0.451580842	0.374275202	0.70819958	0.988585656
Ddx47	-0.031023455	0.121627657	-0.255069081	0.798669744	0.990988839
Gprc5a	-0.467933214	0.170750509	-2.740449893	0.006135514	0.427133716
Hebp1	0.192072182	0.175231568	1.096104914	0.273032864	0.898826892
8430419L09Rik	-0.172826622	0.336977236	-0.512873284	0.608039962	0.974001549
Gsg1	-0.003066058	0.765818294	-0.004003637	0.996805568	0.999449398
Pbp2	-0.344524645	1.461880489	-0.235672237	0.813687015	0.992048071
Emp1	-0.172429427	0.091772178	-1.878885637	0.06026011	0.725221283
Atf7ip	-0.070081614	0.281147708	-0.249269734	0.803152142	0.990988839
Plbd1	0.01770049	0.178077889	0.099397459	0.9208227	0.994688009
Gucy2c	-0.425119302	1.460135658	-0.291150552	0.770936179	0.989990452
Hist4h4	5.4333779	1.881347254	2.888024998	0.003876691	0.376754331
H2afj	0.593864564	0.180939357	3.282119348	0.0010303	0.240510037
Wbp11	-0.191436673	0.215561076	-0.888085532	0.374494745	0.928883222
BC049715	-0.191129755	1.070349988	-0.178567532	0.858277291	0.992886758
Smco3	0.720583614	2.070337244	0.348051322	0.727801641	0.988585656
Art4	-0.219877335	0.206237605	-1.066136002	0.286362167	0.905916508
Mgp	-0.124275253	0.145381001	-0.85482458	0.392648268	0.933734231
Erp27	0.760867405	1.175151764	0.6474631	0.517332269	0.964352198
Arhgdib	0.297972548	0.115490435	2.580062564	0.009878242	0.490219004
Pde6h	1.894482419	1.791318816	1.057590866	0.290242008	0.907334416
Rerg	0.335092943	0.162027365	2.068125608	0.038628213	0.679118604
Ptpro	0.246794028	0.296013427	0.833725788	0.404435523	0.938127315
Eps8	-0.282115273	0.144927856	-1.94659109	0.051583778	0.707153766
Strap	-0.095711794	0.095940858	-0.99761245	0.318467322	0.91640698
Dera	0.123413578	0.158455779	0.778851859	0.436066985	0.946762848
Mgst1	0.05948616	0.240506817	0.24733669	0.804647666	0.990988839
Lmo3	-0.829209359	2.125076574	-0.390202108	0.696387103	0.987490725
Pik3c2g	0.567838787	0.816882458	0.695129123	0.486974385	0.958288569
Plekha5	-0.16019612	0.328852568	-0.487136593	0.626161536	0.976534277
Aebp2	0.105293383	0.136647707	0.770546288	0.440975909	0.948068187
Pde3a	-0.082601273	0.266304913	-0.310175553	0.756427461	0.989266039
Slco1c1	4.457599921	2.290279295	1.946312806	0.051617176	0.707153766
Slco1a4	-1.387921488	1.78475366	-0.777654373	0.436772797	0.946762848
Slco1a5	-0.50642479	0.316811593	-1.598504604	0.109930722	0.798948974
Pyroxd1	-0.318606828	0.32279033	-0.987039568	0.323623242	0.91640698
Recql	-0.026821707	0.163379187	-0.164168444	0.869598542	0.992886758
Golt1b	-0.029545176	0.124368492	-0.237561581	0.812221152	0.991931689

Spx	-0.181425957	1.154370206	-0.157164448	0.875115246	0.992886758
Ldhb	0.114611534	0.153304136	0.747608885	0.454696104	0.951162497
Kcnj8	0.142447272	0.154971727	0.919182327	0.358000214	0.924354588
Abcc9	0.168342779	0.377273055	0.446209388	0.655446002	0.979133069
Cmas	0.204675988	0.119825952	1.708110678	0.087615808	0.766704005
St8sia1	0.304050965	0.826108022	0.368052309	0.71283423	0.988585656
C2cd5	-0.20238222	0.206151702	-0.981715009	0.326240269	0.916800143
Etnk1	-0.203715448	0.128306631	-1.587723455	0.112348887	0.801398667
Sox5	0.20096549	0.461603773	0.43536362	0.663298513	0.980889931
Lrmp	-0.054926705	0.283165672	-0.193973743	0.846196441	0.992886758
Casc1	0.081096547	0.201117937	0.403228813	0.686779905	0.98538669
Lyrm5	0.158932476	0.169736482	0.936348358	0.349093858	0.921552698
Kras	0.059671965	0.108757207	0.548671361	0.583231004	0.972174584
Gm15706	-0.157651158	0.321039633	-0.491064473	0.623380848	0.975691835
Ifltd1	0.690070838	0.588834422	1.171926796	0.241226448	0.896939594
Rassf8	-0.317176714	0.260303506	-1.218488056	0.223038561	0.892391445
Bhlhe41	-0.465664266	0.303892632	-1.532331543	0.125440641	0.822401003
Sspn	-0.051502698	0.107838843	-0.477589487	0.632942415	0.977034429
Itpr2	0.315818965	0.209519824	1.507346461	0.131721874	0.827800491
Asun	-0.027593957	0.180285293	-0.153057169	0.878353192	0.992886758
Fgfr1op2	0.153309402	0.112863897	1.358356445	0.174350608	0.863528862
Tm7sf3	0.047759439	0.129652611	0.368364653	0.71260135	0.988585656
Med21	0.22843876	0.250651777	0.911378975	0.362095728	0.926455466
Gm6654	0.675317726	1.350140484	0.500183302	0.616946014	0.974001549
4930479D17Rik	2.849487345	1.459548301	1.952307671	0.050901687	0.707153766
Stk38l	-0.005940368	0.167182753	-0.035532181	0.971655386	0.996733101
Arntl2	0.898482392	0.705768259	1.273055823	0.202998234	0.882297774
Ppfibp1	-0.268250463	0.122831186	-2.18389541	0.028969934	0.64858361
Rep15	1.480346816	1.328360735	1.11441627	0.265100647	0.89768067
Mrps35	0.2846448	0.185525339	1.534263734	0.124964784	0.821707621
Mansc4	0.853084866	1.390145716	0.613665788	0.539436201	0.966900344
Klhl42	0.070999267	0.297270782	0.238837018	0.811231965	0.991762208
Pthlh	-0.078818705	0.51677707	-0.152519741	0.878777021	0.992886758
Ccdc91	0.190250854	0.122473334	1.553406348	0.120326087	0.81558552
Far2	-0.565511412	1.257234926	-0.449805681	0.652850561	0.979004493
Ergic2	0.081874864	0.149461554	0.547798823	0.583830047	0.972333834
4732416N19Rik	0.468476173	1.391696209	0.336622439	0.736401538	0.988585656
Tmtc1	-0.080234251	0.142838705	-0.561712251	0.574312086	0.971613657
Rps4l	-0.050129251	0.15512251	-0.323159103	0.746574751	0.988585656
Ipo8	-0.333612514	0.226162978	-1.47509781	0.140186249	0.837748392
Caprin2	0.322631731	0.401658879	0.803248099	0.42183135	0.943415794
Fam60a	-0.058924594	0.119539918	-0.49292818	0.622063335	0.97514112
Dennd5b	-0.354229632	0.226490078	-1.563996245	0.11781845	0.814289188
Mettl20	-0.217262348	0.160693347	-1.352030757	0.176365476	0.865862073
Amn1	-0.018780467	0.170890859	-0.109897433	0.912490719	0.994688009
2810474O19Rik	-0.176301233	0.16147621	-1.091809335	0.274916915	0.900302631
Bicd1	-0.57791047	0.263178468	-2.195888111	0.028099953	0.641661442
Nlrp12	0.90294313	0.894613673	1.009310675	0.312825662	0.914819219
Myadm	-0.253598945	0.225398326	-1.125114593	0.260540478	0.897248195
Prkcg	0.29044334	1.462121	0.198645215	0.842540282	0.992886758
Cacng7	0.013638219	0.565036659	0.024136874	0.98074343	0.997296835
Oscar	-1.137698155	4.777181361	-0.238152599	0.81176274	0.991816977
Ndufa3	0.265029485	0.17676843	1.49930327	0.133794975	0.827800491
Tfpt	0.485969559	0.194650753	2.496623065	0.012538215	0.525888996
Prpf31	0.092223265	0.143484748	0.642739151	0.520393372	0.964739706
Cnot3	0.166753404	0.482681462	0.345472981	0.729738819	0.988585656
Leng1	-0.065795079	0.248075078	-0.265222445	0.790838106	0.990988839
Tmc4	-0.242315276	0.201817703	-1.200664122	0.229881517	0.895404344
Mboat7	-0.329853954	0.213267036	-1.54667107	0.121942583	0.816233249
Tsen34	-0.103704466	0.14428586	-0.718743097	0.472299224	0.955003345
Rps9	0.425121744	0.246155497	1.7270455	0.084159492	0.76099108
Pirb	0.015715125	0.209200476	0.075119928	0.940119293	0.994688009

Pira6	-0.259761489	0.360996308	-0.71956827	0.471790854	0.95486913
Pira2	0.394908747	0.68228502	0.578803191	0.562721978	0.969658675
Lilra6	0.043537964	0.308996047	0.140901363	0.887947865	0.992886758
Lair1	0.317312384	0.18124425	1.750744559	0.07998992	0.75659671
Ttyh1	0.014345679	0.655912923	0.021871317	0.982550605	0.997390438
D030047H15Rik	-1.047423553	1.571945736	-0.666322971	0.505204686	0.962138588
Leng8	-0.220915944	0.19315781	-1.143707024	0.252745159	0.897044747
Leng9	-0.052902996	0.301705058	-0.175346731	0.860807195	0.992886758
Cdc42ep5	0.186720629	0.218735426	0.85363689	0.393306212	0.933734231
Lilra5	0.3736597	0.184940676	2.020430057	0.043338798	0.686006913
Ncr1	0.875558464	0.258879254	3.382111354	0.00071931	0.195736186
Gp6	1.92350842	0.596666658	3.223757176	0.001265206	0.25997989
Rdh13	0.270186914	0.178223314	1.516002072	0.129518828	0.827800491
Eps8l1	-0.018110975	0.264634846	-0.068437604	0.945437288	0.995012175
Ppp1r12c	-0.0612895	0.1385614	-0.442327376	0.658252324	0.979697875
Tnnt1	1.081054502	0.478784183	2.257916075	0.023950892	0.622011301
Tnni3	0.243658777	0.23201144	1.050201567	0.293625449	0.907334416
Dnaaf3	-0.363078106	0.254629247	-1.425908887	0.153894644	0.854159937
Syt5	-0.393065781	0.180888765	-2.172969571	0.029782608	0.649459041
Tmem86b	2.473962313	1.082509635	2.28539519	0.022289666	0.618917302
Ppp6r1	0.002675756	0.152531659	0.017542297	0.98600399	0.998148029
Hspbp1	-0.025261905	0.174845016	-0.144481699	0.885120107	0.992886758
Brsk1	-0.478649654	0.59822294	-0.800119191	0.423641743	0.94351935
Tmem150b	-0.897916184	0.624246806	-1.438399325	0.150320784	0.849879783
Suv420h2	-0.169069472	0.271118569	-0.62359975	0.532890475	0.96674935
Cox6b2	0.389454659	0.181888784	2.141169181	0.032260397	0.665167506
Il11	-0.316931183	0.503419634	-0.629556659	0.528984688	0.965637248
Tmem190	-0.673269344	1.289272666	-0.522208654	0.601525061	0.974001549
Tmem238	-0.099973097	0.176350286	-0.56690068	0.570781662	0.971613657
Rpl28	0.166499054	0.110033386	1.513168504	0.130236862	0.827800491
Ube2s	0.138929956	0.121518075	1.143286351	0.25291972	0.897044747
Shisa7	0.587864518	1.146196149	0.512882999	0.608033167	0.974001549
Isoc2b	0.10986764	0.167869511	0.654482397	0.512801065	0.964129777
Isoc2a	0.154295729	0.116988789	1.318893298	0.187204787	0.873455432
Zfp628	-0.165396182	0.433054844	-0.38192895	0.702514066	0.988482081
Nat14	0.327918044	0.313931841	1.044551715	0.296230206	0.90895519
Ssc5d	-0.429948759	0.525097052	-0.818798654	0.412901299	0.942708418
Sbk2	1.254129115	1.290251688	0.972003468	0.331048823	0.917115058
Sbk3	-0.42764453	0.387734877	-1.102930264	0.270057451	0.89804647
Zfp579	0.272791164	0.2593629	1.051774038	0.292903233	0.907334416
Fiz1	-0.073378792	0.223014603	-0.329031332	0.742132006	0.988585656
Zfp524	0.164875835	0.171298962	0.962503408	0.335796792	0.918908218
Zfp865	0.174905857	0.301420102	0.580272703	0.561730732	0.969592734
Zfp784	0.133513416	0.25053215	0.53291929	0.594089452	0.974001549
Zfp580	-0.036587095	0.369492531	-0.099019849	0.921122511	0.994688009
Ccdc106	-0.498323121	0.529343426	-0.941398526	0.346500669	0.921552698
U2af2	0.024521795	0.097973922	0.250288997	0.802363865	0.990988839
Epn1	-0.156873373	0.189571613	-0.827515104	0.407945165	0.939557159
Rasl2-9	0.668815883	0.952636252	0.702068478	0.482636459	0.957463006
Zfp787	0.062764366	0.158820744	0.39518998	0.692702687	0.986599035
Zfp444	0.035939503	0.172881079	0.207885696	0.835318218	0.992886758
Gm6792	-2.099160963	2.060787905	-1.018620576	0.308383132	0.9139204
Zfp667	0.138382312	0.267324545	0.517656588	0.604697893	0.974001549
Zfp583	-0.0634881	0.351165126	-0.180792724	0.856530274	0.992886758
Zfp78	-0.012126297	0.33900309	-0.035770463	0.971465385	0.996733101
Zfp28	0.104064912	0.246337343	0.42244879	0.672697472	0.983233856
Peg3	0.501167381	0.765243778	0.654912064	0.512524373	0.963720538
Zfp954	-0.13648273	0.154519678	-0.883270864	0.377089942	0.928883222
Zfp773	0.6450686	0.543954044	1.185888048	0.235666514	0.896002526
Zfp418	-0.470236711	0.419977482	-1.119671247	0.262853882	0.897248195
Zfp772	0.269569138	0.20153048	1.337609768	0.181023678	0.868087032
Vmn2r29	0.413061627	0.221919012	1.861316992	0.062699425	0.727650961

Clcn4-2	0.066267133	0.1048633	0.631938275	0.527427212	0.965637248
2810047C21Rik1	1.245143065	0.39629386	3.141969103	0.001678157	0.284736028
Zik1	-0.429641434	0.480890726	-0.893428405	0.371627797	0.928840865
Zfp551	-0.903724588	0.737506275	-1.225378845	0.220432514	0.889301125
Zfp606	0.295371671	0.154791456	1.908191048	0.056366528	0.717582253
Zscan18	0.295758582	0.433133892	0.682834077	0.494711694	0.959168486
Zfp329	0.051363024	0.200665697	0.255963149	0.797979292	0.990988839
Zfp110	-0.065706446	0.100856095	-0.65148711	0.514732095	0.964352198
Zfp128	0.154087809	0.291916256	0.527849362	0.597603894	0.974001549
Zscan22	-0.601841784	0.241970463	-2.487253101	0.012873378	0.532176722
Rps5	0.219603524	0.169771962	1.293520564	0.195831076	0.879601031
Rnf225	0.063549716	0.730384899	0.087008529	0.930664733	0.994688009
Zfp324	-0.177335624	0.227630424	-0.779050626	0.435949893	0.946762848
Zfp446	-0.170507371	0.240727351	-0.70830078	0.478758493	0.957463006
Slc27a5	0.102872216	2.67801419	0.038413619	0.969357903	0.996600044
Zbtb45	-0.461155111	0.327206737	-1.409369243	0.158726014	0.855986074
Trim28	-0.227802948	0.194609928	-1.170561803	0.241774958	0.896939594
Chmp2a	0.281919536	0.148881442	1.893584123	0.058280233	0.72285319
Ube2m	0.07155555	0.154493515	0.463162158	0.643248138	0.978801899
Mzf1	-0.970086214	1.114440314	-0.87046942	0.384043924	0.931591679
6330408A02Rik	0.224821471	0.476298573	0.472017938	0.636913985	0.977284137
Lig1	-0.058198596	0.216368393	-0.268979192	0.787945695	0.990988839
Pla2g4c	0.641772188	0.897809359	0.714820114	0.474720205	0.956387791
Sult2a2	-1.39450034	1.581741194	-0.881623583	0.377980397	0.928883222
Sult2a1	-0.444409818	0.872223298	-0.509513813	0.610392119	0.974001549
Sult2a8	3.117821788	4.943859751	0.630645274	0.528272489	0.965637248
Nlrp5-ps	0.600698723	0.602240712	0.997439581	0.318551187	0.91640698
Sepw1	0.473433845	0.174986085	2.705551397	0.00681911	0.445342404
Gltscr2	0.104125074	0.135811045	0.766690768	0.443265394	0.948476747
Ehd2	0.089482138	0.096716218	0.925203037	0.354860304	0.924354588
Gltscr1	0.154053302	0.551863345	0.279151176	0.78012881	0.990988839
Napa	-0.122906463	0.146658594	-0.83804474	0.402005577	0.937834293
Kptn	0.165962451	0.199864933	0.830373035	0.406327907	0.939557159
Meis3	-0.012468844	0.289393702	-0.043086092	0.965632906	0.996474415
Dhx34	-0.144486519	0.366783508	-0.393928614	0.693633744	0.986787302
C5ar2	-0.111258197	0.394628421	-0.281931535	0.777996008	0.990988839
C5ar1	0.4403602	0.393377213	1.119434948	0.262954628	0.897248195
Inafm1	0.166322557	0.21837819	0.761626228	0.446283116	0.948476747
Ccdc9	-0.107003936	0.201177847	-0.531887272	0.594804073	0.974001549
Bbc3	0.357724848	0.195683669	1.828077171	0.067537971	0.742385562
Sae1	-0.159664912	0.106415312	-1.500394158	0.133512332	0.827800491
Zc3h4	0.228492626	0.317140394	0.720477839	0.47123084	0.95486913
Tmem160	0.35894578	0.180406129	1.989654016	0.046629062	0.698868643
Npas1	-0.034292385	1.408115712	-0.024353386	0.98057073	0.997296835
Arhgap35	0.272310861	0.180588457	1.507908453	0.131577959	0.827800491
Ap2s1	0.064605579	0.129755628	0.497901943	0.618553158	0.974471036
Slc1a5	0.025642589	0.105424821	0.243231039	0.807826423	0.99099448
Fkrp	-0.415219494	0.181882847	-2.28289529	0.022436537	0.619833063
Strn4	-0.093608727	0.149346991	-0.62678683	0.530798977	0.966026275
Prkd2	-0.298300875	0.171779367	-1.736534951	0.08246929	0.76099108
Dact3	-0.111161986	0.229505041	-0.484355313	0.628133727	0.976968316
Gng8	0.028063656	0.314999164	0.089091209	0.929009424	0.994688009
Ptgir	-0.006103533	0.589274692	-0.010357705	0.991735895	0.998770784
Calm3	0.269150511	0.101420297	2.653813096	0.00795879	0.457546335
Pnmal2	-0.474656766	0.612701292	-0.774695226	0.438519772	0.947816234
Ccdc8	0.124056646	0.449955567	0.275708658	0.782771845	0.990988839
Ppp5c	-0.023178815	0.125096329	-0.185287731	0.853003361	0.992886758
Hif3a	-0.576530559	1.190655322	-0.48421281	0.628234847	0.976968316
Mill2	0.203359342	0.181412359	1.120978432	0.262297043	0.897248195
Pglyrp1	0.147074482	0.178365681	0.824567154	0.409617376	0.940886864
Ccdc61	0.262882461	0.296039093	0.887999144	0.374541212	0.928883222
Nova2	-0.70123686	0.33145873	-2.115608359	0.03437813	0.674629488

Mypop	-0.20279689	0.352307441	-0.575624771	0.564868834	0.969658675
Irf2bp1	-0.055372601	0.228921031	-0.241885164	0.808869146	0.991174313
Foxa3	0.020978936	1.074616615	0.019522252	0.984424486	0.998148029
Sympk	-0.213599085	0.193679063	-1.102850669	0.27009202	0.89804647
Dmwd	-0.251751092	0.217165229	-1.159260594	0.246349979	0.896939594
Dmpk	-0.252362045	0.23998437	-1.051577005	0.292993662	0.907334416
Six5	-0.013994976	0.26224371	-0.053366297	0.957440058	0.996226826
Fbxo46	-0.04385479	0.297361662	-0.147479637	0.882753451	0.992886758
Qpctl	-0.358371064	0.319942787	-1.12010984	0.262666958	0.897248195
Snrpd2	0.354175886	0.180312428	1.964234471	0.049502907	0.707153766
Eml2	0.018625336	0.18828943	0.09891865	0.921202861	0.994688009
Gpr4	-0.194848576	0.411947347	-0.472993885	0.636217542	0.977284137
Opa3	-0.104592107	0.22892027	-0.456893164	0.647747838	0.979004493
Vasp	-0.073116636	0.118324933	-0.61793093	0.53662087	0.96674935
Ppm1n	0.580256954	1.373230464	0.422548851	0.672624452	0.983233856
Rtn2	0.150710905	0.164688259	0.915128414	0.36012422	0.925913281
Fosb	0.688267778	0.785529211	0.876183556	0.380930255	0.930420854
Ercc1	0.231082448	0.191789863	1.204873108	0.228252292	0.894545167
Cd3eap	0.325617543	0.241091839	1.350595458	0.176825057	0.866546104
Ppp1r13l	-0.559272034	0.526133798	-1.062984428	0.287789013	0.907334416
Ercc2	0.114250614	0.42183728	0.270840487	0.786513719	0.990988839
Klc3	0.169717718	0.266350102	0.637197873	0.523995963	0.965637248
Ckm	0.481826939	0.357933594	1.346135002	0.178258982	0.867377413
Mark4	-0.340088508	0.308052782	-1.103994277	0.26959562	0.89804647
Bloc1s3	0.194705723	0.151321323	1.286703811	0.198197544	0.880381196
Trappc6a	0.286281815	0.163941008	1.746248962	0.0807677	0.758307521
Nkpd1	-1.429697027	1.662419659	-0.860009697	0.389783697	0.933734231
Ppp1r37	0.095023148	0.170831853	0.556237882	0.578048246	0.971613657
Gemin7	0.322026974	0.197561772	1.63000651	0.103100121	0.79028905
Zfp296	0.213392753	0.567143704	0.3762587	0.706724579	0.988585656
Clasrp	0.03423104	0.291014291	0.117626662	0.906363478	0.994688009
Relb	-0.019869683	0.27222329	-0.072990385	0.941813768	0.994688009
Clptm1	-0.287227153	0.142052838	-2.021974049	0.043179033	0.685056403
Apoc1	0.09381284	0.271989109	0.344913958	0.730159057	0.988585656
Apoe	-0.053651395	0.121952241	-0.439937756	0.659982189	0.979924124
Tomm40	0.05598842	0.172222137	0.325094216	0.745109766	0.988585656
Pvrl2	-0.121463301	0.179708348	-0.675891259	0.499109688	0.960700483
Bcam	-0.045356477	0.175792304	-0.258011731	0.796397854	0.990988839
Cblc	0.276212445	0.292777411	0.943421295	0.345465455	0.921434283
Bcl3	-0.453919523	0.266778582	-1.701484128	0.088852116	0.767814344
Pvr	-0.213675805	0.183670533	-1.163364644	0.244681588	0.896939594
Igsf23	-0.095820202	0.35295902	-0.271476847	0.786024304	0.990988839
Zfp180	-0.180506087	0.112259212	-1.607940079	0.107848303	0.796929372
Zfp112	-0.257026884	0.502503219	-0.51149301	0.609005882	0.974001549
Zfp235	-0.113375819	0.254400701	-0.44565844	0.65584399	0.979416887
Zfp114	-0.458392124	1.984250734	-0.231015222	0.817302976	0.992678823
Zfp111	-0.496794068	0.21491182	-2.311618164	0.020798735	0.604325792
Zfp109	-0.576136111	0.502580178	-1.146356614	0.251647627	0.896939594
Zfp108	-0.527462414	0.317101242	-1.663388041	0.096234786	0.780927109
Zfp93	-0.13890549	0.219331408	-0.633313265	0.526529092	0.965637248
Zfp61	-0.04312517	0.265908366	-0.162180569	0.871163661	0.992886758
Zfp94	-0.018868438	0.268184627	-0.070356149	0.943910193	0.99486515
Tescl	-0.307056455	1.197996513	-0.256308305	0.797712785	0.990988839
Kcnn4	-0.551794122	0.359243561	-1.535988901	0.124541102	0.821164769
Smg9	0.426815644	0.30469025	1.400818191	0.161268448	0.857316691
Irgc1	-0.640422001	2.086455491	-0.306942565	0.758887089	0.989266039
Plaur	-0.140967796	0.142261843	-0.990903764	0.321732574	0.91640698
Cadm4	-0.017224219	0.248233488	-0.069387169	0.944681442	0.99486515
Zfp428	-0.081389067	0.421675297	-0.1930136	0.846948317	0.992886758
Irgq	0.260020997	0.369240188	0.704205571	0.481304762	0.957463006
Pinlyp	1.342334402	1.766269524	0.759982768	0.447264885	0.948476747
Xrcc1	-0.134594733	0.205926668	-0.653605163	0.513366217	0.964352198

Ethe1	0.083945197	0.231519101	0.362584327	0.71691542	0.988585656
Phldb3	0.343853071	0.508778519	0.675840387	0.49914199	0.960700483
Lypd3	2.254974091	1.896795792	1.188833347	0.234505253	0.89581803
Gm4598	0.746086412	2.276973608	0.327665815	0.743164354	0.988585656
Cd177	-0.752577639	0.436358756	-1.724676379	0.08458581	0.76099108
Ceacam10	1.311484983	1.590281674	0.824687227	0.409549185	0.940886864
Rps19	0.497599408	0.244997683	2.031037199	0.042251221	0.680982912
Cd79a	0.048232446	0.437429843	0.110263272	0.912200584	0.994688009
Arhgef1	-4.065177982	0.220805766	-18.41065136	1.08E-75	1.76E-71
Rabac1	0.216419079	0.102800273	2.105238373	0.035270564	0.674795441
Atp1a3	-0.016188318	0.766546448	-0.021118509	0.98315112	0.997569444
Grik5	0.14062538	0.28537753	0.492769629	0.622175372	0.97514112
Zfp574	-0.271493806	0.475819704	-0.570581261	0.568283524	0.971380162
Pou2f2	-1.935906699	1.355760519	-1.427911989	0.153317192	0.854159937
D930028M14Rik	-0.059066303	1.050077711	-0.056249459	0.955143081	0.99614946
Dedd2	-0.116689409	0.170512782	-0.684344052	0.493757936	0.958916246
Zfp526	-0.325405268	0.461075851	-0.705752138	0.480342291	0.957463006
Gsk3a	-0.140892942	0.221283172	-0.636708793	0.524314545	0.965637248
9130221H12Rik	0.6550382	1.2948423	0.505882608	0.612939062	0.974001549
Erf	0.197229686	0.206846374	0.953508066	0.340332712	0.919850248
Cic	0.090365018	0.224189571	0.403074138	0.686893685	0.98538669
Pafah1b3	0.383402854	0.196649429	1.949676925	0.051214638	0.707153766
Megf8	0.515457437	0.211715517	2.434670088	0.014905373	0.554936045
4732471J01Rik	0.408649201	0.892401678	0.4579207	0.647009415	0.979004493
Lipe	0.413225853	0.286361079	1.443023801	0.149013767	0.848779008
Cxcl17	-0.204963249	0.196296992	-1.044148696	0.296416599	0.909005508
Ceacam1	-0.358799783	0.304941787	-1.176617302	0.239348289	0.896918485
Atp5sl	0.187012156	0.155503231	1.202625531	0.229121263	0.895404344
B3gnt8	0.652584961	0.288230639	2.264106839	0.023567544	0.622011301
Bckdha	-0.135002517	0.15062726	-0.896268822	0.370109207	0.928436166
Exosc5	-0.008083038	0.15200779	-0.053175155	0.957592351	0.996226826
B9d2	0.241388288	0.195065821	1.237470954	0.215912291	0.887633697
Tgfb1	-0.046797881	0.210971146	-0.221821241	0.824453044	0.992886758
Ccdc97	0.068258945	0.256125558	0.266505794	0.789849697	0.990988839
Hnrnpul1	0.011157834	0.221441174	0.050387349	0.959813718	0.996474415
Axl	-0.259178516	0.151421509	-1.711636063	0.086963758	0.765418158
Cyp2s1	-0.421513398	0.221628852	-1.901888647	0.057185714	0.721735927
Cyp2b10	-0.348745471	0.156124512	-2.233765007	0.025498539	0.631530315
Cyp2b9	1.013541909	1.073891268	0.943803102	0.345270275	0.92132235
Cyp2a4	0.939326879	0.877691221	1.070224763	0.284518151	0.905916508
Cyp2b19	-0.127206552	0.56244456	-0.226167272	0.821071325	0.992886758
Cyp2g1	-0.168520354	0.51317162	-0.328389856	0.742616912	0.988585656
Cyp2a5	-0.38424777	0.284481571	-1.350694771	0.176793228	0.866546104
Cyp2a12	-2.929200336	1.959112628	-1.49516689	0.134870892	0.829704995
Cyp2f2	-0.447651292	0.208147565	-2.150643906	0.031504317	0.661395937
Cyp2t4	2.398366011	1.650079895	1.453484779	0.146089203	0.843494228
Egln2	0.136085566	0.13100703	1.038765372	0.29891387	0.909933614
Rab4b	-0.099945938	0.120646697	-0.828418357	0.407433614	0.939557159
Mia	0.392436519	0.286761918	1.368509885	0.17115253	0.86244803
Snrpa	0.039705722	0.236133356	0.168149571	0.866465613	0.992886758
BC024978	0.39274966	0.519176016	0.756486523	0.449357549	0.948476747
Itpkc	-0.585148532	0.255591273	-2.28939167	0.022056606	0.616892692
Adck4	-0.050510195	0.11985439	-0.421429656	0.673441367	0.983375962
Numbl	0.212438154	0.323539022	0.656607519	0.511433313	0.963452765
Ltbp4	0.04953159	0.197575988	0.250696405	0.802048841	0.990988839
Shkbp1	-0.284408696	0.256858645	-1.107257638	0.268182554	0.89804647
Sptbn4	0.443682898	1.208521098	0.367128798	0.713522947	0.988585656
Blvrb	0.299403629	0.206206136	1.45196275	0.146511963	0.843780493
Sertad3	0.222778565	0.197479766	1.128108311	0.25927418	0.897248195
Sertad1	-0.201281763	0.154531461	-1.302529348	0.192735492	0.879588734
Prx	-0.187604568	0.143443132	-1.307867204	0.190918355	0.877906723
Hipk4	0.506707203	1.010718174	0.50133382	0.616136207	0.974001549

Pld3	-0.219192197	0.225644866	-0.97140343	0.331347421	0.917115058
2310022A10Rik	0.048399534	0.338321548	0.143057792	0.886244539	0.992886758
Akt2	0.130214802	0.092440264	1.408637289	0.158942447	0.855986074
Map3k10	0.325209808	0.179462868	1.81212867	0.069966317	0.744227652
C030039L03Rik	-0.213856867	0.245223372	-0.872090069	0.383159243	0.93148317
Zfp60	-0.089415771	0.188754663	-0.473714239	0.635703698	0.977104816
Gm10046	0.383520633	0.535101838	0.716724566	0.473544065	0.955974667
Zfp626	0.172764613	0.120078709	1.438761412	0.150218133	0.849830719
Zfp59	-0.07797843	0.206805418	-0.377061834	0.706127654	0.988585656
Zfp607	0.000131721	0.275260368	0.000478531	0.999618188	0.999950896
1700049G17Rik	0.106727454	0.164854459	0.647404105	0.51737044	0.964352198
Zfp780b	-0.109206068	0.203051989	-0.537823189	0.590699121	0.974001549
Zfp850	-0.108662236	0.262429372	-0.414062784	0.678828127	0.9838639
Psmc4	-0.147391817	0.090580528	-1.627190959	0.103696547	0.79155232
Fcgbp	-0.056703447	0.408260019	-0.138890521	0.889536662	0.993043931
9530053A07Rik	0.261868868	0.21177206	1.236560046	0.21625046	0.887633697
Fbl	0.111310406	0.152476952	0.730014634	0.46538124	0.953083512
Dyrk1b	-0.324746672	0.355007191	-0.914760828	0.360317203	0.925913281
Eid2	-0.042797536	0.224624302	-0.19052941	0.848894299	0.992886758
Eid2b	0.208751989	0.222457707	0.938389556	0.348044251	0.921552698
Timm50	0.026738515	0.141248325	0.189301464	0.849856548	0.992886758
Supt5	0.012154996	0.158496645	0.076689296	0.93887072	0.994688009
Rps16	0.412243082	0.232456361	1.773421381	0.076158909	0.752876039
AF357399	0.540245308	0.858944324	0.628964291	0.529372434	0.965637248
Plekhg2	-0.559848201	0.340648618	-1.643477094	0.100284261	0.788783372
Zfp36	0.105112919	0.498968377	0.210660482	0.833152213	0.992886758
Med29	0.030650075	0.160732709	0.190689718	0.848768694	0.992886758
Paf1	0.016919517	0.157856523	0.107182881	0.914643896	0.994688009
Samd4b	0.040513798	0.269107251	0.15054889	0.880331581	0.992886758
Gmfg	0.502000121	0.195102684	2.573004689	0.010081984	0.497306815
Lrfn1	-1.740791657	1.11666325	-1.558922671	0.119014684	0.814755048
Nccrp1	-1.634930487	1.461069793	-1.118995475	0.263142069	0.897248195
Pak4	-0.200805264	0.202772757	-0.990297054	0.322028946	0.91640698
Papl	-0.281778396	0.747394462	-0.377014294	0.706162982	0.988585656
Fbxo27	0.616693983	0.616722071	0.999954456	0.317332549	0.91640698
Fbxo17	0.174822033	0.200438802	0.872196558	0.383101157	0.931480654
Mrps12	0.335302816	0.159617388	2.100665968	0.035670299	0.674795441
Sars2	-0.420011947	0.48211634	-0.871183803	0.383653802	0.931591679
Nfkbib	-0.178652065	0.149597407	-1.194218992	0.232392316	0.895404344
Sirt2	0.010606559	0.147212299	0.07204941	0.942562586	0.994688009
Gm19897	0.851375722	1.149342878	0.740749987	0.458845045	0.952683168
Rinl	0.236678853	0.18022881	1.313213204	0.189111116	0.875548883
Hnrnpl	0.010537574	0.107033895	0.098450812	0.921574328	0.994688009
Ech1	0.304878786	0.124020725	2.458289014	0.013960078	0.545277966
Lgals4	-0.828498141	1.530170227	-0.541441812	0.588203094	0.973455044
Lgals7	-0.11144885	0.657644696	-0.16946666	0.865429594	0.992886758
Actn4	-0.310228081	0.191997743	-1.615790251	0.10613967	0.79419908
Eif3k	0.092752347	0.123711688	0.749746033	0.453407677	0.950146765
Map4k1	-0.086451022	0.278709889	-0.310182831	0.756421926	0.989266039
Ryr1	0.030312681	0.308583512	0.098231693	0.921748317	0.994688009
Rasgrp4	0.00946956	0.400532872	0.023642405	0.981137848	0.997364526
Fam98c	0.081012052	0.193971395	0.417649477	0.676203416	0.983549699
Spred3	-0.215184671	0.578802801	-0.371775449	0.710060044	0.988585656
Ggn	1.701724825	1.51048881	1.126605383	0.259909362	0.897248195
Psmd8	0.107354996	0.109664158	0.978943326	0.327607983	0.917115058
Catsperg1	-0.562030424	0.676949805	-0.830239435	0.406403423	0.939557159
Kcnk6	-0.340459217	0.248094424	-1.372296932	0.16997102	0.861730117
Yif1b	0.02297553	0.128210866	0.179201116	0.857779787	0.992886758
2200002D01Rik	0.383081046	0.192628821	1.988700575	0.046734263	0.698868643
Spint2	-0.026232786	0.077656386	-0.337805912	0.735509455	0.988585656
Ppp1r14a	0.592153475	0.16726484	3.540214884	0.000399801	0.139568962
Dpf1	0.038963359	1.07182303	0.036352418	0.971001354	0.996733101

Sipa1l3	0.344658297	0.33733159	1.0217196	0.306913632	0.9139204
Zfp84	0.020975176	0.27156784	0.077237335	0.938434741	0.994688009
Zfp30	0.59738682	0.694632405	0.860004249	0.3897867	0.933734231
Zfp790	-0.157018812	0.188041847	-0.83502058	0.403706121	0.937834293
Zfp940	0.204061664	0.353362237	0.577485771	0.563611348	0.969658675
Zfp420	0.202251299	0.243847401	0.829417486	0.40686821	0.939557159
Zfp27	0.151628715	0.178098337	0.851376368	0.394560315	0.933796635
Zfp383	0.22698008	0.188204624	1.206028179	0.227806625	0.894410876
Zfp74	0.253596071	0.272851773	0.929427977	0.352667335	0.923348232
Zfp568	-0.126681147	0.303494891	-0.417407839	0.676380122	0.983549699
Zfp14	0.070237459	0.219808128	0.319539864	0.749317167	0.988585656
Zfp82	-0.214698462	0.308982676	-0.694855985	0.487145559	0.958288569
Zfp566	0.163142671	0.314101663	0.519394483	0.603485678	0.974001549
Zfp260	0.021195528	0.136736834	0.155009645	0.876813713	0.992886758
Zfp382	-0.111050036	0.540283495	-0.205540307	0.83715001	0.992886758
Zfp146	-0.13729371	0.1857784	-0.739018692	0.459895647	0.953083512
Gm5113	-0.349285136	0.35353204	-0.987987218	0.323158908	0.91640698
Cox7a1	0.469459735	0.397552831	1.180873831	0.237652848	0.896609492
Capns1	-0.04259979	0.146209676	-0.291360949	0.770775278	0.989990452
Tbcb	0.107002869	0.10155209	1.053674708	0.292031872	0.907334416
Polr2i	0.406028399	0.176433052	2.301317091	0.021373714	0.60743619
Wdr62	-1.051907454	0.67098644	-1.567702998	0.116950462	0.812679331
Clip3	0.333570389	0.877358917	0.380198323	0.703798204	0.988585656
Alkbh6	-0.002176708	0.128352564	-0.016958817	0.98646947	0.998148029
Syne4	-0.163221578	0.359462733	-0.454070931	0.649777775	0.979004493
Sdhaf1	0.26341936	0.184276274	1.429480604	0.152866146	0.854156592
Lrfn3	-0.174479331	0.542605592	-0.321558299	0.74778734	0.988585656
Tyrobp	0.42707636	0.183377673	2.328944161	0.019862024	0.595248411
Hcst	0.544850678	0.278770429	1.954478029	0.050644711	0.707153766
Nfkbid	0.459318867	0.618076618	0.743142279	0.457395545	0.952102513
Aplp1	-0.223364205	0.368298312	-0.606476321	0.544198502	0.967491297
Arhgap33	-0.404115157	0.669672649	-0.603451787	0.54620818	0.96778188
Proser3	-0.417874507	0.543589106	-0.768732307	0.442052242	0.948476747
Hspb6	-0.31274427	0.373323276	-0.837730433	0.402182117	0.937834293
Lin37	0.028947657	0.149979889	0.193010258	0.846950934	0.992886758
Psenen	-0.154046086	0.09930297	-1.551273695	0.120836108	0.816233249
U2af1l4	0.200715559	0.150747426	1.331469232	0.183034662	0.869576666
Igflr1	0.098390648	0.279831274	0.351607046	0.72513298	0.988585656
Kmt2b	0.151866967	0.229670372	0.661238826	0.508459166	0.963324595
Zbtb32	-0.076741757	0.545973077	-0.140559599	0.888217867	0.992886758
Upk1a	-0.076797563	0.450238094	-0.170571003	0.8645611	0.992886758
Cox6b1	0.096106333	0.172479956	0.557202905	0.577388805	0.971613657
Rbm42	-0.13098215	0.154114072	-0.849903895	0.39537852	0.934066368
Haus5	0.049750232	0.287753377	0.172891913	0.862736388	0.992886758
2200002J24Rik	-2.110584727	2.145315201	-0.983811016	0.325208442	0.91640698
Tmem147	0.244999195	0.202185893	1.211752168	0.225607263	0.89336109
Gapdhs	0.146262573	0.320155897	0.456847975	0.64778032	0.979004493
Sbsn	-0.078608206	0.293252981	-0.268055949	0.788656253	0.990988839
Dmkn	0.050820865	0.184248313	0.275828118	0.782680086	0.990988839
Ffar2	-0.190892608	0.555445953	-0.343674495	0.731091094	0.988585656
Cd22	0.05260051	0.267429515	0.196689245	0.844070727	0.992886758
Mag	-0.032860564	0.332031855	-0.098968108	0.921163592	0.994688009
Hamp	-0.406312631	0.361920322	-1.122657687	0.261582905	0.897248195
Usf2	0.267713546	0.097993916	2.731940498	0.006296251	0.427133716
Lsr	-0.046986815	0.132747913	-0.353955204	0.723372455	0.988585656
Fam187b	-0.248051174	0.236809153	-1.047472915	0.294881518	0.90737175
Fxyd5	-0.006843374	0.138401769	-0.049445709	0.960564102	0.996474415
Fxyd7	-1.422689348	0.840966978	-1.691730336	0.090697401	0.770055369
Fxyd1	0.308339465	0.132519768	2.326743174	0.019978939	0.595248411
Lgi4	-0.06939474	0.437720962	-0.158536478	0.874034079	0.992886758
Fxyd3	-0.288984494	0.207142194	-1.39510202	0.162985077	0.859982992
Hpn	0.115183094	0.178652744	0.644731734	0.519101048	0.96446859

Scn1b	-0.043467768	0.286728501	-0.151599049	0.879503183	0.992886758
Gramd1a	-0.162440611	0.144184887	-1.126613301	0.259906013	0.897248195
Gm10640	0.538628812	1.187714211	0.453500351	0.65018849	0.979004493
Wtip	-0.310878438	0.135580995	-2.292935209	0.021851735	0.614067614
Uba2	-0.115545013	0.089214533	-1.295136669	0.195273087	0.879588734
Pdcd2l	0.24941818	0.135060728	1.846711351	0.064788986	0.733548823
Gpi1	0.05955314	0.117934425	0.504968245	0.61358114	0.974001549
4931406P16Rik	-0.174235681	0.159484379	-1.092493709	0.274616154	0.900302631
Lsm14a	0.003256555	0.079268331	0.041082673	0.967229988	0.996474415
Kctd15	-0.005743561	0.215088333	-0.026703268	0.978696406	0.997262526
Chst8	0.025932044	0.586531596	0.044212527	0.964734997	0.996474415
Pepd	-0.117709528	0.154405902	-0.762338267	0.44585814	0.948476747
Cebpg	-0.030568187	0.127595862	-0.239570363	0.810663343	0.991438232
Cebpa	-0.02985862	0.154043172	-0.193832802	0.846306802	0.992886758
Slc7a10	0.339476546	0.235411394	1.442056564	0.149286417	0.848779008
Lrp3	-0.870916147	0.425788208	-2.045421013	0.040813369	0.679118604
Gpatch1	0.140004114	0.329009297	0.425532393	0.670448613	0.982434419
Rhpn2	-0.018185597	0.190955699	-0.095234641	0.924128455	0.994688009
Faap24	0.197962545	0.271401114	0.729409476	0.465751223	0.953083512
Cep89	-0.006695312	0.221030198	-0.03029139	0.975834664	0.997262526
Tdrd12	1.317652742	2.276491367	0.578808583	0.562718339	0.969658675
Nudt19	0.07822255	0.180923608	0.432351261	0.665486136	0.981727712
Rgs9bp	-0.265422797	4.191863581	-0.063318567	0.949512831	0.995624732
Ankrd27	0.141128024	0.178147208	0.792198911	0.428244704	0.945321484
Pdcd5	0.410814869	0.173646486	2.365811593	0.017990597	0.581648476
Dpy19l3	0.413903825	0.232489163	1.780314491	0.075024507	0.75241101
Zfp507	-0.575034629	0.264905075	-2.170719564	0.029952377	0.65030913
E130304I02Rik	-2.270372735	2.143368581	-1.059254463	0.289483907	0.907334416
Tshz3	0.192895859	0.487005104	0.396085909	0.692041653	0.986464472
Zfp536	-0.910507393	2.33851382	-0.389353009	0.697015028	0.987518602
Uri1	0.034135751	0.119092808	0.286631507	0.774394487	0.990795297
Ccne1	-0.231943396	0.389749462	-0.595108959	0.551770641	0.968926429
1600014C10Rik	-0.149052695	0.169499408	-0.879370004	0.379200691	0.929137643
Plekhf1	0.027166449	0.21892782	0.124088611	0.901245115	0.994688009
Pop4	-0.006696324	0.134820398	-0.049668479	0.960386575	0.996474415
Zfp939	0.443070336	0.322924911	1.372053751	0.170046705	0.861730117
Zfp619	-0.885127968	0.425713401	-2.079163976	0.037602281	0.679118604
Vstm2b	0.204753781	1.346566933	0.152056148	0.879142651	0.992886758
AI987944	0.00908794	0.17675029	0.051416831	0.958993373	0.996340108
Vmn2r57	0.232893013	1.145223947	0.203360237	0.838853477	0.992886758
AW146154	0.279283869	0.217943853	1.281448711	0.200036104	0.880888611
Zfp788	0.080069071	0.15255813	0.524843029	0.599692316	0.974001549
Zfp141	-0.070114018	0.179028941	-0.391635102	0.695327848	0.98715859
9830147E19Rik	0.062690806	0.244532167	0.256370387	0.797664852	0.990988839
Gm5595	-0.046069973	0.225677196	-0.204141019	0.8382433	0.992886758
Zfp715	0.052728756	0.14172506	0.372049625	0.709855902	0.988585656
Siglecf	-0.364558489	0.222640507	-1.637431093	0.101540426	0.788783372
Siglecg	-0.12906399	0.545489416	-0.236602188	0.812965422	0.992048071
Nkg7	1.199860442	0.223420588	5.370411268	7.86E-08	0.000320651
Cldnd2	3.857239949	2.208638459	1.746433389	0.080735672	0.758307521
Etfb	0.267746156	0.142723248	1.875981385	0.060657836	0.725221283
Vsig10l	0.13734469	0.691934706	0.198493715	0.842658802	0.992886758
Cd33	-0.138746441	0.275483219	-0.503647522	0.614509092	0.974001549
Zfp658	0.409133458	0.298707599	1.369678774	0.170787197	0.86244803
Zfp719	-0.091993969	0.148142071	-0.620984766	0.534609646	0.96674935
Zfp819	-0.515824479	1.478027595	-0.348995161	0.727092942	0.988585656
Siglece	0.082512394	0.421099053	0.195945333	0.844652955	0.992886758
Ctu1	-0.274696965	0.264861459	-1.037134531	0.299673164	0.910105802
Klk14	0.192478025	0.802562783	0.239829243	0.810462636	0.991438232
Klk13	0.323321242	0.305874234	1.057039811	0.290493419	0.907334416
2310002F09Rik	0.533194713	0.810978224	0.657471061	0.510878073	0.963452765
Klk11	-0.307214326	0.366345725	-0.838591268	0.401698713	0.937834293

Klk10	0.385615315	0.771507593	0.499820505	0.617201471	0.974001549
Klk9	-1.727184309	1.508088063	-1.145280804	0.252092855	0.896939594
Klk8	0.408551358	0.181038847	2.256705483	0.024026484	0.62266731
Klk1b27	0.088488993	1.62055613	0.05460409	0.95645388	0.996173928
Klk1b21	-0.704937421	2.182346189	-0.323018146	0.746681499	0.988585656
Klk1	-0.059939949	0.642445935	-0.0932996	0.92566555	0.994688009
1700028J19Rik	-0.508921692	0.821933116	-0.619176527	0.535800074	0.96674935
2410002F23Rik	-0.05923421	0.141860452	-0.417552669	0.676274208	0.983549699
Clec11a	-0.180170232	0.176818637	-1.018954985	0.308224338	0.9139204
Shank1	-0.627340217	2.204076379	-0.284627259	0.775929725	0.990861469
1700008O03Rik	-0.80362976	1.211284723	-0.663452403	0.507040854	0.963011338
Syt3	-0.05944202	0.805057404	-0.073835754	0.941141077	0.994688009
Lrrc4b	0.523726299	3.413398572	0.153432507	0.878057212	0.992886758
Josd2	0.050272772	0.16260455	0.309171988	0.757190701	0.989266039
Emc10	-0.012435881	0.109683959	-0.113379214	0.909729918	0.994688009
Fam71e1	0.308954973	0.434823339	0.710529875	0.477375612	0.95716557
Mybpc2	0.025966534	1.005241591	0.025831138	0.979392026	0.997262526
Spib	-0.005719062	0.466786026	-0.012252	0.990224563	0.998417328
Pold1	-0.103938736	0.215475056	-0.482370156	0.629543017	0.977034429
Nr1h2	-0.325178835	0.157723852	-2.061697268	0.039236567	0.679118604
Napsa	-0.196448142	0.18699059	-1.05057769	0.293452592	0.907334416
Kcnc3	-0.006736656	0.403765893	-0.016684559	0.986688266	0.998148029
Myh14	0.200390438	0.192341777	1.04184562	0.29748326	0.909070179
Zfp473	-0.991015252	1.402011504	-0.70685244	0.479658182	0.957463006
Vrk3	0.006657551	0.160341865	0.041520977	0.96688057	0.996474415
Atf5	0.130308648	0.118432831	1.1002747	0.271212452	0.89804647
Nup62	0.164550802	0.209048482	0.787141818	0.431198852	0.945321484
Tbc1d17	-0.039304221	0.154115284	-0.255031302	0.798698923	0.990988839
Akt1s1	0.021974537	0.121727084	0.180522985	0.856742011	0.992886758
Pnkp	0.126690127	0.160404496	0.789816559	0.429634907	0.945321484
Ptov1	-0.283537567	0.120177099	-2.359331093	0.018307913	0.582676994
Med25	-0.428837411	0.243772975	-1.759167158	0.078549121	0.754732183
Fuz	0.042611795	0.14414966	0.295608017	0.767529451	0.989990452
Ap2a1	0.022399592	0.267578323	0.083712283	0.933285191	0.994688009
Tsks	0.877312928	2.415009093	0.363275207	0.716399312	0.988585656
Cpt1c	0.190124765	0.365457911	0.520237104	0.602898327	0.974001549
Prmt1	0.227771989	0.100027432	2.277095236	0.022780539	0.620006604
Gm15545	-0.154244061	0.500734929	-0.308035355	0.75805543	0.989266039
Bcl2l12	0.019481089	0.241695804	0.080601686	0.935758725	0.994688009
Irf3	-0.000240415	0.10643536	-0.002258791	0.998197747	0.999591134
Scaf1	0.008570822	0.218898768	0.039154273	0.96876739	0.996474415
Rras	0.090989115	0.143952255	0.63207843	0.527335629	0.965637248
Prr12	0.263500957	0.360282241	0.731373703	0.464550923	0.953083512
Prrg2	0.113795517	0.126257887	0.901294321	0.367431852	0.928436166
Nosip	0.121898874	0.15270899	0.798242944	0.424729521	0.944280828
Rcn3	0.068174158	0.123983918	0.549862906	0.582413409	0.972017845
Fcgrt	0.220992848	0.134850236	1.638802087	0.101254485	0.788783372
Rps11	0.340445288	0.230370514	1.477816248	0.139456968	0.836535783
Rpl13a	0.311711326	0.311235182	1.001529853	0.316570715	0.91640698
Flt3l	-0.057956813	0.163876053	-0.353662491	0.723591836	0.988585656
Aldh16a1	0.122915525	0.184427724	0.666469889	0.505110804	0.962138588
Pih1d1	0.113307378	0.156908658	0.722123171	0.470218753	0.95486913
Slc17a7	-0.6977429	0.835212563	-0.835407573	0.403488267	0.937834293
Ccdc155	-0.229782185	0.276262612	-0.831752742	0.405548524	0.939557159
Dkkl1	-0.282939019	0.367393477	-0.770125322	0.441225557	0.948068187
Tead2	-0.16084207	0.273867657	-0.587298523	0.557003233	0.968926429
Cd37	-0.11461218	0.331568604	-0.345666565	0.729593314	0.988585656
Trpm4	0.125198235	0.133459314	0.938100394	0.348192819	0.921552698
Rpl14-ps1	0.434272452	0.224928078	1.930716943	0.053518066	0.707153766
Hrc	0.141941004	0.669116461	0.212131986	0.832004066	0.992886758
Ppfia3	0.905326807	0.976887953	0.926745799	0.354058532	0.924354588
Mtag2	3.949093828	1.928229334	2.048041567	0.040555927	0.679118604

Lin7b	0.74229568	1.241072447	0.598108258	0.5497677	0.968926429
Snrnp70	-0.139775057	0.160586717	-0.870402359	0.384080558	0.931591679
Kcna7	0.654933031	1.439628976	0.454931821	0.649158289	0.979004493
Ntf5	-0.023542349	0.230893154	-0.101962092	0.918786764	0.994688009
Lhb	-0.267482273	0.331571939	-0.806709619	0.419833801	0.943415794
Ruvbl2	-0.118090841	0.119621651	-0.987202904	0.323543178	0.91640698
Gys1	-0.323097284	0.23777001	-1.358864746	0.174189452	0.863528862
Ftl1	-0.023359003	0.141305889	-0.165308066	0.86870151	0.992886758
Bax	0.290836638	0.146493637	1.98531925	0.047108964	0.69958699
Dhdh	-0.112558157	0.188827733	-0.596089118	0.551115694	0.968926429
Tulp2	0.018679911	1.013520773	0.018430714	0.985295251	0.998148029
Nucb1	-0.275337813	0.154517124	-1.781924274	0.074761582	0.75241101
Ppp1r15a	-0.031155802	0.178729006	-0.174318669	0.861615028	0.992886758
Plekha4	-0.013069666	0.308694603	-0.0423385	0.966228855	0.996474415
Hsd17b14	-0.747416534	0.921305209	-0.811258339	0.417217329	0.943415794
0610005C13Rik	-0.528697775	1.343122517	-0.39363332	0.693851778	0.986894729
Bcat2	-0.064602561	0.144556268	-0.446902527	0.654945439	0.979133069
Fut1	-0.991446753	1.966411028	-0.504191005	0.614127161	0.974001549
Izumo1	-1.300559322	1.278540029	-1.017222216	0.30904773	0.9139204
Rasip1	-0.210572723	0.128251827	-1.641869187	0.100617117	0.788783372
Mamstr	0.758705509	0.697627155	1.087551571	0.27679312	0.900604936
Fut2	0.70134294	0.760381445	0.922356726	0.356342534	0.924354588
Sec1	-0.207734351	0.587225216	-0.353755843	0.723521868	0.988585656
Ntn5	1.958319769	1.369517277	1.42993433	0.152735868	0.853744627
Car11	-0.235122827	0.656142518	-0.358341093	0.720088069	0.988585656
Dbp	-0.770938575	0.319891028	-2.410003742	0.015952357	0.557717626
Sphk2	-0.04964217	0.184721667	-0.268740372	0.788129482	0.990988839
Rpl18	0.351629515	0.155328473	2.263780155	0.02358764	0.622011301
Fam83e	-0.342612184	0.405515373	-0.844880876	0.398177353	0.935327115
Sult2b1	0.444641492	0.271838102	1.635684948	0.101905541	0.788783372
Lmtk3	-0.445614473	1.886950412	-0.2361559	0.813311698	0.992048071
Cyth2	-0.027316641	0.108534366	-0.251686563	0.801283344	0.990988839
Kcnj14	2.108351015	1.797974663	1.172625543	0.240946002	0.896939594
Grwd1	-0.178219754	0.24702958	-0.721451068	0.470632036	0.95486913
Grin2d	0.282206161	0.630959452	0.447265129	0.654683641	0.979133069
Kdelr1	-0.129565257	0.12055907	-1.074703517	0.282507488	0.905832632
Tmem143	-0.023142245	0.261223884	-0.088591614	0.929406473	0.994688009
Emp3	0.08282577	0.127022102	0.652057941	0.514363795	0.964352198
Ccdc114	-0.347428321	0.204926918	-1.695376694	0.090003984	0.769347579
Abcc6	-0.83280079	2.071205766	-0.402085009	0.687621465	0.98538669
Nomo1	-0.222317762	0.197243046	-1.127125981	0.25968922	0.897248195
Kcnj11	-0.572853988	0.498407594	-1.149368499	0.250404064	0.896939594
Abcc8	-0.690276204	1.455904047	-0.474122045	0.635412879	0.977034429
Kcnc1	-1.650813611	2.355529043	-0.700824987	0.483412242	0.957710647
Sergef	0.311911223	0.152990135	2.038766902	0.041473297	0.679118604
Saal1	0.284851467	0.154310031	1.845968569	0.064896769	0.733773928
Saa3	0.062039644	0.697438699	0.088953545	0.92911883	0.994688009
Saa1	0.126953715	1.045084417	0.121476995	0.903313235	0.994688009
Hps5	-0.097828545	0.16979936	-0.576142014	0.564519195	0.969658675
Gtf2h1	-0.011682132	0.137723795	-0.084822904	0.932402185	0.994688009
Ldha	-0.075275763	0.127666778	-0.589626871	0.555440832	0.968926429
Tsg101	-0.00149845	0.090938786	-0.016477566	0.986853399	0.998148029
Uevld	0.004653379	0.221133202	0.021043329	0.983211092	0.997569444
Spty2d1	-0.214090279	0.154392663	-1.386660961	0.16554517	0.860011234
Tmem86a	-0.183555955	0.2032159	-0.903255875	0.36639011	0.927834635
Ptpn5	-1.460447524	1.027819486	-1.420918308	0.15534051	0.855261441
Zdhhc13	-0.063437925	0.175574439	-0.361316406	0.717862931	0.988585656
Csrp3	0.255838238	0.324092775	0.789398155	0.429879334	0.945321484
E2f8	-0.261724561	1.134570661	-0.230681587	0.817562179	0.992678823
Nav2	-0.310064974	0.292444277	-1.060253176	0.289029435	0.907334416
Htatip2	0.462191036	0.173487386	2.664118967	0.007719026	0.45506991
Prmt3	-0.180117186	0.213290097	-0.844470457	0.398406565	0.935536313

Nell1	-0.18287378	0.84805001	-0.215640325	0.82926812	0.992886758
Ano5	0.882436778	0.908726551	0.971069655	0.331513594	0.917115058
Fancf	0.089025056	0.290422022	0.306536864	0.759195917	0.989266039
Gas2	0.271766458	0.228319686	1.190289206	0.233932742	0.89581803
Svip	0.202854107	0.149743762	1.354674838	0.175521178	0.864596675
Siglech	0.883847285	0.372186406	2.374743597	0.017561144	0.579835303
Tubgcp5	0.134082118	0.198876186	0.674198959	0.500184831	0.9611436
Cyfip1	-0.10420391	0.131101197	-0.794835684	0.42670909	0.944290206
Nipa2	0.130346047	0.136064573	0.957971971	0.338076893	0.919246575
A230056P14Rik	-0.023227664	0.225427814	-0.103038144	0.917932698	0.994688009
Nipa1	0.129970383	0.145837225	0.891201704	0.372820971	0.928840865
Herc2	0.327813929	0.204046915	1.606561557	0.108150581	0.797772336
Atp10a	-0.299998259	0.242420518	-1.237511834	0.215897124	0.887633697
Ube3a	-0.14873528	0.148191206	-1.003671433	0.315537011	0.91586731
C230091D08Rik	-0.517119342	0.324926052	-1.591498553	0.111497421	0.800752782
Snhg14	0.021817413	0.331366055	0.065840821	0.947504557	0.995361401
Snord64	-0.038012294	1.645275729	-0.023103905	0.981567391	0.997390438
A230057D06Rik	0.577509555	0.638592414	0.90434766	0.365811082	0.927134049
A330076H08Rik	0.359997775	0.376457996	0.956276076	0.338932773	0.919246575
Ndn	0.191588622	0.165345097	1.158719704	0.246570457	0.896939594
Mkrn3	0.039726813	0.351187778	0.113121285	0.9099344	0.994688009
Peg12	0.149966073	0.546875581	0.274223386	0.783912958	0.990988839
Chrna7	0.18478145	1.218116	0.151694461	0.879427926	0.992886758
Otud7a	0.970317596	1.522347207	0.637382584	0.52387567	0.965637248
Klf13	-0.112665148	0.185900704	-0.606050144	0.544481456	0.967491297
Trpm1	-0.060135526	1.174442453	-0.051203468	0.959163388	0.996367077
Mtmr10	-0.43216568	0.170154916	-2.539836576	0.011090428	0.505791657
Fan1	-0.582497413	0.433695849	-1.343101195	0.179239209	0.867377413
Mphosph10	-0.061234062	0.126763818	-0.483056311	0.629055754	0.977034429
Mcee	0.162279015	0.142315832	1.140273807	0.254172247	0.897044747
Apba2	-1.407920883	0.645442663	-2.181326034	0.02915931	0.64858361
Ndnl2	0.092458165	0.183461319	0.503965445	0.61428566	0.974001549
Tjp1	0.086514644	0.132747743	0.651722149	0.514580431	0.964352198
Tarsl2	0.117384369	0.292869078	0.400808341	0.688561237	0.98538669
Tm2d3	-0.090667355	0.123414923	-0.734654715	0.462549807	0.953083512
1810008I18Rik	-0.090138787	0.332117414	-0.271406385	0.786078492	0.990988839
Pcsk6	-0.167977338	0.312481502	-0.537559302	0.590881333	0.974001549
Snrpa1	0.041289421	0.118827083	0.347474836	0.72823462	0.988585656
Vimp	0.023620194	0.116124537	0.203403991	0.83881928	0.992886758
Chsy1	0.110018174	0.152785456	0.72008277	0.471474035	0.95486913
Lrrk1	0.25492473	0.139713748	1.824621658	0.068058143	0.742385562
Aldh1a3	0.036363962	0.724534087	0.050189443	0.959971424	0.996474415
Asb7	0.244097496	0.188578222	1.294409789	0.195523911	0.879601031
Lins	0.085020759	0.218794587	0.388587124	0.697581593	0.987550046
Cers3	0.073768615	0.731772951	0.100808065	0.919702824	0.994688009
Adamts17	0.217153508	0.437105654	0.49679867	0.619331033	0.974471036
1700112J16Rik	-0.141743397	1.372825275	-0.103249408	0.917765027	0.994688009
Lysmd4	0.257093765	0.168727701	1.523719959	0.127578681	0.824941438
Mef2a	-0.100904501	0.142359686	-0.708799688	0.478448791	0.957463006
Lrrc28	-0.507157696	0.324126103	-1.564692542	0.117655018	0.813618585
Ttc23	0.185395495	0.164259329	1.128675584	0.259034713	0.897248195
Synm	0.10488007	0.208711157	0.502513	0.61530671	0.974001549
4833412C05Rik	-0.190228288	1.442531256	-0.131871172	0.895086191	0.993604681
Igf1r	0.151106089	0.243795548	0.619806597	0.535385125	0.96674935
Pgpep1l	-0.181488457	0.417545913	-0.43465509	0.663812801	0.981154469
Gm16157	-0.271627756	1.543879971	-0.17593839	0.860342345	0.992886758
Fam169b	-0.352291894	0.356818278	-0.987314595	0.323488438	0.91640698
4933436H12Rik	-0.370039905	2.141809123	-0.172769786	0.862832387	0.992886758
Arrdc4	0.139855958	0.157619926	0.887298716	0.374918097	0.928883222
Gm29683	-0.261208382	0.601284791	-0.434417078	0.663985598	0.981154469
Nr2f2	0.279641036	0.156194331	1.790340497	0.07339919	0.748833631
B130024G19Rik	0.190536471	0.295795928	0.64414839	0.519479214	0.964665379

Mctp2	-0.212910181	0.316476738	-0.672751439	0.501105437	0.96121484
A730056A06Rik	0.169035962	0.994925357	0.169898134	0.865090248	0.992886758
Rgma	0.329758431	0.426931162	0.772392508	0.43988199	0.947849924
Chd2	0.193233239	0.240872543	0.802221942	0.422424586	0.943509323
1810026B05Rik	0.142791848	0.124343663	1.148364498	0.250818123	0.896939594
Fam174b	-0.11020801	0.105203018	-1.047574601	0.294834644	0.90737175
St8sia2	0.289654713	0.408363353	0.709306335	0.478134398	0.957438234
Slco3a1	-0.151033955	0.152320484	-0.991553801	0.321415235	0.91640698
Sv2b	-0.368423528	1.401657705	-0.262848431	0.792667405	0.990988839
Akap13	0.002856207	0.194009223	0.014722018	0.988253953	0.998148029
Klhl25	0.201118499	0.179237619	1.122077498	0.261829491	0.897248195
Ntrk3	-0.254655948	1.129361024	-0.225486751	0.821600631	0.992886758
Mrpl46	0.32394328	0.1929859	1.67858522	0.093232909	0.775558829
Mrps11	0.153838942	0.198329838	0.7756722	0.437942558	0.947663582
Det1	0.175862114	0.226745891	0.775591184	0.437990408	0.947663582
Aen	0.172859565	0.187875396	0.920075585	0.357533262	0.924354588
Isg20	0.164106487	0.117948698	1.391337842	0.164123004	0.859982992
Hapln3	-0.734662768	1.097136512	-0.669618375	0.503101097	0.961729397
Mfge8	-0.36798975	0.126699658	-2.904425758	0.003679275	0.376311503
Abhd2	-0.271722463	0.250295562	-1.085606397	0.277653168	0.901241208
Rlbp1	-0.291795661	0.693667574	-0.420656338	0.674006049	0.983375962
Fanci	0.160194625	0.504850152	0.317311234	0.751007454	0.988585656
Polg	0.221653468	0.13786747	1.607728548	0.107894643	0.796929372
Ticrr	-1.089649465	1.68228215	-0.647720993	0.517165424	0.964352198
Kif7	-0.11321564	0.525837986	-0.215305176	0.829529394	0.992886758
Plin1	-0.542214606	0.499784473	-1.084896862	0.277967338	0.901315669
Pex11a	0.072560567	0.250235556	0.289969051	0.771839914	0.990398482
Wdr93	-0.476552662	0.25454584	-1.872168337	0.061183318	0.725221283
Mesp1	1.128221823	0.837011339	1.347917011	0.177685076	0.866855369
Mesp2	-0.168733798	0.495101949	-0.340806168	0.733249506	0.988585656
Anpep	-0.068816769	0.203191573	-0.338679249	0.734851374	0.988585656
Ap3s2	0.09799608	0.161746168	0.605863378	0.54460548	0.967491297
Arpin	0.049292113	0.107446275	0.458760554	0.646406125	0.979004493
Zfp710	-0.054469224	0.264935845	-0.205594015	0.837108053	0.992886758
Gm21057	0.225839772	0.486834813	0.463894047	0.642723658	0.978681333
Idh2	0.003856047	0.104299643	0.036970852	0.970508246	0.996733101
Sema4b	-0.05502977	0.342722241	-0.160566673	0.872434706	0.992886758
Cib1	0.163402156	0.127768657	1.278890772	0.200935523	0.881109608
Gdpgp1	0.145542491	0.303284329	0.479887939	0.631307078	0.977034429
Ttll13	0.580225808	0.472300108	1.228510853	0.219255258	0.888847883
Ngrn	0.043665449	0.112143389	0.389371586	0.697001288	0.987518602
Vps33b	0.295659444	0.288703264	1.024094567	0.30579061	0.91333012
Prc1	0.343626632	0.429142943	0.800727678	0.423289316	0.943509323
Rccd1	-0.520727484	0.252284311	-2.064050206	0.039012956	0.679118604
Unc45a	-0.088191566	0.164560289	-0.535922525	0.592012096	0.974001549
Hddc3	0.177660484	0.203366327	0.873598332	0.382337035	0.930775109
Man2a2	-0.175631156	0.205495781	-0.854670373	0.392733657	0.933734231
Fes	-0.123133385	0.1853579	-0.66430071	0.506497867	0.962475637
Furin	-0.134785154	0.210076933	-0.641599017	0.521133571	0.964784335
Blm	0.32986968	0.631379197	0.522458899	0.601350856	0.974001549
Crtc3	0.034207045	0.298925526	0.114433334	0.908894291	0.994688009
Gm15880	-0.758711949	2.041957801	-0.371561033	0.710219706	0.988585656
Iqgap1	-0.143188573	0.117538398	-1.218228041	0.223137327	0.892421271
Zscan2	-0.02904661	0.327091002	-0.08880284	0.929238601	0.994688009
Wdr73	0.164120242	0.134752689	1.217936676	0.223248037	0.892500173
Nmb	0.305801756	0.328315885	0.931425405	0.351633555	0.923082865
Sec11a	0.187799334	0.129315482	1.452257159	0.146430115	0.843780493
Zfp592	0.016199798	0.259958313	0.062316908	0.950310464	0.99573416
Alpk3	-0.371042376	0.975885122	-0.38021112	0.703788705	0.988585656
Pde8a	-0.035847659	0.2273621	-0.157667699	0.874718654	0.992886758
Rps17	0.369727278	0.141545294	2.612077503	0.008999385	0.475600561
Cpeb1	-0.269076578	0.214093952	-1.25681541	0.208820485	0.884258007

2900076A07Rik	0.49276412	0.299114204	1.6474113	0.099473534	0.788783372
Mir1839	-1.835559076	2.160935889	-0.849427827	0.395643275	0.934066368
Fsd2	-0.22235551	0.350257	-0.634835308	0.525535833	0.965637248
Whamm	-0.221738646	0.213587092	-1.038165011	0.299193239	0.910105802
Homer2	-0.114211002	0.259235722	-0.440568146	0.659525668	0.979924124
Fam103a1	0.084258904	0.087388333	0.964189386	0.334950985	0.918190441
3110040N11Rik	0.063989687	0.163665186	0.390979222	0.695812594	0.98718563
Btbd1	-0.026089406	0.101319583	-0.25749618	0.796795764	0.990988839
Tm6sf1	0.135525216	0.129096424	1.049798375	0.293810823	0.907334416
Hdgfrp3	-0.058031074	0.170554544	-0.340249357	0.733668751	0.988585656
Bnc1	-0.191879982	0.283964806	-0.675717474	0.499220039	0.960700483
Gm20744	1.737134689	1.607026694	1.080961937	0.279714045	0.903083096
Sh3gl3	-0.411936758	0.595224009	-0.692070132	0.488893288	0.958560421
Adamtsl3	0.078070344	0.412939596	0.18905996	0.850045823	0.992886758
Saxo2	-0.300565863	0.163656846	-1.836561506	0.066274654	0.738254074
Eftud1	-0.435614918	0.276810518	-1.573693518	0.115558321	0.810389074
Mex3b	-0.013020313	0.388871634	-0.03348229	0.973289989	0.997107714
Tmc3	-0.810287037	0.705612316	-1.148345938	0.250825782	0.896939594
Stard5	0.06263434	0.175111158	0.357683318	0.720580318	0.988585656
Il16	0.288591157	0.167681453	1.721067843	0.085238514	0.761635307
1700026D08Rik	-0.341186482	0.167412184	-2.038002698	0.041549663	0.679118604
Mesdc1	-0.114293222	0.121042542	-0.944240097	0.345046969	0.92132235
Mesdc2	0.053774512	0.096837001	0.555309557	0.578682943	0.971613657
Cemip	-0.173331321	1.886716701	-0.091869289	0.926801892	0.994688009
Abhd17c	-0.164584389	0.126961075	-1.296337388	0.194859273	0.879588734
Arnt2	0.355733805	1.156096573	0.307702499	0.758308718	0.989266039
Gm2115	-0.620838609	0.894139952	-0.694341649	0.487467978	0.958288569
Fah	-0.13650158	0.248387589	-0.549550727	0.582627562	0.972086107
Zfand6	0.113376147	0.112055528	1.011785397	0.311640674	0.914819219
2610206C17Rik	1.321852287	2.13675027	0.618627411	0.53616184	0.96674935
Folh1	1.774907439	1.355556928	1.309356621	0.190413578	0.877513044
Nox4	0.030192382	0.125866311	0.239876594	0.810425926	0.991438232
Tyr	-3.004699235	1.55093658	-1.937345004	0.052703184	0.707153766
Ctsc	-0.293652273	0.138981897	-2.112881463	0.034610915	0.674795441
Rab38	-0.139320041	0.215222601	-0.647329977	0.517418404	0.964352198
Tmem135	-0.251102132	0.151954884	-1.652478192	0.098437107	0.786907305
Fzd4	-0.3694083	0.286465975	-1.289536391	0.197211673	0.879755417
Prss23	-0.133081153	0.156867038	-0.848369133	0.396232428	0.934066368
Me3	0.078461224	0.281002621	0.279218835	0.78007689	0.990988839
Ccdc81	-0.104490285	0.375717969	-0.278108298	0.780929226	0.990988839
l7Rn6	0.190981549	0.15374026	1.242235114	0.214149828	0.886067976
Eed	0.149745454	0.115193916	1.299942393	0.193620714	0.879588734
E230029C05Rik	0.064475325	0.862567961	0.07474811	0.940415128	0.994688009
2310010J17Rik	0.277010068	0.303218933	0.913564549	0.360945701	0.926101223
Picalm	-0.140992448	0.111453287	-1.265036249	0.205858334	0.883765092
Ccdc83	-0.389871518	0.455668103	-0.855604146	0.392216776	0.933734231
Sytl2	-0.261934923	0.238937807	-1.096247292	0.272970569	0.898826892
Ccdc89	2.039896424	0.670275416	3.043370495	0.002339441	0.31402518
Crebzf	0.092664541	0.164938402	0.561813012	0.574243426	0.971613657
Tmem126a	0.351148377	0.221531135	1.585097173	0.112944258	0.804555365
Tmem126b	0.04685527	0.165245855	0.283548835	0.776756152	0.990861469
Dlg2	0.175056503	1.181259703	0.148194764	0.882189064	0.992886758
Ccdc90b	0.348158413	0.25943667	1.341978422	0.179602992	0.868087032
Ankrd42	-0.275860297	0.290252741	-0.950414098	0.341901883	0.919850248
Pcf11	-0.076742525	0.138154186	-0.555484618	0.578563229	0.971613657
4632427E13Rik	0.643234301	0.624654205	1.029744609	0.303129909	0.911623139
Rab30	0.006478689	0.286172763	0.022639082	0.981938169	0.997390438
Ddias	0.239023624	0.628249671	0.380459609	0.703604273	0.988585656
Prcp	-0.142711568	0.131703503	-1.083582172	0.278550099	0.901682903
Fam181b	0.235038723	0.442919624	0.530657731	0.595655982	0.974001549
Tenm4	-2.127704511	1.777119412	-1.197277176	0.231198537	0.895404344
Nars2	0.041613674	0.179282638	0.232112123	0.816450929	0.992676801

Gab2	-0.186275799	0.215465068	-0.864528993	0.38729736	0.932858077
Usp35	-0.02089955	0.343226811	-0.060891368	0.951445724	0.996023481
Kctd21	-0.372644151	0.266151031	-1.400122892	0.161476521	0.857374685
Alg8	-0.372690585	0.418204956	-0.891167308	0.37283942	0.928840865
Ndufc2	0.144476107	0.15274325	0.945875562	0.34421206	0.920669678
Thrsp	-0.344768804	0.479787188	-0.718586932	0.472395467	0.955003345
Kctd14	-0.19801775	0.224176341	-0.883312435	0.377067487	0.928883222
Ints4	0.065402737	0.199695371	0.327512536	0.743280263	0.988585656
Aamdc	0.127247394	0.170441487	0.746575239	0.45532	0.951224746
Rsf1	-0.191037348	0.167966958	-1.137350768	0.255391679	0.897044747
Clns1a	0.037621265	0.153897229	0.244457064	0.806876851	0.99099448
Aqp11	-0.055023774	0.331763858	-0.165852225	0.868273246	0.992886758
Pak1	0.055276872	0.265676126	0.208061117	0.835181247	0.992886758
Myo7a	-0.08038073	0.303848617	-0.264542032	0.791362281	0.990988839
Capn5	0.290085986	0.262326649	1.105819737	0.268804551	0.89804647
Omp	0.065059047	1.886710297	0.034482796	0.972492161	0.996790728
Gm16938	0.110016838	0.588432993	0.186965788	0.851687457	0.992886758
B3gnt6	-1.058994189	1.451912543	-0.729378773	0.465769999	0.953083512
Acer3	0.232134581	0.143194478	1.621114059	0.104993179	0.793869912
Tsku	-0.293827986	0.344651431	-0.852536679	0.39391629	0.933796635
Lrrc32	0.047257349	0.280448857	0.16850612	0.866185129	0.992886758
2210018M11Rik	-0.305089811	0.258698459	-1.179325968	0.238268402	0.896609492
Prkrir	-0.084422413	0.145366974	-0.580753739	0.561406438	0.96944293
Wnt11	-0.228779177	0.20749012	-1.102602751	0.270199715	0.89804647
Uvrag	-0.120756505	0.158764681	-0.760600561	0.446895685	0.948476747
Dgat2	-0.185888823	0.236483103	-0.786055411	0.431835025	0.9459793
Mogat2	-1.465494938	1.675698288	-0.874557758	0.38181458	0.930635634
Gm26705	0.318726208	1.585284982	0.201052941	0.840657178	0.992886758
Map6	-0.047351138	0.216008395	-0.21920971	0.826486692	0.992886758
Serpinh1	-0.316370989	0.114590382	-2.760886072	0.005764478	0.423948802
Gdpd5	0.196111964	0.332148771	0.590434109	0.554899649	0.968926429
Klhl35	-2.070468762	1.704336902	-1.214823641	0.224433362	0.892989918
Rps3	0.419047623	0.288314378	1.45343991	0.146101652	0.843494228
Snord15b	-0.3509024	1.101266775	-0.318635238	0.750003129	0.988585656
Arrb1	-0.129537731	0.125871825	-1.029124119	0.303421354	0.911786415
Gm4980	0.326120758	0.407292	0.800705041	0.423302424	0.943509323
Slco2b1	-0.249675402	0.267311227	-0.934025125	0.350290933	0.922338606
Neu3	-0.195827858	0.250976848	-0.780262639	0.435236299	0.946762848
Spcs2	0.087640416	0.111269048	0.787644164	0.430904874	0.945321484
Xrra1	-1.049390337	0.677863502	-1.548085027	0.121601826	0.816233249
Rnf169	0.207843591	0.296330728	0.701390611	0.483059279	0.957463006
Pold3	-0.032845197	0.135013024	-0.243274286	0.807792923	0.99099448
Lipt2	-0.032650471	0.269144123	-0.121312219	0.903443742	0.994688009
Kcne3	0.294829862	0.252822058	1.166155615	0.243551534	0.896939594
Pgm2l1	0.183965721	0.204802979	0.898257058	0.36904852	0.928436166
P4ha3	1.564122291	1.518845232	1.029810186	0.303099118	0.911623139
Ppme1	-0.250466281	0.225677903	-1.109839631	0.267068138	0.897760242
C2cd3	-0.180527119	0.30287593	-0.596043137	0.551146409	0.968926429
Ucp3	0.184932648	0.361821117	0.511116237	0.609269667	0.974001549
Ucp2	0.001323131	0.191648539	0.006903944	0.994491493	0.999285699
Dnajb13	-0.266920814	0.238910464	-1.117242041	0.263890849	0.897248195
Coa4	0.415174989	0.214880366	1.932121562	0.053344504	0.707153766
Mrpl48	0.28042182	0.17543967	1.598394592	0.109955187	0.798948974
Rab6a	-0.213080658	0.099012353	-2.152061349	0.031392523	0.661395937
Plekhb1	0.084346544	0.117575685	0.717380846	0.473139135	0.955584198
Fam168a	-0.22048205	0.168129447	-1.311382708	0.189728498	0.876293406
Relt	-0.086382537	0.26106877	-0.330880392	0.740734833	0.988585656
Arhgef17	-0.045729842	0.222228299	-0.205778662	0.83696381	0.992886758
P2ry6	-0.358531641	0.377998654	-0.948499781	0.342875084	0.919850248
P2ry2	0.239158981	0.269464686	0.887533667	0.374791649	0.928883222
Fchsd2	-0.129838143	0.159156313	-0.815790091	0.414620202	0.943415794
Atg16l2	0.265203797	0.24866703	1.066501646	0.286196935	0.905916508

Stard10	0.239347933	0.127103146	1.883099988	0.059686819	0.725079384
Arap1	-0.325297117	0.144946839	-2.244251192	0.024816244	0.627796923
Pde2a	0.24178852	0.474472179	0.509594726	0.610335419	0.974001549
Art2b	0.827623941	0.971396446	0.851993997	0.394217424	0.933796635
Clpb	-0.016932663	0.183509422	-0.092271357	0.926482445	0.994688009
Inppl1	0.177058751	0.157393447	1.1249436	0.260612935	0.897248195
Folr2	-0.1722451	0.25093107	-0.686423965	0.492445796	0.958916246
Folr1	-0.378067865	0.642910626	-0.588056643	0.556494274	0.968926429
Anapc15	0.301989545	0.229895216	1.313596473	0.188982036	0.87553356
Tomt	0.436355312	0.367931864	1.185967714	0.23563505	0.896002526
Lamtor1	0.129743777	0.145741497	0.890232219	0.373341208	0.928840865
Lrrc51	0.018033262	0.197623808	0.091250452	0.927293588	0.994688009
Numa1	0.280284741	0.165437369	1.694204535	0.090226423	0.769347579
Il18bp	-0.412125084	0.2244909	-1.835820891	0.066384151	0.738254074
Rnf121	0.037583007	0.132032579	0.284649498	0.775912685	0.990861469
Xndc1	1.222764457	0.591726184	2.066436285	0.038787303	0.679118604
Art5	-0.309517638	1.062135636	-0.291410652	0.770737269	0.989990452
Art1	-0.402530237	0.568308341	-0.708295494	0.478761775	0.957463006
Chrna10	0.608930946	1.635756291	0.372262634	0.709697316	0.988585656
Nup98	-0.23309101	0.17429372	-1.337346006	0.18110972	0.868087032
Pgap2	-0.16734721	0.099841995	-1.676120452	0.093714602	0.775558829
Rhog	-0.298893489	0.17192349	-1.738526179	0.082118141	0.76099108
Stim1	-0.365630256	0.161658627	-2.261742926	0.023713291	0.622011301
Rrm1	-0.130002798	0.172647114	-0.752997227	0.45145158	0.949134998
Trim21	0.183734948	0.148006558	1.241397347	0.214458999	0.886400046
Olfr550	-1.162481686	0.80848282	-1.437855768	0.150474982	0.849879783
Trim68	0.137334595	0.187382109	0.732911997	0.463612105	0.953083512
Olfr558	-3.921387927	3.814358776	-1.028059539	0.303921821	0.911786415
Trim6	0.691846102	0.783489628	0.883031603	0.377219197	0.928883222
Trim34a	0.155688843	0.138431078	1.124666839	0.260730239	0.897248195
Trim12a	0.192380021	0.143094091	1.344430224	0.178809306	0.867377413
Gm15133	0.093248539	0.589548222	0.158169486	0.874323248	0.992886758
Trim34b	0.721697457	1.510752288	0.47770734	0.632858519	0.977034429
Trim12c	0.074004414	0.158594289	0.466627231	0.640766601	0.977921835
Trim30b	0.599864616	0.519191473	1.155382257	0.247933924	0.896939594
Trim30a	0.181163166	0.132536396	1.366893713	0.171658623	0.86244803
Trim30d	0.131360375	0.177607903	0.739608841	0.459537376	0.952865981
Fam160a2	-0.042801606	0.244362442	-0.175156237	0.860956871	0.992886758
Prkcdbp	0.119287388	0.119701654	0.996539175	0.318988242	0.91640698
Smpd1	-0.370094499	0.127905865	-2.893491234	0.003809849	0.376754331
Apbb1	-0.143622093	0.132883535	-1.080811797	0.27978084	0.90312016
Hpx	-0.475248954	0.452673568	-1.049871226	0.293777322	0.907334416
Trim3	-0.070996095	0.133195356	-0.5330223	0.594018143	0.974001549
Arfip2	0.096490313	0.12343351	0.781718944	0.434379766	0.946762848
Timm10b	0.152340993	0.169817724	0.897085351	0.369673375	0.928436166
Rrp8	-0.096383842	0.161820975	-0.595620205	0.551428976	0.968926429
Ilk	-0.060965767	0.121287846	-0.502653561	0.615207865	0.974001549
Taf10	0.146958172	0.150390102	0.97717982	0.328480136	0.917115058
Tpp1	-0.302063576	0.188382857	-1.603455753	0.108834071	0.798400564
Dchs1	0.507840109	0.411975649	1.232694482	0.217689771	0.887633697
Mrpl17	0.105033185	0.162562643	0.646108994	0.518208771	0.96446859
Gm4759	0.285496637	1.930140311	0.147914965	0.882409877	0.992886758
Gm1966	-0.34277999	0.268052494	-1.278779335	0.200974773	0.881109608
Syt9	-0.118984527	0.659067234	-0.180534733	0.85673279	0.992886758
Olfml1	0.000983373	0.140390009	0.007004582	0.994411198	0.999285699
Ppfibp2	-0.054301253	0.172857721	-0.314138431	0.753415902	0.988585656
Cyb5r2	1.030917082	1.778660192	0.579603168	0.562182256	0.969658675
Nlrp10	0.40997475	0.818631107	0.500805243	0.616508196	0.974001549
Eif3f	0.046493357	0.118776219	0.39143658	0.695474558	0.98715859
Tub	-0.052766303	1.034843566	-0.050989642	0.959333775	0.996367077
Ric3	-0.080731904	0.251535129	-0.320956777	0.748243147	0.988585656
Lmo1	0.532798635	0.304386176	1.750403524	0.080048708	0.75659671

Stk33	-0.230192885	0.148867063	-1.546298289	0.122032545	0.816233249
Trim66	2.160713766	1.710699349	1.263058741	0.20656807	0.883765092
Rpl27a	0.180934327	0.186407849	0.970636846	0.331729151	0.917115058
Snora3	0.032398456	0.924386571	0.035048601	0.972040986	0.996750504
St5	-0.050533718	0.197575431	-0.255769239	0.798129028	0.990988839
Akip1	0.043146748	0.151808197	0.284218828	0.776242688	0.990861469
BC051019	-0.057812737	0.158485215	-0.36478316	0.715273276	0.988585656
Tmem9b	0.013924477	0.099361782	0.140139159	0.888550042	0.992886758
Nrip3	-0.454706423	0.801648757	-0.567214032	0.570568776	0.971613657
Scube2	-0.168836169	0.353508756	-0.477601093	0.632934153	0.977034429
Dennd5a	-0.234667392	0.147325518	-1.592849593	0.111193937	0.800468873
Tmem41b	-0.09628997	0.130705651	-0.736693247	0.461308915	0.953083512
1600010M07Rik	0.274286455	0.321819432	0.852299233	0.394048031	0.933796635
Ipo7	-0.172642586	0.130388063	-1.324067412	0.185480657	0.871716376
Zfp143	0.281364069	0.238206189	1.181178667	0.237531754	0.896609492
Wee1	-0.451568765	0.230328043	-1.960546179	0.049931986	0.707153766
Swap70	-0.169261487	0.143681419	-1.178033234	0.238783357	0.896609492
Sbf2	-0.231919554	0.225349028	-1.02915711	0.303405853	0.911786415
Adm	0.136607236	0.229884808	0.59424212	0.552350185	0.968926429
Ampd3	-0.277161791	0.267564656	-1.035868471	0.300263509	0.910285132
Rnf141	0.118782376	0.123439932	0.962268639	0.335914678	0.918908218
Lyve1	-0.571114454	0.267190204	-2.137482762	0.032558746	0.665167506
Mrvi1	0.032938027	0.404085943	0.081512429	0.935034441	0.994688009
Ctr9	-0.257605973	0.203899361	-1.263397646	0.20644631	0.883765092
Eif4g2	-0.225986567	0.064081733	-3.526536434	0.000421033	0.139568962
1700012D14Rik	0.657681181	0.600379199	1.095442984	0.273322611	0.89897004
Galnt18	-0.180349521	0.146329934	-1.232485493	0.217767782	0.887633697
Usp47	-0.028681704	0.187312225	-0.153122435	0.878301724	0.992886758
Dkk3	0.003969695	0.135149278	0.029372667	0.976567372	0.997262526
Mical2	-0.251284812	0.25393929	-0.989546798	0.322395685	0.91640698
Micalcl	0.493976569	0.369552736	1.336687624	0.181324623	0.868178042
Parva	-0.12399684	0.116999518	-1.059806417	0.289232677	0.907334416
Tead1	-0.295675793	0.250592595	-1.179906344	0.238037466	0.896609492
Rassf10	-0.097447738	0.494379482	-0.197111211	0.843740511	0.992886758
Arntl	0.327460612	0.291783739	1.122271628	0.261746966	0.897248195
Btbd10	0.165271487	0.179730728	0.919550537	0.357807685	0.924354588
Far1	-0.079357059	0.102757923	-0.772271929	0.439953388	0.947849924
Far1os	-0.46115608	1.215394852	-0.379429022	0.704369303	0.988585656
Spon1	0.027043695	0.163904981	0.164996176	0.868946992	0.992886758
Rras2	0.06574598	0.13959409	0.470979681	0.637655245	0.977468848
Copb1	-0.145518738	0.120923205	-1.203397959	0.228822357	0.895404344
Psma1	0.064768625	0.102362515	0.632737728	0.526904928	0.965637248
Pde3b	-0.098135449	0.23505213	-0.41750504	0.676309039	0.983549699
Cyp2r1	0.318917369	0.371110643	0.859359264	0.390142337	0.933734231
Calca	-0.129498975	0.406822503	-0.318318121	0.75024364	0.988585656
Calcb	0.778993219	0.405883435	1.919253538	0.054952255	0.711503145
Insc	0.726281609	0.714881865	1.015946332	0.309654944	0.9139204
Sox6	-0.451193252	0.411555554	-1.096311901	0.272942303	0.898826892
1110004F10Rik	0.279449658	0.137359983	2.034432822	0.041907975	0.679118604
Plekha7	-0.311278758	0.342645075	-0.908458289	0.363636142	0.926455466
Rps13	0.347436089	0.26124969	1.329900483	0.183551062	0.869657049
Pik3c2a	-0.255964342	0.125716416	-2.036045483	0.041745788	0.679118604
Nucb2	-0.009379409	0.155946721	-0.06014496	0.952040182	0.996023481
Xylt1	0.375753676	1.550665738	0.242317649	0.808534039	0.991020605
Rps15a	0.278321265	0.241181544	1.153990724	0.248503972	0.896939594
Arl6ip1	-0.029922343	0.075617599	-0.395706063	0.692321882	0.986599035
Smg1	-0.080404974	0.166611154	-0.482590584	0.629386466	0.977034429
Syt17	-0.286409222	0.637601233	-0.449198037	0.653288803	0.979004493
Itpripl2	-0.039551851	0.155513375	-0.254330865	0.799239956	0.990988839
Coq7	0.189141857	0.129966217	1.455315558	0.145581921	0.842832198
Tmc7	-0.654266678	0.564247453	-1.159538558	0.246236729	0.896939594
Tmc5	-0.47693347	0.345610116	-1.379975434	0.167594209	0.860403806

Gde1	-0.057103231	0.110859278	-0.515096544	0.606485558	0.974001549
Ccp110	-0.107785686	0.193583818	-0.556790783	0.577670382	0.971613657
9030624J02Rik	-0.090085847	0.183150091	-0.49186897	0.622811975	0.975486913
Knop1	-0.170226616	0.165854383	-1.026361877	0.304721041	0.912429485
Iqck	-0.195310192	0.22950653	-0.851000588	0.394769026	0.933796635
Gprc5b	-0.127393169	0.216170964	-0.589316746	0.555648813	0.968926429
Gp2	-0.300134196	0.587740941	-0.510657289	0.609591053	0.974001549
Acsm5	1.37274155	1.181299218	1.162060831	0.245210756	0.896939594
Acsm1	0.458583414	0.26390868	1.737659459	0.082270836	0.76099108
Thumpd1	0.052039055	0.220691114	0.235800407	0.813587552	0.992048071
Acsm3	-0.997746138	0.5511494	-1.810300687	0.070249171	0.744227652
Eri2	-0.250623952	0.213534638	-1.173692259	0.240518312	0.896939594
2610020H08Rik	0.023806041	0.313527593	0.075929651	0.939475067	0.994688009
Dcun1d3	-0.152613241	0.144321996	-1.057449628	0.290306432	0.907334416
Lyrm1	0.226360592	0.212151687	1.066975214	0.285983029	0.905916508
Tmem159	0.2506846	0.104449557	2.400054218	0.016392644	0.564647031
Crym	0.123994631	0.279393802	0.443798789	0.657188064	0.979614766
Abca14	-0.058290266	0.637979147	-0.091367039	0.927200951	0.994688009
E130201H02Rik	-0.810889487	1.06778792	-0.759410621	0.447606962	0.948476747
Uqcrc2	0.073438062	0.149627644	0.490805444	0.623564059	0.975691835
BC030336	-0.039905545	0.155951449	-0.255884416	0.798040089	0.990988839
Vwa3a	-0.372258922	0.242554538	-1.534743172	0.124846927	0.821400908
Eef2k	-0.098848298	0.255503744	-0.386876125	0.698847917	0.987770663
Polr3e	-0.076740952	0.209868623	-0.365661863	0.714617407	0.988585656
Cdr2	-0.247966619	0.134352325	-1.845644425	0.064943852	0.733798108
4933427G17Rik	0.529473622	1.505876945	0.35160484	0.725134635	0.988585656
Mettl9	0.122675495	0.123890523	0.99019273	0.322079925	0.91640698
Igsf6	0.39348479	0.223532474	1.760302575	0.078356518	0.754732183
Usp31	-0.235771697	0.251456488	-0.937624235	0.348437553	0.921552698
Scnn1g	-0.16486384	0.116872366	-1.410631498	0.158353299	0.855986074
Scnn1b	0.025064869	0.150300024	0.166765569	0.867554511	0.992886758
Cog7	0.130794026	0.204859328	0.638457754	0.523175746	0.965637248
Gga2	0.01264984	0.174306227	0.072572509	0.942146303	0.994688009
Ears2	-0.320671522	0.309103691	-1.037423787	0.299538397	0.910105802
Ubfd1	0.040567388	0.137148892	0.295790853	0.767389809	0.98990072
Ndufab1	0.081863011	0.146078437	0.560404481	0.575203576	0.971613657
Palb2	0.565477423	0.511539093	1.105443222	0.268967584	0.89804647
Dctn5	0.118869341	0.161571991	0.735705121	0.461910172	0.953083512
Plk1	-0.826608754	0.775625471	-1.065731832	0.286544884	0.906143389
Ern2	2.926666439	1.657560569	1.765646754	0.077455131	0.754090591
Chp2	0.175686892	0.36250882	0.484641703	0.627930526	0.976968316
Prkcb	0.183702421	0.347520224	0.528609295	0.597076511	0.974001549
4930413G21Rik	-0.049812772	0.230022504	-0.216556081	0.828554316	0.992886758
Rbbp6	0.037008321	0.138970344	0.266303733	0.790005298	0.990988839
Tnrc6a	-0.216186154	0.21826738	-0.990464787	0.321946992	0.91640698
Slc5a11	-0.188462909	2.001480711	-0.094161741	0.924980676	0.994688009
Arhgap17	-0.20735419	0.206460304	-1.004329578	0.315219782	0.91586731
Lcmt1	0.19087966	0.137000788	1.393274176	0.163536897	0.859982992
Zkscan2	-1.095654073	1.296187982	-0.845289486	0.39794923	0.935327115
Kdm8	-0.326550203	0.415304414	-0.786291193	0.431696911	0.945955639
Nsmce1	0.20844902	0.183621842	1.135208199	0.256288095	0.897248195
Il4ra	-0.012270303	0.121563675	-0.100937251	0.919600272	0.994688009
Il21r	0.077800187	0.331405101	0.234758567	0.814396128	0.992048071
Gtf3c1	-0.466949532	0.244637397	-1.908741415	0.056295458	0.717514394
D430042O09Rik	0.0325837	0.366686887	0.088859736	0.929193383	0.994688009
Gsg1l	-0.028782508	0.604490719	-0.047614475	0.962023496	0.996474415
Xpo6	-0.260194773	0.242468685	-1.073106715	0.283223239	0.905916508
Sbk1	-0.345403091	0.332154369	-1.039887245	0.298392289	0.909779403
Lat	0.517077531	0.233231629	2.217012904	0.02662221	0.633489138
Spns1	0.067009761	0.134774149	0.4972004	0.61904774	0.974471036
Nfatc2ip	-0.078103025	0.196341459	-0.397791811	0.690783654	0.986048673
Cd19	0.397879452	0.35602291	1.117566991	0.263751973	0.897248195

Rabep2	0.066002513	0.207685166	0.317800807	0.750636038	0.988585656
Atp2a1	1.354055167	0.896621726	1.510174389	0.130998932	0.827800491
Sh2b1	-0.03967304	0.138648794	-0.286140532	0.774770489	0.990861469
Tufm	-0.070126321	0.133849254	-0.523920149	0.60033408	0.974001549
Atxn2l	-0.104493596	0.218544827	-0.478133466	0.632555214	0.977034429
Eif3c	-0.189513898	0.160320382	-1.182094855	0.237168066	0.896609492
Cln3	0.031698209	0.230277986	0.137651929	0.890515512	0.993043931
Apobr	0.191873411	0.213261369	0.899710115	0.368274539	0.928436166
Il27	1.241907766	1.270083143	0.977816116	0.328165279	0.917115058
Nupr1	0.300520107	0.101129469	2.971637358	0.002962163	0.345591998
Ccdc101	0.181028426	0.173940347	1.040750056	0.297991565	0.909779403
Sult1a1	0.097836505	0.1781223	0.549265898	0.582822987	0.972086107
Slx1b	0.172945751	0.162709556	1.062910843	0.287822385	0.907334416
Bola2	0.298066953	0.276626638	1.077506327	0.281254126	0.904300139
Coro1a	0.570956924	0.151858649	3.759791946	0.000170055	0.079328099
Mapk3	-0.022237253	0.142533739	-0.156013961	0.876022017	0.992886758
Gdpd3	0.239328585	0.340694316	0.702473078	0.482384186	0.957463006
Ypel3	0.023213919	0.128962861	0.180004683	0.857148892	0.992886758
Tbx6	1.096792191	1.208514434	0.907554068	0.364113871	0.926556975
Ppp4c	0.080974353	0.099201618	0.816260408	0.414351214	0.943415794
Aldoa	-0.188453955	0.126803885	-1.486184387	0.137230356	0.833085238
Fam57b	0.06523097	0.576046031	0.113239162	0.909840949	0.994688009
Doc2a	-0.269748883	0.37028276	-0.728494307	0.466311052	0.953083512
Ino80e	0.152349697	0.190515989	0.799668822	0.423902701	0.943823729
Hirip3	0.704956085	0.269317978	2.617560445	0.00885608	0.475600561
Taok2	0.144382839	0.154731335	0.93311958	0.350758231	0.922338606
Tmem219	0.175039375	0.180269317	0.970988173	0.331554168	0.917115058
Kctd13	-0.026270604	0.348690592	-0.075340731	0.939943615	0.994688009
Sez6l2	0.461189749	1.455425519	0.316876228	0.75133752	0.988585656
Cdipt	-0.136220585	0.137471155	-0.990903035	0.32173293	0.91640698
Mvp	-0.048713912	0.171704641	-0.283707605	0.776634467	0.990861469
Pagr1a	0.029026946	0.123760378	0.234541508	0.814564613	0.992048071
Prrt2	-0.133741666	0.733609111	-0.182306441	0.855342244	0.992886758
Maz	-0.028072033	0.127242124	-0.220619021	0.825389089	0.992886758
Kif22	0.061144479	0.534871553	0.114316192	0.908987148	0.994688009
AI467606	-0.167619098	0.128971265	-1.299662352	0.193716719	0.879588734
Qprt	0.772598346	1.387676457	0.556756831	0.577693582	0.971613657
Spn	0.573510266	0.310607411	1.846415269	0.064831932	0.733548823
Cd2bp2	0.128616837	0.145819679	0.882026614	0.377762415	0.928883222
Tbc1d10b	-0.285520836	0.256929411	-1.111281246	0.266447309	0.89768067
Mylpf	-0.009078579	0.288773503	-0.031438407	0.974919912	0.997262526
Sep-01	0.111337101	0.27510153	0.404712765	0.685688658	0.98538669
Gm4532	-0.391958923	0.945268037	-0.414653736	0.678395405	0.983786595
Zfp553	0.000878169	0.451404259	0.001945415	0.998447784	0.999708875
Zfp771	-0.319831437	0.1760526	-1.816681135	0.069265952	0.743426713
Dctpp1	0.338650822	0.230431888	1.469635232	0.141660573	0.840741274
Sephs2	0.133235083	0.147155405	0.905403938	0.36525143	0.926556975
Itgal	-0.08483444	0.22700615	-0.373709875	0.708620178	0.988585656
Zfp768	-0.235166018	0.197236469	-1.192304947	0.233141695	0.89581803
Zfp747	0.01919574	0.20195655	0.095048859	0.924276018	0.994688009
9130019O22Rik	-0.183755212	0.25645626	-0.716516773	0.473672315	0.955974667
E430018J23Rik	0.192156686	0.245771316	0.781851558	0.434301816	0.946762848
Zfp764	0.310199299	0.203302953	1.525798295	0.127060105	0.824450458
Zfp688	0.245726603	0.2065795	1.189501395	0.234242423	0.89581803
Zfp689	-0.108097253	0.389843323	-0.277283838	0.781562168	0.990988839
Prr14	-0.121117437	0.170486401	-0.710422863	0.47744195	0.95716557
Fbrs	-0.434145692	0.202266033	-2.146409294	0.031840339	0.661395937
1700008J07Rik	0.04223401	0.449642333	0.09392801	0.925166343	0.994688009
Srcap	0.104708675	0.212446576	0.492870618	0.622104009	0.97514112
Tmem265	0.533528794	0.353376972	1.509800682	0.131094291	0.827800491
Phkg2	0.07425783	0.223432804	0.33234972	0.7396252	0.988585656
Gm166	-0.028586757	0.189163964	-0.151121581	0.879879809	0.992886758

Rnf40	0.024621173	0.206148839	0.119433963	0.904931556	0.994688009
1700120K04Rik	4.215307349	1.894672152	2.224821505	0.026093223	0.631530315
Zfp629	0.034870024	0.345629974	0.100888311	0.919639122	0.994688009
Bcl7c	0.369264241	0.161499829	2.286468314	0.022226876	0.618422027
Ctf1	-0.27181629	0.233836433	-1.162420614	0.245064654	0.896939594
Fbxl19	-0.184165271	0.314095971	-0.586334394	0.557650822	0.968926429
Orai3	-0.04481646	0.116816567	-0.383648155	0.701239243	0.988482081
Setd1a	-0.190982417	0.281108541	-0.679390306	0.49689059	0.960475196
Hsd3b7	0.102889303	0.113216197	0.908786074	0.363463059	0.926455466
Stx1b	-1.476005184	1.07578527	-1.372025836	0.170055395	0.861730117
Stx4a	-0.069163147	0.137165002	-0.504233193	0.614097518	0.974001549
Zfp668	-0.258264558	0.670803521	-0.385007756	0.700231669	0.988482081
Zfp646	0.255703578	0.281556487	0.908178604	0.363783866	0.926455466
Prss53	-0.246118604	0.489791211	-0.50249698	0.615317976	0.974001549
Vkorc1	-0.029627438	0.12909792	-0.229495862	0.818483536	0.992734473
Bckdk	-0.025240423	0.138811558	-0.181832289	0.855714344	0.992886758
Kat8	0.089543719	0.166622298	0.537405377	0.59098763	0.974001549
Prss8	-0.224267475	0.187141162	-1.198386673	0.230766518	0.895404344
Prss36	0.124267414	0.199572761	0.622667205	0.533503238	0.96674935
Fus	-0.099423419	0.314264927	-0.316368168	0.751723075	0.988585656
Pycard	0.347998971	0.203881045	1.70687261	0.087845733	0.7667678
Trim72	0.365127789	0.69078593	0.528568653	0.59710471	0.974001549
Itgam	-0.029079921	0.307987932	-0.094419027	0.924776302	0.994688009
Itgax	-0.45388043	0.275967626	-1.644687226	0.100034328	0.788783372
Itgad	0.421643091	0.926694732	0.45499675	0.649111577	0.979004493
Cox6a2	-0.117180824	0.215546741	-0.543644612	0.586686054	0.972963251
9130023H24Rik	-0.36547956	0.22311292	-1.638092315	0.101402438	0.788783372
Armc5	-0.02118663	0.220904821	-0.095908411	0.923593314	0.994688009
Tgfb1i1	-0.099973308	0.244716416	-0.408527182	0.682886679	0.984738223
BC017158	0.363270694	0.286994227	1.265777009	0.205592929	0.883765092
Rgs10	0.069389716	0.147862423	0.46928567	0.638865456	0.977795535
Tial1	0.137265561	0.136013878	1.009202612	0.312877474	0.914819219
Bag3	-0.210095387	0.169667495	-1.238277184	0.215613301	0.887633697
Inpp5f	-0.027078945	0.179011311	-0.151269461	0.879763158	0.992886758
Mcmbp	-0.209991703	0.144095398	-1.45731027	0.145030753	0.842729381
Sec23ip	0.014669895	0.185139541	0.079236961	0.936844147	0.994688009
Wdr11	-0.150908845	0.176027742	-0.857301487	0.391278287	0.933734231
Fgfr2	-0.051766302	0.16296286	-0.317657054	0.750745091	0.988585656
Ate1	-0.05994826	0.158037203	-0.379330051	0.704442786	0.988585656
Nsmce4a	0.089034689	0.097961984	0.908869801	0.363418856	0.926455466
Tacc2	-0.101618821	0.202244073	-0.50245636	0.615346542	0.974001549
Btbd16	-0.195219398	1.712674965	-0.113985083	0.909249618	0.994688009
Plekha1	-0.214445884	0.154856951	-1.384799863	0.166113673	0.860082
Htra1	0.235557253	0.129581602	1.817829452	0.069090204	0.743426713
2310057M21Rik	0.03679486	0.312674593	0.117677804	0.906322954	0.994688009
Pstk	0.365578105	0.236010126	1.548993301	0.12138333	0.816233249
Ikzf5	-0.04184905	0.118919362	-0.351911157	0.724904892	0.988585656
Acadsb	-0.006264594	0.112987296	-0.055445116	0.955783853	0.99614946
Bub3	0.007002599	0.133265299	0.052546304	0.958093401	0.996226826
Cpxm2	-0.248518249	0.483646393	-0.513842865	0.607361856	0.974001549
Chst15	-0.233131593	0.244199169	-0.954678074	0.339740521	0.919246575
Gm10584	2.785714193	1.832494058	1.520176385	0.128466651	0.826336015
Oat	-0.270777984	0.167455255	-1.617016939	0.105874624	0.79419908
Lhpp	0.012022265	0.211569821	0.056824101	0.954685315	0.996070133
Fam53b	-0.118284937	0.172811718	-0.684472897	0.493676598	0.958916246
Mettl10	0.417846422	0.14674216	2.847487204	0.004406587	0.385355746
Fam175b	-0.106775266	0.135928947	-0.785522648	0.432147197	0.9459793
Zranb1	-0.121188955	0.172671223	-0.701848012	0.482773953	0.957463006
Ctbp2	-0.08981326	0.111196851	-0.807696072	0.419265565	0.943415794
4930483O08Rik	0.271254178	1.634115104	0.165994536	0.86816125	0.992886758
Edrf1	-0.483074288	0.160466078	-3.010444905	0.002608653	0.327099208
Uros	0.228113891	0.217518635	1.048709645	0.294311774	0.907334416

Bccip	0.291691039	0.121987042	2.391164128	0.016795043	0.56772809
Dhx32	-0.108151808	0.155501241	-0.695504472	0.48673921	0.958288569
Fank1	-0.016805588	0.19995305	-0.08404767	0.93301853	0.994688009
Adam12	-1.399718899	0.975273256	-1.435206892	0.15122815	0.850827708
D7Ertd443e	0.941769802	1.764597234	0.533702413	0.59354744	0.974001549
Dock1	-0.128533973	0.196046962	-0.655628488	0.512063191	0.963452765
Fam196a	0.589793359	2.108719409	0.279692669	0.779713304	0.990988839
Ptpre	0.152421691	0.20671127	0.73736517	0.46090031	0.953083512
5830432E09Rik	0.633479121	0.547430999	1.15718533	0.24719665	0.896939594
Mki67	-0.350078356	0.483346337	-0.724280561	0.468893509	0.954315695
Mgmt	0.338787814	0.312383728	1.084524523	0.2781323	0.901362854
9430038I01Rik	0.42718252	0.213373105	2.002044821	0.045279911	0.693980041
Glrx3	-0.029664094	0.842051237	-0.035228372	0.971897639	0.996733101
Tcerg1l	-0.221372765	2.08126418	-0.106364568	0.915293104	0.994688009
Mapk1ip1	-0.328205241	0.327705594	-1.001524683	0.316573213	0.91640698
Ppp2r2d	0.012828364	0.140890233	0.091052191	0.927451121	0.994688009
Bnip3	0.037730829	0.195816769	0.192684361	0.847206172	0.992886758
Stk32c	0.093614333	0.729024714	0.128410369	0.897824226	0.994101834
Lrrc27	0.050285649	0.281770625	0.178463063	0.858359327	0.992886758
Pwwp2b	-0.026299475	0.137855519	-0.190775642	0.848701373	0.992886758
Inpp5a	0.090501409	0.154256956	0.58669256	0.557410205	0.968926429
Nkx6-2	-0.135612399	0.386875684	-0.350532237	0.725939302	0.988585656
Cfap46	-0.140186328	0.36715831	-0.381814394	0.702599041	0.988482081
Kndc1	0.276959055	0.39966102	0.692984908	0.488319023	0.958481967
6430531B16Rik	0.163985318	0.179257362	0.914803811	0.360294634	0.925913281
Adam8	-0.501762732	0.241782411	-2.075265652	0.037961921	0.679118604
Tubgcp2	-0.213388471	0.194235124	-1.098609084	0.271938614	0.89804647
Zfp511	-0.123651728	0.212379442	-0.582220797	0.560417967	0.968926429
Fuom	0.055404259	0.226188331	0.244947466	0.806497108	0.99099448
Echs1	0.031417905	0.16894044	0.185970304	0.852468049	0.992886758
Paox	-0.214106011	0.239810688	-0.892812628	0.371957523	0.928840865
Mtg1	0.002302537	0.183048001	0.012578871	0.989963778	0.998417328
Cd163l1	-1.56168818	1.768060978	-0.88327733	0.377086449	0.928883222
5830411N06Rik	0.14898613	2.161679877	0.068921458	0.945052138	0.99486515
Cyp2e1	-0.358050913	0.321742093	-1.112850699	0.265772557	0.89768067
Syce1	0.179096096	3.438908188	0.052079348	0.958465469	0.996226826
Zfp941	-1.850238777	1.087020907	-1.702118851	0.088733091	0.767814344
Urah	0.252624775	0.273544233	0.923524406	0.355733988	0.924354588
Scgb1c1	0.013601121	0.275510293	0.049367016	0.960626814	0.996474415
Bet1l	-0.054606355	0.127306744	-0.428935284	0.667970322	0.981863224
Ric8	0.12486778	0.094929509	1.315373704	0.188384339	0.874826736
Sirt3	0.264778186	0.162593288	1.628469351	0.103425403	0.790925787
Psmd13	0.148783659	0.127288542	1.168869226	0.242456324	0.896939594
Cox8b	0.175261052	0.349060047	0.502094278	0.615601205	0.974001549
Nlrp6	3.665342725	2.162306737	1.695107666	0.090054998	0.769347579
Athl1	-0.082207617	0.242688549	-0.338737105	0.734807786	0.988585656
Ifitm5	0.306194549	1.19031124	0.257239064	0.79699423	0.990988839
Ifitm2	0.121551081	0.106212438	1.144414757	0.252451671	0.897013805
Ifitm1	-0.089081425	0.208756858	-0.426723344	0.669580845	0.981863224
Ifitm3	0.06433747	0.122760198	0.524090633	0.600215503	0.974001549
Ifitm6	0.733817506	0.358780816	2.045308647	0.040824439	0.679118604
B4galnt4	-0.012679555	1.191796364	-0.010639028	0.991511444	0.998770784
Pkp3	0.24922247	0.220607001	1.129712424	0.25859742	0.897248195
Sigirr	0.022325798	0.177467343	0.125802287	0.899888432	0.994508803
Ano9	0.159445189	0.349630888	0.456038625	0.648362203	0.979004493
Ptdss2	0.144671323	0.131828627	1.097419634	0.272457995	0.898460682
Rnh1	0.009315776	0.126096419	0.073878198	0.941107304	0.994688009
Hras	0.086662655	0.145454127	0.595807469	0.551303854	0.968926429
Lrrc56	0.035350585	0.22872274	0.154556494	0.877170971	0.992886758
Lmntd2	1.012389624	0.953041097	1.062272789	0.288111864	0.907334416
Rassf7	-0.042861669	0.137037885	-0.312772408	0.754453579	0.98902053
Phrf1	-0.016995644	0.209774449	-0.081018655	0.935427117	0.994688009

Irf7	0.016466117	0.219801288	0.074913649	0.940283416	0.994688009
Deaf1	-0.224832155	0.212695741	-1.057059977	0.290484216	0.907334416
Tmem80	0.037235531	0.138377912	0.269085798	0.787863659	0.990988839
Eps8l2	0.103053967	0.295407713	0.348853339	0.727199417	0.988585656
Taldo1	0.146123136	0.105329935	1.387289717	0.165353438	0.860011234
Pddc1	0.020445297	0.148353439	0.137814785	0.890386799	0.993043931
Cend1	0.120099702	1.279934359	0.093832704	0.925242052	0.994688009
Slc25a22	-0.216201828	0.316145016	-0.683869167	0.494057785	0.958916246
Pidd1	0.467253015	1.090877086	0.42832783	0.66841246	0.981863224
Rplp2	0.257073799	0.156385465	1.643847132	0.100207783	0.788783372
Pnpla2	-0.275310658	0.233358838	-1.179773862	0.238090168	0.896609492
Cracr2b	0.137143568	0.097059153	1.412989534	0.157658802	0.855986074
Cd151	0.047125821	0.111807641	0.421490167	0.67339719	0.983375962
Polr2l	0.1895435	0.161700002	1.17219232	0.241119851	0.896939594
Tspan4	-0.27718827	0.155936807	-1.777567951	0.075474841	0.752876039
Chid1	0.080146858	0.15126699	0.529837064	0.596224905	0.974001549
Ap2a2	0.06123416	0.152485658	0.40157324	0.687998127	0.98538669
Muc5ac	-0.148956748	0.856452631	-0.173922926	0.861926035	0.992886758
Muc5b	-0.164387986	0.329211178	-0.499339015	0.617540574	0.974150208
Tollip	-0.144364289	0.112794795	-1.279884315	0.200585825	0.880888611
Mob2	0.195118135	0.122297889	1.595433387	0.110615355	0.800111791
Dusp8	-0.467124613	0.308779779	-1.512808302	0.130328359	0.827800491
Ifitm10	0.58874459	0.281515082	2.09134298	0.03649733	0.677009445
Ctsd	-0.242924025	0.168153931	-1.444652669	0.148555471	0.84832848
Syt8	-1.203300111	1.339374806	-0.898404319	0.368970034	0.928436166
Tnni2	0.675498814	0.332736221	2.030133096	0.042343012	0.68118841
Lsp1	0.130829839	0.142444031	0.91846487	0.358375543	0.924798085
Prr33	-0.217970769	1.103384762	-0.19754738	0.843399208	0.992886758
Tnnt3	-0.26100544	0.584836274	-0.446288048	0.655389188	0.979133069
Mrpl23	0.150771808	0.139632676	1.079774535	0.280242593	0.903192609
H19	-1.857881526	1.050095256	-1.76925047	0.076852085	0.752876039
Igf2	0.336793841	0.248720119	1.35410775	0.175702004	0.864843721
Igf2os	-0.549878533	1.597423913	-0.34422831	0.730674594	0.988585656
Ascl2	0.034822662	1.388499111	0.025079355	0.979991667	0.997296835
Tspan32	0.048197174	0.240831604	0.200128111	0.841380388	0.992886758
R74862	-0.08551247	0.326997006	-0.261508417	0.793700459	0.990988839
Cd81	-0.003194296	0.090835369	-0.035165774	0.971947553	0.996733101
Tssc4	0.208103301	0.124997015	1.664866161	0.095939461	0.780081462
Kcnq1	-0.141328817	0.224492997	-0.629546664	0.528991229	0.965637248
Kcnq1ot1	0.722887095	0.50503581	1.431358095	0.152327612	0.852974739
4933417O13Rik	1.266778282	1.746291709	0.725410466	0.468200254	0.953657709
Cdkn1c	-0.028572773	0.127092579	-0.224818578	0.822120413	0.992886758
Slc22a18	-0.40387698	0.239413724	-1.686941638	0.091614575	0.772516176
Phlda2	0.305766813	0.438697887	0.696987203	0.485810803	0.957710647
Nap1l4	0.097254914	0.089037629	1.092290022	0.274705645	0.900302631
Cars	0.075516305	0.1285564	0.587417702	0.556923208	0.968926429
Tnfrsf26	-0.650389171	0.506493498	-1.28410172	0.199106366	0.88073954
Tnfrsf22	-0.88536151	0.702117032	-1.260988511	0.207312987	0.883765092
Tnfrsf23	0.082181965	0.808922058	0.101594417	0.919078611	0.994688009
Osbpl5	-0.365751746	0.159188455	-2.297602215	0.021584439	0.60865137
Mrgpre	-0.634797283	0.540312627	-1.174870346	0.240046591	0.896939594
Nadsyn1	0.069800597	0.274277487	0.254488978	0.799117818	0.990988839
Dhcr7	-0.09847296	0.245986488	-0.400318573	0.68892189	0.98538669
Shank2	-0.249257418	0.617144153	-0.403888486	0.686294723	0.98538669
Cttn	-0.043079887	0.115497455	-0.372994257	0.709152718	0.988585656
Ppfia1	-0.235250706	0.136983656	-1.717363315	0.08591281	0.763596872
Fadd	0.146708073	0.202166589	0.725679123	0.468035503	0.953657709
Ano1	-0.06782122	0.21713167	-0.31235066	0.754774043	0.98902053
Oraov1	0.03808255	0.156407631	0.243482686	0.807631497	0.99099448
Ccnd1	-0.113151414	0.139805109	-0.809351067	0.41831324	0.943415794
1810010D01Rik	1.227768514	0.434632244	2.824844522	0.004730356	0.391906015
Tpcn2	0.306002707	0.217186191	1.408941817	0.158852373	0.855986074

Mrgprf	-0.610177251	0.526698321	-1.158494771	0.246662184	0.896939594
Insr	-0.170619242	0.198703253	-0.858663556	0.390526163	0.933734231
A430078G23Rik	-0.85603783	0.691092788	-1.238672786	0.215466701	0.887633697
Arhgef18	-0.006581111	0.182313075	-0.036097853	0.971204334	0.996733101
Pex11g	-0.286948033	0.258208311	-1.111304407	0.266437343	0.89768067
Zfp358	-0.523970565	0.293634726	-1.784429831	0.074353847	0.751093915
Mcoln1	0.278197044	0.177116717	1.570698965	0.11625259	0.812176308
Pnpla6	-0.333696077	0.214075935	-1.558774357	0.119049796	0.814755048
C330021F23Rik	0.171614579	0.285182902	0.601770224	0.547327098	0.968115622
Camsap3	-0.662561665	0.428426434	-1.546500431	0.121983756	0.816233249
Xab2	0.168253693	0.173390793	0.970372706	0.331860748	0.917115058
Pet100	0.600902068	0.305443288	1.967311419	0.049147319	0.707153766
Stxbp2	-0.03143845	0.159421662	-0.197203126	0.843668584	0.992886758
Retn	-0.309641476	0.424867734	-0.728794989	0.466127077	0.953083512
Mcemp1	0.037963794	0.190671115	0.199106164	0.8421797	0.992886758
Trappc5	0.176000937	0.089356468	1.969649666	0.048878535	0.707153766
Fcer2a	-0.256753425	0.565876653	-0.453726838	0.650025448	0.979004493
Clec4g	-0.365138668	0.293288245	-1.244982281	0.213138263	0.884945675
Cd209a	-0.260197898	0.221301435	-1.175762363	0.23968985	0.896939594
Cd209e	-1.592389981	1.159444282	-1.373407938	0.169625563	0.861597949
Cd209d	-1.556197627	0.561535344	-2.771326227	0.005582846	0.416936884
Cd209b	-0.12388907	0.49166624	-0.251977987	0.801058079	0.990988839
Cd209c	0.835633368	1.151917945	0.725427858	0.468189588	0.953657709
Cd209f	-0.732180357	0.342141246	-2.139994417	0.032355218	0.665167506
Cd209g	-0.279300594	0.359058022	-0.777870362	0.436645442	0.946762848
Evi5l	0.057955651	0.430920432	0.134492697	0.893012985	0.99333638
Prr36	-0.317353405	0.5072929	-0.625582193	0.531589022	0.966606934
Lrrc8e	-0.19801158	0.206579605	-0.958524343	0.337798423	0.919246575
Map2k7	0.028694877	0.149465832	0.191982857	0.847755634	0.992886758
Tgfbr3l	0.663468516	0.801606526	0.827673547	0.407855404	0.939557159
Snapc2	-0.231758838	0.256381461	-0.903960987	0.366016088	0.927408779
Ctxn1	-0.053476442	0.169676115	-0.315167766	0.752634278	0.988585656
Timm44	0.165243309	0.122818877	1.34542273	0.178488759	0.867377413
Elavl1	-0.295535088	0.170949748	-1.728783412	0.083847866	0.76099108
Ccl25	0.145882325	0.278636453	0.523557931	0.600586049	0.974001549
Cers4	0.146857102	0.143917783	1.020423599	0.307527607	0.9139204
Zfp958	0.152817267	0.228447146	0.668939272	0.503534217	0.961769205
Shcbp1	0.378065	0.465941859	0.811399518	0.417136276	0.943415794
Efnb2	-0.101351735	0.146723233	-0.690768142	0.489711259	0.958560421
Arglu1	-0.037247598	0.105807312	-0.352032366	0.72481399	0.988585656
Lig4	-0.064094469	0.346884365	-0.184771859	0.853407981	0.992886758
Abhd13	-0.14892162	0.098833479	-1.50679327	0.131863655	0.827800491
Tnfsf13b	0.198704994	0.176030074	1.128812766	0.258976826	0.897248195
B930025P03Rik	-0.106494958	1.158507611	-0.091924263	0.926758214	0.994688009
4833411C07Rik	0.150398817	0.734623866	0.20472901	0.837783853	0.992886758
3930402G23Rik	0.027553646	0.386382926	0.07131176	0.943149636	0.994819052
Irs2	-0.725087201	0.579681934	-1.250836292	0.210994211	0.884390587
9530052E02Rik	3.296751737	2.468679539	1.335431224	0.181735251	0.868546072
Col4a1	0.105157978	0.17349964	0.606099112	0.544448941	0.967491297
Col4a2	-0.051547422	0.210580154	-0.244787655	0.806620853	0.99099448
Rab20	0.359914544	0.279509908	1.287662921	0.197863326	0.880239572
E230013L22Rik	0.647451232	0.57818828	1.119793074	0.262801951	0.897248195
Carkd	0.223853503	0.136795221	1.636413184	0.101753142	0.788783372
Cars2	0.167746654	0.143799478	1.166531732	0.243399527	0.896939594
Ing1	0.060272081	0.095187508	0.63319318	0.526607499	0.965637248
Ankrd10	-0.231171029	0.122974429	-1.879830073	0.06013124	0.725221283
Arhgef7	-0.18598499	0.202776188	-0.917193445	0.359041281	0.925199968
Tubgcp3	0.112239783	0.11384035	0.98594025	0.324162434	0.91640698
Atp11a	-0.132775782	0.160535334	-0.827081357	0.40819095	0.939990641
Mcf2l	-0.038067527	0.335099454	-0.113600684	0.909554345	0.994688009
F7	-0.59617278	0.268639297	-2.219231457	0.026470982	0.632784368
F10	-0.016774022	0.260679591	-0.064347276	0.94869371	0.99560893

Proz	-0.131414984	0.441827737	-0.29743489	0.76613451	0.989884648
Pcid2	-0.154145701	0.284615432	-0.541592915	0.588098974	0.973455044
Cul4a	-0.082381075	0.100434827	-0.820244109	0.412076961	0.942121471
Lamp1	-0.12824325	0.069944762	-1.833493277	0.066729247	0.738254074
Grtp1	0.106357758	0.162971282	0.652616565	0.514003504	0.964352198
Adprhl1	0.087504967	0.866730131	0.100959877	0.919582311	0.994688009
Dcun1d2	-0.124928317	0.176341672	-0.708444667	0.478669163	0.957463006
Tmco3	-0.073727687	0.131767349	-0.559529262	0.575800567	0.971613657
Tfdp1	-0.137597489	0.131826232	-1.043779275	0.296587522	0.909070179
Tmem255b	-0.458579161	0.544958301	-0.841494038	0.400071224	0.936631983
Gas6	0.077583107	0.12873752	0.602645656	0.546744441	0.967902958
Rasa3	0.205627179	0.148998043	1.380066304	0.167566231	0.860403806
4932443I19Rik	0.037431928	0.276944033	0.135160624	0.892484879	0.993300147
Cdc16	0.119553549	0.121298383	0.985615363	0.324321896	0.91640698
Upf3a	-0.109652459	0.214563699	-0.511048514	0.609317087	0.974001549
Champ1	-0.508404161	0.274961863	-1.848998821	0.064457983	0.73299549
Coprs	0.257455265	0.212961812	1.208926908	0.226690927	0.89398447
Fbxo25	0.031688254	0.136942849	0.231397652	0.817005889	0.992678823
Tdrp	0.041855897	0.175741025	0.238168051	0.811750756	0.991816977
Erich1	0.433922644	0.162490603	2.670447623	0.007575019	0.454518821
Cln8	-0.033175307	0.173262217	-0.19147456	0.84815381	0.992886758
Arhgef10	-0.118061505	0.264353126	-0.446605294	0.655160073	0.979133069
Kbtbd11	0.212146121	0.220504178	0.962095697	0.336001535	0.918908218
Myom2	0.109582497	0.678077156	0.161607711	0.871614785	0.992886758
Mcph1	-0.36565427	0.421524174	-0.867457413	0.385691436	0.931855682
Angpt2	-0.167260812	0.253687899	-0.659317266	0.509692057	0.963452765
Agpat5	0.081686399	0.117011912	0.698103275	0.48511261	0.957710647
Xkr5	-0.445743527	1.670894888	-0.26676934	0.789646762	0.990988839
Spag11b	0.574867393	0.408112554	1.40860012	0.158953443	0.855986074
6820431F20Rik	-0.203300641	0.133070596	-1.527765312	0.126570818	0.823315433
2610005L07Rik	-0.126186546	0.526409177	-0.239711904	0.810553606	0.991438232
Defb1	-0.673667148	0.778280954	-0.865583495	0.38671861	0.932626359
Atp7b	-0.244782435	0.277269725	-0.882831455	0.377327342	0.928883222
Alg11	-0.025260934	0.156224973	-0.161695878	0.871545351	0.992886758
Nek5	-0.203957142	0.179494006	-1.136289436	0.255835451	0.897044747
Nek3	-0.14392409	0.352015793	-0.40885691	0.682644673	0.984738223
Ckap2	0.596861293	0.514755778	1.159503823	0.246250879	0.896939594
Vps36	0.107812994	0.115905383	0.930181078	0.352277337	0.923152465
Thsd1	-0.120666728	0.215692783	-0.559437949	0.575862869	0.971613657
Slc25a15	0.075608059	0.17634812	0.428743209	0.668110112	0.981863224
1810012K16Rik	0.458983912	1.163570802	0.394461524	0.693240328	0.986698113
Mrps31	-0.045162798	0.161592928	-0.279484989	0.779872656	0.990988839
Smim19	-0.015926143	0.167495336	-0.095084097	0.924248029	0.994688009
Slc20a2	-0.09223296	0.233375556	-0.395212602	0.692685993	0.986599035
Vdac3	0.068421008	0.097008432	0.705309907	0.480617397	0.957463006
Polb	0.122192366	0.167108116	0.731217425	0.464646358	0.953083512
A930013F10Rik	-2.183709065	1.708922016	-1.277828388	0.201309942	0.881187967
Ikbkb	-0.161405181	0.121693157	-1.326329147	0.184730697	0.871716376
Plat	-0.35669903	0.136676003	-2.609814605	0.00905913	0.475600561
Ap3m2	-0.107994337	0.246081232	-0.438856455	0.660765551	0.979924124
Kat6a	0.045341099	0.17964338	0.252395047	0.800735728	0.990988839
Ank1	0.154368243	0.323719002	0.476858764	0.633462697	0.977034429
Gm15816	0.609630851	0.617836268	0.986719107	0.32378036	0.91640698
Agpat6	-0.07156814	0.138101297	-0.518229315	0.604298283	0.974001549
Gins4	0.279550826	0.1550583	1.802875597	0.071407754	0.744227652
Golga7	0.233234543	0.117119432	1.991424822	0.046434203	0.698098452
Sfrp1	0.260945051	0.221577059	1.177671785	0.23892748	0.896609492
Zmat4	-0.388369807	0.41203923	-0.942555414	0.345908354	0.921552698
1810011O10Rik	0.305786758	0.270466388	1.130590608	0.258227442	0.897248195
Ido1	0.45440141	0.451048715	1.007433111	0.313726686	0.915639195
Adam3	0.03648699	0.324932263	0.112291065	0.910592626	0.994688009
Adam5	-0.520042711	0.456536646	-1.139103982	0.254659787	0.897044747

Adam32	-0.767718485	1.647639874	-0.465950416	0.641250993	0.977996701
Adam9	-0.242278547	0.101045309	-2.397721866	0.016497387	0.566772949
Tm2d2	0.048590548	0.15481229	0.313867509	0.753621669	0.988585656
Htra4	0.266674326	0.302481246	0.881622678	0.377980887	0.928883222
Plekha2	-0.092052333	0.124044121	-0.742093475	0.458030703	0.952235842
Tacc1	0.02922004	0.201095942	0.145303975	0.884470877	0.992886758
5430421F17Rik	-0.809266382	0.861235696	-0.939657269	0.34739339	0.921552698
Fgfr1	-0.054759271	0.274472155	-0.199507565	0.841865726	0.992886758
Letm2	0.323410779	0.377441908	0.856849152	0.391528257	0.933734231
Whsc1l1	0.019296168	0.112305178	0.171819036	0.863579799	0.992886758
Ppapdc1b	0.002860603	0.170076856	0.016819471	0.986580636	0.998148029
Ddhd2	0.281694288	0.187198039	1.504792942	0.132377319	0.827800491
Bag4	-0.181555844	0.161585617	-1.123589139	0.261187366	0.897248195
Lsm1	0.105350775	0.199284126	0.528646096	0.597050977	0.974001549
Star	-0.500180116	0.389769408	-1.283271867	0.199396845	0.880831248
Ash2l	0.166517316	0.124253015	1.340147089	0.18019753	0.868087032
Kcnu1	-0.05141848	1.011158427	-0.050851062	0.959444202	0.996367077
Hgsnat	-0.087449518	0.132625136	-0.659373631	0.509655871	0.963452765
Pomk	0.137174631	0.153080402	0.896095318	0.370201857	0.928436166
Fnta	0.140789993	0.121267231	1.160989597	0.24564613	0.896939594
Hook3	-0.27764837	0.149369489	-1.858802432	0.063055148	0.727650961
Rnf170	-0.086694908	0.167669211	-0.517059199	0.605114834	0.974001549
Thap1	-0.076544794	0.201603225	-0.379680405	0.704182667	0.988585656
2310008N11Rik	1.513491933	1.521984539	0.994420044	0.320018414	0.91640698
Zfp703	-0.14667311	0.228766579	-0.641147454	0.521426885	0.964946923
Erlin2	-0.148458402	0.133533776	-1.111766674	0.266238484	0.89768067
Prosc	-0.218152159	0.110107661	-1.981262309	0.047561865	0.700850692
Adgra3	0.060110378	0.184569688	0.325678493	0.744667618	0.988585656
Brf2	0.465290826	0.263060931	1.768756855	0.07693446	0.752876039
Rab11fip1	0.069001565	0.214449476	0.321761404	0.747633455	0.988585656
Got1l1	0.096796649	0.308408357	0.313858708	0.753628353	0.988585656
Adrb3	0.129374858	0.281120083	0.46021208	0.645364001	0.979004493
Eif4ebp1	-0.042009954	0.13981642	-0.300465093	0.763822419	0.989331656
Unc5d	-1.119246601	1.763014741	-0.634848124	0.525527473	0.965637248
Dusp26	0.027495231	0.431192629	0.06376554	0.949156917	0.995624732
Rnf122	-0.056890057	0.242518497	-0.234580281	0.814534516	0.992048071
Tti2	0.015617946	0.19237636	0.081184331	0.935295361	0.994688009
Mak16	-0.01823573	0.146412527	-0.124550342	0.900879544	0.994688009
Fut10	0.139251794	0.190069939	0.732634495	0.463781386	0.953083512
Nrg1	-2.050297293	0.543350653	-3.773433014	0.000161016	0.077321054
Wrn	-0.478693496	0.238365137	-2.008236197	0.044618198	0.689849737
Purg	0.140093881	0.231734308	0.604545274	0.545481178	0.967491297
Tex15	-0.179474959	0.406389979	-0.441632344	0.658755275	0.979924124
Ppp2cb	-0.26027361	0.101101008	-2.574391833	0.010041648	0.49681814
Ubxn8	0.108282276	0.119606811	0.905318644	0.365296602	0.926556975
Gsr	0.022964888	0.130928959	0.175399608	0.860765648	0.992886758
Gtf2e2	-0.084199259	0.106863472	-0.787914315	0.430746827	0.945321484
Rbpms	-0.21264168	0.140353042	-1.515048596	0.129760098	0.827800491
Dctn6	0.062330744	0.112343165	0.554824535	0.579014684	0.971613657
Leprotl1	-0.102989654	0.09117961	-1.129525054	0.258676407	0.897248195
Saraf	-0.029801099	0.106636057	-0.279465501	0.77988761	0.990988839
Dusp4	0.097825887	0.353363864	0.276841798	0.781901585	0.990988839
Tnks	0.097715136	0.366433651	0.26666529	0.789726881	0.990988839
Ppp1r3b	-0.276592954	0.366886635	-0.75389215	0.450913984	0.949134998
Eri1	0.061097213	0.15422263	0.396162438	0.6919852	0.986464472
Gm16793	0.105923617	0.627504205	0.168801446	0.865952821	0.992886758
Mfhas1	0.320294781	0.268159217	1.194420181	0.232313646	0.895404344
Cldn23	-0.265448897	0.360515148	-0.73630442	0.461545457	0.953083512
D8Ertd82e	-1.191739887	0.772172212	-1.543360235	0.122743396	0.817788082
Lonrf1	-0.282467053	0.368340996	-0.766862925	0.443163019	0.948476747
6430573F11Rik	0.187197187	0.61962361	0.302114355	0.76256489	0.989285682
Dlc1	-0.018352545	0.162412937	-0.112999282	0.910031124	0.994688009

G630064G18Rik	-2.220175823	2.40103327	-0.92467516	0.355134904	0.924354588
AI429214	0.158784379	0.334688066	0.474424978	0.635196885	0.977034429
Tusc3	-0.02315609	0.094126726	-0.246009726	0.805674704	0.99099448
Gm6213	0.38352732	0.347216712	1.104576209	0.269343265	0.89804647
Msr1	-0.359211786	0.351185312	-1.022855382	0.306376228	0.913641886
Mir7666	0.162164861	0.601603351	0.269554451	0.787503047	0.990988839
Micu3	0.005988468	0.163610753	0.036601925	0.970802409	0.996733101
Zdhhc2	-0.245620118	0.244878734	-1.003027555	0.315847566	0.915957942
Cnot7	-0.031055893	0.104958785	-0.295886553	0.767316721	0.989884648
Vps37a	-0.074581118	0.146489365	-0.509123089	0.610665949	0.974001549
Mtmr7	0.466007838	0.35977418	1.295278713	0.1952241	0.879588734
Slc7a2	-0.480320861	0.19185213	-2.503599313	0.012293717	0.525888996
Pdgfrl	0.357435134	0.28612645	1.249220872	0.2115843	0.884755882
Mtus1	-0.19316927	0.127101493	-1.519803309	0.128560417	0.826380289
B430010I23Rik	0.578622679	0.208593258	2.773927999	0.005538393	0.416706681
Fgl1	0.446739498	0.905048708	0.493608238	0.621582882	0.97514112
Pcm1	-0.219187359	0.154033848	-1.422981776	0.154741438	0.854973821
Asah1	-0.129285044	0.090584048	-1.427238528	0.153511153	0.854159937
Frg1	0.156792786	0.135019891	1.161256939	0.245537425	0.896939594
Fat1	0.252468176	0.236786297	1.066227986	0.286320594	0.905916508
AY512931	-1.080359807	1.939178698	-0.557122357	0.577443833	0.971613657
Gm6329	0.753337274	0.727458908	1.035573647	0.300401092	0.910285132
F11	0.805296956	1.624368202	0.495760108	0.620063673	0.974471036
Klkb1	0.467415575	0.838712432	0.557301355	0.57732155	0.971613657
Cyp4v3	0.017107111	0.16430069	0.104120749	0.917073525	0.994688009
Fam149a	-0.237595875	0.173887789	-1.366374702	0.171821385	0.86244803
Tlr3	-0.171690096	0.218822585	-0.784608665	0.432683048	0.94605734
Sorbs2	0.008360776	0.317697118	0.026316815	0.979004643	0.997262526
Sorbs2os	0.658289677	0.821817614	0.801016753	0.423121947	0.943509323
Pdlim3	-0.171494731	0.198964275	-0.861937306	0.388722018	0.933332999
Ccdc110	-1.653202211	2.166614994	-0.763034603	0.44544276	0.948476747
1700029J07Rik	-0.136296709	0.230534744	-0.591219815	0.554373148	0.968926429
Ufsp2	-0.050170225	0.149078464	-0.336535701	0.736466934	0.988585656
Ankrd37	0.185455006	0.220161656	0.842358337	0.399587406	0.936395796
Lrp2bp	-0.743202188	0.669263281	-1.110478057	0.266793079	0.89768067
Snx25	-0.052006509	0.137628116	-0.377877073	0.705521915	0.988585656
Cfap97	0.093279921	0.139585201	0.668265122	0.503964373	0.96179897
Slc25a4	0.08800576	0.098855922	0.890242671	0.373335597	0.928840865
Acsl1	-0.312094047	0.189067997	-1.650697382	0.098800383	0.786907305
Cenpu	0.052498564	0.478780207	0.109650657	0.912686434	0.994688009
Primpol	0.216579046	0.353439225	0.612775918	0.540024516	0.966900344
Casp3	0.279105321	0.164392591	1.697797439	0.089545999	0.769347579
Gm16675	0.042270909	0.475722659	0.088856202	0.929196192	0.994688009
Irf2	0.048371848	0.2357356	0.205195347	0.837419505	0.992886758
Enpp6	-0.274482012	0.486880423	-0.563756519	0.572919847	0.971613657
Stox2	-0.440071712	0.242069039	-1.817959508	0.069070322	0.743426713
Trappc11	-0.063189458	0.161564031	-0.391110925	0.695715246	0.98715859
Rwdd4a	-0.04815624	0.143080809	-0.336566728	0.736443541	0.988585656
Ing2	0.21863382	0.137355468	1.591737287	0.111443746	0.800752782
Cdkn2aip	0.021561142	0.133673612	0.161296923	0.871859546	0.992886758
Cldn22	-0.085607961	0.62040524	-0.137987167	0.89025056	0.993043931
Wwc2	-0.245971316	0.192581262	-1.2772339	0.201519682	0.881220006
Dctd	-1.492204201	1.249400914	-1.194335769	0.232346651	0.895404344
Tenm3	1.011283196	1.385659264	0.729820976	0.465499621	0.953083512
Aga	-0.018105251	0.131666669	-0.137508235	0.890629084	0.993043931
Neil3	-0.485914093	1.088582289	-0.446373322	0.6553276	0.979133069
Vegfc	-0.314593433	0.166954746	-1.884303627	0.059523917	0.724178089
Spcs3	-0.10389241	0.138667926	-0.749217312	0.453726236	0.950351284
Asb5	0.170739901	0.477558353	0.357526781	0.720697481	0.988585656
Gpm6a	-0.170537483	0.174497769	-0.977304659	0.328418347	0.917115058
Hpgd	-0.029513723	0.129957654	-0.22710261	0.820343955	0.992886758
Cep44	0.166442283	0.156791243	1.061553439	0.288438461	0.907334416

Fbxo8	0.171991051	0.107844843	1.594800877	0.110756771	0.800111791
LOC102636514	0.867448597	0.489385481	1.772526222	0.076307247	0.752876039
Hand2	-0.726795197	1.371879647	-0.529780581	0.59626407	0.974001549
Scrg1	-1.081303366	0.981045387	-1.102195046	0.270376885	0.89804647
Sap30	0.144601488	0.164290206	0.880158905	0.378773232	0.929137643
2500002B13Rik	0.011391287	0.536654644	0.021226476	0.983064994	0.997545193
Hmgb2	0.310606134	0.126822269	2.449145065	0.014319576	0.5540183
Galnt7	-0.327939998	0.215541602	-1.521469617	0.12814203	0.826336015
AW046200	-0.313740207	1.031877371	-0.304047958	0.761091355	0.989266039
Galntl6	0.126141931	0.593261985	0.212624329	0.831619994	0.992886758
BC030500	0.781880889	0.823192463	0.949815412	0.342206053	0.919850248
Mfap3l	-0.384725256	0.195595974	-1.966938521	0.049190299	0.707153766
2700029M09Rik	0.026071898	0.152752647	0.1706805	0.864474996	0.992886758
Clcn3	-0.235735698	0.161229753	-1.462110397	0.14371096	0.841934045
Nek1	-0.378020135	0.175036007	-2.159670694	0.030798171	0.659455637
Sh3rf1	0.014724277	0.239163878	0.061565638	0.950908742	0.99573416
Cbr4	0.319076664	0.183928788	1.734783702	0.082779124	0.76099108
Palld	0.031421938	0.195108858	0.161048239	0.872055407	0.992886758
Ddx60	-0.216604371	0.232747416	-0.930641358	0.352039113	0.923082865
Spock3	-0.077228938	0.600548624	-0.12859731	0.897676295	0.994101834
Tll1	-0.117480721	0.479513585	-0.244999777	0.806456604	0.99099448
Cpe	-0.246190217	0.16527405	-1.48958785	0.136332639	0.832457116
Msmo1	-0.115074228	0.140907872	-0.816662877	0.414121112	0.943415794
Klhl2	-0.285263146	0.15522709	-1.837714965	0.066104418	0.738254074
Tmem192	-0.004832035	0.18782678	-0.025726016	0.979475873	0.997262526
Apela	-2.655182411	2.240290715	-1.185195472	0.23594017	0.896067726
BC030870	0.05050589	0.202994039	0.248804795	0.803511782	0.990988839
Mar-01	0.149052296	0.172323607	0.864955759	0.387063072	0.932858077
Tma16	0.067497866	0.324354361	0.208099147	0.835151554	0.992886758
Npy1r	-0.126603022	0.362791593	-0.348969006	0.727112578	0.988585656
Naf1	-0.193381995	0.196025179	-0.986516095	0.323879921	0.91640698
Nat1	0.17106959	0.97837385	0.174850942	0.861196759	0.992886758
Nat2	0.168969815	0.207253684	0.815280155	0.414911965	0.943415794
Psd3	-0.048989212	0.173130535	-0.282961131	0.777206633	0.990861469
Sh2d4a	0.004576849	0.142295706	0.032164351	0.974340985	0.997262526
Csgalnact1	0.008715398	0.171658982	0.050771581	0.959507537	0.996367077
Ints10	-0.359422159	0.198999827	-1.806143073	0.070895994	0.744227652
Lpl	0.037948631	0.151986209	0.2496847	0.802831191	0.990988839
Slc18a1	-0.065154185	0.710546395	-0.091695891	0.926939662	0.994688009
Atp6v1b2	-0.258128166	0.138300154	-1.866434403	0.061980614	0.725730283
Lzts1	0.350489157	1.347119357	0.260176766	0.794727426	0.990988839
Zfp930	0.019372776	0.1844271	0.105043003	0.916341686	0.994688009
D130040H23Rik	0.141133359	0.433568343	0.32551583	0.744790704	0.988585656
Gm10033	0.053302993	0.292520063	0.182219956	0.855410112	0.992886758
Zfp868	0.040815607	0.129221677	0.315857279	0.752110839	0.988585656
Zfp964	0.301366839	0.303876534	0.991741068	0.321323852	0.91640698
Zfp869	-0.015057721	0.10631275	-0.14163608	0.887367466	0.992886758
Zfp963	-0.280713316	0.253838205	-1.105874967	0.268780641	0.89804647
Zfp866	-0.078984998	0.197321265	-0.400286294	0.688945661	0.98538669
Atp13a1	-0.143158859	0.184489322	-0.775973683	0.437764525	0.94749626
Gmip	0.157476125	0.272765219	0.577332131	0.563715112	0.969658675
Lpar2	-0.275603181	0.542308514	-0.508203678	0.611310512	0.974001549
Pbx4	0.009330853	0.427633663	0.021819734	0.982591753	0.997390438
Ndufa13	0.171084568	0.144863696	1.18100375	0.237601234	0.896609492
Tssk6	0.266683154	0.468766427	0.568904124	0.569421207	0.97144955
Gatad2a	-0.326513572	0.164277477	-1.98757357	0.046858872	0.698868643
Mau2	-0.190746814	0.136037743	-1.402160969	0.160867184	0.857316691
Sugp1	0.443086638	0.166552243	2.660346266	0.007806035	0.45506991
Tm6sf2	-0.474740011	0.403986094	-1.175139487	0.239938915	0.896939594
Rfxank	0.212428876	0.198969719	1.067644246	0.285681017	0.905916508
Nr2c2ap	0.143114458	0.338655554	0.422595927	0.672590099	0.983233856
2310045N01Rik	0.280093588	0.148880782	1.881328031	0.05992731	0.725221283

Tmem161a	0.097705831	0.160345059	0.609347317	0.54229425	0.967491297
Slc25a42	0.205981319	0.333946589	0.616809173	0.537360601	0.96674935
Armc6	0.170769006	0.216536659	0.788637856	0.430323698	0.945321484
Sugp2	-0.145393405	0.266473943	-0.545619596	0.585327451	0.972904784
Homer3	0.112860053	0.15248068	0.740159691	0.459203103	0.952683168
Ddx49	0.172792424	0.216244794	0.799059348	0.424256	0.94406679
Cope	0.154169451	0.121746039	1.266320056	0.205398519	0.883765092
Upf1	-0.140893067	0.204406411	-0.6892791	0.490647648	0.958896244
Comp	1.229304041	2.072012315	0.59328993	0.552987137	0.968926429
Crtc1	-0.005568184	0.460151119	-0.012100772	0.990345217	0.998417328
Klhl26	-0.530188496	0.318654875	-1.663832996	0.096145808	0.780593042
Tmem59l	-0.390771659	0.309967846	-1.260684498	0.207422541	0.883765092
Crlf1	-0.235863515	0.196648989	-1.199413818	0.230367077	0.895404344
2810428I15Rik	0.227825517	0.146639767	1.553640742	0.120270135	0.81558552
Uba52	0.23608476	0.137455797	1.71753222	0.085881973	0.763596872
Kxd1	0.352161355	0.129277816	2.724066398	0.006448354	0.429927454
Fkbp8	0.012404336	0.105416137	0.117670185	0.906328992	0.994688009
Ell	0.004535651	0.23577788	0.019236966	0.984652068	0.998148029
Isyna1	-0.247406459	0.196866765	-1.256720295	0.208854936	0.884258007
Ssbp4	-0.036087454	0.145656338	-0.247757524	0.804322022	0.990988839
Lrrc25	-0.191375012	0.335421442	-0.570550919	0.568304097	0.971380162
Gdf15	0.103637922	0.430041019	0.240995435	0.809558655	0.991295569
Pgpep1	-0.109530807	0.153961475	-0.71141698	0.47682588	0.95716557
Lsm4	0.5073073	0.172612441	2.938996157	0.003292772	0.357710224
Jund	-0.042762046	0.176783983	-0.241888688	0.808866415	0.991174313
Gm3336	0.241951838	0.48432131	0.499568845	0.6173787	0.974001549
Pde4c	0.439182344	0.654089365	0.671440888	0.501939705	0.961378071
Rab3a	-0.201465349	0.17562336	-1.147144374	0.251321957	0.896939594
Mpv17l2	0.161545371	0.149876619	1.07785572	0.281098149	0.904300139
Ifi30	-0.249104869	0.178996878	-1.391671586	0.16402187	0.859982992
Pik3r2	-0.389443459	0.244900386	-1.590211702	0.111787094	0.801067615
2010320M18Rik	0.312656695	0.187033661	1.671660023	0.094591381	0.777748075
Mast3	0.185963279	0.311728661	0.59655496	0.55080455	0.968926429
Il12rb1	0.718115147	1.388181365	0.517306431	0.604942266	0.974001549
Arrdc2	-0.14901025	0.286640578	-0.519850508	0.603167773	0.974001549
Kcnn1	-0.044836893	0.672956374	-0.066626745	0.946878854	0.995216367
Ccdc124	0.377682902	0.141165386	2.675463949	0.00746259	0.452943141
Slc5a5	-1.015372086	0.918769975	-1.105142869	0.269097688	0.89804647
Rpl18a	0.245376316	0.108165757	2.268521223	0.023297457	0.622011301
Map1s	-0.154661022	0.325864496	-0.474617591	0.635059566	0.977034429
Haus8	0.397914769	0.217843262	1.826610407	0.067758368	0.742385562
Myo9b	0.235478689	0.154415582	1.524967145	0.127267293	0.824450458
Use1	0.368098955	0.191054394	1.92667097	0.054020648	0.707153766
Ocel1	0.440093727	0.178667411	2.463200895	0.013770274	0.545277966
Nr2f6	0.052552298	0.112176696	0.46847786	0.6394429	0.977843986
Ushbp1	0.306286169	0.13203722	2.319695688	0.020357345	0.601921946
Babam1	0.218793167	0.096421763	2.269126398	0.023260641	0.622011301
Ankle1	0.269680846	1.453214992	0.185575326	0.852777805	0.992886758
Abhd8	0.322537751	0.256226598	1.258798866	0.20810299	0.884258007
Mrpl34	0.25489464	0.140233641	1.817642605	0.069118775	0.743426713
Dda1	0.079718986	0.110279887	0.722878746	0.469754383	0.95486913
Ano8	0.360572181	0.337212875	1.069271691	0.284947263	0.905916508
Gtpbp3	0.089308257	0.170171521	0.524813181	0.599713067	0.974001549
Plvap	-0.359457171	0.160306361	-2.242313828	0.024941097	0.627917508
Bst2	0.322954038	0.146118113	2.210225899	0.027089488	0.635474249
Mvb12a	-0.032659838	0.133139783	-0.245304878	0.806220375	0.99099448
Tmem221	0.339999551	0.210003063	1.619021864	0.105442559	0.793982106
Nxnl1	-0.929750951	1.223331944	-0.760015264	0.447245461	0.948476747
Slc27a1	-0.212834894	0.233314431	-0.912223448	0.361651103	0.926455466
Pgls	-0.040611498	0.143885373	-0.28224897	0.777752611	0.990988839
Fam129c	0.6141001	0.611905843	1.003585939	0.315578235	0.91586731
Colgalt1	-0.148332533	0.097700886	-1.518231185	0.128956128	0.827295367

Unc13a	0.075501714	1.363848733	0.055359302	0.955852218	0.99614946
Jak3	0.149951844	0.293312645	0.511235525	0.609186146	0.974001549
Insl3	-2.750359682	1.826001977	-1.506219444	0.13201085	0.827800491
B3gnt3	0.017148059	0.239006657	0.071747204	0.94280309	0.994744476
Fcho1	0.600033915	0.647011183	0.927393423	0.353722304	0.92416951
Zfp709	-0.128673837	0.195203705	-0.659177224	0.509781971	0.963452765
Zfp882	-0.15908398	0.19850042	-0.801428936	0.422883369	0.943509323
Zfp617	0.029097379	0.144988034	0.200688139	0.840942435	0.992886758
Zfp961	-0.071370684	0.150361509	-0.474660601	0.635028904	0.977034429
Cyp4f18	-0.205941299	0.298401099	-0.690149263	0.490100326	0.958560421
Tpm4	0.0186797	0.096823537	0.192925196	0.847017552	0.992886758
Rab8a	0.094428098	0.094434231	0.999935055	0.317341939	0.91640698
Hsh2d	-0.259165156	0.66995867	-0.386837528	0.698876493	0.987770663
Cib3	-0.539804075	1.027866809	-0.525169283	0.599465517	0.974001549
Fam32a	0.047244152	0.097954195	0.482308614	0.629586729	0.977034429
Ap1m1	-0.091450893	0.114118087	-0.801370712	0.422917065	0.943509323
Klf2	-0.238003821	0.206902375	-1.150319424	0.250012333	0.896939594
Eps15l1	-0.289804384	0.194737282	-1.488181314	0.136703084	0.832506992
Calr3	0.338162609	0.352003573	0.960679479	0.336713352	0.919242242
1700030K09Rik	0.079249975	0.244942054	0.32354581	0.74628192	0.988585656
Cherp	0.071081957	0.203576149	0.349166429	0.726964368	0.988585656
Slc35e1	-0.257853484	0.144021867	-1.790377317	0.073393274	0.748833631
Med26	-0.009869611	0.419921966	-0.023503441	0.981248694	0.997375797
Smim7	0.142825659	0.117454933	1.216003917	0.22398343	0.892989918
Tmem38a	0.024492367	0.169671752	0.144351468	0.885222938	0.992886758
Nwd1	0.060719019	0.73936029	0.082123722	0.93454833	0.994688009
Sin3b	0.249648569	0.121324561	2.05769193	0.039619716	0.679118604
F2rl3	0.730882331	0.815255483	0.896507103	0.369981988	0.928436166
Large	-0.076516566	0.234141839	-0.326795784	0.743822348	0.988585656
Hmgxb4	0.069772548	0.182451305	0.382417369	0.702151809	0.988482081
Tom1	0.34334269	0.393874927	0.871704864	0.383369406	0.931591679
Hmox1	-0.267604237	0.203429822	-1.315462176	0.188354621	0.874826736
Mcm5	0.180333527	0.250982177	0.718511287	0.472442091	0.955003345
Rasd2	-0.1845744	0.639062519	-0.288820568	0.772718691	0.990678393
Gm10649	-0.496771876	1.705055835	-0.291352263	0.77078192	0.989990452
Nr3c2	-0.15653478	0.387212948	-0.40426019	0.686021396	0.98538669
Arhgap10	-0.010606672	0.217294997	-0.048812317	0.961068866	0.996474415
0610038B21Rik	0.897573814	1.029850932	0.871557025	0.383450084	0.931591679
Prmt10	-0.004968708	0.181989519	-0.027302165	0.97821873	0.997262526
Tmem184c	0.071297147	0.161315932	0.441972136	0.658509369	0.979746192
Ednra	-0.406156106	0.186483946	-2.177968204	0.029408404	0.64858361
Ttc29	-0.085546467	0.175412663	-0.487686955	0.625771593	0.97639266
Rbmxl1	0.061873202	0.137716297	0.449280176	0.653229557	0.979004493
Slc10a7	-0.273909135	0.160995105	-1.7013507	0.088877153	0.767814344
Lsm6	0.088416903	0.160031552	0.552496693	0.5806081	0.971613657
Zfp827	0.11929418	0.474677462	0.251316293	0.801569579	0.990988839
Mmaa	0.178175765	0.178415004	0.998659089	0.317959865	0.91640698
Gm4890	0.387996606	0.479822362	0.808625518	0.418730582	0.943415794
Smad1	-0.091631891	0.193848249	-0.472699091	0.636427875	0.977284137
Otud4	-0.064627283	0.225975248	-0.285992751	0.774883674	0.990861469
Abce1	-0.073715784	0.0947669	-0.777864252	0.436649045	0.946762848
Anapc10	0.382964129	0.192801132	1.986316806	0.046998158	0.698868643
Hhip	0.123915508	0.344608065	0.359583889	0.719158334	0.988585656
Gypa	-0.205319371	0.72191245	-0.284410347	0.77609593	0.990861469
Frem3	-0.651312882	1.604061948	-0.406039731	0.684713408	0.985105247
Smarca5	-0.191005075	0.152569078	-1.251925208	0.210597118	0.884340413
Gab1	-0.015616809	0.158898552	-0.098281632	0.921708663	0.994688009
Usp38	-0.130185036	0.170723542	-0.76254882	0.445732517	0.948476747
Inpp4b	-0.399030346	0.194884169	-2.04752571	0.040606495	0.679118604
Il15	0.045680389	0.222961824	0.204879866	0.837665986	0.992886758
Zfp330	0.209151914	0.135255154	1.546350787	0.122019873	0.816233249
Rnf150	0.003734969	0.225007288	0.016599324	0.986756264	0.998148029

Tbc1d9	0.062385778	0.25242607	0.247144751	0.804796202	0.990988839
Ucp1	-1.592840853	1.381261587	-1.153178274	0.248837219	0.896939594
Elmod2	0.01132703	0.157963077	0.071706821	0.942835228	0.994744476
Clgn	0.047638952	1.933093366	0.024643896	0.980339006	0.997296835
Scoc	-0.146010985	0.118428984	-1.232899079	0.217613419	0.887633697
Ndufb7	0.275229177	0.179102435	1.536713765	0.124363419	0.821164769
Tecr	0.02745851	0.173753005	0.158031858	0.874431695	0.992886758
Dnajb1	-0.111581219	0.199527833	-0.559226338	0.57600726	0.971613657
Gipc1	0.150501178	0.129117601	1.165613182	0.243770876	0.896939594
Ptger1	-0.126943511	0.224605283	-0.565184883	0.571948018	0.971613657
Pkn1	0.214732975	0.151516291	1.417226976	0.156416577	0.855455553
Ddx39	0.026135898	0.126179405	0.207132841	0.835906115	0.992886758
Adgre5	0.04178417	0.126082911	0.331402331	0.740340605	0.988585656
Adgrl1	0.084090308	0.199340993	0.42184152	0.673140698	0.983375962
Asf1b	-0.166085159	0.491942937	-0.337610617	0.73565664	0.988585656
Prkaca	-0.172371656	0.183178311	-0.941004724	0.346702438	0.921552698
Samd1	-0.022417129	0.19475012	-0.115107139	0.908360202	0.994688009
1700067K01Rik	-0.170010926	0.769479332	-0.22094281	0.825136964	0.992886758
2210011C24Rik	0.097673828	0.164425775	0.594029909	0.552492109	0.968926429
Palm3	0.353110814	1.24561231	0.283483722	0.776806058	0.990861469
Il27ra	-0.512142056	0.339784623	-1.507254953	0.131745319	0.827800491
Rln3	-0.31508386	1.339232687	-0.235271931	0.81399768	0.992048071
Rfx1	0.010421861	0.375753416	0.027735906	0.977872786	0.997262526
Dcaf15	0.039292536	0.195820444	0.200655942	0.840967612	0.992886758
Podnl1	0.19781791	1.204845364	0.16418531	0.869585265	0.992886758
Cc2d1a	0.243701486	0.273426751	0.891286186	0.372775658	0.928840865
4930432K21Rik	-0.157671334	0.804730716	-0.195930552	0.844664524	0.992886758
Nanos3	0.670951249	0.685239343	0.979148755	0.327506485	0.917115058
Mir24-2	0.522994887	0.390548583	1.33912888	0.18052872	0.868087032
Zswim4	0.64322925	0.346482344	1.85645607	0.063388579	0.728651743
D8Ertd738e	0.186763157	0.127269479	1.46746226	0.142250349	0.840741274
Mri1	-0.024412559	0.181353268	-0.134613286	0.892917636	0.993300147
Ccdc130	0.112622058	0.211965827	0.53132177	0.595195822	0.974001549
Cacna1a	-0.098075466	0.39927727	-0.245632481	0.805966743	0.99099448
Ier2	0.423785105	0.355571131	1.191843396	0.233322657	0.89581803
Nacc1	-0.10962098	0.217084065	-0.504970183	0.613579778	0.974001549
Trmt1	0.071503041	0.148440805	0.481693972	0.630023359	0.977034429
Lyl1	0.567948722	0.305982413	1.856148252	0.06343243	0.728651743
Nfix	0.122258736	0.214565619	0.569796489	0.568815737	0.97144955
Dand5	0.33245017	0.217465337	1.528750167	0.12632639	0.822708806
Gadd45gip1	0.228088659	0.179916987	1.267743879	0.20488943	0.883765092
Rad23a	-0.005190323	0.166360361	-0.031199275	0.975110618	0.997262526
Calr	-0.221806835	0.085385366	-2.597714886	0.009384638	0.478229167
Farsa	0.129553904	0.177367666	0.730425712	0.465130007	0.953083512
Syce2	0.16663172	0.297425699	0.560246544	0.575311284	0.971613657
Gcdh	-0.092897253	0.125202072	-0.741978557	0.458100327	0.952235842
Klf1	-1.811702129	1.92072576	-0.943238315	0.34555902	0.921434283
Dnase2a	-0.252445307	0.225880869	-1.117603754	0.263736265	0.897248195
Mast1	0.31692768	0.422818652	0.749559364	0.453520132	0.950163378
Gm38426	0.337119236	0.24290488	1.387865224	0.165178089	0.860011234
Rnaseh2a	0.16489145	0.161961438	1.0180908	0.308634807	0.9139204
Prdx2	0.257506989	0.194182222	1.326110016	0.184803259	0.871716376
Junb	0.372901084	0.414645275	0.899325535	0.36847929	0.928436166
Hook2	-0.096077051	0.225486527	-0.426087766	0.670043894	0.982177283
Asna1	0.07586203	0.131388414	0.577387514	0.563677707	0.969658675
2310036O22Rik	0.274834777	0.120543041	2.27997216	0.02260934	0.620006604
Tnpo2	-0.100515693	0.170852856	-0.588317311	0.556319328	0.968926429
Fbxw9	-0.001568939	0.129657974	-0.012100597	0.990345356	0.998417328
Dhps	0.087426257	0.162006687	0.539645977	0.589441203	0.974001549
Wdr83	0.150085872	0.212117858	0.707558874	0.47921924	0.957463006
Wdr83os	0.035721645	0.141736916	0.252027811	0.801019568	0.990988839
Man2b1	0.038216006	0.180038087	0.212266232	0.831899338	0.992886758

Zfp791	-0.25954541	0.319691584	-0.811861877	0.416870892	0.943415794
Vps35	0.076043326	0.129306851	0.588084274	0.556475728	0.968926429
Orc6	0.019845191	0.142935637	0.138840052	0.889576543	0.993043931
Mylk3	-0.224027855	0.325373172	-0.688525897	0.491121668	0.958916246
4921524J17Rik	-0.06950676	0.174982593	-0.397220997	0.6912045	0.986191149
Gpt2	-0.33860035	0.179285707	-1.888607607	0.058944427	0.72285319
Dnaja2	0.006771165	0.101981684	0.066395897	0.947062637	0.995217331
Neto2	-0.120736291	0.398925739	-0.302653549	0.762153901	0.989285682
Itfg1	-0.247563684	0.102383047	-2.418014424	0.015605458	0.557315333
Phkb	0.032799406	0.165216263	0.198524074	0.842635051	0.992886758
Abcc12	-1.729782095	1.758421319	-0.983713105	0.325256595	0.91640698
Lonp2	-0.040939735	0.129011462	-0.317334089	0.750990113	0.988585656
Siah1a	-0.05063662	0.134217804	-0.377272003	0.705971476	0.988585656
Gm10638	0.027118253	0.648311727	0.041829034	0.966634989	0.996474415
N4bp1	-0.237250154	0.190136901	-1.24778595	0.212109455	0.884945675
Zfp423	0.378448205	0.694755664	0.544721296	0.585945216	0.972904784
Cnep1r1	-0.196298764	0.11018833	-1.781484146	0.074833393	0.75241101
Heatr3	0.024868362	0.184024917	0.135135843	0.892504472	0.993300147
Papd5	-0.245004212	0.223823377	-1.094631917	0.273677927	0.899400109
Adcy7	0.104670289	0.160356473	0.65273504	0.513927109	0.964352198
Brd7	-0.040515728	0.126401061	-0.320533133	0.748564218	0.988585656
Nkd1	-0.106766398	0.13792801	-0.774073355	0.438887413	0.947838535
Snx20	0.046949958	0.214735892	0.218640477	0.826930119	0.992886758
Nod2	-0.081421363	0.445817401	-0.182633883	0.855085297	0.992886758
Cyld	-0.065832767	0.1619125	-0.406594719	0.684305677	0.985105247
Tox3	-0.666095315	0.327468537	-2.034074237	0.041944111	0.679118604
Chd9	0.209619404	0.211754575	0.989916767	0.322214803	0.91640698
Rbl2	-0.359678157	0.19897706	-1.807636303	0.070663124	0.744227652
Aktip	0.102546703	0.130821022	0.783870219	0.433116266	0.946239431
Rpgrip1l	0.107053045	0.149369348	0.716700223	0.473559088	0.955974667
Fto	-0.28136697	0.212543237	-1.323810508	0.185565985	0.87179632
Irx3	-0.025299918	0.216452305	-0.116884494	0.906951586	0.994688009
Crnde	0.196112534	0.201437398	0.973565666	0.330272241	0.917115058
Irx5	-0.399266228	0.203367822	-1.963271393	0.049614648	0.707153766
Mmp2	-0.472172966	0.262822399	-1.796547662	0.07240746	0.74633396
Lpcat2	-0.44351507	0.237543133	-1.867092788	0.061888631	0.725730283
Slc6a2	-0.123106961	0.257668759	-0.477772168	0.632812372	0.977034429
Ces1b	1.015331364	0.624797844	1.625055806	0.104150668	0.79155232
Ces1c	0.115991412	4.03051846	0.028778286	0.977041419	0.997262526
Ces1d	-0.030435683	0.163036464	-0.18668022	0.851911366	0.992886758
Ces1e	0.140430265	0.234463744	0.598942349	0.54921133	0.968926429
Ces1f	0.387471154	0.640063405	0.605363704	0.544937362	0.967491297
Ces1g	1.20424615	0.836115631	1.440286613	0.149786327	0.848779008
Gnao1	-0.171485791	0.389446611	-0.440331964	0.659696693	0.979924124
Amfr	-0.119165971	0.09582966	-1.243518669	0.213676766	0.884945675
Nudt21	0.05269446	0.145740996	0.361562373	0.717679087	0.988585656
Ogfod1	-0.095781032	0.215887142	-0.443662512	0.657286603	0.979614766
Bbs2	-0.151846201	0.17737392	-0.856079636	0.391953732	0.933734231
Mt3	0.425714291	0.629386923	0.676395196	0.498789766	0.960700483
Mt2	-0.664105927	0.413043732	-1.607834414	0.107871449	0.796929372
Mt1	-0.20891545	0.2707498	-0.771618114	0.440340643	0.947849924
Nup93	-0.044035967	0.219913606	-0.200242122	0.841291225	0.992886758
Slc12a3	0.550991537	1.637959175	0.336389054	0.736577503	0.988585656
Herpud1	-0.396371606	0.149606078	-2.649435172	0.008062644	0.461890481
9330175E14Rik	3.063467671	1.901781902	1.61084069	0.10721445	0.796401421
Nlrc5	0.479296495	0.279173399	1.716841564	0.086008124	0.763596872
Cpne2	0.481501543	0.347104236	1.387195813	0.165382062	0.860011234
Fam192a	0.102141031	0.12637136	0.808260911	0.4189404	0.943415794
Rspry1	-0.10628238	0.181848062	-0.584457042	0.558912859	0.968926429
Arl2bp	0.12452527	0.093365178	1.333744257	0.182287685	0.868546072
Pllp	0.158054189	0.142460396	1.109460554	0.267231552	0.89804647
Ccl22	-0.978000167	0.346488341	-2.822606279	0.004763503	0.391906015

Cx3cl1	-0.57673679	0.255450895	-2.257720762	0.023963074	0.622011301
Ccl17	-0.655191062	0.396303403	-1.65325621	0.098278731	0.786907305
Ciapin1	0.260170487	0.144382457	1.801953591	0.071552708	0.744227652
Coq9	0.083649182	0.133063917	0.628639105	0.529585353	0.965637248
Polr2c	0.135969385	0.153660324	0.884869832	0.376226841	0.928883222
Dok4	0.278456716	0.147943353	1.882184704	0.059810942	0.725221283
Ccdc102a	-0.049433749	0.263322633	-0.187730724	0.851087744	0.992886758
Adgrg5	0.841789002	0.746503023	1.127643126	0.259470666	0.897248195
Adgrg1	-0.188953667	0.228865519	-0.825610026	0.409025346	0.940768024
Adgrg3	-0.630311359	0.422013697	-1.493580335	0.135285343	0.830377364
Drc7	-0.114140162	0.310555937	-0.367534953	0.713220025	0.988585656
Katnb1	-0.114780376	0.249502382	-0.460037195	0.645489523	0.979004493
Kifc3	-0.044373379	0.144133573	-0.307862896	0.75818666	0.989266039
Tepp	0.789589373	0.526950844	1.498411819	0.134026287	0.827800491
Zfp319	-0.066661401	0.288633087	-0.230955508	0.817349367	0.992678823
Usb1	0.368982856	0.147886097	2.49504764	0.012594022	0.525888996
Mmp15	-0.068526137	0.263224052	-0.260333872	0.794606247	0.990988839
Cfap20	-0.10152884	0.096674812	-1.050209856	0.293621639	0.907334416
Csnk2a2	-0.032345084	0.174362292	-0.18550504	0.852832928	0.992886758
Ccdc113	0.01081577	0.150658761	0.071789853	0.942769148	0.994744476
Gins3	0.331138959	0.386779508	0.856144008	0.391918129	0.933734231
Ndrg4	0.179721708	0.528644412	0.339967101	0.733881303	0.988585656
Setd6	0.117228989	0.232632558	0.503923398	0.614315207	0.974001549
Cnot1	-0.176269757	0.132096157	-1.334404888	0.182071199	0.868546072
4930513N10Rik	0.344866376	0.540478015	0.638076604	0.523423815	0.965637248
Slc38a7	-0.074559628	0.320243127	-0.232821946	0.81589967	0.992524109
Got2	0.021213504	0.161303709	0.13151281	0.895369653	0.993618558
Cdh11	-0.13222766	0.15404539	-0.858368172	0.390689197	0.933734231
Cdh5	-0.206393509	0.161234949	-1.28007923	0.200517273	0.880888611
Bean1	0.298662692	1.593102614	0.18747235	0.8512903	0.992886758
Tk2	0.267327872	0.127834598	2.091201261	0.036510026	0.677009445
Cklf	0.442179719	0.226668078	1.950780732	0.051083135	0.707153766
Cmtm2b	0.635293881	0.816208147	0.77834788	0.436363956	0.946762848
Cmtm3	0.040272358	0.124096551	0.324524397	0.745541055	0.988585656
Cmtm4	-0.311852144	0.128087842	-2.434674035	0.01490521	0.554936045
Dync1li2	-0.134577029	0.13752843	-0.978539702	0.327807465	0.917115058
Ccdc79	-0.564772969	0.873465267	-0.646588926	0.517898027	0.964437658
Nae1	0.02451254	0.106082535	0.231070456	0.817260067	0.992678823
Car7	0.995090714	1.063529785	0.935649126	0.349453872	0.921585102
Pdp2	-0.654256256	0.400647116	-1.63299879	0.102469251	0.789151138
Cdh16	-0.441035765	0.374681946	-1.177093718	0.239158102	0.896609492
Rrad	-0.269907519	0.384556295	-0.701867379	0.482761874	0.957463006
Fam96b	0.327345705	0.174872094	1.871915058	0.061218356	0.725221283
Ces2b	-0.255306117	0.526657715	-0.48476669	0.627841854	0.976968316
Ces2c	0.94047711	1.03236478	0.910993022	0.36229905	0.926455466
Ces2e	-0.411228895	0.286994897	-1.432878771	0.151892485	0.852599143
Ces2g	0.069853516	0.175326544	0.398419511	0.690320978	0.986002801
Cbfb	0.135862152	0.112131236	1.211635192	0.225652057	0.89336109
D230025D16Rik	-0.326661857	0.134424851	-2.430070445	0.015095888	0.555113898
B3gnt9	-0.076094022	0.20801973	-0.365801945	0.714512868	0.988585656
Tradd	0.064638773	0.12725984	0.507927506	0.611504184	0.974001549
Fbxl8	0.126547879	0.309578971	0.408774144	0.682705417	0.984738223
Hsf4	-0.36632322	0.331062386	-1.106508123	0.26850665	0.89804647
Nol3	-0.056466345	0.192807597	-0.292863692	0.769626342	0.989990452
4931428F04Rik	0.396479809	0.478184669	0.829135342	0.407027827	0.939557159
Exoc3l	0.101938634	0.276656877	0.368465934	0.712525842	0.988585656
E2f4	0.0372265	0.185474707	0.200709305	0.840925883	0.992886758
Elmo3	-0.234410265	0.164423314	-1.425651019	0.153969102	0.854159937
Lrrc29	0.567368533	0.558349317	1.016153358	0.309556363	0.9139204
Tmem208	0.29646169	0.197617345	1.500180511	0.13356765	0.827800491
Fhod1	-0.08083035	0.16940013	-0.477156363	0.633250782	0.977034429
Slc9a5	-0.132332683	0.377784626	-0.350286045	0.726124037	0.988585656

Plekhg4	-0.233533845	1.841025994	-0.126849836	0.899059251	0.99447264
Kctd19	-0.920566697	1.860135554	-0.494892265	0.620676171	0.975058198
Lrrc36	-0.254335183	0.212381443	-1.197539579	0.23109631	0.895404344
Tppp3	0.108512896	0.145192978	0.747370135	0.454840168	0.951220112
Zdhhc1	0.127741668	0.158384435	0.806529174	0.419937793	0.943415794
Hsd11b2	-0.794766102	0.884253381	-0.898799054	0.368759704	0.928436166
Atp6v0d1	-0.145511155	0.083823261	-1.735928099	0.082576549	0.76099108
Agrp	0.143607781	0.429854186	0.334084873	0.738315514	0.988585656
Fam65a	-0.143326614	0.168267437	-0.85177867	0.394336947	0.933796635
Ctcf	0.013964002	0.116793027	0.119561952	0.904830161	0.994688009
Rltpr	-0.204982699	0.653676239	-0.313584443	0.753836677	0.988585656
Acd	0.199477394	0.282195203	0.706877339	0.479642708	0.957463006
Pard6a	-0.093353235	0.17449081	-0.535003734	0.592647275	0.974001549
Enkd1	0.042437373	0.199387267	0.212838931	0.831452597	0.992886758
Gfod2	0.046849754	0.175460358	0.267010475	0.789461096	0.990988839
Ranbp10	0.001005758	0.220439084	0.004562523	0.996359646	0.999379819
Tsnaxip1	-0.191333706	0.304144677	-0.6290878	0.529291577	0.965637248
Cenpt	0.089025626	0.284113531	0.313345253	0.754018373	0.988744517
Thap11	-0.075548622	0.142101989	-0.531650705	0.59496794	0.974001549
Nutf2	-0.383649092	0.372142666	-1.030919395	0.302578621	0.911518274
Edc4	-0.041719782	0.188802607	-0.220970371	0.825115503	0.992886758
Nrn1l	-0.628926426	0.820451716	-0.766561168	0.443342471	0.948476747
Pskh1	-0.016562437	0.237419516	-0.069760217	0.944384512	0.99486515
Ctrl	2.410863151	1.692550024	1.424396985	0.154331587	0.854159937
Psmb10	0.064525296	0.12570524	0.513306334	0.607737054	0.974001549
Lcat	-0.395334172	0.684728635	-0.577358901	0.563697032	0.969658675
Slc12a4	0.015666075	0.180322101	0.086878286	0.930768259	0.994688009
Dpep2	-0.034758014	0.179544008	-0.193590497	0.846496539	0.992886758
Ddx28	-0.123567193	0.188205904	-0.656553221	0.511468236	0.963452765
Dus2	-0.12644333	0.250031476	-0.50570965	0.613060493	0.974001549
Nfatc3	-0.043396992	0.159667142	-0.271796632	0.785778395	0.990988839
Esrp2	-0.183871173	0.211391967	-0.869811544	0.384403402	0.931689378
1810019D21Rik	-0.064810338	0.212351094	-0.305203691	0.760211021	0.989266039
Pla2g15	-0.035035548	0.156697338	-0.223587386	0.82307838	0.992886758
Slc7a6	-0.411164351	0.138894632	-2.960260911	0.003073786	0.346777009
Slc7a6os	0.31010498	0.127496539	2.432261946	0.015004851	0.554936045
Prmt7	0.200428072	0.151349416	1.324273833	0.185412118	0.871716376
Smpd3	-0.148533968	0.44231804	-0.335808071	0.737015604	0.988585656
Zfp90	0.245169331	0.213844142	1.146486077	0.251594085	0.896939594
Cdh3	-0.776738056	0.822955093	-0.943840147	0.345251341	0.92132235
Cdh1	-0.213740157	0.097163023	-2.199809671	0.027820402	0.637954629
Tango6	-0.487908762	0.241197863	-2.022856904	0.043087903	0.685056403
Has3	-0.394874682	0.768518505	-0.513812848	0.607382844	0.974001549
Chtf8	-0.134405499	0.130154729	-1.03265936	0.301763339	0.911518274
Cirh1a	-0.071542098	0.228106506	-0.313634622	0.753798561	0.988585656
Sntb2	-0.150744676	0.253359029	-0.594984422	0.551853884	0.968926429
Vps4a	0.067926914	0.108321605	0.627085552	0.530603157	0.966026275
Pdf	0.116829634	0.172815269	0.676037683	0.49901672	0.960700483
Cog8	-0.151040317	0.190890078	-0.791242369	0.428802572	0.945321484
Nip7	0.1560916	0.149840459	1.041718645	0.297542142	0.909070179
Tmed6	0.384347749	0.70915636	0.541978851	0.587833075	0.973455044
Terf2	-0.178762263	0.226262116	-0.79006714	0.429488559	0.945321484
Cyb5b	-0.056831077	0.093422306	-0.608324491	0.542972281	0.967491297
Nfat5	-0.078071785	0.206403213	-0.378248885	0.705245713	0.988585656
Nqo1	0.377615678	0.611623895	0.617398503	0.536971909	0.96674935
Nob1	0.20161494	0.291288823	0.692147875	0.488844469	0.958560421
Wwp2	0.093079902	0.133080629	0.699424874	0.484286547	0.957710647
Psmd7	-0.065990203	0.095533612	-0.69075377	0.489720292	0.958560421
Lncbate1	-1.024465599	0.963845116	-1.062894423	0.287829833	0.907334416
Zfhx3	0.366190755	0.296723716	1.23411354	0.217160599	0.887633697
Gm1943	-0.177653984	0.345534712	-0.514142219	0.607152562	0.974001549
Dhx38	-0.174577	0.246843696	-0.707237018	0.479419198	0.957463006

Txnl4b	0.178449134	0.202632974	0.880652002	0.378506203	0.929137643
Hp	-0.062922719	0.167479903	-0.375703102	0.707137631	0.988585656
Dhodh	-0.030009012	0.173091379	-0.17337092	0.862359881	0.992886758
Pkd1l3	-1.077740146	0.954116007	-1.129569296	0.258657755	0.897248195
Ist1	-0.198166778	0.10504894	-1.886423391	0.059237923	0.72285319
Zfp821	-0.146578174	0.183996573	-0.796635348	0.425662842	0.944290206
Atxn1l	0.189118266	0.207576252	0.911078526	0.362254	0.926455466
Ap1g1	-0.123457382	0.124515137	-0.991505008	0.321439048	0.91640698
Phlpp2	-0.083963542	0.3052729	-0.275044203	0.783282278	0.990988839
Marveld3	0.021962812	0.173574598	0.126532409	0.899310497	0.99447264
Tat	-0.419432119	0.780168324	-0.537617468	0.590841168	0.974001549
Chst4	-0.383485572	0.344204485	-1.11412137	0.265227122	0.89768067
Zfp612	-0.026683996	0.335251577	-0.079593945	0.936560211	0.994688009
Cmtr2	-0.401900402	0.171226596	-2.347184442	0.018915888	0.590246276
Hydin	0.098143611	0.344134504	0.285189685	0.775498823	0.990861469
Vac14	-0.038135602	0.221926256	-0.171839073	0.863564047	0.992886758
Mtss1l	0.037921249	0.26898606	0.140978493	0.887886933	0.992886758
Il34	-0.06037112	0.183072165	-0.32976679	0.741576183	0.988585656
Sf3b3	-0.294597204	0.19847729	-1.484286706	0.137732876	0.833085238
Cog4	-0.119755145	0.11398966	-1.050579016	0.293451983	0.907334416
Fuk	0.158881491	0.269829783	0.58882118	0.555981236	0.968926429
St3gal2	0.024304079	0.192571636	0.126207988	0.899567288	0.99447264
Ddx19a	0.027770527	0.132531817	0.209538568	0.834027831	0.992886758
Ddx19b	-0.042710575	0.202921243	-0.210478577	0.83329417	0.992886758
Aars	-0.192489192	0.22015947	-0.87431711	0.381945584	0.930635634
Exosc6	-0.018332888	0.194253223	-0.094376236	0.924810292	0.994688009
Pdpr	0.122220434	0.510863848	0.239242677	0.810917412	0.991600404
9430091E24Rik	-0.1888345	0.502846462	-0.375531129	0.7072655	0.988585656
Glg1	-0.140519629	0.278447261	-0.504654375	0.613801611	0.974001549
Rfwd3	0.227570672	0.139673873	1.629300219	0.10324948	0.790843776
Mlkl	0.193099232	0.256318791	0.753355738	0.451236173	0.949134998
Fa2h	4.222154418	3.001040537	1.40689683	0.159457984	0.856630464
Wdr59	-0.07675581	0.229984673	-0.333743154	0.738573383	0.988585656
Znrf1	0.025399062	0.170342836	0.14910555	0.881470347	0.992886758
Ldhd	-0.261799024	0.258766994	-1.011717225	0.311673277	0.914819219
Zfp1	-0.208447406	0.263886771	-0.789912298	0.429578989	0.945321484
Bcar1	-0.076237328	0.200718953	-0.379821271	0.704078092	0.988585656
Cfdp1	0.124238747	0.130142313	0.954637617	0.339760987	0.919246575
Tmem170	-0.122576555	0.219567003	-0.558264921	0.576663495	0.971613657
Chst5	0.412757981	0.507996895	0.812520677	0.416492927	0.943415794
Tmem231	-0.032151726	0.261140674	-0.12312033	0.902011813	0.994688009
Gabarapl2	0.176803637	0.170492103	1.037019508	0.299726765	0.910105802
Adat1	0.245472317	0.349469079	0.702414981	0.482420405	0.957463006
Kars	0.038227223	0.113777951	0.335980941	0.736885239	0.988585656
Terf2ip	0.111184601	0.112380417	0.989359208	0.322487425	0.91640698
Mon1b	-0.01646671	0.245076984	-0.067189947	0.946430488	0.995065399
Nudt7	0.20344463	0.198607478	1.024355332	0.305667472	0.91333012
Vat1l	0.214833898	1.987753539	0.10807874	0.913933232	0.994688009
Wwox	0.084102233	0.37129277	0.226511906	0.8208033	0.992886758
Maf	0.146371091	0.221110198	0.661982543	0.50798241	0.963106419
Dynlrb2	-0.079534215	0.145909967	-0.545091037	0.585690907	0.972904784
Cdyl2	-0.000466841	0.433862249	-0.001076011	0.999141468	0.999886097
Cmc2	0.117519828	0.157222457	0.74747482	0.454776997	0.951209842
Cenpn	1.489536661	0.754211279	1.974959408	0.048272755	0.706226941
Atmin	0.367094258	0.238658124	1.538159488	0.124009624	0.820382953
1700030J22Rik	-0.101219827	0.490406713	-0.206399758	0.83647866	0.992886758
Gcsh	0.067259288	0.173203605	0.388324988	0.697775548	0.987577127
Bco1	-0.685885149	0.763634837	-0.89818473	0.369087073	0.928436166
Gan	0.245739349	0.339398652	0.724043386	0.4690391	0.954387011
Cmip	-0.288511584	0.260616585	-1.107034629	0.268278957	0.89804647
Plcg2	0.179711095	0.359659654	0.499669875	0.617307548	0.974001549
Sdr42e1	-0.399853862	0.297425481	-1.344383339	0.178824459	0.867377413

Hsd17b2	-1.210782833	2.047091258	-0.591465001	0.554208899	0.968926429
Mphosph6	0.163323063	0.134449001	1.214758477	0.224458222	0.892989918
Cdh13	-0.946728841	0.483184305	-1.959353461	0.050071407	0.707153766
Hsbp1	0.391608277	0.147562742	2.653842498	0.007958096	0.457546335
Mlycd	0.160195788	0.137214166	1.167487245	0.243013656	0.896939594
Osgin1	-0.397190553	0.239297469	-1.659819282	0.096950813	0.782676152
Necab2	-0.627943577	1.23403783	-0.508852777	0.610855422	0.974001549
Mbtps1	0.007165786	0.16884061	0.042441131	0.96614704	0.996474415
Hsdl1	0.072781035	0.114326454	0.636607121	0.524380785	0.965637248
Dnaaf1	-0.107012785	0.297577192	-0.359613531	0.719136164	0.988585656
Taf1c	-0.559517948	0.349370443	-1.601503388	0.10926547	0.798400564
Wfdc1	-0.165584199	0.179522065	-0.922361265	0.356340167	0.924354588
Atp2c2	-0.417887567	0.335763319	-1.244589698	0.213282609	0.884945675
Tldc1	-0.275591665	0.233297543	-1.181288329	0.237488202	0.896609492
Cotl1	0.008273837	0.154919407	0.053407366	0.957407337	0.996226826
Klhl36	0.22634886	0.271731751	0.832986426	0.404852385	0.938874266
Usp10	-0.122622922	0.150156536	-0.816633932	0.414137658	0.943415794
Crispld2	-0.052833198	0.175121796	-0.301694014	0.762885332	0.989285682
Zdhhc7	-0.050440611	0.145778707	-0.346008081	0.729336642	0.988585656
6430548M08Rik	0.109391719	0.17888501	0.611519766	0.540855537	0.966989525
Fam92b	-0.312480808	0.160362926	-1.948585097	0.051344993	0.707153766
Gse1	0.238589731	0.275280246	0.866715771	0.386097762	0.932379553
Gins2	0.334027698	0.286479155	1.165975577	0.24362432	0.896939594
Emc8	0.083577416	0.160495693	0.520745538	0.602544047	0.974001549
Cox4i1	0.372017294	0.240987406	1.543720894	0.122655962	0.817788082
Irf8	0.310525928	0.153808767	2.018909162	0.043496662	0.687169821
Fendrr	-0.012758588	0.166905327	-0.076442069	0.939067402	0.994688009
Foxf1	-0.186812794	0.107686766	-1.734779507	0.082779867	0.76099108
Mthfsd	0.233036163	0.194235699	1.199759697	0.230232681	0.895404344
Foxc2	-0.093962411	1.323572927	-0.070991487	0.94340453	0.99486515
Foxl1	0.562203639	0.490302753	1.146645896	0.251528	0.896939594
Fbxo31	-0.157195229	0.17060852	-0.921379714	0.356852216	0.924354588
Map1lc3b	0.096959389	0.132262573	0.733082585	0.463508061	0.953083512
Zcchc14	-0.124333582	0.233135336	-0.533310757	0.593818483	0.974001549
Klhdc4	0.300513255	0.168537322	1.78306651	0.074575477	0.75241101
Slc7a5	-0.430923575	0.551700789	-0.781082034	0.434754248	0.946762848
Banp	-0.213484867	0.235964592	-0.904732633	0.365607048	0.926760795
Zfpm1	-0.156071047	0.314415957	-0.49638399	0.619623518	0.974471036
Zc3h18	0.174901302	0.178833157	0.978013833	0.328067482	0.917115058
Cyba	0.316684083	0.174819315	1.81149367	0.070064468	0.744227652
Mvd	-0.103190264	0.178620367	-0.577707156	0.563461847	0.969658675
9330133O14Rik	-0.121403817	0.320824894	-0.378411462	0.705124954	0.988585656
Snai3	-0.749621386	1.662665308	-0.450855252	0.652093877	0.979004493
Rnf166	0.157996742	0.130761007	1.20828637	0.226937129	0.89398447
Ctu2	0.319114623	0.175577571	1.817513597	0.069138508	0.743426713
Piezo1	0.146072838	0.321871852	0.453822963	0.649956254	0.979004493
Cdt1	-0.48421966	0.404282406	-1.197726274	0.231023597	0.895404344
Aprt	0.040002967	0.145066133	0.27575676	0.782734897	0.990988839
Galns	-0.368676498	0.252155913	-1.462097375	0.143714528	0.841934045
Trappc2l	0.14382536	0.197069324	0.729821145	0.465499518	0.953083512
Pabpn1l	2.215816301	1.673345685	1.324183234	0.185442198	0.871716376
Cbfa2t3	-0.24358148	0.301378354	-0.808224867	0.418961145	0.943415794
Acsf3	-0.072607432	0.218790833	-0.331857744	0.739996679	0.988585656
Cdh15	-0.288943922	0.970590141	-0.297699214	0.765932744	0.989884648
Ankrd11	-0.231317098	0.153987086	-1.502185044	0.133049326	0.827800491
2810013P06Rik	0.415041649	0.18623181	2.228629193	0.025838587	0.631530315
Spg7	0.015573383	0.155322607	0.100264754	0.920134138	0.994688009
Rpl13	0.146898142	0.127212132	1.154749469	0.248193035	0.896939594
Cpne7	0.025868321	1.165919663	0.022187053	0.982298745	0.997390438
Sult5a1	-0.134112843	0.294022119	-0.456131815	0.648295194	0.979004493
Dpep1	0.002540443	0.172561474	0.01472196	0.988254	0.998148029
Chmp1a	0.021781209	0.101125986	0.215386868	0.829465708	0.992886758

Spata33	0.368863931	0.403684964	0.913742061	0.360852397	0.926066817
Cdk10	0.115712396	0.131581535	0.879396915	0.379186104	0.929137643
Spata2l	0.649961843	0.64551422	1.006890045	0.313987616	0.91586731
Vps9d1	0.175474959	0.178228325	0.984551471	0.324844437	0.91640698
Zfp276	0.032295349	0.237117191	0.136199948	0.891663216	0.993043931
Fanca	-0.041148931	0.288215249	-0.142771528	0.886470624	0.992886758
Spire2	-0.211075	0.35317544	-0.597649144	0.550074066	0.968926429
Tcf25	-0.095200999	0.099583178	-0.955994782	0.339074871	0.919246575
Tubb3	-2.025154692	1.1314425	-1.789887416	0.073472012	0.748833631
Def8	0.065294248	0.226050634	0.288847884	0.772697787	0.990678393
Afg3l1	0.003982807	0.148864733	0.026754536	0.978655516	0.997262526
Dbndd1	-0.430625035	0.372788914	-1.155144421	0.24803129	0.896939594
Gas8	0.056554103	0.281560144	0.200859761	0.840808233	0.992886758
Rhou	0.176006822	0.235231496	0.748228128	0.454322568	0.950990329
6030466F02Rik	1.246067293	1.252187313	0.995112536	0.319681534	0.91640698
Rab4a	0.050191416	0.24346953	0.206150707	0.836673191	0.992886758
Ccsap	0.326079197	0.435795075	0.748239748	0.45431556	0.950990329
Acta1	-0.626466077	1.567961421	-0.399541767	0.689494055	0.985586538
Nup133	0.039516511	0.244164577	0.161843751	0.871428899	0.992886758
Abcb10	0.089515593	0.13438206	0.666127553	0.505329575	0.962138588
Taf5l	-0.135582944	0.162086058	-0.836487393	0.402880766	0.937834293
Urb2	-0.528095944	0.3527602	-1.497039473	0.134382987	0.828263887
Galnt2	-0.115639845	0.113613726	-1.017833394	0.308757139	0.9139204
Pgbd5	-1.568809936	1.80460815	-0.869335504	0.384663649	0.931689378
Cog2	0.003682541	0.123159131	0.02990067	0.976146271	0.997262526
Agt	-0.680718005	1.118797406	-0.608437239	0.542897519	0.967491297
Capn9	-0.428135077	2.290717797	-0.186899965	0.851739066	0.992886758
2310022B05Rik	-0.423491752	0.204751122	-2.068324451	0.038609524	0.679118604
Ttc13	0.012540277	0.176848234	0.070909825	0.943469524	0.99486515
Arv1	0.297135615	0.303049463	0.980485538	0.326846505	0.916882624
Fam89a	-0.275010195	0.363688795	-0.756169008	0.449547872	0.948476747
Trim67	3.345134209	2.152410594	1.554133871	0.120152487	0.81558552
2810004N23Rik	0.267923943	0.19971815	1.341510238	0.179754848	0.868087032
Gnpat	-0.150288378	0.118552646	-1.267693159	0.204907549	0.883765092
Exoc8	0.168265382	0.175710354	0.957629293	0.338249723	0.919246575
Sprtn	0.041304916	0.291501135	0.141697272	0.88731913	0.992886758
Egln1	-0.097552663	0.1244235	-0.784037286	0.433018232	0.946184245
Tsnax	-0.006955127	0.08030392	-0.086610058	0.93098147	0.994688009
Disc1	0.510359749	0.42011959	1.21479636	0.224443769	0.892989918
Sipa1l2	0.387872359	0.263783704	1.470418197	0.141448526	0.840741274
4930567H12Rik	0.695102732	0.787774304	0.882362789	0.377580652	0.928883222
Map10	-0.228541004	0.284527696	-0.803229376	0.421842169	0.943415794
Ntpcr	0.121390882	0.147080389	0.825336966	0.409180311	0.940809748
Pcnxl2	0.004320808	0.537292672	0.008041815	0.993583629	0.999270171
BC021891	0.536669368	0.916658221	0.585462886	0.558236515	0.968926429
Kcnk1	-0.312010855	0.235178962	-1.326695436	0.184609451	0.871716376
Slc35f3	0.402876661	0.573674051	0.702274507	0.482507989	0.957463006
Coa6	0.339921732	0.187154491	1.816262762	0.069330075	0.743426713
Gm17296	0.084323189	0.445816226	0.189143383	0.849980441	0.992886758
Irf2bp2	0.108546754	0.186227639	0.582871341	0.559979915	0.968926429
Tomm20	0.354611223	0.28092754	1.262287146	0.20684548	0.883765092
Rbm34	0.134985092	0.122724177	1.099906276	0.27137296	0.89804647
Pard3	0.123108062	0.239029259	0.515033439	0.606529653	0.974001549
Nrp1	-0.160115547	0.1410368	-1.135274962	0.256260129	0.897248195
Itgb1	-0.122321306	0.116685215	-1.048301675	0.294499638	0.907334416
Alkbh8	0.073794467	0.282195674	0.261501055	0.793706136	0.990988839
Cwf19l2	-0.130377705	0.160950846	-0.810046718	0.417913326	0.943415794
Gucy1a2	-0.376222775	0.171973783	-2.187675172	0.028693272	0.647064985
Aasdhppt	0.123227529	0.12897541	0.955434287	0.339358122	0.919246575
Kbtbd3	0.339821091	0.186189795	1.825132742	0.067981	0.742385562
Msantd4	0.09468813	0.115901561	0.81697028	0.413945412	0.943415794
Gria4	-0.078784013	1.354573891	-0.058161473	0.953620009	0.996070133

Casp1	0.169192272	0.189217917	0.894166237	0.371232954	0.928840865
Casp4	0.385990247	0.18227952	2.117573309	0.034211221	0.673783594
Casp12	0.054854941	0.138089234	0.397242707	0.691188492	0.986191149
Pdgfd	-0.046664142	0.145351896	-0.321042541	0.748178153	0.988585656
Dync2h1	-0.033449011	0.198491867	-0.168515777	0.866177533	0.992886758
Dcun1d5	0.238185935	0.184150973	1.293427514	0.195863239	0.879601031
Mmp13	-1.152235018	0.967373482	-1.191096344	0.233615765	0.89581803
Mmp3	-0.141067825	0.204737541	-0.689017873	0.490812021	0.958896244
Mmp8	-0.022252132	0.459375526	-0.04843996	0.961365613	0.996474415
Mmp27	-0.764147482	0.478606501	-1.596609072	0.110352875	0.800111791
Tmem123	-0.056801101	0.078680412	-0.72192175	0.470342588	0.95486913
Birc2	-0.023529723	0.124279661	-0.189328828	0.849835103	0.992886758
Birc3	-0.261058873	0.143789031	-1.815568761	0.069436549	0.743426713
Yap1	-0.241412874	0.123809879	-1.949867621	0.051191899	0.707153766
9230110C19Rik	-0.089677656	0.184676854	-0.485592285	0.627256267	0.976752533
Cep126	-0.369600391	0.189620803	-1.949155296	0.051276882	0.707153766
Trpc6	0.452601276	0.201572687	2.245350213	0.024745659	0.627796923
Pgr	0.111731659	0.489814013	0.228110377	0.819560433	0.992886758
Arhgap42	-0.018811706	0.170438988	-0.110372082	0.912114293	0.994688009
Maml2	-0.025352072	0.221834612	-0.114283662	0.909012934	0.994688009
Mtmr2	0.030637131	0.099237521	0.308725278	0.757530513	0.989266039
Cep57	0.03164657	0.137170317	0.230710044	0.81754007	0.992678823
Fam76b	0.160354738	0.137373682	1.167288635	0.243093827	0.896939594
Sesn3	0.061193233	0.276186293	0.221565061	0.824652483	0.992886758
Endod1	-0.242517875	0.14877538	-1.630094139	0.103081602	0.79028905
Cwc15	0.081070649	0.105396633	0.769195816	0.441777074	0.948476447
Amotl1	-0.170857693	0.209527213	-0.815443925	0.41481825	0.943415794
Piwil4	-0.385213521	0.280409188	-1.373754988	0.169517759	0.861597949
Fut4	-0.039200572	0.287665715	-0.136271267	0.891606837	0.993043931
1700012B09Rik	0.406222872	0.248325169	1.635850578	0.101870864	0.788783372
Ankrd49	0.117477014	0.182736236	0.642877496	0.520303593	0.964682803
Mre11a	-0.179091013	0.312968537	-0.572233281	0.567163943	0.970862413
Izumo1r	1.390608689	0.621317179	2.238162305	0.025210473	0.629375214
Panx1	0.037062005	0.246547881	0.150323761	0.880509188	0.992886758
Vstm5	0.48995959	0.432181437	1.133689576	0.256924784	0.897248195
Med17	-0.162121609	0.121637206	-1.332829113	0.182587889	0.869129
4931406C07Rik	-0.059246136	0.116799675	-0.507245727	0.611982414	0.974001549
Taf1d	0.269539275	0.181282113	1.486849812	0.137054482	0.833085238
Cep295	-0.199503475	0.310786906	-0.641930117	0.520918558	0.964784335
Smco4	0.243193672	0.15562893	1.562650798	0.118134753	0.814755048
Ccdc67	-0.187825044	0.320262193	-0.586472734	0.557557879	0.968926429
Slc36a4	-0.517416932	0.274000207	-1.888381534	0.058974749	0.72285319
Fat3	-0.29674336	0.417516128	-0.710735083	0.477248415	0.95716557
Chordc1	-0.269944697	0.194475017	-1.388068769	0.165116106	0.860011234
Naalad2	0.29364179	0.420861723	0.697715602	0.485355069	0.957710647
Zfp558	0.575946094	0.474824476	1.212966312	0.225142701	0.893255444
Zfp317	0.1365852	0.155645484	0.877540394	0.380193188	0.929640874
Zfp560	0.252943023	0.206805123	1.223098435	0.22129252	0.891315805
Zfp26	-0.249513684	0.187823942	-1.328444504	0.184031305	0.870921485
Zfp426	-0.006478068	0.153450622	-0.042215979	0.966326524	0.996474415
Zfp266	0.036856384	0.1253651	0.293992379	0.768763727	0.989990452
Zfp846	0.033956812	0.294146046	0.115442014	0.908094779	0.994688009
Fbxl12os	0.177263402	0.265884246	0.666693889	0.504967683	0.962138588
Fbxl12	0.07744708	0.167090494	0.463503807	0.643003287	0.978681333
Ubl5	0.277836059	0.155718078	1.784224815	0.074387142	0.751093915
Pin1	0.2707429	0.141169416	1.917858046	0.055129013	0.712244081
Olfm2	-0.396209524	0.413851193	-0.957371952	0.33837955	0.919246575
Col5a3	0.590974022	0.750814209	0.787110865	0.431216969	0.945321484
A230050P20Rik	0.154051342	0.22817308	0.675151259	0.49957967	0.960847835
Angptl6	0.649139084	0.315706084	2.056150063	0.039768054	0.679118604
Ppan	0.00228102	0.169869445	0.013428078	0.989286266	0.998182772
Eif3g	0.100093001	0.110121684	0.908930894	0.363386605	0.926455466

Dnmt1	-0.071910992	0.22574001	-0.318556697	0.750062695	0.988585656
S1pr2	-0.096440718	0.148665976	-0.648707395	0.516527523	0.964352198
Mrpl4	-0.026272094	0.124940234	-0.210277291	0.833451258	0.992886758
Icam1	-0.041592189	0.149074082	-0.27900349	0.780242146	0.990988839
Icam4	-0.610659936	0.511013384	-1.194997929	0.232087839	0.895404344
Icam5	-0.305883358	0.909420532	-0.336349738	0.736607147	0.988585656
Zglp1	-0.450493588	1.259359809	-0.357716345	0.7205556	0.988585656
Raver1	-0.342879883	0.388586166	-0.882377999	0.377572429	0.928883222
Tyk2	0.323770656	0.145743881	2.221504283	0.026316825	0.632301593
Cdc37	0.184703311	0.089416145	2.06565951	0.038860641	0.679118604
Pde4a	-0.21647742	0.369843679	-0.585321398	0.558331629	0.968926429
Keap1	0.0535724	0.143435441	0.373494859	0.708780171	0.988585656
S1pr5	1.108064836	0.52961309	2.092215727	0.036419222	0.677009445
Atg4d	0.138786005	0.12193618	1.138185607	0.255042987	0.897044747
Kri1	-0.370614626	0.339618601	-1.091267159	0.275155344	0.900302631
Cdkn2d	0.471749903	0.236732661	1.99275377	0.046288416	0.698098452
Ap1m2	-0.188636905	0.15875859	-1.188199675	0.234754752	0.89581803
Slc44a2	-0.10272648	0.173641545	-0.591600819	0.554117925	0.968926429
Ilf3	0.290747233	0.168015707	1.730476503	0.083545176	0.76099108
Gm16853	1.95052624	1.630935423	1.195955531	0.231713913	0.895404344
Qtrt1	-0.045293139	0.214959835	-0.210705125	0.833117375	0.992886758
Dnm2	-0.25133753	0.208629875	-1.204705368	0.228317063	0.894584278
Tmed1	0.295478726	0.163933101	1.802434796	0.071477025	0.744227652
AB124611	0.289424912	0.251066916	1.152779971	0.249000707	0.896939594
Carm1	-0.377585928	0.389202122	-0.970153827	0.331969822	0.917115058
Yipf2	-0.138151375	0.171732347	-0.804457502	0.421132803	0.943415794
1810026J23Rik	-0.044936362	0.123335811	-0.364341562	0.715602968	0.988585656
Smarca4	-0.004768586	0.167523024	-0.028465256	0.977291079	0.997262526
Ldlr	-0.266775787	0.265517247	-1.004739955	0.315022084	0.91586731
Spc24	0.263555504	0.264499376	0.996431477	0.319040546	0.91640698
Kank2	0.113622774	0.177433157	0.640369456	0.521932436	0.965291218
Dock6	-0.005512882	0.165077387	-0.033395743	0.973359004	0.997115853
Gm6484	0.057954369	0.353811663	0.163800052	0.869888551	0.992886758
Rab3d	-0.069869377	0.153864147	-0.454097839	0.649758409	0.979004493
Tmem205	0.08083754	0.146041787	0.553523356	0.579905092	0.971613657
Ccdc159	0.020981454	0.314278169	0.066760773	0.946772152	0.995195989
Lppr2	0.197761526	0.489647465	0.40388553	0.686296897	0.98538669
LOC100049077	1.930466468	1.666234453	1.158580334	0.246627289	0.896939594
Swsap1	-0.08350114	0.266079617	-0.313820128	0.753657656	0.988585656
Epor	0.685651374	0.396895622	1.727535743	0.084071492	0.76099108
Rgl3	-0.134794484	0.303269235	-0.444471343	0.656701843	0.979614766
Ccdc151	-0.221705416	0.316818657	-0.699786489	0.484060654	0.957710647
Prkcsh	-0.082629707	0.162519463	-0.50842961	0.611152092	0.974001549
Zfp653	0.032948521	0.295837427	0.111373742	0.911319982	0.994688009
Gm16845	0.103131847	0.839077129	0.122911046	0.902177539	0.994688009
Ecsit	0.085587074	0.196187219	0.436252038	0.662653876	0.980760274
Cnn1	0.277845142	0.208772694	1.330850008	0.183238369	0.869576666
Elof1	0.125196312	0.235301255	0.532068188	0.59467877	0.974001549
Acp5	-0.344265839	0.204154374	-1.686301554	0.091737732	0.772516176
Pigyl	0.245507526	0.190752108	1.287050128	0.198076817	0.880239572
Zfp872	0.743123562	0.333774535	2.226423782	0.025985809	0.631530315
1810064F22Rik	0.060940353	0.232007983	0.262664898	0.792808874	0.990988839
Zfp809	-0.324487631	0.23931205	-1.355918481	0.175125107	0.86434934
Zfp599	-0.402488101	0.695220731	-0.578935701	0.56263256	0.969658675
Zfp810	0.095460852	0.171576367	0.556375299	0.577954322	0.971613657
Anln	-0.167808734	0.237118092	-0.707701095	0.479130897	0.957463006
9530077C05Rik	0.180474144	0.353162168	0.511023435	0.609334647	0.974001549
Rp9	0.005692374	0.1128705	0.050432791	0.959777506	0.996474415
Bbs9	-0.240585363	0.17759955	-1.354650745	0.175528858	0.864596675
Bmper	0.130868131	0.243752475	0.536889443	0.591343984	0.974001549
Dpy19l1	-0.161818203	0.181044971	-0.893801151	0.371428293	0.928840865
Dpy19l2	-0.633272592	0.69735172	-0.908110747	0.363819713	0.926455466

Tbx20	-1.170734223	0.924870308	-1.265836099	0.205571768	0.883765092
Herpud2	-0.163388401	0.11134655	-1.467386292	0.142271001	0.840741274
Sep-07	-0.03882207	0.106067001	-0.366014589	0.714354189	0.988585656
Eepd1	0.156868823	0.326007603	0.481181488	0.630387517	0.977034429
Glb1l2	-0.191262869	0.110832974	-1.725685616	0.084403987	0.76099108
Glb1l3	-1.391080473	0.485666886	-2.864268728	0.004179733	0.38338479
Acad8	-0.005713743	0.124918549	-0.045739748	0.963517683	0.996474415
Thyn1	0.067703218	0.328801326	0.2059092	0.836861839	0.992886758
Vps26b	0.051803152	0.158146256	0.327564831	0.743240717	0.988585656
Ncapd3	-0.310736155	0.264573464	-1.17447967	0.24020295	0.896939594
Jam3	-0.011555587	0.178848796	-0.06461093	0.948483782	0.99560893
AI414108	-0.300379152	1.111359761	-0.270280752	0.786944272	0.990988839
Opcml	0.249777359	1.158641297	0.215577815	0.82931685	0.992886758
Ntm	0.002575585	2.226815192	0.001156623	0.999077149	0.999886097
Snx19	-0.187327919	0.123741669	-1.513862883	0.13006062	0.827800491
Adamts15	-0.141786557	0.39195535	-0.361741604	0.717545134	0.988585656
Adamts8	0.162109781	0.275381935	0.588672533	0.556080967	0.968926429
Zbtb44	0.242966381	0.17975745	1.351634554	0.176492251	0.865862073
St14	-0.130166376	0.201932308	-0.644604014	0.519183833	0.96446859
Aplp2	-0.22055628	0.176919968	-1.246644358	0.212527929	0.884945675
Prdm10	0.396952705	0.545108044	0.728209223	0.466485521	0.953083512
Nfrkb	0.144106997	0.207729042	0.693725805	0.48785418	0.958288569
Tmem45b	-0.795646809	0.698792887	-1.138601756	0.254869296	0.897044747
Barx2	-0.462735873	0.21780627	-2.124529619	0.033625882	0.671818913
Arhgap32	-0.24252284	0.282975504	-0.857045353	0.39141982	0.933734231
Kcnj5	-0.357534084	0.443229648	-0.806656517	0.419864403	0.943415794
Fli1	0.146780615	0.146285486	1.003384672	0.315675297	0.915946432
Ets1	0.088622795	0.208500668	0.425048016	0.670801673	0.982434419
Gm27162	1.721983405	1.870447951	0.920626209	0.357245614	0.924354588
Kirrel3	0.144064469	1.280347389	0.112519829	0.910411248	0.994688009
St3gal4	0.038373769	0.104399379	0.367567025	0.713196106	0.988585656
4930581F22Rik	0.667870165	0.330032838	2.023647615	0.043006422	0.685039858
Dcps	0.05454054	0.189513322	0.287792642	0.77350547	0.990730604
Tirap	-0.207134782	0.211139943	-0.981030772	0.326577567	0.916882624
Foxred1	0.141452993	0.151989998	0.930673042	0.352022719	0.923082865
Srpr	-0.14588504	0.088378563	-1.650683553	0.098803208	0.786907305
Fam118b	-0.00448148	0.149792452	-0.029917929	0.976132507	0.997262526
Rpusd4	0.08976874	0.162044799	0.553974828	0.579596073	0.971613657
Cdon	0.012976819	0.325326374	0.039888615	0.968181928	0.996474415
Ddx25	-0.011984445	1.184394067	-0.010118629	0.99192664	0.998783765
Pus3	0.148476816	0.192280643	0.772188058	0.440003053	0.947849924
Hyls1	0.145268504	0.254287003	0.571277737	0.567811391	0.971054423
Chek1	0.096676449	0.534353657	0.180922218	0.856428629	0.992886758
Stt3a	-0.193669368	0.133868337	-1.446715268	0.147976686	0.847427345
Ei24	0.016580009	0.087281566	0.18996003	0.849340452	0.992886758
Fez1	0.125186703	0.281392379	0.444883062	0.656404263	0.979614766
Pknox2	-0.0795937	0.364456525	-0.218390108	0.827125172	0.992886758
Tmem218	0.366088463	0.180832225	2.024464736	0.042922357	0.685039858
Slc37a2	-0.59252801	0.476331077	-1.243941532	0.213521082	0.884945675
Ccdc15	0.392291261	0.562037467	0.697980623	0.485189312	0.957710647
Hepacam	-0.122447884	0.479144465	-0.255555251	0.798294275	0.990988839
Robo4	-0.202211049	0.196163575	-1.030828732	0.302621142	0.911518274
Robo3	-1.357184265	2.087316979	-0.650205157	0.51555971	0.964352198
Msantd2	0.184592542	0.196439772	0.939690267	0.347376459	0.921552698
Esam	-0.257439372	0.105836804	-2.432418223	0.014998378	0.554936045
Vsig2	-0.373715584	0.199306827	-1.875076685	0.060782175	0.725221283
Nrgn	0.229406355	0.408845788	0.561107296	0.574724395	0.971613657
Spa17	0.026886074	0.160142016	0.167888942	0.86667065	0.992886758
Siae	-0.005689452	0.202306708	-0.028122904	0.977564126	0.997262526
Tbrg1	-0.112046186	0.109485271	-1.0233905	0.30612325	0.91333012
Vwa5a	-0.215819794	0.099181796	-2.176002075	0.029555105	0.64858361
Olfr920	-0.553957339	1.337126661	-0.414289353	0.67866221	0.983853811

AW551984	0.485043655	0.691535813	0.70140063	0.483053029	0.957463006
Zfp202	0.000136791	0.307161157	0.000445341	0.999644669	0.999950896
Scn3b	-0.095082301	0.171618857	-0.554031781	0.579557096	0.971613657
Gramd1b	-0.258521047	0.444379236	-0.58175771	0.560729894	0.968926429
Clmp	0.125769179	0.34241505	0.367300383	0.713394968	0.988585656
Hspa8	-0.255108647	0.175852481	-1.45069689	0.146864282	0.844560298
4931429I11Rik	-0.088747129	0.547718296	-0.162030609	0.87128175	0.992886758
Crtam	-0.406060619	1.105618664	-0.367270047	0.713417594	0.988585656
Ubash3b	0.2555841	0.292007007	0.875267009	0.38142864	0.930635634
3110039I08Rik	-0.399138316	0.428142532	-0.9322557	0.351204396	0.92247654
Sorl1	0.412830102	0.192310639	2.146683634	0.031818477	0.661395937
4930546K05Rik	1.166124322	1.534824057	0.759777199	0.447387775	0.948476747
Sc5d	-0.130524684	0.130950146	-0.996750956	0.318885409	0.91640698
Tbcel	-0.122913992	0.209464995	-0.586799679	0.557338252	0.968926429
Grik4	2.021406114	1.418833229	1.424696062	0.154245078	0.854159937
Arhgef12	-0.187212023	0.187008717	-1.001087145	0.316784679	0.91640698
Tmem136	0.153431396	0.293820431	0.522194444	0.601534954	0.974001549
Pou2f3	-1.398201107	1.984930213	-0.704408194	0.481178604	0.957463006
Oaf	0.008262085	0.200361487	0.041235896	0.967107837	0.996474415
Trim29	-0.32335395	0.462920621	-0.698508416	0.484859296	0.957710647
Pvrl1	0.092811183	0.313421044	0.296123011	0.767136141	0.989884648
Thy1	0.061314767	0.194545498	0.315169295	0.752633117	0.988585656
Usp2	-0.382638756	0.358565201	-1.067138569	0.285909267	0.905916508
Rnf26	-0.193911582	0.382929445	-0.506389845	0.612583002	0.974001549
Mcam	-0.333407562	0.138341806	-2.410027532	0.015951317	0.557717626
Cbl	0.36870291	0.594677939	0.620004352	0.535254922	0.96674935
Ccdc153	0.229900051	0.173320139	1.326447416	0.184691542	0.871716376
Pdzd3	0.66245651	1.619622967	0.409018965	0.682525743	0.984738223
Nlrx1	-0.379758457	0.295788132	-1.283886728	0.199181592	0.880831248
Hinfp	-0.305788411	0.263606691	-1.160017637	0.246041626	0.896939594
C2cd2l	-0.294493911	0.192399441	-1.530638081	0.125858865	0.822401003
Dpagt1	-0.268409089	0.13464682	-1.993430592	0.046214317	0.698098452
H2afx	-0.123473992	0.199361198	-0.619348163	0.535687022	0.96674935
Hmbs	0.119540319	0.138104561	0.865578362	0.386721426	0.932626359
Vps11	-0.150200166	0.235141174	-0.638765909	0.522975229	0.965637248
Hyou1	-0.298794236	0.164617762	-1.815078951	0.069511777	0.743426713
Slc37a4	0.05930958	0.190310698	0.311646062	0.755309524	0.989245836
Trappc4	-0.00474891	0.127512572	-0.037242682	0.970291507	0.996628386
Rps25	0.301088448	0.263212566	1.143898455	0.252665752	0.897044747
Ccdc84	0.101642988	0.265155052	0.383334154	0.701472018	0.988482081
Upk2	0.699057292	0.641121379	1.090366529	0.27555172	0.900302631
Bcl9l	0.02634864	0.328174912	0.080288403	0.936007881	0.994688009
Cxcr5	0.101827166	0.539015338	0.188913299	0.85016077	0.992886758
Ddx6	0.1026578	0.107963768	0.950854176	0.341678406	0.919850248
Phldb1	-0.096071191	0.172327371	-0.557492348	0.577191086	0.971613657
Arcn1	-0.165679257	0.099471957	-1.665587584	0.095795586	0.7796882
Ift46	0.078651205	0.13281688	0.59217778	0.553731547	0.968926429
Tmem25	0.375318111	0.697026061	0.538456354	0.59026203	0.974001549
Ttc36	0.404665003	0.217410736	1.861292642	0.062702862	0.727650961
Kmt2a	-0.323570343	0.280820437	-1.152232175	0.24922568	0.896939594
Atp5l	0.238522269	0.252599771	0.944269539	0.345031928	0.92132235
Ube4a	-0.138906071	0.162736184	-0.853565985	0.393345512	0.933734231
Cd3g	0.351741443	0.289368536	1.215548337	0.224157025	0.892989918
Cd3d	0.660589652	0.23203188	2.846977983	0.004413641	0.385355746
Cd3e	-0.244796027	0.482922817	-0.506905076	0.612221424	0.974001549
Mpzl2	-0.237135949	0.246848716	-0.960652958	0.336726692	0.919242242
Mpzl3	0.325541419	0.433263802	0.751369991	0.452430018	0.94958784
Amica1	-0.017487243	0.304409987	-0.05744635	0.954189642	0.996070133
Scn2b	1.348245162	1.397363483	0.964849288	0.334620305	0.918030088
Scn4b	-1.1724997	0.9559367	-1.226545336	0.219993526	0.888847883
Tmprss4	0.010957499	0.150554623	0.072780888	0.94198048	0.994688009
BC049352	2.320348886	1.484918723	1.562610027	0.118144348	0.814755048

Il10ra	0.121549006	0.195487155	0.621774899	0.534089894	0.96674935
Tmprss13	0.263267494	0.338294294	0.778220321	0.436439139	0.946762848
Fxyd6	0.07948122	0.167481936	0.474565925	0.635096398	0.977034429
Fxyd2	0.2256808	0.505470991	0.446476264	0.655253254	0.979133069
Cep164	0.08962292	0.276500919	0.32413245	0.745837761	0.988585656
Bace1	0.08518661	0.24833876	0.343025833	0.731579026	0.988585656
Rnf214	0.003669107	0.213679023	0.017171116	0.986300105	0.998148029
Pcsk7	-0.371760743	0.210285808	-1.767883187	0.077080434	0.752876039
Tagln	0.329851454	0.136504181	2.416420151	0.015673964	0.557315333
Sidt2	-0.264380415	0.184393001	-1.433787691	0.151632859	0.85193038
Pafah1b2	-0.038553752	0.090667323	-0.425222122	0.670674759	0.982434419
Sik3	0.112116722	0.229506465	0.488512259	0.625187045	0.976218048
Apoa1	3.418351428	2.844814324	1.201607922	0.229515471	0.895404344
Apoc3	3.012936969	2.696258469	1.117451092	0.2638015	0.897248195
Zpr1	0.083333986	0.159726031	0.521730778	0.601857792	0.974001549
Bud13	-0.073679674	0.244048014	-0.301906466	0.762723366	0.989285682
Cadm1	-0.024714778	0.123924352	-0.199434397	0.841922956	0.992886758
Nxpe2	0.529242711	0.342241907	1.546399493	0.122008117	0.816233249
Nxpe4	0.311639708	0.312810948	0.996255758	0.319125894	0.91640698
Rexo2	0.288560406	0.141525812	2.038924225	0.041457591	0.679118604
Rbm7	0.222871551	0.18663648	1.194147849	0.232420139	0.895404344
Gm5617	0.410042782	0.311769967	1.31520937	0.188439547	0.874826736
Nnmt	0.245781191	0.230411569	1.066705078	0.286105034	0.905916508
Zbtb16	-0.487101161	0.988927146	-0.492555153	0.622326943	0.97514112
Usp28	-0.183880745	0.203685071	-0.902769872	0.366648044	0.927866786
Zw10	0.104194006	0.18226534	0.571661105	0.567551589	0.970955746
Tmprss5	0.645727454	0.635580363	1.01596508	0.309646016	0.9139204
Ttc12	-0.375180628	0.200113847	-1.874835918	0.0608153	0.725221283
Ncam1	0.187349439	0.968202438	0.193502342	0.846565572	0.992886758
Plet1os	-0.243130872	1.360162854	-0.178751295	0.85813299	0.992886758
Plet1	-0.402696123	0.170623465	-2.360145028	0.018267792	0.582676994
Pts	0.105857576	0.246807385	0.428907651	0.667990432	0.981863224
Bco2	-0.308759627	0.480928882	-0.642006831	0.520868747	0.964784335
Il18	0.209981381	0.211985965	0.990543788	0.321908397	0.91640698
Sdhd	-0.071434397	0.141944022	-0.503257521	0.614783228	0.974001549
Timm8b	0.424675763	0.307299649	1.381959806	0.166984043	0.860403806
AU019823	0.18099808	0.158535308	1.141689394	0.253583151	0.897044747
Pih1d2	-0.396408535	0.182102489	-2.176843035	0.029492281	0.64858361
Dlat	0.151847749	0.142470582	1.065818268	0.286505801	0.906143389
Dixdc1	-0.336437702	0.197497194	-1.703506241	0.088473371	0.767814344
2310030G06Rik	-0.140824476	0.1494041	-0.942574369	0.345898655	0.921552698
Hspb2	-0.040903589	0.520901059	-0.078524679	0.9374107	0.994688009
Cryab	-0.059888291	0.100021898	-0.598751792	0.549338414	0.968926429
1110032A03Rik	0.080312439	0.11714937	0.685555874	0.492993215	0.958916246
Fdxacb1	0.263326076	0.208223906	1.264629413	0.206004204	0.883765092
Alg9	0.027607009	0.15725353	0.175557323	0.860641733	0.992886758
Ppp2r1b	-0.195436566	0.201450779	-0.970145497	0.331973974	0.917115058
Sik2	-0.816268088	0.499188394	-1.635190438	0.102009132	0.788783372
Layn	0.150070242	0.173635887	0.864281256	0.387433404	0.932858077
4833427G06Rik	-0.345840965	0.152516093	-2.267570322	0.023355408	0.622011301
Btg4	0.658548889	0.331496637	1.986592968	0.046967521	0.698868643
Pou2af1	0.171848815	0.395298219	0.434732074	0.663756914	0.981154469
Gm684	-0.365661166	0.268941359	-1.359631586	0.173946538	0.863528862
Arhgap20	0.044040984	0.470012189	0.093701791	0.925346047	0.994688009
Fdx1	-0.079272561	0.174271537	-0.454879565	0.649195885	0.979004493
Gm6981	0.349987983	0.756509263	0.462635423	0.643625713	0.97889195
Rdx	-0.100509496	0.101610383	-0.989165609	0.322582121	0.91640698
Zc3h12c	-0.214339772	0.181726991	-1.179460298	0.238214937	0.896609492
Ddx10	0.086766496	0.243484299	0.356353558	0.721575805	0.988585656
Exph5	0.296199311	0.196453279	1.507734117	0.13162259	0.827800491
Kdelc2	-0.04696867	0.10335944	-0.454420707	0.649526051	0.979004493
4930550C14Rik	0.272143962	0.257488902	1.056915309	0.290550241	0.907334416

Atm	0.078902726	0.319318944	0.247096915	0.804833222	0.990988839
Npat	0.143348517	0.21436767	0.668703991	0.503684321	0.96179897
Acat1	0.127097765	0.108152951	1.17516687	0.239927962	0.896939594
Cul5	-0.123870513	0.117575591	-1.053539358	0.292093866	0.907334416
Rab39	-0.364552757	0.34724858	-1.049832246	0.293795247	0.907334416
Slc35f2	-0.5051211	1.070815771	-0.471716157	0.637129403	0.977284137
Sln	-0.066963512	0.324911483	-0.206097707	0.83671459	0.992886758
Elmod1	-0.458219677	0.177046967	-2.588124979	0.009649996	0.483161941
Tnfaip8l3	0.020740621	0.280781617	0.073867445	0.94111586	0.994688009
Dmxl2	-0.11952716	0.187055916	-0.638991608	0.522828391	0.965637248
Sh2d7	0.121368434	0.336389845	0.360796963	0.718251234	0.988585656
Cib2	0.435996426	0.20018422	2.177975999	0.029407824	0.64858361
Idh3a	0.118608982	0.109233583	1.085828904	0.277554696	0.901241208
Acsbg1	-1.07578658	0.834630765	-1.288937126	0.197419946	0.87989762
Dnaja4	-0.176941916	0.137580782	-1.286094708	0.198410011	0.880661448
Wdr61	0.148483701	0.13160087	1.12828814	0.259198251	0.897248195
Crabp1	0.864640438	0.733778392	1.178340012	0.238661083	0.896609492
Ireb2	-0.099426738	0.128682318	-0.772652683	0.439727957	0.947849924
Hykk	-0.392811993	0.386352726	-1.016718575	0.309287327	0.9139204
Psma4	0.117815969	0.13390232	0.879865037	0.378932427	0.929137643
Ube2q2	0.068871927	0.119898908	0.574416631	0.565685901	0.970061308
Fbxo22	-0.073256024	0.112484886	-0.65125215	0.514883731	0.964352198
Nrg4	-0.411203718	0.637610515	-0.64491364	0.518983152	0.96446859
AI118078	-0.035076811	0.889543852	-0.039432357	0.968545683	0.996474415
Etfa	0.192319262	0.111070887	1.731500185	0.083362592	0.76099108
Scaper	-0.217951914	0.221160372	-0.98549262	0.324382155	0.91640698
Rcn2	-0.06315657	0.107317618	-0.588501415	0.556195784	0.968926429
Pstpip1	0.636173292	0.200878808	3.166950745	0.001540464	0.28259727
Tspan3	-0.105943217	0.107335082	-0.987032526	0.323626694	0.91640698
Peak1	0.061802013	0.274411288	0.22521673	0.821810675	0.992886758
Hmg20a	-0.200618488	0.225573498	-0.889370824	0.37380382	0.928840865
Lingo1	0.088966204	0.819942761	0.108502945	0.913596745	0.994688009
Cspg4	0.17800244	0.372581777	0.477754016	0.632825294	0.977034429
Snx33	-0.314526308	0.159038238	-1.97767727	0.047965126	0.703094165
Imp3	-0.00607815	0.110364994	-0.055073169	0.956080172	0.99614946
Snupn	0.097156446	0.178303286	0.544894308	0.585826211	0.972904784
Ptpn9	-0.287036636	0.224629804	-1.277820802	0.201312618	0.881187967
Gm10658	-0.25892635	1.329695967	-0.19472598	0.845607472	0.992886758
Sin3a	0.169019485	0.140066435	1.206709409	0.227544074	0.894410876
Man2c1os	1.624689025	0.779021256	2.085551598	0.03701926	0.678243111
Man2c1	-0.154263032	0.191189377	-0.80685985	0.419747233	0.943415794
Neil1	0.394464789	0.293974742	1.34183225	0.179650393	0.868087032
Commd4	0.178390179	0.144583936	1.233817423	0.217270945	0.887633697
1700017B05Rik	-0.125151278	0.297819995	-0.420224566	0.67432141	0.983375962
Ppcdc	0.189071806	0.122080534	1.548746556	0.121442658	0.816233249
Scamp5	-0.048432126	0.29271747	-0.165456902	0.868584369	0.992886758
Rpp25	-0.34490565	0.518740411	-0.664890652	0.506120435	0.962318428
Cox5a	-0.094010352	0.12139049	-0.774445773	0.438667224	0.947831419
Fam219b	0.202220177	0.151660242	1.333376333	0.182408335	0.86878089
Mpi	0.14191373	0.198935252	0.713366424	0.475619044	0.957156188
Scamp2	-0.359476409	0.240524393	-1.494552815	0.135031188	0.829809681
Ulk3	-0.770245161	0.403688286	-1.908019599	0.056388683	0.717582253
Csk	0.122940329	0.111054563	1.107026359	0.268282532	0.89804647
Cyp1a1	0.095044522	1.121620287	0.08473859	0.932469216	0.994688009
Edc3	-0.114605452	0.16916162	-0.677490859	0.498094568	0.960700483
Clk3	0.049420446	0.115506652	0.42785801	0.668754499	0.981863224
Arid3b	0.053709913	0.302920245	0.177307111	0.859267166	0.992886758
Ubl7	-0.120903928	0.196159759	-0.616354384	0.537660653	0.96674935
Sema7a	0.050645638	0.311559695	0.162555165	0.87086869	0.992886758
Cyp11a1	1.117625617	1.123101575	0.995124254	0.319675836	0.91640698
Ccdc33	-0.49312019	0.190602338	-2.587167585	0.009676852	0.483161941
Stra6	-0.251117283	2.085650585	-0.120402374	0.904164411	0.994688009

Islr	-0.084352593	0.19420902	-0.434339215	0.664042131	0.981154469
Pml	0.093900102	0.379322281	0.247547024	0.804484904	0.990988839
Stoml1	0.288364765	0.166708736	1.72975197	0.083674599	0.76099108
Loxl1	0.019054577	0.209291881	0.091043079	0.927458361	0.994688009
6030419C18Rik	-0.170357527	0.301759418	-0.564547506	0.572381579	0.971613657
Cd276	-0.116858758	0.526114959	-0.222116394	0.824223276	0.992886758
Nptn	-0.207213148	0.082292797	-2.517998615	0.011802379	0.520803876
Rec114	0.458623094	0.35928307	1.276495145	0.201780542	0.881220006
Neo1	0.148369738	0.192625531	0.770249597	0.441151848	0.948068187
Adpgk	-0.181426073	0.137199575	-1.322351567	0.186051109	0.872305715
Bbs4	-0.067838673	0.150234254	-0.451552631	0.651591301	0.979004493
Arih1	-0.234838896	0.116032647	-2.023903642	0.042980067	0.685039858
Tmem202	0.072164689	0.42863881	0.168357804	0.866301801	0.992886758
Hexa	-0.125914897	0.11787372	-1.068218573	0.285421929	0.905916508
Parp6	-0.06683778	0.138131687	-0.483870001	0.628478132	0.977034429
Pkm	0.079939957	0.123371836	0.647959536	0.517011122	0.964352198
Gramd2	-0.166468246	0.133838969	-1.243795042	0.213575006	0.884945675
Senp8	-0.162765691	0.231747136	-0.702341757	0.482466058	0.957463006
Myo9a	-0.21198211	0.203041237	-1.044034767	0.296469304	0.909005508
Thsd4	-0.377253036	0.289340235	-1.303838839	0.192288537	0.879588734
9230112J17Rik	0.478413787	0.379228369	1.261545353	0.207112431	0.883765092
Lrrc49	0.047738625	0.249439494	0.191383586	0.848225079	0.992886758
Larp6	0.078851339	0.505968395	0.155842419	0.876157233	0.992886758
Uaca	-0.01219699	0.310713822	-0.039254738	0.968687292	0.996474415
Tle3	-0.016544294	0.240347963	-0.068834759	0.94512115	0.99486515
Rplp1	0.274014815	0.128805053	2.12736075	0.033390121	0.670819369
Kif23	-0.201787579	0.436280791	-0.462517681	0.643710126	0.97889195
Paqr5	-0.112209547	0.38189323	-0.293824396	0.768892092	0.989990452
Glce	-0.284454891	0.18844505	-1.509484546	0.131175002	0.827800491
Mir5133	-0.788181148	1.311432672	-0.60100771	0.547834851	0.968648431
Anp32a	0.237259539	0.11602301	2.044935221	0.040861245	0.679118604
Coro2b	-0.194530457	0.271660477	-0.716079348	0.473942355	0.955974667
Itga11	-0.704445912	0.504357196	-1.396720255	0.162497711	0.859982992
Fem1b	-0.11129612	0.146315841	-0.760656668	0.446862163	0.948476747
Cln6	0.145241667	0.236988328	0.612864218	0.539966125	0.966900344
Calml4	0.254715909	0.197772507	1.28792375	0.197772507	0.880239572
Pias1	-0.162543584	0.226175383	-0.718661692	0.472349392	0.955003345
Map2k5	0.123550335	0.205478758	0.601280327	0.54765329	0.96847584
2300009A05Rik	-0.053612043	0.224910921	-0.238370119	0.811594041	0.991816977
Iqch	-0.862379643	0.630521395	-1.367724632	0.171398287	0.86244803
Aagab	-0.167535021	0.113977803	-1.469891653	0.1415911	0.840741274
Smad3	-0.454102984	0.268396233	-1.691912657	0.090662628	0.770055369
Smad6	0.09594235	0.178670656	0.536978774	0.591282276	0.974001549
Scarletltr	0.224633047	0.67470094	0.332937208	0.739181682	0.988585656
Lctl	-1.325924251	0.969183619	-1.368083638	0.171285898	0.86244803
Zwilch	0.007187602	0.485772319	0.014796237	0.988194742	0.998148029
Rpl4	0.074264286	0.074799118	0.992849767	0.320783173	0.91640698
Snapc5	0.048816368	0.109066918	0.4475818	0.65445504	0.979133069
Map2k1	-0.076891467	0.109212248	-0.704055344	0.481398308	0.957463006
Tipin	0.026145705	0.171888812	0.152108245	0.879101561	0.992886758
Dis3l	0.098412967	0.141169586	0.697125847	0.48572404	0.957710647
Megf11	0.398425553	0.84037135	0.474106539	0.635423936	0.977034429
Rab11a	0.197625048	0.137682658	1.435366306	0.151182742	0.850827708
Dennd4a	0.311236489	0.173106557	1.797947427	0.072185339	0.74633396
Slc24a1	0.405339945	1.701250274	0.238260032	0.811679418	0.991816977
Vwa9	0.060838014	0.190445598	0.31945088	0.749384634	0.988585656
Hacd3	0.035746865	0.108033959	0.33088545	0.740731013	0.988585656
Dpp8	-0.218285402	0.151454559	-1.441260029	0.149511235	0.848779008
Igdcc4	-0.55420549	0.795490913	-0.696683621	0.486000811	0.957710647
Parp16	-0.112670507	0.250498055	-0.449785954	0.652864786	0.979004493
Cilp	0.280713182	0.78041897	0.359695488	0.719074867	0.988585656
Clpx	-0.124189046	0.134256388	-0.925014053	0.354958598	0.924354588

Pdcd7	-0.030282135	0.234652402	-0.129051033	0.897317268	0.994101834
Ubap1l	0.073415756	1.71616423	0.04277898	0.96587772	0.996474415
Kbtbd13	-0.463029792	0.623725073	-0.742361998	0.457868037	0.952235842
Rasl12	-0.00766369	0.122017247	-0.062808252	0.949919195	0.995727234
Mtfmt	0.032497773	0.175988776	0.184658213	0.853497123	0.992886758
Spg21	0.0317535	0.095678618	0.331876659	0.739982396	0.988585656
Plekho2	-0.025268785	0.28244877	-0.089463252	0.928713757	0.994688009
Pif1	0.532326739	1.280080651	0.415854062	0.677516796	0.983670411
Rbpms2	0.185678464	0.537524634	0.345432475	0.729769267	0.988585656
Oaz2	-0.011737298	0.094019381	-0.124839131	0.900650908	0.994688009
Zfp609	-0.701869752	0.226626237	-3.09703661	0.001954657	0.298625366
Trip4	0.153602145	0.112261436	1.368253882	0.171232621	0.86244803
2810417H13Rik	0.044001178	0.270469957	0.16268416	0.87076712	0.992886758
Csnk1g1	0.110860445	0.292308002	0.379259015	0.704495532	0.988585656
Ppib	0.359535722	0.125036788	2.875439523	0.004034654	0.378480793
Snx22	-0.291496448	0.258644383	-1.12701635	0.259735568	0.897248195
Snx1	0.004774322	0.111782292	0.042710897	0.965931993	0.996474415
Fam96a	0.288459354	0.256869629	1.122979602	0.261446158	0.897248195
Dapk2	-0.101815082	0.168570679	-0.60399046	0.545849985	0.967759008
Herc1	-0.061373686	0.254767237	-0.240901017	0.809631834	0.991295569
Fbxl22	0.282898921	0.344508064	0.821167777	0.411550708	0.941762685
Usp3	0.177562488	0.129620606	1.369863121	0.170729634	0.86244803
Car12	0.130696865	1.958660921	0.066727662	0.946798512	0.995195989
Aph1b	-0.225405586	0.157531613	-1.430859379	0.152470521	0.853113844
Aph1c	0.055102277	0.186725886	0.295097152	0.767919666	0.989990452
Rab8b	-0.045804413	0.110336771	-0.415132799	0.67804469	0.983670411
Rps27l	0.333577538	0.276539472	1.206256511	0.227718601	0.894410876
Lactb	0.05800569	0.215378728	0.269319495	0.787683831	0.990988839
Tpm1	0.040483205	0.085953479	0.470989718	0.637648077	0.977468848
Tln2	-0.069265416	0.254740647	-0.27190563	0.785694582	0.990988839
C2cd4b	0.113330124	0.47605907	0.238058954	0.81183537	0.991817851
C2cd4a	0.306032953	1.331907827	0.229770369	0.818270211	0.992734473
Vps13c	-0.327708244	0.25404057	-1.289983895	0.19705625	0.879755417
4930502A04Rik	0.86706779	1.139558195	0.760880659	0.446728352	0.948476747
Rora	-0.090456333	0.205267778	-0.440674784	0.659448454	0.979924124
Ice2	-0.131296325	0.196141502	-0.669395936	0.503242943	0.961729397
Anxa2	-0.259228985	0.120121198	-2.158061927	0.030923017	0.659973993
Bnip2	0.081885771	0.092819236	0.882206904	0.377664929	0.928883222
Gtf2a2	0.285345319	0.186750522	1.527949246	0.12652514	0.823315433
Fam81a	-0.232965178	0.219740648	-1.060182445	0.289061606	0.907334416
Myo1e	-0.253201944	0.246224583	-1.028337388	0.30379115	0.911786415
Ccnb2	0.386301661	0.30187192	1.279687296	0.200655134	0.880907872
Rnf111	0.046172462	0.155733432	0.296483944	0.766860527	0.989884648
Sltm	-0.302707819	0.244946542	-1.235811767	0.21652854	0.887633697
Fam63b	-0.228369347	0.125057776	-1.826110736	0.067833583	0.742385562
Adam10	-0.197862312	0.116151683	-1.7034821	0.088477885	0.767814344
Lipc	-0.029048575	1.000694075	-0.029028427	0.976841919	0.997262526
Aqp9	-0.545683447	0.609592919	-0.895160409	0.370701345	0.928436166
Aldh1a2	-0.207638746	0.253127448	-0.820293285	0.412048934	0.942121471
Polr2m	0.043068285	0.081252511	0.530054819	0.596073924	0.974001549
Myzap	-0.00876746	0.13840856	-0.063344779	0.949491959	0.995624732
Cgnl1	-0.078601795	0.197100926	-0.398789576	0.690048258	0.986002801
Tcf12	-0.004823307	0.166088096	-0.029040653	0.976832168	0.997262526
Zfp280d	-0.119267669	0.176381395	-0.676191888	0.498918822	0.960700483
Mns1	-0.446900031	0.30471489	-1.466616978	0.142480279	0.840741274
Tex9	-0.001197848	0.268596978	-0.004459648	0.996441727	0.999379819
4930509E16Rik	0.309032778	1.08817754	0.283991139	0.776417172	0.990861469
Rfx7	0.032081885	0.224130045	0.1431396	0.88617993	0.992886758
Nedd4	-0.242483058	0.180286942	-1.344984029	0.178630392	0.867377413
Pygo1	0.159650569	0.436587852	0.365677991	0.714605371	0.988585656
Dyx1c1	-0.070841162	0.204324093	-0.346709784	0.728809359	0.988585656
Ccpg1os	-0.017822122	0.131328925	-0.135705988	0.892053714	0.993218834

Ccpg1	-0.173253228	0.133612538	-1.296683909	0.194739968	0.879588734
Pigb	-1.314898951	0.719038	-1.828691879	0.06744578	0.742385562
2310009A05Rik	0.073030285	0.185845296	0.392962785	0.694346968	0.987010101
Rab27a	0.121723948	0.115211556	1.056525512	0.290728192	0.907334416
Khdc3	0.514073331	1.139073079	0.451308472	0.65176724	0.979004493
Rsl24d1	0.354741485	0.10812334	3.280896474	0.001034777	0.240510037
Wdr72	0.187441047	0.349704526	0.535998343	0.591959695	0.974001549
Fam214a	-0.133648825	0.290496191	-0.460070833	0.645465379	0.979004493
Arpp19	0.21235915	0.125807694	1.687966311	0.091417696	0.772516176
Myo5a	-0.066723806	0.193259987	-0.345254115	0.729903339	0.988585656
Myo5c	-0.235146344	0.154818071	-1.518855919	0.128798767	0.827261001
Gnb5	0.023036739	0.169863346	0.135619248	0.892122288	0.993227453
Mapk6	0.001310269	0.203030615	0.006453556	0.994850843	0.999285699
4933433G15Rik	0.954826741	1.971863118	0.484225671	0.62822572	0.976968316
Leo1	0.072340636	0.259978052	0.278256706	0.780815308	0.990988839
Tmod3	-0.03449553	0.092427038	-0.37321903	0.708985434	0.988585656
Tmod2	0.03781245	0.136749269	0.276509336	0.78215689	0.990988839
Lysmd2	0.029816227	0.155371382	0.191902955	0.847818223	0.992886758
Scg3	0.949319645	0.94006234	1.009847544	0.312568338	0.914819219
Bmp5	0.208492573	0.157681237	1.322240857	0.18608796	0.872305715
Hmgcll1	-0.147839038	0.138350972	-1.068579684	0.285259107	0.905916508
Hcrtr2	-0.046908544	1.627338798	-0.028825309	0.977003915	0.997262526
Fam83b	-0.594254124	0.795431704	-0.74708378	0.45501299	0.951224746
Tinag	-0.161538018	0.263441016	-0.613184766	0.539754176	0.966900344
Mlip	0.06588442	0.97069456	0.067873482	0.945886348	0.995065399
Lrrc1	-0.162612715	0.148887492	-1.092185198	0.274751707	0.900302631
Klhl31	0.212078684	1.190914301	0.178080559	0.85865971	0.992886758
Gclc	-0.125434994	0.156210019	-0.802989429	0.421980845	0.943415794
Elovl5	-0.121604516	0.095630215	-1.271611862	0.20351106	0.883178549
Fbxo9	0.007628212	0.110135534	0.069262041	0.94478104	0.99486515
Ick	-0.020960277	0.22035019	-0.095122574	0.924217468	0.994688009
C920006O11Rik	-0.049103599	0.285342753	-0.172086372	0.863369626	0.992886758
Gsta4	0.120403333	0.189900202	0.634034781	0.526058124	0.965637248
Gsta1	-0.858858215	1.145435936	-0.749809036	0.453369726	0.950146765
Gm3776	-1.735920932	2.144873971	-0.8093347	0.418322652	0.943415794
Gsta2	0.692294486	0.424145972	1.632208087	0.102635658	0.789323778
Dppa5a	-0.820097552	1.267890925	-0.646820271	0.517748271	0.964437658
Ooep	-0.033523913	0.29067908	-0.115329638	0.908183847	0.994688009
Ddx43	0.218243921	1.613107064	0.135294133	0.892379324	0.993300147
Mb21d1	-0.578932035	0.248091841	-2.333539193	0.019619864	0.593385684
Mto1	-0.272944257	0.245791498	-1.1104707	0.266796247	0.89768067
Eef1a1	0.175680556	0.17285374	1.016353802	0.309460936	0.9139204
Slc17a5	0.062391583	0.164153237	0.380081347	0.703885031	0.988585656
Cd109	1.187286143	1.178361536	1.007573742	0.31365914	0.915639195
Gm10635	-1.467459033	1.816505666	-0.807847209	0.419178544	0.943415794
Col12a1	-0.043691904	0.229362647	-0.190492673	0.848923084	0.992886758
Cox7a2	0.167107625	0.19228543	0.869060253	0.384814175	0.931689378
Tmem30a	-0.045376267	0.132647875	-0.342080621	0.732290221	0.988585656
4930429F24Rik	-0.041437328	0.641677117	-0.064576603	0.948511114	0.99560893
Filip1	0.265449878	0.392992776	0.675457398	0.499385209	0.960700483
Senp6	-0.163399623	0.137099463	-1.191832697	0.233326852	0.89581803
Myo6	-0.083154675	0.120420266	-0.690537213	0.489856416	0.958560421
Htr1b	-0.157061236	0.803086967	-0.19557189	0.844945264	0.992886758
D430036J16Rik	-0.330042218	0.207584588	-1.589916774	0.111853566	0.801067615
Mei4	0.194969373	0.517872692	0.376481278	0.706559132	0.988585656
Irak1bp1	-0.109295286	0.173458421	-0.630095011	0.528632424	0.965637248
Phip	-0.035017878	0.160459385	-0.21823515	0.8272459	0.992886758
Hmgn3	-0.042318504	0.158109173	-0.267653693	0.788965897	0.990988839
Lca5	-0.409132583	0.194728875	-2.101037059	0.035637713	0.674795441
Sh3bgrl2	-0.308583155	0.138952576	-2.220780388	0.02636584	0.632301593
Elovl4	1.229127736	0.954958677	1.287100442	0.198059281	0.880239572
Ttk	0.578280973	1.164166638	0.496733847	0.619376751	0.974471036

Bckdhb	-0.056255367	0.189659404	-0.29661259	0.766762298	0.989884648
Fam46a	-0.47283898	0.212606484	-2.224010156	0.026147761	0.631530315
Ibtk	-0.210739255	0.141040665	-1.494173719	0.13513022	0.829809681
Tpbg	0.096449531	0.532565029	0.181103764	0.85628613	0.992886758
Ube2cbp	1.000888668	0.886582595	1.128928848	0.25892785	0.897248195
Dopey1	-0.074487803	0.273873724	-0.271978641	0.785638443	0.990988839
Pgm3	-0.342621639	0.210716063	-1.625987285	0.10395236	0.79155232
Rwdd2a	0.200706118	0.331775079	0.604946336	0.545214654	0.967491297
Me1	-0.130573144	0.153300812	-0.851744633	0.394355842	0.933796635
A330041J22Rik	0.252969638	0.763310896	0.331411014	0.740334046	0.988585656
Prss35	-0.247046527	1.088714884	-0.226915725	0.820489274	0.992886758
Cyb5r4	-0.066315053	0.099697786	-0.665160736	0.505947691	0.96224682
Cep162	-0.209176325	0.475373037	-0.440025641	0.659918537	0.979924124
Tbx18	-0.71278575	0.79630936	-0.895111606	0.37072743	0.928436166
Nt5e	-0.23887595	0.088891427	-2.687277694	0.007203704	0.452031612
Snx14	-0.13828255	0.138257931	-1.000178065	0.317224343	0.91640698
Syncrip	-0.162007198	0.091455198	-1.771437837	0.076487921	0.752876039
Snhg5	0.072224596	0.169313374	0.426573484	0.669690014	0.981863224
Zfp949	0.192683171	0.176690696	1.090511132	0.275488053	0.900302631
Trim43a	1.209570417	2.181017235	0.554590031	0.579175109	0.971613657
9430037G07Rik	0.122718023	0.524085442	0.234156519	0.81486347	0.992116023
Mthfsl	0.416430095	0.223761955	1.861040653	0.062738436	0.727650961
Bcl2a1d	0.161677049	0.238163989	0.678847588	0.497234438	0.960475196
9330159M07Rik	0.206458213	0.470586079	0.438725713	0.660860294	0.979924124
Bcl2a1a	-0.144963086	0.252409496	-0.574317087	0.565753249	0.97007491
Bcl2a1b	0.095770268	0.232043168	0.412726084	0.679807312	0.983903958
Mthfs	0.497267968	0.218703685	2.273706396	0.022983645	0.620006604
Tmed3	0.055611713	0.150371837	0.369827979	0.711510667	0.988585656
Rasgrf1	0.13923952	0.796918565	0.174722394	0.86129777	0.992886758
Ctsh	0.082047983	0.160450141	0.511361238	0.609098132	0.974001549
Morf4l1	0.162055202	0.096991658	1.670815877	0.094758052	0.777748075
Adamts7	1.374248125	1.103034881	1.245879118	0.212808777	0.884945675
Tbc1d2b	0.150319598	0.182079405	0.825571668	0.409047112	0.940768024
Plscr1	0.112447786	0.147303008	0.763377392	0.445238359	0.948476747
Mir6386	-0.904839124	1.429109793	-0.633148782	0.526636489	0.965637248
Plscr2	0.230776292	0.144205212	1.600332531	0.109524834	0.798400564
Plscr4	-0.148672316	0.144911702	-1.025951074	0.304914646	0.912452608
B430319G15Rik	0.061154247	0.542705194	0.112684101	0.910281006	0.994688009
Plod2	-0.233882257	0.181284938	-1.290136177	0.197003381	0.879755417
1190002N15Rik	-0.182642994	0.143782823	-1.270269917	0.2039885	0.883661513
Slc9a9	0.007661759	0.338923843	0.022606136	0.981964449	0.997390438
Chst2	0.254506861	0.247059165	1.030145393	0.302941759	0.911623139
U2surp	-0.044668822	0.138796396	-0.321829839	0.747581607	0.988585656
Paqr9	0.752961234	1.226621696	0.613849597	0.53931472	0.966900344
Pcolce2	0.033649798	0.114508552	0.293862745	0.768862787	0.989990452
Trpc1	-0.619004856	0.45031611	-1.374600734	0.169255261	0.861597949
Pls1	-0.25937319	0.247029265	-1.049969483	0.293732144	0.907334416
Atr	-0.10263172	0.255889116	-0.401078879	0.688362051	0.98538669
Xrn1	-0.458972233	0.354064576	-1.296295264	0.19487378	0.879588734
Gk5	-0.106791058	0.600002125	-0.177984466	0.858735176	0.992886758
Tfdp2	0.07540029	0.128944294	0.584750886	0.558715233	0.968926429
Atp1b3	0.004515989	0.090031165	0.050160285	0.959994659	0.996474415
Rnf7	0.306649633	0.250939903	1.222004271	0.221706012	0.89157489
Rasa2	0.028259754	0.171329503	0.164943885	0.868988151	0.992886758
Zbtb38	-0.375057103	0.416437026	-0.90063342	0.36778326	0.928436166
E030011O05Rik	-0.051327997	0.655609162	-0.078290543	0.93759694	0.994688009
Pxylp1	-0.13720992	0.219897879	-0.623971092	0.53264657	0.96674935
Spsb4	-1.416604541	0.901587294	-1.571233923	0.116128323	0.811920148
Slc25a36	-0.33919257	0.144950523	-2.340057586	0.019280767	0.592187315
Clstn2	-0.086202322	0.341803382	-0.252198564	0.800887588	0.990988839
Nmnat3	0.074054136	0.189104879	0.391603519	0.695351187	0.98715859
Rbp1	0.150098439	0.139637942	1.074911565	0.282414324	0.90579922

Rbp2	-0.120169275	0.493379075	-0.243563785	0.807568679	0.99099448
4930579K19Rik	0.525638658	0.328186519	1.601646099	0.109233891	0.798400564
Copb2	-0.150906778	0.135411201	-1.1144335	0.265093259	0.89768067
Mrps22	0.070208185	0.161633458	0.434366658	0.664022206	0.981154469
Faim	0.359281527	0.171273853	2.097702136	0.035931466	0.674795441
Gm1123	-0.090706019	1.973422689	-0.045963807	0.963339098	0.996474415
Pik3cb	0.054287315	0.2144441	0.253153692	0.80014945	0.990988839
Cep70	0.124640704	0.154343378	0.807554593	0.419347035	0.943415794
Esyt3	0.212649745	0.189985518	1.1192945	0.263014521	0.897248195
Mras	-0.039044431	0.229482846	-0.170140955	0.864899285	0.992886758
Nme9	0.037984335	0.144316238	0.263202084	0.792394824	0.990988839
1600029I14Rik	0.009030964	0.305527553	0.029558592	0.97641909	0.997262526
Armc8	0.009752292	0.118662604	0.08218505	0.934499562	0.994688009
Dbr1	-0.107707119	0.178388483	-0.603778435	0.545990958	0.96778188
Dzip1l	-0.394689037	0.262175127	-1.505440434	0.13221088	0.827800491
Cldn18	-0.060555227	0.103500889	-0.585069628	0.5585009	0.968926429
Il20rb	-0.228593625	0.38468561	-0.594234927	0.552354995	0.968926429
Nck1	0.151504467	0.158708198	0.954610213	0.33977485	0.919246575
Slc35g2	0.046515753	0.163997818	0.283636412	0.77668903	0.990861469
Stag1	-0.120752518	0.161348804	-0.748394251	0.454222389	0.950990329
Pccb	0.042845726	0.118844638	0.360518793	0.718459206	0.988585656
Msl2	-0.008808471	0.117848002	-0.074744334	0.940418132	0.994688009
Ppp2r3a	0.021721156	0.254642564	0.085300572	0.932022437	0.994688009
Ephb1	0.019507573	0.524288604	0.037207701	0.970319399	0.996628386
Ky	0.065717586	0.679217628	0.09675483	0.922921096	0.994688009
Cep63	0.118386058	0.16075505	0.736437564	0.461464451	0.953083512
Anapc13	0.420097975	0.163554856	2.568544804	0.010212651	0.499427958
Amotl2	-0.106695128	0.19552551	-0.545683924	0.585283225	0.972904784
Ryk	-0.154421794	0.100586387	-1.535215636	0.124730869	0.821164769
Slco2a1	-0.126775229	0.204461194	-0.620045428	0.535227879	0.96674935
Rab6b	0.076790571	0.150993525	0.508568634	0.611054619	0.974001549
Srprb	-0.092521296	0.108516668	-0.852599856	0.393881243	0.933796635
Trf	-0.418951072	0.137824652	-3.039739736	0.002367827	0.31402518
1300017J02Rik	-1.152485142	1.521032926	-0.757699009	0.448631192	0.948476747
Topbp1	-0.213048907	0.161544974	-1.318821016	0.187228957	0.873455432
Cdv3	-0.103646259	0.122637643	-0.845142294	0.398031397	0.935327115
Bfsp2	0.322848801	1.192508049	0.27073092	0.786597994	0.990988839
Tmem108	-0.244930613	0.477611837	-0.512823582	0.608074732	0.974001549
Nphp3	-0.351148973	0.193506154	-1.814665657	0.069575306	0.743426713
Uba5	0.047895834	0.099748427	0.480166314	0.631109138	0.977034429
Acad11	-0.299384406	0.230043887	-1.301423003	0.193113704	0.879588734
Ackr4	0.015704365	0.332681548	0.047205399	0.962349524	0.996474415
Dnajc13	-0.102207696	0.172215532	-0.593487094	0.552855218	0.968926429
Acpp	-0.238401797	0.366726377	-0.650080856	0.515639994	0.964352198
Mrpl3	-0.049046572	0.165403376	-0.296527034	0.766827625	0.989884648
Nudt16	0.129506327	0.170992882	0.757378467	0.448823153	0.948476747
Nek11	-0.479168193	0.182989876	-2.618550293	0.008830427	0.475600561
Aste1	0.158356387	0.246105366	0.64344955	0.51993244	0.964665379
Atp2c1	-0.079558179	0.10869188	-0.731960648	0.464192587	0.953083512
Pik3r4	0.061843345	0.226835943	0.272634681	0.78513405	0.990988839
Col6a5	0.500149083	2.023180932	0.247209271	0.804746271	0.990988839
Glyctk	0.874481274	0.696506818	1.255524355	0.209288472	0.884258007
Wdr82	-0.16335831	0.132300812	-1.234749107	0.216923894	0.887633697
Ppm1m	0.129067757	0.149553216	0.863022279	0.388125218	0.933269543
Twf2	0.264937563	0.13009036	2.036565685	0.041693584	0.679118604
Tlr9	-0.508768659	1.545469177	-0.329200133	0.742004422	0.988585656
Alas1	-0.563955857	0.141314243	-3.990792765	6.59E-05	0.03839924
Poc1a	-0.15343422	0.213174778	-0.719757852	0.471674099	0.95486913
Dusp7	-0.289276833	0.172573396	-1.676253928	0.093688465	0.775558829
Rpl29	-0.021246625	0.10231657	-0.20765576	0.835497763	0.992886758
Acy1	0.436534259	0.354072368	1.232895585	0.217614723	0.887633697
Abhd14a	-0.128968774	0.168198807	-0.766763902	0.443221902	0.948476747

Abhd14b	-0.083170387	0.109675075	-0.758334446	0.448250791	0.948476747
Pcbp4	0.042503161	0.197472862	0.215235452	0.829583752	0.992886758
Gpr62	-0.17398191	1.320442331	-0.131760325	0.895173869	0.993604681
Parp3	-0.062325506	0.124042758	-0.502451792	0.615349755	0.974001549
Rrp9	0.054164797	0.150147922	0.3607429	0.718291651	0.988585656
Tex264	-0.267609174	0.156307959	-1.712063647	0.08688494	0.765418158
Rad54l2	-0.428006643	0.281756308	-1.519066766	0.128745691	0.827245532
Vprbp	-0.160407197	0.199615919	-0.803579185	0.421640048	0.943415794
Rbm15b	-0.307201632	0.238624563	-1.287384783	0.197960206	0.880239572
Manf	0.088634314	0.17302172	0.512272757	0.608460129	0.974001549
Dock3	0.413270794	0.830693349	0.497501027	0.618835779	0.974471036
Mapkapk3	0.072906303	0.130285835	0.559587334	0.575760947	0.971613657
Cish	-0.25569658	0.177358132	-1.441696396	0.149388041	0.848779008
Hemk1	0.233811129	0.195197405	1.197818838	0.230987552	0.895404344
6430571L13Rik	2.865808267	1.398311797	2.049477286	0.040415467	0.679118604
Tmem115	-0.454127858	0.215847886	-2.103925435	0.035384952	0.674795441
Cyb561d2	0.055335621	0.152234223	0.363490024	0.716238864	0.988585656
Nprl2	-0.024869735	0.167664443	-0.148330405	0.882082021	0.992886758
Zmynd10	-0.426687281	0.173365122	-2.461206015	0.013847083	0.545277966
Rassf1	-0.192923305	0.103941739	-1.856071557	0.063443359	0.728651743
Tusc2	0.129374006	0.176841248	0.731582749	0.464423281	0.953083512
Hyal2	-0.17647595	0.144711457	-1.219502272	0.222653614	0.892391445
Hyal1	0.150650391	0.450184993	0.334641077	0.737895855	0.988585656
Nat6	0.052491532	0.237896737	0.220648391	0.825366219	0.992886758
Hyal3	0.595385357	0.587796794	1.012910181	0.311103065	0.914819219
Ifrd2	-0.259975597	0.167613844	-1.551038932	0.120892354	0.816233249
Sema3b	-0.245796182	0.233891954	-1.050896266	0.293306236	0.907334416
Gnai2	-0.110125264	0.133854618	-0.822722934	0.41066557	0.941195652
Sema3f	0.052800851	0.144045276	0.366557323	0.713949247	0.988585656
Rbm5	0.023130161	0.105689531	0.218850065	0.826766846	0.992886758
Rbm6	-0.093193198	0.22393247	-0.416166525	0.677288153	0.983670411
Mon1a	0.196780341	0.244396572	0.805168171	0.420722641	0.943415794
Mst1r	-0.293421192	0.334028883	-0.878430599	0.379710085	0.929185635
Traip	-0.220128058	0.492715036	-0.446765457	0.655044414	0.979133069
Uba7	0.167248801	0.174026433	0.961054004	0.336525015	0.919242242
Fam212a	0.321971303	0.255233611	1.261476897	0.207137079	0.883765092
Ip6k1	-0.188790835	0.102858188	-1.835447798	0.066439367	0.738254074
Gmppb	0.041501254	0.16866958	0.246050616	0.805643051	0.99099448
Rnf123	0.024750993	0.367980239	0.067261745	0.946373331	0.995065399
Amigo3	0.120872752	0.739756934	0.163395226	0.870207261	0.992886758
Mst1	-0.507012116	0.535447359	-0.946894419	0.343692582	0.920669678
Apeh	-0.022136497	0.138099702	-0.16029359	0.872649808	0.992886758
Bsn	-0.321800361	0.908710409	-0.354128618	0.723242496	0.988585656
Dag1	-0.121015389	0.139841433	-0.865375785	0.386832567	0.93263664
Nicn1	-0.119612286	0.138497203	-0.86364405	0.387783458	0.933137881
Amt	0.080238121	0.255091481	0.314546456	0.753106039	0.988585656
Tcta	0.089959666	0.115098425	0.78158903	0.434456136	0.946762848
Rhoa	0.068765307	0.086320398	0.796628703	0.425666702	0.944290206
Gpx1	0.048375645	0.181577184	0.266419181	0.789916395	0.990988839
Usp4	-0.154901862	0.142481438	-1.087172228	0.276960702	0.900604936
1700102P08Rik	0.038216022	0.908955061	0.042043907	0.966463696	0.996474415
Ccdc36	-0.3874975	1.733389984	-0.223548944	0.823108295	0.992886758
Klhdc8b	0.249374913	0.21236998	1.174247473	0.240295916	0.896939594
Ccdc71	-0.04860628	0.151622892	-0.320573491	0.748533629	0.988585656
Lamb2	0.233394563	0.173375692	1.34617812	0.17824508	0.867377413
Usp19	-0.007628066	0.172440228	-0.044236	0.964716286	0.996474415
Qars	0.056403904	0.121260531	0.46514644	0.641826591	0.97813956
Qrich1	0.025356772	0.145802861	0.173911344	0.861935137	0.992886758
Impdh2	0.040910875	0.124873398	0.32761882	0.74319989	0.988585656
Ndufaf3	-0.20036458	0.132291349	-1.514570543	0.129881197	0.827800491
Mir425	1.222845873	1.747519149	0.699761072	0.484076529	0.957710647
Dalrd3	0.011907228	0.121919869	0.097664378	0.922198803	0.994688009

Wdr6	-0.380739627	0.225452387	-1.68878064	0.091261475	0.771949327
P4htm	-0.305736369	0.203729544	-1.500697261	0.133433882	0.827800491
Arih2	-0.16360271	0.114882259	-1.42409029	0.154420337	0.854361518
Slc25a20	0.366432164	0.153125275	2.393022076	0.016710236	0.56772809
Prkar2a	-0.174335015	0.192306635	-0.906547061	0.364646367	0.926556975
Ip6k2	-0.147445483	0.176820799	-0.833869566	0.404354489	0.938127315
Nckipsd	-0.184971874	0.295761979	-0.625407886	0.531703388	0.966606934
Celsr3	-0.633925164	1.525916858	-0.415438863	0.677820662	0.983670411
Slc26a6	0.256667607	0.508411249	0.504842503	0.613669461	0.974001549
Uqcrc1	-0.098188683	0.108293739	-0.906688455	0.36457157	0.926556975
Col7a1	1.154517616	0.874226532	1.32061608	0.186629408	0.872512868
Pfkfb4	-0.112710415	0.137776577	-0.818066598	0.41331916	0.943143495
Shisa5	0.052694835	0.173753457	0.303273593	0.761681369	0.989285682
Trex1	-0.091835608	0.172066908	-0.533720334	0.593535039	0.974001549
Atrip	-0.095041911	0.266042659	-0.357243125	0.720909804	0.988585656
Tma7	0.22757431	0.19983971	1.138784226	0.254793162	0.897044747
Ccdc51	-0.116776594	0.244465184	-0.477681901	0.632876628	0.977034429
Plxnb1	-0.062614118	0.15620306	-0.400850776	0.688529993	0.98538669
Fbxw13	-0.012605853	2.161908523	-0.005830891	0.995347648	0.999285699
3000002C10Rik	0.558161394	0.578049273	0.965594838	0.334246959	0.918030088
Nme6	0.124781425	0.214392092	0.582024381	0.560550259	0.968926429
Camp	2.629842633	1.057729506	2.486309229	0.012907575	0.532176722
Cdc25a	0.10760853	0.32739763	0.328678402	0.742398781	0.988585656
Map4	-0.183842875	0.187658175	-0.979668886	0.327249589	0.916882624
Dhx30	-0.191196092	0.211797333	-0.902731349	0.366668494	0.927866786
Smarcc1	-0.202546983	0.287415949	-0.704717269	0.480986202	0.957463006
Cspg5	-0.336992349	0.740500262	-0.455087414	0.649046352	0.979004493
Mir6236	-0.020988037	0.333619783	-0.062910049	0.949838133	0.995727234
Elp6	-0.353759136	0.34717646	-1.018960606	0.308221669	0.9139204
Scap	0.035370707	0.162041115	0.218282301	0.827209164	0.992886758
Ptpn23	-0.204439545	0.322766959	-0.633396759	0.526474581	0.965637248
Ngp	3.209957124	1.08459735	2.959584149	0.003080546	0.346777009
Klhl18	-0.432534455	0.331403105	-1.305161141	0.191837983	0.878912371
Kif9	-0.238542589	0.209821724	-1.136882226	0.255587523	0.897044747
Setd2	-0.243955702	0.221118867	-1.103278545	0.269906221	0.89804647
Nradd	0.093061863	0.146683611	0.634439407	0.525794099	0.965637248
Nbeal2	0.100613504	0.196981993	0.510775138	0.60950852	0.974001549
Ccdc12	0.361188781	0.110264868	3.275646985	0.001054202	0.240510037
Pth1r	0.059786235	0.470136708	0.127167767	0.898807616	0.994364545
Myl3	0.835590929	0.350998915	2.380608299	0.01728408	0.578272886
Tmie	0.473972554	0.351612801	1.347995731	0.177659756	0.866855369
Als2cl	0.099457336	0.143105728	0.694992	0.487060315	0.958288569
Tdgf1	0.611000046	0.354841243	1.721896926	0.085088192	0.761635307
Lrrc2	0.216900735	0.486834025	0.445533229	0.655934452	0.979462392
Rtp3	0.393500384	0.312241333	1.260244378	0.207581218	0.883846885
Ltf	-0.223034856	0.427523981	-0.521689697	0.601886399	0.974001549
Ccrl2	-0.064982053	0.14077511	-0.461601859	0.644366861	0.97889195
Lrrfip2	-0.104557811	0.153002364	-0.683373824	0.494370656	0.958962778
Mlh1	0.029533514	0.21670086	0.136287017	0.891594387	0.993043931
Epm2aip1	-0.408963568	0.19751444	-2.07055022	0.038400848	0.679118604
Dclk3	-1.062344707	0.980901977	-1.083028408	0.278795814	0.90176568
Stac	1.104339775	1.244129198	0.88764075	0.374734026	0.928883222
Pdcd6ip	-0.319265637	0.150382957	-2.12301742	0.033752392	0.671818913
Clasp2	0.093849554	0.214146392	0.438249522	0.661205413	0.979955675
Ubp1	-0.012480959	0.125175354	-0.0997078	0.920576308	0.994688009
Fbxl2	-0.294483181	0.231751891	-1.270682972	0.203841456	0.88349335
Susd5	-0.554989609	1.801232629	-0.308116564	0.757993638	0.989266039
4930520O04Rik	-0.304361437	0.979407344	-0.310760828	0.755982451	0.989266039
Crtap	-0.136184908	0.162996406	-0.835508654	0.403431376	0.937834293
Glb1	-0.263519301	0.237913665	-1.107625746	0.26802348	0.89804647
Tmppe	-0.471215418	0.418605882	-1.125677968	0.260301853	0.897248195
Ccr4	-1.543064357	1.131574543	-1.363643576	0.17267977	0.86244803

Cnot10	0.000455323	0.151373858	0.003007937	0.997600017	0.99957296
Dync1li1	-0.173576263	0.124666442	-1.392325475	0.163823861	0.859982992
Cmtm6	-0.047524192	0.122613783	-0.387592571	0.698317567	0.987736745
Cmtm7	-0.053711271	0.168375792	-0.318996394	0.749729247	0.988585656
Cmtm8	0.040528224	0.122060724	0.33203329	0.739864122	0.988585656
Gpd1l	-0.172919703	0.118736116	-1.456336191	0.145299705	0.842729381
Osbpl10	-0.603752695	0.43933786	-1.374233249	0.169369282	0.861597949
Stt3b	-0.255642827	0.136513647	-1.872654004	0.061116179	0.725221283
Tgfbr2	-0.155726959	0.132161424	-1.178308722	0.238673552	0.896609492
Rbms3	-0.187229956	0.202975919	-0.922424473	0.356307209	0.924354588
Azi2	-0.030955634	0.091744252	-0.337412242	0.735806158	0.988585656
Cmc1	0.201681577	0.200775421	1.004513284	0.315131272	0.91586731
Eomes	0.775379159	0.899139531	0.862356878	0.388491162	0.933269543
Golga4	0.076624612	0.186354602	0.411176389	0.6809432	0.984395221
Itga9	-0.184082191	0.152372834	-1.208103744	0.22700736	0.89398447
Ctdspl	-0.174709168	0.182318335	-0.958264386	0.337929458	0.919246575
Vill	-0.044478003	0.283245832	-0.157029682	0.875221456	0.992886758
Plcd1	0.008407121	0.290388581	0.028951281	0.976903447	0.997262526
Dlec1	0.236250592	0.465184742	0.507864018	0.61154871	0.974001549
Acaa1b	0.053491536	0.382271933	0.139930588	0.888714833	0.992886758
Slc22a14	0.687971052	0.572668897	1.201341745	0.229618664	0.895404344
Slc22a13b-ps	0.681942412	1.15283475	0.591535267	0.554161832	0.968926429
Oxsr1	-0.011556065	0.111868793	-0.103300169	0.917724741	0.994688009
Myd88	-0.03978747	0.106089994	-0.375035085	0.707634374	0.988585656
Acaa1a	0.073889924	0.129857855	0.569006197	0.569351935	0.97144955
Xylb	0.167648146	0.484961632	0.345693627	0.729572974	0.988585656
Acvr2b	-0.789048223	0.53801104	-1.466602289	0.142484277	0.840741274
Exog	-0.153713479	0.245113429	-0.627111616	0.530586074	0.966026275
Scn5a	-0.827101249	0.438150554	-1.887710152	0.059064873	0.72285319
Scn10a	0.872656484	1.921704425	0.454105466	0.649752919	0.979004493
Wdr48	-0.188653416	0.202246027	-0.932791699	0.350927528	0.922414914
Gorasp1	-0.156526585	0.15484013	-1.010891589	0.31206832	0.914819219
Ttc21a	-0.102587739	0.274215101	-0.374114113	0.708319419	0.988585656
Csrnp1	-0.103415917	0.395804794	-0.261280103	0.793876509	0.990988839
Xirp1	0.432418103	1.824805258	0.236966712	0.812682615	0.992000477
Cx3cr1	0.249416846	0.348405489	0.715880932	0.474064873	0.955974667
Ccr8	0.446845635	1.06571098	0.419293451	0.675001682	0.983549699
Slc25a38	-0.091663931	0.124725164	-0.734927322	0.462383758	0.953083512
Rpsa	0.079600035	0.133631969	0.595666112	0.551398301	0.968926429
Myrip	0.12055528	0.40436958	0.298131426	0.765602857	0.989884648
Eif1b	0.145946904	0.119376621	1.222575261	0.221490162	0.891315805
Entpd3	0.093380537	0.46373644	0.201365537	0.840412761	0.992886758
Rpl14	0.296720002	0.163449064	1.815366784	0.069467562	0.743426713
5830454E08Rik	0.862330374	0.44035177	1.958276165	0.050197616	0.707153766
Ctnnb1	-0.242166507	0.160889712	-1.505170865	0.132280154	0.827800491
Ulk4	-0.221793099	0.367594863	-0.603362889	0.546267304	0.96778188
Trak1	-0.232492806	0.160760451	-1.446206478	0.148119297	0.847572129
Lyzl4	-0.373963734	0.523200416	-0.714761922	0.474756168	0.956387791
Vipr1	-0.067723142	0.595141119	-0.113793418	0.909401557	0.994688009
Sec22c	-0.236270042	0.295659613	-0.799128566	0.424215867	0.94406679
Deb1	0.189971705	0.196834651	0.965133447	0.334477976	0.918030088
Nktr	-0.340222644	0.159246897	-2.136447557	0.032642951	0.665167506
E530011L22Rik	0.08184715	0.556882686	0.146973775	0.88315272	0.992886758
Zfp651	-0.037270251	0.173359395	-0.214988357	0.829776396	0.992886758
Klhl40	-0.62837765	1.859416299	-0.337943499	0.735405767	0.988585656
Hhatl	-0.499058037	0.365182188	-1.366600161	0.171750667	0.86244803
Ccdc13	-0.15796989	0.47611056	-0.331792451	0.740045985	0.988585656
Higd1a	0.299606118	0.182321077	1.643288443	0.100323268	0.788783372
Ackr2	-0.513438814	0.27648222	-1.857040982	0.063305324	0.728651743
Cyp8b1	0.504916744	0.917100157	0.550557909	0.58193677	0.971998122
1700048O20Rik	0.403416324	0.441202369	0.91435666	0.360529467	0.925913281
Fam198a	0.193445064	0.380206521	0.508789443	0.61089982	0.974001549

Pomgnt2	0.117822363	0.24350278	0.483864549	0.628482001	0.977034429
Snrk	-0.341800467	0.180833166	-1.890142577	0.058738894	0.72285319
Ano10	-0.246110277	0.217541652	-1.131324854	0.257918387	0.897248195
Abhd5	0.019713529	0.103044016	0.191311726	0.848281375	0.992886758
Topaz1	-0.46929414	0.91220518	-0.514461165	0.606929605	0.974001549
Tcaim	-0.261274664	0.170059866	-1.536368755	0.124447965	0.821164769
Zfp445	-0.207910245	0.188858072	-1.100880907	0.270948493	0.89804647
Zkscan7	-0.40786963	0.325466049	-1.253186412	0.210137874	0.884258007
Zfp105	-0.064239737	0.163568489	-0.392739076	0.694512207	0.987010101
1110059G10Rik	0.252562566	0.14115433	1.789265449	0.073572076	0.748833631
Kif15	0.334968303	0.397413178	0.842871654	0.399300228	0.936273856
Tmem42	0.306167786	0.189837606	1.612787853	0.106790606	0.794908043
Tgm4	0.210072018	0.263077269	0.798518319	0.424569767	0.944280828
Zdhhc3	-0.047265159	0.123056582	-0.384092896	0.700909596	0.988482081
Exosc7	0.258594318	0.117463109	2.201493892	0.027701079	0.637644759
Clec3b	-0.031160362	0.149436742	-0.208518743	0.834823949	0.992886758
Cdcp1	-0.153464575	0.310861873	-0.493674486	0.621536087	0.97514112
Tmem158	-0.050037271	0.251656053	-0.19883198	0.842394179	0.992886758
Lars2	-0.018878866	0.183707735	-0.102765767	0.918148875	0.994688009
Limd1	0.005773518	0.130041547	0.044397491	0.964587561	0.996474415
Sacm1l	-0.077720573	0.09154555	-0.84898254	0.395891008	0.934066368
Slc6a20a	-1.143786732	0.372926589	-3.067055999	0.002161784	0.308737709
Lztfl1	-0.080546088	0.131225777	-0.613797762	0.539348976	0.966900344
Ccr9	0.829791756	0.621664149	1.334791074	0.181944736	0.868546072
Fyco1	0.258634542	0.205875408	1.256267291	0.209019077	0.884258007
Cxcr6	-0.519827275	0.258652449	-2.009751995	0.044457444	0.689308953
Xcr1	0.05726949	0.418372853	0.136886248	0.891120709	0.993043931
Ccr1	-0.325587168	0.260815966	-1.248340635	0.211906339	0.884945675
Ccr3	-0.40759103	0.864372808	-0.47154541	0.6372513	0.977284137
Ccr2	-0.006389996	0.22433619	-0.028484016	0.977276116	0.997262526
Ccr5	0.169475958	0.261158955	0.648937954	0.516378481	0.964352198
Nudt11	-0.094753145	0.330811921	-0.28642603	0.77455184	0.99084133
Nudt10	-0.251987398	0.475975104	-0.529412981	0.596518995	0.974001549
Shroom4	0.360013342	0.593733179	0.606355438	0.544278752	0.967491297
Clcn5	0.287538186	0.911648893	0.315404525	0.752454532	0.988585656
Usp27x	-0.018382228	0.578905887	-0.031753397	0.974668712	0.997262526
Gm14379	0.580197327	0.583190409	0.994867745	0.319800592	0.91640698
2010204K13Rik	0.162275035	0.214968597	0.754877861	0.45032227	0.949134998
Ppp1r3f	0.258681805	0.488825374	0.529190626	0.596673219	0.974001549
Ppp1r3fos	-0.110773955	1.500432973	-0.073827993	0.941147252	0.994688009
Ccdc22	-0.009832014	0.21095576	-0.046606995	0.962826457	0.996474415
Cacna1f	-0.591571842	0.542317046	-1.09082288	0.275350827	0.900302631
Syp	0.289314397	0.312815644	0.924871893	0.355032549	0.924354588
Prickle3	-0.271032676	0.376900119	-0.719110083	0.472073095	0.955003345
Plp2	-0.080341466	0.130341829	-0.616390506	0.537636818	0.96674935
Magix	-0.000943348	0.169142435	-0.005577242	0.995550028	0.999285699
Gpkow	-0.084299479	0.099981903	-0.843147376	0.399146025	0.936273856
Wdr45	-0.016509201	0.150408627	-0.109762326	0.91259787	0.994688009
Praf2	-0.126288961	0.196698879	-0.642042097	0.52084585	0.964784335
Ccdc120	-0.011245506	0.268995089	-0.041805618	0.966653656	0.996474415
Tfe3	-0.056589393	0.224401183	-0.252179566	0.800902271	0.990988839
Gripap1	0.170801897	0.196754038	0.86809856	0.38534038	0.931689378
Kcnd1	-0.838146672	1.862034499	-0.450124137	0.652620934	0.979004493
Otud5	-0.025731553	0.110876774	-0.232073426	0.816480984	0.992676801
Pim2	-0.082780666	0.28467104	-0.290794125	0.771208779	0.989990452
Slc35a2	-0.126654124	0.189293272	-0.669089409	0.503438445	0.961729397
Pqbp1	-0.132058747	0.156443636	-0.844129873	0.398596836	0.93579994
Timm17b	0.456202307	0.15899122	2.869355353	0.004113094	0.38069687
Pcsk1n	-3.005405939	1.862649817	-1.613510984	0.106633539	0.794908043
Hdac6	-0.155445712	0.356124845	-0.436492185	0.662479668	0.980647283
Gata1	0.361007369	1.096063457	0.329367215	0.741878145	0.988585656
Suv39h1	-0.410073218	0.21204727	-1.93387643	0.053128323	0.707153766

Was	0.27260722	0.251016056	1.086015071	0.277472324	0.901191693
Gm6787	2.48210433	1.211257495	2.049196261	0.040442928	0.679118604
Wdr13	-0.052440419	0.128162974	-0.409169806	0.682415051	0.984738223
Rbm3	0.395747039	0.17161822	2.305973332	0.021112122	0.60743619
Rbm3os	0.746643737	1.827150938	0.408638236	0.682805166	0.984738223
Tbc1d25	-0.38330296	0.300274294	-1.276509403	0.201775505	0.881220006
Ebp	-0.470844602	0.116195036	-4.052192049	5.07E-05	0.03405023
Porcn	-0.452750275	0.212275066	-2.132847179	0.032937265	0.665167506
Ftsj1	-0.110614094	0.150361693	-0.735653421	0.461941643	0.953083512
Slc38a5	-0.196272902	0.254967365	-0.769796173	0.44142081	0.94817492
B630019K06Rik	0.021960857	0.486828044	0.04511009	0.964019559	0.996474415
Lancl3	-1.232698518	1.358328403	-0.907511406	0.364136421	0.926556975
Xk	-0.5429391	0.579623527	-0.936709907	0.348907799	0.921552698
Cybb	0.001584717	0.152960112	0.010360329	0.991733801	0.998770784
Dynlt3	0.137213158	0.153981045	0.891104218	0.372873262	0.928840865
Sytl5	-0.736729496	0.899975705	-0.818610426	0.413008716	0.942708418
Srpx	-0.229604961	0.196756358	-1.166950655	0.243230298	0.896939594
Rpgr	-0.094809511	0.161040002	-0.588732674	0.556040616	0.968926429
Otc	-0.191576482	0.717186725	-0.267122181	0.78937509	0.990988839
Tspan7	-0.131502984	0.137238274	-0.958209251	0.337957253	0.919246575
Mid1ip1	-0.013240948	0.162276435	-0.081595015	0.934968765	0.994688009
Bcor	-0.161805377	0.288676508	-0.5605076	0.575133257	0.971613657
2900008C10Rik	0.118747676	0.41836857	0.283835078	0.776536771	0.990861469
Atp6ap2	-0.200348121	0.098934316	-2.025061962	0.042861002	0.685039858
1810030O07Rik	-0.174356148	0.12880064	-1.353690073	0.175835276	0.865082036
Med14	-0.227931591	0.164658796	-1.384266108	0.166276988	0.860082
Gm14634	3.378866418	2.184109268	1.547022609	0.121857794	0.816233249
AA414768	-0.34773253	0.344419025	-1.009620565	0.312677113	0.914819219
5730405O15Rik	0.494772451	0.58044713	0.852398823	0.393992773	0.933796635
Usp9x	0.01388554	0.224323593	0.061899597	0.950642788	0.99573416
Ddx3x	-0.062738339	0.106782112	-0.587536036	0.556843756	0.968926429
Nyx	-0.282504311	0.511429634	-0.552381583	0.580686946	0.971613657
Cask	0.013781818	0.161445798	0.085364981	0.931971232	0.994688009
Gpr34	0.045502174	0.236652716	0.192274041	0.847527551	0.992886758
Gpr82	-1.159981245	0.56685127	-2.046359083	0.040721055	0.679118604
Maoa	-0.065658318	0.133111877	-0.493256644	0.621831259	0.97514112
Maob	-0.065175679	0.214999069	-0.303144007	0.761780118	0.989285682
Ndp	-0.245921374	1.126723465	-0.218262405	0.827224664	0.992886758
Efhc2	-0.769135116	0.445711064	-1.72563613	0.084412895	0.76099108
Fundc1	0.075197945	0.157852292	0.476381711	0.633802462	0.977034429
Kdm6a	-0.039865699	0.193749844	-0.205758614	0.836979471	0.992886758
4930578C19Rik	1.031051187	0.589426485	1.749244755	0.080248721	0.757353102
Chst7	-0.213523341	0.265338045	-0.804721922	0.420980166	0.943415794
Slc9a7	-0.117573156	0.390918271	-0.300761475	0.763596387	0.989331656
Rp2h	0.050836382	0.125261171	0.405843105	0.684857885	0.985105247
Jade3	-0.263460186	0.201991245	-1.304314877	0.192126245	0.879588734
Rgn	0.043331887	0.583721621	0.074233823	0.940824333	0.994688009
Ndufb11	0.159337036	0.146017971	1.091215247	0.275178181	0.900302631
Rbm10	-0.24151009	0.127520461	-1.893892854	0.058239233	0.72285319
Uba1	-0.328373999	0.183909691	-1.785517649	0.074177391	0.750704981
Cdk16	-0.029566941	0.130070357	-0.227314982	0.820178824	0.992886758
Usp11	-0.555569119	0.315924919	-1.75854795	0.07865432	0.754946439
Araf	0.323868858	0.128244123	2.525408964	0.011556372	0.516933925
Syn1	0.374021639	2.263612146	0.165232211	0.868761213	0.992886758
Timp1	-1.02241083	0.413842056	-2.470533905	0.013491153	0.540715407
Cfp	-0.383578945	0.233836486	-1.640372516	0.100927736	0.788783372
Elk1	-0.233644925	0.188387709	-1.240234441	0.214888693	0.886877576
Uxt	-0.126428971	0.206567708	-0.612046153	0.540507222	0.966989525
A230072C01Rik	0.175320475	0.24007155	0.730284263	0.465216446	0.953083512
Zfp182	0.042273724	0.203990613	0.207233673	0.835827371	0.992886758
Zfp300	0.188332542	0.296925517	0.63427537	0.525901127	0.965637248
Slc6a14	-0.013909198	0.126639562	-0.109832964	0.912541848	0.994688009

Klhl13	0.031595438	0.149318226	0.211598	0.83242067	0.992886758
Wdr44	-0.091584453	0.210870944	-0.434315185	0.664059579	0.981154469
Dock11	-0.150166031	0.247409812	-0.60695261	0.543882363	0.967491297
Il13ra1	-0.187223489	0.131130062	-1.427769394	0.153358245	0.854159937
Zcchc12	-0.421345462	0.534742738	-0.787940503	0.430731509	0.945321484
Lonrf3	-0.186338063	0.306595868	-0.607764432	0.543343723	0.967491297
Pgrmc1	0.158902346	0.119448217	1.330303207	0.183418391	0.869576666
Akap17b	-0.464679241	0.418049895	-1.111540144	0.266335921	0.89768067
Slc25a43	-0.293140973	0.55147971	-0.531553506	0.595035274	0.974001549
Slc25a5	-0.067839388	0.118327747	-0.573317667	0.566429625	0.970426802
2310010G23Rik	-1.153240198	0.59418416	-1.940880077	0.052272825	0.707153766
C330007P06Rik	-0.034019295	0.114257733	-0.297741727	0.765900294	0.989884648
Ube2a	0.071300096	0.142924835	0.498864289	0.617874993	0.974153
Nkrf	-0.55586726	0.418304614	-1.328857585	0.18389496	0.87052856
Sep-06	0.154341436	0.19982522	0.772382161	0.439888116	0.947849924
Rpl39	0.00536871	0.236563101	0.02269462	0.981893867	0.997390438
Snora69	-0.120490919	1.768937211	-0.068114865	0.945694197	0.995032756
Upf3b	-0.283107063	0.318710728	-0.888288464	0.374385604	0.928883222
Nkap	0.186872515	0.155060085	1.205161951	0.228140788	0.894468681
Akap14	-0.676682519	0.348966108	-1.939106701	0.052488347	0.707153766
Ndufa1	0.3896045	0.184202725	2.115085432	0.034422667	0.674692531
Rnf113a1	0.486918057	0.368322288	1.321989118	0.186171775	0.872305715
Zbtb33	-0.24825458	0.187414989	-1.324625002	0.185295559	0.871716376
Tmem255a	0.721876282	0.579895152	1.244839312	0.213190822	0.884945675
Lamp2	-0.078250696	0.116266042	-0.673031388	0.500927323	0.961175273
Cul4b	-0.160941884	0.148985567	-1.08025151	0.280030196	0.903192609
Mcts1	-0.170868584	0.139021558	-1.229079765	0.219041901	0.888847883
C1galt1c1	-0.109264551	0.125827152	-0.868370216	0.385191694	0.931689378
Gria3	0.427769009	0.283897784	1.506771218	0.131869309	0.827800491
Thoc2	-0.043365435	0.144350316	-0.300418015	0.763858324	0.989331656
Xiap	-0.093925364	0.127317772	-0.737723909	0.46068224	0.953083512
Stag2	-0.220465717	0.093076106	-2.368660737	0.017852621	0.581000616
Sh2d1a	0.528856305	0.428780889	1.233395232	0.217428343	0.887633697
Dcaf12l1	0.440371583	0.776384879	0.567207831	0.570572988	0.971613657
Smarca1	0.28556821	0.895977899	0.318722382	0.74993704	0.988585656
Ocrl	-0.575957837	0.280133755	-2.056010129	0.03978154	0.679118604
Apln	-0.451632991	0.226218323	-1.996447432	0.045885242	0.696900792
Xpnpep2	-0.125333409	0.348399265	-0.35974074	0.719041023	0.988585656
Sash3	0.076253739	0.280479011	0.27186968	0.785722225	0.990988839
Zdhhc9	-0.308009028	0.281542658	-1.094004833	0.273952859	0.899464226
Utp14a	-0.082084624	0.187325346	-0.438192832	0.661246504	0.979955675
9530027J09Rik	0.174740058	1.688409483	0.103493886	0.917571002	0.994688009
Bcorl1	-0.310493577	0.598796258	-0.518529587	0.604088822	0.974001549
Elf4	-0.446554017	0.377665785	-1.182405275	0.237044931	0.896609492
Aifm1	0.32153216	0.251042019	1.28079021	0.200267364	0.880888611
Rab33a	0.968124987	0.956285073	1.012381156	0.311355845	0.914819219
Zfp280c	-0.155857948	0.276145573	-0.564405023	0.572478521	0.971613657
Slc25a14	0.202774812	0.179438389	1.130052563	0.258454077	0.897248195
Rbmx2	0.285693822	0.211577341	1.350304437	0.17691835	0.866546104
Enox2	0.196197279	0.150935183	1.299877704	0.193642888	0.879588734
Igsf1	1.781447014	1.032950516	1.724619899	0.084595995	0.76099108
Gm35612	1.288305958	0.604275132	2.131985729	0.03300802	0.665335727
Firre	0.001179174	0.178942031	0.006589696	0.994742221	0.999285699
Stk26	0.010196033	0.245100842	0.041599339	0.9668181	0.996474415
Frmd7	-0.349198472	0.27883274	-1.252358216	0.210439365	0.884340413
Rap2c	0.000838401	0.100512254	0.008341278	0.9933447	0.999270171
Mbnl3	-0.157635892	0.332031488	-0.474761876	0.634956709	0.977034429
Hs6st2	-0.605307862	0.265400494	-2.280733745	0.022564207	0.620006604
Gpc4	0.190925305	0.283002539	0.674641666	0.499903454	0.961017744
Gpc3	-0.100145828	0.201020929	-0.498186079	0.618352893	0.974471036
Ccdc160	0.317742243	0.244504021	1.299537904	0.193759394	0.879588734
Phf6	0.09244255	0.17002228	0.543708445	0.58664212	0.972963251

Hprt	0.189808379	0.164852657	1.151381979	0.249575124	0.896939594
C430049B03Rik	0.280781702	0.197714789	1.420135048	0.155568369	0.855261441
Fam122b	0.138816239	0.249931145	0.555417929	0.578608832	0.971613657
Mospd1	0.220029536	0.127544187	1.725123988	0.08450513	0.76099108
Cxx1c	-0.235006722	0.208264142	-1.12840703	0.25914806	0.897248195
Cxx1a	-0.111746249	0.196178358	-0.56961558	0.568938459	0.97144955
Cxx1b	-0.08356209	0.210014367	-0.397887494	0.690713119	0.986039532
AW822252	-0.201373952	1.276036476	-0.157812066	0.874604891	0.992886758
4930502E18Rik	-0.616198179	0.680724351	-0.905209544	0.365354387	0.926556975
Xlr	-0.182403728	0.240044326	-0.759875192	0.447329192	0.948476747
3830403N18Rik	0.093411789	0.603440228	0.154798744	0.876979981	0.992886758
Zfp449	0.026505978	0.274317033	0.096625345	0.923023928	0.994688009
Smim10l2a	-0.119088465	0.33820693	-0.352117163	0.724750398	0.988585656
Ddx26b	0.034431245	0.146366245	0.235240338	0.814022199	0.992048071
Mmgt1	-0.348819789	0.123047042	-2.834849044	0.004584734	0.390758702
Slc9a6	-0.034081744	0.153110067	-0.222596364	0.823849667	0.992886758
Fhl1	-0.336910824	0.123770685	-2.722056721	0.006487701	0.430588162
Mtap7d3	-0.199656123	0.662582718	-0.30133011	0.763162783	0.989285682
Htatsf1	0.096942103	0.165062167	0.587306618	0.556997797	0.968926429
Cd40lg	-0.555672853	1.811803097	-0.30669605	0.759074737	0.989266039
Arhgef6	-0.297807806	0.197624339	-1.506938913	0.131826316	0.827800491
Rbmx	-0.104224108	0.152309638	-0.684290959	0.493791455	0.958916246
Fgf13	-1.268739753	0.866724952	-1.463832038	0.143239846	0.840741274
F9	1.428775713	1.474040243	0.969292202	0.332399419	0.917115058
Atp11c	-0.158533695	0.203712938	-0.778221047	0.436438711	0.946762848
Gm715	-0.006518279	1.431267178	-0.004554202	0.996366285	0.999379819
Slitrk4	0.518915991	1.449933837	0.357889428	0.720426063	0.988585656
Fmr1	0.023853737	0.116092008	0.205472693	0.83720283	0.992886758
Fmr1nb	1.278016454	2.305780182	0.554266388	0.57939655	0.971613657
Aff2	-0.922041894	1.495655198	-0.616480252	0.537577601	0.96674935
Ids	-0.385892456	0.118008736	-3.270032948	0.00107535	0.240510037
1110012L19Rik	0.258863208	0.169758801	1.524888289	0.127286963	0.824450458
BC023829	-0.096856926	0.159213605	-0.608345788	0.542958158	0.967491297
Mamld1	-0.940610041	0.778430465	-1.208341764	0.22691583	0.89398447
Mtm1	-0.081336973	0.139665552	-0.582369606	0.56031775	0.968926429
Mtmr1	-0.12190863	0.20133948	-0.605487952	0.544854827	0.967491297
Cd99l2	-0.019911339	0.109587489	-0.181693545	0.855823232	0.992886758
Hmgb3	0.288645851	0.198336785	1.455331905	0.145577398	0.842832198
Vma21	-0.114887621	0.11990679	-0.958141074	0.337991626	0.919246575
Gm1141	-0.389872761	1.924568177	-0.202576747	0.839465866	0.992886758
Prrg3	-0.318378184	0.191742018	-1.660450779	0.096823802	0.782250977
Fate1	0.347234612	2.037412984	0.170429174	0.86467263	0.992886758
Cnga2	-0.001992067	0.393317376	-0.005064784	0.995958905	0.999323659
Gabre	0.187590376	1.071614857	0.175053915	0.861037269	0.992886758
Gabra3	-0.04251273	0.205196232	-0.207180851	0.835868622	0.992886758
Gabrq	-0.372756203	0.766428641	-0.48635474	0.626715674	0.976601842
Cetn2	0.167524355	0.174019471	0.962675926	0.335710181	0.918908218
Nsdhl	-0.309269196	0.202190441	-1.52959356	0.126117364	0.822401003
Zfp185	-0.170054715	0.180963869	-0.939716397	0.347363052	0.921552698
Xlr4a	1.869645457	0.504570047	3.705422999	0.000211038	0.091541268
Xlr3a	0.625502248	0.563433954	1.110160727	0.266929773	0.89768067
Xlr3b	1.80918104	0.638296253	2.834390822	0.004591314	0.390758702
Xlr4b	0.59383519	0.4574614	1.298109939	0.19424956	0.879588734
F8a	-0.322641571	0.301160969	-1.071325983	0.284022881	0.905916508
Xlr4c	-1.204710566	0.707944214	-1.701702679	0.088811118	0.767814344
Zfp275	-0.379560929	0.210091226	-1.806648169	0.070817154	0.744227652
Haus7	0.02148893	0.137977688	0.155742065	0.876236338	0.992886758
Bgn	-0.122099244	0.187499884	-0.65119637	0.514919733	0.964352198
Atp2b3	1.635529761	1.049215647	1.558811828	0.119040924	0.814755048
Dusp9	0.855746916	1.064241923	0.80409059	0.421344659	0.943415794
Pnck	-0.082011658	0.256591832	-0.31961913	0.749257071	0.988585656
Slc6a8	-0.15586772	0.256477986	-0.607723578	0.543370822	0.967491297

Bcap31	-0.049609558	0.116943266	-0.424219019	0.671406091	0.9828788
Abcd1	-0.040609074	0.293808445	-0.138216157	0.890069586	0.993043931
Plxnb3	-0.050026185	0.670650561	-0.074593519	0.940538131	0.994688009
Srpk3	-0.087444042	0.451605259	-0.19362937	0.846466099	0.992886758
Idh3g	0.01755449	0.110076251	0.159475723	0.873294083	0.992886758
Ssr4	0.229347653	0.18652884	1.229555994	0.218863417	0.888671255
Pdzd4	0.310061338	0.254303461	1.219257251	0.222746569	0.892391445
L1cam	-0.495529108	0.747001804	-0.663357311	0.50710174	0.963011338
Avpr2	-1.812938564	1.886980415	-0.960761728	0.336671986	0.919242242
Arhgap4	0.466002103	0.204875036	2.274567527	0.022931886	0.620006604
Naa10	0.066880013	0.142610699	0.468969112	0.639091715	0.977832483
Renbp	0.306925896	0.181630566	1.689836153	0.091059305	0.771538719
Hcfc1	0.214914536	0.206073132	1.042904208	0.296992661	0.909070179
Irak1	-0.197031538	0.148043741	-1.330900833	0.183221643	0.869576666
Mecp2	0.071543249	0.139917992	0.511322727	0.609125093	0.974001549
Tktl1	-0.259683242	1.10186989	-0.235675051	0.813684831	0.992048071
Flna	-0.065386553	0.11364095	-0.575378445	0.565035379	0.969658675
Emd	0.031285296	0.112084041	0.279123558	0.780150004	0.990988839
Rpl10	0.102695457	0.118296996	0.868115505	0.385331104	0.931689378
Snora70	0.632295969	0.720448999	0.877641541	0.380138278	0.929640874
Dnase1l1	0.053351059	0.118769744	0.449197387	0.653289272	0.979004493
Taz	0.310328638	0.117589004	2.639095736	0.00831275	0.468007836
Atp6ap1	-0.193273095	0.104187315	-1.855054004	0.063588515	0.729199878
Gdi1	-0.089235335	0.130891164	-0.681752171	0.495395673	0.959696861
Fam50a	0.049662511	0.122702796	0.404738215	0.685669949	0.98538669
Plxna3	-0.170789101	0.598986568	-0.285130101	0.775544469	0.990861469
Lage3	0.275051059	0.209512547	1.312814257	0.189245545	0.875548883
Ubl4a	-0.084905032	0.144640786	-0.587006156	0.557199572	0.968926429
Slc10a3	-0.276798079	0.282559914	-0.979608451	0.327279432	0.916882624
Fam3a	-0.112683139	0.156073446	-0.721987897	0.470301919	0.95486913
Ikbkg	0.032022222	0.151407934	0.211496331	0.832499995	0.992886758
G6pdx	-0.500621438	0.231255761	-2.164795533	0.030403347	0.657477414
Gab3	0.10741227	0.518055767	0.207337272	0.835746468	0.992886758
Dkc1	-0.04556757	0.134065762	-0.339889691	0.733939601	0.988585656
Mpp1	0.102349242	0.124969554	0.818993414	0.412790171	0.942708418
F8	-0.916117319	0.657302749	-1.393752454	0.16339237	0.859982992
Fundc2	0.244012905	0.16053142	1.520032061	0.128502918	0.826336015
Cmc4	0.090032391	0.289149563	0.311369626	0.755519642	0.989266039
Mtcp1	-0.004631206	0.323922181	-0.01429728	0.98859281	0.998182772
Brcc3	-0.0526052	0.134651955	-0.390675354	0.696037218	0.987418513
Vbp1	0.148391568	0.150293947	0.987342279	0.323474871	0.91640698
Rab39b	0.430192874	0.384678854	1.118316926	0.263431661	0.897248195
4933407K13Rik	-0.821644565	0.507930568	-1.617631655	0.105742003	0.79419908
Pls3	-0.094188255	0.191880654	-0.49086895	0.623519139	0.975691835
Tbl1x	-0.218679633	0.30523683	-0.716426102	0.473728282	0.955974667
Prkx	-0.383109232	0.216926079	-1.766081946	0.077382102	0.753829104
Prrg1	0.073131765	0.156226485	0.468113744	0.639703251	0.977843986
Gm8787	-0.591975893	0.700328167	-0.845283569	0.397952533	0.935327115
Gm7173	-2.778858233	1.363218546	-2.038453952	0.041504555	0.679118604
Tmem47	0.064520858	0.135788583	0.47515672	0.634675273	0.977034429
Dmd	0.010772578	0.174184098	0.061845934	0.950685524	0.99573416
Tab3	-0.563539271	0.254779148	-2.211873597	0.0269754	0.634942608
Gyk	-0.135394855	0.168667886	-0.802730496	0.422130523	0.943415794
5430427O19Rik	0.313558568	0.381545018	0.82181277	0.411183465	0.941701842
Pola1	0.150494489	0.331779421	0.453598021	0.650118178	0.979004493
Pcyt1b	-0.570998037	0.590357085	-0.967207901	0.333440113	0.917554801
Pdk3	-0.098557413	0.13640238	-0.72254907	0.469956968	0.95486913
Zfx	-0.15388039	0.114124384	-1.34835681	0.177543648	0.866855369
Eif2s3x	0.057260993	0.119016839	0.481116731	0.630433537	0.977034429
Klhl15	0.053076076	0.316920011	0.167474676	0.866996571	0.992886758
Apoo	-0.4522521	0.449571939	-1.005961584	0.31443405	0.91586731
Maged1	-0.385639017	0.144427179	-2.670127745	0.007582239	0.454518821

Gspt2	0.850373663	0.628672266	1.352650196	0.176167408	0.865308446
Zxdb	0.129861451	0.247423901	0.52485411	0.599684612	0.974001549
Zxda	-0.242290402	0.368715361	-0.657120446	0.511103473	0.963452765
Spin4	0.290764145	0.215828747	1.347198409	0.17791634	0.867377413
Arhgef9	0.008844301	0.335405156	0.026369006	0.978963015	0.997262526
Amer1	-0.26243798	0.258689431	-1.01449054	0.310348741	0.914704835
Asb12	-2.934607492	2.338639804	-1.254835177	0.2095386	0.884258007
Zc4h2	0.090077906	0.248403009	0.362628078	0.716882733	0.988585656
Zc3h12b	-0.847408674	1.071722528	-0.790697828	0.429120344	0.945321484
Las1l	-0.040262335	0.192152893	-0.209532805	0.834032329	0.992886758
Msn	-0.198365097	0.16898727	-1.173846391	0.240456558	0.896939594
F630028O10Rik	-0.036465467	0.331481736	-0.110007469	0.912403452	0.994688009
Vsig4	-1.105931238	1.78574894	-0.619309475	0.535712504	0.96674935
Hsf3	4.468731456	1.612816701	2.770762142	0.005592526	0.416936884
Heph	-0.25846418	0.305424307	-0.846246269	0.397415375	0.935327115
Eda2r	-0.105474855	0.289457708	-0.364387792	0.715568451	0.988585656
Ar	0.48629564	0.266876518	1.822174704	0.068428481	0.743426713
Ophn1	0.011909214	0.284701254	0.041830563	0.96663377	0.996474415
Yipf6	-0.093188102	0.128316752	-0.726234889	0.467694787	0.953657709
Stard8	-0.124910505	0.179133815	-0.69730277	0.485613335	0.957710647
Efnb1	-0.166120233	0.182287853	-0.911307205	0.362133531	0.926455466
Gm14812	-0.952525827	1.649921766	-0.577315753	0.563726174	0.969658675
Pja1	-0.081085103	0.10590596	-0.765633049	0.443894674	0.948476747
Tmem28	0.285210548	1.12758274	0.252939796	0.800314736	0.990988839
Eda	0.167265838	0.513901214	0.325482473	0.744815945	0.988585656
Awat2	-0.061648012	0.30618453	-0.201342675	0.840430636	0.992886758
Igbp1	0.292916528	0.119145891	2.458469412	0.013953066	0.545277966
Pdzd11	0.146161701	0.12577243	1.162112404	0.24518981	0.896939594
Kif4	0.133764147	0.539346085	0.248011714	0.804125343	0.990988839
Gdpd2	0.171619743	0.261455008	0.656402585	0.511565128	0.963452765
Dlg3	-0.053991285	0.174571528	-0.309278871	0.757109402	0.989266039
Tex11	0.044674712	0.275152694	0.162363345	0.871019734	0.992886758
Slc7a3	0.113537886	0.90772921	0.125079026	0.900460987	0.994688009
Snx12	0.057996103	0.143078675	0.405344143	0.685224563	0.985349783
Foxo4	-0.050186004	0.179237921	-0.279996574	0.779480133	0.990988839
Gm614	-2.008101493	1.806045524	-1.111877561	0.266190798	0.89768067
Il2rg	0.222066437	0.222785795	0.996771078	0.31887564	0.91640698
Med12	0.057687755	0.356029241	0.162030947	0.871281483	0.992886758
Nlgn3	-0.28184435	1.344302666	-0.209658403	0.833934293	0.992886758
Gjb1	-0.279668596	0.324135005	-0.86281516	0.388239103	0.933269543
Zmym3	-0.263549792	0.2143698	-1.229416604	0.218915648	0.888671255
Nono	-0.071457176	0.134160078	-0.532626223	0.594292346	0.974001549
Itgb1bp2	0.303634211	0.40152009	0.756211754	0.449522247	0.948476747
Taf1	0.037499526	0.160322697	0.233900292	0.815062387	0.992210229
Ogt	-0.02943709	0.1381288	-0.213113342	0.831238558	0.992886758
Cxcr3	-0.249846854	0.744193579	-0.335728312	0.737075754	0.988585656
Nhsl2	-0.03243031	0.429386683	-0.075527052	0.939795375	0.994688009
Rgag4	-0.346849624	0.352494433	-0.983986105	0.325122345	0.91640698
Pin4	0.159483233	0.151054911	1.055796407	0.29106124	0.907334416
Ercc6l	-0.062740383	0.615241099	-0.101976905	0.918775007	0.994688009
Rps4x	0.070004792	0.089031054	0.786296346	0.431693893	0.945955639
Hdac8	0.189184859	0.244780099	0.772876797	0.439595298	0.947849924
Phka1	-0.217886961	0.196903968	-1.106564599	0.26848222	0.89804647
Dmrtc1a	-0.111566657	0.819989938	-0.136058568	0.891774981	0.993043931
Gm5126	-0.825065454	1.376684312	-0.599313471	0.548963866	0.968926429
Nap1l2	-0.370421388	1.018880542	-0.363557231	0.716188669	0.988585656
Chic1	0.731496958	0.392281429	1.864724924	0.062219971	0.726656275
Tsix	1.707003393	1.210210513	1.41050121	0.158391739	0.855986074
Xist	-0.465875855	0.221984525	-2.098686176	0.035844574	0.674795441
Jpx	0.213116053	0.313874769	0.678984342	0.497147783	0.960475196
Ftx	0.518086561	0.281455828	1.840738438	0.065659898	0.737296531
Slc16a2	0.066407372	0.242717513	0.273599423	0.784392477	0.990988839

Rlim	-0.017242562	0.144680559	-0.119176774	0.905135307	0.994688009
C77370	-0.412509066	0.290073931	-1.422082517	0.155002297	0.854973821
Abcb7	0.003197489	0.127705731	0.025037941	0.9800247	0.997296835
Uprt	-0.471522116	0.405128398	-1.16388315	0.244471369	0.896939594
Zdhhc15	-0.114871152	0.310089214	-0.370445493	0.711050582	0.988585656
Magee2	-1.24618889	2.111121655	-0.590297052	0.554991515	0.968926429
5530601H04Rik	0.269522292	0.21132015	1.275421638	0.202160044	0.881588418
Pbdc1	-0.042178771	0.116590596	-0.361768209	0.717525251	0.988585656
Magee1	-0.320936265	0.482580681	-0.665041676	0.506023837	0.96224682
Fgf16	-0.188162668	0.917113041	-0.205168457	0.837440512	0.992886758
Atrx	-0.122339295	0.203903052	-0.59998756	0.548514526	0.968887406
Magt1	-0.09685257	0.114924331	-0.84275078	0.399367841	0.936273856
Cox7b	0.344488773	0.250618098	1.374556648	0.169268937	0.861597949
Atp7a	-0.155000268	0.163405785	-0.948560466	0.342844206	0.919850248
Tlr13	0.113415637	0.326644496	0.347214289	0.728430336	0.988585656
Pgk1	-0.069311001	0.115436051	-0.600427689	0.548221239	0.968738549
Taf9b	-0.019806899	0.193033295	-0.102608717	0.918273524	0.994688009
Cysltr1	-0.383436835	0.310563266	-1.234649674	0.216960913	0.887633697
Gm5127	1.346078249	0.984410613	1.3673951	0.171501497	0.86244803
Zcchc5	-1.250466992	2.207805306	-0.56638463	0.571132341	0.971613657
Lpar4	-0.369737312	0.432918107	-0.854058322	0.393072675	0.933734231
P2ry10	-0.095040899	0.198227746	-0.479453059	0.631616355	0.977034429
A630033H20Rik	0.171448615	0.289555	0.592110704	0.55377646	0.968926429
Gpr174	0.559743513	0.278567815	2.009361752	0.044498783	0.689308953
Itm2a	-0.340736552	0.192749667	-1.767767271	0.077099819	0.752876039
2610002M06Rik	-0.058298976	0.138503317	-0.420921151	0.67381266	0.983375962
Brwd3	-1.474960964	0.50697611	-2.90933031	0.00362204	0.374400632
2810403D21Rik	0.331676614	0.318309622	1.041993678	0.29741461	0.909070179
Hmgn5	-0.272990142	0.241059311	-1.132460474	0.257440893	0.897248195
Sh3bgrl	0.052165626	0.12930281	0.403437685	0.686626269	0.98538669
Gm6377	0.790299192	0.528936555	1.49412852	0.135142031	0.829809681
Rps6ka6	0.335068669	0.442028709	0.758024675	0.448436211	0.948476747
Hdx	0.653258013	0.831806323	0.785348699	0.432249151	0.9459793
4933403O08Rik	-0.66122443	1.375064142	-0.480868063	0.630610273	0.977034429
Apool	0.090924876	0.150572809	0.603859865	0.545936815	0.96778188
Zfp711	0.478747212	0.453945722	1.054635365	0.291592123	0.907334416
Pof1b	0.031159988	0.155278302	0.200671875	0.840955153	0.992886758
Chm	-0.229894235	0.154025698	-1.49257064	0.135549613	0.831062162
Dach2	1.257327554	1.716524642	0.732484418	0.46387295	0.953083512
Klhl4	-0.047976885	0.24825555	-0.193256041	0.846758451	0.992886758
Pcdh11x	0.578872535	1.422539147	0.406929072	0.684060083	0.985105247
Nap1l3	-0.473837277	0.352184183	-1.345424638	0.178488143	0.867377413
Cldn34c1	0.769685185	0.517879858	1.486223442	0.137220029	0.833085238
Diap2	-0.618943789	0.276845408	-2.235701843	0.025371308	0.631459375
Pcdh19	1.539906242	0.535491531	2.875687386	0.004031488	0.378480793
Tspan6	0.131395251	0.18635928	0.705064168	0.480770304	0.957463006
Srpx2	0.466683036	0.371530762	1.256108737	0.209076549	0.884258007
Sytl4	-0.081048375	0.194719401	-0.416231635	0.677240513	0.983670411
Cstf2	-0.104444868	0.145923557	-0.715750567	0.474145381	0.955974667
Nox1	0.682462713	0.818597428	0.833697602	0.40445141	0.938127315
Xkrx	-0.878529526	1.140980311	-0.769977814	0.441313053	0.948068187
Trmt2b	0.163977602	0.119389089	1.373472261	0.169605579	0.861597949
Tmem35	0.039120198	0.267753221	0.146105425	0.883838163	0.992886758
Cenpi	0.259049369	0.32555017	0.795727951	0.426190177	0.944290206
Timm8a1	0.167856019	0.271117036	0.619127525	0.535832352	0.96674935
Btk	0.075759851	0.261785277	0.289396914	0.772277656	0.99050004
Rpl36a	0.246342483	0.158747065	1.551792363	0.120711914	0.816092513
Gla	-0.104973835	0.203270327	-0.516424785	0.605557759	0.974001549
Hnrnph2	-0.164822069	0.141644969	-1.163628119	0.244574751	0.896939594
Armcx4	0.773465397	0.247005957	3.131363334	0.001739967	0.284736028
Armcx1	0.163617981	0.160464873	1.019649837	0.30789456	0.9139204
Armcx6	0.190578539	0.239557731	0.795543264	0.426297555	0.944290206

Armcx3	-0.087551545	0.162920704	-0.53738747	0.590999997	0.974001549
Armcx2	0.029225697	0.202468488	0.144346894	0.88522655	0.992886758
Nxf2	1.861314127	1.113949776	1.670913866	0.094738692	0.777748075
Zmat1	-0.127380101	0.186576445	-0.682723375	0.494781656	0.959190228
AV320801	1.710667005	2.255312319	0.758505592	0.448148367	0.948476747
Nxf7	-0.717121216	0.97063337	-0.738817805	0.460017638	0.953083512
Armcx5	0.370976565	0.377850452	0.981807918	0.326194487	0.916800143
Gprasp1	-0.027566562	0.257631168	-0.107000104	0.914788897	0.994688009
Gprasp2	0.203263361	0.488923963	0.41573614	0.677603093	0.983670411
Bhlhb9	0.234546394	0.239262258	0.980289979	0.326943	0.916882624
Arxes2	0.151847863	0.218889018	0.693720795	0.487857322	0.958288569
Arxes1	0.68042501	0.787376118	0.864167701	0.387495772	0.932858077
Bex2	0.736728688	0.185611003	3.969208056	7.21E-05	0.040598999
Bex4	0.621653079	0.176665064	3.518822944	0.000433466	0.139568962
Tceal8	0.008697424	0.123959071	0.070163679	0.944063383	0.99486515
Tceal5	-1.333003706	2.065206018	-0.645457981	0.51863044	0.96446859
Bex1	0.396393302	0.468817834	0.845516687	0.39782242	0.935327115
Wbp5	0.055658276	0.125583307	0.443198046	0.657622492	0.979614766
Ngfrap1	0.174149481	0.154425768	1.127722943	0.259436945	0.897248195
Tceal3	0.476776122	0.750813679	0.635012567	0.525420219	0.965637248
Tceal1	0.07384462	0.178791084	0.413021825	0.679590623	0.983903958
Morf4l2	-0.039028395	0.078855772	-0.494933904	0.620646777	0.975058198
BC065397	0.159563737	0.371728485	0.429248076	0.6677427	0.981863224
Plp1	0.345763028	0.43812621	0.7891859	0.430003362	0.945321484
Tmsb15b1	3.544679131	1.802430242	1.966610994	0.049228075	0.707153766
Slc25a53	0.198088078	0.287457303	0.689104351	0.490757602	0.958896244
Zcchc18	0.465231527	0.486523843	0.956235822	0.338953106	0.919246575
Fam199x	-0.120693994	0.41767831	-0.288963998	0.772608929	0.990678393
Serpina7	-0.342511118	1.157305631	-0.295955631	0.767263966	0.989884648
Mum1l1	-0.305741368	0.26586972	-1.149966863	0.25015752	0.896939594
Platr21	-0.124627192	0.366613225	-0.339941889	0.73390029	0.988585656
D330045A20Rik	-0.632156047	0.979206603	-0.645579845	0.518551494	0.96446859
Rnf128	-0.07756123	0.179431067	-0.432261987	0.665551012	0.981727712
Tbc1d8b	-0.086469064	0.155213748	-0.557096685	0.577461372	0.971613657
Cldn2	0.383140606	0.866774123	0.442030509	0.658467129	0.979746192
Morc4	-0.434637378	0.401257387	-1.083188477	0.278724773	0.90176568
Rbm41	-0.373750623	0.18265446	-2.046216792	0.040735046	0.679118604
Nup62cl	-0.579125283	0.385468676	-1.502392593	0.132995748	0.827800491
Pih1h3b	-0.188435428	0.263484819	-0.715166167	0.474506372	0.956387791
Frmpd3	-2.900174875	3.664167668	-0.791496225	0.428654478	0.945321484
Prps1	-0.09763836	0.147362928	-0.662570709	0.507605535	0.963011338
Tsc22d3	-0.327226539	0.341484018	-0.958248473	0.33793748	0.919246575
Mid2	-0.320159266	0.183078668	-1.748752437	0.080333822	0.75763999
Vsig1	0.974516706	1.208514004	0.806376014	0.420026073	0.943415794
Psmd10	0.164965258	0.183526375	0.898864037	0.368725086	0.928436166
Atg4a	0.140661535	0.135061184	1.04146529	0.297659654	0.909070179
Col4a6	-0.085825182	0.344613801	-0.249047431	0.803324093	0.990988839
Col4a5	-0.046668834	0.122868717	-0.37982682	0.704073973	0.988585656
Nxt2	0.162052088	0.136302931	1.188911249	0.234474593	0.89581803
Acsl4	-0.276526318	0.148795309	-1.858434382	0.063107354	0.727650961
Tmem164	0.056707245	0.184294456	0.307699136	0.758311277	0.989266039
Ammecr1	-0.516643567	0.538875827	-0.958743258	0.3376881	0.919246575
Chrdl1	-0.15669582	0.20688861	-0.7573922	0.448814928	0.948476747
Pak3	-0.20211955	0.67440727	-0.299699543	0.764406347	0.989417128
Capn6	-1.645491411	0.737439595	-2.231357554	0.025657454	0.631530315
A730046J19Rik	-0.823340397	0.607060285	-1.35627452	0.175011839	0.864111192
Alg13	-0.109012048	0.178930705	-0.609241705	0.542364241	0.967491297
Lhfpl1	-0.095969204	1.294871493	-0.074114848	0.940919	0.994688009
Amot	0.115992939	0.41404568	0.280145269	0.779366055	0.990988839
Htr2c	-0.268081408	0.51129668	-0.524316738	0.600058257	0.974001549
Il13ra2	-1.469090567	1.639725787	-0.895936734	0.370286553	0.928436166
Lrch2	0.27054867	0.497374478	0.543953664	0.586473359	0.972904784

Tmem29	0.039082462	0.226366467	0.172651287	0.862925536	0.992886758
Alas2	0.440773485	0.215263984	2.04759513	0.040599687	0.679118604
Apex2	-0.484547898	0.400579197	-1.209618228	0.22642542	0.89398447
Pfkfb1	-0.042185548	0.376445236	-0.112062908	0.910773527	0.994688009
Tro	0.20916003	0.793561571	0.263571268	0.7921103	0.990988839
Maged2	-0.100943973	0.098119376	-1.028787356	0.303579609	0.911786415
Gnl3l	-0.335949012	0.170489346	-1.970498561	0.04878126	0.707153766
Fgd1	0.65998207	0.809783762	0.815010255	0.41506644	0.943415794
Tsr2	-0.074624762	0.132265286	-0.564205197	0.572614491	0.971613657
Fam120c	-0.048528524	0.313652154	-0.154720837	0.877041402	0.992886758
Phf8	-0.011435597	0.269054327	-0.04250293	0.966097776	0.996474415
Huwe1	0.01796883	0.119963026	0.149786405	0.880933135	0.992886758
Hsd17b10	0.119612691	0.182841536	0.654187736	0.512990862	0.964264541
Ribc1	-0.247658535	0.32279563	-0.767230135	0.442944699	0.948476747
Smc1a	0.10125629	0.207159205	0.488784893	0.624993994	0.976148792
Iqsec2	-0.020093631	0.186110575	-0.107966087	0.914022594	0.994688009
Kdm5c	0.027550106	0.181940823	0.151423442	0.879641699	0.992886758
Kantr	-0.381820553	0.381658557	-1.000424453	0.317105141	0.91640698
Tspyl2	-0.17566437	0.181852151	-0.965973562	0.334057411	0.918030088
Gpr173	0.351705795	0.948542518	0.370785482	0.710797316	0.988585656
3010001F23Rik	1.001453152	0.959196834	1.044053854	0.296460474	0.909005508
Shroom2	-0.147524952	0.366133493	-0.402926679	0.687002164	0.98538669
Mageh1	0.186491398	0.144261688	1.292729902	0.196104492	0.879755417
9530051G07Rik	0.070702849	0.68463194	0.103271327	0.917747632	0.994688009
Rragb	-0.20000291	0.363800781	-0.549759432	0.582484387	0.972017845
Klf8	-0.334524661	0.323745271	-1.033295901	0.301465445	0.911518274
Ubqln2	0.067798637	0.233055186	0.290912374	0.771118338	0.989990452
Kctd12b	-0.338616988	0.238631768	-1.418993749	0.155900838	0.855455553
2210013O21Rik	-0.090791763	0.164106813	-0.55324798	0.580093616	0.971613657
Spin2c	0.580710854	0.680766547	0.853024956	0.393645465	0.933796635
Sat1	0.216012096	0.185988766	1.161425504	0.245468902	0.896939594
Acot9	0.041269296	0.142607958	0.289389853	0.772283059	0.99050004
Prdx4	-0.136271289	0.131397818	-1.037089439	0.299694177	0.910105802
Ptchd1	2.76344653	1.230882265	2.245094116	0.024762091	0.627796923
Phex	0.530331434	0.338871433	1.564993042	0.117584541	0.813475762
Sms	0.073213938	0.134616862	0.543869001	0.586531622	0.972904784
Mbtps2	-0.224500975	0.16944904	-1.324887854	0.18520835	0.871716376
Yy2	-0.265332515	0.286961369	-0.924627994	0.355159446	0.924354588
Smpx	-0.072965889	0.319837307	-0.228134392	0.819541764	0.992886758
Cnksr2	-0.240623778	0.49892605	-0.482283453	0.629604601	0.977034429
Rps6ka3	-0.281281039	0.155440415	-1.809574683	0.070361769	0.744227652
Eif1ax	0.242551669	0.15733246	1.541650522	0.123158541	0.818400287
A830080D01Rik	0.020202017	0.222217295	0.090911093	0.927563236	0.994688009
Sh3kbp1	0.006888293	0.329682097	0.020893743	0.983330418	0.997628517
Map3k15	0.307890134	0.512962298	0.600219812	0.548359752	0.968738549
Pdha1	0.076426766	0.108057739	0.707277116	0.479394284	0.957463006
Adgrg2	-0.260095333	0.598170487	-0.434818064	0.663694492	0.981154469
Phka2	-0.001994834	0.303892238	-0.00656428	0.9947625	0.999285699
Rs1	-0.346838145	2.030541114	-0.170810698	0.864372617	0.992886758
Cdkl5	-0.520089375	0.303991616	-1.710867493	0.087105577	0.765418158
Gja6	2.084108573	1.295845083	1.608300714	0.107769334	0.796929372
Scml2	1.227817474	1.479324812	0.829985047	0.406547238	0.939557159
Rai2	0.063137746	0.349499443	0.180651923	0.856640798	0.992886758
Reps2	-0.040419191	0.127959339	-0.315875272	0.752097182	0.988585656
Rbbp7	-0.068184776	0.117228908	-0.581637905	0.560810605	0.968926429
Txlng	-0.22155425	0.182484911	-1.214096273	0.224710963	0.892989918
Syap1	0.02011493	0.087020111	0.23115266	0.817196206	0.992678823
Ctps2	-0.085405307	0.15021956	-0.568536529	0.569670709	0.97144955
S100g	0.193093895	0.255270215	0.756429395	0.449391789	0.948476747
Rnf138rt1	0.257039868	0.651785896	0.394362427	0.693313479	0.986698113
Ap1s2	-0.107823868	0.133231372	-0.809297883	0.418343823	0.943415794
Zrsr2	-0.086520477	0.155657893	-0.55583739	0.578322022	0.971613657

Car5b	0.040956016	0.205744903	0.199062115	0.842214156	0.992886758
Siah1b	-0.116207038	0.27111316	-0.428629278	0.668193035	0.981863224
Tmem27	-0.0049668	1.449093771	-0.003427521	0.997265239	0.99957296
Ace2	-0.058532119	0.143975908	-0.406541066	0.68434509	0.985105247
Bmx	-0.425615871	0.194821045	-2.184650386	0.028914491	0.64858361
Pir	0.191206517	0.144780862	1.32066155	0.18661424	0.872512868
Figf	0.021283521	0.159553643	0.133394139	0.89388168	0.993422442
Piga	0.053715719	0.22732581	0.236293976	0.813204561	0.992048071
Asb11	-0.404031326	0.568056694	-0.711251765	0.476928236	0.95716557
Mospd2	-0.218578124	0.117440878	-1.861175828	0.062719351	0.727650961
Fancb	-0.116829847	0.25438544	-0.459263105	0.646045239	0.979004493
Gemin8	0.178284535	0.252782626	0.70528793	0.48063107	0.957463006
Gpm6b	-0.067414114	0.180078982	-0.374358591	0.708137546	0.988585656
Ofd1	0.159933951	0.174312435	0.917513149	0.358873806	0.925199968
Trappc2	0.024008364	0.153805811	0.156095296	0.875957906	0.992886758
Rab9	0.144982303	0.193828888	0.747991202	0.454465464	0.951045582
Tceanc	0.208299033	0.222706843	0.935305939	0.349630656	0.921902408
Egfl6	-0.172971984	0.231771203	-0.746304899	0.455483253	0.951224746
Gm8817	0.587391658	1.710896653	0.343323869	0.731354826	0.988585656
Tmsb4x	0.141997393	0.22990863	0.617625331	0.536822343	0.96674935
Tlr8	-0.57654911	0.371047994	-1.553839716	0.120222654	0.81558552
Tlr7	0.157761053	0.335336151	0.470456443	0.638028946	0.977764089
Prps2	0.092791725	0.139167911	0.666760923	0.504924857	0.962138588
Msl3	-0.153597158	0.110558232	-1.389287398	0.164745374	0.859982992
Arhgap6	-0.379001611	0.183338306	-2.067225446	0.038712916	0.679118604
Hccs	-0.064390291	0.11627283	-0.553786217	0.579725162	0.971613657
Mid1	-1.436752678	0.380641648	-3.774554589	0.000160294	0.077321054
G530011O06Rik	-1.275234694	0.675899031	-1.88672366	0.059197504	0.72285319
