# Supplementary material for: Aspirin prevents metastasis by limiting platelet TXA2 suppression of T cell immunity
Source: Nature. 2025 Mar 5;640(8060):1052–61. doi: 10.1038/s41586-025-08626-7 (PMC12018268; doi:10.1038/s41586-025-08626-7)
Supplement: Supplementary file 4 — Differentially expressed genes in TXA2 analogue-treated CD8 T cells (Arhgef1 KO versus WT) [file 41586_2025_8626_MOESM4_ESM.pdf]

Suppementary Table 2. Expression of genes significantly up- or down-regulated between TXA2 analogue U46619 treated Arhgef 1 KO vs WT CD8 T cells. Naïve WT and Arhgef1 KO CD8+ T cells were stimulated in vitro with anti-CD3/28 antibodies and rhIL-2 in the presence of TXA2 analogue or vehicle control for 5 days. Data are representative of three to four biological replicates per group.

Gene name	WT_d5_Veh_1	WT_d5_Veh_2	WT_d5_Veh_3	WT_d5_Veh_4	KO_d5_Veh_1	KO_d5_Veh_2	KO_d5_Veh_3	WT_d5_U46619_1	WT_d5_U46619_2	WT_d5_U46619_3	WT_d5_U46619_4	KO_d5_U46619_1	KO_d5_U46619_2	KO_d5_U46619_3	KO_d5_U46619_4	Cluster
B3gat2	1.831392963	0	2.349483883	0	3.800179466	1.402825611	0.902064715	0	12.79104008	0.873901969	8.512836289	0.867528106	4.260421576	0.903554652	2.874262688	1
Igfbp2	0.915696482	0	3.915806472	8.626094197	3.166816221	2.805651223	1.80412943	14.24725822	18.05793894	3.495607876	26.48437957	0	0	7.228437213	2.155697016	1
Ptprn	31.13368037	30.10595632	19.57903236	23.29045433	11.4005384	14.02825611	13.53097072	71.23629111	50.41174621	46.31680435	56.75224193	14.74797781	5.112505891	12.64976512	20.83840449	1
Serpine2	401.9907554	505.4631614	513.7538092	484.7864938	252.7119345	226.5563362	219.2017257	1086.981995	983.4052582	1064.412598	799.2607405	378.2422543	357.8754124	313.5334641	344.9115225	1
Htr2b	1.831392963	1.584524017	0	1.725218839	0	0.701412806	1.80412943	4.190370065	10.53379772	5.243411813	6.621094891	6.072696744	0.852084315	0.903554652	2.874262688	1
Tnfrsf11a	17.39823315	26.14464628	20.36219366	19.84001665	5.700269198	4.208476834	8.118582433	36.87525657	35.36346376	55.05582404	45.40179354	8.675281063	14.48543336	4.517773258	4.311394032	1
Cd55	14.65114371	36.44405239	42.2907099	51.75656518	39.90188439	41.38335554	49.61355931	107.2734737	74.48899814	83.02068705	90.80358708	54.6542707	21.30210788	33.43152211	55.32955674	1
Phlda3	304.0112319	318.4893274	393.9301311	426.1290533	259.0455669	276.3566455	268.815285	1226.102281	1003.72044	885.2626945	821.0157665	236.835173	259.0336318	345.1578769	268.0249956	1
Pla2g4a	0	0	0	0	3.800179466	0	1.80412943	4.190370065	0	4.369509845	6.621094891	0.867528106	0.852084315	0	0.718565672	1
Tnfsf4	215.1886732	246.3934846	238.8641948	260.5080447	154.5406316	185.1729807	185.8253312	613.4701776	501.1078056	522.5933774	400.1033056	255.9207914	234.3231867	199.685578	217.0068329	1
Fam78b	0	0.792262009	0	0	5.066905954	2.104238417	5.412388289	7.542666117	1.504828245	7.86511772	1.891741398	11.27786538	4.260421576	2.710663955	4.311394032	1
Marcks	39.37494871	34.85952837	75.18348427	56.9322217	41.16861088	49.80030921	40.59291217	217.8992434	133.1772997	171.2847859	114.4503546	62.46202365	46.86463733	30.72085815	40.9582433	1
Gja1	8.241268334	2.376786026	8.614774239	8.626094197	12.03390164	11.22260489	7.216517718	34.36103454	7.524141226	38.45168663	22.70089677	5.205168638	3.408337261	4.517773258	1.437131344	1
Chst3	2.747089445	6.338096068	4.698967767	11.21392246	11.4005384	9.118366474	4.510323574	20.95185033	15.04828245	9.612921658	9.458706988	5.205168638	9.372927467	9.035546516	6.467091048	1
Col6a2	32.96507334	41.98988645	54.82129061	37.09220505	5.700269198	11.22260489	6.314453003	77.1028092	72.98416989	104.8682363	81.34488009	19.08561834	16.18960199	12.64976512	26.58692986	1
Kcnmb4os2	0	0	0	1.725218839	1.900089733	5.611302446	0.902064715	6.704592104	0	2.621705907	4.729353494	3.470112425	7.668758836	4.517773258	0.718565672	1
Coro6	5.494178889	7.130358077	12.53058071	5.175656518	5.066905954	4.90988964	6.314453003	25.14222039	21.06759543	35.82998073	20.80915537	9.542809169	7.668758836	9.939101167	5.748525376	1
Ccl1	215.1886732	291.5524191	283.5043886	336.4176737	437.0206386	394.1939968	314.8205855	1207.664653	582.3685309	525.2150833	585.4939625	377.3747262	366.3962555	403.8889293	380.1212405	1
Ccl9	20.14532259	12.67619214	28.9769679	45.71829924	22.16771355	25.25086101	25.25781201	173.4813207	54.92623095	58.55143192	88.91184568	49.44910206	23.85836082	43.37062328	18.68270747	1
Wnt3	6.409875371	16.63750218	8.614774239	16.38957897	4.43354271	3.507064029	4.510323574	46.93214473	18.81035306	29.71266694	17.97154328	6.94022485	9.372927467	13.55331977	5.748525376	1
Cacng4	0	0	0	1.725218839	1.900089733	0	0	0	6.771727103	9.612921658	6.621094891	0	1.70416863	3.614218606	1.437131344	1
Abca5	25.63950148	35.65179038	30.54329049	27.60350143	14.56735462	30.16075065	9.922711863	87.15969736	84.27038173	91.75970673	80.3990094	13.8804497	20.45002356	14.45687443	12.21561642	1
Smim5	9.156964816	3.961310043	15.66322589	0.86260942	1.900089733	3.507064029	7.216517718	29.33259046	35.36346376	31.46047088	19.86328467	4.337640531	10.22501178	11.74621047	5.029959704	1
Dcxr	47.61621704	57.83512662	61.08658097	55.20700286	44.96879034	50.50172201	49.61355931	105.5973256	88.03245234	126.7157855	144.7182169	39.03876478	41.75213144	43.37062328	47.42533435	1
Zfp750	5.494178889	7.130358077	9.397935534	3.450437679	1.266726489	1.402825611	3.608258859	42.74177467	39.12553437	23.59535316	5.675224193	4.337640531	2.556252945	7.228437213	1.437131344	1
Fam84a	0.915696482	4.753572051	4.698967767	3.450437679	11.4005384	10.52119209	9.922711863	16.76148026	12.79104008	3.495607876	13.24218978	6.072696744	11.92918041	6.324882561	5.748525376	1
Twist1	8.241268334	10.29940611	7.831612945	3.450437679	15.20071786	18.23673295	17.13922958	95.54043749	44.39243323	52.43411813	25.53850887	26.02584319	3.408337261	14.45687443	5.029959704	1
Trim9	0.915696482	0.792262009	2.349483883	0	0	0	0	5.028444078	2.257242368	6.991215751	10.40457769	0.867528106	0	0	1.437131344	1
Acot1	5.494178889	3.169048034	14.0969033	3.450437679	7.600358931	7.014128057	5.412388289	29.33259046	9.028969471	16.60413741	17.97154328	2.602584319	3.408337261	5.421327909	2.874262688	1
Asb2	12.81975074	17.42976419	10.96425812	17.25218839	17.1008076	17.53532014	11.72684129	71.23629111	33.10622139	15.73023544	92.69532848	7.807752957	4.260421576	10.84265582	10.77848508	1
D430019H16Rik	0.915696482	0.792262009	3.132645178	1.725218839	9.500448664	1.402825611	6.314453003	2.514222039	15.80069657	6.991215751	1.891741398	2.602584319	2.556252945	3.614218606	0.718565672	1
Idi2	32.04937685	17.42976419	29.76012919	14.66436013	38.00179466	17.53532014	29.76813559	72.07436512	57.93588744	73.40776539	45.40179354	39.90629289	26.41461377	34.33507676	25.14979852	1
C230035I16Rik	0.915696482	6.338096068	1.566322589	0.86260942	4.43354271	2.104238417	0.902064715	7.542666117	9.028969471	11.3607256	8.512836289	7.807752957	3.408337261	0	7.18565672	1
Hist1h2ad	1.831392963	0	3.132645178	1.725218839	6.333632443	11.9240177	7.216517718	0	12.79104008	12.23462756	6.621094891	1.735056213	15.33751767	4.517773258	10.05991941	1
Dsp	22.89241204	25.35238427	26.62748401	26.74089201	13.30062813	23.84803539	31.57226502	93.86428946	49.65933209	80.39898114	30.26786236	29.49595561	23.85836082	18.07109303	29.46119255	1
5033403F01Rik	0	0	0	0.86260942	0.633363244	0.701412806	3.608258859	0.838074013	0	6.991215751	0.945870699	0.867528106	0.852084315	0	0.718565672	1
Gm2762	0.915696482	0.792262009	1.566322589	0.86260942	0.633363244	0	1.80412943	6.704592104	3.00965649	1.747803938	3.783482795	0	1.70416863	1.807109303	0	1
Il9	12.81975074	2.376786026	1.566322589	8.626094197	2.533452977	4.208476834	9.020647148	98.05465953	28.59173666	67.29045161	38.78069865	0	0.852084315	1.807109303	2.155697016	1
Mir7-1	0	0.792262009	0.783161294	6.900875357	12.03390164	5.611302446	5.412388289	5.028444078	2.257242368	8.739019689	8.512836289	4.337640531	4.260421576	2.710663955	5.029959704	1
Zfp85os	0.915696482	0.792262009	3.915806472	4.313047098	1.266726489	2.104238417	5.412388289	3.352296052	10.53379772	7.86511772	10.40457769	5.205168638	4.260421576	4.517773258	1.437131344	1
Ahrr	45.78482408	43.57441047	47.77283896	54.34439344	29.76807248	28.75792503	37.88671802	97.21658551	66.21244279	106.6160402	228.9007091	24.29078698	34.08337261	35.23863141	17.9641418	1
Snord69	4.578482408	5.54583406	3.915806472	1.725218839	5.066905954	5.611302446	5.412388289	11.73303618	8.276555348	20.97364725	7.56696559	12.14539349	8.520843152	8.131991864	10.05991941	1
Pnp2	12.81975074	15.84524017	11.74741942	9.488703616	10.76717515	6.312715251	18.94335901	14.24725822	30.0965649	25.3431571	31.21373306	20.82067455	10.22501178	17.16753838	16.52701046	1
Gzmf	0	0	0.783161294	6.038265938	1.900089733	6.312715251	4.510323574	14.24725822	6.771727103	7.86511772	8.512836289	1.735056213	6.816674521	3.614218606	0.718565672	1
Bmp1	8.241268334	9.507144102	10.96425812	8.626094197	12.03390164	9.118366474	15.33510015	54.47481085	21.06759543	15.73023544	22.70089677	11.27786538	3.408337261	3.614218606	10.05991941	1
Enox1	1.831392963	0.792262009	3.132645178	3.450437679	1.266726489	7.014128057	2.706194144	6.704592104	18.81035306	5.243411813	5.675224193	1.735056213	1.70416863	7.228437213	0.718565672	1
Rai14	21.06101908	22.97559825	29.76012919	15.52696955	23.43444004	29.45933784	27.06194144	45.2559967	41.38277674	41.94729451	56.75224193	34.70112425	35.78754124	16.26398373	28.02406121	1
Aard	5.494178889	1.584524017	7.04845165	5.175656518	3.800179466	4.208476834	0	51.96058881	24.82966605	18.35194135	30.26786236	2.602584319	0.852084315	2.710663955	0.718565672	1
Prr5	109.8835778	106.1631091	136.2700652	144.0557731	47.50224332	64.52997813	54.12388289	214.5469473	140.7014409	295.3788655	401.995047	62.46202365	58.79381775	66.86304422	39.52111196	1
Cela1	9.156964816	8.714882094	10.96425812	8.626094197	8.233722176	6.312715251	16.23716487	17.59955427	26.33449429	17.47803938	17.02567258	8.675281063	8.520843152	3.614218606	9.341353736	1
Zfp385a	17.39823315	19.80655021	41.50754861	36.22959563	13.30062813	5.611302446	15.33510015	119.0065099	93.2993512	101.3726284	81.34488009	18.21809023	14.48543336	29.8173035	7.904222392	1
Ypel1	2.747089445	3.169048034	7.04845165	2.587828259	3.800179466	9.118366474	10.82477658	10.05688816	3.762070613	6.117313782	19.86328467	3.470112425	11.92918041	15.36042908	2.874262688	1
Rimbp3	0.915696482	3.961310043	0	0.86260942	5.700269198	6.312715251	6.314453003	7.542666117	4.514484735	5.243411813	4.729353494	0	2.556252945	0.903554652	0.718565672	1
Ccr6	3.662785926	11.88393013	16.44638718	6.900875357	13.93399137	33.66781467	26.15987673	25.9802944	26.33449429	19.22584332	6.621094891	26.89337129	16.18960199	11.74621047	10.05991941	1
Hspa1a	4.578482408	6.338096068	10.18109683	3.450437679	14.56735462	14.02825611	5.412388289	27.65644243	15.80069657	11.3607256	10.40457769	11.27786538	8.520843152	7.228437213	12.9341821	1
Vwa7	0.915696482	0	1.566322589	2.587828259	7.600358931	0	1.80412943	3.352296052	12.03862596	0.873901969	7.56696559	4.337640531	2.556252945	0	1.437131344	1
Apom	1.831392963	1.584524017	0.783161294	0.86260942	1.266726489	1.402825611	4.510323574	10.05688816	1.504828245	5.243411813	3.783482795	1.735056213	0	3.614218606	0	1
Cd70	17.39823315	26.93690829	19.57903236	28.46611085	20.90098706	19.63955856	19.84542373	78.77895723	37.62070613	57.67752995	15.13393118	29.49595561	28.97086672	18.07109303	22.27553583	1
Mir1949	0	2.376786026	2.349483883	5.175656518	1.900089733	5.611302446	8.118582433	3.352296052	9.028969471	5.243411813	18.91741398	6.94022485	3.408337261	4.517773258	7.18565672	1
A930007I19Rik	0	0	0.783161294	0	3.166816221	3.507064029	0	0	1.504828245	6.991215751	1.891741398	3.470112425	0	0	2.155697016	1
Papss2	62.26736075	58.62738863	60.30341968	57.79483112	36.73506817	33.66781467	29.76813559	213.7088733	198.6373284	162.5457662	127.6925443	58.99191123	49.42089028	52.40616979	36.64684927	1
Shtn1	5.494178889	6.338096068	9.397935534	10.35131304	10.13381191	13.32684331	14.43303544	41.90370065	8.276555348	26.21705907	34.99721585	15.61550591	8.520843152	18.97464768	14.37131344	1
Ak1	9.156964816	9.507144102	15.66322589	18.97740723	3.800179466	3.507064029	5.412388289	43.57984868	66.96485691	31.46047088	41.61831075	6.94022485	1.70416863	4.517773258	12.21561642	1
Gm13546	52.19469945	37.2363144	39.15806472	30.19132969	29.76807248	16.13249453	20.74748844	53.63673684	58.68830156	112.733354	106.883389	13.01292159	18.74585493	23.49242094	12.9341821	1
Zak	62.26736075	55.4583406	61.86974226	83.67311371	29.13470924	36.4734659	55.0259476	141.6345082	101.5759065	159.9240603	153.2310532	27.7608994	53.68131186	43.37062328	46.70676868	1
Ell3	3.662785926	1.584524017	1.566322589	4.313047098	0	3.507064029	2.706194144	10.05688816	12.03862596	9.612921658	2.837612096	6.94022485	2.556252945	2.710663955	7.18565672	1
Sulf2	10.98835778	12.67619214	11.74741942	8.626094197	6.966995687	3.507064029	5.412388289	72.07436512	44.39243323	34.08217679	52.02288843	13.01292159	0.852084315	10.84265582	5.748525376	1
Car2	31.13368037	41.98988645	35.24225825	31.05393911	24.70116653	14.72966892	9.020647148	109.7876957	31.60139315	73.40776539	89.85771638	29.49595561	21.30210788	23.49242094	12.9341821	1
Ccdc39	0.915696482	6.338096068	5.482129061	3.450437679	2.533452977	4.90988964	1.80412943	10.05688816	18.05793894	6.991215751	10.40457769	4.337640531	4.260421576	11.74621047	1.437131344	1
Trim2	3.662785926	17.42976419	5.482129061	7.763484777	12.03390164	8.416953669	1.80412943	29.33259046	17.30552482	20.97364725	17.02567258	17.35056213	10.22501178	13.55331977	3.59282836	1
Fhdc1	14.65114371	7.922620085	10.18109683	9.488703616	5.066905954	5.611302446	8.118582433	34.36103454	52.66898858	27.964863	30.26786236	22.55573076	3.408337261	16.26398373	5.748525376	1
Fam160a1	1.831392963	2.376786026	0	0.86260942	0.633363244	1.402825611	0.902064715	12.5711102	7.524141226	3.495607876	7.56696559	0	1.70416863	0.903554652	2.155697016	1
Fcrl1	7.325571853	9.507144102	10.96425812	7.763484777	5.700269198	5.611302446	5.412388289	86.32162334	31.60139315	39.3255886	35.94308655	6.94022485	6.816674521	4.517773258	8.622788064	1
Sort1	27.47089445	22.18333624	34.45909696	13.80175071	10.76717515	24.5494482	24.3557473	48.60829276	73.73658401	42.82119648	46.34766424	29.49595561	17.89377062	7.228437213	15.08987911	1
Npnt	8.241268334	7.130358077	3.915806472	4.313047098	10.76717515	8.416953669	6.314453003	27.65644243	11.28621184	21.84754922	11.35044839	11.27786538	4.260421576	6.324882561	10.05991941	1
Ddit4l	0	0	0.783161294	0.86260942	1.266726489	0	0	6.704592104	6.771727103	7.86511772	11.35044839	0.867528106	1.70416863	1.807109303	0.718565672	1
Col27a1	11.90405426	26.14464628	24.27800013	8.626094197	13.93399137	8.416953669	6.314453003	53.63673684	38.37312025	42.82119648	47.29353494	21.68820266	11.92918041	8.131991864	11.49705075	1
Ppap2b	32.96507334	30.10595632	32.89277437	44.85568982	17.1008076	16.83390734	10.82477658	80.45510525	55.67864507	87.39019689	81.34488009	16.48303402	11.0770961	23.49242094	28.02406121	1
Azin2	0.915696482	7.922620085	3.132645178	3.450437679	2.533452977	3.507064029	2.706194144	31.8468125	9.028969471	12.23462756	7.56696559	1.735056213	3.408337261	5.421327909	2.155697016	1
Tnfrsf8	136.4387758	109.3321572	85.3645811	76.77223835	26.60125626	37.87629151	45.10323574	192.757023	188.1035306	254.305473	199.5787174	30.36348372	46.86463733	28.0101942	23.71266717	1
9230114K14Rik	34.7964663	65.75774671	57.1707745	57.79483112	44.3354271	30.16075065	36.08258859	124.0349539	130.1676432	90.0119028	112.5586132	44.24393342	39.1958785	47.88839653	33.05402091	1
C130083M11Rik	33.88076982	44.36667248	55.60445191	43.13047098	46.86888008	32.26498906	24.3557473	134.0918421	99.31866418	113.607256	72.83204381	39.03876478	30.67503535	28.91374885	28.74262688	1
Igfbp7	47.61621704	45.95119649	71.2676778	47.44351808	36.10170492	69.43986777	44.20117102	152.5294704	139.9490268	113.607256	79.4531387	55.5217988	57.94173343	42.46706862	40.9582433	1
Arhgap24	1.831392963	24.56012226	14.0969033	14.66436013	4.43354271	7.014128057	14.43303544	54.47481085	15.80069657	26.21705907	30.26786236	12.14539349	11.92918041	9.035546516	10.05991941	1
P2rx2	1.831392963	3.961310043	0	0.86260942	2.533452977	2.104238417	3.608258859	10.05688816	6.771727103	9.612921658	1.891741398	1.735056213	0	1.807109303	8.622788064	1
Hnf1a	0	0.792262009	0	0	1.266726489	4.90988964	2.706194144	3.352296052	2.257242368	0	4.729353494	0.867528106	0	0.903554652	1.437131344	1
D5Ertd605e	7.325571853	6.338096068	5.482129061	6.038265938	13.30062813	8.416953669	13.53097072	22.62799835	11.28621184	20.97364725	17.97154328	5.205168638	3.408337261	5.421327909	7.18565672	1
Mest	3.662785926	2.376786026	5.482129061	1.725218839	1.266726489	4.208476834	1.80412943	18.43762829	9.028969471	10.48682363	5.675224193	9.542809169	7.668758836	9.035546516	8.622788064	1
D630045J12Rik	61.35166426	85.56429692	65.78554874	76.77223835	63.96968767	38.57770431	40.59291217	153.3675444	133.9297138	157.3023544	182.5530449	52.91921448	31.52711966	49.69550584	47.42533435	1
P3h3	0	0	3.132645178	0	3.166816221	2.805651223	3.608258859	7.542666117	4.514484735	0.873901969	4.729353494	2.602584319	0	6.324882561	1.437131344	1
Olr1	0	5.54583406	6.265290356	2.587828259	1.266726489	0	9.020647148	36.87525657	22.57242368	4.369509845	19.86328467	8.675281063	10.22501178	1.807109303	4.311394032	1
Dact3	10.0726613	11.88393013	22.71167754	10.35131304	17.73417084	19.63955856	17.13922958	75.42666117	58.68830156	57.67752995	30.26786236	19.08561834	11.0770961	20.78175699	11.49705075	1
Bcam	29.30228741	30.89821833	21.92851625	45.71829924	27.2346195	35.07064029	23.45368258	87.15969736	53.4214027	75.15556933	54.86050053	23.42325887	21.30210788	25.29953024	38.08398061	1
Ryr1	14.65114371	28.52143231	20.36219366	31.91654853	26.60125626	16.83390734	25.25781201	41.06562664	54.17381683	63.79484373	43.51005214	21.68820266	22.15419219	19.87820233	32.33545524	1
Syne4	4.578482408	3.169048034	2.349483883	0	4.43354271	0.701412806	5.412388289	11.73303618	2.257242368	6.991215751	6.621094891	1.735056213	4.260421576	1.807109303	4.311394032	1
Siglecf	6.409875371	9.507144102	9.397935534	8.626094197	10.76717515	7.715540863	8.118582433	42.74177467	20.31518131	31.46047088	20.80915537	4.337640531	6.816674521	20.78175699	9.341353736	1
Spib	4.578482408	28.52143231	18.79587107	21.56523549	20.26762382	13.32684331	14.43303544	149.1771743	70.72692752	65.54264767	33.10547446	24.29078698	19.59793925	16.26398373	10.77848508	1
Cpt1c	1.831392963	6.338096068	3.132645178	2.587828259	1.900089733	2.805651223	1.80412943	20.11377631	3.762070613	10.48682363	15.13393118	0.867528106	1.70416863	0.903554652	5.748525376	1
Fam83e	2.747089445	5.54583406	1.566322589	0	6.333632443	5.611302446	3.608258859	11.73303618	3.00965649	17.47803938	4.729353494	10.41033728	5.964590206	7.228437213	6.467091048	1
Kctd14	3.662785926	7.130358077	14.0969033	8.626094197	16.46744435	6.312715251	5.412388289	31.00873848	9.028969471	31.46047088	19.86328467	8.675281063	5.964590206	5.421327909	4.311394032	1
Ppfibp2	1.831392963	0	3.915806472	3.450437679	8.86708542	4.90988964	7.216517718	25.14222039	14.29586833	10.48682363	32.15960376	7.807752957	6.816674521	9.939101167	5.748525376	1
Trim66	12.81975074	21.39107423	14.8800646	44.85568982	17.1008076	9.81977928	6.314453003	98.05465953	88.03245234	55.05582404	52.02288843	17.35056213	16.18960199	6.324882561	15.80844478	1
H19	95.23243408	107.7476332	107.2930973	108.6887869	27.86798275	56.11302446	54.12388289	263.9933141	198.6373284	349.5607876	175.93195	78.07752957	68.16674521	50.59906049	12.21561642	1
Wwc2	6.409875371	10.29940611	1.566322589	0	4.43354271	8.416953669	0.902064715	7.542666117	7.524141226	11.3607256	20.80915537	11.27786538	11.0770961	3.614218606	3.59282836	1
Psd3	1.831392963	10.29940611	2.349483883	9.488703616	9.500448664	7.014128057	11.72684129	14.24725822	21.06759543	11.3607256	26.48437957	10.41033728	4.260421576	3.614218606	7.18565672	1
Csgalnact1	0	1.584524017	1.566322589	2.587828259	0.633363244	0	3.608258859	7.542666117	4.514484735	11.3607256	11.35044839	3.470112425	0.852084315	0	2.155697016	1
Nqo1	42.12203815	26.14464628	43.0738712	42.26786156	27.86798275	31.56357626	37.88671802	126.549176	81.26072524	96.12921658	150.3934411	78.07752957	37.49170987	49.69550584	35.9282836	1
Ldhd	0.915696482	3.169048034	6.265290356	3.450437679	5.066905954	4.208476834	3.608258859	10.05688816	11.28621184	6.991215751	14.18806048	6.072696744	7.668758836	2.710663955	7.18565672	1
Crispld2	8.241268334	18.2220262	12.53058071	0.86260942	10.76717515	4.90988964	14.43303544	71.23629111	35.36346376	31.46047088	31.21373306	5.205168638	13.63334904	7.228437213	2.874262688	1
Eepd1	1.831392963	0	1.566322589	3.450437679	5.700269198	5.611302446	6.314453003	8.380740131	13.54345421	4.369509845	0	11.27786538	2.556252945	2.710663955	1.437131344	1
Tmem136	4.578482408	3.961310043	2.349483883	9.488703616	3.166816221	7.715540863	4.510323574	15.08533223	14.29586833	6.991215751	10.40457769	4.337640531	6.816674521	8.131991864	7.18565672	1
Oaf	35.71216278	36.44405239	35.24225825	28.46611085	40.53524763	30.16075065	13.53097072	132.4156941	101.5759065	108.3638441	77.5613973	32.09853993	29.82295103	31.62441281	35.20971793	1
Dmxl2	7.325571853	5.54583406	2.349483883	12.07653188	15.20071786	9.118366474	7.216517718	10.05688816	5.266898858	13.10852953	40.67244005	7.807752957	12.78126473	4.517773258	6.467091048	1
Myo1e	72.34002204	57.83512662	49.33916155	61.2452688	62.06959794	39.27911712	64.04659475	160.0721365	110.604876	117.9767658	104.9916476	39.03876478	57.94173343	46.98484188	38.08398061	1
Gsta4	1.831392963	0	0	2.587828259	10.76717515	7.715540863	2.706194144	6.704592104	3.00965649	0.873901969	12.29631908	6.072696744	3.408337261	1.807109303	5.748525376	1
Mst1r	3.662785926	10.29940611	5.482129061	9.488703616	5.700269198	2.805651223	4.510323574	25.14222039	12.79104008	6.991215751	31.21373306	6.94022485	3.408337261	9.939101167	3.59282836	1
Ccr8	8.241268334	12.67619214	7.831612945	13.80175071	29.13470924	32.96640187	27.96400616	31.8468125	7.524141226	34.08217679	14.18806048	17.35056213	24.71044514	27.10663955	28.02406121	1
Fam198a	15.56684019	12.67619214	14.8800646	18.11479781	12.03390164	14.02825611	10.82477658	49.44636677	36.11587788	48.06460829	27.43025026	4.337640531	12.78126473	9.035546516	2.874262688	1
Timp1	4.578482408	9.507144102	5.482129061	6.900875357	2.533452977	1.402825611	0	24.30414638	10.53379772	26.21705907	33.10547446	1.735056213	6.816674521	4.517773258	1.437131344	1
Gria3	6.409875371	3.961310043	9.397935534	8.626094197	3.166816221	3.507064029	7.216517718	19.2757023	19.56276719	21.84754922	27.43025026	4.337640531	1.70416863	3.614218606	4.311394032	1
Zfp711	0.915696482	4.753572051	1.566322589	0.86260942	1.266726489	0.701412806	0	23.46607237	13.54345421	13.10852953	13.24218978	0.867528106	0	0	0	1
Drp2	2.747089445	8.714882094	3.915806472	6.900875357	12.03390164	9.118366474	9.020647148	7.542666117	25.58208017	4.369509845	19.86328467	16.48303402	12.78126473	7.228437213	8.622788064	1
Gprasp2	12.81975074	7.922620085	8.614774239	8.626094197	5.066905954	4.208476834	13.53097072	22.62799835	22.57242368	30.58656891	25.53850887	9.542809169	10.22501178	11.74621047	7.904222392	1
Rftn2	21.97671556	11.88393013	8.614774239	6.038265938	8.86708542	17.53532014	20.74748844	3.352296052	3.00965649	4.369509845	0.945870699	7.807752957	13.63334904	15.36042908	16.52701046	2
Fzd7	186.8020822	111.7089432	122.9563232	85.39833255	70.93668336	108.7189849	122.6808012	18.43762829	14.29586833	34.08217679	36.88895725	58.12438312	80.94800994	59.634607	87.66501198	2
Glrp1	31.13368037	10.29940611	13.31374201	23.29045433	20.26762382	14.72966892	14.43303544	11.73303618	6.019312981	9.612921658	1.891741398	6.94022485	1.70416863	11.74621047	11.49705075	2
Bok	10.0726613	7.922620085	5.482129061	6.038265938	6.966995687	4.208476834	7.216517718	1.676148026	0	6.117313782	0.945870699	3.470112425	4.260421576	7.228437213	8.622788064	2
Cxcr4	450.5226689	389.0006462	376.7005826	363.1585657	303.380994	298.8018552	359.9238212	123.1968799	153.492481	128.4635894	136.2053806	315.7802307	282.8919926	261.1272943	293.8933598	2
Gm19705	40.29064519	33.27500436	20.36219366	19.84001665	28.50134599	10.52119209	23.45368258	12.5711102	11.28621184	18.35194135	11.35044839	27.7608994	17.89377062	20.78175699	10.05991941	2
Fasl	487.1505282	413.5607684	348.506776	414.9151309	352.1499638	343.6922748	375.2589213	160.9102105	151.2352386	151.1850406	192.0117519	292.3569718	310.1586907	343.3507676	298.2047539	2
Sell	3483.309416	3300.563527	3145.95892	3291.717545	3210.518285	3469.88915	3796.790385	1142.29488	1768.925602	1355.421954	1476.504161	3348.65849	3179.12658	3111.84222	3303.246394	2
Selp	79.6655939	35.65179038	46.98967767	55.20700286	9.500448664	23.84803539	24.3557473	12.5711102	2.257242368	28.83876497	24.59263817	19.95314644	25.56252945	15.36042908	30.17975822	2
Slamf7	1914.721343	1559.963895	1553.008847	1481.100374	1681.579414	1740.906584	2205.548228	805.3891265	726.8320424	741.0688696	751.9672055	1658.713739	1691.387366	1507.129159	1700.844946	2
BC094916	384.5925223	316.9048034	270.9738079	278.6228425	434.4871856	462.231039	538.5326347	89.6739194	117.3766031	97.00311855	127.6925443	347.0112425	330.6087143	366.8431885	411.73813	2
Pydc4	137.3544722	134.6845414	140.969033	146.6436013	185.5754306	227.2577491	298.5834206	28.49451644	41.38277674	43.69509845	57.69811262	186.5185429	167.8606101	197.8784687	196.8869941	2
Pyhin1	2234.299415	1924.404419	1545.960395	1465.573404	2059.063907	2295.724113	2651.168197	349.4768634	564.3105919	425.5902589	562.7930658	2070.78959	1768.927038	2070.043707	2006.953922	2
Pydc3	695.0136295	625.8869867	445.6187766	480.4734467	568.7601934	726.6636667	739.6930661	127.38725	180.5793894	188.7628253	188.2282691	539.6024821	529.996444	524.0616979	505.8702331	2
Ifi204	297.6013565	209.9494323	149.5838072	171.6592745	128.5727386	202.7083009	237.24302	58.66518091	79.00348287	45.44290238	64.31920752	157.0225872	149.9668395	172.5789385	168.8629329	2
Mnda	293.9385706	199.6500261	183.2597429	163.0331803	151.3738154	194.99276	183.1191371	94.70236348	79.75589699	113.607256	106.883389	132.7318003	126.1084786	99.39101167	145.1502657	2
Tlr5	24.723805	19.0142882	14.0969033	31.91654853	16.46744435	0.701412806	9.922711863	3.352296052	3.762070613	6.991215751	13.24218978	6.94022485	5.112505891	8.131991864	11.49705075	2
Slc35d3	83.32837982	39.61310043	49.33916155	26.74089201	38.6351579	70.84269338	79.3816949	9.218814144	25.58208017	30.58656891	8.512836289	45.11146153	37.49170987	28.0101942	41.67680897	2
Samd3	23.80810852	34.06726637	32.10961307	44.85568982	109.5718413	87.67660071	82.08788905	1.676148026	5.266898858	12.23462756	7.56696559	78.94505767	94.58135898	93.96968376	78.32365824	2
Prdm1	59.5202713	69.71905675	29.76012919	68.14614415	79.80376878	90.48225194	97.4229892	6.704592104	8.276555348	42.82119648	56.75224193	78.07752957	87.76468446	102.1016756	91.25784034	2
P4ha1	2808.441109	2646.155108	2612.626078	2502.429926	2451.749119	2889.119347	2855.034822	1001.498446	1344.564037	1337.943914	1478.395902	2600.849263	2588.632149	2403.455373	2587.554985	2
Ddit4	4671.883449	3725.215964	3656.580084	3032.07211	3067.378192	3360.468752	3308.773374	1426.40197	1887.807034	2013.470136	1854.85244	2468.98499	2603.117583	2302.257252	2544.441044	2
Spock2	43.03773463	27.7291703	26.62748401	22.42784491	24.70116653	30.86216345	41.49497688	17.59955427	3.00965649	34.95607876	2.837612096	45.11146153	24.71044514	28.91374885	24.43123285	2
Ppap2c	103.4737024	87.14882094	79.09929074	83.67311371	65.23641416	91.88507755	95.61885977	30.17066447	27.83932254	41.07339254	29.32199166	77.21000146	76.68758836	83.12702794	93.41353736	2
Zfr2	7.325571853	3.961310043	0.783161294	2.587828259	1.266726489	5.611302446	0.902064715	0	3.00965649	0	0	1.735056213	5.112505891	5.421327909	0.718565672	2
Matk	125.450418	107.7476332	93.19619404	88.84877022	73.47013634	75.75258302	85.6961479	25.14222039	48.15450385	27.09096104	46.34766424	97.1631479	83.50426289	96.68034772	84.07218362	2
Smim24	30.21798389	41.98988645	39.94122602	25.87828259	41.16861088	36.4734659	29.76813559	13.40918421	19.56276719	9.612921658	19.86328467	13.01292159	36.63962555	39.75640467	35.20971793	2
Slc41a2	13.73544722	3.169048034	18.01270977	22.42784491	8.233722176	11.22260489	9.922711863	1.676148026	0	2.621705907	9.458706988	6.94022485	10.22501178	9.035546516	10.77848508	2
Gm17745	16.48253667	11.09166812	13.31374201	11.21392246	15.20071786	9.81977928	14.43303544	0.838074013	9.781383594	1.747803938	2.837612096	12.14539349	15.33751767	7.228437213	10.05991941	2
Tmtc2	20.14532259	14.26071615	8.614774239	11.21392246	13.93399137	9.81977928	4.510323574	5.028444078	4.514484735	7.86511772	7.56696559	8.675281063	6.816674521	7.228437213	9.341353736	2
Tmevpg1	1.831392963	4.753572051	7.831612945	7.763484777	15.83408111	4.208476834	6.314453003	0.838074013	1.504828245	4.369509845	0	4.337640531	12.78126473	18.97464768	10.05991941	2
Itga7	59.5202713	40.40536243	41.50754861	34.50437679	29.13470924	53.30737323	47.80942988	0.838074013	6.771727103	0.873901969	4.729353494	68.5347204	40.04796281	35.23863141	51.01816271	2
Lif	5160.86537	4144.322566	3898.576924	3596.218671	3320.090126	3921.598997	4287.513589	1733.137059	2290.348589	1991.622587	2292.790574	3094.472755	3027.455572	3082.024917	3474.265024	2
Gck	5.494178889	9.507144102	6.265290356	6.900875357	3.800179466	3.507064029	3.608258859	0	0	1.747803938	0	6.072696744	4.260421576	5.421327909	2.874262688	2
Ramp3	137.3544722	48.32798252	70.4845165	56.9322217	30.40143573	51.20313482	48.7114946	11.73303618	12.79104008	43.69509845	36.88895725	28.62842751	25.56252945	20.78175699	47.42533435	2
1700042O10Rik	13.73544722	14.26071615	12.53058071	13.80175071	5.700269198	7.014128057	9.020647148	0.838074013	5.266898858	6.991215751	9.458706988	8.675281063	13.63334904	16.26398373	17.9641418	2

Stc2	303.0955354	219.4565764	227.8999367	187.1862441	198.2426955	270.745343	276.9338674	31.8468125	41.38277674	48.06460829	62.42746612	185.6510147	181.4939591	160.832728	211.9768732	2
Gm5431	4.578482408	0	7.831612945	2.587828259	4.43354271	4.208476834	2.706194144	2.514222039	0	0	0	3.470112425	0	1.807109303	5.748525376	2
9930111J21Rik1	244.4909606	206.7803842	169.9460009	203.575823	171.008076	171.8461374	220.1037904	86.32162334	119.6338455	76.90337326	88.91184568	197.7964082	208.7606572	159.9291733	230.6595807	2
P4ha2	131.8602933	81.60298688	88.49722628	69.87136299	60.80287145	89.07942633	82.08788905	14.24725822	5.266898858	17.47803938	15.13393118	78.07752957	80.94800994	71.38081747	76.8865269	2
Slc36a3	13.73544722	5.54583406	14.0969033	6.038265938	12.66726489	6.312715251	8.118582433	0	1.504828245	10.48682363	0	9.542809169	11.92918041	4.517773258	4.311394032	2
Fam183b	13.73544722	19.0142882	9.397935534	15.52696955	12.03390164	16.83390734	14.43303544	0	3.00965649	0	4.729353494	3.470112425	10.22501178	13.55331977	12.9341821	2
4933439C10Rik	4.578482408	9.507144102	4.698967767	7.763484777	2.533452977	5.611302446	8.118582433	0.838074013	3.762070613	2.621705907	1.891741398	6.94022485	8.520843152	4.517773258	14.37131344	2
Myo15	2.747089445	0	2.349483883	5.175656518	0.633363244	0	0	0	0	0	0	2.602584319	11.92918041	0	2.874262688	2
Tmem220	3.662785926	5.54583406	3.915806472	1.725218839	0.633363244	0.701412806	4.510323574	0	0	0	0.945870699	4.337640531	0	1.807109303	0.718565672	2
Tmem88	6.409875371	7.922620085	4.698967767	9.488703616	5.066905954	2.104238417	6.314453003	0	0.752414123	0.873901969	1.891741398	1.735056213	5.112505891	1.807109303	6.467091048	2
Xaf1	821.379744	648.862585	538.0318093	468.3969149	599.1616291	752.6159405	775.7756547	195.271245	277.6408112	231.5840218	259.1685715	525.7220324	435.415085	549.3612282	587.7867197	2
Smtnl2	82.41268334	49.91250654	50.12232285	42.26786156	39.90188439	61.0229141	67.65485361	1.676148026	14.29586833	9.612921658	9.458706988	52.05168638	52.82922754	43.37062328	40.9582433	2
Rap1gap2	8.241268334	20.59881222	25.06116142	25.87828259	20.90098706	16.83390734	21.64955315	10.89496217	4.514484735	10.48682363	6.621094891	32.09853993	36.63962555	36.14218606	25.86836419	2
Aldoc	1043.893989	1006.172751	840.332069	862.6094197	868.9743711	1059.133337	1240.338983	71.23629111	137.6917844	207.1147666	232.6841919	927.3875456	1017.388672	946.0217202	1049.824447	2
Slfn5	340.6390911	155.2833537	111.2089038	87.9861608	151.3738154	257.4184997	310.3102619	26.81836842	56.43105919	34.08217679	58.64398332	97.1631479	126.960563	113.8478861	157.3658822	2
Slfn8	1275.565199	877.8263054	775.3296815	739.2562726	936.110875	1151.018414	1308.895901	349.4768634	477.0305537	382.7690624	484.2857978	923.0499051	932.1802408	887.2906678	926.2311512	2
Slfn1	2637.205867	2233.386602	2105.13756	1725.218839	2309.242389	2842.826102	2901.040123	895.0630459	1149.688779	940.3185185	1180.446632	2500.216002	2251.206761	2550.734781	2411.506395	2
Ccl5	249.069443	202.8190742	188.741872	158.7201332	286.2801864	303.7117449	386.0836979	100.5688816	62.45037217	71.65996145	84.18249219	269.8012411	254.7732102	232.2135455	294.6119255	2
Ttc25	8.241268334	3.169048034	12.53058071	0	6.333632443	4.90988964	2.706194144	1.676148026	0	0	0.945870699	0	4.260421576	0.903554652	5.748525376	2
Dhx58	1130.885155	1037.070969	778.4623267	696.9884111	818.9386748	1021.257045	1058.12191	295.8401266	414.5801815	282.270336	370.7813139	764.2922616	693.5966325	799.6458666	892.4585646	2
Plekhh3	14.65114371	39.61310043	15.66322589	18.97740723	29.76807248	28.75792503	26.15987673	1.676148026	10.53379772	4.369509845	2.837612096	19.95314644	29.82295103	15.36042908	32.33545524	2
Ccr10	46.70052056	23.76786026	39.94122602	24.15306375	30.40143573	44.89041957	64.94865946	5.866518091	5.266898858	13.9824315	16.07980188	32.96606804	31.52711966	43.37062328	33.05402091	2
Cntnap1	56.77318186	39.61310043	54.82129061	46.58090866	53.20251252	56.11302446	46.00530045	13.40918421	16.5531107	21.84754922	22.70089677	55.5217988	40.90004713	41.56351397	58.9223851	2
Ramp2	9.156964816	1.584524017	2.349483883	18.11479781	4.43354271	4.90988964	2.706194144	1.676148026	2.257242368	2.621705907	0.945870699	12.14539349	11.0770961	0	6.467091048	2
Nags	0.915696482	4.753572051	3.915806472	0	5.066905954	2.805651223	4.510323574	0	0	0.873901969	0	3.470112425	2.556252945	1.807109303	2.155697016	2
Itgb3	420.304685	461.888751	417.42497	483.9238844	513.6575911	459.4253877	616.1102002	171.8051727	181.3318035	188.7628253	182.5530449	564.7607972	535.9610342	543.0363456	462.0377271	2
Fam20a	165.7410632	149.7375196	145.6680008	162.1705709	88.03749095	84.87094949	88.40234205	54.47481085	61.69795805	82.14678508	104.0457769	102.3683165	131.2209845	97.58390237	119.2819015	2
Cd300lf	4.578482408	11.09166812	9.397935534	1.725218839	11.4005384	4.90988964	8.118582433	0	0	1.747803938	2.837612096	3.470112425	10.22501178	6.324882561	5.029959704	2
Bahcc1	21.97671556	4.753572051	9.397935534	4.313047098	3.800179466	10.52119209	0.902064715	0	0.752414123	5.243411813	4.729353494	3.470112425	2.556252945	1.807109303	3.59282836	2
0610009L18Rik	9.156964816	20.59881222	6.265290356	5.175656518	2.533452977	4.90988964	7.216517718	7.542666117	0.752414123	0.873901969	4.729353494	7.807752957	16.18960199	2.710663955	3.59282836	2
Slc16a3	5712.114652	5282.803073	5202.540479	4513.172484	4701.455362	5024.92134	5075.016085	762.6473519	1443.130287	1931.323351	2098.887081	4913.679194	5107.393385	4678.605986	5092.474917	2
Mboat2	54.02609241	41.19762444	44.64019379	49.16873692	33.56825195	44.89041957	36.98465331	18.43762829	7.524141226	13.9824315	16.07980188	30.36348372	20.45002356	43.37062328	31.61688957	2
Rsad2	371.7727715	154.4910917	140.969033	72.45919125	161.5076273	274.252407	323.8412326	12.5711102	33.85863552	3.495607876	23.64676747	135.3343846	133.7772375	100.2945663	211.2583076	2
Cmpk2	682.1938788	547.4530479	470.679938	344.1811584	519.9912236	793.2978833	725.2600307	129.063398	283.6601242	166.9152761	231.7383212	535.2648416	408.148387	471.6555281	501.558839	2
Egln3	4612.363178	3100.913501	3088.004984	2536.934303	2689.260335	3889.334008	4329.910631	286.6213125	540.23334	605.6140645	742.5084985	2771.7523	3312.051733	2454.957988	3393.785669	2
Pygl	152.9213124	111.7089432	109.6425812	105.2383492	69.66995687	120.6430026	134.4076425	0	7.524141226	17.47803938	12.29631908	87.62033873	78.39175699	108.4265582	102.0363254	2
Ifi27l2a	4567.49405	4460.435108	3946.349763	3854.138887	4662.820204	5002.47613	5638.806532	1531.999296	2242.194085	1783.633919	1973.086278	4357.593678	4199.923589	4280.138385	4355.945103	2
Jag2	85.15977279	42.78214846	57.95393579	46.58090866	38.6351579	61.7243269	67.65485361	20.95185033	19.56276719	29.71266694	17.02567258	47.71404585	74.98341973	25.29953024	40.23967763	2
Sugct	1.831392963	9.507144102	3.915806472	4.313047098	5.700269198	2.104238417	1.80412943	0.838074013	0	0.873901969	0.945870699	2.602584319	0.852084315	1.807109303	5.029959704	2
Nkapl	3.662785926	1.584524017	3.132645178	6.900875357	1.900089733	7.014128057	0.902064715	0.838074013	0	0	0	6.072696744	2.556252945	0.903554652	6.467091048	2
Hist1h2bg	21.97671556	23.76786026	14.8800646	8.626094197	29.76807248	15.43108173	18.94335901	6.704592104	1.504828245	18.35194135	6.621094891	16.48303402	17.0416863	11.74621047	17.24557613	2
Ppp1r3g	19.22962611	10.29940611	15.66322589	21.56523549	11.4005384	18.23673295	20.74748844	0	0.752414123	5.243411813	2.837612096	0.867528106	17.89377062	8.131991864	7.18565672	2
Gcm2	8.241268334	9.507144102	8.614774239	6.900875357	1.900089733	0	3.608258859	0	0.752414123	0	0	4.337640531	4.260421576	0.903554652	4.311394032	2
Shc3	8.241268334	13.46845414	5.482129061	2.587828259	1.266726489	2.104238417	6.314453003	0.838074013	0	3.495607876	2.837612096	6.072696744	3.408337261	6.324882561	3.59282836	2
4921525O09Rik	36.62785926	56.2506026	36.80858084	28.46611085	43.06870061	35.07064029	37.88671802	24.30414638	15.80069657	18.35194135	14.18806048	32.96606804	49.42089028	35.23863141	27.30549553	2
Nfil3	1059.460829	790.6774845	836.4162625	760.8215081	716.3338293	894.3013273	862.3738673	272.3740542	348.3677388	381.8951604	470.0977373	619.4150679	699.5612227	541.2292363	622.2778719	2
Nkd2	23.80810852	15.05297816	25.06116142	26.74089201	15.20071786	25.95227381	33.37639445	9.218814144	6.771727103	19.22584332	5.675224193	16.48303402	27.26669809	20.78175699	30.17975822	2
Arsb	1120.812493	1089.360262	939.0103921	1059.284367	1335.129719	1189.596118	1523.587303	310.9254588	461.9822713	415.1034352	490.9068927	1312.570025	1251.711859	1365.271079	1310.663786	2
F2rl1	300.348446	255.9006287	256.8769046	315.7150476	363.5505022	376.6586767	392.3981509	98.05465953	133.1772997	121.4723737	209.9832951	368.6994452	334.8691359	361.4218606	382.9955032	2
Tmem171	38.45925223	38.02857641	21.14535495	19.84001665	21.53435031	16.13249453	23.45368258	10.05688816	3.00965649	13.10852953	14.18806048	21.68820266	11.92918041	19.87820233	19.40127314	2
Gzma	27.47089445	24.56012226	18.79587107	35.36698621	19.63426057	27.35509942	41.49497688	5.866518091	10.53379772	13.10852953	16.07980188	19.08561834	15.33751767	34.33507676	38.08398061	2
Gm6904	24.723805	47.53572051	25.84432272	25.01567317	21.53435031	21.04238417	34.27845916	6.704592104	8.276555348	10.48682363	15.13393118	15.61550591	25.56252945	19.87820233	17.24557613	2
Phf11a	88.82255871	87.94108294	58.73709709	71.59658183	77.90367905	84.87094949	97.4229892	43.57984868	27.83932254	36.70388269	34.99721585	54.6542707	47.71672165	58.73105235	58.9223851	2
Dleu7	39.37494871	38.02857641	28.9769679	42.26786156	27.2346195	47.69607079	31.57226502	7.542666117	35.36346376	4.369509845	25.53850887	36.43618046	24.71044514	24.39597559	26.58692986	2
Neil2	2.747089445	14.26071615	3.132645178	1.725218839	1.900089733	2.104238417	3.608258859	4.190370065	0	0	0.945870699	2.602584319	7.668758836	0.903554652	6.467091048	2
Zfp395	617.1794286	606.0804365	614.7816162	629.7048763	768.9029786	776.4639759	839.8222495	288.2974605	320.5284162	287.5137478	277.1401147	871.8657468	822.2613641	795.1280934	757.3682183	2
Bnip3l	2575.854203	2442.543772	2498.284529	2191.890535	2067.930993	2195.422082	2328.229029	955.4043749	1157.965335	1240.066894	1304.355694	2416.933304	2528.986247	2267.922175	2246.236291	2
Stc1	22.89241204	19.0142882	10.96425812	24.15306375	11.4005384	7.014128057	22.55161787	4.190370065	0	3.495607876	1.891741398	8.675281063	13.63334904	5.421327909	0.718565672	2
Pou4f1	15.56684019	8.714882094	7.04845165	10.35131304	4.43354271	7.715540863	7.216517718	3.352296052	3.00965649	1.747803938	7.56696559	3.470112425	19.59793925	4.517773258	6.467091048	2
Cldn10	0	0.792262009	7.04845165	0	1.266726489	0.701412806	1.80412943	0	0	0	0	0	0	0	2.155697016	2
Clybl	217.9357626	193.3119301	214.5861947	176.834931	191.909063	182.3673295	188.5315254	72.07436512	94.80417945	79.52507917	99.31642337	159.6251716	184.9022964	187.0358129	206.2283479	2
Ndrg1	2281.915632	1746.145467	1712.773751	1458.672529	1552.373312	1885.397622	1945.75359	729.1243914	723.8223859	727.9603401	755.7506883	1440.096656	1647.931066	1363.463969	1767.671553	2
2010109I03Rik	3.662785926	7.130358077	4.698967767	6.900875357	3.800179466	4.90988964	6.314453003	0	0	1.747803938	1.891741398	4.337640531	4.260421576	0.903554652	5.748525376	2
Ly6c2	3736.957341	3340.96889	2874.985112	2712.044015	3567.101792	3523.196523	4057.487087	318.468125	775.7389604	853.8022236	977.0844318	2837.684436	2709.628122	2923.902852	3273.785201	2
Mafa	6.409875371	2.376786026	0	0.86260942	3.166816221	4.90988964	0.902064715	0	0	0	0	0	11.0770961	0	2.155697016	2
Chadl	0.915696482	6.338096068	1.566322589	0.86260942	1.900089733	1.402825611	2.706194144	0.838074013	0	0	0	0	0	5.421327909	2.155697016	2
Fam109b	32.96507334	45.15893448	30.54329049	24.15306375	24.70116653	19.63955856	20.74748844	10.89496217	27.08690841	15.73023544	9.458706988	31.23101183	27.26669809	32.52796746	15.80844478	2
Endou	98.89522001	104.5785851	104.9436135	85.39833255	81.07049527	70.14128057	75.77343604	17.59955427	46.6496756	43.69509845	47.29353494	55.5217988	67.3146609	47.88839653	82.63505228	2
Pou6f1	107.1364883	120.4238253	133.9205814	146.6436013	212.8100501	171.8461374	181.3150077	66.20784703	58.68830156	34.95607876	75.6696559	189.1211272	209.6127415	224.9851082	196.1684284	2
C330013E15Rik	5.494178889	0	2.349483883	14.66436013	2.533452977	2.104238417	2.706194144	1.676148026	1.504828245	1.747803938	0	4.337640531	2.556252945	1.807109303	2.874262688	2
Acvrl1	272.8775515	181.4279999	242.7800013	210.4766984	229.9108577	250.4043716	296.7792912	36.03718256	56.43105919	50.6863142	69.99443171	190.8561834	232.619018	201.4926873	251.4979852	2
Abat	2.747089445	0.792262009	5.482129061	0	3.800179466	1.402825611	0	0	0	0.873901969	0	3.470112425	4.260421576	9.035546516	6.467091048	2
Prm1	8.241268334	6.338096068	7.04845165	3.450437679	0.633363244	4.208476834	5.412388289	0	2.257242368	1.747803938	0	0.867528106	2.556252945	7.228437213	7.904222392	2
Pkp2	43.95343112	28.52143231	39.94122602	21.56523549	22.80107679	25.25086101	30.6702003	5.866518091	8.276555348	16.60413741	16.07980188	36.43618046	28.1187824	27.10663955	17.9641418	2
Scarf2	2.747089445	1.584524017	0	2.587828259	0.633363244	0	0	0	0	0	0	0.867528106	0.852084315	1.807109303	0	2
St6gal1	1114.402618	1147.195388	1098.775296	1098.964401	1661.945153	1818.061992	1871.784283	480.2164095	546.252653	542.6931227	635.6251096	1702.090145	1756.145774	1460.144317	1835.935292	2
Rtp4	650.1445019	583.1048383	525.5012286	508.9395576	657.4310476	811.5346162	841.6263789	125.711102	209.9235402	173.9064918	283.7612096	585.5814717	565.7839853	639.7166933	565.5111838	2
Cd86	127.2818109	138.6458515	121.3900006	152.6818673	249.5451182	256.7170869	260.6967026	61.17940295	82.76555348	48.93851026	75.6696559	245.5104541	193.4231395	211.4317885	229.2224494	2
Ildr1	32.04937685	31.69048034	19.57903236	40.54264272	43.70206386	49.80030921	55.92801232	12.5711102	10.53379772	4.369509845	20.80915537	45.11146153	38.34379418	56.92394305	45.98820301	2
Mx1	804.8972073	598.1578164	462.0651637	318.3028759	464.8886213	735.7820332	733.3786131	85.48354933	121.8910879	110.1116481	147.555829	467.5976493	380.0296046	363.2289699	519.5229808	2
Mx2	290.2757847	222.6256244	158.1985815	98.33747384	143.1400932	204.1111265	198.4542373	25.14222039	43.64001911	40.19949057	46.34766424	116.2487662	108.214708	114.7514407	134.3717807	2
Mir692-1	3.662785926	4.753572051	7.831612945	5.175656518	5.700269198	4.90988964	0	0	0	2.621705907	0	2.602584319	5.964590206	0	7.18565672	2
Kremen2	71.42432556	54.66607859	52.47180673	47.44351808	46.23551683	78.55823424	73.0672419	20.11377631	24.07725192	16.60413741	24.59263817	39.90629289	78.39175699	55.11683375	65.38947615	2
Prss41	4.578482408	0.792262009	5.482129061	4.313047098	1.266726489	4.90988964	1.80412943	0.838074013	0.752414123	0	0.945870699	1.735056213	1.70416863	1.807109303	0	2
Syngr3	38.45925223	18.2220262	37.59174214	35.36698621	12.66726489	30.16075065	38.78878274	3.352296052	21.06759543	18.35194135	13.24218978	30.36348372	38.34379418	21.68531164	23.71266717	2
Rgs11	54.94178889	72.09584277	82.23193592	41.40525214	58.26941847	70.84269338	64.04659475	13.40918421	20.31518131	56.80362798	20.80915537	70.26977661	82.65217857	100.2945663	90.53927467	2
Gm6402	20.14532259	20.59881222	27.41064531	15.52696955	27.86798275	23.84803539	15.33510015	4.190370065	18.05793894	6.991215751	7.56696559	9.542809169	10.22501178	13.55331977	12.21561642	2
Rsph1	18.31392963	14.26071615	29.76012919	19.84001665	14.56735462	8.416953669	27.96400616	8.380740131	12.79104008	5.243411813	9.458706988	23.42325887	20.45002356	28.0101942	16.52701046	2
H2-K2	169.4038491	176.6744279	151.9332911	119.9027093	138.7065505	185.8743935	194.8459784	43.57984868	42.88760499	97.00311855	88.91184568	130.1292159	142.2980806	169.8682745	163.8329732	2
H2-Ob	36.62785926	41.19762444	38.37490343	31.05393911	29.76807248	30.86216345	42.39704159	10.05688816	12.03862596	12.23462756	5.675224193	21.68820266	38.34379418	37.94929537	45.26963733	2
H2-Ab1	412.9791132	262.2387248	309.3487113	271.7219672	233.7110371	281.9679479	248.0677966	118.1684358	136.9393703	153.8067465	119.179708	209.0742736	253.0690416	168.0611652	204.7912165	2
Notch4	0	2.376786026	4.698967767	0.86260942	3.166816221	2.104238417	8.118582433	0	0.752414123	0	0	0.867528106	3.408337261	6.324882561	8.622788064	2
Msh5	19.22962611	27.7291703	28.9769679	31.05393911	18.36753408	26.65368662	30.6702003	15.08533223	20.31518131	7.86511772	9.458706988	19.08561834	19.59793925	37.94929537	17.24557613	2
H2-Q2	10.98835778	10.29940611	12.53058071	3.450437679	3.166816221	6.312715251	4.510323574	0	0	2.621705907	0	2.602584319	6.816674521	4.517773258	6.467091048	2
H2-Q10	146.5114371	115.6702532	83.79825851	71.59658183	67.13650389	75.75258302	81.18582433	36.03718256	21.06759543	32.33437285	28.37612096	81.54764199	98.84178056	70.47726282	82.63505228	2
Ier3	461.5110267	211.5339563	266.2748401	219.965402	119.0722899	153.6094045	186.727396	100.5688816	97.81383594	93.50751067	148.5016997	116.2487662	128.6647316	131.9189791	137.964609	2
Gm6623	2.747089445	3.961310043	2.349483883	2.587828259	1.900089733	1.402825611	1.80412943	0.838074013	0	0	0	2.602584319	1.70416863	2.710663955	2.155697016	2
Trim15	0.915696482	4.753572051	3.132645178	0.86260942	6.966995687	1.402825611	3.608258859	0	0	0	0	4.337640531	2.556252945	2.710663955	2.155697016	2
Olfr99	9.156964816	18.2220262	7.831612945	3.450437679	0.633363244	4.208476834	5.412388289	0	0	1.747803938	8.512836289	3.470112425	3.408337261	3.614218606	5.029959704	2
Vegfa	1222.454803	972.8977464	1062.749877	859.158982	803.1045937	974.9637999	1074.359075	222.9276875	320.5284162	390.6341801	442.667487	766.0273178	970.524035	746.3361422	877.3686855	2
Emilin2	853.4291208	563.298288	515.3201318	428.7168816	398.3854806	417.3406194	472.6819105	102.2450296	85.77520997	173.0325898	202.4163295	380.8448387	425.1900733	349.6756502	458.4448987	2
Camk2a	9.156964816	2.376786026	3.915806472	11.21392246	2.533452977	0.701412806	0.902064715	1.676148026	0	1.747803938	2.837612096	3.470112425	5.112505891	0.903554652	2.155697016	2
B430212C06Rik	9.156964816	13.46845414	13.31374201	7.763484777	8.233722176	11.22260489	10.82477658	4.190370065	3.762070613	6.117313782	1.891741398	14.74797781	15.33751767	12.64976512	12.9341821	2
Myo5b	1.831392963	3.169048034	1.566322589	3.450437679	0.633363244	1.402825611	0	0	0	0	0	0	2.556252945	0	1.437131344	2
Ovol1	8.241268334	4.753572051	3.915806472	6.900875357	8.233722176	16.83390734	18.94335901	3.352296052	0	0.873901969	0	3.470112425	4.260421576	11.74621047	10.77848508	2
Ms4a4c	1313.108755	1403.096017	1243.660136	1188.67578	1710.714123	1807.5408	1963.794884	469.3214473	644.8189031	490.2590046	450.2344526	1480.002949	1415.312047	1476.408301	1415.574374	2
Vldlr	72.34002204	38.82083842	67.35187133	42.26786156	29.76807248	52.60596043	56.83007703	7.542666117	11.28621184	16.60413741	34.99721585	57.25685501	46.86463733	42.46706862	50.29959704	2
Slc1a1	7.325571853	3.961310043	0	5.175656518	3.166816221	0.701412806	5.412388289	0.838074013	0	0	0	6.072696744	0	6.324882561	0.718565672	2
Ifit3	769.1850445	619.5488907	472.2462606	390.7620671	691.6326627	972.8595615	871.3945145	116.4922878	144.4635115	102.2465304	180.6613035	562.1582129	473.7588792	435.5133421	601.4394674	2
Ifit1bl1	380.0140398	411.1839824	292.9023241	257.0576071	481.3560656	556.2203549	573.7131586	101.4069556	97.06142181	88.26409886	93.64119918	445.0419185	444.7880125	393.9498281	428.2651405	2
Ifit3b	171.2352421	112.5012052	117.4741942	100.9253021	159.6075376	230.7648131	228.2223728	43.57984868	37.62070613	25.3431571	26.48437957	128.3941597	92.87719035	130.1118698	139.4017404	2
Ifit1bl2	16.48253667	17.42976419	17.22954848	10.35131304	8.233722176	11.22260489	12.62890601	3.352296052	7.524141226	1.747803938	0	10.41033728	9.372927467	4.517773258	8.622788064	2
Ifit1	854.3448173	555.375668	429.9555507	415.7777403	586.4943642	931.476206	751.4199074	87.15969736	121.8910879	73.40776539	125.8008029	454.5847277	403.0358811	445.4524432	556.1698301	2
Dntt	30.21798389	30.10595632	51.68864544	46.58090866	16.46744435	32.96640187	23.45368258	8.380740131	12.79104008	10.48682363	12.29631908	18.21809023	34.08337261	28.91374885	26.58692986	2
Pik3ap1	548.5021925	512.5935195	403.3280667	379.5481446	447.1544505	528.1638427	576.4193527	108.9496217	151.2352386	142.4460209	105.9375183	347.8787706	410.7046399	409.3102572	478.5647375	2
Mxi1	872.6587469	629.0560348	628.0953582	504.6265105	482.6227921	549.9076397	608.8936825	250.5841299	329.5573857	389.7602781	331.0547446	558.6881004	510.3985048	455.3915444	551.1398704	2
5830416P10Rik	10.0726613	15.05297816	4.698967767	6.900875357	6.333632443	14.02825611	12.62890601	0.838074013	3.00965649	0.873901969	0	15.61550591	12.78126473	5.421327909	20.83840449	2
Itih5	237.1653887	247.1857467	238.8641948	260.5080447	278.6798275	309.3230473	325.645362	45.2559967	121.8910879	88.26409886	122.9631908	271.5362973	293.1170044	254.8024117	283.1148748	2
Grin1	10.98835778	7.922620085	10.18109683	2.587828259	3.166816221	9.118366474	6.314453003	0	0.752414123	2.621705907	0	4.337640531	3.408337261	1.807109303	2.155697016	2
Lcn4	23.80810852	37.2363144	32.10961307	31.05393911	30.40143573	39.27911712	37.88671802	13.40918421	15.04828245	3.495607876	17.02567258	34.70112425	63.05423932	25.29953024	39.52111196	2
Ier5l	169.4038491	129.9309694	137.0532265	98.33747384	107.6717515	111.5246361	125.3869954	36.03718256	60.94554393	52.43411813	68.10269031	126.6591035	121.8480571	96.68034772	114.2519418	2
C1qtnf4	15.56684019	3.961310043	16.44638718	14.66436013	13.30062813	19.63955856	8.118582433	2.514222039	3.762070613	3.495607876	2.837612096	16.48303402	11.0770961	18.97464768	7.904222392	2
Lrp4	8.241268334	12.67619214	3.132645178	1.725218839	1.266726489	4.90988964	2.706194144	3.352296052	0	1.747803938	0.945870699	5.205168638	1.70416863	0	3.59282836	2
Chac1	138.2701687	85.56429692	95.54567793	69.87136299	83.60394824	96.09355438	100.1291833	35.19910855	54.92623095	34.95607876	36.88895725	56.38932691	75.83550405	66.86304422	67.54517316	2
Smox	314.9995897	205.9881222	243.5631626	188.9114629	190.6423365	232.8690515	248.9698613	77.94088321	97.81383594	139.824315	132.4218978	196.9288801	207.0564886	163.5433919	236.4081061	2
Cd93	18.31392963	10.29940611	28.9769679	25.01567317	18.36753408	15.43108173	13.53097072	10.89496217	6.019312981	2.621705907	11.35044839	17.35056213	10.22501178	5.421327909	15.08987911	2
3300002I08Rik	5.494178889	14.26071615	14.8800646	10.35131304	8.86708542	9.118366474	5.412388289	1.676148026	8.276555348	1.747803938	3.783482795	9.542809169	12.78126473	17.16753838	3.59282836	2
Zfp442	8.241268334	11.88393013	13.31374201	11.21392246	4.43354271	6.312715251	10.82477658	9.218814144	1.504828245	1.747803938	3.783482795	5.205168638	13.63334904	0.903554652	5.748525376	2
E130215H24Rik	0.915696482	6.338096068	3.132645178	0.86260942	0	2.104238417	4.510323574	1.676148026	0	0	0	0	0	2.710663955	3.59282836	2
Slc52a3	67.76153964	83.18751089	75.18348427	74.18441009	103.8715721	99.60061841	98.32505391	22.62799835	40.63036262	27.964863	37.83482795	79.81258578	69.01882953	85.8376919	71.13800152	2
Tgm2	58.60457482	31.69048034	18.01270977	45.71829924	17.73417084	35.77205309	41.49497688	7.542666117	11.28621184	15.73023544	27.43025026	19.95314644	17.0416863	26.2030849	13.65274777	2
Nespas	16.48253667	14.26071615	30.54329049	25.87828259	8.86708542	12.6254305	17.13922958	5.028444078	3.00965649	16.60413741	17.02567258	19.08561834	19.59793925	13.55331977	9.341353736	2
Lama5	233.5026028	207.5726462	138.6195491	169.0714463	145.0401829	176.0546142	168.6861017	62.01747697	57.18347332	124.0940796	98.37055267	169.1679807	202.796067	183.4215943	184.6713777	2
4930577N17Rik	9.156964816	11.88393013	11.74741942	21.56523549	13.93399137	8.416953669	10.82477658	10.05688816	4.514484735	6.117313782	3.783482795	11.27786538	13.63334904	3.614218606	5.748525376	2
Mgarp	157.4997948	110.1244192	101.8109683	100.0626927	122.8724694	93.98931597	106.4436363	12.5711102	17.30552482	18.35194135	17.97154328	111.9111257	164.4522728	127.4012059	100.5991941	2
Lrrc71	0.915696482	2.376786026	1.566322589	3.450437679	3.166816221	2.805651223	0.902064715	0	0	0	0	4.337640531	0.852084315	6.324882561	1.437131344	2
Muc1	15.56684019	3.169048034	14.0969033	9.488703616	3.166816221	4.208476834	4.510323574	0	1.504828245	6.117313782	1.891741398	7.807752957	0.852084315	0	10.05991941	2
Selenbp1	28.38659093	14.26071615	14.8800646	13.80175071	14.56735462	15.43108173	24.3557473	1.676148026	6.019312981	0	11.35044839	11.27786538	23.85836082	4.517773258	11.49705075	2
Ciart	228.9241204	289.1756331	297.6012919	210.4766984	261.5790199	266.5368662	255.2843143	89.6739194	95.55659357	116.2289619	133.3677685	252.4506789	247.9565357	225.8886629	239.2823688	2
Tspan2	70.50862908	96.65596504	86.14774239	56.9322217	44.3354271	59.62008849	56.83007703	29.33259046	27.83932254	41.94729451	33.10547446	65.06460797	58.79381775	78.60925469	66.82660749	2
Fndc7	2.747089445	5.54583406	4.698967767	6.038265938	1.266726489	0.701412806	2.706194144	0	1.504828245	0.873901969	0	6.072696744	3.408337261	3.614218606	6.467091048	2
F3	25.63950148	17.42976419	20.36219366	27.60350143	13.30062813	11.22260489	20.74748844	8.380740131	6.019312981	17.47803938	12.29631908	18.21809023	14.48543336	9.939101167	13.65274777	2
Prss12	10.98835778	9.507144102	17.22954848	8.626094197	21.53435031	12.6254305	35.18052388	4.190370065	3.00965649	2.621705907	3.783482795	19.08561834	25.56252945	21.68531164	25.14979852	2
Ifi44	100.726613	66.55000871	46.98967767	63.83309705	48.76896981	66.63421654	69.45898304	20.11377631	16.5531107	12.23462756	16.07980188	33.83359614	34.93545692	35.23863141	71.13800152	2
Tdrd7	109.8835778	99.03275106	87.71406498	73.32180067	91.83767042	101.7048568	112.7580893	34.36103454	63.95520042	34.95607876	49.18527634	78.07752957	94.58135898	104.8123396	86.94644631	2
Gm12596	11.90405426	4.753572051	9.397935534	17.25218839	8.233722176	18.23673295	9.922711863	0.838074013	0	0	1.891741398	11.27786538	5.112505891	9.939101167	10.05991941	2
Ak4	1119.896797	1073.515022	975.0358116	809.1276356	805.0046835	879.5716584	977.8381508	119.0065099	285.9173666	385.3907683	408.6161419	839.7672069	892.132278	755.3716887	971.5007885	2
Slc2a1	6466.648553	5836.594217	5865.878096	5364.567981	5667.33431	6173.835516	6519.221694	1869.743123	2225.640975	3007.970577	2968.142253	5462.824485	5701.296153	5323.744007	6104.933949	2
Sdc3	944.998769	663.9155631	475.3789058	464.9464772	519.9912236	753.3173533	826.2912787	153.3675444	207.6662978	138.9504131	228.9007091	461.5249525	457.5692772	411.1173665	584.1938913	2
Ephb2	21.06101908	20.59881222	16.44638718	11.21392246	10.76717515	16.13249453	11.72684129	1.676148026	6.771727103	3.495607876	17.02567258	7.807752957	14.48543336	13.55331977	17.24557613	2
Padi4	2.747089445	5.54583406	0	0	3.800179466	1.402825611	3.608258859	0	0	0	0	4.337640531	0.852084315	1.807109303	0.718565672	2
Padi3	12.81975074	11.09166812	14.8800646	11.21392246	6.966995687	17.53532014	23.45368258	0	6.771727103	0.873901969	0	13.8804497	9.372927467	14.45687443	5.029959704	2
Gm13032	1.831392963	0	4.698967767	6.900875357	0.633363244	1.402825611	0.902064715	0	0.752414123	0	0	0	0.852084315	0	0.718565672	2
Isg15	1808.500551	1239.890043	957.0231019	860.8842008	1149.554288	1510.843184	1553.355439	253.936426	376.9594754	331.2088462	425.6418144	938.665411	775.3967268	927.0470725	1076.411377	2
AW011738	62.26736075	52.28929256	28.9769679	40.54264272	47.50224332	64.52997813	64.94865946	5.028444078	12.79104008	20.09974528	14.18806048	31.23101183	39.1958785	33.43152211	27.30549553	2
Fosl2	1623.529862	1173.340035	1133.234393	1035.131304	892.4088112	1080.877134	1246.653436	419.8750805	407.0560403	468.4114553	521.174755	844.9723755	973.9323722	970.4176958	1026.11178	2
Yes1	45.78482408	35.65179038	39.94122602	43.13047098	36.10170492	39.98052993	33.37639445	21.78992434	12.79104008	22.72145119	16.07980188	33.83359614	43.45630007	18.97464768	28.74262688	2
Ablim2	2.747089445	9.507144102	5.482129061	2.587828259	3.800179466	2.805651223	3.608258859	0.838074013	0	0.873901969	0	1.735056213	0	4.517773258	2.874262688	2
Cxcl10	825.9582264	441.2899387	340.6751631	347.6315961	384.4514893	657.9252118	534.0223112	249.7460559	230.2387215	194.8801391	216.60439	386.0500073	360.4316653	356.9040874	494.3731823	2
Ccng2	973.3853599	862.7733273	958.5894245	872.9607327	794.8708716	840.2925412	840.7243142	271.5359802	358.1491223	436.9509845	493.7445048	870.1306906	921.955229	848.4378178	848.6260586	2
Plac8	1800.259283	1762.782969	1522.465556	1498.352562	1525.138692	1563.449144	1809.541818	545.5861825	1008.987338	775.1510464	817.2322837	1510.366433	1344.589049	1386.95639	1385.394616	2
Hpse	119.9562391	148.1529956	131.5710975	94.88703616	93.10439691	132.5670203	100.1291833	35.19910855	61.69795805	57.67752995	62.42746612	104.9709009	112.4751296	89.45191051	76.8865269	2
Plcxd1	100.726613	99.03275106	76.74980686	62.97048763	81.70385851	107.3161593	92.91266562	10.89496217	18.05793894	20.09974528	34.99721585	81.54764199	109.0667923	89.45191051	95.56923437	2
Wscd2	43.03773463	36.44405239	33.67593566	43.13047098	52.56914927	45.59183237	67.65485361	14.24725822	9.781383594	15.73023544	4.729353494	44.24393342	80.94800994	74.99503608	43.11394032	2
Oasl2	632.7462688	430.9905326	382.965873	357.9829092	445.887724	589.1867568	644.0742064	113.9780658	115.1193608	94.38141264	146.6099583	399.0629289	325.4962084	435.5133421	358.5642703	2

Oasl1	204.2003154	132.3077554	102.5941296	87.9861608	133.0062813	201.3054752	205.670755	10.05688816	9.028969471	21.84754922	60.53572472	86.75281063	70.72299816	94.87323842	125.0304269	2
Cabp1	12.81975074	11.88393013	10.18109683	11.21392246	5.066905954	7.715540863	2.706194144	5.028444078	3.762070613	0.873901969	4.729353494	19.08561834	1.70416863	3.614218606	4.311394032	2
Oas2	190.4648682	171.1285938	115.9078716	101.7879115	142.50673	144.491038	175.9026194	17.59955427	37.62070613	28.83876497	55.80637123	141.4070813	94.58135898	118.3656594	140.120306	2
Oas3	3129.850574	2668.338445	2232.009689	2125.46961	2229.43862	2638.714975	3088.669583	971.3277811	1411.528894	1039.069441	1387.592315	2278.996335	2096.9795	2265.211511	2364.799626	2
Oas1a	282.9502128	209.9494323	141.7521943	196.6749477	167.8412597	183.0687423	222.8099846	64.53169901	97.06142181	69.03825554	91.74945778	142.2746094	128.6647316	105.7158942	143.7131344	2
Tmem116	3.662785926	2.376786026	4.698967767	6.038265938	1.900089733	3.507064029	1.80412943	0	1.504828245	1.747803938	0	5.205168638	1.70416863	4.517773258	0.718565672	2
Abcb9	1399.184224	1266.03469	1083.11207	1117.941808	849.3401106	765.241371	904.7709089	362.0479736	562.0533496	697.3737712	723.5910846	843.2373193	827.37387	781.5747736	819.164866	2
Alox5ap	5.494178889	20.59881222	1.566322589	6.900875357	7.600358931	0.701412806	3.608258859	2.514222039	0	0	0.945870699	5.205168638	5.112505891	1.807109303	1.437131344	2
Stard13	16.48253667	9.507144102	17.22954848	11.21392246	5.066905954	8.416953669	4.510323574	8.380740131	10.53379772	2.621705907	2.837612096	2.602584319	5.964590206	6.324882561	10.77848508	2
Samd9l	1836.887142	1662.957956	1546.743557	1361.197664	1733.5152	2124.579389	2348.074453	458.4264851	589.8926721	565.4145739	781.2891972	1882.535991	1842.206289	1854.094145	2078.810489	2
Hilpda	846.103549	645.6935369	652.3733583	527.0543554	626.3962486	650.2096709	693.6877657	142.4725822	188.8559448	231.5840218	246.8722524	612.474843	689.336211	611.7064991	691.9787421	2
Tmem140	0	10.29940611	6.265290356	0.86260942	0	0	3.608258859	0.838074013	0	0	0	11.27786538	2.556252945	0	3.59282836	2
Try5	0	3.961310043	9.397935534	0	2.533452977	2.805651223	4.510323574	1.676148026	0	0	0	3.470112425	1.70416863	0	0	2
Gpnmb	30.21798389	30.89821833	32.10961307	26.74089201	38.6351579	37.87629151	52.31975346	1.676148026	4.514484735	6.991215751	21.75502607	42.50887721	36.63962555	41.56351397	58.20381943	2
Particl	15.56684019	24.56012226	37.59174214	22.42784491	17.1008076	20.34097137	35.18052388	20.11377631	4.514484735	9.612921658	13.24218978	21.68820266	28.1187824	28.0101942	22.27553583	2
Rab11fip5	10.98835778	5.54583406	10.18109683	18.97740723	3.800179466	6.312715251	6.314453003	2.514222039	3.762070613	1.747803938	3.783482795	4.337640531	7.668758836	2.710663955	4.311394032	2
Aldh1l1	10.98835778	28.52143231	28.9769679	32.77915795	15.20071786	12.6254305	21.64955315	2.514222039	12.03862596	13.10852953	22.70089677	14.74797781	9.372927467	15.36042908	15.80844478	2
Bhlhe40	6538.072878	5020.564348	5261.277576	4743.489199	4900.964784	5046.665137	5610.842526	2189.887396	2302.387215	2862.028948	3027.732107	4324.62761	4744.405467	4365.976076	4940.138995	2
Rasgef1a	39.37494871	57.04286461	68.13503262	59.52004996	81.70385851	79.96105985	68.55691832	35.19910855	20.31518131	26.21705907	22.70089677	100.6332603	83.50426289	82.22347329	68.26373884	2
Ret	0.915696482	3.961310043	3.915806472	5.175656518	3.166816221	1.402825611	3.608258859	0	0	0	0	2.602584319	3.408337261	7.228437213	6.467091048	2
Usp18	1334.169774	672.6304452	581.1056805	463.2212584	829.70585	1158.032542	1129.385023	116.4922878	179.0745612	137.2026091	221.3337435	585.5814717	511.2505891	643.3309119	716.409975	2
Slc2a3	4387.101843	3329.877222	3084.089178	2778.464941	2998.341598	3592.636391	4209.936024	793.6560904	992.4342277	1027.708715	1098.155881	3273.183545	3690.377169	2796.501647	3543.965894	2
Pianp	13.73544722	3.169048034	4.698967767	2.587828259	2.533452977	4.90988964	2.706194144	0	0.752414123	2.621705907	1.891741398	7.807752957	11.92918041	7.228437213	2.155697016	2
Plekhg6	9.156964816	3.961310043	5.482129061	10.35131304	7.600358931	4.208476834	9.020647148	1.676148026	2.257242368	0.873901969	2.837612096	4.337640531	9.372927467	2.710663955	4.311394032	2
Apold1	51.27900297	30.10595632	37.59174214	21.56523549	43.06870061	24.5494482	34.27845916	14.24725822	6.771727103	5.243411813	16.07980188	7.807752957	19.59793925	16.26398373	20.11983882	2
Bhlhe41	25.63950148	10.29940611	5.482129061	15.52696955	12.03390164	14.02825611	6.314453003	5.866518091	1.504828245	2.621705907	4.729353494	1.735056213	5.112505891	7.228437213	12.9341821	2
Mansc4	0.915696482	3.169048034	2.349483883	1.725218839	1.900089733	4.90988964	3.608258859	0.838074013	0	0	0	4.337640531	0	2.710663955	1.437131344	2
Syt5	10.0726613	11.88393013	1.566322589	5.175656518	5.700269198	17.53532014	14.43303544	0.838074013	1.504828245	0.873901969	2.837612096	9.542809169	5.964590206	9.939101167	20.11983882	2
Pglyrp1	185.8863858	154.4910917	144.8848395	137.1548977	207.1097809	197.7984112	250.7739907	50.28444078	67.71727103	48.93851026	67.15681961	157.8901153	185.7543807	206.0104606	238.5638031	2
Irgc1	38.45925223	22.18333624	27.41064531	12.93914129	14.56735462	16.83390734	26.15987673	5.028444078	12.03862596	12.23462756	12.29631908	11.27786538	16.18960199	24.39597559	7.18565672	2
D930028M14Rik	2.747089445	1.584524017	0	7.763484777	1.266726489	1.402825611	3.608258859	0.838074013	1.504828245	0	0	8.675281063	5.112505891	9.035546516	2.874262688	2
9130221H12Rik	34.7964663	19.0142882	23.49483883	21.56523549	18.36753408	16.13249453	14.43303544	13.40918421	7.524141226	6.117313782	8.512836289	16.48303402	28.97086672	34.33507676	20.83840449	2
Cyp2s1	33.88076982	16.63750218	25.84432272	20.70262607	20.90098706	37.87629151	27.06194144	3.352296052	9.781383594	24.46925513	5.675224193	30.36348372	16.18960199	37.04574071	28.74262688	2
Aplp1	86.07546927	51.49703055	54.03812932	43.13047098	43.70206386	51.20313482	35.18052388	10.89496217	33.85863552	28.83876497	34.99721585	32.09853993	42.60421576	43.37062328	41.67680897	2
Cd22	1.831392963	3.169048034	1.566322589	2.587828259	6.333632443	4.90988964	6.314453003	0	0	0	0.945870699	4.337640531	4.260421576	3.614218606	14.37131344	2
Kcnc1	11.90405426	12.67619214	17.22954848	18.97740723	15.83408111	13.32684331	15.33510015	2.514222039	3.762070613	1.747803938	4.729353494	32.96606804	16.18960199	11.74621047	9.341353736	2
Tmem86a	18.31392963	11.88393013	6.265290356	12.93914129	17.1008076	14.72966892	12.62890601	3.352296052	6.771727103	2.621705907	11.35044839	12.14539349	9.372927467	16.26398373	12.9341821	2
Slc17a6	70.50862908	79.22620085	55.60445191	56.06961228	43.06870061	42.78618115	50.51562403	25.14222039	16.5531107	17.47803938	20.80915537	58.12438312	46.86463733	45.17773258	51.01816271	2
Tjp1	12.81975074	13.46845414	11.74741942	12.07653188	7.600358931	4.90988964	4.510323574	2.514222039	2.257242368	1.747803938	3.783482795	8.675281063	10.22501178	12.64976512	12.21561642	2
Arrdc4	56.77318186	44.36667248	41.50754861	43.9930804	44.3354271	56.11302446	52.31975346	24.30414638	27.08690841	21.84754922	12.29631908	29.49595561	48.56880596	45.17773258	31.61688957	2
Isg20	979.7952353	732.8423579	621.8300678	602.1013749	687.8324833	956.0256542	1012.11661	273.2121283	325.7953151	284.0181399	293.2199166	601.1969777	612.6486226	714.7117294	666.8289436	2
Lrrc32	1438.559173	1272.372786	1268.721297	1091.200916	1431.400932	1403.527024	1635.443328	562.3476628	704.2596187	556.6755542	664.9471012	1078.337436	1161.390922	1097.818902	1305.633826	2
Arrb1	921.1906605	721.7506897	744.7863911	633.155314	591.5612702	629.8686995	725.2600307	155.8817664	176.0649047	223.718904	322.5419083	704.4328223	745.5737758	639.7166933	713.5357123	2
Xrra1	7.325571853	4.753572051	12.53058071	6.038265938	6.333632443	9.118366474	4.510323574	0	6.771727103	1.747803938	0	0.867528106	1.70416863	7.228437213	7.18565672	2
Trim34a	531.1039593	513.3857815	400.1954215	395.9377236	474.38907	455.9183237	520.4913404	206.1662072	261.8401147	221.0971981	214.7126486	422.4861878	387.6983634	468.0413095	444.7921509	2
Trim30a	4135.285311	3333.838532	2780.222595	2505.017755	2785.531548	3259.465308	3730.037596	1278.900944	1775.697329	1490.002857	1685.541585	2770.884771	2474.452851	2662.775558	2853.424283	2
Trim30d	716.0746486	523.6851876	409.593357	379.5481446	492.1232408	624.2573971	664.8216948	159.2340625	212.9331967	186.1411194	209.9832951	395.5928165	396.2192066	456.295099	426.8280091	2
Apbb1	71.42432556	84.77203491	83.79825851	75.90962893	58.26941847	85.5723623	88.40234205	28.49451644	38.37312025	27.964863	38.78069865	85.88528252	86.06051583	79.51280934	120.0004672	2
Btbd16	2.747089445	3.169048034	0.783161294	0	0.633363244	0	0	0	0	0	0	1.735056213	1.70416863	0	0	2
Glrx3	5.494178889	5.54583406	10.18109683	7.763484777	5.066905954	3.507064029	4.510323574	1.676148026	0	2.621705907	0	0.867528106	0	2.710663955	4.311394032	2
Bnip3	2693.063352	2444.920558	2290.746786	2044.384325	1919.723993	2134.399168	2114.439691	615.9843996	984.1576723	1232.201776	1363.945548	2226.944649	2189.85669	2070.043707	2245.517725	2
Lmntd2	9.156964816	26.14464628	12.53058071	14.66436013	10.76717515	8.416953669	25.25781201	3.352296052	9.028969471	11.3607256	4.729353494	8.675281063	15.33751767	13.55331977	12.9341821	2
Irf7	1594.227574	1369.821013	996.9643279	929.8929544	1150.187652	1533.288393	1527.195562	258.96487	471.0112407	308.487395	460.6390303	999.3923784	978.1927938	1201.727687	1146.830812	2
Muc2	9.156964816	6.338096068	0	6.900875357	3.800179466	5.611302446	6.314453003	0	0	0	0	4.337640531	1.70416863	0.903554652	7.18565672	2
Dusp8	10.0726613	10.29940611	10.96425812	3.450437679	6.333632443	14.02825611	9.922711863	0	0	4.369509845	5.675224193	2.602584319	5.112505891	1.807109303	12.9341821	2
Lrrc8e	4.578482408	8.714882094	3.132645178	2.587828259	3.800179466	0.701412806	1.80412943	0	0	0	0	1.735056213	6.816674521	6.324882561	0	2
Atp7b	31.13368037	14.26071615	21.92851625	18.97740723	13.93399137	19.63955856	15.33510015	14.24725822	14.29586833	3.495607876	3.783482795	12.14539349	17.0416863	4.517773258	17.9641418	2
Rnf122	9.156964816	8.714882094	15.66322589	15.52696955	13.93399137	11.22260489	15.33510015	11.73303618	6.019312981	0	5.675224193	20.82067455	11.0770961	19.87820233	12.9341821	2
Ppp1r3b	358.9530208	239.2631266	289.769679	180.2853687	175.4416187	233.5704643	236.3409553	75.42666117	31.60139315	78.6511772	84.18249219	169.1679807	213.0210788	185.2287036	168.8629329	2
Ankrd37	332.3978228	299.4750392	339.1088405	295.8750309	381.9180363	308.6216345	367.1403389	62.01747697	101.5759065	147.6894327	169.3108551	295.8270842	351.9108222	364.1325246	368.6241897	2
Ddx60	165.7410632	165.5827598	115.1247103	113.001834	149.4737256	117.1359386	224.614114	15.08533223	48.15450385	40.19949057	58.64398332	105.838429	142.2980806	117.4621047	94.13210303	2
B3gnt3	279.2874269	207.5726462	198.1398075	213.9271361	205.8430544	210.4238417	217.3975963	60.34132894	111.3572901	90.88580477	90.80358708	203.869105	192.5710552	170.7718291	185.3899434	2
2210011C24Rik	5.494178889	0	4.698967767	2.587828259	3.800179466	5.611302446	3.608258859	0	0	0	0.945870699	2.602584319	7.668758836	1.807109303	9.341353736	2
Disc1	38.45925223	36.44405239	41.50754861	24.15306375	30.40143573	37.87629151	52.31975346	6.704592104	17.30552482	14.85633347	27.43025026	39.03876478	38.34379418	24.39597559	54.61099107	2
Kdm4d	69.5929326	47.53572051	46.98967767	52.6191746	42.43533737	35.77205309	60.43833589	20.95185033	34.61104964	41.07339254	3.783482795	32.09853993	22.15419219	19.87820233	35.9282836	2
Gm27162	20.14532259	32.48274235	21.14535495	24.15306375	19.00089733	11.9240177	26.15987673	5.866518091	7.524141226	6.991215751	5.675224193	26.02584319	13.63334904	12.64976512	20.11983882	2
Il18	2.747089445	11.09166812	21.14535495	15.52696955	8.86708542	11.22260489	12.62890601	5.866518091	7.524141226	4.369509845	2.837612096	4.337640531	8.520843152	10.84265582	5.029959704	2
Islr	0	0.792262009	1.566322589	4.313047098	0	1.402825611	1.80412943	0	0	0	0	0	0	4.517773258	3.59282836	2
Parp16	124.5347215	72.88810478	96.32883922	75.90962893	82.970585	114.3302873	106.4436363	33.52296052	48.90691797	39.3255886	61.48159542	156.1550591	167.0085258	113.8478861	107.0662851	2
Kbtbd13	6.409875371	4.753572051	18.01270977	6.038265938	8.233722176	7.014128057	4.510323574	0	0	3.495607876	0	6.94022485	7.668758836	2.710663955	4.311394032	2
Myo5c	7.325571853	3.169048034	4.698967767	2.587828259	4.43354271	4.208476834	2.706194144	0	0.752414123	0	0.945870699	1.735056213	0.852084315	4.517773258	0	2
Cspg5	33.88076982	22.18333624	14.8800646	12.07653188	11.4005384	20.34097137	6.314453003	7.542666117	9.781383594	7.86511772	10.40457769	8.675281063	16.18960199	11.74621047	23.71266717	2
Ccrl2	318.6623756	223.4178864	173.0786461	151.8192579	187.4755203	249.001546	241.7533436	34.36103454	60.94554393	51.56021617	30.26786236	141.4070813	178.9377062	133.7260884	175.330024	2
Ccr2	257.3107113	299.4750392	288.9865177	371.7846599	450.9546299	385.7770431	451.0323574	96.3785115	130.1676432	123.2201776	174.0402086	412.0758505	450.7526027	414.7315851	399.5225136	2
Ccr5	724.3159169	767.7018862	709.5441328	707.3397241	941.8111442	824.1600467	992.2711863	281.5928684	350.6249811	285.7659438	294.1657873	793.7882172	812.8884367	859.2804736	859.4045437	2
Apln	8.241268334	19.0142882	18.79587107	13.80175071	16.46744435	18.23673295	22.55161787	0.838074013	1.504828245	1.747803938	3.783482795	24.29078698	16.18960199	16.26398373	18.68270747	2
Xpnpep2	5.494178889	1.584524017	6.265290356	3.450437679	3.166816221	3.507064029	0.902064715	0	0	0.873901969	0	2.602584319	0	0.903554652	0	2
Gm14718	10.98835778	4.753572051	5.482129061	5.175656518	8.86708542	9.81977928	9.922711863	0	0.752414123	0.873901969	0.945870699	4.337640531	4.260421576	1.807109303	4.311394032	2
Pnck	0.915696482	2.376786026	1.566322589	0.86260942	1.900089733	2.104238417	0	0	0	0	0	0	1.70416863	2.710663955	2.155697016	2
Chic1	10.0726613	11.09166812	7.831612945	11.21392246	3.800179466	4.208476834	10.82477658	4.190370065	1.504828245	4.369509845	4.729353494	0.867528106	6.816674521	4.517773258	2.874262688	2
Rnf128	1.831392963	8.714882094	3.915806472	9.488703616	3.800179466	7.715540863	10.82477658	0	0	1.747803938	0	8.675281063	1.70416863	14.45687443	0.718565672	2
Tsc22d3	438.6186147	345.4262357	396.279615	325.2037512	326.1820708	351.4078157	368.0424036	144.1487302	131.6724715	180.8977076	174.9860793	292.3569718	310.1586907	274.6806141	296.0490568	2
Scml2	21.06101908	11.09166812	10.18109683	20.70262607	6.966995687	10.52119209	9.922711863	5.866518091	6.019312981	13.10852953	5.675224193	5.205168638	1.70416863	2.710663955	4.311394032	2
