# Supplementary material for: Aspirin prevents metastasis by limiting platelet TXA2 suppression of T cell immunity
Source: Nature. 2025 Mar 5;640(8060):1052–61. doi: 10.1038/s41586-025-08626-7 (PMC12018268; doi:10.1038/s41586-025-08626-7)
Supplement: Supplementary file 5 — Gene expression profiles in treated CD8 T cells [file 41586_2025_8626_MOESM5_ESM.pdf]

**Suppementary Table 3. Gene expression profiles of Vehicle or TXA2 analogue U46619 treated Arhgef 1 KO vs WT CD8 T cells. Naïve WT and Arhgef1 KO CD8+ T cells were stimulated in vitro with anti-CD3/28 antibodies and rhIL-2 in the presence of TXA2 analogue or vehicle control for 5 days. FPM values for all expressed genes shown. Data are representative of three to four biological replicates per group.**

| Gene name | WT_d5_Veh_1 | WT_d5_Veh_2 | WT_d5_Veh_3 | WT_d5_Veh_4 | KO_d5_Veh_1 | KO_d5_Veh_2 | KO_d5_Veh_3 | WT_d5_U46619_1 | WT_d5_U46619_2 | WT_d5_U46619_3 | WT_d5_U46619_4 | KO_d5_U46619_1 | KO_d5_U46619_2 | KO_d5_U46619_3 | KO_d5_U46619_4 |
|---|---|---|---|---|---|---|---|---|---|---|---|---|---|---|---|
| **Mrpl15** | 1340.579649 | 1361.898393 | 1449.631556 | 1217.141891 | 1349.06371 | 1364.247907 | 1177.194453 | 1405.45012 | 1379.175087 | 1377.269503 | 1397.051022 | 1243.167776 | 1291.759822 | 1216.184561 | 1235.21439 |
| **Lypla1** | 1257.251269 | 1350.806725 | 1415.172459 | 1270.623675 | 1184.389267 | 1339.698459 | 1404.514761 | 1399.583602 | 1527.400669 | 1415.72119 | 1500.150928 | 1239.697664 | 1428.945397 | 1276.722723 | 1294.855341 |
| **Tcea1** | 2616.144848 | 2528.900331 | 2503.766658 | 2504.155145 | 2413.113961 | 2252.937932 | 2477.069707 | 2619.819365 | 2795.97088 | 2577.136906 | 2708.027811 | 2413.463192 | 2508.536224 | 2486.582401 | 2485.518659 |
| **Atp6v1h** | 960.5656092 | 912.6858338 | 884.9722628 | 874.6859515 | 894.9422642 | 892.8985017 | 832.6057317 | 887.5203798 | 911.1735024 | 915.8492634 | 943.0330867 | 905.699343 | 849.5280622 | 870.1231295 | 822.7576944 |
| **Rb1cc1** | 496.307493 | 409.5994584 | 476.9452283 | 463.2212584 | 421.8199207 | 418.743445 | 451.9344221 | 490.2732976 | 532.7091988 | 478.898279 | 489.961022 | 466.7301212 | 438.8234223 | 445.4524432 | 425.3908778 |
| **Pcmtd1** | 323.240858 | 304.2286113 | 393.1469698 | 252.74456 | 295.7806351 | 263.0298021 | 296.7792912 | 324.3346431 | 280.6504677 | 271.7835123 | 347.1345464 | 326.190568 | 313.567028 | 315.3405734 | 336.2887345 |
| **Rrs1** | 2153.718125 | 2063.05027 | 2235.925496 | 2131.507876 | 2318.742837 | 2405.845924 | 2369.724006 | 2424.54812 | 2344.522406 | 2304.479492 | 2253.064004 | 2409.125551 | 2359.421469 | 2183.891593 | 2121.205864 |
| **Adhfe1** | 0 | 1.584524017 | 0 | 0.86260942 | 0 | 1.402825611 | 2.706194144 | 0 | 0 | 0 | 0.945870699 | 0.867528106 | 0 | 0.903554652 | 0 |
| **Mybl1** | 76.00280797 | 94.27917901 | 109.6425812 | 86.26094197 | 76.63695256 | 58.91867568 | 73.96930661 | 139.9583602 | 121.8910879 | 142.4460209 | 104.9916476 | 113.6461819 | 106.5105394 | 81.31991864 | 86.22788064 |
| **Vcpip1** | 1021.917273 | 1056.085257 | 1065.882522 | 966.12255 | 1021.614913 | 935.6846828 | 1032.864098 | 982.2227433 | 1040.588732 | 959.5443619 | 945.8706988 | 983.7768725 | 1061.697057 | 915.300862 | 902.518484 |
| **1700034P13Rik** | 0.915696482 | 0.792262009 | 1.566322589 | 0 | 3.166816221 | 0.701412806 | 4.510323574 | 0.838074013 | 0.752414123 | 0.873901969 | 0 | 0 | 3.408337261 | 0 | 1.437131344 |
| **Sgk3** | 403.8221484 | 469.811371 | 413.5091635 | 400.2507707 | 361.6504125 | 354.2134669 | 366.2382742 | 459.2645592 | 507.8795327 | 443.9422002 | 561.8471951 | 412.0758505 | 338.2774731 | 419.2493583 | 334.8516031 |
| **Mcmdc2** | 0.915696482 | 2.376786026 | 0 | 1.725218839 | 0.633363244 | 1.402825611 | 0 | 0 | 0 | 0 | 1.891741398 | 0 | 0 | 0 | 0 |
| **Snhg6** | 687.6880577 | 607.6649605 | 561.5266481 | 554.6578568 | 608.6620777 | 627.0630483 | 592.6565176 | 745.8858716 | 708.7741035 | 824.9634586 | 652.6507822 | 571.701022 | 551.2985519 | 635.1989201 | 601.4394674 |
| **Cops5** | 1808.500551 | 1635.228786 | 1842.778526 | 1758.860607 | 1749.982644 | 1816.659167 | 1672.427981 | 2233.467245 | 1869.749095 | 2049.300117 | 1978.761502 | 1724.645875 | 1801.306242 | 1752.896024 | 1774.138644 |
| **Cspp1** | 243.5752641 | 195.6887161 | 256.0937433 | 223.4158397 | 232.4443106 | 188.6800447 | 247.1657318 | 200.2996891 | 201.6469849 | 220.2232962 | 240.2511575 | 228.159892 | 264.998222 | 225.8886629 | 219.8810956 |
| **Arfgef1** | 3033.702443 | 3255.404593 | 3178.851694 | 2916.482448 | 2750.69657 | 2794.428618 | 3159.030631 | 3099.1977 | 3032.228914 | 3062.152499 | 3026.786236 | 3085.797474 | 3101.586907 | 2809.151412 | 2987.077498 |
| **Slco5a1** | 1.831392963 | 6.338096068 | 3.132645178 | 8.626094197 | 6.333632443 | 7.715540863 | 9.922711863 | 5.866518091 | 2.257242368 | 8.739019689 | 6.621094891 | 5.205168638 | 3.408337261 | 0.903554652 | 2.155697016 |
| **Ncoa2** | 1542.948571 | 1643.151406 | 1583.552137 | 1569.086534 | 1542.872863 | 1604.131087 | 1771.6551 | 1553.78922 | 1672.616594 | 1309.979051 | 1530.418791 | 1693.414863 | 1729.73116 | 1539.657126 | 1754.018805 |
| **Tram1** | 2387.220727 | 2497.209851 | 2628.289304 | 2502.429926 | 2700.660874 | 2689.91811 | 2971.40117 | 2248.552577 | 2567.9894 | 2331.570453 | 2390.215256 | 2970.416236 | 2889.417913 | 2922.095743 | 2844.08293 |
| **Lactb2** | 415.7262026 | 456.3429169 | 515.3201318 | 464.9464772 | 480.7227024 | 472.7522311 | 490.7232048 | 445.0173009 | 452.2008877 | 489.3851026 | 558.0637123 | 505.768886 | 511.2505891 | 509.6048235 | 483.5946972 |
| **Msc** | 10.98835778 | 2.376786026 | 2.349483883 | 5.175656518 | 1.900089733 | 2.104238417 | 4.510323574 | 34.36103454 | 13.54345421 | 15.73023544 | 12.29631908 | 0 | 0.852084315 | 0 | 0 |
| **Terf1** | 324.1565545 | 413.5607684 | 345.3741309 | 339.8681113 | 365.4505919 | 348.6021644 | 411.3415099 | 293.3259046 | 298.7084067 | 352.1824935 | 312.1373306 | 376.5071981 | 378.3254359 | 409.3102572 | 345.6300882 |
| **Sbspon** | 1.831392963 | 0 | 4.698967767 | 0 | 0 | 2.805651223 | 0.902064715 | 0 | 0.752414123 | 1.747803938 | 0 | 0 | 2.556252945 | 2.710663955 | 6.467091048 |
| **Rpl7** | 25751.21645 | 24124.37816 | 24971.88104 | 24256.57688 | 25173.02214 | 25281.02176 | 25508.586 | 25604.83725 | 24675.42115 | 25484.72922 | 25454.32637 | 25034.25856 | 24260.54462 | 24852.27069 | 24762.49162 |
| **Rdh10** | 117.2091496 | 126.7619214 | 114.341549 | 130.2540224 | 103.8715721 | 95.39214158 | 101.9333128 | 190.242801 | 125.6531585 | 157.3023544 | 163.6356309 | 118.8513506 | 94.58135898 | 111.1372221 | 99.16206273 |
| **Stau2** | 5.494178889 | 7.922620085 | 8.614774239 | 4.313047098 | 3.166816221 | 2.104238417 | 9.020647148 | 10.89496217 | 13.54345421 | 7.86511772 | 15.13393118 | 0.867528106 | 0.852084315 | 10.84265582 | 7.904222392 |
| **Ube2w** | 215.1886732 | 183.804786 | 184.0429042 | 221.6906209 | 216.6102295 | 167.6376606 | 193.9439137 | 258.96487 | 230.2387215 | 235.0796296 | 242.1428989 | 146.61225 | 214.7252474 | 177.0967117 | 173.1743269 |
| **Tceb1** | 1143.704905 | 1064.007877 | 1124.619619 | 1076.536556 | 1035.548904 | 1027.56976 | 1052.709522 | 1173.303618 | 987.9197429 | 1205.110815 | 1186.121856 | 1099.158111 | 1059.992888 | 1157.453509 | 1046.231618 |
| **Tmem70** | 703.2548978 | 795.4310565 | 704.845165 | 820.3415581 | 715.0671028 | 803.1176625 | 747.8116486 | 874.9492696 | 852.4852009 | 838.9458901 | 830.4744735 | 746.9416995 | 771.9883895 | 803.2600852 | 852.9374526 |
| **Ly96** | 12.81975074 | 13.46845414 | 6.265290356 | 9.488703616 | 12.66726489 | 11.9240177 | 5.412388289 | 12.5711102 | 1.504828245 | 16.60413741 | 21.75502607 | 9.542809169 | 11.0770961 | 12.64976512 | 11.49705075 |
| **Il17a** | 0 | 2.376786026 | 0 | 0 | 1.900089733 | 0.701412806 | 11.72684129 | 0 | 0 | 0 | 0 | 0 | 0 | 0 | 0.718565672 |
| **Il17f** | 8.241268334 | 4.753572051 | 11.74741942 | 8.626094197 | 9.500448664 | 21.74379698 | 58.63420646 | 5.866518091 | 13.54345421 | 4.369509845 | 4.729353494 | 20.82067455 | 7.668758836 | 22.58886629 | 7.904222392 |
| **Mcm3** | 9693.562954 | 9553.095299 | 9779.335084 | 9824.25868 | 9711.991988 | 9362.458131 | 9670.133742 | 10284.84429 | 10203.48792 | 10118.037 | 10505.78585 | 10002.59907 | 9822.827985 | 9930.969175 | 9511.6538 |
| **Paqr8** | 34.7964663 | 46.7434585 | 46.20651637 | 56.9322217 | 40.53524763 | 37.87629151 | 42.39704159 | 54.47481085 | 33.10622139 | 42.82119648 | 34.99721585 | 45.97898963 | 42.60421576 | 44.27417793 | 33.77258658 |
| **Efhc1** | 5.494178889 | 0 | 2.349483883 | 0 | 3.166816221 | 0.701412806 | 0 | 0 | 0 | 1.747803938 | 0.945870699 | 0 | 0.852084315 | 0 | 2.155697016 |
| **Tram2** | 32.04937685 | 29.31369431 | 32.10961307 | 36.22959563 | 42.43533737 | 32.96640187 | 36.98465331 | 26.81836842 | 27.08690841 | 28.83876497 | 25.53850887 | 35.56865236 | 41.75213144 | 28.91374885 | 27.30549553 |
| **Tmem14a** | 18.31392963 | 15.05297816 | 20.36219366 | 6.900875357 | 6.966995687 | 14.02825611 | 4.510323574 | 10.89496217 | 15.80069657 | 21.84754922 | 18.91741398 | 13.01292159 | 11.92918041 | 9.035546516 | 14.37131344 |
| **Gsta3** | 0 | 0 | 0 | 1.725218839 | 0.633363244 | 1.402825611 | 0 | 0 | 4.514484735 | 0 | 0 | 0 | 0 | 0 | 0 |
| **Khdc1a** | 6.409875371 | 1.584524017 | 3.132645178 | 7.763484777 | 2.533452977 | 0.701412806 | 0 | 0.838074013 | 0.752414123 | 0 | 3.783482795 | 0 | 0 | 2.710663955 | 5.029959704 |
| **Khdc1c** | 0.915696482 | 0.792262009 | 8.614774239 | 2.587828259 | 0 | 0 | 0 | 0 | 0 | 0 | 3.783482795 | 0 | 0 | 0.903554652 | 0 |
| **Kcnq5** | 409.3163273 | 339.0881396 | 370.4352923 | 335.5550642 | 272.346195 | 290.3849016 | 326.5474268 | 372.1048618 | 359.6539506 | 309.361297 | 335.7840981 | 328.7931523 | 280.3357397 | 312.6299094 | 333.4144718 |
| **Ogfrl1** | 427.6302569 | 419.1066025 | 375.1342601 | 381.2733635 | 328.0821605 | 377.3600895 | 357.2176271 | 299.1924227 | 354.3870517 | 361.7954151 | 365.1060897 | 326.190568 | 355.3191594 | 382.2036176 | 402.3967763 |
| **B3gat2** | 1.831392963 | 0 | 2.349483883 | 0 | 3.800179466 | 1.402825611 | 0.902064715 | 0 | 12.79104008 | 0.873901969 | 8.512836289 | 0.867528106 | 4.260421576 | 0.903554652 | 2.874262688 |
| **Smap1** | 415.7262026 | 431.7827946 | 433.0881959 | 477.0230091 | 385.7182158 | 396.999648 | 439.3055161 | 409.8181924 | 390.5029296 | 360.0476112 | 460.6390303 | 386.0500073 | 457.5692772 | 423.7671316 | 447.6664136 |
| **Sdhaf4** | 72.34002204 | 35.65179038 | 75.96664557 | 92.2992079 | 77.2703158 | 56.11302446 | 61.34040061 | 60.34132894 | 66.96485691 | 79.52507917 | 63.37333682 | 63.32955176 | 59.64590206 | 83.12702794 | 89.10214332 |
| **Fam135a** | 7.325571853 | 7.130358077 | 1.566322589 | 9.488703616 | 0.633363244 | 7.014128057 | 7.216517718 | 0.838074013 | 6.019312981 | 8.739019689 | 4.729353494 | 1.735056213 | 8.520843152 | 1.807109303 | 5.029959704 |
| **Lmbrd1** | 311.3368037 | 315.3202794 | 324.2287759 | 339.8681113 | 343.9162416 | 319.1428266 | 348.1969799 | 230.4703536 | 275.3835689 | 271.7835123 | 269.5731491 | 320.1178712 | 311.010775 | 322.5690106 | 324.7916837 |
| **Phf3** | 2023.689224 | 2132.769327 | 2016.640333 | 2009.017338 | 1634.710533 | 1681.987908 | 1994.465084 | 1957.740894 | 2045.813999 | 1921.71043 | 1721.484672 | 2121.10622 | 1903.55636 | 1751.088915 | 1867.552181 |
| **Ptp4a1** | 16.48253667 | 22.18333624 | 35.24225825 | 32.77915795 | 34.83497844 | 32.96640187 | 32.47432973 | 34.36103454 | 18.05793894 | 23.59535316 | 25.53850887 | 38.17123668 | 47.71672165 | 35.23863141 | 31.61688957 |
| **Prim2** | 841.5250666 | 898.4251176 | 866.1763917 | 858.2963726 | 846.8066576 | 759.6300686 | 752.3219721 | 945.3474867 | 940.5176532 | 859.9195374 | 930.7367676 | 888.3487808 | 809.4800994 | 851.1484818 | 751.6196929 |
| **1700001G17Rik** | 0 | 0.792262009 | 3.132645178 | 2.587828259 | 1.900089733 | 5.611302446 | 0.902064715 | 1.676148026 | 0.752414123 | 4.369509845 | 0 | 0 | 0 | 0.903554652 | 4.311394032 |
| **Rab23** | 140.1015617 | 180.6357379 | 191.8745171 | 169.9340557 | 145.6735462 | 162.0263581 | 115.4642835 | 215.3850214 | 242.2773475 | 191.3845312 | 235.521804 | 164.8303402 | 145.7064179 | 173.4824931 | 163.8329732 |
| **Bag2** | 446.859883 | 429.4060086 | 353.2057438 | 370.059441 | 386.351579 | 444.6957188 | 393.3002156 | 465.9691513 | 428.8760499 | 489.3851026 | 491.8527634 | 414.6784348 | 396.2192066 | 376.7822897 | 469.2233838 |
| **Zfp451** | 590.6242306 | 578.3512662 | 527.8507125 | 540.8561061 | 516.1910441 | 537.983622 | 553.8677349 | 520.4439621 | 482.2974526 | 509.4848479 | 479.5564443 | 538.734954 | 495.9130714 | 549.3612282 | 505.1516674 |
| **Gm15455** | 0.915696482 | 5.54583406 | 3.915806472 | 4.313047098 | 5.700269198 | 8.416953669 | 3.608258859 | 5.028444078 | 3.00965649 | 9.612921658 | 0 | 2.602584319 | 3.408337261 | 0.903554652 | 7.904222392 |
| **Bend6** | 12.81975074 | 12.67619214 | 3.132645178 | 7.763484777 | 8.86708542 | 7.715540863 | 13.53097072 | 4.190370065 | 13.54345421 | 6.117313782 | 4.729353494 | 5.205168638 | 2.556252945 | 12.64976512 | 10.77848508 |
| **Dst** | 181.3079033 | 141.8148995 | 178.5607751 | 179.4227593 | 91.83767042 | 103.1076824 | 102.8353775 | 159.2340625 | 188.1035306 | 172.1586879 | 167.4191137 | 104.1033728 | 84.3563472 | 94.87323842 | 120.0004672 |
| **Mir5103** | 0 | 0 | 2.349483883 | 3.450437679 | 0 | 0.701412806 | 0 | 3.352296052 | 2.257242368 | 0 | 4.729353494 | 0.867528106 | 0 | 0 | 1.437131344 |
| **Ccdc115** | 745.376936 | 809.6917727 | 833.2836173 | 772.89804 | 923.4436102 | 843.7996053 | 840.7243142 | 858.1877894 | 827.6555348 | 905.3624398 | 896.6854224 | 835.4295663 | 809.4800994 | 927.0470725 | 855.0931496 |
| **Imp4** | 1821.320302 | 1856.269886 | 1846.694332 | 1879.625925 | 1707.547307 | 1808.943626 | 1713.922958 | 2213.353468 | 2027.75606 | 2142.807628 | 2117.804495 | 1878.19835 | 1893.331348 | 1841.44438 | 1879.767798 |
| **Ptpn18** | 1094.257295 | 1087.775738 | 1225.647426 | 1240.432345 | 1397.199317 | 1423.166583 | 1424.360185 | 924.3956364 | 1097.019791 | 1041.691147 | 928.8450262 | 1286.544182 | 1406.791204 | 1486.347402 | 1360.244817 |
| **Prss39** | 0 | 2.376786026 | 0 | 0 | 0 | 0.701412806 | 0 | 0 | 0 | 0 | 0 | 0 | 0 | 0.903554652 | 5.748525376 |
| **Cfc1** | 1.831392963 | 0 | 2.349483883 | 0 | 0 | 1.402825611 | 0 | 0 | 0 | 0 | 3.783482795 | 0 | 1.70416863 | 4.517773258 | 0 |
| **Arhgef4** | 69.5929326 | 61.00417465 | 62.65290356 | 76.77223835 | 88.6708542 | 108.0175721 | 110.9539599 | 51.96058881 | 52.66898858 | 88.26409886 | 52.96875913 | 85.88528252 | 72.42716679 | 90.35546516 | 85.50931496 |
| **Fam168b** | 3472.321058 | 3889.2142 | 3917.372795 | 3657.463939 | 3816.013547 | 3760.975464 | 3975.399198 | 3831.674388 | 3919.325165 | 3777.00431 | 3917.796434 | 4192.763338 | 4181.177734 | 4168.097608 | 3893.188811 |
| **Plekhb2** | 1265.492538 | 1212.953135 | 1258.5402 | 1192.988827 | 1192.622989 | 1217.652631 | 1336.859907 | 1283.929388 | 1171.508789 | 1206.858619 | 1246.657581 | 1302.159688 | 1180.136776 | 1242.387646 | 1287.669684 |
| **Hs6st1** | 442.2814006 | 449.2125588 | 460.4988412 | 376.097707 | 500.356963 | 526.7610171 | 528.6099229 | 436.6365608 | 397.2746567 | 379.2734545 | 398.2115642 | 471.9352898 | 535.9610342 | 457.1986537 | 440.4807569 |
| **Uggt1** | 2954.952546 | 2775.293816 | 2931.372725 | 2910.444182 | 2655.692083 | 2617.672591 | 2882.096764 | 3129.368365 | 3016.428217 | 2861.155046 | 3104.347633 | 2913.159381 | 2776.942783 | 2710.663955 | 2793.064767 |
| **Neurl3** | 1282.890771 | 1384.081729 | 1153.596587 | 1183.500124 | 1310.428552 | 1308.134883 | 1436.087026 | 833.883643 | 637.2947618 | 794.3768897 | 1131.261356 | 1076.60238 | 1228.705582 | 1138.478861 | 1208.62746 |
| **Arid5a** | 3709.486447 | 3737.099894 | 3510.912083 | 3478.04118 | 2548.020332 | 2606.449986 | 2881.194699 | 2928.230602 | 2722.234295 | 3192.363892 | 3304.872222 | 2409.125551 | 2458.263249 | 2355.566977 | 2543.003913 |
| **Kansl3** | 1368.050543 | 1285.048978 | 1264.022329 | 1197.301874 | 1125.486485 | 1195.207421 | 1270.107118 | 1049.268664 | 1115.830144 | 1144.811579 | 998.8394579 | 1230.154855 | 1309.653592 | 1168.296164 | 1325.753665 |
| **Fer1l5** | 10.98835778 | 35.65179038 | 18.79587107 | 18.11479781 | 28.50134599 | 16.13249453 | 21.64955315 | 14.24725822 | 22.57242368 | 17.47803938 | 21.75502607 | 17.35056213 | 23.00627651 | 13.55331977 | 33.77258658 |
| **Lman2l** | 226.1770309 | 220.2488384 | 179.3439364 | 155.2696955 | 197.6093322 | 207.6181905 | 189.4335901 | 227.9561316 | 190.360773 | 171.2847859 | 202.4163295 | 233.3650606 | 138.8897434 | 207.8175699 | 179.641418 |
| **Cnnm4** | 316.8309826 | 350.1798078 | 335.9761953 | 307.0889534 | 303.380994 | 290.3849016 | 337.3722033 | 260.6410181 | 291.1842654 | 236.8274336 | 351.8638999 | 316.6477588 | 311.010775 | 292.7517071 | 328.3845121 |
| **Cnnm3** | 496.307493 | 606.8726985 | 559.9603256 | 590.024843 | 580.794095 | 568.1443726 | 565.5945762 | 526.3104802 | 583.8733591 | 507.7370439 | 495.6362462 | 548.2777632 | 626.2819716 | 521.351034 | 596.4095077 |
| **Ankrd23** | 24.723805 | 42.78214846 | 35.24225825 | 35.36698621 | 46.23551683 | 23.84803539 | 40.59291217 | 60.34132894 | 36.11587788 | 41.94729451 | 46.34766424 | 49.44910206 | 51.97714322 | 36.14218606 | 63.9523448 |
| **Ankrd39** | 163.9096702 | 171.9208558 | 176.9944526 | 115.5896622 | 158.3408111 | 160.6235325 | 167.7840369 | 195.271245 | 148.9779963 | 173.9064918 | 183.4989156 | 130.996744 | 166.1564415 | 168.0611652 | 162.3958419 |
| **Sema4c** | 84.2440763 | 94.27917901 | 70.4845165 | 86.26094197 | 44.3354271 | 77.15540863 | 56.83007703 | 161.7482845 | 107.5952195 | 152.0589426 | 101.2081648 | 48.58157395 | 60.49798638 | 55.11683375 | 43.83250599 |
| **Fam178b** | 0 | 2.376786026 | 0.783161294 | 0 | 0 | 0 | 0.902064715 | 0.838074013 | 6.019312981 | 7.86511772 | 0.945870699 | 0.867528106 | 0 | 3.614218606 | 0 |
| **Cox5b** | 2597.830918 | 2700.028925 | 2701.906466 | 2523.132552 | 2552.453874 | 2625.388132 | 2591.631926 | 2746.368541 | 2734.272921 | 2703.852692 | 2463.99317 | 2763.077018 | 2493.198706 | 2682.653761 | 2441.686153 |
| **Actr1b** | 1304.867486 | 1382.497205 | 1457.463169 | 1277.524551 | 1276.226937 | 1226.069584 | 1244.849306 | 1640.110844 | 1367.136461 | 1652.548623 | 1609.871929 | 1254.445642 | 1269.60563 | 1238.773427 | 1271.861239 |
| **4933424G06Rik** | 0 | 0 | 0 | 0 | 0 | 1.402825611 | 0.902064715 | 2.514222039 | 0 | 1.747803938 | 1.891741398 | 0.867528106 | 0 | 0 | 0.718565672 |
| **Zap70** | 3278.193404 | 3760.075492 | 3637.001052 | 3611.74564 | 3938.886016 | 4045.749063 | 3862.641109 | 3167.081695 | 3086.402731 | 3231.689481 | 3020.165141 | 3851.824792 | 4084.892207 | 3952.148046 | 3938.458448 |
| **Tmem131** | 1810.331944 | 1757.237135 | 1797.355171 | 1904.641599 | 1553.640038 | 1564.150557 | 1757.222064 | 2081.775848 | 1995.402253 | 1873.645821 | 1955.114734 | 1703.825201 | 1849.875048 | 1801.687975 | 1923.600304 |
| **Inpp4a** | 134.6073828 | 162.4137117 | 142.5353556 | 169.0714463 | 226.7440415 | 182.3673295 | 184.0212018 | 192.757023 | 170.0455917 | 184.3933154 | 191.0658812 | 167.4329245 | 253.0690416 | 205.1069059 | 211.9768732 |
| **Coa5** | 2227.88954 | 2444.128296 | 2268.035109 | 2362.6872 | 2262.373509 | 2188.407954 | 2477.971772 | 2387.672863 | 2361.827931 | 2293.118766 | 2373.189583 | 2386.56982 | 2302.33182 | 2314.907017 | 2187.313905 |
| **Unc50** | 430.3773463 | 446.8357728 | 510.621164 | 509.802167 | 486.4229716 | 483.2734231 | 455.542681 | 429.0938947 | 422.8567369 | 473.6548671 | 473.8812201 | 524.8545043 | 472.0547106 | 479.78752 | 517.3672838 |
| **Mgat4a** | 695.0136295 | 646.4857989 | 601.4678742 | 684.9118792 | 580.1607318 | 603.9164257 | 647.6824652 | 738.3432055 | 732.0989413 | 652.8047708 | 704.6736706 | 595.1242809 | 623.7257187 | 578.274977 | 608.6251242 |
| **4930594C11Rik** | 6.409875371 | 3.961310043 | 4.698967767 | 1.725218839 | 3.166816221 | 2.104238417 | 2.706194144 | 7.542666117 | 0.752414123 | 0.873901969 | 2.837612096 | 1.735056213 | 0.852084315 | 0.903554652 | 4.311394032 |
| **2010300C02Rik** | 51.27900297 | 45.95119649 | 37.59174214 | 50.89395576 | 39.26852115 | 32.26498906 | 30.6702003 | 31.8468125 | 38.37312025 | 60.29923585 | 39.72656935 | 39.03876478 | 30.67503535 | 44.27417793 | 34.49115225 |
| **Tsga10** | 10.98835778 | 11.09166812 | 12.53058071 | 9.488703616 | 3.800179466 | 6.312715251 | 11.72684129 | 16.76148026 | 6.771727103 | 17.47803938 | 26.48437957 | 7.807752957 | 14.48543336 | 16.26398373 | 6.467091048 |
| **Lipt1** | 76.91850445 | 95.07144102 | 45.42335508 | 55.20700286 | 77.90367905 | 64.52997813 | 76.67550076 | 68.72206907 | 68.46968515 | 59.42533389 | 78.507268 | 86.75281063 | 77.53967268 | 57.8274977 | 58.20381943 |
| **Mitd1** | 395.58088 | 396.1310043 | 357.9047116 | 319.1654853 | 392.0518482 | 436.2787652 | 415.8518335 | 300.0304967 | 319.7760021 | 327.7132383 | 298.8951408 | 397.3278727 | 394.5150379 | 364.1325246 | 363.59423 |
| **Mrpl30** | 1569.503769 | 1526.68889 | 1511.501298 | 1428.481199 | 1444.068197 | 1541.705347 | 1600.262804 | 1400.421676 | 1331.772997 | 1341.439522 | 1502.04267 | 1416.673398 | 1353.109892 | 1430.327013 | 1472.341062 |
| **Txndc9** | 925.7691429 | 1003.795965 | 1005.579102 | 992.863442 | 989.9467508 | 985.484992 | 1025.647581 | 1107.095771 | 1060.151499 | 1049.556265 | 1048.970605 | 1067.927099 | 927.0677349 | 934.2755097 | 942.7581616 |
| **Eif5b** | 5102.260795 | 4554.714287 | 4552.516605 | 4523.523797 | 4257.467728 | 4154.468048 | 5009.165361 | 4855.800832 | 4561.134411 | 4769.756946 | 4756.783744 | 4518.953906 | 4182.029819 | 4017.203981 | 4165.5252 |
| **Rev1** | 254.5636219 | 296.3059912 | 303.8665823 | 257.0576071 | 252.7119345 | 238.4803539 | 220.1037904 | 370.4287138 | 324.2904868 | 360.0476112 | 309.2997185 | 261.12596 | 272.6669809 | 229.5028815 | 234.2524091 |
| **Aff3** | 1133.632244 | 1106.790026 | 1149.68078 | 1149.858356 | 765.7361623 | 793.2978833 | 943.5596917 | 1125.5334 | 1311.457816 | 1129.081344 | 1009.244036 | 884.0111403 | 892.132278 | 732.7828224 | 862.997372 |
| **Chst10** | 140.1015617 | 153.6988296 | 183.2597429 | 153.5444767 | 133.6396445 | 143.7896252 | 156.9592604 | 161.7482845 | 159.511794 | 123.2201776 | 151.3393118 | 144.0096656 | 169.5647787 | 133.7260884 | 184.6713777 |
| **Pdcl3** | 686.7723612 | 694.0215194 | 677.4345197 | 735.805835 | 649.1973254 | 748.4074637 | 718.9455777 | 759.2950558 | 739.6230825 | 741.0688696 | 695.2149636 | 685.347204 | 623.7257187 | 686.7015352 | 744.4340362 |
| **Npas2** | 109.8835778 | 167.9595458 | 146.4511621 | 169.9340557 | 143.1400932 | 119.240177 | 134.4076425 | 138.2822122 | 153.492481 | 140.698217 | 154.1769239 | 121.4539349 | 126.1084786 | 131.9189791 | 120.7190329 |
| **Rpl31** | 11147.68897 | 10594.12758 | 10478.69812 | 9958.82575 | 11086.39023 | 11912.79509 | 11668.20709 | 10569.78945 | 10446.51768 | 10409.92025 | 9674.365507 | 10955.14493 | 10591.40804 | 11485.98673 | 10810.82053 |
| **Tbc1d8** | 3.662785926 | 1.584524017 | 5.482129061 | 4.313047098 | 1.900089733 | 0 | 2.706194144 | 4.190370065 | 11.28621184 | 7.86511772 | 1.891741398 | 4.337640531 | 0.852084315 | 2.710663955 | 1.437131344 |
| **Cnot11** | 1047.556775 | 1118.673956 | 1077.629941 | 1117.941808 | 1087.48469 | 1099.815279 | 1030.157904 | 1257.949094 | 1352.840592 | 1136.946462 | 1306.247435 | 1132.991707 | 1013.980335 | 1147.514407 | 1138.208024 |
| **Snord89** | 1.831392963 | 3.961310043 | 1.566322589 | 0 | 0 | 0 | 3.608258859 | 5.866518091 | 0 | 0 | 0.945870699 | 0.867528106 | 0 | 1.807109303 | 0 |
| **Rnf149** | 684.9409682 | 729.6733098 | 749.4853588 | 771.1728212 | 682.1322141 | 709.8297594 | 698.1980892 | 999.8222976 | 918.6976437 | 856.4239295 | 905.1982587 | 733.9287779 | 711.4904032 | 736.397041 | 666.1103779 |
| **Rfx8** | 1.831392963 | 1.584524017 | 2.349483883 | 3.450437679 | 1.266726489 | 2.805651223 | 3.608258859 | 5.866518091 | 1.504828245 | 0 | 0 | 2.602584319 | 4.260421576 | 1.807109303 | 0 |
| **Map4k4** | 3167.39413 | 3493.083195 | 3636.21789 | 3445.262022 | 3349.224836 | 3366.080055 | 3673.207519 | 3977.499266 | 3678.552645 | 3607.467328 | 3648.223285 | 3757.264228 | 3808.816889 | 3876.249455 | 3707.798867 |
| **Il1r2** | 10.98835778 | 13.46845414 | 21.14535495 | 19.84001665 | 6.333632443 | 9.81977928 | 9.020647148 | 25.9802944 | 21.06759543 | 13.9824315 | 19.86328467 | 12.14539349 | 11.92918041 | 8.131991864 | 4.311394032 |
| **Il1r1** | 0 | 3.961310043 | 2.349483883 | 3.450437679 | 1.900089733 | 0.701412806 | 1.80412943 | 0 | 0 | 5.243411813 | 0 | 0 | 0 | 2.710663955 | 0 |
| **Il1rl2** | 86.99116575 | 71.30358077 | 72.05083909 | 65.55831589 | 23.43444004 | 36.4734659 | 54.12388289 | 29.33259046 | 37.62070613 | 48.06460829 | 47.29353494 | 35.56865236 | 39.1958785 | 51.50261514 | 49.58103137 |
| **Il1rl1** | 59.5202713 | 56.2506026 | 90.06354887 | 78.49745719 | 36.73506817 | 30.86216345 | 44.20117102 | 93.86428946 | 65.46002866 | 76.90337326 | 76.6155266 | 49.44910206 | 33.23128829 | 43.37062328 | 51.01816271 |
| **Il18r1** | 1955.011988 | 2207.241956 | 2375.328206 | 2154.79833 | 2195.870368 | 2177.185349 | 2371.528135 | 1725.594393 | 1696.693846 | 1873.645821 | 1595.683869 | 2417.800832 | 2433.552804 | 2228.165771 | 2389.949425 |
| **Il18rap** | 955.9871268 | 1052.123947 | 1119.13749 | 1065.322633 | 1391.499048 | 1203.624375 | 1286.344283 | 975.5181512 | 867.5334833 | 898.371224 | 822.9075079 | 1314.305081 | 1319.02652 | 1344.489322 | 1291.981078 |
| **Slc9a2** | 11.90405426 | 7.130358077 | 16.44638718 | 17.25218839 | 0 | 12.6254305 | 10.82477658 | 3.352296052 | 11.28621184 | 19.22584332 | 3.783482795 | 11.27786538 | 0.852084315 | 5.421327909 | 15.08987911 |
| **Mfsd9** | 9.156964816 | 10.29940611 | 10.96425812 | 8.626094197 | 2.533452977 | 7.014128057 | 5.412388289 | 1.676148026 | 9.781383594 | 6.991215751 | 5.675224193 | 11.27786538 | 11.0770961 | 7.228437213 | 7.904222392 |
| **Mrps9** | 999.9405579 | 1140.06503 | 1075.280457 | 1118.804417 | 1022.88164 | 1117.3506 | 1133.895346 | 1072.734737 | 1137.650153 | 1098.494775 | 1137.882451 | 1073.132267 | 1057.436635 | 1198.113468 | 1053.417275 |
| **Gpr45** | 4.578482408 | 1.584524017 | 0.783161294 | 0 | 0.633363244 | 0 | 0 | 0.838074013 | 3.00965649 | 0 | 0 | 0 | 0 | 2.710663955 | 0 |
| **Tgfbrap1** | 397.412273 | 423.0679125 | 539.5981319 | 431.3047098 | 444.6209975 | 458.0225621 | 543.945023 | 446.693449 | 515.403674 | 489.3851026 | 425.6418144 | 475.4054022 | 538.5172872 | 472.5590828 | 505.8702331 |
| **AI597479** | 476.1621704 | 400.0923143 | 454.2335508 | 414.9151309 | 419.2864677 | 397.7010608 | 408.6353158 | 377.9713799 | 382.9787884 | 417.7251411 | 375.5106674 | 408.6057381 | 387.6983634 | 364.1325246 | 347.7857852 |
| **Fhl2** | 178.5608139 | 160.0369257 | 145.6680008 | 199.2627759 | 163.407717 | 143.7896252 | 165.9799075 | 166.7767286 | 149.7304104 | 169.536982 | 139.9888634 | 151.8174186 | 185.7543807 | 183.4215943 | 186.108509 |
| **Nck2** | 295.7699635 | 282.045275 | 268.624324 | 258.7828259 | 374.9510406 | 331.7682571 | 359.0217565 | 346.9626414 | 237.7628627 | 267.4140025 | 237.4135454 | 418.1485472 | 320.3837025 | 299.9801443 | 367.905624 |
| **1500015O10Rik** | 0 | 0 | 0 | 0 | 0 | 0.701412806 | 0 | 0 | 0 | 1.747803938 | 3.783482795 | 1.735056213 | 8.520843152 | 0 | 0 |
| **Uxs1** | 423.967471 | 453.9661309 | 465.1978089 | 437.3429758 | 475.0224332 | 451.0084341 | 476.2901694 | 528.8247022 | 445.4291606 | 514.7282597 | 476.7188322 | 485.8157395 | 432.0067478 | 486.1124025 | 458.4448987 |
| **Tpp2** | 3210.431864 | 3035.155755 | 3078.607049 | 3131.272193 | 2885.602941 | 2861.764247 | 3069.726224 | 3108.416514 | 3201.522092 | 3109.343205 | 3198.934703 | 2960.873427 | 3013.822223 | 2958.237929 | 2997.855983 |
| **Tex30** | 758.1966867 | 732.8423579 | 722.0747135 | 677.1483944 | 734.0680001 | 701.4128057 | 694.5898304 | 701.4679489 | 750.1568802 | 707.8605948 | 688.5938687 | 659.3213608 | 691.0403796 | 716.5188387 | 678.3259943 |
| **Kdelc1** | 299.4327495 | 343.0494497 | 334.4098727 | 295.0124215 | 229.2774944 | 225.1535106 | 270.6194144 | 328.5250131 | 291.1842654 | 385.3907683 | 332.946486 | 194.3262958 | 276.0753181 | 327.0867839 | 244.3123285 |
| **Bivm** | 251.8165324 | 209.9494323 | 207.537743 | 192.3619006 | 167.2078965 | 190.7842832 | 193.9439137 | 186.8905049 | 172.3028341 | 215.8537863 | 303.6244943 | 171.770565 | 212.1689945 | 170.7718291 | 175.330024 |
| **Ercc5** | 324.1565545 | 278.083965 | 331.2772276 | 286.3863273 | 287.5469129 | 311.4272857 | 297.6813559 | 457.5884111 | 381.4739601 | 454.4290238 | 483.3399271 | 294.9595561 | 321.2357868 | 327.9903385 | 295.3304912 |
| **Wdr75** | 1997.134026 | 2039.28241 | 1928.926268 | 1923.619006 | 1938.724891 | 1962.55303 | 2040.470385 | 2185.697026 | 2064.624352 | 2136.690314 | 2187.798926 | 1858.245204 | 1970.871021 | 1873.068793 | 1962.40285 |
| **Slc39a10** | 936.7575006 | 846.1358251 | 845.0310368 | 732.3553973 | 808.8048629 | 804.5204882 | 1023.843451 | 552.2907746 | 592.9023286 | 651.9308688 | 671.5681961 | 826.7542853 | 915.1385545 | 810.4885225 | 977.2493139 |
| **Nabp1** | 1571.335162 | 1503.713292 | 1354.869039 | 1325.830678 | 1027.948545 | 972.1581487 | 1138.40567 | 1076.925107 | 1116.582558 | 1165.785227 | 1116.127425 | 939.5329391 | 935.588578 | 882.7728946 | 942.0395959 |
| **Myo1b** | 6.409875371 | 2.376786026 | 3.915806472 | 6.038265938 | 2.533452977 | 4.90988964 | 2.706194144 | 5.028444078 | 1.504828245 | 2.621705907 | 5.675224193 | 2.602584319 | 2.556252945 | 2.710663955 | 9.341353736 |
| **Stat4** | 1835.055749 | 1617.006759 | 1585.11846 | 1616.530052 | 1542.2395 | 1543.108173 | 1716.629152 | 1220.235763 | 1233.206747 | 1301.240032 | 1224.902555 | 1654.376099 | 1561.018465 | 1412.25592 | 1476.652456 |
| **Stat1** | 12676.90209 | 13791.69704 | 12988.73007 | 13727.5663 | 12672.96515 | 12740.4622 | 14049.65793 | 12689.27863 | 12981.40086 | 10931.63973 | 11243.565 | 12759.60339 | 12240.19119 | 12598.26251 | 12598.61193 |
| **Gls** | 3558.396527 | 3996.961833 | 3846.105117 | 3578.966482 | 3347.324746 | 3321.189635 | 3484.675993 | 4019.402967 | 4066.798333 | 4107.339254 | 4217.637446 | 3772.879734 | 3732.1293 | 3646.746574 | 3545.403025 |
| **Nab1** | 1759.052941 | 1671.672838 | 1756.630784 | 1710.554479 | 1537.172594 | 1679.88367 | 1783.381941 | 1609.940179 | 1578.564829 | 1601.862309 | 1734.726862 | 1654.376099 | 1657.303993 | 1577.606422 | 1687.192198 |
| **Tmem194b** | 234.4182993 | 264.6155108 | 260.0095498 | 252.74456 | 240.6780328 | 225.8549234 | 213.7893374 | 177.6716908 | 161.7690364 | 224.592806 | 244.0346403 | 283.6816908 | 255.6252945 | 272.8735048 | 245.7494598 |
| **Mfsd6** | 555.8277643 | 566.4673361 | 541.1644545 | 467.5343055 | 469.322164 | 540.0878604 | 481.7025577 | 372.1048618 | 437.9050193 | 382.7690624 | 436.9922628 | 525.7220324 | 579.4173343 | 480.6910746 | 497.9660107 |
| **Inpp1** | 295.7699635 | 245.6012226 | 285.0707112 | 283.7984991 | 248.2783918 | 294.5933784 | 272.4235439 | 213.7088733 | 221.209752 | 241.1969434 | 230.7924505 | 294.092028 | 281.187824 | 274.6806141 | 242.8751971 |
| **Hibch** | 240.8281747 | 240.0553886 | 225.5504528 | 219.1027926 | 233.0776739 | 243.3902436 | 238.1450847 | 294.1639786 | 272.3739124 | 280.522532 | 334.8382274 | 258.5233757 | 260.7378004 | 312.6299094 | 236.4081061 |
| **1700019D03Rik** | 6.409875371 | 0 | 1.566322589 | 1.725218839 | 0 | 2.805651223 | 6.314453003 | 0 | 9.028969471 | 3.495607876 | 0.945870699 | 2.602584319 | 1.70416863 | 9.035546516 | 8.622788064 |
| **Pms1** | 242.6595676 | 257.4851528 | 310.9150339 | 255.3323882 | 260.3122934 | 237.0775283 | 281.444191 | 300.8685707 | 226.4766509 | 189.6367273 | 274.3025026 | 294.9595561 | 228.3585965 | 272.8735048 | 296.7676225 |
| **Ormdl1** | 316.8309826 | 355.7256418 | 322.6624533 | 359.708128 | 366.7173184 | 361.2275949 | 392.3981509 | 339.4199753 | 387.4932731 | 388.8863762 | 354.701512 | 399.0629289 | 373.21293 | 413.8280304 | 425.3908778 |
| **Osgepl1** | 92.48534464 | 115.6702532 | 135.4869039 | 132.8418506 | 112.7386575 | 92.58649035 | 105.5415716 | 152.5294704 | 137.6917844 | 129.3374914 | 144.7182169 | 102.3683165 | 84.3563472 | 126.4976512 | 114.2519418 |
| **Asnsd1** | 935.8418042 | 922.9852399 | 1026.724457 | 932.4807826 | 950.6782297 | 960.9355438 | 1000.389769 | 1139.780658 | 1095.514962 | 1119.468422 | 1090.588916 | 1058.38429 | 1000.346986 | 949.6359388 | 1032.578871 |
| **Dnah7a** | 1.831392963 | 7.130358077 | 7.831612945 | 10.35131304 | 15.20071786 | 9.81977928 | 11.72684129 | 24.30414638 | 9.028969471 | 10.48682363 | 9.458706988 | 7.807752957 | 14.48543336 | 2.710663955 | 8.622788064 |
| **Stk17b** | 3182.045273 | 3514.47427 | 3490.54989 | 3236.510543 | 3352.391652 | 3288.924646 | 3412.510816 | 2811.738314 | 3017.933046 | 2858.53334 | 3218.797988 | 3395.505008 | 3360.620539 | 3591.62974 | 3496.54056 |
| **Hecw2** | 1.831392963 | 11.09166812 | 0 | 4.313047098 | 6.966995687 | 4.90988964 | 1.80412943 | 3.352296052 | 1.504828245 | 3.495607876 | 3.783482795 | 4.337640531 | 7.668758836 | 0.903554652 | 0.718565672 |
| **Ccdc150** | 0 | 0 | 3.132645178 | 0.86260942 | 0 | 0 | 0 | 0 | 0 | 5.243411813 | 0 | 0 | 2.556252945 | 0 | 5.029959704 |

| | | | | | | | | | | | | | | | |
|---|---|---|---|---|---|---|---|---|---|---|---|---|---|---|---|
| **Gtf3c3** | 501.8016719 | 511.0089955 | 483.99368 | 495.1378069 | 391.418485 | 425.0561603 | 460.9550693 | 492.7875197 | 647.8285595 | 501.6197302 | 514.5536601 | 444.1743904 | 414.9650615 | 473.4626374 | 461.3191614 |
| **Pgap1** | 65.93014667 | 52.28929256 | 66.56871003 | 72.45919125 | 114.005384 | 68.73845496 | 113.6601541 | 152.5294704 | 93.2993512 | 65.54264767 | 104.9916476 | 103.2358446 | 89.46885309 | 96.68034772 | 99.16206273 |
| **Ankrd44** | 2973.266476 | 3356.021868 | 3386.389437 | 3245.999246 | 3349.224836 | 3315.578333 | 3792.280061 | 2965.943932 | 3005.142006 | 2950.293047 | 3005.03121 | 3680.921755 | 3750.023071 | 3513.92404 | 3620.133855 |
| **Sf3b1** | 7733.972483 | 7804.573046 | 8115.900495 | 7482.274106 | 7854.337592 | 7923.159053 | 8061.752356 | 7853.591576 | 7925.930367 | 7928.912564 | 7871.535955 | 8297.906337 | 8433.078467 | 8184.398034 | 8246.259651 |
| **Coq10b** | 229.8398169 | 203.6113362 | 223.9841302 | 265.6837013 | 179.2417981 | 221.6464466 | 221.0058551 | 291.6497565 | 284.4125383 | 232.4579237 | 250.6557352 | 191.7237115 | 228.3585965 | 195.1678047 | 193.2941658 |
| **Hspd1** | 15901.9851 | 15061.69304 | 15775.21795 | 15249.20932 | 14188.6034 | 13488.86967 | 13450.68696 | 20207.6406 | 18638.05023 | 19010.86343 | 19143.47707 | 13971.54015 | 13997.18905 | 13654.51789 | 13698.73597 |
| **Hspe1** | 3428.367627 | 3340.96889 | 3187.466469 | 3083.828675 | 3373.292639 | 3356.260275 | 3275.396979 | 4218.026508 | 3578.481567 | 3935.180566 | 3751.323191 | 3143.054329 | 2985.70344 | 3222.075888 | 2978.45471 |
| **Mob4** | 434.0401323 | 397.7155283 | 488.6926478 | 456.320383 | 437.0206386 | 462.9324518 | 469.9757164 | 442.5030789 | 422.1043228 | 473.6548671 | 481.4481857 | 465.8625931 | 509.5464205 | 541.2292363 | 448.3849793 |
| **Rftn2** | 21.97671556 | 11.88393013 | 8.614774239 | 6.038265938 | 8.86708542 | 17.53532014 | 20.74748844 | 3.352296052 | 3.00965649 | 4.369509845 | 0.945870699 | 7.807752957 | 13.63334904 | 15.36042908 | 16.52701046 |
| **Mars2** | 440.4500076 | 423.8601745 | 492.6084542 | 497.7256351 | 406.6192028 | 401.9095377 | 469.0736517 | 617.6605476 | 572.5871473 | 575.9013975 | 578.8728676 | 451.9821434 | 474.6109635 | 453.5844351 | 403.115342 |
| **Boll** | 4.578482408 | 6.338096068 | 3.132645178 | 12.93914129 | 5.066905954 | 4.90988964 | 14.43303544 | 7.542666117 | 14.29586833 | 0.873901969 | 4.729353494 | 3.470112425 | 6.816674521 | 6.324882561 | 4.311394032 |
| **Plcl1** | 28.38659093 | 15.05297816 | 32.89277437 | 19.84001665 | 12.66726489 | 13.32684331 | 9.020647148 | 21.78992434 | 35.36346376 | 27.09096104 | 36.88895725 | 16.48303402 | 14.48543336 | 11.74621047 | 17.9641418 |
| **9130024F11Rik** | 0.915696482 | 0 | 0 | 0 | 0 | 0 | 0.902064715 | 3.352296052 | 1.504828245 | 0.873901969 | 4.729353494 | 0 | 0 | 0 | 0 |
| **4930558J18Rik** | 43.03773463 | 41.19762444 | 48.55600026 | 24.15306375 | 27.86798275 | 35.07064029 | 42.39704159 | 27.65644243 | 30.84897903 | 31.46047088 | 28.37612096 | 53.78674259 | 43.45630007 | 32.52796746 | 30.17975822 |
| **1700066M21Rik** | 123.619025 | 174.2976419 | 132.3542588 | 124.2157564 | 164.0410803 | 121.3444154 | 135.3097072 | 174.3193947 | 145.2159257 | 158.1762564 | 141.8806048 | 173.5056213 | 191.7189709 | 160.832728 | 153.7730538 |
| **Tyw5** | 166.6567596 | 152.9065676 | 121.3900006 | 138.8801166 | 133.0062813 | 133.9698459 | 142.5262249 | 150.0152483 | 148.9779963 | 120.5984717 | 151.3393118 | 161.3602278 | 144.0022493 | 149.0865175 | 154.4916195 |
| **9430016H08Rik** | 365.3628961 | 382.6625501 | 395.4964537 | 421.8160062 | 374.3176774 | 368.241723 | 382.4754391 | 496.1398157 | 408.5608686 | 401.1210037 | 432.2629093 | 360.0241641 | 355.3191594 | 344.2543222 | 382.9955032 |
| **Spats2l** | 0.915696482 | 4.753572051 | 3.132645178 | 2.587828259 | 2.533452977 | 8.416953669 | 4.510323574 | 1.676148026 | 2.257242368 | 0.873901969 | 2.837612096 | 6.072696744 | 3.408337261 | 6.324882561 | 5.748525376 |
| **Kctd18** | 244.4909606 | 246.3934846 | 227.8999367 | 192.3619006 | 232.4443106 | 218.1393826 | 238.1450847 | 211.1946513 | 257.3256299 | 199.2496489 | 220.3878728 | 232.4975325 | 259.0336318 | 234.9242094 | 232.8152777 |
| **Sgol2a** | 673.0369139 | 736.0114059 | 693.0977456 | 706.4771147 | 712.5336498 | 692.2944392 | 682.8629891 | 685.5445427 | 732.0989413 | 646.687457 | 576.0352556 | 776.4376551 | 645.0278266 | 757.178798 | 653.1761958 |
| **Aox2** | 0 | 0.792262009 | 0 | 0 | 1.266726489 | 0 | 0 | 0.838074013 | 0 | 0 | 0 | 1.735056213 | 0 | 0.903554652 | 0 |
| **Bzw1** | 8363.971663 | 8350.44157 | 8779.238111 | 8621.781149 | 8306.558949 | 8379.077377 | 8726.574051 | 9888.43528 | 9367.555826 | 9154.123124 | 9752.872775 | 8863.534662 | 8836.966433 | 8637.078914 | 8666.62057 |
| **Clk1** | 722.484524 | 661.5387771 | 692.3145843 | 583.1239677 | 611.828894 | 602.5136001 | 620.6205238 | 682.1922466 | 689.2113363 | 683.3913397 | 594.9526695 | 592.5216966 | 635.6548991 | 502.3763863 | 571.2597092 |
| **Ppil3** | 442.2814006 | 450.0048208 | 408.0270344 | 401.9759896 | 440.1874548 | 450.3070213 | 391.4960862 | 474.3498914 | 471.0112407 | 528.7106912 | 466.3142545 | 391.2551759 | 344.2420633 | 406.5995932 | 399.5225136 |
| **Nif3l1** | 568.6475151 | 511.0089955 | 568.5750998 | 550.3448097 | 532.6584884 | 533.7751451 | 470.8777811 | 604.2513634 | 528.1947141 | 571.5318877 | 543.8756518 | 465.8625931 | 440.5275909 | 440.93467 | 485.0318286 |
| **Orc2** | 1525.550338 | 1521.935318 | 1644.638718 | 1560.46044 | 1475.736359 | 1437.194839 | 1547.040986 | 1734.813207 | 1765.163532 | 1817.716095 | 1831.205673 | 1507.763849 | 1445.134999 | 1440.266115 | 1397.610232 |
| **Fam126b** | 204.2003154 | 186.973834 | 129.2216136 | 186.3236346 | 164.6744435 | 164.8320093 | 168.6861017 | 217.0611694 | 109.8524619 | 202.7452568 | 193.9034932 | 159.6251716 | 163.6001885 | 182.5180396 | 177.485721 |
| **Ndufb3** | 712.4118627 | 703.5286636 | 741.6537459 | 662.4840343 | 752.4355342 | 674.7591191 | 740.5951308 | 677.1638025 | 572.5871473 | 702.617183 | 668.730584 | 724.3859687 | 680.8153678 | 751.7574701 | 678.3259943 |
| **Gm20257** | 25.63950148 | 15.05297816 | 25.84432272 | 29.32872027 | 17.1008076 | 28.05651223 | 20.74748844 | 29.33259046 | 33.10622139 | 34.08217679 | 11.35044839 | 18.21809023 | 27.26669809 | 25.29953024 | 27.30549553 |
| **Als2cr12** | 0.915696482 | 4.753572051 | 0 | 1.725218839 | 0 | 0 | 0.902064715 | 0 | 4.514484735 | 0 | 1.891741398 | 0 | 0 | 0 | 4.311394032 |
| **Cflar** | 1541.117178 | 1357.937083 | 1271.853942 | 1235.256689 | 1190.722899 | 1331.281505 | 1444.205608 | 1164.922878 | 1155.708092 | 931.5794989 | 1106.668718 | 1213.671821 | 1284.943147 | 1142.09308 | 1212.220289 |
| **Casp8** | 2021.857831 | 1956.887161 | 2015.074011 | 1846.846767 | 1706.28058 | 1927.48239 | 1946.655654 | 1817.782534 | 1762.153875 | 1843.059252 | 1819.855224 | 1771.492393 | 1832.833362 | 1770.967117 | 1821.563978 |
| **Trak2** | 1415.66676 | 1316.739458 | 1440.233621 | 1318.067193 | 1295.861198 | 1259.035986 | 1500.133621 | 1481.714855 | 1397.233026 | 1519.715524 | 1490.692221 | 1425.348679 | 1431.501649 | 1490.865175 | 1440.005607 |
| **Stradb** | 132.7759898 | 174.2976419 | 161.3312267 | 139.742726 | 134.2730078 | 140.9839739 | 147.9386132 | 124.0349539 | 127.1579867 | 160.7979623 | 153.2310532 | 166.5653964 | 147.4105865 | 135.5331977 | 148.0245284 |
| **Tmem237** | 274.7089445 | 316.1125414 | 301.5170984 | 270.8593578 | 263.4791096 | 274.9538198 | 303.9958089 | 259.802944 | 272.3739124 | 243.8186493 | 206.1998123 | 264.5960724 | 288.8565828 | 271.0663955 | 222.0367926 |
| **Mpp4** | 0 | 0 | 0.783161294 | 0 | 1.900089733 | 1.402825611 | 0 | 0 | 0 | 0 | 0 | 0.867528106 | 0 | 1.807109303 | 4.311394032 |
| **Als2** | 459.6796337 | 434.9518427 | 498.0905833 | 458.0456018 | 421.1865574 | 439.0844164 | 441.1096455 | 595.0325493 | 545.5002389 | 500.7458282 | 467.2601252 | 489.2858519 | 504.4339146 | 485.2088479 | 467.0676868 |
| **Fzd7** | 186.8020822 | 111.7089432 | 122.9563232 | 85.39833255 | 70.93668336 | 108.7189849 | 122.6808012 | 18.43762829 | 14.29586833 | 34.08217679 | 36.88895725 | 58.12438312 | 80.94800994 | 59.634607 | 87.66501198 |
| **Gm973** | 1.831392963 | 5.54583406 | 1.566322589 | 4.313047098 | 0 | 0.701412806 | 0 | 3.352296052 | 0 | 0 | 0 | 2.602584319 | 0 | 4.517773258 | 0.718565672 |
| **Sumo1** | 1756.305852 | 1911.728227 | 2002.54343 | 1909.817255 | 1917.823904 | 1976.581286 | 1973.717596 | 2119.489179 | 1917.903598 | 2000.361607 | 2179.28609 | 2163.615097 | 1970.871021 | 2029.383747 | 2001.205396 |
| **Nop58** | 6267.02672 | 5964.1484 | 6139.201387 | 5660.443012 | 5870.643911 | 6128.243684 | 6335.200492 | 6786.723358 | 6473.771111 | 6703.702003 | 7126.189845 | 5919.144269 | 5625.460649 | 5783.653325 | 5678.10594 |
| **Snord70** | 2.747089445 | 0.792262009 | 4.698967767 | 2.587828259 | 1.266726489 | 1.402825611 | 1.80412943 | 3.352296052 | 5.266898858 | 0.873901969 | 0.945870699 | 6.072696744 | 0 | 2.710663955 | 0 |
| **Snord11** | 0.915696482 | 0 | 0 | 0 | 0 | 0 | 0 | 1.676148026 | 0 | 6.117313782 | 4.729353494 | 0 | 0 | 0 | 0 |
| **Bmpr2** | 117.2091496 | 110.9166812 | 146.4511621 | 93.16181732 | 63.96968767 | 77.15540863 | 79.3816949 | 124.0349539 | 117.3766031 | 131.0852953 | 112.5586132 | 78.94505767 | 101.3980335 | 81.31991864 | 99.8806284 |
| **Fam117b** | 1100.667171 | 938.8304801 | 1000.096973 | 928.1677355 | 881.641636 | 875.3631815 | 959.7968565 | 749.2381677 | 776.4913745 | 751.5556933 | 695.2149636 | 935.1952986 | 944.9615055 | 786.0925469 | 1003.836244 |
| **Ica1l** | 2.747089445 | 4.753572051 | 4.698967767 | 6.038265938 | 8.233722176 | 2.805651223 | 10.82477658 | 2.514222039 | 6.019312981 | 0 | 2.837612096 | 5.205168638 | 9.372927467 | 3.614218606 | 4.311394032 |
| **Wdr12** | 1287.469253 | 1295.348384 | 1303.180394 | 1335.319382 | 1276.8603 | 1268.855766 | 1229.514206 | 1526.970852 | 1407.766823 | 1519.715524 | 1538.931627 | 1191.983618 | 1164.799259 | 1253.230302 | 1191.381884 |
| **Carf** | 35.71216278 | 45.15893448 | 62.65290356 | 56.9322217 | 55.10260225 | 51.90454762 | 46.00530045 | 35.19910855 | 43.64001911 | 46.31680435 | 45.40179354 | 45.11146153 | 56.2375648 | 47.88839653 | 56.76668809 |
| **Nbeal1** | 130.0289004 | 111.7089432 | 107.2930973 | 114.7270528 | 113.3720207 | 83.46812388 | 111.8560246 | 97.21658551 | 91.04210883 | 83.02068705 | 103.0999062 | 117.1162943 | 120.9959728 | 106.6194489 | 136.5274777 |
| **Cyp20a1** | 378.1826469 | 434.1595807 | 393.1469698 | 426.9916627 | 439.5540915 | 439.0844164 | 442.0117102 | 409.8181924 | 394.2650002 | 381.0212584 | 409.5620126 | 457.187312 | 455.0130243 | 425.5742409 | 483.5946972 |
| **Abi2** | 567.7318186 | 476.9417291 | 527.0675512 | 543.4439344 | 516.1910441 | 541.490686 | 588.146194 | 561.5095887 | 592.9023286 | 486.7633967 | 555.2261002 | 551.7478756 | 485.6880596 | 552.9754468 | 559.7626585 |
| **Raph1** | 616.2637321 | 564.8828121 | 610.0826484 | 570.1848264 | 490.2231511 | 504.3158073 | 581.831741 | 542.2338864 | 485.3071091 | 506.863142 | 444.5592284 | 580.3763031 | 602.4236108 | 587.3105235 | 587.7867197 |
| **Cd28** | 2997.990281 | 3030.402183 | 3145.175759 | 2990.666858 | 2883.069488 | 2773.386234 | 3214.958643 | 3058.132074 | 3361.7863 | 2826.198967 | 3154.47878 | 3202.913768 | 3355.508033 | 3084.73558 | 2942.526427 |
| **Ctla4** | 5246.025143 | 4957.975649 | 4765.536477 | 4066.340804 | 2271.873957 | 2370.775283 | 2384.157041 | 2152.174066 | 3060.820651 | 3485.994954 | 3078.809124 | 2481.997912 | 2712.184375 | 2169.434718 | 2603.36343 |
| **Icos** | 5373.306954 | 4357.441047 | 4932.349833 | 4357.902788 | 3736.209778 | 3788.330564 | 4013.285916 | 4886.80957 | 4690.54964 | 4715.575024 | 5007.439479 | 3571.613214 | 3669.075061 | 3496.756502 | 3682.649069 |
| **Pard3b** | 4.578482408 | 0 | 1.566322589 | 5.175656518 | 5.700269198 | 4.90988964 | 8.118582433 | 2.514222039 | 2.257242368 | 4.369509845 | 0 | 3.470112425 | 10.22501178 | 1.807109303 | 0.718565672 |
| **Nrp2** | 1.831392963 | 2.376786026 | 3.915806472 | 0.86260942 | 7.600358931 | 8.416953669 | 2.706194144 | 1.676148026 | 9.781383594 | 1.747803938 | 3.783482795 | 2.602584319 | 9.372927467 | 0 | 6.467091048 |
| **Ino80d** | 1278.312288 | 1287.425764 | 1273.420265 | 1218.86711 | 1066.583703 | 998.1104225 | 1242.143112 | 1319.128497 | 1298.666776 | 1105.485991 | 1338.407039 | 1278.736429 | 1304.541087 | 1191.788585 | 1156.172166 |
| **Ino80dos** | 10.0726613 | 7.130358077 | 9.397935534 | 7.763484777 | 16.46744435 | 4.208476834 | 11.72684129 | 13.40918421 | 9.781383594 | 8.739019689 | 13.24218978 | 11.27786538 | 7.668758836 | 11.74621047 | 4.311394032 |
| **Ndufs1** | 1331.422684 | 1394.381135 | 1524.81504 | 1410.366401 | 1442.168107 | 1365.650733 | 1446.911803 | 1563.846108 | 1553.735163 | 1629.827172 | 1714.863577 | 1499.956096 | 1441.726661 | 1478.21541 | 1417.730071 |
| **Eef1b2** | 12339.01009 | 11677.14974 | 11676.15174 | 11918.67435 | 12241.64479 | 12429.03492 | 12546.81812 | 12654.9176 | 12420.85234 | 12293.179 | 12257.53839 | 12727.50485 | 11935.99709 | 11634.16969 | 11380.64311 |
| **Snora41** | 0.915696482 | 0.792262009 | 0.783161294 | 2.587828259 | 1.900089733 | 1.402825611 | 0 | 3.352296052 | 1.504828245 | 0 | 0 | 0.867528106 | 3.408337261 | 1.807109303 | 0.718565672 |
| **Zdbf2** | 2.747089445 | 3.169048034 | 3.132645178 | 0.86260942 | 0 | 1.402825611 | 0.902064715 | 0 | 0 | 3.495607876 | 0 | 0 | 0.852084315 | 3.614218606 | 0.718565672 |
| **Fastkd2** | 462.4267232 | 516.5548295 | 523.934906 | 498.5882446 | 431.9537326 | 461.5296262 | 406.8311864 | 586.6518091 | 616.9795805 | 569.7840837 | 543.8756518 | 507.5039422 | 452.4567714 | 496.9550584 | 449.103545 |
| **4933402D24Rik** | 0 | 1.584524017 | 0 | 0 | 0.633363244 | 0.701412806 | 0 | 2.514222039 | 0 | 0 | 2.837612096 | 0 | 0 | 0 | 1.437131344 |
| **Klf7** | 154.7527054 | 196.4809781 | 155.8490976 | 139.742726 | 130.4728283 | 131.1641947 | 203.8666255 | 146.6629523 | 150.4828245 | 125.8418835 | 169.3108551 | 140.5395532 | 161.0439356 | 88.54835585 | 138.6831747 |
| **Creb1** | 1472.439942 | 1586.108541 | 1571.021557 | 1469.023842 | 1604.309098 | 1586.595767 | 1786.088135 | 1359.356049 | 1306.190917 | 1274.149071 | 1463.261971 | 1605.794525 | 1622.368536 | 1597.484624 | 1593.77866 |
| **Mettl21a** | 184.0549928 | 167.9595458 | 184.8260655 | 184.5984158 | 162.7743538 | 171.1447246 | 165.9799075 | 155.0436924 | 161.7690364 | 146.8155308 | 118.2338373 | 190.8561834 | 159.3397669 | 191.5535861 | 165.2701046 |
| **2810408I11Rik** | 94.3167376 | 111.7089432 | 88.49722628 | 107.8261775 | 74.10349958 | 114.3302873 | 81.18582433 | 87.99777137 | 81.26072524 | 87.39019689 | 93.64119918 | 117.1162943 | 112.4751296 | 120.1727687 | 108.5034165 |
| **Ccnyl1** | 208.7787978 | 213.1184803 | 180.1270977 | 171.6592745 | 175.4416187 | 160.6235325 | 194.8459784 | 199.4616151 | 173.0552482 | 162.5457662 | 193.9034932 | 209.0742736 | 146.5585022 | 178.0002664 | 137.964609 |
| **Fzd5** | 228.9241204 | 221.8333624 | 188.741872 | 221.6906209 | 178.6084349 | 187.2772191 | 207.4748844 | 168.4528766 | 144.4635115 | 166.0413741 | 131.4760271 | 189.1211272 | 184.0502121 | 148.1829629 | 189.7013374 |
| **Plekhm3** | 78.74989741 | 92.694655 | 97.89516181 | 95.74964558 | 58.90278172 | 66.63421654 | 56.83007703 | 75.42666117 | 63.95520042 | 77.77727523 | 57.69811262 | 49.44910206 | 69.87091384 | 64.15238026 | 63.9523448 |
| **D630023F18Rik** | 0 | 2.376786026 | 3.132645178 | 8.626094197 | 0 | 0 | 0 | 0.838074013 | 0 | 2.621705907 | 2.837612096 | 0 | 2.556252945 | 0 | 0.718565672 |
| **Idh1** | 190.4648682 | 148.9452576 | 158.1985815 | 170.7966651 | 177.9750716 | 180.9645039 | 179.5108782 | 128.225324 | 176.0649047 | 194.8801391 | 167.4191137 | 223.8222514 | 225.8023435 | 184.3251489 | 193.2941658 |
| **Pikfyve** | 369.0256821 | 380.2857641 | 383.7490343 | 408.8768649 | 414.852925 | 388.5826944 | 397.8105392 | 362.0479736 | 421.3519086 | 339.9478659 | 359.4308655 | 417.2810191 | 452.4567714 | 440.0311153 | 444.0735853 |
| **Map2** | 10.0726613 | 30.10595632 | 23.49483883 | 21.56523549 | 24.06780328 | 7.715540863 | 21.64955315 | 31.8468125 | 12.03862596 | 7.86511772 | 24.59263817 | 13.01292159 | 16.18960199 | 29.8173035 | 16.52701046 |
| **Unc80** | 0 | 1.584524017 | 7.04845165 | 0.86260942 | 0 | 0 | 1.80412943 | 2.514222039 | 1.504828245 | 2.621705907 | 5.675224193 | 4.337640531 | 0 | 2.710663955 | 0.718565672 |
| **Rpe** | 846.103549 | 702.7364015 | 808.2224559 | 808.2650262 | 730.9011839 | 736.483446 | 828.0954082 | 885.0061578 | 753.9189508 | 754.1773992 | 836.1496977 | 763.4247335 | 777.1008954 | 872.8337934 | 740.1226421 |
| **Kansl1l** | 58.60457482 | 57.83512662 | 36.80858084 | 36.22959563 | 44.3354271 | 41.38335554 | 59.53627118 | 32.68488651 | 38.37312025 | 38.45168663 | 55.80637123 | 59.85943933 | 48.56880596 | 44.27417793 | 46.70676868 |
| **Acadl** | 2523.659503 | 2483.741397 | 2319.723754 | 2368.725466 | 1924.790899 | 2181.393826 | 2228.099846 | 1844.600903 | 2213.602349 | 2183.007118 | 2217.120918 | 2108.960826 | 2160.033739 | 2189.312921 | 2235.457805 |
| **Lancl1** | 456.9325443 | 380.2857641 | 404.8943892 | 345.9063773 | 378.1178568 | 415.9377938 | 405.9291217 | 357.8576036 | 347.6153246 | 415.1034352 | 289.4364338 | 385.1824792 | 387.6983634 | 487.0159572 | 390.1811599 |
| **Ikzf2** | 324.1565545 | 301.0595632 | 364.9531632 | 379.5481446 | 198.2426955 | 180.9645039 | 209.2790138 | 305.0589408 | 261.8401147 | 318.1003167 | 385.9152451 | 177.8432618 | 183.1981278 | 154.5078454 | 168.1443672 |
| **Bard1** | 580.5515693 | 593.4042444 | 593.6362612 | 580.5361394 | 514.9243176 | 521.8511275 | 635.9556239 | 583.2995131 | 554.5292083 | 485.8894947 | 525.9041085 | 577.7737188 | 604.9798638 | 533.0972444 | 561.9183555 |
| **Atic** | 4734.15081 | 4500.048208 | 4863.431639 | 4551.989907 | 4683.721191 | 4667.200809 | 4638.416763 | 5199.411177 | 4971.952522 | 4964.637085 | 5172.020981 | 4582.283457 | 4717.990853 | 4297.305923 | 4465.885651 |
| **Fn1** | 7.325571853 | 0.792262009 | 0 | 2.587828259 | 3.166816221 | 0.701412806 | 0.902064715 | 2.514222039 | 0 | 2.621705907 | 2.837612096 | 0.867528106 | 10.22501178 | 0 | 0 |
| **Mreg** | 11.90405426 | 8.714882094 | 11.74741942 | 13.80175071 | 5.066905954 | 8.416953669 | 4.510323574 | 16.76148026 | 11.28621184 | 20.09974528 | 8.512836289 | 6.072696744 | 4.260421576 | 1.807109303 | 5.748525376 |
| **Pecr** | 141.0172582 | 99.03275106 | 132.3542588 | 110.4140057 | 109.5718413 | 125.5528922 | 101.9333128 | 119.8445839 | 146.7207539 | 162.5457662 | 125.8008029 | 113.6461819 | 103.9542864 | 99.39101167 | 107.7848508 |
| **Xrcc5** | 1106.16135 | 1061.631091 | 1134.800716 | 1070.49829 | 1056.449891 | 1057.029098 | 1142.915994 | 1214.369245 | 1180.537758 | 1187.632776 | 1354.486841 | 1065.324515 | 1048.915792 | 1121.311323 | 1042.63879 |
| **Smarcal1** | 515.5371191 | 470.6036331 | 491.8252929 | 479.6108373 | 475.6557965 | 437.6815908 | 458.2488751 | 476.0260394 | 471.0112407 | 507.7370439 | 399.1574349 | 440.704278 | 447.3442655 | 523.1581433 | 462.7562927 |
| **Rpl37a** | 7942.751281 | 7563.725395 | 7765.044235 | 7345.119208 | 8306.558949 | 8430.981925 | 8153.762957 | 7711.957068 | 7072.692752 | 7348.641657 | 7079.84218 | 7333.215082 | 7029.6956 | 7758.823793 | 7225.177832 |
| **Igfbp2** | 0.915696482 | 0 | 3.915806472 | 8.626094197 | 3.166816221 | 2.805651223 | 1.80412943 | 14.24725822 | 18.05793894 | 3.495607876 | 26.48437957 | 0 | 0 | 7.228437213 | 2.155697016 |
| **Tns1** | 757.2809903 | 867.5268993 | 792.55923 | 762.546727 | 712.5336498 | 814.3402674 | 876.8069028 | 1040.04985 | 917.9452295 | 897.4973221 | 923.169802 | 832.826982 | 849.5280622 | 790.6103201 | 836.4104422 |
| **Rufy4** | 0 | 0 | 0.783161294 | 0 | 0.633363244 | 0 | 0.902064715 | 0 | 0 | 0 | 0 | 0 | 0 | 0.903554652 | 0 |
| **Arpc2** | 9878.533643 | 9967.448329 | 10135.67347 | 10083.90412 | 10358.0225 | 10984.82595 | 10684.05448 | 9704.897071 | 9861.891905 | 9943.256602 | 9891.915768 | 10942.99953 | 10474.67249 | 11101.07245 | 10764.83233 |
| **Aamp** | 2431.174159 | 2297.559825 | 2317.37427 | 2064.224341 | 2317.476111 | 2344.121597 | 2347.172388 | 2040.710222 | 2230.155459 | 2150.672745 | 2154.693452 | 2437.753979 | 2273.360953 | 2314.003463 | 2223.960755 |
| **Pnkd** | 478.9092599 | 484.8643492 | 469.1136154 | 486.5117127 | 365.4505919 | 356.3177053 | 350.903174 | 613.4701776 | 554.5292083 | 582.0187113 | 545.7673932 | 411.2083224 | 380.8816889 | 398.4676013 | 375.0912808 |
| **Tmbim1** | 415.7262026 | 406.4304104 | 353.9889051 | 391.6246765 | 297.6807248 | 314.9343498 | 318.4288443 | 345.2864934 | 442.4195041 | 450.059514 | 415.2372368 | 254.1857351 | 332.3128829 | 281.9090513 | 270.8992583 |
| **Slc11a1** | 0.915696482 | 0.792262009 | 0 | 0 | 1.900089733 | 1.402825611 | 8.118582433 | 1.676148026 | 0 | 0 | 0 | 0 | 0 | 0 | 0 |
| **Ctdsp1** | 1595.143271 | 1805.565117 | 1827.1153 | 1729.531886 | 1832.953229 | 1790.00548 | 1845.624406 | 1555.465368 | 1580.069657 | 1566.90623 | 1627.843473 | 1853.907563 | 1811.531254 | 1874.875902 | 1833.779595 |
| **Vil1** | 0 | 0.792262009 | 0 | 2.587828259 | 0.633363244 | 0 | 0 | 0 | 0 | 0 | 0 | 0 | 2.556252945 | 0 | 0 |
| **Usp37** | 670.2898245 | 735.2191439 | 724.4241974 | 651.2701118 | 660.5978638 | 723.1566027 | 755.0281663 | 785.2753502 | 747.8996378 | 617.848692 | 732.1039208 | 661.056417 | 653.5486697 | 718.325948 | 666.1103779 |
| **Rqcd1** | 2605.15649 | 2815.699178 | 2714.437047 | 2534.346475 | 2468.849926 | 2377.087999 | 2672.81775 | 2982.705412 | 2935.167492 | 2985.249126 | 2885.851502 | 2690.204658 | 2653.390557 | 2592.298295 | 2542.285347 |
| **Plcd4** | 10.0726613 | 0.792262009 | 7.04845165 | 1.725218839 | 4.43354271 | 0.701412806 | 3.608258859 | 3.352296052 | 10.53379772 | 6.117313782 | 4.729353494 | 0.867528106 | 3.408337261 | 6.324882561 | 1.437131344 |
| **Zfp142** | 735.3042747 | 795.4310565 | 830.9341335 | 778.9363059 | 770.169705 | 735.0806204 | 800.131402 | 982.2227433 | 891.6107353 | 820.5939488 | 793.5855163 | 739.1339465 | 790.7342445 | 750.8539155 | 765.9910063 |
| **Bcs1l** | 154.7527054 | 168.7518078 | 182.4765816 | 148.3688202 | 162.1409905 | 167.6376606 | 147.9386132 | 181.0239868 | 178.3221471 | 185.2672174 | 142.8264755 | 150.0823624 | 141.4459963 | 169.8682745 | 169.5814986 |
| **Rnf25** | 304.0112319 | 297.0982532 | 286.6370338 | 292.4245933 | 280.5799172 | 281.2665351 | 258.8925731 | 310.9254588 | 270.8690841 | 265.6661985 | 326.3253911 | 293.2244999 | 250.5127887 | 332.5081118 | 270.1806927 |
| **Stk36** | 2.747089445 | 0.792262009 | 0 | 0.86260942 | 1.266726489 | 0.701412806 | 0.902064715 | 0 | 8.276555348 | 1.747803938 | 5.675224193 | 0 | 0.852084315 | 0 | 0 |
| **Ttll4** | 700.5078084 | 686.8911614 | 805.872972 | 781.5241342 | 559.8931079 | 590.5895824 | 581.831741 | 874.9492696 | 844.2086455 | 918.4709693 | 908.9817415 | 590.7866404 | 597.3111049 | 543.0363456 | 645.9905391 |
| **Cyp27a1** | 0 | 2.376786026 | 7.04845165 | 0.86260942 | 0 | 0.701412806 | 0 | 0 | 2.257242368 | 4.369509845 | 11.35044839 | 0.867528106 | 0 | 4.517773258 | 2.874262688 |
| **Wnt6** | 5.494178889 | 1.584524017 | 14.0969033 | 5.175656518 | 5.700269198 | 11.9240177 | 12.62890601 | 4.190370065 | 2.257242368 | 8.739019689 | 15.13393118 | 21.68820266 | 9.372927467 | 3.614218606 | 17.24557613 |
| **Wnt10a** | 143.7643476 | 180.6357379 | 241.99684 | 214.7897455 | 191.2756998 | 259.5227381 | 189.4335901 | 152.5294704 | 194.1228436 | 221.0971981 | 148.5016997 | 188.2535991 | 172.1210317 | 181.614485 | 180.3599837 |
| **Ihh** | 0 | 0 | 1.566322589 | 0 | 0.633363244 | 2.805651223 | 0 | 4.190370065 | 4.514484735 | 0 | 0 | 3.470112425 | 0 | 1.807109303 | 1.437131344 |
| **Nhej1** | 26.55519797 | 41.19762444 | 33.67593566 | 50.89395576 | 41.80197412 | 52.60596043 | 47.80942988 | 54.47481085 | 66.21244279 | 27.09096104 | 60.53572472 | 36.43618046 | 39.1958785 | 28.91374885 | 59.64095077 |
| **Slc23a3** | 0.915696482 | 1.584524017 | 0.783161294 | 0.86260942 | 1.266726489 | 2.805651223 | 0 | 0 | 0.752414123 | 1.747803938 | 0 | 3.470112425 | 5.112505891 | 4.517773258 | 2.155697016 |
| **Cnppd1** | 1154.693263 | 1323.077554 | 1311.795168 | 1285.288035 | 1210.990523 | 1272.36283 | 1277.323636 | 1354.327605 | 1300.171604 | 1248.805914 | 1301.518082 | 1381.972273 | 1298.576496 | 1448.398106 | 1320.005139 |
| **Fam134a** | 845.1878525 | 1010.134061 | 953.1072954 | 919.5416413 | 935.4775118 | 927.2677292 | 985.9567333 | 968.8135591 | 909.6686742 | 1007.60897 | 895.7395517 | 1040.166199 | 1000.346986 | 1042.702068 | 1017.488992 |
| **Zfand2b** | 267.3833726 | 325.6196855 | 259.2263885 | 272.5845766 | 314.1481692 | 288.982076 | 308.5061325 | 227.9561316 | 292.6890937 | 266.5401005 | 243.0887696 | 275.8739378 | 324.6441241 | 298.173035 | 308.9832389 |
| **Abcb6** | 408.4006308 | 381.8702881 | 400.1954215 | 425.2664439 | 350.2498741 | 356.3177053 | 375.2589213 | 401.4374523 | 338.5863552 | 412.4817293 | 451.1803233 | 399.0629289 | 454.16094 | 356.9040874 | 428.9837062 |
| **Atg9a** | 380.0140398 | 450.0048208 | 414.2923248 | 380.4107541 | 359.7503227 | 394.8954096 | 402.3208628 | 300.8685707 | 320.5284162 | 321.5959246 | 379.2941502 | 471.0677617 | 430.3025792 | 426.4777955 | 449.103545 |
| **Ankzf1** | 179.4765104 | 181.4279999 | 191.8745171 | 209.614089 | 209.0098706 | 182.3673295 | 206.5728197 | 139.9583602 | 153.492481 | 171.2847859 | 178.7695621 | 155.287531 | 157.6355983 | 187.0358129 | 218.4439643 |
| **Glb1l** | 19.22962611 | 24.56012226 | 26.62748401 | 21.56523549 | 15.20071786 | 28.05651223 | 15.33510015 | 17.59955427 | 12.03862596 | 10.48682363 | 17.02567258 | 16.48303402 | 9.372927467 | 16.26398373 | 24.43123285 |
| **Stk16** | 404.7378449 | 507.8399475 | 477.7283896 | 474.4351808 | 469.9555273 | 472.0508182 | 451.0323574 | 535.5292943 | 529.6995423 | 580.2709074 | 563.7389365 | 492.7559644 | 501.0255773 | 482.4981839 | 462.0377271 |
| **Tuba4a** | 5115.996242 | 5209.914968 | 5161.032931 | 5355.941887 | 5203.712415 | 5017.205799 | 5045.24795 | 5589.953667 | 5312.043705 | 5785.231034 | 5671.44071 | 4707.207505 | 4740.99713 | 5133.99753 | 4880.498044 |
| **A630095N17Rik** | 7.325571853 | 11.09166812 | 3.915806472 | 6.038265938 | 3.166816221 | 13.32684331 | 7.216517718 | 15.92340625 | 1.504828245 | 11.3607256 | 0 | 6.072696744 | 11.92918041 | 7.228437213 | 9.341353736 |
| **Dnajb2** | 220.6828521 | 174.2976419 | 227.1167754 | 169.0714463 | 221.6771355 | 234.9732899 | 202.0624961 | 221.2515394 | 200.1421566 | 209.7364725 | 201.4704588 | 205.6041612 | 219.8377533 | 199.685578 | 219.8810956 |
| **Ptprn** | 31.13368037 | 30.10595632 | 19.57903236 | 23.29045433 | 11.4005384 | 14.02825611 | 13.53097072 | 71.23629111 | 50.41174621 | 46.31680435 | 56.75224193 | 14.74797781 | 5.112505891 | 12.64976512 | 20.83840449 |
| **Dnpep** | 725.2316134 | 728.0887858 | 711.8936167 | 719.416256 | 765.7361623 | 737.1848588 | 677.4506008 | 704.820245 | 707.2692752 | 693.8781633 | 695.2149636 | 735.6638341 | 670.590356 | 800.5494213 | 686.9487824 |
| **Gmppa** | 607.1067673 | 598.1578164 | 610.8658097 | 569.322217 | 592.1946334 | 566.0401342 | 552.0636054 | 548.1004045 | 534.214027 | 513.8543577 | 565.6306779 | 510.1065265 | 591.3465147 | 468.0413095 | 605.7508615 |
| **Chpf** | 846.103549 | 899.2173797 | 790.2097461 | 878.9989986 | 824.0055808 | 902.718281 | 852.4511555 | 735.8289835 | 747.8996378 | 769.0337326 | 778.4515851 | 811.1387794 | 855.4926524 | 793.3209841 | 830.6619168 |
| **Tmem198** | 9.156964816 | 10.29940611 | 13.31374201 | 7.763484777 | 13.30062813 | 9.118366474 | 16.23716487 | 2.514222039 | 5.266898858 | 20.97364725 | 9.458706988 | 11.27786538 | 12.78126473 | 25.29953024 | 25.86836419 |
| **Obsl1** | 42.12203815 | 19.0142882 | 22.71167754 | 23.29045433 | 12.66726489 | 20.34097137 | 19.84542373 | 13.40918421 | 9.781383594 | 15.73023544 | 17.97154328 | 23.42325887 | 33.23128829 | 20.78175699 | 9.341353736 |
| **Inha** | 1.831392963 | 7.922620085 | 1.566322589 | 0.86260942 | 1.266726489 | 0 | 9.020647148 | 0 | 0 | 0 | 1.891741398 | 0 | 0 | 0.903554652 | 0.718565672 |
| **Stk11ip** | 523.7783875 | 513.3857815 | 530.9833577 | 483.9238844 | 501.6236895 | 531.6709067 | 543.945023 | 535.5292943 | 474.7733113 | 565.4145739 | 517.3912722 | 668.8641699 | 574.3048284 | 568.3358758 | 559.7626585 |
| **Sgpp2** | 0.915696482 | 1.584524017 | 0 | 0 | 0 | 0.701412806 | 3.608258859 | 1.676148026 | 5.266898858 | 5.243411813 | 6.621094891 | 0.867528106 | 0 | 0 | 0.718565672 |
| **Farsb** | 2424.764283 | 2238.140174 | 2340.869109 | 2313.518464 | 2287.074675 | 2279.591619 | 2267.790693 | 2366.721013 | 2244.451328 | 2310.596806 | 2248.334651 | 2057.776668 | 2050.966947 | 2208.287568 | 2125.517258 |
| **Acsl3** | 619.0108215 | 671.8381832 | 712.676778 | 653.8579401 | 715.700466 | 677.5647703 | 700.0022187 | 582.4614391 | 531.9567847 | 585.5143192 | 613.8700835 | 708.7704628 | 812.8884367 | 698.4477457 | 753.0568242 |
| **Utp14b** | 263.7205867 | 256.6928908 | 300.7339371 | 258.7828259 | 236.8778534 | 300.9060937 | 313.9185207 | 249.7460559 | 283.6601242 | 256.9271789 | 293.2199166 | 308.8400058 | 329.75663 | 304.4979176 | 268.7435613 |
| **Scg2** | 1.831392963 | 0 | 0 | 0 | 0 | 0 | 0 | 1.676148026 | 0 | 0 | 0 | 0 | 2.556252945 | 0 | 0.718565672 |
| **Ap1s3** | 695.0136295 | 706.6977116 | 725.2073587 | 636.6057517 | 723.300825 | 742.7961612 | 806.445855 | 531.3389243 | 624.5037217 | 486.7633967 | 544.8215225 | 760.8221492 | 757.5029562 | 780.671219 | 719.2842376 |
| **Wdfy1** | 533.8510488 | 412.7685064 | 403.3280667 | 348.4942055 | 219.1436825 | 284.7735991 | 274.2276733 | 272.3740542 | 314.5091032 | 304.9917871 | 284.7070803 | 255.0532632 | 245.4002828 | 219.5637803 | 293.1747942 |
| **Mrpl44** | 685.8566647 | 672.6304452 | 642.1922615 | 667.6596908 | 651.0974151 | 638.2856532 | 615.2081355 | 760.9712039 | 647.0761454 | 686.0130456 | 670.6223254 | 726.121025 | 627.134056 | 581.8891956 | 634.4934883 |
| **Serpine2** | 401.9907554 | 505.4631614 | 513.7538092 | 484.7864938 | 252.7119345 | 226.5563362 | 219.2017257 | 1086.981995 | 983.4052582 | 1064.412598 | 799.2607405 | 378.2422543 | 357.8754124 | 313.5334641 | 344.9115225 |
| **Fam124b** | 18.31392963 | 15.84524017 | 35.24225825 | 14.66436013 | 31.66816221 | 25.25086101 | 17.13922958 | 31.00873848 | 21.82000955 | 15.73023544 | 32.15960376 | 33.83359614 | 22.15419219 | 19.87820233 | 28.02406121 |
| **Cul3** | 2971.435083 | 3085.860523 | 3191.382275 | 3003.605999 | 3076.878641 | 3193.532504 | 3340.345639 | 3265.974429 | 3236.885555 | 3253.53703 | 3442.023473 | 3247.157702 | 3232.807892 | 3404.593927 | 3329.833324 |
| **1700016L21Rik** | 6.409875371 | 0 | 0 | 0 | 1.266726489 | 3.507064029 | 0 | 1.676148026 | 0.752414123 | 0 | 2.837612096 | 0 | 1.70416863 | 4.517773258 | 0 |
| **Dock10** | 5108.670671 | 4874.788138 | 4715.414154 | 4984.157227 | 4600.750606 | 4792.052289 | 5373.599506 | 4445.982639 | 4364.001911 | 4518.073179 | 4458.834474 | 4942.307621 | 5230.093526 | 4585.539857 | 5060.139462 |
| **Rhbdd1** | 508.2115473 | 478.5262531 | 424.4734216 | 470.9847431 | 381.2846731 | 406.8194273 | 469.0736517 | 360.3718256 | 366.4256777 | 333.8305521 | 339.5675809 | 347.0112425 | 382.5858575 | 384.0107269 | 408.8638673 |
| **Col4a3** | 0 | 2.376786026 | 0 | 0 | 0 | 0 | 0.902064715 | 1.676148026 | 1.504828245 | 0 | 0 | 0 | 0 | 3.614218606 | 0 |
| **Mff** | 997.1934684 | 854.8507072 | 981.301102 | 899.7016247 | 896.2089907 | 963.741195 | 943.5596917 | 1059.325553 | 975.881117 | 1030.330421 | 1026.269708 | 1084.410133 | 926.2156506 | 1038.184295 | 982.2792736 |
| **Tm4sf20** | 0 | 0 | 0 | 0 | 0 | 0 | 0.902064715 | 1.676148026 | 3.00965649 | 0 | 0 | 0.867528106 | 0 | 0 | 0 |
| **Agfg1** | 3855.082187 | 3620.637379 | 3793.63331 | 3670.403081 | 3607.003676 | 3901.959438 | 3856.326656 | 4001.803412 | 3611.587788 | 3730.687505 | 4249.79705 | 3742.51625 | 3754.283493 | 3958.472929 | 3648.157917 |
| **Ccl20** | 2.747089445 | 2.376786026 | 29.76012919 | 11.21392246 | 10.76717515 | 9.81977928 | 9.020647148 | 26.81836842 | 27.83932254 | 4.369509845 | 12.29631908 | 10.41033728 | 10.22501178 | 1.807109303 | 1.437131344 |
| **Pid1** | 0 | 3.169048034 | 1.566322589 | 0.86260942 | 0 | 1.402825611 | 0 | 0.838074013 | 0 | 1.747803938 | 7.56696559 | 0 | 0 | 2.710663955 | 0 |
| **Trip12** | 6089.381602 | 5812.826356 | 5666.955127 | 6003.761561 | 5331.65179 | 5323.723195 | 5880.559876 | 6030.780598 | 5846.257732 | 5762.509583 | 6045.059636 | 5584.27842 | 5656.987768 | 5282.180493 | 5477.626117 |
| **Fbxo36** | 18.31392963 | 12.67619214 | 10.96425812 | 24.15306375 | 15.20071786 | 7.014128057 | 28.86607087 | 21.78992434 | 21.06759543 | 7.86511772 | 20.80915537 | 22.55573076 | 19.59793925 | 24.39597559 | 19.40127314 |
| **A530032D15Rik** | 0.915696482 | 0.792262009 | 0 | 0 | 1.900089733 | 0.701412806 | 0 | 0 | 0.752414123 | 0 | 0 | 1.735056213 | 2.556252945 | 0 | 1.437131344 |

| | | | | | | | | | | | | | | | |
|---|---|---|---|---|---|---|---|---|---|---|---|---|---|---|---|
| **Gm7609** | 2.747089445 | 0.792262009 | 0 | 1.725218839 | 0 | 7.715540863 | 8.118582433 | 0 | 2.257242368 | 0 | 0 | 8.675281063 | 9.372927467 | 3.614218606 | 6.467091048 |
| **C130026I21Rik** | 7.325571853 | 3.961310043 | 5.482129061 | 12.93914129 | 3.800179466 | 18.93814575 | 15.33510015 | 5.028444078 | 5.266898858 | 1.747803938 | 8.512836289 | 4.337640531 | 17.0416863 | 9.035546516 | 14.37131344 |
| **Sp110** | 3024.545479 | 3050.208733 | 2978.362403 | 2817.282365 | 2880.536035 | 2985.212901 | 3167.149214 | 2825.985572 | 2836.601242 | 2640.057848 | 2776.130501 | 2969.548708 | 2872.376226 | 3000.704998 | 2982.766104 |
| **Sp140** | 1213.297838 | 1118.673956 | 1025.158134 | 985.0999572 | 1128.019938 | 1113.843535 | 1205.158459 | 806.2272006 | 908.9162601 | 874.7758709 | 792.6396456 | 1087.012717 | 1033.578274 | 1087.8798 | 1135.333762 |
| **Sp100** | 3629.820853 | 3596.077257 | 3150.657888 | 3314.14539 | 3638.038475 | 3707.668091 | 4165.734853 | 2014.729927 | 2450.612797 | 2155.916157 | 2323.058436 | 3658.366024 | 3435.603959 | 3657.58923 | 3431.869649 |
| **A630001G21Rik** | 258.2264078 | 311.3589693 | 367.3026471 | 339.8681113 | 376.2177671 | 339.483798 | 318.4288443 | 398.9232302 | 369.4353342 | 372.2822388 | 313.0832013 | 373.9046138 | 356.1712437 | 368.6502978 | 379.4026748 |
| **Cab39** | 4225.023566 | 4328.127352 | 4420.162346 | 4181.067857 | 4151.696066 | 4252.665841 | 4373.209737 | 4140.923699 | 4402.375031 | 4410.583237 | 4406.811586 | 4662.096043 | 4677.090806 | 4566.565209 | 4334.388133 |
| **Itm2c** | 1011.844612 | 932.492384 | 893.587037 | 900.5642341 | 898.1090804 | 908.3295834 | 998.5856393 | 996.4700015 | 834.4272619 | 935.9490087 | 979.9220439 | 972.4990071 | 976.4886252 | 913.4937527 | 942.7581616 |
| **4933407L21Rik** | 1.831392963 | 1.584524017 | 0 | 0.86260942 | 4.43354271 | 0 | 0.902064715 | 3.352296052 | 0 | 0 | 1.891741398 | 0 | 0 | 1.807109303 | 2.155697016 |
| **Gpr55** | 377.2669504 | 381.0780261 | 469.8967767 | 353.6698621 | 307.8145367 | 368.241723 | 421.2642218 | 402.2755263 | 417.589838 | 513.8543577 | 623.3287905 | 357.4215798 | 345.946232 | 371.3609618 | 367.905624 |
| **2810459M11Rik** | 1.831392963 | 3.169048034 | 0 | 0.86260942 | 0.633363244 | 0.701412806 | 0 | 2.514222039 | 0 | 0 | 0 | 0.867528106 | 0.852084315 | 8.131991864 | 2.155697016 |
| **Psmd1** | 5172.769424 | 4788.431579 | 5164.948737 | 4795.245764 | 4717.922807 | 4803.976306 | 4942.412572 | 5768.463432 | 5774.025977 | 5512.57362 | 5974.119333 | 4974.406161 | 5006.847436 | 4988.525231 | 4828.761316 |
| **Htr2b** | 1.831392963 | 1.584524017 | 0 | 1.725218839 | 0 | 0.701412806 | 1.80412943 | 4.190370065 | 10.53379772 | 5.243411813 | 6.621094891 | 6.072696744 | 0.852084315 | 0.903554652 | 2.874262688 |
| **Armc9** | 45.78482408 | 38.02857641 | 53.25496803 | 75.90962893 | 40.53524763 | 55.41161165 | 75.77343604 | 46.93214473 | 77.49865463 | 76.90337326 | 91.74945778 | 62.46202365 | 63.90632364 | 61.44171631 | 64.67091048 |
| **B3gnt7** | 9.156964816 | 12.67619214 | 10.18109683 | 12.07653188 | 17.1008076 | 16.13249453 | 14.43303544 | 5.866518091 | 15.04828245 | 6.991215751 | 12.29631908 | 16.48303402 | 16.18960199 | 8.131991864 | 11.49705075 |
| **Ncl** | 50810.16637 | 47192.67106 | 48925.65239 | 47123.48999 | 47203.29587 | 46000.05462 | 49417.81127 | 56237.2805 | 52742.72516 | 53435.60979 | 55528.28524 | 46982.71965 | 44849.45793 | 44236.22863 | 43217.41377 |
| **Snora75** | 0 | 0 | 3.132645178 | 0.86260942 | 0.633363244 | 2.104238417 | 0 | 1.676148026 | 4.514484735 | 2.621705907 | 0.945870699 | 0 | 0.852084315 | 0 | 0 |
| **Gm24148** | 1.831392963 | 0.792262009 | 3.132645178 | 6.038265938 | 3.166816221 | 2.805651223 | 4.510323574 | 1.676148026 | 1.504828245 | 0.873901969 | 2.837612096 | 2.602584319 | 0.852084315 | 1.807109303 | 2.874262688 |
| **C130036L24Rik** | 32.04937685 | 20.59881222 | 37.59174214 | 32.77915795 | 27.86798275 | 23.84803539 | 44.20117102 | 36.03718256 | 24.07725192 | 27.964863 | 24.59263817 | 26.89337129 | 17.89377062 | 24.39597559 | 17.9641418 |
| **Ptma** | 46976.1452 | 45494.06131 | 47191.73328 | 45097.22046 | 44857.95178 | 44407.84756 | 49230.18181 | 44359.25751 | 45072.6156 | 45546.02282 | 44980.88108 | 47516.24944 | 45251.64173 | 46540.29299 | 44950.59418 |
| **Pde6d** | 150.174223 | 208.3649082 | 182.4765816 | 211.3393078 | 233.7110371 | 257.4184997 | 258.8925731 | 145.8248783 | 142.2062692 | 172.1586879 | 139.0429927 | 209.9418017 | 234.3231867 | 233.1171001 | 267.30643 |
| **Cops7b** | 417.5575956 | 456.3429169 | 455.7998734 | 464.0838678 | 418.0197412 | 448.9041957 | 461.857134 | 578.271069 | 558.291279 | 562.792868 | 436.0463921 | 520.5168638 | 419.2254831 | 444.5488886 | 436.1693629 |
| **Dis3l2** | 497.2231895 | 442.8744628 | 453.4503895 | 472.709962 | 492.1232408 | 425.7575731 | 511.4706933 | 436.6365608 | 438.6574335 | 411.6078274 | 466.3142545 | 432.896525 | 487.3922283 | 454.4879897 | 491.4989196 |
| **Ecel1** | 0 | 0 | 0 | 0 | 0 | 0 | 3.608258859 | 0 | 0 | 0 | 0 | 4.337640531 | 1.70416863 | 0 | 0 |
| **Eif4e2** | 2661.929672 | 2390.25448 | 2444.2464 | 2531.758647 | 2463.149657 | 2427.589721 | 2523.075007 | 2599.705588 | 2417.506576 | 2383.130669 | 2470.614265 | 2345.795999 | 2342.379782 | 2357.374086 | 2404.320738 |
| **Efhd1** | 0 | 0 | 0 | 0 | 0 | 0 | 0 | 0 | 0 | 0.873901969 | 0.945870699 | 0 | 0 | 1.807109303 | 0 |
| **Gigyf2** | 1459.620192 | 1466.476978 | 1575.720525 | 1475.062108 | 1377.565056 | 1346.712587 | 1685.958952 | 1563.008034 | 1657.568312 | 1448.929464 | 1568.253619 | 1470.46014 | 1414.459963 | 1454.722989 | 1514.017871 |
| **Kcnj13** | 1.831392963 | 0 | 1.566322589 | 1.725218839 | 0 | 0 | 2.706194144 | 3.352296052 | 1.504828245 | 1.747803938 | 4.729353494 | 0 | 0 | 0.903554652 | 1.437131344 |
| **Inpp5d** | 1907.395771 | 1965.602043 | 1955.553752 | 2040.071277 | 2072.997899 | 1980.789763 | 2261.47624 | 2013.053779 | 2024.746404 | 1920.836528 | 1810.396517 | 1976.229026 | 2129.358704 | 2014.023318 | 2128.39152 |
| **Atg16l1** | 346.13327 | 488.8256592 | 449.534583 | 336.4176737 | 405.9858396 | 418.0420322 | 435.6972572 | 414.8466365 | 441.66709 | 543.5670247 | 450.2344526 | 424.221244 | 521.4756009 | 404.7924839 | 489.3432226 |
| **Scarna6** | 0 | 0 | 0.783161294 | 1.725218839 | 0 | 0 | 0.902064715 | 0 | 0 | 0 | 3.783482795 | 0 | 0 | 0.903554652 | 0 |
| **Sag** | 14.65114371 | 14.26071615 | 17.22954848 | 9.488703616 | 8.86708542 | 6.312715251 | 11.72684129 | 8.380740131 | 15.04828245 | 7.86511772 | 5.675224193 | 15.61550591 | 10.22501178 | 5.421327909 | 19.40127314 |
| **Dgkd** | 3437.524592 | 3428.909973 | 3440.427567 | 3319.321047 | 3843.88153 | 3870.395862 | 4122.435747 | 2624.847809 | 2738.034992 | 2883.002595 | 2837.612096 | 3768.542094 | 4095.969303 | 3830.168168 | 4026.12346 |
| **Usp40** | 165.7410632 | 206.7803842 | 185.6092268 | 200.9879948 | 194.442516 | 190.0828703 | 193.041849 | 239.6891677 | 258.078044 | 272.6574143 | 228.9007091 | 200.3989926 | 217.2815004 | 171.6753838 | 194.7312971 |
| **Ugt1a7c** | 2.747089445 | 3.961310043 | 1.566322589 | 0.86260942 | 0.633363244 | 0.701412806 | 0 | 6.704592104 | 3.00965649 | 3.495607876 | 7.56696559 | 0 | 3.408337261 | 1.807109303 | 0 |
| **Dnajb3** | 6.409875371 | 3.961310043 | 1.566322589 | 1.725218839 | 5.700269198 | 2.805651223 | 4.510323574 | 3.352296052 | 12.03862596 | 4.369509845 | 0.945870699 | 1.735056213 | 5.112505891 | 2.710663955 | 13.65274777 |
| **Mroh2a** | 0.915696482 | 0 | 0 | 0.86260942 | 0.633363244 | 0 | 1.80412943 | 0 | 0 | 0 | 0 | 0 | 0 | 0.903554652 | 0 |
| **Hjurp** | 37.54355574 | 59.41965064 | 54.82129061 | 56.06961228 | 39.26852115 | 42.78618115 | 55.92801232 | 41.06562664 | 36.86829201 | 25.3431571 | 37.83482795 | 68.5347204 | 57.08964912 | 39.75640467 | 53.8924254 |
| **Glrp1** | 31.13368037 | 10.29940611 | 13.31374201 | 23.29045433 | 20.26762382 | 14.72966892 | 14.43303544 | 11.73303618 | 6.019312981 | 9.612921658 | 1.891741398 | 6.94022485 | 1.70416863 | 11.74621047 | 11.49705075 |
| **Arl4c** | 2051.160119 | 2295.975301 | 2228.093883 | 2344.572403 | 2366.878444 | 2445.826454 | 2473.461448 | 1475.010263 | 1692.179362 | 1715.469565 | 2021.325683 | 2351.001168 | 2419.919455 | 2466.704199 | 2376.296677 |
| **Sh3bp4** | 91.56964816 | 59.41965064 | 66.56871003 | 83.67311371 | 93.10439691 | 91.18366474 | 86.59821262 | 127.38725 | 91.04210883 | 67.29045161 | 122.9631908 | 78.07752957 | 98.84178056 | 95.77679307 | 96.28780004 |
| **Agap1** | 4.578482408 | 5.54583406 | 3.132645178 | 0.86260942 | 3.166816221 | 2.805651223 | 5.412388289 | 10.89496217 | 8.276555348 | 5.243411813 | 7.56696559 | 4.337640531 | 3.408337261 | 0 | 2.155697016 |
| **Cops8** | 1334.169774 | 1352.391249 | 1354.085878 | 1368.09854 | 1259.759493 | 1277.974132 | 1194.333682 | 1408.802416 | 1368.641289 | 1480.389935 | 1259.899771 | 1278.736429 | 1250.00769 | 1221.605889 | 1176.292005 |
| **Lrrfip1** | 3265.373653 | 3136.565292 | 3276.746856 | 2836.259772 | 3184.550392 | 3235.617273 | 3684.032295 | 2856.156236 | 2874.221948 | 2723.078535 | 2821.532294 | 3111.823317 | 3317.164239 | 3205.811904 | 3130.072067 |
| **Rbm44** | 22.89241204 | 61.00417465 | 54.03812932 | 58.65744054 | 20.90098706 | 32.26498906 | 26.15987673 | 51.1225148 | 29.34415078 | 30.58656891 | 38.78069865 | 14.74797781 | 25.56252945 | 31.62441281 | 21.55697016 |
| **Ramp1** | 32.04937685 | 20.59881222 | 30.54329049 | 26.74089201 | 32.9348887 | 44.18900676 | 26.15987673 | 17.59955427 | 13.54345421 | 25.3431571 | 25.53850887 | 25.15831508 | 46.01255302 | 13.55331977 | 43.83250599 |
| **Ube2f** | 383.6768258 | 427.8214846 | 442.4861314 | 400.2507707 | 367.3506817 | 411.0279041 | 443.8158397 | 437.4746348 | 452.2008877 | 484.1416908 | 425.6418144 | 406.0031537 | 402.1837968 | 427.3813502 | 428.2651405 |
| **Scly** | 347.964663 | 322.4506375 | 373.5679375 | 394.2125048 | 376.2177671 | 357.7205309 | 350.0011093 | 435.7984868 | 416.8374239 | 418.5990431 | 403.8867884 | 395.5928165 | 388.5504477 | 390.3356095 | 405.271039 |
| **Espnl** | 0.915696482 | 0 | 3.915806472 | 0.86260942 | 0 | 0 | 0 | 1.676148026 | 0 | 0 | 0 | 1.735056213 | 0 | 0 | 1.437131344 |
| **Klhl30** | 1.831392963 | 3.169048034 | 7.831612945 | 12.93914129 | 13.93399137 | 5.611302446 | 15.33510015 | 3.352296052 | 2.257242368 | 3.495607876 | 3.783482795 | 8.675281063 | 10.22501178 | 7.228437213 | 8.622788064 |
| **Fam132b** | 22.89241204 | 32.48274235 | 23.49483883 | 40.54264272 | 27.86798275 | 23.84803539 | 28.86607087 | 31.8468125 | 24.07725192 | 20.09974528 | 28.37612096 | 27.7608994 | 15.33751767 | 26.2030849 | 32.33545524 |
| **Ilkap** | 750.8711149 | 773.2477203 | 816.8372302 | 779.7989154 | 855.673743 | 859.9320998 | 789.3066254 | 816.2840887 | 814.8644948 | 943.8141264 | 839.9331805 | 804.1985545 | 780.5092327 | 885.4835585 | 767.4281377 |
| **1700020N18Rik** | 10.0726613 | 2.376786026 | 1.566322589 | 1.725218839 | 3.166816221 | 3.507064029 | 1.80412943 | 1.676148026 | 0 | 6.117313782 | 3.783482795 | 0 | 6.816674521 | 2.710663955 | 2.874262688 |
| **Hes6** | 357.1216278 | 425.4446986 | 400.9785828 | 334.6924548 | 380.0179466 | 401.2081249 | 396.0064098 | 296.6782006 | 367.9305059 | 374.0300427 | 374.5647967 | 439.8367499 | 408.148387 | 387.6249455 | 400.2410793 |
| **Per2** | 93.40104112 | 106.9553711 | 115.1247103 | 123.353147 | 91.20430718 | 125.5528922 | 178.6088135 | 112.3019177 | 97.06142181 | 126.7157855 | 85.12836289 | 127.5266316 | 140.593912 | 131.0154245 | 171.0186299 |
| **Traf3ip1** | 195.0433506 | 212.3262183 | 166.8133557 | 186.3236346 | 209.0098706 | 196.3955856 | 199.356302 | 194.433171 | 182.8366318 | 193.1323351 | 222.2796142 | 238.5702292 | 197.6835611 | 218.6602257 | 222.0367926 |
| **Asb1** | 257.3107113 | 205.9881222 | 295.251808 | 264.8210918 | 228.0107679 | 206.9167777 | 233.6347611 | 241.3653158 | 203.1518131 | 248.1881592 | 217.5502607 | 229.0274201 | 250.5127887 | 215.9495617 | 222.7553583 |
| **Hdac4** | 537.5138347 | 446.0435108 | 497.307422 | 445.96907 | 376.8511303 | 408.2222529 | 478.9963635 | 414.8466365 | 361.1587788 | 370.5344348 | 401.0491763 | 399.930457 | 462.6817831 | 354.1934234 | 479.2833032 |
| **Ndufa10** | 2125.331534 | 2056.712174 | 2129.41556 | 2205.692286 | 2124.933685 | 1970.268571 | 2152.326409 | 2314.760424 | 2330.978952 | 2225.828315 | 2342.921721 | 2254.705548 | 2092.719078 | 2152.26718 | 2156.415582 |
| **Myeov2** | 537.5138347 | 620.3411527 | 590.503616 | 565.8717793 | 652.3641416 | 596.2008849 | 570.1048997 | 527.9866282 | 597.4168133 | 619.596496 | 503.2032117 | 521.3843919 | 495.0609871 | 650.5593491 | 508.0259301 |
| **Otos** | 0.915696482 | 2.376786026 | 3.132645178 | 12.07653188 | 1.266726489 | 1.402825611 | 3.608258859 | 2.514222039 | 0 | 1.747803938 | 0.945870699 | 6.072696744 | 6.816674521 | 2.710663955 | 0 |
| **Gpc1** | 136.4387758 | 148.1529956 | 158.1985815 | 178.5601499 | 208.3765074 | 233.5704643 | 216.4955315 | 119.8445839 | 103.8331489 | 81.27288311 | 108.7751304 | 155.287531 | 218.985669 | 202.396242 | 178.2042866 |
| **Ankmy1** | 2.747089445 | 0.792262009 | 3.915806472 | 4.313047098 | 3.800179466 | 1.402825611 | 2.706194144 | 5.028444078 | 0 | 2.621705907 | 0.945870699 | 2.602584319 | 0 | 6.324882561 | 0 |
| **Dusp28** | 58.60457482 | 76.05715282 | 61.08658097 | 86.26094197 | 69.03659363 | 76.45399582 | 83.89201847 | 64.53169901 | 69.22209928 | 83.02068705 | 62.42746612 | 70.26977661 | 58.79381775 | 58.73105235 | 82.63505228 |
| **Rnpepl1** | 1018.254487 | 956.2602443 | 939.7935534 | 896.251187 | 1064.683614 | 1054.223447 | 1040.080616 | 806.2272006 | 881.8293517 | 841.5675961 | 864.5258187 | 1038.431143 | 1100.040851 | 1052.641169 | 1108.028266 |
| **9430060I03Rik** | 1.831392963 | 0.792262009 | 0.783161294 | 0 | 0 | 1.402825611 | 0 | 0 | 0 | 1.747803938 | 0 | 0 | 0 | 0 | 0 |
| **Capn10** | 240.8281747 | 209.9494323 | 238.8641948 | 213.0645267 | 187.4755203 | 236.3761155 | 172.2943605 | 202.8139112 | 276.135983 | 218.4754922 | 279.0318561 | 204.7366331 | 223.2460906 | 208.7211245 | 233.5338434 |
| **Kif1a** | 0 | 2.376786026 | 2.349483883 | 0 | 0 | 0 | 0 | 0 | 2.257242368 | 0 | 0 | 0 | 0 | 0 | 7.18565672 |
| **Sned1** | 0 | 0 | 0 | 0.86260942 | 0 | 0 | 0.902064715 | 0.838074013 | 0 | 0 | 0.945870699 | 0 | 1.70416863 | 0 | 0 |
| **Mterf4** | 187.7177787 | 179.8434759 | 190.3081946 | 188.9114629 | 173.5415289 | 190.7842832 | 174.09849 | 192.757023 | 187.3511165 | 183.5194135 | 207.145683 | 171.770565 | 191.7189709 | 171.6753838 | 153.0544881 |
| **Pask** | 426.7145604 | 484.0720872 | 502.7895511 | 432.1673192 | 469.322164 | 418.0420322 | 463.6612634 | 516.253592 | 468.0015842 | 457.9246317 | 419.9665903 | 456.3197839 | 476.3151322 | 489.7266212 | 533.8942943 |
| **Ppp1r7** | 1376.291812 | 1466.476978 | 1544.394073 | 1499.215171 | 1412.400035 | 1402.124199 | 1540.726533 | 1512.723594 | 1531.915154 | 1562.53672 | 1743.239698 | 1451.374522 | 1370.151579 | 1469.179863 | 1450.784092 |
| **Ano7** | 1.831392963 | 0 | 0 | 2.587828259 | 1.266726489 | 3.507064029 | 0.902064715 | 3.352296052 | 6.019312981 | 4.369509845 | 1.891741398 | 0 | 0 | 0 | 0.718565672 |
| **Hdlbp** | 8400.599522 | 7780.012924 | 8132.346882 | 8157.697282 | 6843.489854 | 6969.93905 | 7386.105885 | 8985.829568 | 8490.240959 | 8729.406767 | 8948.882681 | 7279.42834 | 7370.529326 | 7149.827958 | 7324.339894 |
| **Sept2** | 3110.620948 | 2982.0742 | 3193.731759 | 2916.482448 | 2741.829484 | 2857.55577 | 3144.597596 | 3328.82998 | 3073.611691 | 3103.225892 | 3168.666841 | 3032.87826 | 2856.186624 | 2828.126059 | 2901.568183 |
| **Farp2** | 22.89241204 | 34.06726637 | 42.2907099 | 43.13047098 | 24.70116653 | 30.86216345 | 41.49497688 | 41.90370065 | 38.37312025 | 31.46047088 | 59.58985402 | 48.58157395 | 37.49170987 | 36.14218606 | 38.08398061 |
| **Stk25** | 727.0630064 | 716.2048557 | 715.0262619 | 659.896206 | 642.8636929 | 689.488788 | 686.4712479 | 630.2316578 | 613.969924 | 685.1391436 | 635.6251096 | 722.6509125 | 817.1488582 | 678.5695433 | 775.3323601 |
| **Bok** | 10.0726613 | 7.922620085 | 5.482129061 | 6.038265938 | 6.966995687 | 4.208476834 | 7.216517718 | 1.676148026 | 0 | 6.117313782 | 0.945870699 | 3.470112425 | 4.260421576 | 7.228437213 | 8.622788064 |
| **Thap4** | 622.6736075 | 745.51855 | 760.4496169 | 734.9432255 | 706.8333806 | 762.4357198 | 793.816949 | 878.3015657 | 821.6362219 | 838.9458901 | 793.5855163 | 790.3181048 | 733.6445954 | 664.1126689 | 760.9610466 |
| **Atg4b** | 885.4784977 | 804.9382006 | 851.2963271 | 835.0059182 | 746.1019018 | 846.6052565 | 856.961479 | 826.3409769 | 818.6265654 | 819.7200468 | 891.9560689 | 725.2534968 | 731.9404267 | 836.6916074 | 822.7576944 |
| **Dtymk** | 1345.158131 | 1517.974008 | 1605.480654 | 1506.116047 | 1635.343897 | 1750.726363 | 1510.958397 | 1832.029793 | 1839.65253 | 1935.692861 | 1690.270939 | 1734.188684 | 1624.072705 | 1838.733716 | 1562.161771 |
| **Ing5** | 629.9991793 | 691.6447334 | 655.5060035 | 640.0561894 | 580.794095 | 596.9022977 | 621.5225885 | 685.5445427 | 635.7899336 | 574.1535936 | 553.3343588 | 534.3973135 | 593.0506834 | 502.3763863 | 524.5529405 |
| **D2hgdh** | 161.1625808 | 183.804786 | 188.741872 | 166.483618 | 193.8091527 | 167.6376606 | 217.3975963 | 156.7198404 | 188.8559448 | 141.572119 | 149.4475704 | 148.3473062 | 156.783514 | 164.4469466 | 168.1443672 |
| **Pdcd1** | 787.4989741 | 680.5530653 | 718.9420683 | 653.8579401 | 483.8895186 | 490.988964 | 506.058305 | 902.6057121 | 727.5844565 | 711.3562027 | 733.9956622 | 533.5297854 | 551.2985519 | 446.3559979 | 506.5887987 |
| **Fam174a** | 173.9823315 | 165.5827598 | 166.8133557 | 124.2157564 | 155.8073581 | 186.5758063 | 171.3922958 | 168.4528766 | 190.360773 | 194.8801391 | 167.4191137 | 177.8432618 | 180.6418748 | 189.7464768 | 188.2642061 |
| **St8sia4** | 610.7695532 | 636.1863928 | 617.1311001 | 585.7117959 | 685.2990303 | 697.2043289 | 722.5538365 | 607.6036595 | 517.6609163 | 575.0274955 | 520.2288843 | 778.1727113 | 635.6548991 | 714.7117294 | 666.8289436 |
| **D1Ertd622e** | 634.5776617 | 534.7768557 | 556.8276804 | 552.0700286 | 578.8940053 | 570.248611 | 611.5998766 | 523.7962582 | 542.4905824 | 487.6372986 | 495.6362462 | 508.3714703 | 533.4047813 | 500.569277 | 592.0981137 |
| **Ppip5k2** | 1169.344407 | 1252.566235 | 1307.0962 | 1216.279282 | 1262.926309 | 1246.410556 | 1374.746625 | 1304.043164 | 1302.428846 | 1249.679816 | 1416.914307 | 1274.398788 | 1397.418277 | 1244.194755 | 1303.478129 |
| **Gin1** | 82.41268334 | 76.05715282 | 106.5099361 | 127.6661941 | 127.9393753 | 105.2119209 | 119.9746071 | 113.9780658 | 85.77520997 | 132.8330993 | 150.3934411 | 101.5007884 | 114.1792982 | 116.5585501 | 112.0962448 |
| **Pam** | 48.53191352 | 33.27500436 | 51.68864544 | 43.13047098 | 43.06870061 | 49.80030921 | 44.20117102 | 19.2757023 | 23.3248378 | 39.3255886 | 54.86050053 | 36.43618046 | 57.08964912 | 28.91374885 | 46.70676868 |
| **B230216N24Rik** | 0 | 3.169048034 | 0 | 3.450437679 | 0 | 1.402825611 | 0 | 0 | 0.752414123 | 1.747803938 | 0 | 0.867528106 | 0 | 0 | 0 |
| **1810006J02Rik** | 0.915696482 | 9.507144102 | 1.566322589 | 6.900875357 | 2.533452977 | 9.118366474 | 2.706194144 | 0 | 3.00965649 | 0 | 3.783482795 | 11.27786538 | 9.372927467 | 9.939101167 | 9.341353736 |
| **Rnf152** | 4.578482408 | 8.714882094 | 4.698967767 | 5.175656518 | 6.333632443 | 4.90988964 | 6.314453003 | 10.89496217 | 0 | 5.243411813 | 13.24218978 | 4.337640531 | 9.372927467 | 4.517773258 | 6.467091048 |
| **Pign** | 545.755103 | 514.1780435 | 461.2820025 | 455.4577736 | 414.852925 | 499.4059177 | 450.1302927 | 558.1572927 | 509.384361 | 489.3851026 | 539.1462983 | 475.4054022 | 476.3151322 | 457.1986537 | 491.4989196 |
| **2310035C23Rik** | 494.4761 | 493.5792313 | 472.2462606 | 393.3498954 | 404.7191131 | 450.3070213 | 375.2589213 | 474.3498914 | 483.8022808 | 490.2590046 | 501.3114703 | 488.4183238 | 497.6172401 | 410.2138118 | 508.0259301 |
| **Tnfrsf11a** | 17.39823315 | 26.14464628 | 20.36219366 | 19.84001665 | 5.700269198 | 4.208476834 | 8.118582433 | 36.87525657 | 35.36346376 | 55.05582404 | 45.40179354 | 8.675281063 | 14.48543336 | 4.517773258 | 4.311394032 |
| **Zcchc2** | 1476.102728 | 1410.226375 | 1311.012007 | 1431.069027 | 1283.193933 | 1449.820269 | 1511.860462 | 1158.218286 | 1238.473646 | 1199.867403 | 1301.518082 | 1335.993284 | 1433.205818 | 1381.535062 | 1412.700111 |
| **Phlpp1** | 358.9530208 | 457.9274409 | 404.111228 | 331.2420171 | 346.4496946 | 364.0332462 | 398.7126039 | 397.2470822 | 431.1332922 | 398.4992978 | 407.6702712 | 374.7721419 | 456.7171929 | 367.7467432 | 352.8157449 |
| **Bcl2** | 5380.632526 | 5164.756033 | 5326.279964 | 4723.649182 | 4021.223238 | 4416.095025 | 4930.685731 | 3386.657087 | 4009.614859 | 3643.297308 | 3844.01852 | 4079.984684 | 4378.861296 | 3916.00586 | 4343.729487 |
| **Kdsr** | 346.13327 | 356.5179038 | 368.0858084 | 357.1202997 | 339.4826989 | 320.5456522 | 352.7073035 | 324.3346431 | 322.7856586 | 353.0563954 | 328.2171325 | 364.3618046 | 364.6920869 | 362.3254153 | 332.6959061 |
| **Vps4b** | 1834.140053 | 1865.77703 | 1924.227301 | 1847.709377 | 1817.119148 | 1716.357136 | 1862.763636 | 1821.972904 | 1729.800068 | 1743.434428 | 1758.373629 | 1926.779924 | 1865.212566 | 1910.114533 | 1743.958886 |
| **Serpinb5** | 0 | 0 | 0.783161294 | 0.86260942 | 3.800179466 | 0.701412806 | 0 | 2.514222039 | 0 | 0 | 0 | 0 | 0 | 0 | 0 |
| **Dsel** | 25.63950148 | 22.18333624 | 28.1938066 | 32.77915795 | 19.63426057 | 18.23673295 | 27.96400616 | 41.06562664 | 19.56276719 | 24.46925513 | 30.26786236 | 20.82067455 | 24.71044514 | 34.33507676 | 30.89832389 |
| **Tsn** | 2856.973022 | 2947.214672 | 3114.632468 | 2956.162481 | 2966.040073 | 2926.995638 | 2990.344529 | 3272.679021 | 3067.592378 | 3194.985598 | 3175.287936 | 3152.597138 | 3067.503535 | 3102.806674 | 3029.472873 |
| **Nifk** | 1941.276541 | 1941.834183 | 2091.823818 | 2128.057438 | 1916.557177 | 1928.183803 | 2042.274514 | 2178.992434 | 2231.660288 | 2071.147666 | 2180.231961 | 1984.904307 | 1991.321045 | 1962.520703 | 1815.815453 |
| **Clasp1** | 1784.692443 | 1782.589519 | 1756.630784 | 1677.775321 | 1591.00847 | 1700.926054 | 1767.144776 | 1795.154536 | 1658.320726 | 1697.991526 | 1637.30218 | 1699.48756 | 1634.297716 | 1508.936268 | 1652.701046 |
| **Tfcp2l1** | 0.915696482 | 2.376786026 | 1.566322589 | 3.450437679 | 5.700269198 | 1.402825611 | 1.80412943 | 5.028444078 | 1.504828245 | 1.747803938 | 3.783482795 | 2.602584319 | 1.70416863 | 6.324882561 | 2.155697016 |
| **Ralb** | 205.1160119 | 155.2833537 | 150.3669685 | 141.4679448 | 143.7734565 | 176.756027 | 176.8046841 | 102.2450296 | 151.9876528 | 137.2026091 | 118.2338373 | 144.0096656 | 142.2980806 | 128.3047605 | 168.1443672 |
| **Tmem185b** | 278.3717304 | 278.876227 | 256.8769046 | 268.2715295 | 287.5469129 | 316.3371754 | 284.1503852 | 277.4024983 | 235.5056204 | 240.3230414 | 199.5787174 | 278.4765221 | 304.1941005 | 277.391278 | 324.7916837 |
| **Epb4.1l5** | 24.723805 | 7.922620085 | 11.74741942 | 12.07653188 | 13.93399137 | 20.34097137 | 20.74748844 | 17.59955427 | 24.07725192 | 19.22584332 | 8.512836289 | 14.74797781 | 18.74585493 | 13.55331977 | 17.24557613 |
| **Ptpn4** | 265.5519797 | 210.7416943 | 235.7315496 | 239.8054187 | 196.3426057 | 203.4097137 | 188.5315254 | 214.5469473 | 243.0297616 | 207.9886686 | 293.2199166 | 222.0871952 | 218.985669 | 165.3505012 | 231.3781464 |
| **Tmem177** | 131.8602933 | 87.94108294 | 130.0047749 | 130.2540224 | 133.0062813 | 143.7896252 | 141.6241602 | 146.6629523 | 151.2352386 | 150.3111387 | 140.9347341 | 115.3812381 | 147.4105865 | 130.1118698 | 120.0004672 |
| **Tmem37** | 9.156964816 | 13.46845414 | 7.831612945 | 12.07653188 | 13.30062813 | 11.22260489 | 17.13922958 | 3.352296052 | 15.80069657 | 6.991215751 | 3.783482795 | 13.01292159 | 4.260421576 | 18.97464768 | 5.748525376 |
| **Dbi** | 1672.977472 | 1726.338917 | 1734.702267 | 1638.095288 | 1864.621391 | 1857.34111 | 1805.933559 | 1454.896487 | 1739.581451 | 1699.73933 | 1725.268155 | 1976.229026 | 1931.675142 | 2012.216209 | 1897.013374 |
| **3110009E18Rik** | 54.94178889 | 69.71905675 | 59.52025838 | 52.6191746 | 68.40323038 | 61.0229141 | 45.10323574 | 36.03718256 | 41.38277674 | 35.82998073 | 34.99721585 | 45.11146153 | 52.82922754 | 60.53816166 | 37.36541494 |
| **Steap3** | 31.13368037 | 34.06726637 | 23.49483883 | 50.89395576 | 48.13560656 | 21.74379698 | 41.49497688 | 45.2559967 | 47.40208972 | 33.20827482 | 51.07701773 | 28.62842751 | 22.15419219 | 31.62441281 | 51.01816271 |
| **C1ql2** | 0.915696482 | 0 | 0.783161294 | 1.725218839 | 0 | 0 | 0 | 0 | 0 | 0 | 0 | 0 | 0 | 0 | 0 |
| **Insig2** | 244.4909606 | 231.3405065 | 230.2494206 | 219.965402 | 228.0107679 | 199.9026496 | 220.1037904 | 158.3959885 | 183.5890459 | 202.7452568 | 186.3365277 | 215.1469704 | 222.3940063 | 212.3353431 | 186.108509 |
| **Ccdc93** | 516.4528156 | 525.2697116 | 546.6465836 | 519.2908706 | 472.4889802 | 472.7522311 | 532.2181817 | 582.4614391 | 480.0402102 | 550.5582404 | 507.9325652 | 538.734954 | 518.9193479 | 438.224006 | 495.8103137 |
| **Ddx18** | 2724.197033 | 2745.980121 | 2829.561757 | 2877.665024 | 2835.567245 | 2756.552326 | 2774.751063 | 2998.628819 | 3027.714429 | 3022.82691 | 2967.196382 | 2710.157804 | 2831.476179 | 2600.430287 | 2698.932664 |
| **Actr3** | 14374.60337 | 14591.88167 | 14667.04472 | 14533.2435 | 14557.22081 | 14575.3581 | 15311.64647 | 14284.97155 | 14492.24842 | 14728.74378 | 14478.44279 | 15317.07624 | 15303.4343 | 15736.30781 | 14516.46371 |
| **Slc35f5** | 35.71216278 | 61.79643666 | 47.77283896 | 54.34439344 | 44.3354271 | 37.87629151 | 36.98465331 | 62.01747697 | 48.15450385 | 54.18192207 | 42.56418144 | 51.18415827 | 43.45630007 | 35.23863141 | 47.42533435 |
| **Mgat5** | 216.1043696 | 267.7845589 | 241.2136787 | 250.1567317 | 194.442516 | 189.3814575 | 284.1503852 | 271.5359802 | 259.5828723 | 284.0181399 | 260.1144422 | 221.2196671 | 241.9919455 | 186.1322582 | 267.30643 |
| **Tmem163** | 48.53191352 | 68.13453273 | 58.73709709 | 60.38265938 | 61.43623469 | 86.97518791 | 73.96930661 | 52.79866282 | 23.3248378 | 41.94729451 | 34.99721585 | 49.44910206 | 63.05423932 | 71.38081747 | 92.69497168 |
| **Ccnt2** | 425.7988639 | 497.5405413 | 434.6545184 | 410.6020838 | 449.0545402 | 413.8335554 | 424.8724807 | 527.9866282 | 458.2202007 | 475.4026711 | 432.2629093 | 464.1275369 | 521.4756009 | 450.8737711 | 465.6305554 |
| **Map3k19** | 13.73544722 | 14.26071615 | 18.01270977 | 12.93914129 | 10.76717515 | 4.208476834 | 7.216517718 | 20.11377631 | 20.31518131 | 20.97364725 | 15.13393118 | 14.74797781 | 2.556252945 | 4.517773258 | 11.49705075 |
| **Rab3gap1** | 1160.187442 | 1171.755511 | 1196.670458 | 1169.698373 | 1096.985139 | 1061.938988 | 1217.787365 | 1224.426133 | 1234.711575 | 1169.280834 | 1258.9539 | 1194.586202 | 1178.432608 | 1189.077921 | 1081.441336 |
| **Zranb3** | 130.0289004 | 138.6458515 | 128.4384523 | 154.4070861 | 127.9393753 | 125.5528922 | 141.6241602 | 171.8051727 | 151.2352386 | 140.698217 | 156.0686653 | 135.3343846 | 186.606465 | 167.1576105 | 150.1802254 |
| **R3hdm1** | 1578.660734 | 1528.273414 | 1585.11846 | 1412.09162 | 1311.061916 | 1292.703801 | 1499.231556 | 1467.467597 | 1333.277825 | 1559.041113 | 1534.202273 | 1453.977106 | 1477.514202 | 1356.235532 | 1575.814519 |
| **Ubxn4** | 1732.497743 | 1775.459161 | 1664.217751 | 1740.745809 | 1563.140487 | 1699.523228 | 1786.088135 | 1859.686235 | 1862.224953 | 1881.510939 | 1932.413838 | 1792.313068 | 1837.093783 | 1635.433919 | 1738.21036 |
| **Lct** | 3.662785926 | 5.54583406 | 3.132645178 | 0.86260942 | 1.266726489 | 0.701412806 | 3.608258859 | 4.190370065 | 9.781383594 | 0 | 5.675224193 | 3.470112425 | 2.556252945 | 8.131991864 | 0 |
| **Mcm6** | 9185.351407 | 9703.62508 | 9450.407341 | 9313.593904 | 9319.306776 | 9141.513097 | 9305.699598 | 9937.043573 | 9599.299376 | 9544.757304 | 9959.072587 | 9633.89962 | 9047.431258 | 9168.36905 | 8973.448112 |
| **Dars** | 3020.882693 | 2768.95572 | 2824.079628 | 2655.974403 | 2627.190737 | 2659.757359 | 2772.946933 | 2597.191366 | 2579.275612 | 2446.925513 | 2710.865423 | 2726.640838 | 2681.50934 | 2665.486222 | 2571.74654 |
| **Cxcr4** | 450.5226689 | 389.0006462 | 376.7005826 | 363.1585657 | 303.380994 | 298.8018552 | 359.9238212 | 123.1968799 | 153.492481 | 128.4635894 | 136.2053806 | 315.7802307 | 282.8919926 | 261.1272943 | 293.8933598 |
| **Cd55** | 14.65114371 | 36.44405239 | 42.2907099 | 51.75656518 | 39.90188439 | 41.38335554 | 49.61355931 | 107.2734737 | 74.48899814 | 83.02068705 | 90.80358708 | 54.6542707 | 21.30210788 | 33.43152211 | 55.32955674 |
| **Zp3r** | 2.747089445 | 0 | 0 | 0 | 0 | 0.701412806 | 0 | 0 | 0.752414123 | 3.495607876 | 0 | 0 | 0 | 0 | 0.718565672 |
| **Pfkfb2** | 73.25571853 | 104.5785851 | 79.09929074 | 104.3757398 | 86.13740122 | 73.6483446 | 72.16517718 | 77.94088321 | 101.5759065 | 83.89458901 | 61.48159542 | 79.81258578 | 98.84178056 | 67.76659887 | 78.32365824 |
| **Yod1** | 38.45925223 | 68.92679474 | 88.49722628 | 77.63484777 | 55.7359655 | 44.89041957 | 48.7114946 | 91.35006742 | 84.27038173 | 92.6336087 | 71.88617311 | 52.91921448 | 54.53339617 | 46.08128723 | 77.60509257 |
| **AA986860** | 11.90405426 | 3.169048034 | 3.132645178 | 2.587828259 | 3.800179466 | 2.104238417 | 9.020647148 | 4.190370065 | 5.266898858 | 8.739019689 | 4.729353494 | 6.072696744 | 5.964590206 | 0 | 4.311394032 |
| **Fcmr** | 57.68887834 | 47.53572051 | 47.77283896 | 53.48178402 | 20.90098706 | 23.84803539 | 23.45368258 | 41.06562664 | 59.44071568 | 43.69509845 | 45.40179354 | 15.61550591 | 38.34379418 | 19.87820233 | 25.14979852 |
| **Il24** | 230.7555134 | 81.60298688 | 108.0762586 | 98.33747384 | 5.700269198 | 22.44520978 | 14.43303544 | 76.26473519 | 88.03245234 | 213.2320804 | 145.6640876 | 9.542809169 | 6.816674521 | 15.36042908 | 14.37131344 |
| **Il19** | 2.747089445 | 0 | 1.566322589 | 2.587828259 | 0 | 1.402825611 | 3.608258859 | 0 | 0 | 0 | 0.945870699 | 0 | 0 | 0 | 0.718565672 |
| **Il10** | 41.20634167 | 45.15893448 | 21.14535495 | 20.70262607 | 0.633363244 | 3.507064029 | 9.922711863 | 14.24725822 | 18.81035306 | 51.56021617 | 63.37333682 | 6.072696744 | 0.852084315 | 1.807109303 | 3.59282836 |
| **Mapkapk2** | 6979.438582 | 6221.633553 | 6285.65255 | 6075.358143 | 5345.585782 | 5964.113087 | 6082.622372 | 5232.934138 | 5364.712694 | 5793.970054 | 5867.235944 | 5450.679092 | 5843.594233 | 5296.637368 | 5783.016528 |
| **Dyrk3** | 224.345638 | 193.3119301 | 155.0659363 | 142.3305542 | 146.3069094 | 167.6376606 | 176.8046841 | 215.3850214 | 238.5152769 | 249.9359631 | 202.4163295 | 125.7915754 | 201.9439827 | 166.2540559 | 168.1443672 |
| **Eif2d** | 1229.780375 | 1285.048978 | 1281.251878 | 1181.774905 | 1114.71931 | 1066.848877 | 1086.987981 | 1532.83737 | 1463.445468 | 1520.589426 | 1463.261971 | 1119.978785 | 1019.944925 | 1135.768197 | 1247.430007 |
| **Rassf5** | 2659.182582 | 2955.929554 | 3293.976405 | 3188.204415 | 3144.648508 | 3043.430164 | 3262.768073 | 3105.064218 | 3217.322788 | 3312.088462 | 3197.042962 | 3401.577705 | 3393.851827 | 3582.594193 | 3212.707119 |
| **Ikbke** | 568.6475151 | 656.7852051 | 633.5774872 | 605.5518126 | 737.8681796 | 733.6777948 | 810.9561786 | 638.6123979 | 514.6512598 | 571.5318877 | 576.9811262 | 723.5184406 | 758.3550405 | 857.4733643 | 771.020966 |
| **Srgap2** | 934.9261077 | 900.8019037 | 930.3956179 | 870.3729044 | 801.8378673 | 866.9462279 | 964.3071801 | 795.3322384 | 677.9251244 | 824.0895567 | 765.2093953 | 936.9303548 | 837.5988818 | 899.0368783 | 953.5366467 |
| **Fam72a** | 249.069443 | 263.8232488 | 222.4178076 | 228.5914962 | 217.2435928 | 194.2913472 | 232.7326964 | 270.6979062 | 243.7821757 | 239.4491395 | 217.5502607 | 179.578318 | 160.1918513 | 267.4521769 | 210.5397419 |
| **Ctse** | 117.2091496 | 131.5154934 | 180.1270977 | 130.2540224 | 179.2417981 | 183.7701551 | 153.3510015 | 98.89273354 | 87.28003822 | 86.51629492 | 92.69532848 | 172.6380931 | 172.973116 | 197.8784687 | 196.8869941 |
| **Slc41a1** | 392.8337906 | 388.2083842 | 416.6418087 | 345.0437679 | 281.2132805 | 309.3230473 | 401.4187981 | 335.2296052 | 322.0332445 | 282.270336 | 294.1657873 | 314.0451745 | 311.8628593 | 342.4472129 | 373.6541494 |
| **Rab29** | 201.4532259 | 269.3690829 | 212.2367108 | 196.6749477 | 238.1445798 | 234.9732899 | 212.8872727 | 200.2996891 | 240.0201051 | 235.0796296 | 229.8465798 | 266.3311286 | 209.6127415 | 266.5486222 | 237.8452374 |
| **Nucks1** | 6545.39845 | 6586.074077 | 6887.120424 | 6634.329047 | 6075.853602 | 6021.628937 | 7365.358396 | 7427.011904 | 7646.032314 | 6964.12479 | 7132.810939 | 7153.636764 | 6871.207917 | 7075.736476 | 6542.540443 |
| **Slc45a3** | 0 | 3.961310043 | 0.783161294 | 0.86260942 | 0.633363244 | 0 | 0 | 13.40918421 | 0 | 0 | 2.837612096 | 0 | 0.852084315 | 0.903554652 | 0 |
| **Elk4** | 562.2376397 | 651.239371 | 670.3860681 | 619.3535633 | 563.6932874 | 570.248611 | 733.3786131 | 630.2316578 | 647.0761454 | 570.6579857 | 721.6993432 | 722.6509125 | 756.6508719 | 622.5491549 | 659.6432869 |

| | | | | | | | | | | | | | | | |
|---|---|---|---|---|---|---|---|---|---|---|---|---|---|---|---|
| **Mfsd4** | 232.5869063 | 276.499441 | 255.310582 | 219.1027926 | 258.4122037 | 277.0580583 | 317.5267796 | 228.7942056 | 248.2966605 | 231.5840218 | 258.2227008 | 268.0661848 | 309.3066064 | 241.249092 | 270.1806927 |
| **Nuak2** | 41.20634167 | 11.09166812 | 46.20651637 | 36.22959563 | 32.9348887 | 43.48759395 | 28.86607087 | 35.19910855 | 33.85863552 | 21.84754922 | 20.80915537 | 33.83359614 | 25.56252945 | 28.91374885 | 43.83250599 |
| **Tmcc2** | 13.73544722 | 22.18333624 | 12.53058071 | 6.900875357 | 10.13381191 | 17.53532014 | 15.33510015 | 28.49451644 | 30.84897903 | 27.09096104 | 15.13393118 | 8.675281063 | 7.668758836 | 8.131991864 | 15.80844478 |
| **Dstyk** | 86.99116575 | 85.56429692 | 73.61716168 | 88.84877022 | 96.90457637 | 94.69072877 | 94.71679505 | 111.4638437 | 103.0807348 | 83.02068705 | 73.7779145 | 72.87236093 | 76.68758836 | 73.18792678 | 76.16796123 |
| **Rbbp5** | 1082.353241 | 1137.688244 | 1144.198651 | 1142.094872 | 1034.282178 | 1006.527376 | 1115.854052 | 1140.618732 | 1145.174295 | 1060.043088 | 1078.292597 | 1067.927099 | 1082.999165 | 994.8136714 | 1097.249781 |
| **Tmem81** | 79.6655939 | 83.18751089 | 90.84671016 | 106.1009586 | 87.40412771 | 82.76671107 | 89.30440676 | 93.86428946 | 72.23175577 | 87.39019689 | 97.42468197 | 83.2826982 | 95.4334433 | 97.58390237 | 56.76668809 |
| **Mdm4** | 1749.895976 | 1935.496087 | 1946.155817 | 1656.210086 | 1691.713225 | 1576.074574 | 1787.892265 | 1915.837194 | 1832.880803 | 1979.38796 | 2022.271554 | 1816.603855 | 2026.256501 | 1798.073757 | 1915.696081 |
| **Pik3c2b** | 44.8691276 | 26.93690829 | 39.94122602 | 18.11479781 | 41.16861088 | 35.07064029 | 51.41768874 | 50.28444078 | 60.94554393 | 38.45168663 | 67.15681961 | 33.83359614 | 23.85836082 | 48.79195118 | 31.61688957 |
| **Ppp1r15b** | 2988.833316 | 3135.77303 | 3136.560984 | 3087.279113 | 3078.145367 | 3030.804733 | 3373.722033 | 3379.114421 | 3257.200737 | 3065.648107 | 3215.960376 | 3211.589049 | 3137.374448 | 3080.217807 | 2905.161012 |
| **Plekha6** | 10.98835778 | 13.46845414 | 12.53058071 | 6.038265938 | 17.73417084 | 9.118366474 | 12.62890601 | 15.08533223 | 6.771727103 | 13.10852953 | 11.35044839 | 17.35056213 | 12.78126473 | 12.64976512 | 5.748525376 |
| **Etnk2** | 0.915696482 | 2.376786026 | 0.783161294 | 1.725218839 | 1.266726489 | 3.507064029 | 2.706194144 | 0 | 4.514484735 | 0.873901969 | 0.945870699 | 1.735056213 | 3.408337261 | 2.710663955 | 0.718565672 |
| **Sox13** | 0 | 0.792262009 | 2.349483883 | 2.587828259 | 3.166816221 | 0 | 2.706194144 | 0 | 0 | 0 | 0 | 1.735056213 | 0 | 3.614218606 | 0 |
| **Snrpe** | 1896.407413 | 1819.825834 | 1956.336914 | 1757.997997 | 1947.591976 | 1957.643141 | 2006.191926 | 2102.727699 | 1930.694639 | 2066.778156 | 1794.316716 | 2051.703971 | 1867.768819 | 2010.4091 | 1911.384687 |
| **Zc3h11a** | 2058.485691 | 2211.995528 | 2156.043044 | 2036.62084 | 2051.463548 | 2101.432766 | 2131.578921 | 2278.723241 | 2185.763026 | 2179.51151 | 2114.966882 | 2263.380829 | 2254.615098 | 2033.901521 | 2122.642995 |
| **Zbed6** | 643.7346265 | 605.2881745 | 646.8912292 | 639.19358 | 524.4247663 | 554.8175293 | 610.6978119 | 661.2403963 | 729.8416989 | 605.6140645 | 777.5057144 | 689.6848445 | 655.2528384 | 604.4780619 | 611.4993868 |
| **Lax1** | 232.5869063 | 369.986358 | 312.4813565 | 288.9741556 | 354.6834168 | 310.0244601 | 301.2896147 | 282.4309424 | 288.1746089 | 330.3349442 | 295.111658 | 438.9692218 | 342.5378947 | 483.4017386 | 374.3727151 |
| **Atp2b4** | 655.6386808 | 640.1477029 | 588.9372935 | 595.2004996 | 461.0884418 | 462.231039 | 531.316117 | 673.8115065 | 702.7547905 | 607.3618684 | 806.8277061 | 594.2567528 | 620.3173814 | 580.0820863 | 600.0023361 |
| **Btg2** | 708.7490767 | 686.8911614 | 648.4575518 | 592.6126713 | 488.3230613 | 515.5384122 | 438.4034514 | 363.7241217 | 486.8119373 | 319.8481206 | 401.995047 | 424.221244 | 475.4630479 | 403.8889293 | 441.1993226 |
| **Chit1** | 9.156964816 | 4.753572051 | 4.698967767 | 7.763484777 | 8.233722176 | 13.32684331 | 5.412388289 | 2.514222039 | 0 | 1.747803938 | 14.18806048 | 19.08561834 | 11.92918041 | 3.614218606 | 14.37131344 |
| **Ppfia4** | 3.662785926 | 13.46845414 | 5.482129061 | 2.587828259 | 0.633363244 | 2.104238417 | 0.902064715 | 4.190370065 | 1.504828245 | 6.117313782 | 0.945870699 | 1.735056213 | 0.852084315 | 5.421327909 | 2.155697016 |
| **Tmem183a** | 1185.826944 | 1234.344209 | 1157.512393 | 1120.529636 | 1018.448097 | 1139.094396 | 1073.457011 | 1228.616503 | 1039.083903 | 1157.920109 | 1192.742951 | 1165.090247 | 1162.243006 | 1149.321517 | 1051.980144 |
| **Cyb5r1** | 484.4034387 | 444.4589868 | 491.8252929 | 463.2212584 | 416.7530147 | 463.6338646 | 491.6252696 | 383.837898 | 338.5863552 | 444.8161022 | 358.4849948 | 451.9821434 | 436.2671694 | 505.9906049 | 525.2715062 |
| **Adipor1** | 2159.212304 | 1989.369903 | 1946.155817 | 1887.38941 | 1895.65619 | 1994.818019 | 1977.325855 | 1854.657791 | 1862.977368 | 2023.083058 | 1731.889249 | 1931.985093 | 1934.231395 | 1905.59676 | 1788.509958 |
| **Klhl12** | 434.0401323 | 438.1208907 | 455.7998734 | 404.5638178 | 463.6218948 | 492.3917896 | 473.5839753 | 502.0063338 | 516.9085022 | 514.7282597 | 526.8499792 | 412.9433786 | 535.9610342 | 559.3003293 | 502.9959704 |
| **Rabif** | 597.034106 | 611.6262706 | 632.7943259 | 634.0179234 | 616.2624367 | 690.1902008 | 680.1567949 | 637.7743239 | 682.4396092 | 623.0921038 | 692.3773515 | 665.3940575 | 730.2362581 | 637.909584 | 625.1521346 |
| **Mgat4e** | 0.915696482 | 0 | 0 | 0 | 0 | 1.402825611 | 0 | 0 | 1.504828245 | 0 | 2.837612096 | 0 | 0 | 0 | 0 |
| **Kdm5b** | 711.4961662 | 691.6447334 | 626.5290356 | 578.8109206 | 613.7289837 | 558.3245933 | 800.131402 | 336.9057532 | 374.702233 | 332.0827482 | 334.8382274 | 600.3294495 | 610.944454 | 482.4981839 | 702.7572272 |
| **Ppp1r12b** | 43.03773463 | 72.09584277 | 59.52025838 | 64.69570647 | 39.90188439 | 58.21726287 | 51.41768874 | 63.69362499 | 54.92623095 | 92.6336087 | 48.23940564 | 57.25685501 | 73.2792511 | 66.86304422 | 68.98230451 |
| **Ube2t** | 200.5375295 | 198.8577641 | 235.7315496 | 208.7514796 | 238.1445798 | 228.6605747 | 185.8253312 | 189.404727 | 227.229065 | 203.6191588 | 180.6613035 | 266.3311286 | 195.9793925 | 235.8277641 | 240.0009344 |
| **Lgr6** | 3.662785926 | 0 | 0 | 0 | 0 | 0 | 0.902064715 | 3.352296052 | 0 | 0 | 0 | 0 | 0 | 0 | 0 |
| **Ptprv** | 27.47089445 | 66.55000871 | 68.91819391 | 42.26786156 | 68.40323038 | 49.80030921 | 49.61355931 | 43.57984868 | 57.93588744 | 42.82119648 | 59.58985402 | 55.5217988 | 52.82922754 | 55.11683375 | 63.23377913 |
| **Ptpn7** | 3530.925633 | 3722.839178 | 3802.248085 | 3886.918045 | 3959.15364 | 3900.556613 | 4121.533682 | 3623.832032 | 4111.94318 | 3755.15676 | 3787.266278 | 3881.320747 | 3961.339981 | 4122.919875 | 3850.07487 |
| **Arl8a** | 637.3247512 | 630.6405588 | 668.8197455 | 573.6352641 | 628.2963383 | 625.6602227 | 692.785701 | 599.2229193 | 559.0436931 | 593.3794369 | 610.0866007 | 709.6379909 | 632.2465618 | 574.6607584 | 656.0504585 |
| **Rnpep** | 1641.843791 | 1572.640087 | 1606.263815 | 1626.018756 | 1440.268017 | 1399.318547 | 1464.953097 | 1711.347135 | 1719.26627 | 1725.082487 | 1812.288259 | 1506.028793 | 1648.78315 | 1465.565645 | 1508.987911 |
| **Timm17a** | 1367.134847 | 1333.37696 | 1395.593427 | 1414.679448 | 1408.599855 | 1395.811483 | 1296.266995 | 1684.528766 | 1408.519237 | 1588.753779 | 1689.325068 | 1354.211374 | 1319.02652 | 1382.438617 | 1330.783624 |
| **Ipo9** | 1706.858242 | 2025.813956 | 1971.216978 | 1871.862441 | 1781.017443 | 1886.099035 | 1891.629707 | 2281.237464 | 2126.32231 | 2182.133216 | 2190.636538 | 2027.413184 | 2011.771068 | 2044.744177 | 2071.624832 |
| **Nav1** | 49.44761 | 35.65179038 | 67.35187133 | 40.54264272 | 36.10170492 | 51.20313482 | 51.41768874 | 31.8468125 | 34.61104964 | 38.45168663 | 48.23940564 | 32.09853993 | 60.49798638 | 37.04574071 | 39.52111196 |
| **Csrp1** | 2695.810442 | 2707.159283 | 2732.449756 | 2775.014503 | 2703.194327 | 2818.276653 | 3022.818859 | 2591.324848 | 2482.966605 | 2357.787512 | 2216.175047 | 2891.471178 | 2776.942783 | 2676.328878 | 2650.788764 |
| **Phlda3** | 304.0112319 | 318.4893274 | 393.9301311 | 426.1290533 | 259.0455669 | 276.3566455 | 268.815285 | 1226.102281 | 1003.72044 | 885.2626945 | 821.0157665 | 236.835173 | 259.0336318 | 345.1578769 | 268.0249956 |
| **Tnni1** | 33.88076982 | 37.2363144 | 29.76012919 | 26.74089201 | 22.80107679 | 14.72966892 | 17.13922958 | 35.19910855 | 30.84897903 | 35.82998073 | 17.97154328 | 20.82067455 | 17.89377062 | 17.16753838 | 15.80844478 |
| **Lad1** | 2373.48528 | 2063.05027 | 2238.27498 | 2038.346059 | 2034.996104 | 2341.315945 | 2120.754144 | 3672.440325 | 2642.478399 | 2925.823792 | 2713.703035 | 2017.870375 | 2089.310741 | 1818.855514 | 2021.325235 |
| **Tnnt2** | 0.915696482 | 3.169048034 | 2.349483883 | 0 | 2.533452977 | 9.118366474 | 0.902064715 | 5.028444078 | 0.752414123 | 3.495607876 | 8.512836289 | 2.602584319 | 1.70416863 | 9.035546516 | 6.467091048 |
| **Tmem9** | 314.9995897 | 330.3732575 | 317.1803243 | 306.226344 | 273.6129215 | 350.0049901 | 361.7279506 | 315.1158289 | 297.2035784 | 314.6047088 | 299.8410115 | 372.1695576 | 399.6275438 | 361.4218606 | 344.1929569 |
| **Cacna1s** | 7.325571853 | 2.376786026 | 3.915806472 | 9.488703616 | 3.800179466 | 6.312715251 | 2.706194144 | 5.028444078 | 5.266898858 | 7.86511772 | 0 | 6.072696744 | 2.556252945 | 3.614218606 | 3.59282836 |
| **Gm15850** | 2.747089445 | 0 | 1.566322589 | 0 | 0 | 0 | 0 | 0 | 0 | 2.621705907 | 0 | 0 | 1.70416863 | 0 | 0 |
| **Kif21b** | 2088.703674 | 2563.75986 | 2515.514078 | 2437.73422 | 2527.752708 | 2546.128485 | 2715.214791 | 1440.649228 | 1753.87732 | 1683.135192 | 1749.860793 | 3150.862082 | 3089.657727 | 3157.923507 | 2880.729779 |
| **Gpr25** | 1.831392963 | 2.376786026 | 0.783161294 | 0.86260942 | 4.43354271 | 0.701412806 | 0.902064715 | 7.542666117 | 7.524141226 | 3.495607876 | 2.837612096 | 4.337640531 | 0 | 0 | 0 |
| **Camsap2** | 750.8711149 | 735.2191439 | 761.2327782 | 609.8648597 | 591.5612702 | 603.9164257 | 646.7804005 | 774.3803881 | 726.8320424 | 672.0306141 | 771.8304902 | 617.6800117 | 598.1631892 | 551.1683375 | 635.9306197 |
| **9230116N13Rik** | 5.494178889 | 2.376786026 | 9.397935534 | 10.35131304 | 7.600358931 | 7.014128057 | 16.23716487 | 15.92340625 | 6.771727103 | 10.48682363 | 6.621094891 | 10.41033728 | 9.372927467 | 6.324882561 | 8.622788064 |
| **Ddx59** | 97.97952353 | 112.5012052 | 104.1604522 | 96.612255 | 118.4389267 | 115.7331129 | 106.4436363 | 126.549176 | 114.3669466 | 108.3638441 | 97.42468197 | 104.9709009 | 115.8834669 | 107.5230035 | 146.5873971 |
| **Kif14** | 831.4524053 | 1044.993589 | 967.2041987 | 996.3138797 | 875.9413668 | 847.3066693 | 902.0647148 | 965.461263 | 1042.09356 | 946.4358323 | 1089.643045 | 1036.696087 | 1056.584551 | 983.9710156 | 1115.213923 |
| **Zfp281** | 1401.931313 | 1386.458515 | 1413.606137 | 1348.258523 | 1113.452583 | 1088.592674 | 1301.679383 | 1107.095771 | 1174.518445 | 1003.23946 | 990.3266216 | 1113.03856 | 1172.468018 | 1100.529566 | 1197.848975 |
| **Gm19705** | 40.29064519 | 33.27500436 | 20.36219366 | 19.84001665 | 28.50134599 | 10.52119209 | 23.45368258 | 12.5711102 | 11.28621184 | 18.35194135 | 11.35044839 | 27.7608994 | 17.89377062 | 20.78175699 | 10.05991941 |
| **Ptprc** | 22750.47908 | 23555.53404 | 23555.92542 | 23273.20214 | 24869.00779 | 24267.48025 | 28125.47574 | 20189.20297 | 20610.12765 | 18822.97451 | 20326.76132 | 27519.72659 | 26756.29958 | 26678.35464 | 26987.88951 |
| **Atp6v1g3** | 0.915696482 | 4.753572051 | 2.349483883 | 0 | 0 | 0 | 0.902064715 | 0 | 0 | 0 | 0 | 3.470112425 | 0 | 0 | 3.59282836 |
| **Nek7** | 1707.773938 | 1876.868698 | 1793.439364 | 1789.914546 | 1771.516994 | 1606.936738 | 1886.217319 | 1888.180751 | 2011.20295 | 1610.601329 | 1659.057206 | 1956.27588 | 1964.906431 | 1858.611918 | 1822.282544 |
| **2310009B15Rik** | 144.6800441 | 80.81072487 | 90.06354887 | 119.0400999 | 99.43802935 | 123.4486538 | 118.1704776 | 107.2734737 | 120.3862596 | 90.88580477 | 118.2338373 | 102.3683165 | 119.2918041 | 84.93413725 | 107.7848508 |
| **Dennd1b** | 651.9758949 | 692.4369954 | 652.3733583 | 602.1013749 | 553.5594755 | 605.3192513 | 695.4918951 | 691.4110608 | 638.0471759 | 619.596496 | 563.7389365 | 681.0095634 | 616.9090442 | 737.3005957 | 648.8648018 |
| **4930596I21Rik** | 1.831392963 | 1.584524017 | 3.132645178 | 1.725218839 | 1.266726489 | 1.402825611 | 0.902064715 | 3.352296052 | 1.504828245 | 0 | 2.837612096 | 0.867528106 | 0 | 0.903554652 | 2.155697016 |
| **Zbtb41** | 251.8165324 | 302.6440872 | 314.8308404 | 268.2715295 | 258.4122037 | 306.5173961 | 335.5680739 | 355.3433815 | 376.9594754 | 262.1705907 | 367.9437018 | 347.8787706 | 341.6858104 | 299.9801443 | 315.45033 |
| **Aspm** | 1568.588073 | 1586.900803 | 1559.274137 | 1775.250186 | 1515.638244 | 1371.262035 | 1797.814977 | 1737.327429 | 1813.318035 | 1433.199229 | 1502.04267 | 1669.991605 | 1731.435328 | 1718.560947 | 1598.090054 |
| **Cdc73** | 976.1324493 | 966.5596504 | 1021.242328 | 956.6338464 | 969.679127 | 1024.062696 | 1040.982681 | 1186.712802 | 1099.277033 | 1094.999167 | 1125.586132 | 1022.815637 | 996.9386487 | 1011.077655 | 1045.513053 |
| **Glrx2** | 286.6129987 | 253.5238427 | 314.0476791 | 257.0576071 | 243.844849 | 277.7594711 | 238.1450847 | 272.3740542 | 263.3449429 | 269.1618064 | 294.1657873 | 237.7027011 | 274.3711495 | 286.4268245 | 259.4022076 |
| **Trove2** | 95.23243408 | 105.3708471 | 94.76251663 | 107.8261775 | 76.63695256 | 78.55823424 | 77.57756547 | 120.6826579 | 125.6531585 | 110.9855501 | 111.6127425 | 78.94505767 | 108.214708 | 83.12702794 | 66.82660749 |
| **Uchl5** | 1879.00918 | 1990.954427 | 1749.582332 | 1757.997997 | 1658.778337 | 1651.125745 | 1688.665146 | 1966.959709 | 1972.829829 | 1894.619469 | 1968.356924 | 1593.649131 | 1611.29144 | 1488.154511 | 1539.886235 |
| **Rgs2** | 269.2147656 | 218.6643143 | 216.1525173 | 192.3619006 | 149.4737256 | 176.0546142 | 175.9026194 | 239.6891677 | 216.6952673 | 148.5633347 | 165.5273723 | 163.9628121 | 163.6001885 | 142.7616349 | 182.5156807 |
| **Rgs1** | 992.614986 | 861.9810653 | 863.0437465 | 763.4093364 | 877.2080933 | 930.0733804 | 937.2452387 | 1177.493988 | 1057.141842 | 956.9226559 | 709.4030241 | 906.5668711 | 956.8906859 | 814.1027411 | 908.2670094 |
| **Rgs18** | 17.39823315 | 32.48274235 | 36.02541955 | 33.64176737 | 25.96789302 | 31.56357626 | 19.84542373 | 41.90370065 | 30.84897903 | 15.73023544 | 19.86328467 | 31.23101183 | 18.74585493 | 37.04574071 | 20.11983882 |
| **Pla2g4a** | 0 | 0 | 0 | 0 | 3.800179466 | 0 | 1.80412943 | 4.190370065 | 0 | 4.369509845 | 6.621094891 | 0.867528106 | 0.852084315 | 0 | 0.718565672 |
| **Ptgs2** | 1.831392963 | 8.714882094 | 0.783161294 | 6.900875357 | 0.633363244 | 0 | 2.706194144 | 14.24725822 | 2.257242368 | 3.495607876 | 11.35044839 | 0 | 3.408337261 | 0.903554652 | 2.874262688 |
| **BC003331** | 391.0023976 | 369.986358 | 423.6902603 | 363.1585657 | 351.5166006 | 425.0561603 | 371.6506625 | 357.8576036 | 361.1587788 | 407.2383175 | 427.5335558 | 422.4861878 | 368.1004241 | 363.2289699 | 384.4326345 |
| **Tpr** | 7124.118627 | 6976.659247 | 6995.979844 | 7092.374648 | 6465.371998 | 6397.586201 | 7455.564868 | 7783.193359 | 7333.028039 | 7131.913968 | 7274.691544 | 6983.601256 | 7210.337475 | 6875.147344 | 7229.489226 |
| **Prg4** | 0.915696482 | 3.961310043 | 3.132645178 | 0.86260942 | 1.266726489 | 0 | 0.902064715 | 0 | 8.276555348 | 6.117313782 | 1.891741398 | 0.867528106 | 0.852084315 | 0 | 6.467091048 |
| **Hmcn1** | 0 | 0 | 1.566322589 | 0 | 0 | 0 | 0.902064715 | 0 | 5.266898858 | 0 | 0 | 2.602584319 | 3.408337261 | 1.807109303 | 0 |
| **Ivns1abp** | 2731.522605 | 2788.76227 | 2898.479951 | 2646.485699 | 2581.588584 | 2757.253739 | 2844.210046 | 3032.151779 | 2929.148179 | 3130.316853 | 3043.811909 | 2698.879939 | 3011.26597 | 2669.100441 | 2772.944928 |
| **Swt1** | 247.23805 | 290.7601571 | 268.624324 | 230.316715 | 212.8100501 | 199.9026496 | 230.0265023 | 235.4987977 | 221.9621662 | 237.7013355 | 250.6557352 | 240.3052854 | 248.80862 | 257.5130757 | 224.1924897 |
| **Trmt1l** | 619.926518 | 694.0215194 | 666.4702616 | 640.9187988 | 662.4979535 | 605.3192513 | 647.6824652 | 706.496393 | 689.9637504 | 596.0011428 | 738.7250157 | 592.5216966 | 582.8256716 | 580.985641 | 668.9846406 |
| **Rnf2** | 639.1561441 | 629.8482968 | 619.4805839 | 669.3849096 | 641.5969665 | 662.1336886 | 729.7703543 | 691.4110608 | 680.9347809 | 720.0952224 | 600.6278937 | 658.4538327 | 655.2528384 | 634.2953654 | 658.9247212 |
| **Fam129a** | 2281.915632 | 2043.24372 | 2106.703882 | 1870.137222 | 1654.978157 | 1755.636253 | 1832.093436 | 2486.565597 | 2248.213398 | 1940.936273 | 2070.51096 | 1802.723405 | 1894.183433 | 1678.804543 | 1862.522222 |
| **Edem3** | 886.3941942 | 944.3763141 | 902.9849725 | 814.3032922 | 725.8342779 | 803.8190753 | 834.4098612 | 893.3868979 | 789.2824146 | 793.5029878 | 855.0671117 | 890.083837 | 837.5988818 | 879.158676 | 837.8475735 |
| **1700025G04Rik** | 639.1561441 | 637.7709168 | 642.1922615 | 670.2475191 | 525.0581295 | 458.7239749 | 541.2388289 | 459.2645592 | 576.3492179 | 458.7985337 | 540.092169 | 555.217988 | 528.2922754 | 552.9754468 | 563.3554868 |
| **Tsen15** | 184.9706893 | 150.5297816 | 152.7164524 | 156.132305 | 190.6423365 | 190.0828703 | 180.412943 | 168.4528766 | 190.360773 | 182.6455115 | 174.9860793 | 207.3392174 | 187.4585493 | 252.9953024 | 155.2101851 |
| **Rgl1** | 6.409875371 | 23.76786026 | 14.8800646 | 12.07653188 | 17.1008076 | 9.81977928 | 5.412388289 | 18.43762829 | 8.276555348 | 20.09974528 | 19.86328467 | 22.55573076 | 4.260421576 | 12.64976512 | 17.24557613 |
| **Arpc5** | 5467.623691 | 5289.141169 | 5800.875708 | 5239.489615 | 5828.841937 | 5888.360504 | 5780.430692 | 4730.08973 | 5252.60299 | 4959.393674 | 5157.83292 | 5835.861571 | 5791.61709 | 6093.57257 | 5860.621621 |
| **Ncf2** | 48.53191352 | 38.82083842 | 47.77283896 | 54.34439344 | 16.46744435 | 40.68194273 | 32.47432973 | 98.89273354 | 72.23175577 | 89.13800083 | 75.6696559 | 21.68820266 | 33.23128829 | 24.39597559 | 20.83840449 |
| **Smg7** | 2510.839752 | 2346.680069 | 2376.894529 | 2258.311461 | 2075.531351 | 2105.641243 | 2409.414853 | 2338.226496 | 2254.985125 | 2124.455686 | 2254.009875 | 2252.970492 | 2431.848635 | 2054.683278 | 2230.427846 |
| **Nmnat2** | 8.241268334 | 9.507144102 | 7.04845165 | 12.93914129 | 8.233722176 | 4.208476834 | 15.33510015 | 17.59955427 | 7.524141226 | 17.47803938 | 4.729353494 | 12.14539349 | 5.964590206 | 9.035546516 | 7.904222392 |
| **Lamc2** | 1.831392963 | 1.584524017 | 6.265290356 | 0 | 1.266726489 | 2.104238417 | 2.706194144 | 2.514222039 | 18.81035306 | 0.873901969 | 6.621094891 | 7.807752957 | 10.22501178 | 1.807109303 | 7.18565672 |
| **Lamc1** | 1066.786401 | 812.8608207 | 730.6894878 | 734.0806161 | 478.1892494 | 498.7045049 | 623.3267179 | 751.7523897 | 663.6292561 | 622.2182019 | 692.3773515 | 475.4054022 | 555.5589735 | 440.0311153 | 555.4512644 |
| **E330020D12Rik** | 11.90405426 | 24.56012226 | 17.22954848 | 5.175656518 | 27.2346195 | 14.02825611 | 18.94335901 | 25.9802944 | 15.80069657 | 20.97364725 | 31.21373306 | 20.82067455 | 39.1958785 | 11.74621047 | 22.9941015 |
| **Shcbp1l** | 2.747089445 | 9.507144102 | 1.566322589 | 4.313047098 | 3.800179466 | 2.104238417 | 2.706194144 | 5.028444078 | 7.524141226 | 12.23462756 | 7.56696559 | 2.602584319 | 6.816674521 | 3.614218606 | 5.748525376 |
| **Dhx9** | 8955.51159 | 8631.694583 | 8654.715465 | 8469.099282 | 8562.437699 | 8351.020865 | 8927.734482 | 9520.520788 | 9211.053689 | 9231.026498 | 9843.676362 | 9156.759162 | 8905.985262 | 8580.154971 | 8769.375461 |
| **Npl** | 5.494178889 | 7.130358077 | 3.915806472 | 3.450437679 | 1.266726489 | 3.507064029 | 3.608258859 | 30.17066447 | 6.771727103 | 6.991215751 | 3.783482795 | 10.41033728 | 4.260421576 | 8.131991864 | 5.029959704 |
| **Rgs8** | 54.94178889 | 28.52143231 | 36.80858084 | 49.16873692 | 31.03479897 | 31.56357626 | 46.00530045 | 20.11377631 | 48.90691797 | 27.964863 | 31.21373306 | 30.36348372 | 41.75213144 | 36.14218606 | 38.80254629 |
| **C230024C17Rik** | 0 | 0 | 0.783161294 | 1.725218839 | 2.533452977 | 0 | 0.902064715 | 1.676148026 | 0 | 0 | 0 | 0 | 0 | 0.903554652 | 0 |
| **Rgs16** | 1661.989114 | 1276.334096 | 1461.378976 | 1362.060274 | 1277.493664 | 1389.498768 | 1298.071125 | 1846.277051 | 1406.261995 | 1151.802795 | 1250.441064 | 1425.348679 | 1340.328628 | 1351.717759 | 1326.47223 |
| **Rnasel** | 376.3512539 | 408.0149344 | 436.220841 | 401.9759896 | 345.1829681 | 315.6357626 | 388.7898921 | 302.5447187 | 332.5670422 | 383.6429643 | 347.1345464 | 406.8706818 | 422.6338203 | 380.3965083 | 380.1212405 |
| **Glul** | 1356.146489 | 1292.179336 | 1325.892072 | 1261.134972 | 1307.895099 | 1259.035986 | 1405.416826 | 1191.741247 | 1138.402567 | 1196.371795 | 1145.449416 | 1277.001372 | 1382.932844 | 1294.793816 | 1244.555744 |
| **Ier5** | 1340.579649 | 1305.64779 | 1284.384523 | 1374.999415 | 1474.469633 | 1607.638151 | 1556.963698 | 1376.117529 | 1306.190917 | 1198.119599 | 1261.791512 | 1626.615199 | 1603.622681 | 1626.398373 | 1480.96385 |
| **Mr1** | 65.93014667 | 54.66607859 | 88.49722628 | 61.2452688 | 97.53793962 | 93.28790316 | 80.28375962 | 65.36977302 | 77.49865463 | 83.02068705 | 79.4531387 | 77.21000146 | 94.58135898 | 87.6448012 | 102.7548911 |
| **Stx6** | 1226.117589 | 1246.228139 | 1267.154974 | 1122.254855 | 1275.593574 | 1314.447598 | 1362.117719 | 1266.329834 | 1276.094352 | 1145.685481 | 1062.212795 | 1313.437553 | 1285.795232 | 1189.077921 | 1226.591602 |
| **Xpr1** | 839.6936736 | 862.7733273 | 868.5258756 | 853.9833255 | 789.1706024 | 735.7820332 | 863.275932 | 775.2184621 | 754.6713649 | 817.0983409 | 852.2294996 | 782.5103519 | 891.2801937 | 712.9046201 | 765.2724406 |
| **Gm5532** | 1.831392963 | 0 | 0 | 3.450437679 | 1.900089733 | 1.402825611 | 2.706194144 | 0 | 0 | 5.243411813 | 2.837612096 | 7.807752957 | 1.70416863 | 0 | 2.874262688 |
| **Acbd6** | 1379.038901 | 1274.749572 | 1389.328136 | 1372.411587 | 1285.094023 | 1374.067686 | 1306.189707 | 1267.167908 | 1219.663293 | 1313.474659 | 1170.987925 | 1220.612046 | 1150.313825 | 1169.199719 | 1222.280208 |
| **Qsox1** | 478.9092599 | 472.1881571 | 532.5496803 | 581.3987488 | 625.1295221 | 544.99775 | 602.5792295 | 426.5796726 | 481.5450385 | 510.3587498 | 458.7472889 | 556.9530442 | 685.9278737 | 629.7775921 | 518.0858495 |
| **Cep350** | 1461.451585 | 1434.786497 | 1319.626781 | 1428.481199 | 1333.229629 | 1242.903492 | 1532.60795 | 1387.850566 | 1389.708884 | 1258.418835 | 1253.278676 | 1589.311491 | 1542.27261 | 1360.753305 | 1555.69468 |
| **Tor1aip1** | 1607.047325 | 1649.489502 | 1642.289235 | 1524.230845 | 1562.507124 | 1635.694663 | 1781.577812 | 1407.964342 | 1388.95647 | 1389.504131 | 1518.122472 | 1484.34059 | 1577.208067 | 1655.312122 | 1580.125913 |
| **Tor1aip2** | 2038.340368 | 1959.263947 | 1873.321816 | 1813.205 | 1721.481298 | 1907.842832 | 1910.573066 | 1563.008034 | 1704.970402 | 1642.935702 | 1827.42219 | 1872.993181 | 1694.795703 | 1850.479926 | 1713.060562 |
| **Tdrd5** | 0 | 2.376786026 | 0 | 0 | 1.900089733 | 0 | 0 | 0 | 0 | 0 | 3.783482795 | 0 | 0 | 0 | 0 |
| **Soat1** | 442.2814006 | 427.8214846 | 372.7847762 | 414.0525214 | 328.0821605 | 408.9236657 | 415.8518335 | 447.531523 | 409.3132827 | 404.6166116 | 417.1289782 | 458.0548401 | 426.8942419 | 394.8533827 | 444.7921509 |
| **Abl2** | 832.3681017 | 732.0500959 | 724.4241974 | 719.416256 | 699.2330217 | 601.1107745 | 650.3886594 | 862.3781594 | 799.0637982 | 736.6993598 | 790.7479042 | 692.2874288 | 630.5423932 | 581.8891956 | 635.9306197 |
| **Tor3a** | 499.0545825 | 391.3774322 | 377.4837439 | 289.836765 | 359.7503227 | 375.2558511 | 400.5167334 | 255.612574 | 302.4704773 | 290.1354537 | 220.3878728 | 332.2632647 | 344.2420633 | 367.7467432 | 313.294633 |
| **Fam20b** | 722.484524 | 770.0786723 | 812.1382624 | 753.9206328 | 781.5702434 | 832.5770004 | 849.7449613 | 910.9864522 | 823.8934642 | 877.3975768 | 729.2663088 | 840.634735 | 904.9135427 | 878.2551213 | 845.0332302 |
| **Ralgps2** | 238.9967817 | 221.8333624 | 220.851485 | 240.6680281 | 233.7110371 | 260.9255637 | 260.6967026 | 187.7285789 | 170.0455917 | 209.7364725 | 182.5530449 | 205.6041612 | 196.8314768 | 260.2237397 | 229.2224494 |
| **Rasal2** | 26.55519797 | 15.05297816 | 14.0969033 | 6.900875357 | 19.00089733 | 12.6254305 | 12.62890601 | 25.9802944 | 24.82966605 | 20.09974528 | 13.24218978 | 12.14539349 | 6.816674521 | 13.55331977 | 16.52701046 |
| **2810025M15Rik** | 10.0726613 | 26.93690829 | 19.57903236 | 9.488703616 | 17.73417084 | 4.208476834 | 6.314453003 | 15.08533223 | 18.81035306 | 7.86511772 | 18.91741398 | 7.807752957 | 8.520843152 | 5.421327909 | 10.05991941 |
| **BC026585** | 12.81975074 | 15.05297816 | 15.66322589 | 19.84001665 | 8.86708542 | 5.611302446 | 9.922711863 | 20.11377631 | 11.28621184 | 25.3431571 | 23.64676747 | 3.470112425 | 21.30210788 | 6.324882561 | 8.622788064 |
| **Rfwd2** | 580.5515693 | 635.3941308 | 720.5083909 | 692.675364 | 720.1340087 | 688.7873752 | 761.3426193 | 723.2578733 | 724.5748 | 752.4295952 | 745.3461106 | 761.6896773 | 669.7382717 | 683.0873166 | 656.0504585 |
| **4930523C07Rik** | 586.9614447 | 702.7364015 | 612.4321323 | 565.0091699 | 488.9564246 | 533.0737323 | 607.9916178 | 390.5424901 | 493.5836644 | 441.3204943 | 409.5620126 | 550.8803475 | 528.2922754 | 528.5794712 | 554.0141331 |
| **4930562F07Rik** | 1.831392963 | 2.376786026 | 0.783161294 | 6.038265938 | 2.533452977 | 0 | 0.902064715 | 0.838074013 | 6.771727103 | 0 | 1.891741398 | 0.867528106 | 0 | 0.903554652 | 1.437131344 |
| **Tnn** | 0.915696482 | 0.792262009 | 0.783161294 | 3.450437679 | 1.266726489 | 1.402825611 | 2.706194144 | 2.514222039 | 0 | 0 | 6.621094891 | 0 | 5.964590206 | 2.710663955 | 5.029959704 |
| **Mrps14** | 831.4524053 | 729.6733098 | 798.0413591 | 765.1345552 | 710.0001968 | 836.7854772 | 814.5644374 | 943.6713387 | 802.8258688 | 752.4295952 | 774.6681023 | 766.0273178 | 737.9050169 | 762.6001259 | 721.4399347 |
| **Cacybp** | 3469.573969 | 3344.9302 | 3361.328276 | 3307.244515 | 3372.659276 | 3447.44394 | 3474.753281 | 4347.089906 | 4058.521777 | 4280.371844 | 4208.178739 | 3359.068828 | 3190.203676 | 3218.461669 | 3342.767506 |
| **Rabgap1l** | 1097.004385 | 986.3662006 | 1014.193876 | 889.3503117 | 825.2723073 | 876.7660071 | 934.5390445 | 1092.010439 | 998.4535407 | 1171.028638 | 1164.36683 | 962.956198 | 897.2447839 | 831.2702794 | 891.7399989 |
| **Gpr52** | 2.747089445 | 0 | 0 | 6.900875357 | 3.166816221 | 2.104238417 | 4.510323574 | 5.028444078 | 1.504828245 | 3.495607876 | 2.837612096 | 0.867528106 | 0 | 5.421327909 | 5.748525376 |
| **Rc3h1** | 1118.065404 | 1089.360262 | 1098.775296 | 1135.193996 | 1052.649712 | 1026.166935 | 1166.369676 | 1053.459034 | 1030.054934 | 1000.617754 | 1037.620157 | 1089.615301 | 1163.09509 | 1112.275776 | 1136.052327 |
| **Serpinc1** | 30.21798389 | 49.12024453 | 46.20651637 | 48.3061275 | 32.30152546 | 33.66781467 | 33.37639445 | 114.8161398 | 77.49865463 | 53.3080201 | 60.53572472 | 33.83359614 | 40.90004713 | 20.78175699 | 26.58692986 |
| **Zbtb37** | 80.58129038 | 81.60298688 | 80.66561333 | 72.45919125 | 72.20340985 | 77.15540863 | 77.57756547 | 97.21658551 | 109.1000478 | 88.26409886 | 82.29075079 | 62.46202365 | 84.3563472 | 75.89859073 | 68.26373884 |
| **Gas5** | 1372.629026 | 1535.403772 | 1549.876202 | 1255.959315 | 1468.769363 | 1372.664861 | 1337.761972 | 1537.02774 | 1408.519237 | 1536.319661 | 1557.849041 | 1434.02396 | 1422.128722 | 1450.205216 | 1457.969748 |
| **Dars2** | 314.0838932 | 323.2428995 | 361.8205181 | 319.1654853 | 352.1499638 | 295.2947912 | 332.8618798 | 273.2121283 | 298.7084067 | 373.1561407 | 321.5960376 | 307.1049496 | 351.0587378 | 342.4472129 | 363.59423 |
| **Cenpl** | 348.8803595 | 371.570882 | 393.1469698 | 389.0368483 | 304.0143573 | 279.1622967 | 352.7073035 | 402.2755263 | 324.2904868 | 360.0476112 | 364.160219 | 381.7123668 | 274.3711495 | 354.1934234 | 333.4144718 |
| **Klhl20** | 449.6069724 | 503.8786374 | 549.7792287 | 494.2751975 | 442.0875445 | 425.7575731 | 497.0376578 | 543.0719605 | 547.0050671 | 562.792868 | 525.9041085 | 506.6364141 | 519.7714322 | 501.4728316 | 507.3073644 |
| **Prdx6** | 2019.110742 | 2090.77944 | 2115.318656 | 2027.132136 | 1902.623186 | 1912.752721 | 1894.335901 | 2698.598322 | 2444.593484 | 2631.318828 | 2242.659427 | 2056.041612 | 1954.681419 | 2004.084217 | 1834.498161 |
| **Tnfsf4** | 215.1886732 | 246.3934846 | 238.8641948 | 260.5080447 | 154.5406316 | 185.1729807 | 185.8253312 | 613.4701776 | 501.1078056 | 522.5933774 | 400.1033056 | 255.9207914 | 234.3231867 | 199.685578 | 217.0068329 |
| **Fasl** | 487.1505282 | 413.5607684 | 348.506776 | 414.9151309 | 352.1499638 | 343.6922748 | 375.2589213 | 160.9102105 | 151.2352386 | 151.1850406 | 192.0117519 | 292.3569718 | 310.1586907 | 343.3507676 | 298.2047539 |
| **Suco** | 718.821738 | 677.3840173 | 736.1716168 | 703.026677 | 661.231227 | 657.9252118 | 749.615778 | 525.4724062 | 604.1885404 | 564.5406719 | 687.647998 | 667.9966418 | 749.8341973 | 740.0112596 | 697.7272675 |
| **4930558K02Rik** | 0 | 0.792262009 | 0 | 0 | 1.266726489 | 4.90988964 | 1.80412943 | 0 | 0 | 0 | 0 | 0.867528106 | 5.964590206 | 0 | 3.59282836 |
| **Pigc** | 165.7410632 | 141.8148995 | 198.9229688 | 177.6975404 | 170.3747127 | 188.6800447 | 180.412943 | 186.8905049 | 140.7014409 | 143.3199229 | 189.1741398 | 146.61225 | 135.4814061 | 158.122064 | 140.8388717 |
| **Dnm3** | 0.915696482 | 1.584524017 | 6.265290356 | 16.38957897 | 3.800179466 | 0.701412806 | 4.510323574 | 10.89496217 | 11.28621184 | 10.48682363 | 15.13393118 | 4.337640531 | 0.852084315 | 0 | 3.59282836 |
| **Mettl13** | 850.6820314 | 812.0685587 | 817.6203914 | 816.8911204 | 742.9350855 | 784.1795168 | 865.0800615 | 1039.211776 | 1039.836317 | 965.6616756 | 996.9477165 | 785.1129362 | 767.727968 | 801.4529759 | 725.7513287 |
| **Vamp4** | 351.6274489 | 335.9190916 | 352.4225825 | 306.226344 | 306.5478102 | 335.9767339 | 323.8412326 | 316.7919769 | 322.7856586 | 265.6661985 | 301.7327529 | 347.8787706 | 368.9525085 | 309.9192455 | 300.3604509 |
| **Prrc2c** | 9374.900578 | 8991.381535 | 8473.805206 | 9403.305284 | 7464.819197 | 7385.876844 | 9388.689551 | 9395.64776 | 9092.172257 | 7825.792132 | 8928.073526 | 8408.949934 | 8469.718093 | 7524.803138 | 8333.924663 |
| **Gorab** | 74.17141501 | 82.39524888 | 60.30341968 | 64.69570647 | 67.76986714 | 70.84269338 | 59.53627118 | 84.64547532 | 84.27038173 | 74.28166736 | 94.58706988 | 58.12438312 | 89.46885309 | 49.69550584 | 77.60509257 |
| **Kifap3** | 161.1625808 | 133.8922794 | 146.4511621 | 156.9949144 | 167.8412597 | 176.0546142 | 163.2737134 | 163.4244325 | 180.5793894 | 187.8889233 | 190.1200105 | 168.3004526 | 187.4585493 | 146.3758536 | 143.7131344 |
| **Scyl3** | 810.3913862 | 838.213205 | 973.469489 | 836.7311371 | 792.9707818 | 812.9374418 | 886.7296146 | 836.397865 | 863.7714127 | 771.6554385 | 754.8048176 | 764.2922616 | 783.0654856 | 755.3716887 | 862.2788064 |
| **BC055324** | 407.4849343 | 469.019109 | 478.5115509 | 453.7325547 | 431.3203694 | 401.9095377 | 460.0530045 | 444.1792269 | 490.5740079 | 526.0889853 | 481.4481857 | 493.6234925 | 439.6755066 | 438.224006 | 430.4208375 |
| **Mettl18** | 104.3893989 | 78.43393884 | 80.66561333 | 97.47486442 | 101.9714823 | 75.75258302 | 81.18582433 | 103.0831036 | 104.585563 | 94.38141264 | 111.6127425 | 72.87236093 | 121.8480571 | 76.80214538 | 98.44349706 |
| **Sele** | 3.662785926 | 0.792262009 | 4.698967767 | 1.725218839 | 7.600358931 | 10.52119209 | 1.80412943 | 0 | 3.00965649 | 0 | 0.945870699 | 7.807752957 | 3.408337261 | 5.421327909 | 2.155697016 |
| **Sell** | 3483.309416 | 3300.563527 | 3145.95892 | 3291.717545 | 3210.518285 | 3469.88915 | 3796.790385 | 1142.29488 | 1768.925602 | 1355.421954 | 1476.504161 | 3348.65849 | 3179.12658 | 3111.84222 | 3303.246394 |
| **Selp** | 79.6655939 | 35.65179038 | 46.98967767 | 55.20700286 | 9.500448664 | 23.84803539 | 24.3557473 | 12.5711102 | 2.257242368 | 28.83876497 | 24.59263817 | 19.95314644 | 25.56252945 | 15.36042908 | 30.17975822 |
| **Slc19a2** | 421.2203815 | 472.9804191 | 516.8864544 | 557.2456851 | 434.4871856 | 485.3776616 | 496.1355931 | 874.9492696 | 809.5975959 | 843.3154 | 773.7222316 | 537.8674259 | 512.1026734 | 442.7417793 | 555.4512644 |
| **Ccdc181** | 100.726613 | 95.86370303 | 95.54567793 | 77.63484777 | 111.471931 | 89.07942633 | 96.52092448 | 103.0831036 | 87.28003822 | 120.5984717 | 98.37055267 | 111.9111257 | 83.50426289 | 83.12702794 | 92.69497168 |
| **Blzf1** | 295.7699635 | 324.8274235 | 327.3614211 | 262.2332636 | 286.2801864 | 333.1710827 | 315.7226502 | 274.0502023 | 267.1070135 | 308.487395 | 307.4079771 | 289.7543875 | 311.8628593 | 301.7872536 | 282.3963091 |
| **Nme7** | 201.4532259 | 226.5869344 | 241.99684 | 213.0645267 | 229.9108577 | 203.4097137 | 239.9492141 | 231.3084276 | 209.9235402 | 224.592806 | 226.063097 | 178.7107899 | 243.6961141 | 234.9242094 | 242.8751971 |
| **Atp1b1** | 119.0405426 | 105.3708471 | 116.6910329 | 118.1774905 | 92.47103366 | 111.5246361 | 140.7220955 | 115.6542138 | 167.0359352 | 129.3374914 | 103.0999062 | 130.1292159 | 112.4751296 | 149.0865175 | 114.9705075 |
| **Xcl1** | 2955.868242 | 3007.426584 | 3365.244082 | 3396.955895 | 2552.453874 | 2282.39727 | 2341.76 | 7259.397101 | 4835.013152 | 4109.96096 | 4308.441033 | 2445.561732 | 2358.569384 | 2655.547121 | 2539.411085 |

| | | | | | | | | | | | | | | |
|---|---|---|---|---|---|---|---|---|---|---|---|---|---|---|
| **Tbx19** | 2.747089445 | 3.169048034 | 0.783161294 | 2.587828259 | 2.533452977 | 0 | 5.412388289 | 0 | 0 | 5.243411813 | 0 | 0.867528106 | 4.260421576 | 1.807109303 | 0.718565672 |
| **Sft2d2** | 1394.605741 | 1233.551947 | 1313.361491 | 1251.646268 | 1167.288459 | 1219.055456 | 1349.488813 | 886.6823058 | 917.9452295 | 1005.861166 | 951.545923 | 1177.23564 | 1216.776402 | 1133.961088 | 1165.51352 |
| **Tiprl** | 1046.641078 | 982.4048905 | 1014.193876 | 973.0234254 | 921.5435204 | 1000.214661 | 1024.745516 | 885.8442318 | 1024.788035 | 1093.251363 | 1041.403639 | 996.7897941 | 972.2282036 | 986.6816795 | 981.5607079 |
| **Gpr161** | 0 | 0 | 2.349483883 | 0 | 0 | 0 | 1.80412943 | 1.676148026 | 0 | 0.873901969 | 1.891741398 | 0 | 0 | 0 | 0 |
| **Dcaf6** | 193.2119576 | 199.6500261 | 217.7188399 | 165.6210086 | 201.4095117 | 185.8743935 | 168.6861017 | 198.6235411 | 230.2387215 | 219.3493942 | 183.4989156 | 242.9078698 | 175.5293689 | 173.4824931 | 188.9827717 |
| **Mpc2** | 448.691276 | 474.5649431 | 498.8737446 | 458.9082113 | 447.1544505 | 462.9324518 | 442.0117102 | 615.1463256 | 550.7671377 | 557.5494562 | 439.8298749 | 453.7171996 | 448.1963498 | 503.2799409 | 439.0436256 |
| **Mpzl1** | 12.81975074 | 25.35238427 | 13.31374201 | 22.42784491 | 22.80107679 | 24.5494482 | 22.55161787 | 53.63673684 | 24.82966605 | 33.20827482 | 32.15960376 | 19.08561834 | 23.85836082 | 29.8173035 | 25.86836419 |
| **Rcsd1** | 2678.412209 | 2816.49144 | 2874.985112 | 2867.313711 | 3437.262327 | 3356.961688 | 3646.145577 | 2189.887396 | 2355.808618 | 2278.262433 | 2129.154943 | 3640.147934 | 3598.352063 | 3630.48259 | 3605.762542 |
| **Creg1** | 253.6479254 | 283.629799 | 285.0707112 | 295.8750309 | 218.5103193 | 302.3089193 | 286.8565793 | 317.6300509 | 251.3063169 | 276.1530222 | 241.1970282 | 256.7883195 | 242.8440298 | 268.3557315 | 256.5279449 |
| **Cd247** | 2778.223125 | 2839.467038 | 2710.52124 | 2738.784907 | 2862.801864 | 2918.578685 | 3151.814113 | 2150.497917 | 2370.104486 | 2362.157022 | 2486.694067 | 2899.278931 | 2770.126109 | 2990.765897 | 3109.233663 |
| **Pou2f1** | 912.0336956 | 865.9423753 | 778.4623267 | 870.3729044 | 707.4667439 | 711.9339978 | 838.01812 | 809.5794966 | 787.0251722 | 740.1949677 | 731.1580501 | 868.3956344 | 807.7759308 | 750.8539155 | 721.4399347 |
| **Dusp27** | 0.915696482 | 0 | 0 | 0 | 0 | 0.701412806 | 0 | 0.838074013 | 1.504828245 | 0 | 0 | 0.867528106 | 0.852084315 | 0 | 3.59282836 |
| **Tada1** | 510.9586367 | 513.3857815 | 525.5012286 | 527.9169648 | 561.1598344 | 556.2203549 | 575.517288 | 615.1463256 | 562.0533496 | 571.5318877 | 581.7104797 | 545.6751788 | 530.8485283 | 575.5643131 | 569.1040122 |
| **Pogk** | 288.4443917 | 313.7357554 | 328.9277437 | 333.8298454 | 291.9804556 | 285.4750119 | 355.4134976 | 320.982347 | 313.004275 | 276.1530222 | 297.0033994 | 326.190568 | 317.8274496 | 290.9445978 | 302.5161479 |
| **Fam78b** | 0 | 0.792262009 | 0 | 0 | 5.066905954 | 2.104238417 | 5.412388289 | 7.542666117 | 1.504828245 | 7.86511772 | 1.891741398 | 11.27786538 | 4.260421576 | 2.710663955 | 4.311394032 |
| **Uck2** | 3671.942891 | 3647.574287 | 3621.337826 | 3286.541889 | 3330.857302 | 3457.965132 | 3371.015839 | 3483.873672 | 3525.060164 | 3656.405838 | 3779.699312 | 3342.585793 | 3429.639369 | 3333.21311 | 3313.306313 |
| **Tmco1** | 872.6587469 | 945.9608382 | 931.9619404 | 885.0372646 | 880.3749095 | 897.8083913 | 957.0906624 | 891.7107499 | 821.6362219 | 971.7789894 | 1034.782544 | 904.8318148 | 923.6593976 | 895.4226597 | 891.0214332 |
| **Aldh9a1** | 2193.093073 | 2171.590165 | 2198.333754 | 2165.149643 | 1965.95951 | 2087.40451 | 2063.022003 | 2097.699255 | 2300.129973 | 2238.062942 | 2325.896048 | 2114.165995 | 2101.239921 | 2158.592063 | 2014.139579 |
| **Mgst3** | 46.70052056 | 80.81072487 | 86.14774239 | 95.74964558 | 93.10439691 | 98.1977928 | 86.59821262 | 81.29317927 | 69.9745134 | 56.80362798 | 69.99443171 | 91.95797927 | 69.87091384 | 89.45191051 | 68.98230451 |
| **Nuf2** | 900.1296414 | 1145.610864 | 1006.362263 | 973.8860348 | 1094.451686 | 1033.882476 | 1101.421017 | 1054.297108 | 1192.576384 | 1133.450854 | 1136.93658 | 1104.363279 | 1068.513731 | 1189.981476 | 1112.33966 |
| **Hsd17b7** | 776.5106164 | 669.4613972 | 740.8705846 | 717.6910371 | 674.5318552 | 713.3368234 | 738.7910014 | 781.0849802 | 793.7968993 | 799.6203015 | 896.6854224 | 692.2874288 | 757.5029562 | 702.9655189 | 692.6973078 |
| **3110045C21Rik** | 10.98835778 | 7.922620085 | 14.0969033 | 9.488703616 | 4.43354271 | 11.22260489 | 7.216517718 | 19.2757023 | 4.514484735 | 5.243411813 | 20.80915537 | 13.01292159 | 6.816674521 | 11.74621047 | 5.029959704 |
| **Uap1** | 1411.088278 | 1346.845414 | 1394.810265 | 1323.24285 | 1374.39824 | 1420.360932 | 1454.12832 | 968.8135591 | 1043.598388 | 1053.925774 | 1104.776976 | 1362.019127 | 1404.234951 | 1414.966584 | 1436.412778 |
| **Uhmk1** | 778.3420093 | 819.9911788 | 862.2605852 | 804.8145885 | 689.0992098 | 709.8297594 | 828.9974729 | 888.3584538 | 943.5273097 | 779.5205563 | 814.3946716 | 802.4634983 | 829.930123 | 765.3107899 | 815.5720377 |
| **Nos1ap** | 2.747089445 | 0 | 0.783161294 | 0 | 1.266726489 | 0 | 0 | 2.514222039 | 0.752414123 | 0 | 0 | 0 | 0 | 0.903554652 | 0 |
| **Atf6** | 1107.992743 | 1081.437642 | 1082.328909 | 923.8546884 | 964.612221 | 986.8878176 | 1084.281787 | 718.2294292 | 821.6362219 | 981.3919111 | 1037.620157 | 1018.477997 | 1009.719913 | 1008.366991 | 1095.81265 |
| **Dusp12** | 206.9474048 | 143.3994235 | 147.2343234 | 175.9723216 | 183.6753408 | 179.5616783 | 181.3150077 | 183.5382089 | 148.9779963 | 246.4403552 | 201.4704588 | 171.770565 | 208.7606572 | 225.8886629 | 160.2401448 |
| **Fcgr3** | 0 | 3.961310043 | 0 | 0 | 5.066905954 | 6.312715251 | 0.902064715 | 0 | 0.752414123 | 0.873901969 | 5.675224193 | 6.94022485 | 5.964590206 | 8.131991864 | 0 |
| **Cfap126** | 3.662785926 | 0.792262009 | 6.265290356 | 6.038265938 | 1.900089733 | 0 | 4.510323574 | 0.838074013 | 0 | 4.369509845 | 1.891741398 | 12.14539349 | 4.260421576 | 1.807109303 | 2.155697016 |
| **Sdhc** | 1556.684019 | 1598.784733 | 1650.904009 | 1604.453521 | 1610.64273 | 1572.56751 | 1456.834514 | 1666.091138 | 1650.044171 | 1697.991526 | 1651.49024 | 1636.158008 | 1581.468489 | 1664.347668 | 1602.401448 |
| **Mpz** | 1.831392963 | 0 | 0 | 2.587828259 | 1.266726489 | 0.701412806 | 2.706194144 | 0 | 0 | 2.621705907 | 0 | 0 | 0 | 0 | 1.437131344 |
| **Nr1i3** | 1.831392963 | 0 | 0.783161294 | 0 | 0 | 0.701412806 | 0 | 0.838074013 | 0 | 0 | 1.891741398 | 1.735056213 | 0 | 5.421327909 | 0 |
| **Tomm40l** | 357.1216278 | 343.8417117 | 335.193034 | 327.7915795 | 334.415793 | 343.6922748 | 314.8205855 | 365.4002697 | 365.6732636 | 455.3029258 | 337.6758395 | 296.6946123 | 294.821173 | 368.6502978 | 354.2528763 |
| **Apoa2** | 4.578482408 | 3.961310043 | 9.397935534 | 0 | 2.533452977 | 8.416953669 | 9.020647148 | 7.542666117 | 6.771727103 | 7.86511772 | 5.675224193 | 0.867528106 | 5.964590206 | 8.131991864 | 0.718565672 |
| **Fcer1g** | 0 | 0.792262009 | 0 | 1.725218839 | 1.900089733 | 0 | 0 | 0.838074013 | 0 | 0 | 0 | 2.602584319 | 0 | 0 | 0 |
| **Ndufs2** | 2378.063763 | 2650.90868 | 2577.38382 | 2556.77432 | 2317.476111 | 2652.743231 | 2445.497442 | 2865.375051 | 2791.456395 | 2955.536459 | 2947.333097 | 2568.750723 | 2484.677863 | 2510.074822 | 2428.033406 |
| **Adamts4** | 0 | 0 | 0 | 3.450437679 | 0 | 0 | 0.902064715 | 0 | 4.514484735 | 1.747803938 | 0 | 2.602584319 | 0 | 0.903554652 | 1.437131344 |
| **B4galt3** | 293.0228741 | 270.1613449 | 278.0222595 | 252.74456 | 284.3800967 | 223.0492722 | 226.4182434 | 227.9561316 | 249.8014887 | 212.3581784 | 261.0603129 | 250.7156227 | 210.4648258 | 271.9699501 | 269.462127 |
| **Ppox** | 228.9241204 | 262.2387248 | 231.0325819 | 237.2175904 | 212.1766868 | 233.5704643 | 215.5934668 | 265.6694621 | 206.1614696 | 228.0884139 | 240.2511575 | 213.4119141 | 229.2106808 | 284.6197152 | 260.8393389 |
| **Usp21** | 259.1421043 | 329.5809955 | 366.5194858 | 342.4559396 | 326.815434 | 335.2753211 | 308.5061325 | 281.5928684 | 300.2132349 | 339.9478659 | 334.8382274 | 340.0710177 | 339.1295574 | 331.6045571 | 364.3127957 |
| **Ufc1** | 919.3592675 | 878.6185674 | 955.4567793 | 962.6721123 | 913.9431615 | 930.7747932 | 974.229892 | 1100.391179 | 929.2314414 | 1027.708715 | 966.6798541 | 965.5587823 | 928.7719035 | 1077.037145 | 861.5602407 |
| **Dedd** | 791.1617601 | 739.9727159 | 762.0159395 | 668.5223002 | 751.1688077 | 714.739649 | 868.6883203 | 751.7523897 | 629.7706206 | 694.7520653 | 670.6223254 | 731.3261936 | 706.3778973 | 703.8690736 | 703.4757929 |
| **Nit1** | 232.5869063 | 229.7559825 | 207.537743 | 207.8888701 | 187.4755203 | 216.0351442 | 231.8306317 | 268.1836842 | 215.9428532 | 222.8450021 | 275.2483733 | 189.1211272 | 228.3585965 | 199.685578 | 209.1026105 |
| **Pfdn2** | 943.167376 | 828.7060609 | 918.6481984 | 828.1050429 | 842.3731149 | 887.988612 | 860.5697379 | 1009.879186 | 807.3403535 | 920.2187733 | 934.5202504 | 818.9465323 | 868.2739171 | 928.8541818 | 778.2066227 |
| **Klhdc9** | 69.5929326 | 56.2506026 | 54.03812932 | 70.73397241 | 78.53704229 | 93.98931597 | 75.77343604 | 70.3982171 | 69.22209928 | 65.54264767 | 78.507268 | 50.31663016 | 88.61676878 | 69.57370817 | 45.98820301 |
| **Pvrl4** | 0 | 0 | 2.349483883 | 4.313047098 | 0.633363244 | 0 | 0 | 3.352296052 | 0 | 0.873901969 | 0 | 1.735056213 | 1.70416863 | 1.807109303 | 0.718565672 |
| **Arhgap30** | 5164.528156 | 4949.260767 | 5078.017833 | 4913.423254 | 4766.691776 | 4901.472686 | 5347.439629 | 4415.811975 | 4379.050193 | 4557.398768 | 4672.601252 | 5234.664593 | 5122.730903 | 4996.657223 | 5093.193483 |
| **Usf1** | 730.7257923 | 780.3780784 | 837.1994238 | 771.1728212 | 727.7343677 | 758.9286558 | 789.3066254 | 763.4854259 | 783.2631016 | 729.708144 | 686.7021273 | 806.8011388 | 693.5966325 | 799.6458666 | 707.7871869 |
| **Tstd1** | 4.578482408 | 7.130358077 | 9.397935534 | 9.488703616 | 7.600358931 | 7.715540863 | 9.922711863 | 5.028444078 | 8.276555348 | 3.495607876 | 1.891741398 | 8.675281063 | 3.408337261 | 6.324882561 | 15.80844478 |
| **F11r** | 0.915696482 | 3.961310043 | 0 | 5.175656518 | 3.166816221 | 0.701412806 | 0 | 0.838074013 | 3.762070613 | 1.747803938 | 2.837612096 | 0 | 0 | 4.517773258 | 6.467091048 |
| **Alyref2** | 47.61621704 | 47.53572051 | 57.95393579 | 53.48178402 | 47.50224332 | 66.63421654 | 50.51562403 | 46.93214473 | 33.10622139 | 32.33437285 | 39.72656935 | 56.38932691 | 46.01255302 | 42.46706862 | 50.29959704 |
| **Cd244** | 11.90405426 | 13.46845414 | 10.96425812 | 14.66436013 | 18.36753408 | 3.507064029 | 13.53097072 | 20.11377631 | 9.781383594 | 21.84754922 | 21.75502607 | 13.8804497 | 16.18960199 | 3.614218606 | 16.52701046 |
| **Ly9** | 2195.840163 | 2405.307458 | 2244.54027 | 2131.507876 | 2786.164912 | 2656.951708 | 3077.844807 | 1446.515747 | 1758.391804 | 1452.425072 | 1527.581179 | 2702.350051 | 2700.255195 | 2747.709695 | 2803.843252 |
| **Slamf7** | 1914.721343 | 1559.963895 | 1553.008847 | 1481.100374 | 1681.579414 | 1740.906584 | 2205.548228 | 805.3891265 | 726.8320424 | 741.0688696 | 751.9672055 | 1658.713739 | 1691.387366 | 1507.129159 | 1700.844946 |
| **Cd48** | 1742.570404 | 1722.377606 | 1837.296397 | 1829.594579 | 1806.985336 | 1871.369366 | 1853.742989 | 1401.25975 | 1579.317243 | 1573.897446 | 1729.051637 | 1908.561834 | 1726.322823 | 1846.865708 | 1808.629796 |
| **Slamf1** | 883.6471047 | 679.7608033 | 864.6100691 | 860.0215914 | 813.2384057 | 817.8473315 | 883.1213558 | 707.334467 | 773.481718 | 663.2915944 | 617.6535663 | 792.9206891 | 793.2904974 | 865.6053562 | 861.5602407 |
| **Cd84** | 515.5371191 | 589.4429343 | 549.7792287 | 632.2927046 | 600.4283556 | 629.1672867 | 635.0535592 | 521.2820361 | 504.8698763 | 476.2765731 | 509.8243066 | 654.1161921 | 650.1403325 | 630.6811468 | 639.523448 |
| **Slamf6** | 456.0168478 | 543.4917378 | 563.876132 | 628.8422669 | 570.0269198 | 502.9129817 | 530.4140523 | 444.1792269 | 637.2947618 | 488.5112006 | 449.2885819 | 535.2648416 | 558.9673107 | 527.6759165 | 480.7204345 |
| **Vangl2** | 1.831392963 | 3.169048034 | 7.04845165 | 2.587828259 | 5.066905954 | 2.104238417 | 0 | 9.218814144 | 6.771727103 | 0.873901969 | 2.837612096 | 2.602584319 | 0 | 3.614218606 | 1.437131344 |
| **Ncstn** | 1237.105947 | 1288.218026 | 1370.532265 | 1242.157564 | 1226.824604 | 1171.359386 | 1328.741325 | 1317.452349 | 1288.885392 | 1230.453972 | 1448.12804 | 1237.962608 | 1235.522257 | 1175.524602 | 1253.897098 |
| **Copa** | 5404.440634 | 5353.314391 | 5316.098867 | 5506.035926 | 4976.968374 | 5120.313482 | 5613.54872 | 5612.581665 | 5411.36237 | 5210.203539 | 5719.680115 | 5478.439991 | 5391.989546 | 5441.206112 | 5481.937511 |
| **Pex19** | 746.2926325 | 791.4697465 | 750.2685201 | 689.2249263 | 685.9323936 | 728.7679051 | 752.3219721 | 812.0937186 | 819.3789795 | 784.7639681 | 812.5029302 | 693.1549569 | 705.525813 | 773.4427817 | 775.3323601 |
| **Dcaf8** | 1630.855434 | 1734.261537 | 1760.54659 | 1563.910878 | 1559.973671 | 1517.155899 | 1556.061633 | 1557.141516 | 1499.561346 | 1660.413741 | 1625.00586 | 1581.503738 | 1594.249754 | 1533.332244 | 1592.341529 |
| **Pea15a** | 556.7434608 | 496.7482793 | 580.3225192 | 545.1691532 | 480.7227024 | 511.3299354 | 617.9143296 | 540.5577384 | 500.3553915 | 462.2941415 | 517.3912722 | 614.2098992 | 587.0860931 | 631.5847014 | 608.6251242 |
| **Casq1** | 0 | 0 | 3.915806472 | 0 | 0.633363244 | 1.402825611 | 0 | 0 | 1.504828245 | 0 | 0 | 1.735056213 | 0 | 0 | 1.437131344 |
| **Atp1a2** | 5.494178889 | 0 | 0 | 0.86260942 | 0 | 0 | 4.510323574 | 0 | 0 | 0.873901969 | 1.891741398 | 0.867528106 | 0 | 0.903554652 | 0.718565672 |
| **Igsf8** | 1037.484114 | 883.3721395 | 915.5155533 | 844.4946218 | 835.4061192 | 865.5434022 | 865.0800615 | 965.461263 | 854.7424433 | 835.4502823 | 961.9505006 | 785.9804643 | 794.994666 | 786.9961015 | 772.4580974 |
| **Kcnj9** | 0 | 0 | 0 | 0 | 0 | 0 | 1.80412943 | 0 | 3.00965649 | 0 | 0 | 0.867528106 | 0 | 4.517773258 | 0 |
| **Kcnj10** | 7.325571853 | 6.338096068 | 0 | 5.175656518 | 0.633363244 | 3.507064029 | 2.706194144 | 0 | 8.276555348 | 0.873901969 | 1.891741398 | 1.735056213 | 6.816674521 | 9.939101167 | 3.59282836 |
| **Pigm** | 629.0834828 | 667.8768732 | 681.3503262 | 715.9658183 | 654.8975946 | 635.480002 | 721.6517718 | 736.6670575 | 766.7099909 | 740.1949677 | 750.0754641 | 700.9627099 | 645.8799109 | 578.274977 | 694.8530048 |
| **Slamf9** | 1.831392963 | 2.376786026 | 2.349483883 | 4.313047098 | 0 | 1.402825611 | 0 | 0 | 0 | 0 | 3.783482795 | 0 | 10.22501178 | 0 | 2.874262688 |
| **Igsf9** | 2.747089445 | 13.46845414 | 9.397935534 | 4.313047098 | 3.800179466 | 9.118366474 | 9.922711863 | 20.11377631 | 8.276555348 | 15.73023544 | 2.837612096 | 2.602584319 | 5.112505891 | 4.517773258 | 12.9341821 |
| **Tagln2** | 13019.37257 | 11925.12775 | 11994.89839 | 11430.43742 | 10434.02609 | 10075.09354 | 10870.78188 | 10237.07407 | 10849.81165 | 10563.727 | 9941.101044 | 10527.45357 | 10087.82621 | 9884.887888 | 9926.984758 |
| **Cfap45** | 0 | 3.169048034 | 0 | 3.450437679 | 0 | 0.701412806 | 3.608258859 | 0 | 0.752414123 | 0 | 0 | 0 | 0 | 0.903554652 | 0 |
| **Dusp23** | 4.578482408 | 19.80655021 | 19.57903236 | 13.80175071 | 17.1008076 | 16.13249453 | 12.62890601 | 12.5711102 | 14.29586833 | 13.10852953 | 9.458706988 | 21.68820266 | 10.22501178 | 14.45687443 | 13.65274777 |
| **Aim2** | 404.7378449 | 386.6238602 | 354.7720664 | 320.0280947 | 353.4166903 | 334.5739083 | 381.5733744 | 399.7613042 | 380.721546 | 388.8863762 | 401.0491763 | 443.3068623 | 414.9650615 | 397.5640467 | 350.6600479 |
| **BC094916** | 384.5925223 | 316.9048034 | 270.9738079 | 278.6228425 | 434.4871856 | 462.231039 | 538.5326347 | 89.6739194 | 117.3766031 | 97.00311855 | 127.6925443 | 347.0112425 | 330.6087143 | 366.8431885 | 411.73813 |
| **Pydc4** | 137.3544722 | 134.6845414 | 140.969033 | 146.6436013 | 185.5754306 | 227.2577491 | 298.5834206 | 28.49451644 | 41.38277674 | 43.69509845 | 57.69811262 | 186.5185429 | 167.8606101 | 197.8784687 | 196.8869941 |
| **Pyhin1** | 2234.299415 | 1924.404419 | 1545.960395 | 1465.573404 | 2059.063907 | 2295.724113 | 2651.168197 | 349.4768634 | 564.3105919 | 425.5902589 | 562.7930658 | 2070.78959 | 1768.927038 | 2070.043707 | 2006.953922 |
| **Pydc3** | 695.0136295 | 625.8869867 | 445.6187766 | 480.4734467 | 568.7601934 | 726.6636667 | 739.6930661 | 127.38725 | 180.5793894 | 188.7628253 | 188.2282691 | 539.6024821 | 529.996444 | 524.0616979 | 505.8702331 |
| **AI607873** | 9.156964816 | 20.59881222 | 6.265290356 | 6.900875357 | 8.86708542 | 8.416953669 | 9.922711863 | 0.838074013 | 8.276555348 | 2.621705907 | 12.29631908 | 1.735056213 | 3.408337261 | 3.614218606 | 7.904222392 |
| **Ifi204** | 297.6013565 | 209.9494323 | 149.5838072 | 171.6592745 | 128.5727386 | 202.7083009 | 237.24302 | 58.66518091 | 79.00348287 | 45.44290238 | 64.31920752 | 157.0225872 | 149.9668395 | 172.5789385 | 168.8629329 |
| **Mndal** | 2194.00877 | 2360.940785 | 2184.23685 | 1999.528635 | 2587.288853 | 2620.478242 | 3022.818859 | 1382.822122 | 1568.783446 | 1360.665366 | 1608.926059 | 2522.771733 | 2470.19243 | 2622.115599 | 2591.147813 |
| **Mnda** | 293.9385706 | 199.6500261 | 183.2597429 | 163.0331803 | 151.3738154 | 194.99276 | 183.1191371 | 94.70236348 | 79.75589699 | 113.607256 | 106.883389 | 132.7318003 | 126.1084786 | 99.39101167 | 145.1502657 |
| **Ifi203** | 2695.810442 | 2781.631912 | 2536.659433 | 2272.975821 | 3049.010658 | 3170.385882 | 3510.83587 | 1341.756495 | 1577.812415 | 1344.93513 | 1537.039886 | 3099.677924 | 2921.797117 | 3022.39031 | 3041.688489 |
| **Ifi205** | 1.831392963 | 0 | 1.566322589 | 1.725218839 | 0.633363244 | 2.104238417 | 3.608258859 | 2.514222039 | 0 | 6.991215751 | 0 | 0 | 0 | 0 | 0 |
| **Fh1** | 1783.776746 | 1874.491912 | 1970.433817 | 1728.669277 | 1868.421571 | 1848.924156 | 1906.964807 | 1832.867867 | 1787.735955 | 1777.516605 | 1838.772638 | 1833.086889 | 1771.483291 | 1699.5863 | 1743.958886 |
| **Kmo** | 49.44761 | 68.13453273 | 43.85703249 | 52.6191746 | 50.66905954 | 40.68194273 | 41.49497688 | 59.50325493 | 57.93588744 | 66.41654964 | 51.07701773 | 26.02584319 | 52.82922754 | 28.91374885 | 30.17975822 |
| **Opn3** | 53.11039593 | 49.12024453 | 62.65290356 | 80.22267603 | 55.10260225 | 46.99465798 | 68.55691832 | 71.23629111 | 72.98416989 | 49.81241223 | 99.31642337 | 59.85943933 | 48.56880596 | 57.8274977 | 44.55107166 |
| **Chml** | 206.0317084 | 245.6012226 | 252.1779368 | 250.1567317 | 215.9768663 | 202.006888 | 277.8359322 | 237.1749457 | 240.7725192 | 196.627943 | 262.9520543 | 256.7883195 | 239.4356926 | 212.3353431 | 224.1924897 |
| **Exo1** | 834.1994947 | 921.4007159 | 947.6251663 | 968.7103783 | 790.4373289 | 733.6777948 | 911.9874266 | 1079.439329 | 1065.418398 | 1113.351108 | 1034.782544 | 834.5620382 | 822.2613641 | 815.9098504 | 890.3028676 |
| **Cep170** | 965.1440916 | 898.4251176 | 930.3956179 | 900.5642341 | 694.1661157 | 776.4639759 | 888.5337441 | 709.8486891 | 811.8548383 | 713.1040066 | 806.8277061 | 741.7365309 | 775.3967268 | 672.2446608 | 825.6319571 |
| **Sdccag8** | 190.4648682 | 212.3262183 | 169.1628396 | 193.22451 | 184.3087041 | 192.8885216 | 199.356302 | 152.5294704 | 176.0649047 | 139.824315 | 196.7411053 | 180.4458461 | 201.0918984 | 171.6753838 | 204.7912165 |
| **Akt3** | 429.4616499 | 354.9333798 | 374.3510988 | 332.1046266 | 323.0152546 | 324.754129 | 393.3002156 | 329.3630871 | 338.5863552 | 287.5137478 | 342.405193 | 322.7204555 | 396.2192066 | 331.6045571 | 339.1629972 |
| **Zbtb18** | 386.4239152 | 504.6708994 | 457.366196 | 447.6942888 | 499.0902365 | 415.9377938 | 387.8878274 | 532.1769983 | 416.8374239 | 458.7985337 | 425.6418144 | 471.9352898 | 437.971338 | 487.0159572 | 421.0794838 |
| **Adss** | 2508.092663 | 2617.633676 | 2697.99066 | 2662.875278 | 2545.486879 | 2537.010118 | 2655.67852 | 2975.162746 | 2833.591586 | 2672.392221 | 2656.004922 | 2757.87185 | 2580.963391 | 2547.120563 | 2576.057934 |
| **Desi2** | 1027.411452 | 1062.423353 | 1061.966715 | 1031.680866 | 953.8450459 | 1047.910732 | 1090.59624 | 1013.231482 | 1023.283207 | 1096.746971 | 1073.563243 | 1043.636312 | 1019.092841 | 1104.143784 | 1089.345559 |
| **Cox20** | 214.2729767 | 236.8863405 | 245.9126465 | 229.4541056 | 234.9777636 | 285.4750119 | 271.5214791 | 266.5075362 | 268.6118418 | 221.0971981 | 238.3594161 | 271.5362973 | 322.9399554 | 281.0054966 | 267.30643 |
| **Hnrnpu** | 24960.05469 | 23354.29949 | 24586.56568 | 24414.4344 | 24990.61353 | 24507.36343 | 26004.7216 | 24895.82663 | 24586.63628 | 24119.69434 | 24606.82623 | 25865.35049 | 25368.25423 | 24486.33106 | 24721.53338 |
| **Efcab2** | 74.17141501 | 63.38096068 | 53.25496803 | 58.65744054 | 54.46923901 | 65.93280374 | 54.12388289 | 57.8271069 | 44.39243323 | 94.38141264 | 54.86050053 | 73.73988903 | 34.08337261 | 69.57370817 | 50.29959704 |
| **Smyd3** | 354.3745384 | 345.4262357 | 423.6902603 | 391.6246765 | 350.8832373 | 371.0473742 | 379.7692449 | 410.6562664 | 369.4353342 | 396.7514939 | 419.9665903 | 365.2293327 | 352.7629065 | 290.9445978 | 334.8516031 |
| **Tfb2m** | 376.3512539 | 339.8804016 | 388.4480021 | 334.6924548 | 338.8493357 | 359.8247693 | 292.2689676 | 477.7021874 | 395.7698285 | 505.98924 | 480.502315 | 334.865849 | 346.7983163 | 285.5232699 | 316.8874613 |
| **Cnst** | 438.6186147 | 448.4202968 | 476.162067 | 445.1064605 | 378.1178568 | 387.1798688 | 421.2642218 | 564.0238108 | 468.0015842 | 471.9070632 | 456.8555475 | 411.2083224 | 420.9296517 | 449.9702165 | 400.2410793 |
| **Sccpdh** | 54.94178889 | 52.28929256 | 47.77283896 | 67.28353473 | 42.43533737 | 63.12715251 | 54.12388289 | 41.06562664 | 21.06759543 | 33.20827482 | 29.32199166 | 46.84651774 | 46.01255302 | 43.37062328 | 59.64095077 |
| **Ahctf1** | 3570.300582 | 3359.983178 | 3489.766728 | 3536.698621 | 2987.574423 | 3048.340054 | 3355.680739 | 3832.512462 | 4023.158313 | 3810.212584 | 4043.597237 | 3200.311184 | 3312.051733 | 2925.709962 | 3117.137885 |
| **Cdc42bpa** | 58.60457482 | 35.65179038 | 40.72438731 | 44.85568982 | 29.76807248 | 31.56357626 | 39.69084745 | 25.14222039 | 27.08690841 | 38.45168663 | 40.67244005 | 46.84651774 | 46.01255302 | 21.68531164 | 38.08398061 |
| **Adck3** | 130.9445969 | 120.4238253 | 96.32883922 | 100.9253021 | 58.26941847 | 88.37801352 | 79.3816949 | 93.86428946 | 85.02279585 | 78.6511772 | 49.18527634 | 65.93213608 | 78.39175699 | 59.634607 | 92.69497168 |
| **Psen2** | 276.5403374 | 339.0881396 | 321.879292 | 302.7759063 | 317.3149854 | 338.7823852 | 343.6866563 | 290.8116825 | 252.0587311 | 353.0563954 | 303.6244943 | 319.2503431 | 304.1941005 | 328.8938932 | 338.4444315 |
| **Gm5069** | 5.494178889 | 3.169048034 | 7.04845165 | 3.450437679 | 1.900089733 | 3.507064029 | 0.902064715 | 3.352296052 | 4.514484735 | 1.747803938 | 0 | 2.602584319 | 0.852084315 | 0.903554652 | 0 |
| **Itpkb** | 1227.033285 | 1247.020401 | 1198.236781 | 1268.898456 | 1083.051148 | 1176.970688 | 1288.148413 | 967.1374111 | 971.3666323 | 938.5707146 | 950.6000523 | 1299.557103 | 1330.9557 | 1231.54499 | 1321.442271 |
| **Parp1** | 4904.470355 | 4989.66613 | 5128.140156 | 4792.657936 | 4479.778227 | 4606.879308 | 4752.076917 | 5848.080463 | 5705.556292 | 5644.532817 | 5659.144391 | 4753.186494 | 4965.095304 | 4765.347232 | 4758.34188 |
| **Lin9** | 327.8193404 | 310.5667073 | 390.0143247 | 275.1724049 | 327.4487973 | 342.2894492 | 335.5680739 | 368.7525657 | 361.1587788 | 335.5783561 | 363.2143483 | 385.1824792 | 299.9336789 | 374.0716258 | 341.3186942 |
| **Acbd3** | 1028.327149 | 986.3662006 | 990.6990375 | 923.8546884 | 917.1099777 | 952.5185902 | 997.6835745 | 947.8617088 | 945.7845521 | 956.048754 | 899.5230345 | 1050.576537 | 963.7073604 | 918.011526 | 968.6265258 |
| **H3f3a** | 6150.733267 | 6566.267526 | 7096.224489 | 7042.343302 | 7120.902955 | 7113.728676 | 7167.806224 | 6779.180692 | 7105.04656 | 6569.1211 | 6674.063651 | 7417.365309 | 7390.127265 | 7666.661219 | 7498.232787 |
| **Sde2** | 1142.789209 | 1164.625153 | 1097.992135 | 1139.507043 | 1109.652404 | 1106.127995 | 1154.642835 | 1190.903173 | 1102.28669 | 1212.102031 | 1140.720063 | 1129.521594 | 1006.311576 | 1019.209647 | 1028.986042 |
| **Pycr2** | 1215.129231 | 1257.319807 | 1206.851555 | 1277.524551 | 1237.591779 | 1230.979474 | 1217.787365 | 1319.966571 | 1274.589524 | 1307.357345 | 1335.569427 | 1135.594291 | 1313.06193 | 1167.39261 | 1223.717339 |
| **Lefty1** | 0.915696482 | 0 | 0 | 0 | 0 | 4.208476834 | 0 | 2.514222039 | 0 | 2.621705907 | 0 | 0 | 0 | 0 | 2.874262688 |
| **Tmem63a** | 99.81091649 | 122.0083493 | 146.4511621 | 162.1705709 | 177.9750716 | 183.7701551 | 191.2377195 | 108.1115477 | 125.6531585 | 102.2465304 | 118.2338373 | 197.7964082 | 168.7126944 | 215.9495617 | 176.0485896 |
| **Ephx1** | 170.3195456 | 209.1571702 | 236.5147109 | 154.4070861 | 231.8109474 | 288.982076 | 303.9958089 | 156.7198404 | 169.2931776 | 106.6160402 | 173.0943379 | 262.8610162 | 237.7315239 | 258.4166304 | 248.6237225 |
| **Nvl** | 1258.166966 | 1235.136471 | 1271.853942 | 1192.126218 | 1165.388369 | 1166.449496 | 1254.772018 | 1475.010263 | 1407.014409 | 1392.999738 | 1414.076695 | 1148.607213 | 1215.072233 | 1054.448278 | 1226.591602 |
| **Cnih4** | 576.8887834 | 608.4572225 | 536.4654867 | 539.9934967 | 607.3953513 | 653.0153221 | 611.5998766 | 628.5555098 | 572.5871473 | 553.1799463 | 548.6050053 | 585.5814717 | 512.9547577 | 622.5491549 | 561.1997898 |
| **Wdr26** | 3647.219086 | 3423.364139 | 3659.712729 | 3469.415086 | 3695.67453 | 3864.784559 | 4056.585022 | 3507.339745 | 3634.160212 | 3555.907111 | 3544.177508 | 3889.1285 | 4089.152628 | 3658.492784 | 3832.829294 |
| **Cnih3** | 3.662785926 | 1.584524017 | 3.132645178 | 1.725218839 | 0.633363244 | 1.402825611 | 1.80412943 | 5.866518091 | 5.266898858 | 7.86511772 | 0 | 0 | 0.852084315 | 7.228437213 | 1.437131344 |
| **Lbr** | 5528.059659 | 5873.830531 | 5884.673967 | 5612.999494 | 5714.20319 | 5543.265404 | 6117.802896 | 4927.037123 | 5447.478247 | 5270.502774 | 5562.665579 | 6306.929333 | 6363.365666 | 6100.801007 | 5845.531741 |
| **Enah** | 146.5114371 | 115.6702532 | 131.5710975 | 102.6505209 | 52.56914927 | 81.36388546 | 83.89201847 | 113.9780658 | 124.1483302 | 139.824315 | 114.4503546 | 68.5347204 | 54.53339617 | 76.80214538 | 66.82660749 |
| **Srp9** | 1570.419466 | 1657.412122 | 1644.638718 | 1616.530052 | 1566.940666 | 1705.835943 | 1560.571957 | 1821.972904 | 1745.600764 | 1860.537292 | 1697.837904 | 1725.513403 | 1660.71233 | 1875.779457 | 1611.742802 |
| **Degs1** | 2168.369268 | 1967.978829 | 1934.408397 | 1906.366817 | 2061.59736 | 1897.321639 | 1969.207272 | 1751.574687 | 1808.051137 | 1864.0329 | 1865.257018 | 1935.455205 | 2021.99608 | 1816.14485 | 1925.037435 |
| **Fbxo28** | 759.1123832 | 704.3209256 | 691.531423 | 770.3102117 | 659.9645005 | 691.5930264 | 764.0488134 | 786.1134242 | 768.9672333 | 773.4032425 | 817.2322837 | 715.7106877 | 693.5966325 | 686.7015352 | 666.8289436 |
| **Trp53bp2** | 477.9935634 | 394.5464802 | 412.7260022 | 451.1447265 | 355.31678 | 375.2558511 | 402.3208628 | 427.4177467 | 493.5836644 | 462.2941415 | 460.6390303 | 341.8060739 | 495.9130714 | 328.8938932 | 394.4925539 |
| **Capn2** | 2291.072597 | 2032.944314 | 1988.446527 | 1971.062524 | 1952.025519 | 1874.175017 | 2129.774792 | 1683.690692 | 1729.800068 | 1801.111958 | 2082.807279 | 1872.125653 | 2048.410694 | 1962.520703 | 2011.983882 |
| **Susd4** | 3.662785926 | 3.169048034 | 0 | 0 | 1.266726489 | 0 | 0 | 5.866518091 | 0 | 0 | 2.837612096 | 0 | 1.70416863 | 0.903554652 | 3.59282836 |
| **Tlr5** | 24.723805 | 19.0142882 | 14.0969033 | 31.91654853 | 16.46744435 | 0.701412806 | 9.922711863 | 3.352296052 | 3.762070613 | 6.991215751 | 13.24218978 | 6.94022485 | 5.112505891 | 8.131991864 | 11.49705075 |
| **1700056E22Rik** | 14.65114371 | 9.507144102 | 8.614774239 | 5.175656518 | 3.166816221 | 15.43108173 | 9.020647148 | 7.542666117 | 27.08690841 | 2.621705907 | 11.35044839 | 6.94022485 | 11.92918041 | 1.807109303 | 7.18565672 |
| **Dusp10** | 113.5463637 | 109.3321572 | 126.0889684 | 141.4679448 | 144.4068197 | 131.1641947 | 218.299661 | 117.3303618 | 144.4635115 | 96.12921658 | 131.4760271 | 178.7107899 | 150.8189238 | 181.614485 | 145.1502657 |
| **Hlx** | 0.915696482 | 0 | 0 | 3.450437679 | 0 | 0 | 2.706194144 | 0 | 0 | 3.495607876 | 0 | 1.735056213 | 0 | 0 | 0 |
| **Marc2** | 991.6992895 | 1040.240017 | 1044.737167 | 943.6947051 | 989.9467508 | 1107.53082 | 1021.137257 | 826.3409769 | 826.1507066 | 848.5588118 | 915.6028364 | 956.8835012 | 918.5468917 | 1004.752773 | 1041.920224 |
| **Rab3gap2** | 726.1473099 | 781.1703404 | 790.2097461 | 674.5605662 | 772.0697948 | 788.3879936 | 749.615778 | 872.4350476 | 797.5589699 | 751.5556933 | 865.4716894 | 766.894846 | 850.3801465 | 795.1280934 | 794.0150675 |
| **Iars2** | 1967.831739 | 2032.944314 | 2112.186011 | 2104.766984 | 1977.360049 | 1905.738593 | 2014.310508 | 2208.325024 | 2151.904391 | 2342.057277 | 2500.882128 | 2206.991502 | 2163.442076 | 2082.693472 | 2042.882205 |
| **Mir194-1** | 2.747089445 | 1.584524017 | 3.915806472 | 0.86260942 | 0 | 2.104238417 | 0 | 1.676148026 | 0 | 0 | 0 | 0.867528106 | 0.852084315 | 0 | 10.77848508 |
| **Mir215** | 0 | 1.584524017 | 1.566322589 | 0 | 1.266726489 | 0 | 0.902064715 | 0 | 0.752414123 | 0 | 0 | 0 | 8.520843152 | 0 | 0 |
| **Bpnt1** | 482.5720458 | 526.8542357 | 576.4067127 | 498.5882446 | 487.0563348 | 467.8423414 | 507.8624344 | 516.253592 | 541.7381683 | 598.6228487 | 673.4599375 | 523.9869762 | 490.8005655 | 485.2088479 | 475.6904748 |
| **Eprs** | 7589.292439 | 7051.131876 | 6838.564423 | 6634.329047 | 6432.437109 | 6538.570175 | 7092.934852 | 7401.031609 | 6976.383745 | 6997.333065 | 7508.321607 | 6488.242707 | 6349.732317 | 5601.135285 | 6402.420137 |
| **Tgfb2** | 4.578482408 | 0 | 1.566322589 | 3.450437679 | 0 | 0 | 0 | 5.028444078 | 4.514484735 | 2.621705907 | 0.945870699 | 0 | 0.852084315 | 0 | 2.874262688 |
| **Rrp15** | 1332.338381 | 1321.49303 | 1378.363878 | 1142.094872 | 1324.995907 | 1266.751527 | 1286.344283 | 1458.248783 | 1370.898531 | 1454.172876 | 1397.051022 | 1285.676654 | 1190.361788 | 1129.443314 | 1205.753198 |
| **Gpatch2** | 191.3805646 | 228.9637205 | 222.4178076 | 232.9045433 | 197.6093322 | 208.3196033 | 170.4902311 | 173.4813207 | 210.6759543 | 229.8362178 | 224.1713556 | 219.4846109 | 178.0856219 | 187.9393675 | 178.9228523 |
| **Kctd3** | 396.4965765 | 400.0923143 | 433.8713571 | 370.9220505 | 401.5522969 | 419.4448578 | 369.8465331 | 382.16175 | 379.9691319 | 428.2119648 | 426.5876851 | 399.0629289 | 391.958785 | 361.4218606 | 420.3609181 |
| **Cenpf** | 1932.119576 | 2158.121711 | 2019.772978 | 2115.118297 | 1958.359151 | 1901.530116 | 2302.971217 | 2198.268136 | 2197.049238 | 2066.778156 | 2025.109166 | 2330.180493 | 2125.950366 | 2136.906751 | 2246.236291 |
| **Ptpn14** | 240.8281747 | 205.9881222 | 201.2724527 | 224.2784491 | 57.00269198 | 61.7243269 | 68.55691832 | 420.7131546 | 307.7373761 | 377.5256506 | 264.8437957 | 63.32955176 | 58.79381775 | 65.05593491 | 69.70087018 |
| **Smyd2** | 1007.26613 | 1106.790026 | 1153.596587 | 1089.475697 | 974.7460329 | 1010.735853 | 969.7195684 | 1457.410709 | 1264.055726 | 1438.442641 | 1394.21341 | 982.0418163 | 969.6719507 | 1029.148748 | 975.8121825 |
| **Rps6kc1** | 258.2264078 | 245.6012226 | 249.8284529 | 287.2489367 | 239.4113063 | 230.7648131 | 235.4388906 | 324.3346431 | 301.7180632 | 296.2527675 | 302.6786236 | 225.5573076 | 235.175271 | 224.9851082 | 207.6654792 |
| **Angel2** | 743.545543 | 810.4840347 | 914.732392 | 786.6997907 | 858.207196 | 827.6671107 | 932.7349151 | 938.6428946 | 891.6107353 | 879.1453807 | 1026.269708 | 909.1694554 | 891.2801937 | 942.4075016 | 911.8598377 |
| **Vash2** | 6.409875371 | 7.922620085 | 7.04845165 | 0.86260942 | 6.966995687 | 2.104238417 | 0.902064715 | 8.380740131 | 26.33449429 | 6.117313782 | 7.56696559 | 3.470112425 | 5.112505891 | 0.903554652 | 0 |
| **Mfsd7b** | 663.8799491 | 687.6834234 | 661.7712938 | 634.0179234 | 561.7931977 | 626.3616355 | 647.6824652 | 742.5335756 | 776.4913745 | 762.9164189 | 842.7707926 | 622.0176522 | 709.7862345 | 720.1330573 | 625.1521346 |
| **A230020J21Rik** | 0.915696482 | 3.169048034 | 1.566322589 | 3.450437679 | 0 | 1.402825611 | 2.706194144 | 0.838074013 | 0.752414123 | 0.873901969 | 0 | 4.337640531 | 0.852084315 | 2.710663955 | 1.437131344 |
| **Tatdn3** | 52.19469945 | 53.87381658 | 48.55600026 | 52.6191746 | 50.66905954 | 43.48759395 | 46.90736517 | 68.72206907 | 30.0965649 | 44.56900041 | 63.37333682 | 56.38932691 | 69.01882953 | 54.21327909 | 60.35951645 |
| **Nsl1** | 676.6996999 | 689.2679474 | 716.5925845 | 642.6440176 | 665.6647697 | 653.7167349 | 705.414607 | 648.6692861 | 719.3079012 | 630.9572215 | 649.8131701 | 590.7866404 | 610.944454 | 688.5086445 | 572.6968406 |

| | | | | | | | | | | | | | | | |
|---|---|---|---|---|---|---|---|---|---|---|---|---|---|---|---|
| **Batf3** | 921.1906605 | 668.6691352 | 657.0723261 | 631.4300952 | 305.2810837 | 382.2699791 | 327.4494915 | 747.5620196 | 710.2789317 | 974.4006953 | 809.6653181 | 373.9046138 | 345.0941476 | 341.5436583 | 352.0971793 |
| **Atf3** | 1238.021643 | 836.628681 | 849.7300045 | 646.0944553 | 596.6281761 | 666.3421654 | 660.3113712 | 447.531523 | 415.3325957 | 542.6931227 | 512.6619187 | 609.8722587 | 622.8736344 | 516.8332607 | 595.6909421 |
| **Nenf** | 188.6334752 | 199.6500261 | 186.3923881 | 156.132305 | 188.7422468 | 200.6040624 | 194.8459784 | 201.9758371 | 173.0552482 | 168.66308 | 154.1769239 | 208.2067455 | 220.6898376 | 159.9291733 | 197.6055598 |
| **Tmem206** | 410.2320237 | 313.7357554 | 409.593357 | 352.8072526 | 377.4844936 | 404.0137761 | 411.3415099 | 386.35212 | 398.0270708 | 373.1561407 | 361.3226069 | 379.9773106 | 378.3254359 | 384.0107269 | 342.0372599 |
| **Ppp2r5a** | 1788.355228 | 1944.210969 | 1874.104978 | 1951.222507 | 1910.856908 | 2201.033384 | 2047.686903 | 1842.086681 | 2014.212606 | 1698.865428 | 1884.174432 | 2024.8106 | 1971.723105 | 2061.911715 | 2006.953922 |
| **Dtl** | 1590.564788 | 1685.933554 | 1688.495751 | 1663.110961 | 1441.534744 | 1502.42623 | 1557.865762 | 1777.554982 | 1817.83252 | 1809.850978 | 1698.783775 | 1657.846211 | 1480.070455 | 1494.479394 | 1397.610232 |
| **Ints7** | 1755.390155 | 1957.679423 | 1918.745171 | 1885.664191 | 1901.356459 | 1872.772191 | 1895.237966 | 2353.311829 | 2374.618971 | 2286.127551 | 2201.986987 | 1928.51498 | 2088.458656 | 1958.00293 | 1858.929393 |
| **Lpgat1** | 1273.733806 | 1218.498969 | 1224.081103 | 1142.094872 | 1173.622092 | 1243.604905 | 1378.354884 | 1207.664653 | 1202.357768 | 1233.94958 | 1346.919875 | 1165.957775 | 1231.261835 | 1223.412998 | 1282.639724 |
| **Nek2** | 1321.350023 | 1495.790672 | 1537.345621 | 1588.926551 | 1409.233219 | 1408.436914 | 1321.524807 | 1656.872324 | 1701.960745 | 1666.531055 | 1681.758102 | 1655.243627 | 1479.218371 | 1706.814737 | 1516.892134 |
| **1700034H15Rik** | 0 | 2.376786026 | 0.783161294 | 1.725218839 | 0 | 1.402825611 | 0.902064715 | 4.190370065 | 0 | 1.747803938 | 0 | 0 | 1.70416863 | 0 | 2.155697016 |
| **Slc30a1** | 160.2468843 | 202.0268122 | 192.6576784 | 187.1862441 | 171.008076 | 216.736557 | 195.7480431 | 181.8620608 | 206.9138837 | 213.2320804 | 251.6016059 | 169.1679807 | 172.973116 | 199.685578 | 185.3899434 |
| **Rd3** | 9.156964816 | 11.88393013 | 1.566322589 | 12.07653188 | 13.30062813 | 13.32684331 | 13.53097072 | 2.514222039 | 6.771727103 | 1.747803938 | 6.621094891 | 5.205168638 | 16.18960199 | 8.131991864 | 22.9941015 |
| **Traf5** | 305.8426248 | 311.3589693 | 322.6624533 | 363.1585657 | 319.2150751 | 357.7205309 | 349.0990446 | 386.35212 | 339.3387693 | 319.8481206 | 288.4905631 | 398.1954008 | 346.7983163 | 463.5235363 | 356.4085733 |
| **Mir3473c** | 0 | 1.584524017 | 1.566322589 | 0 | 0 | 0 | 1.80412943 | 2.514222039 | 0 | 2.621705907 | 0 | 0.867528106 | 2.556252945 | 0 | 0 |
| **Rcor3** | 187.7177787 | 220.2488384 | 210.6703882 | 231.1793245 | 150.1070889 | 197.7984112 | 185.8253312 | 215.3850214 | 208.418712 | 232.4579237 | 201.4704588 | 180.4458461 | 178.9377062 | 196.974914 | 191.1384687 |
| **Gm10516** | 0 | 4.753572051 | 5.482129061 | 6.038265938 | 0 | 7.014128057 | 1.80412943 | 8.380740131 | 0 | 5.243411813 | 5.675224193 | 4.337640531 | 2.556252945 | 1.807109303 | 4.311394032 |
| **Hhat** | 48.53191352 | 40.40536243 | 19.57903236 | 21.56523549 | 26.60125626 | 18.23673295 | 42.39704159 | 22.62799835 | 24.07725192 | 31.46047088 | 29.32199166 | 24.29078698 | 28.97086672 | 16.26398373 | 38.08398061 |
| **Syt14** | 0.915696482 | 0.792262009 | 0 | 0.86260942 | 0 | 0.701412806 | 0.902064715 | 5.028444078 | 0.752414123 | 0.873901969 | 2.837612096 | 1.735056213 | 7.668758836 | 5.421327909 | 0 |
| **Diexf** | 912.9493921 | 849.3048731 | 890.4543918 | 899.7016247 | 777.770064 | 859.230687 | 910.1832972 | 985.5750394 | 923.9645425 | 914.1014595 | 950.6000523 | 804.1985545 | 924.511482 | 805.9707492 | 872.3387258 |
| **Irf6** | 43.03773463 | 49.91250654 | 46.98967767 | 59.52004996 | 67.13650389 | 70.84269338 | 42.39704159 | 41.90370065 | 32.35380727 | 25.3431571 | 25.53850887 | 93.69303548 | 69.01882953 | 84.93413725 | 79.04222392 |
| **A130010J15Rik** | 106.2207919 | 114.8779912 | 114.341549 | 86.26094197 | 85.50403798 | 97.49637999 | 107.3457011 | 155.0436924 | 108.3476337 | 139.824315 | 139.0429927 | 103.2358446 | 98.84178056 | 110.2336675 | 86.22788064 |
| **Traf3ip3** | 1024.664363 | 1147.195388 | 1226.430587 | 1227.493204 | 1107.118951 | 1165.04667 | 1251.163759 | 1076.925107 | 1057.894256 | 1127.33354 | 1096.26414 | 1243.167776 | 1221.888908 | 1245.09831 | 1198.567541 |
| **Hsd11b1** | 62.26736075 | 97.44822705 | 104.1604522 | 119.0400999 | 94.3711234 | 101.7048568 | 95.61885977 | 86.32162334 | 99.31866418 | 58.55143192 | 157.9604067 | 100.6332603 | 85.20843152 | 137.340307 | 132.9346493 |
| **G0s2** | 0.915696482 | 0 | 0 | 2.587828259 | 1.266726489 | 0.701412806 | 3.608258859 | 0 | 0 | 0.873901969 | 0 | 0 | 0 | 3.614218606 | 0 |
| **Lamb3** | 133.6916863 | 110.1244192 | 131.5710975 | 133.70446 | 110.8385677 | 111.5246361 | 97.4229892 | 139.9583602 | 129.4152291 | 119.7245697 | 96.47881127 | 113.6461819 | 108.214708 | 123.7869873 | 107.0662851 |
| **A330023F24Rik** | 0 | 0 | 0 | 0 | 0 | 0.701412806 | 3.608258859 | 1.676148026 | 0 | 0 | 0.945870699 | 0.867528106 | 0 | 0 | 1.437131344 |
| **Cd46** | 0 | 3.169048034 | 0 | 10.35131304 | 1.266726489 | 3.507064029 | 2.706194144 | 0.838074013 | 5.266898858 | 0 | 4.729353494 | 0 | 0 | 0.903554652 | 1.437131344 |
| **Cr1l** | 975.2167529 | 1056.085257 | 1076.063619 | 1109.315714 | 1037.448994 | 1007.930202 | 1021.137257 | 1057.649404 | 1077.457024 | 936.8229107 | 1031.944932 | 1027.153278 | 1043.803286 | 923.4328539 | 1051.980144 |
| **Cr2** | 0 | 0 | 1.566322589 | 0.86260942 | 0 | 0.701412806 | 0 | 0 | 0 | 0 | 0 | 0 | 1.70416863 | 0.903554652 | 0 |
| **Cnksr3** | 254.5636219 | 274.1226549 | 299.9507758 | 233.7671527 | 297.0473616 | 328.9626059 | 322.0371032 | 203.6519852 | 260.3352864 | 248.1881592 | 232.6841919 | 285.416747 | 306.7503535 | 211.4317885 | 292.4562285 |
| **Oprm1** | 1.831392963 | 0.792262009 | 8.614774239 | 1.725218839 | 5.700269198 | 2.805651223 | 3.608258859 | 1.676148026 | 3.00965649 | 0 | 1.891741398 | 6.94022485 | 0.852084315 | 2.710663955 | 0 |
| **Ipcef1** | 1340.579649 | 1660.58117 | 1748.016009 | 1819.243266 | 1870.32166 | 1851.028394 | 1912.377195 | 1979.530819 | 1804.289066 | 1403.486562 | 1489.746351 | 2162.747569 | 2124.246198 | 2108.896557 | 1889.827717 |
| **Mtrf1l** | 182.2235998 | 219.4565764 | 229.4662593 | 211.3393078 | 202.0428749 | 164.1305965 | 192.1397842 | 175.1574687 | 203.9042272 | 173.9064918 | 210.9291658 | 207.3392174 | 208.7606572 | 224.0815536 | 219.8810956 |
| **Fbxo5** | 1005.434737 | 996.6656067 | 1004.795941 | 1009.253021 | 1024.781729 | 930.7747932 | 928.2245915 | 1068.544367 | 1005.225268 | 1097.620873 | 974.2468197 | 923.0499051 | 1031.874106 | 967.7070318 | 987.3092333 |
| **Syne1** | 358.0373243 | 407.2226724 | 444.8356153 | 495.1378069 | 391.418485 | 347.9007516 | 419.4600924 | 410.6562664 | 443.9243323 | 335.5783561 | 366.0519604 | 415.5459629 | 427.7463262 | 434.6097874 | 398.0853823 |
| **Esr1** | 16.48253667 | 24.56012226 | 16.44638718 | 18.11479781 | 19.63426057 | 16.13249453 | 16.23716487 | 17.59955427 | 17.30552482 | 27.964863 | 29.32199166 | 13.8804497 | 11.92918041 | 9.035546516 | 22.27553583 |
| **Ccdc170** | 0 | 0 | 0 | 4.313047098 | 3.800179466 | 0 | 3.608258859 | 0 | 0 | 0 | 0 | 0.867528106 | 0.852084315 | 0 | 2.155697016 |
| **Armt1** | 330.5664298 | 423.8601745 | 396.279615 | 370.9220505 | 359.7503227 | 379.4643279 | 373.4547919 | 329.3630871 | 346.1104964 | 385.3907683 | 424.6959437 | 402.5330413 | 351.0587378 | 402.08182 | 439.7621912 |
| **Rmnd1** | 0 | 7.922620085 | 0 | 0 | 0 | 1.402825611 | 0 | 0 | 0 | 0 | 0 | 0 | 0.852084315 | 0 | 1.437131344 |
| **Gm5512** | 0 | 0 | 0 | 0 | 2.533452977 | 0 | 0.902064715 | 0 | 0.752414123 | 0.873901969 | 0 | 0 | 0 | 0 | 0.718565672 |
| **Zbtb2** | 428.5459534 | 438.1208907 | 463.6314863 | 488.2369315 | 480.0893392 | 451.0084341 | 507.8624344 | 530.5008503 | 522.9278152 | 526.9628872 | 563.7389365 | 542.2050664 | 444.7880125 | 448.1631072 | 465.6305554 |
| **Akap12** | 105.3050954 | 107.7476332 | 117.4741942 | 109.5513963 | 108.3051148 | 91.88507755 | 110.0518952 | 90.51199341 | 150.4828245 | 113.607256 | 104.0457769 | 104.1033728 | 97.13761193 | 93.06612911 | 130.0603866 |
| **Mthfd1l** | 5189.251961 | 4797.938724 | 4857.166348 | 4956.553725 | 4736.290341 | 4945.661693 | 4844.989583 | 3968.280452 | 4233.081854 | 4333.679864 | 4316.953869 | 4598.766491 | 4797.234694 | 4403.021817 | 4697.263798 |
| **Plekhg1** | 0.915696482 | 0.792262009 | 0 | 0 | 0 | 0.701412806 | 0 | 0 | 0 | 1.747803938 | 0 | 0 | 2.556252945 | 0.903554652 | 6.467091048 |
| **Ppp1r14c** | 1.831392963 | 0 | 0.783161294 | 0.86260942 | 2.533452977 | 2.104238417 | 2.706194144 | 0.838074013 | 3.762070613 | 0 | 2.837612096 | 0 | 0.852084315 | 0.903554652 | 3.59282836 |
| **B020014A21Rik** | 0 | 3.169048034 | 1.566322589 | 2.587828259 | 0.633363244 | 0 | 0.902064715 | 3.352296052 | 0 | 0.873901969 | 2.837612096 | 4.337640531 | 2.556252945 | 4.517773258 | 0 |
| **Lrp11** | 57.68887834 | 94.27917901 | 101.027807 | 83.67311371 | 88.03749095 | 96.09355438 | 93.81473034 | 149.1771743 | 133.9297138 | 131.0852953 | 115.3962252 | 86.75281063 | 88.61676878 | 121.0763233 | 86.94644631 |
| **Pcmt1** | 1371.713329 | 1356.352559 | 1426.136717 | 1326.693287 | 1404.799676 | 1524.170027 | 1524.489368 | 1258.787168 | 1382.184743 | 1392.125836 | 1336.515297 | 1446.169353 | 1490.295467 | 1460.144317 | 1396.891666 |
| **A630066F11Rik** | 9.156964816 | 8.714882094 | 26.62748401 | 19.84001665 | 16.46744435 | 19.63955856 | 9.922711863 | 13.40918421 | 1.504828245 | 16.60413741 | 10.40457769 | 10.41033728 | 15.33751767 | 24.39597559 | 17.24557613 |
| **Nup43** | 663.8799491 | 709.0744976 | 673.5187133 | 603.8265938 | 631.4631545 | 621.4517459 | 670.2340831 | 810.4175706 | 775.7389604 | 623.9660058 | 714.1323776 | 635.8981019 | 625.4298873 | 647.8486852 | 646.7091048 |
| **BC020402** | 10.98835778 | 14.26071615 | 10.96425812 | 8.626094197 | 5.700269198 | 7.014128057 | 4.510323574 | 8.380740131 | 7.524141226 | 5.243411813 | 7.56696559 | 4.337640531 | 11.92918041 | 5.421327909 | 4.311394032 |
| **Lats1** | 792.0774566 | 763.7405762 | 758.1001331 | 747.8823668 | 715.700466 | 662.8351014 | 744.2033897 | 931.1002285 | 903.6493612 | 859.0456354 | 814.3946716 | 717.4457439 | 741.3133542 | 761.6965713 | 767.4281377 |
| **Katna1** | 638.2404477 | 593.4042444 | 666.4702616 | 607.2770314 | 696.0662055 | 704.9198697 | 675.6464714 | 662.0784703 | 596.6643992 | 541.8192207 | 569.4141607 | 694.8900131 | 665.4778501 | 680.3766526 | 640.9605794 |
| **Ginm1** | 231.6712098 | 263.8232488 | 284.2875499 | 266.5463107 | 253.978661 | 303.7117449 | 260.6967026 | 197.7854671 | 262.5925288 | 207.9886686 | 236.4676747 | 267.1986567 | 272.6669809 | 284.6197152 | 291.7376628 |
| **Ppil4** | 1114.402618 | 1121.050742 | 1195.887297 | 1029.093038 | 1024.781729 | 1053.522034 | 1130.287088 | 1051.782886 | 1144.42188 | 1104.612089 | 1059.375183 | 1085.277661 | 963.7073604 | 1173.717492 | 1038.327396 |
| **Zc3h12d** | 1153.777567 | 1105.205502 | 1152.030264 | 930.7555638 | 1087.48469 | 1020.555632 | 1124.874699 | 1009.041112 | 989.4245712 | 1047.808461 | 925.0615434 | 1063.589458 | 1067.661647 | 888.1942225 | 1052.698709 |
| **Tab2** | 2793.789965 | 2703.990235 | 2744.980337 | 2577.476946 | 2291.508218 | 2259.250647 | 2380.548782 | 2388.510937 | 2414.496919 | 2439.934297 | 2522.637154 | 2403.920383 | 2448.890322 | 2256.175965 | 2611.986218 |
| **Ust** | 193.2119576 | 200.4422882 | 214.5861947 | 244.9810752 | 198.8760587 | 213.9309057 | 260.6967026 | 146.6629523 | 141.453855 | 144.1938249 | 174.9860793 | 268.0661848 | 233.4711024 | 272.8735048 | 242.8751971 |
| **Stxbp5** | 934.0104112 | 1002.211441 | 1010.27807 | 1062.734805 | 1031.748725 | 939.1917468 | 946.2658858 | 1112.962289 | 1041.341146 | 974.4006953 | 1048.970605 | 1029.755862 | 1061.697057 | 1002.945663 | 1012.459032 |
| **Rab32** | 31.13368037 | 47.53572051 | 35.24225825 | 45.71829924 | 31.03479897 | 44.89041957 | 34.27845916 | 57.8271069 | 56.43105919 | 66.41654964 | 46.34766424 | 48.58157395 | 52.82922754 | 34.33507676 | 50.29959704 |
| **Shprh** | 566.8161221 | 587.8584103 | 631.2280034 | 564.1465605 | 530.1250355 | 506.4200457 | 627.8370415 | 647.8312121 | 527.4422999 | 585.5143192 | 576.0352556 | 642.8383268 | 549.5943833 | 493.3408398 | 571.2597092 |
| **Fbxo30** | 235.3339958 | 183.804786 | 184.8260655 | 195.8123383 | 155.8073581 | 126.254305 | 144.3303544 | 250.5841299 | 208.418712 | 179.1499036 | 171.2025965 | 163.9628121 | 167.0085258 | 171.6753838 | 122.8747299 |
| **Epm2a** | 6.409875371 | 1.584524017 | 0 | 1.725218839 | 3.800179466 | 0.701412806 | 4.510323574 | 0 | 3.762070613 | 0 | 3.783482795 | 1.735056213 | 0 | 2.710663955 | 6.467091048 |
| **Utrn** | 1013.676005 | 1198.692419 | 1018.892844 | 1104.140057 | 875.3080036 | 843.0981925 | 1165.467611 | 942.8332647 | 1014.254237 | 927.209989 | 922.2239313 | 1159.885078 | 1040.394949 | 940.6003923 | 1101.561175 |
| **B230208H11Rik** | 3.662785926 | 0 | 3.132645178 | 0 | 0 | 8.416953669 | 2.706194144 | 0 | 0 | 5.243411813 | 0 | 0.867528106 | 0 | 1.807109303 | 5.029959704 |
| **Stx11** | 1089.678813 | 945.1685761 | 883.4059402 | 823.7919958 | 812.6050424 | 915.3437115 | 1018.431063 | 689.7349127 | 755.4237791 | 706.1127909 | 664.0012305 | 906.5668711 | 874.2385074 | 813.1991864 | 960.0037377 |
| **Sf3b5** | 2005.375295 | 2158.913973 | 2148.211431 | 2146.172236 | 2033.096014 | 2093.717225 | 1931.320554 | 2215.029617 | 2213.602349 | 2209.224177 | 2167.935642 | 2072.524646 | 2033.073176 | 2170.338273 | 1983.95982 |
| **Plagl1** | 44.8691276 | 36.44405239 | 44.64019379 | 37.95481446 | 44.96879034 | 41.38335554 | 58.63420646 | 68.72206907 | 48.15450385 | 60.29923585 | 52.96875913 | 43.37640531 | 37.49170987 | 26.2030849 | 39.52111196 |
| **Ltv1** | 1256.335573 | 1140.857292 | 1201.369426 | 1256.821924 | 1132.453481 | 1154.525478 | 1194.333682 | 1407.964342 | 1263.303312 | 1352.800248 | 1414.076695 | 1237.962608 | 1091.520008 | 1178.235266 | 1142.519418 |
| **Phactr2** | 181.3079033 | 217.0797903 | 177.7776138 | 222.5532303 | 227.3774047 | 208.3196033 | 216.4955315 | 286.6213125 | 246.7918322 | 211.4842765 | 206.1998123 | 241.1728135 | 201.0918984 | 198.7820233 | 194.7312971 |
| **Fuca2** | 581.4672658 | 537.9459038 | 539.5981319 | 536.543059 | 503.5237792 | 474.8564695 | 478.0942988 | 457.5884111 | 471.0112407 | 505.115338 | 462.5307717 | 484.9482114 | 442.2317596 | 536.711463 | 532.4571629 |
| **Pex3** | 256.3950148 | 298.6827772 | 291.3360015 | 302.7759063 | 262.2123831 | 245.494482 | 234.5368258 | 320.144273 | 298.7084067 | 277.9008261 | 325.3795204 | 249.8480946 | 255.6252945 | 253.8988571 | 298.9233195 |
| **Adat2** | 208.7787978 | 225.7946724 | 263.1421949 | 184.5984158 | 199.5094219 | 225.1535106 | 246.2636671 | 218.7373174 | 234.7532062 | 232.4579237 | 250.6557352 | 176.9757337 | 185.7543807 | 180.7109303 | 194.0127314 |
| **Aig1** | 100.726613 | 72.88810478 | 97.89516181 | 99.20008326 | 90.57094393 | 78.55823424 | 97.4229892 | 112.3019177 | 56.43105919 | 104.8682363 | 71.88617311 | 108.4410133 | 66.46257658 | 79.51280934 | 74.01226421 |
| **Hivep2** | 787.4989741 | 795.4310565 | 700.1461973 | 716.8284277 | 684.0323038 | 736.483446 | 733.3786131 | 617.6605476 | 575.5968038 | 498.9980242 | 607.2489886 | 740.0014747 | 677.4070306 | 702.0619643 | 691.9787421 |
| **Vta1** | 678.5310928 | 724.1274758 | 636.7101324 | 678.0110038 | 677.6986714 | 669.8492295 | 694.5898304 | 730.8005394 | 779.501031 | 733.2037519 | 666.8388426 | 640.2357424 | 714.0466561 | 708.3868468 | 655.3318928 |
| **Cited2** | 731.6414888 | 750.2721221 | 781.5949719 | 807.4024168 | 735.3347266 | 817.8473315 | 820.8788905 | 633.5839539 | 732.8513554 | 688.6347515 | 676.2975496 | 787.7155205 | 792.4384131 | 887.2906678 | 742.9969048 |
| **Txlnb** | 6.409875371 | 3.961310043 | 6.265290356 | 10.35131304 | 8.86708542 | 4.208476834 | 6.314453003 | 5.866518091 | 12.79104008 | 0.873901969 | 2.837612096 | 5.205168638 | 5.112505891 | 3.614218606 | 9.341353736 |
| **Heca** | 662.0485562 | 643.3167509 | 707.9778102 | 609.0022503 | 623.8627956 | 657.223799 | 773.9715253 | 543.0719605 | 576.3492179 | 526.9628872 | 542.9297811 | 751.27934 | 757.5029562 | 712.0010654 | 708.5057526 |
| **Abracl** | 1158.356049 | 1072.72276 | 1178.657748 | 1092.063525 | 1250.892407 | 1277.974132 | 1174.488259 | 1028.316814 | 1160.974991 | 1169.280834 | 1177.60902 | 1252.710585 | 1112.822116 | 1298.408034 | 1212.938854 |
| **Reps1** | 1259.998359 | 1205.030515 | 1207.634716 | 1108.453104 | 1105.218861 | 1164.345257 | 1127.580893 | 1048.43059 | 1085.733579 | 1089.755755 | 1212.606236 | 1143.402044 | 1158.834669 | 1160.164173 | 1199.286107 |
| **Ect2l** | 3.662785926 | 0 | 1.566322589 | 1.725218839 | 3.800179466 | 5.611302446 | 1.80412943 | 0.838074013 | 0 | 7.86511772 | 4.729353494 | 6.94022485 | 15.33751767 | 13.55331977 | 0 |
| **Ccdc28a** | 54.02609241 | 76.05715282 | 81.44877463 | 50.03134634 | 60.80287145 | 86.2737751 | 69.45898304 | 79.61703124 | 65.46002866 | 83.89458901 | 57.69811262 | 62.46202365 | 67.3146609 | 74.09148143 | 97.00636572 |
| **Nhsl1** | 4.578482408 | 4.753572051 | 2.349483883 | 1.725218839 | 5.700269198 | 1.402825611 | 0.902064715 | 0.838074013 | 4.514484735 | 6.117313782 | 7.56696559 | 6.072696744 | 9.372927467 | 3.614218606 | 5.029959704 |
| **Arfgef3** | 123.619025 | 152.1143056 | 132.3542588 | 134.5670695 | 74.73686282 | 70.84269338 | 94.71679505 | 178.5097648 | 79.75589699 | 115.3550599 | 132.4218978 | 65.93213608 | 100.5459492 | 55.11683375 | 68.26373884 |
| **Perp** | 406.5692378 | 324.8274235 | 350.8562599 | 345.9063773 | 335.6825195 | 408.9236657 | 396.0064098 | 365.4002697 | 343.853254 | 317.2264147 | 305.5162357 | 406.8706818 | 433.7109164 | 335.2187757 | 417.4866554 |
| **Tnfaip3** | 1548.44275 | 1330.207912 | 1317.277297 | 1206.790578 | 1403.532949 | 1502.42623 | 1745.495223 | 813.7698667 | 786.2727581 | 797.8724976 | 873.9845257 | 1368.959352 | 1618.960199 | 1326.418229 | 1460.125445 |
| **Gm20139** | 1.831392963 | 0 | 0 | 3.450437679 | 0.633363244 | 1.402825611 | 0.902064715 | 0 | 0 | 0 | 0 | 0 | 0.852084315 | 0.903554652 | 0 |
| **Ifngr1** | 2407.36605 | 2797.477152 | 2672.929498 | 2706.005749 | 1862.087938 | 1725.475502 | 1818.562465 | 1308.233534 | 1627.471747 | 1387.756327 | 1403.672117 | 2018.737903 | 1964.054346 | 1988.723788 | 1971.744204 |
| **Slc35d3** | 83.32837982 | 39.61310043 | 49.33916155 | 26.74089201 | 38.6351579 | 70.84269338 | 79.3816949 | 9.218814144 | 25.58208017 | 30.58656891 | 8.512836289 | 45.11146153 | 37.49170987 | 28.0101942 | 41.67680897 |
| **Pex7** | 333.3135193 | 361.2714759 | 309.3487113 | 341.5933302 | 340.7494254 | 307.9202217 | 331.0577503 | 331.0392352 | 297.9559925 | 404.6166116 | 324.4336497 | 317.5152869 | 334.8691359 | 318.954792 | 324.7916837 |
| **Map3k5** | 604.3596778 | 639.3554409 | 644.5417454 | 671.1101285 | 519.9912236 | 451.7098469 | 571.9090292 | 709.8486891 | 708.7741035 | 582.8926133 | 600.6278937 | 500.5637173 | 455.8651086 | 481.5946293 | 449.103545 |
| **Map7** | 321.409465 | 369.986358 | 342.2414857 | 301.0506875 | 323.0152546 | 305.8159833 | 349.0990446 | 337.7438273 | 271.6214983 | 270.9096104 | 307.4079771 | 287.1518032 | 299.9336789 | 324.3761199 | 275.929218 |
| **Gm6251** | 17.39823315 | 10.29940611 | 7.04845165 | 4.313047098 | 18.36753408 | 14.72966892 | 9.020647148 | 3.352296052 | 9.028969471 | 4.369509845 | 6.621094891 | 10.41033728 | 10.22501178 | 18.97464768 | 9.341353736 |
| **Bclaf1** | 4422.814006 | 4323.37378 | 4442.090862 | 3992.156394 | 3941.419469 | 3818.491314 | 4246.018612 | 4959.722009 | 4858.33799 | 4941.915634 | 5194.721878 | 4405.307724 | 4347.334176 | 4233.153543 | 4046.961865 |
| **Mtfr2** | 276.5403374 | 374.73993 | 286.6370338 | 321.7533135 | 310.9813529 | 277.0580583 | 329.2536209 | 352.8291595 | 418.3422522 | 335.5783561 | 332.946486 | 346.1437144 | 329.75663 | 416.5386944 | 319.0431584 |
| **Ahi1** | 307.6740178 | 286.0065851 | 288.2033564 | 229.4541056 | 226.7440415 | 195.6941728 | 211.985208 | 353.6672335 | 349.872567 | 355.6781013 | 370.7813139 | 226.4248357 | 237.7315239 | 209.6246792 | 171.0186299 |
| **Myb** | 1040.231203 | 1212.160873 | 1195.104135 | 1175.736639 | 1168.555186 | 1047.910732 | 1174.488259 | 951.2140048 | 1103.791518 | 982.265813 | 949.6541816 | 1175.500584 | 1222.740992 | 1147.514407 | 1056.291538 |
| **Hbs1l** | 1278.312288 | 1269.996 | 1214.683168 | 1249.05844 | 1250.892407 | 1234.486538 | 1196.137812 | 1316.614275 | 1276.094352 | 1355.421954 | 1269.358478 | 1227.55227 | 1203.143053 | 1187.270812 | 1220.124511 |
| **4930455C13Rik** | 0.915696482 | 0 | 1.566322589 | 0 | 0 | 0.701412806 | 1.80412943 | 0.838074013 | 4.514484735 | 0.873901969 | 0 | 0 | 0 | 0.903554652 | 0 |
| **Aldh8a1** | 0 | 0 | 2.349483883 | 0 | 0 | 0 | 0.902064715 | 7.542666117 | 0 | 0 | 0 | 1.735056213 | 0 | 2.710663955 | 2.155697016 |
| **Sgk1** | 107.1364883 | 105.3708471 | 130.0047749 | 119.0400999 | 230.5442209 | 181.6659167 | 218.299661 | 128.225324 | 85.77520997 | 131.9591973 | 121.0714494 | 226.4248357 | 216.4294161 | 283.7161606 | 293.8933598 |
| **E030030I06Rik** | 1.831392963 | 7.922620085 | 6.265290356 | 7.763484777 | 15.20071786 | 10.52119209 | 5.412388289 | 12.5711102 | 5.266898858 | 8.739019689 | 5.675224193 | 7.807752957 | 11.0770961 | 14.45687443 | 8.622788064 |
| **H60b** | 1.831392963 | 7.922620085 | 2.349483883 | 0.86260942 | 1.900089733 | 1.402825611 | 5.412388289 | 1.676148026 | 0.752414123 | 0.873901969 | 4.729353494 | 0.867528106 | 6.816674521 | 9.939101167 | 2.155697016 |
| **C920009B18Rik** | 130.9445969 | 138.6458515 | 142.5353556 | 127.6661941 | 129.8394651 | 123.4486538 | 163.2737134 | 93.02621545 | 74.48899814 | 98.75092249 | 102.1540355 | 134.4668565 | 167.8606101 | 135.5331977 | 151.6173568 |
| **Gm10825** | 2.747089445 | 3.169048034 | 4.698967767 | 0 | 0 | 1.402825611 | 1.80412943 | 0 | 0 | 0 | 0 | 0.867528106 | 10.22501178 | 0 | 3.59282836 |
| **Tbpl1** | 623.5893039 | 511.0089955 | 569.3582611 | 504.6265105 | 481.3560656 | 539.3864476 | 543.945023 | 524.6343322 | 498.0981491 | 554.0538483 | 493.7445048 | 510.9740546 | 478.0193008 | 469.8484188 | 498.6845763 |
| **Rps12** | 15844.29622 | 16513.90931 | 15719.6135 | 15974.66384 | 16767.02517 | 16863.36667 | 17266.42071 | 18069.7138 | 17067.00954 | 17422.10965 | 16989.72949 | 16794.47661 | 15870.07037 | 17195.54857 | 16647.72949 |
| **Snora33** | 0 | 0 | 2.349483883 | 2.587828259 | 0 | 0.701412806 | 1.80412943 | 0 | 0 | 4.369509845 | 0 | 0 | 0 | 0 | 0.718565672 |
| **Snord100** | 0 | 0 | 0 | 0 | 0.633363244 | 0 | 0.902064715 | 0 | 0 | 0 | 1.891741398 | 3.470112425 | 2.556252945 | 3.614218606 | 0 |
| **Stx7** | 283.8659093 | 267.7845589 | 247.4789691 | 189.7740723 | 231.1775842 | 271.4467558 | 285.0524499 | 156.7198404 | 196.380086 | 216.7276883 | 227.9548384 | 258.5233757 | 234.3231867 | 245.7668652 | 254.3722479 |
| **Enpp1** | 96.14813056 | 85.56429692 | 83.79825851 | 70.73397241 | 65.8697774 | 81.36388546 | 87.50027733 | 37.71333059 | 55.67864507 | 49.81241223 | 50.13114703 | 56.38932691 | 67.3146609 | 37.04574071 | 42.39537465 |
| **Med23** | 738.9670606 | 726.5042618 | 739.304262 | 734.9432255 | 656.1643211 | 639.6884788 | 714.4352541 | 807.9033486 | 786.2727581 | 788.259576 | 713.1865069 | 628.9578771 | 633.9507305 | 753.5645794 | 692.6973078 |
| **Arg1** | 0 | 0 | 4.698967767 | 0 | 1.900089733 | 0 | 0 | 2.514222039 | 0.752414123 | 3.495607876 | 0 | 0.867528106 | 0 | 0 | 0.718565672 |
| **Akap7** | 3.662785926 | 29.31369431 | 12.53058071 | 21.56523549 | 6.333632443 | 16.83390734 | 17.13922958 | 25.14222039 | 7.524141226 | 15.73023544 | 13.24218978 | 14.74797781 | 15.33751767 | 9.035546516 | 10.05991941 |
| **Epb4.1l2** | 1281.975074 | 1354.768035 | 1273.420265 | 1285.288035 | 1344.630168 | 1367.053558 | 1477.582003 | 1417.183156 | 1161.727405 | 1184.137168 | 1271.250219 | 1339.463396 | 1450.247504 | 1356.235532 | 1364.556211 |
| **Samd3** | 23.80810852 | 34.06726637 | 32.10961307 | 44.85568982 | 109.5718413 | 87.67660071 | 82.08788905 | 1.676148026 | 5.266898858 | 12.23462756 | 7.56696559 | 78.94505767 | 94.58135898 | 93.96968376 | 78.32365824 |
| **L3mbtl3** | 341.5547876 | 298.6827772 | 325.0119372 | 264.8210918 | 325.5487076 | 351.4078157 | 410.4394452 | 235.4987977 | 303.2228914 | 262.1705907 | 280.9235975 | 392.9902321 | 396.2192066 | 345.1578769 | 390.8997255 |
| **Arhgap18** | 325.072251 | 315.3202794 | 323.4456146 | 328.6541889 | 367.3506817 | 364.734659 | 371.6506625 | 241.3653158 | 246.7918322 | 270.0357084 | 210.9291658 | 314.0451745 | 405.592134 | 402.9853746 | 356.4085733 |
| **Ptprk** | 74.17141501 | 41.19762444 | 66.56871003 | 67.28353473 | 71.5700466 | 62.42573971 | 64.94865946 | 79.61703124 | 51.16416034 | 47.19070632 | 65.26507822 | 89.35539495 | 103.1022021 | 55.11683375 | 63.23377913 |
| **Themis** | 3607.844137 | 3161.125414 | 3177.285372 | 3077.790409 | 2376.378893 | 2662.56301 | 2917.277288 | 2127.869919 | 2440.079 | 2620.832005 | 2638.97925 | 2718.833085 | 2703.663532 | 2427.851349 | 2634.261753 |
| **Echdc1** | 98.89522001 | 86.35655893 | 94.76251663 | 113.001834 | 84.87067473 | 95.39214158 | 97.4229892 | 108.1115477 | 127.1579867 | 126.7157855 | 104.9916476 | 118.8513506 | 116.7355512 | 114.7514407 | 100.5991941 |
| **Rnf146** | 380.9297363 | 380.2857641 | 379.8332278 | 420.0907874 | 362.917139 | 431.3688755 | 380.6713096 | 431.6081167 | 437.9050193 | 432.5814746 | 424.6959437 | 435.4991094 | 430.3025792 | 448.1631072 | 389.4625942 |
| **Cenpw** | 427.6302569 | 391.3774322 | 388.4480021 | 347.6315961 | 396.4853909 | 408.9236657 | 396.9084745 | 460.1026332 | 392.760172 | 415.1034352 | 422.8042024 | 415.5459629 | 367.2483398 | 490.6301758 | 342.7558255 |
| **Trmt11** | 305.8426248 | 358.1024278 | 388.4480021 | 380.4107541 | 320.4818016 | 317.0385882 | 312.1143913 | 461.7787812 | 417.589838 | 445.6900041 | 432.2629093 | 311.4425902 | 339.1295574 | 367.7467432 | 303.2347136 |
| **Gm20300** | 29.30228741 | 41.98988645 | 22.71167754 | 19.84001665 | 37.36843141 | 36.4734659 | 37.88671802 | 30.17066447 | 32.35380727 | 27.09096104 | 18.91741398 | 33.83359614 | 46.01255302 | 44.27417793 | 48.14390002 |
| **Hint3** | 187.7177787 | 194.8964541 | 203.6219366 | 200.9879948 | 162.1409905 | 203.4097137 | 171.3922958 | 164.2625066 | 145.2159257 | 135.4548052 | 136.2053806 | 159.6251716 | 199.3877297 | 189.7464768 | 207.6654792 |
| **Ncoa7** | 829.6210123 | 852.4739212 | 738.5211007 | 748.7449763 | 815.1384954 | 907.6281706 | 975.1319567 | 587.4898832 | 618.4844088 | 616.97479 | 603.4655058 | 779.9077675 | 748.982113 | 730.0721585 | 839.2847049 |
| **Hddc2** | 367.1942891 | 403.2613623 | 382.965873 | 365.7463939 | 386.9849423 | 352.8106413 | 388.7898921 | 335.2296052 | 289.6794372 | 437.8248864 | 349.0262878 | 432.0289969 | 404.7400497 | 414.7315851 | 396.6482509 |
| **Gm3258** | 10.0726613 | 2.376786026 | 4.698967767 | 3.450437679 | 6.333632443 | 11.22260489 | 2.706194144 | 0.838074013 | 6.019312981 | 13.10852953 | 9.458706988 | 6.94022485 | 5.964590206 | 7.228437213 | 7.18565672 |
| **Zufsp** | 299.4327495 | 292.3446811 | 231.8157432 | 185.4610252 | 255.8787507 | 290.3849016 | 275.129738 | 201.9758371 | 221.209752 | 235.0796296 | 162.6897602 | 270.6687692 | 218.985669 | 293.6552618 | 251.4979852 |
| **A830082N09Rik** | 10.98835778 | 3.961310043 | 8.614774239 | 7.763484777 | 12.66726489 | 6.312715251 | 16.23716487 | 10.89496217 | 10.53379772 | 10.48682363 | 13.24218978 | 13.8804497 | 6.816674521 | 4.517773258 | 4.311394032 |
| **Rwdd1** | 2031.014796 | 1893.5062 | 2120.017624 | 2009.879948 | 1793.051345 | 1887.50186 | 1883.511124 | 2186.5351 | 2268.52858 | 2300.109982 | 2150.909969 | 1839.159585 | 1730.583244 | 1881.200785 | 1738.21036 |
| **Trappc3l** | 0 | 1.584524017 | 4.698967767 | 0.86260942 | 0.633363244 | 0.701412806 | 0 | 3.352296052 | 0 | 4.369509845 | 0 | 0 | 5.112505891 | 3.614218606 | 0.718565672 |
| **Fam26e** | 6.409875371 | 0.792262009 | 10.96425812 | 10.35131304 | 5.066905954 | 2.104238417 | 3.608258859 | 15.08533223 | 3.762070613 | 12.23462756 | 5.675224193 | 10.41033728 | 0.852084315 | 0 | 2.874262688 |
| **Fam26f** | 338.8076982 | 429.4060086 | 372.0016149 | 439.0681946 | 363.5505022 | 417.3406194 | 388.7898921 | 277.4024983 | 322.0332445 | 290.1354537 | 307.4079771 | 365.2293327 | 362.9879183 | 403.8889293 | 312.5760673 |
| **Dse** | 498.138886 | 542.6994758 | 545.8634223 | 546.0317626 | 521.8913133 | 486.7804872 | 513.2748227 | 502.8444078 | 528.9471282 | 450.933416 | 505.0949531 | 536.9998978 | 557.2631421 | 615.3207177 | 561.1997898 |
| **Tspyl1** | 1218.792017 | 1250.981711 | 1295.348781 | 1375.862024 | 1300.928104 | 1300.419342 | 1388.277596 | 1230.292651 | 1173.013617 | 1109.855501 | 1268.412607 | 1339.463396 | 1280.682726 | 1260.458739 | 1307.789523 |
| **Tspyl4** | 208.7787978 | 209.9494323 | 180.1270977 | 182.873197 | 184.9420673 | 190.7842832 | 204.7686903 | 231.3084276 | 212.1807826 | 165.1674721 | 161.7438895 | 221.2196671 | 184.9022964 | 208.7211245 | 201.9169538 |
| **Nt5dc1** | 481.6563493 | 505.4631614 | 545.080261 | 536.543059 | 589.0278172 | 564.6373086 | 587.2441293 | 564.0238108 | 618.4844088 | 588.1360251 | 480.502315 | 547.4102351 | 587.0860931 | 546.6505642 | 606.4694271 |
| **Col10a1** | 0.915696482 | 5.54583406 | 0 | 1.725218839 | 1.266726489 | 0.701412806 | 0.902064715 | 1.676148026 | 0 | 0 | 3.783482795 | 4.337640531 | 1.70416863 | 3.614218606 | 2.155697016 |
| **Hdac2** | 1582.32352 | 1411.810899 | 1542.044589 | 1575.98741 | 1569.474119 | 1616.756517 | 1601.164869 | 1678.662248 | 1676.378665 | 1560.788916 | 1673.245266 | 1528.584523 | 1526.935093 | 1605.616616 | 1570.065993 |
| **Marcks** | 39.37494871 | 34.85952837 | 75.18348427 | 56.9322217 | 41.16861088 | 49.80030921 | 40.59291217 | 217.8992434 | 133.1772997 | 171.2847859 | 114.4503546 | 62.46202365 | 46.86463733 | 30.72085815 | 40.9582433 |
| **Fam229b** | 11.90405426 | 8.714882094 | 6.265290356 | 21.56523549 | 12.03390164 | 10.52119209 | 13.53097072 | 15.92340625 | 12.03862596 | 20.97364725 | 16.07980188 | 12.14539349 | 11.0770961 | 14.45687443 | 10.77848508 |
| **Tube1** | 259.1421043 | 276.499441 | 236.5147109 | 263.095873 | 291.9804556 | 260.2241509 | 243.557473 | 289.1355345 | 275.3835689 | 242.0708454 | 251.6016059 | 278.4765221 | 248.80862 | 223.1779989 | 278.8034807 |
| **Wisp3** | 9.156964816 | 2.376786026 | 6.265290356 | 0.86260942 | 3.166816221 | 1.402825611 | 1.80412943 | 19.2757023 | 0 | 6.991215751 | 5.675224193 | 5.205168638 | 10.22501178 | 4.517773258 | 3.59282836 |
| **Fyn** | 4107.814416 | 4431.121414 | 4450.705637 | 4504.546389 | 4838.261823 | 4738.744915 | 4767.412018 | 4455.201453 | 4649.166863 | 4076.752685 | 4099.403608 | 4645.613009 | 4594.438627 | 4678.605986 | 4857.503942 |
| **Traf3ip2** | 619.926518 | 624.3024627 | 621.8300678 | 573.6352641 | 647.9305989 | 575.1585007 | 638.6618181 | 496.9778897 | 522.9278152 | 508.6109459 | 509.8243066 | 627.2228208 | 590.4944304 | 589.1176328 | 609.3436898 |
| **E130307A14Rik** | 30.21798389 | 33.27500436 | 49.33916155 | 25.01567317 | 48.76896981 | 40.68194273 | 55.0259476 | 31.00873848 | 30.84897903 | 39.3255886 | 38.78069865 | 53.78674259 | 46.86463733 | 45.17773258 | 48.86246569 |
| **Rev3l** | 940.4202866 | 943.5840521 | 855.2121336 | 990.2756138 | 753.7022607 | 753.3173533 | 811.8582433 | 1328.347311 | 1151.946022 | 1084.512343 | 1104.776976 | 830.2243977 | 806.0717621 | 766.2143445 | 717.1285406 |
| **AA474331** | 20.14532259 | 26.14464628 | 28.9769679 | 37.09220505 | 38.00179466 | 42.08476834 | 29.76813559 | 36.87525657 | 40.63036262 | 34.95607876 | 35.94308655 | 29.49595561 | 37.49170987 | 20.78175699 | 22.9941015 |
| **Slc16a10** | 137.3544722 | 141.0226375 | 146.4511621 | 120.7653188 | 187.4755203 | 149.4009276 | 181.3150077 | 281.5928684 | 224.2194085 | 181.7716095 | 205.2539416 | 191.7237115 | 176.3814532 | 196.974914 | 198.3241255 |
| **Rpf2** | 1270.986716 | 1161.456104 | 1271.070781 | 1140.369653 | 1179.955724 | 1304.627819 | 1233.122465 | 1386.174418 | 1326.506098 | 1376.395601 | 1403.672117 | 1139.064404 | 1061.697057 | 1183.656594 | 1092.219821 |
| **Gtf3c6** | 485.3191352 | 430.9905326 | 468.3304541 | 468.3969149 | 417.386378 | 396.2982352 | 428.4807395 | 478.5402615 | 513.1464316 | 502.4936321 | 518.3371429 | 458.9223682 | 425.1900733 | 425.5742409 | 426.8280091 |
| **Cdk19** | 722.484524 | 818.4066548 | 732.2558103 | 746.157148 | 895.5756274 | 714.739649 | 875.0027733 | 636.0981759 | 767.462405 | 674.65232 | 614.8159542 | 849.310016 | 775.3967268 | 860.1840283 | 864.4345034 |
| **Cdc40** | 1156.524656 | 1171.755511 | 1134.800716 | 1111.040933 | 1122.953032 | 1228.875236 | 1322.426872 | 1107.933845 | 1105.296346 | 1145.685481 | 1186.121856 | 1155.547438 | 1188.65762 | 1187.270812 | 1273.298371 |

| | | | | | | | | | | | | | | | | |
|---|---|---|---|---|---|---|---|---|---|---|---|---|---|---|---|---|
| **Wasf1** | 0 | 0.792262009 | 0 | 0 | 0.633363244 | 2.104238417 | 1.80412943 | 2.514222039 | 3.00965649 | 0 | 0 | 1.735056213 | 0 | 0 | 2.155697016 |
| **Fig4** | 656.5543773 | 663.9155631 | 653.1565196 | 683.1866604 | 682.7655773 | 605.3192513 | 637.7597533 | 729.1243914 | 610.9602675 | 726.2125362 | 681.9727738 | 676.6719229 | 606.6840324 | 583.6963049 | 617.9664779 |
| **Zbtb24** | 280.2031234 | 316.1125414 | 274.8896144 | 285.5237179 | 256.5121139 | 232.1676387 | 272.4235439 | 279.0786463 | 249.0490746 | 255.1793749 | 265.7896664 | 255.9207914 | 282.8919926 | 252.9953024 | 278.8034807 |
| **Mical1** | 1787.439532 | 1859.438934 | 1708.857945 | 1771.799748 | 1947.591976 | 1852.43122 | 2036.862126 | 1395.393232 | 1443.882701 | 1371.152189 | 1438.669333 | 1916.369587 | 2025.404417 | 1942.642501 | 2039.289377 |
| **Smpd2** | 115.3777567 | 95.07144102 | 83.01509722 | 81.94789487 | 100.7047558 | 86.97518791 | 94.71679505 | 97.21658551 | 99.31866418 | 92.6336087 | 84.18249219 | 111.0435976 | 119.2918041 | 159.0256187 | 93.41353736 |
| **Cd164** | 4184.732921 | 4128.477326 | 4149.971699 | 4177.617419 | 4057.324943 | 4012.081249 | 4344.343666 | 3866.035422 | 3914.81068 | 4056.65294 | 4158.993462 | 4153.724573 | 4025.246305 | 4249.417526 | 4248.878818 |
| **Ccdc162** | 2.747089445 | 1.584524017 | 2.349483883 | 0.86260942 | 1.266726489 | 3.507064029 | 5.412388289 | 3.352296052 | 6.019312981 | 1.747803938 | 2.837612096 | 2.602584319 | 10.22501178 | 5.421327909 | 2.874262688 |
| **Cep57l1** | 215.1886732 | 208.3649082 | 225.5504528 | 180.2853687 | 217.2435928 | 220.9450338 | 230.0265023 | 217.0611694 | 211.4283684 | 184.3933154 | 188.2282691 | 188.2535991 | 210.4648258 | 241.249092 | 251.4979852 |
| **Sesn1** | 396.4965765 | 568.8441221 | 462.0651637 | 502.0386822 | 502.2570527 | 486.0790744 | 506.058305 | 451.721893 | 512.3940175 | 489.3851026 | 470.0977373 | 520.5168638 | 525.7360225 | 566.5287665 | 484.3132629 |
| **Armc2** | 9.156964816 | 2.376786026 | 2.349483883 | 0 | 1.900089733 | 0.701412806 | 2.706194144 | 2.514222039 | 0.752414123 | 0.873901969 | 0 | 8.675281063 | 0.852084315 | 0.903554652 | 2.155697016 |
| **Foxo3** | 423.967471 | 508.6322095 | 437.0040023 | 391.6246765 | 377.4844936 | 393.492584 | 497.9397226 | 434.1223388 | 504.8698763 | 435.2031805 | 398.2115642 | 444.1743904 | 474.6109635 | 356.0005327 | 490.780354 |
| **Lace1** | 47.61621704 | 57.04286461 | 40.72438731 | 55.20700286 | 37.36843141 | 48.39748359 | 52.31975346 | 35.19910855 | 54.17381683 | 50.6863142 | 70.94030241 | 29.49595561 | 50.27297459 | 34.33507676 | 35.9282836 |
| **Snx3** | 3019.966996 | 2742.018811 | 2824.079628 | 2762.937971 | 2883.702851 | 3050.444292 | 2876.684375 | 2708.65521 | 2705.681185 | 2660.157593 | 2760.050699 | 2829.009155 | 2788.871964 | 2875.110901 | 2880.011213 |
| **Ostm1** | 566.8161221 | 614.0030566 | 552.9118739 | 547.7569815 | 528.858309 | 604.6178385 | 571.9090292 | 512.063222 | 526.6898858 | 646.687457 | 578.8728676 | 571.701022 | 540.2214558 | 554.7825561 | 605.0322958 |
| **Sec63** | 1796.596497 | 1685.141292 | 1724.52117 | 1668.286618 | 1719.581208 | 1679.182257 | 1863.665701 | 1631.730103 | 1508.590316 | 1549.428191 | 1753.644276 | 1759.347 | 1828.57294 | 1630.012591 | 1697.252117 |
| **Scml4** | 150.174223 | 156.8678777 | 209.8872269 | 198.4001665 | 235.6111269 | 234.9732899 | 223.7120493 | 120.6826579 | 124.1483302 | 112.733354 | 107.8292597 | 245.5104541 | 210.4648258 | 271.9699501 | 214.1325702 |
| **Sobp** | 12.81975074 | 9.507144102 | 6.265290356 | 1.725218839 | 0 | 8.416953669 | 4.510323574 | 9.218814144 | 6.019312981 | 4.369509845 | 7.56696559 | 0.867528106 | 1.70416863 | 5.421327909 | 7.904222392 |
| **Pdss2** | 187.7177787 | 151.3220436 | 169.9460009 | 144.0557731 | 135.5397343 | 136.7754971 | 135.3097072 | 216.2230954 | 170.0455917 | 185.2672174 | 166.473243 | 123.1889911 | 129.5168159 | 161.7362826 | 162.3958419 |
| **Bend3** | 634.5776617 | 625.0947247 | 629.6616808 | 633.155314 | 525.0581295 | 589.1867568 | 640.4659475 | 698.9537269 | 659.1147714 | 677.2740259 | 616.7076956 | 515.3116951 | 560.6714794 | 617.127827 | 587.068154 |
| **1700021F05Rik** | 145.5957406 | 134.6845414 | 118.2573555 | 138.8801166 | 144.4068197 | 152.9079916 | 138.9179661 | 149.1771743 | 134.6821279 | 119.7245697 | 112.5586132 | 147.4797781 | 117.5876355 | 126.4976512 | 121.4375986 |
| **Cd24a** | 302.1798389 | 336.7113536 | 360.2541955 | 326.92897 | 257.7788404 | 190.7842832 | 198.4542373 | 560.6715147 | 551.5195519 | 431.7075726 | 335.7840981 | 204.7366331 | 178.9377062 | 247.5739745 | 211.2583076 |
| **Qrsl1** | 255.4793184 | 294.7214672 | 318.7466469 | 260.5080447 | 288.1802761 | 291.0863144 | 291.3669029 | 336.9057532 | 328.0525574 | 277.9008261 | 387.8069865 | 297.5621405 | 287.1524142 | 286.4268245 | 233.5338434 |
| **Rtn4ip1** | 184.9706893 | 225.0024104 | 195.0071623 | 193.22451 | 164.0410803 | 216.0351442 | 228.2223728 | 212.0327253 | 200.8945707 | 202.7452568 | 249.7098645 | 173.5056213 | 209.6127415 | 165.3505012 | 178.2042866 |
| **Aim1** | 2267.264488 | 1799.227021 | 1833.38059 | 1657.072695 | 1955.825698 | 2049.528218 | 2207.352357 | 1121.343029 | 1252.0171 | 1275.022973 | 1471.774807 | 1849.569923 | 2014.327321 | 1963.424258 | 2137.732874 |
| **Atg5** | 584.2143552 | 623.5102007 | 644.5417454 | 641.7814082 | 651.7307784 | 675.4605319 | 723.4559013 | 666.2688404 | 649.3333878 | 643.1918491 | 728.3204381 | 602.0645058 | 701.2653914 | 725.5543852 | 690.5416108 |
| **Prdm1** | 59.5202713 | 69.71905675 | 29.76012919 | 68.14614415 | 79.80376878 | 90.48225194 | 97.4229892 | 6.704592104 | 8.276555348 | 42.82119648 | 56.75224193 | 78.07752957 | 87.76468446 | 102.1016756 | 91.25784034 |
| **Prep** | 2698.557531 | 2496.417589 | 2511.598271 | 2498.116879 | 2259.206692 | 2409.352988 | 2412.121047 | 2451.366488 | 2439.326585 | 2413.717238 | 2476.289489 | 2348.398584 | 2233.31299 | 2260.693738 | 2431.626234 |
| **Lin28b** | 0 | 0 | 3.132645178 | 3.450437679 | 0 | 0.701412806 | 1.80412943 | 0 | 0 | 0 | 2.837612096 | 0 | 0 | 0 | 0 |
| **Hace1** | 326.9036439 | 241.6399126 | 328.1445824 | 234.6297621 | 326.815434 | 302.3089193 | 293.1710323 | 269.8598322 | 245.287004 | 285.7659438 | 256.3309594 | 331.3957366 | 295.6732574 | 313.5334641 | 314.0131986 |
| **Ascc3** | 1389.111563 | 1182.054917 | 1148.114458 | 1157.621841 | 1133.720207 | 1113.843535 | 1301.679383 | 1211.855023 | 1075.952195 | 1063.538696 | 1207.876882 | 1088.747773 | 1116.230453 | 1018.306092 | 1261.80132 |
| **Lilr4b** | 151.0899195 | 83.9797729 | 129.2216136 | 69.00875357 | 64.60305092 | 50.50172201 | 83.89201847 | 46.93214473 | 69.22209928 | 66.41654964 | 82.29075079 | 57.25685501 | 68.16674521 | 55.11683375 | 65.38947615 |
| **Lilrb4a** | 409.3163273 | 245.6012226 | 272.5401305 | 278.6228425 | 157.7074478 | 166.234835 | 243.557473 | 212.8707993 | 166.2835211 | 226.3406099 | 204.3080709 | 161.3602278 | 184.0502121 | 206.9140152 | 228.5038837 |
| **Dcbld1** | 13.73544722 | 11.09166812 | 13.31374201 | 11.21392246 | 28.50134599 | 14.02825611 | 25.25781201 | 11.73303618 | 7.524141226 | 11.3607256 | 15.13393118 | 15.61550591 | 11.92918041 | 7.228437213 | 18.68270747 |
| **Gopc** | 701.4235049 | 728.0887858 | 738.5211007 | 714.2405995 | 721.4007352 | 709.1283466 | 781.188043 | 722.4197993 | 662.876842 | 621.3442999 | 635.6251096 | 773.8350708 | 718.3070777 | 745.4325875 | 736.5298138 |
| **Nus1** | 2324.03767 | 2300.728873 | 2383.159819 | 2331.633261 | 2214.237902 | 2438.110913 | 2239.826687 | 2335.712274 | 2293.358246 | 2481.00769 | 2451.696851 | 2565.28061 | 2535.802922 | 2349.242094 | 2264.918998 |
| **Zfa-ps** | 0 | 0 | 0 | 0 | 0 | 3.507064029 | 0.902064715 | 0.838074013 | 0 | 0 | 0.945870699 | 0 | 0.852084315 | 0 | 1.437131344 |
| **Cep85l** | 77.83420093 | 61.79643666 | 55.60445191 | 41.40525214 | 72.83677309 | 49.0988964 | 75.77343604 | 37.71333059 | 57.18347332 | 45.44290238 | 52.02288843 | 61.59449555 | 80.09592563 | 44.27417793 | 61.07808212 |
| **Gm20597** | 0 | 0 | 0 | 0 | 1.266726489 | 2.104238417 | 0 | 1.676148026 | 2.257242368 | 0 | 5.675224193 | 0.867528106 | 0 | 0 | 0 |
| **Mcm9** | 247.23805 | 273.3303929 | 255.310582 | 258.7828259 | 241.3113961 | 236.3761155 | 221.0058551 | 273.2121283 | 275.3835689 | 210.6103745 | 216.60439 | 264.5960724 | 270.9628122 | 245.7668652 | 228.5038837 |
| **Asf1a** | 803.9815108 | 796.2233185 | 866.959553 | 772.89804 | 780.9368802 | 838.8897156 | 847.9408319 | 922.7194884 | 804.330697 | 837.1980862 | 839.9331805 | 929.9901299 | 821.4092798 | 918.9150807 | 817.7277347 |
| **Man1a** | 3196.696417 | 2691.314043 | 2608.710272 | 2697.379655 | 2332.676829 | 2511.759257 | 2677.328073 | 2230.953023 | 2371.609314 | 2304.479492 | 2571.82243 | 2409.993079 | 2429.292383 | 2069.140152 | 2305.877241 |
| **Gm16998** | 5.494178889 | 5.54583406 | 3.132645178 | 5.175656518 | 1.900089733 | 1.402825611 | 6.314453003 | 7.542666117 | 7.524141226 | 11.3607256 | 3.783482795 | 0.867528106 | 11.92918041 | 6.324882561 | 4.311394032 |
| **Tbc1d32** | 34.7964663 | 24.56012226 | 50.90548414 | 39.6800333 | 46.23551683 | 43.48759395 | 38.78878274 | 42.74177467 | 25.58208017 | 48.06460829 | 29.32199166 | 39.03876478 | 36.63962555 | 35.23863141 | 42.39537465 |
| **Msl3l2** | 92.48534464 | 63.38096068 | 48.55600026 | 81.94789487 | 88.6708542 | 56.81443726 | 73.96930661 | 42.74177467 | 57.18347332 | 57.67752995 | 51.07701773 | 65.06460797 | 62.20215501 | 86.74124655 | 63.9523448 |
| **Gja1** | 8.241268334 | 2.376786026 | 8.614774239 | 8.626094197 | 12.03390164 | 11.22260489 | 7.216517718 | 34.36103454 | 7.524141226 | 38.45168663 | 22.70089677 | 5.205168638 | 3.408337261 | 4.517773258 | 1.437131344 |
| **Hsf2** | 524.6940839 | 418.3143405 | 418.2081313 | 343.318549 | 418.6531045 | 460.8282134 | 442.0117102 | 491.9494457 | 434.1429487 | 416.8512392 | 411.453754 | 401.6655132 | 445.6400968 | 402.08182 | 457.0077674 |
| **Serinc1** | 1718.762296 | 1550.456751 | 1626.626009 | 1593.239598 | 1324.362544 | 1435.792013 | 1451.422126 | 1366.898715 | 1416.043379 | 1404.360464 | 1519.068342 | 1364.621711 | 1429.797481 | 1262.265848 | 1488.149507 |
| **Pkib** | 6.409875371 | 3.961310043 | 7.04845165 | 4.313047098 | 0.633363244 | 3.507064029 | 1.80412943 | 2.514222039 | 3.00965649 | 0 | 8.512836289 | 0 | 1.70416863 | 4.517773258 | 2.155697016 |
| **Smpdl3a** | 115.3777567 | 118.8393013 | 103.3772909 | 102.6505209 | 170.3747127 | 189.3814575 | 224.614114 | 64.53169901 | 91.04210883 | 73.40776539 | 66.21094891 | 179.578318 | 198.5356454 | 171.6753838 | 169.5814986 |
| **Gcc2** | 674.8683069 | 680.5530653 | 674.3018746 | 571.0474358 | 632.0965178 | 608.8263154 | 684.6671185 | 672.9734325 | 671.1533973 | 644.9396531 | 649.8131701 | 746.9416995 | 707.2299816 | 658.691341 | 647.4276704 |
| **Lims1** | 1318.602933 | 1381.704943 | 1444.932588 | 1324.105459 | 1376.29833 | 1441.403316 | 1537.118274 | 1231.968799 | 1376.16543 | 1197.245697 | 1338.407039 | 1496.485983 | 1433.205818 | 1513.454041 | 1524.07779 |
| **Ranbp2** | 6093.044388 | 5404.811422 | 5558.095707 | 5355.079277 | 4888.930883 | 4807.48337 | 5435.841971 | 5619.286258 | 5613.009354 | 5091.352871 | 5489.833536 | 5494.923025 | 5251.395634 | 4853.895588 | 5336.787246 |
| **Ccdc138** | 43.03773463 | 45.15893448 | 43.0738712 | 50.03134634 | 36.10170492 | 54.00878604 | 41.49497688 | 33.52296052 | 56.43105919 | 50.6863142 | 54.86050053 | 51.18415827 | 59.64590206 | 72.28437213 | 52.45529405 |
| **Sept10** | 77.83420093 | 72.88810478 | 65.78554874 | 57.79483112 | 61.43623469 | 61.0229141 | 59.53627118 | 61.17940295 | 41.38277674 | 54.18192207 | 34.99721585 | 73.73988903 | 58.79381775 | 38.85285002 | 51.01816271 |
| **Sowahc** | 108.9678813 | 85.56429692 | 92.41303275 | 79.36006661 | 50.66905954 | 63.12715251 | 75.77343604 | 60.34132894 | 97.06142181 | 86.51629492 | 111.6127425 | 63.32955176 | 93.72927467 | 64.15238026 | 51.01816271 |
| **P4ha1** | 2808.441109 | 2646.155108 | 2612.626078 | 2502.429926 | 2451.749119 | 2889.119347 | 2855.034822 | 1001.498446 | 1344.564037 | 1337.943914 | 1478.395902 | 2600.849263 | 2588.632149 | 2403.455373 | 2587.554985 |
| **Oit3** | 4.578482408 | 0.792262009 | 8.614774239 | 1.725218839 | 1.900089733 | 1.402825611 | 7.216517718 | 0.838074013 | 4.514484735 | 2.621705907 | 9.458706988 | 7.807752957 | 11.0770961 | 7.228437213 | 4.311394032 |
| **Mcu** | 240.8281747 | 191.7274061 | 223.9841302 | 186.3236346 | 219.7770458 | 199.9026496 | 245.3616024 | 191.918949 | 237.7628627 | 151.1850406 | 174.0402086 | 201.2665207 | 218.1335847 | 180.7109303 | 203.3540852 |
| **Micu1** | 401.9907554 | 409.5994584 | 406.4607118 | 444.2438511 | 357.2168698 | 374.5544383 | 405.0270569 | 453.3980411 | 330.3097998 | 415.1034352 | 297.0033994 | 438.1016937 | 336.5733045 | 390.3356095 | 367.905624 |
| **Dnajb12** | 640.0718406 | 629.8482968 | 625.7458743 | 636.6057517 | 566.8601036 | 536.5807964 | 633.2494298 | 653.6977302 | 625.2561359 | 644.0657511 | 655.4883942 | 584.7139436 | 616.9090442 | 547.5541189 | 617.2479122 |
| **Ddit4** | 4671.883449 | 3725.215964 | 3656.580084 | 3032.07211 | 3067.378192 | 3360.468752 | 3308.773374 | 1426.40197 | 1887.807034 | 2013.470136 | 1854.85244 | 2468.98499 | 2603.117583 | 2302.257252 | 2544.441044 |
| **Anapc16** | 837.8622806 | 850.0971351 | 787.077101 | 786.6997907 | 826.5390338 | 796.1035345 | 759.5384898 | 917.6910443 | 957.0707639 | 877.3975768 | 865.4716894 | 859.7203533 | 813.740521 | 866.5089109 | 890.3028676 |
| **Ascc1** | 358.9530208 | 383.4548121 | 356.338389 | 299.3254686 | 325.5487076 | 338.0809724 | 387.8878274 | 297.5162746 | 313.7566891 | 330.3349442 | 311.1914599 | 355.6865236 | 312.7149437 | 318.954792 | 312.5760673 |
| **Spock2** | 43.03773463 | 27.7291703 | 26.62748401 | 22.42784491 | 24.70116653 | 30.86216345 | 41.49497688 | 17.59955427 | 3.00965649 | 34.95607876 | 2.837612096 | 45.11146153 | 24.71044514 | 28.91374885 | 24.43123285 |
| **Chst3** | 2.747089445 | 6.338096068 | 4.698967767 | 11.21392246 | 11.4005384 | 9.118366474 | 4.510323574 | 20.95185033 | 15.04828245 | 9.612921658 | 9.458706988 | 5.205168638 | 9.372927467 | 9.035546516 | 6.467091048 |
| **Psap** | 4279.965355 | 4506.386304 | 4587.758863 | 4539.050766 | 4596.317064 | 4558.481824 | 4670.891093 | 4326.976129 | 4149.563886 | 4192.981647 | 4107.916445 | 4907.606497 | 4621.705325 | 4754.504577 | 4561.454886 |
| **Cdh23** | 152.0056159 | 130.7232314 | 152.7164524 | 164.7583992 | 136.8064608 | 140.9839739 | 123.5828659 | 149.1771743 | 130.9200573 | 125.8418835 | 127.6925443 | 117.1162943 | 163.6001885 | 135.5331977 | 147.3059628 |
| **4632428N05Rik** | 1271.902413 | 1581.354969 | 1476.25904 | 1507.841266 | 1645.477709 | 1738.802345 | 1900.650354 | 1254.596798 | 1211.386737 | 1079.268932 | 1161.529218 | 1602.324412 | 1648.78315 | 1794.459538 | 1851.025171 |
| **Gm17455** | 5.494178889 | 1.584524017 | 1.566322589 | 6.900875357 | 6.966995687 | 5.611302446 | 0 | 3.352296052 | 3.00965649 | 6.991215751 | 0.945870699 | 3.470112425 | 3.408337261 | 0 | 5.748525376 |
| **Slc29a3** | 680.3624858 | 689.2679474 | 684.4829714 | 667.6596908 | 623.2294324 | 638.2856532 | 720.7497071 | 900.09149 | 802.8258688 | 744.5644775 | 832.3662149 | 692.2874288 | 656.1049227 | 578.274977 | 680.4816914 |
| **Unc5b** | 64.09875371 | 62.58869867 | 92.41303275 | 85.39833255 | 89.93758069 | 115.7331129 | 80.28375962 | 124.8730279 | 70.72692752 | 97.87702052 | 272.4107612 | 95.42809169 | 77.53967268 | 77.70570004 | 71.13800152 |
| **Sgpl1** | 836.9465841 | 796.2233185 | 899.8523274 | 841.0441842 | 865.8075549 | 861.3349254 | 946.2658858 | 914.3387482 | 975.1287029 | 921.9665772 | 837.0955684 | 969.0288947 | 1019.092841 | 902.6510969 | 991.6206273 |
| **Adamts14** | 15.56684019 | 21.39107423 | 14.0969033 | 2.587828259 | 10.13381191 | 4.90988964 | 2.706194144 | 13.40918421 | 8.276555348 | 8.739019689 | 26.48437957 | 12.14539349 | 21.30210788 | 12.64976512 | 14.37131344 |
| **Prf1** | 988.9522001 | 652.823895 | 691.531423 | 641.7814082 | 646.0305092 | 620.0489202 | 696.3939598 | 675.4876545 | 526.6898858 | 541.8192207 | 677.2434203 | 685.347204 | 743.0175228 | 585.5034142 | 712.8171466 |
| **Pald1** | 2.747089445 | 0.792262009 | 5.482129061 | 2.587828259 | 3.166816221 | 4.208476834 | 9.922711863 | 3.352296052 | 0 | 0 | 1.891741398 | 5.205168638 | 4.260421576 | 2.710663955 | 1.437131344 |
| **Eif4ebp2** | 2240.70929 | 2291.221729 | 2334.603819 | 2188.440098 | 2083.13171 | 2280.994444 | 2350.780647 | 2215.029617 | 2349.036891 | 2147.177138 | 2427.104213 | 2453.369485 | 2465.932008 | 2244.429755 | 2245.517725 |
| **Lrrc20** | 186.8020822 | 170.3363318 | 186.3923881 | 161.3079615 | 196.3426057 | 171.8461374 | 175.0005547 | 140.7964342 | 149.7304104 | 124.0940796 | 174.0402086 | 168.3004526 | 196.8314768 | 154.5078454 | 147.3059628 |
| **Ppa1** | 4277.218265 | 4103.124942 | 4131.95899 | 4091.356477 | 3726.075966 | 3776.406546 | 3550.526717 | 4888.485718 | 4540.81923 | 4672.753828 | 4736.920459 | 3757.264228 | 3643.512532 | 3664.817667 | 3640.97226 |
| **1700022H01Rik** | 0 | 0 | 0 | 0 | 1.266726489 | 0 | 0 | 1.676148026 | 0 | 0.873901969 | 0 | 0 | 0 | 0 | 0 |
| **Sar1a** | 2869.792773 | 2688.937257 | 2711.304402 | 2550.736054 | 2645.558271 | 2856.152945 | 2762.122157 | 2833.528238 | 2896.794372 | 2827.946771 | 2880.176278 | 2714.495445 | 2871.524142 | 2706.146181 | 2652.225895 |
| **Tysnd1** | 787.4989741 | 880.9953535 | 894.3701983 | 841.9067936 | 841.1063884 | 956.727067 | 932.7349151 | 985.5750394 | 960.0804204 | 945.5619304 | 862.6340773 | 870.1306906 | 901.5052054 | 917.1079713 | 855.8117153 |
| **Aifm2** | 32.96507334 | 32.48274235 | 36.80858084 | 25.87828259 | 26.60125626 | 40.68194273 | 29.76813559 | 15.92340625 | 15.04828245 | 33.20827482 | 31.21373306 | 26.02584319 | 24.71044514 | 27.10663955 | 20.83840449 |
| **H2afy2** | 5.494178889 | 0 | 0.783161294 | 0 | 0 | 0 | 0.902064715 | 2.514222039 | 3.762070613 | 1.747803938 | 0 | 0 | 1.70416863 | 0 | 4.311394032 |
| **Gm5424** | 11.90405426 | 7.922620085 | 12.53058071 | 22.42784491 | 25.33452977 | 32.26498906 | 36.08258859 | 27.65644243 | 12.03862596 | 20.97364725 | 17.97154328 | 20.82067455 | 25.56252945 | 17.16753838 | 30.17975822 |
| **2010107G23Rik** | 0.915696482 | 2.376786026 | 0.783161294 | 3.450437679 | 2.533452977 | 4.208476834 | 6.314453003 | 4.190370065 | 0 | 0.873901969 | 3.783482795 | 5.205168638 | 3.408337261 | 1.807109303 | 1.437131344 |
| **Tspan15** | 1.831392963 | 4.753572051 | 7.04845165 | 0 | 1.900089733 | 0 | 0 | 0 | 0 | 0 | 0 | 0.867528106 | 3.408337261 | 0.903554652 | 0 |
| **Hk1** | 4963.990627 | 4549.960715 | 4450.705637 | 4553.715126 | 4149.162613 | 4461.686857 | 4670.891093 | 3258.431763 | 3494.211185 | 3536.681268 | 3363.516205 | 4556.257614 | 4676.238722 | 4375.011623 | 4463.729954 |
| **Hk1os** | 8.241268334 | 9.507144102 | 6.265290356 | 11.21392246 | 15.20071786 | 8.416953669 | 7.216517718 | 15.08533223 | 26.33449429 | 3.495607876 | 6.621094891 | 17.35056213 | 12.78126473 | 12.64976512 | 9.341353736 |
| **Supv3l1** | 790.2460636 | 724.9197378 | 769.0643912 | 709.064943 | 706.2000174 | 668.4464038 | 709.0228658 | 772.70424 | 716.2982447 | 787.385674 | 708.4571534 | 652.3811359 | 637.3590677 | 630.6811468 | 707.0686212 |
| **4930507D05Rik** | 7.325571853 | 4.753572051 | 5.482129061 | 6.038265938 | 1.266726489 | 2.805651223 | 0.902064715 | 5.866518091 | 2.257242368 | 6.991215751 | 2.837612096 | 5.205168638 | 7.668758836 | 2.710663955 | 3.59282836 |
| **Vps26a** | 544.8394065 | 633.8096068 | 623.3963904 | 653.8579401 | 613.0956205 | 567.4429598 | 566.4966409 | 694.7633568 | 679.4299527 | 651.0569668 | 716.024119 | 633.2955176 | 599.0152736 | 645.1380212 | 597.1280734 |
| **Srgn** | 3411.88509 | 2680.222375 | 2882.033564 | 2706.868359 | 3262.454071 | 3699.251137 | 3542.408135 | 2448.014192 | 2262.509267 | 2356.039708 | 2551.959145 | 3213.324106 | 3164.641147 | 3121.781321 | 3310.432051 |
| **Kif1bp** | 393.7494871 | 527.6464977 | 411.9428409 | 444.2438511 | 396.4853909 | 444.6957188 | 438.4034514 | 511.225148 | 535.7188553 | 479.7721809 | 400.1033056 | 471.9352898 | 439.6755066 | 484.3052932 | 444.7921509 |
| **Ddx21** | 12776.71301 | 12192.12005 | 12390.39484 | 11934.20132 | 11703.91939 | 11386.03408 | 12255.45122 | 14500.35657 | 13599.13285 | 13521.01126 | 13996.9946 | 12003.98641 | 11600.27587 | 11174.26038 | 11483.398 |
| **Ddx50** | 822.2954404 | 846.1358251 | 821.5361979 | 797.9137132 | 858.207196 | 858.5292742 | 894.8481971 | 812.9317927 | 826.9031207 | 832.8285764 | 814.3946716 | 880.5410279 | 877.6468446 | 955.9608214 | 895.3328273 |
| **Stox1** | 3.662785926 | 0 | 0 | 0.86260942 | 6.333632443 | 2.805651223 | 0.902064715 | 4.190370065 | 4.514484735 | 3.495607876 | 2.837612096 | 0.867528106 | 3.408337261 | 5.421327909 | 1.437131344 |
| **Ccar1** | 2592.336739 | 2685.768209 | 2887.515693 | 2748.273611 | 2577.788404 | 2484.404158 | 2712.508597 | 2981.867338 | 2999.875107 | 3140.803676 | 3262.30804 | 2952.198146 | 2798.244891 | 2829.029614 | 2667.315774 |
| **Tet1** | 43.03773463 | 52.28929256 | 57.95393579 | 78.49745719 | 93.10439691 | 67.33562935 | 82.08788905 | 32.68488651 | 50.41174621 | 33.20827482 | 28.37612096 | 91.09045116 | 75.83550405 | 106.6194489 | 66.10804182 |
| **Slc25a16** | 302.1798389 | 351.7643318 | 408.0270344 | 345.9063773 | 357.850233 | 321.247065 | 376.1609861 | 393.8947861 | 435.647777 | 286.6398458 | 419.0207196 | 408.6057381 | 399.6275438 | 387.6249455 | 374.3727151 |
| **Dna2** | 490.8133141 | 551.4143579 | 483.2105187 | 475.2977902 | 520.6245868 | 451.0084341 | 466.3674575 | 655.3738782 | 618.4844088 | 664.1654964 | 723.5910846 | 641.1032705 | 518.0672636 | 590.9247421 | 529.5829002 |
| **Rufy2** | 193.2119576 | 144.1916855 | 164.4638718 | 119.0400999 | 157.0740846 | 169.741899 | 168.6861017 | 149.1771743 | 103.8331489 | 114.4811579 | 148.5016997 | 193.4587677 | 163.6001885 | 208.7211245 | 174.6114583 |
| **Hnrnph3** | 174.898028 | 170.3363318 | 184.8260655 | 169.9340557 | 143.1400932 | 188.6800447 | 153.3510015 | 212.8707993 | 209.9235402 | 178.2760017 | 174.9860793 | 182.1809023 | 167.8606101 | 162.6398373 | 135.808912 |
| **Pbld2** | 1.831392963 | 6.338096068 | 3.915806472 | 0 | 1.266726489 | 2.104238417 | 0 | 0 | 0.752414123 | 1.747803938 | 0 | 4.337640531 | 0 | 0.903554652 | 10.05991941 |
| **Herc4** | 1140.957816 | 1221.668017 | 1091.726845 | 1100.689619 | 1117.252763 | 1205.0272 | 1092.40037 | 1069.382441 | 1124.106699 | 1131.70305 | 1009.244036 | 1186.778449 | 1167.355512 | 1157.453509 | 1184.914793 |
| **Sirt1** | 640.0718406 | 622.7179387 | 689.1819391 | 710.7901618 | 663.1313168 | 663.5365142 | 670.2340831 | 765.1615739 | 799.0637982 | 728.8342421 | 701.8360585 | 682.7446196 | 696.1528855 | 655.0771224 | 571.9782749 |
| **Dnajc12** | 36.62785926 | 53.08155457 | 21.92851625 | 39.6800333 | 42.43533737 | 25.25086101 | 33.37639445 | 75.42666117 | 45.89726148 | 29.71266694 | 47.29353494 | 52.91921448 | 51.12505891 | 46.98484188 | 25.86836419 |
| **Gm31763** | 1.831392963 | 0.792262009 | 3.132645178 | 2.587828259 | 2.533452977 | 6.312715251 | 6.314453003 | 0 | 0 | 8.739019689 | 1.891741398 | 6.94022485 | 1.70416863 | 0.903554652 | 7.18565672 |
| **Reep3** | 1350.65231 | 1264.450166 | 1295.348781 | 1259.409753 | 1307.895099 | 1233.083712 | 1413.535408 | 1418.859304 | 1402.499924 | 1247.05811 | 1255.170417 | 1377.634633 | 1344.589049 | 1286.661824 | 1274.735502 |
| **Jmjd1c** | 1235.274554 | 1306.440052 | 1249.925426 | 1275.799332 | 1262.926309 | 1256.931748 | 1488.406779 | 1352.651457 | 1191.071556 | 1246.184208 | 1363.945548 | 1511.233961 | 1440.874577 | 1292.083152 | 1360.244817 |
| **Nrbf2** | 282.0345163 | 371.570882 | 371.2184536 | 316.577657 | 335.6825195 | 352.8106413 | 341.8825269 | 352.8291595 | 394.2650002 | 363.5432191 | 295.111658 | 289.7543875 | 290.5607515 | 296.3659257 | 365.0313614 |
| **Egr2** | 575.0573904 | 513.3857815 | 548.2129061 | 519.2908706 | 544.6923901 | 554.1161165 | 700.0022187 | 489.4352236 | 460.477443 | 396.7514939 | 454.9638061 | 611.6073149 | 679.1111992 | 657.7877863 | 645.9905391 |
| **Ado** | 563.1533362 | 573.5976942 | 577.9730353 | 587.4370148 | 602.9618086 | 570.9500239 | 592.6565176 | 642.802768 | 588.3878439 | 612.6052802 | 512.6619187 | 532.6622573 | 521.4756009 | 580.0820863 | 549.7027391 |
| **Zfp365** | 160.2468843 | 137.0613275 | 141.7521943 | 150.9566484 | 127.9393753 | 100.3020312 | 119.9746071 | 243.0414638 | 302.4704773 | 289.2615517 | 225.1172263 | 114.51371 | 138.0376591 | 100.2945663 | 94.8506687 |
| **Rtkn2** | 19.22962611 | 24.56012226 | 20.36219366 | 35.36698621 | 33.56825195 | 32.26498906 | 18.0412943 | 23.46607237 | 41.38277674 | 19.22584332 | 11.35044839 | 30.36348372 | 33.23128829 | 33.43152211 | 31.61688957 |
| **Arid5b** | 141.0172582 | 156.8678777 | 157.4154202 | 194.0871194 | 145.0401829 | 156.4150557 | 159.6654545 | 161.7482845 | 154.2448951 | 119.7245697 | 125.8008029 | 157.0225872 | 182.3460434 | 146.3758536 | 186.8270747 |
| **1700040L02Rik** | 26.55519797 | 10.29940611 | 22.71167754 | 12.93914129 | 9.500448664 | 9.118366474 | 15.33510015 | 8.380740131 | 13.54345421 | 27.09096104 | 6.621094891 | 16.48303402 | 11.92918041 | 11.74621047 | 9.341353736 |
| **Rhobtb1** | 3.662785926 | 5.54583406 | 0 | 3.450437679 | 3.166816221 | 4.90988964 | 4.510323574 | 0 | 0 | 0 | 1.891741398 | 0 | 0 | 0 | 0 |
| **Cdk1** | 2577.685596 | 2650.90868 | 2694.074853 | 2667.188326 | 2559.42087 | 2463.361774 | 2532.095654 | 2626.523957 | 2701.1667 | 2631.318828 | 2843.287321 | 2593.04151 | 2462.523671 | 2679.039542 | 2596.896338 |
| **Ccdc6** | 1678.471651 | 1497.375196 | 1494.27175 | 1361.197664 | 1292.694382 | 1243.604905 | 1385.571402 | 1417.183156 | 1412.281308 | 1339.691718 | 1331.785944 | 1342.933509 | 1316.470267 | 1247.808974 | 1406.951586 |
| **Tfam** | 1055.798043 | 1055.292995 | 1076.063619 | 955.771237 | 1097.618502 | 1105.426582 | 1142.915994 | 1154.027916 | 1024.788035 | 1054.799676 | 1065.996278 | 1114.773617 | 1099.188767 | 1054.448278 | 1088.626993 |
| **Ube2d1** | 324.1565545 | 338.2958776 | 404.111228 | 339.8681113 | 381.9180363 | 385.0756303 | 440.2075808 | 305.8970148 | 304.7277196 | 325.0915324 | 388.7528572 | 416.413491 | 380.8816889 | 452.6808804 | 439.7621912 |
| **Cisd1** | 846.103549 | 873.0727334 | 910.0334242 | 885.899874 | 738.5015428 | 776.4639759 | 764.9508781 | 998.9842236 | 954.8135216 | 1014.600186 | 987.4890095 | 702.6977661 | 794.1425817 | 800.5494213 | 796.1707645 |
| **Ipmk** | 826.8739229 | 744.726288 | 837.9825851 | 774.6232588 | 679.5987611 | 669.1478166 | 799.2293373 | 810.4175706 | 759.1858497 | 862.5412433 | 834.2579563 | 751.27934 | 817.1488582 | 756.2752434 | 785.3922795 |
| **Zwint** | 1729.750654 | 1632.852 | 1694.761041 | 1575.1248 | 1642.310892 | 1627.979122 | 1529.901756 | 1812.75409 | 1771.935259 | 1744.30833 | 1804.721293 | 1719.440707 | 1702.464462 | 1698.682745 | 1567.910296 |
| **Gnaz** | 5.494178889 | 2.376786026 | 3.915806472 | 0.86260942 | 0 | 2.805651223 | 0 | 0 | 2.257242368 | 2.621705907 | 0 | 6.072696744 | 6.816674521 | 5.421327909 | 5.029959704 |
| **Rab36** | 3.662785926 | 6.338096068 | 0 | 0 | 0 | 2.104238417 | 0 | 2.514222039 | 0 | 0 | 0.945870699 | 0.867528106 | 2.556252945 | 1.807109303 | 0.718565672 |
| **Bcr** | 662.9642527 | 556.16793 | 533.3328415 | 523.6039177 | 445.2543607 | 498.0030921 | 560.1821879 | 660.4023223 | 585.3781874 | 572.4057896 | 707.5112827 | 465.8625931 | 570.0444068 | 491.5337305 | 480.0018689 |
| **Specc1l** | 1033.821328 | 1147.98765 | 1139.499683 | 1061.009586 | 1071.650609 | 1085.787023 | 1173.586194 | 1085.305847 | 1048.865287 | 1035.573833 | 1101.939364 | 1129.521594 | 1143.497151 | 1051.737614 | 1102.279741 |
| **Adora2a** | 784.7518847 | 755.0256941 | 744.7863911 | 765.9971647 | 824.0055808 | 752.6159405 | 741.4971955 | 872.4350476 | 674.1630538 | 636.2006334 | 589.2774453 | 744.3391152 | 769.4321366 | 736.397041 | 681.9188227 |
| **Upb1** | 3.662785926 | 4.753572051 | 3.132645178 | 11.21392246 | 4.43354271 | 4.208476834 | 3.608258859 | 10.05688816 | 8.276555348 | 11.3607256 | 8.512836289 | 3.470112425 | 3.408337261 | 6.324882561 | 6.467091048 |
| **Gucd1** | 301.2641424 | 343.0494497 | 306.9992274 | 310.5393911 | 310.9813529 | 297.3990296 | 303.9958089 | 271.5359802 | 333.3194563 | 249.0620611 | 233.6300626 | 271.5362973 | 294.821173 | 310.8228001 | 314.7317643 |
| **Snrpd3** | 2798.368448 | 2641.401536 | 2824.862789 | 2739.647517 | 2891.30321 | 2944.530958 | 2828.874946 | 2913.145269 | 2810.266748 | 2945.049635 | 2959.629416 | 2871.518032 | 2759.901097 | 2781.141218 | 2635.698885 |
| **Lrrc75b** | 2.747089445 | 2.376786026 | 3.915806472 | 6.038265938 | 3.800179466 | 5.611302446 | 6.314453003 | 6.704592104 | 2.257242368 | 3.495607876 | 5.675224193 | 6.072696744 | 0.852084315 | 11.74621047 | 11.49705075 |
| **Ggt1** | 19.22962611 | 17.42976419 | 13.31374201 | 14.66436013 | 23.43444004 | 27.35509942 | 26.15987673 | 2.514222039 | 15.04828245 | 11.3607256 | 2.837612096 | 25.15831508 | 38.34379418 | 32.52796746 | 36.64684927 |
| **Ggt5** | 1.831392963 | 3.961310043 | 1.566322589 | 2.587828259 | 6.333632443 | 5.611302446 | 1.80412943 | 6.704592104 | 0.752414123 | 2.621705907 | 0 | 7.807752957 | 4.260421576 | 0 | 2.874262688 |
| **Susd2** | 10.0726613 | 11.88393013 | 17.22954848 | 9.488703616 | 11.4005384 | 16.13249453 | 23.45368258 | 16.76148026 | 5.266898858 | 20.97364725 | 18.91741398 | 16.48303402 | 18.74585493 | 23.49242094 | 6.467091048 |
| **Cabin1** | 2251.697648 | 2404.515196 | 2474.789691 | 2361.824591 | 2342.177277 | 2240.312501 | 2459.028412 | 2475.670635 | 2604.105278 | 2455.664533 | 2435.617049 | 2349.266112 | 2574.9988 | 2367.313187 | 2442.404719 |
| **Ddt** | 792.993153 | 732.8423579 | 785.5107784 | 737.5310538 | 831.6059397 | 788.3879936 | 786.6004313 | 879.9777137 | 826.9031207 | 846.8110079 | 861.6882066 | 818.0790042 | 823.9655328 | 750.8539155 | 829.9433511 |
| **Gstt3** | 34.7964663 | 61.79643666 | 38.37490343 | 21.56523549 | 42.43533737 | 42.78618115 | 46.90736517 | 34.36103454 | 45.14484735 | 36.70388269 | 55.80637123 | 45.97898963 | 46.01255302 | 30.72085815 | 38.80254629 |
| **Gstt1** | 249.069443 | 212.3262183 | 175.42813 | 217.3775738 | 211.5433236 | 207.6181905 | 252.5781201 | 113.1399918 | 118.8814314 | 166.9152761 | 176.8778207 | 203.869105 | 184.9022964 | 221.3708896 | 232.8152777 |
| **Gstt4** | 2.747089445 | 12.67619214 | 3.915806472 | 6.900875357 | 2.533452977 | 0.701412806 | 0.902064715 | 2.514222039 | 4.514484735 | 9.612921658 | 1.891741398 | 5.205168638 | 0.852084315 | 2.710663955 | 2.874262688 |
| **Gstt2** | 564.9847291 | 508.6322095 | 523.1517447 | 496.8630257 | 495.9234203 | 537.983622 | 494.3314637 | 563.1857368 | 461.9822713 | 561.045064 | 490.9068927 | 497.0936049 | 536.8131186 | 539.422127 | 451.259242 |
| **Mif** | 13820.607 | 12870.29633 | 12966.01839 | 11977.33179 | 13242.35871 | 13396.98459 | 12469.24055 | 9238.92792 | 9509.009681 | 10103.18066 | 9711.254464 | 12737.91518 | 12505.18941 | 12858.48625 | 12626.63599 |
| **Derl3** | 11.90405426 | 18.2220262 | 22.71167754 | 24.15306375 | 10.76717515 | 17.53532014 | 9.020647148 | 15.92340625 | 16.5531107 | 27.09096104 | 17.97154328 | 22.55573076 | 17.89377062 | 17.16753838 | 7.18565672 |
| **Smarcb1** | 1760.884334 | 1817.449048 | 1882.719752 | 1781.288452 | 1893.7561 | 1733.892456 | 1895.237966 | 1743.193947 | 1872.758751 | 1849.176566 | 1621.222378 | 1796.650708 | 1876.289662 | 1748.378251 | 1836.653858 |
| **Mmp11** | 51.27900297 | 30.89821833 | 59.52025838 | 43.13047098 | 34.83497844 | 48.39748359 | 36.98465331 | 31.8468125 | 28.59173666 | 33.20827482 | 26.48437957 | 47.71404585 | 37.49170987 | 50.59906049 | 50.29959704 |
| **Chchd10** | 2478.790376 | 2146.237781 | 2137.247173 | 1967.612086 | 2063.49745 | 2478.792855 | 2574.492696 | 1441.487302 | 1926.932568 | 1795.868546 | 1472.720678 | 2095.947905 | 2192.412943 | 2268.82573 | 2283.601706 |
| **Gm867** | 7.325571853 | 9.507144102 | 5.482129061 | 0.86260942 | 5.066905954 | 4.90988964 | 9.922711863 | 11.73303618 | 0.752414123 | 7.86511772 | 1.891741398 | 1.735056213 | 5.964590206 | 4.517773258 | 7.18565672 |
| **Vpreb3** | 5.494178889 | 15.84524017 | 2.349483883 | 4.313047098 | 3.166816221 | 9.81977928 | 12.62890601 | 5.028444078 | 5.266898858 | 0.873901969 | 9.458706988 | 7.807752957 | 1.70416863 | 2.710663955 | 8.622788064 |
| **Gm5134** | 9.156964816 | 7.922620085 | 3.132645178 | 6.900875357 | 10.13381191 | 9.118366474 | 11.72684129 | 9.218814144 | 12.79104008 | 4.369509845 | 7.56696559 | 12.14539349 | 2.556252945 | 6.324882561 | 20.11983882 |
| **Zfp280b** | 1716.930903 | 1907.766916 | 2011.941366 | 1743.333637 | 1787.984439 | 1702.328879 | 1894.335901 | 1552.951146 | 1785.478713 | 1848.302664 | 1774.453431 | 2089.00768 | 1983.652286 | 1750.18536 | 2018.450973 |
| **Prmt2** | 161.1625808 | 141.0226375 | 137.0532265 | 115.5896622 | 106.405025 | 147.2966892 | 128.0931895 | 150.0152483 | 142.9586833 | 99.62482445 | 157.014536 | 164.8303402 | 146.5585022 | 161.7362826 | 146.5873971 |
| **Dip2a** | 90.65395167 | 74.4726288 | 86.14774239 | 59.52004996 | 82.33722176 | 76.45399582 | 103.7374422 | 70.3982171 | 102.3283207 | 96.12921658 | 88.91184568 | 84.15022631 | 98.84178056 | 70.47726282 | 69.70087018 |
| **Pcnt** | 1922.962611 | 2056.712174 | 2100.438592 | 2035.75823 | 2001.427852 | 1865.758063 | 2000.779537 | 2016.406075 | 2091.711261 | 2037.939391 | 2137.667779 | 2165.350153 | 2039.88985 | 2216.41956 | 2170.786895 |
| **Ybey** | 84.2440763 | 91.90239299 | 83.01509722 | 71.59658183 | 77.2703158 | 68.73845496 | 87.50027733 | 72.91243914 | 94.05176532 | 95.25531461 | 72.83204381 | 71.13730471 | 61.35007069 | 85.8376919 | 72.57513287 |
| **Mcm3ap** | 1837.802838 | 1873.69965 | 1850.610139 | 1822.693704 | 1693.613315 | 1660.945524 | 1789.696394 | 1971.150079 | 1868.99668 | 1900.736782 | 2070.51096 | 1904.224193 | 1824.312519 | 1781.809773 | 1831.623898 |

| | | | | | | | | | | | | | | | |
|---|---|---|---|---|---|---|---|---|---|---|---|---|---|---|---|
| **Lss** | 2371.653887 | 2305.482445 | 2371.4124 | 2352.335887 | 2309.242389 | 2323.079213 | 2386.863235 | 2425.386194 | 2587.552168 | 2755.412908 | 2869.7717 | 2302.419594 | 2479.565357 | 2287.800378 | 2354.739707 |
| **4930483K19Rik** | 0 | 0 | 0 | 1.725218839 | 0 | 2.805651223 | 1.80412943 | 0 | 0 | 2.621705907 | 3.783482795 | 3.470112425 | 0 | 1.807109303 | 1.437131344 |
| **Spatc1l** | 7.325571853 | 7.130358077 | 3.915806472 | 0 | 3.166816221 | 2.104238417 | 0.902064715 | 18.43762829 | 6.771727103 | 12.23462756 | 4.729353494 | 1.735056213 | 7.668758836 | 0.903554652 | 7.18565672 |
| **Col6a2** | 32.96507334 | 41.98988645 | 54.82129061 | 37.09220505 | 5.700269198 | 11.22260489 | 6.314453003 | 77.1028092 | 72.98416989 | 104.8682363 | 81.34488009 | 19.08561834 | 16.18960199 | 12.64976512 | 26.58692986 |
| **Col6a1** | 12.81975074 | 12.67619214 | 10.96425812 | 12.93914129 | 2.533452977 | 3.507064029 | 11.72684129 | 56.98903289 | 6.771727103 | 24.46925513 | 10.40457769 | 4.337640531 | 5.964590206 | 6.324882561 | 2.874262688 |
| **Pcbp3** | 59.5202713 | 56.2506026 | 66.56871003 | 62.97048763 | 62.06959794 | 73.6483446 | 75.77343604 | 108.9496217 | 73.73658401 | 67.29045161 | 53.91462983 | 79.81258578 | 92.02510604 | 82.22347329 | 63.9523448 |
| **Slc19a1** | 2168.369268 | 2084.441344 | 2307.976335 | 2122.019172 | 2062.864087 | 2100.02994 | 2024.23322 | 2445.49997 | 2308.406528 | 2280.884139 | 2178.340219 | 1912.899474 | 1917.189709 | 1754.703133 | 2038.570811 |
| **Col18a1** | 1.831392963 | 4.753572051 | 10.18109683 | 6.900875357 | 7.600358931 | 6.312715251 | 7.216517718 | 1.676148026 | 2.257242368 | 1.747803938 | 6.621094891 | 10.41033728 | 3.408337261 | 9.035546516 | 6.467091048 |
| **Pofut2** | 414.8105061 | 441.2899387 | 422.1239377 | 350.2194244 | 375.5844039 | 337.3795595 | 425.7745454 | 393.0567121 | 397.2746567 | 443.0682982 | 423.750073 | 349.6138268 | 384.2900261 | 340.6401036 | 370.7798867 |
| **Adarb1** | 127.2818109 | 110.9166812 | 132.3542588 | 94.88703616 | 105.1382985 | 112.2260489 | 132.6035131 | 144.9868043 | 147.473168 | 131.0852953 | 132.4218978 | 120.5864068 | 103.9542864 | 115.6549954 | 104.1920224 |
| **Fam207a** | 1286.553557 | 1364.275179 | 1439.450459 | 1462.122966 | 1245.825501 | 1296.210865 | 1294.462866 | 1598.207143 | 1398.737854 | 1554.671603 | 1608.926059 | 1214.539349 | 1301.984834 | 1233.352099 | 1320.723705 |
| **Itgb2** | 4012.581982 | 4221.171981 | 4218.889893 | 4384.64368 | 4681.821102 | 4700.167211 | 4995.63439 | 3807.370241 | 3908.791367 | 3765.643584 | 3682.27463 | 4724.558067 | 4955.722377 | 4896.362657 | 4983.252935 |
| **Pttg1ip** | 716.9903451 | 677.3840173 | 722.8578748 | 765.9971647 | 692.8993892 | 702.1142185 | 715.3373188 | 760.9712039 | 762.9479203 | 762.9164189 | 648.8672994 | 825.0192291 | 844.4155563 | 738.2041503 | 753.0568242 |
| **Sumo3** | 1471.524246 | 1581.354969 | 1539.695105 | 1456.0847 | 1493.47053 | 1532.58698 | 1622.814422 | 1751.574687 | 1589.851041 | 1584.38427 | 1619.330636 | 1614.469806 | 1716.097811 | 1638.144583 | 1391.861707 |
| **Ube2g2** | 1219.707713 | 1328.623388 | 1329.807878 | 1314.616756 | 1408.599855 | 1280.07837 | 1298.973189 | 1495.962113 | 1435.606146 | 1475.146524 | 1345.028134 | 1283.074069 | 1405.93912 | 1246.001865 | 1193.537581 |
| **Tspear** | 5.494178889 | 0 | 1.566322589 | 4.313047098 | 3.166816221 | 4.90988964 | 2.706194144 | 3.352296052 | 3.762070613 | 3.495607876 | 0.945870699 | 5.205168638 | 1.70416863 | 3.614218606 | 2.155697016 |
| **Trpm2** | 0 | 1.584524017 | 0 | 0 | 0 | 1.402825611 | 0 | 0 | 0.752414123 | 0 | 4.729353494 | 0 | 0 | 0 | 0 |
| **1810043G02Rik** | 28.38659093 | 45.15893448 | 99.4614844 | 54.34439344 | 68.40323038 | 48.39748359 | 57.73214175 | 68.72206907 | 54.92623095 | 54.18192207 | 70.94030241 | 59.85943933 | 62.20215501 | 62.34527096 | 68.26373884 |
| **Pfkl** | 5219.469945 | 5270.919143 | 5250.313318 | 5035.913792 | 4951.633844 | 5118.910656 | 4980.29929 | 2909.792973 | 3845.588581 | 4090.735116 | 4136.292566 | 5102.800321 | 4995.77034 | 4743.661921 | 5096.786311 |
| **Icosl** | 78.74989741 | 70.51131876 | 53.25496803 | 63.83309705 | 60.80287145 | 79.96105985 | 72.16517718 | 62.01747697 | 51.16416034 | 39.3255886 | 33.10547446 | 57.25685501 | 58.79381775 | 50.59906049 | 84.07218362 |
| **D10Jhu81e** | 428.5459534 | 508.6322095 | 462.848325 | 451.1447265 | 504.7905057 | 475.5578823 | 470.8777811 | 533.0150723 | 489.8215938 | 478.024377 | 434.1546507 | 480.6105709 | 518.9193479 | 534.0007991 | 471.3790808 |
| **Pwp2** | 1568.588073 | 1598.784733 | 1582.768976 | 1660.523133 | 1531.472325 | 1559.240667 | 1623.716487 | 1844.600903 | 1841.909772 | 1708.478349 | 1928.630355 | 1567.623288 | 1460.472516 | 1420.387912 | 1452.221223 |
| **Trappc10** | 1723.340778 | 1841.216908 | 1857.658591 | 1786.464108 | 1784.817622 | 1745.816473 | 1931.320554 | 1800.18298 | 1783.973885 | 1761.786369 | 1851.068957 | 1720.308235 | 1872.02924 | 1914.632307 | 1851.025171 |
| **Agpat3** | 3675.605677 | 3531.904034 | 3648.748471 | 3503.919463 | 3331.490665 | 3603.858996 | 3456.711987 | 3371.571755 | 3500.230498 | 3513.085915 | 3330.41073 | 3169.9477 | 3104.14316 | 3036.847184 | 3227.078433 |
| **Rrp1** | 5749.658208 | 5365.990584 | 5533.034546 | 5358.529715 | 5363.953316 | 5510.299002 | 5421.408936 | 5456.699899 | 5527.986559 | 5476.743639 | 5491.725277 | 5447.208979 | 5374.095776 | 5509.876265 | 5259.182153 |
| **Cstb** | 552.1649784 | 480.1107772 | 585.8046483 | 526.191746 | 551.0260225 | 550.6090525 | 548.4553466 | 513.73937 | 490.5740079 | 486.7633967 | 609.14073 | 567.3633815 | 517.2151793 | 559.3003293 | 546.1099107 |
| **Pdxk** | 992.614986 | 1079.060856 | 1015.760199 | 986.8251761 | 963.9788578 | 904.8225194 | 948.0700152 | 1144.809102 | 1109.058417 | 1001.491656 | 1003.568811 | 900.4941743 | 941.5531683 | 850.2449271 | 985.8721019 |
| **Ilvbl** | 491.7290106 | 482.4875632 | 411.9428409 | 376.9603164 | 412.319472 | 369.6445486 | 417.6559629 | 363.7241217 | 392.0077579 | 368.7866309 | 400.1033056 | 427.6913564 | 393.6629536 | 427.3813502 | 406.7081703 |
| **Syde1** | 48.53191352 | 72.88810478 | 53.25496803 | 38.81742388 | 40.53524763 | 42.78618115 | 64.94865946 | 36.03718256 | 27.08690841 | 48.93851026 | 35.94308655 | 65.06460797 | 37.49170987 | 44.27417793 | 54.61099107 |
| **2610008E11Rik** | 61.35166426 | 33.27500436 | 78.31612945 | 59.52004996 | 56.36932874 | 77.15540863 | 57.73214175 | 51.1225148 | 56.43105919 | 52.43411813 | 50.13114703 | 56.38932691 | 68.16674521 | 65.95948956 | 66.82660749 |
| **Ppap2c** | 103.4737024 | 87.14882094 | 79.09929074 | 83.67311371 | 65.23641416 | 91.88507755 | 95.61885977 | 30.17066447 | 27.83932254 | 41.07339254 | 29.32199166 | 77.21000146 | 76.68758836 | 83.12702794 | 93.41353736 |
| **Mier2** | 342.4704841 | 363.6482619 | 353.9889051 | 320.8907041 | 359.7503227 | 350.0049901 | 330.1556856 | 338.5819013 | 352.8822235 | 296.2527675 | 358.4849948 | 318.382815 | 384.2900261 | 371.3609618 | 385.8697658 |
| **Madcam1** | 4.578482408 | 3.961310043 | 3.132645178 | 13.80175071 | 7.600358931 | 2.805651223 | 0.902064715 | 5.866518091 | 12.79104008 | 2.621705907 | 4.729353494 | 0 | 2.556252945 | 0.903554652 | 2.874262688 |
| **Tpgs1** | 473.415081 | 484.8643492 | 390.7974859 | 458.0456018 | 511.7575014 | 476.2592951 | 475.3881047 | 545.5861825 | 638.0471759 | 430.8336707 | 459.6931596 | 475.4054022 | 466.9422047 | 517.7368154 | 425.3908778 |
| **Cdc34** | 3102.37968 | 3142.903388 | 3149.091565 | 3333.985407 | 3003.408504 | 3110.064381 | 3006.581694 | 3763.790393 | 3801.196147 | 3823.321114 | 3419.322576 | 2805.585896 | 2868.967889 | 2951.009492 | 2763.603574 |
| **Gzmm** | 24.723805 | 41.98988645 | 45.42335508 | 31.91654853 | 57.00269198 | 29.45933784 | 41.49497688 | 30.17066447 | 44.39243323 | 26.21705907 | 32.15960376 | 27.7608994 | 39.1958785 | 41.56351397 | 48.86246569 |
| **Bsg** | 8337.416465 | 7543.126583 | 7566.904427 | 7167.421668 | 6749.118731 | 7102.506071 | 7368.966655 | 4996.597266 | 5140.493285 | 5750.274955 | 5293.09243 | 6797.082713 | 6958.972602 | 6550.771224 | 7105.177364 |
| **Hcn2** | 23.80810852 | 26.93690829 | 29.76012919 | 24.15306375 | 34.83497844 | 17.53532014 | 26.15987673 | 35.19910855 | 30.84897903 | 37.57778466 | 31.21373306 | 19.95314644 | 27.26669809 | 23.49242094 | 30.89832389 |
| **Polrmt** | 723.4002204 | 827.9137989 | 722.0747135 | 842.769403 | 646.6638724 | 756.1230046 | 736.0848073 | 919.3671923 | 777.9962027 | 829.3329685 | 964.7881127 | 765.1597897 | 835.0426289 | 785.1889922 | 799.0450272 |
| **Fgf22** | 5.494178889 | 0 | 4.698967767 | 0 | 2.533452977 | 1.402825611 | 2.706194144 | 0 | 0 | 0 | 0 | 0 | 0 | 0.903554652 | 0 |
| **Rnf126** | 2404.618961 | 2423.529484 | 2463.042271 | 2368.725466 | 2243.372611 | 2422.679831 | 2210.058551 | 2261.961761 | 2327.969295 | 2467.89916 | 2263.468582 | 2161.880041 | 2250.354676 | 2108.896557 | 2221.086492 |
| **Fstl3** | 0 | 1.584524017 | 1.566322589 | 5.175656518 | 0 | 1.402825611 | 1.80412943 | 0.838074013 | 0 | 6.117313782 | 0 | 0.867528106 | 0 | 0 | 0 |
| **Prss57** | 3.662785926 | 0.792262009 | 1.566322589 | 4.313047098 | 0.633363244 | 0 | 3.608258859 | 1.676148026 | 1.504828245 | 6.117313782 | 2.837612096 | 0 | 0 | 0 | 0 |
| **Palm** | 64.09875371 | 79.22620085 | 89.28038757 | 75.90962893 | 60.80287145 | 51.90454762 | 55.92801232 | 72.07436512 | 44.39243323 | 84.76849098 | 85.12836289 | 64.19707986 | 34.08337261 | 48.79195118 | 53.8924254 |
| **E130317F20Rik** | 17.39823315 | 22.18333624 | 21.92851625 | 21.56523549 | 15.83408111 | 21.74379698 | 23.45368258 | 21.78992434 | 21.06759543 | 14.85633347 | 17.97154328 | 15.61550591 | 33.23128829 | 24.39597559 | 25.14979852 |
| **Ptbp1** | 11207.20924 | 11363.41399 | 11277.52264 | 11439.06351 | 11398.63831 | 11182.62436 | 11587.92333 | 12361.59169 | 11743.67963 | 11991.68282 | 12253.7549 | 11586.70539 | 11739.16561 | 11831.14461 | 11760.76435 |
| **Lppr3** | 23.80810852 | 42.78214846 | 49.33916155 | 14.66436013 | 30.40143573 | 26.65368662 | 38.78878274 | 31.8468125 | 9.781383594 | 46.31680435 | 59.58985402 | 42.50887721 | 23.00627651 | 35.23863141 | 35.20971793 |
| **Prtn3** | 0 | 0 | 0 | 0 | 0 | 0 | 0 | 0 | 0 | 0 | 0 | 0 | 0 | 0 | 0 |
| **Cfd** | 0.915696482 | 0 | 4.698967767 | 0 | 0.633363244 | 0 | 0 | 0 | 1.504828245 | 0.873901969 | 0 | 0 | 0 | 0 | 0.718565672 |
| **Med16** | 792.0774566 | 827.9137989 | 870.8753595 | 861.7468102 | 909.5096188 | 944.1016365 | 939.9514328 | 906.7960821 | 972.8714605 | 866.0368512 | 822.9075079 | 923.9174332 | 896.3926995 | 919.8186353 | 941.3210303 |
| **R3hdm4** | 932.1790182 | 910.3090478 | 969.5536826 | 885.0372646 | 1065.95034 | 959.5327182 | 1069.848752 | 983.8988913 | 923.9645425 | 862.5412433 | 854.121241 | 937.7978829 | 1050.619961 | 1171.006828 | 1054.854406 |
| **Kiss1r** | 1.831392963 | 5.54583406 | 0.783161294 | 0 | 8.233722176 | 5.611302446 | 4.510323574 | 1.676148026 | 4.514484735 | 0.873901969 | 0.945870699 | 3.470112425 | 7.668758836 | 1.807109303 | 5.748525376 |
| **Arid3a** | 448.691276 | 439.7054147 | 401.7617441 | 398.5255519 | 326.1820708 | 344.3936876 | 349.0990446 | 380.4856019 | 343.853254 | 387.1385722 | 347.1345464 | 339.2034896 | 310.1586907 | 315.3405734 | 329.1030778 |
| **Wdr18** | 2052.991512 | 2089.987178 | 2274.300399 | 2267.800164 | 2042.596463 | 2021.471706 | 1875.392542 | 2737.987801 | 2641.725984 | 2600.732259 | 2573.714171 | 1898.151497 | 1941.900154 | 1985.10957 | 1872.582141 |
| **Grin3b** | 8.241268334 | 6.338096068 | 16.44638718 | 23.29045433 | 17.1008076 | 12.6254305 | 11.72684129 | 10.89496217 | 12.79104008 | 20.09974528 | 8.512836289 | 10.41033728 | 10.22501178 | 16.26398373 | 17.9641418 |
| **Tmem259** | 1868.020822 | 1783.381781 | 1927.359946 | 1677.775321 | 1841.186951 | 2016.561816 | 1848.330601 | 1707.994839 | 1811.060793 | 1933.945057 | 1781.074526 | 1899.019025 | 1902.704276 | 1791.748874 | 1886.234889 |
| **Cnn2** | 5125.153207 | 5688.441221 | 5662.256159 | 6048.617251 | 6630.679804 | 6232.754192 | 6317.159198 | 4976.48349 | 5173.599507 | 4890.355418 | 4757.729615 | 6129.953599 | 6044.686132 | 6662.812001 | 6251.521346 |
| **Abca7** | 814.9698686 | 963.3906023 | 935.0945856 | 810.8528545 | 881.0082728 | 887.2871992 | 903.8688442 | 662.9165443 | 681.6871951 | 773.4032425 | 789.8020335 | 954.2809169 | 1021.649094 | 1181.849484 | 1054.854406 |
| **Hmha1** | 3728.716073 | 4524.608331 | 4684.087702 | 4704.671775 | 5069.439407 | 4702.972862 | 4915.350631 | 3974.14697 | 4019.396243 | 3911.585213 | 4056.839427 | 5201.698525 | 5301.668609 | 5864.069689 | 5358.344216 |
| **Polr2e** | 1903.732985 | 1868.946078 | 1895.250333 | 1751.959731 | 1936.824801 | 1947.121949 | 1765.340647 | 2346.607237 | 2115.036099 | 2386.626277 | 2086.590761 | 1886.873631 | 1764.666617 | 1943.546056 | 1807.911231 |
| **Gpx4** | 2774.560339 | 2609.711056 | 2616.541885 | 2790.541473 | 2481.517191 | 2620.478242 | 2472.559383 | 2355.826051 | 2400.953465 | 2556.163259 | 2399.673963 | 2553.135217 | 2520.465404 | 2562.480992 | 2642.884541 |
| **Sbno2** | 858.9232997 | 868.3191613 | 815.2709076 | 829.8302617 | 791.0706921 | 793.9992961 | 789.3066254 | 820.4744588 | 777.2437886 | 735.8254578 | 846.5542754 | 811.1387794 | 794.1425817 | 796.031648 | 806.2306839 |
| **Stk11** | 1965.084649 | 2054.335388 | 2061.280527 | 1945.184241 | 2066.030903 | 2097.224289 | 2033.253867 | 1992.940003 | 2050.328484 | 2169.898589 | 2002.408269 | 2047.366331 | 2034.777345 | 2012.216209 | 1958.810022 |
| **Dos** | 354.3745384 | 357.3101658 | 321.879292 | 307.9515628 | 306.5478102 | 287.5792503 | 290.4648382 | 315.1158289 | 223.4669944 | 285.7659438 | 219.4420021 | 256.7883195 | 316.9753652 | 270.1628408 | 326.9473807 |
| **Atp5d** | 5040.909131 | 4942.130409 | 4664.50867 | 4869.430174 | 4847.128908 | 4853.075203 | 4869.34533 | 4906.085272 | 4865.109717 | 4730.431358 | 4578.014182 | 4650.818178 | 4602.107386 | 4524.09814 | 4519.778077 |
| **Midn** | 1923.878308 | 1848.347266 | 1887.41872 | 1911.542474 | 1738.582106 | 1699.523228 | 1864.567765 | 1675.309952 | 1679.388322 | 1602.736211 | 1490.692221 | 1654.376099 | 1755.293689 | 1582.124195 | 1662.760965 |
| **Cirbp** | 696.8450225 | 799.3923666 | 776.8960041 | 693.5379734 | 770.169705 | 763.8385454 | 718.9455777 | 557.3192187 | 580.1112885 | 675.526222 | 663.0553598 | 722.6509125 | 731.9404267 | 773.4427817 | 750.1825615 |
| **1600002K03Rik** | 186.8020822 | 177.4666899 | 154.282775 | 152.6818673 | 156.4407213 | 128.3585434 | 111.8560246 | 169.2909506 | 204.6566413 | 188.7628253 | 180.6613035 | 138.804497 | 159.3397669 | 175.2896024 | 180.3599837 |
| **Efna2** | 6.409875371 | 0 | 1.566322589 | 10.35131304 | 3.166816221 | 7.715540863 | 3.608258859 | 3.352296052 | 6.019312981 | 5.243411813 | 15.13393118 | 7.807752957 | 2.556252945 | 0.903554652 | 2.874262688 |
| **Mum1** | 596.1184095 | 702.7364015 | 660.2049713 | 593.4752807 | 765.1027991 | 714.0382362 | 662.1155006 | 610.1178815 | 610.2078534 | 637.0745353 | 621.4370491 | 759.9546211 | 788.1779915 | 799.6458666 | 755.2125212 |
| **Ndufs7** | 1237.105947 | 1189.977537 | 1300.047749 | 1195.576656 | 1231.89151 | 1202.922962 | 1096.008628 | 1226.102281 | 1336.287482 | 1334.448307 | 1181.392503 | 1178.970696 | 1129.863802 | 1206.24546 | 1090.78269 |
| **Gamt** | 5.494178889 | 0 | 7.04845165 | 3.450437679 | 5.066905954 | 12.6254305 | 5.412388289 | 4.190370065 | 2.257242368 | 6.991215751 | 3.783482795 | 6.072696744 | 18.74585493 | 0.903554652 | 2.874262688 |
| **Dazap1** | 4644.412554 | 4383.585693 | 4478.899443 | 4292.344472 | 4272.035083 | 4329.119837 | 4279.395007 | 4343.73761 | 4394.098476 | 4324.940844 | 4322.629093 | 4568.403008 | 4309.842466 | 4457.235096 | 4232.351808 |
| **Rps15** | 16111.67959 | 15190.83175 | 15614.66989 | 14792.88894 | 16687.2214 | 17673.49847 | 17040.90453 | 14077.1292 | 13731.55774 | 14083.80413 | 13395.42084 | 15859.28131 | 15714.99102 | 15861.90191 | 15674.073 |
| **Apc2** | 1.831392963 | 3.961310043 | 1.566322589 | 1.725218839 | 6.333632443 | 6.312715251 | 1.80412943 | 2.514222039 | 9.028969471 | 7.86511772 | 5.675224193 | 0 | 2.556252945 | 0.903554652 | 6.467091048 |
| **2310011J03Rik** | 981.6266282 | 1088.568 | 1043.954006 | 1115.35398 | 1084.317874 | 1020.555632 | 1031.962034 | 1159.05636 | 1092.505306 | 1035.573833 | 1122.748519 | 1050.576537 | 952.6302644 | 1012.884764 | 957.8480407 |
| **Pcsk4** | 241.7438711 | 240.8476506 | 220.851485 | 176.834931 | 181.7752511 | 178.8602655 | 189.4335901 | 223.7657615 | 205.4090555 | 241.1969434 | 262.0061836 | 220.352139 | 217.2815004 | 165.3505012 | 204.0726508 |
| **Reep6** | 31.13368037 | 15.05297816 | 30.54329049 | 19.84001665 | 29.76807248 | 23.14662259 | 21.64955315 | 32.68488651 | 30.0965649 | 26.21705907 | 33.10547446 | 18.21809023 | 17.0416863 | 12.64976512 | 21.55697016 |
| **Adamtsl5** | 6.409875371 | 1.584524017 | 0 | 7.763484777 | 3.166816221 | 12.6254305 | 3.608258859 | 3.352296052 | 0.752414123 | 1.747803938 | 3.783482795 | 3.470112425 | 3.408337261 | 0.903554652 | 10.77848508 |
| **Mex3d** | 412.0634167 | 351.7643318 | 349.2899373 | 358.8455186 | 362.2837757 | 411.0279041 | 372.5527272 | 337.7438273 | 294.1939219 | 326.8393364 | 324.4336497 | 360.0241641 | 345.946232 | 402.9853746 | 399.5225136 |
| **Mbd3** | 2956.783939 | 3108.043859 | 2954.867564 | 3045.011251 | 3034.443303 | 3168.281643 | 2746.787057 | 3260.107911 | 3362.538714 | 3264.023854 | 3372.974912 | 3019.865338 | 3093.918148 | 3114.552884 | 2900.131052 |
| **Uqcr11** | 923.9377499 | 881.7876155 | 868.5258756 | 873.8233421 | 867.0742814 | 1054.92486 | 911.9874266 | 822.9886808 | 975.1287029 | 942.9402244 | 967.6257248 | 910.0369835 | 837.5988818 | 962.2857039 | 898.9256556 |
| **Tcf3** | 4468.59883 | 4763.871457 | 4568.962992 | 4522.661187 | 4747.690879 | 4926.022135 | 4817.025577 | 4094.829628 | 4510.722665 | 4691.979671 | 4353.842826 | 5003.902117 | 5144.033011 | 4949.672381 | 4905.647843 |
| **Atp8b3** | 0 | 1.584524017 | 0 | 0 | 0 | 0.701412806 | 0 | 0 | 0 | 0 | 2.837612096 | 2.602584319 | 11.0770961 | 1.807109303 | 0.718565672 |
| **Rexo1** | 1512.730588 | 1712.870462 | 1727.653816 | 1607.041349 | 1544.772953 | 1564.150557 | 1749.103482 | 1733.137059 | 1691.426948 | 1808.103174 | 1732.83512 | 1715.103066 | 1808.975001 | 1813.434186 | 1733.898966 |
| **Klf16** | 1693.122794 | 1452.216262 | 1307.879362 | 1393.976822 | 1352.230527 | 1341.101285 | 1444.205608 | 1457.410709 | 1403.252339 | 1382.512915 | 1394.21341 | 1306.497328 | 1244.895184 | 1210.763233 | 1228.028733 |
| **Abhd17a** | 1517.30907 | 1685.933554 | 1726.087493 | 1659.660523 | 1629.010264 | 1815.256341 | 1594.850416 | 1624.187437 | 1756.886976 | 1753.04735 | 1555.957299 | 1605.794525 | 1682.866522 | 1674.286769 | 1591.622963 |
| **Adat3** | 119.9562391 | 149.7375196 | 126.8721297 | 116.4522717 | 132.3729181 | 140.2825611 | 138.0159014 | 167.6148026 | 149.7304104 | 123.2201776 | 132.4218978 | 91.09045116 | 139.7418277 | 150.8936268 | 130.0603866 |
| **Scamp4** | 1263.661145 | 1308.816838 | 1297.698265 | 1208.515797 | 1223.657788 | 1261.140225 | 1160.055223 | 1413.83086 | 1391.966127 | 1351.052444 | 1442.452816 | 1149.474741 | 1264.493124 | 1171.910383 | 1222.280208 |
| **Csnk1g2** | 2478.790376 | 2832.33668 | 2818.597499 | 2743.960564 | 2776.664463 | 3145.836434 | 2990.344529 | 2093.508885 | 2366.342416 | 2291.370962 | 2363.730876 | 3032.010731 | 2903.051262 | 3106.420892 | 2873.544122 |
| **Btbd2** | 301.2641424 | 312.9434934 | 288.2033564 | 305.3637346 | 295.7806351 | 328.9626059 | 328.3515562 | 289.1355345 | 257.3256299 | 273.5313163 | 224.1713556 | 303.6348372 | 299.0815946 | 306.3050269 | 349.2229166 |
| **Mknk2** | 8091.094111 | 8272.007631 | 8460.491464 | 8013.641509 | 7306.478386 | 6972.043289 | 7410.461632 | 9847.369653 | 9575.222124 | 9389.202754 | 8221.508114 | 7417.365309 | 7589.514995 | 7415.473025 | 7174.159669 |
| **Mob3a** | 3678.352766 | 3599.246305 | 3691.039181 | 3646.250017 | 3352.391652 | 3391.330916 | 3665.088936 | 3536.672335 | 3406.178733 | 3594.358798 | 3444.861085 | 3180.358038 | 3563.416606 | 3348.573539 | 3409.594113 |
| **Izumo4** | 53.11039593 | 56.2506026 | 75.18348427 | 59.52004996 | 45.60215359 | 42.08476834 | 58.63420646 | 42.74177467 | 47.40208972 | 37.57778466 | 50.13114703 | 35.56865236 | 46.86463733 | 65.95948956 | 58.9223851 |
| **Ap3d1** | 2066.726959 | 2106.624681 | 2093.39014 | 2042.659106 | 2016.62857 | 1936.600757 | 2017.016702 | 1807.725646 | 1907.369801 | 1878.015331 | 2046.864192 | 2035.220937 | 2022.848164 | 2047.45484 | 2089.588974 |
| **Dot1l** | 3312.98987 | 3165.086724 | 3103.66821 | 2736.197079 | 3047.110568 | 3003.449634 | 3124.752172 | 2848.61357 | 2698.909458 | 2494.990121 | 2791.264432 | 2948.728033 | 3146.747376 | 2875.110901 | 3115.700754 |
| **Plekhj1** | 1146.451995 | 1021.225729 | 1154.379748 | 1073.086118 | 1083.684511 | 1205.0272 | 1114.951987 | 1388.68864 | 1202.357768 | 1383.386817 | 1171.933796 | 1133.859235 | 1175.876355 | 1086.976246 | 1107.3097 |
| **Sf3a2** | 3676.521373 | 3344.137938 | 3352.713502 | 3359.86369 | 3460.696767 | 3506.362616 | 3521.660647 | 3404.256641 | 3026.962015 | 3028.070322 | 3401.351033 | 3360.803884 | 3330.797588 | 3185.030147 | 3357.138819 |
| **Amh** | 6.409875371 | 10.29940611 | 11.74741942 | 11.21392246 | 11.4005384 | 8.416953669 | 9.020647148 | 12.5711102 | 13.54345421 | 11.3607256 | 5.675224193 | 6.072696744 | 19.59793925 | 8.131991864 | 15.08987911 |
| **Jsrp1** | 0 | 0.792262009 | 2.349483883 | 0 | 2.533452977 | 1.402825611 | 2.706194144 | 0.838074013 | 3.762070613 | 2.621705907 | 8.512836289 | 1.735056213 | 0 | 0 | 0 |
| **Lingo3** | 4.578482408 | 0 | 0.783161294 | 0 | 5.700269198 | 3.507064029 | 4.510323574 | 0 | 0.752414123 | 3.495607876 | 0.945870699 | 1.735056213 | 1.70416863 | 0 | 2.155697016 |
| **Lsm7** | 933.0947147 | 861.9810653 | 1007.145425 | 894.5259682 | 984.2464816 | 976.3666256 | 891.2399382 | 1130.561844 | 1068.428054 | 1087.134049 | 1027.215579 | 947.3406921 | 936.4406624 | 1014.691874 | 887.4286049 |
| **Sppl2b** | 330.5664298 | 370.77862 | 358.6878729 | 389.0368483 | 367.3506817 | 364.0332462 | 372.5527272 | 410.6562664 | 376.2070613 | 355.6781013 | 459.6931596 | 366.964389 | 364.6920869 | 417.442249 | 421.0794838 |
| **Tmprss9** | 0 | 0.792262009 | 1.566322589 | 1.725218839 | 1.266726489 | 0 | 0 | 2.514222039 | 0 | 0 | 1.891741398 | 1.735056213 | 0.852084315 | 4.517773258 | 0.718565672 |
| **Timm13** | 1945.855023 | 1813.487737 | 1823.199494 | 1820.968485 | 1829.153049 | 1961.150205 | 1713.922958 | 2023.948742 | 2024.746404 | 2184.754922 | 2035.513744 | 1676.064301 | 1767.22287 | 1860.419028 | 1720.246219 |
| **Lmnb2** | 1074.111973 | 1186.808489 | 1018.892844 | 1174.01142 | 1063.416887 | 1068.251703 | 998.5856393 | 1107.933845 | 1128.621184 | 1089.755755 | 1131.261356 | 1154.679909 | 1019.092841 | 1121.311323 | 1132.459499 |
| **Gadd45b** | 1681.21874 | 1427.656139 | 1339.988975 | 1142.957481 | 1210.990523 | 1423.867996 | 1366.628043 | 1249.568353 | 1026.292863 | 1147.433285 | 1146.395287 | 1096.555526 | 1154.574247 | 1213.473897 | 1225.154471 |
| **Gng7** | 4.578482408 | 2.376786026 | 4.698967767 | 6.900875357 | 3.166816221 | 4.90988964 | 6.314453003 | 5.028444078 | 10.53379772 | 19.22584332 | 6.621094891 | 6.072696744 | 3.408337261 | 4.517773258 | 5.029959704 |
| **Slc39a3** | 312.2525002 | 389.7929082 | 348.506776 | 373.5098787 | 405.9858396 | 385.7770431 | 380.6713096 | 412.3324144 | 394.2650002 | 393.255886 | 377.4024088 | 473.670346 | 389.402532 | 393.0462734 | 386.5883315 |
| **Sgta** | 1780.11396 | 1679.595458 | 1906.997752 | 1849.434596 | 1712.614213 | 1745.115061 | 1713.020893 | 2096.861181 | 2056.347797 | 2105.229843 | 2096.995339 | 1781.90273 | 1771.483291 | 1856.804809 | 1772.701513 |
| **Thop1** | 1515.477677 | 1399.134707 | 1498.187556 | 1486.27603 | 1474.469633 | 1412.645391 | 1384.669337 | 1922.541786 | 1649.291757 | 2017.839646 | 1887.012044 | 1323.84789 | 1400.826614 | 1336.35733 | 1483.119547 |
| **Map2k2** | 2424.764283 | 2447.297344 | 2462.25911 | 2574.026508 | 2546.120242 | 2430.395372 | 2423.847889 | 2699.436396 | 2822.305374 | 2854.16383 | 2738.295673 | 2437.753979 | 2413.102781 | 2437.79045 | 2320.248555 |
| **Zbtb7a** | 2463.223535 | 2341.134235 | 2344.001754 | 2296.266275 | 2117.333326 | 2179.991 | 2311.991864 | 2368.397161 | 2369.352072 | 2367.400434 | 2217.120918 | 2154.939816 | 2140.4358 | 2112.510775 | 2073.061964 |
| **Pias4** | 626.3363934 | 549.8298339 | 631.2280034 | 549.4822003 | 537.7253944 | 561.8316574 | 528.6099229 | 648.6692861 | 501.1078056 | 589.883829 | 618.599437 | 582.9788874 | 521.4756009 | 578.274977 | 516.6487181 |
| **Eef2** | 109397.343 | 104122.2422 | 106483.3086 | 104377.465 | 103871.5721 | 107482.3941 | 109665.8115 | 101929.9138 | 101633.8424 | 100368.515 | 104117.663 | 104518.9187 | 106119.4327 | 100534.9119 | 104836.5758 |
| **Dapk3** | 594.2870165 | 638.5631789 | 601.4678742 | 618.4909539 | 621.9627059 | 725.9622539 | 598.9709706 | 664.5926924 | 666.6389126 | 635.3267314 | 551.4426174 | 644.573383 | 638.2111521 | 705.6761829 | 602.8765988 |
| **Atcay** | 52.19469945 | 47.53572051 | 55.60445191 | 50.89395576 | 66.50314065 | 55.41161165 | 64.94865946 | 62.01747697 | 53.4214027 | 70.78605948 | 46.34766424 | 46.84651774 | 31.52711966 | 53.30972444 | 74.73082988 |
| **Zfr2** | 7.325571853 | 3.961310043 | 0.783161294 | 2.587828259 | 1.266726489 | 5.611302446 | 0.902064715 | 0 | 3.00965649 | 0 | 0 | 1.735056213 | 5.112505891 | 5.421327909 | 0.718565672 |
| **Matk** | 125.450418 | 107.7476332 | 93.19619404 | 88.84877022 | 73.47013634 | 75.75258302 | 85.6961479 | 25.14222039 | 48.15450385 | 27.09096104 | 46.34766424 | 97.1631479 | 83.50426289 | 96.68034772 | 84.07218362 |
| **Mrpl54** | 1050.303864 | 1099.659668 | 1108.956393 | 1081.712212 | 1064.683614 | 1120.857664 | 989.5649921 | 1041.725998 | 1204.61501 | 1115.972814 | 997.8935872 | 1060.986874 | 982.4532154 | 957.7679307 | 1049.105881 |
| **Apba3** | 487.1505282 | 494.3714933 | 458.1493573 | 496.8630257 | 431.3203694 | 408.2222529 | 460.0530045 | 344.4484194 | 363.4160212 | 401.1210037 | 384.9693744 | 387.7850635 | 396.2192066 | 369.5538525 | 505.8702331 |
| **Tjp3** | 88.82255871 | 76.84941482 | 111.9920651 | 99.20008326 | 99.43802935 | 100.3020312 | 94.71679505 | 92.18814144 | 52.66898858 | 83.02068705 | 57.69811262 | 143.1421375 | 126.1084786 | 99.39101167 | 94.8506687 |
| **Pip5k1c** | 1500.826533 | 1813.487737 | 1856.092268 | 1778.700623 | 1536.539231 | 1586.595767 | 1731.062188 | 2071.71896 | 1941.228436 | 2002.983313 | 1975.92389 | 1780.167674 | 1738.252003 | 1694.164972 | 1825.875372 |
| **Cactin** | 1737.991922 | 1729.507965 | 1798.138332 | 1929.657272 | 1839.286861 | 1743.010822 | 1707.608505 | 2040.710222 | 1940.476022 | 1882.384841 | 1914.442294 | 1898.151497 | 1689.683197 | 1837.830161 | 1851.743737 |
| **Tbxa2r** | 659.3014667 | 871.4882094 | 863.0437465 | 899.7016247 | 750.5354445 | 700.0099801 | 796.5231432 | 746.7239456 | 902.8969471 | 835.4502823 | 773.7222316 | 822.4166448 | 731.0883424 | 846.6307085 | 769.5838347 |
| **Gipc3** | 24.723805 | 16.63750218 | 21.14535495 | 22.42784491 | 10.76717515 | 9.118366474 | 15.33510015 | 29.33259046 | 40.63036262 | 36.70388269 | 38.78069865 | 20.82067455 | 9.372927467 | 13.55331977 | 16.52701046 |
| **Hmg20b** | 497.2231895 | 472.1881571 | 477.7283896 | 423.541225 | 407.8859293 | 502.2115689 | 406.8311864 | 532.1769983 | 440.1622617 | 492.8807105 | 496.5821169 | 493.6234925 | 464.3859518 | 430.9955688 | 487.1875256 |
| **Mfsd12** | 64.09875371 | 102.2017991 | 115.1247103 | 124.2157564 | 143.1400932 | 101.7048568 | 114.5622188 | 154.2056184 | 154.2448951 | 126.7157855 | 144.7182169 | 137.9369689 | 120.1438884 | 114.7514407 | 103.4734568 |
| **4930404N11Rik** | 86.07546927 | 120.4238253 | 100.2446457 | 102.6505209 | 96.27121313 | 98.89920561 | 85.6961479 | 91.35006742 | 147.473168 | 124.9679816 | 87.02010429 | 98.03067601 | 89.46885309 | 103.9087849 | 88.38357765 |
| **Fzr1** | 2573.107113 | 2757.864052 | 2946.25279 | 2785.365816 | 2756.396839 | 2799.338508 | 2717.018921 | 2781.567649 | 2981.817168 | 2617.336397 | 2619.115965 | 2938.317696 | 2834.884517 | 2873.303792 | 2874.262688 |
| **Dohh** | 1502.657926 | 1403.888279 | 1458.24633 | 1505.253437 | 1343.363441 | 1365.650733 | 1250.261695 | 1647.65351 | 1587.593799 | 1519.715524 | 1624.05999 | 1185.043393 | 1351.405724 | 1399.606155 | 1302.759563 |
| **Mir6913** | 0 | 0 | 0 | 0.86260942 | 1.266726489 | 0 | 2.706194144 | 0 | 2.257242368 | 0 | 0.945870699 | 2.602584319 | 0.852084315 | 0.903554652 | 0 |
| **Smim24** | 30.21798389 | 41.98988645 | 39.94122602 | 25.87828259 | 41.16861088 | 36.4734659 | 29.76813559 | 13.40918421 | 19.56276719 | 9.612921658 | 19.86328467 | 13.01292159 | 36.63962555 | 39.75640467 | 35.20971793 |
| **Nfic** | 373.6041645 | 447.6280348 | 407.2438731 | 397.6629425 | 354.0500536 | 340.1852108 | 406.8311864 | 305.8970148 | 323.5380727 | 313.7308068 | 354.701512 | 381.7123668 | 412.4088085 | 379.4929537 | 388.0254629 |
| **Celf5** | 0 | 6.338096068 | 7.831612945 | 14.66436013 | 1.266726489 | 0 | 1.80412943 | 9.218814144 | 6.019312981 | 7.86511772 | 9.458706988 | 0.867528106 | 0 | 7.228437213 | 5.748525376 |
| **Ncln** | 2259.938917 | 2423.529484 | 2278.999367 | 2245.372319 | 2144.567945 | 2212.957402 | 2141.501633 | 2352.473755 | 2406.972778 | 2469.646964 | 2471.560136 | 2287.671616 | 2171.110835 | 2187.505811 | 2217.493664 |
| **S1pr4** | 1324.097112 | 1552.041275 | 1632.108138 | 1679.50054 | 1502.970979 | 1537.49687 | 1489.308844 | 1107.933845 | 1294.904705 | 1171.028638 | 1321.381366 | 1597.986772 | 1587.433079 | 1712.236065 | 1587.311569 |
| **Gna15** | 51.27900297 | 40.40536243 | 78.31612945 | 83.67311371 | 50.0356963 | 48.39748359 | 55.92801232 | 62.85555098 | 66.96485691 | 55.05582404 | 60.53572472 | 66.79966418 | 43.45630007 | 56.0203884 | 35.9282836 |
| **Gna11** | 212.4415837 | 221.8333624 | 225.5504528 | 225.1410585 | 221.0437723 | 214.6323185 | 254.3822496 | 258.96487 | 258.078044 | 242.0708454 | 291.3281752 | 249.8480946 | 247.9565357 | 230.4064362 | 245.0308941 |
| **Aes** | 5158.118281 | 5148.910793 | 5285.555576 | 5113.54864 | 5042.204788 | 5281.638427 | 5159.810169 | 4340.385314 | 4849.30902 | 4702.466495 | 4355.734568 | 5252.015155 | 4965.095304 | 5195.439247 | 5135.588858 |
| **Tle2** | 2.747089445 | 7.130358077 | 7.04845165 | 5.175656518 | 1.900089733 | 5.611302446 | 0 | 1.676148026 | 3.00965649 | 13.10852953 | 13.24218978 | 5.205168638 | 10.22501178 | 5.421327909 | 2.155697016 |
| **Tle6** | 19.22962611 | 7.922620085 | 12.53058071 | 6.038265938 | 6.333632443 | 5.611302446 | 2.706194144 | 13.40918421 | 9.781383594 | 12.23462756 | 9.458706988 | 7.807752957 | 7.668758836 | 4.517773258 | 12.9341821 |
| **BC025920** | 11.90405426 | 21.39107423 | 23.49483883 | 22.42784491 | 28.50134599 | 22.44520978 | 22.55161787 | 22.62799835 | 26.33449429 | 25.3431571 | 17.97154328 | 37.30370857 | 34.93545692 | 29.8173035 | 36.64684927 |
| **Sirt6** | 385.5082187 | 392.9619562 | 399.4122602 | 350.2194244 | 344.5496049 | 381.5685663 | 359.9238212 | 302.5447187 | 342.3484258 | 343.4434738 | 292.2740459 | 346.1437144 | 342.5378947 | 373.1680711 | 326.9473807 |
| **Ankrd24** | 21.06101908 | 31.69048034 | 28.9769679 | 21.56523549 | 19.00089733 | 16.83390734 | 21.64955315 | 23.46607237 | 12.03862596 | 17.47803938 | 13.24218978 | 15.61550591 | 24.71044514 | 15.36042908 | 15.80844478 |
| **Gm10778** | 0 | 2.376786026 | 4.698967767 | 3.450437679 | 1.266726489 | 4.90988964 | 3.608258859 | 5.028444078 | 0 | 4.369509845 | 0 | 1.735056213 | 2.556252945 | 1.807109303 | 0.718565672 |
| **Zfp433** | 20.14532259 | 31.69048034 | 43.0738712 | 29.32872027 | 24.06780328 | 23.14662259 | 17.13922958 | 31.00873848 | 16.5531107 | 21.84754922 | 19.86328467 | 19.95314644 | 23.00627651 | 23.49242094 | 20.83840449 |
| **Zfp781** | 10.0726613 | 11.09166812 | 14.0969033 | 12.93914129 | 12.03390164 | 14.72966892 | 11.72684129 | 18.43762829 | 6.771727103 | 7.86511772 | 5.675224193 | 3.470112425 | 16.18960199 | 9.939101167 | 18.68270747 |
| **Zfp873** | 49.44761 | 43.57441047 | 55.60445191 | 38.81742388 | 55.7359655 | 45.59183237 | 70.36104775 | 51.1225148 | 56.43105919 | 43.69509845 | 18.91741398 | 45.97898963 | 91.17302172 | 50.59906049 | 61.07808212 |
| **AU041133** | 36.62785926 | 44.36667248 | 49.33916155 | 24.15306375 | 24.06780328 | 17.53532014 | 32.47432973 | 46.93214473 | 8.276555348 | 27.964863 | 31.21373306 | 25.15831508 | 22.15419219 | 11.74621047 | 25.14979852 |
| **Zfp938** | 51.27900297 | 74.4726288 | 92.41303275 | 66.42092531 | 58.26941847 | 67.33562935 | 63.14453003 | 74.58858716 | 71.47934165 | 66.41654964 | 59.58985402 | 58.99191123 | 63.90632364 | 66.86304422 | 48.86246569 |
| **1190007I07Rik** | 197.79044 | 222.6256244 | 194.224001 | 201.8506042 | 184.9420673 | 184.4715679 | 158.7633898 | 200.2996891 | 215.1904391 | 159.0501583 | 186.3365277 | 203.0015769 | 141.4459963 | 166.2540559 | 155.9287508 |
| **Glt8d2** | 9.156964816 | 4.753572051 | 2.349483883 | 3.450437679 | 0 | 0.701412806 | 0.902064715 | 4.190370065 | 3.762070613 | 4.369509845 | 0.945870699 | 6.94022485 | 2.556252945 | 0.903554652 | 1.437131344 |
| **Hcfc2** | 285.6973022 | 294.7214672 | 285.8538725 | 269.1341389 | 283.1133702 | 267.9396918 | 257.0884437 | 284.9451644 | 279.1456395 | 261.2966887 | 318.7584255 | 307.1049496 | 263.2940534 | 279.1983873 | 303.2347136 |
| **Nfyb** | 773.7635269 | 793.8465325 | 837.1994238 | 797.9137132 | 818.3053116 | 826.9656979 | 862.3738673 | 821.3125328 | 837.4369184 | 779.5205563 | 839.9331805 | 939.5329391 | 867.4218328 | 868.3160202 | 849.3446243 |
| **Txnrd1** | 4454.863383 | 4364.571405 | 4597.93996 | 4522.661187 | 3686.174082 | 3769.392418 | 3900.527827 | 6505.968563 | 5801.865299 | 5863.882211 | 6511.37389 | 4399.235027 | 4235.711131 | 4142.798077 | 4262.531566 |

| | | | | | | | | | | | | | | | |
|---|---|---|---|---|---|---|---|---|---|---|---|---|---|---|---|
| **Eid3** | 8.241268334 | 7.922620085 | 15.66322589 | 8.626094197 | 9.500448664 | 11.9240177 | 6.314453003 | 6.704592104 | 4.514484735 | 8.739019689 | 19.86328467 | 5.205168638 | 4.260421576 | 8.131991864 | 7.18565672 |
| **Chst11** | 383.6768258 | 250.3547947 | 324.2287759 | 250.1567317 | 241.3113961 | 235.6747027 | 311.2123266 | 231.3084276 | 218.9525097 | 291.8832576 | 326.3253911 | 334.865849 | 324.6441241 | 287.3303792 | 275.2106524 |
| **Slc41a2** | 13.73544722 | 3.169048034 | 18.01270977 | 22.42784491 | 8.233722176 | 11.22260489 | 9.922711863 | 1.676148026 | 0 | 2.621705907 | 9.458706988 | 6.94022485 | 10.22501178 | 9.035546516 | 10.77848508 |
| **D10Wsu102e** | 1344.242435 | 1311.193624 | 1336.85633 | 1263.7228 | 1331.962903 | 1453.327333 | 1529.901756 | 1402.097824 | 1525.895841 | 1336.19611 | 1379.079479 | 1453.977106 | 1451.951673 | 1364.367524 | 1399.047363 |
| **Aldh1l2** | 61.35166426 | 41.98988645 | 67.35187133 | 41.40525214 | 29.13470924 | 37.87629151 | 43.29910631 | 22.62799835 | 43.64001911 | 56.80362798 | 56.75224193 | 38.17123668 | 24.71044514 | 20.78175699 | 37.36541494 |
| **A230046K03Rik** | 804.8972073 | 762.1560522 | 867.7427143 | 798.7763226 | 727.7343677 | 740.6919228 | 792.0128196 | 759.2950558 | 880.3245234 | 818.8461449 | 859.7964652 | 812.8738356 | 803.5155092 | 774.3463364 | 779.6437541 |
| **Appl2** | 97.06382705 | 104.5785851 | 90.84671016 | 86.26094197 | 81.07049527 | 98.89920561 | 118.1704776 | 67.04592104 | 65.46002866 | 93.50751067 | 76.6155266 | 110.1760695 | 112.4751296 | 108.4265582 | 94.13210303 |
| **1500009L16Rik** | 305.8426248 | 342.2571877 | 284.2875499 | 277.7602331 | 224.2105885 | 258.8213253 | 264.3049614 | 242.2033898 | 249.0490746 | 262.1705907 | 243.0887696 | 281.9466345 | 230.9148494 | 300.883699 | 286.7077031 |
| **Nuak1** | 4.578482408 | 1.584524017 | 7.04845165 | 9.488703616 | 4.43354271 | 2.104238417 | 4.510323574 | 10.05688816 | 3.762070613 | 6.117313782 | 6.621094891 | 5.205168638 | 3.408337261 | 7.228437213 | 7.18565672 |
| **Ckap4** | 90.65395167 | 101.4095371 | 97.89516181 | 106.963568 | 96.90457637 | 83.46812388 | 104.6395069 | 104.7592516 | 115.1193608 | 87.39019689 | 92.69532848 | 104.1033728 | 87.76468446 | 83.12702794 | 87.66501198 |
| **Tcp11l2** | 77.83420093 | 80.01846286 | 74.40032298 | 101.7879115 | 109.5718413 | 101.7048568 | 127.1911248 | 72.91243914 | 60.19312981 | 36.70388269 | 67.15681961 | 130.1292159 | 132.9251532 | 118.3656594 | 104.9105881 |
| **Polr3b** | 1169.344407 | 1371.405537 | 1326.675233 | 1283.562816 | 1181.855814 | 1107.53082 | 1200.648135 | 1435.620784 | 1467.207539 | 1365.034875 | 1392.321669 | 1204.129012 | 1324.139026 | 1177.331711 | 1113.058226 |
| **Rfx4** | 4.578482408 | 1.584524017 | 7.831612945 | 4.313047098 | 4.43354271 | 1.402825611 | 6.314453003 | 0 | 0 | 7.86511772 | 2.837612096 | 3.470112425 | 0 | 0.903554652 | 2.155697016 |
| **Ric8b** | 220.6828521 | 247.1857467 | 222.4178076 | 201.8506042 | 172.2748024 | 168.3390734 | 174.09849 | 246.3937598 | 141.453855 | 256.0532769 | 208.0915537 | 196.061352 | 164.4522728 | 209.6246792 | 206.9469135 |
| **Fhl4** | 16.48253667 | 5.54583406 | 14.0969033 | 18.11479781 | 4.43354271 | 9.118366474 | 11.72684129 | 16.76148026 | 4.514484735 | 2.621705907 | 8.512836289 | 14.74797781 | 5.964590206 | 1.807109303 | 17.24557613 |
| **Tmem263** | 501.8016719 | 400.0923143 | 480.8610348 | 444.2438511 | 409.1526558 | 437.6815908 | 455.542681 | 406.4658963 | 374.702233 | 392.381984 | 386.8611158 | 442.4393342 | 475.4630479 | 403.8889293 | 442.6364539 |
| **Mterf2** | 75.08711149 | 71.30358077 | 75.96664557 | 61.2452688 | 86.77076447 | 75.75258302 | 73.96930661 | 108.9496217 | 85.77520997 | 95.25531461 | 54.86050053 | 77.21000146 | 81.80009426 | 78.60925469 | 96.28780004 |
| **Cry1** | 734.3885782 | 726.5042618 | 707.9778102 | 654.7205495 | 701.1331114 | 723.1566027 | 752.3219721 | 740.0193535 | 665.1340844 | 781.2683602 | 721.6993432 | 696.6250693 | 693.5966325 | 640.620248 | 675.4517316 |
| **Btbd11** | 133.6916863 | 130.7232314 | 116.6910329 | 119.0400999 | 167.2078965 | 187.2772191 | 201.1604314 | 114.8161398 | 78.25106875 | 122.3462756 | 108.7751304 | 151.8174186 | 182.3460434 | 176.1931571 | 153.0544881 |
| **Pwp1** | 1967.831739 | 1934.703825 | 1975.915946 | 1802.853687 | 1820.285964 | 1808.943626 | 1883.511124 | 2150.497917 | 2081.177463 | 2125.329588 | 2022.271554 | 1746.334078 | 1662.416499 | 1654.408567 | 1675.695147 |
| **Prdm4** | 1011.844612 | 1041.824541 | 917.8650371 | 960.0842841 | 855.673743 | 904.1211066 | 949.8741447 | 897.577268 | 1017.263894 | 1028.582617 | 944.9248281 | 935.1952986 | 935.588578 | 878.2551213 | 873.7758571 |
| **Ascl4** | 0 | 4.753572051 | 4.698967767 | 3.450437679 | 1.266726489 | 1.402825611 | 4.510323574 | 4.190370065 | 9.781383594 | 7.86511772 | 4.729353494 | 0 | 6.816674521 | 1.807109303 | 4.311394032 |
| **Rtcb** | 4029.980215 | 4229.094601 | 4217.323571 | 4114.646932 | 3865.41588 | 3922.30041 | 3909.548474 | 4601.864406 | 4354.972941 | 4357.275217 | 4268.714464 | 3792.832881 | 3669.927145 | 3485.010291 | 3792.589617 |
| **Fbxo7** | 326.9036439 | 396.1310043 | 319.5298082 | 379.5481446 | 378.1178568 | 325.4555419 | 346.3928505 | 355.3433815 | 331.8146281 | 400.2471018 | 325.3795204 | 354.8189955 | 387.6983634 | 302.6908083 | 308.2646733 |
| **Syn3** | 6.409875371 | 21.39107423 | 16.44638718 | 25.01567317 | 20.90098706 | 14.02825611 | 16.23716487 | 25.9802944 | 13.54345421 | 22.72145119 | 12.29631908 | 24.29078698 | 13.63334904 | 20.78175699 | 17.24557613 |
| **Timp3** | 9.156964816 | 2.376786026 | 0.783161294 | 3.450437679 | 1.900089733 | 1.402825611 | 0.902064715 | 13.40918421 | 3.762070613 | 0 | 10.40457769 | 0 | 0 | 0 | 2.155697016 |
| **1810014B01Rik** | 86.99116575 | 64.9654847 | 77.53296815 | 89.71137964 | 79.17040553 | 86.97518791 | 67.65485361 | 102.2450296 | 115.1193608 | 76.02947129 | 59.58985402 | 58.99191123 | 82.65217857 | 58.73105235 | 98.44349706 |
| **Hsp90b1** | 13445.17144 | 12633.40999 | 12594.79994 | 12244.74071 | 12239.7447 | 11713.59386 | 12299.65239 | 12416.90458 | 11910.71556 | 12718.76926 | 13542.03079 | 12432.54529 | 11907.8783 | 12188.95225 | 12268.07172 |
| **Ttc41** | 17.39823315 | 23.76786026 | 13.31374201 | 23.29045433 | 32.30152546 | 22.44520978 | 21.64955315 | 24.30414638 | 18.05793894 | 22.72145119 | 13.24218978 | 15.61550591 | 25.56252945 | 20.78175699 | 17.9641418 |
| **Nt5dc3** | 522.862691 | 518.1393536 | 548.9960674 | 514.9778235 | 431.9537326 | 415.9377938 | 453.7385515 | 585.8137351 | 529.6995423 | 614.3530841 | 552.3884881 | 479.7430428 | 503.5818303 | 453.5844351 | 454.1335047 |
| **Stab2** | 2.747089445 | 0 | 3.915806472 | 2.587828259 | 1.266726489 | 1.402825611 | 0.902064715 | 5.866518091 | 3.762070613 | 0 | 5.675224193 | 0 | 5.112505891 | 0.903554652 | 5.029959704 |
| **Tyms-ps** | 1.831392963 | 0.792262009 | 0 | 0 | 0 | 0 | 0.902064715 | 0 | 0.752414123 | 0 | 0 | 0.867528106 | 0 | 0 | 0 |
| **Pmch** | 0.915696482 | 0 | 2.349483883 | 0.86260942 | 0.633363244 | 3.507064029 | 0 | 0.838074013 | 0.752414123 | 5.243411813 | 0 | 5.205168638 | 0 | 7.228437213 | 1.437131344 |
| **Parpbp** | 371.7727715 | 441.2899387 | 495.7410994 | 426.1290533 | 435.7539121 | 424.3547475 | 391.4960862 | 420.7131546 | 424.3615651 | 461.4202396 | 503.2032117 | 462.3924806 | 444.7880125 | 467.1377549 | 439.7621912 |
| **Nup37** | 781.0890988 | 854.0584452 | 853.645811 | 793.6006661 | 798.671051 | 869.0504663 | 773.0694606 | 886.6823058 | 927.7266131 | 913.2275575 | 850.3377582 | 814.6088918 | 793.2904974 | 902.6510969 | 819.164866 |
| **Ccdc53** | 493.5604036 | 487.2411352 | 497.307422 | 419.228178 | 440.820818 | 438.3830036 | 379.7692449 | 455.0741891 | 445.4291606 | 519.9716715 | 410.5078833 | 496.2260768 | 412.4088085 | 502.3763863 | 485.7503942 |
| **Dram1** | 7.325571853 | 18.2220262 | 9.397935534 | 7.763484777 | 12.03390164 | 20.34097137 | 14.43303544 | 3.352296052 | 2.257242368 | 8.739019689 | 17.97154328 | 17.35056213 | 16.18960199 | 15.36042908 | 13.65274777 |
| **Gnptab** | 2033.761886 | 1974.316925 | 1986.880204 | 1900.328551 | 2019.795386 | 2002.53356 | 2187.506933 | 1657.710398 | 1644.024858 | 1716.343467 | 2061.052253 | 2280.731391 | 2269.952616 | 2112.510775 | 2275.697483 |
| **Sycp3** | 1.831392963 | 3.961310043 | 0.783161294 | 9.488703616 | 0.633363244 | 0 | 0 | 4.190370065 | 0 | 0 | 0 | 0.867528106 | 4.260421576 | 2.710663955 | 0.718565672 |
| **Chpt1** | 91.56964816 | 80.81072487 | 74.40032298 | 89.71137964 | 107.0383883 | 110.1218105 | 116.3663482 | 73.75051315 | 66.21244279 | 80.39898114 | 105.9375183 | 124.9240473 | 106.5105394 | 129.2083152 | 101.3177597 |
| **Gm4925** | 1.831392963 | 6.338096068 | 7.831612945 | 1.725218839 | 1.900089733 | 0.701412806 | 0.902064715 | 6.704592104 | 0 | 1.747803938 | 12.29631908 | 0 | 0.852084315 | 2.710663955 | 3.59282836 |
| **Arl1** | 1282.890771 | 1320.700768 | 1348.603749 | 1256.821924 | 1204.656891 | 1223.965346 | 1267.400924 | 1377.793677 | 1434.101318 | 1470.777014 | 1472.720678 | 1296.086991 | 1265.345208 | 1184.560148 | 1217.250248 |
| **Utp20** | 2751.667927 | 2591.48903 | 2581.299627 | 2885.428509 | 2454.915935 | 2451.437756 | 2564.569984 | 3781.389947 | 3427.998742 | 3345.296737 | 3704.975527 | 2525.374317 | 2562.217536 | 2431.465567 | 2479.770134 |
| **Gas2l3** | 259.1421043 | 251.1470567 | 227.1167754 | 201.8506042 | 236.2444901 | 241.987418 | 288.6607087 | 187.7285789 | 211.4283684 | 173.0325898 | 217.5502607 | 195.1938239 | 235.175271 | 267.4521769 | 273.0549553 |
| **Scyl2** | 1241.684429 | 1086.191214 | 1082.328909 | 933.3433921 | 957.0118621 | 1054.92486 | 1105.029276 | 1108.771919 | 1005.977682 | 1017.221892 | 952.4917937 | 1057.516762 | 1086.407502 | 1002.945663 | 1016.770426 |
| **1500026H17Rik** | 0 | 1.584524017 | 0 | 0 | 0 | 0 | 0 | 3.352296052 | 0 | 0 | 0 | 0 | 0 | 0 | 0 |
| **Actr6** | 204.2003154 | 205.1958602 | 197.3566462 | 190.6366817 | 191.909063 | 211.1252545 | 206.5728197 | 184.3762829 | 188.8559448 | 215.8537863 | 258.2227008 | 204.7366331 | 194.2752239 | 182.5180396 | 212.6954389 |
| **Uhrf1bp1l** | 1176.669979 | 1155.91027 | 1074.497296 | 1063.597414 | 1007.680922 | 1094.90539 | 1222.297689 | 1102.067327 | 1069.932882 | 1080.142834 | 1130.315485 | 1106.965864 | 1053.176214 | 1026.438084 | 1108.028266 |
| **Apaf1** | 954.1557338 | 1075.891808 | 1137.933361 | 1144.6827 | 993.113567 | 971.4567359 | 1030.157904 | 1349.299161 | 1267.817797 | 1268.905659 | 1108.560459 | 1099.158111 | 1154.574247 | 1040.894959 | 1059.1658 |
| **Ikbip** | 49.44761 | 34.85952837 | 43.85703249 | 34.50437679 | 35.46834168 | 33.66781467 | 36.08258859 | 30.17066447 | 32.35380727 | 24.46925513 | 31.21373306 | 33.83359614 | 45.1604687 | 56.0203884 | 42.39537465 |
| **Slc25a3** | 9675.249024 | 8866.204137 | 9382.272308 | 9362.762641 | 8880.386048 | 8891.810138 | 8398.222495 | 11305.61844 | 10645.15501 | 11172.83667 | 10664.69213 | 9311.179165 | 9049.987511 | 9335.52666 | 8890.813059 |
| **Tmpo** | 7085.659374 | 7261.081308 | 7552.024363 | 7165.696449 | 7385.015428 | 7341.687837 | 7967.937626 | 7016.355637 | 7394.725997 | 7287.468519 | 7307.797019 | 8184.260155 | 7740.333919 | 7855.504141 | 7867.575542 |
| **Nedd1** | 924.8534464 | 926.94655 | 974.2526503 | 871.2355138 | 810.7049527 | 830.472762 | 865.0800615 | 1019.936074 | 991.6818136 | 1036.447735 | 966.6798541 | 875.3358592 | 930.4760722 | 802.3565306 | 873.7758571 |
| **Cdk17** | 1393.690045 | 1523.519842 | 1574.937363 | 1409.503792 | 1579.607931 | 1542.40676 | 1712.118829 | 1775.878834 | 1802.031824 | 1593.123289 | 1643.923274 | 1794.915652 | 1801.306242 | 1656.215676 | 1663.479531 |
| **Elk3** | 1042.062596 | 1099.659668 | 1264.805491 | 1172.286201 | 1468.769363 | 1326.371616 | 1491.112974 | 1271.358278 | 1295.657119 | 1081.016736 | 981.8137853 | 1429.686319 | 1416.164132 | 1570.377984 | 1471.622496 |
| **Gm17745** | 16.48253667 | 11.09166812 | 13.31374201 | 11.21392246 | 15.20071786 | 9.81977928 | 14.43303544 | 0.838074013 | 9.781383594 | 1.747803938 | 2.837612096 | 12.14539349 | 15.33751767 | 7.228437213 | 10.05991941 |
| **Mir7688** | 4.578482408 | 0.792262009 | 2.349483883 | 0 | 3.166816221 | 4.90988964 | 2.706194144 | 4.190370065 | 0 | 5.243411813 | 0 | 0 | 2.556252945 | 2.710663955 | 0 |
| **Lta4h** | 3014.472817 | 3028.025397 | 3273.614211 | 3246.861856 | 3959.787003 | 3653.659305 | 3780.55322 | 2820.957128 | 2960.749572 | 2768.521437 | 2653.16731 | 3959.398277 | 3767.916842 | 3977.447576 | 3758.81703 |
| **Hal** | 1.831392963 | 0 | 0.783161294 | 0 | 1.266726489 | 0.701412806 | 0 | 0 | 0 | 0 | 0 | 0.867528106 | 0.852084315 | 2.710663955 | 0 |
| **Amdhd1** | 10.98835778 | 5.54583406 | 7.04845165 | 3.450437679 | 6.966995687 | 11.9240177 | 6.314453003 | 0.838074013 | 11.28621184 | 7.86511772 | 0 | 3.470112425 | 4.260421576 | 9.939101167 | 2.155697016 |
| **Ccdc38** | 6.409875371 | 7.130358077 | 7.04845165 | 5.175656518 | 13.30062813 | 7.014128057 | 2.706194144 | 10.89496217 | 0 | 9.612921658 | 25.53850887 | 1.735056213 | 1.70416863 | 5.421327909 | 3.59282836 |
| **Snrpf** | 0.915696482 | 5.54583406 | 5.482129061 | 4.313047098 | 8.233722176 | 4.90988964 | 4.510323574 | 0 | 2.257242368 | 3.495607876 | 0.945870699 | 1.735056213 | 4.260421576 | 3.614218606 | 3.59282836 |
| **4933408J17Rik** | 3.662785926 | 3.169048034 | 2.349483883 | 0.86260942 | 2.533452977 | 3.507064029 | 6.314453003 | 1.676148026 | 8.276555348 | 6.117313782 | 1.891741398 | 0.867528106 | 9.372927467 | 0 | 2.874262688 |
| **Usp44** | 11.90405426 | 20.59881222 | 22.71167754 | 17.25218839 | 10.13381191 | 12.6254305 | 9.922711863 | 21.78992434 | 9.028969471 | 13.9824315 | 25.53850887 | 13.01292159 | 9.372927467 | 1.807109303 | 12.9341821 |
| **Metap2** | 4703.932826 | 4719.504785 | 4814.875639 | 4676.205664 | 4508.279573 | 4358.579175 | 4658.262187 | 5365.349832 | 5093.091196 | 5416.444403 | 5509.69682 | 4714.14773 | 4643.859518 | 4857.509807 | 4251.034515 |
| **Vezt** | 451.4383654 | 491.9947073 | 427.6060668 | 482.1986656 | 326.1820708 | 392.7911712 | 376.1609861 | 478.5402615 | 449.1912312 | 486.7633967 | 467.2601252 | 373.0370857 | 325.4962084 | 389.4320548 | 382.9955032 |
| **Fgd6** | 89.73825519 | 115.6702532 | 114.341549 | 109.5513963 | 96.27121313 | 89.07942633 | 79.3816949 | 99.73080755 | 138.4441986 | 156.4284524 | 160.7980188 | 137.9369689 | 86.91260015 | 79.51280934 | 95.56923437 |
| **Nr2c1** | 184.0549928 | 208.3649082 | 162.8975493 | 147.5062108 | 150.1070889 | 157.1164685 | 135.3097072 | 179.3478388 | 158.7593799 | 168.66308 | 168.3649844 | 167.4329245 | 149.9668395 | 149.9900722 | 153.0544881 |
| **Ndufa12** | 1490.753872 | 1445.878166 | 1567.888912 | 1452.634263 | 1549.839859 | 1562.046318 | 1463.148967 | 1531.161222 | 1433.348904 | 1528.454544 | 1572.037101 | 1451.374522 | 1456.212095 | 1477.311855 | 1399.765929 |
| **Tmcc3** | 42.12203815 | 66.55000871 | 43.85703249 | 60.38265938 | 27.86798275 | 45.59183237 | 44.20117102 | 67.04592104 | 112.1097043 | 65.54264767 | 85.12836289 | 29.49595561 | 34.08337261 | 17.16753838 | 72.57513287 |
| **Cep83os** | 109.8835778 | 111.7089432 | 108.8594199 | 73.32180067 | 102.6048456 | 110.8232233 | 102.8353775 | 67.04592104 | 139.9490268 | 112.733354 | 90.80358708 | 104.1033728 | 123.5522257 | 123.7869873 | 82.63505228 |
| **Cep83** | 1003.603344 | 951.5066722 | 1066.665683 | 900.5642341 | 1033.648815 | 981.977928 | 1040.982681 | 1073.572811 | 1063.161155 | 1096.746971 | 1127.477873 | 951.6783326 | 1020.79701 | 949.6359388 | 985.8721019 |
| **Plxnc1** | 1279.227985 | 1100.45193 | 1063.533038 | 979.9243007 | 604.228535 | 642.49413 | 718.043513 | 780.2469062 | 795.3017276 | 934.2012048 | 1036.674286 | 753.8819244 | 864.8655799 | 623.4527096 | 729.344157 |
| **Gm29684** | 0 | 0 | 0 | 0 | 0 | 1.402825611 | 0 | 2.514222039 | 0 | 1.747803938 | 0.945870699 | 1.735056213 | 0 | 0 | 0 |
| **Cradd** | 150.174223 | 190.142882 | 220.851485 | 198.4001665 | 170.3747127 | 175.3532014 | 171.3922958 | 148.3391003 | 142.9586833 | 171.2847859 | 218.4961314 | 173.5056213 | 194.2752239 | 170.7718291 | 160.2401448 |
| **Socs2** | 1574.997948 | 1750.899039 | 1769.161364 | 1625.156147 | 1461.169005 | 1447.716031 | 1499.231556 | 1987.911559 | 2143.627835 | 2047.552313 | 2037.405485 | 1375.032048 | 1403.382867 | 1340.875103 | 1387.550313 |
| **5730420D15Rik** | 4.578482408 | 2.376786026 | 0.783161294 | 0 | 0 | 0 | 0 | 1.676148026 | 0 | 0.873901969 | 3.783482795 | 0.867528106 | 0.852084315 | 0 | 0 |
| **Mrpl42** | 897.3825519 | 903.1786897 | 989.9158762 | 887.6250928 | 963.9788578 | 999.5132481 | 886.7296146 | 1235.321095 | 1220.415707 | 1178.893756 | 1135.990709 | 1028.888334 | 1038.69078 | 972.2248051 | 1003.117678 |
| **Ube2n** | 2582.264078 | 2329.250305 | 2600.878659 | 2378.21417 | 2377.012256 | 2598.734445 | 2402.198335 | 2742.178171 | 2592.066652 | 2601.606161 | 2721.27 | 2479.395328 | 2366.238143 | 2557.963219 | 2569.590843 |
| **Nudt4** | 1165.681621 | 1205.030515 | 1292.216136 | 1181.774905 | 1169.821912 | 1127.170379 | 1183.508906 | 1266.329834 | 1321.991613 | 1336.19611 | 1323.273108 | 1224.949686 | 1216.776402 | 1204.438351 | 1199.286107 |
| **Mir3058** | 0 | 0.792262009 | 0.783161294 | 0 | 0 | 0.701412806 | 0.902064715 | 4.190370065 | 2.257242368 | 1.747803938 | 0.945870699 | 1.735056213 | 2.556252945 | 4.517773258 | 2.155697016 |
| **Eea1** | 1371.713329 | 1139.272768 | 1212.333684 | 1107.590495 | 998.8138362 | 1021.257045 | 1126.678829 | 1474.172189 | 1380.679915 | 1247.05811 | 1254.224547 | 1058.38429 | 1131.567971 | 936.082619 | 1062.758629 |
| **4930459C07Rik** | 0 | 0 | 0 | 0 | 0 | 0 | 0.902064715 | 7.542666117 | 4.514484735 | 4.369509845 | 0 | 0.867528106 | 0 | 0 | 0.718565672 |
| **Btg1** | 3007.147245 | 2977.320628 | 3048.063758 | 3030.346891 | 3551.267711 | 3674.000276 | 3683.13023 | 3693.392176 | 3413.702874 | 2810.468732 | 2676.814078 | 3665.306249 | 3701.454265 | 3461.51787 | 3552.588682 |
| **Atp2b1** | 1557.599715 | 1469.646026 | 1676.748331 | 1605.31613 | 1378.831783 | 1528.378504 | 1648.072234 | 1490.095595 | 1368.641289 | 1360.665366 | 1503.934411 | 1561.550591 | 1620.664367 | 1505.32205 | 1421.322899 |
| **Poc1b** | 384.5925223 | 461.0964889 | 418.2081313 | 396.800333 | 407.8859293 | 399.1038865 | 377.0630508 | 377.9713799 | 388.2456873 | 356.5520033 | 344.2969344 | 402.5330413 | 353.6149908 | 340.6401036 | 418.2052211 |
| **Galnt4** | 176.7294209 | 185.38931 | 221.6346463 | 196.6749477 | 242.5781226 | 196.3955856 | 199.356302 | 201.9758371 | 228.7338933 | 170.4108839 | 235.521804 | 202.1340488 | 241.9919455 | 220.467335 | 237.8452374 |
| **Dusp6** | 414.8105061 | 460.3042269 | 473.8125832 | 508.0769482 | 599.7949923 | 665.6407526 | 675.6464714 | 493.6255937 | 495.0884927 | 415.1034352 | 432.2629093 | 601.1969777 | 655.2528384 | 647.8486852 | 645.2719734 |
| **Csl** | 2.747089445 | 0 | 0 | 0.86260942 | 1.900089733 | 1.402825611 | 0.902064715 | 0 | 0.752414123 | 0 | 0 | 1.735056213 | 4.260421576 | 0 | 0.718565672 |
| **Tmtc3** | 52.19469945 | 86.35655893 | 67.35187133 | 71.59658183 | 46.23551683 | 47.69607079 | 52.31975346 | 84.64547532 | 58.68830156 | 109.2377461 | 90.80358708 | 50.31663016 | 40.04796281 | 55.11683375 | 45.98820301 |
| **Cep290** | 91.56964816 | 114.8779912 | 84.5814198 | 89.71137964 | 84.23731149 | 63.12715251 | 72.16517718 | 107.2734737 | 107.5952195 | 110.1116481 | 108.7751304 | 65.93213608 | 79.24384131 | 80.41636399 | 77.60509257 |
| **4930430F08Rik** | 87.90686223 | 48.32798252 | 85.3645811 | 69.00875357 | 41.16861088 | 54.71019885 | 71.26311247 | 67.88399506 | 70.72692752 | 69.03825554 | 82.29075079 | 65.06460797 | 47.71672165 | 72.28437213 | 53.17385973 |
| **Nts** | 0 | 0 | 0 | 0 | 0.633363244 | 0 | 1.80412943 | 0 | 0 | 0 | 1.891741398 | 0 | 0 | 0 | 0.718565672 |
| **Tmtc2** | 20.14532259 | 14.26071615 | 8.614774239 | 11.21392246 | 13.93399137 | 9.81977928 | 4.510323574 | 5.028444078 | 4.514484735 | 7.86511772 | 7.56696559 | 8.675281063 | 6.816674521 | 7.228437213 | 9.341353736 |
| **Mettl25** | 125.450418 | 116.4625153 | 119.8236781 | 115.5896622 | 83.60394824 | 119.240177 | 138.0159014 | 147.5010263 | 156.5021375 | 82.14678508 | 136.2053806 | 119.7188787 | 140.593912 | 115.6549954 | 135.808912 |
| **Ccdc59** | 606.1910708 | 626.6792487 | 649.2407131 | 636.6057517 | 628.2963383 | 622.8545715 | 612.5019413 | 696.4395048 | 652.3430443 | 657.1742806 | 664.9471012 | 574.3036064 | 565.7839853 | 579.1785317 | 644.5534078 |
| **Ppp1r12a** | 2214.154092 | 2056.712174 | 2009.591882 | 1966.749477 | 1847.520584 | 1879.084907 | 2136.991309 | 1706.318691 | 1893.073932 | 1711.100055 | 1844.447863 | 1986.639363 | 2125.098282 | 1965.231367 | 1924.31887 |
| **Pawr** | 0 | 0 | 0 | 1.725218839 | 0 | 2.104238417 | 0.902064715 | 0 | 0 | 2.621705907 | 0.945870699 | 5.205168638 | 4.260421576 | 6.324882561 | 0 |
| **E2f7** | 332.3978228 | 343.0494497 | 394.7132924 | 333.8298454 | 284.3800967 | 302.3089193 | 348.1969799 | 386.35212 | 291.9366796 | 371.4083368 | 360.3767362 | 335.7333771 | 379.1775202 | 364.1325246 | 370.7798867 |
| **Csrp2** | 93.40104112 | 69.71905675 | 87.71406498 | 74.18441009 | 46.86888008 | 86.2737751 | 83.89201847 | 36.87525657 | 36.86829201 | 56.80362798 | 64.31920752 | 72.00483282 | 62.20215501 | 84.93413725 | 76.16796123 |
| **Zdhhc17** | 193.2119576 | 242.4321746 | 223.2009689 | 196.6749477 | 169.1079862 | 173.9503758 | 221.9079198 | 196.1093191 | 181.3318035 | 173.9064918 | 175.93195 | 175.2406775 | 201.0918984 | 197.8784687 | 154.4916195 |
| **Osbpl8** | 2422.017194 | 2196.94255 | 2164.657818 | 2056.460856 | 1719.581208 | 1851.729807 | 2078.357103 | 2101.051551 | 2072.900908 | 1908.6019 | 1968.356924 | 1970.156329 | 2059.48779 | 1797.170202 | 1752.581674 |
| **Bbs10** | 29.30228741 | 32.48274235 | 26.62748401 | 34.50437679 | 36.73506817 | 23.84803539 | 31.57226502 | 37.71333059 | 32.35380727 | 24.46925513 | 36.88895725 | 31.23101183 | 30.67503535 | 36.14218606 | 29.46119255 |
| **Nap1l1** | 11025.90133 | 10852.40499 | 11184.32645 | 11022.42316 | 11278.93265 | 10448.24515 | 10981.73584 | 13172.84734 | 12159.01222 | 13269.3275 | 13222.3265 | 11510.36291 | 11272.22341 | 11577.24575 | 11284.35531 |
| **Gm5176** | 1.831392963 | 1.584524017 | 0 | 3.450437679 | 0 | 1.402825611 | 0 | 0 | 0 | 0 | 0 | 3.470112425 | 0 | 0.903554652 | 2.155697016 |
| **Phlda1** | 176.7294209 | 147.3607336 | 154.282775 | 143.1931637 | 195.0758792 | 173.248963 | 203.8666255 | 158.3959885 | 148.2255821 | 145.0677268 | 110.6668718 | 198.6639363 | 220.6898376 | 198.7820233 | 214.1325702 |
| **Krr1** | 984.3737177 | 938.0382181 | 866.1763917 | 963.5347218 | 917.743341 | 892.1970889 | 985.0546685 | 1087.820069 | 936.0031685 | 935.0751067 | 1051.808217 | 894.4214776 | 912.5823015 | 927.0470725 | 941.3210303 |
| **Glipr1** | 423.967471 | 443.6667248 | 492.6084542 | 488.2369315 | 388.2516687 | 417.3406194 | 407.7332511 | 707.334467 | 589.140258 | 687.7608495 | 650.7590408 | 425.0887721 | 443.9359282 | 534.9043537 | 469.2233838 |
| **Atxn7l3b** | 1045.725382 | 973.6900085 | 1016.54336 | 1047.207835 | 1083.684511 | 1025.465522 | 1119.462311 | 1157.380212 | 1072.190125 | 943.8141264 | 1020.594484 | 1015.875412 | 1157.982584 | 1004.752773 | 1095.094084 |
| **Tbc1d15** | 1354.315096 | 1235.928733 | 1252.27491 | 1288.738473 | 1150.187652 | 1249.216207 | 1267.400924 | 1392.040936 | 1333.277825 | 1212.102031 | 1254.224547 | 1197.188787 | 1222.740992 | 1155.646399 | 1161.920692 |
| **Rab21** | 1477.018425 | 1481.529956 | 1415.172459 | 1364.648102 | 1336.396445 | 1362.143669 | 1360.31359 | 1761.631575 | 1527.400669 | 1520.589426 | 1380.02535 | 1402.792948 | 1392.305771 | 1355.331977 | 1342.999241 |
| **Tmem19** | 369.0256821 | 339.0881396 | 411.1596796 | 393.3498954 | 370.5174979 | 406.8194273 | 370.7485978 | 505.3586299 | 520.6705728 | 390.6341801 | 470.0977373 | 396.4603446 | 402.1837968 | 401.1782653 | 377.2469778 |
| **Thap2** | 184.9706893 | 205.1958602 | 189.5250333 | 212.2019172 | 230.5442209 | 201.3054752 | 213.7893374 | 196.1093191 | 217.4476814 | 210.6103745 | 227.0089677 | 203.869105 | 207.0564886 | 212.3353431 | 237.8452374 |
| **Zfc3h1** | 868.995961 | 820.7834408 | 813.704585 | 778.0736965 | 689.732573 | 756.8244174 | 791.1107549 | 910.9864522 | 907.4114318 | 923.7143811 | 931.6826383 | 752.1468681 | 829.930123 | 733.6863771 | 776.0509257 |
| **Kcnmb4** | 0 | 0 | 5.482129061 | 0 | 0.633363244 | 0 | 0 | 1.676148026 | 0 | 0 | 0 | 0 | 0 | 4.517773258 | 1.437131344 |
| **Kcnmb4os2** | 0 | 0 | 0 | 1.725218839 | 1.900089733 | 5.611302446 | 0.902064715 | 6.704592104 | 0 | 2.621705907 | 4.729353494 | 3.470112425 | 7.668758836 | 4.517773258 | 0.718565672 |
| **Cnot2** | 1863.44234 | 1796.850235 | 1870.189171 | 1765.761482 | 1732.881836 | 1726.176915 | 1841.114083 | 1814.430238 | 1793.755268 | 1839.563645 | 1882.282691 | 1860.847788 | 1792.785399 | 1729.403603 | 1812.94119 |
| **Myrfl** | 0.915696482 | 0 | 0 | 0 | 0 | 0.701412806 | 1.80412943 | 0 | 0 | 0 | 0 | 0 | 3.408337261 | 0 | 0 |
| **Rab3ip** | 510.9586367 | 518.9316156 | 551.3455513 | 505.4891199 | 424.9867369 | 467.1409286 | 476.2901694 | 524.6343322 | 531.9567847 | 580.2709074 | 567.5224193 | 497.0936049 | 479.7234694 | 498.7621677 | 492.936051 |
| **4933412E12Rik** | 130.9445969 | 118.8393013 | 145.6680008 | 119.0400999 | 146.9402727 | 140.2825611 | 133.5055778 | 100.5688816 | 136.1869562 | 109.2377461 | 111.6127425 | 164.8303402 | 151.6710081 | 168.9647198 | 155.9287508 |
| **D630029K05Rik** | 0 | 0.792262009 | 0 | 0 | 0 | 0 | 2.706194144 | 2.514222039 | 3.762070613 | 0 | 0.945870699 | 0 | 2.556252945 | 0 | 2.155697016 |
| **Cct2** | 12232.7893 | 11471.95388 | 12308.1629 | 11527.91228 | 11474.00853 | 11543.15054 | 11366.01541 | 13552.49487 | 12321.53367 | 13124.25977 | 12867.62499 | 11626.61168 | 11245.80879 | 11538.3929 | 11448.18829 |
| **Frs2** | 282.0345163 | 345.4262357 | 318.7466469 | 287.2489367 | 296.4139983 | 284.0721863 | 299.4854853 | 326.0107911 | 245.287004 | 304.1178852 | 355.6473827 | 280.2115783 | 328.9045457 | 291.8481525 | 318.3245927 |
| **Yeats4** | 927.6005358 | 859.6042792 | 1050.219296 | 992.863442 | 974.7460329 | 1008.631615 | 916.4977502 | 1047.592516 | 1147.431537 | 1093.251363 | 986.5431388 | 1106.965864 | 973.9323722 | 1038.184295 | 955.6923437 |
| **Lyz2** | 1.831392963 | 0 | 0 | 0 | 0 | 6.312715251 | 0 | 0 | 0 | 0 | 0 | 0.867528106 | 0 | 0 | 0 |
| **Cpsf6** | 2934.807223 | 3034.363493 | 3048.063758 | 3009.644265 | 2874.835766 | 3004.151047 | 2925.39587 | 3432.751157 | 3349.747674 | 3115.460519 | 3233.931919 | 3051.963878 | 3159.528641 | 3213.943896 | 2960.490568 |
| **Cpm** | 33.88076982 | 33.27500436 | 28.1938066 | 29.32872027 | 49.40233305 | 56.81443726 | 54.12388289 | 20.95185033 | 14.29586833 | 20.97364725 | 17.02567258 | 34.70112425 | 60.49798638 | 57.8274977 | 40.23967763 |
| **Mdm2** | 1495.332354 | 1395.965659 | 1490.355943 | 1382.7629 | 1323.095817 | 1245.709143 | 1433.380832 | 2018.082223 | 1726.037997 | 1735.56931 | 1681.758102 | 1412.335757 | 1393.157855 | 1359.849751 | 1370.304736 |
| **Slc35e3** | 168.4881526 | 193.3119301 | 160.5480654 | 178.5601499 | 192.5424263 | 159.9221197 | 174.09849 | 216.2230954 | 212.1807826 | 211.4842765 | 227.0089677 | 184.7834866 | 184.0502121 | 164.4469466 | 174.6114583 |
| **Nup107** | 2765.403374 | 2625.556296 | 2881.250402 | 2855.237179 | 2750.063207 | 2724.287337 | 2725.137503 | 2817.604832 | 2845.630212 | 2933.68891 | 2863.150605 | 2786.500277 | 2800.801144 | 2888.664221 | 2701.088361 |
| **Rap1b** | 5593.074109 | 5493.544767 | 5787.561966 | 5690.634341 | 5792.106869 | 6332.35481 | 6463.293681 | 5974.629639 | 6115.621988 | 5888.351466 | 5802.916737 | 6368.523828 | 6255.150958 | 6429.694901 | 5982.777785 |
| **Mdm1** | 118.1248461 | 173.5053799 | 202.055614 | 134.5670695 | 145.0401829 | 189.3814575 | 156.9592604 | 171.8051727 | 163.2738646 | 179.1499036 | 149.4475704 | 175.2406775 | 218.985669 | 204.2033513 | 189.7013374 |
| **Ifng** | 14029.38579 | 8981.082128 | 9300.823533 | 8213.766894 | 6052.419162 | 6221.531587 | 7619.740646 | 7018.869859 | 6868.036111 | 5839.412956 | 6604.069219 | 5811.570784 | 6411.082387 | 5324.647562 | 6279.545407 |
| **Tmevpg1** | 1.831392963 | 4.753572051 | 7.831612945 | 7.763484777 | 15.83408111 | 4.208476834 | 6.314453003 | 0.838074013 | 1.504828245 | 4.369509845 | 0 | 4.337640531 | 12.78126473 | 18.97464768 | 10.05991941 |
| **Dyrk2** | 614.4323391 | 617.9643666 | 578.7561966 | 639.19358 | 573.8270993 | 754.7201789 | 657.6051771 | 397.2470822 | 490.5740079 | 511.2326518 | 496.5821169 | 690.5523726 | 630.5423932 | 725.5543852 | 660.3618525 |
| **Cand1** | 3892.625743 | 3638.859405 | 3627.603116 | 3541.874277 | 3501.232014 | 3400.449282 | 3909.548474 | 3680.821065 | 3615.349859 | 3457.156189 | 3524.314224 | 3644.485574 | 3663.110471 | 3174.187491 | 3407.438416 |
| **Grip1** | 3.662785926 | 1.584524017 | 0.783161294 | 1.725218839 | 3.800179466 | 0 | 0 | 0 | 0 | 0 | 0 | 0 | 0 | 0.903554652 | 1.437131344 |
| **Helb** | 990.7835931 | 1028.356087 | 982.0842633 | 985.0999572 | 948.7781399 | 916.0451243 | 1027.45171 | 672.9734325 | 673.4106397 | 713.1040066 | 725.482826 | 981.1742882 | 1001.19907 | 1011.98121 | 1062.758629 |
| **Irak3** | 0.915696482 | 9.507144102 | 5.482129061 | 2.587828259 | 2.533452977 | 0 | 2.706194144 | 9.218814144 | 4.514484735 | 2.621705907 | 5.675224193 | 3.470112425 | 0.852084315 | 0.903554652 | 2.874262688 |
| **Tmbim4** | 518.2842086 | 469.811371 | 492.6084542 | 518.4282612 | 431.3203694 | 465.0366902 | 467.2695223 | 453.3980411 | 476.2781396 | 493.7546124 | 499.419729 | 483.2131552 | 474.6109635 | 473.4626374 | 447.6664136 |
| **Llph** | 801.2344214 | 769.2864103 | 848.9468432 | 791.0128378 | 815.7718586 | 783.478104 | 803.7396609 | 838.9120871 | 902.8969471 | 942.9402244 | 888.1725861 | 761.6896773 | 736.2008483 | 785.1889922 | 710.6614496 |
| **Msrb3** | 0.915696482 | 0 | 4.698967767 | 1.725218839 | 3.166816221 | 0 | 0 | 0.838074013 | 8.276555348 | 2.621705907 | 0 | 2.602584319 | 0.852084315 | 0 | 4.311394032 |
| **Lemd3** | 587.8771412 | 592.6119824 | 663.3376164 | 644.3692365 | 666.298133 | 701.4128057 | 708.1208011 | 516.253592 | 576.3492179 | 472.7809652 | 634.6792389 | 640.2357424 | 678.2591149 | 627.9704828 | 608.6251242 |
| **Tbc1d30** | 211.5258872 | 208.3649082 | 163.6807105 | 187.1862441 | 212.8100501 | 192.1871088 | 249.871926 | 128.225324 | 142.9586833 | 109.2377461 | 134.3136392 | 173.5056213 | 172.973116 | 214.1424524 | 177.485721 |
| **Gns** | 653.8072878 | 628.2637727 | 567.7919385 | 547.7569815 | 571.2936463 | 620.0489202 | 678.3526655 | 598.3848453 | 574.8443897 | 514.7282597 | 566.5765486 | 618.5475398 | 649.2882482 | 480.6910746 | 595.6909421 |
| **Rassf3** | 636.4090547 | 602.1191265 | 671.1692294 | 675.4231756 | 657.4310476 | 667.744991 | 715.3373188 | 697.2775789 | 720.0603153 | 622.2182019 | 665.8929719 | 761.6896773 | 822.2613641 | 679.473098 | 778.2066227 |
| **Tbk1** | 734.3885782 | 845.3435631 | 845.0310368 | 801.3641509 | 773.3365213 | 789.0894064 | 802.8375962 | 851.4831973 | 768.9672333 | 927.209989 | 1001.67707 | 848.4424879 | 791.5863288 | 814.1027411 | 759.5239153 |
| **Xpot** | 4539.107459 | 4287.72199 | 4184.430796 | 4017.172067 | 3662.106278 | 3972.100719 | 4111.61097 | 3689.201805 | 3819.254086 | 4039.1749 | 4005.762409 | 3685.259395 | 3989.458764 | 3287.131822 | 3909.715821 |
| **D930020B18Rik** | 0.915696482 | 0 | 0.783161294 | 0 | 0.633363244 | 0 | 0 | 0 | 2.257242368 | 0 | 0 | 0.867528106 | 0.852084315 | 0 | 0 |
| **BC048403** | 280.2031234 | 245.6012226 | 248.2621304 | 245.8436846 | 225.477315 | 227.2577491 | 242.6554083 | 229.6322796 | 258.078044 | 228.9623159 | 239.3052868 | 249.8480946 | 246.2523671 | 196.974914 | 233.5338434 |
| **Srgap1** | 0.915696482 | 3.961310043 | 3.132645178 | 1.725218839 | 0 | 2.805651223 | 2.706194144 | 0 | 3.762070613 | 1.747803938 | 0.945870699 | 2.602584319 | 0 | 0 | 0 |
| **Gm9079** | 0 | 7.130358077 | 0 | 5.175656518 | 4.43354271 | 2.805651223 | 2.706194144 | 9.218814144 | 0 | 1.747803938 | 3.783482795 | 10.41033728 | 0.852084315 | 1.807109303 | 3.59282836 |
| **Tmem5** | 364.4471997 | 355.7256418 | 300.7339371 | 321.7533135 | 307.1811735 | 356.3177053 | 320.2329737 | 399.7613042 | 415.3325957 | 415.1034352 | 384.9693744 | 333.9983209 | 297.377426 | 306.3050269 | 358.5642703 |
| **A130077B15Rik** | 0.915696482 | 0 | 0 | 0 | 0 | 0 | 0 | 0 | 0 | 0 | 0 | 0 | 0 | 0 | 0 |
| **Ppm1h** | 792.0774566 | 928.531074 | 939.0103921 | 941.9694863 | 944.3445972 | 897.1069785 | 904.7709089 | 812.9317927 | 880.3245234 | 757.673007 | 929.7908969 | 888.3487808 | 862.3093269 | 923.4328539 | 901.7999183 |
| **Mirlet7i** | 0 | 3.169048034 | 1.566322589 | 0.86260942 | 0 | 2.805651223 | 0 | 0.838074013 | 0.752414123 | 0 | 0.945870699 | 0 | 0.852084315 | 2.710663955 | 0 |

| | | | | | | | | | | | | | | |
|---|---|---|---|---|---|---|---|---|---|---|---|---|---|---|---|
| **Mon2** | 1153.777567 | 1304.063266 | 1300.047749 | 1225.767985 | 1092.551596 | 1222.56252 | 1302.581448 | 1205.988505 | 1183.547415 | 1202.489109 | 1139.774192 | 1318.642722 | 1301.132749 | 1192.69214 | 1230.902996 |
| **Usp15** | 1801.174979 | 1905.39013 | 1898.382978 | 1852.022424 | 1653.078068 | 1720.565612 | 1808.639753 | 1883.152307 | 1893.826347 | 1892.871665 | 1932.413838 | 1763.68464 | 1812.383338 | 1752.896024 | 1714.497693 |
| **Lrig3** | 0.915696482 | 1.584524017 | 2.349483883 | 1.725218839 | 1.900089733 | 5.611302446 | 0.902064715 | 0.838074013 | 0 | 0 | 0 | 3.470112425 | 1.70416863 | 0 | 6.467091048 |
| **Xrcc6bp1** | 133.6916863 | 200.4422882 | 161.3312267 | 156.9949144 | 222.943862 | 169.0404862 | 171.3922958 | 153.3675444 | 201.6469849 | 207.9886686 | 214.7126486 | 196.061352 | 192.5710552 | 206.9140152 | 187.5456404 |
| **Ctdsp2** | 1223.370499 | 1461.723406 | 1483.307492 | 1494.039515 | 1473.202906 | 1297.613691 | 1498.329491 | 1159.05636 | 1386.699228 | 1133.450854 | 1139.774192 | 1617.939918 | 1588.285163 | 1667.058332 | 1430.664253 |
| **Avil** | 2.747089445 | 3.961310043 | 9.397935534 | 3.450437679 | 5.066905954 | 4.208476834 | 4.510323574 | 1.676148026 | 2.257242368 | 6.991215751 | 0 | 2.602584319 | 1.70416863 | 0 | 0.718565672 |
| **Tsfm** | 708.7490767 | 720.9584277 | 740.0874233 | 779.7989154 | 680.8654876 | 709.8297594 | 645.8783358 | 702.3060229 | 690.7161645 | 779.5205563 | 770.8846195 | 689.6848445 | 673.9986933 | 698.4477457 | 676.1702973 |
| **Mettl21b** | 1.831392963 | 1.584524017 | 4.698967767 | 6.038265938 | 0.633363244 | 0 | 0.902064715 | 3.352296052 | 10.53379772 | 6.991215751 | 3.783482795 | 3.470112425 | 5.964590206 | 0 | 0 |
| **Mettl1** | 499.0545825 | 447.6280348 | 507.4885188 | 472.709962 | 484.5228819 | 455.9183237 | 478.9963635 | 648.6692861 | 549.2623095 | 647.561359 | 571.3059021 | 515.3116951 | 514.6589264 | 496.0515037 | 441.1993226 |
| **Cyp27b1** | 6.409875371 | 7.130358077 | 2.349483883 | 0.86260942 | 0 | 2.104238417 | 1.80412943 | 5.866518091 | 1.504828245 | 3.495607876 | 0 | 1.735056213 | 2.556252945 | 2.710663955 | 1.437131344 |
| **March9** | 229.8398169 | 188.558358 | 216.1525173 | 179.4227593 | 180.5085246 | 199.9026496 | 202.0624961 | 147.5010263 | 188.8559448 | 149.4372367 | 108.7751304 | 225.5573076 | 217.2815004 | 192.4571408 | 184.6713777 |
| **Cdk4** | 5752.405297 | 6062.388889 | 6143.900355 | 6125.389489 | 5960.581492 | 6010.406332 | 5876.049552 | 6381.095535 | 6374.452447 | 6008.076036 | 6094.244912 | 5925.216966 | 5934.767255 | 5750.221803 | 5742.77685 |
| **Tspan31** | 1151.946174 | 1192.354323 | 1232.695878 | 1155.896622 | 1048.216169 | 1165.748083 | 1178.998582 | 1272.196352 | 1144.42188 | 1193.75009 | 1213.552107 | 1095.687998 | 1122.195043 | 1127.636205 | 1171.262045 |
| **LOC100504703** | 7.325571853 | 11.88393013 | 3.132645178 | 3.450437679 | 3.166816221 | 7.014128057 | 9.922711863 | 5.866518091 | 14.29586833 | 4.369509845 | 2.837612096 | 5.205168638 | 12.78126473 | 6.324882561 | 6.467091048 |
| **Agap2** | 1260.914055 | 1518.76627 | 1362.700652 | 1576.850019 | 1572.640936 | 1407.034088 | 1463.148967 | 1463.277227 | 1634.243474 | 1371.152189 | 1345.028134 | 1523.379355 | 1467.289191 | 1497.190058 | 1438.568475 |
| **Os9** | 2439.415427 | 2314.989589 | 2402.738851 | 2291.090619 | 2395.37979 | 2204.540448 | 2552.843143 | 1924.217934 | 1998.41191 | 1979.38796 | 1969.302795 | 2325.842853 | 2344.936035 | 2327.556782 | 2352.58401 |
| **B4galnt1** | 8474.770937 | 9370.875037 | 8888.880692 | 8967.687527 | 10333.32133 | 10225.89729 | 10520.78077 | 6940.090902 | 7704.720615 | 6951.890163 | 6844.320376 | 10175.23716 | 10313.62855 | 10165.89338 | 10179.20131 |
| **Slc26a10** | 165.7410632 | 154.4910917 | 155.8490976 | 159.5827426 | 172.9081657 | 211.1252545 | 211.985208 | 160.0721365 | 117.3766031 | 146.8155308 | 112.5586132 | 192.5912396 | 254.7732102 | 252.9953024 | 217.0068329 |
| **Arhgef25** | 48.53191352 | 42.78214846 | 25.06116142 | 38.81742388 | 27.2346195 | 41.38335554 | 31.57226502 | 32.68488651 | 39.8779485 | 41.07339254 | 32.15960376 | 37.30370857 | 34.08337261 | 28.0101942 | 40.9582433 |
| **Dtx3** | 423.967471 | 414.3530304 | 454.2335508 | 380.4107541 | 423.7200104 | 474.1550567 | 444.7179044 | 377.1333059 | 339.3387693 | 395.8775919 | 397.2656935 | 464.995065 | 447.3442655 | 450.8737711 | 416.7680897 |
| **F420014N23Rik** | 1.831392963 | 0.792262009 | 3.132645178 | 3.450437679 | 7.600358931 | 3.507064029 | 2.706194144 | 4.190370065 | 7.524141226 | 0.873901969 | 3.783482795 | 7.807752957 | 7.668758836 | 7.228437213 | 5.748525376 |
| **Pip4k2c** | 745.376936 | 743.934026 | 750.2685201 | 735.805835 | 749.268718 | 678.9675959 | 717.1414483 | 765.9996479 | 748.652052 | 843.3154 | 718.8617311 | 694.022485 | 741.3133542 | 704.7726282 | 781.7994511 |
| **Kif5a** | 4.578482408 | 19.0142882 | 10.18109683 | 11.21392246 | 7.600358931 | 9.81977928 | 5.412388289 | 5.028444078 | 10.53379772 | 14.85633347 | 4.729353494 | 15.61550591 | 9.372927467 | 0.903554652 | 7.18565672 |
| **Dctn2** | 1821.320302 | 1668.50379 | 1867.839687 | 1825.281532 | 1602.409008 | 1563.449144 | 1658.89701 | 1845.438977 | 1677.883493 | 1886.754351 | 1617.438895 | 1640.495649 | 1750.181183 | 1777.292 | 1700.12638 |
| **Mbd6** | 612.6009462 | 561.713764 | 590.503616 | 552.0700286 | 577.6272788 | 534.476558 | 648.5845299 | 497.8159638 | 480.0402102 | 505.115338 | 502.257341 | 534.3973135 | 553.8548049 | 571.0465398 | 565.5111838 |
| **Ddit3** | 188.6334752 | 188.558358 | 205.9714205 | 165.6210086 | 176.0749819 | 204.8125393 | 205.670755 | 103.9211776 | 157.2545516 | 124.0940796 | 121.0714494 | 149.2148343 | 185.7543807 | 138.2438617 | 174.6114583 |
| **Mars** | 4073.01795 | 3958.140994 | 4087.318796 | 3836.886699 | 3589.902869 | 3826.206855 | 3826.55852 | 4055.440149 | 3963.717598 | 3924.693742 | 3975.494547 | 3679.186699 | 3631.583351 | 3384.715725 | 3669.714887 |
| **Arhgap9** | 2051.160119 | 2385.500908 | 2286.047819 | 2296.266275 | 2648.725088 | 2683.605395 | 2753.10151 | 1637.596622 | 1970.572587 | 2023.083058 | 1850.123087 | 2490.673193 | 2639.757208 | 2798.308756 | 2568.872277 |
| **Gli1** | 8.241268334 | 6.338096068 | 4.698967767 | 10.35131304 | 7.600358931 | 0.701412806 | 2.706194144 | 2.514222039 | 2.257242368 | 13.9824315 | 12.29631908 | 2.602584319 | 2.556252945 | 13.55331977 | 13.65274777 |
| **R3hdm2** | 955.0714303 | 888.1257115 | 866.959553 | 872.9607327 | 879.1081831 | 814.3402674 | 903.8688442 | 931.1002285 | 876.5624528 | 786.511772 | 748.1837227 | 837.1646226 | 905.765627 | 844.8235992 | 922.6383228 |
| **Stac3** | 1.831392963 | 0 | 10.96425812 | 0.86260942 | 6.966995687 | 2.104238417 | 1.80412943 | 5.028444078 | 0 | 2.621705907 | 6.621094891 | 2.602584319 | 4.260421576 | 8.131991864 | 7.904222392 |
| **Ndufa4l2** | 1.831392963 | 0.792262009 | 0 | 0 | 0.633363244 | 4.208476834 | 0.902064715 | 0 | 0 | 0 | 0 | 4.337640531 | 1.70416863 | 0 | 1.437131344 |
| **Shmt2** | 10263.12617 | 9527.742914 | 9533.422438 | 9201.454679 | 9103.32991 | 9403.140073 | 9457.24647 | 9393.971612 | 9107.972954 | 9193.448713 | 9068.062389 | 8557.29724 | 9098.556317 | 8409.383142 | 9521.713719 |
| **Nxph4** | 4.578482408 | 3.169048034 | 0 | 0.86260942 | 2.533452977 | 0.701412806 | 2.706194144 | 0 | 0 | 0.873901969 | 0 | 0.867528106 | 1.70416863 | 1.807109303 | 0.718565672 |
| **Lrp1** | 24.723805 | 30.10595632 | 28.1938066 | 31.05393911 | 12.03390164 | 14.72966892 | 7.216517718 | 61.17940295 | 36.11587788 | 32.33437285 | 33.10547446 | 7.807752957 | 11.0770961 | 14.45687443 | 5.029959704 |
| **Stat6** | 3724.137591 | 3760.075492 | 3799.898601 | 3635.898704 | 3634.238296 | 3586.323676 | 3766.120184 | 3503.149375 | 3329.432492 | 3281.501893 | 3162.045746 | 3643.618046 | 3756.839746 | 3556.391109 | 3818.457981 |
| **Nab2** | 2696.726138 | 2717.458689 | 2678.411627 | 2553.323882 | 2557.52078 | 2864.569899 | 2740.472604 | 2415.329306 | 2327.969295 | 2053.669627 | 2079.969667 | 2579.16106 | 2741.155242 | 2400.744709 | 2583.962156 |
| **1700012D01Rik** | 6.409875371 | 7.130358077 | 7.831612945 | 3.450437679 | 7.600358931 | 7.014128057 | 3.608258859 | 3.352296052 | 17.30552482 | 7.86511772 | 0 | 5.205168638 | 2.556252945 | 9.035546516 | 3.59282836 |
| **Tmem194** | 1240.768733 | 1373.782323 | 1351.736394 | 1326.693287 | 1243.925412 | 1294.808039 | 1423.45812 | 1082.791625 | 1097.772205 | 957.7965579 | 1077.346726 | 1421.878566 | 1433.205818 | 1361.65686 | 1401.20306 |
| **Myo1a** | 0 | 10.29940611 | 1.566322589 | 5.175656518 | 3.800179466 | 1.402825611 | 3.608258859 | 3.352296052 | 6.019312981 | 0.873901969 | 2.837612096 | 1.735056213 | 1.70416863 | 0 | 3.59282836 |
| **Zbtb39** | 233.5026028 | 193.3119301 | 227.8999367 | 194.9497288 | 156.4407213 | 187.2772191 | 186.727396 | 230.4703536 | 245.287004 | 166.9152761 | 207.145683 | 176.1082056 | 192.5710552 | 130.1118698 | 196.8869941 |
| **Gpr182** | 0 | 1.584524017 | 1.566322589 | 2.587828259 | 1.266726489 | 1.402825611 | 2.706194144 | 4.190370065 | 0 | 0.873901969 | 1.891741398 | 0 | 1.70416863 | 0.903554652 | 0 |
| **Rdh1** | 0 | 3.169048034 | 5.482129061 | 1.725218839 | 0 | 0 | 0.902064715 | 0.838074013 | 1.504828245 | 3.495607876 | 0 | 0 | 3.408337261 | 4.517773258 | 5.748525376 |
| **Rdh9** | 9.156964816 | 0 | 0 | 0.86260942 | 2.533452977 | 0 | 2.706194144 | 0 | 0 | 3.495607876 | 2.837612096 | 2.602584319 | 0.852084315 | 0 | 0 |
| **Prim1** | 903.7924273 | 1005.380489 | 1080.762586 | 975.6112536 | 1050.116259 | 1005.124551 | 977.8381508 | 1271.358278 | 1182.795001 | 1200.741305 | 1233.415391 | 1048.84148 | 1045.507455 | 1105.047339 | 1027.548911 |
| **Naca** | 10822.61672 | 10262.1698 | 10487.31289 | 9575.827168 | 10806.44367 | 10244.83544 | 11164.85497 | 10178.40889 | 9870.920874 | 10333.01688 | 10315.66584 | 10782.50683 | 10285.50977 | 10707.12262 | 10238.84226 |
| **Ptges3** | 4388.933236 | 4291.6833 | 4367.690539 | 4123.273026 | 4056.69158 | 4117.29317 | 4335.323019 | 4363.013312 | 4474.606787 | 4296.975981 | 4515.586716 | 4272.575923 | 4039.731738 | 3971.122694 | 3949.955499 |
| **Atp5b** | 32866.17812 | 32300.52209 | 33527.13502 | 31827.69976 | 30753.58569 | 31198.14019 | 31031.92825 | 35261.9641 | 34877.40424 | 35303.89174 | 34912.08749 | 32270.3105 | 31438.50289 | 31397.62059 | 30297.60299 |
| **Baz2a** | 2749.836534 | 2715.081903 | 2582.082788 | 2641.310043 | 2446.048849 | 2335.00323 | 2615.987673 | 2634.066623 | 2785.437082 | 2494.116219 | 2658.842534 | 2584.366229 | 2609.082173 | 2438.694005 | 2553.063832 |
| **Rbms2** | 426.7145604 | 435.7441047 | 422.1239377 | 416.6403497 | 396.4853909 | 352.8106413 | 396.9084745 | 451.721893 | 400.2843132 | 357.4259053 | 390.6445986 | 406.8706818 | 397.0712909 | 374.0716258 | 354.971442 |
| **Gls2** | 265.5519797 | 155.2833537 | 174.6449687 | 163.8957897 | 129.2061018 | 112.2260489 | 116.3663482 | 119.8445839 | 123.3959161 | 124.9679816 | 178.7695621 | 145.7447219 | 149.1147552 | 141.8580803 | 165.9886702 |
| **Spryd4** | 223.4299415 | 240.0553886 | 228.683098 | 199.2627759 | 205.2096911 | 208.3196033 | 211.0831433 | 265.6694621 | 246.0394181 | 294.5049635 | 319.7042962 | 196.061352 | 225.8023435 | 215.0460071 | 194.0127314 |
| **Mip** | 1.831392963 | 0 | 3.132645178 | 0.86260942 | 1.900089733 | 2.805651223 | 0 | 0.838074013 | 6.019312981 | 4.369509845 | 7.56696559 | 4.337640531 | 1.70416863 | 0 | 0 |
| **Timeless** | 2075.883924 | 2164.459807 | 2130.981882 | 2102.179156 | 2151.534941 | 2051.632457 | 2045.882773 | 2106.918069 | 2111.274028 | 2047.552313 | 2120.642107 | 2043.02869 | 2018.587743 | 2159.495617 | 2017.013841 |
| **Apon** | 7.325571853 | 0.792262009 | 8.614774239 | 7.763484777 | 10.13381191 | 14.02825611 | 9.020647148 | 0 | 2.257242368 | 4.369509845 | 10.40457769 | 13.01292159 | 4.260421576 | 4.517773258 | 7.18565672 |
| **Apof** | 0.915696482 | 0.792262009 | 7.04845165 | 3.450437679 | 3.166816221 | 1.402825611 | 4.510323574 | 0.838074013 | 0.752414123 | 0 | 1.891741398 | 3.470112425 | 1.70416863 | 2.710663955 | 1.437131344 |
| **Stat2** | 1496.248051 | 1498.95972 | 1182.573555 | 1167.110545 | 1069.117156 | 1212.742741 | 1274.617442 | 649.5073601 | 877.3148669 | 759.420811 | 868.3093015 | 889.2163089 | 960.2990232 | 1077.940699 | 1095.094084 |
| **Il23a** | 38.45925223 | 19.0142882 | 13.31374201 | 25.87828259 | 1.900089733 | 7.014128057 | 5.412388289 | 31.00873848 | 7.524141226 | 25.3431571 | 24.59263817 | 13.01292159 | 9.372927467 | 7.228437213 | 11.49705075 |
| **Pan2** | 314.0838932 | 400.0923143 | 342.2414857 | 359.708128 | 386.351579 | 396.999648 | 400.5167334 | 362.8860477 | 373.1974048 | 312.8569049 | 368.8895725 | 478.8755147 | 416.6692301 | 462.6199816 | 428.2651405 |
| **Cnpy2** | 976.1324493 | 897.6328556 | 960.9389083 | 896.251187 | 824.0055808 | 933.5804444 | 881.3172263 | 780.2469062 | 789.2824146 | 785.63787 | 867.3634308 | 807.6686669 | 823.9655328 | 880.9657853 | 744.4340362 |
| **Cs** | 6874.133487 | 6592.412173 | 6570.723261 | 6618.802077 | 5968.181851 | 5942.36929 | 6290.999321 | 6842.036243 | 6890.608535 | 6979.855026 | 6946.474412 | 6357.245963 | 6330.986462 | 6119.775655 | 6425.414239 |
| **Coq10a** | 163.9096702 | 140.2303755 | 160.5480654 | 206.1636513 | 162.1409905 | 156.4150557 | 144.3303544 | 162.5863585 | 118.8814314 | 125.8418835 | 132.4218978 | 153.5524748 | 138.8897434 | 138.2438617 | 160.9587105 |
| **Ankrd52** | 3806.550274 | 3772.751684 | 3766.222665 | 3669.540471 | 3435.362237 | 3409.567649 | 3794.986255 | 3936.433639 | 3674.038161 | 3557.654915 | 3592.416914 | 3654.895912 | 3975.825415 | 3520.248923 | 3760.254161 |
| **Slc39a5** | 25.63950148 | 16.63750218 | 17.22954848 | 27.60350143 | 21.53435031 | 28.75792503 | 20.74748844 | 13.40918421 | 30.84897903 | 17.47803938 | 20.80915537 | 24.29078698 | 17.89377062 | 21.68531164 | 15.08987911 |
| **Nabp2** | 851.5977279 | 841.382253 | 793.3423913 | 900.5642341 | 834.1393927 | 827.6671107 | 869.590385 | 936.9667466 | 963.0900769 | 1067.034304 | 1000.731199 | 870.1306906 | 880.2030976 | 890.9048865 | 862.2788064 |
| **Rnf41** | 381.8454328 | 381.8702881 | 419.7744538 | 377.8229258 | 422.4532839 | 365.4360718 | 365.3362095 | 474.3498914 | 425.8663934 | 411.6078274 | 522.1206257 | 392.122704 | 367.2483398 | 409.3102572 | 330.5402091 |
| **Mir8105** | 2.747089445 | 1.584524017 | 7.831612945 | 5.175656518 | 2.533452977 | 1.402825611 | 2.706194144 | 1.676148026 | 5.266898858 | 1.747803938 | 10.40457769 | 0 | 0 | 0 | 1.437131344 |
| **Smarcc2** | 2504.429877 | 2745.187859 | 2838.959693 | 2795.717129 | 2546.120242 | 2564.365218 | 2988.5404 | 2681.836842 | 2721.481881 | 2624.327613 | 2422.37486 | 2821.201402 | 2851.926203 | 2792.887428 | 2852.705718 |
| **Myl6** | 4970.400502 | 5037.994112 | 5377.968609 | 5171.343471 | 5647.066686 | 5399.475778 | 5462.903913 | 4968.102749 | 4846.299364 | 4923.563693 | 4872.179969 | 5666.69359 | 5447.375027 | 5793.592426 | 5504.931613 |
| **Myl6b** | 42.12203815 | 31.69048034 | 40.72438731 | 41.40525214 | 25.96789302 | 38.57770431 | 20.74748844 | 73.75051315 | 48.90691797 | 41.07339254 | 48.23940564 | 40.773821 | 29.82295103 | 37.94929537 | 28.02406121 |
| **A430046D13Rik** | 14.65114371 | 5.54583406 | 24.27800013 | 18.97740723 | 16.46744435 | 18.93814575 | 25.25781201 | 22.62799835 | 13.54345421 | 17.47803938 | 22.70089677 | 28.62842751 | 23.00627651 | 31.62441281 | 22.27553583 |
| **Esyt1** | 9443.577814 | 9315.416696 | 9832.590052 | 9481.802741 | 9810.796654 | 9911.664358 | 10407.12061 | 8526.565009 | 8726.498994 | 8532.778824 | 8886.455215 | 10413.80739 | 10559.88092 | 10804.70652 | 10549.9812 |
| **Zc3h10** | 437.7029182 | 444.4589868 | 442.4861314 | 388.1742388 | 399.0188439 | 418.0420322 | 390.5940215 | 346.9626414 | 319.023588 | 402.8688077 | 364.160219 | 387.7850635 | 398.7754595 | 409.3102572 | 388.7440285 |
| **Rpl41** | 18964.98983 | 17746.66899 | 17826.31739 | 17035.67343 | 19224.47455 | 20080.0458 | 18131.50077 | 18292.64148 | 17615.51944 | 18310.86795 | 16913.11396 | 16635.71897 | 15868.3662 | 17483.78251 | 16175.63184 |
| **Pa2g4** | 14439.61782 | 13973.91731 | 14230.82388 | 14115.74054 | 13021.9483 | 12668.9181 | 13381.22798 | 16168.12386 | 15596.03993 | 15758.2003 | 16216.00726 | 12952.19463 | 12579.32074 | 12679.58243 | 12683.40268 |
| **Erbb3** | 124.5347215 | 118.8393013 | 144.1016782 | 116.4522717 | 109.5718413 | 119.240177 | 129.8973189 | 143.3106562 | 134.6821279 | 147.6894327 | 102.1540355 | 122.321463 | 120.1438884 | 140.050971 | 132.9346493 |
| **Rps26** | 20996.92032 | 19892.90677 | 20633.95063 | 20505.95112 | 21338.0077 | 20460.91296 | 20355.09029 | 21349.93548 | 19981.10944 | 20388.13293 | 19275.89897 | 20335.72634 | 20340.95677 | 21827.16972 | 20892.29691 |
| **Ikzf4** | 568.6475151 | 443.6667248 | 443.2692927 | 464.0838678 | 279.3131907 | 298.1004424 | 303.9958089 | 439.9888569 | 430.3808781 | 439.5726904 | 463.4766424 | 272.4038254 | 350.2066535 | 295.4623711 | 315.45033 |
| **Suox** | 324.1565545 | 361.2714759 | 339.8920018 | 376.9603164 | 331.88234 | 310.0244601 | 367.1403389 | 419.8750805 | 390.5029296 | 352.1824935 | 385.9152451 | 367.8319171 | 318.6795339 | 354.1934234 | 329.1030778 |
| **Rab5b** | 715.1589521 | 800.9768906 | 812.9214237 | 676.285785 | 735.3347266 | 724.5594283 | 764.9508781 | 733.3147614 | 723.8223859 | 700.8693791 | 731.1580501 | 771.2324865 | 764.3196307 | 884.5800039 | 799.0450272 |
| **Cdk2** | 1061.292222 | 1155.91027 | 1159.078716 | 1197.301874 | 1174.255455 | 1145.407112 | 1127.580893 | 1420.535452 | 1270.075039 | 1261.040541 | 1263.683254 | 1209.33418 | 1214.220149 | 1273.108504 | 1245.27431 |
| **Pmel** | 14.65114371 | 27.7291703 | 32.10961307 | 15.52696955 | 27.2346195 | 23.14662259 | 26.15987673 | 16.76148026 | 12.79104008 | 34.08217679 | 29.32199166 | 26.89337129 | 33.23128829 | 21.68531164 | 22.9941015 |
| **Dgka** | 2018.195045 | 2457.59675 | 2348.700722 | 2373.038513 | 2252.239697 | 2243.118153 | 2270.496887 | 1718.051727 | 1997.659495 | 1958.414312 | 1844.447863 | 2552.267689 | 2367.090228 | 2526.338806 | 2444.560416 |
| **Wibg** | 1041.1469 | 1149.572174 | 1129.318587 | 1044.620007 | 1169.821912 | 1085.787023 | 1133.895346 | 984.7369653 | 1115.07773 | 1057.421382 | 1047.078864 | 1140.79946 | 1133.272139 | 1123.118432 | 1069.944286 |
| **Mmp19** | 2.747089445 | 4.753572051 | 0 | 0 | 0 | 0 | 0 | 0 | 6.019312981 | 3.495607876 | 0 | 0 | 2.556252945 | 4.517773258 | 2.155697016 |
| **Tmem198b** | 43.95343112 | 70.51131876 | 46.98967767 | 61.2452688 | 37.36843141 | 48.39748359 | 34.27845916 | 58.66518091 | 48.90691797 | 42.82119648 | 44.45592284 | 49.44910206 | 52.82922754 | 46.08128723 | 48.14390002 |
| **Dnajc14** | 874.4901399 | 1002.211441 | 986.0000698 | 903.1520624 | 857.5738327 | 900.6140425 | 969.7195684 | 916.0148963 | 840.4465749 | 940.3185185 | 854.121241 | 895.2890057 | 899.8010368 | 943.3110562 | 977.9678795 |
| **Ormdl2** | 190.4648682 | 210.7416943 | 217.7188399 | 213.0645267 | 235.6111269 | 213.9309057 | 203.8666255 | 206.1662072 | 228.7338933 | 212.3581784 | 213.7667779 | 207.3392174 | 288.0044985 | 219.5637803 | 211.2583076 |
| **Sarnp** | 1317.687237 | 1224.044803 | 1382.279685 | 1164.522717 | 1323.729181 | 1287.793911 | 1397.298243 | 1392.040936 | 1340.801966 | 1447.181661 | 1374.350125 | 1384.574858 | 1299.428581 | 1358.042641 | 1230.902996 |
| **Gdf11** | 73.25571853 | 64.9654847 | 64.21922615 | 77.63484777 | 59.53614496 | 79.25964705 | 82.08788905 | 104.7592516 | 72.23175577 | 55.92972601 | 87.02010429 | 83.2826982 | 57.08964912 | 96.68034772 | 64.67091048 |
| **Cd63** | 15.56684019 | 5.54583406 | 10.18109683 | 14.66436013 | 10.76717515 | 8.416953669 | 9.020647148 | 19.2757023 | 6.771727103 | 10.48682363 | 20.80915537 | 22.55573076 | 14.48543336 | 15.36042908 | 11.49705075 |
| **Rdh5** | 0 | 0 | 0 | 4.313047098 | 3.800179466 | 0.701412806 | 0 | 0 | 0.752414123 | 0.873901969 | 1.891741398 | 0 | 11.0770961 | 0.903554652 | 0 |
| **Bloc1s1** | 262.8048902 | 273.3303929 | 303.8665823 | 263.9584824 | 297.6807248 | 299.503268 | 258.8925731 | 236.3368717 | 221.209752 | 293.6310616 | 244.980511 | 331.3957366 | 246.2523671 | 309.9192455 | 243.5937628 |
| **Itga7** | 59.5202713 | 40.40536243 | 41.50754861 | 34.50437679 | 29.13470924 | 53.30737323 | 47.80942988 | 0.838074013 | 6.771727103 | 0.873901969 | 4.729353494 | 68.5347204 | 40.04796281 | 35.23863141 | 51.01816271 |
| **Mettl7b** | 0 | 0 | 0 | 0 | 0 | 0 | 0 | 2.514222039 | 1.504828245 | 0 | 0 | 0 | 0 | 0 | 0 |
| **Mir684-1** | 0 | 7.130358077 | 3.132645178 | 7.763484777 | 3.800179466 | 4.208476834 | 7.216517718 | 4.190370065 | 6.019312981 | 3.495607876 | 3.783482795 | 6.94022485 | 5.112505891 | 4.517773258 | 9.341353736 |
| **Tespa1** | 271.0461585 | 300.2673012 | 328.9277437 | 304.5011251 | 351.5166006 | 345.7965132 | 396.0064098 | 289.1355345 | 267.8594276 | 242.9447474 | 232.6841919 | 291.4894437 | 283.7440769 | 315.3405734 | 322.6359867 |
| **Vmn2r84** | 0 | 5.54583406 | 2.349483883 | 0 | 1.900089733 | 0 | 4.510323574 | 5.028444078 | 0 | 1.747803938 | 0.945870699 | 6.072696744 | 3.408337261 | 1.807109303 | 0 |
| **Sfi1** | 199.621833 | 207.5726462 | 205.1882592 | 210.4766984 | 195.0758792 | 190.0828703 | 179.5108782 | 303.3827927 | 294.9463361 | 267.4140025 | 204.3080709 | 209.0742736 | 198.5356454 | 209.6246792 | 193.2941658 |
| **Eif4enif1** | 3994.268053 | 3867.030864 | 3742.727826 | 3593.630842 | 3313.123131 | 3347.843322 | 3363.799321 | 3747.028912 | 4023.158313 | 3758.652368 | 3872.394641 | 3559.46782 | 3542.114498 | 3446.157441 | 3575.582784 |
| **Drg1** | 1844.212714 | 1723.169868 | 1924.227301 | 1725.218839 | 1846.253857 | 1745.115061 | 1798.717041 | 1940.979414 | 2023.241576 | 1946.179685 | 2086.590761 | 1750.671718 | 1759.554111 | 1700.489854 | 1817.97115 |
| **Patz1** | 952.3243408 | 1003.795965 | 898.2860048 | 1024.779991 | 927.2437896 | 1005.124551 | 1032.864098 | 998.1461495 | 1089.495649 | 988.3831268 | 1052.754088 | 974.2340634 | 1015.684504 | 909.8795341 | 957.8480407 |
| **Gm11944** | 10.0726613 | 15.05297816 | 14.0969033 | 3.450437679 | 19.00089733 | 1.402825611 | 13.53097072 | 15.92340625 | 12.03862596 | 4.369509845 | 5.675224193 | 11.27786538 | 10.22501178 | 5.421327909 | 10.05991941 |
| **Pik3ip1** | 223.4299415 | 278.876227 | 210.6703882 | 248.4315129 | 215.3435031 | 277.0580583 | 262.500832 | 112.3019177 | 197.1325001 | 173.9064918 | 155.1227946 | 286.2842751 | 270.1107279 | 322.5690106 | 186.108509 |
| **Limk2** | 694.097933 | 672.6304452 | 725.2073587 | 686.637098 | 734.0680001 | 747.7060509 | 764.9508781 | 584.9756611 | 692.9734069 | 660.6698885 | 803.0442233 | 717.4457439 | 731.0883424 | 839.4022713 | 781.7994511 |
| **Rnf185** | 287.5286952 | 252.7315807 | 299.9507758 | 314.8524382 | 266.6459258 | 275.6552326 | 294.073097 | 387.190194 | 370.9401624 | 389.7602781 | 350.9180292 | 314.0451745 | 252.2169573 | 319.8583467 | 252.2165509 |
| **8430429K09Rik** | 42.12203815 | 38.82083842 | 50.90548414 | 28.46611085 | 43.06870061 | 30.16075065 | 39.69084745 | 44.41792269 | 63.2027863 | 21.84754922 | 61.48159542 | 26.89337129 | 25.56252945 | 28.0101942 | 27.30549553 |
| **Selm** | 10.98835778 | 7.922620085 | 3.915806472 | 1.725218839 | 8.233722176 | 2.805651223 | 6.314453003 | 1.676148026 | 6.771727103 | 10.48682363 | 2.837612096 | 6.072696744 | 9.372927467 | 4.517773258 | 0 |
| **Smtn** | 65.01445019 | 58.62738863 | 89.28038757 | 75.04701951 | 62.70296118 | 55.41161165 | 66.75278889 | 71.23629111 | 30.0965649 | 48.93851026 | 61.48159542 | 83.2826982 | 57.08964912 | 74.09148143 | 72.57513287 |
| **Tug1** | 861.6703892 | 923.7775019 | 957.0231019 | 939.381658 | 928.5105161 | 916.0451243 | 1021.137257 | 1083.629699 | 1033.06459 | 971.7789894 | 1016.811001 | 956.8835012 | 962.8552761 | 920.72219 | 937.7282019 |
| **Morc2a** | 1384.53308 | 1319.908506 | 1288.300329 | 1246.470611 | 1195.789805 | 1209.93709 | 1340.468166 | 1512.723594 | 1323.496442 | 1308.231247 | 1246.657581 | 1208.466652 | 1270.457714 | 1252.326747 | 1298.448169 |
| **Dusp18** | 14.65114371 | 12.67619214 | 1.566322589 | 1.725218839 | 4.43354271 | 3.507064029 | 5.412388289 | 11.73303618 | 18.81035306 | 6.991215751 | 11.35044839 | 3.470112425 | 4.260421576 | 9.035546516 | 7.904222392 |
| **Slc35e4** | 23.80810852 | 20.59881222 | 27.41064531 | 21.56523549 | 23.43444004 | 18.23673295 | 13.53097072 | 31.00873848 | 64.70761454 | 28.83876497 | 44.45592284 | 21.68820266 | 33.23128829 | 23.49242094 | 15.08987911 |
| **Tcn2** | 76.00280797 | 91.90239299 | 69.70135521 | 92.2992079 | 72.83677309 | 79.25964705 | 50.51562403 | 68.72206907 | 45.14484735 | 62.92094176 | 68.10269031 | 91.09045116 | 60.49798638 | 43.37062328 | 82.63505228 |
| **Pes1** | 3544.66108 | 3388.50461 | 3416.149567 | 3327.084532 | 3301.722592 | 3389.226677 | 3421.531463 | 3741.162394 | 3468.629105 | 3696.605328 | 3619.847164 | 3318.295007 | 3304.382974 | 3350.380648 | 3171.748876 |
| **Gal3st1** | 0 | 0 | 0 | 0 | 2.533452977 | 0 | 1.80412943 | 0 | 0 | 0.873901969 | 2.837612096 | 0 | 0 | 2.710663955 | 3.59282836 |
| **Mtfp1** | 91.56964816 | 107.7476332 | 86.93090369 | 123.353147 | 78.53704229 | 96.79496719 | 85.6961479 | 111.4638437 | 92.54693708 | 142.4460209 | 113.5044839 | 102.3683165 | 102.2501178 | 119.269214 | 79.76078959 |
| **Sec14l2** | 6.409875371 | 13.46845414 | 18.01270977 | 4.313047098 | 8.233722176 | 12.6254305 | 12.62890601 | 6.704592104 | 9.781383594 | 19.22584332 | 8.512836289 | 9.542809169 | 10.22501178 | 13.55331977 | 10.05991941 |
| **Rnf215** | 180.3922069 | 224.2101484 | 194.224001 | 222.5532303 | 186.2087938 | 202.7083009 | 208.3769491 | 159.2340625 | 141.453855 | 146.8155308 | 207.145683 | 149.2148343 | 174.6772846 | 189.7464768 | 178.9228523 |
| **Mir3060** | 0 | 0 | 0.783161294 | 0 | 0.633363244 | 0.701412806 | 0.902064715 | 0 | 4.514484735 | 0 | 0 | 0.867528106 | 0 | 1.807109303 | 0 |
| **Ccdc157** | 92.48534464 | 105.3708471 | 79.09929074 | 81.94789487 | 75.37022607 | 77.85682143 | 66.75278889 | 115.6542138 | 79.75589699 | 102.2465304 | 89.85771638 | 89.35539495 | 116.7355512 | 110.2336675 | 84.79074929 |
| **Sf3a1** | 4740.560685 | 4516.68571 | 4579.92725 | 4562.341221 | 4527.913833 | 4411.185135 | 4821.535901 | 4870.04809 | 4537.057159 | 4249.785275 | 4623.415976 | 4562.330311 | 4539.905231 | 4403.021817 | 4524.089471 |
| **Tbc1d10a** | 766.4379551 | 808.8995107 | 744.0032298 | 759.9588987 | 811.3383159 | 803.8190753 | 777.5797841 | 802.0368305 | 754.6713649 | 768.1598307 | 722.6452139 | 779.9077675 | 766.0237993 | 799.6458666 | 756.6496526 |
| **Gatsl3** | 172.1509385 | 191.7274061 | 180.910259 | 165.6210086 | 163.407717 | 207.6181905 | 229.1244376 | 165.1005806 | 111.3572901 | 131.0852953 | 150.3934411 | 179.578318 | 176.3814532 | 184.3251489 | 210.5397419 |
| **Osm** | 785.6675812 | 792.2620085 | 783.9444558 | 691.8127546 | 675.1652184 | 762.4357198 | 686.4712479 | 584.1375871 | 591.3975003 | 579.3970054 | 653.5966529 | 550.8803475 | 613.5007069 | 590.0211875 | 644.5534078 |
| **Lif** | 5160.86537 | 4144.322566 | 3898.576924 | 3596.218671 | 3320.090126 | 3921.598997 | 4287.513589 | 1733.137059 | 2290.348589 | 1991.622587 | 2292.790574 | 3094.472755 | 3027.455572 | 3082.024917 | 3474.265024 |
| **Hormad2** | 0.915696482 | 10.29940611 | 2.349483883 | 2.587828259 | 1.900089733 | 1.402825611 | 3.608258859 | 0 | 0 | 14.85633347 | 0 | 0 | 0.852084315 | 0 | 2.874262688 |
| **Mtmr3** | 1670.230382 | 1861.023458 | 1724.52117 | 1660.523133 | 1531.472325 | 1685.494972 | 1740.082835 | 1751.574687 | 1805.793894 | 1748.67784 | 1729.051637 | 1827.88172 | 1674.345679 | 1783.616882 | 1803.599837 |
| **Ascc2** | 1256.335573 | 1129.765624 | 1214.683168 | 1077.399165 | 1137.520387 | 1116.649187 | 1166.369676 | 1221.073837 | 1303.18126 | 1134.324756 | 1207.876882 | 1080.94002 | 1124.751296 | 1127.636205 | 1142.519418 |
| **Uqcr10** | 1540.201482 | 1572.640087 | 1665.784073 | 1590.65177 | 1595.442012 | 1637.798901 | 1517.27285 | 1686.204914 | 1610.166222 | 1656.918133 | 1660.948947 | 1528.584523 | 1430.649565 | 1708.621846 | 1498.927992 |
| **Zmat5** | 363.5315032 | 356.5179038 | 363.3868406 | 398.5255519 | 354.0500536 | 378.7629151 | 445.6199691 | 425.7415986 | 452.2008877 | 424.7163569 | 450.2344526 | 412.9433786 | 389.402532 | 413.8280304 | 345.6300882 |
| **Nf2** | 1448.631834 | 1578.185921 | 1476.25904 | 1530.26911 | 1309.795189 | 1252.723271 | 1409.927149 | 1821.972904 | 1707.980058 | 1790.625134 | 1816.071742 | 1377.634633 | 1390.601602 | 1290.276042 | 1390.424575 |
| **Nipsnap1** | 540.2609241 | 583.8971003 | 632.0111647 | 607.2770314 | 535.8253047 | 516.239825 | 520.4913404 | 614.3082516 | 562.8057637 | 530.4584951 | 558.0637123 | 516.1792232 | 529.996444 | 618.9349363 | 559.7626585 |
| **Thoc5** | 1146.451995 | 1007.757275 | 1041.604522 | 1008.390412 | 1093.818323 | 1000.214661 | 986.858798 | 1030.831036 | 1130.878426 | 1004.113362 | 957.2211472 | 938.665411 | 975.6365409 | 1047.219841 | 972.2193542 |
| **AA413626** | 0.915696482 | 0 | 0 | 1.725218839 | 1.900089733 | 0.701412806 | 0 | 1.676148026 | 2.257242368 | 0 | 0.945870699 | 2.602584319 | 4.260421576 | 5.421327909 | 0 |
| **Nefh** | 1396.437134 | 1283.464454 | 1303.180394 | 1274.074113 | 1460.535641 | 1368.456384 | 1515.468721 | 1435.620784 | 1389.708884 | 1347.556836 | 1402.726246 | 1489.545758 | 1451.099589 | 1320.093346 | 1488.868072 |
| **Ap1b1** | 1515.477677 | 1475.19186 | 1595.299557 | 1646.721382 | 1492.203804 | 1555.733603 | 1581.319445 | 1780.907278 | 1843.4146 | 1721.586879 | 1770.669948 | 1536.392276 | 1657.303993 | 1564.956657 | 1669.946622 |
| **Gas2l1** | 56.77318186 | 42.78214846 | 46.20651637 | 45.71829924 | 24.70116653 | 28.05651223 | 32.47432973 | 92.18814144 | 61.69795805 | 90.0119028 | 49.18527634 | 52.05168638 | 42.60421576 | 30.72085815 | 42.39537465 |
| **Ewsr1** | 8986.64527 | 8911.363072 | 9029.849725 | 8733.920374 | 9002.625154 | 8520.762764 | 9246.163327 | 8545.002637 | 8500.022343 | 8472.479589 | 8853.34974 | 9063.066126 | 8968.187417 | 9117.769989 | 8678.836186 |
| **Rhbdd3** | 142.8486511 | 122.8006113 | 181.6934203 | 130.2540224 | 139.973277 | 125.5528922 | 105.5415716 | 169.2909506 | 160.2642081 | 158.1762564 | 180.6613035 | 149.2148343 | 157.6355983 | 165.3505012 | 176.7671553 |
| **Emid1** | 6.409875371 | 4.753572051 | 3.132645178 | 0.86260942 | 3.166816221 | 10.52119209 | 5.412388289 | 7.542666117 | 8.276555348 | 6.117313782 | 9.458706988 | 4.337640531 | 7.668758836 | 0.903554652 | 6.467091048 |
| **Kremen1** | 491.7290106 | 541.1149518 | 548.9960674 | 507.2143388 | 463.6218948 | 547.1019885 | 585.4399999 | 481.8925575 | 485.3071091 | 448.31171 | 478.6105736 | 511.8415827 | 528.2922754 | 566.5287665 | 571.2597092 |
| **Znrf3** | 368.1099856 | 383.4548121 | 364.9531632 | 373.5098787 | 324.2819811 | 320.5456522 | 331.0577503 | 326.8488651 | 415.3325957 | 391.5080821 | 395.3739521 | 327.0580961 | 383.4379418 | 351.4827595 | 380.1212405 |
| **Xbp1** | 3142.670325 | 2604.165222 | 2689.375885 | 2628.370902 | 2442.882033 | 2630.999434 | 2698.977627 | 2261.123687 | 2461.146595 | 2336.813865 | 2450.750981 | 2359.676449 | 2580.963391 | 2354.663422 | 2512.824155 |
| **Ccdc117** | 564.9847291 | 534.7768557 | 562.3098094 | 496.0004163 | 404.7191131 | 457.3211493 | 515.9810169 | 462.6168552 | 425.1139793 | 488.5112006 | 503.2032117 | 401.6655132 | 500.173493 | 459.9093177 | 449.103545 |
| **Mrps24** | 871.7430504 | 848.5126111 | 854.4289723 | 922.1294696 | 818.9386748 | 920.2536011 | 821.7809552 | 827.1790509 | 1041.341146 | 959.5443619 | 820.0698958 | 779.9077675 | 850.3801465 | 839.4022713 | 772.4580974 |
| **Urgcp** | 687.6880577 | 725.7119998 | 744.0032298 | 778.9363059 | 685.2990303 | 764.5399582 | 724.357966 | 772.70424 | 611.7126817 | 686.0130456 | 728.3204381 | 772.9675427 | 731.9404267 | 777.0570004 | 725.032763 |
| **2210015D19Rik** | 66.84584315 | 66.55000871 | 56.3876132 | 81.94789487 | 77.90367905 | 40.68194273 | 60.43833589 | 66.20784703 | 54.92623095 | 66.41654964 | 59.58985402 | 82.4151701 | 75.83550405 | 57.8274977 | 68.98230451 |
| **Dbnl** | 3847.756616 | 4090.44875 | 3947.132924 | 3998.19466 | 3901.517585 | 4031.019394 | 3950.141386 | 4130.86681 | 4223.30047 | 4026.940273 | 4046.434849 | 4001.907154 | 3851.421105 | 4057.86394 | 3785.40396 |
| **Pgam2** | 38.45925223 | 37.2363144 | 56.3876132 | 49.16873692 | 62.70296118 | 31.56357626 | 35.18052388 | 57.8271069 | 69.9745134 | 65.54264767 | 56.75224193 | 48.58157395 | 75.83550405 | 49.69550584 | 65.38947615 |
| **Polm** | 295.7699635 | 282.837537 | 339.8920018 | 316.577657 | 364.8172287 | 327.5597803 | 342.7845916 | 219.5753914 | 266.3545994 | 232.4579237 | 196.7411053 | 352.2164111 | 335.7212202 | 319.8583467 | 334.1330375 |
| **Aebp1** | 43.03773463 | 32.48274235 | 50.90548414 | 33.64176737 | 50.0356963 | 39.98052993 | 55.92801232 | 46.93214473 | 29.34415078 | 51.56021617 | 65.26507822 | 37.30370857 | 41.75213144 | 37.94929537 | 17.24557613 |
| **Pold2** | 2449.488088 | 2480.572349 | 2495.935046 | 2375.626342 | 2487.850824 | 2729.89864 | 2522.172943 | 2426.224268 | 2424.278303 | 2334.192159 | 2369.4061 | 2442.091619 | 2402.877769 | 2425.140685 | 2363.362495 |
| **Gck** | 5.494178889 | 9.507144102 | 6.265290356 | 6.900875357 | 3.800179466 | 3.507064029 | 3.608258859 | 0 | 0 | 1.747803938 | 0 | 6.072696744 | 4.260421576 | 5.421327909 | 2.874262688 |
| **Ykt6** | 1994.386937 | 1916.481799 | 2187.369496 | 1947.77207 | 1976.726685 | 2036.902788 | 1986.346502 | 2015.568001 | 1954.019476 | 2028.32647 | 2013.758718 | 2174.892962 | 2050.966947 | 2006.794881 | 1995.456871 |
| **Camk2b** | 17.39823315 | 30.89821833 | 35.24225825 | 26.74089201 | 26.60125626 | 30.86216345 | 32.47432973 | 15.92340625 | 14.29586833 | 17.47803938 | 17.97154328 | 31.23101183 | 34.08337261 | 32.52796746 | 40.23967763 |

| | | | | | | | | | | | | | | | |
|---|---|---|---|---|---|---|---|---|---|---|---|---|---|---|---|
| **Nudcd3** | 1398.268527 | 1341.29958 | 1418.305104 | 1338.769819 | 1359.197522 | 1392.304419 | 1446.009738 | 1492.609817 | 1467.207539 | 1427.955817 | 1357.324453 | 1574.563513 | 1435.762071 | 1456.530098 | 1426.352859 |
| **Rps15a-ps6** | 21.97671556 | 12.67619214 | 25.06116142 | 18.97740723 | 24.06780328 | 39.27911712 | 27.06194144 | 16.76148026 | 12.79104008 | 17.47803938 | 15.13393118 | 19.08561834 | 19.59793925 | 18.07109303 | 17.24557613 |
| **Ddx56** | 1799.343586 | 1846.762742 | 1885.069236 | 1913.267693 | 1806.985336 | 1809.645039 | 1697.685793 | 2064.176294 | 2008.945707 | 1865.780704 | 2018.488071 | 1669.991605 | 1759.554111 | 1742.053368 | 1781.324301 |
| **Tmed4** | 468.8365986 | 422.2756505 | 516.1032931 | 452.8699453 | 463.6218948 | 417.3406194 | 433.8931278 | 550.6146266 | 567.3202484 | 528.7106912 | 427.5335558 | 424.221244 | 457.5692772 | 463.5235363 | 451.9778077 |
| **Ogdh** | 3156.405772 | 3123.096838 | 3288.494276 | 3303.794077 | 2980.607428 | 3039.9231 | 3239.314391 | 3600.36596 | 3350.500088 | 3554.159308 | 3585.795819 | 3232.409724 | 3266.891264 | 3112.745775 | 3168.156048 |
| **Zmiz2** | 6560.96529 | 6522.693116 | 6288.785195 | 6079.67119 | 5870.643911 | 6304.298298 | 6765.485361 | 7173.913552 | 6486.562151 | 6257.138097 | 6279.635569 | 6168.124836 | 6325.873956 | 5961.653591 | 6324.096479 |
| **Ppia** | 37344.84961 | 37155.50367 | 38135.25607 | 36644.51076 | 36799.67122 | 36006.32497 | 36858.36425 | 36605.39674 | 36846.472 | 37155.69001 | 37371.35131 | 36999.2062 | 36618.32344 | 38624.25069 | 37358.94785 |
| **H2afv** | 767.3536516 | 831.0828469 | 803.5234881 | 905.7398906 | 1081.151058 | 1081.578546 | 1063.534299 | 615.1463256 | 658.3623573 | 625.7138097 | 575.0893849 | 1135.594291 | 971.3761193 | 1139.382416 | 1028.986042 |
| **Purb** | 3781.826469 | 3788.596925 | 3706.702407 | 3818.771901 | 3572.168698 | 3493.035772 | 3894.213374 | 4135.895254 | 4050.997636 | 3604.845622 | 3932.930365 | 3691.332092 | 3639.25211 | 3629.579035 | 3477.857852 |
| **Myo1g** | 7951.908246 | 7381.505133 | 7631.123653 | 6798.224836 | 6913.159811 | 7308.721436 | 7635.075746 | 7080.887336 | 6445.931788 | 6852.265338 | 7029.711033 | 6797.950241 | 7269.131293 | 6677.268875 | 7311.405712 |
| **Gm11974** | 310.4211073 | 371.570882 | 357.1215503 | 321.7533135 | 309.7146265 | 364.0332462 | 369.8465331 | 441.6650049 | 273.1263265 | 414.2295333 | 371.7271846 | 297.5621405 | 255.6252945 | 299.9801443 | 325.5102494 |
| **Ccm2** | 1005.434737 | 1109.959074 | 1101.12478 | 1097.239182 | 1271.793395 | 1201.520136 | 1224.101818 | 1171.62747 | 1138.402567 | 1028.582617 | 1080.184338 | 1173.765528 | 1056.584551 | 1203.534796 | 1090.064124 |
| **Nacad** | 11.90405426 | 0.792262009 | 6.265290356 | 9.488703616 | 5.066905954 | 10.52119209 | 9.922711863 | 11.73303618 | 11.28621184 | 10.48682363 | 6.621094891 | 15.61550591 | 15.33751767 | 10.84265582 | 7.904222392 |
| **Tbrg4** | 2773.644643 | 2675.468803 | 2637.68724 | 2594.729134 | 2639.224639 | 2521.579037 | 2453.616024 | 3111.76881 | 2887.765402 | 3092.739068 | 3030.569719 | 2481.997912 | 2529.838332 | 2422.430021 | 2468.991649 |
| **Snora5c** | 1.831392963 | 6.338096068 | 8.614774239 | 8.626094197 | 7.600358931 | 4.90988964 | 3.608258859 | 10.05688816 | 6.771727103 | 8.739019689 | 3.783482795 | 15.61550591 | 2.556252945 | 17.16753838 | 2.155697016 |
| **Ramp3** | 137.3544722 | 48.32798252 | 70.4845165 | 56.9322217 | 30.40143573 | 51.20313482 | 48.7114946 | 11.73303618 | 12.79104008 | 43.69509845 | 36.88895725 | 28.62842751 | 25.56252945 | 20.78175699 | 47.42533435 |
| **Adcy1** | 0 | 2.376786026 | 0 | 0 | 0.633363244 | 0.701412806 | 2.706194144 | 0 | 0 | 0 | 0 | 0.867528106 | 0.852084315 | 0 | 0 |
| **Tns3** | 8.241268334 | 10.29940611 | 4.698967767 | 9.488703616 | 4.43354271 | 4.208476834 | 4.510323574 | 4.190370065 | 9.028969471 | 12.23462756 | 7.56696559 | 8.675281063 | 2.556252945 | 3.614218606 | 10.05991941 |
| **Hus1** | 568.6475151 | 580.7280522 | 610.8658097 | 482.1986656 | 533.2918517 | 566.741547 | 564.6925115 | 663.7546183 | 628.2657924 | 545.3148286 | 693.3232222 | 508.3714703 | 537.6652029 | 474.3661921 | 559.0440928 |
| **Gm11992** | 1.831392963 | 0 | 0.783161294 | 1.725218839 | 1.900089733 | 1.402825611 | 0 | 0 | 0 | 0 | 2.837612096 | 2.602584319 | 0.852084315 | 0.903554652 | 0 |
| **Upp1** | 173.9823315 | 108.5398952 | 109.6425812 | 107.8261775 | 80.43713202 | 90.48225194 | 92.01060091 | 66.20784703 | 54.92623095 | 70.78605948 | 89.85771638 | 65.06460797 | 74.98341973 | 58.73105235 | 66.10804182 |
| **Zpbp** | 20.14532259 | 46.7434585 | 50.90548414 | 29.32872027 | 33.56825195 | 46.99465798 | 42.39704159 | 15.08533223 | 32.35380727 | 20.97364725 | 35.94308655 | 58.99191123 | 41.75213144 | 40.65995932 | 43.83250599 |
| **4930415F15Rik** | 5.494178889 | 8.714882094 | 4.698967767 | 11.21392246 | 4.43354271 | 7.715540863 | 15.33510015 | 10.05688816 | 4.514484735 | 8.739019689 | 2.837612096 | 5.205168638 | 14.48543336 | 5.421327909 | 12.9341821 |
| **Ikzf1** | 7722.984126 | 8378.17074 | 8541.157078 | 8438.045343 | 8591.572409 | 8209.335478 | 9335.467733 | 7849.401206 | 8184.008411 | 7201.826126 | 7710.737936 | 9301.636356 | 8797.770554 | 8716.591724 | 8604.823922 |
| **Fignl1** | 1553.936929 | 1666.127004 | 1761.329751 | 1618.255271 | 1744.915738 | 1724.774089 | 1800.521171 | 1530.323148 | 1537.934467 | 1447.181661 | 1552.173817 | 1698.620032 | 1796.193736 | 1713.139619 | 1724.557613 |
| **Ddc** | 154.7527054 | 135.4768035 | 151.1501298 | 106.963568 | 114.6387472 | 111.5246361 | 120.8766718 | 93.02621545 | 103.8331489 | 118.8506678 | 75.6696559 | 124.0565192 | 113.3272139 | 143.6651896 | 94.8506687 |
| **1700042O10Rik** | 13.73544722 | 14.26071615 | 12.53058071 | 13.80175071 | 5.700269198 | 7.014128057 | 9.020647148 | 0.838074013 | 5.266898858 | 6.991215751 | 9.458706988 | 8.675281063 | 13.63334904 | 16.26398373 | 17.9641418 |
| **Grb10** | 21.06101908 | 13.46845414 | 10.96425812 | 8.626094197 | 9.500448664 | 8.416953669 | 16.23716487 | 4.190370065 | 0.752414123 | 12.23462756 | 15.13393118 | 8.675281063 | 12.78126473 | 4.517773258 | 2.155697016 |
| **Sec61g** | 1092.425903 | 1049.747161 | 1080.762586 | 882.4494363 | 1026.048456 | 942.6988109 | 939.0493681 | 896.739194 | 977.3859452 | 959.5443619 | 902.3606466 | 916.1096802 | 858.0489054 | 998.42789 | 945.6324243 |
| **Egfr** | 3.662785926 | 3.961310043 | 3.915806472 | 6.038265938 | 0.633363244 | 2.104238417 | 4.510323574 | 3.352296052 | 1.504828245 | 0 | 0 | 2.602584319 | 0.852084315 | 0.903554652 | 6.467091048 |
| **Eldr** | 19.22962611 | 16.63750218 | 6.265290356 | 16.38957897 | 15.20071786 | 12.6254305 | 3.608258859 | 11.73303618 | 3.00965649 | 20.97364725 | 25.53850887 | 24.29078698 | 13.63334904 | 27.10663955 | 20.83840449 |
| **Fbxo48** | 57.68887834 | 68.13453273 | 61.86974226 | 64.69570647 | 42.43533737 | 47.69607079 | 64.04659475 | 56.98903289 | 58.68830156 | 53.3080201 | 50.13114703 | 80.68011388 | 83.50426289 | 46.98484188 | 48.14390002 |
| **Plek** | 2447.656695 | 1981.447283 | 2047.183624 | 1939.145975 | 1803.185156 | 1649.021506 | 1914.181325 | 2000.482669 | 1994.649839 | 1727.704193 | 1531.364661 | 1800.120821 | 1774.891628 | 1641.758802 | 1731.743269 |
| **Cnrip1** | 15.56684019 | 23.76786026 | 26.62748401 | 29.32872027 | 24.06780328 | 20.34097137 | 34.27845916 | 20.95185033 | 15.04828245 | 37.57778466 | 11.35044839 | 23.42325887 | 18.74585493 | 25.29953024 | 24.43123285 |
| **Ppp3r1** | 1986.145669 | 1966.394305 | 2213.213818 | 2079.751311 | 2031.195924 | 1994.818019 | 2155.032604 | 2294.646648 | 2254.232711 | 2322.831433 | 2335.354755 | 2207.85903 | 2126.802451 | 2136.003196 | 2141.325702 |
| **Pno1** | 1665.6519 | 1702.571056 | 1760.54659 | 1801.991078 | 1598.608829 | 1727.57974 | 1513.664591 | 2236.819541 | 2090.958847 | 2157.663961 | 2254.955746 | 1711.632954 | 1538.012189 | 1742.956923 | 1700.844946 |
| **Wdr92** | 425.7988639 | 475.3572051 | 475.3789058 | 455.4577736 | 424.9867369 | 436.2787652 | 457.3468104 | 535.5292943 | 533.4616129 | 523.4672794 | 558.0637123 | 481.478099 | 486.540144 | 454.4879897 | 434.7322315 |
| **C1d** | 551.2492819 | 644.1090129 | 691.531423 | 680.5988321 | 632.0965178 | 627.0630483 | 602.5792295 | 736.6670575 | 774.2341321 | 753.3034972 | 764.2635246 | 713.9756315 | 653.5486697 | 697.544191 | 620.8407406 |
| **Etaa1** | 363.5315032 | 457.9274409 | 408.0270344 | 394.2125048 | 380.6513098 | 396.999648 | 462.7591987 | 475.1879654 | 468.7539984 | 453.5551219 | 481.4481857 | 412.0758505 | 348.5024849 | 385.8178362 | 436.1693629 |
| **Etaa1os** | 0.915696482 | 3.169048034 | 2.349483883 | 4.313047098 | 3.166816221 | 1.402825611 | 0 | 10.89496217 | 3.762070613 | 10.48682363 | 2.837612096 | 3.470112425 | 4.260421576 | 0 | 3.59282836 |
| **Spred2** | 324.1565545 | 270.1613449 | 281.938066 | 354.5324715 | 334.415793 | 324.754129 | 383.3775038 | 217.0611694 | 277.6408112 | 208.8625706 | 232.6841919 | 360.8916922 | 322.9399554 | 282.8126059 | 301.0790166 |
| **Actr2** | 10573.54727 | 10449.93589 | 10546.04999 | 10240.89903 | 10340.92169 | 10399.14626 | 10813.04974 | 10527.88575 | 10574.42808 | 10093.56774 | 10641.99123 | 10755.61346 | 10587.9997 | 10556.22899 | 10218.00386 |
| **Rab1** | 2183.936109 | 2160.498497 | 2287.614141 | 2260.899289 | 1943.158433 | 2099.328527 | 2147.816086 | 2343.25494 | 2367.847244 | 2445.177709 | 2530.204119 | 2211.329143 | 2244.390086 | 2258.886629 | 2116.175904 |
| **Cep68** | 935.8418042 | 1064.800139 | 1069.015167 | 941.9694863 | 974.1126697 | 951.1157645 | 1040.080616 | 865.7304555 | 874.3052104 | 994.5004406 | 836.1496977 | 981.1742882 | 1081.294996 | 1031.859412 | 1011.0219 |
| **Slc1a4** | 714.2432556 | 657.5774671 | 700.1461973 | 637.4683611 | 539.6254841 | 647.4040197 | 627.8370415 | 450.045745 | 434.8953629 | 470.1592593 | 424.6959437 | 504.0338297 | 565.7839853 | 468.0413095 | 632.3377913 |
| **Sertad2** | 403.8221484 | 495.1637553 | 381.3995504 | 351.0820338 | 308.4479 | 355.6162925 | 376.1609861 | 295.0020526 | 361.911193 | 358.2998073 | 390.6445986 | 357.4215798 | 418.3733987 | 310.8228001 | 404.5524733 |
| **Aftph** | 1135.463637 | 1195.523371 | 1244.443297 | 1191.263609 | 1176.788908 | 1104.725169 | 1103.225146 | 1211.016949 | 1297.161947 | 1237.445188 | 1153.962252 | 1204.129012 | 1175.024271 | 1076.13359 | 1177.010571 |
| **Lgalsl** | 243.5752641 | 236.8863405 | 228.683098 | 226.0036679 | 247.6450285 | 258.8213253 | 262.500832 | 263.9933141 | 190.360773 | 243.8186493 | 241.1970282 | 236.835173 | 217.2815004 | 263.8379583 | 256.5279449 |
| **Peli1** | 1558.515412 | 1533.819248 | 1582.768976 | 1482.825592 | 1265.459762 | 1495.412102 | 1455.030385 | 1231.968799 | 1396.480612 | 1224.336658 | 1260.845641 | 1414.070813 | 1403.382867 | 1388.763499 | 1291.262513 |
| **Vps54** | 1300.289004 | 1174.132297 | 1251.491749 | 1063.597414 | 1042.5159 | 1091.398326 | 1165.467611 | 1122.181103 | 1039.083903 | 1036.447735 | 1260.845641 | 1160.752606 | 1241.486847 | 1141.189525 | 1236.651521 |
| **Ugp2** | 630.9148758 | 666.2923492 | 682.1334875 | 676.285785 | 662.4979535 | 657.9252118 | 751.4199074 | 720.7436512 | 692.2209928 | 770.7815366 | 698.0525757 | 683.6121477 | 705.525813 | 712.0010654 | 702.0386615 |
| **Mdh1** | 3384.414196 | 3416.233781 | 3455.307631 | 3482.354227 | 3793.845833 | 3671.194625 | 3535.191617 | 3307.87813 | 3491.201529 | 3309.466756 | 3424.9978 | 3883.923332 | 3609.429159 | 4028.046637 | 3713.547393 |
| **Wdpcp** | 17.39823315 | 34.06726637 | 33.67593566 | 34.50437679 | 22.80107679 | 29.45933784 | 20.74748844 | 26.81836842 | 21.82000955 | 38.45168663 | 18.91741398 | 32.09853993 | 28.1187824 | 28.91374885 | 40.9582433 |
| **Otx1** | 0.915696482 | 3.961310043 | 3.132645178 | 2.587828259 | 3.166816221 | 0.701412806 | 3.608258859 | 17.59955427 | 13.54345421 | 5.243411813 | 3.783482795 | 0.867528106 | 6.816674521 | 1.807109303 | 2.155697016 |
| **Ehbp1** | 7.325571853 | 1.584524017 | 4.698967767 | 2.587828259 | 1.900089733 | 9.81977928 | 4.510323574 | 8.380740131 | 9.781383594 | 0 | 3.783482795 | 12.14539349 | 1.70416863 | 9.035546516 | 10.05991941 |
| **Tmem17** | 9.156964816 | 7.922620085 | 12.53058071 | 6.038265938 | 8.86708542 | 14.72966892 | 9.020647148 | 10.05688816 | 3.00965649 | 8.739019689 | 8.512836289 | 6.94022485 | 8.520843152 | 12.64976512 | 17.24557613 |
| **B3gnt2** | 1661.989114 | 1593.238899 | 1546.743557 | 1463.848185 | 1723.381388 | 1902.932942 | 1967.403143 | 1295.662424 | 1362.621976 | 1296.870522 | 1348.811616 | 1724.645875 | 1767.22287 | 1595.677515 | 1697.970683 |
| **Commd1** | 710.5804697 | 703.5286636 | 684.4829714 | 693.5379734 | 708.7334703 | 731.5735564 | 718.043513 | 755.9427598 | 808.0927677 | 726.2125362 | 733.9956622 | 740.8690028 | 740.4612699 | 667.7268875 | 700.6015302 |
| **Zrsr1** | 77.83420093 | 81.60298688 | 59.52025838 | 80.22267603 | 85.50403798 | 71.54410618 | 86.59821262 | 108.9496217 | 94.05176532 | 66.41654964 | 79.4531387 | 88.48786684 | 74.13133542 | 73.18792678 | 92.69497168 |
| **Cct4** | 7584.713957 | 7349.022391 | 7695.34288 | 7247.644344 | 7342.580091 | 7257.518301 | 6998.218057 | 8264.247843 | 7839.402743 | 8267.112626 | 8126.921044 | 7207.423507 | 6842.237051 | 7114.589326 | 6843.61946 |
| **Fam161a** | 2.747089445 | 0.792262009 | 0.783161294 | 0 | 3.166816221 | 7.715540863 | 2.706194144 | 4.190370065 | 3.00965649 | 4.369509845 | 0.945870699 | 7.807752957 | 0.852084315 | 0 | 2.874262688 |
| **Xpo1** | 6285.340649 | 6313.535946 | 6354.570743 | 5921.813666 | 6042.918714 | 6032.851542 | 6583.268288 | 6468.255233 | 6637.044975 | 6632.915944 | 6684.468228 | 6338.160344 | 6160.569599 | 6035.745073 | 6385.893127 |
| **Usp34** | 2530.985075 | 2556.629501 | 2618.891369 | 2570.576071 | 2335.843645 | 2344.823009 | 2735.96228 | 3001.981115 | 2768.883971 | 2835.811889 | 2879.230407 | 2777.824996 | 2774.38653 | 2643.800911 | 2725.519594 |
| **Ahsa2** | 206.9474048 | 251.9393187 | 237.2978722 | 241.5306375 | 200.7761484 | 220.9450338 | 229.1244376 | 241.3653158 | 239.267691 | 311.9830029 | 323.487779 | 258.5233757 | 241.1398612 | 250.2846385 | 266.5878643 |
| **0610010F05Rik** | 259.1421043 | 289.1756331 | 332.8435502 | 283.7984991 | 262.8457464 | 289.6834888 | 248.0677966 | 336.0676792 | 332.5670422 | 453.5551219 | 401.0491763 | 327.9256242 | 276.0753181 | 320.7619013 | 267.30643 |
| **Pex13** | 233.5026028 | 231.3405065 | 219.2851625 | 209.614089 | 279.3131907 | 278.4608839 | 236.3409553 | 295.0020526 | 287.4221948 | 285.7659438 | 245.9263817 | 248.9805665 | 276.0753181 | 288.2339339 | 231.3781464 |
| **Pus10** | 771.932134 | 861.9810653 | 835.6331012 | 809.1276356 | 702.3998379 | 649.5082581 | 788.4045607 | 1115.476511 | 963.842491 | 956.9226559 | 946.8165695 | 726.9885531 | 616.9090442 | 650.5593491 | 823.4762601 |
| **Rel** | 1107.077046 | 957.0525063 | 1004.01278 | 887.6250928 | 740.4016326 | 789.7908192 | 989.5649921 | 905.9580081 | 761.4430921 | 854.6761256 | 803.0442233 | 736.5313622 | 864.8655799 | 684.8944259 | 843.5960989 |
| **Papolg** | 388.2553082 | 358.8946899 | 401.7617441 | 419.228178 | 400.2855704 | 403.3123633 | 395.1043451 | 428.2558207 | 392.760172 | 329.4610423 | 424.6959437 | 406.8706818 | 335.7212202 | 346.0614316 | 368.6241897 |
| **Fancl** | 228.9241204 | 242.4321746 | 223.2009689 | 226.0036679 | 217.876956 | 217.4379698 | 225.5161787 | 254.7745 | 247.5442463 | 202.7452568 | 244.980511 | 234.2325887 | 230.9148494 | 219.5637803 | 194.7312971 |
| **Vrk2** | 360.7844137 | 286.0065851 | 317.1803243 | 286.3863273 | 301.4809043 | 368.241723 | 349.0990446 | 283.2690164 | 306.2325479 | 274.4052182 | 267.6814078 | 362.6267484 | 323.7920398 | 312.6299094 | 299.6418852 |
| **Gm12070** | 18.31392963 | 15.05297816 | 3.915806472 | 12.93914129 | 15.83408111 | 13.32684331 | 10.82477658 | 11.73303618 | 8.276555348 | 13.10852953 | 7.56696559 | 7.807752957 | 13.63334904 | 10.84265582 | 16.52701046 |
| **Pnpt1** | 1037.484114 | 972.8977464 | 871.6585208 | 920.4042508 | 805.0046835 | 824.1600467 | 887.6316793 | 879.1396397 | 954.8135216 | 1045.186755 | 1078.292597 | 878.8059717 | 845.2676406 | 924.3364086 | 891.0214332 |
| **Smek2** | 2173.863447 | 2398.969362 | 2513.947755 | 2267.800164 | 2155.33512 | 2220.672943 | 2422.945824 | 2960.915488 | 2668.060479 | 2726.574143 | 2975.709218 | 2474.190159 | 2611.638426 | 2404.358928 | 2455.338901 |
| **Cfap36** | 411.1477202 | 316.9048034 | 363.3868406 | 358.8455186 | 290.7137291 | 328.2611931 | 382.4754391 | 382.999824 | 394.2650002 | 358.2998073 | 319.7042962 | 358.2891079 | 299.9336789 | 368.6502978 | 308.2646733 |
| **Ccdc88a** | 35.71216278 | 30.10595632 | 43.85703249 | 38.81742388 | 37.36843141 | 27.35509942 | 36.08258859 | 36.87525657 | 29.34415078 | 33.20827482 | 43.51005214 | 60.72696744 | 28.1187824 | 43.37062328 | 28.02406121 |
| **Prorsd1** | 170.3195456 | 163.9982358 | 137.8363878 | 140.6053354 | 141.8733667 | 166.234835 | 181.3150077 | 203.6519852 | 176.8173188 | 166.0413741 | 162.6897602 | 137.0694408 | 149.1147552 | 154.5078454 | 140.8388717 |
| **Mtif2** | 528.3568699 | 441.2899387 | 444.8356153 | 437.3429758 | 411.0527455 | 436.2787652 | 514.1768874 | 569.0522549 | 443.9243323 | 520.8455735 | 559.9554537 | 492.7559644 | 440.5275909 | 493.3408398 | 402.3967763 |
| **Rps27a** | 13827.01687 | 13148.38029 | 13540.07562 | 12481.9583 | 13804.15191 | 13024.53439 | 14663.06194 | 13568.41827 | 12418.59509 | 13327.00503 | 12661.42517 | 14150.25094 | 12686.68337 | 13244.30408 | 12825.67868 |
| **Clhc1** | 0 | 0.792262009 | 0 | 0.86260942 | 0.633363244 | 1.402825611 | 0 | 0 | 1.504828245 | 1.747803938 | 0 | 2.602584319 | 0 | 0 | 0 |
| **Rtn4** | 2304.808044 | 2179.512785 | 2340.085948 | 2236.746225 | 2201.570637 | 2408.651575 | 2358.899229 | 2404.434343 | 2248.213398 | 2263.406099 | 2061.052253 | 2400.45027 | 2413.954865 | 2348.338539 | 2387.793728 |
| **Eml6** | 0.915696482 | 3.961310043 | 0.783161294 | 1.725218839 | 2.533452977 | 3.507064029 | 6.314453003 | 0 | 0.752414123 | 2.621705907 | 3.783482795 | 3.470112425 | 0.852084315 | 0 | 2.874262688 |
| **4931440F15Rik** | 0 | 0 | 0.783161294 | 0 | 0.633363244 | 2.104238417 | 4.510323574 | 3.352296052 | 0 | 0.873901969 | 0.945870699 | 2.602584319 | 0.852084315 | 0 | 3.59282836 |
| **Sptbn1** | 2499.851395 | 2616.049152 | 2752.81195 | 2783.640597 | 2700.660874 | 2612.762701 | 2980.421818 | 2501.650929 | 2524.349381 | 2216.215393 | 2478.181231 | 3283.593882 | 3116.072341 | 2864.268245 | 3024.442913 |
| **Acyp2** | 5.494178889 | 11.09166812 | 8.614774239 | 7.763484777 | 0.633363244 | 6.312715251 | 6.314453003 | 8.380740131 | 7.524141226 | 9.612921658 | 8.512836289 | 1.735056213 | 6.816674521 | 2.710663955 | 7.904222392 |
| **Psme4** | 1737.076226 | 1579.770445 | 1670.483041 | 1535.444767 | 1444.70156 | 1529.781329 | 1630.933004 | 1700.452172 | 1647.034514 | 1700.613231 | 1869.986371 | 1538.994861 | 1574.651814 | 1574.895758 | 1503.957951 |
| **Erlec1** | 458.7639373 | 454.7583929 | 476.162067 | 433.0299287 | 442.0875445 | 438.3830036 | 466.3674575 | 355.3433815 | 404.7987979 | 378.3995525 | 372.6730553 | 438.1016937 | 477.1672165 | 449.0666618 | 434.0136659 |
| **Asb3** | 245.4066571 | 296.3059912 | 294.4686467 | 326.0663606 | 307.1811735 | 260.9255637 | 264.3049614 | 242.2033898 | 270.11667 | 239.4491395 | 284.7070803 | 295.8270842 | 316.9753652 | 244.8633106 | 273.0549553 |
| **Chac2** | 91.56964816 | 87.94108294 | 92.41303275 | 66.42092531 | 93.73776015 | 90.48225194 | 89.30440676 | 77.1028092 | 100.0710783 | 76.90337326 | 92.69532848 | 97.1631479 | 86.06051583 | 66.86304422 | 56.76668809 |
| **Stc2** | 303.0955354 | 219.4565764 | 227.8999367 | 187.1862441 | 198.2426955 | 270.745343 | 276.9338674 | 31.8468125 | 41.38277674 | 48.06460829 | 62.42746612 | 185.6510147 | 181.4939591 | 160.832728 | 211.9768732 |
| **Bod1** | 212.4415837 | 217.0797903 | 245.1294852 | 232.0419339 | 266.6459258 | 232.8690515 | 234.5368258 | 293.3259046 | 264.097357 | 298.0005714 | 285.652951 | 248.9805665 | 230.0627651 | 288.2339339 | 245.7494598 |
| **Cpeb4** | 380.9297363 | 359.6869519 | 335.9761953 | 340.7307208 | 246.378302 | 246.8973076 | 296.7792912 | 419.8750805 | 392.0077579 | 310.235199 | 387.8069865 | 325.3230399 | 347.6504006 | 285.5232699 | 280.9591777 |
| **Nsg2** | 489.8976176 | 679.7608033 | 537.248648 | 646.9570647 | 948.1447767 | 930.0733804 | 1023.843451 | 258.126796 | 358.1491223 | 304.9917871 | 428.4794265 | 980.3067601 | 901.5052054 | 966.8034772 | 915.4526661 |
| **Il9r** | 14.65114371 | 37.2363144 | 52.47180673 | 51.75656518 | 29.13470924 | 19.63955856 | 22.55161787 | 31.8468125 | 48.90691797 | 48.93851026 | 43.51005214 | 32.09853993 | 23.85836082 | 19.87820233 | 21.55697016 |
| **Snrnp25** | 629.0834828 | 718.5816417 | 680.5671649 | 696.1258017 | 737.8681796 | 719.6495387 | 680.1567949 | 709.010615 | 593.6547427 | 732.3298499 | 776.5598437 | 707.0354066 | 636.5069834 | 779.7676643 | 661.0804182 |
| **Rhbdf1** | 10.98835778 | 16.63750218 | 7.04845165 | 11.21392246 | 13.93399137 | 6.312715251 | 11.72684129 | 11.73303618 | 33.10622139 | 15.73023544 | 18.91741398 | 13.01292159 | 9.372927467 | 14.45687443 | 15.80844478 |
| **Mpg** | 190.4648682 | 194.1041921 | 219.2851625 | 199.2627759 | 209.0098706 | 220.243621 | 221.9079198 | 183.5382089 | 215.9428532 | 224.592806 | 210.9291658 | 209.0742736 | 193.4231395 | 209.6246792 | 201.9169538 |
| **Nprl3** | 325.9879474 | 301.8518252 | 306.2160661 | 292.4245933 | 249.5451182 | 315.6357626 | 324.7432973 | 312.6016069 | 315.2615174 | 316.3525127 | 253.4933473 | 315.7802307 | 282.0399083 | 288.2339339 | 296.7676225 |
| **Sh3pxd2b** | 5.494178889 | 15.05297816 | 9.397935534 | 12.93914129 | 5.700269198 | 4.208476834 | 6.314453003 | 14.24725822 | 6.771727103 | 20.09974528 | 10.40457769 | 1.735056213 | 3.408337261 | 3.614218606 | 4.311394032 |
| **Ubtd2** | 173.066635 | 193.3119301 | 173.8618074 | 212.2019172 | 217.876956 | 213.2294929 | 234.5368258 | 222.0896135 | 189.6083589 | 159.9240603 | 182.5530449 | 172.6380931 | 247.9565357 | 229.5028815 | 219.8810956 |
| **Efcab9** | 7.325571853 | 3.169048034 | 3.915806472 | 3.450437679 | 10.76717515 | 4.208476834 | 4.510323574 | 5.028444078 | 6.771727103 | 6.991215751 | 1.891741398 | 3.470112425 | 9.372927467 | 6.324882561 | 11.49705075 |
| **Stk10** | 3211.347561 | 3558.840942 | 3478.019309 | 3497.018587 | 3418.894793 | 3400.449282 | 3721.919013 | 3300.335463 | 3510.011882 | 3226.446069 | 3229.202566 | 3712.152767 | 3727.016795 | 3735.29493 | 3685.523332 |
| **Fbxw11** | 1731.582047 | 1643.151406 | 1840.429042 | 1673.462274 | 1514.371517 | 1601.325435 | 1711.216764 | 1665.253064 | 1594.365526 | 1619.340348 | 1723.376413 | 1656.111155 | 1779.15205 | 1638.144583 | 1764.078725 |
| **Npm1** | 38476.65046 | 35105.1296 | 37526.73975 | 34058.40772 | 34629.13538 | 34729.75366 | 35440.31851 | 41203.07078 | 38366.34852 | 41303.22876 | 42167.86162 | 35448.93348 | 33627.5075 | 33550.79132 | 33137.37453 |
| **Ranbp17** | 0 | 0.792262009 | 6.265290356 | 2.587828259 | 0 | 1.402825611 | 1.80412943 | 6.704592104 | 0 | 7.86511772 | 3.783482795 | 0.867528106 | 0 | 1.807109303 | 0 |
| **Kcnip1** | 0 | 0 | 2.349483883 | 0 | 2.533452977 | 2.805651223 | 1.80412943 | 0 | 0 | 1.747803938 | 0 | 0 | 1.70416863 | 0 | 0 |
| **Kcnmb1** | 16.48253667 | 23.76786026 | 21.92851625 | 21.56523549 | 23.43444004 | 18.23673295 | 10.82477658 | 31.00873848 | 16.5531107 | 21.84754922 | 19.86328467 | 13.8804497 | 21.30210788 | 21.68531164 | 22.9941015 |
| **Lcp2** | 6476.721214 | 6195.488907 | 6426.621583 | 6037.403328 | 6493.23998 | 6423.538475 | 6963.939598 | 5105.546888 | 5585.922446 | 4877.246888 | 5698.87096 | 6203.693488 | 6230.440512 | 6231.816432 | 6324.815045 |
| **Dock2** | 5366.897078 | 5485.622147 | 5372.48648 | 5714.787405 | 5386.754393 | 5280.937014 | 6154.787549 | 5063.643187 | 5251.098161 | 4858.894947 | 5138.915506 | 5532.226734 | 5616.087721 | 5594.810403 | 5660.860364 |
| **Fam196b** | 2.747089445 | 3.961310043 | 0.783161294 | 4.313047098 | 3.800179466 | 1.402825611 | 0.902064715 | 5.028444078 | 1.504828245 | 2.621705907 | 0.945870699 | 4.337640531 | 3.408337261 | 1.807109303 | 4.311394032 |
| **Spdl1** | 766.4379551 | 827.9137989 | 816.8372302 | 767.7223835 | 737.8681796 | 695.8015033 | 796.5231432 | 948.6997828 | 831.4176055 | 873.9019689 | 778.4515851 | 763.4247335 | 834.1905445 | 809.5849678 | 676.1702973 |
| **Pank3** | 1336.916863 | 1177.301345 | 1170.826135 | 1032.543475 | 1038.082357 | 1112.44071 | 1197.941941 | 1072.734737 | 968.3569758 | 1046.060657 | 991.2724923 | 1010.670244 | 1109.413778 | 972.2248051 | 1099.405478 |
| **Rars** | 3446.681557 | 3098.536715 | 3098.186081 | 3074.339972 | 2959.706441 | 2884.209457 | 3143.695531 | 3363.191014 | 3197.760021 | 3327.818698 | 3459.995016 | 2855.902526 | 2837.440769 | 2720.603056 | 2920.250891 |
| **Wwc1** | 10.98835778 | 16.63750218 | 7.831612945 | 12.07653188 | 2.533452977 | 2.805651223 | 5.412388289 | 5.028444078 | 9.028969471 | 7.86511772 | 19.86328467 | 9.542809169 | 8.520843152 | 5.421327909 | 12.9341821 |
| **Mat2b** | 1119.896797 | 1259.696594 | 1269.504458 | 1245.608002 | 1292.061018 | 1264.647289 | 1259.282342 | 1249.568353 | 1203.862596 | 1233.075678 | 1291.113504 | 1444.434297 | 1298.576496 | 1371.595961 | 1332.220756 |
| **Hmmr** | 1869.852215 | 1894.298462 | 2066.762656 | 1919.305959 | 1745.549101 | 1672.869542 | 1900.650354 | 2182.34473 | 2187.267854 | 2215.341491 | 2172.664995 | 1926.779924 | 1934.231395 | 1958.906485 | 1976.055598 |
| **Nudcd2** | 1380.870294 | 1392.796611 | 1495.054911 | 1349.983742 | 1333.229629 | 1341.802697 | 1252.065824 | 1839.572459 | 1601.137253 | 1824.707311 | 1624.05999 | 1416.673398 | 1476.662118 | 1367.078188 | 1412.700111 |
| **Ccng1** | 3309.327084 | 3452.677833 | 3658.146407 | 3606.569984 | 2895.10339 | 2858.958596 | 3119.339784 | 6451.493752 | 5877.106711 | 6201.208371 | 6200.18243 | 3336.513097 | 2999.336789 | 3382.908615 | 3063.24546 |
| **Mir146** | 3.662785926 | 0 | 1.566322589 | 1.725218839 | 1.900089733 | 0 | 0 | 0.838074013 | 0 | 0 | 0 | 0 | 0 | 1.807109303 | 0.718565672 |
| **Pttg1** | 381.8454328 | 346.2184977 | 321.879292 | 277.7602331 | 323.0152546 | 312.1286985 | 289.5627734 | 312.6016069 | 238.5152769 | 309.361297 | 295.111658 | 280.2115783 | 288.8565828 | 291.8481525 | 268.0249956 |
| **Slu7** | 1257.251269 | 1109.959074 | 1316.494136 | 1141.232262 | 1179.322361 | 1182.58199 | 1326.035131 | 1133.91414 | 1173.013617 | 1120.342324 | 1090.588916 | 1256.180698 | 1094.928345 | 1156.549954 | 1174.854874 |
| **Ccnjl** | 0.915696482 | 5.54583406 | 0.783161294 | 5.175656518 | 3.166816221 | 4.208476834 | 1.80412943 | 0.838074013 | 2.257242368 | 3.495607876 | 3.783482795 | 2.602584319 | 0 | 0.903554652 | 2.155697016 |
| **Pwwp2a** | 369.0256821 | 409.5994584 | 399.4122602 | 362.2959563 | 342.6495152 | 427.1603987 | 393.3002156 | 364.5621957 | 408.5608686 | 459.6724356 | 382.1317623 | 455.4522558 | 394.5150379 | 382.2036176 | 442.6364539 |
| **Ttc1** | 628.1677864 | 631.4328208 | 707.9778102 | 640.9187988 | 690.9992995 | 672.6548807 | 712.6311247 | 739.1812795 | 793.7968993 | 747.1861834 | 681.0269031 | 688.8173164 | 693.5966325 | 674.0517701 | 646.7091048 |
| **Ublcp1** | 176.7294209 | 173.5053799 | 202.055614 | 204.4384325 | 162.7743538 | 192.1871088 | 210.1810785 | 163.4244325 | 199.3897425 | 184.3933154 | 212.8209072 | 179.578318 | 213.8731631 | 268.3557315 | 165.2701046 |
| **Rnf145** | 2355.171351 | 2669.130707 | 2600.095498 | 2715.494453 | 2912.204197 | 2872.285439 | 2950.653682 | 2037.357926 | 2178.238885 | 2109.599353 | 2210.499823 | 2779.560053 | 2914.980442 | 2941.070391 | 2984.921801 |
| **Ebf1** | 0.915696482 | 0 | 2.349483883 | 2.587828259 | 0 | 0.701412806 | 0 | 6.704592104 | 12.03862596 | 8.739019689 | 0.945870699 | 0 | 0 | 0.903554652 | 2.155697016 |
| **Clint1** | 3245.228331 | 3233.221257 | 3110.716662 | 3115.745224 | 3073.711824 | 3020.984954 | 3205.937996 | 3131.044513 | 3254.19108 | 3121.577833 | 2977.60096 | 3257.568039 | 3209.801615 | 3080.217807 | 3008.634469 |
| **Lsm11** | 490.8133141 | 434.9518427 | 473.0294219 | 461.4960395 | 426.8868266 | 472.7522311 | 490.7232048 | 425.7415986 | 461.2298571 | 411.6078274 | 374.5647967 | 492.7559644 | 502.7297459 | 524.0616979 | 454.1335047 |
| **Thg1l** | 434.9558287 | 446.8357728 | 449.534583 | 455.4577736 | 394.5853012 | 420.8476834 | 415.8518335 | 530.5008503 | 477.0305537 | 557.5494562 | 473.8812201 | 427.6913564 | 463.5338674 | 405.6960386 | 365.0313614 |
| **Adam19** | 2103.354818 | 1962.432995 | 2069.11214 | 2147.034846 | 1778.48399 | 1841.910028 | 2281.321664 | 1667.767286 | 1826.109076 | 1563.410622 | 1869.986371 | 2083.802511 | 1958.089756 | 2116.124994 | 2082.403317 |
| **Nipal4** | 0.915696482 | 0 | 1.566322589 | 0 | 1.266726489 | 1.402825611 | 0 | 1.676148026 | 0 | 1.747803938 | 0 | 0 | 0.852084315 | 0 | 0 |
| **Cyfip2** | 7911.617601 | 7702.371247 | 7863.722558 | 8069.711121 | 7389.448971 | 7362.028809 | 8184.433157 | 6951.823938 | 7541.446751 | 6925.673104 | 6925.665256 | 7707.987224 | 7818.725676 | 7731.717154 | 7685.778427 |
| **Fndc9** | 101.6423095 | 102.2017991 | 103.3772909 | 113.8644434 | 92.47103366 | 99.60061841 | 95.61885977 | 104.7592516 | 107.5952195 | 106.6160402 | 99.31642337 | 96.2956198 | 114.1792982 | 103.9087849 | 140.8388717 |
| **Itk** | 11648.57494 | 11453.73186 | 11676.15174 | 10343.54955 | 10624.03506 | 11050.75875 | 11670.91328 | 8505.613158 | 8798.730749 | 8384.21549 | 9352.769469 | 10282.81064 | 10495.12251 | 9865.009686 | 10863.27583 |
| **Fam71b** | 1047.556775 | 903.1786897 | 950.7578115 | 793.6006661 | 753.0688974 | 681.7732472 | 1005.802157 | 463.4549292 | 559.7961072 | 544.4409266 | 701.8360585 | 714.8431596 | 789.0300758 | 691.2193085 | 828.5062198 |
| **Med7** | 246.3223535 | 282.045275 | 278.0222595 | 244.9810752 | 296.4139983 | 301.6075065 | 274.2276733 | 299.1924227 | 342.3484258 | 219.3493942 | 309.2997185 | 289.7543875 | 225.8023435 | 318.0512374 | 247.9051568 |
| **Havcr2** | 460.5953302 | 296.3059912 | 314.8308404 | 313.1272193 | 360.383686 | 369.6445486 | 408.6353158 | 283.2690164 | 238.5152769 | 269.1618064 | 325.3795204 | 383.447423 | 400.4796281 | 356.0005327 | 425.3908778 |
| **Timd2** | 4.578482408 | 9.507144102 | 3.915806472 | 1.725218839 | 3.166816221 | 0.701412806 | 0.902064715 | 10.05688816 | 15.04828245 | 4.369509845 | 8.512836289 | 1.735056213 | 0.852084315 | 0 | 2.874262688 |
| **Gnb2l1** | 33836.81639 | 32448.67508 | 32729.09366 | 31749.2023 | 33290.20548 | 33860.00178 | 33572.14249 | 30809.27687 | 31349.33442 | 31630.88176 | 31457.7677 | 32941.77725 | 32560.69794 | 33262.55739 | 32979.29008 |
| **Snord96a** | 17.39823315 | 51.49703055 | 41.50754861 | 39.6800333 | 41.16861088 | 31.56357626 | 34.27845916 | 51.96058881 | 24.07725192 | 48.06460829 | 57.69811262 | 51.18415827 | 46.01255302 | 50.59906049 | 33.05402091 |
| **Snord95** | 1.831392963 | 3.961310043 | 10.18109683 | 2.587828259 | 12.03390164 | 7.014128057 | 2.706194144 | 14.24725822 | 11.28621184 | 1.747803938 | 3.783482795 | 11.27786538 | 13.63334904 | 2.710663955 | 8.622788064 |
| **Trim41** | 572.310301 | 533.1923317 | 559.1771643 | 523.6039177 | 502.2570527 | 505.7186329 | 497.9397226 | 522.1201101 | 516.1560881 | 545.3148286 | 466.3142545 | 574.3036064 | 512.1026734 | 552.9754468 | 604.3137301 |
| **Trim7** | 23.80810852 | 30.89821833 | 31.32645178 | 18.97740723 | 21.53435031 | 32.26498906 | 27.96400616 | 36.03718256 | 42.88760499 | 34.95607876 | 28.37612096 | 30.36348372 | 33.23128829 | 49.69550584 | 30.89832389 |
| **Irgm1** | 2869.792773 | 2636.647964 | 2638.470401 | 2674.089201 | 2413.113961 | 2786.713077 | 2939.828905 | 2598.02944 | 2623.668045 | 2257.288786 | 2445.075756 | 2716.230501 | 2522.169573 | 2857.039808 | 2677.375694 |
| **Gm5431** | 4.578482408 | 0 | 7.831612945 | 2.587828259 | 4.43354271 | 4.208476834 | 2.706194144 | 2.514222039 | 0 | 0 | 0 | 3.470112425 | 0 | 1.807109303 | 5.748525376 |
| **Gm12185** | 323.240858 | 340.6726637 | 306.2160661 | 355.3950809 | 286.9135497 | 307.2188089 | 374.3568566 | 222.0896135 | 250.5539028 | 212.3581784 | 193.9034932 | 299.2971967 | 250.5127887 | 306.3050269 | 260.1207733 |
| **Psme2b** | 427.6302569 | 457.1351789 | 433.0881959 | 402.838599 | 426.8868266 | 433.4731139 | 493.429399 | 443.3411529 | 340.8435975 | 374.0300427 | 404.8326591 | 453.7171996 | 411.5567242 | 391.2391641 | 387.3068972 |
| **9930111J21Rik1** | 244.4909606 | 206.7803842 | 169.9460009 | 203.575823 | 171.008076 | 171.8461374 | 220.1037904 | 86.32162334 | 119.6338455 | 76.90337326 | 88.91184568 | 197.7964082 | 208.7606572 | 159.9291733 | 230.6595807 |
| **9930111J21Rik2** | 30.21798389 | 24.56012226 | 23.49483883 | 30.19132969 | 30.40143573 | 35.07064029 | 26.15987673 | 12.5711102 | 26.33449429 | 3.495607876 | 30.26786236 | 26.02584319 | 28.1187824 | 12.64976512 | 34.49115225 |
| **Tgtp2** | 1430.317904 | 1600.369257 | 1558.490976 | 1510.429094 | 1478.903175 | 1503.829055 | 1501.035685 | 1475.848337 | 1534.172396 | 1350.178542 | 1351.649229 | 1667.38902 | 1474.95795 | 1617.362826 | 1511.143608 |
| **Olfr56** | 29.30228741 | 37.2363144 | 38.37490343 | 30.19132969 | 29.13470924 | 35.07064029 | 28.86607087 | 20.11377631 | 36.86829201 | 18.35194135 | 29.32199166 | 22.55573076 | 27.26669809 | 36.14218606 | 21.55697016 |
| **Ifi47** | 3985.111088 | 4143.530304 | 4142.140087 | 4311.321879 | 4097.226827 | 4206.372596 | 4254.137195 | 3868.549644 | 4258.663934 | 3806.716977 | 3554.582086 | 4211.848956 | 4118.975579 | 4318.08768 | 4118.818432 |
| **Olfr1396** | 0.915696482 | 0.792262009 | 1.566322589 | 0 | 0 | 0.701412806 | 0 | 0 | 0 | 0 | 0 | 0.867528106 | 3.408337261 | 0 | 0.718565672 |
| **Btnl9** | 2.747089445 | 1.584524017 | 0.783161294 | 6.900875357 | 0.633363244 | 0 | 1.80412943 | 9.218814144 | 1.504828245 | 1.747803938 | 3.783482795 | 0 | 2.556252945 | 0 | 0.718565672 |
| **Zfp62** | 771.932134 | 883.3721395 | 818.4035527 | 703.8892864 | 749.9020812 | 714.0382362 | 813.6623727 | 761.8092779 | 691.4685787 | 694.7520653 | 780.3433265 | 711.3730471 | 789.0300758 | 657.7877863 | 730.0627227 |

| | | | | | | | | | | | | | | | |
|---|---|---|---|---|---|---|---|---|---|---|---|---|---|---|---|
| **Mgat1** | 2367.991101 | 2720.627737 | 2552.322659 | 2517.094287 | 2418.180867 | 2415.665703 | 2426.554083 | 2432.92886 | 2488.985917 | 2471.394768 | 2370.351971 | 2366.616674 | 2273.360953 | 2268.82573 | 2350.428313 |
| **Flt4** | 0 | 7.130358077 | 3.915806472 | 1.725218839 | 0.633363244 | 2.104238417 | 7.216517718 | 0 | 0 | 0 | 5.675224193 | 1.735056213 | 2.556252945 | 0 | 3.59282836 |
| **Cnot6** | 2816.682377 | 2826.790846 | 2889.865177 | 2876.802415 | 2715.861591 | 2649.236167 | 2943.437164 | 3138.587179 | 3018.68546 | 2926.697694 | 3162.991617 | 3143.054329 | 2953.324236 | 2977.212577 | 3024.442913 |
| **Gfpt2** | 5.494178889 | 12.67619214 | 0.783161294 | 3.450437679 | 1.900089733 | 5.611302446 | 6.314453003 | 7.542666117 | 0.752414123 | 2.621705907 | 2.837612096 | 1.735056213 | 1.70416863 | 0.903554652 | 0.718565672 |
| **Mapk9** | 848.8506384 | 915.0626198 | 809.0056172 | 835.0059182 | 824.0055808 | 845.9038437 | 856.0594143 | 873.2731216 | 929.2314414 | 830.2068705 | 770.8846195 | 940.4004672 | 821.4092798 | 864.7018016 | 845.7517959 |
| **Rasgef1c** | 2.747089445 | 1.584524017 | 0.783161294 | 0 | 0 | 0.701412806 | 0 | 0 | 0 | 0 | 0 | 0 | 0 | 0 | 0 |
| **Rnf130** | 148.34283 | 102.9940611 | 171.5123235 | 160.4453521 | 143.7734565 | 126.9557178 | 128.9952542 | 109.7876957 | 94.80417945 | 116.2289619 | 103.0999062 | 105.838429 | 115.0313825 | 112.0407768 | 121.4375986 |
| **Tbc1d9b** | 961.4813056 | 1010.926323 | 1006.362263 | 898.8390153 | 867.7076447 | 835.3826516 | 951.6782741 | 723.2578733 | 697.4878916 | 778.6466543 | 694.2690929 | 818.9465323 | 896.3926995 | 833.0773888 | 885.9914735 |
| **3010026O09Rik** | 86.07546927 | 78.43393884 | 96.32883922 | 73.32180067 | 111.471931 | 86.97518791 | 69.45898304 | 72.07436512 | 79.75589699 | 90.88580477 | 66.21094891 | 105.838429 | 86.06051583 | 106.6194489 | 115.6890732 |
| **Sqstm1** | 3094.138411 | 2915.524191 | 2657.266272 | 2591.278697 | 2577.155041 | 2644.326278 | 2815.343975 | 2422.871972 | 2549.179047 | 2441.682101 | 2434.671179 | 2635.550387 | 2707.923954 | 2742.288368 | 2479.051568 |
| **Mgat4b** | 232.5869063 | 182.220262 | 195.7903236 | 193.22451 | 196.3426057 | 191.485696 | 165.0778428 | 136.6060641 | 145.9683398 | 172.1586879 | 188.2282691 | 134.4668565 | 177.2335376 | 189.7464768 | 158.8030135 |
| **Ltc4s** | 0.915696482 | 1.584524017 | 3.132645178 | 0.86260942 | 1.900089733 | 0.701412806 | 0 | 0 | 5.266898858 | 5.243411813 | 2.837612096 | 0.867528106 | 5.112505891 | 0 | 3.59282836 |
| **Maml1** | 726.1473099 | 710.6590216 | 704.0620037 | 759.0962893 | 676.4319449 | 687.3845496 | 750.5178427 | 564.0238108 | 483.0498667 | 483.2677888 | 521.174755 | 722.6509125 | 660.3653443 | 703.8690736 | 675.4517316 |
| **Canx** | 10652.29717 | 10365.95612 | 11122.4567 | 10246.07469 | 10155.97962 | 9994.431069 | 11309.18533 | 10942.73239 | 11119.1759 | 10977.95653 | 11229.37694 | 10904.8283 | 10962.06471 | 10427.92423 | 10274.05198 |
| **Hnrnph1** | 5329.353523 | 5744.691824 | 5994.316548 | 5389.583654 | 5596.397626 | 5675.131011 | 5572.053743 | 7112.734149 | 6668.646368 | 6914.312378 | 6883.101075 | 6131.688655 | 5957.773532 | 6100.801007 | 5813.914852 |
| **Rufy1** | 191.3805646 | 178.2589519 | 213.8030334 | 222.5532303 | 186.8421571 | 172.5475502 | 230.928567 | 235.4987977 | 190.360773 | 234.2057277 | 215.6585193 | 181.3133742 | 200.2398141 | 193.3606954 | 165.9886702 |
| **Zfp354c** | 26.55519797 | 30.89821833 | 27.41064531 | 27.60350143 | 22.16771355 | 16.83390734 | 40.59291217 | 18.43762829 | 15.80069657 | 28.83876497 | 24.59263817 | 30.36348372 | 11.92918041 | 28.91374885 | 25.86836419 |
| **Zfp879** | 0 | 1.584524017 | 0.783161294 | 2.587828259 | 0 | 2.104238417 | 0.902064715 | 1.676148026 | 0 | 0 | 3.783482795 | 0 | 3.408337261 | 2.710663955 | 1.437131344 |
| **Grm6** | 0 | 3.169048034 | 0 | 0 | 0 | 0.701412806 | 0 | 0 | 0 | 0 | 3.783482795 | 0 | 0 | 0.903554652 | 0 |
| **Zfp2** | 7.325571853 | 1.584524017 | 10.18109683 | 2.587828259 | 1.266726489 | 7.715540863 | 4.510323574 | 0.838074013 | 2.257242368 | 0.873901969 | 8.512836289 | 6.94022485 | 7.668758836 | 6.324882561 | 2.874262688 |
| **BC049762** | 2.747089445 | 3.169048034 | 1.566322589 | 0 | 7.600358931 | 6.312715251 | 4.510323574 | 0.838074013 | 3.762070613 | 0 | 0 | 0 | 0.852084315 | 0.903554652 | 0.718565672 |
| **Clk4** | 375.4355574 | 328.7887335 | 389.2311634 | 370.9220505 | 344.5496049 | 364.0332462 | 388.7898921 | 376.2952319 | 301.7180632 | 336.452258 | 387.8069865 | 333.9983209 | 355.3191594 | 357.807642 | 370.7798867 |
| **Col23a1** | 22.89241204 | 38.82083842 | 13.31374201 | 25.01567317 | 27.2346195 | 28.05651223 | 41.49497688 | 20.95185033 | 19.56276719 | 15.73023544 | 29.32199166 | 27.7608994 | 26.41461377 | 30.72085815 | 31.61688957 |
| **Phykpl** | 256.3950148 | 244.8089606 | 278.8054208 | 256.1949976 | 271.0794686 | 218.8407954 | 229.1244376 | 323.496569 | 310.7470326 | 267.4140025 | 277.1401147 | 255.0532632 | 265.8503063 | 252.9953024 | 243.5937628 |
| **Hnrnpab** | 29365.47047 | 27232.42202 | 28210.25299 | 27570.72227 | 26833.70057 | 29667.65744 | 28073.15599 | 29630.10673 | 31704.47388 | 29221.53404 | 29283.21096 | 27962.16592 | 26480.22426 | 25960.93225 | 25657.10588 |
| **Nhp2** | 3707.655054 | 3418.610567 | 3705.919246 | 3513.408166 | 3593.703048 | 3702.056789 | 3455.809922 | 4270.825171 | 4230.824611 | 4126.565097 | 4017.112858 | 3364.273996 | 3402.37267 | 3260.928738 | 3329.114758 |
| **Rmnd5b** | 840.6093701 | 826.3292749 | 837.9825851 | 807.4024168 | 897.4757171 | 899.2112169 | 864.1779968 | 866.5685295 | 896.8776341 | 823.2156547 | 881.5514913 | 910.0369835 | 912.5823015 | 905.3617609 | 965.0336975 |
| **N4bp3** | 278.3717304 | 358.8946899 | 339.1088405 | 329.5167983 | 323.0152546 | 305.8159833 | 317.5267796 | 295.8401266 | 316.7663456 | 260.4227867 | 271.4648905 | 255.9207914 | 311.8628593 | 308.1121362 | 345.6300882 |
| **D930048N14Rik** | 7.325571853 | 17.42976419 | 7.04845165 | 12.07653188 | 9.500448664 | 10.52119209 | 21.64955315 | 12.5711102 | 24.82966605 | 32.33437285 | 10.40457769 | 24.29078698 | 22.15419219 | 9.939101167 | 21.55697016 |
| **0610009B22Rik** | 85.15977279 | 96.65596504 | 98.67832311 | 75.90962893 | 74.73686282 | 81.36388546 | 127.1911248 | 129.901472 | 89.53728059 | 157.3023544 | 122.9631908 | 111.0435976 | 89.46885309 | 112.0407768 | 81.9164866 |
| **Sec24a** | 996.2777719 | 1083.022166 | 1043.954006 | 961.8095029 | 758.1358034 | 741.3933356 | 833.5077965 | 1144.809102 | 1154.203264 | 968.2833815 | 1069.77976 | 870.9982187 | 839.3030504 | 959.57504 | 892.4585646 |
| **Sar1b** | 1031.989935 | 1156.702532 | 1178.657748 | 1082.574822 | 1083.051148 | 1120.857664 | 982.3484744 | 1366.898715 | 1191.071556 | 1303.861738 | 1211.660365 | 1001.994963 | 1050.619961 | 1101.43312 | 1024.674648 |
| **Jade2** | 2381.726549 | 2643.778322 | 2505.332981 | 2530.033428 | 2395.37979 | 2310.453782 | 2548.332819 | 3001.981115 | 2855.411595 | 2549.172043 | 2341.97585 | 2403.920383 | 2534.098753 | 2426.947794 | 2271.386089 |
| **Cdkn2aipnl** | 1737.076226 | 1821.410358 | 1961.819043 | 1843.39633 | 1889.955921 | 1982.894002 | 1857.351248 | 1939.303266 | 1963.048446 | 2033.569882 | 1989.16608 | 1865.185429 | 1864.360482 | 1922.764299 | 1771.264381 |
| **Ube2b** | 1004.51904 | 975.2745325 | 1053.351941 | 998.0390985 | 1058.983344 | 1044.403668 | 1066.240493 | 922.7194884 | 987.1673288 | 1085.386245 | 965.7339834 | 1078.337436 | 1137.532561 | 1152.935735 | 1051.980144 |
| **Cdkl3** | 37.54355574 | 79.22620085 | 74.40032298 | 63.83309705 | 40.53524763 | 47.69607079 | 42.39704159 | 72.07436512 | 59.44071568 | 53.3080201 | 70.94030241 | 44.24393342 | 34.93545692 | 60.53816166 | 34.49115225 |
| **Ppp2ca** | 5543.626499 | 5181.393536 | 5424.175126 | 5230.863521 | 5241.080846 | 5593.767126 | 5451.177071 | 5422.338864 | 5364.712694 | 5350.901756 | 5638.335235 | 5342.238078 | 5352.793668 | 5447.530994 | 5200.259768 |
| **Mir3061** | 10.98835778 | 14.26071615 | 14.0969033 | 24.15306375 | 12.03390164 | 17.53532014 | 12.62890601 | 16.76148026 | 1.504828245 | 12.23462756 | 26.48437957 | 10.41033728 | 11.0770961 | 7.228437213 | 16.52701046 |
| **Olfr1372-ps1** | 0 | 0.792262009 | 0 | 0 | 1.266726489 | 0.701412806 | 0.902064715 | 0 | 0 | 0 | 0 | 0 | 1.70416863 | 0.903554652 | 0 |
| **Skp1a** | 2270.927274 | 2285.675895 | 2425.450529 | 2398.916796 | 2556.254054 | 2259.95206 | 2315.600123 | 2731.283209 | 2683.861175 | 2774.638751 | 2700.460845 | 2586.968813 | 2652.538473 | 2432.369122 | 2435.937628 |
| **Tcf7** | 4096.826059 | 4929.454217 | 5370.136996 | 5496.547222 | 5854.176467 | 5608.496794 | 5939.194082 | 4779.536096 | 4915.521463 | 4403.592021 | 4414.378551 | 6565.452708 | 6330.986462 | 6936.58906 | 6014.394674 |
| **Vdac1** | 5926.387629 | 5514.935841 | 5774.248224 | 5563.830757 | 5385.487666 | 5421.920988 | 5608.136332 | 5100.518443 | 5282.699555 | 5251.276931 | 5256.203473 | 5513.141115 | 5448.227111 | 5464.698533 | 5596.189453 |
| **9530068E07Rik** | 1987.977061 | 2083.649082 | 2052.665753 | 1855.472862 | 2064.130813 | 1985.699653 | 2046.784838 | 1902.42801 | 1865.23461 | 1932.197253 | 2009.975235 | 2003.122397 | 2130.210788 | 2118.835658 | 2121.924429 |
| **Fstl4** | 2.747089445 | 0 | 3.915806472 | 1.725218839 | 3.166816221 | 2.104238417 | 3.608258859 | 13.40918421 | 6.019312981 | 3.495607876 | 4.729353494 | 0 | 0 | 0 | 0 |
| **Hspa4** | 8493.084867 | 7520.943247 | 8257.652689 | 8239.645177 | 7578.824581 | 7540.187661 | 8076.185391 | 8752.006918 | 8617.398946 | 8383.341588 | 8507.161065 | 7731.410483 | 7650.865066 | 7310.660686 | 7333.681248 |
| **Zcchc10** | 375.4355574 | 369.986358 | 397.8459376 | 365.7463939 | 353.4166903 | 359.1233565 | 373.4547919 | 459.2645592 | 366.4256777 | 393.255886 | 447.3968405 | 381.7123668 | 319.5316182 | 364.1325246 | 365.749927 |
| **Aff4** | 1468.777156 | 1336.546008 | 1393.243943 | 1390.526384 | 1088.751417 | 1179.776339 | 1257.478212 | 1340.918421 | 1330.268169 | 1307.357345 | 1440.561074 | 1337.72834 | 1264.493124 | 1288.468933 | 1400.484495 |
| **Uqcrq** | 2601.493704 | 2515.431877 | 2557.021627 | 2275.563649 | 2418.81423 | 2477.39003 | 2399.492141 | 2411.138936 | 2549.931461 | 2598.984456 | 2351.434557 | 2201.786334 | 2132.767041 | 2276.054167 | 2281.446008 |
| **Gdf9** | 0 | 0 | 2.349483883 | 2.587828259 | 4.43354271 | 1.402825611 | 2.706194144 | 3.352296052 | 2.257242368 | 3.495607876 | 0.945870699 | 0.867528106 | 2.556252945 | 1.807109303 | 1.437131344 |
| **Sept8** | 47.61621704 | 55.4583406 | 65.00238744 | 56.9322217 | 49.40233305 | 54.00878604 | 58.63420646 | 86.32162334 | 59.44071568 | 39.3255886 | 68.10269031 | 50.31663016 | 44.30838439 | 59.634607 | 49.58103137 |
| **Kif3a** | 191.3805646 | 106.9553711 | 115.1247103 | 122.4905376 | 82.970585 | 112.2260489 | 125.3869954 | 117.3303618 | 113.6145325 | 139.824315 | 135.2595099 | 120.5864068 | 87.76468446 | 106.6194489 | 114.2519418 |
| **Il13** | 87.90686223 | 37.2363144 | 43.0738712 | 69.00875357 | 33.56825195 | 50.50172201 | 37.88671802 | 113.9780658 | 12.79104008 | 86.51629492 | 79.4531387 | 40.773821 | 33.23128829 | 29.8173035 | 23.71266717 |
| **Rad50** | 1613.457201 | 1601.953781 | 1586.684783 | 1557.872612 | 1420.633757 | 1336.191395 | 1578.613251 | 1604.073661 | 1558.249648 | 1606.231819 | 1673.245266 | 1512.101489 | 1497.112142 | 1549.596227 | 1372.460433 |
| **Irf1** | 2338.688814 | 2959.098602 | 2748.896144 | 2754.311877 | 2427.681315 | 2280.293031 | 2463.538736 | 2755.587355 | 2680.099105 | 2856.785536 | 2521.691283 | 2750.931625 | 2673.840581 | 3015.161872 | 2900.849618 |
| **Gm12216** | 28.38659093 | 34.06726637 | 46.98967767 | 50.89395576 | 41.16861088 | 30.16075065 | 13.53097072 | 26.81836842 | 31.60139315 | 13.10852953 | 19.86328467 | 39.03876478 | 31.52711966 | 34.33507676 | 27.30549553 |
| **Slc22a5** | 173.9823315 | 235.3018165 | 191.8745171 | 207.0262607 | 185.5754306 | 200.6040624 | 201.1604314 | 182.7001348 | 276.8883971 | 199.2496489 | 176.8778207 | 200.3989926 | 209.6127415 | 216.8531164 | 206.9469135 |
| **Slc22a21** | 9.156964816 | 7.922620085 | 1.566322589 | 0 | 0.633363244 | 7.715540863 | 5.412388289 | 5.028444078 | 4.514484735 | 7.86511772 | 2.837612096 | 2.602584319 | 5.964590206 | 9.035546516 | 7.904222392 |
| **Pdlim4** | 1.831392963 | 0.792262009 | 1.566322589 | 0 | 1.900089733 | 0.701412806 | 2.706194144 | 1.676148026 | 0.752414123 | 0 | 6.621094891 | 0 | 0 | 0 | 1.437131344 |
| **P4ha2** | 131.8602933 | 81.60298688 | 88.49722628 | 69.87136299 | 60.80287145 | 89.07942633 | 82.08788905 | 14.24725822 | 5.266898858 | 17.47803938 | 15.13393118 | 78.07752957 | 80.94800994 | 71.38081747 | 76.8865269 |
| **4933405E24Rik** | 1.831392963 | 3.169048034 | 0 | 3.450437679 | 1.900089733 | 2.805651223 | 0 | 0 | 0 | 2.621705907 | 0 | 0 | 1.70416863 | 0 | 0.718565672 |
| **Csf2** | 475.2464739 | 267.7845589 | 260.0095498 | 227.7288868 | 147.5736359 | 208.3196033 | 191.2377195 | 179.3478388 | 88.78486646 | 194.0062371 | 160.7980188 | 141.4070813 | 143.1501649 | 138.2438617 | 94.8506687 |
| **Il3** | 489.8976176 | 321.6583755 | 314.8308404 | 273.447186 | 136.8064608 | 173.9503758 | 208.3769491 | 315.9539029 | 246.7918322 | 338.200062 | 400.1033056 | 166.5653964 | 190.0148023 | 210.5282338 | 156.6473165 |
| **Acsl6** | 70.50862908 | 34.06726637 | 39.15806472 | 52.6191746 | 27.2346195 | 36.4734659 | 17.13922958 | 77.94088321 | 40.63036262 | 90.88580477 | 95.53294058 | 32.09853993 | 21.30210788 | 21.68531164 | 16.52701046 |
| **Fnip1** | 716.0746486 | 593.4042444 | 674.3018746 | 505.4891199 | 559.8931079 | 506.4200457 | 535.8264406 | 610.1178815 | 601.9312981 | 629.2094176 | 665.8929719 | 567.3633815 | 633.9507305 | 612.6100538 | 584.1938913 |
| **Rapgef6** | 3380.75141 | 3398.804016 | 3627.603116 | 3552.22559 | 3150.98214 | 2991.525616 | 3737.254113 | 3279.383613 | 3401.664248 | 3222.950461 | 3482.695913 | 3379.021974 | 3450.941476 | 3367.548186 | 3329.833324 |
| **Cdc42se2** | 2167.453572 | 2494.040803 | 2741.064531 | 2728.433594 | 2468.849926 | 2419.87418 | 2555.549337 | 2826.823646 | 2722.98671 | 2769.395339 | 2649.383827 | 2815.128705 | 2732.634399 | 2830.836723 | 2689.59131 |
| **Lyrm7** | 43.03773463 | 53.87381658 | 50.12232285 | 48.3061275 | 66.50314065 | 58.91867568 | 44.20117102 | 77.1028092 | 61.69795805 | 51.56021617 | 54.86050053 | 34.70112425 | 58.79381775 | 48.79195118 | 46.70676868 |
| **Hint1** | 3478.730933 | 3416.233781 | 3491.333051 | 3349.512377 | 3562.668249 | 3631.915508 | 3367.40758 | 3551.757667 | 3689.086443 | 3769.139192 | 3522.422482 | 3427.603548 | 3153.56405 | 3713.609618 | 3354.264557 |
| **Gpx3** | 7.325571853 | 6.338096068 | 6.265290356 | 12.07653188 | 10.76717515 | 9.118366474 | 2.706194144 | 5.866518091 | 14.29586833 | 27.964863 | 5.675224193 | 11.27786538 | 5.112505891 | 5.421327909 | 17.24557613 |
| **Tnip1** | 2172.032054 | 2306.274707 | 2181.104205 | 2197.928801 | 2260.473419 | 2292.217049 | 2468.04906 | 2241.009911 | 2229.403045 | 2011.722332 | 2030.78439 | 2199.183749 | 2209.454629 | 2285.089714 | 2239.7692 |
| **Anxa6** | 10840.93065 | 10974.41334 | 10882.80935 | 11059.51537 | 12522.2247 | 12337.14984 | 12757.90126 | 8622.105446 | 9188.481265 | 8847.383533 | 9628.017843 | 12317.16405 | 12004.16383 | 12267.5615 | 12143.04129 |
| **Ccdc69** | 163.9096702 | 140.2303755 | 177.7776138 | 166.483618 | 191.2756998 | 187.9786319 | 217.3975963 | 186.8905049 | 129.4152291 | 194.0062371 | 195.7952346 | 212.544386 | 234.3231867 | 245.7668652 | 200.4798225 |
| **Gm2a** | 1931.20388 | 1752.483563 | 1789.523558 | 1773.524967 | 1988.127224 | 1994.116607 | 2244.33701 | 916.0148963 | 1091.752892 | 1240.940796 | 1332.731815 | 1842.629698 | 1862.656313 | 1797.170202 | 1912.821819 |
| **Slc36a3** | 13.73544722 | 5.54583406 | 14.0969033 | 6.038265938 | 12.66726489 | 6.312715251 | 8.118582433 | 0 | 1.504828245 | 10.48682363 | 0 | 9.542809169 | 11.92918041 | 4.517773258 | 4.311394032 |
| **Slc36a1** | 108.0521848 | 120.4238253 | 159.7649041 | 131.1166318 | 136.1730975 | 95.39214158 | 120.8766718 | 129.063398 | 120.3862596 | 175.6542957 | 210.9291658 | 152.6849467 | 140.593912 | 187.0358129 | 168.1443672 |
| **Atox1** | 538.4295312 | 499.1250654 | 599.1183903 | 577.9483112 | 594.0947231 | 559.727419 | 549.3574113 | 579.947217 | 620.7416511 | 611.7313782 | 668.730584 | 562.1582129 | 636.5069834 | 578.274977 | 522.3972435 |
| **G3bp1** | 11546.01694 | 11410.15745 | 11609.58303 | 11451.14005 | 11207.36261 | 11322.90692 | 11476.0673 | 12108.49334 | 11970.90869 | 12088.68594 | 12083.49818 | 11475.66179 | 11538.07371 | 11223.05233 | 11141.36074 |
| **Gm12238** | 3.662785926 | 12.67619214 | 0.783161294 | 0.86260942 | 2.533452977 | 5.611302446 | 3.608258859 | 15.92340625 | 2.257242368 | 4.369509845 | 6.621094891 | 6.072696744 | 5.112505891 | 0.903554652 | 2.874262688 |
| **Fam114a2** | 771.0164375 | 855.6429692 | 786.2939397 | 778.9363059 | 767.0028888 | 746.3032253 | 797.4252079 | 745.8858716 | 735.1085978 | 740.1949677 | 746.2919813 | 794.6557454 | 912.5823015 | 777.960555 | 790.4222392 |
| **Mfap3** | 704.1705943 | 701.9441395 | 639.8427776 | 650.4075024 | 590.2945437 | 605.3192513 | 625.1308473 | 621.0128437 | 677.9251244 | 657.1742806 | 606.3031179 | 589.0515842 | 614.3527912 | 588.2140782 | 582.75676 |
| **Galnt10** | 106.2207919 | 133.8922794 | 85.3645811 | 125.9409753 | 138.0731873 | 116.4345257 | 150.6448074 | 222.9276875 | 154.9973093 | 181.7716095 | 138.097122 | 167.4329245 | 133.7772375 | 195.1678047 | 138.6831747 |
| **2010001A14Rik** | 25.63950148 | 15.84524017 | 17.22954848 | 19.84001665 | 20.26762382 | 11.9240177 | 18.94335901 | 12.5711102 | 6.019312981 | 23.59535316 | 2.837612096 | 21.68820266 | 15.33751767 | 27.10663955 | 10.77848508 |
| **Sap30l** | 422.136078 | 423.0679125 | 390.7974859 | 398.5255519 | 489.5897878 | 485.3776616 | 441.1096455 | 437.4746348 | 493.5836644 | 348.6868856 | 371.7271846 | 514.444167 | 443.9359282 | 466.2342002 | 488.6246569 |
| **Larp1** | 17272.78273 | 16291.28368 | 16084.56667 | 15745.20974 | 14790.93184 | 15060.03435 | 16293.09288 | 14031.03513 | 14692.39057 | 14311.89254 | 15070.55784 | 14545.84376 | 15357.11561 | 14115.33077 | 14949.04024 |
| **Cnot8** | 1072.28058 | 1090.152524 | 1129.318587 | 1106.727885 | 1055.816528 | 1096.308215 | 1017.528998 | 1156.542138 | 1008.234924 | 1136.07256 | 1058.429312 | 1130.389122 | 918.5468917 | 1094.204683 | 1128.148105 |
| **Gemin5** | 2866.129987 | 2657.246777 | 2649.434659 | 2586.10304 | 2528.386071 | 2558.753915 | 2698.075562 | 3184.68125 | 2850.89711 | 2948.545243 | 2896.25608 | 2557.472857 | 2616.750932 | 2212.805342 | 2581.087894 |
| **Mrpl22** | 498.138886 | 400.0923143 | 466.7641315 | 420.9533968 | 437.0206386 | 413.1321426 | 443.8158397 | 479.3783355 | 456.7153724 | 478.024377 | 511.716048 | 410.3407943 | 423.4859046 | 424.6706862 | 426.1094435 |
| **Gm12250** | 2454.066571 | 2394.21579 | 2294.662593 | 2306.617588 | 1832.319866 | 2149.128837 | 2211.862681 | 2241.847985 | 2516.072826 | 2155.042255 | 2194.420021 | 2069.054533 | 1816.64376 | 2023.96242 | 1843.839514 |
| **Igtp** | 8511.398796 | 8408.276696 | 8602.243659 | 8364.723542 | 7019.564836 | 7306.617197 | 7957.112849 | 8563.440265 | 8763.367286 | 8094.953938 | 7466.703296 | 7819.89835 | 7463.406517 | 7734.427817 | 7229.489226 |
| **Irgm2** | 1357.062186 | 1297.72517 | 1255.407555 | 1228.355814 | 996.9137465 | 1094.203977 | 1173.586194 | 1083.629699 | 1069.932882 | 956.048754 | 953.4376644 | 1071.397211 | 962.0031918 | 982.1639063 | 967.1893945 |
| **Zfp692** | 124.5347215 | 137.0613275 | 147.2343234 | 110.4140057 | 139.973277 | 107.3161593 | 135.3097072 | 152.5294704 | 105.3379772 | 138.9504131 | 126.7466736 | 131.8642722 | 152.5230924 | 134.6296431 | 144.4317001 |
| **Zfp672** | 240.8281747 | 228.9637205 | 222.4178076 | 246.706294 | 259.6789302 | 232.1676387 | 240.8512788 | 330.2011611 | 294.1939219 | 289.2615517 | 237.4135454 | 220.352139 | 224.0981749 | 238.538428 | 240.0009344 |
| **Sh3bp5l** | 434.0401323 | 448.4202968 | 498.8737446 | 458.0456018 | 481.3560656 | 430.6674627 | 489.8211401 | 451.721893 | 494.3360785 | 512.1065538 | 471.043608 | 495.3585487 | 466.9422047 | 508.7012688 | 444.7921509 |
| **Gm12253** | 0.915696482 | 3.961310043 | 1.566322589 | 0.86260942 | 3.166816221 | 1.402825611 | 1.80412943 | 1.676148026 | 0 | 0 | 0.945870699 | 1.735056213 | 0 | 0.903554652 | 2.155697016 |
| **Fam183b** | 13.73544722 | 19.0142882 | 9.397935534 | 15.52696955 | 12.03390164 | 16.83390734 | 14.43303544 | 0 | 3.00965649 | 0 | 4.729353494 | 3.470112425 | 10.22501178 | 13.55331977 | 12.9341821 |
| **2810021J22Rik** | 56.77318186 | 69.71905675 | 72.83400039 | 88.84877022 | 84.87067473 | 54.00878604 | 85.6961479 | 46.93214473 | 61.69795805 | 57.67752995 | 80.3990094 | 72.87236093 | 86.91260015 | 72.28437213 | 68.26373884 |
| **Zfp39** | 140.1015617 | 139.4381135 | 150.3669685 | 115.5896622 | 109.5718413 | 139.5811483 | 140.7220955 | 121.5207319 | 100.0710783 | 103.1204323 | 114.4503546 | 120.5864068 | 141.4459963 | 131.9189791 | 146.5873971 |
| **Rnf187** | 3089.559929 | 3277.587929 | 3263.433114 | 3235.647933 | 3182.650302 | 3401.852108 | 3124.752172 | 3654.002697 | 3593.529849 | 3589.989288 | 3521.476612 | 3480.522762 | 3271.151686 | 3133.527532 | 3161.688957 |
| **Hist3h2ba** | 18.31392963 | 22.97559825 | 20.36219366 | 31.05393911 | 13.93399137 | 31.56357626 | 14.43303544 | 16.76148026 | 19.56276719 | 32.33437285 | 18.91741398 | 26.02584319 | 34.08337261 | 49.69550584 | 23.71266717 |
| **Hist3h2bb-ps** | 0 | 0 | 0.783161294 | 0 | 2.533452977 | 1.402825611 | 2.706194144 | 0 | 1.504828245 | 0 | 0 | 5.205168638 | 5.112505891 | 5.421327909 | 0 |
| **Hist3h2a** | 38.45925223 | 37.2363144 | 43.85703249 | 47.44351808 | 55.10260225 | 41.38335554 | 45.10323574 | 16.76148026 | 38.37312025 | 24.46925513 | 18.91741398 | 31.23101183 | 52.82922754 | 44.27417793 | 37.36541494 |
| **Trim17** | 6.409875371 | 15.84524017 | 13.31374201 | 8.626094197 | 4.43354271 | 7.715540863 | 9.922711863 | 9.218814144 | 11.28621184 | 14.85633347 | 11.35044839 | 5.205168638 | 15.33751767 | 11.74621047 | 9.341353736 |
| **Trim11** | 391.9180941 | 439.7054147 | 393.9301311 | 364.8837845 | 362.2837757 | 384.3742175 | 386.0836979 | 440.8269309 | 471.0112407 | 443.9422002 | 419.9665903 | 351.348883 | 421.781736 | 349.6756502 | 416.7680897 |
| **Obscn** | 0 | 2.376786026 | 0 | 0 | 0.633363244 | 0 | 0 | 0 | 0 | 0 | 0 | 0 | 0 | 5.421327909 | 0 |
| **Iba57** | 220.6828521 | 195.6887161 | 226.3336141 | 252.74456 | 228.6441312 | 194.2913472 | 198.4542373 | 238.8510937 | 266.3545994 | 325.0915324 | 202.4163295 | 235.1001168 | 206.2044043 | 222.2744443 | 218.4439643 |
| **Guk1** | 671.205521 | 640.1477029 | 694.6640682 | 636.6057517 | 628.9297016 | 730.1707307 | 622.4246532 | 740.8574275 | 750.1568802 | 763.7903208 | 803.990094 | 626.3552927 | 686.779958 | 661.402005 | 664.6732466 |
| **2610507I01Rik** | 62.26736075 | 51.49703055 | 46.20651637 | 41.40525214 | 45.60215359 | 46.99465798 | 42.39704159 | 46.93214473 | 53.4214027 | 57.67752995 | 48.23940564 | 69.4022485 | 51.12505891 | 46.98484188 | 63.9523448 |
| **Mrpl55** | 504.5487613 | 463.473275 | 431.5218733 | 433.0299287 | 423.7200104 | 403.3123633 | 393.3002156 | 349.4768634 | 406.3036262 | 425.5902589 | 401.995047 | 387.7850635 | 375.769183 | 418.3458037 | 398.8039479 |
| **2310033P09Rik** | 407.4849343 | 446.0435108 | 454.2335508 | 433.8925381 | 384.4514893 | 471.3494054 | 464.5633281 | 453.3980411 | 441.66709 | 512.1065538 | 387.8069865 | 470.2002336 | 451.604687 | 477.076856 | 428.2651405 |
| **Arf1** | 7277.039939 | 6848.312802 | 7378.162555 | 7218.315624 | 7112.03587 | 7174.75159 | 6944.996239 | 7614.740483 | 7451.90947 | 7498.078893 | 7809.108489 | 7348.830588 | 7226.527077 | 7324.214006 | 6972.242715 |
| **Snap47** | 282.9502128 | 268.5768209 | 263.1421949 | 271.7219672 | 277.413101 | 241.2860052 | 248.0677966 | 282.4309424 | 274.6311547 | 274.4052182 | 279.0318561 | 246.3779822 | 213.0210788 | 221.3708896 | 234.2524091 |
| **Jmjd4** | 236.2496922 | 280.460751 | 263.9253562 | 288.1115462 | 236.8778534 | 217.4379698 | 243.557473 | 306.7350888 | 295.6987502 | 308.487395 | 332.0006153 | 281.9466345 | 277.7794867 | 243.9597559 | 245.7494598 |
| **Zfp867** | 33.88076982 | 38.82083842 | 26.62748401 | 41.40525214 | 23.43444004 | 30.16075065 | 46.90736517 | 41.06562664 | 25.58208017 | 38.45168663 | 34.99721585 | 28.62842751 | 30.67503535 | 38.85285002 | 26.58692986 |
| **Zkscan17** | 1027.411452 | 954.6757202 | 923.3471662 | 935.9312203 | 949.4115032 | 900.6140425 | 1039.178551 | 760.9712039 | 828.407949 | 700.8693791 | 776.5598437 | 962.956198 | 915.9906388 | 930.6612911 | 1046.950184 |
| **4933439C10Rik** | 4.578482408 | 9.507144102 | 4.698967767 | 7.763484777 | 2.533452977 | 5.611302446 | 8.118582433 | 0.838074013 | 3.762070613 | 2.621705907 | 1.891741398 | 6.94022485 | 8.520843152 | 4.517773258 | 14.37131344 |
| **Mprip** | 862.5860856 | 908.7245238 | 992.2653601 | 949.732971 | 902.5426231 | 911.8366474 | 961.600986 | 1151.513694 | 1085.733579 | 1125.585736 | 1053.699958 | 915.2421521 | 978.1927938 | 997.5243353 | 945.6324243 |
| **1700007J10Rik** | 0 | 0 | 0 | 0 | 2.533452977 | 0 | 0.902064715 | 3.352296052 | 3.00965649 | 0 | 0.945870699 | 2.602584319 | 1.70416863 | 0 | 0 |
| **Pld6** | 0 | 0 | 2.349483883 | 3.450437679 | 6.966995687 | 0 | 0 | 0 | 2.257242368 | 0 | 0 | 0 | 0 | 2.710663955 | 2.874262688 |
| **Flcn** | 476.1621704 | 518.1393536 | 461.2820025 | 493.412588 | 488.3230613 | 466.4395158 | 442.0117102 | 591.6802532 | 581.6161168 | 499.8719262 | 566.5765486 | 514.444167 | 514.6589264 | 506.8941595 | 511.6187584 |
| **Gm16062** | 0 | 2.376786026 | 0 | 0 | 0.633363244 | 0.701412806 | 0 | 0 | 1.504828245 | 1.747803938 | 0 | 4.337640531 | 0 | 0 | 2.155697016 |
| **Cops3** | 1217.87632 | 1123.427528 | 1261.672845 | 1180.049686 | 1112.81922 | 1138.392984 | 1168.173806 | 1299.852794 | 1313.715058 | 1340.56562 | 1261.791512 | 1140.79946 | 1198.882631 | 1122.214877 | 1303.478129 |
| **Nt5m** | 219.7671556 | 194.1041921 | 185.6092268 | 206.1636513 | 192.5424263 | 204.1111265 | 236.3409553 | 207.0042812 | 261.8401147 | 272.6574143 | 186.3365277 | 206.4716893 | 245.4002828 | 188.8429222 | 196.1684284 |
| **Med9os** | 0 | 2.376786026 | 1.566322589 | 0.86260942 | 0.633363244 | 0.701412806 | 1.80412943 | 5.866518091 | 0 | 0 | 2.837612096 | 0 | 0 | 0 | 1.437131344 |
| **Med9** | 655.6386808 | 728.8810478 | 715.0262619 | 722.8666937 | 647.2972357 | 700.7113929 | 669.3320184 | 793.6560904 | 746.3948096 | 674.65232 | 716.024119 | 582.1113593 | 782.2134013 | 665.9197782 | 638.0863167 |
| **Rasd1** | 25.63950148 | 15.05297816 | 15.66322589 | 12.07653188 | 13.30062813 | 23.84803539 | 14.43303544 | 11.73303618 | 9.028969471 | 12.23462756 | 5.675224193 | 19.95314644 | 16.18960199 | 4.517773258 | 30.89832389 |
| **Pemt** | 55.85748538 | 45.95119649 | 80.66561333 | 69.87136299 | 76.00358931 | 55.41161165 | 72.16517718 | 76.26473519 | 103.8331489 | 70.78605948 | 109.7210011 | 72.87236093 | 53.68131186 | 56.92394305 | 73.29369854 |
| **Rai1** | 1551.18984 | 1483.11448 | 1468.427427 | 1494.902124 | 1466.869274 | 1434.389188 | 1547.040986 | 1278.06287 | 1378.422673 | 1263.662247 | 1476.504161 | 1520.77677 | 1532.047599 | 1384.245726 | 1526.952053 |
| **Srebf1** | 4645.328251 | 4994.419702 | 5000.484865 | 5129.075609 | 5125.808736 | 4994.760589 | 5083.134668 | 4380.612866 | 4797.392446 | 4599.346062 | 4488.156466 | 5052.483691 | 5099.724626 | 5115.022883 | 5118.343281 |
| **Tom1l2** | 187.7177787 | 194.8964541 | 208.3209043 | 160.4453521 | 164.0410803 | 152.2065788 | 200.2583667 | 186.8905049 | 170.0455917 | 194.8801391 | 185.390657 | 183.0484304 | 167.8606101 | 151.7971815 | 183.2342464 |
| **Lrrc48** | 1.831392963 | 1.584524017 | 3.132645178 | 8.626094197 | 8.233722176 | 4.208476834 | 12.62890601 | 0.838074013 | 3.00965649 | 0 | 2.837612096 | 6.072696744 | 5.964590206 | 2.710663955 | 2.155697016 |
| **Atpaf2** | 640.9875371 | 780.3780784 | 688.3987779 | 766.8597741 | 637.1634237 | 647.4040197 | 653.0948535 | 879.9777137 | 856.2472715 | 813.602733 | 777.5057144 | 698.3601256 | 683.3716208 | 733.6863771 | 677.6074287 |
| **Gid4** | 835.1151912 | 757.4024801 | 751.8348427 | 671.9727379 | 687.8324833 | 775.0611503 | 803.7396609 | 624.3651397 | 647.0761454 | 683.3913397 | 599.682023 | 650.6460797 | 742.1654385 | 698.4477457 | 700.6015302 |
| **Drg2** | 1420.245243 | 1345.26089 | 1580.419492 | 1497.489953 | 1311.695279 | 1290.599563 | 1427.066379 | 1631.730103 | 1710.989715 | 1714.595663 | 1713.917706 | 1316.907665 | 1203.143053 | 1313.768463 | 1309.226654 |
| **Myo15** | 2.747089445 | 0 | 2.349483883 | 5.175656518 | 0.633363244 | 0 | 0 | 0 | 0 | 0 | 0 | 2.602584319 | 11.92918041 | 0 | 2.874262688 |
| **Alkbh5** | 4732.319417 | 4647.408942 | 4930.78351 | 4703.809165 | 4448.110065 | 4645.457012 | 4953.237349 | 3484.711746 | 3997.576233 | 3935.180566 | 4056.839427 | 4711.545145 | 5054.564158 | 4479.823963 | 4677.862524 |
| **Llgl1** | 1045.725382 | 1035.486445 | 988.3495536 | 980.7869101 | 1023.515003 | 1031.778237 | 1068.044622 | 1082.791625 | 922.4597143 | 942.9402244 | 946.8165695 | 1040.166199 | 1062.549141 | 977.646133 | 1016.770426 |
| **Flii** | 3131.681967 | 3194.400418 | 3246.203566 | 3277.053185 | 3129.44779 | 3150.04491 | 3213.154514 | 3081.598146 | 2886.260574 | 2973.014498 | 3084.484349 | 3281.858826 | 3368.289298 | 3260.025183 | 3386.600012 |
| **Mief2** | 111.7149708 | 121.2160873 | 136.2700652 | 119.0400999 | 122.2391061 | 129.7613691 | 113.6601541 | 127.38725 | 145.2159257 | 123.2201776 | 117.2879666 | 104.1033728 | 120.1438884 | 108.4265582 | 158.8030135 |
| **Top3a** | 523.7783875 | 518.9316156 | 465.1978089 | 477.8856185 | 461.7218051 | 474.1550567 | 513.2748227 | 495.3017417 | 533.4616129 | 506.863142 | 478.6105736 | 544.8076507 | 534.2568656 | 464.4270909 | 479.2833032 |
| **Smcr8** | 894.6354625 | 972.1054844 | 960.155747 | 917.8164225 | 867.7076447 | 805.221901 | 970.6216331 | 1132.237992 | 1069.180468 | 963.9138717 | 1101.939364 | 829.3568696 | 978.1927938 | 943.3110562 | 903.9556153 |
| **Shmt1** | 2110.68039 | 2074.141938 | 2040.918333 | 2073.713045 | 2094.532249 | 1963.955856 | 2059.413744 | 2647.475807 | 2238.432015 | 2328.948747 | 2527.366507 | 1964.083633 | 1948.716829 | 1776.388445 | 1933.660223 |
| **Dhrs7b** | 477.0778669 | 540.3226898 | 479.2947122 | 398.5255519 | 480.0893392 | 467.1409286 | 494.3314637 | 489.4352236 | 572.5871473 | 526.9628872 | 485.2316685 | 405.1356256 | 487.3922283 | 482.4981839 | 457.726333 |
| **Tmem11** | 844.272156 | 804.9382006 | 865.3932304 | 784.1119625 | 719.5006455 | 849.4109077 | 799.2293373 | 817.1221627 | 832.9224337 | 748.9339874 | 794.531387 | 748.6767557 | 783.0654856 | 730.0721585 | 668.2660749 |
| **Natd1** | 139.1858652 | 150.5297816 | 172.2954848 | 117.3148811 | 155.1739948 | 152.9079916 | 167.7840369 | 142.4725822 | 128.662815 | 140.698217 | 114.4503546 | 176.1082056 | 172.973116 | 170.7718291 | 155.9287508 |
| **Map2k3os** | 37.54355574 | 32.48274235 | 23.49483883 | 26.74089201 | 33.56825195 | 33.66781467 | 32.47432973 | 39.38947861 | 19.56276719 | 30.58656891 | 39.72656935 | 23.42325887 | 15.33751767 | 47.88839653 | 25.86836419 |
| **Map2k3** | 1875.346394 | 1880.830008 | 1830.247945 | 1895.152895 | 1900.723096 | 1960.448792 | 1896.14003 | 2282.075538 | 1946.495335 | 2001.235509 | 2009.029364 | 1964.083633 | 1828.57294 | 1911.921643 | 1969.588507 |
| **Tnfrsf13b** | 97.06382705 | 95.07144102 | 96.32883922 | 114.7270528 | 95.00448664 | 86.97518791 | 93.81473034 | 115.6542138 | 131.6724715 | 134.5809032 | 141.8806048 | 117.9838225 | 82.65217857 | 116.5585501 | 93.41353736 |
| **Usp22** | 1248.094304 | 1132.14241 | 1311.012007 | 1192.126218 | 1120.419579 | 1193.804595 | 1358.50946 | 1035.021406 | 1036.074247 | 987.5092249 | 1140.720063 | 1195.45373 | 1266.197292 | 1189.981476 | 1297.011038 |
| **Aldh3a1** | 2.747089445 | 2.376786026 | 0 | 0 | 1.266726489 | 0.701412806 | 0 | 10.05688816 | 3.00965649 | 7.86511772 | 6.621094891 | 0.867528106 | 0.852084315 | 2.710663955 | 0.718565672 |
| **Aldh3a2** | 395.58088 | 439.7054147 | 468.3304541 | 426.1290533 | 413.5861985 | 415.9377938 | 439.3055161 | 420.7131546 | 411.5705251 | 476.2765731 | 420.912461 | 389.5201197 | 356.1712437 | 373.1680711 | 415.3309584 |
| **Mapk7** | 497.2231895 | 507.0476854 | 514.5369705 | 439.0681946 | 541.5255739 | 510.6285226 | 621.5225885 | 347.8007154 | 372.4449907 | 374.9039447 | 439.8298749 | 506.6364141 | 564.931901 | 479.78752 | 533.1757286 |
| **B9d1** | 43.03773463 | 38.02857641 | 34.45909696 | 29.32872027 | 42.43533737 | 35.07064029 | 35.18052388 | 29.33259046 | 54.17381683 | 48.93851026 | 42.56418144 | 44.24393342 | 24.71044514 | 46.98484188 | 22.9941015 |
| **Epn2** | 68.67723612 | 80.01846286 | 58.73709709 | 76.77223835 | 57.00269198 | 76.45399582 | 71.26311247 | 44.41792269 | 81.26072524 | 110.9855501 | 80.3990094 | 61.59449555 | 74.98341973 | 61.44171631 | 56.04812241 |
| **Grap** | 2330.447546 | 2504.340209 | 2528.82782 | 2251.410585 | 2804.532446 | 2887.716521 | 2984.932141 | 2358.340273 | 2288.091347 | 2192.62004 | 2131.992555 | 2752.666681 | 2660.207232 | 2673.618214 | 2668.752906 |
| **Slc5a10** | 0 | 0.792262009 | 0.783161294 | 0.86260942 | 1.266726489 | 0.701412806 | 3.608258859 | 3.352296052 | 0 | 3.495607876 | 0.945870699 | 0.867528106 | 0 | 0.903554652 | 7.904222392 |
| **Fam83g** | 55.85748538 | 52.28929256 | 50.90548414 | 41.40525214 | 49.40233305 | 56.81443726 | 52.31975346 | 72.91243914 | 40.63036262 | 43.69509845 | 45.40179354 | 42.50887721 | 58.79381775 | 46.98484188 | 54.61099107 |

| | | | | | | | | | | | | | | | |
|---|---|---|---|---|---|---|---|---|---|---|---|---|---|---|---|
| **Prpsap2** | 522.862691 | 592.6119824 | 559.1771643 | 503.7639011 | 471.2222537 | 504.3158073 | 495.2335284 | 605.0894374 | 613.2175099 | 598.6228487 | 601.5737644 | 513.5766389 | 493.3568185 | 529.4830258 | 466.3491211 |
| **Ulk2** | 396.4965765 | 433.3673187 | 362.6036793 | 391.6246765 | 381.2846731 | 366.8388974 | 441.1096455 | 331.0392352 | 373.1974048 | 322.4698265 | 362.2684776 | 429.4264126 | 403.8879654 | 333.4116664 | 455.570636 |
| **Akap10** | 521.031298 | 494.3714933 | 522.3685834 | 496.0004163 | 425.6201002 | 405.4166017 | 471.7798458 | 517.9297401 | 480.7926243 | 487.6372986 | 515.4995308 | 415.5459629 | 470.350542 | 445.4524432 | 522.3972435 |
| **Specc1** | 104.3893989 | 117.2547773 | 114.341549 | 108.6887869 | 120.3390164 | 91.18366474 | 118.1704776 | 176.8336168 | 179.0745612 | 156.4284524 | 164.5815016 | 128.3941597 | 87.76468446 | 123.7869873 | 131.497518 |
| **Adora2b** | 76.91850445 | 50.70476854 | 63.43606485 | 35.36698621 | 27.86798275 | 41.38335554 | 39.69084745 | 62.85555098 | 37.62070613 | 47.19070632 | 69.04856101 | 37.30370857 | 38.34379418 | 27.10663955 | 38.80254629 |
| **Zswim7** | 102.5580059 | 97.44822705 | 81.44877463 | 92.2992079 | 66.50314065 | 88.37801352 | 116.3663482 | 113.1399918 | 103.0807348 | 140.698217 | 113.5044839 | 81.54764199 | 93.72927467 | 92.16257446 | 86.94644631 |
| **Ttc19** | 220.6828521 | 197.2732401 | 208.3209043 | 169.0714463 | 209.0098706 | 221.6464466 | 230.0265023 | 187.7285789 | 226.4766509 | 229.8362178 | 232.6841919 | 178.7107899 | 210.4648258 | 209.6246792 | 201.1983882 |
| **Ncor1** | 4624.267232 | 4961.144697 | 4837.587316 | 4746.077027 | 4337.90486 | 4348.057983 | 5131.846162 | 4997.43534 | 5206.705728 | 4702.466495 | 5286.471335 | 5166.997401 | 5092.907952 | 4885.520001 | 5031.396835 |
| **Pigl** | 153.8370089 | 174.2976419 | 178.5607751 | 154.4070861 | 169.7413495 | 175.3532014 | 159.6654545 | 188.5666529 | 168.5407635 | 145.9416288 | 158.9062774 | 174.3731494 | 195.1273082 | 189.7464768 | 156.6473165 |
| **Cenpv** | 232.5869063 | 236.8863405 | 215.369356 | 249.2941223 | 226.7440415 | 236.3761155 | 240.8512788 | 176.8336168 | 165.531107 | 184.3933154 | 225.1172263 | 244.642926 | 234.3231867 | 232.2135455 | 245.7494598 |
| **Ubb** | 18381.69117 | 16606.60396 | 17354.07112 | 16881.26634 | 17057.10553 | 18330.02085 | 17878.02058 | 14930.28854 | 15031.72934 | 14970.81463 | 14512.49413 | 16823.97257 | 16692.33173 | 16829.60894 | 16912.88022 |
| **Trpv2** | 1944.939327 | 2083.649082 | 1997.844462 | 2043.521715 | 1885.522378 | 2052.33387 | 2067.532326 | 1502.666705 | 1598.127596 | 1673.52227 | 2143.343003 | 1984.036779 | 1943.604323 | 2094.439682 | 2062.283479 |
| **2410006H16Rik** | 1017.338791 | 945.1685761 | 956.2399406 | 774.6232588 | 915.8432512 | 973.5609743 | 985.0546685 | 706.496393 | 831.4176055 | 769.9076346 | 694.2690929 | 872.7332749 | 853.7884838 | 907.1688702 | 944.195293 |
| **Snord49a** | 8.241268334 | 5.54583406 | 14.0969033 | 2.587828259 | 3.166816221 | 5.611302446 | 1.80412943 | 5.028444078 | 4.514484735 | 0 | 1.891741398 | 6.94022485 | 0.852084315 | 1.807109303 | 9.341353736 |
| **Lrrc75a** | 24.723805 | 26.93690829 | 27.41064531 | 34.50437679 | 17.73417084 | 28.05651223 | 36.08258859 | 25.9802944 | 12.03862596 | 27.09096104 | 23.64676747 | 34.70112425 | 42.60421576 | 21.68531164 | 16.52701046 |
| **Mmgt2** | 223.4299415 | 258.2774148 | 252.1779368 | 213.9271361 | 245.7449388 | 216.736557 | 238.1450847 | 232.9845756 | 231.7435498 | 276.1530222 | 317.8125548 | 217.7495547 | 197.6835611 | 259.320185 | 226.3481867 |
| **Zfp287** | 145.5957406 | 173.5053799 | 171.5123235 | 191.4992912 | 131.1061916 | 126.254305 | 142.5262249 | 118.1684358 | 115.8717749 | 109.2377461 | 172.1484672 | 148.3473062 | 101.3980335 | 133.7260884 | 173.1743269 |
| **Zfp286** | 30.21798389 | 30.89821833 | 31.32645178 | 13.80175071 | 25.33452977 | 23.14662259 | 18.0412943 | 12.5711102 | 20.31518131 | 29.71266694 | 31.21373306 | 18.21809023 | 22.15419219 | 31.62441281 | 20.83840449 |
| **Trim16** | 97.97952353 | 78.43393884 | 86.93090369 | 81.08528545 | 52.56914927 | 62.42573971 | 79.3816949 | 44.41792269 | 37.62070613 | 80.39898114 | 58.64398332 | 50.31663016 | 59.64590206 | 67.76659887 | 66.10804182 |
| **Fbxw10** | 1.831392963 | 0 | 10.96425812 | 4.313047098 | 3.166816221 | 0 | 1.80412943 | 1.676148026 | 5.266898858 | 6.991215751 | 0 | 2.602584319 | 2.556252945 | 0.903554652 | 0.718565672 |
| **Tvp23b** | 295.7699635 | 224.2101484 | 274.8896144 | 251.8819505 | 266.0125626 | 324.754129 | 279.6400616 | 311.7635329 | 246.0394181 | 276.1530222 | 279.0318561 | 244.642926 | 280.3357397 | 286.4268245 | 266.5878643 |
| **Hs3st3b1** | 347.0489665 | 263.0309868 | 303.8665823 | 320.8907041 | 221.0437723 | 270.0439302 | 307.6040677 | 155.8817664 | 172.3028341 | 188.7628253 | 204.3080709 | 254.1857351 | 304.1941005 | 271.0663955 | 237.8452374 |
| **Cox10** | 792.993153 | 709.8667596 | 762.0159395 | 827.2424334 | 748.0019915 | 742.7961612 | 797.4252079 | 896.739194 | 850.9803726 | 1032.078225 | 969.5174662 | 686.2147321 | 703.8216443 | 722.8437213 | 779.6437541 |
| **9630013K17Rik** | 14.65114371 | 1.584524017 | 7.831612945 | 9.488703616 | 6.333632443 | 4.90988964 | 5.412388289 | 1.676148026 | 4.514484735 | 7.86511772 | 0 | 1.735056213 | 4.260421576 | 8.131991864 | 8.622788064 |
| **2810001G20Rik** | 21.06101908 | 29.31369431 | 19.57903236 | 55.20700286 | 50.0356963 | 54.00878604 | 54.12388289 | 27.65644243 | 21.06759543 | 47.19070632 | 30.26786236 | 53.78674259 | 36.63962555 | 46.98484188 | 30.89832389 |
| **Elac2** | 1623.529862 | 1657.412122 | 1594.516396 | 1579.437847 | 1603.042371 | 1411.242565 | 1551.551309 | 1877.285789 | 1659.825554 | 1854.419978 | 1882.282691 | 1560.683063 | 1491.999636 | 1532.428689 | 1583.000175 |
| **Arhgap44** | 0 | 4.753572051 | 0.783161294 | 0.86260942 | 1.266726489 | 0 | 0 | 2.514222039 | 0 | 0 | 0 | 0.867528106 | 0 | 0 | 1.437131344 |
| **Map2k4** | 1414.751064 | 1238.305519 | 1386.978653 | 1335.319382 | 1193.889715 | 1228.875236 | 1307.993836 | 1479.200633 | 1417.548207 | 1442.812151 | 1529.47292 | 1382.839801 | 1355.666145 | 1290.276042 | 1386.113181 |
| **Zkscan6** | 214.2729767 | 247.1857467 | 218.5020012 | 230.316715 | 209.0098706 | 190.0828703 | 263.4028967 | 179.3478388 | 153.492481 | 175.6542957 | 190.1200105 | 216.0144985 | 208.7606572 | 215.9495617 | 240.0009344 |
| **Dnah9** | 0 | 1.584524017 | 0 | 2.587828259 | 0.633363244 | 1.402825611 | 2.706194144 | 0 | 0 | 0.873901969 | 0.945870699 | 0 | 1.70416863 | 0.903554652 | 0 |
| **Tmem220** | 3.662785926 | 5.54583406 | 3.915806472 | 1.725218839 | 0.633363244 | 0.701412806 | 4.510323574 | 0 | 0 | 0 | 0.945870699 | 4.337640531 | 0 | 1.807109303 | 0.718565672 |
| **Adprm** | 456.0168478 | 365.2327859 | 360.2541955 | 357.9829092 | 290.0803659 | 356.3177053 | 339.1763328 | 261.4790921 | 298.7084067 | 378.3995525 | 380.2400209 | 345.2761863 | 307.6024378 | 312.6299094 | 311.138936 |
| **Sco1** | 1248.094304 | 825.5370129 | 941.359876 | 793.6006661 | 659.3311373 | 786.2837552 | 843.4305083 | 553.9669226 | 647.0761454 | 706.1127909 | 803.990094 | 673.2018105 | 656.957007 | 554.7825561 | 695.5715705 |
| **Myh3** | 4.578482408 | 0 | 0 | 2.587828259 | 0.633363244 | 1.402825611 | 0.902064715 | 2.514222039 | 0 | 0 | 2.837612096 | 0 | 0 | 1.807109303 | 0 |
| **Stx8** | 379.0983434 | 347.8030217 | 311.6981952 | 292.4245933 | 355.9501433 | 325.4555419 | 323.8412326 | 324.3346431 | 319.7760021 | 353.9302974 | 283.7612096 | 332.2632647 | 322.0878711 | 332.5081118 | 316.1688957 |
| **Ntn1** | 2.747089445 | 1.584524017 | 2.349483883 | 1.725218839 | 6.966995687 | 4.208476834 | 18.0412943 | 0.838074013 | 0 | 2.621705907 | 2.837612096 | 10.41033728 | 7.668758836 | 8.131991864 | 5.029959704 |
| **Pik3r5** | 3246.144027 | 3135.77303 | 2992.459306 | 3059.675612 | 2264.906962 | 2327.287689 | 2367.919876 | 2757.263503 | 2810.266748 | 2851.542125 | 2742.079156 | 2285.93656 | 2495.754959 | 2407.973146 | 2520.009812 |
| **Pik3r6** | 14.65114371 | 13.46845414 | 12.53058071 | 12.93914129 | 6.333632443 | 7.715540863 | 4.510323574 | 20.95185033 | 5.266898858 | 21.84754922 | 41.61831075 | 5.205168638 | 0 | 7.228437213 | 5.748525376 |
| **Mfsd6l** | 0 | 7.130358077 | 0 | 0.86260942 | 1.900089733 | 0.701412806 | 3.608258859 | 2.514222039 | 5.266898858 | 0.873901969 | 0 | 0 | 0 | 1.807109303 | 0 |
| **Myh10** | 30.21798389 | 64.9654847 | 38.37490343 | 30.19132969 | 24.70116653 | 25.95227381 | 27.96400616 | 42.74177467 | 33.85863552 | 56.80362798 | 36.88895725 | 30.36348372 | 35.78754124 | 27.10663955 | 26.58692986 |
| **Ndel1** | 844.272156 | 760.5715282 | 778.4623267 | 778.9363059 | 712.5336498 | 758.227243 | 760.4405546 | 824.6648288 | 705.0120329 | 780.3944582 | 703.7277999 | 728.7236093 | 753.2425346 | 688.5086445 | 765.9910063 |
| **Arhgef15** | 0.915696482 | 1.584524017 | 0 | 0 | 0 | 0 | 0.902064715 | 0 | 1.504828245 | 2.621705907 | 2.837612096 | 0 | 1.70416863 | 2.710663955 | 0 |
| **Slc25a35** | 42.12203815 | 33.27500436 | 31.32645178 | 22.42784491 | 24.70116653 | 24.5494482 | 22.55161787 | 44.41792269 | 18.05793894 | 18.35194135 | 20.80915537 | 25.15831508 | 28.97086672 | 25.29953024 | 16.52701046 |
| **Rangrf** | 272.8775515 | 262.2387248 | 246.6958078 | 261.3706542 | 241.3113961 | 254.6128485 | 156.0571957 | 376.2952319 | 339.3387693 | 429.9597687 | 333.8923567 | 199.5314644 | 214.7252474 | 240.3455373 | 226.3481867 |
| **Pfas** | 2302.060955 | 2277.753274 | 2286.83098 | 2304.02976 | 2185.103193 | 2057.243759 | 2353.486841 | 2857.832385 | 2596.581137 | 2509.846455 | 2537.771085 | 2313.697459 | 2295.515145 | 2063.718824 | 2237.613503 |
| **Ctc1** | 941.335983 | 1066.384663 | 989.1327149 | 874.6859515 | 941.177781 | 875.3631815 | 950.7762094 | 1083.629699 | 1104.543932 | 972.6528914 | 972.3550783 | 950.8108045 | 973.9323722 | 897.229769 | 1017.488992 |
| **Aurkb** | 2577.685596 | 2809.361082 | 2798.235305 | 2935.459855 | 2589.188943 | 2713.766145 | 2704.390015 | 2693.569878 | 2736.530164 | 2841.929203 | 2770.455277 | 2790.837918 | 2665.319738 | 2974.501913 | 2900.131052 |
| **2310047M10Rik** | 331.4821263 | 310.5667073 | 268.624324 | 297.6002498 | 291.3470924 | 291.7877272 | 294.9751617 | 191.918949 | 293.4415078 | 224.592806 | 244.0346403 | 281.9466345 | 302.4899319 | 281.9090513 | 285.9891374 |
| **Tmem107** | 179.4765104 | 156.0756157 | 179.3439364 | 175.1097122 | 162.1409905 | 167.6376606 | 159.6654545 | 171.8051727 | 148.2255821 | 129.3374914 | 213.7667779 | 146.61225 | 157.6355983 | 149.9900722 | 122.1561642 |
| **Vamp2** | 360.7844137 | 362.0637379 | 395.4964537 | 408.8768649 | 435.7539121 | 396.2982352 | 412.2435747 | 377.1333059 | 378.4643037 | 346.0651797 | 411.453754 | 424.221244 | 419.2254831 | 410.2138118 | 449.103545 |
| **Per1** | 1043.893989 | 911.1013098 | 929.6124566 | 911.7781566 | 787.2705126 | 718.246713 | 869.590385 | 667.1069144 | 605.6933687 | 664.1654964 | 690.4856101 | 805.0660826 | 779.6571484 | 757.178798 | 809.1049466 |
| **Aloxe3** | 0 | 0.792262009 | 0 | 2.587828259 | 0 | 2.805651223 | 0.902064715 | 0 | 0 | 1.747803938 | 0 | 0.867528106 | 0 | 0 | 0 |
| **Alox12b** | 0 | 0.792262009 | 0 | 0 | 0 | 1.402825611 | 0 | 2.514222039 | 9.781383594 | 0 | 0 | 0 | 0 | 0 | 0 |
| **Alox8** | 1.831392963 | 0 | 0.783161294 | 10.35131304 | 0 | 2.104238417 | 0.902064715 | 4.190370065 | 1.504828245 | 5.243411813 | 0.945870699 | 2.602584319 | 2.556252945 | 3.614218606 | 2.155697016 |
| **Gucy2e** | 8.241268334 | 25.35238427 | 26.62748401 | 30.19132969 | 24.70116653 | 16.13249453 | 13.53097072 | 28.49451644 | 27.83932254 | 28.83876497 | 42.56418144 | 22.55573076 | 46.01255302 | 24.39597559 | 34.49115225 |
| **Cntrob** | 212.4415837 | 219.4565764 | 253.7442594 | 243.2558563 | 275.5130113 | 281.2665351 | 233.6347611 | 193.595097 | 229.4863074 | 228.9623159 | 235.521804 | 298.4296686 | 317.8274496 | 344.2543222 | 331.2587748 |
| **Trappc1** | 240.8281747 | 282.837537 | 291.3360015 | 272.5845766 | 325.5487076 | 317.0385882 | 305.7999383 | 283.2690164 | 294.1939219 | 264.7922966 | 327.2712618 | 341.8060739 | 320.3837025 | 297.2694804 | 300.3604509 |
| **Kcnab3** | 4.578482408 | 2.376786026 | 6.265290356 | 3.450437679 | 1.900089733 | 4.90988964 | 8.118582433 | 2.514222039 | 0 | 2.621705907 | 0.945870699 | 6.072696744 | 8.520843152 | 2.710663955 | 9.341353736 |
| **Chd3os** | 37.54355574 | 57.04286461 | 43.0738712 | 78.49745719 | 54.46923901 | 51.90454762 | 54.12388289 | 45.2559967 | 50.41174621 | 59.42533389 | 40.67244005 | 58.12438312 | 63.90632364 | 57.8274977 | 67.54517316 |
| **Chd3** | 2858.804415 | 3643.612977 | 3548.503825 | 3429.735053 | 4031.35705 | 3762.37829 | 4220.7608 | 3121.825699 | 3172.930355 | 2987.870832 | 2970.033994 | 4257.827946 | 4222.929866 | 4214.178895 | 4044.806167 |
| **Cyb5d1** | 105.3050954 | 101.4095371 | 151.9332911 | 152.6818673 | 119.0722899 | 96.79496719 | 108.2477658 | 199.4616151 | 139.1966127 | 148.5633347 | 173.0943379 | 105.838429 | 115.0313825 | 111.1372221 | 123.5932956 |
| **Naa38** | 161.1625808 | 186.181572 | 169.9460009 | 124.2157564 | 164.0410803 | 174.6517886 | 155.1551309 | 173.4813207 | 148.2255821 | 158.1762564 | 210.9291658 | 155.287531 | 164.4522728 | 152.7007361 | 153.7730538 |
| **Tmem88** | 6.409875371 | 7.922620085 | 4.698967767 | 9.488703616 | 5.066905954 | 2.104238417 | 6.314453003 | 0 | 0.752414123 | 0.873901969 | 1.891741398 | 1.735056213 | 5.112505891 | 1.807109303 | 6.467091048 |
| **Kdm6b** | 2419.270104 | 2080.480034 | 2009.591882 | 1846.846767 | 1787.984439 | 1953.434664 | 2183.898674 | 1468.305671 | 1278.351594 | 1056.54748 | 1251.386934 | 1686.474639 | 1747.62493 | 1605.616616 | 1838.090989 |
| **Dnah2** | 0 | 0 | 0 | 0 | 0.633363244 | 0.701412806 | 0.902064715 | 0.838074013 | 0 | 0 | 0 | 1.735056213 | 0 | 0 | 3.59282836 |
| **Efnb3** | 1.831392963 | 14.26071615 | 10.18109683 | 7.763484777 | 7.600358931 | 5.611302446 | 2.706194144 | 11.73303618 | 12.03862596 | 12.23462756 | 12.29631908 | 3.470112425 | 4.260421576 | 9.939101167 | 10.77848508 |
| **Wrap53** | 360.7844137 | 503.8786374 | 418.9912926 | 395.0751142 | 423.0866472 | 399.1038865 | 401.4187981 | 428.2558207 | 401.0367273 | 415.9773372 | 461.584901 | 372.1695576 | 377.4733516 | 366.8431885 | 417.4866554 |
| **Trp53** | 4632.5085 | 4804.27682 | 5021.63022 | 4791.795326 | 4848.395635 | 4552.169109 | 4986.613743 | 4789.592985 | 4267.692903 | 4615.9502 | 4650.846226 | 4571.87312 | 4855.176428 | 4658.727784 | 4810.797174 |
| **Atp1b2** | 18.31392963 | 16.63750218 | 13.31374201 | 12.07653188 | 11.4005384 | 18.93814575 | 10.82477658 | 11.73303618 | 16.5531107 | 11.3607256 | 16.07980188 | 6.94022485 | 9.372927467 | 4.517773258 | 16.52701046 |
| **Fxr2** | 2201.334342 | 1951.341327 | 1894.467171 | 1822.693704 | 1828.519686 | 1840.507202 | 1838.407889 | 2021.434519 | 1928.437396 | 1857.041684 | 1812.288259 | 1659.581267 | 1836.241699 | 1710.428955 | 1841.683817 |
| **Sox15** | 2.747089445 | 0.792262009 | 2.349483883 | 0 | 1.266726489 | 0 | 0 | 0 | 6.019312981 | 0 | 0 | 0.867528106 | 0 | 1.807109303 | 0.718565672 |
| **Mpdu1** | 1173.007193 | 1267.619214 | 1242.876974 | 1224.905376 | 1176.155545 | 1255.528922 | 1212.374977 | 1406.288194 | 1338.544724 | 1343.187326 | 1458.532618 | 1204.99654 | 1276.422304 | 1353.524868 | 1245.992875 |
| **Cd68** | 34.7964663 | 28.52143231 | 54.82129061 | 37.95481446 | 31.03479897 | 37.1748787 | 50.51562403 | 31.8468125 | 38.37312025 | 34.95607876 | 38.78069865 | 36.43618046 | 42.60421576 | 46.98484188 | 45.26963733 |
| **Eif4a1** | 26716.36055 | 25074.30031 | 26254.69924 | 25655.72936 | 24076.67037 | 24437.92356 | 24141.95796 | 29750.78939 | 28079.34264 | 28472.60005 | 29465.76401 | 24032.2636 | 23863.47333 | 23955.04092 | 22902.84366 |
| **Senp3** | 2487.94734 | 2416.399126 | 2596.179691 | 2418.756813 | 2304.175483 | 2345.524422 | 2316.502188 | 2862.022755 | 2798.228122 | 2886.498203 | 2672.084724 | 2393.510045 | 2349.196457 | 2298.643034 | 2516.416983 |
| **Polr2a** | 7118.624448 | 7180.270583 | 7086.043393 | 7910.990988 | 6393.801951 | 6020.226111 | 7368.06459 | 8530.755379 | 7817.582734 | 7342.524343 | 8252.721847 | 6986.20384 | 7011.801829 | 6923.939295 | 7044.817848 |
| **Slc35g3** | 0 | 0 | 0.783161294 | 0 | 0 | 0 | 0 | 0 | 0 | 0.873901969 | 0 | 0 | 0 | 0.903554652 | 2.155697016 |
| **Zbtb4** | 223.4299415 | 233.7172925 | 209.1040656 | 181.1479781 | 204.5763279 | 213.2294929 | 219.2017257 | 220.4134654 | 166.2835211 | 133.7070012 | 192.0117519 | 192.5912396 | 215.5773317 | 236.7313187 | 238.5638031 |
| **Chrnb1** | 24.723805 | 14.26071615 | 28.1938066 | 24.15306375 | 13.93399137 | 25.95227381 | 22.55161787 | 18.43762829 | 40.63036262 | 20.97364725 | 25.53850887 | 25.15831508 | 12.78126473 | 14.45687443 | 9.341353736 |
| **Fgf11** | 5.494178889 | 3.169048034 | 0.783161294 | 3.450437679 | 1.266726489 | 0 | 1.80412943 | 0.838074013 | 0 | 0.873901969 | 0.945870699 | 0.867528106 | 5.964590206 | 0 | 0 |
| **Spem1** | 0 | 0 | 1.566322589 | 6.900875357 | 5.700269198 | 0 | 0.902064715 | 5.866518091 | 0 | 0.873901969 | 0 | 0 | 0 | 0 | 0 |
| **Nlgn2** | 654.7229843 | 560.12924 | 620.2637452 | 504.6265105 | 450.3212667 | 448.9041957 | 402.3208628 | 542.2338864 | 537.9760976 | 506.863142 | 554.2802295 | 438.9692218 | 405.592134 | 362.3254153 | 360.7199673 |
| **Tmem256** | 607.1067673 | 612.4185326 | 586.5878096 | 529.6421837 | 670.0983124 | 596.2008849 | 690.9815715 | 460.9407072 | 486.8119373 | 519.9716715 | 483.3399271 | 553.4829318 | 548.742299 | 566.5287665 | 526.7086376 |
| **Plscr3** | 353.4588419 | 301.8518252 | 325.0119372 | 309.6767817 | 240.0446696 | 300.2046808 | 330.1556856 | 382.16175 | 294.9463361 | 306.7395911 | 255.3850887 | 271.5362973 | 276.9274024 | 313.5334641 | 316.8874613 |
| **Tmem95** | 0 | 0.792262009 | 0 | 0.86260942 | 1.266726489 | 0 | 1.80412943 | 0 | 0 | 0 | 0 | 0 | 0 | 2.710663955 | 0 |
| **Kctd11** | 74.17141501 | 61.79643666 | 68.13503262 | 66.42092531 | 59.53614496 | 43.48759395 | 24.3557473 | 114.8161398 | 62.45037217 | 69.91215751 | 87.02010429 | 45.97898963 | 37.49170987 | 23.49242094 | 72.57513287 |
| **Acap1** | 2476.958983 | 2753.11048 | 2606.360788 | 2515.369068 | 2554.353964 | 2599.435858 | 2585.317473 | 2178.992434 | 2116.540927 | 2272.145119 | 2499.936257 | 2555.737801 | 2611.638426 | 2636.572473 | 2825.400222 |
| **Mir6924** | 0.915696482 | 0 | 0 | 0.86260942 | 1.900089733 | 0 | 0 | 0 | 0 | 0.873901969 | 0 | 0 | 0 | 1.807109303 | 0 |
| **2810408A11Rik** | 8.241268334 | 3.169048034 | 7.04845165 | 12.93914129 | 12.66726489 | 11.9240177 | 6.314453003 | 10.89496217 | 14.29586833 | 9.612921658 | 10.40457769 | 18.21809023 | 5.964590206 | 12.64976512 | 10.05991941 |
| **Neurl4** | 735.3042747 | 800.9768906 | 755.7506492 | 715.9658183 | 664.3980432 | 634.7785892 | 663.9196301 | 782.7611282 | 723.8223859 | 741.9427716 | 752.9130762 | 670.5992262 | 748.1300287 | 721.9401666 | 714.2542779 |
| **Gps2** | 768.269348 | 724.1274758 | 680.5671649 | 718.5536466 | 748.0019915 | 730.8721436 | 758.6364251 | 742.5335756 | 600.4264698 | 698.2476732 | 763.3176539 | 766.894846 | 790.7342445 | 833.0773888 | 752.3382585 |
| **Eif5a** | 40145.96514 | 39225.6843 | 39628.74466 | 38354.20263 | 37978.36022 | 39079.91588 | 37037.87512 | 44898.1391 | 43515.11837 | 43643.53823 | 45401.79354 | 37433.83779 | 37173.03033 | 37449.62964 | 36069.84104 |
| **Ybx2** | 1.831392963 | 2.376786026 | 0.783161294 | 2.587828259 | 0 | 2.104238417 | 3.608258859 | 0 | 0 | 1.747803938 | 0.945870699 | 1.735056213 | 6.816674521 | 4.517773258 | 0 |
| **Cldn7** | 0.915696482 | 0.792262009 | 0.783161294 | 2.587828259 | 0.633363244 | 2.805651223 | 0 | 2.514222039 | 0 | 4.369509845 | 0 | 5.205168638 | 0 | 0 | 4.311394032 |
| **Elp5** | 983.4580212 | 1002.211441 | 1040.82136 | 932.4807826 | 977.2794859 | 941.2959853 | 932.7349151 | 1259.625242 | 1081.219094 | 1139.568167 | 1021.540355 | 947.3406921 | 921.955229 | 991.1994528 | 911.8598377 |
| **Ctdnep1** | 1904.648682 | 1999.669309 | 1910.130397 | 1954.672945 | 1791.784618 | 1814.554928 | 1918.691648 | 2056.633628 | 2019.479505 | 1947.053587 | 2066.727477 | 1805.325989 | 1785.11664 | 1945.353165 | 1817.97115 |
| **Gabarap** | 1441.306262 | 1370.613275 | 1378.363878 | 1478.512545 | 1428.867479 | 1418.958106 | 1564.180215 | 1386.174418 | 1295.657119 | 1367.656581 | 1389.484056 | 1484.34059 | 1405.93912 | 1516.164705 | 1321.442271 |
| **Phf23** | 1285.63786 | 1304.063266 | 1220.948458 | 1248.19583 | 1305.361646 | 1258.334573 | 1311.602095 | 1262.977538 | 1282.113665 | 1173.650344 | 1146.395287 | 1159.01755 | 1186.953451 | 1134.864642 | 1070.662851 |
| **Dvl2** | 878.1529258 | 907.1399997 | 964.0715535 | 907.4651095 | 922.1768837 | 904.1211066 | 994.0753157 | 988.9273354 | 925.4693708 | 948.1836363 | 908.0358708 | 979.439232 | 908.32188 | 1034.570076 | 967.1893945 |
| **Acadvl** | 1288.38495 | 1304.063266 | 1423.004072 | 1324.968069 | 1336.396445 | 1319.357488 | 1327.83926 | 1310.747756 | 1258.036413 | 1311.726855 | 1381.917091 | 1343.801037 | 1401.678698 | 1481.829629 | 1376.771827 |
| **Dlg4** | 496.307493 | 422.2756505 | 457.366196 | 409.7394743 | 506.0572322 | 453.1126725 | 432.0889984 | 336.0676792 | 326.5477292 | 342.5695718 | 311.1914599 | 374.7721419 | 442.2317596 | 429.1884595 | 418.2052211 |
| **Asgr2** | 0 | 0.792262009 | 0.783161294 | 0 | 3.166816221 | 0.701412806 | 2.706194144 | 0.838074013 | 4.514484735 | 0 | 0 | 0.867528106 | 0 | 0.903554652 | 0 |
| **Slc16a11** | 30.21798389 | 19.0142882 | 28.1938066 | 30.19132969 | 10.13381191 | 21.74379698 | 9.020647148 | 33.52296052 | 15.80069657 | 15.73023544 | 43.51005214 | 24.29078698 | 17.89377062 | 14.45687443 | 15.08987911 |
| **Slc16a13** | 206.0317084 | 212.3262183 | 210.6703882 | 187.1862441 | 182.4086144 | 202.006888 | 179.5108782 | 222.0896135 | 140.7014409 | 224.592806 | 223.2254849 | 221.2196671 | 196.8314768 | 230.4064362 | 194.7312971 |
| **Bcl6b** | 7.325571853 | 20.59881222 | 14.0969033 | 10.35131304 | 11.4005384 | 11.9240177 | 13.53097072 | 11.73303618 | 4.514484735 | 11.3607256 | 4.729353494 | 16.48303402 | 25.56252945 | 13.55331977 | 10.77848508 |
| **0610010K14Rik** | 925.7691429 | 968.1441744 | 1032.989747 | 899.7016247 | 1033.015451 | 1011.437266 | 998.5856393 | 967.1374111 | 925.4693708 | 877.3975768 | 857.9047238 | 997.6573222 | 910.0260486 | 1029.148748 | 1041.920224 |
| **Rnasek** | 559.4905502 | 496.7482793 | 594.4194225 | 539.9934967 | 554.826202 | 536.5807964 | 535.8264406 | 604.2513634 | 493.5836644 | 500.7458282 | 478.6105736 | 510.9740546 | 466.0901204 | 546.6505642 | 536.0499913 |
| **Alox15** | 0 | 5.54583406 | 7.04845165 | 6.038265938 | 6.966995687 | 7.014128057 | 8.118582433 | 11.73303618 | 9.781383594 | 9.612921658 | 9.458706988 | 7.807752957 | 1.70416863 | 0.903554652 | 4.311394032 |
| **Pelp1** | 2522.743807 | 2353.018165 | 2365.147109 | 2345.435012 | 2406.780328 | 2460.556122 | 2570.884437 | 2450.528414 | 2289.596175 | 2374.39165 | 2438.454661 | 2424.741057 | 2379.871492 | 2211.901787 | 2447.434679 |
| **Arrb2** | 1646.422274 | 1935.496087 | 1783.258268 | 1733.844933 | 2021.695476 | 1987.102479 | 1983.640308 | 1568.036478 | 1665.092453 | 1673.52227 | 1604.196705 | 1865.185429 | 1936.787648 | 1939.931837 | 1958.091456 |
| **Mir7115** | 0 | 0.792262009 | 2.349483883 | 0 | 0 | 0 | 0.902064715 | 0 | 1.504828245 | 0 | 0 | 0 | 0 | 1.807109303 | 0 |
| **Med11** | 666.6270386 | 810.4840347 | 710.3272941 | 722.8666937 | 765.1027991 | 860.6335126 | 774.87359 | 858.1877894 | 878.0672811 | 743.6905755 | 713.1865069 | 765.1597897 | 735.348764 | 783.3818829 | 728.6255914 |
| **Cxcl16** | 8.241268334 | 1.584524017 | 8.614774239 | 9.488703616 | 14.56735462 | 4.208476834 | 6.314453003 | 11.73303618 | 12.03862596 | 11.3607256 | 4.729353494 | 15.61550591 | 4.260421576 | 4.517773258 | 1.437131344 |
| **Zmynd15** | 0 | 0 | 0 | 0.86260942 | 1.900089733 | 0.701412806 | 0 | 5.028444078 | 0 | 3.495607876 | 0 | 0 | 0 | 0 | 0 |
| **Tm4sf5** | 28.38659093 | 45.15893448 | 17.22954848 | 11.21392246 | 28.50134599 | 34.36922748 | 25.25781201 | 40.22755263 | 19.56276719 | 26.21705907 | 27.43025026 | 20.82067455 | 23.00627651 | 18.07109303 | 12.21561642 |
| **Psmb6** | 3224.167312 | 3236.390305 | 3250.902533 | 3270.15231 | 3226.98573 | 3284.716169 | 3082.35513 | 3505.663597 | 3485.182216 | 3485.121052 | 3265.145652 | 3033.745788 | 3066.65145 | 3025.100973 | 3097.018046 |
| **Pld2** | 76.91850445 | 89.52560696 | 79.09929074 | 80.22267603 | 76.00358931 | 56.11302446 | 73.96930661 | 57.8271069 | 25.58208017 | 66.41654964 | 57.69811262 | 85.88528252 | 82.65217857 | 91.25901981 | 98.44349706 |
| **Mink1** | 1520.971856 | 1606.707353 | 1581.202654 | 1454.359482 | 1396.565954 | 1522.065788 | 1543.432727 | 1376.955603 | 1358.107491 | 1389.504131 | 1421.64366 | 1481.738006 | 1508.189238 | 1574.895758 | 1535.574841 |
| **Chrne** | 8.241268334 | 1.584524017 | 3.132645178 | 1.725218839 | 1.900089733 | 0.701412806 | 3.608258859 | 0.838074013 | 1.504828245 | 0.873901969 | 8.512836289 | 1.735056213 | 0 | 0 | 3.59282836 |
| **Gp1ba** | 4.578482408 | 7.130358077 | 3.132645178 | 6.038265938 | 3.166816221 | 9.118366474 | 4.510323574 | 6.704592104 | 1.504828245 | 3.495607876 | 5.675224193 | 6.072696744 | 4.260421576 | 3.614218606 | 8.622788064 |
| **Slc25a11** | 1131.800851 | 1159.87158 | 1177.091426 | 1112.766151 | 1037.448994 | 1096.308215 | 1086.085917 | 1140.618732 | 1105.296346 | 1112.477206 | 1126.532002 | 1134.726763 | 979.8969624 | 1129.443314 | 1080.722771 |
| **Rnf167** | 217.9357626 | 263.0309868 | 305.4329049 | 261.3706542 | 264.7458361 | 280.5651223 | 246.2636671 | 290.8116825 | 263.3449429 | 233.3318257 | 289.4364338 | 322.7204555 | 311.8628593 | 372.2645164 | 296.7676225 |
| **Pfn1** | 30211.57402 | 30098.0337 | 29991.94493 | 30836.56153 | 32937.42216 | 33874.03004 | 33508.0959 | 27513.96985 | 28398.36623 | 27423.91769 | 27207.02478 | 31582.36071 | 31406.97577 | 32334.60676 | 31044.19273 |
| **Eno3** | 932.1790182 | 965.7673884 | 913.1660694 | 783.249353 | 1043.149263 | 1168.553734 | 1143.818058 | 583.2995131 | 653.8478725 | 826.7112626 | 882.497362 | 1030.62339 | 1037.838696 | 1049.930505 | 1008.147638 |
| **Spag7** | 923.9377499 | 830.2905849 | 865.3932304 | 830.6928711 | 832.239303 | 803.1176625 | 775.7756547 | 859.8639374 | 874.3052104 | 903.6146358 | 979.9220439 | 784.2454081 | 779.6571484 | 912.5901981 | 718.565672 |
| **Camta2** | 331.4821263 | 412.7685064 | 422.1239377 | 326.0663606 | 355.31678 | 310.0244601 | 365.3362095 | 371.2667878 | 395.0174144 | 360.0476112 | 357.5391241 | 414.6784348 | 410.7046399 | 392.1427188 | 389.4625942 |
| **Inca1** | 5.494178889 | 0 | 3.132645178 | 11.21392246 | 3.800179466 | 3.507064029 | 3.608258859 | 5.866518091 | 5.266898858 | 3.495607876 | 4.729353494 | 1.735056213 | 4.260421576 | 2.710663955 | 4.311394032 |
| **Kif1c** | 1418.41385 | 1386.458515 | 1505.236008 | 1674.324884 | 1414.933488 | 1440.00049 | 1375.64869 | 1544.570406 | 1434.101318 | 1566.90623 | 1701.621387 | 1575.431041 | 1546.533032 | 1469.179863 | 1499.646557 |
| **Zfp3** | 62.26736075 | 52.28929256 | 43.0738712 | 38.81742388 | 48.76896981 | 42.08476834 | 65.85072418 | 31.8468125 | 63.2027863 | 48.93851026 | 85.12836289 | 52.91921448 | 51.97714322 | 46.98484188 | 48.14390002 |
| **Scimp** | 10.98835778 | 8.714882094 | 5.482129061 | 8.626094197 | 8.233722176 | 9.118366474 | 4.510323574 | 7.542666117 | 5.266898858 | 9.612921658 | 14.18806048 | 2.602584319 | 4.260421576 | 4.517773258 | 4.311394032 |
| **Rabep1** | 865.3331751 | 791.4697465 | 866.959553 | 798.7763226 | 732.8012736 | 744.1989869 | 802.8375962 | 1066.868219 | 927.7266131 | 828.4590665 | 808.7194474 | 759.9546211 | 728.5320895 | 658.691341 | 813.4163407 |
| **Nup88** | 2259.938917 | 2223.087196 | 2341.652271 | 2117.706125 | 2254.139786 | 2098.627115 | 2190.213127 | 2411.138936 | 2402.458293 | 2439.934297 | 2410.07854 | 2285.93656 | 2320.22559 | 2176.663156 | 2254.859079 |
| **Rpain** | 141.0172582 | 130.7232314 | 142.5353556 | 153.5444767 | 124.1391959 | 145.8938636 | 158.7633898 | 136.6060641 | 92.54693708 | 195.754041 | 120.1255787 | 131.8642722 | 147.4105865 | 174.3860478 | 153.0544881 |
| **C1qbp** | 4890.734908 | 4378.832121 | 4743.607961 | 4487.294201 | 4526.013744 | 4619.504738 | 4478.751309 | 5473.461379 | 5050.203591 | 5405.083678 | 5441.59413 | 4425.26087 | 4338.813333 | 4349.712093 | 4331.513871 |
| **Dhx33** | 1160.187442 | 1159.079318 | 1165.344006 | 1236.981908 | 1090.651507 | 1131.378856 | 1203.35433 | 1366.060641 | 1349.078522 | 1247.05811 | 1389.484056 | 1183.308337 | 1192.918041 | 1031.859412 | 1154.735035 |
| **Derl2** | 626.3363934 | 697.1905675 | 722.8578748 | 624.5292198 | 678.3320346 | 685.981724 | 634.1514945 | 735.8289835 | 752.4141226 | 671.1567121 | 662.1094891 | 655.8512483 | 631.3944775 | 676.762434 | 604.3137301 |
| **Mis12** | 669.374128 | 607.6649605 | 608.5163258 | 589.1622336 | 555.4595652 | 600.4093617 | 510.5686286 | 628.5555098 | 547.7574812 | 536.5758089 | 659.271877 | 495.3585487 | 499.3214087 | 498.7621677 | 505.8702331 |
| **6330403K07Rik** | 5.494178889 | 0 | 4.698967767 | 7.763484777 | 2.533452977 | 4.90988964 | 9.020647148 | 13.40918421 | 3.00965649 | 2.621705907 | 4.729353494 | 5.205168638 | 12.78126473 | 7.228437213 | 1.437131344 |
| **Nlrp1a** | 5.494178889 | 9.507144102 | 6.265290356 | 9.488703616 | 4.43354271 | 7.014128057 | 5.412388289 | 8.380740131 | 5.266898858 | 6.117313782 | 11.35044839 | 5.205168638 | 5.112505891 | 9.939101167 | 0 |
| **Nlrp1b** | 0.915696482 | 2.376786026 | 6.265290356 | 4.313047098 | 3.166816221 | 2.104238417 | 6.314453003 | 3.352296052 | 0 | 3.495607876 | 11.35044839 | 3.470112425 | 4.260421576 | 9.939101167 | 5.748525376 |
| **Wscd1** | 1.831392963 | 3.169048034 | 0 | 0.86260942 | 0 | 0 | 0.902064715 | 0 | 1.504828245 | 0 | 1.891741398 | 0 | 0 | 0 | 0.718565672 |
| **Aipl1** | 29.30228741 | 43.57441047 | 23.49483883 | 38.81742388 | 32.30152546 | 30.16075065 | 29.76813559 | 37.71333059 | 16.5531107 | 27.964863 | 31.21373306 | 26.89337129 | 22.15419219 | 36.14218606 | 33.77258658 |
| **Fam64a** | 888.2255871 | 941.9995281 | 1045.520328 | 1002.352146 | 1044.41599 | 1041.598016 | 1052.709522 | 804.5510525 | 954.0611074 | 997.9960485 | 863.579948 | 1110.435976 | 1189.509704 | 1206.24546 | 1108.028266 |
| **4933427D14Rik** | 315.9152861 | 410.3917204 | 331.2772276 | 365.7463939 | 332.5157032 | 290.3849016 | 346.3928505 | 310.9254588 | 276.8883971 | 355.6781013 | 308.3538478 | 318.382815 | 345.0941476 | 331.6045571 | 316.1688957 |
| **Txndc17** | 1039.315507 | 1041.032279 | 1002.446457 | 909.1903283 | 923.4436102 | 972.1581487 | 952.5803388 | 1071.896663 | 1099.277033 | 1096.746971 | 1127.477873 | 962.0886699 | 855.4926524 | 955.9608214 | 927.6682825 |
| **Med31** | 103.4737024 | 98.24048905 | 115.1247103 | 100.9253021 | 93.73776015 | 122.0458282 | 93.81473034 | 133.2537681 | 126.4055726 | 103.9943343 | 82.29075079 | 113.6461819 | 120.1438884 | 112.9443314 | 117.8447702 |
| **4930563E22Rik** | 1.831392963 | 0.792262009 | 0 | 0 | 0 | 0 | 0.902064715 | 2.514222039 | 0 | 0 | 0 | 1.735056213 | 0.852084315 | 0 | 2.874262688 |
| **Xaf1** | 821.379744 | 648.862585 | 538.0318093 | 468.3969149 | 599.1616291 | 752.6159405 | 775.7756547 | 195.271245 | 277.6408112 | 231.5840218 | 259.1685715 | 525.7220324 | 435.415085 | 549.3612282 | 587.7867197 |
| **Fbxo39** | 1.831392963 | 2.376786026 | 0 | 0 | 1.266726489 | 2.104238417 | 3.608258859 | 0 | 0 | 0 | 0.945870699 | 0 | 0.852084315 | 9.035546516 | 2.874262688 |
| **Smtnl2** | 82.41268334 | 49.91250654 | 50.12232285 | 42.26786156 | 39.90188439 | 61.0229141 | 67.65485361 | 1.676148026 | 14.29586833 | 9.612921658 | 9.458706988 | 52.05168638 | 52.82922754 | 43.37062328 | 40.9582433 |
| **Mybbp1a** | 14631.91408 | 13577.7863 | 13717.07007 | 14077.78573 | 12903.50938 | 12703.98874 | 13640.12055 | 16581.29435 | 15679.5579 | 15389.41367 | 16043.85879 | 12857.63406 | 12982.35663 | 11950.41382 | 13279.09362 |
| **Spns2** | 0 | 0 | 1.566322589 | 3.450437679 | 1.266726489 | 1.402825611 | 3.608258859 | 7.542666117 | 5.266898858 | 2.621705907 | 5.675224193 | 0 | 0 | 5.421327909 | 0 |
| **Spns3** | 2.747089445 | 2.376786026 | 4.698967767 | 1.725218839 | 3.166816221 | 4.208476834 | 1.80412943 | 0 | 3.00965649 | 3.495607876 | 2.837612096 | 2.602584319 | 5.112505891 | 2.710663955 | 0.718565672 |
| **Ube2g1** | 1134.547941 | 1103.620978 | 1180.224071 | 1059.284367 | 1103.952135 | 1141.900048 | 1181.704776 | 1130.561844 | 1098.524619 | 1014.600186 | 1072.617372 | 1127.786538 | 1108.561694 | 1088.783355 | 1156.172166 |
| **Ankfy1** | 2538.310647 | 2335.588401 | 2195.201108 | 2075.438264 | 1943.158433 | 2147.726011 | 2369.724006 | 2190.72547 | 2207.583036 | 2038.813293 | 2079.969667 | 2140.191838 | 2128.506619 | 2128.774759 | 2095.337499 |
| **Cyb5d2** | 35.71216278 | 39.61310043 | 52.47180673 | 33.64176737 | 55.10260225 | 33.66781467 | 44.20117102 | 53.63673684 | 49.65933209 | 52.43411813 | 41.61831075 | 38.17123668 | 28.97086672 | 36.14218606 | 36.64684927 |

| | | | | | | | | | | | | | | | |
|---|---|---|---|---|---|---|---|---|---|---|---|---|---|---|---|
| **Zzef1** | 1150.114781 | 1113.128122 | 1135.583877 | 1160.209669 | 1067.217067 | 918.8507755 | 1147.426317 | 1102.067327 | 1253.521928 | 1037.321637 | 1195.580563 | 1164.222719 | 1138.384645 | 1075.230035 | 1146.112247 |
| **Atp2a3** | 3067.583213 | 3416.233781 | 3548.503825 | 3483.216837 | 3738.109868 | 3794.643279 | 3985.32191 | 2608.924403 | 2905.070927 | 2872.515772 | 3140.29072 | 3922.962097 | 3794.331455 | 4196.107802 | 4235.226071 |
| **P2rx1** | 0 | 3.169048034 | 1.566322589 | 1.725218839 | 0.633363244 | 1.402825611 | 2.706194144 | 0 | 0 | 0.873901969 | 3.783482795 | 5.205168638 | 0.852084315 | 0.903554652 | 2.155697016 |
| **Camkk1** | 25.63950148 | 31.69048034 | 56.3876132 | 36.22959563 | 37.36843141 | 37.1748787 | 33.37639445 | 31.8468125 | 31.60139315 | 30.58656891 | 41.61831075 | 44.24393342 | 41.75213144 | 46.08128723 | 34.49115225 |
| **1200014J11Rik** | 1270.07102 | 1285.048978 | 1430.835685 | 1287.013254 | 1191.989626 | 1306.030644 | 1373.844561 | 1460.763005 | 1556.74482 | 1280.266384 | 1407.4556 | 1397.587779 | 1329.251532 | 1309.25069 | 1261.80132 |
| **Itgae** | 418.4732921 | 578.3512662 | 617.9142614 | 761.6841176 | 718.8672823 | 599.0065361 | 601.6771648 | 1231.130725 | 774.2341321 | 807.4854193 | 1376.241867 | 653.248664 | 680.8153678 | 711.0975108 | 607.1879928 |
| **Gsg2** | 547.586496 | 640.1477029 | 539.5981319 | 600.3761561 | 521.8913133 | 542.1920988 | 555.6718643 | 698.1156529 | 635.0375195 | 693.8781633 | 676.2975496 | 565.6283253 | 593.0506834 | 465.3306456 | 546.8284764 |
| **P2rx5** | 2.747089445 | 3.961310043 | 3.132645178 | 6.900875357 | 5.700269198 | 0 | 12.62890601 | 9.218814144 | 3.762070613 | 1.747803938 | 16.07980188 | 3.470112425 | 0 | 6.324882561 | 2.874262688 |
| **Emc6** | 1060.376526 | 966.5596504 | 1039.255038 | 1046.345226 | 949.4115032 | 1137.691571 | 991.3691215 | 1200.121987 | 1119.592214 | 1163.163521 | 1019.648613 | 1041.901256 | 1052.324129 | 1129.443314 | 977.2493139 |
| **Tax1bp3** | 61.35166426 | 76.05715282 | 83.01509722 | 71.59658183 | 80.43713202 | 84.87094949 | 78.47963019 | 76.26473519 | 73.73658401 | 71.65996145 | 76.6155266 | 53.78674259 | 56.2375648 | 62.34527096 | 76.8865269 |
| **Ctns** | 75.08711149 | 94.27917901 | 128.4384523 | 87.9861608 | 103.2382088 | 65.93280374 | 70.36104775 | 92.18814144 | 108.3476337 | 97.00311855 | 101.2081648 | 91.09045116 | 74.13133542 | 93.06612911 | 84.79074929 |
| **Shpk** | 52.19469945 | 22.18333624 | 49.33916155 | 20.70262607 | 29.76807248 | 35.07064029 | 40.59291217 | 43.57984868 | 30.84897903 | 24.46925513 | 22.70089677 | 39.03876478 | 39.1958785 | 44.27417793 | 32.33545524 |
| **Rap1gap2** | 8.241268334 | 20.59881222 | 25.06116142 | 25.87828259 | 20.90098706 | 16.83390734 | 21.64955315 | 10.89496217 | 4.514484735 | 10.48682363 | 6.621094891 | 32.09853993 | 36.63962555 | 36.14218606 | 25.86836419 |
| **Cluh** | 3589.530208 | 3699.86358 | 3781.885891 | 3694.556144 | 3550.000984 | 3524.599349 | 3792.280061 | 4330.328425 | 4230.824611 | 4419.322257 | 4485.318854 | 3747.721419 | 3812.225226 | 3629.579035 | 4049.836127 |
| **Pafah1b1** | 3958.55589 | 3845.639789 | 3804.597569 | 3650.563064 | 3463.863583 | 3662.076259 | 3839.187426 | 3812.398685 | 3688.334029 | 3666.892662 | 3791.049761 | 3745.986363 | 3454.349814 | 3509.406267 | 3525.283187 |
| **Mettl16** | 1258.166966 | 1226.421589 | 1206.068394 | 1192.126218 | 1241.391959 | 1227.47241 | 1214.179106 | 1336.728051 | 1285.123321 | 1375.521699 | 1357.324453 | 1175.500584 | 1175.024271 | 1166.489055 | 1180.603399 |
| **Mnt** | 885.4784977 | 753.4411701 | 847.3805206 | 840.1815747 | 644.1304194 | 655.8209733 | 707.2187364 | 548.9384786 | 620.7416511 | 582.0187113 | 633.7333682 | 621.1501241 | 684.2237051 | 645.1380212 | 646.7091048 |
| **Sgsm2** | 139.1858652 | 155.2833537 | 158.9817428 | 169.9340557 | 134.2730078 | 159.2207069 | 130.7993836 | 160.0721365 | 175.3124906 | 166.0413741 | 184.4447863 | 113.6461819 | 143.1501649 | 132.8225338 | 128.6232553 |
| **Tsr1** | 1407.425492 | 1412.603161 | 1423.787233 | 1366.373321 | 1335.129719 | 1398.617135 | 1433.380832 | 1635.920473 | 1704.970402 | 1624.58376 | 1674.191137 | 1367.224295 | 1314.766098 | 1238.773427 | 1328.627927 |
| **Snord91a** | 1.831392963 | 2.376786026 | 0 | 0 | 0.633363244 | 0.701412806 | 3.608258859 | 2.514222039 | 0.752414123 | 5.243411813 | 1.891741398 | 5.205168638 | 0 | 1.807109303 | 0 |
| **Srr** | 32.04937685 | 28.52143231 | 28.1938066 | 41.40525214 | 43.06870061 | 35.07064029 | 39.69084745 | 37.71333059 | 30.0965649 | 33.20827482 | 19.86328467 | 37.30370857 | 38.34379418 | 35.23863141 | 46.70676868 |
| **Smg6** | 781.0890988 | 907.1399997 | 816.0540689 | 845.3572313 | 888.6086317 | 782.7766912 | 936.3431739 | 762.6473519 | 825.3982925 | 716.5996145 | 694.2690929 | 957.7510293 | 921.1031447 | 896.3262144 | 1001.680547 |
| **Hic1** | 41.20634167 | 30.10595632 | 28.9769679 | 16.38957897 | 15.20071786 | 25.95227381 | 24.3557473 | 87.99777137 | 36.11587788 | 42.82119648 | 37.83482795 | 26.02584319 | 23.00627651 | 12.64976512 | 27.30549553 |
| **Ovca2** | 151.0899195 | 179.8434759 | 156.6322589 | 137.1548977 | 143.1400932 | 130.4627819 | 184.9232665 | 136.6060641 | 163.2738646 | 124.9679816 | 155.1227946 | 144.0096656 | 144.8543336 | 136.4367524 | 161.6772762 |
| **Dph1** | 258.2264078 | 249.5625327 | 255.310582 | 263.095873 | 238.7779431 | 277.7594711 | 206.5728197 | 282.4309424 | 266.3545994 | 259.5488848 | 249.7098645 | 203.869105 | 232.619018 | 267.4521769 | 245.0308941 |
| **Rtn4rl1** | 25.63950148 | 15.05297816 | 28.9769679 | 14.66436013 | 28.50134599 | 23.14662259 | 27.06194144 | 51.1225148 | 18.05793894 | 22.72145119 | 34.99721585 | 26.02584319 | 18.74585493 | 28.91374885 | 38.80254629 |
| **Rpa1** | 4524.456315 | 4810.614916 | 4894.758091 | 5037.639011 | 5113.774834 | 4931.633437 | 4803.494606 | 5486.870563 | 5549.806568 | 5299.341539 | 5491.725277 | 5186.950547 | 5309.337368 | 5296.637368 | 4890.557963 |
| **Smyd4** | 188.6334752 | 180.6357379 | 183.2597429 | 188.9114629 | 220.410409 | 166.234835 | 197.5521725 | 206.1662072 | 176.8173188 | 184.3933154 | 249.7098645 | 199.5314644 | 213.8731631 | 191.5535861 | 207.6654792 |
| **Serpinf1** | 12.81975074 | 6.338096068 | 16.44638718 | 18.11479781 | 3.800179466 | 7.014128057 | 6.314453003 | 11.73303618 | 21.06759543 | 18.35194135 | 15.13393118 | 11.27786538 | 2.556252945 | 7.228437213 | 15.08987911 |
| **Wdr81** | 357.1216278 | 408.0149344 | 396.279615 | 452.8699453 | 379.3845833 | 382.2699791 | 359.9238212 | 340.2580493 | 359.6539506 | 411.6078274 | 351.8638999 | 378.2422543 | 403.8879654 | 444.5488886 | 419.6423524 |
| **Mir22hg** | 12.81975074 | 22.97559825 | 18.79587107 | 20.70262607 | 18.36753408 | 9.118366474 | 18.94335901 | 11.73303618 | 15.04828245 | 31.46047088 | 16.07980188 | 23.42325887 | 24.71044514 | 20.78175699 | 12.21561642 |
| **Tlcd2** | 89.73825519 | 76.84941482 | 73.61716168 | 70.73397241 | 65.8697774 | 62.42573971 | 62.24246532 | 103.9211776 | 106.0903913 | 77.77727523 | 94.58706988 | 83.2826982 | 67.3146609 | 78.60925469 | 51.73672838 |
| **Prpf8** | 12313.37059 | 11498.89079 | 11500.72361 | 12639.81583 | 11276.3992 | 11071.09973 | 12245.5285 | 14140.82282 | 13553.988 | 12882.18892 | 13844.70942 | 11728.98 | 12149.87025 | 11224.85944 | 11936.09438 |
| **Rilp** | 3.662785926 | 2.376786026 | 1.566322589 | 1.725218839 | 0 | 2.805651223 | 0.902064715 | 0.838074013 | 4.514484735 | 0 | 1.891741398 | 0.867528106 | 5.112505891 | 2.710663955 | 1.437131344 |
| **Scarf1** | 2.747089445 | 2.376786026 | 1.566322589 | 3.450437679 | 0.633363244 | 6.312715251 | 2.706194144 | 10.89496217 | 9.028969471 | 1.747803938 | 0 | 0 | 0.852084315 | 1.807109303 | 0 |
| **Slc43a2** | 693.1822365 | 873.8649954 | 805.0898107 | 855.7085443 | 879.1081831 | 714.739649 | 896.6523265 | 598.3848453 | 721.5651436 | 620.4703979 | 781.2891972 | 893.5539495 | 863.1614113 | 1005.656327 | 875.9315541 |
| **Pitpna** | 4062.945289 | 3848.808837 | 4079.487183 | 4164.678278 | 4106.093913 | 4146.051095 | 4163.028659 | 4446.820713 | 4186.432178 | 4116.078274 | 4211.016351 | 4254.357833 | 4215.261107 | 4225.021551 | 3998.817964 |
| **Gm12338** | 42.12203815 | 29.31369431 | 27.41064531 | 42.26786156 | 41.80197412 | 29.45933784 | 39.69084745 | 31.8468125 | 33.85863552 | 56.80362798 | 26.48437957 | 30.36348372 | 51.97714322 | 33.43152211 | 30.17975822 |
| **Inpp5k** | 499.9702789 | 533.9845937 | 661.7712938 | 609.8648597 | 683.3989406 | 639.6884788 | 614.3060708 | 605.9275114 | 552.271966 | 533.080201 | 484.2857978 | 679.2745072 | 597.3111049 | 733.6863771 | 651.7390645 |
| **Myo1c** | 1449.54753 | 1399.134707 | 1435.534653 | 1225.767985 | 1225.557878 | 1193.804595 | 1332.349584 | 1594.016773 | 1355.097835 | 1351.052444 | 1266.520866 | 1248.372945 | 1274.718135 | 1256.84452 | 1266.83128 |
| **Crk** | 811.3070827 | 741.55724 | 811.3551011 | 723.7293031 | 658.0644108 | 753.3173533 | 769.4612017 | 877.4634917 | 878.0672811 | 853.8022236 | 854.121241 | 771.2324865 | 763.4675464 | 721.036612 | 741.5597735 |
| **Ywhae** | 12131.14699 | 11655.75867 | 11965.13826 | 11671.10545 | 11349.23597 | 11008.67399 | 12063.31143 | 12307.11688 | 11763.99481 | 12433.00331 | 12148.76326 | 11699.48404 | 11244.95671 | 11678.44387 | 11654.41663 |
| **Rph3al** | 27.47089445 | 35.65179038 | 49.33916155 | 40.54264272 | 34.83497844 | 31.56357626 | 36.08258859 | 37.71333059 | 40.63036262 | 38.45168663 | 26.48437957 | 62.46202365 | 43.45630007 | 30.72085815 | 25.86836419 |
| **1700016K19Rik** | 0.915696482 | 0 | 0 | 0 | 0 | 0 | 0 | 0 | 0 | 0 | 0 | 0.867528106 | 0 | 0 | 0.718565672 |
| **Fam101b** | 31.13368037 | 27.7291703 | 31.32645178 | 32.77915795 | 31.03479897 | 42.08476834 | 46.90736517 | 51.96058881 | 67.71727103 | 41.94729451 | 62.42746612 | 36.43618046 | 38.34379418 | 37.04574071 | 30.17975822 |
| **Vps53** | 809.4756897 | 767.7018862 | 911.5997468 | 910.0529377 | 810.7049527 | 859.230687 | 781.188043 | 834.721717 | 869.0383116 | 866.9107532 | 906.1441294 | 822.4166448 | 858.9009897 | 925.2399632 | 804.7935526 |
| **Fam57a** | 101.6423095 | 94.27917901 | 97.11200052 | 80.22267603 | 81.70385851 | 87.67660071 | 98.32505391 | 84.64547532 | 94.80417945 | 88.26409886 | 83.23662149 | 60.72696744 | 74.98341973 | 70.47726282 | 102.0363254 |
| **Gemin4** | 1187.658337 | 1043.409065 | 1182.573555 | 1215.416672 | 1169.821912 | 1080.877134 | 1289.952542 | 1340.080347 | 1196.338455 | 1211.228129 | 1266.520866 | 1142.534516 | 1136.680476 | 1052.641169 | 1095.81265 |
| **Dbil5** | 0 | 0.792262009 | 2.349483883 | 0 | 0 | 0 | 2.706194144 | 0.838074013 | 0 | 2.621705907 | 0 | 1.735056213 | 0 | 0.903554652 | 1.437131344 |
| **Glod4** | 1011.844612 | 853.2661832 | 917.8650371 | 902.289453 | 891.7754479 | 872.5575303 | 931.8328504 | 961.270893 | 875.8100387 | 902.7407339 | 1073.563243 | 863.1904657 | 904.0614584 | 913.4937527 | 891.7399989 |
| **Rnmtl1** | 293.0228741 | 298.6827772 | 323.4456146 | 282.9358896 | 258.4122037 | 303.7117449 | 257.9905084 | 324.3346431 | 349.1201529 | 378.3995525 | 352.8097706 | 254.1857351 | 327.200377 | 255.7059664 | 298.2047539 |
| **Nxn** | 590.6242306 | 594.1965064 | 584.2383257 | 603.8265938 | 489.5897878 | 657.9252118 | 557.4759937 | 587.4898832 | 731.3465271 | 602.9923585 | 619.5453077 | 552.6154037 | 500.173493 | 577.3714224 | 518.8044152 |
| **Timm22** | 619.0108215 | 678.9685413 | 639.8427776 | 645.2318459 | 704.2999276 | 656.5223861 | 613.4040061 | 775.2184621 | 695.9830634 | 651.9308688 | 633.7333682 | 608.1372025 | 602.4236108 | 690.3157538 | 638.0863167 |
| **Abr** | 940.4202866 | 1113.920384 | 1202.152587 | 1117.079198 | 936.7442383 | 959.5327182 | 1027.45171 | 1154.027916 | 1189.566728 | 1165.785227 | 1310.030918 | 1073.999796 | 1129.863802 | 1138.478861 | 1111.621095 |
| **Gosr1** | 814.0541721 | 852.4739212 | 823.1025205 | 772.0354306 | 787.2705126 | 756.8244174 | 852.4511555 | 734.1528354 | 866.7810692 | 780.3944582 | 825.74512 | 834.5620382 | 737.0529326 | 725.5543852 | 784.6737138 |
| **Cpd** | 581.4672658 | 520.5161396 | 593.6362612 | 544.3065438 | 516.8244073 | 512.0313482 | 564.6925115 | 1019.098 | 771.2244756 | 709.6083987 | 657.3801356 | 567.3633815 | 585.3819245 | 478.8839653 | 524.5529405 |
| **Blmh** | 2054.822905 | 1960.056209 | 2052.665753 | 1916.71813 | 2102.765971 | 2142.114709 | 2116.243821 | 2136.250659 | 2116.540927 | 1850.92437 | 1962.6817 | 2132.384085 | 2079.937813 | 1995.952225 | 1962.40285 |
| **Ccdc55** | 582.3829623 | 593.4042444 | 537.248648 | 548.6195909 | 553.5594755 | 530.2680811 | 650.3886594 | 561.5095887 | 538.7285118 | 476.2765731 | 557.1178416 | 568.2309096 | 482.2797224 | 580.985641 | 491.4989196 |
| **Efcab5** | 6.409875371 | 17.42976419 | 8.614774239 | 14.66436013 | 12.03390164 | 14.02825611 | 11.72684129 | 8.380740131 | 15.80069657 | 10.48682363 | 10.40457769 | 7.807752957 | 11.0770961 | 16.26398373 | 15.08987911 |
| **Ssh2** | 1227.033285 | 1385.666253 | 1451.98104 | 1361.197664 | 1052.016349 | 1082.279959 | 1307.091772 | 1168.275174 | 1303.18126 | 1052.177971 | 1169.096184 | 1329.920587 | 1396.566193 | 1177.331711 | 1307.789523 |
| **Coro6** | 5.494178889 | 7.130358077 | 12.53058071 | 5.175656518 | 5.066905954 | 4.90988964 | 6.314453003 | 25.14222039 | 21.06759543 | 35.82998073 | 20.80915537 | 9.542809169 | 7.668758836 | 9.939101167 | 5.748525376 |
| **Ankrd13b** | 978.8795388 | 983.1971526 | 1092.510006 | 973.8860348 | 934.8441486 | 866.2448151 | 1007.606286 | 1396.231306 | 1154.203264 | 1326.583189 | 1192.742951 | 887.4812527 | 1013.980335 | 965.8999225 | 1081.441336 |
| **Git1** | 2203.165735 | 2179.512785 | 2176.405237 | 2370.450685 | 2007.128121 | 2072.674841 | 2173.975963 | 2686.027212 | 2342.265164 | 2210.098079 | 2397.782221 | 1984.036779 | 2038.185682 | 1976.074023 | 1998.331134 |
| **Trp53i13** | 448.691276 | 400.8845763 | 411.1596796 | 439.930804 | 456.0215359 | 479.7663591 | 396.9084745 | 406.4658963 | 392.760172 | 407.2383175 | 428.4794265 | 418.1485472 | 464.3859518 | 420.152913 | 479.2833032 |
| **Abhd15** | 89.73825519 | 125.1773973 | 105.7267748 | 138.8801166 | 109.5718413 | 93.98931597 | 96.52092448 | 149.1771743 | 112.1097043 | 151.1850406 | 116.3420959 | 101.5007884 | 110.770961 | 131.9189791 | 102.0363254 |
| **Taok1** | 830.5367088 | 908.7245238 | 819.186714 | 792.7380567 | 646.0305092 | 606.0206641 | 795.6210784 | 907.6341561 | 927.7266131 | 807.4854193 | 829.5286028 | 894.4214776 | 901.5052054 | 822.2347329 | 736.5298138 |
| **Nufip2** | 1173.922889 | 1138.480506 | 1210.767361 | 1144.6827 | 1088.118054 | 979.8736896 | 1193.431618 | 1268.005982 | 1208.377081 | 1192.876188 | 1184.230115 | 1177.23564 | 1109.413778 | 1083.362027 | 1155.453601 |
| **Myo18a** | 884.5628012 | 1108.37455 | 1061.183554 | 1005.802583 | 996.2803832 | 932.1776188 | 1053.611587 | 952.0520788 | 910.4210883 | 979.6441071 | 946.8165695 | 978.5717039 | 1001.19907 | 986.6816795 | 1097.968347 |
| **Pipox** | 9.156964816 | 7.922620085 | 8.614774239 | 2.587828259 | 12.03390164 | 4.90988964 | 9.020647148 | 2.514222039 | 22.57242368 | 15.73023544 | 12.29631908 | 8.675281063 | 5.112505891 | 3.614218606 | 7.18565672 |
| **Phf12** | 1563.093894 | 1705.740104 | 1738.618074 | 1605.31613 | 1698.046858 | 1832.791661 | 1700.391987 | 1759.955427 | 1654.558656 | 1836.941939 | 1689.325068 | 1699.48756 | 1763.814532 | 1777.292 | 1746.114583 |
| **Dhrs13** | 389.1710047 | 418.3143405 | 432.3050346 | 382.1359729 | 409.786019 | 405.4166017 | 371.6506625 | 343.6103454 | 369.4353342 | 401.9949057 | 338.6217102 | 434.6315812 | 452.4567714 | 371.3609618 | 365.749927 |
| **Flot2** | 912.9493921 | 871.4882094 | 884.1891015 | 899.7016247 | 843.6398414 | 852.9179717 | 902.0647148 | 807.9033486 | 838.9417467 | 910.6058516 | 879.6597499 | 845.8399036 | 920.2510604 | 885.4835585 | 854.374584 |
| **Eral1** | 547.586496 | 616.3798426 | 541.9476158 | 493.412588 | 516.1910441 | 474.8564695 | 523.1975346 | 531.3389243 | 545.5002389 | 561.918966 | 495.6362462 | 537.8674259 | 512.9547577 | 553.8790014 | 520.2415465 |
| **Fam222b** | 449.6069724 | 427.0292226 | 456.5830347 | 465.8090866 | 388.885032 | 371.0473742 | 375.2589213 | 472.6737434 | 478.535382 | 425.5902589 | 524.0123671 | 373.0370857 | 382.5858575 | 381.300063 | 344.9115225 |
| **Traf4** | 3401.812429 | 2791.931318 | 2957.217048 | 2863.863273 | 2197.137094 | 2369.372458 | 2532.997719 | 2578.753738 | 2479.956948 | 2732.691457 | 2905.714787 | 2132.384085 | 2161.737908 | 2023.058865 | 2121.924429 |
| **Nek8** | 282.9502128 | 301.0595632 | 274.8896144 | 314.8524382 | 232.4443106 | 253.9114357 | 248.9698613 | 282.4309424 | 278.3932254 | 354.8041994 | 300.7868822 | 272.4038254 | 295.6732574 | 228.5993268 | 274.4920867 |
| **Tlcd1** | 85.15977279 | 91.90239299 | 91.62987146 | 69.87136299 | 55.7359655 | 61.0229141 | 64.04659475 | 54.47481085 | 74.48899814 | 81.27288311 | 74.7237852 | 39.03876478 | 63.90632364 | 44.27417793 | 66.82660749 |
| **Rpl23a** | 23201.00175 | 20487.89554 | 20911.18972 | 20496.46242 | 22050.54135 | 22288.09331 | 23415.79587 | 20669.41938 | 20233.92058 | 20196.7484 | 19288.19529 | 21485.20108 | 20496.8882 | 21117.87932 | 20954.81213 |
| **Snord4a** | 7.325571853 | 3.961310043 | 6.265290356 | 10.35131304 | 1.266726489 | 2.104238417 | 1.80412943 | 9.218814144 | 6.771727103 | 6.117313782 | 15.13393118 | 3.470112425 | 0.852084315 | 4.517773258 | 0.718565672 |
| **Snord42b** | 2.747089445 | 0 | 6.265290356 | 0.86260942 | 1.900089733 | 3.507064029 | 1.80412943 | 5.028444078 | 6.019312981 | 1.747803938 | 3.783482795 | 2.602584319 | 0 | 0.903554652 | 2.874262688 |
| **Rab34** | 127.2818109 | 117.2547773 | 106.5099361 | 82.81050429 | 76.63695256 | 72.94693179 | 49.61355931 | 165.1005806 | 127.9104008 | 163.4196682 | 91.74945778 | 110.1760695 | 70.72299816 | 96.68034772 | 80.47935526 |
| **Proca1** | 2.747089445 | 1.584524017 | 14.8800646 | 0.86260942 | 0.633363244 | 0 | 2.706194144 | 3.352296052 | 1.504828245 | 0 | 0.945870699 | 4.337640531 | 4.260421576 | 3.614218606 | 0 |
| **Supt6** | 4672.799145 | 4237.017221 | 4150.754861 | 4196.594827 | 3867.31597 | 3819.89414 | 4320.889984 | 4363.013312 | 4549.095785 | 4055.779038 | 4313.170386 | 4151.989517 | 4060.181762 | 3608.797278 | 4093.668633 |
| **Sdf2** | 480.7406528 | 497.5405413 | 514.5369705 | 471.8473525 | 405.3524763 | 453.1126725 | 489.8211401 | 408.9801184 | 521.4229869 | 456.1768278 | 422.8042024 | 458.0548401 | 427.7463262 | 473.4626374 | 435.4507972 |
| **2610507B11Rik** | 5487.769014 | 5710.624557 | 5852.564354 | 5631.976901 | 4736.923704 | 4941.453216 | 5080.428474 | 6366.010203 | 5959.119851 | 6109.448665 | 6423.407915 | 5181.745379 | 5192.601817 | 5092.434016 | 5132.714595 |
| **BC030499** | 5.494178889 | 16.63750218 | 23.49483883 | 5.175656518 | 11.4005384 | 14.72966892 | 9.020647148 | 20.95185033 | 2.257242368 | 2.621705907 | 11.35044839 | 5.205168638 | 8.520843152 | 10.84265582 | 8.622788064 |
| **Spag5** | 1425.739422 | 1562.340681 | 1493.488589 | 1614.804834 | 1411.133308 | 1392.304419 | 1559.669892 | 1600.721365 | 1585.336556 | 1551.175995 | 1415.968436 | 1597.119244 | 1689.683197 | 1655.312122 | 1537.730538 |
| **Aldoc** | 1043.893989 | 1006.172751 | 840.332069 | 862.6094197 | 868.9743711 | 1059.133337 | 1240.338983 | 71.23629111 | 137.6917844 | 207.1147666 | 232.6841919 | 927.3875456 | 1017.388672 | 946.0217202 | 1049.824447 |
| **Pigs** | 1640.928095 | 1599.576995 | 1614.878589 | 1598.415255 | 1450.401829 | 1598.519784 | 1615.597904 | 1471.657967 | 1545.458608 | 1538.941367 | 1554.065558 | 1598.8543 | 1715.245726 | 1639.951693 | 1743.24032 |
| **Unc119** | 314.9995897 | 320.0738514 | 332.0603889 | 285.5237179 | 357.2168698 | 416.6392066 | 384.2795685 | 288.2974605 | 274.6311547 | 320.7220226 | 277.1401147 | 322.7204555 | 344.2420633 | 337.025885 | 364.3127957 |
| **Slc46a1** | 9.156964816 | 13.46845414 | 7.04845165 | 15.52696955 | 8.233722176 | 4.208476834 | 10.82477658 | 13.40918421 | 0 | 22.72145119 | 21.75502607 | 7.807752957 | 10.22501178 | 7.228437213 | 6.467091048 |
| **Sarm1** | 6.409875371 | 1.584524017 | 3.915806472 | 5.175656518 | 3.166816221 | 2.104238417 | 10.82477658 | 21.78992434 | 7.524141226 | 7.86511772 | 2.837612096 | 6.072696744 | 7.668758836 | 1.807109303 | 6.467091048 |
| **Tmem199** | 326.9036439 | 303.4363493 | 334.4098727 | 313.9898288 | 289.4470026 | 355.6162925 | 319.330909 | 366.2383437 | 361.911193 | 357.4259053 | 358.4849948 | 270.6687692 | 349.3545692 | 287.3303792 | 353.5343106 |
| **Poldip2** | 967.891181 | 1028.356087 | 1007.928586 | 1101.552229 | 904.4427128 | 929.3719676 | 976.9360861 | 1114.638437 | 1263.303312 | 1072.277716 | 1163.420959 | 884.8786684 | 896.3926995 | 976.7425784 | 939.1653333 |
| **Tnfaip1** | 773.7635269 | 802.5614146 | 715.0262619 | 690.9501451 | 567.4934669 | 645.2997813 | 656.7031124 | 807.0652746 | 698.9927199 | 815.350537 | 822.9075079 | 607.2696744 | 639.0632364 | 624.3562642 | 615.0922152 |
| **Ift20** | 382.7611293 | 352.5565938 | 324.2287759 | 344.1811584 | 300.2141778 | 362.6304206 | 318.4288443 | 346.9626414 | 376.9594754 | 393.255886 | 312.1373306 | 340.0710177 | 322.0878711 | 347.8685409 | 291.0190971 |
| **Tmem97** | 2537.39495 | 2559.798549 | 2513.947755 | 2397.191577 | 2472.650106 | 2469.674489 | 2491.502742 | 2202.458506 | 2332.48378 | 2349.048492 | 2528.312378 | 2349.266112 | 2384.983998 | 2399.841155 | 2364.081061 |
| **Nlk** | 497.2231895 | 444.4589868 | 544.2970997 | 470.9847431 | 452.2213564 | 502.2115689 | 510.5686286 | 533.0150723 | 533.4616129 | 475.4026711 | 470.0977373 | 458.9223682 | 475.4630479 | 473.4626374 | 486.4689599 |
| **Fam58b** | 328.7350369 | 281.253013 | 303.8665823 | 306.226344 | 333.1490665 | 334.5739083 | 316.6247149 | 322.658495 | 314.5091032 | 325.9654344 | 259.1685715 | 333.9983209 | 294.821173 | 334.3152211 | 334.1330375 |
| **Lyrm9** | 124.5347215 | 75.26489081 | 122.1731619 | 119.9027093 | 132.3729181 | 129.0599563 | 120.8766718 | 125.711102 | 134.6821279 | 129.3374914 | 137.1512513 | 104.1033728 | 112.4751296 | 115.6549954 | 104.1920224 |
| **Nos2** | 20.14532259 | 14.26071615 | 3.132645178 | 9.488703616 | 2.533452977 | 5.611302446 | 9.922711863 | 10.89496217 | 6.019312981 | 1.747803938 | 0.945870699 | 4.337640531 | 9.372927467 | 2.710663955 | 6.467091048 |
| **Lgals9** | 2648.194225 | 2498.794375 | 2289.963625 | 2278.151477 | 2456.182661 | 2783.206013 | 2984.932141 | 1806.049498 | 1984.868455 | 1712.847859 | 1922.00926 | 2418.66836 | 2335.563108 | 2361.891859 | 2466.117386 |
| **Ksr1** | 472.4993845 | 375.532192 | 336.7593566 | 352.8072526 | 327.4487973 | 321.247065 | 349.0990446 | 434.1223388 | 461.9822713 | 400.2471018 | 422.8042024 | 343.5411301 | 323.7920398 | 381.300063 | 359.282836 |
| **Gm11201** | 0 | 4.753572051 | 4.698967767 | 0 | 3.800179466 | 2.805651223 | 1.80412943 | 0 | 1.504828245 | 0.873901969 | 1.891741398 | 0.867528106 | 0 | 2.710663955 | 2.874262688 |
| **Wsb1** | 1082.353241 | 1240.682305 | 1238.178007 | 1239.569736 | 1164.121643 | 1239.396428 | 1152.838705 | 1103.743475 | 1161.727405 | 1147.433285 | 1151.12464 | 1153.812381 | 1215.924318 | 1221.605889 | 1241.681481 |
| **Nf1** | 314.9995897 | 286.7988471 | 265.4916788 | 295.8750309 | 191.2756998 | 248.3001332 | 253.4801849 | 284.9451644 | 259.5828723 | 250.8098651 | 215.6585193 | 261.12596 | 241.1398612 | 190.6500315 | 265.150733 |
| **Omg** | 0.915696482 | 2.376786026 | 0 | 0 | 0 | 0 | 1.80412943 | 1.676148026 | 6.771727103 | 0 | 4.729353494 | 0 | 1.70416863 | 2.710663955 | 0.718565672 |
| **Evi2a** | 175.8137245 | 186.181572 | 196.5734849 | 175.1097122 | 212.1766868 | 223.750685 | 206.5728197 | 168.4528766 | 115.8717749 | 151.1850406 | 182.5530449 | 194.3262958 | 201.9439827 | 248.4775292 | 237.8452374 |
| **Rab11fip4** | 237.1653887 | 251.1470567 | 234.9483883 | 238.0801998 | 257.7788404 | 267.9396918 | 254.3822496 | 252.2602779 | 171.5504199 | 194.8801391 | 208.0915537 | 243.7753979 | 221.5419219 | 278.2948327 | 235.6895404 |
| **Rab11fip4os1** | 3.662785926 | 3.961310043 | 2.349483883 | 0.86260942 | 6.333632443 | 0 | 3.608258859 | 0.838074013 | 1.504828245 | 0 | 0.945870699 | 1.735056213 | 11.0770961 | 3.614218606 | 7.904222392 |
| **Utp6** | 2208.659914 | 2182.681833 | 2315.024787 | 2160.836596 | 2100.232518 | 2163.157093 | 2211.862681 | 2722.902468 | 2403.963122 | 2465.277454 | 2644.654474 | 2131.516557 | 2176.223341 | 2026.673083 | 1965.277113 |
| **Suz12** | 4675.546235 | 4748.026217 | 4973.857381 | 4779.718794 | 4614.051235 | 4680.527653 | 4807.102865 | 5012.520672 | 4991.515289 | 4880.742496 | 5166.345757 | 5047.278522 | 5129.547577 | 4803.296528 | 5027.085441 |
| **Crlf3** | 1813.99473 | 1849.93179 | 1921.094655 | 1844.258939 | 1892.489374 | 1920.468262 | 1812.248012 | 1863.876605 | 1866.739438 | 1836.068037 | 1748.914922 | 1889.476215 | 1903.55636 | 1918.246525 | 1885.516323 |
| **Atad5** | 966.9754845 | 1032.317397 | 1027.507618 | 1004.077364 | 887.3419052 | 769.4498479 | 999.487704 | 1226.940355 | 1244.492959 | 1095.873069 | 1089.643045 | 937.7978829 | 975.6365409 | 882.7728946 | 873.7758571 |
| **Tefm** | 194.1276541 | 198.8577641 | 177.7776138 | 181.1479781 | 191.909063 | 180.2630911 | 201.1604314 | 242.2033898 | 167.7883493 | 244.6925513 | 232.6841919 | 203.869105 | 180.6418748 | 156.3149547 | 194.7312971 |
| **Rnf135** | 75.08711149 | 80.81072487 | 81.44877463 | 71.59658183 | 63.33632443 | 69.43986777 | 71.26311247 | 75.42666117 | 91.79452295 | 61.17313782 | 66.21094891 | 78.07752957 | 95.4334433 | 64.15238026 | 51.73672838 |
| **Rhot1** | 748.1240254 | 759.7792662 | 827.018327 | 709.9275524 | 768.9029786 | 783.478104 | 836.2139906 | 834.721717 | 816.369323 | 871.280263 | 943.9789574 | 872.7332749 | 891.2801937 | 862.8946923 | 907.5484437 |
| **Rhbdl3** | 26.55519797 | 24.56012226 | 28.1938066 | 28.46611085 | 27.2346195 | 34.36922748 | 29.76813559 | 62.01747697 | 23.3248378 | 53.3080201 | 52.96875913 | 45.11146153 | 33.23128829 | 24.39597559 | 26.58692986 |
| **5730455P16Rik** | 332.3978228 | 294.7214672 | 325.0119372 | 296.7376404 | 317.9483486 | 302.3089193 | 301.2896147 | 278.2405723 | 313.004275 | 312.8569049 | 315.9208134 | 346.1437144 | 312.7149437 | 282.8126059 | 320.4802897 |
| **Zfp207** | 4181.070135 | 3982.701117 | 4450.705637 | 4261.290533 | 4135.228622 | 4186.031624 | 4580.684622 | 4234.787988 | 4145.049401 | 4144.917039 | 4186.423713 | 4368.871543 | 4239.119468 | 4155.447843 | 4311.394032 |
| **Psmd11** | 2767.234767 | 2526.523545 | 2651.000982 | 2568.850852 | 2544.220152 | 2539.114357 | 2514.956425 | 2986.895783 | 2952.473017 | 2758.034614 | 2878.284536 | 2483.732968 | 2609.934257 | 2449.53666 | 2640.728844 |
| **Cdk5r1** | 26.55519797 | 37.2363144 | 32.89277437 | 8.626094197 | 14.56735462 | 28.75792503 | 17.13922958 | 45.2559967 | 15.04828245 | 27.964863 | 24.59263817 | 15.61550591 | 21.30210788 | 18.07109303 | 20.11983882 |
| **Myo1d** | 2.747089445 | 6.338096068 | 6.265290356 | 0.86260942 | 6.333632443 | 9.118366474 | 10.82477658 | 2.514222039 | 3.762070613 | 5.243411813 | 1.891741398 | 8.675281063 | 4.260421576 | 9.035546516 | 5.029959704 |
| **Ccl1** | 215.1886732 | 291.5524191 | 283.5043886 | 336.4176737 | 437.0206386 | 394.1939968 | 314.8205855 | 1207.664653 | 582.3685309 | 525.2150833 | 585.4939625 | 377.3747262 | 366.3962555 | 403.8889293 | 380.1212405 |
| **Cct6b** | 6.409875371 | 6.338096068 | 1.566322589 | 5.175656518 | 3.800179466 | 2.805651223 | 0.902064715 | 4.190370065 | 4.514484735 | 1.747803938 | 3.783482795 | 3.470112425 | 3.408337261 | 0.903554652 | 2.874262688 |
| **Zfp830** | 361.7001102 | 316.1125414 | 379.0500665 | 357.9829092 | 306.5478102 | 397.7010608 | 310.3102619 | 401.4374523 | 357.3967082 | 321.5959246 | 401.0491763 | 343.5411301 | 334.0170515 | 283.7161606 | 299.6418852 |
| **Gm11423** | 3.662785926 | 9.507144102 | 10.18109683 | 8.626094197 | 8.86708542 | 13.32684331 | 3.608258859 | 7.542666117 | 6.019312981 | 5.243411813 | 7.56696559 | 7.807752957 | 5.112505891 | 11.74621047 | 4.311394032 |
| **Lig3** | 988.9522001 | 1008.549537 | 998.5306505 | 920.4042508 | 885.4418155 | 977.7694512 | 1130.287088 | 1107.933845 | 1092.505306 | 1008.482872 | 1102.885235 | 914.374624 | 1036.134527 | 890.9048865 | 1024.674648 |
| **Rffl** | 272.8775515 | 315.3202794 | 311.6981952 | 228.5914962 | 253.978661 | 272.8495814 | 250.7739907 | 280.7547944 | 275.3835689 | 274.4052182 | 281.8694682 | 284.5492189 | 240.2877769 | 273.7770594 | 257.2465106 |
| **Rad51d** | 419.3889886 | 466.642323 | 448.7514217 | 401.9759896 | 398.3854806 | 399.1038865 | 453.7385515 | 558.1572927 | 498.8505633 | 519.0977695 | 433.20878 | 387.7850635 | 452.4567714 | 426.4777955 | 437.6064942 |
| **Fndc8** | 0 | 0 | 3.132645178 | 0 | 0 | 0 | 0.902064715 | 0 | 0 | 1.747803938 | 1.891741398 | 3.470112425 | 0 | 5.421327909 | 5.029959704 |
| **Nle1** | 1005.434737 | 1027.563825 | 1065.099361 | 951.4581899 | 989.3133876 | 1062.640401 | 986.858798 | 1112.124215 | 1064.665983 | 1070.529912 | 1092.480657 | 862.3229376 | 956.0386016 | 891.8084411 | 1026.830345 |
| **Slfn5** | 340.6390911 | 155.2833537 | 111.2089038 | 87.9861608 | 151.3738154 | 257.4184997 | 310.3102619 | 26.81836842 | 56.43105919 | 34.08217679 | 58.64398332 | 97.1631479 | 126.960563 | 113.8478861 | 157.3658822 |
| **Slfn9** | 355.2902348 | 339.0881396 | 288.9865177 | 325.2037512 | 300.847541 | 366.1374846 | 400.5167334 | 295.8401266 | 281.4028818 | 282.270336 | 302.6786236 | 325.3230399 | 291.4128358 | 307.2085815 | 335.5701688 |
| **Slfn8** | 1275.565199 | 877.8263054 | 775.3296815 | 739.2562726 | 936.110875 | 1151.018414 | 1308.895901 | 349.4768634 | 477.0305537 | 382.7690624 | 484.2857978 | 923.0499051 | 932.1802408 | 887.2906678 | 926.2311512 |
| **Slfn10-ps** | 291.1914811 | 221.8333624 | 247.4789691 | 220.8280114 | 195.0758792 | 216.736557 | 280.5421263 | 201.9758371 | 252.8111452 | 213.2320804 | 201.4704588 | 209.9418017 | 270.9628122 | 169.8682745 | 224.1924897 |
| **Slfn2** | 4049.209841 | 4050.043387 | 3849.237762 | 3726.472693 | 4674.220743 | 4850.970964 | 4949.62909 | 3489.74019 | 3391.882865 | 2833.190183 | 3207.44754 | 4473.842444 | 4287.688274 | 4568.372318 | 4366.723589 |
| **Slfn1** | 2637.205867 | 2233.386602 | 2105.13756 | 1725.218839 | 2309.242389 | 2842.826102 | 2901.040123 | 895.0630459 | 1149.688779 | 940.3185185 | 1180.446632 | 2500.216002 | 2251.206761 | 2550.734781 | 2411.506395 |
| **Slfn4** | 4.578482408 | 3.961310043 | 1.566322589 | 2.587828259 | 2.533452977 | 3.507064029 | 3.608258859 | 3.352296052 | 0 | 0 | 0 | 3.470112425 | 5.112505891 | 0 | 2.155697016 |
| **Slfn3** | 471.583688 | 462.681013 | 409.593357 | 418.3655685 | 386.9849423 | 504.3158073 | 580.0276116 | 265.6694621 | 290.4318513 | 270.9096104 | 357.5391241 | 415.5459629 | 382.5858575 | 385.8178362 | 434.0136659 |
| **AI662270** | 2027.35201 | 2074.141938 | 2130.981882 | 1931.382491 | 2254.139786 | 2209.450338 | 2229.00191 | 2356.664125 | 2483.719019 | 2380.508963 | 2372.243713 | 2324.107797 | 2142.992053 | 2221.840888 | 2195.218128 |
| **AA465934** | 10.0726613 | 10.29940611 | 12.53058071 | 10.35131304 | 5.066905954 | 4.90988964 | 9.020647148 | 20.11377631 | 16.5531107 | 10.48682363 | 12.29631908 | 6.94022485 | 9.372927467 | 10.84265582 | 3.59282836 |
| **AI450353** | 7.325571853 | 7.130358077 | 6.265290356 | 7.763484777 | 5.066905954 | 7.014128057 | 6.314453003 | 18.43762829 | 10.53379772 | 20.09974528 | 6.621094891 | 5.205168638 | 8.520843152 | 3.614218606 | 5.029959704 |
| **Pex12** | 156.5840983 | 192.5196681 | 169.1628396 | 155.2696955 | 173.5415289 | 163.4291837 | 205.670755 | 215.3850214 | 172.3028341 | 183.5194135 | 185.390657 | 150.0823624 | 198.5356454 | 204.2033513 | 191.1384687 |
| **Ap2b1** | 3651.797568 | 3309.27841 | 3196.081243 | 3409.895036 | 3293.48887 | 3305.057141 | 3577.588659 | 3146.129845 | 3145.843447 | 3137.308068 | 3248.11998 | 3404.180289 | 3387.887237 | 3202.197685 | 3384.444315 |
| **Rasl10b** | 0 | 0 | 0 | 1.725218839 | 0 | 0 | 0 | 0 | 0 | 4.369509845 | 0 | 0 | 0 | 0 | 0.718565672 |
| **1700020L24Rik** | 0.915696482 | 6.338096068 | 6.265290356 | 8.626094197 | 8.233722176 | 11.22260489 | 7.216517718 | 7.542666117 | 16.5531107 | 13.9824315 | 1.891741398 | 13.8804497 | 5.112505891 | 9.035546516 | 2.874262688 |
| **Taf15** | 2934.807223 | 2879.872401 | 2913.360015 | 2679.264857 | 2786.164912 | 2695.529412 | 2932.612388 | 2871.241569 | 2593.571481 | 2574.5152 | 2645.600344 | 2873.253088 | 2724.113556 | 2783.851881 | 2692.465573 |
| **Heatr9** | 5.494178889 | 2.376786026 | 3.132645178 | 2.587828259 | 9.500448664 | 6.312715251 | 3.608258859 | 2.514222039 | 0 | 1.747803938 | 2.837612096 | 6.072696744 | 2.556252945 | 7.228437213 | 4.311394032 |
| **Ccl5** | 249.069443 | 202.8190742 | 188.741872 | 158.7201332 | 286.2801864 | 303.7117449 | 386.0836979 | 100.5688816 | 62.45037217 | 71.65996145 | 84.18249219 | 269.8012411 | 254.7732102 | 232.2135455 | 294.6119255 |
| **Ccl9** | 20.14532259 | 12.67619214 | 28.9769679 | 45.71829924 | 22.16771355 | 25.25086101 | 25.25781201 | 173.4813207 | 54.92623095 | 58.55143192 | 88.91184568 | 49.44910206 | 23.85836082 | 43.37062328 | 18.68270747 |
| **Ccl3** | 4856.854138 | 3630.144523 | 4056.775505 | 4090.493868 | 2531.552887 | 2501.238065 | 2871.271987 | 5071.185853 | 4176.650794 | 3440.552052 | 4069.135746 | 2427.343641 | 2575.850885 | 2517.303259 | 2762.166443 |
| **Ccl4** | 3964.965765 | 2803.815248 | 2903.96208 | 2915.619838 | 2501.784815 | 2335.00323 | 2893.823605 | 2922.364084 | 2574.008713 | 2134.068608 | 2621.007706 | 2258.175661 | 2304.035988 | 2325.749673 | 2506.357064 |
| **Heatr6** | 698.6764154 | 740.764978 | 738.5211007 | 644.3692365 | 658.0644108 | 671.2520551 | 706.3166717 | 780.2469062 | 739.6230825 | 703.491085 | 693.3232222 | 686.2147321 | 728.5320895 | 693.0264178 | 717.8471063 |
| **Ddx52** | 1513.646284 | 1498.95972 | 1534.212976 | 1450.909044 | 1458.002188 | 1343.906936 | 1434.282897 | 1801.021054 | 1704.217988 | 1684.882996 | 1612.709541 | 1414.938341 | 1515.857997 | 1573.088648 | 1307.789523 |
| **Synrg** | 803.0658143 | 783.5471264 | 822.3193592 | 821.2041675 | 772.0697948 | 763.8385454 | 880.4151616 | 930.2621545 | 900.6397047 | 740.1949677 | 832.3662149 | 818.9465323 | 855.4926524 | 814.1027411 | 811.2606436 |

| | | | | | | | | | | | | | | | |
|---|---|---|---|---|---|---|---|---|---|---|---|---|---|---|---|
| **Dusp14** | 76.00280797 | 47.53572051 | 48.55600026 | 56.06961228 | 39.90188439 | 42.08476834 | 37.88671802 | 79.61703124 | 45.14484735 | 78.6511772 | 74.7237852 | 59.85943933 | 40.04796281 | 51.50261514 | 35.20971793 |
| **Tada2a** | 362.6158067 | 325.6196855 | 346.1572922 | 343.318549 | 302.7476308 | 319.8442394 | 327.4494915 | 367.9144917 | 386.740859 | 395.0036899 | 455.9096768 | 321.8529274 | 337.4253888 | 361.4218606 | 375.0912808 |
| **Acaca** | 3288.266065 | 3224.506375 | 3246.203566 | 3363.314127 | 2857.734958 | 2755.149501 | 3159.932696 | 3748.70506 | 3674.790575 | 3348.792345 | 3595.254526 | 3077.989721 | 3096.474401 | 2682.653761 | 2903.72388 |
| **Aatf** | 1081.437545 | 1044.201327 | 1194.320974 | 1096.376572 | 1015.914644 | 1056.327685 | 1131.189152 | 1277.224796 | 1093.25772 | 1138.694265 | 1168.150313 | 1083.542605 | 1054.880382 | 983.0674609 | 1041.920224 |
| **Mrm1** | 380.9297363 | 360.4792139 | 339.1088405 | 326.92897 | 292.6138189 | 305.1145705 | 281.444191 | 303.3827927 | 326.5477292 | 275.2791202 | 322.5419083 | 299.2971967 | 315.2711966 | 250.2846385 | 273.0549553 |
| **Dhrs11** | 160.2468843 | 161.6214497 | 217.7188399 | 199.2627759 | 211.5433236 | 187.2772191 | 173.1964252 | 212.8707993 | 212.9331967 | 196.627943 | 235.521804 | 163.095284 | 173.8252003 | 179.8073757 | 198.3241255 |
| **4930502E09Rik** | 0 | 0 | 0.783161294 | 1.725218839 | 0 | 2.805651223 | 0.902064715 | 2.514222039 | 0 | 0 | 1.891741398 | 0 | 0 | 0 | 2.155697016 |
| **Ggnbp2** | 1927.541094 | 1891.921676 | 1910.913559 | 1662.248352 | 1806.985336 | 1847.52133 | 1976.42379 | 1716.375579 | 1793.002854 | 1857.041684 | 1846.339604 | 1834.821945 | 1782.560387 | 1803.495085 | 1764.078725 |
| **Ggnbp2os** | 6.409875371 | 8.714882094 | 5.482129061 | 8.626094197 | 12.03390164 | 10.52119209 | 13.53097072 | 7.542666117 | 9.028969471 | 5.243411813 | 9.458706988 | 5.205168638 | 9.372927467 | 10.84265582 | 12.21561642 |
| **Pigw** | 89.73825519 | 103.7863231 | 122.1731619 | 83.67311371 | 90.57094393 | 98.89920561 | 89.30440676 | 108.9496217 | 109.8524619 | 96.12921658 | 66.21094891 | 91.95797927 | 81.80009426 | 57.8274977 | 63.23377913 |
| **Myo19** | 314.9995897 | 324.8274235 | 309.3487113 | 311.4020005 | 267.9126523 | 232.1676387 | 263.4028967 | 384.675972 | 428.8760499 | 389.7602781 | 392.53634 | 255.9207914 | 278.6315711 | 282.8126059 | 295.3304912 |
| **Znhit3** | 319.5780721 | 255.1083667 | 339.1088405 | 310.5393911 | 271.0794686 | 282.6693607 | 299.4854853 | 346.9626414 | 376.2070613 | 369.6605328 | 412.3996247 | 255.9207914 | 257.3294632 | 291.8481525 | 344.9115225 |
| **Usp32** | 423.967471 | 472.1881571 | 466.7641315 | 499.450854 | 422.4532839 | 430.6674627 | 464.5633281 | 484.4067795 | 504.8698763 | 522.5933774 | 496.5821169 | 583.8464155 | 490.8005655 | 488.8230665 | 500.8402734 |
| **Appbp2** | 872.6587469 | 850.0971351 | 945.2756824 | 919.5416413 | 723.9341882 | 792.5964705 | 819.074761 | 1040.887924 | 928.4790273 | 917.5970673 | 978.0303025 | 873.600803 | 889.576025 | 702.0619643 | 830.6619168 |
| **Appbp2os** | 4.578482408 | 5.54583406 | 5.482129061 | 9.488703616 | 1.900089733 | 4.208476834 | 7.216517718 | 5.028444078 | 11.28621184 | 2.621705907 | 1.891741398 | 1.735056213 | 5.964590206 | 0.903554652 | 7.18565672 |
| **Ppm1d** | 511.8743332 | 548.2453099 | 552.9118739 | 541.7187155 | 492.1232408 | 466.4395158 | 480.800493 | 612.6321035 | 682.4396092 | 630.0833196 | 558.0637123 | 450.2470872 | 510.3985048 | 454.4879897 | 430.4208375 |
| **Bcas3** | 247.23805 | 222.6256244 | 206.7545817 | 192.3619006 | 209.0098706 | 205.5139521 | 244.4595377 | 199.4616151 | 252.0587311 | 209.7364725 | 198.6328467 | 214.2794423 | 202.796067 | 242.1526466 | 241.4380658 |
| **Tbx2** | 1.831392963 | 0 | 0.783161294 | 3.450437679 | 0 | 0.701412806 | 0 | 2.514222039 | 0 | 0.873901969 | 1.891741398 | 0.867528106 | 0 | 0 | 5.748525376 |
| **Brip1** | 645.5660195 | 640.9399649 | 643.7585841 | 692.675364 | 661.8645903 | 645.2997813 | 663.0175654 | 737.5051315 | 687.706508 | 659.7959865 | 675.3516789 | 628.0903489 | 679.1111992 | 700.254855 | 704.1943585 |
| **Brip1os** | 1489.838176 | 1621.760331 | 1557.707815 | 1431.931637 | 1508.671248 | 1461.744287 | 1482.092326 | 1560.493812 | 1522.13377 | 1507.480896 | 1475.55829 | 1411.468229 | 1328.399447 | 1419.484358 | 1351.622029 |
| **Ints2** | 825.9582264 | 843.7590391 | 785.5107784 | 770.3102117 | 747.3686282 | 727.3650795 | 758.6364251 | 895.0630459 | 889.3534929 | 830.2068705 | 933.5743797 | 768.6299022 | 836.7467975 | 808.6814132 | 796.8893302 |
| **Med13** | 1502.657926 | 1401.511493 | 1551.442524 | 1494.039515 | 1311.695279 | 1272.36283 | 1498.329491 | 1438.97308 | 1479.246165 | 1219.967149 | 1308.139176 | 1471.327668 | 1443.43083 | 1335.453775 | 1232.340127 |
| **Rnft1** | 481.6563493 | 507.8399475 | 559.9603256 | 560.6961228 | 520.6245868 | 529.5666683 | 517.7851463 | 566.5380328 | 630.5230347 | 555.8016522 | 577.9269969 | 570.8334939 | 601.5715265 | 607.1887259 | 538.2056883 |
| **Rps6kb1** | 1262.745448 | 1267.619214 | 1394.027104 | 1324.968069 | 1228.724694 | 1308.836295 | 1334.153713 | 1211.016949 | 1201.605354 | 1384.260719 | 1300.572211 | 1309.099912 | 1368.44741 | 1344.489322 | 1348.747766 |
| **Tubd1** | 122.7033285 | 149.7375196 | 137.8363878 | 125.0783658 | 165.3078068 | 147.998102 | 144.3303544 | 155.0436924 | 106.0903913 | 95.25531461 | 135.2595099 | 147.4797781 | 140.593912 | 149.0865175 | 122.8747299 |
| **Vmp1** | 1049.388168 | 1075.891808 | 1052.56878 | 1048.933054 | 1055.816528 | 1010.735853 | 957.0906624 | 1071.896663 | 1100.029447 | 1070.529912 | 1012.081648 | 1041.901256 | 1037.838696 | 1056.255388 | 979.4050109 |
| **Ptrh2** | 433.1244358 | 404.8458863 | 377.4837439 | 441.6560229 | 426.2534634 | 413.8335554 | 423.9704159 | 455.0741891 | 435.647777 | 458.7985337 | 447.3968405 | 400.7979851 | 414.9650615 | 384.0107269 | 352.8157449 |
| **Cltc** | 8150.614382 | 7631.859928 | 7750.947332 | 8312.966977 | 7018.931473 | 6826.850838 | 7711.751247 | 9185.291183 | 8881.496303 | 8303.816509 | 8892.130439 | 7729.675427 | 7937.165396 | 7247.41186 | 7493.202827 |
| **Dhx40** | 552.1649784 | 564.0905501 | 561.5266481 | 559.8335134 | 505.4238689 | 524.6567787 | 510.5686286 | 473.5118174 | 592.1499145 | 503.3675341 | 558.0637123 | 549.1452913 | 581.1215029 | 564.7216572 | 533.8942943 |
| **Ypel2** | 35.71216278 | 30.89821833 | 43.85703249 | 40.54264272 | 25.96789302 | 25.95227381 | 27.96400616 | 31.8468125 | 29.34415078 | 34.08217679 | 45.40179354 | 42.50887721 | 27.26669809 | 34.33507676 | 42.39537465 |
| **Gdpd1** | 27.47089445 | 32.48274235 | 28.1938066 | 20.70262607 | 19.00089733 | 14.72966892 | 21.64955315 | 55.31288486 | 39.12553437 | 42.82119648 | 19.86328467 | 18.21809023 | 17.89377062 | 29.8173035 | 27.30549553 |
| **Smg8** | 897.3825519 | 773.2477203 | 814.4877463 | 802.2267603 | 828.4391235 | 765.9427838 | 887.6316793 | 851.4831973 | 942.7748956 | 894.0017142 | 953.4376644 | 749.5442838 | 832.4863759 | 771.6356724 | 845.7517959 |
| **Prr11** | 884.5628012 | 945.9608382 | 986.0000698 | 954.9086276 | 938.644328 | 913.239473 | 1015.724869 | 916.8529703 | 925.4693708 | 954.30095 | 960.0587593 | 971.631479 | 1025.909515 | 983.0674609 | 947.7881213 |
| **Ska2** | 373.6041645 | 518.9316156 | 488.6926478 | 445.96907 | 490.8565143 | 477.6621207 | 502.4500461 | 516.253592 | 472.516069 | 464.9158475 | 465.3683838 | 490.15338 | 476.3151322 | 491.5337305 | 472.0976465 |
| **Mir301** | 0.915696482 | 0.792262009 | 0 | 0 | 0.633363244 | 0.701412806 | 0 | 1.676148026 | 0 | 0 | 0 | 0 | 1.70416863 | 0.903554652 | 0 |
| **Trim37** | 975.2167529 | 1014.095371 | 1077.629941 | 1057.559148 | 981.7130286 | 859.9320998 | 938.1473034 | 1304.881238 | 1179.03293 | 1289.879306 | 1240.036486 | 975.9691196 | 1028.465768 | 1016.498983 | 1051.261578 |
| **Ppm1e** | 4.578482408 | 12.67619214 | 5.482129061 | 12.07653188 | 9.500448664 | 5.611302446 | 4.510323574 | 5.028444078 | 16.5531107 | 20.97364725 | 26.48437957 | 9.542809169 | 5.964590206 | 0 | 1.437131344 |
| **Rad51c** | 43.03773463 | 61.79643666 | 58.73709709 | 43.9930804 | 58.26941847 | 58.21726287 | 49.61355931 | 64.53169901 | 63.95520042 | 52.43411813 | 54.86050053 | 68.5347204 | 54.53339617 | 66.86304422 | 65.38947615 |
| **Tex14** | 3.662785926 | 1.584524017 | 5.482129061 | 1.725218839 | 3.166816221 | 0.701412806 | 3.608258859 | 8.380740131 | 0 | 0 | 4.729353494 | 0.867528106 | 0 | 3.614218606 | 2.874262688 |
| **Sept4** | 0 | 3.961310043 | 2.349483883 | 1.725218839 | 5.700269198 | 3.507064029 | 0.902064715 | 5.028444078 | 4.514484735 | 5.243411813 | 3.783482795 | 5.205168638 | 10.22501178 | 0 | 8.622788064 |
| **Mtmr4** | 545.755103 | 457.9274409 | 554.4781965 | 609.0022503 | 529.4916722 | 561.1302446 | 543.945023 | 694.7633568 | 687.706508 | 668.5350062 | 685.7562566 | 602.9320339 | 616.0569599 | 557.49322 | 538.2056883 |
| **Rnf43** | 131.8602933 | 139.4381135 | 140.969033 | 147.5062108 | 110.2052045 | 112.9274617 | 133.5055778 | 93.86428946 | 93.2993512 | 105.7421382 | 189.1741398 | 131.8642722 | 138.8897434 | 150.8936268 | 176.0485896 |
| **Supt4a** | 1404.678403 | 1376.159109 | 1348.603749 | 1481.962983 | 1387.698868 | 1393.707245 | 1290.854607 | 1725.594393 | 1686.912463 | 1732.073702 | 1488.80048 | 1539.862389 | 1357.370314 | 1394.184827 | 1272.579805 |
| **Mir142b** | 10.98835778 | 9.507144102 | 21.14535495 | 16.38957897 | 12.03390164 | 9.81977928 | 18.0412943 | 12.5711102 | 11.28621184 | 12.23462756 | 17.97154328 | 17.35056213 | 17.89377062 | 11.74621047 | 9.341353736 |
| **Bzrap1** | 30.21798389 | 39.61310043 | 46.98967767 | 33.64176737 | 46.86888008 | 43.48759395 | 73.96930661 | 62.01747697 | 47.40208972 | 61.17313782 | 47.29353494 | 71.13730471 | 77.53967268 | 59.634607 | 77.60509257 |
| **Mks1** | 112.6306672 | 97.44822705 | 116.6910329 | 124.2157564 | 88.6708542 | 108.0175721 | 120.8766718 | 119.0065099 | 118.8814314 | 143.3199229 | 134.3136392 | 129.2616878 | 97.98969624 | 127.4012059 | 104.9105881 |
| **Dynll2** | 2572.191417 | 2501.963423 | 2534.309949 | 2437.73422 | 2367.511807 | 2429.693959 | 2416.631371 | 2664.237287 | 2826.067444 | 2776.386555 | 2543.446309 | 2547.930048 | 2450.59449 | 2644.704465 | 2376.296677 |
| **Srsf1** | 9885.859215 | 10136.9924 | 10078.5027 | 10182.24159 | 10035.64061 | 10049.84268 | 10442.30114 | 11270.41933 | 10901.72822 | 10901.92706 | 11106.41374 | 10858.84931 | 10910.08757 | 10318.59412 | 10222.31525 |
| **Vezf1** | 703.2548978 | 750.2721221 | 764.3654234 | 690.9501451 | 661.8645903 | 738.5876844 | 839.8222495 | 597.5467713 | 629.7706206 | 547.9365345 | 597.7902816 | 820.6815885 | 696.1528855 | 791.5138748 | 773.8952287 |
| **Cuedc1** | 69.5929326 | 83.18751089 | 64.21922615 | 59.52004996 | 57.00269198 | 103.8090952 | 91.10853619 | 42.74177467 | 78.25106875 | 48.06460829 | 66.21094891 | 73.73988903 | 58.79381775 | 74.99503608 | 65.38947615 |
| **Mrps23** | 1210.550749 | 1188.393013 | 1252.27491 | 1255.959315 | 1274.326847 | 1227.47241 | 1240.338983 | 1333.375755 | 1324.248856 | 1260.166639 | 1311.922659 | 1270.928676 | 1213.368065 | 1238.773427 | 1184.914793 |
| **Msi2** | 1285.63786 | 1164.625153 | 1297.698265 | 1186.950561 | 1160.954827 | 1199.415898 | 1393.689984 | 1138.10451 | 1199.348111 | 1104.612089 | 1188.959468 | 1249.240473 | 1315.618183 | 1212.570342 | 1245.992875 |
| **Mir378b** | 4.578482408 | 1.584524017 | 4.698967767 | 3.450437679 | 0 | 0 | 0.902064715 | 0 | 0 | 0 | 0 | 0 | 0 | 0 | 0 |
| **0610039H22Rik** | 3.662785926 | 1.584524017 | 11.74741942 | 7.763484777 | 5.700269198 | 4.90988964 | 6.314453003 | 3.352296052 | 1.504828245 | 6.991215751 | 4.729353494 | 2.602584319 | 4.260421576 | 1.807109303 | 9.341353736 |
| **C030037D09Rik** | 18.31392963 | 18.2220262 | 13.31374201 | 1.725218839 | 8.233722176 | 7.014128057 | 14.43303544 | 5.866518091 | 12.79104008 | 13.10852953 | 2.837612096 | 16.48303402 | 5.112505891 | 25.29953024 | 17.9641418 |
| **Akap1** | 1004.51904 | 989.5352486 | 1051.002457 | 853.9833255 | 924.7103366 | 945.5044621 | 989.5649921 | 1184.19858 | 1220.415707 | 1121.216226 | 1153.016382 | 954.2809169 | 956.0386016 | 954.1537121 | 914.7341004 |
| **Scpep1os** | 0 | 2.376786026 | 1.566322589 | 0 | 0 | 0 | 4.510323574 | 0 | 0 | 0 | 0 | 1.735056213 | 0 | 4.517773258 | 0.718565672 |
| **Scpep1** | 1713.268117 | 1714.454986 | 1678.314654 | 1641.545726 | 1717.047755 | 1804.735149 | 1701.294052 | 1350.975309 | 1324.248856 | 1376.395601 | 1419.751919 | 1755.009359 | 1699.056124 | 1743.860478 | 1667.072359 |
| **Coil** | 452.3540619 | 456.3429169 | 459.7156799 | 476.1603996 | 459.8217153 | 429.2646371 | 467.2695223 | 508.7109259 | 498.0981491 | 456.1768278 | 485.2316685 | 433.7640531 | 437.1192537 | 361.4218606 | 411.0195644 |
| **Trim25** | 4010.750589 | 3390.089134 | 3329.218663 | 2996.705124 | 3343.524567 | 3636.825398 | 4223.466995 | 2099.375403 | 2652.259782 | 2481.00769 | 2517.9078 | 3364.273996 | 3323.980913 | 3490.431619 | 3489.354903 |
| **Dgke** | 439.5343112 | 447.6280348 | 406.4607118 | 442.5186323 | 433.8538223 | 385.7770431 | 379.7692449 | 400.5993782 | 394.2650002 | 411.6078274 | 370.7813139 | 404.2680975 | 461.8296988 | 438.224006 | 422.5166151 |
| **Pctp** | 44.8691276 | 49.91250654 | 33.67593566 | 30.19132969 | 36.10170492 | 35.77205309 | 30.6702003 | 15.08533223 | 24.07725192 | 43.69509845 | 49.18527634 | 38.17123668 | 29.82295103 | 35.23863141 | 40.23967763 |
| **Mmd** | 526.5254769 | 591.8197204 | 621.0469065 | 585.7117959 | 656.1643211 | 669.1478166 | 674.7444067 | 741.6955016 | 792.2920711 | 776.0249484 | 712.2406362 | 673.2018105 | 708.9341502 | 744.5290329 | 692.6973078 |
| **Hlf** | 8.241268334 | 10.29940611 | 9.397935534 | 6.900875357 | 13.30062813 | 8.416953669 | 4.510323574 | 3.352296052 | 3.00965649 | 6.117313782 | 5.675224193 | 1.735056213 | 17.0416863 | 0 | 10.05991941 |
| **Stxbp4** | 33.88076982 | 19.80655021 | 37.59174214 | 21.56523549 | 39.90188439 | 32.96640187 | 34.27845916 | 48.60829276 | 33.85863552 | 55.92972601 | 41.61831075 | 33.83359614 | 46.01255302 | 30.72085815 | 32.33545524 |
| **Cox11** | 261.8891937 | 281.253013 | 265.4916788 | 217.3775738 | 248.2783918 | 278.4608839 | 263.4028967 | 274.8882763 | 255.8208017 | 289.2615517 | 260.1144422 | 255.0532632 | 308.4545221 | 250.2846385 | 237.1266717 |
| **Tom1l1** | 2.747089445 | 0.792262009 | 1.566322589 | 0.86260942 | 1.266726489 | 3.507064029 | 0.902064715 | 0.838074013 | 0.752414123 | 0.873901969 | 0 | 3.470112425 | 2.556252945 | 3.614218606 | 1.437131344 |
| **Utp18** | 1903.732985 | 1716.03951 | 1802.8373 | 1686.401415 | 1767.716815 | 1797.019608 | 1759.928259 | 1983.721189 | 1824.604247 | 1911.223606 | 1865.257018 | 1750.671718 | 1858.395891 | 1605.616616 | 1700.844946 |
| **Mbtd1** | 602.5282849 | 644.9012749 | 637.4932937 | 547.7569815 | 624.4961589 | 568.1443726 | 663.0175654 | 609.2798075 | 606.4457828 | 539.1975148 | 630.8957561 | 700.0951818 | 673.9986933 | 611.7064991 | 592.8166794 |
| **Nme2** | 8475.686633 | 8464.527299 | 8759.659079 | 8656.285526 | 8616.906938 | 8329.978481 | 8311.624282 | 10065.2689 | 9486.437257 | 10270.09594 | 9864.485517 | 8661.400613 | 8087.98432 | 8464.499976 | 8067.336799 |
| **Nme1** | 3360.606087 | 3612.714759 | 3653.447439 | 3561.714294 | 3404.327438 | 3467.083499 | 3063.411771 | 4440.116121 | 4140.534917 | 4393.9791 | 4337.763025 | 3648.823215 | 3245.589156 | 3613.315052 | 3162.407522 |
| **Spag9** | 1765.462816 | 1533.819248 | 1482.52433 | 1451.771653 | 1156.521284 | 1209.235677 | 1430.674638 | 1440.649228 | 1307.695745 | 1359.791464 | 1378.133608 | 1209.33418 | 1327.547363 | 1202.631241 | 1319.286574 |
| **Tob1** | 264.6362832 | 254.3161047 | 242.7800013 | 213.9271361 | 222.3104987 | 227.9591619 | 202.0624961 | 236.3368717 | 238.5152769 | 214.1059824 | 212.8209072 | 229.0274201 | 227.5065121 | 227.6957722 | 229.941015 |
| **Wfikkn2** | 28.38659093 | 19.80655021 | 27.41064531 | 18.11479781 | 25.96789302 | 23.84803539 | 21.64955315 | 29.33259046 | 22.57242368 | 22.72145119 | 30.26786236 | 21.68820266 | 13.63334904 | 28.91374885 | 16.52701046 |
| **Luc7l3** | 2217.816878 | 2025.021694 | 2195.201108 | 2118.568735 | 2282.007769 | 2194.019256 | 2351.682711 | 2391.025159 | 2178.238885 | 2220.584903 | 2236.984203 | 2398.715214 | 2189.004606 | 2239.008427 | 2162.164107 |
| **Ankrd40** | 1476.102728 | 1404.680541 | 1481.741169 | 1507.841266 | 1454.202009 | 1433.687775 | 1492.015038 | 1485.067151 | 1476.988923 | 1565.158426 | 1403.672117 | 1495.618455 | 1566.983056 | 1399.606155 | 1397.610232 |
| **Abcc3** | 4.578482408 | 3.169048034 | 0 | 0 | 3.800179466 | 2.104238417 | 6.314453003 | 0 | 0 | 0 | 3.783482795 | 0.867528106 | 0.852084315 | 4.517773258 | 2.155697016 |
| **Cacna1g** | 1.831392963 | 0.792262009 | 0 | 0 | 0 | 0 | 0 | 5.866518091 | 0.752414123 | 0 | 0.945870699 | 1.735056213 | 0 | 2.710663955 | 2.155697016 |
| **Rsad1** | 207.8631013 | 224.2101484 | 167.596517 | 148.3688202 | 157.7074478 | 202.7083009 | 148.8406779 | 183.5382089 | 135.4345421 | 175.6542957 | 139.0429927 | 170.9030369 | 164.4522728 | 175.2896024 | 194.0127314 |
| **Acsf2** | 401.0750589 | 304.2286113 | 379.0500665 | 400.2507707 | 274.2462848 | 305.8159833 | 309.4081972 | 232.9845756 | 290.4318513 | 282.270336 | 322.5419083 | 346.1437144 | 316.9753652 | 325.2796746 | 362.8756643 |
| **Chad** | 0 | 0.792262009 | 0.783161294 | 4.313047098 | 0 | 1.402825611 | 3.608258859 | 0 | 5.266898858 | 3.495607876 | 1.891741398 | 0.867528106 | 0.852084315 | 2.710663955 | 0 |
| **Lrrc59** | 5488.68471 | 5295.479265 | 5496.225965 | 5387.858435 | 4902.864874 | 5083.840016 | 4969.474514 | 5951.163567 | 5498.642408 | 5696.966935 | 5989.253265 | 4787.02009 | 5144.885095 | 4738.240593 | 4784.210244 |
| **Eme1** | 288.4443917 | 355.7256418 | 318.7466469 | 376.097707 | 295.7806351 | 303.0103321 | 284.1503852 | 437.4746348 | 379.9691319 | 364.417121 | 346.1886757 | 364.3618046 | 364.6920869 | 320.7619013 | 300.3604509 |
| **Mrpl27** | 659.3014667 | 659.9542531 | 627.3121969 | 682.3240509 | 681.4988508 | 675.4605319 | 659.4093065 | 679.6780246 | 748.652052 | 678.1479279 | 628.058144 | 615.9449555 | 657.8090913 | 721.9401666 | 639.523448 |
| **Xylt2** | 346.13327 | 325.6196855 | 328.1445824 | 324.3411418 | 338.2159724 | 324.0527162 | 411.3415099 | 266.5075362 | 260.3352864 | 258.6749828 | 262.0061836 | 368.6994452 | 396.2192066 | 435.5133421 | 357.8457046 |
| **Gm11541** | 0.915696482 | 0 | 0 | 0 | 0 | 0.701412806 | 0 | 0 | 0 | 0 | 0 | 0 | 1.70416863 | 0 | 0 |
| **Col1a1** | 6.409875371 | 7.130358077 | 2.349483883 | 3.450437679 | 10.13381191 | 9.118366474 | 2.706194144 | 0.838074013 | 1.504828245 | 5.243411813 | 5.675224193 | 6.94022485 | 4.260421576 | 9.035546516 | 0.718565672 |
| **Ppp1r9b** | 2422.93289 | 2513.055091 | 2510.031949 | 2761.212752 | 2579.688494 | 2562.260979 | 2827.070816 | 2666.75151 | 2756.092931 | 2245.92806 | 2284.277738 | 2750.064097 | 2891.974166 | 2960.948593 | 2865.6399 |
| **Samd14** | 9.156964816 | 8.714882094 | 10.96425812 | 9.488703616 | 1.900089733 | 4.208476834 | 6.314453003 | 12.5711102 | 21.06759543 | 2.621705907 | 1.891741398 | 1.735056213 | 7.668758836 | 3.614218606 | 0 |
| **Pdk2** | 20.14532259 | 33.27500436 | 30.54329049 | 32.77915795 | 34.20161519 | 32.26498906 | 51.41768874 | 15.92340625 | 36.86829201 | 40.19949057 | 21.75502607 | 39.03876478 | 15.33751767 | 21.68531164 | 20.11983882 |
| **Itga3** | 136.4387758 | 106.1631091 | 121.3900006 | 113.001834 | 100.0713926 | 91.18366474 | 75.77343604 | 120.6826579 | 88.03245234 | 63.79484373 | 132.4218978 | 75.47494525 | 78.39175699 | 81.31991864 | 76.8865269 |
| **Tac4** | 0 | 0.792262009 | 0 | 0 | 0.633363244 | 0.701412806 | 0 | 0 | 0 | 1.747803938 | 0.945870699 | 1.735056213 | 9.372927467 | 0 | 2.155697016 |
| **Kat7** | 2023.689224 | 2024.229432 | 1983.747559 | 1858.923299 | 1833.586592 | 1904.335768 | 1973.717596 | 1891.533047 | 1815.575278 | 1901.610684 | 1954.168864 | 1949.335655 | 1999.841888 | 1876.683011 | 1915.696081 |
| **Fam117a** | 271.0461585 | 312.9434934 | 302.3002597 | 313.1272193 | 340.7494254 | 322.6498906 | 327.4494915 | 217.0611694 | 245.287004 | 232.4579237 | 229.8465798 | 360.8916922 | 395.3671222 | 496.0515037 | 334.8516031 |
| **Slc35b1** | 716.0746486 | 711.4512836 | 703.2788424 | 675.4231756 | 682.1322141 | 692.995852 | 646.7804005 | 749.2381677 | 689.9637504 | 779.5205563 | 812.5029302 | 642.8383268 | 769.4321366 | 726.4579399 | 658.9247212 |
| **Spop** | 1498.079444 | 1569.471039 | 1547.526718 | 1427.61859 | 1343.996804 | 1282.182609 | 1364.823913 | 1625.025511 | 1446.892358 | 1443.686053 | 1582.441679 | 1429.686319 | 1346.293218 | 1309.25069 | 1400.484495 |
| **Phb** | 3803.803184 | 3928.035038 | 3933.819182 | 3856.726715 | 3758.377492 | 3911.077805 | 3656.970354 | 4627.006626 | 4486.645413 | 4498.847336 | 4452.213379 | 3732.105913 | 3889.764899 | 3828.361059 | 3850.793436 |
| **Zfp652os** | 0.915696482 | 0 | 0 | 0 | 0 | 0.701412806 | 1.80412943 | 0 | 3.00965649 | 0.873901969 | 0 | 0 | 0 | 0 | 0 |
| **B130006D01Rik** | 0 | 2.376786026 | 0.783161294 | 2.587828259 | 0 | 0 | 0 | 0 | 7.524141226 | 0 | 0.945870699 | 0 | 1.70416863 | 0 | 0 |
| **Zfp652** | 253.6479254 | 305.0208733 | 286.6370338 | 289.836765 | 280.5799172 | 262.3283893 | 313.9185207 | 231.3084276 | 258.078044 | 235.9535316 | 264.8437957 | 294.9595561 | 328.0524613 | 345.1578769 | 331.9773404 |
| **Phospho1** | 0.915696482 | 4.753572051 | 6.265290356 | 1.725218839 | 0.633363244 | 4.90988964 | 0.902064715 | 5.028444078 | 0 | 3.495607876 | 4.729353494 | 1.735056213 | 2.556252945 | 0 | 5.748525376 |
| **Abi3** | 99.81091649 | 142.6071615 | 158.1985815 | 144.9183825 | 131.1061916 | 120.6430026 | 128.9952542 | 279.9167204 | 173.8076623 | 229.8362178 | 268.6272785 | 137.0694408 | 128.6647316 | 135.5331977 | 137.964609 |
| **Gngt2** | 30.21798389 | 30.89821833 | 35.24225825 | 33.64176737 | 40.53524763 | 35.07064029 | 29.76813559 | 31.8468125 | 28.59173666 | 29.71266694 | 42.56418144 | 24.29078698 | 28.1187824 | 27.10663955 | 20.83840449 |
| **4833417C18Rik** | 10.0726613 | 11.88393013 | 6.265290356 | 10.35131304 | 7.600358931 | 8.416953669 | 2.706194144 | 8.380740131 | 12.79104008 | 12.23462756 | 3.783482795 | 10.41033728 | 15.33751767 | 9.939101167 | 14.37131344 |
| **B4galnt2** | 10.98835778 | 30.89821833 | 18.79587107 | 18.97740723 | 13.93399137 | 14.72966892 | 19.84542373 | 22.62799835 | 7.524141226 | 48.93851026 | 32.15960376 | 18.21809023 | 19.59793925 | 16.26398373 | 14.37131344 |
| **Snf8** | 977.9638423 | 1100.45193 | 1089.377361 | 1090.338306 | 1062.783524 | 1076.668657 | 1050.003328 | 1170.789396 | 1182.795001 | 1200.741305 | 1212.606236 | 1111.303504 | 1125.60338 | 1217.99167 | 1032.578871 |
| **Ube2z** | 2976.929262 | 2992.373606 | 3044.147952 | 2789.678863 | 2859.635048 | 2970.483232 | 2849.622434 | 2835.204386 | 3074.364105 | 3041.178852 | 2846.124933 | 2884.530953 | 2970.365923 | 2896.796213 | 2843.364364 |
| **Atp5g1** | 2533.732164 | 2432.244366 | 2488.886594 | 2550.736054 | 2520.785712 | 2441.617977 | 2437.378859 | 3125.177995 | 2750.073618 | 2719.582927 | 2846.124933 | 2391.774989 | 2386.688167 | 2765.780788 | 2269.230392 |
| **Hoxb9** | 4.578482408 | 0 | 0.783161294 | 0 | 0.633363244 | 0.701412806 | 0 | 4.190370065 | 4.514484735 | 0.873901969 | 5.675224193 | 0 | 0 | 0 | 0 |
| **Hoxb4** | 19.22962611 | 16.63750218 | 14.0969033 | 5.175656518 | 9.500448664 | 16.13249453 | 9.922711863 | 31.00873848 | 27.08690841 | 27.964863 | 18.91741398 | 15.61550591 | 12.78126473 | 19.87820233 | 20.11983882 |
| **Gm11529** | 4.578482408 | 3.169048034 | 5.482129061 | 0.86260942 | 0.633363244 | 2.104238417 | 3.608258859 | 4.190370065 | 1.504828245 | 2.621705907 | 5.675224193 | 9.542809169 | 0 | 8.131991864 | 2.874262688 |
| **Skap1** | 2023.689224 | 2048.789554 | 2124.716592 | 2210.867943 | 2400.446696 | 2390.414842 | 2579.905084 | 1857.172013 | 1936.713952 | 1726.830291 | 1962.6817 | 2511.493868 | 2481.269526 | 2450.440215 | 2480.4887 |
| **Snx11** | 290.2757847 | 360.4792139 | 370.4352923 | 336.4176737 | 350.2498741 | 330.3654315 | 327.4494915 | 464.2930032 | 437.9050193 | 436.0770825 | 458.7472889 | 380.8448387 | 370.6566771 | 383.1071723 | 405.9896047 |
| **Cbx1** | 988.9522001 | 1003.795965 | 1075.280457 | 1057.559148 | 1130.553391 | 1065.446052 | 1115.854052 | 1031.66911 | 1097.772205 | 1055.673578 | 998.8394579 | 1154.679909 | 1121.342959 | 1082.458473 | 1138.92659 |
| **Gm11517** | 1.831392963 | 1.584524017 | 0.783161294 | 1.725218839 | 0.633363244 | 2.104238417 | 1.80412943 | 1.676148026 | 1.504828245 | 0 | 1.891741398 | 1.735056213 | 3.408337261 | 5.421327909 | 0.718565672 |
| **Nfe2l1** | 3133.51336 | 2744.395597 | 2865.587177 | 2560.224758 | 2606.923113 | 2666.771487 | 3056.195254 | 2717.03595 | 2715.462568 | 2736.187065 | 2589.793973 | 2435.151394 | 2653.390557 | 2541.699235 | 2633.543188 |
| **Copz2** | 5.494178889 | 0 | 0 | 3.450437679 | 3.166816221 | 4.208476834 | 3.608258859 | 6.704592104 | 3.00965649 | 0 | 3.783482795 | 0.867528106 | 0 | 8.131991864 | 0.718565672 |
| **Cdk5rap3** | 1044.809685 | 907.9322617 | 885.7554241 | 950.5955805 | 961.4454048 | 883.7801352 | 938.1473034 | 899.253416 | 844.2086455 | 877.3975768 | 890.0643275 | 909.1694554 | 886.1676878 | 925.2399632 | 807.6678153 |
| **Pnpo** | 308.5897143 | 357.3101658 | 347.7236148 | 369.1968316 | 332.5157032 | 368.9431358 | 296.7792912 | 497.8159638 | 346.1104964 | 400.2471018 | 430.3711679 | 320.9853993 | 364.6920869 | 370.4574071 | 354.971442 |
| **D030028A08Rik** | 106.2207919 | 81.60298688 | 89.28038757 | 119.9027093 | 104.5049353 | 102.4062696 | 119.0725424 | 98.89273354 | 130.1676432 | 121.4723737 | 155.1227946 | 77.21000146 | 80.09592563 | 88.54835585 | 90.53927467 |
| **Sp2** | 1111.655529 | 1028.356087 | 1037.688715 | 973.0234254 | 952.5783194 | 1026.868348 | 1113.147858 | 948.6997828 | 1116.582558 | 869.5324591 | 900.4689052 | 1044.50384 | 1072.774153 | 1067.098044 | 1107.3097 |
| **Sp6** | 8.241268334 | 1.584524017 | 3.915806472 | 2.587828259 | 5.700269198 | 1.402825611 | 4.510323574 | 6.704592104 | 3.762070613 | 9.612921658 | 7.56696559 | 11.27786538 | 4.260421576 | 0.903554652 | 7.18565672 |
| **Scrn2** | 174.898028 | 257.4851528 | 202.055614 | 182.873197 | 224.2105885 | 206.9167777 | 212.8872727 | 259.802944 | 208.418712 | 228.9623159 | 231.7383212 | 193.4587677 | 211.3169102 | 207.8175699 | 184.6713777 |
| **Lrrc46** | 0.915696482 | 2.376786026 | 3.132645178 | 2.587828259 | 6.333632443 | 2.104238417 | 1.80412943 | 13.40918421 | 1.504828245 | 6.991215751 | 0 | 0.867528106 | 9.372927467 | 0 | 0 |
| **Mrpl10** | 992.614986 | 1108.37455 | 1099.558457 | 1004.077364 | 951.9449561 | 977.0680384 | 946.2658858 | 1123.019177 | 984.9100865 | 1140.442069 | 1007.352294 | 1007.200131 | 885.3156035 | 971.3212504 | 960.7223034 |
| **Osbpl7** | 314.0838932 | 363.6482619 | 363.3868406 | 406.2890367 | 324.9153443 | 337.3795595 | 359.9238212 | 432.4461907 | 386.740859 | 425.5902589 | 365.1060897 | 353.9514674 | 361.2837496 | 414.7315851 | 349.9414822 |
| **Tbx21** | 895.551159 | 864.3578513 | 749.4853588 | 823.7919958 | 472.4889802 | 562.5330702 | 500.6459167 | 1217.721541 | 1045.85563 | 935.9490087 | 915.6028364 | 627.2228208 | 580.2694186 | 564.7216572 | 488.6246569 |
| **Tbkbp1** | 216.1043696 | 209.1571702 | 205.9714205 | 196.6749477 | 134.906371 | 141.6853868 | 144.3303544 | 236.3368717 | 239.267691 | 234.2057277 | 233.6300626 | 153.5524748 | 140.593912 | 124.6905419 | 107.7848508 |
| **Kpnb1** | 16870.79198 | 16616.90337 | 17114.42377 | 16728.58448 | 15806.84649 | 15598.71939 | 16126.21091 | 18742.68723 | 18347.61838 | 18568.66904 | 18740.53615 | 16055.34266 | 15875.18288 | 15567.34309 | 15765.33084 |
| **Npepps** | 1731.582047 | 1669.296052 | 1755.064461 | 1629.469194 | 1588.475017 | 1645.514442 | 1697.685793 | 1764.145797 | 1722.275927 | 1733.821506 | 1852.014828 | 1778.432618 | 1720.358232 | 1686.03298 | 1784.917129 |
| **Mrpl45** | 1122.643886 | 1137.688244 | 1285.950846 | 1260.272362 | 1155.887921 | 1035.285301 | 1145.622188 | 1539.541962 | 1385.946814 | 1614.970839 | 1366.78316 | 1154.679909 | 1117.082537 | 1175.524602 | 1143.237984 |
| **Gpr179** | 26.55519797 | 23.76786026 | 21.92851625 | 18.97740723 | 19.63426057 | 11.22260489 | 13.53097072 | 25.14222039 | 27.83932254 | 24.46925513 | 37.83482795 | 27.7608994 | 28.1187824 | 19.87820233 | 25.86836419 |
| **Socs7** | 989.8678966 | 978.4435805 | 964.0715535 | 898.8390153 | 898.1090804 | 866.2448151 | 971.5236978 | 843.9405311 | 913.4307448 | 890.5061063 | 982.759656 | 983.7768725 | 927.9198192 | 875.5444574 | 870.1830288 |
| **Arhgap23** | 1.831392963 | 2.376786026 | 7.831612945 | 6.038265938 | 6.966995687 | 4.208476834 | 5.412388289 | 6.704592104 | 12.03862596 | 6.117313782 | 3.783482795 | 2.602584319 | 5.964590206 | 7.228437213 | 8.622788064 |
| **2410003L11Rik** | 0.915696482 | 0.792262009 | 0 | 1.725218839 | 1.900089733 | 1.402825611 | 4.510323574 | 4.190370065 | 2.257242368 | 5.243411813 | 1.891741398 | 3.470112425 | 1.70416863 | 0 | 0 |
| **E130012A19Rik** | 1432.149297 | 1304.063266 | 1314.927813 | 1475.924717 | 1328.162723 | 1324.267377 | 1298.973189 | 1307.39546 | 1398.737854 | 1495.246269 | 1438.669333 | 1334.258227 | 1260.232702 | 1199.017023 | 1266.112714 |
| **Mllt6** | 5109.586367 | 5139.403649 | 5038.076607 | 5083.35731 | 4726.789892 | 5076.124475 | 5278.882711 | 5213.658435 | 4855.328333 | 5171.751852 | 5364.978603 | 5014.312454 | 5274.401911 | 4638.849581 | 5142.055949 |
| **Cisd3** | 371.7727715 | 400.8845763 | 389.2311634 | 394.2125048 | 325.5487076 | 373.8530254 | 359.9238212 | 429.0938947 | 384.4836166 | 443.0682982 | 431.3170386 | 412.0758505 | 385.1421105 | 392.1427188 | 360.0014017 |
| **Pcgf2** | 142.8486511 | 186.973834 | 158.1985815 | 150.9566484 | 141.2400035 | 140.2825611 | 171.3922958 | 154.2056184 | 124.9007443 | 146.8155308 | 142.8264755 | 177.8432618 | 149.9668395 | 122.8834326 | 153.0544881 |
| **Psmb3** | 3773.585201 | 3787.012401 | 3915.806472 | 3710.945723 | 3838.814624 | 3652.256479 | 3669.59926 | 3728.591284 | 3690.591271 | 3880.124742 | 3634.035225 | 3710.417711 | 3965.600403 | 3784.086881 | 3565.522864 |
| **Pip4k2b** | 2129.910016 | 2202.488384 | 2264.119302 | 2159.973987 | 2088.198616 | 2157.54579 | 2300.265023 | 2168.935546 | 2273.795478 | 2293.992668 | 2474.397748 | 2234.752402 | 2349.196457 | 2344.724321 | 2219.649361 |
| **Mir8102** | 9.156964816 | 0.792262009 | 0 | 3.450437679 | 0 | 0 | 4.510323574 | 0.838074013 | 4.514484735 | 1.747803938 | 0 | 1.735056213 | 0 | 3.614218606 | 1.437131344 |
| **Cwc25** | 448.691276 | 403.2613623 | 485.5600026 | 415.7777403 | 417.386378 | 424.3547475 | 469.9757164 | 402.2755263 | 422.1043228 | 323.3437285 | 455.9096768 | 388.6525916 | 417.5213144 | 461.716427 | 455.570636 |
| **1700001P01Rik** | 1.831392963 | 0 | 3.915806472 | 2.587828259 | 0.633363244 | 0.701412806 | 0 | 0 | 6.019312981 | 0 | 0 | 0.867528106 | 5.112505891 | 0 | 2.874262688 |
| **Rpl23** | 17137.25965 | 15380.18237 | 15886.42686 | 14477.17389 | 16734.09028 | 16924.38959 | 16717.96536 | 15475.87473 | 14668.31332 | 14530.36804 | 14575.86747 | 15723.94693 | 15416.76151 | 16229.64865 | 15270.95766 |
| **Lasp1** | 3040.112319 | 3349.683772 | 3436.51176 | 3572.928216 | 3515.166006 | 3488.125883 | 3850.012203 | 3578.576036 | 3572.462254 | 3310.340658 | 3284.063066 | 3889.996029 | 3701.454265 | 3994.615115 | 3702.050342 |
| **B230217C12Rik** | 12.81975074 | 6.338096068 | 11.74741942 | 11.21392246 | 4.43354271 | 9.118366474 | 12.62890601 | 26.81836842 | 21.82000955 | 6.117313782 | 17.02567258 | 6.94022485 | 11.92918041 | 5.421327909 | 2.874262688 |
| **Fbxo47** | 13.73544722 | 8.714882094 | 5.482129061 | 13.80175071 | 5.700269198 | 10.52119209 | 3.608258859 | 5.028444078 | 3.762070613 | 5.243411813 | 11.35044839 | 8.675281063 | 11.92918041 | 3.614218606 | 6.467091048 |
| **Plxdc1** | 72.34002204 | 83.18751089 | 65.00238744 | 78.49745719 | 98.80466611 | 74.34975741 | 92.01060091 | 42.74177467 | 49.65933209 | 40.19949057 | 38.78069865 | 89.35539495 | 109.0667923 | 106.6194489 | 86.22788064 |
| **Arl5c** | 301.2641424 | 396.1310043 | 378.2669052 | 420.0907874 | 443.354271 | 372.4501998 | 477.1922341 | 196.1093191 | 276.8883971 | 206.2408647 | 232.6841919 | 412.9433786 | 409.8525556 | 394.8533827 | 350.6600479 |
| **Cacnb1** | 45.78482408 | 61.00417465 | 52.47180673 | 38.81742388 | 41.16861088 | 51.20313482 | 52.31975346 | 39.38947861 | 39.8779485 | 29.71266694 | 52.02288843 | 26.02584319 | 39.1958785 | 33.43152211 | 40.23967763 |
| **Rpl19** | 22312.77617 | 21880.69215 | 21128.1254 | 20430.0415 | 22051.80808 | 21855.32161 | 22601.23143 | 20909.10855 | 19875.01905 | 20390.75464 | 19218.20086 | 21206.72456 | 20302.61298 | 21259.7374 | 20783.07493 |
| **Stac2** | 80.58129038 | 110.9166812 | 97.11200052 | 87.12355138 | 64.60305092 | 61.0229141 | 59.53627118 | 27.65644243 | 57.18347332 | 38.45168663 | 88.91184568 | 60.72696744 | 75.83550405 | 103.9087849 | 72.57513287 |
| **Fbxl20** | 358.0373243 | 396.9232663 | 422.1239377 | 445.1064605 | 332.5157032 | 340.8866236 | 350.903174 | 369.5906398 | 309.2422044 | 340.8217679 | 343.3510637 | 410.3407943 | 418.3733987 | 441.8382246 | 405.9896047 |
| **Med1** | 1277.396592 | 1303.271004 | 1401.075556 | 1242.157564 | 1176.788908 | 1117.3506 | 1289.050477 | 1432.268488 | 1370.146117 | 1246.184208 | 1352.595099 | 1211.936764 | 1226.14933 | 1096.011792 | 1332.939321 |
| **Cdk12** | 1561.262501 | 1552.041275 | 1386.195491 | 1437.107293 | 1252.159134 | 1210.638503 | 1342.272296 | 1557.97959 | 1667.349696 | 1476.894327 | 1612.709541 | 1380.237217 | 1453.655842 | 1269.494285 | 1304.91526 |

| | | | | | | | | | | | | | | | |
|---|---|---|---|---|---|---|---|---|---|---|---|---|---|---|---|
| **Ppp1r1b** | 6.409875371 | 7.130358077 | 2.349483883 | 2.587828259 | 9.500448664 | 4.208476834 | 2.706194144 | 5.028444078 | 6.771727103 | 4.369509845 | 14.18806048 | 4.337640531 | 7.668758836 | 11.74621047 | 5.029959704 |
| **Stard3** | 436.7872217 | 444.4589868 | 422.907099 | 338.1428925 | 432.5870958 | 387.1798688 | 434.7951925 | 408.9801184 | 352.8822235 | 320.7220226 | 350.9180292 | 385.1824792 | 404.7400497 | 420.152913 | 423.2351808 |
| **Tcap** | 0 | 0.792262009 | 0 | 0 | 0 | 0 | 0 | 5.028444078 | 0 | 2.621705907 | 0 | 0 | 0.852084315 | 0 | 0 |
| **Pgap3** | 178.5608139 | 180.6357379 | 159.7649041 | 187.1862441 | 210.2765971 | 160.6235325 | 198.4542373 | 125.711102 | 167.7883493 | 172.1586879 | 148.5016997 | 176.9757337 | 208.7606572 | 181.614485 | 180.3599837 |
| **Erbb2** | 0 | 0 | 0.783161294 | 0 | 0 | 0 | 0.902064715 | 8.380740131 | 0 | 1.747803938 | 10.40457769 | 0 | 0.852084315 | 0 | 0 |
| **Mien1** | 421.2203815 | 442.8744628 | 425.2565829 | 442.5186323 | 483.2561554 | 401.2081249 | 419.4600924 | 436.6365608 | 449.1912312 | 512.9804557 | 425.6418144 | 437.2341656 | 422.6338203 | 411.1173665 | 441.1993226 |
| **Grb7** | 11.90405426 | 15.84524017 | 11.74741942 | 19.84001665 | 18.36753408 | 14.72966892 | 22.55161787 | 12.5711102 | 9.781383594 | 24.46925513 | 15.13393118 | 15.61550591 | 10.22501178 | 9.939101167 | 7.18565672 |
| **Ikzf3** | 1127.222369 | 1449.839476 | 1325.892072 | 1473.336889 | 1542.872863 | 1402.124199 | 1557.865762 | 1476.686411 | 1647.786928 | 1353.67415 | 1320.435495 | 1781.90273 | 1632.593548 | 1557.728219 | 1480.96385 |
| **Zpbp2** | 7.325571853 | 10.29940611 | 9.397935534 | 8.626094197 | 7.600358931 | 12.6254305 | 8.118582433 | 9.218814144 | 9.781383594 | 5.243411813 | 6.621094891 | 8.675281063 | 3.408337261 | 6.324882561 | 10.05991941 |
| **Ormdl3** | 357.1216278 | 369.986358 | 433.0881959 | 401.9759896 | 378.1178568 | 385.7770431 | 372.5527272 | 346.9626414 | 399.5318991 | 412.4817293 | 351.8638999 | 419.8836034 | 397.9233752 | 425.5742409 | 379.4026748 |
| **Psmd3** | 5702.957687 | 5514.143579 | 5724.125901 | 5509.486363 | 5434.256636 | 5359.495248 | 5346.537565 | 6063.465484 | 5950.843295 | 6105.079155 | 5952.364307 | 5388.217068 | 5530.87929 | 5314.708461 | 5347.565731 |
| **Med24** | 1238.021643 | 1402.303755 | 1317.277297 | 1336.181991 | 1291.427655 | 1228.173823 | 1296.266995 | 1542.894258 | 1401.74751 | 1490.876759 | 1475.55829 | 1449.639466 | 1411.051626 | 1402.316819 | 1403.358757 |
| **Thra** | 75.08711149 | 75.26489081 | 75.96664557 | 69.00875357 | 74.10349958 | 69.43986777 | 98.32505391 | 57.8271069 | 61.69795805 | 47.19070632 | 46.34766424 | 91.09045116 | 62.20215501 | 80.41636399 | 93.41353736 |
| **Nr1d1** | 41.20634167 | 58.62738863 | 56.3876132 | 50.89395576 | 38.6351579 | 54.00878604 | 50.51562403 | 48.60829276 | 44.39243323 | 38.45168663 | 53.91462983 | 46.84651774 | 59.64590206 | 43.37062328 | 42.39537465 |
| **Msl1** | 1324.097112 | 1432.409711 | 1434.751492 | 1619.98049 | 1402.266223 | 1508.738945 | 1443.303544 | 1418.02123 | 1689.922119 | 1577.393054 | 1541.769239 | 1630.085312 | 1653.043571 | 1571.281539 | 1659.886702 |
| **Gm12359** | 4.578482408 | 8.714882094 | 3.132645178 | 2.587828259 | 11.4005384 | 1.402825611 | 0.902064715 | 1.676148026 | 6.771727103 | 2.621705907 | 1.891741398 | 3.470112425 | 5.112505891 | 9.939101167 | 3.59282836 |
| **Casc3** | 1777.366871 | 1766.744279 | 1836.513236 | 1746.784075 | 1664.478606 | 1684.092147 | 1722.041541 | 1754.926983 | 1747.858007 | 1731.1998 | 1879.445078 | 1746.334078 | 1711.837389 | 1576.702867 | 1780.605735 |
| **Rapgefl1** | 9.156964816 | 5.54583406 | 3.132645178 | 4.313047098 | 7.600358931 | 4.90988964 | 6.314453003 | 1.676148026 | 6.019312981 | 11.3607256 | 0.945870699 | 12.14539349 | 6.816674521 | 9.035546516 | 7.18565672 |
| **Wipf2** | 945.9144655 | 888.1257115 | 913.9492307 | 857.4337631 | 799.3044143 | 800.3120113 | 927.3225268 | 862.3781594 | 993.9390559 | 847.6849098 | 945.8706988 | 884.8786684 | 855.4926524 | 861.0875829 | 897.4885243 |
| **Cdc6** | 1537.454393 | 1445.085904 | 1449.631556 | 1483.688202 | 1350.330437 | 1344.608349 | 1359.411525 | 1692.909506 | 1549.220678 | 1609.727427 | 1641.085662 | 1363.754183 | 1307.097339 | 1330.936002 | 1225.154471 |
| **Rara** | 1150.114781 | 1062.423353 | 1032.989747 | 994.5886609 | 1337.029809 | 1466.654177 | 1583.123574 | 803.7129785 | 665.1340844 | 727.9603401 | 812.5029302 | 1184.175865 | 1358.222398 | 1323.707565 | 1403.358757 |
| **Gjd3** | 5.494178889 | 7.922620085 | 3.915806472 | 0.86260942 | 6.966995687 | 9.81977928 | 4.510323574 | 4.190370065 | 0 | 3.495607876 | 2.837612096 | 2.602584319 | 9.372927467 | 0.903554652 | 1.437131344 |
| **Top2a** | 17160.15206 | 18843.95187 | 18542.12681 | 19212.03699 | 17519.4607 | 16457.24866 | 18675.44579 | 20003.15054 | 20308.40958 | 18975.90735 | 19098.07528 | 19371.90261 | 18747.5591 | 19278.24205 | 18351.4487 |
| **Igfbp4** | 558.5748538 | 778.0012924 | 711.8936167 | 811.7154639 | 1029.215272 | 1149.615589 | 1118.560246 | 825.5029029 | 753.9189508 | 540.9453188 | 569.4141607 | 1146.872157 | 1092.372092 | 1320.093346 | 1100.124044 |
| **Tns4** | 7.325571853 | 2.376786026 | 6.265290356 | 15.52696955 | 15.20071786 | 16.83390734 | 15.33510015 | 6.704592104 | 0.752414123 | 6.991215751 | 5.675224193 | 13.01292159 | 27.26669809 | 9.035546516 | 17.9641418 |
| **Ccr7** | 14130.11241 | 12609.64213 | 12532.14703 | 11587.43233 | 8782.214745 | 8865.857864 | 9218.19932 | 7308.005394 | 7892.824146 | 7915.804034 | 7319.147467 | 8979.783428 | 9025.277066 | 7871.768125 | 8601.949659 |
| **Smarce1** | 2602.409401 | 2514.639615 | 2542.924723 | 2390.290702 | 2344.71073 | 2439.513738 | 2560.059661 | 2394.377455 | 2491.24316 | 2514.215965 | 2503.71974 | 2606.054431 | 2593.744655 | 2565.191656 | 2414.380658 |
| **Krt222** | 14.65114371 | 11.88393013 | 6.265290356 | 10.35131304 | 17.73417084 | 27.35509942 | 18.94335901 | 11.73303618 | 8.276555348 | 13.10852953 | 7.56696559 | 7.807752957 | 15.33751767 | 17.16753838 | 18.68270747 |
| **Krt10** | 11.90405426 | 17.42976419 | 21.14535495 | 21.56523549 | 29.13470924 | 20.34097137 | 23.45368258 | 22.62799835 | 11.28621184 | 34.08217679 | 23.64676747 | 19.08561834 | 12.78126473 | 23.49242094 | 22.9941015 |
| **Krt16** | 1.831392963 | 1.584524017 | 0.783161294 | 0 | 6.966995687 | 10.52119209 | 1.80412943 | 3.352296052 | 0 | 0.873901969 | 0 | 0.867528106 | 6.816674521 | 0 | 0.718565672 |
| **Krt17** | 0 | 0.792262009 | 0.783161294 | 0 | 0 | 1.402825611 | 0 | 0.838074013 | 6.771727103 | 4.369509845 | 2.837612096 | 0 | 1.70416863 | 0 | 0 |
| **Eif1** | 8309.029874 | 8020.860574 | 7920.110171 | 7521.954139 | 7986.077147 | 7923.860466 | 8079.79365 | 8283.523545 | 7762.656503 | 7763.745092 | 7752.356247 | 7618.631829 | 7795.719399 | 7916.042302 | 7989.013141 |
| **Hap1** | 4.578482408 | 0 | 1.566322589 | 0.86260942 | 3.166816221 | 2.104238417 | 0 | 0 | 0 | 0.873901969 | 0 | 1.735056213 | 1.70416863 | 0 | 0 |
| **Jup** | 12.81975074 | 7.922620085 | 12.53058071 | 4.313047098 | 10.13381191 | 20.34097137 | 5.412388289 | 15.92340625 | 22.57242368 | 13.10852953 | 13.24218978 | 19.08561834 | 14.48543336 | 4.517773258 | 10.77848508 |
| **P3h4** | 2.747089445 | 1.584524017 | 2.349483883 | 3.450437679 | 2.533452977 | 1.402825611 | 2.706194144 | 4.190370065 | 4.514484735 | 3.495607876 | 0 | 7.807752957 | 0 | 3.614218606 | 0.718565672 |
| **Fkbp10** | 2.747089445 | 0.792262009 | 0.783161294 | 3.450437679 | 8.233722176 | 4.90988964 | 3.608258859 | 1.676148026 | 3.762070613 | 1.747803938 | 1.891741398 | 6.072696744 | 5.964590206 | 3.614218606 | 4.311394032 |
| **Nt5c3b** | 1280.143681 | 1380.120419 | 1423.004072 | 1472.474279 | 1345.263531 | 1388.095943 | 1446.911803 | 1237.835317 | 1366.384047 | 1370.278287 | 1406.509729 | 1317.775193 | 1325.843194 | 1401.413265 | 1266.112714 |
| **Klhl10** | 0 | 5.54583406 | 3.915806472 | 0.86260942 | 0.633363244 | 2.104238417 | 0.902064715 | 1.676148026 | 1.504828245 | 5.243411813 | 0.945870699 | 1.735056213 | 1.70416863 | 3.614218606 | 3.59282836 |
| **Klhl11** | 140.1015617 | 167.9595458 | 148.800646 | 135.4296789 | 145.6735462 | 140.9839739 | 159.6654545 | 184.3762829 | 155.7497234 | 173.0325898 | 191.0658812 | 163.095284 | 147.4105865 | 153.6042908 | 144.4317001 |
| **Acly** | 11023.15425 | 10773.17879 | 10861.66399 | 10608.37064 | 10323.18752 | 10354.95725 | 11052.09689 | 9776.133362 | 10630.85914 | 10512.16678 | 10860.48736 | 10321.84941 | 11084.76486 | 10427.92423 | 11208.90592 |
| **Ttc25** | 8.241268334 | 3.169048034 | 12.53058071 | 0 | 6.333632443 | 4.90988964 | 2.706194144 | 1.676148026 | 0 | 0 | 0.945870699 | 0 | 4.260421576 | 0.903554652 | 5.748525376 |
| **Cnp** | 2697.641835 | 2627.933082 | 2729.317111 | 2746.548392 | 2655.05872 | 2601.540096 | 2693.565238 | 2114.460735 | 2337.750679 | 2052.795725 | 2223.742013 | 2626.007578 | 2514.500814 | 2821.801177 | 2606.237692 |
| **Dnajc7** | 3060.257641 | 2899.678951 | 3090.354468 | 2882.84068 | 3055.34429 | 2908.057492 | 2893.823605 | 3219.042284 | 3183.464153 | 3245.671913 | 3141.236591 | 3023.33545 | 2961.84508 | 3130.816868 | 3161.688957 |
| **Nkiras2** | 256.3950148 | 240.0553886 | 249.8284529 | 227.7288868 | 234.9777636 | 230.0634003 | 245.3616024 | 167.6148026 | 191.8656013 | 211.4842765 | 208.0915537 | 230.7624763 | 239.4356926 | 235.8277641 | 254.3722479 |
| **Zfp385c** | 0 | 0.792262009 | 1.566322589 | 0 | 0 | 0 | 0.902064715 | 0 | 0 | 0 | 0 | 0 | 2.556252945 | 0 | 1.437131344 |
| **Dhx58** | 1130.885155 | 1037.070969 | 778.4623267 | 696.9884111 | 818.9386748 | 1021.257045 | 1058.12191 | 295.8401266 | 414.5801815 | 282.270336 | 370.7813139 | 764.2922616 | 693.5966325 | 799.6458666 | 892.4585646 |
| **Kat2a** | 2086.872281 | 2101.871109 | 1951.637946 | 2001.253854 | 1999.527762 | 2116.863848 | 2085.573621 | 1950.198228 | 2362.580345 | 1940.062371 | 2110.237529 | 1958.878464 | 1991.321045 | 1904.693206 | 1991.864043 |
| **Hspb9** | 5.494178889 | 7.922620085 | 5.482129061 | 2.587828259 | 8.86708542 | 7.715540863 | 11.72684129 | 8.380740131 | 6.771727103 | 9.612921658 | 5.675224193 | 11.27786538 | 11.0770961 | 1.807109303 | 7.904222392 |
| **Rab5c** | 2844.153272 | 3038.324803 | 2908.661048 | 2974.277279 | 2655.692083 | 2817.575241 | 2944.339229 | 2671.779954 | 2752.33086 | 2951.166949 | 2799.777268 | 2813.393649 | 2875.784564 | 2899.506877 | 2887.915436 |
| **Kcnh4** | 38.45925223 | 25.35238427 | 32.89277437 | 25.87828259 | 34.83497844 | 53.30737323 | 34.27845916 | 73.75051315 | 63.2027863 | 22.72145119 | 36.88895725 | 32.09853993 | 45.1604687 | 46.98484188 | 54.61099107 |
| **Ghdc** | 127.2818109 | 114.0857292 | 98.67832311 | 123.353147 | 111.471931 | 110.8232233 | 131.7014484 | 144.1487302 | 134.6821279 | 144.1938249 | 155.1227946 | 84.15022631 | 107.3626237 | 134.6296431 | 128.6232553 |
| **Stat5b** | 3092.307018 | 3063.677187 | 3033.183694 | 2921.658104 | 2690.527062 | 2853.347294 | 3048.978736 | 3025.447187 | 2950.968189 | 3046.422264 | 2881.122148 | 2770.884771 | 2855.33454 | 2583.262749 | 2891.508264 |
| **Stat5a** | 3210.431864 | 2718.250951 | 2778.656273 | 2680.127467 | 2437.815127 | 2794.428618 | 2833.385269 | 3793.961057 | 3096.184114 | 3117.208323 | 2906.660657 | 2642.490612 | 2621.863438 | 2469.414863 | 2811.747474 |
| **Stat3** | 6940.063634 | 6022.775789 | 5772.681902 | 5655.267355 | 4121.294631 | 4375.413082 | 4605.040369 | 5290.761244 | 4977.971835 | 5636.667699 | 5516.317915 | 4365.401431 | 4645.563686 | 4371.397404 | 4791.395901 |
| **Ptrf** | 13.73544722 | 8.714882094 | 8.614774239 | 10.35131304 | 6.333632443 | 4.90988964 | 4.510323574 | 12.5711102 | 3.00965649 | 2.621705907 | 5.675224193 | 6.072696744 | 3.408337261 | 6.324882561 | 5.029959704 |
| **Atp6v0a1** | 76.00280797 | 95.86370303 | 63.43606485 | 61.2452688 | 92.47103366 | 93.28790316 | 82.08788905 | 98.05465953 | 85.02279585 | 70.78605948 | 82.29075079 | 89.35539495 | 106.5105394 | 81.31991864 | 81.19792093 |
| **Naglu** | 210.6101908 | 205.9881222 | 243.5631626 | 232.9045433 | 202.0428749 | 238.4803539 | 296.7792912 | 121.5207319 | 167.7883493 | 131.0852953 | 173.0943379 | 207.3392174 | 203.6481513 | 159.0256187 | 237.1266717 |
| **Hsd17b1** | 1.831392963 | 1.584524017 | 3.915806472 | 4.313047098 | 0.633363244 | 0.701412806 | 1.80412943 | 2.514222039 | 0 | 2.621705907 | 0 | 2.602584319 | 0.852084315 | 1.807109303 | 4.311394032 |
| **Coasy** | 517.3685121 | 466.642323 | 541.1644545 | 482.1986656 | 494.0233305 | 472.7522311 | 440.2075808 | 437.4746348 | 442.4195041 | 476.2765731 | 492.7986341 | 487.5507957 | 539.3693715 | 485.2088479 | 500.1217077 |
| **Mlx** | 684.0252717 | 789.0929605 | 704.845165 | 793.6006661 | 684.0323038 | 721.7537771 | 678.3526655 | 807.9033486 | 626.7609641 | 752.4295952 | 712.2406362 | 745.2066433 | 736.2008483 | 703.8690736 | 768.865269 |
| **Psmc3ip** | 259.1421043 | 209.9494323 | 234.1652271 | 214.7897455 | 248.2783918 | 242.6888308 | 239.0471494 | 289.9736085 | 294.9463361 | 242.0708454 | 249.7098645 | 241.1728135 | 248.80862 | 238.538428 | 219.1625299 |
| **Fam134c** | 1147.367691 | 1243.059091 | 1191.97149 | 1226.630595 | 1096.351776 | 1207.832851 | 1188.019229 | 1122.181103 | 1209.129495 | 1018.095794 | 1094.372398 | 1184.175865 | 1264.493124 | 1276.722723 | 1205.753198 |
| **Tubg1** | 1371.713329 | 1401.511493 | 1359.568007 | 1266.310628 | 1201.490074 | 1327.073028 | 1299.875254 | 1382.822122 | 1552.982749 | 1473.39872 | 1372.458384 | 1407.130588 | 1308.801508 | 1312.864909 | 1319.286574 |
| **Tubg2** | 18.31392963 | 19.0142882 | 15.66322589 | 15.52696955 | 15.83408111 | 25.25086101 | 15.33510015 | 12.5711102 | 10.53379772 | 8.739019689 | 5.675224193 | 26.02584319 | 13.63334904 | 22.58886629 | 12.21561642 |
| **Plekhh3** | 14.65114371 | 39.61310043 | 15.66322589 | 18.97740723 | 29.76807248 | 28.75792503 | 26.15987673 | 1.676148026 | 10.53379772 | 4.369509845 | 2.837612096 | 19.95314644 | 29.82295103 | 15.36042908 | 32.33545524 |
| **Ccr10** | 46.70052056 | 23.76786026 | 39.94122602 | 24.15306375 | 30.40143573 | 44.89041957 | 64.94865946 | 5.866518091 | 5.266898858 | 13.9824315 | 16.07980188 | 32.96606804 | 31.52711966 | 43.37062328 | 33.05402091 |
| **Cntnap1** | 56.77318186 | 39.61310043 | 54.82129061 | 46.58090866 | 53.20251252 | 56.11302446 | 46.00530045 | 13.40918421 | 16.5531107 | 21.84754922 | 22.70089677 | 55.5217988 | 40.90004713 | 41.56351397 | 58.9223851 |
| **Ezh1** | 395.58088 | 419.1066025 | 440.9198088 | 388.1742388 | 407.2525661 | 403.3123633 | 389.6919568 | 375.4571578 | 369.4353342 | 339.9478659 | 352.8097706 | 385.1824792 | 497.6172401 | 409.3102572 | 402.3967763 |
| **Ramp2** | 9.156964816 | 1.584524017 | 2.349483883 | 18.11479781 | 4.43354271 | 4.90988964 | 2.706194144 | 1.676148026 | 2.257242368 | 2.621705907 | 0.945870699 | 12.14539349 | 11.0770961 | 0 | 6.467091048 |
| **Vps25** | 1227.948982 | 1304.855528 | 1297.698265 | 1189.53839 | 1342.730078 | 1283.585434 | 1214.179106 | 1407.964342 | 1262.550898 | 1272.401267 | 1270.304348 | 1305.6298 | 1245.747269 | 1270.39784 | 1251.022835 |
| **Wnk4** | 5.494178889 | 10.29940611 | 2.349483883 | 11.21392246 | 4.43354271 | 6.312715251 | 2.706194144 | 15.08533223 | 4.514484735 | 2.621705907 | 6.621094891 | 5.205168638 | 11.92918041 | 8.131991864 | 5.748525376 |
| **Coa3** | 572.310301 | 594.9887684 | 622.6132291 | 609.0022503 | 595.3614496 | 565.3387214 | 557.4759937 | 605.0894374 | 601.1788839 | 658.9220846 | 611.9783421 | 537.8674259 | 511.2505891 | 581.8891956 | 531.0200316 |
| **Cntd1** | 4.578482408 | 8.714882094 | 4.698967767 | 6.900875357 | 6.333632443 | 5.611302446 | 5.412388289 | 10.89496217 | 18.05793894 | 8.739019689 | 11.35044839 | 6.072696744 | 5.112505891 | 3.614218606 | 3.59282836 |
| **Becn1** | 1524.634642 | 1523.519842 | 1487.223298 | 1488.001249 | 1380.098509 | 1484.89091 | 1498.329491 | 1417.183156 | 1487.52272 | 1568.654034 | 1515.284859 | 1486.075646 | 1549.941369 | 1437.555451 | 1476.652456 |
| **Psme3** | 6560.049594 | 6260.454391 | 6630.243519 | 6481.647179 | 6238.627956 | 6125.438032 | 6402.855346 | 7566.970264 | 7227.690062 | 6932.664319 | 6871.750627 | 6398.887312 | 6364.21775 | 6206.516902 | 6493.677978 |
| **Aoc2** | 126.3661145 | 125.9696594 | 115.9078716 | 131.1166318 | 130.4728283 | 140.9839739 | 138.0159014 | 187.7285789 | 144.4635115 | 156.4284524 | 174.0402086 | 167.4329245 | 178.9377062 | 151.7971815 | 190.4199031 |
| **Aoc3** | 9.156964816 | 2.376786026 | 4.698967767 | 9.488703616 | 5.700269198 | 3.507064029 | 1.80412943 | 17.59955427 | 3.00965649 | 4.369509845 | 4.729353494 | 9.542809169 | 10.22501178 | 10.84265582 | 1.437131344 |
| **Aarsd1** | 1174.838586 | 1258.904332 | 1170.042974 | 1119.667027 | 1190.722899 | 1125.06614 | 994.0753157 | 1165.760952 | 1191.82397 | 1179.767658 | 1311.922659 | 1164.222719 | 1120.490874 | 1188.174367 | 1105.154003 |
| **Ptges3l** | 33.88076982 | 39.61310043 | 30.54329049 | 19.84001665 | 25.33452977 | 29.45933784 | 25.25781201 | 51.96058881 | 30.0965649 | 30.58656891 | 35.94308655 | 25.15831508 | 17.0416863 | 29.8173035 | 37.36541494 |
| **Rundc1** | 391.0023976 | 351.7643318 | 405.6775505 | 373.5098787 | 367.3506817 | 359.1233565 | 431.1869337 | 374.6190838 | 399.5318991 | 358.2998073 | 280.9235975 | 343.5411301 | 380.8816889 | 357.807642 | 365.749927 |
| **Rpl27** | 16137.31909 | 15050.60138 | 15427.49434 | 14282.22416 | 15792.27913 | 16269.97144 | 15607.5237 | 14518.7942 | 13852.69641 | 13988.54882 | 13647.02244 | 14845.14095 | 15065.70278 | 15654.08434 | 15214.19097 |
| **Ifi35** | 1602.468843 | 1329.41565 | 1389.328136 | 1319.792412 | 1302.19483 | 1358.636605 | 1429.772573 | 1217.721541 | 1332.525411 | 1200.741305 | 1143.557675 | 1242.300248 | 1143.497151 | 1316.479127 | 1377.490393 |
| **Vat1** | 1167.513014 | 997.4578687 | 1079.196264 | 976.473863 | 889.8753582 | 935.6846828 | 981.4464097 | 1000.660372 | 928.4790273 | 836.3241842 | 913.711095 | 991.5846255 | 1100.040851 | 888.1942225 | 976.5307482 |
| **Rnd2** | 7.325571853 | 7.922620085 | 6.265290356 | 20.70262607 | 5.700269198 | 9.81977928 | 2.706194144 | 15.08533223 | 9.028969471 | 11.3607256 | 12.29631908 | 10.41033728 | 11.0770961 | 18.97464768 | 20.11983882 |
| **Brca1** | 1319.51863 | 1387.250777 | 1390.111298 | 1334.456772 | 1221.124335 | 1073.863006 | 1263.792665 | 1383.660196 | 1393.470955 | 1267.157855 | 1415.022565 | 1328.185531 | 1227.853498 | 1237.869873 | 1216.531683 |
| **Nbr1** | 840.6093701 | 872.2804714 | 765.1485847 | 841.0441842 | 659.9645005 | 664.237927 | 692.785701 | 754.2666117 | 731.3465271 | 708.7344968 | 733.0497915 | 667.9966418 | 719.159162 | 708.3868468 | 678.3259943 |
| **Tmem106a** | 60.43596778 | 57.83512662 | 52.47180673 | 54.34439344 | 46.86888008 | 55.41161165 | 58.63420646 | 25.9802944 | 51.91657446 | 48.06460829 | 42.56418144 | 37.30370857 | 51.12505891 | 65.05593491 | 74.01226421 |
| **Rdm1** | 290.2757847 | 218.6643143 | 263.1421949 | 260.5080447 | 244.4782123 | 284.7735991 | 234.5368258 | 170.9670987 | 165.531107 | 186.1411194 | 192.9576225 | 225.5573076 | 192.5710552 | 261.1272943 | 266.5878643 |
| **Arl4d** | 23.80810852 | 16.63750218 | 28.1938066 | 13.80175071 | 24.70116653 | 28.05651223 | 32.47432973 | 26.81836842 | 34.61104964 | 8.739019689 | 19.86328467 | 36.43618046 | 57.08964912 | 42.46706862 | 33.77258658 |
| **Dhx8** | 1287.469253 | 1175.716821 | 1281.251878 | 1364.648102 | 1227.457967 | 1104.023756 | 1253.869954 | 1290.63398 | 1308.448159 | 1247.05811 | 1247.603452 | 1178.103168 | 1227.001414 | 1110.468667 | 1103.716872 |
| **Etv4** | 66.84584315 | 45.95119649 | 70.4845165 | 43.9930804 | 56.36932874 | 57.51585007 | 50.51562403 | 44.41792269 | 43.64001911 | 34.08217679 | 49.18527634 | 78.94505767 | 54.53339617 | 29.8173035 | 55.32955674 |
| **Dusp3** | 33.88076982 | 15.84524017 | 32.10961307 | 19.84001665 | 6.966995687 | 9.118366474 | 15.33510015 | 25.9802944 | 11.28621184 | 29.71266694 | 46.34766424 | 5.205168638 | 6.816674521 | 5.421327909 | 9.341353736 |
| **Mpp2** | 290.2757847 | 324.0351615 | 386.0985182 | 373.5098787 | 266.0125626 | 321.9484778 | 333.7639445 | 429.0938947 | 352.8822235 | 461.4202396 | 787.9102921 | 326.190568 | 345.946232 | 273.7770594 | 363.59423 |
| **Nags** | 0.915696482 | 4.753572051 | 3.915806472 | 0 | 5.066905954 | 2.805651223 | 4.510323574 | 0 | 0 | 0.873901969 | 0 | 3.470112425 | 2.556252945 | 1.807109303 | 2.155697016 |
| **Tmem101** | 270.1304621 | 264.6155108 | 252.9610981 | 228.5914962 | 237.5112166 | 251.1057844 | 225.5161787 | 255.612574 | 274.6311547 | 296.2527675 | 223.2254849 | 243.7753979 | 266.7023906 | 275.5841687 | 260.8393389 |
| **Lsm12** | 1342.411042 | 1192.354323 | 1239.744329 | 1048.070445 | 1249.625681 | 1194.506008 | 1288.148413 | 1272.196352 | 1196.338455 | 1215.597639 | 1169.096184 | 1120.846313 | 1134.124223 | 1164.681946 | 1214.375986 |
| **G6pc3** | 524.6940839 | 503.0863754 | 498.0905833 | 538.2682779 | 420.5531942 | 447.50137 | 461.857134 | 454.2361151 | 492.0788362 | 521.7194754 | 485.2316685 | 425.0887721 | 432.0067478 | 461.716427 | 496.5288793 |
| **Hdac5** | 662.9642527 | 635.3941308 | 732.2558103 | 709.9275524 | 772.703158 | 712.6354106 | 746.9095838 | 512.063222 | 613.2175099 | 601.2445546 | 597.7902816 | 746.0741714 | 842.7113877 | 855.666255 | 751.6196929 |
| **BC030867** | 543.92371 | 587.0661483 | 568.5750998 | 560.6961228 | 549.1259328 | 512.732761 | 545.7491524 | 532.1769983 | 577.101632 | 505.115338 | 575.0893849 | 560.4231567 | 513.806842 | 531.2901351 | 573.4154062 |
| **Asb16** | 1.831392963 | 3.961310043 | 2.349483883 | 1.725218839 | 2.533452977 | 4.90988964 | 3.608258859 | 5.028444078 | 0 | 0.873901969 | 5.675224193 | 2.602584319 | 0.852084315 | 0 | 1.437131344 |
| **Tmub2** | 400.1593624 | 444.4589868 | 415.0754861 | 370.059441 | 378.1178568 | 389.2841072 | 394.2022804 | 351.1530115 | 438.6574335 | 415.1034352 | 384.0235037 | 474.5378741 | 446.4921811 | 429.1884595 | 432.5765345 |
| **Atxn7l3** | 2207.744217 | 2262.700296 | 2306.410012 | 2236.746225 | 2263.640235 | 2122.47515 | 2286.734052 | 2272.856723 | 2376.123799 | 2231.945629 | 2381.70242 | 2305.889706 | 2226.496316 | 2361.891859 | 2274.260352 |
| **Ubtf** | 5533.553838 | 5667.050147 | 5807.140999 | 5727.726546 | 5813.007856 | 5632.34483 | 5818.31741 | 6746.495805 | 6106.593019 | 6396.962412 | 6193.561336 | 5833.258987 | 5756.681633 | 5692.394305 | 5662.297495 |
| **Rundc3a** | 53.11039593 | 91.11013098 | 78.31612945 | 62.10787821 | 92.47103366 | 78.55823424 | 90.20647148 | 63.69362499 | 60.19312981 | 49.81241223 | 60.53572472 | 88.48786684 | 65.61049227 | 84.0305826 | 78.32365824 |
| **Slc25a39** | 1688.544312 | 1654.243074 | 1706.508461 | 1632.057022 | 1682.212777 | 1752.830601 | 1628.22681 | 1795.99261 | 1657.568312 | 1811.598782 | 1547.444463 | 1581.503738 | 1613.847693 | 1739.342704 | 1638.329732 |
| **Grn** | 595.202713 | 603.7036505 | 564.6592933 | 574.4978735 | 473.7557067 | 472.0508182 | 497.0376578 | 562.3476628 | 550.0147236 | 546.1887306 | 516.4454015 | 503.1663016 | 489.0963969 | 523.1581433 | 472.8162122 |
| **Itga2b** | 2.747089445 | 6.338096068 | 5.482129061 | 2.587828259 | 7.600358931 | 7.014128057 | 2.706194144 | 6.704592104 | 0 | 10.48682363 | 9.458706988 | 17.35056213 | 0.852084315 | 9.035546516 | 5.748525376 |
| **Gpatch8** | 1224.286196 | 1308.024576 | 1154.379748 | 1288.738473 | 1087.48469 | 1034.583888 | 1353.999137 | 1337.566125 | 1154.203264 | 1125.585736 | 1246.657581 | 1246.637889 | 1293.46399 | 1148.417962 | 1363.11908 |
| **Gm1564** | 0 | 0 | 0 | 3.450437679 | 0.633363244 | 0 | 1.80412943 | 0 | 0 | 0.873901969 | 0 | 0 | 0 | 1.807109303 | 0 |
| **Ccdc43** | 635.4933582 | 587.8584103 | 596.7689064 | 546.8943721 | 545.9591166 | 556.2203549 | 629.6411709 | 694.7633568 | 578.6064603 | 712.2301047 | 597.7902816 | 549.1452913 | 593.0506834 | 634.2953654 | 581.3196286 |
| **Gjc1** | 7.325571853 | 2.376786026 | 8.614774239 | 1.725218839 | 4.43354271 | 2.805651223 | 9.020647148 | 6.704592104 | 1.504828245 | 0.873901969 | 0.945870699 | 5.205168638 | 0 | 0 | 2.874262688 |
| **Eftud2** | 5149.877012 | 5181.393536 | 5375.619125 | 5188.595659 | 5024.470617 | 4954.78006 | 5231.073281 | 6045.027856 | 5596.456244 | 6003.706526 | 5878.586393 | 5133.163805 | 5150.849685 | 5131.286866 | 5147.804474 |
| **Ccdc103** | 1.831392963 | 0 | 0 | 0 | 0 | 0 | 0 | 0 | 0 | 0 | 0 | 0 | 0 | 0 | 0 |
| **Kif18b** | 1178.501372 | 1343.676366 | 1300.047749 | 1346.533304 | 1264.826399 | 1178.373514 | 1305.287642 | 1248.730279 | 1367.136461 | 1277.644679 | 1275.979573 | 1350.741261 | 1224.445161 | 1378.824398 | 1231.621562 |
| **C1ql1** | 1.831392963 | 1.584524017 | 0 | 0 | 3.800179466 | 0 | 0 | 0 | 0 | 0 | 4.729353494 | 0 | 0 | 1.807109303 | 0 |
| **Dcakd** | 410.2320237 | 415.1452925 | 421.3407764 | 455.4577736 | 478.8226127 | 463.6338646 | 453.7385515 | 408.9801184 | 408.5608686 | 348.6868856 | 401.0491763 | 443.3068623 | 433.7109164 | 478.8839653 | 429.7022718 |
| **Nmt1** | 2813.019591 | 2359.356261 | 2523.345691 | 2473.963816 | 2346.61082 | 2393.921906 | 2448.203636 | 2625.685883 | 2566.484572 | 2550.045945 | 2734.51219 | 2426.476113 | 2297.219314 | 2349.242094 | 2250.547685 |
| **Plcd3** | 8.241268334 | 0.792262009 | 8.614774239 | 4.313047098 | 3.166816221 | 8.416953669 | 6.314453003 | 10.05688816 | 5.266898858 | 0.873901969 | 0 | 6.072696744 | 17.0416863 | 4.517773258 | 8.622788064 |
| **Acbd4** | 119.9562391 | 96.65596504 | 111.9920651 | 125.9409753 | 95.00448664 | 99.60061841 | 119.0725424 | 105.5973256 | 97.81383594 | 108.3638441 | 89.85771638 | 130.1292159 | 106.5105394 | 100.2945663 | 114.9705075 |
| **Hexim1** | 981.6266282 | 1086.191214 | 996.1811666 | 1178.324467 | 1033.015451 | 1013.541504 | 1003.095963 | 1214.369245 | 1357.355077 | 1085.386245 | 1142.611804 | 1195.45373 | 1142.645067 | 1279.433387 | 1143.237984 |
| **Hexim2** | 79.6655939 | 86.35655893 | 101.027807 | 104.3757398 | 84.23731149 | 79.96105985 | 95.61885977 | 87.99777137 | 57.93588744 | 69.91215751 | 63.37333682 | 76.34247335 | 74.98341973 | 79.51280934 | 93.41353736 |
| **Fmnl1** | 9225.642052 | 8802.030914 | 8664.113401 | 8917.65618 | 8978.557351 | 9073.476055 | 9547.452941 | 8997.562604 | 8838.608698 | 8430.532294 | 8860.916706 | 8886.957921 | 8895.76025 | 9143.069519 | 9391.653333 |
| **1700023F06Rik** | 2.747089445 | 0 | 0 | 0 | 1.266726489 | 0.701412806 | 0 | 1.676148026 | 0 | 0 | 0 | 0 | 0 | 0 | 0.718565672 |
| **Spata32** | 0 | 4.753572051 | 0 | 1.725218839 | 1.266726489 | 3.507064029 | 1.80412943 | 0 | 0 | 0 | 1.891741398 | 0.867528106 | 0 | 1.807109303 | 0.718565672 |
| **Map3k14** | 944.998769 | 900.0096417 | 786.2939397 | 847.0824501 | 852.5069268 | 794.7007089 | 856.961479 | 736.6670575 | 850.2279585 | 772.5293405 | 785.07268 | 760.8221492 | 856.3447367 | 835.7880527 | 899.6442213 |
| **Arhgap27** | 777.4263128 | 820.7834408 | 816.8372302 | 857.4337631 | 862.6407387 | 855.723623 | 948.9720799 | 860.7020114 | 874.3052104 | 817.0983409 | 793.5855163 | 897.89159 | 916.8427231 | 896.3262144 | 947.0695557 |
| **Arhgap27os3** | 65.93014667 | 133.1000174 | 68.91819391 | 77.63484777 | 59.53614496 | 75.05117021 | 77.57756547 | 82.13125328 | 66.21244279 | 116.2289619 | 91.74945778 | 95.42809169 | 86.91260015 | 129.2083152 | 135.0903463 |
| **Plekhm1** | 653.8072878 | 700.3596155 | 746.3527136 | 699.5762393 | 631.4631545 | 620.0489202 | 677.4506008 | 650.3454341 | 717.0506588 | 621.3442999 | 775.613973 | 689.6848445 | 743.8696071 | 655.0771224 | 714.2542779 |
| **Gosr2** | 1555.768322 | 1534.61151 | 1730.786461 | 1651.034429 | 1667.645422 | 1667.959652 | 1743.691094 | 1656.872324 | 1650.044171 | 1712.847859 | 1618.384766 | 1727.24846 | 1774.039544 | 1778.195554 | 1703.719208 |
| **C130046K22Rik** | 44.8691276 | 66.55000871 | 46.98967767 | 69.00875357 | 54.46923901 | 61.0229141 | 68.55691832 | 67.88399506 | 42.88760499 | 62.92094176 | 85.12836289 | 59.85943933 | 61.35007069 | 56.0203884 | 47.42533435 |
| **Wnt3** | 6.409875371 | 16.63750218 | 8.614774239 | 16.38957897 | 4.43354271 | 3.507064029 | 4.510323574 | 46.93214473 | 18.81035306 | 29.71266694 | 17.97154328 | 6.94022485 | 9.372927467 | 13.55331977 | 5.748525376 |
| **Nsf** | 1185.826944 | 1144.818602 | 1147.331296 | 1113.628761 | 1019.714823 | 1029.673999 | 1075.26114 | 1406.288194 | 1243.740545 | 1351.052444 | 1340.29878 | 1137.329347 | 1088.11167 | 1150.225071 | 1217.968814 |
| **Arf2** | 304.9269284 | 298.6827772 | 316.397163 | 330.3794077 | 347.7164211 | 310.7258729 | 314.8205855 | 278.2405723 | 312.2518609 | 278.7747281 | 331.0547446 | 333.1307928 | 282.0399083 | 279.1983873 | 289.5819658 |
| **Mapt** | 0 | 2.376786026 | 1.566322589 | 5.175656518 | 1.900089733 | 0 | 0 | 0 | 0 | 1.747803938 | 0 | 0 | 1.70416863 | 0 | 4.311394032 |
| **Kansl1** | 1509.067802 | 1636.81331 | 1567.888912 | 1568.223925 | 1362.997702 | 1451.924508 | 1540.726533 | 1600.721365 | 1628.224161 | 1532.824053 | 1570.14536 | 1542.464973 | 1737.399919 | 1723.078721 | 1668.50949 |
| **Cdc27** | 1569.503769 | 1604.330567 | 1697.110525 | 1554.422174 | 1397.199317 | 1322.163139 | 1526.293497 | 1789.288018 | 1830.62356 | 1774.894899 | 1841.610251 | 1531.187108 | 1496.260057 | 1457.433653 | 1468.029668 |
| **Myl4** | 72.34002204 | 53.87381658 | 60.30341968 | 65.55831589 | 54.46923901 | 54.71019885 | 59.53627118 | 39.38947861 | 42.88760499 | 46.31680435 | 53.91462983 | 72.87236093 | 74.98341973 | 51.50261514 | 73.29369854 |
| **Itgb3** | 420.304685 | 461.888751 | 417.42497 | 483.9238844 | 513.6575911 | 459.4253877 | 616.1102002 | 171.8051727 | 181.3318035 | 188.7628253 | 182.5530449 | 564.7607972 | 535.9610342 | 543.0363456 | 462.0377271 |
| **Mettl2** | 657.4700738 | 682.1375893 | 683.6998101 | 636.6057517 | 633.3632443 | 707.0241082 | 650.3886594 | 722.4197993 | 669.6485691 | 681.6435357 | 697.106705 | 639.3682143 | 646.7319952 | 659.5948956 | 597.1280734 |
| **Tlk2** | 1480.681211 | 1294.556122 | 1300.83091 | 1151.583575 | 1153.987831 | 1267.45294 | 1290.854607 | 1231.968799 | 1232.454333 | 1138.694265 | 1198.418175 | 1169.427887 | 1178.432608 | 1212.570342 | 1217.968814 |
| **1700052K11Rik** | 76.00280797 | 66.55000871 | 81.44877463 | 69.00875357 | 68.40323038 | 78.55823424 | 80.28375962 | 75.42666117 | 51.16416034 | 66.41654964 | 55.80637123 | 65.93213608 | 102.2501178 | 80.41636399 | 76.8865269 |
| **Mrc2** | 3.662785926 | 0 | 0.783161294 | 3.450437679 | 2.533452977 | 1.402825611 | 0 | 0 | 0.752414123 | 2.621705907 | 0 | 1.735056213 | 0 | 1.807109303 | 0.718565672 |
| **Tanc2** | 132.7759898 | 123.5928733 | 106.5099361 | 87.12355138 | 62.70296118 | 57.51585007 | 60.43833589 | 87.99777137 | 70.72692752 | 45.44290238 | 75.6696559 | 64.19707986 | 92.87719035 | 47.88839653 | 61.79664779 |
| **Cyb561** | 20.14532259 | 22.97559825 | 19.57903236 | 31.91654853 | 12.66726489 | 16.83390734 | 15.33510015 | 14.24725822 | 25.58208017 | 13.9824315 | 31.21373306 | 11.27786538 | 22.15419219 | 9.035546516 | 14.37131344 |
| **Kcnh6** | 1.831392963 | 0 | 0 | 0.86260942 | 0.633363244 | 0.701412806 | 0 | 2.514222039 | 3.762070613 | 0 | 0 | 0 | 2.556252945 | 1.807109303 | 0 |
| **Dcaf7** | 3616.085406 | 3672.926671 | 3895.444279 | 3774.77882 | 3728.609419 | 3589.83074 | 3839.187426 | 4048.735557 | 4268.445317 | 3663.397054 | 3832.668071 | 4067.83929 | 3842.048177 | 3897.934767 | 3833.54786 |
| **Taco1** | 326.9036439 | 320.8661134 | 376.7005826 | 315.7150476 | 338.8493357 | 327.5597803 | 335.5680739 | 315.1158289 | 390.5029296 | 385.3907683 | 328.2171325 | 320.1178712 | 323.7920398 | 393.9498281 | 333.4144718 |
| **Map3k3** | 943.167376 | 1132.934672 | 1141.066006 | 1029.093038 | 999.4471995 | 951.1157645 | 1177.194453 | 1097.876957 | 1058.64667 | 1073.151618 | 1042.34951 | 1028.020806 | 1065.105394 | 1114.98644 | 985.1535363 |
| **Limd2** | 6787.142321 | 7276.926548 | 7050.017973 | 7361.508787 | 8024.078942 | 7908.429384 | 8132.113404 | 6814.3798 | 7308.198373 | 6768.370749 | 6127.350387 | 7901.445992 | 7656.829656 | 7980.194683 | 7590.927759 |
| **Strada** | 543.92371 | 577.5590042 | 600.6847129 | 624.5292198 | 573.1937361 | 479.0649463 | 550.259476 | 537.2054424 | 556.7864507 | 542.6931227 | 667.7847133 | 514.444167 | 553.8548049 | 522.2545886 | 538.924254 |
| **Ccdc47** | 2142.729767 | 2060.673484 | 2261.769818 | 1911.542474 | 1953.925609 | 2048.826805 | 2109.929368 | 1935.95097 | 2098.482988 | 2016.091842 | 2042.134839 | 2161.012513 | 1971.723105 | 2117.028549 | 2097.493196 |
| **Ddx42** | 2661.013975 | 2669.130707 | 2669.013692 | 2568.850852 | 2506.218358 | 2645.729103 | 2763.024221 | 2496.622485 | 2505.539028 | 2423.33016 | 2602.090292 | 2673.721624 | 2682.361424 | 2523.628142 | 2463.961689 |
| **Ftsj3** | 3854.166491 | 3492.290933 | 3636.21789 | 3589.317795 | 3569.001881 | 3438.325574 | 3757.099537 | 4332.842647 | 3991.55692 | 4114.33047 | 4194.936549 | 3575.950854 | 3448.385223 | 3414.533028 | 3370.791567 |
| **Psmc5** | 3452.175735 | 3239.559353 | 3369.94305 | 3298.618421 | 3300.455866 | 3457.263719 | 3071.530354 | 3912.967567 | 3605.568475 | 3699.227034 | 3481.750042 | 3217.661746 | 3250.701662 | 3268.157175 | 2962.646266 |
| **Smarcd2** | 2452.235178 | 2676.261065 | 2849.140789 | 2611.981323 | 2596.789302 | 2697.633651 | 2781.96758 | 2666.75151 | 2927.643351 | 2670.644417 | 2562.363723 | 2717.965557 | 2627.828028 | 2864.268245 | 2767.914968 |
| **Tcam1** | 21.06101908 | 22.18333624 | 15.66322589 | 30.19132969 | 20.90098706 | 16.83390734 | 15.33510015 | 28.49451644 | 14.29586833 | 32.33437285 | 19.86328467 | 26.02584319 | 8.520843152 | 12.64976512 | 20.83840449 |

| | | | | | | | | | | | | | | | |
|---|---|---|---|---|---|---|---|---|---|---|---|---|---|---|---|
| **Gh** | 0 | 0 | 0 | 0 | 0 | 0 | 0 | 0 | 0 | 0 | 0 | 0 | 0 | 0 | 0 |
| **Cd79b** | 71.42432556 | 52.28929256 | 85.3645811 | 73.32180067 | 82.970585 | 59.62008849 | 80.28375962 | 68.72206907 | 75.24141226 | 65.54264767 | 55.80637123 | 57.25685501 | 57.08964912 | 73.18792678 | 85.50931496 |
| **Prr29** | 0 | 1.584524017 | 0.783161294 | 0 | 1.266726489 | 0.701412806 | 1.80412943 | 4.190370065 | 0.752414123 | 0 | 0 | 0.867528106 | 0 | 0 | 0 |
| **Icam2** | 349.796056 | 331.9577816 | 303.083421 | 284.6611085 | 316.0482589 | 305.1145705 | 335.5680739 | 227.1180575 | 319.023588 | 262.1705907 | 272.4107612 | 320.9853993 | 339.9816417 | 277.391278 | 293.1747942 |
| **Ern1** | 50.36330649 | 82.39524888 | 96.32883922 | 110.4140057 | 50.0356963 | 70.14128057 | 74.87137133 | 90.51199341 | 71.47934165 | 62.92094176 | 101.2081648 | 57.25685501 | 72.42716679 | 67.76659887 | 73.29369854 |
| **Snord104** | 9.156964816 | 7.922620085 | 10.18109683 | 10.35131304 | 10.13381191 | 10.52119209 | 8.118582433 | 1.676148026 | 6.019312981 | 19.22584332 | 2.837612096 | 6.94022485 | 11.0770961 | 9.939101167 | 8.622788064 |
| **Tex2** | 2544.720522 | 2461.55806 | 2478.705497 | 2404.955062 | 1754.416187 | 1879.084907 | 2005.289861 | 1986.235411 | 2113.53127 | 2137.564216 | 2529.258249 | 1613.602278 | 1824.312519 | 1692.357862 | 1761.923028 |
| **Pecam1** | 649.2288054 | 701.9441395 | 660.2049713 | 717.6910371 | 708.1001071 | 707.0241082 | 747.8116486 | 404.7897483 | 527.4422999 | 423.8424549 | 413.3454954 | 811.1387794 | 645.8799109 | 727.3614945 | 610.0622555 |
| **Milr1** | 293.9385706 | 282.837537 | 209.8872269 | 210.4766984 | 167.2078965 | 173.9503758 | 216.4955315 | 164.2625066 | 194.8752577 | 193.1323351 | 185.390657 | 185.6510147 | 212.1689945 | 176.1931571 | 188.9827717 |
| **Polg2** | 83.32837982 | 121.2160873 | 119.8236781 | 101.7879115 | 95.63784989 | 101.7048568 | 96.52092448 | 81.29317927 | 128.662815 | 128.4635894 | 151.3393118 | 99.76573222 | 92.02510604 | 106.6194489 | 119.2819015 |
| **Ddx5** | 16027.43552 | 15824.64136 | 16733.80738 | 15048.22133 | 16451.61027 | 15699.02142 | 16492.44918 | 16336.57674 | 15271.74945 | 16135.72595 | 15894.41122 | 17073.82066 | 17357.80958 | 17129.58908 | 16679.34638 |
| **Mir3064** | 12.81975074 | 6.338096068 | 6.265290356 | 10.35131304 | 13.30062813 | 11.9240177 | 16.23716487 | 10.89496217 | 3.762070613 | 7.86511772 | 5.675224193 | 7.807752957 | 33.23128829 | 13.55331977 | 12.9341821 |
| **Cep95** | 166.6567596 | 173.5053799 | 219.2851625 | 166.483618 | 191.909063 | 178.1588527 | 175.0005547 | 191.080875 | 179.0745612 | 184.3933154 | 184.4447863 | 201.2665207 | 194.2752239 | 175.2896024 | 180.3599837 |
| **Smurf2** | 445.9441865 | 396.9232663 | 428.3892281 | 387.3116294 | 362.2837757 | 430.6674627 | 446.5220338 | 463.4549292 | 379.9691319 | 453.5551219 | 473.8812201 | 452.8496715 | 425.1900733 | 364.1325246 | 400.2410793 |
| **Kpna2** | 7221.182454 | 7566.894443 | 7520.697911 | 7433.105369 | 7133.57022 | 6772.140639 | 7011.749028 | 8120.099112 | 8305.147085 | 8228.660939 | 8221.508114 | 7519.733625 | 7403.760614 | 7606.123057 | 7123.141506 |
| **1810010H24Rik** | 9.156964816 | 6.338096068 | 7.04845165 | 10.35131304 | 3.800179466 | 7.014128057 | 3.608258859 | 9.218814144 | 12.79104008 | 0.873901969 | 1.891741398 | 3.470112425 | 0 | 0 | 3.59282836 |
| **Bptf** | 4031.811608 | 3732.346322 | 3947.132924 | 3767.877945 | 3096.512901 | 3114.97427 | 3637.12493 | 3817.427129 | 3965.97484 | 4011.210037 | 4263.039239 | 3585.493663 | 3819.893985 | 3278.999831 | 3462.049408 |
| **Nol11** | 1731.582047 | 1772.290113 | 1892.117687 | 1840.808502 | 1803.81852 | 1804.735149 | 1897.042095 | 2199.944284 | 2121.055412 | 2022.209156 | 2205.77047 | 1780.167674 | 1768.927038 | 1657.119231 | 1697.970683 |
| **Pitpnc1** | 558.5748538 | 675.0072312 | 689.9651004 | 715.9658183 | 663.1313168 | 662.1336886 | 668.4299537 | 628.5555098 | 540.23334 | 536.5758089 | 565.6306779 | 704.4328223 | 744.7216915 | 680.3766526 | 621.5593063 |
| **Psmd12** | 2507.176967 | 2484.533659 | 2488.886594 | 2398.054187 | 2389.679521 | 2316.065084 | 2403.1004 | 2603.895959 | 2616.896318 | 2451.295023 | 2461.155558 | 2287.671616 | 2325.338096 | 2526.338806 | 2350.428313 |
| **Helz** | 1366.21915 | 1423.694829 | 1394.810265 | 1260.272362 | 1179.955724 | 1235.187951 | 1395.494114 | 1394.555158 | 1353.593007 | 1273.275169 | 1355.432711 | 1393.250139 | 1381.228675 | 1405.027483 | 1408.388717 |
| **Cacng4** | 0 | 0 | 0 | 1.725218839 | 1.900089733 | 0 | 0 | 0 | 6.771727103 | 9.612921658 | 6.621094891 | 0 | 1.70416863 | 3.614218606 | 1.437131344 |
| **Prkca** | 1132.716548 | 1011.718585 | 1110.522716 | 1077.399165 | 932.3106956 | 1012.840091 | 1105.93134 | 1048.43059 | 1114.325316 | 1044.312853 | 984.6513974 | 1008.935188 | 1001.19907 | 941.5039469 | 991.6206273 |
| **Axin2** | 130.9445969 | 160.0369257 | 173.0786461 | 154.4070861 | 155.1739948 | 166.234835 | 163.2737134 | 135.7679901 | 112.8621184 | 137.2026091 | 108.7751304 | 193.4587677 | 195.1273082 | 141.8580803 | 164.5515389 |
| **Rgs9** | 32.04937685 | 50.70476854 | 34.45909696 | 34.50437679 | 44.3354271 | 56.11302446 | 60.43833589 | 32.68488651 | 21.06759543 | 16.60413741 | 46.34766424 | 47.71404585 | 52.82922754 | 32.52796746 | 44.55107166 |
| **1700096J18Rik** | 2.747089445 | 2.376786026 | 2.349483883 | 1.725218839 | 0.633363244 | 1.402825611 | 0 | 4.190370065 | 0 | 0 | 0 | 1.735056213 | 0.852084315 | 0.903554652 | 1.437131344 |
| **Gm11696** | 14.65114371 | 15.84524017 | 9.397935534 | 12.07653188 | 22.16771355 | 14.72966892 | 15.33510015 | 16.76148026 | 21.82000955 | 27.09096104 | 14.18806048 | 15.61550591 | 5.112505891 | 20.78175699 | 15.80844478 |
| **Gna13** | 4522.624922 | 4271.084488 | 4149.188538 | 3959.377236 | 3778.645115 | 4156.572287 | 4944.216702 | 3614.613218 | 3328.680078 | 3253.53703 | 3582.958207 | 4387.089633 | 4346.482092 | 4111.173665 | 4202.17205 |
| **Amz2** | 417.5575956 | 399.3000523 | 411.1596796 | 434.7551475 | 427.5201899 | 459.4253877 | 509.6665639 | 339.4199753 | 267.8594276 | 319.8481206 | 309.2997185 | 471.0677617 | 489.9484812 | 419.2493583 | 447.6664136 |
| **Slc16a6** | 551.2492819 | 602.9113885 | 581.1056805 | 527.0543554 | 572.5603728 | 609.5277282 | 624.2287826 | 465.9691513 | 435.647777 | 388.0124742 | 467.2601252 | 564.7607972 | 611.7965383 | 544.8434549 | 597.1280734 |
| **Arsg** | 5.494178889 | 6.338096068 | 12.53058071 | 10.35131304 | 12.03390164 | 16.83390734 | 8.118582433 | 10.89496217 | 4.514484735 | 10.48682363 | 5.675224193 | 11.27786538 | 5.964590206 | 23.49242094 | 11.49705075 |
| **Wipi1** | 12.81975074 | 12.67619214 | 18.01270977 | 12.07653188 | 11.4005384 | 12.6254305 | 9.922711863 | 19.2757023 | 11.28621184 | 13.10852953 | 15.13393118 | 20.82067455 | 18.74585493 | 11.74621047 | 11.49705075 |
| **Prkar1a** | 9267.76409 | 9322.547054 | 9487.215921 | 9279.952137 | 9220.50211 | 9173.778086 | 9498.741447 | 9563.262563 | 9387.871007 | 9231.900399 | 9365.065789 | 9459.526471 | 9671.156977 | 9732.187152 | 9259.437249 |
| **Fam20a** | 165.7410632 | 149.7375196 | 145.6680008 | 162.1705709 | 88.03749095 | 84.87094949 | 88.40234205 | 54.47481085 | 61.69795805 | 82.14678508 | 104.0457769 | 102.3683165 | 131.2209845 | 97.58390237 | 119.2819015 |
| **Abca5** | 25.63950148 | 35.65179038 | 30.54329049 | 27.60350143 | 14.56735462 | 30.16075065 | 9.922711863 | 87.15969736 | 84.27038173 | 91.75970673 | 80.3990094 | 13.8804497 | 20.45002356 | 14.45687443 | 12.21561642 |
| **Map2k6** | 25.63950148 | 28.52143231 | 47.77283896 | 39.6800333 | 55.7359655 | 38.57770431 | 50.51562403 | 31.8468125 | 51.91657446 | 53.3080201 | 27.43025026 | 52.05168638 | 57.94173343 | 72.28437213 | 51.01816271 |
| **Kcnj16** | 0 | 0.792262009 | 0 | 0 | 0 | 0 | 0 | 0 | 0 | 0 | 0 | 0 | 1.70416863 | 0 | 2.155697016 |
| **2610035D17Rik** | 50.36330649 | 53.08155457 | 50.90548414 | 55.20700286 | 65.8697774 | 57.51585007 | 72.16517718 | 31.8468125 | 40.63036262 | 40.19949057 | 48.23940564 | 62.46202365 | 76.68758836 | 42.46706862 | 43.83250599 |
| **4732490B19Rik** | 0 | 2.376786026 | 0 | 0.86260942 | 1.900089733 | 1.402825611 | 0 | 0 | 0 | 0 | 0 | 1.735056213 | 0 | 0.903554652 | 0 |
| **Slc39a11** | 319.5780721 | 260.6542008 | 288.2033564 | 283.7984991 | 288.1802761 | 242.6888308 | 237.24302 | 299.1924227 | 331.8146281 | 370.5344348 | 301.7327529 | 292.3569718 | 253.9211259 | 262.9344036 | 227.785318 |
| **Cog1** | 617.1794286 | 635.3941308 | 682.9166488 | 582.2613583 | 589.6611804 | 567.4429598 | 666.6258242 | 683.0303206 | 743.3851531 | 644.9396531 | 598.7361523 | 611.6073149 | 743.8696071 | 580.985641 | 678.3259943 |
| **Fam104a** | 818.6326545 | 823.9524888 | 781.5949719 | 741.8441009 | 796.1375981 | 911.8366474 | 901.1626501 | 855.6735673 | 977.3859452 | 810.1071252 | 809.6653181 | 809.4037232 | 769.4321366 | 800.5494213 | 866.5902004 |
| **D11Wsu47e** | 307.6740178 | 266.2000349 | 296.8181306 | 342.4559396 | 262.8457464 | 300.2046808 | 277.8359322 | 336.9057532 | 323.5380727 | 301.4961793 | 302.6786236 | 285.416747 | 320.3837025 | 242.1526466 | 291.0190971 |
| **Cpsf4l** | 3.662785926 | 5.54583406 | 0 | 1.725218839 | 0.633363244 | 0 | 2.706194144 | 6.704592104 | 2.257242368 | 5.243411813 | 0.945870699 | 2.602584319 | 6.816674521 | 2.710663955 | 2.874262688 |
| **Cdc42ep4** | 91.56964816 | 99.82501307 | 75.18348427 | 69.00875357 | 53.20251252 | 87.67660071 | 81.18582433 | 62.85555098 | 57.93588744 | 80.39898114 | 64.31920752 | 76.34247335 | 74.98341973 | 48.79195118 | 71.8565672 |
| **Rpl38** | 4559.252782 | 3977.947545 | 4250.216345 | 3601.394327 | 4329.671138 | 4289.139307 | 4236.997965 | 3448.674564 | 3317.393866 | 3377.63111 | 3214.068634 | 3621.929844 | 3275.412107 | 3884.381447 | 3684.0862 |
| **Ttyh2** | 4.578482408 | 4.753572051 | 5.482129061 | 10.35131304 | 5.700269198 | 3.507064029 | 6.314453003 | 12.5711102 | 3.00965649 | 6.117313782 | 6.621094891 | 11.27786538 | 4.260421576 | 2.710663955 | 1.437131344 |
| **Rab37** | 177.6451174 | 209.1571702 | 240.4305174 | 244.9810752 | 280.5799172 | 237.0775283 | 289.5627734 | 165.1005806 | 206.9138837 | 185.2672174 | 192.9576225 | 258.5233757 | 259.8857161 | 286.4268245 | 262.9950359 |
| **Cd300lf** | 4.578482408 | 11.09166812 | 9.397935534 | 1.725218839 | 11.4005384 | 4.90988964 | 8.118582433 | 0 | 0 | 1.747803938 | 2.837612096 | 3.470112425 | 10.22501178 | 6.324882561 | 5.029959704 |
| **Slc9a3r1** | 3013.557121 | 3353.645082 | 3303.37434 | 3438.361147 | 3258.653892 | 3361.170165 | 3371.015839 | 3206.471174 | 3588.262951 | 3052.539577 | 3187.584255 | 3371.214221 | 3335.05801 | 3406.401036 | 3185.401624 |
| **Nat9** | 82.41268334 | 103.7863231 | 104.1604522 | 61.2452688 | 84.23731149 | 78.55823424 | 79.3816949 | 72.07436512 | 78.25106875 | 84.76849098 | 69.04856101 | 60.72696744 | 83.50426289 | 74.99503608 | 70.41943585 |
| **Tmem104** | 425.7988639 | 472.1881571 | 418.2081313 | 382.9985823 | 364.1838655 | 351.4078157 | 356.3155623 | 350.3149375 | 388.9981014 | 344.3173757 | 366.9978311 | 326.190568 | 384.2900261 | 403.8889293 | 362.1570987 |
| **Grin2c** | 0 | 3.169048034 | 0 | 5.175656518 | 0 | 2.104238417 | 0 | 4.190370065 | 0 | 3.495607876 | 7.56696559 | 0 | 0.852084315 | 0 | 1.437131344 |
| **Fdxr** | 398.3279695 | 442.0822007 | 404.8943892 | 402.838599 | 369.8841347 | 364.0332462 | 440.2075808 | 453.3980411 | 395.0174144 | 366.164925 | 454.0179354 | 378.2422543 | 382.5858575 | 387.6249455 | 390.8997255 |
| **Fads6** | 7.325571853 | 15.84524017 | 10.18109683 | 5.175656518 | 7.600358931 | 6.312715251 | 4.510323574 | 9.218814144 | 15.80069657 | 10.48682363 | 14.18806048 | 7.807752957 | 8.520843152 | 10.84265582 | 10.77848508 |
| **Hid1** | 410.2320237 | 404.8458863 | 441.7029701 | 502.0386822 | 350.2498741 | 352.1092285 | 378.8671802 | 332.7153832 | 334.0718704 | 403.7427096 | 530.633462 | 419.0160753 | 475.4630479 | 415.6351397 | 395.9296853 |
| **Cdr2l** | 12.81975074 | 16.63750218 | 24.27800013 | 13.80175071 | 22.16771355 | 11.22260489 | 13.53097072 | 27.65644243 | 14.29586833 | 20.97364725 | 32.15960376 | 14.74797781 | 17.0416863 | 27.10663955 | 18.68270747 |
| **Ict1** | 736.2199712 | 791.4697465 | 720.5083909 | 737.5310538 | 758.1358034 | 755.4215918 | 661.2134359 | 775.2184621 | 700.4975481 | 720.9691243 | 726.4286967 | 708.7704628 | 775.3967268 | 668.6304422 | 686.2302167 |
| **Atp5h** | 2566.697238 | 2449.67413 | 2456.776981 | 2611.118713 | 2687.360245 | 2588.213253 | 2636.735161 | 2567.020702 | 2397.191395 | 2363.904826 | 2426.158342 | 2684.131961 | 2577.555053 | 2639.283137 | 2548.752438 |
| **Kctd2** | 237.1653887 | 243.2244366 | 343.8078083 | 265.6837013 | 268.5460156 | 313.5315242 | 299.4854853 | 226.2799835 | 220.4573379 | 215.8537863 | 256.3309594 | 282.8141626 | 291.4128358 | 271.9699501 | 293.1747942 |
| **Slc16a5** | 37.54355574 | 56.2506026 | 54.03812932 | 64.69570647 | 41.16861088 | 29.45933784 | 44.20117102 | 43.57984868 | 61.69795805 | 50.6863142 | 77.5613973 | 55.5217988 | 23.85836082 | 21.68531164 | 39.52111196 |
| **Armc7** | 559.4905502 | 542.6994758 | 585.021487 | 587.4370148 | 637.796787 | 600.4093617 | 643.1721416 | 464.2930032 | 537.2236835 | 516.4760636 | 474.8270908 | 573.4360783 | 545.3339617 | 530.3865805 | 530.3014659 |
| **Nt5c** | 1472.439942 | 1471.23055 | 1399.509233 | 1435.382074 | 1257.859403 | 1357.935192 | 1237.632789 | 1317.452349 | 1133.888083 | 1496.120171 | 1296.788728 | 1204.99654 | 1264.493124 | 1367.981742 | 1314.97518 |
| **Hn1** | 4833.961726 | 4850.228016 | 5390.49919 | 5137.701703 | 5514.693768 | 5290.756793 | 5166.124622 | 5012.520672 | 5097.60568 | 5091.352871 | 5149.320084 | 5373.46909 | 5389.433293 | 5672.516103 | 5341.09864 |
| **Sumo2** | 2434.836944 | 2377.578288 | 2331.471174 | 2350.610669 | 2480.883828 | 2386.907778 | 2453.616024 | 2633.228549 | 2499.519715 | 2377.013355 | 2371.297842 | 2320.637684 | 2320.22559 | 2437.79045 | 2279.290311 |
| **Nup85** | 2094.197853 | 2200.90386 | 2265.685625 | 2190.165316 | 2231.972073 | 2180.692413 | 2040.470385 | 2477.346783 | 2300.882387 | 2581.506416 | 2597.360939 | 2260.778245 | 2246.946339 | 2295.028815 | 2197.373825 |
| **Gga3** | 595.202713 | 629.0560348 | 595.9857451 | 569.322217 | 528.2249457 | 559.727419 | 602.5792295 | 577.432995 | 516.1560881 | 585.5143192 | 604.4113765 | 601.1969777 | 535.1089499 | 562.9145479 | 540.3613853 |
| **Mrps7** | 1420.245243 | 1455.38531 | 1452.764201 | 1461.260357 | 1442.80147 | 1407.735501 | 1396.396178 | 1709.670987 | 1577.060001 | 1801.98586 | 1571.091231 | 1421.878566 | 1365.891157 | 1230.641435 | 1427.071425 |
| **Mif4gd** | 599.7811954 | 591.0274583 | 592.8530999 | 539.1308873 | 609.9288042 | 699.3085673 | 639.5638828 | 589.1660312 | 593.6547427 | 582.8926133 | 538.2004276 | 550.0128194 | 668.0341031 | 671.3411061 | 666.1103779 |
| **Slc25a19** | 543.0080136 | 620.3411527 | 610.8658097 | 554.6578568 | 556.7262917 | 521.8511275 | 533.1202464 | 646.1550641 | 568.0726625 | 578.5231034 | 628.058144 | 551.7478756 | 548.742299 | 509.6048235 | 513.0558898 |
| **Grb2** | 2861.551505 | 2994.750392 | 3042.581629 | 2910.444182 | 2870.402223 | 2818.978066 | 2947.045423 | 2842.747052 | 2929.148179 | 3001.853263 | 2948.278968 | 3069.31444 | 3070.059788 | 3073.892925 | 3027.317176 |
| **2310067B10Rik** | 983.4580212 | 835.836419 | 909.2502629 | 892.8007493 | 841.7397516 | 844.5010181 | 918.3018796 | 836.397865 | 823.8934642 | 810.9810271 | 820.0698958 | 829.3568696 | 861.4572426 | 846.6307085 | 872.3387258 |
| **Caskin2** | 89.73825519 | 87.94108294 | 61.08658097 | 92.2992079 | 113.3720207 | 67.33562935 | 80.28375962 | 113.1399918 | 121.1386737 | 117.1028638 | 82.29075079 | 106.7059571 | 62.20215501 | 101.198121 | 87.66501198 |
| **Tsen54** | 477.0778669 | 562.506026 | 545.8634223 | 546.8943721 | 552.9261123 | 560.4288318 | 523.1975346 | 648.6692861 | 629.7706206 | 618.722594 | 637.516851 | 536.1323697 | 616.0569599 | 537.6150177 | 576.2896689 |
| **Llgl2** | 141.9329546 | 151.3220436 | 183.2597429 | 159.5827426 | 185.5754306 | 125.5528922 | 143.4282897 | 201.9758371 | 159.511794 | 166.0413741 | 180.6613035 | 154.4200029 | 200.2398141 | 179.8073757 | 192.5756001 |
| **Recql5** | 364.4471997 | 423.0679125 | 415.0754861 | 392.4872859 | 376.2177671 | 365.4360718 | 390.5940215 | 388.028268 | 431.8857064 | 330.3349442 | 391.5904693 | 379.9773106 | 440.5275909 | 453.5844351 | 430.4208375 |
| **Smim5** | 9.156964816 | 3.961310043 | 15.66322589 | 0.86260942 | 1.900089733 | 3.507064029 | 7.216517718 | 29.33259046 | 35.36346376 | 31.46047088 | 19.86328467 | 4.337640531 | 10.22501178 | 11.74621047 | 5.029959704 |
| **Smim6** | 0 | 3.169048034 | 5.482129061 | 4.313047098 | 6.966995687 | 4.208476834 | 0.902064715 | 5.028444078 | 0 | 1.747803938 | 16.07980188 | 3.470112425 | 1.70416863 | 0 | 2.155697016 |
| **Sap30bp** | 901.0453379 | 886.5411875 | 975.0358116 | 909.1903283 | 882.9083625 | 960.234131 | 893.0440676 | 897.577268 | 906.6590177 | 963.0399697 | 970.4633369 | 885.7461965 | 913.4343859 | 886.3871132 | 848.6260586 |
| **Itgb4** | 7.325571853 | 11.09166812 | 8.614774239 | 5.175656518 | 13.30062813 | 2.805651223 | 3.608258859 | 5.028444078 | 0.752414123 | 11.3607256 | 6.621094891 | 10.41033728 | 4.260421576 | 6.324882561 | 7.904222392 |
| **Galk1** | 3756.186967 | 3452.677833 | 3349.580857 | 3443.536803 | 3529.73336 | 3677.50734 | 3601.042341 | 2201.620432 | 2411.487263 | 2537.811318 | 2456.426205 | 3190.768375 | 3322.276745 | 3135.334641 | 3339.174678 |
| **H3f3b** | 10692.58782 | 10937.17703 | 11120.89038 | 10592.84367 | 11372.67041 | 10793.34025 | 10989.85442 | 10252.1594 | 10581.19981 | 10477.21071 | 10060.28075 | 11339.45988 | 11639.47175 | 11896.20054 | 11257.76838 |
| **Unk** | 555.8277643 | 677.3840173 | 659.42181 | 563.283951 | 615.6290734 | 700.0099801 | 629.6411709 | 602.5752154 | 625.2561359 | 580.2709074 | 632.7874975 | 580.3763031 | 690.1882953 | 631.5847014 | 648.8648018 |
| **Unc13d** | 2092.36646 | 2099.494323 | 2062.063688 | 1906.366817 | 1974.826596 | 1970.969984 | 2022.429091 | 1978.692745 | 1939.723608 | 1958.414312 | 1951.331252 | 2024.8106 | 2064.600296 | 2186.602257 | 1944.438708 |
| **Wbp2** | 1194.983908 | 1139.272768 | 1134.017554 | 1189.53839 | 1076.084152 | 1099.815279 | 1040.982681 | 1047.592516 | 999.2059548 | 956.048754 | 941.1413453 | 1055.781705 | 1065.105394 | 1180.042375 | 1108.746832 |
| **Trim47** | 10.98835778 | 9.507144102 | 0.783161294 | 3.450437679 | 5.700269198 | 7.715540863 | 6.314453003 | 8.380740131 | 12.79104008 | 12.23462756 | 4.729353494 | 17.35056213 | 3.408337261 | 2.710663955 | 7.18565672 |
| **Trim65** | 161.1625808 | 167.9595458 | 157.4154202 | 162.1705709 | 140.6066402 | 174.6517886 | 167.7840369 | 173.4813207 | 215.9428532 | 129.3374914 | 175.93195 | 189.9886553 | 182.3460434 | 169.8682745 | 190.4199031 |
| **Mrpl38** | 862.5860856 | 823.1602268 | 863.8269078 | 1027.367819 | 844.9065679 | 884.481548 | 732.4765484 | 1044.24022 | 1023.283207 | 1016.34799 | 998.8394579 | 757.3520368 | 904.9135427 | 792.4174294 | 806.9492496 |
| **Fbf1** | 268.2990691 | 343.0494497 | 292.9023241 | 280.3480614 | 260.3122934 | 211.1252545 | 218.299661 | 241.3653158 | 256.5732158 | 344.3173757 | 313.0832013 | 240.3052854 | 292.2649201 | 254.8024117 | 273.773521 |
| **Acox1** | 323.240858 | 316.1125414 | 321.0961307 | 320.0280947 | 306.5478102 | 301.6075065 | 320.2329737 | 382.16175 | 425.1139793 | 358.2998073 | 378.3482795 | 353.9514674 | 374.0650144 | 323.4725653 | 324.7916837 |
| **Ten1** | 289.3600882 | 240.0553886 | 299.9507758 | 276.8976237 | 292.6138189 | 313.5315242 | 288.6607087 | 224.6038355 | 243.0297616 | 242.0708454 | 296.0575287 | 303.6348372 | 270.9628122 | 281.9090513 | 293.1747942 |
| **Cdk3-ps** | 0 | 0 | 2.349483883 | 0 | 3.166816221 | 1.402825611 | 0.902064715 | 0 | 0 | 1.747803938 | 8.512836289 | 1.735056213 | 4.260421576 | 9.035546516 | 0 |
| **Evpl** | 4.578482408 | 6.338096068 | 21.14535495 | 17.25218839 | 12.03390164 | 7.715540863 | 9.922711863 | 26.81836842 | 11.28621184 | 11.3607256 | 24.59263817 | 13.01292159 | 20.45002356 | 9.035546516 | 10.77848508 |
| **Srp68** | 2644.531439 | 2726.965833 | 2775.523628 | 2634.409168 | 2656.325446 | 2800.03992 | 2751.29738 | 2450.528414 | 2573.256299 | 2414.59114 | 2565.201335 | 2601.716791 | 2576.702969 | 2620.30849 | 2651.50733 |
| **Galr2** | 1.831392963 | 2.376786026 | 3.915806472 | 5.175656518 | 6.333632443 | 4.90988964 | 3.608258859 | 1.676148026 | 2.257242368 | 3.495607876 | 2.837612096 | 3.470112425 | 7.668758836 | 0 | 2.155697016 |
| **Exoc7** | 652.8915914 | 615.5875806 | 646.108068 | 659.896206 | 707.4667439 | 711.9339978 | 746.9095838 | 638.6123979 | 660.6195996 | 630.0833196 | 664.0012305 | 701.830238 | 692.7445482 | 692.1228631 | 689.8230451 |
| **Foxj1** | 21.97671556 | 10.29940611 | 10.96425812 | 4.313047098 | 6.966995687 | 14.72966892 | 11.72684129 | 14.24725822 | 2.257242368 | 1.747803938 | 11.35044839 | 7.807752957 | 14.48543336 | 7.228437213 | 8.622788064 |
| **Rnf157** | 802.1501179 | 610.0417465 | 643.7585841 | 539.1308873 | 639.6968767 | 724.5594283 | 736.986872 | 316.7919769 | 310.7470326 | 371.4083368 | 496.5821169 | 606.4021463 | 673.146609 | 587.3105235 | 686.2302167 |
| **Qrich2** | 0 | 0 | 0.783161294 | 5.175656518 | 1.900089733 | 0 | 3.608258859 | 1.676148026 | 0 | 0 | 4.729353494 | 0 | 5.964590206 | 1.807109303 | 0 |
| **Prpsap1** | 1242.600125 | 1224.837065 | 1370.532265 | 1258.547143 | 1297.127924 | 1268.154353 | 1337.761972 | 1513.561668 | 1528.153083 | 1493.498465 | 1470.828937 | 1316.907665 | 1319.878604 | 1293.890261 | 1342.280675 |
| **Sphk1** | 10.98835778 | 15.84524017 | 9.397935534 | 24.15306375 | 8.233722176 | 8.416953669 | 1.80412943 | 22.62799835 | 12.03862596 | 16.60413741 | 17.97154328 | 10.41033728 | 11.0770961 | 6.324882561 | 8.622788064 |
| **Ube2o** | 1445.884744 | 1335.753746 | 1391.67762 | 1361.197664 | 1330.062813 | 1221.861108 | 1467.659291 | 1217.721541 | 1169.251546 | 1211.228129 | 1345.974004 | 1283.074069 | 1376.116169 | 1302.022253 | 1358.807686 |
| **Rhbdf2** | 433.1244358 | 435.7441047 | 433.0881959 | 355.3950809 | 316.0482589 | 353.5120541 | 431.1869337 | 452.559967 | 466.496756 | 337.32616 | 542.9297811 | 331.3957366 | 351.0587378 | 341.5436583 | 396.6482509 |
| **1810032O08Rik** | 175.8137245 | 194.8964541 | 225.5504528 | 156.9949144 | 200.7761484 | 176.0546142 | 184.0212018 | 174.3193947 | 109.8524619 | 200.9974528 | 175.93195 | 184.7834866 | 191.7189709 | 206.0104606 | 237.1266717 |
| **St6galnac2** | 10.0726613 | 5.54583406 | 7.04845165 | 5.175656518 | 14.56735462 | 4.90988964 | 12.62890601 | 5.028444078 | 6.771727103 | 6.117313782 | 13.24218978 | 7.807752957 | 8.520843152 | 8.131991864 | 5.748525376 |
| **Mxra7** | 2.747089445 | 2.376786026 | 1.566322589 | 0 | 3.800179466 | 2.104238417 | 0 | 3.352296052 | 1.504828245 | 6.117313782 | 0 | 0.867528106 | 0 | 5.421327909 | 4.311394032 |
| **Jmjd6** | 1534.707303 | 1514.012698 | 1424.570395 | 1466.436013 | 1499.804162 | 1453.327333 | 1482.994391 | 1242.025687 | 1233.206747 | 1323.087581 | 1278.817185 | 1538.994861 | 1438.318324 | 1395.991937 | 1503.239386 |
| **Mettl23** | 467.9209021 | 457.9274409 | 459.7156799 | 418.3655685 | 490.8565143 | 500.8087433 | 464.5633281 | 342.7722713 | 373.1974048 | 403.7427096 | 367.9437018 | 453.7171996 | 435.415085 | 492.4372851 | 389.4625942 |
| **Srsf2** | 11510.30477 | 11372.12887 | 11781.87851 | 11738.38898 | 11620.31544 | 12295.76648 | 11368.7216 | 12107.65527 | 12942.27532 | 12253.85341 | 12167.68067 | 12175.75697 | 12122.60355 | 11814.88062 | 11512.14063 |
| **Mfsd11** | 130.9445969 | 127.5541834 | 109.6425812 | 132.8418506 | 117.8055634 | 104.5105081 | 157.8613251 | 133.2537681 | 137.6917844 | 130.2113934 | 104.0457769 | 139.6720251 | 113.3272139 | 121.979878 | 114.2519418 |
| **Snhg20** | 33.88076982 | 64.9654847 | 36.02541955 | 43.9930804 | 41.16861088 | 42.08476834 | 42.39704159 | 30.17066447 | 28.59173666 | 41.07339254 | 46.34766424 | 53.78674259 | 72.42716679 | 53.30972444 | 37.36541494 |
| **Mir6516** | 0 | 5.54583406 | 0.783161294 | 2.587828259 | 1.900089733 | 3.507064029 | 2.706194144 | 2.514222039 | 1.504828245 | 3.495607876 | 3.783482795 | 3.470112425 | 4.260421576 | 0.903554652 | 0.718565672 |
| **Sec14l1** | 384.5925223 | 399.3000523 | 478.5115509 | 437.3429758 | 407.8859293 | 335.2753211 | 439.3055161 | 447.531523 | 488.3167656 | 413.3556313 | 487.1234099 | 424.221244 | 398.7754595 | 448.1631072 | 395.2111196 |
| **Sept9** | 8566.340585 | 8814.707107 | 8561.519271 | 8898.678773 | 8219.154821 | 7981.376316 | 8237.654975 | 10114.71526 | 9482.675187 | 9929.274171 | 10065.95598 | 8135.678581 | 8472.274346 | 8581.058526 | 8520.751738 |
| **2900041M22Rik** | 10.98835778 | 15.05297816 | 14.8800646 | 5.175656518 | 10.13381191 | 10.52119209 | 9.922711863 | 8.380740131 | 1.504828245 | 14.85633347 | 6.621094891 | 13.01292159 | 21.30210788 | 9.035546516 | 15.80844478 |
| **Tnrc6c** | 977.9638423 | 1193.146585 | 1151.247103 | 1161.072279 | 1056.449891 | 965.1440207 | 1219.591494 | 1252.082576 | 1131.63084 | 1114.22501 | 1187.067727 | 1152.077325 | 1032.72619 | 1144.803744 | 1069.944286 |
| **Tmc6** | 611.6852497 | 724.1274758 | 712.676778 | 692.675364 | 708.1001071 | 721.7537771 | 680.1567949 | 624.3651397 | 686.9540939 | 622.2182019 | 579.8187383 | 752.1468681 | 801.8113406 | 705.6761829 | 762.398178 |
| **Tmc8** | 624.5050004 | 658.3697291 | 700.1461973 | 697.8510205 | 759.4025299 | 798.2077729 | 870.4924498 | 652.0215822 | 607.950611 | 545.3148286 | 604.4113765 | 790.3181048 | 834.1905445 | 890.0013318 | 893.8956959 |
| **6030468B19Rik** | 2.747089445 | 0 | 0 | 1.725218839 | 0 | 0 | 0 | 0 | 0 | 0 | 0 | 0 | 5.964590206 | 0 | 1.437131344 |
| **Syngr2** | 2852.39454 | 2825.998584 | 2831.12808 | 2630.096121 | 2554.353964 | 2473.181553 | 2465.342865 | 3259.269837 | 3017.180632 | 3090.117362 | 2916.119364 | 2406.522967 | 2584.371728 | 2438.694005 | 2423.003446 |
| **Tk1** | 1481.596907 | 1559.963895 | 1530.297169 | 1634.64485 | 1534.005778 | 1500.321991 | 1525.391433 | 1749.060465 | 1740.333866 | 1757.416859 | 1761.211241 | 1636.158008 | 1540.568442 | 1695.972081 | 1445.754132 |
| **Afmid** | 127.2818109 | 149.7375196 | 131.5710975 | 121.6279282 | 141.2400035 | 143.0882124 | 140.7220955 | 92.18814144 | 98.56625006 | 93.50751067 | 127.6925443 | 130.996744 | 130.3689002 | 143.6651896 | 142.9945687 |
| **Birc5** | 1875.346394 | 1986.200855 | 2138.813495 | 2135.820923 | 2146.468035 | 1974.477048 | 1956.578366 | 2109.432291 | 2225.640975 | 2236.315138 | 2264.414453 | 2205.256446 | 1960.646009 | 2421.526466 | 2154.97845 |
| **Tha1** | 41.20634167 | 47.53572051 | 29.76012919 | 32.77915795 | 22.16771355 | 22.44520978 | 18.0412943 | 28.49451644 | 32.35380727 | 32.33437285 | 18.91741398 | 30.36348372 | 27.26669809 | 21.68531164 | 11.49705075 |
| **Socs3** | 316.8309826 | 261.4464628 | 268.624324 | 214.7897455 | 148.2069992 | 183.7701551 | 185.8253312 | 223.7657615 | 213.6856108 | 219.3493942 | 237.4135454 | 208.2067455 | 178.9377062 | 191.5535861 | 219.1625299 |
| **Pgs1** | 1002.687647 | 1075.099546 | 955.4567793 | 996.3138797 | 848.7067473 | 870.4532919 | 887.6316793 | 1087.820069 | 1012.749409 | 1011.104578 | 1152.070511 | 834.5620382 | 873.386423 | 880.9657853 | 973.6564855 |
| **Dnah17** | 35.71216278 | 40.40536243 | 25.84432272 | 38.81742388 | 33.56825195 | 28.05651223 | 42.39704159 | 41.06562664 | 38.37312025 | 21.84754922 | 52.02288843 | 40.773821 | 28.97086672 | 22.58886629 | 34.49115225 |
| **Cyth1** | 2353.339958 | 2608.126532 | 2378.460851 | 2376.488951 | 2668.359348 | 2754.448088 | 2776.555192 | 2241.847985 | 2253.480297 | 2065.904254 | 2181.177831 | 2685.867017 | 2668.728075 | 2701.628408 | 2802.406121 |
| **Usp36** | 1962.33756 | 1971.147877 | 1961.035881 | 1838.220673 | 1736.048653 | 1849.625569 | 2032.351802 | 1940.979414 | 1969.067759 | 1907.727998 | 2034.567873 | 1790.578011 | 1789.377062 | 1612.845053 | 1792.821352 |
| **Timp2** | 73.25571853 | 83.18751089 | 110.4257425 | 116.4522717 | 148.2069992 | 134.6712587 | 138.0159014 | 129.901472 | 97.81383594 | 90.0119028 | 87.96597499 | 131.8642722 | 117.5876355 | 104.8123396 | 120.7190329 |
| **BC100451** | 1.831392963 | 0 | 0 | 0.86260942 | 0 | 2.104238417 | 1.80412943 | 0 | 1.504828245 | 0 | 0 | 0 | 0 | 4.517773258 | 0 |
| **Lgals3bp** | 4098.657451 | 4063.511842 | 3608.024084 | 3784.267524 | 3522.133001 | 3948.252683 | 4298.338366 | 2360.016421 | 2630.439773 | 2568.397887 | 3012.598176 | 3371.214221 | 3164.641147 | 3479.588963 | 3596.421188 |
| **Cant1** | 934.0104112 | 1018.848943 | 979.7347794 | 848.8076689 | 977.2794859 | 894.3013273 | 996.7815098 | 988.0892614 | 1016.51148 | 982.265813 | 1032.890803 | 1015.007884 | 985.0094683 | 1036.377185 | 998.0877184 |
| **C1qtnf1** | 43.95343112 | 64.9654847 | 65.78554874 | 45.71829924 | 32.30152546 | 39.98052993 | 31.57226502 | 58.66518091 | 81.26072524 | 60.29923585 | 62.42746612 | 19.95314644 | 32.37920398 | 27.10663955 | 25.14979852 |
| **Engase** | 132.7759898 | 96.65596504 | 123.7394845 | 119.9027093 | 141.8733667 | 139.5811483 | 136.2117719 | 160.0721365 | 179.8269753 | 136.3287071 | 180.6613035 | 154.4200029 | 115.8834669 | 149.0865175 | 121.4375986 |
| **Cbx2** | 351.6274489 | 347.0107597 | 434.6545184 | 313.9898288 | 370.5174979 | 308.6216345 | 324.7432973 | 382.16175 | 340.8435975 | 305.8656891 | 338.6217102 | 392.122704 | 312.7149437 | 318.0512374 | 321.1988554 |
| **Cbx8** | 89.73825519 | 86.35655893 | 119.0405168 | 140.6053354 | 116.5388369 | 103.8090952 | 107.3457011 | 76.26473519 | 110.604876 | 124.0940796 | 93.64119918 | 128.3941597 | 97.13761193 | 91.25901981 | 81.9164866 |
| **Cbx4** | 2042.91885 | 2114.547301 | 1953.98743 | 2168.600081 | 1941.258344 | 2132.996342 | 2175.780092 | 1637.596622 | 1899.093245 | 1798.490252 | 1834.989156 | 2150.602175 | 1923.154299 | 2184.795148 | 2033.540852 |
| **Tbc1d16** | 84.2440763 | 124.3851353 | 158.9817428 | 79.36006661 | 163.407717 | 157.8178813 | 149.7427427 | 88.83584538 | 106.8428054 | 96.12921658 | 90.80358708 | 149.2148343 | 189.162718 | 194.2642501 | 178.9228523 |
| **Ccdc40** | 120.8719356 | 102.9940611 | 104.1604522 | 109.5513963 | 74.10349958 | 86.2737751 | 82.08788905 | 108.9496217 | 126.4055726 | 125.8418835 | 148.5016997 | 68.5347204 | 60.49798638 | 88.54835585 | 87.66501198 |
| **Gaa** | 741.7141501 | 702.7364015 | 722.8578748 | 695.2631922 | 587.7610907 | 579.3669775 | 579.1255469 | 761.8092779 | 680.9347809 | 788.259576 | 879.6597499 | 577.7737188 | 670.590356 | 550.2647828 | 537.4871226 |
| **Eif4a3** | 4293.700802 | 4313.866636 | 4222.022539 | 4300.107957 | 4349.305398 | 4307.37604 | 4229.781448 | 4381.45094 | 4175.89838 | 4227.937726 | 4234.663118 | 4204.041203 | 4065.294268 | 4380.432951 | 4114.507038 |
| **Card14** | 1.831392963 | 0 | 1.566322589 | 0.86260942 | 0 | 0 | 1.80412943 | 0 | 6.019312981 | 6.991215751 | 0 | 0.867528106 | 0.852084315 | 3.614218606 | 1.437131344 |
| **Sgsh** | 94.3167376 | 105.3708471 | 129.2216136 | 110.4140057 | 114.005384 | 122.0458282 | 106.4436363 | 108.1115477 | 110.604876 | 104.8682363 | 108.7751304 | 118.8513506 | 99.69386487 | 133.7260884 | 117.1262045 |
| **Slc26a11** | 17.39823315 | 28.52143231 | 34.45909696 | 26.74089201 | 24.70116653 | 32.26498906 | 22.55161787 | 15.08533223 | 18.81035306 | 14.85633347 | 27.43025026 | 30.36348372 | 47.71672165 | 35.23863141 | 57.48525376 |
| **Mir1932** | 0 | 0 | 0 | 1.725218839 | 0 | 0.701412806 | 0.902064715 | 0 | 0 | 0 | 0 | 0 | 0 | 0 | 1.437131344 |
| **Endov** | 224.345638 | 227.3791964 | 270.1906466 | 215.6523549 | 297.6807248 | 264.4326278 | 285.9545146 | 192.757023 | 197.8849142 | 196.627943 | 293.2199166 | 279.3440502 | 260.7378004 | 271.0663955 | 272.3363897 |
| **Rptor** | 926.6848393 | 1038.655493 | 1051.785618 | 973.8860348 | 919.6434307 | 942.6988109 | 1005.802157 | 1026.640666 | 1093.25772 | 1044.312853 | 991.2724923 | 978.5717039 | 962.8552761 | 1021.016756 | 1034.734568 |
| **Rptoros** | 0 | 0 | 3.132645178 | 0 | 0.633363244 | 0 | 0 | 0 | 0 | 0 | 0 | 0 | 0 | 2.710663955 | 1.437131344 |
| **Chmp6** | 622.6736075 | 665.5000871 | 642.1922615 | 597.7883278 | 637.796787 | 637.5842404 | 635.9556239 | 615.1463256 | 660.6195996 | 655.4264767 | 594.9526695 | 655.8512483 | 651.8445011 | 574.6607584 | 588.5052853 |
| **Baiap2** | 315.9152861 | 313.7357554 | 397.0627763 | 377.8229258 | 339.4826989 | 348.6021644 | 347.2949152 | 364.5621957 | 382.2263743 | 393.255886 | 404.8326591 | 375.63967 | 387.6983634 | 415.6351397 | 420.3609181 |
| **Cep131** | 58.60457482 | 77.64167683 | 58.73709709 | 37.95481446 | 72.83677309 | 40.68194273 | 56.83007703 | 37.71333059 | 32.35380727 | 38.45168663 | 68.10269031 | 55.5217988 | 71.57508247 | 53.30972444 | 44.55107166 |
| **Enthd2** | 150.174223 | 179.0512139 | 185.6092268 | 147.5062108 | 156.4407213 | 159.9221197 | 161.4695839 | 213.7088733 | 148.9779963 | 151.1850406 | 186.3365277 | 158.7576434 | 175.5293689 | 198.7820233 | 159.5215792 |
| **1810043H04Rik** | 310.4211073 | 258.2774148 | 302.3002597 | 278.6228425 | 271.0794686 | 336.6781467 | 320.2329737 | 289.9736085 | 309.2422044 | 292.7571596 | 311.1914599 | 308.8400058 | 287.1524142 | 334.3152211 | 313.294633 |
| **Slc38a10** | 2602.409401 | 2750.733694 | 2858.538725 | 2689.61617 | 2516.35217 | 2633.103673 | 2666.503297 | 2628.200105 | 2656.774267 | 2698.60928 | 2630.466413 | 2539.254767 | 2701.107279 | 2646.511574 | 2604.800561 |
| **2810410L24Rik** | 0.915696482 | 2.376786026 | 3.915806472 | 1.725218839 | 0 | 0 | 0.902064715 | 1.676148026 | 5.266898858 | 0 | 0 | 0 | 1.70416863 | 0.903554652 | 2.155697016 |

| | | | | | | | | | | | | | | | |
|---|---|---|---|---|---|---|---|---|---|---|---|---|---|---|---|
| **Bahcc1** | 21.97671556 | 4.753572051 | 9.397935534 | 4.313047098 | 3.800179466 | 10.52119209 | 0.902064715 | 0 | 0.752414123 | 5.243411813 | 4.729353494 | 3.470112425 | 2.556252945 | 1.807109303 | 3.59282836 |
| **Actg1** | 45049.5198 | 40586.79043 | 42322.03635 | 42762.99937 | 37921.35752 | 37653.94365 | 38958.3709 | 41291.90662 | 40136.77895 | 42318.70284 | 44158.91944 | 37985.58566 | 39302.38904 | 38945.91615 | 39753.92724 |
| **0610009L18Rik** | 9.156964816 | 20.59881222 | 6.265290356 | 5.175656518 | 2.533452977 | 4.90988964 | 7.216517718 | 7.542666117 | 0.752414123 | 0.873901969 | 4.729353494 | 7.807752957 | 16.18960199 | 2.710663955 | 3.59282836 |
| **Fscn2** | 8.241268334 | 6.338096068 | 0.783161294 | 0.86260942 | 6.333632443 | 3.507064029 | 5.412388289 | 8.380740131 | 12.79104008 | 3.495607876 | 8.512836289 | 4.337640531 | 2.556252945 | 0.903554652 | 0 |
| **Faap100** | 556.7434608 | 482.4875632 | 464.4146476 | 460.6334301 | 494.0233305 | 507.8228713 | 543.0429583 | 424.0654506 | 471.7636549 | 353.9302974 | 425.6418144 | 458.9223682 | 485.6880596 | 493.3408398 | 542.5170823 |
| **Nploc4** | 3183.876666 | 3104.874811 | 3410.667437 | 2988.941639 | 2826.066796 | 2756.552326 | 3013.798212 | 3318.773092 | 3383.606309 | 3402.974267 | 3429.727154 | 2947.860505 | 2847.665781 | 2956.43082 | 3026.59861 |
| **Pde6g** | 0 | 4.753572051 | 0 | 0.86260942 | 2.533452977 | 0.701412806 | 0 | 0 | 0 | 0 | 0 | 0.867528106 | 0 | 8.131991864 | 2.155697016 |
| **Oxld1** | 62.26736075 | 66.55000871 | 60.30341968 | 61.2452688 | 44.3354271 | 43.48759395 | 77.57756547 | 79.61703124 | 54.92623095 | 55.92972601 | 65.26507822 | 55.5217988 | 49.42089028 | 60.53816166 | 64.67091048 |
| **Ccdc137** | 588.7928376 | 617.1721046 | 565.4424546 | 523.6039177 | 530.1250355 | 615.1390306 | 588.146194 | 639.450472 | 647.8285595 | 696.4998692 | 716.9699897 | 551.7478756 | 583.6777559 | 573.7572037 | 561.9183555 |
| **Arl16** | 132.7759898 | 198.0655021 | 183.2597429 | 156.9949144 | 158.3408111 | 184.4715679 | 202.9645608 | 163.4244325 | 168.5407635 | 190.5106292 | 172.1484672 | 159.6251716 | 193.4231395 | 185.2287036 | 182.5156807 |
| **Hgs** | 833.2837982 | 802.5614146 | 876.3574885 | 846.2198407 | 843.6398414 | 825.5628723 | 858.7656085 | 847.2928272 | 881.0769375 | 851.1805177 | 942.087216 | 848.4424879 | 854.6405681 | 892.7119958 | 891.7399989 |
| **Mrpl12** | 2832.249217 | 2888.587283 | 2774.740466 | 2787.091035 | 2728.528856 | 2793.025792 | 2900.138058 | 3358.16257 | 2925.386109 | 3219.454853 | 2918.011106 | 2744.858928 | 2566.477957 | 2628.440481 | 2615.579046 |
| **Slc25a10** | 374.519861 | 352.5565938 | 386.0985182 | 402.838599 | 386.9849423 | 375.2558511 | 343.6866563 | 502.8444078 | 516.9085022 | 471.9070632 | 542.9297811 | 425.0887721 | 366.3962555 | 404.7924839 | 375.0912808 |
| **Fam195b** | 630.9148758 | 689.2679474 | 666.4702616 | 687.4997075 | 787.9038759 | 777.1653887 | 765.8529428 | 643.640842 | 619.2368229 | 558.4233581 | 636.5709803 | 651.5136078 | 735.348764 | 720.1330573 | 786.8294108 |
| **P4hb** | 10095.55371 | 9318.585744 | 10011.15083 | 9579.277605 | 8900.653672 | 9134.498969 | 9523.999259 | 8802.291359 | 8596.33135 | 9089.454379 | 9523.026195 | 9373.641188 | 9198.250182 | 9019.282532 | 9460.635637 |
| **Arhgdia** | 17097.8847 | 16929.0546 | 17125.38803 | 16952.00031 | 16677.08759 | 16727.994 | 17168.09565 | 16803.38396 | 16614.05624 | 16355.94925 | 17383.2117 | 16974.05493 | 16904.50073 | 16825.99472 | 17096.83303 |
| **Alyref** | 3386.245589 | 3252.235545 | 3319.820727 | 3324.496703 | 3373.292639 | 3716.786457 | 3347.562157 | 3641.431587 | 3792.167178 | 3574.259053 | 3403.242774 | 3358.201299 | 3404.076839 | 3396.461935 | 3183.964492 |
| **Anapc11** | 1042.978293 | 1113.920384 | 1199.019942 | 1076.536556 | 1043.782627 | 1101.919518 | 1073.457011 | 957.9185969 | 961.5852487 | 999.7438524 | 1036.674286 | 1039.298671 | 1048.063708 | 1133.057533 | 1077.848508 |
| **Npb** | 4.578482408 | 3.169048034 | 6.265290356 | 2.587828259 | 3.166816221 | 6.312715251 | 9.020647148 | 0.838074013 | 0.752414123 | 0 | 16.07980188 | 3.470112425 | 5.112505891 | 10.84265582 | 10.05991941 |
| **Pcyt2** | 1892.744627 | 2010.760978 | 1986.097043 | 1896.015504 | 1951.392156 | 2102.134179 | 2008.89812 | 1821.13483 | 1731.304896 | 1850.050468 | 1869.986371 | 1977.096554 | 2066.304464 | 1914.632307 | 2082.403317 |
| **Sirt7** | 1016.423095 | 1011.718585 | 992.2653601 | 973.0234254 | 996.2803832 | 1028.271173 | 1002.193898 | 1049.268664 | 1052.627357 | 1195.497893 | 1081.130209 | 1090.48283 | 1056.584551 | 1033.666521 | 1035.453133 |
| **Mafg** | 412.0634167 | 408.8071964 | 343.8078083 | 354.5324715 | 348.9831476 | 359.8247693 | 320.2329737 | 324.3346431 | 339.3387693 | 366.164925 | 356.5932534 | 360.8916922 | 335.7212202 | 262.030849 | 334.8516031 |
| **Pycr1** | 159.3311878 | 99.82501307 | 122.9563232 | 101.7879115 | 112.1052942 | 122.0458282 | 135.3097072 | 103.0831036 | 91.04210883 | 83.89458901 | 92.69532848 | 92.82550737 | 94.58135898 | 93.96968376 | 107.0662851 |
| **Aspscr1** | 652.8915914 | 769.2864103 | 757.3169718 | 671.9727379 | 696.6995687 | 714.739649 | 744.2033897 | 690.5729868 | 732.0989413 | 699.9954771 | 780.3433265 | 727.8560812 | 682.5195364 | 701.1584096 | 702.0386615 |
| **Stra13** | 603.4439814 | 625.0947247 | 582.6720031 | 668.5223002 | 687.8324833 | 693.6972648 | 630.5432356 | 663.7546183 | 618.4844088 | 683.3913397 | 661.1636184 | 673.2018105 | 614.3527912 | 699.3513003 | 714.9728436 |
| **Lrrc45** | 232.5869063 | 266.2000349 | 261.5758724 | 254.4697788 | 254.6120242 | 218.8407954 | 256.186379 | 281.5928684 | 253.5635593 | 319.8481206 | 320.6501669 | 275.0064097 | 250.5127887 | 276.4877234 | 272.3363897 |
| **Rac3** | 16.48253667 | 22.18333624 | 20.36219366 | 11.21392246 | 12.03390164 | 17.53532014 | 3.608258859 | 10.89496217 | 0 | 19.22584332 | 3.783482795 | 12.14539349 | 11.0770961 | 16.26398373 | 21.55697016 |
| **Dcxr** | 47.61621704 | 57.83512662 | 61.08658097 | 55.20700286 | 44.96879034 | 50.50172201 | 49.61355931 | 105.5973256 | 88.03245234 | 126.7157855 | 144.7182169 | 39.03876478 | 41.75213144 | 43.37062328 | 47.42533435 |
| **Rfng** | 334.2292158 | 382.6625501 | 381.3995504 | 431.3047098 | 359.7503227 | 359.8247693 | 362.6300153 | 367.9144917 | 443.1719182 | 352.1824935 | 429.4252972 | 335.7333771 | 379.1775202 | 335.2187757 | 398.8039479 |
| **Gps1** | 1970.578828 | 1925.988943 | 2033.08672 | 1991.76515 | 1839.286861 | 1895.918814 | 1967.403143 | 2298.837018 | 2007.440879 | 2166.402981 | 2027.946778 | 1907.694306 | 1878.845915 | 1887.525667 | 1901.324768 |
| **Dus1l** | 1302.120397 | 1239.097781 | 1338.422652 | 1281.837598 | 1175.522181 | 1253.424684 | 1148.328382 | 1516.913964 | 1611.671051 | 1641.187898 | 1568.253619 | 1244.035304 | 1215.072233 | 1241.484091 | 1174.854874 |
| **Fasn** | 19545.5414 | 19413.58826 | 18348.68597 | 20590.48685 | 18448.60458 | 18421.20452 | 20242.3322 | 20574.71702 | 20416.75722 | 19373.53275 | 20862.12413 | 18728.19676 | 19982.22927 | 18077.41791 | 20274.33043 |
| **Ccdc57** | 54.94178889 | 39.61310043 | 72.83400039 | 40.54264272 | 44.96879034 | 46.29324518 | 44.20117102 | 46.93214473 | 29.34415078 | 34.95607876 | 35.94308655 | 52.91921448 | 48.56880596 | 51.50261514 | 60.35951645 |
| **Slc16a3** | 5712.114652 | 5282.803073 | 5202.540479 | 4513.172484 | 4701.455362 | 5024.92134 | 5075.016085 | 762.6473519 | 1443.130287 | 1931.323351 | 2098.887081 | 4913.679194 | 5107.393385 | 4678.605986 | 5092.474917 |
| **Csnk1d** | 3370.678749 | 3321.954602 | 3363.67776 | 3352.100205 | 3116.147162 | 3354.85745 | 3478.36154 | 3328.82998 | 3494.211185 | 3361.026972 | 3169.612712 | 3438.881413 | 3349.543443 | 3270.867839 | 3263.725282 |
| **Cd7** | 78.74989741 | 85.56429692 | 119.8236781 | 106.963568 | 105.7716618 | 113.6288745 | 123.5828659 | 61.17940295 | 45.14484735 | 72.53386342 | 62.42746612 | 131.8642722 | 135.4814061 | 167.1576105 | 111.3776792 |
| **Sectm1a** | 2.747089445 | 1.584524017 | 0 | 0.86260942 | 0.633363244 | 2.805651223 | 2.706194144 | 0 | 0 | 0.873901969 | 1.891741398 | 4.337640531 | 0 | 1.807109303 | 2.155697016 |
| **Ogfod3** | 116.2934532 | 99.82501307 | 76.74980686 | 86.26094197 | 91.20430718 | 82.06529827 | 82.08788905 | 87.99777137 | 69.22209928 | 73.40776539 | 81.34488009 | 86.75281063 | 90.32093741 | 87.6448012 | 80.47935526 |
| **Hexdc** | 34.7964663 | 48.32798252 | 39.15806472 | 43.13047098 | 41.80197412 | 37.1748787 | 53.22181817 | 52.79866282 | 68.46968515 | 50.6863142 | 52.96875913 | 42.50887721 | 57.94173343 | 59.634607 | 56.76668809 |
| **BC017643** | 853.4291208 | 766.1173622 | 827.018327 | 866.9224667 | 745.4685385 | 770.1512607 | 722.5538365 | 775.2184621 | 685.4492657 | 681.6435357 | 686.7021273 | 795.5232735 | 750.6862817 | 772.5392271 | 822.0391287 |
| **Narf** | 400.1593624 | 418.3143405 | 533.3328415 | 554.6578568 | 494.0233305 | 480.4677719 | 515.9810169 | 417.3608585 | 404.7987979 | 398.4992978 | 385.9152451 | 586.4489998 | 549.5943833 | 575.5643131 | 507.3073644 |
| **Foxk2** | 3011.725728 | 3012.180156 | 2956.433887 | 2963.925966 | 2719.661771 | 2862.46566 | 3065.215901 | 2880.460383 | 3087.155145 | 3058.656891 | 3101.510021 | 2776.08994 | 2933.726297 | 2772.105671 | 2865.6399 |
| **Wdr45b** | 673.9526104 | 609.2494845 | 613.9984549 | 619.3535633 | 609.295441 | 586.3811056 | 592.6565176 | 616.8224736 | 645.5713172 | 669.4089082 | 730.2121795 | 623.7527084 | 610.944454 | 546.6505642 | 591.379548 |
| **Fn3krp** | 65.93014667 | 81.60298688 | 89.28038757 | 87.12355138 | 89.93758069 | 65.93280374 | 73.96930661 | 113.1399918 | 88.03245234 | 83.02068705 | 65.26507822 | 91.95797927 | 103.1022021 | 97.58390237 | 91.25784034 |
| **Fn3k** | 0 | 0 | 0 | 0 | 0 | 0 | 0 | 0 | 0 | 0 | 0 | 0 | 0 | 2.710663955 | 0 |
| **Tbcd** | 1428.486511 | 1449.839476 | 1521.682395 | 1493.176905 | 1425.0673 | 1326.371616 | 1419.849861 | 1465.791449 | 1522.13377 | 1483.011641 | 1463.261971 | 1448.771937 | 1287.4994 | 1340.875103 | 1448.628395 |
| **Zfp750** | 5.494178889 | 7.130358077 | 9.397935534 | 3.450437679 | 1.266726489 | 1.402825611 | 3.608258859 | 42.74177467 | 39.12553437 | 23.59535316 | 5.675224193 | 4.337640531 | 2.556252945 | 7.228437213 | 1.437131344 |
| **B3gntl1** | 55.85748538 | 38.82083842 | 65.78554874 | 76.77223835 | 48.76896981 | 49.0988964 | 48.7114946 | 61.17940295 | 49.65933209 | 49.81241223 | 60.53572472 | 42.50887721 | 55.38548049 | 59.634607 | 54.61099107 |
| **Metrnl** | 173.9823315 | 146.5684716 | 129.2216136 | 158.7201332 | 52.56914927 | 42.08476834 | 59.53627118 | 106.4353997 | 140.7014409 | 153.8067465 | 157.014536 | 39.90629289 | 49.42089028 | 60.53816166 | 34.49115225 |
| **Rab10os** | 176.7294209 | 163.2059738 | 159.7649041 | 188.0488535 | 118.4389267 | 160.6235325 | 138.9179661 | 155.8817664 | 153.492481 | 228.9623159 | 192.0117519 | 120.5864068 | 167.0085258 | 125.5940966 | 120.0004672 |
| **Rab10** | 3051.100677 | 2955.929554 | 2994.80879 | 2874.214586 | 2682.926703 | 2781.803187 | 2828.874946 | 2820.119054 | 2982.569582 | 2911.84136 | 2893.418468 | 3001.647248 | 2909.015852 | 2714.278173 | 2744.920867 |
| **Kif3c** | 256.3950148 | 209.1571702 | 267.0580014 | 263.095873 | 244.4782123 | 207.6181905 | 267.9132203 | 209.5185033 | 192.6180154 | 214.1059824 | 205.2539416 | 279.3440502 | 250.5127887 | 166.2540559 | 249.3422882 |
| **1110002L01Rik** | 33.88076982 | 26.93690829 | 41.50754861 | 32.77915795 | 31.03479897 | 38.57770431 | 43.29910631 | 32.68488651 | 62.45037217 | 37.57778466 | 38.78069865 | 40.773821 | 45.1604687 | 34.33507676 | 26.58692986 |
| **Asxl2** | 1158.356049 | 1184.431703 | 1150.463942 | 1138.644434 | 939.9110545 | 921.6564267 | 1061.730169 | 1123.019177 | 1046.608045 | 1010.230676 | 1076.400855 | 1083.542605 | 1190.361788 | 1032.762967 | 1113.776792 |
| **Dtnb** | 380.0140398 | 405.6381484 | 426.0397442 | 375.2350975 | 421.1865574 | 426.4589859 | 403.2229275 | 443.3411529 | 337.833941 | 398.4992978 | 401.995047 | 404.2680975 | 411.5567242 | 446.3559979 | 472.8162122 |
| **Dnmt3a** | 2810.272502 | 3090.614095 | 3062.160661 | 2970.826841 | 3658.939462 | 3313.474094 | 3739.058243 | 3482.197524 | 3298.583513 | 2844.550909 | 3068.404547 | 3549.057483 | 3727.868879 | 3380.197952 | 3425.402558 |
| **Dnmt3aos** | 0.915696482 | 0 | 0 | 0 | 0 | 0 | 0.902064715 | 6.704592104 | 3.762070613 | 0 | 0 | 4.337640531 | 0.852084315 | 0 | 0 |
| **Pomc** | 14.65114371 | 7.922620085 | 18.01270977 | 12.93914129 | 13.30062813 | 15.43108173 | 13.53097072 | 11.73303618 | 16.5531107 | 20.09974528 | 14.18806048 | 33.83359614 | 17.0416863 | 10.84265582 | 25.14979852 |
| **Efr3b** | 16.48253667 | 11.88393013 | 21.92851625 | 7.763484777 | 19.63426057 | 23.14662259 | 22.55161787 | 16.76148026 | 15.80069657 | 30.58656891 | 28.37612096 | 19.08561834 | 22.15419219 | 28.91374885 | 14.37131344 |
| **Dnajc27** | 314.0838932 | 371.570882 | 330.4940663 | 340.7307208 | 342.0161519 | 366.8388974 | 401.4187981 | 347.8007154 | 328.0525574 | 337.32616 | 272.4107612 | 320.9853993 | 339.1295574 | 378.589399 | 370.0613211 |
| **Adcy3** | 600.6968919 | 640.1477029 | 655.5060035 | 624.5292198 | 623.8627956 | 687.3845496 | 693.6877657 | 678.8399506 | 688.4589222 | 623.9660058 | 665.8929719 | 596.8593371 | 591.3465147 | 643.3309119 | 625.1521346 |
| **Cenpo** | 357.1216278 | 389.7929082 | 361.0373568 | 419.228178 | 373.0509509 | 384.3742175 | 366.2382742 | 362.8860477 | 359.6539506 | 355.6781013 | 344.2969344 | 399.0629289 | 379.1775202 | 402.9853746 | 382.2769375 |
| **Ptrhd1** | 79.6655939 | 77.64167683 | 86.93090369 | 71.59658183 | 84.23731149 | 92.58649035 | 75.77343604 | 74.58858716 | 126.4055726 | 119.7245697 | 81.34488009 | 79.81258578 | 80.09592563 | 72.28437213 | 78.32365824 |
| **Ncoa1** | 434.0401323 | 439.7054147 | 415.0754861 | 471.8473525 | 429.4202796 | 458.7239749 | 514.1768874 | 486.9210016 | 474.7733113 | 436.9509845 | 464.4225131 | 449.3795591 | 501.8776616 | 431.8991235 | 474.2533435 |
| **Itsn2** | 1736.160529 | 1887.168104 | 1747.232848 | 1620.8431 | 1533.372414 | 1597.116959 | 1971.011402 | 1784.259574 | 1843.4146 | 1749.551742 | 1828.368061 | 1884.271047 | 1843.058374 | 1836.023052 | 1821.563978 |
| **Fam228b** | 1.831392963 | 3.169048034 | 13.31374201 | 6.038265938 | 3.166816221 | 5.611302446 | 0.902064715 | 5.028444078 | 4.514484735 | 1.747803938 | 0.945870699 | 3.470112425 | 7.668758836 | 4.517773258 | 7.18565672 |
| **Pfn4** | 7.325571853 | 0.792262009 | 1.566322589 | 0 | 0 | 3.507064029 | 1.80412943 | 1.676148026 | 2.257242368 | 1.747803938 | 0.945870699 | 0.867528106 | 3.408337261 | 8.131991864 | 2.874262688 |
| **Gm6682** | 5.494178889 | 3.169048034 | 6.265290356 | 7.763484777 | 3.166816221 | 14.72966892 | 4.510323574 | 11.73303618 | 6.771727103 | 6.991215751 | 5.675224193 | 4.337640531 | 9.372927467 | 9.939101167 | 7.904222392 |
| **Sf3b6** | 1383.617384 | 1377.743633 | 1379.14704 | 1374.136806 | 1375.031603 | 1343.205523 | 1383.767272 | 1361.032197 | 1386.699228 | 1252.301521 | 1388.538186 | 1464.387443 | 1498.81631 | 1422.195022 | 1427.78999 |
| **Fkbp1b** | 3.662785926 | 0 | 3.915806472 | 1.725218839 | 1.900089733 | 4.90988964 | 1.80412943 | 1.676148026 | 0.752414123 | 2.621705907 | 2.837612096 | 2.602584319 | 11.0770961 | 5.421327909 | 7.18565672 |
| **BC068281** | 104.3893989 | 106.9553711 | 126.0889684 | 121.6279282 | 113.3720207 | 114.3302873 | 85.6961479 | 113.9780658 | 83.51796761 | 101.3726284 | 87.02010429 | 95.42809169 | 90.32093741 | 97.58390237 | 94.13210303 |
| **Mfsd2b** | 8.241268334 | 10.29940611 | 3.915806472 | 6.038265938 | 6.966995687 | 4.208476834 | 5.412388289 | 7.542666117 | 0.752414123 | 7.86511772 | 8.512836289 | 15.61550591 | 10.22501178 | 9.035546516 | 2.155697016 |
| **Ubxn2a** | 871.7430504 | 837.420943 | 854.4289723 | 850.5328878 | 748.0019915 | 840.993954 | 891.2399382 | 982.2227433 | 939.012825 | 1012.852382 | 947.7624402 | 816.343948 | 933.8844094 | 861.9911376 | 799.0450272 |
| **Atad2b** | 957.8185197 | 947.5453622 | 936.6609082 | 891.9381399 | 962.7121313 | 845.9038437 | 1000.389769 | 947.8617088 | 908.9162601 | 859.9195374 | 873.038655 | 968.1613666 | 979.0448781 | 861.9911376 | 925.5125855 |
| **Ldah** | 510.9586367 | 458.7197029 | 539.5981319 | 458.0456018 | 466.1553478 | 455.9183237 | 490.7232048 | 625.2032137 | 489.8215938 | 544.4409266 | 511.716048 | 501.4312454 | 500.173493 | 475.2697467 | 533.8942943 |
| **Hs1bp3** | 59.5202713 | 63.38096068 | 70.4845165 | 73.32180067 | 50.66905954 | 51.20313482 | 88.40234205 | 38.5514046 | 48.90691797 | 44.56900041 | 48.23940564 | 57.25685501 | 57.08964912 | 73.18792678 | 48.14390002 |
| **Rhob** | 318.6623756 | 278.083965 | 205.9714205 | 251.0193411 | 262.2123831 | 303.7117449 | 315.7226502 | 194.433171 | 191.1131871 | 179.1499036 | 200.5245881 | 290.6219156 | 270.1107279 | 210.5282338 | 285.2705718 |
| **Pum2** | 5529.891052 | 5245.566758 | 5624.664417 | 5314.536634 | 5052.971963 | 4928.827786 | 5754.270816 | 5398.872792 | 5551.311396 | 5340.414932 | 5615.634339 | 5544.372127 | 5789.060837 | 5237.002761 | 5415.110904 |
| **Sdc1** | 40.29064519 | 34.85952837 | 35.24225825 | 44.85568982 | 46.23551683 | 28.75792503 | 20.74748844 | 47.77021874 | 57.93588744 | 42.82119648 | 39.72656935 | 52.05168638 | 44.30838439 | 33.43152211 | 43.11394032 |
| **Laptm4a** | 879.0686223 | 789.8852225 | 823.8856818 | 737.5310538 | 805.0046835 | 824.8614595 | 814.5644374 | 873.2731216 | 942.0224815 | 779.5205563 | 790.7479042 | 759.087093 | 885.3156035 | 868.3160202 | 732.9369854 |
| **Wdr35** | 95.23243408 | 95.86370303 | 90.84671016 | 82.81050429 | 75.37022607 | 57.51585007 | 90.20647148 | 161.7482845 | 100.0710783 | 124.9679816 | 104.0457769 | 98.89820412 | 54.53339617 | 73.18792678 | 97.72493139 |
| **Ttc32** | 92.48534464 | 99.82501307 | 127.655291 | 118.1774905 | 106.405025 | 97.49637999 | 92.91266562 | 125.711102 | 97.06142181 | 134.5809032 | 114.4503546 | 143.1421375 | 76.68758836 | 146.3758536 | 88.38357765 |
| **Rdh14** | 114.4620602 | 128.3464454 | 111.2089038 | 114.7270528 | 120.3390164 | 108.0175721 | 102.8353775 | 112.3019177 | 138.4441986 | 77.77727523 | 106.883389 | 139.6720251 | 127.8126473 | 131.0154245 | 129.341821 |
| **9530020I12Rik** | 0 | 0 | 0 | 0 | 3.800179466 | 0 | 2.706194144 | 0 | 0 | 0 | 0.945870699 | 1.735056213 | 5.964590206 | 0 | 0 |
| **Msgn1** | 3.662785926 | 1.584524017 | 3.915806472 | 2.587828259 | 1.266726489 | 6.312715251 | 3.608258859 | 0 | 3.00965649 | 1.747803938 | 0.945870699 | 3.470112425 | 1.70416863 | 2.710663955 | 7.18565672 |
| **Gen1** | 544.8394065 | 567.2595981 | 593.6362612 | 620.2161727 | 627.0296118 | 625.6602227 | 582.7338057 | 610.9559555 | 622.2464794 | 576.7752995 | 563.7389365 | 674.0693386 | 618.6132128 | 608.0922805 | 595.6909421 |
| **Smc6** | 2970.519386 | 2942.4611 | 2968.181306 | 2806.931052 | 3021.142675 | 2906.654667 | 3149.107919 | 2993.600375 | 3070.602034 | 3002.727165 | 3104.347633 | 3239.349949 | 3270.299602 | 3069.375151 | 3127.91637 |
| **Fam49a** | 310.4211073 | 382.6625501 | 420.5576151 | 383.8611917 | 466.1553478 | 384.3742175 | 444.7179044 | 266.5075362 | 282.9077101 | 208.8625706 | 239.3052868 | 489.2858519 | 416.6692301 | 459.005763 | 471.3790808 |
| **Mycn** | 68.67723612 | 54.66607859 | 62.65290356 | 75.90962893 | 51.30242279 | 72.94693179 | 55.0259476 | 71.23629111 | 21.82000955 | 53.3080201 | 97.42468197 | 85.88528252 | 73.2792511 | 61.44171631 | 89.820709 |
| **Ddx1** | 3839.515347 | 3716.501082 | 4005.870021 | 3774.77882 | 3757.744128 | 3671.194625 | 3754.393343 | 4596.835962 | 4679.263428 | 4366.014237 | 4595.985725 | 3778.952431 | 3565.120775 | 3700.959853 | 3539.6545 |
| **Nbas** | 831.4524053 | 797.0155806 | 783.1612945 | 696.1258017 | 625.1295221 | 733.6777948 | 764.0488134 | 797.8464604 | 777.2437886 | 801.3681055 | 839.9331805 | 716.5782158 | 732.792511 | 714.7117294 | 691.2601764 |
| **Fam84a** | 0.915696482 | 4.753572051 | 4.698967767 | 3.450437679 | 11.4005384 | 10.52119209 | 9.922711863 | 16.76148026 | 12.79104008 | 3.495607876 | 13.24218978 | 6.072696744 | 11.92918041 | 6.324882561 | 5.748525376 |
| **Trib2** | 333.3135193 | 337.5036156 | 353.9889051 | 309.6767817 | 339.4826989 | 342.2894492 | 403.2229275 | 253.936426 | 224.2194085 | 219.3493942 | 302.6786236 | 385.1824792 | 326.3482927 | 346.0614316 | 398.0853823 |
| **Lpin1** | 887.3098906 | 834.251895 | 825.4520044 | 778.0736965 | 813.2384057 | 791.1936448 | 844.332573 | 414.0085624 | 611.7126817 | 536.5758089 | 562.7930658 | 705.3003504 | 795.8467504 | 704.7726282 | 770.3024003 |
| **E2f6** | 493.5604036 | 557.752454 | 574.0572289 | 557.2456851 | 545.3257533 | 613.0347922 | 624.2287826 | 572.4045509 | 556.7864507 | 582.8926133 | 559.009583 | 609.0047306 | 561.5235637 | 588.2140782 | 615.8107809 |
| **Rock2** | 1650.08506 | 1687.518078 | 1600.781686 | 1506.978656 | 1435.834475 | 1440.00049 | 1572.298798 | 1687.881062 | 1820.842177 | 1587.879877 | 1714.863577 | 1632.687896 | 1595.953922 | 1586.641968 | 1580.844478 |
| **Pqlc3** | 458.7639373 | 483.2798252 | 524.7180673 | 502.0386822 | 483.8895186 | 434.1745267 | 443.8158397 | 481.8925575 | 398.779485 | 437.8248864 | 481.4481857 | 493.6234925 | 494.2089028 | 528.5794712 | 435.4507972 |
| **2410004P03Rik** | 21.97671556 | 19.80655021 | 21.92851625 | 15.52696955 | 16.46744435 | 11.22260489 | 11.72684129 | 13.40918421 | 18.81035306 | 21.84754922 | 17.97154328 | 19.08561834 | 29.82295103 | 18.07109303 | 16.52701046 |
| **Pdia6** | 4752.464739 | 4460.435108 | 4715.414154 | 4644.289115 | 4333.471317 | 4519.202707 | 4378.622126 | 5062.805113 | 4857.585575 | 5196.221107 | 5323.360293 | 4375.811768 | 4261.27366 | 4539.45857 | 4457.262863 |
| **Nol10** | 1694.038491 | 1507.674602 | 1614.095428 | 1577.712629 | 1559.340307 | 1430.882124 | 1596.654545 | 1830.353645 | 1684.65522 | 1594.871093 | 1717.701189 | 1471.327668 | 1466.437106 | 1430.327013 | 1354.496292 |
| **Odc1** | 13385.65117 | 13283.8571 | 13271.4513 | 13492.07393 | 12323.34864 | 13108.70393 | 12669.49892 | 14663.78101 | 14626.93054 | 15013.63583 | 13945.91758 | 11939.78933 | 12156.68692 | 12047.99772 | 12147.35268 |
| **Hpcal1** | 500.8859754 | 571.2209081 | 656.2891648 | 671.1101285 | 644.1304194 | 599.0065361 | 628.7391062 | 615.1463256 | 641.0568324 | 528.7106912 | 505.0949531 | 668.8641699 | 650.9924168 | 666.8233329 | 664.6732466 |
| **5730507C01Rik** | 0.915696482 | 0 | 0 | 0 | 1.266726489 | 1.402825611 | 5.412388289 | 5.028444078 | 0 | 0 | 0 | 0 | 3.408337261 | 0 | 3.59282836 |
| **1700030C10Rik** | 13.73544722 | 7.922620085 | 6.265290356 | 18.11479781 | 10.76717515 | 9.118366474 | 13.53097072 | 10.05688816 | 7.524141226 | 13.10852953 | 7.56696559 | 16.48303402 | 5.964590206 | 3.614218606 | 10.77848508 |
| **Asap2** | 59.5202713 | 57.83512662 | 61.08658097 | 61.2452688 | 52.56914927 | 55.41161165 | 51.41768874 | 77.1028092 | 46.6496756 | 55.05582404 | 34.99721585 | 69.4022485 | 51.97714322 | 58.73105235 | 66.82660749 |
| **Itgb1bp1** | 484.4034387 | 552.2066199 | 528.6338738 | 539.9934967 | 545.9591166 | 575.8599135 | 597.1668412 | 635.2601019 | 580.1112885 | 589.009927 | 611.0324714 | 556.0855161 | 599.8673579 | 589.1176328 | 603.5951645 |
| **Cpsf3** | 1632.686827 | 1643.943668 | 1783.258268 | 1695.02751 | 1682.84614 | 1731.086804 | 1713.922958 | 1852.143569 | 1824.604247 | 1823.833409 | 1733.780991 | 1659.581267 | 1782.560387 | 1680.611652 | 1679.287975 |
| **Iah1** | 307.6740178 | 280.460751 | 248.2621304 | 231.1793245 | 271.0794686 | 265.8354534 | 270.6194144 | 222.9276875 | 270.8690841 | 246.4403552 | 210.9291658 | 252.4506789 | 328.0524613 | 269.2592862 | 237.8452374 |
| **Adam17** | 1455.041709 | 1407.057327 | 1521.682395 | 1376.724634 | 1473.202906 | 1357.935192 | 1578.613251 | 1371.927159 | 1392.718541 | 1320.465875 | 1370.566643 | 1455.712162 | 1460.472516 | 1568.570875 | 1470.185365 |
| **Ywhaq** | 2615.229151 | 2595.45034 | 2599.312336 | 2730.158813 | 2586.022126 | 2685.709633 | 2751.29738 | 2907.278751 | 2877.984019 | 2842.803105 | 2790.318561 | 2814.261177 | 2845.961613 | 2758.552351 | 2903.72388 |
| **2410018L13Rik** | 1.831392963 | 3.961310043 | 9.397935534 | 6.038265938 | 0 | 2.805651223 | 3.608258859 | 2.514222039 | 1.504828245 | 6.117313782 | 0.945870699 | 5.205168638 | 5.964590206 | 3.614218606 | 5.748525376 |
| **9030624G23Rik** | 7.325571853 | 2.376786026 | 5.482129061 | 1.725218839 | 1.900089733 | 2.104238417 | 7.216517718 | 2.514222039 | 3.00965649 | 0 | 2.837612096 | 1.735056213 | 5.112505891 | 0 | 2.155697016 |
| **Taf1b** | 478.9092599 | 504.6708994 | 484.7768413 | 506.3517293 | 499.7235997 | 498.7045049 | 502.4500461 | 475.1879654 | 473.2684831 | 472.7809652 | 477.6647029 | 464.1275369 | 450.7526027 | 431.8991235 | 485.0318286 |
| **Grhl1** | 27.47089445 | 40.40536243 | 28.9769679 | 34.50437679 | 27.2346195 | 29.45933784 | 20.74748844 | 30.17066447 | 48.90691797 | 30.58656891 | 44.45592284 | 29.49595561 | 35.78754124 | 23.49242094 | 57.48525376 |
| **Klf11** | 144.6800441 | 112.5012052 | 104.1604522 | 150.9566484 | 95.63784989 | 126.254305 | 156.9592604 | 70.3982171 | 79.00348287 | 73.40776539 | 81.34488009 | 129.2616878 | 140.593912 | 125.5940966 | 176.7671553 |
| **Rrm2** | 5936.46029 | 6081.403177 | 6268.423001 | 6197.84868 | 5690.135387 | 5436.650657 | 5726.306809 | 8110.880298 | 7555.742619 | 7446.518677 | 7619.934349 | 5700.527186 | 5678.289876 | 6022.191753 | 5749.962507 |
| **Mboat2** | 54.02609241 | 41.19762444 | 44.64019379 | 49.16873692 | 33.56825195 | 44.89041957 | 36.98465331 | 18.43762829 | 7.524141226 | 13.9824315 | 16.07980188 | 30.36348372 | 20.45002356 | 43.37062328 | 31.61688957 |
| **Kidins220** | 2035.593279 | 2101.078847 | 2305.626851 | 2254.861023 | 2122.400232 | 2273.980316 | 2484.286225 | 1945.169784 | 1969.820173 | 1833.446331 | 2045.918321 | 2283.333976 | 2477.009104 | 2288.703932 | 2341.805525 |
| **Id2** | 843.3564595 | 816.8221308 | 897.5028435 | 902.289453 | 1437.101201 | 1346.712587 | 1344.97849 | 636.9362499 | 683.9444374 | 693.0042613 | 787.9102921 | 1053.179121 | 1373.559916 | 1417.677248 | 1391.143141 |
| **Rnf144a** | 40.29064519 | 61.79643666 | 51.68864544 | 44.85568982 | 69.66995687 | 70.14128057 | 80.28375962 | 25.9802944 | 40.63036262 | 15.73023544 | 30.26786236 | 60.72696744 | 71.57508247 | 84.93413725 | 66.10804182 |
| **Rsad2** | 371.7727715 | 154.4910917 | 140.969033 | 72.45919125 | 161.5076273 | 274.252407 | 323.8412326 | 12.5711102 | 33.85863552 | 3.495607876 | 23.64676747 | 135.3343846 | 133.7772375 | 100.2945663 | 211.2583076 |
| **Cmpk2** | 682.1938788 | 547.4530479 | 470.679938 | 344.1811584 | 519.9912236 | 793.2978833 | 725.2600307 | 129.063398 | 283.6601242 | 166.9152761 | 231.7383212 | 535.2648416 | 408.148387 | 471.6555281 | 501.558839 |
| **Colec11** | 0.915696482 | 0 | 0 | 0 | 2.533452977 | 1.402825611 | 0 | 0 | 0 | 0 | 0 | 0 | 0 | 0 | 0 |
| **Rps7** | 16982.50695 | 15709.76337 | 16646.87648 | 15873.73854 | 17770.27254 | 18198.85666 | 18485.11014 | 16054.14579 | 15127.28593 | 15353.58369 | 15455.52722 | 17104.18414 | 16736.64012 | 17650.94012 | 17175.87526 |
| **Rnaseh1** | 428.5459534 | 495.9560173 | 422.1239377 | 402.838599 | 454.7548094 | 435.5773523 | 394.2022804 | 465.9691513 | 410.0656968 | 468.4114553 | 413.3454954 | 435.4991094 | 411.5567242 | 414.7315851 | 395.2111196 |
| **Adi1** | 511.8743332 | 454.7583929 | 483.99368 | 457.1829924 | 491.4898776 | 509.9271098 | 525.9037287 | 542.2338864 | 504.1174621 | 493.7546124 | 473.8812201 | 459.7898963 | 491.6526498 | 526.7723619 | 478.5647375 |
| **Trappc12** | 850.6820314 | 783.5471264 | 834.8499399 | 821.2041675 | 857.5738327 | 822.0558083 | 760.4405546 | 790.3037943 | 838.1893326 | 787.385674 | 789.8020335 | 832.826982 | 829.930123 | 792.4174294 | 869.4644631 |
| **Tssc1** | 527.4411734 | 579.1435282 | 582.6720031 | 500.3134634 | 545.9591166 | 505.7186329 | 555.6718643 | 598.3848453 | 662.876842 | 547.0626325 | 574.1435142 | 543.0725945 | 547.0381303 | 549.3612282 | 521.6786778 |
| **Tmem18** | 304.0112319 | 305.8131353 | 369.652131 | 344.1811584 | 426.2534634 | 339.483798 | 368.9444683 | 301.7066447 | 278.3932254 | 304.1178852 | 334.8382274 | 345.2761863 | 374.9170987 | 414.7315851 | 397.3668166 |
| **Acp1** | 1623.529862 | 1463.30793 | 1568.672073 | 1616.530052 | 1603.675735 | 1548.018062 | 1578.613251 | 1613.292475 | 1597.375182 | 1580.888662 | 1742.293827 | 1688.209695 | 1598.510175 | 1452.91588 | 1464.436839 |
| **Sh3yl1** | 16.48253667 | 29.31369431 | 11.74741942 | 26.74089201 | 18.36753408 | 30.16075065 | 28.86607087 | 21.78992434 | 25.58208017 | 27.09096104 | 10.40457769 | 8.675281063 | 28.1187824 | 17.16753838 | 21.55697016 |
| **Fam110c** | 1.831392963 | 0 | 2.349483883 | 1.725218839 | 0 | 1.402825611 | 0 | 0 | 0 | 0 | 3.783482795 | 0 | 0 | 1.807109303 | 2.155697016 |
| **Dld** | 2237.046504 | 2158.913973 | 2209.298012 | 2093.553061 | 2068.564356 | 2118.266673 | 2103.614915 | 2241.847985 | 2169.96233 | 2104.355941 | 2212.391564 | 2082.934983 | 2002.398141 | 1832.408833 | 1931.504526 |
| **Cbll1** | 480.7406528 | 485.6566112 | 480.8610348 | 466.671696 | 497.1901468 | 479.7663591 | 504.2541756 | 495.3017417 | 457.4677865 | 484.1416908 | 495.6362462 | 442.4393342 | 420.9296517 | 416.5386944 | 477.8461719 |
| **Bcap29** | 1326.844202 | 1472.815074 | 1539.695105 | 1312.891537 | 1517.538333 | 1426.673647 | 1541.628598 | 1547.084628 | 1644.777272 | 1451.55117 | 1544.606851 | 1386.309914 | 1459.620432 | 1440.266115 | 1426.352859 |
| **Dus4l** | 260.0578008 | 251.9393187 | 255.310582 | 264.8210918 | 266.0125626 | 244.0916564 | 251.6760554 | 248.9079819 | 229.4863074 | 261.2966887 | 292.2740459 | 222.0871952 | 227.5065121 | 272.8735048 | 208.3840449 |
| **Cog5** | 766.4379551 | 669.4613972 | 725.99052 | 642.6440176 | 606.1286248 | 596.9022977 | 727.9662248 | 720.7436512 | 718.5554871 | 699.9954771 | 710.3488948 | 642.8383268 | 739.6091856 | 512.3154874 | 666.1103779 |
| **Gpr22** | 1.831392963 | 0 | 0 | 0 | 1.266726489 | 0 | 1.80412943 | 0 | 2.257242368 | 0 | 0 | 0.867528106 | 0 | 0 | 0 |
| **Hbp1** | 415.7262026 | 400.8845763 | 438.5703249 | 408.8768649 | 396.4853909 | 347.1993388 | 468.171587 | 267.3456102 | 328.8049716 | 312.8569049 | 349.9721585 | 367.8319171 | 383.4379418 | 369.5538525 | 358.5642703 |
| **Prkar2b** | 88.82255871 | 72.09584277 | 70.4845165 | 97.47486442 | 83.60394824 | 82.06529827 | 101.9333128 | 136.6060641 | 136.1869562 | 142.4460209 | 122.9631908 | 60.72696744 | 60.49798638 | 90.35546516 | 91.25784034 |
| **Pik3cg** | 898.2982484 | 694.0215194 | 806.6561333 | 827.2424334 | 768.9029786 | 820.6529827 | 890.3378735 | 825.5029029 | 698.2403058 | 790.0073799 | 922.2239313 | 856.2502409 | 864.0134956 | 832.1738341 | 770.3024003 |
| **Ccdc71l** | 508.2115473 | 472.1881571 | 534.1160028 | 499.450854 | 466.788711 | 490.2875512 | 487.114946 | 605.9275114 | 639.5520042 | 596.8750448 | 573.1976435 | 530.0596729 | 547.0381303 | 476.1733014 | 449.103545 |
| **Nampt** | 4433.802364 | 4194.235073 | 3947.916086 | 3673.853518 | 3465.763673 | 3761.676877 | 3876.172079 | 3001.981115 | 3632.655384 | 3606.593426 | 3560.25731 | 3544.719842 | 3551.487426 | 3391.040607 | 3611.511067 |
| **Gdap10** | 36.62785926 | 36.44405239 | 34.45909696 | 19.84001665 | 36.73506817 | 21.74379698 | 32.47432973 | 31.00873848 | 18.05793894 | 20.97364725 | 19.86328467 | 45.11146153 | 32.37920398 | 34.33507676 | 22.27553583 |
| **Sypl** | 422.136078 | 406.4304104 | 388.4480021 | 350.2194244 | 403.4523866 | 463.6338646 | 454.6406162 | 313.4396809 | 376.9594754 | 318.1003167 | 339.5675809 | 441.5718061 | 411.5567242 | 415.6351397 | 429.7022718 |
| **Cdhr3** | 1.831392963 | 0 | 3.132645178 | 0 | 0.633363244 | 0 | 3.608258859 | 0 | 0 | 0 | 0 | 2.602584319 | 0 | 0 | 4.311394032 |
| **F730043M19Rik** | 15.56684019 | 10.29940611 | 8.614774239 | 3.450437679 | 6.966995687 | 13.32684331 | 15.33510015 | 9.218814144 | 9.781383594 | 8.739019689 | 6.621094891 | 3.470112425 | 11.0770961 | 6.324882561 | 7.18565672 |
| **Atxn7l1** | 311.3368037 | 316.1125414 | 326.5782598 | 297.6002498 | 264.7458361 | 284.0721863 | 319.330909 | 235.4987977 | 242.2773475 | 208.8625706 | 211.8750365 | 294.9595561 | 311.010775 | 240.3455373 | 255.0908135 |
| **Atxn7l1os2** | 3.662785926 | 4.753572051 | 0.783161294 | 1.725218839 | 0.633363244 | 2.805651223 | 0 | 1.676148026 | 3.00965649 | 0.873901969 | 9.458706988 | 5.205168638 | 2.556252945 | 0.903554652 | 0 |
| **Twistnb** | 809.4756897 | 742.349502 | 802.7403268 | 735.805835 | 754.3356239 | 726.6636667 | 792.9148843 | 838.0740131 | 877.3148669 | 833.7024783 | 748.1837227 | 836.2970945 | 740.4612699 | 749.9503608 | 715.6914093 |
| **Twist1** | 8.241268334 | 10.29940611 | 7.831612945 | 3.450437679 | 15.20071786 | 18.23673295 | 17.13922958 | 95.54043749 | 44.39243323 | 52.43411813 | 25.53850887 | 26.02584319 | 3.408337261 | 14.45687443 | 5.029959704 |
| **Hdac9** | 1.831392963 | 0 | 0 | 0.86260942 | 0 | 2.805651223 | 0 | 2.514222039 | 3.00965649 | 0 | 1.891741398 | 0 | 0 | 0.903554652 | 3.59282836 |
| **Snx13** | 263.7205867 | 319.2815894 | 256.0937433 | 281.2106708 | 281.2132805 | 326.1569547 | 296.7792912 | 305.8970148 | 293.4415078 | 302.3700812 | 308.3538478 | 361.7592203 | 351.0587378 | 321.665456 | 328.3845121 |
| **Ahr** | 54.94178889 | 68.92679474 | 46.98967767 | 89.71137964 | 51.93578603 | 54.00878604 | 54.12388289 | 152.5294704 | 110.604876 | 94.38141264 | 124.8549322 | 42.50887721 | 76.68758836 | 63.24882561 | 51.73672838 |
| **Tspan13** | 1189.48973 | 1112.33586 | 1240.52749 | 1225.767985 | 1061.516797 | 1086.488436 | 965.2092448 | 1449.868043 | 1455.921327 | 1392.999738 | 1385.700574 | 1129.521594 | 1070.2179 | 1068.905153 | 1016.770426 |
| **Bzw2** | 3337.713675 | 3380.58199 | 3439.644405 | 3344.33672 | 3358.725284 | 3354.85745 | 3316.891956 | 3778.875725 | 3671.028504 | 3828.564526 | 3786.320407 | 3396.372536 | 3272.00377 | 3312.431353 | 3211.988554 |

| | | | | | | | | | | | | | | | |
|---|---|---|---|---|---|---|---|---|---|---|---|---|---|---|---|
| **Ankmy2** | 232.5869063 | 232.9250305 | 262.3590337 | 267.4089201 | 286.9135497 | 241.2860052 | 296.7792912 | 292.4878306 | 254.3159734 | 240.3230414 | 281.8694682 | 295.8270842 | 297.377426 | 226.7922175 | 279.5220464 |
| **Ispd** | 3.662785926 | 7.130358077 | 2.349483883 | 2.587828259 | 1.266726489 | 2.104238417 | 2.706194144 | 1.676148026 | 0.752414123 | 5.243411813 | 5.675224193 | 1.735056213 | 4.260421576 | 1.807109303 | 0 |
| **Etv1** | 2.747089445 | 7.922620085 | 10.18109683 | 7.763484777 | 0.633363244 | 2.805651223 | 0.902064715 | 5.028444078 | 5.266898858 | 3.495607876 | 0 | 4.337640531 | 1.70416863 | 4.517773258 | 0.718565672 |
| **Arl4a** | 338.8076982 | 381.0780261 | 317.9634856 | 301.9132969 | 287.5469129 | 307.9202217 | 306.702003 | 370.4287138 | 346.8629105 | 336.452258 | 317.8125548 | 289.7543875 | 288.8565828 | 298.173035 | 268.7435613 |
| **Scin** | 517.3685121 | 434.9518427 | 551.3455513 | 478.7482279 | 247.0116653 | 284.0721863 | 208.3769491 | 1045.078294 | 661.3720137 | 1004.113362 | 826.6909907 | 239.4377573 | 260.7378004 | 228.5993268 | 234.9709747 |
| **Ifrd1** | 2083.209496 | 2044.828244 | 2146.645108 | 1921.893787 | 1914.023724 | 1900.127291 | 1862.763636 | 2006.349187 | 2120.302997 | 2271.271217 | 2182.123702 | 1821.809023 | 1889.070927 | 1773.677781 | 1874.019272 |
| **Mir1938** | 0.915696482 | 0 | 0 | 1.725218839 | 1.900089733 | 0.701412806 | 4.510323574 | 0 | 3.00965649 | 12.23462756 | 0 | 0.867528106 | 0 | 1.807109303 | 2.155697016 |
| **Gm7008** | 0.915696482 | 1.584524017 | 0.783161294 | 0.86260942 | 1.900089733 | 2.805651223 | 0.902064715 | 5.866518091 | 0 | 1.747803938 | 0 | 0.867528106 | 4.260421576 | 0.903554652 | 2.874262688 |
| **Zfp277** | 381.8454328 | 419.8988645 | 478.5115509 | 392.4872859 | 422.4532839 | 435.5773523 | 471.7798458 | 387.190194 | 384.4836166 | 333.8305521 | 371.7271846 | 430.2939407 | 434.5630007 | 451.7773258 | 408.8638673 |
| **Dock4** | 10.98835778 | 7.130358077 | 20.36219366 | 16.38957897 | 19.00089733 | 18.23673295 | 5.412388289 | 20.11377631 | 15.04828245 | 13.9824315 | 13.24218978 | 16.48303402 | 20.45002356 | 5.421327909 | 23.71266717 |
| **Immp2l** | 36.62785926 | 30.10595632 | 30.54329049 | 43.13047098 | 24.70116653 | 34.36922748 | 26.15987673 | 31.8468125 | 30.0965649 | 30.58656891 | 34.99721585 | 16.48303402 | 28.1187824 | 39.75640467 | 40.23967763 |
| **Dnajb9** | 90.65395167 | 95.07144102 | 111.2089038 | 83.67311371 | 107.6717515 | 117.1359386 | 131.7014484 | 114.8161398 | 128.662815 | 93.50751067 | 115.3962252 | 132.7318003 | 159.3397669 | 127.4012059 | 152.3359225 |
| **Gm2027** | 0 | 0.792262009 | 0 | 0 | 0.633363244 | 2.104238417 | 2.706194144 | 0.838074013 | 1.504828245 | 1.747803938 | 0 | 0 | 0.852084315 | 0.903554652 | 0 |
| **Pnpla8** | 330.5664298 | 299.4750392 | 277.2390982 | 303.6385157 | 273.6129215 | 356.3177053 | 270.6194144 | 263.1552401 | 269.3642559 | 261.2966887 | 314.9749427 | 316.6477588 | 319.5316182 | 314.4370187 | 283.1148748 |
| **Nrcam** | 4.578482408 | 0.792262009 | 8.614774239 | 6.900875357 | 2.533452977 | 0.701412806 | 3.608258859 | 5.866518091 | 4.514484735 | 1.747803938 | 0 | 1.735056213 | 0 | 0.903554652 | 4.311394032 |
| **Stxbp6** | 32.96507334 | 27.7291703 | 26.62748401 | 30.19132969 | 16.46744435 | 18.23673295 | 7.216517718 | 46.93214473 | 21.82000955 | 45.44290238 | 22.70089677 | 16.48303402 | 0.852084315 | 19.87820233 | 2.874262688 |
| **G2e3** | 446.859883 | 601.3268645 | 501.2232285 | 524.4665271 | 452.2213564 | 443.994306 | 518.687211 | 439.9888569 | 513.1464316 | 427.3380628 | 398.2115642 | 485.8157395 | 498.4693244 | 445.4524432 | 402.3967763 |
| **Scfd1** | 901.0453379 | 1086.191214 | 986.7832311 | 1019.604334 | 893.6755377 | 991.7977073 | 995.8794451 | 1213.531171 | 1151.946022 | 1122.96403 | 1120.856778 | 1085.277661 | 1055.732466 | 1090.590464 | 926.2311512 |
| **Coch** | 5.494178889 | 1.584524017 | 6.265290356 | 3.450437679 | 1.266726489 | 1.402825611 | 4.510323574 | 3.352296052 | 6.019312981 | 2.621705907 | 0 | 1.735056213 | 0 | 3.614218606 | 2.874262688 |
| **Strn3** | 757.2809903 | 770.8709343 | 790.2097461 | 789.287619 | 710.0001968 | 676.8633575 | 748.7137133 | 893.3868979 | 873.5527963 | 884.3887925 | 841.8249219 | 711.3730471 | 698.7091384 | 710.1939561 | 679.7631257 |
| **Ap4s1** | 234.4182993 | 239.2631266 | 245.1294852 | 272.5845766 | 278.6798275 | 267.9396918 | 294.073097 | 313.4396809 | 322.7856586 | 306.7395911 | 279.0318561 | 234.2325887 | 272.6669809 | 297.2694804 | 251.4979852 |
| **Hectd1** | 4544.601638 | 4489.748802 | 4469.501508 | 4206.94614 | 3942.052832 | 3805.865884 | 4389.446902 | 4650.472698 | 4887.68214 | 4639.545553 | 4865.558874 | 4509.411096 | 4343.925839 | 4088.584798 | 4177.022251 |
| **Heatr5a** | 578.7201763 | 535.5691177 | 548.9960674 | 547.7569815 | 463.6218948 | 446.7999572 | 544.8470877 | 501.1682598 | 562.0533496 | 539.1975148 | 585.4939625 | 463.2600088 | 526.5881068 | 462.6199816 | 523.1158092 |
| **Dtd2** | 171.2352421 | 217.0797903 | 212.2367108 | 214.7897455 | 253.978661 | 242.6888308 | 213.7893374 | 233.8226496 | 240.7725192 | 245.5664533 | 227.0089677 | 240.3052854 | 233.4711024 | 225.8886629 | 229.941015 |
| **Nubpl** | 118.1248461 | 104.5785851 | 115.9078716 | 106.1009586 | 105.1382985 | 104.5105081 | 121.7787365 | 155.0436924 | 104.585563 | 89.13800083 | 115.3962252 | 111.0435976 | 134.6293218 | 108.4265582 | 102.7548911 |
| **Arhgap5** | 162.0782772 | 152.9065676 | 143.3185169 | 124.2157564 | 109.5718413 | 117.8373514 | 135.3097072 | 175.9955427 | 124.9007443 | 169.536982 | 175.93195 | 132.7318003 | 141.4459963 | 106.6194489 | 132.9346493 |
| **Egln3** | 4612.363178 | 3100.913501 | 3088.004984 | 2536.934303 | 2689.260335 | 3889.334008 | 4329.910631 | 286.6213125 | 540.23334 | 605.6140645 | 742.5084985 | 2771.7523 | 3312.051733 | 2454.957988 | 3393.785669 |
| **Sptssa** | 608.0224638 | 769.2864103 | 719.7252296 | 722.8666937 | 693.5327525 | 750.5117021 | 755.0281663 | 710.6867631 | 752.4141226 | 693.0042613 | 679.1351617 | 785.1129362 | 710.6383188 | 825.8489515 | 699.8829645 |
| **Eapp** | 455.1011513 | 425.4446986 | 442.4861314 | 415.7777403 | 453.4880829 | 444.6957188 | 386.9857626 | 381.3236759 | 384.4836166 | 414.2295333 | 366.0519604 | 397.3278727 | 463.5338674 | 473.4626374 | 462.7562927 |
| **Snx6** | 1134.547941 | 1246.228139 | 1397.159749 | 1192.126218 | 1074.184062 | 1077.37007 | 1136.601541 | 1273.034426 | 1305.438503 | 1393.87364 | 1238.144745 | 1195.45373 | 1214.220149 | 1091.494019 | 1118.806751 |
| **Cfl2** | 94.3167376 | 95.07144102 | 87.71406498 | 99.20008326 | 88.6708542 | 70.14128057 | 45.10323574 | 84.64547532 | 71.47934165 | 92.6336087 | 85.12836289 | 80.68011388 | 63.90632364 | 71.38081747 | 74.01226421 |
| **Baz1a** | 3476.89954 | 3342.553414 | 3510.128922 | 3418.52113 | 3123.114158 | 3183.712725 | 3593.825824 | 3751.219282 | 3525.812578 | 3519.203229 | 3518.638999 | 3343.453322 | 3432.195621 | 3056.725386 | 3426.83969 |
| **2700097O09Rik** | 82.41268334 | 110.9166812 | 75.96664557 | 88.84877022 | 70.93668336 | 96.09355438 | 95.61885977 | 133.2537681 | 92.54693708 | 110.1116481 | 109.7210011 | 78.07752957 | 88.61676878 | 100.2945663 | 64.67091048 |
| **Srp54a** | 217.9357626 | 171.9208558 | 162.8975493 | 163.8957897 | 157.0740846 | 162.7277709 | 199.356302 | 180.1859128 | 133.1772997 | 160.7979623 | 170.2567258 | 176.9757337 | 144.8543336 | 140.050971 | 150.1802254 |
| **Srp54c** | 199.621833 | 170.3363318 | 242.7800013 | 152.6818673 | 190.0089733 | 178.1588527 | 192.1397842 | 161.7482845 | 173.0552482 | 193.1323351 | 240.2511575 | 172.6380931 | 145.7064179 | 154.5078454 | 162.3958419 |
| **Ppp2r3c** | 381.8454328 | 353.3488558 | 447.9682604 | 364.8837845 | 408.5192926 | 364.734659 | 419.4600924 | 441.6650049 | 500.3553915 | 436.0770825 | 457.8014182 | 409.4732662 | 403.8879654 | 401.1782653 | 352.8157449 |
| **1110008L16Rik** | 279.2874269 | 264.6155108 | 220.851485 | 242.3932469 | 248.911755 | 223.750685 | 260.6967026 | 243.0414638 | 208.418712 | 287.5137478 | 261.0603129 | 199.5314644 | 214.7252474 | 210.5282338 | 204.0726508 |
| **Psma6** | 3420.126359 | 3405.934375 | 3329.218663 | 3252.900122 | 3308.056225 | 3298.043012 | 3232.999938 | 3493.092486 | 3377.586996 | 3388.117933 | 3293.521773 | 3199.443656 | 3120.332762 | 3436.21834 | 3154.5033 |
| **Nfkbia** | 2425.67998 | 2208.034218 | 2371.4124 | 2092.690452 | 2020.428749 | 2218.568704 | 2306.579476 | 2179.830508 | 1851.691156 | 1893.745567 | 1878.499208 | 2121.10622 | 2153.217064 | 2337.495884 | 2291.505928 |
| **Aldoart2** | 0.915696482 | 2.376786026 | 6.265290356 | 2.587828259 | 8.233722176 | 4.208476834 | 3.608258859 | 0 | 0 | 0 | 1.891741398 | 4.337640531 | 0.852084315 | 0 | 0 |
| **Ralgapa1** | 930.3476253 | 993.4965587 | 1012.627554 | 896.251187 | 890.5087215 | 922.3578395 | 1019.333128 | 996.4700015 | 981.90043 | 1046.060657 | 1129.369614 | 891.8188933 | 909.1739643 | 974.0319144 | 965.0336975 |
| **Brms1l** | 173.066635 | 159.2446637 | 178.5607751 | 175.1097122 | 214.0767766 | 204.1111265 | 185.8253312 | 171.8051727 | 226.4766509 | 207.9886686 | 175.93195 | 220.352139 | 218.985669 | 210.5282338 | 217.7253986 |
| **Mbip** | 211.5258872 | 240.8476506 | 175.42813 | 171.6592745 | 197.6093322 | 191.485696 | 165.0778428 | 163.4244325 | 146.7207539 | 208.8625706 | 167.4191137 | 184.7834866 | 214.7252474 | 177.0967117 | 220.5996613 |
| **Prps1l3** | 1164.765925 | 1164.625153 | 1234.2622 | 1192.988827 | 1244.558775 | 1204.325787 | 1289.952542 | 1325.833089 | 1164.737062 | 1229.58007 | 1292.059375 | 1265.723507 | 1278.126473 | 1158.357063 | 1174.136308 |
| **Mipol1** | 9.156964816 | 15.05297816 | 16.44638718 | 5.175656518 | 17.73417084 | 22.44520978 | 14.43303544 | 16.76148026 | 7.524141226 | 13.9824315 | 28.37612096 | 17.35056213 | 21.30210788 | 18.07109303 | 26.58692986 |
| **Sec23a** | 430.3773463 | 420.6911265 | 426.0397442 | 414.9151309 | 431.9537326 | 396.2982352 | 397.8105392 | 419.0370065 | 387.4932731 | 381.8951604 | 477.6647029 | 392.9902321 | 438.8234223 | 420.152913 | 426.8280091 |
| **Gemin2** | 353.4588419 | 322.4506375 | 310.1318726 | 320.8907041 | 335.0491562 | 303.7117449 | 295.8772264 | 340.2580493 | 335.5766987 | 360.9215132 | 308.3538478 | 288.0193313 | 311.8628593 | 280.101942 | 306.8275419 |
| **Trappc6b** | 702.3392014 | 636.9786548 | 673.5187133 | 608.1396409 | 652.9975048 | 607.4234897 | 638.6618181 | 622.6889917 | 562.0533496 | 593.3794369 | 587.3857039 | 746.0741714 | 650.1403325 | 608.0922805 | 651.7390645 |
| **Pnn** | 2748.005141 | 2719.043213 | 2756.727757 | 2622.332636 | 2539.153246 | 2614.165527 | 2857.741016 | 2840.23283 | 2954.730259 | 2957.284263 | 2861.258864 | 2792.572974 | 2767.569856 | 2695.303526 | 2650.070198 |
| **Ctage5** | 3048.353587 | 2925.031335 | 2983.844532 | 2749.99883 | 2616.423562 | 2882.105219 | 2956.06607 | 2747.206615 | 2683.108761 | 2903.102341 | 2878.284536 | 2937.450168 | 2866.411636 | 2782.044772 | 2979.173276 |
| **Fbxo33** | 827.7896193 | 774.0399823 | 794.9087139 | 777.2110871 | 765.7361623 | 750.5117021 | 823.5850846 | 813.7698667 | 924.7169567 | 806.6115173 | 884.3891034 | 884.0111403 | 933.0323251 | 811.3920771 | 821.3205631 |
| **Gm527** | 10.98835778 | 8.714882094 | 3.915806472 | 6.900875357 | 4.43354271 | 8.416953669 | 0.902064715 | 10.05688816 | 9.028969471 | 16.60413741 | 7.56696559 | 13.01292159 | 13.63334904 | 18.07109303 | 9.341353736 |
| **Klhl28** | 127.2818109 | 117.2547773 | 141.7521943 | 119.9027093 | 136.8064608 | 119.240177 | 159.6654545 | 131.57762 | 126.4055726 | 121.4723737 | 130.5301564 | 136.2019127 | 122.7001414 | 122.8834326 | 135.808912 |
| **Fam179b** | 794.824546 | 810.4840347 | 905.3344564 | 805.677198 | 749.268718 | 705.6212825 | 741.4971955 | 931.9383025 | 911.9259166 | 842.441498 | 806.8277061 | 824.151701 | 794.994666 | 874.6409027 | 776.0509257 |
| **Prpf39** | 588.7928376 | 610.8340086 | 612.4321323 | 546.0317626 | 615.6290734 | 613.736205 | 666.6258242 | 679.6780246 | 645.5713172 | 619.596496 | 668.730584 | 662.7914732 | 685.9278737 | 685.7979805 | 686.9487824 |
| **Fkbp3** | 1341.495345 | 1283.464454 | 1366.616459 | 1340.495038 | 1310.428552 | 1390.200181 | 1316.112419 | 1391.202862 | 1386.699228 | 1320.465875 | 1446.236298 | 1371.561936 | 1371.003663 | 1428.519904 | 1358.08912 |
| **Fancm** | 477.9935634 | 544.2839998 | 534.8991641 | 558.1082945 | 418.0197412 | 522.5525403 | 516.8830816 | 669.6211364 | 604.9409546 | 676.4001239 | 587.3857039 | 552.6154037 | 552.1506362 | 535.8079084 | 594.9723764 |
| **Mis18bp1** | 543.92371 | 625.8869867 | 559.1771643 | 541.7187155 | 556.0929285 | 554.1161165 | 607.089553 | 548.9384786 | 575.5968038 | 553.1799463 | 606.3031179 | 574.3036064 | 656.1049227 | 547.5541189 | 630.90066 |
| **Rps29** | 5788.11746 | 4627.602392 | 5348.20848 | 3846.375402 | 5337.985423 | 5034.741119 | 5033.521108 | 3938.947861 | 3595.034678 | 3674.757779 | 3602.821492 | 4178.01536 | 3800.296046 | 4540.362124 | 4186.363605 |
| **Lrr1** | 180.3922069 | 253.5238427 | 230.2494206 | 211.3393078 | 227.3774047 | 199.2012368 | 176.8046841 | 280.7547944 | 242.2773475 | 253.431571 | 253.4933473 | 198.6639363 | 213.0210788 | 223.1779989 | 194.0127314 |
| **Rpl36al** | 3949.398925 | 3629.352261 | 3553.985954 | 3339.161063 | 3550.000984 | 3723.800586 | 3660.578613 | 3193.900064 | 3163.901385 | 2917.958674 | 2988.951408 | 3527.36928 | 3182.534917 | 3437.121895 | 3298.935 |
| **Mgat2** | 1959.590471 | 2155.744925 | 2035.436204 | 2166.012253 | 2070.464446 | 2126.683627 | 2120.754144 | 2315.598498 | 2340.007921 | 2251.171472 | 2253.064004 | 2071.657118 | 2145.548306 | 2201.059131 | 2139.888571 |
| **Dnaaf2** | 509.1272437 | 505.4631614 | 553.6950352 | 543.4439344 | 613.0956205 | 551.3104653 | 548.4553466 | 571.5664769 | 546.252653 | 538.3236128 | 591.1691867 | 596.8593371 | 535.1089499 | 521.351034 | 484.3132629 |
| **9330151L19Rik** | 15.56684019 | 8.714882094 | 23.49483883 | 9.488703616 | 6.966995687 | 7.014128057 | 4.510323574 | 9.218814144 | 12.03862596 | 13.9824315 | 9.458706988 | 13.01292159 | 5.964590206 | 5.421327909 | 10.77848508 |
| **Pole2** | 431.2930428 | 429.4060086 | 398.6290989 | 383.8611917 | 418.6531045 | 372.4501998 | 382.4754391 | 445.8553749 | 431.8857064 | 478.898279 | 465.3683838 | 415.5459629 | 396.2192066 | 460.8128723 | 420.3609181 |
| **Klhdc1** | 81.49698686 | 64.17322269 | 59.52025838 | 50.89395576 | 53.20251252 | 64.52997813 | 72.16517718 | 48.60829276 | 61.69795805 | 65.54264767 | 78.507268 | 75.47494525 | 86.06051583 | 57.8274977 | 58.20381943 |
| **Klhdc2** | 692.2665401 | 666.2923492 | 674.3018746 | 693.5379734 | 649.1973254 | 650.2096709 | 662.1155006 | 655.3738782 | 673.4106397 | 765.5381248 | 632.7874975 | 612.474843 | 685.0757894 | 646.9451305 | 679.7631257 |
| **Nemf** | 1086.931724 | 1109.166812 | 1163.777684 | 1148.133138 | 1065.316977 | 993.9019457 | 1019.333128 | 1225.264207 | 1137.650153 | 1181.515462 | 1206.931012 | 1126.91901 | 1097.484598 | 1106.854448 | 1047.66875 |
| **Arf6** | 6943.72642 | 6631.233011 | 6701.511197 | 6313.438342 | 6718.083932 | 7550.708853 | 7202.986748 | 6455.684123 | 6847.72093 | 6362.006334 | 6351.521742 | 6885.57058 | 6777.478643 | 6849.847814 | 6889.607663 |
| **5830428M24Rik** | 20.14532259 | 22.18333624 | 12.53058071 | 7.763484777 | 13.93399137 | 15.43108173 | 18.0412943 | 15.92340625 | 7.524141226 | 18.35194135 | 5.675224193 | 8.675281063 | 21.30210788 | 17.16753838 | 11.49705075 |
| **Vcpkmt** | 325.9879474 | 305.8131353 | 349.2899373 | 346.7689867 | 310.3479897 | 359.1233565 | 377.0630508 | 359.5337516 | 337.833941 | 380.1473565 | 309.2997185 | 311.4425902 | 303.3420162 | 295.4623711 | 310.4203703 |
| **Sos2** | 663.8799491 | 637.7709168 | 641.4091002 | 642.6440176 | 551.6593858 | 591.2909952 | 586.3420646 | 509.5489999 | 489.8215938 | 450.933416 | 449.2885819 | 628.0903489 | 613.5007069 | 552.0718921 | 574.8525376 |
| **L2hgdh** | 218.8514591 | 225.0024104 | 272.5401305 | 216.5149643 | 218.5103193 | 221.6464466 | 241.7533436 | 304.2208667 | 321.2808303 | 283.1442379 | 273.3566319 | 235.1001168 | 222.3940063 | 243.0562013 | 264.4321673 |
| **Atp5s** | 44.8691276 | 44.36667248 | 38.37490343 | 41.40525214 | 41.80197412 | 41.38335554 | 57.73214175 | 21.78992434 | 31.60139315 | 51.56021617 | 17.97154328 | 27.7608994 | 38.34379418 | 30.72085815 | 42.39537465 |
| **Map4k5** | 30.21798389 | 30.89821833 | 22.71167754 | 27.60350143 | 30.40143573 | 46.29324518 | 47.80942988 | 25.14222039 | 30.84897903 | 27.964863 | 33.10547446 | 48.58157395 | 43.45630007 | 23.49242094 | 30.89832389 |
| **Atl1** | 0 | 0 | 2.349483883 | 0 | 1.266726489 | 0.701412806 | 0 | 0 | 0 | 0 | 0 | 0 | 0 | 5.421327909 | 1.437131344 |
| **Gm3086** | 0 | 0 | 0 | 1.725218839 | 0 | 6.312715251 | 2.706194144 | 0 | 0 | 0.873901969 | 0 | 4.337640531 | 0 | 6.324882561 | 0 |
| **Sav1** | 915.6964816 | 884.9566635 | 873.2248434 | 757.3710705 | 818.3053116 | 882.3773096 | 823.5850846 | 817.1221627 | 787.7775863 | 907.1102437 | 802.0983526 | 924.7849613 | 887.0197721 | 908.0724248 | 924.7940198 |
| **Nin** | 1808.500551 | 2113.755039 | 1977.482269 | 2093.553061 | 1699.946948 | 1865.05665 | 2119.85208 | 2139.602955 | 2143.627835 | 1897.241174 | 2135.776038 | 2125.44386 | 2191.560859 | 2034.805075 | 2016.295276 |
| **Pygl** | 152.9213124 | 111.7089432 | 109.6425812 | 105.2383492 | 69.66995687 | 120.6430026 | 134.4076425 | 0 | 7.524141226 | 17.47803938 | 12.29631908 | 87.62033873 | 78.39175699 | 108.4265582 | 102.0363254 |
| **Trim9** | 0.915696482 | 0.792262009 | 2.349483883 | 0 | 0 | 0 | 0 | 5.028444078 | 2.257242368 | 6.991215751 | 10.40457769 | 0.867528106 | 0 | 0 | 1.437131344 |
| **Tmx1** | 1454.126013 | 1347.637676 | 1401.858717 | 1344.808085 | 1252.792497 | 1280.07837 | 1294.462866 | 1356.841827 | 1306.190917 | 1432.325327 | 1536.094015 | 1282.206541 | 1381.228675 | 1354.428423 | 1330.065059 |
| **Frmd6** | 589.7085341 | 556.960192 | 540.3812932 | 559.8335134 | 452.8547197 | 490.2875512 | 476.2901694 | 577.432995 | 486.8119373 | 520.8455735 | 510.7701773 | 530.927201 | 496.7651557 | 498.7621677 | 516.6487181 |
| **Actr10** | 1205.05657 | 1234.344209 | 1162.211361 | 1199.889703 | 1243.292049 | 1203.624375 | 1216.8853 | 1169.951322 | 1176.775688 | 1096.746971 | 1123.69439 | 1116.508673 | 1105.153357 | 1176.428156 | 1268.268411 |
| **Psma3** | 3279.1091 | 3502.59034 | 3541.455374 | 3532.385573 | 3388.493357 | 3494.438598 | 3156.324437 | 3826.645944 | 3628.140899 | 3706.21825 | 3809.021304 | 3511.753774 | 3249.849578 | 3374.776624 | 3318.336273 |
| **3110056K07Rik** | 19.22962611 | 34.85952837 | 24.27800013 | 20.70262607 | 20.90098706 | 25.95227381 | 25.25781201 | 23.46607237 | 39.8779485 | 44.56900041 | 33.10547446 | 28.62842751 | 49.42089028 | 30.72085815 | 15.08987911 |
| **Arid4a** | 776.5106164 | 865.9423753 | 939.0103921 | 822.9293863 | 849.3401106 | 803.8190753 | 906.5750384 | 856.5116413 | 856.2472715 | 811.8549291 | 793.5855163 | 851.9126004 | 907.4697956 | 847.5342632 | 862.997372 |
| **Timm9** | 391.0023976 | 416.7298165 | 418.2081313 | 436.4803663 | 382.5513995 | 401.2081249 | 386.0836979 | 478.5402615 | 430.3808781 | 480.6460829 | 480.502315 | 458.9223682 | 416.6692301 | 424.6706862 | 379.4026748 |
| **2700049A03Rik** | 338.8076982 | 352.5565938 | 284.2875499 | 330.3794077 | 264.7458361 | 278.4608839 | 325.645362 | 330.2011611 | 264.8497711 | 318.1003167 | 325.3795204 | 323.5879836 | 359.579581 | 286.4268245 | 319.0431584 |
| **Dact1** | 2.747089445 | 1.584524017 | 0 | 1.725218839 | 0 | 0.701412806 | 2.706194144 | 7.542666117 | 4.514484735 | 0 | 0.945870699 | 0 | 0 | 2.710663955 | 2.874262688 |
| **Daam1** | 148.34283 | 147.3607336 | 106.5099361 | 115.5896622 | 129.2061018 | 140.2825611 | 151.5468721 | 124.8730279 | 125.6531585 | 97.00311855 | 111.6127425 | 124.0565192 | 126.960563 | 112.9443314 | 151.6173568 |
| **Gpr135** | 0 | 0 | 1.566322589 | 0 | 0 | 1.402825611 | 0 | 0.838074013 | 0 | 0 | 0 | 3.470112425 | 3.408337261 | 1.807109303 | 2.155697016 |
| **L3hypdh** | 0.915696482 | 0 | 5.482129061 | 0 | 1.266726489 | 0 | 1.80412943 | 1.676148026 | 3.762070613 | 1.747803938 | 0.945870699 | 6.072696744 | 4.260421576 | 1.807109303 | 0 |
| **Jkamp** | 152.0056159 | 186.973834 | 166.8133557 | 153.5444767 | 184.3087041 | 180.9645039 | 175.9026194 | 188.5666529 | 173.8076623 | 208.8625706 | 213.7667779 | 176.1082056 | 151.6710081 | 181.614485 | 173.8928926 |
| **Pcnxl4** | 115.3777567 | 152.9065676 | 141.7521943 | 140.6053354 | 133.0062813 | 155.0122301 | 150.6448074 | 201.1377631 | 173.0552482 | 177.4020997 | 154.1769239 | 158.7576434 | 115.0313825 | 176.1931571 | 145.1502657 |
| **Dhrs7** | 418.4732921 | 326.4119475 | 385.3153569 | 362.2959563 | 390.1517585 | 425.0561603 | 382.4754391 | 278.2405723 | 246.0394181 | 311.1091009 | 258.2227008 | 388.6525916 | 413.2608929 | 396.660492 | 383.7140688 |
| **Ppm1a** | 1238.021643 | 1260.488856 | 1216.24949 | 1317.204584 | 1268.626578 | 1338.997046 | 1344.97849 | 1230.292651 | 1562.011718 | 1372.899993 | 1389.484056 | 1368.959352 | 1348.849471 | 1196.306359 | 1272.579805 |
| **Six1** | 1.831392963 | 1.584524017 | 3.915806472 | 3.450437679 | 1.900089733 | 0 | 3.608258859 | 5.028444078 | 8.276555348 | 0 | 2.837612096 | 0 | 5.112505891 | 2.710663955 | 0 |
| **Mnat1** | 511.8743332 | 563.298288 | 520.8022608 | 505.4891199 | 481.3560656 | 550.6090525 | 479.8984283 | 518.7678141 | 540.23334 | 575.0274955 | 540.092169 | 493.6234925 | 527.4401911 | 515.0261514 | 560.4812241 |
| **Trmt5** | 382.7611293 | 381.0780261 | 384.5321956 | 372.6472693 | 331.88234 | 401.2081249 | 373.4547919 | 410.6562664 | 331.8146281 | 392.381984 | 437.9381335 | 372.1695576 | 352.7629065 | 390.3356095 | 356.4085733 |
| **Slc38a6** | 45.78482408 | 40.40536243 | 28.9769679 | 38.81742388 | 29.76807248 | 35.77205309 | 27.96400616 | 56.98903289 | 54.92623095 | 44.56900041 | 51.07701773 | 53.78674259 | 44.30838439 | 27.10663955 | 55.32955674 |
| **Tmem30b** | 3.662785926 | 10.29940611 | 0 | 0 | 1.900089733 | 2.805651223 | 1.80412943 | 1.676148026 | 0 | 0.873901969 | 13.24218978 | 1.735056213 | 5.112505891 | 2.710663955 | 0 |
| **Prkch** | 2453.150874 | 2767.371196 | 2924.324274 | 2840.572819 | 3017.342496 | 3036.416036 | 3211.350385 | 2970.134302 | 2959.997158 | 2628.697122 | 2302.249281 | 3318.295007 | 3170.605737 | 3516.634704 | 3150.191906 |
| **Hif1a** | 8288.884551 | 7028.948539 | 6822.901198 | 6248.742636 | 4933.26631 | 5102.076749 | 5775.920369 | 4466.93449 | 5263.136787 | 4951.528556 | 4909.068927 | 5348.310775 | 5556.441819 | 4974.971912 | 5111.87619 |
| **Snapc1** | 597.9498025 | 475.3572051 | 538.8149706 | 470.9847431 | 452.8547197 | 518.3440634 | 501.5479814 | 473.5118174 | 436.4001911 | 461.4202396 | 494.6903755 | 501.4312454 | 443.0838439 | 436.4168967 | 473.5347778 |
| **Kcnh5** | 2.747089445 | 4.753572051 | 7.831612945 | 0 | 5.066905954 | 0.701412806 | 0 | 1.676148026 | 4.514484735 | 0.873901969 | 4.729353494 | 1.735056213 | 0 | 2.710663955 | 2.155697016 |
| **Ppp2r5e** | 1195.899605 | 1105.997764 | 1144.981813 | 1157.621841 | 1138.787113 | 1210.638503 | 1222.297689 | 1134.752214 | 1128.621184 | 1050.430167 | 1145.449416 | 1210.201708 | 1101.74502 | 1175.524602 | 1220.124511 |
| **Wdr89** | 235.3339958 | 239.2631266 | 207.537743 | 257.9202165 | 207.7431441 | 197.0969984 | 231.8306317 | 257.288722 | 251.3063169 | 248.1881592 | 216.60439 | 229.8949482 | 219.8377533 | 202.396242 | 188.2642061 |
| **Sgpp1** | 1031.989935 | 1188.393013 | 1253.058071 | 1211.966235 | 1173.622092 | 1232.3823 | 1228.612142 | 1448.191895 | 1468.712367 | 1399.117052 | 1468.937195 | 1288.279238 | 1261.936871 | 1195.402804 | 1310.663786 |
| **Syne2** | 163.9096702 | 254.3161047 | 187.1755494 | 230.316715 | 209.0098706 | 211.1252545 | 252.5781201 | 195.271245 | 201.6469849 | 216.7276883 | 170.2567258 | 211.6768579 | 207.0564886 | 222.2744443 | 253.6536822 |
| **Mthfd1** | 3434.777502 | 3642.028453 | 3636.21789 | 3642.799579 | 3472.097305 | 3440.429812 | 3426.041787 | 4683.157585 | 4285.750842 | 4444.665414 | 4462.617957 | 3501.343437 | 3577.902039 | 3532.898688 | 3423.246861 |
| **Akap5** | 1.831392963 | 3.169048034 | 1.566322589 | 0 | 1.900089733 | 0.701412806 | 0.902064715 | 11.73303618 | 0.752414123 | 9.612921658 | 1.891741398 | 4.337640531 | 11.0770961 | 1.807109303 | 0.718565672 |
| **Zbtb25** | 509.1272437 | 472.1881571 | 483.2105187 | 405.4264272 | 438.287365 | 429.2646371 | 478.9963635 | 416.5227845 | 416.0850098 | 393.255886 | 349.0262878 | 468.4651774 | 428.5984105 | 448.1631072 | 403.8339076 |
| **Zbtb1** | 859.8389962 | 774.8322443 | 947.6251663 | 795.3258849 | 773.3365213 | 813.6388546 | 882.2192911 | 714.0390591 | 743.3851531 | 750.6817913 | 716.9699897 | 803.3310264 | 806.9238465 | 660.4984503 | 761.6796123 |
| **Hspa2** | 74.17141501 | 53.08155457 | 65.00238744 | 37.95481446 | 65.8697774 | 56.11302446 | 92.01060091 | 41.06562664 | 51.91657446 | 44.56900041 | 34.99721585 | 86.75281063 | 58.79381775 | 64.15238026 | 68.98230451 |
| **Gm10451** | 17.39823315 | 15.05297816 | 12.53058071 | 17.25218839 | 8.233722176 | 7.014128057 | 9.922711863 | 18.43762829 | 7.524141226 | 6.991215751 | 18.91741398 | 10.41033728 | 9.372927467 | 10.84265582 | 15.08987911 |
| **Plekhg3** | 103.4737024 | 106.1631091 | 111.9920651 | 135.4296789 | 113.3720207 | 92.58649035 | 131.7014484 | 90.51199341 | 91.79452295 | 124.9679816 | 119.179708 | 110.1760695 | 140.593912 | 142.7616349 | 108.5034165 |
| **Sptb** | 10.98835778 | 6.338096068 | 14.0969033 | 18.97740723 | 12.66726489 | 15.43108173 | 22.55161787 | 27.65644243 | 15.80069657 | 27.09096104 | 4.729353494 | 13.01292159 | 5.964590206 | 9.035546516 | 17.9641418 |
| **Churc1** | 356.2059313 | 382.6625501 | 352.4225825 | 373.5098787 | 430.6870061 | 370.3459614 | 400.5167334 | 364.5621957 | 418.3422522 | 352.1824935 | 349.9721585 | 402.5330413 | 368.9525085 | 479.78752 | 363.59423 |
| **Rab15** | 0 | 0 | 3.915806472 | 1.725218839 | 0 | 0 | 0 | 3.352296052 | 0.752414123 | 0 | 3.783482795 | 0 | 0.852084315 | 0 | 3.59282836 |
| **Fntb** | 538.4295312 | 604.4959125 | 677.4345197 | 630.5674858 | 621.9627059 | 578.6655647 | 584.5379352 | 651.1835081 | 681.6871951 | 587.2621231 | 666.8388426 | 633.2955176 | 642.4715736 | 691.2193085 | 643.1162764 |
| **Max** | 2803.862627 | 2909.978357 | 2849.923951 | 2788.816254 | 2695.593968 | 2785.310251 | 2707.998274 | 2820.119054 | 2723.739124 | 2850.668223 | 2628.574672 | 2696.277354 | 2723.261471 | 2734.156376 | 2807.43608 |
| **Fut8** | 293.9385706 | 309.7744453 | 360.2541955 | 313.9898288 | 283.7467334 | 277.7594711 | 315.7226502 | 290.8116825 | 304.7277196 | 311.1091009 | 378.3482795 | 260.2584319 | 230.9148494 | 278.2948327 | 262.2764703 |
| **Gphn** | 566.8161221 | 556.16793 | 578.7561966 | 541.7187155 | 468.6888008 | 479.7663591 | 504.2541756 | 683.8683947 | 689.2113363 | 620.4703979 | 551.4426174 | 458.0548401 | 478.0193008 | 487.9195118 | 490.780354 |
| **Mpp5** | 251.8165324 | 236.8863405 | 254.5274207 | 188.0488535 | 203.9429647 | 176.0546142 | 196.6501078 | 226.2799835 | 192.6180154 | 228.9623159 | 231.7383212 | 175.2406775 | 232.619018 | 168.9647198 | 190.4199031 |
| **Atp6v1d** | 884.5628012 | 944.3763141 | 977.3852955 | 1009.253021 | 993.113567 | 914.6422986 | 927.3225268 | 975.5181512 | 1008.234924 | 946.4358323 | 893.8478103 | 910.9045116 | 1010.571998 | 1016.498983 | 960.0037377 |
| **Eif2s1** | 4432.886667 | 4322.581518 | 4640.23067 | 4393.269774 | 4118.761178 | 4204.268357 | 4487.771956 | 5066.157409 | 4807.926243 | 4787.234986 | 4823.940564 | 4164.13491 | 4107.046399 | 4003.650661 | 4011.752147 |
| **Plek2** | 0 | 0 | 6.265290356 | 0 | 0 | 1.402825611 | 0 | 0 | 5.266898858 | 0 | 0.945870699 | 5.205168638 | 0 | 1.807109303 | 0 |
| **Tmem229b** | 914.7807851 | 952.2989342 | 889.6712305 | 920.4042508 | 1003.247379 | 977.7694512 | 1051.807457 | 813.7698667 | 934.4983402 | 729.708144 | 793.5855163 | 976.8366477 | 935.588578 | 974.935469 | 931.9796765 |
| **Plekhh1** | 0.915696482 | 2.376786026 | 7.04845165 | 4.313047098 | 1.266726489 | 3.507064029 | 1.80412943 | 0.838074013 | 3.762070613 | 4.369509845 | 1.891741398 | 0 | 1.70416863 | 0.903554652 | 7.18565672 |
| **Pigh** | 44.8691276 | 45.95119649 | 39.94122602 | 49.16873692 | 42.43533737 | 65.23139093 | 40.59291217 | 49.44636677 | 54.17381683 | 34.95607876 | 30.26786236 | 71.13730471 | 64.75840795 | 33.43152211 | 53.8924254 |
| **9230116L04Rik** | 0 | 0 | 1.566322589 | 0 | 0 | 1.402825611 | 0 | 5.028444078 | 0 | 2.621705907 | 0 | 0 | 1.70416863 | 0 | 1.437131344 |
| **Arg2** | 0 | 0 | 0 | 0.86260942 | 0 | 0 | 0 | 1.676148026 | 0 | 1.747803938 | 2.837612096 | 0.867528106 | 0 | 3.614218606 | 0 |
| **Vti1b** | 749.0397219 | 808.1072487 | 822.3193592 | 888.4877022 | 864.5408284 | 950.4143517 | 847.9408319 | 790.3037943 | 899.1348765 | 845.9371059 | 828.5827321 | 828.4893415 | 883.6114348 | 824.9453969 | 852.218887 |
| **Rdh11** | 1457.788799 | 1269.996 | 1316.494136 | 1259.409753 | 1233.158237 | 1258.334573 | 1344.97849 | 1077.763181 | 1217.40605 | 1274.149071 | 1244.76584 | 1224.082158 | 1310.505677 | 1181.849484 | 1320.005139 |
| **Rdh12** | 37.54355574 | 53.87381658 | 42.2907099 | 26.74089201 | 40.53524763 | 51.90454762 | 47.80942988 | 39.38947861 | 64.70761454 | 54.18192207 | 20.80915537 | 53.78674259 | 47.71672165 | 46.08128723 | 27.30549553 |
| **Zfyve26** | 765.5222586 | 696.3983055 | 666.4702616 | 640.0561894 | 559.8931079 | 531.6709067 | 611.5998766 | 609.2798075 | 671.9058115 | 647.561359 | 689.5397394 | 594.2567528 | 658.6611756 | 602.6709526 | 625.8707003 |
| **Rad51b** | 82.41268334 | 102.9940611 | 79.88245204 | 104.3757398 | 109.5718413 | 103.8090952 | 92.01060091 | 98.89273354 | 57.18347332 | 112.733354 | 114.4503546 | 114.51371 | 123.5522257 | 99.39101167 | 84.79074929 |
| **9430078K24Rik** | 0 | 0 | 0 | 0 | 0 | 0 | 0 | 0 | 0 | 0 | 0 | 6.072696744 | 0 | 0 | 0.718565672 |
| **Zfp36l1** | 2514.502538 | 2458.389012 | 2525.695175 | 2249.685366 | 2765.263925 | 2940.322482 | 3191.504961 | 1802.697202 | 1847.929085 | 1630.701074 | 1882.282691 | 2591.306453 | 2675.54475 | 2770.298562 | 2874.262688 |
| **2310015A10Rik** | 25.63950148 | 32.48274235 | 26.62748401 | 22.42784491 | 25.33452977 | 23.84803539 | 16.23716487 | 10.05688816 | 21.06759543 | 12.23462756 | 18.91741398 | 29.49595561 | 28.97086672 | 33.43152211 | 15.80844478 |
| **Actn1** | 790.2460636 | 744.726288 | 879.4901337 | 824.6546052 | 618.1625264 | 650.2096709 | 699.100154 | 1490.933669 | 1204.61501 | 1011.104578 | 1020.594484 | 727.8560812 | 635.6548991 | 660.4984503 | 667.5475093 |
| **Dcaf5** | 917.5278745 | 945.9608382 | 967.98736 | 935.0686109 | 942.4445075 | 933.5804444 | 1047.297134 | 817.1221627 | 887.0962505 | 887.8844004 | 839.9331805 | 969.8964228 | 962.8552761 | 974.935469 | 1023.237517 |
| **Exd2** | 311.3368037 | 320.8661134 | 286.6370338 | 330.3794077 | 288.8136394 | 291.7877272 | 322.0371032 | 291.6497565 | 358.1491223 | 314.6047088 | 320.6501669 | 288.8868594 | 334.8691359 | 313.5334641 | 334.1330375 |
| **Erh** | 3952.146014 | 4073.811248 | 4087.318796 | 4093.944306 | 4171.96369 | 4376.114495 | 3888.800985 | 4968.940823 | 4655.938591 | 4805.586927 | 4721.786528 | 4368.871543 | 4166.692301 | 4479.823963 | 4214.387666 |
| **Slc39a9** | 754.5339008 | 907.9322617 | 953.1072954 | 847.0824501 | 811.3383159 | 759.6300686 | 911.0853619 | 962.947041 | 957.823178 | 796.1246937 | 841.8249219 | 946.473164 | 858.0489054 | 824.9453969 | 809.8235123 |
| **Susd6** | 644.650323 | 741.55724 | 705.6283263 | 709.9275524 | 720.767372 | 730.1707307 | 737.8889367 | 835.559791 | 707.2692752 | 757.673007 | 587.3857039 | 695.7575412 | 718.3070777 | 770.7321178 | 750.1825615 |
| **Gm20337** | 6.409875371 | 2.376786026 | 7.04845165 | 0 | 4.43354271 | 1.402825611 | 4.510323574 | 6.704592104 | 0 | 1.747803938 | 0 | 6.072696744 | 3.408337261 | 0 | 5.029959704 |
| **Srsf5** | 4411.825648 | 4448.551178 | 4584.626218 | 4112.921713 | 4770.491956 | 4815.198911 | 4651.947734 | 4797.135651 | 4643.14755 | 5478.491443 | 5061.354109 | 5400.362462 | 5203.678913 | 5393.317715 | 5318.823104 |
| **Slc10a1** | 0 | 1.584524017 | 2.349483883 | 4.313047098 | 0 | 0 | 0.902064715 | 0 | 0.752414123 | 2.621705907 | 2.837612096 | 2.602584319 | 0 | 0.903554652 | 1.437131344 |
| **Smoc1** | 40.29064519 | 12.67619214 | 17.22954848 | 15.52696955 | 13.93399137 | 30.16075065 | 28.86607087 | 8.380740131 | 26.33449429 | 20.09974528 | 51.07701773 | 30.36348372 | 22.15419219 | 35.23863141 | 15.08987911 |
| **Cox16** | 10.98835778 | 3.961310043 | 10.96425812 | 5.175656518 | 6.333632443 | 21.74379698 | 14.43303544 | 6.704592104 | 15.04828245 | 12.23462756 | 0 | 17.35056213 | 1.70416863 | 5.421327909 | 7.904222392 |
| **Gm4787** | 2.747089445 | 0 | 0.783161294 | 3.450437679 | 1.900089733 | 0 | 0 | 0 | 1.504828245 | 5.243411813 | 2.837612096 | 0.867528106 | 0.852084315 | 0 | 1.437131344 |
| **Adam4** | 1.831392963 | 8.714882094 | 6.265290356 | 0 | 3.800179466 | 3.507064029 | 0.902064715 | 10.89496217 | 0 | 5.243411813 | 5.675224193 | 6.072696744 | 5.964590206 | 1.807109303 | 2.874262688 |
| **Synj2bp** | 530.1882628 | 555.375668 | 543.5139384 | 483.9238844 | 511.1241381 | 540.0878604 | 562.888382 | 508.7109259 | 525.9374717 | 434.3292785 | 535.3628155 | 547.4102351 | 568.3402382 | 546.6505642 | 564.0740525 |
| **Med6** | 218.8514591 | 240.0553886 | 216.1525173 | 161.3079615 | 181.1418879 | 184.4715679 | 164.1757781 | 244.7176118 | 258.078044 | 279.64863 | 260.1144422 | 182.1809023 | 201.9439827 | 196.0713594 | 195.4498628 |

| | | | | | | | | | | | | | | | |
|---|---|---|---|---|---|---|---|---|---|---|---|---|---|---|---|
| **Map3k9** | 21.97671556 | 8.714882094 | 10.96425812 | 18.11479781 | 15.20071786 | 22.44520978 | 20.74748844 | 7.542666117 | 12.03862596 | 14.85633347 | 7.56696559 | 20.82067455 | 29.82295103 | 22.58886629 | 24.43123285 |
| **Pcnx** | 458.7639373 | 477.7339911 | 445.6187766 | 454.5951642 | 468.6888008 | 533.0737323 | 533.1202464 | 458.4264851 | 401.7891415 | 405.4905136 | 360.3767362 | 471.0677617 | 477.1672165 | 487.9195118 | 467.0676868 |
| **Sipa1l1** | 2235.215111 | 1988.577641 | 1879.587107 | 1979.688618 | 1767.716815 | 1655.334221 | 1938.537072 | 1542.056184 | 1674.121423 | 1660.413741 | 1679.866361 | 1734.188684 | 1940.195986 | 1790.845319 | 1919.28891 |
| **Dpf3** | 0.915696482 | 0 | 1.566322589 | 0 | 2.533452977 | 0 | 1.80412943 | 1.676148026 | 1.504828245 | 0 | 0 | 4.337640531 | 0.852084315 | 0 | 5.748525376 |
| **Dcaf4** | 281.1188198 | 247.9780087 | 317.9634856 | 289.836765 | 258.4122037 | 248.3001332 | 252.5781201 | 338.5819013 | 385.2360308 | 356.5520033 | 325.3795204 | 271.5362973 | 261.5898848 | 284.6197152 | 294.6119255 |
| **Zfyve1** | 341.5547876 | 388.2083842 | 372.7847762 | 389.8994577 | 364.8172287 | 346.497926 | 431.1869337 | 383.837898 | 291.1842654 | 354.8041994 | 386.8611158 | 367.8319171 | 372.3608457 | 329.7974478 | 382.2769375 |
| **Rbm25** | 4742.392078 | 4184.727929 | 4478.899443 | 4469.179403 | 4154.229519 | 4023.303854 | 4475.14305 | 4575.884111 | 4537.057159 | 4620.31971 | 4858.93778 | 4450.419185 | 4347.334176 | 4239.478425 | 4053.428956 |
| **Psen1** | 1722.425082 | 1869.73834 | 1784.041429 | 1786.464108 | 1724.014751 | 1756.337666 | 1757.222064 | 1629.215881 | 1726.790411 | 1653.422525 | 1702.567258 | 1738.526325 | 1697.351956 | 1652.601458 | 1685.036501 |
| **Papln** | 39.37494871 | 43.57441047 | 21.14535495 | 44.85568982 | 37.36843141 | 38.57770431 | 43.29910631 | 51.1225148 | 36.11587788 | 22.72145119 | 26.48437957 | 39.90629289 | 38.34379418 | 21.68531164 | 43.83250599 |
| **Numb** | 366.2785926 | 353.3488558 | 385.3153569 | 357.1202997 | 375.5844039 | 338.7823852 | 368.0424036 | 312.6016069 | 300.965649 | 277.9008261 | 358.4849948 | 363.4942765 | 379.1775202 | 365.9396339 | 362.1570987 |
| **2410016O06Rik** | 1004.51904 | 1030.732873 | 975.0358116 | 937.6564392 | 1045.682716 | 1033.181063 | 1106.833405 | 1082.791625 | 1045.85563 | 991.0048327 | 1013.027518 | 1002.862491 | 1002.903239 | 993.9101167 | 979.4050109 |
| **Acot2** | 108.9678813 | 125.9696594 | 134.7037427 | 141.4679448 | 162.1409905 | 147.2966892 | 150.6448074 | 170.9670987 | 143.7110974 | 215.8537863 | 152.2851825 | 151.8174186 | 147.4105865 | 138.2438617 | 184.6713777 |
| **Acot1** | 5.494178889 | 3.169048034 | 14.0969033 | 3.450437679 | 7.600358931 | 7.014128057 | 5.412388289 | 29.33259046 | 9.028969471 | 16.60413741 | 17.97154328 | 2.602584319 | 3.408337261 | 5.421327909 | 2.874262688 |
| **Acot4** | 0.915696482 | 0 | 1.566322589 | 0.86260942 | 0 | 4.208476834 | 0 | 0 | 1.504828245 | 0.873901969 | 0 | 1.735056213 | 4.260421576 | 2.710663955 | 0.718565672 |
| **Acot6** | 20.14532259 | 19.0142882 | 27.41064531 | 30.19132969 | 13.93399137 | 15.43108173 | 11.72684129 | 21.78992434 | 34.61104964 | 33.20827482 | 21.75502607 | 13.01292159 | 12.78126473 | 13.55331977 | 28.02406121 |
| **Dnal1** | 33.88076982 | 26.93690829 | 23.49483883 | 25.87828259 | 17.73417084 | 18.93814575 | 18.94335901 | 24.30414638 | 24.82966605 | 50.6863142 | 53.91462983 | 18.21809023 | 30.67503535 | 34.33507676 | 29.46119255 |
| **Pnma1** | 4.578482408 | 7.130358077 | 7.831612945 | 12.07653188 | 6.966995687 | 3.507064029 | 4.510323574 | 0.838074013 | 7.524141226 | 3.495607876 | 2.837612096 | 5.205168638 | 1.70416863 | 1.807109303 | 2.874262688 |
| **Elmsan1** | 1000.856254 | 1196.315633 | 1187.272522 | 1041.16957 | 1193.889715 | 1146.809937 | 1118.560246 | 889.1965279 | 1081.971508 | 955.174852 | 1156.799865 | 1130.389122 | 1221.888908 | 1136.671752 | 1172.699177 |
| **Ptgr2** | 392.8337906 | 396.1310043 | 398.6290989 | 411.4646932 | 365.4505919 | 355.6162925 | 398.7126039 | 429.9319687 | 408.5608686 | 441.3204943 | 420.912461 | 386.0500073 | 375.769183 | 385.8178362 | 376.5284121 |
| **Zfp410** | 557.6591573 | 653.616157 | 636.7101324 | 581.3987488 | 564.9600139 | 579.3669775 | 598.0689059 | 652.8596562 | 638.0471759 | 658.9220846 | 681.9727738 | 629.8254052 | 596.4590206 | 671.3411061 | 647.4276704 |
| **Fam161b** | 10.98835778 | 15.05297816 | 10.96425812 | 7.763484777 | 17.1008076 | 9.118366474 | 8.118582433 | 3.352296052 | 12.79104008 | 13.9824315 | 25.53850887 | 18.21809023 | 17.0416863 | 11.74621047 | 10.05991941 |
| **Coq6** | 331.4821263 | 320.0738514 | 343.8078083 | 371.7846599 | 284.3800967 | 302.3089193 | 304.8978736 | 368.7525657 | 395.0174144 | 325.0915324 | 324.4336497 | 307.9724777 | 340.8337261 | 312.6299094 | 326.9473807 |
| **Entpd5** | 760.0280797 | 906.3477377 | 920.9976823 | 877.2737798 | 924.7103366 | 831.1741748 | 947.1679505 | 763.4854259 | 869.7907257 | 871.280263 | 929.7908969 | 977.7041758 | 904.9135427 | 936.082619 | 901.7999183 |
| **Ccdc176** | 7.325571853 | 7.922620085 | 2.349483883 | 8.626094197 | 10.13381191 | 7.014128057 | 13.53097072 | 12.5711102 | 5.266898858 | 7.86511772 | 7.56696559 | 6.072696744 | 9.372927467 | 8.131991864 | 7.18565672 |
| **Rnf113a2** | 321.409465 | 310.5667073 | 289.769679 | 282.0732802 | 310.9813529 | 324.754129 | 326.5474268 | 233.8226496 | 267.1070135 | 230.7101198 | 237.4135454 | 297.5621405 | 259.0336318 | 283.7161606 | 303.2347136 |
| **Aldh6a1** | 35.71216278 | 32.48274235 | 28.1938066 | 27.60350143 | 37.36843141 | 32.96640187 | 27.96400616 | 52.79866282 | 38.37312025 | 37.57778466 | 27.43025026 | 45.97898963 | 22.15419219 | 30.72085815 | 47.42533435 |
| **Lin52** | 104.3893989 | 151.3220436 | 151.1501298 | 128.5288035 | 125.4059224 | 115.7331129 | 119.0725424 | 110.6257697 | 122.643502 | 131.0852953 | 183.4989156 | 130.996744 | 142.2980806 | 121.0763233 | 116.4076389 |
| **Abcd4** | 122.7033285 | 133.8922794 | 162.114388 | 135.4296789 | 127.3060121 | 134.6712587 | 132.6035131 | 131.57762 | 148.9779963 | 126.7157855 | 153.2310532 | 150.0823624 | 144.8543336 | 159.9291733 | 153.0544881 |
| **Vrtn** | 4.578482408 | 2.376786026 | 0.783161294 | 0.86260942 | 2.533452977 | 0.701412806 | 2.706194144 | 0 | 0 | 0 | 0 | 0.867528106 | 0 | 0.903554652 | 0 |
| **Syndig1l** | 1.831392963 | 0 | 0 | 0 | 1.266726489 | 1.402825611 | 0 | 0 | 3.762070613 | 0 | 0 | 1.735056213 | 2.556252945 | 1.807109303 | 0 |
| **Npc2** | 3193.949328 | 3125.473624 | 3225.058211 | 3222.708792 | 3413.827887 | 3520.390872 | 3591.11963 | 2383.482493 | 2550.683876 | 2370.896042 | 2189.690668 | 3322.632647 | 3186.795339 | 3441.639668 | 3311.869182 |
| **Isca2** | 261.8891937 | 255.9006287 | 218.5020012 | 234.6297621 | 245.1115755 | 263.0298021 | 246.2636671 | 189.404727 | 194.1228436 | 262.1705907 | 229.8465798 | 217.7495547 | 243.6961141 | 233.1171001 | 235.6895404 |
| **Arel1** | 1309.445969 | 1165.417415 | 1246.792781 | 1105.865276 | 1163.48828 | 1057.029098 | 1215.983236 | 1024.964518 | 1007.48251 | 977.8963032 | 1048.970605 | 1338.595868 | 1145.20132 | 1242.387646 | 1199.286107 |
| **Fcf1** | 989.8678966 | 968.1441744 | 964.0715535 | 942.8320957 | 946.8780502 | 958.1298926 | 959.7968565 | 941.1571167 | 891.6107353 | 1072.277716 | 998.8394579 | 973.3665352 | 892.9843623 | 894.5191051 | 893.8956959 |
| **Ylpm1** | 1674.808865 | 1695.440698 | 1670.483041 | 1662.248352 | 1685.379593 | 1611.846628 | 1811.345947 | 1784.259574 | 1720.771098 | 1645.557407 | 1805.667164 | 1879.065878 | 1882.254252 | 1576.702867 | 1729.587572 |
| **Prox2** | 38.45925223 | 45.95119649 | 65.78554874 | 52.6191746 | 53.20251252 | 49.80030921 | 50.51562403 | 55.31288486 | 45.14484735 | 73.40776539 | 66.21094891 | 52.91921448 | 32.37920398 | 44.27417793 | 64.67091048 |
| **Dlst** | 2623.47042 | 2741.226549 | 2719.136014 | 2539.522131 | 2524.585892 | 2364.462568 | 2582.611278 | 2743.016245 | 2639.468742 | 2844.550909 | 3004.085339 | 2492.408249 | 2668.728075 | 2573.323648 | 2621.327571 |
| **Rps6kl1** | 15.56684019 | 10.29940611 | 25.84432272 | 17.25218839 | 24.06780328 | 11.22260489 | 14.43303544 | 14.24725822 | 19.56276719 | 8.739019689 | 34.99721585 | 25.15831508 | 7.668758836 | 18.07109303 | 28.74262688 |
| **Pgf** | 3.662785926 | 5.54583406 | 4.698967767 | 1.725218839 | 1.266726489 | 2.104238417 | 4.510323574 | 9.218814144 | 12.03862596 | 8.739019689 | 8.512836289 | 1.735056213 | 0 | 4.517773258 | 0 |
| **Eif2b2** | 1259.998359 | 1407.057327 | 1231.129555 | 1248.19583 | 1261.659583 | 1264.647289 | 1278.225701 | 1290.63398 | 1229.444676 | 1327.457091 | 1362.999677 | 1182.440809 | 1246.599353 | 1290.276042 | 1320.005139 |
| **Mlh3** | 173.066635 | 247.1857467 | 210.6703882 | 175.1097122 | 209.0098706 | 188.6800447 | 190.3356548 | 197.7854671 | 163.2738646 | 212.3581784 | 213.7667779 | 205.6041612 | 197.6835611 | 186.1322582 | 183.2342464 |
| **Acyp1** | 70.50862908 | 95.07144102 | 97.11200052 | 66.42092531 | 119.7056532 | 66.63421654 | 99.22711863 | 82.96932729 | 79.00348287 | 76.02947129 | 60.53572472 | 91.09045116 | 104.8063708 | 103.0052303 | 88.38357765 |
| **Zc2hc1c** | 0 | 7.130358077 | 5.482129061 | 7.763484777 | 10.76717515 | 7.014128057 | 1.80412943 | 12.5711102 | 3.762070613 | 2.621705907 | 0 | 5.205168638 | 2.556252945 | 7.228437213 | 5.029959704 |
| **Nek9** | 2379.895156 | 2227.048506 | 2246.106593 | 2246.234929 | 2109.732967 | 2188.407954 | 2259.672111 | 1976.178523 | 2058.605039 | 2137.564216 | 2223.742013 | 2235.61993 | 2241.833833 | 2074.56148 | 2143.481399 |
| **Tmed10** | 3645.387693 | 3455.846881 | 3575.914471 | 3410.757645 | 3178.21676 | 3117.779921 | 3297.948597 | 3303.687759 | 3497.973256 | 3521.824935 | 3488.371137 | 3475.317594 | 3409.189345 | 3172.380382 | 3297.497869 |
| **Fos** | 274.7089445 | 202.8190742 | 246.6958078 | 196.6749477 | 194.442516 | 175.3532014 | 223.7120493 | 139.9583602 | 132.4248856 | 145.0677268 | 127.6925443 | 144.0096656 | 196.8314768 | 168.0611652 | 145.1502657 |
| **Jdp2** | 88.82255871 | 94.27917901 | 67.35187133 | 91.43659848 | 69.66995687 | 86.97518791 | 79.3816949 | 54.47481085 | 46.6496756 | 78.6511772 | 59.58985402 | 68.5347204 | 88.61676878 | 88.54835585 | 70.41943585 |
| **Batf** | 564.0690326 | 503.8786374 | 563.0929707 | 512.3899953 | 500.356963 | 467.8423414 | 453.7385515 | 491.1113716 | 463.4870995 | 571.5318877 | 586.4398332 | 442.4393342 | 406.4442183 | 459.9093177 | 388.7440285 |
| **Mfsd7c** | 2.747089445 | 8.714882094 | 3.915806472 | 0.86260942 | 3.800179466 | 0.701412806 | 0.902064715 | 4.190370065 | 2.257242368 | 2.621705907 | 3.783482795 | 4.337640531 | 3.408337261 | 2.710663955 | 0.718565672 |
| **0610007P14Rik** | 1201.393784 | 1198.692419 | 1159.861877 | 1120.529636 | 1203.390164 | 1178.373514 | 1341.370231 | 1293.986276 | 1232.454333 | 1226.958364 | 1266.520866 | 1219.744517 | 1199.734716 | 1152.935735 | 1100.124044 |
| **Ttll5** | 299.4327495 | 305.8131353 | 331.2772276 | 296.7376404 | 277.413101 | 307.2188089 | 268.815285 | 232.1465016 | 274.6311547 | 313.7308068 | 406.7244005 | 333.9983209 | 303.3420162 | 290.0410432 | 288.1448345 |
| **Tgfb3** | 14.65114371 | 22.97559825 | 3.132645178 | 15.52696955 | 1.266726489 | 2.104238417 | 3.608258859 | 28.49451644 | 3.00965649 | 7.86511772 | 24.59263817 | 4.337640531 | 5.964590206 | 2.710663955 | 7.904222392 |
| **Ift43** | 6.409875371 | 20.59881222 | 4.698967767 | 6.900875357 | 9.500448664 | 7.715540863 | 7.216517718 | 10.89496217 | 0 | 5.243411813 | 3.783482795 | 11.27786538 | 0.852084315 | 4.517773258 | 2.155697016 |
| **Gpatch2l** | 424.8831674 | 450.0048208 | 505.1390349 | 461.4960395 | 466.1553478 | 534.476558 | 532.2181817 | 416.5227845 | 398.0270708 | 417.7251411 | 329.1630032 | 443.3068623 | 488.2443126 | 466.2342002 | 488.6246569 |
| **Vash1** | 316.8309826 | 353.3488558 | 273.3232918 | 323.4785324 | 209.0098706 | 266.5368662 | 249.871926 | 202.8139112 | 179.0745612 | 277.0269241 | 297.0033994 | 189.9886553 | 215.5773317 | 222.2744443 | 241.4380658 |
| **Angel1** | 203.2846189 | 213.1184803 | 235.7315496 | 223.4158397 | 219.7770458 | 163.4291837 | 191.2377195 | 252.2602779 | 184.34146 | 216.7276883 | 250.6557352 | 208.2067455 | 232.619018 | 221.3708896 | 174.6114583 |
| **Irf2bpl** | 1212.382142 | 1159.079318 | 1205.285232 | 1381.037681 | 1342.730078 | 1394.408658 | 1452.324191 | 1351.813383 | 1326.506098 | 1291.62711 | 1269.358478 | 1405.395532 | 1423.832891 | 1393.281273 | 1414.855808 |
| **Cipc** | 580.5515693 | 606.8726985 | 544.2970997 | 531.3674025 | 514.9243176 | 519.746889 | 521.3934051 | 447.531523 | 593.6547427 | 455.3029258 | 551.4426174 | 478.0079866 | 573.4527441 | 542.1327909 | 515.9301525 |
| **Pomt2** | 198.7061365 | 181.4279999 | 193.4408397 | 172.5218839 | 194.442516 | 177.4574398 | 200.2583667 | 186.8905049 | 178.3221471 | 169.536982 | 263.897925 | 176.9757337 | 149.1147552 | 198.7820233 | 177.485721 |
| **Gstz1** | 122.7033285 | 167.1672838 | 155.0659363 | 121.6279282 | 128.5727386 | 173.9503758 | 126.2890601 | 113.9780658 | 168.5407635 | 158.1762564 | 159.8521481 | 133.5993284 | 141.4459963 | 147.2794082 | 132.9346493 |
| **Tmed8** | 534.7667452 | 640.9399649 | 565.4424546 | 547.7569815 | 453.4880829 | 463.6338646 | 556.573929 | 548.1004045 | 588.3878439 | 503.3675341 | 564.6848072 | 513.5766389 | 472.0547106 | 512.3154874 | 527.4272032 |
| **Samd15** | 4.578482408 | 9.507144102 | 9.397935534 | 2.587828259 | 2.533452977 | 6.312715251 | 2.706194144 | 2.514222039 | 14.29586833 | 14.85633347 | 4.729353494 | 9.542809169 | 7.668758836 | 8.131991864 | 10.77848508 |
| **Noxred1** | 10.98835778 | 12.67619214 | 15.66322589 | 18.11479781 | 7.600358931 | 7.014128057 | 18.0412943 | 11.73303618 | 8.276555348 | 15.73023544 | 1.891741398 | 5.205168638 | 13.63334904 | 17.16753838 | 6.467091048 |
| **Vipas39** | 520.1156015 | 581.5203142 | 575.6235514 | 571.9100452 | 526.324856 | 575.8599135 | 539.4346994 | 615.1463256 | 630.5230347 | 592.5055349 | 701.8360585 | 538.734954 | 565.7839853 | 552.0718921 | 495.8103137 |
| **Ahsa1** | 2918.324687 | 3041.493851 | 3108.367178 | 3026.896454 | 2807.065899 | 2710.259081 | 2755.807704 | 3684.173361 | 3434.018055 | 3640.675602 | 3575.391241 | 2923.569718 | 3004.449295 | 3100.09601 | 2798.813292 |
| **Sptlc2** | 917.5278745 | 915.8548818 | 1041.604522 | 876.4111704 | 1004.514105 | 960.234131 | 1000.389769 | 863.2162334 | 922.4597143 | 883.5148906 | 900.4689052 | 1015.007884 | 979.0448781 | 1031.859412 | 1018.207557 |
| **Alkbh1** | 309.5054108 | 320.0738514 | 325.7950985 | 285.5237179 | 312.8814427 | 287.5792503 | 254.3822496 | 331.0392352 | 288.1746089 | 358.2998073 | 343.3510637 | 280.2115783 | 301.6378476 | 336.1223304 | 293.8933598 |
| **Mir3068** | 0.915696482 | 14.26071615 | 0 | 5.175656518 | 3.800179466 | 2.805651223 | 1.80412943 | 4.190370065 | 5.266898858 | 0.873901969 | 1.891741398 | 1.735056213 | 0 | 2.710663955 | 8.622788064 |
| **Nrp** | 5.494178889 | 3.961310043 | 0.783161294 | 2.587828259 | 8.86708542 | 5.611302446 | 9.020647148 | 1.676148026 | 0.752414123 | 2.621705907 | 7.56696559 | 5.205168638 | 5.964590206 | 2.710663955 | 4.311394032 |
| **Slirp** | 64.09875371 | 55.4583406 | 73.61716168 | 61.2452688 | 67.76986714 | 61.7243269 | 61.34040061 | 56.15095887 | 62.45037217 | 50.6863142 | 52.02288843 | 33.83359614 | 57.94173343 | 62.34527096 | 57.48525376 |
| **Snw1** | 2525.490896 | 2420.360436 | 2362.014464 | 2335.946308 | 2347.244183 | 2379.192237 | 2589.827796 | 2158.040584 | 2320.445154 | 2368.274336 | 2212.391564 | 2419.535888 | 2285.290133 | 2263.404402 | 2310.188635 |
| **Adck1** | 173.066635 | 169.5440698 | 212.2367108 | 166.483618 | 194.442516 | 118.5387642 | 195.7480431 | 170.9670987 | 176.8173188 | 182.6455115 | 236.4676747 | 209.0742736 | 170.416863 | 171.6753838 | 219.8810956 |
| **3200001D21Rik** | 5.494178889 | 4.753572051 | 0.783161294 | 3.450437679 | 0 | 5.611302446 | 5.412388289 | 8.380740131 | 1.504828245 | 0 | 0.945870699 | 3.470112425 | 5.964590206 | 1.807109303 | 0 |
| **Dio2** | 2.747089445 | 3.961310043 | 6.265290356 | 6.038265938 | 3.166816221 | 6.312715251 | 8.118582433 | 10.05688816 | 12.79104008 | 5.243411813 | 4.729353494 | 2.602584319 | 0 | 1.807109303 | 1.437131344 |
| **Cep128** | 358.9530208 | 397.7155283 | 353.9889051 | 334.6924548 | 290.7137291 | 374.5544383 | 357.2176271 | 300.0304967 | 260.3352864 | 326.8393364 | 334.8382274 | 340.0710177 | 384.2900261 | 326.1832292 | 302.5161479 |
| **Gtf2a1** | 564.0690326 | 540.3226898 | 504.3558736 | 515.840433 | 479.4559759 | 526.7610171 | 530.4140523 | 457.5884111 | 525.9374717 | 575.9013975 | 502.257341 | 517.9142794 | 510.3985048 | 431.8991235 | 439.7621912 |
| **Ston2** | 54.94178889 | 49.12024453 | 53.25496803 | 37.09220505 | 29.76807248 | 30.86216345 | 43.29910631 | 36.87525657 | 75.99382638 | 32.33437285 | 33.10547446 | 42.50887721 | 34.93545692 | 34.33507676 | 41.67680897 |
| **Sel1l** | 1694.038491 | 1780.212733 | 1589.817428 | 1536.307376 | 1579.607931 | 1541.705347 | 1619.206163 | 1685.36684 | 1699.703503 | 1604.484015 | 1874.715725 | 1688.209695 | 1673.493595 | 1626.398373 | 1623.958419 |
| **Galc** | 188.6334752 | 161.6214497 | 162.114388 | 154.4070861 | 99.43802935 | 121.3444154 | 123.5828659 | 147.5010263 | 154.9973093 | 127.5896875 | 144.7182169 | 117.1162943 | 131.2209845 | 103.0052303 | 134.3717807 |
| **Gpr65** | 1391.858652 | 1156.702532 | 1107.39007 | 1069.63568 | 982.3463919 | 1001.617487 | 1154.642835 | 989.7654094 | 954.8135216 | 988.3831268 | 1113.289812 | 927.3875456 | 972.2282036 | 1019.209647 | 1054.854406 |
| **Kcnk10** | 43.03773463 | 41.98988645 | 28.9769679 | 38.81742388 | 74.73686282 | 52.60596043 | 59.53627118 | 51.1225148 | 48.90691797 | 35.82998073 | 47.29353494 | 48.58157395 | 43.45630007 | 65.05593491 | 78.32365824 |
| **Spata7** | 28.38659093 | 28.52143231 | 32.10961307 | 50.03134634 | 35.46834168 | 30.16075065 | 49.61355931 | 39.38947861 | 49.65933209 | 37.57778466 | 42.56418144 | 40.773821 | 44.30838439 | 26.2030849 | 38.80254629 |
| **Ptpn21** | 88.82255871 | 72.88810478 | 80.66561333 | 81.08528545 | 82.970585 | 70.84269338 | 104.6395069 | 64.53169901 | 65.46002866 | 75.15556933 | 106.883389 | 75.47494525 | 63.90632364 | 63.24882561 | 83.35361795 |
| **Zc3h14** | 1444.969048 | 1521.143056 | 1601.564847 | 1417.267276 | 1501.704252 | 1465.952764 | 1533.510015 | 1702.128321 | 1623.709677 | 1768.777585 | 1590.954515 | 1616.204862 | 1656.451909 | 1560.438883 | 1595.215792 |
| **Eml5** | 148.34283 | 169.5440698 | 151.1501298 | 139.742726 | 156.4407213 | 161.3249453 | 154.2530662 | 188.5666529 | 142.2062692 | 138.0765111 | 115.3962252 | 152.6849467 | 164.4522728 | 156.3149547 | 153.0544881 |
| **Ttc8** | 12.81975074 | 30.10595632 | 25.06116142 | 21.56523549 | 7.600358931 | 15.43108173 | 9.922711863 | 6.704592104 | 18.81035306 | 19.22584332 | 18.91741398 | 14.74797781 | 20.45002356 | 14.45687443 | 14.37131344 |
| **Foxn3** | 306.7583213 | 287.5911091 | 264.7085175 | 241.5306375 | 244.4782123 | 275.6552326 | 305.7999383 | 292.4878306 | 324.2904868 | 213.2320804 | 243.0887696 | 289.7543875 | 366.3962555 | 204.2033513 | 260.1207733 |
| **Efcab11** | 92.48534464 | 87.94108294 | 106.5099361 | 84.53572313 | 82.33722176 | 88.37801352 | 94.71679505 | 85.48354933 | 102.3283207 | 95.25531461 | 103.0999062 | 77.21000146 | 93.72927467 | 99.39101167 | 102.0363254 |
| **Tdp1** | 771.932134 | 780.3780784 | 831.7172947 | 738.3936632 | 849.3401106 | 801.0134241 | 815.4665022 | 847.2928272 | 774.2341321 | 820.5939488 | 862.6340773 | 778.1727113 | 802.6634249 | 790.6103201 | 871.6201601 |
| **Psmc1** | 4248.831674 | 4026.275527 | 4124.910538 | 4350.139303 | 4050.99131 | 3991.038865 | 4056.585022 | 4958.045861 | 4876.395928 | 4874.625183 | 4835.291012 | 4242.21244 | 3980.93792 | 4189.782919 | 3837.140688 |
| **Nrde2** | 515.5371191 | 517.3470916 | 475.3789058 | 514.1152141 | 525.6914927 | 491.6903768 | 506.058305 | 610.9559555 | 428.8760499 | 573.2796916 | 502.257341 | 558.6881004 | 484.8359753 | 476.1733014 | 520.9601122 |
| **Gm10433** | 0 | 0.792262009 | 0 | 0 | 0.633363244 | 0 | 0 | 0 | 2.257242368 | 0 | 0 | 2.602584319 | 0 | 0 | 0 |
| **Calm1** | 14663.96346 | 14380.34772 | 14828.37595 | 14393.50078 | 14643.99157 | 15191.19855 | 15189.86773 | 15242.89015 | 14964.01207 | 15260.95008 | 15210.54671 | 15578.2022 | 15074.22362 | 15410.12458 | 14955.50733 |
| **Ttc7b** | 574.1416939 | 550.6220959 | 633.5774872 | 620.2161727 | 627.0296118 | 641.7927172 | 591.7544529 | 580.785291 | 555.2816225 | 630.0833196 | 646.0296873 | 544.8076507 | 548.742299 | 611.7064991 | 612.2179525 |
| **Rps6ka5** | 110.7992743 | 111.7089432 | 130.0047749 | 133.70446 | 134.906371 | 122.747241 | 110.0518952 | 111.4638437 | 114.3669466 | 149.4372367 | 135.2595099 | 124.9240473 | 138.0376591 | 101.198121 | 122.1561642 |
| **9030617O03Rik** | 278.3717304 | 322.4506375 | 368.8689697 | 363.1585657 | 256.5121139 | 255.3142613 | 292.2689676 | 574.0806989 | 536.4712694 | 537.4497109 | 525.9041085 | 346.1437144 | 253.0690416 | 325.2796746 | 308.2646733 |
| **Gpr68** | 1246.262911 | 1190.769799 | 1209.9842 | 1394.839432 | 1142.587293 | 1291.300975 | 1246.653436 | 1531.999296 | 1321.991613 | 1311.726855 | 1390.429927 | 1266.591035 | 1392.305771 | 1366.174633 | 1325.753665 |
| **Ccdc88c** | 1745.317494 | 1997.292523 | 1809.10259 | 1803.716296 | 1828.519686 | 1904.335768 | 2092.790138 | 1661.900768 | 1769.678016 | 1593.997191 | 1762.157112 | 2044.763747 | 2111.464933 | 1863.129692 | 1991.864043 |
| **Smek1** | 1945.855023 | 1911.728227 | 1925.793623 | 1949.497288 | 1860.821212 | 1869.96654 | 1934.026748 | 2237.657615 | 2133.094038 | 2264.280001 | 2220.904401 | 2059.511724 | 2036.481513 | 2036.612185 | 1902.043334 |
| **D130020L05Rik** | 10.98835778 | 10.29940611 | 2.349483883 | 5.175656518 | 17.1008076 | 11.22260489 | 11.72684129 | 14.24725822 | 6.019312981 | 3.495607876 | 2.837612096 | 10.41033728 | 10.22501178 | 11.74621047 | 3.59282836 |
| **Fbln5** | 7.325571853 | 7.130358077 | 21.92851625 | 17.25218839 | 11.4005384 | 7.715540863 | 11.72684129 | 6.704592104 | 9.028969471 | 6.117313782 | 14.18806048 | 6.072696744 | 12.78126473 | 0.903554652 | 2.155697016 |
| **Trip11** | 576.8887834 | 579.1435282 | 495.7410994 | 463.2212584 | 438.9207283 | 432.7717011 | 513.2748227 | 546.4242565 | 550.7671377 | 575.9013975 | 541.9839104 | 511.8415827 | 524.0318538 | 483.4017386 | 467.7862524 |
| **Atxn3** | 592.4556236 | 568.8441221 | 580.3225192 | 582.2613583 | 498.4568732 | 527.4624299 | 574.6152233 | 631.0697318 | 610.2078534 | 525.2150833 | 592.1150574 | 547.4102351 | 522.3276852 | 461.716427 | 589.223851 |
| **Cpsf2** | 2301.145258 | 2321.327685 | 2383.94298 | 2341.121965 | 2259.206692 | 2398.831796 | 2326.424899 | 2534.335815 | 2702.671528 | 2538.68522 | 2594.523327 | 2363.146562 | 2324.486012 | 2273.343503 | 2268.511826 |
| **Rin3** | 820.4640475 | 936.4536941 | 834.8499399 | 862.6094197 | 910.142982 | 885.1829608 | 980.544345 | 734.1528354 | 674.915468 | 687.7608495 | 705.6195413 | 905.699343 | 890.4281093 | 904.4582062 | 967.9079601 |
| **Lgmn** | 39.37494871 | 27.7291703 | 32.10961307 | 35.36698621 | 26.60125626 | 32.96640187 | 31.57226502 | 20.95185033 | 15.04828245 | 13.9824315 | 45.40179354 | 30.36348372 | 31.52711966 | 27.10663955 | 30.89832389 |
| **Golga5** | 622.6736075 | 636.9786548 | 619.4805839 | 581.3987488 | 567.4934669 | 556.2203549 | 567.3987056 | 615.1463256 | 634.2851053 | 574.1535936 | 559.9554537 | 673.2018105 | 666.3299345 | 646.0415759 | 633.7749227 |
| **Chga** | 1.831392963 | 0 | 1.566322589 | 0 | 0.633363244 | 2.805651223 | 2.706194144 | 1.676148026 | 0 | 0 | 1.891741398 | 0 | 4.260421576 | 0.903554652 | 2.155697016 |
| **Itpk1** | 905.6238203 | 882.5798775 | 877.1406498 | 812.5780733 | 778.4034272 | 859.9320998 | 947.1679505 | 848.1309012 | 853.237615 | 836.3241842 | 841.8249219 | 842.3697912 | 859.753074 | 890.0013318 | 854.374584 |
| **Tmem251** | 368.1099856 | 331.9577816 | 319.5298082 | 368.3342222 | 354.0500536 | 363.3318334 | 368.0424036 | 391.3805641 | 408.5608686 | 408.1122195 | 413.3454954 | 371.3020295 | 362.9879183 | 398.4676013 | 372.2170181 |
| **AK010878** | 68.67723612 | 67.34227072 | 104.1604522 | 74.18441009 | 87.40412771 | 82.06529827 | 73.96930661 | 79.61703124 | 114.3669466 | 90.0119028 | 117.2879666 | 117.1162943 | 96.28552761 | 105.7158942 | 82.63505228 |
| **Ubr7** | 1482.512604 | 1496.582934 | 1592.950073 | 1506.116047 | 1437.734565 | 1494.710689 | 1405.416826 | 1453.220339 | 1438.615802 | 1542.436975 | 1432.048238 | 1532.922164 | 1370.151579 | 1458.337208 | 1432.101384 |
| **Btbd7** | 457.8482408 | 430.1982706 | 398.6290989 | 448.5568982 | 376.8511303 | 359.1233565 | 409.5373805 | 380.4856019 | 409.3132827 | 399.3731998 | 432.2629093 | 414.6784348 | 392.8108693 | 362.3254153 | 434.7322315 |
| **Unc79** | 4.578482408 | 0.792262009 | 0 | 0.86260942 | 1.266726489 | 0 | 0 | 0 | 0 | 0 | 0 | 0.867528106 | 0.852084315 | 0 | 1.437131344 |
| **Asb2** | 12.81975074 | 17.42976419 | 10.96425812 | 17.25218839 | 17.1008076 | 17.53532014 | 11.72684129 | 71.23629111 | 33.10622139 | 15.73023544 | 92.69532848 | 7.807752957 | 4.260421576 | 10.84265582 | 10.77848508 |
| **Otub2** | 33.88076982 | 17.42976419 | 28.1938066 | 23.29045433 | 13.93399137 | 18.23673295 | 26.15987673 | 12.5711102 | 10.53379772 | 28.83876497 | 17.97154328 | 48.58157395 | 27.26669809 | 36.14218606 | 18.68270747 |
| **Ddx24** | 4828.467547 | 4279.007108 | 4227.504668 | 4519.21075 | 4377.173381 | 4613.893436 | 4773.726471 | 3837.540906 | 3971.241739 | 3997.227606 | 3890.366184 | 4249.152665 | 4190.550662 | 4257.549518 | 4278.340011 |
| **Ifi27l2a** | 4567.49405 | 4460.435108 | 3946.349763 | 3854.138887 | 4662.820204 | 5002.47613 | 5638.806532 | 1531.999296 | 2242.194085 | 1783.633919 | 1973.086278 | 4357.593678 | 4199.923589 | 4280.138385 | 4355.945103 |
| **Ifi27** | 1268.239627 | 1129.765624 | 1248.359103 | 1080.849603 | 1098.885229 | 1200.117311 | 1106.833405 | 698.1156529 | 884.8390082 | 852.9283216 | 913.711095 | 1133.859235 | 1076.18249 | 1078.844254 | 1031.141739 |
| **Ppp4r4** | 0.915696482 | 0 | 0.783161294 | 0.86260942 | 0 | 0.701412806 | 0.902064715 | 0.838074013 | 0 | 0.873901969 | 0.945870699 | 0 | 0 | 5.421327909 | 0 |
| **Serpina3g** | 5417.260385 | 5305.778671 | 5280.073447 | 5267.093116 | 4848.395635 | 5088.048493 | 5486.357595 | 3222.39458 | 3828.283056 | 3924.693742 | 3612.280199 | 4913.679194 | 4760.595069 | 4963.225701 | 5046.486714 |
| **Serpina3f** | 1257.251269 | 1274.749572 | 1212.333684 | 1312.028927 | 1151.454378 | 1111.037884 | 1263.792665 | 642.802768 | 826.1507066 | 623.0921038 | 580.764609 | 1065.324515 | 1056.584551 | 1067.098044 | 1092.938387 |
| **Dicer1** | 1872.599305 | 1740.599633 | 1701.026332 | 1732.119715 | 1654.344794 | 1609.742389 | 1730.160123 | 1995.454225 | 1865.23461 | 1860.537292 | 1916.334036 | 1817.471383 | 1703.316546 | 1706.814737 | 1665.635228 |
| **Clmn** | 8.241268334 | 2.376786026 | 0.783161294 | 1.725218839 | 1.266726489 | 0 | 0 | 0 | 0 | 0 | 0 | 0 | 2.556252945 | 0 | 1.437131344 |
| **Syne3** | 1568.588073 | 1612.253187 | 1772.294009 | 1640.683116 | 1523.871966 | 1606.235325 | 1837.505824 | 1729.784763 | 1819.337348 | 1627.205466 | 1664.73243 | 1684.739582 | 1791.08123 | 1664.347668 | 1746.114583 |
| **Snhg10** | 7.325571853 | 4.753572051 | 9.397935534 | 3.450437679 | 2.533452977 | 7.715540863 | 7.216517718 | 10.89496217 | 6.771727103 | 13.10852953 | 7.56696559 | 4.337640531 | 7.668758836 | 9.939101167 | 10.77848508 |
| **Scarna13** | 0 | 3.169048034 | 0.783161294 | 0 | 0.633363244 | 0 | 0 | 2.514222039 | 3.00965649 | 0 | 0.945870699 | 0 | 0 | 0 | 0 |
| **Glrx5** | 1679.387347 | 1712.0782 | 1709.641106 | 1636.370069 | 1823.45278 | 2006.742037 | 1673.330046 | 2023.110668 | 2133.846452 | 2023.95696 | 1941.872545 | 1728.115988 | 1812.383338 | 1823.373287 | 1720.964784 |
| **D430019H16Rik** | 0.915696482 | 0.792262009 | 3.132645178 | 1.725218839 | 9.500448664 | 1.402825611 | 6.314453003 | 2.514222039 | 15.80069657 | 6.991215751 | 1.891741398 | 2.602584319 | 2.556252945 | 3.614218606 | 0.718565672 |
| **Atg2b** | 1323.181416 | 1378.535895 | 1274.203426 | 1243.882783 | 1095.718413 | 1090.696913 | 1210.570847 | 1325.833089 | 1223.425363 | 1126.459638 | 1187.067727 | 1254.445642 | 1227.853498 | 1121.311323 | 1250.304269 |
| **Gskip** | 304.0112319 | 296.3059912 | 285.0707112 | 338.1428925 | 328.7155238 | 321.247065 | 310.3102619 | 269.0217582 | 285.1649525 | 324.2176305 | 214.7126486 | 386.9175354 | 295.6732574 | 273.7770594 | 334.1330375 |
| **Ak7** | 3.662785926 | 13.46845414 | 5.482129061 | 2.587828259 | 11.4005384 | 8.416953669 | 2.706194144 | 10.89496217 | 9.781383594 | 8.739019689 | 11.35044839 | 3.470112425 | 9.372927467 | 7.228437213 | 7.18565672 |
| **Papola** | 4170.997474 | 4295.64461 | 4572.095637 | 4212.984406 | 4152.962793 | 4273.006812 | 4512.127703 | 4745.175062 | 4604.77443 | 5031.927537 | 4771.917675 | 4565.800423 | 4671.126216 | 4244.899753 | 4494.628278 |
| **Vrk1** | 1734.329136 | 1595.615685 | 1746.449687 | 1607.903958 | 1569.474119 | 1630.784773 | 1629.128875 | 1810.239868 | 1669.606938 | 1926.953841 | 1842.556121 | 1727.24846 | 1621.516452 | 1771.870672 | 1563.598902 |
| **Bcl11b** | 2249.866255 | 2490.079493 | 2460.692787 | 2375.626342 | 2540.419973 | 2482.299919 | 2800.008875 | 2653.342325 | 2869.707464 | 2323.705335 | 2321.166695 | 2662.443758 | 2639.757208 | 2595.008959 | 2665.878643 |
| **Setd3** | 1835.971446 | 1815.864523 | 1869.40601 | 1847.709377 | 1711.347486 | 1862.250999 | 1881.706995 | 1847.115125 | 1765.163532 | 1767.029781 | 1794.316716 | 1889.476215 | 1805.566664 | 1838.733716 | 1871.863575 |
| **Ccnk** | 1750.811673 | 1812.695475 | 1831.814268 | 1809.754562 | 1755.682913 | 1619.562168 | 1741.886964 | 1853.819717 | 1996.907081 | 1739.93882 | 1916.334036 | 1708.162841 | 1715.245726 | 1695.972081 | 1733.898966 |
| **Ccdc85c** | 141.9329546 | 174.2976419 | 177.7776138 | 169.9340557 | 171.008076 | 202.7083009 | 171.3922958 | 170.1290246 | 145.9683398 | 196.627943 | 145.6640876 | 156.1550591 | 199.3877297 | 143.6651896 | 161.6772762 |
| **Eml1** | 2.747089445 | 2.376786026 | 2.349483883 | 0.86260942 | 0 | 1.402825611 | 0.902064715 | 0 | 0 | 0.873901969 | 0 | 5.205168638 | 0.852084315 | 0 | 0 |
| **Evl** | 2155.549518 | 2563.75986 | 2433.282142 | 2287.640181 | 2665.825895 | 2609.95705 | 2692.663174 | 1690.395284 | 1869.749095 | 1895.493371 | 1839.718509 | 3032.87826 | 2833.180348 | 3037.750739 | 2797.376161 |
| **Degs2** | 73.25571853 | 86.35655893 | 56.3876132 | 54.34439344 | 63.33632443 | 105.9133337 | 101.9333128 | 63.69362499 | 47.40208972 | 62.92094176 | 61.48159542 | 91.95797927 | 111.6230453 | 122.8834326 | 85.50931496 |
| **Yy1** | 2601.493704 | 2631.894392 | 2814.681692 | 2745.685783 | 2643.024818 | 2870.882614 | 2832.483204 | 2859.508533 | 2811.771576 | 2882.128693 | 2786.535079 | 2955.668258 | 2897.938756 | 2800.115865 | 2678.812825 |
| **Slc25a29** | 47.61621704 | 31.69048034 | 41.50754861 | 28.46611085 | 34.20161519 | 34.36922748 | 18.94335901 | 33.52296052 | 36.86829201 | 49.81241223 | 39.72656935 | 35.56865236 | 34.08337261 | 18.07109303 | 42.39537465 |
| **Slc25a47** | 10.98835778 | 16.63750218 | 14.0969033 | 14.66436013 | 19.63426057 | 13.32684331 | 8.118582433 | 15.08533223 | 7.524141226 | 13.10852953 | 16.07980188 | 15.61550591 | 9.372927467 | 13.55331977 | 22.9941015 |
| **Wars** | 3277.277708 | 3202.323038 | 3035.533177 | 3068.301706 | 2939.438817 | 3027.999082 | 3080.551001 | 3162.053251 | 3178.197254 | 3104.973696 | 2944.495485 | 3021.600394 | 2900.495009 | 3022.39031 | 2941.089295 |
| **Wdr25** | 146.5114371 | 85.56429692 | 118.2573555 | 74.18441009 | 78.53704229 | 89.78083913 | 92.91266562 | 76.26473519 | 64.70761454 | 90.88580477 | 92.69532848 | 92.82550737 | 90.32093741 | 115.6549954 | 78.32365824 |
| **Begain** | 37.54355574 | 33.27500436 | 50.12232285 | 29.32872027 | 31.03479897 | 42.78618115 | 43.29910631 | 52.79866282 | 21.82000955 | 28.83876497 | 18.91741398 | 44.24393342 | 31.52711966 | 30.72085815 | 28.74262688 |
| **Dlk1** | 20.14532259 | 11.09166812 | 7.831612945 | 10.35131304 | 14.56735462 | 10.52119209 | 9.922711863 | 0 | 6.771727103 | 5.243411813 | 14.18806048 | 9.542809169 | 4.260421576 | 9.939101167 | 11.49705075 |
| **Dio3** | 4.578482408 | 1.584524017 | 7.04845165 | 5.175656518 | 2.533452977 | 11.9240177 | 5.412388289 | 27.65644243 | 3.00965649 | 5.243411813 | 2.837612096 | 0 | 0.852084315 | 0 | 0.718565672 |
| **Ppp2r5c** | 2614.313455 | 2794.308104 | 2856.972402 | 2860.412836 | 3006.575321 | 2792.32438 | 3009.287889 | 2913.145269 | 2920.871624 | 2881.254791 | 2977.60096 | 3110.088261 | 2947.359646 | 3044.075621 | 3099.173743 |
| **B930059L03Rik** | 0 | 0.792262009 | 0.783161294 | 0 | 0 | 1.402825611 | 2.706194144 | 4.190370065 | 0 | 2.621705907 | 4.729353494 | 0 | 8.520843152 | 0 | 2.874262688 |
| **Dync1h1** | 5648.015898 | 5705.078723 | 5341.160028 | 6070.182486 | 5197.378783 | 5081.735777 | 5830.044252 | 6300.64043 | 6062.953 | 5423.435619 | 6169.914568 | 6052.743598 | 5935.619339 | 5386.089278 | 6038.107342 |
| **1700001K19Rik** | 1.831392963 | 7.130358077 | 3.915806472 | 2.587828259 | 3.800179466 | 11.9240177 | 7.216517718 | 10.89496217 | 9.781383594 | 6.117313782 | 9.458706988 | 6.94022485 | 0.852084315 | 12.64976512 | 10.05991941 |
| **Hsp90aa1** | 28417.72461 | 24708.27526 | 25974.32749 | 25096.75846 | 23305.8673 | 24012.8674 | 24582.16554 | 27878.53204 | 26718.22549 | 27831.156 | 29063.76896 | 23889.12146 | 22839.26798 | 22089.20057 | 23392.90545 |
| **Wdr20** | 291.1914811 | 248.7702707 | 295.251808 | 247.5689034 | 272.346195 | 260.2241509 | 278.7379969 | 334.3915312 | 311.4994467 | 243.8186493 | 358.4849948 | 282.8141626 | 249.6607043 | 232.2135455 | 235.6895404 |
| **Mok** | 5.494178889 | 7.130358077 | 0 | 0 | 0 | 0 | 0.902064715 | 1.676148026 | 3.762070613 | 0 | 0 | 0 | 0 | 0 | 0 |

| | | | | | | | | | | | | | | | |
|---|---|---|---|---|---|---|---|---|---|---|---|---|---|---|---|
| **Zfp839** | 95.23243408 | 112.5012052 | 128.4384523 | 107.8261775 | 99.43802935 | 102.4062696 | 104.6395069 | 101.4069556 | 105.3379772 | 85.64239295 | 113.5044839 | 109.3085414 | 106.5105394 | 112.9443314 | 120.0004672 |
| **Cinp** | 960.5656092 | 970.5209604 | 1005.579102 | 958.3590652 | 879.1081831 | 980.5751024 | 980.544345 | 1057.649404 | 1015.006651 | 998.8699505 | 1105.722847 | 962.0886699 | 910.8781329 | 946.9252748 | 980.8421422 |
| **Tecpr2** | 228.9241204 | 266.2000349 | 251.3947755 | 201.8506042 | 186.2087938 | 230.7648131 | 201.1604314 | 251.4222039 | 218.9525097 | 254.305473 | 230.7924505 | 151.8174186 | 212.1689945 | 178.903821 | 223.473924 |
| **Ankrd9** | 87.90686223 | 64.9654847 | 93.19619404 | 104.3757398 | 67.76986714 | 75.75258302 | 73.0672419 | 94.70236348 | 86.5276241 | 104.8682363 | 76.6155266 | 97.1631479 | 80.94800994 | 88.54835585 | 76.16796123 |
| **Rcor1** | 930.3476253 | 1045.785851 | 1008.711747 | 1008.390412 | 909.5096188 | 913.239473 | 1002.193898 | 863.2162334 | 901.3921189 | 779.5205563 | 952.4917937 | 1072.264739 | 1017.388672 | 957.7679307 | 1061.321497 |
| **4930595D18Rik** | 1.831392963 | 4.753572051 | 0.783161294 | 6.038265938 | 0.633363244 | 1.402825611 | 0.902064715 | 0 | 11.28621184 | 1.747803938 | 0 | 2.602584319 | 1.70416863 | 6.324882561 | 5.029959704 |
| **Traf3** | 1324.097112 | 1295.348384 | 1340.772136 | 1444.008168 | 1462.435731 | 1375.470512 | 1548.845115 | 1331.699607 | 1305.438503 | 1247.932012 | 1429.210626 | 1479.135421 | 1554.201791 | 1456.530098 | 1436.412778 |
| **Amn** | 0.915696482 | 0 | 5.482129061 | 2.587828259 | 0 | 0.701412806 | 4.510323574 | 0 | 3.762070613 | 0.873901969 | 5.675224193 | 6.94022485 | 5.964590206 | 0 | 2.155697016 |
| **Cdc42bpb** | 271.0461585 | 193.3119301 | 227.8999367 | 211.3393078 | 165.3078068 | 247.5987204 | 219.2017257 | 193.595097 | 169.2931776 | 186.1411194 | 296.0575287 | 202.1340488 | 208.7606572 | 251.1881931 | 247.1865912 |
| **A230065H16Rik** | 0 | 0 | 3.132645178 | 1.725218839 | 2.533452977 | 0 | 0 | 0 | 1.504828245 | 0 | 1.891741398 | 0.867528106 | 0 | 1.807109303 | 0 |
| **Tnfaip2** | 0 | 0.792262009 | 3.915806472 | 4.313047098 | 0 | 0 | 2.706194144 | 3.352296052 | 8.276555348 | 3.495607876 | 3.783482795 | 0 | 0.852084315 | 0 | 0 |
| **Eif5** | 5679.149579 | 5687.648959 | 5957.507967 | 5759.643095 | 5573.59655 | 5720.722843 | 6028.498489 | 6302.316578 | 6226.226864 | 6285.10296 | 6411.111596 | 5778.604716 | 6048.094469 | 5649.927236 | 5875.7115 |
| **Snora28** | 25.63950148 | 21.39107423 | 25.06116142 | 26.74089201 | 20.90098706 | 21.04238417 | 26.15987673 | 30.17066447 | 41.38277674 | 19.22584332 | 36.88895725 | 39.03876478 | 43.45630007 | 35.23863141 | 20.11983882 |
| **2810029C07Rik** | 0 | 2.376786026 | 0 | 0.86260942 | 6.966995687 | 4.90988964 | 0 | 1.676148026 | 5.266898858 | 5.243411813 | 1.891741398 | 2.602584319 | 5.112505891 | 7.228437213 | 5.748525376 |
| **Mark3** | 1029.242845 | 1062.423353 | 982.0842633 | 975.6112536 | 1050.749622 | 1091.398326 | 1158.251094 | 1041.725998 | 1159.470163 | 1137.820364 | 1147.341158 | 1081.807549 | 1100.040851 | 1120.407768 | 1109.465398 |
| **Ckb** | 55.85748538 | 41.98988645 | 62.65290356 | 72.45919125 | 60.16950821 | 56.81443726 | 49.61355931 | 99.73080755 | 79.75589699 | 79.52507917 | 89.85771638 | 57.25685501 | 60.49798638 | 45.17773258 | 43.11394032 |
| **Trmt61a** | 487.1505282 | 640.9399649 | 583.4551644 | 576.2230923 | 580.1607318 | 591.992408 | 662.1155006 | 795.3322384 | 745.6423955 | 682.5174377 | 716.9699897 | 593.3892247 | 569.1923225 | 570.1429851 | 541.079951 |
| **Bag5** | 574.1416939 | 644.9012749 | 559.1771643 | 614.1779068 | 551.0260225 | 574.4570879 | 584.5379352 | 627.7174358 | 615.4747523 | 578.5231034 | 555.2261002 | 576.9061907 | 566.6360696 | 532.1936898 | 578.4453659 |
| **Apopt1** | 496.307493 | 415.1452925 | 469.8967767 | 421.8160062 | 502.890416 | 500.1073305 | 502.4500461 | 482.7306315 | 438.6574335 | 421.220749 | 434.1546507 | 474.5378741 | 524.0318538 | 507.7977142 | 452.6963733 |
| **Klc1** | 1161.103139 | 1226.421589 | 1211.550523 | 1128.293121 | 1229.358057 | 1360.03943 | 1234.926595 | 1174.979766 | 1141.412224 | 1228.706168 | 1250.441064 | 1295.219463 | 1353.109892 | 1323.707565 | 1299.166735 |
| **Xrcc3** | 202.3689224 | 276.499441 | 242.7800013 | 263.095873 | 237.5112166 | 257.4184997 | 267.0111556 | 272.3740542 | 212.1807826 | 270.0357084 | 272.4107612 | 243.7753979 | 289.7086672 | 272.8735048 | 255.0908135 |
| **Zfyve21** | 83.32837982 | 98.24048905 | 113.5583877 | 113.001834 | 79.80376878 | 94.69072877 | 77.57756547 | 97.21658551 | 82.76555348 | 147.6894327 | 100.2622941 | 85.88528252 | 88.61676878 | 88.54835585 | 90.53927467 |
| **Ppp1r13b** | 457.8482408 | 483.2798252 | 463.6314863 | 486.5117127 | 488.3230613 | 472.0508182 | 550.259476 | 416.5227845 | 368.6829201 | 485.0155927 | 469.1518666 | 473.670346 | 477.1672165 | 561.1074386 | 510.9001928 |
| **5033406O09Rik** | 3.662785926 | 0.792262009 | 0.783161294 | 0.86260942 | 0 | 0 | 1.80412943 | 0 | 5.266898858 | 0 | 0 | 0 | 0 | 0.903554652 | 0.718565672 |
| **2010107E04Rik** | 955.0714303 | 1003.795965 | 933.528263 | 847.0824501 | 933.5774221 | 986.1864048 | 959.7968565 | 902.6057121 | 790.0348287 | 879.1453807 | 806.8277061 | 852.7801285 | 904.9135427 | 1021.920311 | 920.4826258 |
| **Inf2** | 1577.745038 | 1544.910917 | 1379.930201 | 1330.143725 | 1230.624784 | 1353.025302 | 1495.623297 | 1261.30139 | 1104.543932 | 1108.107697 | 1001.67707 | 1231.889911 | 1427.241228 | 1285.758269 | 1329.346493 |
| **Adssl1** | 57.68887834 | 57.04286461 | 41.50754861 | 47.44351808 | 42.43533737 | 42.08476834 | 46.90736517 | 49.44636677 | 20.31518131 | 25.3431571 | 22.70089677 | 39.03876478 | 34.93545692 | 49.69550584 | 45.26963733 |
| **Siva1** | 657.4700738 | 749.47986 | 729.9063265 | 747.0197574 | 724.5675515 | 766.6441966 | 658.5072418 | 787.7895723 | 957.0707639 | 858.1717335 | 863.579948 | 763.4247335 | 666.3299345 | 777.960555 | 741.5597735 |
| **Akt1** | 4751.549043 | 5090.283405 | 4859.515832 | 5003.997243 | 4885.130703 | 4700.868624 | 4850.401971 | 5596.658259 | 5264.641616 | 5576.368464 | 5551.315131 | 4823.456271 | 4926.75151 | 4999.367887 | 5065.169422 |
| **Zbtb42** | 109.8835778 | 97.44822705 | 98.67832311 | 100.0626927 | 82.970585 | 54.00878604 | 99.22711863 | 100.5688816 | 63.95520042 | 50.6863142 | 77.5613973 | 97.1631479 | 103.1022021 | 84.93413725 | 74.73082988 |
| **Cep170b** | 597.034106 | 632.2250828 | 663.3376164 | 650.4075024 | 532.0251252 | 592.6938208 | 622.4246532 | 650.3454341 | 644.0664889 | 686.0130456 | 708.4571534 | 605.5346182 | 703.8216443 | 588.2140782 | 732.9369854 |
| **Pld4** | 172.1509385 | 200.4422882 | 166.0301944 | 193.22451 | 141.2400035 | 135.3726715 | 156.9592604 | 88.83584538 | 142.9586833 | 203.6191588 | 189.1741398 | 148.3473062 | 132.0730689 | 147.2794082 | 128.6232553 |
| **BC022687** | 25.63950148 | 12.67619214 | 27.41064531 | 27.60350143 | 17.73417084 | 14.02825611 | 18.94335901 | 25.9802944 | 35.36346376 | 46.31680435 | 36.88895725 | 10.41033728 | 20.45002356 | 21.68531164 | 12.21561642 |
| **Cdca4** | 1910.142861 | 1956.887161 | 2096.522785 | 1896.878114 | 1823.45278 | 1895.918814 | 1790.598459 | 2090.156589 | 2006.688465 | 2105.229843 | 2010.921106 | 1917.237115 | 1914.633456 | 2008.60199 | 1861.08509 |
| **Gpr132** | 1569.503769 | 1491.0371 | 1358.001685 | 1299.952395 | 1103.318772 | 1037.38954 | 1106.833405 | 1082.791625 | 1034.569419 | 1114.22501 | 1195.580563 | 1084.410133 | 1111.970031 | 1045.412732 | 1072.099983 |
| **Jag2** | 85.15977279 | 42.78214846 | 57.95393579 | 46.58090866 | 38.6351579 | 61.7243269 | 67.65485361 | 20.95185033 | 19.56276719 | 29.71266694 | 17.02567258 | 47.71404585 | 74.98341973 | 25.29953024 | 40.23967763 |
| **Nudt14** | 150.174223 | 202.8190742 | 176.2112913 | 148.3688202 | 178.6084349 | 169.741899 | 148.8406779 | 111.4638437 | 110.604876 | 107.4899422 | 136.2053806 | 160.4926997 | 178.0856219 | 162.6398373 | 149.4616598 |
| **Brf1** | 643.7346265 | 661.5387771 | 749.4853588 | 650.4075024 | 718.233919 | 702.1142185 | 690.9815715 | 721.5817252 | 776.4913745 | 728.8342421 | 748.1837227 | 753.0143963 | 705.525813 | 772.5392271 | 672.577469 |
| **Btbd6** | 208.7787978 | 217.0797903 | 242.7800013 | 252.74456 | 258.4122037 | 288.2806631 | 255.2843143 | 265.6694621 | 290.4318513 | 221.9711001 | 236.4676747 | 233.3650606 | 259.0336318 | 265.6450676 | 285.2705718 |
| **Pacs2** | 703.2548978 | 705.9054496 | 731.4726491 | 690.9501451 | 738.5015428 | 709.8297594 | 794.7190137 | 697.2775789 | 604.1885404 | 677.2740259 | 723.5910846 | 761.6896773 | 769.4321366 | 775.2498911 | 781.0808854 |
| **Tex22** | 2.747089445 | 1.584524017 | 1.566322589 | 0 | 3.800179466 | 0 | 0 | 0 | 0 | 1.747803938 | 0 | 0 | 0 | 0 | 0.718565672 |
| **Mta1** | 3236.071366 | 3070.015283 | 2961.916016 | 3006.193827 | 3009.108774 | 3110.765793 | 3208.64419 | 3234.96569 | 3035.990985 | 3201.102912 | 3239.607143 | 2793.440502 | 2788.019879 | 3085.639135 | 3067.556854 |
| **Crip2** | 101.6423095 | 127.5541834 | 89.28038757 | 81.08528545 | 68.40323038 | 66.63421654 | 64.94865946 | 93.02621545 | 133.9297138 | 111.859452 | 93.64119918 | 72.87236093 | 92.02510604 | 70.47726282 | 84.79074929 |
| **Crip1** | 1609.794415 | 1657.412122 | 1758.980267 | 1732.119715 | 1956.459062 | 1832.090249 | 1865.46983 | 979.7085213 | 1219.663293 | 1084.512343 | 1182.338373 | 1797.518236 | 1780.856219 | 1900.175432 | 1810.066928 |
| **4930427A07Rik** | 483.4877423 | 642.5244889 | 576.4067127 | 570.1848264 | 526.324856 | 591.992408 | 557.4759937 | 583.2995131 | 607.1981969 | 596.0011428 | 521.174755 | 576.9061907 | 586.2340088 | 627.0669282 | 573.4154062 |
| **Tmem121** | 8.241268334 | 7.922620085 | 7.04845165 | 10.35131304 | 4.43354271 | 15.43108173 | 10.82477658 | 4.190370065 | 11.28621184 | 7.86511772 | 12.29631908 | 9.542809169 | 15.33751767 | 13.55331977 | 9.341353736 |
| **Adam6b** | 0 | 0.792262009 | 0.783161294 | 3.450437679 | 0 | 0 | 1.80412943 | 1.676148026 | 4.514484735 | 0.873901969 | 1.891741398 | 3.470112425 | 0 | 1.807109303 | 0.718565672 |
| **Zfp386** | 253.6479254 | 211.5339563 | 251.3947755 | 239.8054187 | 227.3774047 | 216.736557 | 221.9079198 | 232.9845756 | 206.1614696 | 241.1969434 | 272.4107612 | 263.7285443 | 264.1461377 | 288.2339339 | 230.6595807 |
| **Vipr2** | 0.915696482 | 0 | 0 | 0 | 3.800179466 | 0 | 0 | 5.028444078 | 0.752414123 | 0 | 0.945870699 | 2.602584319 | 0 | 0 | 0 |
| **Wdr60** | 41.20634167 | 35.65179038 | 51.68864544 | 51.75656518 | 40.53524763 | 41.38335554 | 45.10323574 | 54.47481085 | 46.6496756 | 67.29045161 | 55.80637123 | 47.71404585 | 50.27297459 | 56.0203884 | 41.67680897 |
| **Esyt2** | 1263.661145 | 1541.741869 | 1495.054911 | 1579.437847 | 1446.60165 | 1592.908482 | 1725.649799 | 1295.662424 | 1379.927501 | 1358.04366 | 1413.130824 | 1749.80419 | 1757.849942 | 1726.692939 | 1617.491328 |
| **D430020J02Rik** | 85.15977279 | 127.5541834 | 122.1731619 | 103.5131304 | 152.6405419 | 106.6147465 | 132.6035131 | 117.3303618 | 125.6531585 | 118.8506678 | 120.1255787 | 120.5864068 | 120.9959728 | 91.25901981 | 135.0903463 |
| **Ncapg2** | 3001.653067 | 3066.846235 | 3197.647565 | 3174.402664 | 2853.934779 | 2808.456874 | 2921.787611 | 3295.307019 | 3324.918008 | 3295.484325 | 3474.183077 | 3190.768375 | 3266.891264 | 3040.461403 | 2805.280383 |
| **Rapgef5** | 22.89241204 | 14.26071615 | 18.01270977 | 34.50437679 | 6.966995687 | 12.6254305 | 20.74748844 | 28.49451644 | 14.29586833 | 5.243411813 | 0.945870699 | 12.14539349 | 15.33751767 | 11.74621047 | 14.37131344 |
| **Cdca7l** | 1054.882347 | 1182.847179 | 1182.573555 | 1142.094872 | 1099.518592 | 1127.871792 | 1121.26644 | 1068.544367 | 1063.913569 | 1094.125265 | 988.4348802 | 1081.807549 | 1199.734716 | 1032.762967 | 1139.645156 |
| **Dnah11** | 0 | 2.376786026 | 0.783161294 | 8.626094197 | 3.800179466 | 2.805651223 | 3.608258859 | 5.866518091 | 5.266898858 | 4.369509845 | 2.837612096 | 13.01292159 | 7.668758836 | 2.710663955 | 5.029959704 |
| **Sp4** | 882.7314082 | 957.0525063 | 910.0334242 | 853.120716 | 982.9797551 | 925.1634907 | 1101.421017 | 634.4220279 | 770.4720615 | 740.1949677 | 803.0442233 | 1031.490918 | 1038.69078 | 964.0928132 | 1050.543012 |
| **Itgb8** | 0.915696482 | 0 | 2.349483883 | 0 | 0 | 2.805651223 | 2.706194144 | 0 | 0 | 0 | 0 | 1.735056213 | 0 | 3.614218606 | 3.59282836 |
| **2810429I04Rik** | 10.0726613 | 4.753572051 | 5.482129061 | 9.488703616 | 1.900089733 | 1.402825611 | 3.608258859 | 8.380740131 | 6.771727103 | 6.117313782 | 4.729353494 | 6.94022485 | 6.816674521 | 5.421327909 | 5.029959704 |
| **1700024F13Rik** | 0.915696482 | 0 | 0 | 0.86260942 | 0 | 2.104238417 | 0 | 0 | 1.504828245 | 0 | 0.945870699 | 0 | 0 | 0 | 1.437131344 |
| **Gdi2** | 6908.014257 | 6731.850286 | 7278.701071 | 6964.708454 | 6699.716398 | 6708.312074 | 6804.274144 | 6422.999236 | 6279.648267 | 6615.437905 | 6470.70145 | 6843.92923 | 6725.5015 | 6706.182624 | 6693.439234 |
| **Fam208b** | 741.7141501 | 822.3679648 | 816.8372302 | 757.3710705 | 756.2357137 | 660.730863 | 857.8635438 | 874.9492696 | 777.2437886 | 820.5939488 | 794.531387 | 765.1597897 | 812.8884367 | 702.0619643 | 753.0568242 |
| **Asb13** | 219.7671556 | 214.7030043 | 256.0937433 | 244.1184658 | 246.378302 | 244.0916564 | 285.0524499 | 243.0414638 | 210.6759543 | 237.7013355 | 260.1144422 | 224.6897795 | 241.9919455 | 243.0562013 | 288.1448345 |
| **Net1** | 1162.934532 | 1187.600751 | 1157.512393 | 1027.367819 | 1066.583703 | 1083.682785 | 1129.385023 | 1115.476511 | 1116.582558 | 1208.606423 | 1170.042054 | 1211.936764 | 1150.313825 | 1101.43312 | 1171.980611 |
| **Akr1c18** | 2.747089445 | 6.338096068 | 25.06116142 | 0 | 20.26762382 | 14.72966892 | 7.216517718 | 12.5711102 | 12.03862596 | 28.83876497 | 31.21373306 | 5.205168638 | 11.0770961 | 9.939101167 | 10.77848508 |
| **Akr1c13** | 124.5347215 | 104.5785851 | 101.027807 | 97.47486442 | 77.2703158 | 124.8514794 | 118.1704776 | 78.77895723 | 54.17381683 | 66.41654964 | 179.7154328 | 108.4410133 | 102.2501178 | 126.4976512 | 127.1861239 |
| **Akr1c12** | 34.7964663 | 24.56012226 | 46.98967767 | 18.97740723 | 20.26762382 | 30.16075065 | 38.78878274 | 19.2757023 | 14.29586833 | 22.72145119 | 19.86328467 | 38.17123668 | 39.1958785 | 25.29953024 | 40.23967763 |
| **Akr1e1** | 149.2585265 | 143.3994235 | 154.282775 | 138.0175071 | 117.1722002 | 132.5670203 | 131.7014484 | 154.2056184 | 196.380086 | 195.754041 | 162.6897602 | 133.5993284 | 149.9668395 | 91.25901981 | 130.7789523 |
| **Klf6** | 3750.692788 | 3474.861169 | 3460.006599 | 3142.486116 | 2915.371013 | 3143.030782 | 3196.015284 | 2995.276523 | 2868.955049 | 2648.796868 | 2648.437957 | 2842.889604 | 3080.284799 | 2772.105671 | 3231.389827 |
| **Pitrm1** | 1801.174979 | 1840.424646 | 1940.673688 | 1806.304125 | 1750.616007 | 1700.224641 | 1796.010847 | 2437.957304 | 2190.277511 | 2358.661414 | 2396.836351 | 1847.834866 | 1808.975001 | 1760.124461 | 1761.204462 |
| **Pfkp** | 9417.022616 | 8329.842757 | 7913.06172 | 7705.689946 | 7659.895076 | 8270.358392 | 8370.258488 | 3845.921646 | 4734.942073 | 4880.742496 | 5298.767655 | 7626.439582 | 7912.454951 | 6864.304688 | 8047.935526 |
| **Wdr37** | 613.5166426 | 573.5976942 | 617.9142614 | 619.3535633 | 558.6263815 | 568.1443726 | 525.9037287 | 555.6430707 | 577.8540461 | 596.8750448 | 580.764609 | 549.1452913 | 583.6777559 | 587.3105235 | 556.8883958 |
| **Idi1** | 1954.096292 | 1858.646672 | 2209.298012 | 1959.848601 | 2052.096911 | 2140.711883 | 2233.512234 | 2381.806345 | 2385.152769 | 2535.189612 | 2608.711387 | 2326.710381 | 2315.113084 | 2311.292799 | 2162.164107 |
| **Idi2** | 32.04937685 | 17.42976419 | 29.76012919 | 14.66436013 | 38.00179466 | 17.53532014 | 29.76813559 | 72.07436512 | 57.93588744 | 73.40776539 | 45.40179354 | 39.90629289 | 26.41461377 | 34.33507676 | 25.14979852 |
| **Gtpbp4** | 3376.172928 | 3149.241484 | 3170.23692 | 2975.139888 | 3077.512004 | 3163.371754 | 3375.526163 | 3456.21723 | 3092.422044 | 3133.81246 | 3325.681377 | 2815.128705 | 2891.974166 | 2733.252821 | 2900.131052 |
| **Larp4b** | 2733.353997 | 2560.590811 | 2551.539497 | 2532.621256 | 2397.27988 | 2359.552678 | 2731.451956 | 2615.628995 | 2552.188704 | 2410.22163 | 2571.82243 | 2429.078698 | 2530.690416 | 2320.328345 | 2571.027974 |
| **Dip2c** | 267.3833726 | 281.253013 | 212.2367108 | 236.354981 | 213.4434133 | 184.4715679 | 237.24302 | 191.080875 | 202.399399 | 197.501845 | 250.6557352 | 202.1340488 | 187.4585493 | 142.7616349 | 201.1983882 |
| **Zmynd11** | 1127.222369 | 1225.629327 | 1303.963555 | 1276.661941 | 1222.391061 | 1205.728613 | 1326.937195 | 1264.653686 | 1249.007443 | 1275.022973 | 1403.672117 | 1284.809125 | 1353.109892 | 1423.098576 | 1257.489926 |
| **Chrm3** | 0 | 0 | 0.783161294 | 0 | 0 | 0 | 0.902064715 | 0 | 0 | 0 | 0 | 1.735056213 | 5.112505891 | 1.807109303 | 0 |
| **Gm10336** | 136.4387758 | 156.8678777 | 119.8236781 | 140.6053354 | 148.2069992 | 113.6288745 | 104.6395069 | 179.3478388 | 155.7497234 | 163.4196682 | 153.2310532 | 130.1292159 | 140.593912 | 159.0256187 | 127.9046896 |
| **Mtr** | 843.3564595 | 831.8751089 | 825.4520044 | 859.158982 | 833.5060295 | 745.6018125 | 828.9974729 | 1132.237992 | 1042.845974 | 1010.230676 | 1060.321053 | 845.8399036 | 794.994666 | 830.3667248 | 837.8475735 |
| **Actn2** | 28.38659093 | 33.27500436 | 28.1938066 | 49.16873692 | 39.26852115 | 40.68194273 | 32.47432973 | 41.90370065 | 27.08690841 | 37.57778466 | 56.75224193 | 39.03876478 | 35.78754124 | 22.58886629 | 30.17975822 |
| **Heatr1** | 3868.817635 | 3797.311807 | 3531.274277 | 3749.763147 | 3547.467531 | 3246.138465 | 3872.563821 | 4224.7311 | 3927.60172 | 3770.886996 | 3994.411961 | 3434.543773 | 3614.541665 | 3119.070657 | 3620.133855 |
| **Lgals8** | 248.1537465 | 287.5911091 | 244.3463239 | 213.0645267 | 291.9804556 | 281.2665351 | 316.6247149 | 152.5294704 | 197.8849142 | 179.1499036 | 274.3025026 | 278.4765221 | 352.7629065 | 369.5538525 | 321.1988554 |
| **Edaradd** | 7.325571853 | 5.54583406 | 3.915806472 | 12.07653188 | 22.16771355 | 9.81977928 | 26.15987673 | 4.190370065 | 3.762070613 | 4.369509845 | 6.621094891 | 2.602584319 | 13.63334904 | 15.36042908 | 13.65274777 |
| **Ero1lb** | 155.6684019 | 237.6786026 | 153.4996137 | 171.6592745 | 162.1409905 | 171.1447246 | 176.8046841 | 164.2625066 | 156.5021375 | 198.3757469 | 169.3108551 | 160.4926997 | 150.8189238 | 203.2997966 | 165.9886702 |
| **Gpr137b-ps** | 30.21798389 | 15.84524017 | 19.57903236 | 22.42784491 | 37.36843141 | 21.04238417 | 24.3557473 | 9.218814144 | 18.05793894 | 20.97364725 | 13.24218978 | 21.68820266 | 15.33751767 | 20.78175699 | 12.9341821 |
| **Gpr137b** | 0 | 0 | 0.783161294 | 0 | 0 | 2.104238417 | 0 | 4.190370065 | 0 | 0 | 0 | 0 | 0 | 0 | 2.155697016 |
| **Nid1** | 55.85748538 | 25.35238427 | 52.47180673 | 50.03134634 | 34.83497844 | 49.80030921 | 46.00530045 | 64.53169901 | 24.07725192 | 46.31680435 | 81.34488009 | 30.36348372 | 40.90004713 | 37.94929537 | 42.39537465 |
| **Lyst** | 1078.690455 | 1281.087668 | 1179.440909 | 1287.875864 | 1202.756801 | 924.4620779 | 1204.256394 | 818.7983108 | 896.8776341 | 672.0306141 | 783.1809386 | 1152.077325 | 1143.497151 | 1128.53976 | 1104.435438 |
| **Gng4** | 2.747089445 | 3.169048034 | 2.349483883 | 7.763484777 | 3.800179466 | 0.701412806 | 0.902064715 | 0 | 9.028969471 | 2.621705907 | 1.891741398 | 2.602584319 | 0.852084315 | 1.807109303 | 2.874262688 |
| **B3galnt2** | 426.7145604 | 439.7054147 | 485.5600026 | 453.7325547 | 437.6540018 | 451.0084341 | 454.6406162 | 546.4242565 | 490.5740079 | 514.7282597 | 539.1462983 | 525.7220324 | 447.3442655 | 428.2849048 | 474.2533435 |
| **Tbce** | 389.1710047 | 472.9804191 | 455.0167121 | 359.708128 | 430.0536429 | 420.8476834 | 446.5220338 | 451.721893 | 457.4677865 | 403.7427096 | 447.3968405 | 418.1485472 | 483.1318067 | 456.295099 | 376.5284121 |
| **Ggps1** | 474.3307775 | 502.2941134 | 418.2081313 | 497.7256351 | 479.4559759 | 448.2027829 | 494.3314637 | 480.2164095 | 468.7539984 | 464.9158475 | 540.092169 | 419.0160753 | 501.0255773 | 421.0564676 | 401.6782106 |
| **Arid4b** | 705.0862908 | 715.4125937 | 761.2327782 | 667.6596908 | 666.298133 | 620.7503331 | 702.7084128 | 659.5642483 | 645.5713172 | 585.5143192 | 551.4426174 | 641.9707986 | 657.8090913 | 625.2598189 | 649.5833675 |
| **Mrpl32** | 422.136078 | 420.6911265 | 458.1493573 | 453.7325547 | 461.7218051 | 410.3264913 | 479.8984283 | 418.1989325 | 501.1078056 | 470.1592593 | 488.0692806 | 437.2341656 | 448.1963498 | 491.5337305 | 450.5406763 |
| **Psma2** | 4331.244358 | 4194.235073 | 4359.858926 | 4272.504456 | 4220.73266 | 4279.319528 | 4103.492388 | 4797.973725 | 4630.35651 | 4568.759493 | 4421.945517 | 4151.121989 | 4153.911036 | 4309.955688 | 4143.249665 |
| **AW209491** | 268.2990691 | 232.9250305 | 260.7927111 | 270.8593578 | 205.8430544 | 257.4184997 | 250.7739907 | 306.7350888 | 309.9946185 | 257.8010808 | 254.439218 | 201.2665207 | 213.8731631 | 258.4166304 | 193.2941658 |
| **Inhba** | 11.90405426 | 11.88393013 | 8.614774239 | 4.313047098 | 1.900089733 | 0.701412806 | 7.216517718 | 17.59955427 | 8.276555348 | 6.991215751 | 17.02567258 | 2.602584319 | 0.852084315 | 1.807109303 | 10.05991941 |
| **Sugct** | 1.831392963 | 9.507144102 | 3.915806472 | 4.313047098 | 5.700269198 | 2.104238417 | 1.80412943 | 0.838074013 | 0 | 0.873901969 | 0.945870699 | 2.602584319 | 0.852084315 | 1.807109303 | 5.029959704 |
| **Mplkip** | 308.5897143 | 293.1369431 | 299.9507758 | 314.8524382 | 257.7788404 | 335.9767339 | 286.8565793 | 330.2011611 | 310.7470326 | 292.7571596 | 333.8923567 | 312.3101183 | 352.7629065 | 349.6756502 | 260.8393389 |
| **Cdk13** | 2043.834547 | 2162.875283 | 2150.560915 | 2049.559981 | 1977.993412 | 2035.499962 | 2122.558274 | 2184.020878 | 2379.133456 | 2150.672745 | 2249.280522 | 2207.85903 | 2203.490039 | 1968.845586 | 2085.996146 |
| **Mir466i** | 0 | 0 | 0.783161294 | 2.587828259 | 0.633363244 | 1.402825611 | 0 | 2.514222039 | 2.257242368 | 0 | 3.783482795 | 0.867528106 | 0 | 4.517773258 | 2.874262688 |
| **Rala** | 709.6647732 | 735.2191439 | 687.6156166 | 734.9432255 | 654.2642313 | 680.3704215 | 712.6311247 | 643.640842 | 657.6099431 | 619.596496 | 663.0553598 | 733.0612498 | 694.4487169 | 701.1584096 | 725.032763 |
| **Yae1d1** | 161.1625808 | 196.4809781 | 166.0301944 | 169.0714463 | 210.2765971 | 178.1588527 | 172.2943605 | 181.8620608 | 220.4573379 | 187.8889233 | 209.9832951 | 156.1550591 | 157.6355983 | 138.2438617 | 155.2101851 |
| **Vps41** | 666.6270386 | 735.2191439 | 725.2073587 | 627.9796575 | 608.6620777 | 587.7839312 | 703.6104775 | 698.1156529 | 816.369323 | 714.8518106 | 711.2947655 | 634.1630457 | 714.8987404 | 609.8993898 | 592.0981137 |
| **Stard3nl** | 426.7145604 | 369.986358 | 396.279615 | 376.9603164 | 357.850233 | 366.1374846 | 331.959815 | 427.4177467 | 440.1622617 | 391.5080821 | 343.3510637 | 393.8577603 | 357.0233281 | 403.8889293 | 408.8638673 |
| **Epdr1** | 0 | 0 | 0 | 0.86260942 | 0 | 1.402825611 | 1.80412943 | 5.028444078 | 3.00965649 | 6.117313782 | 1.891741398 | 0 | 0.852084315 | 0 | 6.467091048 |
| **Gpr141** | 1.831392963 | 5.54583406 | 4.698967767 | 0.86260942 | 0 | 1.402825611 | 0 | 0 | 0 | 1.747803938 | 4.729353494 | 1.735056213 | 1.70416863 | 0 | 0 |
| **Elmo1** | 3149.0802 | 3103.290287 | 3181.98434 | 3148.524382 | 2704.461053 | 2739.017006 | 3062.509707 | 2912.307195 | 3147.348275 | 2910.967458 | 3101.510021 | 2868.915447 | 3000.188874 | 2860.654027 | 3015.820125 |
| **Trim27** | 2959.531028 | 3028.817659 | 2857.755564 | 3073.477362 | 2625.290648 | 2941.725307 | 2806.323328 | 3260.107911 | 3149.605517 | 3118.956127 | 2967.196382 | 2721.435669 | 2792.280301 | 2600.430287 | 2630.668925 |
| **Zscan12** | 184.0549928 | 231.3405065 | 222.4178076 | 204.4384325 | 153.2739051 | 175.3532014 | 175.9026194 | 186.8905049 | 167.0359352 | 200.1235509 | 183.4989156 | 174.3731494 | 139.7418277 | 159.0256187 | 181.0785493 |
| **Zkscan3** | 332.3978228 | 354.1411178 | 364.1700019 | 310.5393911 | 369.8841347 | 341.5880364 | 364.4341448 | 299.1924227 | 347.6153246 | 331.2088462 | 387.8069865 | 314.0451745 | 351.9108222 | 391.2391641 | 354.2528763 |
| **Mir6942** | 0 | 0 | 2.349483883 | 0 | 0 | 0 | 1.80412943 | 0 | 0 | 2.621705907 | 7.56696559 | 2.602584319 | 1.70416863 | 1.807109303 | 0.718565672 |
| **Pgbd1** | 42.12203815 | 44.36667248 | 38.37490343 | 13.80175071 | 36.10170492 | 15.43108173 | 29.76813559 | 25.9802944 | 32.35380727 | 41.07339254 | 31.21373306 | 19.08561834 | 40.04796281 | 30.72085815 | 10.77848508 |
| **Zscan26** | 291.1914811 | 323.2428995 | 305.4329049 | 315.7150476 | 272.346195 | 291.7877272 | 300.38755 | 284.1070904 | 252.8111452 | 271.7835123 | 238.3594161 | 274.1388816 | 269.2586436 | 275.5841687 | 311.138936 |
| **Nkapl** | 3.662785926 | 1.584524017 | 3.132645178 | 6.900875357 | 1.900089733 | 7.014128057 | 0.902064715 | 0.838074013 | 0 | 0 | 0 | 6.072696744 | 2.556252945 | 0.903554652 | 6.467091048 |
| **Zfp389** | 0 | 0 | 0 | 0 | 0 | 2.104238417 | 0 | 0 | 0 | 0 | 0 | 0 | 0 | 1.807109303 | 0 |
| **Zkscan8** | 222.514245 | 249.5625327 | 201.2724527 | 207.0262607 | 164.6744435 | 193.5899344 | 230.0265023 | 204.4900592 | 202.399399 | 187.0150213 | 197.686976 | 168.3004526 | 223.2460906 | 181.614485 | 168.8629329 |
| **Hist1h2bl** | 6.409875371 | 3.961310043 | 6.265290356 | 9.488703616 | 4.43354271 | 9.118366474 | 6.314453003 | 2.514222039 | 12.03862596 | 9.612921658 | 3.783482795 | 9.542809169 | 5.964590206 | 13.55331977 | 10.77848508 |
| **Hist1h2ai** | 21.97671556 | 31.69048034 | 47.77283896 | 38.81742388 | 58.90278172 | 66.63421654 | 49.61355931 | 21.78992434 | 39.8779485 | 40.19949057 | 15.13393118 | 38.17123668 | 54.53339617 | 61.44171631 | 51.73672838 |
| **Hist1h3h** | 24.723805 | 37.2363144 | 26.62748401 | 43.13047098 | 28.50134599 | 45.59183237 | 27.06194144 | 29.33259046 | 21.06759543 | 45.44290238 | 52.96875913 | 38.17123668 | 63.05423932 | 48.79195118 | 15.80844478 |
| **Hist1h2bm** | 22.89241204 | 12.67619214 | 14.8800646 | 16.38957897 | 20.26762382 | 21.04238417 | 18.0412943 | 7.542666117 | 12.03862596 | 11.3607256 | 8.512836289 | 23.42325887 | 28.97086672 | 27.10663955 | 28.02406121 |
| **Hist1h4j** | 12.81975074 | 12.67619214 | 4.698967767 | 2.587828259 | 10.13381191 | 28.75792503 | 12.62890601 | 5.866518091 | 4.514484735 | 3.495607876 | 20.80915537 | 3.470112425 | 23.00627651 | 11.74621047 | 10.05991941 |
| **Hist1h4k** | 15.56684019 | 4.753572051 | 8.614774239 | 11.21392246 | 15.83408111 | 24.5494482 | 20.74748844 | 20.11377631 | 17.30552482 | 17.47803938 | 14.18806048 | 12.14539349 | 23.85836082 | 8.131991864 | 10.77848508 |
| **Hist1h2ak** | 22.89241204 | 26.93690829 | 9.397935534 | 31.91654853 | 20.90098706 | 31.56357626 | 30.6702003 | 13.40918421 | 20.31518131 | 20.09974528 | 16.07980188 | 13.8804497 | 41.75213144 | 31.62441281 | 28.74262688 |
| **Hist1h2bn** | 4.578482408 | 3.961310043 | 7.04845165 | 3.450437679 | 3.800179466 | 9.118366474 | 9.922711863 | 9.218814144 | 6.771727103 | 16.60413741 | 11.35044839 | 1.735056213 | 8.520843152 | 3.614218606 | 10.77848508 |
| **Hist1h1b** | 21.06101908 | 22.18333624 | 12.53058071 | 9.488703616 | 25.96789302 | 23.84803539 | 13.53097072 | 15.08533223 | 6.771727103 | 18.35194135 | 7.56696559 | 26.02584319 | 9.372927467 | 19.87820233 | 18.68270747 |
| **Hist1h3i** | 11.90405426 | 22.18333624 | 14.8800646 | 22.42784491 | 33.56825195 | 26.65368662 | 27.06194144 | 11.73303618 | 15.04828245 | 26.21705907 | 15.13393118 | 36.43618046 | 14.48543336 | 37.94929537 | 34.49115225 |
| **Hist1h2an** | 16.48253667 | 7.922620085 | 3.132645178 | 12.07653188 | 13.93399137 | 12.6254305 | 18.0412943 | 2.514222039 | 8.276555348 | 6.117313782 | 1.891741398 | 16.48303402 | 7.668758836 | 16.26398373 | 21.55697016 |
| **Hist1h2bp** | 0 | 3.169048034 | 0.783161294 | 1.725218839 | 0 | 0.701412806 | 1.80412943 | 1.676148026 | 6.771727103 | 0.873901969 | 2.837612096 | 5.205168638 | 5.964590206 | 8.131991864 | 2.874262688 |
| **Hist1h4n** | 14.65114371 | 7.922620085 | 7.831612945 | 8.626094197 | 17.73417084 | 16.13249453 | 15.33510015 | 2.514222039 | 6.771727103 | 4.369509845 | 20.80915537 | 15.61550591 | 15.33751767 | 22.58886629 | 12.9341821 |
| **Zfp184** | 3.662785926 | 8.714882094 | 4.698967767 | 5.175656518 | 1.266726489 | 2.805651223 | 0.902064715 | 2.514222039 | 11.28621184 | 2.621705907 | 3.783482795 | 0.867528106 | 4.260421576 | 6.324882561 | 5.748525376 |
| **Pom121l2** | 34.7964663 | 36.44405239 | 30.54329049 | 31.05393911 | 2.533452977 | 7.014128057 | 20.74748844 | 7.542666117 | 12.79104008 | 28.83876497 | 18.91741398 | 16.48303402 | 5.112505891 | 5.421327909 | 8.622788064 |
| **Prss16** | 253.6479254 | 191.7274061 | 253.7442594 | 194.9497288 | 136.1730975 | 167.6376606 | 123.5828659 | 118.1684358 | 93.2993512 | 190.5106292 | 192.0117519 | 125.7915754 | 140.593912 | 148.1829629 | 155.2101851 |
| **Hist1h2ah** | 2.747089445 | 6.338096068 | 3.915806472 | 5.175656518 | 10.13381191 | 9.118366474 | 10.82477658 | 17.59955427 | 7.524141226 | 6.117313782 | 1.891741398 | 13.01292159 | 10.22501178 | 9.035546516 | 7.18565672 |
| **Hist1h2bk** | 28.38659093 | 53.08155457 | 61.86974226 | 51.75656518 | 57.00269198 | 56.81443726 | 55.0259476 | 36.87525657 | 36.86829201 | 37.57778466 | 55.80637123 | 37.30370857 | 36.63962555 | 77.70570004 | 49.58103137 |
| **Hist1h4i** | 229.8398169 | 259.8619388 | 234.1652271 | 256.1949976 | 246.378302 | 298.1004424 | 269.7173497 | 262.3171661 | 255.0683876 | 266.5401005 | 222.2796142 | 292.3569718 | 261.5898848 | 263.8379583 | 274.4920867 |
| **Hist1h2ag** | 62.26736075 | 57.04286461 | 25.06116142 | 46.58090866 | 53.83587576 | 83.46812388 | 51.41768874 | 45.2559967 | 50.41174621 | 47.19070632 | 54.86050053 | 51.18415827 | 62.20215501 | 53.30972444 | 63.9523448 |
| **Hist1h2bj** | 65.93014667 | 95.86370303 | 94.76251663 | 61.2452688 | 104.5049353 | 110.8232233 | 99.22711863 | 72.07436512 | 73.73658401 | 75.15556933 | 50.13114703 | 118.8513506 | 136.3334904 | 123.7869873 | 99.16206273 |
| **Zfp322a** | 261.8891937 | 213.9107423 | 252.1779368 | 202.7132136 | 313.5148059 | 256.0156741 | 299.4854853 | 251.4222039 | 252.8111452 | 230.7101198 | 283.7612096 | 232.4975325 | 247.1044514 | 265.6450676 | 223.473924 |
| **Abt1** | 610.7695532 | 631.4328208 | 639.0596163 | 635.7431423 | 676.4319449 | 626.3616355 | 616.1102002 | 652.0215822 | 626.00855 | 591.6316329 | 554.2802295 | 589.0515842 | 570.0444068 | 602.6709526 | 594.9723764 |
| **C230035I16Rik** | 0.915696482 | 6.338096068 | 1.566322589 | 0.86260942 | 4.43354271 | 2.104238417 | 0.902064715 | 7.542666117 | 9.028969471 | 11.3607256 | 8.512836289 | 7.807752957 | 3.408337261 | 0 | 7.18565672 |
| **Hist1h4h** | 6.409875371 | 11.88393013 | 24.27800013 | 10.35131304 | 19.63426057 | 16.83390734 | 28.86607087 | 7.542666117 | 22.57242368 | 15.73023544 | 6.621094891 | 19.08561834 | 9.372927467 | 12.64976512 | 19.40127314 |
| **Hist1h2af** | 10.98835778 | 11.88393013 | 2.349483883 | 18.11479781 | 10.13381191 | 13.32684331 | 20.74748844 | 4.190370065 | 15.80069657 | 12.23462756 | 12.29631908 | 14.74797781 | 25.56252945 | 21.68531164 | 12.9341821 |
| **Hist1h3g** | 27.47089445 | 20.59881222 | 14.0969033 | 20.70262607 | 40.53524763 | 18.93814575 | 25.25781201 | 13.40918421 | 10.53379772 | 32.33437285 | 22.70089677 | 23.42325887 | 14.48543336 | 38.85285002 | 24.43123285 |
| **Hist1h2bh** | 20.14532259 | 11.88393013 | 19.57903236 | 6.038265938 | 10.13381191 | 14.02825611 | 15.33510015 | 5.866518091 | 21.82000955 | 9.612921658 | 0.945870699 | 15.61550591 | 25.56252945 | 5.421327909 | 14.37131344 |
| **Hist1h3f** | 40.29064519 | 27.7291703 | 39.94122602 | 18.97740723 | 27.2346195 | 30.16075065 | 32.47432973 | 9.218814144 | 23.3248378 | 39.3255886 | 22.70089677 | 27.7608994 | 21.30210788 | 20.78175699 | 35.9282836 |
| **Hist1h4f** | 21.97671556 | 10.29940611 | 7.04845165 | 12.07653188 | 16.46744435 | 10.52119209 | 15.33510015 | 13.40918421 | 13.54345421 | 14.85633347 | 9.458706988 | 6.072696744 | 21.30210788 | 17.16753838 | 9.341353736 |
| **Hist1h1d** | 17.39823315 | 19.0142882 | 29.76012919 | 31.91654853 | 20.90098706 | 24.5494482 | 49.61355931 | 21.78992434 | 24.07725192 | 24.46925513 | 6.621094891 | 34.70112425 | 28.1187824 | 40.65995932 | 23.71266717 |
| **Hist1h3e** | 41.20634167 | 41.98988645 | 39.94122602 | 20.70262607 | 53.83587576 | 71.54410618 | 51.41768874 | 48.60829276 | 82.01313936 | 48.93851026 | 46.34766424 | 47.71404585 | 51.12505891 | 55.11683375 | 49.58103137 |
| **Hist1h2ae** | 66.84584315 | 87.94108294 | 54.03812932 | 87.12355138 | 73.47013634 | 70.14128057 | 56.83007703 | 67.04592104 | 85.02279585 | 78.6511772 | 63.37333682 | 75.47494525 | 88.61676878 | 76.80214538 | 70.41943585 |
| **Hist1h2bg** | 21.97671556 | 23.76786026 | 14.8800646 | 8.626094197 | 29.76807248 | 15.43108173 | 18.94335901 | 6.704592104 | 1.504828245 | 18.35194135 | 6.621094891 | 16.48303402 | 17.0416863 | 11.74621047 | 17.24557613 |
| **Hist1h2bf** | 0.915696482 | 3.961310043 | 1.566322589 | 3.450437679 | 0.633363244 | 1.402825611 | 3.608258859 | 0 | 0 | 1.747803938 | 2.837612096 | 0.867528106 | 1.70416863 | 0 | 0.718565672 |
| **Hist1h2ad** | 1.831392963 | 0 | 3.132645178 | 1.725218839 | 6.333632443 | 11.9240177 | 7.216517718 | 0 | 12.79104008 | 12.23462756 | 6.621094891 | 1.735056213 | 15.33751767 | 4.517773258 | 10.05991941 |
| **Hist1h3d** | 5.494178889 | 6.338096068 | 3.132645178 | 5.175656518 | 6.333632443 | 8.416953669 | 6.314453003 | 2.514222039 | 12.03862596 | 0.873901969 | 10.40457769 | 9.542809169 | 5.112505891 | 6.324882561 | 3.59282836 |

| | | | | | | | | | | | | | | | |
|---|---|---|---|---|---|---|---|---|---|---|---|---|---|---|---|
| **Hist1h4d** | 44.8691276 | 49.91250654 | 41.50754861 | 50.89395576 | 45.60215359 | 61.0229141 | 55.0259476 | 24.30414638 | 42.88760499 | 35.82998073 | 28.37612096 | 34.70112425 | 44.30838439 | 67.76659887 | 38.08398061 |
| **Hist1h2be** | 12.81975074 | 22.18333624 | 17.22954848 | 25.01567317 | 18.36753408 | 29.45933784 | 23.45368258 | 16.76148026 | 23.3248378 | 15.73023544 | 19.86328467 | 15.61550591 | 12.78126473 | 17.16753838 | 27.30549553 |
| **Hist1h1e** | 28.38659093 | 39.61310043 | 28.1938066 | 36.22959563 | 51.30242279 | 54.71019885 | 63.14453003 | 31.00873848 | 24.82966605 | 39.3255886 | 52.96875913 | 54.6542707 | 41.75213144 | 64.15238026 | 41.67680897 |
| **Hist1h2ac** | 3.662785926 | 11.09166812 | 6.265290356 | 18.11479781 | 13.93399137 | 19.63955856 | 17.13922958 | 4.190370065 | 2.257242368 | 11.3607256 | 0.945870699 | 21.68820266 | 28.97086672 | 25.29953024 | 16.52701046 |
| **Hist1h2bc** | 42.12203815 | 48.32798252 | 56.3876132 | 56.9322217 | 69.66995687 | 53.30737323 | 57.73214175 | 24.30414638 | 63.95520042 | 33.20827482 | 41.61831075 | 50.31663016 | 57.94173343 | 36.14218606 | 38.80254629 |
| **Hist1h4c** | 27.47089445 | 26.14464628 | 13.31374201 | 26.74089201 | 20.26762382 | 44.18900676 | 35.18052388 | 12.5711102 | 27.08690841 | 14.85633347 | 9.458706988 | 20.82067455 | 30.67503535 | 10.84265582 | 28.02406121 |
| **Hfe** | 10.98835778 | 3.169048034 | 1.566322589 | 8.626094197 | 3.800179466 | 7.715540863 | 2.706194144 | 7.542666117 | 0 | 6.991215751 | 4.729353494 | 3.470112425 | 4.260421576 | 4.517773258 | 1.437131344 |
| **Hist1h1c** | 124.5347215 | 114.8779912 | 110.4257425 | 108.6887869 | 138.0731873 | 139.5811483 | 128.9952542 | 124.0349539 | 133.1772997 | 120.5984717 | 111.6127425 | 125.7915754 | 144.8543336 | 127.4012059 | 137.964609 |
| **Hist1h3c** | 141.9329546 | 141.8148995 | 101.8109683 | 109.5513963 | 150.7404521 | 180.9645039 | 131.7014484 | 134.9299161 | 146.7207539 | 102.2465304 | 87.96597499 | 141.4070813 | 174.6772846 | 172.5789385 | 158.8030135 |
| **Hist1h2bb** | 13.73544722 | 14.26071615 | 18.01270977 | 6.038265938 | 20.26762382 | 19.63955856 | 17.13922958 | 3.352296052 | 11.28621184 | 13.10852953 | 6.621094891 | 13.01292159 | 15.33751767 | 14.45687443 | 15.08987911 |
| **Hist1h2ab** | 48.53191352 | 32.48274235 | 18.01270977 | 27.60350143 | 27.2346195 | 27.35509942 | 38.78878274 | 41.06562664 | 12.79104008 | 20.09974528 | 18.91741398 | 19.08561834 | 24.71044514 | 26.2030849 | 25.14979852 |
| **Hist1h3b** | 12.81975074 | 3.961310043 | 14.0969033 | 6.900875357 | 4.43354271 | 21.04238417 | 7.216517718 | 10.89496217 | 16.5531107 | 13.9824315 | 7.56696559 | 9.542809169 | 14.48543336 | 15.36042908 | 17.9641418 |
| **Hist1h4b** | 30.21798389 | 35.65179038 | 25.84432272 | 31.05393911 | 41.16861088 | 54.00878604 | 40.59291217 | 36.87525657 | 26.33449429 | 32.33437285 | 38.78069865 | 24.29078698 | 36.63962555 | 51.50261514 | 25.86836419 |
| **Hist1h4a** | 11.90405426 | 3.169048034 | 3.132645178 | 6.900875357 | 10.13381191 | 9.81977928 | 11.72684129 | 5.028444078 | 15.80069657 | 6.991215751 | 0 | 10.41033728 | 16.18960199 | 13.55331977 | 2.155697016 |
| **Hist1h3a** | 40.29064519 | 46.7434585 | 51.68864544 | 33.64176737 | 53.83587576 | 49.0988964 | 61.34040061 | 43.57984868 | 47.40208972 | 40.19949057 | 35.94308655 | 58.12438312 | 48.56880596 | 37.94929537 | 63.23377913 |
| **Hist1h1a** | 11.90405426 | 8.714882094 | 4.698967767 | 6.900875357 | 20.90098706 | 23.14662259 | 27.06194144 | 3.352296052 | 18.81035306 | 7.86511772 | 11.35044839 | 7.807752957 | 16.18960199 | 22.58886629 | 33.77258658 |
| **Lrrc16a** | 36.62785926 | 36.44405239 | 39.94122602 | 31.05393911 | 27.86798275 | 21.04238417 | 40.59291217 | 20.95185033 | 30.0965649 | 16.60413741 | 23.64676747 | 27.7608994 | 20.45002356 | 21.68531164 | 30.17975822 |
| **Cmah** | 626.3363934 | 721.7506897 | 790.2097461 | 771.1728212 | 817.0385851 | 746.3032253 | 945.3638211 | 534.6912203 | 547.0050671 | 575.0274955 | 540.092169 | 1029.755862 | 955.1865173 | 1023.72742 | 906.1113123 |
| **Fam65b** | 370.857075 | 517.3470916 | 400.9785828 | 478.7482279 | 584.5942745 | 522.5525403 | 594.460647 | 347.8007154 | 384.4836166 | 280.522532 | 291.3281752 | 550.8803475 | 593.0506834 | 587.3105235 | 550.4213047 |
| **Gm11346** | 10.98835778 | 5.54583406 | 7.831612945 | 10.35131304 | 8.86708542 | 11.22260489 | 5.412388289 | 10.89496217 | 7.524141226 | 5.243411813 | 5.675224193 | 4.337640531 | 12.78126473 | 3.614218606 | 11.49705075 |
| **Gmnn** | 1249.010001 | 1326.246602 | 1429.269362 | 1295.639348 | 1379.465146 | 1370.560622 | 1239.436918 | 1392.040936 | 1333.277825 | 1371.152189 | 1249.495193 | 1389.780026 | 1347.145302 | 1416.773694 | 1301.322432 |
| **BC005537** | 4768.03158 | 4920.739335 | 4812.526155 | 4656.365647 | 4391.740736 | 4421.004914 | 4940.608443 | 4508.83819 | 4681.520671 | 4470.882473 | 4708.544338 | 4791.357731 | 4569.728182 | 4590.05763 | 4636.185716 |
| **Acot13** | 188.6334752 | 204.4035982 | 217.7188399 | 211.3393078 | 207.7431441 | 215.3337314 | 203.8666255 | 192.757023 | 221.9621662 | 161.6718642 | 179.7154328 | 194.3262958 | 202.796067 | 186.1322582 | 258.6836419 |
| **Tdp2** | 711.4961662 | 744.726288 | 800.390843 | 616.7657351 | 736.6014531 | 737.8862716 | 636.8576886 | 761.8092779 | 672.6582256 | 687.7608495 | 715.0782483 | 753.8819244 | 689.336211 | 700.254855 | 731.4998541 |
| **D130043K22Rik** | 0 | 0 | 0 | 3.450437679 | 0.633363244 | 1.402825611 | 1.80412943 | 1.676148026 | 3.00965649 | 5.243411813 | 0.945870699 | 0 | 1.70416863 | 1.807109303 | 2.155697016 |
| **Aldh5a1** | 11.90405426 | 11.09166812 | 15.66322589 | 9.488703616 | 17.73417084 | 21.74379698 | 19.84542373 | 11.73303618 | 18.05793894 | 26.21705907 | 8.512836289 | 7.807752957 | 22.15419219 | 11.74621047 | 8.622788064 |
| **Gpld1** | 301.2641424 | 288.3833711 | 358.6878729 | 326.0663606 | 310.9813529 | 300.9060937 | 362.6300153 | 341.9341973 | 355.89188 | 326.8393364 | 422.8042024 | 299.2971967 | 351.9108222 | 286.4268245 | 342.0372599 |
| **Mrs2** | 458.7639373 | 465.850061 | 516.8864544 | 452.0073359 | 405.3524763 | 416.6392066 | 497.9397226 | 458.4264851 | 478.535382 | 502.4936321 | 523.0664964 | 429.4264126 | 462.6817831 | 496.9550584 | 446.947848 |
| **2610307P16Rik** | 0 | 0 | 3.915806472 | 0 | 1.266726489 | 0 | 0.902064715 | 1.676148026 | 0 | 0 | 0 | 1.735056213 | 0.852084315 | 0.903554652 | 0 |
| **Sox4** | 52.19469945 | 88.73334495 | 61.86974226 | 69.00875357 | 36.10170492 | 44.89041957 | 38.78878274 | 167.6148026 | 130.1676432 | 97.00311855 | 106.883389 | 42.50887721 | 34.08337261 | 40.65995932 | 38.08398061 |
| **Cdkal1** | 202.3689224 | 185.38931 | 231.0325819 | 196.6749477 | 206.4764176 | 196.3955856 | 184.9232665 | 240.5272417 | 227.229065 | 213.2320804 | 207.145683 | 191.7237115 | 187.4585493 | 160.832728 | 222.0367926 |
| **E2f3** | 652.8915914 | 639.3554409 | 668.0365842 | 696.9884111 | 578.260642 | 531.6709067 | 544.8470877 | 709.8486891 | 748.652052 | 763.7903208 | 719.8076018 | 580.3763031 | 507.8422518 | 589.1176328 | 571.9782749 |
| **Mboat1** | 325.072251 | 396.9232663 | 387.6648408 | 322.6159229 | 399.6522071 | 454.5154981 | 406.8311864 | 320.144273 | 361.1587788 | 317.2264147 | 317.8125548 | 437.2341656 | 434.5630007 | 468.9448642 | 380.1212405 |
| **Uqcrfs1** | 3429.283323 | 3118.343265 | 3391.088405 | 3377.115878 | 3214.318465 | 3179.504248 | 3135.576949 | 3529.967743 | 3431.760813 | 3599.60221 | 3529.989448 | 3411.988042 | 3250.701662 | 3335.020219 | 3255.102494 |
| **Dusp22** | 294.8542671 | 356.5179038 | 349.2899373 | 310.5393911 | 356.5835065 | 334.5739083 | 303.0937442 | 241.3653158 | 250.5539028 | 298.8744734 | 332.0006153 | 315.7802307 | 341.6858104 | 314.4370187 | 293.1747942 |
| **Irf4** | 9049.828327 | 7381.505133 | 7378.945717 | 6774.934382 | 7113.302596 | 7223.149073 | 8034.690415 | 7125.305259 | 6904.151989 | 6785.848789 | 7158.349448 | 7140.623843 | 7462.554432 | 6553.481888 | 7350.926824 |
| **Exoc2** | 1618.951379 | 1511.635912 | 1553.008847 | 1612.217005 | 1524.505329 | 1442.104729 | 1615.597904 | 1395.393232 | 1442.377873 | 1317.844169 | 1403.672117 | 1512.101489 | 1526.935093 | 1497.190058 | 1492.460901 |
| **Foxq1** | 0 | 0.792262009 | 1.566322589 | 0 | 0.633363244 | 0 | 0 | 0.838074013 | 0.752414123 | 0 | 0 | 0 | 5.112505891 | 0 | 2.155697016 |
| **Gmds** | 450.5226689 | 434.9518427 | 463.6314863 | 468.3969149 | 502.890416 | 416.6392066 | 442.0117102 | 525.4724062 | 507.8795327 | 503.3675341 | 456.8555475 | 471.0677617 | 410.7046399 | 388.5285002 | 453.414939 |
| **Wrnip1** | 1590.564788 | 1696.23296 | 1816.934203 | 1743.333637 | 1620.143179 | 1827.881772 | 1569.592604 | 1849.629347 | 1848.681499 | 1873.645821 | 1775.399302 | 1755.009359 | 1859.247976 | 1765.545789 | 1755.455937 |
| **Serpinb1a** | 36.62785926 | 32.48274235 | 22.71167754 | 20.70262607 | 13.30062813 | 18.93814575 | 17.13922958 | 31.8468125 | 20.31518131 | 28.83876497 | 26.48437957 | 16.48303402 | 24.71044514 | 11.74621047 | 24.43123285 |
| **Serpinb6b** | 2326.78476 | 1646.320454 | 1626.626009 | 1674.324884 | 1096.351776 | 1302.52358 | 1512.762527 | 1752.412761 | 1429.586833 | 1356.295856 | 1398.942763 | 1190.248562 | 1381.228675 | 1118.600659 | 1248.148572 |
| **Serpinb9** | 3432.030413 | 2453.63544 | 2394.907239 | 2228.120131 | 1735.415289 | 1618.860756 | 1899.748289 | 2890.517271 | 2133.846452 | 2346.426787 | 2451.696851 | 1663.05138 | 1849.875048 | 1604.713061 | 1726.71331 |
| **Serpinb9b** | 27.47089445 | 17.42976419 | 10.18109683 | 6.038265938 | 0 | 0.701412806 | 5.412388289 | 23.46607237 | 6.019312981 | 16.60413741 | 24.59263817 | 3.470112425 | 0 | 0 | 7.904222392 |
| **Serpinb1b** | 1.831392963 | 10.29940611 | 7.831612945 | 4.313047098 | 0.633363244 | 0.701412806 | 9.020647148 | 4.190370065 | 1.504828245 | 0 | 1.891741398 | 0.867528106 | 0.852084315 | 8.131991864 | 0 |
| **Gm6093** | 27.47089445 | 30.89821833 | 30.54329049 | 23.29045433 | 29.76807248 | 31.56357626 | 44.20117102 | 62.01747697 | 31.60139315 | 36.70388269 | 34.99721585 | 36.43618046 | 30.67503535 | 36.14218606 | 64.67091048 |
| **Serpinb6a** | 1096.088688 | 945.9608382 | 996.9643279 | 1099.82701 | 1060.883434 | 1132.080268 | 1122.168505 | 1053.459034 | 900.6397047 | 1143.937677 | 1051.808217 | 1121.713841 | 1190.361788 | 1120.407768 | 1128.148105 |
| **1110046J04Rik** | 7.325571853 | 4.753572051 | 4.698967767 | 6.038265938 | 3.800179466 | 2.805651223 | 3.608258859 | 2.514222039 | 3.00965649 | 3.495607876 | 5.675224193 | 8.675281063 | 5.112505891 | 9.035546516 | 3.59282836 |
| **Nqo2** | 74.17141501 | 95.86370303 | 108.8594199 | 91.43659848 | 112.1052942 | 97.49637999 | 113.6601541 | 78.77895723 | 88.03245234 | 83.02068705 | 124.8549322 | 130.1292159 | 97.13761193 | 116.5585501 | 107.0662851 |
| **Ripk1** | 1173.922889 | 1207.407301 | 1157.512393 | 1190.400999 | 963.3454945 | 1043.702255 | 1030.157904 | 1197.607765 | 1188.814314 | 1232.201776 | 1208.822753 | 1021.948109 | 1075.330406 | 960.4785946 | 1031.141739 |
| **Bphl** | 67.76153964 | 74.4726288 | 61.08658097 | 72.45919125 | 71.5700466 | 80.66247266 | 100.1291833 | 62.85555098 | 59.44071568 | 70.78605948 | 66.21094891 | 97.1631479 | 66.46257658 | 103.0052303 | 82.63505228 |
| **Tubb2a** | 619.0108215 | 630.6405588 | 687.6156166 | 665.934472 | 601.0617188 | 582.1726287 | 549.3574113 | 965.461263 | 805.8355253 | 720.0952224 | 788.8561628 | 669.731698 | 598.1631892 | 756.2752434 | 635.212054 |
| **4930447K03Rik** | 0 | 0 | 0 | 0 | 0 | 2.104238417 | 0.902064715 | 0 | 0 | 0 | 0 | 2.602584319 | 0.852084315 | 0 | 5.029959704 |
| **Tubb2b** | 428.5459534 | 412.7685064 | 395.4964537 | 420.0907874 | 350.2498741 | 446.7999572 | 369.8465331 | 353.6672335 | 306.984962 | 336.452258 | 335.7840981 | 378.2422543 | 369.8045928 | 328.8938932 | 415.3309584 |
| **Psmg4** | 502.7173684 | 526.8542357 | 549.7792287 | 500.3134634 | 516.8244073 | 540.0878604 | 523.1975346 | 558.9953667 | 580.8637026 | 620.4703979 | 454.0179354 | 532.6622573 | 412.4088085 | 571.0465398 | 531.0200316 |
| **Slc22a23** | 7.325571853 | 0 | 4.698967767 | 2.587828259 | 2.533452977 | 3.507064029 | 1.80412943 | 10.05688816 | 2.257242368 | 10.48682363 | 1.891741398 | 2.602584319 | 1.70416863 | 0.903554652 | 5.748525376 |
| **Pxdc1** | 7.325571853 | 9.507144102 | 6.265290356 | 5.175656518 | 0.633363244 | 4.90988964 | 0.902064715 | 10.89496217 | 2.257242368 | 6.991215751 | 8.512836289 | 5.205168638 | 0.852084315 | 1.807109303 | 4.311394032 |
| **Prpf4b** | 1753.558762 | 1758.029397 | 1787.957235 | 1834.770236 | 1720.847935 | 1774.574398 | 1847.428536 | 1792.640314 | 1761.401461 | 1846.55486 | 1755.536017 | 1915.502059 | 1842.206289 | 1779.099109 | 1850.306605 |
| **Eci2** | 527.4411734 | 520.5161396 | 606.9500032 | 521.0160895 | 528.858309 | 582.8740415 | 541.2388289 | 522.9581841 | 478.535382 | 573.2796916 | 506.9866945 | 580.3763031 | 633.9507305 | 573.7572037 | 509.4630614 |
| **Cdyl** | 542.0923171 | 467.434585 | 563.0929707 | 517.5656518 | 455.3881726 | 512.0313482 | 516.8830816 | 522.9581841 | 438.6574335 | 475.4026711 | 516.4454015 | 428.5588845 | 503.5818303 | 484.3052932 | 417.4866554 |
| **Rpp40** | 186.8020822 | 190.935144 | 206.7545817 | 152.6818673 | 214.0767766 | 207.6181905 | 221.0058551 | 248.9079819 | 167.7883493 | 209.7364725 | 205.2539416 | 189.9886553 | 148.2626708 | 138.2438617 | 196.1684284 |
| **Ppp1r3g** | 19.22962611 | 10.29940611 | 15.66322589 | 21.56523549 | 11.4005384 | 18.23673295 | 20.74748844 | 0 | 0.752414123 | 5.243411813 | 2.837612096 | 0.867528106 | 17.89377062 | 8.131991864 | 7.18565672 |
| **Lyrm4** | 228.9241204 | 207.5726462 | 252.1779368 | 182.873197 | 233.0776739 | 245.494482 | 231.8306317 | 244.7176118 | 311.4994467 | 273.5313163 | 228.9007091 | 191.7237115 | 181.4939591 | 227.6957722 | 221.318227 |
| **Fars2** | 217.0200661 | 290.7601571 | 265.4916788 | 276.0350143 | 286.2801864 | 305.8159833 | 240.8512788 | 305.8970148 | 234.7532062 | 265.6661985 | 267.6814078 | 252.4506789 | 248.80862 | 228.5993268 | 268.0249956 |
| **Nrn1** | 3991.520963 | 3895.552296 | 4252.565829 | 3683.342222 | 4082.026109 | 4060.478732 | 4335.323019 | 2685.189138 | 2969.026128 | 2386.626277 | 2155.639322 | 4203.173675 | 4186.29024 | 4189.782919 | 4338.699527 |
| **F13a1** | 3.662785926 | 5.54583406 | 2.349483883 | 8.626094197 | 12.66726489 | 3.507064029 | 8.118582433 | 5.866518091 | 6.771727103 | 2.621705907 | 1.891741398 | 2.602584319 | 5.964590206 | 4.517773258 | 1.437131344 |
| **Ly86** | 3.662785926 | 1.584524017 | 0 | 4.313047098 | 3.800179466 | 4.208476834 | 6.314453003 | 0.838074013 | 22.57242368 | 1.747803938 | 4.729353494 | 5.205168638 | 0 | 0 | 3.59282836 |
| **Rreb1** | 280.2031234 | 297.8905152 | 313.2645178 | 345.9063773 | 323.6486178 | 300.2046808 | 339.1763328 | 315.9539029 | 286.6697807 | 333.8305521 | 284.7070803 | 372.1695576 | 330.6087143 | 319.8583467 | 295.3304912 |
| **Ssr1** | 4680.124717 | 4662.46192 | 4825.056735 | 4815.08578 | 4448.110065 | 4188.837276 | 4514.833897 | 4653.824994 | 4573.173037 | 4515.451473 | 4754.892003 | 4724.558067 | 4604.663639 | 4359.651194 | 4556.424926 |
| **Cage1** | 21.97671556 | 10.29940611 | 10.96425812 | 11.21392246 | 13.93399137 | 10.52119209 | 19.84542373 | 11.73303618 | 6.019312981 | 13.10852953 | 28.37612096 | 19.08561834 | 17.0416863 | 9.939101167 | 7.904222392 |
| **Riok1** | 1580.492127 | 1581.354969 | 1535.779298 | 1518.192579 | 1331.962903 | 1344.608349 | 1421.65399 | 1113.800363 | 1269.322625 | 1254.923227 | 1271.250219 | 1493.883399 | 1369.299494 | 1347.199985 | 1388.268878 |
| **Dsp** | 22.89241204 | 25.35238427 | 26.62748401 | 26.74089201 | 13.30062813 | 23.84803539 | 31.57226502 | 93.86428946 | 49.65933209 | 80.39898114 | 30.26786236 | 29.49595561 | 23.85836082 | 18.07109303 | 29.46119255 |
| **Snrnp48** | 275.624641 | 282.837537 | 299.9507758 | 259.6454353 | 311.6147162 | 291.7877272 | 287.758644 | 266.5075362 | 261.0877005 | 322.4698265 | 324.4336497 | 317.5152869 | 316.9753652 | 346.0614316 | 316.1688957 |
| **Txndc5** | 2103.354818 | 2171.590165 | 2251.588722 | 2103.904375 | 1933.657985 | 2018.666055 | 2123.460339 | 2183.182804 | 2140.618179 | 2185.628824 | 2286.169479 | 1990.109476 | 2087.606572 | 2062.81527 | 2172.942592 |
| **Bloc1s5** | 195.9590471 | 152.1143056 | 173.0786461 | 174.2471028 | 174.1748922 | 195.6941728 | 169.5881664 | 160.9102105 | 200.8945707 | 194.8801391 | 156.0686653 | 200.3989926 | 183.1981278 | 140.050971 | 181.0785493 |
| **Eef1e1** | 1225.201892 | 1174.132297 | 1165.344006 | 1166.247935 | 1072.917336 | 1244.306317 | 1146.524252 | 1314.938126 | 1140.65981 | 1279.392482 | 1287.330021 | 1087.012717 | 1132.420055 | 1098.722456 | 1050.543012 |
| **Slc35b3** | 231.6712098 | 293.9292052 | 260.7927111 | 253.6071694 | 238.1445798 | 263.7312149 | 285.9545146 | 284.9451644 | 307.7373761 | 283.1442379 | 289.4364338 | 286.2842751 | 275.2232338 | 267.4521769 | 244.3123285 |
| **5033403F01Rik** | 0 | 0 | 0 | 0.86260942 | 0.633363244 | 0.701412806 | 3.608258859 | 0.838074013 | 0 | 6.991215751 | 0.945870699 | 0.867528106 | 0.852084315 | 0 | 0.718565672 |
| **Tfap2a** | 4.578482408 | 3.961310043 | 10.18109683 | 8.626094197 | 4.43354271 | 2.104238417 | 1.80412943 | 1.676148026 | 3.762070613 | 2.621705907 | 11.35044839 | 4.337640531 | 2.556252945 | 1.807109303 | 1.437131344 |
| **Gcnt2** | 8.241268334 | 1.584524017 | 3.915806472 | 5.175656518 | 6.333632443 | 1.402825611 | 1.80412943 | 5.028444078 | 3.00965649 | 11.3607256 | 12.29631908 | 6.94022485 | 0.852084315 | 0.903554652 | 2.874262688 |
| **Pak1ip1** | 1468.777156 | 1546.495441 | 1431.618846 | 1423.305542 | 1438.367928 | 1511.544596 | 1403.612696 | 1516.913964 | 1553.735163 | 1579.140858 | 1621.222378 | 1460.049803 | 1399.122445 | 1482.733183 | 1372.460433 |
| **Tmem14c** | 1784.692443 | 1769.913327 | 1914.046204 | 1978.826009 | 1782.284169 | 1855.938284 | 1735.572511 | 1978.692745 | 2137.608522 | 2183.88102 | 1990.11195 | 1833.954417 | 1980.243948 | 2084.500581 | 1876.17497 |
| **Gcm2** | 8.241268334 | 9.507144102 | 8.614774239 | 6.900875357 | 1.900089733 | 0 | 3.608258859 | 0 | 0.752414123 | 0 | 0 | 4.337640531 | 4.260421576 | 0.903554652 | 4.311394032 |
| **Elovl2** | 1.831392963 | 0 | 0 | 0 | 0 | 1.402825611 | 0 | 4.190370065 | 0 | 0 | 0.945870699 | 0.867528106 | 0 | 1.807109303 | 0.718565672 |
| **Smim13** | 293.0228741 | 245.6012226 | 286.6370338 | 266.5463107 | 273.6129215 | 277.0580583 | 281.444191 | 241.3653158 | 268.6118418 | 280.522532 | 210.9291658 | 287.1518032 | 281.187824 | 260.2237397 | 224.1924897 |
| **Nedd9** | 821.379744 | 794.6387945 | 767.4980686 | 734.0806161 | 798.0376878 | 824.1600467 | 954.3844682 | 1017.421852 | 990.9293994 | 841.5675961 | 873.9845257 | 945.6056358 | 869.1260015 | 932.4684004 | 871.6201601 |
| **Tmem170b** | 569.5632115 | 479.3185151 | 492.6084542 | 420.9533968 | 423.7200104 | 422.250509 | 519.5892757 | 391.3805641 | 367.9305059 | 424.7163569 | 327.2712618 | 530.927201 | 576.8610814 | 455.3915444 | 444.0735853 |
| **Adtrp** | 0 | 3.961310043 | 0 | 0 | 0 | 1.402825611 | 0 | 0 | 1.504828245 | 0 | 0 | 4.337640531 | 0 | 0 | 0 |
| **Hivep1** | 1520.971856 | 1262.865642 | 1329.807878 | 1268.898456 | 1279.393753 | 1209.235677 | 1471.26755 | 1064.353997 | 993.1866418 | 977.8963032 | 900.4689052 | 1211.936764 | 1261.084786 | 1196.306359 | 1289.825381 |
| **Tbc1d7** | 222.514245 | 198.0655021 | 248.2621304 | 213.9271361 | 221.0437723 | 200.6040624 | 222.8099846 | 220.4134654 | 196.380086 | 240.3230414 | 204.3080709 | 218.6170828 | 203.6481513 | 198.7820233 | 210.5397419 |
| **Gfod1** | 787.4989741 | 785.9239124 | 829.3678109 | 895.3885776 | 765.7361623 | 823.4586339 | 856.961479 | 841.4263091 | 797.5589699 | 680.7696338 | 659.271877 | 836.2970945 | 810.3321837 | 784.2854376 | 850.0631899 |
| **Sirt5** | 54.94178889 | 50.70476854 | 44.64019379 | 70.73397241 | 44.3354271 | 66.63421654 | 49.61355931 | 56.15095887 | 90.28969471 | 59.42533389 | 80.3990094 | 47.71404585 | 44.30838439 | 58.73105235 | 33.77258658 |
| **Nol7** | 2449.488088 | 2391.839004 | 2507.682465 | 2411.855937 | 2310.509115 | 2388.310603 | 2476.167642 | 2539.36426 | 2643.230813 | 2548.298141 | 2350.488686 | 2376.159483 | 2405.434022 | 2546.217008 | 2392.105122 |
| **Ranbp9** | 1146.451995 | 1081.437642 | 1271.853942 | 1191.263609 | 1267.993215 | 1191.700357 | 1365.725978 | 1248.730279 | 1205.367424 | 1230.453972 | 1325.164849 | 1156.414966 | 1263.641039 | 1311.057799 | 1408.388717 |
| **Mcur1** | 593.3713201 | 606.8726985 | 642.9754228 | 597.7883278 | 651.7307784 | 730.8721436 | 754.1261016 | 669.6211364 | 686.9540939 | 758.546909 | 666.8388426 | 696.6250693 | 762.6154621 | 653.2700131 | 657.4875898 |
| **Cd83** | 727.9787028 | 645.6935369 | 703.2788424 | 672.8353473 | 803.737957 | 875.3631815 | 851.5490908 | 831.3694209 | 523.6802293 | 656.3003786 | 650.7590408 | 703.5652942 | 720.0112463 | 640.620248 | 708.5057526 |
| **Jarid2** | 1553.021233 | 1280.295406 | 1395.593427 | 1261.134972 | 1038.082357 | 1045.105081 | 1226.808012 | 911.8245262 | 934.4983402 | 813.602733 | 943.9789574 | 979.439232 | 1041.247033 | 861.9911376 | 1118.806751 |
| **Dtnbp1** | 370.857075 | 411.9762444 | 402.5449054 | 388.1742388 | 394.5853012 | 406.8194273 | 392.3981509 | 377.9713799 | 382.2263743 | 419.4729451 | 376.4565381 | 386.0500073 | 363.8400026 | 423.7671316 | 434.7322315 |
| **Mylip** | 88.82255871 | 85.56429692 | 72.83400039 | 77.63484777 | 77.90367905 | 79.96105985 | 107.3457011 | 92.18814144 | 54.92623095 | 50.6863142 | 65.26507822 | 91.09045116 | 106.5105394 | 97.58390237 | 97.00636572 |
| **Gmpr** | 0 | 0 | 0 | 2.587828259 | 0.633363244 | 0 | 0 | 1.676148026 | 1.504828245 | 6.117313782 | 0 | 0 | 0 | 0 | 0.718565672 |
| **Atxn1** | 463.3424197 | 633.8096068 | 569.3582611 | 484.7864938 | 487.6896981 | 441.8900676 | 577.3214175 | 652.0215822 | 522.9278152 | 404.6166116 | 484.2857978 | 591.6541685 | 599.0152736 | 606.2851712 | 570.5411435 |
| **Cap2** | 4.578482408 | 3.169048034 | 3.132645178 | 0 | 0 | 2.104238417 | 0 | 0 | 0 | 0 | 6.621094891 | 9.542809169 | 1.70416863 | 0 | 2.155697016 |
| **Fam8a1** | 167.5724561 | 143.3994235 | 159.7649041 | 134.5670695 | 98.80466611 | 145.1924508 | 116.3663482 | 152.5294704 | 201.6469849 | 166.0413741 | 136.2053806 | 153.5524748 | 134.6293218 | 149.9900722 | 112.0962448 |
| **Nup153** | 4046.462752 | 4225.133291 | 4043.461763 | 4018.897286 | 3598.769954 | 3716.085045 | 4247.822742 | 4559.122631 | 4468.587474 | 4255.902589 | 4484.372983 | 3965.470974 | 4167.544385 | 3987.386677 | 4128.878351 |
| **Kif13a** | 34.7964663 | 34.06726637 | 43.0738712 | 21.56523549 | 29.76807248 | 37.87629151 | 36.08258859 | 61.17940295 | 28.59173666 | 34.08217679 | 16.07980188 | 55.5217988 | 39.1958785 | 40.65995932 | 25.14979852 |
| **Nhlrc1** | 27.47089445 | 35.65179038 | 14.0969033 | 28.46611085 | 19.00089733 | 32.26498906 | 44.20117102 | 21.78992434 | 12.79104008 | 29.71266694 | 15.13393118 | 32.96606804 | 23.85836082 | 22.58886629 | 36.64684927 |
| **Tpmt** | 29.30228741 | 16.63750218 | 26.62748401 | 17.25218839 | 26.60125626 | 24.5494482 | 10.82477658 | 26.81836842 | 36.86829201 | 13.10852953 | 35.94308655 | 20.82067455 | 21.30210788 | 17.16753838 | 23.71266717 |
| **Kdm1b** | 402.9064519 | 405.6381484 | 426.0397442 | 364.8837845 | 357.850233 | 385.7770431 | 449.228228 | 515.415518 | 474.7733113 | 435.2031805 | 428.4794265 | 379.1097824 | 433.7109164 | 380.3965083 | 334.8516031 |
| **Dek** | 5311.95529 | 5812.826356 | 5815.755773 | 5444.790657 | 5303.150444 | 5539.056927 | 5849.889675 | 5884.117646 | 6217.197895 | 5827.178329 | 6085.732076 | 6444.866302 | 5889.606786 | 6054.71972 | 5989.244876 |
| **Rnf144b** | 5.494178889 | 3.961310043 | 3.132645178 | 4.313047098 | 5.700269198 | 9.118366474 | 9.020647148 | 5.028444078 | 0.752414123 | 8.739019689 | 10.40457769 | 12.14539349 | 1.70416863 | 2.710663955 | 4.311394032 |
| **Zfp169** | 76.00280797 | 62.58869867 | 56.3876132 | 64.69570647 | 48.76896981 | 44.89041957 | 54.12388289 | 58.66518091 | 40.63036262 | 27.964863 | 62.42746612 | 71.13730471 | 81.80009426 | 56.0203884 | 50.29959704 |
| **Mirlet7d** | 5.494178889 | 0 | 0 | 0 | 0 | 0 | 3.608258859 | 1.676148026 | 5.266898858 | 1.747803938 | 0 | 4.337640531 | 0.852084315 | 0.903554652 | 0 |
| **Ptpdc1** | 13.73544722 | 10.29940611 | 7.04845165 | 11.21392246 | 4.43354271 | 16.83390734 | 21.64955315 | 20.95185033 | 8.276555348 | 13.10852953 | 16.07980188 | 9.542809169 | 6.816674521 | 24.39597559 | 10.05991941 |
| **Phf2** | 1847.8755 | 2029.775266 | 2008.025559 | 1988.314712 | 1765.183362 | 1875.577842 | 1952.970107 | 1991.263855 | 1923.922911 | 1882.384841 | 1834.043285 | 1966.686217 | 1916.337625 | 2014.023318 | 2024.199498 |
| **Fam120a** | 7216.603971 | 7305.44798 | 7465.87662 | 7234.705203 | 6874.524653 | 6840.879094 | 7180.43513 | 7698.547884 | 7545.208821 | 7634.4076 | 7730.601221 | 7271.620587 | 7387.571012 | 7113.685772 | 7447.214624 |
| **Fam120aos** | 76.00280797 | 66.55000871 | 79.88245204 | 78.49745719 | 96.90457637 | 93.28790316 | 95.61885977 | 69.56014308 | 122.643502 | 88.26409886 | 99.31642337 | 52.05168638 | 77.53967268 | 93.06612911 | 70.41943585 |
| **Wnk2** | 1.831392963 | 1.584524017 | 5.482129061 | 2.587828259 | 1.900089733 | 13.32684331 | 4.510323574 | 6.704592104 | 5.266898858 | 6.991215751 | 4.729353494 | 4.337640531 | 10.22501178 | 7.228437213 | 5.029959704 |
| **Ninj1** | 355.2902348 | 343.8417117 | 366.5194858 | 321.7533135 | 292.6138189 | 339.483798 | 325.645362 | 470.9975953 | 307.7373761 | 439.5726904 | 376.4565381 | 309.7075339 | 330.6087143 | 318.0512374 | 317.606027 |
| **1110007C09Rik** | 173.066635 | 232.1327685 | 172.2954848 | 162.1705709 | 191.2756998 | 198.499824 | 153.3510015 | 212.8707993 | 178.3221471 | 200.1235509 | 178.7695621 | 207.3392174 | 178.0856219 | 238.538428 | 190.4199031 |
| **Susd3** | 371.7727715 | 426.2369606 | 421.3407764 | 489.9621504 | 287.5469129 | 315.6357626 | 284.1503852 | 534.6912203 | 492.0788362 | 534.828005 | 579.8187383 | 345.2761863 | 322.0878711 | 322.5690106 | 343.4743912 |
| **Fgd3** | 373.6041645 | 347.8030217 | 396.279615 | 372.6472693 | 329.9822503 | 315.6357626 | 364.4341448 | 291.6497565 | 305.4801338 | 301.4961793 | 366.9978311 | 360.8916922 | 356.1712437 | 386.7213909 | 348.5043509 |
| **Bicd2** | 1480.681211 | 1430.825187 | 1436.317814 | 1437.969903 | 1383.265325 | 1383.186053 | 1442.401479 | 1387.012492 | 1431.844075 | 1505.733092 | 1520.960084 | 1507.763849 | 1614.699777 | 1471.890527 | 1514.017871 |
| **Ippk** | 394.6651836 | 479.3185151 | 421.3407764 | 460.6334301 | 343.9162416 | 380.8671535 | 362.6300153 | 476.0260394 | 430.3808781 | 411.6078274 | 465.3683838 | 425.0887721 | 431.1546635 | 361.4218606 | 375.8098464 |
| **Cenpp** | 252.7322289 | 219.4565764 | 266.2748401 | 262.2332636 | 235.6111269 | 251.1057844 | 260.6967026 | 221.2515394 | 252.0587311 | 241.1969434 | 209.0374244 | 216.0144985 | 264.998222 | 270.1628408 | 235.6895404 |
| **Ecm2** | 0 | 1.584524017 | 2.349483883 | 0 | 2.533452977 | 0 | 0 | 1.676148026 | 9.028969471 | 0 | 2.837612096 | 0 | 1.70416863 | 0 | 3.59282836 |
| **Aspn** | 12.81975074 | 13.46845414 | 11.74741942 | 12.07653188 | 5.700269198 | 6.312715251 | 8.118582433 | 3.352296052 | 27.83932254 | 18.35194135 | 14.18806048 | 7.807752957 | 7.668758836 | 14.45687443 | 14.37131344 |
| **Omd** | 2.747089445 | 0 | 0 | 0 | 0.633363244 | 0 | 0 | 4.190370065 | 1.504828245 | 2.621705907 | 0 | 0 | 0 | 0 | 0 |
| **Ogn** | 0 | 0.792262009 | 0 | 0 | 0.633363244 | 0.701412806 | 0 | 2.514222039 | 5.266898858 | 0 | 0.945870699 | 1.735056213 | 0 | 0 | 0 |
| **Nol8** | 1089.678813 | 1132.14241 | 1138.716522 | 1068.773071 | 1122.953032 | 1015.645743 | 1124.874699 | 1348.461087 | 1139.154982 | 1168.406932 | 1172.879666 | 1123.448898 | 1017.388672 | 989.3923435 | 1067.070023 |
| **Iars** | 6263.363934 | 5419.8644 | 5363.088545 | 5148.053016 | 4996.602634 | 5113.299354 | 5165.222557 | 5188.516215 | 5305.271978 | 5120.191636 | 5104.864161 | 4980.478858 | 5065.641254 | 4688.545087 | 5027.804007 |
| **Fbxw17** | 199.621833 | 214.7030043 | 198.1398075 | 171.6592745 | 142.50673 | 194.2913472 | 184.9232665 | 151.6913964 | 111.3572901 | 220.2232962 | 172.1484672 | 163.095284 | 182.3460434 | 162.6398373 | 177.485721 |
| **Spin1** | 1166.597318 | 1161.456104 | 1176.308264 | 1079.124384 | 1020.348187 | 1133.483094 | 1177.194453 | 1066.030145 | 1083.476337 | 1112.477206 | 1001.67707 | 1113.03856 | 1163.09509 | 1157.453509 | 1177.729136 |
| **S1pr3** | 26.55519797 | 26.14464628 | 22.71167754 | 28.46611085 | 16.46744435 | 9.81977928 | 17.13922958 | 30.17066447 | 24.82966605 | 36.70388269 | 17.02567258 | 13.01292159 | 17.89377062 | 1.807109303 | 9.341353736 |
| **Shc3** | 8.241268334 | 13.46845414 | 5.482129061 | 2.587828259 | 1.266726489 | 2.104238417 | 6.314453003 | 0.838074013 | 0 | 3.495607876 | 2.837612096 | 6.072696744 | 3.408337261 | 6.324882561 | 3.59282836 |
| **Cks2** | 1690.375705 | 1693.856174 | 1756.630784 | 1636.370069 | 1606.842551 | 1738.100933 | 1637.247457 | 1622.511289 | 1819.337348 | 1945.305783 | 1992.003692 | 1757.611943 | 1691.387366 | 1897.464768 | 1810.785493 |
| **Secisbp2** | 634.5776617 | 729.6733098 | 664.1207777 | 664.2092531 | 658.0644108 | 628.4658739 | 599.8730353 | 696.4395048 | 684.6968515 | 653.6786727 | 655.4883942 | 650.6460797 | 732.792511 | 727.3614945 | 692.6973078 |
| **Sema4d** | 7793.492755 | 7840.224836 | 7881.735268 | 7708.277774 | 6779.520167 | 6608.010043 | 7515.101139 | 7353.261391 | 7724.283382 | 7046.271575 | 7911.262525 | 6961.913053 | 7101.270683 | 7040.497845 | 7120.985809 |
| **Gadd45g** | 1432.149297 | 1139.272768 | 1060.400393 | 1064.460024 | 1326.262634 | 1417.55528 | 1369.334237 | 938.6428946 | 701.2499622 | 880.8931847 | 802.0983526 | 947.3406921 | 1089.815839 | 1124.021987 | 915.4526661 |
| **4921525O09Rik** | 36.62785926 | 56.2506026 | 36.80858084 | 28.46611085 | 43.06870061 | 35.07064029 | 37.88671802 | 24.30414638 | 15.80069657 | 18.35194135 | 14.18806048 | 32.96606804 | 49.42089028 | 35.23863141 | 27.30549553 |
| **Syk** | 0.915696482 | 3.169048034 | 3.132645178 | 3.450437679 | 0.633363244 | 0.701412806 | 0 | 1.676148026 | 1.504828245 | 0.873901969 | 2.837612096 | 0 | 0.852084315 | 1.807109303 | 0 |
| **Auh** | 244.4909606 | 240.0553886 | 251.3947755 | 222.5532303 | 196.975969 | 244.7930692 | 203.8666255 | 186.8905049 | 182.8366318 | 240.3230414 | 246.8722524 | 209.0742736 | 230.0627651 | 197.8784687 | 214.1325702 |
| **Nfil3** | 1059.460829 | 790.6774845 | 836.4162625 | 760.8215081 | 716.3338293 | 894.3013273 | 862.3738673 | 272.3740542 | 348.3677388 | 381.8951604 | 470.0977373 | 619.4150679 | 699.5612227 | 541.2292363 | 622.2778719 |
| **Sptlc1** | 1235.274554 | 1455.38531 | 1365.050136 | 1459.535138 | 1290.794292 | 1268.154353 | 1376.550755 | 1414.668934 | 1422.062692 | 1365.034875 | 1377.187737 | 1288.279238 | 1243.191016 | 1323.707565 | 1283.35829 |
| **Gm2762** | 0.915696482 | 0.792262009 | 1.566322589 | 0.86260942 | 0.633363244 | 0 | 1.80412943 | 6.704592104 | 3.00965649 | 1.747803938 | 3.783482795 | 0 | 1.70416863 | 1.807109303 | 0 |
| **Sfxn1** | 2735.18539 | 2684.975947 | 2725.401305 | 2636.996996 | 2539.78661 | 2466.167425 | 2643.049614 | 2998.628819 | 2925.386109 | 2895.237223 | 2918.956976 | 2482.86544 | 2599.709246 | 2655.547121 | 2559.530924 |
| **Hrh2** | 105.3050954 | 103.7863231 | 108.0762586 | 104.3757398 | 101.3381191 | 119.240177 | 117.2684129 | 56.15095887 | 73.73658401 | 102.2465304 | 118.2338373 | 124.0565192 | 117.5876355 | 118.3656594 | 127.1861239 |
| **Thoc3** | 1412.003975 | 1478.360908 | 1610.962783 | 1577.712629 | 1532.105688 | 1493.307863 | 1437.891155 | 1771.688464 | 1701.960745 | 1498.741877 | 1681.758102 | 1612.73475 | 1460.472516 | 1509.839823 | 1434.975647 |
| **Simc1** | 425.7988639 | 362.8559999 | 363.3868406 | 332.967236 | 326.1820708 | 326.8583675 | 379.7692449 | 418.1989325 | 393.5125861 | 363.5432191 | 360.3767362 | 357.4215798 | 364.6920869 | 341.5436583 | 382.2769375 |
| **4833439L19Rik** | 1047.556775 | 1094.113834 | 1072.930973 | 949.732971 | 960.1786783 | 979.8736896 | 1066.240493 | 1039.211776 | 989.4245712 | 1025.960911 | 1001.67707 | 1046.238896 | 1027.613684 | 929.7577365 | 996.650587 |
| **Arl10** | 41.20634167 | 28.52143231 | 37.59174214 | 26.74089201 | 22.80107679 | 35.77205309 | 28.86607087 | 22.62799835 | 21.06759543 | 29.71266694 | 24.59263817 | 47.71404585 | 27.26669809 | 40.65995932 | 28.02406121 |
| **Nop16** | 1605.215932 | 1587.693065 | 1566.322589 | 1597.552645 | 1706.913943 | 1698.821815 | 1580.41738 | 1831.191719 | 1668.10211 | 1788.003428 | 1746.07731 | 1545.935085 | 1566.130971 | 1526.103807 | 1578.688781 |
| **Higd2a** | 504.5487613 | 446.0435108 | 507.4885188 | 517.5656518 | 495.9234203 | 497.3016793 | 444.7179044 | 528.8247022 | 447.6864029 | 536.5758089 | 490.9068927 | 501.4312454 | 473.7588792 | 506.8941595 | 515.2115868 |
| **Cltb** | 348.8803595 | 400.8845763 | 415.0754861 | 453.7325547 | 354.6834168 | 352.1092285 | 327.4494915 | 428.2558207 | 525.1850576 | 489.3851026 | 492.7986341 | 377.3747262 | 408.148387 | 374.0716258 | 335.5701688 |
| **Faf2** | 1564.009591 | 1508.466864 | 1495.838072 | 1417.267276 | 1493.47053 | 1449.118857 | 1607.479322 | 1348.461087 | 1520.628942 | 1375.521699 | 1376.241867 | 1427.083735 | 1365.891157 | 1395.088382 | 1371.023302 |
| **Rnf44** | 1913.805646 | 1968.771091 | 1943.806333 | 1940.871194 | 1786.717712 | 1887.50186 | 1979.129984 | 1899.075714 | 1794.507682 | 1683.135192 | 1748.914922 | 1862.582844 | 1856.691723 | 1758.317352 | 1877.612101 |
| **Sncb** | 12.81975074 | 5.54583406 | 4.698967767 | 4.313047098 | 1.266726489 | 4.208476834 | 5.412388289 | 1.676148026 | 9.781383594 | 0.873901969 | 0.945870699 | 3.470112425 | 0 | 4.517773258 | 5.748525376 |

| | | | | | | | | | | | | | | | |
|---|---|---|---|---|---|---|---|---|---|---|---|---|---|---|---|
| **Tspan17** | 1.831392963 | 5.54583406 | 2.349483883 | 8.626094197 | 18.36753408 | 11.9240177 | 8.118582433 | 4.190370065 | 3.00965649 | 0 | 0.945870699 | 1.735056213 | 2.556252945 | 1.807109303 | 6.467091048 |
| **Unc5a** | 28.38659093 | 16.63750218 | 38.37490343 | 32.77915795 | 21.53435031 | 27.35509942 | 16.23716487 | 19.2757205 | 36.86829201 | 31.46047088 | 31.21373306 | 43.37640531 | 40.04796281 | 46.08128723 | 45.26923733 |
| **Hk3** | 21.06101908 | 14.26071615 | 17.22954848 | 9.488703616 | 13.93399137 | 11.22260489 | 17.13922958 | 11.73303618 | 3.762070613 | 10.48682363 | 11.35044839 | 8.675281063 | 1.70416863 | 10.84265582 | 14.37131344 |
| **Uimc1** | 481.6563498 | 627.4715107 | 531.766519 | 473.5725714 | 499.0902365 | 541.490686 | 468.171587 | 558.1572927 | 452.9533018 | 548.8104365 | 561.8471951 | 523.1194481 | 504.4339146 | 583.6963049 | 536.0499913 |
| **Zfp346** | 337.8920017 | 411.9762444 | 447.1850992 | 377.8229258 | 433.8538223 | 396.999648 | 460.9550693 | 338.5819013 | 451.4484735 | 344.3173757 | 342.405193 | 380.8448387 | 432.0067478 | 410.2138118 | 412.4566957 |
| **Nsd1** | 2935.72292 | 2981.281938 | 3243.07092 | 2887.153728 | 2707.627869 | 2665.368662 | 3029.133312 | 3459.569526 | 3105.213084 | 2960.779871 | 3170.558582 | 2926.172302 | 3080.284799 | 2773.009226 | 2836.897273 |
| **Rab24** | 462.4267232 | 434.1595807 | 435.4376797 | 481.3360562 | 404.7191131 | 420.1462706 | 413.1456946 | 384.675972 | 404.7987979 | 478.024377 | 480.502315 | 412.0758505 | 472.9067949 | 520.4474793 | 424.6723121 |
| **Prelid1** | 10587.28272 | 10515.69364 | 10605.57025 | 10676.51679 | 10575.26609 | 10721.09474 | 10188.82095 | 7841.85854 | 8666.305864 | 8761.74114 | 8519.457384 | 10329.65716 | 10605.04139 | 10520.08681 | 10361.71699 |
| **Mxd3** | 293.9385706 | 367.6095719 | 363.3868406 | 425.2664439 | 468.0554375 | 411.729317 | 368.9444683 | 330.2011611 | 395.0174144 | 330.3349442 | 338.6217102 | 449.3795591 | 442.2317596 | 450.8737711 | 446.947848 |
| **Lman2** | 4667.304967 | 4793.977413 | 4752.222735 | 5024.699869 | 4446.209975 | 4629.324518 | 4648.339475 | 4601.864406 | 4743.218629 | 4844.038614 | 4685.843442 | 4538.907052 | 4370.340452 | 4599.093177 | 4508.281026 |
| **Rgs14** | 395.58088 | 490.4101833 | 437.7871636 | 489.0995409 | 622.5960691 | 476.2592951 | 500.6459167 | 407.3039703 | 454.45813 | 472.7809652 | 444.5592284 | 554.3504599 | 556.4110578 | 509.6048235 | 622.9964376 |
| **Grk6** | 3551.070956 | 3970.024925 | 3968.278279 | 3987.843347 | 4051.624674 | 4025.408092 | 4067.409799 | 3367.381384 | 3593.529849 | 3522.698837 | 3700.246174 | 4034.005694 | 4136.86935 | 4343.38721 | 3983.728085 |
| **Prr7** | 162.9939737 | 113.2934672 | 135.4869039 | 162.1705709 | 140.6066402 | 176.0546142 | 165.0778428 | 85.48354933 | 70.72692752 | 99.62482445 | 56.75224193 | 138.804497 | 190.8668866 | 162.6398373 | 204.0726508 |
| **Dbn1** | 4.578482408 | 3.961310043 | 12.53058071 | 4.313047098 | 7.600358931 | 9.81977928 | 5.412388289 | 0.838074013 | 0.752414123 | 6.991215751 | 11.35044839 | 6.072696744 | 0 | 1.807109303 | 7.18565672 |
| **Pdlim7** | 91.56964816 | 87.14882094 | 115.1247103 | 97.47486442 | 100.7047558 | 92.58649035 | 82.98995376 | 54.47481085 | 79.75589699 | 79.52507917 | 100.2622941 | 80.68011388 | 86.91260015 | 114.7514407 | 97.00636572 |
| **Dok3** | 0 | 3.169048034 | 10.96425812 | 1.725218839 | 3.800179466 | 3.507064029 | 3.608258859 | 4.190370065 | 7.524141226 | 13.9824315 | 5.675224193 | 0.867528106 | 0 | 0 | 5.029959704 |
| **Ddx41** | 1428.486511 | 1461.723406 | 1452.764201 | 1402.602916 | 1409.233219 | 1454.028746 | 1419.849861 | 1503.504779 | 1378.422673 | 1499.615779 | 1510.555506 | 1424.481151 | 1306.245255 | 1477.311855 | 1511.143608 |
| **Fam193b** | 368.1099856 | 466.642323 | 489.4758091 | 401.9759896 | 466.788711 | 476.9607079 | 459.1509398 | 421.5512356 | 425.8663934 | 500.7458282 | 439.8298749 | 494.4910206 | 576.8610814 | 501.4728316 | 566.2297495 |
| **Tmed9** | 3067.583213 | 3180.139702 | 3225.058211 | 3220.120964 | 3296.022323 | 3224.394668 | 3104.004684 | 3297.821241 | 3039.753055 | 3043.800558 | 3278.387842 | 3161.272419 | 3185.943254 | 3319.65979 | 3052.466975 |
| **B4galt7** | 294.8542671 | 373.155406 | 382.965873 | 314.8524382 | 332.5157032 | 347.1993388 | 351.8052388 | 329.3630871 | 340.0911834 | 308.487395 | 343.3510637 | 344.4086582 | 331.4607986 | 346.9649862 | 366.4684927 |
| **Caml** | 337.8920017 | 328.7887335 | 366.5194858 | 370.9220505 | 323.6486178 | 324.0527162 | 334.6660092 | 358.6956776 | 326.5477292 | 343.4434738 | 325.3795204 | 365.2293327 | 279.4836554 | 302.6908083 | 361.438533 |
| **Ddx46** | 4632.5085 | 4385.170217 | 4851.684219 | 4514.035093 | 4707.788995 | 4458.881206 | 4874.757719 | 5131.527182 | 4752.247598 | 4781.117672 | 5200.397102 | 4924.957059 | 4798.086779 | 4777.093443 | 4484.568359 |
| **B230219D22Rik** | 925.7691429 | 937.2459561 | 946.0588437 | 954.0460181 | 889.8753582 | 948.3101133 | 966.1113095 | 1035.021406 | 1011.244581 | 953.4270481 | 949.6541816 | 939.5329391 | 1010.571998 | 992.1030074 | 980.1235766 |
| **Txndc15** | 317.7466791 | 347.8030217 | 427.6060668 | 347.6315961 | 359.7503227 | 374.5544383 | 337.3722033 | 403.1136003 | 445.4291606 | 418.5990431 | 429.4252972 | 372.1695576 | 323.7920398 | 345.1578769 | 356.4085733 |
| **Pcbd2** | 163.9096702 | 217.0797903 | 190.3081946 | 231.1793245 | 209.0098706 | 235.6747027 | 253.4801849 | 211.1946513 | 221.9621662 | 182.6455115 | 164.5815016 | 241.1728135 | 215.5773317 | 217.756671 | 228.5038837 |
| **H2afy** | 7310.920709 | 7266.627142 | 7659.31746 | 7141.543385 | 7493.320543 | 7938.590135 | 7929.148843 | 5610.905517 | 6424.864193 | 6041.284311 | 6760.137884 | 7855.467002 | 7707.954715 | 7687.442976 | 7685.778427 |
| **Tifab** | 1.831392963 | 0 | 3.132645178 | 8.626094197 | 5.700269198 | 2.104238417 | 5.412388289 | 3.352296052 | 3.00965649 | 5.243411813 | 0 | 2.602584319 | 2.556252945 | 1.807109303 | 5.029959704 |
| **Il9** | 12.81975074 | 2.376786026 | 1.566322589 | 8.626094197 | 2.533452977 | 4.208476834 | 9.020647148 | 98.05465953 | 28.59173666 | 67.29045161 | 38.78069865 | 0 | 0.852084315 | 1.807109303 | 2.155697016 |
| **Smad5** | 303.0955354 | 305.8131353 | 287.4201951 | 244.9810752 | 304.6477205 | 298.8018552 | 320.2329737 | 235.4987977 | 270.11667 | 236.8274336 | 277.1401147 | 338.3359614 | 328.0524613 | 287.3303792 | 315.45033 |
| **Klhl3** | 19.22962611 | 38.82083842 | 25.84432272 | 27.60350143 | 26.60125626 | 28.05651223 | 26.15987673 | 34.36103454 | 46.6496756 | 55.05582404 | 44.45592284 | 47.71404585 | 34.93545692 | 42.46706862 | 32.33545524 |
| **Hnrnpa0** | 15903.81649 | 15370.67523 | 15856.66673 | 15944.47251 | 17200.87899 | 20249.08629 | 16760.3624 | 16125.38209 | 17124.94543 | 15543.22042 | 15239.8687 | 17026.97414 | 17547.82439 | 17307.58935 | 16129.64364 |
| **Idnk** | 335.1449123 | 331.9577816 | 404.111228 | 387.3116294 | 341.3827887 | 363.3318334 | 309.4081972 | 496.9778897 | 420.5994945 | 430.8336707 | 349.9721585 | 324.4555117 | 345.0941476 | 377.6858444 | 358.5642703 |
| **Ubqln1** | 3447.597253 | 3103.290287 | 3345.66505 | 3190.792243 | 3230.785909 | 3145.135021 | 3407.098428 | 3606.232478 | 3547.632588 | 3594.358798 | 3503.505068 | 3233.277252 | 3185.09117 | 2991.669451 | 3178.215967 |
| **Gkap1** | 76.91850445 | 58.62738863 | 38.37490343 | 55.20700286 | 61.43623469 | 48.39748359 | 53.22181817 | 59.50325493 | 81.26072524 | 73.40776539 | 68.10269031 | 49.44910206 | 61.35007069 | 47.88839653 | 55.32955674 |
| **2210016F16Rik** | 930.3476253 | 943.5840521 | 957.8062632 | 998.0390985 | 942.4445075 | 986.8878176 | 1000.389769 | 939.4809686 | 930.7362696 | 919.3448713 | 887.2267154 | 995.0547379 | 950.9260957 | 950.5394935 | 963.5965661 |
| **Hnrnpk** | 15078.77396 | 15083.08412 | 15980.40621 | 15195.72754 | 14968.27355 | 15245.90874 | 15337.80635 | 16439.65984 | 16109.18636 | 16379.5446 | 16950.94879 | 15660.61737 | 15626.37426 | 15658.60211 | 15185.44835 |
| **Mir7-1** | 0 | 0.792262009 | 0.783161294 | 6.900875357 | 12.03390164 | 5.611302446 | 5.412388289 | 5.028444078 | 2.257242368 | 8.739019689 | 8.512836289 | 4.337640531 | 4.260421576 | 2.710663955 | 5.029959704 |
| **Rmi1** | 467.9209021 | 435.7441047 | 529.4170351 | 483.9238844 | 510.4907749 | 443.994306 | 540.3367642 | 437.4746348 | 477.0305537 | 513.8543577 | 478.6105736 | 517.9142794 | 471.2026263 | 534.9043537 | 480.7204345 |
| **Agtpbp1** | 362.6158067 | 373.155406 | 398.6290989 | 331.2420171 | 352.1499638 | 392.7911712 | 382.4754391 | 382.999824 | 415.3325957 | 405.4905136 | 419.0207196 | 416.413491 | 372.3608457 | 368.6502978 | 402.3967763 |
| **Naa35** | 680.3624858 | 674.2149692 | 711.8936167 | 626.2544387 | 651.0974151 | 690.1902008 | 674.7444067 | 814.6079407 | 714.7934165 | 684.2652417 | 773.7222316 | 718.313272 | 686.779958 | 677.6659887 | 675.4517316 |
| **Golm1** | 326.9036439 | 366.8173099 | 350.0730986 | 403.7012084 | 374.3176774 | 310.7258729 | 360.8258859 | 420.7131546 | 427.3712216 | 429.0858667 | 417.1289782 | 379.1097824 | 369.8045928 | 355.0969781 | 357.127139 |
| **Isca1** | 683.1095752 | 598.9500784 | 725.99052 | 765.9971647 | 755.6023504 | 676.1619447 | 668.4299537 | 726.6101693 | 809.5975959 | 737.5732618 | 776.5598437 | 822.4166448 | 651.8445011 | 787.8996562 | 666.1103779 |
| **Etohd2** | 10.0726613 | 1.584524017 | 0 | 7.763484777 | 4.43354271 | 4.208476834 | 2.706194144 | 6.704592104 | 4.514484735 | 7.86511772 | 5.675224193 | 6.94022485 | 11.0770961 | 8.131991864 | 6.467091048 |
| **Zcchc6** | 1203.225177 | 1248.604925 | 1190.405168 | 1110.178323 | 1079.250968 | 1060.536162 | 1273.715377 | 1079.439329 | 1062.408741 | 1097.620873 | 1079.238467 | 1154.679909 | 1198.882631 | 1082.458473 | 1213.65742 |
| **Dapk1** | 31.13368037 | 46.7434585 | 35.24225825 | 43.9930804 | 13.93399137 | 32.96640187 | 36.98465331 | 79.61703124 | 75.24141226 | 65.54264767 | 50.13114703 | 32.09853993 | 32.37920398 | 19.87820233 | 22.27553583 |
| **4930486L24Rik** | 55.85748538 | 97.44822705 | 111.9920651 | 83.67311371 | 114.6387472 | 79.96105985 | 88.40234205 | 40.22755263 | 63.95520042 | 46.31680435 | 65.26507822 | 84.15022631 | 143.1501649 | 109.3301128 | 110.6591135 |
| **Ctla2b** | 117.2091496 | 96.65596504 | 106.5099361 | 119.9027093 | 114.6387472 | 98.89920561 | 85.6961479 | 44.41792269 | 24.07725192 | 80.39898114 | 89.85771638 | 103.2358446 | 92.87719035 | 93.96968376 | 114.9705075 |
| **Ctla2a** | 945.9144655 | 960.2215543 | 962.5052309 | 1031.68066 | 867.0742814 | 873.9603559 | 891.2399382 | 587.4898832 | 752.4141226 | 689.5086535 | 724.5369553 | 918.7122645 | 876.7947603 | 939.6968376 | 924.0754541 |
| **Zfp808** | 50.36330649 | 86.35655893 | 58.73709709 | 35.36698621 | 56.36932874 | 46.29324518 | 47.80942988 | 56.98903289 | 70.72692752 | 57.67752995 | 65.26507822 | 46.84651774 | 62.20215501 | 60.53816166 | 63.23377913 |
| **Gm3604** | 51.27900297 | 53.87381658 | 37.59174214 | 36.22959563 | 23.43444004 | 35.77205309 | 44.20117102 | 37.71333059 | 40.63036262 | 50.6863142 | 54.86050053 | 40.773821 | 34.93545692 | 56.92394305 | 51.01816271 |
| **Zfp935** | 140.1015617 | 110.1244192 | 130.7879362 | 111.2766151 | 108.938478 | 115.7331129 | 148.8406779 | 115.6542138 | 134.6821279 | 97.87702052 | 107.8292597 | 129.2616878 | 132.9251532 | 131.0154245 | 113.5333762 |
| **Zfp934** | 21.06101908 | 17.42976419 | 18.01270977 | 28.46611085 | 28.50134599 | 23.14662259 | 22.55161787 | 41.90370065 | 14.29586833 | 23.59535316 | 27.43025026 | 22.55573076 | 28.97086672 | 24.39597559 | 21.55697016 |
| **6720489N17Rik** | 24.723805 | 29.31369431 | 28.9769679 | 31.05393911 | 19.00089733 | 31.56357626 | 27.96400616 | 36.03718256 | 39.8779485 | 27.964863 | 38.78069865 | 27.7608994 | 22.15419219 | 41.56351397 | 39.52111196 |
| **Gm5141** | 44.8691276 | 46.7434585 | 46.20651637 | 29.32872027 | 43.06870061 | 35.07064029 | 28.86607087 | 41.90370065 | 23.3248378 | 30.58656891 | 36.88895725 | 39.03876478 | 32.37920398 | 34.33507676 | 57.48525376 |
| **2010111I01Rik** | 462.4267232 | 445.2512488 | 490.2589703 | 433.0299287 | 502.890416 | 471.3494054 | 478.0942988 | 473.5118174 | 510.8891892 | 495.5024164 | 503.2032117 | 452.8496715 | 466.0901204 | 536.711463 | 477.1276062 |
| **Gm16907** | 37.54355574 | 76.84941482 | 48.55600026 | 67.28353473 | 50.66905954 | 49.0988964 | 47.80942988 | 49.44636677 | 53.4214027 | 57.67752995 | 77.5613973 | 72.00483282 | 69.01882953 | 48.79195118 | 53.17385973 |
| **Fancc** | 139.1858652 | 160.0369257 | 166.0301944 | 148.3688202 | 181.1418879 | 154.3108173 | 201.1604314 | 210.3565773 | 182.8366318 | 200.1235509 | 181.6071742 | 180.4458461 | 156.783514 | 177.0967117 | 164.5515389 |
| **Ptch1** | 742.6298465 | 813.6530827 | 694.6640682 | 785.8371813 | 708.1001071 | 706.3226954 | 821.7809552 | 741.6955016 | 750.9092943 | 690.3825554 | 741.5626278 | 785.9804643 | 835.0426289 | 705.6761829 | 801.2007242 |
| **Ercc6l2** | 437.7029182 | 440.4976767 | 473.0294219 | 413.189912 | 412.319472 | 439.7858292 | 437.5013867 | 461.7787812 | 385.2360308 | 450.059514 | 443.6133577 | 427.6913564 | 450.7526027 | 447.2595525 | 435.4507772 |
| **Slc35d2** | 98.89522001 | 83.97977729 | 80.66561333 | 102.6505209 | 109.5718413 | 71.54410618 | 71.2634422 | 71.23629111 | 114.3669466 | 88.26409886 | 89.85771638 | 109.3085414 | 117.5876355 | 102.1016756 | 64.67091048 |
| **Zfp367** | 477.0778669 | 502.2941134 | 472.2462606 | 474.4351808 | 393.9519379 | 423.6533347 | 442.0117102 | 456.7503371 | 484.5546949 | 487.6372986 | 498.4738583 | 477.1404585 | 574.3048284 | 566.5287665 | 562.6369211 |
| **Habp4** | 166.6567596 | 176.6744279 | 171.5123235 | 210.4766984 | 188.7422468 | 179.5616783 | 173.1964252 | 233.8226496 | 265.6021853 | 188.7628253 | 168.3649844 | 212.544386 | 224.0981749 | 231.3099908 | 211.2583076 |
| **Cdc14b** | 202.3689224 | 179.0512139 | 196.5734849 | 189.7740723 | 169.7413495 | 140.2825611 | 167.7840369 | 234.6607237 | 218.9525097 | 225.466708 | 265.7896664 | 181.3133742 | 161.8960199 | 164.4469466 | 168.1443672 |
| **1810034E14Rik** | 20.14532259 | 27.7291703 | 21.14535495 | 41.40525214 | 33.65825195 | 24.5494482 | 22.55161787 | 45.2559967 | 36.11587788 | 40.19949057 | 44.45592284 | 36.43618046 | 32.37920398 | 27.10663955 | 32.33545524 |
| **Aaed1** | 138.2701687 | 137.0613275 | 148.0174847 | 116.4522717 | 117.1722002 | 129.0599563 | 142.5262249 | 71.23629111 | 77.49865463 | 80.39898114 | 89.85771638 | 131.8642722 | 165.3043571 | 143.6651896 | 135.808912 |
| **Ctsl** | 78.74989741 | 73.68036679 | 86.93090369 | 114.7270528 | 58.90278172 | 61.0229141 | 59.53627118 | 119.0065099 | 72.23175577 | 63.79484373 | 111.612425 | 65.93213608 | 64.75840795 | 76.80214538 | 77.60509257 |
| **Cdk20** | 45.78482408 | 36.44405239 | 39.15806472 | 20.70262607 | 24.06780328 | 32.26498906 | 46.00530045 | 23.46607237 | 51.16416034 | 60.29923585 | 54.86050053 | 33.83359614 | 56.2375648 | 42.46706862 | 53.8924254 |
| **Hiatl1** | 908.3709097 | 922.1929779 | 1061.966715 | 986.8251761 | 947.5114134 | 1003.020312 | 1058.12191 | 883.3300098 | 908.163846 | 953.4270481 | 930.7367676 | 1033.225975 | 1094.928345 | 1052.641169 | 1082.159902 |
| **Zfp369** | 110.7992743 | 126.7619214 | 133.1374201 | 83.67311371 | 96.27121313 | 86.2737751 | 131.7014484 | 105.5973256 | 110.604876 | 106.6160402 | 121.0714494 | 99.76573222 | 134.6293218 | 80.41636399 | 99.16206273 |
| **Uqcrb** | 872.6587469 | 831.8751089 | 872.4416821 | 880.7242175 | 821.4722278 | 873.9603559 | 845.2346377 | 913.5006742 | 863.0189986 | 809.2332232 | 822.9075079 | 819.8140604 | 828.3491738 | 937.8897283 | 720.721369 |
| **Gm10767** | 1.831392963 | 11.88393013 | 4.698967767 | 8.626094197 | 10.13381191 | 7.014128057 | 3.608258859 | 5.028444078 | 5.266898858 | 7.86511772 | 12.29631908 | 3.470112425 | 13.63334904 | 7.228437213 | 6.467091048 |
| **Mterf3** | 244.4909606 | 248.7702707 | 195.0071623 | 196.6749477 | 209.0098706 | 212.5280801 | 230.928567 | 272.3740542 | 292.6890937 | 255.1793749 | 234.5759333 | 254.1857351 | 238.5836082 | 230.4064362 | 238.5638031 |
| **Ptdss1** | 841.5250666 | 860.3965412 | 960.9389083 | 930.7555638 | 944.9779605 | 962.3383694 | 994.0753157 | 916.8529703 | 938.2604109 | 851.1805177 | 978.9761732 | 943.8705796 | 953.4823487 | 867.4125445 | 987.3092333 |
| **4933433G19Rik** | 10.0726613 | 2.376786026 | 3.915806472 | 16.38957897 | 12.03390164 | 5.611302446 | 8.118582433 | 16.76148026 | 15.04828245 | 11.3607256 | 14.18806048 | 8.675281063 | 6.816674521 | 8.131991864 | 7.18565672 |
| **Zfp712** | 32.04937685 | 22.97559825 | 42.2907099 | 20.70262607 | 37.36843141 | 23.84803539 | 36.98465331 | 12.5711102 | 5.266898858 | 19.22584332 | 30.26786236 | 28.62842751 | 17.89377062 | 30.72085815 | 27.30549553 |
| **Zfp708** | 14.65114371 | 12.67619214 | 16.44638718 | 17.25218839 | 15.20071786 | 18.23673295 | 12.62890601 | 15.08533223 | 4.514484735 | 14.85633347 | 27.43025026 | 13.8804497 | 12.78126473 | 12.64976512 | 18.68270747 |
| **Rslcan18** | 7.325571853 | 0 | 2.349483883 | 0.86260942 | 4.43354271 | 7.014128057 | 3.608258859 | 6.704592104 | 0 | 6.991215751 | 6.621094891 | 6.072696744 | 5.112505891 | 2.710663955 | 2.155697016 |
| **Zfp759** | 36.62785926 | 45.95119649 | 39.15806472 | 26.74089201 | 31.66816221 | 37.87629151 | 28.86607087 | 41.06562664 | 19.56276719 | 25.3431571 | 16.07980188 | 26.89337129 | 24.71044514 | 21.68531164 | 30.17975822 |
| **Rsl1** | 99.81091649 | 65.75774671 | 81.44877463 | 102.6505209 | 91.83767042 | 78.55823424 | 101.0312481 | 74.58858716 | 68.46968515 | 88.26409886 | 68.10269031 | 90.22292305 | 68.16674521 | 84.0305826 | 75.44939556 |
| **Zfp455** | 30.21798389 | 31.69048034 | 25.06116142 | 30.19132969 | 40.53524763 | 25.95227381 | 23.45368258 | 32.68488651 | 13.54345421 | 21.84754922 | 7.56696559 | 27.7608994 | 30.67503535 | 13.55331977 | 13.65274777 |
| **Zfp458** | 23.80810852 | 26.95690829 | 32.10961307 | 13.80175071 | 6.333632443 | 36.4734659 | 23.45368258 | 24.30414638 | 11.28621184 | 17.47803938 | 21.75502607 | 19.95314644 | 17.0416863 | 9.939101167 | 17.24557613 |
| **Zfp457** | 0 | 0 | 4.698967767 | 0 | 0 | 0.701412806 | 0 | 0 | 0 | 0 | 0 | 2.602584319 | 2.556252945 | 0 | 0 |
| **Zfp595** | 61.35166426 | 69.71905675 | 88.49722628 | 56.06961228 | 62.06959794 | 74.34975741 | 62.24246532 | 38.5514046 | 51.16416034 | 49.81241223 | 51.07701773 | 56.38932691 | 66.46257658 | 62.34527096 | 63.9523448 |
| **Zfp953** | 87.90686223 | 83.18751089 | 97.11200052 | 113.001834 | 86.77076447 | 81.36388546 | 94.71679505 | 99.73080755 | 112.8621184 | 104.8682363 | 62.42746612 | 91.95797927 | 88.61676878 | 105.7158942 | 115.6890732 |
| **Zfp456** | 65.01445019 | 65.75774671 | 78.31612945 | 75.04701951 | 53.20251252 | 46.99465798 | 70.36104775 | 65.36977302 | 34.61104964 | 36.70388269 | 43.51005214 | 65.93213608 | 58.79381775 | 41.56351397 | 58.20381943 |
| **Zfp429** | 88.82255871 | 90.31786897 | 93.97935534 | 60.38265938 | 84.23731149 | 95.39214158 | 117.2684129 | 81.29317927 | 64.70761454 | 77.77727523 | 48.23940564 | 91.95797927 | 67.3146609 | 82.22347329 | 76.16796123 |
| **Zfp459** | 11.90405426 | 24.56012226 | 14.8800646 | 23.29045433 | 15.83408111 | 17.53532014 | 14.43303544 | 15.92340625 | 27.83932254 | 16.60413741 | 7.56696559 | 10.41033728 | 14.48543336 | 0.903554652 | 15.80844478 |
| **Zfp874a** | 72.34002204 | 83.18751089 | 94.76251663 | 90.57398906 | 78.53704229 | 63.12715251 | 93.81473034 | 85.48354933 | 102.3283207 | 76.90337326 | 89.85771638 | 68.5347204 | 95.4334433 | 92.16257446 | 58.20381943 |
| **Zfp874b** | 65.93014667 | 84.77203491 | 77.53296815 | 85.39833255 | 94.3711234 | 63.12715251 | 56.83007703 | 76.26473519 | 69.9745134 | 57.67752995 | 47.29353494 | 66.79966418 | 62.20215501 | 58.73105235 | 75.44939556 |
| **Zfp58** | 68.67723612 | 66.55000871 | 67.35187133 | 78.49745719 | 62.06959794 | 76.45399582 | 68.55691832 | 41.06562664 | 60.94554393 | 53.3080201 | 74.7237852 | 58.99191123 | 73.2792511 | 48.79195118 | 82.63505228 |
| **Zfp87** | 182.2235998 | 156.8678777 | 128.4384523 | 124.2157564 | 155.8073581 | 118.5387642 | 143.4282897 | 124.0349539 | 106.0903913 | 103.1204323 | 98.37055267 | 117.9838225 | 127.8126473 | 116.5585501 | 106.3477195 |
| **Zfp748** | 113.5463637 | 118.0470393 | 119.8236781 | 113.8644434 | 100.7047558 | 121.3444154 | 129.8973189 | 139.9583602 | 86.5276241 | 76.90337326 | 124.8549322 | 108.4410133 | 116.7355512 | 112.0407768 | 110.6591135 |
| **9430065F17Rik** | 0 | 2.349483883 | 0 | 0 | 0 | 2.805651223 | 0.902064715 | 2.514222039 | 3.00965649 | 0 | 1.891741398 | 0 | 0.852084315 | 3.614218606 | 0.718565672 |
| **Zfp729b** | 182.2235998 | 160.8291877 | 184.0429042 | 187.1862441 | 159.6075376 | 182.3673295 | 223.7102493 | 176.0536168 | 145.2159759 | 165.1674721 | 169.3108551 | 229.0274201 | 201.9439827 | 194.2642501 | 181.0785493 |
| **Zfp729a** | 165.7410632 | 167.9595458 | 188.741872 | 175.1097122 | 184.3087041 | 171.8461374 | 211.985208 | 194.433171 | 206.9138837 | 147.6894327 | 173.0943379 | 146.61225 | 157.6355983 | 178.90321 | 194.7312971 |
| **Zfp738** | 118.1248461 | 109.3321572 | 154.282775 | 120.7653188 | 105.7716618 | 136.0740843 | 128.0931895 | 141.6345082 | 151.9876528 | 193.1323351 | 132.4218978 | 134.4668565 | 132.9251532 | 131.9189791 | 160.2401448 |
| **Zfp65** | 174.898028 | 152.1143056 | 169.1628396 | 159.5827426 | 167.8412597 | 137.4769099 | 180.412943 | 184.3762829 | 167.7883493 | 216.7276883 | 200.5245881 | 163.9628121 | 188.3106337 | 160.832728 | 165.9886702 |
| **Zfp85os** | 0.915696482 | 0.792262009 | 3.915806472 | 4.313047098 | 1.266726489 | 2.104238417 | 5.412388289 | 3.352296052 | 10.53379772 | 7.86511772 | 10.40457769 | 5.205168638 | 4.260421576 | 4.517773258 | 1.437131344 |
| **Zfp85** | 39.37494871 | 41.98988645 | 28.1938066 | 54.34439344 | 41.16861088 | 47.69607079 | 27.06194144 | 36.03718256 | 40.63036262 | 39.3255886 | 36.88895725 | 31.23101183 | 26.41461377 | 34.33507676 | 20.83840449 |
| **Zfp493** | 21.97671556 | 22.18333624 | 23.49483883 | 14.66436013 | 16.46744435 | 11.22260489 | 15.33510015 | 11.73303618 | 23.3248378 | 20.97364725 | 26.48437957 | 19.95314644 | 36.63962555 | 13.55331977 | 15.08987911 |
| **4930525G20Rik** | 0.915696482 | 1.584524017 | 0 | 0 | 0 | 2.805651223 | 0.902064715 | 0.838074013 | 0 | 0 | 2.837612096 | 0 | 0 | 1.807109303 | 1.437131344 |
| **Zfp273** | 17.39823315 | 26.95690829 | 14.0969033 | 30.19132969 | 24.06780328 | 19.63955856 | 13.53097072 | 11.73303618 | 24.07725192 | 21.84754922 | 17.97154328 | 13.8804497 | 17.0416863 | 15.36042908 | 31.61688957 |
| **Gm10037** | 0 | 0 | 0 | 0 | 2.533452977 | 0.701412806 | 0 | 0 | 0 | 1.747803938 | 0 | 0 | 1.70416863 | 4.517773258 | 0 |
| **Mtrr** | 626.3363934 | 601.3268645 | 640.6259389 | 595.2004996 | 637.1634237 | 631.2715251 | 687.3733127 | 649.5073601 | 619.989237 | 710.4823007 | 682.9186445 | 575.1711345 | 606.6840324 | 622.5491549 | 579.1639316 |
| **Fastkd3** | 434.0401323 | 365.2327859 | 441.7029701 | 444.2438511 | 390.1517585 | 348.6021644 | 352.7073035 | 574.0806989 | 452.2008877 | 473.6548671 | 517.3912722 | 386.0500073 | 393.6629536 | 370.4574071 | 398.8039479 |
| **Papd7** | 1022.83297 | 858.0197552 | 893.587037 | 757.3710705 | 746.735265 | 800.6140425 | 912.8894914 | 732.4766874 | 868.2858975 | 775.1510464 | 870.2010429 | 896.1565338 | 969.6719507 | 905.3617609 | 923.3568885 |
| **Srd5a1** | 60.43596778 | 70.51131876 | 61.86974226 | 84.53572313 | 55.10260225 | 77.15540863 | 87.50027733 | 57.8271069 | 82.01313936 | 59.42533389 | 77.5613973 | 76.34247335 | 82.65217857 | 65.95948956 | 91.25784034 |
| **Nsun2** | 7947.329763 | 7583.531945 | 7942.821849 | 7468.472355 | 7385.648792 | 7685.380112 | 7668.45214 | 8731.055068 | 8351.044347 | 8581.717335 | 8674.580178 | 7478.959804 | 7268.279208 | 7143.503075 | 7208.650821 |
| **Med10** | 796.655939 | 781.1703404 | 811.3551011 | 785.8371813 | 770.8030683 | 816.4445058 | 785.6983666 | 835.559791 | 797.5589699 | 728.8342421 | 786.9644214 | 796.3908016 | 783.9175699 | 895.4226597 | 795.4521989 |
| **Ice1** | 1836.887142 | 1940.249659 | 1986.097043 | 1684.676197 | 1823.45278 | 1851.028394 | 2031.449738 | 2023.948742 | 1872.758751 | 1915.593116 | 1904.037717 | 1971.891386 | 1858.395891 | 1880.29723 | 1898.450505 |
| **Ndufs6** | 977.0481458 | 985.5739386 | 946.0588437 | 829.8302617 | 893.0421744 | 936.3860956 | 938.1473034 | 847.2928272 | 899.8872906 | 951.6792441 | 832.3662149 | 897.89159 | 878.4989289 | 998.42789 | 824.9133914 |
| **Mrpl36** | 936.7575006 | 738.3881919 | 889.6712305 | 789.287619 | 889.8753582 | 816.4445058 | 818.1726963 | 894.2249719 | 774.2341321 | 912.3536555 | 797.3689991 | 766.894846 | 852.0843152 | 890.9048865 | 794.0150675 |
| **Lpcat1** | 485.3191352 | 520.5161396 | 538.8149706 | 523.6039177 | 562.4265609 | 655.1195605 | 662.1155006 | 460.9407072 | 483.0498667 | 408.9861216 | 327.2712618 | 643.7058549 | 662.0695129 | 589.1176328 | 628.744963 |
| **Clptm1l** | 2710.461585 | 2601.788436 | 2849.923951 | 2807.793661 | 2635.424459 | 2765.670693 | 2849.622434 | 2876.270013 | 2901.308857 | 2896.985027 | 2957.737675 | 2809.056008 | 2756.49276 | 2775.71989 | 2816.058868 |
| **Tert** | 22.89241204 | 53.08155457 | 30.54329049 | 37.09220505 | 34.83497844 | 26.65368662 | 31.57226502 | 38.5514046 | 39.12553437 | 19.22584332 | 21.75502607 | 25.15831508 | 38.34379418 | 30.72085815 | 23.71266717 |
| **Slc6a19** | 80.58129038 | 106.1631091 | 102.5941296 | 132.8418506 | 88.03749095 | 90.48225194 | 92.01060091 | 82.13125328 | 90.28969471 | 43.69509845 | 69.99443171 | 154.4200029 | 89.46885309 | 121.979878 | 90.53927467 |
| **Slc6a19os** | 2.747089445 | 0 | 0 | 0 | 0.633363244 | 0 | 3.608258859 | 0 | 0 | 0 | 1.891741398 | 0 | 0 | 0 | 0 |
| **Slc12a7** | 302.1798389 | 434.9518427 | 455.0167121 | 469.2595243 | 460.4550786 | 465.738103 | 538.5326347 | 470.9975953 | 495.8409068 | 415.1034352 | 355.6473827 | 540.4700102 | 574.3048284 | 602.6709526 | 520.2415465 |
| **Nkd2** | 23.80810852 | 15.05297816 | 25.06116142 | 26.74089201 | 15.20071786 | 25.95227381 | 33.37639445 | 9.218814144 | 6.771727103 | 19.22584332 | 5.675224193 | 16.48303402 | 27.26669809 | 20.78175699 | 30.17975822 |
| **Trip13** | 1033.821328 | 1230.382899 | 1231.912716 | 1174.87403 | 1122.319669 | 1130.677443 | 1034.668228 | 1515.237816 | 1372.40336 | 1458.542386 | 1365.837289 | 1107.833392 | 1109.413778 | 1229.737881 | 1146.830812 |
| **Brd9** | 727.9787028 | 819.1989168 | 863.8269078 | 930.7555638 | 843.6398414 | 857.1264486 | 946.2658858 | 1009.879186 | 946.5369662 | 990.1309308 | 935.4661211 | 923.0499051 | 968.8198663 | 920.72219 | 952.0995154 |
| **Tppp** | 0.915696482 | 0.792262009 | 3.132645178 | 1.725218839 | 5.700269198 | 0.701412806 | 4.510323574 | 0.838074013 | 0 | 1.747803938 | 2.837612096 | 6.94022485 | 5.964590206 | 0.903554652 | 3.59282836 |
| **Cep72** | 134.6073828 | 172.7131179 | 142.5353556 | 161.3079615 | 165.3078068 | 190.7842832 | 151.5468721 | 176.8336168 | 133.1772997 | 149.4372367 | 175.93195 | 161.3602278 | 165.3043571 | 202.396242 | 145.8688314 |
| **Exoc3** | 1044.809685 | 976.0667945 | 1045.520328 | 1018.741725 | 1001.980652 | 972.8595615 | 974.229892 | 1155.704064 | 990.9293994 | 1027.708715 | 1216.389719 | 979.439232 | 1017.388672 | 1030.955857 | 903.9556153 |
| **Ahrr** | 45.78482408 | 43.57441047 | 47.77283896 | 54.34439344 | 29.76807248 | 28.75792503 | 37.88671802 | 97.21658551 | 66.21244279 | 106.6160402 | 228.9007091 | 24.29078698 | 34.08337261 | 35.23863141 | 17.9641418 |
| **Pdcd6** | 1223.370499 | 1285.84124 | 1328.241555 | 1165.385326 | 1259.759493 | 1258.334573 | 1221.395624 | 1185.874728 | 1198.595697 | 1334.448307 | 1345.028134 | 1372.429464 | 1346.293218 | 1323.707565 | 1302.040998 |
| **Sdha** | 4764.368794 | 4919.154811 | 5039.64293 | 4719.336135 | 4792.659669 | 4779.426858 | 4894.603142 | 5579.058705 | 5534.758286 | 5198.842813 | 5437.810647 | 4852.952227 | 5001.73493 | 5054.484721 | 4614.628745 |
| **Ccdc127** | 760.9437762 | 770.8709343 | 747.1355749 | 788.4250096 | 776.5033375 | 691.5930024 | 752.3219721 | 871.5969736 | 750.1568802 | 867.7846551 | 817.2322837 | 728.7236093 | 677.4070306 | 712.0016654 | 702.7572722 |
| **Lrrc14b** | 11.90405426 | 6.338096068 | 10.96425812 | 5.175656518 | 5.066905954 | 0.701412806 | 2.706194144 | 4.190370065 | 0 | 1.747803938 | 7.56696559 | 9.542809169 | 11.92918041 | 0.903554652 | 6.467091048 |
| **Zfp72** | 23.80810852 | 7.130358077 | 15.66322589 | 12.93914129 | 12.03390164 | 15.43108173 | 19.84542373 | 18.43762829 | 12.79104008 | 14.85633347 | 6.621094891 | 7.807752957 | 13.63334904 | 13.55331977 | 25.14979852 |
| **Zfp825** | 74.17141501 | 109.3321572 | 87.71406498 | 80.22267603 | 86.77076447 | 92.58649035 | 92.01060091 | 90.51199341 | 91.79452295 | 69.91215751 | 102.1540355 | 110.1760695 | 76.68758836 | 88.54835585 | 87.66501198 |
| **Erap1** | 3508.033221 | 3520.020104 | 3642.483181 | 3552.22559 | 3296.022323 | 3320.488222 | 3498.206964 | 3598.689812 | 3786.147865 | 3573.385151 | 3449.590438 | 3483.992875 | 3350.395527 | 3324.177563 | 3357.857385 |
| **Cast** | 1936.698059 | 1843.593694 | 1873.321816 | 1801.128468 | 1842.453678 | 1842.611441 | 2114.439691 | 1590.664477 | 1705.722816 | 1752.173448 | 1695.946163 | 1992.71206 | 2154.921233 | 2078.175699 | 1899.887637 |
| **Pcsk1** | 1.831392963 | 0 | 3.132645178 | 6.038265938 | 1.266726489 | 0 | 0 | 1.676148026 | 0 | 6.117313782 | 1.891741398 | 0 | 1.70416863 | 0 | 0.718565672 |
| **Mir682** | 120.8719356 | 68.92679474 | 125.3058071 | 108.6887869 | 117.1722002 | 127.6571306 | 126.2890601 | 97.21658551 | 89.53728059 | 91.75970673 | 141.8806048 | 113.6461819 | 128.6647316 | 107.5230035 | 104.9105881 |
| **Ell2** | 340.6390911 | 302.6440872 | 314.0476791 | 276.8976237 | 255.8787507 | 249.7029588 | 331.0577503 | 449.207671 | 388.9981014 | 350.4346895 | 440.7757456 | 306.2374215 | 254.7732102 | 285.5232699 | 288.1448345 |
| **Glrx** | 445.9441865 | 537.9459038 | 554.4781965 | 626.2544387 | 490.8565143 | 491.6903768 | 454.6406162 | 741.6955016 | 711.0313458 | 683.3913397 | 764.2635246 | 592.5216966 | 593.9027677 | 643.3309119 | 518.0858495 |
| **Rhobtb3** | 0 | 0.792262009 | 0.783161294 | 0.86260942 | 0 | 1.402825611 | 0 | 0 | 2.257242368 | 0.873901969 | 3.783482795 | 0 | 0.852084315 | 0.903554652 | 0 |
| **Spata9** | 0.915696482 | 2.376786026 | 2.349483883 | 5.175656518 | 0 | 2.104238417 | 0.902064715 | 0 | 0.752414123 | 5.243411813 | 4.729353494 | 0.867528106 | 0.852084315 | 0.903554652 | 0.718565672 |
| **Rfesd** | 84.24440763 | 117.2547773 | 82.23193592 | 56.06961228 | 82.33722176 | 75.75258302 | 90.20647148 | 55.31288486 | 66.21244279 | 69.03825554 | 67.15733682 | 66.79966418 | 76.68758836 | 86.74124655 | 86.22788064 |
| **Gpr150** | 9.156964816 | 7.130358077 | 7.04845165 | 18.11479781 | 6.966995687 | 5.611302446 | 9.020647148 | 5.866518091 | 6.771727103 | 8.739019689 | 3.783482795 | 3.470112425 | 0.852084315 | 0.903554652 | 17.9641418 |
| **Arsk** | 77.83420093 | 88.73334495 | 65.00238744 | 73.32180067 | 63.96968767 | 65.93280374 | 44.20117102 | 63.69362499 | 55.67864507 | 44.56900041 | 85.12836289 | 58.99191123 | 50.27297459 | 58.73105235 | 68.98230451 |
| **Ttc37** | 977.9638423 | 960.2215543 | 1047.869812 | 908.3277189 | 955.7451356 | 940.5945725 | 973.3278272 | 1208.502727 | 1182.795001 | 1119.468422 | 1097.210011 | 910.9045516 | 957.7427702 | 987.5852342 | 955.6923437 |
| **Mctp1** | 0 | 0.792262009 | 5.482129061 | 0 | 4.43354271 | 2.104238417 | 10.82477658 | 2.514222039 | 7.524141226 | 0.873901969 | 2.837612096 | 4.337640531 | 1.70416863 | 0 | 4.311394032 |
| **Ankrd32** | 532.9353523 | 552.9988819 | 496.5242067 | 510.6647764 | 503.5237792 | 466.4395158 | 520.4913404 | 539.7196644 | 510.8891892 | 587.2621231 | 583.6022211 | 516.1792232 | 486.540144 | 524.9652526 | 476.4090405 |
| **2210408I21Rik** | 19.22962611 | 41.98988645 | 35.24225825 | 36.22959563 | 29.76807248 | 24.5494482 | 43.29910631 | 22.62799835 | 40.63036262 | 34.08217679 | 33.10547446 | 30.36348372 | 40.90004713 | 9.035546516 | 45.26963733 |
| **Fam172a** | 794.824546 | 860.3965412 | 982.0842633 | 785.8371813 | 900.6425334 | 887.9881612 | 921.0080738 | 906.7960821 | 923.9645425 | 944.6880284 | 886.2808447 | 875.3358592 | 881.9072662 | 922.5292993 | 916.8897974 |
| **Arrdc3** | 277.4560339 | 239.2631266 | 249.8284529 | 213.9271361 | 333.1490665 | 336.6781467 | 311.2123266 | 170.9670987 | 212.9331691 | 194.8801391 | 208.0915537 | 315.7802307 | 310.1586907 | 295.4623711 | 297.4861882 |
| **Adgrv1** | 1.831392963 | 0 | 2.349483883 | 0 | 0 | 2.104238417 | 1.80412943 | 0 | 1.504828245 | 0.873901969 | 2.837612096 | 0 | 0 | 0.903554652 | 0 |
| **Lysmd3** | 231.6712098 | 221.8333624 | 224.7672915 | 169.9340557 | 183.0419776 | 177.4574398 | 184.9232665 | 185.2143569 | 253.5636593 | 206.2408647 | 194.8493639 | 196.9288801 | 258.1815475 | 210.5282338 | 222.0367926 |
| **Polr3g** | 510.0429402 | 483.2798252 | 491.8252929 | 420.0907874 | 435.7539121 | 422.250509 | 443.8158397 | 489.4352236 | 463.4870995 | 498.9980242 | 425.6418144 | 487.5507957 | 501.0255773 | 446.3559979 | 476.4090405 |
| **Mblac2** | 63.18305723 | 93.486917 | 83.01509722 | 66.42092531 | 88.03749095 | 65.93280374 | 62.24246532 | 94.70236348 | 67.71727103 | 103.9943343 | 118.2338373 | 72.87236093 | 92.02510604 | 87.6448012 | 82.63505228 |
| **Cetn3** | 776.5106164 | 791.4697465 | 872.4416821 | 703.8892864 | 845.5399311 | 909.0309962 | 868.6883203 | 948.6997828 | 972.8714605 | 927.209989 | 867.3634308 | 877.0709154 | 725.1237522 | 824.9453969 | 836.4104422 |
| **Mef2c** | 0 | 0 | 2.349483883 | 1.725218839 | 0 | 0.701412806 | 0.902064715 | 0 | 0 | 0 | 0.945870699 | 2.602584319 | 0.852084315 | 1.807109303 | 1.437131344 |
| **Tmem161b** | 170.3195456 | 194.1041921 | 202.055614 | 208.7514796 | 169.7413495 | 197.7984112 | 176.8046841 | 151.6913964 | 171.5504199 | 196.627943 | 181.6071742 | 151.8174186 | 178.9377062 | 185.2287036 | 155.9287508 |
| **Ccnh** | 821.379744 | 854.8570587 | 881.0564563 | 836.7311371 | 862.6407387 | 890.0928504 | 785.6983666 | 878.3015657 | 905.1541895 | 864.2890472 | 814.3946716 | 897.89159 | 829.930123 | 779.7676643 | 756.6496526 |

| | | | | | | | | | | | | | | | |
|---|---|---|---|---|---|---|---|---|---|---|---|---|---|---|---|
| **Rasa1** | 1803.922069 | 1865.77703 | 1885.069236 | 1784.738889 | 1945.691886 | 1961.851618 | 2071.140585 | 1659.386546 | 1739.581451 | 1672.648368 | 1656.219594 | 2007.460038 | 1967.462684 | 2014.023318 | 1958.810022 |
| **Cox7c** | 1928.45679 | 1785.758567 | 1854.525945 | 1535.444767 | 1758.216366 | 1702.328879 | 1720.237411 | 1739.003577 | 1638.757959 | 1712.847859 | 1703.513128 | 1579.768682 | 1491.147552 | 1660.73345 | 1554.976114 |
| **Vcan** | 10.98835778 | 0.792262009 | 5.482129061 | 6.038265938 | 1.900089733 | 4.208476834 | 7.216517718 | 15.08533223 | 6.771727103 | 13.10852953 | 10.40457769 | 3.470112425 | 6.816674521 | 3.614218606 | 4.311394032 |
| **Xrcc4** | 178.5608139 | 181.4279999 | 208.3209043 | 137.1548977 | 152.0071786 | 172.5475502 | 160.5675192 | 139.1202862 | 151.2352386 | 177.4020997 | 205.2539416 | 189.1211272 | 179.7897905 | 149.0865175 | 154.4916195 |
| **Tmem167** | 1207.803659 | 1169.378725 | 1274.986587 | 1212.828844 | 1188.82281 | 1197.311659 | 1189.823359 | 1344.270717 | 1221.920535 | 1162.289619 | 1281.654797 | 1183.308337 | 1091.520008 | 1170.103274 | 1107.3097 |
| **Rps23** | 14687.77156 | 14098.30244 | 14298.95891 | 12860.64384 | 14317.8095 | 15379.87859 | 14821.82533 | 13858.39188 | 12946.78981 | 13262.33628 | 12661.42517 | 14022.72431 | 13078.64215 | 14487.59528 | 13410.59114 |
| **Atg10** | 50.36330649 | 36.44405239 | 54.03812932 | 37.95481446 | 60.80287145 | 41.38335554 | 39.69084745 | 48.60829276 | 51.91657446 | 51.56021617 | 76.6155266 | 24.29078698 | 44.30838439 | 45.17773258 | 23.71266717 |
| **Ssbp2** | 432.2087393 | 484.0720872 | 481.6441961 | 487.3743221 | 427.5201899 | 396.999648 | 408.6353158 | 420.7131546 | 507.8795327 | 427.3380628 | 465.3683838 | 428.5588845 | 451.604687 | 468.9448642 | 450.5406763 |
| **4833422C13Rik** | 0 | 0 | 0.783161294 | 0 | 1.900089733 | 0 | 0 | 0 | 0 | 1.747803938 | 1.891741398 | 0.867528106 | 4.260421576 | 0 | 0 |
| **Acot12** | 0 | 0 | 0 | 1.725218839 | 2.533452977 | 0 | 0 | 0 | 0 | 1.747803938 | 1.891741398 | 2.602584319 | 0.852084315 | 0 | 0 |
| **Zcchc9** | 495.3917965 | 501.5018514 | 463.6314863 | 479.6108373 | 446.5210872 | 516.9412378 | 556.573929 | 547.2623305 | 494.3360785 | 519.9716715 | 564.6848072 | 483.2131552 | 489.0963969 | 505.9906049 | 429.7022718 |
| **Rasgrf2** | 35.71216278 | 29.31369431 | 24.27800013 | 32.77915795 | 34.20161519 | 28.75792503 | 35.18052388 | 31.8468125 | 18.81035306 | 25.3431571 | 34.05134516 | 49.44910206 | 32.37920398 | 30.72085815 | 33.05402091 |
| **Msh3** | 353.4588419 | 365.2327859 | 383.7490343 | 348.4942055 | 356.5835065 | 362.6304206 | 388.7898921 | 386.35212 | 382.9787884 | 438.6987884 | 382.1317623 | 340.0710177 | 394.5150379 | 374.9751804 | 382.2769375 |
| **Dhfr** | 1247.178608 | 1293.76386 | 1252.27491 | 1202.477531 | 1248.992318 | 1144.004286 | 1151.936641 | 1369.412937 | 1323.496442 | 1383.386817 | 1401.780376 | 1261.385867 | 1209.959728 | 1262.265848 | 1168.387783 |
| **Ankrd34b** | 6.409875371 | 4.753572051 | 5.482129061 | 3.450437679 | 3.800179466 | 3.507064029 | 10.82477658 | 5.866518091 | 0 | 0 | 1.891741398 | 3.470112425 | 0.852084315 | 7.228437213 | 2.874262688 |
| **Fam151b** | 10.0726613 | 16.63750218 | 13.31374201 | 12.07653188 | 15.20071786 | 16.13249453 | 10.82477658 | 13.40918421 | 26.33449429 | 18.35194135 | 10.40457769 | 12.14539349 | 14.48543336 | 6.324882561 | 14.37131344 |
| **Zfyve16** | 258.2264078 | 228.1714584 | 228.683098 | 189.7740723 | 208.3765074 | 216.736557 | 187.6294607 | 258.96487 | 241.5249333 | 165.1674721 | 230.7924505 | 199.5314644 | 226.6544278 | 266.5486222 | 211.9768732 |
| **Serinc5** | 398.3279695 | 389.0006462 | 387.6648408 | 375.2350975 | 223.5772252 | 277.0580583 | 332.8618798 | 580.785291 | 476.2781396 | 440.4465923 | 388.7528572 | 266.3311286 | 297.377426 | 263.8379583 | 329.8216434 |
| **Mtx3** | 134.6073828 | 118.8393013 | 165.2470331 | 138.0175071 | 133.6396445 | 112.2260489 | 111.8560246 | 153.3675444 | 112.1097043 | 160.7979623 | 167.4191137 | 119.7188787 | 153.3751767 | 147.2794082 | 125.0304269 |
| **Cmya5** | 19.22962611 | 17.42976419 | 15.66322589 | 15.52696955 | 17.73417084 | 23.84803539 | 20.74748844 | 17.59955427 | 20.31518131 | 15.73023544 | 11.35044839 | 34.70112425 | 27.26669809 | 27.10663955 | 35.20971793 |
| **A630019I02Rik** | 2.747089445 | 0.792262009 | 1.566322589 | 0 | 1.900089733 | 0 | 0.902064715 | 0.838074013 | 0 | 1.747803938 | 0 | 0 | 1.70416863 | 2.710663955 | 0 |
| **Papd4** | 1168.42871 | 1132.14241 | 1266.371813 | 1159.34706 | 1182.489177 | 1162.241019 | 1238.534853 | 1092.010439 | 1056.389428 | 1136.946462 | 1065.050407 | 1124.316426 | 1238.930594 | 1137.575306 | 1197.848975 |
| **Homer1** | 212.4415837 | 194.1041921 | 177.7776138 | 197.5375571 | 159.6075376 | 152.2065788 | 186.727396 | 287.4593865 | 229.4863074 | 229.8362178 | 201.4704588 | 197.7964082 | 195.1273082 | 168.9647198 | 204.7912165 |
| **Jmy** | 426.7145604 | 411.1839824 | 382.965873 | 401.1133801 | 317.9483486 | 350.7064029 | 381.5733744 | 372.9429358 | 329.5573857 | 351.3085915 | 296.0575287 | 390.3876478 | 361.2837496 | 290.0410432 | 344.9115225 |
| **Arsb** | 1120.812493 | 1089.360262 | 939.0103921 | 1059.284367 | 1335.129719 | 1189.596118 | 1523.587303 | 310.9254588 | 461.9822713 | 415.1034352 | 490.9068927 | 1312.570025 | 1251.711859 | 1365.271079 | 1310.663786 |
| **Lhfpl2** | 21.97671556 | 14.26071615 | 11.74741942 | 16.38957897 | 10.13381191 | 9.81977928 | 7.216517718 | 12.5711102 | 7.524141226 | 18.35194135 | 9.458706988 | 7.807752957 | 3.408337261 | 6.324882561 | 7.904222392 |
| **Scamp1** | 101.6423095 | 113.2934672 | 111.9920651 | 107.8261775 | 101.3381191 | 102.4062696 | 88.40234205 | 116.4922878 | 112.1097043 | 117.9767658 | 122.9631908 | 119.7188787 | 115.0313825 | 115.6549954 | 112.8148105 |
| **Gm9776** | 35.71216278 | 42.78214846 | 34.45909696 | 31.91654853 | 31.03479897 | 35.77205309 | 32.47432973 | 43.57984868 | 45.89726148 | 20.97364725 | 61.48159542 | 43.37640531 | 28.97086672 | 52.40616979 | 25.86836419 |
| **Ap3b1** | 2296.566776 | 2014.722288 | 2205.382205 | 2191.890535 | 1948.858703 | 2098.627115 | 2210.960616 | 2185.697026 | 2288.091347 | 2118.338373 | 2158.476935 | 2222.607008 | 2175.371257 | 2137.810306 | 2339.649828 |
| **Tbca** | 1017.338791 | 900.8019037 | 1123.836458 | 929.8929544 | 1132.453481 | 1108.933646 | 988.6629274 | 1076.087033 | 1048.865287 | 1085.386245 | 1012.081648 | 1120.846313 | 946.6656741 | 1025.53453 | 1023.956083 |
| **Wdr41** | 198.7061365 | 251.9393187 | 198.9229688 | 195.8123383 | 215.9768663 | 224.4520978 | 237.24302 | 216.2230954 | 223.4669944 | 241.1969434 | 227.0089677 | 221.2196671 | 201.9439827 | 255.7059664 | 202.6355195 |
| **Pde8b** | 3.662785926 | 7.130358077 | 5.482129061 | 6.900875357 | 5.700269198 | 8.416953669 | 8.118582433 | 3.352296052 | 10.53379772 | 1.747803938 | 2.837612096 | 4.337640531 | 4.260421576 | 8.131991864 | 9.341353736 |
| **Zbed3** | 501.8016719 | 526.0619736 | 483.99368 | 512.3899953 | 424.9867369 | 478.3635335 | 470.8777811 | 558.1572927 | 527.4422999 | 475.4026711 | 506.0408238 | 402.5330413 | 431.1546635 | 369.5538525 | 424.6723121 |
| **Snora47** | 4.578482408 | 1.584524017 | 2.349483883 | 3.450437679 | 4.43354271 | 0 | 0 | 0.838074013 | 4.514484735 | 0 | 1.891741398 | 0.867528106 | 3.408337261 | 4.517773258 | 2.155697016 |
| **Aggf1** | 1525.550338 | 1368.236489 | 1638.373428 | 1397.42726 | 1450.401829 | 1509.440358 | 1542.530662 | 1656.03425 | 1679.388322 | 1663.035447 | 1586.225162 | 1578.901153 | 1532.047599 | 1516.164705 | 1492.460901 |
| **F2rl1** | 300.348446 | 255.9006287 | 256.8769046 | 315.7150476 | 363.5505022 | 376.6586767 | 392.3981509 | 98.05465953 | 133.1772997 | 121.4723737 | 209.9832951 | 368.6994452 | 334.8691359 | 361.4218606 | 382.9955032 |
| **F2r** | 1773.704085 | 1908.559178 | 1862.357558 | 1880.488535 | 2020.428749 | 2131.593517 | 2207.352357 | 1716.375579 | 1799.022167 | 1715.469565 | 1651.49024 | 2010.062622 | 2209.454629 | 2201.962686 | 2055.097822 |
| **Iqgap2** | 1313.108755 | 1225.629327 | 1280.468716 | 1289.601082 | 1372.49815 | 1269.557178 | 1470.365485 | 976.3562252 | 1063.913569 | 1118.59452 | 1156.799865 | 1470.46014 | 1419.572469 | 1612.845053 | 1505.395083 |
| **F2rl2** | 148.34283 | 109.3321572 | 131.5710975 | 144.9183825 | 158.3408111 | 147.998102 | 149.7427427 | 106.4353997 | 102.3283207 | 83.89458901 | 104.9916476 | 144.8771937 | 118.4397198 | 137.340307 | 148.0245284 |
| **Sv2c** | 37.54355574 | 19.80655021 | 21.14535495 | 17.25218839 | 24.06780328 | 16.83390734 | 18.94335901 | 20.95185033 | 4.514484735 | 24.46925513 | 35.94308655 | 23.42325887 | 25.56252945 | 25.29953024 | 14.37131344 |
| **Poc5** | 346.13327 | 447.6280348 | 358.6878729 | 393.3498954 | 380.0179466 | 336.6781467 | 352.7073035 | 455.0741891 | 443.9243323 | 505.115338 | 431.3170386 | 405.1356256 | 337.4253888 | 364.1325246 | 380.8398061 |
| **Ankdd1b** | 9.156964816 | 5.54583406 | 14.8800646 | 12.93914129 | 19.00089733 | 15.43108173 | 2.706194144 | 13.40918421 | 16.5531107 | 3.495607876 | 14.18806048 | 8.675281063 | 17.89377062 | 12.64976512 | 14.37131344 |
| **Polk** | 315.9152861 | 270.9536069 | 342.2414857 | 245.8436846 | 259.6789302 | 233.5704643 | 258.8925731 | 359.5337516 | 279.1456395 | 326.8393364 | 362.2684776 | 273.2713535 | 245.4002828 | 221.3708896 | 236.4081061 |
| **Col4a3bp** | 1379.954598 | 1296.932908 | 1412.822975 | 1324.968069 | 1361.730975 | 1347.414 | 1502.839815 | 1166.599026 | 1206.872253 | 1187.632776 | 1235.307133 | 1494.750927 | 1476.662118 | 1311.057799 | 1402.640192 |
| **Hmgcr** | 7433.624037 | 7116.09736 | 7595.098234 | 7121.703369 | 7568.690769 | 7770.952474 | 8389.201847 | 6350.086797 | 7123.856913 | 6816.435357 | 6973.904662 | 7821.633406 | 7970.396684 | 7621.483486 | 7739.670853 |
| **Gcnt4** | 100.726613 | 90.31786897 | 76.74980686 | 82.81050429 | 48.13560656 | 73.6483446 | 57.73214175 | 72.07436512 | 48.15450385 | 85.64239295 | 125.8008029 | 58.12438312 | 63.05423932 | 71.38081747 | 66.82660749 |
| **Fam169a** | 24.723805 | 10.29940611 | 21.92851625 | 8.626094197 | 15.20071786 | 11.9240177 | 16.23716487 | 14.24725822 | 36.86829201 | 27.964863 | 9.458706988 | 18.21809023 | 18.74585493 | 18.97464768 | 12.21561642 |
| **Nsa2** | 3463.164093 | 3378.997466 | 3316.688082 | 3228.747058 | 3199.117747 | 3352.753211 | 3639.831124 | 3148.644067 | 3092.422044 | 3250.041422 | 3361.624463 | 3321.765119 | 3318.016323 | 3233.822098 | 3298.935 |
| **Gfm2** | 611.6852497 | 560.12924 | 578.7561966 | 586.5744054 | 527.5915825 | 582.1726287 | 634.1514945 | 640.288546 | 613.2175099 | 670.2828101 | 640.3544631 | 576.0386626 | 534.2568656 | 490.6301758 | 534.6128599 |
| **Hexb** | 401.9907554 | 362.0637379 | 468.3304541 | 426.9916627 | 448.4211769 | 427.8618115 | 428.4807395 | 241.3653158 | 252.0587311 | 282.270336 | 303.6244943 | 432.896525 | 414.9650615 | 435.5133421 | 435.4507972 |
| **Enc1** | 370.857075 | 305.8131353 | 337.5425179 | 338.1428925 | 293.2471821 | 349.3035772 | 379.7692449 | 529.6627763 | 442.4195041 | 453.5551219 | 440.7757456 | 275.8739378 | 315.2711966 | 318.0512374 | 345.6300882 |
| **Utp15** | 1693.122794 | 1678.010934 | 1758.980267 | 1741.608418 | 1727.181567 | 1696.016164 | 1859.155377 | 1960.255117 | 1826.109076 | 1909.475802 | 1947.547769 | 1712.500482 | 1759.554111 | 1651.697903 | 1743.958886 |
| **Ankra2** | 158.4154913 | 183.804786 | 178.5607751 | 163.8957897 | 182.4086144 | 197.7984112 | 196.6501078 | 161.7482845 | 187.3511165 | 214.1059824 | 182.5530449 | 169.1679807 | 218.985669 | 233.1171001 | 197.6055598 |
| **Btf3** | 10504.87004 | 10266.92337 | 10380.0198 | 9769.051678 | 10233.8833 | 10554.8599 | 10325.03273 | 10307.47229 | 9992.811962 | 10645.87379 | 10235.26683 | 10298.42615 | 9651.559038 | 10089.99479 | 10018.2426 |
| **Tmem171** | 38.45925223 | 38.02857641 | 21.14535495 | 19.84001665 | 21.53435031 | 16.13249453 | 23.45368258 | 10.05688816 | 3.00965649 | 13.10852953 | 14.18806048 | 21.68820266 | 11.92918041 | 19.87820233 | 19.40127314 |
| **Fcho2** | 373.6041645 | 423.8601745 | 424.4734216 | 388.1742388 | 291.9804556 | 324.0527162 | 349.0990446 | 288.2974605 | 285.9173666 | 361.7954151 | 388.7528572 | 307.9724777 | 443.9359282 | 397.5640467 | 415.3309584 |
| **Tnpo1** | 4627.014321 | 4748.818479 | 4874.395897 | 4759.878778 | 3898.350769 | 3855.666193 | 4414.704714 | 5436.586123 | 5385.780289 | 5228.55548 | 5664.819615 | 4472.974916 | 4609.776145 | 4003.650661 | 4321.453951 |
| **Ptcd2** | 825.9582264 | 857.2274932 | 831.7172947 | 865.1972479 | 815.1384954 | 846.6052565 | 811.8582433 | 920.2052663 | 931.4886838 | 893.1278122 | 920.3321899 | 842.3697912 | 785.6217386 | 759.889462 | 760.9610466 |
| **Mrps27** | 552.1649784 | 528.4387597 | 574.8403902 | 559.8335134 | 527.5915825 | 541.490686 | 535.8264406 | 607.6036595 | 576.3492179 | 669.4089082 | 615.7618249 | 533.5297854 | 547.0381303 | 543.9399002 | 525.2715062 |
| **6430562O15Rik** | 0 | 0 | 0 | 2.587828259 | 2.533452977 | 0.701412806 | 2.706194144 | 0 | 0 | 4.369509845 | 2.837612096 | 0 | 3.408337261 | 1.807109303 | 0 |
| **Map1b** | 0 | 4.753572051 | 0.783161294 | 0.86260942 | 0 | 4.208476834 | 0 | 0.838074013 | 0.752414123 | 2.621705907 | 6.621094891 | 0.867528106 | 0 | 0 | 0 |
| **Mccc2** | 183.1392963 | 198.8577641 | 198.1398075 | 167.3462274 | 188.1088835 | 204.8125393 | 181.3150077 | 216.2230954 | 256.5732158 | 290.1354537 | 239.3052868 | 189.1211272 | 208.7606572 | 177.0967117 | 206.9469135 |
| **Bdp1** | 2060.317084 | 2014.722288 | 1803.620461 | 1699.340557 | 1510.571338 | 1562.747731 | 1879.902866 | 1725.594393 | 1730.552482 | 1624.58376 | 1693.108551 | 1715.970594 | 1722.062401 | 1558.631774 | 1693.659289 |
| **Serf1** | 44.8691276 | 34.06726637 | 27.41064531 | 31.05393911 | 25.96789302 | 44.18900676 | 29.76813559 | 36.03718256 | 41.38277674 | 33.20827482 | 33.10547446 | 44.24393342 | 34.93545692 | 25.29953024 | 22.9941015 |
| **Smn1** | 1094.257295 | 1076.68407 | 1177.874587 | 1043.757398 | 1120.419579 | 958.1298926 | 1050.905393 | 1232.806873 | 1094.010134 | 1123.837932 | 1118.019166 | 976.8366477 | 999.4949017 | 1026.438084 | 1010.303335 |
| **Naip2** | 32.04937685 | 24.56012226 | 49.33916155 | 66.42092531 | 62.06959794 | 51.20313482 | 56.83007703 | 41.90370065 | 45.89726148 | 45.44290238 | 52.02288843 | 57.25685501 | 52.82922754 | 66.86304422 | 71.8565672 |
| **Naip5** | 0.915696482 | 7.130358077 | 6.265290356 | 5.175656518 | 4.43354271 | 5.611302446 | 5.412388289 | 0 | 3.00965649 | 0 | 2.837612096 | 2.602584319 | 1.70416863 | 1.807109303 | 5.029959704 |
| **Gtf2h2** | 530.1882628 | 551.4143579 | 577.9730353 | 509.802167 | 513.6575911 | 566.741547 | 497.0376578 | 591.6802532 | 588.3878439 | 656.3003786 | 565.6306779 | 546.542707 | 506.1380832 | 462.6199816 | 543.9542137 |
| **Ocln** | 8.241268334 | 4.753572051 | 3.915806472 | 7.763484777 | 5.066905954 | 4.90988964 | 2.706194144 | 2.514222039 | 1.504828245 | 13.10852953 | 0.945870699 | 2.602584319 | 10.22501178 | 2.710663955 | 0 |
| **Marveld2** | 130.9445969 | 141.8148995 | 109.6425812 | 126.8035847 | 67.76986714 | 56.81443726 | 63.14453003 | 85.48354933 | 135.4345421 | 154.6806485 | 132.4218978 | 72.87236093 | 75.83550405 | 66.86304422 | 85.50931496 |
| **Rad17** | 501.8016719 | 575.1822182 | 528.6338738 | 502.9012917 | 514.9243176 | 512.732761 | 563.7904467 | 544.7481085 | 551.5195519 | 602.1184566 | 641.3003338 | 468.4651774 | 537.6652029 | 591.8282968 | 472.8162122 |
| **Ak6** | 376.3512539 | 325.6196855 | 359.4710342 | 328.6541889 | 340.7494254 | 284.7735991 | 307.6040677 | 349.4768634 | 376.9594754 | 382.7690624 | 332.946486 | 347.8787706 | 341.6858104 | 315.3405734 | 295.3304912 |
| **Taf9** | 1007.26613 | 1035.486445 | 1087.027877 | 1112.766151 | 1128.653301 | 1080.877134 | 1055.415716 | 1289.795906 | 1274.589524 | 1312.600757 | 1342.190522 | 1131.256651 | 1207.403475 | 1222.509444 | 1210.783157 |
| **Ccdc125** | 119.0405426 | 111.7089432 | 159.7649041 | 110.4140057 | 125.4059224 | 129.0599563 | 128.0931895 | 127.38725 | 111.3572901 | 97.87702052 | 114.4503546 | 123.1889911 | 108.214708 | 130.1118698 | 133.653215 |
| **Cdk7** | 695.929326 | 689.2679474 | 698.5798747 | 642.6440176 | 674.5318552 | 660.0294502 | 668.4299537 | 741.6955016 | 672.6582256 | 857.2978315 | 788.8561628 | 649.7785516 | 712.3424875 | 705.6761829 | 730.7812884 |
| **Mrps36** | 523.7783875 | 461.0964889 | 482.4273574 | 468.3969149 | 478.1892494 | 476.9607079 | 487.114946 | 441.6650049 | 389.7505155 | 466.6636514 | 454.0179354 | 454.5847277 | 422.6338203 | 421.9600223 | 406.7081703 |
| **Cenph** | 362.6158067 | 404.0536243 | 457.366196 | 388.1742388 | 417.386378 | 411.729317 | 367.1403389 | 487.7590756 | 391.2553437 | 478.898279 | 363.2143483 | 438.9692218 | 443.9359282 | 461.716427 | 421.0794838 |
| **Ccnb1** | 3450.344343 | 3713.332034 | 3986.290989 | 3792.893618 | 3389.760083 | 3313.474094 | 3403.490169 | 4407.431235 | 4019.396243 | 4120.447783 | 4274.389688 | 3748.588947 | 3636.695857 | 3863.59969 | 3536.061672 |
| **Slc30a5** | 1297.541914 | 1305.64779 | 1372.881749 | 1315.479365 | 1295.227835 | 1318.656075 | 1335.957843 | 1308.233534 | 1328.010926 | 1358.917562 | 1438.669333 | 1350.741261 | 1324.99111 | 1264.072958 | 1371.741868 |
| **Pik3r1** | 1291.132039 | 1331.792436 | 1393.243943 | 1468.161232 | 1293.961108 | 1237.292189 | 1401.808567 | 1380.3079 | 1246.750201 | 1242.6886 | 1459.478488 | 1355.078902 | 1301.132749 | 1504.418495 | 1293.41821 |
| **Mast4** | 39.37494871 | 27.7291703 | 32.89277437 | 33.64176737 | 19.00089733 | 25.95227381 | 23.45368258 | 56.98903289 | 25.58208017 | 28.83876497 | 49.18527634 | 19.95314644 | 17.0416863 | 27.10663955 | 15.08987911 |
| **Srek1** | 970.6382705 | 764.5328382 | 939.0103921 | 887.6250928 | 892.4088112 | 857.8278614 | 959.7968565 | 937.8048206 | 967.6045616 | 941.1924205 | 942.087216 | 923.9174332 | 844.4155563 | 914.3973074 | 926.2311512 |
| **Erbb2ip** | 2651.857011 | 2654.86999 | 2871.069306 | 2739.647517 | 2484.050644 | 2537.010118 | 2869.467858 | 2945.830156 | 2639.468742 | 2719.582927 | 2819.640553 | 2790.837918 | 2838.292854 | 2620.30849 | 2748.513695 |
| **Nln** | 513.7057262 | 504.6708994 | 509.0548414 | 483.9238844 | 514.2909544 | 469.9465798 | 450.1302927 | 477.7021874 | 489.0691797 | 568.9101818 | 547.6591346 | 442.4393342 | 443.0838439 | 437.3204514 | 472.8162122 |
| **Sgtb** | 3.662785926 | 7.130358077 | 8.614774239 | 13.80175071 | 5.700269198 | 2.805651223 | 2.706194144 | 2.514222039 | 9.028969471 | 4.369509845 | 1.891741398 | 4.337640531 | 8.520843152 | 9.939101167 | 1.437131344 |
| **Trappc13** | 729.8100958 | 812.0685587 | 769.0643912 | 727.1797408 | 713.1670131 | 687.3845496 | 744.2033897 | 926.0717844 | 942.0224815 | 968.2833815 | 897.6312931 | 766.894846 | 764.3196307 | 782.4783283 | 732.9369854 |
| **Trim23** | 281.1188198 | 279.668489 | 304.6497436 | 256.1949976 | 227.3774047 | 225.1535106 | 250.7739907 | 263.9933141 | 268.6118418 | 332.0827482 | 240.2511575 | 294.092028 | 264.998222 | 258.4166304 | 228.5038837 |
| **Ppwd1** | 276.5403374 | 285.2143231 | 267.0580014 | 273.447186 | 286.2801864 | 273.5509942 | 300.38755 | 336.0676792 | 290.4318513 | 276.1530222 | 301.7327529 | 298.4296686 | 252.2169573 | 294.5588164 | 245.0308941 |
| **Cenpk** | 226.1770309 | 200.4422882 | 245.9126465 | 210.4766984 | 231.1775842 | 231.4662259 | 234.5368258 | 290.8116825 | 261.8401147 | 335.5783561 | 285.652951 | 244.642926 | 272.6669809 | 227.6957722 | 247.9051568 |
| **Adamts6** | 154.7527054 | 176.6744279 | 186.3923881 | 113.8644434 | 148.8403624 | 159.2207069 | 186.727396 | 175.1574687 | 122.643502 | 111.859452 | 122.9631908 | 154.4200029 | 152.5230924 | 163.5433919 | 192.5756001 |
| **Cwc27** | 676.6996999 | 554.583406 | 605.3836806 | 496.8630257 | 583.9609112 | 554.8175293 | 588.146194 | 599.2229193 | 601.9312981 | 616.97479 | 606.3031179 | 523.9869762 | 516.363095 | 585.5034142 | 602.1580331 |
| **Srek1ip1** | 271.0461585 | 314.5280174 | 288.9865177 | 280.3480614 | 278.0464642 | 283.3707735 | 318.4288443 | 317.6300509 | 306.984962 | 298.0005714 | 296.0575287 | 347.8787706 | 307.6024378 | 278.2948327 | 294.6119255 |
| **Fam159b** | 3.662785926 | 11.88393013 | 1.566322589 | 0 | 1.266726489 | 0.701412806 | 0 | 5.866518091 | 4.514484735 | 6.117313782 | 0.945870699 | 0 | 0 | 0.903554652 | 2.155697016 |
| **Ipo11** | 1312.193058 | 1248.604925 | 1271.853942 | 1197.301874 | 1164.755006 | 1076.668657 | 1199.746071 | 1578.093367 | 1400.995096 | 1563.410622 | 1530.418791 | 1180.705753 | 1233.818088 | 1135.768197 | 1139.645156 |
| **Dimt1** | 485.3191352 | 441.2899387 | 489.4758091 | 389.0368483 | 423.7200104 | 454.5154981 | 466.3674575 | 568.2141809 | 507.8795327 | 524.3411813 | 545.7673932 | 445.0419185 | 463.5338674 | 414.7315851 | 443.3550196 |
| **Kif2a** | 1247.178608 | 1285.84124 | 1300.83091 | 1308.57849 | 1290.794292 | 1385.991704 | 1491.112974 | 1191.741247 | 1221.920535 | 1194.623991 | 1225.848426 | 1421.011038 | 1342.884881 | 1350.814204 | 1519.766396 |
| **3830408C21Rik** | 2.747089445 | 3.169048034 | 4.698967767 | 15.52696955 | 10.76717515 | 4.90988964 | 10.82477658 | 9.218814144 | 6.771727103 | 7.86511772 | 10.40457769 | 13.8804497 | 4.260421576 | 15.36042908 | 2.874262688 |
| **Zswim6** | 304.0112319 | 232.9250305 | 278.8054208 | 280.3480614 | 281.2132805 | 258.1199125 | 291.3669029 | 260.6410181 | 249.0490746 | 231.5840218 | 243.0887696 | 251.5831508 | 247.9565357 | 222.2744443 | 270.1806927 |
| **Smim15** | 760.0280797 | 762.1560522 | 855.2121336 | 747.0197574 | 850.6068371 | 742.0947484 | 833.5077965 | 762.6473519 | 806.5879394 | 744.5644775 | 873.038655 | 865.7930501 | 835.0426289 | 779.7676643 | 799.0450272 |
| **Ndufaf2** | 231.6712098 | 244.0166986 | 223.2009689 | 230.316715 | 243.844849 | 226.5563362 | 195.7480431 | 274.8882763 | 237.7628627 | 284.0181399 | 289.4364338 | 190.8561834 | 187.4585493 | 233.1171001 | 199.7612568 |
| **Ercc8** | 241.7438711 | 273.3303929 | 285.0707112 | 248.4315129 | 226.1106782 | 274.9538198 | 284.1503852 | 258.96487 | 300.965649 | 286.6398458 | 341.4593223 | 266.3311286 | 233.4711024 | 268.3557315 | 304.6718449 |
| **Elovl7** | 20.14532259 | 35.65179038 | 28.1938066 | 17.25218839 | 27.86798275 | 28.05651223 | 29.76813559 | 19.2757023 | 13.54345421 | 16.60413741 | 19.86328467 | 27.7608994 | 26.41461377 | 28.91374885 | 13.65274777 |
| **Depdc1b** | 230.7555134 | 342.2571877 | 366.5194858 | 369.1968316 | 361.6504125 | 347.9007516 | 311.2123266 | 278.2405723 | 366.4256777 | 277.9008261 | 309.2997185 | 418.1485472 | 362.1358339 | 379.4929537 | 379.4026748 |
| **Pde4d** | 1556.684019 | 1625.721641 | 1695.544203 | 1588.926551 | 946.2446869 | 1035.285301 | 1076.163205 | 983.0608173 | 1237.721232 | 1216.471541 | 1222.064943 | 1064.456986 | 1025.057431 | 1065.290934 | 1117.36962 |
| **Plk2** | 948.6615549 | 786.7161744 | 811.3551011 | 667.6596908 | 514.2909544 | 537.2822092 | 594.460647 | 1001.498446 | 823.1410501 | 674.65232 | 582.6563504 | 428.5588845 | 525.7360225 | 501.4728316 | 506.5887987 |
| **Actbl2** | 0.915696482 | 7.130358077 | 1.566322589 | 0 | 0 | 1.402825611 | 1.80412943 | 5.866518091 | 4.514484735 | 0.873901969 | 2.837612096 | 0 | 1.70416863 | 0 | 0 |
| **Gpbp1** | 3018.135603 | 2661.208087 | 2833.477563 | 2446.360314 | 2360.544811 | 2422.679831 | 2815.343975 | 2431.252712 | 2752.33086 | 2682.005143 | 2688.164526 | 2531.447014 | 2495.754959 | 2379.059398 | 2453.183204 |
| **Mier3** | 245.4066571 | 240.8476506 | 234.9483883 | 188.0488535 | 240.6780328 | 253.9114357 | 283.2483204 | 263.9933141 | 240.7725192 | 206.2408647 | 215.6585193 | 235.1001168 | 298.2295103 | 215.9495617 | 283.1148748 |
| **Map3k1** | 1180.332765 | 1103.620978 | 1079.979425 | 1061.872196 | 994.3802935 | 996.0061841 | 1117.658182 | 603.4132894 | 671.9058115 | 633.5789275 | 631.8416268 | 982.9093444 | 1006.311576 | 960.4785946 | 954.973778 |
| **Gm15326** | 8.241268334 | 7.922620085 | 7.831612945 | 12.07653188 | 7.600358931 | 1.402825611 | 7.216517718 | 6.704592104 | 0 | 4.369509845 | 5.675224193 | 9.542809169 | 5.112505891 | 9.939101167 | 12.9341821 |
| **Ankrd55** | 3.662785926 | 3.961310043 | 4.698967767 | 6.900875357 | 4.43354271 | 7.014128057 | 10.82477658 | 7.542666117 | 5.266898858 | 8.739019689 | 4.729353494 | 12.14539349 | 9.372927467 | 8.131991864 | 2.874262688 |
| **Il6st** | 469.752295 | 537.1536418 | 545.080261 | 464.9464772 | 684.6656671 | 697.2043289 | 773.0694606 | 415.6847105 | 484.5546949 | 389.7602781 | 445.5050991 | 727.8560812 | 796.6988347 | 740.0112596 | 744.4340362 |
| **Ddx4** | 0 | 1.584524017 | 4.698967767 | 0.86260942 | 1.266726489 | 2.104238417 | 1.80412943 | 0 | 2.257242368 | 2.621705907 | 0 | 2.602584319 | 1.70416863 | 0 | 0.718565672 |
| **Slc38a9** | 89.73825519 | 85.56429692 | 126.0889684 | 129.3914129 | 111.471931 | 74.34975741 | 88.40234205 | 105.5973256 | 112.1097043 | 62.92094176 | 122.9631908 | 111.0435976 | 125.2563943 | 117.4621047 | 90.53927467 |
| **Ppap2a** | 43.03773463 | 64.17322269 | 58.73709709 | 45.71829924 | 45.60215359 | 60.32150129 | 37.88671802 | 53.63673684 | 51.16416034 | 76.90337326 | 63.37333682 | 53.78674259 | 59.64590206 | 42.46706862 | 52.45529405 |
| **Skiv2l2** | 2678.412209 | 2725.381309 | 2842.875499 | 2859.550226 | 2680.39325 | 2691.320936 | 2798.204745 | 2987.733857 | 2729.758437 | 2898.732831 | 3046.649521 | 2745.726456 | 2658.503063 | 2541.699235 | 2754.980786 |
| **Dhx29** | 1078.690455 | 1080.64538 | 1099.558457 | 1153.308794 | 949.4115032 | 1077.37007 | 1114.049923 | 1329.185385 | 1171.508789 | 1148.307187 | 1203.147529 | 1100.025639 | 1038.69078 | 1052.641169 | 1072.099983 |
| **Ccno** | 9.156964816 | 4.753572051 | 10.18109683 | 9.488703616 | 12.66726489 | 7.715540863 | 4.510323574 | 25.14222039 | 7.524141226 | 12.23462756 | 13.24218978 | 12.14539349 | 11.92918041 | 2.710663955 | 8.622788064 |
| **Mcidas** | 0 | 0 | 2.349483883 | 0 | 0 | 0.701412806 | 0 | 0 | 0 | 0 | 0 | 1.735056213 | 0 | 0 | 0 |
| **Cdc20b** | 2.747089445 | 1.584524017 | 0.783161294 | 1.725218839 | 0 | 0.701412806 | 0 | 0.838074013 | 3.762070613 | 0 | 0 | 0.867528106 | 3.408337261 | 1.807109303 | 0.718565672 |
| **Gzma** | 27.47089445 | 24.56012226 | 18.79587107 | 35.36698621 | 19.63426057 | 27.35509942 | 41.49497688 | 5.866518091 | 10.53379772 | 13.10852953 | 16.07980188 | 19.08561834 | 15.33751767 | 34.33507676 | 38.08398061 |
| **Gzmk** | 6.409875371 | 10.29940611 | 8.614774239 | 23.29045433 | 12.03390164 | 9.118366474 | 15.33510015 | 2.514222039 | 6.771727103 | 5.243411813 | 7.56696559 | 24.29078698 | 5.964590206 | 7.228437213 | 25.86836419 |
| **Esm1** | 0.915696482 | 1.584524017 | 3.132645178 | 6.900875357 | 0.633363244 | 1.402825611 | 0 | 7.542666117 | 3.762070613 | 2.621705907 | 5.675224193 | 0 | 0 | 0 | 2.874262688 |
| **Snx18** | 745.376936 | 697.1905675 | 688.3987779 | 656.4457684 | 594.0947231 | 633.3757636 | 643.1721416 | 599.2229193 | 547.0050671 | 585.5143192 | 573.1976435 | 619.4150679 | 590.4944304 | 494.2443944 | 515.2115868 |
| **Arl15** | 78.74989741 | 119.6315633 | 158.9817428 | 101.7879115 | 110.2052045 | 101.7048568 | 102.8353775 | 112.3019177 | 121.1386737 | 79.52507917 | 135.2595099 | 127.5266316 | 112.4751296 | 129.2083152 | 127.9046896 |
| **Ndufs4** | 809.4756897 | 731.2578338 | 774.5465202 | 761.6841176 | 749.268718 | 784.1795168 | 769.4612017 | 823.8267548 | 742.632739 | 807.4854193 | 773.7222316 | 761.6896773 | 699.5612227 | 671.3411061 | 792.5779362 |
| **Mocs2** | 287.5286952 | 274.914917 | 296.8181306 | 246.706294 | 272.9795583 | 324.754129 | 270.6194144 | 403.9516743 | 294.9463361 | 362.6693171 | 336.7299688 | 291.4894437 | 337.4253888 | 316.2441281 | 292.4562285 |
| **Itga2** | 3.662785926 | 3.169048034 | 0 | 3.450437679 | 8.86708542 | 4.90988964 | 11.72684129 | 0.838074013 | 0 | 0.873901969 | 12.29631908 | 3.470112425 | 11.0770961 | 11.74621047 | 6.467091048 |
| **Pelo** | 605.2753743 | 489.6179213 | 504.3558736 | 549.4822003 | 533.9252149 | 505.7186329 | 530.4140523 | 511.225148 | 510.1367751 | 457.0507297 | 514.5536601 | 491.0209082 | 461.8296988 | 527.6759165 | 460.6005957 |
| **Parp8** | 405.6535413 | 392.9619562 | 380.6163891 | 401.1133801 | 361.0170492 | 402.6109505 | 395.1043451 | 305.0589408 | 388.9981014 | 288.3876497 | 273.3566319 | 360.8916922 | 375.769183 | 440.0311153 | 366.4684927 |
| **Emb** | 3533.672722 | 3341.761152 | 3563.38389 | 3366.764565 | 2724.728677 | 2882.105219 | 2981.323882 | 2795.814908 | 3184.216567 | 3161.777323 | 3435.402378 | 3191.635903 | 3128.001521 | 3150.69507 | 2880.729779 |
| **Mrps30** | 1341.495345 | 1220.083493 | 1401.075556 | 1325.830678 | 1232.524873 | 1439.299077 | 1352.195007 | 1324.995015 | 1438.615802 | 1363.287071 | 1259.899771 | 1265.723507 | 1222.740992 | 1205.341905 | 1230.902996 |
| **Nnt** | 383.6768258 | 396.9232663 | 395.4964537 | 372.6472693 | 378.7512201 | 385.7770431 | 448.3261632 | 543.0719605 | 507.1271186 | 498.1241223 | 459.6931596 | 387.7850635 | 434.5630007 | 389.4320548 | 397.3668166 |
| **Paip1** | 631.8305723 | 616.3798426 | 694.6640682 | 616.7657351 | 614.9957102 | 627.7644611 | 655.8010476 | 762.6473519 | 740.3754966 | 593.3794369 | 576.9811262 | 569.0984377 | 570.8964912 | 532.1936898 | 536.768557 |
| **4833420G17Rik** | 432.2087393 | 496.7482793 | 538.0318093 | 483.9238844 | 487.6896981 | 447.50137 | 474.48604 | 378.8094539 | 438.6574335 | 541.8192207 | 448.3427112 | 523.1194481 | 485.6880596 | 522.2545886 | 493.6546166 |
| **3110070M22Rik** | 10.98835778 | 6.338096068 | 6.265290356 | 1.725218839 | 5.066905954 | 2.104238417 | 5.412388289 | 7.542666117 | 12.79104008 | 11.3607256 | 12.29631908 | 6.94022485 | 5.112505891 | 1.807109303 | 5.748525376 |
| **Gm7120** | 92.48534464 | 100.6172751 | 111.9920651 | 115.5896622 | 40.53524763 | 46.99465798 | 40.59291217 | 108.9496217 | 130.1676432 | 109.2377461 | 107.8292597 | 33.83359614 | 38.34379418 | 30.72085815 | 37.36541494 |
| **LOC100861615** | 0 | 0 | 0.783161294 | 0.86260942 | 0 | 2.805651223 | 0 | 0 | 0 | 0 | 2.837612096 | 0.867528106 | 0 | 0.903554652 | 0 |
| **Gm3636** | 19.22962611 | 11.88393013 | 25.84432272 | 16.38957897 | 23.43444004 | 20.34097137 | 12.62890601 | 19.2757023 | 21.06759543 | 20.97364725 | 16.07980188 | 19.95314644 | 23.00627651 | 28.0101942 | 28.02406121 |
| **Gm3558** | 0 | 0 | 0 | 0 | 0 | 0 | 3.608258859 | 0 | 0 | 0 | 0.945870699 | 1.735056213 | 0 | 0 | 0.718565672 |
| **Flnb** | 1510.899195 | 1380.120419 | 1493.488589 | 1682.088368 | 1282.56057 | 1303.926406 | 1560.571957 | 2144.631399 | 1437.863388 | 1816.842193 | 2201.041116 | 1421.011038 | 1456.212095 | 1201.727687 | 1421.322899 |
| **Dnase1l3** | 4.578482408 | 5.54583406 | 3.915806472 | 6.900875357 | 0 | 4.90988964 | 4.510323574 | 7.542666117 | 0.752414123 | 3.495607876 | 2.837612096 | 0.867528106 | 6.816674521 | 0 | 0.718565672 |
| **Abhd6** | 74.17141501 | 65.75774671 | 63.43606485 | 100.0626927 | 91.83767042 | 105.9133337 | 104.6395069 | 95.54043749 | 80.50831112 | 68.16435357 | 88.91184568 | 93.69303548 | 67.3146609 | 108.4265582 | 79.04222392 |
| **Rpp14** | 701.4235049 | 747.103074 | 801.1740043 | 748.7449763 | 757.5024402 | 742.7961612 | 739.6930661 | 883.3300098 | 860.7617562 | 874.7758709 | 852.2294996 | 737.3988903 | 719.159162 | 665.0162236 | 768.1467033 |
| **Pxk** | 613.5166426 | 604.4959125 | 615.5647775 | 635.7431423 | 573.1937361 | 620.0489202 | 672.9402772 | 452.559967 | 516.1560881 | 557.5494562 | 550.4967467 | 589.0515842 | 643.3236579 | 665.0162236 | 617.9664779 |
| **Pdhb** | 1957.759078 | 1914.105013 | 1841.212203 | 1830.457188 | 1783.550896 | 1786.498416 | 1758.124129 | 2086.804293 | 1879.530478 | 1933.945057 | 2051.593546 | 1968.421273 | 1918.041793 | 2025.769529 | 1869.707878 |
| **Kctd6** | 56.77318186 | 52.28929256 | 66.56871003 | 62.97048763 | 58.26941847 | 60.32150129 | 58.63420646 | 45.2559967 | 45.14484735 | 44.56900041 | 52.96875913 | 79.81258578 | 82.65217857 | 72.28437213 | 79.76078959 |
| **4930452B06Rik** | 9.156964816 | 0 | 3.915806472 | 5.175656518 | 0 | 0 | 3.608258859 | 2.514222039 | 0 | 7.86511772 | 4.729353494 | 6.072696744 | 3.408337261 | 0.903554652 | 5.029959704 |
| **Fhit** | 12.81975074 | 7.922620085 | 14.0969033 | 16.38957897 | 13.30062813 | 12.6254305 | 9.020647148 | 13.40918421 | 24.07725192 | 10.48682363 | 16.07980188 | 7.807752957 | 11.0770961 | 3.614218606 | 9.341353736 |
| **Mnd1-ps** | 1.831392963 | 0 | 0.783161294 | 0 | 0 | 0.701412806 | 0 | 0 | 0 | 0.873901969 | 0.945870699 | 0.867528106 | 0.852084315 | 0 | 0.718565672 |
| **3830406C13Rik** | 248.1537465 | 246.3934846 | 258.4432272 | 251.8819505 | 260.9456566 | 272.1481686 | 275.129738 | 266.5075362 | 220.4573379 | 208.8625706 | 195.7952346 | 274.1388816 | 256.4773789 | 262.9344036 | 296.7676225 |
| **Thoc7** | 1114.402618 | 1062.423353 | 1101.907941 | 1019.604334 | 1092.551596 | 1053.522034 | 1093.302434 | 1085.305847 | 1063.161155 | 1168.406932 | 1069.77976 | 1078.337436 | 1047.211623 | 1145.707298 | 1123.836711 |
| **Atxn7** | 436.7872217 | 466.642323 | 424.4734216 | 338.1428925 | 299.5808145 | 378.7629151 | 363.5320801 | 354.5053075 | 429.628464 | 410.7339254 | 433.20878 | 424.221244 | 442.2317596 | 390.3356095 | 423.2351808 |
| **Psmd6** | 2349.677172 | 2326.081257 | 2412.919948 | 2320.419339 | 2321.909654 | 2200.331972 | 2347.172388 | 2607.248255 | 2574.761127 | 2618.210299 | 2519.799542 | 2334.518134 | 2181.335847 | 2325.749673 | 2239.050634 |
| **Il3ra** | 345.2175735 | 341.4649257 | 298.3844532 | 284.6611085 | 273.6129215 | 308.6216345 | 303.0937442 | 168.4528766 | 164.0262787 | 222.8450021 | 218.4961314 | 261.9934881 | 249.6607043 | 237.6348734 | 258.6836419 |
| **Slc4a7** | 1211.466445 | 1051.331685 | 975.8189729 | 978.1990819 | 678.9653979 | 798.9091857 | 922.8122032 | 1118.828807 | 964.5949051 | 845.0632039 | 910.8734829 | 766.894846 | 821.4092798 | 694.8335271 | 811.9792093 |

| | | | | | | | | | | | | | | | |
|---|---|---|---|---|---|---|---|---|---|---|---|---|---|---|---|
| **Oxsm** | 216.1043696 | 219.4565764 | 210.6703882 | 233.7671527 | 223.5772252 | 216.736557 | 207.4748844 | 234.6607237 | 237.7628627 | 233.3318257 | 216.60439 | 275.8739378 | 208.7606572 | 235.8277641 | 286.7077031 |
| **Ngly1** | 748.1240254 | 677.3840173 | 848.1636819 | 740.1188821 | 782.2036067 | 812.9374418 | 849.7449613 | 666.2688404 | 774.9865463 | 700.8693791 | 708.4571534 | 835.4295663 | 777.9529797 | 774.3463364 | 763.8353093 |
| **Top2b** | 4078.512129 | 4176.805309 | 4411.547572 | 4321.673192 | 4373.373202 | 4486.937718 | 4667.282834 | 4583.426777 | 4642.395136 | 4379.122766 | 4367.085016 | 4665.566156 | 4909.709824 | 4607.225168 | 4450.795772 |
| **Rarb** | 3.662785926 | 0 | 3.132645178 | 1.725218839 | 2.533452977 | 1.402825611 | 2.706194144 | 0 | 0.752414123 | 0 | 1.891741398 | 0 | 0 | 0.903554652 | 0 |
| **Thrb** | 83.32837982 | 119.6315633 | 71.2676778 | 84.53572313 | 61.43623469 | 62.42573971 | 82.08788905 | 93.02621545 | 79.75589699 | 86.51629492 | 65.26507822 | 74.60741714 | 73.2792511 | 50.59906049 | 70.41943585 |
| **Nr1d2** | 230.7555134 | 179.0512139 | 221.6346463 | 203.575823 | 209.0098706 | 227.2577491 | 227.3203081 | 207.0042812 | 205.4090555 | 196.627943 | 201.4704588 | 217.7495547 | 237.7315239 | 221.3708896 | 220.5996613 |
| **Rpl15** | 16480.70528 | 14579.99774 | 15791.66434 | 14766.14805 | 15218.45203 | 15085.98663 | 15666.1579 | 14430.79643 | 14292.10626 | 14361.70496 | 14292.10626 | 14495.52713 | 14424.08329 | 15176.10393 | 14973.47147 |
| **Nkiras1** | 91.56964816 | 58.62738863 | 61.08658097 | 72.45919125 | 77.2703158 | 63.82856532 | 55.0259476 | 79.61703124 | 60.19312981 | 58.55143192 | 74.7237852 | 89.35539495 | 72.42716679 | 74.99503608 | 70.41943585 |
| **Ube2e1** | 432.2087393 | 476.9417291 | 511.4043253 | 438.2055852 | 445.2543607 | 470.6479926 | 480.800493 | 457.5884111 | 436.4001911 | 451.8073179 | 447.3968405 | 442.4393342 | 407.2963026 | 445.4524432 | 456.2892017 |
| **Ube2e2** | 10.0726613 | 11.09166812 | 10.18109683 | 12.07653188 | 5.066905954 | 9.118366474 | 19.84542373 | 27.65644243 | 15.04828245 | 20.97364725 | 15.13393118 | 9.542809169 | 5.964590206 | 14.45687443 | 11.49705075 |
| **Nid2** | 12.81975074 | 0 | 7.831612945 | 6.038265938 | 2.533452977 | 14.02825611 | 2.706194144 | 4.190370065 | 0 | 6.991215751 | 2.837612096 | 9.542809169 | 1.70416863 | 1.807109303 | 5.748525376 |
| **2700060E02Rik** | 2075.883924 | 2072.557414 | 2042.484656 | 2103.904375 | 2080.598257 | 2086.001684 | 2109.929368 | 2171.449768 | 2209.087864 | 2215.341491 | 2085.644891 | 2079.464871 | 2030.516923 | 2184.795148 | 1971.025638 |
| **Gng2** | 1813.99473 | 1742.184157 | 1742.53388 | 1655.347476 | 1523.238602 | 1524.87144 | 1663.407334 | 1614.130549 | 1692.931776 | 1551.175995 | 1415.968436 | 1618.807446 | 1594.249754 | 1759.220907 | 1645.515389 |
| **Saysd1** | 183.1392963 | 194.1041921 | 214.5861947 | 152.6818673 | 202.6762382 | 185.1729807 | 195.7480431 | 137.4441381 | 143.7110974 | 166.0413741 | 142.8264755 | 157.8901153 | 164.4522728 | 198.7820233 | 191.8570344 |
| **Kcnk5** | 1119.896797 | 1138.480506 | 1164.560845 | 1065.322633 | 1102.052045 | 1100.516692 | 1214.179106 | 726.6101693 | 878.8196952 | 996.2482445 | 906.1441294 | 1060.986874 | 1197.178463 | 1198.113468 | 1063.477195 |
| **Nudt13** | 80.58129038 | 115.6702532 | 80.66561333 | 75.04701951 | 85.50403798 | 75.75258302 | 119.0725424 | 113.9780658 | 64.70761454 | 111.859452 | 92.69532848 | 82.4151701 | 97.98969624 | 106.6194489 | 92.69497168 |
| **Ecd** | 1808.500551 | 1850.724052 | 1852.176461 | 1757.135388 | 1667.012059 | 1724.072676 | 1861.861571 | 2158.878658 | 1990.135354 | 2032.69598 | 1967.411053 | 1676.931829 | 1575.503899 | 1721.271611 | 1669.228056 |
| **Fam149b** | 282.0345163 | 346.2184977 | 361.0373568 | 346.7689867 | 304.6477205 | 373.1516126 | 347.2949152 | 377.9713799 | 356.6442941 | 375.7778466 | 367.9437018 | 290.6219156 | 412.4088085 | 368.6502978 | 331.9773404 |
| **Dnajc9** | 2111.596086 | 2288.844943 | 2420.751561 | 2349.748059 | 2309.875752 | 2378.490824 | 2390.471494 | 2615.628995 | 2503.281786 | 2590.245436 | 2448.859239 | 2432.54881 | 2419.919455 | 2617.597826 | 2341.805525 |
| **Mrps16** | 918.443571 | 895.2560696 | 946.842005 | 908.3277189 | 936.7442383 | 961.6369566 | 863.275932 | 965.461263 | 933.7459261 | 866.0368512 | 879.6597499 | 897.89159 | 823.9655328 | 1013.788319 | 901.7999183 |
| **Anxa7** | 1618.035683 | 1685.933554 | 1598.432202 | 1657.072695 | 1618.876452 | 1568.359034 | 1603.871063 | 1598.207143 | 1495.799276 | 1660.413741 | 1743.239698 | 1594.516659 | 1582.320573 | 1595.677515 | 1720.246219 |
| **Mss51** | 5.494178889 | 17.42976419 | 13.31374201 | 27.60350143 | 19.63426057 | 16.83390734 | 14.43303544 | 16.76148026 | 17.30552482 | 8.739019689 | 26.48437957 | 19.08561834 | 11.92918041 | 14.45687443 | 15.80844478 |
| **Ppp3cb** | 2169.284965 | 2245.270532 | 2216.346463 | 2250.547976 | 2150.901578 | 2170.872634 | 2383.254976 | 2194.91584 | 2244.451328 | 2137.564216 | 2323.058436 | 2188.773412 | 2217.975472 | 2221.840888 | 2285.038837 |
| **1810062O18Rik** | 0 | 0 | 9.397935534 | 0.86260942 | 0 | 0 | 2.706194144 | 7.542666117 | 3.00965649 | 0.873901969 | 0.945870699 | 1.735056213 | 0 | 2.710663955 | 0 |
| **Usp54** | 108.9678813 | 89.52560696 | 102.5941296 | 86.26094197 | 73.47013634 | 99.60061841 | 67.65485361 | 92.18814144 | 139.1966127 | 110.9855501 | 124.8549322 | 89.35539495 | 77.53967268 | 103.9087849 | 91.25784034 |
| **Synpo2l** | 4.578482408 | 14.26071615 | 6.265290356 | 10.35131304 | 15.20071786 | 9.118366474 | 13.53097072 | 4.190370065 | 18.05793894 | 11.3607256 | 12.29631908 | 9.542809169 | 10.22501178 | 15.36042908 | 4.311394032 |
| **Sec24c** | 3540.998294 | 3431.286759 | 3405.96847 | 3571.202997 | 3224.452277 | 3293.133123 | 3371.917904 | 3866.035422 | 3563.433285 | 3489.490562 | 3890.366184 | 3303.547029 | 3414.301851 | 3090.156908 | 3273.785201 |
| **Fut11** | 209.6944943 | 279.668489 | 230.2494206 | 222.5532303 | 247.6450285 | 251.8071973 | 224.614114 | 191.080875 | 181.3318035 | 251.683767 | 235.521804 | 275.8739378 | 251.364873 | 309.0156908 | 240.0009344 |
| **6230400D17Rik** | 5.494178889 | 0.792262009 | 2.349483883 | 0 | 5.066905954 | 7.715540863 | 1.80412943 | 6.704592104 | 1.504828245 | 6.991215751 | 4.729353494 | 3.470112425 | 1.70416863 | 0.903554652 | 1.437131344 |
| **Chchd1** | 572.310301 | 554.583406 | 570.9245837 | 556.3830757 | 541.5255739 | 575.1585007 | 458.2488751 | 667.1069144 | 623.7513076 | 578.5231034 | 605.3572472 | 499.6961892 | 488.2443126 | 509.6048235 | 508.7444958 |
| **Zswim8** | 1070.449187 | 1217.706707 | 1186.489361 | 1243.020174 | 1098.251866 | 1180.477752 | 1234.02453 | 1287.281684 | 1196.338455 | 1150.928893 | 1200.309917 | 1150.342269 | 1244.0431 | 1124.925541 | 1214.375986 |
| **Ndst2** | 329.6507334 | 276.499441 | 329.710905 | 276.0350143 | 333.1490665 | 342.2894492 | 347.2949152 | 347.8007154 | 274.6311547 | 311.1091009 | 311.1914599 | 327.9256242 | 322.9399554 | 379.4929537 | 399.5225136 |
| **Camk2g** | 672.1212175 | 768.4941482 | 736.1716168 | 762.546727 | 678.9653979 | 650.9110837 | 756.8322957 | 679.6780246 | 736.613426 | 780.3944582 | 777.5057144 | 769.4974303 | 751.538366 | 786.0925469 | 740.1226421 |
| **Plau** | 2.747089445 | 0 | 0 | 1.725218839 | 6.333632443 | 6.312715251 | 3.608258859 | 4.190370065 | 0.752414123 | 3.495607876 | 4.729353494 | 4.337640531 | 3.408337261 | 7.228437213 | 0.718565672 |
| **Vcl** | 509.1272437 | 403.2613623 | 433.8713571 | 452.0073359 | 421.8199207 | 460.8282134 | 456.4447457 | 360.3718256 | 413.0753533 | 341.6956698 | 371.7271846 | 437.2341656 | 516.363095 | 428.2849048 | 487.9060913 |
| **Ap3m1** | 1075.027669 | 1029.148349 | 1109.739554 | 1012.703459 | 1091.918233 | 962.3383694 | 1119.462311 | 1238.673391 | 1142.164638 | 1192.876188 | 1236.253003 | 1144.269572 | 1025.057431 | 1109.565112 | 1094.375518 |
| **Adk** | 569.5632115 | 542.6994758 | 646.8912292 | 569.322217 | 501.6236895 | 502.2115689 | 479.8984283 | 719.9055772 | 667.3913267 | 689.5086535 | 722.6452139 | 598.5943933 | 550.4464676 | 559.3003293 | 541.7985167 |
| **Kat6b** | 210.6101908 | 222.6256244 | 185.6092268 | 208.7514796 | 167.2078965 | 141.6853868 | 211.985208 | 165.1005806 | 182.0842177 | 165.1674721 | 174.0402086 | 191.7237115 | 205.35232 | 159.0256187 | 199.0426911 |
| **Samd8** | 277.4560339 | 212.3262183 | 238.8641948 | 235.4923716 | 181.7752511 | 166.234835 | 220.1037904 | 261.4790921 | 238.5152769 | 200.9974528 | 246.8722524 | 192.5912396 | 245.4002828 | 193.3606954 | 242.8751971 |
| **Vdac2** | 5256.097804 | 4765.455981 | 5110.910608 | 5139.426922 | 4794.559759 | 4833.435644 | 4646.535346 | 5579.896779 | 5136.731215 | 5475.869737 | 5372.545569 | 4659.493459 | 4814.276381 | 4871.966681 | 4736.78491 |
| **Comtd1** | 287.5286952 | 301.8518252 | 296.0349693 | 304.5011251 | 271.7128318 | 311.4272857 | 297.6813559 | 379.6475279 | 325.042901 | 342.5695718 | 345.2428051 | 272.4038254 | 277.7794867 | 255.7059664 | 273.0549553 |
| **Zfp503** | 2.747089445 | 1.584524017 | 0 | 0 | 0 | 0.701412806 | 0 | 0 | 2.257242368 | 0 | 0 | 0.867528106 | 0 | 0 | 0 |
| **Dlg5** | 14.65114371 | 16.63750218 | 20.36219366 | 25.01567317 | 15.20071786 | 18.23673295 | 29.76813559 | 5.866518091 | 2.257242368 | 13.9824315 | 32.15960376 | 14.74797781 | 21.30210788 | 26.2030849 | 20.11983882 |
| **Polr3a** | 840.6093701 | 733.6346199 | 826.2351657 | 828.9676523 | 696.6995687 | 744.1989869 | 775.7756547 | 922.7194884 | 831.4176055 | 841.5675961 | 913.711095 | 741.7365309 | 761.7633778 | 675.8588794 | 807.6678153 |
| **Rps24** | 17615.25322 | 16643.84027 | 17120.68906 | 16433.57205 | 17253.44814 | 17042.22694 | 18095.41818 | 17545.07946 | 16281.4892 | 17277.04193 | 16013.59093 | 17048.66234 | 16947.10494 | 17086.21846 | 17248.45039 |
| **Zmiz1** | 3847.756616 | 3928.035038 | 3823.39344 | 3660.051768 | 3833.114354 | 4148.155333 | 4767.412018 | 2963.42971 | 2669.565307 | 2905.724047 | 3185.692513 | 4120.758505 | 4753.778394 | 4008.168434 | 4667.802605 |
| **4931406H21Rik** | 0 | 0 | 3.915806472 | 3.450437679 | 0 | 1.402825611 | 0 | 0 | 0 | 0 | 3.783482795 | 0 | 0 | 2.710663955 | 3.59282836 |
| **Ppif** | 593.3713201 | 666.2923492 | 704.0620037 | 644.3692365 | 638.4301502 | 681.0718343 | 664.8216948 | 811.2556446 | 753.1665367 | 672.0306141 | 776.5598437 | 622.8851803 | 681.6674521 | 568.3358758 | 691.2601764 |
| **Ppifos** | 11.90405426 | 10.29940611 | 10.96425812 | 7.763484777 | 10.76717515 | 17.53532014 | 11.72684129 | 12.5711102 | 9.781383594 | 10.48682363 | 9.458706988 | 18.21809023 | 6.816674521 | 9.035546516 | 10.05991941 |
| **Zcchc24** | 10.0726613 | 26.14464628 | 10.18109683 | 17.25218839 | 18.36753408 | 25.25086101 | 23.45368258 | 6.704592104 | 18.05793894 | 24.46925513 | 18.91741398 | 16.48303402 | 13.63334904 | 18.97464768 | 21.55697016 |
| **Anxa11** | 1209.635052 | 1181.262655 | 1163.777684 | 1276.661941 | 1216.057429 | 1231.680887 | 1292.658736 | 1269.68213 | 1235.463989 | 1169.280834 | 1253.278676 | 1203.261483 | 1280.682726 | 1270.39784 | 1258.927057 |
| **4933413J09Rik** | 0 | 0 | 0.783161294 | 0 | 0.633363244 | 1.402825611 | 0 | 0 | 1.504828245 | 0 | 0 | 0.867528106 | 0.852084315 | 0 | 0 |
| **Slmap** | 1316.77154 | 1327.038864 | 1337.639491 | 1405.190745 | 1254.692587 | 1219.055456 | 1282.736024 | 1423.887748 | 1487.52272 | 1427.955817 | 1422.589531 | 1325.582946 | 1353.109892 | 1332.743111 | 1304.196695 |
| **Dennd6a** | 443.1970971 | 436.5363667 | 444.8356153 | 420.0907874 | 376.2177671 | 403.3123633 | 423.0683512 | 382.999824 | 374.702233 | 387.1385722 | 389.6987279 | 490.15338 | 443.9359282 | 397.5640467 | 375.8098464 |
| **Arf4** | 2279.168543 | 2021.852646 | 2012.724527 | 2021.09387 | 1818.385874 | 2022.173119 | 2092.790138 | 1749.060465 | 1878.778064 | 1822.085605 | 1783.912138 | 1978.83161 | 1906.964697 | 1948.967383 | 1835.216726 |
| **Pde12** | 1160.187442 | 1123.427528 | 1132.451232 | 1082.574822 | 1047.582806 | 1038.090952 | 1058.12191 | 1189.227025 | 1212.891566 | 1159.667913 | 1095.318269 | 1159.885078 | 1067.661647 | 1009.270546 | 981.5607079 |
| **Asb14** | 0.915696482 | 1.584524017 | 3.915806472 | 3.450437679 | 6.333632443 | 2.805651223 | 0.902064715 | 5.866518091 | 6.771727103 | 3.495607876 | 2.837612096 | 7.807752957 | 6.816674521 | 4.517773258 | 2.874262688 |
| **Appl1** | 1412.919671 | 1433.201973 | 1408.907169 | 1387.075947 | 1376.29833 | 1497.51634 | 1472.169615 | 1249.568353 | 1267.817797 | 1301.240032 | 1273.141961 | 1525.981939 | 1501.372563 | 1348.10354 | 1447.909829 |
| **Hesx1** | 7.325571853 | 4.753572051 | 15.66322589 | 9.488703616 | 5.700269198 | 13.32684331 | 12.62890601 | 8.380740131 | 14.29586833 | 13.10852953 | 10.40457769 | 5.205168638 | 15.33751767 | 12.64976512 | 8.622788064 |
| **Il17rd** | 10.98835778 | 6.338096068 | 7.831612945 | 6.900875357 | 16.46744435 | 14.72966892 | 13.53097072 | 13.40918421 | 3.00965649 | 7.86511772 | 14.18806048 | 19.95314644 | 11.92918041 | 12.64976512 | 8.622788064 |
| **Arhgef3** | 1792.933711 | 1959.263947 | 1936.757881 | 1932.2451 | 2204.10409 | 2046.021154 | 2408.512788 | 1814.430238 | 1695.189018 | 1524.958936 | 1640.139792 | 2113.298467 | 2119.985776 | 2151.363625 | 2117.613035 |
| **Fam208a** | 422.136078 | 441.2899387 | 444.8356153 | 406.2890367 | 355.9501433 | 377.3600895 | 372.5527272 | 393.0567121 | 396.5222426 | 390.6341801 | 314.029072 | 393.8577603 | 429.4504948 | 319.8583467 | 382.2769375 |
| **Ccdc66** | 240.8281747 | 337.5036156 | 338.3256792 | 275.1724049 | 290.7137291 | 250.4043716 | 294.073097 | 290.8116825 | 330.3097998 | 269.1618064 | 270.5190198 | 321.8529274 | 305.8982691 | 282.8126059 | 340.6001285 |
| **Selk** | 1117.149708 | 1244.643615 | 1186.489361 | 1103.277448 | 1152.087741 | 1186.089054 | 1165.467611 | 1012.393408 | 1109.058417 | 1056.54748 | 1016.811001 | 1226.684742 | 1125.60338 | 1074.326481 | 1128.148105 |
| **Actr8** | 462.4267232 | 471.3958951 | 473.0294219 | 475.2977902 | 509.2240484 | 434.8759395 | 468.171587 | 440.8269309 | 500.3553915 | 443.0682982 | 576.0352556 | 489.2858519 | 478.8713851 | 461.716427 | 431.1394032 |
| **Il17rb** | 0 | 7.130358077 | 3.915806472 | 5.175656518 | 0 | 1.402825611 | 0.902064715 | 2.514222039 | 4.514484735 | 0 | 0 | 0 | 0 | 2.710663955 | 0 |
| **Cacna1d** | 5.494178889 | 0.792262009 | 5.482129061 | 1.725218839 | 0 | 0.701412806 | 0.902064715 | 7.542666117 | 1.504828245 | 11.3607256 | 2.837612096 | 2.602584319 | 3.408337261 | 0 | 0.718565672 |
| **Dcp1a** | 1300.289004 | 1126.596576 | 1249.142265 | 1028.230428 | 1048.849533 | 1023.361284 | 1140.209799 | 1143.132954 | 1203.110182 | 1042.565049 | 1025.323837 | 1045.371368 | 1174.172186 | 1106.854448 | 1129.585236 |
| **Tkt** | 14928.59974 | 13934.30421 | 14337.33382 | 13963.05868 | 13860.52124 | 14022.64481 | 14052.36413 | 12834.26544 | 12465.99718 | 12477.57231 | 12950.86161 | 13450.15576 | 14169.31008 | 13364.47685 | 13876.22169 |
| **Prkcd** | 1173.922889 | 1139.272768 | 1240.52749 | 1227.493204 | 1140.05384 | 1243.604905 | 1242.143112 | 1302.367016 | 1158.717749 | 1114.22501 | 1124.640261 | 1264.855979 | 1175.024271 | 1184.560148 | 1158.327863 |
| **Rft1** | 188.6334752 | 225.0024104 | 216.9356786 | 222.5532303 | 235.6111269 | 246.8973076 | 213.7893374 | 228.7942056 | 211.4283684 | 190.5106292 | 285.652951 | 224.6897795 | 186.606465 | 231.3099908 | 204.7912165 |
| **Sfmbt1** | 427.6302569 | 487.2411352 | 409.593357 | 428.7168816 | 359.7503227 | 345.7965132 | 404.1249922 | 607.6036595 | 460.477443 | 480.6460829 | 476.7188322 | 406.8706818 | 413.2608929 | 410.2138118 | 381.5583718 |
| **Tmem110** | 369.9413786 | 380.2857641 | 360.2541955 | 422.6786156 | 333.7824297 | 318.4414138 | 305.7999383 | 354.5053075 | 385.2360308 | 421.220749 | 401.0491763 | 326.190568 | 342.5378947 | 279.1983873 | 277.3663494 |
| **Mustn1** | 3.662785926 | 1.584524017 | 2.349483883 | 4.313047098 | 1.900089733 | 0.701412806 | 3.608258859 | 0 | 2.257242368 | 7.86511772 | 6.621094891 | 4.337640531 | 2.556252945 | 7.228437213 | 1.437131344 |
| **Nek4** | 190.4648682 | 186.973834 | 169.9460009 | 129.3914129 | 156.4407213 | 157.1164685 | 161.4695839 | 242.2033898 | 185.8462883 | 229.8362178 | 228.9007091 | 164.8303402 | 166.1564415 | 149.0865175 | 189.7013374 |
| **Spcs1** | 1132.716548 | 1092.52931 | 1197.453619 | 1019.604334 | 1245.192138 | 1218.354044 | 1145.622188 | 1087.820069 | 1104.543932 | 1108.107697 | 1047.078864 | 1210.201708 | 1161.390922 | 1253.230302 | 1087.189862 |
| **Glt8d1** | 124.5347215 | 119.6315633 | 101.8109683 | 88.84877022 | 85.50403798 | 88.37801352 | 85.6961479 | 68.72206907 | 63.2027863 | 83.89458901 | 65.26507822 | 112.7786538 | 100.5459492 | 119.269214 | 84.07218362 |
| **Gnl3** | 2510.839752 | 2401.346148 | 2459.909626 | 2140.99658 | 2332.676829 | 2149.83025 | 2060.315809 | 2717.03595 | 2416.001748 | 2768.521437 | 2867.879959 | 2193.978581 | 2128.506619 | 2208.287568 | 2209.589441 |
| **Snord69** | 4.578482408 | 5.54583406 | 3.915806472 | 1.725218839 | 5.066905954 | 5.611302446 | 5.412388289 | 11.73303618 | 8.276555348 | 20.97364725 | 7.56696559 | 12.14539349 | 8.520843152 | 8.131991864 | 10.05991941 |
| **Snord19** | 4.578482408 | 3.961310043 | 1.566322589 | 1.725218839 | 4.43354271 | 2.104238417 | 3.608258859 | 3.352296052 | 15.80069657 | 6.117313782 | 5.675224193 | 0.867528106 | 2.556252945 | 6.324882561 | 2.155697016 |
| **Pbrm1** | 2574.938506 | 2631.894392 | 2901.612596 | 2681.852686 | 2470.116653 | 2503.342304 | 2948.849553 | 2748.044689 | 2719.977053 | 2459.16014 | 2835.720355 | 2907.086684 | 2978.034681 | 2632.958255 | 2840.490101 |
| **Smim4** | 76.00280797 | 99.82501307 | 81.44877463 | 76.77223835 | 93.10439691 | 85.5723623 | 59.53627118 | 94.70236348 | 86.5276241 | 103.9943343 | 81.34488009 | 116.2487662 | 72.42716679 | 78.60925469 | 99.8806284 |
| **Nt5dc2** | 559.4905502 | 602.1191265 | 548.2129061 | 575.3604829 | 582.0608215 | 529.5666683 | 533.1202464 | 472.6737434 | 486.0595232 | 455.3029258 | 543.8756518 | 607.2696744 | 627.134056 | 505.0870502 | 574.1339719 |
| **Stab1** | 10.98835778 | 8.714882094 | 4.698967767 | 2.587828259 | 3.166816221 | 4.208476834 | 9.020647148 | 18.43762829 | 8.276555348 | 4.369509845 | 7.56696559 | 6.072696744 | 2.556252945 | 0.903554652 | 3.59282836 |
| **Nisch** | 2140.898374 | 2284.883633 | 2170.923108 | 1990.902541 | 1896.922917 | 1902.231529 | 2022.429091 | 2123.679549 | 1986.373284 | 2151.546647 | 2328.73366 | 2061.246781 | 2076.529476 | 2070.947261 | 2160.00841 |
| **Tnnc1** | 0.915696482 | 7.130358077 | 0.783161294 | 0 | 1.266726489 | 0 | 2.706194144 | 0 | 0 | 1.747803938 | 1.891741398 | 0 | 0 | 0 | 2.874262688 |
| **Phf7** | 195.9590471 | 217.8720523 | 198.9229688 | 200.1253854 | 215.3435031 | 202.7083009 | 218.299661 | 219.5753914 | 155.7497234 | 244.6925513 | 167.4191137 | 165.6978683 | 184.0502121 | 210.5282338 | 194.0127314 |
| **Bap1** | 1784.692443 | 1883.999056 | 1830.247945 | 1614.804834 | 1692.346589 | 1702.328879 | 1832.093436 | 1826.163274 | 1854.700812 | 1991.622587 | 1965.519312 | 1822.676551 | 1779.15205 | 1893.85055 | 1833.779595 |
| **Dnah1** | 1.831392963 | 2.376786026 | 1.566322589 | 3.450437679 | 10.13381191 | 1.402825611 | 7.216517718 | 3.352296052 | 3.762070613 | 2.621705907 | 0 | 6.94022485 | 0.852084315 | 2.710663955 | 1.437131344 |
| **Capn7** | 671.205521 | 674.2149692 | 760.4496169 | 767.7223835 | 697.3329319 | 683.8774856 | 690.9815715 | 774.3803881 | 791.539657 | 741.9427716 | 878.7138792 | 811.1387794 | 870.8301701 | 739.107705 | 776.0509257 |
| **Sh3bp5** | 971.5539669 | 1098.075144 | 1079.196264 | 1088.613088 | 967.145674 | 890.7942633 | 926.4204621 | 954.5663009 | 1005.977682 | 977.0224012 | 929.7908969 | 1027.153278 | 1009.719913 | 1009.270546 | 929.8239795 |
| **Mettl6** | 551.2492819 | 532.4000697 | 556.8276804 | 492.5499786 | 501.6236895 | 547.1019885 | 521.3934051 | 669.6211364 | 538.7285118 | 627.4616137 | 611.0324714 | 540.4700102 | 472.0547106 | 455.3915444 | 481.4390002 |
| **Eaf1** | 1953.180595 | 1925.988943 | 1954.770591 | 1885.664191 | 1680.94605 | 1747.920712 | 1820.366594 | 2077.585478 | 1889.311862 | 1995.118195 | 2113.075141 | 1776.697562 | 1851.579217 | 1641.758802 | 1797.132746 |
| **Colq** | 9.156964816 | 18.2220262 | 4.698967767 | 32.77915795 | 15.20071786 | 14.02825611 | 25.25781201 | 10.89496217 | 12.03862596 | 5.243411813 | 5.675224193 | 6.94022485 | 23.00627651 | 18.97464768 | 18.68270747 |
| **Hacl1** | 189.5491717 | 184.597048 | 213.0198721 | 172.5218839 | 177.9750716 | 176.0546142 | 191.2377195 | 138.2822122 | 101.5759065 | 158.1762564 | 147.555829 | 199.5314644 | 218.1335847 | 169.8682745 | 199.7612568 |
| **Btd** | 281.1188198 | 316.9048034 | 351.6394212 | 271.7219672 | 299.5808145 | 288.982076 | 319.330909 | 270.6979062 | 245.287004 | 250.8098651 | 257.2768301 | 315.7802307 | 348.5024849 | 318.954792 | 327.6659464 |
| **Ankrd28** | 1063.123615 | 1036.278707 | 1054.918264 | 1039.444351 | 931.0439691 | 960.9355438 | 1107.73547 | 1147.323324 | 1191.82397 | 1146.559383 | 1151.12464 | 1171.162943 | 1108.561694 | 978.5496877 | 1060.602932 |
| **Dph3** | 587.8771412 | 622.7179387 | 647.6743905 | 597.7883278 | 583.327548 | 589.1867568 | 580.9296763 | 739.1812795 | 710.2789317 | 648.4352609 | 660.2177477 | 629.8254052 | 556.4110578 | 594.5389607 | 560.4812241 |
| **Oxnad1** | 252.7322289 | 305.0208733 | 272.5401305 | 250.1567317 | 229.9108577 | 260.9255637 | 244.4595377 | 238.0130197 | 274.6311547 | 291.0093556 | 259.1685715 | 247.2455103 | 219.8377533 | 226.7922175 | 245.7494598 |
| **Ncoa4** | 789.3303671 | 778.7935544 | 806.6561333 | 753.9206328 | 727.7343677 | 708.4269338 | 772.1673959 | 760.1331298 | 761.4430921 | 679.0218298 | 715.0782483 | 808.5361951 | 766.8758836 | 754.4681341 | 752.3382585 |
| **Timm23** | 2778.223125 | 2654.077728 | 2713.653885 | 2754.311877 | 2432.114858 | 2497.731001 | 2425.652018 | 2916.497565 | 2889.270231 | 3125.947343 | 2951.11658 | 2393.510045 | 2433.552804 | 2554.349 | 2387.075162 |
| **Parg** | 1054.882347 | 1003.795965 | 1021.242328 | 1102.414838 | 1018.448097 | 1078.071482 | 1136.601541 | 1020.774148 | 887.0962505 | 1019.843598 | 1001.67707 | 1142.534516 | 1077.034574 | 1149.321517 | 920.4826258 |
| **Ogdhl** | 21.97671556 | 23.76786026 | 25.06116142 | 8.626094197 | 5.066905954 | 9.81977928 | 13.53097072 | 11.73303618 | 17.30552482 | 25.3431571 | 8.512836289 | 7.807752957 | 17.0416863 | 16.26398373 | 12.21561642 |
| **Ercc6** | 508.2115473 | 436.5363667 | 488.6926478 | 452.8699453 | 427.5201899 | 443.994306 | 476.2901694 | 507.0347779 | 467.2491701 | 496.3763183 | 504.1490824 | 478.0079866 | 507.8422518 | 437.3204514 | 506.5887987 |
| **Wdfy4** | 39.37494871 | 22.18333624 | 40.72438731 | 55.20700286 | 19.00089733 | 7.715540863 | 19.84542373 | 85.48354933 | 54.17381683 | 80.39898114 | 49.18527634 | 19.08561834 | 18.74585493 | 8.131991864 | 19.40127314 |
| **Mapk8** | 354.3745384 | 319.2815894 | 307.7823887 | 298.4628592 | 323.0152546 | 303.7117449 | 328.3515562 | 308.4112368 | 288.1746089 | 343.4434738 | 313.0832013 | 331.3957366 | 383.4379418 | 289.1374885 | 331.9773404 |
| **Syt15** | 3.662785926 | 1.584524017 | 1.566322589 | 0 | 0 | 0 | 0 | 0 | 0 | 0 | 0 | 0 | 4.260421576 | 3.614218606 | 1.437131344 |
| **Fam35a** | 190.4648682 | 232.1327685 | 199.7061301 | 210.4766984 | 187.4755203 | 150.1023404 | 199.356302 | 240.5272417 | 236.2580345 | 200.1235509 | 253.4933473 | 191.7237115 | 198.5356454 | 190.6500315 | 204.0726508 |
| **Glud1** | 6139.744909 | 5353.314391 | 5699.847901 | 5364.567981 | 4881.963887 | 5088.749905 | 5220.248504 | 5250.533692 | 5308.281635 | 5642.785013 | 5797.241513 | 5196.493357 | 5126.991324 | 4968.647029 | 4991.875723 |
| **Gm3219** | 10.98835778 | 14.26071615 | 10.18109683 | 12.93914129 | 15.20071786 | 14.02825611 | 11.72684129 | 15.92340625 | 13.54345421 | 13.10852953 | 17.02567258 | 19.08561834 | 22.15419219 | 16.26398373 | 4.311394032 |
| **Mmrn2** | 0 | 0 | 0 | 0 | 0 | 0 | 1.80412943 | 4.190370065 | 3.762070613 | 1.747803938 | 6.621094891 | 0 | 0 | 0 | 0 |
| **Bmpr1a** | 105.3050954 | 56.2506026 | 64.21922615 | 45.71829924 | 50.66905954 | 66.63421654 | 61.34040061 | 58.66518091 | 36.86829201 | 55.05582404 | 66.21094891 | 65.06460797 | 63.05423932 | 60.53816166 | 73.29369854 |
| **Wapal** | 3243.396938 | 3240.351615 | 3167.104275 | 3001.018171 | 3156.682409 | 3076.396566 | 3346.660092 | 3235.803764 | 3279.77316 | 3259.654344 | 3051.378874 | 3229.80714 | 3217.470374 | 3027.811637 | 3334.144718 |
| **Ccser2** | 771.932134 | 809.6917727 | 780.8118106 | 712.5153806 | 720.1340087 | 662.8351014 | 815.4665022 | 765.9996479 | 790.0348287 | 732.3298499 | 657.3801356 | 817.2114761 | 868.2739171 | 693.0264178 | 781.0808854 |
| **Ghitm** | 4983.220253 | 4670.38454 | 5048.257704 | 4449.339387 | 4387.307193 | 4607.580721 | 4742.154206 | 4593.483666 | 4385.069506 | 4525.938297 | 4263.039239 | 4485.987838 | 4365.227947 | 4227.732215 | 4343.010921 |
| **Sh2d4b** | 2.747089445 | 2.376786026 | 2.349483883 | 0 | 0 | 2.104238417 | 0.902064715 | 0.838074013 | 0 | 3.495607876 | 6.621094891 | 3.470112425 | 0.852084315 | 5.421327909 | 0.718565672 |
| **Tspan14** | 689.5194506 | 885.7489255 | 883.4059402 | 878.1363892 | 840.4730252 | 913.239473 | 852.4511555 | 596.7086973 | 689.9637504 | 637.9484373 | 713.1865069 | 921.3148489 | 916.8427231 | 923.4328539 | 977.2493139 |
| **Fam213a** | 171.2352421 | 190.142882 | 180.1270977 | 175.9723216 | 181.7752511 | 162.7277709 | 167.7840369 | 230.4703536 | 167.0359352 | 208.8625706 | 214.7126486 | 193.4587677 | 165.3043571 | 146.3758536 | 142.9945687 |
| **4930431P03Rik** | 0 | 11.09166812 | 16.44638718 | 6.038265938 | 7.600358931 | 11.9240177 | 8.118582433 | 0.838074013 | 7.524141226 | 3.495607876 | 4.729353494 | 5.205168638 | 9.372927467 | 6.324882561 | 1.437131344 |
| **Ptger2** | 175.8137245 | 225.7946724 | 140.969033 | 175.9723216 | 165.3078068 | 225.1535106 | 185.8253312 | 174.3193947 | 187.3511165 | 144.1938249 | 117.2879666 | 147.4797781 | 183.1981278 | 157.2185094 | 139.4017404 |
| **Txndc16** | 328.7350369 | 418.3143405 | 375.1342601 | 387.3116294 | 403.4523866 | 418.743445 | 412.2435747 | 325.1727171 | 337.0815269 | 302.3700812 | 315.9208134 | 415.5459629 | 391.958785 | 402.9853746 | 370.7798867 |
| **Gpr137c** | 0 | 1.584524017 | 0 | 3.450437679 | 1.266726489 | 0 | 2.706194144 | 0 | 0 | 1.747803938 | 0.945870699 | 1.735056213 | 0 | 1.807109303 | 0 |
| **Ero1l** | 4830.29894 | 3643.612977 | 3723.148794 | 3613.470859 | 3272.587883 | 3957.37105 | 4161.224529 | 1785.097648 | 1881.787721 | 2144.555432 | 2143.343003 | 3457.967032 | 3827.562744 | 3214.84745 | 3669.714887 |
| **Psmc6** | 2443.078213 | 2528.900331 | 2422.317884 | 2279.876696 | 2275.040773 | 2211.554576 | 2336.347611 | 2725.41669 | 2519.834897 | 2815.712144 | 2776.130501 | 2427.343641 | 2418.215286 | 2487.485956 | 2420.847749 |
| **Styx** | 395.58088 | 412.7685064 | 398.6290989 | 351.0820338 | 308.4479 | 380.8671535 | 379.7692449 | 391.3805641 | 398.0270708 | 451.8073179 | 424.6959437 | 350.4813549 | 404.7400497 | 345.1578769 | 364.3127957 |
| **Gnpnat1** | 711.4961662 | 590.2351963 | 611.648971 | 571.9100452 | 481.9894289 | 580.7698031 | 580.9296763 | 618.4986216 | 532.7091988 | 623.9660058 | 489.961022 | 559.5556286 | 537.6652029 | 470.7519735 | 528.1457689 |
| **Fermt2** | 0 | 0 | 0.783161294 | 0 | 0 | 0 | 4.510323574 | 0.838074013 | 1.504828245 | 4.369509845 | 0.945870699 | 2.602584319 | 2.556252945 | 2.710663955 | 0 |
| **Ddhd1** | 243.5752641 | 308.9821833 | 289.769679 | 296.7376404 | 267.2792891 | 239.1817667 | 289.5627734 | 368.7525657 | 307.7373761 | 345.1912777 | 306.4621064 | 250.7156227 | 262.4419691 | 224.0815536 | 277.3663494 |
| **Gm1821** | 334.2292158 | 391.3774322 | 360.2541955 | 461.4960395 | 388.2516687 | 429.9660499 | 438.4034514 | 409.8181924 | 421.3519086 | 399.3731998 | 365.1060897 | 491.8884363 | 554.7068892 | 456.295099 | 533.8942943 |
| **Cdkn3** | 241.7438711 | 246.3934846 | 224.7672915 | 238.9428092 | 250.1784815 | 234.9732899 | 262.500832 | 194.433171 | 173.0552482 | 237.7013355 | 255.3850887 | 300.1647248 | 312.7149437 | 306.3050269 | 268.0249956 |
| **Cnih1** | 1165.681621 | 1260.488856 | 1216.24949 | 1173.148811 | 1216.690792 | 1263.945876 | 1183.508906 | 1296.500498 | 1241.483302 | 1203.363011 | 1268.412607 | 1228.419798 | 1255.972281 | 1317.382682 | 1279.046896 |
| **Gmfb** | 2004.459598 | 2113.755039 | 2144.295624 | 2102.179156 | 1964.05942 | 1950.629013 | 2048.588967 | 2149.659843 | 1931.447053 | 2148.05104 | 2109.291658 | 2036.088465 | 2000.693972 | 1879.393675 | 1893.420546 |
| **Cgrrf1** | 107.1364883 | 127.5541834 | 111.9920651 | 128.5288035 | 98.17130286 | 105.9133337 | 108.2477658 | 120.6826579 | 92.54693708 | 110.9855501 | 135.2595099 | 126.6591035 | 99.69386487 | 110.2336675 | 120.0004672 |
| **Samd4** | 12.81975074 | 4.753572051 | 19.57903236 | 17.25218839 | 24.70116653 | 23.84803539 | 10.82477658 | 30.17066447 | 23.3248378 | 15.73023544 | 9.458706988 | 19.08561834 | 26.41461377 | 2.710663955 | 15.08987911 |
| **Gch1** | 489.8976176 | 419.1066025 | 471.4630993 | 433.8925381 | 355.9501433 | 474.8564695 | 442.0117102 | 517.0916661 | 498.0981491 | 391.5080821 | 392.53634 | 400.7979851 | 467.794289 | 384.9142816 | 400.959645 |
| **Wdhd1** | 1301.2047 | 1449.839476 | 1501.320202 | 1500.077781 | 1273.060121 | 1188.894706 | 1404.514761 | 1742.355873 | 1490.532377 | 1703.234937 | 1657.165464 | 1379.369689 | 1466.437106 | 1320.996901 | 1330.065059 |
| **Socs4** | 380.0140398 | 453.1738689 | 443.2692927 | 395.0751142 | 345.8163314 | 354.2134669 | 396.9084745 | 493.6255937 | 472.516069 | 484.1416908 | 489.0151513 | 450.2470872 | 397.0712909 | 407.5031479 | 393.7739882 |
| **Mapk1ip1l** | 3239.734152 | 3409.895685 | 3323.736534 | 3273.602748 | 3112.980346 | 3171.087295 | 3350.268351 | 3265.136355 | 3421.979429 | 3372.387698 | 3488.371137 | 3176.887925 | 3081.988968 | 3163.344835 | 3112.826491 |
| **Lgals3** | 5207.565891 | 4289.306514 | 4980.122672 | 5182.557393 | 3793.845833 | 4151.662397 | 3931.198027 | 6201.747697 | 3722.192664 | 6249.27298 | 10404.57769 | 4257.827946 | 4569.728182 | 4762.636568 | 4830.198447 |
| **Dlgap5** | 1824.983088 | 1962.432995 | 2000.193946 | 1855.472862 | 1786.084349 | 1803.332323 | 1969.207272 | 1980.368893 | 1984.868455 | 1794.120742 | 1872.823984 | 1933.720149 | 1896.739686 | 1935.414064 | 1900.606202 |
| **Fbxo34** | 540.2609241 | 572.0131701 | 526.2843899 | 583.1239677 | 490.2231511 | 563.234483 | 526.8057934 | 639.450472 | 636.5423477 | 673.778418 | 601.5737644 | 553.4829318 | 547.0381303 | 590.9247421 | 527.4272032 |
| **Atg14** | 108.0521848 | 153.6988296 | 137.8363878 | 116.4522717 | 120.9723797 | 106.6147465 | 119.9746071 | 127.38725 | 152.7400669 | 121.4723737 | 109.7210011 | 110.1760695 | 94.58135898 | 96.68034772 | 117.8447702 |
| **Ktn1** | 1017.338791 | 938.0382181 | 949.1914889 | 890.2129211 | 820.2054013 | 845.9038437 | 923.7142679 | 998.1461495 | 937.5079967 | 948.1836363 | 954.3835351 | 937.7978829 | 845.2676406 | 900.8439876 | 870.1830288 |
| **Peli2** | 6.409875371 | 3.961310043 | 1.566322589 | 3.450437679 | 1.266726489 | 0.701412806 | 2.706194144 | 5.028444078 | 4.514484735 | 0.873901969 | 0 | 0 | 0 | 1.807109303 | 0 |
| **Gm6498** | 26.55519797 | 9.507144102 | 20.36219366 | 6.900875357 | 12.66726489 | 19.63955856 | 12.62890601 | 5.028444078 | 6.771727103 | 9.612921658 | 10.40457769 | 6.072696744 | 12.78126473 | 10.84265582 | 9.341353736 |
| **Tmem260** | 298.517053 | 352.5565938 | 364.1700019 | 335.5550642 | 378.7512201 | 345.7965132 | 365.3362095 | 282.4309424 | 300.2132349 | 283.1442379 | 263.897925 | 343.5411301 | 371.5087614 | 344.2543222 | 274.4920867 |
| **Exoc5** | 1486.17539 | 1479.945432 | 1543.610911 | 1490.589077 | 1320.562364 | 1477.175369 | 1487.504715 | 1661.062694 | 1594.365526 | 1637.69229 | 1677.97462 | 1496.485983 | 1604.474765 | 1472.794082 | 1480.245284 |
| **Ap5m1** | 245.4066571 | 290.7601571 | 310.9150339 | 266.5463107 | 280.5799172 | 240.5845924 | 268.815285 | 287.4593865 | 273.8787406 | 297.1266694 | 344.2969344 | 291.4894437 | 336.5733045 | 304.4979176 | 229.941015 |
| **Naa30** | 868.0802645 | 815.2376068 | 921.7808436 | 825.5172146 | 805.0046835 | 838.1883028 | 880.4151616 | 893.3868979 | 881.0769375 | 933.3273028 | 869.2551722 | 887.4812527 | 920.2510604 | 777.0570004 | 760.9610466 |
| **1700011H14Rik** | 0 | 0 | 0 | 4.313047098 | 0 | 0.701412806 | 0 | 0 | 0 | 0 | 0 | 0 | 0.852084315 | 0 | 0 |
| **Ttc5** | 542.0923171 | 530.8155457 | 572.4909063 | 553.7952474 | 540.8922106 | 540.0878604 | 561.0842526 | 559.8334407 | 514.6512598 | 564.5406719 | 504.1490824 | 553.4829318 | 645.0278266 | 589.1176328 | 597.1280734 |
| **Rpph1** | 54.94178889 | 66.55000871 | 43.85703249 | 40.54264272 | 70.30332011 | 56.81443726 | 52.31975346 | 68.72206907 | 43.64001911 | 92.6336087 | 84.18249219 | 64.19707986 | 98.84178056 | 79.51280934 | 69.70087018 |
| **Parp2** | 678.5310928 | 759.7792662 | 762.7991008 | 745.2945386 | 761.9359829 | 714.739649 | 756.8322957 | 850.6451232 | 811.1024241 | 823.2156547 | 788.8561628 | 724.3859687 | 734.4966797 | 750.8539155 | 819.8834317 |
| **Tep1** | 548.5021925 | 633.8096068 | 629.6616808 | 686.637098 | 580.794095 | 573.7556751 | 649.4865946 | 688.8968387 | 646.3237313 | 679.0218298 | 594.9526695 | 607.2696744 | 644.1757423 | 605.3816166 | 609.3436898 |
| **Osgep** | 594.2870165 | 614.0030566 | 643.7585841 | 634.8805329 | 682.1322141 | 721.0523643 | 691.8836362 | 559.8334407 | 588.3878439 | 645.813555 | 579.8187383 | 608.1372025 | 710.6383188 | 740.0112596 | 582.75676 |
| **Apex1** | 465.1738126 | 412.7685064 | 415.8586474 | 307.0889534 | 367.9840449 | 361.9290077 | 359.9238212 | 362.8860477 | 314.5091032 | 352.1824935 | 445.5050991 | 266.3311286 | 313.567028 | 365.9396339 | 311.8575016 |

| | | | | | | | | | | | | | | | |
|---|---|---|---|---|---|---|---|---|---|---|---|---|---|---|---|
| **Tmem55b** | 316.8309826 | 375.532192 | 328.1445824 | 390.7620671 | 290.7137291 | 346.497926 | 322.0371032 | 357.8576036 | 348.3677388 | 377.5256506 | 307.4079771 | 349.6138268 | 316.1232809 | 346.0614316 | 344.9115225 |
| **Pnp** | 1390.027259 | 1372.990061 | 1361.13433 | 1428.481199 | 1307.895099 | 1357.233779 | 1279.127766 | 1449.029969 | 1491.284791 | 1436.694837 | 1537.039886 | 1336.860812 | 1255.972281 | 1318.286237 | 1170.54348 |
| **Pnp2** | 12.81975074 | 15.84524017 | 11.74741942 | 9.488703616 | 10.76717515 | 6.312715251 | 18.94335901 | 14.24725822 | 30.0965649 | 25.3431571 | 31.21373306 | 20.82067455 | 10.22501178 | 17.16753838 | 16.52701046 |
| **Ang** | 2.747089445 | 2.376786026 | 1.566322589 | 0 | 2.533452977 | 0 | 1.80412943 | 0 | 0 | 0.873901969 | 1.891741398 | 0.867528106 | 0 | 3.614218606 | 2.874262688 |
| **Rnase4** | 3.662785926 | 10.29940611 | 9.397935534 | 17.25218839 | 11.4005384 | 7.014128057 | 9.020647148 | 2.514222039 | 4.514484735 | 4.369509845 | 7.56696559 | 9.542809169 | 23.85836082 | 13.55331977 | 9.341353736 |
| **Rnase6** | 0 | 0 | 0 | 0 | 0 | 0 | 0 | 0 | 0 | 0 | 0 | 0 | 0 | 0 | 0 |
| **Mettl17** | 264.6362832 | 231.3405065 | 267.0580014 | 236.354981 | 274.879648 | 213.9309057 | 210.1810785 | 226.2799835 | 255.8208017 | 221.9711001 | 227.9548384 | 242.9078698 | 178.9377062 | 234.9242094 | 191.1384687 |
| **Slc39a2** | 3.662785926 | 1.584524017 | 8.614774239 | 1.725218839 | 0.633363244 | 0 | 2.706194144 | 5.028444078 | 12.79104008 | 5.243411813 | 7.56696559 | 0.867528106 | 3.408337261 | 1.807109303 | 4.311394032 |
| **Ndrg2** | 406.5692378 | 393.7542182 | 377.4837439 | 345.0437679 | 299.5808145 | 330.3654315 | 329.2536209 | 207.0042812 | 202.399399 | 213.2320804 | 235.521804 | 324.4555117 | 330.6087143 | 273.7770594 | 348.5043509 |
| **Arhgef40** | 10.0726613 | 11.88393013 | 0.783161294 | 3.450437679 | 3.800179466 | 4.208476834 | 7.216517718 | 5.866518091 | 6.019312981 | 20.09974528 | 10.40457769 | 6.072696744 | 0 | 3.614218606 | 2.874262688 |
| **Zfp219** | 546.6707995 | 479.3185151 | 525.5012286 | 536.543059 | 425.6201002 | 458.7239749 | 507.8624344 | 471.8356693 | 467.2491701 | 479.7721809 | 439.8298749 | 382.5798949 | 490.8005655 | 464.4270909 | 407.426736 |
| **Tmem253** | 9.156964816 | 9.507144102 | 17.22954848 | 4.313047098 | 11.4005384 | 5.611302446 | 9.020647148 | 13.40918421 | 5.266898858 | 11.3607256 | 10.40457769 | 5.205168638 | 5.964590206 | 15.36042908 | 5.029959704 |
| **Snord58b** | 2.747089445 | 2.376786026 | 1.566322589 | 2.587828259 | 2.533452977 | 2.104238417 | 4.510323574 | 2.514222039 | 5.266898858 | 5.243411813 | 3.783482795 | 0.867528106 | 0.852084315 | 5.421327909 | 5.029959704 |
| **Hnrnpc** | 9121.252653 | 8742.611264 | 9291.425598 | 8718.393404 | 8938.655466 | 8585.292742 | 8932.244806 | 9438.389535 | 9161.394357 | 9339.390342 | 9956.234975 | 8874.812527 | 8533.624416 | 8937.059059 | 8487.697717 |
| **Rpgrip1** | 100.726613 | 89.52560696 | 88.49722628 | 81.08528545 | 95.00448664 | 60.32150129 | 111.8560246 | 100.5688816 | 83.51796761 | 97.87702052 | 93.64119918 | 87.62033873 | 93.72927467 | 108.4265582 | 89.820709 |
| **Supt16** | 4464.936044 | 4571.351789 | 4284.675442 | 4179.342638 | 4082.026109 | 4085.02818 | 4512.127703 | 4549.903817 | 4538.561987 | 4532.055611 | 4609.227915 | 4577.945817 | 4273.202841 | 3958.472929 | 4149.716756 |
| **Chd8** | 2799.284144 | 2860.065851 | 2813.898531 | 2885.428509 | 2784.264822 | 2619.776829 | 2916.375223 | 2746.368541 | 2721.481881 | 2541.306926 | 2647.492086 | 2937.450168 | 2871.524142 | 2774.816335 | 2987.077498 |
| **Snord8** | 5.494178889 | 0.792262009 | 1.566322589 | 0 | 0 | 0 | 0.902064715 | 0 | 0 | 0.873901969 | 0.945870699 | 0 | 1.70416863 | 0 | 1.437131344 |
| **Rab2b** | 92.48534464 | 94.27917901 | 121.3900006 | 93.16181732 | 119.7056532 | 105.9133337 | 139.8200308 | 99.73080755 | 106.8428054 | 84.76849098 | 83.23662149 | 95.42809169 | 101.3980335 | 99.39101167 | 120.0004672 |
| **Tox4** | 2296.566776 | 2143.068733 | 2102.788076 | 2152.210502 | 1934.291348 | 2080.390382 | 2164.053251 | 2292.9705 | 2227.898217 | 2293.992668 | 2235.092461 | 2091.610264 | 2073.973223 | 2103.475229 | 2147.074228 |
| **Mettl3** | 353.4588419 | 377.116716 | 348.506776 | 353.6698621 | 393.3185747 | 401.2081249 | 367.1403389 | 401.4374523 | 334.8242845 | 415.9773372 | 415.2372368 | 360.8916922 | 403.8879654 | 379.4929537 | 384.4326345 |
| **Sall2** | 173.9823315 | 204.4035982 | 169.1628396 | 193.22451 | 224.2105885 | 220.243621 | 222.8099846 | 175.1574687 | 158.7593799 | 179.1499036 | 184.4447863 | 226.4248357 | 186.606465 | 215.0460071 | 171.7371956 |
| **Dad1** | 2037.424671 | 2185.058619 | 2128.632398 | 2093.553061 | 2198.403821 | 2009.547688 | 2069.336456 | 1931.7606 | 1991.640182 | 1908.6019 | 1852.960699 | 2134.119141 | 1970.871021 | 2053.779723 | 2059.409216 |
| **Abhd4** | 142.8486511 | 144.9839476 | 146.4511621 | 146.6436013 | 92.47103366 | 87.67660071 | 112.7580893 | 232.1465016 | 185.0938742 | 195.754041 | 212.8209072 | 91.95797927 | 130.3689002 | 135.5331977 | 132.2160836 |
| **Oxa1l** | 1058.545133 | 1124.21979 | 1164.560845 | 1098.101791 | 1100.151955 | 1065.446052 | 1161.859353 | 1108.771919 | 1162.479819 | 1150.054991 | 1180.446632 | 1082.675077 | 1048.063708 | 1168.296164 | 1045.513053 |
| **Slc7a7** | 4.578482408 | 12.67619214 | 16.44638718 | 11.21392246 | 9.500448664 | 18.93814575 | 15.33510015 | 16.76148026 | 8.276555348 | 4.369509845 | 6.621094891 | 18.21809023 | 9.372927467 | 9.035546516 | 12.21561642 |
| **Mrpl52** | 745.376936 | 659.1619911 | 749.4853588 | 661.6214249 | 663.76468 | 650.2096709 | 679.2547302 | 703.144097 | 709.5265176 | 708.7344968 | 647.9214287 | 634.1630457 | 589.6423461 | 705.6761829 | 659.6432869 |
| **Mmp14** | 1.831392963 | 2.376786026 | 0.783161294 | 0 | 2.533452977 | 2.805651223 | 9.020647148 | 0 | 1.504828245 | 1.747803938 | 0.945870699 | 0.867528106 | 3.408337261 | 4.517773258 | 5.748525376 |
| **Lrp10** | 3508.033221 | 3617.468331 | 3712.184536 | 3635.898704 | 3959.15364 | 4025.408092 | 4094.47174 | 3144.453697 | 3136.814477 | 2881.254791 | 3098.672409 | 4014.920076 | 3969.860824 | 3976.544022 | 3899.655902 |
| **Rem2** | 6.409875371 | 7.130358077 | 5.482129061 | 5.175656518 | 17.73417084 | 8.416953669 | 9.922711863 | 2.514222039 | 24.82966605 | 8.739019689 | 0.945870699 | 7.807752957 | 12.78126473 | 10.84265582 | 8.622788064 |
| **Prmt5** | 3791.89913 | 3916.151108 | 3967.495118 | 3872.253685 | 3966.120636 | 3751.155685 | 3826.55852 | 4114.943404 | 3814.739601 | 4232.307235 | 3983.061513 | 3686.994452 | 3700.602181 | 3699.152744 | 3894.625942 |
| **Haus4** | 920.274964 | 981.6126285 | 1067.448844 | 936.7938297 | 972.8459432 | 958.1298926 | 929.1266562 | 791.9799423 | 1018.768722 | 1129.081344 | 1056.537571 | 989.8495693 | 1001.19907 | 1011.077655 | 1014.614729 |
| **Ajuba** | 20.14532259 | 23.76786026 | 28.9769679 | 28.46611085 | 32.9348887 | 25.95227381 | 22.55161787 | 26.81836842 | 9.781383594 | 16.60413741 | 42.56418144 | 45.11146153 | 30.67503535 | 18.97464768 | 20.83840449 |
| **4931414P19Rik** | 95.23243408 | 106.9553711 | 90.84671016 | 107.8261775 | 91.20430718 | 92.58649035 | 84.79408319 | 93.86428946 | 97.06142181 | 94.38141264 | 85.12836289 | 69.4022485 | 115.0313825 | 84.0305826 | 89.10214332 |
| **Psmb5** | 1173.007193 | 1122.635266 | 1233.479039 | 1123.980074 | 1065.95034 | 1175.567862 | 1152.838705 | 1171.62747 | 1214.396394 | 1289.879306 | 1296.788728 | 1047.106424 | 953.4823487 | 1053.544724 | 971.5007885 |
| **Cdh24** | 21.06101908 | 21.39107423 | 41.50754861 | 19.84001665 | 40.53524763 | 30.16075065 | 44.20117102 | 30.17066447 | 48.90691797 | 34.95607876 | 35.94308655 | 38.17123668 | 28.97086672 | 25.29953024 | 35.9282836 |
| **Acin1** | 4634.339893 | 4664.046444 | 4696.618283 | 4633.075193 | 4728.689982 | 4550.766283 | 5015.479814 | 4965.588527 | 4758.266911 | 4918.320281 | 5165.399886 | 5156.587064 | 5069.901675 | 5125.865538 | 5009.121299 |
| **Mir6948** | 0 | 0 | 3.915806472 | 0 | 0 | 0.701412806 | 0 | 0 | 0 | 0 | 0 | 0 | 0 | 0 | 2.155697016 |
| **4930579G18Rik** | 43.03773463 | 57.04286461 | 79.09929074 | 62.10787821 | 61.43623469 | 46.99465798 | 37.88671802 | 76.26473519 | 109.1000478 | 90.88580477 | 71.88617311 | 52.05168638 | 51.12505891 | 83.12702794 | 74.01226421 |
| **1700123O20Rik** | 393.7494871 | 386.6238602 | 444.052454 | 426.1290533 | 409.1526558 | 380.1657407 | 396.0064098 | 419.8750805 | 410.0656968 | 403.7427096 | 471.043608 | 430.2939407 | 414.1129772 | 384.0107269 | 382.2769375 |
| **Slc7a8** | 0 | 3.961310043 | 1.566322589 | 0.86260942 | 0 | 0.701412806 | 0.902064715 | 0 | 3.00965649 | 0 | 1.891741398 | 0.867528106 | 0 | 3.614218606 | 2.155697016 |
| **Homez** | 177.6451174 | 133.8922794 | 184.8260655 | 145.7809919 | 141.2400035 | 172.5475502 | 167.7840369 | 150.0152483 | 133.1772997 | 187.0150213 | 122.9631908 | 132.7318003 | 119.2918041 | 104.8123396 | 178.9228523 |
| **Ppp1r3e** | 169.4038491 | 176.6744279 | 155.0659363 | 165.6210086 | 119.7056532 | 173.248963 | 196.6501078 | 177.6716908 | 142.9586833 | 120.5984717 | 162.6897602 | 144.8771937 | 141.4459963 | 147.2794082 | 158.0844478 |
| **Bcl2l2** | 176.7294209 | 167.9595458 | 180.1270977 | 153.5444767 | 92.47103366 | 108.7189849 | 119.0725424 | 215.3850214 | 179.0745612 | 222.8450021 | 155.1227946 | 139.6720251 | 113.3272139 | 118.3656594 | 136.5274777 |
| **Pabpn1** | 3755.271271 | 3523.981414 | 3857.069375 | 3584.142139 | 3607.637039 | 3622.797142 | 3815.733744 | 4087.286962 | 3803.45339 | 3942.171782 | 3909.283598 | 3719.092992 | 3692.933422 | 3691.924306 | 3550.432985 |
| **Slc22a17** | 4.578482408 | 16.63750218 | 7.04845165 | 3.450437679 | 5.700269198 | 9.118366474 | 1.80412943 | 0 | 12.03862596 | 11.3607256 | 6.621094891 | 0 | 0.852084315 | 9.035546516 | 6.467091048 |
| **Ngdn** | 1167.513014 | 1289.80255 | 1087.811038 | 1137.781825 | 1097.618502 | 1188.193293 | 1080.673528 | 1101.229253 | 1266.312968 | 1159.667913 | 1192.742951 | 1150.342269 | 1154.574247 | 1207.149015 | 1043.357356 |
| **Zfhx2** | 34.7964663 | 18.2220262 | 54.03812932 | 62.10787821 | 39.90188439 | 42.08476834 | 51.41768874 | 61.17940295 | 53.4214027 | 34.95607876 | 30.26786236 | 67.66719229 | 74.98341973 | 60.53816166 | 45.98820301 |
| **Thtpa** | 134.6073828 | 142.6071615 | 148.0174847 | 145.7809919 | 138.0731873 | 155.0122301 | 128.0931895 | 147.5010263 | 118.8814314 | 155.5545505 | 124.8549322 | 139.6720251 | 140.593912 | 148.1829629 | 154.4916195 |
| **Ap1g2** | 1160.187442 | 1089.360262 | 1098.775296 | 1015.291287 | 1057.716618 | 1072.46018 | 1067.142558 | 1079.439329 | 877.3148669 | 1019.843598 | 1056.537571 | 949.0757483 | 1110.265863 | 1125.829096 | 1133.178065 |
| **Jph4** | 3.662785926 | 4.753572051 | 1.566322589 | 1.725218839 | 3.800179466 | 5.611302446 | 1.80412943 | 5.866518091 | 5.266898858 | 1.747803938 | 2.837612096 | 2.602584319 | 0 | 2.710663955 | 3.59282836 |
| **Dhrs4** | 279.2874269 | 343.8417117 | 297.6012919 | 343.318549 | 305.2810837 | 319.8442394 | 286.8565793 | 412.3324144 | 328.0525574 | 328.5871403 | 325.3795204 | 366.964389 | 324.6441241 | 325.2796746 | 311.8575016 |
| **Lrrc16b** | 0 | 1.584524017 | 2.349483883 | 1.725218839 | 4.43354271 | 0.701412806 | 6.314453003 | 6.704592104 | 7.524141226 | 2.621705907 | 1.891741398 | 2.602584319 | 5.112505891 | 0.903554652 | 3.59282836 |
| **Pck2** | 1391.858652 | 1198.692419 | 1147.331296 | 954.9086276 | 1055.183165 | 1296.912278 | 1147.426317 | 781.9230542 | 762.9479203 | 891.3800083 | 730.2121795 | 959.4860855 | 1028.465768 | 919.8186353 | 1060.602932 |
| **Dcaf11** | 770.100741 | 754.2334321 | 762.0159395 | 755.6458516 | 633.3632443 | 741.3933356 | 739.6930661 | 666.2688404 | 741.1279107 | 692.1303594 | 719.8076018 | 786.8479924 | 687.6320423 | 697.544191 | 745.8711675 |
| **Psme1** | 6279.846471 | 6207.372837 | 6183.841581 | 6104.686863 | 6384.934866 | 6084.054677 | 6377.597533 | 6107.045333 | 5966.643992 | 5892.720976 | 6236.125517 | 6156.84697 | 6067.692408 | 6206.516902 | 6062.538574 |
| **Emc9** | 49.44761 | 59.41965064 | 55.60445191 | 58.65744054 | 71.5700466 | 77.85682143 | 41.49497688 | 53.63673684 | 45.14484735 | 40.19949057 | 30.26786236 | 50.31663016 | 74.98341973 | 49.69550584 | 52.45529405 |
| **Psme2** | 5865.035964 | 5953.848994 | 5699.06474 | 5610.411665 | 5844.676018 | 5875.033661 | 6113.292572 | 5925.183272 | 5571.626578 | 5510.825816 | 5625.093046 | 5979.871237 | 5343.42074 | 5788.171098 | 5327.445892 |
| **Rnf31** | 1183.079854 | 1179.678131 | 1090.943683 | 1065.322633 | 1152.087741 | 1248.514794 | 1096.008628 | 974.6800772 | 1050.370115 | 942.9402244 | 957.2211472 | 1110.435976 | 1085.555418 | 1004.752773 | 1149.705075 |
| **Irf9** | 759.1123832 | 752.6489081 | 620.2637452 | 612.452688 | 697.3329319 | 703.5170441 | 747.8116486 | 442.5030789 | 491.326422 | 471.9070632 | 549.550876 | 667.9966418 | 702.96956 | 592.7318514 | 714.2542779 |
| **Rec8** | 1.831392963 | 0 | 1.566322589 | 0 | 2.533452977 | 2.104238417 | 0.902064715 | 0.838074013 | 5.266898858 | 0 | 0 | 0 | 0 | 0 | 0 |
| **Ipo4** | 1772.788388 | 1795.265711 | 1822.416332 | 1925.344225 | 1751.882734 | 1618.159343 | 1757.222064 | 2258.609465 | 2147.389906 | 2314.966316 | 2371.297842 | 1700.355088 | 1710.133221 | 1647.18013 | 1746.114583 |
| **Tm9sf1** | 1104.329957 | 1170.170987 | 1168.476651 | 1103.277448 | 1026.048456 | 1066.147465 | 1146.524252 | 1242.863761 | 1096.267377 | 1336.19611 | 1221.119072 | 1198.923843 | 1129.011718 | 1298.408034 | 1193.537581 |
| **Tssk4** | 8.241268334 | 13.46845414 | 9.397935534 | 10.35131304 | 5.700269198 | 10.52119209 | 8.118582433 | 4.190370065 | 5.266898858 | 5.243411813 | 7.56696559 | 8.675281063 | 7.668758836 | 3.614218606 | 5.029959704 |
| **Mdp1** | 428.5459534 | 435.7441047 | 441.7029701 | 414.0525214 | 475.6557965 | 542.1920988 | 465.4653928 | 415.6847105 | 468.7539984 | 481.5199849 | 364.160219 | 406.0031537 | 421.781736 | 500.569277 | 438.3250599 |
| **Nedd8** | 2123.500141 | 2094.74075 | 2096.522785 | 1989.177322 | 2128.733864 | 2234.701199 | 2168.563574 | 2220.058061 | 2195.54441 | 2279.136335 | 2048.755934 | 2235.61993 | 2171.962919 | 2266.115066 | 2098.930328 |
| **Gmpr2** | 756.3652938 | 667.8768732 | 693.8809069 | 736.6684444 | 609.295441 | 637.5842404 | 578.2234822 | 782.7611282 | 717.8030729 | 830.2068705 | 771.8304902 | 663.6590013 | 665.4778501 | 722.8437213 | 610.0622555 |
| **Tinf2** | 527.4411734 | 488.0333972 | 581.1056805 | 476.1603996 | 494.6566938 | 547.8034013 | 565.5945762 | 502.8444078 | 517.6609163 | 464.9158475 | 531.5793327 | 556.9530442 | 535.9610342 | 558.3967747 | 572.6968406 |
| **Tgm1** | 21.06101908 | 15.84524017 | 18.01270977 | 12.93914129 | 4.43354271 | 12.6254305 | 18.0412943 | 16.76148026 | 21.06759543 | 38.45168663 | 12.29631908 | 5.205168638 | 5.112505891 | 9.035546516 | 12.21561642 |
| **Rabggta** | 362.6158067 | 369.194096 | 375.1342601 | 325.2037512 | 348.3497844 | 330.3654315 | 340.9804622 | 269.8598322 | 324.2904868 | 353.9302974 | 327.2712618 | 343.5411301 | 339.9816417 | 377.6858444 | 370.0613211 |
| **Dhrs1** | 318.6623756 | 311.3589693 | 338.3256792 | 314.8524382 | 274.879648 | 298.1004424 | 328.3515562 | 292.4878306 | 331.8146281 | 317.2264147 | 278.0859854 | 340.9385458 | 345.0941476 | 396.660492 | 324.0731181 |
| **Nop9** | 1828.645874 | 1639.982358 | 1847.477494 | 1864.098956 | 1572.640936 | 1667.258239 | 1631.835069 | 2006.349187 | 2141.370593 | 1919.088724 | 1999.570657 | 1656.111155 | 1641.966475 | 1548.692673 | 1598.80862 |
| **Cideb** | 1.831392963 | 11.88393013 | 7.04845165 | 4.313047098 | 5.700269198 | 7.715540863 | 0.902064715 | 9.218814144 | 4.514484735 | 6.991215751 | 2.837612096 | 6.94022485 | 4.260421576 | 3.614218606 | 3.59282836 |
| **Ltb4r1** | 0.915696482 | 0.792262009 | 0 | 4.313047098 | 0.633363244 | 4.208476834 | 2.706194144 | 5.866518091 | 9.028969471 | 6.117313782 | 1.891741398 | 5.205168638 | 1.70416863 | 3.614218606 | 3.59282836 |
| **Adcy4** | 0 | 0 | 3.132645178 | 0 | 3.166816221 | 0 | 0 | 0 | 2.257242368 | 4.369509845 | 2.837612096 | 0 | 1.70416863 | 0 | 2.155697016 |
| **Ripk3** | 681.2781823 | 676.5917553 | 737.7379394 | 747.0197574 | 667.5648595 | 673.3562935 | 696.3939598 | 656.2119522 | 707.2692752 | 646.687457 | 570.3600314 | 749.5442838 | 694.4487169 | 651.4629038 | 690.5416108 |
| **Nynrin** | 0 | 0 | 1.566322589 | 1.725218839 | 1.266726489 | 0 | 1.80412943 | 0 | 0 | 0 | 1.891741398 | 0 | 0 | 0 | 0 |
| **Cbln3** | 0 | 0 | 0 | 0 | 1.266726489 | 0.701412806 | 2.706194144 | 0 | 0.752414123 | 0 | 0 | 0 | 0 | 0 | 3.59282836 |
| **Khnyn** | 503.6330649 | 549.8298339 | 527.0675512 | 496.8630257 | 532.0251252 | 518.3440634 | 605.2854236 | 373.7810098 | 387.4932731 | 323.3437285 | 392.53634 | 537.8674259 | 562.375648 | 525.8688072 | 565.5111838 |
| **Sdr39u1** | 837.8622806 | 938.0382181 | 863.8269078 | 836.7311371 | 836.0394824 | 786.2837552 | 801.0334667 | 763.4854259 | 761.4430921 | 854.6761256 | 724.5369553 | 876.2033873 | 859.753074 | 834.8844981 | 794.7336332 |
| **Gm5801** | 10.98835778 | 6.338096068 | 11.74741942 | 0.86260942 | 16.46744435 | 7.014128057 | 9.020647148 | 3.352296052 | 4.514484735 | 4.369509845 | 5.675224193 | 5.205168638 | 4.260421576 | 12.64976512 | 2.155697016 |
| **Gzme** | 0.915696482 | 5.54583406 | 5.482129061 | 2.587828259 | 0 | 1.402825611 | 4.510323574 | 0 | 0 | 0 | 0 | 0 | 1.70416863 | 1.807109303 | 1.437131344 |
| **Gzmf** | 0 | 0 | 0.783161294 | 6.038265938 | 1.900089733 | 6.312715251 | 4.510323574 | 14.24725822 | 6.771727103 | 7.86511772 | 8.512836289 | 1.735056213 | 6.816674521 | 3.614218606 | 0.718565672 |
| **Gzmc** | 1260.914055 | 1098.075144 | 772.9801977 | 1038.581741 | 364.8172287 | 460.1268005 | 366.2382742 | 1687.042988 | 929.2314414 | 1582.636466 | 1600.413222 | 336.6009052 | 534.2568656 | 384.0107269 | 324.7916837 |
| **Gzmb** | 84305.42797 | 63997.34052 | 62713.20698 | 59678.77009 | 45376.67627 | 46877.52204 | 52403.64548 | 59493.19804 | 55200.8621 | 64004.5802 | 73278.49478 | 42504.53957 | 45960.57588 | 43136.60262 | 49433.00684 |
| **Rnf17** | 0 | 3.169048034 | 0.783161294 | 5.175656518 | 0 | 2.104238417 | 0.902064715 | 1.676148026 | 3.762070613 | 0 | 0.945870699 | 0 | 0 | 1.807109303 | 0.718565672 |
| **Cenpj** | 423.967471 | 429.4060086 | 408.0270344 | 399.3881613 | 385.0848525 | 369.6445486 | 394.2022804 | 383.837898 | 360.4063647 | 390.6341801 | 403.8867884 | 399.930457 | 399.6275438 | 399.371156 | 361.438533 |
| **Parp4** | 1034.737024 | 987.9507246 | 958.5894245 | 932.4807826 | 944.9779605 | 918.8507755 | 1118.560246 | 835.559791 | 729.8416989 | 699.1215751 | 777.5057144 | 937.7978829 | 1013.980335 | 1039.087849 | 1078.567074 |
| **Mphosph8** | 767.3536516 | 781.1703404 | 832.500456 | 765.1345552 | 810.7049527 | 799.6105985 | 850.647026 | 1015.745704 | 931.4886838 | 853.8022236 | 998.8394579 | 857.117769 | 808.6280151 | 778.8641097 | 754.4939556 |
| **Gm16973** | 18.31392963 | 19.80655021 | 16.44638718 | 18.11479781 | 20.90098706 | 18.93814575 | 19.84542373 | 20.95185033 | 15.80069657 | 13.10852953 | 16.07980188 | 13.01292159 | 17.89377062 | 11.74621047 | 22.9941015 |
| **Pspc1** | 1281.975074 | 1403.888279 | 1401.858717 | 1305.128052 | 1297.127924 | 1302.52358 | 1351.292943 | 1705.480617 | 1591.355869 | 1618.466446 | 1584.33342 | 1316.907665 | 1375.264085 | 1433.037677 | 1310.663786 |
| **Zmym5** | 269.2147656 | 328.7887335 | 372.0016149 | 330.3794077 | 389.5183952 | 321.9484778 | 326.5474268 | 382.16175 | 346.1104964 | 401.9949057 | 344.2969344 | 376.5071981 | 442.2317596 | 402.9853746 | 396.6482509 |
| **Zmym2** | 782.9204917 | 795.4310565 | 820.7530366 | 781.5241342 | 786.0037861 | 745.6018125 | 826.2912787 | 816.2840887 | 759.9382638 | 779.5205563 | 736.8332743 | 798.1258578 | 785.6217386 | 789.7067655 | 796.1707645 |
| **Gjb2** | 2.747089445 | 6.338096068 | 6.265290356 | 2.587828259 | 17.1008076 | 7.014128057 | 12.62890601 | 9.218814144 | 5.266898858 | 6.991215751 | 3.783482795 | 18.21809023 | 10.22501178 | 8.131991864 | 9.341353736 |
| **Cryl1** | 173.9823315 | 180.6357379 | 191.8745171 | 166.483618 | 234.3444004 | 186.5758063 | 180.412943 | 164.2625066 | 139.9490268 | 146.8155308 | 138.097122 | 198.6639363 | 202.796067 | 178.0002664 | 234.9709747 |
| **Ift88** | 65.93014667 | 92.694655 | 98.67832311 | 75.04701951 | 62.70296118 | 68.73845496 | 61.34040061 | 106.4353997 | 81.26072524 | 93.50751067 | 116.3420959 | 73.73988903 | 84.3563472 | 86.74124655 | 68.26373884 |
| **Il17d** | 12.81975074 | 6.338096068 | 8.614774239 | 4.313047098 | 8.233722176 | 12.6254305 | 12.62890601 | 6.704592104 | 4.514484735 | 17.47803938 | 7.56696559 | 6.072696744 | 5.112505891 | 14.45687443 | 15.80844478 |
| **N6amt2** | 484.4034387 | 530.8155457 | 484.7768413 | 466.671696 | 489.5897878 | 450.3070213 | 423.9704159 | 562.3476628 | 566.5678343 | 557.5494562 | 501.3114703 | 456.3197839 | 518.0672636 | 486.1124025 | 441.9178883 |
| **Xpo4** | 1086.931724 | 1171.755511 | 1193.537813 | 1142.094872 | 923.4436102 | 986.1864048 | 1116.756117 | 1202.636209 | 1345.316451 | 1254.923227 | 1296.788728 | 1129.521594 | 1192.918041 | 1017.402538 | 1135.333762 |
| **Lats2** | 322.3251615 | 331.1655196 | 326.5782598 | 364.0211751 | 342.0161519 | 357.0191181 | 362.6300153 | 224.6038355 | 264.8497711 | 225.466708 | 291.3281752 | 445.9094466 | 368.9525085 | 404.7924839 | 441.9178883 |
| **Sap18** | 195.9590471 | 192.5196681 | 165.2470331 | 195.8123383 | 177.9750716 | 194.99276 | 218.299661 | 178.5097648 | 196.380086 | 187.0150213 | 151.3393118 | 205.6041612 | 173.8252003 | 184.3251489 | 172.4557613 |
| **Ska3** | 480.7406528 | 522.8929256 | 577.189874 | 496.0004163 | 587.1277274 | 493.7946152 | 536.7285053 | 621.0128437 | 607.1981969 | 548.8104365 | 595.8985402 | 532.6622573 | 508.6943362 | 590.0211875 | 505.1516674 |
| **Mrpl57** | 1122.643886 | 1094.113834 | 1058.050909 | 1185.225343 | 1034.915541 | 1192.40177 | 1064.436363 | 1028.316814 | 1070.685296 | 1121.216226 | 1079.238467 | 1032.358446 | 990.1219742 | 1077.037145 | 1046.231618 |
| **Zdhhc20** | 1458.704495 | 1559.171633 | 1599.998525 | 1516.46736 | 1542.2395 | 1573.970336 | 1696.783729 | 1521.942408 | 1504.075831 | 1457.668484 | 1583.38755 | 1653.508571 | 1737.399919 | 1682.418761 | 1662.042399 |
| **Micu2** | 1531.960214 | 1472.815074 | 1466.861105 | 1443.145559 | 1429.500842 | 1537.49687 | 1630.933004 | 1444.001524 | 1428.082005 | 1365.908777 | 1441.506945 | 1539.862389 | 1501.372563 | 1522.489588 | 1381.083222 |
| **Rcbtb1** | 1088.763117 | 1176.509083 | 1112.872199 | 1138.644434 | 1124.853122 | 1030.375412 | 1169.977935 | 1164.922878 | 1242.235716 | 1174.524246 | 1244.76584 | 1149.474741 | 1121.342959 | 1187.270812 | 1147.549378 |
| **Gm6904** | 24.723805 | 47.53572051 | 25.84432272 | 25.01567317 | 21.53435031 | 21.04238417 | 34.27845916 | 6.704592104 | 8.276555348 | 10.48682363 | 15.13393118 | 15.61550591 | 25.56252945 | 19.87820233 | 17.24557613 |
| **Phf11a** | 88.82255871 | 87.94108294 | 58.73709709 | 71.59658183 | 77.90367905 | 84.87094949 | 97.4229892 | 43.57984868 | 27.83932254 | 36.70388269 | 34.99721585 | 54.6542707 | 47.71672165 | 58.73105235 | 58.9223851 |
| **Phf11b** | 684.0252717 | 594.9887684 | 574.8403902 | 508.9395576 | 585.861001 | 633.3757636 | 664.8216948 | 316.7919769 | 303.2228914 | 346.0651797 | 315.9208134 | 539.6024821 | 598.1631892 | 605.3816166 | 612.2179525 |
| **Phf11d** | 5.494178889 | 10.29940611 | 4.698967767 | 3.450437679 | 2.533452977 | 6.312715251 | 3.608258859 | 0.838074013 | 5.266898858 | 1.747803938 | 4.729353494 | 6.94022485 | 0.852084315 | 11.74621047 | 5.748525376 |
| **Phf11c** | 682.1938788 | 663.1233011 | 509.8380027 | 525.3291366 | 551.6593858 | 703.5170441 | 730.672419 | 263.9933141 | 415.3325957 | 271.7835123 | 310.2455892 | 543.9401226 | 525.7360225 | 582.7927503 | 600.0023361 |
| **Setdb2** | 515.5371191 | 426.2369606 | 397.0627763 | 386.44902 | 334.415793 | 345.0951004 | 377.0630508 | 289.9736085 | 355.1394659 | 382.7690624 | 402.9409177 | 390.3876478 | 369.8045928 | 326.1832292 | 346.3486539 |
| **Cab39l** | 535.6824417 | 576.7667422 | 577.9730353 | 516.7030424 | 623.8627956 | 589.8881696 | 631.4453003 | 539.7196644 | 518.4133305 | 557.5494562 | 562.7930658 | 612.474843 | 615.2048755 | 637.0060294 | 594.2538107 |
| **Cdadc1** | 247.23805 | 234.5095545 | 271.7569692 | 291.5619838 | 275.5130113 | 288.982076 | 267.0111556 | 224.6038355 | 252.0587311 | 213.2320804 | 294.1657873 | 268.9337129 | 239.4356926 | 312.6299094 | 330.5402091 |
| **Shisa2** | 77.83420093 | 108.5398952 | 92.41303275 | 112.1392246 | 76.63695256 | 91.88507755 | 98.32505391 | 90.51199341 | 74.48899814 | 96.12921658 | 160.7980188 | 105.838429 | 98.84178056 | 121.0763233 | 100.5991941 |
| **Atp8a2** | 21.97671556 | 26.93690829 | 23.49483883 | 42.26786156 | 27.86798275 | 42.08476834 | 33.37639445 | 18.43762829 | 21.06759543 | 19.22584332 | 39.72656935 | 43.37640531 | 33.23128829 | 42.46706862 | 35.20971793 |
| **Nupl1** | 1145.536298 | 1134.519196 | 1137.1502 | 1043.757398 | 1090.018143 | 1100.516692 | 1182.606841 | 1187.550876 | 1137.650153 | 1055.673578 | 1181.392503 | 1138.196875 | 1161.390922 | 1212.570342 | 1185.633359 |
| **Mtmr6** | 948.6615549 | 1034.694183 | 1098.775296 | 972.1608159 | 969.679127 | 1049.313557 | 1132.091217 | 983.0608173 | 1025.540449 | 1032.952127 | 1003.568811 | 1115.641145 | 1100.892935 | 1168.296164 | 1014.614729 |
| **Spata13** | 2711.377282 | 2820.45275 | 2909.444209 | 2932.009417 | 2886.236304 | 2849.84023 | 3177.97399 | 2235.143393 | 2492.747988 | 2233.693433 | 2073.348572 | 3117.028486 | 3368.289298 | 3084.73558 | 3176.06027 |
| **C1qtnf9** | 0 | 2.376786026 | 0 | 0.86260942 | 0 | 0.701412806 | 0.902064715 | 0 | 0 | 0 | 0 | 0 | 2.556252945 | 3.614218606 | 0 |
| **Mipep** | 560.4062467 | 606.0804365 | 526.2843899 | 538.2682779 | 581.4274582 | 537.983622 | 561.9863173 | 608.4417335 | 619.989237 | 597.7489467 | 549.550876 | 504.0338297 | 610.944454 | 519.5439247 | 505.1516674 |
| **Tnfrsf19** | 5.494178889 | 3.169048034 | 7.04845165 | 0.86260942 | 3.800179466 | 1.402825611 | 8.118582433 | 13.40918421 | 1.504828245 | 4.369509845 | 5.675224193 | 3.470112425 | 5.112505891 | 5.421327909 | 2.874262688 |
| **Sacs** | 165.7410632 | 134.6845414 | 170.7291622 | 175.1097122 | 97.53793962 | 117.8373514 | 126.2890601 | 186.8905049 | 215.9428532 | 146.8155308 | 156.0686653 | 149.2148343 | 132.0730689 | 135.5331977 | 149.4616598 |
| **Arl11** | 14.65114371 | 9.507144102 | 16.44638718 | 7.763484777 | 6.333632443 | 6.312715251 | 12.62890601 | 0.838074013 | 3.762070613 | 0.873901969 | 19.86328467 | 11.27786538 | 11.0770961 | 13.55331977 | 23.71266717 |
| **Ebpl** | 138.2701687 | 152.1143056 | 144.8848395 | 126.8035847 | 141.2400035 | 127.6571306 | 159.6654545 | 160.9102105 | 154.2448951 | 115.3550599 | 148.5016997 | 102.3683165 | 145.7064179 | 141.8580803 | 163.8329732 |
| **Kpna3** | 2344.182993 | 2407.684244 | 2261.769818 | 2385.115045 | 2101.499245 | 2151.233075 | 2494.208936 | 2688.541434 | 2613.134248 | 2515.963768 | 2481.964714 | 2275.526223 | 2324.486012 | 2138.71386 | 2290.787362 |
| **Spryd7** | 249.069443 | 237.6786026 | 252.1779368 | 249.2941223 | 211.5433236 | 218.1393826 | 238.1450847 | 293.3259046 | 266.3545994 | 264.7922966 | 314.9749427 | 200.3989926 | 226.6544278 | 277.391278 | 194.7312971 |
| **Trim13** | 77.83420093 | 97.44822705 | 86.93090369 | 113.8644434 | 84.23731149 | 91.88507755 | 59.53627118 | 82.96932729 | 94.80417945 | 92.6336087 | 108.7751304 | 120.5864068 | 104.8063708 | 93.96968376 | 87.66501198 |
| **Dleu2** | 24.723805 | 19.80655021 | 38.37490343 | 16.38957897 | 29.76807248 | 30.16075065 | 38.78878274 | 14.24725822 | 24.07725192 | 20.09974528 | 33.10547446 | 35.56865236 | 38.34379418 | 40.65995932 | 56.04812241 |
| **Kcnrg** | 4.578482408 | 0.792262009 | 3.132645178 | 1.725218839 | 0 | 0.701412806 | 0.902064715 | 2.514222039 | 5.266898858 | 1.747803938 | 0.945870699 | 6.072696744 | 1.70416863 | 5.421327909 | 2.155697016 |
| **Mir16-1** | 2.747089445 | 1.584524017 | 0 | 0 | 0 | 0 | 0 | 0 | 0 | 1.747803938 | 0.945870699 | 2.602584319 | 2.556252945 | 4.517773258 | 1.437131344 |
| **Dleu7** | 39.37494871 | 38.02857641 | 28.9769679 | 42.26786156 | 27.2346195 | 47.69607079 | 31.57226502 | 7.542666117 | 35.36346376 | 4.369509845 | 25.53850887 | 36.43618046 | 24.71044514 | 24.39597559 | 26.58692986 |
| **Rnaseh2b** | 1075.027669 | 1175.716821 | 1215.466329 | 1117.941808 | 1232.524873 | 1158.032542 | 1123.07057 | 1309.071608 | 1223.425363 | 1128.207442 | 1222.064943 | 1165.090247 | 1219.332655 | 1216.184561 | 1130.303802 |
| **Fam124a** | 15.56684019 | 11.88393013 | 20.36219366 | 28.46611085 | 32.30152546 | 29.45933784 | 30.6702003 | 19.2757023 | 16.5531107 | 9.612921658 | 17.02567258 | 37.30370857 | 27.26669809 | 28.0101942 | 30.89832389 |
| **Serpine3** | 8.241268334 | 6.338096068 | 8.614774239 | 20.70262607 | 12.66726489 | 14.72966892 | 17.13922958 | 16.76148026 | 16.5531107 | 4.369509845 | 9.458706988 | 14.74797781 | 13.63334904 | 10.84265582 | 8.622788064 |
| **Ints6** | 836.0308877 | 816.0298688 | 849.7300045 | 792.7380567 | 777.770064 | 712.6354106 | 853.3532202 | 844.7786052 | 826.1507066 | 785.63787 | 696.1608343 | 775.570127 | 721.7154149 | 696.6406364 | 756.6496526 |
| **Wdfy2** | 52.19469945 | 73.68036679 | 58.73709709 | 50.03134634 | 50.66905954 | 52.60596043 | 46.00530045 | 52.79866282 | 45.14484735 | 69.03825554 | 50.13114703 | 62.46202365 | 58.79381775 | 45.17773258 | 54.61099107 |
| **Ctsb** | 1851.538286 | 1926.781205 | 1974.349623 | 1856.335471 | 2071.097809 | 1957.643141 | 2095.496332 | 1881.476159 | 1979.601557 | 1868.40241 | 1818.909354 | 2055.174084 | 2085.902404 | 2085.404136 | 2062.283479 |
| **Fdft1** | 1994.386937 | 1982.239545 | 1955.553752 | 1853.747643 | 2009.028211 | 2102.835592 | 2064.826132 | 1924.217934 | 1984.116041 | 1948.801391 | 2199.149375 | 2160.144985 | 2085.050319 | 1916.439416 | 1965.995678 |
| **Neil2** | 2.747089445 | 14.26071615 | 3.132645178 | 1.725218839 | 1.900089733 | 2.104238417 | 3.608258859 | 4.190370065 | 0 | 0 | 0.945870699 | 2.602584319 | 7.668758836 | 0.903554652 | 6.467091048 |
| **Mtmr9** | 784.7518847 | 763.7405762 | 758.1001331 | 858.2963726 | 710.6335601 | 730.1707307 | 799.2293373 | 714.0390591 | 756.9286073 | 723.5908303 | 739.6708864 | 765.1597897 | 757.5029562 | 682.1837619 | 750.9011272 |
| **Pinx1** | 522.862691 | 540.3226898 | 526.2843899 | 530.5047931 | 501.6236895 | 446.7999572 | 506.9603697 | 454.2361151 | 519.9181587 | 491.1329065 | 507.9325652 | 469.3327055 | 429.4504948 | 427.3813502 | 478.5647375 |
| **Msra** | 205.1160119 | 225.7946724 | 240.4305174 | 219.965402 | 228.0107679 | 239.8831796 | 228.2223728 | 218.7373174 | 170.7980058 | 241.1969434 | 192.9576225 | 197.7964082 | 210.4648258 | 232.2135455 | 208.3840449 |
| **Kif13b** | 213.3572802 | 201.2345502 | 278.8054208 | 276.0350143 | 190.0089733 | 192.8885216 | 194.8459784 | 372.9429358 | 290.4318513 | 270.0357084 | 250.6557352 | 249.8480946 | 232.619018 | 229.5028815 | 226.3481867 |
| **Hmbox1** | 243.5752641 | 240.8476506 | 232.5989045 | 214.7897455 | 187.4755203 | 193.5899344 | 216.4955315 | 265.6694621 | 227.229065 | 191.3845312 | 235.521804 | 240.3052854 | 261.5898848 | 190.6500315 | 230.6595807 |
| **Ints9** | 924.8534464 | 957.0525063 | 940.5767147 | 1006.665193 | 941.177781 | 820.6529827 | 782.0901077 | 990.6034834 | 957.0707639 | 913.2275575 | 1046.132993 | 890.9513651 | 907.4697956 | 972.2248051 | 954.973778 |
| **Extl3** | 1453.210316 | 1466.476978 | 1376.797556 | 1539.757814 | 1448.50174 | 1421.062344 | 1542.530662 | 1952.71245 | 1694.436604 | 1517.093818 | 1640.139792 | 1544.200029 | 1573.79973 | 1540.560681 | 1509.706477 |
| **Fzd3** | 1.831392963 | 0 | 1.566322589 | 0.86260942 | 1.266726489 | 0.701412806 | 0 | 6.704592104 | 2.257242368 | 4.369509845 | 0 | 1.735056213 | 1.70416863 | 1.807109303 | 5.748525376 |
| **Zfp395** | 617.1794286 | 606.0804365 | 614.7816162 | 629.7048763 | 768.9029786 | 776.4639759 | 839.8222495 | 288.2974605 | 320.5284162 | 287.5137478 | 277.1401147 | 871.8657468 | 822.2613641 | 795.1280934 | 757.3682183 |
| **Pnoc** | 0 | 0 | 0 | 0.86260942 | 1.900089733 | 0.701412806 | 0 | 0 | 3.00965649 | 0.873901969 | 0 | 0.867528106 | 0 | 0 | 0 |
| **Elp3** | 1025.580059 | 1060.046567 | 1085.461554 | 981.6495196 | 1019.08146 | 998.1104225 | 1140.209799 | 1024.964518 | 1093.25772 | 955.174852 | 1076.400855 | 1005.465075 | 989.2698899 | 1183.656594 | 1030.423174 |
| **Pbk** | 711.4961662 | 682.1375893 | 790.9929074 | 624.5292198 | 687.19912 | 655.8209733 | 658.5072418 | 611.7940295 | 723.8223859 | 693.8781633 | 699.9443171 | 666.2615856 | 592.198599 | 710.1939561 | 615.8107809 |
| **Esco2** | 638.2404477 | 757.4024801 | 838.7657464 | 783.249353 | 814.5051321 | 714.0382362 | 774.87359 | 859.8639374 | 895.3728059 | 767.2859287 | 814.3946716 | 831.9594539 | 771.1363052 | 765.3107899 | 761.6796123 |
| **Ccdc25** | 743.545543 | 769.2864103 | 838.7657464 | 791.0128378 | 759.4025299 | 765.241371 | 827.1933435 | 944.5094127 | 907.4114318 | 814.476635 | 879.6597499 | 808.5361951 | 794.1425817 | 839.4022713 | 828.5062198 |
| **Chrna2** | 5.494178889 | 0 | 2.349483883 | 2.587828259 | 6.333632443 | 10.52119209 | 0.902064715 | 5.028444078 | 11.28621184 | 5.243411813 | 1.891741398 | 1.735056213 | 16.18960199 | 2.710663955 | 7.18565672 |
| **Ptk2b** | 2038.340368 | 2321.327685 | 2177.188399 | 2191.890535 | 2292.774944 | 2196.123495 | 2368.821941 | 2209.163098 | 2176.734057 | 2031.822078 | 2111.1834 | 2332.783078 | 2412.250696 | 2304.967916 | 2269.948958 |

| | | | | | | | | | | | | | | | |
|---|---|---|---|---|---|---|---|---|---|---|---|---|---|---|---|
| **1700001G11Rik** | 0.915696482 | 3.961310043 | 0.783161294 | 5.175656518 | 4.43354271 | 2.805651223 | 2.706194144 | 0 | 2.257242368 | 6.117313782 | 1.891741398 | 1.735056213 | 3.408337261 | 3.614218606 | 0 |
| **Trim35** | 2646.362832 | 2746.772383 | 2645.518853 | 2537.796913 | 2287.708038 | 2399.533208 | 2528.487396 | 2603.895959 | 2589.056996 | 2728.321947 | 2533.041731 | 2435.151394 | 2514.500814 | 2250.754637 | 2424.440577 |
| **Dpysl2** | 627.2520899 | 620.3411527 | 575.6235514 | 582.2613583 | 626.3962486 | 578.6655647 | 627.8370415 | 573.2426249 | 610.2078534 | 489.3851026 | 538.2004276 | 695.7575412 | 622.8736344 | 611.7064991 | 602.1580331 |
| **Gm5464** | 0 | 0 | 0.783161294 | 1.725218839 | 0 | 1.402825611 | 6.314453003 | 0 | 0 | 0.873901969 | 1.891741398 | 6.072696744 | 0.852084315 | 1.807109303 | 0.718565672 |
| **Bnip3l** | 2575.854203 | 2442.543772 | 2498.284529 | 2191.890535 | 2067.930993 | 2195.422082 | 2328.229029 | 955.4043749 | 1157.965335 | 1240.066894 | 1304.355694 | 2416.933304 | 2528.986247 | 2267.922175 | 2246.236291 |
| **Ppp2r2a** | 2399.124782 | 2127.223493 | 2212.430657 | 2180.676613 | 2105.932787 | 2122.47515 | 2377.842588 | 2051.605184 | 2075.910564 | 2106.103745 | 2027.000907 | 2070.78959 | 2050.114862 | 1951.678047 | 2067.313438 |
| **Cdca2** | 1322.265719 | 1440.332331 | 1301.614071 | 1426.75598 | 1318.662275 | 1255.528922 | 1477.582003 | 1346.784939 | 1382.937157 | 1294.248816 | 1170.987925 | 1412.335757 | 1382.932844 | 1424.002131 | 1328.627927 |
| **Kctd9** | 581.4672658 | 568.8441221 | 593.6362612 | 565.0091699 | 595.3614496 | 603.9164257 | 603.4812942 | 521.2820361 | 531.9567847 | 528.7106912 | 611.9783421 | 622.8851803 | 602.4236108 | 627.9704828 | 596.4095077 |
| **Gnrh1** | 4.578482408 | 2.376786026 | 12.53058071 | 7.763484777 | 5.700269198 | 6.312715251 | 4.510323574 | 8.380740131 | 0.752414123 | 6.991215751 | 10.40457769 | 10.41033728 | 6.816674521 | 3.614218606 | 12.21561642 |
| **Dock5** | 286.6129987 | 179.0512139 | 186.3923881 | 236.354981 | 179.2417981 | 182.3673295 | 165.9799075 | 128.225324 | 167.7883493 | 166.9152761 | 204.3080709 | 202.1340488 | 200.2398141 | 181.614485 | 176.7671553 |
| **Stc1** | 22.89241204 | 19.0142882 | 10.96425812 | 24.15306375 | 11.4005384 | 7.014128057 | 22.55161787 | 4.190370065 | 0 | 3.495607876 | 1.891741398 | 8.675281063 | 13.63334904 | 5.421327909 | 0.718565672 |
| **Slc25a37** | 550.3335854 | 595.7810304 | 629.6616808 | 583.9865771 | 532.0251252 | 514.8369994 | 563.7904467 | 554.8049966 | 577.8540461 | 628.3355156 | 571.3059021 | 545.6751788 | 556.4110578 | 545.7470096 | 629.4635286 |
| **Gm16677** | 1.831392963 | 0 | 2.349483883 | 0 | 1.266726489 | 0 | 1.80412943 | 3.352296052 | 2.257242368 | 0 | 0 | 1.735056213 | 0 | 0.903554652 | 0 |
| **Entpd4** | 543.92371 | 487.2411352 | 533.3328415 | 534.8178402 | 784.7370597 | 908.3295834 | 799.2293373 | 591.6802532 | 592.1499145 | 551.4321424 | 575.0893849 | 854.5151847 | 771.9883895 | 791.5138748 | 806.2306839 |
| **Loxl2** | 6.409875371 | 7.130358077 | 6.265290356 | 9.488703616 | 39.26852115 | 32.26498906 | 33.37639445 | 10.05688816 | 6.771727103 | 9.612921658 | 4.729353494 | 30.36348372 | 34.93545692 | 53.30972444 | 13.65274777 |
| **R3hcc1** | 535.6824417 | 509.4244715 | 521.5854221 | 487.3743221 | 473.7557067 | 500.1073305 | 461.857134 | 542.2338864 | 461.2298571 | 510.3587498 | 437.9381335 | 500.5637173 | 455.8651086 | 461.716427 | 508.0259301 |
| **Chmp7** | 765.5222586 | 802.5614146 | 781.5949719 | 730.6301784 | 639.0635135 | 796.1035345 | 766.7550076 | 874.1111956 | 800.5686264 | 904.4885378 | 801.1524819 | 737.3988903 | 833.3384602 | 855.666255 | 776.0509257 |
| **4930480K23Rik** | 0.915696482 | 10.29940611 | 8.614774239 | 11.21392246 | 4.43354271 | 8.416953669 | 9.922711863 | 14.24725822 | 12.79104008 | 4.369509845 | 17.97154328 | 6.072696744 | 14.48543336 | 7.228437213 | 7.904222392 |
| **Tnfrsf10b** | 383.6768258 | 379.4935021 | 371.2184536 | 359.708128 | 338.2159724 | 331.0668443 | 423.0683512 | 590.0041052 | 442.4195041 | 478.898279 | 462.5307717 | 295.8270842 | 270.9628122 | 307.2085815 | 340.6001285 |
| **Rhobtb2** | 433.1244358 | 506.2554234 | 426.0397442 | 421.8160062 | 440.1874548 | 420.8476834 | 441.1096455 | 466.8072253 | 529.6995423 | 470.1592593 | 438.8840042 | 449.3795591 | 484.8359753 | 460.8128723 | 446.2292823 |
| **Egr3** | 3078.571571 | 2525.731283 | 2711.304402 | 2627.508292 | 2784.898185 | 2811.963938 | 3278.103174 | 1874.771567 | 1928.437396 | 1732.073702 | 1866.202889 | 2631.212746 | 2669.580159 | 2656.450676 | 2721.2082 |
| **Bin3** | 614.4323391 | 613.2107946 | 604.6005193 | 615.0405162 | 562.4265609 | 573.7556751 | 635.9556239 | 603.4132894 | 511.6416034 | 567.1623778 | 586.4398332 | 549.1452913 | 533.4047813 | 595.4425154 | 554.7326988 |
| **Ccar2** | 1593.311878 | 1515.597222 | 1426.919879 | 1454.359482 | 1489.670351 | 1530.482742 | 1534.41208 | 1332.537681 | 1331.020583 | 1512.724308 | 1404.617988 | 1736.791269 | 1581.468489 | 1639.951693 | 1570.065993 |
| **9930012K11Rik** | 9.156964816 | 20.59881222 | 20.36219366 | 25.87828259 | 31.66816221 | 23.84803539 | 35.18052388 | 46.93214473 | 21.06759543 | 31.46047088 | 20.80915537 | 38.17123668 | 30.67503535 | 28.91374885 | 23.71266717 |
| **Pdlim2** | 595.202713 | 614.0030566 | 715.8094232 | 657.3083778 | 770.169705 | 775.0611503 | 791.1107549 | 545.5861825 | 582.3685309 | 579.3970054 | 628.058144 | 730.4586655 | 717.4549934 | 712.9046201 | 720.0028033 |
| **Sorbs3** | 2.747089445 | 0.792262009 | 4.698967767 | 2.587828259 | 3.800179466 | 1.402825611 | 3.608258859 | 7.542666117 | 8.276555348 | 7.86511772 | 3.783482795 | 3.470112425 | 5.112505891 | 0.903554652 | 0 |
| **Ppp3cc** | 867.164568 | 854.8507072 | 902.9849725 | 960.9468935 | 907.609529 | 880.974484 | 942.6576269 | 706.496393 | 843.4562314 | 826.7112626 | 815.3405423 | 972.4990071 | 886.1676878 | 965.8999225 | 994.49489 |
| **Slc39a14** | 773.7635269 | 720.9584277 | 817.6203914 | 766.8597741 | 680.8654876 | 659.3280374 | 747.8116486 | 1011.555334 | 747.8996378 | 847.6849098 | 1016.811001 | 613.3423711 | 757.5029562 | 642.4273573 | 775.3323601 |
| **Piwil2** | 47.61621704 | 53.87381658 | 57.95393579 | 62.10787821 | 39.26852115 | 39.98052993 | 57.73214175 | 30.17066447 | 37.62070613 | 50.6863142 | 40.67244005 | 35.56865236 | 33.23128829 | 43.37062328 | 35.20971793 |
| **Polr3d** | 588.7928376 | 570.4286461 | 523.934906 | 507.2143388 | 474.38907 | 477.6621207 | 529.5119876 | 622.6889917 | 622.2464794 | 653.6786727 | 642.2462045 | 514.444167 | 473.7588792 | 505.9906049 | 426.8280091 |
| **Bmp1** | 8.241268334 | 9.507144102 | 10.96425812 | 8.626094197 | 12.03390164 | 9.118366474 | 15.33510015 | 54.47481085 | 21.06759543 | 15.73023544 | 22.70089677 | 11.27786538 | 3.408337261 | 3.614218606 | 10.05991941 |
| **Reep4** | 1476.102728 | 1511.635912 | 1475.475879 | 1547.521299 | 1357.930796 | 1418.958106 | 1433.380832 | 1308.233534 | 1477.741337 | 1377.269503 | 1420.69779 | 1538.127332 | 1567.83514 | 1564.956657 | 1508.987911 |
| **Hr** | 6.409875371 | 0 | 7.04845165 | 2.587828259 | 0.633363244 | 5.611302446 | 4.510323574 | 8.380740131 | 6.019312981 | 1.747803938 | 6.621094891 | 0 | 11.92918041 | 6.324882561 | 9.341353736 |
| **Nudt18** | 151.0899195 | 159.2446637 | 147.2343234 | 148.3688202 | 143.7734565 | 206.9167777 | 156.0571957 | 123.1968799 | 100.0710783 | 116.2289619 | 133.3677685 | 157.8901153 | 175.5293689 | 201.4926873 | 180.3599837 |
| **Fam160b2** | 277.4560339 | 335.9190916 | 273.3232918 | 300.188078 | 284.3800967 | 279.8637095 | 313.9185207 | 297.5162746 | 252.0587311 | 334.7044541 | 339.5675809 | 324.4555117 | 334.8691359 | 255.7059664 | 337.0073002 |
| **Dmtn** | 0 | 1.584524017 | 0.783161294 | 0 | 0 | 3.507064029 | 0.902064715 | 1.676148026 | 3.762070613 | 0.873901969 | 0 | 0 | 2.556252945 | 0 | 0 |
| **Xpo7** | 2353.339958 | 2324.496733 | 2514.730917 | 2530.033428 | 2216.771355 | 2222.075768 | 2382.352912 | 2603.057885 | 2578.523198 | 2668.022711 | 2673.030595 | 2272.05611 | 2375.611071 | 2281.475495 | 2247.673422 |
| **Dok2** | 1373.544722 | 1686.725816 | 1560.84046 | 1649.30921 | 1622.043269 | 1435.0906 | 1486.60265 | 1399.583602 | 1519.124113 | 1508.354798 | 1477.450031 | 1522.511827 | 1501.372563 | 1591.159741 | 1573.658822 |
| **Gfra2** | 0 | 0 | 3.132645178 | 0 | 0 | 0.701412806 | 0.902064715 | 3.352296052 | 0 | 0.873901969 | 0 | 0.867528106 | 0.852084315 | 0 | 1.437131344 |
| **Fndc3a** | 761.8594727 | 674.2149692 | 720.5083909 | 596.063109 | 494.6566938 | 454.5154981 | 575.517288 | 733.3147614 | 632.027863 | 820.5939488 | 820.0698958 | 496.2260768 | 478.8713851 | 489.7266212 | 538.924254 |
| **Gm9199** | 10.0726613 | 5.54583406 | 11.74741942 | 18.97740723 | 17.1008076 | 16.83390734 | 8.118582433 | 11.73303618 | 6.771727103 | 2.621705907 | 6.621094891 | 12.14539349 | 17.0416863 | 1.807109303 | 11.49705075 |
| **Cysltr2** | 21.97671556 | 22.97559825 | 28.9769679 | 18.11479781 | 20.90098706 | 11.9240177 | 14.43303544 | 16.76148026 | 17.30552482 | 18.35194135 | 28.37612096 | 16.48303402 | 32.37920398 | 15.36042908 | 18.68270747 |
| **Rcbtb2** | 328.7350369 | 411.1839824 | 376.7005826 | 442.5186323 | 411.6861088 | 394.8954096 | 377.9651155 | 351.9910855 | 406.3036262 | 404.6166116 | 372.6730553 | 424.221244 | 467.794289 | 491.5337305 | 362.8756643 |
| **Rb1** | 703.2548978 | 758.1947421 | 845.0310368 | 822.0667769 | 692.266026 | 680.3704215 | 779.3839136 | 949.5378568 | 901.3921189 | 753.3034972 | 856.0129824 | 766.894846 | 777.9529797 | 852.9555911 | 769.5838347 |
| **Lpar6** | 92.48534464 | 87.94108294 | 148.800646 | 106.1009586 | 92.47103366 | 100.3020312 | 100.1291833 | 117.3303618 | 81.26072524 | 101.3726284 | 117.2879666 | 95.42809169 | 126.1084786 | 127.4012059 | 68.26373884 |
| **Itm2b** | 3585.867422 | 3872.576698 | 3732.54673 | 4035.286865 | 4089.626468 | 4251.263015 | 4174.7555 | 3079.921998 | 3301.59317 | 3191.48999 | 3334.194213 | 4280.383676 | 4181.177734 | 4357.844085 | 4047.68043 |
| **Med4** | 572.310301 | 621.1334147 | 658.6386487 | 561.5587322 | 692.266026 | 690.8916136 | 605.2854236 | 710.6867631 | 678.6775386 | 727.9603401 | 688.5938687 | 664.5265294 | 617.7611285 | 672.2446608 | 647.4276704 |
| **Nudt15** | 28.38659093 | 41.19762444 | 46.98967767 | 63.83309705 | 48.13560656 | 55.41161165 | 49.61355931 | 63.69362499 | 85.77520997 | 46.31680435 | 81.34488009 | 64.19707986 | 58.79381775 | 51.50261514 | 56.76668809 |
| **Sucla2** | 1149.199084 | 1048.954899 | 1139.499683 | 1138.644434 | 1017.18137 | 1081.578546 | 1096.910693 | 1229.454577 | 1187.309485 | 1217.345443 | 1183.284244 | 1108.70092 | 1072.774153 | 1092.397574 | 1111.621095 |
| **Esd** | 1921.131218 | 1824.579406 | 1942.24001 | 1776.112795 | 1832.319866 | 1807.5408 | 1886.217319 | 2153.850214 | 1962.296032 | 1980.261862 | 1836.880897 | 1868.655541 | 1839.650036 | 1874.875902 | 1739.647492 |
| **Lrch1** | 739.8827571 | 800.1846286 | 703.2788424 | 675.4231756 | 642.2303297 | 707.0241082 | 749.615778 | 697.2775789 | 731.3465271 | 620.4703979 | 627.1122733 | 741.7365309 | 729.3841738 | 653.2700131 | 704.9129242 |
| **5031414D18Rik** | 0 | 4.753572051 | 0 | 1.725218839 | 0 | 0 | 0 | 0 | 1.504828245 | 0 | 0.945870699 | 0 | 0 | 0 | 1.437131344 |
| **Lcp1** | 39828.21847 | 39212.21585 | 40140.93215 | 40180.34677 | 38999.97513 | 38290.82648 | 41184.66662 | 38380.4375 | 38674.83831 | 36643.58346 | 38536.66401 | 39921.90839 | 39376.52037 | 39661.53143 | 39098.59534 |
| **Zc3h13** | 1757.221548 | 1558.379371 | 1612.529105 | 1694.1649 | 1621.409905 | 1509.440358 | 1832.093436 | 1696.261802 | 1847.176671 | 1508.354798 | 1564.470136 | 1664.786436 | 1586.580995 | 1646.276575 | 1572.940256 |
| **Siah3** | 0.915696482 | 3.169048034 | 2.349483883 | 2.587828259 | 6.966995687 | 2.104238417 | 0.902064715 | 5.028444078 | 0 | 2.621705907 | 2.837612096 | 0 | 7.668758836 | 0.903554652 | 0 |
| **Cog3** | 650.1445019 | 777.2090303 | 810.5719398 | 753.0580234 | 732.8012736 | 765.9427838 | 837.1160553 | 771.866166 | 718.5554871 | 771.6554385 | 759.5341711 | 753.0143963 | 823.1134484 | 737.3005957 | 865.8716347 |
| **Slc25a30** | 302.1798389 | 259.8619388 | 243.5631626 | 273.447186 | 247.6450285 | 234.9732899 | 270.6194144 | 284.1070904 | 246.7918322 | 266.5401005 | 287.5446924 | 261.9934881 | 299.9336789 | 306.3050269 | 265.150733 |
| **Gm4285** | 17.39823315 | 21.39107423 | 30.54329049 | 21.56523549 | 27.2346195 | 18.23673295 | 20.74748844 | 11.73303618 | 23.3248378 | 12.23462756 | 11.35044839 | 23.42325887 | 15.33751767 | 29.8173035 | 12.21561642 |
| **Tpt1** | 30543.97184 | 29231.29907 | 29638.73919 | 28686.07625 | 30884.05852 | 32082.62173 | 33112.99155 | 28726.66295 | 28099.65782 | 28105.56122 | 27900.348 | 30687.93923 | 30179.12227 | 31843.07303 | 31005.39018 |
| **Snora31** | 7.325571853 | 11.09166812 | 10.18109683 | 0 | 5.700269198 | 6.312715251 | 3.608258859 | 0.838074013 | 7.524141226 | 6.991215751 | 8.512836289 | 14.74797781 | 6.816674521 | 6.324882561 | 12.21561642 |
| **Gtf2f2** | 1302.120397 | 1125.012052 | 1139.499683 | 1123.980074 | 1085.584601 | 1122.260489 | 1130.287088 | 1105.419623 | 1100.029447 | 1043.438951 | 1163.420959 | 1145.1371 | 1088.11167 | 1038.184295 | 1180.603399 |
| **Kctd4** | 0 | 0 | 0 | 0 | 4.43354271 | 0 | 0 | 4.190370065 | 0 | 0.873901969 | 1.891741398 | 0 | 0 | 0 | 0.718565672 |
| **Gpalpp1** | 1034.737024 | 1048.954899 | 1017.326522 | 1082.574822 | 963.9788578 | 913.239473 | 1021.137257 | 967.1374111 | 999.9583689 | 1015.474088 | 1009.244036 | 965.5587823 | 1067.661647 | 1004.752773 | 920.4826258 |
| **Nufip1** | 1135.463637 | 1183.639441 | 1173.95878 | 1086.025259 | 1047.582806 | 1103.322343 | 1056.317781 | 1137.266436 | 1146.679123 | 1046.060657 | 1095.318269 | 983.7768725 | 985.8615526 | 919.8186353 | 1072.099983 |
| **Tsc22d1** | 153.8370089 | 174.2976419 | 198.9229688 | 177.6975404 | 114.005384 | 95.39214158 | 93.81473034 | 152.5294704 | 148.2255821 | 177.4020997 | 195.7952346 | 117.9838225 | 112.4751296 | 90.35546516 | 116.4076389 |
| **Serp2** | 32.96507334 | 40.40536243 | 44.64019379 | 60.38265938 | 50.0356963 | 46.29324518 | 61.34040061 | 61.17940295 | 63.2027863 | 54.18192207 | 80.3990094 | 42.50887721 | 65.61049227 | 35.23863141 | 66.10804182 |
| **Lacc1** | 42.12203815 | 61.79643666 | 61.86974226 | 80.22267603 | 69.03659363 | 63.12715251 | 55.92801232 | 90.51199341 | 79.75589699 | 69.03825554 | 54.86050053 | 32.09853993 | 71.57508247 | 58.73105235 | 65.38947615 |
| **Ccdc122** | 39.37494871 | 37.2363144 | 47.77283896 | 32.77915795 | 50.0356963 | 50.50172201 | 39.69084745 | 77.1028092 | 66.96485691 | 42.82119648 | 52.02288843 | 31.23101183 | 35.78754124 | 40.65995932 | 34.49115225 |
| **Enox1** | 1.831392963 | 0.792262009 | 3.132645178 | 3.450437679 | 1.266726489 | 7.014128057 | 2.706194144 | 6.704592104 | 18.81035306 | 5.243411813 | 5.675224193 | 1.735056213 | 1.70416863 | 7.228437213 | 0.718565672 |
| **Dnajc15** | 1454.126013 | 1695.440698 | 1674.398848 | 1713.142307 | 1596.075376 | 1492.606451 | 1690.469275 | 1673.633804 | 1813.318035 | 1628.95327 | 1480.287644 | 1764.552168 | 1615.551862 | 1744.764032 | 1541.323366 |
| **Epsti1** | 2054.822905 | 2371.240191 | 2108.270205 | 2216.043599 | 2451.749119 | 2213.658815 | 2540.214237 | 1288.119758 | 1818.584934 | 1528.454544 | 1477.450031 | 2508.891283 | 2314.261 | 2743.191922 | 2443.123285 |
| **Tnfsf11** | 368.1099856 | 366.8173099 | 420.5576151 | 411.4646932 | 370.5174979 | 422.250509 | 350.903174 | 910.9864522 | 671.9058115 | 720.0952224 | 596.8444109 | 419.8836034 | 414.9650615 | 421.9600223 | 410.3009987 |
| **Akap11** | 993.5306825 | 1158.287056 | 1076.063619 | 948.8703616 | 892.4088112 | 984.7835792 | 1014.822804 | 1185.874728 | 1206.119838 | 1140.442069 | 1147.341158 | 1053.179121 | 1051.472045 | 984.8745702 | 1101.561175 |
| **Dgkh** | 198.7061365 | 172.7131179 | 158.1985815 | 150.094039 | 141.8733667 | 125.5528922 | 140.7220955 | 136.6060641 | 154.9973093 | 152.0589426 | 140.9347341 | 145.7447219 | 163.6001885 | 102.1016756 | 150.1802254 |
| **Vwa8** | 582.3829623 | 565.6750741 | 592.8530999 | 565.8717793 | 476.9225229 | 518.3440634 | 465.4653928 | 752.5904637 | 644.8189031 | 702.617183 | 659.271877 | 492.7559644 | 596.4590206 | 536.711463 | 554.0141331 |
| **Rgcc** | 1271.902413 | 1355.560297 | 1274.986587 | 1363.785492 | 1314.228732 | 1381.783227 | 1269.205054 | 1358.517975 | 1385.1944 | 1291.62711 | 1168.150313 | 1303.894744 | 1341.180712 | 1377.017289 | 1451.502657 |
| **Naa16** | 736.2199712 | 655.200681 | 624.1795517 | 634.8805329 | 736.6014531 | 655.1195605 | 769.4612017 | 821.3125328 | 747.1472237 | 722.7169283 | 785.07268 | 722.6509125 | 721.7154149 | 680.3766526 | 754.4939556 |
| **Mtrf1** | 96.14813056 | 95.86370303 | 102.5941296 | 96.612255 | 90.57094393 | 90.48225194 | 73.0672419 | 91.35006742 | 103.8331489 | 118.8506678 | 118.2338373 | 110.1760695 | 102.2501178 | 132.8225338 | 105.6291538 |
| **Kbtbd7** | 70.50862908 | 100.6172751 | 101.027807 | 109.5513963 | 82.33722176 | 84.87094949 | 83.89201847 | 91.35006742 | 88.03245234 | 50.6863142 | 67.15681961 | 96.2956198 | 92.02510604 | 102.1016756 | 93.41353736 |
| **Kbtbd6** | 51.27900297 | 77.64167683 | 47.77283896 | 70.73397241 | 41.16861088 | 44.18900676 | 43.29910631 | 54.47481085 | 61.69795805 | 48.93851026 | 30.26786236 | 37.30370857 | 65.61049227 | 46.08128723 | 28.02406121 |
| **Wbp4** | 798.4873319 | 724.9197378 | 723.6410361 | 707.3397241 | 727.7343677 | 770.8526735 | 763.1467487 | 823.8267548 | 628.2657924 | 769.0337326 | 730.2121795 | 703.5652942 | 760.0592091 | 789.7067655 | 663.9546809 |
| **Elf1** | 2624.386116 | 2743.603335 | 2955.650725 | 2587.828259 | 2538.519883 | 2569.97652 | 2901.040123 | 2223.410357 | 2637.2115 | 2595.488848 | 2454.534463 | 2802.983311 | 2760.753181 | 2875.110901 | 2789.471939 |
| **Sugt1** | 1516.393373 | 1605.122829 | 1636.807105 | 1578.575238 | 1458.002188 | 1632.187599 | 1611.989645 | 1749.060465 | 1610.918636 | 1663.035447 | 1620.276507 | 1525.114411 | 1564.426803 | 1599.291733 | 1527.670619 |
| **Diap3** | 1077.774759 | 1080.64538 | 1123.836458 | 1095.513963 | 989.3133876 | 958.1298926 | 1039.178551 | 1107.095771 | 983.4052582 | 1062.664794 | 1112.343942 | 1086.145189 | 1059.140804 | 1090.590464 | 1029.704608 |
| **Tdrd3** | 247.23805 | 202.0268122 | 230.2494206 | 240.6680281 | 217.876956 | 204.1111265 | 275.129738 | 207.0042812 | 212.1807826 | 247.3142572 | 218.4961314 | 206.4716893 | 207.0564886 | 241.249092 | 211.9768732 |
| **Gm5088** | 94.3167376 | 63.38096068 | 74.40032298 | 71.59658183 | 48.76896981 | 89.07942633 | 69.45898304 | 107.2734737 | 66.21244279 | 107.4899422 | 86.07423359 | 67.66719229 | 86.91260015 | 90.35546516 | 62.51521346 |
| **Mzt1** | 724.3159169 | 820.7834408 | 832.500456 | 694.4005828 | 685.9323936 | 772.9569119 | 754.1261016 | 797.8464604 | 766.7099909 | 842.441498 | 832.3662149 | 904.8318148 | 838.4509661 | 846.6307085 | 840.0032705 |
| **Bora** | 589.7085341 | 641.7322269 | 719.7252296 | 640.9187988 | 639.0635135 | 621.4517459 | 642.2700769 | 689.7349127 | 715.5458306 | 702.617183 | 671.5681961 | 712.2405753 | 690.1882953 | 663.2091143 | 735.8112481 |
| **Dis3** | 1966.000346 | 1831.709764 | 2013.507688 | 1857.198081 | 1833.586592 | 1800.526672 | 2008.89812 | 2230.114949 | 2036.78503 | 2074.643274 | 2260.63097 | 1899.886553 | 1900.148023 | 1782.713328 | 1853.899434 |
| **Pibf1** | 174.898028 | 149.7375196 | 178.5607751 | 159.5827426 | 153.9072684 | 144.491038 | 129.8973189 | 200.2996891 | 164.7786928 | 157.3023544 | 140.9347341 | 155.287531 | 169.5647787 | 140.050971 | 165.9886702 |
| **Klf5** | 0.915696482 | 0 | 0 | 1.725218839 | 0.633363244 | 3.507064029 | 4.510323574 | 2.514222039 | 3.762070613 | 0.873901969 | 1.891741398 | 1.735056213 | 0 | 4.517773258 | 1.437131344 |
| **Klf12** | 1.831392963 | 3.961310043 | 0.783161294 | 1.725218839 | 0 | 0.701412806 | 0.902064715 | 10.05688816 | 0 | 5.243411813 | 3.783482795 | 2.602584319 | 0 | 2.710663955 | 1.437131344 |
| **Tbc1d4** | 97.97952353 | 141.8148995 | 140.1858717 | 178.5601499 | 107.0383883 | 96.09355438 | 114.5622188 | 76.26473519 | 109.1000478 | 115.3550599 | 139.0429927 | 128.3941597 | 115.0313825 | 126.4976512 | 117.1262045 |
| **Commd6** | 255.4793184 | 294.7214672 | 280.3717434 | 257.0576071 | 264.7458361 | 291.0863144 | 278.7379969 | 310.0873848 | 287.4221948 | 303.2439832 | 296.0575287 | 301.899781 | 279.4836554 | 321.665456 | 283.1148748 |
| **Uchl3** | 792.0774566 | 823.1602268 | 825.4520044 | 852.2581066 | 775.8699742 | 836.0840644 | 819.9768257 | 933.6144505 | 895.3728059 | 1001.491656 | 928.8450262 | 781.6428238 | 765.171715 | 794.2245387 | 862.2788064 |
| **Lmo7** | 4.578482408 | 7.922620085 | 2.349483883 | 1.725218839 | 2.533452977 | 2.104238417 | 2.706194144 | 5.866518091 | 0 | 9.612921658 | 1.891741398 | 0.867528106 | 2.556252945 | 4.517773258 | 2.155697016 |
| **Kctd12** | 236.2496922 | 221.8333624 | 178.5607751 | 200.1253854 | 162.7743538 | 220.243621 | 211.0831433 | 105.5973256 | 133.1772997 | 118.8506678 | 139.9888634 | 233.3650606 | 207.0564886 | 168.0611652 | 209.8211762 |
| **Irg1** | 16.48253667 | 5.54583406 | 10.96425812 | 3.450437679 | 5.700269198 | 4.90988964 | 10.82477658 | 11.73303618 | 4.514484735 | 1.747803938 | 4.729353494 | 7.807752957 | 4.260421576 | 18.97464768 | 5.748525376 |
| **Cln5** | 350.7117524 | 345.4262357 | 379.8332278 | 362.2959563 | 348.3497844 | 389.2841072 | 400.5167334 | 373.7810098 | 352.8822235 | 368.7866309 | 309.2997185 | 314.9127026 | 419.2254831 | 380.3965083 | 347.0672196 |
| **Fbxl3** | 726.1473099 | 820.7834408 | 762.0159395 | 701.3014582 | 689.0992098 | 714.0382362 | 679.2547302 | 721.5817252 | 681.6871951 | 760.2947129 | 716.9699897 | 729.5911374 | 725.1237522 | 632.4882561 | 718.565672 |
| **Mycbp2** | 3877.058903 | 3788.596925 | 3774.054278 | 3924.01025 | 3170.616401 | 3380.809724 | 3943.826933 | 3955.709342 | 4262.426004 | 3653.784132 | 3989.682607 | 3871.777938 | 3953.671222 | 3635.000363 | 4038.339076 |
| **Scel** | 5.494178889 | 7.130358077 | 2.349483883 | 0.86260942 | 2.533452977 | 0 | 4.510323574 | 0 | 3.762070613 | 1.747803938 | 0 | 0 | 1.70416863 | 0 | 0 |
| **Slain1** | 835.1151912 | 941.9995281 | 798.0413591 | 879.861608 | 822.7388543 | 916.0451243 | 920.1060091 | 885.8442318 | 894.6203917 | 949.0575382 | 878.7138792 | 802.4634983 | 881.9072662 | 954.1537121 | 806.2306839 |
| **Slain1os** | 5.494178889 | 4.753572051 | 4.698967767 | 4.313047098 | 2.533452977 | 4.208476834 | 4.510323574 | 3.352296052 | 0.752414123 | 2.621705907 | 5.675224193 | 6.072696744 | 5.112505891 | 7.228437213 | 1.437131344 |
| **Pou4f1** | 15.56684019 | 8.714882094 | 7.04845165 | 10.35131304 | 4.43354271 | 7.715540863 | 7.216517718 | 3.352296052 | 3.00965649 | 1.747803938 | 7.56696559 | 3.470112425 | 19.59793925 | 4.517773258 | 6.467091048 |
| **Rnf219** | 454.1854549 | 457.9274409 | 504.3558736 | 387.3116294 | 431.9537326 | 415.9377938 | 431.1869337 | 477.7021874 | 413.8277674 | 422.9685529 | 498.4738583 | 419.0160753 | 352.7629065 | 373.1680711 | 428.9837062 |
| **Gm17066** | 204.2003154 | 157.6601397 | 192.6576784 | 197.5375571 | 238.1445798 | 167.6376606 | 175.9026194 | 173.4813207 | 212.9331967 | 226.3406099 | 208.0915537 | 193.4587677 | 199.3877297 | 191.5535861 | 209.8211762 |
| **Rbm26** | 1088.763117 | 1114.712646 | 1072.930973 | 987.6877855 | 1116.6194 | 1057.730511 | 1201.5502 | 1148.999472 | 1087.238407 | 1025.960911 | 1106.668718 | 1120.846313 | 1046.359539 | 1010.1741 | 1108.746832 |
| **Ndfip2** | 1008.181826 | 1020.433467 | 1180.224071 | 1160.209669 | 1084.317874 | 1023.361284 | 1065.338428 | 1367.736789 | 1211.386737 | 1281.140286 | 1276.925443 | 1152.077325 | 1131.567971 | 1184.560148 | 1137.489459 |
| **Spry2** | 154.7527054 | 119.6315633 | 119.8236781 | 159.5827426 | 103.2382088 | 102.4062696 | 104.6395069 | 199.4616151 | 131.6724715 | 197.501845 | 174.0402086 | 114.51371 | 134.6293218 | 112.0407768 | 125.7489926 |
| **Mir17** | 0.915696482 | 1.584524017 | 6.265290356 | 0 | 2.533452977 | 2.805651223 | 0 | 2.514222039 | 0.752414123 | 1.747803938 | 0.945870699 | 0 | 1.70416863 | 0 | 1.437131344 |
| **Mir18** | 0 | 4.753572051 | 0 | 0 | 2.533452977 | 0 | 0 | 0 | 0 | 0 | 0 | 0 | 0 | 1.807109303 | 2.874262688 |
| **Mir17hg** | 80.58129038 | 110.9166812 | 126.0889684 | 90.57398906 | 157.7074478 | 130.4627819 | 110.9539599 | 133.2537681 | 95.55659357 | 150.3111387 | 149.4475704 | 142.2746094 | 183.1981278 | 148.1829629 | 156.6473165 |
| **Tgds** | 280.2031234 | 285.2143231 | 293.6854854 | 281.2106708 | 277.413101 | 279.8637095 | 280.5421263 | 352.8291595 | 286.6697807 | 315.4786108 | 295.111658 | 329.6606804 | 286.3003299 | 310.8228001 | 328.3845121 |
| **Gpr180** | 241.7438711 | 265.4077728 | 277.2390982 | 283.7984991 | 294.5139086 | 253.2100229 | 255.2843143 | 298.3543486 | 282.9077101 | 321.5959246 | 245.9263817 | 294.9595561 | 259.8857161 | 273.7770594 | 284.5520061 |
| **Abcc4** | 528.3568699 | 648.862585 | 584.2383257 | 588.2996242 | 554.1928387 | 526.0596043 | 559.2801232 | 636.9362499 | 649.3333878 | 639.6962412 | 615.7618249 | 606.4021463 | 518.0672636 | 544.8434549 | 560.4812241 |
| **Cldn10** | 0 | 0.792262009 | 7.04845165 | 0 | 1.266726489 | 0.701412806 | 1.80412943 | 0 | 0 | 0 | 0 | 0 | 0 | 0 | 2.155697016 |
| **Dzip1** | 57.68887834 | 122.8006113 | 105.7267748 | 110.4140057 | 118.4389267 | 108.7189849 | 132.6035131 | 98.89273354 | 58.68830156 | 106.6160402 | 88.91184568 | 125.7915754 | 109.9188767 | 155.4114001 | 153.7730538 |
| **Dnajc3** | 1933.950969 | 2153.368139 | 2242.973947 | 2270.387993 | 2045.763279 | 2130.190691 | 2326.424899 | 2387.672863 | 2172.219572 | 2241.55855 | 2324.950178 | 2397.847686 | 2296.367229 | 2276.957722 | 2136.295743 |
| **Uggt2** | 8.241268334 | 5.54583406 | 6.265290356 | 0 | 0.633363244 | 1.402825611 | 0.902064715 | 4.190370065 | 0.752414123 | 3.495607876 | 3.783482795 | 0 | 3.408337261 | 3.614218606 | 7.18565672 |
| **Mbnl2** | 483.4877423 | 454.7583929 | 491.8252929 | 469.2595243 | 355.31678 | 351.4078157 | 437.5013867 | 533.8531463 | 419.0946663 | 487.6372986 | 423.750073 | 433.7640531 | 441.3796753 | 401.1782653 | 426.1094435 |
| **Rap2a** | 373.6041645 | 352.5565938 | 412.7260022 | 409.7394743 | 438.287365 | 451.0084341 | 476.2901694 | 325.1727171 | 342.3484258 | 361.7954151 | 338.6217102 | 385.1824792 | 469.4984577 | 397.5640467 | 415.3309584 |
| **Ipo5** | 9377.647668 | 9251.243473 | 9409.682953 | 9240.272103 | 9136.898162 | 8817.460381 | 9181.214667 | 11086.04304 | 10582.70463 | 10474.589 | 10993.85513 | 9279.948153 | 9599.581895 | 8919.89152 | 9428.300182 |
| **Farp1** | 326.9036439 | 302.6440872 | 355.5552277 | 345.0437679 | 343.9162416 | 316.3371754 | 395.1043451 | 372.9429358 | 340.8435975 | 360.9215132 | 325.3795204 | 334.865849 | 333.1649672 | 310.8228001 | 390.8997255 |
| **Stk24** | 3383.498499 | 3364.73675 | 3464.705567 | 3420.246349 | 3414.46125 | 3551.954448 | 3463.928505 | 2970.972376 | 3300.840756 | 3358.405266 | 3306.763963 | 3583.758607 | 3553.191594 | 3443.446777 | 3520.253227 |
| **Slc15a1** | 115.3777567 | 151.3220436 | 163.6807105 | 152.6818673 | 165.94117 | 166.9362478 | 158.7633898 | 117.3303618 | 115.8717749 | 104.8682363 | 162.6897602 | 189.9886553 | 154.227261 | 191.5535861 | 168.8629329 |
| **Dock9** | 712.4118627 | 628.2637727 | 627.3121969 | 577.9483112 | 473.7557067 | 473.4536439 | 557.4759937 | 604.2513634 | 579.3588744 | 645.813555 | 595.8985402 | 556.0855161 | 486.540144 | 477.9804107 | 551.8584361 |
| **1810041H14Rik** | 2.747089445 | 2.376786026 | 3.915806472 | 7.763484777 | 13.30062813 | 7.014128057 | 15.33510015 | 4.190370065 | 23.3248378 | 1.747803938 | 4.729353494 | 0.867528106 | 9.372927467 | 15.36042908 | 21.55697016 |
| **Ubac2** | 1346.073828 | 1145.610864 | 1139.499683 | 1180.049686 | 1215.424066 | 1179.776339 | 1143.818058 | 1026.640666 | 1135.392911 | 1071.403814 | 1048.970605 | 1180.705753 | 1171.615933 | 1169.199719 | 1196.411844 |
| **Gpr18** | 693.1822365 | 667.0846112 | 725.2073587 | 681.4614415 | 599.1616291 | 552.0118781 | 565.5945762 | 574.918773 | 532.7091988 | 546.1887306 | 540.092169 | 511.8415827 | 550.4464676 | 638.8131387 | 543.9542137 |
| **Gpr183** | 120.8719356 | 164.7904978 | 200.4892914 | 143.1931637 | 185.5754306 | 187.2772191 | 184.0212018 | 86.32162334 | 127.1579867 | 129.3374914 | 121.0714494 | 216.0144985 | 137.1855747 | 209.6246792 | 160.2401448 |
| **A330035P11Rik** | 10.98835778 | 0.792262009 | 3.915806472 | 2.587828259 | 7.600358931 | 3.507064029 | 2.706194144 | 10.89496217 | 7.524141226 | 8.739019689 | 4.729353494 | 5.205168638 | 4.260421576 | 0 | 6.467091048 |
| **Tm9sf2** | 2083.209496 | 2112.962777 | 2033.08672 | 2046.109543 | 1943.158433 | 1909.94707 | 2026.037349 | 2087.642367 | 2143.627835 | 2114.842765 | 2231.308978 | 1973.626442 | 2151.512896 | 1979.688242 | 2011.265316 |
| **Clybl** | 217.9357626 | 193.3119301 | 214.5861947 | 176.834931 | 191.909063 | 182.3673295 | 188.5315254 | 72.07436512 | 94.80417945 | 79.52507917 | 99.31642337 | 159.6251716 | 184.9022964 | 187.0358129 | 206.2283479 |
| **Pcca** | 379.0983434 | 315.3202794 | 335.9761953 | 319.1654853 | 320.4818016 | 311.4272857 | 361.7279506 | 370.4287138 | 435.647777 | 344.3173757 | 398.2115642 | 353.0839393 | 354.4670751 | 341.5436583 | 337.0073002 |
| **Ggact** | 221.5985485 | 187.766096 | 218.5020012 | 169.9340557 | 238.1445798 | 226.5563362 | 202.0624961 | 248.9079819 | 224.2194085 | 214.9798844 | 219.4420021 | 183.9159585 | 230.9148494 | 158.122064 | 238.5638031 |
| **Tmtc4** | 137.3544722 | 148.1529956 | 171.5123235 | 139.742726 | 127.9393753 | 115.0317001 | 175.9026194 | 130.739546 | 128.662815 | 151.1850406 | 109.7210011 | 201.2665207 | 107.3626237 | 159.9291733 | 158.8030135 |
| **Sepp1** | 253.6479254 | 298.6827772 | 310.1318726 | 301.0506875 | 381.9180363 | 320.5456522 | 396.0064098 | 244.7176118 | 334.8242845 | 258.6749828 | 166.473243 | 401.6655132 | 377.4733516 | 408.4067025 | 364.3127957 |
| **Ccdc152** | 0 | 0 | 0 | 1.725218839 | 0 | 0 | 0.902064715 | 0.838074013 | 0 | 0.873901969 | 0 | 1.735056213 | 0.852084315 | 0 | 0.718565672 |
| **Fbxo4** | 183.1392963 | 163.9982358 | 200.4892914 | 184.5984158 | 173.5415289 | 212.5280801 | 232.7326964 | 186.0524309 | 179.0745612 | 219.3493942 | 191.0658812 | 186.5185429 | 200.2398141 | 224.9851082 | 179.641418 |
| **AW549877** | 231.6712098 | 263.8232488 | 274.1064531 | 260.5080447 | 252.7119345 | 244.7930692 | 257.9905084 | 270.6979062 | 264.8497711 | 246.4403552 | 253.4933473 | 274.1388816 | 299.0815946 | 237.6348734 | 298.9233195 |
| **A630020A06** | 0.915696482 | 0.792262009 | 0 | 0.86260942 | 0.633363244 | 0.701412806 | 0 | 1.676148026 | 0 | 1.747803938 | 0 | 2.602584319 | 0 | 0 | 0 |
| **BC037032** | 0.915696482 | 1.584524017 | 0.783161294 | 3.450437679 | 0 | 0 | 0 | 0 | 0.752414123 | 0 | 0 | 4.337640531 | 5.964590206 | 7.228437213 | 1.437131344 |
| **Oxct1** | 2021.857831 | 2112.962777 | 2229.660205 | 2368.725466 | 2235.138889 | 2297.126939 | 2216.373004 | 2795.814908 | 2517.577654 | 2706.474398 | 2827.207519 | 2425.608585 | 2367.942312 | 2530.856579 | 2221.805058 |
| **Card6** | 285.6973022 | 398.5077903 | 323.4456146 | 381.2733635 | 276.7797377 | 286.1764247 | 350.903174 | 243.0414638 | 285.1649525 | 246.4403552 | 329.1630032 | 339.2034896 | 309.3066064 | 371.3609618 | 362.1570987 |
| **Rpl37** | 7824.626435 | 7144.618793 | 7393.825781 | 6530.815916 | 7435.051125 | 7759.028457 | 7156.079382 | 6399.533164 | 6152.49028 | 6256.264195 | 6204.911784 | 6408.430121 | 6157.161261 | 7118.203545 | 6707.810548 |
| **Snord72** | 3.662785926 | 7.130358077 | 9.397935534 | 0.86260942 | 13.93399137 | 4.208476834 | 3.608258859 | 5.866518091 | 6.771727103 | 6.117313782 | 15.13393118 | 2.602584319 | 6.816674521 | 7.228437213 | 7.18565672 |
| **Prkaa1** | 814.0541721 | 894.4638076 | 825.4520044 | 848.8076689 | 787.2705126 | 730.8721436 | 862.3738673 | 967.1374111 | 750.9092943 | 849.4327138 | 857.9047238 | 783.37788 | 835.0426289 | 803.2600852 | 782.5180168 |
| **Ttc33** | 311.3368037 | 286.0065851 | 354.7720664 | 310.5393911 | 279.946554 | 307.9202217 | 327.4494915 | 315.9539029 | 302.4704773 | 285.7659438 | 234.5759333 | 338.3359614 | 351.9108222 | 381.300063 | 292.4562285 |
| **Ptger4** | 1286.553557 | 1197.900157 | 1296.131942 | 1306.853271 | 1556.173491 | 1603.429674 | 1662.505269 | 1661.062694 | 1424.319934 | 1221.714953 | 1304.355694 | 1538.994861 | 1666.67692 | 1646.276575 | 1714.497693 |
| **Fyb** | 3102.37968 | 3306.901623 | 3003.423564 | 3368.489784 | 3664.006368 | 3263.673785 | 3937.51248 | 3072.379332 | 2907.32817 | 2646.175162 | 2877.338666 | 3771.144678 | 3823.302322 | 3722.645164 | 3540.373066 |
| **Rictor** | 548.5021925 | 521.3084016 | 560.7434869 | 497.7256351 | 507.3239587 | 488.8847256 | 550.259476 | 549.7765526 | 448.4388171 | 440.4465923 | 535.3628155 | 511.8415827 | 483.1318067 | 522.2545886 | 490.0617883 |
| **Lifr** | 1.831392963 | 0 | 0 | 0 | 2.533452977 | 0 | 0 | 5.866518091 | 0 | 2.621705907 | 0 | 0 | 0 | 0.903554652 | 1.437131344 |
| **Wdr70** | 446.859883 | 404.8458863 | 496.5242607 | 425.2664439 | 404.7191131 | 400.5067121 | 413.1456394 | 470.1595213 | 464.9919278 | 425.5902589 | 488.0692806 | 457.187312 | 437.1192537 | 459.005763 | 437.6064942 |
| **Nup155** | 1744.401797 | 1783.381781 | 1698.676848 | 1889.114629 | 1501.070889 | 1571.164685 | 1704.000246 | 1982.883115 | 1776.449743 | 1909.475802 | 1886.066173 | 1691.679807 | 1720.358232 | 1509.839823 | 1611.024237 |
| **2410089E03Rik** | 319.5780721 | 379.4935021 | 342.2414857 | 376.9603164 | 345.8163314 | 264.4326278 | 314.8205855 | 377.1333059 | 365.6732636 | 351.3085915 | 342.405193 | 347.0112425 | 358.7274967 | 319.8583467 | 390.1811599 |

| | | | | | | | | | | | | | | | |
|---|---|---|---|---|---|---|---|---|---|---|---|---|---|---|---|
| **Nipbl** | 2336.857421 | 2219.125886 | 2376.111367 | 2421.344641 | 2209.170996 | 2152.635901 | 2486.090354 | 2454.718784 | 2544.664563 | 2198.737354 | 2502.773869 | 2428.211169 | 2298.923482 | 2336.592329 | 2318.092858 |
| **Ranbp3l** | 0.915696482 | 0 | 0 | 0 | 2.533452977 | 0 | 2.706194144 | 0 | 0 | 0 | 1.891741398 | 0 | 0 | 9.035546516 | 1.437131344 |
| **Nadk2** | 516.4528156 | 536.3613798 | 540.3812932 | 540.8561061 | 509.2240484 | 501.5101561 | 522.2954699 | 537.2054424 | 525.9374717 | 557.5494562 | 570.3600314 | 517.0467513 | 511.2505891 | 514.1225967 | 506.5887987 |
| **Skp2** | 338.8076982 | 366.8173099 | 343.024647 | 391.6246765 | 312.2480794 | 337.3795595 | 325.645362 | 393.0567121 | 468.7539984 | 478.898279 | 434.1546507 | 344.4086582 | 362.9879183 | 423.7671316 | 331.2587748 |
| **Lmbrd2** | 173.9823315 | 236.8863405 | 230.2494206 | 179.4227593 | 152.6405419 | 143.7896252 | 195.7480431 | 217.8992434 | 230.2387215 | 216.7276883 | 247.8181231 | 206.4716893 | 240.2877769 | 178.0002664 | 185.3899434 |
| **Capsl** | 46.70052056 | 39.61310043 | 35.24225825 | 34.50437679 | 13.93399137 | 14.72966892 | 11.72684129 | 51.1225148 | 35.36346376 | 61.17313782 | 90.80358708 | 23.42325887 | 17.89377062 | 26.2030849 | 15.80844478 |
| **Il7r** | 932.1790182 | 797.8078426 | 897.5028435 | 774.6232588 | 569.3935566 | 562.5330702 | 584.5379352 | 522.1201101 | 592.9023286 | 704.3649869 | 746.2919813 | 545.6751788 | 613.5007069 | 545.7470096 | 541.079951 |
| **Spef2** | 61.35166426 | 45.95119649 | 53.25496803 | 39.6800333 | 27.86798275 | 40.68194273 | 55.92801232 | 56.98903289 | 34.61104964 | 54.18192207 | 44.45592284 | 31.23101183 | 34.08337261 | 43.37062328 | 39.52111196 |
| **Prlr** | 4.578482408 | 8.714882094 | 1.566322589 | 6.038265938 | 1.266726489 | 1.402825611 | 3.608258859 | 11.73303618 | 0 | 1.747803938 | 8.512836289 | 0 | 4.260421576 | 0 | 0.718565672 |
| **Dnajc21** | 1268.239627 | 1090.944786 | 1054.918264 | 1101.552229 | 1006.414195 | 1173.463624 | 1192.529553 | 987.2511874 | 1041.341146 | 1105.485991 | 1035.728415 | 1098.290583 | 1019.944925 | 1083.362027 | 1096.531215 |
| **Brix1** | 1815.826123 | 1720.00082 | 1880.370268 | 1829.594579 | 1887.422468 | 1869.96654 | 1882.60906 | 2141.279103 | 1948.000163 | 2176.015903 | 1977.815631 | 1800.988349 | 1792.785399 | 1659.829895 | 1694.377854 |
| **Rad1** | 391.9180941 | 354.1411178 | 379.0500665 | 366.6090034 | 353.4166903 | 321.247065 | 327.4494915 | 338.5819013 | 376.2070613 | 423.8424549 | 419.9665903 | 376.5071981 | 387.6983634 | 352.3863141 | 338.4444315 |
| **Rai14** | 21.06101908 | 22.97559825 | 29.76012919 | 15.52696955 | 23.43444004 | 29.45933784 | 27.06194144 | 45.2559967 | 41.38277674 | 41.94729451 | 56.75224193 | 34.70112425 | 35.78754124 | 16.26398373 | 28.02406121 |
| **Amacr** | 71.42432556 | 52.28929256 | 40.72438731 | 52.6191746 | 74.73686282 | 65.23139093 | 47.80942988 | 44.41792269 | 42.13519086 | 43.69509845 | 72.83204381 | 45.97898963 | 49.42089028 | 52.40616979 | 49.58103137 |
| **Tars** | 4111.477202 | 3718.085606 | 3734.896213 | 3674.716128 | 3771.67812 | 3730.814714 | 3850.012203 | 3652.326549 | 3740.250603 | 3599.60221 | 3834.559813 | 3534.309505 | 3433.89979 | 3533.802242 | 3413.186942 |
| **Npr3** | 0 | 0.792262009 | 0 | 0 | 0 | 1.402825611 | 0 | 0 | 0 | 0.873901969 | 5.675224193 | 2.602584319 | 0 | 0 | 5.748525376 |
| **Sub1** | 4255.24155 | 4493.710112 | 4451.488798 | 4198.320045 | 4526.013744 | 4699.465798 | 4338.029213 | 3863.5212 | 3918.57275 | 3887.98986 | 4194.936549 | 4616.984582 | 4001.387944 | 4480.727517 | 4267.561526 |
| **Zfr** | 2111.596086 | 2200.90386 | 2215.563302 | 2078.026092 | 2066.030903 | 2098.627115 | 2196.52758 | 2258.609465 | 2146.637492 | 2176.889805 | 2339.138238 | 2416.933304 | 2212.862966 | 2078.175699 | 2293.661625 |
| **Mir1898** | 0 | 0 | 10.18109683 | 0 | 0 | 3.507064029 | 1.80412943 | 4.190370065 | 0 | 2.621705907 | 0.945870699 | 3.470112425 | 0.852084315 | 0 | 2.874262688 |
| **Mtmr12** | 874.4901399 | 893.6715456 | 984.4337472 | 966.12255 | 944.3445972 | 908.3295834 | 920.1060091 | 859.0258634 | 911.1735024 | 796.9985956 | 908.0358708 | 1048.84148 | 987.5657213 | 994.8136714 | 1080.004205 |
| **Golph3** | 2033.761886 | 2076.518724 | 2011.158204 | 1982.276446 | 1913.390361 | 1847.52133 | 2088.279815 | 1956.064746 | 2106.759543 | 2128.825196 | 2120.642107 | 2057.776668 | 2135.323294 | 1987.820233 | 1993.301174 |
| **Pdzd2** | 7.325571853 | 11.88393013 | 5.482129061 | 6.038265938 | 4.43354271 | 2.805651223 | 4.510323574 | 10.05688816 | 6.019312981 | 6.117313782 | 5.675224193 | 9.542809169 | 8.520843152 | 5.421327909 | 9.341353736 |
| **6030458C11Rik** | 548.5021925 | 580.7280522 | 577.9730353 | 627.9796575 | 607.3953513 | 544.2963372 | 630.5432356 | 672.1353585 | 563.5581778 | 604.7401625 | 663.0553598 | 570.8334939 | 610.0923697 | 599.056734 | 577.7268003 |
| **Drosha** | 1304.867486 | 1321.49303 | 1323.542588 | 1292.188911 | 1257.22604 | 1194.506008 | 1231.318336 | 1533.675444 | 1390.461299 | 1538.067465 | 1516.23073 | 1244.902833 | 1209.959728 | 1195.402804 | 1195.693278 |
| **Acot10** | 0.915696482 | 0 | 3.132645178 | 0.86260942 | 3.166816221 | 2.805651223 | 0.902064715 | 0 | 2.257242368 | 0.873901969 | 0.945870699 | 0.867528106 | 1.70416863 | 4.517773258 | 0.718565672 |
| **Gm5803** | 11.90405426 | 18.2220262 | 19.57903236 | 11.21392246 | 17.1008076 | 37.1748787 | 13.53097072 | 6.704592104 | 6.019312981 | 13.10852953 | 17.02567258 | 11.27786538 | 11.92918041 | 9.939101167 | 7.904222392 |
| **Basp1** | 513.7057262 | 377.116716 | 354.7720664 | 269.9967484 | 354.0500536 | 480.4677719 | 432.0889984 | 340.2580493 | 297.9559925 | 293.6310616 | 218.4961314 | 476.2729303 | 384.2900261 | 332.5081118 | 389.4625942 |
| **Myo10** | 572.310301 | 329.5809955 | 345.3741309 | 333.8298454 | 252.0785712 | 279.8637095 | 314.8205855 | 339.4199753 | 291.1842654 | 325.9654344 | 334.8382274 | 231.6300044 | 268.4065593 | 199.685578 | 275.2106524 |
| **Fam134b** | 539.3452276 | 514.9703055 | 635.9269711 | 570.1848264 | 562.4265609 | 521.1497146 | 611.5998766 | 582.4614391 | 633.5326912 | 583.7665152 | 606.3031179 | 596.8593371 | 657.8090913 | 507.7977142 | 630.1820943 |
| **Zfp622** | 1838.718535 | 1864.984768 | 1697.893686 | 1799.403249 | 1710.714123 | 1842.611441 | 1851.938859 | 1959.417043 | 1818.584934 | 1947.053587 | 1682.703973 | 1792.313068 | 1821.756266 | 1455.626544 | 1630.42551 |
| **Ank** | 156.5840983 | 125.1773973 | 171.5123235 | 179.4227593 | 191.909063 | 214.6323185 | 207.4748844 | 208.6804293 | 166.2835211 | 208.8625706 | 179.7154328 | 242.0403417 | 206.2044043 | 234.9242094 | 209.8211762 |
| **Otulin** | 3519.937275 | 2898.094427 | 2980.711887 | 3132.134803 | 2492.284366 | 2656.250295 | 2640.34342 | 3774.685355 | 3159.386901 | 3425.695718 | 3688.895725 | 2441.224091 | 2384.983998 | 2451.34377 | 2349.709747 |
| **Fam105a** | 502.7173684 | 583.8971003 | 626.5290356 | 539.1308873 | 741.668359 | 671.9534679 | 662.1155006 | 554.8049966 | 551.5195519 | 452.6812199 | 490.9068927 | 797.2583297 | 679.9632835 | 757.178798 | 664.6732466 |
| **Trio** | 500.8859754 | 568.8441221 | 443.2692927 | 478.7482279 | 438.9207283 | 474.1550567 | 548.4553466 | 461.7787812 | 395.7698285 | 373.1561407 | 491.8527634 | 515.3116951 | 483.983891 | 459.005763 | 493.6546166 |
| **Ctnnd2** | 17.39823315 | 32.48274235 | 30.54329049 | 30.19132969 | 14.56735462 | 14.72966892 | 17.13922958 | 21.78992434 | 12.79104008 | 27.09096104 | 21.75502607 | 16.48303402 | 33.23128829 | 15.36042908 | 22.9941015 |
| **Dap** | 1498.079444 | 1425.279353 | 1511.501298 | 1484.550811 | 1715.781029 | 1779.484288 | 1773.459229 | 878.3015657 | 956.3183498 | 994.5004406 | 922.2239313 | 1824.411608 | 1750.181183 | 1845.962153 | 1708.749168 |
| **Ankrd33b** | 52.19469945 | 37.2363144 | 42.2907099 | 18.97740723 | 8.86708542 | 16.13249453 | 29.76813559 | 30.17066447 | 13.54345421 | 36.70388269 | 28.37612096 | 12.14539349 | 10.22501178 | 13.55331977 | 7.904222392 |
| **Ropn1l** | 19.22962611 | 12.67619214 | 10.18109683 | 8.626094197 | 13.30062813 | 11.9240177 | 11.72684129 | 4.190370065 | 5.266898858 | 12.23462756 | 1.891741398 | 10.41033728 | 17.0416863 | 13.55331977 | 12.21561642 |
| **March6** | 2166.537875 | 2097.909799 | 2130.981882 | 2153.073111 | 1914.657087 | 2022.173119 | 2097.300462 | 1831.191719 | 1955.524305 | 1741.686624 | 2079.023796 | 2017.002847 | 2152.36498 | 1935.414064 | 1968.869941 |
| **Cmbl** | 0.915696482 | 0.792262009 | 4.698967767 | 0 | 0 | 4.90988964 | 0 | 0 | 2.257242368 | 6.991215751 | 2.837612096 | 0.867528106 | 0.852084315 | 0 | 2.874262688 |
| **Cct5** | 11874.75197 | 11021.1568 | 11543.79748 | 11116.44759 | 11209.89606 | 11005.86833 | 10795.00844 | 11800.92018 | 11502.90711 | 11842.24558 | 12036.20464 | 10806.79762 | 10676.61647 | 10647.48801 | 10458.00479 |
| **Fam173b** | 127.2818109 | 140.2303755 | 148.800646 | 156.9949144 | 153.2739051 | 147.2966892 | 126.2890601 | 191.918949 | 131.6724715 | 171.2847859 | 124.8549322 | 109.3085414 | 182.3460434 | 136.4367524 | 149.4616598 |
| **Cpq** | 16.48253667 | 9.507144102 | 17.22954848 | 11.21392246 | 17.1008076 | 14.02825611 | 13.53097072 | 3.352296052 | 5.266898858 | 20.09974528 | 4.729353494 | 13.8804497 | 12.78126473 | 11.74621047 | 20.11983882 |
| **Mtdh** | 4301.94207 | 4085.695178 | 4483.598411 | 4234.549641 | 3890.75041 | 4136.932728 | 4335.323019 | 4360.49909 | 4258.663934 | 4334.553766 | 4430.458353 | 4307.277048 | 4505.821859 | 4012.686208 | 4291.274193 |
| **Laptm4b** | 29.30228741 | 24.56012226 | 21.92851625 | 12.93914129 | 25.96789302 | 7.014128057 | 15.33510015 | 21.78992434 | 21.82000955 | 25.3431571 | 28.37612096 | 5.205168638 | 10.22501178 | 17.16753838 | 10.05991941 |
| **Matn2** | 4.578482408 | 4.753572051 | 3.915806472 | 9.488703616 | 6.966995687 | 5.611302446 | 9.020647148 | 7.542666117 | 5.266898858 | 10.48682363 | 10.40457769 | 5.205168638 | 3.408337261 | 13.55331977 | 8.622788064 |
| **Rpl30** | 10.98835778 | 16.63750218 | 21.14535495 | 20.70262607 | 19.63426057 | 15.43108173 | 8.118582433 | 14.24725822 | 10.53379772 | 15.73023544 | 13.24218978 | 10.41033728 | 14.48543336 | 7.228437213 | 5.748525376 |
| **Hrsp12** | 114.4620602 | 148.9452576 | 136.2700652 | 138.0175071 | 113.3720207 | 120.6430026 | 123.5828659 | 156.7198404 | 127.9104008 | 160.7979623 | 138.097122 | 146.61225 | 133.7772375 | 149.9900722 | 120.0004672 |
| **Pop1** | 1193.152515 | 1203.445991 | 1150.463942 | 1248.19583 | 1052.649712 | 1079.474308 | 1096.008628 | 1464.115301 | 1412.281308 | 1464.6597 | 1330.840073 | 1163.355191 | 1152.017994 | 1086.072691 | 1137.489459 |
| **Stk3** | 82.41268334 | 110.9166812 | 98.67832311 | 89.71137964 | 73.47013634 | 67.33562935 | 87.50027733 | 87.99777137 | 48.15450385 | 109.2377461 | 125.8008029 | 82.4151701 | 57.94173343 | 103.9087849 | 63.23377913 |
| **Osr2** | 0 | 0 | 0 | 6.038265938 | 0 | 0 | 0.902064715 | 0.838074013 | 3.00965649 | 0 | 0 | 0 | 1.70416863 | 0 | 0 |
| **Vps13b** | 1104.329957 | 1079.853118 | 1101.12478 | 1120.529636 | 891.1420847 | 955.3242414 | 1191.627488 | 977.1942992 | 1082.723922 | 1063.538696 | 1045.187122 | 1055.781705 | 1120.490874 | 986.6816795 | 1128.866671 |
| **Cox6c** | 1773.704085 | 1675.634148 | 1813.018397 | 1611.354396 | 1771.516994 | 1851.729807 | 1787.892265 | 1742.355873 | 1662.082797 | 1762.660271 | 1810.396517 | 1834.821945 | 1795.341652 | 2047.45484 | 1705.15634 |
| **Fbxo43** | 0 | 6.338096068 | 0 | 0 | 0.633363244 | 0 | 0.902064715 | 0.838074013 | 0 | 0 | 0 | 0 | 0 | 0 | 0 |
| **Polr2k** | 219.7671556 | 259.8619388 | 247.4789691 | 238.0801998 | 238.7779431 | 237.0775283 | 232.7326964 | 220.4134654 | 177.5697329 | 291.0093556 | 217.5502607 | 226.4248357 | 201.9439827 | 238.538428 | 207.6654792 |
| **Spag1** | 17.39823315 | 19.0142882 | 17.22954848 | 34.50437679 | 27.2346195 | 23.14662259 | 19.84542373 | 26.81836842 | 29.34415078 | 40.19949057 | 42.56418144 | 18.21809023 | 32.37920398 | 28.0101942 | 23.71266717 |
| **Rnf19a** | 1180.332765 | 1147.195388 | 1201.369426 | 1070.49829 | 1112.81922 | 1122.260489 | 1317.916548 | 1017.421852 | 910.4210883 | 911.4797536 | 801.1524819 | 1127.786538 | 1093.224176 | 1074.326481 | 1113.058226 |
| **Ankrd46** | 619.926518 | 616.3798426 | 675.8681971 | 538.2682779 | 627.0296118 | 677.5647703 | 641.3680122 | 691.4110608 | 638.0471759 | 634.4528294 | 544.8215225 | 669.731698 | 708.0820659 | 639.7166933 | 748.0268645 |
| **Pabpc1** | 36181.91508 | 33439.00259 | 35637.75471 | 35205.67824 | 36733.80144 | 37616.76877 | 38584.01405 | 36637.24355 | 35818.67431 | 34398.5293 | 35048.29287 | 36635.71193 | 36124.96663 | 35470.84496 | 35404.44922 |
| **Ywhaz** | 11079.92743 | 10552.92995 | 10648.64412 | 10575.59148 | 11149.72655 | 10876.80838 | 11757.51149 | 10843.83965 | 10572.17084 | 10427.39829 | 11028.85235 | 11152.07381 | 11158.04411 | 11055.89472 | 10603.15506 |
| **Zfp706** | 2271.842971 | 2373.616977 | 2371.4124 | 2348.88545 | 2446.048849 | 2320.974974 | 2299.362958 | 2712.84558 | 2830.581929 | 2695.987574 | 2631.412284 | 2380.497124 | 2455.706996 | 2326.653228 | 2325.99708 |
| **Grhl2** | 0 | 7.130358077 | 4.698967767 | 2.587828259 | 2.533452977 | 0.701412806 | 2.706194144 | 2.514222039 | 6.771727103 | 0.873901969 | 0.945870699 | 4.337640531 | 0 | 2.710663955 | 0 |
| **Ncald** | 71.42432556 | 87.94108294 | 86.93090369 | 100.0626927 | 60.16950821 | 40.68194273 | 37.88671802 | 138.2822122 | 104.585563 | 158.1762564 | 131.4760271 | 75.47494525 | 70.72299816 | 65.95948956 | 81.9164866 |
| **Rrm2b** | 282.9502128 | 274.1226549 | 232.5989045 | 234.6297621 | 249.5451182 | 249.7029588 | 289.5627734 | 231.3084276 | 243.0297616 | 235.9535316 | 253.4933473 | 245.5104541 | 259.8857161 | 234.9242094 | 260.1207733 |
| **Ubr5** | 4953.917965 | 4799.523248 | 5155.550802 | 4753.840512 | 4210.598848 | 4268.096923 | 4742.154206 | 5413.958124 | 5482.089297 | 5367.505893 | 5326.197905 | 4656.023346 | 4728.215865 | 4272.006393 | 4676.425393 |
| **Klf10** | 357.1216278 | 384.2470741 | 339.1088405 | 286.3863273 | 414.2195618 | 354.9148797 | 436.599322 | 257.288722 | 207.6662978 | 188.7628253 | 238.3594161 | 339.2034896 | 400.4796281 | 401.1782653 | 402.3967763 |
| **Azin1** | 2068.558352 | 2062.258008 | 2069.895301 | 1927.069444 | 1976.093322 | 1874.175017 | 2007.996055 | 2438.795378 | 2245.203742 | 2164.655177 | 2294.682315 | 2046.498803 | 2074.825307 | 1929.992736 | 2087.433277 |
| **Atp6v1c1** | 904.7081238 | 847.7203491 | 988.3495536 | 924.7172979 | 892.4088112 | 826.2642851 | 863.275932 | 967.9754851 | 925.4693708 | 887.0104984 | 902.3606466 | 845.8399036 | 796.6988347 | 866.5089109 | 853.6560183 |
| **Fzd6** | 3.662785926 | 8.714882094 | 10.96425812 | 11.21392246 | 9.500448664 | 18.23673295 | 5.412388289 | 0 | 20.31518131 | 12.23462756 | 20.80915537 | 0.867528106 | 8.520843152 | 14.45687443 | 4.311394032 |
| **Slc25a32** | 630.9148758 | 615.5875806 | 629.6616808 | 519.2908706 | 605.4952615 | 585.6796928 | 611.5998766 | 698.1156529 | 613.969924 | 690.3825554 | 606.3031179 | 643.7058549 | 612.6486226 | 533.0972444 | 605.0322958 |
| **Dcaf13** | 1425.739422 | 1379.328157 | 1465.294782 | 1487.138639 | 1418.733667 | 1419.659519 | 1361.215655 | 1707.994839 | 1443.130287 | 1537.193563 | 1514.338989 | 1378.502161 | 1385.489096 | 1371.595961 | 1320.005139 |
| **Rims2** | 1.831392963 | 3.169048034 | 0.783161294 | 0 | 5.700269198 | 2.805651223 | 0.902064715 | 1.676148026 | 3.00965649 | 0 | 2.837612096 | 0 | 0.852084315 | 0 | 0 |
| **Lrp12** | 227.0927274 | 221.8333624 | 231.8157432 | 183.7358064 | 155.1739948 | 176.756027 | 174.09849 | 203.6519852 | 165.531107 | 168.66308 | 227.9548384 | 175.2406775 | 164.4522728 | 185.2287036 | 196.1684284 |
| **Oxr1** | 369.9413786 | 352.5565938 | 360.2541955 | 374.3724881 | 407.8859293 | 378.7629151 | 387.8878274 | 342.7722713 | 337.833941 | 313.7308068 | 396.3198228 | 448.5120309 | 432.0067478 | 443.6453339 | 415.3309584 |
| **Angpt1** | 0 | 0 | 0.783161294 | 2.587828259 | 1.266726489 | 2.805651223 | 3.608258859 | 0 | 3.00965649 | 0 | 0 | 0 | 0 | 0 | 0 |
| **Eif3e** | 5790.864549 | 5896.806129 | 5771.115579 | 5565.555976 | 5973.248757 | 5994.273838 | 5920.250723 | 6147.272886 | 5931.280528 | 6012.445546 | 5890.882712 | 6049.273485 | 5663.804443 | 5655.348564 | 5642.177656 |
| **Emc2** | 577.8044799 | 557.752454 | 646.108068 | 566.7343887 | 595.3614496 | 605.3192513 | 632.3473651 | 648.6692861 | 611.7126817 | 594.2533389 | 639.4085924 | 645.4409111 | 643.3236579 | 680.3766526 | 619.4036092 |
| **Nudcd1** | 905.6238203 | 823.1602268 | 823.8856818 | 930.7555638 | 761.3026196 | 752.6159405 | 838.9201847 | 911.8245262 | 953.3086933 | 987.5092249 | 911.8193536 | 707.0354066 | 747.2779444 | 801.4529759 | 844.3146646 |
| **Eny2** | 380.9297363 | 434.1595807 | 437.7871636 | 353.6698621 | 396.4853909 | 414.5349682 | 335.5680739 | 500.3301858 | 369.4353342 | 420.346847 | 404.8326591 | 388.6525916 | 432.0067478 | 369.5538525 | 390.8997255 |
| **Pkhd1l1** | 10.98835778 | 15.05297816 | 7.04845165 | 9.488703616 | 1.266726489 | 0 | 2.706194144 | 31.00873848 | 9.781383594 | 24.46925513 | 17.97154328 | 0.867528106 | 0 | 3.614218606 | 1.437131344 |
| **Ebag9** | 260.0578008 | 214.7030043 | 188.741872 | 219.965402 | 203.3096014 | 209.7224289 | 243.557473 | 251.4222039 | 244.5345898 | 218.4754922 | 200.5245881 | 219.4846109 | 192.5710552 | 225.8886629 | 214.1325702 |
| **Trps1** | 184.9706893 | 213.9107423 | 173.0786461 | 167.3462274 | 151.3738154 | 175.3532014 | 184.9232665 | 194.433171 | 203.1518131 | 166.0413741 | 167.4191137 | 189.1211272 | 172.1210317 | 143.6651896 | 154.4916195 |
| **Mir1907** | 0 | 0.792262009 | 0 | 0 | 0 | 0 | 0.902064715 | 1.676148026 | 3.00965649 | 0 | 0 | 0 | 0 | 0 | 0 |
| **Eif3h** | 8118.565006 | 7898.852225 | 7985.112559 | 7955.846677 | 7799.23499 | 8348.215214 | 8440.619536 | 8220.667994 | 7901.853115 | 7651.88564 | 7857.347895 | 7990.801387 | 7676.427595 | 7754.30602 | 8246.978217 |
| **Utp23** | 397.412273 | 391.3774322 | 392.3638085 | 375.2350975 | 353.4166903 | 365.4360718 | 395.1043451 | 395.5709342 | 464.2395136 | 396.7514939 | 350.9180292 | 333.1307928 | 395.3671222 | 430.0920141 | 349.2229166 |
| **Rad21** | 7334.728817 | 7367.244417 | 7794.804364 | 7790.225669 | 7347.013634 | 7108.117373 | 7765.87513 | 7442.93531 | 7831.878602 | 7482.348658 | 7672.903108 | 7967.378128 | 7956.763335 | 8233.189985 | 7784.94049 |
| **Aard** | 5.494178889 | 1.584524017 | 7.04845165 | 5.175656518 | 3.800179466 | 4.208476834 | 0 | 51.96058881 | 24.82966605 | 18.35194135 | 30.26786236 | 2.602584319 | 0.852084315 | 2.710663955 | 0.718565672 |
| **Med30** | 644.650323 | 579.1435282 | 653.9396809 | 595.2004996 | 670.0983124 | 673.3562935 | 620.6205238 | 732.4766874 | 714.0410023 | 804.8637134 | 641.3003338 | 632.4279895 | 645.8799109 | 710.1939561 | 597.8466391 |
| **Ext1** | 168.4881526 | 117.2547773 | 157.4154202 | 165.6210086 | 133.0062813 | 153.6094045 | 141.6241602 | 232.1465016 | 130.9200573 | 127.5896875 | 110.6668718 | 112.7786538 | 133.7772375 | 92.16257446 | 124.3118613 |
| **Tnfrsf11b** | 104.3893989 | 46.7434585 | 89.28038757 | 34.50437679 | 53.83587576 | 69.43986777 | 59.53627118 | 92.18814144 | 82.01313936 | 113.607256 | 43.51005214 | 31.23101183 | 39.1958785 | 51.50261514 | 60.35951645 |
| **Nov** | 82.41268334 | 99.03275106 | 149.5838072 | 78.49745719 | 63.33632443 | 88.37801352 | 85.6961479 | 111.4638437 | 60.19312981 | 111.859452 | 142.8264755 | 101.5007884 | 52.82922754 | 77.70570004 | 45.26963733 |
| **Enpp2** | 4.578482408 | 9.507144102 | 0.783161294 | 6.038265938 | 3.166816221 | 4.90988964 | 6.314453003 | 9.218814144 | 4.514484735 | 2.621705907 | 8.512836289 | 7.807752957 | 4.260421576 | 1.807109303 | 1.437131344 |
| **Taf2** | 960.5656092 | 1087.775738 | 1040.82136 | 905.7398906 | 1055.816528 | 960.234131 | 1037.374422 | 1169.113248 | 1154.203264 | 1078.39503 | 1241.928227 | 1076.60238 | 1013.980335 | 1133.961088 | 1080.722771 |
| **Dscc1** | 246.3223535 | 224.2101484 | 234.9483883 | 276.0350143 | 254.6120242 | 234.2718771 | 214.6914021 | 263.9933141 | 228.7338933 | 227.2145119 | 269.5731491 | 248.1130384 | 269.2586436 | 240.3455373 | 200.4798225 |
| **Deptor** | 1.831392963 | 14.26071615 | 14.8800646 | 15.52696955 | 13.30062813 | 5.611302446 | 16.23716487 | 15.08533223 | 8.276555348 | 19.22584332 | 14.18806048 | 13.8804497 | 24.71044514 | 8.131991864 | 3.59282836 |
| **Mrpl13** | 1260.914055 | 1178.885869 | 1288.300329 | 1186.950561 | 1332.596266 | 1225.368172 | 1188.019229 | 1445.677673 | 1519.876528 | 1538.941367 | 1416.914307 | 1170.295415 | 1105.153357 | 1292.986706 | 1177.010571 |
| **Mtbp** | 984.3737177 | 1051.331685 | 957.8062632 | 957.4964558 | 886.708542 | 850.1123205 | 975.1319567 | 976.3562252 | 1025.540449 | 994.5004406 | 1095.318269 | 916.9772083 | 935.588578 | 851.1484818 | 924.0754541 |
| **Sntb1** | 86.99116575 | 101.4095371 | 111.9920651 | 100.0626927 | 107.6717515 | 101.7048568 | 156.9592604 | 121.5207319 | 122.643502 | 81.27288311 | 98.37055267 | 124.9240473 | 127.8126473 | 156.3149547 | 138.6831747 |
| **Zhx2** | 423.0517745 | 496.7482793 | 408.8101957 | 403.7012084 | 420.5531942 | 519.746889 | 509.6665639 | 476.8641134 | 404.7987979 | 343.4434738 | 312.1373306 | 467.5976493 | 418.3733987 | 391.2391641 | 426.1094435 |
| **Derl1** | 2020.026438 | 2077.310986 | 1961.819043 | 1994.352978 | 1791.784618 | 1862.952412 | 1850.13473 | 1872.257345 | 1854.700812 | 2025.704764 | 1890.795527 | 1828.749248 | 1960.646009 | 1901.078987 | 1874.737838 |
| **Tbc1d31** | 391.9180941 | 376.324454 | 368.0858084 | 400.2507707 | 364.1838655 | 374.5544383 | 349.0990446 | 405.6278223 | 364.9208495 | 407.2383175 | 323.487779 | 406.0031537 | 311.010775 | 401.1782653 | 372.9355838 |
| **9130401M01Rik** | 847.9349419 | 817.6143928 | 842.6815529 | 828.9676523 | 811.3383159 | 842.3967797 | 771.2653311 | 1039.211776 | 1015.759065 | 1026.834813 | 873.038655 | 825.0192291 | 722.5674993 | 800.5494213 | 791.8593705 |
| **Zhx1** | 836.0308877 | 851.6816591 | 822.3193592 | 822.0667769 | 813.2384057 | 795.4021217 | 890.3378735 | 879.9777137 | 815.6169089 | 810.9810271 | 989.3807509 | 838.0321507 | 815.4446896 | 847.5342632 | 904.674181 |
| **Atad2** | 3014.472817 | 3183.30875 | 3172.586404 | 2984.628592 | 2648.091724 | 2853.347294 | 2943.437164 | 3550.919593 | 3307.612483 | 3140.803676 | 3236.769531 | 3224.601971 | 3130.557774 | 3013.354763 | 2887.915436 |
| **Wdyhv1** | 178.5608139 | 204.4035982 | 166.8133557 | 170.7966651 | 189.37561 | 161.3249453 | 179.5108782 | 155.8817664 | 187.3511165 | 191.3845312 | 186.3365277 | 193.4587677 | 156.783514 | 184.3251489 | 197.6055598 |
| **Fbxo32** | 119.9562391 | 122.0083493 | 113.5583877 | 102.6505209 | 88.03749095 | 81.36388546 | 91.10853619 | 115.6542138 | 76.7462405 | 86.51629492 | 69.99443171 | 108.4410133 | 66.46257658 | 106.6194489 | 76.8865269 |
| **D15Ertd621e** | 1215.129231 | 1159.87158 | 1274.986587 | 1200.752312 | 1011.481101 | 1014.94433 | 1077.065269 | 1257.949094 | 1138.402567 | 1265.410051 | 1423.535402 | 1144.269572 | 1094.928345 | 1061.676716 | 1159.046429 |
| **Tmem65** | 537.5138347 | 530.0232837 | 494.1747768 | 494.2751975 | 518.0911338 | 582.1726287 | 598.9709706 | 613.4701776 | 494.3360785 | 522.5933774 | 441.7216163 | 517.9142794 | 568.3402382 | 567.4323212 | 513.0558898 |
| **Trmt12** | 84.2440763 | 136.2690655 | 99.4614844 | 130.2540224 | 145.6735462 | 90.48225194 | 111.8560246 | 186.0524309 | 141.453855 | 156.4284524 | 136.2053806 | 133.5993284 | 92.87719035 | 121.979878 | 78.32365824 |
| **Rnf139** | 702.3392014 | 663.9155631 | 758.8832944 | 605.5518126 | 625.1295221 | 702.1142185 | 665.7237595 | 688.0587647 | 622.2464794 | 718.3474184 | 663.0553598 | 734.796306 | 724.2716679 | 679.473098 | 654.6133272 |
| **Tatdn1** | 124.5347215 | 110.1244192 | 113.5583877 | 96.612255 | 119.7056532 | 113.6288745 | 119.9746071 | 129.063398 | 112.1097043 | 115.3550599 | 115.3962252 | 109.3085414 | 109.0667923 | 115.6549954 | 113.5333762 |
| **Ndufb9** | 1378.123205 | 1425.279353 | 1350.953233 | 1230.081032 | 1333.862992 | 1375.470512 | 1390.081725 | 1203.474283 | 1244.492959 | 1329.204895 | 1204.0934 | 1292.616878 | 1244.895184 | 1395.991937 | 1170.54348 |
| **Mtss1** | 488.9819212 | 432.5750566 | 518.452777 | 422.6786156 | 402.8190234 | 407.5208401 | 482.6046224 | 362.8860477 | 357.3967082 | 345.1912777 | 401.0491763 | 459.7898963 | 457.5692772 | 419.2493583 | 472.0976465 |
| **Sqle** | 4230.517745 | 4342.388069 | 4279.976474 | 4426.048932 | 4395.540915 | 4695.257321 | 4728.623235 | 4422.516567 | 4440.748151 | 4689.357965 | 4650.846226 | 4904.136385 | 4712.878347 | 4546.687007 | 4567.203411 |
| **E430025E21Rik** | 1170.260103 | 1121.050742 | 1222.514781 | 1002.352146 | 1031.115362 | 1086.488436 | 1075.26114 | 1324.995015 | 1182.795001 | 1251.427619 | 1243.819969 | 1066.192043 | 1069.365816 | 1018.306092 | 1110.183963 |
| **Nsmce2** | 512.7900297 | 465.850061 | 535.6823254 | 530.5047931 | 547.8592063 | 556.2203549 | 533.1202464 | 459.2645592 | 474.0208972 | 506.863142 | 494.6903755 | 543.9401226 | 506.9901675 | 510.5083781 | 521.6786778 |
| **Trib1** | 152.0056159 | 114.0857292 | 162.114388 | 140.6053354 | 127.9393753 | 126.9557178 | 134.4076425 | 208.6804293 | 174.5600764 | 189.6367273 | 174.9860793 | 130.1292159 | 147.4105865 | 146.3758536 | 134.3717807 |
| **Gm19510** | 4.578482408 | 2.376786026 | 5.482129061 | 4.313047098 | 1.266726489 | 0.701412806 | 4.510323574 | 7.542666117 | 0 | 2.621705907 | 0 | 2.602584319 | 0 | 0.903554652 | 0.718565672 |
| **Fam84b** | 292.1071776 | 296.3059912 | 278.0222595 | 202.7132136 | 192.5424263 | 237.7789411 | 270.6194144 | 212.0327253 | 195.6276719 | 193.1323351 | 207.145683 | 198.6639363 | 259.8857161 | 183.4215943 | 205.5097822 |
| **9930014A18Rik** | 54.02609241 | 55.4583406 | 41.50754861 | 52.6191746 | 65.23641416 | 53.30737323 | 53.22181817 | 36.87525657 | 67.71727103 | 34.08217679 | 51.07701773 | 32.96606804 | 46.86463733 | 72.28437213 | 37.36541494 |
| **Myc** | 5869.614447 | 5270.126881 | 5159.466608 | 5147.190407 | 5650.866865 | 5640.761784 | 5935.585823 | 5206.953843 | 4793.630375 | 4691.105769 | 4888.259771 | 5212.976391 | 5480.606315 | 5097.855344 | 5576.78818 |
| **Pvt1** | 152.9213124 | 165.5827598 | 152.7164524 | 140.6053354 | 145.6735462 | 124.8514794 | 163.2737134 | 108.9496217 | 124.9007443 | 98.75092249 | 92.69532848 | 109.3085414 | 151.6710081 | 142.7616349 | 148.0245284 |
| **H2afy3** | 6.409875371 | 3.961310043 | 4.698967767 | 9.488703616 | 0 | 1.402825611 | 1.80412943 | 9.218814144 | 0 | 3.495607876 | 5.675224193 | 5.205168638 | 0.852084315 | 0.903554652 | 1.437131344 |
| **Gsdmc4** | 0.915696482 | 0.792262009 | 1.566322589 | 0.86260942 | 0.633363244 | 1.402825611 | 0 | 0.838074013 | 0 | 9.612921658 | 0 | 1.735056213 | 0 | 5.421327909 | 2.874262688 |
| **Fam49b** | 4169.166081 | 3932.78861 | 4154.670667 | 4062.890367 | 3977.521174 | 4188.837276 | 4278.492942 | 3971.632748 | 3861.389277 | 4079.374391 | 3820.371752 | 3951.590524 | 4029.506726 | 4013.589762 | 3977.260994 |
| **Asap1** | 670.2898245 | 838.213205 | 859.9111013 | 793.6006661 | 1008.947648 | 956.727067 | 1094.204499 | 694.7633568 | 717.8030729 | 685.1391436 | 583.6022211 | 1003.730019 | 1031.022021 | 1021.920311 | 1003.117678 |
| **Efr3a** | 1994.386937 | 2067.01158 | 2189.718979 | 2247.097538 | 2003.327942 | 2086.001684 | 2119.85208 | 2092.670811 | 2212.849935 | 2029.200372 | 2093.211856 | 2429.946226 | 2270.8047 | 2296.835924 | 2177.972552 |
| **Kcnq3** | 1.831392963 | 2.376786026 | 0.783161294 | 0 | 2.533452977 | 1.402825611 | 2.706194144 | 0 | 0 | 0 | 1.891741398 | 2.602584319 | 0 | 0 | 0 |
| **Lrrc6** | 1.831392963 | 7.922620085 | 7.831612945 | 2.587828259 | 3.166816221 | 2.805651223 | 0.902064715 | 2.514222039 | 21.82000955 | 2.621705907 | 4.729353494 | 6.072696744 | 1.70416863 | 4.517773258 | 2.874262688 |
| **Tmem71** | 293.9385706 | 388.2083842 | 364.1700019 | 358.8455186 | 446.5210872 | 448.9041957 | 443.8158397 | 217.8992434 | 275.3835689 | 281.396434 | 258.2227008 | 487.5507957 | 464.3859518 | 562.0109933 | 464.1934241 |
| **Phf20l1** | 1219.707713 | 1399.926969 | 1292.999297 | 1260.272362 | 1285.094023 | 1255.528922 | 1378.354884 | 1429.754266 | 1422.062692 | 1358.04366 | 1453.803264 | 1362.886655 | 1422.128722 | 1447.494552 | 1475.215325 |
| **Tg** | 321.409465 | 291.5524191 | 263.9253562 | 234.6297621 | 202.0428749 | 200.6040624 | 191.2377195 | 103.9211776 | 142.9586833 | 181.7716095 | 131.4760271 | 288.0193313 | 255.6252945 | 170.7718291 | 235.6895404 |
| **Sla** | 2893.600882 | 2803.022986 | 2781.005757 | 2385.977655 | 2485.317371 | 2478.792855 | 2665.601232 | 1618.320919 | 1921.665669 | 1725.082487 | 1647.706757 | 2508.023755 | 2459.115334 | 2519.110369 | 2644.321673 |
| **Wisp1** | 3.662785926 | 4.753572051 | 1.566322589 | 0.86260942 | 1.266726489 | 0.701412806 | 1.80412943 | 7.542666117 | 3.00965649 | 4.369509845 | 3.783482795 | 0.867528106 | 3.408337261 | 0 | 0 |
| **Ndrg1** | 2281.915632 | 1746.145467 | 1712.773751 | 1458.672529 | 1552.373312 | 1885.397622 | 1945.75359 | 729.1243914 | 723.8223859 | 727.9603401 | 755.7506883 | 1440.096656 | 1647.931066 | 1363.463969 | 1767.671553 |
| **St3gal1** | 430.3773463 | 430.9905326 | 407.2438731 | 356.2576903 | 444.6209975 | 429.9660499 | 530.4140523 | 489.4352236 | 379.2167178 | 337.32616 | 334.8382274 | 481.478099 | 527.4401911 | 515.0261514 | 488.6246569 |
| **Zfat** | 142.8486511 | 129.9309694 | 133.1374201 | 140.6053354 | 143.7734565 | 143.0882124 | 143.4282897 | 129.063398 | 137.6917844 | 125.8418835 | 113.5044839 | 152.6849467 | 109.0667923 | 122.8834326 | 153.0544881 |
| **Khdrbs3** | 4.578482408 | 3.169048034 | 1.566322589 | 5.175656518 | 3.166816221 | 3.507064029 | 1.80412943 | 5.866518091 | 2.257242368 | 5.243411813 | 8.512836289 | 2.602584319 | 1.70416863 | 5.421327909 | 2.874262688 |
| **Fam135b** | 0.915696482 | 0 | 0.783161294 | 1.725218839 | 0 | 2.104238417 | 0 | 0 | 0 | 0 | 0.945870699 | 0.867528106 | 0 | 1.807109303 | 4.311394032 |
| **Trappc9** | 620.8422145 | 568.8441221 | 621.0469065 | 598.6509372 | 573.8270993 | 564.6373086 | 612.5019413 | 615.1463256 | 569.5774908 | 623.0921038 | 601.5737644 | 540.4700102 | 558.1152264 | 538.5185723 | 593.535245 |
| **Peg13** | 62.26736075 | 115.6702532 | 79.09929074 | 83.67311371 | 96.90457637 | 83.46812388 | 102.8353775 | 104.7592516 | 100.8234924 | 92.6336087 | 116.3420959 | 114.51371 | 81.80009426 | 77.70570004 | 80.47935526 |
| **Chrac1** | 611.6852497 | 696.3983055 | 641.4091002 | 673.6979567 | 651.0974151 | 684.5788984 | 702.7084128 | 828.8551989 | 743.3851531 | 679.8957318 | 706.565412 | 647.1759673 | 605.8319481 | 687.6050898 | 616.5293465 |
| **Ago2** | 4076.680736 | 3811.572523 | 3863.334666 | 3631.585657 | 3173.149854 | 3211.769237 | 4239.704159 | 3425.208491 | 3485.93463 | 3090.117362 | 3624.576518 | 3494.403212 | 3693.785506 | 3123.58843 | 3546.121591 |
| **Ptk2** | 75.08711149 | 81.60298688 | 80.66561333 | 64.69570647 | 78.53704229 | 61.0229141 | 69.45898304 | 129.901472 | 102.3283207 | 70.78605948 | 92.69532848 | 72.00483282 | 54.53339617 | 73.18792678 | 58.9223851 |
| **Dennd3** | 215.1886732 | 149.7375196 | 162.8975493 | 113.8644434 | 86.77076447 | 65.93280374 | 115.4642835 | 222.0896135 | 161.0166222 | 215.8537863 | 243.0887696 | 97.1631479 | 87.76468446 | 91.25901981 | 108.5034165 |
| **Slc45a4** | 324.1565545 | 292.3446811 | 317.1803243 | 309.6767817 | 265.3791994 | 290.3849016 | 274.2276733 | 327.6869391 | 260.3352864 | 254.305473 | 215.6585193 | 303.6348372 | 276.0753181 | 282.8126059 | 315.45033 |
| **Ptp4a3** | 2501.682788 | 2718.250951 | 2900.046273 | 2929.421589 | 2382.079162 | 2607.151399 | 2535.703913 | 3683.335287 | 3763.575441 | 3881.872546 | 3677.545277 | 2540.989823 | 2618.4551 | 2669.100441 | 2513.542721 |
| **Arc** | 184.9706893 | 160.0369257 | 227.8999367 | 211.3393078 | 236.2444901 | 235.6747027 | 235.4388906 | 265.6694621 | 226.4766509 | 234.2057277 | 268.6272785 | 236.835173 | 208.7606572 | 265.6450676 | 201.9169538 |
| **Jrk** | 108.9678813 | 115.6702532 | 127.655291 | 125.0783658 | 136.1730975 | 143.0882124 | 146.1344838 | 167.6148026 | 82.76555348 | 145.9416288 | 148.5016997 | 142.2746094 | 125.2563943 | 122.8834326 | 130.0603866 |
| **4933427E11Rik** | 0 | 0 | 6.265290356 | 1.725218839 | 3.800179466 | 2.805651223 | 1.80412943 | 0 | 0.752414123 | 1.747803938 | 0 | 0 | 0 | 0 | 1.437131344 |
| **Them6** | 1274.649502 | 1438.747807 | 1474.692718 | 1400.015088 | 1413.033398 | 1387.39453 | 1385.571402 | 1434.78271 | 1421.310278 | 1371.152189 | 1321.381366 | 1552.007782 | 1342.032796 | 1308.347135 | 1327.909362 |
| **Lynx1** | 27.47089445 | 27.7291703 | 25.84432272 | 45.71829924 | 26.60125626 | 25.25086101 | 37.88671802 | 36.03718256 | 35.36346376 | 13.9824315 | 44.45592284 | 30.36348372 | 25.56252945 | 25.29953024 | 15.80844478 |
| **Ly6d** | 0 | 3.961310043 | 3.132645178 | 0.86260942 | 0 | 0.701412806 | 0.902064715 | 0.838074013 | 3.762070613 | 0 | 0 | 0 | 1.70416863 | 1.807109303 | 1.437131344 |
| **D730001G18Rik** | 0 | 1.584524017 | 0 | 0 | 0 | 1.402825611 | 0.902064715 | 2.514222039 | 4.514484735 | 0 | 0 | 0 | 0.852084315 | 0 | 0.718565672 |
| **Ly6k** | 36.62785926 | 65.75774671 | 56.3876132 | 59.52004996 | 38.6351579 | 34.36922748 | 29.76813559 | 77.94088321 | 48.90691797 | 65.54264767 | 73.7779145 | 26.89337129 | 35.78754124 | 33.43152211 | 22.9941015 |
| **2010109I03Rik** | 3.662785926 | 7.130358077 | 4.698967767 | 6.900875357 | 3.800179466 | 4.90988964 | 6.314453003 | 0 | 0 | 1.747803938 | 1.891741398 | 4.337640531 | 4.260421576 | 0.903554652 | 5.748525376 |

| | | | | | | | | | | | | | | | |
|---|---|---|---|---|---|---|---|---|---|---|---|---|---|---|---|
| **Ly6e** | 13119.18349 | 13340.89996 | 13041.20188 | 13383.38515 | 13866.22151 | 12814.11055 | 14204.81306 | 9310.164211 | 10369.01902 | 9965.978053 | 10479.30147 | 12907.08317 | 12763.37096 | 13653.61434 | 13123.16487 |
| **Ly6i** | 8.241268334 | 7.130358077 | 3.915806472 | 6.038265938 | 4.43354271 | 2.805651223 | 5.412388289 | 8.380740131 | 9.781383594 | 6.117313782 | 1.891741398 | 4.337640531 | 5.964590206 | 0.903554652 | 3.59282836 |
| **Ly6a** | 9114.842777 | 8992.966059 | 8492.601077 | 8463.061016 | 7455.318748 | 7556.320156 | 8229.536393 | 6490.883231 | 7121.59967 | 6750.89271 | 7347.523588 | 7396.544634 | 7024.583094 | 7880.803671 | 7215.117912 |
| **Ly6c1** | 3.662785926 | 3.169048034 | 4.698967767 | 2.587828259 | 0 | 4.208476834 | 0 | 0 | 9.028969471 | 1.747803938 | 6.621094891 | 2.602584319 | 0.852084315 | 0 | 0.718565672 |
| **Ly6c2** | 3736.957341 | 3340.96889 | 2874.985112 | 2712.044015 | 3567.101792 | 3523.196523 | 4057.487087 | 318.468125 | 775.7389604 | 853.8022236 | 977.0844318 | 2837.684436 | 2709.628122 | 2923.902852 | 3273.785201 |
| **Zfp41** | 97.97952353 | 118.0470393 | 148.0174847 | 106.963568 | 118.4389267 | 127.6571306 | 94.71679505 | 103.0831036 | 85.02279585 | 117.9767658 | 99.31642337 | 121.4539349 | 144.0022493 | 122.8834326 | 117.1262045 |
| **Top1mt** | 328.7350369 | 276.499441 | 357.9047116 | 281.2106708 | 274.2462848 | 298.1004424 | 307.6040677 | 389.7044161 | 288.9270231 | 318.1003167 | 398.2115642 | 235.9676449 | 293.9690887 | 235.8277641 | 345.6300882 |
| **Mafa** | 6.409875371 | 2.376786026 | 0 | 0.86260942 | 3.166816221 | 4.90988964 | 0.902064715 | 0 | 0 | 0 | 0 | 0 | 11.0770961 | 0 | 2.155697016 |
| **Zc3h3** | 359.8687173 | 327.9964715 | 304.6497436 | 303.6385157 | 353.4166903 | 270.0439302 | 294.073097 | 338.5819013 | 260.3352864 | 263.9183946 | 254.439218 | 271.5362973 | 290.5607515 | 292.7517071 | 270.1806927 |
| **Gsdmd** | 432.2087393 | 513.3857815 | 493.3916155 | 465.8090866 | 536.4586679 | 459.4253877 | 484.4087518 | 461.7787812 | 488.3167656 | 429.9597687 | 444.5592284 | 453.7171996 | 511.2505891 | 496.0515037 | 505.1516674 |
| **Mroh6** | 2.747089445 | 0 | 0 | 0 | 0 | 3.507064029 | 0 | 0 | 3.00965649 | 0 | 1.891741398 | 4.337640531 | 3.408337261 | 0 | 0 |
| **Naprt** | 11.90405426 | 12.67619214 | 7.831612945 | 6.900875357 | 10.13381191 | 12.6254305 | 7.216517718 | 10.89496217 | 4.514484735 | 14.85633347 | 8.512836289 | 11.27786538 | 5.112505891 | 5.421327909 | 9.341353736 |
| **Eef1d** | 4555.589996 | 4493.710112 | 4819.574606 | 4715.023088 | 4640.652491 | 4474.312288 | 4386.740708 | 5245.505248 | 4956.90424 | 5295.845932 | 5362.140991 | 4468.637275 | 4485.371835 | 4408.443145 | 4411.993226 |
| **Tigd5** | 42.12203815 | 30.10595632 | 34.45909696 | 40.54264272 | 39.26852115 | 35.77205309 | 25.25781201 | 36.03718256 | 19.56276719 | 23.59535316 | 37.83482795 | 26.02584319 | 42.60421576 | 32.52796746 | 30.89832389 |
| **Pycrl** | 579.6358728 | 733.6346199 | 705.6283263 | 634.0179234 | 713.1670131 | 711.9339978 | 770.3632664 | 864.8923815 | 670.4009832 | 804.8637134 | 768.9928781 | 726.121025 | 740.4612699 | 739.107705 | 684.7930854 |
| **Tsta3** | 823.2111369 | 738.3881919 | 759.6664557 | 650.4075024 | 685.2990303 | 673.3562935 | 626.0329121 | 642.802768 | 738.8706684 | 742.8166736 | 560.9013244 | 706.1678785 | 618.6132128 | 652.3664584 | 623.7150033 |
| **Zfp623** | 130.0289004 | 120.4238253 | 90.06354887 | 134.5670695 | 109.5718413 | 107.3161593 | 129.8973189 | 159.2340625 | 103.8331489 | 107.4899422 | 157.9604067 | 96.2956198 | 117.5876355 | 99.39101167 | 98.44349706 |
| **Zfp707** | 124.5347215 | 115.6702532 | 115.9078716 | 110.4140057 | 145.0401829 | 103.1076824 | 122.6808012 | 143.3106562 | 112.1097043 | 113.607256 | 140.9347341 | 124.9240473 | 115.0313825 | 172.5789385 | 134.3717807 |
| **Ccdc166** | 94.3167376 | 83.9797729 | 108.0762586 | 114.7270528 | 146.3069094 | 119.9415898 | 111.8560246 | 108.9496217 | 110.604876 | 124.9679816 | 99.31642337 | 105.838429 | 108.214708 | 86.74124655 | 101.3177597 |
| **Fam83h** | 10.98835778 | 19.80655021 | 6.265290356 | 6.038265938 | 7.600358931 | 4.90988964 | 7.216517718 | 10.89496217 | 8.276555348 | 7.86511772 | 7.56696559 | 10.41033728 | 11.0770961 | 4.517773258 | 11.49705075 |
| **Scrib** | 1314.024451 | 1350.014462 | 1360.351169 | 1356.022008 | 1476.369722 | 1440.701903 | 1462.246903 | 1347.623013 | 1315.219886 | 1337.070012 | 1383.808832 | 1476.532837 | 1601.918513 | 1464.66209 | 1611.742802 |
| **Puf60** | 3269.036439 | 3242.728401 | 3239.155114 | 3226.159229 | 3174.41658 | 3225.096081 | 3300.654791 | 3312.0685 | 3065.335135 | 3265.771658 | 3165.829229 | 3280.991298 | 3401.520586 | 3283.517604 | 3133.664895 |
| **Nrbp2** | 7.325571853 | 0 | 4.698967767 | 16.38957897 | 1.900089733 | 8.416953669 | 2.706194144 | 1.676148026 | 5.266898858 | 6.117313782 | 3.783482795 | 1.735056213 | 4.260421576 | 3.614218606 | 2.874262688 |
| **Eppk1** | 0.915696482 | 3.961310043 | 0 | 0 | 3.800179466 | 4.90988964 | 1.80412943 | 0.838074013 | 0 | 0 | 0.945870699 | 0 | 1.70416863 | 0 | 1.437131344 |
| **Plec** | 4859.601228 | 4958.767911 | 4667.641315 | 5019.524213 | 5552.695563 | 5471.019885 | 6327.983974 | 4515.542782 | 3932.868619 | 3671.262171 | 4373.706111 | 5521.816396 | 5962.886038 | 5260.495181 | 5772.956609 |
| **Parp10** | 1219.707713 | 1300.894218 | 1086.244715 | 1107.590495 | 1208.45707 | 1255.528922 | 1254.772018 | 885.0061578 | 1039.083903 | 930.7055969 | 1057.483441 | 1213.671821 | 1244.895184 | 1271.301395 | 1286.951118 |
| **Grina** | 749.0397219 | 776.4167683 | 758.1001331 | 728.0423502 | 703.6665644 | 752.6159405 | 839.8222495 | 564.0238108 | 514.6512598 | 525.2150833 | 515.4995308 | 604.6670901 | 697.0049698 | 736.397041 | 769.5838347 |
| **Spatc1** | 0 | 0 | 0 | 0 | 0 | 0.701412806 | 0 | 0 | 0 | 0 | 3.783482795 | 1.735056213 | 0 | 0 | 0 |
| **Smpd5** | 39.37494871 | 50.70476854 | 39.15806472 | 31.91654853 | 41.16861088 | 53.30737323 | 52.31975346 | 39.38947861 | 8.276555348 | 34.08217679 | 22.70089677 | 27.7608994 | 49.42089028 | 36.14218606 | 58.9223851 |
| **Oplah** | 21.97671556 | 13.46845414 | 18.01270977 | 10.35131304 | 9.500448664 | 8.416953669 | 24.3557473 | 13.40918421 | 3.00965649 | 17.47803938 | 17.97154328 | 4.337640531 | 19.59793925 | 10.84265582 | 12.9341821 |
| **Exosc4** | 734.3885782 | 756.6102181 | 732.2558103 | 800.5015414 | 796.1375981 | 872.5575303 | 835.3119259 | 949.5378568 | 753.1665367 | 815.350537 | 863.579948 | 816.343948 | 808.6280151 | 804.1636399 | 736.5298138 |
| **Gpaa1** | 789.3303671 | 869.1114233 | 861.4774239 | 803.0893697 | 886.708542 | 850.1123205 | 700.9042834 | 917.6910443 | 968.3569758 | 1042.565049 | 968.5715955 | 801.5959702 | 1000.346986 | 806.8743039 | 883.8357765 |
| **Cyc1** | 3453.091432 | 3544.580226 | 3704.352923 | 3625.547391 | 3285.255148 | 3410.269061 | 3264.572203 | 4051.249779 | 4012.624516 | 4113.456568 | 3773.078217 | 3381.624558 | 3433.89979 | 3560.908882 | 3351.390294 |
| **Sharpin** | 619.0108215 | 663.9155631 | 640.6259389 | 636.6057517 | 606.761988 | 603.9164257 | 615.2081355 | 603.4132894 | 568.0726625 | 611.7313782 | 629.9498854 | 592.5216966 | 612.6486226 | 684.8944259 | 592.0981137 |
| **Maf1** | 1197.730998 | 1251.773973 | 1181.790393 | 1067.047852 | 1157.154647 | 1270.960004 | 1293.560801 | 1025.802592 | 1051.874943 | 933.3273028 | 858.8505945 | 1321.245306 | 1223.593077 | 1296.600925 | 1241.681481 |
| **Hgh1** | 814.0541721 | 742.349502 | 710.3272941 | 715.9658183 | 677.6986714 | 901.3154553 | 709.9249305 | 750.9143157 | 744.1375672 | 766.4120267 | 781.2891972 | 732.1937217 | 775.3967268 | 627.0669282 | 723.5956317 |
| **Mroh1** | 456.9325443 | 408.0149344 | 404.8943892 | 397.6629425 | 362.2837757 | 333.1710827 | 410.4394452 | 388.8663421 | 325.042901 | 330.3349442 | 348.0804171 | 422.4861878 | 431.1546635 | 468.9448642 | 385.1512002 |
| **Bop1** | 3488.803595 | 3672.134409 | 3535.190083 | 3567.75256 | 3605.103586 | 3432.714271 | 3371.015839 | 4118.2957 | 4257.91152 | 4103.843646 | 4404.919844 | 3504.813549 | 3323.980913 | 3380.197952 | 3430.432518 |
| **Scx** | 1.831392963 | 0 | 3.132645178 | 0 | 1.266726489 | 0 | 0 | 0 | 0 | 2.621705907 | 4.729353494 | 0 | 0 | 0.903554652 | 0 |
| **Hsf1** | 825.9582264 | 917.4394058 | 817.6203914 | 847.9450595 | 841.7397516 | 826.2642851 | 835.3119259 | 971.3277811 | 874.3052104 | 935.0751067 | 830.4744735 | 926.5200175 | 881.9072662 | 902.6510969 | 922.6383228 |
| **Dgat1** | 305.8426248 | 264.6155108 | 239.6473561 | 208.7514796 | 168.474623 | 166.234835 | 165.0778428 | 156.7198404 | 207.6662978 | 176.5281977 | 180.6613035 | 186.5185429 | 198.5356454 | 171.6753838 | 183.2342464 |
| **Scrt1** | 0.915696482 | 0 | 0.783161294 | 3.450437679 | 0 | 0 | 0 | 0 | 0 | 0 | 0 | 4.337640531 | 5.964590206 | 0 | 0 |
| **Fbxl6** | 709.6647732 | 819.1989168 | 711.8936167 | 680.5988321 | 788.5372391 | 785.5823424 | 716.2393835 | 591.6802532 | 756.1761932 | 713.9779086 | 654.5425235 | 851.9126004 | 740.4612699 | 761.6965713 | 797.6078959 |
| **Slc52a2** | 292.1071776 | 310.5667073 | 318.7466469 | 293.2872027 | 336.949246 | 359.8247693 | 329.2536209 | 346.1245674 | 364.1684353 | 339.0739639 | 321.5960376 | 404.2680975 | 341.6858104 | 394.8533827 | 337.0073002 |
| **Adck5** | 258.2264078 | 307.3976593 | 272.5401305 | 332.967236 | 312.8814427 | 341.5880364 | 289.5627734 | 291.6497565 | 265.6021853 | 277.9008261 | 278.0859854 | 329.6606804 | 339.9816417 | 340.6401036 | 366.4684927 |
| **Cpsf1** | 2772.728946 | 2872.742043 | 2809.199563 | 2932.009417 | 2937.538727 | 2780.400362 | 2913.669029 | 3009.523781 | 3187.978637 | 2960.779871 | 3350.274015 | 2957.403314 | 2892.82625 | 2889.567776 | 2899.412486 |
| **Slc39a4** | 508.2115473 | 513.3857815 | 500.4400672 | 446.8316794 | 492.756604 | 518.3440634 | 583.6358705 | 429.9319687 | 392.760172 | 409.8600234 | 495.6362462 | 486.6832676 | 587.0860931 | 616.2242724 | 512.3373241 |
| **Vps28** | 1145.536298 | 1208.199563 | 1211.550523 | 1283.562816 | 1278.127027 | 1283.585434 | 1234.02453 | 1088.658143 | 1141.412224 | 1193.75009 | 1133.153097 | 1243.167776 | 1260.232702 | 1249.616083 | 1263.957017 |
| **Tonsl** | 599.7811954 | 661.5387771 | 593.6362612 | 633.155314 | 711.2669233 | 542.8935116 | 672.9402772 | 739.1812795 | 796.8065558 | 694.7520653 | 626.1664026 | 731.3261936 | 668.0341031 | 640.620248 | 682.6373884 |
| **Cyhr1** | 889.1412836 | 962.5983403 | 1036.905554 | 1035.993913 | 1001.980652 | 1128.573204 | 1050.003328 | 996.4700015 | 1042.09356 | 1035.573833 | 1103.831105 | 995.0547379 | 1125.60338 | 1096.011792 | 1011.740466 |
| **Kifc2** | 3.662785926 | 7.922620085 | 11.74741942 | 4.313047098 | 10.13381191 | 1.402825611 | 8.118582433 | 8.380740131 | 8.276555348 | 6.117313782 | 11.35044839 | 13.01292159 | 6.816674521 | 8.131991864 | 4.311394032 |
| **Foxh1** | 0 | 2.376786026 | 3.132645178 | 0.86260942 | 0 | 0.701412806 | 1.80412943 | 0 | 0 | 5.243411813 | 0 | 1.735056213 | 0.852084315 | 0 | 1.437131344 |
| **Ppp1r16a** | 422.136078 | 422.2756505 | 474.5957445 | 408.0142555 | 412.319472 | 366.8388974 | 423.9704159 | 468.4833733 | 472.516069 | 439.5726904 | 479.5564443 | 434.6315812 | 421.781736 | 438.224006 | 457.726333 |
| **Gpt** | 51.27900297 | 61.00417465 | 57.1707745 | 65.55831589 | 73.47013634 | 44.18900676 | 50.51562403 | 51.1225148 | 81.26072524 | 62.04703979 | 49.18527634 | 51.18415827 | 49.42089028 | 56.0203884 | 49.58103137 |
| **Mfsd3** | 79.6655939 | 88.73334495 | 87.71406498 | 81.94789487 | 84.23731149 | 61.0229141 | 70.36104775 | 93.86428946 | 73.73658401 | 61.17313782 | 86.07423359 | 75.47494525 | 90.32093741 | 53.30972444 | 51.73672838 |
| **Recql4** | 379.0983434 | 456.3429169 | 397.8459376 | 399.3881613 | 364.8172287 | 379.4643279 | 337.3722033 | 471.8356693 | 410.0656968 | 491.1329065 | 540.092169 | 346.1437144 | 473.7588792 | 430.0920141 | 420.3609181 |
| **Lrrc14** | 487.1505282 | 469.811371 | 463.6314863 | 467.5343055 | 464.8886213 | 514.1355866 | 453.7385515 | 583.2995131 | 477.0305537 | 496.3763183 | 487.1234099 | 502.2987735 | 464.3859518 | 395.7569374 | 416.7680897 |
| **Lrrc24** | 21.06101908 | 11.09166812 | 18.79587107 | 12.93914129 | 27.2346195 | 12.6254305 | 19.84542373 | 28.49451644 | 8.276555348 | 19.22584332 | 15.13393118 | 15.61550591 | 17.89377062 | 10.84265582 | 20.11983882 |
| **C030006K11Rik** | 214.2729767 | 205.9881222 | 180.1270977 | 196.6749477 | 169.7413495 | 180.2630911 | 176.8046841 | 220.4134654 | 186.5987024 | 172.1586879 | 231.7383212 | 171.770565 | 155.0793454 | 174.3860478 | 165.2701046 |
| **Arhgap39** | 278.3717304 | 301.0595632 | 277.2390982 | 300.188078 | 270.4461053 | 335.9767339 | 298.5834206 | 194.433171 | 204.6566413 | 219.3493942 | 227.0089677 | 267.1986567 | 343.389979 | 290.0410432 | 309.7018046 |
| **Zfp251** | 449.6069724 | 404.8458863 | 427.6060668 | 445.96907 | 381.9180363 | 459.4253877 | 464.5633281 | 545.5861825 | 451.4484735 | 526.0889853 | 468.2059959 | 460.6574244 | 459.2734459 | 480.6910746 | 447.6664136 |
| **Zfp7** | 249.069443 | 295.5137292 | 263.9253562 | 253.6071694 | 292.6138189 | 230.7648131 | 264.3049614 | 272.3740542 | 270.11667 | 249.0620611 | 284.7070803 | 271.5362973 | 321.2357868 | 252.9953024 | 270.1806927 |
| **Commd5** | 264.6362832 | 233.7172925 | 271.7569692 | 251.8819505 | 285.6468232 | 298.8018552 | 254.3822496 | 266.5075362 | 301.7180632 | 245.5664533 | 266.7355371 | 294.9595561 | 266.7023906 | 275.5841687 | 237.8452374 |
| **Rpl8** | 37536.23017 | 36412.36191 | 36175.00335 | 36431.44623 | 38629.45763 | 40009.28785 | 38328.72973 | 35619.8217 | 34552.36134 | 35115.12891 | 34629.27215 | 37065.13834 | 37299.9909 | 37820.08705 | 36805.65228 |
| **Zfp647** | 25.63950148 | 17.42976419 | 23.49483883 | 20.70262607 | 29.13470924 | 21.74379698 | 21.64955315 | 26.81836842 | 17.30552482 | 29.71266694 | 20.80915537 | 36.43618046 | 37.49170987 | 26.2030849 | 24.43123285 |
| **1110038F14Rik** | 427.6302569 | 361.2714759 | 401.7617441 | 383.8611917 | 456.0215359 | 436.980178 | 458.2488751 | 443.3411529 | 410.0656968 | 435.2031805 | 476.7188322 | 452.8496715 | 443.9359282 | 500.569277 | 477.1276062 |
| **Rbfox2** | 6.409875371 | 6.338096068 | 3.915806472 | 11.21392246 | 0 | 2.805651223 | 4.510323574 | 4.190370065 | 6.019312981 | 8.739019689 | 0 | 1.735056213 | 0.852084315 | 8.131991864 | 0.718565672 |
| **Apol9a** | 2.747089445 | 2.376786026 | 2.349483883 | 0 | 2.533452977 | 0.701412806 | 3.608258859 | 0 | 0 | 0 | 0 | 0 | 0 | 1.807109303 | 0 |
| **Apol7b** | 0 | 0 | 0 | 0 | 0 | 1.402825611 | 0.902064715 | 0 | 0 | 0 | 0 | 0 | 0 | 0 | 0 |
| **Apol10b** | 6.409875371 | 7.922620085 | 8.614774239 | 11.21392246 | 5.700269198 | 4.208476834 | 5.412388289 | 2.514222039 | 9.781383594 | 2.621705907 | 10.40457769 | 5.205168638 | 5.112505891 | 3.614218606 | 5.029959704 |
| **Gm8221** | 3.662785926 | 6.338096068 | 0.783161294 | 1.725218839 | 3.800179466 | 4.208476834 | 3.608258859 | 0 | 0 | 0 | 0 | 0.867528106 | 0 | 0 | 0 |
| **Apol11b** | 0.915696482 | 0 | 3.132645178 | 6.038265938 | 0 | 0 | 1.80412943 | 0 | 5.266898858 | 5.243411813 | 0 | 0 | 1.70416863 | 2.710663955 | 0 |
| **Apol9b** | 7.325571853 | 8.714882094 | 7.831612945 | 4.313047098 | 14.56735462 | 7.715540863 | 6.314453003 | 4.190370065 | 0.752414123 | 0.873901969 | 5.675224193 | 2.602584319 | 1.70416863 | 4.517773258 | 4.311394032 |
| **Apol8** | 0 | 1.584524017 | 0 | 2.587828259 | 0.633363244 | 2.104238417 | 0.902064715 | 2.514222039 | 3.00965649 | 4.369509845 | 4.729353494 | 3.470112425 | 4.260421576 | 3.614218606 | 2.155697016 |
| **Myh9** | 17714.14844 | 19275.73467 | 19907.96011 | 21651.49643 | 21477.34761 | 19773.52841 | 22843.88684 | 21829.31382 | 20222.63437 | 19043.1978 | 20470.53366 | 22426.46908 | 22376.5862 | 22492.18594 | 22493.26123 |
| **Txn2** | 1900.070199 | 2084.441344 | 2080.859559 | 2200.51663 | 2015.995207 | 1995.519432 | 2011.604314 | 2688.541434 | 2443.84107 | 2367.400434 | 2444.129886 | 2117.636107 | 1989.616876 | 2225.455107 | 1964.558547 |
| **Foxred2** | 441.3657041 | 527.6464977 | 464.4146476 | 437.3429758 | 483.2561554 | 434.1745267 | 433.8931278 | 435.7984868 | 480.0402102 | 464.9158475 | 484.2857978 | 424.221244 | 470.350542 | 450.8737711 | 469.9419495 |
| **Eif3d** | 6146.154784 | 6050.504959 | 5832.20216 | 5558.6551 | 5764.238886 | 5729.139797 | 5800.276116 | 5848.080463 | 5401.580986 | 5430.426835 | 5651.577425 | 5376.071675 | 5380.91245 | 5334.586663 | 5472.596158 |
| **Ift27** | 374.519861 | 368.401834 | 408.8101957 | 398.5255519 | 428.7869164 | 338.0809724 | 335.5680739 | 481.8925575 | 422.1043228 | 463.1680435 | 500.3655996 | 400.7979851 | 427.7463262 | 393.9498281 | 388.7440285 |
| **Ncf4** | 759.1123832 | 906.3477377 | 805.0898107 | 835.0059182 | 792.9707818 | 745.6018125 | 701.8063481 | 1032.507184 | 923.2121284 | 866.9107532 | 922.2239313 | 714.8431596 | 747.2779444 | 701.1584096 | 720.0028033 |
| **Tst** | 18.31392963 | 15.05297816 | 14.8800646 | 18.97740723 | 13.93399137 | 14.72966892 | 12.62890601 | 17.59955427 | 12.79104008 | 13.10852953 | 15.13393118 | 19.08561834 | 16.18960199 | 12.64976512 | 9.341353736 |
| **Mpst** | 352.5431454 | 305.0208733 | 367.3026471 | 320.8907041 | 388.2516687 | 402.6109505 | 368.0424036 | 310.0873848 | 310.7470326 | 295.3788655 | 292.2740459 | 407.73821 | 414.9650615 | 401.1782653 | 369.3427554 |
| **Kctd17** | 548.5021925 | 525.2697116 | 508.2716801 | 514.1152141 | 422.4532839 | 406.8194273 | 417.6559629 | 476.8641134 | 579.3588744 | 495.5024164 | 558.0637123 | 414.6784348 | 425.1900733 | 461.716427 | 464.1934241 |
| **Tmprss6** | 16.48253667 | 11.09166812 | 9.397935534 | 18.97740723 | 6.966995687 | 12.6254305 | 10.82477658 | 0 | 3.762070613 | 9.612921658 | 12.29631908 | 5.205168638 | 2.556252945 | 18.07109303 | 10.77848508 |
| **Il2rb** | 17686.67754 | 17128.70462 | 17217.0179 | 16932.1603 | 14219.00483 | 14056.31263 | 15737.42101 | 13303.58688 | 14199.55932 | 15127.24308 | 16719.21047 | 15286.71276 | 15843.65576 | 15420.96724 | 15140.89727 |
| **C1qtnf6** | 124.5347215 | 102.9940611 | 115.1247103 | 123.353147 | 88.6708542 | 64.52997813 | 72.16517718 | 100.5688816 | 130.9200573 | 117.9767658 | 133.3677685 | 109.3085414 | 80.09592563 | 88.54835585 | 81.19792093 |
| **Sstr3** | 0 | 0 | 3.915806472 | 0 | 1.900089733 | 0 | 0 | 4.190370065 | 0 | 0.873901969 | 0.945870699 | 0.867528106 | 0 | 0 | 0.718565672 |
| **Rac2** | 8993.970842 | 9012.772609 | 9480.16747 | 9800.105617 | 9096.996278 | 8750.124751 | 9072.064837 | 9996.546828 | 9730.971847 | 9333.273028 | 9836.109397 | 9468.201752 | 9180.356412 | 9538.826457 | 9117.161246 |
| **Cyth4** | 1067.702098 | 1124.21979 | 1088.594199 | 1134.331387 | 1085.584601 | 934.2818572 | 1030.157904 | 1205.988505 | 1157.965335 | 1099.368677 | 1162.475089 | 1204.129012 | 1049.767876 | 1161.971282 | 1158.327863 |
| **Elfn2** | 5.494178889 | 4.753572051 | 3.915806472 | 4.313047098 | 3.800179466 | 7.715540863 | 2.706194144 | 5.028444078 | 0 | 2.621705907 | 3.783482795 | 0.867528106 | 0.852084315 | 0.903554652 | 9.341353736 |
| **Mfng** | 869.9116575 | 1018.056681 | 1159.861877 | 1009.253021 | 981.7130286 | 1041.598016 | 1044.59094 | 1113.800363 | 1191.071556 | 1203.363011 | 1248.549322 | 1103.495751 | 996.0865644 | 1032.762967 | 998.0877184 |
| **Card10** | 0 | 0 | 0 | 2.587828259 | 5.066905954 | 0 | 2.706194144 | 5.028444078 | 1.504828245 | 0 | 8.512836289 | 8.675281063 | 2.556252945 | 7.228437213 | 0.718565672 |
| **Gga1** | 1338.748256 | 1421.318043 | 1278.902394 | 1480.237764 | 1352.230527 | 1294.808039 | 1208.766718 | 1415.507008 | 1334.782653 | 1436.694837 | 1508.663765 | 1354.211374 | 1315.618183 | 1401.413265 | 1409.825848 |
| **Mir6955** | 0 | 0 | 2.349483883 | 0 | 0.633363244 | 0 | 0 | 0 | 0 | 0 | 0 | 0 | 0 | 2.710663955 | 0 |
| **Sh3bp1** | 2544.720522 | 2866.403947 | 2832.694402 | 3039.835595 | 2584.7554 | 2522.280449 | 2654.776456 | 3469.626414 | 3236.133141 | 3341.801129 | 3415.539093 | 2698.879939 | 3012.118054 | 3074.796479 | 2693.902704 |
| **Pdxp** | 630.9148758 | 545.0762619 | 570.1414224 | 597.7883278 | 613.7289837 | 630.5701123 | 653.0948535 | 400.5993782 | 437.9050193 | 444.8161022 | 477.6647029 | 573.4360783 | 670.590356 | 586.4069689 | 571.2597092 |
| **Lgals1** | 13001.05865 | 13194.33149 | 13138.31388 | 13481.72262 | 11834.39222 | 11804.77752 | 11730.44955 | 15579.7959 | 14410.23528 | 16216.99884 | 15643.75549 | 11920.70371 | 11354.0235 | 12703.07485 | 11241.95994 |
| **Nol12** | 875.4058364 | 770.0786723 | 798.0413591 | 767.7223835 | 844.9065679 | 819.9515699 | 755.930231 | 824.6648288 | 801.3210405 | 828.4590665 | 887.2267154 | 805.0660826 | 729.3841738 | 799.6458666 | 806.2306839 |
| **Triobp** | 1057.629436 | 884.9566635 | 920.9976823 | 954.9086276 | 913.3097982 | 958.8313054 | 1030.157904 | 878.3015657 | 1003.72044 | 873.9019689 | 899.5230345 | 1033.225975 | 1013.128251 | 1051.737614 | 1011.0219 |
| **H1f0** | 983.4580212 | 975.2745325 | 944.4925212 | 860.8842008 | 853.7736533 | 1043.702255 | 1050.003328 | 502.8444078 | 527.4422999 | 751.5556933 | 693.3232222 | 904.8318148 | 927.0677349 | 744.5290329 | 944.9138586 |
| **Gcat** | 1223.370499 | 1071.930498 | 1063.533038 | 1049.795664 | 988.0466611 | 1155.928304 | 1152.838705 | 765.1615739 | 804.330697 | 958.6704599 | 1048.024734 | 1058.38429 | 983.3052997 | 974.935469 | 1023.956083 |
| **Galr3** | 21.06101908 | 10.29940611 | 17.22954848 | 24.15306375 | 19.00089733 | 23.84803539 | 16.23716487 | 25.14222039 | 21.82000955 | 27.09096104 | 28.37612096 | 20.82067455 | 12.78126473 | 28.0101942 | 17.9641418 |
| **Ankrd54** | 533.8510488 | 536.3613798 | 504.3558736 | 549.4822003 | 487.0563348 | 547.1019885 | 483.5066871 | 472.6737434 | 617.7319946 | 632.7050255 | 578.8728676 | 511.8415827 | 565.7839853 | 530.3865805 | 553.2955674 |
| **Eif3l** | 5517.071301 | 5723.300749 | 5649.725578 | 5371.468856 | 5620.46543 | 5511.701827 | 5718.188227 | 6355.953315 | 5958.367437 | 5914.568526 | 6055.464214 | 5624.184713 | 5428.629172 | 5411.388808 | 5488.404602 |
| **Micall1** | 453.2697584 | 459.5119649 | 403.3280667 | 470.9847431 | 440.820818 | 490.988964 | 529.5119876 | 391.3805641 | 461.9822713 | 441.3204943 | 445.5050991 | 431.1614688 | 502.7297459 | 383.1071723 | 496.5288793 |
| **Polr2f** | 1302.120397 | 1271.580524 | 1328.241555 | 1207.653188 | 1352.86389 | 1311.641947 | 1095.106564 | 1314.938126 | 1344.564037 | 1432.325327 | 1308.139176 | 1249.240473 | 1226.14933 | 1232.448545 | 1159.764995 |
| **Gm10863** | 10.0726613 | 4.753572051 | 7.831612945 | 10.35131304 | 5.066905954 | 9.81977928 | 15.33510015 | 5.028444078 | 2.257242368 | 8.739019689 | 10.40457769 | 4.337640531 | 7.668758836 | 5.421327909 | 9.341353736 |
| **Pick1** | 177.6451174 | 212.3262183 | 185.6092268 | 169.0714463 | 200.1427852 | 223.0492722 | 175.9026194 | 155.8817664 | 218.9525097 | 194.0062371 | 160.7980188 | 174.3731494 | 163.6001885 | 238.538428 | 185.3899434 |
| **Slc16a8** | 0 | 4.753572051 | 3.915806472 | 2.587828259 | 3.166816221 | 0.701412806 | 1.80412943 | 2.514222039 | 2.257242368 | 4.369509845 | 0 | 0 | 0 | 4.517773258 | 0.718565672 |
| **Baiap2l2** | 6.409875371 | 11.09166812 | 4.698967767 | 1.725218839 | 8.86708542 | 4.90988964 | 9.020647148 | 4.190370065 | 7.524141226 | 1.747803938 | 7.56696559 | 3.470112425 | 6.816674521 | 9.939101167 | 5.748525376 |
| **Pla2g6** | 178.5608139 | 169.5440698 | 149.5838072 | 140.6053354 | 117.8055634 | 149.4009276 | 129.8973189 | 183.5382089 | 152.7400669 | 158.1762564 | 169.3108551 | 124.9240473 | 170.416863 | 129.2083152 | 149.4616598 |
| **Maff** | 143.7643476 | 159.2446637 | 176.2112913 | 124.2157564 | 119.7056532 | 138.8797355 | 124.4849306 | 45.2559967 | 86.5276241 | 94.38141264 | 89.85771638 | 82.4151701 | 115.0313825 | 121.0763233 | 139.4017404 |
| **Tmem184b** | 1510.899195 | 1120.25848 | 1071.364651 | 1041.16957 | 1003.880742 | 1144.004286 | 1279.127766 | 814.6079407 | 753.1665367 | 766.4120267 | 880.6056206 | 955.148445 | 985.0094683 | 1021.016756 | 1026.11178 |
| **Csnk1e** | 329.6507334 | 316.9048034 | 303.8665823 | 244.9810752 | 266.0125626 | 320.5456522 | 294.073097 | 264.8313881 | 268.6118418 | 267.4140025 | 232.6841919 | 272.4038254 | 293.1170044 | 224.9851082 | 247.9051568 |
| **Kdelr3** | 9.156964816 | 3.169048034 | 2.349483883 | 4.313047098 | 2.533452977 | 7.014128057 | 5.412388289 | 0 | 3.00965649 | 4.369509845 | 0 | 0 | 8.520843152 | 0.903554652 | 2.155697016 |
| **Ddx17** | 3445.76586 | 3668.173099 | 3978.459376 | 3630.723047 | 3497.431835 | 3476.201865 | 3696.661201 | 4290.100873 | 3987.79485 | 4381.744472 | 4499.506914 | 3634.075237 | 3922.996187 | 3781.376217 | 3847.200608 |
| **Dmc1** | 4.578482408 | 0.792262009 | 3.915806472 | 0.86260942 | 2.533452977 | 1.402825611 | 1.80412943 | 3.352296052 | 5.266898858 | 6.991215751 | 0 | 0 | 0.852084315 | 0 | 8.622788064 |
| **Fam227a** | 0 | 0 | 0 | 4.313047098 | 0 | 0 | 1.80412943 | 1.676148026 | 3.00965649 | 0.873901969 | 0.945870699 | 0.867528106 | 2.556252945 | 0 | 2.874262688 |
| **Cby1** | 247.23805 | 243.2244366 | 224.7672915 | 210.4766984 | 204.5763279 | 238.4803539 | 204.7686903 | 278.2405723 | 253.5635593 | 243.8186493 | 255.3850887 | 242.0403417 | 253.9211259 | 234.9242094 | 217.7253986 |
| **Tomm22** | 2071.305441 | 2016.306812 | 2108.270205 | 1991.76515 | 2052.730275 | 1873.473604 | 1888.021448 | 2137.088733 | 1993.897425 | 2349.048492 | 2100.778822 | 1933.720149 | 1803.862495 | 1982.398906 | 1936.534486 |
| **Josd1** | 1370.797633 | 1447.46269 | 1467.644266 | 1510.429094 | 1273.693484 | 1373.366274 | 1395.494114 | 1544.570406 | 1478.493751 | 1583.510368 | 1617.438895 | 1487.810702 | 1495.407973 | 1288.468933 | 1337.250716 |
| **Gtpbp1** | 2840.490486 | 3002.673012 | 3001.07408 | 3014.819922 | 2914.73765 | 2746.732547 | 3019.2106 | 3185.519324 | 3232.371071 | 2942.427929 | 2944.495485 | 2868.047919 | 2938.838803 | 3104.613783 | 3094.143783 |
| **Sun2** | 2961.362421 | 3446.339737 | 3365.244082 | 3435.773318 | 3678.573723 | 3634.721159 | 3951.945515 | 2771.510761 | 2993.10338 | 2834.064085 | 2716.540647 | 3867.440298 | 3692.081338 | 3874.442346 | 3776.062606 |
| **Gm16576** | 38.45925223 | 59.41965064 | 39.15806472 | 34.50437679 | 27.2346195 | 31.56357626 | 35.18052388 | 48.60829276 | 36.86829201 | 31.46047088 | 51.07701773 | 38.17123668 | 57.94173343 | 42.46706862 | 32.33545524 |
| **Dnal4** | 193.2119576 | 186.973834 | 173.8618074 | 163.0331803 | 134.906371 | 159.9221197 | 162.3716487 | 150.8533223 | 206.9138837 | 211.4842765 | 155.1227946 | 218.6170828 | 161.0439356 | 174.3860478 | 141.5574374 |
| **Nptxr** | 0 | 0 | 0.783161294 | 6.900875357 | 6.333632443 | 7.014128057 | 1.80412943 | 1.676148026 | 7.524141226 | 8.739019689 | 6.621094891 | 1.735056213 | 0 | 0 | 4.311394032 |
| **Cbx6** | 1090.59451 | 1193.938847 | 1288.300329 | 1162.797498 | 1201.490074 | 1187.49188 | 1252.065824 | 1105.419623 | 1089.495649 | 1171.028638 | 1083.967821 | 1113.03856 | 1158.834669 | 1247.808974 | 1125.992408 |
| **D730005E14Rik** | 68.67723612 | 68.13453273 | 46.20651637 | 62.10787821 | 77.2703158 | 57.51585007 | 69.45898304 | 81.29317927 | 66.21244279 | 63.79484373 | 46.34766424 | 59.85943933 | 47.71672165 | 65.95948956 | 43.83250599 |
| **Apobec3** | 4478.671491 | 4443.797606 | 4693.485638 | 4401.895868 | 3950.286555 | 4050.658953 | 4406.586132 | 4229.759544 | 4062.283848 | 4059.274646 | 4427.620741 | 4022.727829 | 3938.333705 | 4478.920408 | 4174.147988 |
| **Cbx7** | 479.8249563 | 509.4244715 | 494.9579381 | 441.6560229 | 492.756604 | 414.5349682 | 480.800493 | 441.6650049 | 522.1754011 | 476.2765731 | 552.3884881 | 444.1743904 | 447.3442655 | 436.4168967 | 397.3668166 |
| **Pdgfb** | 306.7583213 | 250.3547947 | 269.4074853 | 244.9810752 | 300.847541 | 295.996204 | 351.8052388 | 352.8291595 | 258.078044 | 281.396434 | 263.897925 | 245.5104541 | 293.1170044 | 262.9344036 | 293.8933598 |
| **Rpl3** | 35089.48917 | 33454.84783 | 33838.05005 | 32139.10176 | 34324.48766 | 35925.6625 | 35713.64412 | 31523.31593 | 30836.9404 | 31034.88062 | 31517.35755 | 34040.06783 | 33106.88398 | 33501.09582 | 33324.92017 |
| **Snord83b** | 1.831392963 | 5.54583406 | 5.482129061 | 4.313047098 | 3.800179466 | 2.104238417 | 0.902064715 | 1.676148026 | 0 | 6.991215751 | 4.729353494 | 8.675281063 | 2.556252945 | 5.421327909 | 2.874262688 |
| **Snord43** | 9.156964816 | 7.130358077 | 12.53058071 | 1.725218839 | 6.333632443 | 10.52119209 | 9.020647148 | 13.40918421 | 12.79104008 | 18.35194135 | 10.40457769 | 4.337640531 | 9.372927467 | 26.2030849 | 7.18565672 |
| **Syngr1** | 69.5929326 | 57.83512662 | 61.86974226 | 38.81742388 | 57.63605523 | 70.84269338 | 68.55691832 | 38.5514046 | 51.91657446 | 45.44290238 | 41.61831075 | 49.44910206 | 63.05423932 | 54.21327909 | 53.17385973 |
| **Tab1** | 380.9297363 | 395.3387422 | 437.0040023 | 390.7620671 | 438.9207283 | 391.3883456 | 420.3621571 | 358.6956776 | 325.7953151 | 306.7395911 | 319.7042962 | 457.187312 | 410.7046399 | 425.5742409 | 430.4208375 |
| **Mgat3** | 8.241268334 | 10.29940611 | 12.53058071 | 2.587828259 | 9.500448664 | 17.53532014 | 16.23716487 | 7.542666117 | 2.257242368 | 12.23462756 | 6.621094891 | 8.675281063 | 9.372927467 | 9.939101167 | 8.622788064 |
| **Mief1** | 799.4030284 | 766.9096242 | 716.5925845 | 667.6596908 | 720.767372 | 707.0241082 | 737.8889367 | 739.1812795 | 705.764447 | 695.6259672 | 684.8103859 | 654.1161921 | 634.8028148 | 617.127827 | 652.4576301 |
| **Atf4** | 6966.618832 | 6361.863928 | 6525.299906 | 5733.764812 | 5939.047142 | 6149.98748 | 6050.148042 | 5563.973373 | 5412.867198 | 6005.45433 | 5898.449678 | 5905.263819 | 5853.819245 | 5813.470628 | 6045.292998 |
| **Rps19bp1** | 619.0108215 | 680.5530653 | 651.590197 | 587.4370148 | 601.6950821 | 679.6690087 | 594.460647 | 670.4592104 | 720.0603153 | 700.8693791 | 704.6736706 | 579.508775 | 579.4173343 | 570.1429851 | 678.3259943 |
| **Cacna1i** | 0 | 0 | 0 | 7.763484777 | 3.800179466 | 0.701412806 | 0 | 0 | 0 | 0 | 3.783482795 | 0.867528106 | 0 | 1.807109303 | 2.874262688 |
| **Enthd1** | 127.2818109 | 106.9553711 | 111.2089038 | 97.47486442 | 94.3711234 | 98.1977928 | 104.6395069 | 126.549176 | 120.3862596 | 117.1028638 | 106.883389 | 72.00483282 | 92.87719035 | 86.74124655 | 97.00636572 |
| **Grap2** | 6721.212175 | 7341.099771 | 7245.808297 | 7038.030255 | 6627.512988 | 6627.649601 | 7530.436239 | 6412.104274 | 6711.533973 | 6312.193921 | 6821.61948 | 6954.1053 | 7237.604173 | 6967.309918 | 6941.344391 |
| **Fam83f** | 4.578482408 | 2.376786026 | 4.698967767 | 3.450437679 | 0.633363244 | 3.507064029 | 4.510323574 | 3.352296052 | 0 | 3.495607876 | 6.621094891 | 0.867528106 | 2.556252945 | 6.324882561 | 0.718565672 |
| **A430088P11Rik** | 14.65114371 | 20.59881222 | 15.66322589 | 22.42784491 | 18.36753408 | 16.83390734 | 27.06194144 | 5.028444078 | 11.28621184 | 12.23462756 | 8.512836289 | 34.70112425 | 27.26669809 | 43.37062328 | 28.74262688 |
| **Tnrc6b** | 1021.001577 | 1092.52931 | 1161.4282 | 1240.432345 | 914.5765247 | 807.3261394 | 1133.895346 | 1178.332062 | 1317.477129 | 1083.638441 | 1096.26414 | 1048.84148 | 1013.128251 | 1017.402538 | 982.2792736 |
| **Adsl** | 3011.725728 | 3043.870637 | 3037.0995 | 3089.004332 | 2921.704646 | 2989.421378 | 2984.030076 | 3491.416338 | 3439.284954 | 3317.331874 | 3371.08317 | 2836.816908 | 2778.646952 | 2701.628408 | 2796.657595 |
| **Sgsm3** | 226.1770309 | 201.2345502 | 236.5147109 | 202.7132136 | 200.1427852 | 225.8549234 | 235.4388906 | 269.8598322 | 300.965649 | 263.9183946 | 296.0575287 | 186.5185429 | 199.3877297 | 206.0104606 | 229.2224494 |
| **Mkl1** | 1058.545133 | 1293.76386 | 1264.022329 | 1264.585409 | 1166.655096 | 1060.536162 | 1208.766718 | 1118.828807 | 1172.261203 | 1232.201776 | 1079.238467 | 1191.11609 | 1181.840945 | 1201.727687 | 1257.489926 |
| **4930483J18Rik** | 0 | 0 | 2.349483883 | 0 | 0.633363244 | 0 | 0 | 0 | 0 | 0 | 0 | 1.735056213 | 0 | 0 | 0.718565672 |
| **Slc25a17** | 813.1384756 | 839.797729 | 845.814198 | 846.2198407 | 751.1688077 | 770.8526735 | 775.7756547 | 919.3671923 | 858.5045139 | 814.476635 | 982.759656 | 794.6557454 | 737.9050169 | 807.7778585 | 724.3141973 |
| **St13** | 3741.535824 | 3603.207615 | 3747.426794 | 3695.418754 | 3553.1678 | 3544.238907 | 3857.22872 | 4004.317634 | 3407.683561 | 3658.153642 | 3589.579302 | 3582.023551 | 3586.422883 | 3410.015255 | 3574.864218 |
| **Xpnpep3** | 191.3805646 | 198.8577641 | 180.1270977 | 183.7358064 | 127.3060121 | 175.3532014 | 195.7480431 | 194.433171 | 189.6083589 | 212.3581784 | 206.1998123 | 191.7237115 | 191.7189709 | 184.3251489 | 193.2941658 |
| **Rbx1** | 1523.718945 | 1495.790672 | 1565.539428 | 1440.557731 | 1542.872863 | 1573.970336 | 1538.922403 | 1596.530995 | 1507.837902 | 1681.387388 | 1776.345172 | 1500.823624 | 1364.186989 | 1548.692673 | 1468.748233 |
| **Ep300** | 2107.933301 | 1976.693711 | 2010.375043 | 2105.629593 | 1641.044166 | 1699.523228 | 1935.830878 | 2138.764881 | 2086.444362 | 1872.771919 | 2005.245881 | 1972.758914 | 1956.385588 | 1885.718558 | 1863.959353 |
| **L3mbtl2** | 1238.021643 | 1302.478742 | 1297.698265 | 1335.319382 | 1295.861198 | 1202.922962 | 1216.8853 | 1456.572635 | 1455.921327 | 1334.448307 | 1321.381366 | 1258.783282 | 1176.728439 | 1266.783622 | 1174.136308 |

| | | | | | | | | | | | | | | | | |
|---|---|---|---|---|---|---|---|---|---|---|---|---|---|---|---|---|
| **Chadl** | 0.915696482 | 6.338096068 | 1.566322589 | 0.86260942 | 1.900089733 | 1.402825611 | 2.706194144 | 0.838074013 | 0 | 0 | 0 | 0 | 0 | 5.421327909 | 2.155697016 |
| **Rangap1** | 7065.514052 | 7022.610443 | 7238.759845 | 7045.79374 | 6730.751197 | 6634.663729 | 7000.924251 | 7608.035891 | 7497.054317 | 7339.028735 | 7642.635246 | 6994.879121 | 6786.85157 | 6789.309652 | 6891.044794 |
| **Zc3h7b** | 2592.336739 | 2648.531894 | 2711.304402 | 2577.476946 | 2299.74194 | 2332.898992 | 2547.430755 | 2897.221863 | 2935.167492 | 2813.96434 | 2906.660657 | 2447.296788 | 2512.796645 | 2630.247591 | 2647.195936 |
| **Tef** | 228.9241204 | 308.9821833 | 260.7927111 | 326.92897 | 274.2462848 | 310.0244601 | 323.8412326 | 318.468125 | 279.8980536 | 313.7308068 | 355.6473827 | 334.865849 | 310.1586907 | 377.6858444 | 283.1148748 |
| **Tob2** | 1863.44234 | 1776.251423 | 1751.931816 | 1773.524967 | 1365.531155 | 1414.749629 | 1592.144222 | 1811.077942 | 1682.397978 | 1570.401838 | 1621.222378 | 1299.557103 | 1567.83514 | 1349.910649 | 1379.64609 |
| **Phf5a** | 1502.657926 | 1455.38531 | 1696.327364 | 1537.169986 | 1561.240397 | 1557.136429 | 1527.195562 | 1710.509061 | 1625.966919 | 1658.665937 | 1549.336205 | 1532.054636 | 1524.37884 | 1549.596227 | 1457.969748 |
| **Aco2** | 7844.771758 | 7299.109884 | 7308.4612 | 7642.719458 | 5861.776826 | 6139.466288 | 6367.674822 | 9536.444195 | 9230.616456 | 9152.37532 | 9241.156727 | 6143.834049 | 6068.544493 | 6223.68444 | 6057.508615 |
| **Polr3h** | 551.2492819 | 663.9155631 | 681.3503262 | 615.9031256 | 572.5603728 | 606.7220769 | 605.2854236 | 808.7414226 | 770.4720615 | 795.2507917 | 729.2663088 | 660.1888889 | 670.590356 | 601.7673979 | 674.733166 |
| **Pmm1** | 390.0867011 | 416.7298165 | 451.1009056 | 402.838599 | 351.5166006 | 364.734659 | 340.9804622 | 570.7284029 | 464.9919278 | 596.0011428 | 601.5737644 | 325.3230399 | 382.5858575 | 359.6147513 | 302.5161479 |
| **Desi1** | 1379.954598 | 1398.342445 | 1408.907169 | 1417.267276 | 1174.255455 | 1214.145567 | 1303.483513 | 1615.806697 | 1530.410325 | 1521.463328 | 1464.207842 | 1273.53126 | 1312.209845 | 1295.69737 | 1239.525784 |
| **Xrcc6** | 1026.495756 | 989.5352486 | 979.7347794 | 920.4042508 | 911.4097085 | 887.2871992 | 976.0340214 | 1050.944812 | 1113.572901 | 1115.972814 | 1282.600668 | 932.5927143 | 985.8615526 | 1041.798513 | 941.3210303 |
| **Nhp2l1** | 3483.309416 | 3306.109361 | 3427.896986 | 3308.107124 | 3238.386268 | 3277.702041 | 3311.479568 | 3950.680898 | 3562.68087 | 3773.508702 | 3541.339896 | 3376.41939 | 3224.287049 | 3317.852681 | 3056.059803 |
| **Ccdc134** | 272.8775515 | 316.1125414 | 336.7593566 | 282.0732802 | 245.1115755 | 263.7312149 | 289.5627734 | 298.3543486 | 326.5477292 | 309.361297 | 292.2740459 | 256.7883195 | 260.7378004 | 309.9192455 | 293.1747942 |
| **Srebf2** | 11605.53721 | 11588.4164 | 11407.52742 | 11345.03909 | 11314.401 | 11989.9505 | 12564.85941 | 9713.277811 | 9794.174634 | 9834.018856 | 10129.32931 | 11978.82809 | 12344.99756 | 11998.30222 | 12256.57467 |
| **Shisa8** | 8.241268334 | 4.753572051 | 3.132645178 | 1.725218839 | 3.800179466 | 3.507064029 | 8.118582433 | 2.514222039 | 4.514484735 | 1.747803938 | 5.675224193 | 5.205168638 | 1.70416863 | 6.324882561 | 9.341353736 |
| **Tnfrsf13c** | 11.90405426 | 24.56012226 | 11.74741942 | 22.42784491 | 19.00089733 | 18.23673295 | 18.94335901 | 16.76148026 | 4.514484735 | 18.35194135 | 21.75502607 | 21.68820266 | 26.41461377 | 13.55331977 | 31.61688957 |
| **Cenpm** | 484.4034387 | 652.031633 | 549.7792287 | 536.543059 | 639.0635135 | 552.0118781 | 500.6459167 | 601.7371414 | 626.7609641 | 576.7752995 | 551.4426174 | 547.4102351 | 497.6172401 | 599.9602886 | 528.1457689 |
| **Sept3** | 1.831392963 | 5.54583406 | 3.132645178 | 1.725218839 | 3.166816221 | 2.805651223 | 3.608258859 | 2.514222039 | 0 | 3.495607876 | 0.945870699 | 0.867528106 | 0 | 5.421327909 | 0.718565672 |
| **Wbp2nl** | 0 | 0 | 3.915806472 | 0 | 1.266726489 | 0 | 0.902064715 | 0 | 4.514484735 | 0 | 0 | 0 | 0 | 0 | 0 |
| **Naga** | 224.345638 | 261.4464628 | 200.4892914 | 210.4766984 | 188.7422468 | 178.1588527 | 225.5161787 | 132.4156941 | 153.492481 | 199.2496489 | 178.7695621 | 212.544386 | 222.3940063 | 211.4317885 | 155.9287508 |
| **Fam109b** | 32.96507334 | 45.15893448 | 30.54329049 | 24.15306375 | 24.70116653 | 19.63955856 | 20.74748844 | 10.89496217 | 27.08690841 | 15.73023544 | 9.458706988 | 31.23101183 | 27.26669809 | 32.52796746 | 15.80844478 |
| **Smdt1** | 1132.716548 | 1322.285292 | 1234.2622 | 1327.555897 | 1282.56057 | 1247.111969 | 1197.941941 | 1353.489531 | 1349.078522 | 1369.404385 | 1259.899771 | 1397.587779 | 1301.132749 | 1421.291467 | 1296.292472 |
| **Ndufa6** | 709.6647732 | 706.6977116 | 671.1692294 | 672.8353473 | 729.0010942 | 716.1424746 | 674.7444067 | 669.6211364 | 640.3044183 | 657.1742806 | 692.3773515 | 594.2567528 | 645.0278266 | 677.6659887 | 650.3019331 |
| **Cyp2d22** | 2.747089445 | 3.169048034 | 0.783161294 | 1.725218839 | 3.800179466 | 0 | 0 | 0 | 0 | 0.873901969 | 4.729353494 | 4.337640531 | 1.70416863 | 2.710663955 | 2.155697016 |
| **Tcf20** | 2794.705662 | 3009.80337 | 2827.995434 | 2944.948559 | 2844.43433 | 2725.690163 | 3205.035932 | 2759.777725 | 2770.388799 | 2800.85581 | 2703.298457 | 3002.514776 | 3081.136884 | 2738.674149 | 3056.059803 |
| **Tbrg3** | 2.747089445 | 17.42976419 | 3.915806472 | 12.07653188 | 6.333632443 | 3.507064029 | 3.608258859 | 13.40918421 | 8.276555348 | 18.35194135 | 4.729353494 | 4.337640531 | 5.112505891 | 0.903554652 | 4.311394032 |
| **Gm20324** | 119.0405426 | 110.9166812 | 133.1374201 | 115.5896622 | 147.5736359 | 108.7189849 | 96.52092448 | 140.7964342 | 160.2642081 | 141.572119 | 168.3649844 | 109.3085414 | 99.69386487 | 132.8225338 | 106.3477195 |
| **Nfam1** | 14.65114371 | 10.29940611 | 27.41064531 | 28.46611085 | 15.20071786 | 21.74379698 | 18.0412943 | 12.5711102 | 25.58208017 | 15.73023544 | 23.64676747 | 13.01292159 | 21.30210788 | 11.74621047 | 28.02406121 |
| **Serhl** | 142.8486511 | 188.558358 | 182.4765816 | 196.6749477 | 163.407717 | 165.5334221 | 188.5315254 | 182.7001348 | 155.7497234 | 181.7716095 | 172.1484672 | 193.4587677 | 167.0085258 | 188.8429222 | 186.108509 |
| **Rrp7a** | 1771.872692 | 1906.182392 | 1787.174074 | 1748.509294 | 1694.880042 | 1633.590425 | 1708.51057 | 1816.106386 | 1756.134562 | 1798.490252 | 1766.886465 | 1616.204862 | 1578.912236 | 1639.048138 | 1588.748701 |
| **Poldip3** | 2522.743807 | 2573.267004 | 2555.455304 | 2398.054187 | 2430.214768 | 2494.223937 | 2591.631926 | 2508.355521 | 2285.08169 | 2225.828315 | 2386.431773 | 2505.421171 | 2430.996551 | 2536.277907 | 2522.884074 |
| **Cyb5r3** | 1369.881936 | 1254.150759 | 1250.708587 | 1084.300041 | 1110.91913 | 1133.483094 | 1183.508906 | 1278.900944 | 1224.177777 | 1083.638441 | 1265.574995 | 1103.495751 | 1043.803286 | 1160.164173 | 1086.471296 |
| **Arfgap3** | 171.2352421 | 155.2833537 | 183.2597429 | 155.2696955 | 133.6396445 | 137.4769099 | 155.1551309 | 147.5010263 | 134.6821279 | 110.1116481 | 129.5842857 | 143.1421375 | 161.0439356 | 118.3656594 | 135.808912 |
| **1700001L05Rik** | 77.83420093 | 80.01846286 | 90.84671016 | 64.69570647 | 40.53524763 | 39.27911712 | 45.10323574 | 56.15095887 | 48.90691797 | 46.31680435 | 48.23940564 | 46.84651774 | 45.1604687 | 38.85285002 | 52.45529405 |
| **Pacsin2** | 1314.940148 | 1330.207912 | 1363.483814 | 1236.981908 | 1093.818323 | 1249.91762 | 1216.8853 | 1279.739018 | 1358.859905 | 1277.644679 | 1474.612419 | 1345.536093 | 1335.216122 | 1254.133856 | 1333.657887 |
| **Ttll1** | 62.26736075 | 66.55000871 | 51.68864544 | 67.28353473 | 50.0356963 | 51.90454762 | 59.53627118 | 68.72206907 | 60.94554393 | 55.05582404 | 65.26507822 | 74.60741714 | 60.49798638 | 69.57370817 | 70.41943585 |
| **Bik** | 18.31392963 | 27.7291703 | 19.57903236 | 22.42784491 | 20.26762382 | 25.25086101 | 31.57226502 | 14.24725822 | 16.5531107 | 21.84754922 | 20.80915537 | 30.36348372 | 10.22501178 | 36.14218606 | 23.71266717 |
| **Mcat** | 451.4383654 | 483.2798252 | 458.9325186 | 489.0995409 | 416.1196515 | 436.2787652 | 398.7126039 | 366.2383437 | 458.2202007 | 425.5902589 | 416.1831075 | 446.7769747 | 455.8651086 | 519.5439247 | 430.4208375 |
| **Tspo** | 1797.512193 | 1590.862113 | 1455.113685 | 1586.338723 | 1647.377798 | 1671.466716 | 1714.825023 | 1083.629699 | 1027.045277 | 1021.591402 | 994.1101044 | 1501.691152 | 1501.372563 | 1602.905952 | 1610.305671 |
| **Ttll12** | 1641.843791 | 1707.324628 | 1735.485429 | 1808.029344 | 1573.907662 | 1531.885568 | 1524.489368 | 1873.095419 | 1843.4146 | 1788.87733 | 2003.35414 | 1629.217784 | 1710.985305 | 1549.596227 | 1672.820884 |
| **Sult4a1** | 2.747089445 | 4.753572051 | 0.783161294 | 4.313047098 | 0 | 5.611302446 | 0 | 4.190370065 | 0 | 5.243411813 | 0 | 2.602584319 | 0 | 0.903554652 | 1.437131344 |
| **Pnpla3** | 8.241268334 | 3.961310043 | 1.566322589 | 4.313047098 | 3.800179466 | 7.014128057 | 9.020647148 | 2.514222039 | 0.752414123 | 1.747803938 | 2.837612096 | 2.602584319 | 5.112505891 | 9.035546516 | 0 |
| **Samm50** | 3077.655875 | 3075.561117 | 2977.579242 | 2963.925966 | 3030.009761 | 3117.078509 | 2879.39057 | 3234.127616 | 3035.23857 | 3268.393364 | 3132.723754 | 2920.099606 | 2841.701191 | 2796.501647 | 2786.597676 |
| **Parvg** | 3669.195802 | 3590.531423 | 3502.297309 | 3625.547391 | 3592.436322 | 3518.286633 | 3716.506625 | 3458.731452 | 3435.522884 | 3416.082796 | 3479.858301 | 3478.787706 | 3428.787284 | 3440.736113 | 3617.978158 |
| **Ldoc1l** | 154.7527054 | 190.142882 | 187.1755494 | 178.5601499 | 133.0062813 | 159.2207069 | 197.5521725 | 153.3675444 | 178.3221471 | 122.3462756 | 131.4760271 | 182.1809023 | 187.4585493 | 161.7362826 | 187.5456404 |
| **Prr5** | 109.8835778 | 106.1631091 | 136.2700652 | 144.0557731 | 47.50224332 | 64.52997813 | 54.12388289 | 214.5469473 | 140.7014409 | 295.3788655 | 401.995047 | 62.46202365 | 58.79381775 | 66.86304422 | 39.52111196 |
| **Arhgap8** | 0 | 1.584524017 | 1.566322589 | 2.587828259 | 1.266726489 | 0.701412806 | 0 | 0 | 0 | 0.873901969 | 1.891741398 | 0.867528106 | 2.556252945 | 0 | 0 |
| **Phf21b** | 33.88076982 | 26.14464628 | 36.80858084 | 39.6800333 | 37.36843141 | 32.26498906 | 49.61355931 | 11.73303618 | 30.84897903 | 27.09096104 | 31.21373306 | 28.62842751 | 43.45630007 | 42.46706862 | 23.71266717 |
| **Nup50** | 2943.964188 | 3005.049798 | 3083.306016 | 2982.040764 | 2755.763476 | 2842.124689 | 2975.009429 | 3167.919769 | 3064.582721 | 3042.052754 | 3147.857686 | 2910.556797 | 2793.132385 | 2829.029614 | 2846.957192 |
| **5031439G07Rik** | 259.1421043 | 264.6155108 | 281.1549047 | 282.9358896 | 283.7467334 | 293.8919656 | 345.4907858 | 260.6410181 | 249.0490746 | 252.557669 | 276.194244 | 301.0322529 | 305.8982691 | 238.538428 | 284.5520061 |
| **Fam118a** | 290.2757847 | 302.6440872 | 272.5401305 | 284.6611085 | 273.6129215 | 294.5933784 | 307.6040677 | 367.0764177 | 354.3870517 | 333.8305521 | 298.8951408 | 325.3230399 | 357.0233281 | 327.9903385 | 283.1148748 |
| **Smc1b** | 0 | 0 | 0 | 0.86260942 | 1.266726489 | 0 | 0.902064715 | 0 | 0 | 1.747803938 | 1.891741398 | 0 | 0 | 0 | 0.718565672 |
| **Fbln1** | 169.4038491 | 133.8922794 | 153.4996137 | 185.4610252 | 149.4737256 | 140.9839739 | 125.3869954 | 107.2734737 | 114.3669466 | 145.0677268 | 114.4503546 | 145.7447219 | 136.3334904 | 178.0002664 | 178.2042866 |
| **Atxn10** | 1871.683608 | 1745.353205 | 1715.123235 | 1732.119715 | 1844.987131 | 1733.191043 | 1881.706995 | 1858.848161 | 1780.964228 | 1735.56931 | 1812.288259 | 1838.292057 | 1745.068677 | 1793.555983 | 1786.354261 |
| **Lincppara** | 0 | 0.792262009 | 0.783161294 | 0.86260942 | 5.066905954 | 0 | 0 | 0 | 2.257242368 | 1.747803938 | 0 | 1.735056213 | 5.112505891 | 0 | 0 |
| **Ppara** | 0 | 1.584524017 | 2.349483883 | 0 | 0.633363244 | 2.104238417 | 0.902064715 | 0 | 0 | 0 | 0 | 0 | 0.852084315 | 0 | 0 |
| **Cdpf1** | 277.4560339 | 262.2387248 | 245.9126465 | 191.4992912 | 273.6129215 | 235.6747027 | 286.8565793 | 287.4593865 | 240.0201051 | 263.0444926 | 270.5190198 | 248.9805665 | 264.998222 | 243.0562013 | 283.8334404 |
| **Pkdrej** | 5.494178889 | 1.584524017 | 0.783161294 | 0 | 1.900089733 | 0 | 0 | 0 | 0 | 0 | 2.837612096 | 0 | 2.556252945 | 0 | 0.718565672 |
| **Ttc38** | 125.450418 | 142.6071615 | 126.0889684 | 152.6818673 | 145.6735462 | 122.747241 | 128.0931895 | 115.6542138 | 142.2062692 | 125.8418835 | 128.638415 | 151.8174186 | 145.7064179 | 152.7007361 | 116.4076389 |
| **Gtse1** | 1609.794415 | 1701.778794 | 1867.839687 | 1788.189327 | 1613.809546 | 1595.01272 | 1645.36604 | 2710.331358 | 2416.754162 | 2626.075417 | 2462.101429 | 1763.68464 | 1709.281136 | 1751.992469 | 1766.234422 |
| **Trmu** | 105.3050954 | 126.7619214 | 171.5123235 | 116.4522717 | 183.0419776 | 137.4769099 | 125.3869954 | 138.2822122 | 174.5600764 | 181.7716095 | 151.3393118 | 118.8513506 | 126.960563 | 163.5433919 | 140.8388717 |
| **Celsr1** | 1308.530272 | 1472.815074 | 1440.233621 | 1446.595997 | 1416.200214 | 1361.442256 | 1539.824468 | 1516.07589 | 1342.306795 | 1281.140286 | 1191.79708 | 1303.894744 | 1490.295467 | 1491.76873 | 1504.676517 |
| **Gramd4** | 1150.114781 | 1287.425764 | 1369.749104 | 1221.454938 | 1376.931693 | 1317.253249 | 1410.829214 | 1299.852794 | 1135.392911 | 1136.946462 | 1205.985141 | 1505.161264 | 1516.710081 | 1561.342438 | 1560.006074 |
| **Cerk** | 368.1099856 | 496.7482793 | 490.2589703 | 482.1986656 | 537.0920311 | 461.5296262 | 473.5839753 | 476.0260394 | 433.3905346 | 396.7514939 | 288.4905631 | 524.8545043 | 535.9610342 | 483.4017386 | 500.1217077 |
| **Tbc1d22a** | 680.3624858 | 739.9727159 | 723.6410361 | 705.6145053 | 716.9671925 | 679.6690087 | 649.4865946 | 667.1069144 | 680.1823668 | 719.2213204 | 632.7874975 | 646.3084392 | 677.4070306 | 676.762434 | 685.5116511 |
| **Brd1** | 2320.374884 | 2400.553886 | 2291.529948 | 2292.815837 | 2429.581405 | 2285.202921 | 2447.301571 | 1934.274822 | 1962.296032 | 1959.288214 | 2045.918321 | 2319.770156 | 2450.59449 | 2403.455373 | 2389.230859 |
| **Zbed4** | 585.1300517 | 617.9643666 | 572.4909063 | 565.8717793 | 497.1901468 | 514.1355866 | 607.089553 | 577.432995 | 659.1147714 | 575.0274955 | 651.7049115 | 602.0645058 | 621.1694658 | 655.980677 | 587.7867197 |
| **Alg12** | 192.2962611 | 209.1571702 | 222.4178076 | 149.2314296 | 153.9072684 | 194.99276 | 180.412943 | 139.1202862 | 149.7304104 | 180.0238056 | 172.1484672 | 181.3133742 | 179.7897905 | 196.0713594 | 190.4199031 |
| **Creld2** | 856.1762103 | 819.1989168 | 785.5107784 | 787.5624001 | 789.1706024 | 939.1917468 | 773.0694606 | 922.7194884 | 795.3017276 | 930.7055969 | 916.5487071 | 759.9546211 | 858.9009897 | 834.8844981 | 834.2547452 |
| **Pim3** | 1689.460008 | 1418.941257 | 1399.509233 | 1357.747227 | 1268.626578 | 1418.256693 | 1357.607396 | 1224.426133 | 1130.878426 | 1220.841051 | 1190.85121 | 1199.791371 | 1301.132749 | 1090.590464 | 1261.082754 |
| **1810021B22Rik** | 7.325571853 | 0 | 4.698967767 | 1.725218839 | 5.066905954 | 0.701412806 | 4.510323574 | 0 | 0 | 5.243411813 | 0.945870699 | 0.867528106 | 8.520843152 | 3.614218606 | 3.59282836 |
| **Trabd** | 1109.824136 | 1174.924559 | 1162.994522 | 1154.171403 | 1118.519489 | 1181.880578 | 1145.622188 | 1113.800363 | 1058.64667 | 1185.884972 | 1071.671502 | 1184.175865 | 1271.309798 | 1215.281006 | 1164.794954 |
| **Selo** | 154.7527054 | 154.4910917 | 176.9944526 | 158.7201332 | 142.50673 | 156.4150557 | 175.9026194 | 102.2450296 | 169.2931776 | 136.3287071 | 137.1512513 | 107.5734852 | 206.2044043 | 177.0967117 | 170.3000643 |
| **Tubgcp6** | 673.0369139 | 798.6001046 | 738.5211007 | 773.7606494 | 646.0305092 | 716.8438874 | 785.6983666 | 722.4197993 | 669.6485691 | 743.6905755 | 743.4543692 | 818.9465323 | 859.753074 | 719.2295027 | 793.2965019 |
| **Hdac10** | 187.7177787 | 210.7416943 | 205.9714205 | 207.0262607 | 190.0089733 | 178.8602655 | 182.2170724 | 202.8139112 | 173.0552482 | 232.4579237 | 209.9832951 | 190.8561834 | 240.2877769 | 255.7059664 | 214.8511359 |
| **Mapk12** | 5.494178889 | 7.922620085 | 14.8800646 | 14.66436013 | 20.26762382 | 13.32684331 | 16.23716487 | 12.5711102 | 3.762070613 | 5.243411813 | 10.40457769 | 6.94022485 | 13.63334904 | 6.324882561 | 10.77848508 |
| **Mapk11** | 237.1653887 | 276.499441 | 273.3232918 | 213.0645267 | 269.8127421 | 307.9202217 | 303.9958089 | 193.595097 | 252.8111452 | 225.466708 | 214.7126486 | 310.575062 | 297.377426 | 309.9192455 | 295.3304912 |
| **Plxnb2** | 99.81091649 | 85.56429692 | 73.61716168 | 103.5131304 | 57.63605523 | 84.87094949 | 82.08788905 | 37.71333059 | 57.18347332 | 80.39898114 | 55.80637123 | 57.25685501 | 52.82922754 | 91.25901981 | 76.8865269 |
| **Dennd6b** | 64.09875371 | 87.14882094 | 109.6425812 | 62.97048763 | 76.63695256 | 73.6483446 | 76.67550076 | 90.51199341 | 63.2027863 | 94.38141264 | 69.04856101 | 80.68011388 | 57.08964912 | 74.99503608 | 102.7548911 |
| **Ppp6r2** | 174.898028 | 249.5625327 | 234.1652271 | 219.1027926 | 180.5085246 | 206.9167777 | 254.3822496 | 212.0327253 | 184.34146 | 219.3493942 | 139.0429927 | 204.7366331 | 220.6898376 | 180.7109303 | 227.0667523 |
| **Sbf1** | 2737.016783 | 2836.29799 | 2948.602274 | 2939.772902 | 3009.108774 | 3063.069723 | 3010.189953 | 2705.302914 | 2727.501194 | 2758.034614 | 2822.478165 | 3142.186801 | 3215.766205 | 3252.796746 | 3246.479706 |
| **Adm2** | 0.915696482 | 0.792262009 | 2.349483883 | 0 | 0 | 0.701412806 | 3.608258859 | 0 | 0 | 1.747803938 | 0 | 0 | 0.852084315 | 1.807109303 | 2.155697016 |
| **Lmf2** | 1606.131629 | 1900.636558 | 1776.209816 | 1872.72505 | 1789.251165 | 1611.846628 | 1725.649799 | 1653.520028 | 1624.462091 | 1735.56931 | 1830.259802 | 1797.518236 | 1815.791676 | 1721.271611 | 1859.647959 |
| **Ncaph2** | 4240.590406 | 4366.948191 | 4523.539637 | 4187.968732 | 4263.167997 | 4409.080897 | 4294.730107 | 3859.33083 | 3883.961701 | 3929.937154 | 3804.29195 | 4350.653453 | 4259.569491 | 4504.219938 | 4383.969165 |
| **Tymp** | 7.325571853 | 4.753572051 | 10.18109683 | 7.763484777 | 6.333632443 | 7.014128057 | 7.216517718 | 6.704592104 | 3.00965649 | 4.369509845 | 4.729353494 | 6.072696744 | 11.0770961 | 3.614218606 | 5.029959704 |
| **Odf3b** | 2.747089445 | 0 | 3.132645178 | 0 | 1.900089733 | 1.402825611 | 0 | 0.838074013 | 1.504828245 | 0 | 1.891741398 | 0.867528106 | 13.63334904 | 6.324882561 | 2.155697016 |
| **Klhdc7b** | 0 | 0 | 0 | 0 | 0.633363244 | 0 | 1.80412943 | 0 | 0 | 0.873901969 | 0 | 0 | 0 | 0 | 2.155697016 |
| **Cpt1b** | 0 | 3.169048034 | 1.566322589 | 0 | 0 | 0 | 1.80412943 | 0 | 0 | 0 | 0 | 5.205168638 | 2.556252945 | 5.421327909 | 0 |
| **Mapk8ip2** | 0 | 2.376786026 | 0.783161294 | 2.587828259 | 0 | 0.701412806 | 0.902064715 | 0 | 3.00965649 | 3.495607876 | 0 | 4.337640531 | 0.852084315 | 0 | 5.029959704 |
| **Arsa** | 145.5957406 | 139.4381135 | 147.2343234 | 180.2853687 | 136.8064608 | 131.8656075 | 126.2890601 | 126.549176 | 116.624189 | 143.3199229 | 141.8806048 | 138.804497 | 146.5585022 | 130.1118698 | 148.0245284 |
| **C230037L18Rik** | 9.156964816 | 0 | 0.783161294 | 0 | 3.166816221 | 0.701412806 | 3.608258859 | 0.838074013 | 0.752414123 | 0 | 0.945870699 | 0 | 1.70416863 | 0 | 0 |
| **Rabl2** | 41.20634167 | 51.49703055 | 50.90548414 | 24.15306375 | 43.70206386 | 39.98052993 | 25.25781201 | 69.56014308 | 70.72692752 | 64.6687457 | 65.26507822 | 35.56865236 | 49.42089028 | 58.73105235 | 47.42533435 |
| **Alg10b** | 813.1384756 | 805.7304626 | 912.3829081 | 807.4024168 | 752.4355342 | 776.4639759 | 845.2346377 | 842.2643831 | 924.7169567 | 926.336087 | 909.9276122 | 955.148445 | 927.9198192 | 860.1840283 | 852.218887 |
| **Abcd2** | 2.747089445 | 0 | 0 | 0 | 0 | 0 | 0.902064715 | 6.704592104 | 4.514484735 | 0 | 1.891741398 | 0 | 0 | 1.807109303 | 0.718565672 |
| **Lrrk2** | 5.494178889 | 6.338096068 | 7.04845165 | 3.450437679 | 3.166816221 | 2.104238417 | 3.608258859 | 10.89496217 | 3.762070613 | 12.23462756 | 8.512836289 | 3.470112425 | 3.408337261 | 0 | 3.59282836 |
| **Cntn1** | 6.409875371 | 8.714882094 | 4.698967767 | 2.587828259 | 0.633363244 | 0 | 0 | 19.2757023 | 12.03862596 | 27.964863 | 17.97154328 | 0 | 0 | 0 | 2.874262688 |
| **Gxylt1** | 581.4672658 | 671.8381832 | 660.2049713 | 608.1396409 | 505.4238689 | 552.0118781 | 609.7957472 | 545.5861825 | 565.8154202 | 512.9804557 | 575.0893849 | 561.2906848 | 639.9153207 | 560.203884 | 581.3196286 |
| **Yaf2** | 469.752295 | 496.7482793 | 491.8252929 | 475.2977902 | 450.9546299 | 505.0172201 | 570.1048997 | 464.2930032 | 416.0850098 | 454.4290238 | 453.0720647 | 522.25192 | 488.2443126 | 484.3052932 | 565.5111838 |
| **Zcrb1** | 651.0601984 | 518.9316156 | 706.4114876 | 600.3761561 | 636.5300605 | 636.8828276 | 596.2647765 | 590.8421792 | 681.6871951 | 654.5525747 | 667.7847133 | 587.316528 | 638.2111521 | 571.9500944 | 603.5951645 |
| **Pphln1** | 1386.364473 | 1331.000174 | 1495.838072 | 1415.542058 | 1290.160929 | 1369.85921 | 1515.468721 | 1655.196176 | 1483.008236 | 1441.064347 | 1648.652628 | 1434.891488 | 1407.643289 | 1361.65686 | 1350.903463 |
| **Prickle1** | 76.91850445 | 82.39524888 | 89.28038757 | 44.85568982 | 55.7359655 | 51.90454762 | 56.83007703 | 133.2537681 | 70.72692752 | 118.8506678 | 85.12836289 | 58.99191123 | 55.38548049 | 43.37062328 | 47.42533435 |
| **Adamts20** | 0 | 2.376786026 | 0 | 3.450437679 | 0 | 0 | 1.80412943 | 0 | 0 | 0 | 4.729353494 | 2.602584319 | 4.260421576 | 0 | 0 |
| **Pus7l** | 394.6651836 | 402.4691003 | 376.7005826 | 370.059441 | 396.4853909 | 404.7151889 | 375.2589213 | 467.6452993 | 476.2781396 | 430.8336707 | 475.7729615 | 382.5798949 | 413.2608929 | 300.883699 | 413.1752614 |
| **Irak4** | 250.9008359 | 220.2488384 | 245.9126465 | 255.3323882 | 241.3113961 | 208.3196033 | 249.871926 | 228.7942056 | 241.5249333 | 265.6661985 | 265.7896664 | 243.7753979 | 236.8794396 | 230.4064362 | 227.0667523 |
| **Twf1** | 619.0108215 | 671.8381832 | 590.503616 | 629.7048763 | 562.4265609 | 628.4658739 | 591.7544529 | 790.3037943 | 639.5520042 | 757.673007 | 850.3377582 | 624.6202365 | 616.9090442 | 645.1380212 | 673.2960346 |
| **Ano6** | 1299.373307 | 1183.639441 | 1222.514781 | 1283.562816 | 1226.191241 | 1078.772895 | 1146.524252 | 1298.176646 | 1183.547415 | 1154.424501 | 1179.500761 | 1228.419798 | 1261.084786 | 1214.377452 | 1163.357823 |
| **Gm4371** | 3.662785926 | 2.376786026 | 0 | 0.86260942 | 0 | 2.805651223 | 1.80412943 | 1.676148026 | 3.762070613 | 0.873901969 | 0.945870699 | 0.867528106 | 3.408337261 | 0.903554652 | 0 |
| **E330033B04Rik** | 0.915696482 | 7.922620085 | 5.482129061 | 0.86260942 | 1.266726489 | 2.805651223 | 5.412388289 | 4.190370065 | 1.504828245 | 0 | 8.512836289 | 1.735056213 | 4.260421576 | 0 | 0 |
| **Arid2** | 1661.073418 | 1754.068087 | 1766.028719 | 1680.363149 | 1619.509816 | 1636.396076 | 1844.722342 | 1506.857075 | 1562.764133 | 1427.955817 | 1541.769239 | 1795.78318 | 1863.508397 | 1686.936534 | 1839.52812 |
| **Scaf11** | 4953.002269 | 4743.272645 | 4708.365702 | 4771.0927 | 4489.912039 | 4565.495952 | 4987.515808 | 4861.66735 | 4955.399411 | 4629.058729 | 4787.997477 | 4806.105709 | 4634.48659 | 4668.666885 | 4787.084507 |
| **Slc38a1** | 6660.776207 | 6907.732452 | 7013.209392 | 6928.478859 | 6305.131097 | 6383.557945 | 6721.28419 | 6568.824114 | 6503.115261 | 6215.190803 | 6825.402962 | 6599.286304 | 6630.920141 | 6357.410528 | 6569.845939 |
| **Slc38a2** | 3108.789555 | 3095.367667 | 3037.0995 | 2807.793661 | 2366.878444 | 2443.722215 | 2803.617134 | 2505.841299 | 2450.612797 | 2397.113101 | 2477.23536 | 2618.199825 | 2636.348871 | 2516.399705 | 2624.201834 |
| **Amigo2** | 39.37494871 | 45.15893448 | 43.0738712 | 59.52004996 | 49.40233305 | 43.48759395 | 40.59291217 | 69.56014308 | 69.22209928 | 48.93851026 | 48.23940564 | 40.773821 | 42.60421576 | 43.37062328 | 22.9941015 |
| **Pced1b** | 748.1240254 | 814.4453447 | 736.9547781 | 732.3553973 | 774.6032477 | 701.4128057 | 693.6877657 | 674.6495805 | 597.4168133 | 549.6843384 | 624.2746612 | 731.3261936 | 702.96956 | 652.3664584 | 600.0023361 |
| **Rpap3** | 756.3652938 | 737.5959299 | 767.4980686 | 747.0197574 | 708.1001071 | 681.0718343 | 700.0022187 | 850.6451232 | 840.4465749 | 765.5381248 | 766.155266 | 746.9416995 | 828.2259543 | 722.8437213 | 721.4399347 |
| **Endou** | 98.89522001 | 104.5785851 | 104.9436135 | 85.39833255 | 81.07049527 | 70.14128057 | 75.77343604 | 17.59955427 | 46.6496756 | 43.69509845 | 47.29353494 | 55.5217988 | 67.3146609 | 47.88839653 | 82.63505228 |
| **Rapgef3** | 1.831392963 | 9.507144102 | 5.482129061 | 3.450437679 | 0 | 2.805651223 | 2.706194144 | 0.838074013 | 2.257242368 | 0 | 1.891741398 | 0 | 0 | 0.903554652 | 7.18565672 |
| **Slc48a1** | 746.2926325 | 866.7346373 | 772.1970364 | 778.9363059 | 698.5996584 | 752.6159405 | 738.7910014 | 882.4919357 | 793.0444852 | 913.2275575 | 756.696559 | 731.3261936 | 761.7633778 | 826.7525062 | 840.7218362 |
| **Hdac7** | 4220.445084 | 4555.506549 | 4491.430024 | 3955.926799 | 5408.922106 | 5711.604477 | 5764.193527 | 2665.913436 | 3007.399248 | 3120.703931 | 3301.088739 | 5492.320441 | 5528.323037 | 5506.262047 | 5949.723764 |
| **Vdr** | 13.73544722 | 17.42976419 | 28.1938066 | 16.38957897 | 17.73417084 | 16.13249453 | 27.06194144 | 35.19910855 | 25.58208017 | 11.3607256 | 26.48437957 | 19.08561834 | 32.37920398 | 32.52796746 | 22.9941015 |
| **Tmem106c** | 251.8165324 | 253.5238427 | 223.2009689 | 222.5532303 | 250.1784815 | 252.5086101 | 218.299661 | 195.271245 | 234.0007921 | 194.0062371 | 235.521804 | 226.4248357 | 268.4065593 | 238.538428 | 203.3540852 |
| **Col2a1** | 0 | 0 | 0 | 0 | 1.266726489 | 1.402825611 | 0 | 0 | 0 | 0 | 2.837612096 | 0.867528106 | 0 | 1.807109303 | 0.718565672 |
| **Senp1** | 1491.669568 | 1567.094253 | 1509.934976 | 1527.681282 | 1485.236808 | 1402.825611 | 1505.546009 | 1728.108615 | 1610.166222 | 1788.87733 | 1555.957299 | 1563.285648 | 1634.297716 | 1396.895491 | 1445.035566 |
| **Pfkm** | 369.9413786 | 385.0393361 | 375.9174214 | 353.6698621 | 293.2471821 | 328.9626059 | 386.0836979 | 366.2383437 | 272.3739124 | 370.5344348 | 355.6473827 | 382.5798949 | 281.187824 | 331.6045571 | 360.7199673 |
| **Asb8** | 406.5692378 | 479.3185151 | 456.5830347 | 418.3655685 | 431.9537326 | 415.9377938 | 403.2229275 | 421.5512286 | 437.9050193 | 469.2853573 | 436.0463921 | 426.8238283 | 428.5984105 | 496.9550584 | 411.0195644 |
| **Ccdc184** | 412.9791132 | 307.3976593 | 279.5885821 | 284.6611085 | 189.37561 | 266.5368662 | 240.8512788 | 227.9561316 | 99.31866418 | 300.6222773 | 296.0575287 | 203.0015769 | 236.0273553 | 208.7211245 | 227.785318 |
| **Lalba** | 1.831392963 | 0 | 2.349483883 | 0 | 0.633363244 | 0.701412806 | 0 | 4.190370065 | 0 | 2.621705907 | 0 | 1.735056213 | 0 | 0 | 0 |
| **Kansl2** | 1293.879128 | 1243.059091 | 1282.8182 | 1155.896622 | 1181.222451 | 1171.359386 | 1261.988536 | 1267.167908 | 1224.177777 | 1254.923227 | 1310.030918 | 1251.843057 | 1279.830641 | 1292.986706 | 1213.65742 |
| **Mir1291** | 0 | 0 | 1.566322589 | 0 | 3.166816221 | 1.402825611 | 0 | 0 | 0 | 0 | 0 | 0 | 0 | 3.614218606 | 0 |
| **Snora2b** | 2.747089445 | 7.922620085 | 0 | 0.86260942 | 1.900089733 | 0 | 1.80412943 | 2.514222039 | 0.752414123 | 6.117313782 | 13.24218978 | 4.337640531 | 0 | 1.807109303 | 5.029959704 |
| **Ccnt1** | 435.8715252 | 387.4161222 | 404.8943892 | 394.2125048 | 333.7824297 | 375.9572639 | 407.7332511 | 401.4374523 | 391.2553437 | 429.9597687 | 439.8298749 | 416.413491 | 414.9650615 | 438.224006 | 360.7199673 |
| **9330020H09Rik** | 32.96507334 | 26.14464628 | 29.76012919 | 26.74089201 | 22.16771355 | 32.96640187 | 29.76813559 | 28.49451644 | 53.4214027 | 38.45168663 | 32.15960376 | 38.17123668 | 13.63334904 | 35.23863141 | 30.89832389 |
| **Adcy6** | 148.34283 | 127.5541834 | 136.2700652 | 128.5288035 | 94.3711234 | 117.8373514 | 132.6035131 | 147.5010263 | 126.4055726 | 133.7070012 | 137.1512513 | 98.03067601 | 110.770961 | 111.1372221 | 121.4375986 |
| **Cacnb3** | 97.06382705 | 72.09584277 | 73.61716168 | 82.81050429 | 43.70206386 | 61.7243269 | 67.65485361 | 95.54043749 | 107.5952195 | 110.1116481 | 104.0457769 | 99.76573222 | 82.65217857 | 54.21327909 | 69.70087018 |
| **Ddx23** | 2315.796402 | 2220.71041 | 2375.328206 | 2390.290702 | 2518.252259 | 2384.803539 | 2463.538736 | 2732.959357 | 2520.587311 | 2494.990121 | 2695.731491 | 2394.377573 | 2373.054818 | 2470.318417 | 2326.715646 |
| **Rnd1** | 34.7964663 | 17.42976419 | 28.9769679 | 23.29045433 | 19.00089733 | 25.95227381 | 30.6702003 | 31.00873848 | 39.8779485 | 42.82119648 | 25.53850887 | 20.82067455 | 21.30210788 | 9.035546516 | 16.52701046 |
| **Fkbp11** | 26.55519797 | 19.0142882 | 14.8800646 | 15.52696955 | 22.80107679 | 29.45933784 | 20.74748844 | 20.95185033 | 30.84897903 | 26.21705907 | 29.32199166 | 16.48303402 | 37.49170987 | 14.45687443 | 21.55697016 |
| **Arf3** | 3727.800376 | 3698.279056 | 3926.770731 | 3829.985823 | 3758.377492 | 3866.187385 | 4036.739599 | 4122.48607 | 3888.476185 | 3918.576429 | 3715.380105 | 4065.236706 | 4142.83394 | 4169.001162 | 3882.410326 |
| **Wnt10b** | 471.583688 | 367.6095719 | 384.5321956 | 310.5393911 | 278.6798275 | 263.7312149 | 292.2689676 | 460.9407072 | 425.8663934 | 480.6460829 | 360.3767362 | 289.7543875 | 392.8108693 | 259.320185 | 324.0731181 |
| **Wnt1** | 0 | 0 | 0 | 2.587828259 | 8.233722176 | 0.701412806 | 0 | 4.190370065 | 8.276555348 | 0 | 5.675224193 | 2.602584319 | 0 | 3.614218606 | 0.718565672 |
| **Ddn** | 37.54355574 | 40.40536243 | 47.77283896 | 13.80175071 | 24.06780328 | 33.66781467 | 27.06194144 | 26.81836842 | 49.65933209 | 49.81241223 | 52.02288843 | 29.49595561 | 32.37920398 | 27.10663955 | 43.83250599 |
| **Prkag1** | 1104.329957 | 1140.06503 | 1282.035039 | 1228.355814 | 1248.992318 | 1151.719827 | 1082.477658 | 1315.7762 | 1318.981957 | 1326.583189 | 1321.381366 | 1300.424631 | 1211.663896 | 1219.79878 | 1279.765462 |
| **Kmt2d** | 3400.896733 | 3516.851056 | 3202.346533 | 3639.349142 | 2957.806351 | 2765.670693 | 3403.490169 | 3382.466717 | 3405.426319 | 2790.368987 | 3268.929135 | 3283.593882 | 3152.711966 | 3029.618747 | 3205.521463 |
| **Rhebl1** | 105.3050954 | 82.39524888 | 85.3645811 | 79.36006661 | 88.6708542 | 73.6483446 | 74.87137133 | 103.0831036 | 79.75589699 | 90.0119028 | 89.85771638 | 91.95797927 | 86.06051583 | 98.48745702 | 84.07218362 |
| **Dhh** | 4.578482408 | 11.88393013 | 9.397935534 | 1.725218839 | 1.266726489 | 3.507064029 | 2.706194144 | 0.838074013 | 1.504828245 | 4.369509845 | 2.837612096 | 0 | 2.556252945 | 9.035546516 | 4.311394032 |
| **Lmbr1l** | 284.7816058 | 293.1369431 | 314.8308404 | 253.6071694 | 327.4487973 | 341.5880364 | 322.0371032 | 253.936426 | 270.11667 | 304.1178852 | 302.6786236 | 335.7333771 | 348.5024849 | 344.2543222 | 344.1929569 |
| **Tuba1b** | 30149.30666 | 30838.79868 | 31819.8434 | 32514.33685 | 31434.45118 | 31299.14363 | 31234.89281 | 32927.92797 | 32185.26651 | 32124.63638 | 32376.20815 | 32661.56567 | 32471.22908 | 31117.51865 | 31414.25405 |
| **Tuba1a** | 6266.111023 | 6000.592452 | 6190.890033 | 5987.371982 | 5548.895383 | 5603.586905 | 5763.291463 | 5279.028208 | 5464.031358 | 5642.785013 | 5660.090261 | 5909.60146 | 5952.661026 | 5349.043537 | 5706.130001 |
| **Tuba1c** | 2968.687993 | 2898.886689 | 3154.573694 | 3155.425257 | 3123.114158 | 3011.165175 | 3026.427118 | 3342.239164 | 3195.502779 | 3259.654344 | 3324.735506 | 3164.742532 | 3115.220256 | 3119.974212 | 3179.653098 |
| **Prph** | 7.325571853 | 0.792262009 | 6.265290356 | 4.313047098 | 1.900089733 | 2.805651223 | 12.62890601 | 2.514222039 | 2.257242368 | 0 | 0 | 8.675281063 | 6.816674521 | 10.84265582 | 5.748525376 |
| **Troap** | 413.8948097 | 455.5506549 | 493.3916155 | 466.671696 | 439.5540915 | 417.3406194 | 416.7538982 | 365.4002697 | 446.1815747 | 429.9597687 | 418.0748489 | 471.0677617 | 484.8359753 | 477.9804107 | 457.0077674 |
| **Spats2** | 49.44761 | 49.91250654 | 36.80858084 | 45.71829924 | 21.53435031 | 43.48759395 | 39.69084745 | 54.47481085 | 35.36346376 | 42.82119648 | 36.88895725 | 24.29078698 | 33.23128829 | 30.72085815 | 37.36541494 |
| **Kcnh3** | 0 | 0 | 0 | 0.86260942 | 0 | 0.701412806 | 1.80412943 | 0 | 3.762070613 | 1.747803938 | 0 | 0 | 0 | 0 | 0 |
| **Mcrs1** | 1112.571225 | 1167.001939 | 1206.068394 | 1140.369653 | 1157.788011 | 1097.711041 | 1143.818058 | 1275.548648 | 1202.357768 | 1358.04366 | 1127.477873 | 1092.217886 | 1125.60338 | 1096.011792 | 1133.89663 |
| **1700120C14Rik** | 0 | 0 | 1.566322589 | 0 | 1.266726489 | 0.701412806 | 0 | 0 | 0 | 0 | 0 | 0 | 1.70416863 | 0 | 0 |
| **Prpf40b** | 39.37494871 | 26.93690829 | 14.8800646 | 24.15306375 | 27.2346195 | 30.16075065 | 34.27845916 | 47.77021874 | 34.61104964 | 36.70388269 | 39.72656935 | 48.58157395 | 51.97714322 | 28.0101942 | 25.14979852 |

| | | | | | | | | | | | | | | | |
|---|---|---|---|---|---|---|---|---|---|---|---|---|---|---|---|
| **Fmnl3** | 287.5286952 | 292.3446811 | 249.0452916 | 297.6002498 | 292.6138189 | 291.7877272 | 330.1556856 | 357.0195296 | 327.3001433 | 343.4434738 | 231.7383212 | 352.2164111 | 346.7983163 | 317.1476827 | 352.8157449 |
| **Tmbim6** | 9861.13541 | 9774.928661 | 10139.58928 | 9974.352719 | 9928.602217 | 9947.436411 | 9965.108904 | 8266.762065 | 8591.064452 | 8145.640252 | 8389.873098 | 10037.30019 | 10435.47661 | 10471.29486 | 9786.145887 |
| **Nckap5l** | 200.5375295 | 204.4035982 | 217.7188399 | 244.9810752 | 210.9099603 | 172.5475502 | 230.0265023 | 183.5382089 | 181.3318035 | 185.2672174 | 195.7952346 | 180.4458461 | 142.2980806 | 167.1576105 | 163.8329732 |
| **Bcdin3d** | 108.9678813 | 118.8393013 | 98.67832311 | 138.0175071 | 100.7047558 | 95.39214158 | 92.01060091 | 112.3019177 | 59.44071568 | 71.65996145 | 89.85771638 | 104.9709009 | 138.0376591 | 87.6448012 | 79.04222392 |
| **Racgap1** | 4203.04685 | 4507.970828 | 4517.274347 | 4388.094118 | 4604.550786 | 4567.600191 | 4576.174298 | 4141.761773 | 4537.809573 | 4316.201824 | 4513.694975 | 4717.617842 | 4752.074226 | 4796.971645 | 4764.090405 |
| **Asic1** | 21.06101908 | 15.84524017 | 32.10961307 | 17.25218839 | 20.90098706 | 9.118366474 | 20.74748844 | 9.218814144 | 15.80069657 | 18.35194135 | 12.29631908 | 13.8804497 | 18.74585493 | 25.29953024 | 19.40127314 |
| **Smarcd1** | 1322.265719 | 1343.676366 | 1255.407555 | 1311.166318 | 1296.494561 | 1258.334573 | 1257.478212 | 1468.305671 | 1365.631632 | 1260.166639 | 1220.173201 | 1227.55227 | 1366.743242 | 1278.529832 | 1361.681948 |
| **Gpd1** | 3.662785926 | 7.130358077 | 3.132645178 | 0 | 0 | 2.104238417 | 0.902064715 | 14.24725822 | 8.276555348 | 6.117313782 | 4.729353494 | 3.470112425 | 5.964590206 | 7.228437213 | 3.59282836 |
| **Cox14** | 392.8337906 | 459.5119649 | 451.1009056 | 438.2055852 | 406.6192028 | 547.8034013 | 471.7798458 | 465.1310772 | 475.5257255 | 450.933416 | 401.0491763 | 436.3666375 | 480.5755538 | 419.2493583 | 421.0794838 |
| **Cers5** | 1083.268938 | 1110.751336 | 1236.611684 | 1080.849603 | 1161.58819 | 1060.536162 | 1130.287088 | 1222.749985 | 1142.164638 | 1039.069441 | 1166.258572 | 1124.316426 | 1050.619961 | 1197.209913 | 1230.18443 |
| **Lima1** | 0.915696482 | 0 | 0.783161294 | 0 | 0 | 2.805651223 | 6.314453003 | 1.676148026 | 0 | 0 | 1.891741398 | 0 | 1.70416863 | 0.903554652 | 0 |
| **Larp4** | 4048.294145 | 3721.254654 | 3915.806472 | 3624.684781 | 3226.352366 | 3300.848664 | 3787.769737 | 4258.25406 | 3915.563094 | 3889.737664 | 4246.013567 | 3628.00254 | 3543.818667 | 3288.035377 | 3282.40799 |
| **2310068J16Rik** | 0.915696482 | 3.961310043 | 0 | 6.038265938 | 7.600358931 | 4.208476834 | 2.706194144 | 2.514222039 | 0 | 3.495607876 | 4.729353494 | 3.470112425 | 0 | 4.517773258 | 6.467091048 |
| **Dip2b** | 1068.617794 | 1202.653729 | 1292.216136 | 1254.234096 | 1084.951237 | 1086.488436 | 1123.972635 | 1394.555158 | 1263.303312 | 1237.445188 | 1246.657581 | 1157.282494 | 1245.747269 | 1126.732651 | 1125.273842 |
| **Atf1** | 1739.823315 | 1869.73834 | 1937.541043 | 1922.756396 | 1720.214571 | 1692.5091 | 1949.361849 | 2062.500146 | 2019.479505 | 1919.962626 | 2113.075141 | 1985.771835 | 1771.483291 | 1980.591796 | 1919.28891 |
| **Mettl7a1** | 64.09875371 | 58.62738863 | 86.93090369 | 66.42092531 | 39.26852115 | 47.69607079 | 31.57226502 | 85.48354933 | 68.46968515 | 64.6687457 | 85.12836289 | 55.5217988 | 51.12505891 | 77.70570004 | 28.02406121 |
| **Slc11a2** | 538.4295312 | 575.1822182 | 625.7458743 | 558.9709039 | 592.8279966 | 546.4005757 | 571.9090292 | 537.2054424 | 570.3299049 | 539.1975148 | 544.8215225 | 577.7737188 | 523.1797695 | 648.7522398 | 574.1339719 |
| **Letmd1** | 510.0429402 | 457.1351789 | 495.7410994 | 485.6491033 | 497.1901468 | 415.236381 | 434.7951925 | 612.6321035 | 649.3333878 | 623.9660058 | 562.7930658 | 465.8625931 | 500.173493 | 482.4981839 | 507.3073644 |
| **Csrnp2** | 52.19469945 | 61.79643666 | 39.94122602 | 66.42092531 | 36.73506817 | 38.57770431 | 58.63420646 | 62.85555098 | 58.68830156 | 48.06460829 | 55.80637123 | 39.90629289 | 48.56880596 | 46.98484188 | 38.08398061 |
| **Tfcp2** | 55.85748538 | 62.58869867 | 79.88245204 | 53.48178402 | 56.36932874 | 53.30737323 | 40.59291217 | 59.50325493 | 33.10622139 | 75.15556933 | 56.75224193 | 58.99191123 | 74.98341973 | 66.86304422 | 68.98230451 |
| **Pou6f1** | 107.1364883 | 120.4238253 | 133.9205814 | 146.6436013 | 212.8100501 | 171.8461374 | 181.3150077 | 66.20784703 | 58.68830156 | 34.95607876 | 75.6696559 | 189.1211272 | 209.6127415 | 224.9851082 | 196.1684284 |
| **C330013E15Rik** | 5.494178889 | 0 | 2.349483883 | 14.66436013 | 2.533452977 | 2.104238417 | 2.706194144 | 1.676148026 | 1.504828245 | 1.747803938 | 0 | 4.337640531 | 2.556252945 | 1.807109303 | 2.874262688 |
| **Dazap2** | 4605.037606 | 4696.529186 | 4813.309316 | 4672.755226 | 4709.055721 | 4886.743017 | 4988.417873 | 4342.899536 | 4499.436453 | 4576.624611 | 4539.233483 | 5229.459425 | 5098.020458 | 5147.55085 | 5167.205747 |
| **Smagp** | 0 | 0.792262009 | 7.04845165 | 0.86260942 | 2.533452977 | 2.805651223 | 2.706194144 | 0.838074013 | 0 | 2.621705907 | 6.621094891 | 4.337640531 | 3.408337261 | 3.614218606 | 3.59282836 |
| **Bin2** | 2393.630603 | 2520.185449 | 2595.39653 | 2500.704708 | 2777.297826 | 2801.442746 | 2954.261941 | 2403.596269 | 2385.905183 | 2370.896042 | 2361.839135 | 2753.534209 | 2749.676085 | 3125.39554 | 3015.820125 |
| **Cela1** | 9.156964816 | 8.714882094 | 10.96425812 | 8.626094197 | 8.233722176 | 6.312715251 | 16.23716487 | 17.59955427 | 26.33449429 | 17.47803938 | 17.02567258 | 8.675281063 | 8.520843152 | 3.614218606 | 9.341353736 |
| **Galnt6** | 2264.517399 | 2383.916384 | 2348.700722 | 2275.563649 | 2231.972073 | 2330.794753 | 2244.33701 | 1837.896311 | 1841.157358 | 2008.226725 | 2189.690668 | 2304.15465 | 2295.515145 | 2264.307957 | 2479.051568 |
| **I730030J21Rik** | 49.44761 | 43.57441047 | 86.93090369 | 59.52004996 | 81.07049527 | 66.63421654 | 92.91266562 | 57.8271069 | 54.92623095 | 56.80362798 | 59.58985402 | 66.79966418 | 62.20215501 | 82.22347329 | 73.29369854 |
| **Slc4a8** | 131.8602933 | 146.5684716 | 122.1731619 | 155.2696955 | 107.6717515 | 118.5387642 | 105.5415716 | 122.3588059 | 104.585563 | 116.2289619 | 122.9631908 | 137.0694408 | 113.3272139 | 109.3301128 | 132.2160836 |
| **Scn8a** | 0 | 0.792262009 | 0 | 0.86260942 | 4.43354271 | 0.701412806 | 0.902064715 | 3.352296052 | 0 | 0 | 2.837612096 | 0.867528106 | 1.70416863 | 0 | 2.874262688 |
| **Acvrl1** | 272.8775515 | 181.4279999 | 242.7800013 | 210.4766984 | 229.9108577 | 250.4043716 | 296.7792912 | 36.03718256 | 56.43105919 | 50.6863142 | 69.99443171 | 190.8561834 | 232.619018 | 201.4926873 | 251.4979852 |
| **Acvr1b** | 184.9706893 | 184.597048 | 202.055614 | 196.6749477 | 214.7101398 | 194.99276 | 217.3975963 | 147.5010263 | 186.5987024 | 125.8418835 | 120.1255787 | 226.4248357 | 215.5773317 | 200.5891326 | 201.1983882 |
| **A330009N23Rik** | 0 | 0 | 3.132645178 | 0.86260942 | 0 | 3.507064029 | 2.706194144 | 4.190370065 | 0 | 4.369509845 | 1.891741398 | 1.735056213 | 5.112505891 | 5.421327909 | 0.718565672 |
| **Grasp** | 176.7294209 | 144.9839476 | 208.3209043 | 176.834931 | 142.50673 | 141.6853868 | 165.0778428 | 118.1684358 | 153.492481 | 115.3550599 | 141.8806048 | 129.2616878 | 156.783514 | 141.8580803 | 131.497518 |
| **Nr4a1** | 6000.559044 | 4835.175038 | 4937.831962 | 4389.819337 | 5034.604429 | 4942.154629 | 5499.888566 | 2758.101577 | 2771.893628 | 2487.998905 | 2710.865423 | 4247.417608 | 4391.64256 | 4141.894523 | 4524.089471 |
| **Atg101** | 438.6186147 | 453.9661309 | 437.7871636 | 502.9012917 | 406.6192028 | 474.8564695 | 426.6766101 | 463.4549292 | 540.23334 | 418.5990431 | 445.5050991 | 417.2810191 | 412.4088085 | 420.152913 | 435.4507972 |
| **Krt81** | 1.831392963 | 3.169048034 | 0.783161294 | 0 | 0 | 0.701412806 | 0 | 1.676148026 | 1.504828245 | 6.117313782 | 0 | 4.337640531 | 0 | 0 | 0 |
| **Krt18** | 0 | 0 | 0 | 0 | 5.700269198 | 2.104238417 | 0.902064715 | 0 | 1.504828245 | 0 | 0.945870699 | 1.735056213 | 2.556252945 | 0 | 0 |
| **Eif4b** | 13500.11323 | 13287.02614 | 14233.17337 | 13815.55247 | 13617.30975 | 13469.93152 | 14482.649 | 14615.17271 | 14115.28894 | 14325.00107 | 14338.45392 | 13905.60802 | 13870.22848 | 13537.05579 | 13968.91666 |
| **Tns2** | 16.48253667 | 7.922620085 | 18.01270977 | 19.84001665 | 31.66816221 | 6.312715251 | 18.0412943 | 19.2757023 | 14.29586833 | 21.84754922 | 29.32199166 | 34.70112425 | 31.52711966 | 21.68531164 | 30.89832389 |
| **Spryd3** | 553.9963713 | 582.3125763 | 523.934906 | 542.581325 | 532.6584884 | 549.9076397 | 532.2181817 | 555.6430707 | 489.0691797 | 561.045064 | 649.8131701 | 560.4231567 | 580.2694186 | 566.5287665 | 569.8225779 |
| **Igfbp6** | 4.578482408 | 3.961310043 | 3.915806472 | 1.725218839 | 6.966995687 | 1.402825611 | 5.412388289 | 5.866518091 | 1.504828245 | 1.747803938 | 13.24218978 | 6.072696744 | 0.852084315 | 0.903554652 | 4.311394032 |
| **Soat2** | 212.4415837 | 167.1672838 | 207.537743 | 150.094039 | 164.0410803 | 189.3814575 | 144.3303544 | 147.5010263 | 154.2448951 | 162.5457662 | 132.4218978 | 162.2277559 | 161.0439356 | 198.7820233 | 209.1026105 |
| **Csad** | 86.07546927 | 93.486917 | 86.14774239 | 69.00875357 | 60.16950821 | 89.07942633 | 74.87137133 | 93.86428946 | 76.7462405 | 97.00311855 | 76.6155266 | 83.2826982 | 70.72299816 | 107.5230035 | 63.23377913 |
| **Zfp740** | 1282.890771 | 1391.212087 | 1348.603749 | 1349.983742 | 1356.664069 | 1263.945876 | 1292.658736 | 1304.881238 | 1330.268169 | 1161.415717 | 1286.38415 | 1366.356767 | 1369.299494 | 1322.80401 | 1411.26298 |
| **Itgb7** | 1935.782362 | 2498.794375 | 2364.363948 | 2492.078613 | 2456.182661 | 2680.799743 | 2644.853744 | 2508.355521 | 2707.938427 | 2266.027805 | 2201.041116 | 2998.177135 | 2752.232338 | 2685.364424 | 2729.830988 |
| **Rarg** | 232.5869063 | 262.2387248 | 234.9483883 | 269.9967484 | 274.879648 | 272.1481686 | 285.0524499 | 388.028268 | 309.9946185 | 273.5313163 | 231.7383212 | 288.0193313 | 320.3837025 | 309.0156908 | 261.5579046 |
| **Mfsd5** | 1193.152515 | 1186.016227 | 1235.828523 | 1206.790578 | 1061.516797 | 1238.695015 | 1192.529553 | 1218.559615 | 1195.586041 | 1310.852953 | 1173.825537 | 1129.521594 | 1207.403475 | 1165.585501 | 1146.830812 |
| **Espl1** | 2526.406593 | 2764.99441 | 2795.885821 | 2888.878946 | 2575.254951 | 2516.669147 | 2724.235439 | 2867.889273 | 2773.398456 | 2931.941106 | 2913.281752 | 2921.834662 | 2897.938756 | 2856.136254 | 2957.616306 |
| **Pfdn5** | 1298.457611 | 1319.116244 | 1324.325749 | 1337.90721 | 1449.768466 | 1407.034088 | 1473.071679 | 1238.673391 | 1273.83711 | 1274.149071 | 1190.85121 | 1508.631377 | 1524.37884 | 1449.301661 | 1394.735969 |
| **Myg1** | 1025.580059 | 1029.148349 | 1032.989747 | 938.5190486 | 996.9137465 | 969.3524975 | 993.173251 | 944.5094127 | 1053.379772 | 936.8229107 | 944.9248281 | 912.6395678 | 937.2927467 | 930.6612911 | 945.6324243 |
| **Aaas** | 1684.881526 | 1630.475214 | 1681.447299 | 1520.780407 | 1394.665864 | 1298.315103 | 1363.019784 | 1732.298985 | 1678.635907 | 1776.642703 | 1739.456215 | 1491.280815 | 1524.37884 | 1485.443847 | 1455.814051 |
| **Sp1** | 2520.912414 | 2540.784261 | 2630.638788 | 2536.934303 | 2404.246875 | 2279.591619 | 2735.060215 | 2551.93537 | 2680.851519 | 2469.646964 | 2515.070188 | 2535.784655 | 2831.476179 | 2609.465834 | 2549.471004 |
| **Amhr2** | 0 | 0 | 0 | 0 | 0 | 0.701412806 | 0.902064715 | 4.190370065 | 0 | 0 | 0 | 0 | 0 | 0 | 0 |
| **Prr13** | 1983.398579 | 1910.143703 | 1878.803945 | 1937.420757 | 1696.780131 | 1817.36058 | 1787.892265 | 1924.217934 | 1783.973885 | 1812.472684 | 1787.695621 | 1685.607111 | 1679.458185 | 1786.327546 | 1730.306138 |
| **Pcbp2** | 6744.104587 | 6282.637727 | 6623.978229 | 6475.608913 | 6227.227418 | 6232.052779 | 6285.586933 | 6741.467361 | 6375.204861 | 6289.47247 | 6474.484933 | 6201.958432 | 6238.961356 | 6258.019517 | 6224.934416 |
| **Map3k12** | 45.78482408 | 80.81072487 | 53.25496803 | 64.69570647 | 63.33632443 | 49.80030921 | 59.53627118 | 83.80740131 | 66.21244279 | 61.17313782 | 85.12836289 | 52.05168638 | 57.94173343 | 66.86304422 | 57.48525376 |
| **Tarbp2** | 594.2870165 | 676.5917553 | 673.5187133 | 630.5674858 | 506.0572322 | 600.4093617 | 530.4140523 | 884.1680838 | 866.7810692 | 835.4502823 | 805.8818354 | 612.474843 | 573.4527441 | 650.5593491 | 633.7749227 |
| **Npff** | 9.156964816 | 15.05297816 | 3.915806472 | 6.038265938 | 3.166816221 | 7.014128057 | 12.62890601 | 12.5711102 | 13.54345421 | 10.48682363 | 2.837612096 | 9.542809169 | 5.112505891 | 11.74621047 | 15.08987911 |
| **Atf7** | 771.0164375 | 760.5715282 | 895.1533596 | 765.9971647 | 716.3338293 | 681.0718343 | 809.1520492 | 826.3409769 | 744.1375672 | 803.9898114 | 751.0213348 | 746.0741714 | 741.3133542 | 653.2700131 | 681.200257 |
| **Atp5g2** | 3137.176146 | 2818.075964 | 3037.882661 | 2814.694536 | 2901.437022 | 2860.361422 | 3072.432419 | 2800.005278 | 2683.108761 | 2682.005143 | 2568.038947 | 2847.227245 | 2907.311683 | 2973.598358 | 2818.214565 |
| **Calcoco1** | 241.7438711 | 246.3934846 | 250.6116142 | 269.9967484 | 226.1106782 | 193.5899344 | 270.6194144 | 216.2230954 | 230.2387215 | 229.8362178 | 198.6328467 | 288.8868594 | 227.5065121 | 261.1272943 | 222.0367926 |
| **Smug1** | 65.01445019 | 99.82501307 | 81.44877463 | 62.10787821 | 83.60394824 | 65.93280374 | 73.96930661 | 91.35006742 | 97.06142181 | 81.27288311 | 99.31642337 | 76.34247335 | 81.80009426 | 63.24882561 | 63.9523448 |
| **Cbx5** | 4485.081367 | 4689.398828 | 5046.691382 | 4540.775985 | 4360.072574 | 4152.36381 | 4676.303481 | 4870.04809 | 4950.132512 | 4710.331612 | 4967.71291 | 4750.58391 | 4950.609871 | 4599.996731 | 4537.023653 |
| **Hnrnpa1** | 46.70052056 | 68.92679474 | 65.00238744 | 46.58090866 | 63.96968767 | 42.78618115 | 44.20117102 | 69.56014308 | 38.37312025 | 66.41654964 | 49.18527634 | 78.07752957 | 60.49798638 | 64.15238026 | 40.9582433 |
| **Gm5643** | 18.31392963 | 6.338096068 | 9.397935534 | 5.175656518 | 12.66726489 | 24.5494482 | 27.96400616 | 8.380740131 | 18.81035306 | 10.48682363 | 10.40457769 | 8.675281063 | 10.22501178 | 15.36042908 | 7.904222392 |
| **Nfe2** | 0 | 2.376786026 | 3.915806472 | 8.626094197 | 6.966995687 | 1.402825611 | 10.82477658 | 0.838074013 | 9.781383594 | 3.495607876 | 0.945870699 | 2.602584319 | 1.70416863 | 0 | 6.467091048 |
| **Copz1** | 1962.33756 | 1983.824069 | 1956.336914 | 2038.346059 | 1998.261036 | 1841.208615 | 1922.299907 | 2052.443258 | 2064.624352 | 2035.317686 | 2271.035548 | 2018.737903 | 2033.92526 | 2154.977844 | 2090.30754 |
| **Zfp385a** | 17.39823315 | 19.80655021 | 41.50754861 | 36.22959563 | 13.30062813 | 5.611302446 | 15.33510015 | 119.0065099 | 93.2993512 | 101.3726284 | 81.34488009 | 18.21809023 | 14.48543336 | 29.8173035 | 7.904222392 |
| **Itga5** | 146.5114371 | 151.3220436 | 144.8848395 | 117.3148811 | 81.70385851 | 101.7048568 | 149.7427427 | 137.4441381 | 141.453855 | 78.6511772 | 85.12836289 | 101.5007884 | 105.6584551 | 118.3656594 | 104.1920224 |
| **Nckap1l** | 4078.512129 | 4184.727929 | 4079.487183 | 3986.118128 | 3860.348974 | 3851.457716 | 4247.822742 | 4044.545187 | 4182.670107 | 3894.981075 | 4111.699928 | 4047.018616 | 4140.277687 | 3969.315584 | 4018.219238 |
| **Pde1b** | 42.12203815 | 58.62738863 | 51.68864544 | 45.71829924 | 57.00269198 | 48.39748359 | 45.10323574 | 46.93214473 | 48.90691797 | 50.6863142 | 46.34766424 | 52.05168638 | 64.75840795 | 56.92394305 | 68.98230451 |
| **Ppp1r1a** | 0.915696482 | 0 | 1.566322589 | 5.175656518 | 0 | 0.701412806 | 0 | 0.838074013 | 0 | 1.747803938 | 3.783482795 | 0.867528106 | 0.852084315 | 0 | 0.718565672 |
| **Zfp263** | 476.1621704 | 569.6363841 | 527.0675512 | 458.0456018 | 487.6896981 | 425.7575731 | 538.5326347 | 449.207671 | 454.45813 | 498.1241223 | 460.6390303 | 466.7301212 | 486.540144 | 411.1173665 | 465.6305554 |
| **Zfp174** | 1.831392963 | 7.130358077 | 10.18109683 | 9.488703616 | 3.166816221 | 2.805651223 | 8.118582433 | 5.866518091 | 9.781383594 | 6.991215751 | 10.40457769 | 4.337640531 | 9.372927467 | 6.324882561 | 7.18565672 |
| **Zfp597** | 118.1248461 | 155.2833537 | 131.5710975 | 108.6887869 | 143.7734565 | 119.240177 | 147.0365485 | 141.6345082 | 142.2062692 | 138.0765111 | 173.0943379 | 139.6720251 | 117.5876355 | 136.4367524 | 122.1561642 |
| **Naa60** | 902.8767308 | 920.6084539 | 984.4337472 | 928.1677355 | 1022.248276 | 949.7129389 | 899.3585206 | 974.6800772 | 953.3086933 | 927.209989 | 993.1642337 | 966.4263104 | 990.9740585 | 997.5243353 | 1036.890265 |
| **1700037C18Rik** | 34.7964663 | 37.2363144 | 44.64019379 | 26.74089201 | 41.16861088 | 38.57770431 | 34.27845916 | 43.57984868 | 48.90691797 | 26.21705907 | 69.04856101 | 57.25685501 | 50.27297459 | 54.21327909 | 47.42533435 |
| **Cluap1** | 304.9269284 | 335.1268296 | 336.7593566 | 345.9063773 | 325.5487076 | 306.5173961 | 368.0424036 | 354.5053075 | 326.5477292 | 373.1561407 | 298.8951408 | 333.1307928 | 276.9274024 | 282.8126059 | 291.0190971 |
| **Nlrc3** | 597.034106 | 733.6346199 | 774.5465202 | 720.2788654 | 640.33024 | 620.7503331 | 672.9402772 | 755.1046858 | 756.1761932 | 679.8957318 | 746.2919813 | 734.796306 | 725.9758365 | 684.8944259 | 740.1226421 |
| **Slx4** | 612.6009462 | 694.0215194 | 598.335229 | 580.5361394 | 534.5585782 | 610.229141 | 632.3473651 | 683.0303206 | 686.9540939 | 618.722594 | 636.5709803 | 577.7737188 | 650.1403325 | 682.1837619 | 602.1580331 |
| **Dnase1** | 20.14532259 | 26.14464628 | 30.54329049 | 16.38957897 | 10.76717515 | 18.23673295 | 21.64955315 | 32.68488651 | 31.60139315 | 32.33437285 | 59.58985402 | 45.97898963 | 23.85836082 | 28.0101942 | 37.36541494 |
| **Trap1** | 2339.60451 | 2435.413414 | 2352.616529 | 2316.968901 | 2173.702654 | 2142.114709 | 2179.388351 | 2474.832561 | 2480.709362 | 2713.465613 | 2835.720355 | 2250.367908 | 2261.431772 | 2358.277641 | 2176.53542 |
| **Crebbp** | 1051.219561 | 1149.572174 | 1026.724457 | 1108.453104 | 901.9092598 | 948.3101133 | 1160.957288 | 1056.81133 | 1128.621184 | 894.0017142 | 996.0018458 | 1135.594291 | 1190.361788 | 968.6105865 | 1027.548911 |
| **Adcy9** | 10.98835778 | 39.61310043 | 11.74741942 | 19.84001665 | 13.30062813 | 17.53532014 | 23.45368258 | 30.17066447 | 12.03862596 | 20.09974528 | 4.729353494 | 12.14539349 | 34.93545692 | 13.55331977 | 22.9941015 |
| **Srl** | 33.88076982 | 30.10595632 | 25.84432272 | 27.60350143 | 12.03390164 | 27.35509942 | 34.27845916 | 20.11377631 | 29.34415078 | 32.33437285 | 31.21373306 | 19.95314644 | 15.33751767 | 22.58886629 | 32.33545524 |
| **Tfap4** | 636.4090547 | 560.921502 | 615.5647775 | 621.9413916 | 581.4274582 | 543.5949244 | 612.5019413 | 623.5270657 | 622.2464794 | 511.2326518 | 520.2288843 | 511.8415827 | 567.4881539 | 572.8536491 | 599.2837704 |
| **Glis2** | 50.36330649 | 28.52143231 | 35.24225825 | 22.42784491 | 31.66816221 | 32.26498906 | 13.53097072 | 42.74177467 | 49.65933209 | 31.46047088 | 56.75224193 | 32.96606804 | 40.90004713 | 36.14218606 | 40.9582433 |
| **Pam16** | 551.2492819 | 460.3042269 | 561.5266481 | 466.671696 | 437.0206386 | 503.6143945 | 448.3261632 | 429.0938947 | 534.9664412 | 513.8543577 | 403.8867884 | 444.1743904 | 450.7526027 | 439.1275607 | 432.5765345 |
| **Coro7** | 1113.486922 | 1113.920384 | 1163.777684 | 1194.714046 | 1223.024425 | 1231.680887 | 1367.530108 | 935.2905986 | 941.2700673 | 907.1102437 | 935.4661211 | 1341.198452 | 1341.180712 | 1303.829362 | 1425.634293 |
| **Vasn** | 54.94178889 | 68.13453273 | 54.03812932 | 69.00875357 | 71.5700466 | 40.68194273 | 53.22181817 | 38.5514046 | 27.08690841 | 35.82998073 | 36.88895725 | 70.26977661 | 55.38548049 | 51.50261514 | 68.98230451 |
| **Dnaja3** | 1123.559583 | 1182.847179 | 1231.129555 | 1174.01142 | 1197.056532 | 1153.122653 | 1174.488259 | 1242.863761 | 1237.721232 | 1228.706168 | 1177.60902 | 1096.555526 | 1148.609657 | 1248.712528 | 1115.213923 |
| **Nmral1** | 339.7233947 | 335.9190916 | 352.4225825 | 325.2037512 | 378.1178568 | 367.5403102 | 391.4960862 | 267.3456102 | 252.0587311 | 257.8010808 | 272.4107612 | 368.6994452 | 300.7857633 | 377.6858444 | 356.4085733 |
| **Hmox2** | 533.8510488 | 629.8482968 | 640.6259389 | 523.6039177 | 591.5612702 | 561.1302446 | 598.0689059 | 594.1944753 | 547.7574812 | 598.6228487 | 559.9554537 | 627.2228208 | 576.8610814 | 708.3868468 | 571.9782749 |
| **Cdip1** | 380.9297363 | 362.8559999 | 404.8943892 | 349.356815 | 383.1847628 | 324.754129 | 388.7898921 | 417.3608585 | 370.9401624 | 344.3173757 | 329.1630032 | 366.964389 | 351.0587378 | 370.4574071 | 372.2170181 |
| **4930562C15Rik** | 1.831392963 | 1.584524017 | 0.783161294 | 0 | 1.900089733 | 3.507064029 | 0.902064715 | 0 | 8.276555348 | 0 | 1.891741398 | 0.867528106 | 2.556252945 | 0.903554652 | 5.029959704 |
| **Ubald1** | 586.0457482 | 686.8911614 | 647.6743905 | 728.9049596 | 672.6317654 | 694.3986777 | 728.8682895 | 512.063222 | 556.7864507 | 523.4672794 | 521.174755 | 684.4796759 | 675.7028619 | 761.6965713 | 664.6732466 |
| **Mgrn1** | 274.7089445 | 248.7702707 | 274.8896144 | 236.354981 | 292.6138189 | 281.2665351 | 315.7226502 | 258.126796 | 259.5828723 | 235.0796296 | 227.9548384 | 296.6946123 | 293.9690887 | 309.9192455 | 299.6418852 |
| **Gm16861** | 1.831392963 | 3.961310043 | 0.783161294 | 9.488703616 | 6.333632443 | 2.805651223 | 5.412388289 | 5.866518091 | 2.257242368 | 5.243411813 | 0.945870699 | 1.735056213 | 9.372927467 | 0.903554652 | 1.437131344 |
| **Nudt16l1** | 364.4471997 | 422.2756505 | 392.3638085 | 328.6541889 | 396.4853909 | 397.7010608 | 380.6713096 | 335.2296052 | 306.2325479 | 345.1912777 | 366.0519604 | 365.2293327 | 404.7400497 | 390.3356095 | 384.4326345 |
| **Anks3** | 181.3079033 | 201.2345502 | 188.741872 | 181.1479781 | 195.7092425 | 210.4238417 | 192.1397842 | 212.8707993 | 246.7918322 | 185.2672174 | 238.3594161 | 205.6041612 | 273.5190652 | 181.614485 | 206.2283479 |
| **4930451G09Rik** | 0 | 1.584524017 | 3.132645178 | 12.07653188 | 1.266726489 | 0.701412806 | 1.80412943 | 0.838074013 | 1.504828245 | 6.117313782 | 0.945870699 | 1.735056213 | 3.408337261 | 0 | 2.155697016 |
| **Rogdi** | 81.49698686 | 96.65596504 | 81.44877463 | 87.9861608 | 84.87067473 | 102.4062696 | 94.71679505 | 132.4156941 | 78.25106875 | 160.7979623 | 110.6668718 | 98.89820412 | 79.24384131 | 143.6651896 | 113.5333762 |
| **Glyr1** | 2574.02281 | 2841.843825 | 3000.290919 | 2726.708376 | 2692.427151 | 2593.824556 | 2680.936332 | 3157.024807 | 2984.826824 | 2985.249126 | 3124.210918 | 2914.026909 | 2867.263721 | 3083.832026 | 2776.537756 |
| **Ubn1** | 1996.21833 | 2131.184803 | 2185.803173 | 2361.824591 | 1915.290451 | 1968.865746 | 2099.104591 | 1977.016597 | 2208.33545 | 2045.804509 | 2153.747581 | 2280.731391 | 2140.4358 | 2043.840622 | 2109.708813 |
| **Ppl** | 0 | 2.376786026 | 0 | 0 | 0 | 0 | 0 | 0 | 0 | 0 | 5.675224193 | 3.470112425 | 0 | 1.807109303 | 0 |
| **Nagpa** | 522.862691 | 512.5935195 | 490.2589703 | 586.5744054 | 502.2570527 | 494.496028 | 492.5273343 | 658.7261743 | 612.4650958 | 640.5701432 | 647.9214287 | 458.9223682 | 590.4944304 | 560.203884 | 503.714536 |
| **AU021092** | 0 | 0 | 0 | 0 | 3.166816221 | 0 | 0 | 0 | 0 | 0 | 0 | 0 | 0 | 0 | 0 |
| **Alg1** | 292.1071776 | 298.6827772 | 306.9992274 | 288.1115462 | 274.879648 | 310.0244601 | 263.4028967 | 318.468125 | 337.0815269 | 327.7132383 | 286.5988217 | 294.092028 | 285.4482456 | 318.954792 | 308.9832389 |
| **Eef2kmt** | 174.898028 | 217.8720523 | 237.2978722 | 220.8280114 | 191.909063 | 187.9786319 | 175.0005547 | 158.3959885 | 221.209752 | 246.4403552 | 215.6585193 | 180.4458461 | 172.973116 | 243.0562013 | 188.9827717 |
| **Mettl22** | 224.345638 | 209.1571702 | 202.055614 | 223.4158397 | 212.1766868 | 204.8125393 | 202.9645608 | 197.7854671 | 182.0842177 | 218.4754922 | 188.2282691 | 209.0742736 | 206.2044043 | 186.1322582 | 198.3241255 |
| **Abat** | 2.747089445 | 0.792262009 | 5.482129061 | 0 | 3.800179466 | 1.402825611 | 0 | 0 | 0 | 0.873901969 | 0 | 3.470112425 | 4.260421576 | 9.035546516 | 6.467091048 |
| **Tmem186** | 351.6274489 | 394.5464802 | 397.0627763 | 351.0820338 | 377.4844936 | 328.2611931 | 359.9238212 | 459.2645592 | 439.4098476 | 449.185612 | 460.6390303 | 362.6267484 | 322.0878711 | 390.3356095 | 383.7140688 |
| **Pmm2** | 716.0746486 | 759.7792662 | 838.7657464 | 800.5015414 | 763.2027094 | 758.227243 | 696.3939598 | 988.9273354 | 866.7810692 | 992.7526367 | 949.6541816 | 829.3568696 | 753.2425346 | 832.1738341 | 739.4040765 |
| **Carhsp1** | 792.0774566 | 815.2376068 | 817.6203914 | 853.120716 | 832.8726662 | 829.7713492 | 861.4718026 | 745.8858716 | 879.5721093 | 783.8900661 | 784.1268093 | 933.4602424 | 892.132278 | 888.1942225 | 919.7640601 |
| **Usp7** | 4714.921184 | 4539.661309 | 4832.888348 | 4733.137886 | 4582.383072 | 4671.409286 | 4781.845053 | 4752.717728 | 4871.12903 | 4650.032377 | 5046.220178 | 4965.73088 | 4568.876098 | 4712.941063 | 4605.287392 |
| **1810013L24Rik** | 632.7462688 | 621.9256767 | 604.6005193 | 532.2300119 | 522.5246765 | 507.8228713 | 602.5792295 | 496.1398157 | 577.101632 | 505.98924 | 520.2288843 | 576.9061907 | 488.2443126 | 529.4830258 | 484.3132629 |
| **Rpl39l** | 20.14532259 | 10.29940611 | 8.614774239 | 8.626094197 | 5.066905954 | 12.6254305 | 1.80412943 | 5.028444078 | 3.00965649 | 34.95607876 | 19.86328467 | 0.867528106 | 5.112505891 | 0 | 10.77848508 |
| **Nubp1** | 780.1734023 | 819.1989168 | 770.6307138 | 860.0215914 | 751.1688077 | 828.3685235 | 763.1467487 | 859.8639374 | 733.6037695 | 873.9019689 | 716.9699897 | 681.8770915 | 749.8341973 | 727.3614945 | 768.865269 |
| **Tvp23a** | 4.578482408 | 7.922620085 | 3.132645178 | 5.175656518 | 2.533452977 | 4.208476834 | 2.706194144 | 4.190370065 | 0 | 6.117313782 | 1.891741398 | 1.735056213 | 4.260421576 | 13.55331977 | 7.18565672 |
| **Ciita** | 19.22962611 | 19.0142882 | 32.89277437 | 28.46611085 | 8.233722176 | 28.75792503 | 18.94335901 | 28.49451644 | 53.4214027 | 41.07339254 | 24.59263817 | 15.61550591 | 28.97086672 | 10.84265582 | 26.58692986 |
| **Dexi** | 230.7555134 | 246.3934846 | 258.4432272 | 298.4628592 | 264.1124729 | 238.4803539 | 222.8099846 | 358.6956776 | 343.853254 | 338.200062 | 358.4849948 | 264.5960724 | 207.9085729 | 259.320185 | 237.8452374 |
| **Clec16a** | 469.752295 | 451.5893448 | 498.8737446 | 471.8473525 | 390.7851217 | 380.1657407 | 490.7232048 | 522.1201101 | 556.0340366 | 573.2796916 | 540.092169 | 418.1485472 | 449.0484341 | 375.8787351 | 450.5406763 |
| **Socs1** | 1868.020822 | 1727.923441 | 1695.544203 | 1901.191161 | 1497.904073 | 1593.609895 | 1460.442773 | 1978.692745 | 1814.822864 | 1896.367273 | 1608.926059 | 1404.528004 | 1364.186989 | 1449.301661 | 1396.173101 |
| **Tnp2** | 106.2207919 | 82.39524888 | 50.12232285 | 79.36006661 | 75.37022607 | 71.54410618 | 73.0672419 | 47.77021874 | 82.76555348 | 55.05582404 | 58.64398332 | 51.18415827 | 74.13133542 | 59.634607 | 68.26373884 |
| **Prm2** | 3.662785926 | 3.961310043 | 3.915806472 | 0 | 0 | 1.402825611 | 1.80412943 | 0.838074013 | 0 | 0 | 4.729353494 | 2.602584319 | 0 | 0 | 0 |
| **Prm1** | 8.241268334 | 6.338096068 | 7.04845165 | 3.450437679 | 0.633363244 | 4.208476834 | 5.412388289 | 0 | 2.257242368 | 1.747803938 | 0 | 0.867528106 | 2.556252945 | 7.228437213 | 7.904222392 |
| **Rmi2** | 758.1966867 | 739.1804539 | 747.9190362 | 886.7624834 | 801.8378673 | 808.728965 | 744.2033897 | 905.1199341 | 832.1700196 | 782.1422622 | 888.1725861 | 814.6088918 | 771.9883895 | 774.3463364 | 773.8952287 |
| **Litaf** | 998.1091649 | 953.0911962 | 930.3956179 | 974.7486442 | 953.2116826 | 1008.631615 | 951.6782741 | 803.7129785 | 820.8838077 | 798.7463996 | 869.2551722 | 821.5491166 | 948.3698428 | 929.7577365 | 939.1653333 |
| **Snn** | 14.65114371 | 25.35238427 | 28.1938066 | 22.42784491 | 36.73506817 | 22.44520978 | 31.57226502 | 27.65644243 | 33.10622139 | 46.31680435 | 30.26786236 | 27.7608994 | 28.97086672 | 31.62441281 | 31.61688957 |
| **Txndc11** | 740.7984536 | 793.8465325 | 802.7403268 | 768.5849929 | 758.7691666 | 815.743093 | 797.4252079 | 816.2840887 | 790.7872428 | 825.8373606 | 891.0101982 | 783.37788 | 746.4258601 | 765.3107899 | 807.6678153 |
| **Zc3h7a** | 892.8040695 | 952.2989342 | 845.0310368 | 853.9833255 | 855.673743 | 845.9038437 | 938.1473034 | 838.9120871 | 720.8127294 | 873.0280669 | 887.2267154 | 926.5200175 | 921.955229 | 876.448012 | 916.1712318 |
| **Rsl1d1** | 5346.751756 | 4918.362549 | 5168.864544 | 4917.736301 | 4601.38397 | 4594.253877 | 4846.793713 | 5734.940471 | 5333.863715 | 5596.468209 | 5622.255433 | 4969.200993 | 4708.617926 | 4703.905516 | 4682.892484 |
| **2610020C07Rik** | 11.90405426 | 12.67619214 | 8.614774239 | 11.21392246 | 20.26762382 | 16.13249453 | 10.82477658 | 18.43762829 | 12.79104008 | 14.85633347 | 5.675224193 | 15.61550591 | 12.78126473 | 12.64976512 | 5.748525376 |
| **Gspt1** | 5890.675466 | 5645.659073 | 5551.047255 | 5513.79941 | 5350.019324 | 5381.940458 | 5600.919814 | 6364.334055 | 5895.917065 | 6216.938607 | 6145.32193 | 5485.380216 | 5365.574933 | 5241.520534 | 5407.206682 |
| **Snx29** | 42.12203815 | 18.2220262 | 37.59174214 | 43.13047098 | 31.03479897 | 25.95227381 | 33.37639445 | 28.49451644 | 33.10622139 | 37.57778466 | 52.02288843 | 31.23101183 | 28.97086672 | 28.91374885 | 42.39537465 |
| **Cpped1** | 246.3223535 | 293.1369431 | 332.0603889 | 289.836765 | 273.6129215 | 249.001546 | 285.9545146 | 328.5250131 | 330.3097998 | 349.5607876 | 301.7327529 | 300.1647248 | 283.7440769 | 283.7161606 | 330.5402091 |
| **Ercc4** | 412.9791132 | 493.5792313 | 458.1493573 | 417.5029591 | 428.7869164 | 424.3547475 | 391.4960862 | 526.3104802 | 446.1815747 | 442.1943963 | 412.3996247 | 454.5847277 | 396.2192066 | 458.1022083 | 368.6241897 |
| **Mkl2** | 343.3861806 | 362.0637379 | 306.9992274 | 332.1046266 | 305.2810837 | 308.6216345 | 396.0064098 | 269.8598322 | 252.0587311 | 255.1793749 | 256.3309594 | 343.5411301 | 403.0358811 | 366.8431885 | 388.0254629 |
| **Parn** | 915.6964816 | 959.4292923 | 893.587037 | 925.5799073 | 855.0403798 | 876.0645943 | 914.6936208 | 907.6341561 | 945.032138 | 906.2363418 | 927.8991555 | 870.9982187 | 857.1968211 | 861.0875829 | 857.2488467 |
| **Bfar** | 555.8277643 | 511.8012575 | 521.5854221 | 521.8786989 | 561.7931977 | 549.9076397 | 526.8057934 | 553.1288486 | 463.4870995 | 558.4233581 | 478.6105736 | 512.7091108 | 541.9256244 | 515.929706 | 459.1634644 |
| **3110001I22Rik** | 55.85748538 | 60.21191265 | 75.18348427 | 82.81050429 | 68.40323038 | 63.12715251 | 64.94865946 | 61.17940295 | 63.95520042 | 61.17313782 | 87.96597499 | 63.32955176 | 65.61049227 | 65.05593491 | 76.16796123 |
| **Rrn3** | 1780.11396 | 1700.19427 | 1705.725299 | 1730.394496 | 1603.675735 | 1539.601109 | 1685.056887 | 2095.185033 | 1872.006337 | 1882.384841 | 1908.76707 | 1678.666886 | 1619.812283 | 1576.702867 | 1622.521287 |
| **Ntan1** | 636.4090547 | 575.9744802 | 574.8403902 | 513.2526047 | 557.359655 | 542.8935116 | 606.1874883 | 465.1310772 | 529.6995423 | 454.4290238 | 492.7986341 | 590.7866404 | 562.375648 | 538.5185723 | 510.9001928 |
| **Pdxdc1** | 665.7113421 | 646.4857989 | 675.8681971 | 628.8422669 | 542.1589371 | 582.8740415 | 621.5225885 | 716.5532812 | 784.7679299 | 784.7639681 | 781.2891972 | 586.4489998 | 604.9798638 | 673.1482154 | 626.589266 |
| **Mpv17l** | 6.409875371 | 11.09166812 | 9.397935534 | 3.450437679 | 5.066905954 | 6.312715251 | 0.902064715 | 13.40918421 | 6.019312981 | 5.243411813 | 8.512836289 | 1.735056213 | 3.408337261 | 6.324882561 | 4.311394032 |
| **2900011O08Rik** | 3.662785926 | 3.169048034 | 2.349483883 | 1.725218839 | 0 | 0.701412806 | 0 | 0 | 0 | 5.243411813 | 2.837612096 | 0 | 0 | 0 | 0 |
| **Marf1** | 1037.484114 | 1258.11207 | 1148.114458 | 1117.079198 | 1159.054737 | 1038.792365 | 1157.349029 | 1176.655914 | 1085.733579 | 1098.494775 | 1151.12464 | 1250.108001 | 1175.876355 | 1133.961088 | 1090.064124 |
| **Nde1** | 1508.152105 | 1639.190096 | 1761.329751 | 1582.025676 | 1596.075376 | 1631.486186 | 1590.340092 | 1904.104158 | 1805.793894 | 1898.988978 | 1784.858009 | 1606.662053 | 1692.23945 | 1729.403603 | 1734.617532 |
| **Myh11** | 10.98835778 | 11.09166812 | 7.04845165 | 7.763484777 | 6.966995687 | 3.507064029 | 3.608258859 | 10.05688816 | 3.762070613 | 21.84754922 | 11.35044839 | 6.072696744 | 5.112505891 | 1.807109303 | 5.748525376 |
| **Fopnl** | 467.9209021 | 427.0292226 | 448.7514217 | 457.1829924 | 449.0545402 | 448.9041957 | 451.0323574 | 486.0829276 | 510.1367751 | 533.080201 | 466.3142545 | 452.8496715 | 425.1900733 | 511.4119328 | 456.2892017 |
| **Abcc1** | 2150.971035 | 1899.052034 | 1976.699107 | 1871.862441 | 1552.373312 | 1714.95431 | 1825.778983 | 1827.839422 | 1626.719333 | 1557.293309 | 1720.538801 | 1562.418119 | 1781.708303 | 1527.007361 | 1840.965252 |
| **Snai2** | 0.915696482 | 3.169048034 | 3.132645178 | 6.900875357 | 0 | 1.402825611 | 1.80412943 | 4.190370065 | 0 | 0 | 0.945870699 | 0 | 0 | 0 | 0.718565672 |
| **Ube2v2** | 407.4849343 | 376.324454 | 443.2692927 | 356.2576903 | 423.7200104 | 393.492584 | 442.913775 | 522.1201101 | 502.6126339 | 466.6636514 | 460.6390303 | 395.5928165 | 503.5818303 | 419.2493583 | 389.4625942 |
| **Mcm4** | 5827.492409 | 6053.674007 | 6398.427776 | 6105.549472 | 5973.248757 | 5766.314676 | 5897.699105 | 6976.128085 | 6527.192513 | 6247.525176 | 6823.511221 | 6195.018207 | 6075.361167 | 6126.100538 | 5890.082813 |
| **Prkdc** | 461.5110267 | 459.5119649 | 451.1009056 | 461.4960395 | 392.6852115 | 375.9572639 | 480.800493 | 469.3214473 | 531.2043705 | 473.6548671 | 500.3655996 | 432.896525 | 403.8879654 | 374.9751804 | 444.7921509 |

| | | | | | | | | | | | | | | | |
|---|---|---|---|---|---|---|---|---|---|---|---|---|---|---|---|
| **Mzt2** | 336.9763052 | 383.4548121 | 319.5298082 | 355.3950809 | 329.9822503 | 376.6586767 | 316.6247149 | 391.3805641 | 404.0463838 | 388.8863762 | 375.5106674 | 376.5071981 | 334.8691359 | 364.1325246 | 326.2288151 |
| **Cebpd** | 10.98835778 | 6.338096068 | 28.1938066 | 4.313047098 | 6.333632443 | 17.53532014 | 8.118582433 | 3.352296052 | 21.06759543 | 11.3607256 | 15.13393118 | 6.94022485 | 5.112505891 | 2.710663955 | 2.874262688 |
| **Spidr** | 322.3251615 | 282.045275 | 287.4201951 | 285.5237179 | 267.9126523 | 240.5845924 | 264.3049614 | 258.96487 | 256.5732158 | 289.2615517 | 250.6557352 | 312.3101183 | 275.2232338 | 253.8988571 | 306.8275419 |
| **Pkp2** | 43.95343112 | 28.52143231 | 39.94122602 | 21.56523549 | 22.80107679 | 25.25086101 | 30.6702003 | 5.866518091 | 8.276555348 | 16.60413741 | 16.07980188 | 36.43618046 | 28.1187824 | 27.10663955 | 17.9641418 |
| **Yars2** | 638.2404477 | 655.200681 | 680.5671649 | 589.1622336 | 620.0626161 | 692.2944392 | 617.0122649 | 734.9909094 | 673.4106397 | 825.8373606 | 770.8846195 | 597.7268652 | 680.8153678 | 659.5948956 | 610.7808212 |
| **Dnm1l** | 1470.608549 | 1353.975773 | 1453.547363 | 1317.204584 | 1360.464249 | 1356.532366 | 1352.195007 | 1396.231306 | 1342.306795 | 1456.794582 | 1409.347341 | 1388.04497 | 1462.176685 | 1416.773694 | 1415.574374 |
| **Spag6** | 0 | 0 | 2.349483883 | 0 | 0.633363244 | 0 | 0.902064715 | 0 | 0 | 0 | 1.891741398 | 0 | 0 | 0 | 0 |
| **Rpl31-ps12** | 7.325571853 | 0 | 4.698967767 | 0.86260942 | 6.333632443 | 9.81977928 | 5.412388289 | 3.352296052 | 3.762070613 | 6.991215751 | 0.945870699 | 2.602584319 | 3.408337261 | 0 | 5.748525376 |
| **Vpreb1** | 0 | 0 | 2.349483883 | 0 | 0.633363244 | 0 | 2.706194144 | 0.838074013 | 3.762070613 | 0.873901969 | 0 | 0.867528106 | 0 | 5.421327909 | 2.874262688 |
| **Top3b** | 556.7434608 | 569.6363841 | 598.335229 | 524.4665271 | 544.0590268 | 556.9217677 | 609.7957472 | 638.6123979 | 586.1306015 | 617.848692 | 536.3086862 | 589.9191123 | 615.2048755 | 566.5287665 | 620.8407406 |
| **Ppm1f** | 1460.535888 | 1525.104366 | 1676.748331 | 1583.750894 | 1572.640936 | 1575.373162 | 1643.56191 | 1789.288018 | 1678.635907 | 1804.607566 | 1820.801095 | 1552.007782 | 1534.603852 | 1520.682479 | 1697.970683 |
| **Mapk1** | 4847.697173 | 4666.42323 | 4819.574606 | 4744.351808 | 4423.408898 | 4810.289022 | 4815.221447 | 4884.295348 | 4869.624201 | 4830.930084 | 4818.26534 | 4891.990991 | 4884.147295 | 4642.4638 | 4749.000526 |
| **Ypel1** | 2.747089445 | 3.169048034 | 7.04845165 | 2.587828259 | 3.800179466 | 9.118366474 | 10.82477658 | 10.05688816 | 3.762070613 | 6.117313782 | 19.86328467 | 3.470112425 | 11.92918041 | 15.36042908 | 2.874262688 |
| **Ppil2** | 1114.402618 | 1165.417415 | 1199.803103 | 1177.461858 | 1138.15375 | 1113.142123 | 1177.194453 | 1086.143921 | 1100.029447 | 1210.354227 | 1159.637477 | 1145.1371 | 1157.982584 | 1143.900189 | 1097.249781 |
| **2610318N02Rik** | 287.5286952 | 310.5667073 | 296.8181306 | 338.1428925 | 342.0161519 | 426.4589859 | 360.8258859 | 269.8598322 | 247.5442463 | 309.361297 | 299.8410115 | 346.1437144 | 357.8754124 | 327.0867839 | 396.6482509 |
| **Mir130b** | 1.831392963 | 0 | 0 | 0 | 0 | 0 | 0.902064715 | 0.838074013 | 0 | 0 | 2.837612096 | 0.867528106 | 0 | 1.807109303 | 0.718565672 |
| **Sdf2l1** | 1485.259693 | 1449.047214 | 1430.052524 | 1454.359482 | 1334.496356 | 1377.57475 | 1228.612142 | 1623.349363 | 1633.49106 | 1690.126408 | 1808.504776 | 1305.6298 | 1269.60563 | 1279.433387 | 1266.83128 |
| **Ccdc116** | 124.5347215 | 96.65596504 | 93.97935534 | 83.67311371 | 89.30421744 | 84.87094949 | 79.3816949 | 117.3303618 | 154.9973093 | 134.5809032 | 122.9631908 | 89.35539495 | 91.17302172 | 109.3301128 | 117.1262045 |
| **Ydjc** | 527.4411734 | 503.0863754 | 472.2462606 | 490.8247598 | 425.6201002 | 411.729317 | 364.4341448 | 433.2842647 | 485.3071091 | 548.8104365 | 500.3655996 | 402.5330413 | 423.4859046 | 454.4879897 | 395.9296853 |
| **Ube2l3** | 4199.384064 | 3937.542182 | 4023.882731 | 3996.469441 | 3935.7192 | 3775.00372 | 4007.873528 | 4560.798779 | 4364.001911 | 4185.116529 | 4362.355663 | 4179.750416 | 4066.146352 | 4082.259916 | 4091.512936 |
| **Rimbp3** | 0.915696482 | 3.961310043 | 0 | 0.86260942 | 5.700269198 | 6.312715251 | 6.314453003 | 7.542666117 | 4.514484735 | 5.243411813 | 4.729353494 | 0 | 2.556252945 | 0.903554652 | 0.718565672 |
| **Hic2** | 73.25571853 | 82.39524888 | 66.56871003 | 79.36006661 | 53.83587576 | 86.2737751 | 88.40234205 | 68.72206907 | 56.43105919 | 87.39019689 | 71.88617311 | 70.26977661 | 57.08964912 | 56.0203884 | 72.57513287 |
| **Tmem191c** | 39.37494871 | 19.0142882 | 10.96425812 | 21.56523549 | 35.46834168 | 15.43108173 | 18.94335901 | 23.46607237 | 23.3248378 | 14.85633347 | 12.29631908 | 34.70112425 | 23.00627651 | 7.228437213 | 25.14979852 |
| **Pi4ka** | 2075.883924 | 2084.441344 | 2220.26227 | 2125.46961 | 2030.562561 | 1917.662611 | 2192.017257 | 2311.408128 | 2361.827931 | 2144.555432 | 2347.651074 | 2134.119141 | 2179.631678 | 2223.647998 | 2070.906267 |
| **Serpind1** | 0.915696482 | 0 | 1.566322589 | 0 | 0.633363244 | 0 | 2.706194144 | 2.514222039 | 3.00965649 | 0 | 5.675224193 | 0 | 0 | 0 | 0 |
| **Snap29** | 401.9907554 | 377.116716 | 410.3765183 | 416.6403497 | 403.4523866 | 398.4024736 | 400.5167334 | 380.4856019 | 330.3097998 | 387.1385722 | 435.1005214 | 336.6009052 | 426.8942419 | 395.7569374 | 412.4566957 |
| **Crkl** | 1754.474459 | 1817.449048 | 1804.403622 | 1808.891953 | 1653.078068 | 1647.618681 | 1833.897565 | 1752.412761 | 1856.20564 | 1794.994644 | 1891.741398 | 1751.539247 | 1759.554111 | 1762.835125 | 1649.108217 |
| **Lztr1** | 964.2283951 | 873.8649954 | 961.7220696 | 862.6094197 | 938.0109648 | 879.5716584 | 970.6216331 | 966.2993371 | 903.6493612 | 1084.512343 | 1000.731199 | 917.8447364 | 985.8615526 | 937.8897283 | 952.818081 |
| **Thap7** | 546.6707995 | 576.7667422 | 517.6696157 | 551.2074192 | 503.5237792 | 502.9129817 | 541.2388289 | 615.1463256 | 544.7478247 | 566.2884758 | 507.9325652 | 529.1921448 | 483.983891 | 580.985641 | 457.0077674 |
| **Slc7a4** | 0 | 2.376786026 | 2.349483883 | 0.86260942 | 3.166816221 | 3.507064029 | 0 | 0 | 6.771727103 | 0.873901969 | 2.837612096 | 2.602584319 | 0 | 0.903554652 | 1.437131344 |
| **Smpd4** | 1587.817699 | 1583.731755 | 1662.651428 | 1565.636097 | 1491.57044 | 1444.208967 | 1422.556055 | 1843.762829 | 1926.180154 | 1923.458234 | 1853.90657 | 1466.990028 | 1611.29144 | 1553.210446 | 1493.898032 |
| **Ccdc74a** | 6.409875371 | 6.338096068 | 2.349483883 | 5.175656518 | 4.43354271 | 11.22260489 | 4.510323574 | 3.352296052 | 3.762070613 | 10.48682363 | 12.29631908 | 6.072696744 | 10.22501178 | 7.228437213 | 7.904222392 |
| **Med15** | 1560.346805 | 1586.108541 | 1661.868267 | 1392.251603 | 1493.47053 | 1526.274265 | 1660.70114 | 1397.06938 | 1477.741337 | 1386.882425 | 1408.40147 | 1633.555424 | 1714.393642 | 1573.088648 | 1599.527186 |
| **Klhl22** | 170.3195456 | 183.804786 | 153.4996137 | 154.4070861 | 130.4728283 | 158.5192941 | 182.2170724 | 177.6716908 | 149.7304104 | 116.2289619 | 114.4503546 | 183.0484304 | 151.6710081 | 158.122064 | 186.108509 |
| **Scarf2** | 2.747089445 | 1.584524017 | 0 | 2.587828259 | 0.633363244 | 0 | 0 | 0 | 0 | 0 | 0 | 0.867528106 | 0.852084315 | 1.807109303 | 0 |
| **Car15** | 7.325571853 | 17.42976419 | 8.614774239 | 6.038265938 | 12.03390164 | 4.208476834 | 6.314453003 | 12.5711102 | 2.257242368 | 7.86511772 | 14.18806048 | 10.41033728 | 11.92918041 | 15.36042908 | 7.18565672 |
| **Dgcr2** | 1551.18984 | 1524.312104 | 1603.914331 | 1490.589077 | 1589.741743 | 1576.074574 | 1648.072234 | 1625.863585 | 1702.713159 | 1539.815269 | 1587.171033 | 1584.106322 | 1692.23945 | 1624.591264 | 1702.282077 |
| **Tssk1** | 2.747089445 | 2.376786026 | 0 | 0.86260942 | 0 | 0.701412806 | 0.902064715 | 0 | 0 | 0 | 0 | 0.867528106 | 0.852084315 | 0 | 0 |
| **Tssk2** | 0.915696482 | 0 | 0 | 2.587828259 | 0 | 0 | 0 | 5.028444078 | 0 | 1.747803938 | 0 | 0 | 7.668758836 | 0 | 0 |
| **Dgcr14** | 542.0923171 | 539.5304278 | 566.2256159 | 570.1848264 | 449.6879034 | 498.7045049 | 522.2954699 | 536.3673684 | 616.9795805 | 486.7633967 | 557.1178416 | 513.5766389 | 565.7839853 | 485.2088479 | 526.7086376 |
| **Slc25a1** | 1418.41385 | 1382.497205 | 1444.149427 | 1361.197664 | 1395.93259 | 1392.304419 | 1345.880554 | 1334.213829 | 1170.756375 | 1252.301521 | 1268.412607 | 1230.154855 | 1362.48282 | 1280.336941 | 1341.56211 |
| **Dgcr6** | 273.793248 | 300.2673012 | 269.4074853 | 288.9741556 | 264.1124729 | 305.8159833 | 282.3462557 | 257.288722 | 258.078044 | 242.0708454 | 279.9777268 | 288.8868594 | 275.2232338 | 262.030849 | 250.7794195 |
| **Prodh** | 18.31392963 | 25.35238427 | 32.89277437 | 25.01567317 | 30.40143573 | 11.9240177 | 22.55161787 | 21.78992434 | 26.33449429 | 16.60413741 | 14.18806048 | 19.95314644 | 23.00627651 | 18.07109303 | 30.17975822 |
| **4933432I09Rik** | 0.915696482 | 2.376786026 | 1.566322589 | 6.900875357 | 1.266726489 | 1.402825611 | 0 | 0 | 0 | 9.612921658 | 1.891741398 | 1.735056213 | 0 | 0.903554652 | 6.467091048 |
| **Zdhhc8** | 1672.061775 | 1605.915091 | 1841.212203 | 1720.043183 | 1471.93618 | 1540.302521 | 1492.917103 | 1852.143569 | 1704.217988 | 1718.965173 | 1866.202889 | 1460.049803 | 1482.626708 | 1513.454041 | 1603.120014 |
| **Ranbp1** | 5866.867357 | 5690.025745 | 5777.380869 | 5691.496951 | 5667.967673 | 5957.098959 | 5608.136332 | 6461.550641 | 6147.223381 | 6341.906588 | 6082.894464 | 5803.763031 | 5545.364723 | 5676.130321 | 5553.794079 |
| **Trmt2a** | 1143.704905 | 1258.11207 | 1300.83091 | 1205.065359 | 1091.28487 | 1216.249805 | 1194.333682 | 1368.574863 | 1227.939848 | 1264.536149 | 1279.763055 | 1190.248562 | 1200.5868 | 1096.915347 | 1194.974712 |
| **Dgcr8** | 922.1063569 | 1039.447755 | 996.1811666 | 924.7172979 | 913.9431615 | 930.0733804 | 1009.410416 | 1108.771919 | 1114.325316 | 1067.034304 | 1058.429312 | 1058.38429 | 1015.684504 | 960.4785946 | 991.6206273 |
| **Tango2** | 135.5230793 | 188.558358 | 149.5838072 | 150.094039 | 158.3408111 | 152.9079916 | 148.8406779 | 161.7482845 | 203.1518131 | 164.2935702 | 183.4989156 | 191.7237115 | 184.9022964 | 185.2287036 | 156.6473165 |
| **Arvcf** | 21.06101908 | 13.46845414 | 14.0969033 | 7.763484777 | 16.46744435 | 14.72966892 | 23.45368258 | 14.24725822 | 10.53379772 | 21.84754922 | 5.675224193 | 12.14539349 | 23.85836082 | 11.74621047 | 4.311394032 |
| **Comt** | 966.9754845 | 966.5596504 | 1038.471876 | 1001.489536 | 1010.847738 | 997.4090097 | 1035.570293 | 1128.047622 | 1009.739753 | 1050.430167 | 994.1101044 | 939.5329391 | 875.0905917 | 943.3110562 | 980.1235766 |
| **Txnrd2** | 288.4443917 | 259.0696768 | 278.8054208 | 252.74456 | 307.1811735 | 306.5173961 | 304.8978736 | 336.0676792 | 309.9946185 | 325.0915324 | 293.2199166 | 277.608994 | 302.4899319 | 253.8988571 | 303.2347136 |
| **Gnb1l** | 144.6800441 | 190.142882 | 175.42813 | 200.1253854 | 209.0098706 | 180.9645039 | 203.8666255 | 213.7088733 | 212.1807826 | 189.6367273 | 228.9007091 | 205.6041612 | 228.3585965 | 224.0815536 | 166.7072359 |
| **Tbx1** | 0.915696482 | 0 | 0 | 0 | 0 | 0.701412806 | 0 | 0 | 0 | 0.873901969 | 0 | 0 | 0 | 0 | 4.311394032 |
| **Sept5** | 9.156964816 | 14.26071615 | 19.57903236 | 13.80175071 | 4.43354271 | 4.208476834 | 7.216517718 | 10.89496217 | 9.781383594 | 13.10852953 | 18.91741398 | 3.470112425 | 3.408337261 | 7.228437213 | 7.904222392 |
| **Cdc45** | 1739.823315 | 1708.909152 | 1669.69988 | 1578.575238 | 1585.3082 | 1565.553382 | 1598.458675 | 1836.220163 | 1688.417291 | 1656.044231 | 1792.424974 | 1635.29048 | 1559.314297 | 1618.266381 | 1502.52082 |
| **Ufd1l** | 803.0658143 | 736.0114059 | 913.9492307 | 791.8754472 | 946.8780502 | 757.5258302 | 871.3945145 | 867.4066035 | 899.8872906 | 799.6203015 | 863.579948 | 828.4893415 | 857.1968211 | 853.8591457 | 820.6019974 |
| **2510002D24Rik** | 174.898028 | 187.766096 | 187.1755494 | 169.9340557 | 190.0089733 | 167.6376606 | 184.0212018 | 168.4528766 | 219.7049238 | 191.3845312 | 177.8236914 | 173.5056213 | 207.9085729 | 151.7971815 | 212.6954389 |
| **Mrpl40** | 802.1501179 | 822.3679648 | 892.0207144 | 830.6928711 | 786.0037861 | 808.728965 | 806.445855 | 867.4066035 | 947.2893803 | 910.6058516 | 923.169802 | 837.1646226 | 760.9112934 | 832.1738341 | 770.3024003 |
| **Hira** | 1671.146079 | 1820.618096 | 1839.645881 | 1776.112795 | 1720.214571 | 1629.381948 | 1825.778983 | 1795.99261 | 1957.029133 | 1801.98586 | 1989.16608 | 1842.629698 | 1710.133221 | 1809.819967 | 1799.288443 |
| **Lamp3** | 7.325571853 | 1.584524017 | 2.349483883 | 0 | 0 | 0.701412806 | 0 | 0 | 1.504828245 | 0 | 0 | 2.602584319 | 0 | 0 | 0 |
| **B3gnt5** | 128.1975074 | 122.8006113 | 129.2216136 | 130.2540224 | 137.439824 | 131.1641947 | 167.7840369 | 82.96932729 | 109.8524619 | 103.1204323 | 97.42468197 | 112.7786538 | 168.7126944 | 139.1474163 | 144.4317001 |
| **Klhl6** | 737.1356677 | 847.7203491 | 812.1382624 | 775.4858683 | 741.668359 | 650.2096709 | 737.8889367 | 753.4285377 | 695.2306493 | 769.9076346 | 824.7992493 | 786.8479924 | 823.1134484 | 814.1027411 | 714.2542779 |
| **Klhl24** | 130.0289004 | 199.6500261 | 222.4178076 | 166.483618 | 164.6744435 | 161.3249453 | 175.9026194 | 124.0349539 | 117.3766031 | 117.9767658 | 85.12836289 | 157.8901153 | 182.3460434 | 159.9291733 | 159.5215792 |
| **Yeats2** | 677.6153964 | 652.823895 | 642.1922615 | 684.9118792 | 631.4631545 | 613.736205 | 667.5278889 | 704.820245 | 731.3465271 | 677.2740259 | 751.9672055 | 663.6590013 | 758.3550405 | 679.473098 | 690.5416108 |
| **Map6d1** | 5.494178889 | 8.714882094 | 1.566322589 | 0 | 1.266726489 | 5.611302446 | 1.80412943 | 0.838074013 | 6.019312981 | 3.495607876 | 0.945870699 | 2.602584319 | 4.260421576 | 4.517773258 | 0.718565672 |
| **Parl** | 855.2605138 | 898.4251176 | 939.7935534 | 833.2806994 | 831.6059397 | 860.6335126 | 819.074761 | 964.623189 | 982.6528441 | 998.8699505 | 1001.67707 | 819.8140604 | 777.9529797 | 833.9809434 | 862.2788064 |
| **Abcc5** | 301.2641424 | 325.6196855 | 279.5885821 | 342.4559396 | 283.1133702 | 300.9060937 | 305.7999383 | 322.658495 | 340.0911834 | 340.8217679 | 339.5675809 | 362.6267484 | 337.4253888 | 373.1680711 | 384.4326345 |
| **Eif2b5** | 2131.741409 | 2246.855056 | 2273.517238 | 2083.201748 | 2127.467138 | 2187.005128 | 2106.321109 | 2349.959533 | 2333.236194 | 2431.195277 | 2448.859239 | 2062.981837 | 2137.879547 | 1960.713594 | 2124.798692 |
| **Dvl3** | 320.4937685 | 326.4119475 | 336.7593566 | 301.0506875 | 291.9804556 | 295.2947912 | 312.1143913 | 268.1836842 | 270.11667 | 273.5313163 | 271.4648905 | 284.5492189 | 328.9045457 | 285.5232699 | 344.1929569 |
| **Ap2m1** | 4417.319827 | 4549.168453 | 4569.746153 | 4625.311708 | 4516.513295 | 4493.951846 | 4375.915931 | 5035.14867 | 4788.363476 | 4649.158475 | 4869.342357 | 4626.527391 | 4527.123966 | 4626.199816 | 4720.257899 |
| **Gm15760** | 29.30228741 | 38.82083842 | 34.45909696 | 25.87828259 | 27.2346195 | 40.68194273 | 35.18052388 | 43.57984868 | 37.62070613 | 34.08217679 | 40.67244005 | 32.96606804 | 31.52711966 | 55.11683375 | 52.45529405 |
| **Abcf3** | 738.0513641 | 751.0643841 | 780.0286493 | 813.4406827 | 706.2000174 | 730.8721436 | 700.0022187 | 804.5510525 | 648.5809737 | 737.5732618 | 781.2891972 | 744.3391152 | 760.0592091 | 852.9555911 | 753.7753899 |
| **Vwa5b2** | 0 | 1.584524017 | 7.04845165 | 3.450437679 | 0.633363244 | 1.402825611 | 0.902064715 | 2.514222039 | 1.504828245 | 2.621705907 | 0 | 0 | 0.852084315 | 0 | 5.029959704 |
| **Alg3** | 495.3917965 | 430.1982706 | 471.4630993 | 422.6786156 | 424.3533737 | 380.8671535 | 479.8984283 | 393.8947861 | 386.740859 | 350.4346895 | 420.912461 | 404.2680975 | 383.4379418 | 419.2493583 | 436.1693629 |
| **Ece2** | 295.7699635 | 312.9434934 | 278.8054208 | 295.8750309 | 286.2801864 | 280.5651223 | 258.8925731 | 314.2777549 | 275.3835689 | 297.1266694 | 317.8125548 | 237.7027011 | 244.5481985 | 292.7517071 | 265.150733 |
| **Camk2n2** | 9.156964816 | 26.93690829 | 16.44638718 | 15.52696955 | 16.46744435 | 24.5494482 | 27.06194144 | 15.08533223 | 46.6496756 | 46.31680435 | 24.59263817 | 19.95314644 | 12.78126473 | 11.74621047 | 25.14979852 |
| **Psmd2** | 6832.927145 | 7251.574164 | 7152.612103 | 7000.93805 | 6618.012539 | 6781.259006 | 6937.779721 | 8470.41405 | 8210.342906 | 8090.584428 | 8000.17437 | 7149.299124 | 6924.037145 | 7005.259214 | 6916.913158 |
| **Eif4g1** | 17691.25602 | 17624.66064 | 17569.44048 | 17959.52812 | 16896.86463 | 16563.16199 | 17570.41651 | 20523.59451 | 18942.77795 | 20130.33185 | 20288.92649 | 17722.73168 | 17925.29774 | 17038.33006 | 17415.87619 |
| **Fam131a** | 2.747089445 | 0.792262009 | 2.349483883 | 5.175656518 | 5.066905954 | 4.208476834 | 4.510323574 | 5.866518091 | 0 | 6.117313782 | 1.891741398 | 1.735056213 | 0 | 12.64976512 | 5.029959704 |
| **Clcn2** | 11.90405426 | 9.507144102 | 7.831612945 | 16.38957897 | 8.86708542 | 6.312715251 | 16.23716487 | 18.43762829 | 30.0965649 | 20.09974528 | 13.24218978 | 30.36348372 | 30.67503535 | 18.97464768 | 18.68270747 |
| **Polr2h** | 508.2115473 | 544.2839998 | 483.2105187 | 493.412588 | 544.6923901 | 545.6991628 | 570.1048997 | 600.0609933 | 597.4168133 | 594.2533389 | 556.1719709 | 528.3246167 | 518.9193479 | 487.0159572 | 476.4090405 |
| **Magef1** | 34.7964663 | 28.52143231 | 38.37490343 | 31.91654853 | 35.46834168 | 31.56357626 | 59.53627118 | 33.52296052 | 45.14484735 | 22.72145119 | 34.99721585 | 37.30370857 | 56.2375648 | 45.17773258 | 34.49115225 |
| **Vps8** | 488.9819212 | 419.8988645 | 449.534583 | 393.3498954 | 364.1838655 | 439.0844164 | 442.0117102 | 386.35212 | 407.0560403 | 412.4817293 | 401.0491763 | 414.6784348 | 427.7463262 | 412.9244758 | 426.8280091 |
| **2510009E07Rik** | 21.06101908 | 30.89821833 | 56.3876132 | 55.20700286 | 44.96879034 | 24.5494482 | 40.59291217 | 43.57984868 | 14.29586833 | 54.18192207 | 40.67244005 | 29.49595561 | 31.52711966 | 49.69550584 | 53.8924254 |
| **Ehhadh** | 83.32837982 | 80.01846286 | 62.65290356 | 77.63484777 | 74.73686282 | 51.90454762 | 75.77343604 | 62.01747697 | 91.79452295 | 58.55143192 | 69.99443171 | 67.66719229 | 74.13133542 | 74.09148143 | 78.32365824 |
| **1300002E11Rik** | 65.01445019 | 95.07144102 | 76.74980686 | 70.73397241 | 87.40412771 | 79.96105985 | 82.08788905 | 114.8161398 | 103.0807348 | 102.2465304 | 105.9375183 | 99.76573222 | 95.4334433 | 80.41636399 | 99.16206273 |
| **Map3k13** | 10.98835778 | 12.67619214 | 5.482129061 | 6.038265938 | 3.800179466 | 6.312715251 | 9.020647148 | 14.24725822 | 6.019312981 | 6.991215751 | 17.02567258 | 8.675281063 | 5.112505891 | 4.517773258 | 4.311394032 |
| **Tmem41a** | 246.3223535 | 269.3690829 | 231.8157432 | 244.9810752 | 223.5772252 | 231.4662259 | 239.0471494 | 315.9539029 | 247.5442463 | 250.8098651 | 267.6814078 | 268.9337129 | 245.4002828 | 222.2744443 | 232.096712 |
| **Liph** | 4.578482408 | 7.130358077 | 3.132645178 | 13.80175071 | 0 | 2.104238417 | 2.706194144 | 2.514222039 | 0 | 3.495607876 | 2.837612096 | 0 | 5.112505891 | 0 | 4.311394032 |
| **Senp2** | 1158.356049 | 1269.203738 | 1239.744329 | 1104.140057 | 1187.556083 | 1165.04667 | 1219.591494 | 1459.924931 | 1320.486785 | 1553.797701 | 1248.549322 | 1198.056315 | 1377.820338 | 1288.468933 | 1236.651521 |
| **Igf2bp2** | 174.898028 | 145.7762096 | 166.8133557 | 138.0175071 | 91.83767042 | 110.1218105 | 126.2890601 | 62.85555098 | 72.23175577 | 111.859452 | 124.8549322 | 89.35539495 | 94.58135898 | 101.198121 | 79.76078959 |
| **Tra2b** | 4163.671902 | 4405.769029 | 4440.52454 | 4237.137469 | 4434.176073 | 4618.803326 | 4633.004375 | 4306.024279 | 4596.497875 | 4589.733141 | 4505.182138 | 4628.262447 | 4576.544857 | 4831.306722 | 4588.041816 |
| **Etv5** | 34.7964663 | 50.70476854 | 18.79587107 | 12.93914129 | 30.40143573 | 40.68194273 | 54.12388289 | 24.30414638 | 18.81035306 | 34.08217679 | 26.48437957 | 26.02584319 | 45.1604687 | 34.33507676 | 34.49115225 |
| **Dgkg** | 0.915696482 | 0 | 4.698967767 | 0 | 0 | 3.507064029 | 0.902064715 | 0 | 0.752414123 | 2.621705907 | 0.945870699 | 1.735056213 | 0.852084315 | 0 | 5.748525376 |
| **Tbccd1** | 173.9823315 | 228.9637205 | 238.8641948 | 220.8280114 | 229.2774944 | 232.1676387 | 202.9645608 | 241.3653158 | 282.155296 | 261.2966887 | 286.5988217 | 201.2665207 | 202.796067 | 240.3455373 | 251.4979852 |
| **Dnajb11** | 2735.18539 | 2680.222375 | 2613.40924 | 2582.652602 | 2462.516294 | 2616.971178 | 2527.585331 | 2584.620256 | 2600.343208 | 2518.585474 | 2665.463629 | 2634.682859 | 2648.278052 | 2566.998765 | 2476.177306 |
| **Eif4a2** | 2256.276131 | 2458.389012 | 2481.054981 | 2074.575654 | 2420.71432 | 2323.079213 | 2297.558829 | 2570.372998 | 2333.988608 | 2730.069751 | 2692.893879 | 2449.031844 | 2630.384281 | 2532.663688 | 2614.86048 |
| **Snord2** | 0.915696482 | 8.714882094 | 0 | 0 | 3.166816221 | 2.104238417 | 0.902064715 | 0.838074013 | 8.276555348 | 0 | 6.621094891 | 1.735056213 | 1.70416863 | 0.903554652 | 2.874262688 |
| **Snora81** | 9.156964816 | 23.76786026 | 18.79587107 | 10.35131304 | 16.46744435 | 21.74379698 | 3.608258859 | 4.190370065 | 22.57242368 | 16.60413741 | 17.97154328 | 18.21809023 | 17.89377062 | 9.939101167 | 17.9641418 |
| **Rfc4** | 696.8450225 | 747.895336 | 852.8626497 | 755.6458516 | 813.2384057 | 885.8843736 | 784.7963019 | 853.9974193 | 781.0058592 | 773.4032425 | 819.1240251 | 812.8738356 | 791.5863288 | 890.0013318 | 823.4762601 |
| **St6gal1** | 1114.402618 | 1147.195388 | 1098.775296 | 1098.964401 | 1661.945153 | 1818.061992 | 1871.784283 | 480.2164095 | 546.252653 | 542.6931227 | 635.6251096 | 1702.090145 | 1756.145774 | 1460.144317 | 1835.935292 |
| **Rtp4** | 650.1445019 | 583.1048383 | 525.5012286 | 508.9395576 | 657.4310476 | 811.5346162 | 841.6263789 | 125.711102 | 209.9235402 | 173.9064918 | 283.7612096 | 585.5814717 | 565.7839853 | 639.7166933 | 565.5111838 |
| **Bcl6** | 21.97671556 | 20.59881222 | 15.66322589 | 14.66436013 | 32.30152546 | 25.25086101 | 29.76813559 | 36.03718256 | 10.53379772 | 14.85633347 | 14.18806048 | 38.17123668 | 27.26669809 | 46.98484188 | 32.33545524 |
| **Lppos** | 8.241268334 | 18.2220262 | 18.01270977 | 6.900875357 | 16.46744435 | 10.52119209 | 15.33510015 | 10.89496217 | 8.276555348 | 16.60413741 | 11.35044839 | 6.94022485 | 11.92918041 | 25.29953024 | 17.24557613 |
| **Lpp** | 380.0140398 | 373.947668 | 339.8920018 | 341.5933302 | 258.4122037 | 267.9396918 | 318.4288443 | 341.9341973 | 289.6794372 | 251.683767 | 336.7299688 | 250.7156227 | 333.1649672 | 304.4979176 | 306.8275419 |
| **Morf4l1-ps1** | 16.48253667 | 18.2220262 | 18.01270977 | 24.15306375 | 17.73417084 | 33.66781467 | 34.27845916 | 27.65644243 | 19.56276719 | 20.97364725 | 29.32199166 | 24.29078698 | 24.71044514 | 31.62441281 | 26.58692986 |
| **Il1rap** | 213.3572802 | 213.9107423 | 183.2597429 | 187.1862441 | 233.7110371 | 235.6747027 | 239.9492141 | 226.2799835 | 179.0745612 | 194.8801391 | 157.9604067 | 230.7624763 | 247.1044514 | 268.3557315 | 224.9110553 |
| **Ostn** | 0 | 0.792262009 | 0 | 0 | 0 | 0 | 1.80412943 | 1.676148026 | 0 | 0 | 0 | 2.602584319 | 1.70416863 | 0 | 3.59282836 |
| **Ccdc50** | 4428.308185 | 4241.770794 | 4461.669895 | 4020.622505 | 4260.001181 | 4449.061427 | 4956.845608 | 4657.177291 | 4245.872894 | 4351.157903 | 4298.036455 | 4763.596832 | 4841.543079 | 4536.747906 | 4703.012323 |
| **Opa1** | 1391.858652 | 1506.090078 | 1509.151814 | 1420.717714 | 1253.42586 | 1460.341461 | 1361.215655 | 1605.749809 | 1484.513064 | 1669.152761 | 1591.900386 | 1457.447219 | 1375.264085 | 1310.154245 | 1294.136775 |
| **4632428C04Rik** | 0 | 0 | 0 | 0 | 0 | 1.402825611 | 0 | 1.676148026 | 0 | 3.495607876 | 5.675224193 | 0 | 0 | 0 | 0.718565672 |
| **Gp5** | 0 | 0 | 0 | 1.725218839 | 0 | 0.701412806 | 0.902064715 | 0 | 2.257242368 | 0 | 0.945870699 | 0.867528106 | 0 | 0 | 0.718565672 |
| **Atp13a3** | 4695.691557 | 4671.969064 | 4983.255317 | 4551.127298 | 4274.568536 | 4214.088137 | 4811.613189 | 4891.838014 | 5144.255356 | 5002.21487 | 5338.494224 | 4764.46436 | 4843.247247 | 4576.50431 | 4499.658238 |
| **Tmem44** | 0.915696482 | 1.584524017 | 0 | 2.587828259 | 0 | 1.402825611 | 0 | 4.190370065 | 0.752414123 | 0 | 2.837612096 | 0 | 0 | 0 | 0 |
| **Lsg1** | 1470.608549 | 1358.729345 | 1373.664911 | 1347.395913 | 1388.965595 | 1453.327333 | 1406.31889 | 1532.83737 | 1586.08897 | 1538.941367 | 1519.068342 | 1328.185531 | 1365.891157 | 1292.083152 | 1322.160836 |
| **Fam43a** | 21.06101908 | 18.2220262 | 14.0969033 | 33.64176737 | 22.16771355 | 27.35509942 | 11.72684129 | 33.52296052 | 21.06759543 | 23.59535316 | 26.48437957 | 6.94022485 | 18.74585493 | 5.421327909 | 6.467091048 |
| **Xxylt1** | 656.5543773 | 720.1661657 | 693.0977456 | 788.4250096 | 664.3980432 | 779.97104 | 725.2600307 | 805.3891265 | 752.4141226 | 739.3210657 | 867.3634308 | 790.3181048 | 670.590356 | 743.6254782 | 762.398178 |
| **Acap2** | 1049.388168 | 1094.113834 | 1138.716522 | 1015.291287 | 1047.582806 | 1062.640401 | 1153.74077 | 1111.286141 | 1182.795001 | 1175.398148 | 1076.400855 | 1201.526427 | 1221.888908 | 1284.854715 | 1168.387783 |
| **Ppp1r2** | 1114.402618 | 1135.311458 | 1156.729232 | 1202.477531 | 1048.216169 | 1162.942432 | 1270.107118 | 1335.889977 | 1249.759858 | 1296.870522 | 1296.788728 | 1152.077325 | 1336.92029 | 1152.032181 | 1125.273842 |
| **Bdh1** | 1301.2047 | 1312.778148 | 1379.14704 | 1167.110545 | 1174.255455 | 1183.984816 | 1221.395624 | 1290.63398 | 1243.740545 | 1304.73564 | 1280.708926 | 1210.201708 | 1171.615933 | 1160.164173 | 1184.196227 |
| **Dlg1** | 819.548351 | 940.4150041 | 997.7474892 | 908.3277189 | 798.671051 | 831.8755876 | 952.5803388 | 988.9273354 | 952.5562792 | 874.7758709 | 1065.996278 | 964.6912542 | 887.0197721 | 938.793283 | 921.9197571 |
| **Pigz** | 7.325571853 | 0.792262009 | 6.265290356 | 18.11479781 | 0 | 1.402825611 | 5.412388289 | 13.40918421 | 10.53379772 | 4.369509845 | 6.621094891 | 2.602584319 | 7.668758836 | 2.710663955 | 10.77848508 |
| **0610012G03Rik** | 690.4351471 | 658.3697291 | 697.7967134 | 740.9814915 | 728.3677309 | 761.734307 | 687.3733127 | 706.496393 | 788.5300005 | 807.4854193 | 761.4259125 | 734.796306 | 728.5320895 | 707.4832922 | 707.0686212 |
| **Ncbp2** | 875.4058364 | 935.661432 | 983.6505859 | 948.0077522 | 979.1795757 | 988.2906432 | 985.0546685 | 1223.588059 | 1187.309485 | 1208.606423 | 1188.013598 | 1104.363279 | 1149.461741 | 1028.245193 | 1046.231618 |
| **Senp5** | 1096.088688 | 1128.1811 | 1213.116845 | 1083.437431 | 960.8120416 | 1050.716383 | 1172.684129 | 1146.48525 | 987.1673288 | 1238.31909 | 1187.067727 | 1038.431143 | 1127.307549 | 974.935469 | 1063.477195 |
| **Gm15694** | 21.06101908 | 20.59881222 | 15.66322589 | 10.35131304 | 17.73417084 | 12.6254305 | 19.84542373 | 24.30414638 | 22.57242368 | 11.3607256 | 20.80915537 | 10.41033728 | 20.45002356 | 13.55331977 | 22.27553583 |
| **Pak2** | 4903.554659 | 4538.869047 | 4695.05196 | 4859.078861 | 4667.253747 | 4702.972862 | 4998.340585 | 4717.518619 | 4983.238734 | 4390.483492 | 4541.125225 | 4812.178406 | 5125.287156 | 4694.86997 | 4783.491678 |
| **Pigx** | 681.2781823 | 679.7608033 | 621.0469065 | 585.7117959 | 687.19912 | 610.9305538 | 644.9762711 | 619.3366956 | 657.6099431 | 679.0218298 | 600.6278937 | 640.2357424 | 617.7611285 | 614.4171631 | 632.3377913 |
| **Cep19** | 238.0810852 | 246.3934846 | 267.8411627 | 286.3863273 | 297.0473616 | 244.0916564 | 271.5214791 | 295.8401266 | 328.0525574 | 251.683767 | 295.111658 | 327.0580961 | 322.9399554 | 297.2694804 | 287.4262688 |
| **Nrros** | 1360.724972 | 1292.179336 | 1403.42504 | 1460.397747 | 1585.3082 | 1642.007378 | 1803.227365 | 1105.419623 | 1139.907396 | 1255.797129 | 1286.38415 | 1496.485983 | 1670.937342 | 1712.236065 | 1593.77866 |
| **Bex6** | 0.915696482 | 0.792262009 | 4.698967767 | 6.038265938 | 6.333632443 | 10.52119209 | 2.706194144 | 0 | 0.752414123 | 5.243411813 | 2.837612096 | 4.337640531 | 6.816674521 | 0.903554652 | 11.49705075 |
| **Fbxo45** | 747.208329 | 812.0685587 | 819.9698753 | 803.9519791 | 751.802171 | 749.1088765 | 719.8476424 | 863.2162334 | 929.9838555 | 816.224439 | 789.8020335 | 752.1468681 | 778.8050641 | 718.325948 | 700.6015302 |
| **Wdr53** | 164.8253667 | 158.4524017 | 177.7776138 | 177.6975404 | 189.37561 | 185.8743935 | 180.412943 | 247.2318339 | 209.1711261 | 160.7979623 | 206.1998123 | 193.4587677 | 153.3751767 | 194.2642501 | 165.2701046 |
| **Rnf168** | 748.1240254 | 607.6649605 | 678.217681 | 674.5605662 | 582.0608215 | 657.9252118 | 607.089553 | 604.2513634 | 624.5037217 | 609.9835743 | 574.1435142 | 600.3294495 | 567.4881539 | 612.6100538 | 587.068154 |
| **Ubxn7** | 1198.646694 | 1191.562061 | 1170.042974 | 1110.178323 | 1135.620297 | 1092.801151 | 1109.539599 | 1017.421852 | 1143.669466 | 1011.104578 | 1062.212795 | 1199.791371 | 1059.140804 | 1033.666521 | 1047.66875 |
| **Tm4sf19** | 6.409875371 | 10.29940611 | 6.265290356 | 9.488703616 | 3.166816221 | 0 | 2.706194144 | 14.24725822 | 21.82000955 | 9.612921658 | 2.837612096 | 6.94022485 | 5.112505891 | 4.517773258 | 2.155697016 |
| **Tctex1d2** | 24.723805 | 30.89821833 | 39.94122602 | 29.32872027 | 20.90098706 | 21.04238417 | 14.43303544 | 40.22755263 | 45.14484735 | 47.19070632 | 52.02288843 | 39.03876478 | 25.56252945 | 23.49242094 | 17.24557613 |
| **Pcyt1a** | 951.4086443 | 854.8507072 | 814.4877463 | 783.249353 | 668.1982227 | 615.1390306 | 692.785701 | 1040.04985 | 924.7169567 | 861.6673413 | 944.9248281 | 747.8092276 | 647.5840795 | 613.5136084 | 686.2302167 |
| **Tfrc** | 16838.7426 | 17103.35224 | 17884.27132 | 16504.30603 | 14730.76234 | 14808.22715 | 16579.04739 | 17342.26555 | 18589.89573 | 18117.73562 | 19244.68524 | 16956.70437 | 16554.29407 | 15842.92726 | 15794.07347 |
| **Tnk2** | 841.5250666 | 925.3620259 | 938.2272308 | 903.1520624 | 1147.654199 | 1177.672101 | 1205.158459 | 858.1877894 | 789.2824146 | 898.371224 | 865.4716894 | 1175.500584 | 1251.711859 | 1158.357063 | 1220.124511 |
| **Tnk2os** | 0 | 0 | 1.566322589 | 0.86260942 | 0.633363244 | 1.402825611 | 1.80412943 | 0 | 2.257242368 | 3.495607876 | 0 | 0 | 0 | 6.324882561 | 0.718565672 |
| **1700021K19Rik** | 458.7639373 | 520.5161396 | 530.9833577 | 526.191746 | 449.0545402 | 436.980178 | 481.7025577 | 548.1004045 | 442.4195041 | 530.4584951 | 575.0893849 | 472.8028179 | 505.2859989 | 477.9804107 | 577.7268003 |
| **Fyttd1** | 944.998769 | 972.1054844 | 1122.270135 | 993.7260514 | 984.2464816 | 1016.347155 | 1066.240493 | 1080.277403 | 1054.8846 | 1020.7175 | 1048.970605 | 1017.610469 | 1137.532561 | 989.3923435 | 1069.22572 |
| **Lrch3** | 950.4929479 | 874.6572574 | 928.046134 | 908.3277189 | 904.4427128 | 895.7041529 | 991.3691215 | 856.5116413 | 802.0734547 | 828.4590665 | 740.6167571 | 882.2760841 | 922.8073133 | 880.0622306 | 918.3269288 |
| **Iqcg** | 37.54355574 | 57.04286461 | 28.9769679 | 33.64176737 | 30.40143573 | 23.14662259 | 41.49497688 | 33.52296052 | 33.10622139 | 15.73023544 | 22.70089677 | 19.08561834 | 54.53339617 | 39.75640467 | 33.77258658 |
| **Rpl35a** | 7754.117806 | 7353.775963 | 7409.489007 | 6609.313373 | 7698.530234 | 7602.613401 | 7912.911678 | 7194.865402 | 6820.634021 | 7231.538793 | 6851.887342 | 7609.08902 | 7207.781222 | 7719.970943 | 7390.447936 |
| **Lmln** | 65.93014667 | 70.51131876 | 54.82129061 | 55.20700286 | 67.13650389 | 61.0229141 | 56.83007703 | 37.71333059 | 26.33449429 | 46.31680435 | 27.43025026 | 54.6542707 | 55.38548049 | 38.85285002 | 60.35951645 |
| **Osbpl11** | 684.0252717 | 631.4328208 | 656.2891648 | 579.67353 | 654.8975946 | 618.6460946 | 596.2647765 | 698.9537269 | 694.4782351 | 737.5732618 | 709.4030241 | 624.6202365 | 691.8924639 | 606.2851712 | 602.1580331 |
| **Snx4** | 1480.681211 | 1479.15317 | 1484.873814 | 1497.489953 | 1604.942461 | 1619.562168 | 1676.938305 | 1458.248783 | 1535.677224 | 1542.436975 | 1600.413222 | 1587.576434 | 1762.110364 | 1733.017822 | 1629.706944 |
| **1700007L15Rik** | 20.14532259 | 11.88393013 | 15.66322589 | 14.66436013 | 17.1008076 | 18.93814575 | 23.45368258 | 25.9802944 | 32.35380727 | 15.73023544 | 26.48437957 | 14.74797781 | 23.00627651 | 24.39597559 | 20.11983882 |
| **Zfp148** | 1072.28058 | 981.6126285 | 1023.591812 | 1105.002667 | 894.9422642 | 968.6510847 | 1168.173806 | 1018.259926 | 1033.06459 | 982.265813 | 1037.620157 | 1044.50384 | 1039.542864 | 953.2501574 | 981.5607079 |
| **Slc12a8** | 29.30228741 | 18.2220262 | 25.06116142 | 31.91654853 | 19.63426057 | 16.13249453 | 14.43303544 | 5.866518091 | 23.3248378 | 25.3431571 | 25.53850887 | 16.48303402 | 13.63334904 | 18.97464768 | 19.40127314 |
| **Heg1** | 667.5427351 | 694.0215194 | 665.6871003 | 765.1345552 | 644.7637827 | 648.8068453 | 771.2653311 | 872.4350476 | 738.8706684 | 649.3091629 | 532.5252034 | 688.8173164 | 702.1174757 | 625.2598189 | 648.1462361 |
| **Itgb5** | 18.31392963 | 12.67619214 | 10.18109683 | 18.97740723 | 20.90098706 | 26.65368662 | 16.23716487 | 14.24725822 | 6.771727103 | 20.09974528 | 9.458706988 | 25.15831508 | 9.372927467 | 9.939101167 | 27.30549553 |
| **Umps** | 2785.548697 | 2827.583108 | 2704.25595 | 2685.303123 | 2678.49316 | 2746.732547 | 2611.477349 | 3167.081695 | 3295.573857 | 3075.261029 | 2854.637769 | 2694.542298 | 2649.98222 | 2663.679113 | 2697.495533 |

| | | | | | | | | | | | | | | | |
|---|---|---|---|---|---|---|---|---|---|---|---|---|---|---|---|
| **Kalrn** | 1.831392963 | 6.338096068 | 1.566322589 | 1.725218839 | 6.333632443 | 4.90988964 | 6.314453003 | 3.352296052 | 0 | 0 | 0 | 6.94022485 | 2.556252945 | 9.035546516 | 2.155697016 |
| **Ccdc14** | 160.2468843 | 171.1285938 | 174.6449687 | 141.4679448 | 124.7725591 | 165.5334221 | 145.2324191 | 161.7482845 | 158.7593799 | 146.8155308 | 118.2338373 | 134.4668565 | 154.227261 | 117.4621047 | 120.7190329 |
| **Mylk** | 4.578482408 | 7.922620085 | 3.132645178 | 1.725218839 | 0 | 0.701412806 | 2.706194144 | 1.676148026 | 1.504828245 | 1.747803938 | 5.675224193 | 2.602584319 | 1.70416863 | 0.903554652 | 4.311394032 |
| **Hacd2** | 375.4355574 | 340.6726637 | 390.0143247 | 352.8072526 | 356.5835065 | 347.1993388 | 335.5680739 | 363.7241217 | 388.9981014 | 362.6693171 | 386.8611158 | 350.4813549 | 322.0878711 | 353.2898688 | 331.2587748 |
| **Adcy5** | 0 | 0 | 0 | 2.587828259 | 0 | 2.104238417 | 0 | 7.542666117 | 0 | 3.495607876 | 0 | 0 | 0 | 4.517773258 | 2.155697016 |
| **Sec22a** | 149.2585265 | 153.6988296 | 150.3669685 | 158.7201332 | 154.5406316 | 143.7896252 | 156.9592604 | 165.1005806 | 162.5214505 | 124.0940796 | 136.2053806 | 192.5912396 | 167.8606101 | 130.1118698 | 152.3359225 |
| **Pdia5** | 84.2440763 | 92.694655 | 102.5941296 | 88.84877022 | 81.07049527 | 67.33562935 | 61.34040061 | 120.6826579 | 109.1000478 | 127.5896875 | 114.4503546 | 62.46202365 | 70.72299816 | 83.12702794 | 65.38947615 |
| **Dirc2** | 107.1364883 | 118.8393013 | 120.6068394 | 84.53572313 | 97.53793962 | 114.3302873 | 122.6808012 | 86.32162334 | 139.9490268 | 103.9943343 | 104.0457769 | 96.2956198 | 107.3626237 | 112.0407768 | 86.94644631 |
| **Hspbap1** | 155.6684019 | 166.3750218 | 135.4869039 | 130.2540224 | 157.0740846 | 151.505166 | 169.5881664 | 196.9473931 | 180.5793894 | 196.627943 | 153.2310532 | 157.0225872 | 181.4939591 | 131.9189791 | 146.5873971 |
| **Parp14** | 5071.127115 | 3921.696942 | 3532.057438 | 3395.230676 | 3429.028605 | 3796.747517 | 4444.47285 | 2452.204562 | 2788.446738 | 2335.939963 | 2515.070188 | 3357.333771 | 3301.826721 | 3134.431086 | 3348.516031 |
| **Dtx3l** | 3250.72251 | 3070.015283 | 3080.173371 | 2853.51196 | 2907.137291 | 3026.596257 | 3617.279506 | 1991.263855 | 2294.863074 | 2049.300117 | 2178.340219 | 3032.010731 | 3055.574354 | 2809.151412 | 2862.765637 |
| **Parp9** | 2748.920838 | 2523.354497 | 2285.264657 | 2213.455771 | 2270.607231 | 2539.114357 | 2780.163451 | 1436.458858 | 1803.536652 | 1696.243722 | 1920.117519 | 2468.98499 | 2293.810976 | 2370.023851 | 2320.96712 |
| **Kpna1** | 1760.884334 | 1625.721641 | 1703.375816 | 1678.637931 | 1592.908559 | 1639.201727 | 1731.062188 | 1460.763005 | 1517.619285 | 1358.917562 | 1518.122472 | 1624.012615 | 1637.706054 | 1532.428689 | 1762.641593 |
| **Wdr5b** | 53.11039593 | 82.39524888 | 47.77283896 | 50.03134634 | 42.43533737 | 30.16075065 | 61.34040061 | 55.31288486 | 35.36346376 | 72.53386342 | 69.04856101 | 55.5217988 | 35.78754124 | 49.69550584 | 48.86246569 |
| **Fam162a** | 2326.78476 | 2126.431231 | 2083.209043 | 1905.504208 | 2026.129018 | 2243.819565 | 2044.980708 | 1097.876957 | 1194.081213 | 1485.633347 | 1379.079479 | 2181.833187 | 1961.498094 | 1955.292266 | 2133.42148 |
| **Ccdc58** | 458.7639373 | 458.7197029 | 393.1469698 | 380.4107541 | 402.1856601 | 396.999648 | 429.3828042 | 403.9516743 | 460.477443 | 471.9070632 | 471.043608 | 454.5847277 | 412.4088085 | 440.93467 | 395.9296853 |
| **Gm6815** | 1.831392963 | 0 | 0 | 0 | 0.633363244 | 0 | 0 | 3.352296052 | 0 | 0 | 0 | 0 | 0 | 0 | 0 |
| **2010005H15Rik** | 0 | 0.792262009 | 0 | 0 | 1.900089733 | 2.104238417 | 1.80412943 | 0 | 0 | 3.495607876 | 0 | 3.470112425 | 0 | 0 | 0.718565672 |
| **Cd86** | 127.2818109 | 138.6458515 | 121.3900006 | 152.6818673 | 249.5451182 | 256.7170869 | 260.6967026 | 61.17940295 | 82.76555348 | 48.93851026 | 75.6696559 | 245.5104541 | 193.4231395 | 211.4317885 | 229.2224494 |
| **Ildr1** | 32.04937685 | 31.69048034 | 19.57903236 | 40.54264272 | 43.70206386 | 49.80030921 | 55.92801232 | 12.5711102 | 10.53379772 | 4.369509845 | 20.80915537 | 45.11146153 | 38.34379418 | 56.92394305 | 45.98820301 |
| **Slc15a2** | 34.7964663 | 43.57441047 | 32.89277437 | 27.60350143 | 10.13381191 | 11.9240177 | 11.72684129 | 24.30414638 | 37.62070613 | 34.95607876 | 23.64676747 | 12.14539349 | 29.82295103 | 10.84265582 | 23.71266717 |
| **Eaf2** | 23.80810852 | 26.14464628 | 17.22954848 | 22.42784491 | 17.73417084 | 32.26498906 | 31.57226502 | 28.49451644 | 41.38277674 | 20.09974528 | 20.80915537 | 40.773821 | 25.56252945 | 38.85285002 | 39.52111196 |
| **Iqcb1** | 226.1770309 | 267.7845589 | 205.1882592 | 202.7132136 | 187.4755203 | 172.5475502 | 192.1397842 | 233.8226496 | 247.5442463 | 190.5106292 | 188.2282691 | 215.1469704 | 157.6355983 | 195.1678047 | 202.6355195 |
| **4930565N06Rik** | 15.56684019 | 14.26071615 | 7.831612945 | 9.488703616 | 5.700269198 | 7.715540863 | 9.020647148 | 11.73303618 | 16.5531107 | 4.369509845 | 8.512836289 | 17.35056213 | 14.48543336 | 5.421327909 | 8.622788064 |
| **Golgb1** | 1005.434737 | 1033.109659 | 948.4083276 | 1011.840849 | 963.3454945 | 893.5999145 | 976.9360861 | 1022.450296 | 1109.810831 | 917.5970673 | 1071.671502 | 1006.332603 | 996.9386487 | 950.5394935 | 947.7881213 |
| **Hcls1** | 4999.702789 | 5150.495317 | 4956.627833 | 5296.421837 | 4944.033485 | 4922.51507 | 5229.269152 | 5742.483137 | 5785.312189 | 5178.743068 | 5289.308948 | 5077.642006 | 4869.661861 | 5171.043271 | 4877.623781 |
| **Fbxo40** | 0.915696482 | 0 | 0 | 0 | 2.533452977 | 0 | 0 | 0 | 0 | 2.621705907 | 2.837612096 | 3.470112425 | 0 | 0 | 0 |
| **Polq** | 721.5688275 | 755.8179561 | 759.6664557 | 747.8823668 | 660.5978638 | 646.7026069 | 740.5951308 | 967.9754851 | 874.3052104 | 807.4854193 | 838.0414391 | 740.0014747 | 794.1425817 | 667.7268875 | 731.4998541 |
| **Gtf2e1** | 924.8534464 | 782.7548644 | 916.2987146 | 921.2668602 | 839.8396619 | 895.0027401 | 1001.291833 | 1099.553105 | 963.0900769 | 1048.682363 | 969.5174662 | 870.9982187 | 931.3281565 | 901.7475423 | 898.20709 |
| **Rabl3** | 243.5752641 | 230.5482445 | 238.8641948 | 243.2558563 | 216.6102295 | 227.2577491 | 274.2276733 | 316.7919769 | 267.1070135 | 231.5840218 | 252.5474766 | 213.4119141 | 214.7252474 | 215.9495617 | 242.8751971 |
| **Ndufb4** | 309.5054108 | 300.2673012 | 327.3614211 | 276.0350143 | 368.6174082 | 314.232937 | 267.9132203 | 333.5534572 | 299.4608208 | 299.7483753 | 279.9777268 | 268.9337129 | 293.9690887 | 328.8938932 | 301.7975822 |
| **Fstl1** | 3.662785926 | 8.714882094 | 0 | 0 | 0.633363244 | 1.402825611 | 2.706194144 | 1.676148026 | 0 | 0.873901969 | 3.783482795 | 1.735056213 | 2.556252945 | 0 | 4.311394032 |
| **Lrrc58** | 1259.082662 | 1220.875755 | 1368.182781 | 1151.583575 | 996.9137465 | 1062.640401 | 1262.890601 | 1395.393232 | 1459.683398 | 1306.483444 | 1478.395902 | 1167.692831 | 1266.197292 | 1137.575306 | 1120.243883 |
| **BC031361** | 3.662785926 | 1.584524017 | 3.915806472 | 6.900875357 | 3.166816221 | 2.104238417 | 9.020647148 | 8.380740131 | 7.524141226 | 0.873901969 | 0.945870699 | 2.602584319 | 3.408337261 | 0 | 3.59282836 |
| **Gsk3b** | 1291.132039 | 1250.981711 | 1271.070781 | 1400.015088 | 1127.386575 | 1198.013072 | 1328.741325 | 1305.719312 | 1365.631632 | 1233.075678 | 1204.0934 | 1464.387443 | 1393.157855 | 1300.215144 | 1321.442271 |
| **Maats1** | 1.831392963 | 3.961310043 | 10.18109683 | 9.488703616 | 0 | 0.701412806 | 6.314453003 | 0.838074013 | 5.266898858 | 42.82119648 | 25.53850887 | 1.735056213 | 0 | 0.903554652 | 5.029959704 |
| **Cox17** | 1227.033285 | 1133.726934 | 1137.933361 | 908.3277189 | 849.3401106 | 857.1264486 | 729.7703543 | 1205.988505 | 1055.637014 | 1204.236913 | 1164.36683 | 782.5103519 | 742.1654385 | 769.8285631 | 702.0386615 |
| **Popdc2** | 15.56684019 | 3.961310043 | 9.397935534 | 1.725218839 | 3.800179466 | 4.208476834 | 9.922711863 | 10.89496217 | 9.028969471 | 8.739019689 | 13.24218978 | 1.735056213 | 5.112505891 | 4.517773258 | 1.437131344 |
| **Pla1a** | 32.96507334 | 39.61310043 | 33.67593566 | 15.52696955 | 17.73417084 | 14.72966892 | 12.62890601 | 34.36103454 | 31.60139315 | 44.56900041 | 41.61831075 | 10.41033728 | 9.372927467 | 3.614218606 | 13.65274777 |
| **Adprh** | 1905.564378 | 1806.357379 | 1785.607751 | 1783.87628 | 1599.875555 | 1764.053206 | 1596.654545 | 1790.126092 | 1849.433913 | 1765.281977 | 1818.909354 | 1564.153176 | 1641.114391 | 1661.637004 | 1655.575308 |
| **Cd80** | 341.5547876 | 290.7601571 | 324.2287759 | 245.8436846 | 124.7725591 | 166.9362478 | 149.7427427 | 508.7109259 | 439.4098476 | 495.5024164 | 519.2830136 | 112.7786538 | 145.7064179 | 110.2336675 | 180.3599837 |
| **Timmdc1** | 343.3861806 | 400.8845763 | 392.3638085 | 397.6629425 | 326.815434 | 375.2558511 | 380.6713096 | 470.9975953 | 483.8022808 | 396.7514939 | 480.502315 | 392.122704 | 432.8588321 | 369.5538525 | 357.8457046 |
| **Poglut1** | 372.688468 | 423.0679125 | 447.9682604 | 426.9916627 | 424.9867369 | 413.8335554 | 457.3468104 | 555.6430707 | 532.7091988 | 465.7897494 | 564.6848072 | 431.1614688 | 400.4796281 | 388.5285002 | 428.2651405 |
| **Tmem39a** | 743.545543 | 758.9870041 | 719.7252296 | 653.8579401 | 647.9305989 | 667.744991 | 637.7597533 | 740.0193535 | 692.2209928 | 694.7520653 | 681.0269031 | 625.4877646 | 636.5069834 | 632.4882561 | 679.7631257 |
| **Arhgap31** | 427.6302569 | 435.7441047 | 494.9579381 | 433.0299287 | 383.818126 | 337.3795595 | 437.5013867 | 450.883819 | 424.3615651 | 415.1034352 | 428.4794265 | 380.8448387 | 393.6629536 | 307.2085815 | 395.9296853 |
| **B4galt4** | 90.65395167 | 96.65596504 | 98.67832311 | 117.3148811 | 107.0383883 | 79.25964705 | 101.0312481 | 65.36977302 | 84.27038173 | 80.39898114 | 65.26507822 | 118.8513506 | 51.12505891 | 112.0407768 | 54.61099107 |
| **Gap43** | 4.578482408 | 15.05297816 | 12.53058071 | 12.07653188 | 18.36753408 | 31.56357626 | 36.08258859 | 25.14222039 | 29.34415078 | 13.10852953 | 6.621094891 | 27.7608994 | 20.45002356 | 17.16753838 | 15.08987911 |
| **Gm19522** | 3.662785926 | 0 | 0 | 0.86260942 | 0.633363244 | 4.90988964 | 1.80412943 | 0 | 0 | 0.873901969 | 0 | 0 | 0 | 2.710663955 | 0.718565672 |
| **Zbtb20** | 12.81975074 | 22.18333624 | 10.96425812 | 6.038265938 | 13.30062813 | 7.715540863 | 23.45368258 | 12.5711102 | 1.504828245 | 12.23462756 | 8.512836289 | 16.48303402 | 12.78126473 | 7.228437213 | 13.65274777 |
| **BC002163** | 2.747089445 | 0.792262009 | 4.698967767 | 4.313047098 | 3.800179466 | 11.22260489 | 3.608258859 | 4.190370065 | 5.266898858 | 2.621705907 | 5.675224193 | 2.602584319 | 5.112505891 | 6.324882561 | 4.311394032 |
| **Mir568** | 0 | 1.584524017 | 2.349483883 | 0 | 0 | 0.701412806 | 2.706194144 | 0 | 0 | 1.747803938 | 0 | 0 | 0 | 0 | 0 |
| **Tigit** | 572.310301 | 385.8315981 | 386.0985182 | 389.0368483 | 494.0233305 | 502.2115689 | 580.0276116 | 304.2208667 | 322.7856586 | 297.1266694 | 292.2740459 | 464.995065 | 441.3796753 | 392.1427188 | 448.3849793 |
| **Qtrtd1** | 517.3685121 | 514.9703055 | 544.2970997 | 510.6647764 | 466.1553478 | 421.5490962 | 528.6099229 | 584.1375871 | 645.5713172 | 529.5845932 | 605.3572472 | 481.478099 | 498.4693244 | 474.3661921 | 484.3132629 |
| **2610015P09Rik** | 136.4387758 | 122.8006113 | 81.44877463 | 105.2383492 | 105.7716618 | 126.254305 | 126.2890601 | 104.7592516 | 109.1000478 | 85.64239295 | 90.80358708 | 115.3812381 | 101.3980335 | 113.8478861 | 114.2519418 |
| **Zdhhc23** | 48.53191352 | 45.15893448 | 56.3876132 | 63.83309705 | 56.36932874 | 51.20313482 | 32.47432973 | 55.31288486 | 41.38277674 | 54.18192207 | 38.78069865 | 43.37640531 | 54.53339617 | 48.79195118 | 61.07808212 |
| **Gramd1c** | 18.31392963 | 19.80655021 | 15.66322589 | 27.60350143 | 20.26762382 | 17.53532014 | 21.64955315 | 15.92340625 | 7.524141226 | 27.09096104 | 21.75502607 | 21.68820266 | 18.74585493 | 22.58886629 | 14.37131344 |
| **Atp6v1a** | 1886.334752 | 1861.023458 | 1930.492591 | 1792.502374 | 1560.607034 | 1662.34835 | 1723.84567 | 2274.532871 | 2170.714744 | 2074.643274 | 2321.166695 | 1684.739582 | 1745.068677 | 1669.768996 | 1613.179934 |
| **Naa50** | 5245.109446 | 4855.77385 | 5232.300608 | 4853.903204 | 4623.551683 | 4857.985092 | 4965.866255 | 5657.837662 | 5502.404478 | 5325.558598 | 5415.10975 | 5054.218747 | 4821.945139 | 4542.169233 | 4577.981896 |
| **Gm608** | 1152.86187 | 1112.33586 | 993.0485214 | 1017.879115 | 976.6461227 | 1017.048568 | 1234.926595 | 984.7369653 | 1139.907396 | 962.1660678 | 1071.671502 | 1138.196875 | 1192.065957 | 1063.483825 | 1038.327396 |
| **Sidt1** | 433.1244358 | 496.7482793 | 445.6187766 | 513.2526047 | 516.1910441 | 559.727419 | 552.0636054 | 303.3827927 | 373.9498189 | 360.9215132 | 388.7528572 | 545.6751788 | 483.983891 | 567.4323212 | 582.0381943 |
| **Spice1** | 132.7759898 | 162.4137117 | 111.9920651 | 122.4905376 | 126.0392856 | 121.3444154 | 122.6808012 | 141.6345082 | 158.0069657 | 173.0325898 | 118.2338373 | 113.6461819 | 151.6710081 | 192.4571408 | 162.3958419 |
| **BC027231** | 437.7029182 | 443.6667248 | 418.9912926 | 394.2125048 | 402.1856601 | 387.8812816 | 432.0889984 | 491.9494457 | 406.3036262 | 421.220749 | 431.3170386 | 399.930457 | 426.0421576 | 417.442249 | 413.1752614 |
| **Gtpbp8** | 104.3893989 | 125.1773973 | 115.9078716 | 138.0175071 | 97.53793962 | 105.2119209 | 112.7580893 | 86.32162334 | 103.8331489 | 114.4811579 | 88.91184568 | 79.81258578 | 97.13761193 | 93.96968376 | 91.25784034 |
| **Cd200r1** | 35.71216278 | 35.65179038 | 30.54329049 | 31.91654853 | 22.80107679 | 35.07064029 | 40.59291217 | 33.52296052 | 30.0965649 | 38.45168663 | 27.43025026 | 29.49595561 | 34.08337261 | 37.04574071 | 30.17975822 |
| **Cd200r4** | 24.723805 | 15.05297816 | 13.31374201 | 12.07653188 | 20.90098706 | 28.05651223 | 26.15987673 | 24.30414638 | 27.83932254 | 11.3607256 | 19.86328467 | 27.7608994 | 18.74585493 | 17.16753838 | 22.9941015 |
| **Ccdc80** | 3.662785926 | 2.376786026 | 5.482129061 | 3.450437679 | 9.500448664 | 5.611302446 | 2.706194144 | 9.218814144 | 0.752414123 | 3.495607876 | 1.891741398 | 5.205168638 | 2.556252945 | 0 | 2.155697016 |
| **Slc35a5** | 155.6684019 | 115.6702532 | 138.6195491 | 127.6661941 | 91.83767042 | 98.89920561 | 123.5828659 | 97.21658551 | 106.0903913 | 113.607256 | 89.85771638 | 139.6720251 | 103.1022021 | 121.0763233 | 103.4734568 |
| **Atg3** | 899.2139449 | 895.2560696 | 877.9238111 | 971.2982065 | 879.7415463 | 892.1970889 | 940.8534975 | 839.7501611 | 957.0707639 | 956.9226559 | 895.7395517 | 919.5797927 | 854.6405681 | 842.1129353 | 907.5484437 |
| **Btla** | 428.5459534 | 581.5203142 | 541.9476158 | 542.581325 | 557.359655 | 509.2256969 | 549.3574113 | 678.0018766 | 613.2175099 | 629.2094176 | 559.009583 | 573.4360783 | 483.1318067 | 479.78752 | 512.3373241 |
| **Cd200** | 745.376936 | 593.4042444 | 681.3503262 | 532.2300119 | 554.826202 | 530.2680811 | 570.1048997 | 1189.227025 | 863.7714127 | 662.4176924 | 698.0525757 | 556.0855161 | 489.9484812 | 507.7977142 | 513.0558898 |
| **Tagln3** | 0 | 0 | 0.783161294 | 0 | 0 | 0 | 0 | 0 | 1.504828245 | 2.621705907 | 0 | 0 | 0.852084315 | 3.614218606 | 0 |
| **Abhd10** | 196.8747435 | 157.6601397 | 156.6322589 | 160.4453521 | 139.3399137 | 183.0687423 | 151.5468721 | 192.757023 | 191.8656013 | 159.9240603 | 202.4163295 | 163.095284 | 126.960563 | 132.8225338 | 181.0785493 |
| **Phldb2** | 0.915696482 | 0.792262009 | 4.698967767 | 11.21392246 | 1.900089733 | 4.90988964 | 7.216517718 | 1.676148026 | 3.00965649 | 4.369509845 | 1.891741398 | 1.735056213 | 1.70416863 | 5.421327909 | 6.467091048 |
| **Plcxd2** | 630.9148758 | 532.4000697 | 582.6720031 | 505.4891199 | 467.4220743 | 540.7892732 | 532.2181817 | 480.2164095 | 412.3229392 | 511.2326518 | 561.8471951 | 632.4279895 | 577.7131657 | 492.4372851 | 506.5887987 |
| **Cd96** | 1356.146489 | 1430.825187 | 1636.023944 | 1506.116047 | 1527.038782 | 1596.415546 | 1676.03624 | 1704.642543 | 1434.853732 | 1609.727427 | 1938.089062 | 1579.768682 | 1668.381089 | 1683.322316 | 1550.66472 |
| **Gm4737** | 33.88076982 | 16.63750218 | 31.32645178 | 19.84001665 | 25.96789302 | 29.45933784 | 31.57226502 | 42.74177467 | 34.61104964 | 40.19949057 | 48.23940564 | 25.15831508 | 26.41461377 | 36.14218606 | 29.46119255 |
| **Pvrl3** | 13.73544722 | 19.80655021 | 14.0969033 | 27.60350143 | 24.06780328 | 15.43108173 | 25.25781201 | 21.78992434 | 22.57242368 | 23.59535316 | 28.37612096 | 14.74797781 | 20.45002356 | 11.74621047 | 30.17975822 |
| **Morc1** | 12.81975074 | 3.961310043 | 9.397935534 | 6.900875357 | 5.066905954 | 2.104238417 | 4.510323574 | 0 | 6.019312981 | 0.873901969 | 4.729353494 | 1.735056213 | 0 | 2.710663955 | 0.718565672 |
| **Trat1** | 258.2264078 | 282.837537 | 264.7085175 | 232.0419339 | 251.445208 | 308.6216345 | 319.330909 | 98.05465953 | 162.5214505 | 184.3933154 | 199.5787174 | 263.7285443 | 300.7857633 | 252.0917478 | 281.6777434 |
| **Dzip3** | 368.1099856 | 362.0637379 | 335.193034 | 303.6385157 | 342.0161519 | 271.4467558 | 280.5421263 | 372.1048618 | 360.4063647 | 323.3437285 | 384.9693744 | 286.2842751 | 376.6212673 | 315.3405734 | 342.0372599 |
| **C330027C09Rik** | 1378.123205 | 1600.369257 | 1699.460009 | 1563.910878 | 1471.302816 | 1437.896252 | 1600.262804 | 1887.342677 | 1935.961537 | 1971.522842 | 1801.883681 | 1736.791269 | 1743.364509 | 1785.423992 | 1685.755066 |
| **Ift57** | 447.7755795 | 416.7298165 | 447.1850992 | 392.4872859 | 363.5505022 | 358.4219437 | 414.9497688 | 459.2645592 | 546.252653 | 490.2590046 | 430.3711679 | 392.122704 | 368.9525085 | 349.6756502 | 357.127139 |
| **Cd47** | 5887.928376 | 5741.522776 | 5743.704934 | 5484.47069 | 5505.193319 | 5990.065361 | 6213.421755 | 4269.987097 | 4823.72694 | 4649.158475 | 4706.652597 | 5990.281574 | 5606.714794 | 5782.74977 | 5783.735094 |
| **Gm4827** | 5.494178889 | 9.507144102 | 3.915806472 | 0 | 0.633363244 | 2.104238417 | 1.80412943 | 7.542666117 | 3.00965649 | 4.369509845 | 0 | 3.470112425 | 5.964590206 | 3.614218606 | 2.874262688 |
| **Bbx** | 826.8739229 | 730.4655718 | 758.1001331 | 624.5292198 | 561.7931977 | 532.3723195 | 732.4765484 | 670.4592104 | 647.0761454 | 606.4879664 | 597.7902816 | 568.2309096 | 537.6652029 | 579.1785317 | 486.4689599 |
| **G730013B05Rik** | 0 | 0 | 0 | 0 | 0 | 0 | 0 | 3.352296052 | 2.257242368 | 0 | 0 | 0.867528106 | 0 | 0 | 5.029959704 |
| **5330426P16Rik** | 189.5491717 | 195.6887161 | 224.7672915 | 171.6592745 | 177.9750716 | 183.0687423 | 183.1191371 | 95.54043749 | 151.2352386 | 149.4372367 | 154.1769239 | 183.9159585 | 182.3460434 | 182.5180396 | 188.9827717 |
| **Cblb** | 3804.718881 | 3538.24213 | 3445.126534 | 3147.661772 | 3240.919721 | 3248.944116 | 3770.630508 | 1970.312005 | 2387.410011 | 2119.212275 | 2088.482503 | 3545.58737 | 3615.393749 | 3118.167103 | 3299.653566 |
| **Alcam** | 934.9261077 | 960.2215543 | 991.4821988 | 921.2668602 | 863.9074652 | 774.3597375 | 819.074761 | 1265.49176 | 1226.43502 | 998.8699505 | 1009.244036 | 928.2550737 | 874.2385074 | 760.7930166 | 854.374584 |
| **Nfkbiz** | 206.0317084 | 261.4464628 | 283.5043886 | 260.5080447 | 299.5808145 | 256.0156741 | 288.6607087 | 227.9561316 | 188.8559448 | 204.4930607 | 293.2199166 | 267.1986567 | 279.4836554 | 362.3254153 | 302.5161479 |
| **Nxpe3** | 553.9963713 | 596.5732924 | 624.1795517 | 640.0561894 | 611.1955307 | 654.4181477 | 706.3166717 | 553.9669226 | 515.403674 | 484.1416908 | 539.1462983 | 648.9110235 | 633.0986462 | 713.8081747 | 633.7749227 |
| **Cep97** | 391.0023976 | 411.1839824 | 460.4988412 | 376.097707 | 412.9528353 | 373.1516126 | 405.0270569 | 327.6869391 | 374.702233 | 316.3525127 | 331.0547446 | 440.704278 | 425.1900733 | 447.2595525 | 400.959645 |
| **Rpl24** | 12164.11206 | 11414.11876 | 11748.98574 | 11567.59232 | 12089.00424 | 11959.78975 | 11939.72856 | 11858.74728 | 11189.15042 | 11409.66411 | 11027.90648 | 11177.23212 | 11228.76711 | 11512.18982 | 11446.03259 |
| **Zbtb11os1** | 11.90405426 | 8.714882094 | 7.831612945 | 12.07653188 | 9.500448664 | 16.83390734 | 13.53097072 | 4.190370065 | 8.276555348 | 13.10852953 | 17.02567258 | 9.542809169 | 17.0416863 | 8.131991864 | 12.21561642 |
| **Zbtb11** | 629.0834828 | 621.9256767 | 578.7561966 | 578.8109206 | 547.2258431 | 547.1019885 | 668.4299537 | 610.1178815 | 666.6389126 | 540.9453188 | 580.764609 | 550.8803475 | 593.0506834 | 497.858613 | 554.7326988 |
| **Pcnp** | 1433.98069 | 1464.100192 | 1603.914331 | 1461.260357 | 1472.569543 | 1430.882124 | 1496.525362 | 1547.922702 | 1513.857215 | 1453.298974 | 1631.626955 | 1497.353511 | 1562.722634 | 1434.844787 | 1539.167669 |
| **Trmt10c** | 550.3335854 | 562.506026 | 541.9476158 | 469.2595243 | 485.1562451 | 497.3016793 | 495.2335284 | 508.7109259 | 503.365048 | 543.5670247 | 495.6362462 | 555.217988 | 479.7234694 | 441.8382246 | 492.2174853 |
| **Senp7** | 262.8048902 | 238.4708646 | 264.7085175 | 303.6385157 | 274.2462848 | 279.1622967 | 349.0990446 | 194.433171 | 201.6469849 | 166.0413741 | 203.3622002 | 280.2115783 | 273.5190652 | 287.3303792 | 310.4203703 |
| **Impg2** | 0.915696482 | 0 | 3.132645178 | 2.587828259 | 0 | 2.104238417 | 3.608258859 | 0 | 0 | 6.991215751 | 4.729353494 | 0.867528106 | 4.260421576 | 2.710663955 | 2.874262688 |
| **Abi3bp** | 13.73544722 | 5.54583406 | 10.96425812 | 6.900875357 | 11.4005384 | 34.36922748 | 21.64955315 | 4.190370065 | 8.276555348 | 0.873901969 | 4.729353494 | 22.55573076 | 11.0770961 | 17.16753838 | 20.11983882 |
| **Tfg** | 1498.079444 | 1362.690655 | 1436.317814 | 1295.639348 | 1216.057429 | 1367.754971 | 1418.045732 | 1288.119758 | 1300.171604 | 1382.512915 | 1333.677685 | 1364.621711 | 1350.55364 | 1326.418229 | 1360.244817 |
| **Tmem45a** | 6.409875371 | 3.169048034 | 3.915806472 | 4.313047098 | 10.13381191 | 7.715540863 | 9.020647148 | 0.838074013 | 3.00965649 | 4.369509845 | 3.783482795 | 1.735056213 | 2.556252945 | 0 | 7.18565672 |
| **Tomm70a** | 4370.619307 | 4205.326741 | 4352.027313 | 4236.27486 | 3862.882427 | 4201.462706 | 4403.879938 | 4766.964986 | 4906.492493 | 4878.994692 | 4765.29658 | 4165.869966 | 4341.369586 | 4147.315851 | 4184.926474 |
| **Nit2** | 287.5286952 | 280.460751 | 281.1549047 | 297.6002498 | 291.9804556 | 295.996204 | 313.016456 | 346.1245674 | 258.8304582 | 292.7571596 | 343.3510637 | 287.1518032 | 312.7149437 | 340.6401036 | 337.7258658 |
| **Tbc1d23** | 180.3922069 | 213.9107423 | 225.5504528 | 180.2853687 | 196.3426057 | 180.2630911 | 186.727396 | 229.6322796 | 170.7980058 | 200.9974528 | 255.3850887 | 170.0355088 | 173.8252003 | 189.7464768 | 181.797115 |
| **Tmem30c** | 0.915696482 | 0.792262009 | 0 | 3.450437679 | 1.266726489 | 2.104238417 | 0.902064715 | 0.838074013 | 0 | 0 | 1.891741398 | 0 | 0 | 0 | 0 |
| **Cmss1** | 682.1938788 | 596.5732924 | 562.3098094 | 690.0875357 | 703.6665644 | 681.0718343 | 640.4659475 | 750.9143157 | 879.5721093 | 732.3298499 | 768.9928781 | 634.1630457 | 590.4944304 | 620.7420456 | 594.9723764 |
| **Filip1l** | 477.9935634 | 504.6708994 | 640.6259389 | 580.5361394 | 481.9894289 | 529.5666683 | 615.2081355 | 578.271069 | 673.4106397 | 554.0538483 | 544.8215225 | 598.5943933 | 552.1506362 | 575.5643131 | 587.7867197 |
| **Dcbld2** | 74.17141501 | 66.55000871 | 55.60445191 | 42.26786156 | 72.83677309 | 53.30737323 | 62.24246532 | 64.53169901 | 55.67864507 | 55.92972601 | 69.99443171 | 45.97898963 | 59.64590206 | 44.27417793 | 55.32955674 |
| **St3gal6** | 132.7759898 | 142.6071615 | 144.1016782 | 169.9340557 | 198.8760587 | 216.0351442 | 221.0058551 | 91.35006742 | 87.28003822 | 100.4987264 | 129.5842857 | 209.0742736 | 190.8668866 | 210.5282338 | 222.7553583 |
| **Cpox** | 1235.274554 | 1398.342445 | 1322.759426 | 1355.159398 | 1167.921822 | 1343.205523 | 1275.519507 | 1485.905225 | 1596.622768 | 1707.604447 | 2164.152159 | 1373.296992 | 1383.784928 | 1332.743111 | 1210.064592 |
| **Gpr15** | 23.80810852 | 11.88393013 | 36.80858084 | 26.74089201 | 19.63426057 | 30.16075065 | 19.84542373 | 26.81836842 | 32.35380727 | 53.3080201 | 150.3934411 | 15.61550591 | 24.71044514 | 37.94929537 | 9.341353736 |
| **Cldnd1** | 1512.730588 | 1525.104366 | 1538.911944 | 1448.321216 | 1281.293843 | 1273.064242 | 1333.251648 | 1594.854847 | 1397.98544 | 1795.868546 | 2651.275569 | 1273.53126 | 1225.297245 | 1283.95116 | 1261.082754 |
| **Mina** | 1114.402618 | 1103.620978 | 1055.701425 | 952.3207993 | 1038.715721 | 1038.090952 | 1090.59624 | 1042.564072 | 971.3666323 | 1000.617754 | 1108.560459 | 1000.259907 | 1057.436635 | 1007.463437 | 1039.045962 |
| **Crybg3** | 199.621833 | 189.35062 | 222.4178076 | 255.3323882 | 179.8751614 | 196.3955856 | 199.356302 | 178.5097648 | 162.5214505 | 256.0532769 | 308.3538478 | 230.7624763 | 265.8503063 | 223.1779989 | 239.2823688 |
| **Arl6** | 97.97952353 | 88.73334495 | 90.06354887 | 81.94789487 | 85.50403798 | 83.46812388 | 65.85072418 | 60.34132894 | 73.73658401 | 101.3726284 | 99.31642337 | 55.5217988 | 73.2792511 | 76.80214538 | 74.01226421 |
| **Nsun3** | 139.1858652 | 150.5297816 | 150.3669685 | 150.094039 | 149.4737256 | 119.240177 | 137.1138366 | 176.8336168 | 147.473168 | 167.789178 | 152.2851825 | 143.1421375 | 159.3397669 | 148.1829629 | 148.7430941 |
| **Arl13b** | 315.9152861 | 331.9577816 | 342.2414857 | 260.5080447 | 248.2783918 | 291.0863144 | 331.0577503 | 310.9254588 | 271.6214983 | 325.0915324 | 263.897925 | 315.7802307 | 271.8148965 | 278.2948327 | 329.1030778 |
| **Pros1** | 655.6386808 | 663.1233011 | 661.7712938 | 672.8353473 | 490.8565143 | 501.5101561 | 504.2541756 | 703.144097 | 591.3975003 | 723.5908303 | 632.7874975 | 517.9142794 | 559.8193951 | 524.0616979 | 479.2833032 |
| **4930453N24Rik** | 623.5893039 | 606.8726985 | 710.3272941 | 630.5674858 | 616.8957999 | 695.8015033 | 746.0075191 | 599.2229193 | 601.9312981 | 657.1742806 | 649.8131701 | 656.7187765 | 616.9090442 | 717.4223933 | 652.4576301 |
| **Zfp654** | 312.2525002 | 320.0738514 | 389.2311634 | 323.4785324 | 357.2168698 | 351.4078157 | 368.9444683 | 314.2777549 | 218.2000955 | 252.557669 | 344.2969344 | 395.5928165 | 414.9650615 | 391.2391641 | 393.7739882 |
| **Cggbp1** | 1813.99473 | 1680.38772 | 1807.536268 | 1694.1649 | 1629.010264 | 1723.371264 | 1803.227365 | 1639.27277 | 1651.548999 | 1654.296427 | 1637.30218 | 1728.115988 | 1890.775095 | 1633.62681 | 1746.833149 |
| **Chmp2b** | 455.1011513 | 489.6179213 | 446.4019379 | 464.9464772 | 452.8547197 | 431.3688755 | 457.3468104 | 447.531523 | 392.760172 | 498.1241223 | 464.4225131 | 457.187312 | 497.6172401 | 421.0564676 | 445.5107166 |
| **Gbe1** | 398.3279695 | 337.5036156 | 314.8308404 | 338.1428925 | 342.6495152 | 413.8335554 | 420.3621571 | 138.2822122 | 210.6759543 | 190.5106292 | 193.9034932 | 400.7979851 | 364.6920869 | 323.4725653 | 330.5402091 |
| **Rbm11** | 3.662785926 | 1.584524017 | 1.566322589 | 0 | 3.166816221 | 2.805651223 | 1.80412943 | 0.838074013 | 0 | 0 | 1.891741398 | 2.602584319 | 1.70416863 | 2.710663955 | 0.718565672 |
| **Hspa13** | 453.2697584 | 442.0822007 | 473.8125832 | 426.1290533 | 388.885032 | 397.7010608 | 498.8417873 | 454.2361151 | 458.2202007 | 454.4290238 | 477.6647029 | 505.768886 | 417.5213144 | 474.3661921 | 434.0136659 |
| **Samsn1** | 1013.676005 | 1025.187039 | 939.7935534 | 794.4632755 | 958.2785886 | 963.0397822 | 1055.415716 | 803.7129785 | 826.1507066 | 787.385674 | 750.0754641 | 949.9432764 | 969.6719507 | 967.7070318 | 911.1412721 |
| **Nrip1** | 470.6679915 | 493.5792313 | 495.7410994 | 502.0386822 | 449.6879034 | 476.2592951 | 558.3780584 | 485.2448536 | 466.496756 | 505.98924 | 517.3912722 | 669.731698 | 652.6965854 | 468.9448642 | 567.6668809 |
| **Usp25** | 2970.519386 | 2524.939021 | 2479.488658 | 2432.558563 | 2579.688494 | 2894.730649 | 2973.2053 | 1815.268312 | 1908.122215 | 1933.071155 | 2224.687884 | 2698.879939 | 2644.01763 | 2633.861809 | 2683.842785 |
| **Cxadr** | 24.723805 | 44.36667248 | 27.41064531 | 16.38957897 | 25.33452977 | 14.72966892 | 9.020647148 | 11.73303618 | 9.028969471 | 26.21705907 | 20.80915537 | 21.68820266 | 18.74585493 | 27.10663955 | 18.68270747 |
| **Btg3** | 10.0726613 | 7.922620085 | 3.132645178 | 11.21392246 | 13.30062813 | 10.52119209 | 13.53097072 | 17.59955427 | 12.03862596 | 13.10852953 | 20.80915537 | 11.27786538 | 13.63334904 | 6.324882561 | 14.37131344 |
| **D16Ertd472e** | 2515.418235 | 2311.820541 | 2183.453689 | 2122.019172 | 1788.617802 | 1921.871088 | 2058.511679 | 1617.482845 | 1565.773789 | 1677.89178 | 1677.028749 | 1769.757337 | 1870.325072 | 1739.342704 | 1779.887169 |
| **4930478L05Rik** | 3.662785926 | 0 | 0 | 0.86260942 | 0 | 1.402825611 | 0.902064715 | 1.676148026 | 5.266898858 | 0 | 0.945870699 | 2.602584319 | 0.852084315 | 1.807109303 | 2.874262688 |
| **Mir155hg** | 35.71216278 | 33.27500436 | 41.50754861 | 28.46611085 | 27.86798275 | 25.95227381 | 32.47432973 | 61.17940295 | 47.40208972 | 67.29045161 | 55.80637123 | 46.84651774 | 29.82295103 | 31.62441281 | 17.9641418 |
| **Mrpl39** | 931.2633218 | 804.1459386 | 825.4520044 | 823.7919958 | 781.5702434 | 800.3120113 | 770.3632664 | 952.8901528 | 860.7617562 | 801.3681055 | 821.0157665 | 854.5151847 | 800.1071719 | 757.178798 | 790.4222392 |
| **Jam2** | 7.325571853 | 3.169048034 | 3.915806472 | 1.725218839 | 3.800179466 | 0.701412806 | 0.902064715 | 7.542666117 | 2.257242368 | 8.739019689 | 2.837612096 | 0.867528106 | 0.852084315 | 0 | 1.437131344 |
| **Atp5j** | 1715.09951 | 1845.178218 | 1895.250333 | 1714.867526 | 1964.05942 | 2003.936386 | 1862.763636 | 1850.467421 | 1968.315345 | 1815.094389 | 1830.259802 | 2036.088465 | 1931.675142 | 2024.865974 | 1775.575775 |
| **Gabpa** | 1474.271335 | 1490.244838 | 1578.85317 | 1479.375155 | 1446.60165 | 1520.662963 | 1555.159568 | 1527.808926 | 1430.339247 | 1505.733092 | 1555.957299 | 1608.397109 | 1596.806007 | 1527.007361 | 1588.748701 |
| **App** | 86.07546927 | 76.05715282 | 68.13503262 | 60.38265938 | 62.06959794 | 59.62008849 | 71.26311247 | 142.4725822 | 72.23175577 | 69.91215751 | 65.26507822 | 82.4151701 | 61.35007069 | 58.73105235 | 54.61099107 |
| **N6amt1** | 226.1770309 | 174.2976419 | 229.4662593 | 167.3462274 | 207.1097809 | 211.1252545 | 211.0831433 | 269.8598322 | 233.248378 | 213.2320804 | 261.0603129 | 232.4975325 | 230.9148494 | 216.8531164 | 215.5697016 |
| **Ltn1** | 1689.460008 | 1731.884751 | 1767.595042 | 1660.523133 | 1559.973671 | 1515.05166 | 1630.03094 | 1878.123863 | 1723.780755 | 1763.534173 | 1894.57901 | 1695.14992 | 1628.333126 | 1564.956657 | 1644.078257 |
| **Rwdd2b** | 273.793248 | 244.8089606 | 244.3463239 | 241.5306375 | 248.911755 | 251.8071973 | 254.3822496 | 296.6782006 | 309.9946185 | 294.5049635 | 289.4364338 | 290.6219156 | 244.5481985 | 298.173035 | 291.7376628 |
| **Usp16** | 1033.821328 | 956.2602443 | 971.1200052 | 985.9625667 | 930.4106058 | 899.9126297 | 955.286533 | 1195.093543 | 1168.499132 | 1115.972814 | 1120.856778 | 1000.259907 | 928.7719035 | 866.5089109 | 939.8838989 |
| **Cct8** | 9808.940711 | 9446.932189 | 10169.34941 | 9604.293278 | 9243.303187 | 9201.133185 | 9203.766285 | 11619.89619 | 10653.43156 | 11093.31159 | 11551.91884 | 9502.902876 | 9412.97543 | 9277.699162 | 9224.946097 |
| **B130034C11Rik** | 13.73544722 | 9.507144102 | 2.349483883 | 15.52696955 | 8.233722176 | 10.52119209 | 2.706194144 | 5.866518091 | 9.781383594 | 3.495607876 | 0.945870699 | 4.337640531 | 1.70416863 | 9.939101167 | 2.874262688 |
| **Bach1** | 328.7350369 | 293.9292052 | 356.338389 | 324.3411418 | 353.4166903 | 354.2134669 | 368.9444683 | 281.5928684 | 331.0622139 | 253.431571 | 209.9832951 | 364.3618046 | 326.3482927 | 263.8379583 | 362.8756643 |
| **Tiam1** | 340.6390911 | 421.4833885 | 358.6878729 | 388.1742388 | 359.1169595 | 315.6357626 | 359.0217565 | 622.6889917 | 425.1139793 | 339.0739639 | 388.7528572 | 327.0580961 | 318.6795339 | 317.1476827 | 289.5819658 |
| **Gm10789** | 1.831392963 | 1.584524017 | 2.349483883 | 9.488703616 | 1.266726489 | 0.701412806 | 10.82477658 | 2.514222039 | 9.028969471 | 12.23462756 | 8.512836289 | 6.072696744 | 9.372927467 | 10.84265582 | 5.029959704 |
| **Sod1** | 3590.445904 | 3495.459982 | 3731.763568 | 3585.004748 | 3644.372108 | 3716.085045 | 3529.779229 | 4046.221335 | 3744.012674 | 3849.538173 | 3944.280814 | 3672.246474 | 3646.068785 | 3821.132622 | 3505.163348 |
| **Scaf4** | 2133.572802 | 2147.822305 | 2171.70627 | 2004.704291 | 1892.489374 | 2111.252545 | 2347.172388 | 2231.791097 | 2127.827139 | 1961.036018 | 1942.818415 | 2062.114309 | 2010.066899 | 1978.784687 | 2116.175904 |
| **Hunk** | 19.22962611 | 11.09166812 | 10.18109683 | 4.313047098 | 12.03390164 | 27.35509942 | 10.82477658 | 3.352296052 | 1.504828245 | 11.3607256 | 5.675224193 | 19.08561834 | 24.71044514 | 4.517773258 | 32.33545524 |
| **Mis18a** | 516.4528156 | 562.506026 | 505.9221962 | 561.5587322 | 574.4604626 | 693.6972648 | 593.5585823 | 601.7371414 | 577.8540461 | 667.6611042 | 608.1948593 | 690.5523726 | 646.7319952 | 794.2245387 | 740.8412078 |
| **Urb1** | 1455.957406 | 1416.564471 | 1384.629169 | 1456.94731 | 1431.400932 | 1348.115413 | 1542.530662 | 1602.397513 | 1476.988923 | 1501.363583 | 1557.849041 | 1323.84789 | 1532.047599 | 1536.946462 | 1493.898032 |
| **1110004E09Rik** | 717.9060415 | 720.1661657 | 610.0826484 | 722.0040842 | 640.33024 | 692.995852 | 722.5538365 | 688.0587647 | 725.3272142 | 748.0600854 | 810.6111888 | 667.1291137 | 676.5549462 | 740.0112596 | 662.5175496 |
| **Synj1** | 833.2837982 | 798.6001046 | 805.0898107 | 782.3867436 | 784.7370597 | 721.0523643 | 738.7910014 | 925.2337104 | 918.6976437 | 862.5412433 | 929.7908969 | 837.1646226 | 854.6405681 | 751.7574701 | 832.0990481 |
| **4930404I05Rik** | 0 | 3.169048034 | 3.915806472 | 8.626094197 | 3.800179466 | 4.208476834 | 0.902064715 | 9.218814144 | 6.019312981 | 1.747803938 | 0 | 0 | 0.852084315 | 3.614218606 | 1.437131344 |

| | | | | | | | | | | | | | | | |
|---|---|---|---|---|---|---|---|---|---|---|---|---|---|---|---|
| **Paxbp1** | 890.9726766 | 930.90786 | 998.5306505 | 900.5642341 | 913.3097982 | 979.1722768 | 949.8741447 | 931.1002285 | 880.3245234 | 964.7877737 | 956.2752765 | 1021.948109 | 1031.022021 | 992.1030074 | 1036.171699 |
| **4932438H23Rik** | 21.06101908 | 27.7291703 | 27.41064531 | 37.95481446 | 18.36753408 | 29.45933784 | 24.3557473 | 12.5711102 | 0.752414123 | 15.73023544 | 32.15960376 | 13.8804497 | 23.85836082 | 15.36042908 | 20.11983882 |
| **Ifnar2** | 948.6615549 | 1126.596576 | 985.2169085 | 917.8164225 | 1035.548904 | 1042.299429 | 1006.704222 | 817.1221627 | 899.8872906 | 682.5174377 | 685.7562566 | 977.7041758 | 978.1927938 | 1051.737614 | 906.829878 |
| **Il10rb** | 723.4002204 | 846.1358251 | 842.6815529 | 786.6997907 | 881.641636 | 850.8137333 | 856.0594143 | 575.756847 | 644.8189031 | 668.5350062 | 666.8388426 | 848.4424879 | 829.0780387 | 916.2044167 | 825.6319571 |
| **A930006K02Rik** | 9.156964816 | 25.35238427 | 30.54329049 | 29.32872027 | 15.83408111 | 12.6254305 | 8.118582433 | 16.76148026 | 22.57242368 | 34.95607876 | 17.02567258 | 19.95314644 | 18.74585493 | 15.36042908 | 15.80844478 |
| **Ifnar1** | 2064.895566 | 2238.140174 | 2377.67769 | 2118.568735 | 2117.333326 | 2015.158991 | 2129.774792 | 1696.261802 | 1821.594591 | 1743.434428 | 1633.518697 | 2246.030267 | 2186.448353 | 2090.825464 | 2201.685219 |
| **Ifngr2** | 171.2352421 | 221.8333624 | 178.5607751 | 207.0262607 | 103.8715721 | 117.1359386 | 119.9746071 | 198.6235411 | 182.0842177 | 165.1674721 | 129.5842857 | 124.9240473 | 103.9542864 | 121.0763233 | 130.7789523 |
| **Tmem50b** | 311.3368037 | 336.7113536 | 335.193034 | 315.7150476 | 330.6156135 | 370.3459614 | 353.6093682 | 248.9079819 | 338.5863552 | 256.9271789 | 281.8694682 | 387.7850635 | 424.337989 | 358.7111967 | 391.6182912 |
| **Dnajc28** | 4.578482408 | 7.130358077 | 9.397935534 | 6.038265938 | 5.700269198 | 9.81977928 | 10.82477658 | 10.89496217 | 7.524141226 | 1.747803938 | 0.945870699 | 0.867528106 | 9.372927467 | 1.807109303 | 6.467091048 |
| **Gart** | 6551.808326 | 6343.641902 | 6699.161713 | 6832.729213 | 6104.988312 | 6230.649953 | 5985.199383 | 7708.604772 | 7147.934165 | 7449.140383 | 7523.455538 | 5895.72101 | 5784.800416 | 5652.6379 | 5963.376512 |
| **Son** | 7743.129448 | 7638.990286 | 7542.626427 | 7640.994239 | 7291.911031 | 7042.184569 | 8174.510445 | 8117.58489 | 7859.717924 | 7140.652988 | 7855.456153 | 7919.664082 | 7952.502913 | 7263.675844 | 7961.707645 |
| **Donson** | 771.932134 | 728.8810478 | 791.7760687 | 789.287619 | 836.0394824 | 786.985168 | 820.8788905 | 677.1638025 | 770.4720615 | 825.8373606 | 865.4716894 | 769.4974303 | 811.184268 | 829.4631701 | 794.7336332 |
| **Gm10785** | 6.409875371 | 4.753572051 | 3.132645178 | 11.21392246 | 3.800179466 | 4.208476834 | 9.020647148 | 3.352296052 | 9.028969471 | 2.621705907 | 15.13393118 | 6.072696744 | 6.816674521 | 6.324882561 | 10.77848508 |
| **Cryzl1** | 108.9678813 | 90.31786897 | 75.18348427 | 116.4522717 | 76.00358931 | 119.9415898 | 97.4229892 | 95.54043749 | 96.30900769 | 93.50751067 | 132.4218978 | 126.6591035 | 94.58135898 | 112.0407768 | 124.3118613 |
| **Itsn1** | 29.30228741 | 49.91250654 | 39.94122602 | 50.89395576 | 43.06870061 | 54.71019885 | 57.73214175 | 59.50325493 | 33.85863552 | 106.6160402 | 68.10269031 | 66.79966418 | 36.63962555 | 82.22347329 | 51.01816271 |
| **Atp5o** | 2677.496512 | 3047.831947 | 2894.564144 | 2791.404082 | 2979.340701 | 2877.896742 | 2866.761664 | 3039.694445 | 2930.653007 | 3179.255363 | 2925.578071 | 2932.244999 | 2972.922176 | 3016.065427 | 2791.627636 |
| **Slc5a3** | 310.4211073 | 332.7500436 | 349.2899373 | 295.0124215 | 292.6138189 | 250.4043716 | 288.6607087 | 399.7613042 | 342.3484258 | 324.2176305 | 317.8125548 | 328.7931523 | 282.0399083 | 269.2592862 | 281.6777434 |
| **Mrps6** | 344.3018771 | 341.4649257 | 367.3026471 | 401.9759896 | 376.8511303 | 401.2081249 | 356.3155623 | 488.5971496 | 407.0560403 | 521.7194754 | 437.9381335 | 384.3149511 | 377.4733516 | 327.0867839 | 389.4625942 |
| **Smim11** | 159.3311878 | 151.3220436 | 180.910259 | 164.7583992 | 193.8091527 | 162.7277709 | 183.1191371 | 187.7285789 | 141.453855 | 157.3023544 | 140.9347341 | 178.7107899 | 176.3814532 | 196.0713594 | 205.5097822 |
| **1700048M11Rik** | 1.831392963 | 3.169048034 | 0.783161294 | 0 | 1.266726489 | 0 | 0 | 2.514222039 | 0 | 0 | 0 | 0 | 0.852084315 | 0 | 0 |
| **Rcan1** | 126.3661145 | 135.4768035 | 147.2343234 | 125.9409753 | 176.7083452 | 154.3108173 | 172.2943605 | 133.2537681 | 214.4380249 | 120.5984717 | 159.8521481 | 141.4070813 | 154.227261 | 131.0154245 | 143.7131344 |
| **Runx1** | 167.5724561 | 205.9881222 | 218.5020012 | 205.3010419 | 275.5130113 | 284.0721863 | 311.2123266 | 170.9670987 | 148.9779963 | 130.2113934 | 103.0999062 | 313.1776464 | 269.2586436 | 281.9090513 | 262.2764703 |
| **Setd4** | 99.81091649 | 89.52560696 | 86.14774239 | 98.33747384 | 95.00448664 | 108.0175721 | 111.8560246 | 102.2450296 | 66.21244279 | 114.4811579 | 79.4531387 | 116.2487662 | 86.91260015 | 94.87323842 | 120.7190329 |
| **Cbr1** | 184.0549928 | 192.5196681 | 200.4892914 | 221.6906209 | 205.8430544 | 194.2913472 | 204.7686903 | 220.4134654 | 212.9331967 | 242.0708454 | 242.1428989 | 241.1728135 | 220.6898376 | 209.6246792 | 218.4439643 |
| **Cbr3** | 5.494178889 | 0 | 1.566322589 | 0.86260942 | 0 | 3.507064029 | 4.510323574 | 0 | 5.266898858 | 1.747803938 | 7.56696559 | 3.470112425 | 2.556252945 | 5.421327909 | 2.155697016 |
| **Dopey2** | 539.3452276 | 507.8399475 | 445.6187766 | 385.5864106 | 538.9921209 | 531.6709067 | 589.0482588 | 301.7066447 | 333.3194563 | 305.8656891 | 340.5134516 | 534.3973135 | 607.5361167 | 569.2394305 | 557.6069614 |
| **Morc3** | 1221.539106 | 1055.292995 | 1109.739554 | 1006.665193 | 1068.483793 | 1111.739297 | 1159.153158 | 862.3781594 | 884.086594 | 950.8053422 | 861.6882066 | 1073.132267 | 1126.455465 | 1019.209647 | 1036.171699 |
| **Chaf1b** | 1199.562391 | 1176.509083 | 1058.83407 | 1141.232262 | 1223.657788 | 1125.767553 | 1120.364376 | 1096.200809 | 1100.781861 | 1216.471541 | 1105.722847 | 1153.812381 | 1053.176214 | 1217.088116 | 1057.010103 |
| **Hlcs** | 257.3107113 | 217.8720523 | 241.2136787 | 229.4541056 | 226.7440415 | 209.7224289 | 265.2070261 | 247.2318339 | 261.8401147 | 231.5840218 | 262.0061836 | 274.1388816 | 222.3940063 | 248.4775292 | 232.096712 |
| **Ripply3** | 3.662785926 | 5.54583406 | 0.783161294 | 9.488703616 | 3.166816221 | 4.208476834 | 4.510323574 | 0 | 1.504828245 | 2.621705907 | 6.621094891 | 3.470112425 | 3.408337261 | 5.421327909 | 7.18565672 |
| **Pigp** | 170.3195456 | 146.5684716 | 176.2112913 | 180.2853687 | 168.474623 | 160.6235325 | 180.412943 | 165.1005806 | 174.5600764 | 206.2408647 | 208.0915537 | 169.1679807 | 167.8606101 | 160.832728 | 209.8211762 |
| **Ttc3** | 930.3476253 | 1014.887633 | 906.900779 | 850.5328878 | 845.5399311 | 825.5628723 | 978.7402155 | 1073.572811 | 914.9355731 | 898.371224 | 951.545923 | 971.631479 | 1031.874106 | 885.4835585 | 942.7581616 |
| **Dscr3** | 492.6447071 | 616.3798426 | 570.9245837 | 601.2387655 | 614.9957102 | 561.8316574 | 658.5072418 | 639.450472 | 612.4650958 | 544.4409266 | 591.1691867 | 600.3294495 | 593.9027677 | 631.5847014 | 597.8466391 |
| **Dyrk1a** | 1548.44275 | 1611.460925 | 1644.638718 | 1657.935305 | 1491.57044 | 1548.018062 | 1661.603205 | 1692.071432 | 1683.902806 | 1495.246269 | 1529.47292 | 1662.183852 | 1537.160105 | 1563.149547 | 1649.826783 |
| **Kcnj15** | 12.81975074 | 12.67619214 | 18.79587107 | 14.66436013 | 1.900089733 | 4.208476834 | 2.706194144 | 23.46607237 | 4.514484735 | 16.60413741 | 27.43025026 | 4.337640531 | 2.556252945 | 1.807109303 | 4.311394032 |
| **Erg** | 21.06101908 | 15.05297816 | 10.18109683 | 11.21392246 | 1.900089733 | 0.701412806 | 2.706194144 | 15.92340625 | 12.79104008 | 17.47803938 | 12.29631908 | 5.205168638 | 11.92918041 | 7.228437213 | 0 |
| **Ets2** | 561.3219432 | 536.3613798 | 510.621164 | 472.709962 | 556.0929285 | 558.3245933 | 667.5278889 | 360.3718256 | 314.5091032 | 350.4346895 | 368.8895725 | 528.3246167 | 545.3339617 | 530.3865805 | 612.9365182 |
| **Psmg1** | 806.7286003 | 795.4310565 | 830.9341335 | 758.2336799 | 869.6077344 | 898.5098041 | 893.9461323 | 889.1965279 | 825.3982925 | 787.385674 | 778.4515851 | 842.3697912 | 866.5697485 | 845.7271539 | 817.009169 |
| **Brwd1** | 519.199905 | 487.2411352 | 510.621164 | 552.932638 | 580.794095 | 529.5666683 | 635.0535592 | 521.2820361 | 565.0630061 | 474.5287691 | 532.5252034 | 621.1501241 | 512.9547577 | 486.1124025 | 564.7926182 |
| **Hmgn1** | 3465.911183 | 3501.798078 | 3647.96531 | 3406.444598 | 3660.839552 | 3841.637937 | 3815.733744 | 3928.890973 | 3774.109239 | 3707.092152 | 3565.932534 | 3877.850635 | 3828.414828 | 3794.025982 | 3616.541027 |
| **Wrb** | 208.7787978 | 190.935144 | 235.7315496 | 204.4384325 | 226.1106782 | 245.494482 | 205.670755 | 264.8313881 | 314.5091032 | 278.7747281 | 277.1401147 | 243.7753979 | 213.8731631 | 203.2997966 | 219.1625299 |
| **Lca5l** | 4.578482408 | 3.961310043 | 2.349483883 | 5.175656518 | 7.600358931 | 7.014128057 | 3.608258859 | 0.838074013 | 2.257242368 | 9.612921658 | 4.729353494 | 1.735056213 | 4.260421576 | 0.903554652 | 5.748525376 |
| **B3galt5** | 10.0726613 | 6.338096068 | 4.698967767 | 18.11479781 | 8.86708542 | 6.312715251 | 5.412388289 | 6.704592104 | 0 | 10.48682363 | 2.837612096 | 4.337640531 | 0 | 4.517773258 | 6.467091048 |
| **Igsf5** | 1.831392963 | 0.792262009 | 0 | 3.450437679 | 0 | 0 | 0 | 0 | 0.752414123 | 3.495607876 | 1.891741398 | 0 | 1.70416863 | 0 | 2.874262688 |
| **Dscam** | 11.90405426 | 14.26071615 | 7.831612945 | 18.11479781 | 16.46744435 | 10.52119209 | 12.62890601 | 16.76148026 | 13.54345421 | 19.22584332 | 17.02567258 | 14.74797781 | 5.964590206 | 11.74621047 | 16.52701046 |
| **Bace2** | 10.98835778 | 7.130358077 | 28.9769679 | 16.38957897 | 10.13381191 | 9.81977928 | 10.82477658 | 12.5711102 | 10.53379772 | 19.22584332 | 10.40457769 | 8.675281063 | 10.22501178 | 6.324882561 | 6.467091048 |
| **Mx1** | 804.8972073 | 598.1578164 | 462.0651637 | 318.3028759 | 464.8886213 | 735.7820332 | 733.3786131 | 85.48354933 | 121.8910879 | 110.1116481 | 147.555829 | 467.5976493 | 380.0296046 | 363.2289699 | 519.5229808 |
| **Fam3b** | 9.156964816 | 0.792262009 | 1.566322589 | 2.587828259 | 5.066905954 | 2.104238417 | 2.706194144 | 5.866518091 | 1.504828245 | 0.873901969 | 1.891741398 | 0 | 0.852084315 | 2.710663955 | 2.155697016 |
| **Mx2** | 290.2757847 | 222.6256244 | 158.1985815 | 98.33747384 | 143.1400932 | 204.1111265 | 198.4542373 | 25.14222039 | 43.64001911 | 40.19949057 | 46.34766424 | 116.2487662 | 108.214708 | 114.7514407 | 134.3717807 |
| **Ripk4** | 12.81975074 | 8.714882094 | 15.66322589 | 20.70262607 | 10.76717515 | 16.13249453 | 9.922711863 | 2.514222039 | 0 | 23.59535316 | 28.37612096 | 6.072696744 | 17.89377062 | 9.035546516 | 2.155697016 |
| **Prdm15** | 293.0228741 | 282.837537 | 314.8308404 | 230.316715 | 302.7476308 | 270.0439302 | 271.5214791 | 280.7547944 | 288.9270231 | 293.6310616 | 306.4621064 | 292.3569718 | 328.0524613 | 290.0410432 | 313.294633 |
| **C2cd2** | 141.0172582 | 93.486917 | 93.19619404 | 110.4140057 | 138.7065505 | 115.0317001 | 101.9333128 | 113.1399918 | 97.06142181 | 110.9855501 | 126.7466736 | 120.5864068 | 110.770961 | 134.6296431 | 140.120306 |
| **Zbtb21** | 547.586496 | 630.6405588 | 514.5369705 | 438.2055852 | 476.9225229 | 472.7522311 | 593.5585823 | 486.9210016 | 371.6925766 | 374.9039447 | 457.8014182 | 542.2050664 | 503.5818303 | 430.9955688 | 487.1875256 |
| **B230307C23Rik** | 103.4737024 | 128.3464454 | 122.1731619 | 113.001834 | 146.9402727 | 136.7754971 | 138.9179661 | 155.0436924 | 114.3669466 | 147.6894327 | 159.8521481 | 130.1292159 | 100.5459492 | 155.4114001 | 126.4675583 |
| **A630089N07Rik** | 97.06382705 | 90.31786897 | 101.8109683 | 75.04701951 | 75.37022607 | 68.73845496 | 83.89201847 | 139.1202862 | 137.6917844 | 155.5545505 | 125.8008029 | 97.1631479 | 104.8063708 | 70.47726282 | 80.47935526 |
| **Pisd-ps2** | 46.70052056 | 57.04286461 | 64.21922615 | 60.38265938 | 50.0356963 | 57.51585007 | 40.59291217 | 41.90370065 | 42.13519086 | 48.93851026 | 78.507268 | 58.99191123 | 51.97714322 | 65.95948956 | 51.73672838 |
| **Scaf8** | 1865.273733 | 1633.644262 | 1902.298784 | 1852.022424 | 1822.819417 | 1767.56027 | 1917.789584 | 1899.913788 | 1805.793894 | 1864.906802 | 1915.388165 | 1798.385764 | 1797.045821 | 1844.155044 | 1912.103253 |
| **Tiam2** | 19.22962611 | 11.09166812 | 9.397935534 | 8.626094197 | 4.43354271 | 22.44520978 | 14.43303544 | 15.92340625 | 15.80069657 | 26.21705907 | 14.18806048 | 12.14539349 | 16.18960199 | 14.45687443 | 8.622788064 |
| **Tfb1m** | 133.6916863 | 143.3994235 | 168.3796783 | 156.9949144 | 147.5736359 | 126.9557178 | 153.3510015 | 156.7198404 | 144.4635115 | 132.8330993 | 102.1540355 | 146.61225 | 120.1438884 | 130.1118698 | 160.9587105 |
| **Cldn20** | 0.915696482 | 0 | 3.915806472 | 0 | 0 | 0 | 0 | 0 | 0 | 2.621705907 | 0.945870699 | 0 | 0 | 0.903554652 | 0 |
| **1700102H20Rik** | 0.915696482 | 6.338096068 | 0 | 0 | 4.43354271 | 2.805651223 | 6.314453003 | 4.190370065 | 4.514484735 | 0 | 2.837612096 | 1.735056213 | 0 | 2.710663955 | 0 |
| **Arid1b** | 1453.210316 | 1532.234724 | 1464.511621 | 1466.436013 | 1294.594471 | 1440.701903 | 1667.917658 | 1567.198404 | 1486.770306 | 1337.943914 | 1443.398686 | 1566.75576 | 1495.407973 | 1413.159475 | 1518.329265 |
| **Tmem242** | 661.1328597 | 712.2435456 | 790.9929074 | 803.9519791 | 786.0037861 | 771.5540863 | 751.4199074 | 927.7479325 | 866.7810692 | 755.0513011 | 941.1413453 | 772.9675427 | 709.7862345 | 794.2245387 | 755.9310869 |
| **Ldhal6b** | 1.831392963 | 3.961310043 | 3.915806472 | 7.763484777 | 6.966995687 | 7.014128057 | 2.706194144 | 0.838074013 | 5.266898858 | 1.747803938 | 0.945870699 | 9.542809169 | 4.260421576 | 2.710663955 | 2.874262688 |
| **Zdhhc14** | 30.21798389 | 49.91250654 | 35.24225825 | 44.85568982 | 43.06870061 | 44.18900676 | 49.61355931 | 58.66518091 | 76.7462405 | 45.44290238 | 55.80637123 | 43.37640531 | 24.71044514 | 40.65995932 | 49.58103137 |
| **3300005D01Rik** | 0 | 4.753572051 | 2.349483883 | 4.313047098 | 0 | 2.104238417 | 0.902064715 | 0.838074013 | 5.266898858 | 0 | 4.729353494 | 2.602584319 | 2.556252945 | 0 | 0.718565672 |
| **Snx9** | 679.4467893 | 818.4066548 | 801.1740043 | 767.7223835 | 772.703158 | 767.3456094 | 775.7756547 | 896.739194 | 893.8679776 | 895.7495181 | 729.2663088 | 870.9982187 | 792.4384131 | 821.3311783 | 821.3205631 |
| **Synj2** | 355.2902348 | 316.9048034 | 422.1239377 | 401.1133801 | 395.8520277 | 385.7770431 | 406.8311864 | 381.3236759 | 324.2904868 | 408.1122195 | 398.2115642 | 376.5071981 | 360.4316653 | 384.0107269 | 412.4566957 |
| **Serac1** | 74.17141501 | 72.88810478 | 70.4845165 | 39.6800333 | 58.26941847 | 67.33562935 | 56.83007703 | 46.09407072 | 61.69795805 | 81.27288311 | 83.23662149 | 52.05168638 | 54.53339617 | 75.89859073 | 45.26963733 |
| **Gtf2h5** | 720.653131 | 758.9870041 | 801.1740043 | 755.6458516 | 795.5042348 | 765.9427838 | 686.4712479 | 862.3781594 | 838.1893326 | 905.3624398 | 811.5570595 | 723.5184406 | 801.8113406 | 712.9046201 | 727.18846 |
| **Tulp4** | 251.8165324 | 305.8131353 | 260.0095498 | 270.8593578 | 245.1115755 | 267.238279 | 273.3256086 | 334.3915312 | 316.0139315 | 295.3788655 | 330.1088739 | 284.5492189 | 296.5253417 | 274.6806141 | 270.1806927 |
| **Tmem181a** | 245.4066571 | 282.045275 | 268.624324 | 267.4089201 | 268.5460156 | 283.3707735 | 303.0937442 | 215.3850214 | 212.1807826 | 263.9183946 | 258.2227008 | 244.642926 | 270.1107279 | 330.7010025 | 264.4321673 |
| **Dynlt1a** | 115.3777567 | 108.5398952 | 128.4384523 | 125.0783658 | 103.8715721 | 95.39214158 | 93.81473034 | 93.02621545 | 114.3669466 | 159.0501583 | 127.6925443 | 98.89820412 | 118.4397198 | 109.3301128 | 123.5932956 |
| **Dynlt1b** | 51.27900297 | 52.28929256 | 43.85703249 | 37.09220505 | 39.90188439 | 42.08476834 | 46.00530045 | 59.50325493 | 69.9745134 | 67.29045161 | 47.29353494 | 48.58157395 | 42.60421576 | 77.70570004 | 50.29959704 |
| **Tmem181b-ps** | 37.54355574 | 71.30358077 | 69.70135521 | 60.38265938 | 80.43713202 | 68.03704215 | 80.28375962 | 70.3982171 | 57.18347332 | 61.17313782 | 62.42746612 | 62.46202365 | 75.83550405 | 94.87323842 | 71.8565672 |
| **Sytl3** | 1131.800851 | 1045.785851 | 1133.234393 | 996.3138797 | 889.241995 | 843.7996053 | 884.9254852 | 1221.911911 | 1200.85294 | 1195.497893 | 1188.959468 | 790.3181048 | 812.8884367 | 817.7169597 | 804.7935526 |
| **Ezr** | 12790.44845 | 11947.31109 | 12215.74987 | 12054.96664 | 11481.60889 | 11799.16622 | 12728.13313 | 9286.698139 | 10315.59762 | 10162.606 | 10789.54706 | 11613.59876 | 11866.12617 | 11520.32181 | 11935.37581 |
| **Mir692-1** | 3.662785926 | 4.753572051 | 7.831612945 | 5.175656518 | 5.700269198 | 4.90988964 | 0 | 0 | 0 | 2.621705907 | 0 | 2.602584319 | 5.964590206 | 0 | 7.18565672 |
| **Rsph3b** | 92.48534464 | 113.2934672 | 128.4384523 | 87.9861608 | 107.0383883 | 87.67660071 | 90.20647148 | 96.3785115 | 161.0166222 | 102.2465304 | 129.5842857 | 125.7915754 | 113.3272139 | 89.45191051 | 110.6591135 |
| **Tagap1** | 183.1392963 | 251.9393187 | 246.6958078 | 221.6906209 | 210.2765971 | 201.3054752 | 217.3975963 | 300.8685707 | 246.0394181 | 254.305473 | 248.7639938 | 247.2455103 | 284.5961613 | 235.8277641 | 291.7376628 |
| **Rnaset2b** | 579.6358728 | 507.0476854 | 498.8737446 | 532.2300119 | 500.356963 | 607.4234897 | 616.1102002 | 421.5512286 | 496.5933209 | 493.7546124 | 392.53634 | 540.4700102 | 601.5715265 | 481.5946293 | 531.0200316 |
| **Rps6ka2** | 15.56684019 | 15.84524017 | 10.18109683 | 32.77915795 | 22.80107679 | 17.53532014 | 30.6702003 | 25.14222039 | 31.60139315 | 19.22584332 | 27.43025026 | 38.17123668 | 17.89377062 | 32.52796746 | 25.14979852 |
| **Tagap** | 3088.644232 | 2803.815248 | 2969.747629 | 2809.51888 | 2944.505723 | 2778.997536 | 3305.165115 | 3538.348483 | 3038.248227 | 3083.126146 | 3213.122764 | 3320.897591 | 3235.364145 | 3302.492252 | 3629.475209 |
| **Rsph3a** | 269.2147656 | 288.3833711 | 359.4710342 | 292.4245933 | 276.1463745 | 237.7789411 | 298.5834206 | 319.306199 | 311.4994467 | 304.9917871 | 255.3850887 | 291.4894437 | 285.4482456 | 269.2592862 | 314.7317643 |
| **Fgfr1op** | 577.8044799 | 561.713764 | 540.3812932 | 577.0857017 | 582.0608215 | 556.9217677 | 580.0276116 | 698.9537269 | 619.989237 | 679.0218298 | 646.0296873 | 558.6881004 | 581.1215029 | 584.5998596 | 569.8225779 |
| **Ccr6** | 3.662785926 | 11.88393013 | 16.44638718 | 6.900875357 | 13.93399137 | 33.66781467 | 26.15987673 | 25.9802944 | 26.33449429 | 19.22584332 | 6.621094891 | 26.89337129 | 16.18960199 | 11.74621047 | 10.05991941 |
| **Mpc1** | 60.43596778 | 58.62738863 | 57.1707745 | 77.63484777 | 85.50403798 | 89.07942633 | 70.36104775 | 94.70236348 | 71.47934165 | 71.65996145 | 67.15681961 | 74.60741714 | 68.16674521 | 56.0203884 | 83.35361795 |
| **4930506C21Rik** | 1.831392963 | 1.584524017 | 3.915806472 | 6.900875357 | 1.900089733 | 3.507064029 | 5.412388289 | 1.676148026 | 3.00965649 | 6.117313782 | 0.945870699 | 4.337640531 | 5.964590206 | 7.228437213 | 5.748525376 |
| **Sft2d1** | 395.58088 | 450.0048208 | 448.7514217 | 424.4038345 | 412.9528353 | 523.9553659 | 512.372758 | 434.9604128 | 448.4388171 | 433.4553766 | 470.0977373 | 447.6445028 | 538.5172872 | 627.0669282 | 552.5770017 |
| **Prr18** | 0.915696482 | 3.169048034 | 1.566322589 | 1.725218839 | 0 | 4.208476834 | 1.80412943 | 0 | 0 | 4.369509845 | 3.783482795 | 0 | 0 | 2.710663955 | 0.718565672 |
| **Gm16702** | 0 | 0.792262009 | 4.698967767 | 0 | 5.066905954 | 0.701412806 | 4.510323574 | 5.028444078 | 5.266898858 | 2.621705907 | 0.945870699 | 0 | 2.556252945 | 3.614218606 | 2.874262688 |
| **1700010I14Rik** | 53.11039593 | 55.4583406 | 36.80858084 | 43.9930804 | 58.26941847 | 36.4734659 | 31.57226502 | 33.52296052 | 29.34415078 | 38.45168663 | 23.64676747 | 34.70112425 | 39.1958785 | 46.08128723 | 49.58103137 |
| **Qk** | 1488.922479 | 1545.703179 | 1628.192331 | 1547.521299 | 1421.26712 | 1465.251351 | 1562.376086 | 1676.9861 | 1631.233818 | 1619.340348 | 1622.168248 | 1630.95284 | 1564.426803 | 1533.332244 | 1566.473165 |
| **Cahm** | 2.747089445 | 0 | 0.783161294 | 0.86260942 | 3.800179466 | 2.104238417 | 6.314453003 | 0 | 1.504828245 | 3.495607876 | 0 | 0 | 1.70416863 | 5.421327909 | 2.155697016 |
| **Agpat4** | 999.0248614 | 808.8995107 | 844.2478755 | 817.7537298 | 810.7049527 | 902.718281 | 848.8428966 | 664.5926924 | 653.0954584 | 664.1654964 | 682.9186445 | 822.4166448 | 761.7633778 | 786.9961015 | 786.8294108 |
| **Map3k4** | 573.2259975 | 637.7709168 | 673.5187133 | 678.8736133 | 603.5951718 | 728.7679051 | 800.131402 | 546.4242565 | 673.4106397 | 549.6843384 | 598.7361523 | 641.1032705 | 752.3904503 | 726.4579399 | 691.9787421 |
| **4732491K20Rik** | 3.662785926 | 7.130358077 | 7.04845165 | 7.763484777 | 6.966995687 | 2.805651223 | 11.72684129 | 1.676148026 | 3.762070613 | 0.873901969 | 3.783482795 | 1.735056213 | 13.63334904 | 7.228437213 | 2.874262688 |
| **Igf2r** | 3202.190596 | 3120.720051 | 3110.716662 | 3290.854936 | 2718.395044 | 2621.881068 | 3002.071371 | 3790.608761 | 3661.999535 | 3088.369558 | 3509.180292 | 3181.225566 | 3176.570327 | 2952.816601 | 3207.67716 |
| **Airn** | 14.65114371 | 20.59881222 | 16.44638718 | 17.25218839 | 8.86708542 | 11.22260489 | 23.45368258 | 35.19910855 | 18.81035306 | 21.84754922 | 17.02567258 | 13.01292159 | 11.0770961 | 16.26398373 | 12.9341821 |
| **Pnldc1** | 6.409875371 | 7.922620085 | 4.698967767 | 11.21392246 | 1.900089733 | 1.402825611 | 2.706194144 | 15.92340625 | 9.028969471 | 5.243411813 | 10.40457769 | 4.337640531 | 8.520843152 | 7.228437213 | 7.18565672 |
| **Mrpl18** | 1478.849818 | 1686.725816 | 1729.220138 | 1583.750894 | 1629.010264 | 1618.159343 | 1592.144222 | 2071.71896 | 1951.762234 | 2056.291333 | 2007.137623 | 1701.222616 | 1595.953922 | 1737.535595 | 1567.910296 |
| **Tcp1** | 9555.292785 | 8764.002338 | 9220.15792 | 9057.398906 | 8380.029085 | 8614.75208 | 8297.191247 | 10082.86845 | 9375.079967 | 9778.08913 | 9954.343234 | 8413.287575 | 8490.168116 | 8356.073418 | 7997.635929 |
| **Snora20** | 0 | 0 | 2.349483883 | 0.86260942 | 0 | 1.402825611 | 0 | 3.352296052 | 4.514484735 | 3.495607876 | 0 | 0.867528106 | 2.556252945 | 0 | 0.718565672 |
| **Acat3** | 0.915696482 | 2.376786026 | 0.783161294 | 2.587828259 | 2.533452977 | 0.701412806 | 1.80412943 | 10.05688816 | 0 | 2.621705907 | 1.891741398 | 0.867528106 | 0 | 3.614218606 | 2.874262688 |
| **Acat2** | 1891.828931 | 1913.312751 | 1949.288462 | 1961.57382 | 1794.951434 | 1839.104377 | 1851.938859 | 2138.764881 | 2218.116833 | 2495.864023 | 2501.827998 | 2105.490714 | 1872.02924 | 1957.099375 | 1858.929393 |
| **Wtap** | 1436.72778 | 1477.568646 | 1578.85317 | 1672.599665 | 1534.639141 | 1630.784773 | 1734.670447 | 1528.647 | 1528.153083 | 1600.114505 | 1464.207842 | 1674.329245 | 1636.001885 | 1466.4692 | 1528.389184 |
| **Sod2** | 1701.364063 | 1597.200209 | 1735.485429 | 1519.917797 | 1571.374209 | 1776.678637 | 1685.958952 | 1813.592164 | 1831.375974 | 1773.147095 | 1821.746966 | 1693.414863 | 1609.587271 | 1687.840089 | 1635.455469 |
| **Mllt4** | 155.6684019 | 132.3077554 | 103.3772909 | 127.6661941 | 133.0062813 | 162.0263581 | 147.9386132 | 119.8445839 | 121.1386737 | 112.733354 | 104.9916476 | 148.3473062 | 153.3751767 | 120.1727687 | 158.0844478 |
| **Wdr27** | 0 | 0 | 1.566322589 | 0 | 1.266726489 | 0 | 1.80412943 | 2.514222039 | 0.752414123 | 0 | 0 | 0 | 3.408337261 | 1.807109303 | 5.029959704 |
| **1600012H06Rik** | 162.0782772 | 202.0268122 | 205.9714205 | 175.1097122 | 170.3747127 | 199.2012368 | 181.3150077 | 178.5097648 | 219.7049238 | 212.3581784 | 201.4704588 | 202.1340488 | 170.416863 | 166.2540559 | 240.0009344 |
| **Phf10** | 1442.221958 | 1370.613275 | 1401.858717 | 1101.552229 | 1127.386575 | 1314.447598 | 1211.472912 | 1173.303618 | 1203.862596 | 1142.189873 | 1281.654797 | 1126.051482 | 1219.332655 | 1110.468667 | 1243.118613 |
| **LOC106740** | 43.03773463 | 54.66607859 | 44.64019379 | 53.48178402 | 55.10260225 | 57.51585007 | 45.10323574 | 31.8468125 | 24.82966605 | 48.06460829 | 19.86328467 | 61.59449555 | 42.60421576 | 46.98484188 | 49.58103137 |
| **9030025P20Rik** | 14.65114371 | 11.09166812 | 10.18109683 | 15.52696955 | 18.36753408 | 25.25086101 | 13.53097072 | 21.78992434 | 12.79104008 | 23.59535316 | 20.80915537 | 13.01292159 | 11.0770961 | 9.035546516 | 16.52701046 |
| **Gm3435** | 0 | 8.714882094 | 2.349483883 | 2.587828259 | 3.800179466 | 2.104238417 | 7.216517718 | 9.218814144 | 6.019312981 | 1.747803938 | 16.07980188 | 6.072696744 | 0.852084315 | 10.84265582 | 10.05991941 |
| **Ermard** | 73.25571853 | 73.68036679 | 75.96664557 | 79.36006661 | 76.00358931 | 54.00878604 | 61.34040061 | 59.50325493 | 79.75589699 | 88.26409886 | 86.07423359 | 46.84651774 | 81.80009426 | 73.18792678 | 71.8565672 |
| **Dll1** | 10.0726613 | 4.753572051 | 7.831612945 | 6.038265938 | 10.13381191 | 25.25086101 | 9.020647148 | 10.05688816 | 3.762070613 | 5.243411813 | 5.675224193 | 3.470112425 | 13.63334904 | 9.035546516 | 13.65274777 |
| **Fam120b** | 470.6679915 | 494.3714933 | 578.7561966 | 501.1760728 | 547.8592063 | 528.8652555 | 521.3934051 | 450.883819 | 497.345735 | 603.8662605 | 553.3343588 | 574.3036064 | 608.388201 | 584.5998596 | 540.3613853 |
| **Psmb1** | 4406.331469 | 4229.886863 | 4613.603186 | 4341.513209 | 4405.674727 | 4286.333656 | 4272.178489 | 4368.87983 | 4497.931625 | 4189.486039 | 4454.105121 | 4175.412776 | 4200.775674 | 4029.853746 | 4150.435321 |
| **Tbp** | 578.7201763 | 621.9256767 | 603.0341968 | 651.2701118 | 560.5264712 | 573.7556751 | 638.6618181 | 686.3826167 | 671.1533973 | 669.4089082 | 664.0012305 | 543.9401226 | 620.3173814 | 570.1429851 | 594.2538107 |
| **Pdcd2** | 679.4467893 | 651.239371 | 656.2891648 | 686.637098 | 658.697774 | 735.7820332 | 731.5744837 | 643.640842 | 796.0541417 | 778.6466543 | 659.271877 | 678.4069791 | 690.1882953 | 622.5491549 | 597.1280734 |
| **Prdm9** | 37.54355574 | 40.40536243 | 26.62748401 | 49.16873692 | 30.40143573 | 30.86216345 | 29.76813559 | 6.704592104 | 34.61104964 | 19.22584332 | 20.80915537 | 37.30370857 | 26.41461377 | 45.17773258 | 37.36541494 |
| **Chd1** | 3532.757026 | 3345.722462 | 3291.626921 | 3316.733219 | 2876.735856 | 2915.773033 | 3413.412881 | 3500.635153 | 3497.220842 | 3356.657463 | 3418.376705 | 3090.135115 | 3048.75768 | 2957.334375 | 3061.808328 |
| **Rgmb** | 79.6655939 | 75.26489081 | 109.6425812 | 103.5131304 | 82.33722176 | 95.39214158 | 109.1498305 | 105.5973256 | 50.41174621 | 105.7421382 | 121.0714494 | 98.03067601 | 93.72927467 | 106.6194489 | 95.56923437 |
| **Zfp960** | 0 | 0.792262009 | 0.783161294 | 3.450437679 | 4.43354271 | 0 | 1.80412943 | 0.838074013 | 0 | 4.369509845 | 1.891741398 | 0 | 0 | 0 | 0 |
| **Zfp97** | 45.78482408 | 66.55000871 | 41.50754861 | 48.3061275 | 58.26941847 | 45.59183237 | 58.63420646 | 48.60829276 | 54.17381683 | 53.3080201 | 51.07701773 | 70.26977661 | 58.79381775 | 68.67015352 | 62.51521346 |
| **Riok2** | 1190.405426 | 1189.185275 | 1159.078716 | 1092.926135 | 1087.48469 | 1080.175721 | 1160.957288 | 1296.500498 | 1267.817797 | 1284.635894 | 1270.304348 | 1039.298671 | 1022.501178 | 1087.8798 | 1049.105881 |
| **Lnpep** | 2090.535067 | 2181.889571 | 2243.757109 | 2175.500956 | 1753.782823 | 1880.487732 | 2362.507488 | 2126.193771 | 2289.596175 | 1997.739901 | 2145.234745 | 2281.59892 | 2316.817253 | 2063.718824 | 2259.170473 |
| **Spaca6** | 2.747089445 | 0 | 1.566322589 | 0 | 0 | 0 | 0 | 0 | 0 | 0 | 3.783482795 | 1.735056213 | 0 | 0 | 0 |
| **Vmn2r96** | 0 | 0 | 0 | 2.587828259 | 1.266726489 | 2.104238417 | 0.902064715 | 0 | 0 | 0 | 0 | 0.867528106 | 1.70416863 | 0 | 0 |
| **Vmn2r97** | 0.915696482 | 0 | 0.783161294 | 0 | 0 | 0 | 1.80412943 | 4.190370065 | 0 | 0 | 0 | 0 | 0 | 1.807109303 | 0 |
| **Ppp2r1a** | 5546.373589 | 5346.184033 | 5117.959059 | 5132.526047 | 5144.809633 | 5277.42995 | 5337.516917 | 5413.12005 | 5224.763667 | 5190.103793 | 5597.662795 | 5388.217068 | 5111.653807 | 4940.636835 | 5052.953805 |
| **Zfp160** | 166.6567596 | 165.5827598 | 163.6807105 | 136.2922883 | 139.3399137 | 145.8938636 | 121.7787365 | 154.2056184 | 146.7207539 | 173.0325898 | 164.5815016 | 169.1679807 | 126.960563 | 111.1372221 | 142.276003 |
| **Zfp677** | 43.95343112 | 54.66607859 | 54.03812932 | 51.75656518 | 52.56914927 | 54.71019885 | 59.53627118 | 62.01747697 | 44.39243323 | 56.80362798 | 55.80637123 | 49.44910206 | 55.38548049 | 55.11683375 | 68.98230451 |
| **Zfp54** | 66.84584315 | 76.05715282 | 79.09929074 | 64.69570647 | 58.26941847 | 61.0229141 | 53.22181817 | 77.1028092 | 49.65933209 | 69.91215751 | 69.04856101 | 66.79966418 | 68.16674521 | 65.95948956 | 65.38947615 |
| **Zfp51** | 162.9939737 | 193.3119301 | 162.8975493 | 160.4453521 | 196.3426057 | 180.2630911 | 178.6088135 | 160.9102105 | 172.3028341 | 180.8977076 | 183.4989156 | 170.9030369 | 144.0022493 | 170.7718291 | 167.4258016 |
| **Zfp53** | 314.0838932 | 316.9048034 | 305.4329049 | 276.0350143 | 246.378302 | 278.4608839 | 303.0937442 | 328.5250131 | 309.2422044 | 297.1266694 | 243.0887696 | 270.6687692 | 270.9628122 | 319.8583467 | 273.0549553 |
| **9330136K24Rik** | 14.65114371 | 3.169048034 | 0 | 7.763484777 | 5.700269198 | 5.611302446 | 9.020647148 | 6.704592104 | 8.276555348 | 6.117313782 | 0.945870699 | 10.41033728 | 5.964590206 | 1.807109303 | 3.59282836 |
| **Zfp52** | 501.8016719 | 397.7155283 | 498.8737446 | 395.9377236 | 388.2516687 | 427.1603987 | 473.5839753 | 427.4177467 | 407.8084544 | 433.4553766 | 335.7840981 | 432.0289969 | 473.7588792 | 422.8635769 | 437.6064942 |
| **Zfp948** | 65.93014667 | 101.4095371 | 58.73709709 | 77.63484777 | 53.83587576 | 49.80030921 | 46.00530045 | 67.88399506 | 48.15450385 | 66.41654964 | 76.6155266 | 47.71404585 | 62.20215501 | 69.57370817 | 45.98820301 |
| **3110052M02Rik** | 225.2613345 | 270.9536069 | 243.5631626 | 202.7132136 | 181.1418879 | 199.9026496 | 248.9698613 | 220.4134654 | 222.7145803 | 256.9271789 | 250.6557352 | 209.9418017 | 203.6481513 | 180.7109303 | 225.629621 |
| **Gm10509** | 94.3167376 | 77.64167683 | 78.31612945 | 87.9861608 | 84.23731149 | 75.75258302 | 73.0672419 | 57.8271069 | 84.27038173 | 86.51629492 | 72.83204381 | 53.78674259 | 79.24384131 | 80.41636399 | 74.01226421 |
| **Zfp760** | 46.70052056 | 60.21191265 | 35.24225825 | 43.9930804 | 24.06780328 | 25.95227381 | 34.27845916 | 31.8468125 | 24.07725192 | 44.56900041 | 43.51005214 | 30.36348372 | 23.00627651 | 38.85285002 | 33.05402091 |
| **Zfp229** | 10.0726613 | 11.09166812 | 11.74741942 | 7.763484777 | 3.800179466 | 7.014128057 | 4.510323574 | 5.866518091 | 12.03862596 | 9.612921658 | 17.02567258 | 2.602584319 | 7.668758836 | 2.710663955 | 6.467091048 |
| **Zfp820** | 9.156964816 | 7.130358077 | 3.132645178 | 7.763484777 | 2.533452977 | 6.312715251 | 0.902064715 | 16.76148026 | 13.54345421 | 10.48682363 | 15.13393118 | 3.470112425 | 4.260421576 | 1.807109303 | 2.874262688 |
| **2210404O09Rik** | 32.04937685 | 44.36667248 | 44.64019379 | 53.48178402 | 40.53524763 | 41.38335554 | 52.31975346 | 42.74177467 | 36.86829201 | 47.19070632 | 47.29353494 | 43.37640531 | 47.71672165 | 53.30972444 | 29.46119255 |
| **Zfp942** | 141.9329546 | 157.6601397 | 166.0301944 | 188.0488535 | 189.37561 | 155.0122301 | 160.5675192 | 185.2143569 | 145.2159257 | 130.2113934 | 195.7952346 | 145.7447219 | 213.0210788 | 167.1576105 | 176.7671553 |
| **Zfp943** | 92.48534464 | 80.01846286 | 61.08658097 | 86.26094197 | 83.60394824 | 108.7189849 | 101.9333128 | 74.58858716 | 86.5276241 | 79.52507917 | 73.7779145 | 83.2826982 | 97.98969624 | 95.77679307 | 87.66501198 |
| **Zfp947** | 8.241268334 | 6.338096068 | 14.0969033 | 2.587828259 | 1.266726489 | 9.118366474 | 9.020647148 | 18.43762829 | 6.771727103 | 6.991215751 | 17.02567258 | 6.94022485 | 6.816674521 | 8.131991864 | 6.467091048 |
| **Gm4944** | 55.85748538 | 62.58869867 | 81.44877463 | 65.55831589 | 71.5700466 | 65.23139093 | 44.20117102 | 65.36977302 | 42.88760499 | 61.17313782 | 57.69811262 | 45.11146153 | 57.94173343 | 52.40616979 | 84.07218362 |
| **Zfp944** | 127.2818109 | 112.5012052 | 106.5099361 | 87.12355138 | 119.7056532 | 96.09355438 | 115.4642835 | 142.4725822 | 146.7207539 | 124.9679816 | 155.1227946 | 92.82550737 | 112.4751296 | 121.0763233 | 117.8447702 |
| **Zfp758** | 93.40104112 | 76.05715282 | 123.7394845 | 96.612255 | 102.6048456 | 81.36388546 | 105.5415716 | 105.5973256 | 65.46002866 | 97.00311855 | 108.7751304 | 99.76573222 | 74.13133542 | 73.18792678 | 70.41943585 |
| **Zfp946** | 34.7964663 | 37.2363144 | 32.10961307 | 27.60350143 | 25.96789302 | 24.5494482 | 40.59291217 | 31.8468125 | 40.63036262 | 34.95607876 | 48.23940564 | 27.7608994 | 28.97086672 | 33.43152211 | 25.14979852 |
| **Gm16386** | 2.747089445 | 1.584524017 | 1.566322589 | 6.900875357 | 0 | 3.507064029 | 1.80412943 | 11.73303618 | 0 | 8.739019689 | 1.891741398 | 0 | 0.852084315 | 2.710663955 | 1.437131344 |
| **Zfp945** | 43.03773463 | 44.36667248 | 57.95393579 | 57.79483112 | 38.00179466 | 39.98052993 | 49.61355931 | 24.30414638 | 23.3248378 | 27.964863 | 28.37612096 | 54.6542707 | 61.35007069 | 50.59906049 | 48.86246569 |
| **Zfp40** | 54.02609241 | 45.15893448 | 81.44877463 | 51.75656518 | 50.0356963 | 42.08476834 | 56.83007703 | 74.58858716 | 51.16416034 | 32.33437285 | 49.18527634 | 43.37640531 | 57.08964912 | 53.30972444 | 43.83250599 |
| **6330415G19Rik** | 3.662785926 | 0.792262009 | 2.349483883 | 1.725218839 | 1.266726489 | 2.805651223 | 2.706194144 | 1.676148026 | 2.257242368 | 9.612921658 | 0 | 3.470112425 | 4.260421576 | 0.903554652 | 1.437131344 |

| | | | | | | | | | | | | | | | |
|---|---|---|---|---|---|---|---|---|---|---|---|---|---|---|---|
| **Zfp213** | 198.7061365 | 194.1041921 | 189.5250333 | 221.6906209 | 187.4755203 | 203.4097137 | 186.727396 | 230.4703536 | 196.380086 | 221.0971981 | 228.9007091 | 176.9757337 | 188.3106337 | 196.0713594 | 170.3000643 |
| **Zfp13** | 0 | 0 | 0 | 0 | 0 | 2.805651223 | 0 | 1.676148026 | 4.514484735 | 0 | 0 | 0 | 0 | 0 | 2.155697016 |
| **Zscan10** | 0 | 0 | 0 | 1.725218839 | 0 | 0.701412806 | 0 | 8.380740131 | 0 | 0 | 0 | 0 | 5.964590206 | 0 | 0 |
| **Thoc6** | 438.6186147 | 453.1738689 | 456.5830347 | 472.709962 | 489.5897878 | 490.2875512 | 436.599322 | 419.8750805 | 433.3905346 | 451.8073179 | 518.3371429 | 497.961133 | 462.6817831 | 477.9804107 | 446.947848 |
| **Hcfc1r1** | 230.7555134 | 250.3547947 | 246.6958078 | 226.8662774 | 239.4113063 | 207.6181905 | 245.3616024 | 187.7285789 | 279.8980536 | 243.8186493 | 201.4704588 | 234.2325887 | 218.985669 | 240.3455373 | 206.9469135 |
| **Tnfrsf12a** | 21.97671556 | 16.63750218 | 27.41064531 | 22.42784491 | 23.43444004 | 26.65368662 | 17.13922958 | 17.59955427 | 21.82000955 | 24.46925513 | 49.18527634 | 31.23101183 | 20.45002356 | 26.2030849 | 30.89832389 |
| **Pkmyt1** | 1029.242845 | 1070.345973 | 1003.229618 | 1035.131304 | 1078.617605 | 1052.820621 | 1013.018675 | 910.1483782 | 987.1673288 | 945.5619304 | 1011.135777 | 1022.815637 | 1114.526284 | 1096.915347 | 1207.190329 |
| **Paqr4** | 290.2757847 | 244.8089606 | 270.9738079 | 279.485452 | 328.7155238 | 363.3318334 | 407.7332511 | 160.9102105 | 172.3028341 | 211.4842765 | 174.0402086 | 301.899781 | 357.8754124 | 263.8379583 | 342.7558255 |
| **Kremen2** | 71.42432556 | 54.66607859 | 52.47180673 | 47.44351808 | 46.23551683 | 78.55823424 | 73.0672419 | 20.11377631 | 24.07725192 | 16.60413741 | 24.59263817 | 39.90629289 | 78.39175699 | 55.11683375 | 65.38947615 |
| **9530082P21Rik** | 207.8631013 | 230.5482445 | 211.4535495 | 189.7740723 | 228.0107679 | 206.9167777 | 193.041849 | 178.5097648 | 104.585563 | 158.1762564 | 153.2310532 | 191.7237115 | 205.35232 | 198.7820233 | 177.485721 |
| **Flywch1** | 592.4556236 | 643.3167509 | 679.0008423 | 597.7883278 | 580.1607318 | 602.5136001 | 556.573929 | 441.6650049 | 432.6381205 | 475.4026711 | 527.7958499 | 615.9449555 | 558.1152264 | 456.295099 | 548.2656077 |
| **Flywch2** | 15.56684019 | 7.922620085 | 0.783161294 | 11.21392246 | 11.4005384 | 12.6254305 | 6.314453003 | 6.704592104 | 2.257242368 | 19.22584332 | 12.29631908 | 4.337640531 | 1.70416863 | 3.614218606 | 5.748525376 |
| **Srrm2** | 11127.54364 | 10895.9794 | 10498.27715 | 11007.7588 | 10159.14644 | 9686.510847 | 12034.44536 | 11115.37564 | 10703.09089 | 10279.70886 | 10834.00298 | 11187.64246 | 11717.8635 | 9893.923435 | 11448.18829 |
| **Mir5125** | 27.47089445 | 41.98988645 | 54.82129061 | 40.54264272 | 55.7359655 | 55.41161165 | 29.76813559 | 63.69362499 | 54.92623095 | 54.18192207 | 51.07701773 | 46.84651774 | 60.49798638 | 42.46706862 | 76.16796123 |
| **Tceb2** | 3189.370845 | 3051.793257 | 2904.745241 | 2855.237179 | 2995.808145 | 3061.666897 | 3070.628289 | 3021.256817 | 2829.077101 | 2632.19273 | 2716.540647 | 3038.950956 | 2997.632621 | 3189.54792 | 2912.346668 |
| **Prss41** | 4.578482408 | 0.792262009 | 5.482129061 | 4.313047098 | 1.266726489 | 4.90988964 | 1.80412943 | 0.838074013 | 0.752414123 | 0 | 0.945870699 | 1.735056213 | 1.70416863 | 1.807109303 | 0 |
| **Prss30** | 2.747089445 | 10.29940611 | 19.57903236 | 14.66436013 | 14.56735462 | 9.118366474 | 12.62890601 | 5.866518091 | 25.58208017 | 4.369509845 | 12.29631908 | 8.675281063 | 9.372927467 | 13.55331977 | 14.37131344 |
| **Kctd5** | 966.0597881 | 957.0525063 | 1051.002457 | 886.7624834 | 952.5783194 | 983.3807536 | 902.0647148 | 926.0717844 | 860.0093421 | 916.7231654 | 1036.674286 | 946.473164 | 902.3572898 | 924.3364086 | 949.9438183 |
| **Pdpk1** | 1853.369679 | 1910.143703 | 2033.869882 | 1738.157981 | 1663.845243 | 1899.425878 | 2068.434391 | 1847.115125 | 2000.669152 | 1904.23239 | 1875.661596 | 1843.497226 | 1922.302215 | 1928.185626 | 1856.055131 |
| **Amdhd2** | 293.9385706 | 292.3446811 | 278.8054208 | 270.8593578 | 300.2141778 | 282.6693607 | 300.38755 | 234.6607237 | 259.5828723 | 237.7013355 | 240.2511575 | 272.4038254 | 271.8148965 | 288.2339339 | 265.8692986 |
| **Tbc1d24** | 88.82255871 | 80.81072487 | 113.5583877 | 80.22267603 | 83.60394824 | 94.69072877 | 86.59821262 | 82.13125328 | 96.30900769 | 92.6336087 | 83.23662149 | 97.1631479 | 82.65217857 | 95.77679307 | 99.16206273 |
| **Ntn3** | 0 | 0 | 0 | 1.725218839 | 0 | 4.90988964 | 0 | 2.514222039 | 0.752414123 | 0.873901969 | 0 | 0.867528106 | 3.408337261 | 3.614218606 | 0 |
| **1600002H07Rik** | 146.5114371 | 124.3851353 | 139.4027104 | 106.963568 | 153.2739051 | 151.505166 | 145.2324191 | 196.1093191 | 155.7497234 | 158.1762564 | 126.7466736 | 163.095284 | 178.0856219 | 183.4215943 | 131.497518 |
| **Ccnf** | 1417.498153 | 1666.127004 | 1745.666525 | 1889.114629 | 1653.078068 | 1595.714133 | 1655.288752 | 1841.248607 | 1890.064276 | 1873.645821 | 1800.93781 | 1778.432618 | 1754.441605 | 1733.921376 | 1619.647025 |
| **Abca17** | 2.747089445 | 0 | 0 | 0 | 1.900089733 | 0 | 0 | 2.514222039 | 0 | 6.991215751 | 4.729353494 | 1.735056213 | 6.816674521 | 2.710663955 | 0 |
| **Abca3** | 872.6587469 | 911.8935718 | 962.5052309 | 868.6476856 | 805.6380467 | 811.5346162 | 843.4305083 | 920.2052663 | 872.8003822 | 914.9753614 | 817.2322837 | 831.9594539 | 890.4281093 | 871.9302388 | 923.3568885 |
| **D330041H03Rik** | 15.56684019 | 29.31369431 | 21.14535495 | 21.56523549 | 19.00089733 | 11.22260489 | 25.25781201 | 4.190370065 | 23.3248378 | 15.73023544 | 8.512836289 | 19.95314644 | 14.48543336 | 29.8173035 | 7.904222392 |
| **Rnps1** | 4491.491242 | 4492.91785 | 4612.820025 | 4439.850683 | 4648.25285 | 4484.132067 | 4464.318273 | 4683.995659 | 4286.503256 | 4352.905707 | 4430.458353 | 4568.403008 | 4463.217643 | 4523.194586 | 4479.538399 |
| **Eci1** | 352.5431454 | 375.532192 | 342.2414857 | 360.5707374 | 298.9474513 | 365.4360718 | 325.645362 | 403.1136003 | 378.4643037 | 455.3029258 | 436.9922628 | 323.5879836 | 307.6024378 | 322.5690106 | 321.917421 |
| **Dnase1l2** | 4.578482408 | 3.961310043 | 2.349483883 | 2.587828259 | 6.333632443 | 6.312715251 | 3.608258859 | 1.676148026 | 4.514484735 | 5.243411813 | 4.729353494 | 9.542809169 | 5.964590206 | 13.55331977 | 9.341353736 |
| **E4f1** | 292.1071776 | 358.8946899 | 267.0580014 | 278.6228425 | 368.6174082 | 321.9484778 | 301.2896147 | 285.7832385 | 215.9428532 | 253.431571 | 277.1401147 | 263.7285443 | 280.3357397 | 329.7974478 | 331.2587748 |
| **Pgp** | 1775.535478 | 1690.687126 | 1762.896074 | 1787.326718 | 1879.822109 | 2153.337314 | 1803.227365 | 1421.373526 | 1565.773789 | 1688.378604 | 1517.176601 | 1912.899474 | 1883.106337 | 1838.733716 | 1902.761899 |
| **Mlst8** | 216.1043696 | 240.0553886 | 231.8157432 | 232.9045433 | 215.9768663 | 208.3196033 | 242.6554083 | 236.3368717 | 221.9621662 | 266.5401005 | 300.7868822 | 237.7027011 | 239.4356926 | 234.9242094 | 230.6595807 |
| **Caskin1** | 10.0726613 | 0.792262009 | 3.915806472 | 7.763484777 | 3.800179466 | 5.611302446 | 12.62890601 | 10.89496217 | 9.028969471 | 2.621705907 | 19.86328467 | 7.807752957 | 3.408337261 | 20.78175699 | 8.622788064 |
| **Traf7** | 1953.180595 | 1900.636558 | 1940.673688 | 1826.144141 | 1822.819417 | 1870.667953 | 1911.475131 | 2202.458506 | 2014.96502 | 2140.185922 | 2133.884296 | 2063.849365 | 2151.512896 | 1998.662889 | 2019.169538 |
| **Rab26os** | 10.98835778 | 7.922620085 | 12.53058071 | 2.587828259 | 2.533452977 | 9.81977928 | 12.62890601 | 11.73303618 | 2.257242368 | 6.117313782 | 14.18806048 | 9.542809169 | 12.78126473 | 5.421327909 | 7.18565672 |
| **Rab26** | 12.81975074 | 4.753572051 | 3.915806472 | 2.587828259 | 0.633363244 | 8.416953669 | 5.412388289 | 3.352296052 | 1.504828245 | 8.739019689 | 5.675224193 | 6.072696744 | 11.0770961 | 8.131991864 | 12.9341821 |
| **Pkd1** | 635.4933582 | 690.0602094 | 693.8809069 | 603.8265938 | 595.9948129 | 667.744991 | 758.6364251 | 630.2316578 | 606.4457828 | 575.0274955 | 568.46829 | 658.4538327 | 718.3070777 | 729.1686038 | 770.3024003 |
| **Tsc2** | 1038.39981 | 1050.539423 | 1043.954006 | 995.4512703 | 978.5462124 | 887.2871992 | 888.5337441 | 1154.86599 | 1044.350802 | 1067.034304 | 1083.02195 | 1015.007884 | 964.5594448 | 924.3364086 | 967.9079601 |
| **Nthl1** | 52.19469945 | 48.32798252 | 63.43606485 | 56.9322217 | 52.56914927 | 48.39748359 | 57.73214175 | 88.83584538 | 58.68830156 | 65.54264767 | 54.86050053 | 42.50887721 | 33.23128829 | 55.11683375 | 48.86246569 |
| **Slc9a3r2** | 15.56684019 | 15.84524017 | 4.698967767 | 12.93914129 | 6.333632443 | 9.81977928 | 6.314453003 | 18.43762829 | 6.019312981 | 12.23462756 | 17.97154328 | 9.542809169 | 7.668758836 | 18.07109303 | 10.77848508 |
| **Npw** | 7.325571853 | 12.67619214 | 14.8800646 | 6.900875357 | 12.66726489 | 13.32684331 | 12.62890601 | 13.40918421 | 6.771727103 | 16.60413741 | 7.56696559 | 9.542809169 | 19.59793925 | 12.64976512 | 16.52701046 |
| **Zfp598** | 1423.908029 | 1613.837711 | 1479.391685 | 1398.289869 | 1426.334026 | 1446.313205 | 1442.401479 | 1451.544191 | 1470.217196 | 1642.0618 | 1608.926059 | 1482.605534 | 1595.953922 | 1325.514674 | 1567.191731 |
| **Syngr3** | 38.45925223 | 18.2220262 | 37.59174214 | 35.36698621 | 12.66726489 | 30.16075065 | 38.78878274 | 3.352296052 | 21.06759543 | 18.35194135 | 13.24218978 | 30.36348372 | 38.34379418 | 21.68531164 | 23.71266717 |
| **Gfer** | 541.1766206 | 469.811371 | 579.5393579 | 496.0004163 | 487.6896981 | 480.4677719 | 452.8364868 | 569.8903289 | 501.8602198 | 622.2182019 | 575.0893849 | 520.5168638 | 464.3859518 | 491.5337305 | 536.768557 |
| **Noxo1** | 27.47089445 | 28.52143231 | 16.44638718 | 24.15306375 | 19.00089733 | 12.6254305 | 20.74748844 | 10.89496217 | 12.79104008 | 20.97364725 | 22.70089677 | 31.23101183 | 21.30210788 | 28.0101942 | 14.37131344 |
| **Tbl3** | 1496.248051 | 1474.399598 | 1527.947686 | 1627.743975 | 1475.102996 | 1613.950866 | 1613.793775 | 1849.629347 | 1697.446261 | 1627.205466 | 1826.476319 | 1511.233961 | 1491.999636 | 1486.347402 | 1680.006541 |
| **Rnf151** | 0.915696482 | 0 | 0 | 0 | 0.633363244 | 0 | 0 | 0 | 2.257242368 | 0.873901969 | 0 | 3.470112425 | 1.70416863 | 1.807109303 | 0.718565672 |
| **Rps2** | 60250.99709 | 59113.04524 | 58806.01528 | 59708.96142 | 60046.63574 | 64223.46073 | 62175.71253 | 63208.38014 | 62552.70049 | 62804.7128 | 59858.4813 | 62726.61972 | 60411.92586 | 60345.70451 | 59350.65024 |
| **Snora64** | 0.915696482 | 0 | 0.783161294 | 0 | 0 | 0 | 0 | 1.676148026 | 0.752414123 | 0 | 0.945870699 | 0 | 0 | 0 | 2.155697016 |
| **Ndufb10** | 1034.737024 | 1088.568 | 1058.83407 | 1098.101791 | 1201.490074 | 1141.198635 | 1133.895346 | 1106.257697 | 1069.180468 | 1140.442069 | 1138.828321 | 1100.025639 | 1055.732466 | 1136.671752 | 1084.315599 |
| **Msrb1** | 431.2930428 | 525.2697116 | 447.9682604 | 433.0299287 | 471.855617 | 507.8228713 | 446.5220338 | 459.2645592 | 478.535382 | 498.1241223 | 441.7216163 | 523.9869762 | 542.7777088 | 476.1733014 | 487.1875256 |
| **Hs3st6** | 0 | 0 | 2.349483883 | 0 | 9.500448664 | 3.507064029 | 0 | 0 | 0 | 0 | 0 | 2.602584319 | 0 | 0 | 2.874262688 |
| **Fahd1** | 37.54355574 | 36.44405239 | 32.89277437 | 48.3061275 | 44.3354271 | 58.91867568 | 54.12388289 | 33.52296052 | 17.30552482 | 42.82119648 | 45.40179354 | 22.55573076 | 35.78754124 | 35.23863141 | 33.77258658 |
| **Hagh** | 206.9474048 | 171.9208558 | 231.0325819 | 167.3462274 | 176.0749819 | 175.3532014 | 169.5881664 | 200.2996891 | 194.8752577 | 236.8274336 | 217.5502607 | 179.578318 | 174.6772846 | 203.2997966 | 215.5697016 |
| **Igfals** | 0 | 0.792262009 | 0 | 0.86260942 | 0.633363244 | 3.507064029 | 0.902064715 | 0 | 0.752414123 | 2.621705907 | 0 | 0 | 0 | 0 | 0 |
| **Nubp2** | 1615.288593 | 1685.933554 | 1671.266202 | 1680.363149 | 1511.204701 | 1547.316649 | 1563.278151 | 1894.885344 | 1881.035306 | 1878.889233 | 1936.19732 | 1452.24205 | 1592.545585 | 1620.07349 | 1491.742335 |
| **Spsb3** | 584.2143552 | 650.447109 | 620.2637452 | 664.2092531 | 624.4961589 | 648.8068453 | 657.6051771 | 657.0500262 | 641.8092466 | 557.5494562 | 579.8187383 | 629.8254052 | 586.2340088 | 647.8486852 | 677.6074287 |
| **Eme2** | 77.83420093 | 74.4726288 | 75.18348427 | 81.08528545 | 54.46923901 | 79.25964705 | 72.16517718 | 90.51199341 | 75.24141226 | 76.02947129 | 99.31642337 | 80.68011388 | 89.46885309 | 50.59906049 | 80.47935526 |
| **Mrps34** | 1336.916863 | 1357.937083 | 1345.471104 | 1345.670695 | 1183.755904 | 1291.300975 | 1242.143112 | 1474.172189 | 1295.657119 | 1548.554289 | 1360.162065 | 1279.603957 | 1243.191016 | 1237.869873 | 1240.24435 |
| **Nme3** | 108.9678813 | 125.1773973 | 134.7037427 | 134.5670695 | 191.909063 | 200.6040624 | 158.7633898 | 172.6432467 | 176.0649047 | 176.5281977 | 121.0714494 | 231.6300044 | 172.973116 | 165.3505012 | 165.9886702 |
| **Mapk8ip3** | 633.6619652 | 781.1703404 | 787.8602623 | 734.0806161 | 711.2669233 | 737.1848588 | 750.5178427 | 665.4307664 | 678.6775386 | 787.385674 | 808.7194474 | 827.6218134 | 845.2676406 | 879.158676 | 864.4345034 |
| **Hn1l** | 1063.123615 | 1109.959074 | 1101.907941 | 1044.620007 | 1128.019938 | 1169.255147 | 1328.741325 | 1163.24673 | 1059.399085 | 1025.08701 | 1183.284244 | 1193.718674 | 1175.024271 | 1143.900189 | 1142.519418 |
| **Cramp1l** | 865.3331751 | 923.7775019 | 888.8880692 | 842.769403 | 685.2990303 | 703.5170441 | 834.4098612 | 806.2272006 | 905.1541895 | 865.1629492 | 874.9303964 | 846.7074317 | 822.2613641 | 785.1889922 | 808.386381 |
| **Ift140** | 443.1970971 | 466.642323 | 498.0905833 | 452.8699453 | 438.9207283 | 394.8954096 | 493.429399 | 484.4067795 | 510.8891892 | 491.1329065 | 496.5821169 | 503.1663016 | 506.9901675 | 437.3204514 | 464.1934241 |
| **Tmem204** | 2.747089445 | 0 | 9.397935534 | 11.21392246 | 6.966995687 | 14.02825611 | 6.314453003 | 15.08533223 | 9.028969471 | 14.85633347 | 15.13393118 | 4.337640531 | 12.78126473 | 9.035546516 | 5.748525376 |
| **Telo2** | 473.415081 | 539.5304278 | 487.1263252 | 477.8856185 | 462.9885316 | 435.5773523 | 457.3468104 | 522.1201101 | 509.384361 | 522.5933774 | 560.9013244 | 486.6832676 | 416.6692301 | 452.6808804 | 446.2292823 |
| **Clcn7** | 339.7233947 | 402.4691003 | 311.6981952 | 432.1673192 | 352.1499638 | 331.7682571 | 350.903174 | 414.8466365 | 348.3677388 | 366.164925 | 403.8867884 | 304.5023653 | 361.2837496 | 374.0716258 | 357.8457046 |
| **BC003965** | 392.8337906 | 448.4202968 | 444.8356153 | 358.8455186 | 400.2855704 | 427.1603987 | 488.0170107 | 471.8356693 | 516.9085022 | 537.4497109 | 467.2601252 | 423.3537159 | 425.1900733 | 409.3102572 | 454.1335047 |
| **Unkl** | 294.8542671 | 349.3875458 | 304.6497436 | 294.1498121 | 317.3149854 | 305.1145705 | 271.5214791 | 293.3259046 | 270.8690841 | 321.5959246 | 384.9693744 | 347.0112425 | 313.567028 | 329.7974478 | 329.1030778 |
| **Gnptg** | 364.4471997 | 328.7887335 | 321.879292 | 343.318549 | 340.7494254 | 376.6586767 | 290.4648382 | 305.8970148 | 310.7470326 | 332.0827482 | 339.5675809 | 382.5798949 | 370.6566771 | 337.9294397 | 342.7558255 |
| **Tsr3** | 436.7872217 | 427.8214846 | 431.5218733 | 410.6020838 | 443.354271 | 425.0561603 | 443.8158397 | 410.6562664 | 464.9919278 | 439.5726904 | 496.5821169 | 432.0289969 | 426.8942419 | 459.005763 | 408.1453017 |
| **Baiap3** | 52.19469945 | 71.30358077 | 63.43606485 | 80.22267603 | 74.10349958 | 100.3020312 | 79.3816949 | 33.52296052 | 42.13519086 | 52.43411813 | 53.91462983 | 91.09045116 | 103.9542864 | 71.38081747 | 109.2219821 |
| **Ube2i** | 4221.36078 | 4506.386304 | 4473.417314 | 4464.003747 | 4413.908449 | 4316.494406 | 4255.941324 | 4477.829452 | 4438.490909 | 4538.172925 | 4615.84901 | 4276.046036 | 4496.448931 | 4491.570173 | 4543.490744 |
| **2810468N07Rik** | 2.747089445 | 8.714882094 | 17.22954848 | 12.93914129 | 10.76717515 | 4.208476834 | 17.13922958 | 3.352296052 | 9.028969471 | 3.495607876 | 6.621094891 | 12.14539349 | 6.816674521 | 4.517773258 | 11.49705075 |
| **Lmf1** | 242.6595676 | 247.1857467 | 214.5861947 | 213.9271361 | 262.2123831 | 251.8071973 | 223.7120493 | 130.739546 | 140.7014409 | 170.4108839 | 149.4475704 | 236.835173 | 198.5356454 | 211.4317885 | 227.785318 |
| **Chtf18** | 558.5748538 | 619.5488907 | 577.189874 | 638.3309705 | 620.6959794 | 659.3280374 | 626.9349768 | 650.3454341 | 683.9444374 | 663.2915944 | 587.3857039 | 661.9239451 | 627.134056 | 681.2802073 | 693.4158734 |
| **Rpusd1** | 222.514245 | 239.2631266 | 263.9253562 | 258.7828259 | 263.4791096 | 284.0721863 | 231.8306317 | 293.3259046 | 263.3449429 | 277.0269241 | 312.1373306 | 235.9676449 | 237.7315239 | 285.5232699 | 283.8334404 |
| **Mslnl** | 0 | 0 | 1.566322589 | 2.587828259 | 0.633363244 | 2.104238417 | 0 | 2.514222039 | 0 | 1.747803938 | 0 | 0.867528106 | 0 | 0 | 0 |
| **Narfl** | 251.8165324 | 289.9678951 | 256.8769046 | 288.9741556 | 270.4461053 | 265.8354534 | 267.9132203 | 281.5928684 | 266.3545994 | 254.305473 | 275.2483733 | 233.3650606 | 270.9628122 | 298.173035 | 305.3904106 |
| **Haghl** | 65.01445019 | 72.09584277 | 66.56871003 | 75.90962893 | 82.970585 | 84.87094949 | 66.75278889 | 57.8271069 | 66.96485691 | 83.02068705 | 97.42468197 | 72.00483282 | 92.87719035 | 88.54835585 | 68.98230451 |
| **Ccdc78** | 0 | 5.54583406 | 1.566322589 | 8.626094197 | 12.03390164 | 7.715540863 | 8.118582433 | 8.380740131 | 8.276555348 | 6.117313782 | 4.729353494 | 6.072696744 | 11.0770961 | 11.74621047 | 10.77848508 |
| **Fam173a** | 512.7900297 | 450.0048208 | 463.6314863 | 483.061275 | 518.0911338 | 557.6231805 | 451.9344221 | 414.0085624 | 343.853254 | 446.5639061 | 457.8014182 | 476.2729303 | 461.8296988 | 510.5083781 | 490.780354 |
| **Metrn** | 897.3825519 | 1062.423353 | 971.9031665 | 961.8095029 | 1003.247379 | 1172.762211 | 1056.317781 | 973.8420032 | 1051.122529 | 961.2921658 | 955.3294058 | 1011.537772 | 1134.976308 | 1063.483825 | 1115.213923 |
| **Fbxl16** | 0 | 0 | 0 | 5.175656518 | 4.43354271 | 0.701412806 | 3.608258859 | 0 | 0 | 0 | 0 | 0 | 0 | 0 | 0 |
| **Wdr24** | 390.0867011 | 485.6566112 | 499.6569059 | 454.5951642 | 391.418485 | 418.743445 | 394.2022804 | 484.4067795 | 480.0402102 | 555.8016522 | 428.4794265 | 384.3149511 | 470.350542 | 433.7062328 | 472.8162122 |
| **Jmjd8** | 65.93014667 | 77.64167683 | 72.83400039 | 85.39833255 | 69.03659363 | 89.07942633 | 84.79408319 | 102.2450296 | 72.23175577 | 68.16435357 | 84.18249219 | 76.34247335 | 86.06051583 | 112.0407768 | 97.00636572 |
| **Stub1** | 372.688468 | 416.7298165 | 324.2287759 | 378.6855352 | 360.383686 | 381.5685663 | 340.9804622 | 531.3389243 | 447.6864029 | 443.0682982 | 467.2601252 | 338.3359614 | 381.7337732 | 407.5031479 | 399.5225136 |
| **Rhbdl1** | 11.90405426 | 16.63750218 | 21.14535495 | 7.763484777 | 30.40143573 | 11.9240177 | 13.53097072 | 25.9802944 | 20.31518131 | 25.3431571 | 34.05134516 | 19.95314644 | 24.71044514 | 41.56351397 | 19.40127314 |
| **Rhot2** | 827.7896193 | 945.9608382 | 855.2121336 | 871.2355138 | 900.6425334 | 897.1069785 | 880.4151616 | 1111.286141 | 936.0031685 | 1115.972814 | 1129.369614 | 901.3617024 | 967.967782 | 950.5394935 | 972.2193542 |
| **Wdr90** | 508.2115473 | 525.2697116 | 561.5266481 | 561.5587322 | 448.4211769 | 432.0702883 | 488.0170107 | 450.883819 | 466.496756 | 530.4584951 | 543.8756518 | 511.8415827 | 539.3693715 | 549.3612282 | 550.4213047 |
| **Fam195a** | 191.3805646 | 181.4279999 | 172.2954848 | 138.8801166 | 152.0071786 | 185.1729807 | 196.6501078 | 193.595097 | 188.1035306 | 241.1969434 | 217.5502607 | 188.2535991 | 185.7543807 | 199.685578 | 186.8270747 |
| **0610011F06Rik** | 275.624641 | 331.1655196 | 293.6854854 | 310.5393911 | 308.4479 | 329.6640187 | 336.4701386 | 280.7547944 | 276.8883971 | 377.5256506 | 318.7584255 | 316.6477588 | 362.9879183 | 271.9699501 | 359.282836 |
| **Wfikkn1** | 11.90405426 | 19.80655021 | 17.22954848 | 20.70262607 | 20.26762382 | 18.23673295 | 21.64955315 | 12.5711102 | 9.028969471 | 26.21705907 | 16.07980188 | 12.14539349 | 16.18960199 | 15.36042908 | 20.11983882 |
| **Rab40c** | 216.1043696 | 215.4952663 | 233.3820658 | 220.8280114 | 238.1445798 | 202.006888 | 211.985208 | 222.9276875 | 229.4863074 | 176.5281977 | 224.1713556 | 164.8303402 | 223.2460906 | 157.2185094 | 206.9469135 |
| **Gm10012** | 5.494178889 | 5.54583406 | 3.915806472 | 5.175656518 | 10.13381191 | 4.90988964 | 4.510323574 | 6.704592104 | 2.257242368 | 0 | 2.837612096 | 4.337640531 | 0 | 7.228437213 | 2.155697016 |
| **Pigq** | 1081.437545 | 1127.388838 | 1077.629941 | 1084.300041 | 991.8468405 | 1061.237575 | 1028.353775 | 1152.351768 | 996.9487124 | 1209.480325 | 1217.335589 | 1178.103168 | 1134.124223 | 1165.585501 | 1125.992408 |
| **Capn15** | 466.0895091 | 546.6607859 | 515.3201318 | 474.4351808 | 479.4559759 | 454.5154981 | 487.114946 | 556.4811447 | 469.5064125 | 485.0155927 | 598.7361523 | 506.6364141 | 552.1506362 | 483.4017386 | 529.5829002 |
| **Rab11fip3** | 172.1509385 | 253.5238427 | 256.0937433 | 215.6523549 | 232.4443106 | 272.1481686 | 250.7739907 | 290.8116825 | 241.5249333 | 259.5488848 | 275.2483733 | 206.4716893 | 243.6961141 | 226.7922175 | 273.0549553 |
| **Decr2** | 184.0549928 | 131.5154934 | 186.3923881 | 141.4679448 | 158.3408111 | 150.1023404 | 142.5262249 | 196.9473931 | 224.2194085 | 201.8713548 | 192.0117519 | 165.6978683 | 144.0022493 | 178.0002664 | 174.6114583 |
| **Nme4** | 113.5463637 | 147.3607336 | 130.7879362 | 135.4296789 | 108.938478 | 104.5105081 | 101.0312481 | 155.8817664 | 179.8269753 | 138.0765111 | 128.638415 | 99.76573222 | 124.40431 | 93.06612911 | 87.66501198 |
| **Tmem8** | 110.7992743 | 129.9309694 | 109.6425812 | 113.001834 | 99.43802935 | 122.0458282 | 110.9539599 | 154.2056184 | 123.3959161 | 127.5896875 | 104.0457769 | 126.6591035 | 120.1438884 | 129.2083152 | 135.808912 |
| **Mrpl28** | 962.3970021 | 1008.549537 | 972.6863278 | 967.8477688 | 923.4436102 | 827.6671107 | 851.5490908 | 1091.172365 | 1008.987338 | 1128.207442 | 1032.890803 | 955.148445 | 882.7593505 | 962.2857039 | 982.2792736 |
| **Axin1** | 1761.800031 | 1990.954427 | 1905.431429 | 1785.601499 | 1732.881836 | 1833.493074 | 1950.263913 | 1916.675268 | 1955.524305 | 1917.34092 | 1999.570657 | 1984.90307 | 1975.983527 | 1910.114533 | 1919.28891 |
| **Pdia2** | 4.578482408 | 4.753572051 | 0.783161294 | 7.763484777 | 5.700269198 | 2.104238417 | 1.80412943 | 0.838074013 | 1.504828245 | 6.117313782 | 0 | 2.602584319 | 5.112505891 | 12.64976512 | 4.311394032 |
| **Arhgdig** | 416.6418991 | 367.6095719 | 368.8689697 | 369.1968316 | 280.5799172 | 375.9572639 | 321.1350385 | 298.3543486 | 242.2773475 | 277.0269241 | 267.6814078 | 297.5621405 | 350.2066535 | 320.7619013 | 265.8692986 |
| **Rgs11** | 54.94178889 | 72.09584277 | 82.23193592 | 41.40525214 | 58.26941847 | 70.84269338 | 64.04659475 | 13.40918421 | 20.31518131 | 56.80362798 | 20.80915537 | 70.26977661 | 82.65217857 | 100.2945663 | 90.53927467 |
| **Itfg3** | 143.7643476 | 215.4952663 | 195.0071623 | 172.5218839 | 195.7092425 | 158.5192941 | 199.356302 | 147.5010263 | 181.3318035 | 172.1586879 | 159.8521481 | 189.1211272 | 214.7252474 | 159.9291733 | 169.5814986 |
| **Luc7l** | 1564.925287 | 1542.534131 | 1564.756266 | 1440.557731 | 1521.338513 | 1559.94208 | 1573.200863 | 1576.417219 | 1547.71585 | 1568.654034 | 1538.931627 | 1553.742838 | 1605.32685 | 1564.053102 | 1572.22169 |
| **Neurl1b** | 97.06382705 | 108.5398952 | 93.97935534 | 107.8261775 | 103.2382088 | 119.240177 | 133.5055778 | 80.45510525 | 138.4441986 | 62.04703979 | 61.48159542 | 111.9111257 | 139.7418277 | 111.1372221 | 135.0903463 |
| **Dusp1** | 300.348446 | 259.8619388 | 259.2263885 | 285.5237179 | 292.6138189 | 273.5509942 | 289.5627734 | 259.802944 | 203.9042272 | 187.0150213 | 253.4933473 | 329.6606804 | 323.7920398 | 328.8938932 | 311.8575016 |
| **Ergic1** | 2002.628205 | 2068.596104 | 1981.398075 | 1897.740723 | 1661.31179 | 1841.910028 | 1824.876918 | 2106.079995 | 2276.805135 | 2176.015903 | 2284.277738 | 1682.136998 | 1730.583244 | 1704.104073 | 1680.725107 |
| **Atp6v0e** | 1368.96624 | 1337.33827 | 1506.019169 | 1372.411587 | 1218.590882 | 1221.861108 | 1344.076425 | 1440.649228 | 1466.455125 | 1398.24315 | 1462.3161 | 1438.3616 | 1285.795232 | 1344.489322 | 1342.999241 |
| **Crebrf** | 191.3805646 | 179.0512139 | 145.6680008 | 191.4992912 | 126.0392856 | 127.6571306 | 187.6294607 | 117.3303618 | 199.3897425 | 110.1116481 | 86.07423359 | 131.8642722 | 155.0793454 | 137.340307 | 134.3717807 |
| **Bnip1** | 390.0867011 | 337.5036156 | 361.0373568 | 364.8837845 | 367.9840449 | 342.990862 | 296.7792912 | 349.4768634 | 370.9401624 | 295.3788655 | 354.701512 | 301.0322529 | 316.9753652 | 312.6299094 | 360.0014017 |
| **Kifc5b** | 327.8193404 | 488.8256592 | 429.1723894 | 425.2664439 | 398.3854806 | 395.5968224 | 405.9291217 | 465.9691513 | 409.3132827 | 465.7897494 | 424.6959437 | 418.1485472 | 474.6109635 | 443.6453339 | 424.6723121 |
| **Phf1** | 77.83420093 | 95.07144102 | 109.6425812 | 82.81050429 | 103.8715721 | 81.36388546 | 91.10853619 | 103.0831036 | 103.0807348 | 78.6511772 | 81.34488009 | 110.1760695 | 106.5105394 | 103.0052303 | 142.9945687 |
| **Cuta** | 1026.495756 | 980.0281045 | 833.2836173 | 925.5799073 | 1026.681819 | 909.732409 | 930.9307857 | 859.0258634 | 819.3789795 | 823.2156547 | 864.5258187 | 857.117769 | 922.8073133 | 914.3973074 | 893.1771303 |
| **Syngap1** | 16.48253667 | 31.69048034 | 24.27800013 | 19.84001665 | 6.966995687 | 19.63955856 | 21.64955315 | 25.14222039 | 5.266898858 | 20.09974528 | 22.70089677 | 19.95314644 | 21.30210788 | 20.78175699 | 23.71266717 |
| **Zbtb9** | 255.4793184 | 289.1756331 | 281.1549047 | 292.4245933 | 241.3113961 | 217.4379698 | 283.2483204 | 307.5731628 | 234.7532062 | 288.3876497 | 259.1685715 | 230.7624763 | 224.9502592 | 224.9851082 | 195.4498628 |
| **Ggnbp1** | 7.325571853 | 1.584524017 | 6.265290356 | 0.86260942 | 12.03390164 | 7.014128057 | 2.706194144 | 6.704592104 | 6.019312981 | 3.495607876 | 1.891741398 | 6.072696744 | 0.852084315 | 4.517773258 | 2.874262688 |
| **Bak1** | 2680.243602 | 2765.786672 | 2664.314724 | 2582.652602 | 2506.218358 | 2599.435858 | 2686.348721 | 2459.747228 | 2524.349381 | 2623.453711 | 2585.06462 | 2507.156227 | 2456.559081 | 2549.831227 | 2502.764235 |
| **Itpr3** | 1194.068212 | 1346.053152 | 1346.254265 | 1381.90029 | 1414.300124 | 1323.565964 | 1359.411525 | 978.0323732 | 1273.084695 | 1095.873069 | 1162.475089 | 1385.442386 | 1528.639261 | 1338.164439 | 1560.006074 |
| **Uqcc2** | 771.932134 | 669.4613972 | 701.7125199 | 574.4978735 | 696.6995687 | 754.0187661 | 724.357966 | 558.1572927 | 603.4361263 | 654.5525747 | 655.4883942 | 595.991809 | 625.4298873 | 696.6406364 | 658.9247212 |
| **Lemd2** | 623.5893039 | 629.8482968 | 664.903939 | 582.2613583 | 688.4658465 | 737.8862716 | 776.6777194 | 592.5183272 | 582.3685309 | 549.6843384 | 572.2517728 | 648.0434954 | 721.7154149 | 768.0214538 | 694.8530048 |
| **Hmga1** | 18.31392963 | 15.84524017 | 24.27800013 | 20.70262607 | 13.30062813 | 21.04238417 | 9.922711863 | 8.380740131 | 15.80069657 | 12.23462756 | 15.13393118 | 10.41033728 | 16.18960199 | 17.16753838 | 22.27553583 |
| **AI413582** | 241.7438711 | 247.1857467 | 267.8411627 | 230.316715 | 236.2444901 | 241.987418 | 223.7120493 | 255.612574 | 225.7242368 | 210.6103745 | 228.9007091 | 246.3779822 | 272.6669809 | 318.954792 | 229.2224494 |
| **Nudt3** | 1735.244833 | 1649.489502 | 1805.969945 | 1660.523133 | 1680.312687 | 1754.93484 | 1675.134175 | 1636.758547 | 1665.092453 | 1510.976504 | 1540.823368 | 1736.791269 | 1686.27486 | 1629.109037 | 1589.467266 |
| **Rps10** | 15102.58207 | 14446.10546 | 14203.41324 | 14120.9162 | 15028.44306 | 15950.1272 | 15811.39032 | 13175.36156 | 13909.87988 | 13541.11101 | 12682.23433 | 14924.95354 | 14527.18549 | 14918.59085 | 14640.77557 |
| **Pacsin1** | 191.3805646 | 198.0655021 | 267.8411627 | 224.2784491 | 274.2462848 | 267.238279 | 296.7792912 | 158.3959885 | 164.7786928 | 115.3550599 | 158.9062774 | 307.1049496 | 264.1461377 | 256.609521 | 251.4979852 |
| **D17Wsu92e** | 3776.33229 | 3805.234427 | 3795.199633 | 3540.149058 | 3453.729771 | 3543.537494 | 3863.543173 | 3384.980939 | 3538.603618 | 3333.936011 | 3467.561982 | 3679.186699 | 3531.037402 | 3403.690372 | 3681.930503 |
| **Snrpc** | 2150.055339 | 2231.009816 | 2185.803173 | 2195.340973 | 2195.870368 | 2201.734797 | 2170.367704 | 2224.248431 | 2392.67691 | 2262.532197 | 2275.764901 | 2052.571499 | 2088.458656 | 2151.363625 | 2111.86451 |
| **Uhrf1bp1** | 796.655939 | 735.2191439 | 758.1001331 | 653.8579401 | 644.7637827 | 681.0718343 | 726.1620954 | 672.9734325 | 671.1533973 | 591.6316329 | 675.3516789 | 641.1032705 | 686.779958 | 547.5541189 | 658.9247212 |
| **Taf11** | 538.4295312 | 561.713764 | 525.5012286 | 502.9012917 | 508.5906852 | 500.8087433 | 543.945023 | 648.6692861 | 626.00855 | 585.5143192 | 623.3287905 | 540.4700102 | 565.7839853 | 593.6354061 | 528.1457689 |
| **Anks1** | 912.9493921 | 835.044157 | 877.1406498 | 835.8685276 | 751.802171 | 765.9427838 | 822.6830199 | 789.4657203 | 898.3824624 | 789.1334779 | 770.8846195 | 763.4247335 | 740.4612699 | 827.6560608 | 810.542078 |
| **Tcp11** | 15.56684019 | 7.130358077 | 5.482129061 | 3.450437679 | 5.066905954 | 5.611302446 | 12.62890601 | 0 | 6.019312981 | 6.117313782 | 6.621094891 | 13.01292159 | 2.556252945 | 5.421327909 | 7.18565672 |
| **Zfp523** | 183.1392963 | 182.220262 | 205.1882592 | 144.0557731 | 156.4407213 | 159.2207069 | 170.4902311 | 162.5863585 | 136.9393703 | 145.9416288 | 139.0429927 | 176.1082056 | 186.606465 | 188.8429222 | 168.8629329 |
| **Def6** | 2436.668337 | 2490.079493 | 2575.817498 | 2712.044015 | 2640.491365 | 2689.216697 | 2599.750508 | 2758.101577 | 2677.089448 | 2612.092985 | 2882.068019 | 2669.383983 | 2539.211259 | 2693.496416 | 2616.297612 |
| **Ppard** | 576.8887834 | 529.2310217 | 589.7204547 | 549.4822003 | 490.2231511 | 445.3971316 | 505.1562403 | 565.6999588 | 470.2588266 | 423.8424549 | 568.46829 | 450.2470872 | 472.0547106 | 474.3661921 | 518.8044152 |
| **Fance** | 344.3018771 | 389.7929082 | 378.2669052 | 376.097707 | 376.8511303 | 342.2894492 | 311.2123266 | 392.2186381 | 388.9981014 | 428.2119648 | 379.2941502 | 325.3230399 | 349.3545692 | 330.7010025 | 368.6241897 |
| **Rpl10a** | 16607.98709 | 15847.61696 | 16075.95189 | 14881.73771 | 16749.29099 | 17084.31171 | 17219.51334 | 14599.24931 | 14204.82622 | 14711.26574 | 14314.80716 | 15850.60603 | 16007.25594 | 16332.65388 | 16387.60871 |
| **Tead3** | 2.747089445 | 3.169048034 | 0.783161294 | 3.450437679 | 0 | 5.611302446 | 1.80412943 | 7.542666117 | 1.504828245 | 2.621705907 | 0 | 1.735056213 | 5.112505891 | 1.807109303 | 2.155697016 |
| **Fkbp5** | 3240.649848 | 3047.039685 | 3073.12492 | 2872.489367 | 2514.45208 | 2520.176211 | 2497.817195 | 2721.22632 | 2922.376452 | 3087.495656 | 3178.125548 | 2660.708702 | 2583.519644 | 2493.810838 | 2583.243591 |
| **Armc12** | 0 | 0 | 0 | 0 | 0 | 0 | 0 | 0 | 0 | 0 | 0 | 0 | 0 | 0 | 0 |
| **Srpk1** | 2163.790786 | 2143.068733 | 2110.619689 | 2040.933887 | 1983.060318 | 1948.524774 | 2136.991309 | 2118.651105 | 2117.293341 | 2113.094961 | 2178.340219 | 2145.397007 | 2049.262778 | 1892.946995 | 2023.480932 |
| **Slc26a8** | 0 | 0.792262009 | 0 | 1.725218839 | 1.266726489 | 2.104238417 | 0.902064715 | 1.676148026 | 0 | 0.873901969 | 0.945870699 | 0.867528106 | 0 | 5.421327909 | 0 |
| **Mapk14** | 1551.18984 | 1617.006759 | 1734.702267 | 1651.897039 | 1734.148563 | 1721.968438 | 1782.479876 | 1599.045217 | 1800.526995 | 1590.501583 | 1642.031533 | 1794.915652 | 1888.218842 | 1780.906218 | 1820.126847 |
| **Mapk13** | 2.747089445 | 2.376786026 | 3.915806472 | 3.450437679 | 6.333632443 | 2.104238417 | 5.412388289 | 3.352296052 | 6.771727103 | 2.621705907 | 1.891741398 | 3.470112425 | 0.852084315 | 0.903554652 | 2.874262688 |
| **Brpf3** | 411.1477202 | 454.7583929 | 452.6672282 | 483.9238844 | 449.0545402 | 393.492584 | 416.7538982 | 496.1398157 | 466.496756 | 462.2941415 | 443.6133577 | 488.4183238 | 453.3088557 | 489.7266212 | 516.6487181 |
| **4930539E08Rik** | 90.65395167 | 49.91250654 | 43.85703249 | 54.34439344 | 48.76896981 | 63.12715251 | 72.16517718 | 46.09407072 | 63.95520042 | 50.6863142 | 30.26786236 | 50.31663016 | 67.3146609 | 71.38081747 | 54.61099107 |
| **Pxt1** | 0 | 0 | 2.349483883 | 0 | 0 | 0 | 0 | 0 | 0 | 0 | 0 | 0 | 0 | 0 | 0 |
| **Kctd20** | 1009.097523 | 1113.128122 | 1175.525103 | 1061.872196 | 1132.453481 | 1058.431924 | 1138.40567 | 1020.774148 | 1089.495649 | 1045.186755 | 999.7853286 | 1191.11609 | 1076.18249 | 1126.732651 | 1064.914326 |
| **Stk38** | 1086.931724 | 1311.193624 | 1308.662523 | 1289.601082 | 1274.326847 | 1294.808039 | 1314.308289 | 1164.084804 | 1239.22606 | 1224.336658 | 1148.287028 | 1404.528004 | 1433.205818 | 1501.707831 | 1360.963383 |
| **Srsf3** | 8399.683825 | 8614.264818 | 9074.489919 | 8394.914872 | 8873.419052 | 9402.438661 | 8922.322094 | 9162.663185 | 9302.848212 | 9058.86781 | 9476.678531 | 9370.171076 | 9115.598004 | 9162.947722 | 8962.669626 |
| **Trp53cor1** | 16.48253667 | 11.09166812 | 6.265290356 | 4.313047098 | 5.066905954 | 3.507064029 | 4.510323574 | 13.40918421 | 11.28621184 | 3.495607876 | 11.35044839 | 6.072696744 | 0.852084315 | 2.710663955 | 5.748525376 |
| **Cdkn1a** | 3995.183749 | 2594.658078 | 2890.648338 | 2800.030176 | 1668.278785 | 2183.498064 | 2278.61547 | 3440.293824 | 2795.97088 | 3202.850716 | 3370.1373 | 1750.671718 | 1654.74774 | 1596.581069 | 1821.563978 |

| | | | | | | | | | | | | | | | |
|---|---|---|---|---|---|---|---|---|---|---|---|---|---|---|---|
| **Cpne5** | 8.241268334 | 8.714882094 | 1.566322589 | 0.86260942 | 1.900089733 | 1.402825611 | 7.216517718 | 0 | 1.504828245 | 1.747803938 | 2.837612096 | 0 | 0 | 3.614218606 | 1.437131344 |
| **Ppil1** | 1771.872692 | 1943.418707 | 2009.591882 | 1977.963399 | 1994.460856 | 1860.146761 | 1860.057442 | 2182.34473 | 1970.572587 | 2220.584903 | 2116.858624 | 1952.805767 | 1770.631207 | 1945.353165 | 1870.426444 |
| **BC004004** | 986.2051106 | 865.1501133 | 899.0691661 | 851.3954972 | 833.5060295 | 883.7801352 | 807.3479197 | 791.1418683 | 803.5782829 | 849.4327138 | 943.9789574 | 856.2502409 | 787.3259072 | 886.3871132 | 820.6019974 |
| **Mtch1** | 2195.840163 | 2486.910445 | 2458.343303 | 2646.485699 | 2397.913243 | 2465.466012 | 2458.126348 | 2603.895959 | 2573.256299 | 2697.735378 | 2482.910584 | 2460.309709 | 2284.438049 | 2481.161073 | 2322.404252 |
| **Fgd2** | 17.39823315 | 11.09166812 | 14.8800646 | 23.29045433 | 5.066905954 | 10.52119209 | 18.94335901 | 3.352296052 | 12.79104008 | 12.23462756 | 9.458706988 | 0.867528106 | 5.112505891 | 2.710663955 | 14.37131344 |
| **Pim1** | 7590.208136 | 6746.903264 | 6556.626357 | 5914.912791 | 6111.321944 | 6860.518653 | 7285.074637 | 5454.185677 | 5302.262322 | 5019.692909 | 5012.168833 | 6202.82596 | 6330.986462 | 5756.546685 | 6250.084215 |
| **Tmem217** | 2.747089445 | 0 | 0 | 3.450437679 | 6.333632443 | 0 | 0.902064715 | 2.514222039 | 0 | 0 | 3.783482795 | 0 | 0 | 0 | 0 |
| **Tbc1d22b** | 579.6358728 | 803.3536766 | 836.4162625 | 748.7449763 | 822.1054911 | 726.6636667 | 837.1160553 | 664.5926924 | 869.7907257 | 761.1686149 | 776.5598437 | 805.0660826 | 829.0780387 | 930.6612911 | 809.1049466 |
| **Tbc1d22bos** | 2.747089445 | 12.67619214 | 0 | 0 | 1.900089733 | 0 | 0 | 0 | 0 | 2.621705907 | 2.837612096 | 2.602584319 | 0 | 0.903554652 | 0 |
| **Rnf8** | 36.62785926 | 45.15893448 | 55.60445191 | 56.06961228 | 42.43533737 | 36.4734659 | 39.69084745 | 36.87525657 | 36.86829201 | 59.42533389 | 54.86050053 | 39.90629289 | 49.42089028 | 28.0101942 | 48.86246569 |
| **Rnf8-cmtr1** | 0.915696482 | 2.376786026 | 0 | 4.313047098 | 2.533452977 | 1.402825611 | 0 | 0.838074013 | 5.266898858 | 1.747803938 | 0 | 0 | 3.408337261 | 0 | 1.437131344 |
| **Cmtr1** | 32.04937685 | 25.35238427 | 47.77283896 | 34.50437679 | 32.9348887 | 36.4734659 | 26.15987673 | 22.62799835 | 33.85863552 | 27.964863 | 23.64676747 | 16.48303402 | 26.41461377 | 20.78175699 | 21.55697016 |
| **Ccdc167** | 24.723805 | 22.18333624 | 37.59174214 | 28.46611085 | 31.66816221 | 38.57770431 | 25.25781201 | 35.19910855 | 38.37312025 | 44.56900041 | 53.91462983 | 48.58157395 | 35.78754124 | 16.26398373 | 28.02406121 |
| **Mdga1** | 0 | 0 | 0.783161294 | 2.587828259 | 0 | 0 | 0 | 0.838074013 | 1.504828245 | 0 | 0 | 0 | 0 | 0 | 2.155697016 |
| **Zfand3** | 804.8972073 | 690.8524714 | 762.0159395 | 709.064943 | 770.169705 | 700.7113929 | 785.6983666 | 661.2403963 | 616.9795805 | 579.3970054 | 701.8360585 | 712.2405753 | 701.2653914 | 682.1837619 | 773.8952287 |
| **Btbd9** | 411.1477202 | 437.3286287 | 407.2438731 | 417.5029591 | 385.7182158 | 396.999648 | 433.8931278 | 444.1792269 | 443.9243323 | 384.5168663 | 405.7785298 | 419.0160753 | 409.8525556 | 388.5285002 | 446.2292823 |
| **Gm6402** | 20.14532259 | 20.59881222 | 27.41064531 | 15.52696955 | 27.86798275 | 23.84803539 | 15.33510015 | 4.190370065 | 18.05793894 | 6.991215751 | 7.56696559 | 9.542809169 | 10.22501178 | 13.55331977 | 12.21561642 |
| **Glo1** | 893.719766 | 861.1888032 | 910.0334242 | 951.4581899 | 869.6077344 | 901.3154553 | 921.9101385 | 1081.953551 | 1072.942539 | 1012.852382 | 1115.181554 | 951.6783326 | 887.0197721 | 862.8946923 | 943.4767273 |
| **1700097N02Rik** | 182.2235998 | 189.35062 | 172.2954848 | 156.9949144 | 195.0758792 | 145.8938636 | 182.2170724 | 99.73080755 | 122.643502 | 167.789178 | 108.7751304 | 165.6978683 | 202.796067 | 187.9393675 | 188.9827717 |
| **Dnah8** | 267.3833726 | 283.629799 | 283.5043886 | 320.0280947 | 291.9804556 | 245.494482 | 303.9958089 | 211.1946513 | 200.8945707 | 218.4754922 | 218.4961314 | 328.7931523 | 282.8919926 | 327.0867839 | 305.3904106 |
| **Abcg1** | 679.4467893 | 867.5268993 | 868.5258756 | 954.0460181 | 642.2303297 | 591.992408 | 700.9042834 | 744.2097236 | 807.3403535 | 743.6905755 | 726.4286967 | 735.6638341 | 764.3196307 | 884.5800039 | 738.6855108 |
| **Tmprss3** | 0.915696482 | 1.584524017 | 9.397935534 | 10.35131304 | 3.800179466 | 5.611302446 | 4.510323574 | 6.704592104 | 4.514484735 | 16.60413741 | 3.783482795 | 0 | 1.70416863 | 17.16753838 | 1.437131344 |
| **Ubash3a** | 1769.125602 | 1884.791318 | 1913.263042 | 2129.782657 | 1770.883631 | 1718.461374 | 1918.691648 | 2054.95748 | 2117.293341 | 2231.945629 | 1928.630355 | 1794.915652 | 1887.366758 | 1728.500048 | 1838.090989 |
| **Rsph1** | 18.31392963 | 14.26071615 | 29.76012919 | 19.84001665 | 14.56735462 | 8.416953669 | 27.96400616 | 8.380740131 | 12.79104008 | 5.243411813 | 9.458706988 | 23.42325887 | 20.45002356 | 28.0101942 | 16.52701046 |
| **Slc37a1** | 634.5776617 | 715.4125937 | 636.7101324 | 688.3623169 | 606.1286248 | 672.6548807 | 645.8783358 | 607.6036595 | 614.7223381 | 629.2094176 | 575.0893849 | 642.8383268 | 760.9112934 | 653.2700131 | 654.6133272 |
| **Wdr4** | 957.8185197 | 1084.60669 | 1060.400393 | 1086.025259 | 1022.88164 | 981.977928 | 979.6422803 | 1361.032197 | 1224.930192 | 1265.410051 | 1256.116288 | 1051.444065 | 976.4886252 | 982.1639063 | 1088.626993 |
| **Ndufv3** | 1212.382142 | 1170.963249 | 1174.741942 | 1098.964401 | 1216.057429 | 1174.86645 | 1259.282342 | 699.7918009 | 767.462405 | 736.6993598 | 821.9616372 | 1270.928676 | 1189.509704 | 1274.012059 | 1289.825381 |
| **Pknox1** | 1437.643476 | 1426.071615 | 1491.139105 | 1432.794246 | 1520.071786 | 1529.781329 | 1621.010292 | 1242.025687 | 1583.831728 | 1350.178542 | 1427.318884 | 1703.825201 | 1615.551862 | 1530.62158 | 1651.263914 |
| **Cbs** | 13.73544722 | 6.338096068 | 6.265290356 | 4.313047098 | 6.333632443 | 8.416953669 | 9.020647148 | 4.190370065 | 14.29586833 | 5.243411813 | 13.24218978 | 14.74797781 | 10.22501178 | 3.614218606 | 2.874262688 |
| **U2af1** | 3441.187378 | 3565.179038 | 3571.215503 | 3327.947141 | 3508.832373 | 3323.293873 | 3394.469522 | 3633.050847 | 3703.382311 | 3587.367582 | 3516.747258 | 3392.034896 | 3549.783257 | 3572.655092 | 3217.737079 |
| **Cryaa** | 4.578482408 | 2.376786026 | 0.783161294 | 3.450437679 | 1.266726489 | 0 | 1.80412943 | 0 | 6.019312981 | 1.747803938 | 5.675224193 | 1.735056213 | 0 | 2.710663955 | 0 |
| **Sik1** | 851.5977279 | 701.1518775 | 684.4829714 | 631.4300952 | 601.6950821 | 647.4040197 | 669.3320184 | 538.8815904 | 615.4747523 | 600.3706526 | 562.7930658 | 661.9239451 | 739.6091856 | 602.6709526 | 651.7390645 |
| **Hsf2bp** | 4.578482408 | 0 | 3.915806472 | 11.21392246 | 10.13381191 | 2.805651223 | 4.510323574 | 0.838074013 | 5.266898858 | 0 | 0 | 8.675281063 | 7.668758836 | 5.421327909 | 3.59282836 |
| **Rrp1b** | 2693.979049 | 2753.902742 | 2710.52124 | 2626.645683 | 2319.376201 | 2384.803539 | 2482.482095 | 2928.230602 | 2797.475708 | 2867.27236 | 2881.122148 | 2458.574653 | 2692.586436 | 2338.399438 | 2500.608538 |
| **Pdxk-ps** | 1.831392963 | 0 | 0 | 0 | 3.800179466 | 2.104238417 | 0.902064715 | 0 | 1.504828245 | 0 | 1.891741398 | 1.735056213 | 0 | 3.614218606 | 3.59282836 |
| **Notch3** | 0.915696482 | 0 | 7.04845165 | 4.313047098 | 3.166816221 | 0 | 0 | 10.05688816 | 5.266898858 | 4.369509845 | 2.837612096 | 4.337640531 | 1.70416863 | 1.807109303 | 0 |
| **Ephx3** | 0 | 0 | 0 | 0 | 3.800179466 | 0 | 0 | 0.838074013 | 0 | 0 | 0 | 5.205168638 | 0 | 6.324882561 | 0 |
| **Brd4** | 5646.184505 | 5162.379247 | 5157.117124 | 5170.480861 | 5007.369809 | 5161.696837 | 5787.64721 | 4809.706761 | 4889.186969 | 4684.988455 | 4945.957884 | 5366.528865 | 5327.231138 | 5299.348031 | 5410.080944 |
| **Gm4432** | 3.662785926 | 0.792262009 | 0.783161294 | 3.450437679 | 1.900089733 | 1.402825611 | 0 | 6.704592104 | 1.504828245 | 6.117313782 | 6.621094891 | 7.807752957 | 0.852084315 | 1.807109303 | 2.155697016 |
| **Akap8** | 2360.665529 | 2621.594986 | 2582.865949 | 2525.720381 | 2422.614409 | 2356.747027 | 2652.070261 | 2818.442906 | 2825.31503 | 2821.829458 | 2815.85707 | 2628.610162 | 2534.950838 | 2569.709429 | 2533.662559 |
| **Akap8l** | 325.9879474 | 366.8173099 | 329.710905 | 340.7307208 | 386.351579 | 411.729317 | 445.6199691 | 375.4571578 | 322.7856586 | 343.4434738 | 301.7327529 | 360.0241641 | 342.5378947 | 400.2747106 | 397.3668166 |
| **Wiz** | 1518.224766 | 1601.161519 | 1536.56246 | 1493.176905 | 1342.096715 | 1433.687775 | 1546.138921 | 1342.594569 | 1580.822072 | 1473.39872 | 1449.073911 | 1410.600701 | 1431.501649 | 1482.733183 | 1519.047831 |
| **Rasal3** | 1705.026849 | 2005.215144 | 2023.688785 | 2012.467776 | 2232.605436 | 2028.485834 | 2149.620215 | 2120.327253 | 2410.734849 | 2216.215393 | 2138.61365 | 2142.794423 | 2265.692194 | 2405.262482 | 2144.918531 |
| **A530088E08Rik** | 1.831392963 | 0 | 2.349483883 | 1.725218839 | 0 | 0 | 0 | 0 | 0.752414123 | 0 | 1.891741398 | 0 | 0 | 1.807109303 | 2.155697016 |
| **Pglyrp2** | 502.7173684 | 699.5673535 | 668.8197455 | 693.5379734 | 761.3026196 | 704.9198697 | 747.8116486 | 696.4395048 | 671.1533973 | 643.1918491 | 562.7930658 | 785.9804643 | 692.7445482 | 819.524069 | 776.7694914 |
| **Cyp4f39** | 1.831392963 | 0.792262009 | 4.698967767 | 3.450437679 | 2.533452977 | 6.312715251 | 2.706194144 | 0 | 3.00965649 | 1.747803938 | 0 | 0 | 4.260421576 | 8.131991864 | 0.718565672 |
| **Cyp4f17** | 31.13368037 | 22.18333624 | 27.41064531 | 31.05393911 | 41.80197412 | 28.05651223 | 32.47432973 | 32.68488651 | 38.37312025 | 25.3431571 | 36.88895725 | 19.95314644 | 23.00627651 | 34.33507676 | 17.24557613 |
| **Cyp4f16** | 17.39823315 | 19.80655021 | 19.57903236 | 25.87828259 | 1.900089733 | 12.6254305 | 9.020647148 | 7.542666117 | 10.53379772 | 15.73023544 | 9.458706988 | 9.542809169 | 8.520843152 | 15.36042908 | 21.55697016 |
| **Zfp871** | 1018.254487 | 1130.557886 | 1137.933361 | 1096.376572 | 906.3428026 | 873.2589431 | 972.4257625 | 1064.353997 | 1029.30252 | 943.8141264 | 1087.751304 | 1064.456986 | 1059.992888 | 1011.077655 | 1021.800386 |
| **Zfp811** | 0.915696482 | 0 | 0 | 0 | 0 | 2.805651223 | 0.902064715 | 1.676148026 | 0 | 2.621705907 | 0 | 0 | 0 | 2.710663955 | 0.718565672 |
| **Zfp799** | 81.49698686 | 65.75774671 | 76.74980686 | 80.22267603 | 77.2703158 | 79.25964705 | 66.75278889 | 59.50325493 | 68.46968515 | 74.28166736 | 67.15681961 | 66.79966418 | 56.2375648 | 98.48745702 | 70.41943585 |
| **Zfp870** | 28.38659093 | 32.48274235 | 39.94122602 | 32.77915795 | 32.30152546 | 30.16075065 | 46.00530045 | 36.87525657 | 37.62070613 | 25.3431571 | 34.05134516 | 23.42325887 | 28.97086672 | 44.27417793 | 27.30549553 |
| **Cyp4f13** | 78.74989741 | 68.13453273 | 70.4845165 | 86.26094197 | 58.26941847 | 74.34975741 | 79.3816949 | 50.28444078 | 42.88760499 | 58.55143192 | 62.42746612 | 70.26977661 | 52.82922754 | 45.17773258 | 81.19792093 |
| **Cyp4f41-ps** | 12.81975074 | 11.88393013 | 3.915806472 | 13.80175071 | 2.533452977 | 9.118366474 | 5.412388289 | 6.704592104 | 5.266898858 | 7.86511772 | 5.675224193 | 5.205168638 | 1.70416863 | 10.84265582 | 7.18565672 |
| **Zfp472** | 158.4154913 | 182.220262 | 223.2009689 | 202.7132136 | 209.0098706 | 167.6376606 | 232.7326964 | 186.8905049 | 158.0069657 | 247.3142572 | 226.063097 | 198.6639363 | 218.985669 | 206.9140152 | 203.3540852 |
| **Zfp952** | 126.3661145 | 125.1773973 | 133.1374201 | 108.6887869 | 106.405025 | 118.5387642 | 133.5055778 | 132.4156941 | 85.77520997 | 83.89458901 | 127.6925443 | 111.9111257 | 115.0313825 | 95.77679307 | 105.6291538 |
| **Zfp763** | 57.68887834 | 61.00417465 | 57.1707745 | 38.81742388 | 32.9348887 | 43.48759395 | 53.22181817 | 54.47481085 | 68.46968515 | 55.05582404 | 37.83482795 | 89.35539495 | 55.38548049 | 47.88839653 | 52.45529405 |
| **Zfp563** | 43.95343112 | 28.52143231 | 31.32645178 | 25.87828259 | 38.6351579 | 25.25086101 | 29.76813559 | 16.76148026 | 34.61104964 | 37.57778466 | 17.97154328 | 26.02584319 | 39.1958785 | 27.10663955 | 38.80254629 |
| **Zfp955a** | 146.5114371 | 158.4524017 | 144.8848395 | 125.0783658 | 149.4737256 | 135.3726715 | 122.6808012 | 140.7964342 | 155.7497234 | 138.9504131 | 104.0457769 | 138.804497 | 109.9188767 | 160.832728 | 135.0903463 |
| **Zfp955b** | 65.01445019 | 76.84941482 | 88.49722628 | 60.38265938 | 65.8697774 | 69.43986777 | 94.71679505 | 62.85555098 | 54.17381683 | 76.90337326 | 79.4531387 | 75.47494525 | 62.20215501 | 77.70570004 | 63.23377913 |
| **Zfp81** | 92.48534464 | 118.0470393 | 123.7394845 | 74.18441009 | 116.5388369 | 147.998102 | 136.2117719 | 84.64547532 | 88.03245234 | 82.14678508 | 91.74945778 | 89.35539495 | 129.5168159 | 140.050971 | 110.6591135 |
| **Zfp101** | 94.3167376 | 94.27917901 | 92.41303275 | 75.04701951 | 54.46923901 | 78.55823424 | 79.3816949 | 62.85555098 | 85.02279585 | 79.52507917 | 49.18527634 | 78.94505767 | 70.72299816 | 67.76659887 | 71.13800152 |
| **Adamts10** | 198.7061365 | 266.2000349 | 249.8284529 | 231.1793245 | 291.9804556 | 296.6976168 | 256.186379 | 286.6213125 | 289.6794372 | 289.2615517 | 289.4364338 | 345.2761863 | 343.389979 | 395.7569374 | 347.7857852 |
| **Myo1f** | 674.8683069 | 755.0256941 | 793.3423913 | 840.1815747 | 981.7130286 | 905.5239322 | 930.0287209 | 531.3389243 | 624.5037217 | 701.743281 | 800.2066112 | 1030.62339 | 978.1927938 | 1161.067727 | 980.1235766 |
| **Zfp414** | 338.8076982 | 354.9333798 | 350.8562599 | 377.8229258 | 408.5192926 | 353.5120541 | 370.7485978 | 362.0479736 | 369.4353342 | 360.9215132 | 312.1373306 | 370.4345014 | 320.3837025 | 368.6502978 | 371.4984524 |
| **Pram1** | 11.90405426 | 8.714882094 | 3.915806472 | 11.21392246 | 3.800179466 | 6.312715251 | 9.922711863 | 3.352296052 | 5.266898858 | 4.369509845 | 4.729353494 | 16.48303402 | 6.816674521 | 5.421327909 | 10.77848508 |
| **Hnrnpm** | 10657.79135 | 10907.07107 | 11093.47974 | 11282.93121 | 11304.90055 | 11080.9195 | 10836.50342 | 11755.66418 | 11638.34165 | 11384.32095 | 11930.26712 | 11499.08505 | 10750.7478 | 11338.70732 | 10637.64621 |
| **March2** | 375.4355574 | 377.9089781 | 423.6902603 | 412.3273026 | 414.852925 | 409.6250785 | 467.2695223 | 383.837898 | 379.9691319 | 393.255886 | 454.9638061 | 499.6961892 | 417.5213144 | 426.4777955 | 442.6364539 |
| **Rab11b** | 1732.497743 | 1727.131179 | 1886.635558 | 1801.991078 | 1926.057626 | 1941.510646 | 1902.454483 | 1859.686235 | 1793.002854 | 1658.665937 | 1715.809448 | 1827.014192 | 2055.227368 | 1893.85055 | 1910.666122 |
| **Angptl4** | 241.7438711 | 270.9536069 | 305.4329049 | 292.4245933 | 282.4800069 | 272.8495814 | 317.5267796 | 250.5841299 | 279.1456395 | 303.2439832 | 262.0061836 | 347.0112425 | 302.4899319 | 264.7415129 | 325.5102494 |
| **Kank3** | 72.34002204 | 93.486917 | 101.8109683 | 69.00875357 | 63.33632443 | 63.12715251 | 77.57756547 | 113.1399918 | 92.54693708 | 103.1204323 | 157.9604067 | 98.89820412 | 70.72299816 | 74.09148143 | 57.48525376 |
| **Rps28** | 5032.667863 | 4369.324977 | 4496.912153 | 3748.900538 | 4732.490161 | 4701.570037 | 4313.673466 | 3886.149199 | 3785.395451 | 3607.467328 | 3445.806956 | 3855.294904 | 3578.754124 | 4288.270376 | 3698.457514 |
| **Ndufa7** | 1067.702098 | 1032.317397 | 1105.823748 | 1100.689619 | 1139.420476 | 1176.269275 | 1188.019229 | 933.6144505 | 1123.354285 | 1079.268932 | 963.842242 | 1000.259907 | 1065.105394 | 1011.98121 | 1051.980144 |
| **Cd320** | 278.3717304 | 284.4220611 | 314.0476791 | 268.2715295 | 290.7137291 | 290.3849016 | 269.7173497 | 333.5534572 | 304.7277196 | 332.0827482 | 329.1630032 | 274.1388816 | 214.7252474 | 265.6450676 | 334.8516031 |
| **Kifc1** | 1825.898784 | 1987.785379 | 1904.648268 | 1882.213754 | 2022.328839 | 1955.538902 | 2026.939414 | 2090.156589 | 2115.036099 | 2199.611256 | 1930.522096 | 2114.165995 | 1964.906431 | 2223.647998 | 2180.128249 |
| **Platr17** | 4.578482408 | 9.507144102 | 8.614774239 | 8.626094197 | 6.966995687 | 23.84803539 | 13.53097072 | 8.380740131 | 6.019312981 | 5.243411813 | 9.458706988 | 7.807752957 | 28.1187824 | 9.035546516 | 11.49705075 |
| **BC051226** | 63.18305723 | 57.83512662 | 46.20651637 | 44.85568982 | 46.86888008 | 55.41161165 | 53.22181817 | 44.41792269 | 45.14484735 | 47.19070632 | 27.43025026 | 44.24393342 | 34.08337261 | 74.09148143 | 45.26963733 |
| **Daxx** | 2483.368858 | 2159.706235 | 1847.477494 | 1751.097122 | 2123.666958 | 2361.656917 | 2483.38416 | 1340.080347 | 1442.377873 | 1270.653463 | 1524.743566 | 1821.809023 | 1883.106337 | 1933.606954 | 2052.942125 |
| **Zbtb22** | 459.6796337 | 473.7726811 | 486.3431639 | 412.3273026 | 473.7557067 | 474.8564695 | 471.7798458 | 530.5008503 | 480.7926243 | 461.4202396 | 538.2004276 | 484.0806833 | 480.5755538 | 519.5439247 | 477.8461719 |
| **Tapbp** | 11730.07193 | 11791.23547 | 10773.16677 | 11064.69103 | 10681.67111 | 11253.46705 | 12174.26539 | 9479.455162 | 9439.035168 | 8581.717335 | 9288.450262 | 10847.57144 | 10904.97507 | 11258.29096 | 10743.27536 |
| **Rgl2** | 200.5375295 | 248.7702707 | 237.2978722 | 198.4001665 | 269.1793788 | 259.5227381 | 294.073097 | 214.5469473 | 230.9911356 | 275.2791202 | 307.4079771 | 270.6687692 | 277.7794867 | 311.7263548 | 326.2288151 |
| **Pfdn6** | 1174.838586 | 1193.146585 | 1207.634716 | 1114.49137 | 1190.722899 | 1116.649187 | 1114.049923 | 989.7654094 | 1124.859113 | 1144.811579 | 1008.298165 | 1161.620134 | 1085.555418 | 1119.504213 | 1000.961981 |
| **Wdr46** | 1279.227985 | 1298.517432 | 1339.205814 | 1296.501958 | 1221.124335 | 1334.087156 | 1298.071125 | 1793.478388 | 1607.156566 | 1677.017878 | 1643.923274 | 1154.679909 | 1278.126473 | 1309.25069 | 1340.843544 |
| **B3galt4** | 108.0521848 | 114.0857292 | 126.8721297 | 136.2922883 | 145.0401829 | 136.0740843 | 139.8200308 | 124.0349539 | 88.78486646 | 95.25531461 | 107.8292597 | 105.838429 | 115.8834669 | 170.7718291 | 131.497518 |
| **Rps18** | 22804.50518 | 21700.05641 | 21532.23663 | 20314.45183 | 22592.06692 | 24212.77005 | 23815.41053 | 19548.07635 | 18844.96411 | 19983.51632 | 19546.41799 | 22031.74379 | 20966.38666 | 21550.68199 | 21905.47451 |
| **Vps52** | 1258.166966 | 1373.782323 | 1260.106523 | 1389.663775 | 1263.559672 | 1167.150909 | 1327.83926 | 1569.712626 | 1549.220678 | 1462.037994 | 1532.310532 | 1207.599124 | 1296.872328 | 1279.433387 | 1243.118613 |
| **H2-K2** | 169.4038491 | 176.6744279 | 151.9332911 | 119.9027093 | 138.7065505 | 185.8743935 | 194.8459784 | 43.57984868 | 42.88760499 | 97.00311855 | 88.91184568 | 130.1292159 | 142.2980806 | 169.8682745 | 163.8329732 |
| **AA388235** | 38.45925223 | 37.2363144 | 35.24225825 | 45.71829924 | 47.50224332 | 35.07064029 | 30.6702003 | 25.9802944 | 30.0965649 | 38.45168663 | 38.78069865 | 61.59449555 | 45.1604687 | 46.98484188 | 37.36541494 |
| **H2-K1** | 39851.11088 | 38571.27588 | 38165.0162 | 37350.12526 | 39677.04044 | 41078.24097 | 40618.16998 | 32546.6043 | 31893.32983 | 31561.84351 | 31742.47478 | 37858.92656 | 38067.71886 | 39085.06356 | 37378.34912 |
| **Ring1** | 363.5315032 | 419.8988645 | 344.5909696 | 406.2890367 | 397.1187542 | 395.5968224 | 410.4394452 | 430.7700427 | 361.911193 | 385.3907683 | 278.0859854 | 349.6138268 | 360.4316653 | 455.3915444 | 415.3309584 |
| **Mir219a-1** | 0 | 0 | 0 | 0 | 0 | 0.701412806 | 6.314453003 | 6.704592104 | 0 | 0 | 0 | 0.867528106 | 5.112505891 | 0 | 0 |
| **H2-Ke6** | 368.1099856 | 379.4935021 | 379.0500665 | 328.6541889 | 286.9135497 | 372.4501998 | 345.4907858 | 342.7722713 | 382.9787884 | 392.381984 | 310.2455892 | 380.8448387 | 421.781736 | 337.9294397 | 352.8157449 |
| **Slc39a7** | 2501.682788 | 2371.240191 | 2371.4124 | 2436.871611 | 2315.576021 | 2350.434312 | 2363.409553 | 2541.040408 | 2171.467158 | 2304.479492 | 2374.135454 | 2249.50038 | 2217.123388 | 2215.516006 | 2267.793261 |
| **Rxrb** | 1356.146489 | 1292.179336 | 1248.359103 | 1284.425426 | 1179.955724 | 1209.235677 | 1206.060524 | 1169.113248 | 1197.090869 | 1196.371795 | 1134.098968 | 1269.193619 | 1163.947175 | 1204.438351 | 1204.316066 |
| **Col11a2** | 55.85748538 | 84.77203491 | 52.47180673 | 38.81742388 | 86.77076447 | 58.91867568 | 46.90736517 | 50.28444078 | 34.61104964 | 65.54264767 | 52.02288843 | 96.2956198 | 88.61676878 | 103.9087849 | 80.47935526 |
| **BC051537** | 0.915696482 | 0.792262009 | 0 | 0 | 1.900089733 | 0 | 6.314453003 | 1.676148026 | 0 | 0 | 0 | 1.735056213 | 0 | 0 | 0 |
| **H2-Oa** | 60.43596778 | 72.88810478 | 67.35187133 | 80.22267603 | 66.50314065 | 76.45399582 | 77.57756547 | 32.68488651 | 33.10622139 | 40.19949057 | 47.29353494 | 72.00483282 | 72.42716679 | 65.05593491 | 46.70676868 |
| **Brd2** | 6528.000217 | 6955.268173 | 6839.347585 | 6533.403744 | 5918.779518 | 6246.782448 | 6696.026378 | 6681.126032 | 6874.055424 | 6837.409005 | 6725.140668 | 6804.022938 | 6696.530633 | 6669.136883 | 6936.314431 |
| **H2-DMa** | 387.3396117 | 342.2571877 | 368.0858084 | 460.6334301 | 397.1187542 | 349.3035772 | 318.4288443 | 388.028268 | 338.5863552 | 329.4610423 | 289.4364338 | 360.0241641 | 417.5213144 | 371.3609618 | 334.1330375 |
| **H2-DMb2** | 54.94178889 | 41.19762444 | 54.82129061 | 46.58090866 | 22.16771355 | 29.45933784 | 33.37639445 | 39.38947861 | 46.6496756 | 48.93851026 | 32.15960376 | 38.17123668 | 28.1187824 | 20.78175699 | 22.27553583 |
| **H2-DMb1** | 24.723805 | 6.338096068 | 3.132645178 | 18.97740723 | 5.700269198 | 2.104238417 | 2.706194144 | 7.542666117 | 6.771727103 | 8.739019689 | 13.24218978 | 6.94022485 | 3.408337261 | 0 | 9.341353736 |
| **Psmb9** | 4062.029592 | 4557.091073 | 4321.484023 | 4232.824422 | 4159.929788 | 4236.533347 | 4234.291771 | 3629.698551 | 3982.527951 | 3536.681268 | 3459.995016 | 4083.454796 | 3882.09614 | 4364.168967 | 3934.147054 |
| **Tap1** | 9960.03063 | 10272.4692 | 9820.059472 | 10114.09545 | 9554.917903 | 9981.104225 | 10294.36253 | 8484.661308 | 8831.084557 | 8212.056802 | 8085.302733 | 9915.846255 | 9646.446532 | 9576.775752 | 9505.905274 |
| **Psmb8** | 9721.033848 | 10089.45668 | 9990.788634 | 10037.32321 | 9504.248844 | 9812.765152 | 9327.349151 | 9776.133362 | 9967.229882 | 9329.77742 | 9211.834735 | 9591.390743 | 9245.966904 | 10087.28413 | 9142.311044 |
| **Tap2** | 5014.353933 | 5434.125116 | 5070.969382 | 5499.99766 | 5103.007659 | 5180.634983 | 5189.578304 | 4942.122455 | 5190.152618 | 4712.953318 | 5041.490824 | 5205.168638 | 5126.991324 | 5423.135019 | 5114.031887 |
| **H2-Ob** | 36.62785926 | 41.19762444 | 38.37490343 | 31.05393911 | 29.76807248 | 30.86216345 | 42.39704159 | 10.05688816 | 12.03862596 | 12.23462756 | 5.675224193 | 21.68820266 | 38.34379418 | 37.94929537 | 45.26963733 |
| **H2-Ab1** | 412.9791132 | 262.2387248 | 309.3487113 | 271.7219672 | 233.7110371 | 281.9679479 | 248.0677966 | 118.1684358 | 136.9393703 | 153.8067465 | 119.179708 | 209.0742736 | 253.0690416 | 168.0611652 | 204.7912165 |
| **H2-Aa** | 43.03773463 | 66.55000871 | 54.03812932 | 56.9322217 | 22.16771355 | 11.22260489 | 19.84542373 | 81.29317927 | 66.21244279 | 62.92094176 | 104.0457769 | 16.48303402 | 16.18960199 | 21.68531164 | 25.86836419 |
| **H2-Eb1** | 38.45925223 | 52.28929256 | 74.40032298 | 72.45919125 | 39.26852115 | 18.23673295 | 14.43303544 | 67.88399506 | 89.53728059 | 61.17313782 | 58.64398332 | 29.49595561 | 21.30210788 | 23.49242094 | 10.77848508 |
| **Btnl2** | 4.578482408 | 0 | 0 | 0 | 1.900089733 | 0 | 0 | 0 | 0 | 0 | 0 | 0 | 0 | 0 | 0 |
| **BC051142** | 0 | 0.792262009 | 0 | 0 | 0 | 0 | 0.902064715 | 0 | 0 | 0 | 4.729353494 | 0 | 0 | 0 | 0 |
| **Notch4** | 0 | 2.376786026 | 4.698967767 | 0.86260942 | 3.166816221 | 2.104238417 | 8.118582433 | 0 | 0.752414123 | 0 | 0 | 0.867528106 | 3.408337261 | 6.324882561 | 8.622788064 |
| **Gpsm3** | 936.7575006 | 1061.631091 | 1101.907941 | 1059.284367 | 1107.118951 | 989.6934689 | 1137.503605 | 906.7960821 | 899.1348765 | 926.336087 | 963.842242 | 1060.119346 | 1082.14708 | 1114.082885 | 1074.974245 |
| **Pbx2** | 452.3540619 | 389.0006462 | 444.052454 | 413.189912 | 419.2864677 | 423.6533347 | 405.0270569 | 395.5709342 | 399.5318991 | 401.1210037 | 382.1317623 | 440.704278 | 439.6755066 | 443.6453339 | 462.7562927 |
| **Ager** | 9.156964816 | 12.67619214 | 10.18109683 | 11.21392246 | 10.76717515 | 5.611302446 | 8.118582433 | 7.542666117 | 10.53379772 | 9.612921658 | 18.91741398 | 19.95314644 | 12.78126473 | 11.74621047 | 6.467091048 |
| **Rnf5** | 469.752295 | 409.5994584 | 409.593357 | 386.44902 | 424.9867369 | 378.0615023 | 412.2435747 | 459.2645592 | 340.0911834 | 415.1034352 | 399.1574349 | 434.6315812 | 342.5378947 | 477.076856 | 368.6241897 |
| **Agpat1** | 1059.460829 | 1164.625153 | 1007.928586 | 1037.719132 | 944.9779605 | 918.1493627 | 997.6835745 | 1180.00821 | 1149.688779 | 1092.377461 | 1057.483441 | 929.9901299 | 1052.324129 | 949.6359388 | 1055.572972 |
| **Egfl8** | 0.915696482 | 3.169048034 | 0 | 0 | 0 | 3.507064029 | 1.80412943 | 0.838074013 | 0 | 3.495607876 | 1.891741398 | 3.470112425 | 2.556252945 | 1.807109303 | 2.874262688 |
| **Ppt2** | 93.40104112 | 104.5785851 | 126.8721297 | 119.9027093 | 86.13740122 | 113.6288745 | 98.32505391 | 88.83584538 | 102.3283207 | 103.9943343 | 95.53294058 | 108.4410133 | 89.46885309 | 129.2083152 | 103.4734568 |
| **Prrt1** | 2.747089445 | 0 | 4.698967767 | 6.038265938 | 1.900089733 | 6.312715251 | 2.706194144 | 1.676148026 | 8.276555348 | 0.873901969 | 3.783482795 | 6.94022485 | 2.556252945 | 0 | 5.748525376 |
| **Fkbpl** | 52.19469945 | 51.49703055 | 40.72438731 | 44.85568982 | 48.76896981 | 49.80030921 | 39.69084745 | 44.41792269 | 61.69795805 | 20.97364725 | 32.15960376 | 39.90629289 | 31.52711966 | 41.56351397 | 50.29959704 |
| **Atf6b** | 1165.681621 | 1219.291231 | 1222.514781 | 1189.53839 | 1104.585498 | 1131.378856 | 1181.704776 | 1298.176646 | 1211.386737 | 1221.714953 | 1206.931012 | 1083.542605 | 1040.394949 | 1128.53976 | 1088.626993 |
| **C4a** | 0.915696482 | 3.169048034 | 0 | 0 | 6.966995687 | 4.90988964 | 1.80412943 | 5.028444078 | 0 | 1.747803938 | 7.56696559 | 3.470112425 | 9.372927467 | 3.614218606 | 2.155697016 |
| **Stk19** | 406.5692378 | 416.7298165 | 402.5449054 | 366.6090034 | 416.1196515 | 420.1462706 | 410.4394452 | 461.7787812 | 483.0498667 | 415.9773372 | 425.6418144 | 417.2810191 | 447.3442655 | 419.2493583 | 426.8280091 |
| **Dxo** | 282.0345163 | 350.1798078 | 328.1445824 | 296.7376404 | 338.8493357 | 345.0951004 | 328.3515562 | 295.0020526 | 279.1456395 | 332.0827482 | 247.8181231 | 351.348883 | 320.3837025 | 355.0969781 | 298.2047539 |
| **Skiv2l** | 1509.983498 | 1622.552593 | 1636.807105 | 1611.354396 | 1551.739948 | 1519.260137 | 1517.27285 | 1500.152483 | 1431.091661 | 1553.797701 | 1690.270939 | 1645.700818 | 1596.806007 | 1709.525401 | 1690.06646 |
| **Nelfe** | 1400.09992 | 1426.863877 | 1433.96833 | 1431.069027 | 1392.765774 | 1420.360932 | 1507.350138 | 1313.261978 | 1319.734371 | 1417.468994 | 1429.210626 | 1386.309914 | 1412.755795 | 1497.190058 | 1355.933423 |
| **Cfb** | 64.09875371 | 47.53572051 | 50.90548414 | 95.74964558 | 53.20251252 | 46.99465798 | 43.29910631 | 51.96058881 | 57.93588744 | 71.65996145 | 37.83482795 | 43.37640531 | 56.2375648 | 50.59906049 | 61.79664779 |
| **C2** | 1.831392963 | 4.753572051 | 5.482129061 | 6.900875357 | 4.43354271 | 2.104238417 | 3.608258859 | 7.542666117 | 2.257242368 | 4.369509845 | 2.837612096 | 0.867528106 | 11.0770961 | 7.228437213 | 2.874262688 |
| **Zbtb12** | 145.5957406 | 110.9166812 | 122.9563232 | 129.3914129 | 111.471931 | 113.6288745 | 127.1911248 | 99.73080755 | 85.02279585 | 117.1028638 | 74.7237852 | 104.9709009 | 138.8897434 | 119.269214 | 140.120306 |
| **Ehmt2** | 3261.710867 | 3302.148051 | 3176.50221 | 3172.677445 | 3106.01335 | 3240.527162 | 3278.103174 | 2826.823646 | 2552.188704 | 2806.973124 | 2778.968113 | 3079.724777 | 3334.205925 | 3082.024917 | 3165.281785 |
| **Neu1** | 448.691276 | 405.6381484 | 457.366196 | 430.4421004 | 411.6861088 | 389.98552 | 451.9344221 | 499.4921118 | 464.2395136 | 497.2502203 | 466.3142545 | 417.2810191 | 460.9776145 | 384.9142816 | 382.2769375 |
| **1110038B12Rik** | 499.9702789 | 432.5750566 | 454.2335508 | 404.5638178 | 374.3176774 | 459.4253877 | 451.9344221 | 344.4484194 | 513.1464316 | 557.5494562 | 450.2344526 | 433.7640531 | 432.8588321 | 421.9600223 | 454.1335047 |
| **Snord52** | 9.156964816 | 6.338096068 | 12.53058071 | 4.313047098 | 4.43354271 | 9.118366474 | 3.608258859 | 4.190370065 | 7.524141226 | 22.72145119 | 14.18806048 | 4.337640531 | 7.668758836 | 7.228437213 | 6.467091048 |
| **Hspa1b** | 53.11039593 | 34.06726637 | 34.45909696 | 23.29045433 | 29.76807248 | 34.36922748 | 38.78878274 | 56.15095887 | 38.37312025 | 46.31680435 | 35.94308655 | 30.36348372 | 36.63962555 | 44.27417793 | 19.40127314 |
| **Hspa1a** | 4.578482408 | 6.338096068 | 10.18109683 | 3.450437679 | 14.56735462 | 14.02825611 | 5.412388289 | 27.65644243 | 15.80069657 | 11.3607256 | 10.40457769 | 11.27786538 | 8.520843152 | 7.228437213 | 12.9341821 |
| **Hspa1l** | 4.578482408 | 11.09166812 | 18.79587107 | 1.725218839 | 20.90098706 | 9.81977928 | 27.06194144 | 14.24725822 | 20.31518131 | 7.86511772 | 22.70089677 | 8.675281063 | 9.372927467 | 9.939101167 | 11.49705075 |
| **Lsm2** | 1037.484114 | 1100.45193 | 1050.219296 | 1063.597414 | 1026.048456 | 1024.764109 | 1086.085917 | 1207.664653 | 1153.45085 | 1175.398148 | 1178.554891 | 1081.807549 | 1117.082537 | 1014.691874 | 1037.60883 |
| **D17H6S56E-5** | 3506.201828 | 3640.443929 | 3714.53402 | 3561.714294 | 3498.698561 | 3324.696699 | 3778.74909 | 3466.274118 | 3448.313924 | 3714.95727 | 3933.876236 | 3333.042984 | 3207.245362 | 3547.355562 | 3419.654033 |
| **Vars** | 11655.90051 | 11413.32649 | 11334.69342 | 11215.64767 | 11242.19759 | 11581.02684 | 11632.1245 | 9442.579905 | 9885.216742 | 10080.45921 | 10168.11001 | 10977.70066 | 11181.90247 | 10669.17333 | 11417.28996 |
| **Vwa7** | 0.915696482 | 0 | 1.566322589 | 2.587828259 | 7.600358931 | 0 | 1.80412943 | 3.352296052 | 12.03862596 | 0.873901969 | 7.56696559 | 4.337640531 | 2.556252945 | 0 | 1.437131344 |
| **Sapcd1** | 4.578482408 | 0 | 0 | 0 | 0 | 0 | 0 | 0 | 3.00965649 | 0 | 0 | 0 | 0 | 4.517773258 | 0 |
| **Msh5** | 19.22962611 | 27.7291703 | 28.9769679 | 31.05393911 | 18.36753408 | 26.65368662 | 30.6702003 | 15.08533223 | 20.31518131 | 7.86511772 | 9.458706988 | 19.08561834 | 19.59793925 | 37.94929537 | 17.24557613 |
| **Clic1** | 7118.624448 | 6881.587806 | 7187.0712 | 6880.172731 | 7373.61489 | 7374.654239 | 7388.812079 | 6356.791389 | 6242.027561 | 6135.665724 | 6047.897248 | 7400.014747 | 7106.383188 | 7526.610248 | 7380.388017 |
| **Ddah2** | 91.56964816 | 57.04286461 | 93.19619404 | 74.18441009 | 81.70385851 | 94.69072877 | 100.1291833 | 40.22755263 | 77.49865463 | 69.91215751 | 61.48159542 | 109.3085414 | 121.8480571 | 88.54835585 | 103.4734568 |
| **Abhd16a** | 625.4206969 | 734.4268819 | 708.7609715 | 565.0091699 | 671.3650389 | 715.4410618 | 695.4918951 | 450.045745 | 492.8312503 | 530.4584951 | 541.9839104 | 713.9756315 | 667.1820188 | 737.3005957 | 788.2665421 |
| **Ly6g5b** | 40.29064519 | 58.62738863 | 68.91819391 | 78.49745719 | 37.36843141 | 44.18900676 | 32.47432973 | 89.6739194 | 69.9745134 | 59.42533389 | 80.3990094 | 43.37640531 | 57.94173343 | 44.27417793 | 38.80254629 |
| **Csnk2b** | 3198.52781 | 3008.218846 | 3129.512533 | 3135.58524 | 2907.770654 | 2881.403806 | 2884.802958 | 3073.217406 | 3151.110345 | 2929.3194 | 3140.29072 | 2865.445335 | 2990.815946 | 3000.704998 | 2913.065234 |
| **Gpank1** | 446.859883 | 408.0149344 | 447.9682604 | 421.8160062 | 392.0518482 | 396.999648 | 355.4134976 | 398.9232302 | 374.702233 | 333.8305521 | 255.3850887 | 422.4861878 | 286.3003299 | 342.4472129 | 368.6241897 |
| **D17H6S53E** | 166.6567596 | 190.935144 | 164.4638718 | 131.9792412 | 150.7404521 | 155.7136429 | 128.0931895 | 184.3762829 | 147.473168 | 142.4460209 | 150.3934411 | 139.6720251 | 147.4105865 | 136.4367524 | 132.9346493 |
| **Apom** | 1.831392963 | 1.584524017 | 0.783161294 | 0.86260942 | 1.266726489 | 1.402825611 | 4.510323574 | 10.05688816 | 1.504828245 | 5.243411813 | 3.783482795 | 1.735056213 | 0 | 3.614218606 | 0 |
| **Bag6** | 5517.986998 | 5403.226898 | 5382.667577 | 5413.736718 | 5219.546496 | 5316.709067 | 5544.991802 | 5222.877249 | 5379.760976 | 5266.133265 | 5222.152128 | 5352.648416 | 5353.645752 | 5281.276938 | 5442.416399 |
| **Prrc2a** | 17174.80321 | 16892.61055 | 16145.65325 | 16725.13404 | 15639.00523 | 15707.43837 | 17312.42601 | 16646.66412 | 16153.5788 | 15895.40291 | 17002.02581 | 16201.95491 | 16472.49398 | 15546.56133 | 16446.5311 |
| **Aif1** | 8.241268334 | 3.169048034 | 3.132645178 | 3.450437679 | 0.633363244 | 7.715540863 | 5.412388289 | 0 | 3.00965649 | 1.747803938 | 0.945870699 | 5.205168638 | 5.964590206 | 6.324882561 | 5.029959704 |
| **Lst1** | 36.62785926 | 23.76786026 | 25.84432272 | 21.56523549 | 28.50134599 | 24.5494482 | 42.39704159 | 25.14222039 | 39.12553437 | 34.08217679 | 24.59263817 | 14.74797781 | 39.1958785 | 15.36042908 | 15.80844478 |
| **Ltb** | 8586.485908 | 9323.339316 | 9187.265146 | 9646.56114 | 9745.56024 | 9957.25619 | 9773.871185 | 8244.97214 | 9244.15991 | 7896.578191 | 7046.736706 | 9469.06928 | 9276.641939 | 9757.486682 | 9155.245227 |

| | | | | | | | | | | | | | | | |
|---|---|---|---|---|---|---|---|---|---|---|---|---|---|---|---|
| **Mir6973a** | 1.831392963 | 6.338096068 | 4.698967767 | 6.038265938 | 7.600358931 | 1.402825611 | 18.0412943 | 4.190370065 | 22.57242368 | 0.873901969 | 1.891741398 | 6.94022485 | 1.70416863 | 17.16753838 | 5.748525376 |
| **Tnf** | 1316.77154 | 953.0911962 | 889.6712305 | 831.5554805 | 677.0653081 | 629.1672867 | 689.1774421 | 695.6014308 | 572.5871473 | 603.8662605 | 592.1150574 | 536.9998978 | 568.3402382 | 516.8332607 | 507.3073644 |
| **Lta** | 3605.097048 | 3169.048034 | 3209.394985 | 3234.785324 | 3231.419272 | 3254.555419 | 3387.253004 | 4709.137879 | 3568.700183 | 3615.332445 | 3564.986664 | 2821.201402 | 2975.478429 | 2750.420359 | 2896.538224 |
| **Nfkbil1** | 430.3773463 | 405.6381484 | 386.8816795 | 443.3812417 | 395.2186644 | 415.236381 | 350.903174 | 289.1355345 | 391.2553437 | 333.8305521 | 369.8354432 | 364.3618046 | 375.769183 | 423.7671316 | 353.5343106 |
| **Atp6v1g2** | 10.98835778 | 13.46845414 | 14.0969033 | 15.52696955 | 10.13381191 | 11.9240177 | 10.82477658 | 18.43762829 | 12.03862596 | 13.10852953 | 11.35044839 | 17.35056213 | 11.0770961 | 18.07109303 | 12.9341821 |
| **Ddx39b** | 3943.904746 | 4116.593396 | 4247.866861 | 3924.872859 | 4038.957409 | 4128.515774 | 3898.723697 | 4342.899536 | 3990.052092 | 4303.093295 | 4325.466705 | 3944.650299 | 4019.281715 | 3977.447576 | 4321.453951 |
| **H2-D1** | 5485.021925 | 5095.829239 | 5126.573834 | 5086.807748 | 5232.213761 | 4966.002664 | 5407.877965 | 4161.037475 | 4083.351443 | 3957.028115 | 4442.754672 | 4792.225259 | 4746.96172 | 5257.784518 | 4751.156223 |
| **H2-Q1** | 7.325571853 | 0.792262009 | 3.915806472 | 0.86260942 | 5.700269198 | 5.611302446 | 0.902064715 | 0 | 1.504828245 | 2.621705907 | 7.56696559 | 0 | 3.408337261 | 6.324882561 | 2.155697016 |
| **H2-Q2** | 10.98835778 | 10.29940611 | 12.53058071 | 3.450437679 | 3.166816221 | 6.312715251 | 4.510323574 | 0 | 0 | 2.621705907 | 0 | 2.602584319 | 6.816674521 | 4.517773258 | 6.467091048 |
| **H2-Q4** | 5507.914337 | 6292.937134 | 5653.641385 | 5826.06402 | 6063.186337 | 6027.941652 | 5784.941016 | 5163.373994 | 5425.658238 | 5136.795773 | 4799.347926 | 5870.562695 | 6003.786085 | 6374.578067 | 6007.209018 |
| **H2-Q5** | 380.9297363 | 441.2899387 | 409.593357 | 373.5098787 | 369.8841347 | 361.9290077 | 430.284869 | 285.7832385 | 334.0718704 | 336.452258 | 266.7355371 | 360.0241641 | 401.3317124 | 354.1934234 | 395.9296853 |
| **H2-Q10** | 146.5114371 | 115.6702532 | 83.79825851 | 71.59658183 | 67.13650389 | 75.75258302 | 81.18582433 | 36.03718256 | 21.06759543 | 32.33437285 | 28.37612096 | 81.54764199 | 98.84178056 | 70.47726282 | 82.63505228 |
| **Pou5f1** | 0.915696482 | 0 | 3.132645178 | 0.86260942 | 6.966995687 | 6.312715251 | 6.314453003 | 0.838074013 | 0 | 0.873901969 | 0 | 1.735056213 | 2.556252945 | 0 | 0 |
| **Tcf19** | 879.0686223 | 1149.572174 | 1036.905554 | 1124.842683 | 1082.417784 | 1023.361284 | 953.4824035 | 1155.704064 | 1186.557071 | 1178.019854 | 1195.580563 | 1110.435976 | 1118.786706 | 1189.077921 | 1074.974245 |
| **Cchcr1** | 297.6013565 | 367.6095719 | 314.0476791 | 302.7759063 | 305.2810837 | 300.9060937 | 292.2689676 | 374.6190838 | 352.1298094 | 291.8832576 | 319.7042962 | 316.6477588 | 356.1712437 | 336.1223304 | 303.9532792 |
| **Psors1c2** | 0 | 1.584524017 | 3.915806472 | 1.725218839 | 0.633363244 | 2.104238417 | 0 | 0.838074013 | 0 | 0.873901969 | 0 | 0 | 0 | 0 | 0.718565672 |
| **Dpcr1** | 3.662785926 | 0.792262009 | 0 | 0 | 3.800179466 | 0 | 0 | 4.190370065 | 0.752414123 | 0 | 0 | 0 | 0 | 1.807109303 | 2.874262688 |
| **Vars2** | 601.6125884 | 560.921502 | 569.3582611 | 567.5969981 | 539.6254841 | 547.1019885 | 568.3007703 | 534.6912203 | 553.0243801 | 536.5758089 | 561.8471951 | 495.3585487 | 572.6006598 | 545.7470096 | 559.0440928 |
| **Gtf2h4** | 683.1095752 | 675.0072312 | 694.6640682 | 633.155314 | 657.4310476 | 651.6124965 | 650.3886594 | 763.4854259 | 678.6775386 | 706.1127909 | 643.1920752 | 642.8383268 | 678.2591149 | 611.7064991 | 661.0804182 |
| **Ddr1** | 170.3195456 | 167.9595458 | 173.8618074 | 144.9183825 | 165.3078068 | 175.3532014 | 176.8046841 | 131.57762 | 133.1772997 | 178.2760017 | 151.3393118 | 157.0225872 | 161.0439356 | 186.1322582 | 128.6232553 |
| **Ier3** | 461.5110267 | 211.5339563 | 266.2748401 | 219.965402 | 119.0722899 | 153.6094045 | 186.727396 | 100.5688816 | 97.81383594 | 93.50751067 | 148.5016997 | 116.2487662 | 128.6647316 | 131.9189791 | 137.964609 |
| **Flot1** | 499.0545825 | 416.7298165 | 416.6418087 | 422.6786156 | 336.949246 | 383.6728047 | 385.1816332 | 329.3630871 | 377.7118895 | 357.4259053 | 343.3510637 | 382.5798949 | 324.6441241 | 384.0107269 | 385.1512002 |
| **Tubb5** | 37755.99733 | 38336.76633 | 39612.29828 | 39375.53218 | 38937.90553 | 38645.74136 | 40017.39488 | 38775.17036 | 38162.4443 | 37168.79854 | 38206.55514 | 39682.47064 | 39680.71447 | 39807.90728 | 39521.11196 |
| **Mdc1** | 875.4058364 | 981.6126285 | 947.6251663 | 1017.879115 | 880.3749095 | 854.3207974 | 976.0340214 | 1007.364964 | 966.0997334 | 893.1278122 | 954.3835351 | 970.7639509 | 916.8427231 | 843.9200446 | 949.9438183 |
| **Nrm** | 581.4672658 | 670.2536592 | 573.2740676 | 629.7048763 | 659.9645005 | 595.499472 | 663.9196301 | 391.3805641 | 502.6126339 | 450.059514 | 473.8812201 | 644.573383 | 610.944454 | 583.6963049 | 677.6074287 |
| **Ppp1r18** | 2720.534247 | 2692.106305 | 2724.618144 | 2832.809334 | 3138.314875 | 3059.562659 | 3367.40758 | 2550.259222 | 2508.548685 | 2152.420549 | 2595.469197 | 2951.330618 | 3033.420162 | 3176.898155 | 3058.2155 |
| **Dhx16** | 2143.645463 | 2131.977065 | 1990.012849 | 2087.514796 | 2095.798975 | 2153.337314 | 1949.361849 | 1978.692745 | 1956.276719 | 2173.394197 | 2118.750365 | 2079.464871 | 2054.375284 | 2160.399172 | 2116.89447 |
| **2310061I04Rik** | 363.5315032 | 387.4161222 | 328.1445824 | 341.5933302 | 317.9483486 | 357.0191181 | 350.0011093 | 438.3127088 | 388.2456873 | 399.3731998 | 420.912461 | 402.5330413 | 385.1421105 | 332.5081118 | 386.5883315 |
| **Atat1** | 36.62785926 | 34.06726637 | 36.02541955 | 25.01567317 | 41.80197412 | 26.65368662 | 33.37639445 | 23.46607237 | 18.05793894 | 22.72145119 | 47.29353494 | 29.49595561 | 40.90004713 | 18.97464768 | 23.71266717 |
| **Mrps18b** | 967.891181 | 935.661432 | 967.98736 | 942.8320957 | 881.641636 | 817.8473315 | 812.760308 | 1073.572811 | 865.276241 | 1056.54748 | 1083.967821 | 913.5070959 | 809.4800994 | 851.1484818 | 897.4885243 |
| **Ppp1r10** | 1476.102728 | 1355.560297 | 1341.555297 | 1295.639348 | 1311.695279 | 1275.869894 | 1397.298243 | 1438.97308 | 1361.869562 | 1393.87364 | 1409.347341 | 1299.557103 | 1221.888908 | 1227.930771 | 1207.190329 |
| **Mir1894** | 4.578482408 | 2.376786026 | 3.132645178 | 2.587828259 | 0 | 0 | 0 | 0 | 0 | 1.747803938 | 1.891741398 | 0.867528106 | 0 | 1.807109303 | 1.437131344 |
| **Gm8801** | 0 | 0 | 0 | 0 | 5.066905954 | 0 | 0 | 0.838074013 | 1.504828245 | 1.747803938 | 0 | 1.735056213 | 0 | 5.421327909 | 0 |
| **Abcf1** | 5185.589175 | 5397.681064 | 5216.637383 | 5236.039177 | 5008.636536 | 4954.078647 | 5153.495716 | 5894.174534 | 5543.787255 | 5454.022188 | 5642.118718 | 4993.49178 | 4999.178677 | 4838.535159 | 5170.08001 |
| **Prr3** | 405.6535413 | 455.5506549 | 449.534583 | 421.8160062 | 479.4559759 | 420.1462706 | 442.913775 | 455.0741891 | 419.0946663 | 436.9509845 | 474.8270908 | 404.2680975 | 398.7754595 | 391.2391641 | 466.3491211 |
| **Gnl1** | 2079.54671 | 2149.406829 | 2165.440979 | 2107.354812 | 2150.901578 | 2166.664157 | 2231.708104 | 2262.799835 | 2183.505784 | 2164.655177 | 2191.582409 | 2299.81701 | 2240.129665 | 2259.790184 | 2170.068329 |
| **A930015D03Rik** | 43.95343112 | 34.06726637 | 32.10961307 | 31.05393911 | 43.06870061 | 46.29324518 | 58.63420646 | 33.52296052 | 28.59173666 | 23.59535316 | 26.48437957 | 35.56865236 | 48.56880596 | 35.23863141 | 36.64684927 |
| **H2-T24** | 456.0168478 | 415.9375545 | 383.7490343 | 415.7777403 | 420.5531942 | 498.7045049 | 598.9709706 | 293.3259046 | 273.8787406 | 200.1235509 | 277.1401147 | 405.1356256 | 420.0775674 | 412.0209211 | 477.8461719 |
| **H2-T23** | 6994.089726 | 6525.069902 | 6241.795517 | 5880.408414 | 5931.446783 | 6448.087923 | 6528.242341 | 5299.980059 | 5268.403686 | 4999.593164 | 4773.809417 | 5822.848649 | 5970.554796 | 5578.546419 | 6089.125504 |
| **Gm6034** | 0 | 0 | 0.783161294 | 4.313047098 | 1.266726489 | 0.701412806 | 0 | 3.352296052 | 1.504828245 | 0 | 0 | 2.602584319 | 0.852084315 | 1.807109303 | 0 |
| **H2-Bl** | 4.578482408 | 7.130358077 | 8.614774239 | 4.313047098 | 0.633363244 | 11.22260489 | 12.62890601 | 5.866518091 | 0.752414123 | 3.495607876 | 9.458706988 | 5.205168638 | 11.92918041 | 7.228437213 | 10.05991941 |
| **C920025E04Rik** | 673.0369139 | 615.5875806 | 646.8912292 | 614.1779068 | 626.3962486 | 644.5983684 | 760.4405546 | 450.883819 | 539.4809259 | 545.3148286 | 417.1289782 | 672.3342824 | 662.0695129 | 617.127827 | 624.4335689 |
| **H2-T10** | 304.9269284 | 408.8071964 | 325.0119372 | 326.0663606 | 386.9849423 | 369.6445486 | 414.0477041 | 320.982347 | 268.6118418 | 368.7866309 | 253.4933473 | 377.3747262 | 423.4859046 | 422.8635769 | 462.0377271 |
| **Gm7030** | 2.747089445 | 7.922620085 | 2.349483883 | 0 | 3.166816221 | 2.805651223 | 1.80412943 | 3.352296052 | 5.266898858 | 0.873901969 | 0.945870699 | 3.470112425 | 5.112505891 | 5.421327909 | 2.155697016 |
| **2410017I17Rik** | 0.915696482 | 2.376786026 | 3.132645178 | 0 | 0 | 2.104238417 | 0 | 0 | 2.257242368 | 0 | 0 | 0 | 0 | 0 | 1.437131344 |
| **Gm8909** | 0 | 2.376786026 | 0.783161294 | 0.86260942 | 1.900089733 | 2.805651223 | 5.412388289 | 0 | 0 | 1.747803938 | 0 | 5.205168638 | 1.70416863 | 5.421327909 | 4.311394032 |
| **Gm6623** | 2.747089445 | 3.961310043 | 2.349483883 | 2.587828259 | 1.900089733 | 1.402825611 | 1.80412943 | 0.838074013 | 0 | 0 | 0 | 2.602584319 | 1.70416863 | 2.710663955 | 2.155697016 |
| **H2-T3** | 0.915696482 | 0 | 0 | 0 | 0 | 0 | 0 | 0 | 2.257242368 | 0.873901969 | 5.675224193 | 0 | 0.852084315 | 1.807109303 | 0 |
| **Rpp21** | 318.6623756 | 344.6339737 | 325.0119372 | 308.8141722 | 345.8163314 | 355.6162925 | 376.1609861 | 419.8750805 | 294.1939219 | 306.7395911 | 314.029072 | 383.447423 | 334.0170515 | 333.4116664 | 339.1629972 |
| **Trim39** | 238.9967817 | 253.5238427 | 272.5401305 | 257.0576071 | 283.7467334 | 237.0775283 | 248.0677966 | 310.0873848 | 266.3545994 | 271.7835123 | 209.0374244 | 275.8739378 | 340.8337261 | 306.3050269 | 295.3304912 |
| **Trim26** | 1069.53349 | 1033.109659 | 1030.640264 | 944.5573145 | 1014.014554 | 1067.55029 | 1215.983236 | 804.5510525 | 894.6203917 | 867.7846551 | 892.9019396 | 1083.542605 | 1017.388672 | 1068.001598 | 1176.292005 |
| **Trim15** | 0.915696482 | 4.753572051 | 3.132645178 | 0.86260942 | 6.966995687 | 1.402825611 | 3.608258859 | 0 | 0 | 0 | 0 | 4.337640531 | 2.556252945 | 2.710663955 | 2.155697016 |
| **Rnf39** | 8.241268334 | 14.26071615 | 22.71167754 | 15.52696955 | 12.66726489 | 4.208476834 | 10.82477658 | 21.78992434 | 7.524141226 | 13.10852953 | 14.18806048 | 11.27786538 | 2.556252945 | 3.614218606 | 7.18565672 |
| **Ppp1r11** | 908.3709097 | 944.3763141 | 880.273295 | 921.2668602 | 898.7424436 | 942.6988109 | 1003.095963 | 829.6932729 | 741.8803249 | 807.4854193 | 850.3377582 | 792.053161 | 731.0883424 | 832.1738341 | 725.032763 |
| **Znrd1** | 436.7872217 | 401.6768383 | 466.7641315 | 357.9829092 | 424.9867369 | 457.3211493 | 382.4754391 | 339.4199753 | 366.4256777 | 376.6517486 | 345.2428051 | 389.5201197 | 443.0838439 | 411.1173665 | 411.0195644 |
| **Znrd1as** | 44.8691276 | 31.69048034 | 25.06116142 | 43.13047098 | 36.10170492 | 48.39748359 | 19.84542373 | 21.78992434 | 24.82966605 | 38.45168663 | 34.05134516 | 32.09853993 | 26.41461377 | 34.33507676 | 35.9282836 |
| **Gabbr1** | 126.3661145 | 171.1285938 | 148.800646 | 124.2157564 | 118.4389267 | 146.5952764 | 156.0571957 | 99.73080755 | 113.6145325 | 64.6687457 | 143.7723462 | 157.8901153 | 150.8189238 | 144.5687443 | 153.7730538 |
| **Ubd** | 22.89241204 | 34.06726637 | 40.72438731 | 15.52696955 | 16.46744435 | 21.04238417 | 26.15987673 | 26.81836842 | 33.10622139 | 44.56900041 | 23.64676747 | 17.35056213 | 16.18960199 | 14.45687443 | 15.80844478 |
| **H2-M3** | 522.862691 | 491.2024453 | 588.1541322 | 512.3899953 | 465.5219845 | 507.1214585 | 551.1615407 | 361.2098996 | 431.1332922 | 453.5551219 | 426.5876851 | 366.0968608 | 471.2026263 | 448.1631072 | 519.5229808 |
| **Olfr99** | 9.156964816 | 18.2220262 | 7.831612945 | 3.450437679 | 0.633363244 | 4.208476834 | 5.412388289 | 0 | 0 | 1.747803938 | 8.512836289 | 3.470112425 | 3.408337261 | 3.614218606 | 5.029959704 |
| **Rn45s** | 90221.74294 | 105817.6829 | 94304.36728 | 72189.1945 | 83888.9617 | 125322.1274 | 119990.8442 | 116072.4127 | 203682.2651 | 122530.669 | 139920.7607 | 85832.36331 | 121555.7921 | 103314.246 | 76666.6458 |
| **Cenpq** | 353.4588419 | 362.0637379 | 401.7617441 | 289.836765 | 374.9510406 | 385.7770431 | 334.6660092 | 396.4090082 | 363.4160212 | 399.3731998 | 405.7785298 | 342.673602 | 362.1358339 | 337.9294397 | 336.2887345 |
| **Mut** | 252.7322289 | 347.0107597 | 278.8054208 | 361.4333468 | 257.7788404 | 270.0439302 | 266.1090909 | 284.1070904 | 325.042901 | 280.522532 | 318.7584255 | 313.1776464 | 295.6732574 | 248.4775292 | 285.2705718 |
| **Cd2ap** | 1211.466445 | 1166.209677 | 1188.055684 | 1287.013254 | 1100.151955 | 1101.919518 | 1251.163759 | 1344.270717 | 1249.759858 | 1338.817816 | 1474.612419 | 1200.658899 | 1134.124223 | 1125.829096 | 1183.477662 |
| **Tnfrsf21** | 200.5375295 | 203.6113362 | 191.0913559 | 204.4384325 | 149.4737256 | 155.0122301 | 179.5108782 | 199.4616151 | 161.7690364 | 207.9886686 | 198.6328467 | 219.4846109 | 192.5710552 | 172.5789385 | 179.641418 |
| **Slc25a27** | 14.65114371 | 7.130358077 | 18.79587107 | 6.900875357 | 1.900089733 | 4.90988964 | 8.118582433 | 16.76148026 | 10.53379772 | 11.3607256 | 4.729353494 | 16.48303402 | 12.78126473 | 11.74621047 | 12.21561642 |
| **Cyp39a1** | 24.723805 | 29.31369431 | 21.14535495 | 29.32872027 | 12.66726489 | 13.32684331 | 11.72684129 | 13.40918421 | 43.64001911 | 25.3431571 | 34.05134516 | 15.61550591 | 10.22501178 | 5.421327909 | 12.21561642 |
| **Enpp5** | 1.831392963 | 8.714882094 | 5.482129061 | 4.313047098 | 3.166816221 | 6.312715251 | 4.510323574 | 4.190370065 | 2.257242368 | 2.621705907 | 0 | 6.94022485 | 3.408337261 | 5.421327909 | 2.874262688 |
| **Enpp4** | 173.066635 | 160.8291877 | 119.8236781 | 185.4610252 | 106.405025 | 126.9557178 | 140.7220955 | 273.2121283 | 261.8401147 | 233.3318257 | 176.8778207 | 116.2487662 | 122.7001414 | 117.4621047 | 143.7131344 |
| **Clic5** | 9.156964816 | 10.29940611 | 12.53058071 | 7.763484777 | 7.600358931 | 8.416953669 | 8.118582433 | 1.676148026 | 6.771727103 | 13.9824315 | 2.837612096 | 6.072696744 | 6.816674521 | 4.517773258 | 2.874262688 |
| **Runx2** | 1703.195456 | 1435.578759 | 1329.024717 | 1437.107293 | 993.113567 | 1074.564418 | 1149.230447 | 1459.086857 | 1334.030239 | 1202.489109 | 1403.672117 | 1120.846313 | 1211.663896 | 1122.214877 | 1074.25568 |
| **Supt3** | 243.5752641 | 205.1958602 | 176.9944526 | 187.1862441 | 162.7743538 | 171.8461374 | 175.9026194 | 163.4244325 | 189.6083589 | 194.0062371 | 164.5815016 | 144.0096656 | 185.7543807 | 152.7007361 | 163.8329732 |
| **Cdc5l** | 2151.886732 | 2121.677659 | 2111.40285 | 2243.647101 | 2060.330634 | 2098.627115 | 2266.888628 | 2264.475983 | 2035.280202 | 2259.03659 | 2253.064004 | 2125.44386 | 2073.121139 | 2143.231634 | 2229.70928 |
| **B230354K17Rik** | 159.3311878 | 198.8577641 | 203.6219366 | 166.483618 | 172.2748024 | 139.5811483 | 149.7427427 | 201.9758371 | 197.1325001 | 155.5545505 | 185.390657 | 151.8174186 | 132.9251532 | 160.832728 | 122.8747299 |
| **Spats1** | 6.409875371 | 12.67619214 | 4.698967767 | 3.450437679 | 2.533452977 | 2.805651223 | 2.706194144 | 6.704592104 | 3.762070613 | 3.495607876 | 6.621094891 | 1.735056213 | 0 | 6.324882561 | 3.59282836 |
| **Aars2** | 288.4443917 | 335.1268296 | 287.4201951 | 244.9810752 | 237.5112166 | 301.6075065 | 289.5627734 | 462.6168552 | 330.3097998 | 374.9039447 | 366.0519604 | 283.6816908 | 299.0815946 | 264.7415129 | 283.1148748 |
| **Tmem151b** | 4.578482408 | 4.753572051 | 3.132645178 | 11.21392246 | 1.900089733 | 3.507064029 | 8.118582433 | 12.5711102 | 12.03862596 | 9.612921658 | 5.675224193 | 0 | 2.556252945 | 5.421327909 | 2.874262688 |
| **Nfkbie** | 831.4524053 | 841.382253 | 737.7379394 | 808.2650262 | 752.4355342 | 796.8049473 | 799.2293373 | 681.3541726 | 556.0340366 | 626.5877117 | 546.7132639 | 659.3213608 | 633.0986462 | 674.9553247 | 697.0087018 |
| **Slc35b2** | 737.1356677 | 786.7161744 | 824.6688431 | 851.3954972 | 856.3071063 | 761.0328942 | 797.4252079 | 871.5969736 | 794.5493134 | 823.2156547 | 780.3433265 | 805.9336107 | 808.6280151 | 768.9250085 | 797.6078959 |
| **Hsp90ab1** | 90267.52776 | 86366.85833 | 87548.03479 | 86890.64684 | 83337.93568 | 83259.80428 | 88000.02119 | 96189.94485 | 91875.78368 | 95150.44637 | 97946.8026 | 86528.12085 | 84779.83311 | 83342.97751 | 84074.33932 |
| **Slc29a1** | 2852.39454 | 2920.277763 | 3100.535565 | 2860.412836 | 2840.634151 | 2685.709633 | 2586.219537 | 3089.140812 | 3201.522092 | 2900.480635 | 2995.572503 | 2681.529377 | 2818.694915 | 2940.166836 | 2779.412019 |
| **Capn11** | 5.494178889 | 10.29940611 | 6.265290356 | 0.86260942 | 0 | 1.402825611 | 0 | 5.028444078 | 3.762070613 | 3.495607876 | 2.837612096 | 0 | 0 | 0 | 0 |
| **Tmem63b** | 140.1015617 | 137.0613275 | 122.9563232 | 134.5670695 | 134.906371 | 103.8090952 | 160.5675192 | 185.2143569 | 180.5793894 | 216.7276883 | 157.014536 | 140.5395532 | 126.1084786 | 143.6651896 | 115.6890732 |
| **Mrpl14** | 571.3946045 | 536.3613798 | 519.2359382 | 446.8316794 | 469.322164 | 528.1638427 | 489.8211401 | 502.8444078 | 400.2843132 | 470.1592593 | 406.7244005 | 459.7898963 | 473.7588792 | 444.5488886 | 492.936051 |
| **1600014C23Rik** | 0.915696482 | 2.376786026 | 3.915806472 | 0.86260942 | 0 | 1.402825611 | 1.80412943 | 0 | 0 | 0 | 0 | 0 | 4.260421576 | 1.807109303 | 7.18565672 |
| **Vegfa** | 1222.454803 | 972.8977464 | 1062.749877 | 859.158982 | 803.1045937 | 974.9637999 | 1074.359075 | 222.9276875 | 320.5284162 | 390.6341801 | 442.667487 | 766.0273178 | 970.524035 | 746.3361422 | 877.3686855 |
| **Mrps18a** | 583.2986588 | 630.6405588 | 625.7458743 | 696.1258017 | 633.9966075 | 595.499472 | 642.2700769 | 674.6495805 | 771.9768898 | 676.4001239 | 667.7847133 | 591.6541685 | 622.0215501 | 666.8233329 | 630.1820943 |
| **Rsph9** | 18.31392963 | 13.46845414 | 7.04845165 | 6.900875357 | 5.700269198 | 6.312715251 | 6.314453003 | 4.190370065 | 3.762070613 | 9.612921658 | 5.675224193 | 8.675281063 | 5.964590206 | 3.614218606 | 10.05991941 |
| **Mad2l1bp** | 440.4500076 | 450.7970828 | 433.0881959 | 503.7639011 | 426.2534634 | 454.5154981 | 411.3415099 | 424.9035246 | 408.5608686 | 408.1122195 | 430.3711679 | 458.0548401 | 472.9067949 | 485.2088479 | 454.1335047 |
| **Gtpbp2** | 915.6964816 | 1016.472157 | 929.6124566 | 785.8371813 | 942.4445075 | 957.4284798 | 984.1526038 | 689.7349127 | 735.8610119 | 758.546909 | 761.4259125 | 949.0757483 | 1038.69078 | 1059.869606 | 1058.447235 |
| **Polh** | 1075.943366 | 1135.311458 | 1097.208974 | 1148.133138 | 1122.953032 | 981.977928 | 1011.214545 | 1192.579321 | 1234.711575 | 1031.204323 | 1053.699958 | 993.3196817 | 1038.69078 | 1019.209647 | 1085.75273 |
| **Xpo5** | 2441.24682 | 2318.158637 | 2407.437819 | 2416.168984 | 2131.267317 | 2187.706541 | 2284.027858 | 2441.3096 | 2521.339725 | 2463.52965 | 2582.227008 | 2203.52139 | 2153.217064 | 2184.795148 | 2287.9131 |
| **Polr1c** | 1253.588483 | 1151.94896 | 1173.175619 | 1135.193996 | 1085.584601 | 1214.846979 | 1112.245793 | 1337.566125 | 1241.483302 | 1287.2576 | 1153.016382 | 972.4990071 | 1107.70961 | 1024.630975 | 1004.554809 |
| **Yipf3** | 1068.617794 | 1052.123947 | 1195.104135 | 1118.804417 | 1109.019041 | 988.9920561 | 1053.611587 | 1004.012668 | 828.407949 | 983.139715 | 990.3266216 | 1041.901256 | 1100.040851 | 1044.509177 | 1028.267477 |
| **Lrrc73** | 43.03773463 | 34.85952837 | 32.10961307 | 66.42092531 | 28.50134599 | 28.05651223 | 40.59291217 | 41.06562664 | 29.34415078 | 62.92094176 | 54.86050053 | 25.15831508 | 17.89377062 | 25.29953024 | 32.33545524 |
| **Tjap1** | 813.1384756 | 800.9768906 | 827.8014883 | 729.767569 | 753.0688974 | 829.7713492 | 801.9355314 | 790.3037943 | 720.0603153 | 797.8724976 | 666.8388426 | 746.9416995 | 782.2134013 | 908.0724248 | 840.7218362 |
| **Dlk2** | 71.42432556 | 120.4238253 | 119.8236781 | 116.4522717 | 126.6726489 | 138.1783227 | 139.8200308 | 156.7198404 | 115.8717749 | 145.9416288 | 112.5586132 | 122.321463 | 115.8834669 | 142.7616349 | 114.9705075 |
| **Abcc10** | 97.06382705 | 128.3464454 | 115.1247103 | 147.5062108 | 106.405025 | 146.5952764 | 132.6035131 | 97.21658551 | 96.30900769 | 116.2289619 | 101.2081648 | 95.42809169 | 157.6355983 | 84.0305826 | 117.8447702 |
| **Zfp318** | 477.0778669 | 476.9417291 | 527.8507125 | 477.0230091 | 533.2918517 | 574.4570879 | 612.5019413 | 527.1485542 | 498.8505633 | 595.1272408 | 627.1122733 | 544.8076507 | 558.1152264 | 453.5844351 | 560.4812241 |
| **Ttbk1** | 2.747089445 | 0 | 5.482129061 | 1.725218839 | 1.266726489 | 3.507064029 | 3.608258859 | 0.838074013 | 3.00965649 | 2.621705907 | 0 | 1.735056213 | 0 | 0 | 0 |
| **Dnph1** | 365.3628961 | 366.8173099 | 387.6648408 | 345.0437679 | 349.6165108 | 357.0191181 | 340.9804622 | 535.5292943 | 407.8084544 | 458.7985337 | 374.5647967 | 318.382815 | 351.0587378 | 356.9040874 | 347.7857852 |
| **Cul9** | 104.3893989 | 139.4381135 | 148.0174847 | 129.3914129 | 130.4728283 | 142.3867996 | 109.1498305 | 136.6060641 | 121.8910879 | 143.3199229 | 135.2595099 | 130.996744 | 108.214708 | 106.6194489 | 109.2219821 |
| **Srf** | 1932.119576 | 2059.881222 | 2154.476721 | 2102.179156 | 2295.308397 | 2353.239963 | 2416.631371 | 2284.58976 | 2220.374076 | 2244.180256 | 2090.374244 | 2370.086786 | 2358.569384 | 2424.23713 | 2218.930795 |
| **Ptk7** | 7.325571853 | 11.09166812 | 7.04845165 | 13.80175071 | 6.333632443 | 3.507064029 | 9.922711863 | 17.59955427 | 14.29586833 | 10.48682363 | 32.15960376 | 9.542809169 | 11.0770961 | 14.45687443 | 12.9341821 |
| **Klc4** | 79.6655939 | 87.14882094 | 86.14774239 | 89.71137964 | 90.57094393 | 108.7189849 | 102.8353775 | 93.86428946 | 97.81383594 | 63.79484373 | 113.5044839 | 81.54764199 | 88.61676878 | 101.198121 | 92.69497168 |
| **Mrpl2** | 1126.306672 | 1226.421589 | 1026.724457 | 1065.322633 | 1076.084152 | 1219.756869 | 1124.874699 | 1111.286141 | 1126.363942 | 1186.758874 | 1142.611804 | 997.6573222 | 1029.317853 | 1135.768197 | 1039.045962 |
| **Cul7** | 779.2577058 | 736.8036679 | 741.6537459 | 784.9745719 | 568.1268301 | 564.6373086 | 653.0948535 | 1013.231482 | 858.5045139 | 967.4094796 | 803.0442233 | 582.9788874 | 626.2819716 | 589.1176328 | 623.7150033 |
| **Rrp36** | 510.9586367 | 572.0131701 | 527.8507125 | 518.4282612 | 495.290057 | 547.1019885 | 546.6512172 | 683.8683947 | 601.1788839 | 646.687457 | 652.6507822 | 505.768886 | 624.577803 | 530.3865805 | 561.1997898 |
| **Klhdc3** | 1586.902003 | 1529.857938 | 1461.378976 | 1515.60475 | 1466.869274 | 1592.207069 | 1484.798521 | 1361.032197 | 1372.40336 | 1393.87364 | 1303.409823 | 1432.288903 | 1466.437106 | 1578.509976 | 1545.63476 |
| **Mea1** | 742.6298465 | 626.6792487 | 609.2994871 | 681.4614415 | 590.9279069 | 654.4181477 | 677.4506008 | 605.0894374 | 513.1464316 | 598.6228487 | 533.4710741 | 651.5136078 | 553.8548049 | 646.0415759 | 598.5652047 |
| **Ppp2r5d** | 2137.235588 | 2081.272296 | 2191.285302 | 2121.156563 | 2033.729377 | 2135.100581 | 2145.109892 | 2397.729751 | 2206.830622 | 2391.869689 | 2416.699635 | 2157.5424 | 2131.062872 | 2075.465035 | 2149.229925 |
| **Pex6** | 660.2171632 | 670.2536592 | 636.7101324 | 599.5135467 | 590.9279069 | 677.5647703 | 670.2340831 | 567.3761068 | 579.3588744 | 603.8662605 | 632.7874975 | 616.8124836 | 639.0632364 | 637.0060294 | 643.8348421 |
| **Gnmt** | 0 | 8.714882094 | 1.566322589 | 0.86260942 | 3.166816221 | 7.014128057 | 1.80412943 | 8.380740131 | 8.276555348 | 6.991215751 | 11.35044839 | 6.072696744 | 8.520843152 | 3.614218606 | 0 |
| **Cnpy3** | 608.0224638 | 532.4000697 | 624.1795517 | 576.2230923 | 554.826202 | 528.8652555 | 529.5119876 | 741.6955016 | 678.6775386 | 642.3179471 | 726.4286967 | 475.4054022 | 531.7006127 | 514.1225967 | 533.8942943 |
| **2310039H08Rik** | 195.9590471 | 190.142882 | 184.0429042 | 156.132305 | 184.3087041 | 180.9645039 | 195.7480431 | 196.9473931 | 165.531107 | 217.6015903 | 149.4475704 | 174.3731494 | 149.1147552 | 203.2997966 | 148.0245284 |
| **Rpl7l1** | 2608.819276 | 2501.963423 | 2679.97795 | 2514.506458 | 2467.5832 | 2545.427072 | 2605.162896 | 3186.357398 | 3034.486156 | 3119.830029 | 3093.943056 | 2530.579486 | 2498.311212 | 2578.744976 | 2394.260819 |
| **Gltscr1l** | 584.2143552 | 547.4530479 | 542.7307771 | 494.2751975 | 533.9252149 | 549.9076397 | 622.4246532 | 542.2338864 | 534.9664412 | 480.6460829 | 533.4710741 | 612.474843 | 679.1111992 | 595.4425154 | 668.9846406 |
| **Tbcc** | 274.7089445 | 316.1125414 | 304.6497436 | 272.5845766 | 312.8814427 | 329.6640187 | 305.7999383 | 311.7635329 | 292.6890937 | 294.5049635 | 268.6272785 | 289.7543875 | 275.2232338 | 338.8329943 | 318.3245927 |
| **Ubr2** | 2006.290991 | 2076.518724 | 2074.594269 | 1951.222507 | 1943.791797 | 2055.139521 | 2061.217873 | 1827.839422 | 1760.649047 | 1783.633919 | 1935.25145 | 2054.306556 | 2107.204511 | 1916.439416 | 2023.480932 |
| **Trerf1** | 226.1770309 | 223.4178864 | 259.2263885 | 262.2332636 | 214.0767766 | 214.6323185 | 253.4801849 | 225.4419095 | 251.3063169 | 309.361297 | 237.4135454 | 248.1130384 | 264.998222 | 252.0917478 | 222.7553583 |
| **Mrps10** | 724.3159169 | 666.2923492 | 704.0620037 | 750.4701951 | 606.761988 | 691.5930264 | 654.8989829 | 651.1835081 | 710.2789317 | 769.9076346 | 707.5112827 | 648.9110235 | 643.3236579 | 601.7673979 | 592.8166794 |
| **Guca1b** | 0.915696482 | 7.922620085 | 3.915806472 | 0.86260942 | 6.333632443 | 3.507064029 | 7.216517718 | 6.704592104 | 7.524141226 | 1.747803938 | 2.837612096 | 0 | 1.70416863 | 9.939101167 | 6.467091048 |
| **Guca1a** | 3.662785926 | 6.338096068 | 5.482129061 | 1.725218839 | 5.066905954 | 0.701412806 | 4.510323574 | 0 | 2.257242368 | 2.621705907 | 1.891741398 | 4.337640531 | 4.260421576 | 0.903554652 | 2.155697016 |
| **AI661453** | 9.156964816 | 7.130358077 | 5.482129061 | 3.450437679 | 1.900089733 | 3.507064029 | 7.216517718 | 0 | 3.762070613 | 0 | 5.675224193 | 5.205168638 | 6.816674521 | 7.228437213 | 2.874262688 |
| **Taf8** | 428.5459534 | 459.5119649 | 477.7283896 | 444.2438511 | 455.3881726 | 478.3635335 | 465.4653928 | 447.531523 | 486.8119373 | 437.8248864 | 399.1574349 | 483.2131552 | 510.3985048 | 477.9804107 | 484.3132629 |
| **Ccnd3** | 3863.323456 | 3760.075492 | 3778.753246 | 3654.013502 | 3392.293536 | 3571.594007 | 3696.661201 | 4068.011259 | 3967.479668 | 3957.028115 | 4151.426497 | 3340.850737 | 3423.674778 | 3529.284469 | 3515.941833 |
| **Bysl** | 1900.985896 | 1739.807371 | 1880.370268 | 1814.06761 | 1775.950537 | 1844.715679 | 1954.774237 | 2155.526362 | 1980.353971 | 2013.470136 | 2003.35414 | 1753.274303 | 1843.058374 | 1884.815003 | 1820.126847 |
| **Med20** | 529.2725663 | 612.4185326 | 570.1414224 | 562.4213416 | 558.6263815 | 493.0932024 | 561.9863173 | 534.6912203 | 622.2464794 | 607.3618684 | 645.0838166 | 573.4360783 | 643.3236579 | 494.2443944 | 569.8225779 |
| **Usp49** | 269.2147656 | 222.6256244 | 247.4789691 | 263.095873 | 245.7449388 | 189.3814575 | 233.6347611 | 246.3937598 | 296.4511643 | 241.1969434 | 227.9548384 | 215.1469704 | 224.9502592 | 215.0460071 | 262.2764703 |
| **Tomm6** | 620.8422145 | 683.7221133 | 529.4170351 | 539.1308873 | 710.0001968 | 666.3421654 | 576.4193527 | 506.1967039 | 495.8409068 | 424.7163569 | 471.9894787 | 536.1323697 | 485.6880596 | 636.1024747 | 513.0558898 |
| **Tomm6os** | 1.831392963 | 0 | 1.566322589 | 0 | 0 | 2.104238417 | 0.902064715 | 1.676148026 | 0 | 0.873901969 | 0 | 2.602584319 | 0 | 0 | 0 |
| **Frs3** | 7.325571853 | 3.961310043 | 8.614774239 | 12.07653188 | 5.700269198 | 7.014128057 | 9.020647148 | 8.380740131 | 0 | 5.243411813 | 0 | 1.735056213 | 0 | 5.421327909 | 5.748525376 |
| **Tfeb** | 442.2814006 | 425.4446986 | 427.6060668 | 372.6472693 | 516.1910441 | 564.6373086 | 562.888382 | 363.7241217 | 319.7760021 | 310.235199 | 378.3482795 | 532.6622573 | 531.7006127 | 541.2292363 | 553.2955674 |
| **Mdfi** | 0.915696482 | 1.584524017 | 0.783161294 | 1.725218839 | 2.533452977 | 0 | 0.902064715 | 0 | 0 | 0 | 2.837612096 | 2.602584319 | 0.852084315 | 0.903554652 | 1.437131344 |
| **Foxp4** | 2010.869474 | 1968.771091 | 1992.362333 | 1904.641599 | 1976.093322 | 2037.604201 | 2132.480986 | 2103.565773 | 2057.100211 | 1904.23239 | 1782.020396 | 2000.519813 | 2068.860717 | 1988.723788 | 1953.780062 |
| **Treml2** | 1104.329957 | 971.3132224 | 859.1279401 | 822.0667769 | 583.327548 | 621.4517459 | 661.2134359 | 924.3956364 | 914.1831589 | 904.4885378 | 954.3835351 | 664.5265294 | 649.2882482 | 575.5643131 | 610.0622555 |
| **B430306N03Rik** | 5.494178889 | 8.714882094 | 3.132645178 | 11.21392246 | 6.333632443 | 10.52119209 | 4.510323574 | 1.676148026 | 3.762070613 | 13.9824315 | 7.56696559 | 0 | 8.520843152 | 8.131991864 | 3.59282836 |
| **Nfya** | 1174.838586 | 1123.427528 | 1211.550523 | 1165.385326 | 1170.455275 | 1280.07837 | 1291.756672 | 1139.780658 | 1157.965335 | 1143.063775 | 1113.289812 | 1256.180698 | 1246.599353 | 1178.235266 | 1272.579805 |
| **Oard1** | 336.0606087 | 369.194096 | 383.7490343 | 307.0889534 | 314.7815324 | 334.5739083 | 295.8772264 | 408.1420444 | 322.7856586 | 385.3907683 | 378.3482795 | 390.3876478 | 343.389979 | 349.6756502 | 380.1212405 |
| **Apobec2** | 3.662785926 | 0.792262009 | 5.482129061 | 11.21392246 | 14.56735462 | 6.312715251 | 11.72684129 | 20.11377631 | 0 | 6.117313782 | 3.783482795 | 6.072696744 | 11.92918041 | 12.64976512 | 10.77848508 |
| **Unc5cl** | 89.73825519 | 144.1916855 | 142.5353556 | 148.3688202 | 129.8394651 | 107.3161593 | 135.3097072 | 111.4638437 | 106.8428054 | 104.8682363 | 112.5586132 | 115.3812381 | 129.5168159 | 152.7007361 | 144.4317001 |
| **Mocs1** | 171.2352421 | 217.0797903 | 209.1040656 | 180.2853687 | 198.8760587 | 176.0546142 | 228.2223728 | 182.7001348 | 201.6469849 | 221.9711001 | 189.1741398 | 208.2067455 | 257.3294632 | 231.3099908 | 206.9469135 |
| **Daam2** | 0.915696482 | 4.753572051 | 8.614774239 | 12.07653188 | 6.333632443 | 1.402825611 | 11.72684129 | 5.028444078 | 2.257242368 | 2.621705907 | 3.783482795 | 1.735056213 | 6.816674521 | 22.58886629 | 7.904222392 |
| **Rftn1** | 1151.030477 | 1168.586463 | 1126.969103 | 1010.11563 | 1297.761288 | 1195.908834 | 1148.328382 | 965.461263 | 1090.248064 | 985.7614209 | 1008.298165 | 1296.954519 | 1217.628486 | 1195.402804 | 1167.669217 |
| **Dazl** | 2.747089445 | 0.792262009 | 1.566322589 | 0 | 1.266726489 | 2.104238417 | 0.902064715 | 0 | 0.752414123 | 0 | 0.945870699 | 0 | 5.112505891 | 0 | 1.437131344 |
| **Plcl2** | 1176.669979 | 1148.779912 | 1181.007232 | 1212.828844 | 1205.923617 | 1309.537708 | 1258.380277 | 1188.388951 | 1207.624667 | 1094.999167 | 1179.500761 | 1303.894744 | 1156.278416 | 1281.240496 | 1311.382351 |
| **Tbc1d5** | 473.415081 | 443.6667248 | 375.1342601 | 388.1742388 | 365.4505919 | 364.0332462 | 369.8465331 | 402.2755263 | 442.4195041 | 382.7690624 | 380.2400209 | 371.3020295 | 350.2066535 | 352.3863141 | 375.0912808 |
| **Satb1** | 10352.86442 | 8786.185674 | 9071.357274 | 8337.120041 | 7009.431024 | 7197.898212 | 7938.16949 | 4631.196996 | 5456.507217 | 6129.54841 | 6526.507822 | 7301.116542 | 7218.858318 | 7110.975108 | 7304.938621 |
| **Gm20098** | 0.915696482 | 0 | 0 | 0 | 1.900089733 | 0.701412806 | 0.902064715 | 0 | 0 | 1.747803938 | 0 | 0 | 0 | 3.614218606 | 0 |
| **Rab5a** | 917.5278745 | 912.6858338 | 917.8650371 | 904.0146718 | 813.2384057 | 896.4055657 | 998.5856393 | 1006.52689 | 929.2314414 | 940.3185185 | 883.4432327 | 933.4602424 | 932.1802408 | 899.0368783 | 942.7581616 |
| **Pp2d1** | 0 | 0 | 0 | 0.86260942 | 0 | 0 | 0 | 0 | 5.266898858 | 2.621705907 | 0 | 0 | 0 | 1.807109303 | 2.874262688 |
| **Kat2b** | 291.1914811 | 372.363144 | 388.4480021 | 354.5324715 | 343.2828784 | 353.5120541 | 379.7692449 | 327.6869391 | 400.2843132 | 325.9654344 | 314.029072 | 395.5928165 | 362.9879183 | 402.9853746 | 381.5583718 |
| **Sgol1** | 854.3448173 | 930.115598 | 960.155747 | 815.1659016 | 883.5417258 | 934.98327 | 909.2812325 | 936.1286726 | 895.3728059 | 978.7702052 | 889.1184568 | 1006.332603 | 985.0094683 | 1002.042109 | 939.8838989 |
| **Pot1b** | 121.787632 | 114.0857292 | 133.9205814 | 98.33747384 | 122.2391061 | 109.4203977 | 100.1291833 | 134.0918421 | 97.06142181 | 122.3462756 | 129.5842857 | 126.6591035 | 124.40431 | 103.9087849 | 102.0363254 |
| **Zfp119a** | 38.45925223 | 43.57441047 | 39.15806472 | 18.11479781 | 31.66816221 | 22.44520978 | 36.98465331 | 31.8468125 | 39.12553437 | 49.81241223 | 33.10547446 | 30.36348372 | 32.37920398 | 38.85285002 | 45.98820301 |
| **Zfp959** | 92.48534464 | 111.7089432 | 106.5099361 | 95.74964558 | 91.83767042 | 97.49637999 | 116.3663482 | 74.58858716 | 114.3669466 | 104.8682363 | 128.638415 | 85.01775442 | 92.87719035 | 115.6549954 | 117.8447702 |

| | | | | | | | | | | | | | | | |
|---|---|---|---|---|---|---|---|---|---|---|---|---|---|---|---|
| **Zfp119b** | 39.37494871 | 32.48274235 | 31.32645178 | 59.52004996 | 31.66816221 | 28.75792503 | 44.20117102 | 22.62799835 | 49.65933209 | 48.93851026 | 23.64676747 | 51.18415827 | 19.59793925 | 57.8274977 | 22.27553583 |
| **Ebi3** | 42.12203815 | 28.52143231 | 25.06116142 | 11.21392246 | 20.90098706 | 25.25086101 | 14.43303544 | 21.78992434 | 14.29586833 | 32.33437285 | 30.26786236 | 20.82067455 | 36.63962555 | 28.91374885 | 25.14979852 |
| **Gm16712** | 1.831392963 | 1.584524017 | 3.915806472 | 9.488703616 | 3.166816221 | 8.416953669 | 0.902064715 | 9.218814144 | 2.257242368 | 8.739019689 | 7.56696559 | 1.735056213 | 9.372927467 | 7.228437213 | 11.49705075 |
| **Ccdc94** | 252.7322289 | 254.3161047 | 260.0095498 | 203.575823 | 286.2801864 | 267.238279 | 259.7946379 | 271.5359802 | 277.6408112 | 241.1969434 | 258.2227008 | 268.9337129 | 220.6898376 | 263.8379583 | 262.2764703 |
| **Shd** | 0 | 3.169048034 | 0 | 1.725218839 | 1.266726489 | 3.507064029 | 3.608258859 | 1.676148026 | 0 | 2.621705907 | 1.891741398 | 2.602584319 | 4.260421576 | 6.324882561 | 2.874262688 |
| **Stap2** | 0.915696482 | 4.753572051 | 0 | 3.450437679 | 3.800179466 | 4.90988964 | 0.902064715 | 0 | 0.752414123 | 1.747803938 | 0.945870699 | 6.94022485 | 0 | 0 | 0 |
| **Mpnd** | 1021.917273 | 1014.887633 | 1009.494909 | 874.6859515 | 1055.183165 | 1060.536162 | 1105.93134 | 710.6867631 | 750.1568802 | 790.0073799 | 784.1268093 | 935.1952986 | 1087.259586 | 951.4430481 | 993.0577587 |
| **Sh3gl1** | 1155.60896 | 1261.281118 | 1271.070781 | 1186.950561 | 1269.259942 | 1330.580092 | 1230.416271 | 1291.472054 | 1107.553588 | 1266.283953 | 1297.734599 | 1386.309914 | 1285.795232 | 1289.372488 | 1274.016936 |
| **Chaf1a** | 2273.674364 | 2470.272943 | 2293.09627 | 2341.121965 | 2270.607231 | 2268.369014 | 2298.460893 | 2542.716556 | 2517.577654 | 2593.741044 | 2499.936257 | 2322.372741 | 2393.504841 | 2304.064362 | 2220.367926 |
| **Ubxn6** | 803.0658143 | 762.1560522 | 791.7760687 | 803.0893697 | 687.19912 | 732.2749692 | 819.074761 | 705.658319 | 772.7293039 | 733.2037519 | 771.8304902 | 764.2922616 | 843.563472 | 773.4427817 | 707.7871869 |
| **Hdgfrp2** | 1405.594099 | 1528.273414 | 1448.065234 | 1316.341974 | 1509.304611 | 1434.389188 | 1410.829214 | 1330.861533 | 1321.239199 | 1241.814698 | 1463.261971 | 1381.104745 | 1376.968253 | 1476.408301 | 1388.268878 |
| **Plin4** | 0.915696482 | 0.792262009 | 0 | 0 | 0.633363244 | 0.701412806 | 0 | 0 | 0 | 4.369509845 | 0 | 0 | 0 | 1.807109303 | 3.59282836 |
| **Sema6b** | 59.5202713 | 36.44405239 | 44.64019379 | 52.6191746 | 41.16861088 | 35.77205309 | 25.25781201 | 56.98903289 | 36.11587788 | 75.15556933 | 74.7237852 | 32.96606804 | 52.82922754 | 31.62441281 | 42.39537465 |
| **Tnfaip8l1** | 201.4532259 | 225.0024104 | 290.5528403 | 263.9584824 | 328.7155238 | 264.4326278 | 304.8978736 | 281.5928684 | 231.7435498 | 207.9886686 | 231.7383212 | 298.4296686 | 290.5607515 | 368.6502978 | 273.773521 |
| **Mydgf** | 1236.19025 | 1198.692419 | 1248.359103 | 1213.691453 | 1280.66048 | 1321.461726 | 1128.482958 | 1349.299161 | 1249.759858 | 1485.633347 | 1365.837289 | 1146.872157 | 1324.139026 | 1199.920577 | 1234.495824 |
| **Dpp9** | 2292.90399 | 2364.902095 | 2330.688012 | 2433.421173 | 2116.066599 | 2189.109367 | 2221.785393 | 2338.226496 | 2668.060479 | 2451.295023 | 2531.14999 | 2246.030267 | 2256.319267 | 2222.744443 | 2321.685686 |
| **Fem1a** | 1192.236819 | 1252.566235 | 1142.632329 | 1292.188911 | 1185.02263 | 1202.922962 | 1317.916548 | 1473.334115 | 1419.053035 | 1248.805914 | 1342.190522 | 1191.983618 | 1229.557667 | 1167.39261 | 1172.699177 |
| **Ticam1** | 249.069443 | 267.7845589 | 242.7800013 | 264.8210918 | 225.477315 | 220.9450338 | 285.9545146 | 227.1180575 | 223.4669944 | 251.683767 | 258.2227008 | 247.2455103 | 244.5481985 | 225.8886629 | 288.8634001 |
| **Plin3** | 687.6880577 | 693.2292574 | 657.8554874 | 579.67353 | 654.2642313 | 627.0630483 | 668.4299537 | 468.4833733 | 547.0050671 | 571.5318877 | 590.223316 | 622.0176522 | 714.0466561 | 721.036612 | 688.3859137 |
| **Uhrf1** | 9509.507961 | 10172.64419 | 9322.75205 | 9565.475855 | 8972.857082 | 8835.697114 | 9681.860584 | 10138.18134 | 10062.03406 | 9986.077799 | 9879.619449 | 9460.393999 | 9501.592198 | 9115.96288 | 9276.682825 |
| **Kdm4b** | 755.4495973 | 778.0012924 | 854.4289723 | 881.5868269 | 841.7397516 | 860.6335126 | 830.8016023 | 595.0325493 | 641.0568324 | 651.0569668 | 627.1122733 | 857.9852971 | 885.3156035 | 806.8743039 | 957.8480407 |
| **Ptprs** | 994.446379 | 949.9221482 | 1016.54336 | 936.7938297 | 1075.450789 | 1121.559076 | 1171.782064 | 835.559791 | 880.3245234 | 808.3593212 | 865.4716894 | 1052.311593 | 1134.976308 | 1126.732651 | 1141.082287 |
| **Gm20219** | 0 | 1.584524017 | 2.349483883 | 2.587828259 | 3.166816221 | 5.611302446 | 4.510323574 | 3.352296052 | 0.752414123 | 2.621705907 | 6.621094891 | 4.337640531 | 10.22501178 | 2.710663955 | 0.718565672 |
| **Safb2** | 1910.142861 | 1998.877047 | 1914.829365 | 1948.634679 | 1995.094219 | 1900.828703 | 1906.062742 | 1889.856899 | 1982.611213 | 1797.61635 | 1904.983587 | 1950.203183 | 1985.356454 | 1945.353165 | 1996.175437 |
| **Safb** | 3338.629372 | 3451.885571 | 3318.254405 | 3400.406332 | 3672.24009 | 3591.934978 | 3648.851771 | 3438.617676 | 3524.30775 | 3450.164973 | 3510.126163 | 3719.092992 | 3746.614734 | 3692.827861 | 3449.115225 |
| **2410015M20Rik** | 722.484524 | 724.1274758 | 764.3654234 | 740.1188821 | 682.7655773 | 739.99051 | 731.5744837 | 716.5532812 | 609.4554393 | 634.4528294 | 673.4599375 | 713.1081034 | 650.1403325 | 770.7321178 | 681.200257 |
| **Rpl36** | 9693.562954 | 9141.911316 | 9149.673403 | 8557.948052 | 9628.388039 | 9322.477601 | 9243.457132 | 7722.013956 | 7661.83301 | 7993.58131 | 7904.64143 | 8572.912746 | 8537.884838 | 9102.40956 | 8752.84845 |
| **Lonp1** | 5700.210598 | 5213.876278 | 5096.813705 | 4645.151725 | 4409.474907 | 4873.416174 | 4586.09701 | 4555.770335 | 4842.537293 | 4519.820983 | 4493.83169 | 4684.651774 | 4579.953194 | 4458.138651 | 4505.406763 |
| **Catsperd** | 0 | 10.29940611 | 5.482129061 | 3.450437679 | 1.266726489 | 0 | 0.902064715 | 1.676148026 | 3.00965649 | 0 | 0 | 0.867528106 | 0 | 2.710663955 | 0 |
| **Ranbp3** | 2187.598894 | 2299.144349 | 2286.83098 | 2387.702874 | 2304.808846 | 2241.013914 | 2343.564129 | 2360.016421 | 2349.789305 | 2419.834552 | 2428.995954 | 2298.081954 | 2292.958892 | 2318.521236 | 2287.9131 |
| **Vmac** | 99.81091649 | 65.75774671 | 65.78554874 | 106.963568 | 78.53704229 | 71.54410618 | 69.45898304 | 67.04592104 | 66.96485691 | 104.8682363 | 98.37055267 | 94.56056358 | 77.53967268 | 60.53816166 | 66.82660749 |
| **Ndufa11** | 926.6848393 | 918.2316679 | 980.5179407 | 929.8929544 | 917.743341 | 993.2005329 | 969.7195684 | 919.3671923 | 1037.579075 | 1051.304069 | 1009.244036 | 923.0499051 | 942.4052526 | 1039.087849 | 1031.860305 |
| **Nrtn** | 1.831392963 | 1.584524017 | 6.265290356 | 8.626094197 | 1.900089733 | 4.90988964 | 1.80412943 | 5.866518091 | 6.771727103 | 13.9824315 | 11.35044839 | 0.867528106 | 0 | 6.324882561 | 0.718565672 |
| **Dus3l** | 640.9875371 | 751.0643841 | 715.0262619 | 769.4476023 | 697.3329319 | 622.8545715 | 708.1208011 | 931.1002285 | 842.7038173 | 765.5381248 | 722.6452139 | 747.8092276 | 662.0695129 | 739.107705 | 719.2842376 |
| **Prr22** | 5.494178889 | 1.584524017 | 13.31374201 | 0 | 2.533452977 | 4.208476834 | 2.706194144 | 2.514222039 | 6.019312981 | 7.86511772 | 16.07980188 | 6.94022485 | 3.408337261 | 9.035546516 | 4.311394032 |
| **Rfx2** | 64.09875371 | 45.15893448 | 64.21922615 | 56.9322217 | 52.56914927 | 54.00878604 | 48.7114946 | 30.17066447 | 22.57242368 | 56.80362798 | 52.02288843 | 97.1631479 | 71.57508247 | 97.58390237 | 74.01226421 |
| **Acsbg2** | 0 | 0 | 1.566322589 | 0 | 1.266726489 | 0 | 3.608258859 | 0 | 0 | 1.747803938 | 0 | 0 | 0 | 3.614218606 | 0.718565672 |
| **1700061G19Rik** | 3.662785926 | 4.753572051 | 5.482129061 | 6.900875357 | 12.66726489 | 7.014128057 | 2.706194144 | 1.676148026 | 1.504828245 | 13.10852953 | 6.621094891 | 1.735056213 | 6.816674521 | 5.421327909 | 5.029959704 |
| **Mllt1** | 1647.33797 | 1716.831772 | 1748.016009 | 1695.02751 | 1726.548204 | 1571.866098 | 1706.70644 | 2016.406075 | 1842.662186 | 1808.977076 | 1780.128655 | 1671.726661 | 1663.268583 | 1608.32728 | 1731.024704 |
| **Clpp** | 667.5427351 | 788.3006985 | 787.8602623 | 714.2405995 | 693.5327525 | 648.8068453 | 604.3833589 | 986.4131134 | 920.2024719 | 843.3154 | 949.6541816 | 677.539451 | 656.957007 | 612.6100538 | 668.2660749 |
| **Alkbh7** | 100.726613 | 109.3321572 | 90.84671016 | 110.4140057 | 117.8055634 | 81.36388546 | 100.1291833 | 125.711102 | 89.53728059 | 102.2465304 | 91.74945778 | 78.07752957 | 103.1022021 | 79.51280934 | 105.6291538 |
| **Pspn** | 0.915696482 | 7.130358077 | 0 | 0 | 6.333632443 | 1.402825611 | 6.314453003 | 4.190370065 | 4.514484735 | 0 | 11.35044839 | 3.470112425 | 1.70416863 | 0 | 5.029959704 |
| **Gtf2f1** | 2515.418235 | 2453.63544 | 2455.99382 | 2517.094287 | 2426.414589 | 2471.778727 | 2468.04906 | 2561.154184 | 2424.278303 | 2456.538435 | 2313.599729 | 2377.894539 | 2464.227839 | 2395.323381 | 2359.769667 |
| **Khsrp** | 7175.39763 | 6876.041972 | 6949.773327 | 7151.032089 | 7170.938652 | 7093.387704 | 7295.899413 | 6622.460851 | 7070.43551 | 6825.174377 | 7037.277999 | 7158.841933 | 7297.250075 | 7230.244322 | 7253.920458 |
| **Slc25a41** | 0.915696482 | 0 | 2.349483883 | 3.450437679 | 2.533452977 | 0 | 3.608258859 | 0 | 0 | 2.621705907 | 0 | 0 | 1.70416863 | 0 | 2.874262688 |
| **Slc25a23** | 63.18305723 | 82.39524888 | 63.43606485 | 88.84877022 | 67.76986714 | 79.25964705 | 74.87137133 | 70.3982171 | 110.604876 | 70.78605948 | 104.9916476 | 87.62033873 | 68.16674521 | 102.1016756 | 81.9164866 |
| **Crb3** | 0 | 0 | 1.566322589 | 1.725218839 | 1.900089733 | 5.611302446 | 0.902064715 | 1.676148026 | 0 | 5.243411813 | 0 | 1.735056213 | 3.408337261 | 0 | 0 |
| **Dennd1c** | 312.2525002 | 471.3958951 | 454.2335508 | 402.838599 | 477.5558862 | 493.7946152 | 446.5220338 | 352.8291595 | 393.5125861 | 391.5080821 | 375.5106674 | 425.9563002 | 451.604687 | 540.3256816 | 489.3432226 |
| **Tubb4a** | 40.29064519 | 34.06726637 | 38.37490343 | 38.81742388 | 34.20161519 | 46.99465798 | 35.18052388 | 28.49451644 | 17.30552482 | 16.60413741 | 21.75502607 | 39.03876478 | 34.93545692 | 26.2030849 | 38.80254629 |
| **Gm11110** | 56.77318186 | 34.85952837 | 65.00238744 | 51.75656518 | 48.76896981 | 42.78618115 | 45.10323574 | 26.81836842 | 31.60139315 | 34.08217679 | 52.96875913 | 52.91921448 | 40.04796281 | 64.15238026 | 33.77258658 |
| **Tnfsf9** | 169.4038491 | 144.9839476 | 132.3542588 | 116.4522717 | 125.4059224 | 117.1359386 | 111.8560246 | 108.1115477 | 96.30900769 | 114.4811579 | 78.507268 | 109.3085414 | 92.02510604 | 110.2336675 | 84.79074929 |
| **Cd70** | 17.39823315 | 26.93690829 | 19.57903236 | 28.46611085 | 20.90098706 | 19.63955856 | 19.84542373 | 78.77895723 | 37.62070613 | 57.67752995 | 15.13393118 | 29.49595561 | 28.97086672 | 18.07109303 | 22.27553583 |
| **Tnfsf14** | 664.7956456 | 518.9316156 | 591.2867773 | 599.5135467 | 648.5639621 | 585.6796928 | 686.4712479 | 539.7196644 | 393.5125861 | 388.0124742 | 447.3968405 | 566.4958534 | 603.2756951 | 634.2953654 | 606.4694271 |
| **C3** | 10.98835778 | 11.09166812 | 10.96425812 | 4.313047098 | 2.533452977 | 9.118366474 | 3.608258859 | 10.05688816 | 9.028969471 | 11.3607256 | 3.783482795 | 2.602584319 | 4.260421576 | 0.903554652 | 0 |
| **Gpr108** | 495.3917965 | 514.9703055 | 481.6441961 | 475.2977902 | 492.1232408 | 500.8087433 | 522.2954699 | 382.16175 | 421.3519086 | 407.2383175 | 403.8867884 | 510.9740546 | 557.2631421 | 551.1683375 | 517.3672838 |
| **Trip10** | 347.964663 | 325.6196855 | 328.9277437 | 232.0419339 | 222.3104987 | 274.252407 | 287.758644 | 266.5075362 | 292.6890937 | 228.9623159 | 256.3309594 | 259.3909038 | 218.1335847 | 246.6704199 | 273.0549553 |
| **Vav1** | 2699.473228 | 2926.615859 | 2897.69679 | 2849.198913 | 2850.767962 | 3030.103321 | 2982.225947 | 2709.493284 | 2637.963914 | 2730.943653 | 3005.977081 | 2855.902526 | 2799.94906 | 2927.517071 | 2917.376628 |
| **Nudt12** | 2.747089445 | 0 | 5.482129061 | 4.313047098 | 3.800179466 | 2.104238417 | 3.608258859 | 0.838074013 | 12.79104008 | 14.85633347 | 5.675224193 | 4.337640531 | 1.70416863 | 3.614218606 | 1.437131344 |
| **Fbxl17** | 343.3861806 | 345.4262357 | 321.0961307 | 351.9446432 | 334.415793 | 349.3035772 | 385.1816332 | 346.1245674 | 306.2325479 | 337.32616 | 387.8069865 | 367.8319171 | 339.1295574 | 315.3405734 | 337.0073002 |
| **Fer** | 1.831392963 | 5.54583406 | 4.698967767 | 0.86260942 | 2.533452977 | 0 | 0.902064715 | 2.514222039 | 2.257242368 | 0.873901969 | 0 | 0 | 0.852084315 | 0.903554652 | 2.874262688 |
| **Pja2** | 569.5632115 | 513.3857815 | 515.3201318 | 545.1691532 | 480.7227024 | 462.9324518 | 571.9090292 | 596.7086973 | 541.7381683 | 507.7370439 | 603.4655058 | 502.2987735 | 556.4110578 | 587.3105235 | 523.8343749 |
| **Man2a1** | 1952.264899 | 2154.952663 | 2115.318656 | 1939.145975 | 1815.852421 | 1862.250999 | 2158.640862 | 1858.010087 | 2034.527787 | 1760.912467 | 2058.214641 | 2002.254869 | 2093.571162 | 1976.977578 | 2018.450973 |
| **Vapa** | 1923.878308 | 1748.522253 | 1854.525945 | 1770.937139 | 1728.448294 | 1914.155547 | 1742.789029 | 1869.743123 | 1840.404944 | 1788.003428 | 1768.778207 | 1925.912396 | 1705.020715 | 1592.966851 | 1756.174502 |
| **Rab31** | 8.241268334 | 17.42976419 | 23.49483883 | 16.38957897 | 17.1008076 | 14.02825611 | 19.84542373 | 20.95185033 | 10.53379772 | 4.369509845 | 12.29631908 | 19.08561834 | 24.71044514 | 28.0101942 | 16.52701046 |
| **Ppp4r1** | 701.4235049 | 706.6977116 | 715.0262619 | 813.4406827 | 723.300825 | 782.7766912 | 782.0901077 | 659.5642483 | 631.2754488 | 722.7169283 | 648.8672994 | 730.4586655 | 728.5320895 | 802.3565306 | 679.04456 |
| **Ralbp1** | 2229.720933 | 2177.928261 | 2224.178076 | 2198.791411 | 1926.690989 | 2132.294929 | 2206.450292 | 2132.898363 | 2069.138837 | 2206.602471 | 2175.502607 | 2179.230603 | 2181.335847 | 2147.749407 | 2246.236291 |
| **Twsg1** | 732.5571853 | 755.0256941 | 830.1509722 | 706.4771147 | 756.2357137 | 700.0099801 | 733.3786131 | 973.0039292 | 920.2024719 | 913.2275575 | 871.1469136 | 810.2712513 | 826.5217857 | 674.9553247 | 748.0268645 |
| **Ankrd12** | 460.5953302 | 546.6607859 | 527.0675512 | 563.283951 | 538.9921209 | 491.6903768 | 497.0376578 | 611.7940295 | 571.082319 | 573.2796916 | 609.14073 | 533.5297854 | 498.4693244 | 537.6150177 | 553.2955674 |
| **Ndufv2** | 1556.684019 | 1600.369257 | 1531.863492 | 1582.025676 | 1591.641833 | 1651.827157 | 1609.283451 | 1679.500322 | 1582.3269 | 1802.859762 | 1759.3195 | 1594.516659 | 1636.001885 | 1468.276309 | 1631.144075 |
| **Wash1** | 811.3070827 | 1018.056681 | 953.1072954 | 935.9312203 | 1019.714823 | 1002.318899 | 942.6576269 | 1056.81133 | 951.0514509 | 1034.699931 | 880.6056206 | 985.5119287 | 930.4760722 | 995.717226 | 900.362787 |
| **Ddx11** | 394.6651836 | 448.4202968 | 461.2820025 | 406.2890367 | 447.1544505 | 433.4731139 | 442.0117102 | 502.0063338 | 545.5002389 | 485.8894947 | 418.0748489 | 470.2002336 | 479.7234694 | 484.3052932 | 478.5647375 |
| **Mtcl1** | 0 | 0 | 0.783161294 | 0 | 1.266726489 | 0 | 0 | 0 | 0 | 0.873901969 | 0.945870699 | 0.867528106 | 2.556252945 | 0.903554652 | 0 |
| **Rab12** | 202.3689224 | 192.5196681 | 173.8618074 | 202.7132136 | 202.0428749 | 204.1111265 | 200.2583667 | 214.5469473 | 246.0394181 | 224.592806 | 196.7411053 | 212.544386 | 204.5002356 | 138.2438617 | 187.5456404 |
| **Zbtb14** | 364.4471997 | 377.116716 | 411.1596796 | 333.8298454 | 366.7173184 | 365.4360718 | 383.3775038 | 376.2952319 | 328.0525574 | 329.4610423 | 370.7813139 | 360.8916922 | 375.769183 | 365.0360792 | 342.7558255 |
| **C030034I22Rik** | 16.48253667 | 11.09166812 | 12.53058071 | 36.22959563 | 21.53435031 | 21.04238417 | 18.94335901 | 19.2757023 | 15.80069657 | 36.70388269 | 15.13393118 | 24.29078698 | 17.0416863 | 22.58886629 | 23.71266717 |
| **Tgif1** | 464.2581162 | 462.681013 | 415.0754861 | 389.0368483 | 414.852925 | 404.0137761 | 419.4600924 | 367.0764177 | 353.6346376 | 393.255886 | 383.077633 | 438.9692218 | 443.9359282 | 382.2036176 | 405.9896047 |
| **Myl12b** | 2260.854613 | 2271.415178 | 2290.746786 | 2253.135804 | 2467.5832 | 2269.070426 | 2397.688012 | 1905.780306 | 2004.431223 | 2073.769372 | 2012.812847 | 2485.468024 | 2406.286106 | 2599.526733 | 2509.949892 |
| **Myl12a** | 6253.291273 | 6060.804365 | 6377.282421 | 5991.685029 | 6321.598541 | 6631.156665 | 6504.788658 | 6115.426073 | 5832.714278 | 5905.829506 | 5893.720324 | 6421.443043 | 6126.486226 | 6546.253451 | 6357.869066 |
| **Myom1** | 1.831392963 | 3.169048034 | 1.566322589 | 0 | 3.800179466 | 0.701412806 | 0 | 0 | 0 | 2.621705907 | 1.891741398 | 8.675281063 | 0 | 2.710663955 | 1.437131344 |
| **Lpin2** | 504.5487613 | 638.5631789 | 565.4424546 | 528.7795742 | 476.2891597 | 539.3864476 | 577.3214175 | 583.2995131 | 517.6609163 | 488.5112006 | 630.8957561 | 613.3423711 | 633.0986462 | 552.9754468 | 610.0622555 |
| **Emilin2** | 853.4291208 | 563.298288 | 515.3201318 | 428.7168816 | 398.3854806 | 417.3406194 | 472.6819105 | 102.2450296 | 85.77520997 | 173.0325898 | 202.4163295 | 380.8448387 | 425.1900733 | 349.6756502 | 458.4448987 |
| **Smchd1** | 4699.354343 | 4330.504138 | 4144.48957 | 4118.959979 | 3834.381081 | 3913.883456 | 4532.875192 | 3566.004926 | 3945.659659 | 3485.994954 | 3756.052545 | 4079.984684 | 4011.612956 | 3910.584532 | 4021.812066 |
| **Ndc80** | 951.4086443 | 1010.134061 | 1059.617231 | 937.6564392 | 884.8084523 | 988.2906432 | 1043.688875 | 943.6713387 | 1039.836317 | 1016.34799 | 1035.728415 | 1140.79946 | 1037.838696 | 1182.753039 | 988.027799 |
| **Spdya** | 0 | 0.792262009 | 0 | 1.725218839 | 0 | 0.701412806 | 0 | 0 | 0 | 2.621705907 | 6.621094891 | 1.735056213 | 0 | 1.807109303 | 0 |
| **Trmt61b** | 75.08711149 | 82.39524888 | 65.00238744 | 49.16873692 | 87.40412771 | 82.06529827 | 75.77343604 | 76.26473519 | 70.72692752 | 95.25531461 | 104.0457769 | 60.72696744 | 90.32093741 | 71.38081747 | 76.8865269 |
| **Wdr43** | 5333.016309 | 5214.66854 | 5214.287899 | 5127.35039 | 4849.028998 | 5002.47613 | 4937.902249 | 5230.419915 | 5188.647789 | 5029.305831 | 5543.748165 | 4670.771324 | 4761.447153 | 4416.575137 | 4694.389535 |
| **Snord53** | 4.578482408 | 5.54583406 | 3.132645178 | 10.35131304 | 3.166816221 | 3.507064029 | 8.118582433 | 13.40918421 | 7.524141226 | 7.86511772 | 19.86328467 | 9.542809169 | 16.18960199 | 1.807109303 | 8.622788064 |
| **Ypel5** | 231.6712098 | 168.7518078 | 231.0325819 | 198.4001665 | 174.1748922 | 198.499824 | 230.928567 | 164.2625066 | 154.9973093 | 148.5633347 | 177.8236914 | 187.386071 | 167.8606101 | 213.2388978 | 189.7013374 |
| **Lbh** | 1305.783183 | 1626.513903 | 1636.023944 | 1718.317964 | 1991.29404 | 1839.104377 | 2056.70755 | 1316.614275 | 1296.409533 | 1364.160973 | 1367.72903 | 2129.781501 | 1994.729382 | 2158.592063 | 1988.98978 |
| **Lclat1** | 691.3508436 | 714.6203317 | 715.0262619 | 619.3535633 | 675.7985816 | 704.2184569 | 675.6464714 | 681.3541726 | 668.1437409 | 575.0274955 | 581.7104797 | 645.4409111 | 725.1237522 | 702.0619643 | 614.3736495 |
| **Galnt14** | 18.31392963 | 14.26071615 | 15.66322589 | 21.56523549 | 14.56735462 | 7.014128057 | 11.72684129 | 30.17066447 | 31.60139315 | 24.46925513 | 26.48437957 | 20.82067455 | 13.63334904 | 6.324882561 | 20.11983882 |
| **Ehd3** | 895.551159 | 1134.519196 | 1027.507618 | 1023.054772 | 1283.193933 | 1177.672101 | 1288.148413 | 667.1069144 | 784.7679299 | 780.3944582 | 768.9928781 | 1389.780026 | 1138.384645 | 1256.84452 | 1223.717339 |
| **Xdh** | 776.5106164 | 693.2292574 | 646.8912292 | 739.2562726 | 730.9011839 | 797.5063601 | 921.9101385 | 441.6650049 | 455.9629583 | 388.8863762 | 542.9297811 | 760.8221492 | 946.6656741 | 752.6610248 | 850.0631899 |
| **Memo1** | 1216.044928 | 1218.498969 | 1240.52749 | 1246.470611 | 1247.092228 | 1190.998944 | 1218.68943 | 1326.671163 | 1318.981957 | 1410.477778 | 1323.273108 | 1303.894744 | 1263.641039 | 1152.032181 | 1286.951118 |
| **Dpy30** | 740.7984536 | 929.323336 | 888.1049079 | 867.7850762 | 843.0064781 | 885.1829608 | 756.8322957 | 978.8704472 | 974.3762887 | 1082.764539 | 962.8963713 | 835.4295663 | 805.2196778 | 842.1129353 | 816.2906034 |
| **Spast** | 731.6414888 | 863.5655893 | 831.7172947 | 725.4545219 | 720.767372 | 762.4357198 | 749.615778 | 720.7436512 | 727.5844565 | 684.2652417 | 715.0782483 | 737.3988903 | 762.6154621 | 765.3107899 | 753.7753899 |
| **Slc30a6** | 337.8920017 | 262.2387248 | 317.9634856 | 313.1272193 | 313.5148059 | 317.740001 | 291.3669029 | 265.6694621 | 324.2904868 | 268.2879045 | 263.897925 | 326.190568 | 328.0524613 | 282.8126059 | 280.2406121 |
| **Nlrc4** | 3.662785926 | 2.376786026 | 1.566322589 | 3.450437679 | 1.266726489 | 4.90988964 | 5.412388289 | 4.190370065 | 0.752414123 | 0.873901969 | 5.675224193 | 1.735056213 | 0 | 0.903554652 | 0 |
| **Yipf4** | 732.5571853 | 687.6834234 | 715.0262619 | 596.9257184 | 585.2276377 | 621.4517459 | 660.3113712 | 683.8683947 | 698.2403058 | 631.8311235 | 679.1351617 | 613.3423711 | 646.7319952 | 710.1939561 | 675.4517316 |
| **Birc6** | 4509.805172 | 4308.320802 | 4377.871636 | 4824.574484 | 3901.517585 | 3812.178599 | 4563.545392 | 4751.879654 | 5032.898066 | 4265.51551 | 4661.250804 | 4682.04919 | 4483.667666 | 4079.549252 | 4261.094435 |
| **Ttc27** | 1077.774759 | 1015.679895 | 1022.808651 | 1054.97132 | 1126.119848 | 1094.90539 | 1093.302434 | 1341.756495 | 1219.663293 | 1251.427619 | 1307.193306 | 1047.106424 | 977.3407095 | 1099.626011 | 1023.956083 |
| **Ltbp1** | 0 | 3.961310043 | 0 | 0 | 0.633363244 | 0.701412806 | 0 | 2.514222039 | 15.04828245 | 1.747803938 | 3.783482795 | 6.072696744 | 1.70416863 | 0 | 2.155697016 |
| **Rasgrp3** | 1.831392963 | 3.169048034 | 7.831612945 | 2.587828259 | 1.266726489 | 0 | 0 | 20.11377631 | 21.82000955 | 6.991215751 | 9.458706988 | 0 | 0 | 1.807109303 | 2.155697016 |
| **Fam98a** | 1125.390976 | 1076.68407 | 1144.198651 | 1186.087952 | 1055.183165 | 996.0061841 | 1142.915994 | 1327.509237 | 1330.268169 | 1189.38058 | 1251.386934 | 1177.23564 | 1202.290969 | 1028.245193 | 1071.381417 |
| **Crim1** | 2090.535067 | 1811.903213 | 1693.97788 | 1540.620423 | 1361.097612 | 1534.691219 | 1575.004992 | 1332.537681 | 1526.648255 | 1507.480896 | 1619.330636 | 1331.655643 | 1478.366287 | 1343.585767 | 1461.562577 |
| **Fez2** | 295.7699635 | 273.3303929 | 262.3590337 | 277.7602331 | 246.378302 | 273.5509942 | 311.2123266 | 164.2625066 | 158.7593799 | 220.2232962 | 190.1200105 | 231.6300044 | 227.5065121 | 241.249092 | 241.4380658 |
| **Strn** | 697.760719 | 747.103074 | 741.6537459 | 767.7223835 | 743.5684488 | 630.5701123 | 794.7190137 | 750.0762417 | 729.8416989 | 694.7520653 | 766.155266 | 727.8560812 | 796.6988347 | 762.6001259 | 758.0867839 |
| **Heatr5b** | 769.1850445 | 759.7792662 | 744.7863911 | 717.6910371 | 746.1019018 | 708.4269338 | 821.7809552 | 764.3234999 | 761.4430921 | 618.722594 | 826.6909907 | 757.3520368 | 794.1425817 | 800.5494213 | 825.6319571 |
| **Gpatch11** | 198.7061365 | 216.2875283 | 218.5020012 | 156.9949144 | 200.7761484 | 208.3196033 | 191.2377195 | 210.3565773 | 218.9525097 | 212.3581784 | 216.60439 | 209.9418017 | 243.6961141 | 218.6602257 | 162.3958419 |
| **Gm6548** | 636.4090547 | 586.2738863 | 525.5012286 | 486.5117127 | 541.5255739 | 547.8034013 | 596.2647765 | 373.7810098 | 397.2746567 | 459.6724356 | 417.1289782 | 460.6574244 | 489.9484812 | 495.1479491 | 448.3849793 |
| **Eif2ak2** | 2226.973843 | 1792.888925 | 1601.564847 | 1476.787326 | 1406.066402 | 1657.43846 | 1844.722342 | 1167.4371 | 1424.319934 | 1291.62711 | 1324.218978 | 1392.382611 | 1445.987083 | 1413.159475 | 1520.484962 |
| **Sult6b1** | 24.723805 | 22.18333624 | 17.22954848 | 12.07653188 | 16.46744435 | 14.02825611 | 24.3557473 | 24.30414638 | 9.028969471 | 10.48682363 | 32.15960376 | 25.15831508 | 19.59793925 | 18.07109303 | 18.68270747 |
| **Cebpzos** | 15.56684019 | 11.88393013 | 10.18109683 | 13.80175071 | 17.73417084 | 21.04238417 | 17.13922958 | 16.76148026 | 18.81035306 | 21.84754922 | 9.458706988 | 18.21809023 | 17.0416863 | 14.45687443 | 15.80844478 |
| **Cebpz** | 1802.090676 | 1643.943668 | 1701.026332 | 1643.270944 | 1631.543717 | 1615.353692 | 1722.943605 | 1935.112896 | 1780.211814 | 1748.67784 | 1760.26537 | 1751.539247 | 1757.849942 | 1637.241029 | 1707.312037 |
| **Ndufaf7** | 394.6651836 | 409.5994584 | 331.2772276 | 383.8611917 | 363.5505022 | 375.9572639 | 353.6093682 | 398.9232302 | 433.3905346 | 354.8041994 | 402.9409177 | 354.8189955 | 383.4379418 | 400.2747106 | 384.4326345 |
| **Prkd3** | 979.7952353 | 922.9852399 | 970.3368439 | 1042.894788 | 967.7790373 | 1019.85422 | 1074.359075 | 1033.345258 | 1002.968025 | 909.7319496 | 989.3807509 | 1125.183954 | 1042.951202 | 1086.072691 | 1138.92659 |
| **Qpct** | 10.98835778 | 30.89821833 | 14.0969033 | 12.07653188 | 15.83408111 | 15.43108173 | 14.43303544 | 26.81836842 | 15.80069657 | 22.72145119 | 17.97154328 | 38.17123668 | 19.59793925 | 26.2030849 | 27.30549553 |
| **Cdc42ep3** | 131.8602933 | 142.6071615 | 105.7267748 | 145.7809919 | 124.7725591 | 122.0458282 | 127.1911248 | 165.9386546 | 121.1386737 | 98.75092249 | 100.2622941 | 138.804497 | 178.0856219 | 124.6905419 | 130.7789523 |
| **Rmdn2** | 32.96507334 | 34.85952837 | 46.98967767 | 40.54264272 | 31.66816221 | 39.27911712 | 29.76813559 | 29.33259046 | 69.9745134 | 44.56900041 | 32.15960376 | 23.42325887 | 40.90004713 | 35.23863141 | 18.68270747 |
| **Cyp1b1** | 0 | 0 | 0 | 0 | 1.900089733 | 2.104238417 | 0 | 2.514222039 | 4.514484735 | 4.369509845 | 21.75502607 | 0 | 1.70416863 | 0.903554652 | 0 |
| **Atl2** | 898.2982484 | 831.8751089 | 841.1152303 | 744.4319292 | 773.3365213 | 768.0470223 | 785.6983666 | 799.5226085 | 737.3658401 | 803.1159094 | 870.2010429 | 849.310016 | 697.0049698 | 745.4325875 | 796.1707645 |
| **Hnrnpll** | 996.2777719 | 1044.201327 | 1063.533038 | 1033.406085 | 927.2437896 | 1071.758767 | 966.1113095 | 1518.590112 | 1387.451642 | 1243.562502 | 1190.85121 | 1125.183954 | 1106.005441 | 1045.412732 | 1051.261578 |
| **Galm** | 39.37494871 | 55.4583406 | 46.20651637 | 38.81742388 | 50.0356963 | 42.08476834 | 44.20117102 | 61.17940295 | 50.41174621 | 79.52507917 | 38.78069865 | 58.12438312 | 44.30838439 | 41.56351397 | 45.98820301 |
| **Srsf7** | 3900.867011 | 4058.75827 | 4135.091635 | 3953.33897 | 4197.29822 | 4392.948402 | 4294.730107 | 4248.197172 | 4170.631481 | 4321.445236 | 4087.107289 | 4288.191429 | 4248.492395 | 4191.590029 | 4269.717223 |
| **Gemin6** | 332.3978228 | 320.0738514 | 347.7236148 | 326.92897 | 343.9162416 | 326.8583675 | 277.8359322 | 362.0479736 | 385.2360308 | 381.8951604 | 377.4024088 | 321.8529274 | 342.5378947 | 313.5334641 | 324.0731181 |
| **Dhx57** | 356.2059313 | 436.5363667 | 381.3995504 | 390.7620671 | 318.5817119 | 375.2558511 | 379.7692449 | 291.6497565 | 314.5091032 | 330.3349442 | 308.3538478 | 362.6267484 | 401.3317124 | 357.807642 | 390.1811599 |
| **Morn2** | 25.63950148 | 11.88393013 | 15.66322589 | 6.038265938 | 10.76717515 | 11.9240177 | 18.0412943 | 2.514222039 | 19.56276719 | 29.71266694 | 12.29631908 | 9.542809169 | 7.668758836 | 11.74621047 | 10.77848508 |
| **Arhgef33** | 3.662785926 | 0.792262009 | 2.349483883 | 0 | 0 | 1.402825611 | 0.902064715 | 0 | 0.752414123 | 0 | 0 | 3.470112425 | 5.112505891 | 0 | 0.718565672 |
| **Gm10190** | 1.831392963 | 0 | 0.783161294 | 0 | 0 | 0 | 0 | 2.514222039 | 0 | 0 | 0.945870699 | 0 | 0 | 0 | 0 |
| **Sos1** | 739.8827571 | 645.6935369 | 592.0699386 | 607.2770314 | 494.0233305 | 561.1302446 | 571.0069645 | 555.6430707 | 663.6292561 | 608.2357704 | 594.9526695 | 523.1194481 | 583.6777559 | 638.8131387 | 592.8166794 |
| **Cdkl4** | 18.31392963 | 28.52143231 | 14.8800646 | 17.25218839 | 13.93399137 | 16.83390734 | 16.23716487 | 9.218814144 | 15.80069657 | 9.612921658 | 9.458706988 | 8.675281063 | 10.22501178 | 18.97464768 | 10.05991941 |
| **Map4k3** | 15.56684019 | 20.59881222 | 26.62748401 | 6.038265938 | 17.1008076 | 28.75792503 | 23.45368258 | 9.218814144 | 6.771727103 | 10.48682363 | 17.02567258 | 12.14539349 | 23.00627651 | 15.36042908 | 18.68270747 |
| **Thumpd2** | 86.99116575 | 98.24048905 | 96.32883922 | 113.001834 | 132.3729181 | 105.9133337 | 107.3457011 | 113.1399918 | 105.3379772 | 136.3287071 | 119.179708 | 111.9111257 | 107.3626237 | 98.48745702 | 98.44349706 |
| **Pkdcc** | 1.831392963 | 4.753572051 | 1.566322589 | 0.86260942 | 1.900089733 | 2.805651223 | 0.902064715 | 0.838074013 | 9.028969471 | 1.747803938 | 0.945870699 | 1.735056213 | 1.70416863 | 0.903554652 | 0 |
| **Eml4** | 2196.755859 | 2452.050916 | 2384.726142 | 2417.894203 | 2330.143376 | 2265.563362 | 2562.765855 | 2641.609289 | 2516.072826 | 2363.030924 | 2433.725308 | 2469.852519 | 2370.498565 | 2164.013391 | 2216.056532 |
| **Cox7a2l** | 2305.723741 | 2160.498497 | 2461.475949 | 2381.664608 | 2390.946247 | 2627.49237 | 2770.240739 | 2248.552577 | 2367.847244 | 2121.83398 | 2252.118134 | 2450.7669 | 2467.636177 | 2683.557315 | 2499.889973 |
| **Kcng3** | 0 | 1.584524017 | 3.132645178 | 0.86260942 | 1.900089733 | 1.402825611 | 3.608258859 | 4.190370065 | 0 | 0 | 6.621094891 | 0.867528106 | 5.112505891 | 0 | 1.437131344 |
| **Mta3** | 1043.893989 | 940.4150041 | 996.9643279 | 1108.453104 | 917.743341 | 921.6564267 | 936.3431739 | 973.0039292 | 1026.292863 | 1063.538696 | 1092.480657 | 969.8964228 | 996.9386487 | 1027.341639 | 941.3210303 |
| **Haao** | 104.3893989 | 81.60298688 | 77.53296815 | 93.16181732 | 84.87067473 | 110.1218105 | 101.9333128 | 55.31288486 | 69.9745134 | 73.40776539 | 79.4531387 | 100.6332603 | 110.770961 | 112.0407768 | 102.0363254 |
| **8430430B14Rik** | 0 | 0 | 0 | 0.86260942 | 0 | 0 | 0.902064715 | 0 | 0 | 1.747803938 | 1.891741398 | 1.735056213 | 0 | 0.903554652 | 0 |
| **4933433H22Rik** | 2.747089445 | 1.584524017 | 2.349483883 | 0 | 0 | 0 | 1.80412943 | 0 | 0 | 0 | 0 | 0 | 0 | 1.807109303 | 0 |
| **Zfp36l2** | 3743.367217 | 4088.071964 | 4079.487183 | 4032.699037 | 3813.480094 | 4143.946856 | 4246.920677 | 3987.556154 | 3914.058266 | 4094.230724 | 4159.939333 | 4471.23986 | 4526.271882 | 4213.27534 | 4202.17205 |
| **Thada** | 791.1617601 | 786.7161744 | 844.2478755 | 852.2581066 | 748.0019915 | 662.8351014 | 755.0281663 | 835.559791 | 781.0058592 | 778.6466543 | 862.6340773 | 723.5184406 | 717.4549934 | 650.5593491 | 674.0146003 |
| **Dync2li1** | 17.39823315 | 13.46845414 | 19.57903236 | 18.11479781 | 12.66726489 | 8.416953669 | 9.020647148 | 6.704592104 | 7.524141226 | 12.23462756 | 26.48437957 | 6.072696744 | 15.33751767 | 13.55331977 | 3.59282836 |
| **Lrpprc** | 2476.958983 | 2393.423528 | 2265.685625 | 2363.54981 | 2193.970278 | 2204.540448 | 2223.589522 | 2874.593865 | 2948.710946 | 2956.410361 | 2994.626632 | 2248.632851 | 2357.7173 | 2349.242094 | 2277.85318 |
| **1110020A21Rik** | 1.831392963 | 3.961310043 | 1.566322589 | 0.86260942 | 1.900089733 | 1.402825611 | 2.706194144 | 0.838074013 | 0.752414123 | 1.747803938 | 0 | 3.470112425 | 0 | 0 | 2.155697016 |
| **Ppm1b** | 1579.576431 | 1418.941257 | 1566.322589 | 1410.366401 | 1386.432142 | 1604.131087 | 1580.41738 | 1356.841827 | 1577.812415 | 1465.533602 | 1360.162065 | 1493.883399 | 1613.847693 | 1469.179863 | 1461.562577 |
| **Prepl** | 210.6101908 | 215.4952663 | 202.8387753 | 177.6975404 | 218.5103193 | 197.7984112 | 245.3616024 | 259.802944 | 252.0587311 | 234.2057277 | 201.4704588 | 176.1082056 | 236.8794396 | 192.4571408 | 173.1743269 |
| **Camkmt** | 20.14532259 | 20.59881222 | 19.57903236 | 22.42784491 | 12.66726489 | 14.72966892 | 13.53097072 | 13.40918421 | 26.33449429 | 21.84754922 | 34.05134516 | 17.35056213 | 28.97086672 | 21.68531164 | 13.65274777 |
| **Srbd1** | 505.4644578 | 471.3958951 | 534.8991641 | 567.5969981 | 478.8226127 | 534.476558 | 565.5945762 | 517.0916661 | 439.4098476 | 528.7106912 | 529.6875913 | 531.7947291 | 533.4047813 | 571.0465398 | 502.2774047 |
| **Prkce** | 214.2729767 | 207.5726462 | 179.3439364 | 175.1097122 | 176.7083452 | 216.0351442 | 202.9645608 | 125.711102 | 149.7304104 | 155.5545505 | 185.390657 | 207.3392174 | 155.0793454 | 175.2896024 | 181.0785493 |
| **Epas1** | 8.241268334 | 7.922620085 | 4.698967767 | 5.175656518 | 7.600358931 | 6.312715251 | 9.922711863 | 4.190370065 | 3.762070613 | 5.243411813 | 6.621094891 | 12.14539349 | 10.22501178 | 4.517773258 | 8.622788064 |
| **Rhoq** | 746.2926325 | 518.1393536 | 563.0929707 | 571.9100452 | 434.4871856 | 453.8140853 | 460.9550693 | 346.1245674 | 377.7118895 | 475.4026711 | 429.4252972 | 429.4264126 | 455.0130243 | 354.1934234 | 434.0136659 |
| **Pigf** | 162.9939737 | 162.4137117 | 173.0786461 | 179.4227593 | 136.8064608 | 188.6800447 | 161.4695839 | 204.4900592 | 167.7883493 | 169.536982 | 144.7182169 | 158.7576434 | 184.0502121 | 168.0611652 | 172.4557613 |
| **Cript** | 401.9907554 | 371.570882 | 394.7132924 | 352.8072526 | 372.4175876 | 420.1462706 | 400.5167334 | 350.3149375 | 405.5512121 | 339.0739639 | 428.4794265 | 390.3876478 | 363.8400026 | 388.5285002 | 332.6959061 |
| **Socs5** | 149.2585265 | 145.7762096 | 160.5480654 | 125.9409753 | 161.5076273 | 157.8178813 | 163.2737134 | 207.0042812 | 164.0262787 | 157.3023544 | 166.473243 | 140.5395532 | 181.4939591 | 157.2185094 | 178.9228523 |

| | | | | | | | | | | | | | | | |
|---|---|---|---|---|---|---|---|---|---|---|---|---|---|---|---|
| **Mcfd2** | 340.6390911 | 326.4119475 | 346.1572922 | 349.356815 | 365.4505919 | 349.3035772 | 367.1403389 | 392.2186381 | 385.2360308 | 366.164925 | 447.3968405 | 372.1695576 | 308.4545221 | 384.9142816 | 399.5225136 |
| **4833418N02Rik** | 6.409875371 | 7.130358077 | 11.74741942 | 1.725218839 | 13.93399137 | 7.014128057 | 9.922711863 | 0.838074013 | 7.524141226 | 18.35194135 | 11.35044839 | 5.205168638 | 9.372927467 | 9.939101167 | 1.437131344 |
| **Ttc7** | 2554.793184 | 2818.868226 | 2679.97795 | 2996.705124 | 2959.073077 | 2861.764247 | 3085.963389 | 2519.250483 | 2707.938427 | 2475.764278 | 2502.773869 | 3010.322529 | 2971.218007 | 3112.745775 | 3001.448812 |
| **Calm2** | 4093.163273 | 4073.018986 | 4375.522152 | 4168.991325 | 4075.059114 | 4166.392066 | 4034.935469 | 3985.041932 | 4002.843132 | 4041.796606 | 4328.304318 | 4465.167163 | 4289.392443 | 4480.727517 | 4140.375402 |
| **Epcam** | 44.8691276 | 57.83512662 | 60.30341968 | 54.34439344 | 65.8697774 | 75.05117021 | 81.18582433 | 79.61703124 | 51.16416034 | 47.19070632 | 35.94308655 | 88.48786684 | 70.72299816 | 77.70570004 | 81.9164866 |
| **Msh2** | 1428.486511 | 1483.906742 | 1537.345621 | 1626.018756 | 1441.534744 | 1474.369718 | 1484.798521 | 1640.110844 | 1592.108283 | 1542.436975 | 1724.322284 | 1640.495649 | 1511.597575 | 1415.870139 | 1461.562577 |
| **Kcnk12** | 8.241268334 | 3.169048034 | 2.349483883 | 5.175656518 | 0 | 0 | 0.902064715 | 3.352296052 | 0 | 2.621705907 | 0 | 1.735056213 | 0.852084315 | 2.710663955 | 1.437131344 |
| **Msh6** | 2501.682788 | 2319.743161 | 2539.008917 | 2583.515212 | 2316.209384 | 2296.425526 | 2330.935223 | 2626.523957 | 2574.761127 | 2514.215965 | 2232.254849 | 2462.912294 | 2482.973694 | 2331.171001 | 2196.655259 |
| **Fbxo11** | 845.1878525 | 873.8649954 | 859.1279401 | 720.2788654 | 777.1367007 | 772.9569119 | 911.0853619 | 815.4460147 | 781.0058592 | 755.0513011 | 757.6424297 | 755.6169806 | 818.0009426 | 768.9250085 | 764.553875 |
| **Foxn2** | 947.7458584 | 931.700122 | 924.9134888 | 914.3659848 | 929.7772426 | 934.2818572 | 1090.59624 | 908.4722302 | 984.1576723 | 956.9226559 | 994.1101044 | 966.4263104 | 976.4886252 | 923.4328539 | 1004.554809 |
| **Ppp1r21** | 396.4965765 | 413.5607684 | 427.6060668 | 400.2507707 | 392.6852115 | 390.6869328 | 439.3055161 | 465.1310772 | 437.9050193 | 555.8016522 | 440.7757456 | 480.6105709 | 455.8651086 | 425.5742409 | 466.3491211 |
| **Ston1** | 105.3050954 | 91.90239299 | 90.06354887 | 69.87136299 | 34.20161519 | 28.05651223 | 23.45368258 | 152.5294704 | 113.6145325 | 123.2201776 | 111.6127425 | 28.62842751 | 40.90004713 | 31.62441281 | 35.9282836 |
| **Gtf2a1l** | 0 | 2.376786026 | 0 | 0.86260942 | 0 | 0 | 0 | 0 | 0 | 0.873901969 | 0.945870699 | 0 | 1.70416863 | 0 | 0 |
| **Mettl4** | 284.7816058 | 288.3833711 | 263.1421949 | 262.2332636 | 245.7449388 | 307.9202217 | 281.444191 | 305.8970148 | 364.1684353 | 279.64863 | 350.9180292 | 234.2325887 | 292.2649201 | 240.3455373 | 279.5220464 |
| **2700099C18Rik** | 58.60457482 | 75.26489081 | 68.13503262 | 51.75656518 | 72.20340985 | 70.84269338 | 72.16517718 | 57.8271069 | 62.45037217 | 71.65996145 | 56.75224193 | 75.47494525 | 76.68758836 | 76.80214538 | 63.9523448 |
| **Gm1976** | 16.48253667 | 26.14464628 | 32.10961307 | 16.38957897 | 22.80107679 | 17.53532014 | 17.13922958 | 34.36103454 | 31.60139315 | 23.59535316 | 25.53850887 | 32.09853993 | 17.89377062 | 18.07109303 | 20.83840449 |
| **2610044O15Rik8** | 49.44761 | 77.64167683 | 75.18348427 | 67.28353473 | 69.03659363 | 67.33562935 | 41.49497688 | 76.26473519 | 76.7462405 | 55.05582404 | 54.86050053 | 61.59449555 | 62.20215501 | 62.34527096 | 62.51521346 |
| **Crem** | 826.8739229 | 674.2149692 | 592.8530999 | 620.2161727 | 411.0527455 | 479.0649463 | 482.6046224 | 327.6869391 | 371.6925766 | 506.863142 | 498.4738583 | 402.5330413 | 402.1837968 | 396.660492 | 428.2651405 |
| **Gm6225** | 2.747089445 | 0.792262009 | 4.698967767 | 4.313047098 | 1.266726489 | 3.507064029 | 7.216517718 | 4.190370065 | 2.257242368 | 10.48682363 | 11.35044839 | 7.807752957 | 0 | 4.517773258 | 2.874262688 |
| **Cul2** | 1407.425492 | 1414.979947 | 1400.292395 | 1356.884617 | 1235.69169 | 1350.921064 | 1317.916548 | 1544.570406 | 1419.805449 | 1538.941367 | 1555.957299 | 1281.339013 | 1282.386894 | 1339.971548 | 1287.669684 |
| **Bambi** | 28.38659093 | 32.48274235 | 36.02541955 | 15.52696955 | 24.06780328 | 27.35509942 | 18.0412943 | 27.65644243 | 26.33449429 | 28.83876497 | 18.91741398 | 19.08561834 | 13.63334904 | 21.68531164 | 30.17975822 |
| **Map3k8** | 589.7085341 | 572.8054321 | 700.9293586 | 519.2908706 | 614.9957102 | 569.5471982 | 655.8010476 | 486.9210016 | 404.0463838 | 488.5112006 | 480.502315 | 569.9659658 | 622.0215501 | 588.2140782 | 681.200257 |
| **4833419F23Rik** | 54.94178889 | 41.98988645 | 52.47180673 | 31.91654853 | 34.83497844 | 28.75792503 | 42.39704159 | 38.5514046 | 15.04828245 | 39.3255886 | 46.34766424 | 30.36348372 | 20.45002356 | 56.92394305 | 40.23967763 |
| **Mtpap** | 1024.664363 | 947.5453622 | 909.2502629 | 886.7624834 | 881.641636 | 893.5999145 | 963.4051154 | 931.9383025 | 926.2217849 | 902.7407339 | 1022.486225 | 805.0660826 | 879.3510132 | 834.8844981 | 832.8176138 |
| **9430020K01Rik** | 103.4737024 | 65.75774671 | 76.74980686 | 69.00875357 | 62.06959794 | 50.50172201 | 68.55691832 | 112.3019177 | 65.46002866 | 96.12921658 | 85.12836289 | 46.84651774 | 34.93545692 | 52.40616979 | 39.52111196 |
| **Svil** | 692.2665401 | 720.9584277 | 702.4956812 | 659.896206 | 622.5960691 | 658.6266246 | 735.1827425 | 652.8596562 | 713.2885882 | 568.9101818 | 661.1636184 | 666.2615856 | 697.8570541 | 638.8131387 | 760.9610466 |
| **Zfp438** | 37.54355574 | 53.08155457 | 41.50754861 | 39.6800333 | 29.13470924 | 29.45933784 | 33.37639445 | 52.79866282 | 33.85863552 | 6.117313782 | 34.05134516 | 19.08561834 | 31.52711966 | 39.75640467 | 14.37131344 |
| **Gm10125** | 0.915696482 | 0 | 0 | 3.450437679 | 1.900089733 | 0.701412806 | 1.80412943 | 0 | 2.257242368 | 0 | 0 | 2.602584319 | 0 | 0 | 0 |
| **Zeb1** | 770.100741 | 638.5631789 | 691.531423 | 647.8196742 | 490.2231511 | 547.1019885 | 634.1514945 | 609.2798075 | 507.8795327 | 545.3148286 | 630.8957561 | 555.217988 | 499.3214087 | 491.5337305 | 563.3554868 |
| **Arhgap12** | 307.6740178 | 323.2428995 | 301.5170984 | 294.1498121 | 278.6798275 | 259.5227381 | 279.6400616 | 243.0414638 | 273.1263265 | 359.1737092 | 304.570365 | 311.4425902 | 308.4545221 | 309.9192455 | 348.5043509 |
| **Kif5b** | 3884.384475 | 3804.442165 | 3677.725439 | 3668.677862 | 3304.256045 | 3572.295419 | 4017.79624 | 4192.884287 | 4071.312817 | 3957.902017 | 4246.013567 | 4028.800526 | 4134.313097 | 3771.437116 | 3745.882848 |
| **Epc1** | 414.8105061 | 457.1351789 | 430.738712 | 414.0525214 | 492.756604 | 462.9324518 | 519.5892757 | 487.7590756 | 439.4098476 | 456.1768278 | 471.043608 | 528.3246167 | 454.16094 | 529.4830258 | 463.4748584 |
| **Rab18** | 935.8418042 | 1006.965013 | 1004.795941 | 1034.268694 | 1041.882537 | 1071.057354 | 1083.379722 | 1117.990733 | 945.7845521 | 1087.134049 | 1129.369614 | 1024.550694 | 1086.407502 | 1083.362027 | 1123.836711 |
| **Mpp7** | 221.5985485 | 202.8190742 | 197.3566462 | 180.2853687 | 165.3078068 | 204.1111265 | 203.8666255 | 189.404727 | 176.0649047 | 221.9711001 | 244.980511 | 222.9547233 | 216.4294161 | 171.6753838 | 190.4199031 |
| **Wac** | 2339.60451 | 2374.409239 | 2459.909626 | 2260.036679 | 2105.299424 | 2287.307159 | 2336.347611 | 2271.180575 | 2286.586519 | 2379.635061 | 2475.343619 | 2312.829931 | 2533.246669 | 2147.749407 | 2360.488232 |
| **Ccny** | 1278.312288 | 1277.91862 | 1415.95562 | 1421.580324 | 1235.69169 | 1306.732057 | 1347.684684 | 1487.581373 | 1405.509581 | 1415.72119 | 1497.313316 | 1398.455307 | 1280.682726 | 1351.717759 | 1271.861239 |
| **Thoc1** | 928.5162323 | 972.1054844 | 962.5052309 | 942.8320957 | 959.5453151 | 928.6705548 | 961.600986 | 1162.408656 | 1074.447367 | 1076.647226 | 1114.235683 | 977.7041758 | 943.2573369 | 1040.894959 | 886.7100392 |
| **Usp14** | 1851.538286 | 2033.736576 | 1969.650656 | 1861.511128 | 1896.289553 | 1746.517886 | 1846.526471 | 1899.913788 | 1901.350488 | 1927.827743 | 2010.921106 | 1879.065878 | 1867.768819 | 1789.03821 | 1927.911698 |
| **Rock1** | 2598.746615 | 2387.085432 | 2461.475949 | 2400.642015 | 2330.776739 | 2238.909676 | 2713.410662 | 2354.987977 | 2506.291442 | 2343.805081 | 2580.335266 | 2550.532632 | 2638.05304 | 2554.349 | 2506.357064 |
| **Greb1l** | 0 | 8.714882094 | 0 | 1.725218839 | 0 | 0 | 4.510323574 | 1.676148026 | 0 | 0 | 10.40457769 | 3.470112425 | 0.852084315 | 0.903554652 | 0 |
| **Esco1** | 705.0862908 | 824.7447509 | 654.7228422 | 671.9727379 | 772.703158 | 775.0611503 | 834.4098612 | 650.3454341 | 633.5326912 | 672.9045161 | 760.4800418 | 775.570127 | 785.6217386 | 815.0062957 | 784.6737138 |
| **Snrpd1** | 1895.491717 | 2036.113362 | 2080.076398 | 1898.603333 | 1953.925609 | 1926.079564 | 1982.738243 | 2070.042812 | 2021.736747 | 2134.94251 | 1992.003692 | 1950.203183 | 2076.529476 | 2028.480193 | 2009.109619 |
| **Mib1** | 563.1533362 | 550.6220959 | 524.7180673 | 588.2996242 | 352.7833271 | 411.729317 | 546.6512172 | 579.109143 | 610.2078534 | 568.0362798 | 578.8728676 | 467.5976493 | 514.6589264 | 491.5337305 | 464.1934241 |
| **Rbbp8** | 1040.231203 | 989.5352486 | 986.7832311 | 973.0234254 | 850.6068371 | 925.8649035 | 983.2505391 | 905.1199341 | 878.0672811 | 865.1629492 | 997.8935872 | 1003.730019 | 1002.903239 | 866.5089109 | 924.0754541 |
| **Gm6277** | 14.65114371 | 14.26071615 | 10.18109683 | 12.07653188 | 3.800179466 | 6.312715251 | 11.72684129 | 4.190370065 | 3.762070613 | 6.117313782 | 17.02567258 | 3.470112425 | 9.372927467 | 2.710663955 | 5.748525376 |
| **Cables1** | 1829.56157 | 1714.454986 | 1621.14388 | 1592.376989 | 1014.647917 | 1268.154353 | 1271.911248 | 571.5664769 | 903.6493612 | 1032.952127 | 1372.458384 | 1149.474741 | 1126.455465 | 1048.123396 | 1113.776792 |
| **Tmem241** | 86.99116575 | 114.0857292 | 118.2573555 | 81.08528545 | 105.1382985 | 143.0882124 | 120.8766718 | 77.94088321 | 68.46968515 | 103.9943343 | 86.07423359 | 87.62033873 | 113.3272139 | 72.28437213 | 102.7548911 |
| **Riok3** | 2045.66594 | 1813.487737 | 1925.010462 | 1970.199914 | 1675.879144 | 1872.772191 | 1900.650354 | 1832.029793 | 1793.755268 | 1776.642703 | 2086.590761 | 1793.180596 | 1647.931066 | 1831.505279 | 1811.504059 |
| **3110002H16Rik** | 406.5692378 | 476.9417291 | 453.4503895 | 382.9985823 | 388.885032 | 376.6586767 | 377.0630508 | 370.4287138 | 409.3132827 | 409.8600234 | 347.1345464 | 396.4603446 | 421.781736 | 390.3356095 | 403.8339076 |
| **Npc1** | 1922.962611 | 1719.208558 | 1798.921493 | 1827.006751 | 1434.567748 | 1455.431572 | 1577.711186 | 1488.419447 | 1586.841385 | 1452.425072 | 1466.099583 | 1479.135421 | 1450.247504 | 1463.758536 | 1625.39555 |
| **Ankrd29** | 30.21798389 | 24.56012226 | 28.1938066 | 32.77915795 | 31.66816221 | 19.63955856 | 41.49497688 | 25.9802944 | 16.5531107 | 18.35194135 | 36.88895725 | 29.49595561 | 25.56252945 | 46.08128723 | 18.68270747 |
| **Ttc39c** | 1205.05657 | 804.1459386 | 762.7991008 | 708.2023335 | 380.0179466 | 452.4112597 | 501.5479814 | 505.3586299 | 437.9050193 | 661.5437905 | 621.4370491 | 464.995065 | 547.0381303 | 427.3813502 | 518.0858495 |
| **Cabyr** | 1.831392963 | 6.338096068 | 2.349483883 | 6.038265938 | 2.533452977 | 0 | 1.80412943 | 0 | 1.504828245 | 0.873901969 | 0.945870699 | 4.337640531 | 1.70416863 | 0 | 5.029959704 |
| **Osbpl1a** | 26.55519797 | 28.52143231 | 32.89277437 | 35.36698621 | 32.30152546 | 30.16075065 | 28.86607087 | 30.17066447 | 33.10622139 | 41.07339254 | 34.05134516 | 19.95314644 | 35.78754124 | 9.939101167 | 26.58692986 |
| **Impact** | 144.6800441 | 189.35062 | 193.4408397 | 223.4158397 | 164.6744435 | 143.7896252 | 149.7427427 | 220.4134654 | 184.34146 | 222.8450021 | 299.8410115 | 147.4797781 | 161.8960199 | 154.5078454 | 144.4317001 |
| **Ss18** | 1863.44234 | 2032.152052 | 2027.604591 | 1985.726884 | 1736.048653 | 1667.258239 | 1905.160678 | 2089.318515 | 2097.730574 | 2202.232962 | 2023.217425 | 1919.839699 | 2014.327321 | 1802.59153 | 1877.612101 |
| **Psma8** | 0 | 4.753572051 | 0.783161294 | 2.587828259 | 0 | 0 | 0 | 0.838074013 | 0 | 0.873901969 | 0 | 0 | 1.70416863 | 0 | 0 |
| **Taf4b** | 633.6619652 | 641.7322269 | 603.817358 | 574.4978735 | 548.4925695 | 615.1390306 | 615.2081355 | 661.2403963 | 642.5616607 | 633.5789275 | 633.7333682 | 576.0386626 | 618.6132128 | 560.203884 | 586.3495883 |
| **Kctd1** | 60.43596778 | 82.39524888 | 75.96664557 | 94.88703616 | 76.63695256 | 69.43986777 | 79.3816949 | 53.63673684 | 60.19312981 | 83.02068705 | 52.02288843 | 53.78674259 | 89.46885309 | 72.28437213 | 79.04222392 |
| **Trappc8** | 1117.149708 | 1296.932908 | 1226.430587 | 1181.774905 | 1026.048456 | 1118.052012 | 1285.442219 | 1269.68213 | 1287.380564 | 1216.471541 | 1382.862962 | 1244.902833 | 1307.949424 | 1246.905419 | 1145.393681 |
| **Rnf125** | 566.8161221 | 514.9703055 | 538.8149706 | 497.7256351 | 393.3185747 | 406.8194273 | 440.2075808 | 396.4090082 | 474.7733113 | 531.3323971 | 555.2261002 | 388.6525916 | 421.781736 | 393.9498281 | 367.905624 |
| **Rnf138** | 631.8305723 | 679.7608033 | 671.1692294 | 609.8648597 | 589.0278172 | 662.8351014 | 651.2907241 | 695.6014308 | 733.6037695 | 756.7991051 | 669.6764547 | 709.6379909 | 648.4361638 | 648.7522398 | 574.1339719 |
| **Mapre2** | 812.2227791 | 918.2316679 | 960.9389083 | 884.1746551 | 839.2062987 | 796.1035345 | 873.1986439 | 1049.268664 | 1037.579075 | 1056.54748 | 914.6569657 | 903.0967586 | 825.6697014 | 863.7982469 | 868.7458974 |
| **Zfp397** | 390.0867011 | 406.4304104 | 525.5012286 | 444.2438511 | 404.7191131 | 400.5067121 | 414.9497688 | 432.4461907 | 367.1780918 | 410.7339254 | 435.1005214 | 385.1824792 | 435.415085 | 367.7467432 | 365.0313614 |
| **Zfp35** | 264.6362832 | 292.3446811 | 306.2160661 | 224.2784491 | 275.5130113 | 259.5227381 | 286.8565793 | 291.6497565 | 343.853254 | 419.4729451 | 332.0006153 | 298.4296686 | 266.7023906 | 297.2694804 | 264.4321673 |
| **Zfp191** | 627.2520899 | 588.6506723 | 577.9730353 | 576.2230923 | 557.359655 | 594.0966464 | 635.9556239 | 625.2032137 | 664.3816702 | 613.4791822 | 610.0866007 | 510.1065265 | 578.56525 | 531.2901351 | 638.8048824 |
| **Ino80c** | 547.586496 | 626.6792487 | 657.8554874 | 648.6822836 | 613.0956205 | 556.9217677 | 602.5792295 | 714.8771331 | 796.8065558 | 670.2828101 | 638.4627217 | 622.8851803 | 669.7382717 | 693.0264178 | 626.589266 |
| **Galnt1** | 1890.913234 | 1962.432995 | 2010.375043 | 1981.413837 | 1958.992515 | 1987.803891 | 2081.063297 | 2039.872148 | 2154.914047 | 2137.564216 | 2043.080709 | 2066.451949 | 2183.8921 | 1967.038476 | 2126.954389 |
| **2700062C07Rik** | 208.7787978 | 200.4422882 | 173.8618074 | 182.873197 | 182.4086144 | 183.0687423 | 191.2377195 | 222.0896135 | 166.2835211 | 199.2496489 | 224.1713556 | 190.8561834 | 202.796067 | 196.974914 | 197.6055598 |
| **Rprd1a** | 108.0521848 | 120.4238253 | 112.7752264 | 144.0557731 | 106.405025 | 118.5387642 | 117.2684129 | 171.8051727 | 133.9297138 | 111.859452 | 109.7210011 | 130.1292159 | 123.5522257 | 121.979878 | 131.497518 |
| **Slc39a6** | 2892.685185 | 2535.238427 | 2604.794465 | 2600.7674 | 2109.732967 | 2239.611089 | 2355.29097 | 2967.62008 | 2692.890145 | 2989.618636 | 3291.630032 | 2177.495547 | 2162.589992 | 2093.536128 | 2054.379256 |
| **Elp2** | 2327.700456 | 2307.066969 | 2193.634786 | 2147.034846 | 1992.560766 | 1988.505304 | 2076.552973 | 2521.764705 | 2327.216881 | 2362.157022 | 2622.899448 | 2093.34532 | 2056.931537 | 2024.865974 | 2006.235356 |
| **Mocos** | 47.61621704 | 49.91250654 | 30.54329049 | 53.48178402 | 32.9348887 | 35.77205309 | 52.31975346 | 45.2559967 | 40.63036262 | 55.92972601 | 34.99721585 | 26.89337129 | 36.63962555 | 40.65995932 | 39.52111196 |
| **Fhod3** | 0.915696482 | 1.584524017 | 0 | 0 | 0 | 0.701412806 | 0 | 0 | 0 | 4.369509845 | 2.837612096 | 3.470112425 | 0.852084315 | 0.903554652 | 0.718565672 |
| **Tpgs2** | 216.1043696 | 289.1756331 | 267.8411627 | 277.7602331 | 248.2783918 | 233.5704643 | 246.2636671 | 315.1158289 | 298.7084067 | 275.2791202 | 299.8410115 | 274.1388816 | 224.9502592 | 273.7770594 | 234.2524091 |
| **AW554918** | 51.27900297 | 76.84941482 | 66.56871003 | 82.81050429 | 63.33632443 | 77.15540863 | 73.96930661 | 71.23629111 | 50.41174621 | 64.6687457 | 82.29075079 | 70.26977661 | 90.32093741 | 79.51280934 | 63.9523448 |
| **Celf4** | 0 | 0 | 0 | 0 | 1.900089733 | 0.701412806 | 0 | 0 | 0 | 0 | 0 | 0 | 0.852084315 | 0 | 4.311394032 |
| **Pik3c3** | 695.929326 | 677.3840173 | 698.5798747 | 665.934472 | 644.7637827 | 713.3368234 | 692.785701 | 758.4569818 | 601.1788839 | 679.0218298 | 711.2947655 | 699.2276537 | 778.8050641 | 667.7268875 | 652.4576301 |
| **Slc25a46** | 994.446379 | 1012.510847 | 1042.387683 | 1086.025259 | 944.9779605 | 972.8595615 | 1057.219846 | 995.6319275 | 986.4149147 | 1056.54748 | 1109.50633 | 1006.332603 | 1063.401225 | 988.4887888 | 1067.070023 |
| **Sap130** | 1021.917273 | 1067.969187 | 1108.173232 | 1211.966235 | 1075.450789 | 1028.271173 | 1150.132511 | 1133.076066 | 1069.180468 | 999.7438524 | 1209.768624 | 1082.675077 | 1175.876355 | 1128.53976 | 1182.759096 |
| **Ammecr1l** | 666.6270386 | 770.8709343 | 707.9778102 | 614.1779068 | 633.9966075 | 610.229141 | 746.0075191 | 683.0303206 | 664.3816702 | 706.1127909 | 669.6764547 | 613.3423711 | 654.400754 | 609.8993898 | 625.1521346 |
| **Polr2d** | 892.8040695 | 964.9751264 | 953.8904567 | 1007.527802 | 1065.316977 | 1099.113867 | 1014.822804 | 1014.069556 | 939.7652391 | 981.3919111 | 1066.942148 | 1003.730019 | 1115.378369 | 1077.940699 | 1015.333294 |
| **Wdr33** | 2530.985075 | 2497.209851 | 2530.394142 | 2485.177738 | 2339.010461 | 2300.634003 | 2492.404807 | 2737.149727 | 2591.314238 | 2433.816983 | 2515.070188 | 2393.510045 | 2431.848635 | 2145.942297 | 2400.009344 |
| **Sft2d3** | 77.83420093 | 91.90239299 | 87.71406498 | 151.8192579 | 86.77076447 | 95.39214158 | 102.8353775 | 107.2734737 | 75.99382638 | 97.00311855 | 110.6668718 | 101.5007884 | 87.76468446 | 69.57370817 | 87.66501198 |
| **Lims2** | 10.98835778 | 5.54583406 | 6.265290356 | 0 | 1.900089733 | 0 | 5.412388289 | 0 | 0 | 6.991215751 | 3.783482795 | 0.867528106 | 5.112505891 | 1.807109303 | 0.718565672 |
| **Iws1** | 1566.75668 | 1638.397834 | 1537.345621 | 1508.703875 | 1432.667659 | 1472.265479 | 1529.901756 | 1638.434696 | 1668.10211 | 1552.049897 | 1527.581179 | 1533.789692 | 1537.160105 | 1497.190058 | 1457.251183 |
| **Proc** | 0 | 0 | 0.783161294 | 0 | 0.633363244 | 0 | 0 | 3.352296052 | 0 | 0.873901969 | 0 | 1.735056213 | 3.408337261 | 0 | 0 |
| **Map3k2** | 547.586496 | 486.4488732 | 554.4781965 | 518.4282612 | 467.4220743 | 461.5296262 | 509.6665639 | 496.1398157 | 408.5608686 | 435.2031805 | 463.4766424 | 534.3973135 | 515.5110107 | 486.1124025 | 457.0077674 |
| **Ercc3** | 958.7342162 | 954.6757202 | 1007.145425 | 1013.566068 | 936.7442383 | 976.3666256 | 1008.508351 | 1192.579321 | 993.1866418 | 1085.386245 | 1007.352294 | 1001.127435 | 1005.459492 | 1030.052303 | 971.5007885 |
| **Bin1** | 1312.193058 | 1338.922794 | 1460.595814 | 1381.90029 | 1542.2395 | 1501.724817 | 1476.679938 | 1235.321095 | 1417.548207 | 1164.037423 | 1189.905339 | 1484.34059 | 1371.003663 | 1553.210446 | 1455.814051 |
| **Gypc** | 814.0541721 | 807.3149867 | 769.0643912 | 848.8076689 | 663.76468 | 760.3314814 | 752.3219721 | 831.3694209 | 744.1375672 | 792.6290858 | 824.7992493 | 609.0047306 | 715.7508247 | 633.3918108 | 677.6074287 |
| **Wdr36** | 2546.551915 | 2492.456279 | 2549.190014 | 2461.887284 | 2273.774047 | 2431.798197 | 2403.1004 | 2837.718608 | 2634.201843 | 2751.043398 | 2670.192983 | 2463.779822 | 2373.054818 | 2349.242094 | 2275.697483 |
| **Camk4** | 2328.616153 | 2179.512785 | 2030.737237 | 1926.206834 | 1879.188746 | 1870.667953 | 2123.460339 | 1770.012316 | 2011.955364 | 1980.261862 | 1967.411053 | 1952.805767 | 1819.200013 | 1747.474696 | 1915.696081 |
| **Stard4** | 2306.639437 | 2116.924087 | 2211.647496 | 2299.716713 | 2040.696373 | 2170.171221 | 2488.796548 | 1864.714679 | 2045.813999 | 2072.89547 | 2148.072357 | 2161.012513 | 2231.608821 | 2117.028549 | 2219.649361 |
| **Nrep** | 0.915696482 | 3.961310043 | 11.74741942 | 3.450437679 | 10.13381191 | 4.90988964 | 7.216517718 | 3.352296052 | 14.29586833 | 8.739019689 | 1.891741398 | 4.337640531 | 6.816674521 | 1.807109303 | 2.874262688 |
| **Epb4.1l4aos** | 212.4415837 | 200.4422882 | 252.9610981 | 176.834931 | 229.2774944 | 306.5173961 | 238.1450847 | 213.7088733 | 197.1325001 | 224.592806 | 292.2740459 | 209.0742736 | 198.5356454 | 233.1171001 | 227.0667523 |
| **Epb4.1l4a** | 0.915696482 | 0 | 6.265290356 | 0 | 2.533452977 | 0.701412806 | 0.902064715 | 1.676148026 | 0 | 0 | 2.837612096 | 0.867528106 | 0 | 0 | 0 |
| **Gm10548** | 13.73544722 | 11.09166812 | 24.27800013 | 10.35131304 | 23.43444004 | 18.93814575 | 15.33510015 | 12.5711102 | 21.06759543 | 18.35194135 | 43.51005214 | 13.8804497 | 11.0770961 | 13.55331977 | 20.83840449 |
| **Apc** | 804.8972073 | 836.628681 | 739.304262 | 769.4476023 | 746.735265 | 708.4269338 | 836.2139906 | 945.3474867 | 860.0093421 | 817.9722429 | 937.3578625 | 878.8059717 | 784.7696543 | 764.4072352 | 814.853472 |
| **Srp19** | 947.7458584 | 982.4048905 | 1044.737167 | 985.0999572 | 927.2437896 | 937.0875084 | 989.5649921 | 1113.800363 | 990.1769853 | 1201.615207 | 1030.053191 | 1023.683165 | 975.6365409 | 987.5852342 | 1021.800386 |
| **Reep5** | 2766.319071 | 2786.385484 | 2796.668983 | 2694.791827 | 2586.65549 | 2739.017006 | 2729.647827 | 2499.136707 | 2491.995574 | 2525.57669 | 2411.970282 | 2730.978479 | 2786.315711 | 2666.389777 | 2759.29218 |
| **Pkd2l2** | 44.8691276 | 52.28929256 | 71.2676778 | 71.59658183 | 74.73686282 | 51.20313482 | 77.57756547 | 79.61703124 | 69.22209928 | 64.6687457 | 91.74945778 | 78.07752957 | 68.16674521 | 70.47726282 | 67.54517316 |
| **Fam13b** | 290.2757847 | 350.9720698 | 353.2057438 | 377.8229258 | 365.4505919 | 348.6021644 | 377.9651155 | 440.8269309 | 387.4932731 | 401.9949057 | 383.077633 | 475.4054022 | 359.579581 | 427.3813502 | 451.259242 |
| **Wnt8a** | 1.831392963 | 0 | 0 | 0 | 0.633363244 | 0 | 0.902064715 | 0 | 0 | 0 | 7.56696559 | 4.337640531 | 0 | 2.710663955 | 2.155697016 |
| **4933408B17Rik** | 0.915696482 | 0 | 3.915806472 | 0 | 0.633363244 | 0.701412806 | 1.80412943 | 0.838074013 | 2.257242368 | 3.495607876 | 0 | 0.867528106 | 0 | 0 | 0 |
| **Brd8** | 1220.62341 | 1208.199563 | 1155.946071 | 1177.461858 | 1125.486485 | 1118.052012 | 1069.848752 | 1247.892205 | 1255.026756 | 1266.283953 | 1186.121856 | 1263.988451 | 1210.811812 | 1320.996901 | 1266.112714 |
| **Kif20a** | 2256.276131 | 2362.525309 | 2491.236078 | 2296.266275 | 2263.006872 | 2016.561816 | 2122.558274 | 2515.898187 | 2614.639076 | 2578.88471 | 2569.930689 | 2290.274201 | 2375.611071 | 2435.983341 | 2290.787362 |
| **Cdc23** | 1084.184634 | 1147.98765 | 1214.683168 | 974.7486442 | 1130.553391 | 1068.251703 | 1130.287088 | 1223.588059 | 1157.212921 | 1081.016736 | 1112.343942 | 1044.50384 | 1191.213873 | 1074.326481 | 1085.75273 |
| **Cdc25c** | 242.6595676 | 297.0982532 | 253.7442594 | 316.577657 | 307.1811735 | 270.0439302 | 297.6813559 | 302.5447187 | 287.4221948 | 318.1003167 | 315.9208134 | 320.1178712 | 282.0399083 | 337.025885 | 314.0131986 |
| **Fam53c** | 1056.71374 | 1123.427528 | 1047.086651 | 1145.545309 | 950.0448664 | 977.0680384 | 1159.153158 | 1095.362735 | 1205.367424 | 1046.060657 | 1094.372398 | 1060.986874 | 1088.11167 | 1037.28074 | 1147.549378 |
| **Kdm3b** | 1596.974664 | 1709.701414 | 1652.470331 | 1611.354396 | 1509.304611 | 1466.654177 | 1695.881664 | 1604.911735 | 1568.031031 | 1514.472112 | 1627.843473 | 1632.687896 | 1756.997858 | 1587.545523 | 1656.293874 |
| **Reep2** | 36.62785926 | 40.40536243 | 21.14535495 | 26.74089201 | 29.13470924 | 36.4734659 | 24.3557473 | 79.61703124 | 36.86829201 | 29.71266694 | 45.40179354 | 25.15831508 | 40.04796281 | 40.65995932 | 55.32955674 |
| **Egr1** | 1724.256475 | 1436.371021 | 1571.804718 | 1557.010002 | 1358.564159 | 1513.648835 | 1664.309399 | 1198.445839 | 1199.348111 | 1018.095794 | 1141.665933 | 1292.616878 | 1330.9557 | 1379.727953 | 1307.789523 |
| **Etf1** | 8514.145886 | 8281.514775 | 8557.603465 | 8024.855431 | 7748.56593 | 7789.89062 | 8292.680923 | 9177.748517 | 8836.351456 | 9024.785633 | 9114.410053 | 7905.783633 | 7785.494388 | 7626.001259 | 7584.460668 |
| **Hspa9** | 14935.00961 | 13635.62143 | 14105.51808 | 12992.62308 | 12541.85896 | 12579.83867 | 12895.91716 | 15364.41088 | 14792.46165 | 15508.26434 | 15176.49536 | 12673.7181 | 12438.72683 | 11566.40309 | 11904.47749 |
| **Ctnna1** | 1402.84701 | 1258.904332 | 1314.144652 | 1318.067193 | 1193.889715 | 1310.940534 | 1271.009183 | 1309.071608 | 1114.325316 | 1297.744424 | 1302.463952 | 1272.663732 | 1359.074483 | 1208.956124 | 1271.142674 |
| **Lrrtm2** | 3.662785926 | 0 | 0.783161294 | 2.587828259 | 0 | 1.402825611 | 1.80412943 | 2.514222039 | 7.524141226 | 0 | 0 | 2.602584319 | 0 | 0 | 2.155697016 |
| **Sil1** | 415.7262026 | 382.6625501 | 421.3407764 | 380.4107541 | 410.4193823 | 415.236381 | 438.4034514 | 312.6016069 | 358.1491223 | 307.6134931 | 320.6501669 | 390.3876478 | 391.958785 | 402.08182 | 393.7739882 |
| **Snhg4** | 107.1364883 | 162.4137117 | 128.4384523 | 94.02442674 | 150.7404521 | 121.3444154 | 114.5622188 | 175.9955427 | 118.8814314 | 143.3199229 | 151.3393118 | 143.1421375 | 140.593912 | 151.7971815 | 135.0903463 |
| **Mir1949** | 0 | 2.376786026 | 2.349483883 | 5.175656518 | 1.900089733 | 5.611302446 | 8.118582433 | 3.352296052 | 9.028969471 | 5.243411813 | 18.91741398 | 6.94022485 | 3.408337261 | 4.517773258 | 7.18565672 |
| **Snora74a** | 0.915696482 | 5.54583406 | 3.132645178 | 0.86260942 | 0 | 0.701412806 | 2.706194144 | 0 | 3.00965649 | 4.369509845 | 1.891741398 | 0 | 3.408337261 | 0 | 2.874262688 |
| **Matr3** | 4581.229497 | 4539.661309 | 4631.615896 | 4499.370733 | 4351.205488 | 4377.51732 | 4848.597842 | 4928.713271 | 4934.331816 | 4802.965221 | 4886.36803 | 4802.635596 | 4627.669916 | 4406.636036 | 4651.99416 |
| **Paip2** | 1240.768733 | 1183.639441 | 1162.211361 | 1135.193996 | 1278.127027 | 1302.52358 | 1365.725978 | 1126.371474 | 1069.180468 | 1126.459638 | 1177.60902 | 1422.746094 | 1353.961977 | 1350.814204 | 1185.633359 |
| **Slc23a1** | 1.831392963 | 5.54583406 | 6.265290356 | 4.313047098 | 3.166816221 | 2.104238417 | 5.412388289 | 0.838074013 | 7.524141226 | 0 | 8.512836289 | 0 | 5.112505891 | 3.614218606 | 6.467091048 |
| **Mzb1** | 0 | 0 | 3.132645178 | 0 | 0 | 0 | 0 | 5.866518091 | 4.514484735 | 0 | 0 | 0 | 0 | 0 | 0 |
| **Prob1** | 12.81975074 | 12.67619214 | 13.31374201 | 21.56523549 | 15.20071786 | 4.208476834 | 21.64955315 | 25.14222039 | 16.5531107 | 20.09974528 | 20.80915537 | 16.48303402 | 10.22501178 | 9.939101167 | 20.83840449 |
| **Spata24** | 231.6712098 | 187.766096 | 189.5250333 | 176.834931 | 211.5433236 | 194.2913472 | 182.2170724 | 204.4900592 | 179.0745612 | 166.0413741 | 173.0943379 | 196.061352 | 190.0148023 | 205.1069059 | 154.4916195 |
| **Dnajc18** | 272.8775515 | 286.0065851 | 290.5528403 | 301.9132969 | 253.3452977 | 248.3001332 | 312.1143913 | 291.6497565 | 291.1842654 | 320.7220226 | 336.7299688 | 286.2842751 | 293.1170044 | 265.6450676 | 283.8334404 |
| **Ecscr** | 0 | 0.792262009 | 1.566322589 | 2.587828259 | 1.900089733 | 0 | 0 | 0 | 1.504828245 | 0 | 0 | 0 | 0.852084315 | 6.324882561 | 0.718565672 |
| **1700066B19Rik** | 35.71216278 | 42.78214846 | 54.03812932 | 56.06961228 | 51.30242279 | 56.81443726 | 46.90736517 | 62.01747697 | 58.68830156 | 50.6863142 | 38.78069865 | 52.05168638 | 37.49170987 | 46.98484188 | 45.98820301 |
| **Tmem173** | 2948.542671 | 3079.522427 | 2935.288532 | 3010.506875 | 2834.933881 | 2809.158287 | 2864.957534 | 3057.294 | 2920.871624 | 2944.175733 | 2926.523942 | 2793.440502 | 2762.45735 | 2830.836723 | 2780.130585 |
| **Ube2d2a** | 2688.48487 | 2777.670602 | 2847.574467 | 2842.298038 | 2755.763476 | 2821.082305 | 2688.15285 | 2862.860829 | 2820.048131 | 2860.281144 | 2792.210303 | 2852.432413 | 2835.736601 | 2692.592862 | 2986.358933 |
| **Cxxc5** | 86.07546927 | 87.14882094 | 77.53296815 | 89.71137964 | 109.5718413 | 125.5528922 | 82.98995376 | 130.739546 | 91.04210883 | 114.4811579 | 90.80358708 | 103.2358446 | 113.3272139 | 91.25901981 | 118.5633359 |
| **Psd2** | 0.915696482 | 0 | 1.566322589 | 2.587828259 | 2.533452977 | 0.701412806 | 1.80412943 | 0 | 1.504828245 | 1.747803938 | 0 | 1.735056213 | 3.408337261 | 1.807109303 | 2.874262688 |
| **Nrg2** | 0 | 1.584524017 | 2.349483883 | 0 | 1.900089733 | 2.805651223 | 0 | 6.704592104 | 3.762070613 | 1.747803938 | 0.945870699 | 3.470112425 | 0 | 1.807109303 | 0 |
| **Pura** | 510.9586367 | 568.0518601 | 569.3582611 | 512.3899953 | 507.3239587 | 545.6991628 | 589.9503235 | 547.2623305 | 568.8250767 | 669.4089082 | 583.6022211 | 609.0047306 | 640.767405 | 597.2496247 | 624.4335689 |
| **Igip** | 35.71216278 | 22.97559825 | 42.2907099 | 26.74089201 | 23.43444004 | 28.05651223 | 23.45368258 | 30.17066447 | 30.0965649 | 40.19949057 | 41.61831075 | 35.56865236 | 32.37920398 | 22.58886629 | 25.86836419 |
| **Cystm1** | 21.06101908 | 27.7291703 | 27.41064531 | 21.56523549 | 24.06780328 | 28.75792503 | 21.64955315 | 15.08533223 | 21.06759543 | 7.86511772 | 19.86328467 | 18.21809023 | 25.56252945 | 17.16753838 | 15.80844478 |
| **Pfdn1** | 1121.72819 | 996.6656067 | 963.2883922 | 1054.108711 | 970.3124902 | 1007.228789 | 1040.080616 | 1088.658143 | 1083.476337 | 1042.565049 | 1048.970605 | 1054.914177 | 988.4178056 | 1112.275776 | 969.3450915 |
| **Hbegf** | 127.2818109 | 87.94108294 | 82.23193592 | 65.55831589 | 95.63784989 | 91.18366474 | 105.5415716 | 84.64547532 | 49.65933209 | 72.53386342 | 60.53572472 | 84.15022631 | 69.01882953 | 87.6448012 | 77.60509257 |
| **Ankhd1** | 1936.698059 | 2014.722288 | 1867.839687 | 1839.083283 | 1585.3082 | 1695.314751 | 2042.274514 | 1733.975133 | 1735.819381 | 1816.842193 | 1926.738613 | 1836.557001 | 1926.562637 | 1611.941498 | 1764.078725 |
| **Eif4ebp3** | 102.5580059 | 89.52560696 | 92.41303275 | 104.3757398 | 86.77076447 | 75.05117021 | 104.6395069 | 85.48354933 | 73.73658401 | 76.90337326 | 65.26507822 | 85.01775442 | 58.79381775 | 84.93413725 | 86.94644631 |
| **Sra1** | 938.5888936 | 867.5268993 | 902.2018112 | 911.7781566 | 915.8432512 | 855.0222102 | 894.8481971 | 768.51387 | 758.4334356 | 899.245126 | 713.1865069 | 888.3487808 | 871.6822544 | 1027.341639 | 873.7758571 |
| **Apbb3** | 108.9678813 | 115.6702532 | 126.0889684 | 91.43659848 | 108.938478 | 122.0458282 | 127.1911248 | 95.54043749 | 82.76555348 | 107.4899422 | 112.5586132 | 121.4539349 | 102.2501178 | 104.8123396 | 114.9705075 |
| **Slc35a4** | 2628.964599 | 2764.202148 | 2777.873112 | 2644.760481 | 2668.992711 | 2672.38279 | 2667.405362 | 3043.884815 | 3034.486156 | 3058.656891 | 3102.455892 | 2602.584319 | 2740.303158 | 2613.983607 | 2605.519127 |
| **Tmco6** | 218.8514591 | 239.2631266 | 278.0222595 | 256.1949976 | 272.346195 | 260.9255637 | 282.3462557 | 211.1946513 | 238.5152769 | 233.3318257 | 222.2796142 | 265.4636005 | 240.2877769 | 276.4877234 | 252.2165509 |
| **Ndufa2** | 903.7924273 | 865.1501133 | 952.3241341 | 831.5554805 | 976.0127594 | 958.8313054 | 959.7968565 | 853.9974193 | 969.861804 | 894.8756162 | 814.3946716 | 835.4295663 | 835.8947132 | 902.6510969 | 896.0513929 |
| **Ik** | 2506.26127 | 2410.853292 | 2446.595884 | 2524.857771 | 2413.113961 | 2445.125041 | 2636.735161 | 2421.195824 | 2106.007129 | 2295.740472 | 2494.261033 | 2408.258023 | 2471.044514 | 2522.724587 | 2438.811891 |
| **Wdr55** | 921.1906605 | 884.1644015 | 898.2860048 | 924.7172979 | 901.2758966 | 871.1547047 | 870.4924498 | 1096.200809 | 1007.48251 | 1052.177971 | 969.5174662 | 764.2922616 | 809.4800994 | 819.524069 | 756.6496526 |
| **Dnd1** | 16.48253667 | 20.59881222 | 18.01270977 | 33.64176737 | 23.43444004 | 16.13245453 | 6.314453003 | 25.9802944 | 7.524141226 | 10.48682363 | 20.80915537 | 15.61550591 | 13.63334904 | 12.64976512 | 27.30549553 |
| **Hars** | 2921.071776 | 2742.811073 | 2793.536337 | 2681.852686 | 2728.528856 | 2677.994092 | 2670.111556 | 3202.280804 | 3047.277196 | 3152.164402 | 3194.20535 | 2585.233757 | 2548.584187 | 2638.379583 | 2451.027507 |
| **Hars2** | 424.8831674 | 453.9661309 | 469.1136154 | 456.320383 | 456.6548991 | 423.6533347 | 429.3828042 | 502.0063338 | 452.9533018 | 406.3644155 | 431.3170386 | 359.156636 | 388.5504477 | 361.4218606 | 446.947848 |
| **Zmat2** | 1262.745448 | 1277.126358 | 1292.999297 | 1311.166318 | 1248.992318 | 1328.475854 | 1368.432172 | 1387.012492 | 1413.786136 | 1391.251934 | 1372.458384 | 1447.036881 | 1274.718135 | 1395.991937 | 1318.568008 |
| **Gm6756** | 2.747089445 | 0.792262009 | 0.783161294 | 0 | 4.43354271 | 2.104238417 | 0 | 0 | 0 | 0 | 0 | 0 | 0 | 0.903554652 | 0.718565672 |
| **Slc25a2** | 1.831392963 | 2.376786026 | 4.698967767 | 0 | 0.633363244 | 0.701412806 | 0 | 9.218814144 | 0 | 3.495607876 | 0 | 0.867528106 | 0 | 0 | 0 |
| **Taf7** | 144.6800441 | 130.7232314 | 136.2700652 | 160.4453521 | 188.7422468 | 199.9026496 | 162.3716487 | 174.3193947 | 188.1035306 | 128.4635894 | 162.6897602 | 169.1679807 | 165.3043571 | 167.1576105 | 171.0186299 |

| | | | | | | | | | | | | | | | |
|---|---|---|---|---|---|---|---|---|---|---|---|---|---|---|---|
| **Pcdhga4** | 0 | 3.169048034 | 0.783161294 | 0 | 0 | 0 | 1.80412943 | 0 | 0 | 0 | 0 | 0.867528106 | 5.112505891 | 0 | 1.437131344 |
| **Pcdhgb2** | 2.747089445 | 0 | 0 | 0 | 0.633363244 | 0.701412806 | 1.80412943 | 0 | 0 | 0 | 0 | 2.602584319 | 0 | 1.807109303 | 0.718565672 |
| **Pcdhga7** | 0 | 0 | 0 | 0 | 0 | 0 | 0 | 3.352296052 | 0 | 0 | 0 | 0 | 0 | 0.903554652 | 0 |
| **Pcdhgb4** | 0 | 0 | 0 | 2.587828259 | 0 | 0.701412806 | 1.80412943 | 0 | 3.762070613 | 0 | 1.891741398 | 0 | 0 | 4.517773258 | 1.437131344 |
| **Pcdhga8** | 4.578482408 | 2.376786026 | 0.783161294 | 0.86260942 | 5.066905954 | 4.90988964 | 0.902064715 | 1.676148026 | 6.019312981 | 0 | 0 | 0.867528106 | 0 | 0 | 0 |
| **Pcdhgb5** | 0 | 0 | 0.783161294 | 0 | 0.633363244 | 4.90988964 | 2.706194144 | 0 | 0.752414123 | 3.495607876 | 0 | 0 | 0 | 0 | 2.155697016 |
| **Pcdhgb6** | 6.409875371 | 10.29940611 | 3.915806472 | 7.763484777 | 0 | 4.90988964 | 5.412388289 | 5.028444078 | 0.752414123 | 5.243411813 | 5.675224193 | 9.542809169 | 8.520843152 | 1.807109303 | 4.311394032 |
| **Pcdhga10** | 1.831392963 | 0.792262009 | 1.566322589 | 1.725218839 | 1.900089733 | 0 | 0.902064715 | 5.028444078 | 3.762070613 | 0.873901969 | 0.945870699 | 4.337640531 | 5.112505891 | 0.903554652 | 0 |
| **Pcdhgb7** | 6.409875371 | 3.961310043 | 2.349483883 | 3.450437679 | 1.266726489 | 4.90988964 | 3.608258859 | 2.514222039 | 4.514484735 | 4.369509845 | 6.621094891 | 2.602584319 | 10.22501178 | 4.517773258 | 0.718565672 |
| **Pcdhga11** | 0 | 3.961310043 | 0 | 0 | 0.633363244 | 2.104238417 | 0 | 0 | 0 | 2.621705907 | 0.945870699 | 1.735056213 | 0 | 0.903554652 | 2.155697016 |
| **Pcdhga12** | 4.578482408 | 2.376786026 | 14.0969033 | 3.450437679 | 3.800179466 | 5.611302446 | 0.902064715 | 0 | 0.752414123 | 2.621705907 | 7.56696559 | 5.205168638 | 0.852084315 | 6.324882561 | 5.748525376 |
| **Pcdhgc3** | 174.898028 | 196.4809781 | 175.42813 | 152.6818673 | 132.3729181 | 141.6853868 | 139.8200308 | 217.8992434 | 215.9428532 | 215.8537863 | 159.8521481 | 179.578318 | 160.1918513 | 115.6549954 | 146.5873971 |
| **Pcdhgc4** | 21.06101908 | 32.48274235 | 25.84432272 | 22.42784491 | 24.06780328 | 17.53532014 | 15.33510015 | 22.62799835 | 28.59173666 | 7.86511772 | 12.29631908 | 14.74797781 | 23.00627651 | 25.29953024 | 43.11394032 |
| **Pcdhgc5** | 10.98835778 | 11.09166812 | 3.132645178 | 3.450437679 | 6.966995687 | 7.715540863 | 9.020647148 | 12.5711102 | 0.752414123 | 4.369509845 | 5.675224193 | 7.807752957 | 10.22501178 | 7.228437213 | 8.622788064 |
| **Diap1** | 7301.763744 | 6445.051439 | 6844.829714 | 6830.141385 | 6297.530738 | 6282.554501 | 6764.583296 | 6334.163391 | 6265.352399 | 6410.944844 | 6589.881158 | 6540.294393 | 6478.397048 | 6151.400068 | 6528.16913 |
| **Hdac3** | 1196.815301 | 1198.692419 | 1380.713362 | 1235.256689 | 1295.861198 | 1265.348702 | 1290.854607 | 1413.83086 | 1338.544724 | 1443.686053 | 1388.538186 | 1387.177442 | 1340.328628 | 1298.408034 | 1316.412311 |
| **Rell2** | 11.90405426 | 22.97559825 | 17.22954848 | 17.25218839 | 12.03390164 | 7.014128057 | 8.118582433 | 10.05688816 | 9.028969471 | 6.117313782 | 14.18806048 | 6.94022485 | 18.74585493 | 9.035546516 | 14.37131344 |
| **Fchsd1** | 83.32837982 | 93.486917 | 118.2573555 | 100.9253021 | 93.10439691 | 99.60061841 | 115.4642835 | 109.7876957 | 83.51796761 | 92.6336087 | 84.18249219 | 78.94505767 | 76.68758836 | 117.4621047 | 93.41353736 |
| **Arap3** | 3.662785926 | 0.792262009 | 3.915806472 | 1.725218839 | 3.166816221 | 1.402825611 | 9.922711863 | 6.704592104 | 6.771727103 | 2.621705907 | 6.621094891 | 0 | 3.408337261 | 9.035546516 | 5.748525376 |
| **1700086O06Rik** | 3.662785926 | 16.63750218 | 16.44638718 | 16.38957897 | 10.76717515 | 14.72966892 | 12.62890601 | 3.352296052 | 22.57242368 | 9.612921658 | 18.91741398 | 8.675281063 | 15.33751767 | 4.517773258 | 10.77848508 |
| **0610009O20Rik** | 540.2609241 | 600.5346024 | 593.6362612 | 594.3378901 | 514.2909544 | 562.5330702 | 580.9296763 | 543.0719605 | 562.0533496 | 546.1887306 | 490.9068927 | 561.2906848 | 562.375648 | 509.6048235 | 565.5111838 |
| **Rnf14** | 782.9204917 | 826.3292749 | 749.4853588 | 786.6997907 | 784.7370597 | 669.8492295 | 882.2192911 | 842.2643831 | 822.388636 | 866.0368512 | 834.2579563 | 815.4764199 | 881.9072662 | 702.9655189 | 843.5960989 |
| **Gnpda1** | 537.5138347 | 594.1965064 | 634.3606485 | 620.2161727 | 601.0617188 | 605.3192513 | 653.0948535 | 693.0872088 | 726.0796283 | 781.2683602 | 678.189291 | 590.7866404 | 643.3236579 | 605.3816166 | 682.6373884 |
| **Ndfip1** | 4040.968573 | 4014.391597 | 4063.823957 | 3827.397995 | 4151.696066 | 4356.474936 | 4527.462803 | 3515.720485 | 3619.864344 | 3573.385151 | 3595.254526 | 4309.879632 | 4475.998908 | 4368.68674 | 4334.388133 |
| **Spry4** | 10.0726613 | 12.67619214 | 5.482129061 | 7.763484777 | 10.13381191 | 23.84803539 | 27.06194144 | 15.08533223 | 9.028969471 | 12.23462756 | 14.18806048 | 16.48303402 | 9.372927467 | 7.228437213 | 12.9341821 |
| **Arhgap26** | 232.5869063 | 260.6542008 | 268.624324 | 266.5463107 | 262.8457464 | 330.3654315 | 371.6506625 | 160.9102105 | 209.9235402 | 198.3757469 | 221.3337435 | 307.1049496 | 344.2420633 | 314.4370187 | 307.5461076 |
| **Nr3c1** | 740.7984536 | 910.3090478 | 783.9444558 | 725.4545219 | 712.5336498 | 716.8438874 | 877.7089675 | 652.8596562 | 815.6169089 | 718.3474184 | 724.5369553 | 781.6428238 | 794.1425817 | 724.6508306 | 730.7812884 |
| **Yipf5** | 659.3014667 | 614.7953186 | 700.1461973 | 647.8196742 | 644.1304194 | 648.1054325 | 707.2187364 | 706.496393 | 777.2437886 | 723.5908303 | 750.0754641 | 763.4247335 | 703.8216443 | 670.4375515 | 668.2660749 |
| **Prelid2** | 790.2460636 | 707.4899736 | 863.0437465 | 679.7362227 | 556.0929285 | 639.6884788 | 705.414607 | 476.0260394 | 541.7381683 | 559.2972601 | 643.1920752 | 587.316528 | 599.8673579 | 552.0718921 | 555.4512644 |
| **Lars** | 4164.587598 | 3394.050444 | 3785.801698 | 3275.327966 | 3280.821605 | 3367.48288 | 3569.470076 | 3151.158289 | 3083.393074 | 3341.801129 | 3364.462076 | 3328.705344 | 3070.059788 | 2987.151678 | 3199.772937 |
| **Gm4013** | 3.662785926 | 4.753572051 | 3.132645178 | 1.725218839 | 1.900089733 | 2.805651223 | 1.80412943 | 2.514222039 | 1.504828245 | 3.495607876 | 4.729353494 | 5.205168638 | 0.852084315 | 5.421327909 | 0.718565672 |
| **Rbm27** | 1963.253256 | 1934.703825 | 1940.673688 | 1843.39633 | 1742.382285 | 1738.100933 | 2068.434391 | 2149.659843 | 2059.357454 | 1992.496489 | 2000.516528 | 1851.304979 | 1944.456407 | 1772.774226 | 1741.084623 |
| **Tcerg1** | 4247.915978 | 4302.774968 | 4249.433184 | 4038.737303 | 3889.483683 | 3929.314538 | 4295.632172 | 4190.370065 | 4188.68942 | 4262.893804 | 4117.375152 | 4252.622777 | 4332.848743 | 4087.681244 | 4146.123927 |
| **Gpr151** | 0.915696482 | 3.169048034 | 1.566322589 | 6.900875357 | 0 | 4.208476834 | 8.118582433 | 0 | 0 | 1.747803938 | 0 | 4.337640531 | 0 | 0 | 0 |
| **Dcp2** | 1693.122794 | 1589.277589 | 1426.919879 | 1525.956063 | 1448.50174 | 1438.597665 | 1655.288752 | 1243.701835 | 1361.117148 | 1291.62711 | 1317.597883 | 1567.623288 | 1451.099589 | 1437.555451 | 1415.574374 |
| **Ythdc2** | 412.9791132 | 377.116716 | 418.2081313 | 446.8316794 | 395.8520277 | 345.7965132 | 391.4960862 | 414.8466365 | 389.7505155 | 408.1122195 | 453.0720647 | 471.0677617 | 434.5630007 | 429.1884595 | 384.4326345 |
| **Trim36** | 47.61621704 | 42.78214846 | 50.12232285 | 53.48178402 | 50.0356963 | 39.98052993 | 44.20117102 | 31.8468125 | 22.57242368 | 70.78605948 | 61.48159542 | 41.6413491 | 52.82922754 | 36.14218606 | 42.39537465 |
| **Pggt1b** | 556.7434608 | 514.9703055 | 480.8610348 | 501.1760728 | 497.82351 | 435.5773523 | 575.517288 | 516.253592 | 490.5740079 | 481.5199849 | 521.174755 | 548.2777632 | 564.931901 | 476.1733014 | 553.2955674 |
| **Ccdc112** | 32.96507334 | 20.59881222 | 18.01270977 | 12.07653188 | 12.03390164 | 17.53532014 | 21.64955315 | 18.43762829 | 19.56276719 | 30.58656891 | 29.32199166 | 14.74797781 | 27.26669809 | 16.26398373 | 17.24557613 |
| **Fem1c** | 654.7229843 | 652.031633 | 630.4448421 | 656.4457684 | 697.9662952 | 643.1955428 | 671.1361478 | 683.0303206 | 717.0506588 | 678.1479279 | 672.5140668 | 651.5136078 | 677.4070306 | 680.3766526 | 661.0804182 |
| **Ticam2** | 311.3368037 | 303.4363493 | 317.1803243 | 332.967236 | 269.1793788 | 270.745343 | 251.6760554 | 409.8181924 | 376.9594754 | 343.4434738 | 307.4079771 | 308.8400058 | 289.7086672 | 242.1526466 | 233.5338434 |
| **Tmed7** | 1983.398579 | 1986.993117 | 2127.849237 | 1987.452103 | 1958.992515 | 1976.581286 | 2107.223174 | 2023.948742 | 1985.620869 | 1977.640156 | 2035.513744 | 1924.17734 | 2082.494066 | 1995.952225 | 2036.415114 |
| **Eif1a** | 3066.667517 | 2936.915266 | 2945.469629 | 2779.32755 | 2418.81423 | 2539.815769 | 2623.204191 | 2752.235059 | 2829.077101 | 3014.087891 | 2839.503838 | 2462.044766 | 2633.792618 | 2321.2319 | 2526.476903 |
| **Cdo1** | 0 | 0 | 0 | 2.587828259 | 2.533452977 | 0.701412806 | 0 | 0 | 0.752414123 | 3.495607876 | 5.675224193 | 4.337640531 | 0.852084315 | 4.517773258 | 1.437131344 |
| **Atg12** | 549.4178889 | 484.0720872 | 467.5472928 | 497.7256351 | 557.359655 | 485.3776616 | 488.0170107 | 408.1420444 | 402.5415556 | 429.0858667 | 445.5050991 | 549.1452913 | 447.3442655 | 540.3256816 | 530.3014659 |
| **Ap3s1** | 1141.873513 | 1145.610864 | 1155.946071 | 1034.268694 | 1090.018143 | 1070.355942 | 1022.039322 | 1108.771919 | 1212.139151 | 1046.934559 | 1184.230115 | 1160.752606 | 1238.930594 | 1193.595695 | 1188.507621 |
| **Commd10** | 625.4206969 | 603.7036505 | 578.7561966 | 584.8491865 | 625.7628853 | 589.1867568 | 617.0122649 | 564.8618848 | 592.1499145 | 592.5055349 | 611.9783421 | 602.0645058 | 611.7965383 | 537.6150177 | 663.2361152 |
| **Eno1b** | 61.35166426 | 45.95119649 | 58.73709709 | 62.97048763 | 46.86888008 | 65.93280374 | 66.75278889 | 46.93214473 | 51.16416034 | 39.3255886 | 71.88617311 | 82.4151701 | 62.20215501 | 44.27417793 | 47.42533435 |
| **Dtwd2** | 86.07546927 | 106.9553711 | 71.2676778 | 89.71137964 | 91.20430718 | 95.39214158 | 98.32505391 | 87.15969736 | 90.28969471 | 117.1028638 | 74.7237852 | 116.2487662 | 92.02510604 | 86.74124655 | 84.07218362 |
| **Dmxl1** | 706.9176838 | 773.2477203 | 659.42181 | 743.5693197 | 659.3311373 | 669.8492295 | 800.131402 | 803.7129785 | 914.9355731 | 719.2213204 | 717.9158604 | 831.9594539 | 835.0426289 | 707.4832922 | 704.1943585 |
| **Tnfaip8** | 1186.74264 | 1161.456104 | 1177.091426 | 1224.042766 | 1301.561467 | 1341.802697 | 1245.751371 | 1216.045393 | 1216.653636 | 1294.248816 | 1298.680469 | 1299.557103 | 1267.901461 | 1367.981742 | 1333.657887 |
| **Hsd17b4** | 780.1734023 | 701.1518775 | 757.3169718 | 749.6075857 | 670.0983124 | 702.1142185 | 729.7703543 | 891.7107499 | 807.3403535 | 921.0926752 | 866.4175601 | 757.3520368 | 802.6634249 | 779.7676643 | 760.2424809 |
| **Srfbp1** | 471.583688 | 444.4589868 | 478.5115509 | 502.0386822 | 453.4880829 | 451.0084341 | 490.7232048 | 496.9778897 | 406.3036262 | 442.1943963 | 454.9638061 | 469.3327055 | 389.402532 | 409.3102572 | 379.4026748 |
| **Snx2** | 1052.135257 | 964.9751264 | 1112.089038 | 989.4130043 | 1062.783524 | 1115.246361 | 1109.539599 | 988.0892614 | 1118.8398 | 1075.773324 | 1079.238467 | 1121.713841 | 1114.526284 | 1110.468667 | 1100.842609 |
| **Ppic** | 168.4881526 | 198.8577641 | 213.8030334 | 186.3236346 | 133.0062813 | 150.1023404 | 167.7840369 | 220.4134654 | 213.6856108 | 181.7716095 | 182.5530449 | 157.8901153 | 140.593912 | 126.4976512 | 120.0004672 |
| **Cep120** | 705.0862908 | 618.7566286 | 664.903939 | 749.6075857 | 611.828894 | 549.9076397 | 654.8989829 | 700.6298749 | 633.5326912 | 597.7489467 | 585.4939625 | 638.5006862 | 601.5715265 | 589.1176328 | 637.367751 |
| **Csnk1g3** | 879.9843188 | 808.8995107 | 738.5211007 | 742.7067103 | 765.1027991 | 779.97104 | 786.6004313 | 895.0630459 | 835.1796761 | 834.5763803 | 824.7992493 | 764.2922616 | 773.6925582 | 767.1178992 | 786.8294108 |
| **Zfp608** | 849.7663349 | 736.0114059 | 794.1255526 | 747.8823668 | 418.6531045 | 454.5154981 | 545.7491524 | 733.3147614 | 718.5554871 | 727.0864381 | 812.5029302 | 488.4183238 | 569.1923225 | 524.9652526 | 532.4571629 |
| **Gramd3** | 1810.331944 | 1923.612157 | 2098.089108 | 2019.368651 | 1836.120045 | 1637.097489 | 1833.897565 | 1376.955603 | 1504.828245 | 1406.98217 | 1378.133608 | 1776.697562 | 1775.743713 | 1829.698169 | 1765.515856 |
| **Aldh7a1** | 398.3279695 | 391.3774322 | 400.1954215 | 391.6246765 | 319.8484384 | 299.503268 | 338.274268 | 388.8663421 | 483.8022808 | 452.6812199 | 415.2372368 | 363.4942765 | 380.0296046 | 442.7417793 | 399.5225136 |
| **Phax** | 760.0280797 | 784.3393884 | 703.2788424 | 790.1502284 | 771.4364315 | 745.6018125 | 776.6777194 | 863.2162334 | 831.4176055 | 805.7376153 | 766.155266 | 824.151701 | 743.8696071 | 770.7321178 | 702.0386615 |
| **Lmnb1** | 7640.571442 | 8131.777255 | 8379.04269 | 8235.332129 | 8731.545686 | 8921.269476 | 8657.115068 | 8088.2523 | 8263.011894 | 8001.446427 | 8023.821138 | 9443.910965 | 9379.744141 | 9601.171728 | 8613.44671 |
| **March3** | 39.37494871 | 45.15893448 | 44.64019379 | 46.58090866 | 46.23551683 | 33.66781467 | 15.33510015 | 69.56014308 | 63.95520042 | 44.56900041 | 25.53850887 | 45.11146153 | 45.1604687 | 35.23863141 | 34.49115225 |
| **C330018D20Rik** | 84.2440763 | 72.09584277 | 81.44877463 | 88.84877022 | 98.17130286 | 90.48225194 | 93.81473034 | 89.6739194 | 82.01313936 | 106.6160402 | 66.21094891 | 89.35539495 | 78.39175699 | 90.35546516 | 93.41353736 |
| **Megf10** | 0 | 0 | 0 | 1.725218839 | 1.266726489 | 2.104238417 | 1.80412943 | 27.65644243 | 0 | 0 | 0 | 1.735056213 | 2.556252945 | 0.903554652 | 0 |
| **Prrc1** | 1193.152515 | 1063.215615 | 1025.941296 | 1062.734805 | 1040.61581 | 1134.184507 | 1174.488259 | 853.9974193 | 806.5879394 | 855.5500276 | 863.579948 | 958.6185574 | 1084.703333 | 978.5496877 | 1021.800386 |
| **Slc12a2** | 777.4263128 | 705.1131876 | 770.6307138 | 787.5624001 | 692.8993892 | 735.7820332 | 798.3272726 | 833.883643 | 928.4790273 | 870.406361 | 851.2836289 | 772.1000146 | 874.2385074 | 707.4832922 | 695.5715705 |
| **Slc27a6** | 5.494178889 | 15.84524017 | 16.44638718 | 5.175656518 | 8.86708542 | 10.52119209 | 9.020647148 | 5.866518091 | 15.04828245 | 5.243411813 | 6.621094891 | 10.41033728 | 10.22501178 | 7.228437213 | 15.80844478 |
| **Isoc1** | 1612.541504 | 1604.330567 | 1659.518783 | 1592.376989 | 1487.770261 | 1637.097489 | 1671.525916 | 1390.364788 | 1822.347005 | 1483.885543 | 1665.678301 | 1598.8543 | 1534.603852 | 1558.631774 | 1564.317468 |
| **Gm4951** | 477.0778669 | 472.1881571 | 471.4630993 | 432.1673192 | 312.2480794 | 404.7151889 | 429.3828042 | 330.2011611 | 393.5125861 | 361.7954151 | 342.405193 | 369.5669733 | 365.5441712 | 351.4827595 | 377.9655435 |
| **Gm4841** | 136.4387758 | 116.4625153 | 144.8848395 | 121.6279282 | 105.1382985 | 155.7136429 | 130.7993836 | 112.3019177 | 124.9007443 | 142.4460209 | 102.1540355 | 106.7059571 | 139.7418277 | 111.1372221 | 99.16206273 |
| **F830016B08Rik** | 29.30228741 | 40.40536243 | 27.41064531 | 37.09220505 | 27.86798275 | 26.65368662 | 38.78878274 | 15.92340625 | 40.63036262 | 34.95607876 | 19.86328467 | 19.08561834 | 21.30210788 | 24.39597559 | 27.30549553 |
| **Iigp1** | 3195.780721 | 3534.28082 | 3429.463309 | 3393.505457 | 2480.883828 | 2621.179655 | 2855.936887 | 3626.346254 | 3850.855479 | 3597.854406 | 3409.863869 | 2711.025332 | 2621.011353 | 2714.278173 | 2488.392922 |
| **Smim3** | 653.8072878 | 581.5203142 | 633.5774872 | 624.5292198 | 452.2213564 | 460.1268005 | 453.7385515 | 637.7743239 | 708.7741035 | 699.9954771 | 849.3918875 | 497.961133 | 447.3442655 | 430.0920141 | 504.4331017 |
| **Dctn4** | 1092.425903 | 1018.848943 | 1022.025489 | 1087.750478 | 892.4088112 | 906.225345 | 1020.235192 | 914.3387482 | 981.1480158 | 940.3185185 | 874.9303964 | 859.7203533 | 879.3510132 | 923.4328539 | 900.362787 |
| **Rbm22** | 1888.166145 | 1775.459161 | 1968.867494 | 1874.450269 | 1917.823904 | 1905.738593 | 1946.655654 | 2024.786816 | 1983.363627 | 1917.34092 | 1764.048853 | 1891.211272 | 1793.637483 | 1915.535861 | 1872.582141 |
| **Synpo** | 284.7816058 | 362.0637379 | 335.193034 | 297.6002498 | 405.9858396 | 479.7663591 | 507.8624344 | 222.9276875 | 231.7435498 | 251.683767 | 303.6244943 | 482.3456271 | 504.4339146 | 515.0261514 | 458.4448987 |
| **Ndst1** | 749.0397219 | 740.764978 | 776.1128428 | 706.4771147 | 638.4301502 | 612.3333794 | 705.414607 | 688.8968387 | 814.8644948 | 613.4791822 | 704.6736706 | 691.4199007 | 679.1111992 | 634.2953654 | 645.2719734 |
| **Rps14** | 20493.28726 | 19568.87161 | 19126.36513 | 19062.80556 | 19876.8387 | 20815.82784 | 20199.03309 | 17444.51058 | 17388.29037 | 18104.62709 | 16947.16531 | 18982.38249 | 18166.4376 | 18701.77418 | 18602.22812 |
| **Cd74** | 2098.776336 | 2093.948488 | 2173.272592 | 2102.179156 | 1490.937077 | 1681.286495 | 1693.17547 | 2924.878306 | 2225.640975 | 2537.811318 | 2388.323514 | 1507.763849 | 1474.95795 | 1567.66732 | 1355.933423 |
| **Tcof1** | 7943.666978 | 7906.774845 | 7472.141911 | 7405.501868 | 7706.130593 | 7796.203335 | 8372.964683 | 7305.491172 | 7105.798974 | 7215.808557 | 7664.390272 | 7787.79981 | 7778.677713 | 7541.067122 | 8188.774398 |
| **Camk2a** | 9.156964816 | 2.376786026 | 3.915806472 | 11.21392246 | 2.533452977 | 0.701412806 | 0.902064715 | 1.676148026 | 0 | 1.747803938 | 2.837612096 | 3.470112425 | 5.112505891 | 0.903554652 | 2.155697016 |
| **Pdgfrb** | 0 | 6.338096068 | 6.265290356 | 2.587828259 | 1.266726489 | 1.402825611 | 0 | 7.542666117 | 0.752414123 | 1.747803938 | 2.837612096 | 6.072696744 | 0 | 1.807109303 | 2.874262688 |
| **Hmgxb3** | 1357.977882 | 1209.784087 | 1199.019942 | 1071.360899 | 988.0466611 | 1104.023756 | 1234.926595 | 1205.150431 | 1122.601871 | 1185.884972 | 1096.26414 | 1042.768784 | 1076.18249 | 958.6714853 | 1031.860305 |
| **Slc26a2** | 169.4038491 | 151.3220436 | 223.2009689 | 227.7288868 | 174.8082554 | 205.5139521 | 199.356302 | 211.1946513 | 261.0877005 | 202.7452568 | 183.4989156 | 214.2794423 | 200.2398141 | 154.5078454 | 192.5756001 |
| **Pde6a** | 0.915696482 | 0 | 0 | 0 | 0 | 0 | 0 | 5.866518091 | 0 | 0 | 0 | 0 | 0.852084315 | 0 | 1.437131344 |
| **Ppargc1b** | 122.7033285 | 187.766096 | 195.0071623 | 175.1097122 | 132.3729181 | 166.234835 | 167.7840369 | 167.6148026 | 165.531107 | 138.9504131 | 174.9860793 | 165.6978683 | 212.1689945 | 151.7971815 | 145.1502657 |
| **Csnk1a1** | 5467.623691 | 5649.620383 | 5922.265709 | 5841.59099 | 5526.72767 | 5541.862578 | 5614.450785 | 5831.318983 | 6038.123334 | 5828.926133 | 6048.843119 | 5905.263819 | 6051.502806 | 5941.775389 | 5814.633418 |
| **Pcyox1l** | 160.2468843 | 137.0613275 | 193.4408397 | 218.2401832 | 180.5085246 | 206.2153649 | 150.6448074 | 202.8139112 | 191.8656013 | 241.1969434 | 202.4163295 | 207.3392174 | 184.9022964 | 177.0967117 | 220.5996613 |
| **Grpel2** | 366.2785926 | 326.4119475 | 382.965873 | 282.9358896 | 385.0848525 | 387.1798688 | 341.8825269 | 414.0085624 | 334.0718704 | 362.6693171 | 342.405193 | 311.4425902 | 304.1941005 | 346.0614316 | 300.3604509 |
| **1500015A07Rik** | 25.63950148 | 15.05297816 | 14.8800646 | 11.21392246 | 20.90098706 | 23.14662259 | 16.23716487 | 16.76148026 | 14.29586833 | 27.09096104 | 25.53850887 | 37.30370857 | 10.22501178 | 28.91374885 | 25.14979852 |
| **Adrb2** | 9.156964816 | 15.05297816 | 16.44638718 | 14.66436013 | 17.73417084 | 20.34097137 | 18.0412943 | 10.89496217 | 8.276555348 | 9.612921658 | 11.35044839 | 13.01292159 | 14.48543336 | 27.10663955 | 18.68270747 |
| **Fbxo38** | 1152.86187 | 1067.969187 | 1047.869812 | 1042.032179 | 1109.019041 | 1066.147465 | 1116.756117 | 1055.135182 | 1187.309485 | 935.0751067 | 963.842242 | 1087.012717 | 1085.555418 | 998.42789 | 1062.040063 |
| **Spink13** | 0 | 0 | 0.783161294 | 0 | 0 | 0 | 1.80412943 | 0 | 0 | 0 | 0 | 0 | 0 | 3.614218606 | 2.874262688 |
| **Apcdd1** | 8.241268334 | 19.80655021 | 6.265290356 | 7.763484777 | 6.333632443 | 4.90988964 | 6.314453003 | 28.49451644 | 21.06759543 | 11.3607256 | 19.86328467 | 6.94022485 | 2.556252945 | 0.903554652 | 8.622788064 |
| **Napg** | 502.7173684 | 461.888751 | 489.4758091 | 486.5117127 | 419.2864677 | 480.4677719 | 483.5066871 | 439.1507828 | 386.740859 | 415.1034352 | 419.0207196 | 451.9821434 | 529.1443597 | 449.0666618 | 516.6487181 |
| **Txnl1** | 2556.624577 | 2527.315807 | 2663.531563 | 2493.803832 | 2553.087238 | 2429.693959 | 2503.229584 | 2802.5195 | 2650.754954 | 2795.612399 | 2808.290105 | 2504.553643 | 2524.725826 | 2660.968449 | 2522.165509 |
| **Wdr7** | 398.3279695 | 422.2756505 | 420.5576151 | 384.7238012 | 403.4523866 | 378.0615023 | 398.7126039 | 479.3783355 | 543.2429965 | 475.4026711 | 491.8527634 | 382.5798949 | 420.0775674 | 443.6453339 | 376.5284121 |
| **Onecut2** | 2.747089445 | 3.961310043 | 0.783161294 | 6.038265938 | 0.633363244 | 0 | 1.80412943 | 0 | 3.762070613 | 4.369509845 | 1.891741398 | 0 | 0 | 0 | 0 |
| **Fech** | 317.7466791 | 366.8173099 | 432.3050346 | 370.059441 | 333.1490665 | 349.3035772 | 311.2123266 | 332.7153832 | 427.3712216 | 407.2383175 | 369.8354432 | 370.4345014 | 351.0587378 | 432.8026781 | 352.8157449 |
| **Nars** | 7679.946391 | 6901.394356 | 7016.342037 | 6408.325379 | 6488.173074 | 7103.908896 | 6938.681786 | 5858.137351 | 6114.869574 | 5909.325114 | 6251.259448 | 6399.75484 | 6288.382246 | 6133.328975 | 6354.994803 |
| **Nedd4l** | 349.796056 | 280.460751 | 320.3129694 | 264.8210918 | 242.5781226 | 298.8018552 | 350.903174 | 263.9933141 | 243.0297616 | 274.4052182 | 256.3309594 | 290.6219156 | 268.4065593 | 251.1881931 | 254.3722479 |
| **Alpk2** | 24.723805 | 32.48274235 | 23.49483883 | 39.6800333 | 46.86888008 | 25.25086101 | 32.47432973 | 10.89496217 | 18.05793894 | 32.33437285 | 11.35044839 | 26.89337129 | 32.37920398 | 24.39597559 | 45.26963733 |
| **Malt1** | 3473.236755 | 3001.88075 | 2945.469629 | 2500.704708 | 2637.324549 | 2987.31714 | 3285.319691 | 1716.375579 | 1868.99668 | 1751.299546 | 2007.137623 | 2569.618251 | 2793.984469 | 2445.922442 | 2910.190971 |
| **Sec11c** | 1094.257295 | 1182.054917 | 1155.162909 | 1144.6827 | 1142.587293 | 1092.099738 | 1201.5502 | 1295.662424 | 1255.026756 | 1202.489109 | 1135.044839 | 1257.048226 | 1184.397198 | 1327.321783 | 1127.429539 |
| **Lman1** | 1742.570404 | 1591.654375 | 1626.626009 | 1530.26911 | 1435.834475 | 1411.242565 | 1621.912357 | 1574.741071 | 1565.773789 | 1607.979623 | 1732.83512 | 1457.447219 | 1506.485069 | 1316.479127 | 1397.610232 |
| **Pmaip1** | 394.6651836 | 337.5036156 | 382.1827117 | 330.3794077 | 285.0134599 | 374.5544383 | 374.3568566 | 442.5030789 | 406.3036262 | 404.6166116 | 452.126194 | 288.0193313 | 325.4962084 | 334.3152211 | 343.4743912 |
| **Gnal** | 74.17141501 | 58.62738863 | 88.49722628 | 59.52004996 | 59.53614496 | 69.43986777 | 67.65485361 | 39.38947861 | 45.14484735 | 62.04703979 | 60.53572472 | 52.91921448 | 65.61049227 | 62.34527096 | 56.76668809 |
| **Chmp1b** | 658.3857702 | 651.239371 | 621.0469065 | 615.0405162 | 635.263334 | 619.3475074 | 653.0948535 | 538.0435164 | 571.082319 | 579.3970054 | 575.0893849 | 610.7397868 | 705.525813 | 659.5948956 | 603.5951645 |
| **Mppe1** | 333.3135193 | 396.9232663 | 390.0143247 | 350.2194244 | 344.5496049 | 371.748787 | 384.2795685 | 329.3630871 | 341.5960117 | 322.4698265 | 307.4079771 | 403.4005694 | 365.5441712 | 383.1071723 | 357.8457046 |
| **Impa2** | 1457.788799 | 1350.806725 | 1290.649813 | 1219.729719 | 1189.456173 | 1488.397974 | 1364.823913 | 870.7588996 | 1010.492167 | 1045.186755 | 939.2496039 | 1381.104745 | 1457.064179 | 1316.479127 | 1419.885768 |
| **B430212C06Rik** | 9.156964816 | 13.46845414 | 13.31374201 | 7.763484777 | 8.233722176 | 11.22260489 | 10.82477658 | 4.190370065 | 3.762070613 | 6.117313782 | 1.891741398 | 14.74797781 | 15.33751767 | 12.64976512 | 12.9341821 |
| **Tubb6** | 831.4524053 | 571.2209081 | 541.1644545 | 561.5587322 | 447.1544505 | 482.5720103 | 442.913775 | 434.1223388 | 379.2167178 | 498.9980242 | 538.2004276 | 433.7640531 | 487.3922283 | 505.0870502 | 564.7926182 |
| **Afg3l2** | 2373.48528 | 2257.946724 | 2371.4124 | 2204.829677 | 2228.171893 | 2149.83025 | 2311.089799 | 2001.320743 | 2048.823656 | 2015.21794 | 2134.830167 | 2115.033523 | 2224.792147 | 2198.348467 | 2131.265783 |
| **Slmo1** | 13.73544722 | 4.753572051 | 10.18109683 | 10.35131304 | 6.333632443 | 4.208476834 | 6.314453003 | 10.89496217 | 20.31518131 | 7.86511772 | 3.783482795 | 3.470112425 | 5.964590206 | 8.131991864 | 7.18565672 |
| **Spire1** | 379.0983434 | 243.2244366 | 335.193034 | 288.1115462 | 195.7092425 | 240.5845924 | 272.4235439 | 324.3346431 | 369.4353342 | 326.8393364 | 347.1345464 | 234.2325887 | 293.9690887 | 211.4317885 | 249.3422882 |
| **Cep76** | 498.138886 | 482.4875632 | 545.080261 | 514.1152141 | 458.5549889 | 474.1550567 | 487.114946 | 536.3673684 | 573.3395614 | 579.3970054 | 631.8416268 | 487.5507957 | 516.363095 | 533.0972444 | 538.924254 |
| **Psmg2** | 689.5194506 | 667.8768732 | 689.1819391 | 589.1622336 | 777.1367007 | 718.9481259 | 644.9762711 | 829.6932729 | 706.5168611 | 741.9427716 | 726.4286967 | 650.6460797 | 665.4778501 | 674.9553247 | 658.2061555 |
| **Ptpn2** | 1402.84701 | 1302.478742 | 1355.652201 | 1294.776739 | 1331.329539 | 1342.50411 | 1297.16906 | 1188.388951 | 1310.705402 | 1237.445188 | 1130.315485 | 1226.684742 | 1338.624459 | 1364.367524 | 1424.197162 |
| **Seh1l** | 1372.629026 | 1454.593048 | 1625.842847 | 1510.429094 | 1317.395548 | 1409.138327 | 1477.582003 | 1624.187437 | 1566.526203 | 1597.492799 | 1539.877498 | 1484.34059 | 1541.420526 | 1373.40307 | 1421.322899 |
| **4930549G23Rik** | 7.325571853 | 11.88393013 | 6.265290356 | 12.07653188 | 11.4005384 | 3.507064029 | 2.706194144 | 1.676148026 | 4.514484735 | 5.243411813 | 5.675224193 | 4.337640531 | 5.112505891 | 10.84265582 | 6.467091048 |
| **Cep192** | 1986.145669 | 2056.712174 | 2039.352011 | 2167.737472 | 1889.322558 | 1820.867644 | 2084.671556 | 2323.141164 | 2213.602349 | 2021.335254 | 2087.536632 | 2071.657118 | 2175.371257 | 1976.977578 | 2083.121883 |
| **Ldlrad4** | 20.14532259 | 38.82083842 | 43.0738712 | 50.89395576 | 33.56825195 | 37.1748787 | 37.88671802 | 69.56014308 | 51.16416034 | 56.80362798 | 32.15960376 | 55.5217988 | 34.08337261 | 46.98484188 | 38.80254629 |
| **Fam210a** | 895.551159 | 880.2030914 | 903.7681338 | 859.158982 | 767.6362521 | 823.4586339 | 929.1266562 | 912.6626002 | 772.7293039 | 808.3593212 | 816.286413 | 792.9206891 | 852.9363995 | 762.6001259 | 754.4939556 |
| **Rnmt** | 1135.463637 | 1137.688244 | 1159.861877 | 1098.101791 | 1118.519489 | 1056.327685 | 1105.93134 | 1274.710574 | 1267.065382 | 1219.093247 | 1151.12464 | 1131.256651 | 1082.14708 | 1075.230035 | 1133.178065 |
| **Tcf4** | 106.2207919 | 110.9166812 | 108.8594199 | 107.8261775 | 62.70296118 | 89.07942633 | 79.3816949 | 147.5010263 | 124.9007443 | 110.9855501 | 105.9375183 | 83.2826982 | 97.13761193 | 75.89859073 | 75.44939556 |
| **Rab27b** | 8.241268334 | 10.29940611 | 8.614774239 | 4.313047098 | 6.333632443 | 4.208476834 | 3.608258859 | 11.73303618 | 20.31518131 | 15.73023544 | 11.35044839 | 3.470112425 | 14.48543336 | 9.939101167 | 9.341353736 |
| **4930503L19Rik** | 247.23805 | 320.8661134 | 304.6497436 | 259.6454353 | 291.3470924 | 293.1905528 | 268.815285 | 311.7635329 | 299.4608208 | 295.3788655 | 256.3309594 | 322.7204555 | 360.4316653 | 290.9445978 | 296.7676225 |
| **Stard6** | 23.80810852 | 22.18333624 | 15.66322589 | 19.84001665 | 13.93399137 | 13.32684331 | 9.922711863 | 16.76148026 | 16.5531107 | 11.3607256 | 25.53850887 | 7.807752957 | 17.0416863 | 8.131991864 | 20.83840449 |
| **Poli** | 251.8165324 | 255.9006287 | 219.2851625 | 245.8436846 | 228.0107679 | 250.4043716 | 248.0677966 | 191.918949 | 191.1131871 | 260.4227867 | 219.4420021 | 236.835173 | 235.175271 | 227.6957722 | 243.5937628 |
| **Mbd2** | 4084.922004 | 4281.383894 | 4441.307701 | 4335.474943 | 4118.127814 | 4308.077453 | 4192.796794 | 4256.577912 | 4414.413657 | 4409.709335 | 4560.042639 | 4151.989517 | 4128.348507 | 4449.103104 | 4072.111663 |
| **Mex3c** | 1227.033285 | 1057.669781 | 1235.045361 | 1083.437431 | 1058.983344 | 1123.663315 | 1096.008628 | 1287.281684 | 1299.41919 | 1257.544933 | 1225.848426 | 1193.718674 | 1165.651343 | 1191.788585 | 1188.507621 |
| **Smad4** | 1133.632244 | 1135.311458 | 1054.918264 | 1098.964401 | 966.5123108 | 1033.882476 | 1106.833405 | 936.9667466 | 968.3569758 | 931.5794989 | 1015.86513 | 985.5119287 | 1087.259586 | 1025.53453 | 928.3868482 |
| **Elac1** | 186.8020822 | 194.8964541 | 236.5147109 | 198.4001665 | 170.3747127 | 199.2012368 | 213.7893374 | 144.9868043 | 210.6759543 | 177.4020997 | 171.2025965 | 202.1340488 | 177.2335376 | 199.685578 | 176.0485896 |
| **Me2** | 2919.240383 | 3155.57958 | 3056.678532 | 2943.22334 | 2901.437022 | 3131.808178 | 3051.68493 | 2765.644243 | 2649.250126 | 2701.230986 | 2740.187414 | 3091.870171 | 3135.67028 | 2896.796213 | 2960.490568 |
| **Ska1** | 410.2320237 | 364.4405239 | 372.0016149 | 378.6855352 | 342.6495152 | 359.8247693 | 349.0990446 | 342.7722713 | 376.9594754 | 412.4817293 | 334.8382274 | 422.4861878 | 397.9233752 | 363.2289699 | 375.0912808 |
| **Cxxc1** | 1270.07102 | 1422.110305 | 1386.195491 | 1407.778573 | 1383.265325 | 1418.256693 | 1448.715932 | 1484.229077 | 1476.236509 | 1446.307759 | 1485.962868 | 1418.408454 | 1302.836918 | 1509.839823 | 1481.682416 |
| **Mbd1** | 611.6852497 | 667.0846112 | 635.9269711 | 709.9275524 | 673.2651287 | 620.7503331 | 661.2134359 | 608.4417335 | 650.838216 | 685.1391436 | 615.7618249 | 581.2438312 | 601.5715265 | 671.3411061 | 653.8947615 |
| **Cfap53** | 0 | 0 | 0 | 0 | 6.333632443 | 0 | 1.80412943 | 0 | 0 | 0 | 0 | 0.867528106 | 0 | 0 | 0 |
| **Myo5b** | 1.831392963 | 3.169048034 | 1.566322589 | 3.450437679 | 0.633363244 | 1.402825611 | 0 | 0 | 0 | 0 | 0 | 0 | 2.556252945 | 0 | 1.437131344 |
| **Acaa2** | 716.0746486 | 853.2661832 | 780.8118106 | 774.6232588 | 752.4355342 | 776.4639759 | 778.4818489 | 754.2666117 | 857.7520997 | 776.0249484 | 722.6452139 | 789.4505767 | 780.5092327 | 715.615284 | 832.0990481 |
| **Lipg** | 409.3163273 | 346.2184977 | 316.397163 | 347.6315961 | 226.1106782 | 260.2241509 | 278.7379969 | 372.9429358 | 420.5994945 | 384.5168663 | 438.8840042 | 210.8093298 | 229.2106808 | 243.9597559 | 291.0190971 |
| **Rpl17** | 21179.14392 | 19620.36864 | 19571.98391 | 18452.94071 | 20361.99494 | 21323.65071 | 22147.49288 | 17063.18691 | 17185.13856 | 17492.89571 | 16331.40348 | 19017.08362 | 18905.1947 | 18746.04836 | 19073.6072 |
| **BC031181** | 726.1473099 | 673.4227072 | 646.8912292 | 654.7205495 | 599.7949923 | 646.0011941 | 654.8989829 | 701.4679489 | 702.7547905 | 655.4264767 | 657.3801356 | 681.8770915 | 579.4173343 | 641.5238026 | 597.1280734 |
| **Dym** | 563.1533362 | 544.2839998 | 522.3685834 | 516.7030424 | 437.0206386 | 474.8564695 | 478.0942988 | 542.2338864 | 603.4361263 | 559.2972601 | 637.516851 | 464.995065 | 470.350542 | 488.8230665 | 436.8879286 |
| **Smad7** | 344.3018771 | 386.6238602 | 375.9174214 | 398.5255519 | 322.3818913 | 364.0332462 | 424.8724807 | 352.8291595 | 384.4836166 | 284.0181399 | 313.0832013 | 403.4005694 | 396.2192066 | 384.9142816 | 347.7857852 |
| **Ctif** | 32.96507334 | 29.31369431 | 22.71167754 | 20.70262607 | 27.2346195 | 22.44520978 | 22.55161787 | 23.46607237 | 5.266898858 | 33.20827482 | 21.75502607 | 22.55573076 | 12.78126473 | 16.26398373 | 18.68270747 |
| **Smad2** | 953.2400373 | 996.6656067 | 1012.627554 | 974.7486442 | 1003.247379 | 959.5327182 | 997.6835745 | 970.4897071 | 1023.283207 | 931.5794989 | 987.4890095 | 983.7768725 | 949.2219271 | 986.6816795 | 996.650587 |
| **Ier3ip1** | 551.2492819 | 564.0905501 | 549.7792287 | 552.932638 | 532.6584884 | 562.5330702 | 519.5892757 | 459.2645592 | 484.5546949 | 535.7019069 | 588.3315746 | 606.4021463 | 513.806842 | 591.8282968 | 542.5170823 |
| **Hdhd2** | 357.1216278 | 300.2673012 | 340.6751631 | 350.2194244 | 317.9483486 | 382.9713919 | 343.6866563 | 328.5250131 | 350.6249811 | 379.2734545 | 393.4822107 | 319.2503431 | 349.3545692 | 321.665456 | 339.1629972 |
| **Katnal2** | 2.747089445 | 0 | 2.349483883 | 2.587828259 | 6.966995687 | 0.701412806 | 6.314453003 | 5.028444078 | 1.504828245 | 3.495607876 | 5.675224193 | 0 | 1.70416863 | 3.614218606 | 5.029959704 |
| **Pias2** | 750.8711149 | 793.0542705 | 765.931746 | 694.4005828 | 630.8297913 | 724.5594283 | 754.1261016 | 838.0740131 | 835.9320902 | 827.5851646 | 688.5938687 | 729.5911374 | 685.9278737 | 707.4832922 | 744.4340362 |
| **8030462N17Rik** | 300.348446 | 366.0250479 | 335.9761953 | 359.708128 | 317.9483486 | 295.996204 | 368.0424036 | 377.9713799 | 366.4256777 | 296.2527675 | 368.8895725 | 344.4086582 | 341.6858104 | 315.3405734 | 302.5161479 |
| **Haus1** | 362.6158067 | 448.4202968 | 457.366196 | 460.6334301 | 414.852925 | 462.231039 | 402.3208628 | 466.8072253 | 416.8374239 | 510.3587498 | 425.6418144 | 472.8028179 | 501.0255773 | 442.7417793 | 487.1875256 |
| **Atp5a1** | 20753.34506 | 20652.68604 | 21062.33985 | 21240.89435 | 20209.3544 | 20733.06112 | 20110.63075 | 22571.84739 | 22372.28152 | 22315.96068 | 22732.1105 | 21513.82951 | 20651.11546 | 20863.0769 | 20214.68948 |
| **Pstpip2** | 13.73544722 | 15.05297816 | 7.831612945 | 6.038265938 | 8.86708542 | 18.23673295 | 11.72684129 | 8.380740131 | 8.276555348 | 3.495607876 | 12.29631908 | 16.48303402 | 17.0416863 | 15.36042908 | 12.9341821 |

| | | | | | | | | | | | | | | | |
|---|---|---|---|---|---|---|---|---|---|---|---|---|---|---|---|
| **Epg5** | 445.02849 | 415.1452925 | 436.220841 | 480.4734467 | 419.919831 | 368.9431358 | 442.0117102 | 403.1136003 | 445.4291606 | 383.6429643 | 406.7244005 | 366.0968608 | 400.4796281 | 452.6808804 | 413.8938271 |
| **Siglec15** | 0 | 0.792262009 | 0.783161294 | 0 | 0 | 0.701412806 | 0 | 6.704592104 | 0 | 0.873901969 | 0 | 0 | 0 | 5.421327909 | 1.437131344 |
| **Slc14a1** | 640.9875371 | 561.713764 | 501.2232285 | 535.6804496 | 527.5915825 | 551.3104653 | 709.9249305 | 222.9276875 | 303.2228914 | 291.0093556 | 340.5134516 | 538.734954 | 549.5943833 | 520.4474793 | 469.2233838 |
| **Setbp1** | 627.2520899 | 643.3167509 | 553.6950352 | 578.8109206 | 550.3926593 | 562.5330702 | 691.8836362 | 325.1727171 | 395.0174144 | 292.7571596 | 324.4336497 | 602.9320339 | 585.3819245 | 546.6505642 | 658.9247212 |
| **Pard6g** | 88.82255871 | 88.73334495 | 92.41303275 | 78.49745719 | 76.63695256 | 92.58649035 | 92.91266562 | 105.5973256 | 91.04210883 | 100.4987264 | 85.12836289 | 98.89820412 | 98.84178056 | 93.96968376 | 89.820709 |
| **Adnp2** | 505.4644578 | 521.3084016 | 526.2843899 | 535.6804496 | 498.4568732 | 502.9129817 | 581.831741 | 497.8159638 | 547.0050671 | 463.1680435 | 475.7729615 | 464.995065 | 501.0255773 | 440.93467 | 477.8461719 |
| **LOC105246506** | 0 | 0.792262009 | 1.566322589 | 3.450437679 | 0.633363244 | 0 | 1.80412943 | 0 | 3.762070613 | 4.369509845 | 0 | 1.735056213 | 0 | 2.710663955 | 0.718565672 |
| **Rbfa** | 412.9791132 | 360.4792139 | 375.1342601 | 397.6629425 | 326.1820708 | 342.990862 | 397.8105392 | 302.5447187 | 400.2843132 | 340.8217679 | 340.5134516 | 407.73821 | 308.4545221 | 293.6552618 | 360.7199673 |
| **Txnl4a** | 3756.186967 | 4010.430287 | 3825.742924 | 4005.095535 | 3820.447089 | 4000.157231 | 3922.17738 | 4616.111664 | 4195.461148 | 4335.427668 | 4217.637446 | 3883.923332 | 3886.356561 | 3816.614848 | 3822.769375 |
| **Pqlc1** | 119.0405426 | 94.27917901 | 99.4614844 | 127.6661941 | 101.9714823 | 98.1977928 | 107.3457011 | 96.3785115 | 85.02279585 | 110.9855501 | 103.0999062 | 99.76573222 | 115.0313825 | 84.0305826 | 94.13210303 |
| **Ctdp1** | 1308.530272 | 1399.926969 | 1349.38691 | 1280.112379 | 1447.868376 | 1388.797355 | 1320.622742 | 1219.397689 | 1428.082005 | 1397.369248 | 1364.891418 | 1362.886655 | 1321.582773 | 1315.575573 | 1311.382351 |
| **Nfatc1** | 3156.405772 | 3147.65696 | 3228.974017 | 3042.423423 | 3696.307894 | 3727.30765 | 4092.667611 | 2901.412233 | 2783.932254 | 2425.077964 | 2596.415068 | 3736.443554 | 3900.841995 | 3513.020485 | 3760.972727 |
| **Atp9b** | 893.719766 | 900.0096417 | 942.9261986 | 924.7172979 | 773.3365213 | 881.6758968 | 900.2605854 | 896.739194 | 902.8969471 | 945.5619304 | 941.1413453 | 1003.730019 | 923.6593976 | 1003.849218 | 987.3092333 |
| **Mbp** | 2465.054928 | 2453.63544 | 2446.595884 | 2410.130719 | 2415.647414 | 2397.42897 | 2594.33812 | 2383.482493 | 2449.860383 | 2271.271217 | 1980.653243 | 2217.40184 | 2505.127887 | 2454.054434 | 2382.045203 |
| **Zfp236** | 757.2809903 | 792.2620085 | 820.7530366 | 802.2267603 | 887.9752685 | 874.6617687 | 906.5750384 | 715.7152071 | 789.2824146 | 688.6347515 | 828.5827321 | 895.2890057 | 944.1094212 | 858.376919 | 998.0877184 |
| **Zfp516** | 118.1248461 | 110.9166812 | 105.7267748 | 86.26094197 | 98.17130286 | 63.12715251 | 105.5415716 | 52.79866282 | 51.91657446 | 50.6863142 | 66.21094891 | 72.00483282 | 80.94800994 | 66.86304422 | 100.5991941 |
| **Tshz1** | 941.335983 | 1003.003703 | 1069.015167 | 942.8320957 | 924.0769734 | 974.9637999 | 1139.307735 | 917.6910443 | 934.4983402 | 872.154165 | 907.0900001 | 1031.490918 | 1081.294996 | 986.6816795 | 1080.722771 |
| **Zadh2** | 779.2577058 | 808.8995107 | 848.1636819 | 878.9989986 | 696.0662055 | 773.6583247 | 842.5284436 | 758.4569818 | 794.5493134 | 694.7520653 | 790.7479042 | 725.2534968 | 803.5155092 | 765.3107899 | 753.7753899 |
| **Zfp407** | 437.7029182 | 484.8643492 | 415.0754861 | 433.0299287 | 451.5879932 | 394.1939968 | 515.9810169 | 434.1223388 | 505.6222904 | 361.7954151 | 476.7188322 | 489.2858519 | 487.3922283 | 473.4626374 | 434.0136659 |
| **Cndp2** | 5632.449058 | 5674.180505 | 5732.740676 | 5518.975067 | 4643.185944 | 4733.835026 | 4609.550693 | 6503.454341 | 6341.346225 | 6412.692648 | 6392.194182 | 4491.193006 | 4449.584294 | 4442.778222 | 4578.700462 |
| **Cyb5a** | 1443.137655 | 1286.633502 | 1386.978653 | 1312.028927 | 1135.620297 | 1209.235677 | 1183.508906 | 1553.78922 | 1469.464781 | 1640.313996 | 1524.743566 | 1274.398788 | 1094.928345 | 1158.357063 | 1220.843077 |
| **Fbxo15** | 0 | 0.792262009 | 2.349483883 | 0 | 0 | 0 | 0.902064715 | 0 | 0 | 0.873901969 | 0 | 0 | 0 | 0 | 0 |
| **Timm21** | 289.3600882 | 267.7845589 | 333.6267115 | 266.5463107 | 275.5130113 | 285.4750119 | 264.3049614 | 283.2690164 | 337.0815269 | 278.7747281 | 329.1630032 | 340.9385458 | 253.9211259 | 272.8735048 | 269.462127 |
| **Socs6** | 265.5519797 | 343.0494497 | 347.7236148 | 286.3863273 | 280.5799172 | 291.0863144 | 290.4648382 | 349.4768634 | 342.3484258 | 369.6605328 | 349.0262878 | 353.0839393 | 297.377426 | 334.3152211 | 290.3005315 |
| **Rttn** | 335.1449123 | 362.0637379 | 364.9531632 | 338.1428925 | 351.5166006 | 338.7823852 | 350.0011093 | 300.0304967 | 370.9401624 | 321.5959246 | 307.4079771 | 386.9175354 | 366.3962555 | 359.6147513 | 382.2769375 |
| **Cd226** | 769.1850445 | 880.9953535 | 852.8626497 | 907.4651095 | 981.7130286 | 955.3242414 | 987.7608627 | 1022.450296 | 976.6335311 | 853.8022236 | 788.8561628 | 1040.166199 | 965.4115291 | 997.5243353 | 939.8838989 |
| **Tmx3** | 1067.702098 | 1143.234078 | 1121.486974 | 1066.185243 | 1019.714823 | 1066.848877 | 1155.5449 | 1112.962289 | 912.6783307 | 1077.521128 | 1156.799865 | 1182.440809 | 1243.191016 | 1142.996634 | 1060.602932 |
| **Ighmbp2** | 467.9209021 | 465.057799 | 439.3534862 | 406.2890367 | 393.3185747 | 411.0279041 | 412.2435747 | 444.1792269 | 410.0656968 | 439.5726904 | 388.7528572 | 380.8448387 | 387.6983634 | 422.8635769 | 420.3609181 |
| **Mrpl21** | 773.7635269 | 850.8893971 | 802.7403268 | 728.0423502 | 775.8699742 | 790.492232 | 810.0541139 | 927.7479325 | 811.8548383 | 886.1365965 | 824.7992493 | 766.894846 | 840.1551347 | 821.3311783 | 727.9070257 |
| **Cpt1a** | 695.929326 | 739.9727159 | 745.5695524 | 744.4319292 | 750.5354445 | 785.5823424 | 883.1213558 | 692.2491348 | 764.4527485 | 745.4383795 | 814.3946716 | 1074.867324 | 928.7719035 | 1028.245193 | 1035.453133 |
| **Mtl5** | 7.325571853 | 16.63750218 | 10.96425812 | 10.35131304 | 4.43354271 | 3.507064029 | 9.020647148 | 10.89496217 | 3.00965649 | 1.747803938 | 14.18806048 | 2.602584319 | 2.556252945 | 4.517773258 | 3.59282836 |
| **Ppp6r3** | 2816.682377 | 2772.91703 | 2859.321886 | 2731.884032 | 2642.391455 | 2600.137271 | 2860.447211 | 3011.199929 | 2741.797063 | 2758.034614 | 2712.757164 | 2894.941291 | 2955.028405 | 2875.110901 | 2875.699819 |
| **Lrp5** | 271.0461585 | 280.460751 | 293.6854854 | 362.2959563 | 335.6825195 | 303.0103321 | 305.7999383 | 325.1727171 | 333.3194563 | 307.6134931 | 305.5162357 | 290.6219156 | 328.0524613 | 260.2237397 | 331.2587748 |
| **1810055G02Rik** | 302.1798389 | 244.0166986 | 288.2033564 | 242.3932469 | 235.6111269 | 249.7029588 | 224.614114 | 274.0502023 | 247.5442463 | 237.7013355 | 211.8750365 | 242.9078698 | 297.377426 | 219.5637803 | 229.2224494 |
| **Suv420h1** | 659.3014667 | 713.0358077 | 712.676778 | 698.7136299 | 730.9011839 | 701.4128057 | 770.3632664 | 600.8990674 | 680.1823668 | 533.080201 | 712.2406362 | 733.0612498 | 762.6154621 | 732.7828224 | 757.3682183 |
| **Chka** | 141.0172582 | 203.6113362 | 187.9587107 | 203.575823 | 202.0428749 | 178.1588527 | 217.3975963 | 238.0130197 | 200.1421566 | 271.7835123 | 224.1713556 | 238.5702292 | 210.4648258 | 219.5637803 | 226.3481867 |
| **Tcirg1** | 595.202713 | 800.1846286 | 690.7482617 | 681.4614415 | 656.1643211 | 601.8121873 | 581.831741 | 709.8486891 | 654.6002866 | 766.4120267 | 637.516851 | 661.9239451 | 623.7257187 | 646.0415759 | 666.1103779 |
| **Ndufs8** | 1262.745448 | 1346.845414 | 1385.41233 | 1324.968069 | 1300.928104 | 1341.802697 | 1215.983236 | 1429.754266 | 1467.207539 | 1391.251934 | 1263.683254 | 1273.53126 | 1224.445161 | 1432.134123 | 1348.029201 |
| **Aldh3b1** | 0 | 0 | 3.132645178 | 1.725218839 | 2.533452977 | 4.90988964 | 4.510323574 | 0 | 0 | 2.621705907 | 0 | 0 | 0.852084315 | 2.710663955 | 0.718565672 |
| **Unc93b1** | 640.9875371 | 736.8036679 | 721.2915522 | 744.4319292 | 561.7931977 | 550.6090525 | 630.5432356 | 451.721893 | 531.2043705 | 578.5231034 | 468.2059959 | 618.5475398 | 540.2214558 | 582.7927503 | 584.912457 |
| **Acy3** | 1.831392963 | 3.169048034 | 1.566322589 | 2.587828259 | 0 | 1.402825611 | 3.608258859 | 6.704592104 | 0.752414123 | 0 | 0 | 0 | 0 | 6.324882561 | 0 |
| **Nudt8** | 96.14813056 | 117.2547773 | 111.9920651 | 122.4905376 | 107.6717515 | 142.3867996 | 114.5622188 | 112.3019177 | 165.531107 | 130.2113934 | 100.2622941 | 126.6591035 | 118.4397198 | 109.3301128 | 132.9346493 |
| **Doc2g** | 1.831392963 | 14.26071615 | 6.265290356 | 6.038265938 | 6.333632443 | 6.312715251 | 11.72684129 | 5.028444078 | 17.30552482 | 11.3607256 | 11.35044839 | 9.542809169 | 11.0770961 | 29.8173035 | 18.68270747 |
| **Ndufv1** | 1941.276541 | 1970.355615 | 1914.046204 | 1931.382491 | 1930.491169 | 1915.558372 | 2020.624961 | 2034.00563 | 2090.206433 | 2137.564216 | 2086.590761 | 1792.313068 | 1917.189709 | 2001.373553 | 1852.462302 |
| **Gstp1** | 1392.774348 | 1396.757921 | 1418.305104 | 1423.305542 | 1378.831783 | 1361.442256 | 1291.756672 | 1465.791449 | 1363.37439 | 1408.729974 | 1485.016997 | 1341.198452 | 1399.122445 | 1367.981742 | 1276.172633 |
| **Gstp2** | 1.831392963 | 0 | 0 | 0 | 0.633363244 | 0 | 0 | 2.514222039 | 0 | 0 | 0.945870699 | 0.867528106 | 0.852084315 | 4.517773258 | 1.437131344 |
| **BC021614** | 190.4648682 | 179.8434759 | 248.2621304 | 225.1410585 | 331.2489768 | 360.5261821 | 352.7073035 | 146.6629523 | 139.1966127 | 191.3845312 | 202.4163295 | 287.1518032 | 292.2649201 | 285.5232699 | 281.6777434 |
| **Cdk2ap2** | 1059.460829 | 1163.83289 | 1213.116845 | 1216.279282 | 1280.66048 | 1229.576648 | 1269.205054 | 1052.62096 | 1032.312176 | 1047.808461 | 1122.748519 | 1356.813958 | 1377.820338 | 1481.829629 | 1350.184898 |
| **Pitpnm1** | 1196.815301 | 1307.232314 | 1286.734007 | 1285.288035 | 1253.42586 | 1233.785125 | 1279.127766 | 1236.159169 | 1166.994304 | 1224.336658 | 1200.309917 | 1325.582946 | 1336.92029 | 1271.301395 | 1459.40688 |
| **Aip** | 1584.154913 | 1649.489502 | 1632.891299 | 1674.324884 | 1674.612418 | 1756.337666 | 1636.345393 | 1590.664477 | 1640.262787 | 1469.903112 | 1604.196705 | 1593.649131 | 1566.983056 | 1641.758802 | 1651.263914 |
| **Tmem134** | 959.6499127 | 934.86917 | 1006.362263 | 1073.086118 | 993.7469303 | 1104.023756 | 997.6835745 | 952.0520788 | 932.993512 | 880.0192827 | 841.8249219 | 1016.742941 | 1146.053404 | 1039.991404 | 988.027799 |
| **Cabp4** | 0.915696482 | 3.169048034 | 3.132645178 | 1.725218839 | 4.43354271 | 1.402825611 | 9.020647148 | 5.028444078 | 5.266898858 | 0 | 3.783482795 | 6.072696744 | 12.78126473 | 13.55331977 | 2.155697016 |
| **Gpr152** | 1.831392963 | 0 | 0 | 4.313047098 | 1.266726489 | 0.701412806 | 1.80412943 | 0 | 0 | 0 | 0 | 0 | 3.408337261 | 1.807109303 | 0 |
| **Coro1b** | 2423.848587 | 2417.191388 | 2332.254335 | 2415.306375 | 2316.209384 | 2374.282347 | 2342.662064 | 2274.532871 | 2126.32231 | 2366.526532 | 2219.95853 | 2272.923638 | 2361.977722 | 2261.597293 | 2305.877241 |
| **Ptprcap** | 5523.481177 | 6358.69488 | 6270.772485 | 6582.572481 | 6905.559452 | 7011.322406 | 6698.732572 | 5972.115417 | 6033.608849 | 6206.451783 | 5783.053452 | 6706.85979 | 6611.322201 | 6648.355126 | 6543.259009 |
| **Rps6kb2** | 228.9241204 | 291.5524191 | 231.8157432 | 233.7671527 | 215.3435031 | 232.8690515 | 211.0831433 | 236.3368717 | 241.5249333 | 255.1793749 | 271.4648905 | 194.3262958 | 204.5002356 | 273.7770594 | 296.0490568 |
| **Carns1** | 82.41268334 | 131.5154934 | 121.3900006 | 104.3757398 | 130.4728283 | 115.0317001 | 107.3457011 | 79.61703124 | 80.50831112 | 106.6160402 | 144.7182169 | 128.3941597 | 126.960563 | 118.3656594 | 145.1502657 |
| **Tbc1d10c** | 1488.922479 | 1876.868698 | 1659.518783 | 1776.975404 | 1855.754306 | 1639.201727 | 1777.067488 | 1564.684182 | 1738.076623 | 1772.273193 | 1660.003076 | 1807.061045 | 1849.022964 | 1992.338007 | 1966.714244 |
| **Ppp1ca** | 8783.360651 | 9074.569045 | 9228.772694 | 8997.016247 | 9043.160402 | 9459.954511 | 9272.323203 | 8820.728987 | 9013.921189 | 8483.840314 | 8689.71411 | 9141.143656 | 8956.258237 | 9461.120757 | 9056.801729 |
| **Rad9a** | 592.4556236 | 735.2191439 | 635.9269711 | 597.7883278 | 623.2294324 | 627.7644611 | 637.7597533 | 667.9449884 | 679.4299527 | 670.2828101 | 636.5709803 | 618.5475398 | 617.7611285 | 640.620248 | 746.5897332 |
| **Clcf1** | 120.8719356 | 160.0369257 | 153.4996137 | 126.8035847 | 152.6405419 | 140.9839739 | 148.8406779 | 125.711102 | 87.28003822 | 95.25531461 | 92.69532848 | 135.3343846 | 181.4939591 | 174.3860478 | 132.9346493 |
| **Pold4** | 11.90405426 | 15.84524017 | 9.397935534 | 10.35131304 | 8.233722176 | 15.43108173 | 10.82477658 | 11.73303618 | 6.019312981 | 11.3607256 | 7.56696559 | 13.01292159 | 9.372927467 | 14.45687443 | 20.83840449 |
| **Ssh3** | 107.1364883 | 118.0470393 | 103.3772909 | 112.1392246 | 89.93758069 | 82.06529827 | 101.0312481 | 107.2734737 | 88.78486646 | 113.607256 | 135.2595099 | 96.2956198 | 90.32093741 | 102.1016756 | 120.0004672 |
| **Ankrd13d** | 52.19469945 | 65.75774671 | 51.68864544 | 56.06961228 | 43.06870061 | 44.18900676 | 27.06194144 | 61.17940295 | 52.66898858 | 69.03825554 | 63.37333682 | 63.32955176 | 70.72299816 | 56.92394305 | 51.01816271 |
| **Adrbk1** | 8578.244639 | 8722.012452 | 9042.380306 | 8903.85443 | 8393.963076 | 8577.577201 | 8473.093866 | 9735.067736 | 9743.010473 | 9718.663796 | 9421.81803 | 8589.39578 | 8487.611863 | 8466.307085 | 8452.487999 |
| **Kdm2a** | 2186.683198 | 2214.372314 | 2154.476721 | 2191.890535 | 2173.069291 | 2091.612987 | 2286.734052 | 1770.85039 | 1859.967711 | 1701.487133 | 1807.558905 | 2076.862286 | 2208.602545 | 2016.733982 | 2124.798692 |
| **Rhod** | 10.98835778 | 7.922620085 | 1.566322589 | 8.626094197 | 3.166816221 | 1.402825611 | 4.510323574 | 30.17066447 | 23.3248378 | 12.23462756 | 7.56696559 | 1.735056213 | 3.408337261 | 4.517773258 | 2.155697016 |
| **A930001C03Rik** | 0 | 0 | 0.783161294 | 0 | 0 | 0 | 0.902064715 | 10.05688816 | 0.752414123 | 1.747803938 | 0 | 0 | 0 | 0 | 0 |
| **Syt12** | 3.662785926 | 5.54583406 | 9.397935534 | 13.80175071 | 3.800179466 | 7.014128057 | 2.706194144 | 14.24725822 | 9.028969471 | 6.991215751 | 17.02567258 | 2.602584319 | 1.70416863 | 7.228437213 | 10.77848508 |
| **Pcx** | 400.1593624 | 408.8071964 | 374.3510988 | 369.1968316 | 329.348887 | 321.9484778 | 308.5061325 | 385.514046 | 356.6442941 | 407.2383175 | 408.6161419 | 317.5152869 | 297.377426 | 268.3557315 | 308.9832389 |
| **Lrfn4** | 192.2962611 | 160.8291877 | 156.6322589 | 130.2540224 | 157.7074478 | 180.9645039 | 195.7480431 | 153.3675444 | 155.7497234 | 168.66308 | 170.2567258 | 185.6510147 | 162.7481042 | 148.1829629 | 221.318227 |
| **Rce1** | 596.1184095 | 625.8869867 | 626.5290356 | 489.9621504 | 594.7280864 | 600.4093617 | 552.9656702 | 507.8728519 | 583.8733591 | 514.7282597 | 491.8527634 | 542.2050664 | 680.8153678 | 623.4527096 | 588.5052853 |
| **Gm960** | 0 | 0.792262009 | 3.132645178 | 0 | 1.900089733 | 4.208476834 | 0 | 2.514222039 | 0 | 0.873901969 | 0 | 1.735056213 | 2.556252945 | 0 | 0 |
| **Sptbn2** | 17.39823315 | 17.42976419 | 12.53058071 | 14.66436013 | 13.30062813 | 7.715540863 | 10.82477658 | 17.59955427 | 27.08690841 | 9.612921658 | 9.458706988 | 10.41033728 | 28.97086672 | 18.97464768 | 12.21561642 |
| **Rbm4b** | 130.9445969 | 141.8148995 | 164.4638718 | 138.8801166 | 149.4737256 | 139.5811483 | 120.8766718 | 194.433171 | 171.5504199 | 155.5545505 | 149.4475704 | 163.9628121 | 132.0730689 | 131.9189791 | 155.2101851 |
| **Rbm4** | 1.831392963 | 3.961310043 | 2.349483883 | 1.725218839 | 3.166816221 | 2.104238417 | 0.902064715 | 6.704592104 | 1.504828245 | 0 | 1.891741398 | 5.205168638 | 0.852084315 | 2.710663955 | 6.467091048 |
| **Rbm14** | 1779.198264 | 1812.695475 | 1671.266202 | 1877.038097 | 1791.784618 | 1668.661065 | 1758.124129 | 1764.983871 | 1957.029133 | 1861.411194 | 1879.445078 | 1669.124076 | 1886.514674 | 1748.378251 | 1804.318402 |
| **Ccs** | 431.2930428 | 411.9762444 | 449.534583 | 375.2350975 | 383.818126 | 425.0561603 | 384.2795685 | 378.8094539 | 340.0911834 | 367.0388269 | 409.5620126 | 377.3747262 | 375.769183 | 449.0666618 | 372.2170181 |
| **Ccdc87** | 0 | 0 | 0 | 0.86260942 | 0.633363244 | 0 | 0 | 0.838074013 | 0 | 0.873901969 | 0 | 0 | 2.556252945 | 1.807109303 | 3.59282836 |
| **Ctsf** | 8.241268334 | 1.584524017 | 0.783161294 | 6.900875357 | 1.900089733 | 0 | 9.020647148 | 1.676148026 | 0.752414123 | 9.612921658 | 12.29631908 | 2.602584319 | 3.408337261 | 3.614218606 | 2.155697016 |
| **Actn3** | 30.21798389 | 29.31369431 | 18.01270977 | 35.36698621 | 29.13470924 | 11.9240177 | 18.0412943 | 39.38947861 | 33.10622139 | 34.95607876 | 31.21373306 | 20.82067455 | 34.93545692 | 26.2030849 | 29.46119255 |
| **Zdhhc24** | 163.9096702 | 88.73334495 | 95.54567793 | 79.36006661 | 91.20430718 | 93.98931597 | 109.1498305 | 102.2450296 | 104.585563 | 98.75092249 | 106.883389 | 93.69303548 | 96.28552761 | 117.4621047 | 111.3776792 |
| **Bbs1** | 14.65114371 | 30.89821833 | 29.76012919 | 27.60350143 | 31.03479897 | 20.34097137 | 10.82477658 | 23.46607237 | 39.12553437 | 20.09974528 | 21.75502607 | 24.29078698 | 18.74585493 | 5.421327909 | 20.11983882 |
| **Dpp3** | 2136.319891 | 2006.799668 | 2087.12485 | 2170.3253 | 2065.39754 | 2013.054752 | 2117.145886 | 2222.572283 | 2273.043064 | 2238.936844 | 2314.5456 | 2147.132063 | 1992.173129 | 2188.409366 | 2086.714711 |
| **Peli3** | 1.831392963 | 0 | 7.831612945 | 0 | 0.633363244 | 1.402825611 | 0.902064715 | 0 | 1.504828245 | 0 | 0 | 0.867528106 | 0 | 0 | 1.437131344 |
| **Mrpl11** | 734.3885782 | 701.9441395 | 808.2224559 | 745.2945386 | 692.266026 | 699.3085673 | 695.4918951 | 793.6560904 | 839.6941608 | 880.0192827 | 906.1441294 | 740.0014747 | 696.1528855 | 753.5645794 | 634.4934883 |
| **Npas4** | 13.73544722 | 18.2220262 | 13.31374201 | 18.11479781 | 10.76717515 | 18.23673295 | 9.922711863 | 28.49451644 | 12.03862596 | 24.46925513 | 16.07980188 | 19.08561834 | 10.22501178 | 30.72085815 | 12.9341821 |
| **Slc29a2** | 123.619025 | 99.03275106 | 118.2573555 | 129.3914129 | 127.9393753 | 112.2260489 | 97.4229892 | 118.1684358 | 83.51796761 | 106.6160402 | 90.80358708 | 95.42809169 | 118.4397198 | 103.9087849 | 122.8747299 |
| **B4gat1** | 183.1392963 | 228.1714584 | 281.938066 | 173.3844933 | 233.0776739 | 239.1817667 | 243.557473 | 205.3281332 | 274.6311547 | 238.5752375 | 188.2282691 | 263.7285443 | 214.7252474 | 212.3353431 | 240.7195001 |
| **Brms1** | 815.8855651 | 853.2661832 | 827.018327 | 884.1746551 | 817.0385851 | 796.8049473 | 775.7756547 | 1071.058589 | 842.7038173 | 937.6968126 | 961.9505006 | 812.0063075 | 823.1134484 | 888.1942225 | 820.6019974 |
| **Rin1** | 8.241268334 | 3.961310043 | 7.831612945 | 1.725218839 | 6.333632443 | 4.90988964 | 3.608258859 | 0.838074013 | 8.276555348 | 3.495607876 | 7.56696559 | 11.27786538 | 5.964590206 | 2.710663955 | 13.65274777 |
| **Cd248** | 2.747089445 | 3.961310043 | 3.132645178 | 0 | 3.800179466 | 3.507064029 | 5.412388289 | 4.190370065 | 0 | 5.243411813 | 8.512836289 | 1.735056213 | 11.92918041 | 6.324882561 | 2.155697016 |
| **Tmem151a** | 5.494178889 | 0 | 0 | 0 | 1.900089733 | 0 | 0 | 0 | 0 | 0 | 0 | 0 | 4.260421576 | 0.903554652 | 2.155697016 |
| **Yif1a** | 567.7318186 | 480.9030392 | 528.6338738 | 557.2456851 | 593.4613599 | 535.8793836 | 486.2128813 | 496.1398157 | 446.1815747 | 490.2590046 | 443.6133577 | 535.2648416 | 546.186046 | 533.0972444 | 556.8883958 |
| **Cnih2** | 76.91850445 | 49.12024453 | 61.08658097 | 90.57398906 | 72.20340985 | 67.33562935 | 67.65485361 | 59.50325493 | 59.44071568 | 46.31680435 | 52.96875913 | 72.87236093 | 74.13133542 | 64.15238026 | 66.82660749 |
| **Rab1b** | 2148.223946 | 2290.429467 | 2567.985885 | 2344.572403 | 2396.013153 | 2355.344202 | 2503.229584 | 2466.45182 | 2262.509267 | 2396.239199 | 2481.018843 | 2547.06252 | 2556.252945 | 2510.074822 | 2497.01571 |
| **Klc2** | 244.4909606 | 245.6012226 | 245.9126465 | 211.3393078 | 232.4443106 | 242.6888308 | 235.4388906 | 188.5666529 | 237.7628627 | 266.5401005 | 257.2768301 | 203.869105 | 222.3940063 | 228.5993268 | 231.3781464 |
| **Pacs1** | 1155.60896 | 1094.906096 | 1235.045361 | 1150.720966 | 1060.250071 | 1191.700357 | 1278.225701 | 1108.771919 | 1065.418398 | 980.5180091 | 972.3550783 | 1113.906088 | 1128.159633 | 1056.255388 | 1215.813117 |
| **Sf3b2** | 7043.537336 | 7113.720574 | 7085.260231 | 7226.079108 | 7252.64251 | 6766.529337 | 7382.497626 | 7716.985512 | 7655.061283 | 7436.031853 | 7304.959407 | 7484.164973 | 7115.756116 | 7428.122791 | 7231.644923 |
| **Gal3st3** | 0 | 0 | 0 | 3.450437679 | 0 | 0.701412806 | 0 | 0 | 0 | 0.873901969 | 0 | 0 | 2.556252945 | 1.807109303 | 0 |
| **Cst6** | 3.662785926 | 6.338096068 | 9.397935534 | 6.900875357 | 6.966995687 | 2.805651223 | 3.608258859 | 12.5711102 | 11.28621184 | 3.495607876 | 2.837612096 | 2.602584319 | 5.112505891 | 0.903554652 | 2.155697016 |
| **Banf1** | 4377.029182 | 4458.850584 | 4362.991572 | 4174.166982 | 4377.806744 | 4545.154981 | 4059.291217 | 4580.074481 | 4254.901863 | 4249.785275 | 4081.432065 | 4041.813447 | 3865.054454 | 4192.493583 | 4059.177481 |
| **Eif1ad** | 1799.343586 | 1698.609746 | 1789.523558 | 1700.203166 | 1606.842551 | 1602.728261 | 1679.644499 | 1784.259574 | 1804.289066 | 1726.830291 | 1750.806663 | 1613.602278 | 1708.429052 | 1622.784154 | 1572.22169 |
| **Sart1** | 2497.104305 | 2460.765798 | 2486.53711 | 2409.268109 | 2579.055131 | 2440.215151 | 2544.72456 | 2314.760424 | 2175.981643 | 2232.819531 | 2348.596945 | 2462.912294 | 2423.327792 | 2417.008693 | 2456.776032 |
| **D330050I16Rik** | 4.578482408 | 6.338096068 | 15.66322589 | 12.07653188 | 5.700269198 | 4.208476834 | 8.118582433 | 14.24725822 | 10.53379772 | 22.72145119 | 18.91741398 | 6.072696744 | 11.0770961 | 12.64976512 | 18.68270747 |
| **4930481A15Rik** | 17.39823315 | 31.69048034 | 37.59174214 | 32.77915795 | 34.20161519 | 25.25086101 | 32.47432973 | 20.11377631 | 26.33449429 | 19.22584332 | 19.86328467 | 26.89337129 | 27.26669809 | 33.43152211 | 41.67680897 |
| **Drap1** | 1670.230382 | 1699.402008 | 1667.350396 | 1622.568318 | 1728.448294 | 1728.281153 | 1740.082835 | 1600.721365 | 1635.748302 | 1557.293309 | 1555.957299 | 1649.17093 | 1641.966475 | 1676.997433 | 1442.879869 |
| **AI837181** | 631.8305723 | 619.5488907 | 613.9984549 | 638.3309705 | 632.729881 | 613.0347922 | 626.0329121 | 601.7371414 | 576.3492179 | 535.7019069 | 513.6077894 | 651.5136078 | 531.7006127 | 580.0820863 | 554.7326988 |
| **Fosl1** | 10.98835778 | 4.753572051 | 3.915806472 | 2.587828259 | 4.43354271 | 1.402825611 | 3.608258859 | 0.838074013 | 15.80069657 | 23.59535316 | 9.458706988 | 8.675281063 | 1.70416863 | 8.131991864 | 4.311394032 |
| **Ccdc85b** | 543.92371 | 557.752454 | 547.4297448 | 606.414422 | 597.2615394 | 634.7785892 | 611.5998766 | 595.8706233 | 640.3044183 | 647.561359 | 546.7132639 | 667.1291137 | 669.7382717 | 554.7825561 | 619.4036092 |
| **Fibp** | 353.4588419 | 293.1369431 | 367.3026471 | 284.6611085 | 319.8484384 | 312.8301113 | 257.9905084 | 313.4396809 | 339.3387693 | 343.4434738 | 309.2997185 | 289.7543875 | 319.5316182 | 383.1071723 | 354.971442 |
| **Ctsw** | 1207.803659 | 1502.92103 | 1352.519556 | 1328.418506 | 1535.272504 | 1540.302521 | 1540.726533 | 1197.607765 | 1088.743235 | 1149.181089 | 1258.008029 | 1491.280815 | 1618.960199 | 1641.758802 | 1539.886235 |
| **Efemp2** | 27.47089445 | 19.80655021 | 16.44638718 | 29.32872027 | 10.13381191 | 11.22260489 | 17.13922958 | 17.59955427 | 5.266898858 | 13.9824315 | 25.53850887 | 10.41033728 | 4.260421576 | 13.55331977 | 9.341353736 |
| **Mus81** | 199.621833 | 215.4952663 | 221.6346463 | 257.9202165 | 229.9108577 | 258.1199125 | 245.3616024 | 285.7832385 | 198.6373284 | 225.466708 | 229.8465798 | 240.3052854 | 194.2752239 | 225.8886629 | 266.5878643 |
| **Cfl1** | 30625.46883 | 30999.62787 | 31190.18171 | 30726.14753 | 32574.50502 | 32074.20478 | 32090.95223 | 30029.86804 | 29912.22344 | 29463.60488 | 30044.63688 | 32549.65455 | 31245.07975 | 33202.01923 | 31140.48053 |
| **Snx32** | 35.71216278 | 31.69048034 | 28.9769679 | 12.07653188 | 21.53435031 | 13.32684331 | 17.13922958 | 32.68488651 | 16.5531107 | 33.20827482 | 9.458706988 | 18.21809023 | 21.30210788 | 26.2030849 | 22.27553583 |
| **1700020D05Rik** | 0 | 3.169048034 | 0 | 0 | 5.700269198 | 0.701412806 | 4.510323574 | 3.352296052 | 0.752414123 | 5.243411813 | 3.783482795 | 0.867528106 | 0 | 0.903554652 | 0 |
| **Ovol1** | 8.241268334 | 4.753572051 | 3.915806472 | 6.900875357 | 8.233722176 | 16.83390734 | 18.94335901 | 3.352296052 | 0 | 0.873901969 | 0 | 3.470112425 | 4.260421576 | 11.74621047 | 10.77848508 |
| **Ap5b1** | 169.4038491 | 211.5339563 | 171.5123235 | 198.4001665 | 196.3426057 | 196.3955856 | 231.8306317 | 186.8905049 | 139.1966127 | 146.8155308 | 185.390657 | 173.5056213 | 192.5710552 | 170.7718291 | 201.9169538 |
| **Rnaseh2c** | 774.6792234 | 785.1316504 | 866.959553 | 824.6546052 | 906.3428026 | 864.1405766 | 835.3119259 | 847.2928272 | 819.3789795 | 853.8022236 | 732.1039208 | 884.0111403 | 749.8341973 | 838.4987167 | 822.7576944 |
| **Kat5** | 644.650323 | 778.0012924 | 770.6307138 | 761.6841176 | 690.9992995 | 698.6071545 | 706.3166717 | 676.3257285 | 774.2341321 | 776.0249484 | 752.9130762 | 753.0143963 | 717.4549934 | 728.2650492 | 763.8353093 |
| **Rela** | 2490.69443 | 2483.741397 | 2424.667368 | 2325.594995 | 2440.981943 | 2487.911222 | 2615.987673 | 2618.143217 | 2421.268646 | 2325.453139 | 2201.041116 | 2425.608585 | 2551.992524 | 2369.120296 | 2494.141447 |
| **Sipa1** | 1999.881116 | 2288.052681 | 2316.591109 | 2436.871611 | 2723.46195 | 2672.38279 | 2659.286779 | 2106.079995 | 2050.328484 | 2050.174019 | 2364.676747 | 2722.303198 | 2693.43852 | 2860.654027 | 2829.711616 |
| **Pcnxl3** | 1389.111563 | 1411.018637 | 1376.797556 | 1287.875864 | 1240.758596 | 1293.405214 | 1447.813867 | 1419.697378 | 1335.535068 | 1287.2576 | 1540.823368 | 1377.634633 | 1397.418277 | 1456.530098 | 1526.952053 |
| **Map3k11** | 1480.681211 | 1475.19186 | 1473.909556 | 1480.237764 | 1505.504432 | 1430.882124 | 1466.757226 | 1493.447891 | 1348.326108 | 1377.269503 | 1519.068342 | 1448.771937 | 1461.324601 | 1454.722989 | 1399.047363 |
| **Kcnk7** | 121.787632 | 106.1631091 | 105.7267748 | 125.0783658 | 112.7386575 | 114.3302873 | 99.22711863 | 56.15095887 | 50.41174621 | 59.42533389 | 88.91184568 | 96.2956198 | 78.39175699 | 96.68034772 | 135.808912 |
| **Ehbp1l1** | 1011.844612 | 840.589991 | 937.4440695 | 916.0912037 | 998.8138362 | 1030.375412 | 965.2092448 | 915.1768223 | 838.9417467 | 783.8900661 | 976.1385611 | 1003.730019 | 1092.372092 | 1010.1741 | 1030.423174 |
| **Fam89b** | 483.4877423 | 526.8542357 | 508.2716801 | 542.581325 | 542.7923003 | 496.6002664 | 552.9656702 | 614.3082516 | 597.4168133 | 573.2796916 | 565.6306779 | 598.5943933 | 564.931901 | 564.7216572 | 566.9483152 |
| **Sssca1** | 702.3392014 | 734.4268819 | 714.2431006 | 683.1866604 | 697.9662952 | 730.1707307 | 770.3632664 | 791.9799423 | 787.7775863 | 774.2771444 | 769.9387488 | 622.0176522 | 619.4652971 | 731.8792678 | 663.9546809 |
| **Ltbp3** | 3.662785926 | 5.54583406 | 3.915806472 | 6.038265938 | 0.633363244 | 4.90988964 | 8.118582433 | 5.866518091 | 1.504828245 | 16.60413741 | 2.837612096 | 4.337640531 | 2.556252945 | 0 | 2.874262688 |
| **Scyl1** | 864.4174786 | 903.9709517 | 855.9952949 | 859.158982 | 858.8405592 | 826.9656979 | 882.2192911 | 879.9777137 | 852.4852009 | 1031.204323 | 993.1642337 | 881.408556 | 945.8135898 | 952.3466028 | 905.3927467 |
| **Malat1** | 769.1850445 | 1092.52931 | 1129.318587 | 1001.489536 | 936.110875 | 810.8332034 | 1026.549645 | 1244.539909 | 1185.804657 | 1145.685481 | 1158.691606 | 1275.266316 | 1063.401225 | 1098.722456 | 1077.848508 |
| **Neat1** | 199.621833 | 322.4506375 | 288.2033564 | 238.0801998 | 300.847541 | 239.1817667 | 204.7686903 | 222.0896135 | 155.7497234 | 214.9798844 | 262.0061836 | 224.6897795 | 269.2586436 | 227.6957722 | 249.3422882 |
| **Frmd8** | 2211.407003 | 2575.64379 | 2629.072466 | 2708.593578 | 2512.55199 | 2484.404158 | 2561.86379 | 2750.558911 | 2668.060479 | 2556.163259 | 2789.372691 | 2884.530953 | 2709.628122 | 2932.938399 | 2616.297612 |
| **Slc25a45** | 103.4737024 | 154.4910917 | 153.4996137 | 127.6661941 | 167.2078965 | 139.5811483 | 147.0365485 | 121.5207319 | 158.7593799 | 146.8155308 | 156.0686653 | 188.2535991 | 176.3814532 | 172.5789385 | 163.8329732 |
| **Tigd3** | 17.39823315 | 19.80655021 | 21.92851625 | 18.11479781 | 24.70116653 | 28.75792503 | 23.45368258 | 29.33259046 | 21.06759543 | 22.72145119 | 28.37612096 | 31.23101183 | 33.23128829 | 23.49242094 | 25.14979852 |
| **Dpf2** | 1812.163337 | 1845.97048 | 1888.201881 | 1869.274612 | 1834.853319 | 1856.639697 | 1958.382496 | 1901.589936 | 1955.524305 | 1849.176566 | 1900.254234 | 1926.779924 | 1955.533503 | 1948.063829 | 1907.791859 |
| **Cdc42ep2** | 3.662785926 | 1.584524017 | 1.566322589 | 0.86260942 | 5.700269198 | 4.90988964 | 5.412388289 | 4.190370065 | 4.514484735 | 0.873901969 | 0.945870699 | 5.205168638 | 0 | 0 | 2.155697016 |
| **Pola2** | 1990.724151 | 1964.017519 | 2073.811108 | 1936.558147 | 1839.286861 | 1864.355238 | 1999.877473 | 2331.521904 | 2277.557549 | 2264.280001 | 2223.742013 | 1925.044868 | 1837.093783 | 1938.124728 | 1913.540384 |
| **Capn1** | 1989.808454 | 1983.824069 | 1968.867494 | 2022.819089 | 2060.330634 | 1988.505304 | 2160.444992 | 1994.616151 | 1957.781547 | 1933.071155 | 1868.09463 | 2035.220937 | 1993.877297 | 2164.916945 | 2055.816387 |
| **Gm10814** | 6.409875371 | 1.584524017 | 1.566322589 | 4.313047098 | 3.166816221 | 2.805651223 | 4.510323574 | 0 | 0 | 4.369509845 | 0 | 0.867528106 | 0 | 6.324882561 | 3.59282836 |
| **Syvn1** | 783.8361882 | 754.2334321 | 776.8960041 | 721.1414748 | 751.802171 | 714.739649 | 785.6983666 | 768.51387 | 805.0831112 | 799.6203015 | 822.9075079 | 763.4247335 | 803.5155092 | 933.3719551 | 768.1467033 |
| **Mrpl49** | 615.3480356 | 762.9483142 | 739.304262 | 602.1013749 | 750.5354445 | 668.4464038 | 677.4506008 | 870.7588996 | 881.8293517 | 858.1717335 | 790.7479042 | 692.2874288 | 651.8445011 | 681.2802073 | 645.9905391 |
| **Fau** | 14993.61419 | 14439.76737 | 13665.38143 | 13551.59398 | 15300.15589 | 16685.90924 | 15771.69947 | 12216.60489 | 11702.29685 | 12278.32266 | 11690.01597 | 13790.22678 | 13448.44675 | 13778.30488 | 13616.81948 |
| **Znhit2** | 390.0867011 | 520.5161396 | 496.5242607 | 470.9847431 | 500.9903262 | 517.6426506 | 493.429399 | 522.9581841 | 469.5064125 | 489.3851026 | 425.6418144 | 476.2729303 | 489.9484812 | 477.9804107 | 474.9719092 |
| **Tm7sf2** | 220.6828521 | 192.5196681 | 202.8387753 | 232.0419339 | 222.943862 | 230.7648131 | 199.356302 | 177.6716908 | 180.5793894 | 196.627943 | 193.9034932 | 230.7624763 | 218.1335847 | 222.2744443 | 227.0667523 |
| **Vps51** | 670.2898245 | 722.5429518 | 695.4472295 | 651.2701118 | 756.8690769 | 749.8102893 | 676.5485361 | 633.5839539 | 670.4009832 | 681.6435357 | 593.0609281 | 738.2664184 | 777.9529797 | 736.397041 | 722.1585003 |
| **BC048609** | 0.915696482 | 0.792262009 | 0 | 0 | 0.633363244 | 0.701412806 | 0 | 3.352296052 | 0 | 5.243411813 | 0 | 5.205168638 | 0 | 1.807109303 | 0 |
| **Zfpl1** | 281.1188198 | 381.0780261 | 330.4940663 | 305.3637346 | 336.949246 | 354.9148797 | 326.5474268 | 342.7722713 | 309.2422044 | 338.200062 | 304.570365 | 305.3698934 | 322.9399554 | 352.3863141 | 336.2887345 |
| **Cdca5** | 1209.635052 | 1200.276943 | 1108.173232 | 1268.898456 | 1121.052942 | 1124.364728 | 1114.951987 | 1167.4371 | 1139.154982 | 1226.084462 | 1090.588916 | 1173.765528 | 1174.172186 | 1230.641435 | 1196.411844 |
| **Naaladl1** | 19.22962611 | 8.714882094 | 5.482129061 | 22.42784491 | 8.86708542 | 14.02825611 | 7.216517718 | 10.89496217 | 1.504828245 | 6.117313782 | 14.18806048 | 12.14539349 | 2.556252945 | 11.74621047 | 15.08987911 |
| **Sac3d1** | 539.3452276 | 537.1536418 | 640.6259389 | 621.0787821 | 566.2267404 | 573.7556751 | 569.202835 | 705.658319 | 765.2051627 | 693.8781633 | 716.9699897 | 618.5475398 | 573.4527441 | 564.7216572 | 538.924254 |
| **Snx15** | 277.4560339 | 274.1226549 | 270.9738079 | 288.1115462 | 274.2462848 | 254.6128485 | 247.1657318 | 341.0961233 | 389.7505155 | 315.4786108 | 308.3538478 | 301.899781 | 278.6315711 | 300.883699 | 296.0490568 |
| **Arl2** | 229.8398169 | 202.8190742 | 205.9714205 | 206.1636513 | 202.0428749 | 204.1111265 | 197.5521725 | 212.8707993 | 204.6566413 | 190.5106292 | 168.3649844 | 164.8303402 | 174.6772846 | 171.6753838 | 201.1983882 |
| **Batf2** | 3.662785926 | 5.54583406 | 4.698967767 | 0 | 3.166816221 | 0.701412806 | 2.706194144 | 0 | 0 | 0 | 0.945870699 | 2.602584319 | 3.408337261 | 0 | 0.718565672 |
| **Ppp2r5b** | 122.7033285 | 106.9553711 | 138.6195491 | 93.16181732 | 74.10349958 | 86.97518791 | 105.5415716 | 123.1968799 | 109.1000478 | 117.9767658 | 111.6127425 | 111.9111257 | 90.32093741 | 117.4621047 | 102.7548911 |

| | | | | | | | | | | | | | | | |
|---|---|---|---|---|---|---|---|---|---|---|---|---|---|---|---|
| **Atg2a** | 613.5166426 | 689.2679474 | 617.9142614 | 633.155314 | 665.0314065 | 619.3475074 | 738.7910014 | 740.0193535 | 690.7161645 | 717.4735165 | 656.4342649 | 632.4279895 | 711.4904032 | 707.4832922 | 720.0028033 |
| **Ehd1** | 2287.409811 | 2019.47586 | 1972.00014 | 2094.415671 | 1896.289553 | 1987.803891 | 2104.51698 | 2059.985924 | 1804.289066 | 1870.150213 | 2207.662211 | 1903.356665 | 2016.03149 | 1853.19059 | 1902.761899 |
| **Cdc42bpg** | 120.8719356 | 135.4768035 | 128.4384523 | 130.2540224 | 107.0383883 | 96.09355438 | 95.61885977 | 189.404727 | 176.8173188 | 178.2760017 | 196.7411053 | 126.6591035 | 124.40431 | 128.3047605 | 132.2160836 |
| **Men1** | 1897.32311 | 1784.966305 | 1896.033494 | 1784.738889 | 1858.921122 | 1881.189145 | 1969.207272 | 1589.826403 | 1565.773789 | 1656.918133 | 1671.353525 | 1741.996437 | 1788.524978 | 1763.73868 | 1916.414647 |
| **Map4k2** | 1669.314686 | 1876.868698 | 1988.446527 | 1701.065776 | 1839.286861 | 1715.655723 | 1934.928813 | 1582.283737 | 1853.195984 | 1766.155879 | 1711.080094 | 1947.600599 | 2121.689945 | 1995.048671 | 1914.25895 |
| **Sf1** | 9084.624794 | 8626.941011 | 9024.367596 | 8716.668186 | 8459.199491 | 8546.013625 | 9095.518519 | 8871.851502 | 8191.532553 | 8630.655845 | 8616.882066 | 8835.773762 | 9150.533461 | 8472.631968 | 8814.645098 |
| **Pygm** | 53.11039593 | 39.61310043 | 62.65290356 | 44.85568982 | 51.30242279 | 61.0229141 | 57.73214175 | 15.92340625 | 35.36346376 | 16.60413741 | 48.23940564 | 27.7608994 | 48.56880596 | 44.27417793 | 33.77258658 |
| **Rasgrp2** | 783.8361882 | 1153.533484 | 1081.545748 | 1084.300041 | 1035.548904 | 972.8595615 | 1047.297134 | 751.7523897 | 974.3762887 | 828.4590665 | 888.1725861 | 1028.888334 | 1024.205347 | 1134.864642 | 1017.488992 |
| **Nrxn2** | 0 | 0 | 0 | 2.587828259 | 0.633363244 | 1.402825611 | 1.80412943 | 2.514222039 | 4.514484735 | 0 | 5.675224193 | 0 | 0 | 0.903554652 | 4.311394032 |
| **Rps6ka4** | 834.1994947 | 821.5757028 | 869.3090369 | 735.805835 | 730.2678207 | 772.9569119 | 764.9508781 | 807.0652746 | 645.5713172 | 762.0425169 | 799.2607405 | 756.4845087 | 814.5926053 | 830.3667248 | 916.8897974 |
| **Ccdc88b** | 3621.579585 | 3511.305222 | 3580.613438 | 3560.851684 | 3590.536232 | 3616.484426 | 3781.455284 | 3227.423024 | 3090.917216 | 3319.079678 | 3229.202566 | 3408.51793 | 3491.841524 | 3469.649862 | 3474.98359 |
| **Prdx5** | 1203.225177 | 1138.480506 | 1101.907941 | 1054.97132 | 1110.285767 | 1139.094396 | 1102.323081 | 846.4547532 | 865.276241 | 976.1484993 | 979.9220439 | 1026.28575 | 1041.247033 | 1026.438084 | 1058.447235 |
| **Trmt112** | 1269.155323 | 1366.651965 | 1319.626781 | 1268.035847 | 1273.060121 | 1310.940534 | 1255.674083 | 1276.386722 | 1190.319142 | 1292.501012 | 1247.603452 | 1178.103168 | 1255.120196 | 1289.372488 | 1222.280208 |
| **Esrra** | 492.6447071 | 364.4405239 | 380.6163891 | 357.1202997 | 351.5166006 | 373.1516126 | 349.0990446 | 422.3893026 | 330.3097998 | 379.2734545 | 382.1317623 | 371.3020295 | 330.6087143 | 346.0614316 | 334.8516031 |
| **Mir6990** | 1.831392963 | 0 | 0.783161294 | 1.725218839 | 0 | 0 | 0 | 0 | 0 | 1.747803938 | 0 | 2.602584319 | 0 | 0 | 2.155697016 |
| **Tex40** | 5.494178889 | 1.584524017 | 3.915806472 | 0.86260942 | 4.43354271 | 5.611302446 | 1.80412943 | 5.866518091 | 0 | 6.991215751 | 0 | 6.072696744 | 2.556252945 | 0.903554652 | 3.59282836 |
| **Gpr137** | 63.18305723 | 66.55000871 | 91.62987146 | 82.81050429 | 70.30332011 | 77.85682143 | 58.63420646 | 122.3588059 | 75.99382638 | 99.62482445 | 62.42746612 | 52.91921448 | 83.50426289 | 89.45191051 | 66.10804182 |
| **Bad** | 209.6944943 | 225.0024104 | 193.4408397 | 163.0331803 | 201.4095117 | 185.1729807 | 165.9799075 | 217.8992434 | 213.6856108 | 214.9798844 | 214.7126486 | 198.6639363 | 181.4939591 | 185.2287036 | 200.4798225 |
| **Plcb3** | 193.2119576 | 202.0268122 | 220.0683238 | 208.7514796 | 176.0749819 | 162.0263581 | 170.4902311 | 186.0524309 | 176.0649047 | 223.718904 | 190.1200105 | 168.3004526 | 194.2752239 | 169.8682745 | 225.629621 |
| **Ppp1r14b** | 2688.48487 | 2732.511667 | 2761.426724 | 2963.063357 | 2789.331728 | 2968.378994 | 2627.714514 | 3398.390123 | 3266.98212 | 3361.900874 | 3250.011721 | 2744.858928 | 2703.663532 | 2726.024384 | 2593.30351 |
| **Fkbp2** | 734.3885782 | 725.7119998 | 666.4702616 | 695.2631922 | 720.767372 | 683.8774856 | 709.9249305 | 626.0412878 | 661.3720137 | 654.5525747 | 632.7874975 | 766.0273178 | 626.2819716 | 750.8539155 | 643.8348421 |
| **Vegfb** | 325.9879474 | 331.1655196 | 296.8181306 | 317.4402664 | 316.6816221 | 333.1710827 | 318.4288443 | 213.7088733 | 319.023588 | 249.0620611 | 305.5162357 | 281.9466345 | 311.8628593 | 343.3507676 | 293.8933598 |
| **Dnajc4** | 75.08711149 | 96.65596504 | 91.62987146 | 117.3148811 | 84.23731149 | 80.66247266 | 97.4229892 | 77.94088321 | 75.24141226 | 71.65996145 | 76.6155266 | 95.42809169 | 103.9542864 | 103.9087849 | 117.1262045 |
| **Nudt22** | 100.726613 | 104.5785851 | 105.7267748 | 107.8261775 | 120.3390164 | 83.46812388 | 101.9333128 | 97.21658551 | 94.80417945 | 88.26409886 | 109.7210011 | 78.07752957 | 106.5105394 | 93.06612911 | 84.79074929 |
| **Trpt1** | 10.98835778 | 4.753572051 | 4.698967767 | 6.038265938 | 8.86708542 | 18.23673295 | 13.53097072 | 5.028444078 | 7.524141226 | 2.621705907 | 32.15960376 | 7.807752957 | 15.33751767 | 7.228437213 | 17.9641418 |
| **Fermt3** | 5545.457892 | 5695.571579 | 5714.727966 | 5820.025754 | 5344.952418 | 5332.140149 | 5600.017749 | 5334.341093 | 5539.27277 | 5834.169544 | 5791.566289 | 5363.926281 | 5277.810248 | 5293.023149 | 5497.027391 |
| **Stip1** | 8122.227791 | 7596.208138 | 7547.325395 | 7164.83384 | 6992.330217 | 7159.320508 | 7172.316547 | 7676.75796 | 7156.21072 | 7607.316639 | 7628.447186 | 6741.560914 | 7097.010261 | 7005.259214 | 6674.037961 |
| **Macrod1** | 204.2003154 | 213.1184803 | 219.2851625 | 179.4227593 | 206.4764176 | 237.7789411 | 269.7173497 | 179.3478388 | 203.1518131 | 156.4284524 | 150.3934411 | 245.5104541 | 213.8731631 | 197.8784687 | 200.4798225 |
| **Otub1** | 1777.366871 | 1763.575231 | 1858.441752 | 1967.612086 | 1881.088835 | 1817.36058 | 1841.114083 | 2155.526362 | 2063.871938 | 2061.534745 | 2023.217425 | 1799.253292 | 1711.837389 | 1884.815003 | 1840.965252 |
| **Cox8a** | 3636.230728 | 3371.867108 | 3420.065373 | 3351.237595 | 3316.92331 | 3243.332814 | 3343.953898 | 3269.326725 | 3482.924973 | 3506.094699 | 3197.042962 | 3433.676245 | 3140.782786 | 3363.030413 | 3258.695322 |
| **Naa40** | 1185.826944 | 1297.72517 | 1251.491749 | 1310.303708 | 1250.259044 | 1235.187951 | 1309.797966 | 1542.894258 | 1522.13377 | 1590.501583 | 1401.780376 | 1496.485983 | 1388.897434 | 1430.327013 | 1350.903463 |
| **Rcor2** | 54.02609241 | 36.44405239 | 38.37490343 | 31.91654853 | 50.66905954 | 43.48759395 | 63.14453003 | 23.46607237 | 31.60139315 | 35.82998073 | 35.94308655 | 58.99191123 | 35.78754124 | 57.8274977 | 40.9582433 |
| **Mark2** | 3000.73737 | 3112.005169 | 3098.969242 | 3051.912127 | 2944.505723 | 2976.795947 | 3303.360986 | 2734.635505 | 2915.604725 | 2680.257339 | 2624.791189 | 3097.075339 | 3029.15974 | 3132.623977 | 2984.921801 |
| **AI846148** | 287.5286952 | 251.1470567 | 273.3232918 | 281.2106708 | 253.3452977 | 253.9114357 | 276.9338674 | 217.0611694 | 221.209752 | 228.9623159 | 204.3080709 | 233.3650606 | 236.0273553 | 280.101942 | 279.5220464 |
| **2700081O15Rik** | 389.1710047 | 356.5179038 | 334.4098727 | 350.2194244 | 288.8136394 | 281.9679479 | 306.702003 | 393.0567121 | 369.4353342 | 374.0300427 | 359.4308655 | 351.348883 | 321.2357868 | 280.101942 | 312.5760673 |
| **Rtn3** | 4663.642181 | 4900.140523 | 4946.446736 | 4867.704955 | 4796.459849 | 4786.440986 | 4842.283389 | 4336.194944 | 4492.664726 | 4360.770825 | 4432.350094 | 5107.137962 | 5016.220363 | 4718.362391 | 4856.066811 |
| **Atl3** | 3039.196622 | 3023.271824 | 3161.622146 | 2919.070276 | 2681.026613 | 2908.057492 | 3194.211155 | 2940.801712 | 3087.155145 | 2675.887829 | 2917.065235 | 3094.472755 | 3084.545221 | 2876.918011 | 2923.125154 |
| **Pla2g16** | 676.6996999 | 748.687598 | 785.5107784 | 746.157148 | 693.5327525 | 702.1142185 | 792.0128196 | 767.675796 | 861.5141704 | 730.582046 | 662.1094891 | 776.4376551 | 784.7696543 | 839.4022713 | 747.3082988 |
| **Slc3a2** | 6723.959264 | 5639.320977 | 5955.941645 | 5578.495117 | 5221.446586 | 5662.505581 | 6117.802896 | 4679.805289 | 4943.360785 | 4913.950771 | 5102.026549 | 5186.083019 | 5207.939334 | 5076.170033 | 5676.668809 |
| **Snhg1** | 795.7402425 | 873.0727334 | 930.3956179 | 698.7136299 | 769.5363418 | 838.1883028 | 783.8942371 | 815.4460147 | 749.4044661 | 933.3273028 | 924.1156727 | 884.0111403 | 884.4635191 | 844.8235992 | 964.3151318 |
| **Snord22** | 0.915696482 | 7.130358077 | 6.265290356 | 0.86260942 | 1.266726489 | 1.402825611 | 0 | 0 | 2.257242368 | 8.739019689 | 0 | 0 | 0.852084315 | 2.710663955 | 1.437131344 |
| **Wdr74** | 1479.765514 | 1510.051388 | 1402.641878 | 1389.663775 | 1399.099407 | 1343.205523 | 1306.189707 | 1515.237816 | 1370.146117 | 1509.2287 | 1377.187737 | 1334.258227 | 1253.416028 | 1353.524868 | 1235.932956 |
| **1700092M07Rik** | 0 | 1.584524017 | 3.132645178 | 3.450437679 | 3.166816221 | 5.611302446 | 1.80412943 | 1.676148026 | 0.752414123 | 2.621705907 | 4.729353494 | 6.072696744 | 0.852084315 | 3.614218606 | 0 |
| **Stx5a** | 500.8859754 | 592.6119824 | 578.7561966 | 523.6039177 | 532.6584884 | 499.4059177 | 614.3060708 | 497.8159638 | 478.535382 | 470.1592593 | 520.2288843 | 573.4360783 | 557.2631421 | 525.8688072 | 566.2297495 |
| **Nxf1** | 1813.079033 | 1923.612157 | 1990.796011 | 1933.970319 | 2046.396642 | 2020.770293 | 1980.032049 | 2113.622661 | 1984.868455 | 2153.294451 | 2200.095245 | 2159.277457 | 2144.696221 | 2111.607221 | 2283.601706 |
| **Tmem223** | 388.2553082 | 373.155406 | 344.5909696 | 420.9533968 | 423.0866472 | 422.250509 | 327.4494915 | 473.5118174 | 434.8953629 | 426.4641608 | 489.0151513 | 373.9046138 | 341.6858104 | 405.6960386 | 370.0613211 |
| **Tmem179b** | 146.5114371 | 173.5053799 | 180.1270977 | 156.132305 | 176.0749819 | 136.0740843 | 172.2943605 | 118.1684358 | 133.9297138 | 124.9679816 | 126.7466736 | 183.0484304 | 171.2689473 | 150.8936268 | 175.330024 |
| **Taf6l** | 179.4765104 | 171.9208558 | 165.2470331 | 205.3010419 | 200.7761484 | 179.5616783 | 194.8459784 | 193.595097 | 217.4476814 | 203.6191588 | 167.4191137 | 178.7107899 | 206.2044043 | 221.3708896 | 158.0844478 |
| **Gm2518** | 0 | 4.753572051 | 0 | 0 | 0.633363244 | 0 | 0.902064715 | 0 | 3.00965649 | 0 | 0 | 0 | 0 | 1.807109303 | 2.155697016 |
| **Polr2g** | 829.6210123 | 825.5370129 | 811.3551011 | 709.9275524 | 816.4052219 | 819.9515699 | 735.1827425 | 819.6363848 | 792.2920711 | 890.5061063 | 891.0101982 | 781.6428238 | 725.9758365 | 767.1178992 | 720.721369 |
| **Zbtb3** | 44.8691276 | 45.15893448 | 46.98967767 | 43.9930804 | 43.06870061 | 56.11302446 | 31.57226502 | 27.65644243 | 44.39243323 | 34.95607876 | 32.15960376 | 45.97898963 | 40.04796281 | 43.37062328 | 31.61688957 |
| **Ttc9c** | 1140.04212 | 1084.60669 | 1078.413103 | 999.7643174 | 913.3097982 | 927.2677292 | 995.8794451 | 978.8704472 | 1074.447367 | 1049.556265 | 1064.104536 | 1043.636312 | 1013.980335 | 997.5243353 | 1010.303335 |
| **Hnrnpul2** | 7496.807095 | 7074.107474 | 7390.693136 | 7063.045928 | 6906.826179 | 7059.719889 | 7175.022741 | 6873.883055 | 7301.426646 | 6704.575905 | 6961.608343 | 7278.560812 | 7218.006234 | 7260.96518 | 7116.674415 |
| **Gng3** | 12.81975074 | 10.29940611 | 3.915806472 | 12.07653188 | 13.30062813 | 7.014128057 | 11.72684129 | 13.40918421 | 9.781383594 | 20.09974528 | 17.02567258 | 7.807752957 | 15.33751767 | 10.84265582 | 9.341353736 |
| **Bscl2** | 573.2259975 | 678.9685413 | 704.0620037 | 692.675364 | 639.0635135 | 616.5418562 | 617.9143296 | 597.5467713 | 668.1437409 | 599.4967507 | 621.4370491 | 647.1759673 | 647.5840795 | 637.0060294 | 611.4993868 |
| **Lrrn4cl** | 3.662785926 | 3.169048034 | 3.132645178 | 6.038265938 | 1.900089733 | 0.701412806 | 3.608258859 | 3.352296052 | 1.504828245 | 0 | 2.837612096 | 3.470112425 | 1.70416863 | 6.324882561 | 0 |
| **Ubxn1** | 2346.014386 | 2404.515196 | 2392.557755 | 2329.908042 | 2227.53853 | 2311.155195 | 2225.393651 | 2279.561316 | 2252.727883 | 2224.954413 | 2058.214641 | 2311.094875 | 2319.373506 | 2351.049203 | 2267.074695 |
| **Uqcc3** | 287.5286952 | 255.1083667 | 325.0119372 | 298.4628592 | 310.9813529 | 295.2947912 | 295.8772264 | 341.0961233 | 277.6408112 | 358.2998073 | 279.9777268 | 355.6865236 | 334.0170515 | 340.6401036 | 321.917421 |
| **5730408K05Rik** | 2.747089445 | 2.376786026 | 0.783161294 | 0.86260942 | 1.266726489 | 1.402825611 | 2.706194144 | 0 | 0 | 0.873901969 | 0 | 0 | 2.556252945 | 0.903554652 | 2.155697016 |
| **1810009A15Rik** | 3.662785926 | 0 | 2.349483883 | 0 | 2.533452977 | 0 | 0 | 0 | 3.762070613 | 0 | 0 | 0 | 0 | 4.517773258 | 1.437131344 |
| **Ints5** | 1434.896387 | 1521.143056 | 1519.332911 | 1499.215171 | 1439.001291 | 1469.459828 | 1454.12832 | 1713.861357 | 1486.770306 | 1639.440094 | 1639.193921 | 1612.73475 | 1511.597575 | 1493.575839 | 1478.808153 |
| **Ganab** | 3923.759424 | 4172.051737 | 4008.219505 | 3936.086782 | 3698.207983 | 3685.924294 | 3951.043451 | 3771.333059 | 3844.836166 | 3829.438428 | 3993.46609 | 3760.734341 | 3833.527334 | 3776.858444 | 3959.296853 |
| **B3gat3** | 585.1300517 | 694.8137815 | 656.2891648 | 684.9118792 | 602.9618086 | 659.3280374 | 685.5691832 | 574.0806989 | 683.1920233 | 602.1184566 | 639.4085924 | 704.4328223 | 645.0278266 | 721.036612 | 663.9546809 |
| **Rom1** | 101.6423095 | 83.9797729 | 79.88245204 | 83.67311371 | 67.13650389 | 64.52997813 | 93.81473034 | 146.6629523 | 138.4441986 | 133.7070012 | 114.4503546 | 64.19707986 | 92.87719035 | 93.96968376 | 90.53927467 |
| **Eml3** | 1162.934532 | 1189.185275 | 1187.272522 | 1210.241016 | 1075.450789 | 1089.294087 | 1067.142558 | 1183.360506 | 1196.338455 | 1241.814698 | 1318.543754 | 1158.150022 | 1230.409751 | 1160.164173 | 1138.92659 |
| **Mta2** | 5791.780246 | 6277.091893 | 6168.178355 | 6108.99991 | 6149.957102 | 6231.351366 | 6396.540893 | 6821.922466 | 6742.382952 | 6296.463686 | 6715.681961 | 6424.913155 | 6258.559295 | 6166.760497 | 6158.826374 |
| **Tut1** | 1164.765925 | 1145.610864 | 1075.280457 | 1042.894788 | 1107.118951 | 984.0821664 | 1010.312481 | 1068.544367 | 1018.768722 | 1042.565049 | 1075.454985 | 983.7768725 | 891.2801937 | 930.6612911 | 1006.710506 |
| **Eef1g** | 23189.0977 | 22821.10715 | 23181.57432 | 23135.18464 | 23389.47125 | 23690.21751 | 23434.73923 | 26069.96832 | 24655.85838 | 25482.10751 | 25347.44299 | 23338.24112 | 22495.02592 | 23067.75025 | 22678.65117 |
| **Ahnak** | 3390.824071 | 3116.758741 | 3049.630081 | 2978.590326 | 3877.449781 | 3521.793697 | 4520.246286 | 2101.889625 | 2008.193293 | 1840.437547 | 2114.966882 | 4426.995926 | 4222.077782 | 3880.767229 | 4320.735386 |
| **Asrgl1** | 569.5632115 | 525.2697116 | 572.4909063 | 538.2682779 | 579.5273685 | 514.8369994 | 562.888382 | 492.7875197 | 574.0919755 | 535.7019069 | 559.009583 | 556.0855161 | 558.1152264 | 548.4576735 | 517.3672838 |
| **Stxbp3-ps** | 1.831392963 | 0 | 0.783161294 | 1.725218839 | 0 | 0 | 1.80412943 | 2.514222039 | 0.752414123 | 0 | 0.945870699 | 0 | 0 | 0.903554652 | 1.437131344 |
| **Incenp** | 3725.053287 | 4275.045798 | 4238.468926 | 4330.299287 | 3944.586285 | 3833.220983 | 4086.353158 | 3955.709342 | 4224.805298 | 3942.171782 | 4315.062128 | 4363.666375 | 4288.540358 | 4128.341203 | 4067.081703 |
| **Fth1** | 6505.107805 | 5987.123998 | 6245.711324 | 6380.721877 | 6098.654679 | 6385.662183 | 6448.860646 | 6526.920414 | 6412.825567 | 6774.488063 | 7024.035809 | 6018.910001 | 6242.369693 | 6456.80154 | 6034.514513 |
| **Best1** | 3.662785926 | 2.376786026 | 0 | 0.86260942 | 0 | 1.402825611 | 0.902064715 | 3.352296052 | 1.504828245 | 0 | 0 | 0 | 0 | 0 | 0 |
| **Fads3** | 97.97952353 | 62.58869867 | 85.3645811 | 54.34439344 | 46.86888008 | 68.03704215 | 60.43833589 | 139.9583602 | 72.23175577 | 107.4899422 | 172.1484672 | 82.4151701 | 80.94800994 | 84.93413725 | 61.07808212 |
| **Fads2** | 3225.998705 | 3319.577816 | 3150.657888 | 2984.628592 | 3031.276487 | 3025.894844 | 3241.11852 | 2266.990205 | 2665.050822 | 2622.579809 | 2928.415683 | 3228.939612 | 3254.11 | 3215.751005 | 3186.120189 |
| **Fads1** | 1376.291812 | 1347.637676 | 1263.239168 | 1299.089766 | 1260.392856 | 1289.196737 | 1320.622742 | 1277.224796 | 1317.477129 | 1255.797129 | 1273.141961 | 1365.489239 | 1365.039073 | 1376.113734 | 1281.202593 |
| **Fen1** | 2884.443917 | 2983.658724 | 2979.145564 | 3171.814836 | 3012.27559 | 3041.325926 | 2857.741016 | 3499.797079 | 3203.779334 | 3243.050207 | 3231.094307 | 3015.527697 | 3202.984941 | 3086.54269 | 2971.987619 |
| **Tmem258** | 923.0220534 | 963.3906023 | 913.9492307 | 869.510295 | 953.8450459 | 977.0680384 | 956.1885977 | 902.6057121 | 858.5045139 | 807.4854193 | 802.0983526 | 951.6783326 | 755.7987875 | 888.1942225 | 894.6142616 |
| **Myrf** | 20.14532259 | 5.54583406 | 11.74741942 | 10.35131304 | 2.533452977 | 0.701412806 | 1.80412943 | 3.352296052 | 5.266898858 | 15.73023544 | 17.97154328 | 8.675281063 | 4.260421576 | 4.517773258 | 2.155697016 |
| **Dagla** | 3.662785926 | 1.584524017 | 3.132645178 | 2.587828259 | 8.233722176 | 1.402825611 | 8.118582433 | 7.542666117 | 0 | 12.23462756 | 0 | 3.470112425 | 5.112505891 | 0 | 1.437131344 |
| **Syt7** | 10.98835778 | 2.376786026 | 1.566322589 | 1.725218839 | 1.900089733 | 1.402825611 | 0.902064715 | 1.676148026 | 3.762070613 | 0.873901969 | 0 | 0 | 0 | 0 | 2.874262688 |
| **Ppp1r32** | 0.915696482 | 1.584524017 | 4.698967767 | 2.587828259 | 0 | 0 | 0 | 4.190370065 | 6.019312981 | 0 | 0 | 2.602584319 | 5.964590206 | 5.421327909 | 3.59282836 |
| **Sdhaf2** | 603.4439814 | 663.1233011 | 599.9015516 | 689.2249263 | 624.4961589 | 653.0153221 | 627.8370415 | 694.7633568 | 726.0796283 | 739.3210657 | 648.8672994 | 679.2745072 | 577.7131657 | 668.6304422 | 622.2778719 |
| **Cpsf7** | 1672.061775 | 1586.900803 | 1551.442524 | 1513.879531 | 1527.038782 | 1448.417444 | 1561.474021 | 1505.180927 | 1605.651738 | 1662.161545 | 1609.871929 | 1610.999693 | 1576.355983 | 1529.718025 | 1553.538983 |
| **Tmem216** | 103.4737024 | 95.86370303 | 119.0405168 | 135.4296789 | 83.60394824 | 96.79496719 | 73.96930661 | 130.739546 | 127.1579867 | 151.1850406 | 150.3934411 | 92.82550737 | 101.3980335 | 113.8478861 | 107.7848508 |
| **Tmem138** | 161.1625808 | 179.8434759 | 205.9714205 | 180.2853687 | 170.3747127 | 157.1164685 | 154.2530662 | 139.9583602 | 177.5697329 | 193.1323351 | 213.7667779 | 181.3133742 | 223.2460906 | 203.2997966 | 186.8270747 |
| **Cyb561a3** | 269.2147656 | 346.2184977 | 333.6267115 | 330.3794077 | 288.8136394 | 280.5651223 | 299.4854853 | 351.1530115 | 375.4546472 | 401.9949057 | 395.3739521 | 343.5411301 | 339.1295574 | 340.6401036 | 357.127139 |
| **Tkfc** | 358.9530208 | 399.3000523 | 400.9785828 | 362.2959563 | 384.4514893 | 362.6304206 | 393.3002156 | 314.2777549 | 317.5187597 | 305.8656891 | 361.3226069 | 399.930457 | 423.4859046 | 397.5640467 | 360.7199673 |
| **Ddb1** | 10362.02139 | 10380.21684 | 10549.9658 | 10356.48869 | 9522.616378 | 9532.901442 | 9831.603326 | 12275.27007 | 11773.02378 | 11837.00217 | 11841.35528 | 10006.93671 | 10080.15745 | 9602.075282 | 10177.76418 |
| **Vwce** | 0 | 2.376786026 | 0 | 1.725218839 | 0 | 0.701412806 | 0.902064715 | 4.190370065 | 0 | 3.495607876 | 0 | 0.867528106 | 0 | 0 | 0 |
| **Vps37c** | 301.2641424 | 296.3059912 | 292.1191628 | 269.1341389 | 278.0464642 | 272.1481686 | 276.0318027 | 271.5359802 | 248.2966605 | 311.1091009 | 296.0575287 | 244.642926 | 300.7857633 | 320.7619013 | 279.5220464 |
| **Cd5** | 1758.137245 | 1954.510375 | 2011.941366 | 1867.549394 | 2762.097108 | 2528.593165 | 2910.06077 | 2107.756143 | 1881.035306 | 1538.941367 | 1531.364661 | 2398.715214 | 2432.70072 | 2606.75517 | 2541.566782 |
| **A430093F15Rik** | 259.1421043 | 274.1226549 | 216.9356786 | 243.2558563 | 296.4139983 | 308.6216345 | 373.4547919 | 248.9079819 | 167.7883493 | 187.8889233 | 166.473243 | 296.6946123 | 351.0587378 | 286.4268245 | 302.5161479 |
| **Cd6** | 5107.754974 | 5176.639964 | 4856.383187 | 4977.256351 | 5984.649295 | 5605.691143 | 5968.060153 | 5578.220631 | 5121.682932 | 4775.87426 | 4355.734568 | 5785.544941 | 5908.352641 | 5795.399535 | 6021.580331 |
| **Slc15a3** | 302.1798389 | 271.7458689 | 191.8745171 | 230.316715 | 161.5076273 | 171.1447246 | 178.6088135 | 227.9561316 | 154.2448951 | 199.2496489 | 175.93195 | 149.2148343 | 140.593912 | 106.6194489 | 148.7430941 |
| **Tmem132a** | 57.68887834 | 54.66607859 | 32.89277437 | 40.54264272 | 17.73417084 | 22.44520978 | 21.64955315 | 19.2757023 | 23.3248378 | 39.3255886 | 38.78069865 | 17.35056213 | 5.964590206 | 20.78175699 | 20.11983882 |
| **Tmem109** | 3337.713675 | 3075.561117 | 3079.39021 | 3059.675612 | 2884.336214 | 2904.550428 | 2945.241294 | 3155.348659 | 2996.113036 | 3105.847597 | 3466.616111 | 2791.705446 | 2864.707468 | 3060.339605 | 3020.850085 |
| **Prpf19** | 4733.235113 | 4976.989937 | 5150.068673 | 5204.122629 | 4806.593661 | 4564.093127 | 4533.777256 | 5668.732624 | 5725.119059 | 5631.424288 | 5556.044485 | 4714.14773 | 4788.713851 | 4937.022616 | 4802.892951 |
| **Ptgdr2** | 0.915696482 | 3.961310043 | 0 | 0.86260942 | 2.533452977 | 4.208476834 | 2.706194144 | 1.676148026 | 0 | 0.873901969 | 0.945870699 | 1.735056213 | 15.33751767 | 5.421327909 | 4.311394032 |
| **Ccdc86** | 957.8185197 | 940.4150041 | 1006.362263 | 1161.934888 | 1053.916438 | 1171.359386 | 1092.40037 | 1299.852794 | 1192.576384 | 1207.732521 | 1203.147529 | 1006.332603 | 973.0802879 | 1006.559882 | 1054.854406 |
| **AW112010** | 2733.353997 | 2542.368785 | 2633.771433 | 2361.824591 | 2074.264625 | 2121.072324 | 2252.455593 | 2638.256993 | 2595.076309 | 2734.439261 | 2519.799542 | 2731.846007 | 2460.819502 | 2554.349 | 2435.219062 |
| **Ms4a8a** | 2.747089445 | 2.376786026 | 0 | 0 | 0 | 0 | 0 | 0.838074013 | 2.257242368 | 1.747803938 | 0 | 0 | 1.70416863 | 0 | 0 |
| **Ms4a4c** | 1313.108755 | 1403.096017 | 1243.660136 | 1188.67578 | 1710.714123 | 1807.5408 | 1963.794884 | 469.3214473 | 644.8189031 | 490.2590046 | 450.2344526 | 1480.002949 | 1415.312047 | 1476.408301 | 1415.574374 |
| **Ms4a4b** | 7637.824353 | 7816.456976 | 7898.181655 | 7723.804744 | 8922.821385 | 9143.617335 | 9970.521292 | 4895.19031 | 6034.361263 | 4620.31971 | 4796.510313 | 9317.251861 | 8957.962405 | 9946.329605 | 9084.825791 |
| **Ms4a6c** | 57.68887834 | 88.73334495 | 70.4845165 | 88.84877022 | 57.63605523 | 48.39748359 | 59.53627118 | 87.15969736 | 71.47934165 | 63.79484373 | 52.02288843 | 67.66719229 | 84.3563472 | 78.60925469 | 57.48525376 |
| **Gm8369** | 526.5254769 | 511.0089955 | 465.1978089 | 463.2212584 | 528.2249457 | 615.8404434 | 653.0948535 | 243.0414638 | 297.2035784 | 267.4140025 | 275.2483733 | 501.4312454 | 524.0318538 | 435.5133421 | 480.0018689 |
| **Ms4a6b** | 2879.865435 | 3298.186741 | 3008.905693 | 2988.07903 | 3266.254251 | 3349.246147 | 3633.516671 | 2156.364436 | 2716.214983 | 2098.238627 | 1970.248666 | 3521.296583 | 3210.6537 | 3383.81217 | 3307.557788 |
| **Ms4a4d** | 5.494178889 | 7.130358077 | 9.397935534 | 5.175656518 | 4.43354271 | 5.611302446 | 4.510323574 | 4.190370065 | 5.266898858 | 5.243411813 | 10.40457769 | 15.61550591 | 5.964590206 | 9.035546516 | 9.341353736 |
| **Ms4a6d** | 44.8691276 | 36.44405239 | 37.59174214 | 39.6800333 | 34.83497844 | 37.87629151 | 43.29910631 | 23.46607237 | 33.85863552 | 13.9824315 | 26.48437957 | 26.89337129 | 18.74585493 | 47.88839653 | 28.02406121 |
| **Oosp1** | 0 | 0.792262009 | 1.566322589 | 3.450437679 | 0 | 0.701412806 | 0 | 0.838074013 | 0 | 0.873901969 | 0.945870699 | 0 | 0 | 0.903554652 | 0.718565672 |
| **Mrpl16** | 736.2199712 | 681.3453273 | 769.8475525 | 699.5762393 | 682.7655773 | 669.1478166 | 681.0588597 | 879.1396397 | 759.1858497 | 784.7639681 | 706.565412 | 670.5992262 | 623.7257187 | 661.402005 | 632.3377913 |
| **Stx3** | 47.61621704 | 48.32798252 | 54.82129061 | 41.40525214 | 32.9348887 | 30.86216345 | 47.80942988 | 73.75051315 | 67.71727103 | 72.53386342 | 35.94308655 | 39.90629289 | 46.86463733 | 47.88839653 | 65.38947615 |
| **Patl1** | 1431.233601 | 1221.668017 | 1509.151814 | 1368.961149 | 1293.327745 | 1195.207421 | 1390.081725 | 1461.601079 | 1461.94064 | 1344.93513 | 1527.581179 | 1387.177442 | 1412.755795 | 1252.326747 | 1397.610232 |
| **Osbp** | 1325.928505 | 1417.356733 | 1506.802331 | 1405.190745 | 1355.397343 | 1336.191395 | 1445.107673 | 1306.557386 | 1400.995096 | 1247.932012 | 1421.64366 | 1458.314747 | 1421.276638 | 1278.529832 | 1377.490393 |
| **Pfpl** | 0.915696482 | 3.961310043 | 0.783161294 | 0.86260942 | 3.166816221 | 0 | 0 | 0 | 0 | 0 | 0 | 0.867528106 | 0 | 0 | 1.437131344 |
| **Mpeg1** | 262.8048902 | 248.7702707 | 184.0429042 | 165.6210086 | 149.4737256 | 154.3108173 | 225.5161787 | 84.64547532 | 160.2642081 | 112.733354 | 103.0999062 | 163.9628121 | 149.1147552 | 112.9443314 | 158.0844478 |
| **Dtx4** | 24.723805 | 25.35238427 | 25.06116142 | 22.42784491 | 18.36753408 | 19.63955856 | 34.27845916 | 14.24725822 | 21.06759543 | 12.23462756 | 14.18806048 | 15.61550591 | 27.26669809 | 9.035546516 | 25.14979852 |
| **Fam111a** | 1938.529451 | 2047.20503 | 1980.614914 | 1952.947726 | 2152.801667 | 2058.646585 | 2351.682711 | 1914.161046 | 1776.449743 | 1861.411194 | 1905.929458 | 2273.791167 | 2103.796174 | 2292.318151 | 2063.72061 |
| **A330040F15Rik** | 2.747089445 | 0 | 0 | 0 | 6.333632443 | 2.805651223 | 0.902064715 | 0 | 0 | 0 | 0 | 0.867528106 | 4.260421576 | 0 | 3.59282836 |
| **Gm4952** | 0 | 0.792262009 | 1.566322589 | 0.86260942 | 0 | 0.701412806 | 1.80412943 | 4.190370065 | 1.504828245 | 0.873901969 | 0 | 0 | 0.852084315 | 0 | 0 |
| **Cntf** | 1.831392963 | 4.753572051 | 1.566322589 | 4.313047098 | 1.266726489 | 0.701412806 | 0.902064715 | 0 | 0 | 2.621705907 | 0 | 6.072696744 | 7.668758836 | 0.903554652 | 1.437131344 |
| **Zfp91** | 1209.635052 | 1229.590637 | 1309.445684 | 1232.668861 | 1328.796086 | 1236.590776 | 1404.514761 | 1142.29488 | 1218.910879 | 1233.075678 | 1285.43828 | 1348.138677 | 1418.720385 | 1281.240496 | 1345.873504 |
| **Lpxn** | 1349.736614 | 1248.604925 | 1331.374201 | 1393.114213 | 1277.493664 | 1173.463624 | 1299.875254 | 1537.865814 | 1496.55169 | 1531.950151 | 1417.860177 | 1291.74935 | 1191.213873 | 1303.829362 | 1205.753198 |
| **Olfr1444** | 0.915696482 | 3.169048034 | 0 | 1.725218839 | 0.633363244 | 4.208476834 | 1.80412943 | 0 | 2.257242368 | 1.747803938 | 3.783482795 | 1.735056213 | 2.556252945 | 0 | 0 |
| **Tle4** | 442.2814006 | 591.8197204 | 527.0675512 | 535.6804496 | 558.6263815 | 568.1443726 | 561.9863173 | 597.5467713 | 547.7574812 | 608.2357704 | 599.682023 | 743.4715871 | 665.4778501 | 703.8690736 | 661.7989839 |
| **Psat1** | 7113.130269 | 6457.727631 | 6631.809842 | 6266.857434 | 6293.730558 | 6661.317416 | 6641.00043 | 6470.769455 | 6461.732485 | 6558.634277 | 6053.572472 | 6234.9245 | 6537.190866 | 5800.820863 | 6214.874497 |
| **Cep78** | 218.8514591 | 229.7559825 | 271.7569692 | 208.7514796 | 221.6771355 | 235.6747027 | 237.24302 | 284.1070904 | 263.3449429 | 309.361297 | 245.9263817 | 331.3957366 | 282.8919926 | 271.0663955 | 285.2705718 |
| **Gnaq** | 403.8221484 | 301.0595632 | 339.8920018 | 357.1202997 | 196.975969 | 219.5422082 | 240.8512788 | 408.1420444 | 406.3036262 | 426.4641608 | 438.8840042 | 232.4975325 | 276.0753181 | 224.0815536 | 217.7253986 |
| **E030024N20Rik** | 11.90405426 | 7.922620085 | 16.44638718 | 7.763484777 | 15.83408111 | 15.43108173 | 9.922711863 | 12.5711102 | 3.762070613 | 3.495607876 | 4.729353494 | 6.94022485 | 5.112505891 | 4.517773258 | 12.21561642 |
| **Vps13a** | 1290.216343 | 1213.745397 | 1260.106523 | 1217.141891 | 1109.652404 | 1129.97603 | 1212.374977 | 1247.892205 | 1416.043379 | 1275.896875 | 1356.378582 | 1311.702497 | 1251.711859 | 1130.346869 | 1276.172633 |
| **Gcnt1** | 152.9213124 | 125.9696594 | 166.8133557 | 144.9183825 | 157.7074478 | 158.5192941 | 169.5881664 | 149.1771743 | 129.4152291 | 189.6367273 | 270.5190198 | 159.6251716 | 155.9314297 | 160.832728 | 180.3599837 |
| **Rfk** | 985.2894142 | 941.2072661 | 1030.640264 | 908.3277189 | 955.7451356 | 985.484992 | 974.229892 | 1072.734737 | 900.6397047 | 1056.54748 | 1138.828321 | 980.3067601 | 1066.809563 | 943.3110562 | 970.0636572 |
| **Ostf1** | 3974.12273 | 3516.058794 | 3772.487956 | 3530.660355 | 3137.048149 | 3320.488222 | 3037.251895 | 3166.243621 | 3312.879382 | 3448.417169 | 3513.909646 | 3334.778041 | 3344.430937 | 3494.949392 | 3231.389827 |
| **Nmrk1** | 179.4765104 | 151.3220436 | 198.1398075 | 166.483618 | 132.3729181 | 100.3020312 | 124.4849306 | 170.9670987 | 198.6373284 | 240.3230414 | 196.7411053 | 141.4070813 | 149.9668395 | 93.06612911 | 150.1802254 |
| **2410127L17Rik** | 945.9144655 | 861.1888032 | 941.359876 | 871.2355138 | 827.8057603 | 851.5151461 | 887.6316793 | 995.6319275 | 1036.826661 | 1061.790892 | 987.4890095 | 928.2550737 | 910.0260486 | 869.2195748 | 799.0450272 |
| **D030056L22Rik** | 592.4556236 | 498.3328033 | 552.9118739 | 507.2143388 | 533.2918517 | 530.2680811 | 588.146194 | 630.2316578 | 686.9540939 | 678.1479279 | 674.4058082 | 555.217988 | 587.9381775 | 628.8740375 | 625.1521346 |
| **Trpm6** | 94.3167376 | 101.4095371 | 115.1247103 | 110.4140057 | 69.66995687 | 43.48759395 | 65.85072418 | 123.1968799 | 105.3379772 | 152.0589426 | 179.7154328 | 88.48786684 | 50.27297459 | 79.51280934 | 85.50931496 |
| **Anxa1** | 21.06101908 | 25.35238427 | 36.02541955 | 31.91654853 | 36.73506817 | 31.56357626 | 58.63420646 | 60.34132894 | 36.11587788 | 30.58656891 | 31.21373306 | 38.17123668 | 34.93545692 | 54.21327909 | 40.23967763 |
| **Zfand5** | 1088.763117 | 1048.954899 | 1064.316199 | 1006.665193 | 966.5123108 | 1045.105081 | 1054.513652 | 1036.697554 | 1244.492959 | 1119.468422 | 1156.799865 | 940.4004672 | 922.8073133 | 970.4176958 | 926.2311512 |
| **1110059E24Rik** | 477.9935634 | 442.8744628 | 465.9809702 | 459.7708207 | 457.2882624 | 424.3547475 | 423.0683512 | 552.2907746 | 560.5485213 | 590.757731 | 540.092169 | 449.3795591 | 487.3922283 | 440.0311153 | 454.1335047 |
| **Abhd17b** | 540.2609241 | 570.4286461 | 633.5774872 | 577.9483112 | 502.2570527 | 567.4429598 | 587.2441293 | 576.594921 | 510.1367751 | 547.9365345 | 540.092169 | 557.8205723 | 495.0609871 | 515.929706 | 529.5829002 |
| **Tmem2** | 333.3135193 | 447.6280348 | 485.5600026 | 527.9169648 | 460.4550786 | 431.3688755 | 531.316117 | 517.9297401 | 513.1464316 | 459.6724356 | 526.8499792 | 538.734954 | 506.1380832 | 525.8688072 | 464.1934241 |
| **Klf9** | 163.9096702 | 148.1529956 | 119.8236781 | 156.9949144 | 108.938478 | 131.1641947 | 128.9952542 | 162.5863585 | 143.7110974 | 154.6806485 | 148.5016997 | 134.4668565 | 144.0022493 | 103.9087849 | 94.8506687 |
| **Smc5** | 1162.934532 | 1083.814428 | 1024.374973 | 895.3885776 | 1059.616708 | 1042.299429 | 1151.936641 | 995.6319275 | 1154.955678 | 1048.682363 | 1166.258572 | 1117.376201 | 1156.278416 | 1119.504213 | 1251.022835 |
| **Ptar1** | 121.787632 | 124.3851353 | 118.2573555 | 115.5896622 | 110.2052045 | 108.7189849 | 119.9746071 | 151.6913964 | 169.2931776 | 186.1411194 | 139.0429927 | 114.51371 | 139.7418277 | 107.5230035 | 132.2160836 |
| **Tjp2** | 204.2003154 | 218.6643143 | 223.2009689 | 166.483618 | 203.9429647 | 211.1252545 | 213.7893374 | 268.1836842 | 206.1614696 | 245.5664533 | 180.6613035 | 214.2794423 | 199.3877297 | 210.5282338 | 229.2224494 |
| **Fxn** | 428.5459534 | 451.5893448 | 435.4376797 | 420.0907874 | 414.852925 | 459.4253877 | 459.1509398 | 471.8356693 | 494.3360785 | 445.6900041 | 360.3767362 | 442.4393342 | 488.2443126 | 421.0564676 | 413.8938271 |
| **Pip5k1b** | 0 | 3.961310043 | 2.349483883 | 0.86260942 | 0.633363244 | 8.416953669 | 0.902064715 | 1.676148026 | 0 | 1.747803938 | 0.945870699 | 4.337640531 | 4.260421576 | 1.807109303 | 0 |
| **Fam122a** | 241.7438711 | 236.0940785 | 216.9356786 | 226.0036679 | 259.0455669 | 240.5845924 | 226.4182434 | 315.1158289 | 225.7242368 | 244.6925513 | 250.6557352 | 217.7495547 | 237.7315239 | 258.4166304 | 237.1266717 |
| **Cbwd1** | 341.5547876 | 260.6542008 | 316.397163 | 262.2332636 | 294.5139086 | 317.0385882 | 298.5834206 | 357.0195296 | 282.9077101 | 307.6134931 | 339.5675809 | 299.2971967 | 253.9211259 | 271.9699501 | 269.462127 |
| **Dock8** | 2956.783939 | 3196.777204 | 3176.50221 | 3407.307208 | 2992.641329 | 2858.257183 | 3356.582804 | 3791.446835 | 3284.287645 | 3154.786108 | 3714.434234 | 3258.435567 | 3219.174543 | 3283.517604 | 3077.616773 |
| **Kank1** | 0 | 0 | 0.783161294 | 5.175656518 | 0 | 0 | 0 | 0 | 0 | 6.117313782 | 0 | 0.867528106 | 1.70416863 | 0 | 1.437131344 |
| **Smarca2** | 581.4672658 | 641.7322269 | 671.9523907 | 675.4231756 | 627.6629751 | 627.0630483 | 720.7497071 | 607.6036595 | 638.0471759 | 669.4089082 | 639.4085924 | 694.022485 | 729.3841738 | 671.3411061 | 722.1585003 |
| **4931403E22Rik** | 0 | 3.169048034 | 0.783161294 | 0 | 2.533452977 | 2.104238417 | 0.902064715 | 0.838074013 | 1.504828245 | 0 | 0 | 5.205168638 | 0 | 0.903554652 | 3.59282836 |
| **Vldlr** | 72.34002204 | 38.82083842 | 67.35187133 | 42.26786156 | 29.76807248 | 52.60596043 | 56.83007703 | 7.542666117 | 11.28621184 | 16.60413741 | 34.99721585 | 57.25685501 | 46.86463733 | 42.46706862 | 50.29959704 |

| | | | | | | | | | | | | | | | |
|---|---|---|---|---|---|---|---|---|---|---|---|---|---|---|---|
| D19Bwg1357e | 2482.453162 | 2276.16875 | 2458.343303 | 2311.793245 | 2257.939966 | 2322.3778 | 2322.816641 | 2808.386018 | 2529.61628 | 2584.128122 | 2652.221439 | 2321.505212 | 2243.538002 | 2098.957456 | 2148.511359 |
| C030016D13Rik | 0.915696482 | 0 | 0.783161294 | 1.725218839 | 0 | 1.402825611 | 0 | 0 | 0.752414123 | 0.873901969 | 0 | 0.867528106 | 7.668758836 | 0 | 0 |
| Rfx3 | 80.58129038 | 102.2017991 | 102.5941296 | 106.1009586 | 93.10439691 | 89.78083913 | 80.28375962 | 93.02621545 | 72.23175577 | 103.1204323 | 69.99443171 | 91.95797927 | 91.17302172 | 82.22347329 | 82.63505228 |
| Glis3 | 0 | 0 | 0 | 0 | 0 | 0 | 0 | 0.838074013 | 0 | 0 | 0 | 0 | 0 | 0 | 2.155697016 |
| Slc1a1 | 7.325571853 | 3.961310043 | 0 | 5.175656518 | 3.166816221 | 0.701412806 | 5.412388289 | 0.838074013 | 0 | 0 | 0 | 6.072696744 | 0 | 6.324882561 | 0.718565672 |
| 4430402I18Rik | 0.915696482 | 1.584524017 | 0.783161294 | 0 | 0 | 0 | 0.902064715 | 0.838074013 | 3.00965649 | 0 | 0 | 0 | 0 | 0.903554652 | 0 |
| Ppapdc2 | 141.0172582 | 154.4910917 | 166.8133557 | 119.9027093 | 146.9402727 | 162.7277709 | 168.6861017 | 162.5863585 | 164.7786928 | 133.7070012 | 157.9604067 | 158.7576434 | 202.796067 | 165.3505012 | 165.2701046 |
| Cdc37l1 | 413.8948097 | 441.2899387 | 497.307422 | 475.2977902 | 421.1865574 | 380.1657407 | 452.8364868 | 505.3586299 | 395.7698285 | 486.7633967 | 520.2288843 | 482.3456271 | 446.4921811 | 430.0920141 | 462.7562927 |
| Ak3 | 674.8683069 | 745.51855 | 682.1334875 | 733.2180067 | 639.6968767 | 686.6831368 | 668.4299537 | 684.7064687 | 672.6582256 | 591.6316329 | 672.5140668 | 765.1597897 | 725.9758365 | 699.3513003 | 733.6555511 |
| 1700018L02Rik | 0 | 2.376786026 | 0 | 0 | 0 | 0 | 0 | 0 | 0 | 2.621705907 | 0.945870699 | 0 | 0 | 0 | 0 |
| Gm9895 | 2.747089445 | 0 | 7.831612945 | 0.86260942 | 3.800179466 | 0.701412806 | 2.706194144 | 5.866518091 | 0 | 1.747803938 | 0 | 5.205168638 | 1.70416863 | 1.807109303 | 0 |
| Rcl1 | 1536.538696 | 1413.395423 | 1379.930201 | 1519.917797 | 1399.73277 | 1510.843184 | 1333.251648 | 1905.780306 | 1610.166222 | 1786.255624 | 1642.031533 | 1525.981939 | 1400.826614 | 1328.225338 | 1328.627927 |
| Jak2 | 1789.270925 | 1777.835947 | 1627.40917 | 1762.311044 | 1736.048653 | 1872.070778 | 1925.006101 | 2108.594217 | 1888.559448 | 1743.434428 | 1601.359093 | 1981.434195 | 1843.058374 | 1773.677781 | 1970.307073 |
| Insl6 | 119.9562391 | 97.44822705 | 107.7291622 | 107.8261775 | 99.43802935 | 86.97518791 | 116.3663482 | 186.8905049 | 121.8910879 | 170.4108839 | 165.5273723 | 96.2956198 | 71.57508247 | 95.77679307 | 76.8865269 |
| Plgrkt | 279.2874269 | 313.7357554 | 296.0349693 | 303.6385157 | 370.5174979 | 352.1092285 | 329.2536209 | 337.7438273 | 343.1008399 | 304.9917871 | 326.3253911 | 390.3876478 | 378.3254359 | 374.0716258 | 372.9355838 |
| Cd274 | 529.2725663 | 461.0964889 | 465.1978089 | 501.1760728 | 447.7878137 | 543.5949244 | 506.9603697 | 564.0238108 | 538.7285118 | 407.2383175 | 455.9096768 | 440.704278 | 507.8422518 | 521.351034 | 506.5887987 |
| Pdcd1lg2 | 226.1770309 | 194.1041921 | 201.2724527 | 194.0871194 | 173.5415289 | 152.9079916 | 213.7893374 | 286.6213125 | 246.7918322 | 222.8450021 | 207.145683 | 170.9030369 | 175.5293689 | 191.5535861 | 184.6713777 |
| A930007I19Rik | 0 | 0 | 0.783161294 | 0 | 3.166816221 | 3.507064029 | 0 | 0 | 1.504828245 | 6.991215751 | 1.891741398 | 3.470112425 | 0 | 0 | 2.155697016 |
| Ric1 | 640.0718406 | 636.9786548 | 649.2407131 | 635.7431423 | 504.1571424 | 546.4005757 | 618.8163943 | 593.3564012 | 668.1437409 | 611.7313782 | 557.1178416 | 591.6541685 | 589.6423461 | 573.7572037 | 549.7027391 |
| Ermp1 | 763.6908656 | 749.47986 | 811.3551011 | 764.2719458 | 772.0697948 | 748.4074637 | 755.930231 | 845.6166792 | 796.0541417 | 776.0249484 | 866.4175601 | 875.3358592 | 843.563472 | 852.5744911 | 749.4639959 |
| 9930021J03Rik | 548.5021925 | 404.8458863 | 474.5957445 | 397.6629425 | 401.5522969 | 427.8618115 | 508.7644991 | 463.4549292 | 514.6512598 | 438.6987884 | 424.6959437 | 508.3714703 | 483.983891 | 436.4168967 | 433.2951002 |
| Ranbp6 | 216.1043696 | 232.1327685 | 255.310582 | 219.965402 | 214.7101398 | 214.6323185 | 248.9698613 | 296.6782006 | 272.3739124 | 248.1881592 | 267.6814078 | 249.8480946 | 222.3940063 | 251.1881931 | 276.6477837 |
| Il33 | 10.98835778 | 15.05297816 | 3.915806472 | 2.587828259 | 0 | 2.104238417 | 2.706194144 | 9.218814144 | 6.771727103 | 4.369509845 | 4.729353494 | 0 | 6.816674521 | 4.517773258 | 1.437131344 |
| Uhrf2 | 990.7835931 | 973.6900085 | 1022.808651 | 937.6564392 | 864.5408284 | 901.3154553 | 950.7762094 | 1019.098 | 1008.234924 | 987.5092249 | 1000.731199 | 915.2421521 | 916.8427231 | 959.57504 | 946.35099 |
| Gldc | 125.450418 | 166.3750218 | 163.6807105 | 132.8418506 | 53.20251252 | 47.69607079 | 46.90736517 | 121.5207319 | 92.54693708 | 167.789178 | 182.5530449 | 71.13730471 | 51.12505891 | 64.15238026 | 63.9523448 |
| Cstf2t | 574.1416939 | 602.5244089 | 644.5417454 | 699.5762328 | 659.3311137 | 692.2944392 | 736.986872 | 734.3909094 | 687.706008 | 666.1656964 | 588.3315746 | 714.8431596 | 655.2528384 | 694.8335271 | 681.9188227 |
| A1cf | 17.39823315 | 7.130358077 | 7.04845165 | 13.80175071 | 9.500448664 | 13.32684331 | 15.33510015 | 9.218814144 | 3.00965649 | 11.3607256 | 5.675224193 | 4.337640531 | 12.78126473 | 11.74621047 | 6.467091048 |
| Asah2 | 62.26736075 | 72.09584277 | 72.05083909 | 61.2452688 | 59.53614496 | 46.99465798 | 73.0672419 | 46.09407072 | 38.37312025 | 67.29045161 | 69.04856101 | 45.97898963 | 72.42716679 | 45.17773258 | 61.07808212 |
| Sgms1 | 86.99116575 | 66.55000871 | 94.76251663 | 78.49745719 | 87.40412771 | 121.3444154 | 118.1704776 | 76.26473519 | 75.24141226 | 100.4987264 | 78.507268 | 107.5734852 | 155.0793454 | 115.6549954 | 102.0363254 |
| 2700046G09Rik | 0.915696482 | 1.584524017 | 3.132645178 | 0.86260942 | 0 | 0 | 0 | 0 | 3.00965649 | 0 | 0.945870699 | 0 | 0.852084315 | 0 | 0 |
| Minpp1 | 1017.338791 | 1193.938847 | 1140.282845 | 1186.950561 | 1159.6881 | 1159.435368 | 1179.900647 | 1287.281684 | 1260.293655 | 1185.01107 | 1118.019166 | 1180.705753 | 1147.757573 | 1082.458473 | 1148.986509 |
| Papss2 | 62.26736075 | 58.62738863 | 60.30341968 | 57.79483112 | 36.73506817 | 33.66781467 | 29.76813559 | 213.7088733 | 198.6373284 | 162.5457662 | 127.6925443 | 58.99191123 | 49.42089028 | 52.40616979 | 36.64684927 |
| Atad1 | 1971.494525 | 1884.791318 | 2043.267817 | 1792.502374 | 1892.489374 | 1896.620227 | 2085.573621 | 1912.484898 | 1818.584934 | 1839.563645 | 1804.721293 | 1974.49397 | 1926.562637 | 1765.545789 | 1916.414647 |
| Pten | 1348.820917 | 1419.733519 | 1410.473491 | 1578.575238 | 1459.902278 | 1483.488084 | 1583.123574 | 1696.261802 | 1586.08897 | 1377.269503 | 1451.911523 | 1677.799358 | 1526.935093 | 1555.92111 | 1591.622963 |
| Rnls | 16.48253667 | 25.35238427 | 32.89277437 | 25.01567317 | 18.36753408 | 16.83390734 | 23.45368258 | 16.76148026 | 36.86829201 | 25.3431571 | 29.32199166 | 23.42325887 | 40.04796281 | 28.91374885 | 27.30549553 |
| Lipo1 | 72.34002204 | 72.09584277 | 84.5814198 | 84.53572313 | 60.80287145 | 76.45399582 | 77.57756547 | 136.6060641 | 81.26072524 | 120.5984717 | 99.31642337 | 81.54764199 | 103.1022021 | 59.634607 | 76.16796123 |
| Lipm | 0.915696482 | 2.376786026 | 1.566322589 | 6.900875357 | 3.166816221 | 0.701412806 | 1.80412943 | 0.838074013 | 0 | 0.873901969 | 0 | 0.867528106 | 3.408337261 | 1.807109303 | 5.748525376 |
| Stambpl1 | 717.9060415 | 817.6143928 | 794.9087139 | 696.9884111 | 756.8690769 | 740.6919228 | 827.1933435 | 731.6386134 | 768.2148192 | 738.4471637 | 636.5709803 | 774.7025989 | 754.9467032 | 676.762434 | 711.3800152 |
| Acta2 | 0.915696482 | 3.169048034 | 0.783161294 | 3.450437679 | 1.266726489 | 3.507064029 | 4.510323574 | 3.352296052 | 1.504828245 | 0.873901969 | 4.729353494 | 6.94022485 | 0 | 0 | 5.029959704 |
| Fas | 507.2958508 | 528.4387597 | 549.7792287 | 478.7482279 | 502.2570527 | 505.0172201 | 464.5633281 | 703.144097 | 579.3588744 | 569.7840837 | 548.6050053 | 455.4522558 | 449.0484341 | 475.2697467 | 501.558839 |
| Ch25h | 0 | 3.169048034 | 8.614774239 | 0 | 1.900089733 | 5.611302446 | 9.922711863 | 5.028444078 | 3.00965649 | 0 | 0.945870699 | 3.470112425 | 8.520843152 | 3.614218606 | 3.5928236 |
| Lipa | 492.6447071 | 546.6607859 | 559.9603256 | 496.8630257 | 510.4907749 | 580.0683903 | 600.7751 | 493.6255937 | 416.0850098 | 512.1065538 | 546.7132639 | 591.6541685 | 529.1443597 | 637.909584 | 648.1462361 |
| Ifit2 | 950.4929479 | 762.1560522 | 696.2303908 | 587.4370148 | 717.6005558 | 1005.124551 | 905.6729736 | 344.4484194 | 401.0367273 | 358.2998073 | 421.8583317 | 693.1549569 | 657.8090913 | 721.036612 | 771.7395317 |
| Ifit3 | 769.1850445 | 619.5488907 | 472.2462606 | 390.7620671 | 691.6326627 | 972.8595615 | 871.3945145 | 116.4922878 | 144.4635115 | 102.2465304 | 180.6613035 | 562.1582129 | 473.7588792 | 435.5133421 | 601.4394674 |
| Ifit1bl1 | 380.0140398 | 411.1839824 | 292.9023241 | 257.0576071 | 481.3560656 | 556.2203549 | 573.7131586 | 101.4069556 | 97.06142181 | 88.26409886 | 93.64119918 | 445.0419185 | 444.7880125 | 393.9498281 | 428.2651405 |
| Ifit3b | 171.2352421 | 112.5012052 | 117.4741942 | 100.9253021 | 159.6075376 | 230.7648131 | 228.2223728 | 43.57984868 | 37.62070613 | 25.3431571 | 26.48437957 | 128.3941597 | 92.87719035 | 130.1118698 | 139.4017404 |
| Ifit1bl2 | 16.48253667 | 17.42976419 | 17.22954848 | 10.35131304 | 8.233722176 | 11.22260489 | 12.62890601 | 3.352296052 | 7.524141226 | 1.747803938 | 0 | 10.41033728 | 9.372927467 | 4.517773258 | 8.622788064 |
| Ifit1 | 854.3448173 | 555.375668 | 429.9555507 | 415.7774403 | 586.4943642 | 931.476206 | 751.4199074 | 87.15969736 | 121.8910879 | 73.40776539 | 125.8008029 | 454.5847277 | 403.0358811 | 445.4524432 | 556.1698301 |
| Pank1 | 108.0521848 | 122.8006113 | 101.8109683 | 137.1548977 | 130.4728283 | 125.5528922 | 152.4489368 | 129.063398 | 99.31866418 | 93.50751067 | 112.5586132 | 142.2746094 | 154.227261 | 137.340307 | 160.2401448 |
| Kif20b | 1407.425492 | 1550.456751 | 1570.238395 | 1564.773487 | 1417.466941 | 1311.641947 | 1578.613251 | 1523.618556 | 1672.616594 | 1514.472112 | 1625.00586 | 1603.19194 | 1546.533032 | 1613.748608 | 1612.461368 |
| Mir1950 | 0 | 0 | 1.566322589 | 0 | 0.633363244 | 0 | 0 | 0 | 0 | 0.873901969 | 0 | 2.602584319 | 0 | 0 | 0 |
| Rpp30 | 456.9325443 | 487.2411352 | 570.9245837 | 574.4978735 | 528.858309 | 506.4200457 | 496.1355931 | 558.1572927 | 571.082319 | 530.4584951 | 585.4939625 | 530.927201 | 444.7880125 | 530.3865805 | 487.1875256 |
| Pcgf5 | 1487.091086 | 1369.821013 | 1260.889684 | 1267.173237 | 1156.521284 | 1249.216207 | 1288.148413 | 1136.428362 | 1073.694953 | 1125.585736 | 1184.230115 | 1272.663732 | 1227.001414 | 1160.164173 | 1322.879402 |
| Hectd2 | 53.11039593 | 65.75774671 | 51.68864544 | 47.44351808 | 73.47013634 | 59.62008849 | 55.0259476 | 58.66518091 | 79.00348287 | 55.92972601 | 78.507268 | 65.93213608 | 88.61676878 | 57.8274977 | 66.10804182 |
| Tnks2 | 3508.033221 | 3874.953484 | 3961.229828 | 3917.971984 | 3407.494254 | 3310.668443 | 3867.151432 | 4058.792445 | 4254.149449 | 4026.066371 | 4410.595068 | 3878.718163 | 3757.69183 | 3802.157974 | 3693.427554 |
| Fgfbp3 | 37.54355574 | 38.02857641 | 36.80858084 | 54.34439344 | 53.83587576 | 42.78618115 | 55.92801232 | 60.34132894 | 57.93588744 | 82.14678508 | 68.10269031 | 45.11146153 | 41.75213144 | 37.94929537 | 57.48525376 |
| Btaf1 | 1645.506577 | 1735.053799 | 1756.630784 | 1744.196247 | 1484.603445 | 1378.276163 | 1607.479322 | 1988.749633 | 1954.019476 | 1908.6019 | 2057.26877 | 1637.025537 | 1708.429052 | 1540.560681 | 1569.347428 |
| Cpeb3 | 0.915696482 | 0 | 5.482129061 | 4.313047098 | 3.166816221 | 2.104238417 | 4.510323574 | 4.190370065 | 0 | 0 | 0.945870699 | 2.602584319 | 0.852084315 | 4.517773258 | 1.437131344 |
| March5 | 1192.236819 | 1185.223965 | 1162.994522 | 1099.82701 | 1077.984242 | 1362.143669 | 1160.055223 | 1133.076066 | 1228.692262 | 1180.64156 | 1225.848426 | 1104.363279 | 1198.882631 | 1158.357063 | 1154.735035 |
| Ide | 2376.23237 | 2238.932436 | 2401.172529 | 2289.3654 | 2328.876609 | 2344.823009 | 2480.677966 | 2736.311653 | 2554.445946 | 2503.729141 | 2701.406716 | 2390.039933 | 2426.73613 | 2331.171001 | 2429.470537 |
| Kif11 | 4237.843317 | 4635.525012 | 4913.553962 | 4760.741387 | 4910.465233 | 4571.808668 | 4798.984283 | 4681.481437 | 4834.260738 | 4784.61328 | 5014.060574 | 5209.506278 | 5275.253995 | 5329.165335 | 5214.631081 |
| Hhex | 3.662785926 | 11.88393013 | 3.132645178 | 6.038265938 | 3.800179466 | 2.104238417 | 9.922711863 | 2.514222039 | 6.019312981 | 5.243411813 | 9.458706988 | 10.41033728 | 8.520843152 | 9.035546516 | 11.49705075 |
| Exoc6 | 716.9903451 | 709.8667596 | 775.3296815 | 752.1954139 | 735.3347266 | 725.2608411 | 743.301325 | 695.6014308 | 653.8478725 | 712.2301047 | 680.0810324 | 723.5184406 | 691.8924639 | 709.2904015 | 716.409975 |
| Cyp26c1 | 0 | 0 | 0 | 0 | 0 | 0 | 0 | 0 | 0 | 0.873901969 | 0.945870699 | 0 | 0 | 0.903554652 | 1.437131344 |
| Myof | 7.325571853 | 7.922620085 | 10.18109683 | 10.35131304 | 4.43354271 | 7.715540863 | 1.80412943 | 10.05688816 | 2.257242368 | 6.991215751 | 14.18806048 | 1.735056213 | 2.556252945 | 2.710663955 | 3.5928236 |
| Cep55 | 1151.030477 | 1243.059091 | 1233.479039 | 1210.241016 | 1243.292049 | 1183.283403 | 1249.35963 | 1199.283913 | 1248.255029 | 1249.679816 | 1118.965037 | 1192.851146 | 1144.349235 | 1252.326747 | 1177.010571 |
| Ffar4 | 7.325571853 | 5.54583406 | 2.349483883 | 5.175656518 | 10.13381191 | 10.52119209 | 9.020647148 | 16.76148026 | 5.266898858 | 11.3607256 | 7.56696559 | 11.27786538 | 6.816674521 | 1.807109303 | 11.49705075 |
| Rbp4 | 0.915696482 | 0 | 0.783161294 | 0 | 0.633363244 | 0 | 0 | 9.218814144 | 0 | 1.747803938 | 4.729353494 | 1.735056213 | 1.70416863 | 0 | 0 |
| Fra10ac1 | 200.5375295 | 176.6744279 | 225.5504528 | 213.0645267 | 253.978661 | 227.2577491 | 198.4542373 | 251.4222039 | 226.4766509 | 229.8362178 | 213.7667779 | 243.7753979 | 259.8857161 | 209.6246792 | 227.785318 |
| Slc35g1 | 926.6848393 | 997.4578687 | 921.7808436 | 933.3433921 | 1249.625681 | 1304.627819 | 1298.071125 | 1018.259926 | 1051.122529 | 1018.095794 | 954.3835351 | 1293.484406 | 1376.968253 | 1270.37984 | 1285.513987 |
| Plce1 | 0 | 0 | 4.698967767 | 0 | 0.633363244 | 0.701412806 | 0.902064715 | 0 | 0 | 1.747803938 | 5.675224193 | 0 | 2.556252945 | 0 | 1.437131344 |
| Noc3l | 988.0365036 | 946.7531002 | 1060.400393 | 970.4355971 | 988.0466611 | 949.7129389 | 1131.189152 | 1248.730279 | 1146.679123 | 1071.403814 | 1252.332805 | 945.6056358 | 1025.909515 | 1041.798513 | 992.339193 |
| Tbc1d12 | 58.60457482 | 54.66607859 | 72.83400039 | 32.77915795 | 55.10260225 | 43.48759395 | 58.63420646 | 51.1225148 | 48.90691797 | 53.3080201 | 59.58985402 | 55.5217988 | 60.49798638 | 56.0203884 | 45.98820301 |
| Hells | 2183.936109 | 2340.341973 | 2257.070851 | 2366.137638 | 2154.701757 | 2184.09089 | 2216.373004 | 3024.609113 | 2773.398456 | 2772.890947 | 2824.369907 | 2416.065776 | 2229.904653 | 2216.41956 | 2112.583076 |
| Pdlim1 | 1756.305852 | 1854.685362 | 1793.439364 | 1738.157981 | 1839.286861 | 1712.148659 | 1789.696394 | 1288.119758 | 1471.722024 | 1334.448307 | 1539.877498 | 1789.710483 | 1724.618654 | 1766.449344 | 1865.396484 |
| Sorbs1 | 102.5580059 | 64.9654847 | 104.1604522 | 100.9253021 | 93.73776015 | 110.8232233 | 111.8560246 | 91.35006742 | 112.1097043 | 96.12921658 | 71.88617311 | 105.838429 | 78.39175699 | 126.4976512 | 91.97640601 |
| Aldh18a1 | 4169.166081 | 3558.840942 | 3729.414084 | 3678.166565 | 3383.426451 | 3516.883808 | 3667.79513 | 3081.598146 | 3214.313132 | 3445.795463 | 3177.179677 | 3285.328938 | 3318.016323 | 3080.217807 | 3471.390761 |
| Tctn3 | 106.2207919 | 89.52560696 | 86.93090369 | 135.4296789 | 112.1052942 | 99.60061841 | 123.5828659 | 132.4156941 | 116.624189 | 104.8682363 | 111.6127425 | 104.1033728 | 120.9959728 | 105.7158942 | 105.6291538 |
| Entpd1 | 114.4620602 | 111.7089432 | 125.3058071 | 104.3757398 | 130.4728283 | 119.9415898 | 141.6241602 | 42.74177467 | 86.5276241 | 59.42533389 | 70.94030241 | 110.1760695 | 142.2980806 | 118.3656594 | 137.964609 |
| Ccnj | 89.73825519 | 142.6071615 | 84.5814198 | 78.49745719 | 76.63695256 | 83.46812388 | 77.57756547 | 118.1684358 | 86.5276241 | 85.64239295 | 78.507268 | 90.22292305 | 124.40431 | 98.48745702 | 74.73082988 |
| Zfp518a | 206.9474048 | 226.5869344 | 223.9841302 | 202.7132136 | 231.1775842 | 212.5280801 | 233.6197461 | 189.404727 | 222.7145803 | 193.1323351 | 182.5530449 | 178.7107899 | 192.5710552 | 213.2388978 | 196.1684284 |
| Dntt | 30.21798389 | 30.10595632 | 51.68864544 | 46.58090866 | 16.46744435 | 32.96640187 | 23.45368258 | 8.380740131 | 12.79104008 | 10.48682363 | 12.29631908 | 18.21809023 | 34.08337261 | 28.91374885 | 26.58692986 |
| Tm9sf3 | 4680.124717 | 4607.003579 | 5061.571446 | 4573.555143 | 4361.3393 | 4597.059529 | 4945.118766 | 4771.99343 | 4995.27736 | 4800.343515 | 4719.894787 | 4839.071777 | 4611.480314 | 4594.575403 | 4649.119898 |
| Pik3ap1 | 548.5021925 | 512.5935195 | 403.3280667 | 379.5481446 | 447.1544505 | 528.1638427 | 576.4193527 | 108.9496217 | 151.2352386 | 142.4460209 | 105.9375183 | 347.8787706 | 410.7046399 | 409.3102572 | 478.5647375 |
| Lcor | 77.83420093 | 66.55000871 | 77.53296815 | 78.49745719 | 76.63695256 | 60.32150129 | 73.96930661 | 103.9211776 | 74.48899814 | 63.79484373 | 50.13114703 | 65.93213608 | 71.57508247 | 95.77679307 | 58.9223851 |
| Mir8091 | 3.662785926 | 11.09166812 | 11.74741942 | 12.07653188 | 2.533452977 | 7.715540863 | 9.922711863 | 2.514222039 | 7.524141226 | 1.747803938 | 19.86328467 | 10.41033728 | 24.71044514 | 6.324882561 | 9.341353736 |
| Slit1 | 2.747089445 | 0 | 7.831612945 | 0 | 1.266726489 | 2.104238417 | 0 | 0 | 0 | 0 | 7.56696559 | 0 | 0 | 0.903554652 | 1.437131344 |
| Arhgap19 | 1233.443161 | 1548.079965 | 1597.649041 | 1620.8431 | 1352.230527 | 1327.774441 | 1505.546009 | 1536.189666 | 1744.095936 | 1570.401838 | 1727.159896 | 1549.405198 | 1437.46624 | 1606.52017 | 1557.850377 |
| Frat1 | 64.09875371 | 43.57441047 | 50.12232285 | 81.08528545 | 46.86888008 | 54.00878604 | 46.00530045 | 55.31288486 | 42.13519086 | 43.69509845 | 46.34766424 | 64.19707986 | 61.35007069 | 47.88839653 | 57.48525376 |
| Frat2 | 109.8835778 | 83.9797729 | 91.62987146 | 106.963568 | 90.57094393 | 103.8090952 | 91.10853619 | 90.51199341 | 105.3379772 | 86.51629492 | 85.12836289 | 134.4668565 | 91.17302172 | 103.0052303 | 120.0004672 |
| Rrp12 | 2105.186211 | 2045.620506 | 1990.012849 | 1905.504208 | 1965.326147 | 1930.989454 | 2029.645608 | 2255.257169 | 2306.149286 | 2231.945629 | 2338.192367 | 1828.749248 | 1963.202262 | 1833.312388 | 2026.355195 |
| Pgam1 | 7367.693891 | 6984.581867 | 6974.834489 | 6756.819584 | 6223.427238 | 6645.184921 | 6381.205792 | 5339.369537 | 5637.086606 | 6275.490039 | 6247.475965 | 6585.405855 | 6765.549462 | 6511.918374 | 6326.970742 |
| Exosc1 | 646.481716 | 765.3251002 | 720.5083909 | 728.9049596 | 703.6665644 | 711.232585 | 699.100154 | 750.0762417 | 820.8838077 | 785.63787 | 765.2093953 | 635.8981019 | 666.3299345 | 758.9859073 | 731.4998541 |
| Zdhhc16 | 416.6418991 | 461.888751 | 414.2923248 | 410.6020838 | 364.8172287 | 374.5544383 | 396.0064098 | 414.8466365 | 452.9533018 | 528.7106912 | 465.3683838 | 407.73821 | 420.9296517 | 436.4168967 | 452.6953733 |
| Mms19 | 1019.170184 | 1032.317397 | 1033.772909 | 1031.680866 | 999.4471195 | 940.5945725 | 1022.941387 | 974.6800772 | 1030.807348 | 1148.307187 | 1065.996278 | 1044.50384 | 892.9843623 | 969.5141411 | 1000.961981 |
| Ubtd1 | 204.2003154 | 183.012524 | 160.5480654 | 251.8819505 | 152.6405419 | 159.2207069 | 157.8613251 | 231.3084276 | 267.1070135 | 255.1793749 | 230.7924505 | 177.8432618 | 162.7481042 | 159.9291733 | 151.6173568 |
| Hoga1 | 2.747089445 | 0 | 2.349483883 | 1.725218839 | 0.633363244 | 1.402825611 | 0.902064715 | 5.866518091 | 0 | 0.873901969 | 0 | 0.867528106 | 3.408337261 | 0 | 0.718565672 |
| Morn4 | 52.19469945 | 22.18333624 | 36.80858084 | 42.26786156 | 19.63426057 | 22.44520978 | 25.25781201 | 25.9802944 | 39.12553437 | 65.54264767 | 38.78069865 | 36.43618046 | 32.37920398 | 24.39597559 | 22.9941015 |
| Pi4k2a | 1201.393784 | 1187.600751 | 1086.244715 | 973.0234254 | 961.4454048 | 1122.260489 | 981.4464097 | 941.1571167 | 1011.996995 | 1108.107697 | 977.0844318 | 982.9093444 | 906.6177113 | 1046.316287 | 1087.908427 |
| Avpi1 | 309.5054108 | 327.2042095 | 250.6116142 | 281.2106708 | 190.6423365 | 232.1676387 | 211.0831433 | 266.5075362 | 264.8497711 | 276.1530222 | 294.1657873 | 185.6510147 | 167.0085258 | 204.2033513 | 202.6355195 |
| Marveld1 | 314.9995897 | 317.6970654 | 307.7823887 | 292.4245933 | 297.0473616 | 259.5227381 | 202.0624961 | 336.0676792 | 343.1008399 | 414.2295333 | 321.5960376 | 304.5023653 | 297.377426 | 292.7517071 | 271.617824 |
| Zfyve27 | 446.859883 | 554.583406 | 491.8252929 | 506.3517293 | 491.4898776 | 536.5807964 | 480.800493 | 487.7590756 | 516.9085022 | 437.8248864 | 442.667487 | 486.6832676 | 486.540144 | 509.6048235 | 513.7744555 |
| Crtac1 | 1.831392963 | 0.792262009 | 0 | 2.587828259 | 1.266726489 | 0.701412806 | 0 | 0 | 1.504828245 | 0.873901969 | 4.729353494 | 1.735056213 | 0.852084315 | 8.131991864 | 2.874262688 |
| R3hcc1l | 341.5547876 | 366.8173099 | 372.0016149 | 313.9898288 | 332.5157032 | 363.3318334 | 414.0477041 | 371.2667878 | 319.023588 | 367.9127289 | 362.2684776 | 379.1097824 | 368.1004241 | 413.8280304 | 349.9414822 |
| Loxl4 | 1.831392963 | 4.753572051 | 0.783161294 | 0.86260942 | 1.266726489 | 2.104238417 | 1.80412943 | 8.380740131 | 13.54345421 | 5.243411813 | 2.837612096 | 1.735056213 | 4.260421576 | 4.517773258 | 0.718565672 |
| Pyroxd2 | 0 | 0 | 0 | 0 | 1.266726489 | 0 | 0.902064715 | 0 | 0 | 1.747803938 | 0 | 2.602584319 | 0.852084315 | 0.903554652 | 0.718565672 |
| Hps1 | 454.1854549 | 476.9417291 | 451.8840669 | 498.5882446 | 511.7575014 | 493.0932024 | 495.2335284 | 385.514046 | 398.0270708 | 376.6517486 | 418.0748489 | 481.478099 | 500.173493 | 488.8230665 | 496.5288793 |
| Cnnm1 | 1.831392963 | 0 | 0 | 0 | 0 | 1.402825611 | 2.706194144 | 2.514222039 | 0 | 0 | 0 | 2.602584319 | 0 | 1.807109303 | 0.718565672 |
| Got1 | 2129.910016 | 1816.656786 | 1907.780913 | 1859.785909 | 1696.146768 | 1725.475502 | 1761.732388 | 1937.627118 | 1891.569104 | 1851.798272 | 1985.382597 | 1650.038458 | 1764.666617 | 1619.169936 | 1685.755066 |
| Slc25a28 | 380.9297363 | 354.1411178 | 386.0985182 | 370.9220505 | 410.4193823 | 384.3742175 | 359.0217565 | 356.1814555 | 392.0077579 | 348.6868856 | 349.9721585 | 336.6009052 | 350.2066535 | 367.7467432 | 378.6841091 |
| BC037704 | 1.831392963 | 0 | 0 | 0.86260942 | 0.633363244 | 2.104238417 | 0.902064715 | 2.514222039 | 3.762070613 | 0.873901969 | 2.837612096 | 0.867528106 | 5.112505891 | 3.614218606 | 0.718565672 |
| Entpd7 | 200.5375295 | 191.7274061 | 191.0913559 | 198.4001665 | 155.1739948 | 182.3673295 | 231.8306317 | 274.0502023 | 217.4476814 | 228.9623159 | 161.7438895 | 208.2067455 | 207.9085729 | 216.8531164 | 225.629621 |
| Cox15 | 853.4291208 | 755.0256941 | 874.7911659 | 747.0197574 | 794.2375083 | 799.6105985 | 820.8788905 | 841.4263091 | 750.9092943 | 832.8285764 | 828.5827321 | 792.9206891 | 877.6468446 | 786.9961015 | 811.9792093 |
| Cutc | 110.7992743 | 124.3851353 | 93.97935534 | 124.2157564 | 107.0383883 | 113.6288745 | 101.9333128 | 105.5973256 | 112.8621184 | 105.7421382 | 147.555829 | 122.321463 | 120.9959728 | 108.4265582 | 79.04222392 |
| Dnmbp | 186.8020822 | 182.220262 | 188.741872 | 173.3844933 | 148.8403624 | 152.9079916 | 200.2583667 | 113.9780658 | 177.5697329 | 170.4108839 | 159.8521481 | 137.9369689 | 191.7189709 | 158.122064 | 140.8388717 |
| Erlin1 | 319.5780781 | 402.4691003 | 421.3407764 | 359.7081228 | 364.8172287 | 343.6922748 | 331.058815 | 510.3870739 | 490.5740079 | 466.6636514 | 477.6646917 | 358.2891079 | 338.2774731 | 376.7822897 | 429.7022718 |
| Chuk | 1087.84742 | 1246.228139 | 1172.392458 | 1071.360899 | 1121.686306 | 1078.071482 | 1030.157904 | 1203.474283 | 1239.978474 | 1365.907778 | 1426.373014 | 1325.582946 | 1268.753545 | 1330.032447 | 1234.495824 |
| Cwf19l1 | 472.4993845 | 511.0089955 | 500.4400672 | 439.0681946 | 494.6566938 | 443.2928932 | 508.7644991 | 473.5118174 | 431.1332922 | 504.2414361 | 537.2545569 | 493.6234925 | 414.9650615 | 586.4069689 | 470.6605151 |
| Bloc1s2 | 564.0690326 | 694.0215194 | 755.7506492 | 663.3466437 | 648.5639621 | 699.3085673 | 623.3267179 | 988.0892614 | 1002.215611 | 997.1221465 | 945.8706988 | 670.5992262 | 670.590356 | 721.9401666 | 674.0146003 |
| Scd3 | 0.915696482 | 0.792262009 | 0.783161294 | 0.86260942 | 0 | 0 | 1.80412943 | 0 | 0 | 0 | 0 | 4.337640531 | 1.70416863 | 0 | 0.718565672 |
| Scd2 | 35372.43939 | 35582.07133 | 36613.57368 | 35941.48408 | 34086.97644 | 33676.93304 | 36096.11956 | 30070.93366 | 32600.5991 | 33878.55763 | 34756.9647 | 35851.46652 | 36382.29609 | 34433.56422 | 35562.53367 |
| Scd4 | 0 | 8.714882094 | 3.915806472 | 2.587828259 | 6.333632443 | 8.416953669 | 1.80412943 | 4.190370065 | 3.00965649 | 2.621705907 | 0 | 6.072696744 | 5.112505891 | 2.710663955 | 5.029959704 |
| Scd1 | 7120.455841 | 6266.000225 | 6363.185518 | 5885.58407 | 4834.461644 | 4981.433746 | 5623.471432 | 3427.722713 | 4400.870203 | 5023.188517 | 5504.967467 | 5312.742123 | 5608.418962 | 4955.997264 | 5451.039188 |
| Sec31b | 3.662785926 | 1.584524017 | 2.349483883 | 0 | 0.633363244 | 0 | 5.412388289 | 2.514222039 | 2.257242368 | 4.369509845 | 2.837612096 | 3.470112425 | 4.260421576 | 10.84265582 | 3.5928236 |
| Ndufb8 | 1705.026849 | 1638.397834 | 1728.436977 | 1684.676197 | 1729.081657 | 1702.328879 | 1804.12943 | 1744.032021 | 1800.526995 | 1828.202919 | 1625.00586 | 1730.718572 | 1699.056124 | 1694.164972 | 1664.916662 |
| Hif1an | 773.7635269 | 751.8566461 | 812.9214237 | 872.9607327 | 742.9350855 | 748.4074637 | 764.9508781 | 895.90112 | 881.0769375 | 819.7200468 | 761.4259125 | 711.3730471 | 739.6091856 | 749.0468062 | 674.0146003 |
| Fam178a | 828.7053158 | 854.0584452 | 890.4543918 | 872.0981233 | 862.0073755 | 771.5540863 | 847.9408319 | 990.6034834 | 1000.710783 | 970.9050875 | 929.7908969 | 970.7639509 | 936.4406624 | 899.940433 | 852.9374526 |
| Sema4g | 10.0726613 | 15.05297816 | 6.265290356 | 21.56523549 | 14.56735462 | 11.9240177 | 6.314453003 | 5.028444078 | 14.29586833 | 3.495607876 | 2.837612096 | 11.27786538 | 5.964590206 | 9.035546516 | 3.5928236 |
| Mrpl43 | 1020.08588 | 952.2989342 | 949.9746502 | 979.9243007 | 967.145674 | 942.6988109 | 984.1526038 | 950.3759308 | 966.8521475 | 933.3273028 | 886.2808447 | 988.1145131 | 929.6239878 | 993.9101167 | 924.0754541 |
| Peo1 | 417.5575956 | 418.3143405 | 474.5957445 | 452.8699453 | 458.5549889 | 373.1516126 | 467.2695223 | 506.1967039 | 492.8312503 | 425.5902589 | 466.3142545 | 463.2600088 | 441.3796753 | 393.0462734 | 457.0077674 |
| Lzts2 | 249.9851395 | 221.0411004 | 255.310582 | 236.354981 | 171.6414392 | 210.4238417 | 199.356302 | 156.7198404 | 185.0938742 | 191.3845312 | 175.93195 | 189.1211272 | 215.5773317 | 172.5789385 | 183.2342464 |
| Pdzd7 | 0 | 0 | 3.915806472 | 3.450437679 | 0 | 0 | 3.608258859 | 0 | 3.762070613 | 1.747803938 | 1.891741398 | 6.94022485 | 0 | 0 | 2.874262688 |
| Sfxn3 | 391.9180941 | 404.8458863 | 413.5091635 | 378.6855352 | 430.6870061 | 413.1321426 | 407.7332511 | 357.0195296 | 291.1842654 | 288.3876497 | 327.2712618 | 444.1743904 | 439.6755066 | 444.5488886 | 422.5166151 |
| Kazald1 | 0.915696482 | 0.792262009 | 0.783161294 | 1.725218839 | 0 | 2.104238417 | 4.510323574 | 2.514222039 | 5.266898858 | 1.747803938 | 0.945870699 | 1.735056213 | 5.112505891 | 1.807109303 | 2.155697016 |
| Btrc | 193.2119576 | 194.8964541 | 209.1040656 | 167.3462274 | 208.3765074 | 178.8602655 | 193.041849 | 222.9276875 | 240.0201051 | 218.4754922 | 259.1685715 | 161.3602278 | 184.0502121 | 200.5891326 | 158.8030135 |
| Poll | 353.4588419 | 338.2958776 | 295.251808 | 309.6767817 | 319.2150751 | 313.5315242 | 344.588721 | 359.5337516 | 331.8146281 | 311.9830029 | 297.9492701 | 366.964389 | 304.1941005 | 334.3152211 | 320.4802897 |
| Dpcd | 393.7494871 | 384.2470741 | 375.1342601 | 357.1202997 | 410.4193823 | 366.1374846 | 374.3568566 | 338.5819013 | 367.1780918 | 387.1385722 | 376.4565381 | 353.9514674 | 355.3191594 | 354.1934234 | 351.3786136 |
| Fbxw4 | 263.7205867 | 252.7315807 | 280.3717434 | 248.4315129 | 281.2132805 | 321.247065 | 285.9545146 | 243.8795378 | 263.3449429 | 299.7483753 | 229.8465798 | 249.8480946 | 269.2586436 | 303.5943629 | 251.4979852 |
| Npm3 | 2617.060544 | 2618.425938 | 2798.235305 | 2638.722215 | 2700.02751 | 2684.91087 | 2655.67852 | 3564.328778 | 3099.193771 | 3270.141168 | 3288.79242 | 2668.516455 | 2714.740628 | 2753.131023 | 2573.183671 |
| Mgea5 | 3327.641014 | 3168.255772 | 3444.343373 | 3262.388825 | 3169.983038 | 3404.657759 | 3383.644745 | 2939.125564 | 3027.714429 | 2872.515772 | 3082.592607 | 3590.698832 | 3434.751874 | 3340.441547 | 3374.384396 |
| Kcnip2 | 3.662785926 | 5.54583406 | 7.04845165 | 4.313047098 | 9.500448664 | 6.312715251 | 9.922711863 | 1.676148026 | 0 | 6.117313782 | 4.729353494 | 11.27786538 | 9.372927467 | 2.710663955 | 7.904222392 |
| 9130011E15Rik | 214.2729767 | 263.8232488 | 258.4432272 | 215.6523549 | 233.0776739 | 254.6128485 | 251.6760554 | 220.4134654 | 232.4959639 | 245.5664533 | 272.4107612 | 247.2455103 | 278.6315711 | 275.5841687 | 262.9950359 |
| Hps6 | 347.964663 | 358.1024278 | 347.7236148 | 400.2507707 | 359.7503227 | 321.9484778 | 377.9651155 | 352.8291595 | 378.4643037 | 374.0300427 | 343.3510637 | 316.6477588 | 270.9628122 | 290.0410432 | 312.5760673 |
| Ldb1 | 2573.107113 | 2842.636087 | 2968.181306 | 2845.748475 | 2874.835766 | 2829.499258 | 2766.63248 | 3187.195472 | 2866.697807 | 3064.774205 | 3022.056883 | 2780.427581 | 2845.961613 | 3056.725386 | 2924.562285 |
| Pprc1 | 2845.068968 | 2740.434287 | 2526.478336 | 2475.689034 | 2323.17638 | 2275.383142 | 2450.90983 | 2719.550172 | 2509.301099 | 2552.667651 | 2515.070188 | 2121.10622 | 2253.763014 | 2174.856046 | 2273.541786 |
| Nolc1 | 7896.050761 | 7319.708697 | 7460.394491 | 7653.933381 | 7253.909237 | 6866.831368 | 7445.642156 | 8254.190955 | 7876.271035 | 8046.88933 | 8221.508114 | 6964.515637 | 7208.633306 | 6713.411061 | 7061.344858 |
| Gbf1 | 1216.044928 | 1161.456104 | 1349.38691 | 1440.557731 | 1140.05384 | 1072.46018 | 1220.493559 | 1282.25324 | 1358.107491 | 1207.732521 | 1250.441064 | 1185.910921 | 1231.261835 | 1149.321517 | 1261.082754 |
| Nfkb2 | 6479.468304 | 5842.932313 | 5937.928935 | 5665.618668 | 5400.688384 | 5682.846552 | 5889.580523 | 6546.196116 | 5630.314879 | 5500.338992 | 5217.422774 | 5050.748635 | 5530.027205 | 4976.779021 | 5431.637914 |
| Psd | 716.0746485 | 665.5000871 | 727.5568426 | 820.3415581 | 697.3329319 | 665.6407526 | 710.8269952 | 999.8222976 | 768.9672333 | 866.0368512 | 729.2663088 | 721.7833844 | 736.2008483 | 701.1584096 | 720.721369 |
| Fbxl15 | 106.2207919 | 77.64167683 | 72.83400039 | 104.3757398 | 106.405025 | 82.76671107 | 101.0312481 | 95.54043749 | 85.77520997 | 115.3550599 | 120.1255787 | 112.7786538 | 130.3689002 | 95.77679307 | 94.8506687 |
| Cuedc2 | 1019.170184 | 983.1971526 | 1096.425812 | 1014.428678 | 1028.581909 | 960.9355438 | 1024.745516 | 1041.725998 | 910.4210883 | 1018.095794 | 936.4119918 | 904.8318148 | 1022.501178 | 1037.28074 | 983.7164049 |
| 2310034G01Rik | 0 | 5.54583406 | 1.566322589 | 3.450437679 | 3.166816221 | 2.104238417 | 4.510323574 | 10.05688816 | 3.00965649 | 1.747803938 | 2.837612096 | 0.867528106 | 4.260421576 | 5.421327909 | 0 |
| Tmem180 | 505.4644578 | 527.6464977 | 490.2589703 | 459.7708207 | 402.8190234 | 390.6869328 | 449.228228 | 356.1814555 | 415.3325957 | 533.954103 | 475.7729615 | 439.8367499 | 460.9776145 | 425.5742409 | 442.6364539 |
| Actr1a | 2614.313455 | 2629.517606 | 2759.07724 | 2815.557146 | 2560.054233 | 2619.075417 | 2734.15815 | 2846.937422 | 2795.97088 | 2949.419145 | 2952.062451 | 2703.217579 | 2769.274024 | 2833.547387 | 2684.56135 |
| Sufu | 853.4291208 | 868.3191613 | 892.8038757 | 819.4789487 | 905.0760761 | 847.3066693 | 939.9514328 | 885.8442318 | 890.8583211 | 766.4120267 | 891.9560689 | 872.7332749 | 841.0072191 | 786.0925469 | 879.5243825 |
| Trim8 | 1515.477677 | 1645.528192 | 1501.320202 | 1458.672529 | 1610.009367 | 1730.385392 | 1851.036795 | 1687.881062 | 1638.005545 | 1529.328446 | 1555.011429 | 1669.124076 | 1626.628958 | 1703.200518 | 1599.527186 |

| | | | | | | | | | | | | | | | |
|---|---|---|---|---|---|---|---|---|---|---|---|---|---|---|---|
| **Arl3** | 489.8976176 | 450.7970828 | 470.679938 | 474.4351808 | 487.6896981 | 433.4731139 | 401.4187981 | 497.8159638 | 412.3229392 | 504.2414361 | 394.4280814 | 432.896525 | 424.337989 | 368.6502978 | 452.6963733 |
| **Sfxn2** | 282.0345163 | 242.4321746 | 275.6727757 | 276.0350143 | 244.4782123 | 278.4608839 | 246.2636671 | 277.4024983 | 257.3256299 | 284.8920419 | 272.4107612 | 266.3311286 | 272.6669809 | 290.0410432 | 232.096712 |
| **Wbp1l** | 964.2283951 | 1062.423353 | 1076.84678 | 987.6877855 | 1217.957519 | 1127.170379 | 1206.962588 | 983.8988913 | 969.1093899 | 993.6265386 | 1010.189906 | 1140.79946 | 1167.355512 | 1349.910649 | 1156.890732 |
| **2010012O05Rik** | 163.9096702 | 175.0899039 | 157.4154202 | 159.5827426 | 131.1061916 | 166.9362478 | 162.3716487 | 126.549176 | 136.9393703 | 145.0677268 | 129.5842857 | 152.6849467 | 118.4397198 | 153.6042908 | 148.0245284 |
| **As3mt** | 63.18305723 | 41.98988645 | 68.91819391 | 36.22959563 | 43.70206386 | 30.86216345 | 45.10323574 | 62.85555098 | 33.85863552 | 52.43411813 | 46.34766424 | 32.96606804 | 37.49170987 | 56.0203884 | 68.98230451 |
| **Cnnm2** | 49.44761 | 32.48274235 | 38.37490343 | 53.48178402 | 65.23641416 | 46.99465798 | 38.78878274 | 74.58858716 | 63.2027863 | 72.53386342 | 49.18527634 | 76.34247335 | 56.2375648 | 54.21327909 | 65.38947615 |
| **Nt5c2** | 581.4672658 | 626.6792487 | 634.3606485 | 695.2631922 | 604.8618983 | 535.1779708 | 583.6358705 | 789.4657203 | 692.9734069 | 719.2213204 | 658.3260063 | 569.9659658 | 563.2277323 | 600.8638433 | 537.4871226 |
| **Ina** | 6.409875371 | 23.76786026 | 14.8800646 | 11.21392246 | 13.30062813 | 10.52119209 | 13.53097072 | 14.24725822 | 11.28621184 | 18.35194135 | 18.91741398 | 19.95314644 | 11.0770961 | 8.131991864 | 15.80844478 |
| **Pcgf6** | 488.0662247 | 525.2697116 | 607.7331645 | 527.0543554 | 495.9234203 | 550.6090525 | 654.8989829 | 651.1835081 | 610.2078534 | 663.2915944 | 580.764609 | 562.1582129 | 615.2048755 | 565.6252119 | 566.9483152 |
| **Taf5** | 473.415081 | 503.8786374 | 496.5242607 | 514.1152141 | 485.7896084 | 472.7522311 | 492.5273343 | 568.2141809 | 513.1464316 | 546.1887306 | 485.2316685 | 551.7478756 | 471.2026263 | 487.9195118 | 469.9419495 |
| **Usmg5** | 770.100741 | 868.3191613 | 833.2836173 | 748.7449763 | 863.9074652 | 754.7201789 | 828.9974729 | 880.8157877 | 847.218302 | 844.189302 | 970.4633369 | 778.1727113 | 731.0883424 | 820.4276236 | 717.1285406 |
| **Pdcd11** | 3497.96056 | 3360.77544 | 3301.808018 | 3382.291534 | 3074.978551 | 3081.306455 | 3418.825269 | 4173.608585 | 3843.331338 | 3606.593426 | 3791.049761 | 3044.156125 | 3388.739321 | 3064.857378 | 3233.545524 |
| **Calhm2** | 151.0899195 | 158.4524017 | 163.6807105 | 132.8418506 | 104.5049353 | 155.0122301 | 146.1344838 | 293.3259046 | 186.5987024 | 186.1411194 | 152.2851825 | 100.6332603 | 103.9542864 | 133.7260884 | 108.5034165 |
| **Neurl1a** | 0 | 3.169048034 | 8.614774239 | 3.450437679 | 1.900089733 | 0 | 0.902064715 | 3.352296052 | 9.028969471 | 4.369509845 | 2.837612096 | 0 | 6.816674521 | 0.903554652 | 4.311394032 |
| **Sh3pxd2a** | 61.35166426 | 97.44822705 | 92.41303275 | 94.88703616 | 87.40412771 | 110.1218105 | 128.9952542 | 61.17940295 | 93.2993512 | 44.56900041 | 55.80637123 | 98.03067601 | 103.1022021 | 108.4265582 | 106.3477195 |
| **Obfc1** | 356.2059313 | 345.4262357 | 337.5425179 | 385.5864106 | 309.0812632 | 273.5509942 | 350.0011093 | 341.0961233 | 328.8049716 | 381.8951604 | 402.9409177 | 372.1695576 | 334.0170515 | 356.0005327 | 363.59423 |
| **Gm19557** | 11.90405426 | 5.54583406 | 4.698967767 | 2.587828259 | 5.066905954 | 5.611302446 | 1.80412943 | 0.838074013 | 3.00965649 | 14.85633347 | 7.56696559 | 6.94022485 | 0.852084315 | 4.517773258 | 6.467091048 |
| **Slk** | 1794.765104 | 1857.062148 | 1853.742784 | 1699.340557 | 1646.111072 | 1636.396076 | 1801.423235 | 1916.675268 | 1850.938742 | 1715.469565 | 1819.855224 | 1718.573179 | 1794.489568 | 1669.768996 | 1876.893535 |
| **Sfr1** | 1961.421864 | 1766.744279 | 1704.942138 | 1757.135388 | 1688.546409 | 1809.645039 | 1858.253312 | 1854.657791 | 1729.047654 | 1751.299546 | 1701.621387 | 1859.112732 | 1774.039544 | 1867.647465 | 1834.498161 |
| **Cfap43** | 15.56684019 | 12.67619214 | 14.8800646 | 16.38957897 | 14.56735462 | 9.118366474 | 12.62890601 | 10.89496217 | 19.56276719 | 6.991215751 | 9.458706988 | 18.21809023 | 7.668758836 | 11.74621047 | 10.05991941 |
| **Gsto1** | 836.9465841 | 940.4150041 | 909.2502629 | 918.6790319 | 850.6068371 | 857.1264486 | 763.1467487 | 1051.782886 | 978.8907735 | 971.7789894 | 1202.201658 | 900.4941743 | 862.3093269 | 707.4832922 | 854.374584 |
| **Gsto2** | 0.915696482 | 0 | 0 | 0.86260942 | 0 | 0 | 0 | 0 | 0 | 0 | 0 | 0 | 1.70416863 | 0 | 0 |
| **Itprip** | 791.1617601 | 844.5513011 | 706.4114876 | 722.0040842 | 691.6326627 | 702.8156313 | 708.1208011 | 734.1528354 | 715.5458306 | 688.6347515 | 622.3829198 | 705.3003504 | 638.2111521 | 655.0771224 | 659.6432869 |
| **Sorcs1** | 3.662785926 | 0.792262009 | 0 | 0 | 2.533452977 | 4.208476834 | 4.510323574 | 2.514222039 | 0 | 0.873901969 | 11.35044839 | 0 | 4.260421576 | 5.421327909 | 7.904222392 |
| **Xpnpep1** | 1936.698059 | 2049.581816 | 2015.857172 | 1957.260773 | 1772.783721 | 1829.284597 | 1804.12943 | 1718.889801 | 1677.883493 | 1773.147095 | 1937.143191 | 1843.497226 | 1688.831113 | 1802.59153 | 1814.378322 |
| **Add3** | 1559.431108 | 1899.844296 | 2012.724527 | 1850.297205 | 2211.071086 | 2203.137623 | 2275.007211 | 1476.686411 | 1690.674533 | 1585.258172 | 1591.900386 | 2304.15465 | 2476.15702 | 2429.658458 | 2165.756935 |
| **1700001K23Rik** | 0 | 0 | 1.566322589 | 0 | 0 | 0 | 0 | 0 | 0 | 0 | 5.675224193 | 0 | 2.556252945 | 0 | 0 |
| **Mxi1** | 872.6587469 | 629.0560348 | 628.0953582 | 504.6265105 | 482.6227921 | 549.9076397 | 608.8936825 | 250.5841299 | 329.5573857 | 389.7602781 | 331.0547446 | 558.6881004 | 510.3985048 | 455.3915444 | 551.1398704 |
| **Smndc1** | 1682.134437 | 1582.147231 | 1669.69988 | 1628.606584 | 1587.20829 | 1592.207069 | 1694.979599 | 1555.465368 | 1641.767615 | 1701.487133 | 1695.946163 | 1733.321156 | 1576.355983 | 1625.494818 | 1700.844946 |
| **5830416P10Rik** | 10.0726613 | 15.05297816 | 4.698967767 | 6.900875357 | 6.333632443 | 14.02825611 | 12.62890601 | 0.838074013 | 3.00965649 | 0.873901969 | 0 | 15.61550591 | 12.78126473 | 5.421327909 | 20.83840449 |
| **Dusp5** | 1383.617384 | 1130.557886 | 1153.596587 | 1148.133138 | 1240.758596 | 1315.850424 | 1289.050477 | 823.8267548 | 932.993512 | 906.2363418 | 926.9532848 | 1229.287327 | 1200.5868 | 1090.590464 | 1153.297904 |
| **Smc3** | 3924.67512 | 3908.228488 | 3835.92402 | 3792.893618 | 3863.51579 | 3803.761645 | 3890.605115 | 3638.917365 | 3273.753847 | 3665.144858 | 3849.693744 | 4014.052548 | 4050.808834 | 3871.731682 | 3934.147054 |
| **Rbm20** | 0.915696482 | 4.753572051 | 5.482129061 | 12.07653188 | 6.333632443 | 6.312715251 | 1.80412943 | 3.352296052 | 3.762070613 | 5.243411813 | 4.729353494 | 1.735056213 | 2.556252945 | 7.228437213 | 0 |
| **Pdcd4** | 1447.716137 | 1517.181746 | 1574.154202 | 1531.994329 | 1703.113764 | 1587.998592 | 1861.861571 | 1251.244501 | 1355.097835 | 1168.406932 | 1155.853994 | 1632.687896 | 1743.364509 | 1635.433919 | 1570.784559 |
| **Bbip1** | 277.4560339 | 286.0065851 | 322.6624533 | 299.3254686 | 290.7137291 | 277.0580583 | 299.4854853 | 337.7438273 | 318.2711739 | 341.6956698 | 276.194244 | 320.1178712 | 264.998222 | 296.3659257 | 347.0672196 |
| **Shoc2** | 955.9871268 | 916.6471438 | 986.7832311 | 886.7624834 | 881.641636 | 892.1970889 | 963.4051154 | 1000.660372 | 968.3569758 | 895.7495181 | 873.038655 | 978.5717039 | 979.8969624 | 879.158676 | 926.9497168 |
| **Adra2a** | 1.831392963 | 1.584524017 | 0 | 2.587828259 | 1.266726489 | 0 | 5.412388289 | 2.514222039 | 3.762070613 | 0 | 0 | 0 | 0 | 2.710663955 | 0 |
| **Gpam** | 347.964663 | 312.9434934 | 324.2287759 | 344.1811584 | 307.8145367 | 326.8583675 | 323.8412326 | 301.7066447 | 310.7470326 | 318.1003167 | 349.9721585 | 350.4813549 | 351.9108222 | 325.2796746 | 360.7199673 |
| **Acsl5** | 6029.861331 | 6226.387125 | 6291.91784 | 6080.533799 | 5979.582389 | 5848.379974 | 6047.441848 | 6570.500262 | 6635.540147 | 6729.045161 | 6390.302441 | 6155.111914 | 6242.369693 | 6390.842051 | 6109.963909 |
| **Zdhhc6** | 953.2400373 | 923.7775019 | 817.6203914 | 945.4199239 | 905.7094393 | 901.3154553 | 991.3691215 | 968.8135591 | 939.7652391 | 939.4446166 | 916.5487071 | 991.5846255 | 863.1614113 | 953.2501574 | 990.183496 |
| **Vti1a** | 467.0052056 | 521.3084016 | 444.052454 | 459.7708207 | 443.9876342 | 426.4589859 | 483.5066871 | 444.1792269 | 482.2974526 | 492.0068085 | 489.0151513 | 451.1146153 | 471.2026263 | 432.8026781 | 459.1634644 |
| **Tcf7l2** | 8.241268334 | 0 | 0 | 3.450437679 | 0.633363244 | 0.701412806 | 4.510323574 | 0 | 6.019312981 | 0 | 0 | 1.735056213 | 2.556252945 | 4.517773258 | 2.155697016 |
| **Nrap** | 0 | 0 | 0 | 3.450437679 | 2.533452977 | 4.90988964 | 1.80412943 | 0 | 0.752414123 | 0 | 4.729353494 | 6.94022485 | 0 | 1.807109303 | 1.437131344 |
| **Casp7** | 283.8659093 | 320.8661134 | 345.3741309 | 312.2646099 | 310.9813529 | 295.2947912 | 322.0371032 | 382.999824 | 319.023588 | 298.8744734 | 366.9978311 | 326.190568 | 322.9399554 | 338.8329943 | 345.6300882 |
| **Dclre1a** | 148.34283 | 167.9595458 | 166.0301944 | 198.4001665 | 183.0419776 | 173.248963 | 186.727396 | 210.3565773 | 179.0745612 | 182.6455115 | 205.2539416 | 164.8303402 | 211.3169102 | 198.7820233 | 199.7612568 |
| **Nhlrc2** | 618.0951251 | 613.2107946 | 700.1461973 | 610.7274691 | 549.759296 | 589.1867568 | 614.3060708 | 670.4592104 | 644.8189031 | 606.4879664 | 646.975558 | 578.6412469 | 622.0215501 | 612.6100538 | 603.5951645 |
| **Ccdc186** | 151.0899195 | 188.558358 | 141.7521943 | 151.8192579 | 140.6066402 | 158.5192941 | 187.6294607 | 138.2822122 | 158.0069657 | 164.2935702 | 141.8806048 | 139.6720251 | 150.8189238 | 163.5433919 | 183.952812 |
| **Ablim1** | 6608.581507 | 7341.892033 | 7003.811457 | 6730.941302 | 5999.21665 | 5665.311232 | 6475.020523 | 6360.143685 | 6504.62009 | 6562.129884 | 6593.664641 | 6799.685297 | 6720.388994 | 6588.720519 | 6515.234948 |
| **B230217O12Rik** | 10.98835778 | 5.54583406 | 12.53058071 | 7.763484777 | 5.066905954 | 10.52119209 | 6.314453003 | 18.43762829 | 6.771727103 | 18.35194135 | 17.97154328 | 10.41033728 | 12.78126473 | 11.74621047 | 17.24557613 |
| **Fam160b1** | 563.1533362 | 546.6607859 | 542.7307771 | 450.2821171 | 430.0536429 | 414.5349682 | 447.4240985 | 382.16175 | 450.6960594 | 423.8424549 | 428.4794265 | 466.7301212 | 457.5692772 | 477.9804107 | 483.5946972 |
| **Trub1** | 295.7699635 | 298.6827772 | 371.2184536 | 277.7602331 | 283.1133702 | 272.1481686 | 257.9905084 | 390.5424901 | 341.5960117 | 316.3525127 | 383.077633 | 274.1388816 | 274.3711495 | 319.8583467 | 317.606027 |
| **Atrnl1** | 155.6684019 | 167.9595458 | 141.7521943 | 157.8575238 | 166.5745332 | 136.7754971 | 163.2737134 | 162.5863585 | 145.9683398 | 201.8713548 | 200.5245881 | 144.0096656 | 144.0022493 | 148.1829629 | 116.4076389 |
| **Gfra1** | 2.747089445 | 1.584524017 | 2.349483883 | 3.450437679 | 0 | 1.402825611 | 1.80412943 | 0.838074013 | 1.504828245 | 9.612921658 | 0 | 0.867528106 | 0 | 4.517773258 | 5.029959704 |
| **Hspa12a** | 0.915696482 | 3.169048034 | 5.482129061 | 9.488703616 | 6.333632443 | 4.208476834 | 6.314453003 | 5.866518091 | 0 | 3.495607876 | 5.675224193 | 0.867528106 | 4.260421576 | 1.807109303 | 1.437131344 |
| **Eno4** | 0 | 0 | 1.566322589 | 2.587828259 | 1.266726489 | 0.701412806 | 0.902064715 | 5.028444078 | 0 | 2.621705907 | 0.945870699 | 0 | 1.70416863 | 1.807109303 | 1.437131344 |
| **Shtn1** | 5.494178889 | 6.338096068 | 9.397935534 | 10.35131304 | 10.13381191 | 13.32684331 | 14.43303544 | 41.90370065 | 8.276555348 | 26.21705907 | 34.99721585 | 15.61550591 | 8.520843152 | 18.97464768 | 14.37131344 |
| **Slc18a2** | 21.97671556 | 27.7291703 | 24.27800013 | 31.91654853 | 38.00179466 | 18.23673295 | 31.57226502 | 41.90370065 | 32.35380727 | 41.94729451 | 26.48437957 | 27.7608994 | 23.85836082 | 38.85285002 | 28.74262688 |
| **Pdzd8** | 1670.230382 | 1668.50379 | 1722.171687 | 1598.415255 | 1493.47053 | 1543.108173 | 1627.324745 | 1804.37335 | 1642.52003 | 1756.542957 | 1710.134223 | 1735.056213 | 1812.383338 | 1520.682479 | 1493.179466 |
| **Rab11fip2** | 128.1975074 | 130.7232314 | 187.1755494 | 157.8575238 | 79.80376878 | 109.4203977 | 110.0518952 | 94.70236348 | 127.9104008 | 143.3199229 | 103.0999062 | 142.2746094 | 118.4397198 | 117.4621047 | 111.3776792 |
| **Fam204a** | 514.6214226 | 453.9661309 | 494.9579381 | 394.2125048 | 451.5879932 | 483.9748359 | 464.5633281 | 564.0238108 | 475.5257255 | 453.5551219 | 432.2629093 | 445.9094466 | 518.0672636 | 447.2595525 | 466.3491211 |
| **Cacul1** | 1472.439942 | 1494.99841 | 1552.225686 | 1587.201332 | 1411.766671 | 1479.98102 | 1564.180215 | 1532.83737 | 1610.918636 | 1614.970839 | 1569.199489 | 1583.238794 | 1601.066428 | 1576.702867 | 1567.910296 |
| **Nanos1** | 130.9445969 | 141.8148995 | 133.9205814 | 143.1931637 | 146.3069094 | 186.5758063 | 182.2170724 | 114.8161398 | 162.5214505 | 127.5896875 | 114.4503546 | 163.095284 | 179.7897905 | 159.9291733 | 155.2101851 |
| **Eif3a** | 16087.87148 | 14706.75966 | 14595.77705 | 14129.54229 | 14571.1548 | 13641.07625 | 15855.59149 | 15892.39751 | 15219.08046 | 14722.62647 | 15734.55907 | 14701.13129 | 14165.04966 | 13365.38041 | 14129.87537 |
| **Fam45a** | 197.79044 | 228.1714584 | 195.7903236 | 183.7358064 | 235.6111269 | 204.8125393 | 205.670755 | 235.4987977 | 167.7883493 | 235.9535316 | 193.9034932 | 178.7107899 | 206.2044043 | 217.756671 | 217.0068329 |
| **Sfxn4** | 15.56684019 | 22.18333624 | 13.31374201 | 19.84001665 | 12.03390164 | 19.63955856 | 18.94335901 | 16.76148026 | 15.04828245 | 20.09974528 | 8.512836289 | 15.61550591 | 18.74585493 | 18.97464768 | 25.14979852 |
| **Prdx3** | 1768.209906 | 1762.782969 | 1797.355171 | 1865.824175 | 1700.580311 | 1679.182257 | 1620.108228 | 2240.171837 | 1963.80086 | 2081.63449 | 2180.231961 | 1649.17093 | 1554.201791 | 1633.62681 | 1390.424575 |
| **Grk5** | 113.5463637 | 119.6315633 | 109.6425812 | 127.6661941 | 117.1722002 | 94.69072877 | 87.50027733 | 156.7198404 | 105.3379772 | 94.38141264 | 101.2081648 | 130.1292159 | 106.5105394 | 68.67015352 | 101.3177597 |
| **Zfp950** | 58.60457482 | 80.01846286 | 85.3645811 | 70.73397241 | 64.60305092 | 51.20313482 | 54.12388289 | 56.15095887 | 74.48899814 | 94.38141264 | 61.48159542 | 78.94505767 | 83.50426289 | 79.51280934 | 56.04812241 |
| **Csf2ra** | 66.84584315 | 85.56429692 | 79.88245204 | 74.18441009 | 67.76986714 | 77.85682143 | 66.75278889 | 88.83584538 | 63.2027863 | 103.9943343 | 62.42746612 | 85.88528252 | 58.79381775 | 78.60925469 | 79.04222392 |
| **Fam171a1** | 6.409875371 | 10.29940611 | 7.831612945 | 15.52696955 | 5.066905954 | 2.805651223 | 8.118582433 | 5.866518091 | 12.79104008 | 15.73023544 | 16.07980188 | 6.94022485 | 3.408337261 | 5.421327909 | 6.467091048 |
| **Nmt2** | 897.3825519 | 862.7733273 | 798.0413591 | 841.0441842 | 723.9341882 | 741.3933356 | 875.0027733 | 959.5947449 | 830.6651913 | 929.8316949 | 936.4119918 | 785.9804643 | 752.3904503 | 723.7472759 | 768.1467033 |
| **Rpp38** | 141.0172582 | 124.3851353 | 126.0889684 | 120.7653188 | 170.3747127 | 139.5811483 | 127.1911248 | 149.1771743 | 164.7786928 | 161.6718642 | 160.7980188 | 124.9240473 | 99.69386487 | 128.3047605 | 104.1920224 |
| **Acbd7** | 0 | 0 | 5.482129061 | 0 | 0 | 0.701412806 | 0.902064715 | 2.514222039 | 3.762070613 | 2.621705907 | 0 | 2.602584319 | 0 | 0 | 0 |
| **Dclre1c** | 200.5375295 | 252.7315807 | 205.9714205 | 224.2784491 | 217.2435928 | 194.2913472 | 274.2276733 | 236.3368717 | 172.3028341 | 223.718904 | 199.5787174 | 189.1211272 | 172.1210317 | 187.0358129 | 165.2701046 |
| **Suv39h2** | 333.3135193 | 353.3488558 | 318.7466469 | 313.1272193 | 264.7458361 | 268.6411046 | 325.645362 | 320.982347 | 337.0815269 | 296.2527675 | 370.7813139 | 328.7931523 | 286.3003299 | 356.0005327 | 279.5220464 |
| **Hspa14** | 2098.776336 | 2322.119947 | 2331.471174 | 2240.196663 | 2116.699962 | 2081.091795 | 2129.774792 | 2325.655386 | 2286.586519 | 2504.603043 | 2408.186799 | 2206.123974 | 2246.094255 | 2291.414596 | 2147.074228 |
| **Cdnf** | 17.39823315 | 12.67619214 | 18.79587107 | 9.488703616 | 14.56735462 | 9.118366474 | 14.43303544 | 13.40918421 | 9.781383594 | 2.621705907 | 12.29631908 | 7.807752957 | 9.372927467 | 12.64976512 | 10.77848508 |
| **Fam107b** | 9297.066377 | 9229.852399 | 9292.99192 | 9636.209827 | 9126.130987 | 8758.541705 | 9781.989767 | 8710.941292 | 9420.224815 | 8317.79894 | 8379.46852 | 9633.032092 | 9828.792575 | 9629.181922 | 9310.455412 |
| **Frmd4a** | 46.70052056 | 41.98988645 | 78.31612945 | 53.48178402 | 51.93578603 | 44.18900676 | 36.08258859 | 75.42666117 | 48.15450385 | 62.04703979 | 52.02288843 | 35.56865236 | 50.27297459 | 57.8274977 | 33.05402091 |
| **Prpf18** | 915.6964816 | 1040.240017 | 1049.436135 | 1081.712212 | 1001.347289 | 903.4196938 | 1069.848752 | 1097.038883 | 1230.949505 | 1163.163521 | 1026.269708 | 1018.477997 | 1065.957478 | 1042.702068 | 970.7822228 |
| **Bend7** | 0 | 0 | 0 | 0 | 0.633363244 | 2.104238417 | 1.80412943 | 4.190370065 | 0 | 0 | 5.675224193 | 1.735056213 | 0 | 0 | 2.155697016 |
| **Sephs1** | 1693.122794 | 1671.672838 | 1795.788848 | 1703.653604 | 1692.346589 | 1722.669851 | 1746.397288 | 1843.762829 | 1794.507682 | 1686.6308 | 1985.382597 | 1681.26947 | 1755.293689 | 1563.149547 | 1626.832681 |
| **Phyh** | 214.2729767 | 282.045275 | 244.3463239 | 215.6523549 | 229.9108577 | 244.7930692 | 217.3975963 | 196.1093191 | 261.8401147 | 180.0238056 | 225.1172263 | 279.3440502 | 221.5419219 | 238.538428 | 218.4439643 |
| **Mcm10** | 1566.75668 | 1640.77462 | 1707.291622 | 1714.004917 | 1568.207393 | 1385.991704 | 1546.138921 | 1994.616151 | 1934.456709 | 2011.722332 | 1855.798311 | 1507.763849 | 1449.39542 | 1509.839823 | 1447.191263 |
| **Optn** | 82.41268334 | 90.31786897 | 74.40032298 | 83.67311371 | 51.93578603 | 82.06529827 | 48.7114946 | 57.8271069 | 57.18347332 | 57.67752995 | 62.42746612 | 70.26977661 | 75.83550405 | 64.15238026 | 63.9523448 |
| **Camk1d** | 424.8831674 | 452.3816069 | 471.4630993 | 461.4960395 | 399.0188439 | 424.3547475 | 477.1922341 | 454.2361151 | 447.6864029 | 366.164925 | 416.1831075 | 431.1614688 | 389.402532 | 441.8382246 | 471.3790808 |
| **Cdc123** | 1362.556365 | 1378.535895 | 1443.366266 | 1293.914129 | 1284.460659 | 1233.785125 | 1311.602095 | 1588.988329 | 1470.217196 | 1599.240603 | 1513.393118 | 1351.60879 | 1251.711859 | 1281.240496 | 1312.100917 |
| **Nudt5** | 1463.282978 | 1510.84365 | 1625.842847 | 1496.627343 | 1281.927206 | 1404.228437 | 1335.957843 | 1899.075714 | 1841.909772 | 1718.091271 | 1715.809448 | 1398.455307 | 1356.51823 | 1406.834592 | 1358.807686 |
| **Sec61a2** | 142.8486511 | 137.0613275 | 119.0405168 | 102.6505209 | 100.0713926 | 91.88507755 | 114.5622188 | 129.063398 | 129.4152291 | 120.5984717 | 158.9062774 | 121.4539349 | 143.1501649 | 120.1727687 | 123.5932956 |
| **Dhtkd1** | 0 | 2.376786026 | 0 | 0 | 0 | 0 | 0 | 0 | 0 | 0 | 0 | 0 | 0 | 0 | 0 |
| **Upf2** | 993.5306825 | 1052.123947 | 1003.229618 | 905.7398906 | 980.4463021 | 944.1016365 | 1050.905393 | 1003.174594 | 952.5562792 | 999.7438524 | 1092.480657 | 1058.38429 | 1039.542864 | 926.1435179 | 947.7881213 |
| **Proser2** | 3.662785926 | 0 | 3.132645178 | 2.587828259 | 0.633363244 | 2.805651223 | 1.80412943 | 0 | 0 | 0 | 0 | 0.867528106 | 2.556252945 | 1.807109303 | 6.467091048 |
| **Echdc3** | 3.662785926 | 0 | 0.783161294 | 0 | 3.800179466 | 2.805651223 | 0 | 4.190370065 | 0.752414123 | 0 | 0 | 0 | 0 | 0 | 0 |
| **Usp6nl** | 327.8193404 | 245.6012226 | 304.6497436 | 243.2558563 | 235.6111269 | 232.8690515 | 278.7379969 | 424.0654506 | 373.9498189 | 360.0476112 | 303.6244943 | 301.899781 | 237.7315239 | 234.0206548 | 217.0068329 |
| **Celf2** | 4787.261206 | 4834.382776 | 4956.627833 | 4956.553725 | 4353.105578 | 4416.796438 | 5075.016085 | 4738.47047 | 4850.813848 | 4761.891829 | 4734.082847 | 4984.816499 | 5109.097554 | 4864.738244 | 4897.025054 |
| **Gata3** | 316.8309826 | 332.7500436 | 353.9889051 | 418.3655685 | 516.8244073 | 498.0030921 | 573.7131586 | 284.9451644 | 249.0490746 | 275.2791202 | 305.5162357 | 476.2729303 | 512.9547577 | 494.2443944 | 521.6786778 |
| **Taf3** | 676.6996999 | 734.4268819 | 677.4345197 | 682.3240509 | 689.0992098 | 579.3669775 | 722.5538365 | 677.1638025 | 732.0989413 | 669.4089082 | 704.6736706 | 712.2405753 | 692.7445482 | 626.1633735 | 612.2179525 |
| **Atp5c1** | 3810.21306 | 4042.120767 | 4062.257635 | 3932.636344 | 3943.952922 | 3947.551271 | 4067.409799 | 4379.774792 | 4120.972149 | 4402.718119 | 4187.369583 | 4097.335246 | 4221.225697 | 4082.259916 | 3934.86562 |
| **Kin** | 271.0461585 | 297.8905152 | 290.5528403 | 259.6454353 | 261.5790199 | 293.8919656 | 276.0318027 | 304.2208667 | 316.0139315 | 286.6398458 | 336.7299688 | 250.7156227 | 247.9565357 | 282.8126059 | 291.7376628 |
| **Itih5** | 237.1653887 | 247.1857467 | 238.8641948 | 260.5080447 | 278.6798275 | 309.3230473 | 325.645362 | 45.2559967 | 121.8910879 | 88.26409886 | 122.9631908 | 271.5362973 | 293.1170044 | 254.8024117 | 283.1148748 |
| **Sfmbt2** | 175.8137245 | 220.2488384 | 195.0071623 | 232.0419339 | 196.975969 | 186.5758063 | 235.4388906 | 210.3565773 | 234.7532062 | 248.1881592 | 198.6328467 | 205.6041612 | 200.2398141 | 178.0002664 | 210.5397419 |
| **Prkcq** | 2819.429467 | 2673.092017 | 2849.923951 | 2953.574653 | 2912.204197 | 3026.596257 | 3105.808813 | 2233.467245 | 2640.97357 | 2347.300688 | 2595.469197 | 3117.028486 | 3051.313933 | 3112.745775 | 3054.622672 |
| **Gm13293** | 0 | 0 | 2.349483883 | 0 | 0 | 2.805651223 | 0 | 5.028444078 | 0 | 0 | 3.783482795 | 0 | 0.852084315 | 0 | 0 |
| **Pfkfb3** | 2582.264078 | 2530.484855 | 2480.27182 | 2305.754979 | 2453.649208 | 2629.596609 | 2937.122711 | 1014.90763 | 1316.724715 | 1439.316543 | 1467.991324 | 2438.621507 | 2583.519644 | 2392.612717 | 2624.9204 |
| **Rbm17** | 3041.028015 | 2997.127178 | 2912.576854 | 2927.69637 | 3039.510209 | 2984.511488 | 2976.813559 | 3239.994134 | 3075.868933 | 3101.478088 | 3021.111012 | 2819.466345 | 2964.401332 | 2813.669185 | 2882.16691 |
| **Il2ra** | 30887.35802 | 25717.61706 | 26798.99634 | 24297.98213 | 20028.84587 | 21379.76373 | 24091.44234 | 21752.21101 | 22414.41671 | 23491.35883 | 25792.94808 | 20264.58903 | 21269.72868 | 19092.10979 | 21360.80173 |
| **Il15ra** | 139.1858652 | 168.7518078 | 166.8133557 | 146.6436013 | 140.6066402 | 171.8461374 | 144.3303544 | 204.4900592 | 245.287004 | 217.6015903 | 123.9090615 | 183.9159585 | 184.0502121 | 178.0002664 | 191.1384687 |
| **Fbxo18** | 461.5110267 | 467.434585 | 462.0651637 | 425.2664439 | 414.852925 | 421.5490962 | 441.1096455 | 428.2558207 | 448.4388171 | 459.6724356 | 442.667487 | 495.3585487 | 453.3088557 | 456.295099 | 482.1575659 |
| **Ankrd16** | 87.90686223 | 118.0470393 | 82.23193592 | 87.9861608 | 96.27121313 | 89.78083913 | 115.4642835 | 105.5973256 | 82.76555348 | 81.27288311 | 78.507268 | 95.42809169 | 138.0376591 | 119.269214 | 137.964609 |
| **Fam188a** | 783.8361882 | 813.6530827 | 809.7887785 | 832.41809 | 753.7022607 | 750.5117021 | 797.4252079 | 814.6079407 | 810.35001 | 853.8022236 | 835.203827 | 725.2534968 | 760.9112934 | 755.3716887 | 730.0627227 |
| **Pter** | 97.06382705 | 62.58869867 | 81.44877463 | 62.97048763 | 54.46923901 | 59.62008849 | 62.24246532 | 119.8445839 | 60.19312981 | 105.7421382 | 95.53294058 | 69.4022485 | 31.52711966 | 53.30972444 | 68.26373884 |
| **C1ql3** | 10.98835778 | 9.507144102 | 3.132645178 | 17.25218839 | 5.066905954 | 17.53532014 | 14.43303544 | 22.62799835 | 19.56276719 | 7.86511772 | 14.18806048 | 20.82067455 | 18.74585493 | 5.421327909 | 15.08987911 |
| **Rsu1** | 428.5459534 | 450.7970828 | 382.1827117 | 452.8699453 | 417.386378 | 400.5067121 | 443.8158397 | 474.3498914 | 486.0595232 | 450.933416 | 478.6105736 | 472.8028179 | 496.7651557 | 524.0616979 | 464.9119898 |
| **Cubn** | 21.06101908 | 34.85952837 | 36.02541955 | 37.09220505 | 29.13470924 | 18.23673295 | 38.78878274 | 40.22755263 | 25.58208017 | 22.72145119 | 47.29353494 | 29.49595561 | 27.26669809 | 33.43152211 | 19.40127314 |
| **Trdmt1** | 181.3079033 | 176.6744279 | 191.0913559 | 208.7514796 | 202.0428749 | 196.3955856 | 207.4748844 | 212.0327253 | 215.9428532 | 194.0062371 | 186.3365277 | 144.8771937 | 249.6607043 | 211.4317885 | 190.4199031 |
| **Vim** | 44253.77956 | 41152.46551 | 42466.92119 | 44470.96602 | 41089.44047 | 42800.91082 | 42604.51648 | 41646.41193 | 38957.74602 | 39780.89153 | 44165.54054 | 36958.43238 | 39366.29536 | 39849.4708 | 41929.02552 |
| **St8sia6** | 0 | 3.169048034 | 1.566322589 | 0 | 0.633363244 | 2.104238417 | 2.706194144 | 4.190370065 | 0 | 3.495607876 | 1.891741398 | 0 | 3.408337261 | 3.614218606 | 0 |
| **Hacd1** | 62.26736075 | 61.79643666 | 39.94122602 | 24.15306375 | 51.93578603 | 65.23139093 | 46.00530045 | 52.79866282 | 79.75589699 | 71.65996145 | 63.37333682 | 59.85943933 | 57.08964912 | 44.27417793 | 41.67680897 |
| **Stamos** | 0 | 2.376786026 | 4.698967767 | 3.450437679 | 2.533452977 | 2.104238417 | 5.412388289 | 0 | 14.29586833 | 0.873901969 | 6.621094891 | 0.867528106 | 3.408337261 | 0.903554652 | 2.155697016 |
| **Stam** | 287.5286952 | 274.914917 | 299.1676145 | 319.1654853 | 276.7797377 | 246.8973076 | 298.5834206 | 325.1727171 | 355.89188 | 342.5695718 | 343.3510637 | 242.9078698 | 270.1107279 | 236.7313187 | 286.7077031 |
| **Mrc1** | 0 | 1.584524017 | 0 | 3.450437679 | 0 | 0 | 0 | 0 | 0.752414123 | 1.747803938 | 7.56696559 | 0 | 0 | 0 | 0 |
| **Cacnb2** | 0 | 0 | 3.132645178 | 0 | 1.266726489 | 1.402825611 | 0 | 4.190370065 | 1.504828245 | 0 | 5.675224193 | 0 | 0 | 1.807109303 | 0 |
| **Nsun6** | 115.3777567 | 147.3607336 | 155.0659363 | 131.1166318 | 139.973277 | 94.69072877 | 141.6241602 | 115.6542138 | 111.3572901 | 135.4548052 | 96.47881127 | 205.6041612 | 161.8960199 | 125.5940966 | 122.8747299 |
| **Arl5b** | 304.0112319 | 320.0738514 | 249.8284529 | 209.614089 | 209.0098706 | 192.1871088 | 211.0831433 | 190.242801 | 216.6952673 | 152.0589426 | 147.555829 | 196.061352 | 174.6772846 | 186.1322582 | 155.9287508 |
| **Plxdc2** | 206.0317084 | 256.6928908 | 224.7672915 | 287.2489367 | 143.1400932 | 144.491038 | 164.1757781 | 279.9167204 | 233.248378 | 219.3493942 | 237.4135454 | 212.544386 | 131.2209845 | 198.7820233 | 178.9228523 |
| **Nebl** | 7.325571853 | 4.753572051 | 8.614774239 | 10.35131304 | 5.700269198 | 4.90988964 | 3.608258859 | 16.76148026 | 6.771727103 | 7.86511772 | 8.512836289 | 10.41033728 | 10.22501178 | 2.710663955 | 1.437131344 |
| **A930004D18Rik** | 16.48253667 | 19.80655021 | 8.614774239 | 11.21392246 | 8.233722176 | 6.312715251 | 4.510323574 | 13.40918421 | 11.28621184 | 11.3607256 | 11.35044839 | 5.205168638 | 5.964590206 | 5.421327909 | 4.311394032 |
| **Gm17762** | 0 | 0 | 2.349483883 | 0 | 1.266726489 | 0.701412806 | 0 | 0 | 6.771727103 | 0 | 0 | 0 | 0 | 0 | 0 |
| **Skida1** | 11.90405426 | 18.2220262 | 20.36219366 | 23.29045433 | 12.03390164 | 10.52119209 | 25.25781201 | 25.9802944 | 17.30552482 | 30.58656891 | 10.40457769 | 13.01292159 | 31.52711966 | 16.26398373 | 17.9641418 |
| **Mllt10** | 720.653131 | 758.9870041 | 710.3272941 | 624.5292198 | 692.8993892 | 667.744991 | 764.0488134 | 650.3454341 | 667.3913267 | 761.1686149 | 776.5598437 | 763.4247335 | 818.8530269 | 832.1738341 | 860.1231093 |
| **Dnajc1** | 863.5017821 | 802.5614146 | 849.7300045 | 759.9588987 | 776.5033375 | 820.6529827 | 792.9148843 | 671.2972845 | 793.7968993 | 768.1598307 | 738.7250157 | 789.4505767 | 735.348764 | 696.6406364 | 765.2724406 |
| **Commd3** | 716.0746486 | 763.7405762 | 909.2502629 | 790.1502284 | 729.0010942 | 756.8244174 | 709.9249305 | 935.2905986 | 979.6431876 | 924.5882831 | 794.531387 | 647.1759673 | 662.0695129 | 782.4783283 | 737.9669451 |
| **Bmi1** | 727.0630064 | 850.0971351 | 834.0667786 | 911.7781566 | 786.6371494 | 840.993954 | 838.01812 | 1297.338572 | 1264.055726 | 1136.946462 | 1186.121856 | 913.5070959 | 966.2636134 | 890.9048865 | 915.4526661 |
| **Pip4k2a** | 1119.896797 | 1280.295406 | 1285.950846 | 1343.082866 | 1242.658685 | 1221.861108 | 1270.107118 | 1388.68864 | 1365.631632 | 1277.644679 | 1178.554891 | 1316.040137 | 1327.547363 | 1403.220374 | 1278.32833 |
| **4930426L09Rik** | 2.747089445 | 3.961310043 | 4.698967767 | 7.763484777 | 2.533452977 | 1.402825611 | 0.902064715 | 3.352296052 | 1.504828245 | 4.369509845 | 2.837612096 | 1.735056213 | 2.556252945 | 1.807109303 | 0 |
| **Armc3** | 3.662785926 | 4.753572051 | 10.18109683 | 6.900875357 | 15.83408111 | 10.52119209 | 14.43303544 | 8.380740131 | 6.019312981 | 6.117313782 | 1.891741398 | 15.61550591 | 6.816674521 | 14.45687443 | 12.21561642 |
| **Msrb2** | 21.06101908 | 41.19762444 | 35.24225825 | 26.74089201 | 20.90098706 | 24.5494482 | 31.57226502 | 26.81836842 | 37.62070613 | 41.94729451 | 48.23940564 | 33.83359614 | 33.23128829 | 44.27417793 | 28.74262688 |
| **Gm3230** | 0 | 2.376786026 | 9.397935534 | 0 | 5.700269198 | 6.312715251 | 3.608258859 | 0 | 2.257242368 | 0 | 0.945870699 | 7.807752957 | 1.70416863 | 4.517773258 | 6.467091048 |
| **Otud1** | 65.01445019 | 59.41965064 | 54.82129061 | 75.90962893 | 67.13650389 | 87.67660071 | 69.45898304 | 59.50325493 | 73.73658401 | 78.6511772 | 72.83204381 | 90.22292305 | 94.58135898 | 76.80214538 | 70.41943585 |
| **Arhgap21** | 923.0220534 | 923.7775019 | 835.6331012 | 767.7223835 | 767.6362521 | 733.6777948 | 816.3685669 | 946.1855607 | 843.4562314 | 917.5970673 | 994.1101044 | 821.5491166 | 957.7427702 | 810.4885225 | 841.4404019 |
| **Gm13375** | 43.95343112 | 33.27500436 | 41.50754861 | 25.87828259 | 52.56914927 | 35.07064029 | 51.41768874 | 41.06562664 | 42.13519086 | 26.21705907 | 39.72656935 | 41.6413491 | 25.56252945 | 37.04574071 | 35.9282836 |
| **Enkur** | 3.662785926 | 2.376786026 | 2.349483883 | 3.450437679 | 0 | 0 | 1.80412943 | 0.838074013 | 3.762070613 | 2.621705907 | 0 | 0.867528106 | 0.852084315 | 0.903554652 | 0 |
| **Thnsl1** | 76.00280797 | 61.79643666 | 65.00238744 | 81.08528545 | 79.17040553 | 80.66247266 | 70.36104775 | 118.1684358 | 61.69795805 | 66.41654964 | 68.10269031 | 81.54764199 | 99.69386487 | 71.38081747 | 68.26373884 |
| **Gad2** | 23.80810852 | 29.31369431 | 23.49483883 | 12.07653188 | 6.333632443 | 7.014128057 | 11.72684129 | 8.380740131 | 24.82966605 | 21.84754922 | 10.40457769 | 6.072696744 | 11.92918041 | 9.939101167 | 12.21561642 |
| **Apbb1ip** | 2424.764283 | 2236.55565 | 2259.420335 | 2473.963816 | 2537.88652 | 2470.375902 | 2632.224838 | 2224.248431 | 2112.026442 | 2086.877902 | 2211.445694 | 2401.317798 | 2420.771539 | 2386.287835 | 2535.818256 |
| **Pdss1** | 324.1565545 | 358.8946899 | 357.9047116 | 345.9063773 | 330.6156135 | 310.0244601 | 338.274268 | 419.8750805 | 388.9981014 | 396.7514939 | 343.3510637 | 353.0839393 | 301.6378476 | 296.3659257 | 293.1747942 |
| **Abi1** | 1424.823725 | 1525.896628 | 1666.567235 | 1592.376989 | 1573.274299 | 1563.449144 | 1640.855716 | 1484.229077 | 1578.564829 | 1625.457662 | 1598.521481 | 1709.030369 | 1702.464462 | 1824.276842 | 1767.671553 |
| **Acbd5** | 362.6158067 | 349.3875458 | 379.0500665 | 332.1046266 | 343.9162416 | 319.8442394 | 363.5320801 | 331.8773092 | 316.0139315 | 346.9390817 | 283.7612096 | 379.9773106 | 332.3128829 | 382.2036176 | 311.138936 |
| **Mastl** | 354.3745384 | 331.9577816 | 412.7260022 | 313.1272193 | 297.0473616 | 263.0298021 | 348.1969799 | 362.8860477 | 371.6925766 | 311.1091009 | 314.029072 | 308.8400058 | 339.9816417 | 282.8126059 | 334.1330375 |
| **Yme1l1** | 2656.435493 | 2758.656314 | 2746.54666 | 2567.988242 | 2632.257643 | 2470.375902 | 2733.256086 | 2950.020526 | 2881.746089 | 2864.650654 | 2984.222055 | 2583.4987 | 2543.471681 | 2631.151145 | 2714.741109 |
| **Spopl** | 112.6306672 | 161.6214497 | 209.8872269 | 163.8957897 | 152.6405419 | 170.4433118 | 155.1551309 | 180.1859128 | 168.5407635 | 131.0852953 | 156.0686653 | 165.6978683 | 157.6355983 | 135.5331977 | 165.9886702 |
| **Psd4** | 3683.846945 | 3682.433816 | 3629.169439 | 3487.529884 | 3555.06789 | 3361.170165 | 3758.903666 | 3752.89543 | 3407.683561 | 3750.787251 | 3777.807571 | 3568.143101 | 3525.072812 | 3526.573805 | 3489.354903 |
| **Cacna1b** | 5.494178889 | 8.714882094 | 10.96425812 | 12.93914129 | 8.233722176 | 7.715540863 | 11.72684129 | 5.866518091 | 11.28621184 | 9.612921658 | 7.56696559 | 5.205168638 | 16.18960199 | 7.228437213 | 2.874262688 |
| **Ehmt1** | 1155.60896 | 1199.484681 | 1180.224071 | 1222.317548 | 1141.953929 | 1216.951218 | 1212.374977 | 1216.883467 | 1234.711575 | 1108.107697 | 1216.389719 | 1206.731596 | 1225.297245 | 1201.727687 | 1264.675583 |
| **Arrdc1** | 518.2842086 | 564.0905501 | 592.0699386 | 565.0091699 | 568.1268301 | 481.1691847 | 486.2128813 | 494.4636677 | 528.1947141 | 508.6109459 | 468.2059959 | 526.5895605 | 565.7839853 | 625.2598189 | 573.4154062 |
| **Zmynd19** | 1224.286196 | 1217.706707 | 1222.514781 | 1175.736639 | 1124.853122 | 1317.954662 | 1290.854607 | 1449.868043 | 1306.943331 | 1327.457091 | 1268.412607 | 1172.898 | 1140.940898 | 1256.84452 | 1146.830812 |

| | | | | | | | | | | | | | | | |
|---|---|---|---|---|---|---|---|---|---|---|---|---|---|---|---|
| **Dph7** | 291.1914811 | 282.045275 | 275.6727757 | 302.7759063 | 331.88234 | 305.1145705 | 284.1503852 | 303.3827927 | 291.9366796 | 266.5401005 | 346.1886757 | 264.5960724 | 305.0461848 | 290.0410432 | 293.8933598 |
| **Mrpl41** | 436.7872217 | 499.9173274 | 536.4654867 | 514.9778235 | 506.6905954 | 544.2963372 | 479.8984283 | 565.6999588 | 592.9023286 | 554.0538483 | 487.1234099 | 493.6234925 | 443.0838439 | 482.4981839 | 459.1634644 |
| **Pnpla7** | 222.514245 | 232.9250305 | 223.2009689 | 233.7671527 | 212.8100501 | 211.1252545 | 248.0677966 | 203.6519852 | 221.9621662 | 216.7276883 | 254.439218 | 233.3650606 | 237.7315239 | 233.1171001 | 221.318227 |
| **Nsmf** | 159.3311878 | 200.4422882 | 176.2112913 | 114.7270528 | 174.1748922 | 178.1588527 | 193.041849 | 161.7482845 | 111.3572901 | 129.3374914 | 131.4760271 | 170.0355088 | 159.3397669 | 174.3860478 | 191.8570344 |
| **Nrarp** | 62.26736075 | 35.65179038 | 62.65290356 | 61.2452688 | 52.56914927 | 70.84269338 | 66.75278889 | 57.8271069 | 45.14484735 | 53.3080201 | 49.18527634 | 71.13730471 | 69.87091384 | 69.57370817 | 51.73672838 |
| **Tor4a** | 714.2432556 | 812.0685587 | 798.0413591 | 717.6910371 | 767.0028888 | 758.9286558 | 782.9921724 | 705.658319 | 679.4299527 | 686.0130456 | 695.2149636 | 677.539451 | 766.8758836 | 692.1228631 | 750.9011272 |
| **Nelfb** | 1607.047325 | 1620.175807 | 1574.937363 | 1659.660523 | 1476.369722 | 1554.330777 | 1628.22681 | 1571.388774 | 1603.394495 | 1670.026663 | 1508.663765 | 1591.046547 | 1659.860246 | 1665.251223 | 1664.916662 |
| **Tubb4b** | 8054.466252 | 8561.183264 | 8875.56695 | 9008.230169 | 8882.919501 | 8571.264486 | 8539.846655 | 9747.638846 | 9335.954433 | 9507.17952 | 9747.197551 | 9120.322981 | 8724.491303 | 9124.998426 | 9030.933365 |
| **Rnf208** | 24.723805 | 13.46845414 | 14.0969033 | 31.91654853 | 8.233722176 | 13.32684331 | 14.43303544 | 15.08533223 | 12.79104008 | 14.85633347 | 8.512836289 | 13.8804497 | 23.00627651 | 2.710663955 | 11.49705075 |
| **Ndor1** | 640.9875371 | 710.6590216 | 725.99052 | 636.6057517 | 601.0617188 | 548.5048141 | 615.2081355 | 655.3738782 | 717.8030729 | 748.0600854 | 714.1323776 | 656.7187765 | 687.6320423 | 665.9197782 | 654.6133272 |
| **Tmem203** | 146.5114371 | 165.5827598 | 148.0174847 | 163.8957897 | 145.6735462 | 136.7754971 | 151.5468721 | 119.8445839 | 151.9876528 | 143.3199229 | 108.7751304 | 160.4926997 | 146.5585022 | 151.7971815 | 140.8388717 |
| **Tprn** | 627.2520899 | 585.4816243 | 571.707745 | 640.0561894 | 528.858309 | 533.7751451 | 559.2801232 | 690.5729868 | 669.6485691 | 679.8957318 | 684.8103859 | 567.3633815 | 562.375648 | 532.1936898 | 545.391345 |
| **Ssna1** | 808.5599932 | 819.1989168 | 846.5973593 | 913.5033754 | 865.8075549 | 1022.659871 | 878.6110322 | 897.577268 | 844.9610597 | 908.8580477 | 878.7138792 | 953.4133888 | 852.0843152 | 939.6968376 | 893.8956959 |
| **Anapc2** | 1855.201072 | 1822.20262 | 1817.717365 | 1855.472862 | 1896.289553 | 1980.088351 | 1923.201972 | 1927.57023 | 1775.697329 | 1835.194135 | 1817.017612 | 1879.065878 | 1820.052097 | 1956.195821 | 1937.971617 |
| **Grin1** | 10.98835778 | 7.922620085 | 10.18109683 | 2.587828259 | 3.166816221 | 9.118366474 | 6.314453003 | 0 | 0.752414123 | 2.621705907 | 0 | 4.337640531 | 3.408337261 | 1.807109303 | 2.155697016 |
| **AA543186** | 1.831392963 | 0.792262009 | 0.783161294 | 0 | 0 | 0 | 2.706194144 | 0.838074013 | 1.504828245 | 0.873901969 | 3.783482795 | 0 | 6.816674521 | 1.807109303 | 5.029959704 |
| **Man1b1** | 1163.850228 | 1199.484681 | 1174.741942 | 1172.286201 | 1114.71931 | 1066.848877 | 1130.287088 | 1330.023459 | 1216.653636 | 1242.6886 | 1265.574995 | 1078.337436 | 1180.136776 | 1163.778391 | 1218.68738 |
| **Dpp7** | 43.03773463 | 53.87381658 | 77.53296815 | 43.13047098 | 61.43623469 | 56.81443726 | 48.7114946 | 77.94088321 | 44.39243323 | 83.02068705 | 52.02288843 | 70.26977661 | 34.93545692 | 58.73105235 | 51.73672838 |
| **Uap1l1** | 156.5840983 | 202.0268122 | 165.2470331 | 142.3305542 | 172.9081657 | 230.7648131 | 215.5934668 | 108.1115477 | 161.7690364 | 173.0325898 | 166.473243 | 223.8222514 | 158.4876826 | 215.9495617 | 177.485721 |
| **Sapcd2** | 399.243666 | 460.3042269 | 487.1263252 | 460.6334301 | 497.82351 | 533.0737323 | 529.5119876 | 466.8072253 | 525.1850576 | 493.7546124 | 533.4710741 | 540.4700102 | 561.5235637 | 555.6861107 | 612.9365182 |
| **Entpd2** | 0.915696482 | 2.376786026 | 3.915806472 | 6.900875357 | 4.43354271 | 4.208476834 | 2.706194144 | 0 | 1.504828245 | 5.243411813 | 2.837612096 | 0.867528106 | 0 | 0.903554652 | 4.311394032 |
| **Npdc1** | 57.68887834 | 63.38096068 | 54.82129061 | 38.81742388 | 51.30242279 | 25.25086101 | 42.39704159 | 32.68488651 | 42.88760499 | 34.08217679 | 17.97154328 | 45.11146153 | 44.30838439 | 43.37062328 | 40.9582433 |
| **Fut7** | 553.0806749 | 680.5530653 | 556.8276804 | 629.7048763 | 595.9948129 | 631.9729379 | 573.7131586 | 601.7371414 | 489.0691797 | 575.0274955 | 649.8131701 | 575.1711345 | 621.1694658 | 620.7420456 | 561.1997898 |
| **Abca2** | 2056.654298 | 2181.889571 | 2134.114527 | 2117.706125 | 2060.330634 | 1941.510646 | 2164.955315 | 2149.659843 | 2343.769992 | 2233.693433 | 2375.081325 | 2188.773412 | 2303.183904 | 2291.414596 | 2345.398353 |
| **Clic3** | 1.831392963 | 11.88393013 | 14.8800646 | 15.52696955 | 16.46744435 | 6.312715251 | 6.314453003 | 7.542666117 | 12.79104008 | 24.46925513 | 14.18806048 | 11.27786538 | 13.63334904 | 9.939101167 | 12.9341821 |
| **BC029214** | 170.3195456 | 172.7131179 | 150.3669685 | 150.9566484 | 176.0749819 | 190.7842832 | 218.299661 | 150.8533223 | 136.1869562 | 131.0852953 | 143.7723462 | 201.2665207 | 252.2169573 | 213.2388978 | 217.0068329 |
| **C8g** | 11.90405426 | 42.78214846 | 17.22954848 | 28.46611085 | 22.16771355 | 18.93814575 | 32.47432973 | 15.92340625 | 16.5531107 | 22.72145119 | 16.07980188 | 28.62842751 | 32.37920398 | 24.39597559 | 27.30549553 |
| **Fbxw5** | 559.4905502 | 634.6018688 | 599.1183903 | 595.2004996 | 582.6941847 | 622.8545715 | 552.0636054 | 606.7655855 | 533.4616129 | 591.6316329 | 615.7618249 | 596.8593371 | 581.9735873 | 565.6252119 | 603.5951645 |
| **Traf2** | 1019.170184 | 1019.641205 | 898.2860048 | 929.8929544 | 1014.647917 | 946.9072877 | 990.4670568 | 955.4043749 | 934.4983402 | 878.2714787 | 884.3891034 | 916.1096802 | 914.2864702 | 953.2501574 | 972.2193542 |
| **Edf1** | 2991.580405 | 3005.049798 | 2957.217048 | 2859.550226 | 2853.934779 | 2795.130031 | 2801.813004 | 2749.720837 | 2825.31503 | 2965.14938 | 2670.192983 | 2866.312863 | 2826.363673 | 2869.689573 | 2771.507797 |
| **Mamdc4** | 26.55519797 | 40.40536243 | 25.06116142 | 40.54264272 | 36.10170492 | 30.86216345 | 15.33510015 | 29.33259046 | 24.07725192 | 21.84754922 | 25.53850887 | 32.09853993 | 31.52711966 | 20.78175699 | 35.20971793 |
| **Phpt1** | 552.1649784 | 556.960192 | 544.2970997 | 468.3969149 | 680.2321244 | 667.0435782 | 593.5585823 | 577.432995 | 573.3395614 | 568.0362798 | 466.3142545 | 569.9659658 | 532.552697 | 635.1989201 | 561.1997898 |
| **Gm996** | 22.89241204 | 17.42976419 | 9.397935534 | 18.97740723 | 12.03390164 | 9.81977928 | 25.25781201 | 13.40918421 | 15.80069657 | 20.97364725 | 17.97154328 | 6.072696744 | 11.92918041 | 16.26398373 | 20.83840449 |
| **Rabl6** | 1924.794004 | 1955.302637 | 1878.020784 | 1847.709377 | 1740.482195 | 1815.256341 | 1987.248567 | 1862.200457 | 1955.524305 | 2109.599353 | 1958.898217 | 1863.450372 | 1824.312519 | 1842.347935 | 1783.479998 |
| **Ccdc183** | 0 | 1.584524017 | 0 | 0 | 0 | 0.701412806 | 0.902064715 | 0 | 0 | 0.873901969 | 0 | 0.867528106 | 0.852084315 | 0 | 0 |
| **Tmem141** | 10.0726613 | 10.29940611 | 4.698967767 | 7.763484777 | 1.900089733 | 16.13249453 | 5.412388289 | 10.05688816 | 4.514484735 | 8.739019689 | 12.29631908 | 2.602584319 | 11.0770961 | 9.939101167 | 7.18565672 |
| **Bmyc** | 328.7350369 | 373.947668 | 352.4225825 | 320.0280947 | 371.7842244 | 364.734659 | 386.9857626 | 302.5447187 | 341.5960117 | 354.8041994 | 296.0575287 | 338.3359614 | 372.3608457 | 281.9090513 | 311.8575016 |
| **Camsap1** | 749.9554184 | 739.1804539 | 801.9571656 | 700.4388488 | 669.4649492 | 636.1814148 | 733.3786131 | 679.6780246 | 705.0120329 | 868.6585571 | 830.4744735 | 700.0951818 | 664.6257658 | 710.1939561 | 760.9610466 |
| **Ubac1** | 773.7635269 | 875.4495194 | 870.0921982 | 814.3032922 | 834.1393927 | 812.9374418 | 782.0901077 | 882.4919357 | 931.4886838 | 859.0456354 | 902.3606466 | 846.7074317 | 791.5863288 | 797.8387573 | 855.8117153 |
| **Nacc2** | 18.31392963 | 44.36667248 | 35.24225825 | 65.55831589 | 51.93578603 | 58.21726287 | 31.57226502 | 34.36103454 | 39.8779485 | 12.23462756 | 32.15960376 | 53.78674259 | 36.63962555 | 50.59906049 | 45.98820301 |
| **C330006A16Rik** | 145.5957406 | 189.35062 | 202.055614 | 193.22451 | 193.1757895 | 210.4238417 | 181.3150077 | 261.4790921 | 189.6083589 | 190.5106292 | 191.0658812 | 185.6510147 | 213.0210788 | 224.0815536 | 193.2941658 |
| **Qsox2** | 441.3657041 | 431.7827946 | 489.4758091 | 395.0751142 | 378.7512201 | 438.3830036 | 462.7591987 | 437.4746348 | 423.609151 | 416.8512392 | 409.5620126 | 347.8787706 | 356.1712437 | 375.8787351 | 372.2170181 |
| **Gpsm1** | 180.3922069 | 198.0655021 | 170.7291622 | 199.2627759 | 193.1757895 | 180.9645039 | 237.24302 | 153.3675444 | 221.209752 | 129.3374914 | 123.9090615 | 211.6768579 | 178.9377062 | 174.3860478 | 204.0726508 |
| **Dnlz** | 526.5254769 | 468.226847 | 464.4146476 | 475.2977902 | 490.8565143 | 464.3352774 | 474.48604 | 559.8334407 | 503.365048 | 521.7194754 | 553.3343588 | 536.1323697 | 459.2734459 | 524.9652526 | 474.2533435 |
| **Card9** | 6.409875371 | 7.130358077 | 13.31374201 | 5.175656518 | 5.066905954 | 9.81977928 | 7.216517718 | 3.352296052 | 5.266898858 | 5.243411813 | 3.783482795 | 7.807752957 | 10.22501178 | 2.710663955 | 7.904222392 |
| **Snapc4** | 663.8799491 | 756.6102181 | 710.3272941 | 717.6910371 | 748.6353547 | 668.4464038 | 746.9095838 | 682.1922466 | 750.1568802 | 823.2156547 | 803.990094 | 684.4796759 | 707.2299816 | 657.7877863 | 695.5715705 |
| **Sdccag3** | 1121.72819 | 1043.409065 | 1080.762586 | 1105.865276 | 1128.653301 | 1080.175721 | 1226.808012 | 1149.837546 | 1306.943331 | 1224.336658 | 1137.882451 | 1159.885078 | 1255.120196 | 1168.296164 | 1240.962915 |
| **Pmpca** | 2040.171761 | 2047.997292 | 2117.66814 | 1976.23818 | 1957.725788 | 1943.614885 | 1895.237966 | 2354.149903 | 2218.116833 | 2213.593687 | 2313.599729 | 1972.758914 | 2006.658562 | 1909.210979 | 1977.492729 |
| **Inpp5e** | 286.6129987 | 284.4220611 | 303.8665823 | 285.5237179 | 270.4461053 | 294.5933784 | 347.2949152 | 300.8685707 | 234.7532062 | 263.0444926 | 284.7070803 | 334.865849 | 366.3962555 | 299.9801443 | 335.5701688 |
| **Sec16a** | 1883.587663 | 1935.496087 | 1811.452074 | 1929.657272 | 1675.879144 | 1616.756517 | 1886.217319 | 1881.476159 | 1987.125698 | 1734.695408 | 1849.177216 | 1897.283968 | 1874.585493 | 1695.068526 | 1911.384687 |
| **Notch1** | 4054.70402 | 3928.035038 | 3866.467311 | 3790.30579 | 3617.137488 | 3544.94032 | 4339.833343 | 4237.30221 | 3893.743084 | 3678.253387 | 3931.038624 | 3800.640634 | 3778.141853 | 3717.223837 | 3852.230567 |
| **Egfl7** | 194.1276541 | 175.0899039 | 167.596517 | 192.3619006 | 168.474623 | 165.5334221 | 140.7220955 | 141.6345082 | 101.5759065 | 165.1674721 | 164.5815016 | 147.4797781 | 123.5522257 | 158.122064 | 204.0726508 |
| **Agpat2** | 280.2031234 | 335.1268296 | 299.1676145 | 313.9898288 | 288.1802761 | 305.8159833 | 289.5627734 | 262.3171661 | 273.8787406 | 256.0532769 | 307.4079771 | 261.9934881 | 293.9690887 | 282.8126059 | 296.7676225 |
| **Fam69b** | 56.77318186 | 72.09584277 | 50.12232285 | 62.97048763 | 44.3354271 | 66.63421654 | 61.34040061 | 35.19910855 | 43.64001911 | 56.80362798 | 73.7779145 | 78.94505767 | 74.98341973 | 45.17773258 | 61.79664779 |
| **Snhg7** | 26.55519797 | 34.06726637 | 23.49483883 | 9.488703616 | 14.56735462 | 11.9240177 | 31.57226502 | 22.62799835 | 31.60139315 | 16.60413741 | 34.99721585 | 30.36348372 | 23.85836082 | 25.29953024 | 32.33545524 |
| **Snora43** | 5.494178889 | 9.507144102 | 0 | 0.86260942 | 3.800179466 | 0 | 1.80412943 | 0 | 1.504828245 | 1.747803938 | 0.945870699 | 0.867528106 | 1.70416863 | 2.710663955 | 2.155697016 |
| **Snora17** | 3.662785926 | 0.792262009 | 4.698967767 | 0.86260942 | 1.900089733 | 0 | 0.902064715 | 5.028444078 | 3.762070613 | 2.621705907 | 0 | 0.867528106 | 2.556252945 | 0 | 0.718565672 |
| **Lcn4** | 23.80810852 | 37.2363144 | 32.10961307 | 31.05393911 | 30.40143573 | 39.27911712 | 37.88671802 | 13.40918421 | 15.04828245 | 3.495607876 | 17.02567258 | 34.70112425 | 63.05423932 | 25.29953024 | 39.52111196 |
| **Surf6** | 1104.329957 | 1006.172751 | 1134.800716 | 1161.934888 | 1107.118951 | 1117.3506 | 1144.720123 | 1149.837546 | 1038.331489 | 1090.629657 | 1098.155881 | 1083.542605 | 939.8489996 | 1003.849218 | 1001.680547 |
| **Med22** | 968.8068775 | 1010.926323 | 1143.41549 | 1019.604334 | 1029.215272 | 941.2959853 | 1018.431063 | 1194.255469 | 1217.40605 | 1233.075678 | 1263.683254 | 945.6056358 | 1100.040851 | 1059.869606 | 1124.555277 |
| **Rpl7a** | 27368.33644 | 25112.32888 | 26212.40853 | 25605.69801 | 26127.50055 | 25909.48763 | 26678.56394 | 26149.58536 | 24742.38601 | 26284.34952 | 25809.97376 | 26502.11612 | 25712.49629 | 26131.70408 | 26139.98201 |
| **Surf1** | 464.2581162 | 443.6667248 | 454.2335508 | 445.1064605 | 456.6548991 | 401.9095377 | 400.5167334 | 468.4833733 | 424.3615651 | 428.2119648 | 477.6647029 | 387.7850635 | 404.7400497 | 485.2088479 | 387.3068972 |
| **Surf2** | 401.0750589 | 348.5952837 | 397.0627763 | 348.4942055 | 340.1160622 | 317.740001 | 395.1043451 | 419.8750805 | 419.8470804 | 441.3204943 | 437.9381335 | 340.0710177 | 344.2420633 | 354.1934234 | 353.5343106 |
| **Surf4** | 4251.578764 | 4278.214846 | 4313.65241 | 4253.527048 | 4078.859293 | 4216.192375 | 4359.678767 | 3956.547416 | 4130.001119 | 4051.409528 | 4132.509083 | 4266.503227 | 4233.154878 | 4392.179161 | 4327.202477 |
| **Stkld1** | 1.831392963 | 3.169048034 | 4.698967767 | 3.450437679 | 4.43354271 | 6.312715251 | 2.706194144 | 4.190370065 | 3.762070613 | 0 | 4.729353494 | 6.94022485 | 6.816674521 | 8.131991864 | 5.748525376 |
| **Rexo4** | 1120.812493 | 1139.272768 | 1100.341619 | 1029.955647 | 1082.417784 | 1036.688127 | 1005.802157 | 1180.846284 | 1140.65981 | 1237.445188 | 1099.101752 | 1106.098336 | 1059.140804 | 1141.189525 | 1037.60883 |
| **Cacfd1** | 160.2468843 | 179.8434759 | 266.2748401 | 250.1567317 | 274.2462848 | 286.1764247 | 283.2483204 | 242.2033898 | 240.0201051 | 197.501845 | 227.9548384 | 275.8739378 | 300.7857633 | 263.8379583 | 262.2764703 |
| **Slc2a6** | 814.9698686 | 846.1358251 | 823.8856818 | 841.0441842 | 585.861001 | 637.5842404 | 596.2647765 | 1068.544367 | 738.8706684 | 947.3097343 | 1076.400855 | 559.5556286 | 525.7360225 | 566.5287665 | 620.1221749 |
| **Adamtsl2** | 3.662785926 | 15.84524017 | 5.482129061 | 9.488703616 | 11.4005384 | 8.416953669 | 3.608258859 | 23.46607237 | 11.28621184 | 23.59535316 | 9.458706988 | 8.675281063 | 6.816674521 | 9.939101167 | 8.622788064 |
| **Sardh** | 3.662785926 | 0 | 0 | 1.725218839 | 0 | 1.402825611 | 5.412388289 | 0.838074013 | 0 | 0 | 0 | 0 | 4.260421576 | 0.903554652 | 0 |
| **Vav2** | 409.3163273 | 337.5036156 | 429.9555507 | 358.8455186 | 319.2150751 | 416.6392066 | 390.5940215 | 430.7700427 | 474.0208972 | 505.115338 | 462.5307717 | 353.9514674 | 349.3545692 | 370.4574071 | 338.4444315 |
| **Brd3** | 1158.356049 | 1249.397187 | 1261.672845 | 1139.507043 | 1154.621194 | 1160.838193 | 1252.065824 | 1254.596798 | 1216.653636 | 1124.711834 | 1128.423744 | 1138.196875 | 1179.284692 | 1196.306359 | 1258.927057 |
| **Wdr5** | 1819.488909 | 1897.46751 | 1921.094655 | 1902.05377 | 1950.758792 | 1937.302169 | 1861.861571 | 2019.758371 | 1773.440087 | 1961.036018 | 1915.388165 | 1881.668463 | 1947.864744 | 2059.201051 | 1925.756001 |
| **Rxra** | 1234.358857 | 1195.523371 | 1274.986587 | 1407.778573 | 1376.29833 | 1472.265479 | 1537.118274 | 2101.051551 | 1674.873837 | 1869.276311 | 2311.707988 | 1504.293736 | 1434.057902 | 1554.114001 | 1506.832214 |
| **Col5a1** | 20.14532259 | 11.88393013 | 14.0969033 | 22.42784491 | 24.70116653 | 12.6254305 | 18.0412943 | 15.92340625 | 16.5531107 | 2.621705907 | 11.35044839 | 23.42325887 | 13.63334904 | 18.97464768 | 14.37131344 |
| **Olfm1** | 21.97671556 | 6.338096068 | 15.66322589 | 6.900875357 | 22.16771355 | 26.65368662 | 22.55161787 | 11.73303618 | 24.82966605 | 24.46925513 | 28.37612096 | 21.68820266 | 23.00627651 | 16.26398373 | 31.61688957 |
| **Ppp1r26** | 7.325571853 | 21.39107423 | 8.614774239 | 11.21392246 | 10.13381191 | 3.507064029 | 20.74748844 | 1.676148026 | 6.019312981 | 13.9824315 | 5.675224193 | 11.27786538 | 12.78126473 | 11.74621047 | 7.904222392 |
| **1700007K13Rik** | 0.915696482 | 0 | 0 | 1.725218839 | 1.900089733 | 1.402825611 | 0 | 3.352296052 | 3.00965649 | 6.991215751 | 1.891741398 | 0 | 0 | 1.807109303 | 0 |
| **Mrps2** | 679.4467893 | 747.895336 | 722.8578748 | 716.8284277 | 715.700466 | 770.8526735 | 745.1054544 | 858.1877894 | 858.5045139 | 671.1567121 | 802.0983526 | 713.1081034 | 758.3550405 | 699.3513003 | 799.0450272 |
| **Ralgds** | 671.205521 | 610.8340086 | 652.3733583 | 602.9639843 | 524.4247663 | 566.0401342 | 657.6051771 | 711.5248371 | 595.9119851 | 676.4001239 | 606.3031179 | 576.9061907 | 584.5298402 | 572.8536491 | 558.3255271 |
| **Gtf3c5** | 1011.844612 | 1137.688244 | 1081.545748 | 1061.009586 | 1046.31608 | 1051.417796 | 1162.761417 | 1116.314585 | 1187.309485 | 1118.59452 | 1061.266924 | 1106.098336 | 1141.792982 | 1062.58027 | 924.0754541 |
| **Tsc1** | 514.6214226 | 513.3857815 | 557.6108417 | 508.0769482 | 502.890416 | 479.0649463 | 521.3934051 | 625.2032137 | 617.7319946 | 572.4057896 | 536.3086862 | 536.9998978 | 521.4756009 | 481.5946293 | 469.2233838 |
| **Gtf3c4** | 979.7952353 | 1151.94896 | 1030.640264 | 1059.284367 | 939.2776913 | 926.5663163 | 939.0493681 | 974.6800772 | 1057.894256 | 948.1836363 | 996.9477165 | 930.857658 | 966.2636134 | 858.376919 | 908.2670094 |
| **Ddx31** | 565.9004256 | 564.0905501 | 548.9960674 | 572.7726546 | 511.7575014 | 537.2822092 | 512.372758 | 499.4921118 | 648.5809737 | 573.2796916 | 589.2774453 | 530.0596729 | 508.6943362 | 442.7417793 | 500.8402734 |
| **Barhl1** | 0 | 0 | 0.783161294 | 0 | 1.900089733 | 0.701412806 | 0 | 0 | 0 | 0.873901969 | 0 | 0.867528106 | 2.556252945 | 0 | 0 |
| **1700101E01Rik** | 7.325571853 | 5.54583406 | 18.01270977 | 24.15306375 | 12.66726489 | 16.83390734 | 12.62890601 | 6.704592104 | 6.771727103 | 11.3607256 | 22.70089677 | 12.14539349 | 19.59793925 | 14.45687443 | 16.52701046 |
| **Ttf1** | 489.8976176 | 552.2066199 | 461.2820025 | 416.6403497 | 528.858309 | 458.0225621 | 505.1562403 | 388.8663421 | 378.4643037 | 434.3292785 | 436.9922628 | 471.0677617 | 556.4110578 | 458.1022083 | 492.936051 |
| **Setx** | 2815.766681 | 2940.084314 | 2808.416402 | 2999.292952 | 2670.892801 | 2553.142613 | 2970.499106 | 2788.272241 | 2697.404629 | 2525.57669 | 2568.984818 | 3052.831406 | 2961.84508 | 2904.02465 | 2754.262221 |
| **Ntng2** | 60.43596778 | 67.34227072 | 52.47180673 | 65.55831589 | 52.56914927 | 72.94693179 | 84.79408319 | 36.87525657 | 74.48899814 | 48.93851026 | 41.61831075 | 68.5347204 | 67.3146609 | 55.11683375 | 64.67091048 |
| **6530402F18Rik** | 444.1127936 | 491.9947073 | 455.0167121 | 447.6942888 | 377.4844936 | 434.8759395 | 443.8158397 | 436.6365608 | 401.7891415 | 443.0682982 | 437.9381335 | 416.413491 | 431.1546635 | 397.5640467 | 493.6546166 |
| **Med27** | 487.1505282 | 535.5691177 | 494.1747768 | 511.5273859 | 464.2552581 | 459.4253877 | 512.372758 | 513.73937 | 549.2623095 | 498.9980242 | 472.9353494 | 503.1663016 | 494.2089028 | 548.4576735 | 431.8579689 |
| **Rapgef1** | 5117.827635 | 5324.792959 | 5442.970997 | 5309.360978 | 4969.368015 | 4956.884298 | 5376.3057 | 5512.850858 | 5415.12444 | 5356.145167 | 5355.519896 | 5408.170215 | 5292.295681 | 5196.342801 | 4950.91748 |
| **Trub2** | 521.9469945 | 434.9518427 | 552.1287126 | 501.1760728 | 457.9216256 | 501.5101561 | 514.1768874 | 517.0916661 | 563.5581778 | 565.4145739 | 523.0664964 | 512.7091108 | 572.6006598 | 541.2292363 | 526.7086376 |
| **Coq4** | 148.34283 | 145.7762096 | 163.6807105 | 187.1862441 | 148.2069992 | 161.3249453 | 168.6861017 | 154.2056184 | 189.6083589 | 174.7803938 | 183.4989156 | 170.0355088 | 122.7001414 | 141.8580803 | 112.8148105 |
| **Slc27a4** | 297.6013565 | 310.5667073 | 327.3614211 | 307.0889534 | 305.914447 | 324.754129 | 303.0937442 | 256.450648 | 302.4704773 | 280.522532 | 261.0603129 | 298.4296686 | 318.6795339 | 291.8481525 | 311.8575016 |
| **Urm1** | 669.374128 | 751.8566461 | 724.4241974 | 746.157148 | 795.5042348 | 755.4215918 | 721.6517718 | 1006.52689 | 905.9066036 | 887.8844004 | 899.5230345 | 766.0273178 | 829.930123 | 799.6458666 | 758.8053496 |
| **Cercam** | 346.13327 | 347.0107597 | 361.8205181 | 321.7533135 | 247.0116653 | 239.8831796 | 250.7739907 | 424.0654506 | 361.911193 | 394.129788 | 336.7299688 | 258.5233757 | 218.1335847 | 301.7872536 | 254.3722479 |
| **Odf2** | 2169.284965 | 2181.097309 | 2237.491818 | 2059.048685 | 2067.930993 | 1960.448792 | 2253.357658 | 2101.889625 | 2017.222263 | 2144.555432 | 2171.719124 | 2141.926894 | 2211.158798 | 2134.196087 | 2098.211762 |
| **Gle1** | 1219.707713 | 1363.482917 | 1270.28762 | 1192.988827 | 1246.458865 | 1155.226891 | 1197.039877 | 1498.476335 | 1426.577176 | 1398.24315 | 1285.43828 | 1275.266316 | 1330.9557 | 1199.920577 | 1248.867138 |
| **Sptan1** | 5122.406118 | 5047.501256 | 5004.400672 | 5360.254934 | 5247.414479 | 5185.544873 | 5646.02305 | 5714.826695 | 5312.043705 | 4868.507869 | 5189.992524 | 5690.984377 | 5764.350392 | 5435.784784 | 5721.938446 |
| **Wdr34** | 193.2119576 | 176.6744279 | 184.8260655 | 185.4610252 | 166.5745332 | 150.8037532 | 147.9386132 | 166.7767286 | 200.1421566 | 186.1411194 | 146.6099583 | 151.8174186 | 158.4876826 | 173.4824931 | 145.8688314 |
| **Set** | 16064.97907 | 16013.99198 | 16361.0226 | 16079.03958 | 16387.00722 | 16617.87219 | 16962.4249 | 18486.23658 | 17854.03471 | 18046.94956 | 18120.04498 | 16549.83368 | 16175.96864 | 15609.81016 | 15561.97676 |
| **Pkn3** | 32.04937685 | 21.39107423 | 29.76012919 | 15.52696955 | 31.03479897 | 27.35509942 | 34.27845916 | 18.43762829 | 26.33449429 | 13.9824315 | 27.43025026 | 32.96606804 | 34.08337261 | 22.58886629 | 29.46119255 |
| **Zdhhc12** | 195.0433506 | 197.2732401 | 176.2112913 | 208.7514796 | 210.2765971 | 194.99276 | 190.3356548 | 219.5753914 | 240.0201051 | 260.4227867 | 209.0374244 | 148.3473062 | 185.7543807 | 188.8429222 | 176.7671553 |
| **Zer1** | 466.0895091 | 518.1393536 | 527.8507125 | 488.2369315 | 494.6566938 | 509.9271098 | 508.7644991 | 429.9319687 | 506.3747045 | 443.9422002 | 498.4738583 | 515.3116951 | 636.5069834 | 600.8638433 | 519.5229808 |
| **Tbc1d13** | 778.3420093 | 922.1929779 | 841.1152303 | 950.5955805 | 853.1063884 | 853.6193846 | 848.8428966 | 996.4700015 | 866.0286551 | 946.4358323 | 947.7624402 | 834.5620382 | 875.942676 | 892.7119958 | 801.9192899 |
| **Endog** | 246.3223535 | 221.0411004 | 286.6370338 | 262.2332636 | 237.5112166 | 304.4131577 | 263.4028967 | 238.8510937 | 243.7821757 | 276.1530222 | 235.521804 | 259.3909038 | 250.5127887 | 243.9597559 | 280.2406121 |
| **D2Wsu81e** | 455.1011513 | 457.9274409 | 448.7514217 | 479.6108373 | 436.3872753 | 430.6674627 | 445.6199691 | 407.3039703 | 466.496756 | 423.8424549 | 493.7445048 | 401.6655132 | 503.5818303 | 411.1173665 | 378.6841091 |
| **Ccbl1** | 54.02609241 | 51.49703055 | 49.33916155 | 64.69570647 | 49.40233305 | 41.38335554 | 46.90736517 | 38.5514046 | 50.41174621 | 44.56900041 | 33.10547446 | 53.78674259 | 40.04796281 | 33.43152211 | 45.98820301 |
| **1700084E18Rik** | 2.747089445 | 0.792262009 | 1.566322589 | 5.175656518 | 0 | 0 | 0.902064715 | 4.190370065 | 0 | 0.873901969 | 2.837612096 | 1.735056213 | 0 | 7.228437213 | 1.437131344 |
| **Lrrc8a** | 314.0838932 | 298.6827772 | 325.7950985 | 331.2420171 | 386.351579 | 346.497926 | 397.8105392 | 313.4396809 | 257.3256299 | 260.4227867 | 281.8694682 | 360.8916922 | 357.8754124 | 358.7111967 | 347.7857852 |
| **Phyhd1** | 17.39823315 | 10.29940611 | 19.57903236 | 15.52696955 | 11.4005384 | 16.83390734 | 21.64955315 | 20.95185033 | 16.5531107 | 16.60413741 | 8.512836289 | 8.675281063 | 17.89377062 | 18.97464768 | 16.52701046 |
| **Dolk** | 193.2119576 | 236.8863405 | 223.9841302 | 244.9810752 | 189.37561 | 230.0634003 | 193.9439137 | 243.0414638 | 219.7049238 | 274.4052182 | 227.9548384 | 202.1340488 | 216.4294161 | 232.2135455 | 160.9587105 |
| **Nup188** | 3439.355985 | 3211.830182 | 3397.353695 | 3152.837429 | 3087.012453 | 3015.373652 | 3453.103728 | 3401.742419 | 3386.615966 | 3263.149952 | 3415.539093 | 3122.233654 | 3262.630843 | 3020.5832 | 3385.881446 |
| **Sh3glb2** | 650.1445019 | 785.9239124 | 805.872972 | 844.4946218 | 791.7040553 | 779.97104 | 728.8682895 | 753.4285377 | 872.0479681 | 860.7934394 | 814.3946716 | 879.6734998 | 771.9883895 | 850.2449271 | 770.3024003 |
| **Fam73b** | 209.6944943 | 227.3791964 | 220.851485 | 251.0193411 | 253.978661 | 274.252407 | 269.7173497 | 296.6782006 | 284.4125383 | 209.7364725 | 239.3052868 | 266.3311286 | 289.7086672 | 271.9699501 | 291.0190971 |
| **Dolpp1** | 310.4211073 | 350.9720698 | 347.7236148 | 304.5011251 | 350.2498741 | 341.5880364 | 325.645362 | 358.6956776 | 319.023588 | 365.291023 | 357.5391241 | 339.2034896 | 330.6087143 | 384.0107269 | 382.9955032 |
| **Crat** | 76.00280797 | 75.26489081 | 95.54567793 | 74.18441009 | 77.2703158 | 87.67660071 | 65.85072418 | 77.1028092 | 77.49865463 | 102.2465304 | 88.91184568 | 87.62033873 | 66.46257658 | 99.39101167 | 66.82660749 |
| **Ppp2r4** | 3595.024387 | 3890.798724 | 3859.418859 | 3804.107541 | 3907.217854 | 3661.374846 | 3865.347303 | 4039.516743 | 3906.534124 | 3850.412075 | 3813.750657 | 3973.278727 | 3887.208646 | 4003.650661 | 4040.494773 |
| **Ier5l** | 169.4038491 | 129.9309694 | 137.0532265 | 98.33747384 | 107.6717515 | 111.5246361 | 125.3869954 | 36.03718256 | 60.94554393 | 52.43411813 | 68.10269031 | 126.6591035 | 121.8480571 | 96.68034772 | 114.2519418 |
| **Cstad** | 0 | 3.169048034 | 0 | 0 | 0 | 0 | 1.80412943 | 0 | 0 | 0 | 0 | 0 | 1.70416863 | 0.903554652 | 0 |
| **1700001O22Rik** | 34.7964663 | 27.7291703 | 7.831612945 | 19.84001665 | 12.03390164 | 38.57770431 | 17.13922958 | 9.218814144 | 37.62070613 | 14.85633347 | 22.70089677 | 19.08561834 | 18.74585493 | 18.97464768 | 18.68270747 |
| **Ntmt1** | 704.1705943 | 616.3798426 | 588.1541322 | 571.0474358 | 537.7253944 | 569.5471982 | 557.4759937 | 646.1550641 | 520.6705728 | 554.9277503 | 567.5224193 | 491.8884363 | 522.3276852 | 437.3204514 | 497.9660107 |
| **Asb6** | 872.6587469 | 899.2173797 | 783.9444558 | 785.8371813 | 773.9698845 | 786.2837552 | 759.5384898 | 870.7588996 | 918.6976437 | 837.1980862 | 845.6084047 | 772.1000146 | 818.8530269 | 771.6356724 | 758.0867839 |
| **Ptges** | 0 | 0 | 0.783161294 | 0 | 0.633363244 | 0 | 1.80412943 | 0 | 0 | 0 | 0.945870699 | 0 | 0 | 2.710663955 | 2.874262688 |
| **Tor1b** | 482.5720458 | 567.2595981 | 523.1517447 | 524.4665271 | 538.9921209 | 531.6709067 | 570.1048997 | 648.6692861 | 630.5230347 | 605.6140645 | 691.4314808 | 516.1792232 | 589.6423461 | 555.6861107 | 555.4512644 |
| **Tor1a** | 700.5078084 | 798.6001046 | 812.1382624 | 706.4771147 | 707.4667439 | 766.6441966 | 763.1467487 | 738.3432055 | 838.9417467 | 762.0425169 | 753.8589469 | 769.4974303 | 710.6383188 | 728.2650492 | 720.721369 |
| **BC005624** | 641.9032336 | 709.8667596 | 739.304262 | 633.155314 | 738.5015428 | 683.8774856 | 723.4559013 | 815.4460147 | 668.896155 | 769.9076346 | 702.7819292 | 701.830238 | 575.1569127 | 678.5695433 | 636.6491854 |
| **Usp20** | 223.4299415 | 213.1184803 | 238.0810335 | 271.7219672 | 209.0098706 | 265.8354534 | 226.4182434 | 236.3368717 | 203.1518131 | 245.5664533 | 289.4364338 | 161.3602278 | 224.0981749 | 224.9851082 | 241.4380658 |
| **Fnbp1** | 3749.777092 | 3724.423702 | 3719.232988 | 3730.78574 | 3701.3748 | 3870.395862 | 4156.714206 | 3481.35945 | 3710.906453 | 3436.182542 | 3698.354432 | 3916.8894 | 4118.123495 | 3789.508209 | 3887.440285 |
| **D330023K18Rik** | 0 | 0 | 3.132645178 | 2.587828259 | 1.900089733 | 2.104238417 | 0.902064715 | 3.352296052 | 0 | 6.991215751 | 1.891741398 | 2.602584319 | 3.408337261 | 2.710663955 | 3.59282836 |
| **Gpr107** | 573.2259975 | 604.4959125 | 559.9603256 | 553.7952474 | 530.7583987 | 586.3811056 | 599.8730353 | 510.3870739 | 474.7733113 | 455.3029258 | 578.8728676 | 511.8415827 | 613.5007069 | 571.0465398 | 520.9601122 |
| **Ncs1** | 468.8365986 | 332.7500436 | 382.1827117 | 369.1968316 | 297.6807248 | 294.5933784 | 383.3775038 | 510.3870739 | 422.1043228 | 337.32616 | 366.0519604 | 261.9934881 | 311.010775 | 277.391278 | 324.7916837 |
| **Ass1** | 3070.330303 | 3205.492086 | 3039.448984 | 3079.515628 | 3416.36134 | 3540.03043 | 3859.03285 | 3013.714151 | 3000.627521 | 2633.066632 | 2653.16731 | 3316.55995 | 3196.168266 | 3317.852681 | 3413.186942 |
| **Fubp3** | 982.5423247 | 958.6370303 | 1057.267748 | 845.3572313 | 814.5051321 | 866.9462279 | 958.8947918 | 1139.780658 | 1045.85563 | 1048.682363 | 1028.16145 | 884.0111403 | 856.3447367 | 871.0266841 | 858.685978 |
| **Exosc2** | 1322.265719 | 1386.458515 | 1457.463169 | 1323.24285 | 1280.027117 | 1285.689673 | 1289.952542 | 1595.692921 | 1589.851041 | 1624.58376 | 1567.307748 | 1317.775193 | 1299.428581 | 1268.590731 | 1265.394148 |
| **Abl1** | 1397.352831 | 1386.458515 | 1545.960395 | 1544.070861 | 1475.102996 | 1438.597665 | 1483.896456 | 1605.749809 | 1497.304104 | 1398.24315 | 1405.563858 | 1545.935085 | 1417.8683 | 1618.266381 | 1659.168137 |
| **Qrfp** | 1.831392963 | 3.169048034 | 2.349483883 | 0 | 2.533452977 | 7.014128057 | 0.902064715 | 8.380740131 | 0 | 2.621705907 | 0 | 6.072696744 | 2.556252945 | 0 | 0 |
| **Fibcd1** | 3.662785926 | 0.792262009 | 1.566322589 | 1.725218839 | 1.900089733 | 5.611302446 | 2.706194144 | 0 | 0 | 0 | 5.675224193 | 5.205168638 | 1.70416863 | 2.710663955 | 0 |
| **Aif1l** | 5.494178889 | 3.961310043 | 5.482129061 | 3.450437679 | 9.500448664 | 12.6254305 | 9.020647148 | 0.838074013 | 0 | 10.48682363 | 4.729353494 | 11.27786538 | 1.70416863 | 3.614218606 | 7.18565672 |
| **Nup214** | 2660.098279 | 2697.652139 | 2625.156659 | 2758.624924 | 2470.116653 | 2493.522524 | 2637.637226 | 2981.029264 | 2872.71712 | 2549.172043 | 2857.475381 | 2560.075442 | 2677.248918 | 2552.541891 | 2808.154646 |
| **Mir7674** | 0 | 2.376786026 | 2.349483883 | 0 | 1.900089733 | 0 | 0.902064715 | 0.838074013 | 0 | 0 | 0 | 0 | 0 | 0 | 0 |
| **Fam78a** | 2313.965009 | 2857.689065 | 2779.439434 | 2815.557146 | 2722.828587 | 2529.99599 | 2746.787057 | 2490.755967 | 2850.89711 | 2447.799415 | 2524.528895 | 3080.592305 | 3093.918148 | 3078.410698 | 2851.987152 |
| **Prrc2b** | 6903.435775 | 7235.728924 | 6877.722488 | 7279.560892 | 6206.326431 | 6247.48386 | 6745.639937 | 7232.578733 | 7259.291455 | 6611.068395 | 6626.770116 | 6720.740239 | 6894.214194 | 6327.593225 | 6740.146003 |
| **Pomt1** | 170.3195456 | 139.4381135 | 168.3796783 | 201.8506042 | 149.4737256 | 172.5475502 | 168.6861017 | 181.0239868 | 176.0649047 | 195.754041 | 231.7383212 | 157.0225872 | 195.1273082 | 210.5282338 | 139.4017404 |
| **Uck1** | 549.4178889 | 626.6792487 | 668.0365842 | 663.3466437 | 647.9305989 | 621.4517459 | 694.5898304 | 779.4088321 | 708.7741035 | 757.673007 | 663.0553598 | 744.3391152 | 662.0695129 | 701.1584096 | 699.8829645 |
| **Swi5** | 2264.517399 | 2322.119947 | 2241.407625 | 1948.634679 | 2228.805257 | 2109.148307 | 2223.589522 | 2143.793325 | 2201.563723 | 2231.945629 | 2174.556736 | 2293.744313 | 2014.327321 | 2325.749673 | 2075.936226 |
| **Golga2** | 1119.896797 | 1006.172751 | 1097.992135 | 1038.581741 | 1000.080563 | 948.3101133 | 1051.807457 | 1029.154888 | 1051.122529 | 1019.843598 | 1076.400855 | 1080.94002 | 1060.844972 | 992.1030074 | 1068.507154 |
| **Dnm1** | 6.409875371 | 8.714882094 | 8.614774239 | 6.900875357 | 6.333632443 | 5.611302446 | 12.62890601 | 4.190370065 | 0 | 5.243411813 | 0 | 0.867528106 | 3.408337261 | 4.517773258 | 5.029959704 |
| **Ciz1** | 914.7807851 | 830.2905849 | 838.7657464 | 863.4720291 | 862.0073755 | 877.4674199 | 875.9048381 | 771.028092 | 835.1796761 | 736.6993598 | 721.6993432 | 864.925522 | 881.0551819 | 847.5342632 | 885.9914735 |
| **1110008P14Rik** | 188.6334752 | 198.8577641 | 178.5607751 | 175.1097122 | 170.3747127 | 162.7277709 | 178.6088135 | 243.0414638 | 234.7532062 | 239.4491395 | 187.2823984 | 181.3133742 | 177.2335376 | 163.5433919 | 200.4798225 |
| **Ptges2** | 248.1537465 | 286.7988471 | 284.2875499 | 283.7984991 | 258.4122037 | 322.6498906 | 270.6194144 | 328.5250131 | 323.5380727 | 254.305473 | 271.4648905 | 249.8480946 | 280.3357397 | 265.6450676 | 255.0908135 |
| **Slc25a25** | 293.9385706 | 329.5809955 | 320.3129694 | 337.2802831 | 329.348887 | 338.7823852 | 362.6300153 | 304.2208667 | 238.5152769 | 283.1442379 | 293.2199166 | 338.3359614 | 329.75663 | 290.0410432 | 317.606027 |
| **Naif1** | 82.41268334 | 100.6172751 | 93.97935534 | 103.5131304 | 104.5049353 | 69.43986777 | 88.40234205 | 71.23629111 | 63.2027863 | 66.41654964 | 85.12836289 | 77.21000146 | 80.09592563 | 65.95948956 | 95.56923437 |
| **Fam102a** | 4244.253192 | 4258.408296 | 4206.359313 | 4268.191408 | 4089.626468 | 4503.070213 | 4861.226748 | 1904.942232 | 2235.422358 | 2134.94251 | 2300.357539 | 4053.958841 | 4438.507198 | 4300.920141 | 4556.424926 |
| **Dpm2** | 420.304685 | 315.3202794 | 415.0754861 | 456.320383 | 428.7869164 | 355.6162925 | 368.0424036 | 470.9975953 | 437.9050193 | 451.8073179 | 472.9353494 | 431.1614688 | 379.1775202 | 386.7213909 | 407.426736 |

| | | | | | | | | | | | | | | | |
|---|---|---|---|---|---|---|---|---|---|---|---|---|---|---|---|
| Pip5kl1 | 0 | 0.792262009 | 0 | 0 | 1.266726489 | 0.701412806 | 0.902064715 | 3.352296052 | 2.257242368 | 1.747803938 | 6.621094891 | 4.337640531 | 0 | 0 | 0 |
| St6galnac4 | 2612.482062 | 2597.034864 | 2503.766658 | 2575.751727 | 2294.041671 | 2288.709985 | 2535.703913 | 2860.346607 | 2873.469534 | 2430.321376 | 2603.036163 | 2226.077121 | 2446.334069 | 2210.998232 | 2274.260352 |
| St6galnac6 | 195.9590471 | 202.8190742 | 208.3209043 | 191.4992912 | 193.1757895 | 208.3196033 | 179.5108782 | 227.1180575 | 212.1807826 | 216.7276883 | 264.8437957 | 227.2923638 | 208.7606572 | 123.7869873 | 173.8928926 |
| Ak1 | 9.156964816 | 9.507144102 | 15.66322589 | 18.97740723 | 3.800179466 | 3.507064029 | 5.412388289 | 43.57984868 | 66.96485691 | 31.46047088 | 41.61831075 | 6.94022485 | 1.70416863 | 4.517773258 | 12.21561642 |
| Eng | 195.9590471 | 227.3791964 | 233.3820658 | 217.3775738 | 128.5727386 | 114.3302873 | 128.0931895 | 387.190194 | 312.2518609 | 306.7395911 | 336.7299688 | 144.8771937 | 86.06051583 | 134.6296431 | 110.6591135 |
| Fpgs | 711.4961662 | 640.9399649 | 640.6259389 | 677.1483944 | 725.8342779 | 699.3085673 | 702.7084128 | 765.9996479 | 683.1920233 | 812.7288311 | 751.9672055 | 595.991809 | 712.3424875 | 683.9908712 | 600.0023361 |
| Cdk9 | 2031.014796 | 2044.035982 | 2051.882592 | 2070.262607 | 2020.428749 | 2123.176563 | 1988.150631 | 2032.329482 | 2008.945707 | 1995.118195 | 2177.394349 | 1949.335655 | 2073.973223 | 1962.520703 | 2002.642528 |
| Mir3960 | 6.409875371 | 0.792262009 | 7.04845165 | 10.35131304 | 3.800179466 | 16.83390734 | 7.216517718 | 3.352296052 | 3.762070613 | 8.739019689 | 8.512836289 | 5.205168638 | 10.22501178 | 6.324882561 | 14.37131344 |
| Sh2d3c | 871.7430504 | 888.1257115 | 902.2018112 | 984.2373478 | 1127.386575 | 1148.914176 | 1144.720123 | 676.3257285 | 762.1955062 | 699.1215751 | 721.6993432 | 1115.641145 | 1118.786706 | 1088.783355 | 1072.818548 |
| 6330409D20Rik | 0 | 10.29940611 | 8.614774239 | 1.725218839 | 4.43354271 | 0.701412806 | 0.902064715 | 0 | 0.752414123 | 4.369509845 | 0.945870699 | 7.807752957 | 3.408337261 | 2.710663955 | 5.029959704 |
| Ttc16 | 2.747089445 | 1.584524017 | 10.18109683 | 5.175656518 | 7.600358931 | 9.118366474 | 6.314453003 | 2.514222039 | 8.276555348 | 1.747803938 | 5.675224193 | 4.337640531 | 0.852084315 | 9.035546516 | 7.904222392 |
| Tor2a | 439.5343112 | 458.7197029 | 490.2589703 | 469.2595243 | 440.820818 | 414.5349682 | 454.6406162 | 472.6737434 | 478.535382 | 471.9070632 | 525.9041085 | 353.0839393 | 373.21293 | 476.1733014 | 478.5647375 |
| Ptrh1 | 70.50862908 | 67.34227072 | 64.21922615 | 89.71137964 | 55.10260225 | 49.0988964 | 49.61355931 | 92.18814144 | 64.70761454 | 59.42533389 | 77.5613973 | 71.13730471 | 65.61049227 | 51.50261514 | 63.23377913 |
| 1700019L03Rik | 2.747089445 | 13.46845414 | 19.57903236 | 5.175656518 | 8.86708542 | 9.81977928 | 9.020647148 | 10.05688816 | 15.04828245 | 7.86511772 | 8.512836289 | 14.74797781 | 12.78126473 | 9.035546516 | 13.65274777 |
| Stxbp1 | 147.4271335 | 148.1529956 | 140.1858717 | 173.3844933 | 112.1052942 | 143.0882124 | 124.4849306 | 157.5579145 | 187.3511165 | 137.2026091 | 148.5016997 | 143.1421375 | 120.9959728 | 127.4012059 | 150.1802254 |
| Fam129b | 585.1300517 | 635.3941308 | 675.0850358 | 644.3692365 | 713.8003763 | 742.7961612 | 689.1774421 | 885.8442318 | 646.3237313 | 601.2445546 | 725.482826 | 746.9416995 | 698.7091384 | 771.6356724 | 806.9492496 |
| Lrsam1 | 67.76153964 | 78.43393884 | 88.49722628 | 69.87136299 | 70.30332011 | 57.51585007 | 60.43833589 | 38.5514046 | 84.27038173 | 65.54264767 | 75.6696559 | 76.34247335 | 79.24384131 | 63.24882561 | 56.76668809 |
| Rpl12 | 21308.25713 | 20782.61701 | 20754.55747 | 20274.7718 | 21724.35928 | 23397.72837 | 23205.61479 | 20162.38461 | 19692.18242 | 20812.84929 | 19856.66358 | 22212.18963 | 21808.24596 | 22156.06361 | 22523.44099 |
| Snora65 | 11.90405426 | 19.0142882 | 10.96425812 | 10.35131304 | 19.00089733 | 17.53532014 | 15.33510015 | 34.36103454 | 14.29586833 | 16.60413741 | 26.48437957 | 23.42325887 | 11.92918041 | 21.68531164 | 18.68270747 |
| Slc2a8 | 96.14813056 | 68.13453273 | 88.49722628 | 75.04701951 | 95.63784989 | 84.16953669 | 80.28375962 | 72.07436512 | 63.95520042 | 64.6687457 | 67.15681961 | 73.73988903 | 119.2918041 | 91.25901981 | 117.8447702 |
| Garnl3 | 5.494178889 | 7.922620085 | 0.783161294 | 6.038265938 | 8.233722176 | 4.90988964 | 8.118582433 | 5.866518091 | 4.514484735 | 6.991215751 | 5.675224193 | 6.94022485 | 3.408337261 | 4.517773258 | 0 |
| Ralgps1 | 23.80810852 | 9.507144102 | 28.9769679 | 18.97740723 | 28.50134599 | 15.43108173 | 22.55161787 | 41.90370065 | 30.0965649 | 28.83876497 | 22.70089677 | 32.96606804 | 17.0416863 | 36.14218606 | 25.14979852 |
| Angptl2 | 27.47089445 | 37.2363144 | 66.56871003 | 51.75656518 | 64.60305092 | 99.60061841 | 90.20647148 | 48.60829276 | 45.89726148 | 49.81241223 | 36.88895725 | 57.25685501 | 52.82922754 | 40.65995932 | 58.9223851 |
| Zbtb34 | 193.2119576 | 228.1714584 | 218.5020012 | 207.8888701 | 158.3408111 | 187.9786319 | 195.7480431 | 196.1093191 | 177.5697329 | 215.8537863 | 209.0374244 | 192.5912396 | 219.8377533 | 215.0460071 | 191.1384687 |
| Zbtb43 | 203.2846189 | 175.0899039 | 206.7545817 | 175.9723216 | 214.0767766 | 220.243621 | 206.5728197 | 179.3478388 | 265.6021853 | 172.1586879 | 179.7154328 | 209.0742736 | 237.7315239 | 229.5028815 | 204.0726508 |
| Mvb12b | 174.898028 | 201.2345502 | 216.1525173 | 239.8054187 | 250.1784815 | 248.3001332 | 247.1657318 | 219.5753914 | 197.1325001 | 211.4842765 | 250.6557352 | 265.4636005 | 224.0981749 | 203.2997966 | 245.7494598 |
| Pbx3 | 27.47089445 | 37.2363144 | 13.31374201 | 20.70262607 | 8.86708542 | 4.90988964 | 11.72684129 | 25.14222039 | 24.82966605 | 25.3431571 | 21.75502607 | 20.82067455 | 16.18960199 | 13.55331977 | 8.622788064 |
| Mapkap1 | 779.2577058 | 742.349502 | 809.0056172 | 728.9049596 | 722.6674617 | 737.8862716 | 742.3992603 | 769.351944 | 833.6748478 | 843.3154 | 790.7479042 | 700.0951818 | 847.8238936 | 860.1840283 | 736.5298138 |
| Gapvd1 | 2600.578008 | 2756.279528 | 2619.67453 | 2493.803832 | 2387.779431 | 2428.992546 | 2672.81775 | 2877.946161 | 2780.922597 | 2662.779299 | 2609.657258 | 2632.947803 | 2569.886295 | 2342.917212 | 2571.74654 |
| Hspa5 | 16227.05735 | 14192.58162 | 14482.21866 | 14046.73179 | 13502.03764 | 13893.58486 | 14213.83371 | 14244.744 | 13866.99228 | 14276.06256 | 14930.56898 | 14077.37858 | 13493.60721 | 13454.83232 | 14021.37196 |
| Rabepk | 620.8422145 | 516.5548295 | 486.3431639 | 483.061275 | 450.9546299 | 502.9129817 | 490.7232048 | 451.721893 | 527.4422999 | 457.0507297 | 482.3940564 | 500.5637173 | 444.7880125 | 506.8941595 | 535.3314256 |
| Fbxw2 | 974.3010564 | 930.90786 | 1002.446457 | 993.7260514 | 931.0439691 | 1021.257045 | 1010.312481 | 912.6626002 | 893.1155635 | 886.1365965 | 1009.244036 | 936.9303548 | 1063.401225 | 1063.483825 | 974.3750512 |
| Psmd5 | 1072.28058 | 1022.810253 | 1031.423425 | 1058.421758 | 996.9137465 | 972.1581487 | 1021.137257 | 1263.815612 | 1082.723922 | 1120.342324 | 1172.879666 | 1049.709009 | 1078.738743 | 1009.270546 | 1031.141739 |
| Phf19 | 684.0252717 | 819.1989168 | 733.8221329 | 832.41809 | 814.5051321 | 666.3421654 | 732.4765484 | 746.7239456 | 965.3473193 | 832.8285764 | 819.1240251 | 833.6945101 | 806.0717621 | 840.305826 | 876.6501198 |
| Traf1 | 2966.8566 | 2276.961012 | 2289.180464 | 2265.212336 | 2383.345888 | 2342.017358 | 2530.291525 | 2391.025159 | 1846.424257 | 1942.684077 | 1955.114734 | 2125.44386 | 2162.589992 | 2140.52097 | 2180.128249 |
| Hc | 0.915696482 | 0.792262009 | 0.783161294 | 0.86260942 | 0 | 4.208476834 | 0 | 1.676148026 | 3.762070613 | 1.747803938 | 0 | 0 | 0 | 0 | 0 |
| AI182371 | 3.662785926 | 3.169048034 | 7.04845165 | 10.35131304 | 4.43354271 | 1.402825611 | 9.020647148 | 3.352296052 | 4.514484735 | 4.369509845 | 0 | 4.337640531 | 1.70416863 | 2.710663955 | 0.718565672 |
| Cntrl | 2969.60369 | 2957.514078 | 2986.977177 | 2873.351977 | 2777.297826 | 2749.538198 | 3022.818859 | 2717.03595 | 2859.92608 | 2750.169496 | 2853.691898 | 2951.330618 | 2995.928452 | 3247.375418 | 3112.826491 |
| Rab14 | 1708.689635 | 1561.548419 | 1706.508461 | 1653.622257 | 1639.144076 | 1538.198283 | 1680.546564 | 1724.756319 | 1747.105593 | 1799.364154 | 1753.644276 | 1583.238794 | 1557.610128 | 1614.652162 | 1544.916195 |
| Gsn | 300.348446 | 264.6155108 | 257.6600659 | 231.1793245 | 184.9420673 | 241.987418 | 250.7739907 | 160.0721365 | 124.9007443 | 110.1116481 | 164.5815016 | 189.9886553 | 253.0690416 | 229.5028815 | 224.1924897 |
| Stom | 12.81975074 | 7.922620085 | 4.698967767 | 5.175656518 | 8.86708542 | 4.208476834 | 11.72684129 | 11.73303618 | 14.29586833 | 3.495607876 | 25.53850887 | 9.542809169 | 3.408337261 | 6.324882561 | 5.029959704 |
| Ggta1 | 700.5078084 | 669.4613972 | 689.9651004 | 571.9100452 | 505.4238689 | 476.2592951 | 520.4913404 | 617.6605476 | 607.1981969 | 575.9013975 | 683.8645152 | 440.704278 | 478.8713851 | 512.3154874 | 490.0617883 |
| Dab2ip | 7.325571853 | 1.584524017 | 15.66322589 | 7.763484777 | 10.76717515 | 7.014128057 | 11.72684129 | 7.542666117 | 15.04828245 | 5.243411813 | 17.02567258 | 10.41033728 | 10.22501178 | 14.45687443 | 19.40127314 |
| Ttll11 | 31.13368037 | 41.19762444 | 7.831612945 | 23.29045433 | 20.26762382 | 31.56357626 | 15.33510015 | 29.33259046 | 19.56276719 | 9.612921658 | 29.32199166 | 34.70112425 | 32.37920398 | 35.23863141 | 23.71266717 |
| Ndufa8 | 1317.687237 | 1369.028751 | 1332.940523 | 1393.114213 | 1335.129719 | 1525.572852 | 1388.277596 | 1458.248783 | 1421.310278 | 1335.322208 | 1317.597883 | 1353.343846 | 1280.682726 | 1431.230568 | 1394.735969 |
| Lhx6 | 0 | 0.792262009 | 3.132645178 | 0 | 0 | 0 | 0 | 0.838074013 | 0 | 0 | 1.891741398 | 0.867528106 | 0 | 0.903554652 | 1.437131344 |
| Rbm18 | 326.9036439 | 347.8030217 | 328.1445824 | 306.226344 | 319.8484384 | 357.0191181 | 398.7126039 | 338.5819013 | 314.5091032 | 335.5783561 | 297.0033994 | 386.0500073 | 306.7503535 | 290.9445978 | 354.2528763 |
| Mrrf | 206.9474048 | 297.0982532 | 252.9610981 | 245.8436846 | 248.2783918 | 252.5086101 | 241.7533436 | 288.2974605 | 276.135983 | 248.1881592 | 238.3594161 | 233.3650606 | 216.4294161 | 233.1171001 | 279.5220464 |
| Ptgs1 | 0.915696482 | 0 | 0 | 0.86260942 | 0 | 0.701412806 | 0 | 0 | 0 | 4.369509845 | 1.891741398 | 0 | 4.260421576 | 0.903554652 | 1.437131344 |
| Pdcl | 462.4267232 | 440.4976767 | 431.5218733 | 452.0073359 | 448.4211769 | 439.0844164 | 492.5273343 | 563.1857368 | 569.5774908 | 498.9980242 | 459.6931596 | 538.734954 | 509.5464205 | 412.0209211 | 454.1335047 |
| Rc3h2 | 380.9297363 | 399.3000523 | 345.3741309 | 320.0280947 | 319.2150751 | 293.8919656 | 377.0630508 | 382.999824 | 411.5705251 | 380.1473565 | 386.8611158 | 371.3020295 | 333.1649672 | 372.2645164 | 359.282836 |
| Zbtb6 | 290.2757847 | 253.5238427 | 263.1421949 | 234.6297621 | 219.7770458 | 239.8831796 | 237.24302 | 279.0786463 | 258.8304582 | 236.8274336 | 246.8722524 | 250.7156227 | 208.7606572 | 239.4419827 | 275.2106524 |
| Zbtb26 | 70.50862908 | 70.51131876 | 53.25496803 | 43.13047098 | 41.80197412 | 44.89041957 | 55.92801232 | 65.36977302 | 56.43105919 | 68.16435357 | 68.10269031 | 39.03876478 | 32.37920398 | 35.23863141 | 31.61688957 |
| Rabgap1 | 641.9032336 | 657.5774671 | 689.9651004 | 601.2387655 | 594.0947231 | 579.3669775 | 643.1721416 | 646.1550641 | 675.6678821 | 681.6435357 | 629.0040147 | 609.0047306 | 609.2402853 | 613.5136084 | 620.1221749 |
| Gpr21 | 2.747089445 | 0 | 0 | 0 | 0 | 0 | 0 | 0 | 0 | 0.873901969 | 3.783482795 | 2.602584319 | 0.852084315 | 0.903554652 | 0 |
| Strbp | 726.1473099 | 592.6119824 | 721.2915522 | 590.024843 | 685.9323936 | 641.7927172 | 700.9042834 | 802.8749045 | 717.8030729 | 701.743281 | 720.7534725 | 695.7575412 | 611.7965383 | 603.5745073 | 661.0804182 |
| Dennd1a | 367.1942891 | 444.4589868 | 365.7363245 | 333.8298454 | 332.5157032 | 361.2275949 | 379.7692449 | 336.9057532 | 334.8242845 | 319.8481206 | 341.4593223 | 401.6655132 | 430.3025792 | 328.8938932 | 364.3127957 |
| Lhx2 | 13.73544722 | 14.26071615 | 14.8800646 | 9.488703616 | 7.600358931 | 9.81977928 | 3.608258859 | 16.76148026 | 12.79104008 | 6.991215751 | 22.70089677 | 11.27786538 | 6.816674521 | 17.16753838 | 7.18565672 |
| Nek6 | 1383.617384 | 1292.971598 | 1305.529878 | 1287.013254 | 955.7451356 | 1015.645743 | 1091.498305 | 1723.080171 | 1428.082005 | 1557.293309 | 1488.80048 | 969.8964228 | 1036.986612 | 988.4887888 | 1129.585236 |
| Psmb7 | 3857.829277 | 3676.887981 | 3944.78344 | 3684.204831 | 3736.209778 | 3757.4684 | 3604.6506 | 4329.490351 | 3984.785193 | 3916.828625 | 3932.930365 | 3591.56636 | 3709.975108 | 3606.990169 | 3558.337208 |
| Nr6a1 | 19.22962611 | 28.52143231 | 28.1938066 | 18.11479781 | 30.40143573 | 31.56357626 | 21.64955315 | 38.5514046 | 21.06759543 | 12.23462756 | 21.75502607 | 28.62842751 | 37.49170987 | 14.45687443 | 20.83840449 |
| Olfml2a | 2.747089445 | 3.169048034 | 0 | 1.725218839 | 0.633363244 | 0 | 0 | 9.218814144 | 1.504828245 | 5.243411813 | 1.891741398 | 0.867528106 | 1.70416863 | 1.807109303 | 0 |
| Rpl35 | 12389.3734 | 12495.5564 | 11821.81974 | 11371.77998 | 12633.69663 | 13174.63673 | 13127.74779 | 12309.6311 | 11648.87545 | 11957.60064 | 11488.54551 | 12014.39674 | 11311.41928 | 12293.76459 | 11951.90282 |
| Arpc5l | 1256.335573 | 1334.169222 | 1227.213748 | 1121.392246 | 1343.363441 | 1297.613691 | 1337.761972 | 1185.874728 | 1240.730888 | 1140.442069 | 1246.657581 | 1308.232384 | 1307.097339 | 1274.012059 | 1222.998774 |
| Golga1 | 138.2701687 | 165.5827598 | 162.114388 | 158.7201332 | 134.2730078 | 133.2684331 | 161.4695839 | 160.9102105 | 167.7883493 | 166.9152761 | 202.4163295 | 110.1760695 | 163.6001885 | 145.4722989 | 151.6173568 |
| Scai | 77.83420093 | 150.5297816 | 144.8848395 | 136.2922883 | 115.2721105 | 123.4486538 | 151.5468721 | 139.1202862 | 143.7110974 | 150.3111387 | 182.5530449 | 137.0694408 | 169.5647787 | 118.3656594 | 132.9346493 |
| Ppp6c | 952.3243408 | 983.1971526 | 1048.652973 | 866.0598573 | 932.3106956 | 913.9408858 | 962.5030507 | 899.253416 | 893.8679776 | 931.5794989 | 1021.540355 | 928.2550737 | 948.3698428 | 946.9252748 | 860.841675 |
| Kynu | 3.662785926 | 0.792262009 | 16.44638718 | 8.626094197 | 1.900089733 | 2.104238417 | 8.118582433 | 25.9802944 | 3.762070613 | 17.47803938 | 5.675224193 | 0.867528106 | 5.964590206 | 1.807109303 | 4.311394032 |
| Arhgap15 | 604.3596778 | 728.8810478 | 732.2558103 | 609.8648597 | 748.6353547 | 737.1848588 | 826.2912787 | 532.1769983 | 665.8864985 | 581.1448093 | 570.3600314 | 804.1985545 | 764.3196307 | 759.889462 | 811.9792093 |
| Gtdc1 | 141.0172582 | 115.6702532 | 130.7879362 | 126.8035847 | 198.8760587 | 138.8797355 | 167.7840369 | 115.6542138 | 140.7014409 | 128.4635894 | 112.5586132 | 150.0823624 | 155.9314297 | 154.5078454 | 168.1443672 |
| Zeb2 | 336.9763052 | 249.5625327 | 298.3844532 | 270.8593578 | 275.5130113 | 285.4750119 | 391.4960862 | 302.5447187 | 258.8304582 | 242.9447474 | 244.980511 | 333.1307928 | 318.6795339 | 322.5690106 | 341.3186942 |
| Zeb2os | 2.747089445 | 0 | 0.783161294 | 3.450437679 | 0 | 0.701412806 | 0 | 0.838074013 | 6.019312981 | 0.873901969 | 0 | 0 | 5.964590206 | 3.614218606 | 1.437131344 |
| Acvr2a | 50.36330649 | 65.75774671 | 45.42335508 | 46.58090866 | 36.10170492 | 38.57770431 | 40.59291217 | 40.22755263 | 66.96485691 | 62.04703979 | 65.26507822 | 48.58157395 | 40.04796281 | 44.27417793 | 30.17975822 |
| Orc4 | 673.9526104 | 655.992943 | 716.5925845 | 693.5379734 | 640.9636032 | 652.3139093 | 668.4299537 | 777.7326841 | 722.3175577 | 737.5732618 | 760.4800418 | 638.5006862 | 657.8090913 | 681.2802073 | 615.0922152 |
| Mbd5 | 144.6800441 | 121.2160873 | 146.4511621 | 125.0783658 | 95.00448664 | 91.88507755 | 116.3663482 | 126.549176 | 151.9876528 | 145.0677268 | 163.6356309 | 153.5524748 | 110.770961 | 94.87323842 | 110.6591135 |
| Epc2 | 501.8016719 | 606.0804365 | 603.0341968 | 543.4439344 | 555.4595652 | 533.7751451 | 533.1202464 | 561.5095887 | 637.2947618 | 543.5670247 | 636.5709803 | 609.8722587 | 600.7194422 | 547.5541189 | 520.9601122 |
| Kif5c | 36.62785926 | 23.76786026 | 32.89277437 | 26.74089201 | 26.60125626 | 18.93814575 | 9.922711863 | 30.17066447 | 39.12553437 | 29.71266694 | 32.15960376 | 19.95314644 | 28.1187824 | 27.10663955 | 16.52701046 |
| Lypd6b | 29.30228741 | 33.27500436 | 42.2907099 | 39.6800333 | 31.03479897 | 35.77205309 | 25.25781201 | 62.01747697 | 15.80069657 | 17.47803938 | 7.56696559 | 39.03876478 | 51.97714322 | 37.04574071 | 28.74262688 |
| Mmadhc | 1232.527464 | 1121.843004 | 1209.9842 | 1136.919215 | 1114.085947 | 1151.018414 | 1152.838705 | 1277.224796 | 1251.264686 | 1291.62711 | 1244.76584 | 1221.479574 | 1301.984834 | 1096.915347 | 1129.585236 |
| Gm13498 | 0.915696482 | 3.169048034 | 3.915806472 | 0 | 1.900089733 | 11.22260489 | 3.608258859 | 2.514222039 | 2.257242368 | 0 | 0.945870699 | 4.337640531 | 2.556252945 | 3.614218606 | 0.718565672 |
| Rnd3 | 0 | 0 | 0 | 0.86260942 | 1.900089733 | 1.402825611 | 1.80412943 | 0.838074013 | 0 | 0.873901969 | 3.783482795 | 0 | 0.852084315 | 0 | 0 |
| Rbm43 | 452.3540619 | 429.4060086 | 457.366196 | 419.228178 | 461.0884418 | 474.8564695 | 478.0942988 | 344.4484194 | 394.2650002 | 365.291023 | 436.9922628 | 430.2939407 | 380.0296046 | 451.7773258 | 459.8820301 |
| Nmi | 786.5832777 | 888.9179735 | 739.304262 | 809.9902451 | 799.9377775 | 819.9515699 | 800.131402 | 614.3082516 | 802.8258688 | 701.743281 | 669.6764547 | 746.9416995 | 771.9883895 | 759.889462 | 772.4580974 |
| Rif1 | 2731.522605 | 2584.358672 | 2802.934273 | 2631.821339 | 2349.777636 | 2386.907778 | 2686.348721 | 2720.388246 | 2993.855794 | 2619.958103 | 2707.08194 | 2675.45668 | 2746.267748 | 2397.130491 | 2564.560883 |
| Neb | 493.5604036 | 424.6524366 | 391.5806472 | 390.7620671 | 238.1445798 | 324.0527162 | 372.5527272 | 274.8882763 | 349.1201529 | 360.9215132 | 454.9638061 | 362.6267484 | 317.8274496 | 303.5943629 | 433.2951002 |
| 4930573O16Rik | 0 | 3.169048034 | 0 | 3.450437679 | 5.700269198 | 4.208476834 | 0.902064715 | 0.838074013 | 3.762070613 | 2.621705907 | 0 | 1.735056213 | 4.260421576 | 0.903554652 | 0.718565672 |
| Arl5a | 1838.718535 | 1723.169868 | 1827.1153 | 1655.347476 | 1696.146768 | 1736.698107 | 1834.79963 | 1638.434696 | 1657.568312 | 1651.674721 | 1681.758102 | 1807.928573 | 1918.041793 | 1528.81447 | 1830.186766 |
| Cacnb4 | 2.747089445 | 3.169048034 | 5.482129061 | 0.86260942 | 13.30062813 | 2.805651223 | 7.216517718 | 0.838074013 | 0.752414123 | 5.243411813 | 0.945870699 | 6.072696744 | 13.63334904 | 10.84265582 | 8.622788064 |
| Stam2 | 534.7667452 | 551.4143579 | 489.4758091 | 529.6421837 | 525.0581295 | 469.9465798 | 505.1562403 | 509.5489999 | 556.0340366 | 532.2062991 | 525.9041085 | 513.5766389 | 478.0193008 | 543.9399002 | 512.3373241 |
| Fmnl2 | 5.494178889 | 10.29940611 | 0 | 5.175656518 | 4.43354271 | 4.208476834 | 4.510323574 | 12.5711102 | 9.781383594 | 4.369509845 | 7.56696559 | 3.470112425 | 5.964590206 | 2.710663955 | 1.437131344 |
| Prpf40a | 4169.166081 | 4047.666601 | 4224.372022 | 3958.514627 | 3634.238296 | 3796.747517 | 4147.693559 | 4500.45745 | 4339.172245 | 4342.418883 | 4417.216163 | 4152.857045 | 4090.004713 | 3894.320548 | 4003.129359 |
| Arl6ip6 | 587.8771412 | 616.3798426 | 592.0699386 | 588.2996242 | 602.3284453 | 530.9694939 | 551.1615407 | 599.2229193 | 717.8030729 | 560.1711621 | 598.7361523 | 661.056417 | 610.0923697 | 559.3003293 | 638.0863167 |
| Nr4a2 | 1870.767912 | 1494.99841 | 1538.128782 | 1475.924717 | 1326.262634 | 1316.551836 | 1551.551309 | 1094.524661 | 1056.389428 | 843.3154 | 917.4945778 | 1293.484406 | 1201.438884 | 1194.499249 | 1349.466332 |
| Gpd2 | 1197.730998 | 1002.211441 | 998.5306505 | 1048.070445 | 820.8387646 | 901.3154553 | 1048.199199 | 1171.62747 | 1081.971508 | 1186.758874 | 1180.446632 | 1020.213053 | 996.0865644 | 846.6307085 | 1018.926123 |
| Cytip | 4241.506103 | 3470.899859 | 3649.531632 | 3491.842931 | 2600.589481 | 2601.540096 | 2772.946933 | 2996.114597 | 3347.490431 | 3997.227606 | 4143.859531 | 2584.366229 | 2563.921704 | 2489.293065 | 2566.71658 |
| Gm13546 | 52.19469945 | 37.2363144 | 39.15806472 | 30.19132969 | 29.76807248 | 16.13249453 | 20.74748844 | 53.63673684 | 58.68830156 | 112.733354 | 106.883389 | 13.01292159 | 18.74585493 | 23.49242094 | 12.9341821 |
| Acvr1 | 56.77318186 | 77.64167683 | 89.28038757 | 50.89395576 | 58.26941847 | 35.77205309 | 42.39704159 | 103.0831036 | 83.51796761 | 68.16435357 | 77.5613973 | 65.93213608 | 71.57508247 | 60.53816166 | 48.86246569 |
| Upp2 | 0 | 2.376786026 | 0 | 1.725218839 | 0 | 0.701412806 | 0 | 0 | 4.514484735 | 0 | 0 | 2.602584319 | 0 | 1.807109303 | 0 |
| Ccdc148 | 14.65114371 | 15.84524017 | 18.01270977 | 10.35131304 | 19.00089733 | 11.22260489 | 20.74748844 | 19.2757023 | 18.05793894 | 27.964863 | 6.621094891 | 14.74797781 | 12.78126473 | 7.228437213 | 12.9341821 |
| Pkp4 | 1611.625808 | 1659.788908 | 1723.738009 | 1492.314296 | 1485.236808 | 1588.700005 | 1749.103482 | 1982.883115 | 1815.575278 | 1674.396172 | 1685.541585 | 1703.825201 | 1723.76657 | 1592.966851 | 1572.940256 |
| Dapl1 | 671.205521 | 648.862585 | 668.0365842 | 652.1327213 | 962.0787681 | 923.7606651 | 1009.410416 | 538.8815904 | 511.6416034 | 572.4057896 | 491.8527634 | 928.2550737 | 898.9489525 | 1054.448278 | 893.1771303 |
| Tanc1 | 424.8831674 | 409.5994584 | 382.965873 | 411.4646932 | 383.1847628 | 373.8530254 | 475.3881047 | 305.8970148 | 411.5705251 | 323.3437285 | 372.6730553 | 404.2680975 | 431.1546635 | 359.6147513 | 425.3908778 |
| Wdsub1 | 73.25571853 | 61.79643666 | 79.88245204 | 62.10787821 | 67.13650389 | 73.6483446 | 65.85072418 | 80.45510525 | 52.66898858 | 58.55143192 | 60.53572472 | 78.94505767 | 78.39175699 | 46.98484188 | 89.10214332 |
| Baz2b | 110.7992743 | 99.82501307 | 86.93090369 | 131.1166318 | 62.70296118 | 76.45399582 | 97.4229892 | 168.4528766 | 136.9393703 | 128.4635894 | 103.0999062 | 117.1162943 | 74.98341973 | 104.8123396 | 105.6291538 |
| March7 | 1031.989935 | 1040.240017 | 1018.892844 | 917.8164225 | 918.3767042 | 972.8595615 | 961.600986 | 828.0171249 | 938.2604109 | 918.4709693 | 943.0330867 | 1047.106424 | 960.2990232 | 993.9101167 | 964.3151318 |
| Cd302 | 0.915696482 | 3.169048034 | 1.566322589 | 0 | 3.166816221 | 0 | 0 | 5.866518091 | 0 | 0 | 5.675224193 | 0.867528106 | 4.260421576 | 1.807109303 | 7.904222392 |
| Ly75 | 1531.044517 | 1376.159109 | 1258.5402 | 1299.089786 | 1050.749622 | 1085.08561 | 1260.184407 | 1471.657967 | 1306.943331 | 1323.087581 | 1459.478488 | 1118.243729 | 1176.728439 | 1056.255388 | 1122.39958 |
| Rbms1 | 1212.382142 | 1432.409711 | 1386.978653 | 1450.046434 | 1319.929001 | 1379.678989 | 1489.308844 | 1145.647176 | 1224.177777 | 1254.923227 | 1381.917091 | 1417.540926 | 1486.035046 | 1451.10877 | 1457.969748 |
| Tank | 662.0485562 | 746.310812 | 780.0286493 | 634.8805329 | 709.3668336 | 750.5117021 | 773.9715253 | 786.9514983 | 729.0892848 | 769.9076346 | 663.0553598 | 756.4845087 | 723.4195836 | 684.8944259 | 772.4580974 |
| Psmd14 | 2668.339547 | 2813.322392 | 2896.130467 | 2656.837013 | 2557.52078 | 2707.45343 | 2707.096209 | 2806.70987 | 2792.961223 | 2828.820673 | 2921.794589 | 2759.606906 | 2736.89482 | 2795.598092 | 2737.016645 |
| Tbr1 | 0 | 1.584524017 | 0 | 0 | 2.533452977 | 0 | 1.80412943 | 2.514222039 | 0 | 0 | 0 | 0.867528106 | 0 | 5.421327909 | 0 |
| Slc4a10 | 0.915696482 | 2.376786026 | 4.698967767 | 3.450437679 | 0 | 1.402825611 | 0 | 2.514222039 | 6.771727103 | 2.621705907 | 4.729353494 | 1.735056213 | 1.70416863 | 0.903554652 | 3.59282836 |
| Dpp4 | 1646.422274 | 1873.69965 | 1850.610139 | 1833.907626 | 1462.435731 | 1538.899696 | 1697.685793 | 1283.929388 | 1572.545516 | 1328.330993 | 1294.896987 | 1656.111155 | 1652.191487 | 1489.058066 | 1603.120014 |
| Fap | 0.915696482 | 0 | 2.349483883 | 0.86260942 | 0 | 2.104238417 | 1.80412943 | 0 | 1.504828245 | 3.495607876 | 0.945870699 | 0 | 0.852084315 | 0.903554652 | 1.437131344 |
| Ifih1 | 776.5106164 | 585.4816243 | 480.0778735 | 437.3429758 | 525.0581295 | 616.5418562 | 636.8576886 | 389.7044161 | 401.7891415 | 365.291023 | 367.9437018 | 451.1146153 | 368.1004241 | 470.7519735 | 516.6487181 |
| Gca | 25.63950148 | 15.84524017 | 9.397935534 | 9.488703616 | 19.63426057 | 12.6254305 | 14.43303544 | 16.76148026 | 25.58208017 | 10.48682363 | 19.86328467 | 27.7608994 | 25.56252945 | 15.36042908 | 12.21561642 |
| Cobll1 | 396.4965765 | 329.5809955 | 332.8435502 | 365.7463939 | 354.6834168 | 427.1603987 | 447.4240985 | 234.6607237 | 270.8690841 | 226.3406099 | 278.0859854 | 386.0500073 | 391.1067007 | 352.3863141 | 437.6064942 |
| Slc38a11 | 0 | 3.961310043 | 0 | 0 | 0.633363244 | 1.402825611 | 0 | 3.352296052 | 0 | 0 | 0.945870699 | 2.602584319 | 0 | 0 | 3.59282836 |
| Galnt3 | 646.481716 | 609.2494845 | 618.6974226 | 619.3535633 | 534.5585782 | 497.3016793 | 508.7644991 | 634.4220279 | 558.291279 | 576.7752995 | 635.6251096 | 589.0515842 | 604.9798638 | 596.34607 | 626.589266 |
| Ttc21b | 216.1043696 | 190.935144 | 239.6473561 | 261.3706542 | 202.6762382 | 227.2577491 | 237.24302 | 248.0699079 | 272.3739124 | 285.7659438 | 231.7383212 | 268.9337129 | 238.5836082 | 234.0206548 | 260.8393389 |
| Stk39 | 3707.655054 | 3555.671894 | 3558.684922 | 3472.002914 | 3512.632553 | 3794.643279 | 3712.898366 | 3029.637557 | 3059.315822 | 3481.625444 | 3431.618895 | 3677.451643 | 3621.358339 | 3372.969514 | 3612.229633 |
| Cers6 | 203.2846189 | 194.8964541 | 224.7672915 | 200.1253854 | 158.3408111 | 167.6376606 | 184.9232665 | 209.5185033 | 193.3704295 | 182.6455115 | 172.1484672 | 205.6041612 | 177.2335376 | 198.7820233 | 187.5456404 |
| Nostrin | 2.747089445 | 3.169048034 | 6.265290356 | 4.313047098 | 4.43354271 | 2.104238417 | 1.80412943 | 10.89496217 | 0 | 1.747803938 | 0 | 5.205168638 | 0 | 2.710663955 | 0.718565672 |
| Spc25 | 443.1970971 | 534.7768557 | 556.8276804 | 514.9778235 | 559.8931079 | 526.0596043 | 539.4346994 | 688.0587647 | 623.7513076 | 601.2445546 | 563.7389365 | 558.6881004 | 557.2631421 | 629.7775921 | 536.768557 |
| Abcb11 | 0 | 2.376786026 | 0.783161294 | 0.86260942 | 0 | 0 | 0 | 0 | 0 | 0 | 0 | 0 | 0 | 0 | 0 |
| Dhrs9 | 0 | 9.507144102 | 7.831612945 | 12.93914129 | 2.533452977 | 2.104238417 | 2.706194144 | 15.92340625 | 2.257242368 | 6.991215751 | 6.621094891 | 0 | 5.112505891 | 4.517773258 | 2.155697016 |
| Bbs5 | 30.21798389 | 21.39107423 | 28.1938066 | 14.66436013 | 12.66726489 | 28.75792503 | 12.62890601 | 31.8468125 | 27.83932254 | 22.72145119 | 42.56418144 | 26.02584319 | 28.1187824 | 24.39597559 | 17.9641418 |
| Klhl41 | 2.747089445 | 0 | 0 | 0 | 0 | 0.701412806 | 0 | 5.028444078 | 0 | 0 | 0 | 0 | 0 | 0.903554652 | 2.155697016 |
| Fastkd1 | 193.2119576 | 175.8821659 | 139.4027104 | 187.1862441 | 186.2087938 | 159.2207069 | 202.9645608 | 155.0436924 | 200.1421566 | 161.6718642 | 159.8521481 | 168.3004526 | 184.0502121 | 159.9291733 | 175.330024 |
| Ppig | 2790.127179 | 2664.377135 | 2770.82466 | 2606.805666 | 2601.222844 | 2639.416388 | 2954.261941 | 2844.4232 | 2911.842654 | 2750.169496 | 2812.073587 | 2780.427581 | 2747.119832 | 2608.562279 | 2727.675291 |
| Ccdc173 | 18.31392963 | 21.39107423 | 17.22954848 | 12.93914129 | 15.20071786 | 14.02825611 | 18.0412943 | 36.03718256 | 12.03862596 | 23.59535316 | 16.07980188 | 16.48303402 | 15.33751767 | 15.36042908 | 26.58692986 |
| Phospho2 | 260.0578008 | 247.9780087 | 263.1421949 | 226.0036679 | 239.4113063 | 223.750685 | 221.0058551 | 285.7832385 | 349.872567 | 280.522532 | 275.2483733 | 266.3311286 | 235.175271 | 234.9242094 | 194.0127314 |
| Klhl23 | 58.60457482 | 39.61310043 | 64.21922615 | 31.05393911 | 36.10170492 | 31.56357626 | 46.00530045 | 39.38947861 | 74.48899814 | 68.16435357 | 48.23940564 | 35.56865236 | 34.93545692 | 45.17773258 | 51.73672838 |
| Ssb | 3601.434262 | 3569.93261 | 3485.06776 | 3449.575069 | 3629.17139 | 3484.618819 | 3662.382742 | 3965.76623 | 3677.047817 | 3978.875664 | 3967.927581 | 3742.51625 | 3727.016795 | 3752.462468 | 3699.176079 |
| Mettl5 | 223.4299415 | 173.5053799 | 213.0198721 | 196.6749477 | 164.6744435 | 201.3054752 | 172.2943605 | 231.3084276 | 180.5793894 | 220.2232962 | 185.390657 | 177.8432618 | 148.2626708 | 216.8531164 | 143.7131344 |
| Ubr3 | 1183.995551 | 1048.162637 | 1135.583877 | 1021.329553 | 959.5453151 | 984.0821664 | 1143.818058 | 1170.789396 | 1138.402567 | 1167.53303 | 1241.928227 | 1028.020806 | 1091.520008 | 968.6105865 | 1074.974245 |
| Myo3b | 10.0726613 | 21.39107423 | 18.79587107 | 20.70262607 | 24.06780328 | 17.53532014 | 26.15987673 | 15.92340625 | 24.82966605 | 18.35194135 | 12.29631908 | 21.68820266 | 13.63334904 | 35.23863141 | 22.27553583 |
| Gorasp2 | 2575.854203 | 2551.083667 | 2478.705497 | 2536.934303 | 2450.482392 | 2595.227381 | 2525.781201 | 2634.066623 | 2495.00523 | 2633.940534 | 2646.546215 | 2484.600496 | 2516.204983 | 2448.633106 | 2555.21953 |
| Tlk1 | 1814.910426 | 2068.596104 | 1983.747559 | 1939.145975 | 2040.696373 | 2109.148307 | 2153.228474 | 1946.007858 | 1954.77189 | 2066.778156 | 2050.647675 | 2284.201504 | 2258.875519 | 2179.37382 | 2265.637564 |
| Mettl8 | 56.77318186 | 80.01846286 | 74.40032298 | 81.94789487 | 84.23731149 | 89.07942633 | 66.75278889 | 103.9211776 | 106.0903913 | 99.62482445 | 72.83204381 | 74.60741714 | 116.7355512 | 131.0154245 | 94.8506687 |
| Dcaf17 | 268.2990691 | 251.1470567 | 280.3717434 | 331.2420171 | 319.8484384 | 268.6411046 | 265.2070261 | 311.7635329 | 334.0718704 | 335.5783561 | 272.4107612 | 281.9466345 | 330.6087143 | 301.7872536 | 290.3005315 |
| Cybrd1 | 2.747089445 | 0.792262009 | 0 | 3.450437679 | 2.533452977 | 4.90988964 | 2.706194144 | 3.352296052 | 7.524141226 | 1.747803938 | 0 | 5.205168638 | 0 | 0 | 5.029959704 |
| Dync1i2 | 1593.311878 | 1332.584698 | 1516.983427 | 1335.319382 | 1383.898689 | 1371.262035 | 1335.957843 | 1399.583602 | 1422.815106 | 1322.213679 | 1268.412607 | 1450.506994 | 1355.666145 | 1497.190058 | 1442.879869 |
| Slc25a12 | 1124.475279 | 1059.254305 | 1130.884909 | 1154.171403 | 996.9137465 | 1094.203977 | 1088.792111 | 1172.465544 | 1299.41919 | 1066.160402 | 1333.677685 | 1088.747773 | 1048.915792 | 1015.595428 | 1036.890265 |
| Hat1 | 2245.287773 | 2265.077082 | 2268.81827 | 2083.201748 | 2238.305705 | 2228.388484 | 2212.764745 | 2297.16087 | 2354.30379 | 2236.315138 | 2411.970282 | 2127.178917 | 2207.750461 | 2288.703932 | 2177.972552 |
| Metap1d | 44.8691276 | 84.77203491 | 81.44877463 | 84.53572313 | 88.03749095 | 67.33562935 | 52.31975346 | 94.70236348 | 58.68830156 | 96.12921658 | 89.85771638 | 83.2826982 | 81.80009426 | 82.22347329 | 70.41943585 |
| Itga6 | 599.7811954 | 705.9054496 | 717.3757458 | 653.8579401 | 661.231227 | 620.0489202 | 807.3479197 | 380.4856019 | 574.8443897 | 552.3060443 | 572.2517728 | 698.3601256 | 760.9112934 | 753.5645794 | 734.3741167 |
| Pdk1 | 2139.982677 | 2314.989589 | 2318.940593 | 2113.393078 | 2074.897988 | 2227.687071 | 2227.197781 | 1129.72377 | 1507.837902 | 1618.466446 | 1700.675516 | 2329.312965 | 2420.771539 | 2205.576904 | 2456.057467 |
| Rapgef4 | 30.21798389 | 35.65179038 | 46.98967767 | 39.6800333 | 34.20161519 | 31.56357626 | 46.00530045 | 34.36103454 | 24.82966605 | 29.71266694 | 29.32199166 | 45.97898963 | 34.93545692 | 57.8274977 | 30.17975822 |
| Zak | 62.26736075 | 55.4583406 | 61.86974226 | 83.67311371 | 29.13470924 | 36.4734659 | 55.0259476 | 141.6345082 | 101.5759065 | 159.9240603 | 153.2310532 | 27.7608994 | 53.68131186 | 43.37062328 | 46.70676868 |
| Cdca7 | 2210.491306 | 2317.366375 | 2358.098658 | 2261.761898 | 2463.149657 | 2394.623319 | 2376.940523 | 2228.438801 | 2375.371385 | 2260.784394 | 2304.141022 | 2341.458359 | 2382.427745 | 2241.719091 | 2227.553583 |
| Sp3 | 1672.061775 | 1834.878812 | 1844.344849 | 1801.128468 | 1682.212777 | 1691.106275 | 1916.887519 | 1847.953199 | 1882.540135 | 1808.977076 | 1811.342388 | 1931.985093 | 1874.585493 | 1768.256453 | 1757.611634 |
| Sp3os | 12.81975074 | 8.714882094 | 13.31374201 | 2.587828259 | 13.93399137 | 6.312715251 | 13.53097072 | 11.73303618 | 4.514484735 | 7.86511772 | 3.783482795 | 17.35056213 | 8.520843152 | 5.421327909 | 13.65274777 |
| Ola1 | 2073.136834 | 1943.418707 | 1889.768204 | 1836.495454 | 1841.820314 | 1830.687423 | 1866.371895 | 2243.524133 | 2098.482988 | 2227.576119 | 2059.160511 | 1921.574755 | 1892.479264 | 1828.794615 | 1877.612101 |
| Sp9 | 1.831392963 | 0 | 0.783161294 | 2.587828259 | 1.266726489 | 0 | 1.80412943 | 0 | 0 | 1.747803938 | 0 | 0.867528106 | 0 | 3.614218606 | 1.437131344 |
| Cir1 | 321.409465 | 358.8946899 | 332.8435502 | 350.2194244 | 277.413101 | 305.8159833 | 368.0424036 | 358.6956776 | 276.135983 | 362.6693171 | 286.5988217 | 351.348883 | 323.7920398 | 353.2898688 | 357.8457046 |

| | | | | | | | | | | | | | | | |
|---|---|---|---|---|---|---|---|---|---|---|---|---|---|---|---|
| **Scrn3** | 52.19469945 | 33.27500436 | 65.00238744 | 71.59658183 | 57.63605523 | 61.7243269 | 60.43833589 | 54.47481085 | 63.95520042 | 58.55143192 | 34.05134516 | 57.25685501 | 74.13133542 | 65.95948956 | 61.79664779 |
| **Gpr155** | 42.12203815 | 69.71905675 | 83.01509722 | 81.94789487 | 82.970585 | 101.003444 | 110.0518952 | 69.56014308 | 53.4214027 | 88.26409886 | 84.18249219 | 104.9709009 | 84.3563472 | 111.1372221 | 125.0304269 |
| **Wipf1** | 6984.017065 | 6517.147282 | 6662.353132 | 6575.671606 | 6681.982227 | 6589.071897 | 7239.069336 | 5179.297401 | 5375.246492 | 5031.927537 | 5521.047269 | 7113.730471 | 7046.737286 | 6829.066057 | 6796.912691 |
| **Chn1** | 5.494178889 | 2.376786026 | 0 | 0 | 2.533452977 | 1.402825611 | 0 | 6.704592104 | 8.276555348 | 7.86511772 | 0 | 3.470112425 | 0.852084315 | 0.903554652 | 0.718565672 |
| **Atf2** | 748.1240254 | 793.8465325 | 832.500456 | 879.861608 | 706.8333806 | 817.8473315 | 798.3272726 | 1001.498446 | 914.9355731 | 908.8580477 | 794.531387 | 860.5878814 | 812.0363523 | 730.0721585 | 829.2247854 |
| **Atp5g3** | 5625.123486 | 5472.153693 | 5968.472225 | 5556.929881 | 6144.256833 | 6202.593441 | 5734.425392 | 5924.345198 | 5535.5107 | 5551.899208 | 5997.766101 | 6172.462476 | 5758.385802 | 6270.669282 | 5762.178123 |
| **Lnp** | 785.6675812 | 793.0542705 | 824.6688431 | 795.3258849 | 794.8708716 | 792.5964705 | 830.8016023 | 848.1309012 | 828.407949 | 792.6290858 | 926.9532848 | 764.2922616 | 767.727968 | 761.6965713 | 753.0568242 |
| **Mtx2** | 532.9353523 | 479.3185151 | 516.8864544 | 522.7413083 | 473.1223435 | 507.1214585 | 447.4240985 | 599.2229193 | 543.9954106 | 557.5494562 | 542.9297811 | 484.9482114 | 483.983891 | 490.6301758 | 446.2292823 |
| **Hnrnpa3** | 830.5367088 | 854.0584452 | 902.9849725 | 886.7624834 | 951.3115929 | 815.0416802 | 910.1832972 | 916.0148963 | 936.0031685 | 875.6497728 | 905.1982587 | 908.3019273 | 1032.72619 | 993.9101167 | 854.374584 |
| **Nfe2l2** | 970.6382705 | 1022.017991 | 971.9031665 | 943.6947051 | 863.9074652 | 878.1688328 | 890.3378735 | 695.6014308 | 702.0023764 | 783.8900661 | 823.8533786 | 812.0063075 | 792.4384131 | 826.7525062 | 811.9792093 |
| **Agps** | 1908.311468 | 2067.803842 | 2037.785688 | 2052.147809 | 1736.682016 | 1718.461374 | 1926.810231 | 2338.226496 | 2179.743713 | 2237.18904 | 2319.274953 | 2007.460038 | 2021.143996 | 1882.104339 | 1847.432343 |
| **Ttc30b** | 14.65114371 | 9.507144102 | 14.0969033 | 1.725218839 | 10.13381191 | 6.312715251 | 5.412388289 | 12.5711102 | 11.28621184 | 6.991215751 | 8.512836289 | 2.602584319 | 20.45002356 | 9.035546516 | 15.08987911 |
| **Ttc30a2** | 0.915696482 | 0 | 0 | 2.587828259 | 0.633363244 | 0 | 0.902064715 | 0 | 0 | 0 | 5.675224193 | 0 | 0 | 0 | 0 |
| **Ttc30a1** | 73.25571853 | 68.13453273 | 59.52025838 | 65.55831589 | 60.16950821 | 47.69607079 | 55.92801232 | 92.18814144 | 72.23175577 | 77.77727523 | 63.37333682 | 58.99191123 | 55.38548049 | 58.73105235 | 74.73082988 |
| **Pde11a** | 24.723805 | 39.61310043 | 45.42335508 | 37.95481446 | 51.30242279 | 39.27911712 | 47.80942988 | 36.87525657 | 59.44071568 | 39.3255886 | 42.56418144 | 32.09853993 | 28.97086672 | 33.43152211 | 56.76668809 |
| **Rbm45** | 852.5134243 | 773.2477203 | 912.3829081 | 779.7989154 | 848.7067473 | 876.7660071 | 745.1054544 | 869.9208256 | 913.4307448 | 956.9226559 | 866.4175601 | 887.4812527 | 997.7907331 | 831.2702794 | 831.3804825 |
| **Osbpl6** | 0 | 0.792262009 | 3.915806472 | 2.587828259 | 1.266726489 | 0.701412806 | 1.80412943 | 0 | 0 | 3.495607876 | 3.783482795 | 0.867528106 | 0.852084315 | 2.710663955 | 0 |
| **Prkra** | 67.76153964 | 44.36667248 | 44.64019379 | 61.2452688 | 36.73506817 | 30.86216345 | 45.10323574 | 32.68488651 | 54.92623095 | 48.93851026 | 46.34766424 | 30.36348372 | 49.42089028 | 48.79195118 | 38.80254629 |
| **Fkbp7** | 9.156964816 | 17.42976419 | 8.614774239 | 13.80175071 | 7.600358931 | 7.715540863 | 18.0412943 | 25.14222039 | 3.762070613 | 3.495607876 | 7.56696559 | 8.675281063 | 11.0770961 | 11.74621047 | 10.77848508 |
| **Plekha3** | 507.2958508 | 437.3286287 | 513.7538092 | 429.579491 | 432.5870958 | 453.1126725 | 460.9550693 | 517.9297401 | 520.6705728 | 510.3587498 | 436.0463921 | 419.8836034 | 432.0067478 | 449.0666618 | 423.9537465 |
| **Ttn** | 21.97671556 | 23.76786026 | 34.45909696 | 25.87828259 | 18.36753408 | 21.04238417 | 10.82477658 | 23.46607237 | 27.08690841 | 31.46047088 | 22.70089677 | 19.95314644 | 28.97086672 | 3.614218606 | 7.18565672 |
| **Ccdc141** | 131.8602933 | 121.2160873 | 151.1501298 | 103.5131304 | 96.27121313 | 108.0175721 | 101.0312481 | 56.98903289 | 101.5759065 | 87.39019689 | 80.3990094 | 123.1889911 | 104.8063708 | 94.87323842 | 105.6291538 |
| **Sestd1** | 100.726613 | 68.92679474 | 100.2446457 | 77.63484777 | 61.43623469 | 82.06529827 | 65.85072418 | 110.6257697 | 138.4441986 | 121.4723737 | 103.0999062 | 73.73988903 | 72.42716679 | 74.09148143 | 52.45529405 |
| **Cwc22** | 1337.83256 | 1233.551947 | 1236.611684 | 1169.698373 | 1183.755904 | 1132.781681 | 1190.725424 | 1288.119758 | 1290.39022 | 1294.248816 | 1181.392503 | 1141.666988 | 1232.11392 | 1176.428156 | 1077.129942 |
| **Ube2e3** | 500.8859754 | 599.7423404 | 553.6950352 | 480.4734467 | 599.7949923 | 558.3245933 | 573.7131586 | 623.5270657 | 661.3720137 | 609.1096723 | 600.6278937 | 528.3246167 | 506.9901675 | 566.5287665 | 495.091748 |
| **Itga4** | 477.0778669 | 570.4286461 | 550.56239 | 598.6509372 | 432.5870958 | 408.9236657 | 464.5633281 | 607.6036595 | 633.5326912 | 519.0977695 | 449.2885819 | 510.1065265 | 449.0484341 | 489.7266212 | 480.7204345 |
| **Cerkl** | 20.14532259 | 15.84524017 | 19.57903236 | 24.15306375 | 17.1008076 | 25.95227381 | 24.3557473 | 15.92340625 | 30.0965649 | 25.3431571 | 17.97154328 | 32.96606804 | 24.71044514 | 27.10663955 | 18.68270747 |
| **Ssfa2** | 539.3452276 | 595.7810304 | 599.9015516 | 519.2908706 | 491.4898776 | 527.4624299 | 561.0842526 | 583.2995131 | 574.8443897 | 593.3794369 | 554.2802295 | 552.6154037 | 565.7839853 | 465.3306456 | 554.7326988 |
| **Pde1a** | 7.325571853 | 15.05297816 | 7.831612945 | 7.763484777 | 1.900089733 | 0.701412806 | 0 | 0 | 0.752414123 | 13.9824315 | 4.729353494 | 0 | 0 | 3.614218606 | 1.437131344 |
| **Dnajc10** | 1420.245243 | 1495.790672 | 1627.40917 | 1580.300457 | 1466.869274 | 1511.544596 | 1704.000246 | 1659.386546 | 1598.88001 | 1481.263837 | 1458.532618 | 1609.264637 | 1557.610128 | 1621.8806 | 1444.317001 |
| **Nckap1** | 384.5925223 | 419.8988645 | 369.652131 | 364.0211751 | 288.1802761 | 353.5120541 | 351.8052388 | 494.4636677 | 395.7698285 | 405.4905136 | 395.3739521 | 342.673602 | 379.1775202 | 325.2796746 | 372.9355838 |
| **Dusp19** | 169.4038491 | 179.8434759 | 158.9817428 | 144.0557731 | 150.1070889 | 127.6571306 | 133.5055778 | 139.9583602 | 130.9200573 | 172.1586879 | 139.9888634 | 134.4668565 | 163.6001885 | 180.7109303 | 141.5574374 |
| **Nup35** | 545.755103 | 514.1780435 | 524.7180673 | 499.450854 | 471.855617 | 490.988964 | 478.9963635 | 657.8881002 | 556.7864507 | 609.1096723 | 649.8131701 | 496.2260768 | 534.2568656 | 472.5590828 | 484.3132629 |
| **Zc3h15** | 3074.908785 | 2913.939667 | 2988.5435 | 2875.939805 | 2910.937471 | 2987.31714 | 3068.82416 | 2905.602603 | 2756.092931 | 3115.460519 | 3043.811909 | 2800.380727 | 3019.786813 | 2824.511841 | 2807.43608 |
| **Itgav** | 1066.786401 | 992.7042967 | 913.1660694 | 878.1363892 | 940.5444177 | 895.0027401 | 998.5856393 | 1081.953551 | 924.7169567 | 935.0751067 | 938.3037332 | 964.6912542 | 994.3823958 | 930.6612911 | 957.1294751 |
| **Fam171b** | 7.325571853 | 13.46845414 | 4.698967767 | 5.175656518 | 5.700269198 | 6.312715251 | 10.82477658 | 15.92340625 | 6.019312981 | 6.117313782 | 0 | 2.602584319 | 8.520843152 | 6.324882561 | 5.748525376 |
| **Calcrl** | 101.6423095 | 106.9553711 | 94.76251663 | 90.57398906 | 70.93668336 | 82.06529827 | 96.52092448 | 87.15969736 | 93.2993512 | 76.90337326 | 84.18249219 | 111.0435976 | 88.61676878 | 66.86304422 | 76.16796123 |
| **Tfpi** | 160.2468843 | 148.1529956 | 150.3669685 | 102.6505209 | 124.7725591 | 113.6288745 | 103.7374422 | 107.2734737 | 99.31866418 | 194.8801391 | 160.7980188 | 102.3683165 | 101.3980335 | 102.1016756 | 118.5633359 |
| **Ctnnd1** | 8.241268334 | 1.584524017 | 10.96425812 | 4.313047098 | 0.633363244 | 0.701412806 | 7.216517718 | 17.59955427 | 3.762070613 | 13.10852953 | 12.29631908 | 3.470112425 | 5.112505891 | 2.710663955 | 2.874262688 |
| **Btbd18** | 4.578482408 | 4.753572051 | 3.915806472 | 0 | 1.266726489 | 2.805651223 | 1.80412943 | 7.542666117 | 0 | 5.243411813 | 2.837612096 | 2.602584319 | 7.668758836 | 5.421327909 | 5.029959704 |
| **2700094K13Rik** | 1836.887142 | 1839.632384 | 1841.995365 | 1802.853687 | 2042.596463 | 2005.339212 | 1694.077534 | 2129.546067 | 1767.420774 | 1946.179685 | 1854.85244 | 2036.955994 | 2077.38156 | 2117.028549 | 1948.031537 |
| **Tmx2** | 880.9000153 | 877.8263054 | 827.018327 | 840.1815747 | 887.3419052 | 908.3295834 | 888.5337441 | 973.0039292 | 866.0286551 | 866.9107532 | 913.711095 | 1015.007884 | 915.9906388 | 957.7679307 | 903.2370497 |
| **Med19** | 380.0140398 | 425.4446986 | 382.1827117 | 346.7689867 | 392.0518482 | 389.2841072 | 454.6406162 | 440.8269309 | 388.9981014 | 428.2119648 | 442.667487 | 390.3876478 | 422.6338203 | 386.7213909 | 412.4566957 |
| **Zdhhc5** | 2111.596086 | 1990.954427 | 2058.931043 | 2006.42951 | 1796.851524 | 1800.526672 | 2074.748844 | 2299.675092 | 2210.592692 | 2274.766825 | 2413.862023 | 1944.130486 | 2242.685918 | 1969.74914 | 2072.343398 |
| **Clp1** | 743.545543 | 728.0887858 | 849.7300045 | 756.508461 | 699.8663849 | 759.6300686 | 716.2393835 | 735.8289835 | 736.613426 | 755.9252031 | 601.5737644 | 683.6121477 | 685.0757894 | 637.909584 | 666.8289436 |
| **Ypel4** | 0 | 0 | 0 | 1.725218839 | 0 | 0 | 0.902064715 | 1.676148026 | 0 | 0 | 0 | 0 | 0 | 0 | 0 |
| **Ube2l6** | 377.2669504 | 310.5667073 | 310.9150339 | 333.8298454 | 326.815434 | 331.0668443 | 328.3515562 | 218.7373174 | 282.155296 | 218.4754922 | 240.2511575 | 279.3440502 | 252.2169573 | 293.6552618 | 256.5279449 |
| **Timm10** | 333.3135193 | 313.7357554 | 322.6624533 | 269.9967484 | 339.4826989 | 328.2611931 | 307.6040677 | 376.2952319 | 311.4994467 | 281.396434 | 313.0832013 | 290.6219156 | 311.8628593 | 325.2796746 | 296.0490568 |
| **Slc43a1** | 41.20634167 | 39.61310043 | 49.33916155 | 37.95481446 | 27.86798275 | 25.95227381 | 29.76813559 | 41.90370065 | 61.69795805 | 44.56900041 | 34.99721585 | 25.15831508 | 30.67503535 | 32.52796746 | 40.23967763 |
| **Slc43a3** | 959.6499127 | 872.2804714 | 958.5894245 | 868.6476856 | 736.6014531 | 737.1848588 | 779.3839136 | 1061.839775 | 999.9583689 | 918.4709693 | 940.1954746 | 776.4376551 | 839.3030504 | 781.5747736 | 773.176663 |
| **Prg2** | 1.831392963 | 0 | 1.566322589 | 0 | 0 | 0.701412806 | 1.80412943 | 0.838074013 | 0 | 0 | 3.783482795 | 0.867528106 | 0 | 0 | 0 |
| **P2rx3** | 19.22962611 | 5.54583406 | 11.74741942 | 7.763484777 | 5.066905954 | 5.611302446 | 14.43303544 | 2.514222039 | 12.79104008 | 11.3607256 | 18.91741398 | 3.470112425 | 5.964590206 | 9.035546516 | 3.59282836 |
| **Ssrp1** | 9196.339764 | 8844.813063 | 9157.505016 | 8693.377731 | 8387.629444 | 7967.34806 | 8394.614236 | 9637.013076 | 9344.983402 | 9178.592379 | 9563.698635 | 8718.657468 | 8439.043057 | 8535.880793 | 8198.834317 |
| **Tnks1bp1** | 414.8105061 | 358.8946899 | 340.6751631 | 279.485452 | 374.3176774 | 409.6250785 | 434.7951925 | 246.3937598 | 250.5539028 | 206.2408647 | 244.0346403 | 369.5669733 | 357.0233281 | 332.5081118 | 375.0912808 |
| **Ptprj** | 651.9758949 | 719.3739037 | 697.0135521 | 719.416256 | 466.1553478 | 494.496028 | 493.429399 | 941.9951907 | 869.0383116 | 791.7551838 | 810.6111888 | 530.0596729 | 506.9901675 | 475.2697467 | 454.1335047 |
| **Nup160** | 2285.578418 | 2436.997938 | 2266.468786 | 2342.847184 | 1969.126326 | 1959.747379 | 2230.80604 | 2704.46484 | 2587.552168 | 2481.881592 | 2495.206903 | 2091.610264 | 2178.779594 | 2041.129958 | 2106.115985 |
| **Fnbp4** | 2158.296607 | 2309.443755 | 2245.323431 | 2054.735638 | 2219.304808 | 2183.498064 | 2206.450292 | 2150.497917 | 2218.869247 | 2239.810746 | 2278.602513 | 2308.492291 | 2377.315239 | 2231.779989 | 2421.566315 |
| **Mtch2** | 1662.904811 | 1717.624034 | 1886.635558 | 1770.937139 | 1758.849729 | 1787.199829 | 1730.160123 | 2085.966218 | 1938.971194 | 1985.505273 | 2152.80171 | 1794.915652 | 1792.785399 | 1861.322582 | 1828.749635 |
| **C1qtnf4** | 15.56684019 | 3.961310043 | 16.44638718 | 14.66436013 | 13.30062813 | 19.63955856 | 8.118582433 | 2.514222039 | 3.762070613 | 3.495607876 | 2.837612096 | 16.48303402 | 11.0770961 | 18.97464768 | 7.904222392 |
| **Ndufs3** | 1405.594099 | 1407.057327 | 1611.745944 | 1363.785492 | 1448.50174 | 1498.919166 | 1389.179661 | 1371.927159 | 1318.229543 | 1414.847288 | 1406.509729 | 1455.712162 | 1415.312047 | 1388.763499 | 1362.400514 |
| **Kbtbd4** | 482.5720458 | 515.7625675 | 527.8507125 | 434.7551475 | 501.6236895 | 454.5154981 | 478.0942988 | 586.6518091 | 540.9857541 | 499.8719262 | 530.633462 | 471.0677617 | 484.8359753 | 437.3204514 | 476.4090405 |
| **Ptpmt1** | 717.9060415 | 645.6935369 | 646.8912292 | 652.9953307 | 595.3614496 | 655.8209733 | 622.4246532 | 594.1944753 | 575.5968038 | 597.7489467 | 630.8957561 | 670.5992262 | 579.4173343 | 623.4527096 | 615.0922152 |
| **Celf1** | 4043.715663 | 3919.320156 | 4030.931183 | 3954.20158 | 3563.934976 | 3553.357274 | 3971.790939 | 4247.359098 | 4244.368065 | 4231.433333 | 4469.239052 | 4075.647043 | 4071.258858 | 3626.868371 | 3862.290487 |
| **Rapsn** | 22.89241204 | 27.7291703 | 27.41064531 | 29.32872027 | 31.66816221 | 38.57770431 | 40.59291217 | 20.11377631 | 32.35380727 | 31.46047088 | 31.21373306 | 34.70112425 | 32.37920398 | 26.2030849 | 19.40127314 |
| **Psmc3** | 3871.564724 | 3952.59516 | 4147.622216 | 3968.00333 | 3835.647807 | 4057.673081 | 3800.398643 | 4277.529763 | 4284.246014 | 4146.664842 | 4369.922628 | 3909.081647 | 3901.694079 | 4023.528863 | 3773.188343 |
| **Slc39a13** | 92.48534464 | 107.7476332 | 126.8721297 | 72.45919125 | 134.2730078 | 97.49637999 | 92.91266562 | 89.6739194 | 109.8524619 | 100.4987264 | 103.0999062 | 137.0694408 | 92.02510604 | 127.4012059 | 110.6591135 |
| **Madd** | 1949.517809 | 2327.665781 | 2213.213818 | 2348.88545 | 2158.501936 | 1933.795105 | 2164.955315 | 1796.830684 | 2057.852625 | 2049.300117 | 2147.126486 | 2390.039933 | 2274.213037 | 2191.12003 | 2415.817789 |
| **Nr1h3** | 1.831392963 | 1.584524017 | 3.915806472 | 4.313047098 | 7.600358931 | 4.90988964 | 6.314453003 | 0.838074013 | 5.266898858 | 3.495607876 | 5.675224193 | 3.470112425 | 6.816674521 | 7.228437213 | 5.029959704 |
| **Acp2** | 116.2934532 | 109.3321572 | 126.0889684 | 112.1392246 | 117.8055634 | 100.3020312 | 104.6395069 | 117.3303618 | 138.4441986 | 115.3550599 | 114.4503546 | 92.82550737 | 125.2563943 | 102.1016756 | 113.5333762 |
| **Ddb2** | 133.6916863 | 168.7518078 | 131.5710975 | 147.5062108 | 151.3738154 | 153.6094045 | 139.8200308 | 111.4638437 | 143.7110974 | 135.4548052 | 87.02010429 | 167.4329245 | 164.4522728 | 169.8682745 | 158.8030135 |
| **A330069E16Rik** | 6.409875371 | 3.961310043 | 4.698967767 | 6.038265938 | 0 | 4.208476834 | 1.80412943 | 5.028444078 | 27.83932254 | 9.612921658 | 3.783482795 | 7.807752957 | 5.964590206 | 9.035546516 | 7.904222392 |
| **Pacsin3** | 22.89241204 | 26.14464628 | 21.14535495 | 22.42784491 | 14.56735462 | 16.83390734 | 14.43303544 | 31.8468125 | 15.80069657 | 20.09974528 | 26.48437957 | 18.21809023 | 24.71044514 | 36.14218606 | 19.40127314 |
| **Arfgap2** | 2212.322699 | 2368.863405 | 2301.711044 | 2327.320214 | 2313.042568 | 2161.754267 | 2192.017257 | 2736.311653 | 2566.484572 | 2532.567906 | 2533.987602 | 2432.54881 | 2340.675614 | 2201.059131 | 2234.020674 |
| **1110051M20Rik** | 86.99116575 | 100.6172751 | 83.01509722 | 97.47486442 | 74.10349958 | 68.73845496 | 83.89201847 | 140.7964342 | 108.3476337 | 88.26409886 | 86.07423359 | 83.2826982 | 79.24384131 | 87.6448012 | 61.07808212 |
| **Lrp4** | 8.241268334 | 12.67619214 | 3.132645178 | 1.725218839 | 1.266726489 | 4.90988964 | 2.706194144 | 3.352296052 | 0 | 1.747803938 | 0.945870699 | 5.205168638 | 1.70416863 | 0 | 3.59282836 |
| **Ckap5** | 3883.468778 | 3872.576698 | 3933.036021 | 3881.742388 | 3668.439911 | 3546.343146 | 4230.683512 | 4182.827399 | 4111.94318 | 3905.467899 | 3981.169771 | 4028.800526 | 4195.663168 | 3858.178362 | 4053.428956 |
| **Zfp408** | 274.7089445 | 232.1327685 | 273.3232918 | 229.4541056 | 275.5130113 | 257.4184997 | 281.444191 | 274.8882763 | 282.9077101 | 293.6310616 | 234.5759333 | 293.2244999 | 264.998222 | 277.391278 | 299.6418852 |
| **Arhgap1** | 1883.587663 | 1946.587755 | 2026.82143 | 1942.596413 | 1898.823006 | 1843.312853 | 1924.104037 | 1842.924755 | 1740.333866 | 1818.589997 | 2120.642107 | 2161.012513 | 2085.050319 | 1929.992736 | 1999.049699 |
| **Atg13** | 797.5716354 | 742.349502 | 816.0540689 | 684.9118792 | 702.3998379 | 678.9675959 | 718.9455777 | 794.4941644 | 847.218302 | 784.7639681 | 759.5341711 | 724.3859687 | 820.5571955 | 768.9250085 | 760.9610466 |
| **Harbi1** | 126.3661145 | 106.1631091 | 111.9920651 | 130.2540224 | 140.6066402 | 105.9133337 | 129.8973189 | 134.9299161 | 153.492481 | 96.12921658 | 121.0714494 | 112.7786538 | 108.214708 | 159.0256187 | 136.5274777 |
| **Ambra1** | 1433.064994 | 1332.584698 | 1295.348781 | 1250.783658 | 1160.954827 | 1212.742741 | 1341.370231 | 1122.181103 | 1299.41919 | 1105.485991 | 1096.26414 | 1141.666988 | 1234.670173 | 1099.626011 | 1307.070957 |
| **Chrm4** | 116.2934532 | 116.4625153 | 122.9563232 | 121.6279282 | 91.83767042 | 97.49637999 | 104.6395069 | 63.69362499 | 112.1097043 | 84.76849098 | 89.85771638 | 100.6332603 | 103.9542864 | 79.51280934 | 105.6291538 |
| **Mdk** | 10.98835778 | 15.84524017 | 18.01270977 | 14.66436013 | 24.70116653 | 29.45933784 | 28.86607087 | 22.62799835 | 21.82000955 | 31.46047088 | 16.07980188 | 11.27786538 | 15.33751767 | 29.8173035 | 15.80844478 |
| **Dgkz** | 4796.41817 | 5358.067964 | 5498.575449 | 5290.383571 | 5939.680505 | 5998.482314 | 6121.411155 | 4572.531815 | 5019.354612 | 4844.038614 | 4847.587331 | 6274.830793 | 6254.298873 | 6266.151509 | 6250.084215 |
| **Creb3l1** | 28.38659093 | 17.42976419 | 14.0969033 | 25.87828259 | 20.26762382 | 19.63955856 | 10.82477658 | 15.08533223 | 18.81035306 | 19.22584332 | 15.13393118 | 12.14539349 | 20.45002356 | 16.26398373 | 15.80844478 |
| **Phf21a** | 397.412273 | 502.2941134 | 487.1263252 | 508.9395576 | 482.6227921 | 443.2928932 | 448.3261632 | 454.2361151 | 429.628464 | 425.5902589 | 364.160219 | 486.6832676 | 446.4921811 | 515.929706 | 472.8162122 |
| **Gyltl1b** | 0 | 1.584524017 | 2.349483883 | 0 | 3.166816221 | 2.805651223 | 2.706194144 | 0 | 5.266898858 | 5.243411813 | 2.837612096 | 0.867528106 | 4.260421576 | 0.903554652 | 2.155697016 |
| **Pex16** | 361.7001102 | 436.5363667 | 417.42497 | 366.6090034 | 342.6495152 | 365.4360718 | 349.0990446 | 430.7700427 | 266.3545994 | 329.4610423 | 276.194244 | 372.1695576 | 385.1421105 | 346.9649862 | 355.6900076 |
| **1700029I15Rik** | 0 | 10.29940611 | 3.132645178 | 8.626094197 | 1.900089733 | 4.208476834 | 5.412388289 | 0 | 6.771727103 | 3.495607876 | 8.512836289 | 3.470112425 | 7.668758836 | 5.421327909 | 8.622788064 |
| **Mapk8ip1** | 15.56684019 | 7.922620085 | 12.53058071 | 9.488703616 | 12.03390164 | 16.83390734 | 10.82477658 | 19.2757023 | 20.31518131 | 20.09974528 | 18.91741398 | 13.01292159 | 5.112505891 | 5.421327909 | 13.65274777 |
| **Cry2** | 80.58129038 | 135.4768035 | 115.9078716 | 121.6279282 | 143.1400932 | 128.3585434 | 128.0931895 | 138.2822122 | 148.2255821 | 125.8418835 | 129.5842857 | 111.9111257 | 128.6647316 | 120.1727687 | 117.1262045 |
| **D930015M05Rik** | 0.915696482 | 1.584524017 | 1.566322589 | 3.450437679 | 2.533452977 | 2.805651223 | 0 | 0 | 0 | 4.369509845 | 2.837612096 | 0 | 4.260421576 | 0 | 2.155697016 |
| **Slc35c1** | 1278.312288 | 1224.044803 | 1363.483814 | 1309.441099 | 1197.689895 | 1256.931748 | 1306.189707 | 1235.321095 | 1218.910879 | 1193.75009 | 1155.853994 | 1204.129012 | 1278.978557 | 1183.656594 | 1222.998774 |
| **Syt13** | 5.494178889 | 10.29940611 | 0.783161294 | 4.313047098 | 4.43354271 | 1.402825611 | 0 | 1.676148026 | 4.514484735 | 6.117313782 | 1.891741398 | 2.602584319 | 2.556252945 | 0 | 0 |
| **Prdm11** | 45.78482408 | 70.51131876 | 44.64019379 | 64.69570647 | 49.40233305 | 60.32150129 | 46.90736517 | 61.17940295 | 38.37312025 | 58.55143192 | 39.72656935 | 77.21000146 | 40.90004713 | 74.99503608 | 51.73672838 |
| **4631405J19Rik** | 0 | 2.376786026 | 0 | 1.725218839 | 1.266726489 | 0 | 0 | 0 | 0 | 0 | 0.945870699 | 0 | 0 | 0 | 0 |
| **Trp53i11** | 3668.280105 | 3690.356436 | 3743.510988 | 3710.083114 | 3035.71003 | 3108.661555 | 3309.675439 | 3021.256817 | 3345.985603 | 3499.977385 | 3394.729938 | 3183.82815 | 3211.505784 | 3269.060729 | 3390.911406 |
| **Tspan18** | 0 | 2.376786026 | 3.915806472 | 6.038265938 | 3.166816221 | 7.014128057 | 1.80412943 | 3.352296052 | 0 | 0.873901969 | 4.729353494 | 5.205168638 | 3.408337261 | 8.131991864 | 2.874262688 |
| **Gm13807** | 24.723805 | 31.69048034 | 25.06116142 | 33.64176737 | 31.66816221 | 39.27911712 | 37.88671802 | 27.65644243 | 27.83932254 | 29.71266694 | 19.86328467 | 24.29078698 | 39.1958785 | 29.8173035 | 42.39537465 |
| **Cd82** | 8231.195673 | 7781.597448 | 7852.7583 | 7588.375065 | 8059.547283 | 8409.93954 | 8737.398827 | 7596.302854 | 6515.906302 | 6459.883354 | 6735.545246 | 7721.867674 | 7705.398462 | 7674.79321 | 7305.657187 |
| **Gm10804** | 8.241268334 | 1.584524017 | 2.349483883 | 1.725218839 | 0 | 0 | 4.510323574 | 1.676148026 | 2.257242368 | 6.991215751 | 5.675224193 | 0.867528106 | 5.112505891 | 4.517773258 | 4.311394032 |
| **Ext2** | 1011.844612 | 948.3376242 | 1191.188329 | 1038.581741 | 1152.721105 | 1154.525478 | 1147.426317 | 1102.067327 | 1100.781861 | 1159.667913 | 1104.776976 | 1129.521594 | 1130.715886 | 1121.311323 | 1161.920692 |
| **Accs** | 60.43596778 | 87.14882094 | 64.21922615 | 69.87136299 | 66.50314065 | 84.87094949 | 88.40234205 | 46.09407072 | 69.22209928 | 59.42533389 | 77.5613973 | 58.99191123 | 84.3563472 | 74.09148143 | 65.38947615 |
| **Gm13889** | 5.494178889 | 5.54583406 | 5.482129061 | 4.313047098 | 2.533452977 | 2.104238417 | 4.510323574 | 8.380740131 | 0.752414123 | 2.621705907 | 1.891741398 | 0 | 6.816674521 | 0.903554652 | 3.59282836 |
| **4921507L20Rik** | 9.156964816 | 4.753572051 | 0.783161294 | 3.450437679 | 1.266726489 | 2.805651223 | 4.510323574 | 4.190370065 | 5.266898858 | 0 | 0.945870699 | 6.072696744 | 3.408337261 | 8.131991864 | 7.18565672 |
| **Alkbh3** | 419.3889886 | 463.473275 | 433.8713571 | 426.1290533 | 384.4514893 | 441.1886548 | 426.6766101 | 423.2273766 | 385.2360308 | 372.2822388 | 347.1345464 | 404.2680975 | 400.4796281 | 411.1173665 | 440.4807569 |
| **Hsd17b12** | 2040.171761 | 1929.950253 | 1929.70943 | 1833.907626 | 1837.386772 | 1975.879874 | 2003.485732 | 1713.861357 | 1850.938742 | 1756.542957 | 1718.64706 | 1911.164418 | 2054.375284 | 1981.495351 | 1856.773696 |
| **Ttc17** | 393.7494871 | 385.8315981 | 462.0651637 | 399.3881613 | 407.8859293 | 420.1462706 | 424.8724807 | 341.0961233 | 312.2518609 | 360.9215132 | 326.3253911 | 420.7511315 | 371.5087614 | 412.9244758 | 444.0735853 |
| **2810002D19Rik** | 39.37494871 | 45.15893448 | 35.24225825 | 25.01567317 | 39.26852115 | 35.07064029 | 40.59291217 | 46.93214473 | 42.88760499 | 39.3255886 | 43.51005214 | 40.773821 | 41.75213144 | 36.14218606 | 36.64684927 |
| **Api5** | 4317.508911 | 4425.57558 | 4665.291831 | 4379.468024 | 4239.100194 | 4387.3371 | 4604.138304 | 4621.978182 | 4837.270394 | 4535.551219 | 5028.248635 | 4594.428851 | 4674.534553 | 4663.245557 | 4337.262396 |
| **B230118H07Rik** | 11.90405426 | 10.29940611 | 17.22954848 | 21.56523549 | 5.066905954 | 9.81977928 | 4.510323574 | 6.704592104 | 25.58208017 | 22.72145119 | 17.97154328 | 5.205168638 | 13.63334904 | 15.36042908 | 7.18565672 |
| **Traf6** | 1895.491717 | 1800.019283 | 1917.96201 | 1782.151061 | 1623.309995 | 1660.244111 | 1902.454483 | 2005.511113 | 1848.681499 | 1807.229272 | 1904.983587 | 1812.266214 | 1637.706054 | 1688.743644 | 1777.012907 |
| **Prr5l** | 224.345638 | 230.5482445 | 209.1040656 | 182.0105875 | 246.378302 | 237.7789411 | 248.0677966 | 108.9496217 | 176.0649047 | 137.2026091 | 185.390657 | 198.6639363 | 247.1044514 | 198.7820233 | 252.9351165 |
| **Commd9** | 216.1043696 | 215.4952663 | 248.2621304 | 205.3010419 | 234.9777636 | 253.9114357 | 237.24302 | 315.1158289 | 302.4704773 | 298.8744734 | 298.8951408 | 253.318207 | 286.3003299 | 264.7415129 | 206.2283479 |
| **Ldlrad3** | 11.90405426 | 19.80655021 | 12.53058071 | 5.175656518 | 12.66726489 | 13.32684331 | 2.706194144 | 24.30414638 | 27.83932254 | 20.97364725 | 18.91741398 | 15.61550591 | 7.668758836 | 4.517773258 | 15.08987911 |
| **Trim44** | 2465.970625 | 2449.67413 | 2398.039884 | 2287.640181 | 2218.671445 | 2316.065084 | 2461.734607 | 2359.178347 | 2489.738332 | 2566.650083 | 2476.289489 | 2348.398584 | 2385.836082 | 2267.922175 | 2341.805525 |
| **Fjx1** | 13.73544722 | 6.338096068 | 9.397935534 | 18.97740723 | 8.233722176 | 9.81977928 | 5.412388289 | 24.30414638 | 22.57242368 | 19.22584332 | 13.24218978 | 10.41033728 | 5.112505891 | 15.36042908 | 0 |
| **Slc1a2** | 165.7410632 | 115.6702532 | 125.3058071 | 116.4522717 | 51.93578603 | 53.30737323 | 55.92801232 | 149.1771743 | 106.8428054 | 162.5457662 | 145.6640876 | 78.07752957 | 76.68758836 | 74.09148143 | 48.14390002 |
| **Cd44** | 6020.704366 | 5295.479265 | 5459.417384 | 5192.046097 | 5004.836356 | 5269.714409 | 5754.270816 | 6079.388891 | 5163.818123 | 4755.774515 | 5036.761471 | 5081.112118 | 5441.410437 | 4986.718122 | 5297.266134 |
| **Pdhx** | 398.3279695 | 370.77862 | 436.220841 | 418.3655685 | 400.2855704 | 427.8618115 | 415.8518335 | 480.2164095 | 430.3808781 | 505.98924 | 492.7986341 | 402.5330413 | 341.6858104 | 451.7773258 | 406.7081703 |
| **Apip** | 243.5752641 | 304.2286113 | 280.3717434 | 245.8436846 | 248.911755 | 270.745343 | 227.3203081 | 318.468125 | 282.9077101 | 284.8920419 | 268.6272785 | 256.7883195 | 233.4711024 | 262.9344036 | 226.3481867 |
| **Cat** | 1059.460829 | 1150.364436 | 1144.198651 | 1144.6827 | 1170.455275 | 1138.392984 | 1273.715377 | 1077.763181 | 1104.543932 | 1168.406932 | 1131.261356 | 1270.928676 | 1206.55139 | 1299.311589 | 1242.400047 |
| **Abtb2** | 1830.477267 | 1528.273414 | 1376.014394 | 1336.181991 | 1031.115362 | 1066.848877 | 1030.157904 | 1289.795906 | 1119.592214 | 1260.166639 | 1121.802649 | 1007.200131 | 1095.780429 | 1046.316287 | 1095.094084 |
| **Nat10** | 1844.212714 | 2006.007406 | 2124.716592 | 2063.361732 | 2026.762382 | 1999.026496 | 2005.289861 | 2514.222039 | 2297.87273 | 2221.458805 | 2356.163911 | 1999.652285 | 1972.57519 | 2018.541092 | 2012.702447 |
| **Caprin1** | 10799.7243 | 11067.108 | 11169.44638 | 10618.72196 | 10184.48097 | 10603.9588 | 11159.44259 | 11071.79579 | 11514.19332 | 11171.96277 | 11333.42271 | 11081.80403 | 11341.24223 | 10707.12262 | 10845.31169 |
| **Lmo2** | 2.747089445 | 3.169048034 | 0.783161294 | 12.07653188 | 0.633363244 | 11.22260489 | 10.82477658 | 3.352296052 | 10.53379772 | 4.369509845 | 7.56696559 | 4.337640531 | 4.260421576 | 5.421327909 | 2.874262688 |
| **Fbxo3** | 559.4905502 | 538.7381658 | 676.6513584 | 599.5135467 | 602.9618086 | 637.5842404 | 662.1155006 | 669.6211364 | 598.9216416 | 634.4528294 | 717.9158604 | 637.6331581 | 593.9027677 | 685.7979805 | 668.9846406 |
| **Cd59a** | 2.747089445 | 0 | 0.783161294 | 0 | 0 | 1.402825611 | 2.706194144 | 0 | 3.762070613 | 0 | 6.621094891 | 1.735056213 | 1.70416863 | 0.903554652 | 1.437131344 |
| **Hipk3** | 1914.721343 | 1966.394305 | 1899.166139 | 1899.465942 | 1663.845243 | 1629.381948 | 1883.511124 | 2168.097472 | 2013.460192 | 2086.877902 | 1884.174432 | 1924.17734 | 1814.087507 | 1746.571141 | 1866.11505 |
| **Platr14** | 1.831392963 | 0 | 0 | 2.587828259 | 0 | 0 | 4.510323574 | 1.676148026 | 0 | 3.495607876 | 5.675224193 | 0 | 0 | 0 | 0.718565672 |
| **Cstf3** | 994.446379 | 903.9709517 | 954.673618 | 893.6633588 | 990.580114 | 884.481548 | 905.6729736 | 957.0805229 | 966.8521475 | 971.7789894 | 1049.916476 | 916.1096802 | 962.8552761 | 972.2248051 | 910.4227064 |
| **Tcp11l1** | 10.98835778 | 13.46845414 | 9.397935534 | 21.56523549 | 8.233722176 | 9.118366474 | 2.706194144 | 7.542666117 | 17.30552482 | 2.621705907 | 1.891741398 | 13.8804497 | 12.78126473 | 4.517773258 | 5.748525376 |
| **Depdc7** | 0 | 0 | 3.132645178 | 0 | 0 | 0 | 0.902064715 | 2.514222039 | 0 | 5.243411813 | 2.837612096 | 3.470112425 | 0 | 0.903554652 | 1.437131344 |
| **Qser1** | 628.1677864 | 575.9744802 | 589.7204547 | 514.1152141 | 496.5567835 | 505.7186329 | 594.460647 | 507.8728519 | 498.0981491 | 437.8248864 | 459.6931596 | 584.7139436 | 635.6548991 | 445.4524432 | 565.5111838 |
| **Prrg4** | 1042.978293 | 879.4108294 | 888.8880692 | 775.4858683 | 460.4550786 | 582.1726287 | 686.4712479 | 989.7654094 | 1082.723922 | 912.3536555 | 988.4348802 | 634.1630457 | 605.8319481 | 540.3256816 | 517.3672838 |
| **Ccdc73** | 8.241268334 | 9.507144102 | 3.915806472 | 17.25218839 | 12.66726489 | 11.9240177 | 18.94335901 | 10.05688816 | 12.03862596 | 7.86511772 | 11.35044839 | 8.675281063 | 15.33751767 | 2.710663955 | 7.18565672 |
| **Eif3m** | 3843.178133 | 3558.04868 | 3646.398987 | 3531.522964 | 3723.542513 | 3713.279393 | 3745.372696 | 3510.692041 | 3551.394659 | 3783.995525 | 3640.65632 | 3631.472653 | 3404.928923 | 3654.878566 | 3592.82836 |
| **Rcn1** | 1134.547941 | 1163.040628 | 1191.97149 | 1288.738473 | 1288.260839 | 1308.836295 | 1347.684684 | 1188.388951 | 1190.319142 | 1212.975933 | 1221.119072 | 1224.949686 | 1350.55364 | 1262.265848 | 1352.340595 |
| **Elp4** | 316.8309826 | 293.9292052 | 284.2875499 | 288.9741556 | 291.9804556 | 288.2806631 | 280.5421263 | 340.2580493 | 288.1746089 | 290.1354537 | 314.029072 | 301.899781 | 247.9565357 | 263.8379583 | 291.7376628 |
| **Immp1l** | 226.1770309 | 264.6155108 | 263.9253562 | 269.1341389 | 291.9804556 | 247.5987204 | 276.0318027 | 270.6979062 | 307.7373761 | 258.6749828 | 269.5731491 | 271.5362973 | 232.619018 | 260.2237397 | 283.1148748 |
| **Dnajc24** | 238.0810852 | 197.2732401 | 211.4535495 | 197.5375571 | 200.1427852 | 220.243621 | 177.7067488 | 273.2121283 | 180.5793894 | 205.3669627 | 212.8209072 | 196.061352 | 188.3106337 | 177.0967117 | 224.1924897 |
| **Arl14ep** | 830.5367088 | 701.1518775 | 754.9674879 | 662.4840343 | 606.761988 | 618.6460946 | 681.9609244 | 571.5664769 | 546.252653 | 594.2533389 | 630.8957561 | 602.0645058 | 629.6903089 | 597.2496247 | 601.4394674 |
| **Mettl15** | 69.5929326 | 52.28929256 | 68.91819391 | 72.45919125 | 53.83587576 | 61.7243269 | 66.75278889 | 47.77021874 | 69.9745134 | 60.29923585 | 60.53572472 | 54.6542707 | 71.57508247 | 69.57370817 | 71.13800152 |
| **Kif18a** | 325.9879474 | 378.7012401 | 384.5321956 | 378.6855352 | 338.2159724 | 354.2134669 | 359.0217565 | 380.4856019 | 382.9787884 | 456.1768278 | 371.7271846 | 414.6784348 | 368.9525085 | 390.3356095 | 444.0735853 |
| **Lin7c** | 1464.198674 | 1295.348384 | 1444.932588 | 1445.733387 | 1314.862095 | 1450.521682 | 1489.308844 | 1583.959885 | 1410.024066 | 1435.820935 | 1489.746351 | 1389.780026 | 1367.595326 | 1338.164439 | 1367.430474 |
| **Platr8** | 1.831392963 | 0.792262009 | 0 | 0 | 1.266726489 | 0 | 2.706194144 | 0 | 0 | 0 | 0 | 0 | 0 | 0 | 1.437131344 |
| **Lgr4** | 2.747089445 | 4.753572051 | 18.79587107 | 10.35131304 | 16.46744435 | 11.22260489 | 18.94335901 | 12.5711102 | 4.514484735 | 9.612921658 | 7.56696559 | 15.61550591 | 13.63334904 | 19.87820233 | 13.65274777 |
| **Ccdc34** | 448.691276 | 428.6137466 | 463.6314863 | 484.7864938 | 402.1856601 | 432.0702883 | 461.857134 | 439.1507828 | 488.3167656 | 481.5199849 | 465.3683838 | 514.444167 | 497.6172401 | 495.1479491 | 465.6305554 |
| **Lpcat4** | 932.1790182 | 874.6572574 | 925.6966501 | 860.8842008 | 899.3758069 | 966.5468463 | 927.3225268 | 750.0762417 | 714.7934165 | 793.5029878 | 719.8076018 | 858.8528252 | 930.4760722 | 941.5039469 | 972.2193542 |
| **Nutm1** | 0 | 0.792262009 | 1.566322589 | 1.725218839 | 0 | 4.208476834 | 2.706194144 | 0 | 0 | 0 | 0 | 2.602584319 | 3.408337261 | 0 | 2.155697016 |
| **Nop10** | 1118.065404 | 1079.060856 | 1183.356716 | 1007.527802 | 1178.688998 | 1164.345257 | 1074.359075 | 1218.559615 | 1219.663293 | 1337.943914 | 1277.871314 | 1061.854402 | 1037.838696 | 1266.783622 | 1064.914326 |
| **Slc12a6** | 492.6447071 | 488.8256592 | 603.0341968 | 550.3448097 | 540.8922106 | 517.6426506 | 568.3007703 | 562.3476628 | 584.6257732 | 543.5670247 | 582.6563504 | 661.056417 | 540.2214558 | 655.0771224 | 609.3436898 |
| **Emc4** | 973.3853599 | 924.5697639 | 949.9746502 | 907.4651095 | 863.9074652 | 981.977928 | 908.3791678 | 962.108967 | 848.7231303 | 1068.782108 | 907.0900001 | 875.3358592 | 814.5926053 | 959.57504 | 847.9074929 |
| **Katnbl1** | 505.4644578 | 429.4060086 | 447.1850992 | 447.6942888 | 424.9867369 | 448.9041957 | 414.9497688 | 569.0522549 | 527.4422999 | 608.2357704 | 493.7445048 | 438.9692218 | 505.2859989 | 473.4626374 | 456.2892017 |
| **Emc7** | 1226.117589 | 1130.557886 | 1304.746717 | 1180.049686 | 1200.223348 | 1249.91762 | 1158.251094 | 1205.150431 | 1190.319142 | 1184.137168 | 1311.922659 | 1144.269572 | 1118.786706 | 1227.930771 | 1181.321965 |
| **Aven** | 759.1123832 | 804.1459386 | 758.8832944 | 692.675364 | 694.1661157 | 718.9481259 | 672.9402772 | 993.9557795 | 931.4886838 | 984.887519 | 994.1101044 | 667.9966418 | 717.4549934 | 732.7828224 | 665.3918122 |

| | | | | | | | | | | | | | | | |
|---|---|---|---|---|---|---|---|---|---|---|---|---|---|---|---|
| **Ryr3** | 10.0726613 | 6.338096068 | 1.566322589 | 6.900875357 | 3.800179466 | 4.208476834 | 9.922711863 | 2.514222039 | 17.30552482 | 7.86511772 | 5.675224193 | 2.602584319 | 2.556252945 | 6.324882561 | 3.59282836 |
| **Scg5** | 0.915696482 | 0.792262009 | 0 | 0 | 2.533452977 | 0 | 0 | 6.704592104 | 3.762070613 | 0 | 0.945870699 | 0 | 0 | 0 | 0 |
| **Arhgap11a** | 1992.555544 | 2087.610392 | 2262.55298 | 2073.713045 | 2064.130813 | 2108.446894 | 2230.80604 | 2117.813031 | 2079.672635 | 2077.26498 | 2111.1834 | 2257.308133 | 2183.8921 | 2188.409366 | 2178.691117 |
| **Aqr** | 2511.755449 | 2589.112244 | 2586.781756 | 2572.301289 | 2385.245978 | 2262.757711 | 2606.064961 | 2857.832385 | 2589.056996 | 2593.741044 | 2778.968113 | 2582.631172 | 2661.059316 | 2568.805874 | 2560.968055 |
| **Zfp770** | 148.34283 | 190.142882 | 149.5838072 | 165.6210086 | 131.1061916 | 126.9557178 | 152.4489368 | 183.5382089 | 154.9973093 | 158.1762564 | 184.4447863 | 149.2148343 | 115.0313825 | 142.7616349 | 159.5215792 |
| **Dph6** | 279.2874269 | 339.0881396 | 245.1294852 | 368.3342222 | 247.6450285 | 222.3478594 | 274.2276733 | 357.8576036 | 297.2035784 | 278.7747281 | 310.2455892 | 270.6687692 | 342.5378947 | 282.8126059 | 263.7136016 |
| **BC052040** | 167.5724561 | 158.4524017 | 194.224001 | 156.9949144 | 156.4407213 | 152.9079916 | 161.4695839 | 182.7001348 | 181.3318035 | 136.3287071 | 142.8264755 | 151.8174186 | 165.3043571 | 131.0154245 | 152.3359225 |
| **Spred1** | 486.2348317 | 370.77862 | 421.3407764 | 420.9533968 | 391.418485 | 461.5296262 | 501.5479814 | 449.207671 | 425.8663934 | 390.6341801 | 395.3739521 | 418.1485472 | 430.3025792 | 360.518306 | 418.9237868 |
| **Fam98b** | 1762.715727 | 1662.957956 | 1722.954848 | 1691.577072 | 1597.975465 | 1614.652279 | 1556.963698 | 1953.550524 | 1958.533961 | 1771.399291 | 1723.376413 | 1662.183852 | 1631.741464 | 1528.81447 | 1549.227589 |
| **Rasgrp1** | 2511.755449 | 2681.014637 | 2783.355241 | 2662.875278 | 2739.296031 | 2724.287337 | 3002.071371 | 2246.876429 | 2375.371385 | 2066.778156 | 2239.821815 | 2805.585896 | 3037.680584 | 2985.344569 | 3049.592712 |
| **Thbs1** | 8.241268334 | 11.09166812 | 15.66322589 | 12.93914129 | 6.966995687 | 7.715540863 | 16.23716487 | 51.1225148 | 9.781383594 | 26.21705907 | 23.64676747 | 6.94022485 | 11.92918041 | 6.324882561 | 0.718565672 |
| **Fsip1** | 2.747089445 | 3.961310043 | 6.265290356 | 4.313047098 | 1.266726489 | 2.104238417 | 0.902064715 | 0 | 3.762070613 | 10.48682363 | 3.783482795 | 0 | 0.852084315 | 0.903554652 | 5.748525376 |
| **Gpr176** | 0 | 6.338096068 | 1.566322589 | 0 | 0.633363244 | 0.701412806 | 0.902064715 | 1.676148026 | 0 | 0.873901969 | 0 | 1.735056213 | 4.260421576 | 0 | 0 |
| **Eif2ak4** | 531.1039593 | 450.0048208 | 451.8840669 | 452.0073359 | 374.3176774 | 409.6250785 | 496.1355931 | 509.5489999 | 464.2395136 | 603.8662605 | 531.5793327 | 421.6186597 | 552.1506362 | 408.4067025 | 514.4930211 |
| **Srp14** | 1151.030477 | 1145.610864 | 1201.369426 | 1116.216589 | 1152.087741 | 1104.725169 | 1033.766163 | 1172.465544 | 1202.357768 | 1240.066894 | 1118.019166 | 1121.713841 | 1186.953451 | 1141.189525 | 1127.429539 |
| **Bmf** | 58.60457482 | 59.41965064 | 79.09929074 | 79.36006661 | 41.16861088 | 44.89041957 | 46.90736517 | 26.81836842 | 43.64001911 | 48.93851026 | 67.15681961 | 45.11146153 | 46.86463733 | 47.88839653 | 46.70676868 |
| **Bub1b** | 2897.263668 | 3142.903388 | 3317.471243 | 3178.715711 | 3154.148956 | 2906.654667 | 3229.391679 | 3321.287314 | 3377.586996 | 3494.733974 | 3550.798603 | 3373.816805 | 3258.370421 | 3321.466899 | 3238.575484 |
| **Pak6** | 194.1276541 | 184.597048 | 187.1755494 | 195.8123383 | 156.4407213 | 137.4769099 | 162.3716487 | 341.9341973 | 276.135983 | 212.3581784 | 254.439218 | 164.8303402 | 144.0022493 | 159.9291733 | 153.0544881 |
| **Plcb2** | 304.9269284 | 306.6053973 | 321.0961307 | 290.6993744 | 306.5478102 | 279.8637095 | 379.7692449 | 361.2098996 | 303.9753055 | 306.7395911 | 257.2768301 | 286.2842751 | 301.6378476 | 311.7263548 | 358.5642703 |
| **Inafm2** | 248.1537465 | 218.6643143 | 235.7315496 | 241.5306375 | 200.1427852 | 216.0351442 | 224.614114 | 186.0524309 | 144.4635115 | 214.9798844 | 214.7126486 | 226.4248357 | 216.4294161 | 212.3353431 | 207.6654792 |
| **Knstrn** | 808.5599932 | 973.6900085 | 964.0715535 | 853.9833255 | 942.4445075 | 941.9973981 | 945.3638211 | 922.7194884 | 881.8293517 | 911.4797536 | 892.9019396 | 933.4602424 | 938.9969153 | 1067.098044 | 952.0995154 |
| **Ivd** | 443.1970971 | 495.9560173 | 509.8380027 | 476.1603996 | 415.4862882 | 442.5914804 | 451.9344221 | 560.6715147 | 511.6416034 | 576.7752995 | 530.633462 | 492.7559644 | 483.1318067 | 489.7266212 | 469.2233838 |
| **Bahd1** | 1055.798043 | 1106.790026 | 1019.676005 | 1056.696539 | 980.4463021 | 1087.891262 | 1116.756117 | 1050.944812 | 1080.46668 | 871.280263 | 997.8935872 | 990.7170974 | 1092.372092 | 963.1892586 | 1085.75273 |
| **Chst14** | 128.1975074 | 112.5012052 | 96.32883922 | 72.45919125 | 97.53793962 | 90.48225194 | 89.30440676 | 107.2734737 | 129.4152291 | 89.13800083 | 87.02010429 | 98.03067601 | 80.94800994 | 67.76659887 | 95.56923437 |
| **Ccdc32** | 263.7205867 | 273.3303929 | 293.6854854 | 274.3097954 | 284.3800967 | 288.982076 | 263.4028967 | 340.2580493 | 244.5345898 | 335.5783561 | 237.4135454 | 282.8141626 | 298.2295103 | 309.9192455 | 276.6477837 |
| **Rpusd2** | 520.1156015 | 479.3185151 | 455.7998734 | 462.3586489 | 468.6888008 | 467.1409286 | 540.3367642 | 474.3498914 | 521.4229869 | 436.9509845 | 466.3142545 | 431.1614688 | 385.1421105 | 421.0564676 | 377.2469778 |
| **Casc5** | 1214.213535 | 1265.242428 | 1247.575942 | 1307.71588 | 1181.222451 | 1044.403668 | 1298.071125 | 1243.701835 | 1281.361251 | 1213.849835 | 1315.706142 | 1321.245306 | 1335.216122 | 1180.94593 | 1244.555744 |
| **Rad51** | 1659.242025 | 1754.860349 | 1755.847622 | 1693.302291 | 1668.912149 | 1669.362478 | 1578.613251 | 1879.800011 | 1969.067759 | 1881.510939 | 1794.316716 | 1710.765426 | 1676.901932 | 1664.347668 | 1636.892601 |
| **Rmdn3** | 1182.164158 | 1300.894218 | 1270.28762 | 1101.552229 | 1063.416887 | 1052.820621 | 1089.694175 | 1430.59234 | 1439.368216 | 1384.260719 | 1347.865746 | 1082.675077 | 1104.301272 | 1122.214877 | 1171.262045 |
| **Dnajc17** | 193.2119576 | 194.8964541 | 176.2112913 | 169.9340557 | 200.1427852 | 150.1023404 | 193.041849 | 191.080875 | 175.3124906 | 157.3023544 | 160.7980188 | 180.4458461 | 122.7001414 | 162.6398373 | 164.5515389 |
| **Gm14137** | 0.915696482 | 3.961310043 | 7.831612945 | 2.587828259 | 3.166816221 | 3.507064029 | 2.706194144 | 0.838074013 | 3.00965649 | 0 | 0 | 1.735056213 | 8.520843152 | 0 | 3.59282836 |
| **Zfyve19** | 424.8831674 | 404.0536243 | 414.2923248 | 430.4421004 | 400.9189336 | 399.1038865 | 414.9497688 | 451.721893 | 468.7539984 | 432.5814746 | 427.5335558 | 445.0419185 | 415.8171458 | 420.152913 | 433.2951002 |
| **Spint1** | 16.48253667 | 9.507144102 | 6.265290356 | 8.626094197 | 3.166816221 | 8.416953669 | 2.706194144 | 20.95185033 | 17.30552482 | 13.10852953 | 15.13393118 | 0.867528106 | 6.816674521 | 4.517773258 | 9.341353736 |
| **Rhov** | 8.241268334 | 1.584524017 | 2.349483883 | 3.450437679 | 3.800179466 | 3.507064029 | 0.902064715 | 4.190370065 | 1.504828245 | 1.747803938 | 8.512836289 | 4.337640531 | 2.556252945 | 2.710663955 | 0 |
| **Vps18** | 432.2087393 | 436.5363667 | 365.7363245 | 432.1673192 | 413.5861985 | 354.2134669 | 381.5733744 | 422.3893026 | 480.7926243 | 435.2031805 | 425.6418144 | 361.7592203 | 377.4733516 | 365.9396339 | 398.8039479 |
| **Chac1** | 138.2701687 | 85.56429692 | 95.54567793 | 69.87136299 | 83.60394824 | 96.09355438 | 100.1291833 | 35.19910855 | 54.92623095 | 34.95607876 | 36.88895725 | 56.38932691 | 75.83550405 | 66.86304422 | 67.54517316 |
| **Ino80** | 1419.329546 | 1343.676366 | 1340.772136 | 1261.134972 | 1212.890613 | 1211.339915 | 1322.426872 | 1247.892205 | 1274.589524 | 1154.424501 | 1374.350125 | 1202.393955 | 1350.55364 | 1129.443314 | 1250.304269 |
| **Exd1** | 2.747089445 | 3.169048034 | 3.132645178 | 3.450437679 | 0.633363244 | 0 | 1.80412943 | 0.838074013 | 0 | 2.621705907 | 3.783482795 | 4.337640531 | 0.852084315 | 0.903554652 | 1.437131344 |
| **Chp1** | 1541.117178 | 1329.41565 | 1462.945298 | 1443.145559 | 1212.25725 | 1228.173823 | 1271.009183 | 1343.432643 | 1355.097835 | 1474.272622 | 1348.811616 | 1310.834969 | 1371.855747 | 1317.382682 | 1313.538048 |
| **1700020I14Rik** | 632.7462688 | 663.9155631 | 628.0953582 | 628.8422669 | 631.4631545 | 624.9588099 | 673.8423419 | 617.6605476 | 665.1340844 | 624.8399078 | 539.1462983 | 715.7106877 | 655.2528384 | 588.2140782 | 668.9846406 |
| **Oip5** | 270.1304621 | 354.9333798 | 297.6012919 | 270.8593578 | 315.4148956 | 266.5368662 | 275.129738 | 305.0589408 | 302.4704773 | 262.1705907 | 257.2768301 | 260.2584319 | 270.9628122 | 243.9597559 | 280.9591777 |
| **Nusap1** | 2606.072187 | 2662.000349 | 2766.125692 | 2651.661356 | 2677.226434 | 2675.188441 | 2729.647827 | 2514.222039 | 2723.739124 | 2456.538435 | 2495.206903 | 2840.28702 | 2812.730324 | 2867.882464 | 2719.771068 |
| **Ndufaf1** | 318.6623756 | 296.3059912 | 314.0476791 | 296.7376404 | 298.9474513 | 302.3089193 | 291.3669029 | 390.5424901 | 334.8242845 | 377.5256506 | 350.9180292 | 319.2503431 | 295.6732574 | 311.7263548 | 268.7435613 |
| **Rtf1** | 1647.33797 | 1714.454986 | 1822.416332 | 1649.30921 | 1759.483093 | 1647.618681 | 1888.021448 | 1791.80224 | 1604.899323 | 1753.04735 | 1898.362492 | 1882.535991 | 1814.087507 | 1838.733716 | 1902.043334 |
| **Itpka** | 19.22962611 | 33.27500436 | 33.67593566 | 17.25218839 | 44.96879034 | 30.86216345 | 33.37639445 | 42.74177467 | 48.15450385 | 24.46925513 | 15.13393118 | 34.70112425 | 29.82295103 | 37.94929537 | 26.58692986 |
| **Ltk** | 9.156964816 | 5.54583406 | 4.698967767 | 4.313047098 | 5.066905954 | 7.014128057 | 3.608258859 | 0.838074013 | 6.771727103 | 1.747803938 | 9.458706988 | 16.48303402 | 2.556252945 | 6.324882561 | 9.341353736 |
| **Rpap1** | 1017.338791 | 1025.187039 | 933.528263 | 910.9155472 | 1000.080563 | 997.4090097 | 975.1319567 | 1063.515923 | 1119.592214 | 1054.799676 | 917.4945778 | 946.473164 | 1042.951202 | 949.6359388 | 973.6564855 |
| **Tyro3** | 227.0927274 | 209.9494323 | 217.7188399 | 257.9202165 | 231.8109474 | 231.4662259 | 284.1503852 | 209.5185033 | 193.3704295 | 194.8801391 | 183.4989156 | 215.1469704 | 270.1107279 | 302.6908083 | 233.5338434 |
| **Mga** | 1612.541504 | 1651.866288 | 1649.337686 | 1578.575238 | 1259.759493 | 1231.680887 | 1482.092326 | 1453.220339 | 1467.207539 | 1358.04366 | 1383.808832 | 1440.964185 | 1393.157855 | 1286.661824 | 1279.046896 |
| **Mapkbp1** | 316.8309826 | 309.7744453 | 262.3590337 | 270.8593578 | 266.0125626 | 293.8919656 | 318.4288443 | 351.1530115 | 336.3291128 | 309.361297 | 314.9749427 | 314.0451745 | 370.6566771 | 296.3659257 | 347.7857852 |
| **Pla2g4b** | 0.915696482 | 0 | 0 | 0 | 0.633363244 | 0 | 1.80412943 | 0.838074013 | 0 | 0 | 0 | 0.867528106 | 0 | 0 | 4.311394032 |
| **Ehd4** | 1183.079854 | 1126.596576 | 1054.918264 | 1117.079198 | 1054.549802 | 1129.97603 | 1113.147858 | 1065.192071 | 1114.325316 | 975.2745973 | 1170.042054 | 1052.311593 | 1098.336682 | 1087.8798 | 1089.345559 |
| **Pla2g4f** | 101.6423095 | 84.77203491 | 111.2089038 | 72.45919125 | 86.13740122 | 91.18366474 | 77.57756547 | 172.6432467 | 117.3766031 | 131.0852953 | 108.7751304 | 103.2358446 | 79.24384131 | 93.06612911 | 89.10214332 |
| **Vps39** | 860.7546927 | 957.8447683 | 905.3344564 | 877.2737798 | 835.4061192 | 801.0134241 | 901.1626501 | 935.2905986 | 884.086594 | 856.4239295 | 979.9220439 | 830.2243977 | 802.6634249 | 809.5849678 | 847.9074929 |
| **Tmem87a** | 779.2577058 | 786.7161744 | 689.1819391 | 708.2023335 | 737.8681796 | 677.5647703 | 801.0334667 | 746.7239456 | 744.1375672 | 711.3562027 | 695.2149636 | 757.3520368 | 675.7028619 | 694.8335271 | 692.6973078 |
| **Ganc** | 113.5463637 | 95.07144102 | 114.341549 | 74.18441009 | 91.20430718 | 105.9133337 | 121.7787365 | 115.6542138 | 68.46968515 | 107.4899422 | 113.5044839 | 111.9111257 | 98.84178056 | 84.93413725 | 121.4375986 |
| **Capn3** | 51.27900297 | 69.71905675 | 81.44877463 | 103.5131304 | 114.005384 | 86.2737751 | 101.9333128 | 65.36977302 | 98.56625006 | 86.51629492 | 73.7779145 | 116.2487662 | 103.9542864 | 100.2945663 | 91.25784034 |
| **Zfp106** | 5589.411323 | 5699.532889 | 5641.893965 | 5418.912374 | 5047.271694 | 5061.394806 | 5631.590014 | 6183.310068 | 6333.06967 | 6125.1789 | 6373.276768 | 5567.795386 | 5871.713016 | 5270.434283 | 5822.53764 |
| **Snap23** | 371.7727715 | 353.3488558 | 361.0373568 | 413.189912 | 298.3140881 | 336.6781467 | 373.4547919 | 359.5337516 | 348.3677388 | 426.4641608 | 419.0207196 | 362.6267484 | 373.21293 | 353.2898688 | 402.3967763 |
| **Lrrc57** | 152.0056159 | 156.8678777 | 144.1016782 | 163.8957897 | 154.5406316 | 159.2207069 | 130.7993836 | 207.0042812 | 183.5890459 | 199.2496489 | 187.2823984 | 163.9628121 | 165.3043571 | 185.2287036 | 164.5515389 |
| **Haus2** | 541.1766206 | 583.8971003 | 586.5878096 | 551.2074192 | 531.3917619 | 546.4005757 | 601.6771648 | 631.0697318 | 592.1499145 | 564.5406719 | 614.8159542 | 623.7527084 | 598.1631892 | 561.1074386 | 612.9365182 |
| **Cdan1** | 700.5078084 | 763.7405762 | 853.645811 | 734.0806161 | 666.298133 | 693.6972648 | 773.0694606 | 799.5226085 | 790.7872428 | 867.7846551 | 778.4515851 | 777.3051832 | 863.1614113 | 779.7676643 | 792.5779362 |
| **Ttbk2** | 65.93014667 | 59.41965064 | 65.00238744 | 80.22267603 | 74.73686282 | 62.42573971 | 81.18582433 | 94.70236348 | 75.24141226 | 73.40776539 | 69.04856101 | 65.06460797 | 66.46257658 | 84.0305826 | 68.98230451 |
| **AV039307** | 0.915696482 | 0.792262009 | 2.349483883 | 1.725218839 | 2.533452977 | 0.701412806 | 0 | 0 | 0.752414123 | 4.369509845 | 0.945870699 | 0 | 0.852084315 | 1.807109303 | 1.437131344 |
| **Ubr1** | 880.9000153 | 865.9423753 | 863.0437465 | 833.2806994 | 789.1706024 | 677.5647703 | 826.2912787 | 925.2337104 | 908.163846 | 907.9841457 | 1100.993493 | 838.0321507 | 922.8073133 | 800.5494213 | 787.5479765 |
| **Tmem62** | 136.4387758 | 167.1672838 | 131.5710975 | 130.2540224 | 144.4068197 | 150.1023404 | 152.4489368 | 186.0524309 | 187.3511165 | 134.5809032 | 189.1741398 | 166.5653964 | 182.3460434 | 164.4469466 | 167.4258016 |
| **Ccndbp1** | 496.307493 | 522.8929256 | 535.6823254 | 472.709962 | 563.6932874 | 538.6850348 | 495.2335284 | 509.5489999 | 468.0015842 | 535.7019069 | 524.9582378 | 542.2050664 | 510.3985048 | 546.6505642 | 497.247445 |
| **Lcmt2** | 190.4648682 | 198.0655021 | 191.8745171 | 193.22451 | 231.1775842 | 213.9309057 | 222.8099846 | 234.6607237 | 230.9911356 | 165.1674721 | 199.5787174 | 215.1469704 | 217.2815004 | 185.2287036 | 183.2342464 |
| **Adal** | 130.9445969 | 143.3994235 | 145.6680008 | 141.4679448 | 152.6405419 | 129.7613691 | 151.5468721 | 116.4922878 | 167.0359352 | 135.4548052 | 175.93195 | 166.5653964 | 124.40431 | 120.1727687 | 153.0544881 |
| **Zscan29** | 463.3424197 | 468.226847 | 507.4885188 | 498.5882446 | 418.6531045 | 456.6197365 | 493.429399 | 496.1398157 | 515.403674 | 478.024377 | 420.912461 | 432.896525 | 400.4796281 | 467.1377549 | 454.8520704 |
| **Tubgcp4** | 380.0140398 | 404.0536243 | 444.052454 | 356.2576903 | 383.1847628 | 377.3600895 | 417.6559629 | 413.1704884 | 407.8084544 | 370.5344348 | 437.9381335 | 393.8577603 | 334.8691359 | 422.8635769 | 438.3250599 |
| **Trp53bp1** | 845.1878525 | 768.4941482 | 763.5822621 | 705.6145053 | 732.8012736 | 732.976382 | 829.8995376 | 767.675796 | 801.3210405 | 804.8637134 | 819.1240251 | 794.6557454 | 737.9050169 | 802.3565306 | 866.5902004 |
| **Map1a** | 24.723805 | 13.46845414 | 14.0969033 | 22.42784491 | 28.50134599 | 21.04238417 | 28.86607087 | 51.96058881 | 24.82966605 | 16.60413741 | 34.05134516 | 37.30370857 | 19.59793925 | 31.62441281 | 27.30549553 |
| **Ppip5k1** | 140.1015617 | 186.181572 | 158.9817428 | 158.7201332 | 163.407717 | 176.0546142 | 160.5675192 | 214.5469473 | 184.34146 | 169.536982 | 164.5815016 | 177.8432618 | 157.6355983 | 172.5789385 | 160.2401448 |
| **Ckmt1** | 0.915696482 | 9.507144102 | 5.482129061 | 1.725218839 | 3.166816221 | 2.805651223 | 1.80412943 | 19.2757023 | 13.54345421 | 14.85633347 | 7.56696559 | 5.205168638 | 1.70416863 | 0 | 0 |
| **Strc** | 0.915696482 | 3.169048034 | 0 | 2.587828259 | 1.266726489 | 0 | 0.902064715 | 1.676148026 | 0 | 1.747803938 | 0 | 3.470112425 | 3.408337261 | 0 | 0 |
| **Catsper2** | 11.90405426 | 22.97559825 | 17.22954848 | 11.21392246 | 12.66726489 | 16.83390734 | 17.13922958 | 14.24725822 | 14.29586833 | 11.3607256 | 20.80915537 | 18.21809023 | 17.0416863 | 11.74621047 | 12.9341821 |
| **Pdia3** | 11957.16466 | 12108.14028 | 12573.65458 | 12115.3493 | 11682.38504 | 11693.9543 | 11413.82484 | 13621.21693 | 13396.73345 | 13810.27281 | 13920.37907 | 12255.56956 | 12120.0473 | 12631.69403 | 11753.5787 |
| **Ell3** | 3.662785926 | 1.584524017 | 1.566322589 | 4.313047098 | 0 | 3.507064029 | 2.706194144 | 10.05688816 | 12.03862596 | 9.612921658 | 2.837612096 | 6.94022485 | 2.556252945 | 2.710663955 | 7.18565672 |
| **Serf2** | 2345.098689 | 2469.480681 | 2317.37427 | 2365.275029 | 2534.08634 | 2774.789059 | 2624.106255 | 2226.762653 | 2136.103694 | 2325.453139 | 2076.186184 | 2507.156227 | 2371.350649 | 2640.186692 | 2389.230859 |
| **Serinc4** | 4.578482408 | 19.0142882 | 4.698967767 | 8.626094197 | 17.73417084 | 8.416953669 | 9.020647148 | 13.40918421 | 4.514484735 | 3.495607876 | 11.35044839 | 6.072696744 | 17.0416863 | 7.228437213 | 2.874262688 |
| **Hypk** | 1037.484114 | 1035.486445 | 1032.206586 | 1059.284367 | 1213.523976 | 1132.080268 | 1086.085917 | 1114.638437 | 1182.042587 | 1198.993501 | 1017.756872 | 1077.469908 | 1145.20132 | 1158.357063 | 1032.578871 |
| **Mfap1b** | 163.9096702 | 195.6887161 | 197.3566462 | 146.6436013 | 147.5736359 | 174.6517886 | 207.4748844 | 183.5382089 | 151.2352386 | 209.7364725 | 172.1484672 | 195.1938239 | 184.9022964 | 224.9851082 | 202.6355195 |
| **Mfap1a** | 309.5054108 | 370.77862 | 343.024647 | 373.5098787 | 348.3497844 | 325.4555419 | 379.7692449 | 369.5906398 | 346.1104964 | 383.6429643 | 417.1289782 | 347.0112425 | 363.8400026 | 376.7822897 | 393.0554226 |
| **Wdr76** | 893.719766 | 944.3763141 | 1009.494909 | 937.6564392 | 897.4757171 | 813.6388546 | 916.4977502 | 1079.439329 | 937.5079967 | 951.6792441 | 1037.620157 | 887.4812527 | 867.4218328 | 906.2653155 | 917.6083631 |
| **Frmd5** | 0.915696482 | 0 | 0.783161294 | 0 | 1.900089733 | 4.208476834 | 3.608258859 | 0 | 0 | 0 | 3.783482795 | 0 | 0 | 0 | 0 |
| **Casc4** | 447.7755795 | 415.1452925 | 438.5703249 | 459.7708207 | 340.7494254 | 401.9095377 | 407.7332511 | 424.0654506 | 483.0498667 | 443.9422002 | 459.6931596 | 398.1954008 | 423.4859046 | 419.2493583 | 368.6241897 |
| **Ctdspl2** | 879.0686223 | 1064.007877 | 931.1787791 | 817.7537298 | 891.7754479 | 848.0080821 | 1058.12191 | 737.5051315 | 790.7872428 | 797.8724976 | 804.9359647 | 884.8786684 | 898.0968682 | 857.4733643 | 965.7522631 |
| **Spg11** | 710.5804697 | 775.6245063 | 811.3551011 | 787.5624001 | 702.3998379 | 710.5311722 | 834.4098612 | 785.2753502 | 785.520344 | 755.9252031 | 779.3974558 | 787.7155205 | 706.3778973 | 783.3818829 | 758.0867839 |
| **Patl2** | 1.831392963 | 0.792262009 | 0 | 0 | 0 | 2.104238417 | 1.80412943 | 0 | 0 | 0 | 3.783482795 | 4.337640531 | 0 | 3.614218606 | 1.437131344 |
| **B2m** | 34118.8509 | 31144.61182 | 32314.80133 | 30544.13694 | 31702.36383 | 32509.08072 | 33818.40616 | 27105.8278 | 26977.80837 | 25987.22285 | 26584.64186 | 31419.26543 | 31331.14027 | 32780.96276 | 31221.67845 |
| **Trim69** | 2.747089445 | 1.584524017 | 3.915806472 | 2.587828259 | 0 | 0.701412806 | 0 | 3.352296052 | 0 | 0 | 2.837612096 | 0.867528106 | 1.70416863 | 2.710663955 | 4.311394032 |
| **Sord** | 98.89522001 | 86.35655893 | 93.19619404 | 79.36006661 | 89.93758069 | 89.78083913 | 68.55691832 | 79.61703124 | 73.73658401 | 129.3374914 | 105.9375183 | 104.9709009 | 102.2501178 | 74.99503608 | 87.66501198 |
| **Shf** | 67.76153964 | 53.87381658 | 60.30341968 | 63.83309705 | 57.63605523 | 59.62008849 | 65.85072418 | 32.68488651 | 81.26072524 | 59.42533389 | 52.02288843 | 58.99191123 | 43.45630007 | 83.12702794 | 56.04812241 |
| **Slc28a2** | 403.8221484 | 458.7197029 | 506.7053575 | 502.0386822 | 453.4880829 | 425.0561603 | 495.2335284 | 453.3980411 | 337.833941 | 397.6253959 | 397.2656935 | 481.478099 | 556.4110578 | 499.6657223 | 454.1335047 |
| **Bambi-ps1** | 41.20634167 | 52.28929256 | 68.91819391 | 83.67311371 | 58.26941847 | 48.39748359 | 48.7114946 | 41.90370065 | 78.25106875 | 55.05582404 | 47.29353494 | 53.78674259 | 49.42089028 | 91.25901981 | 58.9223851 |
| **Gm14085** | 67.76153964 | 122.8006113 | 101.027807 | 125.9409753 | 104.5049353 | 77.85682143 | 74.87137133 | 67.04592104 | 72.23175577 | 53.3080201 | 75.6696559 | 103.2358446 | 101.3980335 | 113.8478861 | 91.97640601 |
| **Gatm** | 29.30228741 | 18.2220262 | 22.71167754 | 18.11479781 | 10.76717515 | 18.23673295 | 17.13922958 | 19.2757023 | 11.28621184 | 41.07339254 | 24.59263817 | 21.68820266 | 19.59793925 | 9.939101167 | 22.27553583 |
| **Spata5l1** | 487.1505282 | 517.3470916 | 530.9833577 | 526.191746 | 455.3881726 | 520.4483018 | 554.7697996 | 438.3127088 | 545.5002389 | 464.0419455 | 484.2857978 | 429.4264126 | 410.7046399 | 421.0564676 | 508.7444958 |
| **AA467197** | 61.35166426 | 47.53572051 | 57.1707745 | 46.58090866 | 15.20071786 | 38.57770431 | 18.94335901 | 66.20784703 | 54.17381683 | 53.3080201 | 53.91462983 | 19.95314644 | 20.45002356 | 20.78175699 | 22.27553583 |
| **Slc30a4** | 2212.322699 | 2178.720523 | 2258.637173 | 2201.379239 | 1228.091331 | 1327.073028 | 1483.896456 | 2331.521904 | 2608.619763 | 2435.564787 | 2704.244328 | 1514.704074 | 1586.580995 | 1255.037411 | 1499.646557 |
| **Bloc1s6** | 599.7811954 | 561.713764 | 586.5878096 | 532.2300119 | 518.7244971 | 514.1355866 | 527.7078581 | 584.9756611 | 586.1306015 | 656.3003786 | 580.764609 | 501.4312454 | 499.3214087 | 571.0465398 | 521.6786778 |
| **Sqrdl** | 12.81975074 | 15.84524017 | 14.8800646 | 16.38957897 | 6.966995687 | 10.52119209 | 9.020647148 | 21.78992434 | 20.31518131 | 20.97364725 | 36.88895725 | 13.01292159 | 5.112505891 | 13.55331977 | 9.341353736 |
| **Sema6d** | 1334.169774 | 1353.975773 | 1498.187556 | 1350.846351 | 888.6086317 | 1039.493778 | 1116.756117 | 1317.452349 | 946.5369662 | 1474.272622 | 1780.128655 | 985.5119287 | 1058.288719 | 908.0724248 | 1173.417742 |
| **Slc24a5** | 14.65114371 | 28.52143231 | 10.18109683 | 27.60350143 | 24.06780328 | 21.74379698 | 18.0412943 | 13.40918421 | 21.06759543 | 25.3431571 | 13.24218978 | 19.95314644 | 13.63334904 | 11.74621047 | 9.341353736 |
| **Myef2** | 741.7141501 | 643.3167509 | 633.5774872 | 570.1848264 | 584.5942745 | 558.3245933 | 629.6411709 | 607.6036595 | 610.2078534 | 681.6435357 | 695.2149636 | 549.1452913 | 588.7902618 | 530.3865805 | 565.5111838 |
| **Dut** | 3668.280105 | 3744.230252 | 3975.326731 | 3837.749308 | 3629.804753 | 3752.558511 | 3513.542064 | 4235.626062 | 4210.50943 | 4408.835433 | 4166.560428 | 3794.567937 | 3938.333705 | 4103.945227 | 3772.469778 |
| **Fbn1** | 8.241268334 | 16.63750218 | 21.92851625 | 6.038265938 | 8.233722176 | 9.81977928 | 9.020647148 | 31.00873848 | 12.03862596 | 9.612921658 | 28.37612096 | 6.94022485 | 7.668758836 | 1.807109303 | 7.904222392 |
| **Cep152** | 605.2753743 | 622.7179387 | 593.6362612 | 580.5361394 | 555.4595652 | 560.4288318 | 589.0482588 | 567.3761068 | 594.4071568 | 586.3882211 | 559.009583 | 612.474843 | 627.134056 | 546.6505642 | 584.1938913 |
| **Shc4** | 12.81975074 | 11.09166812 | 13.31374201 | 15.52696955 | 16.46744435 | 16.13249453 | 14.43303544 | 10.89496217 | 13.54345421 | 12.23462756 | 17.97154328 | 15.61550591 | 10.22501178 | 10.84265582 | 20.11983882 |
| **Eid1** | 868.995961 | 775.6245063 | 852.0794884 | 809.1276356 | 825.2723073 | 904.8225194 | 812.760308 | 796.1703124 | 818.6265654 | 783.0161641 | 845.6084047 | 859.7203533 | 822.2613641 | 796.031648 | 749.4639959 |
| **Secisbp2l** | 988.0365036 | 1121.050742 | 1246.00962 | 1112.766151 | 1063.416887 | 1108.232233 | 1115.854052 | 1103.743475 | 1018.768722 | 1041.691147 | 962.8963713 | 1119.111257 | 1267.901461 | 1083.362027 | 1049.824447 |
| **Cops2** | 2386.305031 | 2217.541362 | 2414.486271 | 2244.50971 | 2305.442209 | 2257.146409 | 2290.342311 | 2629.038179 | 2528.863866 | 2611.219083 | 2653.16731 | 2309.359819 | 2283.585965 | 2231.779989 | 2281.446008 |
| **Galk2** | 528.3568699 | 549.0375719 | 567.0087772 | 575.3604829 | 487.0563348 | 453.8140853 | 523.1975346 | 633.5839539 | 653.0954584 | 624.8399078 | 642.2462045 | 538.734954 | 495.9130714 | 557.49322 | 531.0200316 |
| **Dtwd1** | 179.4765104 | 171.9208558 | 205.1882592 | 197.5375571 | 174.1748922 | 202.006888 | 182.2170724 | 203.6519852 | 185.0938742 | 208.8625706 | 149.4475704 | 159.6251716 | 178.9377062 | 168.9647198 | 176.7671553 |
| **Atp8b4** | 3596.85578 | 3872.576698 | 3810.862859 | 3488.392493 | 3392.9269 | 3262.270959 | 3873.465885 | 2739.663949 | 2962.254401 | 2925.823792 | 3215.014505 | 3436.278829 | 3571.085365 | 3644.939464 | 3531.031712 |
| **Hdc** | 10.98835778 | 6.338096068 | 4.698967767 | 0.86260942 | 14.56735462 | 3.507064029 | 11.72684129 | 7.542666117 | 5.266898858 | 3.495607876 | 3.783482795 | 5.205168638 | 4.260421576 | 6.324882561 | 8.622788064 |
| **Gabpb1** | 962.3970021 | 1009.341799 | 1072.930973 | 871.2355138 | 902.5426231 | 902.0168681 | 1012.11661 | 1023.28837 | 1100.781861 | 1019.843598 | 1026.269708 | 988.9820412 | 905.765627 | 919.8186353 | 926.2311512 |
| **Usp8** | 1227.948982 | 1212.953135 | 1387.761814 | 1220.592329 | 1086.217964 | 1216.249805 | 1296.266995 | 1396.231306 | 1262.550898 | 1436.694837 | 1311.922659 | 1330.788115 | 1238.930594 | 1326.418229 | 1280.484027 |
| **Usp50** | 0.915696482 | 0 | 0.783161294 | 2.587828259 | 0 | 2.805651223 | 1.80412943 | 0 | 3.762070613 | 1.747803938 | 0 | 2.602584319 | 0 | 1.807109303 | 4.311394032 |
| **Trpm7** | 1093.341599 | 1201.069205 | 1296.915104 | 1190.400999 | 1094.451686 | 1080.175721 | 1206.962588 | 1389.526714 | 1523.638598 | 1492.624563 | 1444.344557 | 1224.949686 | 1324.99111 | 1349.007095 | 1267.549845 |
| **Sppl2a** | 1520.971856 | 1639.982358 | 1606.263815 | 1406.053354 | 1326.262634 | 1374.769099 | 1473.071679 | 1394.555158 | 1553.735163 | 1552.049897 | 1527.581179 | 1573.695985 | 1559.314297 | 1536.946462 | 1531.263447 |
| **Ap4e1** | 565.9004256 | 602.1191265 | 608.5163258 | 453.7325547 | 509.2240484 | 541.490686 | 589.0482588 | 486.0829276 | 517.6609163 | 459.6724356 | 528.7417206 | 569.0984377 | 627.134056 | 547.5541189 | 517.3672838 |
| **Blvra** | 443.1970971 | 499.9173274 | 456.5830347 | 489.9621504 | 481.3560656 | 445.3971316 | 482.6046224 | 420.7131546 | 425.8663934 | 461.4202396 | 528.7417206 | 478.0079866 | 500.173493 | 590.0211875 | 462.0377271 |
| **Ncaph** | 1778.282567 | 2002.838357 | 1925.010462 | 2006.42951 | 1988.760587 | 1973.775635 | 1980.032049 | 1862.200457 | 1873.511165 | 1815.968291 | 2070.51096 | 2131.516557 | 1905.260529 | 2101.66812 | 2037.852246 |
| **Itpripl1** | 881.8157117 | 891.2947596 | 906.1176177 | 979.9243007 | 899.3758069 | 821.3543955 | 857.8635438 | 1123.019177 | 1111.315659 | 1106.359893 | 1171.933796 | 813.7413637 | 881.9072662 | 905.3617609 | 940.6024646 |
| **1810024B03Rik** | 5.494178889 | 12.67619214 | 8.614774239 | 2.587828259 | 11.4005384 | 3.507064029 | 5.412388289 | 11.73303618 | 4.514484735 | 2.621705907 | 13.24218978 | 8.675281063 | 6.816674521 | 3.614218606 | 7.904222392 |
| **Snrnp200** | 6485.878179 | 6441.090129 | 6403.126744 | 6795.637008 | 6138.556564 | 5951.487656 | 6761.877102 | 7458.858716 | 7242.738344 | 6989.467947 | 7355.090554 | 6445.73383 | 6437.497001 | 5949.907381 | 6632.361152 |
| **Ciao1** | 764.6065621 | 767.7018862 | 741.6537459 | 712.5153806 | 729.6344574 | 678.2661831 | 677.4506008 | 782.7611282 | 811.1024241 | 783.0161641 | 778.4515851 | 720.9158563 | 743.0175228 | 793.3209841 | 699.8829645 |
| **Tmem127** | 948.6615549 | 1062.423353 | 1015.760199 | 943.6947051 | 953.2116826 | 942.6988109 | 1077.065269 | 1068.544367 | 1036.074247 | 1032.078225 | 1020.594484 | 1006.332603 | 1168.207596 | 1063.483825 | 985.1535363 |
| **Stard7** | 2165.622179 | 2337.965187 | 2358.881819 | 2415.306375 | 2264.906962 | 2195.422082 | 2205.548228 | 2911.469121 | 2738.034992 | 2685.50075 | 2602.090292 | 2385.702292 | 2352.604794 | 2307.67858 | 2223.242189 |
| **Dusp2** | 1877.177787 | 1834.08655 | 1847.477494 | 1938.283366 | 2287.074675 | 2314.662259 | 2432.868536 | 2087.642367 | 2093.968503 | 1852.672174 | 1908.76707 | 2455.972069 | 2403.729853 | 2576.034312 | 2334.619868 |
| **Astl** | 0 | 0.792262009 | 0 | 1.725218839 | 0 | 0 | 0 | 0 | 0 | 4.369509845 | 0 | 0 | 1.70416863 | 0 | 2.155697016 |
| **Gpat2** | 0 | 0 | 0 | 4.313047098 | 1.900089733 | 1.402825611 | 4.510323574 | 0 | 1.504828245 | 0 | 0 | 3.470112425 | 1.70416863 | 1.807109303 | 2.155697016 |
| **Fahd2a** | 47.61621704 | 92.694655 | 59.52025838 | 81.94789487 | 52.56914927 | 70.14128057 | 64.04659475 | 63.69362499 | 74.48899814 | 68.16435357 | 53.91462983 | 55.5217988 | 56.2375648 | 102.1016756 | 76.16796123 |
| **Kcnip3** | 20.14532259 | 44.36667248 | 29.76012919 | 36.22959563 | 29.13470924 | 23.84803539 | 18.94335901 | 27.65644243 | 56.43105919 | 19.22584332 | 8.512836289 | 26.89337129 | 14.48543336 | 33.43152211 | 26.58692986 |
| **Zfp661** | 44.8691276 | 58.62738863 | 36.80858084 | 33.64176737 | 53.20251252 | 46.99465798 | 45.10323574 | 32.68488651 | 50.41174621 | 35.82998073 | 40.67244005 | 41.6413491 | 42.60421576 | 66.86304422 | 38.80254629 |
| **Mrps5** | 1268.239627 | 1377.743633 | 1296.131942 | 1296.501958 | 1285.094023 | 1247.111969 | 1261.988536 | 1355.165679 | 1325.00127 | 1518.841622 | 1545.552722 | 1267.458563 | 1184.397198 | 1311.057799 | 1194.256147 |
| **Nphp1** | 59.5202713 | 73.68036679 | 54.82129061 | 68.14614415 | 53.20251252 | 56.81443726 | 50.51562403 | 67.88399506 | 61.69795805 | 83.89458901 | 69.99443171 | 39.90629289 | 58.79381775 | 38.85285002 | 57.48525376 |
| **1500011K16Rik** | 184.9706893 | 140.2303755 | 136.2700652 | 150.9566484 | 121.6057429 | 164.1305965 | 138.9179661 | 91.35006742 | 167.7883493 | 150.3111387 | 142.8264755 | 117.9838225 | 132.0730689 | 116.5585501 | 113.5333762 |
| **Bub1** | 1384.53308 | 1401.511493 | 1463.728459 | 1375.862024 | 1416.200214 | 1444.208967 | 1428.870508 | 1549.59885 | 1437.863388 | 1485.633347 | 1609.871929 | 1582.371266 | 1546.533032 | 1624.591264 | 1559.287508 |
| **Acoxl** | 38.45925223 | 21.39107423 | 24.27800013 | 12.93914129 | 16.46744435 | 29.45933784 | 27.06194144 | 5.866518091 | 6.019312981 | 21.84754922 | 19.86328467 | 20.82067455 | 38.34379418 | 37.04574071 | 31.61688957 |
| **Bcl2l11** | 1380.870294 | 1756.444873 | 1644.638718 | 1584.613504 | 1984.960408 | 1816.659167 | 2061.217873 | 908.4722302 | 1095.514962 | 977.0224012 | 947.7624402 | 2043.02869 | 2121.689945 | 2152.26718 | 1988.271214 |
| **Gm14005** | 32.04937685 | 45.15893448 | 45.42335508 | 31.05393911 | 69.03659363 | 42.08476834 | 46.00530045 | 42.74177467 | 36.86829201 | 34.08217679 | 26.48437957 | 48.58157395 | 62.20215501 | 57.8274977 | 76.16796123 |
| **Anapc1** | 4355.052466 | 4277.422584 | 4417.812862 | 4313.909708 | 3737.476504 | 3812.880012 | 4112.513035 | 4721.70899 | 4954.646997 | 4848.408123 | 4798.402055 | 4215.319068 | 4552.686496 | 3996.422224 | 4400.496175 |
| **Tmem87b** | 104.3893989 | 142.6071615 | 107.2930973 | 101.7879115 | 103.2382088 | 113.6288745 | 115.4642835 | 155.8817664 | 121.8910879 | 101.3726284 | 133.3677685 | 146.61225 | 120.1438884 | 131.9189791 | 125.0304269 |
| **Zc3h8** | 108.9678813 | 146.5684716 | 137.8363878 | 97.47486442 | 117.8055634 | 142.3867996 | 116.3663482 | 126.549176 | 127.9104008 | 173.9064918 | 148.5016997 | 130.996744 | 147.4105865 | 126.4976512 | 135.808912 |
| **Zc3h6** | 100.726613 | 112.5012052 | 93.19619404 | 77.63484777 | 92.47103366 | 70.84269338 | 99.22711863 | 108.9496217 | 52.66898858 | 61.17313782 | 62.42746612 | 86.75281063 | 82.65217857 | 96.68034772 | 82.63505228 |
| **Ttl** | 266.4676761 | 315.3202794 | 292.9023241 | 295.8750309 | 277.413101 | 258.8213253 | 266.1090909 | 266.5075362 | 321.2808303 | 283.1442379 | 278.0859854 | 281.0791064 | 292.2649201 | 290.9445978 | 290.3005315 |
| **Polr1b** | 2264.517399 | 2201.696122 | 2040.135172 | 2097.003499 | 2007.128121 | 2083.897446 | 2333.641417 | 2505.003225 | 2047.318828 | 2254.66708 | 2236.038332 | 1920.707227 | 2077.38156 | 1844.155044 | 2138.45144 |
| **Chchd5** | 203.2846189 | 192.5196681 | 194.224001 | 156.132305 | 164.0410803 | 192.8885216 | 192.1397842 | 201.1377631 | 194.1228436 | 189.6367273 | 178.7695621 | 155.287531 | 130.3689002 | 194.2642501 | 157.3658822 |
| **AI847159** | 10.98835778 | 18.2220262 | 25.06116142 | 25.87828259 | 24.70116653 | 17.53532014 | 18.94335901 | 28.49451644 | 16.5531107 | 37.57778466 | 27.43025026 | 18.21809023 | 29.82295103 | 37.94929537 | 24.43123285 |
| **Slc20a1** | 2264.517399 | 2377.578288 | 2269.601431 | 2366.137638 | 2392.846337 | 2247.32663 | 2475.265577 | 2612.276699 | 2496.510059 | 2342.057277 | 2512.232576 | 2286.804088 | 2339.823529 | 2147.749407 | 2388.512294 |
| **Ckap2l** | 1309.445969 | 1296.932908 | 1310.228846 | 1366.373321 | 1298.394651 | 1224.666759 | 1372.942496 | 1367.736789 | 1489.027549 | 1413.973386 | 1427.318884 | 1359.416543 | 1274.718135 | 1396.895491 | 1366.711908 |
| **Gm14023** | 0 | 0 | 1.566322589 | 0 | 1.266726489 | 2.805651223 | 1.80412943 | 1.676148026 | 2.257242368 | 0 | 0 | 0 | 0.852084315 | 0 | 0.718565672 |
| **Il1a** | 10.98835778 | 5.54583406 | 10.96425812 | 4.313047098 | 0.633363244 | 1.402825611 | 4.510323574 | 15.08533223 | 12.79104008 | 18.35194135 | 22.70089677 | 5.205168638 | 6.816674521 | 0 | 3.59282836 |

| | | | | | | | | | | | | | | | |
|---|---|---|---|---|---|---|---|---|---|---|---|---|---|---|---|
| 4932416H05Rik | 20.14532259 | 4.753572051 | 10.18109683 | 9.488703616 | 12.66726489 | 16.83390734 | 2.706194144 | 7.542666117 | 6.019312981 | 4.369509845 | 9.458706988 | 6.072696744 | 16.18960199 | 4.517773258 | 14.37131344 |
| Stk35 | 552.1649784 | 513.3857815 | 541.1644545 | 561.5587322 | 476.9225229 | 408.2222529 | 448.3261632 | 615.9843996 | 619.2368229 | 511.2326518 | 585.4939625 | 504.9013579 | 510.3985048 | 512.3154874 | 487.9060913 |
| Snrpb | 8201.893385 | 8272.799893 | 8095.538301 | 7851.470938 | 7789.101178 | 7970.855124 | 8123.994821 | 7818.392468 | 8167.455301 | 8284.590665 | 8154.351294 | 7995.139027 | 8103.321837 | 8338.002325 | 7789.97045 |
| Nop56 | 6978.522886 | 6850.689588 | 6768.079907 | 6327.240093 | 6654.747608 | 6464.220417 | 6776.310137 | 6676.935662 | 6238.26549 | 7043.649869 | 6870.804756 | 6187.210454 | 6563.60548 | 6220.973776 | 6350.683409 |
| Snord110 | 0 | 0 | 3.915806472 | 1.725218839 | 0.633363244 | 2.104238417 | 0 | 0 | 0 | 1.747803938 | 3.783482795 | 0 | 2.556252945 | 3.614218606 | 0 |
| Idh3b | 1848.791196 | 1815.864523 | 1982.964398 | 1918.443349 | 1753.782823 | 1797.019608 | 1786.088135 | 2278.723241 | 2132.341623 | 2063.282549 | 2021.325683 | 1871.258125 | 1739.104087 | 1800.784421 | 1710.904865 |
| Cpxm1 | 1.831392963 | 5.54583406 | 7.04845165 | 4.313047098 | 3.166816221 | 4.208476834 | 0.902064715 | 3.352296052 | 5.266898858 | 10.48682363 | 6.621094891 | 2.602584319 | 11.92918041 | 0.903554652 | 4.311394032 |
| Tmem239 | 0.915696482 | 0 | 0 | 0 | 1.266726489 | 1.402825611 | 0 | 0 | 0.752414123 | 0 | 0 | 0 | 0 | 4.517773258 | 2.155697016 |
| Pced1a | 251.8165324 | 229.7559825 | 227.8999367 | 222.5532303 | 207.1097809 | 203.4097137 | 199.356302 | 308.4112368 | 203.9042272 | 246.4403552 | 254.439218 | 207.3392174 | 253.0690416 | 234.0206548 | 227.0667523 |
| Vps16 | 1170.260103 | 1237.513257 | 1119.13749 | 1073.086118 | 1081.151058 | 1192.40177 | 1181.704776 | 1154.86599 | 1075.952195 | 1107.233795 | 1199.364046 | 1149.474741 | 1197.178463 | 1225.220108 | 1233.058693 |
| Ptpra | 618.0951251 | 729.6733098 | 730.6894878 | 703.026677 | 627.0296118 | 702.1142185 | 710.8269952 | 723.2578733 | 735.8610119 | 741.9427716 | 676.2975496 | 740.0014747 | 735.348764 | 826.7525062 | 721.4399347 |
| 4930473A02Rik | 0.915696482 | 3.169048034 | 4.698967767 | 3.450437679 | 0 | 0 | 2.706194144 | 10.05688816 | 0 | 5.243411813 | 3.783482795 | 6.94022485 | 0.852084315 | 1.807109303 | 4.311394032 |
| Mrps26 | 1057.629436 | 1273.95731 | 1173.95878 | 1323.24285 | 1211.623886 | 1210.638503 | 1204.256394 | 1062.677849 | 1333.277825 | 1375.521699 | 1217.335589 | 1277.001372 | 1212.51598 | 1261.362294 | 1157.609298 |
| Ubox5 | 306.7583213 | 259.0696768 | 278.0222595 | 289.836765 | 257.7788404 | 214.6323185 | 264.3049614 | 318.468125 | 282.9077101 | 247.3142572 | 261.0603129 | 287.1518032 | 253.0690416 | 234.9242094 | 253.6536822 |
| Fastkd5 | 243.5752641 | 287.5911091 | 337.5425179 | 339.0055019 | 229.2774944 | 251.1057844 | 248.0677966 | 282.4309424 | 297.2035784 | 284.0181399 | 310.2455892 | 225.5573076 | 258.1815475 | 223.1779989 | 209.1026105 |
| Lzts3 | 30.21798389 | 40.40536243 | 39.94122602 | 23.29045433 | 22.16771355 | 23.84803539 | 33.37639445 | 26.81836842 | 24.07725192 | 50.6863142 | 37.83482795 | 32.96606804 | 36.63962555 | 34.33507676 | 37.36541494 |
| Ddrgk1 | 996.2777719 | 1135.311458 | 1054.918264 | 982.512129 | 1060.250071 | 1107.53082 | 1177.194453 | 934.4525246 | 923.2121284 | 908.8580477 | 906.1441294 | 972.4990071 | 1019.092841 | 1021.016756 | 1101.561175 |
| Itpa | 1033.821328 | 1037.863231 | 1152.813425 | 1100.689619 | 1096.985139 | 967.2482591 | 954.3844682 | 1406.288194 | 1325.00127 | 1504.85919 | 1454.749135 | 1055.781705 | 1126.455465 | 1139.382416 | 1093.656953 |
| Slc4a11 | 27.47089445 | 26.14464628 | 25.84432272 | 35.36698621 | 23.43444004 | 11.9240177 | 13.53097072 | 35.19910855 | 54.17381683 | 38.45168663 | 34.05134516 | 22.55573076 | 10.22501178 | 8.131991864 | 11.49705075 |
| 4930402H24Rik | 68.67723612 | 80.01846286 | 100.2446457 | 66.42092531 | 93.10439691 | 90.48225194 | 101.0312481 | 81.29317927 | 66.96485691 | 64.6687457 | 53.91462983 | 85.88528252 | 115.8834669 | 91.25901981 | 94.8506687 |
| Gm14057 | 11.90405426 | 1.584524017 | 13.31374201 | 19.84001665 | 7.600358931 | 5.611302446 | 9.922711863 | 3.352296052 | 12.03862596 | 8.739019689 | 1.891741398 | 6.94022485 | 16.18960199 | 5.421327909 | 2.874262688 |
| A730017L22Rik | 30.21798389 | 35.65179038 | 29.76012919 | 31.05393911 | 28.50134599 | 29.45933784 | 38.78878274 | 42.74177467 | 48.90691797 | 40.19949057 | 21.75502607 | 19.08561834 | 43.45630007 | 28.91374885 | 45.98820301 |
| Atrn | 557.6591573 | 526.0619736 | 569.3582611 | 515.840433 | 488.3230613 | 495.8988536 | 549.3574113 | 582.4614391 | 573.3395614 | 586.3882211 | 530.633462 | 572.5685501 | 596.4590206 | 548.4576735 | 547.547042 |
| Hspa12b | 4.578482408 | 15.84524017 | 7.04845165 | 3.450437679 | 0.633363244 | 1.402825611 | 10.82477658 | 5.866518091 | 1.504828245 | 13.9824315 | 10.40457769 | 0.867528106 | 5.112505891 | 1.807109303 | 8.622788064 |
| 1700037H04Rik | 436.7872217 | 450.7970828 | 455.7998734 | 425.2664439 | 487.0563348 | 468.5437542 | 503.3521108 | 472.6737434 | 473.2684831 | 429.0858667 | 418.0748489 | 500.5637173 | 507.8422518 | 520.4474793 | 513.7744555 |
| Spef1 | 31.13368037 | 43.57441047 | 39.94122602 | 56.9322217 | 39.90188439 | 39.27911712 | 33.37639445 | 15.92340625 | 35.36346376 | 37.57778466 | 17.97154328 | 38.17123668 | 51.12505891 | 45.17773258 | 32.33545524 |
| Cenpb | 2068.558352 | 2243.686008 | 2261.769818 | 2259.17407 | 2070.464446 | 2053.736695 | 2118.950015 | 2315.598498 | 2236.174772 | 2335.066061 | 2098.887081 | 2165.350153 | 2217.123388 | 2031.190857 | 2117.613035 |
| Cdc25b | 1425.739422 | 1822.994882 | 1874.888139 | 1696.752728 | 1788.617802 | 1832.090249 | 1953.872172 | 1487.581373 | 1440.873045 | 1310.852953 | 1449.073911 | 1941.527902 | 1974.279358 | 2012.216209 | 1897.013374 |
| Ap5s1 | 149.2585265 | 128.3464454 | 133.1374201 | 119.9027093 | 110.8385677 | 138.1783227 | 93.81473034 | 117.3303618 | 161.7690364 | 149.4372367 | 148.5016997 | 120.5864068 | 126.960563 | 132.8225338 | 106.3477195 |
| Mavs | 507.2958508 | 557.752454 | 526.2843899 | 561.5587322 | 521.8913133 | 479.7663591 | 560.1821879 | 587.4898832 | 500.3553915 | 540.0714168 | 593.0609281 | 537.8674259 | 506.1380832 | 523.1581433 | 579.8824973 |
| Pank2 | 896.4668555 | 816.8221308 | 852.0794884 | 847.9450595 | 765.7361623 | 871.1547047 | 878.6110322 | 755.1046858 | 822.388636 | 852.0544197 | 816.286413 | 787.7155205 | 836.7467975 | 816.813405 | 773.8952287 |
| Rnf24 | 76.00280797 | 45.95119649 | 56.3876132 | 56.06961228 | 38.6351579 | 35.77205309 | 49.61355931 | 68.72206907 | 66.21244279 | 53.3080201 | 62.42746612 | 45.97898963 | 46.86463733 | 40.65995932 | 43.11394032 |
| Smox | 314.9995897 | 205.9881222 | 243.5631626 | 188.9114629 | 190.6423365 | 232.8690515 | 248.9698613 | 77.94088321 | 97.81383594 | 139.824315 | 132.4218978 | 196.9288801 | 207.0564886 | 163.5433919 | 236.4081061 |
| Prnp | 639.1561441 | 642.5244889 | 637.4932937 | 613.3152974 | 389.5183952 | 481.8705975 | 446.5220338 | 704.820245 | 631.2754488 | 655.4264767 | 703.7277999 | 471.9352898 | 506.1380832 | 483.4017386 | 509.4630614 |
| Rassf2 | 1126.306672 | 1143.234078 | 1233.479039 | 1168.835764 | 1294.594471 | 1248.514794 | 1454.12832 | 944.5094127 | 1045.85563 | 1049.556265 | 1135.044839 | 1294.351935 | 1292.611906 | 1333.646666 | 1261.80132 |
| Slc23a2 | 781.0890988 | 777.2090303 | 824.6688431 | 728.0423502 | 665.6647697 | 671.2520551 | 736.986872 | 718.2294292 | 753.1665367 | 618.722594 | 731.1580501 | 713.1081034 | 762.6154621 | 618.0313817 | 682.6373884 |
| Tmem230 | 209.6944943 | 214.7030043 | 259.2263885 | 226.0036679 | 227.3774047 | 223.0492722 | 203.8666255 | 232.1465016 | 244.5345898 | 210.6103745 | 204.3080709 | 239.4377573 | 211.3169102 | 244.8633106 | 276.6477837 |
| Pcna | 9724.696634 | 10087.07989 | 10099.64805 | 9916.557888 | 10235.15003 | 10491.73275 | 10122.97023 | 9993.194532 | 10140.28513 | 10353.99053 | 10016.7707 | 10759.08357 | 10538.57881 | 10988.12812 | 10210.8182 |
| Cds2 | 2714.124371 | 2665.961659 | 2722.26866 | 2622.332636 | 2589.822306 | 2652.743231 | 2842.405916 | 2577.915664 | 2419.763818 | 2417.212846 | 2621.953577 | 2861.975223 | 2993.372199 | 2702.531963 | 2867.077031 |
| Gpcpd1 | 370.857075 | 507.8399475 | 528.6338738 | 452.8699453 | 500.356963 | 526.7610171 | 508.7644991 | 465.9691513 | 451.4484735 | 437.8248864 | 454.0179354 | 547.4102351 | 535.9610342 | 489.7266212 | 515.2115868 |
| 1110034G24Rik | 70.50862908 | 64.17322269 | 43.85703249 | 77.63484777 | 57.63605523 | 74.34975741 | 64.94865946 | 82.13125328 | 60.94554393 | 61.17313782 | 49.18527634 | 61.59449555 | 62.20215501 | 76.80214538 | 61.07808212 |
| Trmt6 | 1176.669979 | 1292.179336 | 1253.058071 | 1265.448019 | 1181.855814 | 1177.672101 | 1262.890601 | 1622.511289 | 1450.654428 | 1649.926917 | 1546.498592 | 1438.3616 | 1183.545114 | 1268.590731 | 1179.884833 |
| Mcm8 | 299.4327495 | 374.73993 | 357.9047116 | 387.3116294 | 336.3158827 | 344.3936876 | 348.1969799 | 396.4090082 | 395.0174144 | 426.4641608 | 405.7785298 | 314.0451745 | 337.4253888 | 350.5792048 | 368.6241897 |
| Crls1 | 510.0429402 | 505.4631614 | 551.3455513 | 602.9639843 | 533.9252149 | 629.1672867 | 541.2388289 | 667.1069144 | 610.2078534 | 604.7401625 | 721.6993432 | 491.0209082 | 552.1506362 | 511.4119328 | 562.6369211 |
| Lrrn4 | 8.241268334 | 1.584524017 | 5.482129061 | 1.725218839 | 8.233722176 | 6.312715251 | 11.72684129 | 3.352296052 | 7.524141226 | 0.873901969 | 2.837612096 | 6.072696744 | 5.964590206 | 9.939101167 | 12.9341821 |
| Tmx4 | 315.9152861 | 303.4363493 | 286.6370338 | 281.2106708 | 286.2801864 | 295.2947912 | 355.4134976 | 262.3171661 | 296.4511643 | 287.5137478 | 289.4364338 | 281.9466345 | 318.6795339 | 316.2441281 | 277.3663494 |
| Plcb4 | 5.494178889 | 6.338096068 | 1.566322589 | 7.763484777 | 3.166816221 | 3.507064029 | 2.706194144 | 5.866518091 | 9.781383594 | 10.48682363 | 6.621094891 | 6.072696744 | 6.816674521 | 2.710663955 | 2.874262688 |
| Mkks | 234.4182993 | 246.3934846 | 267.0580014 | 275.1724049 | 256.5121139 | 261.6269765 | 255.2843143 | 244.7176118 | 279.8980536 | 260.4227867 | 230.7924505 | 249.8480946 | 225.8023435 | 286.4268245 | 243.5937628 |
| Slx4ip | 106.2207919 | 111.7089432 | 139.4027104 | 134.5670695 | 91.20430718 | 103.1076824 | 116.3663482 | 89.6739194 | 82.01313936 | 111.859452 | 102.1540355 | 63.32955176 | 94.58135898 | 84.0305826 | 114.2519418 |
| Jag1 | 0 | 0 | 0 | 0 | 0 | 1.402825611 | 1.80412943 | 8.380740131 | 2.257242368 | 1.747803938 | 0 | 1.735056213 | 8.520843152 | 0 | 0.718565672 |
| Btbd3 | 0.915696482 | 0 | 0 | 0.86260942 | 0 | 0 | 3.608258859 | 0 | 0 | 5.243411813 | 1.891741398 | 0 | 0 | 0 | 0 |
| Ism1 | 4.578482408 | 3.169048034 | 6.265290356 | 1.725218839 | 1.266726489 | 3.507064029 | 4.510323574 | 3.352296052 | 7.524141226 | 5.243411813 | 8.512836289 | 3.470112425 | 5.112505891 | 2.710663955 | 5.029959704 |
| Tasp1 | 557.6591573 | 610.8340086 | 606.1668419 | 533.0926213 | 462.9885316 | 547.8034013 | 533.1202464 | 700.6298749 | 674.915468 | 658.0481826 | 608.1948593 | 633.2955176 | 604.9798638 | 642.4273573 | 556.1698301 |
| Esf1 | 1292.963432 | 1207.407301 | 1184.139877 | 1164.522717 | 1051.382985 | 1040.896604 | 1263.792665 | 1260.463316 | 1285.123321 | 1213.849835 | 1113.289812 | 1206.731596 | 1123.047127 | 1140.28597 | 1125.992408 |
| Ndufaf5 | 193.2119576 | 223.4178864 | 187.9587107 | 211.3393078 | 181.1418879 | 235.6747027 | 208.3769491 | 230.4703536 | 218.2000955 | 260.4227867 | 216.60439 | 191.7237115 | 193.4231395 | 209.6246792 | 176.7671553 |
| Macrod2 | 7.325571853 | 9.507144102 | 2.349483883 | 7.763484777 | 12.03390164 | 9.118366474 | 1.80412943 | 13.40918421 | 1.504828245 | 10.48682363 | 8.512836289 | 4.337640531 | 12.78126473 | 10.84265582 | 6.467091048 |
| Kif16b | 498.138886 | 499.1250654 | 640.6259389 | 475.2977902 | 452.8547197 | 465.738103 | 489.8211401 | 558.9953667 | 522.9278152 | 512.1065538 | 554.2802295 | 469.3327055 | 476.3151322 | 455.3915444 | 435.4507972 |
| Kif16bos | 1.831392963 | 0 | 0 | 0 | 0.633363244 | 0 | 0 | 0 | 0 | 0 | 0 | 0 | 0 | 0 | 1.437131344 |
| Snrpb2 | 1422.992332 | 1294.556122 | 1491.922266 | 1305.990661 | 1429.500842 | 1425.270821 | 1406.31889 | 1552.951146 | 1421.310278 | 1349.30464 | 1403.672117 | 1290.014294 | 1448.543336 | 1346.296431 | 1325.753665 |
| Dstn | 460.5953302 | 457.9274409 | 460.4988412 | 439.930804 | 473.1223435 | 436.2787652 | 477.1922341 | 576.594921 | 487.5643514 | 501.6197302 | 591.1691867 | 455.4522558 | 415.8171458 | 492.4372851 | 501.558839 |
| Rrbp1 | 2526.406593 | 2215.164576 | 2091.823818 | 2087.514796 | 2023.595565 | 1876.980668 | 2095.496332 | 1976.178523 | 1876.520822 | 1851.798272 | 2108.345788 | 2016.135319 | 1924.006384 | 1919.15008 | 2016.295276 |
| Snx5 | 5485.937621 | 5382.628086 | 5509.539707 | 5104.922545 | 5001.66954 | 5365.807964 | 5224.758828 | 5524.583894 | 5230.030566 | 6072.744782 | 5732.922305 | 5206.903694 | 5290.591513 | 5247.845416 | 5195.948374 |
| Snord17 | 8.241268334 | 10.29940611 | 8.614774239 | 10.35131304 | 6.966995687 | 14.72966892 | 9.020647148 | 10.05688816 | 12.03862596 | 16.60413741 | 12.29631908 | 13.01292159 | 13.63334904 | 9.035546516 | 15.80844478 |
| Mgme1 | 271.0461585 | 302.6440872 | 319.5298082 | 315.7150476 | 290.7137291 | 346.497926 | 333.7639445 | 292.4878306 | 362.6636071 | 280.522532 | 306.4621064 | 320.1178712 | 364.6920869 | 255.7059664 | 298.2047539 |
| Csrp2bp | 734.3885782 | 753.4411701 | 835.6331012 | 775.4858683 | 769.5363418 | 746.3032253 | 792.9148843 | 865.7304555 | 767.462405 | 770.7815366 | 966.6798541 | 794.6557454 | 845.2676406 | 815.9098504 | 734.3741167 |
| Zfp133-ps | 3.662785926 | 0.792262009 | 0.783161294 | 2.587828259 | 1.900089733 | 0 | 5.412388289 | 0 | 0 | 6.117313782 | 2.837612096 | 0 | 0 | 0.903554652 | 0 |
| Dzank1 | 5.494178889 | 4.753572051 | 4.698967767 | 12.07653188 | 8.233722176 | 0 | 1.80412943 | 1.676148026 | 5.266898858 | 1.747803938 | 12.29631908 | 11.27786538 | 5.112505891 | 14.45687443 | 2.874262688 |
| Polr3f | 626.3363934 | 634.6018688 | 644.5417454 | 627.1170481 | 585.2276377 | 562.5330702 | 630.5432356 | 684.7064687 | 692.9734069 | 641.4440452 | 784.1268093 | 623.7527084 | 683.3716208 | 618.0313817 | 572.6968406 |
| Rbbp9 | 88.82255871 | 97.44822705 | 85.3645811 | 89.71137964 | 86.13740122 | 84.87094949 | 73.0672419 | 86.32162334 | 90.28969471 | 85.64239295 | 89.85771638 | 72.00483282 | 66.46257658 | 94.87323842 | 71.13800152 |
| Sec23b | 1862.526644 | 1842.801432 | 1846.694332 | 1863.236346 | 1756.94964 | 1756.337666 | 1801.423235 | 1816.94446 | 1922.418083 | 1877.141429 | 1950.385381 | 1794.915652 | 1764.666617 | 1751.088915 | 1828.031069 |
| Gm561 | 133.6916863 | 87.14882094 | 85.3645811 | 100.0626927 | 89.93758069 | 96.79496719 | 72.16517718 | 98.05465953 | 96.30900769 | 113.607256 | 87.02010429 | 91.09045116 | 101.3980335 | 82.22347329 | 81.19792093 |
| Dtd1 | 405.6535413 | 384.2470741 | 454.2335508 | 418.3655685 | 407.2525661 | 383.6728047 | 413.1456394 | 409.8181924 | 523.6802293 | 428.2119648 | 477.6647029 | 447.6445028 | 405.592134 | 402.08182 | 407.426736 |
| Slc24a3 | 9.156964816 | 20.59881222 | 14.8800646 | 10.35131304 | 8.86708542 | 5.611302446 | 7.216517718 | 16.76148026 | 10.53379772 | 16.60413741 | 34.99721585 | 6.072696744 | 10.22501178 | 7.228437213 | 10.77848508 |
| Rin2 | 4.578482408 | 4.753572051 | 1.566322589 | 0.86260942 | 2.533452977 | 0 | 0 | 7.542666117 | 6.019312981 | 2.621705907 | 1.891741398 | 0.867528106 | 0 | 0.903554652 | 1.437131344 |
| Naa20 | 1400.09992 | 1469.646026 | 1389.328136 | 1309.441099 | 1209.090433 | 1327.073028 | 1382.865208 | 1252.92065 | 1334.030239 | 1212.975933 | 1258.9539 | 1319.51025 | 1288.351485 | 1201.727687 | 1417.730071 |
| Crnkl1 | 1152.86187 | 1083.814428 | 1196.670458 | 1111.903542 | 1091.918233 | 1083.682785 | 1150.132511 | 1091.172365 | 1208.377081 | 1039.943343 | 1106.668718 | 1109.568448 | 1120.490874 | 1136.671752 | 1090.78269 |
| Insm1 | 1.831392963 | 1.584524017 | 2.349483883 | 11.21392246 | 3.800179466 | 7.715540863 | 1.80412943 | 9.218814144 | 0 | 16.60413741 | 6.621094891 | 8.675281063 | 5.112505891 | 12.64976512 | 2.874262688 |
| Ralgapa2 | 371.7727715 | 456.3429169 | 391.5806472 | 392.4872859 | 340.1160622 | 300.9060937 | 387.8878274 | 313.4396809 | 379.2167178 | 366.164925 | 359.4308655 | 355.6865236 | 318.6795339 | 311.7263548 | 321.1988554 |
| A930019D19Rik | 0 | 0 | 0 | 0 | 1.266726489 | 0.701412806 | 0 | 0 | 0.752414123 | 3.495607876 | 0.945870699 | 0 | 0 | 2.710663955 | 0 |
| Kiz | 70.50862908 | 83.18751089 | 78.31612945 | 82.81050429 | 82.970585 | 65.23139093 | 68.55691832 | 57.8271069 | 66.21244279 | 29.71266694 | 50.13114703 | 81.54764199 | 71.57508247 | 94.87323842 | 84.79074929 |
| Xrn2 | 3736.957341 | 3610.337973 | 3820.260795 | 3699.731801 | 3663.373005 | 3675.403102 | 3904.136086 | 3695.906398 | 3467.876691 | 3679.127289 | 3756.052545 | 3971.543671 | 3890.616983 | 3680.178096 | 3783.248263 |
| Cd93 | 18.31392963 | 10.29940611 | 28.9769679 | 25.01567317 | 18.36753408 | 15.43108173 | 13.53097072 | 10.89496217 | 6.019312981 | 2.621705907 | 11.35044839 | 17.35056213 | 10.22501178 | 5.421327909 | 15.08987911 |
| Nxt1 | 757.2809903 | 706.6977116 | 717.3757458 | 781.5241342 | 760.6692564 | 721.7537771 | 697.2960245 | 782.7611282 | 694.4782351 | 750.6817913 | 761.4259125 | 783.37788 | 838.4509661 | 789.7067655 | 701.3200958 |
| Gzf1 | 487.1505282 | 557.752454 | 432.3050346 | 445.1064605 | 400.2855704 | 458.0225621 | 501.5479814 | 391.3805641 | 325.042901 | 413.3556313 | 348.0804171 | 463.2600088 | 466.0901204 | 459.9093177 | 455.570636 |
| Napb | 28.38659093 | 18.2220262 | 32.10961307 | 15.52696955 | 27.86798275 | 21.04238417 | 52.31975346 | 17.59955427 | 31.60139315 | 20.97364725 | 31.21373306 | 32.09853993 | 28.97086672 | 41.56351397 | 40.9582433 |
| Cst3 | 1105.245653 | 1048.954899 | 943.7093599 | 898.8390153 | 946.2446869 | 1015.645743 | 952.5803388 | 823.8267548 | 836.6845043 | 956.9226559 | 861.6882066 | 943.8705796 | 958.5948546 | 936.082619 | 1008.147638 |
| Zfp120 | 215.1886732 | 240.0553886 | 203.6219366 | 210.4766984 | 230.5442209 | 196.3955856 | 212.8872727 | 202.8139112 | 196.380086 | 139.824315 | 178.7695621 | 199.5314644 | 184.9022964 | 234.9242094 | 217.0068329 |
| Gm14139 | 4.578482408 | 1.584524017 | 2.349483883 | 5.175656518 | 0 | 2.805651223 | 4.510323574 | 0 | 1.504828245 | 2.621705907 | 0.945870699 | 4.337640531 | 0.852084315 | 1.807109303 | 0.718565672 |
| Zfp937 | 11.90405426 | 11.09166812 | 17.22954848 | 18.11479781 | 15.20071786 | 16.13249453 | 21.64955315 | 15.08533223 | 24.07725192 | 14.85633347 | 19.86328467 | 17.35056213 | 20.45002356 | 15.36042908 | 25.86836419 |
| Gm14124 | 0 | 0 | 0.783161294 | 0 | 0.633363244 | 0 | 0 | 0 | 0 | 0 | 4.729353494 | 0 | 0 | 0 | 0 |
| 3300002I08Rik | 5.494178889 | 14.26071615 | 14.8800646 | 10.35131304 | 8.86708542 | 9.118366474 | 5.412388289 | 1.676148026 | 8.276555348 | 1.747803938 | 3.783482795 | 9.542809169 | 12.78126473 | 17.16753838 | 3.59282836 |
| Zfp442 | 8.241268334 | 11.88393013 | 13.31374201 | 11.21392246 | 4.43354271 | 6.312715251 | 10.82477658 | 9.218814144 | 1.504828245 | 1.747803938 | 3.783482795 | 5.205168638 | 13.63334904 | 0.903554652 | 5.748525376 |
| Zfp345 | 0.915696482 | 0 | 0 | 0 | 2.533452977 | 0 | 0.902064715 | 0 | 0 | 0 | 0.945870699 | 1.735056213 | 0 | 3.614218606 | 2.874262688 |
| Cst7 | 1062.207919 | 1005.380489 | 1025.158134 | 1021.329553 | 1038.715721 | 941.2959853 | 1050.905393 | 1242.025687 | 1134.640497 | 1091.503559 | 1036.674286 | 893.5539495 | 969.6719507 | 1063.483825 | 944.9138586 |
| Apmap | 599.7811954 | 657.5774671 | 669.6029068 | 602.9639843 | 575.727189 | 609.5277282 | 619.7184591 | 573.2426249 | 594.4071568 | 670.2828101 | 600.6278937 | 674.9368667 | 622.0215501 | 637.0060294 | 586.3495883 |
| Acss1 | 206.0317084 | 211.5339563 | 241.99684 | 174.2471028 | 183.6753408 | 199.2012368 | 217.3975963 | 112.3019177 | 156.5021375 | 119.7245697 | 146.6099583 | 231.6300044 | 227.5065121 | 236.7313187 | 191.8570344 |
| E130215H24Rik | 0.915696482 | 6.338096068 | 3.132645178 | 0.86260942 | 0 | 2.104238417 | 4.510323574 | 1.676148026 | 0 | 0 | 0 | 0 | 0 | 2.710663955 | 3.59282836 |
| Entpd6 | 456.0168478 | 475.3572051 | 551.3455513 | 535.6804496 | 543.4256636 | 500.8087433 | 573.7131586 | 470.1595213 | 524.4326434 | 438.6987884 | 506.9866945 | 545.6751788 | 501.8776616 | 571.0465398 | 536.768557 |
| Pygb | 2465.054928 | 2653.285466 | 2470.873884 | 2436.871611 | 2606.28975 | 2539.815769 | 2564.569984 | 2221.734209 | 2394.181738 | 2184.754922 | 2185.907185 | 2547.06252 | 2619.307185 | 2544.409899 | 2468.991649 |
| Abhd12 | 610.7695532 | 630.6405588 | 644.5417454 | 597.7883278 | 656.1643211 | 631.2715251 | 628.7391062 | 605.0894374 | 740.3754966 | 648.4352609 | 702.7819292 | 651.5136078 | 681.6674521 | 598.1531793 | 618.6850436 |
| Gins1 | 462.4267232 | 501.5018514 | 511.4043253 | 490.8247598 | 476.2891597 | 493.7946152 | 480.800493 | 471.8356693 | 521.4229869 | 485.0155927 | 501.3114703 | 489.2858519 | 441.3796753 | 528.5794712 | 414.6123927 |
| Ninl | 7.325571853 | 14.26071615 | 30.54329049 | 8.626094197 | 22.16771355 | 21.04238417 | 18.0412943 | 23.46607237 | 21.06759543 | 22.72145119 | 15.13393118 | 20.82067455 | 13.63334904 | 15.36042908 | 12.21561642 |
| Nanp | 14.65114371 | 15.05297816 | 15.66322589 | 12.07653188 | 23.43444004 | 25.25086101 | 10.82477658 | 5.028444078 | 9.028969471 | 10.48682363 | 6.621094891 | 13.01292159 | 3.408337261 | 18.07109303 | 7.904222392 |
| Nsfl1c | 2257.191827 | 2184.266357 | 2115.318656 | 1922.756396 | 2053.997001 | 2107.745481 | 2099.104591 | 2277.885167 | 2143.627835 | 2162.033471 | 2143.343003 | 2011.797678 | 2016.03149 | 2220.937334 | 2073.780529 |
| Fkbp1a | 7109.467483 | 6656.585395 | 6688.980616 | 6228.04001 | 6077.120329 | 6301.492647 | 6230.560985 | 6982.832677 | 6607.700825 | 6901.203848 | 6655.146237 | 6265.287984 | 6159.717514 | 6177.603153 | 6224.934416 |
| Sdcbp2 | 54.94178889 | 21.39107423 | 28.9769679 | 41.40525214 | 24.70116653 | 16.13249453 | 40.59291217 | 32.68488651 | 36.11587788 | 27.09096104 | 32.15960376 | 29.49595561 | 24.71044514 | 23.49242094 | 29.46119255 |
| Tmem74b | 8.241268334 | 5.54583406 | 1.566322589 | 5.175656518 | 3.800179466 | 4.208476834 | 4.510323574 | 0 | 0 | 2.621705907 | 4.729353494 | 1.735056213 | 1.70416863 | 0 | 2.155697016 |
| Psmf1 | 1130.885155 | 1230.382899 | 1131.668071 | 1192.988827 | 1161.58819 | 1059.834749 | 1082.477658 | 1114.638437 | 1173.766031 | 1137.820364 | 1048.970605 | 1074.867324 | 1130.715886 | 1096.011792 | 1080.004205 |
| Fam110a | 464.2581162 | 457.9274409 | 437.7871636 | 364.8837845 | 397.1187542 | 416.6392066 | 443.8158397 | 320.144273 | 339.3387693 | 339.9478659 | 360.3767362 | 351.348883 | 432.8588321 | 391.2391641 | 377.9655435 |
| Slc52a3 | 67.76153964 | 83.18751089 | 75.18348427 | 74.18441009 | 103.8715721 | 99.60061841 | 98.32505391 | 22.62799835 | 40.63036262 | 27.964863 | 37.83482795 | 79.81258578 | 69.01882953 | 85.8376919 | 71.13800152 |
| Srxn1 | 273.793248 | 289.9678951 | 300.7339371 | 309.6767817 | 191.2756998 | 157.8178813 | 152.4489368 | 587.4898832 | 543.2429965 | 517.3499656 | 530.633462 | 199.5314644 | 204.5002356 | 175.2896024 | 222.7553583 |
| Csnk2a1 | 2963.193814 | 2810.945606 | 2795.885821 | 2657.699622 | 2829.233612 | 2562.260979 | 2811.735716 | 3046.399037 | 3057.810994 | 2897.858929 | 2863.150605 | 2626.875106 | 2874.932479 | 2795.598092 | 2663.00438 |
| Tbc1d20 | 750.8711149 | 852.4739212 | 949.9746502 | 751.3328045 | 773.3365213 | 751.2131149 | 789.3066254 | 807.0652746 | 901.3921189 | 850.3066157 | 854.121241 | 803.3310264 | 807.7759308 | 788.8032108 | 822.7576944 |
| Rbck1 | 3724.137591 | 3889.2142 | 3571.215503 | 3717.846599 | 3472.730668 | 3606.664647 | 3666.893066 | 3760.438097 | 3673.285746 | 3724.570191 | 3723.892941 | 3578.553438 | 3685.264663 | 3563.619546 | 3786.122526 |
| Trib3 | 1170.260103 | 877.0340434 | 863.0437465 | 781.5241342 | 755.6023504 | 895.7041529 | 800.131402 | 392.2186381 | 515.403674 | 522.5933774 | 594.9526695 | 667.1291137 | 722.5674993 | 588.2140782 | 707.0686212 |
| Sox12 | 198.7061365 | 156.8678777 | 191.0913559 | 199.2627759 | 160.2409008 | 159.9221197 | 181.3150077 | 176.8336168 | 161.7690364 | 163.4196682 | 171.2025965 | 198.6639363 | 167.0085258 | 93.06612911 | 174.6114583 |
| Zcchc3 | 95.23243408 | 94.27917901 | 111.9920651 | 89.71137964 | 83.60394824 | 72.94693179 | 73.96930661 | 67.88399506 | 113.6145325 | 98.75092249 | 119.179708 | 105.838429 | 99.69386487 | 93.06612911 | 85.50931496 |
| H13 | 4507.058082 | 4472.319038 | 4451.488798 | 4576.142971 | 4318.903963 | 4578.121383 | 4518.442156 | 4078.068148 | 4157.840441 | 4191.233843 | 4054.001815 | 4417.453117 | 4585.0657 | 4405.732481 | 4123.848391 |
| Mcts2 | 17.39823315 | 20.59881222 | 24.27800013 | 12.07653188 | 19.00089733 | 11.9240177 | 18.94335901 | 13.40918421 | 10.53379772 | 22.72145119 | 18.91741398 | 19.08561834 | 20.45002356 | 12.64976512 | 22.9941015 |
| Id1 | 0 | 3.169048034 | 3.915806472 | 0 | 4.43354271 | 4.90988964 | 3.608258859 | 2.514222039 | 3.00965649 | 0 | 0.945870699 | 1.735056213 | 3.408337261 | 1.807109303 | 0 |
| Cox4i2 | 0 | 0 | 0 | 1.725218839 | 0 | 1.402825611 | 0 | 0.838074013 | 0 | 7.86511772 | 0 | 1.735056213 | 0.852084315 | 0 | 7.18565672 |
| Bcl2l1 | 2838.659093 | 2774.501554 | 2732.449756 | 2594.729134 | 2755.763476 | 3074.292327 | 3023.720924 | 3677.468769 | 2953.225431 | 2957.284263 | 2920.848718 | 2817.731289 | 2815.286577 | 2701.628408 | 2677.375694 |
| Tpx2 | 4284.543837 | 4378.832121 | 4371.606346 | 4441.575902 | 4132.695169 | 4061.180145 | 4335.323019 | 4316.081167 | 4381.307436 | 4532.055611 | 4742.595684 | 4382.751993 | 4204.184011 | 4392.179161 | 4251.753081 |
| Mylk2 | 0.915696482 | 0 | 1.566322589 | 0 | 0.633363244 | 0 | 3.608258859 | 1.676148026 | 0 | 3.495607876 | 0.945870699 | 1.735056213 | 1.70416863 | 0 | 0 |
| Ttll9 | 5.494178889 | 7.130358077 | 7.04845165 | 11.21392246 | 12.66726489 | 4.90988964 | 6.314453003 | 7.542666117 | 9.028969471 | 2.621705907 | 3.783482795 | 6.072696744 | 10.22501178 | 7.228437213 | 9.341353736 |
| Pdrg1 | 454.1854549 | 446.8357728 | 472.2462606 | 519.2908706 | 499.7235997 | 542.8935116 | 508.7644991 | 596.7086973 | 587.6354297 | 568.9101818 | 598.7361523 | 548.2777632 | 538.5172872 | 583.6963049 | 573.4154062 |
| Hck | 70.50862908 | 111.7089432 | 76.74980686 | 95.74964558 | 14.56735462 | 16.83390734 | 17.13922958 | 113.9780658 | 143.7110974 | 109.2377461 | 140.9347341 | 17.35056213 | 15.33751767 | 9.939101167 | 19.40127314 |
| Tm9sf4 | 1743.486101 | 1739.807371 | 1736.26859 | 1619.117881 | 1470.669453 | 1550.122301 | 1608.381386 | 1842.924755 | 1776.449743 | 1685.756898 | 1640.139792 | 1520.77677 | 1503.928816 | 1412.25592 | 1493.179466 |
| Tspyl3 | 776.5106164 | 801.7691526 | 773.763359 | 776.3484777 | 799.3044143 | 785.5823424 | 863.275932 | 812.9317927 | 813.3596665 | 752.4295952 | 777.5057144 | 792.9206891 | 775.3967268 | 763.5036806 | 704.9129242 |
| Plagl2 | 3210.431864 | 2724.589047 | 2854.622918 | 2869.901539 | 2344.077367 | 2302.036828 | 2505.935778 | 3820.779426 | 3134.557235 | 3134.686362 | 3054.216486 | 2203.52139 | 2436.109057 | 2368.216742 | 2415.099223 |
| Pofut1 | 1088.763117 | 1051.331685 | 1085.461554 | 1084.300041 | 912.676435 | 870.4532919 | 1048.199199 | 1078.601255 | 1083.476337 | 1130.829148 | 1078.292597 | 913.5070959 | 927.0677349 | 980.356797 | 879.5243825 |
| Kif3b | 1241.684429 | 1155.118008 | 1188.838845 | 1194.714046 | 1110.285767 | 1124.364728 | 1210.570847 | 1380.3079 | 1210.634323 | 1219.967149 | 1188.959468 | 1090.48283 | 1129.011718 | 1060.773161 | 1139.645156 |
| 2500004C02Rik | 23.80810852 | 41.19762444 | 17.22954848 | 30.19132969 | 15.83408111 | 11.9240177 | 12.62890601 | 25.9802944 | 31.60139315 | 34.95607876 | 24.59263817 | 14.74797781 | 16.18960199 | 18.97464768 | 18.68270747 |
| Asxl1 | 2814.850984 | 2890.964069 | 2871.852467 | 2854.37457 | 2606.28975 | 2704.647779 | 2912.766964 | 2639.095067 | 2673.327378 | 2500.233533 | 2686.272785 | 2757.004322 | 2799.94906 | 2576.034312 | 2794.501898 |
| Nol4l | 83.32837982 | 68.92679474 | 81.44877463 | 50.89395576 | 75.37022607 | 92.58649035 | 90.20647148 | 133.2537681 | 120.3862596 | 129.3374914 | 104.9916476 | 95.42809169 | 103.9542864 | 83.12702794 | 112.0962448 |
| Commd7 | 683.1095752 | 742.349502 | 776.8960041 | 767.7223835 | 675.7985816 | 727.3650795 | 764.9508781 | 812.0937186 | 738.1182543 | 839.8197921 | 761.4259125 | 756.4845087 | 702.96956 | 750.8539155 | 670.4217719 |
| Dnmt3b | 471.583688 | 522.1006636 | 517.6696157 | 513.2526047 | 478.8226127 | 499.4059177 | 534.0223112 | 514.577444 | 590.6450862 | 507.7370439 | 545.7673932 | 467.5976493 | 496.7651557 | 468.0413095 | 492.936051 |
| Mapre1 | 4189.311403 | 4115.801134 | 4359.075765 | 4196.594827 | 4085.826289 | 4105.369152 | 4347.951925 | 4845.743943 | 4762.781396 | 4539.046826 | 4573.284829 | 4424.393342 | 4266.386166 | 4359.651194 | 4233.070374 |
| Efcab8 | 0 | 0 | 0 | 1.725218839 | 0.633363244 | 0 | 0.902064715 | 1.676148026 | 0 | 0 | 1.891741398 | 0 | 3.408337261 | 0.903554652 | 0 |
| Cdk5rap1 | 212.4415837 | 228.1714584 | 255.310582 | 223.4158397 | 326.815434 | 332.4696699 | 294.073097 | 209.5185033 | 229.4863074 | 210.6103745 | 209.0374244 | 328.7931523 | 288.8565828 | 305.4014722 | 316.8874613 |
| Snta1 | 7.325571853 | 0.792262009 | 0 | 4.313047098 | 0 | 3.507064029 | 5.412388289 | 5.028444078 | 1.504828245 | 0 | 2.837612096 | 6.072696744 | 1.70416863 | 0.903554652 | 5.029959704 |
| Cbfa2t2 | 400.1593624 | 425.4446986 | 411.9428409 | 433.0299287 | 383.1847628 | 404.7151889 | 372.5527272 | 381.3236759 | 395.7698285 | 372.2822388 | 386.8611158 | 378.2422543 | 392.8108693 | 328.8938932 | 393.0554226 |
| Necab3 | 17.39823315 | 21.39107423 | 21.14535495 | 21.56523549 | 20.90098706 | 16.13249453 | 17.13922958 | 8.380740131 | 33.10622139 | 10.48682363 | 13.24218978 | 26.02584319 | 15.33751767 | 19.87820233 | 18.68270747 |
| Actl10 | 0 | 6.338096068 | 0 | 5.175656518 | 0 | 0.701412806 | 0 | 0 | 3.00965649 | 0.873901969 | 0 | 0.867528106 | 1.70416863 | 1.807109303 | 5.748525376 |
| E2f1 | 910.2023027 | 934.86917 | 830.9341335 | 841.9067936 | 785.3704229 | 891.4956761 | 872.2965792 | 923.5575624 | 945.032138 | 949.9314402 | 961.00463 | 878.8059717 | 864.0134956 | 993.9101167 | 838.5661392 |
| Pxmp4 | 196.8747435 | 162.4137117 | 201.2724527 | 207.0262607 | 210.9099603 | 200.6040624 | 248.0677966 | 159.2340625 | 155.7497234 | 137.2026091 | 191.0658812 | 227.2923638 | 206.2044043 | 285.5232699 | 207.6654792 |
| Zfp341 | 129.1132039 | 148.1529956 | 126.0889684 | 122.4905376 | 120.3390164 | 110.8232233 | 134.4076425 | 119.0065099 | 88.03245234 | 120.5984717 | 133.3677685 | 130.1292159 | 128.6647316 | 110.2336675 | 114.2519418 |
| Chmp4b | 4617.857357 | 4359.817833 | 4259.614281 | 4271.641846 | 4353.105578 | 4959.689949 | 4628.494052 | 3585.280628 | 3753.041643 | 3660.775348 | 4145.751273 | 4461.697051 | 4572.284435 | 4518.676813 | 4549.957835 |
| Raly | 7527.940775 | 7806.949832 | 7878.602623 | 7988.625835 | 7550.956598 | 8225.467973 | 7628.761293 | 7823.420912 | 7808.553764 | 7498.952795 | 7362.657519 | 7937.882172 | 7699.433872 | 7704.610514 | 7769.132045 |
| Eif2s2 | 7744.960841 | 6952.891387 | 6970.135521 | 6438.516708 | 6497.673523 | 6815.628233 | 6960.331339 | 6702.915956 | 6326.297943 | 6597.959865 | 6590.827029 | 6293.916411 | 6104.332034 | 6045.684174 | 6280.263973 |
| a | 54.02609241 | 36.44405239 | 54.82129061 | 37.09220505 | 48.76896981 | 58.21726287 | 48.7114946 | 13.40918421 | 21.82000955 | 42.82119648 | 40.67244005 | 44.24393342 | 46.01255302 | 63.24882561 | 61.79664779 |
| Ahcy | 5743.248332 | 5604.461448 | 5821.237902 | 5499.99766 | 5523.560853 | 5666.714057 | 5607.234267 | 6532.786932 | 6343.603467 | 6452.892138 | 6853.779083 | 5788.147525 | 5448.227111 | 5265.012955 | 5570.321089 |
| Itch | 1280.143681 | 1172.547773 | 1320.409943 | 1161.934888 | 1184.389267 | 1176.970688 | 1288.148413 | 1239.511465 | 1185.804657 | 1279.392482 | 1276.925443 | 1195.45373 | 1229.557667 | 1278.529832 | 1261.80132 |
| Dynlrb1 | 1416.582457 | 1307.232314 | 1351.736394 | 1198.164484 | 1395.299227 | 1404.228437 | 1385.571402 | 1429.754266 | 1289.637806 | 1290.753208 | 1226.794296 | 1229.287327 | 1307.097339 | 1313.768463 | 1328.627927 |
| Map1lc3a | 78.74989741 | 68.13453273 | 41.50754861 | 33.64176737 | 69.66995687 | 65.93280374 | 84.79408319 | 43.57984868 | 30.84897903 | 48.93851026 | 40.67244005 | 58.12438312 | 44.30838439 | 57.8274977 | 41.67680897 |
| Pigu | 784.7518847 | 872.2804714 | 787.077101 | 777.2110871 | 801.204504 | 782.0752784 | 717.1414483 | 723.2578733 | 712.5361741 | 682.5174377 | 743.4543692 | 701.830238 | 731.9404267 | 760.7930166 | 723.5956317 |

| | | | | | | | | | | | | | | | |
|---|---|---|---|---|---|---|---|---|---|---|---|---|---|---|---|
| **Trp53inp2** | 65.93014667 | 67.34227072 | 57.1707745 | 56.06961228 | 62.06959794 | 62.42573971 | 65.85072418 | 69.56014308 | 89.53728059 | 69.03825554 | 118.2338373 | 65.06460797 | 57.08964912 | 51.50261514 | 43.83250599 |
| **Ncoa6** | 1478.849818 | 1525.896628 | 1513.067621 | 1555.284784 | 1355.397343 | 1389.498768 | 1593.046286 | 1495.962113 | 1433.348904 | 1324.835385 | 1533.256403 | 1545.067557 | 1465.585022 | 1523.393143 | 1529.826316 |
| **Ggt7** | 105.3050954 | 83.18751089 | 108.0762586 | 73.32180067 | 65.23641416 | 82.76671107 | 79.3816949 | 52.79866282 | 69.9745134 | 98.75092249 | 76.6155266 | 70.26977661 | 68.16674521 | 64.15238026 | 74.73082988 |
| **Acss2** | 220.6828521 | 237.6786026 | 243.5631626 | 211.3393078 | 214.7101398 | 201.3054752 | 190.3356548 | 173.4813207 | 171.5504199 | 159.9240603 | 178.7695621 | 228.159892 | 207.0564886 | 234.0206548 | 214.8511359 |
| **Gss** | 393.7494871 | 385.8315981 | 410.3765183 | 354.5324715 | 350.8832373 | 340.8866236 | 383.3775038 | 527.1485542 | 531.9567847 | 537.4497109 | 402.9409177 | 379.9773106 | 325.4962084 | 376.7822897 | 352.8157449 |
| **Myh7b** | 4.578482408 | 2.376786026 | 8.614774239 | 0 | 0 | 1.402825611 | 0 | 0.838074013 | 0.752414123 | 6.117313782 | 3.783482795 | 6.072696744 | 1.70416863 | 4.517773258 | 0 |
| **Trpc4ap** | 2423.848587 | 2410.853292 | 2496.718207 | 2453.261189 | 2257.939966 | 2305.543892 | 2300.265023 | 2564.50648 | 2712.452912 | 2609.471279 | 2329.679531 | 2441.224091 | 2471.044514 | 2346.53143 | 2422.28488 |
| **Edem2** | 916.612178 | 926.1542879 | 902.2018112 | 815.1659016 | 742.9350855 | 745.6018125 | 798.3272726 | 839.7501611 | 911.1735024 | 894.0017142 | 860.7423359 | 755.6169806 | 742.1654385 | 802.3565306 | 827.7876541 |
| **Procr** | 6.409875371 | 7.922620085 | 3.132645178 | 8.626094197 | 0 | 2.805651223 | 2.706194144 | 25.14222039 | 7.524141226 | 20.09974528 | 17.02567258 | 0 | 10.22501178 | 2.710663955 | 5.748525376 |
| **BC029722** | 237.1653887 | 322.4506375 | 271.7569692 | 269.1341389 | 215.3435031 | 263.7312149 | 221.0058551 | 338.5819013 | 233.248378 | 318.1003167 | 358.4849948 | 255.0532632 | 270.1107279 | 286.4268245 | 242.8751971 |
| **Eif6** | 4918.205802 | 4806.653606 | 4660.592863 | 4915.148473 | 4434.176073 | 4651.068315 | 4340.735408 | 4729.251656 | 4375.288123 | 4498.847336 | 4746.379166 | 4151.989517 | 4294.504948 | 4302.727251 | 4097.261462 |
| **Fam83c** | 0 | 0 | 0 | 0 | 0 | 0 | 0 | 14.24725822 | 0.752414123 | 0 | 0 | 0 | 0 | 2.710663955 | 0 |
| **Uqcc1** | 603.4439814 | 526.8542357 | 570.9245837 | 664.2092531 | 546.5924798 | 523.9553659 | 580.9296763 | 650.3454341 | 622.2464794 | 636.2006334 | 617.6535663 | 586.4489998 | 604.1277794 | 531.2901351 | 611.4993868 |
| **Cep250** | 1956.843381 | 2213.580052 | 2077.726914 | 2203.967067 | 2068.564356 | 1961.150205 | 2039.56832 | 2392.701307 | 2524.349381 | 2353.418002 | 2576.551783 | 2161.880041 | 2162.589992 | 2120.642767 | 2139.170005 |
| **6430550D23Rik** | 1.831392963 | 1.584524017 | 0.783161294 | 0.86260942 | 3.800179466 | 4.208476834 | 0.902064715 | 3.352296052 | 0 | 0.873901969 | 0 | 2.602584319 | 2.556252945 | 5.421327909 | 0 |
| **Ergic3** | 1425.739422 | 1336.546008 | 1367.39962 | 1157.621841 | 1340.196625 | 1310.940534 | 1271.009183 | 1311.58583 | 1209.881909 | 1185.884972 | 1245.71171 | 1309.96744 | 1156.278416 | 1294.793816 | 1229.465865 |
| **Spag4** | 16.48253667 | 12.67619214 | 19.57903236 | 12.93914129 | 7.600358931 | 17.53532014 | 8.118582433 | 20.11377631 | 3.762070613 | 22.72145119 | 17.02567258 | 8.675281063 | 5.112505891 | 12.64976512 | 5.748525376 |
| **Cpne1** | 1874.430698 | 1834.08655 | 1784.82459 | 1739.883199 | 1775.950537 | 1794.91537 | 1906.964807 | 1593.178699 | 1610.918636 | 1672.648368 | 1619.330636 | 1817.471383 | 1898.443854 | 1800.784421 | 1901.324768 |
| **Rbm12** | 1159.271746 | 1109.959074 | 1141.066006 | 1072.223509 | 1196.423168 | 1058.431924 | 1129.385023 | 1215.207319 | 1154.203264 | 1094.999167 | 1146.395287 | 1232.757439 | 1163.947175 | 1092.397574 | 1110.183963 |
| **Nfs1** | 565.9004256 | 654.408419 | 678.217681 | 619.3535633 | 669.4649492 | 608.1249026 | 614.3060708 | 664.5926924 | 631.2754488 | 746.3122814 | 664.0012305 | 609.8722587 | 619.4652971 | 713.8081747 | 656.0504585 |
| **Romo1** | 661.1328597 | 630.6405588 | 615.5647775 | 607.2770314 | 659.9645005 | 672.6548807 | 588.146194 | 615.1463256 | 560.5485213 | 689.5086535 | 598.7361523 | 595.1242809 | 572.6006598 | 609.8993898 | 602.8765988 |
| **Rbm39** | 4942.013911 | 5133.857815 | 5344.292674 | 4908.247598 | 4941.500032 | 5146.265756 | 5521.538119 | 4897.704532 | 5022.364268 | 5045.036066 | 5033.923859 | 5040.338297 | 5357.054089 | 5273.144947 | 4879.779478 |
| **Phf20** | 706.0019873 | 645.6935369 | 639.8427776 | 584.8491865 | 538.9921209 | 610.229141 | 746.0075191 | 568.2141809 | 582.3685309 | 563.6667699 | 667.7847133 | 580.3763031 | 599.0152736 | 493.3408398 | 621.5593063 |
| **Scand1** | 733.4728817 | 850.0971351 | 754.9674879 | 891.9381399 | 767.6362521 | 944.1016365 | 728.8682895 | 833.883643 | 893.1155635 | 785.63787 | 809.6653181 | 805.9336107 | 901.5052054 | 923.4328539 | 738.6855108 |
| **Cnbd2** | 7.325571853 | 4.753572051 | 1.566322589 | 4.313047098 | 2.533452977 | 12.6254305 | 3.608258859 | 10.89496217 | 12.79104008 | 8.739019689 | 3.783482795 | 11.27786538 | 4.260421576 | 7.228437213 | 17.24557613 |
| **2900097C17Rik** | 3019.966996 | 3234.013519 | 3163.97163 | 3166.63918 | 3170.616401 | 2969.781819 | 3380.938551 | 3326.315758 | 3269.239363 | 3172.264147 | 3155.424651 | 3230.674668 | 3106.699413 | 3098.2889 | 3054.622672 |
| **Epb4.1l1** | 16.48253667 | 22.18333624 | 18.79587107 | 17.25218839 | 19.00089733 | 21.74379698 | 14.43303544 | 15.92340625 | 14.29586833 | 20.97364725 | 9.458706988 | 9.542809169 | 7.668758836 | 18.97464768 | 15.08987911 |
| **Aar2** | 545.755103 | 575.9744802 | 552.1287126 | 540.8561061 | 515.5576808 | 450.3070213 | 501.5479814 | 574.0806989 | 578.6064603 | 504.2414361 | 525.9041085 | 398.1954008 | 476.3151322 | 428.2849048 | 451.259242 |
| **Dlgap4** | 463.3424197 | 547.4530479 | 556.0445191 | 492.5499786 | 453.4880829 | 468.5437542 | 559.2801232 | 533.0150723 | 530.4519564 | 498.9980242 | 523.0664964 | 504.9013579 | 455.8651086 | 459.005763 | 482.1575659 |
| **4930405A21Rik** | 3.662785926 | 7.922620085 | 0.783161294 | 2.587828259 | 3.166816221 | 2.805651223 | 1.80412943 | 0.838074013 | 0 | 6.117313782 | 0 | 4.337640531 | 5.112505891 | 5.421327909 | 8.622788064 |
| **Tgif2** | 315.9152861 | 274.1226549 | 270.9738079 | 216.5149643 | 233.7110371 | 241.987418 | 258.8925731 | 232.1465016 | 240.0201051 | 204.4930607 | 244.0346403 | 214.2794423 | 244.5481985 | 234.9242094 | 227.0667523 |
| **5430405H02Rik** | 11.90405426 | 14.26071615 | 14.8800646 | 6.038265938 | 15.20071786 | 7.014128057 | 10.82477658 | 3.352296052 | 7.524141226 | 6.991215751 | 20.80915537 | 2.602584319 | 3.408337261 | 4.517773258 | 20.83840449 |
| **1110008F13Rik** | 799.4030284 | 792.2620085 | 744.0032298 | 808.2650262 | 627.0296118 | 758.227243 | 707.2187364 | 728.2863173 | 875.8100387 | 823.2156547 | 742.5084985 | 660.1888889 | 731.9404267 | 715.615284 | 703.4757929 |
| **Sla2** | 843.3564595 | 938.0382181 | 934.3114243 | 1089.475697 | 555.4595652 | 629.1672867 | 616.1102002 | 865.7304555 | 1094.762548 | 987.5092249 | 994.1101044 | 659.3213608 | 745.5737758 | 660.4984503 | 639.523448 |
| **Ndrg3** | 603.4439814 | 689.2679474 | 768.2812299 | 700.4388488 | 690.9992995 | 659.3280374 | 672.0382125 | 842.2643831 | 720.0603153 | 742.8166736 | 756.696559 | 728.7236093 | 659.5132599 | 756.2752434 | 726.4698944 |
| **Dsn1** | 493.5604036 | 487.2411352 | 502.0063898 | 448.5568982 | 532.6584884 | 496.6002664 | 533.1202464 | 571.5664769 | 495.0884927 | 529.5845932 | 540.092169 | 547.4102351 | 555.5589735 | 522.2545886 | 556.8883958 |
| **Soga1** | 30.21798389 | 37.2363144 | 9.397935534 | 15.52696955 | 18.36753408 | 15.43108173 | 20.74748844 | 15.92340625 | 28.59173666 | 21.84754922 | 24.59263817 | 21.68820266 | 20.45002356 | 13.55331977 | 18.68270747 |
| **Samhd1** | 6664.438993 | 6930.70805 | 6861.276101 | 6946.593656 | 6424.83675 | 6711.819138 | 7170.512418 | 5789.415282 | 6400.034527 | 5598.216013 | 5921.150574 | 6800.552825 | 6509.924168 | 6915.807303 | 6334.156398 |
| **Rbl1** | 1463.282978 | 1685.141292 | 1665.000912 | 1612.217005 | 1487.136898 | 1553.629365 | 1641.757781 | 1387.850566 | 1559.754476 | 1340.56562 | 1468.937195 | 1751.539247 | 1757.849942 | 1659.829895 | 1522.640659 |
| **Mroh8** | 9.156964816 | 7.922620085 | 8.614774239 | 9.488703616 | 10.13381191 | 10.52119209 | 9.020647148 | 3.352296052 | 3.00965649 | 5.243411813 | 13.24218978 | 9.542809169 | 5.112505891 | 6.324882561 | 17.9641418 |
| **Rpn2** | 5908.989396 | 5859.569815 | 5860.395967 | 5733.764812 | 5600.831169 | 5664.609819 | 5489.965854 | 5517.879302 | 5479.832055 | 5964.380938 | 6006.278937 | 5202.566053 | 5653.579431 | 5519.815366 | 5399.302459 |
| **Manbal** | 411.1477202 | 387.4161222 | 378.2669052 | 387.3116294 | 371.7842244 | 359.1233565 | 371.6506625 | 401.4374523 | 392.0077579 | 342.5695718 | 391.5904693 | 406.0031537 | 355.3191594 | 343.3507676 | 368.6241897 |
| **Src** | 41.20634167 | 26.93690829 | 15.66322589 | 25.87828259 | 29.76807248 | 28.05651223 | 35.18052388 | 20.11377631 | 23.3248378 | 26.21705907 | 24.59263817 | 14.74797781 | 12.78126473 | 19.87820233 | 9.341353736 |
| **Blcap** | 227.0927274 | 259.0696768 | 253.7442594 | 227.7288868 | 205.2096911 | 185.8743935 | 196.6501078 | 283.2690164 | 252.8111452 | 326.8393364 | 315.9208134 | 176.1082056 | 184.9022964 | 255.7059664 | 213.4140046 |
| **Nnat** | 8.241268334 | 10.29940611 | 5.482129061 | 12.07653188 | 9.500448664 | 2.805651223 | 2.706194144 | 10.89496217 | 14.29586833 | 3.495607876 | 15.13393118 | 9.542809169 | 0.852084315 | 5.421327909 | 5.748525376 |
| **Ctnnbl1** | 1477.934121 | 1538.572821 | 1610.179621 | 1518.192579 | 1553.006675 | 1543.809585 | 1530.803821 | 1371.927159 | 1428.082005 | 1493.498465 | 1485.962868 | 1430.553847 | 1487.739214 | 1500.804276 | 1503.957951 |
| **Tti1** | 592.4556236 | 707.4899736 | 613.9984549 | 619.3535633 | 511.7575014 | 555.5189421 | 568.3007703 | 578.271069 | 572.5871473 | 581.1448093 | 603.4655058 | 540.4700102 | 553.8548049 | 612.6100538 | 559.0440928 |
| **Rprd1b** | 1216.960624 | 1282.672192 | 1194.320974 | 1281.837598 | 1226.191241 | 1245.709143 | 1233.122465 | 1387.012492 | 1384.441986 | 1371.152189 | 1384.754703 | 1267.458563 | 1232.966004 | 1243.291201 | 1195.693278 |
| **Tgm2** | 58.60457482 | 31.69048034 | 18.01270977 | 45.71829924 | 17.73417084 | 35.77205309 | 41.49497688 | 7.542666117 | 11.28621184 | 15.73023544 | 27.43025026 | 19.95314644 | 17.0416863 | 26.2030849 | 13.65274777 |
| **Lbp** | 3.662785926 | 3.961310043 | 1.566322589 | 0 | 2.533452977 | 0.701412806 | 0 | 0 | 0 | 4.369509845 | 0 | 0 | 1.70416863 | 7.228437213 | 1.437131344 |
| **Snhg17** | 322.3251615 | 360.4792139 | 328.9277437 | 250.1567317 | 386.351579 | 337.3795595 | 348.1969799 | 294.1639786 | 252.8111452 | 306.7395911 | 342.405193 | 327.9256242 | 389.402532 | 333.4116664 | 415.3309584 |
| **Ralgapb** | 1559.431108 | 1408.641851 | 1475.475879 | 1561.32305 | 1278.127027 | 1361.442256 | 1614.695839 | 1617.482845 | 1491.284791 | 1410.477778 | 1431.102367 | 1514.704074 | 1431.501649 | 1503.51494 | 1496.772295 |
| **Slc32a1** | 0 | 0.792262009 | 1.566322589 | 3.450437679 | 1.266726489 | 0 | 0.902064715 | 0 | 9.781383594 | 0 | 2.837612096 | 0 | 0 | 2.710663955 | 0 |
| **Actr5** | 349.796056 | 438.1208907 | 442.4861314 | 393.3498954 | 432.5870958 | 415.9377938 | 375.2589213 | 509.5489999 | 386.740859 | 430.8336707 | 401.995047 | 425.0887721 | 436.2671694 | 442.7417793 | 442.6364539 |
| **Ppp1r16b** | 3431.114716 | 3506.55165 | 3409.101115 | 3220.120964 | 2451.115755 | 2467.57025 | 2830.679075 | 2570.372998 | 2945.70129 | 2541.306926 | 2568.038947 | 2344.928471 | 2430.144467 | 2508.267713 | 2422.28488 |
| **Fam83d** | 324.1565545 | 406.4304104 | 407.2438731 | 426.9916627 | 293.2471821 | 324.754129 | 313.016456 | 477.7021874 | 410.8181109 | 489.3851026 | 487.1234099 | 370.4345014 | 384.2900261 | 365.9396339 | 325.5102494 |
| **Dhx35** | 573.2259975 | 515.7625675 | 563.876132 | 534.8178402 | 513.0242279 | 560.4288318 | 595.3627118 | 595.0325493 | 643.3140748 | 545.3148286 | 634.6792389 | 494.4910206 | 566.6360696 | 527.6759165 | 574.1339719 |
| **Mafb** | 0 | 0 | 3.132645178 | 0 | 1.900089733 | 6.312715251 | 0.902064715 | 0 | 2.257242368 | 0.873901969 | 4.729353494 | 0 | 0 | 0 | 1.437131344 |
| **Top1** | 3328.55671 | 3228.467685 | 3357.412469 | 3170.952227 | 3268.787704 | 2875.792503 | 3589.3155 | 4206.293472 | 3861.389277 | 3646.792916 | 3897.93315 | 3212.456578 | 3292.453794 | 3230.207879 | 3340.611809 |
| **Plcg1** | 1799.343586 | 1891.921676 | 1940.673688 | 1968.474696 | 2002.694578 | 2031.992898 | 2070.23852 | 1575.579145 | 1636.500717 | 1562.53672 | 1698.783775 | 2247.765323 | 2147.252474 | 2153.170735 | 2236.894937 |
| **Zhx3** | 83.32837982 | 113.2934672 | 100.2446457 | 107.8261775 | 116.5388369 | 110.1218105 | 114.5622188 | 153.3675444 | 140.7014409 | 142.4460209 | 132.4218978 | 88.48786684 | 92.87719035 | 136.4367524 | 99.8806284 |
| **Lpin3** | 2.747089445 | 1.584524017 | 2.349483883 | 4.313047098 | 1.900089733 | 11.9240177 | 1.80412943 | 5.866518091 | 0 | 4.369509845 | 1.891741398 | 1.735056213 | 0.852084315 | 1.807109303 | 1.437131344 |
| **Chd6** | 478.9092599 | 533.1923317 | 454.2335508 | 474.4351808 | 464.2552581 | 454.5154981 | 550.259476 | 439.9888569 | 479.2877961 | 460.5463376 | 397.2656935 | 583.8464155 | 545.3339617 | 515.0261514 | 554.0141331 |
| **Srsf6** | 5271.664644 | 5435.70964 | 5665.388804 | 5546.578568 | 5552.062199 | 5534.147037 | 5580.172326 | 6080.226965 | 6157.004765 | 6168.000097 | 6071.544015 | 5940.832472 | 5813.771282 | 5925.511405 | 5746.369679 |
| **Ift52** | 513.7057262 | 499.1250654 | 534.1160028 | 497.7256351 | 526.9582192 | 514.8369994 | 506.9603697 | 476.8641134 | 565.0630061 | 474.5287691 | 453.0720647 | 471.9352898 | 482.2797224 | 537.6150177 | 535.3314256 |
| **Mybl2** | 1895.491717 | 2021.060384 | 1898.382978 | 1880.488535 | 1744.282375 | 1751.427776 | 1793.304653 | 2047.414814 | 2084.939534 | 1980.261862 | 2029.83852 | 1918.104643 | 1752.737436 | 1770.967117 | 1866.833616 |
| **Tox2** | 9.156964816 | 0 | 1.566322589 | 0.86260942 | 3.166816221 | 0 | 2.706194144 | 1.676148026 | 2.257242368 | 0 | 2.837612096 | 5.205168638 | 0.852084315 | 0 | 1.437131344 |
| **Oser1** | 319.5780721 | 385.0393361 | 307.7823887 | 300.188078 | 354.6834168 | 303.7117449 | 356.3155623 | 318.468125 | 262.5925288 | 328.5871403 | 288.4905631 | 376.5071981 | 308.4545221 | 333.4116664 | 302.5161479 |
| **Fitm2** | 90.65395167 | 74.4726288 | 98.67832311 | 100.0626927 | 77.2703158 | 87.67660071 | 107.3457011 | 129.063398 | 145.9683398 | 100.4987264 | 112.5586132 | 90.22292305 | 79.24384131 | 83.12702794 | 97.72493139 |
| **Ttpal** | 947.7458584 | 1028.356087 | 1029.073941 | 1010.97824 | 843.6398414 | 887.2871992 | 865.0800615 | 1061.839775 | 1143.669466 | 985.7614209 | 995.0559751 | 948.2082202 | 974.7844565 | 977.646133 | 898.9256556 |
| **Serinc3** | 6058.247922 | 6690.652662 | 6742.235584 | 6283.247013 | 5970.081941 | 5784.551409 | 6183.65362 | 5213.658435 | 5840.990834 | 5352.64956 | 5739.5434 | 6577.598102 | 6272.192644 | 6691.72575 | 6046.73013 |
| **0610039K10Rik** | 1.831392963 | 8.714882094 | 2.349483883 | 0.86260942 | 1.266726489 | 0 | 1.80412943 | 1.676148026 | 0 | 0 | 3.783482795 | 3.470112425 | 0 | 0.903554652 | 3.59282836 |
| **Pkig** | 164.8253667 | 186.973834 | 207.537743 | 184.5984158 | 138.0731873 | 195.6941728 | 182.2170724 | 207.8423552 | 216.6952673 | 208.8625706 | 206.1998123 | 170.9030369 | 175.5293689 | 180.7109303 | 173.8928926 |
| **Ada** | 284.7816058 | 316.1125414 | 330.4940663 | 323.4785324 | 268.5460156 | 286.1764247 | 237.24302 | 322.658495 | 370.1877483 | 299.7483753 | 288.4905631 | 270.6687692 | 260.7378004 | 273.7770594 | 250.7794195 |
| **Ywhab** | 4483.249974 | 4703.659544 | 4965.242607 | 4945.339803 | 4768.591866 | 4664.395158 | 5003.752973 | 5253.047914 | 5290.97611 | 5232.92499 | 5104.864161 | 4966.598408 | 5015.368279 | 5091.530462 | 4836.665538 |
| **Pabpc1l** | 7.325571853 | 8.714882094 | 12.53058071 | 4.313047098 | 6.333632443 | 11.22260489 | 1.80412943 | 2.514222039 | 3.762070613 | 8.739019689 | 7.56696559 | 11.27786538 | 6.816674521 | 5.421327909 | 10.05991941 |
| **Tomm34** | 1011.844612 | 1022.017991 | 1112.089038 | 966.9851594 | 1097.618502 | 982.6793408 | 1014.822804 | 1042.564072 | 1031.559762 | 1071.403814 | 1188.959468 | 1006.332603 | 1097.484598 | 1131.250424 | 1075.692811 |
| **Stk4** | 4908.133141 | 5062.554234 | 5341.160028 | 5124.762562 | 5092.240484 | 4914.79953 | 5281.588905 | 5132.365256 | 4865.109717 | 4949.780752 | 4785.159865 | 5436.798642 | 5533.435543 | 5233.388542 | 5280.739123 |
| **Kcns1** | 13.73544722 | 5.54583406 | 8.614774239 | 0 | 10.13381191 | 2.104238417 | 13.53097072 | 5.028444078 | 14.29586833 | 7.86511772 | 2.837612096 | 7.807752957 | 7.668758836 | 3.614218606 | 2.874262688 |
| **Slpi** | 0 | 0 | 3.132645178 | 0 | 5.700269198 | 0.701412806 | 0.902064715 | 0 | 0 | 0 | 0 | 2.602584319 | 3.408337261 | 2.710663955 | 1.437131344 |
| **Sdc4** | 242.6595676 | 190.935144 | 174.6449687 | 144.0557731 | 130.4728283 | 161.3249453 | 187.6294607 | 174.3193947 | 141.453855 | 133.7070012 | 117.2879666 | 140.5395532 | 151.6710081 | 141.8580803 | 158.0844478 |
| **Sys1** | 858.0076032 | 831.0828469 | 935.0945856 | 924.7172979 | 908.8762555 | 972.8595615 | 882.2192911 | 895.90112 | 953.3086933 | 935.0751067 | 931.6826383 | 929.1226018 | 919.3989761 | 902.6510969 | 894.6142616 |
| **Trp53tg5** | 0 | 2.376786026 | 2.349483883 | 0 | 4.43354271 | 2.805651223 | 5.412388289 | 2.514222039 | 0.752414123 | 3.495607876 | 3.783482795 | 0 | 7.668758836 | 0.903554652 | 6.467091048 |
| **Dbndd2** | 10.98835778 | 13.46845414 | 20.36219366 | 12.07653188 | 10.13381191 | 11.22260489 | 10.82477658 | 12.5711102 | 10.53379772 | 18.35194135 | 20.80915537 | 16.48303402 | 17.0416863 | 8.131991864 | 7.18565672 |
| **Pigt** | 2073.136834 | 2171.590165 | 2077.726914 | 2066.812169 | 2142.667855 | 2104.93983 | 2142.403698 | 2111.946513 | 1981.106385 | 1921.71043 | 2137.667779 | 1973.626442 | 2102.94409 | 2201.059131 | 2160.726976 |
| **Dnttip1** | 650.1445019 | 626.6792487 | 631.2280034 | 642.6440176 | 616.8957999 | 656.5223861 | 651.2907241 | 551.4527006 | 642.5616607 | 580.2709074 | 584.5480918 | 628.9578771 | 647.5840795 | 647.8486852 | 589.9424167 |
| **Ube2c** | 3646.30339 | 3672.134409 | 3712.967697 | 3857.589325 | 3664.639731 | 3692.938422 | 3557.743235 | 3554.271889 | 3831.292712 | 3678.253387 | 3551.744474 | 3950.722996 | 3783.254359 | 3995.518669 | 3652.469311 |
| **Tnnc2** | 0.915696482 | 1.584524017 | 1.566322589 | 2.587828259 | 1.266726489 | 0.701412806 | 1.80412943 | 2.514222039 | 0 | 0 | 0 | 1.735056213 | 10.22501178 | 1.807109303 | 2.155697016 |
| **Snx21** | 31.13368037 | 38.02857641 | 11.74741942 | 25.87828259 | 32.30152546 | 35.07064029 | 23.45368258 | 26.81836842 | 28.59173666 | 18.35194135 | 26.48437957 | 22.55573076 | 50.27297459 | 26.2030849 | 35.20971793 |
| **Acot8** | 285.6973022 | 286.7988471 | 282.7212273 | 266.5463107 | 275.5130113 | 267.238279 | 228.2223728 | 268.1836842 | 227.9814791 | 256.9271789 | 297.0033994 | 254.1857351 | 270.1107279 | 243.9597559 | 297.4861882 |
| **Zswim3** | 74.17141501 | 68.13453273 | 73.61716168 | 65.55831589 | 65.23641416 | 42.08476834 | 52.31975346 | 88.83584538 | 72.23175577 | 67.29045161 | 64.31920752 | 47.71404585 | 65.61049227 | 56.0203884 | 51.01816271 |
| **Zswim1** | 239.9124782 | 236.0940785 | 262.3590337 | 188.9114629 | 222.3104987 | 240.5845924 | 229.1244376 | 248.9079819 | 224.9718227 | 197.501845 | 207.145683 | 210.8093298 | 223.2460906 | 216.8531164 | 250.0608538 |
| **Spata25** | 0 | 0 | 1.566322589 | 0 | 0 | 0 | 0 | 0 | 3.762070613 | 2.621705907 | 0 | 0.867528106 | 0 | 0 | 1.437131344 |
| **Neurl2** | 2.747089445 | 3.169048034 | 4.698967767 | 0.86260942 | 10.76717515 | 7.014128057 | 2.706194144 | 0 | 6.771727103 | 0 | 0.945870699 | 3.470112425 | 3.408337261 | 3.614218606 | 7.904222392 |
| **Ctsa** | 885.4784977 | 854.0584452 | 1025.158134 | 973.8860348 | 961.4454048 | 899.2112169 | 981.4464097 | 934.4525246 | 828.407949 | 908.8580477 | 978.0303025 | 849.310016 | 980.7490468 | 979.4532423 | 954.2552124 |
| **Pltp** | 31.13368037 | 32.48274235 | 36.80858084 | 27.60350143 | 17.1008076 | 32.96640187 | 28.86607087 | 15.08533223 | 29.34415078 | 25.3431571 | 31.21373306 | 27.7608994 | 40.90004713 | 52.40616979 | 30.89832389 |
| **Pcif1** | 1390.942955 | 1498.167458 | 1387.761814 | 1482.825592 | 1509.304611 | 1512.246009 | 1466.757226 | 1242.863761 | 1322.744027 | 1234.823482 | 1253.278676 | 1486.075646 | 1492.85172 | 1513.454041 | 1381.801787 |
| **Zfp335** | 1072.28058 | 1119.466218 | 1195.887297 | 1167.973154 | 1121.052942 | 1069.654529 | 1091.498305 | 1090.334291 | 1149.688779 | 1085.386245 | 1147.341158 | 1153.812381 | 1172.468018 | 1189.077921 | 1188.507621 |
| **Mmp9** | 0 | 0 | 0 | 0 | 3.166816221 | 0.701412806 | 1.80412943 | 0 | 0 | 2.621705907 | 2.837612096 | 0.867528106 | 0 | 0 | 0 |
| **Slc12a5** | 0.915696482 | 0.792262009 | 0 | 0 | 0.633363244 | 0 | 1.80412943 | 2.514222039 | 4.514484735 | 0 | 0.945870699 | 1.735056213 | 0 | 0.903554652 | 0 |
| **Ncoa5** | 1205.05657 | 1202.653729 | 1362.700652 | 1331.868944 | 1291.427655 | 1282.182609 | 1280.931895 | 1444.001524 | 1337.039896 | 1294.248816 | 1313.814401 | 1315.172609 | 1338.624459 | 1363.463969 | 1284.076856 |
| **Cd40** | 20.14532259 | 21.39107423 | 30.54329049 | 22.42784491 | 13.93399137 | 11.22260489 | 24.3557473 | 68.72206907 | 18.05793894 | 20.97364725 | 19.86328467 | 27.7608994 | 20.45002356 | 9.939101167 | 7.18565672 |
| **Slc35c2** | 736.2199712 | 741.55724 | 816.8372302 | 715.9658183 | 688.4658465 | 701.4128057 | 687.3733127 | 832.207495 | 777.2437886 | 791.7551838 | 822.9075079 | 708.7704628 | 662.0695129 | 721.036612 | 735.0926824 |
| **Elmo2** | 1108.908439 | 1273.165048 | 1313.361491 | 1316.341974 | 1328.796086 | 1347.414 | 1355.803266 | 1356.841827 | 1291.895048 | 1233.94958 | 1229.631908 | 1421.011038 | 1460.472516 | 1407.738147 | 1372.460433 |
| **Slc13a3** | 2.747089445 | 10.29940611 | 3.132645178 | 0 | 0 | 3.507064029 | 4.510323574 | 26.81836842 | 0 | 9.612921658 | 12.29631908 | 7.807752957 | 7.668758836 | 1.807109303 | 7.18565672 |
| **Trp53rka** | 273.793248 | 285.2143231 | 303.083421 | 334.6924548 | 355.9501433 | 360.5261821 | 331.0577503 | 291.6497565 | 285.1649525 | 324.2176305 | 313.0832013 | 315.7802307 | 385.9941948 | 279.1983873 | 339.8815628 |
| **Eya2** | 41.20634167 | 59.41965064 | 44.64019379 | 69.00875357 | 76.00358931 | 65.93280374 | 64.94865946 | 58.66518091 | 48.15450385 | 77.77727523 | 83.23662149 | 78.94505767 | 68.16674521 | 94.87323842 | 97.00636572 |
| **Zmynd8** | 1585.07061 | 1635.228786 | 1546.743557 | 1497.489953 | 1461.169005 | 1455.431572 | 1519.979044 | 1644.301214 | 1652.301413 | 1627.205466 | 1577.712326 | 1496.485983 | 1645.374813 | 1553.210446 | 1492.460901 |
| **Ncoa3** | 2443.078213 | 2348.264593 | 2278.999367 | 2284.189743 | 2218.671445 | 2241.013914 | 2471.657318 | 1931.7606 | 1980.353971 | 1971.522842 | 1957.006476 | 2371.821843 | 2414.806949 | 2423.333576 | 2457.494598 |
| **Sulf2** | 10.98835778 | 12.67619214 | 11.74741942 | 8.626094197 | 6.966995687 | 3.507064029 | 5.412388289 | 72.07436512 | 44.39243323 | 34.08217679 | 52.02288843 | 13.01292159 | 0.852084315 | 10.84265582 | 5.748525376 |
| **5031425F14Rik** | 3.662785926 | 0 | 0 | 0 | 3.800179466 | 0.701412806 | 0 | 0 | 0 | 0 | 0 | 2.602584319 | 3.408337261 | 0 | 0.718565672 |
| **Prex1** | 1901.901592 | 1964.809781 | 2044.83414 | 2103.904375 | 1763.916635 | 1762.650381 | 1917.789584 | 2028.977186 | 1987.878112 | 1981.135764 | 2092.265986 | 1914.634531 | 1938.491817 | 2020.348201 | 1998.331134 |
| **Trp53rkb** | 137.3544722 | 135.4768035 | 94.76251663 | 122.4905376 | 164.6744435 | 117.1359386 | 118.1704776 | 152.5294704 | 107.5952195 | 145.0677268 | 139.9888634 | 134.4668565 | 147.4105865 | 94.87323842 | 126.4675583 |
| **Arfgef2** | 1053.96665 | 1098.075144 | 1106.606909 | 1071.360899 | 1014.647917 | 902.718281 | 1056.317781 | 1115.476511 | 1292.647463 | 1261.914443 | 1151.12464 | 1014.140356 | 962.0031918 | 958.6714853 | 1060.602932 |
| **Cse1l** | 3451.260039 | 3432.871283 | 3637.001052 | 3649.700455 | 3332.757391 | 3216.679127 | 3374.624098 | 4475.31523 | 4122.476978 | 4033.057586 | 4462.617957 | 3502.210965 | 3671.631314 | 3378.390842 | 3444.085266 |
| **Stau1** | 771.932134 | 793.0542705 | 804.3066494 | 735.805835 | 799.3044143 | 705.6212825 | 779.3839136 | 957.0805229 | 768.9672333 | 850.3066157 | 931.6826383 | 814.6088918 | 668.0341031 | 721.036612 | 748.0268645 |
| **Ddx27** | 4091.33188 | 3955.764208 | 4072.438731 | 4018.897286 | 3784.978748 | 3927.210299 | 3867.151432 | 4250.711394 | 4304.561195 | 4280.371844 | 4017.112858 | 3588.096248 | 3816.485648 | 3678.370987 | 3888.158851 |
| **Znfx1** | 1059.460829 | 873.0727334 | 800.390843 | 769.4476023 | 761.3026196 | 891.4956761 | 981.4464097 | 623.5270657 | 676.4202962 | 568.9101818 | 649.8131701 | 669.731698 | 757.5029562 | 792.4174294 | 714.2542779 |
| **1500012F01Rik** | 614.4323391 | 606.8726985 | 668.8197455 | 570.1848264 | 622.5960691 | 648.8068453 | 598.0689059 | 630.2316578 | 592.1499145 | 728.8342421 | 671.5681961 | 627.2228208 | 626.2819716 | 621.6456003 | 621.5593063 |
| **Snord12** | 0 | 0 | 0.783161294 | 0 | 0 | 2.805651223 | 0.902064715 | 0.838074013 | 0 | 0 | 0 | 0.867528106 | 1.70416863 | 0.903554652 | 0.718565672 |
| **Kcnb1** | 15.56684019 | 19.80655021 | 22.71167754 | 9.488703616 | 13.30062813 | 23.14662259 | 18.0412943 | 16.76148026 | 25.58208017 | 22.72145119 | 15.13393118 | 13.8804497 | 23.00627651 | 18.97464768 | 20.83840449 |
| **B4galt5** | 4031.811608 | 3840.093955 | 3729.414084 | 3484.942055 | 3769.77803 | 3876.007164 | 4350.658119 | 3033.827927 | 2960.749572 | 3128.569049 | 3153.53291 | 3735.576026 | 3795.18354 | 3753.366023 | 3975.105297 |
| **Slc9a8** | 758.1966867 | 898.4251176 | 737.7379394 | 693.5379734 | 770.169705 | 779.97104 | 780.2859783 | 671.2972845 | 704.2596187 | 742.8166736 | 759.5341711 | 777.3051832 | 754.9467032 | 777.0570004 | 694.8530048 |
| **Spata2** | 855.2605138 | 854.0584452 | 830.9341335 | 837.5937465 | 730.9011839 | 739.99051 | 763.1467487 | 817.1221627 | 907.4114318 | 900.119028 | 898.5771638 | 798.9933859 | 829.930123 | 741.8183689 | 801.2007242 |
| **Rnf114** | 2779.138822 | 2691.314043 | 2597.746014 | 2518.819505 | 2856.468232 | 2853.347294 | 3108.515007 | 1919.18949 | 2192.534753 | 1957.54041 | 2100.778822 | 2909.689268 | 2646.573883 | 2649.222238 | 2693.184139 |
| **Snai1** | 20.14532259 | 17.42976419 | 16.44638718 | 9.488703616 | 10.76717515 | 10.52119209 | 18.0412943 | 15.92340625 | 8.276555348 | 17.47803938 | 24.59263817 | 17.35056213 | 14.48543336 | 22.58886629 | 14.37131344 |
| **Ube2v1** | 1668.398989 | 1736.638323 | 1780.125622 | 1734.707543 | 1599.242192 | 1536.094045 | 1648.974299 | 1858.010087 | 1803.536652 | 1720.712977 | 1629.735214 | 1669.124076 | 1693.091534 | 1642.662357 | 1627.551247 |
| **Tmem189** | 1219.707713 | 1088.568 | 1184.139877 | 1235.256689 | 1199.589985 | 1170.657973 | 1304.385578 | 1164.084804 | 1035.321833 | 1073.151618 | 1235.307133 | 1227.55227 | 1219.332655 | 1136.671752 | 1236.651521 |
| **Cebpb** | 539.3452276 | 491.9947073 | 443.2692927 | 432.1673192 | 437.6540018 | 573.0542623 | 460.9550693 | 360.3718256 | 437.9050193 | 389.7602781 | 365.1060897 | 370.4345014 | 422.6338203 | 317.1476827 | 367.1870584 |
| **Ptpn1** | 5127.900297 | 5221.006636 | 5155.550802 | 5110.098202 | 4329.037775 | 4547.259219 | 4636.612634 | 5610.067443 | 5300.005079 | 5191.851597 | 4933.661565 | 4276.913564 | 4472.59057 | 4460.849315 | 4273.310051 |
| **Fam65c** | 5.494178889 | 9.507144102 | 6.265290356 | 6.900875357 | 3.166816221 | 8.416953669 | 9.020647148 | 1.676148026 | 3.00965649 | 1.747803938 | 6.621094891 | 0 | 2.556252945 | 9.035546516 | 7.904222392 |
| **Pard6b** | 90.65395167 | 66.55000871 | 85.3645811 | 94.88703616 | 69.03659363 | 110.1218105 | 74.87137133 | 82.13125328 | 83.51796761 | 88.26409886 | 94.58706988 | 77.21000146 | 112.4751296 | 87.6448012 | 104.1920224 |
| **Adnp** | 127.2818109 | 133.8922794 | 137.8363878 | 154.4070861 | 158.9741743 | 171.1447246 | 147.9386132 | 151.6913964 | 200.1421566 | 148.5633347 | 134.3136392 | 142.2746094 | 173.8252003 | 165.3505012 | 160.9587105 |
| **Dpm1** | 2.747089445 | 4.753572051 | 2.349483883 | 8.626094197 | 7.600358931 | 2.805651223 | 2.706194144 | 3.352296052 | 0.752414123 | 1.747803938 | 0.945870699 | 5.205168638 | 11.92918041 | 7.228437213 | 7.18565672 |
| **Mocs3** | 312.2525002 | 357.3101658 | 318.7466469 | 305.3637346 | 392.6852115 | 421.5490962 | 340.9804622 | 390.5424901 | 357.3967082 | 315.4786108 | 339.5675809 | 328.7931523 | 345.946232 | 371.3609618 | 321.917421 |
| **Nfatc2** | 1650.08506 | 1662.165694 | 1668.133557 | 1931.382491 | 1805.085246 | 1790.706893 | 2108.125238 | 1816.94446 | 1809.555965 | 1710.226153 | 1768.778207 | 1863.450372 | 2010.918984 | 2039.322849 | 1930.067395 |
| **Atp9a** | 156.5840983 | 154.4910917 | 130.7879362 | 133.70446 | 127.3060121 | 181.6659167 | 180.412943 | 91.35006742 | 98.56625006 | 65.54264767 | 51.07701773 | 105.838429 | 126.1084786 | 140.9545256 | 140.120306 |
| **Zfp64** | 676.6996999 | 683.7221133 | 688.3987779 | 665.934472 | 641.5969665 | 667.0435782 | 647.6824652 | 739.1812795 | 777.2437886 | 713.1040066 | 673.4599375 | 634.1630457 | 655.2528384 | 659.5948956 | 626.589266 |
| **Zfp217** | 1260.914055 | 1258.904332 | 1253.058071 | 1244.745393 | 1273.060121 | 1172.762211 | 1289.050477 | 1042.564072 | 942.0224815 | 1013.726284 | 1133.153097 | 1137.329347 | 1152.870078 | 1205.341905 | 1131.022368 |
| **Bcas1** | 13.73544722 | 9.507144102 | 15.66322589 | 16.38957897 | 16.46744435 | 17.53532014 | 6.314453003 | 12.5711102 | 21.06759543 | 17.47803938 | 10.40457769 | 16.48303402 | 17.0416863 | 18.07109303 | 6.467091048 |
| **Pfdn4** | 437.7029182 | 433.3673187 | 399.4122602 | 374.3724881 | 432.5870958 | 390.6869328 | 377.0630508 | 397.2470822 | 431.8857064 | 377.5256506 | 453.0720647 | 383.447423 | 322.0878711 | 374.0716258 | 360.7199673 |
| **Gm16796** | 8.241268334 | 0.792262009 | 2.349483883 | 1.725218839 | 1.266726489 | 4.208476834 | 8.118582433 | 6.704592104 | 2.257242368 | 0 | 4.729353494 | 0.867528106 | 0.852084315 | 4.517773258 | 4.311394032 |
| **Fam210b** | 73.25571853 | 101.4095371 | 61.86974226 | 50.89395576 | 63.33632443 | 61.7243269 | 86.59821262 | 64.53169901 | 72.23175577 | 79.52507917 | 77.5613973 | 78.07752957 | 91.17302172 | 101.198121 | 48.14390002 |
| **Aurka** | 1468.777156 | 1475.19186 | 1478.608524 | 1501.803 | 1363.631065 | 1440.701903 | 1468.561356 | 1687.881062 | 1545.458608 | 1730.325898 | 1642.031533 | 1508.631377 | 1368.44741 | 1451.10877 | 1445.035566 |
| **Cstf1** | 955.9871268 | 961.8060783 | 986.7832311 | 879.861608 | 933.5774221 | 951.8171774 | 1003.095963 | 1094.524661 | 933.7459261 | 960.4182638 | 1102.885235 | 915.2421521 | 943.2573369 | 955.0572667 | 931.2611109 |
| **Cass4** | 15.56684019 | 20.59881222 | 14.0969033 | 23.29045433 | 10.76717515 | 17.53532014 | 12.62890601 | 37.71333059 | 17.30552482 | 41.07339254 | 42.56418144 | 21.68820266 | 23.85836082 | 11.74621047 | 13.65274777 |
| **Rtfdc1** | 1329.591291 | 1311.985886 | 1335.290007 | 1456.0847 | 1274.960211 | 1327.073028 | 1352.195007 | 1497.638261 | 1497.304104 | 1503.111387 | 1457.586747 | 1333.390699 | 1214.220149 | 1374.306625 | 1241.681481 |
| **Gcnt7** | 0.915696482 | 0.792262009 | 4.698967767 | 0 | 2.533452977 | 2.805651223 | 2.706194144 | 5.028444078 | 0 | 0 | 12.29631908 | 0.867528106 | 5.112505891 | 0 | 0 |
| **Fam209** | 0 | 7.130358077 | 2.349483883 | 6.900875357 | 11.4005384 | 4.208476834 | 5.412388289 | 13.40918421 | 12.79104008 | 6.117313782 | 3.783482795 | 5.205168638 | 11.0770961 | 1.807109303 | 0.718565672 |
| **Bmp7** | 2.747089445 | 16.63750218 | 18.01270977 | 5.175656518 | 15.20071786 | 8.416953669 | 7.216517718 | 5.866518091 | 5.266898858 | 16.60413741 | 17.97154328 | 8.675281063 | 11.0770961 | 3.614218606 | 5.029959704 |
| **Spo11** | 7.325571853 | 5.54583406 | 6.265290356 | 1.725218839 | 0.633363244 | 4.208476834 | 2.706194144 | 2.514222039 | 4.514484735 | 3.495607876 | 6.621094891 | 6.94022485 | 4.260421576 | 3.614218606 | 1.437131344 |
| **Rae1** | 2168.369268 | 2099.494323 | 2087.12485 | 2125.46961 | 2022.328839 | 2040.409852 | 2007.996055 | 1868.905049 | 1946.495335 | 2072.89547 | 1988.220209 | 1905.959249 | 1915.48554 | 1948.063829 | 1911.384687 |
| **Rbm38** | 3646.30339 | 3917.735632 | 3918.155956 | 4011.996411 | 4192.864677 | 4332.626901 | 4163.930723 | 4078.906222 | 4606.279258 | 4356.401315 | 4411.540939 | 4361.06379 | 4143.686025 | 4386.757833 | 4183.489342 |
| **Zbp1** | 1788.355228 | 1759.613921 | 1395.593427 | 1365.510711 | 1256.592677 | 1509.440358 | 1738.278705 | 914.3387482 | 1103.791518 | 776.0249484 | 926.9532848 | 1251.843057 | 1250.859775 | 1314.672018 | 1325.753665 |
| **Pmepa1** | 413.8948097 | 347.0107597 | 387.6648408 | 414.0525214 | 186.2087938 | 218.1393826 | 199.356302 | 615.9843996 | 543.9954106 | 525.2150833 | 467.2601252 | 208.2067455 | 161.0439356 | 192.4571408 | 183.2342464 |
| **Ppp4r1l-ps** | 31.13368037 | 17.42976419 | 31.32645178 | 36.22959563 | 27.2346195 | 49.80030921 | 29.76813559 | 20.95185033 | 48.15450385 | 35.82998073 | 28.37612096 | 26.02584319 | 35.78754124 | 44.27417793 | 35.9282836 |

| | | | | | | | | | | | | | | | |
|---|---|---|---|---|---|---|---|---|---|---|---|---|---|---|---|
| Rab22a | 522.862691 | 579.9357902 | 560.7434869 | 493.412588 | 513.0242279 | 519.746889 | 499.743852 | 501.1682598 | 574.8443897 | 504.2414361 | 502.257341 | 544.8076507 | 598.1631892 | 431.8991235 | 488.6246569 |
| Vapb | 1195.899605 | 1127.388838 | 1210.767361 | 1109.315714 | 1113.452583 | 1190.998944 | 1097.812758 | 1111.286141 | 1019.521136 | 1197.245697 | 1254.224547 | 1169.427887 | 1183.545114 | 1175.524602 | 1229.465865 |
| Stx16 | 586.0457482 | 662.3310391 | 701.7125199 | 644.3692365 | 583.9609112 | 584.97828 | 661.2134359 | 523.7962582 | 532.7091988 | 592.5055349 | 624.2746612 | 589.0515842 | 645.0278266 | 694.8335271 | 638.8048824 |
| Npepl1 | 1890.913234 | 2089.987178 | 1946.938978 | 1957.260773 | 2026.129018 | 1950.629013 | 1867.27396 | 2373.425605 | 2175.981643 | 2333.318257 | 2157.531064 | 1894.681384 | 1885.662589 | 1836.926607 | 1973.899901 |
| Nespas | 16.48253667 | 14.26071615 | 30.54329049 | 25.87828259 | 8.86708542 | 12.6254305 | 17.13922958 | 5.028444078 | 3.00965649 | 16.60413741 | 17.02567258 | 19.08561834 | 19.59793925 | 13.55331977 | 9.341353736 |
| Gnas | 16504.51338 | 15900.69851 | 16024.26325 | 15872.87593 | 17075.47307 | 17775.20332 | 17232.14225 | 14174.34578 | 14435.06494 | 14204.4026 | 14424.52816 | 17390.46842 | 17440.46176 | 17114.22866 | 17178.74952 |
| Nelfcd | 997.1934684 | 1094.906096 | 1094.85949 | 1105.002667 | 1162.854916 | 1069.654529 | 1051.807457 | 1102.067327 | 1058.64667 | 1132.576952 | 1176.663149 | 1211.069236 | 1115.378369 | 1179.13882 | 1094.375518 |
| Ctsz | 2201.334342 | 1669.296052 | 1853.742784 | 1833.907626 | 1689.813136 | 1764.053206 | 1651.680493 | 1905.780306 | 1940.476022 | 2190.872236 | 2092.265986 | 1533.789692 | 1578.912236 | 1681.515207 | 1631.144075 |
| Atp5e | 2346.930082 | 2195.358026 | 2321.290077 | 2309.205416 | 2574.621588 | 2707.45343 | 2385.059106 | 2371.749457 | 2314.425841 | 2351.670198 | 2133.884296 | 2485.468024 | 2306.592241 | 2486.582401 | 2266.356129 |
| Slmo2 | 2077.715317 | 2268.24613 | 2249.239238 | 2063.361732 | 1996.994309 | 2112.655371 | 2157.738798 | 2638.256993 | 2371.609314 | 2427.69967 | 2295.628186 | 2072.524646 | 1995.581466 | 2162.206281 | 2017.013841 |
| Zfp831 | 92.48534464 | 144.1916855 | 115.9078716 | 141.4679448 | 149.4737256 | 115.0317001 | 159.6654545 | 113.9780658 | 147.473168 | 94.38141264 | 87.96597499 | 174.3731494 | 133.7772375 | 145.4722989 | 120.0004672 |
| 0610010B08Rik | 3.662785926 | 0 | 0 | 0 | 1.266726489 | 0 | 0 | 0 | 0 | 3.495607876 | 0 | 0 | 0 | 0 | 2.155697016 |
| Gm14295 | 21.97671556 | 45.95119649 | 32.10961307 | 40.54264272 | 66.50314065 | 51.20313482 | 33.37639445 | 43.57984868 | 39.8779485 | 55.92972601 | 49.18527634 | 55.5217988 | 29.82295103 | 41.56351397 | 40.9582433 |
| Gm14420 | 13.73544722 | 25.35238427 | 26.62748401 | 22.42784491 | 20.90098706 | 18.93814575 | 17.13922958 | 24.30414638 | 43.64001911 | 26.21705907 | 22.70089677 | 18.21809023 | 28.97086672 | 20.78175699 | 37.36541494 |
| Gm14403 | 2.747089445 | 3.961310043 | 7.831612945 | 11.21392246 | 2.533452977 | 2.805651223 | 2.706194144 | 10.89496217 | 3.762070613 | 13.9824315 | 4.729353494 | 7.807752957 | 2.556252945 | 4.517773258 | 7.904222392 |
| Gm14322 | 0 | 2.376786026 | 1.566322589 | 0 | 0 | 0.701412806 | 2.706194144 | 0 | 0 | 5.243411813 | 1.891741398 | 0 | 2.556252945 | 0 | 0 |
| Gm14325 | 77.83420093 | 70.51131876 | 101.8109683 | 85.39833255 | 79.80376878 | 105.2119209 | 103.7374422 | 134.9299161 | 97.06142181 | 88.26409886 | 67.15681961 | 98.03067601 | 80.09592563 | 98.48745702 | 90.53927467 |
| Gm14327 | 0.915696482 | 5.54583406 | 0 | 0 | 4.43354271 | 0 | 0 | 0.838074013 | 0 | 0 | 0 | 1.735056213 | 0 | 0.903554652 | 2.874262688 |
| C330013J21Rik | 10.0726613 | 3.169048034 | 2.349483883 | 9.488703616 | 3.166816221 | 8.416953669 | 4.510323574 | 3.352296052 | 9.028969471 | 5.243411813 | 16.07980188 | 1.735056213 | 4.260421576 | 4.517773258 | 2.874262688 |
| Gm14326 | 75.08711149 | 64.17322269 | 61.08658097 | 62.97048763 | 63.33632443 | 85.5723623 | 47.80942988 | 39.38947861 | 66.96485691 | 64.6687457 | 75.6696559 | 67.66719229 | 78.39175699 | 74.09148143 | 53.8924254 |
| Etohi1 | 74.17141501 | 79.22620085 | 77.53296815 | 62.97048763 | 69.03659363 | 71.54410618 | 93.81473034 | 69.56014308 | 49.65933209 | 69.91215751 | 63.37333682 | 80.68011388 | 64.75840795 | 70.47726282 | 84.79074929 |
| Zfp931 | 15.56684019 | 27.7291703 | 17.22954848 | 22.42784491 | 22.80107679 | 18.23673295 | 19.84542373 | 34.36103454 | 38.37312025 | 25.3431571 | 20.80915537 | 20.82067455 | 27.26669809 | 30.72085815 | 25.14979852 |
| Fam217b | 1.831392963 | 2.376786026 | 0 | 1.725218839 | 2.533452977 | 2.805651223 | 1.80412943 | 0 | 0 | 2.621705907 | 0 | 2.602584319 | 0 | 0 | 0 |
| Taf4a | 736.2199712 | 729.6733098 | 747.9190362 | 756.508461 | 929.1438794 | 862.0363382 | 918.3018796 | 862.3781594 | 782.5106875 | 812.7288311 | 967.6257248 | 938.665411 | 922.8073133 | 990.2958981 | 1011.0219 |
| 4921531C22Rik | 11.90405426 | 23.76786026 | 16.44638718 | 8.626094197 | 18.36753408 | 25.25086101 | 23.45368258 | 25.9802944 | 30.0965649 | 17.47803938 | 20.80915537 | 18.21809023 | 11.92918041 | 18.07109303 | 32.33545524 |
| Lsm14b | 445.9441865 | 515.7625675 | 523.934906 | 499.450854 | 521.8913133 | 479.7663591 | 537.63057 | 584.1375871 | 556.7864507 | 526.9628872 | 559.9554537 | 501.4312454 | 571.7485755 | 562.0109933 | 548.2656077 |
| Psma7 | 4374.282092 | 4560.260121 | 4674.689767 | 4444.16373 | 4460.143966 | 4561.988888 | 4505.81325 | 5021.739486 | 4737.199316 | 4847.534222 | 4613.957269 | 4642.142897 | 4563.763592 | 4579.214974 | 4560.73632 |
| Ss18l1 | 33.88076982 | 53.08155457 | 46.20651637 | 54.34439344 | 34.20161519 | 35.77205309 | 26.15987673 | 23.46607237 | 47.40208972 | 31.46047088 | 22.70089677 | 30.36348372 | 58.79381775 | 32.52796746 | 32.33545524 |
| Mtg2 | 274.7089445 | 282.045275 | 296.8181306 | 231.1793245 | 276.7797377 | 273.5509942 | 276.9338674 | 299.1924227 | 270.8690841 | 325.0915324 | 275.2483733 | 247.2455103 | 260.7378004 | 284.6197152 | 285.2705718 |
| Osbpl2 | 516.4528156 | 488.8256592 | 486.3431639 | 556.3830757 | 492.1232408 | 447.50137 | 469.0736517 | 527.9866282 | 519.1657446 | 465.7897494 | 481.4481857 | 479.7430428 | 413.2608929 | 437.3204514 | 446.2292823 |
| Adrm1 | 2947.626974 | 2799.853938 | 2834.260725 | 2783.640597 | 2816.566347 | 2928.398464 | 2827.972881 | 2950.8586 | 2627.430116 | 2841.055301 | 2868.825829 | 2818.598817 | 2755.640675 | 2793.790983 | 2731.986685 |
| Lama5 | 233.5026028 | 207.5726462 | 138.6195491 | 169.0714463 | 145.0401829 | 176.0546142 | 168.6861017 | 62.01747697 | 57.18347332 | 124.0940796 | 98.37055267 | 169.1679807 | 202.796067 | 183.4215943 | 184.6713777 |
| Rps21 | 5774.382013 | 5386.589396 | 5252.662802 | 4763.329215 | 5681.901664 | 6257.30364 | 5794.863728 | 4402.402791 | 4877.148343 | 4042.670508 | 3949.010167 | 4828.66144 | 4640.45118 | 5587.581965 | 4967.44449 |
| Cables2 | 443.1970971 | 451.5893448 | 486.3431639 | 468.3969149 | 490.2231511 | 464.3352774 | 523.1975346 | 436.6365608 | 452.9533018 | 411.6078274 | 454.0179354 | 432.896525 | 463.5338674 | 507.7977142 | 495.8103137 |
| Slco4a1 | 443.1970971 | 422.2756505 | 465.1978089 | 406.2890367 | 400.9189336 | 439.7858292 | 345.4907858 | 679.6780246 | 511.6416034 | 568.9101818 | 608.1948593 | 405.1356256 | 457.5692772 | 420.152913 | 476.4090405 |
| Mrgbp | 324.1565545 | 356.5179038 | 392.3638085 | 333.8298454 | 377.4844936 | 383.6728047 | 403.2229275 | 326.0107911 | 340.8435975 | 390.6341801 | 379.2941502 | 392.9902321 | 436.2671694 | 370.4574071 | 388.7440285 |
| Ogfr | 2914.661901 | 3047.831947 | 2770.041499 | 2812.106708 | 2625.924011 | 2811.262525 | 2878.488505 | 2821.795202 | 2910.337826 | 2612.966887 | 2622.899448 | 2781.295109 | 2604.821751 | 2669.100441 | 2759.29218 |
| Col9a3 | 5.494178889 | 10.29940611 | 11.74741942 | 3.450437679 | 5.066905954 | 11.22260489 | 3.608258859 | 5.028444078 | 7.524141226 | 4.369509845 | 6.621094891 | 2.602584319 | 4.260421576 | 10.84265582 | 5.748525376 |
| Dido1 | 1444.053351 | 1621.760331 | 1629.758654 | 1587.201332 | 1625.210085 | 1449.118857 | 1764.438582 | 1825.3252 | 1764.411117 | 1671.774467 | 1710.134223 | 1613.602278 | 1655.599824 | 1471.890527 | 1588.748701 |
| Gid8 | 1204.140873 | 1141.649554 | 1165.344006 | 1105.865276 | 1197.056532 | 1160.136781 | 1243.045177 | 1215.207319 | 1145.174295 | 1198.993501 | 1186.121856 | 1218.876989 | 1163.09509 | 1208.052569 | 1217.250248 |
| Slc17a9 | 249.9851395 | 347.8030217 | 295.251808 | 288.9741556 | 346.4496946 | 324.0527162 | 338.274268 | 286.6213125 | 263.3449429 | 347.8129836 | 341.4593223 | 304.5023653 | 338.2774731 | 365.0360792 | 366.4684927 |
| Ythdf1 | 1954.096292 | 1913.312751 | 1968.867494 | 2049.559981 | 1718.947845 | 1858.042522 | 1986.346502 | 2065.852442 | 2027.003646 | 1931.323351 | 1913.496424 | 1773.227449 | 1897.59177 | 1864.936801 | 1907.073293 |
| Arfgap1 | 706.0019873 | 812.0685587 | 803.5234881 | 721.1414748 | 780.3035169 | 799.6105985 | 824.4871493 | 880.8157877 | 826.9031207 | 802.2420075 | 768.9928781 | 790.3181048 | 748.982113 | 776.1534457 | 777.4880571 |
| Col20a1 | 11.90405426 | 22.97559825 | 6.265290356 | 11.21392246 | 12.66726489 | 7.014128057 | 14.43303544 | 7.542666117 | 0 | 15.73023544 | 1.891741398 | 11.27786538 | 10.22501178 | 12.64976512 | 16.52701046 |
| Ppdpf | 646.481716 | 562.506026 | 608.5163258 | 566.7343887 | 597.2615394 | 622.8545715 | 626.0329121 | 470.9975953 | 492.0788362 | 566.2884758 | 479.5564443 | 688.8173164 | 724.2716679 | 614.4171631 | 655.3318928 |
| Srms | 0.915696482 | 0 | 0.783161294 | 0 | 1.266726489 | 0 | 2.706194144 | 0.838074013 | 0 | 0 | 0 | 0 | 0 | 0 | 0 |
| BC051628 | 0.915696482 | 2.376786026 | 0 | 1.725218839 | 0.633363244 | 0 | 4.510323574 | 0 | 0 | 0 | 0 | 0.867528106 | 1.70416863 | 0 | 0 |
| Helz2 | 5324.77504 | 4553.129763 | 3817.128149 | 3710.945723 | 3842.614803 | 4331.925488 | 5299.630199 | 2103.565773 | 2871.212292 | 2295.740472 | 2538.716955 | 3782.422543 | 4012.46504 | 3863.59969 | 4590.197513 |
| Gmeb2 | 671.205521 | 722.5429518 | 682.1334875 | 646.0944553 | 656.1643211 | 655.1195605 | 693.6877657 | 607.6036595 | 729.8416989 | 629.2094176 | 693.3232222 | 675.8043948 | 646.7319952 | 625.2598189 | 684.0745197 |
| Rtel1 | 885.4784977 | 929.323336 | 928.046134 | 919.5416413 | 953.8450459 | 785.5823424 | 839.8222495 | 934.4525246 | 891.6107353 | 948.1836363 | 921.2780606 | 920.4473208 | 900.6531211 | 871.9302388 | 952.818081 |
| Arfrp1 | 469.752295 | 450.0048208 | 447.9682604 | 456.320383 | 442.7209077 | 482.5720103 | 418.5580277 | 468.4833733 | 546.252653 | 456.1768278 | 485.2316685 | 416.413491 | 479.7234694 | 406.5995932 | 487.1875256 |
| Zgpat | 444.1127936 | 542.6994758 | 404.111228 | 412.3273026 | 464.8886213 | 467.8423414 | 523.1975346 | 520.4439621 | 509.384361 | 502.4936321 | 452.126194 | 570.8334939 | 522.3276852 | 558.3967747 | 567.6668809 |
| Lime1 | 1809.416248 | 1961.640733 | 1946.938978 | 2171.187909 | 1832.953229 | 1951.330425 | 1989.052696 | 1400.421676 | 1604.899323 | 1580.888662 | 1576.766455 | 1783.637787 | 1960.646009 | 1834.215943 | 1873.300707 |
| Slc2a4rg-ps | 42.12203815 | 91.90239299 | 86.93090369 | 66.42092531 | 93.10439691 | 68.73845496 | 76.67550076 | 76.26473519 | 48.90691797 | 71.65996145 | 74.7237852 | 82.4151701 | 77.53967268 | 75.89859073 | 85.50931496 |
| Zbtb46 | 108.9678813 | 77.64167683 | 108.8594199 | 107.8261775 | 77.2703158 | 83.46812388 | 64.94865946 | 101.4069556 | 92.54693708 | 76.02947129 | 81.34488009 | 57.25685501 | 74.13133542 | 78.60925469 | 51.73672838 |
| Abhd16b | 2.747089445 | 2.376786026 | 0.783161294 | 4.313047098 | 1.266726489 | 1.402825611 | 0 | 0.838074013 | 0.752414123 | 0.873901969 | 1.891741398 | 2.602584319 | 1.70416863 | 0.903554652 | 0 |
| Tpd52l2 | 952.3243408 | 1003.795965 | 878.7069724 | 867.7850762 | 905.0760761 | 919.5521883 | 923.7142679 | 919.3671923 | 1018.768722 | 921.0926752 | 876.8221378 | 865.7930501 | 886.1676878 | 847.5342632 | 857.9674123 |
| Dnajc5 | 2863.382898 | 2912.355143 | 3030.051048 | 2856.962398 | 2634.791096 | 2663.264423 | 2977.715623 | 2764.806169 | 2828.324687 | 2758.908516 | 2909.498269 | 2858.50511 | 2812.730324 | 2725.120829 | 2854.861415 |
| Uckl1 | 214.2729767 | 191.7274061 | 181.6934203 | 182.873197 | 183.0419776 | 195.6941728 | 199.356302 | 160.9102105 | 151.2352386 | 188.7628253 | 163.6356309 | 170.9030369 | 208.7606572 | 170.7718291 | 199.0426911 |
| Uckl1os | 0.915696482 | 7.922620085 | 0.783161294 | 8.626094197 | 6.333632443 | 8.416953669 | 3.608258859 | 10.89496217 | 9.028969471 | 2.621705907 | 4.729353494 | 2.602584319 | 2.556252945 | 5.421327909 | 6.467091048 |
| Znf512b | 391.0023976 | 468.226847 | 465.9809702 | 431.3047098 | 461.7218051 | 416.6392066 | 468.171587 | 538.8815904 | 494.3360785 | 518.2238676 | 495.6362462 | 454.5847277 | 484.8359753 | 489.7266212 | 531.0200316 |
| Samd10 | 114.4620602 | 154.4910917 | 169.9460009 | 148.3688202 | 142.50673 | 143.7896252 | 163.2737134 | 112.3019177 | 156.5021375 | 110.1116481 | 143.7723462 | 152.6849467 | 189.162718 | 164.4469466 | 200.4798225 |
| Prpf6 | 1754.474459 | 1597.992471 | 1678.314654 | 1606.178739 | 1549.839859 | 1588.700005 | 1728.355994 | 1717.213653 | 1708.732472 | 1694.495918 | 1792.424974 | 1580.63621 | 1694.795703 | 1659.829895 | 1588.748701 |
| Tcea2 | 43.03773463 | 34.85952837 | 41.50754861 | 34.50437679 | 43.06870061 | 37.87629151 | 36.98465331 | 14.24725822 | 33.85863552 | 34.95607876 | 29.32199166 | 22.55573076 | 50.27297459 | 36.14218606 | 40.23967763 |
| Rgs19 | 1169.344407 | 1185.223965 | 1332.157362 | 1244.745393 | 1221.757698 | 1273.064242 | 1259.282342 | 1128.885696 | 1083.476337 | 1145.685481 | 1140.720063 | 1180.705753 | 1157.1305 | 1197.209913 | 1207.908895 |
| Pcmtd2 | 308.5897143 | 354.9333798 | 339.1088405 | 337.2802831 | 340.1160622 | 326.8583675 | 356.3155623 | 310.9254588 | 275.3835689 | 305.8656891 | 295.111658 | 379.9773106 | 386.8462791 | 323.4725653 | 382.9955032 |
| Polr3k | 1255.419876 | 1242.266829 | 1352.519556 | 1287.875864 | 1147.020835 | 1172.762211 | 1177.194453 | 1171.62747 | 1255.026756 | 1348.430738 | 1241.928227 | 1202.393955 | 1271.309798 | 1255.037411 | 1200.004672 |
| Pex2 | 321.409465 | 321.6583755 | 341.4583244 | 297.6002498 | 318.5817119 | 321.9484778 | 347.2949152 | 331.0392352 | 262.5925288 | 287.5137478 | 297.9492701 | 308.8400058 | 301.6378476 | 319.8583467 | 319.761724 |
| Zc2hc1a | 6.409875371 | 5.54583406 | 9.397935534 | 0.86260942 | 12.03390164 | 8.416953669 | 9.020647148 | 13.40918421 | 3.762070613 | 10.48682363 | 15.13393118 | 6.072696744 | 11.0770961 | 0.903554652 | 10.05991941 |
| Gm6194 | 0 | 0 | 0 | 3.450437679 | 4.43354271 | 0.701412806 | 0 | 0.838074013 | 0 | 1.747803938 | 0.945870699 | 0 | 0 | 0.903554652 | 0 |
| Hey1 | 1.831392963 | 0 | 1.566322589 | 1.725218839 | 0.633363244 | 0.701412806 | 0.902064715 | 0.838074013 | 0.752414123 | 0 | 0.945870699 | 0.867528106 | 1.70416863 | 0 | 0 |
| Mrps28 | 603.4439814 | 567.2595981 | 610.0826484 | 609.8648597 | 609.295441 | 655.1195605 | 529.5119876 | 675.4876545 | 699.745134 | 582.0187113 | 644.1379459 | 624.6202365 | 593.9027677 | 647.8486852 | 578.4453659 |
| Tpd52 | 622.6736075 | 570.4286461 | 599.9015516 | 506.3517293 | 564.9600139 | 585.6796928 | 593.5585823 | 390.5424901 | 340.8435975 | 372.2822388 | 394.4280814 | 540.4700102 | 526.5881068 | 530.3865805 | 499.403142 |
| Zbtb10 | 57.68887834 | 64.17322269 | 43.0738712 | 37.95481446 | 72.83677309 | 56.81443726 | 97.4229892 | 46.93214473 | 39.8779485 | 53.3080201 | 27.43025026 | 55.5217988 | 47.71672165 | 50.59906049 | 55.32955674 |
| C030034L19Rik | 0 | 3.169048034 | 0 | 0 | 3.166816221 | 0 | 1.80412943 | 0.838074013 | 6.019312981 | 6.117313782 | 2.837612096 | 7.807752957 | 10.22501178 | 0.903554652 | 0.718565672 |
| Zfp704 | 5.494178889 | 9.507144102 | 9.397935534 | 10.35131304 | 5.700269198 | 4.90988964 | 12.62890601 | 3.352296052 | 3.762070613 | 3.495607876 | 6.621094891 | 1.735056213 | 11.92918041 | 7.228437213 | 2.874262688 |
| Pag1 | 595.202713 | 682.9298513 | 620.2637452 | 759.9588987 | 727.7343677 | 655.8209733 | 779.3839136 | 672.1353585 | 738.1182543 | 659.7959865 | 707.5112827 | 693.1549569 | 737.9050169 | 715.615284 | 698.4458332 |
| Fabp5 | 2441.24682 | 2192.188978 | 2398.823045 | 2137.546142 | 1983.060318 | 2083.196033 | 2187.506933 | 2500.812855 | 2309.911356 | 2461.781846 | 2495.206903 | 2083.802511 | 2091.014909 | 2089.921909 | 2145.637096 |
| Gm9833 | 101.6423095 | 127.5541834 | 99.4614844 | 100.0626927 | 83.60394824 | 92.58649035 | 98.32505391 | 96.3785115 | 115.8717749 | 113.607256 | 99.31642337 | 102.3683165 | 90.32093741 | 87.6448012 | 94.13210303 |
| Impa1 | 1091.510206 | 1155.118008 | 1246.00962 | 1173.148811 | 1079.250968 | 1176.970688 | 1140.209799 | 1341.756495 | 1415.290965 | 1236.571286 | 1267.466736 | 1176.368112 | 1250.859775 | 1129.443314 | 1114.495357 |
| Zfand1 | 138.2701687 | 175.0899039 | 146.4511621 | 150.094039 | 156.4407213 | 171.8461374 | 128.9952542 | 203.6519852 | 171.5504199 | 150.3111387 | 161.7438895 | 164.8303402 | 168.7126944 | 151.7971815 | 160.9587105 |
| Chmp4c | 0.915696482 | 0 | 0 | 0 | 5.066905954 | 0 | 0 | 0 | 2.257242368 | 0 | 0 | 0 | 0.852084315 | 0.903554652 | 2.155697016 |
| Snx16 | 56.77318186 | 82.39524888 | 61.08658097 | 56.06961228 | 89.30421744 | 71.54410618 | 67.65485361 | 76.26473519 | 94.05176532 | 90.0119028 | 59.58985402 | 75.47494525 | 55.38548049 | 86.74124655 | 66.82660749 |
| Lrrcc1 | 209.6944943 | 268.5768209 | 277.2390982 | 267.4089201 | 238.1445798 | 259.5227381 | 265.2070261 | 232.9845756 | 200.1421566 | 357.4259053 | 287.5446924 | 264.5960724 | 270.9628122 | 270.1628408 | 259.4022076 |
| E2f5 | 130.0289004 | 141.8148995 | 155.0659363 | 130.2540224 | 121.6057429 | 107.3161593 | 148.8406779 | 165.1005806 | 127.1579867 | 161.6718642 | 156.0686653 | 119.7188787 | 111.6230453 | 113.8478861 | 119.2819015 |
| 1810022K09Rik | 445.02849 | 393.7542182 | 493.3916155 | 350.2194244 | 411.6861088 | 389.98552 | 442.913775 | 432.4461907 | 439.4098476 | 468.4114553 | 418.0748489 | 451.1146153 | 440.5275909 | 443.6453339 | 426.1094435 |
| Car13 | 36.62785926 | 30.89821833 | 22.71167754 | 45.71829924 | 24.70116653 | 37.87629151 | 32.47432973 | 33.52296052 | 49.65933209 | 57.67752995 | 28.37612096 | 38.17123668 | 13.63334904 | 18.07109303 | 27.30549553 |
| Car2 | 31.13368037 | 41.98988645 | 35.24225825 | 31.05393911 | 24.70116653 | 14.72966892 | 9.020647148 | 109.7876957 | 31.60139315 | 73.40776539 | 89.85771638 | 29.49595561 | 21.30210788 | 23.49242094 | 12.9341821 |
| Ythdf3 | 788.4146706 | 773.2477203 | 862.2605852 | 791.8754472 | 705.5666541 | 728.0664923 | 795.6210784 | 753.4285377 | 919.4500578 | 814.476635 | 923.169802 | 752.1468681 | 812.0363523 | 742.7219236 | 673.2960346 |
| Armc1 | 945.9144655 | 933.284646 | 1004.01278 | 1072.223509 | 953.8450459 | 939.8931597 | 959.7968565 | 1133.91414 | 1080.46668 | 1235.697384 | 1123.69439 | 1005.465075 | 1107.70961 | 974.935469 | 1019.644689 |
| Mtfr1 | 891.888373 | 813.6530827 | 886.5385854 | 781.5241342 | 811.9716792 | 794.7007089 | 829.8995376 | 947.8617088 | 922.4597143 | 942.9402244 | 905.1982587 | 778.1727113 | 845.2676406 | 776.1534457 | 837.8475735 |
| Pde7a | 1116.234011 | 1091.737048 | 1089.377361 | 1064.460024 | 1056.449891 | 1061.938988 | 1132.091217 | 1547.084628 | 1218.910879 | 1254.923227 | 1106.668718 | 1096.555526 | 1088.11167 | 1083.362027 | 1082.159902 |
| 4632415L05Rik | 168.4881526 | 202.0268122 | 195.7903236 | 163.0331803 | 184.9420673 | 143.7896252 | 184.9232665 | 192.757023 | 141.453855 | 176.5281977 | 152.2851825 | 167.4329245 | 153.3751767 | 180.7109303 | 173.1743269 |
| Cp | 0 | 0.792262009 | 2.349483883 | 0.86260942 | 0 | 0 | 0.902064715 | 1.676148026 | 4.514484735 | 0 | 0 | 0 | 0 | 0.903554652 | 0 |
| Hps3 | 196.8747435 | 217.8720523 | 242.7800013 | 212.2019172 | 264.7458361 | 232.1676387 | 247.1657318 | 256.450648 | 303.9753055 | 249.9359631 | 305.5162357 | 258.5233757 | 256.4773789 | 252.0917478 | 278.8034807 |
| Hltf | 619.926518 | 605.2881745 | 617.9142614 | 615.0405162 | 617.5291632 | 608.1249026 | 624.2287826 | 672.1353585 | 695.9830634 | 644.0657511 | 611.9783421 | 651.5136078 | 639.9153207 | 679.473098 | 614.3736495 |
| Gyg | 595.202713 | 506.2554234 | 624.1795517 | 540.8561061 | 535.1919414 | 563.234483 | 554.7697996 | 481.8925575 | 550.7671377 | 596.0011428 | 626.1664026 | 497.961133 | 505.2859989 | 552.9754468 | 515.2115868 |
| Cpa3 | 0 | 0 | 0 | 0 | 1.266726489 | 0 | 0 | 0 | 0 | 0 | 0 | 0 | 2.556252945 | 8.131991864 | 2.155697016 |
| Tbl1xr1 | 1515.477677 | 1536.196034 | 1645.42188 | 1508.703875 | 1597.342102 | 1684.793559 | 1947.557719 | 1593.178699 | 1801.279409 | 1425.334111 | 1572.982972 | 1984.036779 | 1914.633456 | 2040.226403 | 1814.378322 |
| Ect2 | 1139.126423 | 1144.818602 | 1256.190716 | 1283.562816 | 1082.417784 | 1164.345257 | 1344.076425 | 1249.568353 | 1337.039896 | 1275.022973 | 1325.164849 | 1282.206541 | 1134.976308 | 1210.763233 | 1176.292005 |
| Nceh1 | 367.1942891 | 320.8661134 | 382.1827117 | 333.8298454 | 329.9822503 | 326.8583675 | 377.9651155 | 388.028268 | 394.2650002 | 379.2734545 | 359.4308655 | 345.2761863 | 344.2420633 | 372.2645164 | 316.8874613 |
| Tnfsf10 | 669.374128 | 695.6060435 | 714.2431006 | 881.5868269 | 818.3053116 | 890.0928504 | 1013.920739 | 960.432819 | 826.9031207 | 1053.051873 | 1069.77976 | 1033.225975 | 1029.317853 | 955.0572667 | 983.7164049 |
| Fndc3b | 48.53191352 | 40.40536243 | 41.50754861 | 30.19132969 | 44.96879034 | 34.36922748 | 32.47432973 | 27.65644243 | 36.11587788 | 29.71266694 | 19.86328467 | 36.43618046 | 30.67503535 | 24.39597559 | 50.29959704 |
| Tnik | 390.0867011 | 454.7583929 | 497.307422 | 444.2438511 | 360.383686 | 439.7858292 | 456.4447457 | 421.5512286 | 471.0112407 | 416.8512392 | 422.8042024 | 437.2341656 | 483.983891 | 447.2595525 | 430.4208375 |
| Eif5a2 | 295.7699635 | 268.5768209 | 379.0500665 | 338.1428925 | 309.7146265 | 294.5933784 | 318.4288443 | 407.3039703 | 387.4932731 | 313.7308068 | 346.1886757 | 300.1647248 | 305.0461848 | 322.5690106 | 349.9414822 |
| Rpl22l1 | 2197.671556 | 1988.577641 | 2068.328979 | 1811.479781 | 1933.024622 | 2125.982214 | 2058.511679 | 1862.200457 | 1668.10211 | 1883.258743 | 2004.300011 | 1905.959249 | 1981.096033 | 2037.515739 | 1945.87584 |
| Actrt3 | 2.747089445 | 0 | 0 | 0 | 2.533452977 | 0 | 0.902064715 | 0 | 0 | 0 | 0 | 0 | 0 | 0 | 0.718565672 |
| Mynn | 327.8193404 | 354.1411178 | 339.8920018 | 282.0732802 | 307.1811735 | 342.990862 | 334.6660092 | 357.0195296 | 410.0656968 | 310.235199 | 330.1088739 | 307.1049496 | 320.3837025 | 321.665456 | 339.8815628 |
| Sec62 | 1665.6519 | 1648.69724 | 1676.748331 | 1660.523133 | 1706.28058 | 1673.570954 | 1686.861017 | 1706.318691 | 1571.793102 | 1640.313996 | 1563.524265 | 1637.025537 | 1701.612377 | 1682.418761 | 1650.545349 |
| Gpr160 | 47.6162170 4 | 64.9654847 | 70.4845165 | 41.40525214 | 50.66905954 | 39.98052993 | 47.80942988 | 83.80740131 | 43.64001911 | 67.29045161 | 56.75224193 | 59.85943933 | 43.45630007 | 74.99503608 | 65.38947615 |
| Phc3 | 2648.194225 | 2729.342619 | 2701.123305 | 2592.141306 | 1969.126326 | 1860.146761 | 2349.878582 | 2707.817136 | 2552.941118 | 2537.811318 | 2835.720355 | 2128.046445 | 2252.058845 | 1870.358129 | 2107.553116 |
| Prkci | 622.6736075 | 688.4756854 | 671.9523907 | 640.9187988 | 601.0617188 | 632.6743508 | 640.4659475 | 873.2731216 | 753.1665367 | 854.6761256 | 881.5514913 | 650.6460797 | 689.336211 | 679.473098 | 627.3078316 |
| Skil | 942.2516795 | 911.1013098 | 792.55923 | 811.7154639 | 786.6371494 | 711.232585 | 817.2706316 | 813.7698667 | 780.2534451 | 725.3386342 | 703.7277999 | 785.1129362 | 856.3447367 | 708.3868468 | 717.8471063 |
| Zmat3 | 1200.478087 | 1143.234078 | 1343.904781 | 1312.891537 | 861.3740122 | 828.3685235 | 991.3691215 | 2158.040584 | 1995.402253 | 1936.566763 | 2025.109166 | 1035.828559 | 924.511482 | 851.1484818 | 956.4109094 |
| 4930429B21Rik | 7.325571853 | 2.376786026 | 3.915806472 | 21.56523549 | 10.13381191 | 11.22260489 | 2.706194144 | 26.81836842 | 6.771727103 | 12.23462756 | 15.13393118 | 6.072696744 | 7.668758836 | 3.614218606 | 13.65274777 |
| Pik3ca | 1034.737024 | 1034.694183 | 1090.943683 | 1086.025259 | 894.9422642 | 955.3242414 | 976.0340214 | 1119.666881 | 1061.656327 | 1081.016736 | 1087.751304 | 1033.225975 | 1046.359539 | 1011.077655 | 970.7822228 |
| Zfp639 | 504.5487613 | 591.0274583 | 565.4424546 | 522.7413083 | 561.1598344 | 539.3864476 | 599.8730353 | 595.0325493 | 610.9602675 | 589.883829 | 666.8388426 | 549.1452913 | 545.3339617 | 590.0211875 | 612.2179525 |
| Mfn1 | 551.2492819 | 555.375668 | 561.5266481 | 563.283951 | 580.1607318 | 535.1779708 | 639.5638828 | 580.785291 | 664.3816702 | 568.9101818 | 648.8672994 | 622.8851803 | 646.7319952 | 680.3766526 | 637.367751 |
| Gnb4 | 150.174223 | 214.7030043 | 187.9587107 | 172.5218839 | 149.4737256 | 188.6800447 | 203.8666255 | 177.6716908 | 170.0455917 | 138.0765111 | 146.6099583 | 183.9159585 | 259.8857161 | 174.3860478 | 173.1743269 |
| Actl6a | 2059.401387 | 2019.47586 | 2092.606979 | 2071.987826 | 2169.902475 | 2041.812677 | 2027.841479 | 2215.029617 | 2207.583036 | 2256.414884 | 2346.705204 | 2140.191838 | 2137.027462 | 2304.967916 | 2080.24762 |
| Mrpl47 | 415.7262026 | 430.9905326 | 436.220841 | 412.3273026 | 437.0206386 | 383.6728047 | 393.3002156 | 488.5971496 | 417.589838 | 456.1768278 | 530.633462 | 395.5928165 | 357.0233281 | 352.3863141 | 345.6300882 |
| Ndufb5 | 1237.105947 | 1138.480506 | 1193.537813 | 1153.308794 | 1210.35716 | 1195.908834 | 1126.678829 | 1164.084804 | 1188.0619 | 1312.600757 | 1178.554891 | 1224.949686 | 1213.368065 | 1257.748075 | 1152.579338 |
| Ttc14 | 464.2581162 | 537.9459038 | 439.3534862 | 478.7482279 | 495.9234203 | 443.2928932 | 519.5892757 | 617.6605476 | 536.4712694 | 568.0362798 | 565.6306779 | 541.3375383 | 560.6714794 | 495.1479491 | 472.8162122 |
| Ccdc39 | 0.915696482 | 6.338096068 | 5.482129061 | 3.450437679 | 2.533452977 | 4.90988964 | 1.80412943 | 10.05688816 | 18.05793894 | 6.991215751 | 10.40457769 | 4.337640531 | 4.260421576 | 11.74621047 | 1.437131344 |
| Fxr1 | 1400.09992 | 1227.213851 | 1321.976265 | 1251.646268 | 1159.6881 | 1277.974132 | 1289.952542 | 1279.739018 | 1348.326108 | 1192.876188 | 1246.657581 | 1177.23564 | 1224.445161 | 1211.666788 | 1253.178532 |
| Dnajc19 | 427.6302569 | 387.4161222 | 475.3789058 | 500.3134634 | 535.1919414 | 538.6850348 | 516.8830816 | 410.6562664 | 604.9409546 | 501.6197302 | 534.4169448 | 561.2906848 | 495.0609871 | 564.7216572 | 467.0676868 |
| Sox2 | 0 | 0 | 0 | 1.725218839 | 0 | 0 | 0 | 0 | 0 | 0 | 0 | 0 | 0 | 0 | 0 |
| Atp11b | 4449.369204 | 4319.41247 | 4340.279894 | 4094.806915 | 3616.504125 | 3987.5318 | 4414.704714 | 3401.742419 | 3777.118895 | 3776.130408 | 3891.312055 | 3888.260972 | 4018.42963 | 4007.26488 | 4234.507505 |
| Dcun1d1 | 836.0308877 | 880.2030914 | 820.7530366 | 847.0824501 | 846.1732944 | 856.4250358 | 922.8122032 | 997.3080755 | 924.7169567 | 944.6880284 | 893.8478103 | 844.9723755 | 945.8135898 | 890.9048865 | 939.1653333 |
| Mccc1 | 173.9823315 | 207.5726462 | 135.4869039 | 203.575823 | 184.9420673 | 188.6800447 | 186.727396 | 272.3740542 | 231.7435498 | 225.466708 | 225.1172263 | 196.061352 | 178.9377062 | 177.0967117 | 184.6713777 |
| Mccc1os | 1.831392963 | 0.792262009 | 0.783161294 | 0 | 1.266726489 | 0.701412806 | 0 | 0 | 3.00965649 | 0.873901969 | 0.945870699 | 0 | 0 | 0 | 0.718565672 |
| Acad9 | 744.4612395 | 705.1131876 | 707.1946489 | 709.9275524 | 700.4997482 | 719.6495387 | 794.7190137 | 836.397865 | 812.6072524 | 1007.60897 | 840.8790512 | 740.8690028 | 683.3716208 | 747.2396969 | 740.8412078 |
| D3Ertd254e | 91.56964816 | 89.52560696 | 89.28038757 | 72.45919125 | 66.50314065 | 60.32150129 | 78.47963019 | 67.88399506 | 77.49865463 | 57.67752995 | 87.02010429 | 101.5007884 | 92.87719035 | 55.11683375 | 79.76078959 |
| Anxa5 | 1281.975074 | 1151.156698 | 1288.300329 | 1152.446185 | 1139.420476 | 1120.156251 | 1169.07587 | 1211.016949 | 1129.373598 | 1108.981599 | 1118.019166 | 1137.329347 | 1182.693029 | 1206.24546 | 1155.453601 |
| 1810062G17Rik | 7.325571853 | 6.338096068 | 0.783161294 | 0 | 4.43354271 | 3.507064029 | 0 | 10.05688816 | 6.019312981 | 4.369509845 | 5.675224193 | 1.735056213 | 3.408337261 | 0 | 4.311394032 |
| Exosc9 | 888.2255871 | 882.5798775 | 881.8396176 | 894.5259682 | 897.4757171 | 844.5010181 | 869.590385 | 1029.992962 | 916.4404013 | 945.5619304 | 1009.244036 | 903.9642867 | 968.8198663 | 912.5901981 | 809.8235123 |
| Ccna2 | 4028.148822 | 4404.184505 | 4572.095637 | 4465.728966 | 4248.600643 | 4082.923942 | 4330.812696 | 4715.842471 | 4872.633858 | 4599.346062 | 4700.031502 | 4544.979749 | 4445.323872 | 4923.469296 | 4532.712259 |
| Bbs7 | 33.88076982 | 45.15893448 | 27.41064531 | 31.05393911 | 37.36843141 | 24.5494482 | 30.6702003 | 32.68488651 | 48.90691797 | 37.57778466 | 43.51005214 | 32.09853993 | 38.34379418 | 31.62441281 | 45.26963733 |
| 4932438A13Rik | 1599.721753 | 1652.65855 | 1618.794396 | 1575.1248 | 1431.400932 | 1376.171925 | 1676.03624 | 1615.806697 | 1568.031031 | 1554.671603 | 1622.168248 | 1664.786436 | 1722.914485 | 1648.083684 | 1715.934825 |
| Il2 | 373.6041645 | 232.1327685 | 225.5504528 | 251.0193411 | 133.0062813 | 123.4486538 | 156.9592604 | 331.8773092 | 185.0938742 | 186.1411194 | 185.390657 | 96.2956198 | 123.5522257 | 96.68034772 | 84.79074929 |
| Il21 | 0.915696482 | 0 | 3.132645178 | 0 | 0 | 1.402825611 | 9.020647148 | 0 | 1.504828245 | 1.747803938 | 0 | 0 | 1.70416863 | 0 | 1.437131344 |
| Cetn4 | 88.82255871 | 103.7863231 | 80.66561333 | 87.12355138 | 70.93668336 | 72.94693179 | 77.57756547 | 139.1202862 | 83.51796761 | 117.1028638 | 76.6155266 | 74.60741714 | 63.05423932 | 58.73105235 | 68.98230451 |
| Bbs12 | 116.2934532 | 143.3994235 | 129.2216136 | 127.6661941 | 142.50673 | 100.3020312 | 142.5262249 | 182.7001348 | 151.9876528 | 166.0413741 | 115.3962252 | 130.996744 | 130.3689002 | 103.0052303 | 106.3477195 |
| Fgf2 | 7.325571853 | 0.792262009 | 4.698967767 | 6.038265938 | 6.966995687 | 9.118366474 | 5.412388289 | 12.5711102 | 0.752414123 | 5.243411813 | 1.891741398 | 10.41033728 | 5.964590206 | 2.710663955 | 5.029959704 |
| Nudt6 | 79.6655939 | 102.2017991 | 100.2446457 | 77.63484777 | 79.80376878 | 70.14128057 | 78.47963019 | 87.99777137 | 64.70761454 | 79.52507917 | 83.23662149 | 66.79966418 | 97.13761193 | 76.80214538 | 58.20381943 |
| Spata5 | 1047.556775 | 1050.539423 | 1103.474264 | 1049.795664 | 870.2410976 | 867.6476407 | 858.7656085 | 1169.951322 | 1191.071556 | 1233.94958 | 1159.637477 | 794.6557454 | 895.5406152 | 768.0214538 | 815.5720377 |
| Spry1 | 695.0136295 | 756.6102181 | 606.9500032 | 608.1396409 | 700.4997482 | 645.2997813 | 689.1774421 | 561.5095887 | 476.2781396 | 414.2295333 | 429.4252972 | 530.0596729 | 630.5423932 | 619.838491 | 668.2660749 |
| Gm5148 | 172.1509385 | 133.8922794 | 187.1755494 | 163.8957897 | 186.8421571 | 192.8885216 | 158.7633898 | 197.7854671 | 182.0842177 | 182.6455115 | 195.7952346 | 195.1938239 | 207.9085729 | 219.5637803 | 217.7253986 |
| Ankrd50 | 279.2874269 | 337.5036156 | 343.8078083 | 313.1272193 | 266.0125626 | 284.0721863 | 310.3102619 | 272.3740542 | 348.3677388 | 274.4052182 | 257.2768301 | 321.8529274 | 297.377426 | 299.9801443 | 316.8874613 |
| Fat4 | 0.915696482 | 1.584524017 | 0 | 1.725218839 | 0 | 0.701412806 | 0.902064715 | 0 | 0 | 3.495607876 | 9.458706988 | 2.602584319 | 1.70416863 | 0 | 2.874262688 |
| Intu | 39.37494871 | 22.97559825 | 40.72438731 | 30.19132969 | 30.40143573 | 25.95227381 | 32.47432973 | 37.71333059 | 39.12553437 | 29.71266694 | 37.83482795 | 35.56865236 | 38.34379418 | 26.2030849 | 26.58692986 |
| Hspa4l | 410.2320237 | 374.73993 | 416.6418087 | 385.5864106 | 373.0509509 | 414.5349682 | 452.8364868 | 390.5424901 | 465.7443419 | 375.7778466 | 389.6987279 | 431.1614688 | 426.0421576 | 405.6960386 | 358.5642703 |
| Plk4 | 1299.373307 | 1227.213851 | 1341.555297 | 1216.279282 | 1229.99142 | 1240.09784 | 1329.64339 | 1413.83086 | 1257.283999 | 1295.122718 | 1214.497977 | 1279.603957 | 1353.961977 | 1302.022253 | 1191.381884 |
| Mfsd8 | 97.97952353 | 112.5012052 | 114.341549 | 119.0400999 | 93.10439691 | 101.7048568 | 118.1704776 | 124.8730279 | 111.3572901 | 145.9416288 | 108.7751304 | 88.48786684 | 118.4397198 | 137.340307 | 118.5633359 |
| Gm2011 | 4.578482408 | 3.169048034 | 3.132645178 | 1.725218839 | 1.900089733 | 5.611302446 | 5.412388289 | 1.676148026 | 1.504828245 | 4.369509845 | 1.891741398 | 3.470112425 | 8.520843152 | 6.324882561 | 10.77848508 |
| 3110057O12Rik | 213.3572802 | 250.3547947 | 235.7315496 | 214.7897455 | 171.6414392 | 171.8461374 | 198.4542373 | 153.3675444 | 159.511794 | 172.1586879 | 227.9548384 | 178.7107899 | 181.4939591 | 185.2287036 | 180.3599837 |

| | | | | | | | | | | | | | | | |
|---|---|---|---|---|---|---|---|---|---|---|---|---|---|---|---|
| **Larp1b** | 1101.582867 | 1012.510847 | 1085.461554 | 1001.489536 | 909.5096188 | 895.7041529 | 998.5856393 | 1030.831036 | 1017.263894 | 1101.990383 | 959.1128886 | 869.2631625 | 918.5468917 | 916.2044167 | 855.0931496 |
| **Pgrmc2** | 1512.730588 | 1392.004349 | 1452.764201 | 1394.839432 | 1507.404521 | 1534.691219 | 1443.303544 | 1307.39546 | 1531.915154 | 1400.864856 | 1433.939979 | 1417.540926 | 1509.041322 | 1420.387912 | 1406.23302 |
| **Jade1** | 454.1854549 | 485.6566112 | 491.8252929 | 483.061275 | 493.3899673 | 486.0790744 | 495.2335284 | 515.415518 | 512.3940175 | 488.5112006 | 452.126194 | 526.5895605 | 480.5755538 | 498.7621677 | 505.1516674 |
| **Sclt1** | 138.2701687 | 167.1672838 | 200.4892914 | 150.9566484 | 191.2756998 | 167.6376606 | 158.7633898 | 159.2340625 | 150.4828245 | 195.754041 | 165.5273723 | 182.1809023 | 154.227261 | 143.6651896 | 178.9228523 |
| **D3Ertd751e** | 63.18305723 | 86.35655893 | 71.2676778 | 81.94789487 | 70.30332011 | 79.96105985 | 63.14453003 | 77.1028092 | 66.96485691 | 57.67752995 | 78.507268 | 80.68011388 | 66.46257658 | 66.86304422 | 65.38947615 |
| **Slc7a11** | 59.5202713 | 40.40536243 | 57.1707745 | 25.87828259 | 40.53524763 | 38.57770431 | 33.37639445 | 29.33259046 | 45.14484735 | 37.57778466 | 18.91741398 | 35.56865236 | 26.41461377 | 17.16753838 | 31.61688957 |
| **Ccrn4l** | 700.5078084 | 779.5858164 | 803.5234881 | 803.9519791 | 656.7976843 | 713.3368234 | 669.3320184 | 736.6670575 | 670.4009832 | 680.7696338 | 689.5397394 | 681.0095634 | 686.779958 | 712.0010654 | 700.6015302 |
| **Elf2** | 607.1067673 | 693.2292574 | 699.363036 | 699.5762393 | 630.1964281 | 569.5471982 | 646.7804005 | 705.658319 | 747.8996378 | 754.1773992 | 717.9158604 | 732.1937217 | 733.6445954 | 684.8944259 | 685.5116511 |
| **4930577N17Rik** | 9.156964816 | 11.88393013 | 11.74741942 | 21.56523549 | 13.93399137 | 8.416953669 | 10.82477658 | 10.05688816 | 4.514484735 | 6.117313782 | 3.783482795 | 11.27786538 | 13.63334904 | 3.614218606 | 5.748525376 |
| **Mgarp** | 157.4997948 | 110.1244192 | 101.8109683 | 100.0626927 | 122.8724694 | 93.98931597 | 106.4436363 | 12.5711102 | 17.30552482 | 18.35194135 | 17.97154328 | 111.9111257 | 164.4522728 | 127.4012059 | 100.5991941 |
| **Ndufc1** | 479.8249563 | 495.1637553 | 496.5242607 | 473.5725714 | 497.1901468 | 476.9607079 | 497.0376578 | 463.4549292 | 489.0691797 | 490.2590046 | 444.5592284 | 460.6574244 | 437.1192537 | 498.7621677 | 408.1453017 |
| **Naa15** | 4809.237921 | 4444.589868 | 4508.659572 | 4560.616002 | 4616.584688 | 4417.49785 | 4778.236794 | 5006.654154 | 4706.350337 | 4589.733141 | 4767.188322 | 4470.372332 | 4525.419798 | 4319.894789 | 4427.801671 |
| **Rab33b** | 167.5724561 | 194.8964541 | 174.6449687 | 182.0105875 | 181.1418879 | 169.0404862 | 175.9026194 | 170.9670987 | 176.0649047 | 197.501845 | 167.4191137 | 157.8901153 | 184.0502121 | 176.1931571 | 174.6114583 |
| **Setd7** | 1824.067391 | 1824.579406 | 1858.441752 | 1632.919631 | 1421.900483 | 1376.171925 | 1660.70114 | 1912.484898 | 1857.710469 | 1829.076821 | 1944.710157 | 1751.539247 | 1638.558138 | 1641.758802 | 1653.419611 |
| **5031434O11Rik** | 0.915696482 | 3.169048034 | 0 | 0 | 1.266726489 | 2.104238417 | 0 | 0.838074013 | 0 | 0 | 3.783482795 | 0 | 4.260421576 | 0 | 2.874262688 |
| **Mgst2** | 347.0489665 | 342.2571877 | 314.8308404 | 272.5845766 | 231.8109474 | 281.2665351 | 250.7739907 | 170.1290246 | 249.8014887 | 198.3757469 | 169.3108551 | 251.5831508 | 267.554475 | 224.0815536 | 230.6595807 |
| **Maml3** | 31.13368037 | 42.78214846 | 35.24225825 | 55.20700286 | 22.16771355 | 27.35509942 | 8.118582433 | 53.63673684 | 46.6496756 | 34.08217679 | 56.75224193 | 21.68820266 | 23.85836082 | 18.07109303 | 28.02406121 |
| **Foxo1** | 1721.509385 | 1775.459161 | 1788.740397 | 1857.198081 | 1633.443807 | 1681.987908 | 1789.696394 | 1861.362383 | 1787.735955 | 1694.495918 | 1758.373629 | 1816.603855 | 1803.010411 | 1787.231101 | 1723.120481 |
| **Gm20750** | 0 | 0 | 3.132645178 | 1.725218839 | 1.266726489 | 2.805651223 | 0 | 0 | 0.752414123 | 0 | 0 | 0 | 0 | 0 | 0 |
| **Cog6** | 307.6740178 | 314.5280174 | 306.9992274 | 323.4785324 | 327.4487973 | 300.9060937 | 297.6813559 | 310.9254588 | 296.4511643 | 346.9390817 | 338.6217102 | 298.4296686 | 303.3420162 | 358.7111967 | 307.5461076 |
| **Lhfp** | 0 | 0 | 3.132645178 | 0 | 0 | 2.104238417 | 2.706194144 | 0 | 0 | 0.873901969 | 0 | 0.867528106 | 0 | 0 | 1.437131344 |
| **Nhlrc3** | 142.8486511 | 135.4768035 | 137.8363878 | 108.6887869 | 126.0392856 | 111.5246361 | 114.5622188 | 89.6739194 | 86.5276241 | 115.3550599 | 68.10269031 | 136.2019127 | 123.5522257 | 94.87323842 | 122.8747299 |
| **Proser1** | 723.4002204 | 716.9971177 | 718.9420683 | 705.6145053 | 700.4997482 | 695.1000905 | 752.3219721 | 762.6473519 | 755.4237791 | 679.8957318 | 691.4314808 | 747.8092276 | 761.7633778 | 758.0823527 | 712.0985809 |
| **Ufm1** | 786.5832777 | 827.1215369 | 816.8372302 | 747.8823668 | 797.4043245 | 786.2837552 | 838.9201847 | 832.207495 | 779.501031 | 816.224439 | 832.3662149 | 779.0402394 | 852.9363995 | 831.2702794 | 759.5239153 |
| **Supt20** | 649.2288054 | 785.1316504 | 758.1001331 | 684.0492698 | 737.2348163 | 738.5876844 | 786.6004313 | 824.6648288 | 785.520344 | 796.1246937 | 830.4744735 | 770.3649584 | 833.3384602 | 729.1686038 | 894.6142616 |
| **Exosc8** | 1720.593689 | 1665.334742 | 1836.513236 | 1631.194413 | 1729.081657 | 1869.96654 | 1619.206163 | 1588.150255 | 1510.847558 | 1738.191016 | 1726.214025 | 1757.611943 | 1801.306242 | 1873.972347 | 1736.054663 |
| **Alg5** | 1018.254487 | 984.7816766 | 999.3138118 | 900.5642341 | 958.2785886 | 916.7465371 | 976.9360861 | 928.5860065 | 921.7073002 | 904.4885378 | 943.9789574 | 870.9982187 | 956.8906859 | 968.6105865 | 863.7159377 |
| **Rfxap** | 510.9586367 | 446.8357728 | 423.6902603 | 438.2055852 | 488.3230613 | 465.738103 | 532.2181817 | 336.0676792 | 507.1271186 | 388.8863762 | 443.6133577 | 596.8593371 | 524.0318538 | 435.5133421 | 516.6487181 |
| **Spg20** | 572.310301 | 455.5506549 | 541.9476158 | 576.2230923 | 455.3881726 | 530.2680811 | 540.3367642 | 556.4811447 | 475.5257255 | 547.9365345 | 468.2059959 | 518.7818076 | 535.1089499 | 520.4474793 | 488.6246569 |
| **Dclk1** | 17.39823315 | 6.338096068 | 18.01270977 | 13.80175071 | 1.266726489 | 7.014128057 | 5.412388289 | 8.380740131 | 5.266898858 | 7.86511772 | 16.07980188 | 11.27786538 | 7.668758836 | 3.614218606 | 7.904222392 |
| **Nbea** | 0.915696482 | 0 | 1.566322589 | 2.587828259 | 1.266726489 | 0.701412806 | 0 | 6.704592104 | 0 | 0 | 0 | 0.867528106 | 1.70416863 | 0 | 2.874262688 |
| **Wwtr1** | 5.494178889 | 0 | 0 | 0 | 1.900089733 | 2.104238417 | 0 | 0 | 0 | 0 | 0 | 0 | 0 | 0 | 5.748525376 |
| **Commd2** | 496.307493 | 540.3226898 | 530.2001964 | 489.9621504 | 545.3257533 | 551.3104653 | 534.0223112 | 445.0173009 | 554.5292083 | 529.5845932 | 540.092169 | 557.8205723 | 551.2985519 | 550.2647828 | 533.8942943 |
| **Rnf13** | 447.7755795 | 491.9947073 | 515.3201318 | 464.9464772 | 399.6522071 | 359.8247693 | 492.5273343 | 486.0829276 | 471.7636549 | 574.1535936 | 517.3912722 | 414.6784348 | 458.4213616 | 424.6706862 | 512.3373241 |
| **Pfn2** | 90.65395167 | 79.22620085 | 110.4257425 | 98.33747384 | 74.10349958 | 89.78083913 | 82.08788905 | 91.35006742 | 112.1097043 | 89.13800083 | 59.58985402 | 58.12438312 | 89.46885309 | 68.67015352 | 105.6291538 |
| **Tsc22d2** | 553.0806749 | 523.6851876 | 534.8991641 | 546.0317626 | 546.5924798 | 468.5437542 | 538.5326347 | 569.0522549 | 566.5678343 | 463.1680435 | 617.6535663 | 498.8286611 | 530.8485283 | 494.2443944 | 551.1398704 |
| **Serp1** | 3346.87064 | 3201.530776 | 3503.863632 | 3400.406332 | 3265.620887 | 3321.189635 | 3398.077781 | 3202.280804 | 3379.844239 | 3417.8306 | 3419.322576 | 3361.671412 | 3407.485176 | 3261.832292 | 3198.335806 |
| **Eif2a** | 1915.637039 | 1821.410358 | 1915.612526 | 1816.655438 | 1872.855113 | 1734.593869 | 1877.196671 | 2254.419095 | 2047.318828 | 2009.100627 | 2079.023796 | 1805.325989 | 1802.158327 | 1867.647465 | 1845.995211 |
| **Selt** | 3975.038426 | 4023.106479 | 4172.683377 | 3824.810167 | 3871.749512 | 4090.639483 | 4215.348412 | 3682.497213 | 4060.026605 | 3813.708192 | 4058.731168 | 4460.829522 | 4247.640311 | 4318.08768 | 4042.65047 |
| **Erich6** | 0 | 0 | 0 | 0.86260942 | 1.266726489 | 1.402825611 | 0 | 0 | 0 | 0 | 0 | 0.867528106 | 0 | 0 | 0 |
| **Gm8234** | 0 | 0.792262009 | 0 | 0 | 1.900089733 | 0 | 0 | 0 | 0 | 0 | 0.945870699 | 0.867528106 | 0 | 0 | 0 |
| **Siah2** | 938.5888936 | 918.2316679 | 924.9134888 | 846.2198407 | 811.3383159 | 1005.825963 | 941.7555622 | 732.4766874 | 754.6713649 | 868.6585571 | 840.8790512 | 869.2631625 | 793.2904974 | 817.7169597 | 911.8598377 |
| **4930593A02Rik** | 0 | 0 | 1.566322589 | 0 | 1.266726489 | 0.701412806 | 0 | 5.028444078 | 3.762070613 | 2.621705907 | 4.729353494 | 3.470112425 | 0 | 4.517773258 | 0 |
| **Med12l** | 75.08711149 | 72.88810478 | 75.96664557 | 52.6191746 | 57.63605523 | 53.30737323 | 55.92801232 | 56.15095887 | 46.6496756 | 68.16435357 | 52.96875913 | 62.46202365 | 28.1187824 | 51.50261514 | 56.76668809 |
| **Gpr171** | 1608.878718 | 1307.232314 | 1419.871427 | 1201.614922 | 1407.966492 | 1423.166583 | 1707.608505 | 1024.964518 | 970.6142181 | 1045.186755 | 1285.43828 | 1195.45373 | 1285.795232 | 1245.09831 | 1257.489926 |
| **P2ry14** | 721.5688275 | 523.6851876 | 530.9833577 | 429.579491 | 362.917139 | 374.5544383 | 397.8105392 | 221.2515394 | 286.6697807 | 346.9390817 | 412.3996247 | 281.9466345 | 324.6441241 | 331.6045571 | 363.59423 |
| **P2ry13** | 1.831392963 | 3.169048034 | 2.349483883 | 0 | 0 | 0.701412806 | 0 | 0 | 0 | 0 | 0 | 1.735056213 | 0 | 0 | 1.437131344 |
| **P2ry12** | 6.409875371 | 6.338096068 | 10.96425812 | 9.488703616 | 5.066905954 | 7.715540863 | 7.216517718 | 3.352296052 | 10.53379772 | 11.3607256 | 11.35044839 | 6.072696744 | 8.520843152 | 7.228437213 | 3.59282836 |
| **Igsf10** | 1.831392963 | 3.169048034 | 0 | 1.725218839 | 0 | 1.402825611 | 1.80412943 | 0 | 0 | 0 | 4.729353494 | 0 | 0.852084315 | 0 | 4.311394032 |
| **Gm5538** | 7.325571853 | 3.169048034 | 0.783161294 | 0.86260942 | 1.266726489 | 0 | 1.80412943 | 1.676148026 | 1.504828245 | 0 | 1.891741398 | 0.867528106 | 4.260421576 | 0 | 0.718565672 |
| **Mbnl1** | 7467.504807 | 8526.323736 | 8836.408886 | 9150.560724 | 8734.712502 | 8323.665765 | 9450.029952 | 9554.881823 | 9634.66284 | 9241.513321 | 9473.840919 | 9805.670185 | 9904.628079 | 9833.385273 | 9505.905274 |
| **P2ry1** | 10.0726613 | 9.507144102 | 22.71167754 | 20.70262607 | 8.233722176 | 11.9240177 | 19.84542373 | 41.06562664 | 11.28621184 | 8.739019689 | 25.53850887 | 9.542809169 | 8.520843152 | 13.55331977 | 12.9341821 |
| **Rap2b** | 532.0196558 | 503.0863754 | 594.4194225 | 558.1082945 | 574.4604626 | 558.3245933 | 614.3060708 | 671.2972845 | 715.5458306 | 665.9133003 | 579.8187383 | 591.6541685 | 530.8485283 | 539.422127 | 586.3495883 |
| **Arhgef26** | 0 | 0.792262009 | 1.566322589 | 0 | 0.633363244 | 1.402825611 | 0 | 5.028444078 | 2.257242368 | 0 | 0.945870699 | 1.735056213 | 1.70416863 | 0.903554652 | 0 |
| **Dhx36** | 1379.038901 | 1417.356733 | 1449.631556 | 1322.38024 | 1226.824604 | 1247.813381 | 1272.813313 | 1564.684182 | 1531.162739 | 1397.369248 | 1433.939979 | 1261.385867 | 1271.309798 | 1355.331977 | 1177.729136 |
| **Plch1** | 37.54355574 | 31.69048034 | 33.67593566 | 31.05393911 | 9.500448664 | 29.45933784 | 20.74748844 | 20.11377631 | 33.85863552 | 30.58656891 | 63.37333682 | 19.95314644 | 26.41461377 | 27.10663955 | 15.08987911 |
| **E130311K13Rik** | 35.71216278 | 29.31369431 | 15.66322589 | 34.50437679 | 31.66816221 | 39.27911712 | 35.18052388 | 35.19910855 | 12.79104008 | 18.35194135 | 39.72656935 | 44.24393342 | 36.63962555 | 30.72085815 | 28.74262688 |
| **Slc33a1** | 467.9209021 | 456.3429169 | 484.7768413 | 487.3743221 | 440.820818 | 418.0420322 | 424.8724807 | 572.4045509 | 597.4168133 | 552.3060443 | 626.1664026 | 417.2810191 | 426.0421576 | 429.1884595 | 407.426736 |
| **Gmps** | 3292.844548 | 3218.960541 | 3168.670597 | 3219.258354 | 2895.736753 | 2920.682923 | 3164.443019 | 3493.93056 | 3192.493122 | 3248.293618 | 3480.804171 | 3064.976799 | 2922.649201 | 3025.100973 | 2777.974888 |
| **Kcnab1** | 0.915696482 | 0.792262009 | 0 | 3.450437679 | 1.266726489 | 2.104238417 | 4.510323574 | 0 | 4.514484735 | 0.873901969 | 0 | 0 | 1.70416863 | 0 | 0.718565672 |
| **A730090N16Rik** | 1.831392963 | 0.792262009 | 0.783161294 | 0.86260942 | 1.266726489 | 0.701412806 | 0.902064715 | 2.514222039 | 2.257242368 | 2.621705907 | 0.945870699 | 0.867528106 | 0 | 2.710663955 | 7.18565672 |
| **Ssr3** | 3997.930839 | 3886.045152 | 4138.22428 | 4018.034677 | 3843.248166 | 3913.182043 | 4206.327765 | 4047.897483 | 4166.116997 | 4061.896351 | 4342.492378 | 4108.613111 | 4123.236001 | 4071.41726 | 4100.135724 |
| **4931440P22Rik** | 6.409875371 | 11.88393013 | 5.482129061 | 18.97740723 | 7.600358931 | 9.118366474 | 12.62890601 | 9.218814144 | 0.752414123 | 9.612921658 | 6.621094891 | 6.94022485 | 3.408337261 | 18.07109303 | 8.622788064 |
| **Tiparp** | 227.0927274 | 212.3262183 | 202.055614 | 219.965402 | 200.7761484 | 215.3337314 | 238.1450847 | 134.0918421 | 102.3283207 | 131.9591973 | 230.7924505 | 213.4119141 | 231.7669337 | 188.8429222 | 245.0308941 |
| **Lekr1** | 11.90405426 | 11.09166812 | 9.397935534 | 17.25218839 | 6.333632443 | 10.52119209 | 3.608258859 | 5.866518091 | 11.28621184 | 15.73023544 | 21.75502607 | 6.072696744 | 1.70416863 | 0.903554652 | 10.77848508 |
| **Ccnl1** | 863.5017821 | 852.4739212 | 967.98736 | 839.3189653 | 830.9725765 | 892.1970889 | 810.9561786 | 738.3432055 | 749.4044661 | 843.3154 | 865.4716894 | 922.182377 | 938.144831 | 916.2044167 | 974.3750512 |
| **Rsrc1** | 607.1067673 | 649.654847 | 639.8427776 | 545.1691532 | 549.759296 | 568.8457854 | 582.7338057 | 613.4701776 | 617.7319946 | 609.1096723 | 672.5140668 | 649.7785516 | 592.198599 | 559.3003293 | 561.9183555 |
| **Mlf1** | 31.13368037 | 14.26071615 | 25.84432272 | 22.42784491 | 39.26852115 | 33.66781467 | 37.88671802 | 35.19910855 | 32.35380727 | 17.47803938 | 47.29353494 | 34.70112425 | 40.90004713 | 30.72085815 | 28.02406121 |
| **Gfm1** | 1519.140463 | 1526.68889 | 1658.735622 | 1665.698789 | 1542.872863 | 1318.656075 | 1502.839815 | 1803.535276 | 1895.331175 | 1901.610684 | 1794.316716 | 1509.498905 | 1521.822587 | 1535.139353 | 1401.921626 |
| **Lxn** | 282.0345163 | 255.1083667 | 234.1652271 | 207.8888701 | 178.6084349 | 199.9026496 | 247.1657318 | 265.6694621 | 258.8304582 | 349.5607876 | 267.6814078 | 243.7753979 | 222.3940063 | 238.538428 | 239.2823688 |
| **Mfsd1** | 879.0686223 | 947.5453622 | 964.8547148 | 818.6163392 | 804.3713202 | 893.5999145 | 880.4151616 | 983.0608173 | 854.7424433 | 860.7934394 | 881.5514913 | 909.1694554 | 848.6759779 | 872.8337934 | 812.697775 |
| **1110032F04Rik** | 835.1151912 | 974.4822705 | 1055.701425 | 961.8095029 | 960.8120416 | 1082.279959 | 1056.317781 | 1324.156941 | 1239.22606 | 1041.691147 | 967.6257248 | 1090.48283 | 994.3823958 | 1020.113202 | 1098.686912 |
| **Ift80** | 435.8715252 | 562.506026 | 588.1541322 | 472.709962 | 604.8618983 | 487.4819 | 598.0689059 | 555.6430707 | 604.9409546 | 532.2062991 | 602.5196351 | 667.1291137 | 653.5486697 | 569.2394305 | 592.0981137 |
| **Smc4** | 8306.282784 | 8843.228539 | 9011.053854 | 8513.092363 | 8815.149634 | 8456.232786 | 9658.406901 | 8231.562956 | 8719.727267 | 7982.220584 | 8239.479657 | 9855.986815 | 9511.81721 | 9772.847111 | 9300.395492 |
| **Trim59** | 1571.335162 | 1767.536541 | 1758.980267 | 1700.203166 | 1684.112867 | 1676.376606 | 1823.072789 | 1380.3079 | 1549.220678 | 1530.202348 | 1542.71511 | 1826.146664 | 1653.895656 | 1799.880866 | 1751.863108 |
| **Kpna4** | 2974.182172 | 2766.578934 | 2901.612596 | 2825.908459 | 2659.492263 | 2615.568353 | 2749.493251 | 3092.493108 | 2947.958532 | 2970.392792 | 3003.139469 | 2697.144882 | 2496.607043 | 2564.288101 | 2507.794195 |
| **Ppm1l** | 108.0521848 | 102.9940611 | 73.61716168 | 81.94789487 | 101.3381191 | 93.98931597 | 108.2477658 | 72.07436512 | 78.25106875 | 69.91215751 | 82.29075079 | 84.15022631 | 72.42716679 | 49.69550584 | 53.17385973 |
| **B3galnt1** | 1.831392963 | 1.584524017 | 0 | 0 | 0 | 2.805651223 | 1.80412943 | 0.838074013 | 0 | 10.48682363 | 0.945870699 | 0.867528106 | 2.556252945 | 0 | 0 |
| **Nmd3** | 1516.393373 | 1551.249013 | 1627.40917 | 1351.708961 | 1508.671248 | 1540.302521 | 1553.355439 | 1584.797959 | 1528.905497 | 1542.436975 | 1590.954515 | 1452.24205 | 1468.993359 | 1515.261151 | 1462.281142 |
| **Pdcd10** | 1210.550749 | 1273.95731 | 1268.721297 | 1196.439265 | 1189.456173 | 1226.069584 | 1249.35963 | 1061.001701 | 1279.856423 | 1214.723737 | 1310.976788 | 1275.266316 | 1159.686753 | 1302.925808 | 1253.178532 |
| **Serpini1** | 37.54355574 | 50.70476854 | 50.90548414 | 37.09220505 | 62.06959794 | 42.78618115 | 48.7114946 | 39.38947861 | 34.61104964 | 38.45168663 | 49.18527634 | 37.30370857 | 46.01255302 | 57.8274977 | 48.14390002 |
| **Golim4** | 543.0080136 | 533.1923317 | 564.6592933 | 514.1152141 | 493.3899673 | 460.1268005 | 626.9349768 | 547.2623305 | 559.0436931 | 538.3236128 | 594.9526695 | 582.1113593 | 503.5818303 | 519.5439247 | 494.3731823 |
| **Rapgef2** | 250.9008359 | 266.9922969 | 213.8030334 | 313.9898288 | 250.8118447 | 249.7029588 | 267.9132203 | 217.0611694 | 264.8497711 | 206.2408647 | 246.8722524 | 287.1518032 | 307.6024378 | 261.1272943 | 314.7317643 |
| **4921511C10Rik** | 0 | 3.169048034 | 0 | 1.725218839 | 0 | 2.805651223 | 0 | 0 | 0 | 1.747803938 | 0 | 1.735056213 | 0 | 0 | 0 |
| **Fnip2** | 184.9706893 | 195.6887161 | 195.7903236 | 175.9723216 | 215.3435031 | 197.7984112 | 209.2790138 | 148.3391003 | 157.2545516 | 117.1028638 | 127.6925443 | 198.6639363 | 185.7543807 | 160.832728 | 188.9827717 |
| **Ppid** | 2761.740588 | 2710.328331 | 2801.36795 | 2473.963816 | 2364.978354 | 2420.575593 | 2498.71926 | 3063.998592 | 2977.302683 | 3153.038304 | 3110.968728 | 2383.967236 | 2419.067371 | 2327.556782 | 2284.320271 |
| **Etfdh** | 591.5399271 | 605.2881745 | 632.0111647 | 571.0474358 | 654.8975946 | 543.5949244 | 619.7184591 | 711.5248371 | 672.6582256 | 602.9923585 | 658.3260063 | 661.056417 | 660.3653443 | 616.2242724 | 658.2061555 |
| **4930579G24Rik** | 225.2613345 | 301.0595632 | 300.7339371 | 287.2489367 | 290.0803659 | 333.1710827 | 238.1450847 | 311.7635329 | 290.4318513 | 315.4786108 | 275.2483733 | 351.348883 | 304.1941005 | 379.4929537 | 365.749927 |
| **Tmem144** | 8.241268334 | 14.26071615 | 10.18109683 | 3.450437679 | 2.533452977 | 4.90988964 | 9.020647148 | 6.704592104 | 23.3248378 | 12.23462756 | 10.40457769 | 4.337640531 | 2.556252945 | 9.035546516 | 0.718565672 |
| **Ctso** | 144.6800441 | 177.4666899 | 174.6449687 | 166.483618 | 147.5736359 | 167.6376606 | 224.614114 | 138.2822122 | 141.453855 | 138.9504131 | 113.5044839 | 187.386071 | 168.7126944 | 189.7464768 | 207.6654792 |
| **Gucy1b3** | 24.723805 | 26.93690829 | 26.62748401 | 22.42784491 | 36.10170492 | 19.63955856 | 18.0412943 | 18.43762829 | 26.33449429 | 24.46925513 | 15.13393118 | 25.15831508 | 26.41461377 | 12.64976512 | 10.05991941 |
| **Gucy1a3** | 76.00280797 | 122.8006113 | 188.741872 | 98.33747384 | 93.73776015 | 107.3161593 | 105.5415716 | 133.2537681 | 144.4635115 | 147.6894327 | 123.9090615 | 139.6720251 | 128.6647316 | 112.0407768 | 124.3118613 |
| **Map9** | 127.2818109 | 81.60298688 | 106.5099361 | 98.33747384 | 64.60305092 | 60.32150129 | 82.98995376 | 87.99777137 | 53.4214027 | 94.38141264 | 56.75224193 | 65.06460797 | 60.49798638 | 79.51280934 | 61.79664779 |
| **Plrg1** | 1548.44275 | 1389.627563 | 1451.197879 | 1439.695121 | 1396.565954 | 1356.532366 | 1296.266995 | 1494.285965 | 1395.728197 | 1483.011641 | 1437.723462 | 1356.813958 | 1353.961977 | 1310.154245 | 1330.783624 |
| **Sfrp2** | 14.65114371 | 11.88393013 | 20.36219366 | 23.29045433 | 10.13381191 | 6.312715251 | 7.216517718 | 12.5711102 | 15.80069657 | 14.85633347 | 9.458706988 | 9.542809169 | 11.0770961 | 9.035546516 | 5.748525376 |
| **Tlr2** | 81.49698686 | 94.27917901 | 99.4614844 | 76.77223835 | 77.2703158 | 86.2737751 | 64.94865946 | 108.1115477 | 69.9745134 | 81.27288311 | 85.12836289 | 58.99191123 | 52.82922754 | 37.94929537 | 55.32955674 |
| **D930015E06Rik** | 1563.093894 | 1628.098427 | 1639.156589 | 1539.757814 | 1473.202906 | 1529.079916 | 1641.757781 | 1557.97959 | 1610.918636 | 1412.225582 | 1496.367445 | 1471.327668 | 1508.189238 | 1445.687443 | 1513.299305 |
| **Mnd1** | 117.2091496 | 95.86370303 | 95.54567793 | 90.57398906 | 126.6726489 | 92.58649035 | 96.52092448 | 88.83584538 | 105.3379772 | 117.1028638 | 110.6668718 | 101.5007884 | 116.7355512 | 94.87323842 | 104.9105881 |
| **Trim2** | 3.662785926 | 17.42976419 | 5.482129061 | 7.763484777 | 12.03390164 | 8.416953669 | 1.80412943 | 29.33259046 | 17.30552482 | 20.97364725 | 17.02567258 | 17.35056213 | 10.22501178 | 13.55331977 | 3.59282836 |
| **Gm6525** | 0.915696482 | 3.169048034 | 4.698967767 | 1.725218839 | 1.266726489 | 0.701412806 | 0 | 0 | 1.504828245 | 2.621705907 | 0.945870699 | 0 | 0 | 2.710663955 | 0 |
| **Fhdc1** | 14.65114371 | 7.922620085 | 10.18109683 | 9.488703616 | 5.066905954 | 5.611302446 | 8.118582433 | 34.36103454 | 52.66898858 | 27.964863 | 30.26786236 | 22.55573076 | 3.408337261 | 16.26398373 | 5.748525376 |
| **Arfip1** | 586.9614447 | 664.7078251 | 659.42181 | 666.7970814 | 633.9966075 | 601.1107745 | 644.9762711 | 689.7349127 | 638.7995901 | 589.009927 | 652.6507822 | 638.5006862 | 706.3778973 | 633.3918108 | 556.1698301 |
| **Tmem154** | 456.9325443 | 556.960192 | 509.8380027 | 555.5204663 | 623.8627956 | 603.2150129 | 616.1102002 | 371.2667878 | 337.833941 | 363.5432191 | 419.0207196 | 590.7866404 | 625.4298873 | 669.5339968 | 617.2479122 |
| **Fbxw7** | 352.5431454 | 278.083965 | 327.3614211 | 335.5550642 | 328.0821605 | 270.0439302 | 359.9238212 | 341.9341973 | 359.6539506 | 393.255886 | 324.4336497 | 377.3747262 | 449.0484341 | 349.6756502 | 355.6900076 |
| **Gatb** | 206.9474048 | 215.4952663 | 231.8157432 | 220.8280114 | 215.9768663 | 199.2012368 | 168.6861017 | 296.6782006 | 309.9946185 | 232.4579237 | 227.9548384 | 170.9030369 | 202.796067 | 171.6753838 | 190.4199031 |
| **Fam160a1** | 1.831392963 | 2.376786026 | 0 | 0.86260942 | 0.633363244 | 1.402825611 | 0.902064715 | 12.5711102 | 7.524141226 | 3.495607876 | 7.56696559 | 0 | 1.70416863 | 0.903554652 | 2.155697016 |
| **Sh3d19** | 11.90405426 | 11.09166812 | 14.0969033 | 6.038265938 | 12.03390164 | 14.72966892 | 13.53097072 | 9.218814144 | 15.80069657 | 15.73023544 | 16.07980188 | 10.41033728 | 3.408337261 | 12.64976512 | 10.05991941 |
| **Rps3a1** | 26977.33404 | 25816.64981 | 26332.2322 | 25562.56754 | 27647.57234 | 28590.98879 | 28011.81559 | 25489.18303 | 25265.31382 | 25302.08371 | 25107.19183 | 27524.06423 | 26528.79307 | 27340.6602 | 27083.45874 |
| **Snord73a** | 0 | 2.376786026 | 0 | 1.725218839 | 0.633363244 | 0 | 0.902064715 | 0 | 0.752414123 | 1.747803938 | 0.945870699 | 1.735056213 | 1.70416863 | 0 | 10.05991941 |
| **Rnu73b** | 0.915696482 | 5.54583406 | 1.566322589 | 7.763484777 | 3.800179466 | 7.715540863 | 2.706194144 | 6.704592104 | 3.762070613 | 4.369509845 | 10.40457769 | 6.94022485 | 5.964590206 | 2.710663955 | 6.467091048 |
| **Lrba** | 788.4146706 | 695.6060435 | 727.5568426 | 812.5780733 | 668.831586 | 657.9252118 | 726.1620954 | 750.9143157 | 733.6037695 | 714.8518106 | 917.4945778 | 759.9546211 | 702.96956 | 674.9553247 | 700.6015302 |
| **Dclk2** | 181.3079033 | 171.1285938 | 151.9332911 | 129.3914129 | 126.6726489 | 136.0740843 | 149.7427427 | 88.83584538 | 108.3476337 | 115.3550599 | 172.1484672 | 129.2616878 | 146.5585022 | 118.3656594 | 159.5215792 |
| **Cd1d2** | 0 | 0 | 2.349483883 | 0 | 0 | 0 | 0 | 0 | 0 | 0 | 0 | 0 | 0 | 3.614218606 | 2.155697016 |
| **Cd1d1** | 42.12203815 | 34.06726637 | 70.4845165 | 49.16873692 | 56.36932874 | 62.42573971 | 73.96930661 | 49.44636677 | 77.49865463 | 34.08217679 | 51.07701773 | 70.26977661 | 46.86463733 | 56.92394305 | 64.67091048 |
| **Kirrel** | 0 | 0 | 2.349483883 | 1.725218839 | 0 | 0 | 0 | 1.676148026 | 0 | 0 | 0 | 0 | 0 | 0 | 0 |
| **Fcrl1** | 7.325571853 | 9.507144102 | 10.96425812 | 7.763484777 | 5.700269198 | 5.611302446 | 5.412388289 | 86.32162334 | 31.60139315 | 39.3255886 | 35.94308655 | 6.94022485 | 6.816674521 | 4.517773258 | 8.622788064 |
| **Etv3** | 629.9991793 | 655.992943 | 639.8427776 | 609.8648597 | 625.1295221 | 605.3192513 | 643.1721416 | 536.3673684 | 553.7767942 | 550.5582404 | 583.6022211 | 569.9659658 | 591.3465147 | 620.7420456 | 579.8824973 |
| **Arhgef11** | 96.14813056 | 86.35655893 | 98.67832311 | 102.6505209 | 104.5049353 | 91.88507755 | 122.6808012 | 47.77021874 | 56.43105919 | 77.77727523 | 98.37055267 | 101.5007884 | 98.84178056 | 112.0407768 | 104.9105881 |
| **Lrrc71** | 0.915696482 | 2.376786026 | 1.566322589 | 3.450437679 | 3.166816221 | 2.805651223 | 0.902064715 | 0 | 0 | 0 | 0 | 4.337640531 | 0.852084315 | 6.324882561 | 1.437131344 |
| **Pear1** | 153.8370089 | 171.9208558 | 170.7291622 | 156.9949144 | 203.9429647 | 166.9362478 | 177.7067488 | 108.9496217 | 117.3766031 | 116.2289619 | 143.7723462 | 183.0484304 | 197.6835611 | 205.1069059 | 156.6473165 |
| **Ntrk1** | 1.831392963 | 0.792262009 | 0 | 0 | 2.533452977 | 0 | 0 | 0 | 0 | 0.873901969 | 0.945870699 | 2.602584319 | 0 | 0 | 2.155697016 |
| **Insrr** | 23.80810852 | 17.42976419 | 11.74741942 | 33.64176737 | 13.93399137 | 18.93814575 | 25.25781201 | 6.704592104 | 2.257242368 | 13.10852953 | 24.59263817 | 18.21809023 | 20.45002356 | 10.84265582 | 18.68270747 |
| **Sh2d2a** | 4290.953713 | 4233.848173 | 4067.739764 | 4320.810583 | 4139.028801 | 4005.067121 | 4473.338921 | 3227.423024 | 3499.478084 | 3437.930346 | 3458.103275 | 4173.677719 | 4348.18626 | 3953.955155 | 4174.866554 |
| **Prcc** | 1488.006783 | 1433.201973 | 1293.782458 | 1413.816839 | 1390.865684 | 1397.214309 | 1434.282897 | 1387.012492 | 1383.689571 | 1352.800248 | 1270.304348 | 1414.938341 | 1500.520479 | 1365.271079 | 1437.131344 |
| **Hdgf** | 11128.45934 | 11017.19549 | 10827.2049 | 10265.9147 | 10510.02968 | 10309.36542 | 10861.76123 | 11120.40408 | 11045.43932 | 11126.51987 | 11149.9238 | 10722.64739 | 10319.59314 | 9835.192382 | 9869.499504 |
| **Mrpl24** | 586.0457482 | 581.5203142 | 598.335229 | 495.1378069 | 578.8940053 | 551.3104653 | 561.9863173 | 482.7306315 | 530.4519564 | 519.9716715 | 553.3343588 | 512.7091108 | 531.7006127 | 561.1074386 | 525.9900719 |
| **Rrnad1** | 153.8370089 | 188.558358 | 174.6449687 | 181.1479781 | 146.9402727 | 144.491038 | 143.4282897 | 203.6519852 | 133.1772997 | 173.0325898 | 184.4447863 | 178.7107899 | 155.9314297 | 179.8073757 | 196.1684284 |
| **Isg20l2** | 2085.040889 | 2054.335388 | 2051.882592 | 2034.033012 | 1985.593771 | 2096.522876 | 2342.662064 | 2202.458506 | 2227.145803 | 2094.743019 | 2074.294442 | 2183.568244 | 2120.83786 | 2066.429488 | 2172.942592 |
| **Crabp2** | 89.73825519 | 55.4583406 | 54.82129061 | 117.3148811 | 63.33632443 | 72.94693179 | 62.24246532 | 178.5097648 | 124.1483302 | 110.1116481 | 91.74945778 | 79.81258578 | 49.42089028 | 56.0203884 | 63.9523448 |
| **Nes** | 0 | 4.753572051 | 0 | 2.587828259 | 0 | 0.701412806 | 3.608258859 | 0.838074013 | 0 | 0 | 0.945870699 | 1.735056213 | 9.372927467 | 3.614218606 | 0 |
| **Gpatch4** | 1111.655529 | 1041.032279 | 1141.066006 | 1102.414838 | 1129.286665 | 1098.412454 | 1130.287088 | 1364.384493 | 1256.531585 | 1290.753208 | 1215.443848 | 1103.495751 | 1051.472045 | 1038.184295 | 988.7463646 |
| **Apoa1bp** | 401.0750589 | 408.0149344 | 376.7005826 | 390.7620671 | 430.0536429 | 455.2169109 | 464.5633281 | 262.3171661 | 295.6987502 | 275.2791202 | 317.8125548 | 432.896525 | 449.0484341 | 425.5742409 | 409.582433 |
| **Iqgap3** | 341.5547876 | 379.4935021 | 415.8586474 | 414.9151309 | 347.7164211 | 411.729317 | 394.2022804 | 358.6956776 | 379.2167178 | 387.1385722 | 434.1546507 | 463.2600088 | 373.21293 | 412.9244758 | 464.9119898 |
| **Mef2d** | 2165.622179 | 2372.032453 | 2288.397302 | 2125.46961 | 1884.889015 | 1854.535458 | 2210.960616 | 1895.723418 | 1907.369801 | 1717.217369 | 1793.370845 | 1946.73307 | 2100.387837 | 1977.881132 | 2068.032004 |
| **1700113A16Rik** | 149.2585265 | 147.3607336 | 158.1985815 | 179.4227593 | 157.7074478 | 115.7331129 | 120.8766718 | 133.2537681 | 141.453855 | 112.733354 | 106.883389 | 149.2148343 | 142.2980806 | 175.2896024 | 150.1802254 |
| **Tsacc** | 22.89241204 | 21.39107423 | 19.57903236 | 15.52696955 | 15.20071786 | 18.93814575 | 19.84542373 | 19.2757023 | 15.80069657 | 20.09974528 | 9.458706988 | 24.29078698 | 19.59793925 | 18.97464768 | 17.9641418 |
| **Cct3** | 9418.854009 | 9051.593447 | 9332.149985 | 8695.10295 | 8761.947121 | 9184.299278 | 8792.424775 | 10610.01701 | 9817.499471 | 10402.92904 | 10638.20775 | 8957.227697 | 8458.640997 | 8670.510436 | 8609.853881 |
| **Glmp** | 687.6880577 | 683.7221133 | 697.7967134 | 750.4701951 | 632.729881 | 662.8351014 | 661.2134359 | 591.6802532 | 622.9988935 | 605.6140645 | 641.3003338 | 611.6073149 | 623.7257187 | 797.8387573 | 674.733166 |
| **Tmem79** | 29.30228741 | 26.14464628 | 37.59174214 | 29.32872027 | 22.16771355 | 32.96640187 | 34.27845916 | 25.9802944 | 16.5531107 | 37.57778466 | 17.97154328 | 40.773821 | 19.59793925 | 48.79195118 | 35.9282836 |
| **Smg5** | 2378.979459 | 2510.678305 | 2595.39653 | 2361.824591 | 2335.210282 | 2287.307159 | 2266.888628 | 2850.289718 | 2890.775059 | 2944.175733 | 2999.355986 | 2573.955891 | 2594.59674 | 2410.68381 | 2536.536822 |
| **Pmf1** | 1471.524246 | 1541.741869 | 1390.894459 | 1567.361316 | 1600.508918 | 1548.719475 | 1506.448074 | 1474.172189 | 1431.091661 | 1382.512915 | 1374.350125 | 1584.97385 | 1526.083008 | 1659.829895 | 1450.784092 |
| **Slc25a44** | 571.3946045 | 631.4328208 | 580.3225192 | 618.4909539 | 480.7227024 | 568.1443726 | 570.1048997 | 612.6321035 | 530.4519564 | 658.0481826 | 689.5397394 | 506.6364141 | 544.4818774 | 588.2140782 | 546.8284764 |
| **Sema4a** | 2328.616153 | 2807.776558 | 2792.753176 | 2842.298038 | 2943.238996 | 2789.518728 | 2972.303235 | 2376.777901 | 2637.2115 | 2569.271789 | 2770.455277 | 2794.30803 | 2960.140911 | 3166.959054 | 3023.724348 |
| **Lmna** | 1640.928095 | 1345.26089 | 1454.330524 | 1488.863858 | 1031.115362 | 1179.776339 | 1178.096518 | 1673.633804 | 1134.640497 | 1749.551742 | 2185.907185 | 860.5878814 | 949.2219271 | 1096.915347 | 1071.381417 |
| **Mex3a** | 263.7205867 | 240.0553886 | 250.6116142 | 215.6523549 | 202.0428749 | 213.9309057 | 257.9905084 | 230.4703536 | 203.9042272 | 215.8537863 | 255.3850887 | 142.2746094 | 247.1044514 | 175.2896024 | 237.8452374 |
| **Rab25** | 4.578482408 | 0 | 1.566322589 | 0.86260942 | 0 | 0 | 0.902064715 | 0.838074013 | 1.504828245 | 3.495607876 | 2.837612096 | 5.205168638 | 0.852084315 | 2.710663955 | 2.155697016 |
| **Lamtor2** | 636.4090547 | 616.3798426 | 634.3606485 | 560.6961228 | 684.6656671 | 672.6548807 | 650.3886594 | 503.6824818 | 509.384361 | 600.3706526 | 533.4710741 | 620.282596 | 593.0506834 | 704.7726282 | 600.0023361 |
| **Ubqln4** | 1990.724151 | 2009.968716 | 2091.823818 | 2074.575654 | 1900.089733 | 2046.722567 | 2136.089245 | 2158.040584 | 2148.14232 | 2147.177138 | 2222.796142 | 2108.960826 | 2168.554582 | 2046.551286 | 2187.313905 |
| **Ssr2** | 2825.839342 | 2781.631912 | 2856.972402 | 2726.708376 | 2811.499441 | 2645.729103 | 2623.204191 | 2893.031493 | 2761.35983 | 2775.512653 | 2729.782837 | 2575.690948 | 2572.442547 | 2751.323914 | 2714.022543 |
| **Arhgef2** | 2621.639027 | 2570.097956 | 2651.000982 | 2523.132552 | 2545.486879 | 2606.449986 | 2926.297935 | 2826.823646 | 2532.625937 | 2422.456258 | 2605.873775 | 2659.841174 | 2728.373977 | 2733.252821 | 2652.225895 |
| **2810403A07Rik** | 897.3825519 | 980.8203665 | 875.5743272 | 945.4199239 | 836.6728457 | 908.3295834 | 931.8328504 | 938.6428946 | 905.1541895 | 942.9402244 | 897.6312931 | 901.3617024 | 885.3156035 | 852.0520364 | 912.5784034 |
| **Rit1** | 226.1770309 | 255.9006287 | 232.5989045 | 277.7602331 | 228.6441312 | 269.3425174 | 282.3462557 | 294.1639786 | 240.7725192 | 277.0269241 | 173.0943379 | 280.2115783 | 247.1044514 | 293.6552618 | 247.1865912 |
| **Syt11** | 370.857075 | 402.4691003 | 422.1239377 | 474.4351808 | 328.7155238 | 307.2188089 | 351.8052388 | 501.1682598 | 449.9436453 | 475.4026711 | 421.8583317 | 374.7721419 | 405.592134 | 435.5133421 | 337.0073002 |
| **5830417I10Rik** | 782.0047953 | 831.0828469 | 753.4011653 | 741.8441009 | 763.2027094 | 680.3704215 | 758.6364251 | 794.4941644 | 786.2727581 | 682.5174377 | 677.2434203 | 713.1081034 | 757.5029562 | 629.7775921 | 712.0985809 |
| **1500004A13Rik** | 46.70052056 | 55.4583406 | 53.25496803 | 47.44351808 | 47.50224332 | 29.45933784 | 35.18052388 | 40.22755263 | 35.36346376 | 55.92972601 | 63.37333682 | 58.12438312 | 48.56880596 | 27.10663955 | 48.86246569 |
| **Gon4l** | 964.2283951 | 1127.388838 | 1007.145425 | 1187.813171 | 924.0769734 | 944.1016365 | 1061.730169 | 1092.010439 | 1139.154982 | 1004.987264 | 1045.187122 | 1044.50384 | 1052.324129 | 947.8288295 | 1130.303802 |

| | | | | | | | | | | | | | | | |
|---|---|---|---|---|---|---|---|---|---|---|---|---|---|---|---|
| **Msto1** | 852.5134243 | 822.3679648 | 827.018327 | 872.0981233 | 859.4739225 | 855.0222102 | 795.6210784 | 962.108967 | 838.1893326 | 951.6792441 | 953.4376644 | 778.1727113 | 818.8530269 | 863.7982469 | 824.9133914 |
| **Dap3** | 1668.398989 | 1656.61986 | 1717.472719 | 1607.903958 | 1881.088835 | 1807.5408 | 1713.020893 | 1475.848337 | 1622.957262 | 1631.574976 | 1579.604067 | 1786.240371 | 1758.702026 | 1734.824931 | 1680.006541 |
| **Ash1l** | 495.3917965 | 524.4774496 | 516.8864544 | 521.0160895 | 482.6227921 | 415.236381 | 563.7904467 | 542.2338864 | 499.6029774 | 493.7546124 | 562.7930658 | 491.8884363 | 525.7360225 | 453.5844351 | 561.9183555 |
| **Rusc1** | 297.6013565 | 278.083965 | 315.6140017 | 337.2802831 | 271.0794686 | 253.2100229 | 273.3256086 | 240.5272417 | 286.6697807 | 233.3318257 | 332.946486 | 288.0193313 | 265.8503063 | 326.1832292 | 275.2106524 |
| **Fdps** | 7875.905438 | 7274.549762 | 7244.241974 | 6993.174565 | 7061.36681 | 7539.486249 | 7338.296455 | 5403.901236 | 5898.174307 | 6301.707098 | 6554.883942 | 7089.439685 | 7102.974851 | 6952.853044 | 6870.20639 |
| **Hcn3** | 40.29064519 | 61.00417465 | 52.47180673 | 63.83309705 | 51.93578603 | 56.11302446 | 71.26311247 | 76.26473519 | 29.34415078 | 63.79484373 | 65.26507822 | 75.47494525 | 69.01882953 | 49.69550584 | 78.32365824 |
| **Clk2** | 65.93014667 | 101.4095371 | 110.4257425 | 81.94789487 | 68.40323038 | 83.46812388 | 73.0672419 | 77.94088321 | 72.23175577 | 71.65996145 | 89.85771638 | 83.2826982 | 105.6584551 | 78.60925469 | 102.7548911 |
| **Scamp3** | 3.662785926 | 1.584524017 | 5.482129061 | 0.86260942 | 8.86708542 | 4.208476834 | 5.412388289 | 6.704592104 | 3.762070613 | 6.991215751 | 1.891741398 | 4.337640531 | 4.260421576 | 5.421327909 | 3.59282836 |
| **Fam189b** | 653.8072878 | 688.4756854 | 725.2073587 | 746.157148 | 689.0992098 | 594.7980592 | 667.5278889 | 538.0435164 | 639.5520042 | 557.5494562 | 570.3600314 | 775.570127 | 767.727968 | 749.9503608 | 742.9969048 |
| **Gba** | 517.3685121 | 434.9518427 | 516.8864544 | 463.2212584 | 428.1535531 | 407.5208401 | 485.3108166 | 417.3608585 | 471.0112407 | 451.8073179 | 419.9665903 | 438.9692218 | 409.0004713 | 383.1071723 | 457.726333 |
| **Mtx1** | 1060.376526 | 1109.959074 | 1079.196264 | 1123.117464 | 1019.08146 | 961.6369566 | 973.3278272 | 1189.227025 | 1173.013617 | 1185.01107 | 1141.665933 | 1066.192043 | 973.0802879 | 958.6714853 | 1016.770426 |
| **Thbs3** | 6.409875371 | 7.130358077 | 7.04845165 | 7.763484777 | 2.533452977 | 5.611302446 | 3.608258859 | 7.542666117 | 0.752414123 | 4.369509845 | 1.891741398 | 0.867528106 | 5.112505891 | 8.131991864 | 3.59282836 |
| **Muc1** | 15.56684019 | 3.169048034 | 14.0969033 | 9.488703616 | 3.166816221 | 4.208476834 | 4.510323574 | 0 | 1.504828245 | 6.117313782 | 1.891741398 | 7.807752957 | 0.852084315 | 0 | 10.05991941 |
| **Trim46** | 76.00280797 | 90.31786897 | 107.2930973 | 80.22267603 | 50.0356963 | 77.85682143 | 56.83007703 | 72.91243914 | 77.49865463 | 84.76849098 | 63.37333682 | 45.11146153 | 72.42716679 | 54.21327909 | 66.10804182 |
| **Krtcap2** | 1519.140463 | 1277.126358 | 1413.606137 | 1295.639348 | 1388.965595 | 1284.286847 | 1319.720678 | 1066.868219 | 996.9487124 | 1101.116481 | 1160.583347 | 1293.484406 | 1229.557667 | 1281.240496 | 1232.340127 |
| **Dpm3** | 276.5403374 | 265.4077728 | 252.1779368 | 274.3097954 | 328.7155238 | 261.6269765 | 298.5834206 | 219.5753914 | 188.1035306 | 209.7364725 | 254.439218 | 277.608994 | 253.0690416 | 290.9445978 | 282.3963091 |
| **Slc50a1** | 384.5925223 | 318.4893274 | 367.3026471 | 363.1585657 | 342.6495152 | 419.4448578 | 371.6506625 | 325.1727171 | 316.0139315 | 346.0651797 | 368.8895725 | 406.8706818 | 418.3733987 | 362.3254153 | 472.8162122 |
| **Efna1** | 0 | 2.376786026 | 1.566322589 | 0 | 0 | 0 | 0 | 0.838074013 | 0 | 0 | 0 | 0 | 3.408337261 | 0 | 4.311394032 |
| **Efna3** | 1.831392963 | 0 | 0 | 0 | 0 | 1.402825611 | 0 | 0 | 2.257242368 | 0 | 0 | 0 | 0 | 0 | 0 |
| **Efna4** | 1.831392963 | 6.338096068 | 0.783161294 | 0 | 0 | 4.208476834 | 3.608258859 | 0.838074013 | 0 | 0 | 6.621094891 | 1.735056213 | 1.70416863 | 1.807109303 | 2.874262688 |
| **Adam15** | 128.1975074 | 133.1000174 | 123.7394845 | 116.4522717 | 112.7386575 | 159.9221197 | 131.7014484 | 78.77895723 | 102.3283207 | 83.02068705 | 64.31920752 | 114.51371 | 108.214708 | 126.4976512 | 147.3059628 |
| **Dcst1** | 2.747089445 | 5.54583406 | 1.566322589 | 0 | 0.633363244 | 0 | 0.902064715 | 0 | 1.504828245 | 1.747803938 | 1.891741398 | 2.602584319 | 5.112505891 | 0 | 0 |
| **Zbtb7b** | 226.1770309 | 237.6786026 | 190.3081946 | 193.22451 | 249.5451182 | 307.2188089 | 310.3102619 | 120.6826579 | 97.81383594 | 139.824315 | 189.1741398 | 269.8012411 | 233.4711024 | 250.2846385 | 308.9832389 |
| **Gm15417** | 4.578482408 | 3.169048034 | 2.349483883 | 0 | 5.700269198 | 1.402825611 | 1.80412943 | 2.514222039 | 7.524141226 | 4.369509845 | 0.945870699 | 9.542809169 | 2.556252945 | 2.710663955 | 4.311394032 |
| **Lenep** | 8.241268334 | 15.84524017 | 12.53058071 | 16.38957897 | 10.76717515 | 16.13249453 | 13.53097072 | 20.95185033 | 20.31518131 | 12.23462756 | 22.70089677 | 17.35056213 | 10.22501178 | 16.26398373 | 12.21561642 |
| **Flad1** | 337.8920017 | 312.9434934 | 391.5806472 | 320.8907041 | 298.3140881 | 328.2611931 | 324.7432973 | 413.1704884 | 305.4801338 | 386.2646703 | 398.2115642 | 289.7543875 | 334.8691359 | 327.0867839 | 339.1629972 |
| **Cks1b** | 1608.878718 | 1859.438934 | 1911.69672 | 1751.959731 | 1883.622288 | 1857.34111 | 1777.067488 | 1772.526538 | 1832.128388 | 1869.276311 | 1799.046069 | 1964.083633 | 1768.927038 | 1907.403869 | 1829.468201 |
| **Shc1** | 1508.152105 | 1647.112716 | 1657.169299 | 1538.895205 | 1556.806854 | 1650.424332 | 1672.427981 | 1443.16345 | 1535.677224 | 1447.181661 | 1466.099583 | 1626.615199 | 1663.268583 | 1697.77919 | 1569.347428 |
| **Pygo2** | 1075.027669 | 1141.649554 | 1102.691103 | 1103.277448 | 1171.088639 | 1153.824065 | 1240.338983 | 1056.81133 | 1119.592214 | 1119.468422 | 1135.990709 | 1177.23564 | 1133.272139 | 1180.042375 | 1162.639257 |
| **Pbxip1** | 695.929326 | 830.2905849 | 842.6815529 | 817.7537298 | 780.3035169 | 696.5029161 | 755.930231 | 671.2972845 | 567.3202484 | 631.8311235 | 682.9186445 | 814.6088918 | 843.563472 | 814.1027411 | 734.3741167 |
| **Pmvk** | 912.0336956 | 911.1013098 | 942.9261986 | 896.251187 | 891.7754479 | 929.3719676 | 913.7915561 | 838.9120871 | 835.1796761 | 762.9164189 | 865.4716894 | 984.6444006 | 837.5988818 | 1002.945663 | 788.9851078 |
| **Adar** | 3403.643822 | 3278.380191 | 3021.436274 | 2982.903373 | 3054.710927 | 3246.138465 | 3526.17097 | 2292.9705 | 2585.294925 | 2250.29757 | 2422.37486 | 3119.63107 | 3050.461848 | 3177.80171 | 3100.610875 |
| **Chrnb2** | 35.71216278 | 22.18333624 | 18.79587107 | 27.60350143 | 10.76717515 | 13.32684331 | 16.23716487 | 18.43762829 | 22.57242368 | 21.84754922 | 19.86328467 | 20.82067455 | 36.63962555 | 18.97464768 | 29.46119255 |
| **4632404H12Rik** | 22.89241204 | 22.97559825 | 14.0969033 | 34.50437679 | 13.93399137 | 18.93814575 | 33.37639445 | 33.52296052 | 15.80069657 | 17.47803938 | 34.99721585 | 16.48303402 | 30.67503535 | 28.91374885 | 33.05402091 |
| **Ube2q1** | 1709.605331 | 1927.573467 | 1874.104978 | 1757.997997 | 1836.753408 | 1955.538902 | 1941.243266 | 1731.460911 | 1896.836003 | 1897.241174 | 1866.202889 | 1984.904307 | 1892.479264 | 1988.723788 | 2001.205396 |
| **She** | 22.89241204 | 34.06726637 | 28.9769679 | 21.56523549 | 41.80197412 | 46.29324518 | 57.73214175 | 21.78992434 | 13.54345421 | 23.59535316 | 49.18527634 | 58.12438312 | 45.1604687 | 34.33507676 | 42.39537465 |
| **Il6ra** | 110.7992743 | 147.3607336 | 157.4154202 | 127.6661941 | 231.8109474 | 274.9538198 | 305.7999383 | 117.3303618 | 66.96485691 | 61.17313782 | 127.6925443 | 253.318207 | 253.9211259 | 275.5841687 | 262.9950359 |
| **Atp8b2** | 2563.950148 | 2910.770619 | 3109.9335 | 2888.016337 | 3156.682409 | 3171.788707 | 3403.490169 | 3072.379332 | 3162.396557 | 2992.240342 | 3160.154005 | 3484.860403 | 3581.310377 | 3454.289433 | 3683.367634 |
| **Hax1** | 1706.858242 | 1480.737694 | 1437.884137 | 1321.517631 | 1299.028014 | 1403.527024 | 1368.432172 | 1416.345082 | 1362.621976 | 1397.369248 | 1406.509729 | 1172.030472 | 1282.386894 | 1237.869873 | 1205.753198 |
| **Gm19710** | 1.831392963 | 7.130358077 | 6.265290356 | 3.450437679 | 2.533452977 | 1.402825611 | 0.902064715 | 6.704592104 | 2.257242368 | 7.86511772 | 0.945870699 | 6.072696744 | 5.964590206 | 5.421327909 | 4.311394032 |
| **Ubap2l** | 5958.437006 | 5861.946601 | 5895.638225 | 5761.368314 | 5473.525157 | 5648.477324 | 5999.632418 | 6411.2662 | 5844.752904 | 5749.401053 | 6184.102629 | 5947.772697 | 5854.671329 | 5668.901884 | 5917.388309 |
| **4933434E20Rik** | 664.7956456 | 701.9441395 | 711.1104554 | 783.249353 | 708.1001071 | 762.4357198 | 773.9715253 | 798.6845344 | 784.0155157 | 756.7991051 | 716.024119 | 758.2195649 | 605.8319481 | 777.960555 | 665.3918122 |
| **1700094D03Rik** | 22.89241204 | 23.76786026 | 28.9769679 | 22.42784491 | 32.9348887 | 21.04238417 | 43.29910631 | 34.36103454 | 18.05793894 | 30.58656891 | 28.37612096 | 32.09853993 | 43.45630007 | 11.74621047 | 33.05402091 |
| **Tpm3** | 18842.2865 | 18981.80546 | 18891.41675 | 18952.39156 | 18909.05966 | 18973.91781 | 19361.01497 | 19705.63427 | 19838.15076 | 18912.11251 | 19132.12662 | 19273.87194 | 18981.88229 | 19131.86619 | 18539.7129 |
| **Nup210l** | 12.81975074 | 26.93690829 | 21.14535495 | 17.25218839 | 17.73417084 | 21.04238417 | 25.25781201 | 16.76148026 | 18.05793894 | 18.35194135 | 32.15960376 | 45.11146153 | 42.60421576 | 29.8173035 | 26.58692986 |
| **Rps27** | 0 | 5.54583406 | 0 | 0 | 5.700269198 | 3.507064029 | 4.510323574 | 0 | 0 | 0.873901969 | 0.945870699 | 0 | 3.408337261 | 5.421327909 | 0.718565672 |
| **Rab13** | 7.325571853 | 3.961310043 | 0.783161294 | 4.313047098 | 3.166816221 | 2.104238417 | 0.902064715 | 0 | 0 | 2.621705907 | 2.837612096 | 1.735056213 | 5.112505891 | 2.710663955 | 2.155697016 |
| **Jtb** | 528.3568699 | 575.1822182 | 650.8070357 | 612.452688 | 637.1634237 | 655.1195605 | 561.9863173 | 687.2206907 | 705.764447 | 719.2213204 | 740.6167571 | 577.7737188 | 599.0152736 | 726.4579399 | 612.2179525 |
| **Creb3l4** | 0 | 0 | 0.783161294 | 2.587828259 | 0.633363244 | 2.805651223 | 1.80412943 | 1.676148026 | 0 | 1.747803938 | 2.837612096 | 5.205168638 | 1.70416863 | 0 | 3.59282836 |
| **Slc39a1** | 1438.559173 | 1460.138882 | 1430.052524 | 1354.296789 | 1258.492766 | 1308.836295 | 1323.328937 | 1307.39546 | 1361.869562 | 1367.656581 | 1406.509729 | 1398.455307 | 1349.701555 | 1226.123662 | 1299.166735 |
| **Crtc2** | 1000.856254 | 1108.37455 | 1114.438522 | 1136.056606 | 1091.918233 | 1099.113867 | 1152.838705 | 959.5947449 | 889.3534929 | 1042.565049 | 1128.423744 | 1100.025639 | 1065.105394 | 1139.382416 | 1123.118145 |
| **Dennd4b** | 1013.676005 | 1061.631091 | 1039.255038 | 919.5416413 | 1067.217067 | 1059.834749 | 1177.194453 | 1032.507184 | 923.2121284 | 947.3097343 | 920.3321899 | 1011.537772 | 1133.272139 | 1006.559882 | 1185.633359 |
| **Gatad2b** | 882.7314082 | 825.5370129 | 848.1636819 | 851.3954972 | 683.3989406 | 732.976382 | 825.389214 | 807.9033486 | 802.8258688 | 734.0776539 | 663.0553598 | 787.7155205 | 746.4258601 | 719.2295027 | 777.4880571 |
| **Slc27a3** | 12.81975074 | 4.753572051 | 12.53058071 | 9.488703616 | 4.43354271 | 12.6254305 | 5.412388289 | 7.542666117 | 14.29586833 | 13.10852953 | 10.40457769 | 19.08561834 | 4.260421576 | 18.97464768 | 9.341353736 |
| **Ints3** | 1530.128821 | 1625.721641 | 1607.046976 | 1737.295371 | 1502.337615 | 1365.650733 | 1607.479322 | 1790.964166 | 1773.440087 | 1753.921252 | 1855.798311 | 1580.63621 | 1552.497622 | 1663.444114 | 1648.389651 |
| **Ilf2** | 1513.646284 | 1651.074026 | 1600.781686 | 1630.331803 | 1515.638244 | 1457.53581 | 1547.943051 | 1904.942232 | 1644.777272 | 1772.273193 | 1850.123087 | 1577.166097 | 1399.97453 | 1538.753572 | 1579.407347 |
| **Snapin** | 307.6740178 | 301.0595632 | 322.6624533 | 284.6611085 | 297.0473616 | 293.1905528 | 270.6194144 | 300.0304967 | 255.0683876 | 293.6310616 | 295.111658 | 293.2244999 | 298.2295103 | 405.6960386 | 354.971442 |
| **Chtop** | 3511.696007 | 3703.82489 | 3697.304471 | 3640.211751 | 3667.173184 | 3792.53904 | 3868.053497 | 3797.313353 | 3594.282264 | 3501.725189 | 3550.798603 | 3618.459731 | 3739.798059 | 3869.924573 | 3807.679496 |
| **S100a1** | 46.70052056 | 27.7291703 | 41.50754861 | 44.85568982 | 15.83408111 | 37.1748787 | 30.6702003 | 28.49451644 | 40.63036262 | 28.83876497 | 49.18527634 | 30.36348372 | 27.26669809 | 31.62441281 | 14.37131344 |
| **S100a13** | 399.243666 | 413.5607684 | 445.6187766 | 434.7551475 | 417.386378 | 439.0844164 | 421.2642218 | 336.0676792 | 401.7891415 | 382.7690624 | 340.5134516 | 409.4732662 | 431.1546635 | 432.8026781 | 413.8938271 |
| **S100a2** | 0 | 1.584524017 | 0 | 0 | 1.266726489 | 4.90988964 | 0 | 3.352296052 | 3.00965649 | 0 | 0 | 0 | 0 | 0 | 0.718565672 |
| **S100a3** | 0 | 0.792262009 | 4.698967767 | 2.587828259 | 0 | 1.402825611 | 1.80412943 | 2.514222039 | 4.514484735 | 4.369509845 | 3.783482795 | 2.602584319 | 0.852084315 | 2.710663955 | 3.59282836 |
| **S100a4** | 177.6451174 | 214.7030043 | 187.1755494 | 239.8054187 | 249.5451182 | 253.2100229 | 308.5061325 | 326.0107911 | 224.2194085 | 281.396434 | 226.063097 | 277.608994 | 259.0336318 | 385.8178362 | 260.1207733 |
| **S100a5** | 0.915696482 | 0.792262009 | 3.915806472 | 3.450437679 | 0 | 1.402825611 | 0 | 5.028444078 | 0 | 0.873901969 | 1.891741398 | 0.867528106 | 0 | 3.614218606 | 1.437131344 |
| **S100a6** | 473.415081 | 533.1923317 | 563.0929707 | 572.7726546 | 519.3578603 | 429.2646371 | 555.6718643 | 668.7830624 | 461.9822713 | 533.080201 | 616.7076956 | 517.9142794 | 504.4339146 | 562.0109933 | 523.1158092 |
| **S100a11** | 984.3737177 | 869.1114233 | 958.5894245 | 972.1608159 | 983.6131184 | 982.6793408 | 959.7968565 | 756.7808338 | 825.3982925 | 848.5588118 | 884.3891034 | 1014.140356 | 1002.051155 | 1039.991404 | 1041.920224 |
| **S100a10** | 3931.084995 | 3790.181449 | 3974.54357 | 3917.109375 | 4235.933378 | 4449.762839 | 4511.225639 | 3538.348483 | 3544.622931 | 3642.423406 | 3374.866653 | 4374.076712 | 4308.138297 | 4552.108335 | 4484.568359 |
| **Them4** | 65.93014667 | 72.88810478 | 72.05083909 | 73.32180067 | 88.6708542 | 70.14128057 | 74.87137133 | 58.66518091 | 85.77520997 | 90.0119028 | 89.85771638 | 83.2826982 | 78.39175699 | 69.57370817 | 63.23377913 |
| **Rorc** | 5.494178889 | 30.89821833 | 20.36219366 | 16.38957897 | 25.33452977 | 28.05651223 | 36.08258859 | 53.63673684 | 33.10622139 | 18.35194135 | 9.458706988 | 17.35056213 | 17.89377062 | 16.26398373 | 25.14979852 |
| **Lingo4** | 0 | 1.584524017 | 0.783161294 | 0 | 0 | 0 | 0 | 0 | 4.514484735 | 0 | 1.891741398 | 4.337640531 | 0.852084315 | 0 | 0 |
| **Tdrkh** | 106.2207919 | 87.94108294 | 126.0889684 | 113.8644434 | 131.1061916 | 89.07942633 | 107.3457011 | 112.3019177 | 72.98416989 | 86.51629492 | 96.47881127 | 115.3812381 | 85.20843152 | 100.2945663 | 94.8506687 |
| **Oaz3** | 1.831392963 | 1.584524017 | 5.482129061 | 0.86260942 | 0 | 2.104238417 | 2.706194144 | 4.190370065 | 5.266898858 | 1.747803938 | 1.891741398 | 0.867528106 | 0 | 2.710663955 | 0 |
| **Mrpl9** | 1548.44275 | 1655.035336 | 1663.434589 | 1808.029344 | 1608.109277 | 1662.34835 | 1590.340092 | 1847.115125 | 1835.138045 | 1928.701645 | 1879.445078 | 1682.136998 | 1694.795703 | 1705.911182 | 1787.791392 |
| **Riiad1** | 0.915696482 | 0 | 0 | 0 | 0 | 0.701412806 | 0.902064715 | 0 | 3.762070613 | 1.747803938 | 0.945870699 | 1.735056213 | 0 | 0.903554652 | 4.311394032 |
| **Celf3** | 2.747089445 | 0.792262009 | 0 | 0.86260942 | 0.633363244 | 1.402825611 | 4.510323574 | 0 | 2.257242368 | 4.369509845 | 2.837612096 | 0 | 3.408337261 | 0 | 3.59282836 |
| **Snx27** | 1259.998359 | 1214.537659 | 1330.591039 | 1262.86019 | 1150.187652 | 1114.544948 | 1255.674083 | 1381.984048 | 1410.77648 | 1316.970267 | 1431.102367 | 1288.279238 | 1241.486847 | 1130.346869 | 1200.723238 |
| **Tuft1** | 71.42432556 | 83.9797729 | 61.86974226 | 69.87136299 | 82.33722176 | 103.1076824 | 93.81473034 | 60.34132894 | 91.04210883 | 75.15556933 | 77.5613973 | 68.5347204 | 126.1084786 | 85.8376919 | 75.44939556 |
| **Cgn** | 0 | 0 | 1.566322589 | 2.587828259 | 3.800179466 | 4.208476834 | 7.216517718 | 0 | 0 | 1.747803938 | 0.945870699 | 5.205168638 | 5.964590206 | 0.903554652 | 2.874262688 |
| **Pogz** | 801.2344214 | 719.3739037 | 780.8118106 | 750.4701951 | 787.2705126 | 692.2944392 | 839.8222495 | 828.0171249 | 838.1893326 | 756.7991051 | 704.6736706 | 772.1000146 | 818.8530269 | 771.6356724 | 797.6078959 |
| **Psmb4** | 4714.921184 | 4774.170863 | 4730.294219 | 4746.077027 | 4865.496443 | 5161.696837 | 4707.875746 | 5025.929856 | 4872.633858 | 5072.127028 | 4850.424943 | 4796.5629 | 4823.649308 | 4918.951523 | 4766.246102 |
| **Selenbp1** | 28.38659093 | 14.26071615 | 14.8800646 | 13.80175071 | 14.56735462 | 15.43108173 | 24.3557473 | 1.676148026 | 6.019312981 | 0 | 11.35044839 | 11.27786538 | 23.85836082 | 4.517773258 | 11.49705075 |
| **Rfx5** | 340.6390911 | 376.324454 | 375.1342601 | 414.9151309 | 363.5505022 | 374.5544383 | 453.7385515 | 325.1727171 | 321.2808303 | 277.0269241 | 267.6814078 | 391.2551759 | 437.1192537 | 392.1427188 | 362.8756643 |
| **Pi4kb** | 721.5688275 | 705.1131876 | 752.618004 | 779.7989154 | 747.3686282 | 746.3032253 | 738.7910014 | 797.0083864 | 710.2789317 | 859.9195374 | 788.8561628 | 784.2454081 | 757.5029562 | 739.107705 | 777.4880571 |
| **Zfp687** | 379.0983434 | 350.9720698 | 386.8816795 | 351.9446432 | 324.2819811 | 316.3371754 | 293.1710323 | 313.4396809 | 373.1974048 | 342.5695718 | 344.2969344 | 366.0968608 | 357.8754124 | 291.8481525 | 329.8216434 |
| **Psmd4** | 2505.345574 | 2482.949135 | 2507.682465 | 2459.299455 | 2354.844542 | 2405.144511 | 2333.641417 | 2655.018473 | 2589.056996 | 2505.476945 | 2552.905016 | 2326.710381 | 2276.76929 | 2453.150879 | 2247.673422 |
| **Pip5k1a** | 1249.010001 | 1249.397187 | 1263.239168 | 1279.249769 | 1052.016349 | 1114.544948 | 1251.163759 | 1411.316638 | 1294.152291 | 1312.600757 | 1307.193306 | 1100.025639 | 1248.303522 | 1088.783355 | 1143.237984 |
| **Vps72** | 705.0862908 | 779.5858164 | 747.9190362 | 744.4319292 | 713.8003763 | 766.6441966 | 810.0541139 | 706.496393 | 790.0348287 | 745.4383795 | 713.1865069 | 707.9029347 | 766.8758836 | 730.9757131 | 737.9669451 |
| **Tmod4** | 0 | 1.584524017 | 0.783161294 | 1.725218839 | 1.266726489 | 0 | 4.510323574 | 4.190370065 | 6.019312981 | 2.621705907 | 0.945870699 | 4.337640531 | 3.408337261 | 7.228437213 | 0 |
| **Scnm1** | 346.13327 | 303.4363493 | 349.2899373 | 296.7376404 | 321.1151648 | 263.7312149 | 301.2896147 | 257.288722 | 288.1746089 | 316.3525127 | 284.7070803 | 332.2632647 | 306.7503535 | 331.6045571 | 300.3604509 |
| **Lysmd1** | 42.12203815 | 48.32798252 | 45.42335508 | 56.9322217 | 48.13560656 | 47.69607079 | 58.63420646 | 30.17066447 | 36.11587788 | 50.6863142 | 48.23940564 | 48.58157395 | 63.05423932 | 52.40616979 | 51.73672838 |
| **Tnfaip8l2** | 422.136078 | 445.2512488 | 453.4503895 | 469.2595243 | 445.2543607 | 485.3776616 | 475.3881047 | 403.9516743 | 429.628464 | 346.0651797 | 346.1886757 | 419.8836034 | 495.0609871 | 559.3003293 | 503.714536 |
| **Sema6c** | 11.90405426 | 8.714882094 | 8.614774239 | 5.175656518 | 5.700269198 | 14.02825611 | 2.706194144 | 19.2757023 | 9.028969471 | 3.495607876 | 17.02567258 | 6.94022485 | 1.70416863 | 6.324882561 | 3.59282836 |
| **Gabpb2** | 2876.202649 | 3235.598043 | 3104.451371 | 3030.346891 | 2891.936573 | 2841.423276 | 3426.041787 | 2856.156236 | 3037.495813 | 2973.014498 | 2988.951408 | 3338.248153 | 3320.572576 | 3074.796479 | 3301.809263 |
| **Gm16740** | 2.747089445 | 3.169048034 | 1.566322589 | 1.725218839 | 1.266726489 | 0 | 0 | 1.676148026 | 12.03862596 | 0 | 5.675224193 | 0 | 0.852084315 | 0.903554652 | 1.437131344 |
| **Mllt11** | 111.7149708 | 99.03275106 | 89.28038757 | 87.9861608 | 98.80466611 | 95.39214158 | 106.4436363 | 78.77895723 | 50.41174621 | 72.53386342 | 98.37055267 | 74.60741714 | 80.94800994 | 67.76659887 | 75.44939556 |
| **Cdc42se1** | 3363.353177 | 3633.313571 | 3703.569762 | 3874.841513 | 3983.854806 | 3737.127429 | 3837.383297 | 3079.921998 | 3276.01109 | 3314.710168 | 3448.644568 | 3838.81187 | 3875.279465 | 3997.325779 | 3857.979093 |
| **Gm128** | 0.915696482 | 0 | 0.783161294 | 0 | 1.266726489 | 0 | 1.80412943 | 0 | 0 | 0.873901969 | 2.837612096 | 2.602584319 | 0 | 2.710663955 | 2.155697016 |
| **Bnipl** | 0 | 0 | 0 | 0 | 0 | 0 | 0.902064715 | 0 | 0 | 0 | 0 | 0 | 0 | 0 | 0 |
| **Prune** | 360.7844137 | 343.8417117 | 364.9531632 | 305.3637346 | 320.4818016 | 326.1569547 | 342.7845916 | 292.4878306 | 350.6249811 | 299.7483753 | 304.570365 | 340.9385458 | 336.5733045 | 305.4014722 | 287.4262688 |
| **Fam63a** | 110.7992743 | 115.6702532 | 111.9920651 | 114.7270528 | 134.2730078 | 114.3302873 | 138.9179661 | 103.9211776 | 129.4152291 | 131.9591973 | 123.9090615 | 124.0565192 | 145.7064179 | 133.7260884 | 125.0304269 |
| **Anxa9** | 25.63950148 | 26.93690829 | 35.24225825 | 22.42784491 | 17.73417084 | 20.34097137 | 13.53097072 | 14.24725822 | 24.82966605 | 21.84754922 | 8.512836289 | 19.08561834 | 22.15419219 | 25.29953024 | 14.37131344 |
| **Cers2** | 2144.56116 | 2032.944314 | 2058.931043 | 1937.420757 | 1915.290451 | 1999.727909 | 2095.496332 | 1960.255117 | 1923.922911 | 1959.288214 | 1863.365277 | 1907.694306 | 1929.970974 | 1989.627343 | 2140.607137 |
| **Setdb1** | 1338.748256 | 1343.676366 | 1339.205814 | 1264.585409 | 1345.263531 | 1346.011174 | 1419.849861 | 1366.898715 | 1306.943331 | 1379.017307 | 1428.264755 | 1332.523171 | 1359.926567 | 1381.535062 | 1457.251183 |
| **Gm4349** | 0 | 1.584524017 | 2.349483883 | 0.86260942 | 0 | 3.507064029 | 0 | 0 | 0.752414123 | 0 | 0 | 0 | 0.852084315 | 0 | 2.155697016 |
| **Arnt** | 774.6792234 | 755.8179561 | 798.0413591 | 781.5241342 | 836.0394824 | 882.3773096 | 925.5183974 | 782.7611282 | 789.2824146 | 784.7639681 | 757.6424297 | 810.2712513 | 853.7884838 | 898.1333237 | 857.2488467 |
| **Ctsk** | 0 | 0 | 0 | 0 | 0 | 0 | 0.902064715 | 0 | 0 | 1.747803938 | 2.837612096 | 0 | 0 | 0 | 0 |
| **Ctss** | 1410.172582 | 1316.739458 | 1376.014394 | 1320.655021 | 1641.677529 | 1503.127643 | 1602.066933 | 1180.846284 | 1250.512272 | 1167.53303 | 1226.794296 | 1382.839801 | 1478.366287 | 1601.098843 | 1437.84991 |
| **Golph3l** | 408.4006308 | 377.9089781 | 446.4019379 | 393.3498954 | 347.7164211 | 380.1657407 | 389.6919568 | 411.4943404 | 386.740859 | 394.129788 | 401.995047 | 379.9773106 | 409.8525556 | 404.7924839 | 390.1811599 |
| **Ensa** | 1168.42871 | 1193.938847 | 1211.550523 | 1201.614922 | 1272.426758 | 1230.979474 | 1348.586749 | 1229.454577 | 1199.348111 | 1198.119599 | 1222.064943 | 1288.279238 | 1232.11392 | 1325.514674 | 1314.256614 |
| **Mcl1** | 4226.854959 | 4520.647021 | 4660.592863 | 4803.009249 | 4780.625768 | 4968.106903 | 5099.371833 | 5037.662892 | 4792.877961 | 4747.909397 | 4519.370199 | 5337.03291 | 5247.987297 | 5265.916509 | 5075.947907 |
| **Adamtsl4** | 6.409875371 | 5.54583406 | 8.614774239 | 6.038265938 | 5.700269198 | 9.81977928 | 13.53097072 | 5.866518091 | 3.762070613 | 6.117313782 | 6.621094891 | 1.735056213 | 1.70416863 | 9.939101167 | 8.622788064 |
| **Ecm1** | 52.19469945 | 53.87381658 | 33.67593566 | 41.40525214 | 48.13560656 | 51.20313482 | 40.59291217 | 52.79866282 | 46.6496756 | 44.56900041 | 61.48159542 | 41.6413491 | 20.45002356 | 33.43152211 | 42.39537465 |
| **Tars2** | 925.7691429 | 882.5798775 | 874.7911659 | 887.6250928 | 910.142982 | 876.0645943 | 811.8582433 | 1024.964518 | 1209.881909 | 1069.65601 | 1035.728415 | 806.8011388 | 896.3926995 | 922.5292993 | 942.7581616 |
| **Rprd2** | 1058.545133 | 999.8346547 | 921.7808436 | 972.1608159 | 927.2437896 | 957.4284798 | 1041.884746 | 1096.200809 | 1049.617701 | 940.3185185 | 995.0559751 | 1043.636312 | 968.8198663 | 879.158676 | 982.9978392 |
| **Prpf3** | 1181.248461 | 1197.107895 | 1127.752264 | 1162.797498 | 1187.556083 | 1115.947774 | 1284.540154 | 1250.406427 | 1223.425363 | 1314.348561 | 1141.665933 | 1183.308337 | 1287.4994 | 1200.824132 | 1232.340127 |
| **Mrps21** | 599.7811954 | 502.2941134 | 595.2025838 | 527.9169648 | 609.9288042 | 563.234483 | 561.9863173 | 567.3761068 | 537.2236835 | 583.7665152 | 511.716048 | 598.5943933 | 529.996444 | 621.6456003 | 564.7926182 |
| **C920021L13Rik** | 0.915696482 | 0.792262009 | 3.132645178 | 4.313047098 | 3.166816221 | 2.805651223 | 0 | 13.40918421 | 0 | 0.873901969 | 2.837612096 | 2.602584319 | 1.70416863 | 6.324882561 | 7.18565672 |
| **Ciart** | 228.9241204 | 289.1756331 | 297.6012919 | 210.4766984 | 261.5790199 | 266.5368662 | 255.2843143 | 89.6739194 | 95.55659357 | 116.2289619 | 133.3677685 | 252.4506789 | 247.9565357 | 225.8886629 | 239.2823688 |
| **Aph1a** | 1216.044928 | 1342.091842 | 1238.178007 | 1338.769819 | 1304.09492 | 1193.103183 | 1243.947242 | 1283.929388 | 1277.59918 | 1247.05811 | 1239.090615 | 1383.70733 | 1319.02652 | 1273.108504 | 1246.711441 |
| **Car14** | 0.915696482 | 3.961310043 | 1.566322589 | 4.313047098 | 3.800179466 | 4.208476834 | 5.412388289 | 4.190370065 | 10.53379772 | 3.495607876 | 1.891741398 | 2.602584319 | 0.852084315 | 0 | 1.437131344 |
| **Anp32e** | 6991.342637 | 6361.071666 | 6977.967134 | 6441.104537 | 6583.177561 | 6681.658387 | 7029.790322 | 6727.220103 | 6701.75259 | 6776.235867 | 6955.933119 | 6960.177997 | 6682.897284 | 7008.873432 | 6643.858203 |
| **Plekho1** | 99.81091649 | 124.3851353 | 168.3796783 | 161.3079615 | 134.2730078 | 128.3585434 | 115.4642835 | 216.2230954 | 158.0069657 | 175.6542957 | 155.1227946 | 147.4797781 | 170.416863 | 173.4824931 | 132.2160836 |
| **Vps45** | 316.8309826 | 300.2673012 | 289.769679 | 294.1498121 | 267.9126523 | 259.5227381 | 264.3049614 | 285.7832385 | 285.1649525 | 311.1091009 | 312.1373306 | 325.3230399 | 244.5481985 | 314.4370187 | 290.3005315 |
| **Otud7b** | 1187.658337 | 1062.423353 | 1077.629941 | 998.901708 | 900.6425334 | 915.3437115 | 1018.431063 | 1218.559615 | 1078.209438 | 1003.23946 | 1074.509114 | 946.473164 | 959.4469389 | 913.4937527 | 934.1353736 |
| **Mtmr11** | 21.06101908 | 20.59881222 | 32.10961307 | 31.05393911 | 15.83408111 | 28.05651223 | 28.86607087 | 21.78992434 | 25.58208017 | 35.82998073 | 30.26786236 | 21.68820266 | 12.78126473 | 19.87820233 | 30.89832389 |
| **Sf3b4** | 3328.55671 | 3458.223667 | 3451.391825 | 3443.536803 | 3166.816221 | 3352.753211 | 3417.02114 | 3850.112016 | 3396.397349 | 3370.639894 | 3595.254526 | 3295.739276 | 3270.299602 | 3229.304325 | 3230.671261 |
| **Sv2a** | 40.29064519 | 45.95119649 | 57.95393579 | 48.3061275 | 51.30242279 | 41.38335554 | 53.22181817 | 55.31288486 | 63.2027863 | 63.79484373 | 41.61831075 | 44.24393342 | 64.75840795 | 77.70570004 | 51.73672838 |
| **Bola1** | 234.4182993 | 241.6399126 | 254.5274207 | 240.6680281 | 226.7440415 | 281.2665351 | 230.0265023 | 274.0502023 | 267.1070135 | 247.3142572 | 279.0318561 | 233.3650606 | 192.5710552 | 217.756671 | 260.8393389 |
| **Hist2h2ac** | 10.0726613 | 19.0142882 | 7.831612945 | 15.52696955 | 9.500448664 | 15.43108173 | 10.82477658 | 12.5711102 | 8.276555348 | 8.739019689 | 7.56696559 | 9.542809169 | 29.82295103 | 6.324882561 | 9.341353736 |
| **Hist2h2be** | 20.14532259 | 33.27500436 | 21.14535495 | 25.01567317 | 25.96789302 | 21.04238417 | 27.96400616 | 27.65644243 | 27.08690841 | 14.85633347 | 27.43025026 | 13.8804497 | 28.1187824 | 32.52796746 | 15.80844478 |
| **Hist2h4** | 13.73544722 | 8.714882094 | 7.831612945 | 6.900875357 | 12.03390164 | 26.65368662 | 21.64955315 | 2.514222039 | 15.80069657 | 13.9824315 | 8.512836289 | 8.675281063 | 7.668758836 | 19.87820233 | 9.341353736 |
| **Hist2h3b** | 12.81975074 | 19.0142882 | 6.265290356 | 18.11479781 | 15.20071786 | 17.53532014 | 11.72684129 | 10.89496217 | 13.54345421 | 25.3431571 | 8.512836289 | 13.8804497 | 23.85836082 | 14.45687443 | 17.9641418 |
| **Hist2h2bb** | 11.90405426 | 3.961310043 | 0.783161294 | 3.450437679 | 8.86708542 | 11.22260489 | 14.43303544 | 0.838074013 | 1.504828245 | 3.495607876 | 8.512836289 | 13.8804497 | 9.372927467 | 13.55331977 | 10.05991941 |
| **Terc** | 0 | 0 | 0.783161294 | 0 | 5.700269198 | 0 | 1.80412943 | 0 | 0 | 0 | 0 | 0.867528106 | 0 | 0.903554652 | 0.718565672 |
| **Gm15441** | 0.915696482 | 1.584524017 | 6.265290356 | 3.450437679 | 1.266726489 | 2.805651223 | 1.80412943 | 0 | 1.504828245 | 7.86511772 | 6.621094891 | 7.807752957 | 2.556252945 | 6.324882561 | 4.311394032 |
| **Txnip** | 1614.372897 | 1885.58358 | 1955.553752 | 1669.149227 | 1941.891707 | 1899.425878 | 2160.444992 | 1062.677849 | 1260.293655 | 1243.562502 | 1362.999677 | 2123.708804 | 2298.923482 | 2407.069592 | 2237.613503 |
| **Polr3gl** | 141.9329546 | 134.6845414 | 144.1016782 | 143.1931637 | 179.2417981 | 152.9079916 | 173.1964252 | 136.6060641 | 151.2352386 | 103.1204323 | 134.3136392 | 163.9628121 | 163.6001885 | 149.0865175 | 147.3059628 |
| **Ankrd34a** | 0 | 3.169048034 | 0 | 0 | 0 | 0 | 0 | 0 | 0.752414123 | 3.495607876 | 1.891741398 | 3.470112425 | 0 | 0 | 0.718565672 |
| **Lix1l** | 41.20634167 | 32.48274235 | 29.76012919 | 17.25218839 | 13.93399137 | 18.93814575 | 39.69084745 | 16.76148026 | 43.64001911 | 26.21705907 | 35.94308655 | 30.36348372 | 36.63962555 | 36.14218606 | 20.83840449 |
| **6330549D23Rik** | 13.73544722 | 11.88393013 | 17.22954848 | 28.46611085 | 19.63426057 | 15.43108173 | 12.62890601 | 10.05688816 | 13.54345421 | 9.612921658 | 23.64676747 | 12.14539349 | 11.0770961 | 16.26398373 | 15.80844478 |
| **Rbm8a** | 3220.504526 | 2892.548593 | 2895.347306 | 2678.402248 | 2928.671642 | 2906.654667 | 2914.571093 | 2986.057709 | 2934.415078 | 3018.457401 | 3009.760563 | 3057.169047 | 2751.380254 | 2808.247857 | 2666.597209 |
| **Pex11b** | 304.9269284 | 271.7458689 | 253.7442594 | 295.8750309 | 293.8805453 | 253.9114357 | 324.7432973 | 295.0020526 | 314.5091032 | 334.7044541 | 303.6244943 | 323.5879836 | 319.5316182 | 301.7872536 | 296.7676225 |
| **Itga10** | 10.98835778 | 15.84524017 | 14.0969033 | 12.07653188 | 10.13381191 | 14.02825611 | 3.608258859 | 15.08533223 | 21.82000955 | 13.10852953 | 26.48437957 | 12.14539349 | 4.260421576 | 18.07109303 | 12.21561642 |
| **Pias3** | 107.1364883 | 137.8535895 | 131.5710975 | 160.4453521 | 133.6396445 | 129.0599563 | 120.8766718 | 134.9299161 | 118.8814314 | 160.7979623 | 154.1769239 | 155.287531 | 132.0730689 | 135.5331977 | 166.7072359 |
| **Nudt17** | 0.915696482 | 8.714882094 | 0.783161294 | 0 | 1.900089733 | 1.402825611 | 5.412388289 | 1.676148026 | 0 | 2.621705907 | 1.891741398 | 2.602584319 | 0 | 0 | 4.311394032 |
| **Polr3c** | 1184.911247 | 1248.604925 | 1230.346394 | 1186.087952 | 1231.89151 | 1187.49188 | 1259.282342 | 1370.251011 | 1289.637806 | 1247.932012 | 1424.481272 | 1184.175865 | 1221.888908 | 1225.220108 | 1171.980611 |
| **Rnf115** | 1444.053351 | 1494.206148 | 1516.200266 | 1444.008168 | 1414.300124 | 1513.648835 | 1592.144222 | 1573.902997 | 1576.307587 | 1654.296427 | 1581.495808 | 1675.196773 | 1618.108114 | 1692.357862 | 1569.347428 |
| **Cd160** | 629.9991793 | 675.0072312 | 780.0286493 | 752.1954139 | 973.4793065 | 836.7854772 | 917.3998149 | 833.883643 | 667.3913267 | 729.708144 | 760.4800418 | 852.7801285 | 800.1071719 | 993.0065621 | 915.4526661 |
| **Pdzk1** | 119.9562391 | 158.4524017 | 140.969033 | 135.4296789 | 133.6396445 | 157.8178813 | 187.6294607 | 165.9386546 | 165.531107 | 166.9152761 | 193.9034932 | 158.7576434 | 168.7126944 | 143.6651896 | 140.8388717 |
| **Gpr89** | 529.2725663 | 521.3084016 | 544.2970997 | 540.8561061 | 456.0215359 | 449.6056085 | 502.4500461 | 545.5861825 | 518.4133305 | 631.8311235 | 521.174755 | 507.5039422 | 604.9798638 | 510.5083781 | 528.8643346 |
| **Gja8** | 5.494178889 | 0 | 0.783161294 | 0 | 2.533452977 | 3.507064029 | 7.216517718 | 1.676148026 | 3.762070613 | 0 | 0.945870699 | 0.867528106 | 5.112505891 | 1.807109303 | 2.155697016 |
| **Acp6** | 347.0489665 | 310.5667073 | 346.1572922 | 265.6837013 | 300.847541 | 298.1004424 | 292.2689676 | 249.7460559 | 288.1746089 | 258.6749828 | 271.4648905 | 307.9724777 | 282.0399083 | 281.9090513 | 303.9532792 |
| **Bcl9** | 393.7494871 | 393.7542182 | 403.3280667 | 385.5864106 | 376.2177671 | 361.2275949 | 469.0736517 | 430.7700427 | 316.7663456 | 416.8512392 | 341.4593223 | 396.4603446 | 359.579581 | 335.2187757 | 500.8402734 |
| **Chd1l** | 969.722574 | 1027.563825 | 979.7347794 | 965.2599406 | 1024.148366 | 935.6846828 | 1024.745516 | 1116.314585 | 1027.797691 | 1069.65601 | 1047.078864 | 1015.007884 | 1013.128251 | 981.2603516 | 1026.11178 |
| **Fmo5** | 18.31392963 | 11.09166812 | 21.14535495 | 15.52696955 | 19.00089733 | 20.34097137 | 25.25781201 | 25.9802944 | 24.82966605 | 6.991215751 | 18.91741398 | 26.02584319 | 20.45002356 | 22.58886629 | 28.74262688 |
| **Prkab2** | 86.99116575 | 85.56429692 | 53.25496803 | 52.6191746 | 76.63695256 | 69.43986777 | 70.36104775 | 49.44636677 | 39.12553437 | 41.94729451 | 39.72656935 | 76.34247335 | 86.06051583 | 74.09148143 | 96.28780004 |

| | | | | | | | | | | | | | | | |
|---|---|---|---|---|---|---|---|---|---|---|---|---|---|---|---|
| **Pde4dip** | 742.6298465 | 655.200681 | 617.9142614 | 594.3378901 | 569.3935566 | 631.2715251 | 710.8269952 | 540.5577384 | 512.3940175 | 535.7019069 | 475.7729615 | 626.3552927 | 624.577803 | 491.5337305 | 610.0622555 |
| **Sec22b** | 925.7691429 | 985.5739386 | 1016.54336 | 1009.253021 | 958.9119518 | 1045.105081 | 1050.003328 | 1061.001701 | 1033.06459 | 1042.565049 | 983.7055267 | 1027.153278 | 1036.134527 | 1049.930505 | 939.8838989 |
| **Gm5544** | 0.915696482 | 0.792262009 | 4.698967767 | 0.86260942 | 3.800179466 | 0.701412806 | 0 | 1.676148026 | 0 | 0 | 0 | 5.205168638 | 0 | 1.807109303 | 0 |
| **Notch2** | 2308.47083 | 1949.756803 | 1989.229688 | 2109.080031 | 1803.81852 | 1992.713781 | 2251.553528 | 2165.58325 | 2061.614696 | 1714.595663 | 1748.914922 | 1811.398686 | 2117.429523 | 1668.865441 | 1952.342931 |
| **Phgdh** | 13979.93818 | 13557.18749 | 13771.89136 | 12808.88727 | 12652.69753 | 13120.62794 | 12508.9314 | 12871.14069 | 13317.72997 | 13472.94665 | 12896.94698 | 12298.94596 | 12194.17863 | 11943.18538 | 12281.0059 |
| **Wars2** | 125.450418 | 143.3994235 | 135.4869039 | 135.4296789 | 136.1730975 | 127.6571306 | 135.3097072 | 205.3281332 | 139.1966127 | 162.5457662 | 160.7980188 | 138.804497 | 113.3272139 | 114.7514407 | 131.497518 |
| **Mir6481** | 0 | 3.169048034 | 0 | 1.725218839 | 0 | 2.104238417 | 0 | 1.676148026 | 1.504828245 | 0 | 0 | 0.867528106 | 5.964590206 | 0.903554652 | 0 |
| **Spag17** | 0 | 0 | 0.783161294 | 0 | 0 | 1.402825611 | 0 | 0 | 0.752414123 | 2.621705907 | 1.891741398 | 1.735056213 | 0 | 0 | 0 |
| **Wdr3** | 1943.107934 | 1911.728227 | 1921.877817 | 1906.366817 | 1881.088835 | 1906.440006 | 1941.243266 | 2095.185033 | 2081.929877 | 2095.616921 | 2014.704588 | 1807.061045 | 1762.962448 | 1837.830161 | 1818.689716 |
| **Gdap2** | 465.1738126 | 466.642323 | 453.4503895 | 498.5882446 | 421.1865574 | 450.3070213 | 448.3261632 | 481.8925575 | 535.7188553 | 491.1329065 | 523.0664964 | 458.0548401 | 483.983891 | 426.4777955 | 459.1634644 |
| **Fam46c** | 219.7671556 | 180.6357379 | 234.1652271 | 199.2627759 | 193.8091527 | 208.3196033 | 211.985208 | 141.6345082 | 167.0359352 | 132.8330993 | 226.063097 | 244.642926 | 243.6961141 | 191.5535861 | 191.1384687 |
| **Man1a2** | 1269.155323 | 1358.729345 | 1406.557685 | 1364.648102 | 1117.252763 | 1146.809937 | 1307.993836 | 1447.353821 | 1422.062692 | 1348.430738 | 1353.54097 | 1263.120923 | 1257.676449 | 1259.555184 | 1179.166268 |
| **Trim45** | 65.93014667 | 82.39524888 | 73.61716168 | 71.59658183 | 43.06870061 | 69.43986777 | 59.53627118 | 58.66518091 | 74.48899814 | 76.90337326 | 62.42746612 | 63.32955176 | 83.50426289 | 98.48745702 | 51.73672838 |
| **Ttf2** | 1199.562391 | 1228.798375 | 1249.142265 | 1237.844517 | 1216.057429 | 1180.477752 | 1201.5502 | 1325.833089 | 1181.290172 | 1311.726855 | 1324.218978 | 1198.056315 | 1306.245255 | 1178.235266 | 1304.91526 |
| **Cd101** | 299.4327495 | 325.6196855 | 317.1803243 | 278.6228425 | 352.1499638 | 392.7911712 | 359.0217565 | 234.6607237 | 240.7725192 | 258.6749828 | 210.9291658 | 367.8319171 | 347.6504006 | 421.9600223 | 376.5284121 |
| **Ptgfrn** | 767.3536516 | 710.6590216 | 726.7736813 | 628.8422669 | 666.298133 | 738.5876844 | 744.2033897 | 676.3257285 | 750.1568802 | 718.3474184 | 630.8957561 | 689.6848445 | 700.4133071 | 696.6406364 | 695.5715705 |
| **Cd2** | 5864.120268 | 5094.244715 | 5223.685834 | 4426.911542 | 5001.66954 | 5337.050039 | 5577.466131 | 2881.298457 | 3557.413972 | 3975.380057 | 3952.79365 | 4977.008746 | 4833.022236 | 4611.742942 | 5034.989663 |
| **Igsf3** | 218.8514591 | 295.5137292 | 331.2772276 | 318.3028759 | 263.4791096 | 209.0210161 | 270.6194144 | 259.802944 | 212.9331967 | 263.0444926 | 245.9263817 | 235.9676449 | 277.7794867 | 211.4317885 | 252.2165509 |
| **Atp1a1** | 11926.03098 | 11697.74856 | 12140.56639 | 11857.42908 | 11368.23687 | 12116.20481 | 11973.10496 | 12552.67257 | 12606.69862 | 12079.07301 | 12324.6952 | 11896.41292 | 11951.3346 | 11720.00739 | 11724.1175 |
| **Slc22a15** | 51.27900297 | 48.32798252 | 42.2907099 | 50.03134634 | 57.63605523 | 47.69607079 | 41.49497688 | 51.96058881 | 32.35380727 | 65.54264767 | 52.96875913 | 39.90629289 | 51.97714322 | 54.21327909 | 38.80254629 |
| **Vangl1** | 0.915696482 | 0 | 7.831612945 | 1.725218839 | 3.166816221 | 2.805651223 | 1.80412943 | 0 | 0 | 1.747803938 | 1.891741398 | 3.470112425 | 3.408337261 | 5.421327909 | 7.18565672 |
| **Tspan2** | 70.50862908 | 96.65596504 | 86.14774239 | 56.9322217 | 44.3354271 | 59.62008849 | 56.83007703 | 29.33259046 | 27.83932254 | 41.94729451 | 33.10547446 | 65.06460797 | 58.79381775 | 78.60925469 | 66.82660749 |
| **Sike1** | 428.5459534 | 473.7726811 | 470.679938 | 431.3047098 | 461.7218051 | 453.8140853 | 462.7591987 | 405.6278223 | 354.3870517 | 452.6812199 | 353.7556413 | 467.5976493 | 514.6589264 | 460.8128723 | 461.3191614 |
| **Csde1** | 4563.831264 | 4267.91544 | 4528.238605 | 4217.297453 | 4324.604232 | 4141.141205 | 4487.771956 | 4969.778897 | 4305.313609 | 4479.621493 | 4696.248019 | 4303.806935 | 4061.033846 | 4228.635769 | 4274.028617 |
| **Nras** | 2673.833726 | 2818.868226 | 2912.576854 | 2844.023257 | 2608.18984 | 2605.04716 | 2899.235993 | 2992.762301 | 2910.337826 | 2952.914753 | 2999.355986 | 2804.718368 | 2759.901097 | 2757.648797 | 2677.375694 |
| **Ampd1** | 0 | 0.792262009 | 0 | 5.175656518 | 0 | 2.104238417 | 1.80412943 | 4.190370065 | 4.514484735 | 2.621705907 | 0.945870699 | 4.337640531 | 0.852084315 | 1.807109303 | 0 |
| **Dennd2c** | 76.00280797 | 72.88810478 | 55.60445191 | 60.38265938 | 53.20251252 | 39.98052993 | 54.12388289 | 130.739546 | 75.99382638 | 82.14678508 | 91.74945778 | 67.66719229 | 45.1604687 | 73.18792678 | 69.70087018 |
| **Bcas2** | 1509.067802 | 1435.578759 | 1455.896846 | 1395.702041 | 1518.80506 | 1492.606451 | 1416.241602 | 1651.005806 | 1571.040688 | 1580.01476 | 1556.90317 | 1478.267893 | 1490.295467 | 1482.733183 | 1434.257081 |
| **Trim33** | 1464.198674 | 1563.925205 | 1475.475879 | 1414.679448 | 1481.436628 | 1472.265479 | 1501.93775 | 1510.209372 | 1515.362043 | 1427.955817 | 1494.475704 | 1513.836545 | 1571.243477 | 1552.306891 | 1626.114116 |
| **Syt6** | 21.06101908 | 20.59881222 | 18.79587107 | 12.93914129 | 20.26762382 | 23.84803539 | 12.62890601 | 10.05688816 | 12.03862596 | 9.612921658 | 13.24218978 | 15.61550591 | 17.89377062 | 10.84265582 | 15.80844478 |
| **Olfml3** | 2.747089445 | 0.792262009 | 8.614774239 | 3.450437679 | 3.166816221 | 7.715540863 | 3.608258859 | 3.352296052 | 0 | 1.747803938 | 0 | 1.735056213 | 3.408337261 | 6.324882561 | 0.718565672 |
| **Hipk1** | 3291.013155 | 3403.557589 | 3422.414857 | 3552.22559 | 3138.314875 | 3047.638641 | 3490.088381 | 3113.444958 | 3080.383418 | 2892.615517 | 3241.498885 | 3351.261075 | 3479.060259 | 3096.481791 | 3283.845121 |
| **Dclre1b** | 223.4299415 | 228.1714584 | 190.3081946 | 238.9428092 | 216.6102295 | 190.0828703 | 243.557473 | 194.433171 | 238.5152769 | 238.5752375 | 230.7924505 | 177.8432618 | 176.3814532 | 188.8429222 | 201.9169538 |
| **Gm15471** | 0.915696482 | 0.792262009 | 4.698967767 | 0 | 3.166816221 | 2.104238417 | 0 | 0 | 0 | 0 | 1.891741398 | 5.205168638 | 5.964590206 | 0 | 0 |
| **Ap4b1** | 311.3368037 | 317.6970654 | 397.0627763 | 364.8837845 | 355.9501433 | 325.4555419 | 306.702003 | 341.0961233 | 392.0077579 | 353.9302974 | 435.1005214 | 301.0322529 | 339.1295574 | 374.0716258 | 350.6600479 |
| **Bcl2l15** | 0 | 0 | 1.566322589 | 0.86260942 | 0 | 0 | 0 | 1.676148026 | 4.514484735 | 0 | 0 | 0 | 0.852084315 | 0 | 0 |
| **Ptpn22** | 3097.801197 | 3014.556942 | 3153.790533 | 2960.475528 | 3123.114158 | 2952.947912 | 3293.438274 | 2297.998944 | 2318.187912 | 2336.813865 | 2092.265986 | 3326.102759 | 3187.647423 | 3294.36026 | 3125.042107 |
| **Rsbn1** | 684.0252717 | 694.8137815 | 669.6029068 | 627.1170481 | 609.9288042 | 622.8545715 | 663.9196301 | 555.6430707 | 543.2429965 | 558.4233581 | 526.8499792 | 600.3294495 | 634.8028148 | 534.0007991 | 633.056357 |
| **Phtf1os** | 0.915696482 | 2.376786026 | 3.915806472 | 1.725218839 | 0 | 0.701412806 | 2.706194144 | 0 | 0 | 0.873901969 | 0 | 0.867528106 | 0.852084315 | 0 | 0.718565672 |
| **Phtf1** | 712.4118627 | 772.4554583 | 787.8602623 | 748.7449763 | 752.4355342 | 794.7007089 | 762.244684 | 826.3409769 | 826.9031207 | 817.0983409 | 831.4203442 | 825.0192291 | 813.740521 | 827.6560608 | 856.530281 |
| **Magi3** | 175.8137245 | 202.0268122 | 179.3439364 | 209.614089 | 167.8412597 | 179.5616783 | 188.5315254 | 209.5185033 | 216.6952673 | 174.7803938 | 174.0402086 | 187.386071 | 181.4939591 | 165.3505012 | 196.1684284 |
| **Lrig2** | 213.3572802 | 159.2446637 | 191.8745171 | 150.9566484 | 174.8082554 | 187.9786319 | 151.5468721 | 173.4813207 | 155.7497234 | 167.789178 | 175.93195 | 189.1211272 | 197.6835611 | 200.5891326 | 171.7371956 |
| **Slc16a1** | 4439.296543 | 4079.357082 | 4435.042411 | 4172.441763 | 4418.975355 | 4210.581073 | 4494.988474 | 4171.932437 | 4091.627999 | 3934.306664 | 4137.238436 | 4216.186597 | 4317.511225 | 3850.949925 | 4039.057642 |
| **Fam19a3** | 49.44761 | 39.61310043 | 53.25496803 | 26.74089201 | 32.30152546 | 33.66781467 | 29.76813559 | 47.77021874 | 27.08690841 | 62.04703979 | 54.86050053 | 30.36348372 | 21.30210788 | 33.43152211 | 25.86836419 |
| **Ppm1j** | 237.1653887 | 254.3161047 | 205.9714205 | 253.6071694 | 224.2105885 | 248.3001332 | 269.7173497 | 150.8533223 | 205.4090555 | 197.501845 | 170.2567258 | 231.6300044 | 226.6544278 | 244.8633106 | 292.4562285 |
| **Rhoc** | 79.6655939 | 70.51131876 | 60.30341968 | 75.90962893 | 59.53614496 | 59.62008849 | 55.0259476 | 56.98903289 | 74.48899814 | 55.05582404 | 67.15681961 | 45.97898963 | 47.71672165 | 47.88839653 | 56.76668809 |
| **Mov10** | 2654.6041 | 2406.09972 | 2237.491818 | 2373.038513 | 2096.432339 | 2080.390382 | 2225.393651 | 2142.955251 | 2327.216881 | 1982.883567 | 2041.188968 | 1723.778347 | 1796.193736 | 1883.007894 | 1945.87584 |
| **Capza1** | 5520.734087 | 5775.590042 | 5969.255387 | 5707.02392 | 5524.194217 | 5624.629289 | 5922.956917 | 5995.581489 | 5885.383267 | 6016.815056 | 6008.170679 | 6241.864725 | 6145.232081 | 5910.150976 | 5908.046955 |
| **St7l** | 289.3600882 | 291.5524191 | 229.4662593 | 238.0801998 | 262.2123831 | 286.8778375 | 243.557473 | 227.9561316 | 250.5539028 | 223.718904 | 262.9520543 | 250.7156227 | 279.4836554 | 252.9953024 | 243.5937628 |
| **Wnt2b** | 0 | 8.714882094 | 8.614774239 | 15.52696955 | 12.66726489 | 2.104238417 | 2.706194144 | 2.514222039 | 3.762070613 | 6.117313782 | 8.512836289 | 2.602584319 | 10.22501178 | 2.710663955 | 8.622788064 |
| **Cttnbp2nl** | 7.325571853 | 7.922620085 | 4.698967767 | 0.86260942 | 12.03390164 | 3.507064029 | 6.314453003 | 8.380740131 | 0.752414123 | 3.495607876 | 2.837612096 | 0 | 1.70416863 | 4.517773258 | 2.874262688 |
| **Ddx20** | 1154.693263 | 1216.914445 | 1239.744329 | 1126.567902 | 1036.815631 | 1155.226891 | 1115.854052 | 1353.489531 | 1303.18126 | 1274.149071 | 1225.848426 | 1140.79946 | 1065.957478 | 978.5496877 | 1069.944286 |
| **Fam212b** | 154.7527054 | 209.1571702 | 185.6092268 | 238.9428092 | 127.9393753 | 143.7896252 | 123.5828659 | 445.8553749 | 371.6925766 | 335.5783561 | 303.6244943 | 144.0096656 | 104.8063708 | 164.4469466 | 118.5633359 |
| **Rap1a** | 2690.316263 | 2678.637851 | 2768.475176 | 2537.796913 | 2635.424459 | 2609.255637 | 2691.761109 | 2679.32262 | 2853.906767 | 2730.069751 | 2772.347018 | 2813.393649 | 2908.163768 | 2857.943363 | 2514.979852 |
| **Gm5547** | 43.03773463 | 38.82083842 | 46.98967767 | 22.42784491 | 32.9348887 | 31.56357626 | 34.27845916 | 58.66518091 | 66.21244279 | 57.67752995 | 43.51005214 | 39.90629289 | 34.08337261 | 59.634607 | 28.02406121 |
| **Tmigd3** | 1.831392963 | 4.753572051 | 0 | 3.450437679 | 1.266726489 | 2.104238417 | 0 | 2.514222039 | 0 | 3.495607876 | 0 | 0 | 0 | 0.903554652 | 1.437131344 |
| **Adora3** | 5.494178889 | 4.753572051 | 7.04845165 | 1.725218839 | 0.633363244 | 3.507064029 | 3.608258859 | 10.89496217 | 8.276555348 | 13.9824315 | 16.07980188 | 1.735056213 | 0.852084315 | 0 | 2.155697016 |
| **I830077J02Rik** | 0 | 0 | 1.566322589 | 2.587828259 | 1.900089733 | 1.402825611 | 0.902064715 | 2.514222039 | 0 | 2.621705907 | 0 | 0 | 0 | 0.903554652 | 0 |
| **Atp5f1** | 5151.708405 | 5135.442339 | 5474.297448 | 5281.757477 | 5089.073668 | 4846.061075 | 4998.340585 | 6007.314526 | 6048.657131 | 6050.023331 | 5953.310178 | 5095.860096 | 4990.657834 | 5243.327643 | 5116.187584 |
| **Wdr77** | 1284.722164 | 1290.594812 | 1260.889684 | 1274.936722 | 1337.029809 | 1256.931748 | 1280.931895 | 1784.259574 | 1469.464781 | 1633.32278 | 1656.219594 | 1217.141933 | 1263.641039 | 1180.94593 | 1320.005139 |
| **Ovgp1** | 7.325571853 | 15.84524017 | 6.265290356 | 8.626094197 | 9.500448664 | 0.701412806 | 1.80412943 | 5.028444078 | 6.771727103 | 3.495607876 | 17.02567258 | 14.74797781 | 7.668758836 | 8.131991864 | 9.341353736 |
| **Pifo** | 0 | 3.169048034 | 0 | 0.86260942 | 4.43354271 | 1.402825611 | 0 | 0.838074013 | 1.504828245 | 5.243411813 | 8.512836289 | 0.867528106 | 0 | 3.614218606 | 0 |
| **Chia1** | 0 | 0.792262009 | 0 | 4.313047098 | 0 | 0.701412806 | 0 | 0 | 0 | 0 | 0 | 0 | 0 | 0 | 0 |
| **2010016I18Rik** | 16.48253667 | 20.59881222 | 10.96425812 | 4.313047098 | 2.533452977 | 12.6254305 | 13.53097072 | 18.43762829 | 3.762070613 | 9.612921658 | 15.13393118 | 1.735056213 | 8.520843152 | 7.228437213 | 12.21561642 |
| **Dennd2d** | 1224.286196 | 1258.11207 | 1235.045361 | 1169.698373 | 1150.187652 | 1148.212763 | 1202.452265 | 1122.181103 | 1130.126012 | 1312.600757 | 1210.714494 | 1173.765528 | 1248.303522 | 1136.671752 | 1204.316066 |
| **Cept1** | 808.5599932 | 764.5328382 | 834.0667786 | 701.3014582 | 735.9680898 | 710.5311722 | 761.3426193 | 643.640842 | 807.3403535 | 727.0864381 | 715.0782483 | 713.9756315 | 697.8570541 | 677.6659887 | 762.398178 |
| **Dram2** | 208.7787978 | 194.8964541 | 174.6449687 | 165.6210086 | 165.3078068 | 176.756027 | 147.9386132 | 196.9473931 | 230.2387215 | 165.1674721 | 204.3080709 | 178.7107899 | 170.416863 | 154.5078454 | 206.2283479 |
| **Lrif1** | 202.3689224 | 215.4952663 | 241.2136787 | 237.2175904 | 237.5112166 | 220.243621 | 190.3356548 | 238.8510937 | 227.9814791 | 257.8010808 | 211.8750365 | 253.318207 | 213.0210788 | 227.6957722 | 196.8869941 |
| **Cd53** | 7925.353048 | 6518.731806 | 6790.791585 | 6020.15114 | 6330.465627 | 6599.593089 | 7552.085792 | 3791.446835 | 4230.824611 | 4181.620921 | 4310.332774 | 6544.632034 | 6602.801358 | 6103.511671 | 6349.964843 |
| **Kcna3** | 510.0429402 | 675.0072312 | 594.4194225 | 554.6578568 | 499.0902365 | 584.97828 | 650.3886594 | 506.1967039 | 571.082319 | 503.3675341 | 578.8728676 | 555.217988 | 581.1215029 | 534.0007991 | 538.2056883 |
| **AI504432** | 806.7286003 | 1030.732873 | 922.5640049 | 935.9312203 | 983.6131184 | 939.8931597 | 1043.688875 | 928.5860065 | 810.35001 | 730.582046 | 889.1184568 | 968.1613666 | 949.2219271 | 925.2399632 | 961.4408691 |
| **Kcna2** | 32.04937685 | 41.98988645 | 33.67593566 | 30.19132969 | 39.90188439 | 37.87629151 | 53.22181817 | 32.68488651 | 48.15450385 | 27.964863 | 17.97154328 | 40.773821 | 49.42089028 | 44.27417793 | 22.9941015 |
| **Cym** | 0 | 3.961310043 | 0 | 0.86260942 | 5.700269198 | 2.104238417 | 10.82477658 | 0 | 2.257242368 | 0 | 0 | 6.072696744 | 0.852084315 | 1.807109303 | 5.748525376 |
| **A630076J17Rik** | 1.831392963 | 5.54583406 | 0 | 0.86260942 | 5.066905954 | 0.701412806 | 7.216517718 | 2.514222039 | 6.019312981 | 2.621705907 | 0.945870699 | 1.735056213 | 1.70416863 | 0 | 0.718565672 |
| **Prok1** | 0 | 4.753572051 | 0.783161294 | 1.725218839 | 0 | 1.402825611 | 2.706194144 | 0.838074013 | 0 | 1.747803938 | 2.837612096 | 0.867528106 | 0 | 0 | 2.155697016 |
| **Lamtor5** | 691.3508436 | 827.9137989 | 776.8960041 | 722.8666937 | 759.4025299 | 816.4445058 | 773.0694606 | 750.0762417 | 781.0058592 | 830.2068705 | 777.5057144 | 763.4247335 | 725.1237522 | 817.7169597 | 710.6614496 |
| **Slc16a4** | 0.915696482 | 7.922620085 | 4.698967767 | 0 | 1.900089733 | 2.805651223 | 7.216517718 | 1.676148026 | 7.524141226 | 1.747803938 | 1.891741398 | 4.337640531 | 3.408337261 | 5.421327909 | 2.155697016 |
| **Rbm15** | 808.5599932 | 957.8447683 | 794.9087139 | 844.4946218 | 676.4319449 | 776.4639759 | 770.3632664 | 959.5947449 | 942.0224815 | 994.5004406 | 892.9019396 | 879.6734998 | 825.6697014 | 825.8489515 | 867.3087661 |
| **Strip1** | 655.6386808 | 653.616157 | 685.2661327 | 664.2092531 | 649.8306886 | 688.0859624 | 653.9969182 | 574.0806989 | 644.8189031 | 605.6140645 | 594.9526695 | 670.5992262 | 696.1528855 | 687.6050898 | 664.6732466 |
| **Ahcyl1** | 2044.750243 | 2038.490148 | 2130.981882 | 2047.834762 | 1951.392156 | 1898.023052 | 2255.161787 | 2120.327253 | 1961.543618 | 2088.625706 | 2030.78439 | 2051.703971 | 2025.404417 | 1943.546056 | 2055.097822 |
| **Csf1** | 47.61621704 | 40.40536243 | 62.65290356 | 58.65744054 | 93.10439691 | 83.46812388 | 100.1291833 | 100.5688816 | 82.76555348 | 76.02947129 | 84.18249219 | 107.5734852 | 95.4334433 | 104.8123396 | 112.0962448 |
| **Gstm5** | 1.831392963 | 0 | 2.349483883 | 2.587828259 | 1.266726489 | 6.312715251 | 0.902064715 | 0.838074013 | 1.504828245 | 3.495607876 | 4.729353494 | 2.602584319 | 0.852084315 | 0.903554652 | 0 |
| **Gstm1** | 21.06101908 | 6.338096068 | 10.96425812 | 18.11479781 | 16.46744435 | 11.22260489 | 9.922711863 | 18.43762829 | 14.29586833 | 6.991215751 | 11.35044839 | 16.48303402 | 18.74585493 | 20.78175699 | 19.40127314 |
| **Gstm4** | 92.48534464 | 98.24048905 | 99.4614844 | 104.3757398 | 98.80466611 | 107.3161593 | 126.2890601 | 90.51199341 | 85.77520997 | 90.88580477 | 109.7210011 | 59.85943933 | 112.4751296 | 80.41636399 | 109.2219821 |
| **Ampd2** | 1810.331944 | 1837.255598 | 1878.803945 | 1784.738889 | 1609.376004 | 1655.334221 | 1748.201417 | 2180.668582 | 1938.21878 | 2022.209156 | 2043.080709 | 1604.059469 | 1699.908209 | 1713.139619 | 1735.336098 |
| **Gnat2** | 5.494178889 | 7.130358077 | 3.132645178 | 12.93914129 | 6.966995687 | 6.312715251 | 9.922711863 | 6.704592104 | 12.79104008 | 10.48682363 | 9.458706988 | 4.337640531 | 5.964590206 | 8.131991864 | 6.467091048 |
| **Gnai3** | 3501.623346 | 3499.421292 | 3833.574537 | 3452.162897 | 3634.238296 | 3615.783013 | 3829.264714 | 4024.431411 | 4009.614859 | 3769.139192 | 3791.995631 | 3831.871645 | 3873.575297 | 3903.356095 | 3789.715354 |
| **Amigo1** | 47.61621704 | 34.85952837 | 37.59174214 | 50.03134634 | 29.76807248 | 35.07064029 | 46.00530045 | 53.63673684 | 39.12553437 | 37.57778466 | 41.61831075 | 45.97898963 | 36.63962555 | 33.43152211 | 43.83250599 |
| **Cyb561d1** | 228.0084239 | 244.0166986 | 237.2978722 | 234.6297621 | 226.7440415 | 230.7648131 | 245.3616024 | 201.9758371 | 201.6469849 | 194.8801391 | 198.6328467 | 243.7753979 | 268.4065593 | 257.5130757 | 246.4680255 |
| **Atxn7l2** | 282.9502128 | 285.2143231 | 278.8054208 | 313.1272193 | 303.380994 | 291.7877272 | 310.3102619 | 247.2318339 | 263.3449429 | 226.3406099 | 246.8722524 | 304.5023653 | 307.6024378 | 278.2948327 | 351.3786136 |
| **Psma5** | 2768.150464 | 2799.061676 | 2788.83737 | 2639.584824 | 2680.39325 | 2642.222039 | 2653.874391 | 2825.985572 | 2768.883971 | 2932.815008 | 3004.085339 | 2739.65376 | 2678.101003 | 2671.811105 | 2623.483268 |
| **Sort1** | 27.47089445 | 22.18333624 | 34.45909696 | 13.80175071 | 10.76717515 | 24.5494482 | 24.3557473 | 48.60829276 | 73.73658401 | 42.82119648 | 46.34766424 | 29.49595561 | 17.89377062 | 7.228437213 | 15.08987911 |
| **Psrc1** | 255.4793184 | 313.7357554 | 333.6267115 | 351.9446432 | 205.2096911 | 218.8407954 | 256.186379 | 672.9734325 | 525.1850576 | 554.9277503 | 579.8187383 | 268.9337129 | 242.8440298 | 254.8024117 | 329.8216434 |
| **Celsr2** | 90.65395167 | 80.81072487 | 90.84671016 | 58.65744054 | 77.2703158 | 95.39214158 | 81.18582433 | 97.21658551 | 100.8234924 | 74.28166736 | 78.507268 | 111.9111257 | 106.5105394 | 65.95948956 | 84.79074929 |
| **Sars** | 6324.715598 | 5389.758444 | 5387.366545 | 5160.129548 | 5028.90416 | 5277.42995 | 5425.017195 | 4652.98692 | 4475.359201 | 4650.032377 | 4533.558259 | 4813.913462 | 4825.353477 | 4611.742942 | 4794.270163 |
| **5330417C22Rik** | 0 | 0.792262009 | 0.783161294 | 0.86260942 | 2.533452977 | 1.402825611 | 0.902064715 | 1.676148026 | 0 | 3.495607876 | 0 | 4.337640531 | 1.70416863 | 1.807109303 | 0 |
| **Scarna2** | 0 | 2.376786026 | 0 | 0.86260942 | 3.800179466 | 1.402825611 | 0.902064715 | 0.838074013 | 0 | 0 | 4.729353494 | 2.602584319 | 0 | 0 | 0 |
| **Tmem167b** | 375.4355574 | 437.3286287 | 452.6672282 | 391.6246765 | 445.887724 | 381.5685663 | 460.0530045 | 422.3893026 | 336.3291128 | 319.8481206 | 445.5050991 | 400.7979851 | 345.0941476 | 431.8991235 | 403.8339076 |
| **Taf13** | 255.4793184 | 282.045275 | 267.8411627 | 276.0350143 | 268.5460156 | 296.6976168 | 283.2483204 | 309.2493108 | 307.7373761 | 284.0181399 | 332.0006153 | 307.9724777 | 321.2357868 | 285.5232699 | 311.138936 |
| **Wdr47** | 157.4997948 | 162.4137117 | 184.8260655 | 167.3462274 | 146.3069094 | 161.3249453 | 183.1191371 | 157.5579145 | 209.9235402 | 142.4460209 | 192.0117519 | 196.061352 | 199.3877297 | 183.4215943 | 227.785318 |
| **Clcc1** | 520.1156015 | 552.2066199 | 531.766519 | 517.5656518 | 498.4568732 | 499.4059177 | 484.4087518 | 458.4264851 | 531.2043705 | 504.2414361 | 469.1518666 | 481.478099 | 512.1026734 | 507.7977142 | 526.7086376 |
| **Gpsm2** | 476.1621704 | 512.5935195 | 520.8022608 | 577.9483112 | 601.6950821 | 628.4658739 | 642.2700769 | 424.9035246 | 489.0691797 | 518.2238676 | 415.2372368 | 631.5604614 | 668.0341031 | 653.2700131 | 664.6732466 |
| **Stxbp3** | 370.857075 | 327.2042095 | 288.2033564 | 320.0280947 | 274.879648 | 306.5173961 | 324.7432973 | 309.2493108 | 268.6118418 | 323.3437285 | 272.4107612 | 293.2244999 | 314.4191123 | 296.3659257 | 340.6001285 |
| **Fndc7** | 2.747089445 | 5.54583406 | 4.698967767 | 6.038265938 | 1.266726489 | 0.701412806 | 2.706194144 | 0 | 1.504828245 | 0.873901969 | 0 | 6.072696744 | 3.408337261 | 3.614218606 | 6.467091048 |
| **Prpf38b** | 2214.154092 | 2211.203266 | 2292.313109 | 2020.231261 | 2103.399334 | 2166.664157 | 2287.636117 | 2232.629171 | 2148.894734 | 2095.616921 | 2308.870376 | 2352.736224 | 2275.917206 | 2172.145382 | 2150.667056 |
| **Henmt1** | 0 | 1.584524017 | 2.349483883 | 2.587828259 | 1.900089733 | 1.402825611 | 0.902064715 | 1.676148026 | 4.514484735 | 0 | 1.891741398 | 6.072696744 | 0.852084315 | 6.324882561 | 1.437131344 |
| **Fam102b** | 167.5724561 | 173.5053799 | 209.8872269 | 183.7358064 | 195.7092425 | 164.1305965 | 211.0831433 | 172.6432467 | 179.0745612 | 131.0852953 | 165.5273723 | 208.2067455 | 216.4294161 | 187.0358129 | 186.108509 |
| **Slc25a24** | 387.3396117 | 367.6095719 | 374.3510988 | 380.4107541 | 362.2837757 | 369.6445486 | 402.3208628 | 327.6869391 | 383.7312025 | 292.7571596 | 297.9492701 | 383.447423 | 463.5338674 | 440.0311153 | 364.3127957 |
| **Vav3** | 226.1770309 | 202.0268122 | 228.683098 | 252.74456 | 245.7449388 | 196.3955856 | 253.4801849 | 200.2996891 | 137.6917844 | 175.6542957 | 147.555829 | 221.2196671 | 220.6898376 | 211.4317885 | 244.3123285 |
| **Prmt6** | 739.8827571 | 696.3983055 | 729.9063265 | 667.6596908 | 711.2669233 | 745.6018125 | 777.5797841 | 823.8267548 | 701.2499622 | 703.491085 | 803.0442233 | 753.8819244 | 785.6217386 | 726.4579399 | 710.6614496 |
| **Rnpc3** | 289.3600882 | 323.2428995 | 340.6751631 | 288.9741556 | 362.2837757 | 317.740001 | 321.1350385 | 316.7919769 | 340.0911834 | 264.7922966 | 299.8410115 | 334.865849 | 296.5253417 | 319.8583467 | 370.7798867 |
| **S1pr1** | 173.066635 | 255.9006287 | 231.8157432 | 263.9584824 | 257.7788404 | 215.3337314 | 263.4028967 | 207.0042812 | 180.5793894 | 176.5281977 | 151.3393118 | 274.1388816 | 286.3003299 | 296.3659257 | 255.0908135 |
| **A930005H10Rik** | 90.65395167 | 87.14882094 | 105.7267748 | 90.57398906 | 89.93758069 | 64.52997813 | 88.40234205 | 61.17940295 | 57.18347332 | 71.65996145 | 69.04856101 | 112.7786538 | 66.46257658 | 107.5230035 | 90.53927467 |
| **Dph5** | 477.0778669 | 440.4976767 | 422.907099 | 444.2438511 | 445.887724 | 445.3971316 | 420.3621571 | 486.9210016 | 480.7926243 | 475.4026711 | 450.2344526 | 411.2083224 | 406.4442183 | 482.4981839 | 413.8938271 |
| **Slc30a7** | 643.7346265 | 733.6346199 | 819.186714 | 703.026677 | 713.1670131 | 654.4181477 | 622.4246532 | 595.8706233 | 732.0989413 | 682.5174377 | 733.0497915 | 647.1759673 | 692.7445482 | 693.0264178 | 625.1521346 |
| **Extl2** | 173.066635 | 188.558358 | 146.4511621 | 184.5984158 | 192.5424263 | 137.4769099 | 136.2117719 | 197.7854671 | 145.2159257 | 163.4196682 | 207.145683 | 127.5266316 | 200.2398141 | 160.832728 | 159.5215792 |
| **Cdc14a** | 246.3223535 | 263.0309868 | 315.6140017 | 284.6611085 | 255.2453874 | 272.1481686 | 277.8359322 | 377.1333059 | 314.5091032 | 311.9830029 | 297.9492701 | 286.2842751 | 322.0878711 | 286.4268245 | 307.5461076 |
| **Rtca** | 1090.59451 | 980.8203665 | 975.0358116 | 1023.917381 | 994.3802935 | 960.9355438 | 877.7089675 | 1071.058589 | 1052.627357 | 1085.386245 | 1054.645829 | 888.3487808 | 1010.571998 | 993.0065621 | 983.7164049 |
| **Dbt** | 521.031298 | 501.5018514 | 496.5242607 | 489.0995409 | 454.1214461 | 462.231039 | 532.2181817 | 574.0806989 | 564.3105919 | 533.080201 | 528.7417206 | 503.1663016 | 515.5110107 | 488.8230665 | 518.0858495 |
| **Lrrc39** | 6.409875371 | 6.338096068 | 4.698967767 | 4.313047098 | 9.500448664 | 2.805651223 | 3.608258859 | 9.218814144 | 5.266898858 | 3.495607876 | 8.512836289 | 3.470112425 | 2.556252945 | 8.131991864 | 5.029959704 |
| **Trmt13** | 28.38659093 | 42.78214846 | 56.3876132 | 53.48178402 | 45.60215359 | 39.98052993 | 46.00530045 | 36.03718256 | 43.64001911 | 57.67752995 | 50.13114703 | 56.38932691 | 52.82922754 | 34.33507676 | 59.64095077 |
| **Sass6** | 300.348446 | 279.668489 | 337.5425179 | 296.7376404 | 285.0134599 | 274.252407 | 291.3669029 | 326.8488651 | 300.965649 | 313.7308068 | 276.194244 | 297.5621405 | 264.998222 | 271.9699501 | 313.294633 |
| **Hiat1** | 1064.955008 | 1146.403126 | 1223.297942 | 1180.049686 | 1166.021733 | 1216.951218 | 1175.390323 | 1061.001701 | 1221.168121 | 1247.05811 | 1098.155881 | 1315.172609 | 1215.924318 | 1182.753039 | 1284.795421 |
| **Slc35a3** | 1019.170184 | 1012.510847 | 935.8777469 | 920.4042508 | 844.9065679 | 906.9267578 | 1037.374422 | 1045.916368 | 1043.598388 | 1058.295284 | 1008.298165 | 987.2469849 | 1015.684504 | 834.8844981 | 1086.471296 |
| **Agl** | 276.5403374 | 234.5095545 | 288.9865177 | 257.0576071 | 223.5772252 | 186.5758063 | 224.614114 | 179.3478388 | 199.3897425 | 215.8537863 | 188.2282691 | 232.4975325 | 230.0627651 | 209.6246792 | 279.5220464 |
| **Frrs1** | 234.4182993 | 271.7458689 | 273.3232918 | 272.5845766 | 228.6441312 | 242.6888308 | 244.4595377 | 433.2842647 | 315.2615174 | 303.2439832 | 356.5932534 | 252.4506789 | 265.8503063 | 227.6957722 | 290.3005315 |
| **Ptbp2** | 173.9823315 | 205.1958602 | 155.8490976 | 132.8418506 | 214.7101398 | 173.9503758 | 200.2583667 | 191.918949 | 202.399399 | 181.7716095 | 202.4163295 | 199.5314644 | 181.4939591 | 177.0967117 | 168.8629329 |
| **Rwdd3** | 6.409875371 | 5.54583406 | 5.482129061 | 1.725218839 | 5.700269198 | 3.507064029 | 3.608258859 | 1.676148026 | 5.266898858 | 18.35194135 | 1.891741398 | 6.94022485 | 8.520843152 | 10.84265582 | 3.59282836 |
| **Tmem56** | 0.915696482 | 0 | 1.566322589 | 3.450437679 | 4.43354271 | 0.701412806 | 0.902064715 | 0 | 0 | 0 | 0.945870699 | 1.735056213 | 0 | 1.807109303 | 2.155697016 |
| **Alg14** | 76.91850445 | 49.91250654 | 74.40032298 | 55.20700286 | 58.90278172 | 58.91867568 | 57.73214175 | 59.50325493 | 60.94554393 | 48.06460829 | 65.26507822 | 71.13730471 | 60.49798638 | 70.47726282 | 48.14390002 |
| **Cnn3** | 802.1501179 | 618.7566286 | 650.0238744 | 606.414422 | 497.82351 | 544.99775 | 537.63057 | 707.334467 | 837.4369184 | 727.9603401 | 719.8076018 | 499.6961892 | 485.6880596 | 528.5794712 | 488.6246569 |
| **F3** | 25.63950148 | 17.42976419 | 20.36219366 | 27.60350143 | 13.30062813 | 11.22260489 | 20.74748844 | 8.380740131 | 6.019312981 | 17.47803938 | 12.29631908 | 18.21809023 | 14.48543336 | 9.939101167 | 13.65274777 |
| **Abcd3** | 428.5459534 | 350.9720698 | 459.7156799 | 414.9151309 | 419.2864677 | 434.8759395 | 429.3828042 | 729.1243914 | 535.7188553 | 705.2388889 | 707.5112827 | 436.3666375 | 380.0296046 | 420.152913 | 437.6064942 |
| **Arhgap29** | 23.80810852 | 30.10595632 | 34.45909696 | 29.32872027 | 25.96789302 | 34.36922748 | 43.29910631 | 32.68488651 | 17.30552482 | 44.56900041 | 52.02288843 | 24.29078698 | 31.52711966 | 37.94929537 | 33.77258658 |
| **Gclm** | 586.9614447 | 609.2494845 | 577.9730353 | 654.7205495 | 630.8297913 | 644.5983684 | 646.7804005 | 866.5685295 | 705.0120329 | 769.9076346 | 861.6882066 | 659.3213608 | 569.1923225 | 655.0771224 | 620.8407406 |
| **Dnttip2** | 2052.075815 | 1921.235371 | 1943.806333 | 1864.098956 | 1931.124532 | 1771.067334 | 1999.877473 | 2303.027388 | 2160.93336 | 2257.288786 | 2173.610866 | 1851.304979 | 1861.804229 | 1748.378251 | 1833.779595 |
| **Fnbp1l** | 21.97671556 | 15.05297816 | 9.397935534 | 15.52696955 | 12.66726489 | 3.507064029 | 11.72684129 | 44.41792269 | 15.04828245 | 35.82998073 | 14.18806048 | 10.41033728 | 4.260421576 | 7.228437213 | 2.155697016 |
| **Pde5a** | 28.38659093 | 24.56012226 | 17.22954848 | 11.21392246 | 12.66726489 | 6.312715251 | 5.412388289 | 2.514222039 | 11.28621184 | 8.739019689 | 21.75502607 | 0.867528106 | 9.372927467 | 10.84265582 | 12.9341821 |
| **1810037I17Rik** | 1611.625808 | 1533.819248 | 1500.53704 | 1471.61167 | 1432.034295 | 1479.279607 | 1438.79322 | 1398.745528 | 1427.329591 | 1401.738758 | 1486.908738 | 1356.813958 | 1330.103616 | 1355.331977 | 1404.795889 |
| **Usp53** | 95.23243408 | 86.35655893 | 106.5099361 | 111.2766151 | 86.77076447 | 115.0317001 | 118.1704776 | 93.02621545 | 121.1386737 | 106.6160402 | 140.9347341 | 93.69303548 | 146.5585022 | 131.9189791 | 115.6890732 |
| **Sec24d** | 434.9558287 | 384.2470741 | 406.4607118 | 426.9916627 | 333.1490665 | 347.9007516 | 344.588721 | 389.7044161 | 358.1491223 | 402.8688077 | 437.9381335 | 350.4813549 | 376.6212673 | 341.5436583 | 353.5343106 |
| **Mettl14** | 622.6736075 | 651.239371 | 608.5163258 | 581.3987488 | 602.3284453 | 671.9534679 | 610.6978119 | 516.253592 | 646.3237313 | 659.7959865 | 639.4085924 | 674.0693386 | 677.4070306 | 609.8993898 | 625.1521346 |
| **Prss12** | 10.98835778 | 9.507144102 | 17.22954848 | 8.626094197 | 21.53435031 | 12.6254305 | 35.18052388 | 4.190370065 | 3.00965649 | 2.621705907 | 3.783482795 | 19.08561834 | 25.56252945 | 21.68531164 | 25.14979852 |
| **Snhg8** | 245.4066571 | 180.6357379 | 227.1167754 | 152.6818673 | 192.5424263 | 200.6040624 | 215.5934668 | 200.2996891 | 104.585563 | 166.0413741 | 149.4475704 | 169.1679807 | 153.3751767 | 186.1322582 | 165.9886702 |
| **Camk2d** | 823.2111369 | 894.4638076 | 843.4647142 | 870.3729044 | 950.6782297 | 927.2677292 | 955.286533 | 1066.030145 | 975.1287029 | 819.7200468 | 913.711095 | 1010.670244 | 863.1614113 | 1003.849218 | 827.7876541 |
| **Larp7** | 1477.018425 | 1366.651965 | 1488.789621 | 1475.924717 | 1451.035193 | 1483.488084 | 1495.623297 | 1691.233358 | 1543.95378 | 1604.484015 | 1628.789343 | 1552.007782 | 1385.489096 | 1469.179863 | 1463.718274 |
| **Zgrf1** | 278.3717304 | 289.9678951 | 256.0937433 | 270.8593578 | 255.2453874 | 218.1393826 | 239.0471494 | 280.7547944 | 279.8980536 | 299.7483753 | 267.6814078 | 241.1728135 | 276.0753181 | 227.6957722 | 266.5878643 |
| **Alpk1** | 5.494178889 | 6.338096068 | 3.915806472 | 2.587828259 | 13.30062813 | 2.805651223 | 3.608258859 | 0 | 4.514484735 | 6.117313782 | 0.945870699 | 4.337640531 | 6.816674521 | 11.74621047 | 9.341353736 |
| **Ap1ar** | 882.7314082 | 950.7144102 | 910.0334242 | 936.7938297 | 820.8387646 | 892.8985017 | 953.4824035 | 1049.268664 | 1128.621184 | 1084.512343 | 1147.341158 | 1060.986874 | 1067.661647 | 980.356797 | 952.818081 |
| **5730508B09Rik** | 378.1826469 | 354.1411178 | 408.0270344 | 394.2125048 | 295.7806351 | 459.4253877 | 417.6559629 | 429.9319687 | 429.628464 | 454.4290238 | 340.5134516 | 389.5201197 | 355.3191594 | 360.518306 | 385.1512002 |
| **Elovl6** | 1421.160939 | 1502.92103 | 1449.631556 | 1266.310628 | 1036.815631 | 991.0962945 | 1176.292388 | 953.7282269 | 1203.110182 | 1344.061228 | 1486.908738 | 1059.251818 | 1116.230453 | 1125.829096 | 1194.974712 |
| **Egf** | 5.494178889 | 16.63750218 | 12.53058071 | 8.626094197 | 16.46744435 | 19.63955856 | 9.020647148 | 10.89496217 | 6.019312981 | 18.35194135 | 12.29631908 | 10.41033728 | 7.668758836 | 10.84265582 | 9.341353736 |
| **Gar1** | 1966.916042 | 1846.762742 | 1911.69672 | 1746.784075 | 1963.426057 | 1961.851618 | 1932.222619 | 2080.937774 | 1906.617387 | 2009.974528 | 1882.282691 | 1892.946328 | 1744.216593 | 1915.535861 | 1685.755066 |
| **Cfi** | 0 | 0.792262009 | 0 | 0 | 0 | 0.701412806 | 1.80412943 | 0 | 0 | 0 | 0.945870699 | 1.735056213 | 6.816674521 | 0 | 0 |
| **Pla2g12a** | 819.548351 | 730.4655718 | 706.4114876 | 599.5135467 | 723.9341882 | 727.3650795 | 699.100154 | 564.8618848 | 490.5740079 | 604.7401625 | 590.223316 | 656.7187765 | 649.2882482 | 681.2802073 | 646.7091048 |
| **Casp6** | 201.4532259 | 169.5440698 | 152.7164524 | 170.7966651 | 161.5076273 | 196.3955856 | 182.2170724 | 109.7876957 | 147.473168 | 152.0589426 | 114.4503546 | 183.0484304 | 209.6127415 | 185.2287036 | 194.7312971 |

| | | | | | | | | | | | | | | | |
|---|---|---|---|---|---|---|---|---|---|---|---|---|---|---|---|
| **Ccdc109b** | 163.9096702 | 160.0369257 | 157.4154202 | 136.2922883 | 133.0062813 | 136.0740843 | 142.5262249 | 116.4922878 | 104.585563 | 119.7245697 | 104.0457769 | 119.7188787 | 133.7772375 | 128.3047605 | 164.5515389 |
| **Sec24b** | 1704.111152 | 1810.318689 | 1827.1153 | 1739.02059 | 1762.649909 | 1738.802345 | 2009.800185 | 1642.625066 | 1744.095936 | 1527.580642 | 1663.786559 | 1815.736326 | 1816.64376 | 1804.398639 | 1731.024704 |
| **Ostc** | 2071.305441 | 1929.157991 | 2057.364721 | 2002.979072 | 2110.999693 | 2259.250647 | 2037.764191 | 1941.817488 | 1804.289066 | 2091.247412 | 2063.889865 | 2079.464871 | 2071.41697 | 2084.500581 | 2167.912632 |
| **Rpl34** | 8.241268334 | 12.67619214 | 14.8800646 | 2.587828259 | 14.56735462 | 18.23673295 | 17.13922958 | 10.05688816 | 9.028969471 | 10.48682363 | 14.18806048 | 16.48303402 | 5.964590206 | 13.55331977 | 9.341353736 |
| **Lef1** | 1106.16135 | 1092.52931 | 1213.116845 | 1261.997581 | 1103.952135 | 1099.113867 | 1230.416271 | 1418.859304 | 1379.927501 | 1221.714953 | 1126.532002 | 1247.505417 | 1227.001414 | 1206.24546 | 1103.716872 |
| **Hadh** | 681.2781823 | 694.0215194 | 708.7609715 | 649.544893 | 683.3989406 | 659.3280374 | 692.785701 | 729.1243914 | 741.8803249 | 703.491085 | 718.8617311 | 780.7752957 | 686.779958 | 778.8641097 | 683.355954 |
| **Cyp2u1** | 0 | 1.584524017 | 0 | 2.587828259 | 1.900089733 | 4.208476834 | 1.80412943 | 2.514222039 | 1.504828245 | 1.747803938 | 9.458706988 | 3.470112425 | 0 | 0 | 5.029959704 |
| **Sgms2** | 39.37494871 | 27.7291703 | 22.71167754 | 22.42784491 | 12.03390164 | 9.118366474 | 13.53097072 | 20.95185033 | 15.80069657 | 26.21705907 | 33.10547446 | 14.74797781 | 13.63334904 | 12.64976512 | 10.05991941 |
| **Papss1** | 566.8161221 | 545.8685239 | 522.3685834 | 517.5656518 | 535.8253047 | 575.1585007 | 543.945023 | 397.2470822 | 398.779485 | 380.1473565 | 473.8812201 | 549.1452913 | 600.7194422 | 589.1176328 | 527.4272032 |
| **Aimp1** | 1885.419056 | 1740.599633 | 1781.691945 | 1701.065776 | 1803.185156 | 1793.512544 | 1736.474576 | 1788.449944 | 1699.703503 | 1832.572429 | 1727.159896 | 1700.355088 | 1657.303993 | 1837.830161 | 1699.407814 |
| **Tbck** | 207.8631013 | 205.1958602 | 194.224001 | 205.3010419 | 207.1097809 | 195.6941728 | 202.9645608 | 197.7854671 | 190.360773 | 178.2760017 | 187.2823984 | 178.7107899 | 213.0210788 | 234.9242094 | 150.8987911 |
| **Gm29811** | 3.662785926 | 0 | 0.783161294 | 1.725218839 | 0 | 0 | 0.902064715 | 1.676148026 | 0.752414123 | 4.369509845 | 1.891741398 | 3.470112425 | 0 | 4.517773258 | 0 |
| **Npnt** | 8.241268334 | 7.130358077 | 3.915806472 | 4.313047098 | 10.76717515 | 8.416953669 | 6.314453003 | 27.65644243 | 11.28621184 | 21.84754922 | 11.35044839 | 11.27786538 | 4.260421576 | 6.324882561 | 10.05991941 |
| **Gstcd** | 703.2548978 | 690.8524714 | 718.158907 | 642.6440176 | 653.6308681 | 706.3226954 | 730.672419 | 636.0981759 | 658.3623573 | 673.778418 | 730.2121795 | 701.830238 | 711.4904032 | 673.1482154 | 695.5715705 |
| **Ints12** | 432.2087393 | 419.8988645 | 461.2820025 | 354.5324715 | 393.9519379 | 432.7717011 | 432.0889984 | 422.3893026 | 379.9691319 | 415.1034352 | 462.5307717 | 367.8319171 | 398.7754595 | 386.7213909 | 413.1752614 |
| **Ppa2** | 369.9413786 | 307.3976593 | 340.6751631 | 322.6159229 | 381.9180363 | 358.4219437 | 350.903174 | 279.9167204 | 331.0622139 | 353.0563954 | 375.5106674 | 329.6606804 | 375.769183 | 371.3609618 | 375.8098464 |
| **Tet2** | 538.4295312 | 503.0863754 | 526.2843899 | 422.6786156 | 471.855617 | 513.4341738 | 612.5019413 | 430.7700427 | 369.4353342 | 388.0124742 | 395.3739521 | 484.9482114 | 553.8548049 | 496.0515037 | 525.9900719 |
| **Cenpe** | 2655.519797 | 2505.132471 | 2544.491046 | 2576.614336 | 2425.147862 | 2234.701199 | 2585.317473 | 2859.508533 | 2589.80941 | 2539.559122 | 2476.289489 | 2540.122295 | 2657.650979 | 2422.430021 | 2482.644397 |
| **Bdh2** | 14.65114371 | 11.09166812 | 10.96425812 | 5.175656518 | 17.73417084 | 7.715540863 | 2.706194144 | 19.2757023 | 16.5531107 | 17.47803938 | 8.512836289 | 6.072696744 | 5.964590206 | 8.131991864 | 17.24557613 |
| **Slc9b2** | 61.35166426 | 99.03275106 | 88.49722628 | 113.001834 | 88.03749095 | 94.69072877 | 81.18582433 | 108.9496217 | 108.3476337 | 86.51629492 | 82.29075079 | 63.32955176 | 48.56880596 | 83.12702794 | 58.20381943 |
| **Slc9b1** | 1.831392963 | 3.961310043 | 2.349483883 | 4.313047098 | 1.900089733 | 0.701412806 | 0.902064715 | 2.514222039 | 2.257242368 | 0 | 0.945870699 | 0.867528106 | 0 | 0 | 2.155697016 |
| **Cisd2** | 694.097933 | 822.3679648 | 815.2709076 | 759.0962893 | 787.9038759 | 833.2784132 | 720.7497071 | 940.3190426 | 916.4404013 | 922.8404792 | 1035.728415 | 786.8479924 | 891.2801937 | 839.4022713 | 778.9251884 |
| **4930539J05Rik** | 21.97671556 | 13.46845414 | 21.14535495 | 23.29045433 | 13.93399137 | 34.36922748 | 17.13922958 | 18.43762829 | 15.80069657 | 12.23462756 | 17.02567258 | 18.21809023 | 17.0416863 | 27.10663955 | 15.80844478 |
| **Ube2d3** | 7769.684646 | 7171.555701 | 7440.032298 | 7308.889613 | 7575.024402 | 7910.533623 | 7781.21023 | 7603.007446 | 7452.661884 | 7525.169854 | 7741.951669 | 7800.812732 | 7408.021036 | 7795.869534 | 7529.131111 |
| **Manba** | 269.2147656 | 306.6053973 | 325.7950985 | 253.6071694 | 255.8787507 | 286.8778375 | 249.871926 | 222.9276875 | 279.8980536 | 255.1793749 | 322.5419083 | 245.5104541 | 293.1170044 | 290.0410432 | 319.761724 |
| **Nfkb1** | 12914.06748 | 10662.26211 | 11155.34948 | 10394.44351 | 10580.96636 | 11057.07147 | 11463.4384 | 11302.26614 | 10329.89349 | 9652.247247 | 9770.844318 | 10534.39379 | 11008.07727 | 10213.78178 | 11016.33032 |
| **Slc39a8** | 361.7001102 | 340.6726637 | 336.7593566 | 363.1585657 | 328.0821605 | 349.3035772 | 332.8618798 | 536.3673684 | 484.5546949 | 452.6812199 | 391.5904693 | 372.1695576 | 339.9816417 | 304.4979176 | 312.5760673 |
| **Bank1** | 9.156964816 | 14.26071615 | 6.265290356 | 18.11479781 | 25.33452977 | 14.72966892 | 25.25781201 | 12.5711102 | 10.53379772 | 15.73023544 | 15.13393118 | 18.21809023 | 6.816674521 | 28.0101942 | 22.27553583 |
| **Ppp3ca** | 792.993153 | 856.4352312 | 825.4520044 | 840.1815747 | 986.1465713 | 1038.090952 | 1048.199199 | 709.010615 | 769.7196474 | 815.350537 | 768.0470074 | 975.1015915 | 1035.282443 | 946.0217202 | 990.183496 |
| **Ddit4l** | 0 | 0 | 0.783161294 | 0.86260942 | 1.266726489 | 0 | 0 | 6.704592104 | 6.771727103 | 7.86511772 | 11.35044839 | 0.867528106 | 1.70416863 | 1.807109303 | 0.718565672 |
| **H2afz** | 8323.681017 | 9037.332731 | 9280.46134 | 8906.442258 | 8931.055108 | 9220.071331 | 8619.22835 | 10405.52695 | 10013.87956 | 10055.11605 | 9609.100429 | 9049.185677 | 9035.502078 | 9614.725047 | 8937.519828 |
| **Dnajb14** | 30.21798389 | 29.31369431 | 25.84432272 | 39.6800333 | 31.03479897 | 34.36922748 | 32.47432973 | 40.22755263 | 51.91657446 | 45.44290238 | 64.31920752 | 38.17123668 | 34.08337261 | 28.91374885 | 46.70676868 |
| **Lamtor3** | 452.3540619 | 419.1066025 | 484.7768413 | 527.0543554 | 443.9876342 | 488.8847256 | 470.8777811 | 521.2820361 | 530.4519564 | 561.045064 | 596.8444109 | 539.6024821 | 483.983891 | 517.7368154 | 497.247445 |
| **Dapp1** | 349.796056 | 381.8702881 | 376.7005826 | 374.3724881 | 305.914447 | 352.8106413 | 369.8465331 | 450.045745 | 406.3036262 | 439.5726904 | 437.9381335 | 337.4684333 | 384.2900261 | 342.4472129 | 306.8275419 |
| **Mttp** | 51.27900297 | 63.38096068 | 48.55600026 | 53.48178402 | 48.76896981 | 46.99465798 | 40.59291217 | 62.01747697 | 30.0965649 | 45.44290238 | 52.96875913 | 45.97898963 | 42.60421576 | 74.09148143 | 45.26963733 |
| **Trmt10a** | 270.1304621 | 255.1083667 | 235.7315496 | 224.2784491 | 207.7431441 | 225.8549234 | 216.4955315 | 263.9933141 | 268.6118418 | 243.8186493 | 297.9492701 | 195.1938239 | 207.0564886 | 190.6500315 | 195.4498628 |
| **Adh7** | 1.831392963 | 7.130358077 | 0 | 0.86260942 | 0.633363244 | 2.805651223 | 2.706194144 | 0.838074013 | 3.00965649 | 2.621705907 | 0 | 0 | 0 | 0 | 0 |
| **Adh1** | 5.494178889 | 0.792262009 | 1.566322589 | 2.587828259 | 1.266726489 | 4.208476834 | 0 | 0.838074013 | 1.504828245 | 1.747803938 | 3.783482795 | 0 | 0 | 1.807109303 | 0 |
| **Adh4** | 2.747089445 | 4.753572051 | 6.265290356 | 3.450437679 | 4.43354271 | 4.208476834 | 0.902064715 | 9.218814144 | 10.53379772 | 9.612921658 | 7.56696559 | 2.602584319 | 5.112505891 | 1.807109303 | 3.59282836 |
| **Adh5** | 2317.627795 | 2334.796139 | 2507.682465 | 2350.610669 | 2280.107679 | 2305.543892 | 2109.929368 | 2672.618028 | 2540.150078 | 2515.089867 | 2652.221439 | 2196.581165 | 2113.169102 | 2103.475229 | 2159.289844 |
| **Metap1** | 1606.131629 | 1498.95972 | 1401.075556 | 1448.321216 | 1385.165415 | 1376.873338 | 1400.906502 | 1764.983871 | 1607.90898 | 1604.484015 | 1542.71511 | 1366.356767 | 1355.666145 | 1336.35733 | 1351.622029 |
| **Eif4e** | 2979.676351 | 2901.263475 | 3049.630081 | 2922.520714 | 2803.899082 | 2796.532856 | 2973.2053 | 3061.48437 | 3087.907559 | 3204.59852 | 3118.535694 | 2868.047919 | 2745.415663 | 2807.344302 | 2721.926765 |
| **Mir1956** | 1.831392963 | 0 | 0 | 1.725218839 | 0 | 2.104238417 | 0 | 1.676148026 | 0.752414123 | 0 | 0 | 0 | 0 | 3.614218606 | 1.437131344 |
| **Tspan5** | 378.1826469 | 329.5809955 | 393.9301311 | 447.6942888 | 401.5522969 | 473.4536439 | 497.0376578 | 449.207671 | 468.7539984 | 377.5256506 | 373.618926 | 487.5507957 | 478.8713851 | 428.2849048 | 439.0436256 |
| **Rap1gds1** | 2496.188609 | 2227.048506 | 2243.757109 | 2137.546142 | 2161.668753 | 2229.791309 | 2366.115747 | 2283.751686 | 2332.48378 | 2327.200943 | 2345.759333 | 2141.926894 | 2359.421469 | 2291.414596 | 2235.457805 |
| **Pdlim5** | 514.6214226 | 537.9459038 | 641.4091002 | 553.7952474 | 631.4631545 | 603.9164257 | 631.4453003 | 553.1288486 | 535.7188553 | 540.0714168 | 622.3829198 | 622.8851803 | 677.4070306 | 642.4273573 | 598.5652047 |
| **Gbp5** | 3986.026784 | 4019.937431 | 3628.386277 | 3570.340388 | 3065.478102 | 3102.34884 | 3455.809922 | 2896.383789 | 3219.580031 | 2721.330731 | 2497.098645 | 3110.955789 | 3185.09117 | 2880.532229 | 3088.395258 |
| **Gbp7** | 3177.466791 | 3594.492733 | 3211.744469 | 3095.905207 | 2843.167604 | 2967.677581 | 3802.202773 | 2028.977186 | 2549.931461 | 2079.886686 | 2134.830167 | 3180.358038 | 3266.03918 | 3193.162139 | 3091.988086 |
| **Gbp3** | 1198.646694 | 1382.497205 | 1188.838845 | 1318.929803 | 1221.757698 | 1216.951218 | 1282.736024 | 1038.373702 | 1128.621184 | 1014.600186 | 998.8394579 | 1247.505417 | 1227.853498 | 1180.94593 | 1181.321965 |
| **Gbp2b** | 1.831392963 | 1.584524017 | 6.265290356 | 5.175656518 | 0.633363244 | 0 | 5.412388289 | 7.542666117 | 5.266898858 | 5.243411813 | 0 | 1.735056213 | 5.112505891 | 3.614218606 | 1.437131344 |
| **Gbp2** | 6072.899066 | 7146.203317 | 6928.627972 | 7703.964727 | 5279.716004 | 5212.198559 | 5745.250168 | 7647.425369 | 8305.899499 | 6691.467376 | 5983.57804 | 5778.604716 | 5322.118632 | 5598.424621 | 5394.991065 |
| **Ccbl2** | 199.621833 | 172.7131179 | 167.596517 | 196.6749477 | 136.8064608 | 136.7754971 | 143.4282897 | 199.4616151 | 201.6469849 | 219.3493942 | 209.9832951 | 173.5056213 | 181.4939591 | 157.2185094 | 147.3059628 |
| **Gtf2b** | 776.5106164 | 728.8810478 | 769.0643912 | 747.0197574 | 811.3383159 | 740.6919228 | 723.4559013 | 771.866166 | 794.5493134 | 810.9810271 | 802.0983526 | 791.1856329 | 673.9986933 | 740.9148143 | 722.1585003 |
| **Pkn2** | 1080.521848 | 1015.679895 | 1117.571167 | 1001.489536 | 868.9743711 | 1014.94433 | 1069.848752 | 1026.640666 | 1051.874943 | 1092.377461 | 1051.808217 | 1028.020806 | 1037.838696 | 979.4532423 | 1097.968347 |
| **Lmo4** | 395.58088 | 374.73993 | 438.5703249 | 364.8837845 | 329.9822503 | 384.3742175 | 352.7073035 | 357.0195296 | 416.0850098 | 367.9127289 | 403.8867884 | 385.1824792 | 351.0587378 | 378.589399 | 372.2170181 |
| **Hs2st1** | 379.0983434 | 401.6768383 | 476.9452283 | 440.7934134 | 385.0848525 | 426.4589859 | 453.7385515 | 609.2798075 | 488.3167656 | 477.150475 | 468.2059959 | 451.9821434 | 466.0901204 | 393.9498281 | 423.2351808 |
| **Sep15** | 2390.883513 | 2311.820541 | 2661.96524 | 2390.290702 | 2456.816025 | 2588.914666 | 2569.982372 | 3087.464664 | 2823.057788 | 2867.27236 | 2761.94244 | 2770.884771 | 2787.167795 | 2723.31372 | 2698.214098 |
| **Sh3glb1** | 2635.374474 | 2349.056855 | 2609.493433 | 2350.610669 | 2308.609025 | 2286.605747 | 2536.605978 | 2354.149903 | 2163.943017 | 2282.631943 | 2253.064004 | 2307.624763 | 2263.988025 | 2269.729285 | 2468.991649 |
| **Odf2l** | 112.6306672 | 114.8779912 | 146.4511621 | 71.59658183 | 100.0713926 | 108.7189849 | 103.7374422 | 148.3391003 | 115.8717749 | 159.0501583 | 157.014536 | 107.5734852 | 106.5105394 | 108.4265582 | 139.4017404 |
| **Znhit6** | 530.1882628 | 456.3429169 | 590.503616 | 558.9709039 | 551.0260225 | 545.6991628 | 597.1668412 | 557.3192187 | 528.1947141 | 584.6404172 | 569.4141607 | 504.9013579 | 536.8131186 | 534.0007991 | 553.2955674 |
| **Cyr61** | 10.0726613 | 8.714882094 | 13.31374201 | 6.900875357 | 9.500448664 | 13.32684331 | 16.23716487 | 25.14222039 | 18.81035306 | 11.3607256 | 20.80915537 | 9.542809169 | 7.668758836 | 20.78175699 | 4.311394032 |
| **Bcl10** | 1085.100331 | 1029.940611 | 1075.280457 | 1016.153896 | 1066.583703 | 1127.871792 | 1074.359075 | 1021.612222 | 984.9100865 | 1035.573833 | 1058.429312 | 1159.885078 | 1011.424082 | 1135.768197 | 1180.603399 |
| **2410004B18Rik** | 225.2613345 | 240.8476506 | 265.4916788 | 278.6228425 | 263.4791096 | 252.5086101 | 270.6194144 | 327.6869391 | 264.8497711 | 284.0181399 | 236.4676747 | 332.2632647 | 253.9211259 | 251.1881931 | 238.5638031 |
| **Syde2** | 159.3311878 | 191.7274061 | 228.683098 | 175.9723216 | 161.5076273 | 159.9221197 | 184.9232665 | 186.8905049 | 196.380086 | 132.8330993 | 187.2823984 | 143.1421375 | 148.2626708 | 135.5331977 | 160.9587105 |
| **Mcoln3** | 10.0726613 | 11.88393013 | 19.57903236 | 31.05393911 | 20.26762382 | 15.43108173 | 15.33510015 | 21.78992434 | 28.59173666 | 20.09974528 | 7.56696559 | 15.61550591 | 31.52711966 | 21.68531164 | 5.029959704 |
| **Mcoln2** | 195.0433506 | 262.2387248 | 203.6219366 | 210.4766984 | 209.0098706 | 223.0492722 | 237.24302 | 195.271245 | 169.2931776 | 201.8713548 | 290.3823045 | 229.8949482 | 206.2044043 | 269.2592862 | 278.8034807 |
| **Lpar3** | 0 | 1.584524017 | 5.482129061 | 0.86260942 | 2.533452977 | 2.104238417 | 3.608258859 | 6.704592104 | 3.00965649 | 0 | 0 | 0 | 0.852084315 | 0 | 5.029959704 |
| **Ssx2ip** | 388.2553082 | 381.0780261 | 379.8332278 | 362.2959563 | 348.3497844 | 324.0527162 | 313.9185207 | 419.0370065 | 412.3229392 | 423.8424549 | 363.2143483 | 349.6138268 | 378.3254359 | 339.736549 | 365.0313614 |
| **Ctbs** | 65.01445019 | 78.43393884 | 79.88245204 | 65.55831589 | 70.30332011 | 78.55823424 | 68.55691832 | 57.8271069 | 58.68830156 | 63.79484373 | 63.37333682 | 57.25685501 | 78.39175699 | 50.59906049 | 45.26963733 |
| **Spata1** | 16.48253667 | 12.67619214 | 9.397935534 | 13.80175071 | 8.86708542 | 13.32684331 | 13.53097072 | 13.40918421 | 8.276555348 | 38.45168663 | 9.458706988 | 19.95314644 | 12.78126473 | 10.84265582 | 12.9341821 |
| **Gng5** | 1235.274554 | 1171.755511 | 1219.382136 | 1138.644434 | 1117.886126 | 1010.03444 | 1076.163205 | 1110.448067 | 1042.845974 | 1205.984717 | 1158.691606 | 903.0967586 | 1148.609657 | 1185.463703 | 1039.045962 |
| **Rpf1** | 1132.716548 | 1060.046567 | 1092.510006 | 1145.545309 | 983.6131184 | 1078.071482 | 1004.900092 | 1158.218286 | 1209.129495 | 1181.515462 | 1152.070511 | 1047.106424 | 980.7490468 | 953.2501574 | 979.4050109 |
| **4930503B20Rik** | 0 | 3.961310043 | 0 | 0 | 0 | 0.701412806 | 0 | 0 | 0 | 0 | 0 | 0.867528106 | 5.112505891 | 2.710663955 | 0 |
| **Prkacb** | 1685.797223 | 1972.732401 | 2101.221753 | 2008.154729 | 2116.066599 | 1904.335768 | 2321.914576 | 2074.233182 | 2096.97816 | 2048.426215 | 2135.776038 | 2284.201504 | 2152.36498 | 2437.79045 | 2207.433744 |
| **Adgrl2** | 3.662785926 | 3.169048034 | 0.783161294 | 3.450437679 | 0 | 0.701412806 | 1.80412943 | 6.704592104 | 0 | 0.873901969 | 0 | 0 | 0 | 0 | 3.59282836 |
| **Ifi44** | 100.726613 | 66.55000871 | 46.98967767 | 63.83309705 | 48.76896981 | 66.63421654 | 69.45898304 | 20.11377631 | 16.5531107 | 12.23462756 | 16.07980188 | 33.83359614 | 34.93545692 | 35.23863141 | 71.13800152 |
| **Gipc2** | 0 | 0.792262009 | 0 | 8.626094197 | 0 | 2.104238417 | 2.706194144 | 0 | 0 | 1.747803938 | 0 | 0 | 0 | 0 | 0.718565672 |
| **Dnajb4** | 97.97952353 | 88.73334495 | 92.41303275 | 92.2992079 | 70.30332011 | 80.66247266 | 87.50027733 | 113.1399918 | 79.00348287 | 83.02068705 | 127.6925443 | 68.5347204 | 118.4397198 | 104.8123396 | 119.2819015 |
| **Fubp1** | 4337.654233 | 4232.263649 | 4493.779508 | 3936.086782 | 4280.902168 | 4510.084341 | 4671.793158 | 4610.245146 | 4476.111615 | 4435.052492 | 4559.096768 | 4486.855366 | 4427.430102 | 4638.849581 | 4687.922444 |
| **Nexn** | 1.831392963 | 0.792262009 | 3.915806472 | 0 | 0 | 3.507064029 | 0 | 0.838074013 | 0 | 0 | 0 | 0 | 0.852084315 | 0 | 2.155697016 |
| **Fam73a** | 513.7057262 | 479.3185151 | 466.7641315 | 460.6334301 | 457.9216256 | 483.9748359 | 525.001664 | 529.6627763 | 494.3360785 | 547.0626325 | 611.0324714 | 493.6234925 | 541.9256244 | 484.3052932 | 508.7444958 |
| **Usp33** | 445.02849 | 462.681013 | 530.9833577 | 469.2595243 | 483.2561554 | 538.6850348 | 534.0223112 | 438.3127088 | 519.9181587 | 553.1799463 | 548.6050053 | 533.5297854 | 592.198599 | 581.8891956 | 564.0740525 |
| **Zzz3** | 1234.358857 | 1267.619214 | 1362.700652 | 1249.05844 | 1186.289357 | 1228.173823 | 1266.49886 | 1631.730103 | 1566.526203 | 1618.466446 | 1630.681085 | 1462.652387 | 1376.116169 | 1252.326747 | 1319.286574 |
| **Ak5** | 0 | 3.169048034 | 1.566322589 | 6.900875357 | 3.166816221 | 2.104238417 | 1.80412943 | 4.190370065 | 1.504828245 | 4.369509845 | 6.621094891 | 1.735056213 | 0 | 0 | 0 |
| **Pigk** | 736.2199712 | 762.1560522 | 834.8499399 | 730.6301784 | 666.298133 | 696.5029161 | 707.2187364 | 812.9317927 | 794.5493134 | 819.7200468 | 926.9532848 | 726.121025 | 656.1049227 | 658.691341 | 751.6196929 |
| **St6galnac3** | 10.98835778 | 1.584524017 | 12.53058071 | 12.07653188 | 13.30062813 | 11.22260489 | 13.53097072 | 15.92340625 | 6.019312981 | 4.369509845 | 6.621094891 | 10.41033728 | 12.78126473 | 3.614218606 | 6.467091048 |
| **Msh4** | 0 | 3.169048034 | 0 | 2.587828259 | 1.900089733 | 0 | 0.902064715 | 0 | 0 | 0 | 3.783482795 | 0.867528106 | 0.852084315 | 0 | 2.874262688 |
| **Rabggtb** | 985.2894142 | 840.589991 | 859.1279401 | 728.9049596 | 810.7049527 | 839.5911284 | 813.6623727 | 874.9492696 | 856.9996856 | 920.2187733 | 866.4175601 | 696.6250693 | 847.8238936 | 773.4427817 | 923.3568885 |
| **Snord45b** | 1.831392963 | 0 | 0 | 1.725218839 | 0.633363244 | 0 | 1.80412943 | 1.676148026 | 0 | 0 | 1.891741398 | 0.867528106 | 0 | 0.903554652 | 0.718565672 |
| **Acadm** | 387.3396117 | 491.9947073 | 484.7768413 | 439.930804 | 430.0536429 | 413.1321426 | 478.9963635 | 423.2273766 | 499.6029774 | 526.9628872 | 548.6050053 | 468.4651774 | 447.3442655 | 523.1581433 | 449.8221107 |
| **Tyw3** | 48.53191352 | 41.98988645 | 43.0738712 | 37.95481446 | 44.96879034 | 49.0988964 | 39.69084745 | 66.20784703 | 32.35380727 | 69.91215751 | 72.83204381 | 47.71404585 | 56.2375648 | 51.50261514 | 50.29959704 |
| **Cryz** | 116.2934532 | 109.3321572 | 106.5099361 | 92.2992079 | 85.50403798 | 78.55823424 | 106.4436363 | 69.56014308 | 74.48899814 | 92.6336087 | 104.9916476 | 107.5734852 | 97.98969624 | 59.634607 | 66.10804182 |
| **Fpgt** | 169.4038491 | 154.4910917 | 171.5123235 | 183.7358064 | 165.3078068 | 150.1023404 | 160.5675192 | 203.6519852 | 204.6566413 | 132.8330993 | 153.2310532 | 181.3133742 | 131.2209845 | 159.9291733 | 150.8987911 |
| **Lrriq3** | 0 | 3.169048034 | 0 | 0.86260942 | 1.266726489 | 1.402825611 | 2.706194144 | 1.676148026 | 0 | 6.117313782 | 0 | 0.867528106 | 1.70416863 | 0.903554652 | 4.311394032 |
| **Zranb2** | 1424.823725 | 1437.955545 | 1534.212976 | 1404.328135 | 1437.101201 | 1423.166583 | 1532.60795 | 1462.439153 | 1502.571003 | 1576.519152 | 1471.774807 | 1478.267893 | 1539.716357 | 1377.017289 | 1413.418677 |
| **Mir186** | 1.831392963 | 0.792262009 | 2.349483883 | 0 | 0.633363244 | 0.701412806 | 0 | 3.352296052 | 0 | 0.873901969 | 1.891741398 | 1.735056213 | 5.112505891 | 0 | 0 |
| **Ptger3** | 19.22962611 | 14.26071615 | 30.54329049 | 25.87828259 | 20.90098706 | 21.74379698 | 27.96400616 | 41.90370065 | 6.771727103 | 15.73023544 | 24.59263817 | 7.807752957 | 11.92918041 | 17.16753838 | 22.9941015 |
| **Cth** | 439.5343112 | 272.5381309 | 337.5425179 | 284.6611085 | 163.407717 | 162.7277709 | 174.09849 | 613.4701776 | 376.2070613 | 602.1184566 | 489.0151513 | 156.1550591 | 190.8668866 | 172.5789385 | 163.1144075 |
| **Ankrd13c** | 958.7342162 | 905.5554757 | 920.9976823 | 956.6338464 | 780.9368802 | 839.5911284 | 784.7963019 | 1200.121987 | 1162.479819 | 1095.873069 | 1100.047623 | 818.0790042 | 833.3384602 | 896.3262144 | 814.853472 |
| **Srsf11** | 3392.655464 | 3584.985588 | 3637.784213 | 3333.985407 | 3325.790396 | 3473.396214 | 3661.480677 | 3756.247727 | 3689.086443 | 3846.042565 | 3626.468259 | 3666.173777 | 3678.447989 | 3623.254153 | 3690.553291 |
| **Lrrc40** | 825.9582264 | 882.5798775 | 899.8523274 | 737.5310538 | 853.7736533 | 810.8332034 | 795.6210784 | 907.6341561 | 1034.569419 | 975.2745973 | 993.1642337 | 844.9723755 | 842.7113877 | 828.5596155 | 804.0749869 |
| **Depdc1a** | 259.1421043 | 362.0637379 | 353.2057438 | 367.4716128 | 347.0830579 | 284.7735991 | 365.3362095 | 345.2864934 | 340.0911834 | 455.3029258 | 312.1373306 | 417.2810191 | 359.579581 | 387.6249455 | 391.6182912 |
| **Wls** | 235.3339958 | 241.6399126 | 225.5504528 | 220.8280114 | 201.4095117 | 176.756027 | 198.4542373 | 234.6607237 | 250.5539028 | 252.557669 | 244.980511 | 196.9288801 | 172.1210317 | 194.2642501 | 193.2941658 |
| **Tmem68** | 382.7611293 | 324.0351615 | 357.1215503 | 307.0889534 | 316.0482589 | 275.6552326 | 289.5627734 | 307.5731628 | 289.6794372 | 303.2439832 | 332.0006153 | 269.8012411 | 250.5127887 | 222.2744443 | 294.6119255 |
| **Tgs1** | 1022.83297 | 1023.602515 | 1022.808651 | 1003.214755 | 861.3740122 | 885.1829608 | 1006.704222 | 1122.181103 | 1062.408741 | 1086.260147 | 1044.241251 | 903.9642867 | 843.563472 | 872.8337934 | 906.829878 |
| **Lyn** | 3.662785926 | 5.54583406 | 10.96425812 | 12.07653188 | 7.600358931 | 11.22260489 | 2.706194144 | 22.62799835 | 5.266898858 | 6.117313782 | 13.24218978 | 6.072696744 | 8.520843152 | 5.421327909 | 6.467091048 |
| **Rps20** | 17229.745 | 16085.29556 | 16068.90344 | 15583.90178 | 17230.64706 | 17975.10597 | 17027.37356 | 15729.81115 | 15240.14805 | 15451.46071 | 14911.65157 | 16103.05671 | 15870.92245 | 16931.71062 | 16103.05671 |
| **Plag1** | 15.56684019 | 19.80655021 | 11.74741942 | 11.21392246 | 4.43354271 | 5.611302446 | 10.82477658 | 8.380740131 | 13.54345421 | 5.243411813 | 11.35044839 | 9.542809169 | 5.112505891 | 8.131991864 | 8.622788064 |
| **Chchd7** | 253.6479254 | 278.876227 | 223.2009689 | 218.2401832 | 260.9456566 | 277.0580583 | 221.9079198 | 250.5841299 | 198.6373284 | 254.305473 | 204.3080709 | 244.642926 | 195.9793925 | 229.5028815 | 215.5697016 |
| **Penk** | 296.68566 | 182.220262 | 182.4765816 | 273.447186 | 207.7431441 | 206.2153649 | 134.4076425 | 574.918773 | 209.1711261 | 460.5463376 | 344.2969344 | 228.159892 | 241.1398612 | 141.8580803 | 151.6173568 |
| **Impad1** | 1093.341599 | 1113.128122 | 1132.451232 | 1150.720966 | 1021.614913 | 1124.364728 | 1200.648135 | 1304.043164 | 1148.936365 | 1267.157855 | 1326.11072 | 1320.377778 | 1254.268112 | 1243.291201 | 1177.010571 |
| **Fam110b** | 7.325571853 | 4.753572051 | 18.01270977 | 10.35131304 | 10.76717515 | 2.805651223 | 8.118582433 | 7.542666117 | 20.31518131 | 13.10852953 | 3.783482795 | 8.675281063 | 13.63334904 | 6.324882561 | 8.622788064 |
| **Ubxn2b** | 165.7410632 | 185.38931 | 243.5631626 | 198.4001665 | 186.2087938 | 187.2772191 | 202.0624961 | 207.0042812 | 191.1131871 | 229.8362178 | 196.7411053 | 199.5314644 | 184.9022964 | 228.5993268 | 234.9709747 |
| **Sdcbp** | 2221.479664 | 2234.178864 | 2198.333754 | 2152.210502 | 2239.572432 | 2256.444996 | 2275.909275 | 1804.37335 | 2046.566413 | 1793.24684 | 1962.6817 | 2355.338809 | 2423.327792 | 2478.450409 | 2371.985283 |
| **Nsmaf** | 1156.524656 | 1192.354323 | 1209.9842 | 1231.806251 | 1241.391959 | 1339.698459 | 1403.612696 | 1013.231482 | 1177.528102 | 1120.342324 | 1132.207226 | 1382.839801 | 1521.822587 | 1377.920844 | 1388.268878 |
| **Tox** | 230.7555134 | 280.460751 | 303.083421 | 236.354981 | 393.3185747 | 402.6109505 | 467.2695223 | 252.2602779 | 190.360773 | 212.3581784 | 207.145683 | 354.8189955 | 427.7463262 | 413.8280304 | 422.5166151 |
| **Rab2a** | 1849.706893 | 1662.957956 | 1742.53388 | 1614.804834 | 1772.150357 | 1864.355238 | 1689.567211 | 1698.776024 | 1828.366318 | 1898.115076 | 1652.436111 | 1866.052957 | 1676.901932 | 1820.662623 | 1655.575308 |
| **Chd7** | 1992.555544 | 1617.799021 | 1517.766589 | 1538.032595 | 1348.430347 | 1384.588878 | 1577.711186 | 850.6451232 | 1028.550106 | 1027.708715 | 1328.948332 | 1417.540926 | 1358.222398 | 1352.621313 | 1425.634293 |
| **Asph** | 141.0172582 | 93.486917 | 96.32883922 | 56.06961228 | 49.40233305 | 69.43986777 | 40.59291217 | 66.20784703 | 106.0903913 | 88.26409886 | 108.7751304 | 55.5217988 | 51.97714322 | 89.45191051 | 51.01816271 |
| **2610301B20Rik** | 133.6916863 | 129.1387074 | 160.5480654 | 125.9409753 | 122.8724694 | 138.8797355 | 122.6808012 | 186.0524309 | 112.1097043 | 134.5809032 | 157.014536 | 147.4797781 | 155.0793454 | 148.1829629 | 127.1861239 |
| **Plekhf2** | 626.3363934 | 646.4857989 | 659.42181 | 614.1779068 | 651.0974151 | 653.7167349 | 657.6051771 | 586.6518091 | 703.5072046 | 645.813555 | 739.6708864 | 688.8173164 | 652.6965854 | 672.2446608 | 698.4458332 |
| **Ndufaf6** | 261.8891937 | 242.4321746 | 264.7085175 | 247.5689034 | 221.0437723 | 232.1676387 | 205.670755 | 269.0217582 | 391.2553437 | 341.6956698 | 311.1914599 | 220.352139 | 276.9274024 | 251.1881931 | 266.5878643 |
| **Trp53inp1** | 975.2167529 | 1300.894218 | 1364.266975 | 1247.333221 | 1321.829091 | 1043.702255 | 1245.751371 | 1465.791449 | 1701.960745 | 1391.251934 | 1433.939979 | 1579.768682 | 1401.678698 | 1499.900722 | 1267.549845 |
| **Ccne2** | 221.5985485 | 202.8190742 | 237.2978722 | 290.6993744 | 189.37561 | 243.3902436 | 203.8666255 | 280.7547944 | 224.2194085 | 292.7571596 | 242.1428989 | 208.2067455 | 200.2398141 | 218.6602257 | 211.2583076 |
| **Ints8** | 951.4086443 | 856.4352312 | 917.0818758 | 890.2129211 | 936.7442383 | 901.3154553 | 939.9514328 | 1074.410885 | 1067.67564 | 1072.277716 | 1052.754088 | 930.857658 | 949.2219271 | 949.6359388 | 949.9438183 |
| **Dpy19l4** | 184.0549928 | 162.4137117 | 231.8157432 | 183.7358064 | 155.8073581 | 143.0882124 | 204.7686903 | 225.4419095 | 198.6373284 | 180.8977076 | 197.686976 | 206.4716893 | 169.5647787 | 187.9393675 | 178.9228523 |
| **1110037F02Rik** | 2002.628205 | 1990.162165 | 2123.150269 | 1983.139056 | 1907.690092 | 1805.436562 | 2050.393097 | 2132.898363 | 2087.94919 | 2191.746138 | 2148.072357 | 2071.657118 | 2128.506619 | 1932.7034 | 2129.110086 |
| **Rad54b** | 238.0810852 | 239.2631266 | 229.4662593 | 193.22451 | 218.5103193 | 241.2860052 | 242.6554083 | 236.3368717 | 229.4863074 | 184.3933154 | 254.439218 | 261.12596 | 236.0273553 | 235.8277641 | 277.3663494 |
| **Fsbp** | 3.662785926 | 7.922620085 | 6.265290356 | 1.725218839 | 1.900089733 | 6.312715251 | 8.118582433 | 5.028444078 | 0 | 6.117313782 | 11.35044839 | 12.14539349 | 5.964590206 | 8.131991864 | 7.904222392 |
| **Gem** | 222.514245 | 163.9982358 | 167.596517 | 237.2175904 | 186.2087938 | 213.2294929 | 223.7120493 | 160.0721365 | 149.7304104 | 119.7245697 | 122.0173201 | 156.1550591 | 172.973116 | 149.0865175 | 213.4140046 |
| **Cdh17** | 0 | 3.961310043 | 3.132645178 | 2.587828259 | 5.700269198 | 0.701412806 | 0 | 0.838074013 | 0 | 0.873901969 | 0 | 0.867528106 | 0.852084315 | 0.903554652 | 5.748525376 |
| **Pdp1** | 232.5869063 | 199.6500261 | 187.1755494 | 183.7358064 | 204.5763279 | 213.9309057 | 225.5161787 | 183.5382089 | 161.7690364 | 176.5281977 | 132.4218978 | 195.1938239 | 200.2398141 | 236.7313187 | 160.2401448 |
| **1700123M08Rik** | 19.22962611 | 11.09166812 | 14.8800646 | 18.97740723 | 9.500448664 | 7.715540863 | 14.43303544 | 17.59955427 | 8.276555348 | 15.73023544 | 11.35044839 | 16.48303402 | 6.816674521 | 21.68531164 | 22.9941015 |
| **Tmem67** | 92.48534464 | 91.11013098 | 117.4741942 | 79.36006661 | 72.83677309 | 81.36388546 | 93.81473034 | 79.61703124 | 63.2027863 | 69.03825554 | 69.99443171 | 82.4151701 | 80.94800994 | 68.67015352 | 91.97640601 |
| **Rbm12b2** | 123.619025 | 144.1916855 | 151.1501298 | 127.6661941 | 136.1730975 | 131.8656075 | 123.5828659 | 156.7198404 | 167.7883493 | 131.0852953 | 124.8549322 | 162.2277559 | 110.770961 | 142.7616349 | 135.808912 |
| **Rbm12b1** | 61.35166426 | 66.55000871 | 79.09929074 | 104.3757398 | 77.90367905 | 74.34975741 | 65.85072418 | 83.80740131 | 102.3283207 | 115.3550599 | 110.6668718 | 58.12438312 | 90.32093741 | 116.5585501 | 70.41943585 |
| **Fam92a** | 381.8454328 | 426.2369606 | 467.5472928 | 445.1064605 | 375.5844039 | 381.5685663 | 373.4547919 | 479.3783355 | 471.7636549 | 538.3236128 | 477.6647029 | 401.6655132 | 424.337989 | 402.08182 | 390.8997255 |
| **Otud6b** | 975.2167529 | 996.6656067 | 1082.328909 | 960.9468935 | 1008.947648 | 973.5609743 | 1022.941387 | 1331.699607 | 1265.560554 | 1229.58007 | 1293.005245 | 1021.948109 | 1082.999165 | 1065.290934 | 949.2252527 |
| **Tmem55a** | 219.7671556 | 231.3405065 | 265.4916788 | 238.0801998 | 246.378302 | 214.6323185 | 221.0058551 | 277.4024983 | 232.4959639 | 265.6661985 | 260.1144422 | 288.8868594 | 240.2877769 | 240.3455373 | 246.4680255 |
| **Tmem64** | 577.8044799 | 602.1191265 | 592.8530999 | 649.544893 | 575.727189 | 596.9022977 | 608.8936825 | 387.190194 | 407.0560403 | 403.7427096 | 492.7986341 | 532.6622573 | 622.0215501 | 614.4171631 | 553.2955674 |
| **Decr1** | 267.3833726 | 250.3547947 | 248.2621304 | 193.22451 | 222.3104987 | 203.4097137 | 226.4182434 | 248.0699079 | 204.6566413 | 271.7835123 | 292.2740459 | 231.6300044 | 198.5356454 | 222.2744443 | 222.7553583 |
| **Nbn** | 653.8072878 | 625.8869867 | 726.7736813 | 699.5762393 | 719.5006455 | 686.6831368 | 656.7031124 | 782.7611282 | 726.0796283 | 729.708144 | 720.7534725 | 645.4409111 | 703.8216443 | 685.7979805 | 662.5175496 |
| **Osgin2** | 431.2930428 | 378.7012401 | 359.4710342 | 332.1046266 | 291.3470924 | 254.6128485 | 340.9804622 | 503.6824818 | 440.9146758 | 533.954103 | 428.4794265 | 291.4894437 | 338.2774731 | 281.9090513 | 330.5402091 |
| **Ripk2** | 741.7141501 | 662.3310391 | 605.3836806 | 552.0700286 | 550.3926593 | 473.4536439 | 571.0069645 | 747.5620196 | 662.876842 | 673.778418 | 596.8444109 | 491.0209082 | 441.3796753 | 515.929706 | 492.936051 |
| **A530072M11Rik** | 10.98835778 | 19.0142882 | 4.698967767 | 8.626094197 | 12.66726489 | 9.118366474 | 9.020647148 | 0.838074013 | 18.81035306 | 9.612921658 | 4.729353494 | 7.807752957 | 10.22501178 | 22.58886629 | 11.49705075 |
| **Cpne3** | 2070.389745 | 1856.269886 | 2033.869882 | 1732.119715 | 1456.735462 | 1492.606451 | 1917.789584 | 1993.778077 | 2044.309171 | 2016.965744 | 2128.209072 | 1831.351832 | 1800.454158 | 1737.535595 | 1651.98248 |
| **Rmdn1** | 65.93014667 | 64.17322269 | 57.95393579 | 69.00875357 | 70.30332011 | 77.15540863 | 55.92801232 | 72.07436512 | 54.17381683 | 97.00311855 | 73.7779145 | 66.79966418 | 46.01255302 | 71.38081747 | 66.10804182 |
| **Wwp1** | 728.8943993 | 662.3310391 | 704.845165 | 627.9796575 | 495.9234203 | 483.9748359 | 589.9503235 | 699.7918009 | 762.9479203 | 846.8110079 | 825.74512 | 558.6881004 | 507.8422518 | 496.9550584 | 543.9542137 |
| **Atp6v0d2** | 0 | 0 | 3.915806472 | 0.86260942 | 0 | 1.402825611 | 0 | 0 | 0 | 0 | 0 | 0.867528106 | 0 | 0 | 0.718565672 |
| **Ggh** | 108.0521848 | 133.8922794 | 130.0047749 | 102.6505209 | 120.9723797 | 131.8656075 | 104.6395069 | 77.1028092 | 113.6145325 | 117.1028638 | 96.47881127 | 117.9838225 | 114.1792982 | 119.269214 | 142.9945687 |
| **Ccnc** | 544.8394065 | 498.3328033 | 509.0548414 | 460.6334301 | 473.1223435 | 451.0084341 | 503.3521108 | 562.3476628 | 630.5230347 | 534.828005 | 583.6022211 | 487.5507957 | 547.0381303 | 474.3661921 | 493.6546166 |
| **Tstd3** | 93.40104112 | 144.1916855 | 153.4996137 | 100.0626927 | 91.83767042 | 84.16953669 | 120.8766718 | 119.0065099 | 106.8428054 | 110.9855501 | 130.5301564 | 98.03067601 | 91.17302172 | 80.41636399 | 121.4375986 |
| **Usp45** | 430.3773463 | 449.2125588 | 393.1469698 | 377.8229258 | 396.4853909 | 359.1233565 | 429.3828042 | 450.045745 | 445.4291606 | 402.8688077 | 446.4509698 | 433.7640531 | 445.6400968 | 403.8889293 | 357.8457046 |
| **4930528A17Rik** | 6.409875371 | 0.792262009 | 2.349483883 | 0 | 5.066905954 | 0 | 2.706194144 | 0 | 0 | 0 | 3.783482795 | 0.867528106 | 0 | 0 | 0.718565672 |
| **Pnisr** | 774.6792234 | 847.7203491 | 789.4265848 | 835.8685276 | 810.7049527 | 751.2131149 | 760.4405546 | 959.5947449 | 906.6590177 | 1008.482872 | 942.087216 | 943.0030515 | 960.2990232 | 1050.83406 | 946.35099 |
| **Coq3** | 206.0317084 | 213.1184803 | 216.9356786 | 200.1253854 | 167.8412597 | 214.6323185 | 229.1244376 | 255.612574 | 212.9331967 | 189.6367273 | 255.3850887 | 210.8093298 | 175.5293689 | 175.2896024 | 226.3481867 |

| | | | | | | | | | | | | | | | |
|---|---|---|---|---|---|---|---|---|---|---|---|---|---|---|---|
| **Faxc** | 0.915696482 | 0 | 0 | 0.86260942 | 0 | 0 | 1.80412943 | 0 | 0 | 2.621705907 | 0 | 0 | 4.260421576 | 1.807109303 | 0 |
| **Fbxl4** | 63.18305723 | 49.12024453 | 59.52025838 | 51.75656518 | 49.40233305 | 46.29324518 | 60.43833589 | 57.8271069 | 77.49865463 | 69.91215751 | 34.99721585 | 39.90629289 | 52.82922754 | 44.27417793 | 49.58103137 |
| **Mms22l** | 990.7835931 | 1046.578113 | 1076.063619 | 917.8164225 | 906.3428026 | 925.8649035 | 948.0700152 | 1072.734737 | 1056.389428 | 1154.424501 | 1101.939364 | 1137.329347 | 889.576025 | 1003.849218 | 1063.477195 |
| **Klhl32** | 1.831392963 | 0 | 0 | 1.725218839 | 1.900089733 | 0.701412806 | 1.80412943 | 1.676148026 | 0 | 2.621705907 | 0 | 2.602584319 | 0 | 0.903554652 | 0.718565672 |
| **Ndufaf4** | 531.1039593 | 575.9744802 | 603.817358 | 600.3761561 | 632.0965178 | 606.0206641 | 534.0223112 | 730.8005394 | 613.969924 | 635.3267314 | 698.9984464 | 629.8254052 | 588.7902618 | 580.985641 | 588.5052853 |
| **Ufl1** | 429.4616499 | 476.1494671 | 470.679938 | 447.6942888 | 497.82351 | 491.6903768 | 541.2388289 | 496.1398157 | 431.1332922 | 495.5024164 | 503.2032117 | 503.1663016 | 491.6526498 | 461.716427 | 519.5229808 |
| **Manea** | 312.2525002 | 278.876227 | 292.1191628 | 251.0193411 | 260.9456566 | 256.7170869 | 288.6607087 | 355.3433815 | 308.4897903 | 283.1442379 | 302.6786236 | 299.2971967 | 272.6669809 | 321.665456 | 260.8393389 |
| **Map3k7** | 1671.146079 | 1801.603807 | 1966.51801 | 1892.565067 | 1696.780131 | 1657.43846 | 1816.758336 | 2453.042636 | 2247.460984 | 2200.485158 | 2397.782221 | 1886.006103 | 1880.550084 | 1824.276842 | 1820.126847 |
| **Bach2** | 1027.411452 | 1194.731109 | 1104.257425 | 1130.01834 | 1102.685408 | 1040.896604 | 1169.977935 | 1092.848513 | 1170.003961 | 870.406361 | 774.6681023 | 1345.536093 | 1179.284692 | 1168.296164 | 1216.531683 |
| **Bach2os** | 4.578482408 | 8.714882094 | 7.831612945 | 8.626094197 | 10.13381191 | 3.507064029 | 1.80412943 | 4.190370065 | 3.762070613 | 4.369509845 | 1.891741398 | 7.807752957 | 14.48543336 | 5.421327909 | 3.59282836 |
| **Gja10** | 6.409875371 | 3.169048034 | 0 | 2.587828259 | 0 | 0 | 0 | 0 | 0 | 3.495607876 | 0 | 0 | 1.70416863 | 0 | 0 |
| **Casp8ap2** | 1392.774348 | 1458.554358 | 1448.065234 | 1376.724634 | 1203.390164 | 1142.601461 | 1262.890601 | 1533.675444 | 1486.770306 | 1415.72119 | 1518.122472 | 1427.083735 | 1320.730689 | 1330.936002 | 1286.951118 |
| **Mdn1** | 3097.801197 | 3040.701589 | 2954.867564 | 3269.2897 | 2540.419973 | 2465.466012 | 2823.462557 | 4228.92147 | 4057.769363 | 3636.306093 | 3967.927581 | 3029.408147 | 2882.601238 | 2597.719623 | 3054.622672 |
| **Lyrm2** | 180.3922069 | 174.2976419 | 163.6807105 | 148.3688202 | 136.1730975 | 224.4520978 | 175.9026194 | 207.0042812 | 151.2352386 | 137.2026091 | 194.8493639 | 178.7107899 | 146.5585022 | 181.614485 | 171.7371956 |
| **Ankrd6** | 20.14532259 | 10.29940611 | 11.74741942 | 12.07653188 | 12.66726489 | 19.63955856 | 14.43303544 | 25.9802944 | 24.82966605 | 26.21705907 | 8.512836289 | 19.95314644 | 5.112505891 | 17.16753838 | 14.37131344 |
| **Rragd** | 131.8602933 | 85.56429692 | 93.19619404 | 87.12355138 | 71.5700466 | 105.2119209 | 98.32505391 | 183.5382089 | 103.8331489 | 110.1116481 | 110.6668718 | 65.93213608 | 107.3626237 | 76.80214538 | 101.3177597 |
| **4933421O10Rik** | 16.48253667 | 23.76786026 | 13.31374201 | 21.56523549 | 19.63426057 | 24.5494482 | 27.06194144 | 32.68488651 | 27.83932254 | 14.85633347 | 20.80915537 | 26.02584319 | 13.63334904 | 21.68531164 | 16.52701046 |
| **Ube2j1** | 1944.02363 | 1791.304401 | 2077.726914 | 1849.434596 | 1936.824801 | 1963.254443 | 2080.161232 | 2039.034074 | 1906.617387 | 1932.197253 | 2013.758718 | 1971.023857 | 1935.08348 | 1988.723788 | 2023.480932 |
| **Gabrr2** | 2.747089445 | 5.54583406 | 2.349483883 | 0 | 4.43354271 | 4.90988964 | 5.412388289 | 0.838074013 | 0 | 0.873901969 | 2.837612096 | 6.072696744 | 9.372927467 | 4.517773258 | 14.37131344 |
| **Gabrr1** | 0 | 3.169048034 | 0 | 1.725218839 | 0 | 0 | 0 | 0 | 0 | 0 | 0 | 0 | 0 | 0.903554652 | 0 |
| **Pm20d2** | 1.831392963 | 6.338096068 | 1.566322589 | 1.725218839 | 0.633363244 | 0.701412806 | 10.82477658 | 7.542666117 | 2.257242368 | 0 | 0 | 1.735056213 | 22.15419219 | 1.807109303 | 1.437131344 |
| **Srsf12** | 14.65114371 | 8.714882094 | 1.566322589 | 8.626094197 | 13.30062813 | 12.6254305 | 4.510323574 | 7.542666117 | 1.504828245 | 10.48682363 | 1.891741398 | 6.072696744 | 7.668758836 | 7.228437213 | 13.65274777 |
| **Pnrc1** | 1094.257295 | 1014.887633 | 1076.063619 | 1086.887869 | 1089.38478 | 1078.772895 | 1072.554946 | 801.1987565 | 808.0927677 | 879.1453807 | 815.3405423 | 1127.786538 | 1135.828392 | 1220.702334 | 1164.076389 |
| **Rngtt** | 801.2344214 | 746.310812 | 790.2097461 | 707.3397241 | 686.5657568 | 695.8015033 | 739.6930661 | 821.3125328 | 706.5168611 | 743.6905755 | 706.565412 | 708.7704628 | 718.3070777 | 759.889462 | 690.5416108 |
| **Spaca1** | 5.494178889 | 5.54583406 | 4.698967767 | 4.313047098 | 8.233722176 | 2.104238417 | 5.412388289 | 1.676148026 | 0 | 4.369509845 | 0.945870699 | 3.470112425 | 6.816674521 | 4.517773258 | 11.49705075 |
| **Akirin2** | 1034.737024 | 962.5983403 | 990.6990375 | 853.120716 | 819.5720381 | 1035.285301 | 915.5956855 | 870.7588996 | 1018.768722 | 1008.482872 | 787.9102921 | 933.4602424 | 932.1802408 | 950.5394935 | 855.8117153 |
| **Orc3** | 1249.925697 | 1128.1811 | 1197.453619 | 1201.614922 | 1086.217964 | 1059.133337 | 1106.833405 | 1231.968799 | 1180.537758 | 1097.620873 | 1243.819969 | 1081.807549 | 1064.25331 | 1121.311323 | 1151.860772 |
| **Rars2** | 463.3424197 | 449.2125588 | 453.4503895 | 469.2595243 | 452.8547197 | 443.2928932 | 416.7538982 | 447.531523 | 455.9629583 | 500.7458282 | 509.8243066 | 412.0758505 | 423.4859046 | 447.2595525 | 420.3609181 |
| **Slc35a1** | 215.1886732 | 236.8863405 | 268.624324 | 272.5845766 | 245.1115755 | 239.1817667 | 230.928567 | 298.3543486 | 258.8304582 | 284.0181399 | 290.3823045 | 257.6558476 | 222.3940063 | 249.3810838 | 290.3005315 |
| **Smim8** | 161.1625808 | 146.5684716 | 149.5838072 | 147.5062108 | 183.0419776 | 171.8461374 | 156.9592604 | 134.0918421 | 134.6821279 | 186.1411194 | 182.5530449 | 158.7576434 | 204.5002356 | 210.5282338 | 175.330024 |
| **Zfp292** | 1147.367691 | 1148.779912 | 1141.849167 | 1192.988827 | 1008.947648 | 949.0115261 | 1233.122465 | 902.6057121 | 994.6914701 | 959.5443619 | 929.7908969 | 1146.872157 | 1131.567971 | 1075.230035 | 1093.656953 |
| **Mob3b** | 93.40104112 | 101.4095371 | 94.76251663 | 74.18441009 | 69.66995687 | 65.93280374 | 78.47963019 | 70.3982171 | 85.77520997 | 96.12921658 | 83.23662149 | 88.48786684 | 92.02510604 | 63.24882561 | 81.9164866 |
| **3110043O21Rik** | 137.3544722 | 160.8291877 | 132.3542588 | 106.963568 | 127.9393753 | 150.1023404 | 120.8766718 | 113.9780658 | 104.585563 | 101.3726284 | 110.6668718 | 127.5266316 | 98.84178056 | 94.87323842 | 126.4675583 |
| **Aco1** | 1078.690455 | 888.9179735 | 956.2399406 | 954.0460181 | 875.9413668 | 956.727067 | 1001.291833 | 991.4415574 | 1054.8846 | 996.2482445 | 1041.403639 | 969.8964228 | 929.6239878 | 918.011526 | 965.0336975 |
| **Ddx58** | 1480.681211 | 1273.95731 | 1062.749877 | 1121.392246 | 1330.696176 | 1497.51634 | 1608.381386 | 555.6430707 | 687.706508 | 586.3882211 | 784.1268093 | 1225.817214 | 1088.11167 | 1311.057799 | 1287.669684 |
| **Topors** | 872.6587469 | 817.6143928 | 958.5894245 | 872.9607327 | 800.5711408 | 887.2871992 | 903.8688442 | 917.6910443 | 856.9996856 | 1004.987264 | 973.300949 | 906.5668711 | 871.6822544 | 837.595162 | 949.2252527 |
| **Toporsos** | 0 | 0 | 0 | 0 | 0 | 1.402825611 | 0 | 0 | 0 | 0 | 0 | 0.867528106 | 1.70416863 | 0 | 0 |
| **Ndufb6** | 1012.760309 | 1010.134061 | 1070.58149 | 944.5573145 | 1074.817426 | 1154.525478 | 1065.338428 | 1148.161398 | 1059.399085 | 1021.591402 | 1087.751304 | 967.2938385 | 935.588578 | 1081.554918 | 919.7640601 |
| **Aptx** | 293.0228741 | 257.4851528 | 269.4074853 | 251.8819505 | 267.2792891 | 260.9255637 | 257.9905084 | 248.9079819 | 286.6697807 | 242.9447474 | 308.3538478 | 289.7543875 | 255.6252945 | 269.2592862 | 250.0608538 |
| **Gm6297** | 0.915696482 | 0 | 2.349483883 | 0.86260942 | 0 | 0.701412806 | 1.80412943 | 0 | 0.752414123 | 0.873901969 | 4.729353494 | 0.867528106 | 0 | 3.614218606 | 0 |
| **Dnaja1** | 3701.245178 | 3603.207615 | 3802.248085 | 3773.053602 | 3555.06789 | 3486.021644 | 3630.810477 | 3900.396457 | 3862.141691 | 3943.045684 | 4052.110074 | 3680.054227 | 3694.637591 | 3751.558913 | 3621.570987 |
| **Smu1** | 2360.665529 | 2317.366375 | 2542.141562 | 2381.664608 | 2372.578713 | 2419.87418 | 2290.342311 | 2633.228549 | 2509.301099 | 2496.737925 | 2727.891095 | 2413.463192 | 2324.486012 | 2409.780256 | 2327.434211 |
| **B4galt1** | 2927.481652 | 3202.323038 | 3295.542727 | 3346.061939 | 2810.232715 | 2835.811973 | 3216.762773 | 2807.547944 | 3077.373761 | 3034.187636 | 2813.019458 | 3228.072083 | 3030.863909 | 3058.532496 | 2867.795597 |
| **Spink4** | 1.831392963 | 0 | 0.783161294 | 2.587828259 | 3.166816221 | 0 | 2.706194144 | 0 | 3.762070613 | 0 | 0 | 5.205168638 | 0 | 0.903554652 | 5.748525376 |
| **Bag1** | 2402.787568 | 2346.680069 | 2362.797625 | 2513.643849 | 2449.215666 | 2553.142613 | 2436.476795 | 2692.731804 | 2594.323895 | 2792.116791 | 2586.956361 | 2529.711958 | 2334.711024 | 2410.68381 | 2383.482334 |
| **Chmp5** | 653.8072878 | 600.5346024 | 620.2637452 | 599.5135467 | 549.759296 | 606.7220769 | 597.1668412 | 608.4417335 | 543.2429965 | 662.4176924 | 596.8444109 | 561.2906848 | 632.2465618 | 603.5745073 | 574.1339719 |
| **Nfx1** | 2709.545889 | 2662.792611 | 2848.357628 | 2639.584824 | 2347.877547 | 2386.206365 | 2607.86909 | 2906.440677 | 2772.646042 | 2676.761731 | 2867.879959 | 2514.096452 | 2487.234116 | 2454.957988 | 2645.040239 |
| **Aqp7** | 0 | 1.584524017 | 0 | 1.725218839 | 1.900089733 | 0 | 0 | 8.380740131 | 0 | 0 | 0 | 0.867528106 | 0 | 0.903554652 | 1.437131344 |
| **Aqp3** | 114.4620602 | 108.5398952 | 111.9920651 | 121.6279282 | 152.6405419 | 153.6094045 | 144.3303544 | 45.2559967 | 77.49865463 | 62.04703979 | 74.7237852 | 146.61225 | 148.2626708 | 150.8936268 | 150.1802254 |
| **Nol6** | 2312.133616 | 2276.16875 | 2319.723754 | 2505.880364 | 2367.511807 | 2349.732899 | 2481.58003 | 2958.401266 | 2719.224639 | 2715.213417 | 2746.808509 | 2356.206337 | 2321.929759 | 2269.729285 | 2325.278514 |
| **Ube2r2** | 1455.041709 | 1299.309694 | 1456.680008 | 1413.816839 | 1485.236808 | 1442.806141 | 1470.365485 | 1425.563896 | 1322.744027 | 1421.838503 | 1345.028134 | 1474.797781 | 1359.926567 | 1398.702601 | 1411.981545 |
| **Ubap2** | 3829.442686 | 3697.486794 | 3560.251245 | 3848.100621 | 3465.763673 | 3605.261821 | 3818.439938 | 3793.122983 | 3751.536815 | 3525.320543 | 3784.428666 | 3700.874901 | 3747.466818 | 3474.167635 | 3630.91234 |
| **Dcaf12** | 1560.346805 | 1678.803196 | 1617.228073 | 1553.559565 | 1503.604342 | 1425.972234 | 1602.968998 | 1444.001524 | 1489.027549 | 1476.020425 | 1415.968436 | 1642.230705 | 1386.341181 | 1532.428689 | 1469.466799 |
| **Ubap1** | 539.3452276 | 552.9988819 | 567.0087772 | 542.581325 | 567.4934669 | 569.5471982 | 607.9916178 | 593.3564012 | 565.0630061 | 636.2006334 | 573.1976435 | 595.1242809 | 597.3111049 | 606.2851712 | 527.4272032 |
| **Kif24** | 350.7117524 | 374.73993 | 368.0858084 | 383.8611917 | 332.5157032 | 283.3707735 | 367.1403389 | 372.9429358 | 349.872567 | 342.5695718 | 336.7299688 | 360.0241641 | 315.2711966 | 299.9801443 | 353.5343106 |
| **Nudt2** | 176.7294209 | 207.5726462 | 213.0198721 | 195.8123383 | 183.6753408 | 185.1729807 | 185.8253312 | 223.7657615 | 208.418712 | 218.4754922 | 226.063097 | 190.8561834 | 208.7606572 | 201.4926873 | 223.473924 |
| **AI464131** | 2.747089445 | 6.338096068 | 0 | 5.175656518 | 0 | 3.507064029 | 0 | 0.838074013 | 0 | 2.621705907 | 0 | 0 | 0 | 1.807109303 | 5.029959704 |
| **Fam219aos** | 11.90405426 | 22.97559825 | 29.76012919 | 19.84001665 | 7.600358931 | 13.32684331 | 10.82477658 | 15.92340625 | 10.53379772 | 34.08217679 | 6.621094891 | 15.61550591 | 19.59793925 | 12.64976512 | 17.9641418 |
| **Fam219a** | 42.12203815 | 39.61310043 | 46.98967767 | 52.6191746 | 42.43533737 | 61.7243269 | 62.24246532 | 67.04592104 | 51.91657446 | 60.29923585 | 43.51005214 | 47.71404585 | 67.3146609 | 62.34527096 | 55.32955674 |
| **Rpp25l** | 278.3717304 | 303.4363493 | 273.3232918 | 339.8681113 | 356.5835065 | 314.232937 | 303.9958089 | 257.288722 | 307.7373761 | 279.64863 | 283.7612096 | 284.5492189 | 282.0399083 | 309.9192455 | 303.9532792 |
| **Dctn3** | 879.0686223 | 804.1459386 | 888.1049079 | 913.5033754 | 924.7103366 | 852.9179717 | 872.2965792 | 927.7479325 | 862.2665845 | 839.8197921 | 799.2607405 | 929.1226018 | 777.1008954 | 880.9657853 | 879.5243825 |
| **Arid3c** | 0 | 0 | 0 | 0 | 0 | 0 | 0 | 0 | 0 | 0 | 0.945870699 | 0.867528106 | 0 | 0 | 0 |
| **Sigmar1** | 1412.919671 | 1395.965659 | 1372.881749 | 1440.557731 | 1496.003983 | 1592.908482 | 1647.170169 | 1281.415166 | 1466.455125 | 1426.208013 | 1474.612419 | 1449.639466 | 1446.839167 | 1442.073224 | 1409.107283 |
| **Galt** | 231.6712098 | 209.1571702 | 220.0683238 | 219.1027926 | 261.5790199 | 218.8407954 | 219.2017257 | 245.5556858 | 164.7786928 | 207.1147666 | 152.2851825 | 215.1469704 | 213.8731631 | 194.2642501 | 186.8270747 |
| **Il11ra1** | 5.494178889 | 18.2220262 | 13.31374201 | 15.52696955 | 12.03390164 | 19.63955856 | 11.72684129 | 5.866518091 | 9.028969471 | 20.09974528 | 11.35044839 | 10.41033728 | 7.668758836 | 16.26398373 | 6.467091048 |
| **Ccl27a** | 30.21798389 | 25.35238427 | 26.62748401 | 12.07653188 | 12.03390164 | 20.34097137 | 23.45368258 | 10.05688816 | 13.54345421 | 15.73023544 | 9.458706988 | 12.14539349 | 10.22501178 | 10.84265582 | 15.80844478 |
| **Dnajb5** | 359.8687173 | 298.6827772 | 268.624324 | 291.5619838 | 321.1151648 | 271.4467558 | 272.4235439 | 368.7525657 | 318.2711739 | 275.2791202 | 288.4905631 | 306.2374215 | 302.4899319 | 252.9953024 | 265.8692986 |
| **Vcp** | 12241.03057 | 11612.97652 | 11844.53142 | 12192.12154 | 11766.62235 | 11306.07302 | 11614.98527 | 13779.61292 | 12654.85313 | 12539.61935 | 13211.92192 | 12064.71337 | 11638.61966 | 12170.88116 | 11622.08118 |
| **Fancg** | 260.9734972 | 358.8946899 | 271.7569692 | 246.706294 | 270.4461053 | 281.9679479 | 313.9185207 | 264.8313881 | 237.0104486 | 285.7659438 | 293.2199166 | 302.7673091 | 302.4899319 | 289.1374885 | 275.2106524 |
| **Pigo** | 521.9469945 | 542.6994758 | 575.6235514 | 543.4439344 | 525.0581295 | 511.3299354 | 573.7131586 | 540.5577384 | 471.7636549 | 543.5670247 | 563.7389365 | 602.9320339 | 570.0444068 | 612.6100538 | 584.912457 |
| **Stoml2** | 2128.99432 | 2184.266357 | 2134.897689 | 2090.102624 | 1917.19054 | 1963.955856 | 1940.341201 | 2375.101753 | 2331.731366 | 2603.353965 | 2409.13267 | 1867.788013 | 1839.650036 | 1966.134922 | 1963.839981 |
| **Fam214b** | 58.60457482 | 54.66607859 | 80.66561333 | 75.90962893 | 65.8697774 | 40.68194273 | 50.51562403 | 54.47481085 | 38.37312025 | 41.94729451 | 64.31920752 | 65.06460797 | 70.72299816 | 66.86304422 | 51.01816271 |
| **Unc13b** | 0 | 4.753572051 | 2.349483883 | 2.587828259 | 3.166816221 | 1.402825611 | 3.608258859 | 1.676148026 | 2.257242368 | 0 | 0.945870699 | 3.470112425 | 5.112505891 | 3.614218606 | 5.029959704 |
| **Tesk1** | 334.2292158 | 310.5667073 | 347.7236148 | 353.6698621 | 370.5174979 | 453.8140853 | 466.3674575 | 301.7066447 | 294.1939219 | 270.9096104 | 259.1685715 | 362.6267484 | 426.0421576 | 415.6351397 | 435.4507972 |
| **Cd72** | 36.62785926 | 44.36667248 | 31.32645178 | 37.09220505 | 44.3354271 | 52.60596043 | 46.00530045 | 15.08533223 | 13.54345421 | 25.3431571 | 22.70089677 | 49.44910206 | 57.94173343 | 50.59906049 | 56.04812241 |
| **Sit1** | 294.8542671 | 385.8315981 | 382.965873 | 378.6855352 | 456.0215359 | 415.236381 | 408.6353158 | 315.9539029 | 299.4608208 | 348.6868856 | 366.0519604 | 379.1097824 | 426.0421576 | 430.0920141 | 504.4331017 |
| **Rmrp** | 0.915696482 | 0 | 1.566322589 | 4.313047098 | 3.800179466 | 0.701412806 | 5.412388289 | 0 | 0 | 0 | 0.945870699 | 0 | 2.556252945 | 0 | 0 |
| **Ccdc107** | 325.9879474 | 348.5952837 | 368.8689697 | 375.2350975 | 359.7503227 | 359.8247693 | 303.0937442 | 304.2208667 | 344.6056681 | 317.2264147 | 247.8181231 | 363.4942765 | 317.8274496 | 342.4472129 | 357.127139 |
| **Arhgef39** | 60.43596778 | 74.4726288 | 81.44877463 | 96.612255 | 74.10349958 | 87.67660071 | 83.89201847 | 81.29317927 | 69.9745134 | 70.78605948 | 62.42746612 | 98.03067601 | 95.4334433 | 96.68034772 | 77.60509257 |
| **Car9** | 4.578482408 | 4.753572051 | 3.132645178 | 2.587828259 | 5.066905954 | 1.402825611 | 6.314453003 | 2.514222039 | 4.514484735 | 3.495607876 | 0 | 6.94022485 | 6.816674521 | 4.517773258 | 0.718565672 |
| **Tpm2** | 0 | 4.753572051 | 0 | 0 | 1.266726489 | 0 | 0 | 0 | 0 | 0.873901969 | 0.945870699 | 0 | 0 | 0 | 0 |
| **Tln1** | 8503.157528 | 9244.113115 | 8808.215079 | 10305.59474 | 9500.448664 | 8977.3825 | 10174.38792 | 9936.205499 | 9311.877181 | 8723.289454 | 9933.534078 | 10213.4084 | 10523.24129 | 10063.79171 | 10694.4129 |
| **Creb3** | 399.243666 | 476.1494671 | 401.7617441 | 443.3812417 | 457.9216256 | 476.2592951 | 411.3415099 | 382.16175 | 461.9822713 | 452.6812199 | 418.0748489 | 465.8625931 | 455.0130243 | 496.0515037 | 444.7921509 |
| **Gba2** | 311.3368037 | 414.3530304 | 376.7005826 | 420.9533968 | 383.1847628 | 407.5208401 | 456.4447457 | 363.7241217 | 368.6829201 | 339.9478659 | 389.6987279 | 463.2600088 | 388.5504477 | 402.08182 | 422.5166151 |
| **Rgp1** | 543.0080136 | 667.0846112 | 588.9372935 | 625.3918292 | 563.6932874 | 590.5895824 | 588.146194 | 639.450472 | 592.1499145 | 606.4879664 | 529.6875913 | 552.6154037 | 560.6714794 | 592.7318514 | 540.3613853 |
| **Hint2** | 123.619025 | 116.4625153 | 111.2089038 | 115.5896622 | 90.57094393 | 130.4627819 | 150.6448074 | 75.42666117 | 137.6917844 | 102.2465304 | 83.23662149 | 134.4668565 | 111.6230453 | 127.4012059 | 105.6291538 |
| **Tmem8b** | 10.98835778 | 9.507144102 | 7.831612945 | 7.763484777 | 13.93399137 | 4.90988964 | 21.64955315 | 12.5711102 | 13.54345421 | 9.612921658 | 23.64676747 | 17.35056213 | 9.372927467 | 21.68531164 | 15.80844478 |
| **Reck** | 145.5957406 | 106.1631091 | 164.4638718 | 131.9792412 | 130.4728283 | 127.6571306 | 130.7993836 | 101.4069556 | 122.643502 | 133.7070012 | 101.2081648 | 169.1679807 | 127.8126473 | 162.6398373 | 139.4017404 |
| **Glipr2** | 3382.582803 | 3418.610567 | 3485.06776 | 3412.482864 | 3401.160622 | 3287.52182 | 3638.929059 | 2939.125564 | 2983.321996 | 3086.621754 | 3260.416299 | 3309.619725 | 3295.010047 | 3534.705797 | 3324.084799 |
| **Ccin** | 3.662785926 | 6.338096068 | 6.265290356 | 6.038265938 | 2.533452977 | 1.402825611 | 2.706194144 | 1.676148026 | 2.257242368 | 1.747803938 | 6.621094891 | 4.337640531 | 1.70416863 | 5.421327909 | 4.311394032 |
| **Clta** | 3233.324276 | 3279.964715 | 3381.69047 | 3437.498537 | 3468.930489 | 3417.283189 | 3407.098428 | 3457.055304 | 3425.7415 | 3538.429072 | 3492.15462 | 3707.815126 | 3488.433186 | 3804.868638 | 3369.354436 |
| **Gne** | 639.1561441 | 628.2637727 | 627.3121969 | 705.6145053 | 601.6950821 | 628.4658739 | 610.6978119 | 743.3716496 | 662.1244279 | 687.7608495 | 713.1865069 | 543.0725945 | 585.3819245 | 552.0718921 | 632.3377913 |
| **Gm12504** | 8.241268334 | 6.338096068 | 14.8800646 | 11.21392246 | 12.03390164 | 11.22260489 | 8.118582433 | 1.676148026 | 6.019312981 | 4.369509845 | 17.02567258 | 9.542809169 | 7.668758836 | 10.84265582 | 8.622788064 |
| **Rnf38** | 954.1557338 | 1046.578113 | 1173.95878 | 1072.223509 | 1082.417784 | 1139.795809 | 1223.199753 | 963.785115 | 1069.932882 | 994.5004406 | 978.9761732 | 1127.786538 | 1144.349235 | 1266.783622 | 1179.166268 |
| **Melk** | 1012.760309 | 1043.409065 | 1094.85949 | 1193.851437 | 1026.048456 | 1129.97603 | 1013.920739 | 1328.347311 | 1249.759858 | 1173.650344 | 1089.643045 | 996.7897941 | 1100.892935 | 929.7577365 | 959.2851721 |
| **Zcchc7** | 481.6563493 | 476.1494671 | 496.5242607 | 470.9847431 | 450.3212667 | 489.5861384 | 502.4500461 | 395.5709342 | 398.0270708 | 407.2383175 | 395.3739521 | 422.4861878 | 532.552697 | 489.7266212 | 432.5765345 |
| **Grhpr** | 1307.614576 | 1295.348384 | 1289.866652 | 1304.265443 | 1154.621194 | 1227.47241 | 1194.333682 | 571.5664769 | 892.3631494 | 914.9753614 | 792.6396456 | 1214.539349 | 1169.05968 | 1161.067727 | 1134.615196 |
| **Zbtb5** | 234.4182993 | 237.6786026 | 273.3232918 | 276.0350143 | 226.7440415 | 238.4803539 | 257.0884437 | 258.96487 | 238.5152769 | 242.0708454 | 235.521804 | 248.1130384 | 229.2106808 | 201.4926873 | 219.1625299 |
| **Polr1e** | 578.7201763 | 657.5774671 | 628.0953582 | 630.5674858 | 593.4613599 | 588.485344 | 629.6411709 | 652.8596562 | 765.2051627 | 678.1479279 | 688.5938687 | 609.0047306 | 582.8256716 | 576.4678677 | 640.9605794 |
| **Fbxo10** | 27.47089445 | 25.35238427 | 18.01270977 | 33.64176737 | 24.06780328 | 13.32684331 | 14.43303544 | 18.43762829 | 22.57242368 | 15.73023544 | 6.621094891 | 17.35056213 | 42.60421576 | 12.64976512 | 23.71266717 |
| **Tomm5** | 1474.271335 | 1487.07579 | 1535.779298 | 1495.764734 | 1513.738154 | 1521.364376 | 1449.617997 | 1720.565949 | 1602.642081 | 1868.40241 | 1896.470751 | 1424.481151 | 1423.832891 | 1433.941232 | 1416.292939 |
| **Trmt10b** | 105.3050954 | 86.35655893 | 107.2930973 | 72.45919125 | 113.3720207 | 101.7048568 | 88.40234205 | 87.15969736 | 88.03245234 | 137.2026091 | 73.7779145 | 104.1033728 | 100.5459492 | 74.99503608 | 96.28780004 |
| **Exosc3** | 1338.748256 | 1340.507318 | 1376.014394 | 1286.150645 | 1354.76398 | 1535.392632 | 1437.891155 | 1390.364788 | 1386.699228 | 1492.624563 | 1340.29878 | 1382.839801 | 1358.222398 | 1449.301661 | 1365.274777 |
| **Dcaf10** | 531.1039593 | 518.9316156 | 555.2613578 | 527.0543554 | 503.5237792 | 580.7698031 | 579.1255469 | 547.2623305 | 624.5037217 | 580.2709074 | 635.6251096 | 547.4102351 | 547.0381303 | 493.3408398 | 560.4812241 |
| **Slc25a51** | 1291.132039 | 1142.441816 | 1400.292395 | 1366.373321 | 1233.7916 | 1188.193293 | 1334.153713 | 1348.461087 | 1337.79231 | 1307.357345 | 1297.734599 | 1297.822047 | 1356.51823 | 1429.423459 | 1322.160836 |
| **Shb** | 6.409875371 | 16.63750218 | 16.44638718 | 7.763484777 | 16.46744435 | 21.74379698 | 20.74748844 | 7.542666117 | 9.781383594 | 11.3607256 | 17.02567258 | 24.29078698 | 15.33751767 | 18.07109303 | 20.11983882 |
| **Aldh1b1** | 150.174223 | 170.3363318 | 148.0174847 | 170.7966651 | 176.7083452 | 132.5670203 | 160.5675192 | 156.7198404 | 216.6952673 | 166.9152761 | 147.555829 | 178.7107899 | 141.4459963 | 182.5180396 | 163.8329732 |
| **Ccdc180** | 0 | 2.376786026 | 0 | 3.450437679 | 1.266726489 | 0 | 0 | 0 | 0 | 0 | 0 | 0 | 5.964590206 | 0.903554652 | 2.874262688 |
| **Tdrd7** | 109.8835778 | 99.03275106 | 87.71406498 | 73.32180067 | 91.83767042 | 101.7048568 | 112.7580893 | 34.36103454 | 63.95520042 | 34.95607876 | 49.18527634 | 78.07752957 | 94.58135898 | 104.8123396 | 86.94644631 |
| **Tmod1** | 9.156964816 | 14.26071615 | 17.22954848 | 12.07653188 | 19.63426057 | 17.53532014 | 21.64955315 | 17.59955427 | 9.781383594 | 17.47803938 | 13.24218978 | 21.68820266 | 23.00627651 | 30.72085815 | 16.52701046 |
| **Tstd2** | 477.9935634 | 465.850061 | 493.3916155 | 527.9169648 | 442.7209077 | 476.9607079 | 534.9243759 | 504.5205559 | 474.7733113 | 477.150475 | 523.0664964 | 484.0806833 | 494.2089028 | 421.0564676 | 464.1934241 |
| **Ncbp1** | 2795.621358 | 2823.621798 | 2933.722209 | 2776.739722 | 2758.296929 | 2600.838684 | 2800.910939 | 3317.096944 | 3115.746882 | 3333.062109 | 3349.328144 | 2848.094773 | 2759.901097 | 2833.547387 | 2767.914968 |
| **Xpa** | 199.621833 | 201.2345502 | 223.9841302 | 204.4384325 | 190.0089733 | 220.9450338 | 188.5315254 | 188.5666529 | 189.6083589 | 235.0796296 | 195.7952346 | 260.2584319 | 211.3169102 | 244.8633106 | 207.6654792 |
| **5830415F09Rik** | 152.0056159 | 122.8006113 | 180.910259 | 185.4610252 | 164.6744435 | 134.6712587 | 132.6035131 | 162.5863585 | 186.5987024 | 160.7979623 | 154.1769239 | 115.3812381 | 136.3334904 | 134.6296431 | 113.5333762 |
| **Hemgn** | 557.6591573 | 574.3899562 | 577.9730353 | 695.2631922 | 560.5264712 | 490.2875512 | 564.6925115 | 600.0609933 | 711.78376 | 752.4295952 | 745.3461106 | 616.8124836 | 631.3944775 | 627.9704828 | 553.2955674 |
| **Anp32b** | 12532.22205 | 12211.13434 | 12691.12878 | 12249.91637 | 12657.13107 | 12659.79973 | 13608.54829 | 11911.54595 | 12408.81371 | 12184.81515 | 12188.48982 | 13675.71307 | 12437.87475 | 12920.83152 | 12458.49162 |
| **Nans** | 2632.627384 | 2625.556296 | 2778.656273 | 2692.203999 | 2781.098006 | 2798.637095 | 2581.709214 | 3114.283033 | 2814.028818 | 2841.929203 | 3005.03121 | 2646.828252 | 2752.232338 | 2824.511841 | 2699.65123 |
| **Trim14** | 960.5656092 | 804.1459386 | 781.5949719 | 771.1728212 | 830.3392132 | 896.4055657 | 965.2092448 | 481.0544835 | 614.7223381 | 566.2884758 | 594.0067988 | 890.9513651 | 875.942676 | 809.5849678 | 848.6260586 |
| **Coro2a** | 4457.610472 | 4122.931492 | 4055.209183 | 3678.166565 | 3745.076863 | 3838.130873 | 4365.99322 | 3313.744648 | 3603.311233 | 3765.643584 | 4012.383504 | 3869.175354 | 3999.683775 | 3659.396339 | 4047.68043 |
| **Tbc1d2** | 19.22962611 | 19.80655021 | 22.71167754 | 45.71829924 | 17.1008076 | 14.02825611 | 10.82477658 | 20.95185033 | 30.84897903 | 19.22584332 | 34.05134516 | 13.01292159 | 22.15419219 | 17.16753838 | 27.30549553 |
| **Anks6** | 3.662785926 | 0.792262009 | 0 | 0 | 1.266726489 | 1.402825611 | 2.706194144 | 0 | 1.504828245 | 0 | 0 | 1.735056213 | 0 | 1.807109303 | 0.718565672 |
| **Galnt12** | 478.9092599 | 500.7095894 | 502.0063898 | 527.0543554 | 537.0920311 | 491.6903768 | 549.3574113 | 344.4484194 | 463.4870995 | 473.6548671 | 430.3711679 | 483.2131552 | 426.0421576 | 598.1531793 | 523.8343749 |
| **Col15a1** | 43.03773463 | 41.98988645 | 72.83400039 | 42.26786156 | 50.66905954 | 54.71019885 | 36.98465331 | 38.5514046 | 42.88760499 | 36.70388269 | 20.80915537 | 35.56865236 | 51.12505891 | 44.27417793 | 32.33545524 |
| **Tgfbr1** | 463.3424197 | 549.8298339 | 525.5012286 | 521.0160895 | 547.8592063 | 523.9553659 | 587.2441293 | 661.2403963 | 575.5968038 | 459.6724356 | 578.8728676 | 600.3294495 | 584.5298402 | 578.274977 | 508.0259301 |
| **Alg2** | 442.2814006 | 426.2369606 | 401.7617441 | 371.7846599 | 443.354271 | 342.990862 | 436.599322 | 458.4264851 | 424.3615651 | 422.094651 | 415.2372368 | 354.8189955 | 407.2963026 | 370.4574071 | 368.6241897 |
| **Sec61b** | 1850.622589 | 1915.689537 | 1900.732462 | 1787.326718 | 1901.989823 | 1972.37281 | 1858.253312 | 1946.007858 | 1868.99668 | 1947.053587 | 2027.946778 | 1940.660374 | 1861.804229 | 2062.81527 | 1928.630264 |
| **Nr4a3** | 7607.606369 | 6068.726985 | 6139.984549 | 5758.780486 | 5541.295024 | 5550.980944 | 6622.057071 | 4645.444254 | 4333.905346 | 3801.473565 | 4123.050376 | 5411.640327 | 5409.883317 | 4947.865272 | 5612.716464 |
| **Stx17** | 207.8631013 | 232.1327685 | 233.3820658 | 281.2106708 | 233.0776739 | 230.0634003 | 247.1657318 | 237.1749457 | 282.155296 | 239.4491395 | 263.897925 | 238.5702292 | 240.2877769 | 222.2744443 | 249.3422882 |
| **Erp44** | 1484.343997 | 1330.207912 | 1421.437749 | 1194.714046 | 1335.129719 | 1408.436914 | 1419.849861 | 1293.148202 | 1233.959161 | 1284.635894 | 1292.059375 | 1340.330924 | 1318.174436 | 1312.864909 | 1284.795421 |
| **Invs** | 152.9213124 | 160.8291877 | 173.0786461 | 164.7583992 | 167.8412597 | 194.2913472 | 155.1551309 | 144.9868043 | 112.1097043 | 138.0765111 | 170.2567258 | 153.5524748 | 192.5710552 | 188.8429222 | 170.3000643 |
| **Tex10** | 1199.562391 | 1036.278707 | 1062.749877 | 1121.392246 | 1064.05025 | 1028.271173 | 1148.328382 | 1164.084804 | 1141.412224 | 1045.186755 | 1083.02195 | 973.3665352 | 1092.372092 | 974.0319144 | 1051.980144 |
| **Msantd3** | 0 | 0 | 0 | 6.038265938 | 0.633363244 | 0 | 0 | 2.514222039 | 0 | 0 | 0.945870699 | 0 | 0 | 0 | 4.311394032 |
| **Mrpl50** | 733.4728817 | 676.5917553 | 675.8681971 | 627.1170481 | 642.2303297 | 669.1478166 | 636.8576886 | 749.2381677 | 697.4878916 | 698.2476732 | 764.2635246 | 682.7446196 | 700.4133071 | 695.7370817 | 630.90066 |
| **Zfp189** | 77.83420093 | 49.12024453 | 65.00238744 | 57.79483112 | 71.5700466 | 61.7243269 | 64.94865946 | 46.09407072 | 66.96485691 | 45.44290238 | 45.40179354 | 53.78674259 | 47.71672165 | 56.0203884 | 46.70676868 |
| **Rnf20** | 1571.335162 | 1630.475214 | 1719.039041 | 1498.352562 | 1650.544615 | 1592.908482 | 1822.170724 | 1745.708169 | 1742.591108 | 1705.856643 | 1589.062774 | 1743.731494 | 1690.535281 | 1825.180396 | 1570.784559 |
| **Grin3a** | 0 | 1.584524017 | 0.783161294 | 0.86260942 | 1.900089733 | 0.701412806 | 1.80412943 | 0 | 0 | 0.873901969 | 6.621094891 | 0 | 2.556252945 | 1.807109303 | 4.311394032 |
| **Smc2os** | 0 | 0 | 0 | 0 | 1.266726489 | 0 | 0 | 1.676148026 | 0 | 0 | 0 | 1.735056213 | 0 | 0 | 0 |
| **Smc2** | 4456.694776 | 4628.394654 | 4604.988412 | 4492.469858 | 4481.678316 | 4211.282485 | 4706.071617 | 4932.065567 | 5069.013944 | 4580.994121 | 4754.892003 | 4636.937728 | 4754.630479 | 4613.550051 | 4430.675933 |
| **Nipsnap3b** | 443.1970971 | 449.2125588 | 473.0294219 | 529.6421837 | 487.6896981 | 509.9271098 | 497.9397226 | 456.7503371 | 488.3167656 | 500.7458282 | 563.7389365 | 471.0677617 | 426.0421576 | 511.4119328 | 459.1634644 |
| **Abca1** | 135.5230793 | 156.8678777 | 148.0174847 | 131.9792412 | 81.07049527 | 84.16953669 | 66.75278889 | 89.6739194 | 113.6145325 | 131.0852953 | 139.9888634 | 111.9111257 | 125.2563943 | 104.8123396 | 118.5633359 |
| **Slc44a1** | 188.6334752 | 206.7803842 | 215.369356 | 193.22451 | 190.0089733 | 214.6323185 | 173.1964252 | 231.3084276 | 215.9428532 | 215.8537863 | 194.8493639 | 240.3052854 | 202.796067 | 230.4064362 | 181.0785493 |
| **Fsd1l** | 201.4532259 | 202.8190742 | 216.9356786 | 150.9566484 | 182.4086144 | 131.1641947 | 184.9232665 | 291.6497565 | 208.418712 | 235.0796296 | 175.93195 | 163.9628121 | 182.3460434 | 167.1576105 | 164.5515389 |
| **Fktn** | 66.84584315 | 112.5012052 | 69.70135521 | 85.39833255 | 65.8697774 | 71.54410618 | 90.20647148 | 125.711102 | 127.9104008 | 102.2465304 | 85.12836289 | 60.72696744 | 63.90632364 | 70.47726282 | 76.16796123 |
| **Tal2** | 9.156964816 | 3.961310043 | 1.566322589 | 2.587828259 | 2.533452977 | 2.805651223 | 1.80412943 | 8.380740131 | 2.257242368 | 0.873901969 | 0 | 0.867528106 | 5.964590206 | 0 | 0 |
| **Tmem38b** | 177.6451174 | 162.4137117 | 130.0047749 | 117.3148811 | 146.9402727 | 147.998102 | 151.5468721 | 222.0896135 | 176.0649047 | 149.4372367 | 157.9604067 | 156.1550591 | 185.7543807 | 127.4012059 | 204.0726508 |
| **Rad23b** | 7053.609997 | 6804.738391 | 7140.081522 | 6519.601994 | 6256.99549 | 6371.633927 | 6773.603943 | 6646.764998 | 7020.776178 | 7086.471066 | 7118.622879 | 6621.842035 | 6595.984684 | 6445.958884 | 6320.503651 |
| **Klf4** | 97.06382705 | 88.73334495 | 89.28038757 | 75.04701951 | 63.33632443 | 72.94693179 | 82.98995376 | 83.80740131 | 94.05176532 | 75.15556933 | 77.5613973 | 74.60741714 | 82.65217857 | 46.98484188 | 77.60509257 |
| **Ikbkap** | 1631.77113 | 1658.996646 | 1640.722912 | 1726.944058 | 1563.140487 | 1411.242565 | 1685.958952 | 2043.224444 | 1971.325001 | 1824.707311 | 1933.359708 | 1530.319579 | 1599.36226 | 1545.982009 | 1630.42551 |
| **Fam206a** | 629.9991793 | 671.0459212 | 677.4345197 | 659.0335966 | 602.3284453 | 634.0771764 | 679.2547302 | 678.8399506 | 732.8513554 | 664.1654964 | 743.4543692 | 672.3342824 | 592.198599 | 672.2446608 | 677.6074287 |
| **Ctnnal1** | 76.91850445 | 63.38096068 | 75.96664557 | 66.42092531 | 60.16950821 | 84.16953669 | 70.36104775 | 90.51199341 | 85.02279585 | 83.02068705 | 70.94030241 | 98.03067601 | 86.06051583 | 79.51280934 | 96.28780004 |
| **Tmem245** | 478.9092599 | 578.3512662 | 529.4170351 | 476.1603996 | 420.5531942 | 455.9183237 | 528.6099229 | 603.4132894 | 477.0305537 | 454.4290238 | 585.4939625 | 491.0209082 | 533.4047813 | 474.3661921 | 492.936051 |
| **Frrs1l** | 0 | 0 | 1.566322589 | 3.450437679 | 0 | 0 | 0 | 1.676148026 | 0 | 0 | 0 | 1.735056213 | 3.408337261 | 0 | 0 |
| **Epb4.1l4b** | 3.662785926 | 0 | 0.783161294 | 0.86260942 | 0 | 0.701412806 | 1.80412943 | 0 | 3.00965649 | 2.621705907 | 0 | 1.735056213 | 0 | 0.903554652 | 0 |
| **Ptpn3** | 336.9763052 | 348.5952837 | 299.1676145 | 310.5393911 | 198.2426955 | 179.5616783 | 205.670755 | 396.4090082 | 322.7856586 | 312.8569049 | 309.2997185 | 218.6170828 | 254.7732102 | 165.3505012 | 219.8810956 |
| **D630039A03Rik** | 593.3713201 | 567.2595981 | 596.7689064 | 542.581325 | 523.1580398 | 535.8793836 | 542.1408936 | 314.2777549 | 419.8470804 | 404.6166116 | 411.453754 | 502.2987735 | 577.7131657 | 477.9804107 | 527.4272032 |
| **Txn1** | 8224.785797 | 7805.365308 | 8180.119721 | 7461.57148 | 8156.45186 | 7996.807398 | 7851.571277 | 7996.064159 | 8186.265654 | 7869.48723 | 8151.513682 | 8204.213301 | 7831.506941 | 8349.748535 | 7925.060796 |

| | | | | | | | | | | | | | | | |
|---|---|---|---|---|---|---|---|---|---|---|---|---|---|---|---|
| **AI314180** | 2776.391732 | 2600.996174 | 2658.049433 | 2749.13622 | 2315.576021 | 2347.628661 | 2560.059661 | 2800.843352 | 2808.009505 | 2621.705907 | 2745.862639 | 2596.511622 | 2550.288355 | 2403.455373 | 2546.596741 |
| **Ptgr1** | 35.71216278 | 21.39107423 | 43.0738712 | 31.91654853 | 50.66905954 | 33.66781467 | 51.41768874 | 32.68488651 | 24.07725192 | 28.83876497 | 47.29353494 | 52.05168638 | 63.05423932 | 41.56351397 | 61.79664779 |
| **Dnajc25** | 138.2701687 | 143.3994235 | 126.8721297 | 180.2853687 | 125.4059224 | 117.1359386 | 98.32505391 | 142.4725822 | 141.453855 | 127.5896875 | 113.5044839 | 116.2487662 | 109.0667923 | 111.1372221 | 119.2819015 |
| **Gng10** | 710.5804697 | 866.7346373 | 798.0413591 | 759.9588987 | 856.3071063 | 798.2077729 | 838.01812 | 874.9492696 | 932.993512 | 842.441498 | 798.3148698 | 820.6815885 | 896.3926995 | 839.4022713 | 746.5897332 |
| **Ugcg** | 3411.88509 | 3768.790374 | 3571.215503 | 3497.881197 | 3603.203497 | 3476.201865 | 4355.168443 | 2393.539381 | 2682.356347 | 2545.676435 | 2974.763348 | 3978.483895 | 4084.892207 | 3971.122694 | 3790.43392 |
| **Gm12596** | 11.90405426 | 4.753572051 | 9.397935534 | 17.25218839 | 8.233722176 | 18.23673295 | 9.922711863 | 0.838074013 | 0 | 0 | 1.891741398 | 11.27786538 | 5.112505891 | 9.939101167 | 10.05991941 |
| **Susd1** | 65.01445019 | 86.35655893 | 66.56871003 | 83.67311371 | 56.36932874 | 70.14128057 | 62.24246532 | 16.76148026 | 58.68830156 | 75.15556933 | 55.80637123 | 65.06460797 | 76.68758836 | 66.86304422 | 69.70087018 |
| **Ptbp3** | 5549.120678 | 5234.47509 | 5457.0679 | 5317.987072 | 5018.136984 | 5142.758691 | 6135.84419 | 4837.363203 | 5254.107818 | 4450.782728 | 4710.43608 | 5969.460899 | 5962.033953 | 5442.109666 | 5319.54167 |
| **Hsdl2** | 761.8594727 | 726.5042618 | 897.5028435 | 724.5919125 | 716.3338293 | 746.3032253 | 681.0588597 | 769.351944 | 821.6362219 | 869.5324591 | 831.4203442 | 797.2583297 | 703.8216443 | 786.9961015 | 767.4281377 |
| **E130308A19Rik** | 154.7527054 | 135.4768035 | 127.655291 | 128.5288035 | 137.439824 | 131.8656075 | 122.6808012 | 113.9780658 | 118.8814314 | 102.2465304 | 154.1769239 | 117.9838225 | 127.8126473 | 137.340307 | 143.7131344 |
| **Inip** | 718.821738 | 610.0417465 | 621.0469065 | 646.0944553 | 717.6005558 | 692.2944392 | 612.5019413 | 710.6867631 | 721.5651436 | 665.9133003 | 672.5140668 | 618.5475398 | 669.7382717 | 642.4273573 | 724.3141973 |
| **Snx30** | 95.23243408 | 106.9553711 | 72.83400039 | 128.5288035 | 125.4059224 | 109.4203977 | 89.30440676 | 113.9780658 | 88.03245234 | 81.27288311 | 87.02010429 | 104.9709009 | 103.9542864 | 121.0763233 | 99.8806284 |
| **Slc31a2** | 41.20634167 | 44.36667248 | 54.82129061 | 44.85568982 | 41.80197412 | 44.18900676 | 46.90736517 | 47.77021874 | 40.63036262 | 39.3255886 | 62.42746612 | 47.71404585 | 32.37920398 | 56.0203884 | 50.29959704 |
| **Fkbp15** | 1027.411452 | 1116.29717 | 1167.69349 | 1170.560982 | 1148.287562 | 1107.53082 | 1270.107118 | 1076.087033 | 1106.801174 | 999.7438524 | 1120.856778 | 1207.599124 | 1119.63879 | 1099.626011 | 1115.932489 |
| **Slc31a1** | 972.4696634 | 957.8447683 | 1021.242328 | 935.0686109 | 973.4793065 | 981.2765152 | 1115.854052 | 843.9405311 | 915.6879872 | 931.5794989 | 1019.648613 | 1001.994963 | 980.7490468 | 997.5243353 | 965.7522631 |
| **Cdc26** | 545.755103 | 552.9988819 | 553.6950352 | 550.3448097 | 471.2222537 | 544.2963372 | 539.4346994 | 644.478916 | 514.6512598 | 520.8455735 | 526.8499792 | 497.961133 | 534.2568656 | 575.5643131 | 566.9483152 |
| **Prpf4** | 1417.498153 | 1465.684716 | 1509.151814 | 1360.335055 | 1440.901381 | 1334.087156 | 1464.953097 | 1530.323148 | 1578.564829 | 1590.501583 | 1377.187737 | 1440.964185 | 1449.39542 | 1413.159475 | 1335.095019 |
| **Wdr31** | 29.30228741 | 30.89821833 | 28.9769679 | 25.01567317 | 30.40143573 | 18.23673295 | 27.06194144 | 31.8468125 | 33.10622139 | 27.09096104 | 26.48437957 | 32.96606804 | 36.63962555 | 16.26398373 | 32.33545524 |
| **Bspry** | 76.00280797 | 90.31786897 | 78.31612945 | 68.14614415 | 74.73686282 | 94.69072877 | 86.59821262 | 96.3785115 | 54.92623095 | 84.76849098 | 96.47881127 | 72.00483282 | 78.39175699 | 78.60925469 | 79.04222392 |
| **Hdhd3** | 17.39823315 | 23.76786026 | 14.8800646 | 22.42784491 | 8.86708542 | 14.02825611 | 28.86607087 | 25.14222039 | 24.07725192 | 19.22584332 | 17.02567258 | 14.74797781 | 22.15419219 | 25.29953024 | 21.55697016 |
| **Alad** | 587.8771412 | 608.4572225 | 637.4932937 | 581.3987488 | 623.2294324 | 510.6285226 | 607.9916178 | 724.9340213 | 708.0216893 | 698.2476732 | 629.0040147 | 589.9191123 | 576.8610814 | 546.6505642 | 569.1040122 |
| **Pole3** | 752.7025078 | 828.7060609 | 823.1025205 | 847.0824501 | 779.6701537 | 761.0328942 | 766.7550076 | 938.6428946 | 910.4210883 | 931.5794989 | 908.0358708 | 800.7284421 | 841.8593034 | 805.0671946 | 799.7635929 |
| **4933430I17Rik** | 28.38659093 | 18.2220262 | 25.84432272 | 18.97740723 | 24.06780328 | 14.72966892 | 13.53097072 | 25.9802944 | 16.5531107 | 13.9824315 | 30.26786236 | 8.675281063 | 9.372927467 | 27.10663955 | 20.11983882 |
| **Rgs3** | 624.5050004 | 748.687598 | 682.9166488 | 612.452688 | 884.8084523 | 742.7961612 | 799.2293373 | 680.5160986 | 671.9058115 | 614.3530841 | 653.5966529 | 917.8447364 | 850.3801465 | 858.376919 | 800.4821586 |
| **Zfp618** | 0.915696482 | 3.169048034 | 4.698967767 | 0.86260942 | 0.633363244 | 0 | 5.412388289 | 0 | 0 | 2.621705907 | 4.729353494 | 2.602584319 | 12.78126473 | 0 | 4.311394032 |
| **Col27a1** | 11.90405426 | 26.14464628 | 24.27800013 | 8.626094197 | 13.93399137 | 8.416953669 | 6.314453003 | 53.63673684 | 38.37312025 | 42.82119648 | 47.29353494 | 21.68820266 | 11.92918041 | 8.131991864 | 11.49705075 |
| **Orm1** | 0 | 0 | 0 | 0 | 0.633363244 | 0.701412806 | 1.80412943 | 0 | 0 | 0 | 0 | 0 | 6.816674521 | 0.903554652 | 1.437131344 |
| **Orm3** | 2.747089445 | 7.130358077 | 4.698967767 | 8.626094197 | 3.166816221 | 11.22260489 | 7.216517718 | 3.352296052 | 16.5531107 | 16.60413741 | 12.29631908 | 9.542809169 | 23.85836082 | 8.131991864 | 7.18565672 |
| **Orm2** | 10.98835778 | 17.42976419 | 19.57903236 | 12.93914129 | 15.83408111 | 10.52119209 | 20.74748844 | 15.08533223 | 9.781383594 | 2.621705907 | 19.86328467 | 15.61550591 | 21.30210788 | 7.228437213 | 15.80844478 |
| **Akna** | 6201.096573 | 6415.737745 | 6514.335648 | 6463.532381 | 6813.088419 | 6640.275032 | 7304.017996 | 5825.452465 | 5832.714278 | 5721.43619 | 5964.660626 | 6877.762827 | 7094.454008 | 6943.817497 | 7184.938154 |
| **Whrn** | 165.7410632 | 182.220262 | 144.8848395 | 163.0331803 | 141.2400035 | 126.9557178 | 128.0931895 | 196.9473931 | 201.6469849 | 182.6455115 | 181.6071742 | 144.8771937 | 117.5876355 | 140.050971 | 180.3599837 |
| **Atp6v1g1** | 1146.451995 | 1095.698358 | 1109.739554 | 1024.779991 | 1134.35357 | 1138.392984 | 1135.699476 | 830.5313469 | 932.993512 | 963.9138717 | 961.00463 | 1042.768784 | 1015.684504 | 983.0674609 | 1020.363254 |
| **6330416G13Rik** | 317.7466791 | 373.155406 | 429.1723894 | 382.9985823 | 359.1169595 | 329.6640187 | 322.9391679 | 352.8291595 | 367.9305059 | 339.9478659 | 300.7868822 | 292.3569718 | 357.8754124 | 352.3863141 | 293.8933598 |
| **Tnfsf15** | 1.831392963 | 7.922620085 | 3.915806472 | 4.313047098 | 0 | 1.402825611 | 5.412388289 | 4.190370065 | 3.762070613 | 8.739019689 | 5.675224193 | 8.675281063 | 5.112505891 | 0.903554652 | 3.59282836 |
| **Tnfsf8** | 1270.986716 | 1327.038864 | 1348.603749 | 1225.767985 | 1290.160929 | 1259.737399 | 1508.252203 | 1363.546419 | 1355.850249 | 1130.829148 | 1190.85121 | 1215.406877 | 1286.647316 | 1226.123662 | 1409.825848 |
| **Tnc** | 10.0726613 | 19.0142882 | 7.04845165 | 19.84001665 | 10.76717515 | 3.507064029 | 18.0412943 | 24.30414638 | 29.34415078 | 22.72145119 | 18.91741398 | 1.735056213 | 12.78126473 | 17.16753838 | 12.9341821 |
| **Trim32** | 452.3540619 | 430.9905326 | 433.0881959 | 426.9916627 | 468.6888008 | 496.6002664 | 538.5326347 | 398.0851562 | 415.3325957 | 364.417121 | 357.5391241 | 419.8836034 | 488.2443126 | 472.5590828 | 468.5048181 |
| **Tlr4** | 1.831392963 | 0.792262009 | 7.04845165 | 1.725218839 | 2.533452977 | 4.208476834 | 6.314453003 | 9.218814144 | 0.752414123 | 11.3607256 | 0.945870699 | 3.470112425 | 5.964590206 | 9.035546516 | 2.155697016 |
| **Cdk5rap2** | 650.1445019 | 602.9113885 | 667.2534229 | 727.1797408 | 610.5621675 | 600.4093617 | 670.2340831 | 663.7546183 | 567.3202484 | 640.5701432 | 637.516851 | 603.799562 | 630.5423932 | 601.7673979 | 586.3495883 |
| **Megf9** | 49.44761 | 87.14882094 | 52.47180673 | 69.87136299 | 60.16950821 | 68.73845496 | 81.18582433 | 103.0831036 | 72.98416989 | 87.39019689 | 86.07423359 | 78.94505767 | 119.2918041 | 72.28437213 | 75.44939556 |
| **Tle1** | 335.1449123 | 330.3732575 | 361.0373568 | 326.92897 | 179.2417981 | 259.5227381 | 190.3356548 | 455.0741891 | 482.2974526 | 558.4233581 | 631.8416268 | 236.835173 | 203.6481513 | 252.9953024 | 247.1865912 |
| **C630043F03Rik** | 5.494178889 | 11.09166812 | 2.349483883 | 18.97740723 | 4.43354271 | 6.312715251 | 2.706194144 | 13.40918421 | 12.79104008 | 20.09974528 | 25.53850887 | 4.337640531 | 14.48543336 | 6.324882561 | 2.155697016 |
| **Aldoart1** | 6.409875371 | 7.922620085 | 9.397935534 | 6.038265938 | 8.86708542 | 25.25086101 | 7.216517718 | 6.704592104 | 2.257242368 | 4.369509845 | 5.675224193 | 9.542809169 | 11.92918041 | 9.939101167 | 7.904222392 |
| **Kdm4c** | 480.7406528 | 457.9274409 | 497.307422 | 431.3047098 | 459.8217153 | 460.1268005 | 514.1768874 | 400.5993782 | 385.9884449 | 404.6166116 | 359.4308655 | 486.6832676 | 505.2859989 | 460.8128723 | 418.9237868 |
| **Tmem261** | 593.3713201 | 531.6078077 | 562.3098094 | 632.2927046 | 580.1607318 | 596.9022977 | 596.2647765 | 583.2995131 | 578.6064603 | 516.4760636 | 634.6792389 | 622.8851803 | 617.7611285 | 593.6354061 | 565.5111838 |
| **Zdhhc21** | 1390.942955 | 1403.888279 | 1338.422652 | 1207.653188 | 1202.756801 | 1269.557178 | 1361.215655 | 1147.323324 | 1105.296346 | 1127.33354 | 1222.064943 | 1320.377778 | 1385.489096 | 1237.869873 | 1252.459966 |
| **Ttc39b** | 2090.535067 | 2089.194916 | 2152.127237 | 2097.866109 | 2104.666061 | 2141.413296 | 2370.62607 | 2143.793325 | 2155.666461 | 1933.945057 | 1892.687268 | 2292.009257 | 2349.196457 | 2087.211245 | 2281.446008 |
| **Snapc3** | 240.8281747 | 216.2875283 | 216.1525173 | 225.1410585 | 247.0116653 | 200.6040624 | 239.0471494 | 196.9473931 | 194.1228436 | 220.2232962 | 207.145683 | 215.1469704 | 253.0690416 | 240.3455373 | 259.4022076 |
| **Psip1** | 2574.02281 | 2861.650375 | 2710.52124 | 2530.033428 | 2776.664463 | 2879.299567 | 2837.895593 | 2463.937598 | 2710.19567 | 2679.383437 | 2748.700251 | 2930.509943 | 2978.034681 | 3148.887961 | 2863.484203 |
| **Ccdc171** | 19.22962611 | 30.89821833 | 46.98967767 | 22.42784491 | 29.13470924 | 16.13249453 | 46.00530045 | 26.81836842 | 33.10622139 | 20.97364725 | 41.61831075 | 45.97898963 | 32.37920398 | 32.52796746 | 24.43123285 |
| **Cntln** | 109.8835778 | 118.0470393 | 107.2930973 | 110.4140057 | 115.2721105 | 83.46812388 | 120.8766718 | 110.6257697 | 101.5759065 | 97.87702052 | 63.37333682 | 91.95797927 | 95.4334433 | 107.5230035 | 125.7489926 |
| **Rraga** | 1198.646694 | 1178.093607 | 1241.310652 | 1183.500124 | 1079.250968 | 1198.714485 | 1266.49886 | 1238.673391 | 1126.363942 | 1114.22501 | 1135.044839 | 1224.949686 | 1192.918041 | 1203.534796 | 1176.292005 |
| **Haus6** | 863.5017821 | 920.6084539 | 945.2756824 | 908.3277189 | 788.5372391 | 834.6812388 | 895.7502618 | 973.8420032 | 945.032138 | 894.8756162 | 873.9845257 | 863.1904657 | 951.77818 | 815.9098504 | 843.5960989 |
| **Plin2** | 209.6944943 | 213.9107423 | 188.741872 | 194.0871194 | 164.0410803 | 192.8885216 | 204.7686903 | 196.1093191 | 210.6759543 | 205.3669627 | 221.3337435 | 229.8949482 | 261.5898848 | 202.396242 | 245.0308941 |
| **Dennd4c** | 640.0718406 | 598.9500784 | 541.1644545 | 522.7413083 | 509.2240484 | 521.8511275 | 672.9402772 | 507.8728519 | 564.3105919 | 529.5845932 | 563.7389365 | 577.7737188 | 648.4361638 | 585.5034142 | 630.1820943 |
| **Rps6** | 22948.26952 | 21771.35999 | 21334.09682 | 21047.66984 | 23438.24022 | 23201.33279 | 23934.48308 | 19988.90329 | 19849.43697 | 19668.03771 | 19681.6775 | 22422.99896 | 20917.81785 | 22384.66294 | 21932.78001 |
| **Acer2** | 51.27900297 | 44.36667248 | 44.64019379 | 46.58090866 | 50.66905954 | 53.30737323 | 54.12388289 | 48.60829276 | 44.39243323 | 39.3255886 | 45.40179354 | 34.70112425 | 59.64590206 | 35.23863141 | 45.98820301 |
| **Mllt3** | 729.8100958 | 801.7691526 | 735.3884555 | 731.4927879 | 769.5363418 | 705.6212825 | 836.2139906 | 809.5794966 | 819.3789795 | 740.1949677 | 807.7735767 | 833.6945101 | 885.3156035 | 873.7373481 | 825.6319571 |
| **Focad** | 759.1123832 | 718.5816417 | 855.9952949 | 790.1502284 | 678.9653979 | 665.6407526 | 738.7910014 | 751.7523897 | 781.0058592 | 811.8549291 | 946.8165695 | 803.3310264 | 803.5155092 | 691.2193085 | 681.9188227 |
| **Ifna2** | 3.662785926 | 6.338096068 | 3.915806472 | 1.725218839 | 4.43354271 | 0.701412806 | 5.412388289 | 0 | 0 | 0 | 0 | 0 | 0 | 0.903554652 | 0.718565672 |
| **Klhl9** | 945.9144655 | 968.9364364 | 1002.446457 | 954.9086276 | 884.8084523 | 881.6758968 | 979.6422803 | 1001.498446 | 926.974199 | 895.7495181 | 859.7964652 | 953.4133888 | 900.6531211 | 951.4430481 | 880.9615138 |
| **Gm13271** | 0 | 0 | 0.783161294 | 0 | 1.266726489 | 1.402825611 | 0 | 0 | 0 | 0 | 0 | 0 | 0 | 0 | 1.437131344 |
| **Gm13283** | 2.747089445 | 0 | 3.132645178 | 0.86260942 | 1.900089733 | 2.104238417 | 0 | 5.028444078 | 0 | 0 | 0 | 0 | 3.408337261 | 0.903554652 | 2.155697016 |
| **Ifna4** | 5.494178889 | 0.792262009 | 2.349483883 | 3.450437679 | 3.166816221 | 2.805651223 | 4.510323574 | 0 | 1.504828245 | 0 | 0 | 0.867528106 | 5.112505891 | 5.421327909 | 3.59282836 |
| **Ifna1** | 1.831392963 | 2.376786026 | 0.783161294 | 0.86260942 | 1.900089733 | 8.416953669 | 0.902064715 | 2.514222039 | 0 | 0.873901969 | 0 | 0 | 0.852084315 | 0.903554652 | 0.718565672 |
| **Mir31** | 0 | 4.753572051 | 1.566322589 | 0 | 1.266726489 | 0 | 0 | 0 | 0 | 0 | 1.891741398 | 1.735056213 | 1.70416863 | 3.614218606 | 6.467091048 |
| **Mtap** | 2053.907208 | 1999.669309 | 2083.209043 | 2091.827843 | 1872.855113 | 1950.629013 | 1857.351248 | 2279.561316 | 2304.644457 | 2359.535316 | 2285.223608 | 1798.385764 | 1905.260529 | 1791.748874 | 1876.17497 |
| **Cdkn2a** | 203.2846189 | 147.3607336 | 213.0198721 | 175.9723216 | 114.6387472 | 131.8656075 | 128.9952542 | 160.0721365 | 96.30900769 | 214.1059824 | 294.1657873 | 120.5864068 | 126.960563 | 169.8682745 | 196.1684284 |
| **Cdkn2b** | 60.43596778 | 43.57441047 | 28.1938066 | 41.40525214 | 36.10170492 | 37.87629151 | 36.98465331 | 41.90370065 | 15.80069657 | 55.92972601 | 93.64119918 | 47.71404585 | 52.82922754 | 37.04574071 | 76.8865269 |
| **Tusc1** | 50.36330649 | 35.65179038 | 36.02541955 | 33.64176737 | 40.53524763 | 39.27911712 | 46.90736517 | 25.9802944 | 45.89726148 | 47.19070632 | 33.10547446 | 42.50887721 | 28.1187824 | 59.634607 | 43.11394032 |
| **Caap1** | 247.23805 | 252.7315807 | 307.7823887 | 288.1115462 | 259.0455669 | 274.9538198 | 274.2276733 | 302.5447187 | 276.8883971 | 337.32616 | 308.3538478 | 275.0064097 | 279.4836554 | 240.3455373 | 234.2524091 |
| **Plaa** | 2428.427069 | 2444.128296 | 2470.873884 | 2417.031594 | 2516.35217 | 2271.174665 | 2457.224283 | 2560.31611 | 2458.889353 | 2554.415455 | 2726.945225 | 2362.279033 | 2425.031961 | 2270.632839 | 2509.231327 |
| **Gm12657** | 3.662785926 | 4.753572051 | 8.614774239 | 10.35131304 | 10.76717515 | 13.32684331 | 4.510323574 | 2.514222039 | 7.524141226 | 6.991215751 | 5.675224193 | 4.337640531 | 5.964590206 | 4.517773258 | 9.341353736 |
| **Ift74** | 84.2440763 | 95.86370303 | 128.4384523 | 92.2992079 | 97.53793962 | 83.46812388 | 106.4436363 | 119.0065099 | 121.8910879 | 147.6894327 | 133.3677685 | 113.6461819 | 103.1022021 | 106.6194489 | 110.6591135 |
| **Eqtn** | 1.831392963 | 1.584524017 | 0 | 3.450437679 | 5.066905954 | 0 | 0 | 0.838074013 | 0 | 0 | 3.783482795 | 2.602584319 | 0 | 0 | 0 |
| **Mysm1** | 458.7639373 | 448.4202968 | 483.99368 | 460.6334301 | 426.2534634 | 382.9713919 | 427.5786748 | 476.0260394 | 409.3132827 | 424.7163569 | 445.5050991 | 470.2002336 | 454.16094 | 484.3052932 | 444.0735853 |
| **Jun** | 1211.466445 | 1151.94896 | 1061.183554 | 1073.086118 | 806.27141 | 966.5468463 | 1003.998028 | 1110.448067 | 1101.534275 | 960.4182638 | 970.4633369 | 956.0159731 | 1152.017994 | 993.0065621 | 1039.764527 |
| **Fggy** | 94.3167376 | 82.39524888 | 79.88245204 | 94.02442674 | 65.8697774 | 63.82856532 | 62.24246532 | 81.29317927 | 108.3476337 | 71.65996145 | 94.58706988 | 68.5347204 | 49.42089028 | 48.79195118 | 61.07808212 |
| **9530080O11Rik** | 0 | 1.584524017 | 0 | 0 | 0 | 0 | 2.706194144 | 0 | 0 | 1.747803938 | 0.945870699 | 0.867528106 | 0 | 0 | 3.59282836 |
| **Hook1** | 529.2725663 | 550.6220959 | 642.1922615 | 526.191746 | 590.2945437 | 555.5189421 | 564.6925115 | 631.0697318 | 588.3878439 | 528.7106912 | 587.3857039 | 557.8205723 | 637.3590677 | 580.0820863 | 560.4812241 |
| **Nfia** | 9.156964816 | 16.63750218 | 9.397935534 | 8.626094197 | 7.600358931 | 12.6254305 | 8.118582433 | 4.190370065 | 5.266898858 | 14.85633347 | 4.729353494 | 12.14539349 | 12.78126473 | 4.517773258 | 16.52701046 |
| **Tm2d1** | 196.8747435 | 208.3649082 | 176.2112913 | 153.5444767 | 200.7761484 | 173.248963 | 206.5728197 | 177.6716908 | 164.7786928 | 198.3757469 | 197.686976 | 198.6639363 | 171.2689473 | 206.9140152 | 229.2224494 |
| **Inadl** | 173.9823315 | 183.012524 | 202.8387753 | 218.2401832 | 187.4755203 | 205.5139521 | 238.1450847 | 211.1946513 | 174.5600764 | 153.8067465 | 194.8493639 | 209.0742736 | 190.8668866 | 221.3708896 | 224.9110553 |
| **Usp1** | 3591.361601 | 3690.356436 | 3950.265569 | 3726.472693 | 3467.663762 | 3758.169813 | 3993.440492 | 4293.453169 | 4205.242531 | 4368.635943 | 4071.027488 | 4178.882888 | 4141.981856 | 3868.117463 | 3933.428488 |
| **Dock7** | 3.662785926 | 5.54583406 | 3.915806472 | 12.93914129 | 1.900089733 | 0 | 3.608258859 | 1.676148026 | 6.019312981 | 5.243411813 | 8.512836289 | 2.602584319 | 12.78126473 | 0 | 5.029959704 |
| **Atg4c** | 7.325571853 | 0.792262009 | 11.74741942 | 15.52696955 | 6.966995687 | 10.52119209 | 10.82477658 | 5.866518091 | 6.019312981 | 3.495607876 | 5.675224193 | 4.337640531 | 11.92918041 | 9.035546516 | 3.59282836 |
| **Alg6** | 144.6800441 | 125.1773973 | 138.6195491 | 116.4522717 | 150.1070889 | 112.2260489 | 149.7427427 | 144.9868043 | 116.624189 | 153.8067465 | 129.5842857 | 113.6461819 | 96.28552761 | 109.3301128 | 147.3059628 |
| **Itgb3bp** | 101.6423095 | 108.5398952 | 89.28038757 | 87.12355138 | 86.77076447 | 124.1500666 | 111.8560246 | 93.02621545 | 79.75589699 | 73.40776539 | 86.07423359 | 98.03067601 | 84.3563472 | 96.68034772 | 85.50931496 |
| **Efcab7** | 97.06382705 | 74.4726288 | 82.23193592 | 77.63484777 | 82.33722176 | 76.45399582 | 93.81473034 | 67.88399506 | 82.01313936 | 68.16435357 | 74.7237852 | 78.07752957 | 69.87091384 | 96.68034772 | 68.98230451 |
| **Pgm2** | 3149.995897 | 2804.60751 | 2738.715047 | 2535.209084 | 2356.111269 | 2404.443098 | 2449.105701 | 1322.480793 | 1483.008236 | 1643.809604 | 1776.345172 | 2160.144985 | 2497.459128 | 2109.800111 | 2394.260819 |
| **Cachd1** | 0 | 0 | 2.349483883 | 0 | 0 | 0 | 0 | 1.676148026 | 0 | 0 | 0 | 0 | 0 | 0 | 0 |
| **Raver2** | 24.723805 | 28.52143231 | 43.0738712 | 38.81742388 | 33.56825195 | 44.89041957 | 41.49497688 | 22.62799835 | 22.57242368 | 10.48682363 | 20.80915537 | 29.49595561 | 59.64590206 | 40.65995932 | 48.14390002 |
| **Jak1** | 5711.198956 | 5588.616208 | 5732.740676 | 5783.796159 | 5963.114945 | 5797.878252 | 6219.736208 | 5511.17471 | 5382.018219 | 5351.775658 | 5570.232545 | 6388.476975 | 6284.121824 | 6307.715023 | 6176.790516 |
| **E130102H24Rik** | 0.915696482 | 0.792262009 | 0.783161294 | 4.313047098 | 0.633363244 | 0.701412806 | 2.706194144 | 2.514222039 | 0 | 0 | 0 | 3.470112425 | 0.852084315 | 3.614218606 | 7.18565672 |
| **0610043K17Rik** | 0.915696482 | 0 | 0.783161294 | 0 | 0.633363244 | 0.701412806 | 0 | 0 | 0 | 0 | 0 | 0 | 0 | 3.614218606 | 5.029959704 |
| **Ak4** | 1119.896797 | 1073.515022 | 975.0358116 | 809.1276356 | 805.0046835 | 879.5716584 | 977.8381508 | 119.0065099 | 285.9173666 | 385.3907683 | 408.6161419 | 839.7672069 | 892.132278 | 755.3716887 | 971.5007885 |
| **Dnajc6** | 0 | 7.922620085 | 4.698967767 | 0 | 1.900089733 | 0 | 0 | 2.514222039 | 5.266898858 | 3.495607876 | 5.675224193 | 0 | 0 | 0 | 2.155697016 |
| **Leprot** | 401.0750589 | 393.7542182 | 464.4146476 | 353.6698621 | 440.1874548 | 427.1603987 | 408.6353158 | 371.2667878 | 441.66709 | 478.024377 | 418.0748489 | 461.5249525 | 431.1546635 | 446.3559979 | 405.9896047 |
| **Pde4b** | 402.9064519 | 420.6911265 | 417.42497 | 403.7012084 | 381.9180363 | 353.5120541 | 340.0783975 | 365.4002697 | 282.155296 | 325.9654344 | 318.7584255 | 354.8189955 | 373.21293 | 355.0969781 | 437.6064942 |
| **Sgip1** | 2.747089445 | 0 | 3.132645178 | 2.587828259 | 0 | 2.805651223 | 3.608258859 | 2.514222039 | 0 | 1.747803938 | 8.512836289 | 2.602584319 | 3.408337261 | 0.903554652 | 2.155697016 |
| **Gm12709** | 0.915696482 | 0 | 0 | 0 | 0 | 0 | 1.80412943 | 0 | 2.257242368 | 0 | 0 | 0.867528106 | 0 | 2.710663955 | 1.437131344 |
| **Tctex1d1** | 7.325571853 | 3.169048034 | 0.783161294 | 0.86260942 | 0 | 0.701412806 | 0 | 3.352296052 | 3.762070613 | 4.369509845 | 0 | 0 | 0 | 0.903554652 | 2.155697016 |
| **Insl5** | 3.662785926 | 0 | 0 | 0 | 0 | 0 | 1.80412943 | 0 | 5.266898858 | 6.117313782 | 1.891741398 | 0 | 0 | 0.903554652 | 0 |
| **Wdr78** | 77.83420093 | 101.4095371 | 70.4845165 | 56.06961228 | 55.7359655 | 44.89041957 | 73.96930661 | 62.01747697 | 88.03245234 | 95.25531461 | 99.31642337 | 27.7608994 | 68.16674521 | 61.44171631 | 78.32365824 |
| **Mier1** | 2755.330713 | 2595.45034 | 2676.062143 | 2417.894203 | 1895.65619 | 2048.826805 | 2241.630816 | 2635.742771 | 2859.92608 | 2511.594259 | 2795.047915 | 2158.409928 | 2183.040015 | 2115.221439 | 2269.948958 |
| **Slc35d1** | 342.4704841 | 290.7601571 | 320.3129694 | 309.6767817 | 194.442516 | 206.9167777 | 274.2276733 | 411.4943404 | 432.6381205 | 384.5168663 | 403.8867884 | 242.9078698 | 261.5898848 | 296.3659257 | 250.7794195 |
| **Oma1** | 251.8165324 | 243.2244366 | 205.9714205 | 218.2401832 | 219.7770458 | 246.1958948 | 236.3409553 | 287.4593865 | 197.1325001 | 273.5313163 | 269.5731491 | 183.9159585 | 238.5836082 | 224.9851082 | 227.785318 |
| **Prkaa2** | 0 | 0 | 0 | 1.725218839 | 0.633363244 | 1.402825611 | 0 | 0 | 0.752414123 | 0.873901969 | 0 | 0.867528106 | 0 | 1.807109303 | 0.718565672 |
| **Ppap2b** | 32.96507334 | 30.10595632 | 32.89277437 | 44.85568982 | 17.1008076 | 16.83390734 | 10.82477658 | 80.45510525 | 55.67864507 | 87.39019689 | 81.34488009 | 16.48303402 | 11.0770961 | 23.49242094 | 28.02406121 |
| **Usp24** | 3132.597663 | 3316.408768 | 3201.563372 | 3214.082698 | 2897.636843 | 2856.152945 | 3104.906748 | 3716.020174 | 3654.475393 | 3343.548933 | 3517.693129 | 3072.784552 | 3304.382974 | 3009.740544 | 3112.826491 |
| **Pcsk9** | 11.90405426 | 15.05297816 | 14.0969033 | 13.80175071 | 8.233722176 | 8.416953669 | 12.62890601 | 6.704592104 | 5.266898858 | 8.739019689 | 10.40457769 | 11.27786538 | 4.260421576 | 12.64976512 | 4.311394032 |
| **Dhcr24** | 7177.229022 | 7321.293221 | 7662.450105 | 7273.522626 | 7480.019915 | 7531.069295 | 7638.684005 | 7432.878422 | 7440.623258 | 7680.724405 | 7780.732368 | 7551.832165 | 8057.309284 | 7454.325875 | 7696.556912 |
| **BC055111** | 0 | 2.376786026 | 4.698967767 | 0 | 1.266726489 | 2.805651223 | 4.510323574 | 0 | 3.00965649 | 2.621705907 | 2.837612096 | 0 | 1.70416863 | 0 | 0.718565672 |
| **Pars2** | 195.9590471 | 162.4137117 | 184.0429042 | 172.5218839 | 177.9750716 | 214.6323185 | 153.3510015 | 178.5097648 | 205.4090555 | 186.1411194 | 182.5530449 | 193.4587677 | 161.0439356 | 166.2540559 | 158.0844478 |
| **Ttc4** | 651.0601984 | 598.9500784 | 727.5568426 | 690.0875357 | 708.1001071 | 606.7220769 | 669.3320184 | 838.0740131 | 765.9575768 | 751.5556933 | 771.8304902 | 712.2405753 | 691.0403796 | 673.1482154 | 661.7989839 |
| **Acot11** | 95.23243408 | 62.58869867 | 68.91819391 | 94.02442674 | 83.60394824 | 67.33562935 | 78.47963019 | 68.72206907 | 72.23175577 | 80.39898114 | 95.53294058 | 88.48786684 | 86.91260015 | 104.8123396 | 117.1262045 |
| **Ssbp3** | 2049.328726 | 2192.188978 | 2435.631626 | 2340.259356 | 2016.62857 | 2129.489278 | 2161.347057 | 2226.762653 | 2318.187912 | 2428.573572 | 2469.668394 | 2190.508468 | 2033.92526 | 2243.5262 | 2006.235356 |
| **Mrpl37** | 957.8185197 | 1144.818602 | 1099.558457 | 1052.383492 | 1065.95034 | 975.6652127 | 992.2711863 | 1363.546419 | 1357.355077 | 1219.093247 | 1239.090615 | 1103.495751 | 1047.211623 | 981.2603516 | 954.973778 |
| **Cyb5rl** | 54.02609241 | 35.65179038 | 36.80858084 | 49.16873692 | 38.6351579 | 35.07064029 | 27.06194144 | 56.98903289 | 63.95520042 | 68.16435357 | 59.58985402 | 52.05168638 | 42.60421576 | 43.37062328 | 43.11394032 |
| **Tceanc2** | 447.7755795 | 496.7482793 | 506.7053575 | 478.7482279 | 420.5531942 | 463.6338646 | 432.0889984 | 593.3564012 | 471.0112407 | 461.4202396 | 487.1234099 | 441.5718061 | 473.7588792 | 414.7315851 | 502.9959704 |
| **Tmem59** | 806.7286003 | 846.9280871 | 821.5361979 | 866.0598573 | 851.2402003 | 836.7854772 | 849.7449613 | 848.9689752 | 793.0444852 | 753.3034972 | 804.9359647 | 885.7461965 | 802.6634249 | 837.595162 | 811.2606436 |
| **Lrrc42** | 459.6796337 | 455.5506549 | 470.679938 | 499.450854 | 532.6584884 | 524.6567787 | 587.2441293 | 449.207671 | 372.4449907 | 412.4817293 | 483.3399271 | 575.1711345 | 514.6589264 | 512.3154874 | 462.0377271 |
| **Hspb11** | 98.89522001 | 119.6315633 | 120.6068394 | 116.4522717 | 108.3051148 | 152.9079916 | 147.0365485 | 101.4069556 | 85.02279585 | 91.75970673 | 76.6155266 | 111.0435976 | 96.28552761 | 94.87323842 | 103.4734568 |
| **Yipf1** | 546.6707995 | 572.0131701 | 603.0341968 | 553.7952474 | 559.8931079 | 566.741547 | 552.9656702 | 512.063222 | 568.8250767 | 526.0889853 | 511.716048 | 593.3892247 | 574.3048284 | 500.569277 | 559.0440928 |
| **Ndc1** | 1069.53349 | 1119.466218 | 1065.099361 | 973.0234254 | 1008.947648 | 1012.840091 | 1055.415716 | 1185.036654 | 1097.772205 | 991.8787347 | 1161.529218 | 1085.277661 | 1078.738743 | 1045.412732 | 1087.189862 |
| **Glis1** | 0 | 0 | 0 | 6.038265938 | 0.633363244 | 4.90988964 | 0.902064715 | 1.676148026 | 0 | 0 | 3.783482795 | 3.470112425 | 0 | 0 | 0 |
| **Lrp8** | 758.1966867 | 808.1072487 | 725.2073587 | 727.1797408 | 650.4640519 | 668.4464038 | 757.7343604 | 662.9165443 | 741.8803249 | 756.7991051 | 815.3405423 | 740.0014747 | 753.2425346 | 663.2091143 | 713.5357123 |
| **Magoh** | 1310.361665 | 1290.594812 | 1318.84362 | 1247.333221 | 1323.729181 | 1341.802697 | 1360.31359 | 1358.517975 | 1347.573694 | 1354.548052 | 1462.3161 | 1340.330924 | 1307.097339 | 1359.849751 | 1257.489926 |
| **0610037L13Rik** | 326.9036439 | 382.6625501 | 342.2414857 | 300.188078 | 292.6138189 | 361.9290077 | 309.4081972 | 336.9057532 | 375.4546472 | 373.1561407 | 365.1060897 | 356.5540517 | 389.402532 | 340.6401036 | 345.6300882 |
| **Cpt2** | 225.2613345 | 208.3649082 | 211.4535495 | 196.6749477 | 220.410409 | 197.7984112 | 207.4748844 | 219.5753914 | 204.6566413 | 200.1235509 | 210.9291658 | 212.544386 | 223.2460906 | 200.5891326 | 227.0667523 |
| **Podn** | 0 | 0 | 0.783161294 | 0 | 0.633363244 | 0 | 0 | 6.704592104 | 4.514484735 | 2.621705907 | 2.837612096 | 0 | 0 | 0.903554652 | 0.718565672 |
| **Scp2** | 911.1179992 | 945.1685761 | 1080.762586 | 1003.214755 | 984.2464816 | 981.2765152 | 1056.317781 | 1055.973256 | 1051.874943 | 990.1309308 | 976.1385611 | 1125.183954 | 1009.719913 | 1082.458473 | 1048.387315 |
| **Echdc2** | 44.8691276 | 48.32798252 | 46.98967767 | 50.89395576 | 26.60125626 | 22.44520978 | 26.15987673 | 70.3982171 | 42.13519086 | 62.04703979 | 68.10269031 | 42.50887721 | 30.67503535 | 35.23863141 | 31.61688957 |
| **Zyg11b** | 1260.914055 | 1269.203738 | 1242.876974 | 1302.540224 | 1134.35357 | 1186.089054 | 1301.679383 | 1088.658143 | 957.823178 | 957.7965579 | 1086.805433 | 1259.65081 | 1350.55364 | 1229.737881 | 1264.675583 |
| **Coa7** | 301.2641424 | 304.2286113 | 341.4583244 | 322.6159229 | 296.4139983 | 306.5173961 | 245.3616024 | 398.9232302 | 361.911193 | 356.5520033 | 336.7299688 | 297.5621405 | 229.2106808 | 269.2592862 | 327.6659464 |
| **Gpx7** | 32.04937685 | 45.15893448 | 44.64019379 | 37.95481446 | 30.40143573 | 42.08476834 | 49.61355931 | 16.76148026 | 36.86829201 | 27.964863 | 37.83482795 | 50.31663016 | 31.52711966 | 34.33507676 | 44.55107166 |
| **Zcchc11** | 1811.247641 | 1788.927615 | 1704.942138 | 1770.937139 | 1540.33941 | 1490.502212 | 1725.649799 | 1379.469825 | 1563.516547 | 1424.460209 | 1490.692221 | 1689.944751 | 1663.268583 | 1698.682745 | 1732.461835 |
| **Prpf38a** | 1275.565199 | 1408.641851 | 1347.820588 | 1351.708961 | 1206.55698 | 1231.680887 | 1196.137812 | 1452.382265 | 1361.869562 | 1353.67415 | 1362.999677 | 1225.817214 | 1227.853498 | 1270.39784 | 1182.04053 |
| **Orc1** | 702.3392014 | 716.9971177 | 787.077101 | 622.804001 | 599.1616291 | 655.8209733 | 598.0689059 | 867.4066035 | 774.9865463 | 869.5324591 | 802.0983526 | 605.5346182 | 659.5132599 | 646.0415759 | 588.5052853 |
| **Cc2d1b** | 1418.41385 | 1525.896628 | 1591.38375 | 1570.811753 | 1630.910354 | 1575.373162 | 1483.896456 | 1583.959885 | 1657.568312 | 1790.625134 | 1625.951731 | 1594.516659 | 1592.545585 | 1600.195288 | 1531.263447 |
| **Zfyve9** | 3.662785926 | 7.922620085 | 2.349483883 | 5.175656518 | 0.633363244 | 3.507064029 | 7.216517718 | 5.028444078 | 9.028969471 | 0.873901969 | 0 | 0.867528106 | 0 | 3.614218606 | 2.155697016 |
| **3110021N24Rik** | 0.915696482 | 5.54583406 | 0 | 1.725218839 | 2.533452977 | 0.701412806 | 0 | 0.838074013 | 0 | 0 | 7.56696559 | 0 | 4.260421576 | 0 | 1.437131344 |
| **Btf3l4** | 328.7350369 | 387.4161222 | 362.6036793 | 326.92897 | 329.9822503 | 328.2611931 | 407.7332511 | 348.6387894 | 357.3967082 | 345.1912777 | 302.6786236 | 363.4942765 | 345.0941476 | 326.1832292 | 367.905624 |
| **Txndc12** | 449.6069724 | 507.8399475 | 474.5957445 | 511.5273859 | 416.7530147 | 444.6957188 | 465.4653928 | 395.5709342 | 347.6153246 | 407.2383175 | 400.1033056 | 448.5120309 | 421.781736 | 477.076856 | 452.6963733 |
| **Kti12** | 1424.823725 | 1282.672192 | 1325.10891 | 1387.075947 | 1331.962903 | 1427.37506 | 1392.78792 | 1450.706117 | 1490.532377 | 1386.882425 | 1317.597883 | 1303.027216 | 1349.701555 | 1392.377718 | 1313.538048 |
| **Rab3b** | 0 | 2.376786026 | 0.783161294 | 0 | 1.266726489 | 0.701412806 | 1.80412943 | 0 | 0 | 0 | 0 | 0.867528106 | 1.70416863 | 0 | 1.437131344 |
| **Nrd1** | 2723.281336 | 2517.016401 | 2762.993047 | 2535.209084 | 2577.788404 | 2552.4412 | 2788.282033 | 2594.677144 | 2608.619763 | 2543.928631 | 2747.75438 | 2750.931625 | 2640.609293 | 2519.110369 | 2697.495533 |
| **Mir761** | 0 | 0.792262009 | 0 | 0 | 3.166816221 | 0.701412806 | 0 | 0 | 0 | 0 | 0 | 0 | 1.70416863 | 3.614218606 | 0 |
| **Osbpl9** | 968.8068775 | 966.5596504 | 1019.676005 | 923.8546884 | 1045.682716 | 1089.294087 | 1132.091217 | 832.207495 | 913.4307448 | 836.3241842 | 963.842242 | 1123.448898 | 1108.561694 | 1199.017023 | 998.806284 |
| **Eps15** | 1498.99514 | 1527.481152 | 1589.034267 | 1563.048268 | 1463.702458 | 1453.327333 | 1633.639198 | 1730.622837 | 1614.680707 | 1545.932583 | 1538.931627 | 1568.490816 | 1765.518701 | 1602.905952 | 1577.25165 |
| **Rnf11** | 467.0052056 | 428.6137466 | 435.4376797 | 345.0437679 | 405.3524763 | 396.999648 | 402.3208628 | 560.6715147 | 339.3387693 | 449.185612 | 450.2344526 | 447.6445028 | 473.7588792 | 362.3254153 | 380.8398061 |
| **Cdkn2c** | 696.8450225 | 787.5084365 | 843.4647142 | 842.769403 | 813.8717689 | 833.2784132 | 771.2653311 | 690.5729868 | 794.5493134 | 720.0952224 | 694.2690929 | 925.6524894 | 815.4446896 | 944.2146109 | 814.853472 |
| **Faf1** | 1380.870294 | 1272.372786 | 1193.537813 | 1249.05844 | 1169.188549 | 1195.908834 | 1140.209799 | 1123.857252 | 1173.013617 | 1282.014188 | 1160.583347 | 1129.521594 | 1214.220149 | 1072.519371 | 1089.345559 |
| **Dmrta2** | 6.409875371 | 1.584524017 | 9.397935534 | 4.313047098 | 14.56735462 | 7.715540863 | 8.118582433 | 0.838074013 | 7.524141226 | 13.9824315 | 7.56696559 | 13.01292159 | 5.964590206 | 9.939101167 | 0 |
| **Bend5** | 108.9678813 | 72.88810478 | 71.2676778 | 57.79483112 | 97.53793962 | 102.4062696 | 91.10853619 | 49.44636677 | 66.96485691 | 49.81241223 | 48.23940564 | 96.2956198 | 114.1792982 | 91.25901981 | 86.22788064 |
| **Spata6** | 239.9124782 | 234.5095545 | 238.0810335 | 248.4315129 | 252.7119345 | 232.1676387 | 258.8925731 | 185.2143569 | 208.418712 | 182.6455115 | 191.0658812 | 224.6897795 | 266.7023906 | 294.5588164 | 275.2106524 |

| | | | | | | | | | | | | | | | |
|---|---|---|---|---|---|---|---|---|---|---|---|---|---|---|---|
| **Foxd2** | 26.55519797 | 25.35238427 | 27.41064531 | 12.07653188 | 15.20071786 | 32.26498906 | 21.64955315 | 40.22755263 | 18.81035306 | 26.21705907 | 25.53850887 | 19.08561834 | 17.89377062 | 32.52796746 | 14.37131344 |
| **Foxd2os** | 59.5202713 | 72.09584277 | 85.3645811 | 73.32180067 | 73.47013634 | 80.66247266 | 68.55691832 | 69.56014308 | 76.7462405 | 115.3550599 | 64.31920752 | 82.4151701 | 74.98341973 | 59.634607 | 65.38947615 |
| **Cmpk1** | 1126.306672 | 1316.739458 | 1256.190716 | 1158.484451 | 1235.058326 | 1246.410556 | 1380.159014 | 1335.051903 | 1442.377873 | 1315.222463 | 1353.54097 | 1367.224295 | 1312.209845 | 1258.65163 | 1258.208492 |
| **Stil** | 741.7141501 | 785.9239124 | 754.9674879 | 699.5762393 | 663.1313168 | 687.3845496 | 762.244684 | 658.7261743 | 689.9637504 | 672.9045161 | 627.1122733 | 658.4538327 | 700.4133071 | 665.0162236 | 714.2542779 |
| **Tal1** | 0 | 0 | 2.349483883 | 0 | 1.266726489 | 0.701412806 | 0 | 6.704592104 | 3.00965649 | 5.243411813 | 0 | 4.337640531 | 0 | 0 | 1.437131344 |
| **Pdzk1ip1** | 79.6655939 | 54.66607859 | 66.56871003 | 46.58090866 | 74.73686282 | 89.78083913 | 58.63420646 | 64.53169901 | 44.39243323 | 67.29045161 | 75.6696559 | 60.72696744 | 54.53339617 | 43.37062328 | 47.42533435 |
| **Efcab14** | 586.0457482 | 704.3209256 | 671.1692294 | 600.3761561 | 557.9930182 | 559.727419 | 642.2700769 | 618.4986216 | 612.4650958 | 667.6611042 | 648.8672994 | 613.3423711 | 666.3299345 | 586.4069689 | 600.0023361 |
| **Tex38** | 0 | 0 | 0 | 0 | 0 | 1.402825611 | 0 | 0 | 0 | 0 | 0 | 0.867528106 | 1.70416863 | 0 | 2.155697016 |
| **Atpaf1** | 299.4327495 | 366.8173099 | 321.0961307 | 280.3480614 | 274.2462848 | 316.3371754 | 322.0371032 | 356.1814555 | 363.4160212 | 350.4346895 | 328.2171325 | 324.4555117 | 331.4607986 | 255.7059664 | 314.0131986 |
| **Mob3c** | 131.8602933 | 117.2547773 | 104.9436135 | 108.6887869 | 132.3729181 | 119.9415898 | 143.4282897 | 91.35006742 | 123.3959161 | 93.50751067 | 116.3420959 | 118.8513506 | 120.9959728 | 148.1829629 | 105.6291538 |
| **Mknk1** | 404.7378449 | 458.7197029 | 464.4146476 | 476.1603996 | 478.8226127 | 382.9713919 | 423.9704159 | 448.369597 | 430.3808781 | 435.2031805 | 434.1546507 | 463.2600088 | 420.9296517 | 468.9448642 | 509.4630614 |
| **Faah** | 601.6125884 | 682.1375893 | 623.3963904 | 626.2544387 | 647.9305989 | 695.8015033 | 687.3733127 | 459.2645592 | 483.0498667 | 504.2414361 | 576.0352556 | 686.2147321 | 683.3716208 | 687.6050898 | 754.4939556 |
| **Nsun4** | 264.6362832 | 286.0065851 | 234.1652271 | 231.1793245 | 223.5772252 | 255.3142613 | 238.1450847 | 387.190194 | 279.8980536 | 304.9917871 | 270.5190198 | 248.1130384 | 211.3169102 | 226.7922175 | 206.2283479 |
| **Uqcrh** | 1537.454393 | 1510.84365 | 1577.286847 | 1456.0847 | 1693.613315 | 1576.074574 | 1664.309399 | 1588.988329 | 1567.278617 | 1675.270074 | 1494.475704 | 1705.560257 | 1692.23945 | 1682.418761 | 1542.760498 |
| **Lrrc41** | 1145.536298 | 1110.751336 | 1051.785618 | 1036.856522 | 1003.880742 | 1072.46018 | 1055.415716 | 1116.314585 | 1011.996995 | 1059.169186 | 1067.888019 | 971.631479 | 999.4949017 | 902.6510969 | 1064.19576 |
| **Rad54l** | 615.3480356 | 677.3840173 | 678.217681 | 671.1101285 | 573.8270993 | 626.3616355 | 607.9916178 | 725.7720953 | 732.0989413 | 700.8693791 | 799.2607405 | 690.5523726 | 710.6383188 | 665.0162236 | 695.5715705 |
| **2510003B16Rik** | 3.662785926 | 0 | 0 | 0 | 1.266726489 | 0 | 1.80412943 | 1.676148026 | 0 | 2.621705907 | 0 | 0.867528106 | 0 | 3.614218606 | 0 |
| **Lurap1** | 0.915696482 | 0 | 0.783161294 | 0.86260942 | 1.900089733 | 1.402825611 | 1.80412943 | 5.028444078 | 0 | 0.873901969 | 6.621094891 | 9.542809169 | 2.556252945 | 2.710663955 | 6.467091048 |
| **Pomgnt1** | 407.4849343 | 414.3530304 | 364.1700019 | 408.0142555 | 363.5505022 | 340.8866236 | 359.0217565 | 465.9691513 | 364.1684353 | 414.2295333 | 458.7472889 | 358.2891079 | 364.6920869 | 300.883699 | 436.8879286 |
| **Pik3r3** | 14.65114371 | 19.0142882 | 21.92851625 | 37.95481446 | 6.966995687 | 9.118366474 | 10.82477658 | 33.52296052 | 44.39243323 | 30.58656891 | 36.88895725 | 19.95314644 | 15.33751767 | 17.16753838 | 10.77848508 |
| **Mast2** | 836.9465841 | 860.3965412 | 841.8983916 | 786.6997907 | 894.9422642 | 843.0981925 | 844.332573 | 1062.677849 | 911.9259166 | 965.6616756 | 843.7166633 | 841.5022631 | 911.7302172 | 946.0217202 | 928.3868482 |
| **Ipp** | 215.1886732 | 266.2000349 | 325.0119372 | 257.9202165 | 283.1133702 | 292.48914 | 326.5474268 | 296.6782006 | 270.8690841 | 297.1266694 | 279.9777268 | 314.0451745 | 319.5316182 | 290.0410432 | 279.5220464 |
| **Tmem69** | 165.7410632 | 254.3161047 | 260.0095498 | 269.9967484 | 247.0116653 | 223.0492722 | 243.557473 | 316.7919769 | 262.5925288 | 261.2966887 | 289.4364338 | 237.7027011 | 239.4356926 | 222.2744443 | 245.0308941 |
| **Gpbp1l1** | 546.6707995 | 579.9357902 | 546.6465836 | 515.840433 | 521.8913133 | 506.4200457 | 598.0689059 | 567.3761068 | 573.3395614 | 522.5933774 | 546.7132639 | 576.0386626 | 513.806842 | 520.4474793 | 556.8883958 |
| **C530005A16Rik** | 0 | 3.169048034 | 2.349483883 | 0 | 0.633363244 | 3.507064029 | 0 | 0 | 1.504828245 | 2.621705907 | 2.837612096 | 0 | 2.556252945 | 0 | 0.718565672 |
| **Ccdc17** | 10.98835778 | 7.922620085 | 7.831612945 | 2.587828259 | 7.600358931 | 14.02825611 | 9.922711863 | 7.542666117 | 3.00965649 | 13.10852953 | 8.512836289 | 17.35056213 | 7.668758836 | 16.26398373 | 14.37131344 |
| **Nasp** | 3988.773874 | 3881.29158 | 4227.504668 | 3779.091867 | 4104.193823 | 4036.630697 | 4094.47174 | 4369.717904 | 4176.650794 | 4364.266433 | 4609.227915 | 4100.805358 | 4071.258858 | 4208.757567 | 3809.116627 |
| **Akr1a1** | 5978.582328 | 5932.45792 | 5876.842354 | 5889.034508 | 5752.838348 | 5794.371188 | 5900.405299 | 5672.08492 | 5524.224488 | 5863.882211 | 5609.013244 | 5805.498087 | 5894.719292 | 5786.363989 | 5709.722829 |
| **AV051173** | 1.831392963 | 4.753572051 | 0.783161294 | 2.587828259 | 0 | 2.104238417 | 0.902064715 | 10.05688816 | 3.00965649 | 0 | 1.891741398 | 0 | 4.260421576 | 2.710663955 | 0 |
| **Prdx1** | 4888.903515 | 4941.338147 | 5515.804997 | 5123.037343 | 5011.803352 | 4805.379132 | 4736.741817 | 6005.638378 | 5825.190137 | 6184.604234 | 6258.826414 | 5468.897182 | 4972.764063 | 5514.394039 | 5124.810372 |
| **Mmachc** | 285.6973022 | 274.1226549 | 335.193034 | 309.6767817 | 291.9804556 | 312.1286985 | 314.8205855 | 327.6869391 | 370.9401624 | 325.9654344 | 297.9492701 | 320.1178712 | 339.9816417 | 295.4623711 | 312.5760673 |
| **Ccdc163** | 133.6916863 | 126.7619214 | 101.027807 | 114.7270528 | 107.0383883 | 92.58649035 | 100.1291833 | 155.0436924 | 119.6338455 | 143.3199229 | 122.9631908 | 85.88528252 | 121.8480571 | 121.0763233 | 105.6291538 |
| **Tesk2** | 60.43596778 | 93.486917 | 87.71406498 | 71.59658183 | 93.10439691 | 77.15540863 | 83.89201847 | 62.01747697 | 69.22209928 | 73.40776539 | 57.69811262 | 66.79966418 | 62.20215501 | 67.76659887 | 77.60509257 |
| **Toe1** | 606.1910708 | 644.1090129 | 583.4551644 | 532.2300119 | 543.4256636 | 577.2627391 | 607.089553 | 602.5752154 | 687.706508 | 606.4879664 | 604.4113765 | 601.1969777 | 583.6777559 | 611.7064991 | 598.5652047 |
| **Mutyh** | 166.6567596 | 167.9595458 | 191.8745171 | 199.2627759 | 201.4095117 | 204.1111265 | 211.985208 | 134.0918421 | 134.6821279 | 152.0589426 | 113.5044839 | 208.2067455 | 186.606465 | 215.9495617 | 224.9110553 |
| **Hpdl** | 88.82255871 | 115.6702532 | 75.96664557 | 103.5131304 | 89.93758069 | 83.46812388 | 151.5468721 | 106.4353997 | 87.28003822 | 108.3638441 | 98.37055267 | 55.5217988 | 97.98969624 | 83.12702794 | 86.94644631 |
| **Zswim5** | 2.747089445 | 2.376786026 | 3.132645178 | 1.725218839 | 3.800179466 | 1.402825611 | 9.020647148 | 0.838074013 | 0 | 0 | 6.621094891 | 3.470112425 | 5.964590206 | 0 | 4.311394032 |
| **Urod** | 478.9092599 | 474.5649431 | 551.3455513 | 527.9169648 | 544.0590268 | 516.9412378 | 561.9863173 | 461.7787812 | 520.6705728 | 603.8662605 | 519.2830136 | 589.9191123 | 506.1380832 | 557.49322 | 503.714536 |
| **Hectd3** | 624.5050004 | 618.7566286 | 644.5417454 | 558.1082945 | 592.1946334 | 590.5895824 | 619.7184591 | 668.7830624 | 537.2236835 | 593.3794369 | 607.2489886 | 650.6460797 | 615.2048755 | 747.2396969 | 722.877066 |
| **Eif2b3** | 805.8129038 | 835.044157 | 801.1740043 | 798.7763226 | 767.6362521 | 732.976382 | 719.8476424 | 900.929564 | 699.745134 | 961.2921658 | 836.1496977 | 750.4118119 | 672.2945247 | 693.0264178 | 703.4757929 |
| **Ptch2** | 4.578482408 | 13.46845414 | 4.698967767 | 7.763484777 | 6.966995687 | 10.52119209 | 10.82477658 | 15.08533223 | 14.29586833 | 8.739019689 | 13.24218978 | 23.42325887 | 7.668758836 | 4.517773258 | 17.24557613 |
| **Btbd19** | 295.7699635 | 281.253013 | 324.2287759 | 345.0437679 | 309.7146265 | 315.6357626 | 340.0783975 | 471.8356693 | 365.6732636 | 303.2439832 | 390.6445986 | 363.4942765 | 335.7212202 | 367.7467432 | 360.7199673 |
| **Tctex1d4** | 1.831392963 | 0.792262009 | 3.132645178 | 0.86260942 | 6.966995687 | 0.701412806 | 1.80412943 | 0 | 0 | 0 | 3.783482795 | 0.867528106 | 0.852084315 | 3.614218606 | 6.467091048 |
| **Plk3** | 343.3861806 | 298.6827772 | 296.8181306 | 286.3863273 | 262.2123831 | 276.3566455 | 267.9132203 | 394.7328601 | 359.6539506 | 276.1530222 | 316.8666841 | 228.159892 | 250.5127887 | 234.0206548 | 253.6536822 |
| **Rps8** | 24016.88732 | 23469.96974 | 24141.73006 | 23241.28559 | 24711.30034 | 25309.77968 | 25213.61084 | 23782.02627 | 23206.70878 | 24456.1466 | 23526.64189 | 24022.72079 | 24253.72795 | 24898.35198 | 24840.09671 |
| **Snord55** | 5.494178889 | 22.97559825 | 14.0969033 | 6.038265938 | 8.233722176 | 14.72966892 | 13.53097072 | 13.40918421 | 4.514484735 | 25.3431571 | 12.29631908 | 14.74797781 | 5.964590206 | 14.45687443 | 11.49705075 |
| **Kif2c** | 1131.800851 | 1233.551947 | 1237.394845 | 1280.974988 | 1153.354468 | 1057.730511 | 1216.8853 | 1214.369245 | 1173.766031 | 1251.427619 | 1227.740167 | 1242.300248 | 1156.278416 | 1207.149015 | 1169.106348 |
| **Tmem53** | 0.915696482 | 0 | 2.349483883 | 0 | 0.633363244 | 0 | 0 | 5.028444078 | 0 | 2.621705907 | 0.945870699 | 0.867528106 | 1.70416863 | 0.903554652 | 0 |
| **Rnf220** | 1739.823315 | 1876.868698 | 1713.556912 | 1661.385742 | 1815.852421 | 1803.332323 | 1801.423235 | 1684.528766 | 1835.138045 | 1868.40241 | 1828.368061 | 1873.86071 | 1804.71458 | 1822.469732 | 1845.995211 |
| **Eri3** | 566.8161221 | 538.7381658 | 534.1160028 | 520.15348 | 538.9921209 | 533.0737323 | 515.0789521 | 444.1792269 | 562.8057637 | 498.9980242 | 499.419729 | 491.0209082 | 479.7234694 | 541.2292363 | 495.091748 |
| **Dmap1** | 372.688468 | 420.6911265 | 388.4480021 | 401.1133801 | 366.7173184 | 395.5968224 | 386.0836979 | 426.5796726 | 328.8049716 | 381.8951604 | 387.8069865 | 392.122704 | 402.1837968 | 405.6960386 | 388.0254629 |
| **Slc6a9** | 441.3657041 | 372.363144 | 375.9174214 | 275.1724049 | 242.5781226 | 277.0580583 | 273.3256086 | 162.5863585 | 223.4669944 | 181.7716095 | 198.6328467 | 267.1986567 | 253.0690416 | 195.1678047 | 274.4920867 |
| **Ccdc24** | 3.662785926 | 0.792262009 | 16.44638718 | 6.038265938 | 5.066905954 | 2.104238417 | 9.020647148 | 1.676148026 | 7.524141226 | 4.369509845 | 2.837612096 | 7.807752957 | 1.70416863 | 4.517773258 | 4.311394032 |
| **B4galt2** | 64.09875371 | 56.2506026 | 75.96664557 | 86.26094197 | 53.83587576 | 54.00878604 | 76.67550076 | 58.66518091 | 69.9745134 | 74.28166736 | 69.99443171 | 61.59449555 | 36.63962555 | 75.89859073 | 59.64095077 |
| **Atp6v0b** | 799.4030284 | 784.3393884 | 823.8856818 | 730.6301784 | 792.9707818 | 838.8897156 | 801.0334667 | 760.1331298 | 756.1761932 | 777.7727523 | 636.5709803 | 838.0321507 | 760.9112934 | 738.2041503 | 809.8235123 |
| **Dph2** | 496.307493 | 458.7197029 | 408.0270344 | 493.412588 | 423.0866472 | 448.2027829 | 387.8878274 | 521.2820361 | 463.4870995 | 455.3029258 | 490.9068927 | 420.7511315 | 469.4984577 | 409.3102572 | 441.1993226 |
| **Ipo13** | 782.0047953 | 760.5715282 | 778.4623267 | 691.8127546 | 708.7334703 | 737.1848588 | 760.4405546 | 884.1680838 | 759.1858497 | 801.3681055 | 821.0157665 | 721.7833844 | 787.3259072 | 677.6659887 | 804.0749869 |
| **Artn** | 0.915696482 | 0.792262009 | 1.566322589 | 0 | 0 | 3.507064029 | 0 | 0 | 0 | 0 | 1.891741398 | 0 | 1.70416863 | 0 | 0 |
| **St3gal3** | 93.40104112 | 106.9553711 | 109.6425812 | 86.26094197 | 119.0722899 | 98.1977928 | 106.4436363 | 149.1771743 | 98.56625006 | 119.7245697 | 92.69532848 | 93.69303548 | 114.1792982 | 88.54835585 | 90.53927467 |
| **Kdm4a** | 1560.346805 | 1541.741869 | 1520.899234 | 1431.931637 | 1548.573132 | 1494.009276 | 1568.690539 | 1384.49827 | 1451.406842 | 1315.222463 | 1407.4556 | 1493.883399 | 1596.806007 | 1596.581069 | 1571.503125 |
| **Ptprf** | 13.73544722 | 8.714882094 | 3.132645178 | 9.488703616 | 5.066905954 | 9.81977928 | 6.314453003 | 13.40918421 | 12.03862596 | 8.739019689 | 10.40457769 | 6.94022485 | 5.964590206 | 18.97464768 | 6.467091048 |
| **Hyi** | 84.2440763 | 64.17322269 | 61.86974226 | 52.6191746 | 73.47013634 | 62.42573971 | 78.47963019 | 58.66518091 | 60.94554393 | 50.6863142 | 44.45592284 | 65.93213608 | 58.79381775 | 72.28437213 | 74.01226421 |
| **Szt2** | 594.2870165 | 823.9524888 | 776.8960041 | 660.7588155 | 664.3980432 | 650.2096709 | 674.7444067 | 771.028092 | 689.2113363 | 719.2213204 | 757.6424297 | 708.7704628 | 837.5988818 | 751.7574701 | 774.6137944 |
| **Med8** | 693.1822365 | 694.8137815 | 680.5671649 | 727.1797408 | 732.8012736 | 721.7537771 | 698.1980892 | 691.4110608 | 791.539657 | 834.5763803 | 750.0754641 | 690.5523726 | 674.8507776 | 788.8032108 | 642.3977107 |
| **Elovl1** | 1767.294209 | 1696.23296 | 1769.161364 | 1764.036263 | 1635.97726 | 1679.88367 | 1723.84567 | 1753.250835 | 1659.07314 | 1678.765682 | 1755.536017 | 1679.534414 | 1647.931066 | 1691.454308 | 1726.71331 |
| **Cdc20** | 4101.404541 | 4263.161868 | 4372.389507 | 4377.742805 | 4266.334813 | 4449.061427 | 4217.152542 | 4978.159638 | 5020.107026 | 4805.586927 | 4626.253588 | 4459.961994 | 4493.040594 | 4518.676813 | 4504.688198 |
| **Cfap57** | 0 | 0 | 3.132645178 | 2.587828259 | 0 | 0 | 0 | 1.676148026 | 0 | 0 | 0 | 0 | 1.70416863 | 0.903554652 | 1.437131344 |
| **Ebna1bp2** | 3453.091432 | 3159.54089 | 3246.203566 | 3012.232093 | 3050.910748 | 3065.875374 | 3050.782865 | 3337.21072 | 3180.454496 | 3198.481206 | 3362.570334 | 2906.219156 | 2897.086672 | 2754.034578 | 2826.118788 |
| **D4Ertd617e** | 0 | 0 | 0 | 0 | 2.533452977 | 0 | 0 | 0.838074013 | 9.028969471 | 0 | 0 | 0 | 0 | 2.710663955 | 2.155697016 |
| **Slc2a1** | 6466.648553 | 5836.594217 | 5865.878096 | 5364.567981 | 5667.33431 | 6173.835516 | 6519.221694 | 1869.743123 | 2225.640975 | 3007.970577 | 2968.142253 | 5462.824485 | 5701.296153 | 5323.744007 | 6104.933949 |
| **Zfp691** | 62.26736075 | 61.00417465 | 56.3876132 | 50.89395576 | 67.13650389 | 74.34975741 | 66.75278889 | 50.28444078 | 27.83932254 | 45.44290238 | 52.02288843 | 43.37640531 | 52.82922754 | 85.8376919 | 35.9282836 |
| **Ermap** | 0 | 0.792262009 | 0.783161294 | 0 | 1.900089733 | 1.402825611 | 0.902064715 | 0 | 2.257242368 | 0 | 0 | 0 | 0 | 0 | 0 |
| **Ccdc23** | 418.4732921 | 363.6482619 | 369.652131 | 382.1359729 | 426.8868266 | 428.5632243 | 385.1816332 | 331.0392352 | 382.9787884 | 404.6166116 | 302.6786236 | 393.8577603 | 366.3962555 | 379.4929537 | 362.8756643 |
| **4930538K18Rik** | 1.831392963 | 0.792262009 | 0 | 1.725218839 | 3.166816221 | 0.701412806 | 0.902064715 | 0.838074013 | 0 | 1.747803938 | 0 | 0.867528106 | 0 | 0 | 1.437131344 |
| **AU022252** | 224.345638 | 190.142882 | 172.2954848 | 165.6210086 | 195.0758792 | 214.6323185 | 197.5521725 | 248.0699079 | 217.4476814 | 200.1235509 | 168.3649844 | 220.352139 | 211.3169102 | 225.8886629 | 178.9228523 |
| **P3h1** | 184.9706893 | 215.4952663 | 203.6219366 | 213.0645267 | 247.6450285 | 228.6605747 | 204.7686903 | 309.2493108 | 207.6662978 | 285.7659438 | 203.3622002 | 221.2196671 | 203.6481513 | 197.8784687 | 212.6954389 |
| **Ybx1** | 23848.39917 | 23576.92511 | 23788.52432 | 23540.61106 | 23564.2795 | 25352.56586 | 24381.90718 | 26043.14996 | 26608.37303 | 26758.00439 | 25169.61929 | 24469.49777 | 23728.84401 | 23612.59371 | 22999.85003 |
| **Ppih** | 641.9032336 | 735.2191439 | 740.8705846 | 692.675364 | 714.4337395 | 704.2184569 | 737.8889367 | 839.7501611 | 829.1603631 | 772.5293405 | 791.6937749 | 744.3391152 | 750.6862817 | 673.1482154 | 645.2719734 |
| **Ccdc30** | 31.13368037 | 53.08155457 | 48.55600026 | 56.06961228 | 46.86888008 | 43.48759395 | 30.6702003 | 46.09407072 | 63.95520042 | 41.94729451 | 38.78069865 | 45.11146153 | 41.75213144 | 39.75640467 | 42.39537465 |
| **Ppcs** | 237.1653887 | 270.1613449 | 249.8284529 | 270.8593578 | 245.7449388 | 241.987418 | 241.7533436 | 245.5556858 | 234.7532062 | 221.9711001 | 230.7924505 | 228.159892 | 245.4002828 | 249.3810838 | 222.7553583 |
| **Zmynd12** | 0 | 0.792262009 | 0.783161294 | 0.86260942 | 1.900089733 | 1.402825611 | 1.80412943 | 0 | 0 | 2.621705907 | 0 | 0 | 2.556252945 | 0 | 0 |
| **Rimkla** | 0.915696482 | 3.169048034 | 3.915806472 | 0 | 0 | 0 | 0 | 1.676148026 | 3.00965649 | 0 | 5.675224193 | 0.867528106 | 2.556252945 | 2.710663955 | 0 |
| **AA415398** | 13.73544722 | 8.714882094 | 10.18109683 | 8.626094197 | 8.233722176 | 8.416953669 | 10.82477658 | 25.14222039 | 9.781383594 | 12.23462756 | 17.02567258 | 9.542809169 | 3.408337261 | 9.035546516 | 5.748525376 |
| **Foxj3** | 1184.911247 | 1085.398952 | 1090.160522 | 1037.719132 | 1005.147469 | 1021.958458 | 1092.40037 | 1004.012668 | 952.5562792 | 967.4094796 | 1082.076079 | 995.922266 | 1142.645067 | 1012.884764 | 968.6265258 |
| **Hivep3** | 1310.361665 | 1340.507318 | 1419.871427 | 1342.220257 | 1027.315182 | 1113.843535 | 1376.550755 | 1893.209195 | 1648.539343 | 1136.946462 | 1185.175986 | 1036.696087 | 1248.303522 | 1011.98121 | 1250.304269 |
| **Scmh1** | 130.0289004 | 138.6458515 | 156.6322589 | 191.4992912 | 152.0071786 | 172.5475502 | 146.1344838 | 212.0327253 | 210.6759543 | 130.2113934 | 173.0943379 | 164.8303402 | 159.3397669 | 131.0154245 | 140.120306 |
| **Ctps** | 2137.235588 | 2157.329449 | 2098.872269 | 1965.886867 | 1908.323455 | 1953.434664 | 2029.645608 | 2282.075538 | 2303.139629 | 2370.896042 | 2252.118134 | 1967.553745 | 1975.983527 | 1877.586566 | 1823.719675 |
| **Cited4** | 7.325571853 | 14.26071615 | 2.349483883 | 4.313047098 | 8.86708542 | 18.93814575 | 9.922711863 | 0.838074013 | 9.781383594 | 2.621705907 | 6.621094891 | 5.205168638 | 4.260421576 | 1.807109303 | 10.05991941 |
| **Nfyc** | 1010.928916 | 1003.003703 | 1068.232006 | 1021.329553 | 965.2455843 | 951.1157645 | 1046.395069 | 1047.592516 | 1072.190125 | 1046.060657 | 1152.070511 | 1041.033728 | 1025.909515 | 1083.362027 | 1001.680547 |
| **Rims3** | 32.96507334 | 41.98988645 | 54.03812932 | 29.32872027 | 37.36843141 | 33.66781467 | 37.88671802 | 55.31288486 | 52.66898858 | 56.80362798 | 35.94308655 | 31.23101183 | 44.30838439 | 40.65995932 | 45.26963733 |
| **Exo5** | 206.0317084 | 224.2101484 | 220.0683238 | 220.8280114 | 262.8457464 | 216.736557 | 213.7893374 | 255.612574 | 248.2966605 | 210.6103745 | 280.9235975 | 235.1001168 | 213.0210788 | 226.7922175 | 206.9469135 |
| **Zfp69** | 29.30228741 | 34.85952837 | 25.06116142 | 38.81742388 | 22.80107679 | 23.14662259 | 37.88671802 | 49.44636677 | 27.83932254 | 17.47803938 | 42.56418144 | 30.36348372 | 26.41461377 | 45.17773258 | 31.61688957 |
| **Smap2** | 2882.612524 | 2925.823597 | 2945.469629 | 3159.738304 | 2796.932087 | 2598.033032 | 3025.525053 | 2960.077414 | 2762.864658 | 2912.715262 | 3198.934703 | 2939.185224 | 2827.215758 | 2754.938133 | 2638.573147 |
| **Zmpste24** | 1716.930903 | 1769.913327 | 1903.081946 | 1877.900707 | 1717.047755 | 1606.235325 | 1789.696394 | 2049.090962 | 1956.276719 | 1836.068037 | 1849.177216 | 1766.287224 | 1810.67917 | 1667.058332 | 1689.347895 |
| **Rlf** | 893.719766 | 838.213205 | 841.1152303 | 744.4319292 | 848.0733841 | 808.0275522 | 959.7968565 | 637.7743239 | 670.4009832 | 672.9045161 | 719.8076018 | 902.2292305 | 843.563472 | 894.5191051 | 825.6319571 |
| **Ppt1** | 662.9642527 | 643.3167509 | 644.5417454 | 699.5762393 | 500.356963 | 568.8457854 | 592.6565176 | 673.8115065 | 747.1472237 | 790.8812819 | 937.3578625 | 534.3973135 | 602.4236108 | 559.3003293 | 594.9723764 |
| **Cap1** | 7800.818326 | 7964.609971 | 7976.497784 | 8131.818999 | 8265.390338 | 8404.328238 | 8680.56875 | 7779.002989 | 7990.637982 | 7492.835481 | 8045.576164 | 8428.035553 | 8835.262264 | 8675.02821 | 8564.584244 |
| **Mfsd2a** | 253.6479254 | 234.5095545 | 262.3590337 | 244.1184658 | 297.0473616 | 270.0439302 | 303.9958089 | 212.0327253 | 189.6083589 | 241.1969434 | 317.8125548 | 243.7753979 | 284.5961613 | 288.2339339 | 282.3963091 |
| **Mycl** | 13.73544722 | 5.54583406 | 2.349483883 | 8.626094197 | 10.13381191 | 10.52119209 | 7.216517718 | 1.676148026 | 4.514484735 | 6.991215751 | 1.891741398 | 8.675281063 | 8.520843152 | 2.710663955 | 2.874262688 |
| **Trit1** | 331.4821263 | 305.0208733 | 269.4074853 | 252.74456 | 329.348887 | 293.8919656 | 276.9338674 | 289.1355345 | 218.9525097 | 274.4052182 | 227.0089677 | 290.6219156 | 293.9690887 | 284.6197152 | 265.150733 |
| **Oxct2b** | 0 | 0.792262009 | 0 | 0.86260942 | 0 | 2.104238417 | 0 | 1.676148026 | 0 | 0 | 1.891741398 | 3.470112425 | 0 | 0 | 0.718565672 |
| **Ppie** | 1065.870705 | 1056.877519 | 1060.400393 | 960.9468935 | 1133.720207 | 1104.725169 | 1092.40037 | 1101.229253 | 1088.743235 | 1095.873069 | 1051.808217 | 1020.213053 | 1059.140804 | 1091.494019 | 1054.854406 |
| **Hpcal4** | 2.747089445 | 8.714882094 | 10.96425812 | 7.763484777 | 2.533452977 | 4.90988964 | 13.53097072 | 7.542666117 | 0.752414123 | 2.621705907 | 10.40457769 | 5.205168638 | 3.408337261 | 2.710663955 | 5.029959704 |
| **Pabpc4** | 7011.487959 | 6746.111002 | 6780.610488 | 6844.805745 | 6534.408591 | 6633.260904 | 6607.624036 | 8519.022343 | 7713.749585 | 8294.203587 | 8335.958468 | 6570.657877 | 6822.639111 | 6631.187588 | 6702.062022 |
| **Bmp8a** | 1.831392963 | 0.792262009 | 0.783161294 | 0.86260942 | 0.633363244 | 1.402825611 | 0 | 2.514222039 | 0 | 2.621705907 | 0.945870699 | 0 | 0.852084315 | 0 | 0 |
| **Macf1** | 3636.230728 | 3606.376663 | 3475.669825 | 3866.215419 | 3361.258737 | 2886.313696 | 3840.991556 | 4419.164271 | 4378.297779 | 3778.752114 | 4231.825506 | 4101.672886 | 3943.446211 | 3787.701099 | 3891.751679 |
| **D830031N03Rik** | 167.5724561 | 181.4279999 | 178.5607751 | 184.5984158 | 122.2391061 | 143.0882124 | 173.1964252 | 289.9736085 | 276.135983 | 194.0062371 | 209.0374244 | 194.3262958 | 185.7543807 | 124.6905419 | 150.1802254 |
| **Akirin1** | 1298.457611 | 1334.169222 | 1556.141492 | 1462.985576 | 1368.064608 | 1373.366274 | 1409.025084 | 1485.905225 | 1685.407635 | 1559.041113 | 1543.66098 | 1558.948007 | 1448.543336 | 1482.733183 | 1503.957951 |
| **Mycbp** | 233.5026028 | 255.1083667 | 228.683098 | 288.9741556 | 264.7458361 | 248.3001332 | 245.3616024 | 300.8685707 | 278.3932254 | 314.6047088 | 321.5960376 | 221.2196671 | 218.985669 | 246.6704199 | 254.3722479 |
| **Rragc** | 1309.445969 | 1337.33827 | 1314.927813 | 1280.112379 | 1275.593574 | 1364.94932 | 1422.556055 | 1276.386722 | 1443.130287 | 1418.342896 | 1389.484056 | 1452.24205 | 1493.703804 | 1412.25592 | 1442.879869 |
| **Pou3f1** | 0 | 0 | 3.132645178 | 0 | 1.900089733 | 2.805651223 | 0 | 0 | 0.752414123 | 0 | 0 | 1.735056213 | 4.260421576 | 0.903554652 | 2.874262688 |
| **Utp11l** | 1512.730588 | 1547.287703 | 1478.608524 | 1295.639348 | 1414.933488 | 1383.186053 | 1505.546009 | 1551.274998 | 1372.40336 | 1428.829719 | 1360.162065 | 1266.591035 | 1266.197292 | 1291.179597 | 1221.561642 |
| **Fhl3** | 673.9526104 | 709.8667596 | 613.2152936 | 616.7657351 | 729.6344574 | 700.0099801 | 667.5278889 | 770.190018 | 814.8644948 | 726.2125362 | 646.975558 | 669.731698 | 757.5029562 | 680.3766526 | 679.7631257 |
| **Sf3a3** | 2467.802018 | 2522.562235 | 2566.419562 | 2413.581156 | 2442.24867 | 2307.648131 | 2495.111001 | 2803.357574 | 2741.044649 | 2322.831433 | 2590.739844 | 2571.353307 | 2395.20901 | 2474.836191 | 2348.272616 |
| **Inpp5b** | 1547.527054 | 1368.236489 | 1404.991362 | 1314.616756 | 1310.428552 | 1272.36283 | 1399.102373 | 1236.159169 | 1241.483302 | 1355.421954 | 1333.677685 | 1342.933509 | 1399.122445 | 1401.413265 | 1498.927992 |
| **Mtf1** | 542.0923171 | 519.7238776 | 479.2947122 | 547.7569815 | 520.6245868 | 508.5242841 | 527.7078581 | 498.6540378 | 565.8154202 | 589.009927 | 601.5737644 | 558.6881004 | 609.2402853 | 523.1581433 | 530.3014659 |
| **1110065P20Rik** | 118.1248461 | 122.0083493 | 119.8236781 | 144.9183825 | 119.0722899 | 166.234835 | 119.9746071 | 150.8533223 | 147.473168 | 144.1938249 | 155.1227946 | 98.03067601 | 111.6230453 | 122.8834326 | 84.79074929 |
| **Yrdc** | 1120.812493 | 1151.156698 | 1160.645038 | 1001.489536 | 1064.683614 | 1185.387642 | 1175.390323 | 1314.100052 | 1316.724715 | 1320.465875 | 1212.606236 | 1119.978785 | 1165.651343 | 1074.326481 | 1208.62746 |
| **Maneal** | 11.90405426 | 6.338096068 | 10.18109683 | 9.488703616 | 7.600358931 | 17.53532014 | 26.15987673 | 5.028444078 | 21.06759543 | 21.84754922 | 12.29631908 | 19.08561834 | 25.56252945 | 20.78175699 | 10.77848508 |
| **Cdca8** | 2385.389334 | 2346.680069 | 2478.705497 | 2461.887284 | 2482.783918 | 2525.086101 | 2530.291525 | 2634.066623 | 2617.648732 | 2380.508963 | 2437.508791 | 2639.888027 | 2546.027934 | 2687.171534 | 2525.758337 |
| **9930104L06Rik** | 166.6567596 | 179.8434759 | 179.3439364 | 156.9949144 | 181.7752511 | 195.6941728 | 231.8306317 | 195.271245 | 176.0649047 | 207.1147666 | 207.145683 | 200.3989926 | 186.606465 | 215.0460071 | 201.9169538 |
| **Gnl2** | 2195.840163 | 1968.771091 | 2138.030334 | 2034.895621 | 2057.163817 | 1975.178461 | 2124.362403 | 2149.659843 | 1896.836003 | 2085.130098 | 2001.462399 | 1979.699139 | 1908.668866 | 1944.44961 | 2011.265316 |
| **Dnali1** | 0 | 0 | 0.783161294 | 1.725218839 | 2.533452977 | 0.701412806 | 0 | 1.676148026 | 0.752414123 | 8.739019689 | 0 | 0 | 1.70416863 | 0 | 0.718565672 |
| **Snip1** | 808.5599932 | 696.3983055 | 738.5211007 | 647.8196742 | 713.1670131 | 697.2043289 | 722.5538365 | 715.7152071 | 679.4299527 | 711.3562027 | 713.1865069 | 735.6638341 | 727.6800051 | 699.3513003 | 727.9070257 |
| **Meaf6** | 378.1826469 | 503.0863754 | 402.5449054 | 407.1516461 | 473.1223435 | 504.3158073 | 460.9550693 | 413.1704884 | 551.5195519 | 422.094651 | 473.8812201 | 493.6234925 | 391.1067007 | 469.8484188 | 424.6723121 |
| **Zc3h12a** | 438.6186147 | 363.6482619 | 339.1088405 | 313.9898288 | 411.6861088 | 389.2841072 | 393.3002156 | 300.8685707 | 309.2422044 | 293.6310616 | 306.4621064 | 325.3230399 | 380.0296046 | 378.589399 | 408.1453017 |
| **Mrps15** | 1191.321123 | 1165.417415 | 1091.726845 | 1132.606168 | 1069.117156 | 1107.53082 | 1009.410416 | 1217.721541 | 1321.239199 | 1289.005404 | 1176.663149 | 1129.521594 | 1201.438884 | 1125.829096 | 1113.776792 |
| **Oscp1** | 27.47089445 | 23.76786026 | 16.44638718 | 36.22959563 | 31.66816221 | 33.66781467 | 27.06194144 | 21.78992434 | 23.3248378 | 38.45168663 | 25.53850887 | 42.50887721 | 30.67503535 | 28.0101942 | 17.24557613 |
| **Lsm10** | 183.1392963 | 238.4708646 | 202.055614 | 207.8888701 | 217.2435928 | 225.8549234 | 181.3150077 | 201.1377631 | 218.2000955 | 197.501845 | 186.3365277 | 177.8432618 | 190.0148023 | 189.7464768 | 183.2342464 |
| **Stk40** | 1764.54712 | 1577.393659 | 1570.238395 | 1552.696955 | 1467.502637 | 1597.116959 | 1676.938305 | 1288.119758 | 1224.930192 | 1316.970267 | 1438.669333 | 1550.272726 | 1494.555889 | 1414.966584 | 1514.017871 |
| **Eva1b** | 273.793248 | 242.4321746 | 245.9126465 | 256.1949976 | 236.8778534 | 266.5368662 | 251.6760554 | 227.9561316 | 280.6504677 | 276.1530222 | 247.8181231 | 281.9466345 | 267.554475 | 252.9953024 | 281.6777434 |
| **Sh3d21** | 29.30228741 | 37.2363144 | 27.41064531 | 21.56523549 | 24.06780328 | 30.16075065 | 19.84542373 | 18.43762829 | 27.83932254 | 14.85633347 | 37.83482795 | 24.29078698 | 22.15419219 | 29.8173035 | 30.17975822 |
| **Thrap3** | 8624.94516 | 8644.370775 | 8593.628884 | 8528.619332 | 8114.649886 | 7893.699715 | 8938.559259 | 8819.890913 | 9129.040549 | 8468.983981 | 9002.797311 | 8767.239042 | 8724.491303 | 8323.54545 | 8262.786662 |
| **Map7d1** | 3280.940493 | 3459.015929 | 3188.24963 | 3360.726299 | 3418.894793 | 3566.684117 | 3431.454175 | 3241.670282 | 3376.082168 | 3119.830029 | 2890.580855 | 3631.472653 | 3571.937449 | 3512.116931 | 3597.85832 |
| **Trappc3** | 620.8422145 | 675.7994933 | 620.2637452 | 648.6822836 | 672.6317654 | 648.8068453 | 632.3473651 | 726.6101693 | 696.7354775 | 738.4471637 | 682.9186445 | 566.4958534 | 633.0986462 | 680.3766526 | 600.0023361 |
| **Adprhl2** | 706.9176838 | 680.5530653 | 706.4114876 | 689.2249263 | 682.7655773 | 722.4551899 | 653.0948535 | 785.2753502 | 723.8223859 | 725.3386342 | 767.1011367 | 719.1808001 | 728.5320895 | 691.2193085 | 718.565672 |
| **Tekt2** | 0.915696482 | 1.584524017 | 0 | 1.725218839 | 3.166816221 | 0 | 0 | 0 | 1.504828245 | 1.747803938 | 0 | 3.470112425 | 0 | 1.807109303 | 0.718565672 |
| **Ago3** | 126.3661145 | 163.9982358 | 132.3542588 | 121.6279282 | 107.6717515 | 112.9274617 | 155.1551309 | 170.1290246 | 179.8269753 | 130.2113934 | 214.7126486 | 111.9111257 | 135.4814061 | 121.979878 | 160.2401448 |
| **Ago1** | 1073.196276 | 1046.578113 | 1106.606909 | 1132.606168 | 1084.951237 | 982.6793408 | 1116.756117 | 1211.016949 | 1124.106699 | 1143.063775 | 1041.403639 | 1178.970696 | 1290.907737 | 1071.615817 | 1130.303802 |
| **Ago4** | 4.578482408 | 4.753572051 | 1.566322589 | 4.313047098 | 0.633363244 | 0 | 5.412388289 | 0 | 2.257242368 | 6.117313782 | 1.891741398 | 4.337640531 | 1.70416863 | 0.903554652 | 0.718565672 |
| **Clspn** | 2189.430287 | 2234.971126 | 2217.912786 | 2229.84535 | 2072.364535 | 1954.83749 | 2125.264468 | 2400.243973 | 2472.432807 | 2241.55855 | 2543.446309 | 2311.962403 | 2055.227368 | 2086.30769 | 1935.81592 |
| **5730409E04Rik** | 4.578482408 | 4.753572051 | 10.96425812 | 9.488703616 | 10.13381191 | 5.611302446 | 7.216517718 | 5.866518091 | 4.514484735 | 8.739019689 | 2.837612096 | 0.867528106 | 5.964590206 | 9.035546516 | 5.748525376 |
| **Psmb2** | 2811.188198 | 2885.418235 | 2894.564144 | 3050.186908 | 2713.328138 | 2720.780273 | 2734.15815 | 2989.410005 | 2987.836481 | 2939.806223 | 3027.732107 | 2782.162637 | 2796.540722 | 2876.918011 | 2869.232728 |
| **Ncdn** | 1556.684019 | 1498.167458 | 1594.516396 | 1621.705709 | 1519.438423 | 1519.260137 | 1478.484068 | 1779.23113 | 1663.587625 | 1673.52227 | 1707.296611 | 1548.53767 | 1638.558138 | 1496.286503 | 1565.036034 |
| **AU040320** | 659.3014667 | 621.1334147 | 639.8427776 | 558.9709039 | 549.1259328 | 660.0294502 | 657.6051771 | 460.9407072 | 571.8347332 | 544.4409266 | 625.2205319 | 690.5523726 | 602.4236108 | 670.4375515 | 647.4276704 |
| **Zmym4** | 641.9032336 | 617.9643666 | 676.6513584 | 630.5674858 | 655.5309578 | 573.7556751 | 647.6824652 | 581.6233651 | 683.9444374 | 638.8223393 | 619.5453077 | 571.701022 | 610.0923697 | 515.929706 | 625.1521346 |
| **Sfpq** | 10988.35778 | 10686.02997 | 10790.39632 | 10757.60207 | 11164.29391 | 11931.03183 | 12080.45066 | 11149.73667 | 10933.32962 | 10836.38441 | 11315.45117 | 11600.58584 | 11820.11362 | 11274.55494 | 11155.73206 |
| **Zmym1** | 484.4034387 | 455.5506549 | 475.3789058 | 484.7864938 | 409.1526558 | 436.980178 | 451.0323574 | 512.901296 | 461.9822713 | 566.2884758 | 506.0408238 | 445.0419185 | 483.983891 | 457.1986537 | 487.9060913 |
| **Zmym6** | 63.18305723 | 93.486917 | 83.79825851 | 58.65744054 | 70.93668336 | 42.78618115 | 66.75278889 | 84.64547532 | 93.2993512 | 83.89458901 | 110.6668718 | 82.4151701 | 91.17302172 | 68.67015352 | 69.70087018 |
| **Gm12942** | 41.20634167 | 23.76786026 | 32.10961307 | 25.87828259 | 26.60125626 | 29.45933784 | 42.39704159 | 28.49451644 | 38.37312025 | 31.46047088 | 25.53850887 | 39.90629289 | 34.93545692 | 28.91374885 | 29.46119255 |
| **Dlgap3** | 10.0726613 | 14.26071615 | 6.265290356 | 11.21392246 | 3.800179466 | 4.208476834 | 1.80412943 | 10.05688816 | 16.5531107 | 21.84754922 | 23.64676747 | 12.14539349 | 5.964590206 | 8.131991864 | 2.874262688 |
| **Smim12** | 265.5519797 | 273.3303929 | 323.4456146 | 298.4628592 | 260.9456566 | 321.247065 | 280.5421263 | 247.2318339 | 261.0877005 | 287.5137478 | 295.111658 | 310.575062 | 239.4356926 | 278.2948327 | 287.4262688 |
| **Zscan20** | 109.8835778 | 148.9452576 | 154.282775 | 127.6661941 | 155.8073581 | 129.7613691 | 137.1138366 | 114.8161398 | 147.473168 | 121.4723737 | 101.2081648 | 120.5864068 | 120.9959728 | 121.0763233 | 127.9046896 |
| **Tlr12** | 66.84584315 | 82.39524888 | 100.2446457 | 103.5131304 | 91.83767042 | 69.43986777 | 74.87137133 | 63.69362499 | 64.70761454 | 40.19949057 | 30.26786236 | 87.62033873 | 114.1792982 | 96.68034772 | 75.44939556 |
| **Phc2** | 2619.807634 | 2904.432523 | 2821.730144 | 2844.885866 | 2813.399531 | 2851.243055 | 2891.117411 | 3104.226144 | 2854.659181 | 2950.293047 | 3155.424651 | 2951.330618 | 2756.49276 | 2873.303792 | 2683.842785 |
| **A3galt2** | 4.578482408 | 2.376786026 | 2.349483883 | 6.900875357 | 0 | 2.104238417 | 0.902064715 | 0.838074013 | 1.504828245 | 2.621705907 | 0.945870699 | 0 | 0 | 0.903554652 | 6.467091048 |

| | | | | | | | | | | | | | | | |
|---|---|---|---|---|---|---|---|---|---|---|---|---|---|---|---|
| **Zfp362** | 375.4355574 | 442.8744628 | 432.3050346 | 401.1133801 | 466.1553478 | 438.3830036 | 461.857134 | 539.7196644 | 464.9919278 | 414.2295333 | 385.9152451 | 483.2131552 | 426.0421576 | 507.7977142 | 450.5406763 |
| **Trim62** | 53.11039593 | 39.61310043 | 65.00238744 | 49.16873692 | 72.20340985 | 31.56357626 | 48.7114946 | 59.50325493 | 33.85863552 | 53.3080201 | 45.40179354 | 71.13730471 | 30.67503535 | 61.44171631 | 53.8924254 |
| **Azin2** | 0.915696482 | 7.922620085 | 3.132645178 | 3.450437679 | 2.533452977 | 3.507064029 | 2.706194144 | 31.8468125 | 9.028969471 | 12.23462756 | 7.56696559 | 1.735056213 | 3.408337261 | 5.421327909 | 2.155697016 |
| **Ak2** | 4084.922004 | 4035.782671 | 4178.948667 | 3982.667691 | 3893.283863 | 3798.851756 | 3905.03815 | 4707.461731 | 4394.098476 | 4512.829767 | 4477.751888 | 3708.682654 | 3868.462791 | 3902.45254 | 3717.140221 |
| **Rnf19b** | 4525.372012 | 3824.248715 | 3694.171826 | 3701.45702 | 2568.921319 | 3161.968928 | 3002.973436 | 5042.691337 | 4275.217045 | 4927.933203 | 4630.982941 | 2835.081851 | 2850.222034 | 2825.415395 | 2703.244058 |
| **S100pbp** | 336.0606087 | 353.3488558 | 373.5679375 | 307.0889534 | 328.0821605 | 341.5880364 | 357.2176271 | 336.0676792 | 391.2553437 | 356.5520033 | 306.4621064 | 353.9514674 | 356.1712437 | 305.4014722 | 345.6300882 |
| **Yars** | 3627.98946 | 3503.382602 | 3452.958147 | 3204.593994 | 3090.179269 | 3119.182747 | 3177.97399 | 3138.587179 | 3048.782025 | 3117.208323 | 3059.891711 | 2855.902526 | 2934.578381 | 2698.01419 | 2927.436548 |
| **C77080** | 62.26736075 | 59.41965064 | 46.20651637 | 40.54264272 | 57.00269198 | 71.54410618 | 47.80942988 | 40.22755263 | 39.12553437 | 53.3080201 | 63.37333682 | 46.84651774 | 55.38548049 | 61.44171631 | 40.23967763 |
| **Sync** | 2.747089445 | 0 | 4.698967767 | 6.900875357 | 3.800179466 | 2.104238417 | 0 | 1.676148026 | 6.019312981 | 0.873901969 | 0 | 0 | 4.260421576 | 0 | 3.59282836 |
| **Rbbp4** | 6345.776617 | 6381.670479 | 6827.600165 | 6428.165395 | 6640.180253 | 6408.107393 | 6408.267734 | 6979.480381 | 7059.149298 | 6787.596592 | 7197.130147 | 6810.095634 | 6671.820188 | 6788.406097 | 6652.480991 |
| **Zbtb8os** | 476.1621704 | 453.1738689 | 488.6926478 | 514.1152141 | 514.9243176 | 505.0172201 | 506.058305 | 514.577444 | 531.2043705 | 470.1592593 | 527.7958499 | 515.3116951 | 537.6652029 | 468.0413095 | 480.0018689 |
| **Zbtb8a** | 110.7992743 | 106.1631091 | 144.8848395 | 135.4296789 | 124.1391959 | 120.6430026 | 146.1344838 | 123.1968799 | 109.1000478 | 117.1028638 | 136.2053806 | 136.2019127 | 131.2209845 | 128.3047605 | 133.653215 |
| **Zbtb8b** | 0.915696482 | 0.792262009 | 0.783161294 | 1.725218839 | 0 | 0 | 0.902064715 | 2.514222039 | 0 | 2.621705907 | 0 | 0 | 0 | 0.903554652 | 0.718565672 |
| **Bsdc1** | 423.0517745 | 420.6911265 | 421.3407764 | 386.44902 | 376.8511303 | 331.0668443 | 391.4960862 | 413.1704884 | 372.4449907 | 403.7427096 | 366.9978311 | 397.3278727 | 333.1649672 | 350.5792048 | 339.8815628 |
| **Marcksl1** | 2193.093073 | 2109.793729 | 1999.410785 | 1981.413837 | 1910.223545 | 2029.187247 | 2013.408443 | 2458.07108 | 2073.653322 | 2113.094961 | 1885.120303 | 1917.237115 | 1918.041793 | 1710.428955 | 1965.277113 |
| **Hdac1** | 4228.686352 | 4233.848173 | 4492.996346 | 4502.821171 | 4034.523866 | 4190.240101 | 4157.61627 | 4981.511934 | 4638.633066 | 4768.009142 | 4878.801064 | 4287.323901 | 4187.994409 | 4313.569907 | 4309.9569 |
| **Lck** | 8030.658143 | 8503.348137 | 8615.557401 | 8921.106618 | 8510.501913 | 8684.191948 | 8725.671986 | 8130.156001 | 8281.822247 | 8285.464567 | 8411.628124 | 8673.546007 | 8621.389101 | 8949.708824 | 8632.847983 |
| **Fam167b** | 2.747089445 | 0.792262009 | 0.783161294 | 0 | 0.633363244 | 0 | 0.902064715 | 0 | 0 | 0 | 1.891741398 | 0 | 0 | 3.614218606 | 0 |
| **Eif3i** | 4604.121909 | 4491.333326 | 4709.148864 | 4596.845597 | 4720.45626 | 4592.149639 | 4328.106502 | 5187.678141 | 4853.071091 | 4967.258791 | 4956.362462 | 4443.47896 | 4153.911036 | 4421.996465 | 4498.939672 |
| **Tmem234** | 1194.068212 | 1174.132297 | 1152.030264 | 1088.613088 | 1072.917336 | 1183.984816 | 1196.137812 | 1214.369245 | 1200.100526 | 1152.676697 | 1153.016382 | 1181.573281 | 1147.757573 | 1188.174367 | 1074.25568 |
| **Dcdc2b** | 210.6101908 | 217.8720523 | 227.8999367 | 214.7897455 | 214.7101398 | 241.2860052 | 220.1037904 | 220.4134654 | 218.2000955 | 227.2145119 | 254.439218 | 215.1469704 | 224.0981749 | 215.9495617 | 233.5338434 |
| **Iqcc** | 208.7787978 | 207.5726462 | 213.0198721 | 234.6297621 | 214.7101398 | 193.5899344 | 215.5934668 | 208.6804293 | 178.3221471 | 177.4020997 | 200.5245881 | 200.3989926 | 226.6544278 | 220.467335 | 227.785318 |
| **Ccdc28b** | 76.00280797 | 68.92679474 | 71.2676778 | 70.73397241 | 90.57094393 | 83.46812388 | 80.28375962 | 59.50325493 | 54.92623095 | 62.92094176 | 52.02288843 | 74.60741714 | 74.98341973 | 90.35546516 | 81.9164866 |
| **Txlna** | 3081.31866 | 3230.052209 | 3208.611824 | 3146.799163 | 3055.34429 | 2922.787161 | 3032.741571 | 3739.486246 | 3473.896004 | 3579.502465 | 3783.482795 | 3008.587473 | 3047.905595 | 3106.420892 | 3036.65853 |
| **Kpna6** | 1956.843381 | 1813.487737 | 1690.062074 | 1564.773487 | 1532.105688 | 1484.189497 | 1672.427981 | 1706.318691 | 1659.825554 | 1673.52227 | 1639.193921 | 1525.981939 | 1716.949895 | 1471.890527 | 1594.497226 |
| **Tmem39b** | 403.8221484 | 404.8458863 | 429.1723894 | 454.5951642 | 435.1205488 | 447.50137 | 451.0323574 | 413.1704884 | 444.6767464 | 412.4817293 | 364.160219 | 453.7171996 | 455.0130243 | 449.0666618 | 462.0377271 |
| **Khdrbs1** | 5960.268399 | 5966.525186 | 6096.127516 | 5659.580402 | 6106.888401 | 5981.648407 | 6218.834144 | 6383.609757 | 5928.270872 | 5928.550957 | 5923.988186 | 6293.048883 | 6273.044728 | 6111.643663 | 6214.874497 |
| **Ptp4a2** | 6094.875781 | 6410.984173 | 6418.006808 | 6027.914625 | 6158.824187 | 6290.270042 | 6669.866501 | 6107.045333 | 6225.47445 | 6168.000097 | 6164.239344 | 6686.906643 | 6604.505527 | 6339.339435 | 6393.797349 |
| **1700003M07Rik** | 0 | 0 | 0.783161294 | 1.725218839 | 1.266726489 | 2.104238417 | 0 | 0 | 9.028969471 | 0 | 1.891741398 | 0 | 0 | 0 | 0 |
| **Adgrb2** | 3.662785926 | 0.792262009 | 0.783161294 | 5.175656518 | 10.76717515 | 0.701412806 | 6.314453003 | 3.352296052 | 0 | 7.86511772 | 2.837612096 | 0.867528106 | 5.964590206 | 3.614218606 | 9.341353736 |
| **Pef1** | 691.3508436 | 579.9357902 | 696.2303908 | 677.1483944 | 669.4649492 | 650.2096709 | 601.6771648 | 646.9931381 | 671.1533973 | 708.7344968 | 679.1351617 | 706.1678785 | 599.0152736 | 633.3918108 | 630.1820943 |
| **Fabp3** | 0 | 3.961310043 | 2.349483883 | 7.763484777 | 7.600358931 | 2.805651223 | 5.412388289 | 2.514222039 | 5.266898858 | 0.873901969 | 0.945870699 | 3.470112425 | 5.964590206 | 7.228437213 | 5.029959704 |
| **Zcchc17** | 713.3275591 | 766.1173622 | 884.1891015 | 892.8007493 | 761.3026196 | 798.2077729 | 811.8582433 | 974.6800772 | 890.105907 | 945.5619304 | 881.5514913 | 843.2373193 | 788.1779915 | 871.9302388 | 837.8475735 |
| **Snrnp40** | 2030.0991 | 2204.86517 | 2181.104205 | 2191.890535 | 2155.968484 | 2089.508748 | 2040.470385 | 2334.8742 | 2428.792788 | 2296.614374 | 2249.280522 | 2201.786334 | 2108.90868 | 2128.774759 | 2090.30754 |
| **Nkain1** | 220.6828521 | 211.5339563 | 212.2367108 | 186.3236346 | 125.4059224 | 123.4486538 | 153.3510015 | 334.3915312 | 291.1842654 | 367.9127289 | 314.029072 | 133.5993284 | 150.8189238 | 144.5687443 | 131.497518 |
| **Pum1** | 2326.78476 | 2363.317571 | 2372.195561 | 2295.403666 | 2164.202206 | 2102.134179 | 2319.208382 | 2355.826051 | 2355.808618 | 2259.03659 | 2297.519927 | 2195.713637 | 2368.794396 | 2229.069325 | 2403.602173 |
| **Sdc3** | 944.998769 | 663.9155631 | 475.3789058 | 464.9464772 | 519.9912236 | 753.3173533 | 826.2912787 | 153.3675444 | 207.6662978 | 138.9504131 | 228.9007091 | 461.5249525 | 457.5692772 | 411.1173665 | 584.1938913 |
| **Laptm5** | 14314.1674 | 13894.69111 | 14774.33782 | 14088.13704 | 14272.20735 | 14128.55815 | 15622.8588 | 12468.86517 | 12573.5924 | 12449.60745 | 12756.01224 | 14072.17341 | 13825.06801 | 14540.90501 | 14208.9176 |
| **Matn1** | 0 | 1.584524017 | 0.783161294 | 0 | 0 | 2.104238417 | 0 | 0 | 1.504828245 | 0 | 5.675224193 | 0 | 0 | 0 | 0.718565672 |
| **Ptpru** | 0 | 3.961310043 | 4.698967767 | 0 | 1.266726489 | 3.507064029 | 5.412388289 | 6.704592104 | 2.257242368 | 0 | 3.783482795 | 0.867528106 | 0.852084315 | 1.807109303 | 7.904222392 |
| **Mecr** | 499.9702789 | 465.057799 | 494.9579381 | 577.0857017 | 521.8913133 | 521.1497146 | 502.4500461 | 644.478916 | 625.2561359 | 658.0481826 | 652.6507822 | 514.444167 | 564.0798166 | 578.274977 | 517.3672838 |
| **Srsf4** | 546.6707995 | 579.1435282 | 585.8046483 | 509.802167 | 545.3257533 | 533.7751451 | 537.63057 | 491.1113716 | 442.4195041 | 465.7897494 | 454.9638061 | 496.2260768 | 570.8964912 | 475.2697467 | 564.7926182 |
| **Gm12992** | 2.747089445 | 0.792262009 | 7.04845165 | 6.038265938 | 4.43354271 | 7.014128057 | 2.706194144 | 6.704592104 | 0 | 9.612921658 | 5.675224193 | 2.602584319 | 0.852084315 | 4.517773258 | 5.029959704 |
| **Tmem200b** | 7.325571853 | 7.130358077 | 21.14535495 | 3.450437679 | 10.13381191 | 2.805651223 | 9.020647148 | 5.866518091 | 9.781383594 | 7.86511772 | 17.02567258 | 3.470112425 | 4.260421576 | 10.84265582 | 2.155697016 |
| **Epb4.1** | 2324.03767 | 2201.696122 | 2246.106593 | 2182.401832 | 2008.394848 | 1921.871088 | 2239.826687 | 2148.821769 | 2274.547893 | 2138.438118 | 2335.354755 | 2097.682961 | 2207.750461 | 2170.338273 | 2038.570811 |
| **Ythdf2** | 1889.081841 | 1859.438934 | 1809.885752 | 1830.457188 | 1861.454575 | 1991.310955 | 1967.403143 | 2002.158817 | 1899.093245 | 1843.059252 | 1992.003692 | 1804.458461 | 1814.087507 | 1835.119497 | 1811.504059 |
| **Rps15a-ps4** | 8.241268334 | 6.338096068 | 7.831612945 | 12.07653188 | 15.20071786 | 5.611302446 | 9.020647148 | 6.704592104 | 3.762070613 | 2.621705907 | 5.675224193 | 5.205168638 | 4.260421576 | 0.903554652 | 5.748525376 |
| **Gmeb1** | 646.481716 | 757.4024801 | 726.7736813 | 651.2701118 | 663.76468 | 650.2096709 | 714.4352541 | 676.3257285 | 676.4202962 | 530.4584951 | 691.4314808 | 646.3084392 | 657.8090913 | 642.4273573 | 689.8230451 |
| **Taf12** | 543.0080136 | 598.9500784 | 567.0087772 | 642.6440176 | 587.7610907 | 588.485344 | 540.3367642 | 680.5160986 | 671.9058115 | 693.8781633 | 798.3148698 | 628.9578771 | 610.944454 | 616.2242724 | 557.6069614 |
| **Rab42** | 1.831392963 | 4.753572051 | 3.132645178 | 0 | 1.900089733 | 3.507064029 | 0.902064715 | 3.352296052 | 1.504828245 | 0.873901969 | 14.18806048 | 0.867528106 | 5.112505891 | 6.324882561 | 4.311394032 |
| **Snhg12** | 319.5780721 | 288.3833711 | 266.2748401 | 217.3775738 | 269.8127421 | 243.3902436 | 308.5061325 | 155.8817664 | 171.5504199 | 226.3406099 | 141.8806048 | 223.8222514 | 222.3940063 | 223.1779989 | 226.3481867 |
| **Snora16a** | 1.831392963 | 10.29940611 | 5.482129061 | 3.450437679 | 7.600358931 | 7.715540863 | 9.922711863 | 3.352296052 | 6.771727103 | 6.117313782 | 11.35044839 | 0.867528106 | 4.260421576 | 13.55331977 | 11.49705075 |
| **Snora44** | 0 | 0 | 1.566322589 | 0 | 0 | 0.701412806 | 0.902064715 | 0 | 0 | 0 | 0 | 0.867528106 | 0 | 0 | 0 |
| **Snora61** | 0.915696482 | 0.792262009 | 0 | 0 | 0 | 0 | 1.80412943 | 0 | 0 | 0.873901969 | 0.945870699 | 0 | 0 | 0.903554652 | 0 |
| **Snord99** | 0.915696482 | 0.792262009 | 0.783161294 | 6.038265938 | 3.166816221 | 0 | 0 | 2.514222039 | 0 | 4.369509845 | 0 | 6.072696744 | 1.70416863 | 1.807109303 | 1.437131344 |
| **Trnau1ap** | 592.4556236 | 549.0375719 | 552.9118739 | 548.6195909 | 532.0251252 | 519.0454762 | 531.316117 | 616.8224736 | 578.6064603 | 616.97479 | 616.7076956 | 506.6364141 | 573.4527441 | 511.4119328 | 518.8044152 |
| **Rcc1** | 3497.96056 | 3701.448104 | 3624.470471 | 3559.989075 | 3574.702151 | 3552.655861 | 3472.949152 | 4047.897483 | 3917.067922 | 3938.676174 | 4108.862315 | 3513.48883 | 3562.564522 | 3502.177829 | 3334.144718 |
| **Snhg3** | 231.6712098 | 227.3791964 | 268.624324 | 248.4315129 | 297.6807248 | 223.0492722 | 227.3203081 | 326.8488651 | 309.9946185 | 344.3173757 | 367.9437018 | 269.8012411 | 255.6252945 | 275.5841687 | 265.150733 |
| **Phactr4** | 314.9995897 | 322.4506375 | 327.3614211 | 364.8837845 | 338.8493357 | 306.5173961 | 383.3775038 | 308.4112368 | 314.5091032 | 291.8832576 | 289.4364338 | 349.6138268 | 355.3191594 | 326.1832292 | 362.1570987 |
| **Med18** | 97.06382705 | 104.5785851 | 119.0405168 | 99.20008326 | 70.93668336 | 94.69072877 | 101.0312481 | 134.9299161 | 108.3476337 | 103.9943343 | 85.12836289 | 87.62033873 | 114.1792982 | 87.6448012 | 81.19792093 |
| **Sesn2** | 770.100741 | 701.1518775 | 697.0135521 | 693.5379734 | 588.3944539 | 606.0206641 | 646.7804005 | 1091.172365 | 753.9189508 | 990.1309308 | 1012.081648 | 565.6283253 | 529.1443597 | 524.0616979 | 605.0322958 |
| **Atpif1** | 1435.812083 | 1391.212087 | 1376.797556 | 1354.296789 | 1247.725591 | 1227.47241 | 1390.98379 | 1288.957832 | 1245.245373 | 1388.630229 | 1350.703358 | 1274.398788 | 1273.866051 | 1385.149281 | 1183.477662 |
| **Dnajc8** | 2536.479254 | 2491.664017 | 2445.029561 | 2599.904791 | 2409.313781 | 2554.545438 | 2523.075007 | 2646.637733 | 2732.015679 | 2786.873379 | 2600.198551 | 2517.566564 | 2384.983998 | 2466.704199 | 2318.092858 |
| **Eya3** | 1303.036093 | 1349.2222 | 1329.024717 | 1297.364567 | 1243.925412 | 1208.534264 | 1355.803266 | 1493.447891 | 1343.811623 | 1309.105149 | 1339.352909 | 1344.668565 | 1187.805535 | 1165.585501 | 1282.639724 |
| **Xkr8** | 98.89522001 | 70.51131876 | 83.01509722 | 78.49745719 | 80.43713202 | 86.2737751 | 101.0312481 | 112.3019177 | 54.92623095 | 63.79484373 | 102.1540355 | 67.66719229 | 120.9959728 | 85.8376919 | 83.35361795 |
| **Smpdl3b** | 353.4588419 | 339.8804016 | 346.9404535 | 313.1272193 | 209.6432339 | 175.3532014 | 196.6501078 | 385.514046 | 348.3677388 | 398.4992978 | 358.4849948 | 178.7107899 | 195.1273082 | 179.8073757 | 178.9228523 |
| **Rpa2** | 1955.927685 | 1977.485973 | 1981.398075 | 1947.77207 | 2014.72848 | 1947.823361 | 1864.567765 | 2443.823822 | 2211.345106 | 2180.385412 | 2135.776038 | 2006.59251 | 1895.035517 | 2156.784953 | 1868.989313 |
| **Themis2** | 56.77318186 | 59.41965064 | 69.70135521 | 87.12355138 | 31.03479897 | 37.87629151 | 46.00530045 | 66.20784703 | 60.94554393 | 62.92094176 | 70.94030241 | 53.78674259 | 69.01882953 | 56.92394305 | 38.80254629 |
| **Ppp1r8** | 1931.20388 | 2005.215144 | 2082.425882 | 1958.123383 | 1950.758792 | 1867.862302 | 1860.057442 | 2259.447539 | 2279.062377 | 2273.019021 | 2154.693452 | 2023.943072 | 2063.748211 | 2104.378784 | 1945.87584 |
| **Stx12** | 494.4761 | 553.7911439 | 522.3685834 | 480.4734467 | 494.0233305 | 481.8705975 | 526.8057934 | 609.2798075 | 500.3553915 | 547.0626325 | 601.5737644 | 528.3246167 | 561.5235637 | 524.9652526 | 477.1276062 |
| **Fam76a** | 550.3335854 | 611.6262706 | 657.0723261 | 606.414422 | 592.1946334 | 650.9110837 | 632.3473651 | 492.7875197 | 575.5968038 | 625.7138097 | 496.5821169 | 622.8851803 | 528.2922754 | 592.7318514 | 637.367751 |
| **Fgr** | 54.02609241 | 68.13453273 | 58.73709709 | 50.89395576 | 84.87067473 | 72.24551899 | 62.24246532 | 20.11377631 | 49.65933209 | 30.58656891 | 36.88895725 | 67.66719229 | 71.57508247 | 77.70570004 | 48.14390002 |
| **Ahdc1** | 521.031298 | 564.8828121 | 655.5060035 | 609.8648597 | 616.8957999 | 577.2627391 | 717.1414483 | 766.8377219 | 607.950611 | 653.6786727 | 760.4800418 | 543.0725945 | 612.6486226 | 564.7216572 | 656.7690242 |
| **Wasf2** | 4083.090611 | 3970.024925 | 4117.078925 | 4130.173901 | 3824.247269 | 3871.798688 | 4387.642773 | 3443.64612 | 3686.076787 | 3200.22901 | 3709.704881 | 4039.210863 | 4185.438156 | 4076.838588 | 4153.309584 |
| **Gpr3** | 0 | 0 | 2.349483883 | 0.86260942 | 0 | 0 | 0 | 3.352296052 | 2.257242368 | 0.873901969 | 0 | 0 | 0 | 3.614218606 | 0.718565672 |
| **Map3k6** | 14.65114371 | 14.26071615 | 4.698967767 | 12.07653188 | 4.43354271 | 2.104238417 | 5.412388289 | 3.352296052 | 13.54345421 | 10.48682363 | 8.512836289 | 9.542809169 | 9.372927467 | 4.517773258 | 5.029959704 |
| **Sytl1** | 430.3773463 | 523.6851876 | 386.8816795 | 454.5951642 | 342.0161519 | 354.2134669 | 342.7845916 | 662.0784703 | 640.3044183 | 709.6083987 | 627.1122733 | 340.9385458 | 377.4733516 | 378.589399 | 356.4085733 |
| **Tmem222** | 593.3713201 | 653.616157 | 588.9372935 | 623.6666104 | 661.8645903 | 583.5754544 | 584.5379352 | 659.5642483 | 729.8416989 | 654.5525747 | 630.8957561 | 674.9368667 | 552.1506362 | 660.4984503 | 582.0381943 |
| **Wdtc1** | 676.6996999 | 718.5816417 | 793.3423913 | 699.5762393 | 651.0974151 | 658.6266246 | 685.5691832 | 679.6780246 | 650.838216 | 579.3970054 | 639.4085924 | 690.5523726 | 819.7051112 | 768.9250085 | 796.1707645 |
| **Slc9a1** | 818.6326545 | 846.9280871 | 870.8753595 | 964.3973312 | 799.3044143 | 754.0187661 | 885.8275499 | 861.5400854 | 904.4017753 | 842.441498 | 756.696559 | 857.117769 | 893.8364466 | 905.3617609 | 936.2910706 |
| **Trnp1** | 0 | 1.584524017 | 0 | 0 | 1.900089733 | 1.402825611 | 0 | 0 | 4.514484735 | 0 | 0 | 0.867528106 | 1.70416863 | 0.903554652 | 2.155697016 |
| **Kdf1** | 0 | 2.376786026 | 0.783161294 | 0 | 2.533452977 | 0 | 0 | 0 | 0 | 0 | 1.891741398 | 0 | 0 | 0 | 2.874262688 |
| **Nudc** | 5138.888655 | 4889.048854 | 4830.538864 | 4890.1328 | 4553.248363 | 4610.386372 | 4662.772511 | 5403.063162 | 5273.670585 | 5281.8635 | 5319.57681 | 4598.766491 | 4316.659141 | 4586.443411 | 4304.926941 |
| **Gpatch3** | 150.174223 | 165.5827598 | 171.5123235 | 214.7897455 | 173.5415289 | 126.254305 | 156.9592604 | 204.4900592 | 228.7338933 | 195.754041 | 174.9860793 | 137.0694408 | 151.6710081 | 176.1931571 | 156.6473165 |
| **Gpn2** | 622.6736075 | 696.3983055 | 649.2407131 | 687.4997075 | 637.1634237 | 634.7785892 | 580.0276116 | 624.3651397 | 556.7864507 | 552.3060443 | 665.8929719 | 570.8334939 | 592.198599 | 562.0109933 | 612.2179525 |
| **Sfn** | 806.7286003 | 964.9751264 | 895.9365209 | 866.9224667 | 855.673743 | 869.7518791 | 896.6523265 | 583.2995131 | 744.1375672 | 625.7138097 | 727.3745674 | 807.6686669 | 924.511482 | 852.0520364 | 788.2665421 |
| **Zdhhc18** | 4178.323045 | 4149.076139 | 4019.966925 | 3803.244931 | 3736.843141 | 3784.8235 | 3879.780338 | 3038.018297 | 3245.162111 | 3089.24346 | 3422.160188 | 3588.963776 | 3703.158434 | 3837.396605 | 3684.0862 |
| **Pigv** | 208.7787978 | 284.4220611 | 220.0683238 | 226.0036679 | 272.346195 | 242.6888308 | 274.2276733 | 164.2625066 | 233.248378 | 195.754041 | 206.1998123 | 226.4248357 | 290.5607515 | 226.7922175 | 234.2524091 |
| **Arid1a** | 6555.471112 | 6399.892505 | 6463.430163 | 6668.833423 | 6030.884812 | 6250.990925 | 7197.574359 | 6034.970968 | 6205.911683 | 5905.829506 | 6007.224808 | 6806.625522 | 6951.303843 | 6570.649426 | 6793.319863 |
| **Rps6ka1** | 3077.655875 | 3219.752803 | 3214.877114 | 3096.767817 | 3407.494254 | 3323.293873 | 3540.604006 | 2542.716556 | 2902.813685 | 2931.941106 | 2903.823045 | 3287.063995 | 3277.116276 | 3377.487288 | 3360.013082 |
| **Hmgn2** | 4870.589585 | 5663.881099 | 5675.569901 | 5585.395992 | 6496.406797 | 6039.164257 | 6189.066008 | 6125.482961 | 5995.988143 | 6033.419193 | 5868.181815 | 6702.522149 | 6292.642667 | 6848.944259 | 6153.077849 |
| **Dhdds** | 928.5162323 | 911.1013098 | 935.8777469 | 903.1520624 | 847.4400208 | 851.5151461 | 945.3638211 | 871.5969736 | 950.2990368 | 830.2068705 | 961.9505006 | 919.5797927 | 920.2510604 | 983.9710156 | 891.0214332 |
| **Aim1l** | 93.40104112 | 91.11013098 | 94.76251663 | 92.2992079 | 88.03749095 | 96.09355438 | 80.28375962 | 108.1115477 | 74.48899814 | 94.38141264 | 130.5301564 | 99.76573222 | 74.98341973 | 106.6194489 | 83.35361795 |
| **Cd52** | 3632.567942 | 3745.814776 | 3653.447439 | 3895.544139 | 4690.054824 | 4280.02094 | 4668.184899 | 3906.262975 | 3685.324372 | 3806.716977 | 3720.109458 | 4497.265703 | 4158.171458 | 4727.397937 | 4340.136659 |
| **Ubxn11** | 152.9213124 | 156.0756157 | 137.8363878 | 148.3688202 | 135.5397343 | 154.3108173 | 155.1551309 | 139.9583602 | 140.7014409 | 106.6160402 | 122.9631908 | 105.838429 | 114.1792982 | 182.5180396 | 137.2460433 |
| **Sh3bgrl3** | 3492.466381 | 3694.317746 | 3911.107505 | 3896.406749 | 4547.548094 | 4258.978556 | 4297.436301 | 3717.696322 | 3290.306958 | 3399.478659 | 3361.624463 | 4367.136487 | 4206.740264 | 4405.732481 | 4285.525668 |
| **Cep85** | 745.376936 | 734.4268819 | 671.9523907 | 726.3171313 | 620.0626161 | 586.3811056 | 666.6258242 | 733.3147614 | 786.2727581 | 790.0073799 | 823.8533786 | 721.7833844 | 629.6903089 | 693.9299724 | 722.1585003 |
| **Cnksr1** | 0.915696482 | 0.792262009 | 2.349483883 | 2.587828259 | 0 | 2.104238417 | 0 | 2.514222039 | 3.00965649 | 0.873901969 | 0 | 0 | 0 | 0.903554652 | 0 |
| **Zfp593** | 47.61621704 | 61.00417465 | 49.33916155 | 45.71829924 | 64.60305092 | 54.00878604 | 61.34040061 | 57.8271069 | 26.33449429 | 37.57778466 | 62.42746612 | 50.31663016 | 45.1604687 | 62.34527096 | 37.36541494 |
| **Grrp1** | 0 | 1.584524017 | 1.566322589 | 0 | 0 | 1.402825611 | 1.80412943 | 0 | 0 | 0 | 0 | 2.602584319 | 0 | 0 | 0 |
| **Pdik1l** | 470.6679915 | 414.3530304 | 414.2923248 | 364.0211751 | 344.5496049 | 378.0615023 | 367.1403389 | 346.1245674 | 378.4643037 | 381.0212584 | 344.2969344 | 327.9256242 | 394.5150379 | 351.4827595 | 324.7916837 |
| **Slc30a2** | 4.578482408 | 7.130358077 | 5.482129061 | 4.313047098 | 2.533452977 | 2.805651223 | 1.80412943 | 5.866518091 | 2.257242368 | 21.84754922 | 3.783482795 | 4.337640531 | 1.70416863 | 5.421327909 | 2.874262688 |
| **Extl1** | 31.13368037 | 36.44405239 | 28.1938066 | 50.03134634 | 32.9348887 | 27.35509942 | 37.88671802 | 36.03718256 | 14.29586833 | 21.84754922 | 24.59263817 | 25.15831508 | 25.56252945 | 30.72085815 | 22.27553583 |
| **Pafah2** | 43.03773463 | 61.00417465 | 40.72438731 | 44.85568982 | 32.30152546 | 37.1748787 | 41.49497688 | 73.75051315 | 60.94554393 | 56.80362798 | 60.53572472 | 29.49595561 | 36.63962555 | 37.04574071 | 27.30549553 |
| **Stmn1** | 4668.220663 | 5076.022688 | 5276.940802 | 5379.232341 | 5701.535925 | 5377.030569 | 5630.68795 | 4827.306315 | 5110.396721 | 4871.129575 | 4656.52145 | 5848.874493 | 5443.96669 | 5861.359025 | 5410.79951 |
| **Paqr7** | 231.6712098 | 233.7172925 | 209.1040656 | 226.0036679 | 250.8118447 | 199.9026496 | 230.928567 | 133.2537681 | 139.1966127 | 152.0589426 | 188.2282691 | 234.2325887 | 185.7543807 | 217.756671 | 204.7912165 |
| **Aunip** | 352.5431454 | 314.5280174 | 328.1445824 | 355.3950809 | 321.7485281 | 321.9484778 | 290.4648382 | 287.4593865 | 418.3422522 | 326.8393364 | 327.2712618 | 250.7156227 | 241.9919455 | 281.0054966 | 274.4920867 |
| **Mtfr1l** | 490.8133141 | 566.4673361 | 582.6720031 | 577.9483112 | 544.0590268 | 554.8175293 | 584.5379352 | 643.640842 | 593.6547427 | 520.8455735 | 650.7590408 | 560.4231567 | 558.9673107 | 613.5136084 | 517.3672838 |
| **Sepn1** | 68.67723612 | 53.87381658 | 46.98967767 | 43.9930804 | 53.20251252 | 45.59183237 | 53.22181817 | 35.19910855 | 43.64001911 | 52.43411813 | 60.53572472 | 36.43618046 | 48.56880596 | 38.85285002 | 51.73672838 |
| **Man1c1** | 4.578482408 | 0.792262009 | 5.482129061 | 1.725218839 | 2.533452977 | 2.805651223 | 6.314453003 | 0 | 4.514484735 | 0.873901969 | 5.675224193 | 5.205168638 | 5.964590206 | 3.614218606 | 2.155697016 |
| **Ldlrap1** | 160.2468843 | 287.5911091 | 252.1779368 | 265.6837013 | 264.1124729 | 242.6888308 | 281.444191 | 170.9670987 | 160.2642081 | 157.3023544 | 167.4191137 | 315.7802307 | 275.2232338 | 295.4623711 | 321.917421 |
| **Tmem57** | 814.9698686 | 747.103074 | 759.6664557 | 708.2023335 | 592.1946334 | 675.4605319 | 700.9042834 | 627.7174358 | 604.1885404 | 672.9045161 | 669.6764547 | 632.4279895 | 701.2653914 | 618.0313817 | 665.3918122 |
| **Rhd** | 1.831392963 | 2.376786026 | 4.698967767 | 0 | 0 | 0 | 6.314453003 | 0.838074013 | 1.504828245 | 1.747803938 | 0.945870699 | 0 | 2.556252945 | 0 | 2.155697016 |
| **Tmem50a** | 1960.506167 | 1845.178218 | 1936.757881 | 1892.565067 | 2009.661574 | 2096.522876 | 2194.723451 | 1556.303442 | 1624.462091 | 1615.844741 | 1656.219594 | 2139.32431 | 2010.918984 | 2175.759601 | 2088.870408 |
| **Rsrp1** | 792.993153 | 970.5209604 | 983.6505859 | 960.0842841 | 913.3097982 | 883.7801352 | 837.1160553 | 935.2905986 | 752.4141226 | 957.7965579 | 870.2010429 | 954.2809169 | 952.6302644 | 946.0217202 | 989.4649303 |
| **Syf2** | 1123.559583 | 1056.877519 | 1137.933361 | 1089.475697 | 1055.816528 | 1105.426582 | 1144.720123 | 1075.248959 | 987.9197429 | 1001.491656 | 1014.91926 | 1044.50384 | 972.2282036 | 1067.098044 | 981.5607079 |
| **Runx3** | 3828.526989 | 3644.405239 | 3608.024084 | 3578.103873 | 2886.869667 | 2985.914314 | 3465.732634 | 3112.606884 | 3063.077893 | 2917.084772 | 3228.256695 | 3002.514776 | 3180.830748 | 2951.913047 | 3153.784734 |
| **Clic4** | 7102.141911 | 5690.025745 | 5837.684289 | 5008.31029 | 4562.115449 | 5466.109995 | 5924.761047 | 4473.639082 | 4689.797226 | 4317.075726 | 4440.862931 | 4872.905373 | 5044.339146 | 4539.45857 | 5378.464055 |
| **Srrm1** | 6499.613626 | 6282.637727 | 6222.999646 | 6143.504287 | 6164.524457 | 5946.577767 | 6633.783912 | 6517.7016 | 6407.558668 | 6223.929823 | 6492.456476 | 6086.577194 | 6169.090442 | 6141.460967 | 6373.67751 |
| **Ncmap** | 3.662785926 | 4.753572051 | 0 | 1.725218839 | 0.633363244 | 0.701412806 | 1.80412943 | 0 | 0 | 1.747803938 | 1.891741398 | 1.735056213 | 3.408337261 | 0 | 0.718565672 |
| **Rcan3** | 692.2665401 | 706.6977116 | 733.8221329 | 696.1258017 | 627.0296118 | 672.6548807 | 702.7084128 | 627.7174358 | 612.4650958 | 594.2533389 | 699.9443171 | 599.4619214 | 729.3841738 | 648.7522398 | 684.7930854 |
| **Nipal3** | 362.6158067 | 434.1595807 | 489.4758091 | 374.3724881 | 398.3854806 | 379.4643279 | 436.599322 | 311.7635329 | 317.5187597 | 257.8010808 | 366.0519604 | 406.8706818 | 357.8754124 | 390.3356095 | 362.8756643 |
| **Grhl3** | 2.747089445 | 0 | 3.132645178 | 3.450437679 | 3.800179466 | 0.701412806 | 0 | 0 | 6.019312981 | 3.495607876 | 0.945870699 | 0.867528106 | 3.408337261 | 0.903554652 | 0 |
| **Srsf10** | 2910.999115 | 3082.691475 | 3111.499823 | 2958.750309 | 2989.474513 | 3064.472548 | 3123.850107 | 3244.184505 | 3023.199945 | 3259.654344 | 3247.174109 | 3156.934779 | 3233.659976 | 3026.004528 | 3048.874146 |
| **Pnrc2** | 944.0830725 | 934.86917 | 964.0715535 | 905.7398906 | 922.8102469 | 916.0451243 | 1011.214545 | 846.4547532 | 828.407949 | 922.8404792 | 916.5487071 | 910.9045116 | 971.3761193 | 998.42789 | 930.5425452 |
| **Cnr2** | 96.14813056 | 99.82501307 | 89.28038757 | 119.9027093 | 106.405025 | 103.8090952 | 114.5622188 | 75.42666117 | 112.8621184 | 78.6511772 | 69.99443171 | 104.9709009 | 133.7772375 | 102.1016756 | 81.19792093 |
| **Fuca1** | 868.995961 | 866.7346373 | 844.2478755 | 914.3659848 | 863.2741019 | 846.6052565 | 932.7349151 | 838.0740131 | 838.9417467 | 967.4094796 | 826.6909907 | 843.2373193 | 866.5697485 | 839.4022713 | 789.7036735 |
| **Hmgcl** | 461.5110267 | 464.265537 | 472.2462606 | 429.579491 | 454.7548094 | 482.5720103 | 478.0942988 | 414.0085624 | 416.0850098 | 443.9422002 | 444.5592284 | 469.3327055 | 432.0067478 | 423.7671316 | 436.1693629 |
| **Gale** | 706.0019873 | 680.5530653 | 715.0262619 | 544.3065438 | 589.0278172 | 622.8545715 | 602.5792295 | 500.3301858 | 551.5195519 | 710.4823007 | 599.682023 | 581.2438312 | 626.2819716 | 580.0820863 | 520.2415465 |
| **Lypla2** | 2296.566776 | 2423.529484 | 2457.560142 | 2385.977655 | 2222.471624 | 2233.999786 | 2165.85738 | 2226.762653 | 2294.11066 | 2330.696551 | 2381.70242 | 2166.217681 | 2254.615098 | 2163.109836 | 2221.086492 |
| **Pithd1** | 1048.472471 | 1048.162637 | 1084.678393 | 866.0598573 | 1002.614016 | 1073.863006 | 962.5030507 | 1387.012492 | 1184.299829 | 1258.418835 | 1120.856778 | 1178.103168 | 1118.786706 | 1021.920311 | 1086.471296 |
| **Tceb3** | 2088.703674 | 1934.703825 | 1968.084333 | 1968.474696 | 1728.448294 | 1732.48963 | 1884.413189 | 2308.893906 | 2137.608522 | 2191.746138 | 2106.454046 | 1814.868798 | 1730.583244 | 1723.078721 | 1744.677452 |
| **Rpl11** | 17157.40498 | 16570.15991 | 17105.02583 | 16477.56513 | 18261.12906 | 19103.67918 | 17805.85541 | 16406.97495 | 15881.20489 | 16378.6707 | 15960.62217 | 17529.27292 | 16778.39225 | 17978.93046 | 17033.59925 |
| **Id3** | 160.2468843 | 151.3220436 | 191.0913559 | 169.0714463 | 184.3087041 | 188.6800447 | 182.2170724 | 228.7942056 | 249.0490746 | 248.1881592 | 249.7098645 | 207.3392174 | 202.796067 | 232.2135455 | 196.1684284 |
| **E2f2** | 1540.201482 | 1895.090724 | 1748.799171 | 1711.417089 | 1793.051345 | 1728.982566 | 1750.907611 | 1424.725822 | 1569.53586 | 1592.249387 | 1524.743566 | 1862.582844 | 1797.045821 | 1863.129692 | 1824.438241 |
| **Asap3** | 11.90405426 | 15.84524017 | 5.482129061 | 6.900875357 | 10.13381191 | 9.118366474 | 2.706194144 | 16.76148026 | 8.276555348 | 13.10852953 | 23.64676747 | 5.205168638 | 10.22501178 | 20.78175699 | 9.341353736 |
| **Tcea3** | 1.831392963 | 6.338096068 | 3.915806472 | 0.86260942 | 0 | 0 | 0 | 0.838074013 | 3.762070613 | 0 | 3.783482795 | 0 | 1.70416863 | 0 | 0.718565672 |
| **Zfp46** | 280.2031234 | 257.4851528 | 238.0810335 | 260.5080447 | 171.008076 | 234.2718771 | 242.6554083 | 239.6891677 | 224.9718227 | 219.3493942 | 221.3337435 | 204.7366331 | 241.9919455 | 243.9597559 | 207.6654792 |
| **Hnrnpr** | 2682.990691 | 2780.047388 | 2827.212273 | 2865.588492 | 2874.202403 | 2804.248397 | 2950.653682 | 3060.646296 | 2947.958532 | 3084.87395 | 2914.227623 | 3033.745788 | 2949.063815 | 2968.17703 | 3024.442913 |
| **Luzp1** | 401.0750589 | 488.0333972 | 473.8125832 | 487.3743221 | 423.0866472 | 423.6533347 | 555.6718643 | 451.721893 | 434.1429487 | 486.7633967 | 366.9978311 | 420.7511315 | 411.5567242 | 395.7569374 | 451.259242 |
| **Kdm1a** | 2058.485691 | 1982.239545 | 1974.349623 | 2073.713045 | 2053.997001 | 2017.263229 | 2067.532326 | 2018.082223 | 2087.94919 | 1852.672174 | 1877.553337 | 2112.430939 | 1964.054346 | 1906.500315 | 1914.977516 |
| **Lactbl1** | 0.915696482 | 6.338096068 | 0 | 6.038265938 | 5.700269198 | 2.104238417 | 4.510323574 | 0 | 2.257242368 | 2.621705907 | 3.783482795 | 0.867528106 | 0.852084315 | 2.710663955 | 5.029959704 |
| **Ephb2** | 21.06101908 | 20.59881222 | 16.44638718 | 11.21392246 | 10.76717515 | 16.13249453 | 11.72684129 | 1.676148026 | 6.771727103 | 3.495607876 | 17.02567258 | 7.807752957 | 14.48543336 | 13.55331977 | 17.24557613 |
| **Zbtb40** | 331.4821263 | 342.2571877 | 386.8816795 | 328.6541889 | 333.1490665 | 333.8724955 | 375.2589213 | 413.1704884 | 351.3773952 | 399.3731998 | 395.3739521 | 383.447423 | 336.5733045 | 357.807642 | 407.426736 |
| **Cdc42** | 8829.145475 | 8687.945185 | 8977.377919 | 9013.405826 | 9010.858876 | 8939.506209 | 9353.509028 | 8979.96305 | 9062.075692 | 8849.131337 | 9646.935257 | 9282.550737 | 9271.529433 | 9234.328539 | 8995.723647 |
| **Hspg2** | 10.98835778 | 8.714882094 | 5.482129061 | 3.450437679 | 3.800179466 | 4.208476834 | 3.608258859 | 13.40918421 | 1.504828245 | 19.22584332 | 20.80915537 | 0.867528106 | 8.520843152 | 3.614218606 | 2.155697016 |
| **Usp48** | 1305.783183 | 1157.494794 | 1164.560845 | 1163.660107 | 1109.652404 | 1202.221549 | 1213.277041 | 1189.227025 | 1206.119838 | 1283.761992 | 1297.734599 | 1193.718674 | 1166.503427 | 1140.28597 | 1335.095019 |
| **Rap1gap** | 6.409875371 | 3.169048034 | 9.397935534 | 2.587828259 | 1.900089733 | 5.611302446 | 7.216517718 | 14.24725822 | 9.028969471 | 0.873901969 | 1.891741398 | 0 | 0 | 0.903554652 | 5.748525376 |
| **Alpl** | 14.65114371 | 26.93690829 | 18.79587107 | 18.11479781 | 15.20071786 | 11.9240177 | 16.23716487 | 23.46607237 | 24.07725192 | 11.3607256 | 11.35044839 | 7.807752957 | 7.668758836 | 12.64976512 | 5.029959704 |
| **Ece1** | 795.7402425 | 797.0155806 | 760.4496169 | 740.9814915 | 762.5693461 | 784.8809296 | 926.4204621 | 1118.828807 | 969.1093899 | 918.4709693 | 901.4147759 | 882.2760841 | 896.3926995 | 803.2600852 | 814.1349063 |
| **Eif4g3** | 2713.208675 | 2837.090252 | 2701.906466 | 2609.393494 | 2490.384276 | 2607.151399 | 2735.96228 | 2739.663949 | 2734.272921 | 2771.143143 | 2882.068019 | 2693.67477 | 2885.157491 | 2675.425323 | 2775.819191 |
| **Hp1bp3** | 3693.919607 | 3957.348732 | 4164.068603 | 4086.180821 | 4225.799566 | 3889.334008 | 4466.122403 | 3694.23025 | 3899.762397 | 3821.57331 | 3701.192044 | 4524.159074 | 4429.13427 | 4654.21001 | 4375.346377 |
| **Sh2d5** | 105.3050954 | 140.2303755 | 115.1247103 | 131.1166318 | 119.7056532 | 131.8656075 | 99.22711863 | 118.1684358 | 103.0807348 | 121.4723737 | 127.6925443 | 104.9709009 | 115.8834669 | 102.1016756 | 131.497518 |
| **Kif17** | 1.831392963 | 0 | 0 | 0.86260942 | 2.533452977 | 0 | 0 | 0 | 0 | 0.873901969 | 0 | 0 | 0 | 0.903554652 | 0.718565672 |
| **Ddost** | 3872.480421 | 3821.079667 | 4038.762796 | 4115.509541 | 3724.175876 | 4008.574185 | 3700.26946 | 4104.886516 | 4244.368065 | 4317.075726 | 3989.682607 | 3862.235129 | 3866.758622 | 3819.325512 | 3888.158851 |
| **Pink1** | 516.4528156 | 517.3470916 | 479.2947122 | 563.283951 | 453.4880829 | 519.0454762 | 572.8110939 | 348.6387894 | 307.7373761 | 346.9390817 | 363.2143483 | 475.4054022 | 487.3922283 | 477.076856 | 446.2292823 |

| | | | | | | | | | | | | | | | |
|---|---|---|---|---|---|---|---|---|---|---|---|---|---|---|---|
| **Mul1** | 409.3163273 | 411.1839824 | 405.6775505 | 433.0299287 | 384.4514893 | 441.8900676 | 439.3055161 | 429.0938947 | 435.647777 | 471.0331612 | 417.1289782 | 442.4393342 | 426.8942419 | 378.589399 | 365.0313614 |
| **Camk2n1** | 12.81975074 | 18.2220262 | 7.04845165 | 3.450437679 | 10.13381191 | 8.416953669 | 10.82477658 | 2.514222039 | 3.762070613 | 5.243411813 | 10.40457769 | 12.14539349 | 11.0770961 | 2.710663955 | 6.467091048 |
| **Otud3** | 130.9445969 | 103.7863231 | 133.1374201 | 114.7270528 | 107.0383883 | 129.0599563 | 119.0725424 | 118.1684358 | 96.30900769 | 103.9943343 | 104.9916476 | 115.3812381 | 111.6230453 | 93.06612911 | 117.8447702 |
| **Tmco4** | 1638.181006 | 1778.628209 | 1625.059686 | 1775.250186 | 1582.141384 | 1639.90314 | 1656.190816 | 1578.093367 | 1802.031824 | 1614.096937 | 1603.250834 | 1671.726661 | 1618.960199 | 1637.241029 | 1688.629329 |
| **Nbl1** | 25.63950148 | 30.89821833 | 14.0969033 | 19.84001665 | 29.76807248 | 21.74379698 | 35.18052388 | 19.2757023 | 32.35380727 | 19.22584332 | 18.91741398 | 26.02584319 | 27.26669809 | 32.52796746 | 27.30549553 |
| **Minos1** | 1670.230382 | 1571.847825 | 1747.232848 | 1574.262191 | 1755.04955 | 1717.058548 | 1651.680493 | 1692.071432 | 1676.378665 | 1779.264409 | 1767.832336 | 1789.710483 | 1667.529005 | 1677.900988 | 1585.874438 |
| **Gm16287** | 0.915696482 | 0 | 0 | 0.86260942 | 0 | 0 | 0.902064715 | 0.838074013 | 0 | 0.873901969 | 1.891741398 | 1.735056213 | 1.70416863 | 0 | 0 |
| **Capzb** | 7273.377153 | 7410.818828 | 7777.574816 | 7600.451597 | 7920.20737 | 7614.537419 | 7877.731154 | 7570.32256 | 7388.706684 | 7491.961579 | 7564.127978 | 7976.053409 | 7732.66516 | 7959.412926 | 7608.8919 |
| **Pqlc2** | 46.70052056 | 55.4583406 | 54.82129061 | 51.75656518 | 62.70296118 | 57.51585007 | 55.0259476 | 57.8271069 | 100.8234924 | 76.02947129 | 35.94308655 | 52.05168638 | 74.13133542 | 46.98484188 | 55.32955674 |
| **Akr7a5** | 255.4793184 | 260.6542008 | 223.9841302 | 221.6906209 | 248.911755 | 295.2947912 | 230.0265023 | 263.9933141 | 282.155296 | 273.5313163 | 224.1713556 | 259.3909038 | 249.6607043 | 242.1526466 | 254.3722479 |
| **Mrto4** | 2246.203469 | 2140.691947 | 2077.726914 | 2019.368651 | 1977.993412 | 2020.770293 | 2004.387796 | 2598.02944 | 2428.792788 | 2464.403552 | 2595.469197 | 1883.403519 | 1860.952144 | 1967.942031 | 1887.67202 |
| **Emc1** | 825.9582264 | 821.5757028 | 809.7887785 | 846.2198407 | 785.3704229 | 763.1371326 | 1030.157904 | 895.90112 | 902.8969471 | 913.2275575 | 937.3578625 | 911.7720397 | 935.588578 | 846.6307085 | 928.3868482 |
| **Ubr4** | 7218.435364 | 7061.431282 | 6501.021906 | 6642.092531 | 5460.857892 | 5908.000063 | 6751.95439 | 6506.806637 | 6637.797389 | 5814.069799 | 6229.504422 | 6358.981019 | 6405.117797 | 5877.623008 | 6665.415173 |
| **Iffo2** | 331.4821263 | 312.9434934 | 328.9277437 | 357.9829092 | 326.815434 | 443.2928932 | 402.3208628 | 241.3653158 | 225.7242368 | 242.0708454 | 292.2740459 | 358.2891079 | 415.8171458 | 397.5640467 | 368.6241897 |
| **Aldh4a1** | 247.23805 | 240.8476506 | 321.0961307 | 307.0889534 | 260.9456566 | 251.1057844 | 246.2636671 | 491.9494457 | 340.8435975 | 406.3644155 | 362.2684776 | 288.8868594 | 302.4899319 | 255.7059664 | 283.8334404 |
| **Arhgef10l** | 2.747089445 | 0 | 2.349483883 | 0 | 0 | 1.402825611 | 0 | 2.514222039 | 0 | 7.86511772 | 3.783482795 | 0 | 0 | 0 | 0 |
| **Rcc2** | 15701.44757 | 15362.75261 | 15103.26556 | 15206.94146 | 15011.34225 | 15446.51281 | 15125.82114 | 16133.76283 | 15531.33232 | 15410.38732 | 15482.95747 | 15128.82265 | 15219.93004 | 14684.5702 | 14821.85412 |
| **Padi4** | 2.747089445 | 5.54583406 | 0 | 0 | 3.800179466 | 1.402825611 | 3.608258859 | 0 | 0 | 0 | 0 | 4.337640531 | 0.852084315 | 1.807109303 | 0.718565672 |
| **Padi3** | 12.81975074 | 11.09166812 | 14.8800646 | 11.21392246 | 6.966995687 | 17.53532014 | 23.45368258 | 0 | 6.771727103 | 0.873901969 | 0 | 13.8804497 | 9.372927467 | 14.45687443 | 5.029959704 |
| **Gm13032** | 1.831392963 | 0 | 4.698967767 | 6.900875357 | 0.633363244 | 1.402825611 | 0.902064715 | 0 | 0.752414123 | 0 | 0 | 0 | 0.852084315 | 0 | 0.718565672 |
| **Padi1** | 6.409875371 | 8.714882094 | 3.915806472 | 6.900875357 | 1.266726489 | 0.701412806 | 5.412388289 | 0 | 4.514484735 | 8.739019689 | 3.783482795 | 0.867528106 | 7.668758836 | 0 | 5.748525376 |
| **Padi2** | 1602.468843 | 1361.898393 | 1433.185169 | 1378.449853 | 1330.062813 | 1258.334573 | 1464.953097 | 999.8222976 | 1169.251546 | 1172.776442 | 1270.304348 | 1285.676654 | 1275.57022 | 1247.808974 | 1371.741868 |
| **Gm13031** | 3.662785926 | 1.584524017 | 2.349483883 | 3.450437679 | 4.43354271 | 4.90988964 | 1.80412943 | 0.838074013 | 0.752414123 | 0 | 2.837612096 | 0 | 2.556252945 | 2.710663955 | 2.155697016 |
| **Sdhb** | 3119.777913 | 3308.486148 | 3246.203566 | 3206.319213 | 3144.015145 | 3285.417582 | 3049.880801 | 3281.059761 | 3359.529057 | 3237.806795 | 3209.339281 | 3059.771631 | 3053.870186 | 3156.116398 | 3166.718916 |
| **Atp13a2** | 802.1501179 | 697.1905675 | 700.1461973 | 734.9432255 | 616.2624367 | 698.6071545 | 726.1620954 | 722.4197993 | 689.2113363 | 762.9164189 | 727.3745674 | 674.0693386 | 737.0529326 | 696.6406364 | 730.7812884 |
| **Crocc** | 287.5286952 | 368.401834 | 291.3360015 | 276.8976237 | 285.6468232 | 295.2947912 | 329.2536209 | 244.7176118 | 260.3352864 | 267.4140025 | 249.7098645 | 361.7592203 | 312.7149437 | 317.1476827 | 324.0731181 |
| **Necap2** | 1132.716548 | 1186.016227 | 1139.499683 | 1089.475697 | 1081.151058 | 1108.933646 | 1096.910693 | 1051.782886 | 1073.694953 | 1039.943343 | 1126.532002 | 1092.217886 | 1105.153357 | 1053.544724 | 1116.651054 |
| **Spata21** | 0.915696482 | 0 | 0 | 4.313047098 | 0 | 0.701412806 | 1.80412943 | 1.676148026 | 0 | 0 | 0.945870699 | 0 | 12.78126473 | 0.903554652 | 0 |
| **Szrd1** | 3169.225523 | 3247.481973 | 3109.9335 | 2998.430343 | 2958.439714 | 3075.695153 | 3254.649491 | 3276.031317 | 3365.54837 | 3255.284834 | 3283.117195 | 3033.745788 | 3199.576603 | 3118.167103 | 3032.347136 |
| **Fbxo42** | 1391.858652 | 1304.063266 | 1358.784846 | 1251.646268 | 1185.655993 | 1207.832851 | 1318.818613 | 1262.139464 | 1275.341938 | 1219.967149 | 1209.768624 | 1180.705753 | 1254.268112 | 1267.687176 | 1237.370087 |
| **Rsg1** | 18.31392963 | 20.59881222 | 17.22954848 | 25.87828259 | 19.63426057 | 12.6254305 | 21.64955315 | 43.57984868 | 31.60139315 | 26.21705907 | 25.53850887 | 21.68820266 | 27.26669809 | 19.87820233 | 15.08987911 |
| **Epha2** | 31.13368037 | 22.97559825 | 28.1938066 | 19.84001665 | 14.56735462 | 9.118366474 | 9.922711863 | 36.03718256 | 10.53379772 | 12.23462756 | 14.18806048 | 9.542809169 | 4.260421576 | 8.131991864 | 4.311394032 |
| **Zbtb17** | 757.2809903 | 790.6774845 | 705.6283263 | 666.7970814 | 685.9323936 | 803.8190753 | 780.2859783 | 704.820245 | 726.8320424 | 609.9835743 | 747.237852 | 736.5313622 | 732.792511 | 737.3005957 | 794.7336332 |
| **Spen** | 2726.944122 | 2554.252715 | 2418.402077 | 2610.256104 | 2192.070188 | 2147.726011 | 2658.384714 | 2607.248255 | 2568.741814 | 2344.678983 | 2776.130501 | 2502.818587 | 2652.538473 | 2282.37905 | 2558.093792 |
| **B330016D10Rik** | 15.56684019 | 7.922620085 | 18.79587107 | 19.84001665 | 14.56735462 | 18.93814575 | 19.84542373 | 13.40918421 | 9.781383594 | 27.964863 | 7.56696559 | 3.470112425 | 20.45002356 | 20.78175699 | 12.21561642 |
| **Fblim1** | 0 | 0 | 0.783161294 | 0 | 0.633363244 | 0.701412806 | 0 | 1.676148026 | 5.266898858 | 0 | 1.891741398 | 2.602584319 | 1.70416863 | 0 | 0 |
| **Plekhm2** | 396.4965765 | 404.8458863 | 486.3431639 | 407.1516461 | 470.5888905 | 439.7858292 | 465.4653928 | 431.6081167 | 452.2008877 | 478.024377 | 474.8270908 | 441.5718061 | 485.6880596 | 446.3559979 | 417.4866554 |
| **Ddi2** | 510.9586367 | 539.5304278 | 610.8658097 | 583.1239677 | 526.324856 | 571.6514367 | 569.202835 | 665.4307664 | 577.8540461 | 589.883829 | 639.4085924 | 602.9320339 | 583.6777559 | 534.0007991 | 581.3196286 |
| **Dnajc16** | 339.7233947 | 328.7887335 | 375.9174214 | 293.2872027 | 304.0143573 | 288.982076 | 359.0217565 | 277.4024983 | 342.3484258 | 334.7044541 | 324.4336497 | 314.0451745 | 310.1586907 | 326.1832292 | 326.9473807 |
| **Casp9** | 162.0782772 | 166.3750218 | 173.0786461 | 154.4070861 | 141.8733667 | 155.0122301 | 187.6294607 | 150.0152483 | 149.7304104 | 143.3199229 | 155.1227946 | 151.8174186 | 158.4876826 | 168.9647198 | 188.2642061 |
| **Efhd2** | 2367.991101 | 2467.103894 | 2577.38382 | 2676.677029 | 2538.519883 | 2748.836786 | 2674.621879 | 2649.151955 | 2710.948084 | 2566.650083 | 2599.25268 | 2767.414659 | 2519.61332 | 2821.801177 | 2511.387024 |
| **Tmem51** | 5.494178889 | 2.376786026 | 5.482129061 | 0.86260942 | 0.633363244 | 4.208476834 | 2.706194144 | 8.380740131 | 8.276555348 | 9.612921658 | 2.837612096 | 4.337640531 | 6.816674521 | 1.807109303 | 5.029959704 |
| **Kazn** | 1.831392963 | 2.376786026 | 0 | 0 | 0.633363244 | 0 | 3.608258859 | 0 | 0 | 0.873901969 | 3.783482795 | 0 | 0 | 0 | 0 |
| **Prdm2** | 1431.233601 | 1459.34662 | 1409.69033 | 1381.90029 | 1266.093125 | 1376.873338 | 1495.623297 | 1503.504779 | 1442.377873 | 1358.917562 | 1535.148144 | 1429.686319 | 1517.562165 | 1160.164173 | 1317.849442 |
| **Pdpn** | 5.494178889 | 0 | 1.566322589 | 2.587828259 | 0 | 5.611302446 | 5.412388289 | 0.838074013 | 0 | 0 | 8.512836289 | 0 | 1.70416863 | 1.807109303 | 2.155697016 |
| **Pramef8** | 224.345638 | 252.7315807 | 212.2367108 | 277.7602331 | 257.7788404 | 272.8495814 | 261.5987673 | 273.2121283 | 276.8883971 | 267.4140025 | 242.1428989 | 279.3440502 | 270.1107279 | 283.7161606 | 308.9832389 |
| **Dhrs3** | 1.831392963 | 0 | 0.783161294 | 0 | 3.166816221 | 0 | 0 | 0.838074013 | 0 | 2.621705907 | 0.945870699 | 0.867528106 | 0 | 0 | 0 |
| **Vps13d** | 909.2866062 | 870.6959473 | 969.5536826 | 938.5190486 | 793.6041451 | 730.1707307 | 926.4204621 | 910.9864522 | 852.4852009 | 831.9546744 | 846.5542754 | 970.7639509 | 1041.247033 | 899.940433 | 929.1054139 |
| **Tnfrsf1b** | 2711.377282 | 2837.882514 | 2884.383048 | 2860.412836 | 2708.261233 | 2653.444644 | 2691.761109 | 3103.38807 | 2895.289544 | 2763.278026 | 3077.863254 | 2756.136794 | 2742.007326 | 2912.156642 | 2524.321206 |
| **Tnfrsf8** | 136.4387758 | 109.3321572 | 85.3645811 | 76.77223835 | 26.60125626 | 37.87629151 | 45.10323574 | 192.757023 | 188.1035306 | 254.305473 | 199.5787174 | 30.36348372 | 46.86463733 | 28.0101942 | 23.71266717 |
| **Smarca5-ps** | 10.0726613 | 4.753572051 | 5.482129061 | 17.25218839 | 8.233722176 | 14.02825611 | 5.412388289 | 12.5711102 | 11.28621184 | 6.991215751 | 9.458706988 | 8.675281063 | 5.964590206 | 6.324882561 | 2.874262688 |
| **Gm13212** | 86.07546927 | 83.18751089 | 65.78554874 | 83.67311371 | 70.93668336 | 56.81443726 | 100.1291833 | 91.35006742 | 99.31866418 | 62.04703979 | 81.34488009 | 94.56056358 | 69.01882953 | 65.95948956 | 71.13800152 |
| **Gm13242** | 0 | 0.792262009 | 0 | 0 | 0 | 0 | 0 | 0 | 0 | 0 | 2.837612096 | 0 | 0 | 0 | 1.437131344 |
| **Gm13238** | 1.831392963 | 1.584524017 | 0 | 1.725218839 | 3.166816221 | 0 | 0 | 0.838074013 | 5.266898858 | 1.747803938 | 0 | 1.735056213 | 1.70416863 | 0 | 2.155697016 |
| **Gm13251** | 67.76153964 | 36.44405239 | 38.37490343 | 48.3061275 | 45.60215359 | 35.77205309 | 52.31975346 | 40.22755263 | 52.66898858 | 44.56900041 | 38.78069865 | 39.90629289 | 29.82295103 | 41.56351397 | 43.11394032 |
| **Gm13034** | 2.747089445 | 1.584524017 | 2.349483883 | 1.725218839 | 1.900089733 | 2.805651223 | 5.412388289 | 3.352296052 | 2.257242368 | 2.621705907 | 1.891741398 | 2.602584319 | 3.408337261 | 0.903554652 | 2.155697016 |
| **Gm13051** | 13.73544722 | 11.88393013 | 9.397935534 | 18.97740723 | 14.56735462 | 18.23673295 | 27.96400616 | 3.352296052 | 20.31518131 | 14.85633347 | 10.40457769 | 19.08561834 | 13.63334904 | 13.55331977 | 22.9941015 |
| **Zfp600** | 0 | 4.753572051 | 0 | 0 | 0 | 0 | 0 | 0 | 0 | 0 | 0 | 0 | 0 | 0 | 0 |
| **Gm13139** | 7.325571853 | 3.961310043 | 1.566322589 | 3.450437679 | 1.900089733 | 1.402825611 | 5.412388289 | 5.866518091 | 0 | 0 | 2.837612096 | 0 | 5.112505891 | 2.710663955 | 3.59282836 |
| **Znf41-ps** | 8.241268334 | 22.18333624 | 13.31374201 | 18.97740723 | 17.1008076 | 17.53532014 | 6.314453003 | 19.2757023 | 4.514484735 | 17.47803938 | 37.83482795 | 14.74797781 | 10.22501178 | 15.36042908 | 19.40127314 |
| **Gm13152** | 0.915696482 | 0.792262009 | 3.915806472 | 1.725218839 | 0 | 0 | 1.80412943 | 0 | 0 | 0 | 2.837612096 | 0 | 5.112505891 | 0 | 0 |
| **Gm13154** | 0.915696482 | 0.792262009 | 5.482129061 | 5.175656518 | 6.333632443 | 3.507064029 | 6.314453003 | 2.514222039 | 0 | 8.739019689 | 11.35044839 | 13.01292159 | 7.668758836 | 5.421327909 | 7.18565672 |
| **Gm13157** | 242.6595676 | 312.1512314 | 322.6624533 | 258.7828259 | 264.1124729 | 280.5651223 | 254.3822496 | 362.0479736 | 337.0815269 | 275.2791202 | 277.1401147 | 294.092028 | 267.554475 | 264.7415129 | 260.1207733 |
| **Rps19-ps3** | 10.0726613 | 6.338096068 | 10.18109683 | 4.313047098 | 7.600358931 | 7.014128057 | 7.216517718 | 13.40918421 | 2.257242368 | 5.243411813 | 7.56696559 | 10.41033728 | 9.372927467 | 6.324882561 | 9.341353736 |
| **Zfp933** | 94.3167376 | 103.7863231 | 104.1604522 | 83.67311371 | 93.10439691 | 72.94693179 | 131.7014484 | 128.225324 | 99.31866418 | 101.3726284 | 94.58706988 | 89.35539495 | 100.5459492 | 95.77679307 | 79.76078959 |
| **Miip** | 354.3745384 | 415.1452925 | 418.9912926 | 475.2977902 | 392.6852115 | 366.8388974 | 401.4187981 | 352.8291595 | 424.3615651 | 420.346847 | 348.0804171 | 397.3278727 | 378.3254359 | 489.7266212 | 402.3967763 |
| **Fv1** | 35.71216278 | 44.36667248 | 40.72438731 | 43.9930804 | 57.00269198 | 51.90454762 | 53.22181817 | 37.71333059 | 34.61104964 | 43.69509845 | 42.56418144 | 56.38932691 | 38.34379418 | 43.37062328 | 42.39537465 |
| **Mfn2** | 1265.492538 | 1282.672192 | 1282.8182 | 1259.409753 | 1237.591779 | 1228.173823 | 1271.009183 | 1322.480793 | 1487.52272 | 1358.04366 | 1421.64366 | 1237.09508 | 1410.199542 | 1331.839556 | 1307.070957 |
| **Plod1** | 54.02609241 | 34.06726637 | 57.95393579 | 49.16873692 | 48.13560656 | 56.81443726 | 53.22181817 | 72.91243914 | 39.12553437 | 23.59535316 | 50.13114703 | 73.73988903 | 77.53967268 | 87.6448012 | 42.39537465 |
| **2510039O18Rik** | 2098.776336 | 2121.677659 | 2268.035109 | 2106.492203 | 2172.435928 | 2383.400714 | 2262.378305 | 2200.782358 | 2287.338933 | 2105.229843 | 2033.622002 | 2322.372741 | 2272.508869 | 2404.358928 | 2261.32617 |
| **Nppa** | 0 | 0.792262009 | 3.915806472 | 0 | 1.900089733 | 0 | 0 | 2.514222039 | 4.514484735 | 1.747803938 | 2.837612096 | 4.337640531 | 1.70416863 | 1.807109303 | 2.155697016 |
| **Clcn6** | 385.5082187 | 352.5565938 | 318.7466469 | 310.5393911 | 272.346195 | 359.1233565 | 385.1816332 | 315.9539029 | 314.5091032 | 322.4698265 | 322.5419083 | 329.6606804 | 339.1295574 | 316.2441281 | 362.1570987 |
| **Mthfr** | 629.9991793 | 655.992943 | 608.5163258 | 560.6961228 | 552.292749 | 496.6002664 | 543.0429583 | 636.9362499 | 598.9216416 | 537.4497109 | 571.3059021 | 532.6622573 | 553.0027205 | 504.1834956 | 565.5111838 |
| **Agtrap** | 241.7438711 | 321.6583755 | 317.9634856 | 329.5167983 | 267.2792891 | 249.7029588 | 343.6866563 | 267.3456102 | 284.4125383 | 273.5313163 | 241.1970282 | 314.9127026 | 279.4836554 | 281.0054966 | 262.9950359 |
| **Mad2l2** | 540.2609241 | 527.6464977 | 514.5369705 | 516.7030424 | 475.0224332 | 473.4536439 | 472.6819105 | 434.9604128 | 408.5608686 | 492.8807105 | 419.0207196 | 498.8286611 | 536.8131186 | 479.78752 | 485.7503942 |
| **Fbxo6** | 402.9064519 | 291.5524191 | 344.5909696 | 322.6159229 | 333.1490665 | 321.247065 | 300.38755 | 327.6869391 | 316.7663456 | 341.6956698 | 290.3823045 | 296.6946123 | 313.567028 | 278.2948327 | 331.9773404 |
| **Fbxo44** | 91.56964816 | 144.1916855 | 109.6425812 | 89.71137964 | 82.33722176 | 98.1977928 | 103.7374422 | 79.61703124 | 100.0710783 | 90.0119028 | 98.37055267 | 107.5734852 | 90.32093741 | 106.6194489 | 107.7848508 |
| **Fbxo2** | 4.578482408 | 3.961310043 | 8.614774239 | 9.488703616 | 8.86708542 | 9.118366474 | 2.706194144 | 13.40918421 | 19.56276719 | 8.739019689 | 9.458706988 | 10.41033728 | 5.112505891 | 2.710663955 | 8.622788064 |
| **Ubiad1** | 464.2581162 | 519.7238776 | 477.7283896 | 525.3291366 | 490.2231511 | 488.8847256 | 534.0223112 | 562.3476628 | 535.7188553 | 445.6900041 | 500.3655996 | 459.7898963 | 474.6109635 | 452.6808804 | 506.5887987 |
| **Mtor** | 1263.661145 | 1372.197799 | 1229.563232 | 1228.355814 | 1189.456173 | 1192.40177 | 1235.828659 | 1394.555158 | 1358.859905 | 1295.122718 | 1321.381366 | 1329.053059 | 1339.476543 | 1192.69214 | 1261.80132 |
| **Angptl7** | 1.831392963 | 1.584524017 | 0 | 1.725218839 | 5.066905954 | 0 | 0 | 0 | 0 | 2.621705907 | 1.891741398 | 0.867528106 | 0 | 0.903554652 | 0 |
| **Exosc10** | 2449.488088 | 2497.209851 | 2644.735691 | 2475.689034 | 2501.784815 | 2492.821112 | 2552.843143 | 2841.070904 | 2592.819066 | 2562.280573 | 2691.002138 | 2528.84443 | 2369.64648 | 2520.917478 | 2478.333003 |
| **Srm** | 5469.455084 | 5121.973885 | 5340.376867 | 5138.564313 | 5094.140574 | 5615.510923 | 5177.851463 | 6693.697142 | 5947.081225 | 6372.493157 | 6046.005507 | 4803.503124 | 4863.697271 | 4749.083249 | 4884.090872 |
| **Masp2** | 0 | 1.584524017 | 0 | 0 | 0 | 0.701412806 | 2.706194144 | 1.676148026 | 1.504828245 | 0 | 0 | 2.602584319 | 0 | 0 | 2.155697016 |
| **Tardbp** | 5252.435018 | 5434.917378 | 5558.095707 | 4940.164146 | 5413.989012 | 5257.088979 | 5352.852018 | 5617.61011 | 5731.890786 | 5629.676484 | 5738.597529 | 5518.346284 | 5484.014652 | 5205.378348 | 5492.715997 |
| **Casz1** | 119.0405426 | 90.31786897 | 105.7267748 | 74.18441009 | 105.1382985 | 102.4062696 | 101.9333128 | 101.4069556 | 73.73658401 | 73.40776539 | 143.7723462 | 59.85943933 | 91.17302172 | 89.45191051 | 94.8506687 |
| **Pex14** | 720.653131 | 734.4268819 | 762.7991008 | 735.805835 | 688.4658465 | 712.6354106 | 685.5691832 | 730.8005394 | 623.7513076 | 825.8373606 | 660.2177477 | 681.8770915 | 751.538366 | 648.7522398 | 726.4698944 |
| **Dffa** | 554.9120678 | 552.2066199 | 588.9372935 | 552.932638 | 573.8270993 | 539.3864476 | 601.6771648 | 710.6867631 | 702.0023764 | 656.3003786 | 669.6764547 | 558.6881004 | 604.1277794 | 627.0669282 | 575.5711032 |
| **Cort** | 0 | 3.169048034 | 1.566322589 | 0.86260942 | 2.533452977 | 0 | 1.80412943 | 1.676148026 | 0.752414123 | 3.495607876 | 2.837612096 | 3.470112425 | 3.408337261 | 0.903554652 | 3.59282836 |
| **Apitd1** | 256.3950148 | 278.876227 | 320.3129694 | 272.5845766 | 311.6147162 | 256.0156741 | 304.8978736 | 346.9626414 | 367.1780918 | 332.9566502 | 403.8867884 | 302.7673091 | 311.010775 | 316.2441281 | 296.7676225 |
| **Pgd** | 4540.938852 | 4617.302986 | 4220.456216 | 4336.337553 | 4199.831673 | 4086.431006 | 4375.915931 | 4674.776845 | 4361.744669 | 4462.143453 | 4834.345141 | 4377.546824 | 4277.463262 | 4372.300959 | 4072.111663 |
| **Kif1b** | 1001.771951 | 993.4965587 | 916.2987146 | 1051.520883 | 948.1447767 | 954.6228286 | 1026.549645 | 1339.242273 | 1145.174295 | 1068.782108 | 1110.4522 | 1011.537772 | 951.77818 | 913.4937527 | 870.1830288 |
| **Ube4b** | 1811.247641 | 1934.703825 | 1932.058914 | 1889.977238 | 1711.347486 | 1674.272367 | 1771.6551 | 2192.401618 | 2152.656805 | 2015.21794 | 1897.416622 | 1807.061045 | 1914.633456 | 1779.099109 | 1781.324301 |
| **Ube4bos3** | 1.831392963 | 6.338096068 | 1.566322589 | 6.038265938 | 5.700269198 | 0.701412806 | 0.902064715 | 0.838074013 | 4.514484735 | 4.369509845 | 0 | 4.337640531 | 3.408337261 | 0 | 2.155697016 |
| **Nmnat1** | 87.90686223 | 87.14882094 | 100.2446457 | 79.36006661 | 93.73776015 | 86.2737751 | 108.2477658 | 99.73080755 | 85.02279585 | 116.2289619 | 93.64119918 | 104.9709009 | 124.40431 | 81.31991864 | 108.5034165 |
| **Lzic** | 834.1994947 | 847.7203491 | 811.3551011 | 739.2562726 | 739.1349061 | 773.6583247 | 848.8428966 | 996.4700015 | 906.6590177 | 931.5794989 | 926.0074141 | 810.2712513 | 791.5863288 | 750.8539155 | 760.2424809 |
| **Ctnnbip1** | 94.3167376 | 81.60298688 | 105.7267748 | 82.81050429 | 93.10439691 | 95.39214158 | 101.0312481 | 105.5973256 | 88.03245234 | 83.89458901 | 89.85771638 | 81.54764199 | 107.3626237 | 105.7158942 | 108.5034165 |
| **Clstn1** | 449.6069724 | 418.3143405 | 428.3892281 | 393.3498954 | 354.6834168 | 373.1516126 | 425.7745454 | 432.4461907 | 437.9050193 | 402.8688077 | 391.5904693 | 405.1356256 | 432.8588321 | 418.3458037 | 455.570636 |
| **Pik3cd** | 12252.93462 | 13294.1565 | 13128.91594 | 12886.52212 | 11564.57948 | 11632.93138 | 12453.00339 | 11054.19623 | 11772.27136 | 11356.35609 | 12829.79016 | 12365.74563 | 12439.57892 | 12076.00792 | 12360.04812 |
| **Tmem201** | 960.5656092 | 968.9364364 | 1024.374973 | 914.3659848 | 896.8423539 | 885.1829608 | 948.9720799 | 914.3387482 | 957.823178 | 928.083891 | 917.4945778 | 938.665411 | 1087.259586 | 885.4835585 | 990.183496 |
| **Slc25a33** | 265.5519797 | 294.7214672 | 257.6600659 | 301.0506875 | 252.7119345 | 243.3902436 | 294.073097 | 403.9516743 | 293.4415078 | 311.9830029 | 317.8125548 | 254.1857351 | 278.6315711 | 247.5739745 | 276.6477837 |
| **Spsb1** | 21.97671556 | 31.69048034 | 25.06116142 | 15.52696955 | 12.66726489 | 18.93814575 | 27.96400616 | 51.96058881 | 24.82966605 | 37.57778466 | 17.02567258 | 19.95314644 | 28.97086672 | 28.0101942 | 20.11983882 |
| **H6pd** | 468.8365986 | 508.6322095 | 577.9730353 | 605.5518126 | 441.4541813 | 426.4589859 | 520.4913404 | 626.0412878 | 635.7899336 | 686.0130456 | 679.1351617 | 530.927201 | 489.0963969 | 517.7368154 | 549.7027391 |
| **Gpr157** | 15.56684019 | 14.26071615 | 24.27800013 | 12.93914129 | 22.80107679 | 23.84803539 | 20.74748844 | 18.43762829 | 20.31518131 | 9.612921658 | 15.13393118 | 23.42325887 | 16.18960199 | 25.29953024 | 22.27553583 |
| **Eno1** | 0.915696482 | 2.376786026 | 0 | 0 | 0 | 0 | 0 | 0.838074013 | 0 | 0 | 0 | 2.602584319 | 2.556252945 | 1.807109303 | 0 |
| **Rere** | 513.7057262 | 607.6649605 | 516.1032931 | 571.0474358 | 746.1019018 | 728.0664923 | 900.2605854 | 456.7503371 | 446.9339888 | 340.8217679 | 349.0262878 | 870.9982187 | 735.348764 | 818.6205143 | 737.2483794 |
| **Errfi1** | 358.0373243 | 327.2042095 | 327.3614211 | 320.0280947 | 383.818126 | 425.0561603 | 407.7332511 | 233.8226496 | 221.209752 | 249.9359631 | 358.4849948 | 307.9724777 | 414.9650615 | 369.5538525 | 351.3786136 |
| **Park7** | 3100.548287 | 2916.316453 | 3063.726984 | 2680.127467 | 2902.703749 | 2958.559214 | 2716.116856 | 2739.663949 | 2902.061271 | 2822.70336 | 2710.865423 | 2972.151292 | 2776.942783 | 2894.989104 | 2699.65123 |
| **Tnfrsf9** | 14379.18185 | 11611.392 | 11954.174 | 10607.50803 | 9895.033965 | 11354.4705 | 11974.90909 | 8784.691805 | 8047.821455 | 8604.438786 | 9784.086508 | 9983.513447 | 10948.43137 | 9947.233159 | 10728.90405 |
| **Per3** | 27.47089445 | 46.7434585 | 33.67593566 | 37.95481446 | 25.96789302 | 33.66781467 | 21.64955315 | 33.52296052 | 27.83932254 | 69.91215751 | 35.94308655 | 24.29078698 | 40.04796281 | 38.85285002 | 30.89832389 |
| **Vamp3** | 928.5162323 | 899.2173797 | 983.6505859 | 891.0755305 | 800.5711408 | 872.5575303 | 889.4358088 | 1010.71726 | 1069.180468 | 1018.969696 | 1032.890803 | 878.8059717 | 921.1031447 | 937.8897283 | 931.9796765 |
| **Camta1** | 115.3777567 | 114.8779912 | 112.7752264 | 80.22267603 | 122.2391061 | 143.7896252 | 98.32505391 | 93.02621545 | 78.25106875 | 101.3726284 | 81.34488009 | 105.838429 | 107.3626237 | 132.8225338 | 129.341821 |
| **Dnajc11** | 1861.610947 | 1805.565117 | 1892.900849 | 1785.601499 | 1683.479503 | 1666.556826 | 1743.691094 | 2091.832737 | 1928.437396 | 1922.584332 | 2004.300011 | 1636.158008 | 1772.335376 | 1736.63204 | 1792.821352 |
| **Thap3** | 193.2119576 | 204.4035982 | 185.6092268 | 197.5375571 | 196.975969 | 257.4184997 | 211.985208 | 140.7964342 | 194.1228436 | 132.8330993 | 174.0402086 | 219.4846109 | 178.9377062 | 194.2642501 | 253.6536822 |
| **Phf13** | 402.9064519 | 311.3589693 | 335.9761953 | 360.5707374 | 280.5799172 | 310.7258729 | 277.8359322 | 292.4878306 | 274.6311547 | 252.557669 | 301.7327529 | 277.608994 | 263.2940534 | 280.101942 | 278.0849151 |
[truncated: 1,009,628 more chars]
