# Supplementary material for: Aspirin prevents metastasis by limiting platelet TXA2 suppression of T cell immunity
Source: Nature. 2025 Mar 5;640(8060):1052–61. doi: 10.1038/s41586-025-08626-7 (PMC12018268; doi:10.1038/s41586-025-08626-7)
Supplement: Supplementary file 6 — ssGSEA of gene expression changes in TXA2 analogue-treated CD8 T cells (Arhgef1 KO versus WT) [file 41586_2025_8626_MOESM6_ESM.pdf]

Supplementary Table 4. Single-sample Gene Set Enrichment Analysis (ssGSEA) of changes in global gene expression between TXA2 analogue U46619-treated and vehicle-treated WT and Arhgef1 KO CD8+ T cells. Significantly differentially enriched C7 Immunologic signature gene sets (FDR < 0.2, |FC| > 1.5) are shown. Data are reprentative of three to four biological replicates per group.

Name	WT_d5_Veh_1	WT_d5_Veh_2	WT_d5_Veh_3	WT_d5_Veh_4	KO_d5_Veh_1	KO_d5_Veh_2	KO_d5_Veh_3	WT_d5_U46619_1	WT_d5_U46619_2	WT_d5_U46619_3	WT_d5_U46619_4	KO_d5_U46619_1	KO_d5_U46619_2	KO_d5_U46619_3	KO_d5_U46619_4
GSE10239_NAIVE_VS_DAY4.5_EFF_CD8_TCELL	-348.1712476	-335.5426461	-331.2258643	-343.4268815	-167.4147386	-148.5431295	-150.7330766	-542.2142078	-522.4223564	-574.5689253	-579.02554	-241.018499	-267.7153831	-216.1939514	-236.8470307
GSE11864_UNTREATED_VS_CSF1_PAM3CYS_IN_MAC	326.2420453	408.929295	366.3772136	357.4918193	425.4558723	371.0188743	418.1668556	206.7613406	244.6445902	191.4363467	248.5219985	503.3608445	467.8018709	513.7473609	497.6495454
GSE11924_TFH_VS_TH1_CD4_TCELL	-709.4872668	-583.0739503	-611.8055665	-554.3282935	-546.3494049	-582.4130208	-435.5812787	-866.3523542	-800.856941	-918.9551558	-873.0370914	-474.2982869	-444.6006333	-425.855667	-509.5881892
GSE11924_TH1_VS_TH17_CD4_TCELL_UP	720.7867573	682.750485	641.4248109	674.1881347	738.7092133	784.9689627	804.6408865	374.5522589	431.5936616	428.9522283	392.9654964	702.8321665	708.1408572	755.9071457	724.0886556
GSE1460_INTRATHYMIC_T_PROGENITOR_VS_CD4_THYMOCYTE	-1688.394502	-1693.502135	-1673.422016	-1623.288389	-1710.733163	-1762.825521	-1920.317076	-1069.049542	-1170.210455	-1042.461318	-1160.807722	-1714.388734	-1784.596095	-1764.378462	-1835.134799
GSE15930_NAIVE_VS_24H_IN_VITRO_STIM_IL12_CD8_TCELL_UP	259.1418037	285.1595521	310.4469479	289.0797057	308.2897143	334.1285721	376.7263785	73.946536	81.99895984	116.6975388	119.3636133	379.4248215	319.7081409	380.323859	365.7663219
GSE17580_TREG_VS_TEFF	-125.3541276	-277.2841624	-277.9605688	-391.7123186	-415.7387852	-403.9690491	-425.7808846	-155.4760872	-177.6573637	-149.5841964	-192.0342352	-451.9517421	-443.9567475	-486.7536361	-417.391931
GSE17974_0H_VS_2H_IN_VITRO_ACT_CD4_TCELL_UP	1.477790208	45.2969005	6.24212948	66.91262911	79.9116624	82.20356631	116.0870721	-275.7871586	-233.0248302	-248.3870493	-222.0856365	114.0972104	104.486659	90.52507662	121.7548583
GSE17974_0H_VS_4H_IN_VITRO_ACT_CD4_TCELL_UP	-511.7058389	-473.5309885	-513.9827062	-438.1186198	-444.2450738	-430.4543587	-415.0257486	-668.3450514	-643.2664289	-677.4859363	-638.1338831	-446.5441734	-426.8618561	-410.9858176	-403.1799865
GSE17974_CTRL_VS_ACT_IL4_AND_ANTI_IL12_12H_CD4_TCELL_UP	7.524933237	-84.34916392	-100.5741046	-115.4525722	-172.9032225	-112.8333257	-35.02024204	-396.5205176	-383.971971	-435.2407583	-405.7986863	-103.5263049	-64.65366473	-131.4986556	-139.5146612
GSE2706_2H_VS_8H_R848_STIM_DC_UP	-412.3491894	-438.3345301	-432.3247767	-513.2054554	-471.4017391	-438.8411243	-374.6052448	-702.9294504	-714.4691473	-668.2345974	-688.8703274	-449.7528393	-489.8601676	-455.3400647	-445.7553958
GSE30083_SP1_VS_SP4_THYMOCYTE_UP	500.2600947	423.796332	432.0836438	425.5409189	383.8933513	374.0131084	371.0890642	579.5417713	527.0781598	534.1149703	537.8979122	357.8124152	361.6789329	319.2021486	341.6566269
GSE30962_PRIMARY_VS_SECONDARY_ACUTE_LCMV_INF_CD8_TCELL_DN	-68.86678702	-101.3689531	-110.363805	-93.1394438	-74.01604998	-33.80828252	-19.94166806	-283.8005515	-288.5671437	-323.2052167	-334.6605178	-56.40189146	-47.35685849	-78.02987106	-90.43236619
GSE30962_ACUTE_VS_CHRONIC_LCMV_PRIMARY_INF_CD8_TCELL	-1412.360156	-1191.15719	-1197.305296	-1019.428421	-958.9873054	-1082.210766	-964.5649192	-1603.722616	-1440.76908	-1633.700108	-1562.170994	-934.0668035	-945.6521604	-786.0463669	-872.0188672
GSE30962_ACUTE_VS_CHRONIC_LCMV_PRIMARY_INF_CD8_TCELL_UP	1068.877282	1228.958954	1224.496034	1330.547758	1341.096333	1288.956084	1404.957892	863.485275	976.7577983	831.1900438	885.0777255	1391.901713	1396.318322	1518.847023	1421.911649
GSE360_L_MAJOR_VS_T_GONDII_DC	350.0574621	398.7437702	405.565663	359.3212807	555.8493663	566.7399128	578.3420384	39.9327948	139.3736555	100.0622081	71.11495349	465.58284	481.1984994	557.4068306	476.9742029
GSE360_L_MAJOR_VS_T_GONDII_DC_UP	-263.2822548	-235.3037107	-235.4250129	-219.1350935	-145.819851	-189.8526366	-129.5970603	-412.8879065	-355.4668631	-408.3514149	-416.6219106	-174.7620841	-166.9020703	-108.213162	-147.2312525
GSE36392_TYPE_2_MYELOID_VS_EOSINOPHIL_IL25_TREATED_LUNG_DN	504.7143608	530.2329269	499.3482477	479.9832373	517.783925	513.9664832	611.0472153	236.1333798	267.8843216	217.1384121	274.2850245	565.7249813	522.980093	542.7757199	562.7066978
GSE36392_TYPE_2_MYELOID_VS_NEUTROPHIL_IL25_TREATED_LUNG_UP	343.8458466	280.1482939	259.1838155	290.3060302	272.4658179	349.0060218	427.6753729	-116.4109646	-31.6467344	-84.4501739	-23.13034108	365.182437	352.1041528	303.2153067	387.4490952
GSE36476_CTRL_VS_TSST_ACT_72H_MEMORY_CD4_TCELL_YOUNG_UP	743.3800911	740.5495447	729.7124566	715.3939258	720.9824248	778.8305664	869.3134308	452.6825844	459.3351336	452.398679	477.1109742	764.3157455	819.3795073	772.3025068	805.0885977
GSE36476_CTRL_VS_TSST_ACT_16H_MEMORY_CD4_TCELL_OLD_UP	462.8275658	578.6388357	533.8221045	620.0818924	617.1180136	655.7648174	768.9901813	152.4649485	247.629954	171.3089343	188.5168033	769.1063797	760.2445031	726.168248	717.0502864
GSE36476_CTRL_VS_TSST_ACT_40H_MEMORY_CD4_TCELL_OLD_UP	53.56033813	104.5469337	103.7924806	53.53062452	171.7248997	182.0579493	273.0935472	-302.225903	-249.4664655	-241.5365431	-212.3408887	237.4746368	307.4897437	232.3787412	243.8774183
GSE3982_DC_VS_NEUTROPHIL	670.9992216	620.372253	703.9467921	645.9798479	653.0172118	644.3539897	519.8159455	1084.565911	978.1904377	1060.926746	1035.328986	581.2244243	554.659029	535.208591	505.0741144
GSE7460_CTRL_VS_TGFB_TREATED_ACT_FOXP3_MUT_TCONV	34.55640854	-114.7569035	-82.85094508	-141.5155828	41.40894871	88.36834478	164.6652475	-439.944111	-432.4575043	-360.1319562	-348.6979376	76.03667862	77.50602433	9.079890034	23.45259975
GSE7460_WT_VS_FOXP3_HET_ACT_WITH_TGFB_TCONV	790.7837928	912.6634868	875.3615251	902.0698161	1148.446362	1105.628725	1244.709771	364.326497	526.438851	395.639678	465.0142822	1103.254698	1225.017353	1215.854923	1242.914263
GSE7460_WT_VS_FOXP3_HET_ACT_WITH_TGFB_TCONV_UP	222.158615	304.5224482	281.8716609	319.0292909	463.0854294	449.834216	547.8126943	49.28517726	98.48141916	61.97679801	99.96957781	478.9178761	543.7687901	467.7008378	515.250496
GSE7852_TREG_VS_TCONV	989.7263383	584.9336821	614.2110997	513.5734323	325.9315518	448.3805211	347.744327	1053.203162	844.468154	1005.103514	916.8608069	188.7951563	263.4347831	117.4969345	247.6750769
GSE369_SOCS3_KO_VS_IFNG_KO_LIVER	21.0926131	137.487187	114.6755751	184.1994408	241.5761239	226.2020549	218.2436009	-33.63622254	-44.03466328	-19.22175849	4.004544205	262.9879712	316.6884234	271.2473573	286.1678802
GSE411_UNSTIM_VS_400MIN_IL6_STIM_SOCS3_KO_MACROPHAGE_UP	763.077302	804.5889264	776.1894452	788.0681006	876.8886312	934.7556131	948.3594874	517.6115005	559.3291355	538.5037497	531.6825854	892.6805341	884.0114197	902.2007341	908.1841176
GSE557_WT_VS_I_AB_KO_DC_UP	-173.3036497	-93.66857188	-85.7908133	-124.5060038	-116.7459645	-101.0413726	-75.17127966	-231.2705497	-284.0827359	-270.8482692	-267.1788789	-90.37233769	-38.42706927	-48.33595309	-33.3478827
GSE557_CIITA_KO_VS_I_AB_KO_DC_UP	1292.116767	1290.051962	1207.46303	1204.44552	1235.92055	1308.532328	1338.114342	797.1864835	797.4874932	772.3918865	800.0797365	1182.719745	1204.811644	1176.988131	1215.455895
GSE2770_UNTREATED_VS_ACT_CD4_TCELL_2H	-1140.744936	-1124.36086	-1180.036962	-1145.695095	-1131.179607	-1117.824292	-985.5081188	-1761.149575	-1663.161129	-1725.125777	-1666.274524	-1137.278826	-1086.071415	-1090.825495	-1050.202068
GSE2770_IL12_AND_TGFB_VS_IL4_TREATED_ACT_CD4_TCELL_48H_UP	831.2816857	905.4963115	823.9357234	865.3139132	844.1998653	850.8590209	989.4611623	522.6613878	558.7652352	511.0052414	591.9962971	857.2932963	905.9672215	876.7960575	924.4048564
GSE2770_UNTREATED_VS_ACT_CD4_TCELL_48H_DN	-113.8918107	-210.5722065	-214.9380256	-237.9603559	-283.0176991	-208.9289044	-169.4390928	-461.37723	-448.0517107	-479.9741182	-264.2986652	-231.0877103	-266.5130437	-225.5997098
GSE3039_CD4_TCELL_VS_B2_BCELL_UP	-543.7337538	-518.0046482	-538.4158939	-530.4470802	-440.2176349	-417.0212285	-384.7351112	-829.3919851	-750.8398954	-760.2694379	-771.2403435	-413.5457404	-370.3767137	-413.1817002	-396.140722
GSE5589_LPS_VS_LPS_AND_IL10_STIM_IL6_KO_MACROPHAGE_180MIN	-771.675458	-651.1780998	-676.5570172	-654.0167655	-569.1929176	-598.6094829	-511.045534	-922.8616335	-905.8334252	-946.0029615	-973.5046057	-625.9269424	-569.5891433	-530.5101855	-569.7739833
GSE6875_TCONV_VS_FOXP3_KO_TREG_UP	818.0545515	825.03117	854.105649	864.1225147	890.0025441	889.2902848	925.2508622	532.2578576	603.0283615	597.1615807	605.5293915	960.6372886	907.5169042	931.7263241	923.2026497
GSE7548_NAIVE_VS_DAY28_PCC_IMMUNIZATION_CD4_TCELL_DN	939.6604721	927.4455931	897.1546158	872.1852983	705.0479118	726.2550282	652.1606037	1039.363423	985.2788406	1009.261438	937.8877785	682.3329313	671.7001753	645.830783	603.0565708
GSE9239_CTRL_VS_TNF_INHIBITOR_TREATED_DC_UP	75.86257103	96.54910376	84.02655506	136.3855261	129.0678571	113.4523109	135.4376021	-139.2894188	-90.76671868	-106.1056433	-79.28907636	120.3737487	141.8763382	154.9688647	181.6113184
GSE10273_LOW_IL7_VS_HIGH_IL7_AND_IRF4_IN_IRF4_8_NULL_PRE_BCELL	484.4227464	435.5609858	516.6461472	455.9527539	455.5922026	395.8855324	362.2487811	730.155142	701.549421	679.1663195	678.8889136	437.3507758	453.7941495	389.9888638	419.0013563
GSE10273_HIGH_IL7_VS_HIGH_IL7_AND_IRF4_IN_IRF4_8_NULL_PRE_BCELL	-592.5305051	-687.3229586	-706.6240826	-768.3150511	-724.0083307	-702.8564259	-744.5761154	-459.1832246	-474.3818046	-502.3725021	-426.4387057	-748.8504693	-706.6249523	-773.1447977	-771.5129993
GSE10500_ARTHRITIC_SYNOVIAL_FLUID_VS_HEALTHY_MACROPHAGE_DN	-111.3021018	-31.81697789	-28.00190743	14.5860668	-19.32466692	-54.43095686	17.06916596	-157.8705762	-159.0193411	-165.5235309	-200.3728433	7.923939005	-2.633021032	28.75873651	44.97062318
GSE14415_INDUCED_TREG_VS_FOXP3_KO_INDUCED_TREG_IL2_CULTURE	679.9897582	324.9073605	374.2992473	386.9613664	189.4355808	343.5814149	184.0490856	700.6893774	602.8199015	630.7130395	564.4188391	161.9174372	124.632392	59.42797727	183.7306793
GSE14415_INDUCED_TREG_VS_FOXP3_KO_INDUCED_TREG_IL2_CULTURE_DN	-62.34612048	138.933374	122.0254219	78.99218336	266.1484751	203.6282204	319.4239078	-307.5492993	-255.1806102	-335.1229438	-291.5534458	280.4744955	285.659889	412.5751131	296.9812162
GSE13547_WT_VS_ZFX_KO_BCELL_ANTI_IGM_STIM_12H_DN	652.9294109	685.7447939	665.8955171	692.3623513	855.3815181	818.6265619	882.8277714	482.309294	528.3519331	438.596762	446.9996911	829.0798851	812.2994214	884.5943276	859.4529342
GSE15330_MEGAKARYOCYTE_ERYTHROID_VS_GRANULOCYTE_MONOCYTE_PROGENITOR	446.5592567	328.0805307	328.1121496	313.7184923	412.6447073	360.255029	405.0591202	-21.29822898	3.317760704	-4.270872232	32.54475489	381.1923021	408.0327842	393.9827868	366.8532376
GSE17812_WT_VS_THPOK_KO_MEMORY_CD8_TCELL	1078.554305	1052.834284	1039.771259	1057.340326	1178.046903	1257.819276	1137.255503	677.2758757	722.7158327	778.3685953	738.4466955	1060.487573	1126.551539	1093.823242	1119.017354
GSE17186_BLOOD_VS_CORD_BLOOD_CD21HIGH_TRANSITIONAL_BCELL	191.190862	51.37745049	100.9761002	46.80141348	-32.2981661	-32.59954615	-82.19732811	419.4922093	338.0227139	429.3977534	436.613383	-60.49489787	-2.286029566	-75.90051982	-97.64443279
GSE17186_CD21LOW_VS_CD21HIGH_TRANSITIONAL_BCELL_CORD_BLOOD	-689.8055516	-793.1381852	-727.6434423	-673.2739909	-910.9949619	-884.3975356	-886.0713111	-548.9687082	-589.7582462	-548.1930176	-533.2677311	-836.3504161	-832.8202154	-862.4111936	-855.4039959
GSE17186_NAIVE_VS_CD21HIGH_TRANSITIONAL_BCELL	380.251384	377.91873	302.9598988	322.9459733	368.0536485	403.3074145	404.3570736	33.2412908	-6.90918342	1.437255324	56.25519239	347.0897949	336.7085956	308.2707451	356.6657273
GSE19198_CTRL_VS_IL21_TREATED_TCELL_24H	1121.471884	763.4030108	703.0380953	618.7909311	282.5081829	358.1857796	430.8083938	983.1610877	817.1061756	922.0788087	913.9268496	179.4819911	231.7945374	108.4956933	299.0392178
GSE21670_UNTREATED_VS_IL6_TREATED_CD4_TCELL	111.3930166	9.171133749	48.07434339	25.71145875	-71.87071116	-57.7708656	-126.3832858	253.3942184	203.526402	188.5347027	150.7753243	-172.5011416	-132.6155013	-178.7018026	-113.3682631
GSE21774_CD56_BRIGHT_VS_DIM_CD62L_POSITIVE_NK_CELL	-97.84830798	-108.5503895	-156.0580059	-71.71005143	-219.3726283	-114.6617373	-43.55450824	-592.1743558	-511.4150211	-543.6484067	-504.6088079	-79.56730758	-65.47644196	-145.83897	-70.61488413
GSE21774_CD56_BRIGHT_VS_DIM_CD62L_POSITIVE_NK_CELL_DN	-113.6944226	-135.922644	-123.4810683	-124.8689146	-75.78557299	-110.5173392	-111.6078516	202.6304609	151.7783163	149.1296043	151.4481361	-108.6873401	-110.5659796	-142.6557374	-162.6680468
GSE25123_CTRL_VS_IL4_STIM_PPARG_KO_MACROPHAGE_UP	-410.1738212	-550.9792549	-453.7962001	-493.6484488	-513.008375	-482.8773507	-564.6542285	-313.4883003	-361.3746819	-311.1779594	-350.2509012	-542.9796719	-583.9137715	-579.9681036	-553.9939091
GSE28726_NAIVE_VS_ACTIVATED_VA24NEG_NKTCELL_DN	-110.6298246	-39.5672104	-112.3083727	-67.93167998	-43.09463382	-36.66777502	-45.96116848	-263.6492457	-237.4786974	-236.4362212	-228.045155	-60.30402428	-10.30308656	-10.42143055	-18.50718786
GSE28726_NAIVE_VS_ACTIVATED_NKTCELL_UP	888.1698693	976.5183787	924.419542	929.6877167	1011.633258	1015.048662	1007.537508	633.2911021	671.6576682	677.1679676	642.9790877	1005.408181	1020.837382	1054.777233	1038.687011
GSE26030_UNSTIM_VS_RESTIM_TH1_DAY5_POST_POLARIZATION_UP	448.9464475	447.9971219	518.0788281	538.9468627	519.4459377	529.6833187	650.1446885	295.8510074	308.346362	285.1768169	361.1569347	595.6681468	649.1827773	591.5982053	649.6754133
GSE32986_UNSTIM_VS_CURDLAN_HIGHDOSE_STIM_DC	930.4946211	870.500047	886.5709834	840.7677181	667.5602489	675.9238618	669.608329	1145.492115	1077.299787	1198.898572	1148.943556	709.846469	742.1116183	648.9802493	710.0694912
GSE37301_HEMATOPOIETIC_STEM_CELL_VS_LYMPHOID_PRIMED_MPP	157.410574	224.8274432	203.5372014	197.9232842	258.2617898	266.362742	308.7772097	87.22849487	118.9782057	107.1840554	94.9531808	257.8686849	282.2332289	276.6022795	296.341151
GSE37301_CD4_TCELL_VS_GRANULOCYTE_MONOCYTE_PROGENITOR_UP	-72.52431502	74.9100795	76.41060117	84.53088174	94.8412367	61.53721121	119.1505573	-143.3488096	-77.6117292	-138.2194239	-132.6599362	186.8293211	164.0363464	212.653161	184.9091757
GSE40274_CTRL_VS_LEF1_TRANSDUCED_ACTIVATED_CD4_TCELL_UP	816.3253519	853.2258792	844.4232221	815.5341241	862.1519872	860.3303902	904.9716883	573.1902179	632.4214393	592.2138318	605.6104315	905.3229177	933.6773664	958.5221013	938.6613531
GSE40274_CTRL_VS_XBP1_TRANSDUCED_ACTIVATED_CD4_TCELL_UP	877.5322205	936.3961038	917.8869446	923.2404833	938.2335976	900.1192193	1097.516285	494.2351343	593.9488331	530.8110003	606.5565684	995.4040749	1050.35901	973.8476526	1032.345564
GSE40274_CTRL_VS_FOXP3_TRANSDUCED_ACTIVATED_CD4_TCELL	-338.9340148	-337.5748836	-281.8217242	-308.946381	-319.3490796	-343.7988	-435.6553895	118.3668388	83.38589455	123.8531152	75.94741471	-300.7160294	-354.6388368	-307.0379622	-362.6031744
GSE40666_WT_VS_STAT1_KO_CD8_TCELL_WITH_IFNA_STIM_90MIN_UP	387.4297264	383.956041	383.6446852	385.7040999	470.6555521	482.8428115	492.2329134	227.5428762	199.5908958	225.0522853	198.4927993	451.2439208	485.9250742	472.3833404	471.7051969
GSE39110_UNTREATED_VS_IL2_TREATED_CD8_TCELL_DAY3_POST_IMMUNIZATION_UP	549.8847786	617.3111153	550.2944828	560.776777	626.979032	659.2837693	686.2685803	392.5616397	370.5649832	381.5392739	348.3471753	636.9535359	659.3310525	642.3865448	629.9564551
GSE39110_UNTREATED_VS_IL2_TREATED_CD8_TCELL_DAY6_POST_IMMUNIZATION_DN	290.9224442	272.3171445	318.1522182	288.3725266	395.7673817	446.933567	488.1967763	81.26068342	106.5112836	38.20998122	7.292635775	478.2553146	442.6672189	399.3219626	425.5001166
GSE36078_WT_VS_IL1R_KO_LUNG_DC_AFTER_AD5_INF_DN	285.2652099	200.4067931	253.1572804	211.3740948	207.7100129	274.2384921	324.5386275	-11.72884267	4.839565697	-40.12452327	-20.22242174	277.4924917	284.8000184	251.3109582	254.0800619
GSE11961_FOLLICULAR_BCELL_VS_PLASMA_CELL_DAY7	1055.037691	684.6179568	703.8668635	647.3584339	416.3626206	516.7653439	420.7946409	762.6191191	675.1795669	813.2762751	761.3470758	235.0513688	253.1689421	213.7319553	344.8829929
GSE41978_ID2_KO_VS_BIM_KO_KLRG1_LOW_EFFECTOR_CD8_TCELL	-458.7545455	-445.4663218	-594.0173163	-607.7663094	-605.2857413	-510.2851426	-466.3461452	-1096.081447	-1010.570941	-1066.844406	-1064.585636	-592.4994368	-668.4187326	-580.701889	-544.2593613
GSE43863_DAY6_EFF_VS_DAY150_MEM_LY6C_INT_CXCR5POS_CD4_TCELL_UP	630.3479169	613.3928283	652.9745582	646.9512294	757.7767146	718.2660734	776.762399	306.8329365	358.6258884	241.3711303	297.7614303	689.8711855	771.6200708	703.7221558	800.6687522
GSE45365_NK_CELL_VS_CD11B_DC_MCMV_INFECTION	674.0905638	587.8967815	580.9575769	465.7408143	564.7877034	649.5889907	456.9686157	980.7245476	928.8907186	952.711587	896.6063656	511.1665816	458.674677	486.3253576	497.7820141
THAKAR_PBMC_INACTIVATED_INFLUENZA_AGE_21_30YO_NONRESPONDER_7DY	-262.5577275	-344.6798113	-226.8206082	-281.1916339	-398.4245699	-414.7692387	-346.5950445	107.5163295	102.3160181	92.31629305	46.52172042	-316.6344616	-383.7314897	-400.4085328	-398.9447237
chr18q22	936.1725772	990.6131629	1086.720282	957.8705485	717.0050046	662.2454965	659.9205867	1292.082628	1349.771054	1288.263654	1289.021714	675.3487589	581.0736346	688.7866146	654.9360326
chr4q12	158.374534	168.5721312	296.9389081	164.0005429	113.116095	62.31474126	76.56939711	470.9214674	514.3127624	511.6489944	448.2582272	86.30742916	52.03150095	13.57076709	37.88951907
