# Supplementary material for: Aspirin prevents metastasis by limiting platelet TXA2 suppression of T cell immunity
Source: Nature. 2025 Mar 5;640(8060):1052–61. doi: 10.1038/s41586-025-08626-7 (PMC12018268; doi:10.1038/s41586-025-08626-7)
Supplement: Supplementary file 7 — Geneset enrichment analysis genes significantly downregulated in naive versus memory CD8 T cells [file 41586_2025_8626_MOESM7_ESM.pdf]

Supplementary Table 5. Geneset enrichment analysis of genes significantly downregulated in naive versus memory CD8 T cells (GOLDRATH_Naive_VS_MEMORY_CD8_TCELLS_DN) within the global transcriptional differences between TXA2 analogue U46619 treated WT vs Arhgef1-KO CD8 T cells. Data are reprentative of three to four biological replicates per group.

NAME	GENE SYMBOL	RANK IN GENE LIST	RANK METRIC SCORE	RUNNING ES	CORE ENRICHMENT
row_0	CD22	1	4.699699402	0.058718774	Yes
row_1	NOTCH4	6	4.231089115	0.1112315	Yes
row_2	PRSS12	28	2.756770134	0.1433624	Yes
row_3	AIF1	82	1.948204994	0.16174623	Yes
row_4	POU6F1	94	1.801080585	0.18304646	Yes
row_5	PGLYRP1	103	1.752458453	0.20407787	Yes
row_6	CCL5	108	1.724239588	0.2252092	Yes
row_7	CCR2	112	1.679938674	0.24589925	Yes
row_8	PRDM1	117	1.663737535	0.2662732	Yes
row_9	RNASE4	126	1.577231169	0.28511107	Yes
row_10	GZMK	137	1.527275801	0.303097	Yes
row_11	IL15	139	1.522351503	0.32204092	Yes
row_12	CCR5	154	1.455246687	0.33867204	Yes
row_13	FCGRT	175	1.342759132	0.35321528	Yes
row_14	GZMA	199	1.243755341	0.3661793	Yes
row_15	AHNAK	265	1.062997937	0.3721224	Yes
row_16	ST3GAL6	280	1.031944752	0.3834545	Yes
row_17	ANXA2	326	0.950783849	0.39025867	Yes
row_18	ID2	357	0.902531803	0.39815813	Yes
row_19	RASA4	379	0.870453	0.40667564	Yes
row_20	GPC1	384	0.86668539	0.41707188	Yes
row_21	GATA3	400	0.848206818	0.4259906	Yes
row_22	PTPN13	402	0.846809745	0.43647793	Yes
row_23	EOMES	477	0.753656387	0.43752903	Yes
row_24	KCTD12	517	0.718743265	0.44210815	Yes
row_25	SEMA4F	525	0.710061133	0.45020387	Yes
row_26	MYO1F	595	0.643305659	0.45044002	Yes
row_27	UNC119B	685	0.57077539	0.44750243	Yes
row_28	IL18RAP	697	0.563980162	0.4533163	Yes
row_29	EVI2A	771	0.517597377	0.45152566	Yes
row_30	PTPN22	775	0.515690863	0.45764133	Yes
row_31	CHPT1	789	0.504683435	0.46248633	Yes
row_32	LPIN1	828	0.485948384	0.4642646	Yes
row_33	IL18R1	896	0.458435029	0.46241304	Yes
row_34	TMEM37	899	0.457619458	0.4679151	Yes
row_35	ERRFI1	935	0.44349286	0.46950173	Yes
row_36	CTSW	977	0.423003078	0.47015214	Yes
row_37	IL10RB	985	0.419840425	0.4746148	Yes
row_38	MAP3K8	1027	0.405172259	0.47504202	Yes
row_39	RECK	1078	0.382434905	0.474165	Yes
row_40	CCDC130	1112	0.368938327	0.47504494	Yes
row_41	CXCR3	1148	0.361956447	0.47561088	Yes
row_42	S100A10	1255	0.329540759	0.46772757	Yes
row_43	NBEAL2	1292	0.320279747	0.46765852	Yes
row_44	SOS2	1304	0.317804247	0.47039068	Yes
row_45	STX7	1306	0.317329019	0.4742498	Yes
row_46	BAG3	1314	0.316040784	0.47741306	Yes
row_47	IRF8	1344	0.307646632	0.4779789	Yes
row_48	F2R	1373	0.300394356	0.4785672	Yes
row_49	SOAT2	1407	0.293315142	0.4785005	Yes
row_50	GGH	1413	0.291319817	0.48158085	Yes
row_51	CAPG	1510	0.269742966	0.47408184	Yes
row_52	FGL2	1521	0.268170029	0.476306	Yes
row_53	PPP3CC	1562	0.258829653	0.47501454	Yes
row_54	PLP2	1576	0.255495638	0.47674015	Yes

row_55	CD160	1587	0.251791954	0.47875926	Yes
row_56	MAPK12	1608	0.248275831	0.47960147	Yes
row_57	TRAF3IP2	1630	0.245300353	0.48029312	Yes
row_58	GZMM	1644	0.242609128	0.48185742	Yes
row_59	ST3GAL4	1647	0.242321	0.4846643	Yes
row_60	MYADM	1667	0.238343969	0.48549545	Yes
row_61	RAB3D	1668	0.238085866	0.48847586	Yes
row_62	IER3	1732	0.227353826	0.48418474	No
row_63	KRTCAP2	1777	0.219845638	0.48195213	No
row_64	ATF6	1797	0.216340303	0.48250785	No
row_65	CRTAM	1814	0.214225098	0.48337695	No
row_66	S100A13	1860	0.206816211	0.48086792	No
row_67	SEMA4A	1865	0.206318453	0.48299754	No
row_68	HIP1R	1888	0.203408986	0.4830515	No
row_69	MCOLN2	1890	0.203375503	0.48548412	No
row_70	MGST3	1912	0.200550824	0.4856156	No
row_71	GCAT	2081	0.176848769	0.46879694	No
row_72	DENND4C	2111	0.173507333	0.46768355	No
row_73	PRF1	2120	0.172100857	0.46893165	No
row_74	SAMHD1	2183	0.163782805	0.46395802	No
row_75	KLRK1	2192	0.163301542	0.46509597	No
row_76	S100A4	2209	0.160410553	0.4652914	No
row_77	PGAM1	2240	0.15642193	0.4638509	No
row_78	LITAF	2369	0.144499883	0.4511588	No
row_79	ITGB1	2379	0.14370212	0.4519381	No
row_80	CAPN2	2675	0.113827534	0.41994286	No
row_81	ASAH1	2938	0.086287387	0.39134136	No
row_82	TRAF1	2996	0.081651695	0.38590604	No
row_83	ANXA1	3021	0.079624221	0.38418385	No
row_84	PLEKHA5	3044	0.078025937	0.38266826	No
row_85	ENPP1	3167	0.068928182	0.36970988	No
row_86	CYBB	3175	0.068071626	0.369769	No
row_87	ABHD5	3183	0.06734211	0.369819	No
row_88	TMEM159	3244	0.062069044	0.36379865	No
row_89	MED10	3363	0.054624945	0.3511144	No
row_90	GABARAPL2	3408	0.050885551	0.34676668	No
row_91	SKAP2	3638	0.035080213	0.3212627	No
row_92	PLEKHB2	3697	0.030243205	0.31507054	No
row_93	CTNNA1	3755	0.025410518	0.30893117	No
row_94	ITM2C	3788	0.022701854	0.30559012	No
row_95	LIMD1	3942	0.010889488	0.28839326	No
row_96	AQP9	4043	0.003762366	0.27711147	No
row_97	KLHL7	4078	7.77E-04	0.2732694	No
row_98	DNAJC5	4104	-0.001267865	0.27045304	No
row_99	ANTXR2	4113	-0.002180615	0.26957405	No
row_100	OSTF1	4143	-0.00385756	0.26633695	No
row_101	CD44	4324	-0.015814068	0.24614294	No
row_102	HCFC1R1	4418	-0.022475218	0.23588842	No
row_103	LPGAT1	4465	-0.025211357	0.23099275	No
row_104	PHF13	4537	-0.028592419	0.22330718	No
row_105	STARD10	4618	-0.034368273	0.21467431	No
row_106	CHCHD7	4638	-0.035776749	0.21296968	No
row_107	CISH	5095	-0.064907096	0.16212252	No
row_108	KCNJ8	5328	-0.080776557	0.13685071	No
row_109	TXNDC5	5336	-0.081163332	0.13707371	No
row_110	SMYD1	5396	-0.085538425	0.13146047	No
row_111	PRKCA	5437	-0.088342316	0.12803482	No
row_112	ACY1	5582	-0.098927177	0.11295962	No
row_113	IL15RA	5669	-0.105128832	0.104532816	No
row_114	CYB5R4	5788	-0.112960391	0.09257881	No
row_115	S100A6	5794	-0.113372616	0.09343159	No

row_116	TNFRSF1B	5815	-0.114840843	0.09260343	No
row_117	CCL4	5984	-0.125921756	0.075147234	No
row_118	IFNG	6212	-0.144126192	0.051234894	No
row_119	RNF138	6246	-0.146672294	0.049332447	No
row_120	TK1	6293	-0.149673447	0.045994814	No
row_121	SSX2IP	6591	-0.176074862	0.0145522	No
row_122	POLR1B	6644	-0.180057615	0.010915193	No
row_123	TUSC2	6678	-0.183166593	0.00946959	No
row_124	VKORC1	6816	-0.197356805	-0.003580407	No
row_125	TMEM141	6865	-0.201973483	-0.006489911	No
row_126	DSTN	6929	-0.20874019	-0.01101404	No
row_127	CST7	7029	-0.218917295	-0.019489164	No
row_128	CPNE3	7049	-0.220992923	-0.018875204	No
row_129	CCND3	7089	-0.225265667	-0.02047353	No
row_130	ACOT7	7097	-0.226205572	-0.018434852	No
row_131	MAPRE2	7126	-0.229721233	-0.018731227	No
row_132	CASP4	7160	-0.234156102	-0.01953853	No
row_133	N4BP1	7233	-0.243487701	-0.024647279	No
row_134	NCKAP1	7288	-0.251544535	-0.027615972	No
row_135	ARL6	7322	-0.256415933	-0.02814462	No
row_136	ODC1	7420	-0.270489961	-0.035747565	No
row_137	GLRX	7606	-0.305458963	-0.052882176	No
row_138	EMP1	7724	-0.326025784	-0.062055685	No
row_139	PLSCR1	7743	-0.330806583	-0.05995376	No
row_140	GSTO1	7766	-0.33818379	-0.058212638	No
row_141	DBNDD2	7793	-0.348227233	-0.05679895	No
row_142	GCLM	7817	-0.354700118	-0.05496436	No
row_143	EEA1	7818	-0.354701549	-0.050524116	No
row_144	CYFIP1	7851	-0.365294427	-0.049576506	No
row_145	LGALS1	7938	-0.389408588	-0.05444462	No
row_146	MDFIC	7966	-0.399191976	-0.052506227	No
row_147	GPHN	7999	-0.411107689	-0.050985117	No
row_148	EI24	8062	-0.438938588	-0.05251427	No
row_149	TSPAN4	8082	-0.448203683	-0.049056027	No
row_150	GZMB	8137	-0.477021605	-0.04920214	No
row_151	SNX10	8177	-0.499436289	-0.04736833	No
row_152	SERPINB9	8207	-0.521071732	-0.04413079	No
row_153	ELL2	8209	-0.521785796	-0.037712228	No
row_154	LGALS3	8222	-0.528832972	-0.032451626	No
row_155	CLDND1	8271	-0.560261071	-0.030875992	No
row_156	SOCS2	8300	-0.577311635	-0.02682114	No
row_157	BCL2L2	8334	-0.604336917	-0.022994421	No
row_158	CASP1	8340	-0.613360703	-0.015882656	No
row_159	KLRC1	8390	-0.668770671	-0.01306196	No
row_160	PBX3	8444	-0.723911881	-0.010004147	No
row_161	RPGR	8451	-0.730694413	-0.001536857	No
row_162	SLC35E4	8563	-0.861795783	-0.003323725	No
row_163	DAPK2	8571	-0.880099714	0.006900571	No
row_164	FHIT	8639	-1.005424857	0.011896394	No
row_165	NRP1	8648	-1.026351571	0.02383822	No
row_166	PLCD1	8733	-1.208893061	0.029455204	No
